# Supplementary material for: Two–Dimensional and Doppler trans-thoracic echocardiographic patterns of suspected pediatric heart diseases at Tibebe-—Ghion specialized Teaching Hospital and Adinas General Hospital, Bahir Dar, North-west Ethiopia:–An experience from an LMIC
Source: PLoS One. 2024 Mar 11;19(3):e0292694. doi: 10.1371/journal.pone.0292694 (PMC10927071; doi:10.1371/journal.pone.0292694)
Supplement: S1 Table — (DOCX) [file pone.0292694.s001.docx]

| **S1 Table: Proportion of children with heart diseases against reasons of referral for echocardiography; June 2019 – May 2023.** | | | | | | | | | |
| --- | --- | --- | --- | --- | --- | --- | --- | --- | --- |
| **Reasons of referral for echocardiography** | | **Proportion of children with heart diseases** | | | **Reasons of referral for echocardiography** | | **proportion of children with heart diseases** | | |
|  | **#** | **Yes** | **No** | **%** |  | **#** | **Yes** | **No** | **%** |
| Cyanosis | 212 | 212 | 0 | 100% | Palpitation | 205 | 120 | 85 | 58.5% |
| Clubbing | 44 | 44 | 0 | 100% | Syndromic Child | 553 | 314 | 239 | 56.8% |
| Pericardial Friction rub | 13 | 13 | 0 | 100% | Failure to Thrive | 114 | 59 | 55 | 51.8% |
| Thrombo-embolic events | 6 | 6 | 0 | 100% | Galloping Infection/ abscess | 40 | 20 | 20 | 50% |
| Perinatal Asphyxia | 2 | 2 | 0 | 100% | Easy Fatigability/Diaphoresis | 521 | 255 | 266 | 48.9% |
| Corrective or Palliative Surgery | 11 | 11 | 0 | 100% | Connective tissue Disorders | 18 | 8 | 10 | 44.4% |
| Congestive Heart Failure | 516 | 513 | 3 | 99.4% | Cardiac silhouette abnormality | 71 | 31 | 40 | 43.7% |
| Suspected Infective endocarditis | 49 | 48 | 1 | 98% | Arrhythmia | 41 | 15 | 26 | 36.6% |
| Cardiogenic Shock | 18 | 17 | 1 | 94.4% | Chest Pain | 74 | 25 | 49 | 33.8% |
| Murmurs | 1207 | 1136 | 71 | 94.1% | Pre-Operative Screening | 52 | 11 | 41 | 21.2% |
| Dyspnoea on exertion | 275 | 257 | 18 | 93.5% | Congenital Stridor | 5 | 1 | 4 | 20% |
| Absent/asymmetry/Diminished pulse | 6 | 5 | 1 | 83.3% | Recurrent Chest Infection | 252 | 50 | 202 | 19.8% |
| Acute Rheumatic Fever/Recurrence | 476 | 393 | 83 | 82.6% | PPHTN | 31 | 6 | 25 | 19.4% |
| Family History of Heart Disease | 11 | 9 | 2 | 81.8% | Syncope | 11 | 2 | 9 | 18.2% |
| Suspected Pulmonary Hypertension | 32 | 24 | 8 | 75% | Infant of Diabetic Mother | 6 | 1 | 5 | 16.7% |
| Systemic Hypertension | 15 | 11 | 4 | 73.3% | Foetal Echo Diagnosed CHD | 1 | 0 | 1 | 0 |
| Respiratory Distress | 651 | 417 | 234 | 64% | **Total Studies** | **3647** | **2273** | **1374** | **62%** |
| Remark: The sum and proportion of reasons of referral for echocardiography are greater than 3647 and 100% respectively as a child can present with more than one reason of referral. CHD = Congenital heart defect. PPHTN = Persistent pulmonary Hypertension of the newborn. | | | | | | | | | |
