# Supplementary material for: Two–Dimensional and Doppler trans-thoracic echocardiographic patterns of suspected pediatric heart diseases at Tibebe-—Ghion specialized Teaching Hospital and Adinas General Hospital, Bahir Dar, North-west Ethiopia:–An experience from an LMIC
Source: PLoS One. 2024 Mar 11;19(3):e0292694. doi: 10.1371/journal.pone.0292694 (PMC10927071; doi:10.1371/journal.pone.0292694)
Supplement: S1 File — (ZIP) [file pone.0292694.s002.zip › AGH3 Pediatric Echo report template 12.docx]

| Patient Name: Kalkidan Asmamaw. Patient ID: Pawe Hosp SEX/ Age: 4 9/12/F Date of Report: 21/09/2011_. BP: _____ Weight: ____ Height:____ BSA: ____. Referral Diagnosis: **Diaphoresis. AGH03.1771.** | | | |
| --- | --- | --- | --- |
| **Features** | **Finding** | **Features** | **Finding** |
| **Profile** |  | **Atria** |  |
| Abdominal situs | Solitus | Left atrium |  |
| Cardiac position | Levocardia | Right atrium |  |
| Systemic venous drainage | Normal | **Atrioventricular valves** |  |
| Pulmonary venous drainage | Normal | Mitral valve |  |
| Atrioventricular connection | Concordant | Tricuspid valve |  |
| Ventriculoarterial connection | Concordant | **Ventricles** |  |
| Ventricular loop | d-Loop | Left ventricle |  |
|  |  | Right ventricle |  |
| **Septae** |  | **Coronary arteries** |  |
| Interventricular septum | intact | **Doppler Measurement** |  |
| Interatrial septum | intact | Mitral | No MR/MS |
| **Semilunal valves** |  | Aortic | No AR/AS |
| Aortic valve |  | Tricuspid | No TR/TS |
| Pulmonary valve |  | pulmonic | No PR/PS |
| **Great arteries** | NRGA | **Aortic arch** |  |
| Aorta |  | **PDA** |  |
| Pulmonary artery |  |  |  |
| **M-Mode** | | | |
| AO | mm | PWd | mm |
| LA | mm | EDV | ml |
| LVIDd | mm | ESV | Ml |
| LVIDs | mm | LVEF | % |
| IVSd | mm | FS | % |
| **Additional Information**: |  | | |
| N.B: Child was restless | | | |
| **Final Diagnosis:** | | | |
| Normal Heart study. | | | |

Name Signature Date

Tesfaye T., Pediatric Cardiology _____________________ ______________

**Pediatric Echocardiography Report**

**Name of Patient**: Hassen Adem

**Age**: 8years

**Sex**: Male

**Card Number**: 107534 (Adinas)

Situs Solitus, Levocardia

Pulmonary Veins drain to LA.

Systemic veins drain in to RA.

Mitral Valve is thickened with Moderate Mitral Regurgitation.

Severe Tricuspid Regurgitation with PPG = 100mmHg (Severe TR)

No Aortic Regurgitation/Stenosis.

RV is dilated.

Confluent Branch Pulmonary Arteries.

Left ventricular ejection fraction = 54%

No Pericardial/pleural effusion.

**Conclusion:**

Moderate Mitral Regurgitation Secondary to ?Rheumatic Heart Disease

Severe PAH (Pulmonary Hypertension)

**Performed and Reported By**: Tesfaye T., Pediatrician, Pediatric Cardiology.

| Patient Name: **Baby Melkam Adem**. Patient ID: Marie Stopes SEX/ Age: F/5/12 Date of Report: 28/9/2011. BP: _____ Weight: ____ Height:____ BSA: ____. Referral Diagnosis: **DS. AGH06.1772.** | | | |
| --- | --- | --- | --- |
| **Features** | **Finding** | **Features** | **Finding** |
| **Profile** |  | **Atria** |  |
| Abdominal situs | Solitus | Left atrium | N |
| Cardiac position | Levocardia | Right atrium | N |
| Systemic venous drainage | In to RA | **Atrioventricular valves** |  |
| Pulmonary venous drainage | In to LA | Mitral valve | N |
| Atrioventricular connection | Concordant | Tricuspid valve | N |
| Ventriculoarterial connection | Concordant | **Ventricles** |  |
| Ventricular loop | d loop | Left ventricle | N |
|  |  | Right ventricle | N |
| **Septae** |  | **Coronary arteries** |  |
| Interventricular septum | Peri-membranous VSD, 5mm | **Doppler Measurement** |  |
| Interatrial septum | intact | Mitral |  |
| **Semilunal valves** |  | Aortic |  |
| Aortic valve | Annulus = 14mm | Tricuspid |  |
| Pulmonary valve | Annulus = 16mm | pulmonic |  |
| **Great arteries** |  | **Aortic arch** |  |
| Aorta | N | **PDA** | No PDA |
| Pulmonary artery | N |  |  |
| **M-Mode** | | | |
| AO | mm | PWd | 6.5mm |
| LA | mm | EDV | 33.59ml |
| LVIDd | 29.5mm | ESV | 16.2ml |
| LVIDs | 22mm | LVEF | 55% |
| IVSd | 6mm | FS | 29% |
| **Additional Information**: |  | | |
|  | | | |
| **Final Diagnosis:**   1. **{S,D,S}, Levocardia** 2. **Small Perimembranous VSD** | | | |

Name Signature Date

Tesfaye Taye, Pediatrician, Pediatric Cardiologist ______________

| Patient Name: Surafel Yidnekachew. Patient ID: TGSH SEX/ Age: M/10 Date of Report: 28/9/2011_. BP: _____ Weight: ____ Height:____ BSA: ____. Referral Diagnosis: **CHF. AGH06.1773.** | | | |
| --- | --- | --- | --- |
| **Features** | **Finding** | **Features** | **Finding** |
| **Profile** |  | **Atria** |  |
| Abdominal situs | Solitus | Left atrium | Dilated |
| Cardiac position | Levocardia | Right atrium | Dilated |
| Systemic venous drainage | In to RA | **Atrioventricular valves** |  |
| Pulmonary venous drainage | In to LA | Mitral valve | N |
| Atrioventricular connection | Concordant | Tricuspid valve |  |
| Ventriculoarterial connection | Concordant | **Ventricles** |  |
| Ventricular loop | d loop | Left ventricle | Dilated |
|  |  | Right ventricle | Dilated |
| **Septae** |  | **Coronary arteries** |  |
| Interventricular septum | intact | **Doppler Measurement** |  |
| Interatrial septum | intact | Mitral | No MR/MS |
| **Semilunal valves** |  | Aortic | Trivial AR |
| Aortic valve |  | Tricuspid | Grade II TR, PPG = 94mmHg |
| Pulmonary valve |  | pulmonic |  |
| **Great arteries** | NRGA | **Aortic arch** |  |
| Aorta |  | **PDA** | 2.7mm PDA |
| Pulmonary artery |  |  |  |
| **M-Mode** | | | |
| AO | mm | PWd | mm |
| LA | mm | EDV | ml |
| LVIDd | mm | ESV | Ml |
| LVIDs | mm | LVEF | 46% |
| IVSd | mm | FS | % |
| **Additional Information**: |  | | |
|  | | | |
| **Final Diagnosis:** | | | |
| 1. Large PDA 2. Trivial AR 3. Grade II TR 4. Severe Pulmonary Hypertension secondary to ?   Needs follow up echo | | | |

Name Signature Date

Tesfaye T., Pediatrician, pediatric Cardiologist ______________

| Patient Name: **Abrham Yekoye**. Patient ID: **FHRRH** SEX/ Age: **M/5years** Date of Report: **05/10/2011**. BP: _____ Weight: ____ Height:____ BSA: ____. Referral Diagnosis: **Recurrent Chest Infection. AGH06.1774.** | | | |
| --- | --- | --- | --- |
| **Features** | **Finding** | **Features** | **Finding** |
| **Profile** |  | **Atria** |  |
| Abdominal situs | Solitus | Left atrium | Normal |
| Cardiac position | Levocardia | Right atrium | Normal |
| Systemic venous drainage | To RA | **Atrioventricular valves** |  |
| Pulmonary venous drainage | To LA | Mitral valve |  |
| Atrioventricular connection | Concordant | Tricuspid valve |  |
| Ventriculoarterial connection | Concordant | **Ventricles** |  |
| Ventricular loop | d Loop | Left ventricle | Normal |
|  |  | Right ventricle | Normal |
| **Septae** |  | **Coronary arteries** |  |
| Interventricular septum | Intact | **Doppler Measurement** |  |
| Interatrial septum | PFO, L – R shunt | Mitral | No MR/MS |
| **Semilunal valves** |  | Aortic | No AR/AS |
| Aortic valve |  | Tricuspid | Trivial TR |
| Pulmonary valve |  | pulmonic | Trivial PR |
| **Great arteries** | NRGA | **Aortic arch** |  |
| Aorta |  | **PDA** | No PDA |
| Pulmonary artery |  |  |  |
| **M-Mode** | | | |
| AO | mm | PWd | mm |
| LA | mm | EDV | 26.49ml |
| LVIDd | 26.8mm | ESV | 10.02ml |
| LVIDs | 18.2mm | LVEF | 62% |
| IVSd | 6.8mm | FS | 32% |
| **Additional Information**: |  | | |
|  | | | |
| **Final Diagnosis: PFO** | | | |
|  | | | |

Name Signature Date

Tesfaye T., Pediatrician & pediatric Cardiologist _____________________ ______________

| Patient Name: **Baby Melkam Adem**. Patient ID: MSI SEX/ Age: M/5/12 Date of Report: 28/9/2011.  BP: _____ Weight: ____ Height:____ BSA: ____. Referral Diagnosis: **Incidental Murmur. AGH06.1775.** | | | |
| --- | --- | --- | --- |
| **Features** | **Finding** | **Features** | **Finding** |
| **Profile** |  | **Atria** |  |
| Abdominal situs | Solitus | Left atrium | N |
| Cardiac position | Levocardia | Right atrium | N |
| Systemic venous drainage | In to RA | **Atrioventricular valves** |  |
| Pulmonary venous drainage | In to LA | Mitral valve | N |
| Atrioventricular connection | Concordant | Tricuspid valve | N |
| Ventriculoarterial connection | Concordant | **Ventricles** |  |
| Ventricular loop | L loop | Left ventricle | N |
|  |  | Right ventricle | N |
| **Septae** |  | **Coronary arteries** |  |
| Interventricular septum | 3mm Muscular VSD | **Doppler Measurement** |  |
| Interatrial septum | intact | Mitral |  |
| **Semilunal valves** |  | Aortic |  |
| Aortic valve | N | Tricuspid |  |
| Pulmonary valve | N | pulmonic |  |
| **Great arteries** |  | **Aortic arch** |  |
| Aorta | N | **PDA** | No PDA |
| Pulmonary artery | N |  |  |
| **M-Mode** | | | |
| AO | mm | PWd | mm |
| LA | mm | EDV | ml |
| LVIDd | mm | ESV | Ml |
| LVIDs | mm | LVEF | % |
| IVSd | mm | FS | % |
| **Additional Information**: |  | | |
| - Infant was crying and tachycardic during study | | | |
| **Final Diagnosis:** | | | |
| Small Muscular VSD | | | |

| Patient Name: **Meron Tesfaye**. Patient ID: **FHRRH** SEX/ Age: **F/4/12** Date of Report: **30/09/2011**.  BP: __ Weight: __ Height:__ BSA: __. Referral Diagnosis: **Recurrent Chest Infection. AGH06.1776.** | | | |
| --- | --- | --- | --- |
| **Features** | **Finding** | **Features** | **Finding** |
| **Profile** |  | **Atria** |  |
| Abdominal situs | Solitus | Left atrium |  |
| Cardiac position | Levocardia | Right atrium |  |
| Systemic venous drainage | To RA | **Atrioventricular valves** |  |
| Pulmonary venous drainage | To LA | Mitral valve |  |
| Atrioventricular connection | Concordant | Tricuspid valve |  |
| Ventriculoarterial connection | Concordant | **Ventricles** |  |
| Ventricular loop | d loop | Left ventricle |  |
|  |  | Right ventricle |  |
| **Septae** |  | **Coronary arteries** |  |
| Interventricular septum | intact | **Doppler Measurement** |  |
| Interatrial septum | intact | Mitral | No MS/MR |
| **Semilunal valves** |  | Aortic | No AS/AR |
| Aortic valve |  | Tricuspid | No TS/TR |
| Pulmonary valve |  | pulmonic | No PS/PR |
| **Great arteries** | NRGA | **Aortic arch** |  |
| Aorta |  | **PDA** | NO PDA |
| Pulmonary artery |  |  |  |
| **M-Mode** | | | |
| AO | mm | PWd | mm |
| LA | mm | EDV | ml |
| LVIDd | mm | ESV | Ml |
| LVIDs | mm | LVEF | % |
| IVSd | mm | FS | % |
| **Additional Information**: |  | | |
| - Only subcostal window is accessible. Needs follow up echo | | | |
| **Final Diagnosis:** | | | |
| NHS (Normal Heart Study) | | | |

Name Signature Date

**Tesfaye T., Pediatrician, Pediatric Cardiologist**  30/09/2011

| Patient Name: **Endalamaw Adane**. Patient ID: **KYMC** SEX/ Age: M**/12** Date of Report: **8/10/2011**.  BP: _____ Weight: ____ Height:____ BSA: ____. Referral Diagnosis: **RHD + Recurrence. AGH06.1777.** | | | |
| --- | --- | --- | --- |
| **Features** | **Finding** | **Features** | **Finding** |
| **Profile** |  | **Atria** |  |
| Abdominal situs | Solitus | Left atrium | Dilated |
| Cardiac position | Levocardia | Right atrium | Normal |
| Systemic venous drainage | To RA | **Atrioventricular valves** |  |
| Pulmonary venous drainage | To LA | Mitral valve | Thickened valve |
| Atrioventricular connection | Concordant | Tricuspid valve | Normal |
| Ventriculoarterial connection | Concordant | **Ventricles** |  |
| Ventricular loop | d loop | Left ventricle | Dilated |
|  |  | Right ventricle | Normal |
| **Septae** |  | **Coronary arteries** |  |
| Interventricular septum | Intact | **Doppler Measurement** |  |
| Interatrial septum | intact | Mitral | G-II MR, lateral project |
| **Semilunal valves** |  | Aortic | Moderate AR, PHT = 402 |
| Aortic valve | Thickened valve | Tricuspid | Trivial TR |
| Pulmonary valve | Normal | pulmonic | Trivial PR, PPG = 19mmHg |
| **Great arteries** | NRGA | **Aortic arch** |  |
| Aorta |  | **PDA** | NO PDA |
| Pulmonary artery | Confluent branch PAs |  |  |
| **M-Mode** | | | |
| AO | mm | PWd | 8.5mm |
| LA | mm | EDV | 113.77ml |
| LVIDd | 49.2mm | ESV | 60.57ml |
| LVIDs | 37.6mm | LVEF | 46.76% |
| IVSd | 6.7mm | FS | 23.46% |
| **Additional Information**: |  | | |
| **Final Diagnosis:** | | | |
| 1. Grade II MR 2. Moderate AR 3. ?Rheumatic Heart disease (correlate with the clinical scenario) | | | |

Name Signature Date

**Tesfaye T., Pediatrician, Pediatric Cardiologist**  08/10/2011

| Patient Name: **Fanuel Melkamu**. Patient ID: **104852** SEX/ Age: 1/12/M Date of Report: 10/10/2011. BP: _____ Weight: ____ Height:____ BSA: ____. Referral Diagnosis: **Cyanosis + RD. AGH06.1778.** | | | |
| --- | --- | --- | --- |
| **Features** | **Finding** | **Features** | **Finding** |
| **Profile** |  | **Atria** |  |
| Abdominal situs | Solitus | Left atrium | ------ |
| Cardiac position | Levocardia | Right atrium | ------ |
| Systemic venous drainage | Normal | **Atrioventricular valves** |  |
| Pulmonary venous drainage | Normal | Mitral valve | ------ |
| Atrioventricular connection | Concordant | Tricuspid valve | ------ |
| Ventriculoarterial connection | DisConcordant | **Ventricles** |  |
| Ventricular loop | d-Loop | Left ventricle | ------ |
|  |  | Right ventricle | ------ |
| **Septae** |  | **Coronary arteries** |  |
| Interventricular septum | intact | **Doppler Measurement** |  |
| Interatrial septum | 4mm secundum defect, BD shunt | Mitral | ------ |
| **Semilunal valves** |  | Aortic | ------ |
| Aortic valve | Trileaflet | Tricuspid | ------ |
| Pulmonary valve | Trileaflet | pulmonic | ------ |
| **Great arteries** | d-TGA | **Aortic arch** | ------ |
| Aorta | to the right & anterior | **PDA** | No PDA |
| Pulmonary artery | to the left & posterior |  |  |
| **M-Mode** | | | |
| AO | mm | PWd | 6.7mm |
| LA | mm | EDV | 19.27ml |
| LVIDd | 23.6mm | ESV | 8.76ml |
| LVIDs | 17.3mm | LVEF | 54.56% |
| IVSd | 3.9mm | FS | 26.67% |
| **Additional Information**: |  | | |
|  | | | |
| **Final Diagnosis: d-TGA with Intact IVS** | | | |
|  | | | |

Name Signature Date

Tesfaye T., Pediatric Cardiology _____________________ 10/10/2011Eth.C

| Patient Name: **Yitayal Manalegn**. Patient ID: **FHRRH** SEX/ Age: 7yrs/M Date of Report: 11/10/2011.  BP: _____ Weight: ____ Height:____ BSA: ____. Referral Diagnosis: **_RHD + CHF. AGH06.1779.** | | | |
| --- | --- | --- | --- |
| **Features** | **Finding** | **Features** | **Finding** |
| **Profile** |  | **Atria** |  |
| Abdominal situs | Solitus | Left atrium | Dilated |
| Cardiac position | Levocardia | Right atrium | Dilated |
| Systemic venous drainage | Normal | **AV valves** |  |
| Pulmonary venous drainage | Normal | Mitral valve | Thickened, clubbed |
| Atrioventricular connection | Concordant | Tricuspid valve | TAPSE = 15mm |
| Ventriculoarterial connection | Concordant | **Ventricles** |  |
| Ventricular loop | d-Loop | Left ventricle | Dilated |
|  |  | Right ventricle | Dilated |
| **Septae** |  | **Coronary arteries** |  |
| Interventricular septum | Intact | **Doppler Measurement** |  |
| Interatrial septum | Intact | Mitral | Severe MR |
| **Semilunal valves** |  | Aortic | Mild AR, PHT = 530ms |
| Aortic valve | Trileaflet | Tricuspid | Severe TR, PPG = 70mmHg |
| Pulmonary valve | ……… | pulmonic | Mild PR, PPG = 48mmHg |
| **Great arteries** | NRGA | **Aortic arch** | ------ |
| Aorta |  | **PDA** | No PDA |
| Pulmonary artery |  |  |  |
| **M-Mode** | | | |
| AO | mm | PWd | 9.00mm |
| LA | mm | EDV | 121ml |
| LVIDd | 50.5mm | ESV | 44ml |
| LVIDs | 33.0mm | LVEF | 63% |
| IVSd | 8.5mm | FS | 34% |
|  | | | |
| **Final Diagnosis: Severe MR, Thickened Mitral Valve, severe TR, Mild AR, Severe Pulmonary Hypertension** | | | |

Name Signature Date

Tesfaye T., Pediatric Cardiologist _____________________ 11/10/2011Eth.C

| Patient Name: **Senayit Tilahun**. Patient ID: **105911** SEX/ Age: 11yrs/F Date of Report: 21/10/2011.  BP: _____ Weight: ____ Height:____ BSA: ____. Referral Diagnosis: **Incidental Murmur. AGH06.1780.** | | | |
| --- | --- | --- | --- |
| **Features** | **Finding** | **Features** | **Finding** |
| **Profile** |  | **Atria** |  |
| Abdominal situs | Solitus | Left atrium | Normal |
| Cardiac position | Levocardia | Right atrium | Dilated |
| Systemic venous drainage | Normal | **AV valves** |  |
| Pulmonary venous drainage | Normal | Mitral valve | Normal |
| Atrioventricular connection | Concordant | Tricuspid valve | TAPSE = 21mm |
| Ventriculoarterial connection | Concordant | **Ventricles** |  |
| Ventricular loop | d-Loop | Left ventricle | Normal |
|  |  | Right ventricle | Dilated |
| **Septae** |  | **Coronary arteries** | -------- |
| Interventricular septum | Intact | **Doppler Measurement** |  |
| Interatrial septum | Intact | Mitral | No MR/MS |
| **Semilunal valves** |  | Aortic | No AR/AS |
| Aortic valve | Trileaflet | Tricuspid | Grade II TR, PPG = 35mmHg |
| Pulmonary valve | Trileaflet | pulmonic | Doming valve, PPG/MPG =83/45mmHg |
| **Great arteries** | NRGA | **Aortic arch** | Left |
| Aorta | to the right & Posterior | **PDA** | No PDA |
| Pulmonary artery | to the left & anterior |  |  |
| **M-Mode** | | | |
| AO | mm | PWd | 4.4mm |
| LA | mm | EDV | ml |
| LVIDd | 33.3mm | ESV | ml |
| LVIDs | 23.2mm | LVEF | 58% |
| IVSd | 13.2mm | FS | 30% |
| **Final Diagnosis:**   1. **{S, D, S} Levocardia** 2. **Grade II TR** 3. **Severe Pulmonary Stenosis** 4. **Doming pulmonary Valve** 5. **RA/RV Dilated** 6. **Good biventricular Function** | | | |

Name Signature Date

Tesfaye T., Pediatric Cardiologist _____________________ 21/10/2011Eth.C

| Patient Name: **Tsigereda Firew**. Patient ID: **Addis Alem H.** SEX/ Age: 8/12/F Date of Report: 25/10/2011.  BP: __ Weight: ____ Height:____ BSA: ____. Referral Diagnosis: **Recurrent Chest Infection. AGH06.1781.** | | | | |
| --- | --- | --- | --- | --- |
| **Features** | **Finding** | | **Features** | **Finding** |
| **Profile** |  | | **Atria** |  |
| Abdominal situs | Solitus | | Left atrium | Normal |
| Cardiac position | Levocardia | | Right atrium | Normal |
| Systemic venous drainage | Normal | | **AV valves** |  |
| Pulmonary venous drainage | Normal | | Mitral valve | A= 14mm |
| Atrioventricular connection | Concordant | | Tricuspid valve | A = 12mm |
| Ventriculoarterial connection | Concordant | | **Ventricles** |  |
| Ventricular loop | d-Loop | | Left ventricle | Normal |
|  |  | | Right ventricle | Normal |
| **Septae** |  | | **Coronary arteries** |  |
| Interventricular septum | Intact | | **Doppler Measurement** |  |
| Interatrial septum | Intact | | Mitral | No MS/MR |
| **Semilunal valves** |  | | Aortic | No AS/AR |
| Aortic valve | Trileaflet, A=12mm | | Tricuspid | No TS/TR |
| Pulmonary valve | A = 13mm | | pulmonic | No PS/PR |
| **Great arteries** | NRGA | | **Aortic arch** | -------- |
| Aorta |  | | **PDA** | No PDA |
| Pulmonary artery |  | |  |  |
| **M-Mode** | | | | |
| AO | | mm | PWd | 5mm |
| LA | | mm | EDV | 7.25ml |
| LVIDd | | 16.1mm | ESV | 3.2ml |
| LVIDs | | 11.8mm | LVEF | 55% |
| IVSd | | 5mm | FS | 26.7% |
| **Final Diagnosis:**   1. **{S, D, S} Levocardia** 2. **Good biventricular Function** 3. **Normal Heart Study (NHS)** | | | | |

Name Signature Date

Tesfaye T., Pediatric Cardiologist _____________________ 25/10/2011Eth.C

| Patient Name: Tsion Beminabu. Patient ID: K/Mihret SEX/ Age: 11years/F Date of Report: 26/10/2011. BP: _____ Weight: ____ Height:____ BSA: ____. Referral Diagnosis: **Palpitation. AGH06.1782.** | | | | |
| --- | --- | --- | --- | --- |
| **Features** | **Finding** | **Features** | **Finding** | |
| **Profile** |  | **Atria** |  | |
| Abdominal situs | Solitus | Left atrium | ----- | |
| Cardiac position | Levocardia | Right atrium | ------ | |
| Systemic venous drainage | Normal | **Atrioventricular valves** |  | |
| Pulmonary venous drainage | Normal | Mitral valve | ----- | |
| Atrioventricular connection | Concordant | Tricuspid valve | TAPSE = 22mm | |
| Ventriculoarterial connection | Concordant | **Ventricles** |  | |
| Ventricular loop | d-Loop | Left ventricle | ------ | |
|  |  | Right ventricle | ----- | |
| **Septae** |  | **Coronary arteries** |  | |
| Interventricular septum | Intact | **Doppler Measurement** |  | |
| Interatrial septum | Intact | Mitral | No MR/MS | |
| **Semilunal valves** |  | Aortic | No AR/AS | |
| Aortic valve | Trileaflet | Tricuspid | No TR/TS | |
| Pulmonary valve | ----- | pulmonic | Trivial PR, PPG =11mmHg | |
| **Great arteries** | NRGA | **Aortic arch** | left | |
| Aorta |  | **PDA** | No PDA | |
| Pulmonary artery | Confluent branch PAs |  |  | |
| **M-Mode** | | | | |
| AO | mm | PWd | | 7mm |
| LA | mm | EDV | | 92.45ml |
| LVIDd | 32.1mm | ESV | | 41.41ml |
| LVIDs | 32.1mm | LVEF | | 55.% |
| IVSd | 4.8mm | FS | | 28% |
| **Additional Information**: |  | | | |
|  | | | | |
| **Final Diagnosis:** | | | | |
| 1. {S, D, S} Levocardia 2. Normal Heart study. | | | | |

Name Signature Date

Tesfaye T., Pediatric Cardiology _____________________ 26/10/2011Eth.C

| Patient Name: Yalfal Mengesha. Patient ID: 099922 Sex/ Age: M/12 Date of Report: 01/11/2011_. BP: _____ Weight: ____ Height:____ BSA: ____. Referral Diagnosis: **DOE. AGH06.1783.** | | | | |
| --- | --- | --- | --- | --- |
| **Features** | **Finding** | **Features** | **Finding** | |
| **Profile** |  | **Atria** |  | |
| Abdominal situs | Solitus | Left atrium | Dilated | |
| Cardiac position | Levocardia | Right atrium | Normal | |
| Systemic venous drainage | Normal | **Atrioventricular valves** |  | |
| Pulmonary venous drainage | Normal | Mitral valve | Normal | |
| Atrioventricular connection | Concordant | Tricuspid valve | Normal | |
| Ventriculoarterial connection | Concordant | **Ventricles** |  | |
| Ventricular loop | d-Loop | Left ventricle | Mildly dilated | |
|  |  | Right ventricle | TAPSE = 18mm | |
| **Septae** |  | **Coronary arteries** |  | |
| Interventricular septum | Intact | **Doppler Measurement** |  | |
| Interatrial septum | Intact | Mitral | No MR/MS | |
| **Semilunal valves** |  | Aortic | No AR/AS | |
| Aortic valve | Normal | Tricuspid | No TR/TS | |
| Pulmonary valve | Normal | pulmonic | Trivial PR | |
| **Great arteries** | NRGA | **Aortic arch** | Left | |
| Aorta | ----- | **PDA** | No PDA | |
| Pulmonary artery | ----- |  |  | |
| **M-Mode** | | | | |
| AO | mm | PWd | | 8mm |
| LA | mm | EDV | | 111ml |
| LVIDd | 48.7mm | ESV | | 56ml |
| LVIDs | 36.4mm | LVEF | | 49% |
| IVSd | 5.9mm | FS | | 25% |
| **Additional Information**: |  | | | |
| **Final Diagnosis:** | | | | |
| 1. {S, D, S} Levocardia 2. Normal RV Function 3. Mildly dilated LV 4. Mildly Reduced LV Function | | | | |

Tesfaye T., Pediatric Cardiologist . . Date: 01/11/2011Eth.C

| Patient Name: **Ahmed Suleiman**. Patient ID: **107073** SEX/ Age: 11yrs/M Date of Report: 02/11/2011_. BP: _____ Weight: ____ Height:____ BSA: ____. Referral Diagnosis: **Easy Fatigability. AGH06.1784.** | | | | |
| --- | --- | --- | --- | --- |
| **Features** | **Finding** | **Features** | **Finding** | |
| **Profile** |  | **Atria** |  | |
| Abdominal situs | Solitus | Left atrium | Normal | |
| Cardiac position | Levocardia | Right atrium | Normal | |
| Systemic venous drainage | Normal | **Atrioventricular valves** |  | |
| Pulmonary venous drainage | Normal | Mitral valve | Normal leaflets | |
| Atrioventricular connection | Concordant | Tricuspid valve | TAPSE = 20mm | |
| Ventriculoarterial connection | Concordant | **Ventricles** |  | |
| Ventricular loop | d-Loop | Left ventricle | Normal | |
|  |  | Right ventricle | Normal | |
| **Septae** |  | **Coronary arteries** | ----- | |
| Interventricular septum | Intact | **Doppler Measurement** |  | |
| Interatrial septum | Intact | Mitral | Trivial MR | |
| **Semilunal valves** |  | Aortic | No AR/AS | |
| Aortic valve | Trileaflet, | Tricuspid | No TR/TS | |
| Pulmonary valve | Trileaflet | pulmonic | Trivial PR | |
| **Great arteries** | NRGA | **Aortic arch** | Left | |
| Aorta | ---- | **PDA** | ------- | |
| Pulmonary artery | ------ |  |  | |
| **M-Mode** | | | | |
| AO | mm | PWd | | 8.6mm |
| LA | mm | EDV | | 65ml |
| LVIDd | 38.9mm | ESV | | 32.6ml |
| LVIDs | 29.1mm | LVEF | | 55% |
| IVSd | 6.3mm | FS | | 29% |
| **Additional Information**: |  | | | |
| No pleural/pericardial effusion | | | | |
| **Final Diagnosis:** | | | | |
| 1. {S, D, S} Levocardia 2. Trivial MR 3. Trivial PR 4. Good biventricular function | | | | |
| SIGNATURE  Done by: Tesfaye T., Pediatric Cardiologist _______________ 02/11/2011Eth.C | | | | |

| Patient Name**: Amira Nurhussen**. Patient ID: FHRRH. Age /SEX: 3/12/**F** Date of Report: **06/11/2011_**. BP: _____ Weight: ____ Height:____ BSA: ____. Referral Diagnosis: **Incidental Murmur. AGH06.1785.** | | | | |
| --- | --- | --- | --- | --- |
| **Features** | **Finding** | **Features** | **Finding** | |
| **Profile** |  | **Atria** |  | |
| Abdominal situs | Solitus | Left atrium | Normal | |
| Cardiac position | Levocardia | Right atrium | Normal | |
| Systemic venous drainage | Normal | **Atrioventricular valves** |  | |
| Pulmonary venous drainage | Normal | Mitral valve | Normal leaflets | |
| Atrioventricular connection | Concordant | Tricuspid valve | TAPSE = 11mm | |
| Ventriculoarterial connection | Concordant | **Ventricles** |  | |
| Ventricular loop | d-Loop | Left ventricle | Normal | |
|  |  | Right ventricle | Normal | |
| **Septae** |  | **Coronary arteries** | ----- | |
| Interventricular septum | Intact | **Doppler Measurement** |  | |
| Interatrial septum | Intact | Mitral | No MR/MS | |
| **Semilunal valves** |  | Aortic | No AR/AS | |
| Aortic valve | Trileaflet, | Tricuspid | Trivial TR, PPG = 11mmHg | |
| Pulmonary valve | Annulus = 5mm | pulmonic | Moderate PS with PPG of 54mmHg | |
| **Great arteries** | NRGA | **Aortic arch** | Left | |
| Aorta | ---- | **PDA** | ------- | |
| Pulmonary artery | ------ |  |  | |
| **M-Mode:** Normal function on eye balling | | | | |
| AO | mm | PWd | | mm |
| LA | mm | EDV | | ml |
| LVIDd | mm | ESV | | ml |
| LVIDs | mm | LVEF | | % |
| IVSd | mm | FS | | % |
| **Additional Information**: |  | | | |
| Patient was restless and crying | | | | |
| **Final Diagnosis:** | | | | |
| 1. {S, D, S} Levocardia 2. Trivial TR 3. Moderate PS 4. Good biventricular function | | | | |
| SIGNATURE  Done by: Tesfaye T., Pediatric Cardiologist _______________ 06/11/2011Eth.C | | | | |

| Patient Name**:Yoseph Ejigu**. Patient ID: **FHRRH**. SEX/ Age: M/9/12/**M** Date of Report: **09/11/2011_**.  BP: _____ Weight: ____ Height:____ BSA: ____. Referral Diagnosis: **Incidental Murmur. AGH06.1786.** | | | | |
| --- | --- | --- | --- | --- |
| **Features** | **Finding** | **Features** | **Finding** | |
| **Profile** |  | **Atria** |  | |
| Abdominal situs | Solitus | Left atrium | Normal | |
| Cardiac position | Levocardia | Right atrium | Normal | |
| Systemic venous drainage | Normal | **Atrioventricular valves** |  | |
| Pulmonary venous drainage | Normal | Mitral valve | Normal | |
| Atrioventricular connection | Concordant | Tricuspid valve | TAPSE = 13mm | |
| Ventriculoarterial connection | Concordant | **Ventricles** |  | |
| Ventricular loop | d-Loop | Left ventricle | Normal | |
|  |  | Right ventricle | Normal | |
| **Septae** |  | **Coronary arteries** | ----- | |
| Interventricular septum | Intact | **Doppler Measurement** |  | |
| Interatrial septum | Intact | Mitral | No MR/MS | |
| **Semilunal valves** |  | Aortic | No AR/AS | |
| Aortic valve | Annulus = 12mm, Trileaflet | Tricuspid | Mild TR, PPG = 34mmHg | |
| Pulmonary valve | Annulus = 12mm, Trileaflet | pulmonic | No PR/PS | |
| **Great arteries** | NRGA | **Aortic arch** | Left | |
| Aorta | ---- | **PDA** | ------- | |
| Pulmonary artery | ------ |  |  | |
| **M-Mode** | | | | |
| AO | mm | PWd | | 3.6mm |
| LA | mm | EDV | | 17ml |
| LVIDd | 22.5mm | ESV | | 4.99ml |
| LVIDs | 13.9mm | LVEF | | 70% |
| IVSd | 2.9mm | FS | | 38% |
| **Additional Information**: |  | | | |
| No pleural/pericardial effusion | | | | |
| **Final Diagnosis:** | | | | |
| 1. {S, D, S} Levocardia 2. Mild TR 3. Good biventricular function 4. Normal Heart Study | | | | |
| SIGNATURE  Done by: Tesfaye T., Pediatric Cardiologist _______________ 09/11/2011Eth.C | | | | |

| Name**: Anguach Yasab**. Patient ID: **Yohannes Clinic**. SEX/ Age: F/3/12 Date of Report: **09/11/2011_**.  BP: _____ Weight: ____ Height:____ BSA: ____. Referral Diagnosis: **DS. AGH06.1787.** | | | | |
| --- | --- | --- | --- | --- |
| **Features** | **Finding** | **Features** | **Finding** | |
| **Profile** |  | **Atria** |  | |
| Abdominal situs | Solitus | Left atrium | Normal | |
| Cardiac position | Levocardia | Right atrium | Normal | |
| Systemic venous drainage | Normal | **Atrioventricular valves** |  | |
| Pulmonary venous drainage | Normal | Mitral valve | ----- | |
| Atrioventricular connection | Concordant | Tricuspid valve | ------ | |
| Ventriculoarterial connection | Concordant | **Ventricles** |  | |
| Ventricular loop | d-Loop | Left ventricle | Normal | |
|  |  | Right ventricle | Normal | |
| **Septae** |  | **Coronary arteries** | ----- | |
| Interventricular septum | 5mm Septal defect | **Doppler Measurement** |  | |
| Interatrial septum | 12mm premium defect | Mitral | Mild left AVVR | |
| **Semilunal valves** |  | Aortic | No AR/AS | |
| Aortic valve | Annulus = 11mm | Tricuspid | Moderate right AVVR | |
| Pulmonary valve | Annulus = 12mm | pulmonic | No PR/PS | |
| **Great arteries** | NRGA | **Aortic arch** | Left | |
| Aorta | ---- | **PDA** | ------- | |
| Pulmonary artery | ------ |  |  | |
| **M-Mode (Normal function on eye balling)** | | | | |
| AO | mm | PWd | | mm |
| LA | mm | EDV | | ml |
| LVIDd | mm | ESV | | ml |
| LVIDs | mm | LVEF | | % |
| IVSd | mm | FS | | % |
| **Additional Information**: |  | | | |
| No pleural/pericardial effusion | | | | |
| **Final Diagnosis:** | | | | |
| 1. {S, D, S} Levocardia 2. Intermediate AVSD 3. Moderate right AVVR 4. Mild Left AVVR 5. Good biventricular function | | | | |
| SIGNATURE  Done by: Tesfaye T., Pediatric Cardiologist _______________ 09/11/2011Eth.C | | | | |

| Patient Name**: Tewabech Tesfa**. Patient ID: **ADINAS**. SEX/ Age: F/14years Date of Report: **09/11/2011_**. BP: _____ Weight: ____ Height:____ BSA: ____. Referral Diagnosis: **Incidental Murmur. AGH06.1788.** | | | | |
| --- | --- | --- | --- | --- |
| **Features** | **Finding** | **Features** | **Finding** | |
| **Profile** |  | **Atria** |  | |
| Abdominal situs | Solitus | Left atrium | Normal | |
| Cardiac position | Levocardia | Right atrium | Normal | |
| Systemic venous drainage | Normal | **Atrioventricular valves** |  | |
| Pulmonary venous drainage | Normal | Mitral valve | Normal leaflets | |
| Atrioventricular connection | Concordant | Tricuspid valve | TAPSE = 20mm | |
| Ventriculoarterial connection | Concordant | **Ventricles** |  | |
| Ventricular loop | d-Loop | Left ventricle | Normal | |
|  |  | Right ventricle | Normal | |
| **Septae** |  | **Coronary arteries** | ----- | |
| Interventricular septum | Intact | **Doppler Measurement** |  | |
| Interatrial septum | Intact | Mitral | No MR/MS | |
| **Semilunal valves** |  | Aortic | Mild AR, PHT = 517ms | |
| Aortic valve | Trileaflet, | Tricuspid | No TS/TR | |
| Pulmonary valve |  | pulmonic | No PR/PS | |
| **Great arteries** | NRGA | **Aortic arch** | Left | |
| Aorta | ---- | **PDA** | ------- | |
| Pulmonary artery | ------ |  |  | |
| **M-Mode** | | | | |
| AO | mm | PWd | | 10mm |
| LA | mm | EDV | | 53ml |
| LVIDd | 35.7mm | ESV | | 24ml |
| LVIDs | 25.7mm | LVEF | | 55% |
| IVSd | 6.4mm | FS | | 28% |
| **Additional Information**: |  | | | |
| No pleural/pericardial effusion | | | | |
| **Final Diagnosis:** | | | | |
| 1. {S, D, S} Levocardia 2. Mild AR 3. Good biventricular function | | | | |
| SIGNATURE  Done by: Tesfaye T., Pediatric Cardiologist _______________ 09/11/2011Eth.C | | | | |

| Patient Name**: Zema G/Mariam**. Patient ID: **ADINAS**. SEX/ Age: M/ 4 8/12years Date Report: 1**0/11/2011_**. BP: ___ Weight: ___ Height:___ BSA: ___. Referral Diagnosis: **HTN. AGH06.1789.** | | | | |
| --- | --- | --- | --- | --- |
| **Features** | **Finding** | **Features** | **Finding** | |
| **Profile** |  | **Atria** |  | |
| Abdominal situs | Solitus | Left atrium | Normal | |
| Cardiac position | Levocardia | Right atrium | Normal | |
| Systemic venous drainage | Normal | **Atrioventricular valves** |  | |
| Pulmonary venous drainage | Normal | Mitral valve | ---- | |
| Atrioventricular connection | Concordant | Tricuspid valve | ----- | |
| Ventriculoarterial connection | Concordant | **Ventricles** |  | |
| Ventricular loop | d-Loop | Left ventricle | Hypertrophied | |
|  |  | Right ventricle | Normal | |
| **Septae** |  | **Coronary arteries** | ----- | |
| Interventricular septum | Intact | **Doppler Measurement** |  | |
| Interatrial septum | Intact | Mitral | ---- | |
| **Semilunal valves** |  | Aortic | No LVOTO | |
| Aortic valve | Annulus = 17mm | Tricuspid | ----- | |
| Pulmonary valve | Annulus = 16mm | pulmonic | No RVOTO | |
| **Great arteries** | NRGA | **Aortic arch** | ------ | |
| Aorta | ---- | **PDA** | ------- | |
| Pulmonary artery | ------ |  |  | |
| **M-Mode** | | | | |
| AO | mm | PWs | | 13mm |
| LA | mm | PWd | | 8.8mm |
| LVIDd | 33.9mm | EDV | | 47ml |
| LVIDs | 24.6mm | ESV | | 21.5ml |
| IVSd | **10.2mm** | LVEF | | 54% |
| IVSs | 11.6mm | FS | | 27% |
| **Additional Information**: |  | | | |
| No pleural/pericardial effusion | | | | |
| **Final Diagnosis:** | | | | |
| 1. {S, D, S} Levocardia 2. LHV 3. No LVOTO/RVOTO 4. Good Function | | | | |
| SIGNATURE  Done by: Tesfaye T., Pediatric Cardiologist _______________ 10/11/2011Eth.C | | | | |

| Patient Name**: Kene’an G/Mariam**. Patient ID: **ADINAS**. SEX/ Age: M/ 6 8/12years Date of Report: 1**0/11/2011_**. BP: __ Weight: ___ Height:___ BSA: __. Referral Diagnosis: **HTN. AGH06.1790.** | | | | |
| --- | --- | --- | --- | --- |
| **Features** | **Finding** | **Features** | **Finding** | |
| **Profile** |  | **Atria** |  | |
| Abdominal situs | Solitus | Left atrium | Normal | |
| Cardiac position | Levocardia | Right atrium | Normal | |
| Systemic venous drainage | Normal | **Atrioventricular valves** |  | |
| Pulmonary venous drainage | Normal | Mitral valve | ---- | |
| Atrioventricular connection | Concordant | Tricuspid valve | ----- | |
| Ventriculoarterial connection | Concordant | **Ventricles** |  | |
| Ventricular loop | d-Loop | Left ventricle | Hypertrophied | |
|  |  | Right ventricle | Normal | |
| **Septae** |  | **Coronary arteries** | ----- | |
| Interventricular septum | Intact | **Doppler Measurement** |  | |
| Interatrial septum | Intact | Mitral | ---- | |
| **Semilunal valves** |  | Aortic | No LVOTO | |
| Aortic valve | Normal | Tricuspid | ----- | |
| Pulmonary valve | Normal | pulmonic | No RVOTO | |
| **Great arteries** | NRGA | **Aortic arch** | ------ | |
| Aorta | ---- | **PDA** | ------- | |
| Pulmonary artery | ------ |  |  | |
| **M-Mode** | | | | |
| AO | mm | PWs | | 7mm |
| LA | mm | PWd | | 9.6mm |
| LVIDd | 43.4mm | EDV | | 85ml |
| LVIDs | 35.9mm | ESV | | 54ml |
| IVSd | **11.2mm** | LVEF | | 52% |
| IVSs | 11.3mm | FS | | 27% |
| **Additional Information**: |  | | | |
| No pleural/pericardial effusion | | | | |
| **Final Diagnosis:** | | | | |
| 1. {S, D, S} Levocardia 2. LHV 3. No LVOTO/RVOTO 4. Good Function | | | | |
| SIGNATURE  Done by: Tesfaye T., Pediatric Cardiologist _______________ 10/11/2011Eth.C | | | | |

| Patient Name**: Tekle Ab Asfaw** . Patient ID: **DTH**. SEX/ Age: M/12years Date of Report: 1**2/11/2011_**.  BP: ___ Weight: ___ Height:___ BSA: ___. Referral Diagnosis: **Sydenham’s Chorea. AGH06.1791.** | | | | |
| --- | --- | --- | --- | --- |
| **Features** | **Finding** | **Features** | **Finding** | |
| **Profile** |  | **Atria** |  | |
| Abdominal situs | Solitus | Left atrium | Normal | |
| Cardiac position | Levocardia | Right atrium | Normal | |
| Systemic venous drainage | Normal | **Atrioventricular valves** |  | |
| Pulmonary venous drainage | Normal | Mitral valve | Normal | |
| Atrioventricular connection | Concordant | Tricuspid valve | Normal | |
| Ventriculoarterial connection | Concordant | **Ventricles** |  | |
| Ventricular loop | d-Loop | Left ventricle | Normal | |
|  |  | Right ventricle | Normal | |
| **Septae** |  | **Coronary arteries** | ----- | |
| Interventricular septum | Intact | **Doppler Measurement** |  | |
| Interatrial septum | Intact | Mitral | ----- | |
| **Semilunal valves** |  | Aortic | ----- | |
| Aortic valve | Trileaflet | Tricuspid | Trivial TR, PPG = 14mmHg | |
| Pulmonary valve | Normal | pulmonic | ------ | |
| **Great arteries** | NRGA | **Aortic arch** | Left | |
| Aorta | ---- | **PDA** | No PDA | |
| Pulmonary artery | ------ |  |  | |
| **M-Mode (Normal function on eye balling)** | | | | |
| AO | mm | PWd | | mm |
| LA | mm | EDV | | ml |
| LVIDd | mm | ESV | | ml |
| LVIDs | mm | LVEF | | % |
| IVSd | mm | FS | | % |
| **Additional Information**: |  | | | |
| No pleural/pericardial effusion | | | | |
| **Final Diagnosis:** | | | | |
| 1. Normal Heart Study | | | | |
| SIGNATURE  Done by: Tesfaye T., Pediatric Cardiologist _______________ 12/11/2011Eth.C | | | | |

| Patient Name**: Yeshambel Agaxe** . Patient ID: **FHRRH**. SEX/ Age: M/8years Date of Report: 1**5/11/2011_**.  BP: ___ Weight: ___ Height:___ BSA: ___. Referral Diagnosis: **CHF + DOE. AGH06.1792.** | | | | |
| --- | --- | --- | --- | --- |
| **Features** | **Finding** | **Features** | **Finding** | |
| **Profile** |  | **Atria** |  | |
| Abdominal situs | Solitus | Left atrium | Dilated | |
| Cardiac position | Levocardia | Right atrium | Normal | |
| Systemic venous drainage | Normal | **Atrioventricular valves** |  | |
| Pulmonary venous drainage | Normal | Mitral valve | Normal | |
| Atrioventricular connection | Concordant | Tricuspid valve | Normal | |
| Ventriculoarterial connection | Concordant | **Ventricles** |  | |
| Ventricular loop | d-Loop | Left ventricle | Dilated | |
|  |  | Right ventricle | Normal | |
| **Septae** |  | **Coronary arteries** | ----- | |
| Interventricular septum | 4mm PM VSD, L- R Shunt | **Doppler Measurement** |  | |
| Interatrial septum | 2.5mm PFO, L – R Shunt | Mitral | ---- | |
| **Semilunal valves** |  | Aortic | ----- | |
| Aortic valve | Trileaflet, | Tricuspid | Trivial TR, PPG = 14mmHg | |
| Pulmonary valve |  | pulmonic | ------ | |
| **Great arteries** | NRGA | **Aortic arch** |  | |
| Aorta | ---- | **PDA** | 4mm PDA, L – R Shunt with PPG/MPG = 80/24mmHg | |
| Pulmonary artery | ------ |  |  | |
| **M-Mode** | | | | |
| AO | mm | PWd | | 6.4mm |
| LA | mm | EDV | | 135ml |
| LVIDd | 53mm | ESV | | 50ml |
| LVIDs | 35mm | LVEF | | 63%% |
| IVSd | 8.6mm | FS | | 34% |
| **Additional Information**: |  | | | |
| No pleural/pericardial effusion | | | | |
| **Final Diagnosis:** | | | | |
| 1. {S, D, S} Levocardia 2. PFO, L – R Shunt 3. Small PM VSD, L – R Shunt 4. Large PDA, L – R Shunt 5. Good LV Function | | | | |
| SIGNATURE  Done by: Tesfaye T., Pediatric Cardiologist _______________ 15/11/2011Eth.C | | | | |

| Patient Name**: Welelaw Mandie**. Patient ID: **ADINAS 108513**. SEX/ Age: M/15 years Date of Report: 1**5/11/2011_**. BP: __ Weight: __ Height:__ BSA: ___. Referral Diagnosis: **Easy Fatigability. AGH06.1793.** | | | | |
| --- | --- | --- | --- | --- |
| **Features** | **Finding** | **Features** | **Finding** | |
| **Profile** |  | **Atria** |  | |
| Abdominal situs | Solitus | Left atrium | Normal | |
| Cardiac position | Levocardia | Right atrium | Normal | |
| Systemic venous drainage | Normal | **Atrioventricular valves** |  | |
| Pulmonary venous drainage | Normal | Mitral valve |  | |
| Atrioventricular connection | Concordant | Tricuspid valve |  | |
| Ventriculoarterial connection | Concordant | **Ventricles** |  | |
| Ventricular loop | d-Loop | Left ventricle | Normal | |
|  |  | Right ventricle | Normal | |
| **Septae** |  | **Coronary arteries** | ----- | |
| Interventricular septum | Intact | **Doppler Measurement** |  | |
| Interatrial septum | Intact | Mitral |  | |
| **Semilunal valves** |  | Aortic |  | |
| Aortic valve | Trileaflet, | Tricuspid |  | |
| Pulmonary valve |  | pulmonic |  | |
| **Great arteries** | NRGA | **Aortic arch** |  | |
| Aorta | ---- | **PDA** | ------- | |
| Pulmonary artery | ------ |  |  | |
| **M-Mode** | | | | |
| AO | mm | PWd | | 10mm |
| LA | mm | EDV | | 53ml |
| LVIDd | 35.7mm | ESV | | 24ml |
| LVIDs | 25.7mm | LVEF | | 55% |
| IVSd | 6.4mm | FS | | 28% |
| **Additional Information**: |  | | | |
| No pleural/pericardial effusion | | | | |
| **Final Diagnosis:** | | | | |
| 1. Normal Echocardiography Study | | | | |
| SIGNATURE  Done by: Tesfaye T., Pediatric Cardiologist _______________ 10/11/2011Eth.C | | | | |

| Patient Name**: Melaku Gashaw**. Patient ID: **ADINAS 108629**. SEX/ Age: m/1 6/12years Date of Report: 1**6/11/2011_**. BP: ___ Weight: ___ Height:___ BSA: ___. Referral Diagnosis: **Incidental Murmur. AGH03.1794.** | | | | | | |
| --- | --- | --- | --- | --- | --- | --- |
|  | **Finding** | | | **Features** | | **Finding** |
| **Profile** |  | | | **Atria** | |  |
| Abdominal situs | inversus | | | Left atrium | | Posterior & to the right |
| Cardiac position | Dextrocardia | | | Right atrium | | Anterior & to the left, dilated |
| Systemic venous drainage | to left sided RA | | | **Atrioventricular valves** | |  |
| Pulmonary venous drainage | to Right sided LA | | | Mitral valve | | ----- |
| Atrioventricular connection | Concordant | | | Tricuspid valve | | ----- |
| Ventriculoarterial connection | Discordant | | | **Ventricles** | |  |
| Ventricular loop | l-Loop | | | Left ventricle | | Posterior & right |
|  |  | | | Right ventricle | | Anterior & left |
| **Septae** |  | | | **Coronary arteries** | | ----- |
| Interventricular septum | Intact | | | **Doppler Measurement** | |  |
| Interatrial septum | 10mm OS ASD, BD Shunt | | | Mitral | | Mild MR |
| **Semilunal valves** |  | | | Aortic | | ----- |
| Aortic valve | Trileaflet, | | | Tricuspid | | Moderate TR, PPG = 24mmHg |
| Pulmonary valve |  | | | pulmonic | | ------ |
| **Great arteries** | l-TGA | | | **Aortic arch** | |  |
| Aorta | Anterior & to the left | | | **PDA** | | ------- |
| Pulmonary artery | Posterior & to the right | | |  | |  |
| **M-Mode = fair LV Function (eye balling)** | | | | | | |
| AO | | mm | PWd | | mm | |
| LA | | mm | EDV | | ml | |
| LVIDd | | mm | ESV | | ml | |
| LVIDs | | mm | LVEF | | % | |
| IVSd | | mm | FS | | % | |
| **Additional Information**: | |  | | | | |
| No pleural/pericardial effusion | | | | | | |
| **Final Diagnosis:** | | | | | | |
| 1. {I, L, L} Dextrocardia 2. L-TGA with intact IVS 3. OS ASD, BD Shunt 4. Fair LV Function | | | | | | |
| SIGNATURE  Done by: Tesfaye T., Pediatric Cardiologist _______________ 16/11/2011Eth.C | | | | | | |

| Patient Name**: Abrham Chanie** . Patient ID: **Jawi H.**. SEX/ Age: M/2years Date of Report: 1**9/11/2011_**.  BP: ___ Weight: ___ Height:___ BSA: ___. Referral Diagnosis: **Recurrent Chest Infection. AGH03.1795.** | | | | |
| --- | --- | --- | --- | --- |
| **Features** | **Finding** | **Features** | **Finding** | |
| **Profile** |  | **Atria** |  | |
| Abdominal situs | Solitus | Left atrium | Normal | |
| Cardiac position | Levocardia | Right atrium | Normal | |
| Systemic venous drainage | Normal | **Atrioventricular valves** |  | |
| Pulmonary venous drainage | Normal | Mitral valve | Annulus = 15mm | |
| Atrioventricular connection | Concordant | Tricuspid valve | Annulus = 19mm, TAPSE =18mm | |
| Ventriculoarterial connection | Concordant | **Ventricles** |  | |
| Ventricular loop | d-Loop | Left ventricle | Normal | |
|  |  | Right ventricle | Normal | |
| **Septae** |  | **Coronary arteries** | ----- | |
| Interventricular septum | Intact | **Doppler Measurement** |  | |
| Interatrial septum | Intact | Mitral | ---- | |
| **Semilunal valves** |  | Aortic | ---- | |
| Aortic valve | Annulus= 15mm | Tricuspid | ------ | |
| Pulmonary valve | Annulus = 16mm | pulmonic | Trivial PR, PPG = 16mmHg | |
| **Great arteries** | NRGA | **Aortic arch** | Left | |
| Aorta | ---- | **PDA** | No | |
| Pulmonary artery | Confluent branch PAs. |  |  | |
| **M-Mode** | | | | |
| AO | mm | PWd | | 5.6mm |
| LA | mm | EDV | | 45.5ml |
| LVIDd | 33.4mm | ESV | | 15ml |
| LVIDs | 21.4mm | LVEF | | 66% |
| IVSd | 4.3mm | FS | | 35% |
| **Additional Information**: |  | | | |
| No pleural/pericardial effusion | | | | |
| **Final Diagnosis:** | | | | |
| 1. {S, D, S} Levocardia 2. Normal Heart Study | | | | |
| SIGNATURE  Done by: Tesfaye T., Pediatric Cardiologist _______________ 19/11/2011Eth.C | | | | |

| Patient Name**: Bitanya Bekele** . Patient ID: **ADINAS**. SEX/ Age: F/2 9/12years Date of Report: 1**9/11/2011_**.  BP: ___ Weight: ___ Height:___ BSA: ___. Referral Diagnosis: **Incidental Murmur. AGH03.1796.** | | | | |
| --- | --- | --- | --- | --- |
| **Features** | **Finding** | **Features** | **Finding** | |
| **Profile** |  | **Atria** |  | |
| Abdominal situs | Solitus | Left atrium | Normal | |
| Cardiac position | Levocardia | Right atrium | Normal | |
| Systemic venous drainage | Normal | **Atrioventricular valves** |  | |
| Pulmonary venous drainage | Normal | Mitral valve | Annulus =18mm | |
| Atrioventricular connection | Concordant | Tricuspid valve | Annulus = 19mm  TAPSE = 18mm | |
| Ventriculoarterial connection | Concordant | **Ventricles** |  | |
| Ventricular loop | d-Loop | Left ventricle | Normal | |
|  |  | Right ventricle | Normal | |
| **Septae** |  | **Coronary arteries** | ----- | |
| Interventricular septum | Intact | **Doppler Measurement** |  | |
| Interatrial septum | Intact | Mitral |  | |
| **Semilunal valves** |  | Aortic |  | |
| Aortic valve | Annulus =16mm | Tricuspid | Trivial TR, PPG = 22mmHg | |
| Pulmonary valve | Annulus =16mm | pulmonic |  | |
| **Great arteries** | NRGA | **Aortic arch** |  | |
| Aorta | ---- | **PDA** | <1mm PDA, L – R Shunt | |
| Pulmonary artery | ------ |  |  | |
| **M-Mode** | | | | |
| AO | mm | PWd | | 7mm |
| LA | mm | EDV | | 38ml |
| LVIDd | 31mm | ESV | | 15ml |
| LVIDs | 21.4mm | LVEF | | 60% |
| IVSd | 3.2mm | FS | | 31% |
| **Additional Information**: |  | | | |
| No pleural/pericardial effusion | | | | |
| **Final Diagnosis:** | | | | |
| 1. {S, D, S} Levocardia 2. Tiny/Small PDA, L – R Shunt 3. Good Biventricular Function | | | | |
| SIGNATURE  Done by: Tesfaye T., Pediatric Cardiologist _______________ 19/11/2011Eth.C | | | | |

| Patient Name**: Baby of Kasaa**. Patient ID: **ADINAS**. SEX/ Age: M/3hours Date of Report: 24**/11/2011_**.  BP: ___ Weight: ___ Height:___ BSA: ___. Referral Diagnosis: **Incidental Murmur. AGH03.1797.** | | | | |
| --- | --- | --- | --- | --- |
| **Features** | **Finding** | **Features** | **Finding** | |
| **Profile** |  | **Atria** |  | |
| Abdominal situs | Solitus | Left atrium | Normal | |
| Cardiac position | Levocardia | Right atrium | Normal | |
| Systemic venous drainage | Normal | **Atrioventricular valves** |  | |
| Pulmonary venous drainage | Normal | Mitral valve | ---- | |
| Atrioventricular connection | Concordant | Tricuspid valve | ------ | |
| Ventriculoarterial connection | Aortic override | **Ventricles** |  | |
| Ventricular loop | d-Loop | Left ventricle |  | |
|  |  | Right ventricle | Normal | |
| **Septae** |  | **Coronary arteries** | ----- | |
| Interventricular septum | Mal-aligned VSD, 4.5mm, BD shunt | **Doppler Measurement** |  | |
| Interatrial septum | Intact | Mitral | ---- | |
| **Semilunal valves** |  | Aortic | No LVOTO | |
| Aortic valve | Trileaflet, | Tricuspid | ---- | |
| Pulmonary valve |  | pulmonic | Mild RVOTO, PPG = 25mmHg | |
| **Great arteries** | NRGA | **Aortic arch** |  | |
| Aorta | ---- | **PDA** | 2mm PDA, BD Shunt | |
| Pulmonary artery | ------ |  |  | |
| **M-Mode** | | | | |
| AO | mm | PWd | | **5.1mm** |
| LA | mm | EDV | | 3.2ml |
| LVIDd | 11.8mm | ESV | | 9.3ml |
| LVIDs | 7.5mm | LVEF | | 71% |
| IVSd | **9.8mm** | FS | | 36% |
| **Additional Information**: |  | | | |
| No pleural/pericardial effusion. Needs follow up scanning | | | | |
| **Final Diagnosis:** | | | | |
| 1. {S, D, S} Levocardia 2. TOF 3. Moderate PDA | | | | |
| SIGNATURE  Done by: Tesfaye T., Pediatric Cardiologist _______________ 24/11/2011Eth.C | | | | |

| Patient Name**: Esubalew Chalie** . Patient ID: **FHRRH**. SEX/ Age: M/ 1years Date of Report: 24**/11/2011_**.  BP: ___ Weight: ___ Height:___ BSA: ___. Referral Diagnosis: **Incidental Murmur. AGH03.1798.** | | | | | |
| --- | --- | --- | --- | --- | --- |
| **Features** | **Finding** | | **Features** | **Finding** | |
| **Profile** |  | | **Atria** |  | |
| Abdominal situs | Solitus | | Left atrium | Normal | |
| Cardiac position | Levocardia | | Right atrium | Normal | |
| Systemic venous drainage | Normal | | **Atrioventricular valves** |  | |
| Pulmonary venous drainage | Normal | | Mitral valve | Annulus = 14mm | |
| Atrioventricular connection | Concordant | | Tricuspid valve | Annulus = 14mm | |
| Ventriculoarterial connection | Concordant | | **Ventricles** |  | |
| Ventricular loop | d-Loop | | Left ventricle | Normal | |
|  |  | | Right ventricle | Normal | |
| **Septae** |  | | **Coronary arteries** | ----- | |
| Interventricular septum | 6mm PM VSD with inlet extension, L – R Shunt | | **Doppler Measurement** |  | |
| Interatrial septum | Intact | | Mitral | ----- | |
| **Semilunal valves** |  | | Aortic | ----- | |
| Aortic valve | Trileaflet, Annulus = 13mm | | Tricuspid | Trivial TR, PPG = 10mmHg | |
| Pulmonary valve | Annulus = 14mm | | pulmonic | Trivial PR, PPG = 14mmHg.  Mild RVOTO with PPG = 22mmHg | |
| **Great arteries** | NRGA | | **Aortic arch** |  | |
| Aorta | ---- | | **PDA** | ------- | |
| Pulmonary artery | ------ | |  |  | |
| **M-Mode** | | | | | |
| AO | | mm | PWd | | 6.8mm |
| LA | | mm | EDV | | 39.2ml |
| LVIDd | | 31.4mm | ESV | | 19.27ml |
| LVIDs | | 23.6mm | LVEF | | 51% |
| IVSd | | 3.9mm | FS | | 25% |
| **Additional Information**: | |  | | | |
| No pleural/pericardial effusion | | | | | |
| **Final Diagnosis:** | | | | | |
| 1. {S, D, S} Levocardia 2. Peri-Membranous VSD with inlet extension, L – R Shunt 3. Mild PS 4. Good Function | | | | | |
| SIGNATURE  Done by: Tesfaye T., Pediatric Cardiologist _______________ 24/11/2011Eth.C | | | | | |

| Patient Name**: Abrham Mollalign**. Patient ID: **FHRRH**. SEX/ Age: M/12years Date of Report: 25**/11/2011_**. BP: ___ Weight: ___ Height:___ BSA: ___. Referral Diagnosis: **Easy Fatigability. AGH03.1799.** | | | | |
| --- | --- | --- | --- | --- |
| **Features** | **Finding** | **Features** | **Finding** | |
| **Profile** |  | **Atria** |  | |
| Abdominal situs | Solitus | Left atrium | Normal | |
| Cardiac position | Levocardia | Right atrium | Normal | |
| Systemic venous drainage | Normal | **Atrioventricular valves** |  | |
| Pulmonary venous drainage | Normal | Mitral valve | Annulus = 21mm | |
| Atrioventricular connection | Concordant | Tricuspid valve | Annulus = 26mm | |
| Ventriculoarterial connection | Concordant | **Ventricles** |  | |
| Ventricular loop | d-Loop | Left ventricle | Normal | |
|  |  | Right ventricle | Normal | |
| **Septae** |  | **Coronary arteries** | ----- | |
| Interventricular septum | Intact | **Doppler Measurement** |  | |
| Interatrial septum | Intact | Mitral | ----- | |
| **Semilunal valves** |  | Aortic | ---- | |
| Aortic valve | Trileaflet, Annulus = 17mm | Tricuspid | Trivial TR, PPG = 13mmHg | |
| Pulmonary valve | Annulus = 18mm | pulmonic | ---- | |
| **Great arteries** | NRGA | **Aortic arch** | Left | |
| Aorta | ---- | **PDA** | No | |
| Pulmonary artery | ------ |  |  | |
| **M-Mode** | | | | |
| AO | mm | PWd | | 4.8mm |
| LA | mm | EDV | | 68.5ml |
| LVIDd | 39.6mm | ESV | | 27.8ml |
| LVIDs | 27.3mm | LVEF | | 59% |
| IVSd | 7.5mm | FS | | 31% |
| **Additional Information**: | No Coarctation of Aorta | | | |
| No pleural/pericardial effusion | | | | |
| **Final Diagnosis:** | | | | |
| 1. {S, D, S} Levocardia 2. Normal Heart Study | | | | |
| SIGNATURE  Done by: Tesfaye T., Pediatric Cardiologist _______________ 25/11/2011Eth.C | | | | |

| Patient Name**: Balemlay Nibret**. Patient ID: **ADINAS 109610**. SEX/ Age: F/11yrs Date of Report: 25**/11/2011**.  BP: ___ Weight: ___ Height:___ BSA: ___. Referral Diagnosis: **Palpitation. AGH03.1800.** | | | | |
| --- | --- | --- | --- | --- |
| **Features** | **Finding** | **Features** | **Finding** | |
| **Profile** |  | **Atria** |  | |
| Abdominal situs | Solitus | Left atrium | Normal | |
| Cardiac position | Levocardia | Right atrium | Normal | |
| Systemic venous drainage | Normal | **Atrioventricular valves** |  | |
| Pulmonary venous drainage | Normal | Mitral valve | Annulus = 19mm | |
| Atrioventricular connection | Concordant | Tricuspid valve | Annulus = 20mm  TAPSE = 20mm | |
| Ventriculoarterial connection | Concordant | **Ventricles** |  | |
| Ventricular loop | d-Loop | Left ventricle | Normal | |
|  |  | Right ventricle | Normal | |
| **Septae** |  | **Coronary arteries** | ----- | |
| Interventricular septum | Intact | **Doppler Measurement** |  | |
| Interatrial septum | Intact | Mitral | ----- | |
| **Semilunal valves** |  | Aortic | ------- | |
| Aortic valve | Trileaflet, Annulus = 16mm | Tricuspid | ------ | |
| Pulmonary valve | Annulus = 16mm | pulmonic | ------- | |
| **Great arteries** | NRGA | **Aortic arch** | Left | |
| Aorta |  | **PDA** | No | |
| Pulmonary artery |  |  |  | |
| **M-Mode** | | | | |
| AO | mm | PWd | | 5.5mm |
| LA | mm | EDV | | 78ml |
| LVIDd | 41.9mm | ESV | | 29ml |
| LVIDs | 27.9mm | LVEF | | 62% |
| IVSd | 5.5mm | FS | | 33% |
| **Additional Information**: | No Coarctation of Aorta | | | |
| No pleural/pericardial effusion | | | | |
| **Final Diagnosis:** | | | | |
| 1. {S, D, S} Levocardia 2. Normal Heart Study | | | | |
| SIGNATURE  Done by: Tesfaye T., Pediatric Cardiologist _______________ 25/11/2011Eth.C | | | | |

| Patient Name**: Mareshet Getahun**. Patient ID: **Merawi Hosp**. SEX/ Age: F/11 yrs Date of Report: 3**0/11/2011**. BP: ___ Weight: ___ Height:___ BSA: ___. Referral Diagnosis: **HTN. AGH03.1801.** | | | | |
| --- | --- | --- | --- | --- |
| **Features** | **Finding** | **Features** | **Finding** | |
| **Profile** |  | **Atria** |  | |
| Abdominal situs | Solitus | Left atrium | Normal | |
| Cardiac position | Levocardia | Right atrium | Normal | |
| Systemic venous drainage | Normal | **Atrioventricular valves** |  | |
| Pulmonary venous drainage | Normal | Mitral valve | Annulus = 18mm | |
| Atrioventricular connection | Concordant | Tricuspid valve | Annulus = 23mm  TAPSE = 21mm | |
| Ventriculoarterial connection | Concordant | **Ventricles** |  | |
| Ventricular loop | d-Loop | Left ventricle | Normal | |
|  |  | Right ventricle | Normal | |
| **Septae** |  | **Coronary arteries** | ----- | |
| Interventricular septum | Intact | **Doppler Measurement** |  | |
| Interatrial septum | Intact | Mitral |  | |
| **Semilunal valves** |  | Aortic |  | |
| Aortic valve | Annulus = 18mm | Tricuspid |  | |
| Pulmonary valve | Annulus = 20mm | pulmonic |  | |
| **Great arteries** | NRGA | **Aortic arch** | Left | |
| Aorta |  | **PDA** | No | |
| Pulmonary artery |  |  |  | |
| **M-Mode** | | | | |
| AO | mm | PWd | | 7.9mm |
| LA | mm | EDV | | 58.66ml |
| LVIDd | 37.1mm | ESV | | 19.49ml |
| LVIDs | 23.7mm | LVEF | | 66% |
| IVSd | 5.1mm | FS | | 36% |
| **Additional Information**: | No Coarctation of Aorta | | | |
| No pleural/pericardial effusion | | | | |
| **Final Diagnosis:** | | | | |
| 1. {S, D, S} Levocardia 2. Normal Heart Study | | | | |
| SIGNATURE  Done by: Tesfaye T., Pediatric Cardiologist _______________ 30/11/2011Eth.C | | | | |

| Patient Name**: Zinaye Getie**. Patient ID: **FHRH**. SEX/ Age: F/14years Date of Report: 1**/12/2011_**.  BP: ___ Weight: ___ Height:___ BSA: ___. Referral Diagnosis: **Easy Fatigability. AGH03.1802.** | | | | |
| --- | --- | --- | --- | --- |
| **Features** | **Finding** | **Features** | **Finding** | |
| **Profile** |  | **Atria** |  | |
| Abdominal situs | Solitus | Left atrium | Normal | |
| Cardiac position | Levocardia | Right atrium | Normal | |
| Systemic venous drainage | Normal | **Atrioventricular valves** |  | |
| Pulmonary venous drainage | Normal | Mitral valve | Annulus = 24mm | |
| Atrioventricular connection | Concordant | Tricuspid valve | Annulus = 28mm  TAPSE = 20mm | |
| Ventriculoarterial connection | Concordant | **Ventricles** |  | |
| Ventricular loop | d-Loop | Left ventricle | Normal | |
|  |  | Right ventricle | Normal | |
| **Septae** |  | **Coronary arteries** | ----- | |
| Interventricular septum | Intact | **Doppler Measurement** |  | |
| Interatrial septum | Intact | Mitral |  | |
| **Semilunal valves** |  | Aortic |  | |
| Aortic valve | Annulus = | Tricuspid |  | |
| Pulmonary valve | Annulus = 23mm | pulmonic |  | |
| **Great arteries** | NRGA | **Aortic arch** |  | |
| Aorta | ---- | **PDA** | ------- | |
| Pulmonary artery | ------ |  |  | |
| **M-Mode** | | | | |
| AO | mm | PWd | | 7.1mm |
| LA | mm | EDV | | 83ml |
| LVIDd | 43.1mm | ESV | | 28ml |
| LVIDs | 27.6mm | LVEF | | 65% |
| IVSd | 6.4mm | FS | | 35% |
| **Additional Information**: |  | | | |
| No pleural/pericardial effusion | | | | |
| **Final Diagnosis:** | | | | |
| 1. Normal Echocardiographic Heart Study | | | | |
| SIGNATURE  Done by: Tesfaye T., Pediatric Cardiologist _______________ 1/12/2011Eth.C | | | | |

| Patient Name**: Mastewal Aderaw**. Patient ID: **FHRH**. SEX/ Age: F/7 years Date of Report: 2**/12/2011_**.  BP: ___ Weight: ___ Height:___ BSA: ___. Referral Diagnosis: **CHF. AGH03.1803.** | | | | |
| --- | --- | --- | --- | --- |
| **Features** | **Finding** | **Features** | **Finding** | |
| **Profile** |  | **Atria** |  | |
| Abdominal situs | Solitus | Left atrium | Dilated | |
| Cardiac position | Levocardia | Right atrium | Dilated | |
| Systemic venous drainage | Normal | **Atrioventricular valves** |  | |
| Pulmonary venous drainage | Normal | Mitral valve | Annulus = 28mm | |
| Atrioventricular connection | Concordant | Tricuspid valve | Annulus = 29mm | |
| Ventriculoarterial connection | Concordant | **Ventricles** |  | |
| Ventricular loop | d-Loop | Left ventricle | Dilated | |
|  |  | Right ventricle | Dilated | |
| **Septae** |  | **Coronary arteries** | ----- | |
| Interventricular septum | 13mm Perimembranous VSD with inlet extension, BD shunt | **Doppler Measurement** |  | |
| Interatrial septum | Intact | Mitral | ---- | |
| **Semilunal valves** |  | Aortic | ---- | |
| Aortic valve | Annulus = 13mm | Tricuspid | ---- | |
| Pulmonary valve | Annulus = 18mm | pulmonic | Trivial PR | |
| **Great arteries** | NRGA | **Aortic arch** |  | |
| Aorta | ---- | **PDA** | ------- | |
| Pulmonary artery | Dilated MPA |  |  | |
| **M-Mode** | | | | |
| AO | mm | PWd | | 8mm |
| LA | mm | EDV | | 126ml |
| LVIDd | 51.4mm | ESV | | 63ml |
| LVIDs | 38.3mm | LVEF | | 50% |
| IVSd | 3.4mm | FS | | 25% |
| **Additional Information**: |  | | | |
| No pleural/pericardial effusion | | | | |
| **Final Diagnosis:** | | | | |
| 1. {S, D, S} Levocardia 2. Perimembranous VSD with inlet extension, BD Shunt 3. Good function | | | | |
| SIGNATURE  Done by: Tesfaye T., Pediatric Cardiologist _______________ 2/12/2011Eth.C | | | | |

| Patient Name**: Animut Abebe**. Patient ID: **FHRH**. SEX/ Age: M/14years Date of Report: 03**/12/2011_**.  BP: ___ Weight: ___ Height:___ BSA: ___. Referral Diagnosis: **CHF. AGH03.1804.** | | | | |
| --- | --- | --- | --- | --- |
| **Features** | **Finding** | **Features** | **Finding** | |
| **Profile** |  | **Atria** |  | |
| Abdominal situs | Solitus | Left atrium | Normal | |
| Cardiac position | Levocardia | Right atrium | Hugely Dilated, 85mm X 55mm | |
| Systemic venous drainage | Normal | **Atrioventricular valves** |  | |
| Pulmonary venous drainage | Normal | Mitral valve | Annulus = 19mm | |
| Atrioventricular connection | Concordant | Tricuspid valve | Annulus = 24mm,  PTL displaced down 58mm | |
| Ventriculoarterial connection | Concordant | **Ventricles** |  | |
| Ventricular loop | d-Loop | Left ventricle | Normal | |
|  |  | Right ventricle | smallish | |
| **Septae** |  | **Coronary arteries** | ----- | |
| Interventricular septum | Intact | **Doppler Measurement** |  | |
| Interatrial septum | Intact | Mitral | --- | |
| **Semilunal valves** |  | Aortic | --- | |
| Aortic valve | --- | Tricuspid | Severe TR, PPG = 80mmHg | |
| Pulmonary valve | ------- | pulmonic | ----- | |
| **Great arteries** | NRGA | **Aortic arch** |  | |
| Aorta | ---- | **PDA** | ------- | |
| Pulmonary artery | ------ |  |  | |
| **M-Mode:** reduced function (eye balling) | | | | |
| AO | mm | PWd | | mm |
| LA | mm | EDV | | ml |
| LVIDd | mm | ESV | | ml |
| LVIDs | mm | LVEF | | % |
| IVSd | mm | FS | | % |
| **Additional Information**: |  | | | |
| 20 mm pericardial effusion measured on the LV side | | | | |
| **Final Diagnosis:** | | | | |
| 1. {S, D, S} Levocardia 2. Ebstein anomaly 3. Biventricular failure | | | | |
| SIGNATURE  Done by: Tesfaye T., Pediatric Cardiologist _______________ 03/12/2011Eth.C | | | | |

| Patient Name**: Bisrat Werku**. Patient ID: **FHRH**. SEX/ Age: F/1year Date of Report: 0**6/12/2011_**.  BP: ___ Weight: ___ Height:___ BSA: ___. Referral Diagnosis: **Recurrent Chest Infection. AGH03.1805.** | | | | |
| --- | --- | --- | --- | --- |
| **Features** | **Finding** | **Features** | **Finding** | |
| **Profile** |  | **Atria** |  | |
| Abdominal situs | Solitus | Left atrium | Normal | |
| Cardiac position | Levocardia | Right atrium | Normal | |
| Systemic venous drainage | Normal | **Atrioventricular valves** |  | |
| Pulmonary venous drainage | Normal | Mitral valve | Annulus = 13mm | |
| Atrioventricular connection | Concordant | Tricuspid valve | Annulus = 17mm  TAPSE = 13mm | |
| Ventriculoarterial connection | Concordant | **Ventricles** |  | |
| Ventricular loop | d-Loop | Left ventricle | Normal | |
|  |  | Right ventricle | Normal | |
| **Septae** |  | **Coronary arteries** | ----- | |
| Interventricular septum | Intact | **Doppler Measurement** |  | |
| Interatrial septum | Intact | Mitral | ------ | |
| **Semilunal valves** |  | Aortic | ------ | |
| Aortic valve | ------ | Tricuspid | ------ | |
| Pulmonary valve | ------ | pulmonic | ------ | |
| **Great arteries** | NRGA | **Aortic arch** | Left | |
| Aorta | ---- | **PDA** | ------- | |
| Pulmonary artery | ------ |  |  | |
| **M-Mode =** Normal LV Function on eye balling | | | | |
| AO | mm | PWd | | mm |
| LA | mm | EDV | | ml |
| LVIDd | mm | ESV | | ml |
| LVIDs | mm | LVEF | | % |
| IVSd | mm | FS | | % |
| **Additional Information**: |  | | | |
| Child was crying | | | | |
| **Final Diagnosis:** | | | | |
| 1. {S, D, S} Levocardia 2. Normal Heart Study | | | | |
| SIGNATURE  Done by: Tesfaye T., Pediatric Cardiologist _______________ 06/12/2011Eth.C | | | | |

| Patient Name**: Tedi Adhino**. Patient ID: **FHRH**. SEX/ Age: M/8years Date of Report: 07**/12/2011_**.  BP: ___ Weight: ___ Height:___ BSA: ___. Referral Diagnosis: **Rheumatic Recurrence. AGH03.1806.** | | | | |
| --- | --- | --- | --- | --- |
| **Features** | **Finding** | **Features** | **Finding** | |
| **Profile** |  | **Atria** |  | |
| Abdominal situs | Solitus | Left atrium | Dilated | |
| Cardiac position | Levocardia | Right atrium | Normal | |
| Systemic venous drainage | Normal | **Atrioventricular valves** |  | |
| Pulmonary venous drainage | Normal | Mitral valve | Mildly Thickened leaflet  Annulus = 27mm | |
| Atrioventricular connection | Concordant | Tricuspid valve | Annulus = 22mm | |
| Ventriculoarterial connection | Concordant | **Ventricles** |  | |
| Ventricular loop | d-Loop | Left ventricle | Dilated | |
|  |  | Right ventricle | Normal | |
| **Septae** |  | **Coronary arteries** | ----- | |
| Interventricular septum | Intact | **Doppler Measurement** |  | |
| Interatrial septum | Intact | Mitral | Severe MR, | |
| **Semilunal valves** |  | Aortic | Mild AR | |
| Aortic valve | Annulus = 16mm | Tricuspid | Trivial TR | |
| Pulmonary valve | Annulus = 24mm | pulmonic | ----- | |
| **Great arteries** | NRGA | **Aortic arch** |  | |
| Aorta | ---- | **PDA** | ------- | |
| Pulmonary artery | dilated |  |  | |
| **M-Mode** | | | | |
| AO | mm | PWd | | 9mm |
| LA | mm | EDV | | 160ml |
| LVIDd | 57mm | ESV | | 54ml |
| LVIDs | 36mm | LVEF | | 66% |
| IVSd | 4.5mm | FS | | 36% |
| **Additional Information**: |  | | | |
| No pleural/pericardial effusion | | | | |
| **Final Diagnosis:** | | | | |
| 1. {S, D, S} Levocardia 2. Severe MR 3. Mildly thickened Mitral Valve 4. Mild AR 5. LA/LV Dilated 6. Good Biventricular Function | | | | |
| SIGNATURE  Done by: Tesfaye T., Pediatric Cardiologist _______________ 07/12/2011Eth.C | | | | |

| Patient Name**: Hanna Minlargih**. Patient ID: **Sahate birhan**. SEX/ Age: F/6/12 Date of Report: **08/12/2011**.  BP: ___ Weight: ___ Height:___ BSA: ___. Referral Diagnosis: **CHF. AGH03.1807.** | | | | |
| --- | --- | --- | --- | --- |
| **Features** | **Finding** | **Features** | **Finding** | |
| **Profile** |  | **Atria** |  | |
| Abdominal situs | Solitus | Left atrium | Dilated | |
| Cardiac position | Levocardia | Right atrium | Normal | |
| Systemic venous drainage | Normal | **Atrioventricular valves** |  | |
| Pulmonary venous drainage | Normal | Left AV Valve | Annulus = 15mm | |
| Atrioventricular connection | Concordant | Right AV Valve | Annulus = 16mm | |
| Ventriculoarterial connection | Concordant | **Ventricles** |  | |
| Ventricular loop | d-Loop | Left ventricle | Dilated | |
|  |  | Right ventricle | Normal | |
| **Septae** | No tongue of tissue in b/n | **Coronary arteries** | ----- | |
| Interventricular septum | 10mm inlet VSD, BD Shunt | **Doppler Measurement** |  | |
| Interatrial septum | Very large ASD amounting to single atrium | Mitral |  | |
| **Semilunal valves** |  | Aortic | ---- | |
| Aortic valve | Annulus = 11mm | Tricuspid | Mild right AVVR | |
| Pulmonary valve | Annulus = 9mm | pulmonic | ------ | |
| **Great arteries** | NRGA | **Aortic arch** |  | |
| Aorta | ---- | **PDA** | ------- | |
| Pulmonary artery | ------ |  |  | |
| **M-Mode** | | | | |
| AO | mm | PWd | | 7.1mm |
| LA | mm | EDV | | 7.25ml |
| LVIDd | 16.1mm | ESV | | 2.22ml |
| LVIDs | 10.3mm | LVEF | | 69% |
| IVSd | 5.1mm | FS | | 36% |
| **Additional Information**: | Baby was crying. (gradient not measured) | | | |
| No pleural/pericardial effusion | | | | |
| **Final Diagnosis:** | | | | |
| 1. {S, D, S} Levocardia 2. Complete AVSD 3. Good LV Function | | | | |
| SIGNATURE  Done by: Tesfaye T., Pediatric Cardiologist _______________ 08/12/2011Eth.C | | | | |

| Patient Name**: Yohannes Marew**. Patient ID: **ADINAS**. SEX/ Age: M/12years Date of Report: **09/12/2011_**.  BP: ___ Weight: ___ Height:___ BSA: ___. Referral Diagnosis: **DOE. AGH03.1808.** | | | | |
| --- | --- | --- | --- | --- |
| **Features** | **Finding** | **Features** | **Finding** | |
| **Profile** |  | **Atria** |  | |
| Abdominal situs | Solitus | Left atrium | Normal | |
| Cardiac position | Levocardia | Right atrium | Normal | |
| Systemic venous drainage | Normal | **Atrioventricular valves** |  | |
| Pulmonary venous drainage | Normal | Mitral valve | Annulus = 23mm | |
| Atrioventricular connection | Concordant | Tricuspid valve | Annulus = 25mm  TAPSE = 23mm | |
| Ventriculoarterial connection | Concordant | **Ventricles** |  | |
| Ventricular loop | d-Loop | Left ventricle | Normal | |
|  |  | Right ventricle | Normal | |
| **Septae** |  | **Coronary arteries** | ----- | |
| Interventricular septum | Intact | **Doppler Measurement** |  | |
| Interatrial septum | Intact | Mitral | ---- | |
| **Semilunal valves** |  | Aortic | ---- | |
| Aortic valve | Annulus = 19mm | Tricuspid | ---- | |
| Pulmonary valve | Annulus = 20mm | pulmonic | ---- | |
| **Great arteries** | NRGA | **Aortic arch** | Left | |
| Aorta | ---- | **PDA** | No PDA | |
| Pulmonary artery | ------ |  |  | |
| **M-Mode** | | | | |
| AO | mm | PWd | | 9.6mm |
| LA | mm | EDV | | 92ml |
| LVIDd | 45mm | ESV | | 35ml |
| LVIDs | 30mm | LVEF | | 62% |
| IVSd | 8mm | FS | | 33% |
| **Additional Information**: | No Coarctation of Aorta | | | |
| No pleural/pericardial effusion | | | | |
| **Final Diagnosis:** | | | | |
| 1. {S, D, S} Levocardia 2. Normal Echocardiographic Study | | | | |
| SIGNATURE  Done by: Tesfaye T., Pediatric Cardiologist _______________ 09/12/2011Eth.C | | | | |

| Patient Name**: Sintayehu Bayih**. Patient ID: **ADINAS**. SEX/ Age: F/4 years Date of Report: 1**0/12/2011_**.  BP: ___ Weight: ___ Height:___ BSA: ___. Referral Diagnosis: **Cyanosis. AGH03.1809.** | | | | |
| --- | --- | --- | --- | --- |
| **Features** | **Finding** | **Features** | **Finding** | |
| **Profile** |  | **Atria** |  | |
| Abdominal situs | Solitus | Left atrium | Normal | |
| Cardiac position | Levocardia | Right atrium | Normal | |
| Systemic venous drainage | Normal | **Atrioventricular valves** |  | |
| Pulmonary venous drainage | Normal | Mitral valve | Annulus = 17mm | |
| Atrioventricular connection | Concordant | Tricuspid valve | Annulus = 13mm | |
| Ventriculoarterial connection | DORV | **Ventricles** |  | |
| Ventricular loop | d-Loop | Left ventricle | Normal | |
|  |  | Right ventricle | Normal | |
| **Septae** |  | **Coronary arteries** | ----- | |
| Interventricular septum | 7.5mm PM VSD, BD shunt | **Doppler Measurement** |  | |
| Interatrial septum | Large ASD amounting to single atrium | Mitral | ----- | |
| **Semilunal valves** |  | Aortic | ------ | |
| Aortic valve | Annulus = 17mm | Tricuspid | ----- | |
| Pulmonary valve | Annulus = 12mm | pulmonic | Severe PS, PPG = 67mmHg | |
| **Great arteries** | NRGA (d) | **Aortic arch** |  | |
| Aorta | ---- | **PDA** | ------- | |
| Pulmonary artery | Small confluent Branch PAs |  |  | |
| **M-Mode** | | | | |
| AO | mm | PWd | | 3.5mm |
| LA | mm | EDV | | 28ml |
| LVIDd | 27.5mm | ESV | | 12ml |
| LVIDs | 19.5mm | LVEF | | 57% |
| IVSd | 3mm | FS | | 29% |
| **Additional Information**: |  | | | |
| No pleural/pericardial effusion | | | | |
| **Final Diagnosis:** | | | | |
| 1. {S, D, D} Levocardia 2. Large ASD amounting to single atrium 3. DORV 4. Moderate PM VSD, BD Shunt 5. Severe valvar PS 6. Small confluent branch PAs 7. Good LV Function | | | | |
| SIGNATURE  Done by: Tesfaye T., Pediatric Cardiologist _______________ 10/12/2011Eth.C | | | | |

| Patient Name**: Baby Dejitnu**. Patient ID: **FHRH**. SEX/ Age: F/17days Date of Report: 1**0/12/2011_**.  BP: ___ Weight: ___ Height:___ BSA: ___. Referral Diagnosis: **Incidental Murmur. AGH03.1810.** | | | | |
| --- | --- | --- | --- | --- |
| **Features** | **Finding** | **Features** | **Finding** | |
| **Profile** |  | **Atria** |  | |
| Abdominal situs | Solitus | Left atrium | Normal | |
| Cardiac position | Levocardia | Right atrium | Normal | |
| Systemic venous drainage | Normal | **Atrioventricular valves** |  | |
| Pulmonary venous drainage | Normal | Mitral valve | Annulus = 10mm | |
| Atrioventricular connection | Concordant | Tricuspid valve | Annulus = 11mm  TAPSE = 10mm | |
| Ventriculoarterial connection | Concordant | **Ventricles** |  | |
| Ventricular loop | d-Loop | Left ventricle | Normal | |
|  |  | Right ventricle | Normal | |
| **Septae** |  | **Coronary arteries** | ----- | |
| Interventricular septum | Intact | **Doppler Measurement** |  | |
| Interatrial septum | Intact | Mitral |  | |
| **Semilunal valves** |  | Aortic |  | |
| Aortic valve | Annulus = 10mm | Tricuspid |  | |
| Pulmonary valve | Annulus = 11mm | pulmonic |  | |
| **Great arteries** | NRGA | **Aortic arch** |  | |
| Aorta | ---- | **PDA** | 3mm PDA, BD Shunt | |
| Pulmonary artery | ------ |  |  | |
| **M-Mode** | | | | |
| AO | mm | PWd | | 4.7mm |
| LA | mm | EDV | | 10.79ml |
| LVIDd | 18.8mm | ESV | | 4.37ml |
| LVIDs | 13.3mm | LVEF | | 59% |
| IVSd | 6mm | FS | | 29% |
| **Additional Information**: |  | | | |
| No pleural/pericardial effusion | | | | |
| **Final Diagnosis:** | | | | |
| 1. {S, D, S} Levocardia 2. Large PDA, BD Shunt 3. Good Biventricular Function | | | | |
| SIGNATURE  Done by: Tesfaye T., Pediatric Cardiologist _______________ 10/12/2011Eth.C | | | | |

| Patient Name**: Samson Sisay**. Patient ID: **FHRH**. SEX/ Age: M/6months Date of Report: 1**3/12/2011_**.  BP: ___ Weight: ___ Height:___ BSA: ___. Referral Diagnosis: **DS. AGH03.1811.** | | | | |
| --- | --- | --- | --- | --- |
| **Features** | **Finding** | **Features** | **Finding** | |
| **Profile** |  | **Atria** |  | |
| Abdominal situs | Solitus | Left atrium | Normal | |
| Cardiac position | Levocardia | Right atrium | Normal | |
| Systemic venous drainage | Normal | **Atrioventricular valves** |  | |
| Pulmonary venous drainage | Normal | Mitral valve | Annulus = 12mm | |
| Atrioventricular connection | Concordant | Tricuspid valve | Annulus = 18mm  TAPSE = 13mm | |
| Ventriculoarterial connection | Concordant | **Ventricles** |  | |
| Ventricular loop | d-Loop | Left ventricle | Normal | |
|  |  | Right ventricle | Normal | |
| **Septae** |  | **Coronary arteries** | ----- | |
| Interventricular septum | Intact | **Doppler Measurement** |  | |
| Interatrial septum | 6mm OS ASD, L – R Shunt | Mitral | ---- | |
| **Semilunal valves** |  | Aortic | ----- | |
| Aortic valve | Annulus = 13mm | Tricuspid | Trivial TR, PPG = 16mmHg | |
| Pulmonary valve | Annulus = 14mm | pulmonic | Trivial PR, PPG = 13mmHg | |
| **Great arteries** | NRGA | **Aortic arch** | Left | |
| Aorta | ---- | **PDA** | No | |
| Pulmonary artery | ------ |  |  | |
| **M-Mode** | | | | |
| AO | mm | PWd | | 4.7mm |
| LA | mm | EDV | | 5.61ml |
| LVIDd | 14.6mm | ESV | | 1.18ml |
| LVIDs | 8.1mm | LVEF | | 79% |
| IVSd | 4.3mm | FS | | 44% |
| **Additional Information**: |  | | | |
| No pleural/pericardial effusion | | | | |
| **Final Diagnosis:** | | | | |
| 1. {S, D, S} Levocardia 2. Small OS ASD, L – R Shunt 3. Good Biventricular Function | | | | |
| SIGNATURE  Done by: Tesfaye T., Pediatric Cardiologist _______________ 13/12/2011Eth.C | | | | |

| Patient Name**: Amlakie Haymanot**. Patient ID: **Adinas**. SEX/ Age: F/14Years Date of Report: 1**3/12/2011_**.  BP: ___ Weight: ___ Height:___ BSA: ___. Referral Diagnosis: **Palpitation. AGH03.1812.** | | | | |
| --- | --- | --- | --- | --- |
| **Features** | **Finding** | **Features** | **Finding** | |
| **Profile** |  | **Atria** |  | |
| Abdominal situs | Solitus | Left atrium | Normal | |
| Cardiac position | Levocardia | Right atrium | Normal | |
| Systemic venous drainage | Normal | **Atrioventricular valves** |  | |
| Pulmonary venous drainage | Normal | Mitral valve | Annulus = 23mm | |
| Atrioventricular connection | Concordant | Tricuspid valve | Annulus = 24mm  TAPSE = 20mm | |
| Ventriculoarterial connection | Concordant | **Ventricles** |  | |
| Ventricular loop | d-Loop | Left ventricle | Normal | |
|  |  | Right ventricle | Normal | |
| **Septae** |  | **Coronary arteries** | ----- | |
| Interventricular septum | Intact | **Doppler Measurement** |  | |
| Interatrial septum | Intact | Mitral | ---- | |
| **Semilunal valves** |  | Aortic | ---- | |
| Aortic valve | ---- | Tricuspid | ---- | |
| Pulmonary valve | ---- | pulmonic | ---- | |
| **Great arteries** | NRGA | **Aortic arch** | Left | |
| Aorta | ---- | **PDA** | ---- | |
| Pulmonary artery | Good sized Confluent Branch PAs |  |  | |
| **M-Mode** | | | | |
| AO | mm | PWd | | 9.1mm |
| LA | mm | EDV | | 104.5ml |
| LVIDd | 47.4mm | ESV | | 44.6ml |
| LVIDs | 33.1mm | LVEF | | 57% |
| IVSd | 7.4mm | FS | | 30% |
| **Additional Information**: |  | | | |
| No pleural/pericardial effusion | | | | |
| **Final Diagnosis:** | | | | |
| 1. {S, D, S} Levocardia 2. Normal Echocardiography Study | | | | |
| SIGNATURE  Done by: Tesfaye T., Pediatric Cardiologist _______________ 13/12/2011Eth.C | | | | |

| Patient Name**:_Gizew Alene_**. Patient ID:_FHRH_. SEX/ Age: M/10months_ Date of Report: 1**3/12/2011.**  BP: ___ Weight: ___ Height:___ BSA: ___. Referral Diagnosis: **RD. AGH03.1813.** | | | | |
| --- | --- | --- | --- | --- |
| **Features** | **Finding** | **Features** | **Finding** | |
| **Profile** |  | **Atria** |  | |
| Abdominal situs | Solitus | Left atrium | Normal | |
| Cardiac position | Levocardia | Right atrium | Normal | |
| Systemic venous drainage | Normal | **Atrioventricular valves** |  | |
| Pulmonary venous drainage | Normal | Mitral valve | Annulus = 14mm | |
| Atrioventricular connection | Concordant | Tricuspid valve | Annulus = 14mm  TAPSE = 13mm | |
| Ventriculoarterial connection | Concordant | **Ventricles** |  | |
| Ventricular loop | d-Loop | Left ventricle | Normal | |
|  |  | Right ventricle | Normal | |
| **Septae** |  | **Coronary arteries** | ----- | |
| Interventricular septum | Intact | **Doppler Measurement** |  | |
| Interatrial septum | Intact | Mitral | ---- | |
| **Semilunal valves** |  | Aortic | ---- | |
| Aortic valve | Annulus = 10mm | Tricuspid | ---- | |
| Pulmonary valve | Annulus = 12mm | pulmonic | ---- | |
| **Great arteries** | NRGA | **Aortic arch** | Left | |
| Aorta | ---- | **PDA** | No PDA | |
| Pulmonary artery | ------ |  |  | |
| **M-Mode** | | | | |
| AO | mm | PWd | | 5.1mm |
| LA | mm | EDV | | 15.35ml |
| LVIDd | 21.5mm | ESV | | 6.88ml |
| LVIDs | 15.8mm | LVEF | | 55% |
| IVSd | 2.6mm | FS | | 27% |
| **Additional Information**: |  | | | |
| No pleural/pericardial effusion | | | | |
| **Final Diagnosis:** | | | | |
| 1. {S, D, S} Levocardia 2. Normal Echocardiographic Study | | | | |
| SIGNATURE  Done by: Tesfaye T., Pediatric Cardiologist _______________ 13/12/2011Eth.C | | | | |

| Patient Name**: Kalkidan Aschalew_**. Patient ID:FHRH_. SEX/ Age: F/2 3/12_ Date of Report: 1**5/12/2011**.  BP: ___ Weight: ___ Height:___ BSA: ___. Referral Diagnosis: **Recurrent Chest Infection. AGH03.1814.** | | | | |
| --- | --- | --- | --- | --- |
| **Features** | **Finding** | **Features** | **Finding** | |
| **Profile** |  | **Atria** |  | |
| Abdominal situs | Solitus | Left atrium | Normal | |
| Cardiac position | Levocardia | Right atrium | Normal | |
| Systemic venous drainage | Normal | **Atrioventricular valves** |  | |
| Pulmonary venous drainage | Normal | Mitral valve | Annulus = 17mm | |
| Atrioventricular connection | Concordant | Tricuspid valve | Annulus = 18mm  TAPSE = 16mm | |
| Ventriculoarterial connection | Concordant | **Ventricles** |  | |
| Ventricular loop | d-Loop | Left ventricle | Normal | |
|  |  | Right ventricle | Normal | |
| **Septae** |  | **Coronary arteries** | ----- | |
| Interventricular septum | Intact | **Doppler Measurement** |  | |
| Interatrial septum | Intact | Mitral | ----- | |
| **Semilunal valves** |  | Aortic | ---- | |
| Aortic valve | Annulus = 12mm | Tricuspid | Trivial TR, PPG = 20mmHg | |
| Pulmonary valve | Annulus = 13mm | pulmonic | ---- | |
| **Great arteries** | NRGA | **Aortic arch** | Left | |
| Aorta | ---- | **PDA** | No PDA | |
| Pulmonary artery | ------ |  |  | |
| **M-Mode** | | | | |
| AO | mm | PWd | | 5.5mm |
| LA | mm | EDV | | 22.64ml |
| LVIDd | 25.1mm | ESV | | 8.25ml |
| LVIDs | 16.9mm | LVEF | | 63% |
| IVSd | 5.9mm | FS | | 32% |
| **Additional Information**: |  | | | |
| No pleural/pericardial effusion | | | | |
| **Final Diagnosis:** | | | | |
| 1. {S, D, S} Levocardia 2. Normal Echocardiographic Study | | | | |
| SIGNATURE  Done by: Tesfaye T., Pediatric Cardiologist _______________ 15/12/2011Eth.C | | | | |

| Patient Name**: Baby of Agernesh Guadie** . Patient ID: FHH. SEX/ Age: F/2/12 Date of Report: 1**7/12/2011_**.  BP: ___ Weight: ___ Height:___ BSA: ___. Referral Diagnosis: **RD + Murmur. AGH03.1815.** | | | | |
| --- | --- | --- | --- | --- |
| **Features** | **Finding** | **Features** | **Finding** | |
| **Profile** |  | **Atria** |  | |
| Abdominal situs | Solitus | Left atrium | Dilated | |
| Cardiac position | Levocardia | Right atrium | Normal | |
| Systemic venous drainage | Normal | **Atrioventricular valves** |  | |
| Pulmonary venous drainage | Normal | Mitral valve | Normal | |
| Atrioventricular connection | Concordant | Tricuspid valve | Normal | |
| Ventriculoarterial connection | Concordant | **Ventricles** |  | |
| Ventricular loop | d-Loop | Left ventricle | Dilated | |
|  |  | Right ventricle | Normal | |
| **Septae** |  | **Coronary arteries** | ----- | |
| Interventricular septum | Intact | **Doppler Measurement** |  | |
| Interatrial septum | 5mm OS ASD, L – R Shunt | Mitral | ---- | |
| **Semilunal valves** |  | Aortic | ----- | |
| Aortic valve | Annulus = 10mm | Tricuspid | ---- | |
| Pulmonary valve | Annulus = 12mm | pulmonic | ---- | |
| **Great arteries** | NRGA | **Aortic arch** |  | |
| Aorta | ---- | **PDA** | 2.5mm PDA, L- R Shunt | |
| Pulmonary artery | Normal sized Confluent Branch PAs |  |  | |
| **M-Mode** | | | | |
| AO | mm | PWd | | 5.4mm |
| LA | mm | EDV | | 20.77ml |
| LVIDd | 24.3mm | ESV | | 10.12ml |
| LVIDs | 18.3mm | LVEF | | 51% |
| IVSd | 3.7mm | FS | | 25% |
| **Additional Information**: |  | | | |
| No pleural/pericardial effusion | | | | |
| **Final Diagnosis:** | | | | |
| 1. {S, D, S} Levocardia 2. Small OS ASD, L – R Shunt 3. Moderate PDA, L – R Shunt 4. Good LV Function | | | | |
| SIGNATURE  Done by: Tesfaye T., Pediatric Cardiologist _______________ 17/12/2011Eth.C | | | | |

| Patient Name**: Debrie Alebel** Patient ID: FHH. SEX/ Age: F/2/12 Date of Report: 20**/12/2011_**.  BP: ___ Weight: ___ Height:___ BSA: ___. Referral Diagnosis: **DS. AGH03.1816.** | | | | |
| --- | --- | --- | --- | --- |
| **Features** | **Finding** | **Features** | **Finding** | |
| **Profile** |  | **Atria** |  | |
| Abdominal situs | Solitus | Left atrium | Normal | |
| Cardiac position | Levocardia | Right atrium | Normal | |
| Systemic venous drainage | Normal | **Atrioventricular valves** |  | |
| Pulmonary venous drainage | Normal | Mitral valve | Annulus = 10mm | |
| Atrioventricular connection | Concordant | Tricuspid valve | Annulus = 11mm  TAPSE = 10mm | |
| Ventriculoarterial connection | Concordant | **Ventricles** |  | |
| Ventricular loop | d-Loop | Left ventricle | Normal | |
|  |  | Right ventricle | Normal | |
| **Septae** |  | **Coronary arteries** | ----- | |
| Interventricular septum | Intact | **Doppler Measurement** |  | |
| Interatrial septum | PFO, L – R Shunt | Mitral | ---- | |
| **Semilunal valves** |  | Aortic | ---- | |
| Aortic valve | Annulus = 10mm | Tricuspid | ---- | |
| Pulmonary valve | Annulus = 10mm | pulmonic | ---- | |
| **Great arteries** | NRGA | **Aortic arch** | Left | |
| Aorta | ---- | **PDA** | No | |
| Pulmonary artery | Normal sized Confluent Branch PAs |  |  | |
| **M-Mode =** Normal LV function on eye balling | | | | |
| AO | mm | PWd | | mm |
| LA | mm | EDV | | ml |
| LVIDd | mm | ESV | | ml |
| LVIDs | mm | LVEF | | % |
| IVSd | mm | FS | | % |
| **Additional Information**: |  | | | |
| No pleural/pericardial effusion | | | | |
| **Final Diagnosis:** | | | | |
| 1. {S, D, S} Levocardia 2. PFO, L – R Shunt 3. Good Biventricular Function | | | | |
| SIGNATURE  Done by: Tesfaye T., Pediatric Cardiologist _______________ 20/12/2011Eth.C | | | | |

| Patient Name**: Haile Getasew**. Patient ID:DTGH. SEX/ Age: M/14years Date of Report: 21**/12/2011_**.  BP: ___ Weight: ___ Height:___ BSA: ___. Referral Diagnosis: **Rheumatic Recurrence + CHF. AGH03.1817.** | | | | |
| --- | --- | --- | --- | --- |
| **Features** | **Finding** | **Features** | **Finding** | |
| **Profile** |  | **Atria** |  | |
| Abdominal situs | Solitus | Left atrium | Dilated, 61 X 65mm | |
| Cardiac position | Levocardia | Right atrium | Dilated | |
| Systemic venous drainage | Normal | **Atrioventricular valves** |  | |
| Pulmonary venous drainage | Normal | Mitral valve | Annulus = 19mm. Thickened Clubbed. MVA = 0.8cm2. | |
| Atrioventricular connection | Concordant | Tricuspid valve | Annulus = 38mm, non coapting leaflet  TAPSE = 10mm | |
| Ventriculoarterial connection | Concordant | **Ventricles** |  | |
| Ventricular loop | d-Loop | Left ventricle | Normal | |
|  |  | Right ventricle | Dilated, Dysfunctional | |
| **Septae** |  | **Coronary arteries** | ----- | |
| Interventricular septum | Intact | **Doppler Measurement** |  | |
| Interatrial septum | Intact | Mitral | Severe MS | |
| **Semilunal valves** |  | Aortic | ---- | |
| Aortic valve | Annulus = 15mm | Tricuspid | Severe TR, PPG = 78mmHg | |
| Pulmonary valve | Annulus = 19mm | pulmonic | ---- | |
| **Great arteries** | NRGA | **Aortic arch** |  | |
| Aorta | ---- | **PDA** |  | |
| Pulmonary artery | ------ |  |  | |
| **M-Mode: Normal LV Function** | | | | |
| AO | mm | PWd | | mm |
| LA | mm | EDV | | ml |
| LVIDd | mm | ESV | | ml |
| LVIDs | mm | LVEF | | % |
| IVSd | mm | FS | | % |
| **Additional Information**: |  | | | |
| No pleural/pericardial effusion | | | | |
| **Final Diagnosis:** | | | | |
| 1. {S, D, S} Levocardia 2. Severe MS 3. Severe TR 4. RA Dilated 5. RV Dilated, Dysfunctional 6. Severe Pulmonary Hypertension | | | | |
| SIGNATURE  Done by: Tesfaye T., Pediatric Cardiologist _______________ 21/12/2011Eth.C | | | | |

| Patient Name**: Baby of Hikma Abdu**. Patient ID: FHH. SEX/ Age: M/20days Date of Report: 21**/12/2011_**.  BP: ___ Weight: ___ Height:___ BSA: ___. Referral Diagnosis: **DS. AGH03.1818.** | | | | |
| --- | --- | --- | --- | --- |
| **Features** | **Finding** | **Features** | **Finding** | |
| **Profile** |  | **Atria** |  | |
| Abdominal situs | Solitus | Left atrium | Normal | |
| Cardiac position | Levocardia | Right atrium | Normal | |
| Systemic venous drainage | Normal | **Atrioventricular valves** |  | |
| Pulmonary venous drainage | Normal | Mitral valve | Normal | |
| Atrioventricular connection | Concordant | Tricuspid valve | Normal  TAPSE = 12mm | |
| Ventriculoarterial connection | Concordant | **Ventricles** |  | |
| Ventricular loop | d-Loop | Left ventricle | Normal | |
|  |  | Right ventricle | Normal | |
| **Septae** |  | **Coronary arteries** | ----- | |
| Interventricular septum | Intact | **Doppler Measurement** |  | |
| Interatrial septum | Intact | Mitral | ---- | |
| **Semilunal valves** |  | Aortic | ----- | |
| Aortic valve | NORMAL | Tricuspid | ----- | |
| Pulmonary valve | Normal | pulmonic | ----- | |
| **Great arteries** | NRGA | **Aortic arch** | Left | |
| Aorta | ---- | **PDA** | No PDA | |
| Pulmonary artery | ------ |  |  | |
| **M-Mode** | | | | |
| AO | mm | PWd | | 4.2mm |
| LA | mm | EDV | | 9.72ml |
| LVIDd | 18mm | ESV | | 4.59ml |
| LVIDs | 13.5mm | LVEF | | 52% |
| IVSd | 4.8mm | FS | | 25% |
| **Additional Information**: |  | | | |
| No pleural/pericardial effusion | | | | |
| **Final Diagnosis:** | | | | |
| 1. {S, D, S} Levocardia 2. Normal Echocardiographic Study | | | | |
| SIGNATURE  Done by: Tesfaye T., Pediatric Cardiologist _______________ 21/12/2011Eth.C | | | | |

| Patient Name**: Edomawit Abebaw**. Patient ID: 111417 Adinas. SEX/ Age: F/18d Date of Report: 21**/12/2011_**.  BP: ___ Weight: ___ Height:___ BSA: ___. Referral Diagnosis: **Incidental Murmur. AGH03.1819.** | | | | |
| --- | --- | --- | --- | --- |
| **Features** | **Finding** | **Features** | **Finding** | |
| **Profile** |  | **Atria** |  | |
| Abdominal situs | Solitus | Left atrium | Normal | |
| Cardiac position | Levocardia | Right atrium | Normal | |
| Systemic venous drainage | Normal | **Atrioventricular valves** |  | |
| Pulmonary venous drainage | Normal | Mitral valve | Normal | |
| Atrioventricular connection | Concordant | Tricuspid valve | TAPSE = 10mm | |
| Ventriculoarterial connection | Concordant | **Ventricles** |  | |
| Ventricular loop | d-Loop | Left ventricle | Normal | |
|  |  | Right ventricle | Normal | |
| **Septae** |  | **Coronary arteries** | ----- | |
| Interventricular septum | 3mm PM VSD, L – R Shunt | **Doppler Measurement** |  | |
| Interatrial septum | Intact | Mitral | ---- | |
| **Semilunal valves** |  | Aortic | ---- | |
| Aortic valve | Annulus = 10mm | Tricuspid | ---- | |
| Pulmonary valve | Annulus = 10mm | pulmonic | ---- | |
| **Great arteries** | NRGA | **Aortic arch** |  | |
| Aorta | ---- | **PDA** |  | |
| Pulmonary artery | ------ |  |  | |
| **M-Mode** | | | | |
| AO | mm | PWd | | 3.5mm |
| LA | mm | EDV | | 15.94ml |
| LVIDd | 21.9mm | ESV | | 7.25ml |
| LVIDs | 16.1mm | LVEF | | 55% |
| IVSd | 2.9mm | FS | | 29% |
| **Additional Information**: |  | | | |
| No pleural/pericardial effusion | | | | |
| **Final Diagnosis:** | | | | |
| 1. {S, D, S} Levocardia 2. Small Perimembranous VSD 3. Normal Function | | | | |
| SIGNATURE  Done by: Tesfaye T., Pediatric Cardiologist _______________ 21/12/2011Eth.C | | | | |

| Patient Name**: Baby of Abebech**. Patient ID: FHH. SEX/ Age: F/25days Date of Report: 22**/12/2011_**.  BP: ___ Weight: ___ Height:___ BSA: ___. Referral Diagnosis: **RD. AGH03.1820.** | | | | |
| --- | --- | --- | --- | --- |
| **Features** | **Finding** | **Features** | **Finding** | |
| **Profile** |  | **Atria** |  | |
| Abdominal situs | Solitus | Left atrium | Normal | |
| Cardiac position | Levocardia | Right atrium | Normal | |
| Systemic venous drainage | Normal | **Atrioventricular valves** |  | |
| Pulmonary venous drainage | Normal | Mitral valve | Annulus = 10mm | |
| Atrioventricular connection | Concordant | Tricuspid valve | Annulus = 12mm  TAPSE = 12mm | |
| Ventriculoarterial connection | Concordant | **Ventricles** |  | |
| Ventricular loop | d-Loop | Left ventricle | Normal | |
|  |  | Right ventricle | Normal | |
| **Septae** |  | **Coronary arteries** | ----- | |
| Interventricular septum | Intact | **Doppler Measurement** |  | |
| Interatrial septum | Intact | Mitral | ---- | |
| **Semilunal valves** |  | Aortic | ---- | |
| Aortic valve | Annulus = 9mm | Tricuspid | ---- | |
| Pulmonary valve | Annulus = 8mm | pulmonic | ---- | |
| **Great arteries** | NRGA | **Aortic arch** | Left | |
| Aorta | ---- | **PDA** | No | |
| Pulmonary artery | ------ |  |  | |
| **M-Mode** | | | | |
| AO | mm | PWd | | 2.5mm |
| LA | mm | EDV | | 9.04ml |
| LVIDd | 17.5mm | ESV | | 3.55ml |
| LVIDs | 12.3mm | LVEF | | 60% |
| IVSd | 3.2mm | FS | | 30% |
| **Additional Information**: |  | | | |
| No pleural/pericardial effusion | | | | |
| **Final Diagnosis:** | | | | |
| 1. Normal Echocardiographic Study | | | | |
| SIGNATURE  Done by: Tesfaye T., Pediatric Cardiologist _______________ 22/12/2011Eth.C | | | | |

| Patient Name**: Abeba Demeke.** Patient ID: FHH. SEX/ Age: F/10/12 Date of Report: 25**/12/2011_**.  BP: ___ Weight: ___ Height:___ BSA: ___. Referral Diagnosis: **Recurrent Chest Infection. AGH03.1821.** | | | |
| --- | --- | --- | --- |
| **Features** | **Finding** | **Features** | **Finding** |
| **Profile** |  | **Atria** |  |
| Abdominal situs | Solitus | Left atrium | Normal |
| Cardiac position | Levocardia | Right atrium | Normal |
| Systemic venous drainage | Normal | **Atrioventricular valves** |  |
| Pulmonary venous drainage | Normal | Mitral valve | Annulus = 11mm |
| Atrioventricular connection | Concordant | Tricuspid valve | Annulus = 12mm  TAPSE = 13mm |
| Ventriculoarterial connection | Concordant | **Ventricles** |  |
| Ventricular loop | d-Loop | Left ventricle | Normal |
|  |  | Right ventricle | Normal |
| **Septae** |  | **Coronary arteries** | ----- |
| Interventricular septum | Intact | **Doppler Measurement** |  |
| Interatrial septum | 3mm defect, L – R Shunt | Mitral | ---- |
| **Semilunal valves** |  | Aortic | ---- |
| Aortic valve | Annulus = 11mm | Tricuspid | Trivial TR, PPG = 14mmHg |
| Pulmonary valve | Annulus = 12mm | pulmonic | ---- |
| **Great arteries** | NRGA | **Aortic arch** | Left |
| Aorta | ---- | **PDA** | No |
| Pulmonary artery | Good sized Confluent Branch PAs |  |  |
| **M-Mode** | | | |
| AO | mm | PWd | 3.5mm |
| LA | mm | EDV | 20.09ml |
| LVIDd | 24mm | ESV | 9.82ml |
| LVIDs | 18.1mm | LVEF | 55% |
| IVSd | 4.3mm | FS | 29% |
| **Additional Information**: |  | | |
| No pleural/pericardial effusion | | | |
| **Final Diagnosis:** | | | |
| 1. {S, D, S} Levocardia 2. PFO, L – R Shunt 3. Good Biventricular Function | | | |
| SIGNATURE  Done by: Tesfaye T., Pediatric Cardiologist _______________ 25/12/2011Eth.C | | | |

| Patient Name**: Amare Kindie**. Patient ID: FHH. SEX/ Age: M/4/12 Date of Report: 25**/12/2011_**.  BP: ___ Weight: ___ Height:___ BSA: ___. Referral Diagnosis: **?Duchenne Syndrome. AGH03.1822.** | | | |
| --- | --- | --- | --- |
| **Features** | **Finding** | **Features** | **Finding** |
| **Profile** |  | **Atria** |  |
| Abdominal situs | Solitus | Left atrium | Normal |
| Cardiac position | Levocardia | Right atrium | Normal |
| Systemic venous drainage | Normal | **Atrioventricular valves** |  |
| Pulmonary venous drainage | Normal | Mitral valve | Annulus = 10mm |
| Atrioventricular connection | Concordant | Tricuspid valve | Annulus = 11mm  TAPSE = 11mm |
| Ventriculoarterial connection | Concordant | **Ventricles** |  |
| Ventricular loop | d-Loop | Left ventricle | Hypertrophied |
|  |  | Right ventricle | Normal |
| **Septae** |  | **Coronary arteries** | ----- |
| Interventricular septum | Intact | **Doppler Measurement** |  |
| Interatrial septum | 5mm OS ASD, L – R Shunt | Mitral | ---- |
| **Semilunal valves** |  | Aortic | ---- |
| Aortic valve | Annulus = 11mm | Tricuspid | ---- |
| Pulmonary valve | Annulus = 10mm | pulmonic | ---- |
| **Great arteries** | NRGA | **Aortic arch** | Left |
| Aorta | ---- | **PDA** | No |
| Pulmonary artery | Good sized confluent Branch PAs |  |  |
| **M-Mode** | | | |
| AO | mm | PWd | 4.5mm |
| LA | mm | EDV | 10.17ml |
| LVIDd | 18.3mm | ESV | 2.04ml |
| LVIDs | 10mm | LVEF | 79% |
| IVSd | **6.4mm** | FS | 45% |
| **Additional Information**: |  | | |
| No pleural/pericardial effusion | | | |
| **Final Diagnosis:** | | | |
| 1. {S, D, S} Levocardia 2. OS ASD, L – R Shunt 3. Asymmetric LVH secondary to ? 4. Hyper dynamic LV | | | |
| SIGNATURE  Done by: Tesfaye T., Pediatric Cardiologist _______________ 25/12/2011Eth.C | | | |

| Patient Name**: Roza Anteneh**. Patient ID: Pawi. SEX/ Age: F/6 2/12 Date of Report: 25**/12/2011_**.  BP: ___ Weight: ___ Height:___ BSA: ___. Referral Diagnosis: **ARF. AGH03.1823.** | | | | |
| --- | --- | --- | --- | --- |
| **Features** | **Finding** | **Features** | **Finding** | |
| **Profile** |  | **Atria** |  | |
| Abdominal situs | Solitus | Left atrium | Normal | |
| Cardiac position | Levocardia | Right atrium | Normal | |
| Systemic venous drainage | Normal | **Atrioventricular valves** |  | |
| Pulmonary venous drainage | Normal | Mitral valve | Annulus = 21mm | |
| Atrioventricular connection | Concordant | Tricuspid valve | Annulus = 21mm  TAPSE = 16mm | |
| Ventriculoarterial connection | Concordant | **Ventricles** |  | |
| Ventricular loop | d-Loop | Left ventricle | Normal | |
|  |  | Right ventricle | Normal | |
| **Septae** |  | **Coronary arteries** | ----- | |
| Interventricular septum | Intact | **Doppler Measurement** |  | |
| Interatrial septum | Intact | Mitral | ---- | |
| **Semilunal valves** |  | Aortic | ---- | |
| Aortic valve | Annulus = 15mm | Tricuspid | Trivial TR, PPG = 14mmHg | |
| Pulmonary valve | Annulus = 18mm | pulmonic | ---- | |
| **Great arteries** | NRGA | **Aortic arch** | Left | |
| Aorta | ---- | **PDA** | No | |
| Pulmonary artery | ------ |  |  | |
| **M-Mode** | | | | |
| AO | mm | PWd | | 6.3mm |
| LA | mm | EDV | | 32.6ml |
| LVIDd | 29.1mm | ESV | | 15.68ml |
| LVIDs | 21.7mm | LVEF | | 52% |
| IVSd | 4mm | FS | | 25% |
| **Additional Information**: |  | | | |
| No pleural/pericardial effusion | | | | |
| **Final Diagnosis:** | | | | |
| 1. {S, D, S} Levocardia 2. Normal Echocardiographic Study | | | | |
| SIGNATURE  Done by: Tesfaye T., Pediatric Cardiologist _______________ 25/12/2011Eth.C | | | | |

| Patient Name**: Abrham Hunegnaw**. Patient ID:_FHH. SEX/ Age: M/14yrs Date of Report: 27**/12/2011_**.  BP: ___ Weight: ___ Height:___ BSA: ___. Referral Diagnosis: **Easy Fatigability. AGH03.1824.** | | | | |
| --- | --- | --- | --- | --- |
| **Features** | **Finding** | **Features** | **Finding** | |
| **Profile** |  | **Atria** |  | |
| Abdominal situs | Solitus | Left atrium | Normal | |
| Cardiac position | Levocardia | Right atrium | Normal | |
| Systemic venous drainage | Normal | **Atrioventricular valves** |  | |
| Pulmonary venous drainage | Normal | Mitral valve | Annulus = 22mm | |
| Atrioventricular connection | Concordant | Tricuspid valve | Annulus = 25mm  TAPSE = 20mm | |
| Ventriculoarterial connection | Concordant | **Ventricles** |  | |
| Ventricular loop | d-Loop | Left ventricle | Normal | |
|  |  | Right ventricle | Normal | |
| **Septae** |  | **Coronary arteries** | ----- | |
| Interventricular septum | Intact | **Doppler Measurement** |  | |
| Interatrial septum | Intact | Mitral | ---- | |
| **Semilunal valves** |  | Aortic | ---- | |
| Aortic valve | Annulus = 19mm | Tricuspid | ---- | |
| Pulmonary valve | Annulus = 23mm | pulmonic | Mild PR, PPG = 10mmHg | |
| **Great arteries** | NRGA | **Aortic arch** | Left | |
| Aorta | ---- | **PDA** | No | |
| Pulmonary artery | ------ |  |  | |
| **M-Mode** | | | | |
| AO | mm | PWd | | 6mm |
| LA | mm | EDV | | 126.64ml |
| LVIDd | 51.5mm | ESV | | 50.87ml |
| LVIDs | 35mm | LVEF | | 59% |
| IVSd | 5mm | FS | | 32% |
| **Additional Information**: |  | | | |
| No pleural/pericardial effusion | | | | |
| **Final Diagnosis:** | | | | |
| 1. {S, D, S} Levocardia 2. Normal Echocardiography Study | | | | |
| SIGNATURE  Done by: Tesfaye T., Pediatric Cardiologist _______________ 27/12/2011Eth.C | | | | |

| Patient Name**: Mulugeta Abebe**. Patient ID:FHH. SEX/ Age: M/1 6/12 Date of Report: 27**/12/2011_**.  BP: ___ Weight: ___ Height:___ BSA: ___. Referral Diagnosis: **CHF + Diaphoresis. AGH03.1825.** | | | | |
| --- | --- | --- | --- | --- |
| **Features** | **Finding** | **Features** | **Finding** | |
| **Profile** |  | **Atria** |  | |
| Abdominal situs | Solitus | Left atrium | Dilated | |
| Cardiac position | Levocardia | Right atrium | Dilated | |
| Systemic venous drainage | Normal | **Atrioventricular valves** |  | |
| Pulmonary venous drainage | Normal | Mitral valve | Annulus = 15mm | |
| Atrioventricular connection | Concordant | Tricuspid valve | Annulus = 17mm  TAPSE = 15mm | |
| Ventriculoarterial connection | Concordant | **Ventricles** |  | |
| Ventricular loop | d-Loop | Left ventricle | Dilated | |
|  |  | Right ventricle | Dilated | |
| **Septae** |  | **Coronary arteries** | ----- | |
| Interventricular septum | Intact | **Doppler Measurement** |  | |
| Interatrial septum | Intact | Mitral | ---- | |
| **Semilunal valves** |  | Aortic | ---- | |
| Aortic valve | Annulus = 10mm | Tricuspid | Moderate TR, PPG = 50mmHg | |
| Pulmonary valve | Annulus = 15mm | pulmonic | ---- | |
| **Great arteries** | NRGA | **Aortic arch** | ---- | |
| Aorta | ---- | **PDA** | No | |
| Pulmonary artery | ------ |  |  | |
| **M-Mode** | | | | |
| AO | mm | PWd | | 8.5mm |
| LA | mm | EDV | | 33.59ml |
| LVIDd | 29.5mm | ESV | | 15.29ml |
| LVIDs | 21.5mm | LVEF | | 55% |
| IVSd | 6.5mm | FS | | 27% |
| **Additional Information**: |  | | | |
| 5mm Aorto – Pulmonary Window | | | | |
| **Final Diagnosis:** | | | | |
| 1. {S, D, S} Levocardia 2. Aorto – Pulmonary Window 3. All chambers Dilated 4. Moderate to severe Pulmonary Hyperension 5. Good Biventricular Function | | | | |
| SIGNATURE  Done by: Tesfaye T., Pediatric Cardiologist _______________ 27/12/2011Eth.C | | | | |

| Patient Name**: Temesgen Zelalem**. Patient ID:ADINAS. SEX/ Age: M/14years Date of Report: 27**/12/2011_**.  BP: ___ Weight: ___ Height:___ BSA: ___. Referral Diagnosis: **Rheumatic Recurrence. AGH03.1826.** | | | | |
| --- | --- | --- | --- | --- |
| **Features** | **Finding** | **Features** | **Finding** | |
| **Profile** |  | **Atria** |  | |
| Abdominal situs | Solitus | Left atrium | Normal | |
| Cardiac position | Levocardia | Right atrium | Normal | |
| Systemic venous drainage | Normal | **Atrioventricular valves** |  | |
| Pulmonary venous drainage | Normal | Mitral valve | Annulus = 20mm, thickened valve leaflet, shortened PML | |
| Atrioventricular connection | Concordant | Tricuspid valve | Annulus = 25mm  TAPSE = 20mm | |
| Ventriculoarterial connection | Concordant | **Ventricles** |  | |
| Ventricular loop | d-Loop | Left ventricle | Normal | |
|  |  | Right ventricle | Normal | |
| **Septae** |  | **Coronary arteries** | ----- | |
| Interventricular septum | Intact | **Doppler Measurement** |  | |
| Interatrial septum | Intact | Mitral | Mild MR | |
| **Semilunal valves** |  | Aortic | ---- | |
| Aortic valve | ---- | Tricuspid | Mild TR, PPG = 20mmHg | |
| Pulmonary valve | ---- | pulmonic | ---- | |
| **Great arteries** | NRGA | **Aortic arch** |  | |
| Aorta | ---- | **PDA** |  | |
| Pulmonary artery | ------ |  |  | |
| **M-Mode =** Normal LV Function on eye balling | | | | |
| AO | mm | PWd | | mm |
| LA | mm | EDV | | ml |
| LVIDd | mm | ESV | | ml |
| LVIDs | mm | LVEF | | % |
| IVSd | mm | FS | | % |
| **Additional Information**: |  | | | |
| No pleural/pericardial effusion | | | | |
| **Final Diagnosis:** | | | | |
| 1. {S, D, S} Levocardia 2. Mild MR, thickened Mitral valve leaflet, Shortened PML 3. Mild TR 4. Good Biventricular Function | | | | |
| SIGNATURE  Done by: Tesfaye T., Pediatric Cardiologist _______________ 27/12/2011Eth.C | | | | |

| Patient Name**: Biniyam Zelalem**. Patient ID: ADINAS. SEX/ Age: M/12years Date of Report: 1**/13/2011_**.  BP: ___ Weight: ___ Height:___ BSA: ___. Referral Diagnosis: **Rheumatic Fever. AGH03.1827.** | | | | |
| --- | --- | --- | --- | --- |
| **Features** | **Finding** | **Features** | **Finding** | |
| **Profile** |  | **Atria** |  | |
| Abdominal situs | Solitus | Left atrium | Normal | |
| Cardiac position | Levocardia | Right atrium | Normal | |
| Systemic venous drainage | Normal | **Atrioventricular valves** |  | |
| Pulmonary venous drainage | Normal | Mitral valve | Annulus = 22mm  Thickened MVL | |
| Atrioventricular connection | Concordant | Tricuspid valve | Annulus = 20mm  TAPSE = 21mm | |
| Ventriculoarterial connection | Concordant | **Ventricles** |  | |
| Ventricular loop | d-Loop | Left ventricle | Normal | |
|  |  | Right ventricle | Normal | |
| **Septae** |  | **Coronary arteries** | ----- | |
| Interventricular septum | Intact | **Doppler Measurement** |  | |
| Interatrial septum | Intact | Mitral | Mild MR, Holosystolic, velocity = 3.5m/s, projecting posteriorly | |
| **Semilunal valves** |  | Aortic | Mild AR | |
| Aortic valve | Annulus = 18mm | Tricuspid | ---- | |
| Pulmonary valve | Annulus = 16mm | pulmonic | Trivial PR | |
| **Great arteries** | NRGA | **Aortic arch** | ---- | |
| Aorta | ---- | **PDA** | No | |
| Pulmonary artery | ------ |  |  | |
| **M-Mode** | | | | |
| AO | mm | PWd | | 7.3mm |
| LA | mm | EDV | | 62.93ml |
| LVIDd | 38.2mm | ESV | | 30.96ml |
| LVIDs | 28.5mm | LVEF | | 50% |
| IVSd | 6.7mm | FS | | 25% |
| **Additional Information**: |  | | | |
| No pleural/pericardial effusion | | | | |
| **Final Diagnosis:** | | | | |
| 1. {S, D, S} Levocardia 2. Mild AR 3. Mild MR 4. Thickened Mitral valve leaflet 5. Good Biventricular Function | | | | |
| SIGNATURE  Done by: Tesfaye T., Pediatric Cardiologist _______________ 01/13/2011Eth.C | | | | |

| Patient Name**: Redeat Gebru**. Patient ID: **FHH**. SEX/ Age: **F/2 6/12** Date of Report: 02**/13/2011**.  BP: ___ Weight: ___ Height:___ BSA: ___. Referral Diagnosis: **CHF. AGH03.1828.** | | | | |
| --- | --- | --- | --- | --- |
| **Features** | **Finding** | **Features** | **Finding** | |
| **Profile** |  | **Atria** |  | |
| Abdominal situs | Solitus | Left atrium | Dilated | |
| Cardiac position | Levocardia | Right atrium | Dilated | |
| Systemic venous drainage | Normal | **Atrioventricular valves** |  | |
| Pulmonary venous drainage | Normal | Mitral valve | Annulus = 25mm | |
| Atrioventricular connection | Concordant | Tricuspid valve | Annulus = 20mm  TAPSE = 19mm | |
| Ventriculoarterial connection | Concordant | **Ventricles** |  | |
| Ventricular loop | d-Loop | Left ventricle | Dilated | |
|  |  | Right ventricle | Dilated | |
| **Septae** |  | **Coronary arteries** | ----- | |
| Interventricular septum | 14mm inlet VSD, BD Shunt | **Doppler Measurement** |  | |
| Interatrial septum | Intact | Mitral | ---- | |
| **Semilunal valves** |  | Aortic | ---- | |
| Aortic valve | Annulus = 10mm | Tricuspid | ---- | |
| Pulmonary valve | Annulus = 18mm | pulmonic | Trivial PR | |
| **Great arteries** | NRGA | **Aortic arch** | Left | |
| Aorta | ---- | **PDA** | No | |
| Pulmonary artery | 18mm (Large ) |  |  | |
| **M-Mode** | | | | |
| AO | mm | PWd | | 6.4mm |
| LA | mm | EDV | | 62ml |
| LVIDd | 38mm | ESV | | 30.58ml |
| LVIDs | 28.4mm | LVEF | | 50% |
| IVSd | 6.4mm | FS | | 25% |
| **Additional Information**: | No Coarctation of Aorta | | | |
| No pleural/pericardial effusion | | | | |
| **Final Diagnosis:** | | | | |
| 1. {S, D, S} Levocardia 2. Large Inlet VSD, BD Shunt 3. Reduced LV Systolic Function 4. Severe Pulmonary Hypertension | | | | |
| SIGNATURE  Done by: Tesfaye T., Pediatric Cardiologist _______________ 02/13/2011Eth.C | | | | |

| Patient Name**: Baby of Tirusew Abiyu**. Patient ID:FHH. SEX/ Age: F/28Days Date of Report: 02**/13/2011_**.  BP: ___ Weight: ___ Height:___ BSA: ___. Referral Diagnosis: **RD. AGH03.1829.** | | | | |
| --- | --- | --- | --- | --- |
| **Features** | **Finding** | **Features** | **Finding** | |
| **Profile** |  | **Atria** |  | |
| Abdominal situs | Solitus | Left atrium | Normal | |
| Cardiac position | Levocardia | Right atrium | Normal | |
| Systemic venous drainage | Normal | **Atrioventricular valves** |  | |
| Pulmonary venous drainage | Normal | Mitral valve | Annulus = 10mm | |
| Atrioventricular connection | Concordant | Tricuspid valve | Annulus = 6.5mm  TAPSE = 9mm | |
| Ventriculoarterial connection | Concordant | **Ventricles** |  | |
| Ventricular loop | d-Loop | Left ventricle | Normal | |
|  |  | Right ventricle | Normal | |
| **Septae** |  | **Coronary arteries** | ----- | |
| Interventricular septum | Intact | **Doppler Measurement** |  | |
| Interatrial septum | Intact | Mitral | ---- | |
| **Semilunal valves** |  | Aortic | ---- | |
| Aortic valve | Annulus = 7mm | Tricuspid | ---- | |
| Pulmonary valve | Annulus = 6mm | pulmonic | ---- | |
| **Great arteries** | NRGA | **Aortic arch** | ---- | |
| Aorta | ---- | **PDA** | No | |
| Pulmonary artery | Good sized MPA and confluent branch PAs |  |  | |
| **M-Mode** | | | | |
| AO | mm | PWd | | 3mm |
| LA | mm | EDV | | 2.83ml |
| LVIDd | 11.3mm | ESV | | 1.12ml |
| LVIDs | 8mm | LVEF | | 60% |
| IVSd | 2.5mm | FS | | 28% |
| **Additional Information**: |  | | | |
| No pleural/pericardial effusion | | | | |
| **Final Diagnosis:** | | | | |
| 1. {S, D, S} Levocardia 2. Normal Echocardiographic Study 3. Good ventricular Function | | | | |
| SIGNATURE  Done by: Tesfaye T., Pediatric Cardiologist _______________ 02/13/2011Eth.C | | | | |

| Patient Name**: Baby of Zerfie Nebiyu**. Patient ID: **ADINAS**. SEX/ Age: **M/45days** Date of Report: 04**/13/2011_**.  BP: ___ Weight: ___ Height:___ BSA: ___. Referral Diagnosis: **Incidental Murmur. AGH03.1830.** | | | | | |
| --- | --- | --- | --- | --- | --- |
| **Features** | **Finding** | | | **Features** | **Finding** |
| **Profile** |  | | | **Atria** |  |
| Abdominal situs | Solitus | | | Left atrium | Normal |
| Cardiac position | Levocardia | | | Right atrium | Normal |
| Systemic venous drainage | Normal | | | **Atrioventricular valves** |  |
| Pulmonary venous drainage | Normal | | | Mitral valve | Annulus = 13mm |
| Atrioventricular connection | Concordant | | | Tricuspid valve | Annulus = 14mm  TAPSE = 10mm |
| Ventriculoarterial connection | Concordant | | | **Ventricles** |  |
| Ventricular loop | d-Loop | | | Left ventricle | Normal |
|  |  | | | Right ventricle | Normal |
| **Septae** |  | | | **Coronary arteries** | ----- |
| Interventricular septum | Mal-aligned Sub aortic VSD, L – R Shunt | | | **Doppler Measurement** |  |
| Interatrial septum | PFO, L – R Shunt | | | Mitral | ---- |
| **Semilunal valves** |  | | | Aortic | ---- |
| Aortic valve | Annulus = 10mm | | | Tricuspid | ---- |
| Pulmonary valve | Annulus = 12mm, Doming pulmonary valve | | | pulmonic | Mild PS, PPG = 30mmHg. |
| **Great arteries** | NRGA | | | **Aortic arch** |  |
| Aorta | Overriding aorta | | | **PDA** |  |
| Pulmonary artery | ------ | | |  |  |
| **M-Mode** | | | | | |
| AO | | mm | PWd | | 2.9mm |
| LA | | mm | EDV | | 14.53ml |
| LVIDd | | 21.1mm | ESV | | 5.69ml |
| LVIDs | | 14.6mm | LVEF | | 60% |
| IVSd | | 4.6mm | FS | | 30% |
| **Additional Information**: | |  | | | |
| No pleural/pericardial effusion | | | | | |
| **Final Diagnosis:** | | | | | |
| 1. {S, D, S} Levocardia 2. PFO, L – R Shunt 3. TOF 4. Good Biventricular Function | | | | | |
| SIGNATURE  Done by: Tesfaye T., Pediatric Cardiologist _______________ 04/13/2011Eth.C | | | | | |

| Patient Name **Yiketel Fanta**. Patient ID: **FHH**. SEX/ Age: **M/1 6/12** Date of Report: 05**/13/2011_**.  BP: ___ Weight: ___ Height:___ BSA: ___. Referral Diagnosis: **Incidental Murmur. AGH03.1831.** | | | | |
| --- | --- | --- | --- | --- |
| **Features** | **Finding** | **Features** | **Finding** | |
| **Profile** |  | **Atria** |  | |
| Abdominal situs | Solitus | Left atrium | Normal | |
| Cardiac position | Levocardia | Right atrium | Normal | |
| Systemic venous drainage | Normal | **Atrioventricular valves** |  | |
| Pulmonary venous drainage | Normal | Mitral valve | Annulus = 12mm | |
| Atrioventricular connection | Concordant | Tricuspid valve | Annulus = 17mm  TAPSE = 15mm | |
| Ventriculoarterial connection | Concordant | **Ventricles** |  | |
| Ventricular loop | d-Loop | Left ventricle | Normal | |
|  |  | Right ventricle | Normal | |
| **Septae** |  | **Coronary arteries** | ----- | |
| Interventricular septum | 3mm PM VSD, L – R Shunt | **Doppler Measurement** |  | |
| Interatrial septum | Intact | Mitral | ---- | |
| **Semilunal valves** |  | Aortic | ---- | |
| Aortic valve | Annulus = 14mm | Tricuspid | Trivial TR | |
| Pulmonary valve | Annulus = 16mm | pulmonic | Moderate PR, PPG = 46mmHg | |
| **Great arteries** | NRGA | **Aortic arch** | Left | |
| Aorta | ---- | **PDA** | No | |
| Pulmonary artery | Good sized MPA and confluent Branch PAs |  |  | |
| **M-Mode** | | | | |
| AO | mm | PWd | | 8mm |
| LA | mm | EDV | | 23.45ml |
| LVIDd | 25.5mm | ESV | | 10.43ml |
| LVIDs | 18.5mm | LVEF | | 55% |
| IVSd | 5.5mm | FS | | 27% |
| **Additional Information**: |  | | | |
| No pleural/pericardial effusion | | | | |
| **Final Diagnosis:** | | | | |
| 1. {S, D, S} Levocardia 2. Small PM VSD, L – R Shunt 3. Moderate PR 4. Mild Pulmonary Hypertension 5. Good Biventricular Function | | | | |
| SIGNATURE  Done by: Tesfaye T., Pediatric Cardiologist _______________ 05/13/2011Eth.C | | | | |

| Patient Name**: Baby of Amelewerk Girma**. Patient ID:_FHH. SEX/ Age: M/9days Date of Report: 02**/01/2012_**.  BP: ___ Weight: ___ Height:___ BSA: ___. Referral Diagnosis: **DS. AGH03.1832.** | | | | |
| --- | --- | --- | --- | --- |
| **Features** | **Finding** | **Features** | **Finding** | |
| **Profile** |  | **Atria** |  | |
| Abdominal situs | Solitus | Left atrium | Normal | |
| Cardiac position | Levocardia | Right atrium | Normal | |
| Systemic venous drainage | Normal | **Atrioventricular valves** |  | |
| Pulmonary venous drainage | Normal | Mitral valve | Annulus = 8mm | |
| Atrioventricular connection | Concordant | Tricuspid valve | Annulus = 11mm  TAPSE = 10mm | |
| Ventriculoarterial connection | Concordant | **Ventricles** |  | |
| Ventricular loop | d-Loop | Left ventricle | Normal | |
|  |  | Right ventricle | Normal | |
| **Septae** |  | **Coronary arteries** | ----- | |
| Interventricular septum | Intact | **Doppler Measurement** |  | |
| Interatrial septum | PFO, L – R Shunt | Mitral |  | |
| **Semilunal valves** |  | Aortic |  | |
| Aortic valve | Annulus = 8mm | Tricuspid |  | |
| Pulmonary valve | Annulus = 9mm | pulmonic |  | |
| **Great arteries** | NRGA | **Aortic arch** |  | |
| Aorta | ---- | **PDA** |  | |
| Pulmonary artery | ------ |  |  | |
| **M-Mode** | | | | |
| AO | mm | PWd | | 4.3mm |
| LA | mm | EDV | | 5.58ml |
| LVIDd | 14.5mm | ESV | | 2.18ml |
| LVIDs | 10.2mm | LVEF | | 60% |
| IVSd | 3.5mm | FS | | 29% |
| **Additional Information**: |  | | | |
| No pleural/pericardial effusion | | | | |
| **Final Diagnosis:** | | | | |
| 1. {S, D, S} Levocardia 2. PFO, L – R Shunt | | | | |
| SIGNATURE  Done by: Tesfaye T., Pediatric Cardiologist _______________ 02/01/2012Eth.C | | | | |

| Patient Name**: Baby of Seble Yenew**. Patient ID:_FHH. SEX/ Age: _F/29days Date of Report: 02**/01/2012_**.  BP: ___ Weight: ___ Height:___ BSA: ___. Referral Diagnosis: **PPHTN. AGH03.1833.** | | | | |
| --- | --- | --- | --- | --- |
| **Features** | **Finding** | **Features** | **Finding** | |
| **Profile** |  | **Atria** |  | |
| Abdominal situs | Solitus | Left atrium | Normal | |
| Cardiac position | Levocardia | Right atrium | Dilated | |
| Systemic venous drainage | Normal | **Atrioventricular valves** |  | |
| Pulmonary venous drainage | Normal | Mitral valve | Annulus = 13mm | |
| Atrioventricular connection | Concordant | Tricuspid valve | Annulus = 16mm | |
| Ventriculo arterial connection | Concordant | **Ventricles** |  | |
| Ventricular loop | d-Loop | Left ventricle | Normal | |
|  |  | Right ventricle | Dilated | |
| **Septae** |  | **Coronary arteries** | ----- | |
| Interventricular septum | Intact | **Doppler Measurement** |  | |
| Interatrial septum | PFO, R - L Shunt | Mitral | ---- | |
| **Semilunal valves** |  | Aortic | ---- | |
| Aortic valve | Annulus = 6mm | Tricuspid | Moderate to severe TR, PPG = 50mmHg | |
| Pulmonary valve | Annulus = 11mm | pulmonic | Mild PR | |
| **Great arteries** | NRGA | **Aortic arch** | ----- | |
| Aorta | ---- | **PDA** | No | |
| Pulmonary artery | ------ |  |  | |
| **M-Mode** | | | | |
| AO | mm | PWd | | 4.7mm |
| LA | mm | EDV | | 10.38ml |
| LVIDd | 18.5mm | ESV | | 5.58ml |
| LVIDs | 14.5mm | LVEF | | 46% |
| IVSd | **5.5mm** | FS | | 21% |
| **Additional Information**: | Abnormal septal motion | | | |
| No pleural/pericardial effusion | | | | |
| **Final Diagnosis:** | | | | |
| 1. {S, D, S} Levocardia 2. PFO, L – R Shunt 3. Dilated RA/RV 4. Moderate to Severe PR 5. Moderate to Severe PAH 6. Reduced Biventricular Function (PPHTN) | | | | |
| SIGNATURE  Done by: Tesfaye T., Pediatric Cardiologist _______________ 02/01/2012Eth.C | | | | |

| Patient Name**: Matias Abebe**. Patient ID: FHH. SEX/ Age: M/3years Date of Report: 02**/01/2012_**.  BP: ___ Weight: ___ Height:___ BSA: ___. Referral Diagnosis: **Cyanosis + CHF+ Clubbing. AGH03.1834.** | | | |
| --- | --- | --- | --- |
| **Features** | **Finding** | **Features** | **Finding** |
| **Profile** |  | **Atria** |  |
| Abdominal situs | Solitus | Left atrium | Normal |
| Cardiac position | Levocardia | Right atrium | Normal |
| Systemic venous drainage | Normal | **Atrioventricular valves** |  |
| Pulmonary venous drainage | Normal | Mitral valve | Annulus = 14mm |
| Atrioventricular connection | Concordant | Tricuspid valve | Annulus = Atretic |
| Ventriculoarterial connection | Concordant | **Ventricles** |  |
| Ventricular loop | d-Loop | Left ventricle | Normal |
|  |  | Right ventricle | Good sized RV |
| **Septae** |  | **Coronary arteries** | ----- |
| Interventricular septum | 10mm inlet VSD with PM extension, L – R Shunt | **Doppler Measurement** |  |
| Interatrial septum | 12mm ASD, R – L Shunt | Mitral | Mild MR |
| **Semilunal valves** |  | Aortic | ---- |
| Aortic valve | Annulus = 14mm | Tricuspid | Atretic |
| Pulmonary valve | Annulus = 11mm | pulmonic | ---- |
| **Great arteries** | NRGA | **Aortic arch** | ---- |
| Aorta | ---- | **PDA** | No |
| Pulmonary artery | ------ |  |  |
| **M-Mode** | | | |
| AO | mm | PWd | mm |
| LA | mm | EDV | ml |
| LVIDd | mm | ESV | ml |
| LVIDs | mm | LVEF | % |
| IVSd | mm | FS | % |
| **Additional Information**: |  | | |
| No pleural/pericardial effusion | | | |
| **Final Diagnosis:** | | | |
| 1. {S, D, S} Levocardia 2. Tricuspid Atresia Type IC 3. Large OS ASD, R – L Shunt 4. Large Inlet VSD with PM Extension, L – R Shunt | | | |
| SIGNATURE  Done by: Tesfaye T., Pediatric Cardiologist _______________ 02/01/2012Eth.C | | | |

| Patient Name**: Baby Dejitnu Alebachew**. Patient ID:FHH. SEX/ Age: F/4/12 Date of Report: 05**/01/2012_**.  BP: ___ Weight: ___ Height:___ BSA: ___. Referral Diagnosis: **DS. AGH03.1835.** | | | | |
| --- | --- | --- | --- | --- |
| **Features** | **Finding** | **Features** | **Finding** | |
| **Profile** |  | **Atria** |  | |
| Abdominal situs | Solitus | Left atrium | Normal | |
| Cardiac position | Levocardia | Right atrium | Normal | |
| Systemic venous drainage | Normal | **Atrioventricular valves** |  | |
| Pulmonary venous drainage | Normal | Mitral valve | Annulus = 12mm | |
| Atrioventricular connection | Concordant | Tricuspid valve | Annulus = 14mm  TAPSE = 14mm | |
| Ventriculoarterial connection | Concordant | **Ventricles** |  | |
| Ventricular loop | d-Loop | Left ventricle | Normal | |
|  |  | Right ventricle | Normal | |
| **Septae** |  | **Coronary arteries** | ----- | |
| Interventricular septum | Intact | **Doppler Measurement** |  | |
| Interatrial septum | Intact | Mitral | ---- | |
| **Semilunal valves** |  | Aortic | ---- | |
| Aortic valve | Annulus = 11mm | Tricuspid | ---- | |
| Pulmonary valve | Annulus = 11mm | pulmonic | ---- | |
| **Great arteries** | NRGA | **Aortic arch** |  | |
| Aorta | ---- | **PDA** |  | |
| Pulmonary artery | ------ |  |  | |
| **M-Mode** | | | | |
| AO | mm | PWd | | 3.6mm |
| LA | mm | EDV | | 6.44ml |
| LVIDd | 15.4mm | ESV | | 2.48ml |
| LVIDs | 10.7mm | LVEF | | 61% |
| IVSd | 6.1mm | FS | | 30% |
| **Additional Information**: |  | | | |
| No pleural/pericardial effusion | | | | |
| **Final Diagnosis:** | | | | |
| 1. Normal Echocardiographic Study | | | | |
| SIGNATURE  Done by: Tesfaye T., Pediatric Cardiologist _______________ 05/01/2012Eth.C | | | | |

| Patient Name**: Sobrina Yibeltal**. Patient ID: ADINAS. SEX/ Age: F/9/12 Date of Report: 05**/01/2012_**.  BP: ___ Weight: ___ Height:___ BSA: ___. Referral Diagnosis: **CHF. AGH03.1836.** | | | | | |
| --- | --- | --- | --- | --- | --- |
| **Features** | **Finding** | | **Features** | | **Finding** |
| **Profile** |  | | **Atria** | |  |
| Abdominal situs | Solitus | | Left atrium | | Dilated |
| Cardiac position | Levocardia | | Right atrium | | Normal |
| Systemic venous drainage | Normal | | **Atrioventricular valves** | |  |
| Pulmonary venous drainage | Normal | | Mitral valve | | Annulus = 13mm |
| Atrioventricular connection | Concordant | | Tricuspid valve | | Annulus = 11mm |
| Ventriculoarterial connection | Concordant | | **Ventricles** | |  |
| Ventricular loop | d-Loop | | Left ventricle | | Dilated |
|  |  | | Right ventricle | | Normal |
| **Septae** |  | | **Coronary arteries** | | ----- |
| Interventricular septum | 10mm Inlet VSD with PM extension, L – R Shunt | | **Doppler Measurement** | |  |
| Interatrial septum | Intact | | Mitral | | ---- |
| **Semilunal valves** |  | | Aortic | | ---- |
| Aortic valve | Annulus = 12mm | | Tricuspid | | ---- |
| Pulmonary valve | Annulus = 14mm | | pulmonic | | ---- |
| **Great arteries** | NRGA | | **Aortic arch** | | ---- |
| Aorta | ---- | | **PDA** | | No |
| Pulmonary artery | ------ | |  | |  |
| **M-Mode** | | | | | |
| AO | mm | PWd | | 4.6mm | |
| LA | mm | EDV | | 22.32ml | |
| LVIDd | 25mm | ESV | | 6.84ml | |
| LVIDs | 15.7mm | LVEF | | 69% | |
| IVSd | 3.2mm | FS | | 37% | |
| **Additional Information**: |  | | | | |
| No pleural/pericardial effusion | | | | | |
| **Final Diagnosis:** | | | | | |
| 1. {S, D, S} Levocardia 2. Large Inlet VSD with PM Extension, L – R Shunt 3. Good LV Function | | | | | |
| SIGNATURE  Done by: Tesfaye T., Pediatric Cardiologist _______________ 05/01/2012Eth.C | | | | | |

| Patient Name**: Birhanu Tazeb**. Patient ID: Merawi Hospital. SEX/ Age: M/12Yrs Date of Report: 06**/01/2012_**.  BP: ___ Weight: ___ Height:___ BSA: ___. Referral Diagnosis: **ARF. AGH03.1837.** | | | | |
| --- | --- | --- | --- | --- |
| **Features** | **Finding** | **Features** | **Finding** | |
| **Profile** |  | **Atria** |  | |
| Abdominal situs | Solitus | Left atrium | Normal | |
| Cardiac position | Levocardia | Right atrium | Normal | |
| Systemic venous drainage | Normal | **Atrioventricular valves** |  | |
| Pulmonary venous drainage | Normal | Mitral valve | Annulus = 28mm | |
| Atrioventricular connection | Concordant | Tricuspid valve | Annulus = 26mm | |
| Ventriculoarterial connection | Concordant | **Ventricles** |  | |
| Ventricular loop | d-Loop | Left ventricle | Normal | |
|  |  | Right ventricle | Normal | |
| **Septae** |  | **Coronary arteries** | ----- | |
| Interventricular septum | Intact | **Doppler Measurement** |  | |
| Interatrial septum | Intact | Mitral | ---- | |
| **Semilunal valves** |  | Aortic | ---- | |
| Aortic valve | Annulus = 24mm | Tricuspid | ---- | |
| Pulmonary valve | Annulus = 23mm | pulmonic | ---- | |
| **Great arteries** | NRGA | **Aortic arch** | ---- | |
| Aorta | ---- | **PDA** | No | |
| Pulmonary artery | Good sized MPA & confluent branch PAs |  |  | |
| **M-Mode** | | | | |
| AO | mm | PWd | | 6.9mm |
| LA | mm | EDV | | 90.39ml |
| LVIDd | 44.6mm | ESV | | 35.82ml |
| LVIDs | 30.3mm | LVEF | | 60% |
| IVSd | 8.6mm | FS | | 32% |
| **Additional Information**: |  | | | |
| No pleural/pericardial effusion | | | | |
| **Final Diagnosis:** | | | | |
| 1. Normal Echocardiography Study | | | | |
| SIGNATURE  Done by: Tesfaye T., Pediatric Cardiologist _______________ 06/01/2012Eth.C | | | | |

| Patient Name**: Tigist Yitayih**. Patient ID: FHH. SEX/ Age: F/10Years_ Date of Report: 06**/01/2012_**.  BP: ___ Weight: ___ Height:___ BSA: ___. Referral Diagnosis: **Rheumatic Recurrence. AGH03.1838.** | | | | |
| --- | --- | --- | --- | --- |
| **Features** | **Finding** | **Features** | **Finding** | |
| **Profile** |  | **Atria** |  | |
| Abdominal situs | Solitus | Left atrium | Normal | |
| Cardiac position | Levocardia | Right atrium | Normal | |
| Systemic venous drainage | Normal | **Atrioventricular valves** |  | |
| Pulmonary venous drainage | Normal | Mitral valve | Annulus = 22mm, thickened leaflet | |
| Atrioventricular connection | Concordant | Tricuspid valve | Annulus = 25mm  TAPSE = 25mm | |
| Ventriculoarterial connection | Concordant | **Ventricles** |  | |
| Ventricular loop | d-Loop | Left ventricle | Normal | |
|  |  | Right ventricle | Normal | |
| **Septae** |  | **Coronary arteries** | ----- | |
| Interventricular septum | Intact | **Doppler Measurement** |  | |
| Interatrial septum | Intact | Mitral | Mild MR, seen in two planes, Posterior jet | |
| **Semilunal valves** |  | Aortic | Trivial to mild AR | |
| Aortic valve | Annulus = 19mm | Tricuspid | Mild TR, PPG = 42mmHg | |
| Pulmonary valve | Annulus = 25mm | pulmonic | ---- | |
| **Great arteries** | NRGA | **Aortic arch** | ---- | |
| Aorta | ---- | **PDA** | No | |
| Pulmonary artery | ------ |  |  | |
| **M-Mode** | | | | |
| AO | mm | PWd | | 7.1mm |
| LA | mm | EDV | | 64ml |
| LVIDd | 38.6mm | ESV | | 25ml |
| LVIDs | 26.4mm | LVEF | | 60% |
| IVSd | 5.8mm | FS | | 31% |
| **Additional Information**: |  | | | |
| No pleural/pericardial effusion | | | | |
| **Final Diagnosis:** | | | | |
| 1. {S, D, S} Levocardia 2. Mild MR, thickened Mitral valve leaflet 3. Mild TR 4. Trivial to Mild AR 5. Mild PAH 6. Good Biventricular Function | | | | |
| SIGNATURE  Done by: Tesfaye T., Pediatric Cardiologist _______________ 06/01/2012Eth.C | | | | |

| Patient Name**: Misganaw Bable**. Patient ID:FHH. SEX/ Age: M/3Years Date of Report: 06**/01/2012_**.  BP: ___ Weight: ___ Height:___ BSA: ___. Referral Diagnosis: **Cyanosis. AGH03.1839.** | | | | |
| --- | --- | --- | --- | --- |
| **Features** | **Finding** | **Features** | **Finding** | |
| **Profile** |  | **Atria** |  | |
| Abdominal situs | Solitus | Left atrium | Normal | |
| Cardiac position | Levocardia | Right atrium | Normal | |
| Systemic venous drainage | Normal | **Atrioventricular valves** |  | |
| Pulmonary venous drainage | Normal | Mitral valve | Annulus = 14mm | |
| Atrioventricular connection | Concordant | Tricuspid valve | Annulus = 18mm  TAPSE = 15mm | |
| Ventriculoarterial connection | Concordant | **Ventricles** |  | |
| Ventricular loop | d-Loop | Left ventricle | Normal | |
|  |  | Right ventricle | Hypertrophied | |
| **Septae** |  | **Coronary arteries** | ----- | |
| Interventricular septum | Malaligned sub aortic VSD | **Doppler Measurement** |  | |
| Interatrial septum | Intact | Mitral | ---- | |
| **Semilunal valves** |  | Aortic | ---- | |
| Aortic valve | Annulus = 18mm | Tricuspid | ---- | |
| Pulmonary valve | Annulus = 12mm | pulmonic | Severe PS, PPG = 95mmHg | |
| **Great arteries** | NRGA | **Aortic arch** | ---- | |
| Aorta | Overriding aorta | **PDA** | No | |
| Pulmonary artery | ------ |  |  | |
| **M-Mode** | | | | |
| AO | mm | PWd | | mm |
| LA | mm | EDV | | ml |
| LVIDd | mm | ESV | | ml |
| LVIDs | mm | LVEF | | 52% |
| IVSd | mm | FS | | 25% |
| **Additional Information**: |  | | | |
| No pleural/pericardial effusion | | | | |
| **Final Diagnosis:** | | | | |
| 1. {S, D, S} Levocardia 2. TOF | | | | |
| SIGNATURE  Done by: Tesfaye T., Pediatric Cardiologist _______________ 06/01/2012Eth.C | | | | |

| Patient Name**: Redeate Esubalew**. Patient ID:ADINAS. SEX/ Age: F/5/12 Date of Report: 06**/01/2012_**.  BP: ___ Weight: ___ Height:___ BSA: ___. Referral Diagnosis: **CHF. AGH03.1840.** | | | | |
| --- | --- | --- | --- | --- |
| **Features** | **Finding** | **Features** | **Finding** | |
| **Profile** |  | **Atria** |  | |
| Abdominal situs | Solitus | Left atrium | Mildly Dilated | |
| Cardiac position | Levocardia | Right atrium | Normal | |
| Systemic venous drainage | Normal | **Atrioventricular valves** |  | |
| Pulmonary venous drainage | Normal | Mitral valve | ---- | |
| Atrioventricular connection | Concordant | Tricuspid valve | ---- | |
| Ventriculoarterial connection | Concordant | **Ventricles** |  | |
| Ventricular loop | d-Loop | Left ventricle | Dilated, Dysfunctional LV | |
|  |  | Right ventricle | Normal | |
| **Septae** |  | **Coronary arteries** | ----- | |
| Interventricular septum | Intact | **Doppler Measurement** |  | |
| Interatrial septum | Intact | Mitral | ---- | |
| **Semilunal valves** |  | Aortic | Valvar AS, PPG = 43mmHg (May be under estimated due to reduced LV Function) | |
| Aortic valve | Annulus = 7mm, ?Bicuspid aortic valve | Tricuspid | ---- | |
| Pulmonary valve | Annulus = 11mm | pulmonic | ---- | |
| **Great arteries** | NRGA | **Aortic arch** | ---- | |
| Aorta | ---- | **PDA** | NO | |
| Pulmonary artery | ------ |  |  | |
| **M-Mode** | | | | |
| AO | mm | PWd | | 5mm |
| LA | mm | EDV | | 24.77ml |
| LVIDd | 26mm | ESV | | 15.16ml |
| LVIDs | 21.4mm | LVEF | | 38% |
| IVSd | 4.3mm | FS | | 18% |
| **Additional Information**: |  | | | |
| No pleural/pericardial effusion | | | | |
| **Final Diagnosis:** | | | | |
| 1. {S, D, S} Levocardia 2. ?Bicuspid Aortic valve 3. Severe AS (Under estimated gradient because of dysfunctional LV) 4. Dilated Dysfunctional LV | | | | |
| SIGNATURE  Done by: Tesfaye T., Pediatric Cardiologist    _______________ 06/01/2012Eth.C | | | | |

| Patient Name**: Agernesh Kefale**. Patient ID: Pawi Hosp. SEX/ Age: F/10Years Date of Report: 06**/01/2012_**.  BP: ___ Weight: ___ Height:___ BSA: ___. Referral Diagnosis: **Acute Rheumatic Fever. AGH03.1841.** | | | | |
| --- | --- | --- | --- | --- |
| **Features** | **Finding** | **Features** | **Finding** | |
| **Profile** |  | **Atria** |  | |
| Abdominal situs | Solitus | Left atrium | Dilated | |
| Cardiac position | Levocardia | Right atrium | Normal | |
| Systemic venous drainage | Normal | **Atrioventricular valves** |  | |
| Pulmonary venous drainage | Normal | Mitral valve | Annulus = 24, thickened leaflet | |
| Atrioventricular connection | Concordant | Tricuspid valve | Annulus = 19mm | |
| Ventriculoarterial connection | Concordant | **Ventricles** |  | |
| Ventricular loop | d-Loop | Left ventricle | Dilated | |
|  |  | Right ventricle | Normal | |
| **Septae** |  | **Coronary arteries** | ----- | |
| Interventricular septum | Intact | **Doppler Measurement** |  | |
| Interatrial septum | Intact | Mitral | Moderate MR, Velocity = 4m/sec, Posterior Projection | |
| **Semilunal valves** |  | Aortic | ---- | |
| Aortic valve | Annulus = 17mm, Trileaflet | Tricuspid | ---- | |
| Pulmonary valve | Annulus = 20mm | pulmonic | Trivial PR, PPG = 15mmHg | |
| **Great arteries** | NRGA | **Aortic arch** |  | |
| Aorta | ---- | **PDA** |  | |
| Pulmonary artery | ------ |  |  | |
| **M-Mode** | | | | |
| AO | mm | PWd | | 6.4mm |
| LA | mm | EDV | | 105ml |
| LVIDd | 47.7mm | ESV | | 36ml |
| LVIDs | 3.05mm | LVEF | | 65% |
| IVSd | 8.6mm | FS | | 35% |
| **Additional Information**: |  | | | |
| No pleural/pericardial effusion | | | | |
| **Final Diagnosis:** | | | | |
| 1. {S, D, S} Levocardia 2. Moderate MR 3. Thickened Mitral valve 4. Good LV Function | | | | |
| SIGNATURE  Done by: Tesfaye T., Pediatric Cardiologist _______________ 06/01/2012Eth.C | | | | |

| Patient Name**: Mahlet Alemie**. Patient ID: FHH. SEX/ Age: F/9Years Date of Report: 06**/01/2012_**.  BP: ___ Weight: ___ Height:___ BSA: ___. Referral Diagnosis: **Palpitation. AGH03.1842.** | | | | |
| --- | --- | --- | --- | --- |
| **Features** | **Finding** | **Features** | **Finding** | |
| **Profile** |  | **Atria** |  | |
| Abdominal situs | Solitus | Left atrium | Normal | |
| Cardiac position | Levocardia | Right atrium | Normal | |
| Systemic venous drainage | Normal | **Atrioventricular valves** |  | |
| Pulmonary venous drainage | Normal | Mitral valve | Annulus = 20mm | |
| Atrioventricular connection | Concordant | Tricuspid valve | Annulus = 22mm  TAPSE = 18mm | |
| Ventriculoarterial connection | Concordant | **Ventricles** |  | |
| Ventricular loop | d-Loop | Left ventricle | Normal | |
|  |  | Right ventricle | Normal | |
| **Septae** |  | **Coronary arteries** | ----- | |
| Interventricular septum | Intact | **Doppler Measurement** |  | |
| Interatrial septum | Intact | Mitral | ---- | |
| **Semilunal valves** |  | Aortic | ---- | |
| Aortic valve | Annulus = 19mm | Tricuspid | ---- | |
| Pulmonary valve | Annulus = 16mm | pulmonic | ---- | |
| **Great arteries** | NRGA | **Aortic arch** | Left | |
| Aorta | ---- | **PDA** | No | |
| Pulmonary artery | ------ |  |  | |
| **M-Mode** | | | | |
| AO | mm | PWd | | 6.4mm |
| LA | mm | EDV | | 66ml |
| LVIDd | 39mm | ESV | | 29ml |
| LVIDs | 27.9mm | LVEF | | 56% |
| IVSd | 3.8mm | FS | | 28% |
| **Additional Information**: | No coarctation of aorta | | | |
| No pleural/pericardial effusion | | | | |
| **Final Diagnosis:** | | | | |
| 1. Normal Echocardiography Study | | | | |
| SIGNATURE  Done by: Tesfaye T., Pediatric Cardiologist _______________ 06/01/2012Eth.C | | | | |

| Patient Name**: Haile Habte-Weld**. Patient ID:_ADINAS. SEX/ Age: M/11Years Date of Report: 08**/01/2012_**.  BP: ___ Weight: ___ Height:___ BSA: ___. Referral Diagnosis: **Easy Fatigability. AGH03.1843.** | | | | |
| --- | --- | --- | --- | --- |
| **Features** | **Finding** | **Features** | **Finding** | |
| **Profile** |  | **Atria** |  | |
| Abdominal situs | Solitus | Left atrium | Normal | |
| Cardiac position | Levocardia | Right atrium | Normal | |
| Systemic venous drainage | Normal | **Atrioventricular valves** |  | |
| Pulmonary venous drai  nage | Normal | Mitral valve | Annulus = 22mm | |
| Atrioventricular connection | Concordant | Tricuspid valve | Annulus = 24mm  TAPSE = 20mm | |
| Ventriculoarterial connection | Concordant | **Ventricles** |  | |
| Ventricular loop | d-Loop | Left ventricle | Normal | |
|  |  | Right ventricle | Normal | |
| **Septae** |  | **Coronary arteries** | ----- | |
| Interventricular septum | Intact | **Doppler Measurement** |  | |
| Interatrial septum | Intact | Mitral | ---- | |
| **Semilunal valves** |  | Aortic | ---- | |
| Aortic valve | Annulus = 19mm | Tricuspid | Trivial TR, PPG = 24mmHg | |
| Pulmonary valve | Annulus = 20mm | pulmonic | ---- | |
| **Great arteries** | NRGA | **Aortic arch** | Left | |
| Aorta | ---- | **PDA** | No | |
| Pulmonary artery | ------ |  |  | |
| **M-Mode** | | | | |
| AO | mm | PWd | | 5.1mm |
| LA | mm | EDV | | 83.88ml |
| LVIDd | 43.2mm | ESV | | 34.19ml |
| LVIDs | 29.7mm | LVEF | | 59% |
| IVSd | 8.4mm | FS | | 31% |
| **Additional Information**: | No coarctation of aorta | | | |
| No pleural/pericardial effusion | | | | |
| **Final Diagnosis:** | | | | |
| 1. Normal Echocardiographic Study | | | | |
| SIGNATURE  Done by: Tesfaye T., Pediatric Cardiologist _______________ 08/01/2012Eth.C | | | | |

| Patient Name**: Hosaena Zelalem**. Patient ID:ADINAS. SEX/ Age: F/7/12 Date of Report: 08**/01/2012_**.  BP: ___ Weight: ___ Height:___ BSA: ___. Referral Diagnosis: **Incidental Murmur. AGH03.1844.** | | | | |
| --- | --- | --- | --- | --- |
| **Features** | **Finding** | **Features** | **Finding** | |
| **Profile** |  | **Atria** |  | |
| Abdominal situs | Solitus | Left atrium | Normal | |
| Cardiac position | Levocardia | Right atrium | Dilated | |
| Systemic venous drainage | Normal | **Atrioventricular valves** |  | |
| Pulmonary venous drainage | Normal | Mitral valve | Annulus = 14mm | |
| Atrioventricular connection | Concordant | Tricuspid valve | Annulus = 18mm  TAPSE = 12mm | |
| Ventriculoarterial connection | Concordant | **Ventricles** |  | |
| Ventricular loop | d-Loop | Left ventricle | Normal | |
|  |  | Right ventricle | Dilated | |
| **Septae** |  | **Coronary arteries** | ----- | |
| Interventricular septum | Intact | **Doppler Measurement** |  | |
| Interatrial septum | PFO, L – R Shunt | Mitral | ---- | |
| **Semilunal valves** |  | Aortic | ---- | |
| Aortic valve | Annulus = 10mm, Trileaflet | Tricuspid | Mild TR, PPG = 41mmHg | |
| Pulmonary valve | Annulus = 8mm, doming | pulmonic | Severe Valvar PS, PPG = 67mmHg | |
| **Great arteries** | NRGA | **Aortic arch** | Left | |
| Aorta | ---- | **PDA** | No | |
| Pulmonary artery | ------ |  |  | |
| **M-Mode** | | | | |
| AO | mm | PWd | | 3.9mm |
| LA | mm | EDV | | 25.62ml |
| LVIDd | 26.4mm | ESV | | 8.12ml |
| LVIDs | 16.8mm | LVEF | | 68% |
| IVSd | 4.3mm | FS | | 36% |
| **Additional Information**: |  | | | |
| No pleural/pericardial effusion | | | | |
| **Final Diagnosis:** | | | | |
| 1. {S, D, S} Levocardia 2. PFO, L – R Shunt 3. Severe Valvar PS 4. Good Biventricular Function | | | | |
| SIGNATURE  Done by: Tesfaye T., Pediatric Cardiologist _______________ 08/01/2012Eth.C | | | | |

| Patient Name**: Seble – Wengel Temesgen**. Patient ID: Sahate Birhan. SEX/ Age: F/7Years Date of Report: BP: ___ Weight: ___ Height:___ BSA: ___. Referral Diagnosis: **Cyanosis. AGH03.1845.** | | | | |
| --- | --- | --- | --- | --- |
| **Features** | **Finding** | **Features** | **Finding** | |
| **Profile** |  | **Atria** |  | |
| Abdominal situs | Solitus | Left atrium | Normal | |
| Cardiac position | Levocardia | Right atrium | Normal | |
| Systemic venous drainage | Normal | **Atrioventricular valves** |  | |
| Pulmonary venous drainage | Normal | Mitral valve | Annulus = 17mm | |
| Atrioventricular connection | Concordant | Tricuspid valve | Annulus = 23mm | |
| Ventriculoarterial connection | Aortic overriding | **Ventricles** |  | |
| Ventricular loop | d-Loop | Left ventricle | Normal | |
|  |  | Right ventricle | Hypertrophied | |
| **Septae** |  | **Coronary arteries** | Unclear | |
| Interventricular septum | Malaligned VSD, BD Shunt | **Doppler Measurement** |  | |
| Interatrial septum | Intact | Mitral | --- | |
| **Semilunal valves** |  | Aortic | ---- | |
| Aortic valve | Annulus = 20mm | Tricuspid | ---- | |
| Pulmonary valve | Annulus = 12mm | pulmonic | Moderate RVOTO (sub valvar PS) with PPG = 48mmHg | |
| **Great arteries** | NRGA | **Aortic arch** | ---- | |
| Aorta | Aortic overriding | **PDA** | No | |
| Pulmonary artery | Smallish MPA |  |  | |
| **M-Mode** | | | | |
| AO | mm | PWd | | 6.3mm |
| LA | mm | EDV | | 35.82ml |
| LVIDd | 30.3mm | ESV | | 12.73ml |
| LVIDs | 20mm | LVEF | | 64.47% |
| IVSd | 5.7mm | FS | | 33.96% |
| **Additional Information**: |  | | | |
| No pleural/pericardial effusion | | | | |
| **Final Diagnosis:** | | | | |
| 1. {S, D, S} Levocardia 2. TOF 3. Good LV Function 4. Coronaries unclear | | | | |
| SIGNATURE  Done by: Tesfaye T., Pediatric Cardiologist _______________ 08/01/2012Eth.C | | | | |

| Patient Name**: Mekuanint Misganaw**. Patient ID: ADINAS . SEX/ Age: M/9/12 Date of Report: 10**/01/2012_**.  BP: ___ Weight: ___ Height:___ BSA: ___. Referral Diagnosis: **CHF + DS. AGH03.1846.** | | | |
| --- | --- | --- | --- |
| **Features** | **Finding** | **Features** | **Finding** |
| **Profile** |  | **Atria** |  |
| Abdominal situs | Solitus | Left atrium | Dilated |
| Cardiac position | Levocardia | Right atrium | **Dilated** |
| Systemic venous drainage | Normal | **Atrioventricular valves** |  |
| Pulmonary venous drainage | Normal | Mitral valve | Common AV Valve |
| Atrioventricular connection | Common AV Valve | Tricuspid valve | Common AV Valve  TAPSE = 12mm |
| Ventriculoarterial connection | Concordant | **Ventricles** |  |
| Ventricular loop | d-Loop | Left ventricle | Dilated |
|  |  | Right ventricle | **Dilated** |
| **Septae** | No tongue of tissue in b/n | **Coronary arteries** | ----- |
| Interventricular septum | Large Inlet VSD, L – R Shunt | **Doppler Measurement** |  |
| Interatrial septum | Large Premium ASD, L – R Shunt | Mitral | Mild left AVVR |
| **Semilunal valves** |  | Aortic | ---- |
| Aortic valve | Annulus = 13mm | Tricuspid | Severe right AVVR |
| Pulmonary valve | Annulus = 16mm | pulmonic | ---- |
| **Great arteries** | NRGA | **Aortic arch** | ---- |
| Aorta | ---- | **PDA** | No |
| Pulmonary artery | Confluent branch PAs. MPA = **15mm** |  |  |
| **M-Mode** | | | |
| AO | mm | PWd | 4.3mm |
| LA | mm | EDV | 50.87ml |
| LVIDd | 35mm | ESV | 23.12ml |
| LVIDs | 25.4mm | LVEF | 55% |
| IVSd | 6.4mm | FS | 28% |
| **Additional Information**: |  | | |
| No pleural/pericardial effusion | | | |
| **Final Diagnosis:** | | | |
| 1. {S, D, S} Levocardia 2. Complete Balanced AVSD 3. Severe Right AVVR 4. Mild Left AVVR 5. Severe Pulmonary Hypertension 6. Good Function | | | |
| SIGNATURE  Done by: Tesfaye T., Pediatric Cardiologist _______________ 10/01/2012Eth.C | | | |

| Patient Name**: Mahlet Yaregal**. Patient ID: **Pawi Hospital**. SEX/ Age: **F/8Years** Date of Report: 12**/01/2012_**.  BP: ___ Weight: ___ Height:___ BSA: ___. Referral Diagnosis: **Rheumatic Recurrence. AGH03.1847.** | | | | |
| --- | --- | --- | --- | --- |
| **Features** | **Finding** | **Features** | **Finding** | |
| **Profile** |  | **Atria** |  | |
| Abdominal situs | Solitus | Left atrium | Mildly dilated | |
| Cardiac position | Levocardia | Right atrium | Normal | |
| Systemic venous drainage | Normal | **Atrioventricular valves** |  | |
| Pulmonary venous drainage | Normal | Mitral valve | Annulus = 20mm, Thickened clubbed leaflet | |
| Atrioventricular connection | Concordant | Tricuspid valve | Annulus = 21mm  TAPSE = 19mm | |
| Ventriculoarterial connection | Concordant | **Ventricles** |  | |
| Ventricular loop | d-Loop | Left ventricle | Dilated | |
|  |  | Right ventricle | Normal | |
| **Septae** |  | **Coronary arteries** | ----- | |
| Interventricular septum | Intact | **Doppler Measurement** |  | |
| Interatrial septum | Intact | Mitral | Severe MR, posterior projection with a velocity of 4m/sec | |
| **Semilunal valves** |  | Aortic | ---- | |
| Aortic valve | Annulus = 18mm | Tricuspid | Trivial TR, PPG = 13mmHg | |
| Pulmonary valve | Annulus = 18mm | pulmonic | ---- | |
| **Great arteries** | NRGA | **Aortic arch** | Left | |
| Aorta | ---- | **PDA** | No | |
| Pulmonary artery | Normal sized MPA & Confluent Branch PAs. |  |  | |
| **M-Mode** | | | | |
| AO | mm | PWd | | 5.4mm |
| LA | mm | EDV | | 85ml |
| LVIDd | 43mm | ESV | | 40ml |
| LVIDs | 31mm | LVEF | | 55% |
| IVSd | 7.5mm | FS | | 29% |
| **Additional Information**: |  | | | |
| No pleural/pericardial effusion | | | | |
| **Final Diagnosis:** | | | | |
| 1. {S, D, S} Levocardia 2. Severe MR 3. Thickened clubbed Mitral valve leaflets 4. Good Biventricular Function. | | | | |
| SIGNATURE  Done by: Tesfaye T., Pediatric Cardiologist _______________ 12/01/2012Eth.C | | | | |

| Patient Name**: Tigist Alemnew**. Patient ID: **FHH**. SEX/ Age: _**F/2Years** Date of Report: 12**/01/2012_**.  BP: ___ Weight: ___ Height:___ BSA: ___. Referral Diagnosis: **RD + CHF. AGH03.1848.** | | | | |
| --- | --- | --- | --- | --- |
| **Features** | **Finding** | **Features** | **Finding** | |
| **Profile** |  | **Atria** |  | |
| Abdominal situs | Solitus | Left atrium | Dilated | |
| Cardiac position | Levocardia | Right atrium | Dilated | |
| Systemic venous drainage | Normal | **Atrioventricular valves** |  | |
| Pulmonary venous drainage | Normal | Mitral valve | Annulus = 15mm | |
| Atrioventricular connection | Concordant | Tricuspid valve | Annulus = 19mm  TAPSE = 6mm | |
| Ventriculoarterial connection | Concordant | **Ventricles** |  | |
| Ventricular loop | d-Loop | Left ventricle | Dilated | |
|  |  | Right ventricle | Dilated | |
| **Septae** |  | **Coronary arteries** | ----- | |
| Interventricular septum | Intact | **Doppler Measurement** |  | |
| Interatrial septum | Intact | Mitral | Mild MR | |
| **Semilunal valves** |  | Aortic | ---- | |
| Aortic valve | Annulus = 12mm | Tricuspid | Mild TR, PPG = 30mmHg | |
| Pulmonary valve | Annulus = 11mm | pulmonic | Trivial PR | |
| **Great arteries** | NRGA | **Aortic arch** |  | |
| Aorta | ---- | **PDA** |  | |
| Pulmonary artery | ------ |  |  | |
| **M-Mode** | | | | |
| AO | mm | PWd | | 4.7mm |
| LA | mm | EDV | | 32.6ml |
| LVIDd | 29.1mm | ESV | | 18.4ml |
| LVIDs | 23.1mm | LVEF | | 43% |
| IVSd | 4.7mm | FS | | 21% |
| **Additional Information**: | Abnormal Septal motion | | | |
| No pleural/pericardial effusion | | | | |
| **Final Diagnosis:** | | | | |
| 1. {S, D, S} Levocardia 2. Mild MR 3. Mild TR 4. Reduced Biventricular Function | | | | |
| Remark: DCM | | | | |
| SIGNATURE  Done by: Tesfaye T., Pediatric Cardiologist _______________ 12/01/2012Eth.C | | | | |

| Patient Name**: Hanna Biasazinew**. Patient ID:_ADINAS_. SEX/ Age: F/5 10/12 Date of Report: 18**/01/2012_**.  BP: ___ Weight: ___ Height:___ BSA: ___. Referral Diagnosis: **DS. AGH03.1849.** | | | | |
| --- | --- | --- | --- | --- |
| **Features** | **Finding** | **Features** | **Finding** | |
| **Profile** |  | **Atria** |  | |
| Abdominal situs | Solitus | Left atrium | Normal | |
| Cardiac position | Levocardia | Right atrium | Normal | |
| Systemic venous drainage | Normal | **Atrioventricular valves** |  | |
| Pulmonary venous drainage | Normal | Mitral valve | Annulus = 20mm | |
| Atrioventricular connection | Concordant | Tricuspid valve | Annulus = 23mm | |
| Ventriculoarterial connection | Concordant | **Ventricles** |  | |
| Ventricular loop | d-Loop | Left ventricle | Normal | |
|  |  | Right ventricle | Normal | |
| **Septae** |  | **Coronary arteries** | ----- | |
| Interventricular septum | Intact | **Doppler Measurement** |  | |
| Interatrial septum | Intact | Mitral | ---- | |
| **Semilunal valves** |  | Aortic | ---- | |
| Aortic valve | Annulus = 16mm | Tricuspid | ---- | |
| Pulmonary valve | Annulus = 18mm | pulmonic | ---- | |
| **Great arteries** | NRGA | **Aortic arch** | Left | |
| Aorta | ---- | **PDA** | No | |
| Pulmonary artery | ------ |  |  | |
| **M-Mode** | | | | |
| AO | mm | PWd | | 6mm |
| LA | mm | EDV | | 58ml |
| LVIDd | 37mm | ESV | | 25ml |
| LVIDs | 26mm | LVEF | | 57% |
| IVSd | 9mm | FS | | 30% |
| **Additional Information**: |  | | | |
| No pleural/pericardial effusion | | | | |
| **Final Diagnosis:** | | | | |
| 1. {S, D, S} Levocardia 2. Normal Echocardiography report | | | | |
| SIGNATURE  Done by: Tesfaye T., Pediatric Cardiologist _______________ 18/01/2012Eth.C | | | | |

| Patient Name**: Maritu Mequanint**. Patient ID:_FHH. SEX/ Age: _F/12Yrs Date of Report: 23**/01/2012_**.  BP: ___ Weight: ___ Height:___ BSA: ___. Referral Diagnosis: **Palpitation. AGH03.1850.** | | | | |
| --- | --- | --- | --- | --- |
| **Features** | **Finding** | **Features** | **Finding** | |
| **Profile** |  | **Atria** |  | |
| Abdominal situs | Solitus | Left atrium | Normal | |
| Cardiac position | Levocardia | Right atrium | Normal | |
| Systemic venous drainage | Normal | **Atrioventricular valves** |  | |
| Pulmonary venous drainage | Normal | Mitral valve | Annulus = 26mm | |
| Atrioventricular connection | Concordant | Tricuspid valve | Annulus = 27mm  TAPSE = 21mm | |
| Ventriculoarterial connection | Concordant | **Ventricles** |  | |
| Ventricular loop | d-Loop | Left ventricle | Normal | |
|  |  | Right ventricle | Normal | |
| **Septae** |  | **Coronary arteries** | ----- | |
| Interventricular septum | Intact | **Doppler Measurement** |  | |
| Interatrial septum | Intact | Mitral | ---- | |
| **Semilunal valves** |  | Aortic | ---- | |
| Aortic valve | Annulus = 19mm | Tricuspid | Trivial TR, PPG = 13mmHg | |
| Pulmonary valve | Annulus = 23mm | pulmonic | ---- | |
| **Great arteries** | NRGA | **Aortic arch** | Left | |
| Aorta | ---- | **PDA** | No | |
| Pulmonary artery | ------ |  |  | |
| **M-Mode** | | | | |
| AO | mm | PWd | | 7.5mm |
| LA | mm | EDV | | 64.6ml |
| LVIDd | 38.7mm | ESV | | 27.37ml |
| LVIDs | 27.1mm | LVEF | | 58% |
| IVSd | 8.8mm | FS | | 30% |
| **Additional Information**: | No coarctation | | | |
| No pleural/pericardial effusion | | | | |
| **Final Diagnosis:** | | | | |
| 1. Normal Echocardiography Study | | | | |
| SIGNATURE  Done by: Tesfaye T., Pediatric Cardiologist _______________ 23/01/2012Eth.C | | | | |

| Patient Name**: Medina Aweke**. Patient ID:_ADINAS. SEX/ Age: F/13Yrs_ Date of Report: 24**/01/2012_**.  BP: ___ Weight: ___ Height:___ BSA: ___. Referral Diagnosis: **Easy Fatigability. AGH03.1851.** | | | |
| --- | --- | --- | --- |
| **Features** | **Finding** | **Features** | **Finding** |
| **Profile** |  | **Atria** |  |
| Abdominal situs | Solitus | Left atrium | Normal |
| Cardiac position | Levocardia | Right atrium | Normal |
| Systemic venous drainage | Normal | **Atrioventricular valves** |  |
| Pulmonary venous drainage | Normal | Mitral valve | Annulus = 21mm |
| Atrioventricular connection | Concordant | Tricuspid valve | Annulus = 23mm  TAPSE = 27mm |
| Ventriculoarterial connection | Concordant | **Ventricles** |  |
| Ventricular loop | d-Loop | Left ventricle | Normal |
|  |  | Right ventricle | Normal |
| **Septae** |  | **Coronary arteries** | ----- |
| Interventricular septum | Intact | **Doppler Measurement** |  |
| Interatrial septum | Intact | Mitral | --- |
| **Semilunal valves** |  | Aortic | --- |
| Aortic valve | Annulus = 18mm | Tricuspid | ---- |
| Pulmonary valve | Annulus = 21mm | pulmonic | Trivial PR, PPG = 12mmHg |
| **Great arteries** | NRGA | **Aortic arch** | Left |
| Aorta | ---- | **PDA** | No |
| Pulmonary artery | ------ |  |  |
| **M-Mode** | | | |
| AO | mm | PWd | 8.6mm |
| LA | mm | EDV | 58.6ml |
| LVIDd | 37mm | ESV | 22.6ml |
| LVIDs | 25mm | LVEF | 61% |
| IVSd | 7mm | FS | 32% |
| **Additional Information**: | No coarctation of aorta | | |
| No pleural/pericardial effusion | | | |
| **Final Diagnosis:** | | | |
| 1. Normal Echocardiography Study | | | |
| SIGNATURE  Done by: Tesfaye T., Pediatric Cardiologist _______________ 24/01/2012Eth.C | | | |

| Patient Name**: Abebe Tsega**. Patient ID:_Sehate birhan. SEX/ Age: M/13Yrs Date of Report: 27**/01/2012_**.  BP: ___ Weight: ___ Height:___ BSA: ___. Referral Diagnosis: **CHF. AGH03.1852.** | | | | |
| --- | --- | --- | --- | --- |
| **Features** | **Finding** | **Features** | **Finding** | |
| **Profile** |  | **Atria** |  | |
| Abdominal situs | Solitus | Left atrium | Dilated, 56 X 66mm | |
| Cardiac position | Levocardia | Right atrium | Normal | |
| Systemic venous drainage | Normal | **Atrioventricular valves** |  | |
| Pulmonary venous drainage | Normal | Mitral valve | Annulus = 31mm, Thickened, clubbed leaflets with PML Shortening | |
| Atrioventricular connection | Concordant | Tricuspid valve | Annulus = 37mm  TAPSE = 16mm | |
| Ventriculoarterial connection | Concordant | **Ventricles** |  | |
| Ventricular loop | d-Loop | Left ventricle | Dilated | |
|  |  | Right ventricle | Normal | |
| **Septae** |  | **Coronary arteries** | ----- | |
| Interventricular septum | Intact | **Doppler Measurement** |  | |
| Interatrial septum | Intact | Mitral | Severe MR, Mild MS Annulus 2cm**2** | |
| **Semilunal valves** |  | Aortic | Moderate AR , PHT = 350ms | |
| Aortic valve | Annulus = 20mm, Trileaflet, thickened | Tricuspid | Severe TR, PPG = 55mmHg | |
| Pulmonary valve | Annulus = 24mm | pulmonic | Mild PR, PPG = 31mmHg | |
| **Great arteries** | NRGA | **Aortic arch** | Left | |
| Aorta |  | **PDA** | No | |
| Pulmonary artery | MPA = 25mm, LPA = 16mm, RPA = 16mm |  |  | |
| **M-Mode** | | | | |
| AO | mm | PWd | | 6.8mm |
| LA | mm | EDV | | 151ml |
| LVIDd | 55.6mm | ESV | | 70ml |
| LVIDs | 40mm | LVEF | | 55% |
| IVSd | 9.5mm | FS | | 28% |
| **Additional Information**: |  | | | |
| No pleural/pericardial effusion | | | | |
| **Final Diagnosis:** | | | | |
| 1. {S, D, S} Levocardia 2. Severe TR 3. Severe MR, thickened clubbed mitral valve leaflets with PML Shortening 4. Moderate AR, thickened Aortic valve leaflets 5. MILD PR 6. Good Biventricular Function | | | | |
| SIGNATURE  Done by: Tesfaye T., Pediatric Cardiologist _______________ 27/01/2012Eth.C | | | | |

| Patient Name**: Baby of Habtam Kelkay**. Patient ID:_FHRH. SEX/ Age: M/23days Date of Report: 27**/01/2012_**.  BP: ___ Weight: ___ Height:___ BSA: ___. Referral Diagnosis: **DS. AGH03.1853.** | | | | | |
| --- | --- | --- | --- | --- | --- |
| **Features** | **Finding** | | **Features** | **Finding** | |
| **Profile** |  | | **Atria** |  | |
| Abdominal situs | Solitus | | Left atrium | Normal | |
| Cardiac position | Levocardia | | Right atrium | Normal | |
| Systemic venous drainage | Normal | | **Atrioventricular valves** |  | |
| Pulmonary venous drainage | Normal | | Mitral valve | Annulus = 9mm | |
| Atrioventricular connection | Concordant | | Tricuspid valve | Annulus = 10mm  TAPSE = 9mm | |
| Ventriculoarterial connection | Concordant | | **Ventricles** |  | |
| Ventricular loop | d-Loop | | Left ventricle | Normal | |
|  |  | | Right ventricle | Normal | |
| **Septae** |  | | **Coronary arteries** | ----- | |
| Interventricular septum | Intact | | **Doppler Measurement** |  | |
| Interatrial septum | Intact | | Mitral | ---- | |
| **Semilunal valves** |  | | Aortic | ---- | |
| Aortic valve | Annulus = 7.5, Trileaflet | | Tricuspid | Trivial TR, PPG = 14mmHg | |
| Pulmonary valve | Annulus = 7.5mm | | pulmonic | ---- | |
| **Great arteries** | NRGA | | **Aortic arch** | Left | |
| Aorta | ---- | | **PDA** | No | |
| Pulmonary artery | Good sized confluent Branch PAs. | |  |  | |
| **M-Mode:---- good LV Function on eye balling** | | | | | |
| AO | | mm | PWd | | mm |
| LA | | mm | EDV | | ml |
| LVIDd | | mm | ESV | | ml |
| LVIDs | | mm | LVEF | | % |
| IVSd | | mm | FS | | % |
| **Additional Information**: | |  | | | |
| No pleural/pericardial effusion | | | | | |
| **Final Diagnosis:** | | | | | |
| 1. {S, D, S} Levocardia 2. Normal Echocardiography Study | | | | | |
| SIGNATURE  Done by: Tesfaye T., Pediatric Cardiologist _______________ 27/01/2012Eth.C | | | | | |

| Patient Name**: Blen Wendimnew**. Patient ID: FHH. SEX/ Age: F/6Yrs Date of Report: 28**/01/2012_**.  BP: ___ Weight: ___ Height:___ BSA: ___. Referral Diagnosis: **Incidental Murmur. AGH03.1854.** | | | |
| --- | --- | --- | --- |
| **Features** | **Finding** | **Features** | **Finding** |
| **Profile** |  | **Atria** |  |
| Abdominal situs | Solitus | Left atrium | Normal |
| Cardiac position | Levocardia | Right atrium | Normal |
| Systemic venous drainage | Normal | **Atrioventricular valves** |  |
| Pulmonary venous drainage | Normal | Mitral valve | Annulus = 21mm |
| Atrioventricular connection | Concordant | Tricuspid valve | Annulus = 21mm  TAPSE = 23mm |
| Ventriculoarterial connection | Concordant | **Ventricles** |  |
| Ventricular loop | d-Loop | Left ventricle | Normal |
|  |  | Right ventricle | Normal |
| **Septae** |  | **Coronary arteries** | ----- |
| Interventricular septum | 6mm PM VSD partially covered by STL, L – R Shunt | **Doppler Measurement** |  |
| Interatrial septum | Intact | Mitral | ---- |
| **Semilunal valves** |  | Aortic | ---- |
| Aortic valve | Annulus = 18mm | Tricuspid | ---- |
| Pulmonary valve | Annulus = 24mm | pulmonic | ----- |
| **Great arteries** | NRGA | **Aortic arch** |  |
| Aorta | ---- | **PDA** | No |
| Pulmonary artery | Normal sized MPA and Confluent Branch PAs |  |  |
| **M-Mode** | | | |
| AO | mm | PWd | 5.5mm |
| LA | mm | EDV | 38ml |
| LVIDd | 31mm | ESV | 16ml |
| LVIDs | 22mm | LVEF | 57% |
| IVSd | 4.5mm | FS | 29% |
| **Additional Information**: | No coarctation of aorta | | |
| No pleural/pericardial effusion | | | |
| **Final Diagnosis:** | | | |
| 1. {S, D, S} Levocardia 2. Small Perimembranous VSD, L – R Shunt 3. No PAH 4. Good Biventricular Function | | | |
| SIGNATURE  Done by: Tesfaye T., Pediatric Cardiologist _______________ 28/01/2012Eth.C | | | |

| Patient Name**: Filipos Tsedalu**. Patient ID: ADINAS. SEX/ Age: M/8 8/12 Date of Report: 28**/01/2012_**.  BP: ___ Weight: ___ Height:___ BSA: ___. Referral Diagnosis: **Easy Fatigability. AGH03.1855.** | | | | |
| --- | --- | --- | --- | --- |
| **Features** | **Finding** | **Features** | **Finding** | |
| **Profile** |  | **Atria** |  | |
| Abdominal situs | Solitus | Left atrium | Normal | |
| Cardiac position | Levocardia | Right atrium | Normal | |
| Systemic venous drainage | Normal | **Atrioventricular valves** |  | |
| Pulmonary venous drainage | Normal | Mitral valve | Annulus = 20mm | |
| Atrioventricular connection | Concordant | Tricuspid valve | Annulus = 21mm  TAPSE = 20mm | |
| Ventriculoarterial connection | Concordant | **Ventricles** |  | |
| Ventricular loop | d-Loop | Left ventricle | Normal | |
|  |  | Right ventricle | Normal | |
| **Septae** |  | **Coronary arteries** | ----- | |
| Interventricular septum | Intact | **Doppler Measurement** |  | |
| Interatrial septum | Intact | Mitral | ---- | |
| **Semilunal valves** |  | Aortic | ---- | |
| Aortic valve | Annulus = 18mm | Tricuspid | ---- | |
| Pulmonary valve | Annulus = 18mm | pulmonic | ---- | |
| **Great arteries** | NRGA | **Aortic arch** | Left | |
| Aorta | ---- | **PDA** | No | |
| Pulmonary artery | Good sized MPA and Confluent Branch PAs. |  |  | |
| **M-Mode** | | | | |
| AO | mm | PWd | | 6mm |
| LA | mm | EDV | | 48ml |
| LVIDd | 34mm | ESV | | 19ml |
| LVIDs | 24mm | LVEF | | 60% |
| IVSd | 7mm | FS | | 31% |
| **Additional Information**: |  | | | |
| No pleural/pericardial effusion | | | | |
| **Final Diagnosis:** | | | | |
| 1. {S, D, S} Levocardia 2. Normal Echocardiographic Study | | | | |
| SIGNATURE  Done by: Tesfaye T., Pediatric Cardiologist _______________ 28/01/2012Eth.C | | | | |

| Patient Name**: Arafat Muhamed**. Patient ID: ADINAS. SEX/ Age: M/ 2Months Date of Report: 29**/01/2012_**.  BP: ___ Weight: ___ Height:___ BSA: ___. Referral Diagnosis: **Incidental Murmur. AGH03.1856.** | | | |
| --- | --- | --- | --- |
| **Features** | **Finding** | **Features** | **Finding** |
| **Profile** |  | **Atria** |  |
| Abdominal situs | Solitus | Left atrium | Normal |
| Cardiac position | Levocardia | Right atrium | Normal |
| Systemic venous drainage | Normal | **Atrioventricular valves** |  |
| Pulmonary venous drainage | Normal | Mitral valve | Annulus = 12mm |
| Atrioventricular connection | Concordant | Tricuspid valve | Annulus = 13mm  TAPSE = 15mm |
| Ventriculoarterial connection | Concordant | **Ventricles** |  |
| Ventricular loop | d-Loop | Left ventricle | Normal |
|  |  | Right ventricle | Normal |
| **Septae** |  | **Coronary arteries** | ----- |
| Interventricular septum | 2.5mm PM VSD, L – R Shunt | **Doppler Measurement** |  |
| Interatrial septum | Intact | Mitral | ---- |
| **Semilunal valves** |  | Aortic | ---- |
| Aortic valve | Annulus = 10mm | Tricuspid | ---- |
| Pulmonary valve | Annulus = 10mm | pulmonic | ---- |
| **Great arteries** | NRGA | **Aortic arch** | ---- |
| Aorta | ---- | **PDA** | No |
| Pulmonary artery | Good sized MPA and Confluent Branch PAs. |  |  |
| **M-Mode** | | | |
| AO | mm | PWd | 3.1mm |
| LA | mm | EDV | 28.81ml |
| LVIDd | 27.7mm | ESV | 10.53ml |
| LVIDs | 18.6mm | LVEF | 63% |
| IVSd | 2.9mm | FS | 33% |
| **Additional Information**: |  | | |
| No pleural/pericardial effusion | | | |
| **Final Diagnosis:** | | | |
| 1. {S, D, S} Levocardia 2. Small Perimembranous VSD 3. Good Biventricular Function | | | |
| SIGNATURE  Done by: Tesfaye T., Pediatric Cardiologist _______________ 29/01/2012Eth.C | | | |

| Patient Name**: Zeyneb Gashaw**. Patient ID:Pawi H. SEX/ Age: F/13Yrs Date of Report: 30**/01/2012_**.  BP: ___ Weight: ___ Height:___ BSA: ___. Referral Diagnosis: **CHF + DOE + Rheumatic Recurrence. AGH03.1857.** | | | | |
| --- | --- | --- | --- | --- |
| **Features** | **Finding** | **Features** | **Finding** | |
| **Profile** |  | **Atria** |  | |
| Abdominal situs | Solitus | Left atrium | Dilated, 38 X 44mm | |
| Cardiac position | Levocardia | Right atrium | Dilated | |
| Systemic venous drainage | Normal | **Atrioventricular valves** |  | |
| Pulmonary venous drainage | Normal | Mitral valve | Annulus = 25mm. thickened, clubbed Calcified valve leaflets. valve area = 0.8cm**2** | |
| Atrioventricular connection | Concordant | Tricuspid valve | Annulus = 25mm  TAPSE = 19mm | |
| Ventriculoarterial connection | Concordant | **Ventricles** |  | |
| Ventricular loop | d-Loop | Left ventricle | Dilated | |
|  |  | Right ventricle | Dilated | |
| **Septae** |  | **Coronary arteries** | ----- | |
| Interventricular septum | Intact | **Doppler Measurement** |  | |
| Interatrial septum | Intact | Mitral | Severe MS with valve area of 0.8cm2 and Inflow gradient of PPG by MPG = 45/27mmHg | |
| **Semilunal valves** |  | Aortic | Mild AR | |
| Aortic valve | Annulus = 20, Trileaflet | Tricuspid | Moderate TR, PPG = 66mmHg | |
| Pulmonary valve | Annulus = 18mm | pulmonic | Trivial PR | |
| **Great arteries** | NRGA | **Aortic arch** | ---- | |
| Aorta | ---- | **PDA** | NO | |
| Pulmonary artery | ------ |  |  | |
| **M-Mode** | | | | |
| AO | mm | PWd | | 4.8mm |
| LA | mm | EDV | | 41ml |
| LVIDd | 32mm | ESV | | 20ml |
| LVIDs | 24mm | LVEF | | 51% |
| IVSd | 7mm | FS | | 25% |
| No pleural/pericardial effusion | | | | |
| **Final Diagnosis:** | | | | |
| 1. {S, D, S} Levocardia 2. Severe MS 3. Moderate TR 4. Mild AR 5. Thickened, clubbed and calcified mitral valve 6. All chambers Dilated 7. Severe PAH 8. Reduced LV Systolic function | | | | |
| SIGNATURE  Done by: Tesfaye T., Pediatric Cardiologist _______________ 30/01/2012Eth.C | | | | |

| Name**: Baby of Genet Gedamu**. Patient ID: Marie stopes. SEX/ Age: F/4/12 Date of Report: 30**/01/2012**.  BP: ___ Weight: ___ Height:___ BSA: ___. Referral Diagnosis: **Incidental Murmur. AGH03.1858.** | | | | |
| --- | --- | --- | --- | --- |
| **Features** | **Finding** | **Features** | **Finding** | |
| **Profile** |  | **Atria** |  | |
| Abdominal situs | Solitus | Left atrium | Normal | |
| Cardiac position | Levocardia | Right atrium | Normal | |
| Systemic venous drainage | Normal | **Atrioventricular valves** |  | |
| Pulmonary venous drainage | Normal | Mitral valve | Annulus = 15mm | |
| Atrioventricular connection | Concordant | Tricuspid valve | Annulus = 14mm  TAPSE = 12mm | |
| Ventriculoarterial connection | Concordant | **Ventricles** |  | |
| Ventricular loop | d-Loop | Left ventricle | Normal | |
|  |  | Right ventricle | Normal | |
| **Septae** |  | **Coronary arteries** | ----- | |
| Interventricular septum | 4mm PM VSD, L – R Shunt | **Doppler Measurement** |  | |
| Interatrial septum | Intact | Mitral | ---- | |
| **Semilunal valves** |  | Aortic | ---- | |
| Aortic valve | Annulus = 9mm | Tricuspid | ----- | |
| Pulmonary valve | Annulus = 10mm | pulmonic | Mild PS, PPG = 30mmHg | |
| **Great arteries** | NRGA | **Aortic arch** | --- | |
| Aorta | ---- | **PDA** | No | |
| Pulmonary artery | Good sized MPA and Confluent Branch PAs. |  |  | |
| **M-Mode** | | | | |
| AO | mm | PWd | | 4.3mm |
| LA | mm | EDV | | 13ml |
| LVIDd | 20mm | ESV | | 3.36ml |
| LVIDs | 12mm | LVEF | | 74% |
| IVSd | 7mm | FS | | 40% |
| **Additional Information**: |  | | | |
| No pleural/pericardial effusion | | | | |
| **Final Diagnosis:** | | | | |
| 1. {S, D, S} Levocardia 2. Small PM VSD, L – R Shunt 3. Mild valvar PS 4. Good biventricular Function | | | | |
| SIGNATURE  Done by: Tesfaye T., Pediatric Cardiologist _______________ 30/01/2012Eth.C | | | | |

| Patient Name**: Bantamlak Alemie**. Patient ID:_FHRH. SEX/ Age: M/4/12. Date of Report: 30**/01/2012_**.  BP: ___ Weight: ___ Height:___ BSA: ___. Referral Diagnosis: **DS + Murmur. AGH03.1859.** | | | | |
| --- | --- | --- | --- | --- |
| **Features** | **Finding** | **Features** | **Finding** | |
| **Profile** |  | **Atria** |  | |
| Abdominal situs | Solitus | Left atrium | Normal | |
| Cardiac position | Levocardia | Right atrium | Normal | |
| Systemic venous drainage | Normal | **Atrioventricular valves** |  | |
| Pulmonary venous drainage | Normal | Mitral valve | Annulus = 13mm | |
| Atrioventricular connection | Concordant | Tricuspid valve | Annulus = 14mm  TAPSE = 12mm | |
| Ventriculoarterial connection | Concordant | **Ventricles** |  | |
| Ventricular loop | d-Loop | Left ventricle | Normal | |
|  |  | Right ventricle | Normal | |
| **Septae** | Tongue of tissue in b/n | **Coronary arteries** | ----- | |
| Interventricular septum | 6mm Inlet VSD, L – R Shunt | **Doppler Measurement** |  | |
| Interatrial septum | 5mm Primum ASD, L – R Shunt | Mitral | Mild MR | |
| **Semilunal valves** |  | Aortic | ---- | |
| Aortic valve | Annulus = 9mm | Tricuspid | Mild TR | |
| Pulmonary valve | Annulus = 10mm | pulmonic | ----- | |
| **Great arteries** | NRGA | **Aortic arch** | ----- | |
| Aorta | ---- | **PDA** | NO | |
| Pulmonary artery | ------ |  |  | |
| **M-Mode:- Good LV Function on eye balling** | | | | |
| AO | mm | PWd | | mm |
| LA | mm | EDV | | ml |
| LVIDd | mm | ESV | | ml |
| LVIDs | mm | LVEF | | % |
| IVSd | mm | FS | | % |
| **Additional Information**: | Limited echo window (Only subcostal and apical window) | | | |
| No pleural/pericardial effusion | | | | |
| **Final Diagnosis:** | | | | |
| 1. {S, D, S} Levocardia 2. Intermediate AVSD, L – R Shunt 3. MILD MR 4. Mild TR 5. Good function | | | | |
| SIGNATURE  Done by: Tesfaye T., Pediatric Cardiologist _______________ 30/01/2012Eth.C | | | | |

| Patient Name**: G/Silassie Tirualem**. Patient ID: FHRH. SEX/ Age: M/4Months_ Date of Report: 01**/02/2012_**.  BP: ___ Weight: ___ Height:___ BSA: ___. Referral Diagnosis: **Incidental Murmur + Diaphoresis. AGH03.1860.** | | | | | | |
| --- | --- | --- | --- | --- | --- | --- |
| **Features** | **Finding** | | | **Features** | | **Finding** |
| **Profile** |  | | | **Atria** | |  |
| Abdominal situs | Solitus | | | Left atrium | | Dilated |
| Cardiac position | Levocardia | | | Right atrium | | Normal |
| Systemic venous drainage | Normal | | | **Atrioventricular valves** | |  |
| Pulmonary venous drainage | Normal | | | Mitral valve | | Annulus = 13mm |
| Atrioventricular connection | Concordant | | | Tricuspid valve | | Annulus = 13mm  TAPSE = 13mm |
| Ventriculoarterial connection | Concordant | | | **Ventricles** | |  |
| Ventricular loop | d-Loop | | | Left ventricle | | Dilated |
|  |  | | | Right ventricle | | Normal |
| **Septae** |  | | | **Coronary arteries** | | ----- |
| Interventricular septum | Intact | | | **Doppler Measurement** | |  |
| Interatrial septum | 5mm OS ASD, L – R Shunt | | | Mitral | | ---- |
| **Semilunal valves** |  | | | Aortic | | ---- |
| Aortic valve | Annulus = 11mm | | | Tricuspid | | Trivial TR, PPG = 24mmHg |
| Pulmonary valve | Annulus = 13mm | | | pulmonic | | ---- |
| **Great arteries** | NRGA | | | **Aortic arch** | | ---- |
| Aorta | ---- | | | **PDA** | | 2.5mm PDA, L – R Shunt |
| Pulmonary artery | ------ | | |  | |  |
| **M-Mode** | | | | | | |
| AO | | mm | PWd | | 4.3mm | |
| LA | | mm | EDV | | 16ml | |
| LVIDd | | 22mm | ESV | | 5ml | |
| LVIDs | | 14mm | LVEF | | 70% | |
| IVSd | | 4mm | FS | | 37% | |
| **Additional Information**: | |  | | | | |
| No pleural/pericardial effusion | | | | | | |
| **Final Diagnosis:** | | | | | | |
| 1. {S, D, S} Levocardia 2. Small OS ASD, L – R Shunt 3. Moderate PDA, L – R Shunt 4. Good Biventricular Function | | | | | | |
| SIGNATURE  Done by: Tesfaye T., Pediatric Cardiologist _______________ 01/02/2012Eth.C | | | | | | |

| Patient Name**: Yohannes Minyichil**. Patient ID: ADINAS. SEX/ Age: M/2 4/12 Date of Report: 03**/02/2012_**.  BP: ___ Weight: ___ Height:___ BSA: ___. Referral Diagnosis: **Diaphoresis. AGH03.1861.** | | | | |
| --- | --- | --- | --- | --- |
| **Features** | **Finding** | **Features** | **Finding** | |
| **Profile** |  | **Atria** |  | |
| Abdominal situs | Solitus | Left atrium | Normal | |
| Cardiac position | Levocardia | Right atrium | Normal | |
| Systemic venous drainage | Normal | **Atrioventricular valves** |  | |
| Pulmonary venous drainage | Normal | Mitral valve | Annulus = 14mm | |
| Atrioventricular connection | Concordant | Tricuspid valve | Annulus = 15mm  TAPSE = 17mm | |
| Ventriculoarterial connection | Concordant | **Ventricles** |  | |
| Ventricular loop | d-Loop | Left ventricle | Normal | |
|  |  | Right ventricle | Normal | |
| **Septae** |  | **Coronary arteries** | ----- | |
| Interventricular septum | Intact | **Doppler Measurement** |  | |
| Interatrial septum | PFO, L – R Shunt | Mitral | ---- | |
| **Semilunal valves** |  | Aortic | ---- | |
| Aortic valve | Annulus = 13mm | Tricuspid | ---- | |
| Pulmonary valve | Annulus = 13mm | pulmonic | ---- | |
| **Great arteries** | NRGA | **Aortic arch** | ---- | |
| Aorta | ---- | **PDA** | No | |
| Pulmonary artery | ------ |  |  | |
| **M-Mode** | | | | |
| AO | mm | PWd | | 4mm |
| LA | mm | EDV | | 45ml |
| LVIDd | 33mm | ESV | | 13ml |
| LVIDs | 20mm | LVEF | | 71% |
| IVSd | 3.4mm | FS | | 39% |
| **Additional Information**: |  | | | |
| No pleural/pericardial effusion | | | | |
| **Final Diagnosis:** | | | | |
| 1. {S, D, S} Levocardia 2. PFO, L – R Shunt 3. Good function | | | | |
| SIGNATURE  Done by: Tesfaye T., Pediatric Cardiologist _______________ 03/02/2012Eth.C | | | | |

| Patient Name**: Endrias Dereje**. Patient ID: ADINAS. SEX/ Age: M/13years Date of Report: 03**/02/2012_**.  BP: ___ Weight: ___ Height:___ BSA: ___. Referral Diagnosis: **DOE. AGH03.1862.** | | | | |
| --- | --- | --- | --- | --- |
| **Features** | **Finding** | **Features** | **Finding** | |
| **Profile** |  | **Atria** |  | |
| Abdominal situs | Solitus | Left atrium | Normal | |
| Cardiac position | Levocardia | Right atrium | Normal | |
| Systemic venous drainage | Normal | **Atrioventricular valves** |  | |
| Pulmonary venous drainage | Normal | Mitral valve | Annulus = 19mm | |
| Atrioventricular connection | Concordant | Tricuspid valve | Annulus = 22mm  TAPSE = 23mm | |
| Ventriculoarterial connection | Concordant | **Ventricles** |  | |
| Ventricular loop | d-Loop | Left ventricle | Normal | |
|  |  | Right ventricle | Normal | |
| **Septae** |  | **Coronary arteries** | ----- | |
| Interventricular septum | 5mm PM VSD Partially closed by STL | **Doppler Measurement** |  | |
| Interatrial septum | Intact | Mitral |  | |
| **Semilunal valves** |  | Aortic |  | |
| Aortic valve | Annulus = 14mm | Tricuspid |  | |
| Pulmonary valve | Annulus = 16mm | pulmonic |  | |
| **Great arteries** | NRGA | **Aortic arch** |  | |
| Aorta | ---- | **PDA** |  | |
| Pulmonary artery | ------ |  |  | |
| **M-Mode** | | | | |
| AO | mm | PWd | | 6mm |
| LA | mm | EDV | | 59ml |
| LVIDd | 37mm | ESV | | 22ml |
| LVIDs | 25mm | LVEF | | 62% |
| IVSd | 7mm | FS | | 32% |
| **Additional Information**: |  | | | |
| No pleural/pericardial effusion | | | | |
| **Final Diagnosis:** | | | | |
| 1. {S, D, S} Levocardia 2. Small PM VSD, Partially covered by STL 3. Good biventricular function | | | | |
| SIGNATURE  Done by: Tesfaye T., Pediatric Cardiologist _______________ 03/02/2012Eth.C | | | | |

| Patient Name**: Yilkal Aschale**. Patient ID: FHH. SEX/ Age: M/2Months Date of Report: 03**/02/2012_**.  BP: ___ Weight: ___ Height:___ BSA: ___. Referral Diagnosis: **Incidental Murmur. AGH03.1863.** | | | | |
| --- | --- | --- | --- | --- |
| **Features** | **Finding** | **Features** | **Finding** | |
| **Profile** |  | **Atria** |  | |
| Abdominal situs | Solitus | Left atrium | Dilated | |
| Cardiac position | Levocardia | Right atrium | Normal | |
| Systemic venous drainage | Normal | **Atrioventricular valves** |  | |
| Pulmonary venous drainage | Normal | Mitral valve | Annulus = 11mm | |
| Atrioventricular connection | Concordant | Tricuspid valve | Annulus = 13mm  TAPSE = 14mm | |
| Ventriculoarterial connection | Concordant | **Ventricles** |  | |
| Ventricular loop | d-Loop | Left ventricle | Dilated | |
|  |  | Right ventricle | Normal | |
| **Septae** |  | **Coronary arteries** | ----- | |
| Interventricular septum | Intact | **Doppler Measurement** |  | |
| Interatrial septum | 4mm OS ASD, L – R Shunt | Mitral | ---- | |
| **Semilunal valves** |  | Aortic | ---- | |
| Aortic valve | Annulus = 9mm | Tricuspid | Trivial TR, PPG = 26mmHg | |
| Pulmonary valve | Annulus = 12mm | pulmonic | Trivial PR | |
| **Great arteries** | NRGA | **Aortic arch** |  | |
| Aorta | ---- | **PDA** | 2.5mm PDA, L – R Shunt | |
| Pulmonary artery | ------ |  |  | |
| **M-Mode** | | | | |
| AO | mm | PWd | | 4mm |
| LA | mm | EDV | | 10ml |
| LVIDd | 18mm | ESV | | 2ml |
| LVIDs | 9.6mm | LVEF | | 77% |
| IVSd | 6mm | FS | | 40% |
| **Additional Information**: | Baby was restless during study. Needs follow up echo | | | |
| No pleural/pericardial effusion | | | | |
| **Final Diagnosis:** | | | | |
| 1. {S, D, S} Levocardia 2. Small OS ASD, L – R Shunt 3. Moderate PDA, L – R Shunt 4. Good function | | | | |
| SIGNATURE  Done by: Tesfaye T., Pediatric Cardiologist _______________ 03/02/2012Eth.C | | | | |

| Patient Name**: Desalegn Sintayehu**. Patient ID: ADINAS. SEX/ Age: M/4Months Date of Report: 04**/02/2012_**.  BP: ___ Weight: ___ Height:___ BSA: ___. Referral Diagnosis: **Shock. AGH03.1864.** | | | | |
| --- | --- | --- | --- | --- |
| **Features** | **Finding** | **Features** | **Finding** | |
| **Profile** |  | **Atria** |  | |
| Abdominal situs | Solitus | Left atrium | Normal | |
| Cardiac position | Levocardia | Right atrium | Normal | |
| Systemic venous drainage | Normal | **Atrioventricular valves** |  | |
| Pulmonary venous drainage | Normal | Mitral valve | Annulus = 8mm, smallish | |
| Atrioventricular connection | Concordant | Tricuspid valve | Annulus = 16mm  TAPSE = 11mm | |
| Ventriculoarterial connection | Concordant | **Ventricles** |  | |
| Ventricular loop | d-Loop | Left ventricle | Hypoplastic | |
|  |  | Right ventricle | Normal | |
| **Septae** |  | **Coronary arteries** | ----- | |
| Interventricular septum | 5mm inlet VSD, BD Shunt | **Doppler Measurement** |  | |
| Interatrial septum | Large ASD amounting to single atrium | Mitral | ---- | |
| **Semilunal valves** |  | Aortic | Severe LVOTO | |
| Aortic valve | Annulus = 5mm | Tricuspid | Trivial TR | |
| Pulmonary valve | Annulus = 11mm | pulmonic | ---- | |
| **Great arteries** | NRGA | **Aortic arch** | LEFT | |
| Aorta | Hypoplastic ascending aorta and arch | **PDA** | NO | |
| Pulmonary artery | ------ |  |  | |
| **M-Mode** | | | | |
| AO | mm | PWd | | mm |
| LA | mm | EDV | | ml |
| LVIDd | mm | ESV | | ml |
| LVIDs | mm | LVEF | | % |
| IVSd | mm | FS | | % |
| **Additional Information**: | Smallish ascending aorta and arch | | | |
| No pleural/pericardial effusion | | | | |
| **Final Diagnosis:** | | | | |
| 1. {S, D, S} Levocardia 2. Large ASD Amounting single atrium 3. Inlet VSD, BD Shunt 4. HYPOPLASTIC LEFT HEART SYNDROME | | | | |
| SIGNATURE  Done by: Tesfaye T., Pediatric Cardiologist _______________ 04/02/2012Eth.C | | | | |

| Patient Name**: Belete Getinet**. Patient ID: FHRH. SEX/ Age: M/4yrs. Date of Report: 05**/02/2012_**.  BP: ___ Weight: ___ Height:___ BSA: ___. Referral Diagnosis: **Cyanosis + Clubbing. AGH03.1865.** | | | | | |
| --- | --- | --- | --- | --- | --- |
| **Features** | **Finding** | | **Features** | | **Finding** |
| **Profile** |  | | **Atria** | |  |
| Abdominal situs | Solitus | | Left atrium | | Normal |
| Cardiac position | Levocardia | | Right atrium | | Normal |
| Systemic venous drainage | Normal | | **Atrioventricular valves** | |  |
| Pulmonary venous drainage | Normal | | Mitral valve | | Annulus = 13mm |
| Atrioventricular connection | Concordant | | Tricuspid valve | | Annulus = 18mm |
| Ventriculoarterial connection | Concordant | | **Ventricles** | |  |
| Ventricular loop | d-Loop | | Left ventricle | | Normal |
|  |  | | Right ventricle | | Hypertrophied |
| **Septae** |  | | **Coronary arteries** | | ----- |
| Interventricular septum | 12mm Mal-aligned Sub aortic VSD, R – L Shunt | | **Doppler Measurement** | |  |
| Interatrial septum | Intact | | Mitral | | ---- |
| **Semilunal valves** |  | | Aortic | | ---- |
| Aortic valve | Annulus = 17mm | | Tricuspid | | ---- |
| Pulmonary valve | Annulus = 4mm | | pulmonic | | Valvar and supra valvar PS, PPG = 46mmHg |
| **Great arteries** | NRGA | | **Aortic arch** | |  |
| Aorta | Over-riding aorta | | **PDA** | | No |
| Pulmonary artery | Smallish MPA (5mm), RPA (4mm), LPA (4mm) | |  | |  |
| **M-Mode** | | | | | |
| AO | mm | PWd | | 3.7mm | |
| LA | mm | EDV | | 14ml | |
| LVIDd | 21mm | ESV | | 6ml | |
| LVIDs | 15mm | LVEF | | 58% | |
| IVSd | 5mm | FS | | 29% | |
| **Additional Information**: |  | | | | |
| No pleural/pericardial effusion | | | | | |
| **Final Diagnosis:** | | | | | |
| 1. {S, D, S} Levocardia 2. TOF 3. Hypoplastic MPA and Branch PAs. 4. Good LV Function | | | | | |
| SIGNATURE  Done by: Tesfaye T., Pediatric Cardiologist _______________ 05/02/2012Eth.C | | | | | |

| Patient Name**: Hermon Shegaw**. Patient ID:_ADINAS_. SEX/ Age: F/ 8 1/12 Date of Report: 06**/02/2012_**.  BP: ___ Weight: ___ Height:___ BSA: ___. Referral Diagnosis: **Easy Fatigability. AGH03.1866.** | | | | |
| --- | --- | --- | --- | --- |
| **Features** | **Finding** | **Features** | **Finding** | |
| **Profile** |  | **Atria** |  | |
| Abdominal situs | Solitus | Left atrium | Normal | |
| Cardiac position | Levocardia | Right atrium | Normal | |
| Systemic venous drainage | Normal | **Atrioventricular valves** |  | |
| Pulmonary venous drainage | Normal | Mitral valve | Annulus = 14mm | |
| Atrioventricular connection | Concordant | Tricuspid valve | Annulus = 17mm  TAPSE = 18mm | |
| Ventriculoarterial connection | Concordant | **Ventricles** |  | |
| Ventricular loop | d-Loop | Left ventricle | Normal | |
|  |  | Right ventricle | Normal | |
| **Septae** |  | **Coronary arteries** | ----- | |
| Interventricular septum | Intact | **Doppler Measurement** |  | |
| Interatrial septum | PFO, L – R Shunt | Mitral | ---- | |
| **Semilunal valves** |  | Aortic | ---- | |
| Aortic valve | Annulus = 15mm | Tricuspid | ---- | |
| Pulmonary valve | Annulus = 18mm | pulmonic | Trivial PR | |
| **Great arteries** | NRGA | **Aortic arch** | Left | |
| Aorta | ---- | **PDA** | NO | |
| Pulmonary artery | Normal MPA and confluent Branch PAs. |  |  | |
| **M-Mode** | | | | |
| AO | mm | PWd | | 4mm |
| LA | mm | EDV | | 52ml |
| LVIDd | 35mm | ESV | | 17ml |
| LVIDs | 23mm | LVEF | | 67% |
| IVSd | 6mm | FS | | 36% |
| **Additional Information**: |  | | | |
| No pleural/pericardial effusion | | | | |
| **Final Diagnosis:** | | | | |
| 1. {S, D, S} Levocardia 2. PFO | | | | |
| SIGNATURE  Done by: Tesfaye T., Pediatric Cardiologist _______________ 06/02/2012Eth.C | | | | |

| Patient Name**: Fanuel Gashaw**. Patient ID:_ADINAS_. SEX/ Age: M/ 8/12 Date of Report: 06**/02/2012_**.  BP: ___ Weight: ___ Height:___ BSA: ___. Referral Diagnosis: **Incidental Murmur. AGH03.1867.** | | | | |
| --- | --- | --- | --- | --- |
| **Features** | **Finding** | **Features** | **Finding** | |
| **Profile** |  | **Atria** |  | |
| Abdominal situs | Solitus | Left atrium | Normal | |
| Cardiac position | Levocardia | Right atrium | Normal | |
| Systemic venous drainage | Normal | **Atrioventricular valves** |  | |
| Pulmonary venous drainage | Normal | Mitral valve | Annulus = 13mm | |
| Atrioventricular connection | Concordant | Tricuspid valve | Annulus = 11mm  TAPSE = 13 mm | |
| Ventriculoarterial connection | Concordant | **Ventricles** |  | |
| Ventricular loop | d-Loop | Left ventricle | Normal | |
|  |  | Right ventricle | Normal | |
| **Septae** |  | **Coronary arteries** | ----- | |
| Interventricular septum | Intact | **Doppler Measurement** |  | |
| Interatrial septum | Intact | Mitral | ---- | |
| **Semilunal valves** |  | Aortic | ---- | |
| Aortic valve | Annulus = 11mm | Tricuspid | ---- | |
| Pulmonary valve | Annulus = 12mm | pulmonic | Mild PS, PPG = 30mmHg | |
| **Great arteries** | NRGA | **Aortic arch** | ---- | |
| Aorta | ---- | **PDA** | No | |
| Pulmonary artery | Normal MPA and confluent Branch PAs. |  |  | |
| **M-Mode:** Good LV Function (eye balling) | | | | |
| AO | mm | PWd | | mm |
| LA | mm | EDV | | ml |
| LVIDd | mm | ESV | | ml |
| LVIDs | mm | LVEF | | % |
| IVSd | mm | FS | | % |
| **Additional Information**: |  | | | |
| No pleural/pericardial effusion | | | | |
| **Final Diagnosis:** | | | | |
| 1. {S, D, S} Levocardia 2. Mild PS 3. Good Function | | | | |
| SIGNATURE  Done by: Tesfaye T., Pediatric Cardiologist _______________ 06/02/2012Eth.C | | | | |

| Patient Name**: Ikram Tsegaye**. Patient ID:_ADINAS_. SEX/ Age: F/ 13 4/12 Date of Report: 06**/02/2012_**.  BP: ___ Weight: ___ Height:___ BSA: ___. Referral Diagnosis: **Easy Fatigability. AGH03.1868.** | | | | |
| --- | --- | --- | --- | --- |
| **Features** | **Finding** | **Features** | **Finding** | |
| **Profile** |  | **Atria** |  | |
| Abdominal situs | Solitus | Left atrium | Normal | |
| Cardiac position | Levocardia | Right atrium | Normal | |
| Systemic venous drainage | Normal | **Atrioventricular valves** |  | |
| Pulmonary venous drainage | Normal | Mitral valve | Annulus = 21mm | |
| Atrioventricular connection | Concordant | Tricuspid valve | Annulus = 24mm  TAPSE = mm | |
| Ventriculoarterial connection | Concordant | **Ventricles** |  | |
| Ventricular loop | d-Loop | Left ventricle | Normal | |
|  |  | Right ventricle | Normal | |
| **Septae** |  | **Coronary arteries** | ----- | |
| Interventricular septum | Intact | **Doppler Measurement** |  | |
| Interatrial septum | Intact | Mitral | ---- | |
| **Semilunal valves** |  | Aortic | ---- | |
| Aortic valve | Annulus = 16mm | Tricuspid | ---- | |
| Pulmonary valve | Annulus = 25mm | pulmonic | Trivial PR, PPG = 10mmHg | |
| **Great arteries** | NRGA | **Aortic arch** |  | |
| Aorta | ---- | **PDA** |  | |
| Pulmonary artery | Normal MPA and confluent Branch PAs. |  |  | |
| **M-Mode** | | | | |
| AO | mm | PWd | | 6mm |
| LA | mm | EDV | | 56ml |
| LVIDd | 36mm | ESV | | 26ml |
| LVIDs | 26mm | LVEF | | 54% |
| IVSd | 8mm | FS | | 27% |
| **Additional Information**: |  | | | |
| No pleural/pericardial effusion | | | | |
| **Final Diagnosis:** | | | | |
| 1. {S, D, S} Levocardia 2. Normal Echocardiographic Study | | | | |
| SIGNATURE  Done by: Tesfaye T., Pediatric Cardiologist _______________ 06/02/2012Eth.C | | | | |

| Patient Name**: Tsigie Fantahun** . Patient ID:_FHRH_. SEX/ Age: F/ 10/12 Date of Report: 06**/02/2012_**.  BP: ___ Weight: ___ Height:___ BSA: ___. Referral Diagnosis: **Recurrent Chest Infection. AGH03.1869.** | | | | |
| --- | --- | --- | --- | --- |
| **Features** | **Finding** | **Features** | **Finding** | |
| **Profile** |  | **Atria** |  | |
| Abdominal situs | Solitus | Left atrium | Normal | |
| Cardiac position | Levocardia | Right atrium | Normal | |
| Systemic venous drainage | Normal | **Atrioventricular valves** |  | |
| Pulmonary venous drainage | Normal | Mitral valve | Annulus = 12mm | |
| Atrioventricular connection | Concordant | Tricuspid valve | Annulus = 13mm | |
| Ventriculoarterial connection | Concordant | **Ventricles** |  | |
| Ventricular loop | d-Loop | Left ventricle | Normal | |
|  |  | Right ventricle | Normal | |
| **Septae** |  | **Coronary arteries** | ----- | |
| Interventricular septum | Intact | **Doppler Measurement** |  | |
| Interatrial septum | Intact | Mitral | ---- | |
| **Semilunal valves** |  | Aortic | ---- | |
| Aortic valve | Annulus = 13mm | Tricuspid | ---- | |
| Pulmonary valve | Annulus = 12mm | pulmonic | ---- | |
| **Great arteries** | NRGA | **Aortic arch** | --- | |
| Aorta | ---- | **PDA** | NO | |
| Pulmonary artery | Normal MPA and confluent Branch PAs. |  |  | |
| **M-Mode** | | | | |
| AO | mm | PWd | | 5mm |
| LA | mm | EDV | | 14ml |
| LVIDd | 20mm | ESV | | 6ml |
| LVIDs | 15mm | LVEF | | 60% |
| IVSd | 4mm | FS | | 30% |
| **Additional Information**: |  | | | |
| No pleural/pericardial effusion | | | | |
| **Final Diagnosis:** | | | | |
| 1. {S, D, S} Levocardia 2. Normal Echocardiography Study | | | | |
| SIGNATURE  Done by: Tesfaye T., Pediatric Cardiologist _______________ 06/02/2012Eth.C | | | | |

| Patient Name**: Betelehem Melesse** . Patient ID:_FHH_. SEX/ Age: F/ 7Years Date of Report: 07**/02/2012_**.  BP: ___ Weight: ___ Height:___ BSA: ___. Referral Diagnosis: **FTT. AGH03.1870.** | | | |
| --- | --- | --- | --- |
| **Features** | **Finding** | **Features** | **Finding** |
| **Profile** |  | **Atria** |  |
| Abdominal situs | Solitus | Left atrium | Normal |
| Cardiac position | Levocardia | Right atrium | Normal |
| Systemic venous drainage | Normal | **Atrioventricular valves** |  |
| Pulmonary venous drainage | Normal | Mitral valve | Annulus = 17mm |
| Atrioventricular connection | Concordant | Tricuspid valve | Annulus = 22mm  TAPSE = 23mm |
| Ventriculoarterial connection | Concordant | **Ventricles** |  |
| Ventricular loop | d-Loop | Left ventricle | Normal |
|  |  | Right ventricle | Normal |
| **Septae** |  | **Coronary arteries** | ----- |
| Interventricular septum | Intact | **Doppler Measurement** |  |
| Interatrial septum | Intact | Mitral | ---- |
| **Semilunal valves** |  | Aortic | ---- |
| Aortic valve | Annulus = 16mm | Tricuspid | Trivial TR, PPG = 19mmHg |
| Pulmonary valve | Annulus = 20mm | pulmonic |  |
| **Great arteries** | NRGA | **Aortic arch** | Left |
| Aorta | ---- | **PDA** | No |
| Pulmonary artery | Normal MPA and confluent Branch PAs. |  |  |
| **M-Mode** | | | |
| AO | mm | PWd | 8mm |
| LA | mm | EDV | 56ml |
| LVIDd | 36mm | ESV | 19ml |
| LVIDs | 24mm | LVEF | 65% |
| IVSd | 4mm | FS | 35% |
| **Additional Information**: |  | | |
| No pleural/pericardial effusion | | | |
| **Final Diagnosis:** | | | |
| 1. {S, D, S} Levocardia 2. Normal Echocardiography Study | | | |
| SIGNATURE  Done by: Tesfaye T., Pediatric Cardiologist _______________ 07/02/2012Eth.C | | | |

| Patient Name**: Temesgen Melaku**. Patient ID:_FHRH_. SEX/ Age: M/ 1Year Date of Report: 07**/02/2012_**.  BP: ___ Weight: ___ Height:___ BSA: ___. Referral Diagnosis: **Incidental Murmur. AGH03.1871.** | | | |
| --- | --- | --- | --- |
| **Features** | **Finding** | **Features** | **Finding** |
| **Profile** |  | **Atria** |  |
| Abdominal situs | Solitus | Left atrium | Normal |
| Cardiac position | Levocardia | Right atrium | Normal |
| Systemic venous drainage | Normal | **Atrioventricular valves** |  |
| Pulmonary venous drainage | Normal | Mitral valve | Annulus = 16mm |
| Atrioventricular connection | Concordant | Tricuspid valve | Annulus = 22mm |
| Ventriculoarterial connection | Concordant | **Ventricles** |  |
| Ventricular loop | d-Loop | Left ventricle | Normal |
|  |  | Right ventricle | Normal |
| **Septae** |  | **Coronary arteries** | ----- |
| Interventricular septum | 5mm PM VSD, L – R Shunt | **Doppler Measurement** |  |
| Interatrial septum | Intact | Mitral | ---- |
| **Semilunal valves** |  | Aortic | ---- |
| Aortic valve | Annulus = 14mm | Tricuspid | ---- |
| Pulmonary valve | Annulus = 16mm | pulmonic | ---- |
| **Great arteries** | NRGA | **Aortic arch** |  |
| Aorta | ---- | **PDA** |  |
| Pulmonary artery | Normal MPA and confluent Branch PAs. |  |  |
| **M-Mode** | | | |
| AO | mm | PWd | 5mm |
| LA | mm | EDV | 29ml |
| LVIDd | 28mm | ESV | 11ml |
| LVIDs | 19mm | LVEF | 63% |
| IVSd | 5mm | FS | 32% |
| **Additional Information**: |  | | |
| No pleural/pericardial effusion | | | |
| **Final Diagnosis:** | | | |
| 1. {S, D, S} Levocardia 2. Small PM VSD, L – R Shunt 3. Good ventricular Function | | | |
| SIGNATURE  Done by: Tesfaye T., Pediatric Cardiologist _______________ 07/02/2012Eth.C | | | |

| Patient Name**: Mebea – Tsion Abrham** Patient ID:FHRH. SEX/ Age: F/ 1Year 4/12 Date of Report: 07**/02/2012_**.  BP: ___ Weight: ___ Height:___ BSA: ___. Referral Diagnosis: DS + CHF + **Friction rub. AGH03.1872.** | | | | |
| --- | --- | --- | --- | --- |
| **Features** | **Finding** | **Features** | | **Finding** |
| **Profile** |  | **Atria** | |  |
| Abdominal situs | Solitus | Left atrium | | Normal |
| Cardiac position | Levocardia | Right atrium | | Normal |
| Systemic venous drainage | Normal | **Atrioventricular valves** | |  |
| Pulmonary venous drainage | Normal | Mitral valve | | Annulus = 14mm |
| Atrioventricular connection | Concordant | Tricuspid valve | | Annulus = 15mm  TAPSE = 12 mm |
| Ventriculoarterial connection | Concordant | **Ventricles** | |  |
| Ventricular loop | d-Loop | Left ventricle | | Normal |
|  |  | Right ventricle | | Normal |
| **Septae** |  | **Coronary arteries** | | ----- |
| Interventricular septum | Intact | **Doppler Measurement** | |  |
| Interatrial septum | Intact | Mitral | | ---- |
| **Semilunal valves** |  | Aortic | | ---- |
| Aortic valve | Annulus = 13mm | Tricuspid | | Trivial TR, PPG = 12mmHg |
| Pulmonary valve | Annulus = 15mm | pulmonic | | ---- |
| **Great arteries** | NRGA | **Aortic arch** | | ---- |
| Aorta | ---- | **PDA** | | No |
| Pulmonary artery | Normal MPA and confluent Branch PAs. |  | |  |
| **M-Mode** | | | | |
| AO | mm | | PWd | 4mm |
| LA | mm | | EDV | 14ml |
| LVIDd | 20mm | | ESV | 4.5ml |
| LVIDs | 13mm | | LVEF | 68% |
| IVSd | 7.5mm | | FS | 35% |
| **Additional Information**: |  | | | |
| Circumferential pericardial effusion 8 mm on RV side, 11mm on RA side and 5mm on LV side | | | | |
| **Final Diagnosis:** | | | | |
| 1. {S, D, S} Levocardia 2. Mild to moderate Pericardial Effusion 3. Good Biventricular Function 4. No evidence of Cardiac tamponade so far. | | | | |
| SIGNATURE  Done by: Tesfaye T., Pediatric Cardiologist _______________ 07/02/2012Eth.C | | | | |

| Patient Name**: Alayu Takele** Patient ID:_MERAWI HOSP_. SEX/ Age: F/ 13Years Date of Report: 08**/02/2012_**.  BP: ___ Weight: ___ Height:___ BSA: ___. Referral Diagnosis: **Sydenham’s Chorea. AGH03.1873.** | | | |
| --- | --- | --- | --- |
| **Features** | **Finding** | **Features** | **Finding** |
| **Profile** |  | **Atria** |  |
| Abdominal situs | Solitus | Left atrium | Normal |
| Cardiac position | Levocardia | Right atrium | Normal |
| Systemic venous drainage | Normal | **Atrioventricular valves** |  |
| Pulmonary venous drainage | Normal | Mitral valve | Annulus = 25mm |
| Atrioventricular connection | Concordant | Tricuspid valve | Annulus = 27mm  TAPSE = 28mm |
| Ventriculoarterial connection | Concordant | **Ventricles** |  |
| Ventricular loop | d-Loop | Left ventricle | Normal |
|  |  | Right ventricle | Normal |
| **Septae** |  | **Coronary arteries** | ----- |
| Interventricular septum | Intact | **Doppler Measurement** |  |
| Interatrial septum | Intact | Mitral | ---- |
| **Semilunal valves** |  | Aortic | ---- |
| Aortic valve | Annulus = 16mm | Tricuspid | Trivial PR, PPG = 10mmHg |
| Pulmonary valve | Annulus = 22mm | pulmonic |  |
| **Great arteries** | NRGA | **Aortic arch** | Left |
| Aorta | ---- | **PDA** | No |
| Pulmonary artery | Normal MPA and confluent Branch PAs. |  |  |
| **M-Mode** | | | |
| AO | mm | PWd | 8mm |
| LA | mm | EDV | 71ml |
| LVIDd | 40mm | ESV | 26ml |
| LVIDs | 27mm | LVEF | 62% |
| IVSd | 7.5mm | FS | 33% |
| **Additional Information**: |  | | |
| No pleural/pericardial effusion | | | |
| **Final Diagnosis:** | | | |
| 1. {S, D, S} Levocardia 2. Normal Echocardiography Study | | | |
| SIGNATURE  Done by: Tesfaye T., Pediatric Cardiologist _______________ 08/02/2012Eth.C | | | |

| Patient Name**: Dessie Ayele.** Patient ID:_FHRH_. SEX/ Age: M/ 1 2/12Year Date of Report: 08**/02/2012_**.  BP: ___ Weight: ___ Height:___ BSA: ___. Referral Diagnosis: **Cyanosis + Murmur. AGH03.1874.** | | | |
| --- | --- | --- | --- |
| **Features** | **Finding** | **Features** | **Finding** |
| **Profile** |  | **Atria** |  |
| Abdominal situs | Solitus | Left atrium | Smallish |
| Cardiac position | Levocardia | Right atrium | Normal |
| Systemic venous drainage | Normal | **Atrioventricular valves** |  |
| Pulmonary venous drainage | Normal | Mitral valve | Annulus = 10mm |
| Atrioventricular connection | DIRV | Tricuspid valve | Annulus = 13mm |
| Ventriculoarterial connection | DOLV | **Ventricles** |  |
| Ventricular loop | d-Loop | Left ventricle | Smallish |
|  |  | Right ventricle | Normal |
| **Septae** |  | **Coronary arteries** | ----- |
| Interventricular septum | 8mm Inlet VSD, R – L Shunt | **Doppler Measurement** |  |
| Interatrial septum | Intact | Mitral | ---- |
| **Semilunal valves** |  | Aortic | Mild AS, PPG = 25mmHg |
| Aortic valve | Annulus = 10mm | Tricuspid | ---- |
| Pulmonary valve | Annulus = 15mm | pulmonic | ---- |
| **Great arteries** | NRGA | **Aortic arch** |  |
| Aorta | Smallish | **PDA** |  |
| Pulmonary artery | 17mml MPA |  |  |
| **M-Mode: Good function on eye balling** | | | |
| AO | mm | PWd | mm |
| LA | mm | EDV | ml |
| LVIDd | mm | ESV | ml |
| LVIDs | mm | LVEF | % |
| IVSd | mm | FS | % |
| **Additional Information**: |  | | |
| No pleural/pericardial effusion | | | |
| **Final Diagnosis:** | | | |
| 1. {S, D, D} Levocardia 2. DIRV 3. DOLV 4. Moderate VSD, R – L Shunt 5. Mild AS 6. Good function | | | |
| SIGNATURE  Done by: Tesfaye T., Pediatric Cardiologist _______________ 08/02/2012Eth.C | | | |

| Patient Name**: Kirubel Haymanot** Patient ID:_ADINAS_. SEX/ Age: M/ 4Years Date of Report: 08**/02/2012_**.  BP: ___ Weight: ___ Height:___ BSA: ___. Referral Diagnosis: **DS. AGH03.1875.** | | | |
| --- | --- | --- | --- |
| **Features** | **Finding** | **Features** | **Finding** |
| **Profile** |  | **Atria** |  |
| Abdominal situs | Solitus | Left atrium | Normal |
| Cardiac position | Levocardia | Right atrium | Normal |
| Systemic venous drainage | Normal | **Atrioventricular valves** |  |
| Pulmonary venous drainage | Normal | Mitral valve | Annulus = 14 mm |
| Atrioventricular connection | Concordant | Tricuspid valve | Annulus = 17mm  TAPSE = 22mm |
| Ventriculoarterial connection | Concordant | **Ventricles** |  |
| Ventricular loop | d-Loop | Left ventricle | Normal |
|  |  | Right ventricle | Normal |
| **Septae** |  | **Coronary arteries** | ----- |
| Interventricular septum | Intact | **Doppler Measurement** |  |
| Interatrial septum | 4mm OS ASD, L – R Shunt | Mitral | ---- |
| **Semilunal valves** |  | Aortic | ---- |
| Aortic valve | Annulus = 14mm | Tricuspid | ---- |
| Pulmonary valve | Annulus = 16mm | pulmonic | ---- |
| **Great arteries** | NRGA | **Aortic arch** | Left |
| Aorta | ---- | **PDA** | No |
| Pulmonary artery | Normal MPA and confluent Branch PAs. |  |  |
| **M-Mode** | | | |
| AO | mm | PWd | 9mm |
| LA | mm | EDV | 39ml |
| LVIDd | 31mm | ESV | 14ml |
| LVIDs | 21mm | LVEF | 63% |
| IVSd | 5mm | FS | 33% |
| **Additional Information**: |  | | |
| No pleural/pericardial effusion | | | |
| **Final Diagnosis:** | | | |
| 1. {S, D, S} Levocardia 2. Small OS ASD, L – R Shunt 3. Good Biventricular Function | | | |
| SIGNATURE  Done by: Tesfaye T., Pediatric Cardiologist _______________ 08/02/2012Eth.C | | | |

| Patient Name**: Redeate Asmare**. Patient ID: Sahate Birhan. SEX/ Age: M/ 1Year Date of Report: 08**/02/2012**.  BP: ___ Weight: ___ Height:___ BSA: ___. Referral Diagnosis: **Incidental Murmur. AGH03.1876.** | | | |
| --- | --- | --- | --- |
| **Features** | **Finding** | **Features** | **Finding** |
| **Profile** |  | **Atria** |  |
| Abdominal situs | Solitus | Left atrium | Normal |
| Cardiac position | Levocardia | Right atrium | Dilated |
| Systemic venous drainage | Normal | **Atrioventricular valves** |  |
| Pulmonary venous drainage | Normal | Mitral valve | Annulus = 16mm |
| Atrioventricular connection | Concordant | Tricuspid valve | Annulus =16 mm  TAPSE = 12mm |
| Ventriculoarterial connection | Concordant | **Ventricles** |  |
| Ventricular loop | d-Loop | Left ventricle | Normal |
|  |  | Right ventricle | Dilated |
| **Septae** |  | **Coronary arteries** | ----- |
| Interventricular septum | 7mm PM VSD, L – R Shunt | **Doppler Measurement** |  |
| Interatrial septum | Intact | Mitral | ---- |
| **Semilunal valves** |  | Aortic | ---- |
| Aortic valve | Annulus = 14mm | Tricuspid | ---- |
| Pulmonary valve | Annulus = 6mm | pulmonic | Severe Valvar and sub valvar PS, PPG = 110mmHg |
| **Great arteries** | NRGA | **Aortic arch** | --- |
| Aorta | ---- | **PDA** | No |
| Pulmonary artery | Smallish MPA and confluent Branch PAs. |  |  |
| **M-Mode** | | | |
| AO | mm | PWd | mm |
| LA | mm | EDV | ml |
| LVIDd | mm | ESV | ml |
| LVIDs | mm | LVEF | 60% |
| IVSd | mm | FS | 29% |
| **Additional Information**: |  | | |
| No pleural/pericardial effusion | | | |
| **Final Diagnosis:** | | | |
| 1. {S, D, S} Levocardia 2. Moderate PM VSD, L – R Shunt 3. Severe valvar and sub valvar PS 4. Smallish MPA and Branch PAs 5. Good Biventricular Function | | | |
| SIGNATURE  Done by: Tesfaye T., Pediatric Cardiologist _______________ 08/02/2012Eth.C | | | |

| Patient Name**: Tiwobsta Shimelis.** Patient ID:-ADINAS_. SEX/ Age: F/ 10Years Date of Report: 09**/02/2012_**.  BP: ___ Weight: ___ Height:___ BSA: ___. Referral Diagnosis: **Easy Fatigability. AGH03.1877.** | | | |
| --- | --- | --- | --- |
| **Features** | **Finding** | **Features** | **Finding** |
| **Profile** |  | **Atria** |  |
| Abdominal situs | Solitus | Left atrium | Normal |
| Cardiac position | Levocardia | Right atrium | Normal |
| Systemic venous drainage | Normal | **Atrioventricular valves** |  |
| Pulmonary venous drainage | Normal | Mitral valve | Annulus = 17mm |
| Atrioventricular connection | Concordant | Tricuspid valve | Annulus = 17mm  TAPSE = 21mm |
| Ventriculoarterial connection | Concordant | **Ventricles** |  |
| Ventricular loop | d-Loop | Left ventricle | Normal |
|  |  | Right ventricle | Normal |
| **Septae** |  | **Coronary arteries** | ----- |
| Interventricular septum | Intact | **Doppler Measurement** |  |
| Interatrial septum | Intact | Mitral | ---- |
| **Semilunal valves** |  | Aortic | ---- |
| Aortic valve | Annulus = 16mm | Tricuspid | ---- |
| Pulmonary valve | Annulus = 20mm | pulmonic |  |
| **Great arteries** | NRGA | **Aortic arch** | Left |
| Aorta | ---- | **PDA** | No |
| Pulmonary artery | Normal MPA (17mm) and confluent Branch PAs. LPA = 12mm, RPA = 12mm) |  |  |
| **M-Mode** | | | |
| AO | mm | PWd | 6mm |
| LA | mm | EDV | 56.5ml |
| LVIDd | 36.6mm | ESV | 20ml |
| LVIDs | 24mm | LVEF | 64% |
| IVSd | 7mm | FS | 34% |
| **Additional Information**: |  | | |
| No pleural/pericardial effusion | | | |
| **Final Diagnosis:** | | | |
| 1. {S, D, S} Levocardia 2. Normal Echocardiography Study | | | |
| SIGNATURE  Done by: Tesfaye T., Pediatric Cardiologist _______________ 09/02/2012Eth.C | | | |

| Patient Name**: Motuma Abebe**. Patient ID:_ADINAS_. SEX/ Age: M/ 14Years Date of Report: 11**/02/2012_**.  BP: ___ Weight: ___ Height:___ BSA: ___. Referral Diagnosis: **DOE. AGH03.1878.** | | | |
| --- | --- | --- | --- |
| **Features** | **Finding** | **Features** | **Finding** |
| **Profile** |  | **Atria** |  |
| Abdominal situs | Solitus | Left atrium | Normal |
| Cardiac position | Levocardia | Right atrium | Normal |
| Systemic venous drainage | Normal | **Atrioventricular valves** |  |
| Pulmonary venous drainage | Normal | Mitral valve | Annulus = 24mm |
| Atrioventricular connection | Concordant | Tricuspid valve | Annulus = 27mm  TAPSE = 19mm |
| Ventriculoarterial connection | Concordant | **Ventricles** |  |
| Ventricular loop | d-Loop | Left ventricle | Normal |
|  |  | Right ventricle | Normal |
| **Septae** |  | **Coronary arteries** | ----- |
| Interventricular septum | Intact | **Doppler Measurement** |  |
| Interatrial septum | Intact | Mitral | ---- |
| **Semilunal valves** |  | Aortic | ---- |
| Aortic valve | Annulus = 20mm | Tricuspid | ---- |
| Pulmonary valve | Annulus = 22mm | pulmonic | Trivial PR, PPG = 10mmHg |
| **Great arteries** | NRGA | **Aortic arch** | Left |
| Aorta | ---- | **PDA** | No |
| Pulmonary artery | Normal MPA and confluent Branch PAs. |  |  |
| **M-Mode** | | | |
| AO | mm | PWd | 6mm |
| LA | mm | EDV | 82ml |
| LVIDd | 4mm | ESV | 37ml |
| LVIDs | 3mm | LVEF | 55% |
| IVSd | 6mm | FS | 28% |
| **Additional Information**: |  | | |
| No pleural/pericardial effusion | | | |
| **Final Diagnosis:** | | | |
| 1. {S, D, S} Levocardia 2. Normal Echocardiography Study | | | |
| SIGNATURE  Done by: Tesfaye T., Pediatric Cardiologist _______________ 11/02/2012Eth.C | | | |

| Patient Name**: Baby of Sadya Ahmed**. Patient ID:_FHRH_. SEX/ Age: M/ 1Year Date of Report: 11**/02/2012_**.  BP: ___ Weight: ___ Height:___ BSA: ___. Referral Diagnosis: **Incidental Murmur. AGH03.1879.** | | | |
| --- | --- | --- | --- |
| **Features** | **Finding** | **Features** | **Finding** |
| **Profile** |  | **Atria** |  |
| Abdominal situs | Solitus | Left atrium | Normal |
| Cardiac position | Levocardia | Right atrium | Normal |
| Systemic venous drainage | Normal | **Atrioventricular valves** |  |
| Pulmonary venous drainage | Normal | Mitral valve | Annulus = 17mm |
| Atrioventricular connection | Concordant | Tricuspid valve | Annulus = 17mm |
| Ventriculoarterial connection | Concordant | **Ventricles** |  |
| Ventricular loop | d-Loop | Left ventricle | Normal |
|  |  | Right ventricle | Normal |
| **Septae** |  | **Coronary arteries** | ----- |
| Interventricular septum | Intact | **Doppler Measurement** |  |
| Interatrial septum | Intact | Mitral | Trivial MR |
| **Semilunal valves** |  | Aortic | ---- |
| Aortic valve | Annulus = 14mm | Tricuspid | ---- |
| Pulmonary valve | Annulus = 15mm | pulmonic | Mild PS, PPG = 20mmHg. Moderate PR, PPG = 18mmHg |
| **Great arteries** | NRGA | **Aortic arch** | Left |
| Aorta | ---- | **PDA** | No |
| Pulmonary artery | Normal MPA and confluent Branch PAs. |  |  |
| **M-Mode: Normal LV Function (eye balling)** | | | |
| AO | mm | PWd | mm |
| LA | mm | EDV | ml |
| LVIDd | mm | ESV | ml |
| LVIDs | mm | LVEF | % |
| IVSd | mm | FS | % |
| **Additional Information**: |  | | |
| No pleural/pericardial effusion | | | |
| **Final Diagnosis:** | | | |
| 1. {S, D, S} Levocardia 2. Mild PS 3. Good Biventricular Function | | | |
| SIGNATURE  Done by: Tesfaye T., Pediatric Cardiologist _______________ 11/02/2012Eth.C | | | |

| Patient Name**: Tinsae Aschalew**. Patient ID:_ADINAS_. SEX/ Age: M/ 10Years Date of Report: 13**/02/2012_**.  BP: ___ Weight: ___ Height:___ BSA: ___. Referral Diagnosis: **Palpitation. AGH03.1880.** | | | |
| --- | --- | --- | --- |
| **Features** | **Finding** | **Features** | **Finding** |
| **Profile** |  | **Atria** |  |
| Abdominal situs | Solitus | Left atrium | Normal |
| Cardiac position | Levocardia | Right atrium | Normal |
| Systemic venous drainage | Normal | **Atrioventricular valves** |  |
| Pulmonary venous drainage | Normal | Mitral valve | Annulus = 21mm |
| Atrioventricular connection | Concordant | Tricuspid valve | Annulus = 20mm  TAPSE = 28 mm |
| Ventriculoarterial connection | Concordant | **Ventricles** |  |
| Ventricular loop | d-Loop | Left ventricle | Normal |
|  |  | Right ventricle | Normal |
| **Septae** |  | **Coronary arteries** | ----- |
| Interventricular septum | Intact | **Doppler Measurement** |  |
| Interatrial septum | Intact | Mitral |  |
| **Semilunal valves** |  | Aortic | ---- |
| Aortic valve | Annulus = 14mm | Tricuspid | Mild TR, PPG = 28mmHg |
| Pulmonary valve | Annulus = 15mm | pulmonic | Trivial PR, PPG = 23mmHg |
| **Great arteries** | NRGA | **Aortic arch** | Left |
| Aorta | ---- | **PDA** | No |
| Pulmonary artery | MPA = 14mm and confluent Branch PAs. RPA = 9mm, LPA = 10mm |  |  |
| **M-Mode: Normal LV Function (eye balling)** | | | |
| AO | mm | PWd | 5.4mm |
| LA | mm | EDV | 38ml |
| LVIDd | 31mm | ESV | 18ml |
| LVIDs | 23mm | LVEF | 55% |
| IVSd | 6.4mm | FS | 28% |
| **Additional Information**: |  | | |
| No pleural/pericardial effusion | | | |
| **Final Diagnosis:** | | | |
| 1. {S, D, S} Levocardia 2. Normal Echocardiography Study 3. Good Biventricular Function | | | |
| SIGNATURE  Done by: Tesfaye T., Pediatric Cardiologist _______________ 13/02/2012Eth.C | | | |

| Patient Name**: Tinsae Belay**. Patient ID:_FHRH_. SEX/ Age: M/ 6/12 Date of Report: 13**/02/2012_**.  BP: ___ Weight: ___ Height:___ BSA: ___. Referral Diagnosis: **DS + Murmur + RD. AGH03.1881.** | | | |
| --- | --- | --- | --- |
| **Features** | **Finding** | **Features** | **Finding** |
| **Profile** |  | **Atria** |  |
| Abdominal situs | Solitus | Left atrium | Normal |
| Cardiac position | Levocardia | Right atrium | Dilated |
| Systemic venous drainage | Normal | **Atrioventricular valves** |  |
| Pulmonary venous drainage | Normal | Mitral valve | **Annulus = 8mm** |
| Atrioventricular connection | Concordant | Tricuspid valve | Annulus = 15mm |
| Ventriculoarterial connection | DORV | **Ventricles** |  |
| Ventricular loop | d-Loop | Left ventricle | Smallish |
|  |  | Right ventricle | Dilated |
| **Septae** | Tongue of tissue in b/n | **Coronary arteries** | ----- |
| Interventricular septum | 7mm Inlet VSD, L – R Shunt | **Doppler Measurement** |  |
| Interatrial septum | 7mm primum ASD, L – R Shunt | Mitral | ---- |
| **Semilunal valves** |  | Aortic | ---- |
| Aortic valve | Annulus = 8mm | Tricuspid | ---- |
| Pulmonary valve | Annulus = 12mm | pulmonic | Moderate PR, PPG = 59mmHg |
| **Great arteries** | NRGA | **Aortic arch** | Left |
| Aorta | ---- | **PDA** | No |
| Pulmonary artery | **MPA = 15mm**. Confluent Branch PAs. |  |  |
| **M-Mode: Normal LV Function (eye balling)** | | | |
| AO | mm | PWd | mm |
| LA | mm | EDV | ml |
| LVIDd | mm | ESV | ml |
| LVIDs | mm | LVEF | % |
| IVSd | mm | FS | % |
| **Additional Information**: |  | | |
| No pleural/pericardial effusion | | | |
| **Final Diagnosis:** | | | |
| 1. {S, D, S} Levocardia 2. DORV 3. Intermediate Unbalanced AVSD 4. Severe Pulmonary Hypertension 5. Good Biventricular Function | | | |
| SIGNATURE  Done by: Tesfaye T., Pediatric Cardiologist _______________ 13/02/2012Eth.C | | | |

| Patient Name**: Ruth Birhanu**. Patient ID:_ADINAS_. SEX/ Age: F/ 9Years Date of Report: 17**/02/2012_**.  BP: ___ Weight: ___ Height:___ BSA: ___. Referral Diagnosis: **Chest Pain. AGH03.1882.** | | | |
| --- | --- | --- | --- |
| **Features** | **Finding** | **Features** | **Finding** |
| **Profile** |  | **Atria** |  |
| Abdominal situs | Solitus | Left atrium | Normal |
| Cardiac position | Levocardia | Right atrium | Normal |
| Systemic venous drainage | Normal | **Atrioventricular valves** |  |
| Pulmonary venous drainage | Normal | Mitral valve | Annulus = 19mm |
| Atrioventricular connection | Concordant | Tricuspid valve | Annulus = 21mm  TAPSE = 22mm |
| Ventriculoarterial connection | Concordant | **Ventricles** |  |
| Ventricular loop | d-Loop | Left ventricle | Normal |
|  |  | Right ventricle | Normal |
| **Septae** |  | **Coronary arteries** | ----- |
| Interventricular septum | Intact | **Doppler Measurement** |  |
| Interatrial septum | Intact | Mitral |  |
| **Semilunal valves** |  | Aortic | ---- |
| Aortic valve | Annulus = 20mm | Tricuspid | ---- |
| Pulmonary valve | Annulus = 24mm | pulmonic |  |
| **Great arteries** | NRGA | **Aortic arch** | Left |
| Aorta | ---- | **PDA** | No |
| Pulmonary artery | Normal MPA and confluent Branch PAs. |  |  |
| **M-Mode: Normal LV Function (eye balling)** | | | |
| AO | mm | PWd | 8mm |
| LA | mm | EDV | 38ml |
| LVIDd | 31mm | ESV | 16ml |
| LVIDs | 22mm | LVEF | 58% |
| IVSd | 7mm | FS | 29% |
| **Additional Information**: |  | | |
| No pleural/pericardial effusion | | | |
| **Final Diagnosis:** | | | |
| 1. {S, D, S} Levocardia 2. Normal Echocardiography Study | | | |
| SIGNATURE  Done by: Tesfaye T., Pediatric Cardiologist _______________ 17/02/2012Eth.C | | | |

| Patient Name**: Felegush Abiye**. Patient ID:_FHRH_. SEX/ Age: F/ 3 2/12 Date of Report: 17**/02/2012_**.  BP: ___ Weight: ___ Height:___ BSA: ___. Referral Diagnosis: **Incidental Murmur. AGH03.1883.** | | | |
| --- | --- | --- | --- |
| **Features** | **Finding** | **Features** | **Finding** |
| **Profile** |  | **Atria** |  |
| Abdominal situs | Solitus | Left atrium | Normal |
| Cardiac position | Levocardia | Right atrium | Normal |
| Systemic venous drainage | Normal | **Atrioventricular valves** |  |
| Pulmonary venous drainage | Normal | Mitral valve | Annulus = 17mm |
| Atrioventricular connection | Concordant | Tricuspid valve | Annulus = 18mm  TAPSE = 19mm |
| Ventriculoarterial connection | Concordant | **Ventricles** |  |
| Ventricular loop | d-Loop | Left ventricle | Normal |
|  |  | Right ventricle | Normal |
| **Septae** |  | **Coronary arteries** | ----- |
| Interventricular septum | Intact | **Doppler Measurement** |  |
| Interatrial septum | PFO, L – R Shunt | Mitral |  |
| **Semilunal valves** |  | Aortic | ---- |
| Aortic valve | Annulus = 13mm | Tricuspid | ---- |
| Pulmonary valve | Annulus = 12mm | pulmonic | Mild PS, PPG = 32mmHg |
| **Great arteries** | NRGA | **Aortic arch** | Left |
| Aorta | ---- | **PDA** | No |
| Pulmonary artery | Normal MPA and confluent Branch PAs. |  |  |
| **M-Mode:** | | | |
| AO | mm | PWd | 4mm |
| LA | mm | EDV | 27ml |
| LVIDd | 27mm | ESV | 11ml |
| LVIDs | 19mm | LVEF | 59% |
| IVSd | 7mm | FS | 30% |
| **Additional Information**: |  | | |
| No pleural/pericardial effusion | | | |
| **Final Diagnosis:** | | | |
| 1. {S, D, S} Levocardia 2. PFO, L – R Shunt 3. Mild PS 4. Good Biventricular Function | | | |
| SIGNATURE  Done by: Tesfaye T., Pediatric Cardiologist _______________ 17/02/2012Eth.C | | | |

| Patient Name**: Mebea – Tsion Abrham** Patient ID:FHRH. SEX/ Age: F/ 1Year 4/12 Date of Report: 17**/02/2012_**.  BP: ___ Weight: ___ Height:___ BSA: ___. Referral Diagnosis: **DS + PHTN. SEE AGH03.1872.** | | | | |
| --- | --- | --- | --- | --- |
| **Features** | **Finding** | **Features** | | **Finding** |
| **Profile** |  | **Atria** | |  |
| Abdominal situs | Solitus | Left atrium | | Normal |
| Cardiac position | Levocardia | Right atrium | | Dilated |
| Systemic venous drainage | Normal | **Atrioventricular valves** | |  |
| Pulmonary venous drainage | Normal | Mitral valve | | Annulus = 14mm |
| Atrioventricular connection | Concordant | Tricuspid valve | | Annulus = 15mm  TAPSE = 8mm |
| Ventriculoarterial connection | Concordant | **Ventricles** | |  |
| Ventricular loop | d-Loop | Left ventricle | | Normal |
|  |  | Right ventricle | | Dilated |
| **Septae** |  | **Coronary arteries** | | ----- |
| Interventricular septum | Intact | **Doppler Measurement** | |  |
| Interatrial septum | Intact | Mitral | | ---- |
| **Semilunal valves** |  | Aortic | | ---- |
| Aortic valve | Annulus = 13mm | Tricuspid | | Trivial TR, PPG = 12mmHg |
| Pulmonary valve | Annulus = 15mm | pulmonic | | Moderate PR, PPG = 61mmHg |
| **Great arteries** | NRGA | **Aortic arch** | | ---- |
| Aorta | ---- | **PDA** | | No |
| Pulmonary artery | MPA = **18mm(dilated)** and confluent Branch PAs. |  | |  |
| **M-Mode** | | | | |
| AO | mm | | PWd | 6mm |
| LA | mm | | EDV | 10ml |
| LVIDd | 18mm | | ESV | 4ml |
| LVIDs | 11.5mm | | LVEF | 69% |
| IVSd | **8mm** | | FS | 36% |
| **Additional Information**: |  | | | |
| Circumferential pericardial effusion 5 mm on RV side, 10mm on RA side and trace on LV side | | | | |
| **Final Diagnosis:** | | | | |
| 1. {S, D, S} Levocardia 2. RV dilated and mildly Dysfunctional 3. Severe Pulmonary Hypertension 4. Mild to moderate Pericardial Effusion 5. Good LV Function 6. No evidence of Cardiac tamponade so far. | | | | |
| SIGNATURE  Done by: Tesfaye T., Pediatric Cardiologist _______________ 17/02/2012Eth.C | | | | |

N.B: THERE IS DETERIORATION IN RV Function AND MPA AND RV ARE DILATED. SEARCH FOR RESPIRATORY CAUSE FOR THE SEVERE PULMONARY HYPERTENSION.

| Patient Name**: Baby of Asnakech Mulualem**. Patient ID:FHRH. SEX/ Age: F/ 15days Date of Report: 18**/02/2012**.  BP: ___ Weight: ___ Height:___ BSA: ___. Referral Diagnosis: **Incidental Murmur. AGH03.1884.** | | | |
| --- | --- | --- | --- |
| **Features** | **Finding** | **Features** | **Finding** |
| **Profile** |  | **Atria** |  |
| Abdominal situs | Solitus | Left atrium | Mildly Dilated |
| Cardiac position | Levocardia | Right atrium | Normal |
| Systemic venous drainage | Normal | **Atrioventricular valves** |  |
| Pulmonary venous drainage | Normal | Mitral valve | Annulus = 8mm |
| Atrioventricular connection | Concordant | Tricuspid valve | Annulus = 9 mm  TAPSE = 14mm |
| Ventriculoarterial connection | Concordant | **Ventricles** |  |
| Ventricular loop | d-Loop | Left ventricle | Mildly Dilated |
|  |  | Right ventricle | Normal |
| **Septae** |  | **Coronary arteries** | ----- |
| Interventricular septum | Intact | **Doppler Measurement** |  |
| Interatrial septum | PFO, L – R Shunt | Mitral | ---- |
| **Semilunal valves** |  | Aortic | ---- |
| Aortic valve | Annulus = 10mm | Tricuspid | Mild TR, PPG =30mmHg |
| Pulmonary valve | Annulus = 10mm | pulmonic | ---- |
| **Great arteries** | NRGA | **Aortic arch** | Left |
| Aorta | ---- | **PDA** | 2.5mm, L – R Shunt |
| Pulmonary artery | Normal MPA and confluent Branch PAs. |  |  |
| **M-Mode:** | | | |
| AO | mm | PWd | 3.2mm |
| LA | mm | EDV | 8ml |
| LVIDd | 16.8mm | ESV | 2.26ml |
| LVIDs | 10.4mm | LVEF | 72% |
| IVSd | 4.6mm | FS | 38% |
| **Additional Information**: |  | | |
| No pleural/pericardial effusion | | | |
| **Final Diagnosis:** | | | |
| 1. {S, D, S} Levocardia 2. PFO, L – R Shunt 3. Moderate PDA, L – R Shunt 4. Good Biventricular Function | | | |
| SIGNATURE  Done by: Tesfaye T., Pediatric Cardiologist _______________ 18/02/2012Eth.C | | | |

| Patient Name**: Natnael Dereje**. Patient ID:_ADINAS_. SEX/ Age: M/ 10Years Date of Report: 19**/02/2012_**.  BP: ___ Weight: ___ Height:___ BSA: ___. Referral Diagnosis: **Incidental Murmur. AGH03.1885.** | | | |
| --- | --- | --- | --- |
| **Features** | **Finding** | **Features** | **Finding** |
| **Profile** |  | **Atria** |  |
| Abdominal situs | Solitus | Left atrium | Normal |
| Cardiac position | Levocardia | Right atrium | Normal |
| Systemic venous drainage | Normal | **Atrioventricular valves** |  |
| Pulmonary venous drainage | Normal | Mitral valve | Annulus = 21mm |
| Atrioventricular connection | Concordant | Tricuspid valve | Annulus = 21mm  TAPSE = 20mm |
| Ventriculoarterial connection | Concordant | **Ventricles** |  |
| Ventricular loop | d-Loop | Left ventricle | Normal |
|  |  | Right ventricle | Normal |
| **Septae** |  | **Coronary arteries** | ----- |
| Interventricular septum | Intact | **Doppler Measurement** |  |
| Interatrial septum | Intact | Mitral | ---- |
| **Semilunal valves** |  | Aortic | Mild AR |
| Aortic valve | Annulus = 17mm | Tricuspid | Trivial TR, PPG = 13mmHg |
| Pulmonary valve | Annulus = 22mm | pulmonic |  |
| **Great arteries** | NRGA | **Aortic arch** | Left |
| Aorta | ---- | **PDA** | No |
| Pulmonary artery | Normal MPA and confluent Branch PAs. |  |  |
| **M-Mode:** | | | |
| AO | mm | PWd | 5.6mm |
| LA | mm | EDV | 86ml |
| LVIDd | 43.6mm | ESV | 30.39ml |
| LVIDs | 28.3mm | LVEF | 64% |
| IVSd | 5.6mm | FS | 35% |
| **Additional Information**: |  | | |
| No pleural/pericardial effusion | | | |
| **Final Diagnosis:** | | | |
| 1. {S, D, S} Levocardia 2. Mild AR 3. Good Biventricular Function | | | |
| SIGNATURE  Done by: Tesfaye T., Pediatric Cardiologist _______________ 19 /02/2012Eth.C | | | |

RECOMMENDATION: FOLLOW UP

| Patient Name**: Kalkidan Dehninet**. Patient ID: **SAHATE - BIRHAN**. SEX/ Age: F/ 3/12 Date of Report: 19/02/2012  BP: ___ Weight: ___ Height:___ BSA: ___. Referral Diagnosis: **Cyanosis. AGH03.1886.** | | | |
| --- | --- | --- | --- |
| **Features** | **Finding** | **Features** | **Finding** |
| **Profile** |  | **Atria** |  |
| Abdominal situs | Solitus | Left atrium | Normal |
| Cardiac position | Levocardia | Right atrium | Normal |
| Systemic venous drainage | Normal | **Atrioventricular valves** |  |
| Pulmonary venous drainage | Normal | Mitral valve | Annulus = 18mm |
| Atrioventricular connection | Concordant | Tricuspid valve | Annulus = 15mm  TAPSE = 14mm |
| Ventriculoarterial connection | DORV | **Ventricles** |  |
| Ventricular loop | d-Loop | Left ventricle | Normal |
|  |  | Right ventricle | Normal |
| **Septae** |  | **Coronary arteries** | ----- |
| Interventricular septum | 6cm Sub pulmonic VSD, BD Shunt | **Doppler Measurement** |  |
| Interatrial septum | PFO, L – R Shunt | Mitral | ----- |
| **Semilunal valves** |  | Aortic | ----- |
| Aortic valve | Annulus = 12mm | Tricuspid | ----- |
| Pulmonary valve | Annulus = 11mm | pulmonic | Mild PS, PPG = 21mmHg |
| **Great arteries** | d-TGA | **Aortic arch** | Left |
| Aorta | Anterior and to the right | **PDA** | No |
| Pulmonary artery | Posterior and to the left.  MPA = 13mm. Confluent Branch PAs. |  |  |
| **M-Mode:** | | | |
| AO | mm | PWd | 5.7mm |
| LA | mm | EDV | 21ml |
| LVIDd | 24.3mm | ESV | 8ml |
| LVIDs | 16.8mm | LVEF | 61% |
| IVSd | 4.6mm | FS | 31% |
| **Additional Information**: | No pleural/pericardial effusion | | |
| **Final Diagnosis:** | | | |
| 1. {S, D, D} Levocardia 2. DORV 3. PFO, L – R Shunt 4. Sub pulmonic VSD, BD Shunt 5. Mild PS 6. Taussig-Bing Anomaly 7. Good Biventricular Function | | | |
| SIGNATURE  Done by: Tesfaye T., Pediatric Cardiologist _______________ 19/02/2012Eth.C | | | |

| Patient Name**: Nardos Ageru**. Patient ID:_FHRH_. SEX/ Age: F/ 4 6/12Years Date of Report: 20**/02/2012_**.  BP: ___ Weight: ___ Height:___ BSA: ___. Referral Diagnosis: **Innocent Murmur. AGH03.1887.** | | | |
| --- | --- | --- | --- |
| **Features** | **Finding** | **Features** | **Finding** |
| **Profile** |  | **Atria** |  |
| Abdominal situs | Solitus | Left atrium | Normal |
| Cardiac position | Levocardia | Right atrium | Normal |
| Systemic venous drainage | Normal | **Atrioventricular valves** |  |
| Pulmonary venous drainage | Normal | Mitral valve | Annulus = 15mm |
| Atrioventricular connection | Concordant | Tricuspid valve | Annulus = 19mm  TAPSE = 17mm |
| Ventriculoarterial connection | Concordant | **Ventricles** |  |
| Ventricular loop | d-Loop | Left ventricle | Normal |
|  |  | Right ventricle | Normal |
| **Septae** |  | **Coronary arteries** | ----- |
| Interventricular septum | Intact | **Doppler Measurement** |  |
| Interatrial septum | Intact | Mitral | --- |
| **Semilunal valves** |  | Aortic | ---- |
| Aortic valve | Annulus = 14mm | Tricuspid | ---- |
| Pulmonary valve | Annulus = 14mm | pulmonic | ---- |
| **Great arteries** | NRGA | **Aortic arch** | Left |
| Aorta | ---- | **PDA** | No |
| Pulmonary artery | MPA =15mm. Confluent Branch PAs. |  |  |
| **M-Mode:** | | | |
| AO | mm | PWd | 5.7mm |
| LA | mm | EDV | 26.6ml |
| LVIDd | 27mm | ESV | 13ml |
| LVIDs | 20mm | LVEF | 58% |
| IVSd | 7mm | FS | 29% |
| **Additional Information**: |  | | |
| No pleural/pericardial effusion | | | |
| **Final Diagnosis:** | | | |
| 1. {S, D, S} Levocardia 2. Normal Echocardiography Study | | | |
| SIGNATURE  Done by: Tesfaye T., Pediatric Cardiologist _______________ 20/02/2012Eth.C | | | |

| Patient Name**: Yohannes Addisu**. Patient ID:_FHRH_. SEX/ Age: M/ 8Years Date of Report: 20**/02/2012_**.  BP: ___ Weight: ___ Height:___ BSA: ___. Referral Diagnosis: **Rheumatic Recurrence. AGH03.1888.** | | | |
| --- | --- | --- | --- |
| **Features** | **Finding** | **Features** | **Finding** |
| **Profile** |  | **Atria** |  |
| Abdominal situs | Solitus | Left atrium | Normal |
| Cardiac position | Levocardia | Right atrium | Normal |
| Systemic venous drainage | Normal | **Atrioventricular valves** |  |
| Pulmonary venous drainage | Normal | Mitral valve | Annulus = 16mm. Mildly thickened Mitral valve leaflet |
| Atrioventricular connection | Concordant | Tricuspid valve | Annulus = 18mm  TAPSE = 17mm |
| Ventriculoarterial connection | Concordant | **Ventricles** |  |
| Ventricular loop | d-Loop | Left ventricle | Normal |
|  |  | Right ventricle | Normal |
| **Septae** |  | **Coronary arteries** | ----- |
| Interventricular septum | Intact | **Doppler Measurement** |  |
| Interatrial septum | Intact | Mitral | Moderate MR, Posteriorly projected with velocity of 4m/sec |
| **Semilunal valves** |  | Aortic | ---- |
| Aortic valve | Annulus = 15mm | Tricuspid | Trivial TR |
| Pulmonary valve | Annulus = 15mm | pulmonic | Trivial PR |
| **Great arteries** | NRGA | **Aortic arch** | Left |
| Aorta | ---- | **PDA** | No |
| Pulmonary artery | Normal MPA and confluent Branch PAs. |  |  |
| **M-Mode:** | | | |
| AO | mm | PWd | 6mm |
| LA | mm | EDV | 47ml |
| LVIDd | 34mm | ESV | 19ml |
| LVIDs | 23mm | LVEF | 60% |
| IVSd | 6.5mm | FS | 31% |
| **Additional Information**: |  | | |
| No pleural/pericardial effusion | | | |
| **Final Diagnosis:** | | | |
| 1. {S, D, S} Levocardia 2. Moderate MR 3. Mildly Thickened Mitral valve leaflet 4. Good Biventricular Function | | | |
| SIGNATURE  Done by: Tesfaye T., Pediatric Cardiologist _______________ 20/02/2012Eth.C | | | |

| Patient Name**: MESERET AMDEWERK**. Patient ID:_ADINAS_. SEX/ Age: M/11Years Date of Report: 20**/02/2012_**.  BP: ___ Weight: ___ Height:___ BSA: ___. Referral Diagnosis: **Rheumatic Fever. AGH03.1889.** | | | |
| --- | --- | --- | --- |
| **Features** | **Finding** | **Features** | **Finding** |
| **Profile** |  | **Atria** |  |
| Abdominal situs | Solitus | Left atrium | Normal |
| Cardiac position | Levocardia | Right atrium | Normal |
| Systemic venous drainage | Normal | **Atrioventricular valves** |  |
| Pulmonary venous drainage | Normal | Mitral valve | Annulus = 21mm. Thickened mitral valve leaflet. |
| Atrioventricular connection | Concordant | Tricuspid valve | Annulus = 21mm |
| Ventriculoarterial connection | Concordant | **Ventricles** |  |
| Ventricular loop | d-Loop | Left ventricle | Normal |
|  |  | Right ventricle | Normal |
| **Septae** |  | **Coronary arteries** | ----- |
| Interventricular septum | Intact | **Doppler Measurement** |  |
| Interatrial septum | Intact | Mitral | Moderate MR, Posteriorly projected, Holosystolic with jet velocity of 3.5m/sec |
| **Semilunal valves** |  | Aortic | ---- |
| Aortic valve | Annulus = 19mm | Tricuspid | ---- |
| Pulmonary valve | Annulus = 22mm | pulmonic | ------ |
| **Great arteries** | NRGA | **Aortic arch** | Left |
| Aorta | ---- | **PDA** | No |
| Pulmonary artery | Normal MPA and confluent Branch PAs. |  |  |
| **M-Mode: Normal LV Function (eye balling)** | | | |
| AO | mm | PWd | 9mm |
| LA | mm | EDV | 53ml |
| LVIDd | 35mm | ESV | 18ml |
| LVIDs | 23mm | LVEF | 66% |
| IVSd | 7mm | FS | 36% |
| **Additional Information**: |  | | |
| No pleural/pericardial effusion | | | |
| **Final Diagnosis:** | | | |
| 1. {S, D, S} Levocardia 2. Moderate MR 3. Thickened Mitral valve leaflet 4. Good Biventricular Function | | | |
| SIGNATURE  Done by: Tesfaye T., Pediatric Cardiologist _______________ 20/02/2012Eth.C | | | |
| Patient Name**: Kemal Kassim**. Patient ID:_SAHATE BIRHAN_. SEX/ Age: M/ 7Years Date of Report: 20**/02/2012_**.  BP: ___ Weight: ___ Height:___ BSA: ___. Referral Diagnosis: **DOE + Murmur. AGH03.1890.** | | | |
| **Features** | **Finding** | **Features** | **Finding** |
| **Profile** |  | **Atria** |  |
| Abdominal situs | Solitus | Left atrium | Normal |
| Cardiac position | Levocardia | Right atrium | Dilated |
| Systemic venous drainage | Normal | **Atrioventricular valves** |  |
| Pulmonary venous drainage | Normal | Mitral valve | Annulus = 17mm |
| Atrioventricular connection | Concordant | Tricuspid valve | Annulus = 19mm  TAPSE = 30mm |
| Ventriculoarterial connection | Concordant | **Ventricles** |  |
| Ventricular loop | d-Loop | Left ventricle | Normal |
|  |  | Right ventricle | Dilated |
| **Septae** |  | **Coronary arteries** | ----- |
| Interventricular septum | Intact | **Doppler Measurement** |  |
| Interatrial septum | 22mm Premium ASD | Mitral | Severe MR |
| **Semilunal valves** |  | Aortic | ---- |
| Aortic valve | Annulus = 17mm | Tricuspid | Severe TR |
| Pulmonary valve | Annulus = 25mm | pulmonic | Moderate PR |
| **Great arteries** | NRGA | **Aortic arch** | Left |
| Aorta | ---- | **PDA** | No |
| Pulmonary artery | **MPA = 25mm**. Confluent Branch PAs. |  |  |
| **M-Mode: Normal LV Function (eye balling)** | | | |
| AO | mm | PWd | mm |
| LA | mm | EDV | ml |
| LVIDd | mm | ESV | ml |
| LVIDs | mm | LVEF | % |
| IVSd | mm | FS | % |
| **Additional Information**: |  | | |
| No pleural/pericardial effusion | | | |
| **Final Diagnosis:** | | | |
| 1. {S, D, S} Levocardia 2. Partial AVSD 3. Severe right AVVR 4. Severe Left AVVR 5. Moderate PR 6. RA/RV Dilated 7. Severe Pulmonary Hypertension 8. Good Biventricular Function | | | |
| SIGNATURE  Done by: Tesfaye T., Pediatric Cardiologist _______________ 20/02/2012Eth.C | | | |

| Patient Name**: Yohannes Alemu**. Patient ID:_FHRH_. SEX/ Age: M/ 14 9/12Years Date of Report: 22**/02/2012_**.  BP: ___ Weight: ___ Height:___ BSA: ___. Referral Diagnosis: **Easy Fatigability. AGH03.1891.** | | | |
| --- | --- | --- | --- |
| **Features** | **Finding** | **Features** | **Finding** |
| **Profile** |  | **Atria** |  |
| Abdominal situs | Solitus | Left atrium | Normal |
| Cardiac position | Levocardia | Right atrium | Normal |
| Systemic venous drainage | Normal | **Atrioventricular valves** |  |
| Pulmonary venous drainage | Normal | Mitral valve | Annulus = 20mm |
| Atrioventricular connection | Concordant | Tricuspid valve | Annulus = 24mm |
| Ventriculoarterial connection | Concordant | **Ventricles** |  |
| Ventricular loop | d-Loop | Left ventricle | Normal |
|  |  | Right ventricle | Normal |
| **Septae** |  | **Coronary arteries** | ----- |
| Interventricular septum | Intact | **Doppler Measurement** |  |
| Interatrial septum | Intact | Mitral | ---- |
| **Semilunal valves** |  | Aortic | ---- |
| Aortic valve | Annulus = 17mm | Tricuspid | ---- |
| Pulmonary valve | Annulus = 17mm | pulmonic |  |
| **Great arteries** | NRGA | **Aortic arch** | Left |
| Aorta | ---- | **PDA** | No |
| Pulmonary artery | Normal MPA and confluent Branch PAs. |  |  |
| **M-Mode:** | | | |
| AO | mm | PWd | 5mm |
| LA | mm | EDV | 87ml |
| LVIDd | 44mm | ESV | 40ml |
| LVIDs | 32mm | LVEF | 55% |
| IVSd | 5.4mm | FS | 28% |
| **Additional Information**: |  | | |
| No pleural/pericardial effusion | | | |
| **Final Diagnosis:** | | | |
| 1. Normal Echocardiography Study. | | | |
| SIGNATURE  Done by: Tesfaye T., Pediatric Cardiologist _______________ 22/02/2012Eth.C | | | |

| Patient Name**: Werkinesh Chanie**. Patient ID:_FHRH_. SEX/ Age: F/ 11Years Date of Report: 24**/02/2012_**.  BP: ___ Weight: ___ Height:___ BSA: ___. Referral Diagnosis: **IE + CHF + Murmur + DOE. AGH03.1892.** | | | |
| --- | --- | --- | --- |
| **Features** | **Finding** | **Features** | **Finding** |
| **Profile** |  | **Atria** |  |
| Abdominal situs | Solitus | Left atrium | Dilated, 60 X 56mm |
| Cardiac position | Levocardia | Right atrium | Normal |
| Systemic venous drainage | Normal | **Atrioventricular valves** |  |
| Pulmonary venous drainage | Normal | Mitral valve | Thickened, clubbed. MVA = 1.37m2 |
| Atrioventricular connection | Concordant | Tricuspid valve | TAPSE = mm |
| Ventriculoarterial connection | Concordant | **Ventricles** |  |
| Ventricular loop | d-Loop | Left ventricle | Dilated, Dysfunctional |
|  |  | Right ventricle | Normal |
| **Septae** |  | **Coronary arteries** | ----- |
| Interventricular septum | Intact | **Doppler Measurement** |  |
| Interatrial septum | Intact | Mitral | Severe MR, Posteriorly projected with velocity of 3.8m/sec. Moderate MS, PPG/MPG = 15/8mmHg |
| **Semilunal valves** |  | Aortic | Moderate AR, PHT = 314ms. Moderate AS, PPG/MPG = 36/22mmHg. |
| Aortic valve | Annulus = 21mm | Tricuspid | Mild TR, PPG = 17mmHg |
| Pulmonary valve | Annulus = 19mm | pulmonic |  |
| **Great arteries** | NRGA | **Aortic arch** | Left |
| Aorta | ---- | **PDA** | No |
| Pulmonary artery | MPA = 23mm. Confluent Branch PAs. |  |  |
| **M-Mode: Normal LV Function (eye balling)** | | | |
| AO | mm | PWd | 8mm |
| LA | mm | EDV | 243ml |
| LVIDd | 68.5mm | ESV | 131ml |
| LVIDs | 52mm | LVEF | 46% |
| IVSd | 7.5mm | FS | 24% |
| **Additional Information**: | No evidence of Vegetation so far | | |
| 8mm pericardial effusion on RA/RV Side, localized | | | |
| **Final Diagnosis:** | | | |
| 1. {S, D, S} Levocardia 2. LA/LV Dilated. 3. Severe MR 4. Moderate MS 5. Moderate AR 6. Moderate AS 7. LV Dilated and Dysfunctional | | | |
| SIGNATURE  Done by: Tesfaye T., Pediatric Cardiologist _______________ 24/02/2012Eth.C | | | |

| Patient Name**: Wubetu Setegn**. Patient ID:_FHRH_. SEX/ Age: M/ 1Years Date of Report: 24**/02/2012_**.  BP: ___ Weight: ___ Height:___ BSA: ___. Referral Diagnosis: **Diaphoresis + Murmur. AGH03.1893.** | | | |
| --- | --- | --- | --- |
| **Features** | **Finding** | **Features** | **Finding** |
| **Profile** |  | **Atria** |  |
| Abdominal situs | Solitus | Left atrium | Dilated |
| Cardiac position | Levocardia | Right atrium | Normal |
| Systemic venous drainage | Normal | **Atrioventricular valves** |  |
| Pulmonary venous drainage | Normal | Mitral valve | Annulus = 15mm |
| Atrioventricular connection | Concordant | Tricuspid valve | Annulus = 17mm  TAPSE = 20mm |
| Ventriculoarterial connection | Concordant | **Ventricles** |  |
| Ventricular loop | d-Loop | Left ventricle | Dilated |
|  |  | Right ventricle | Normal |
| **Septae** |  | **Coronary arteries** | ----- |
| Interventricular septum | Intact | **Doppler Measurement** |  |
| Interatrial septum | Intact | Mitral |  |
| **Semilunal valves** |  | Aortic | ---- |
| Aortic valve | Annulus = 12mm | Tricuspid | Mild TR, PPG = 33mmHg |
| Pulmonary valve | Annulus = 13mm | pulmonic |  |
| **Great arteries** | NRGA | **Aortic arch** | Left |
| Aorta | ---- | **PDA** | 3.5mm PDA, L – R Shunt |
| Pulmonary artery | Normal MPA and confluent Branch PAs. |  |  |
| **M-Mode: Normal LV Function (eye balling)** | | | |
| AO | mm | PWd | 5mm |
| LA | mm | EDV | 52ml |
| LVIDd | 35mm | ESV | 9ml |
| LVIDs | 18mm | LVEF | 72% |
| IVSd | 4.5mm | FS | 38% |
| **Additional Information**: |  | | |
| No pleural/pericardial effusion | | | |
| **Final Diagnosis:** | | | |
| 1. {S, D, S} Levocardia 2. Large PDA, L – R Shunt 3. Good Biventricular Function | | | |
| SIGNATURE  Done by: Tesfaye T., Pediatric Cardiologist _______________ 24 /02/2012Eth.C | | | |

| Patient Name**: Tejitu Tazeb**. Patient ID:_FHRH_. SEX/ Age: F/ 2 9/12Years Date of Report: 24**/02/2012_**.  BP: ___ Weight: ___ Height:___ BSA: ___. Referral Diagnosis: **FTT. AGH03.1894.** | | | |
| --- | --- | --- | --- |
| **Features** | **Finding** | **Features** | **Finding** |
| **Profile** |  | **Atria** |  |
| Abdominal situs | Solitus | Left atrium | Normal |
| Cardiac position | Levocardia | Right atrium | Normal |
| Systemic venous drainage | Normal | **Atrioventricular valves** |  |
| Pulmonary venous drainage | Normal | Mitral valve | Annulus = 16mm |
| Atrioventricular connection | Concordant | Tricuspid valve | Annulus = 17mm  TAPSE = 16mm |
| Ventriculoarterial connection | Concordant | **Ventricles** |  |
| Ventricular loop | d-Loop | Left ventricle | Normal |
|  |  | Right ventricle | Normal |
| **Septae** |  | **Coronary arteries** | ----- |
| Interventricular septum | Intact | **Doppler Measurement** |  |
| Interatrial septum | Intact | Mitral | ---- |
| **Semilunal valves** |  | Aortic | ---- |
| Aortic valve | Annulus = 12mm | Tricuspid | ---- |
| Pulmonary valve | Annulus = 13mm | pulmonic |  |
| **Great arteries** | NRGA | **Aortic arch** | Left |
| Aorta | ---- | **PDA** | No |
| Pulmonary artery | Normal MPA and confluent Branch PAs. |  |  |
| **M-Mode: Normal LV Function (eye balling)** | | | |
| AO | mm | PWd | 5mm |
| LA | mm | EDV | 40ml |
| LVIDd | 31.5mm | ESV | 12ml |
| LVIDs | 20mm | LVEF | 68% |
| IVSd | 5.4mm | FS | 37% |
| **Additional Information**: |  | | |
| No pleural/pericardial effusion | | | |
| **Final Diagnosis:** | | | |
| 1. Normal Echocardiography Study | | | |
| SIGNATURE  Done by: Tesfaye T., Pediatric Cardiologist _______________ 24/02/2012Eth.C | | | |

| Patient Name**: KIRUBEL SISAY**. Patient ID:_FHRH_. SEX/ Age: M/ 6Years Date of Report: 25**/02/2012_**.  BP: ___ Weight: ___ Height:___ BSA: ___. Referral Diagnosis: **CHF. AGH03.1895.** | | | |
| --- | --- | --- | --- |
| **Features** | **Finding** | **Features** | **Finding** |
| **Profile** |  | **Atria** |  |
| Abdominal situs | Solitus | Left atrium | Dilated |
| Cardiac position | Levocardia | Right atrium | Dilated |
| Systemic venous drainage | Normal | **Atrioventricular valves** |  |
| Pulmonary venous drainage | Normal | Mitral valve | Annulus = 13mm |
| Atrioventricular connection | Concordant | Tricuspid valve | Annulus =19 mm  TAPSE = 19mm |
| Ventriculoarterial connection | Truncus | **Ventricles** |  |
| Ventricular loop | d-Loop | Left ventricle | Dilated |
|  |  | Right ventricle | Dilated l |
| **Septae** |  | **Coronary arteries** | ----- |
| Interventricular septum | Large (15mm) sub arterial VSD, BD Shunt. | **Doppler Measurement** |  |
| Interatrial septum | Intact | Mitral | ---- |
| **Semilunal valves** |  | Aortic | Moderate truncal regurgitation, PPG = 60mmHg |
| Truncus valve | Annulus = 24mm | Tricuspid | ---- |
|  |  |  |  |
| **Great arteries** | Truncus arteriosus | **Aortic arch** | Left |
| Truncus | Diameter = 22mm | **PDA** | No |
|  | Not able to visualize the branch PAs |  |  |
| **M-Mode: Normal LV Function (eye balling)** | | | |
| AO | mm | PWd | 5mm |
| LA | mm | EDV | 13ml |
| LVIDd | 20mm | ESV | 6ml |
| LVIDs | 15mm | LVEF | 54% |
| IVSd | 6mm | FS | 26% |
| **Additional Information**: |  | | |
| No pleural/pericardial effusion | | | |
| **Final Diagnosis:** | | | |
| 1. {S, D, S} Levocardia 2. All chambers Dilated 3. Large Sub arterial VSD, BD Shunt 4. Truncus arteriosus 5. Severe pulmonary Hypertension. 6. Reduced LV Systolic Function | | | |
| SIGNATURE  Done by: Tesfaye T., Pediatric Cardiologist _______________ 25/02/2012Eth.C | | | |

| Patient Name**: Mebea – Tsion Abrham** Patient ID:FHRH. SEX/ Age: F/ 1Year 4/12 Date of Report: 25**/02/2012_**.  BP: ___ Weight: ___ Height:___ BSA: ___. Referral Diagnosis: **________.SEE AGH03.1872.** | | | | |
| --- | --- | --- | --- | --- |
| **Features** | **Finding** | **Features** | | **Finding** |
| **Profile** |  | **Atria** | |  |
| Abdominal situs | Solitus | Left atrium | | Normal |
| Cardiac position | Levocardia | Right atrium | | Normal |
| Systemic venous drainage | Normal | **Atrioventricular valves** | |  |
| Pulmonary venous drainage | Normal | Mitral valve | | Annulus = 14mm |
| Atrioventricular connection | Concordant | Tricuspid valve | | Annulus = 15mm  TAPSE = 11mm |
| Ventriculoarterial connection | Concordant | **Ventricles** | |  |
| Ventricular loop | d-Loop | Left ventricle | | Normal |
|  |  | Right ventricle | | Dilated |
| **Septae** |  | **Coronary arteries** | | ----- |
| Interventricular septum | Intact | **Doppler Measurement** | |  |
| Interatrial septum | Intact | Mitral | | ---- |
| **Semilunal valves** |  | Aortic | | ---- |
| Aortic valve | Annulus = 13mm | Tricuspid | | Trivial TR, PPG = 12mmHg |
| Pulmonary valve | Annulus = 15mm | pulmonic | | Trivial PR, PPG = 23mmHg |
| **Great arteries** | NRGA | **Aortic arch** | | ---- |
| Aorta | ---- | **PDA** | | No |
| Pulmonary artery | MPA = **11mm** and confluent Branch PAs. |  | |  |
| **M-Mode** | | | | |
| AO | mm | | PWd | 5mm |
| LA | mm | | EDV | 12ml |
| LVIDd | 19mm | | ESV | 4ml |
| LVIDs | 12mm | | LVEF | 71% |
| IVSd | **6mm** | | FS | 37% |
| **Additional Information**: |  | | | |
| Circumferential pericardial effusion 2 mm on RV side, 6mm on RA side and 3mm on LV side | | | | |
| **Final Diagnosis:** | | | | |
| 1. {S, D, S} Levocardia 2. Mild Pericardial Effusion (decreasing in size) 3. Good Biventricular Function 4. No evidence of Pulmonary Hypertension. 5. Significant improvement from the previous echo finding | | | | |
| SIGNATURE  Done by: Tesfaye T., Pediatric Cardiologist _______________ 25/02/2012Eth.C | | | | |

| Patient Name**: Anteneh Agalu**. Patient ID:_ENJIBARA HOSP_. SEX/ Age: M/ 8Years Date of Report: 26**/02/2012_**.  BP: ___ Weight: ___ Height:___ BSA: ___. Referral Diagnosis: **Incidental Murmur IE**  **. AGH03.1896.** | | | |
| --- | --- | --- | --- |
| **Features** | **Finding** | **Features** | **Finding** |
| **Profile** |  | **Atria** |  |
| Abdominal situs | Solitus | Left atrium | Normal |
| Cardiac position | Levocardia | Right atrium | Normal |
| Systemic venous drainage | Normal | **Atrioventricular valves** |  |
| Pulmonary venous drainage | Normal | Mitral valve | Annulus = 19mm |
| Atrioventricular connection | Concordant | Tricuspid valve | Annulus = 19mm  TAPSE = 18mm |
| Ventriculoarterial connection | Concordant | **Ventricles** |  |
| Ventricular loop | d-Loop | Left ventricle | Normal |
|  |  | Right ventricle | Normal |
| **Septae** |  | **Coronary arteries** | ----- |
| Interventricular septum | Intact | **Doppler Measurement** |  |
| Interatrial septum | Intact | Mitral |  |
| **Semilunal valves** |  | Aortic | Moderate AR, PHT = 321ms |
| Aortic valve | Annulus = 15mm | Tricuspid | ---- |
| Pulmonary valve | Annulus = 20mm | pulmonic |  |
| **Great arteries** | NRGA | **Aortic arch** | Left |
| Aorta | ---- | **PDA** | 1.5mm PDA, L – R Shunt |
| Pulmonary artery | MPA = 23mm. Confluent Branch PAs. |  |  |
| **M-Mode: Normal LV Function (eye balling)** | | | |
| AO | mm | PWd | 7mm |
| LA | mm | EDV | 51ml |
| LVIDd | 35mm | ESV | 23ml |
| LVIDs | 25mm | LVEF | 55% |
| IVSd | 6mm | FS | 28% |
| **Additional Information**: |  | | |
| No pleural/pericardial effusion | | | |
| **Final Diagnosis:** | | | |
| 1. {S, D, S} Levocardia 2. Moderate AR 3. Small PDA, L – R Shunt 4. Good Biventricular Function | | | |
| SIGNATURE  Done by: Tesfaye T., Pediatric Cardiologist _______________ 26/02/2012Eth.C | | | |

| Patient Name**: Lealem Ayana**. Patient ID:_Enjibara H_. SEX/ Age: M/ 11Years Date of Report: 27**/02/2012_**.  BP: ___ Weight: ___ Height:___ BSA: ___. Referral Diagnosis: **Easy Fatigability. AGH03.1897.** | | | |
| --- | --- | --- | --- |
| **Features** | **Finding** | **Features** | **Finding** |
| **Profile** |  | **Atria** |  |
| Abdominal situs | Solitus | Left atrium | Normal |
| Cardiac position | Levocardia | Right atrium | Normal |
| Systemic venous drainage | Normal | **Atrioventricular valves** |  |
| Pulmonary venous drainage | Normal | Mitral valve | Annulus = 24mm |
| Atrioventricular connection | Concordant | Tricuspid valve | Annulus = 23mm  TAPSE = 25mm |
| Ventriculoarterial connection | Concordant | **Ventricles** |  |
| Ventricular loop | d-Loop | Left ventricle | Normal |
|  |  | Right ventricle | Normal |
| **Septae** |  | **Coronary arteries** | ----- |
| Interventricular septum | Intact | **Doppler Measurement** |  |
| Interatrial septum | Intact | Mitral |  |
| **Semilunal valves** |  | Aortic | ---- |
| Aortic valve | Annulus = 18mm | Tricuspid | ---- |
| Pulmonary valve | Annulus = 21mm | pulmonic |  |
| **Great arteries** | NRGA | **Aortic arch** | Left |
| Aorta | ---- | **PDA** | No |
| Pulmonary artery | Normal MPA and confluent Branch PAs. |  |  |
| **M-Mode: Normal LV Function (eye balling)** | | | |
| AO | mm | PWd | 6mm |
| LA | mm | EDV | 73ml |
| LVIDd | 41mm | ESV | 31ml |
| LVIDs | 29mm | LVEF | 58% |
| IVSd | 6mm | FS | 30% |
| **Additional Information**: | No coarctation of aorta | | |
| No pleural/pericardial effusion | | | |
| **Final Diagnosis:** | | | |
| 1. Normal Echocardiography Study. | | | |
| SIGNATURE  Done by: Tesfaye T., Pediatric Cardiologist _______________ 27/02/2012Eth.C | | | |

| Patient Name**: Mirtie Gedefa**. Patient ID:_Merawi H_. SEX/ Age: F/ 10Years Date of Report: 27**/02/2012_**.  BP: ___ Weight: ___ Height:___ BSA: ___. Referral Diagnosis: **Palpitation. AGH03.1898.** | | | |
| --- | --- | --- | --- |
| **Features** | **Finding** | **Features** | **Finding** |
| **Profile** |  | **Atria** |  |
| Abdominal situs | Solitus | Left atrium | Normal |
| Cardiac position | Levocardia | Right atrium | Normal |
| Systemic venous drainage | Normal | **Atrioventricular valves** |  |
| Pulmonary venous drainage | Normal | Mitral valve | Annulus = 19mm |
| Atrioventricular connection | Concordant | Tricuspid valve | Annulus = 25mm  TAPSE = 23mm |
| Ventriculoarterial connection | Concordant | **Ventricles** |  |
| Ventricular loop | d-Loop | Left ventricle | Normal |
|  |  | Right ventricle | Normal |
| **Septae** |  | **Coronary arteries** | ----- |
| Interventricular septum | Intact | **Doppler Measurement** |  |
| Interatrial septum | Intact | Mitral |  |
| **Semilunal valves** |  | Aortic | ---- |
| Aortic valve | Annulus = 18mm | Tricuspid | Trivial TR, PPG = 20mmHg |
| Pulmonary valve | Annulus = 24mm | pulmonic | Trivial PR |
| **Great arteries** | NRGA | **Aortic arch** | Left |
| Aorta | ---- | **PDA** | No |
| Pulmonary artery | Normal MPA and confluent Branch PAs. |  |  |
| **M-Mode: Normal LV Function (eye balling)** | | | |
| AO | mm | PWd | 6mm |
| LA | mm | EDV | 59ml |
| LVIDd | 37mm | ESV | 27ml |
| LVIDs | 27mm | LVEF | 55% |
| IVSd | 6mm | FS | 28% |
| **Additional Information**: | No coarctation of aorta | | |
| No pleural/pericardial effusion | | | |
| **Final Diagnosis:** | | | |
| 1. Normal Echocardiography Study. | | | |
| SIGNATURE  Done by: Tesfaye T., Pediatric Cardiologist _______________ 27/02/2012Eth.C | | | |

| Patient Name**: Azagne Tewachew**. Patient ID:_FHRH_. SEX/ Age: F/ 7 6/12Years Date of Report: 27**/02/2012_**.  BP: ___ Weight: ___ Height:___ BSA: ___. Referral Diagnosis: **ARF + CHF. AGH03.1899.** | | | | | | |
| --- | --- | --- | --- | --- | --- | --- |
| **Features** | **Finding** | | **Features** | | **Finding** | |
| **Profile** |  | | **Atria** | |  | |
| Abdominal situs | Solitus | | Left atrium | | Dilated | |
| Cardiac position | Levocardia | | Right atrium | | Normal | |
| Systemic venous drainage | Normal | | **Atrioventricular valves** | |  | |
| Pulmonary venous drainage | Normal | | Mitral valve | | Annulus = 28mm. Thickened, clubbed MV Leaflet. MVA = 4.75m2. | |
| Atrioventricular connection | Concordant | | Tricuspid valve | | Annulus = 22mm  TAPSE = 22mm | |
| Ventriculoarterial connection | Concordant | | **Ventricles** | |  | |
| Ventricular loop | d-Loop | | Left ventricle | | Dilated | |
|  |  | | Right ventricle | | Normal | |
| **Septae** |  | | **Coronary arteries** | | ----- | |
| Interventricular septum | Intact | | **Doppler Measurement** | |  | |
| Interatrial septum | Intact | | Mitral | | Severe MR with velocity of 3.9m/sec. posteriorly projected & Holosystolic | |
| **Semilunal valves** |  | | Aortic | | Moderate AR, PHT = 387ms. | |
| Aortic valve | Annulus = 17mm | | Tricuspid | | Mild TR, PPG = 25mmHg | |
| Pulmonary valve | Annulus = 18mm | | pulmonic | | ---- | |
| **Great arteries** | NRGA | | **Aortic arch** | | Left | |
| Aorta | ---- | | **PDA** | | No | |
| Pulmonary artery | Normal MPA and confluent Branch PAs. | |  | |  | |
| **M-Mode: Normal LV Function (eye balling)** | | | | | | |
| AO | | mm | | PWd | | 6mm |
| LA | | mm | | EDV | | 142ml |
| LVIDd | | 54mm | | ESV | | 43ml |
| LVIDs | | 33mm | | LVEF | | 69% |
| IVSd | | 7mm | | FS | | 39% |
| **Additional Information**: | | No evidence for vegetation so far. | | | | |
| No pleural/pericardial effusion | | | | | | |
| **Final Diagnosis:** | | | | | | |
| 1. {S, D, S} Levocardia 2. LA/LV Dilated 3. Thickened, clubbed MV leaflet 4. Severe MR 5. Moderate AR 6. Mild TR 7. Good Biventricular Function | | | | | | |
| SIGNATURE  Done by: Tesfaye T., Pediatric Cardiologist _______________ 27/02/2012Eth.C | | | | | | |

| Patient Name**: Amen Semahegn**. Patient ID:_ADINAS_. SEX/ Age: F/ 1 10/12Years Date of Report: 28**/02/2012_**.  BP: ___ Weight: ___ Height:___ BSA: ___. Referral Diagnosis: **_Incidental Murmur. AGH03.1900.** | | | |
| --- | --- | --- | --- |
| **Features** | **Finding** | **Features** | **Finding** |
| **Profile** |  | **Atria** |  |
| Abdominal situs | Solitus | Left atrium | Dilated |
| Cardiac position | Levocardia | Right atrium | Normal |
| Systemic venous drainage | Normal | **Atrioventricular valves** |  |
| Pulmonary venous drainage | Normal | Mitral valve | Annulus = 15mm |
| Atrioventricular connection | Concordant | Tricuspid valve | Annulus = 16mm  TAPSE = 16mm |
| Ventriculoarterial connection | Concordant | **Ventricles** |  |
| Ventricular loop | d-Loop | Left ventricle | Dilated |
|  |  | Right ventricle | Normal |
| **Septae** |  | **Coronary arteries** | ----- |
| Interventricular septum | Intact | **Doppler Measurement** |  |
| Interatrial septum | Intact | Mitral | ---- |
| **Semilunal valves** |  | Aortic | ---- |
| Aortic valve | Annulus = 13mm | Tricuspid | ---- |
| Pulmonary valve | Annulus = 13mm | pulmonic | ---- |
| **Great arteries** | NRGA | **Aortic arch** | Left |
| Aorta | ---- | **PDA** | 2.5mm PDA, L – R Shunt |
| Pulmonary artery | Normal MPA and confluent Branch PAs. |  |  |
| **M-Mode: Normal LV Function (eye balling)** | | | |
| AO | mm | PWd | 4.5mm |
| LA | mm | EDV | 51ml |
| LVIDd | 35mm | ESV | 17ml |
| LVIDs | 22.5mm | LVEF | 66% |
| IVSd | 5.5mm | FS | 35% |
| **Additional Information**: |  | | |
| No pleural/pericardial effusion | | | |
| **Final Diagnosis:** | | | |
| 1. {S, D, S} Levocardia 2. Moderate PDA, L – R Shunt 3. No PAH 4. Good Biventricular Function | | | |
| SIGNATURE  Done by: Tesfaye T., Pediatric Cardiologist _______________ 28/02/2012Eth.C | | | |

| Patient Name**: Shiferaw Abebe**. Patient ID:_ADINAS_. SEX/ Age: M/ 8Years Date of Report: 29**/02/2012_**.  BP: ___ Weight: ___ Height:___ BSA: ___. Referral Diagnosis: **CHF + Rheumatic Recurrence + DOE. AGH03.1901.** | | | | | |
| --- | --- | --- | --- | --- | --- |
| **Features** | **Finding** | **Features** | | **Finding** | |
| **Profile** |  | **Atria** | |  | |
| Abdominal situs | Solitus | Left atrium | | Dilated, 57 X 60mm | |
| Cardiac position | Levocardia | Right atrium | | Dilated | |
| Systemic venous drainage | Normal | **Atrioventricular valves** | |  | |
| Pulmonary venous drainage | Normal | Mitral valve | | Annulus = 21mm, Thickened MV Leaflet. MVA = 1.4cm2. | |
| Atrioventricular connection | Concordant | Tricuspid valve | | Annulus = 21mm  TAPSE = 20mm | |
| Ventriculoarterial connection | Concordant | **Ventricles** | |  | |
| Ventricular loop | d-Loop | Left ventricle | | Dilated | |
|  |  | Right ventricle | | Dilated | |
| **Septae** |  | **Coronary arteries** | | ----- | |
| Interventricular septum | Intact | **Doppler Measurement** | |  | |
| Interatrial septum | Intact | Mitral | | Severe MR with velocity of 4.9m/sec, posteriorly projected, Holosystolic. Moderate MS, PPG/MPG = 13/7mmHg | |
| **Semilunal valves** |  | Aortic | | Moderate AR, PHT = 230ms. | |
| Aortic valve | Annulus = 17mm | Tricuspid | | Moderate to severe TR, PPG = 82mmHg | |
| Pulmonary valve | Annulus = 20mm | pulmonic | | Mild PR, PPG = 40mmHg | |
| **Great arteries** | NRGA | **Aortic arch** | | Left | |
| Aorta | ---- | **PDA** | | No | |
| Pulmonary artery | Normal MPA and Branch PAs. |  | |  | |
| **M-Mode: Normal LV Function (eye balling)** | | | | | |
| AO | mm | | PWd | | 6mm |
| LA | mm | | EDV | | 120ml |
| LVIDd | 50mm | | ESV | | 40ml |
| LVIDs | 32mm | | LVEF | | 67% |
| IVSd | 7.9mm | | FS | | 37% |
| **Additional Information**: |  | | | | |
| No pleural/pericardial effusion | | | | | |
| **Final Diagnosis:** | | | | | |
| 1. {S, D, S} Levocardia 2. LA/LV Dilated 3. Severe MR 4. Moderate MS 5. Moderate AR 6. Severe Pulmonary Hypertension 7. Good Biventricular Function | | | | | |
| SIGNATURE  Done by: Tesfaye T., Pediatric Cardiologist _______________ 29/02/2012Eth.C | | | | | |

| Patient Name**: Zelalem Wasihun**. Patient ID:_Chagni H._. SEX/ Age: M/ 12Years Date of Report: 29**/02/2012_**.  BP: ___ Weight: ___ Height:___ BSA: ___. Referral Diagnosis: **Incidental Murmur. AGH03.1902.** | | | |
| --- | --- | --- | --- |
| **Features** | **Finding** | **Features** | **Finding** |
| **Profile** |  | **Atria** |  |
| Abdominal situs | Solitus | Left atrium | Normal |
| Cardiac position | Levocardia | Right atrium | Normal |
| Systemic venous drainage | Normal | **Atrioventricular valves** |  |
| Pulmonary venous drainage | Normal | Mitral valve | Annulus = 25mm |
| Atrioventricular connection | Concordant | Tricuspid valve | Annulus = mm  TAPSE = 26mm |
| Ventriculoarterial connection | Concordant | **Ventricles** |  |
| Ventricular loop | d-Loop | Left ventricle | Normal |
|  |  | Right ventricle | Normal |
| **Septae** |  | **Coronary arteries** | ----- |
| Interventricular septum | 3mm PM VSD. | **Doppler Measurement** |  |
| Interatrial septum | Intact | Mitral |  |
| **Semilunal valves** |  | Aortic | ---- |
| Aortic valve | Annulus = 19mm | Tricuspid | Trivial TR |
| Pulmonary valve | Annulus = 22mm | pulmonic |  |
| **Great arteries** | NRGA | **Aortic arch** | Left |
| Aorta | ---- | **PDA** | No |
| Pulmonary artery | MPA = 20mm. Normal confluent Branch PAs. |  |  |
| **M-Mode: Normal LV Function (eye balling)** | | | |
| AO | mm | PWd | 5.5mm |
| LA | mm | EDV | 90ml |
| LVIDd | 44.5mm | ESV | 28ml |
| LVIDs | 27.5mm | LVEF | 68% |
| IVSd | 5.5mm | FS | 38% |
| **Additional Information**: |  | | |
| No pleural/pericardial effusion | | | |
| **Final Diagnosis:** | | | |
| 1. {S, D, S} Levocardia 2. Small Perimembranous VSD, L – R Shunt 3. Good Biventricular Function | | | |
| SIGNATURE  Done by: Tesfaye T., Pediatric Cardiologist _______________ 29/02/2012Eth.C | | | |

| Patient Name**: Ananya Tessera**. Patient ID:_ADINAS H. SEX/ Age: F/ 4 2/12Years Date of Report: 30**/02/2012_**.  BP: ___ Weight: ___ Height:___ BSA: ___. Referral Diagnosis: **FTT. AGH03.1903.** | | | |
| --- | --- | --- | --- |
| **Features** | **Finding** | **Features** | **Finding** |
| **Profile** |  | **Atria** |  |
| Abdominal situs | Solitus | Left atrium | Normal |
| Cardiac position | Levocardia | Right atrium | Normal |
| Systemic venous drainage | Normal | **Atrioventricular valves** |  |
| Pulmonary venous drainage | Normal | Mitral valve | Annulus = 15mm |
| Atrioventricular connection | Concordant | Tricuspid valve | Annulus = 18mm  TAPSE = 17mm |
| Ventriculoarterial connection | Concordant | **Ventricles** |  |
| Ventricular loop | d-Loop | Left ventricle | Normal |
|  |  | Right ventricle | Normal |
| **Septae** |  | **Coronary arteries** | ----- |
| Interventricular septum | Intact | **Doppler Measurement** |  |
| Interatrial septum | Intact | Mitral | ---- |
| **Semilunal valves** |  | Aortic | ---- |
| Aortic valve | Annulus = 15mm | Tricuspid | ---- |
| Pulmonary valve | Annulus = 18mm | pulmonic | ---- |
| **Great arteries** | NRGA | **Aortic arch** | Left |
| Aorta | ---- | **PDA** | No |
| Pulmonary artery | Normal MPA and confluent Branch PAs. |  | No coarctation of aorta |
| **M-Mode: Normal LV Function (eye balling)** | | | |
| AO | mm | PWd | 7mm |
| LA | mm | EDV | 19ml |
| LVIDd | 23mm | ESV | 6ml |
| LVIDs | 15mm | LVEF | 65% |
| IVSd | 6mm | FS | 34% |
| **Additional Information**: |  | | |
| No pleural/pericardial effusion | | | |
| **Final Diagnosis:** | | | |
| 1. Normal Echocardiography Study | | | |
| SIGNATURE  Done by: Tesfaye T., Pediatric Cardiologist _______________ 30/02/2012Eth.C | | | |

| Patient Name**: Adanech Alem**. Patient ID:_FHRH. SEX/ Age: F/ 3Years Date of Report: 01**/03/2012_**.  BP: ___ Weight: ___ Height:___ BSA: ___. Referral Diagnosis: **Cyanosis. AGH03.1904.** | | | |
| --- | --- | --- | --- |
| **Features** | **Finding** | **Features** | **Finding** |
| **Profile** |  | **Atria** |  |
| Abdominal situs | Solitus | Left atrium | Normal |
| Cardiac position | Levocardia | Right atrium | Normal |
| Systemic venous drainage | Normal | **Atrioventricular valves** |  |
| Pulmonary venous drainage | Normal | Mitral valve | ---- |
| Atrioventricular connection | Concordant | Tricuspid valve | ---- |
| Ventriculoarterial connection | Concordant | **Ventricles** |  |
| Ventricular loop | d-Loop | Left ventricle | Normal |
|  |  | Right ventricle | Hypertrophied |
| **Septae** |  | **Coronary arteries** | ----- |
| Interventricular septum | Large Malaligned Sub aortic VSD, R – L Shunt | **Doppler Measurement** |  |
| Interatrial septum | Intact | Mitral | ---- |
| **Semilunal valves** |  | Aortic | ---- |
| Aortic valve | Annulus = 13mm | Tricuspid | ---- |
| Pulmonary valve | Annulus = 14mm | pulmonic | Mild PR. Valvar PS, PPG = 55mmHg |
| **Great arteries** | NRGA | **Aortic arch** |  |
| Aorta | Overriding Aorta | **PDA** | No |
| Pulmonary artery | Normal MPA and confluent Branch PAs. |  |  |
| **M-Mode: Normal LV Function (eye balling)** | | | |
| AO | mm | PWd | mm |
| LA | mm | EDV | ml |
| LVIDd | mm | ESV | ml |
| LVIDs | mm | LVEF | % |
| IVSd | mm | FS | % |
| **Additional Information**: |  | | |
| No pleural/pericardial effusion | | | |
| **Final Diagnosis:** | | | |
| 1. {S, D, S} Levocardia 2. TOF | | | |
| SIGNATURE  Done by: Tesfaye T., Pediatric Cardiologist _______________ 01/03/2012Eth.C | | | |

| Patient Name: **Natan Simachew**. Patient ID:_FHRH. SEX/ Age: M/ 7Years Date of Report: 01**/03/2012_**.  BP: ___ Weight: ___ Height:___ BSA: ___. Referral Diagnosis: **ARF. AGH03.1905.** | | | |
| --- | --- | --- | --- |
| **Features** | **Finding** | **Features** | **Finding** |
| **Profile** |  | **Atria** |  |
| Abdominal situs | Solitus | Left atrium | Normal |
| Cardiac position | Levocardia | Right atrium | Normal |
| Systemic venous drainage | Normal | **Atrioventricular valves** |  |
| Pulmonary venous drainage | Normal | Mitral valve | Annulus = 20mm. Mildly Thickened Mitral Valve |
| Atrioventricular connection | Concordant | Tricuspid valve | Annulus = 20  TAPSE = 22mm |
| Ventriculoarterial connection | Concordant | **Ventricles** |  |
| Ventricular loop | d-Loop | Left ventricle | Normal |
|  |  | Right ventricle | Normal |
| **Septae** |  | **Coronary arteries** | ----- |
| Interventricular septum | Intact | **Doppler Measurement** |  |
| Interatrial septum | Intact | Mitral | Mild MR, Projected posteriorly |
| **Semilunal valves** |  | Aortic | ---- |
| Aortic valve | Annulus = 17mm | Tricuspid | ---- |
| Pulmonary valve | Annulus = 17mm | pulmonic |  |
| **Great arteries** | NRGA | **Aortic arch** |  |
| Aorta |  | **PDA** | No |
| Pulmonary artery | Normal MPA and confluent Branch PAs. |  |  |
| **M-Mode: Normal LV Function (eye balling)** | | | |
| AO | mm | PWd | 5mm |
| LA | mm | EDV | 80ml |
| LVIDd | 42mm | ESV | 32ml |
| LVIDs | 29mm | LVEF | 60% |
| IVSd | 5mm | FS | 32% |
| **Additional Information**: |  | | |
| No pleural/pericardial effusion | | | |
| **Final Diagnosis:** | | | |
| 1. {S, D, S} Levocardia 2. Mildly thickened Mitral valve 3. Mild MR 4. Good Biventricular Function | | | |
| SIGNATURE  Done by: Tesfaye T., Pediatric Cardiologist _______________ 01/03/2012Eth.C | | | |

| Patient Name: **Yared Mequanint**. Patient ID:_FHRH. SEX/ Age: M/ 12Years Date of Report: 01**/03/2012_**.  BP: ___ Weight: ___ Height:___ BSA: ___. Referral Diagnosis: **Easy Fatigability. AGH03.1906.** | | | |
| --- | --- | --- | --- |
| **Features** | **Finding** | **Features** | **Finding** |
| **Profile** |  | **Atria** |  |
| Abdominal situs | Solitus | Left atrium | Normal |
| Cardiac position | Levocardia | Right atrium | Normal |
| Systemic venous drainage | Normal | **Atrioventricular valves** |  |
| Pulmonary venous drainage | Normal | Mitral valve | Annulus = 21mm |
| Atrioventricular connection | Concordant | Tricuspid valve | Annulus = 22mm  TAPSE = 23mm |
| Ventriculoarterial connection | Concordant | **Ventricles** |  |
| Ventricular loop | d-Loop | Left ventricle | Normal |
|  |  | Right ventricle | Normal |
| **Septae** |  | **Coronary arteries** | ----- |
| Interventricular septum | Intact | **Doppler Measurement** |  |
| Interatrial septum | Intact | Mitral | ---- |
| **Semilunal valves** |  | Aortic | ---- |
| Aortic valve | Annulus = 18mm | Tricuspid | Trivial TR, PPG = 28mmHg |
| Pulmonary valve | Annulus = 20mm | pulmonic |  |
| **Great arteries** | NRGA | **Aortic arch** |  |
| Aorta |  | **PDA** | No |
| Pulmonary artery | Normal MPA and confluent Branch PAs. |  |  |
| **M-Mode:** | | | |
| AO | mm | PWd | 10mm |
| LA | mm | EDV | 48ml |
| LVIDd | 34mm | ESV | 15ml |
| LVIDs | 21mm | LVEF | 68% |
| IVSd | 9mm | FS | 37% |
| **Additional Information**: |  | | |
| No pleural/pericardial effusion | | | |
| **Final Diagnosis:** | | | |
| 1. Normal Echocardiography Study. | | | |
| SIGNATURE  Done by: Tesfaye T., Pediatric Cardiologist _______________ 01/03/2012Eth.C | | | |

| Patient Name: **Bezawit Takele**. Patient ID:_FHRH. SEX/ Age: F/ 14Years Date of Report: 02**/03/2012_**.  BP: ___ Weight: ___ Height:___ BSA: ___. Referral Diagnosis: **Easy Fatigability. AGH03.1907.** | | | |
| --- | --- | --- | --- |
| **Features** | **Finding** | **Features** | **Finding** |
| **Profile** |  | **Atria** |  |
| Abdominal situs | Solitus | Left atrium | Normal |
| Cardiac position | Levocardia | Right atrium | Normal |
| Systemic venous drainage | Normal | **Atrioventricular valves** |  |
| Pulmonary venous drainage | Normal | Mitral valve | Annulus = 22mm |
| Atrioventricular connection | Concordant | Tricuspid valve | Annulus = 23mm  TAPSE = 26mm |
| Ventriculoarterial connection | Concordant | **Ventricles** |  |
| Ventricular loop | d-Loop | Left ventricle | Normal |
|  |  | Right ventricle | Normal |
| **Septae** |  | **Coronary arteries** | ----- |
| Interventricular septum | Intact | **Doppler Measurement** |  |
| Interatrial septum | Intact | Mitral | ---- |
| **Semilunal valves** |  | Aortic | ---- |
| Aortic valve | Annulus = 20mm | Tricuspid | ---- |
| Pulmonary valve | Annulus = 22mm | pulmonic | ---- |
| **Great arteries** | NRGA | **Aortic arch** | Left |
| Aorta | ---- | **PDA** | No |
| Pulmonary artery | Normal MPA and confluent Branch PAs. |  |  |
| **M-Mode: Normal LV Function (eye balling)** | | | |
| AO | mm | PWd | 8mm |
| LA | mm | EDV | 58ml |
| LVIDd | 37mm | ESV | 23ml |
| LVIDs | 25mm | LVEF | 61% |
| IVSd | 7mm | FS | 32% |
| **Additional Information**: |  | | |
| No pleural/pericardial effusion | | | |
| **Final Diagnosis:** | | | |
| 1. Normal Echocardiography Study. | | | |
| SIGNATURE  Done by: Tesfaye T., Pediatric Cardiologist _______________ 02/03/2012Eth.C | | | |

| Patient Name: **Asres Birhanu**. Patient ID:_FHRH. SEX/ Age: F/ 14Years Date of Report: 03**/03/2012_**.  BP: ___ Weight: ___ Height:___ BSA: ___. Referral Diagnosis: **Cor Pulmonale + CHF. AGH03.1908.** | | | | |
| --- | --- | --- | --- | --- |
| **Features** | **Finding** | **Features** | | **Finding** |
| **Profile** |  | **Atria** | |  |
| Abdominal situs | Solitus | Left atrium | | Normal |
| Cardiac position | Levocardia | Right atrium | | Dilated |
| Systemic venous drainage | Normal | **Atrioventricular valves** | |  |
| Pulmonary venous drainage | Normal | Mitral valve | | Annulus = 17mm |
| Atrioventricular connection | Concordant | Tricuspid valve | | Annulus = 18mm.  TAPSE = 9mm. |
| Ventriculoarterial connection | Concordant | **Ventricles** | |  |
| Ventricular loop | d-Loop | Left ventricle | | Normal |
|  |  | Right ventricle | | Dilated |
| **Septae** |  | **Coronary arteries** | | ----- |
| Interventricular septum | Intact | **Doppler Measurement** | |  |
| Interatrial septum | Intact | Mitral | | ---- |
| **Semilunal valves** |  | Aortic | | ---- |
| Aortic valve | Annulus = 18mm | Tricuspid | | Moderate TR, PPG =51mmHg(Underestimated) |
| Pulmonary valve | Annulus = 19mm | pulmonic | | Mild PR |
| **Great arteries** | NRGA | **Aortic arch** | |  |
| Aorta | ---- | **PDA** | | No |
| Pulmonary artery | MPA = 27mm. Good sized confluent Branch PAs. |  | |  |
| **M-Mode: Normal LV Function (eye balling)** | | | | |
| AO | mm | PWd | 6.4mm | |
| LA | mm | EDV | 19ml | |
| LVIDd | 23.6mm | ESV | 5ml | |
| LVIDs | 14mm | LVEF | 74% | |
| IVSd | 6mm | FS | 40% | |
| **Additional Information**: | No pleural/pericardial effusion | | | |
| **Final Diagnosis:** | | | | |
| 1. {S, D, S} Levocardia 2. RA/RV Dilated 3. Moderate TR 4. Mild PR 5. Dysfunctional RV (RV Failure) 6. Severe Pulmonary Hypertension   **N.B: Search for Pulmonary cause for the Pulmonary Hypertension and treat accordingly in addition to the RV Failure Rx.** | | | | |
| SIGNATURE  Done by: Tesfaye T., Pediatric Cardiologist _______________ 03/03/2012Eth.C | | | | |

| Patient Name: **Yoseph Tadesse**. Patient ID:_ADINAS. SEX/ Age: M/ 12Years Date of Report: 03**/03/2012_**.  BP: ___ Weight: ___ Height:___ BSA: ___. Referral Diagnosis: **Syncope. AGH03.1909.** | | | |
| --- | --- | --- | --- |
| **Features** | **Finding** | **Features** | **Finding** |
| **Profile** |  | **Atria** |  |
| Abdominal situs | Solitus | Left atrium | Normal |
| Cardiac position | Levocardia | Right atrium | Normal |
| Systemic venous drainage | Normal | **Atrioventricular valves** |  |
| Pulmonary venous drainage | Normal | Mitral valve | Annulus = 22mm |
| Atrioventricular connection | Concordant | Tricuspid valve | Annulus = 23mm |
| Ventriculoarterial connection | Concordant | **Ventricles** |  |
| Ventricular loop | d-Loop | Left ventricle | Normal |
|  |  | Right ventricle | Normal |
| **Septae** |  | **Coronary arteries** | ----- |
| Interventricular septum | Intact | **Doppler Measurement** |  |
| Interatrial septum | Intact | Mitral | ---- |
| **Semilunal valves** |  | Aortic | ---- |
| Aortic valve | Annulus = 16mm | Tricuspid | ---- |
| Pulmonary valve | Annulus = 19mm | pulmonic | ---- |
| **Great arteries** | NRGA | **Aortic arch** |  |
| Aorta | ---- | **PDA** | No |
| Pulmonary artery | Normal MPA and confluent Branch PAs. | **Arch** | No coarctation |
| **M-Mode:** | | | |
| AO | mm | PWd | 8mm |
| LA | mm | EDV | 59ml |
| LVIDd | 37mm | ESV | 18ml |
| LVIDs | 23mm | LVEF | 69% |
| IVSd | 6.4mm | FS | 38% |
| **Additional Information**: |  | | |
| No pleural/pericardial effusion | | | |
| **Final Diagnosis:** | | | |
| 1. Normal Echocardiography Study. | | | |
| SIGNATURE  Done by: Tesfaye T., Pediatric Cardiologist _______________ 03/03/2012Eth.C | | | |

| Patient Name: **Agerie Birhanu**. Patient ID:_FHRH. SEX/ Age: F/ 14Years Date of Report: 04**/03/2012_**.  BP: ___ Weight: ___ Height:___ BSA: ___. Referral Diagnosis: **CHF + Rheumatic Recurrence + DOE. AGH03.1910.** | | | | | |
| --- | --- | --- | --- | --- | --- |
| **Features** | **Finding** | **Features** | | **Finding** | |
| **Profile** |  | **Atria** | |  | |
| Abdominal situs | Solitus | Left atrium | | Dilated, 46 X 39mm | |
| Cardiac position | Levocardia | Right atrium | | Dilated | |
| Systemic venous drainage | Normal | **Atrioventricular valves** | |  | |
| Pulmonary venous drainage | Normal | Mitral valve | | Annulus = 26mm. MVA = 0.7cm2. Clubbed, deformed, calcified MV leaflet. | |
| Atrioventricular connection | Concordant | Tricuspid valve | | Annulus = 22mm  TAPSE = 17mm | |
| Ventriculoarterial connection | Concordant | **Ventricles** | |  | |
| Ventricular loop | d-Loop | Left ventricle | | Dilated | |
|  |  | Right ventricle | | Dilated | |
| **Septae** |  | **Coronary arteries** | | ----- | |
| Interventricular septum | Intact | **Doppler Measurement** | |  | |
| Interatrial septum | Intact | Mitral | | Moderate MR, Posteriorly projected with velocity of 4m/sec, Holosystolic. Severe MS with PPG/MPG = 38/25mmHg | |
| **Semilunal valves** |  | Aortic | | Moderate AR, PHT = 351ms. | |
| Aortic valve | Annulus = 14mm | Tricuspid | | Severe TR, PPG = 114mmHg. | |
| Pulmonary valve | Annulus = 23mm | pulmonic | | ---- | |
| **Great arteries** | NRGA | **Aortic arch** | |  | |
| Aorta |  | **PDA** | | No | |
| Pulmonary artery | Normal MPA and confluent Branch PAs. |  | |  | |
| **M-Mode:** | | | | | |
| AO | mm | | PWd | | 7.3mm |
| LA | mm | | EDV | | 52ml |
| LVIDd | 35mm | | ESV | | 23ml |
| LVIDs | 25.5mm | | LVEF | | 55% |
| IVSd | 8.5mm | | FS | | 28% |
| **Additional Information**: |  | | | | |
| No pleural/pericardial effusion | | | | | |
| **Final Diagnosis:** | | | | | |
| 1. {S, D, S} Levocardia 2. All chambers dilated 3. Thickened, clubbed, calcified MV. 4. Moderate MR 5. Severe MS 6. Severe TR 7. Moderate AR 8. Severe Pulmonary Hypertension | | | | | |
| SIGNATURE  Done by: Tesfaye T., Pediatric Cardiologist _______________ 04/03/2012Eth.C | | | | | |

| Patient Name: **Emawayish Tiget**. Patient ID:_FHRH. SEX/ Age: F/ 11Years Date of Report: 04**/03/2012_**.  BP: ___ Weight: ___ Height:___ BSA: ___. Referral Diagnosis: **CHF + DOE. AGH03.1911.** | | | |
| --- | --- | --- | --- |
| **Features** | **Finding** | **Features** | **Finding** |
| **Profile** |  | **Atria** |  |
| Abdominal situs | Solitus | Left atrium | Normal |
| Cardiac position | Levocardia | Right atrium | Normal |
| Systemic venous drainage | Normal | **Atrioventricular valves** |  |
| Pulmonary venous drainage | Normal | Mitral valve | Annulus = 22mm. |
| Atrioventricular connection | Concordant | Tricuspid valve | Annulus = 22mm.  TAPSE = 13mm. |
| Ventriculoarterial connection | Concordant | **Ventricles** |  |
| Ventricular loop | d-Loop | Left ventricle | Dilated |
|  |  | Right ventricle | Dilated |
| **Septae** |  | **Coronary arteries** | ----- |
| Interventricular septum | Intact | **Doppler Measurement** |  |
| Interatrial septum | Intact | Mitral | Mild MR, velocity of 2m/sec |
| **Semilunal valves** |  | Aortic | ---- |
| Aortic valve | Annulus = 17mm. Trileaflet | Tricuspid | Trivial TR, PPG = 10mmHg. |
| Pulmonary valve | Annulus = 21mm | pulmonic | ---- |
| **Great arteries** | NRGA | **Aortic arch** | Left |
| Aorta | ---- | **PDA** | No |
| Pulmonary artery | Normal MPA and confluent Branch PAs. |  | No Coarctation of aorta |
| **M-Mode:** | | | |
| AO | mm | PWd | 7.3mm |
| LA | mm | EDV | 68ml |
| LVIDd | 39.5mm | ESV | 42ml |
| LVIDs | 32mm | LVEF | 39% |
| IVSd | 6.7mm | FS | 18% |
| **Additional Information**: |  | | |
| No pleural/pericardial effusion | | | |
| **Final Diagnosis:** | | | |
| 1. {S, D, S} Levocardia 2. Mild MR 3. Mildly Dilated Dysfunctional LV | | | |
| SIGNATURE  Done by: Tesfaye T., Pediatric Cardiologist _______________ 04/03/2012Eth.C | | | |

| Patient Name: **Melaku Tesfaye**. Patient ID:_FHRH. SEX/ Age: M/ 12Years Date of Report: 04**/03/2012_**.  BP: ___ Weight: ___ Height:___ BSA: ___. Referral Diagnosis: **Innocent Murmur. AGH03.1912.** | | | |
| --- | --- | --- | --- |
| **Features** | **Finding** | **Features** | **Finding** |
| **Profile** |  | **Atria** |  |
| Abdominal situs | Solitus | Left atrium | Normal |
| Cardiac position | Levocardia | Right atrium | Normal |
| Systemic venous drainage | Normal | **Atrioventricular valves** |  |
| Pulmonary venous drainage | Normal | Mitral valve | Annulus = 20mm. |
| Atrioventricular connection | Concordant | Tricuspid valve | Annulus = 23mm.  TAPSE = 24mm. |
| Ventriculoarterial connection | Concordant | **Ventricles** |  |
| Ventricular loop | d-Loop | Left ventricle | Normal |
|  |  | Right ventricle | Normal |
| **Septae** |  | **Coronary arteries** | ----- |
| Interventricular septum | Intact | **Doppler Measurement** |  |
| Interatrial septum | Intact | Mitral | ---- |
| **Semilunal valves** |  | Aortic | ---- |
| Aortic valve | Annulus = 18mm | Tricuspid | ---- |
| Pulmonary valve | Annulus = 21mm | pulmonic | Flow acceleration across MPA and Branch PAs with gradient of 6mmHg on both. |
| **Great arteries** | NRGA | **Aortic arch** | Left |
| Aorta |  | **PDA** | No |
| Pulmonary artery | MPA = 17mm. RPA = 9mm. LPA = 11mm. | Aortic Isthmus | No coarctation |
| **M-Mode: Normal LV Function (eye balling)** | | | |
| AO | mm | PWd | 6.4mm |
| LA | mm | EDV | 77.6ml |
| LVIDd | 42mm | ESV | 30ml |
| LVIDs | 28mm | LVEF | 61% |
| IVSd | 7.7mm | FS | 32% |
| **Additional Information**: |  | | |
| No pleural/pericardial effusion | | | |
| **Final Diagnosis:** | | | |
| 1. {S, D, S} Levocardia 2. Flow acceleration across the MPA, LPA and RPA without significant gradient. 3. Normal Echocardiography Study.   **N.B: Needs follow up for the flow acceleration across MPA and Branch PAs.** | | | |
| SIGNATURE  Done by: Tesfaye T., Pediatric Cardiologist _______________ 04/03/2012Eth.C | | | |

| Patient Name: **Samuel Getinet**. Patient ID:_**TGSH**. SEX/ Age: M/ 1 2/12Years Date of Report: 05**/03/2012_**.  BP: ___ Weight: ___ Height:___ BSA: ___. Referral Diagnosis: **Incidental Murmur. AGH03.1913.** | | | |
| --- | --- | --- | --- |
| **Features** | **Finding** | **Features** | **Finding** |
| **Profile** |  | **Atria** |  |
| Abdominal situs | Solitus | Left atrium | Normal |
| Cardiac position | Levocardia | Right atrium | Normal |
| Systemic venous drainage | Normal | **Atrioventricular valves** |  |
| Pulmonary venous drainage | Normal | Mitral valve | Annulus = 17mm |
| Atrioventricular connection | Concordant | Tricuspid valve | Annulus = 17mm.  TAPSE = 15mm. |
| Ventriculoarterial connection | Concordant | **Ventricles** |  |
| Ventricular loop | d-Loop | Left ventricle | Normal |
|  |  | Right ventricle | Normal |
| **Septae** |  | **Coronary arteries** | ----- |
| Interventricular septum | Intact | **Doppler Measurement** |  |
| Interatrial septum | Intact | Mitral | ---- |
| **Semilunal valves** |  | Aortic | ---- |
| Aortic valve | Annulus = 12mm | Tricuspid | ---- |
| Pulmonary valve | Annulus = 9mm | pulmonic | Moderate PS, PPG/MPG = 40/24mmHg |
| **Great arteries** | NRGA | **Aortic arch** |  |
| Aorta | ---- | **PDA** | No |
| Pulmonary artery | Normal MPA and confluent Branch PAs. |  |  |
| **M-Mode: Normal LV Function (eye balling)** | | | |
| AO | mm | PWd | 4.2mm |
| LA | mm | EDV | 25.7ml |
| LVIDd | 26.5mm | ESV | 12ml |
| LVIDs | 19.5mm | LVEF | 54% |
| IVSd | 4.6mm | FS | 26% |
| **Additional Information**: | **ECHO DONE UNDER SEDATION.** | | |
| No pleural/pericardial effusion | | | |
| **Final Diagnosis:** | | | |
| 1. {S, D, S} Levocardia 2. Moderate PS. 3. Good Biventricular Function | | | |
| SIGNATURE  Done by: Tesfaye T., Pediatric Cardiologist _______________ 05/03/2012Eth.C | | | |

| Patient Name: **Tekeste Fekadu**. Patient ID:_FHRH. SEX/ Age: M/ 2monthss Date of Report: 05**/03/2012_**.  BP: ___ Weight: ___ Height:___ BSA: ___. Referral Diagnosis: **DS. AGH03.1914.** | | | |
| --- | --- | --- | --- |
| **Features** | **Finding** | **Features** | **Finding** |
| **Profile** |  | **Atria** |  |
| Abdominal situs | Solitus | Left atrium | Normal |
| Cardiac position | Levocardia | Right atrium | Normal |
| Systemic venous drainage | Normal | **Atrioventricular valves** |  |
| Pulmonary venous drainage | Normal | Mitral valve | Annulus = 10mm |
| Atrioventricular connection | Concordant | Tricuspid valve | Annulus = 11mm |
| Ventriculoarterial connection | Concordant | **Ventricles** |  |
| Ventricular loop | d-Loop | Left ventricle | Normal |
|  |  | Right ventricle | Normal |
| **Septae** |  | **Coronary arteries** | ----- |
| Interventricular septum | Intact | **Doppler Measurement** |  |
| Interatrial septum | Intact | Mitral | ---- |
| **Semilunal valves** |  | Aortic | ---- |
| Aortic valve | Annulus = 9mm | Tricuspid | ---- |
| Pulmonary valve | Annulus = 11mm | pulmonic |  |
| **Great arteries** | NRGA | **Aortic arch** |  |
| Aorta | ---- | **PDA** | No |
| Pulmonary artery | Normal MPA and confluent Branch PAs. |  |  |
| **M-Mode: Normal LV Function (eye balling)** | | | |
| AO | mm | PWd | mm |
| LA | mm | EDV | ml |
| LVIDd | mm | ESV | ml |
| LVIDs | mm | LVEF | % |
| IVSd | mm | FS | % |
| **Additional Information**: |  | | |
| No pleural/pericardial effusion | | | |
| **Final Diagnosis:** | | | |
| 1. {S, D, S} Levocardia 2. Normal Echocardiography Study. | | | |
| SIGNATURE  Done by: Tesfaye T., Pediatric Cardiologist _______________ 05/03/2012Eth.C | | | |

| Patient Name: **Nigistie Muhabaw**. Patient ID:_TGSH. SEX/ Age: F/ 11Years Date of Report: 05**/03/2012_**.  BP: ___ Weight: ___ Height:___ BSA: ___. Referral Diagnosis: **Incidental Murmur. AGH03.1915.** | | | |
| --- | --- | --- | --- |
| **Features** | **Finding** | **Features** | **Finding** |
| **Profile** |  | **Atria** |  |
| Abdominal situs | Solitus | Left atrium | Normal |
| Cardiac position | Levocardia | Right atrium | Normal |
| Systemic venous drainage | Normal | **Atrioventricular valves** |  |
| Pulmonary venous drainage | Normal | Mitral valve | Annulus = 18mm. |
| Atrioventricular connection | Concordant | Tricuspid valve | Annulus = 19mm  TAPSE = 18mm. |
| Ventriculoarterial connection | Concordant | **Ventricles** |  |
| Ventricular loop | d-Loop | Left ventricle | Normal |
|  |  | Right ventricle | Normal |
| **Septae** |  | **Coronary arteries** | ----- |
| Interventricular septum | Intact | **Doppler Measurement** |  |
| Interatrial septum | Intact | Mitral | Mild MR, Incomplete signal. Posterior projection, seen in two planes with jet velocity = 3.5m/sec |
| **Semilunal valves** |  | Aortic | ---- |
| Aortic valve | Annulus = 18mm | Tricuspid | ---- |
| Pulmonary valve | Annulus = 18mm | pulmonic |  |
| **Great arteries** | NRGA | **Aortic arch** |  |
| Aorta |  | **PDA** | No |
| Pulmonary artery | Normal MPA and confluent Branch PAs. |  |  |
| **M-Mode:** | | | |
| AO | mm | PWd | 6mm |
| LA | mm | EDV | 71ml |
| LVIDd | 40mm | ESV | 32ml |
| LVIDs | 29mm | LVEF | 55% |
| IVSd | 4.3mm | FS | 28% |
| **Additional Information**: |  | | |
| No pleural/pericardial effusion | | | |
| **Final Diagnosis:** | | | |
| 1. {S, D, S} Levocardia 2. Mild MR 3. Good Biventricular Function. | | | |
| Remark: Borderline RHD | | | |
| SIGNATURE  Done by: Tesfaye T., Pediatric Cardiologist _______________ 05/03/2012Eth.C | | | |

| Patient Name: **Enatnesh Werkie**. Patient ID:_FHRH. SEX/ Age: F/ 8Years Date of Report: 06**/03/2012_**.  BP: ___ Weight: ___ Height:___ BSA: ___. Referral Diagnosis: **Chest Pain. AGH03.1916.** | | | |
| --- | --- | --- | --- |
| **Features** | **Finding** | **Features** | **Finding** |
| **Profile** |  | **Atria** |  |
| Abdominal situs | Solitus | Left atrium | Normal |
| Cardiac position | Levocardia | Right atrium | Normal |
| Systemic venous drainage | Normal | **Atrioventricular valves** |  |
| Pulmonary venous drainage | Normal | Mitral valve | Annulus = 22 |
| Atrioventricular connection | Concordant | Tricuspid valve | Annulus = 22mm.  TAPSE = 21mm. |
| Ventriculoarterial connection | Concordant | **Ventricles** |  |
| Ventricular loop | d-Loop | Left ventricle | Normal |
|  |  | Right ventricle | Normal |
| **Septae** |  | **Coronary arteries** | ----- |
| Interventricular septum | Intact | **Doppler Measurement** |  |
| Interatrial septum | Intact | Mitral | ---- |
| **Semilunal valves** |  | Aortic | ---- |
| Aortic valve | Annulus = mm | Tricuspid | ---- |
| Pulmonary valve | Annulus = 16mm | pulmonic | ---- |
| **Great arteries** | NRGA | **Aortic arch** |  |
| Aorta | ---- | **PDA** | No |
| Pulmonary artery | Normal MPA and confluent Branch PAs. | Arch | Left arch |
| **M-Mode: Normal LV Function (eye balling)** | | | |
| AO | mm | PWd | 6.4mm |
| LA | mm | EDV | 59ml |
| LVIDd | 37mm | ESV | 18ml |
| LVIDs | 23mm | LVEF | 69% |
| IVSd | 7.7mm | FS | 38% |
| **Additional Information**: |  | | |
| No pleural/pericardial effusion | | | |
| **Final Diagnosis:** | | | |
| 1. Normal Echocardiography Study. | | | |
| SIGNATURE  Done by: Tesfaye T., Pediatric Cardiologist _______________ 06/03/2012Eth.C | | | |

| Patient Name: **Werkinesh Challie**. Patient ID:_FHRH. SEX/ Age: F/ 5Years Date of Report: 08**/03/2012_**.  BP: ___ Weight: ___ Height:___ BSA: ___. Referral Diagnosis: **DOE + CHF. AGH03.1917.** | | | |
| --- | --- | --- | --- |
| **Features** | **Finding** | **Features** | **Finding** |
| **Profile** |  | **Atria** |  |
| Abdominal situs | Solitus | Left atrium | Dilated |
| Cardiac position | Levocardia | Right atrium | Dilated |
| Systemic venous drainage | Impressive. | **Atrioventricular valves** |  |
| Pulmonary venous drainage | Normal | Mitral valve | Annulus = 26mm. |
| Atrioventricular connection | Concordant | Tricuspid valve | Annulus = 22mm.  TAPSE = 20mm |
| Ventriculoarterial connection | Concordant | **Ventricles** |  |
| Ventricular loop | d-Loop | Left ventricle | Dilated |
|  |  | Right ventricle | Dilated |
| **Septae** |  | **Coronary arteries** | ----- |
| Interventricular septum | 16mm mid muscular VSD, L – R Shunt. | **Doppler Measurement** |  |
| Interatrial septum | Intact | Mitral | Mild AR |
| **Semilunal valves** |  | Aortic | ---- |
| Aortic valve | Annulus = 12mm | Tricuspid | Mild TR |
| Pulmonary valve | Annulus = 16mm | pulmonic | Mild PS, PPG = 22mmHg. Moderate PR, PPG = 46mmHg. |
| **Great arteries** | NRGA | **Aortic arch** | Left |
| Aorta | ----- | **PDA** | No |
| Pulmonary artery | MPA = 20mm. Confluent Branch PAs. |  |  |
| **M-Mode:** | | | |
| AO | mm | PWd | 6mm |
| LA | mm | EDV | 47ml |
| LVIDd | 34mm | ESV | 22ml |
| LVIDs | 25mm | LVEF | 55% |
| IVSd | 5.4mm | FS | 28% |
| **Additional Information**: | No pleural/pericardial effusion | | |
| **Final Diagnosis:** | | | |
| 1. {S, D, S} Levocardia 2. LA/LV Dilated 3. Mild TR, Mild MR 4. Impressive pulmonary venous return 5. Large Muscular VSD, L – R Shunt 6. Moderate PR 7. Mild PS 8. Moderate Pulmonary Hypertension 9. Good Biventricular Function. | | | |
| SIGNATURE  Done by: Tesfaye T., Pediatric Cardiologist _______________ 08/03/2012Eth.C | | | |

| Patient Name: **Melkamnesh Adamtie**. Patient ID:_FHRH. SEX/ Age: F/ 7Years Date of Report: 11**/03/2012_**.  BP: ___ Weight: ___ Height:___ BSA: ___. Referral Diagnosis: **Sepsis (Galloping Infection). AGH03.1918.** | | | |
| --- | --- | --- | --- |
| **Features** | **Finding** | **Features** | **Finding** |
| **Profile** |  | **Atria** |  |
| Abdominal situs | Solitus | Left atrium | Normal |
| Cardiac position | Levocardia | Right atrium | Normal |
| Systemic venous drainage | Normal | **Atrioventricular valves** |  |
| Pulmonary venous drainage | Normal | Mitral valve | Annulus = 17mm |
| Atrioventricular connection | Concordant | Tricuspid valve | Annulus = 22mm  TAPSE = 23mm |
| Ventriculoarterial connection | Concordant | **Ventricles** |  |
| Ventricular loop | d-Loop | Left ventricle | Normal |
|  |  | Right ventricle | Normal |
| **Septae** |  | **Coronary arteries** | ----- |
| Interventricular septum | Intact | **Doppler Measurement** |  |
| Interatrial septum | Intact | Mitral | Trivial MR |
| **Semilunal valves** |  | Aortic | ---- |
| Aortic valve | Annulus = 19mm | Tricuspid | Trivial TR, PPG = 20mmHg |
| Pulmonary valve | Annulus = 20mm | pulmonic | ---- |
| **Great arteries** | NRGA | **Aortic arch** |  |
| Aorta | ---- | **PDA** | No |
| Pulmonary artery | Normal MPA and confluent Branch PAs. |  |  |
| **M-Mode: Normal LV Function (eye balling)** | | | |
| AO | mm | PWd | 5mm |
| LA | mm | EDV | 65ml |
| LVIDd | 39mm | ESV | 29ml |
| LVIDs | 28mm | LVEF | 55% |
| IVSd | 5.5mm | FS | 28% |
| **Additional Information**: | No Coarctation of Aorta | | |
| No pleural/pericardial effusion | | | |
| **Final Diagnosis:** | | | |
| 1. {S, D, S} Levocardia 2. Trivial MR 3. Good Biventricular Function | | | |
| SIGNATURE  Done by: Tesfaye T., Pediatric Cardiologist _______________ 11/03/2012Eth.C | | | |

| Patient Name: **Mita Belayneh** . Patient ID:_FHRH. SEX/ Age: F/ 41days Date of Report: 11**/03/2012_**.  BP: ___ Weight: ___ Height:___ BSA: ___. Referral Diagnosis: **RD. AGH03.1919.** | | | |
| --- | --- | --- | --- |
| **Features** | **Finding** | **Features** | **Finding** |
| **Profile** |  | **Atria** |  |
| Abdominal situs | Solitus | Left atrium | Normal |
| Cardiac position | Levocardia | Right atrium | Normal |
| Systemic venous drainage | Normal | **Atrioventricular valves** |  |
| Pulmonary venous drainage | Normal | Mitral valve | Annulus = 12mm |
| Atrioventricular connection | Concordant | Tricuspid valve | Annulus = 13mm.  TAPSE = 13mm |
| Ventriculoarterial connection | Concordant | **Ventricles** |  |
| Ventricular loop | d-Loop | Left ventricle | Normal |
|  |  | Right ventricle | Normal |
| **Septae** |  | **Coronary arteries** | ----- |
| Interventricular septum | Intact | **Doppler Measurement** |  |
| Interatrial septum | Intact | Mitral | ---- |
| **Semilunal valves** |  | Aortic | ---- |
| Aortic valve | Annulus = 9mm | Tricuspid | ---- |
| Pulmonary valve | Annulus = 11mm | pulmonic |  |
| **Great arteries** | NRGA | **Aortic arch** | left |
| Aorta | ---- | **PDA** | No |
| Pulmonary artery | Normal MPA and confluent Branch PAs. |  |  |
| **M-Mode: Normal LV Function (eye balling)** | | | |
| AO | mm | PWd | 5.5mm |
| LA | mm | EDV | 17ml |
| LVIDd | 22mm | ESV | 4.5ml |
| LVIDs | 13mm | LVEF | 73% |
| IVSd | 5.5mm | FS | 40% |
| **Additional Information**: | No coarctation | | |
| No pleural/pericardial effusion | | | |
| **Final Diagnosis:** | | | |
| 1. {S, D, S} Levocardia 2. Normal Echocardiography Study. | | | |
| SIGNATURE  Done by: Tesfaye T., Pediatric Cardiologist _______________ 11/03/2012Eth.C | | | |

| Patient Name: **Efrata Alemu**. Patient ID:_FHRH. SEX/ Age: F/ 5Years Date of Report: 11**/03/2012_**.  BP: ___ Weight: ___ Height:___ BSA: ___. Referral Diagnosis: **CHF. AGH03.1920.** | | | |
| --- | --- | --- | --- |
| **Features** | **Finding** | **Features** | **Finding** |
| **Profile** |  | **Atria** |  |
| Abdominal situs | Solitus | Left atrium | Dilated |
| Cardiac position | Levocardia | Right atrium | Dilated |
| Systemic venous drainage | Normal | **Atrioventricular valves** |  |
| Pulmonary venous drainage | Normal | Mitral valve | Annulus = 21mm |
| Atrioventricular connection | Concordant | Tricuspid valve | Annulus = 25mm. The septal leaflet is attached to the tip of the septal defect.  TAPSE = 17mm |
| Ventriculoarterial connection | Concordant | **Ventricles** |  |
| Ventricular loop | d-Loop | Left ventricle | Dilated |
|  |  | Right ventricle | Dilated |
| **Septae** |  | **Coronary arteries** | ----- |
| Interventricular septum | 19mm inlet VSD, L – R Shunt. | **Doppler Measurement** |  |
| Interatrial septum | Intact | Mitral | ---- |
| **Semilunal valves** |  | Aortic | ---- |
| Aortic valve | Annulus = 20mm | Tricuspid | Mild to moderate TR |
| Pulmonary valve | Annulus = 21mm | pulmonic | ---- |
| **Great arteries** | NRGA | **Aortic arch** |  |
| Aorta |  | **PDA** | 2mm PDA, L – R Shunt |
| Pulmonary artery | Normal MPA and confluent Branch PAs. |  |  |
| **M-Mode: Normal LV Function (eye balling)** | | | |
| AO | mm | PWd | 5mm |
| LA | mm | EDV | 82ml |
| LVIDd | 43mm | ESV | 40ml |
| LVIDs | 32mm | LVEF | 52% |
| IVSd | 6mm | FS | 26% |
| **Additional Information**: |  | | |
| No pleural/pericardial effusion | | | |
| **Final Diagnosis:** | | | |
| 1. {S, D, S} Levocardia 2. Mild to moderate TR 3. Large in let VSD, L – R Shunt 4. Moderate PDA, L – R Shunt 5. Reduced LV Systolic Function. | | | |
| SIGNATURE  Done by: Tesfaye T., Pediatric Cardiologist _______________ 11/03/2012Eth.C | | | |

| Patient Name: **Bantager Yihenew**. Patient ID: Dangila HOSP. SEX/ Age: M/ 11Years Date of Report: 11**/03/2012_**.  BP: ___ Weight: ___ Height:___ BSA: ___. Referral Diagnosis: **Easy Fatigability. AGH03.1921.** | | | |
| --- | --- | --- | --- |
| **Features** | **Finding** | **Features** | **Finding** |
| **Profile** |  | **Atria** |  |
| Abdominal situs | Solitus | Left atrium | Normal |
| Cardiac position | Levocardia | Right atrium | Normal |
| Systemic venous drainage | Normal | **Atrioventricular valves** |  |
| Pulmonary venous drainage | Normal | Mitral valve | Annulus = 19mm |
| Atrioventricular connection | Concordant | Tricuspid valve | Annulus = 24mm |
| Ventriculoarterial connection | Concordant | **Ventricles** |  |
| Ventricular loop | d-Loop | Left ventricle | Normal |
|  |  | Right ventricle | Normal |
| **Septae** |  | **Coronary arteries** | ----- |
| Interventricular septum | Intact | **Doppler Measurement** |  |
| Interatrial septum | Intact | Mitral | ---- |
| **Semilunal valves** |  | Aortic | ---- |
| Aortic valve | Annulus = 16mm | Tricuspid | ---- |
| Pulmonary valve | Annulus = 19mm | pulmonic | ---- |
| **Great arteries** | NRGA | **Aortic arch** |  |
| Aorta | ---- | **PDA** | No |
| Pulmonary artery | Normal MPA and confluent Branch PAs. |  |  |
| **M-Mode: Normal LV Function (eye balling)** | | | |
| AO | mm | PWd | 5mm |
| LA | mm | EDV | 76ml |
| LVIDd | 41mm | ESV | 26ml |
| LVIDs | 27mm | LVEF | 66% |
| IVSd | 6mm | FS | 36% |
| **Additional Information**: |  | | |
| No pleural/pericardial effusion | | | |
| **Final Diagnosis:** | | | |
| 1. {S, D, S} Levocardia 2. Normal Echocardiography Study | | | |
| SIGNATURE  Done by: Tesfaye T., Pediatric Cardiologist _______________ 11/03/2012Eth.C | | | |

| Patient Name: **Tejitu Desalegn**. Patient ID:_FHRH. SEX/ Age: F/ 7Years Date of Report: 14**/03/2012_**.  BP: ___ Weight: ___ Height:___ BSA: ___. Referral Diagnosis: **ARF. AGH03.1922.** | | | |
| --- | --- | --- | --- |
| **Features** | **Finding** | **Features** | **Finding** |
| **Profile** |  | **Atria** |  |
| Abdominal situs | Solitus | Left atrium | Normal |
| Cardiac position | Levocardia | Right atrium | Normal |
| Systemic venous drainage | Normal | **Atrioventricular valves** |  |
| Pulmonary venous drainage | Normal | Mitral valve | Annulus = 21mm |
| Atrioventricular connection | Concordant | Tricuspid valve | Annulus = 20mm.  TAPSE = 20mm. |
| Ventriculoarterial connection | Concordant | **Ventricles** |  |
| Ventricular loop | d-Loop | Left ventricle | Normal |
|  |  | Right ventricle | Normal |
| **Septae** |  | **Coronary arteries** | ----- |
| Interventricular septum | Intact | **Doppler Measurement** |  |
| Interatrial septum | Intact | Mitral | ---- |
| **Semilunal valves** |  | Aortic | ---- |
| Aortic valve | Annulus = 16mm | Tricuspid | ---- |
| Pulmonary valve | Annulus = 18mm | pulmonic | ---- |
| **Great arteries** | NRGA | **Aortic arch** | Left |
| Aorta | ---- | **PDA** | No |
| Pulmonary artery | Normal MPA and confluent Branch PAs. |  | No coarctation. |
| **M-Mode: Normal LV Function (eye balling)** | | | |
| AO | mm | PWd | 7mm |
| LA | mm | EDV | 43ml |
| LVIDd | 33mm | ESV | 17ml |
| LVIDs | 22.5mm | LVEF | 61% |
| IVSd | 9mm | FS | 31% |
| **Additional Information**: |  | | |
| No pleural/pericardial effusion | | | |
| **Final Diagnosis:** | | | |
| 1. Normal Echocardiography Study. | | | |
| SIGNATURE  Done by: Tesfaye T., Pediatric Cardiologist _______________ 14/03/2012Eth.C | | | |

| Patient Name: **Anguach Kassegn**. Patient ID:_FHRH. SEX/ Age: F/ 10Years Date of Report: 15**/03/2012_**.  BP: ___ Weight: ___ Height:___ BSA: ___. Referral Diagnosis: **Incidental Murmur. AGH03.1923.** | | | |
| --- | --- | --- | --- |
| **Features** | **Finding** | **Features** | **Finding** |
| **Profile** |  | **Atria** |  |
| Abdominal situs | Solitus | Left atrium | Normal |
| Cardiac position | Levocardia | Right atrium | Normal |
| Systemic venous drainage | Normal | **Atrioventricular valves** |  |
| Pulmonary venous drainage | Normal | Mitral valve | Annulus = 22mm |
| Atrioventricular connection | Concordant | Tricuspid valve | Annulus = 25mm.  TAPSE = 16mm |
| Ventriculoarterial connection | Concordant | **Ventricles** |  |
| Ventricular loop | d-Loop | Left ventricle | Normal |
|  |  | Right ventricle | Normal |
| **Septae** |  | **Coronary arteries** | ----- |
| Interventricular septum | 6mm Perimembranous VSD, L – R Shunt | **Doppler Measurement** |  |
| Interatrial septum | Intact | Mitral | ---- |
| **Semilunal valves** |  | Aortic | ---- |
| Aortic valve | Annulus = 20mm | Tricuspid | ---- |
| Pulmonary valve | Annulus = 21mm | pulmonic | Mild PR, PPG = 32mmHg |
| **Great arteries** | NRGA | **Aortic arch** | Left |
| Aorta | ----- | **PDA** | No |
| Pulmonary artery | Normal MPA and confluent Branch PAs. |  |  |
| **M-Mode: Normal LV Function (eye balling)** | | | |
| AO | mm | PWd | 7mm |
| LA | mm | EDV | 47ml |
| LVIDd | 34mm | ESV | 18ml |
| LVIDs | 23mm | LVEF | 61% |
| IVSd | 6mm | FS | 32% |
| **Additional Information**: |  | | |
| No pleural/pericardial effusion | | | |
| **Final Diagnosis:** | | | |
| 1. {S, D, S} Levocardia 2. Small Perimembranous VSD, L – R Shunt 3. Good Biventricular Function. | | | |
| SIGNATURE  Done by: Tesfaye T., Pediatric Cardiologist _______________ 15/03/2012Eth.C | | | |

| Patient Name: **Nahom Solomon**. Patient ID:_FHRH. SEX/ Age: M/ 10Years Date of Report: 16**/03/2012_**.  BP: _ Weight: __ Height:___ BSA: ___. Referral Diagnosis: **Post-Op Evaluation(S/P ASD Closure). AGH03.1924.** | | | |
| --- | --- | --- | --- |
| **Features** | **Finding** | **Features** | **Finding** |
| **Profile** |  | **Atria** |  |
| Abdominal situs | Solitus | Left atrium | Normal |
| Cardiac position | Levocardia | Right atrium | Normal |
| Systemic venous drainage | Normal | **Atrioventricular valves** |  |
| Pulmonary venous drainage | Normal | Mitral valve | Annulus = 24mm. |
| Atrioventricular connection | Concordant | Tricuspid valve | Annulus = 26mm.  TAPSE = 24mm. |
| Ventriculoarterial connection | Concordant | **Ventricles** |  |
| Ventricular loop | d-Loop | Left ventricle | Normal |
|  |  | Right ventricle | Normal |
| **Septae** |  | **Coronary arteries** | ----- |
| Interventricular septum | Intact | **Doppler Measurement** |  |
| Interatrial septum | Device in situ. No residual ASD. | Mitral | No MR. good mitral inflow. |
| **Semilunal valves** |  | Aortic | No AR. No LVOTO |
| Aortic valve | Annulus = 18mm | Tricuspid | NO TR |
| Pulmonary valve | Annulus = 18mm | pulmonic | ---- |
| **Great arteries** | NRGA | **Aortic arch** |  |
| Aorta | ---- | **PDA** | No |
| Pulmonary artery | Normal MPA and confluent Branch PAs. |  |  |
| **M-Mode: Normal LV Function (eye balling)** | | | |
| AO | mm | PWd | 8mm |
| LA | mm | EDV | 31ml |
| LVIDd | 28.5mm | ESV | 14ml |
| LVIDs | 21mm | LVEF | 56% |
| IVSd | 6mm | FS | 29% |
| **Additional Information**: |  | | |
| No pleural/pericardial effusion | | | |
| **Final Diagnosis:** | | | |
| 1. {S, D, S} Levocardia 2. S/P ASD Device Closure 3. No residual ASD 4. Good Biventricular Function | | | |
| SIGNATURE  Done by: Tesfaye T., Pediatric Cardiologist _______________ 16/03/2012Eth.C | | | |

| Patient Name: **ANANYA Abebe**. Patient ID:_TGSH. SEX/ Age: M/ 6Months Date of Report: 17**/03/2012_**.  BP: ___ Weight: ___ Height:___ BSA: ___. Referral Diagnosis: **CHF 20 to ? PHTN + DS. AGH03.1925.** | | | | | |
| --- | --- | --- | --- | --- | --- |
| **Features** | **Finding** | | **Features** | | **Finding** |
| **Profile** |  | | **Atria** | |  |
| Abdominal situs | Solitus | | Left atrium | | Normal |
| Cardiac position | Levocardia | | Right atrium | | Dilated |
| Systemic venous drainage | Normal | | **Atrioventricular valves** | |  |
| Pulmonary venous drainage | Normal | | Mitral valve | | Annulus = 12mm. |
| Atrioventricular connection | Concordant | | Tricuspid valve | | Annulus = 15mm.  TAPSE = 11mm. |
| Ventriculoarterial connection | Concordant | | **Ventricles** | |  |
| Ventricular loop | d-Loop | | Left ventricle | | Normal |
|  |  | | Right ventricle | | Dilated |
| **Septae** |  | | **Coronary arteries** | | ----- |
| Interventricular septum | Intact | | **Doppler Measurement** | |  |
| Interatrial septum | Intact | | Mitral | | ---- |
| **Semilunal valves** |  | | Aortic | | ---- |
| Aortic valve | Annulus = 9mm | | Tricuspid | | Mild TR, |
| Pulmonary valve | Annulus = 13mm | | pulmonic | | Mild PR |
| **Great arteries** | NRGA | | **Aortic arch** | |  |
| Aorta | ---- | | **PDA** | | No |
| Pulmonary artery | **MPA = 16mm** and good sized confluent Branch PAs. | |  | |  |
| **M-Mode: Normal LV Function (eye balling)** | | | | | |
| AO | mm | PWd | | mm | |
| LA | mm | EDV | | ml | |
| LVIDd | mm | ESV | | ml | |
| LVIDs | mm | LVEF | | 69% | |
| IVSd | mm | FS | | 35% | |
| **Additional Information**: |  | | | | |
| No pleural/pericardial effusion | | | | | |
| **Final Diagnosis:** | | | | | |
| 1. {S, D, S} Levocardia 2. Dilated RA/RV 3. Severe Pulmonary Hypertension secondary to ? | | | | | |
| SIGNATURE  Done by: Tesfaye T., Pediatric Cardiologist _______________ 17/03/2012Eth.C | | | | | |

| Patient Name: **HIWET GEDIF**. Patient ID:_FHRH. SEX/ Age: F/ 8Months Date of Report: 17**/03/2012_**.  BP: ___ Weight: ___ Height:___ BSA: ___. Referral Diagnosis: **Diaphoresis + RD. AGH03.1926.** | | | |
| --- | --- | --- | --- |
| **Features** | **Finding** | **Features** | **Finding** |
| **Profile** |  | **Atria** |  |
| Abdominal situs | Solitus | Left atrium | Dilated |
| Cardiac position | Levocardia | Right atrium | Normal |
| Systemic venous drainage | Normal | **Atrioventricular valves** |  |
| Pulmonary venous drainage | Normal | Mitral valve | Annulus = 15mm. |
| Atrioventricular connection | Concordant | Tricuspid valve | Annulus = 14mm.  TAPSE = 9mm. |
| Ventriculoarterial connection | Concordant | **Ventricles** |  |
| Ventricular loop | d-Loop | Left ventricle | Dilated |
|  |  | Right ventricle | Normal |
| **Septae** |  | **Coronary arteries** | ----- |
| Interventricular septum | 8mm Inlet VSD with Perimembranous extension, L – R Shunt | **Doppler Measurement** |  |
| Interatrial septum | Intact | Mitral | ---- |
| **Semilunal valves** |  | Aortic | ---- |
| Aortic valve | Annulus = 8mm | Tricuspid | ---- |
| Pulmonary valve | Annulus = 11mm | pulmonic | ---- |
| **Great arteries** | NRGA | **Aortic arch** | Left |
| Aorta | ---- | **PDA** | No |
| Pulmonary artery | Normal MPA and confluent Branch PAs. |  |  |
| **M-Mode: Normal LV Function (eye balling)** | | | |
| AO | mm | PWd | 5.6mm |
| LA | mm | EDV | 12ml |
| LVIDd | 19.7mm | ESV | 5ml |
| LVIDs | 14mm | LVEF | 58% |
| IVSd | 7mm | FS | 28% |
| **Additional Information**: |  | | |
| No pleural/pericardial effusion | | | |
| **Final Diagnosis:** | | | |
| 1. {S, D, S} Levocardia 2. Large Inlet VSD, L – R Shunt 3. Good LV Function. | | | |
| SIGNATURE  Done by: Tesfaye T., Pediatric Cardiologist _______________ 17/03/2012Eth.C | | | |

| Patient Name: **Muluken Melkie**. Patient ID:_Enjibara. SEX/ Age: M/ 3Months Date of Report: 18**/03/2012_**.  BP: ___ Weight: ___ Height:___ BSA: ___. Referral Diagnosis: **DS. AGH03.1927.** | | | |
| --- | --- | --- | --- |
| **Features** | **Finding** | **Features** | **Finding** |
| **Profile** |  | **Atria** |  |
| Abdominal situs | Solitus | Left atrium | Normal |
| Cardiac position | Levocardia | Right atrium | Normal |
| Systemic venous drainage | Normal | **Atrioventricular valves** |  |
| Pulmonary venous drainage | Normal | Mitral valve | ---- |
| Atrioventricular connection | Concordant | Tricuspid valve | ---- |
| Ventriculoarterial connection | Concordant | **Ventricles** |  |
| Ventricular loop | d-Loop | Left ventricle | Normal |
|  |  | Right ventricle | Normal |
| **Septae** |  | **Coronary arteries** | ----- |
| Interventricular septum | Intact | **Doppler Measurement** |  |
| Interatrial septum | 7mm Primum defect and additional 4mm OS ASD, Both L – R Shunt. | Mitral | ---- |
| **Semilunal valves** |  | Aortic | ---- |
| Aortic valve | Annulus = 10mm | Tricuspid | ---- |
| Pulmonary valve | Annulus = 13mm | pulmonic | ---- |
| **Great arteries** | NRGA | **Aortic arch** |  |
| Aorta | ---- | **PDA** | No |
| Pulmonary artery | Good Sized MPA and confluent Branch PAs. |  |  |
| **M-Mode: Normal LV Function on eye balling** | | | |
| AO | mm | PWd | mm |
| LA | mm | EDV | ml |
| LVIDd | mm | ESV | ml |
| LVIDs | mm | LVEF | % |
| IVSd | mm | FS | % |
| **Additional Information**: |  | | |
| No pleural/pericardial effusion | | | |
| **Final Diagnosis:** | | | |
| 1. {S, D, S} Levocardia 2. Ostium Primum ASD, L – R Shunt 3. Additional Small OS ASD, L – R Shunt 4. Good LV Function | | | |
| **REMARK**: Only Subcostal window was accessible (Limited Window). | | | |
| SIGNATURE  Done by: Tesfaye T., Pediatric Cardiologist _______________ 18/03/2012Eth.C | | | |

| Patient Name: **Metadel Abebe**. Patient ID:_FHRH. SEX/ Age: F/ 8Years Date of Report: 18**/03/2012_**.  BP: ___ Weight: ___ Height:___ BSA: ___. Referral Diagnosis: **Rheumatic Recurrence + CHF. AGH03.1928.** | | | |
| --- | --- | --- | --- |
| **Features** | **Finding** | **Features** | **Finding** |
| **Profile** |  | **Atria** |  |
| Abdominal situs | Solitus | Left atrium | Dilated, 47 X 60mm |
| Cardiac position | Levocardia | Right atrium | Dilated |
| Systemic venous drainage | Normal | **Atrioventricular valves** |  |
| Pulmonary venous drainage | Normal | Mitral valve | Annulus = 28mm. thickened MVL. |
| Atrioventricular connection | Concordant | Tricuspid valve | Annulus = 26mm.  TAPSE = 18mm |
| Ventriculoarterial connection | Concordant | **Ventricles** |  |
| Ventricular loop | d-Loop | Left ventricle | Dilated |
|  |  | Right ventricle | Dilated |
| **Septae** |  | **Coronary arteries** | ----- |
| Interventricular septum | Intact | **Doppler Measurement** |  |
| Interatrial septum | Intact | Mitral | Severe MR, Posterior jet with velocity of 4m/sec. Holosystolic |
| **Semilunal valves** |  | Aortic | ---- |
| Aortic valve | Annulus = 14mm | Tricuspid | Moderate TR, PPG = 64mmHg |
| Pulmonary valve | Annulus = 20mm | pulmonic | Mild PR |
| **Great arteries** | NRGA | **Aortic arch** |  |
| Aorta |  | **PDA** | No |
| Pulmonary artery | Good sized MPA and confluent Branch PAs. |  |  |
| **M-Mode:** | | | |
| AO | mm | PWd | 9mm |
| LA | mm | EDV | 105ml |
| LVIDd | 48mm | ESV | 36ml |
| LVIDs | 30mm | LVEF | 66% |
| IVSd | 8mm | FS | 36% |
| **Additional Information**: |  | | |
| No pleural/pericardial effusion | | | |
| **Final Diagnosis:** | | | |
| 1. {S, D, S} Levocardia 2. LA/LV Dilated 3. Thickened Mitral valve leaflet 4. Severe MR 5. Moderate TR 6. Severe Pulmonary Hypertension 7. Good Biventricular Function | | | |
| SIGNATURE  Done by: Tesfaye T., Pediatric Cardiologist _______________ 18/03/2012Eth.C | | | |

| Patient Name: **Yanet Afewerk**. Patient ID:_ADINAS. SEX/ Age: F/ 13Years Date of Report: 18**/03/2012_**.  BP: ___ Weight: ___ Height:___ BSA: ___. Referral Diagnosis: **Palpitation + easy fatigability. AGH03.1929.** | | | |
| --- | --- | --- | --- |
| **Features** | **Finding** | **Features** | **Finding** |
| **Profile** |  | **Atria** |  |
| Abdominal situs | Solitus | Left atrium | Normal |
| Cardiac position | Levocardia | Right atrium | Normal |
| Systemic venous drainage | Normal | **Atrioventricular valves** |  |
| Pulmonary venous drainage | Normal | Mitral valve | Annulus = 22mm. |
| Atrioventricular connection | Concordant | Tricuspid valve | Annulus = 23mm.  TAPSE = 24mm |
| Ventriculoarterial connection | Concordant | **Ventricles** |  |
| Ventricular loop | d-Loop | Left ventricle | Normal |
|  |  | Right ventricle | Normal |
| **Septae** |  | **Coronary arteries** | ----- |
| Interventricular septum | Intact | **Doppler Measurement** |  |
| Interatrial septum | Intact | Mitral | ---- |
| **Semilunal valves** |  | Aortic | ---- |
| Aortic valve | Annulus = 18mm | Tricuspid | ---- |
| Pulmonary valve | Annulus = 19mm | pulmonic |  |
| **Great arteries** | NRGA | **Aortic arch** |  |
| Aorta |  | **PDA** | No |
| Pulmonary artery | Normal MPA and confluent Branch PAs. |  |  |
| **M-Mode:** | | | |
| AO | mm | PWd | 5mm |
| LA | mm | EDV | 54ml |
| LVIDd | 36mm | ESV | 27ml |
| LVIDs | 27mm | LVEF | 55% |
| IVSd | 6mm | FS | 28% |
| **Additional Information**: |  | | |
| No pleural/pericardial effusion | | | |
| **Final Diagnosis:** | | | |
| 1. {S, D, S} Levocardia 2. Normal Echocardiography Study. | | | |
| SIGNATURE  Done by: Tesfaye T., Pediatric Cardiologist _______________ 18/03/2012Eth.C | | | |

| Patient Name: **Anchinalu Bantayehu**. Patient ID: FHRH. SEX/ Age: F/ 3Months Date of Report: 20**/03/2012_**.  BP: ___ Weight: ___ Height:___ BSA: ___. Referral Diagnosis: **CHF + RD. AGH03.1930.** | | | |
| --- | --- | --- | --- |
| **Features** | **Finding** | **Features** | **Finding** |
| **Profile** |  | **Atria** |  |
| Abdominal situs | Solitus | Left atrium | Dilated |
| Cardiac position | Levocardia | Right atrium | Dilated |
| Systemic venous drainage | Normal | **Atrioventricular valves** |  |
| Pulmonary venous drainage | Normal | Mitral valve | Annulus = 13mm |
| Atrioventricular connection | Concordant | Tricuspid valve | Annulus = 16mm.  TAPSE = 9mm |
| Ventriculoarterial connection | Concordant | **Ventricles** |  |
| Ventricular loop | d-Loop | Left ventricle | Dilated |
|  |  | Right ventricle | Dilated |
| **Septae** |  | **Coronary arteries** | ----- |
| Interventricular septum | Intact | **Doppler Measurement** |  |
| Interatrial septum | Intact | Mitral | ---- |
| **Semilunal valves** |  | Aortic | ---- |
| Aortic valve | Annulus = 9mm | Tricuspid | Moderate TR, PPG = 69mmHg |
| Pulmonary valve | Annulus = 12mm | pulmonic |  |
| **Great arteries** | NRGA | **Aortic arch** |  |
| Aorta |  | **PDA** | 3mm PDA |
| Pulmonary artery | **MPA = 13mm**. confluent good sized Branch PAs. |  |  |
| **M-Mode:** | | | |
| AO | mm | PWd | 4.6mm |
| LA | mm | EDV | 19ml |
| LVIDd | 23.6mm | ESV | 3.75ml |
| LVIDs | 12.5mm | LVEF | 80% |
| IVSd | 4.3mm | FS | 46% |
| **Additional Information**: |  | | |
| No pleural/pericardial effusion | | | |
| **Final Diagnosis:** | | | |
| 1. {S, D, S} Levocardia 2. All chambers dilated 3. Large PDA, L – R Shunt 4. Severe Pulmonary Hypertension 5. Good Function. | | | |
| SIGNATURE  Done by: Tesfaye T., Pediatric Cardiologist _______________ 20/03/2012Eth.C | | | |

| Patient Name: **Wengel Samuel**. Patient ID: Pawi H. SEX/ Age: F/ 3Years Date of Report: 19**/03/2012_**.  BP: ___ Weight: ___ Height:___ BSA: ___. Referral Diagnosis: **FTT. AGH03.1931.** | | | |
| --- | --- | --- | --- |
| **Features** | **Finding** | **Features** | **Finding** |
| **Profile** |  | **Atria** |  |
| Abdominal situs | Solitus | Left atrium | Dilated |
| Cardiac position | Levocardia | Right atrium | Normal |
| Systemic venous drainage | Normal | **Atrioventricular valves** |  |
| Pulmonary venous drainage | Normal | Mitral valve | Annulus = 19mm |
| Atrioventricular connection | Concordant | Tricuspid valve | Annulus = 21mm.  TAPSE = 16mm |
| Ventriculoarterial connection | Concordant | **Ventricles** |  |
| Ventricular loop | d-Loop | Left ventricle | Dilated |
|  |  | Right ventricle | Normal |
| **Septae** |  | **Coronary arteries** | ----- |
| Interventricular septum | Intact | **Doppler Measurement** |  |
| Interatrial septum | Intact | Mitral | ---- |
| **Semilunal valves** |  | Aortic | ---- |
| Aortic valve | Annulus = 15mm | Tricuspid | ---- |
| Pulmonary valve | Annulus = 17mm | pulmonic |  |
| **Great arteries** | NRGA | **Aortic arch** |  |
| Aorta |  | **PDA** | 2.5mm, L – R Shunt |
| Pulmonary artery | Normal MPA and confluent Branch PAs. |  |  |
| **M-Mode:** | | | |
| AO | mm | PWd | 6mm |
| LA | mm | EDV | 61ml |
| LVIDd | 38mm | ESV | 26ml |
| LVIDs | 26mm | LVEF | 58% |
| IVSd | 8mm | FS | 30% |
| **Additional Information**: |  | | |
| No pleural/pericardial effusion | | | |
| **Final Diagnosis:** | | | |
| 1. {S, D, S} Levocardia 2. Moderate PDA, L – R Shunt 3. Good Biventricular Function. | | | |
| SIGNATURE  Done by: Tesfaye T., Pediatric Cardiologist _______________ 19/03/2012Eth.C | | | |

| Patient Name: **Zakir Endris**. Patient ID:_ADINAS H. SEX/ Age: M/ 7Years Date of Report: 19**/03/2012_**.  BP: ___ Weight: ___ Height:___ BSA: ___. Referral Diagnosis: **DOE. AGH03.1932.** | | | |
| --- | --- | --- | --- |
| **Features** | **Finding** | **Features** | **Finding** |
| **Profile** |  | **Atria** |  |
| Abdominal situs | Solitus | Left atrium | Normal |
| Cardiac position | Levocardia | Right atrium | Normal |
| Systemic venous drainage | Normal | **Atrioventricular valves** |  |
| Pulmonary venous drainage | Normal | Mitral valve | Annulus = 21mm |
| Atrioventricular connection | Concordant | Tricuspid valve | Annulus = 20mm.  TAPSE = 23mm |
| Ventriculoarterial connection | Concordant | **Ventricles** |  |
| Ventricular loop | d-Loop | Left ventricle | Normal |
|  |  | Right ventricle | Normal |
| **Septae** |  | **Coronary arteries** | ----- |
| Interventricular septum | Intact | **Doppler Measurement** |  |
| Interatrial septum | 7mm High Secundum ASD, L – R Shunt | Mitral | Trivial MR |
| **Semilunal valves** |  | Aortic | ---- |
| Aortic valve | Annulus = 16mm | Tricuspid | Mild TR, PPG = 31mmHg |
| Pulmonary valve | Annulus = 17mm | pulmonic | ---- |
| **Great arteries** | NRGA | **Aortic arch** |  |
| Aorta |  | **PDA** | No |
| Pulmonary artery | Normal MPA and confluent Branch PAs. |  |  |
| **M-Mode:** | | | |
| AO | mm | PWd | 6mm |
| LA | mm | EDV | 84ml |
| LVIDd | 43mm | ESV | 28ml |
| LVIDs | 27mm | LVEF | 66% |
| IVSd | 6mm | FS | 36% |
| **Additional Information**: |  | | |
| No pleural/pericardial effusion | | | |
| **Final Diagnosis:** | | | |
| 1. {S, D, S} Levocardia 2. High Secundum ASD, L – R Shunt. 3. Good Biventricular Function. | | | |
| SIGNATURE  Done by: Tesfaye T., Pediatric Cardiologist _______________ 19/03/2012Eth.C | | | |

| Patient Name: **Amet Bal Girma**. Patient ID:_FHRH. SEX/ Age: F/ 5 2/12Years Date of Report: 20**/03/2012_**.  BP: ___ Weight: ___ Height:___ BSA: ___. Referral Diagnosis: **RD + CHF. AGH03.1933.** | | | | |
| --- | --- | --- | --- | --- |
| **Features** | **Finding** | **Features** | **Finding** | |
| **Profile** |  | **Atria** |  | |
| Abdominal situs | Solitus | Left atrium | More Dilated | |
| Cardiac position | Levocardia | Right atrium | Dilated | |
| Systemic venous drainage | Normal | **Atrioventricular valves** |  | |
| Pulmonary venous drainage | Normal | Mitral valve | Annulus = 24mm. thickened MVL. | |
| Atrioventricular connection | Concordant | Tricuspid valve | Annulus = 21mm.  TAPSE = 19mm | |
| Ventriculoarterial connection | Concordant | **Ventricles** |  | |
| Ventricular loop | d-Loop | Left ventricle | More dilated | |
|  |  | Right ventricle | Dilated | |
| **Septae** |  | **Coronary arteries** | ----- | |
| Interventricular septum | Intact | **Doppler Measurement** |  | |
| Interatrial septum | Intact | Mitral | Moderate MR, Holosystolic with posterior projection and jet velocity of 4.35m/sec | |
| **Semilunal valves** |  | Aortic | Moderate AR, PHT = 272ms | |
| Aortic valve | Annulus = 17mm | Tricuspid | Mild TR, PPG = 52mmHg | |
| Pulmonary valve | Annulus = 19mm | pulmonic | Moderate PR, PPG =63mmHg | |
| **Great arteries** | NRGA | **Aortic arch** | Left | |
| Aorta |  | **PDA** | 5mm PDA, L – R Shunt | |
| Pulmonary artery | MPA = 24mm. |  |  | |
| **M-Mode:** | | | | |
| AO | mm | PWd | | 7.5mm |
| LA | mm | EDV | | 110ml |
| LVIDd | 48.5mm | ESV | | 53ml |
| LVIDs | 35.5mm | LVEF | | 52% |
| IVSd | 9.5mm | FS | | 27% |
| **Additional Information**: | No pleural/pericardial effusion | | | |
| **Final Diagnosis:** | | | | |
| 1. {S, D, S} Levocardia 2. Moderate MR, THICKENED Mitral Valve leaflet 3. Moderate AR 4. Mild TR 5. Moderate PR 6. Large PDA, L – R Shunt 7. Severe Pulmonary Hypertension 8. Mildly reduced LV Function | | | | |
| **Recommendation**: in addition to the CHD, Consider Rheumatic Heart Disease. Hence; correlate with clinical evaluation and other investigation findings. | | | | |
| SIGNATURE  Done by: Tesfaye T., Pediatric Cardiologist _______________ 20/03/2012Eth.C | | | | |

| Patient Name: **Muhamed Ahmed**. Patient ID: ADINAS H. SEX/ Age: M/ 15Years Date of Report: 20**/03/2012_**.  BP: ___ Weight: ___ Height:___ BSA: ___. Referral Diagnosis: **Chest Pain. AGH03.1934.** | | | |
| --- | --- | --- | --- |
| **Features** | **Finding** | **Features** | **Finding** |
| **Profile** |  | **Atria** |  |
| Abdominal situs | Solitus | Left atrium | Normal |
| Cardiac position | Levocardia | Right atrium | Normal |
| Systemic venous drainage | Normal | **Atrioventricular valves** |  |
| Pulmonary venous drainage | Normal | Mitral valve | Annulus = 26mm |
| Atrioventricular connection | Concordant | Tricuspid valve | Annulus = 28mm.  TAPSE = 23mm |
| Ventriculoarterial connection | Concordant | **Ventricles** |  |
| Ventricular loop | d-Loop | Left ventricle | Normal |
| **Septae** |  | Right ventricle | Normal |
| Interventricular septum | Intact | **Coronary arteries** | ----- |
| Interatrial septum | Intact | **Doppler Measurement** |  |
| **Semilunal valves** |  | Mitral | ---- |
| Aortic valve | Annulus = 21mm | Aortic | ---- |
| Pulmonary valve | Annulus = 23mm | Tricuspid | ---- |
| **Great arteries** | NRGA | pulmonic | Trivial PR, PPG = 13mmHg |
| Aorta | ---- | **Aortic arch** | left |
| Pulmonary artery | Normal MPA and confluent Branch PAs. | **PDA** | No |
|  |  | Aortic Arch | No coarctation |
| **M-Mode:** | | | |
| AO | mm | PWd | 7.7mm |
| LA | mm | EDV | 103ml |
| LVIDd | 47mm | ESV | 37ml |
| LVIDs | 31mm | LVEF | 64% |
| IVSd | 8.6mm | FS | 35% |
| **Additional Information**: |  | | |
| No pleural/pericardial effusion | | | |
| **Final Diagnosis:** | | | |
| 1. Normal Echocardiography Study. | | | |
| SIGNATURE  Done by: Tesfaye T., Pediatric Cardiologist _______________ 20/03/2012Eth.C | | | |

| Patient Name: **Tesfahun Bitew**. Patient ID:_FHRH. SEX/ Age: M/ 2 8/12 Date of Report: 22**/03/2012_**.  BP: ___ Weight: ___ Height:___ BSA: ___. Referral Diagnosis: **Recurrent Chest Infection. AGH03.1935.** | | | |
| --- | --- | --- | --- |
| **Features** | **Finding** | **Features** | **Finding** |
| **Profile** |  | **Atria** |  |
| Abdominal situs | Solitus | Left atrium | Normal |
| Cardiac position | Levocardia | Right atrium | Normal |
| Systemic venous drainage | Normal | **Atrioventricular valves** |  |
| Pulmonary venous drainage | Normal | Mitral valve | Annulus = 13mm |
| Atrioventricular connection | Concordant | Tricuspid valve | Annulus =17 mm.  TAPSE = 13mm |
| Ventriculoarterial connection | Concordant | **Ventricles** |  |
| Ventricular loop | d-Loop | Left ventricle | Normal |
|  |  | Right ventricle | Normal |
| **Septae** |  | **Coronary arteries** | ----- |
| Interventricular septum | Intact | **Doppler Measurement** |  |
| Interatrial septum | Intact | Mitral | ---- |
| **Semilunal valves** |  | Aortic | ---- |
| Aortic valve | Annulus = 12mm | Tricuspid | ---- |
| Pulmonary valve | Annulus = 12mm | pulmonic | ---- |
| **Great arteries** | NRGA | **Aortic arch** |  |
| Aorta |  | **PDA** | No |
| Pulmonary artery | Normal MPA and confluent Branch PAs. |  | Left arch |
| **M-Mode:** | | | |
| AO | mm | PWd | 6mm |
| LA | mm | EDV | 35ml |
| LVIDd | 30mm | ESV | 16ml |
| LVIDs | 22mm | LVEF | 59% |
| IVSd | 6mm | FS | 29% |
| **Additional Information**: |  | | |
| No pleural/pericardial effusion | | | |
| **Final Diagnosis:** | | | |
| 1. Normal Echocardiography Study. | | | |
| SIGNATURE  Done by: Tesfaye T., Pediatric Cardiologist _______________ 22/03/2012Eth.C | | | |

| Patient Name: **Yamlakie Guadenew**. Patient ID:_ADINAS H. SEX/ Age: F/ 4 Months Date of Report: 22**/03/2012_**.  BP: ___ Weight: ___ Height:___ BSA: ___. Referral Diagnosis: **CHF + Cyanosis. AGH03.1936.** | | | |
| --- | --- | --- | --- |
| **Features** | **Finding** | **Features** | **Finding** |
| **Profile** |  | **Atria** |  |
| Abdominal situs | Solitus | Left atrium | Normal |
| Cardiac position | Levocardia | Right atrium | Dilated |
| Systemic venous drainage | Normal | **Atrioventricular valves** |  |
| Pulmonary venous drainage | Normal | Mitral valve | Annulus = mm |
| Atrioventricular connection | Concordant | Tricuspid valve | Annulus = mm.  TAPSE = mm |
| Ventriculoarterial connection | DORV | **Ventricles** |  |
| Ventricular loop | d-Loop | Left ventricle | Smallish |
|  |  | Right ventricle | Dilated |
| **Septae** |  | **Coronary arteries** | ----- |
| Interventricular septum | 14mm inlet VSD, BD Shunt. (Doubly Committed) | **Doppler Measurement** |  |
| Interatrial septum | Intact | Mitral | ---- |
| **Semilunal valves** |  | Aortic | ---- |
| Aortic valve | Annulus = 6.5mm | Tricuspid | Moderate TR |
| Pulmonary valve | Annulus = 9mmmm | pulmonic | ---- |
| **Great arteries** | NRGA | **Aortic arch** |  |
| Aorta | Smallish, From RV, Posterior to PA | **PDA** | No |
| Pulmonary artery | From RV, Anterior to Aorta |  |  |
| **M-Mode:** | | | |
| AO | mm | PWd | 5.5mm |
| LA | mm | EDV | 14ml |
| LVIDd | 20mm | ESV | 6.6ml |
| LVIDs | 16mm | LVEF | 51% |
| IVSd | 5mm | FS | 24% |
| **Additional Information**: |  | | |
| No pleural/pericardial effusion | | | |
| **Final Diagnosis:** | | | |
| 1. {S, D, D} Levocardia 2. DORV 3. Large Inlet VSD, BD Shunt 4. Smallish Aorta 5. Smallish LV 6. Good sized MPA and Confluent Branch PAs. | | | |
| SIGNATURE  Done by: Tesfaye T., Pediatric Cardiologist _______________ 22/03/2012Eth.C | | | |

| Patient Name: **Zebideru Asmare**. Patient ID:_FHRH. SEX/ Age: F/ 11Years Date of Report: 23**/03/2012_**.  BP: ___ Weight: ___ Height:___ BSA: ___. Referral Diagnosis: **Rheumatic Fever. AGH03.1937.** | | | |
| --- | --- | --- | --- |
| **Features** | **Finding** | **Features** | **Finding** |
| **Profile** |  | **Atria** |  |
| Abdominal situs | Solitus | Left atrium | Normal |
| Cardiac position | Levocardia | Right atrium | Normal |
| Systemic venous drainage | Normal | **Atrioventricular valves** |  |
| Pulmonary venous drainage | Normal | Mitral valve | Annulus = 16mm. thickened MVL. |
| Atrioventricular connection | Concordant | Tricuspid valve | Annulus = 19mm.  TAPSE = 21mm |
| Ventriculoarterial connection | Concordant | **Ventricles** |  |
| Ventricular loop | d-Loop | Left ventricle | Normal |
|  |  | Right ventricle | Normal |
| **Septae** |  | **Coronary arteries** | ----- |
| Interventricular septum | Intact | **Doppler Measurement** |  |
| Interatrial septum | Intact | Mitral | Mild MR, Holosystolic, Posterior projection with jet velocity = 4m/sec |
| **Semilunal valves** |  | Aortic | ---- |
| Aortic valve | Annulus = 17mm | Tricuspid | Trivial TR, PPG = 26mmHg |
| Pulmonary valve | Annulus = 18mm | pulmonic | ---- |
| **Great arteries** | NRGA | **Aortic arch** |  |
| Aorta |  | **PDA** | No |
| Pulmonary artery | Normal MPA and confluent Branch PAs. |  |  |
| **M-Mode:** | | | |
| AO | mm | PWd | 5mm |
| LA | mm | EDV | 25ml |
| LVIDd | 26mm | ESV | 9ml |
| LVIDs | 18mm | LVEF | 63% |
| IVSd | 5.7mm | FS | 32% |
| **Additional Information**: |  | | |
| No pleural/pericardial effusion | | | |
| **Final Diagnosis:** | | | |
| 1. {S, D, S} Levocardia 2. Thickened Mitral valve leaflet 3. Mild MR 4. Good Biventricular Function | | | |
| SIGNATURE  Done by: Tesfaye T., Pediatric Cardiologist _______________ 23/03/2012Eth.C | | | |

| Patient Name: **Getachew Tagele**. Patient ID: Sehate Birhan. SEX/ Age: M/ 7Months Date of Report: 23**/03/2012_**.  BP: ___ Weight: ___ Height:___ BSA: ___. Referral Diagnosis: **Cyanosis + RD. AGH03.1938.** | | | |
| --- | --- | --- | --- |
| **Features** | **Finding** | **Features** | **Finding** |
| **Profile** |  | **Atria** |  |
| Abdominal situs | Solitus | Left atrium | Normal |
| Cardiac position | Levocardia | Right atrium | Normal |
| Systemic venous drainage | Normal | **Atrioventricular valves** |  |
| Pulmonary venous drainage | Flow acceleration across RUPV | Mitral valve | Atretic |
| Atrioventricular connection | Concordant | Tricuspid valve | Annulus = 14mm.  TAPSE = mm |
| Ventriculoarterial connection | Concordant | **Ventricles** |  |
| Ventricular loop | d-Loop | Left ventricle | Smallish |
|  |  | Right ventricle | Normal |
| **Septae** |  | **Coronary arteries** | ----- |
| Interventricular septum | Intact | **Doppler Measurement** |  |
| Interatrial septum | 18mm primum defect, L – R Shunt | Mitral | Atretic |
| **Semilunal valves** |  | Aortic | ---- |
| Aortic valve | Annulus = 12mm | Tricuspid | Mild TR |
| Pulmonary valve | Annulus = 13mm | pulmonic | Mild PS, PPG = 21mmHg |
| **Great arteries** | NRGA | **Aortic arch** |  |
| Aorta | ---- | **PDA** | 3mm PDA, R – L Shunt |
| Pulmonary artery | Normal MPA and confluent Branch PAs. |  |  |
| **M-Mode:** | | | |
| AO | mm | PWd | mm |
| LA | mm | EDV | ml |
| LVIDd | mm | ESV | ml |
| LVIDs | mm | LVEF | % |
| IVSd | mm | FS | % |
| **Additional Information**: |  | | |
| No pleural/pericardial effusion | | | |
| **Final Diagnosis:** | | | |
| 1. {S, D, S} Levocardia 2. Large Ostium primum ASD, L – R Shunt 3. Mitral Atresia 4. Smallish LV 5. Moderate PDA, R – L Shunt 6. Mild PS. | | | |
| SIGNATURE  Done by: Tesfaye T., Pediatric Cardiologist _______________ 23/03/2012Eth.C | | | |

| Patient Name: **Bilen Abrham**. Patient ID: Guzara Clinic. SEX/ Age: F/ 7Years Date of Report: 23**/03/2012_**.  BP: ___ Weight: ___ Height:___ BSA: ___. Referral Diagnosis: **Incidental Murmur. AGH03.1939.** | | | |
| --- | --- | --- | --- |
| **Features** | **Finding** | **Features** | **Finding** |
| **Profile** |  | **Atria** |  |
| Abdominal situs | Solitus | Left atrium | Normal |
| Cardiac position | Levocardia | Right atrium | Normal |
| Systemic venous drainage | Normal | **Atrioventricular valves** |  |
| Pulmonary venous drainage | Normal | Mitral valve | Annulus = 18mm |
| Atrioventricular connection | Concordant | Tricuspid valve | Annulus = 20mm.  TAPSE = 18mm |
| Ventriculoarterial connection | Concordant | **Ventricles** |  |
| Ventricular loop | d-Loop | Left ventricle | Normal |
|  |  | Right ventricle | Normal |
| **Septae** |  | **Coronary arteries** | ----- |
| Interventricular septum | Intact | **Doppler Measurement** |  |
| Interatrial septum | Intact | Mitral | ---- |
| **Semilunal valves** |  | Aortic | ---- |
| Aortic valve | Annulus = 15mm | Tricuspid | ---- |
| Pulmonary valve | Annulus = 18mm | pulmonic | Mild Valvar PS, PPG = 21mmHg. Turbulence across the valve. |
| **Great arteries** | NRGA | **Aortic arch** | Left |
| Aorta |  | **PDA** | No |
| Pulmonary artery | MPA =14mm, RPA = 10mm, LPA = 10mm. |  |  |
| **M-Mode:** | | | |
| AO | mm | PWd | 5.7mm |
| LA | mm | EDV | 56.5ml |
| LVIDd | 36.6mm | ESV | 26.6ml |
| LVIDs | 27mm | LVEF | 56% |
| IVSd | 5.7mm | FS | 29% |
| **Additional Information**: |  | | |
| No pleural/pericardial effusion | | | |
| **Final Diagnosis:** | | | |
| 1. {S, D, S} Levocardia 2. Mild PS 3. Good Biventricular Function | | | |
| SIGNATURE  Done by: Tesfaye T., Pediatric Cardiologist _______________ 23/03/2012Eth.C | | | |

| Patient Name: **Solomon Adugna**. Patient ID: Sehate Birhan. SEX/ Age: M/ 7months Date of Report: 23**/03/2012_**.  BP: ___ Weight: ___ Height:___ BSA: ___. Referral Diagnosis: **CHF. AGH03.1940.** | | | |
| --- | --- | --- | --- |
| **Features** | **Finding** | **Features** | **Finding** |
| **Profile** |  | **Atria** |  |
| Abdominal situs | Solitus | Left atrium | Dilated |
| Cardiac position | Levocardia | Right atrium | Dilated |
| Systemic venous drainage | Normal | **Atrioventricular valves** |  |
| Pulmonary venous drainage | Normal | Mitral valve | Annulus = 16mm |
| Atrioventricular connection | Concordant | Tricuspid valve | Annulus = 16mm.  TAPSE = 17mm |
| Ventriculoarterial connection | Concordant | **Ventricles** |  |
| Ventricular loop | d-Loop | Left ventricle | Dilated |
|  |  | Right ventricle | Dilated |
| **Septae** |  | **Coronary arteries** | ----- |
| Interventricular septum | Intact | **Doppler Measurement** |  |
| Interatrial septum | Intact | Mitral | Mild to Moderate MR |
| **Semilunal valves** |  | Aortic | ---- |
| Aortic valve | Annulus = 12mm | Tricuspid | Mild TR, PPG = 60mmHg |
| Pulmonary valve | Annulus = mm | pulmonic | Moderate PR, PPG = 59mmHg |
| **Great arteries** | NRGA | **Aortic arch** | Left |
| Aorta |  | **PDA** | 3.5mm PDA, L – R Shunt. |
| Pulmonary artery | MPA = 17mm. normal sized confluent Branch PAs. |  |  |
| **M-Mode:** | | | |
| AO | mm | PWd | 6mm |
| LA | mm | EDV | 44ml |
| LVIDd | 33mm | ESV | 15ml |
| LVIDs | 21mm | LVEF | 66% |
| IVSd | 5.6mm | FS | 35% |
| **Additional Information**: |  | | |
| No pleural/pericardial effusion | | | |
| **Final Diagnosis:** | | | |
| 1. {S, D, S} Levocardia 2. Large PDA, L – R Shunt 3. Mild to Moderate MR 4. Mild TR 5. Moderate PR 6. LA/LV Dilated 7. Severe Pulmonary Hypertension 8. Good Biventricular Function | | | |
| SIGNATURE  Done by: Tesfaye T., Pediatric Cardiologist _______________ 23/03/2012Eth.C | | | |

| Patient Name**: Mebea – Tsion Abrham.** Patient ID:FHRH. SEX/ Age: F/ 1Year 4/12 Date of Report: 24**/03/2012_**.  BP: _______ Weight: ______ Height:____________ BSA: ________ | | | | |
| --- | --- | --- | --- | --- |
| **Features** | **Finding** | **Features** | | **Finding** |
| **Profile** |  | **Atria** | |  |
| Abdominal situs | Solitus | Left atrium | | Normal |
| Cardiac position | Levocardia | Right atrium | | Dilated |
| Systemic venous drainage | Normal | **Atrioventricular valves** | |  |
| Pulmonary venous drainage | Normal | Mitral valve | | Annulus = 14mm |
| Atrioventricular connection | Concordant | Tricuspid valve | | Annulus = 18mm  TAPSE = 8 mm |
| Ventriculoarterial connection | Concordant | **Ventricles** | |  |
| Ventricular loop | d-Loop | Left ventricle | | Normal |
|  |  | Right ventricle | | Dilated, Dysfunctional |
| **Septae** |  | **Coronary arteries** | | ----- |
| Interventricular septum | Intact | **Doppler Measurement** | |  |
| Interatrial septum | Intact | Mitral | | ---- |
| **Semilunal valves** |  | Aortic | | ---- |
| Aortic valve | Annulus = 13mm | Tricuspid | | Moderate TR, PPG = 73mmHg |
| Pulmonary valve | Annulus = 15mm | pulmonic | | Moderate PR, PPG = 50mmHg |
| **Great arteries** | NRGA | **Aortic arch** | | ---- |
| Aorta | ---- | **PDA** | | No |
| Pulmonary artery | Normal MPA and confluent Branch PAs. |  | |  |
| **M-Mode** | | | | |
| AO | mm | | PWd | 4mm |
| LA | mm | | EDV | 12ml |
| LVIDd | 20mm | | ESV | 5ml |
| LVIDs | 14mm | | LVEF | 58% |
| IVSd | 6mm | | FS | 29% |
| **Additional Information**: |  | | | |
| 10mm pericardial effusion on RA/RV Side. Trace effusion on LV Side. | | | | |
| **Final Diagnosis:** | | | | |
| 1. {S, D, S} Levocardia 2. Mild Pericardial Effusion 3. Moderate TR 4. Moderate PR 5. RA/RV Dilated 6. Dilated and dysfunctional RV 7. Severe Pulmonary Hypertension 8. Good LV Function | | | | |
| SIGNATURE  Done by: Tesfaye T., Pediatric Cardiologist _______________ 24/03/2012Eth.C | | | | |

| Patient Name: **Tewodros Kassahun**. Patient ID:_FHRH. SEX/ Age: M/ 23Dayss Date of Report: 24**/03/2012_**.  BP: ___ Weight: ___ Height:___ BSA: ___. Referral Diagnosis: **Incidental Murmur. AGH03.1941.** | | | |
| --- | --- | --- | --- |
| **Features** | **Finding** | **Features** | **Finding** |
| **Profile** |  | **Atria** |  |
| Abdominal situs | Solitus | Left atrium | Normal |
| Cardiac position | Levocardia | Right atrium | Normal |
| Systemic venous drainage | Normal | **Atrioventricular valves** |  |
| Pulmonary venous drainage | Normal | Mitral valve | Annulus = 10mm |
| Atrioventricular connection | Concordant | Tricuspid valve | Annulus = 11mm.  TAPSE = 11mm |
| Ventriculoarterial connection | Concordant | **Ventricles** |  |
| Ventricular loop | d-Loop | Left ventricle | Normal |
|  |  | Right ventricle | Normal |
| **Septae** |  | **Coronary arteries** | ----- |
| Interventricular septum | 8mm Lower Muscular VSD, L – R Shunt. | **Doppler Measurement** |  |
| Interatrial septum | Intact | Mitral | ---- |
| **Semilunal valves** |  | Aortic | ---- |
| Aortic valve | Annulus = 9mm | Tricuspid | ---- |
| Pulmonary valve | Annulus = 11mm | pulmonic | ---- |
| **Great arteries** | NRGA | **Aortic arch** |  |
| Aorta |  | **PDA** | No |
| Pulmonary artery | Normal MPA and confluent Branch PAs. |  |  |
| **M-Mode:** | | | |
| AO | mm | PWd | 4.6mm |
| LA | mm | EDV | 14ml |
| LVIDd | 21mm | ESV | 4ml |
| LVIDs | 13mm | LVEF | 73% |
| IVSd | 4mm | FS | 39% |
| **Additional Information**: |  | | |
| No pleural/pericardial effusion | | | |
| **Final Diagnosis:** | | | |
| 1. {S, D, S} Levocardia 2. Large Muscular VSD, L – R Shunt 3. Good Biventricular Function. | | | |
| SIGNATURE  Done by: Tesfaye T., Pediatric Cardiologist _______________ 24/03/2012Eth.C | | | |

| Patient Name: **Tinsae Birhan**. Patient ID:_SBC. SEX/ Age: M/ 7Monthss Date of Report: 29**/03/2012_**.  BP: ___ Weight: ___ Height:___ BSA: ___. Referral Diagnosis: **RD + Diaphoresis + DS. AGH03.1942.** | | | |
| --- | --- | --- | --- |
| **Features** | **Finding** | **Features** | **Finding** |
| **Profile** |  | **Atria** |  |
| Abdominal situs | Solitus | Left atrium | Dilated |
| Cardiac position | Levocardia | Right atrium | Normal |
| Systemic venous drainage | Normal | **Atrioventricular valves** |  |
| Pulmonary venous drainage | Normal | Mitral valve | Annulus = 12mm |
| Atrioventricular connection | Concordant | Tricuspid valve | Annulus = 14mm. |
| Ventriculoarterial connection | Concordant | **Ventricles** |  |
| Ventricular loop | d-Loop | Left ventricle | Dilated |
|  |  | Right ventricle | Normal |
| **Septae** | Tongue of tissue in b/n | **Coronary arteries** | ----- |
| Interventricular septum | 4mm inlet VSD, L – R Shunt | **Doppler Measurement** |  |
| Interatrial septum | 14mm Primum ASD, L – R Shunt | Mitral | Moderate MR |
| **Semilunal valves** |  | Aortic | ---- |
| Aortic valve | Annulus = 10mm | Tricuspid | Moderate TR |
| Pulmonary valve | Annulus = 11mm | pulmonic | ---- |
| **Great arteries** | NRGA | **Aortic arch** |  |
| Aorta |  | **PDA** | 2mm PDA, L – R Shunt |
| Pulmonary artery | Normal MPA and confluent Branch PAs. |  |  |
| **M-Mode:** | | | |
| AO | mm | PWd | 5mm |
| LA | mm | EDV | 14ml |
| LVIDd | 21mm | ESV | 6ml |
| LVIDs | 15mm | LVEF | 55% |
| IVSd | 7mm | FS | 27% |
| **Additional Information**: |  | | |
| No pleural/pericardial effusion | | | |
| **Final Diagnosis:** | | | |
| 1. {S, D, S} Levocardia 2. Transitional AVSD, L – R Shunt 3. Moderate PDA, L – R Shunt 4. Moderate MR 5. Moderate TR 6. Good LV Function. | | | |
| SIGNATURE  Done by: Tesfaye T., Pediatric Cardiologist _______________ 29/03/2012Eth.C | | | |

| Patient Name: **Ibrahim Adane**. Patient ID:_ADINAS. SEX/ Age: M/ 12 6/12Years Date of Report: 29**/03/2012_**.  BP: ___ Weight: ___ Height:___ BSA: ___. Referral Diagnosis: **Incidental Murmur. AGH03.1943.** | | | |
| --- | --- | --- | --- |
| **Features** | **Finding** | **Features** | **Finding** |
| **Profile** |  | **Atria** |  |
| Abdominal situs | Solitus | Left atrium | Normal |
| Cardiac position | Levocardia | Right atrium | Normal |
| Systemic venous drainage | Normal | **Atrioventricular valves** |  |
| Pulmonary venous drainage | Normal | Mitral valve | Annulus = 20mm |
| Atrioventricular connection | Concordant | Tricuspid valve | Annulus = 22mm.  TAPSE = 17mm |
| Ventriculoarterial connection | Concordant | **Ventricles** |  |
| Ventricular loop | d-Loop | Left ventricle | Normal |
|  |  | Right ventricle | Normal |
| **Septae** |  | **Coronary arteries** | ----- |
| Interventricular septum | 3mm PM VSD, L – R Shunt | **Doppler Measurement** |  |
| Interatrial septum | Intact | Mitral | ---- |
| **Semilunal valves** |  | Aortic | ---- |
| Aortic valve | Annulus = 15mm | Tricuspid | ---- |
| Pulmonary valve | Annulus = 18mm | pulmonic |  |
| **Great arteries** | NRGA | **Aortic arch** |  |
| Aorta |  | **PDA** | No |
| Pulmonary artery | Normal MPA and confluent Branch PAs. |  |  |
| **M-Mode:** | | | |
| AO | mm | PWd | 5mm |
| LA | mm | EDV | 65ml |
| LVIDd | 39mm | ESV | 20ml |
| LVIDs | 24mm | LVEF | 69% |
| IVSd | 6mm | FS | 38% |
| **Additional Information**: |  | | |
| No pleural/pericardial effusion | | | |
| **Final Diagnosis:** | | | |
| 1. {S, D, S} Levocardia 2. Small Perimembranous VSD, L – R Shunt 3. Good Biventricular Function. | | | |
| SIGNATURE  Done by: Tesfaye T., Pediatric Cardiologist _______________ 29/03/2012Eth.C | | | |

| Patient Name: **Selamawit Yenesew**. Patient ID:_ADINAS H. SEX/ Age: F/ 4Monthss Date of Report: 30**/03/2012_**.  BP: ___ Weight: ___ Height:___ BSA: ___. Referral Diagnosis: **RD + DS. AGH03.1944.** | | | |
| --- | --- | --- | --- |
| **Features** | **Finding** | **Features** | **Finding** |
| **Profile** |  | **Atria** |  |
| Abdominal situs | Solitus | Left atrium | Dilated |
| Cardiac position | Levocardia | Right atrium | Normal |
| Systemic venous drainage | Normal | **Atrioventricular valves** |  |
| Pulmonary venous drainage | Normal | Mitral valve | Annulus = 12mm |
| Atrioventricular connection | Concordant | Tricuspid valve | Annulus = 10mm.  TAPSE = 12mm |
| Ventriculoarterial connection | Concordant | **Ventricles** |  |
| Ventricular loop | d-Loop | Left ventricle | Dilated |
|  |  | Right ventricle | Normal |
| **Septae** |  | **Coronary arteries** | ----- |
| Interventricular septum | 5cm PM VSD, L – R Shunt | **Doppler Measurement** |  |
| Interatrial septum | 6mm OS ASD, L – R Shunt | Mitral | ---- |
| **Semilunal valves** |  | Aortic | ---- |
| Aortic valve | Annulus = 9mm | Tricuspid | ---- |
| Pulmonary valve | Annulus = 11mm | pulmonic | Moderate Valvar PS, PPG = 40mmHg |
| **Great arteries** | NRGA | **Aortic arch** |  |
| Aorta |  | **PDA** | No |
| Pulmonary artery | Normal MPA and confluent Branch PAs. |  |  |
| **M-Mode:** | | | |
| AO | mm | PWd | mm |
| LA | mm | EDV | ml |
| LVIDd | mm | ESV | ml |
| LVIDs | mm | LVEF | 72% |
| IVSd | mm | FS | 40% |
| **Additional Information**: |  | | |
| No pleural/pericardial effusion | | | |
| **Final Diagnosis:** | | | |
| 1. {S, D, S} Levocardia 2. Small OS ASD, L – R Shunt 3. Moderate Perimembranous VSD, L – R Shunt 4. Moderate Valvar PS 5. Good Biventricular Function. | | | |
| SIGNATURE  Done by: Tesfaye T., Pediatric Cardiologist _______________ 30/03/2012Eth.C | | | |

| Patient Name: **Kidist Ayana**. Patient ID: Adinas H. SEX/ Age: F/ 12Years Date of Report: 30**/03/2012_**.  BP: ___ Weight: ___ Height:___ BSA: ___. Referral Diagnosis: **DOE. AGH03.1945.** | | | |
| --- | --- | --- | --- |
| **Features** | **Finding** | **Features** | **Finding** |
| **Profile** |  | **Atria** |  |
| Abdominal situs | Solitus | Left atrium | Normal |
| Cardiac position | Levocardia | Right atrium | Normal |
| Systemic venous drainage | Normal | **Atrioventricular valves** |  |
| Pulmonary venous drainage | Normal | Mitral valve | Annulus = 17mm |
| Atrioventricular connection | Concordant | Tricuspid valve | Annulus = 21mm.  TAPSE = 23mm |
| Ventriculoarterial connection | Concordant | **Ventricles** |  |
| Ventricular loop | d-Loop | Left ventricle | Normal |
|  |  | Right ventricle | Normal |
| **Septae** |  | **Coronary arteries** | ----- |
| Interventricular septum | Intact | **Doppler Measurement** |  |
| Interatrial septum | 7 X 5mm OS ASD, L – R Shunt | Mitral | ---- |
| **Semilunal valves** |  | Aortic | ---- |
| Aortic valve | Annulus = 17mm | Tricuspid | ---- |
| Pulmonary valve | Annulus = 17mm | pulmonic | ---- |
| **Great arteries** | NRGA | **Aortic arch** |  |
| Aorta |  | **PDA** | No |
| Pulmonary artery | Normal MPA and confluent Branch PAs. |  |  |
| **M-Mode:** | | | |
| AO | mm | PWd | mm |
| LA | mm | EDV | ml |
| LVIDd | mm | ESV | ml |
| LVIDs | mm | LVEF | 52% |
| IVSd | mm | FS | 26% |
| **Additional Information**: |  | | |
| No pleural/pericardial effusion | | | |
| **Final Diagnosis:** | | | |
| 1. {S, D, S} Levocardia 2. Moderate OS ASD, L – R Shunt 3. Good Function | | | |
| SIGNATURE  Done by: Tesfaye T., Pediatric Cardiologist _______________ 30/03/2012Eth.C | | | |

| Patient Name: **Meaza Tarekegn**. Patient ID:_TGSH. SEX/ Age: F/ 7Years Date of Report: 30**/03/2012_**.  BP: ___ Weight: ___ Height:___ BSA: ___. Referral Diagnosis: **DOE + Chest Pain. AGH03.1946.** | | | |
| --- | --- | --- | --- |
| **Features** | **Finding** | **Features** | **Finding** |
| **Profile** |  | **Atria** |  |
| Abdominal situs | Solitus | Left atrium | Normal |
| Cardiac position | Levocardia | Right atrium | 14 X 16mm Hypo echoic mass, freely mobile. Have smooth surface. |
| Systemic venous drainage | Normal | **Atrioventricular valves** |  |
| Pulmonary venous drainage | Normal | Mitral valve | Annulus = 18mm |
| Atrioventricular connection | Concordant | Tricuspid valve | Annulus = 20mm.  TAPSE = 24mm |
| Ventriculoarterial connection | Concordant | **Ventricles** |  |
| Ventricular loop | d-Loop | Left ventricle | Normal |
|  |  | Right ventricle | Normal |
| **Septae** |  | **Coronary arteries** | ----- |
| Interventricular septum | Intact | **Doppler Measurement** |  |
| Interatrial septum | Intact | Mitral | ---- |
| **Semilunal valves** |  | Aortic | ---- |
| Aortic valve | Annulus = 17mm | Tricuspid | ---- |
| Pulmonary valve | Annulus = 18mm | pulmonic | ----- |
| **Great arteries** | NRGA | **Aortic arch** |  |
| Aorta | ----- | **PDA** | No |
| Pulmonary artery | Normal MPA and confluent Branch PAs. |  |  |
| **M-Mode:** | | | |
| AO | mm | PWd | mm |
| LA | mm | EDV | ml |
| LVIDd | mm | ESV | ml |
| LVIDs | mm | LVEF | 69% |
| IVSd | mm | FS | 38% |
| **Additional Information**: |  | | |
| No pleural/pericardial effusion | | | |
| **Final Diagnosis:** | | | |
| 1. {S, D, S} Levocardia 2. Hypo echoic smooth surfaced, freely mobile mass in the Right Atrium (Highly likely to be thrombus) 3. Normal Heart Structure and Function. | | | |
| SIGNATURE  Done by: Tesfaye T., Pediatric Cardiologist _______________ 30/03/2012Eth.C | | | |

| Patient Name: **Animut Minilik**. Patient ID:_TGSH. SEX/ Age: M/ 13Years Date of Report: 30**/03/2012_**.  BP: ___ Weight: ___ Height:___ BSA: ___. Referral Diagnosis: **CHF + Rheumatic Recurrence. AGH03.1947.** | | | | | | |
| --- | --- | --- | --- | --- | --- | --- |
| **Features** | **Finding** | | **Features** | | **Finding** | |
| **Profile** |  | | **Atria** | |  | |
| Abdominal situs | Solitus | | Left atrium | | Dilated, 54 X 63mm | |
| Cardiac position | Levocardia | | Right atrium | | Normal | |
| Systemic venous drainage | Normal. IVC Dilated | | **Atrioventricular valves** | |  | |
| Pulmonary venous drainage | Normal | | Mitral valve | | Annulus = 27mm. Thickened, clubbed MVL. MVA = 2cm**2**. | |
| Atrioventricular connection | Concordant | | Tricuspid valve | | Annulus = 25mm.  TAPSE = 21mm | |
| Ventriculoarterial connection | Concordant | | **Ventricles** | |  | |
| Ventricular loop | d-Loop | | Left ventricle | | Dilated | |
|  |  | | Right ventricle | | Normal | |
| **Septae** |  | | **Coronary arteries** | | ----- | |
| Interventricular septum | Intact | | **Doppler Measurement** | |  | |
| Interatrial septum | Intact | | Mitral | | Severe MR, Holosystolic, posterior jet with velocity of 3.7m/sec. MS, PPG/MPG = 15/7mmHg | |
| **Semilunal valves** |  | | Aortic | | ---- | |
| Aortic valve | Annulus = 16mm | | Tricuspid | | Mild TR, PPG = 34mmHg | |
| Pulmonary valve | Annulus = 22mm | | pulmonic | | Trivial PR, PPG = 26mmHg | |
| **Great arteries** | NRGA | | **Aortic arch** | |  | |
| Aorta |  | | **PDA** | | No | |
| Pulmonary artery | Normal MPA and confluent Branch PAs. | |  | |  | |
| **M-Mode:** | | | | | | |
| AO | | mm | | PWd | | mm |
| LA | | mm | | EDV | | ml |
| LVIDd | | mm | | ESV | | ml |
| LVIDs | | mm | | LVEF | | 52% |
| IVSd | | mm | | FS | | 27% |
| **Additional Information**: | |  | | | | |
| No pleural/pericardial effusion | | | | | | |
| **Final Diagnosis:** | | | | | | |
| 1. {S, D, S} Levocardia 2. Severe MR 3. Mild MS 4. LA/LV Dilated 5. Thickened, clubbed Mitral valve leaflet 6. Mildly Reduced LV Function. | | | | | | |
| SIGNATURE  Done by: Tesfaye T., Pediatric Cardiologist _______________ 30/03/2012Eth.C | | | | | | |

| Patient Name: **Yosef Amare**. Patient ID:_FHRH. SEX/ Age: M/ 9/12s Date of Report: 01**/04/2012_**.  BP: ___ Weight: ___ Height:___ BSA: ___. Referral Diagnosis: **DS. AGH03.1948.** | | | |
| --- | --- | --- | --- |
| **Features** | **Finding** | **Features** | **Finding** |
| **Profile** |  | **Atria** |  |
| Abdominal situs | Solitus | Left atrium | Normal |
| Cardiac position | Levocardia | Right atrium | Normal |
| Systemic venous drainage | Normal | **Atrioventricular valves** |  |
| Pulmonary venous drainage | Normal | Mitral valve | Annulus = 13mm |
| Atrioventricular connection | Concordant | Tricuspid valve | Annulus = 15mm.  TAPSE = 13mm |
| Ventriculoarterial connection | Concordant | **Ventricles** |  |
| Ventricular loop | d-Loop | Left ventricle | Normal |
|  |  | Right ventricle | Normal |
| **Septae** |  | **Coronary arteries** | ----- |
| Interventricular septum | Intact | **Doppler Measurement** |  |
| Interatrial septum | 4 x 6mm OS ASD, L – R Shunt. | Mitral | ---- |
| **Semilunal valves** |  | Aortic | ---- |
| Aortic valve | Annulus = 10mm | Tricuspid | Trivial TR, PPG = 32mmHg |
| Pulmonary valve | Annulus = 11mm | pulmonic | ---- |
| **Great arteries** | NRGA | **Aortic arch** | Left |
| Aorta | ------ | **PDA** | No |
| Pulmonary artery | Normal MPA and confluent Branch PAs. |  |  |
| **M-Mode:** | | | |
| AO | mm | PWd | 5.6mm |
| LA | mm | EDV | 18ml |
| LVIDd | 23mm | ESV | 5ml |
| LVIDs | 14mm | LVEF | 69% |
| IVSd | 5mm | FS | 37% |
| **Additional Information**: |  | | |
| No pleural/pericardial effusion | | | |
| **Final Diagnosis:** | | | |
| 1. {S, D, S} Levocardia 2. Small OS ASD, L – R Shunt 3. Good Biventricular Function. | | | |
| SIGNATURE  Done by: Tesfaye T., Pediatric Cardiologist _______________ 01/04/2012Eth.C | | | |

| Patient Name: **Sinidu Dememew**. Patient ID:_FHRH. SEX/ Age: F/ 14Years Date of Report: 02**/04/2012_**.  BP: ___ Weight: ___ Height:___ BSA: ___. Referral Diagnosis: **Syncope. AGH03.1949.** | | | |
| --- | --- | --- | --- |
| **Features** | **Finding** | **Features** | **Finding** |
| **Profile** |  | **Atria** |  |
| Abdominal situs | Solitus | Left atrium | Normal |
| Cardiac position | Levocardia | Right atrium | Normal |
| Systemic venous drainage | Normal | **Atrioventricular valves** |  |
| Pulmonary venous drainage | Normal | Mitral valve | Annulus = 23mm |
| Atrioventricular connection | Concordant | Tricuspid valve | Annulus = 25mm.  TAPSE = 21mm |
| Ventriculoarterial connection | Concordant | **Ventricles** |  |
| Ventricular loop | d-Loop | Left ventricle | Normal |
|  |  | Right ventricle | Normal |
| **Septae** |  | **Coronary arteries** | ----- |
| Interventricular septum | Intact | **Doppler Measurement** |  |
| Interatrial septum | Intact | Mitral | ---- |
| **Semilunal valves** |  | Aortic | ---- |
| Aortic valve | Annulus = 18mm | Tricuspid | ---- |
| Pulmonary valve | Annulus = 18mm | pulmonic | ---- |
| **Great arteries** | NRGA | **Aortic arch** | Left |
| Aorta | ---- | **PDA** | No |
| Pulmonary artery | Normal MPA and confluent Branch PAs. |  |  |
| **M-Mode:** | | | |
| AO | mm | PWd | mm |
| LA | mm | EDV | ml |
| LVIDd | mm | ESV | ml |
| LVIDs | mm | LVEF | 58% |
| IVSd | mm | FS | 31% |
| **Additional Information**: |  | | |
| No pleural/pericardial effusion | | | |
| **Final Diagnosis:** | | | |
| 1. **Normal Echocardiography Study** | | | |
| SIGNATURE  Done by: Tesfaye T., Pediatric Cardiologist _______________ 02/04/2012Eth.C | | | |

| Patient Name: **Teraye Tesfa**. Patient ID: Debre Tabour H.. SEX/ Age: F/ 14Years Date of Report: 02**/04/2012_**.  BP: ___ Weight: ___ Height:___ BSA: ___. Referral Diagnosis: **CHF + Rheumatic Recurrence. AGH03.1950.** | | | |
| --- | --- | --- | --- |
| **Features** | **Finding** | **Features** | **Finding** |
| **Profile** |  | **Atria** |  |
| Abdominal situs | Solitus | Left atrium | Dilated |
| Cardiac position | Levocardia | Right atrium | More Dilated |
| Systemic venous drainage | Normal | **Atrioventricular valves** |  |
| Pulmonary venous drainage | Normal | Mitral valve | Annulus = 18mm. thickened, Clubbed, Calcified MVL. Old vegetation on the tip of the septal leaflet. MVA = 0.7cm**2**. |
| Atrioventricular connection | Concordant | Tricuspid valve | Annulus = 27mm.  TAPSE = 23mm |
| Ventriculoarterial connection | Concordant | **Ventricles** |  |
| Ventricular loop | d-Loop | Left ventricle | Normal |
|  |  | Right ventricle | Dilated |
| **Septae** |  | **Coronary arteries** | ----- |
| Interventricular septum | Intact | **Doppler Measurement** |  |
| Interatrial septum | Intact | Mitral | Mild MR. Severe MS, PPG/MPG = 15/10mmHg |
| **Semilunal valves** |  | Aortic | ---- |
| Aortic valve | Annulus = 16mm | Tricuspid | Severe TR, PPG = 75mmHg. |
| Pulmonary valve | Annulus = 20mm | pulmonic | ---- |
| **Great arteries** | NRGA | **Aortic arch** | Left |
| Aorta |  | **PDA** | No |
| Pulmonary artery | Normal MPA and confluent Branch PAs. |  |  |
| **M-Mode:** | | | |
| AO | mm | PWd | mm |
| LA | mm | EDV | ml |
| LVIDd | mm | ESV | ml |
| LVIDs | mm | LVEF | 62% |
| IVSd | mm | FS | 32% |
| **Additional Information**: | No pleural/pericardial effusion | | |
| **Final Diagnosis:** | | | |
| 1. {S, D, S} Levocardia 2. Calcified, thickened, clubbed Mitral Valve 3. Severe MS 4. Mild MR 5. Severe TR 6. Old Calcified Vegetation over the septal MVL. 7. Severe Pulmonary Hypertension | | | |
| SIGNATURE  Done by: Tesfaye T., Pediatric Cardiologist _______________ 02/04/2012Eth.C | | | |

| Patient Name: **Natan Simachew**. Patient ID:_FHRH. SEX/ Age: M/ 7Years Date of Report: 02**/04/2012_**.  BP: ___ Weight: ___ Height:___ BSA: ___. Referral Diagnosis: **Easy Fatigability. AGH03.1951.** | | | |
| --- | --- | --- | --- |
| **Features** | **Finding** | **Features** | **Finding** |
| **Profile** |  | **Atria** |  |
| Abdominal situs | Solitus | Left atrium | Normal |
| Cardiac position | Levocardia | Right atrium | Normal |
| Systemic venous drainage | Normal | **Atrioventricular valves** |  |
| Pulmonary venous drainage | Normal | Mitral valve | Annulus = mm |
| Atrioventricular connection | Concordant | Tricuspid valve | Annulus = mm.  TAPSE = mm |
| Ventriculoarterial connection | Concordant | **Ventricles** |  |
| Ventricular loop | d-Loop | Left ventricle | Normal |
|  |  | Right ventricle | Normal |
| **Septae** |  | **Coronary arteries** | ----- |
| Interventricular septum | Intact | **Doppler Measurement** |  |
| Interatrial septum | Intact | Mitral | ---- |
| **Semilunal valves** |  | Aortic | ---- |
| Aortic valve | Annulus = mm | Tricuspid | ---- |
| Pulmonary valve | Annulus = mm | pulmonic |  |
| **Great arteries** | NRGA | **Aortic arch** |  |
| Aorta |  | **PDA** | No |
| Pulmonary artery | Normal MPA and confluent Branch PAs. |  |  |
| **M-Mode:** | | | |
| AO | mm | PWd | mm |
| LA | mm | EDV | ml |
| LVIDd | mm | ESV | ml |
| LVIDs | mm | LVEF | % |
| IVSd | mm | FS | % |
| **Additional Information**: |  | | |
| No pleural/pericardial effusion | | | |
| **Final Diagnosis:** | | | |
| 1. {S, D, S} Levocardia | | | |
| SIGNATURE  Done by: Tesfaye T., Pediatric Cardiologist _______________ 02/04/2012Eth.C | | | |

| Patient Name: **Birtukan Getachew**. Patient ID: TGSH. SEX/ Age: F/ 7Years Date of Report: 02**/04/2012_**.  BP: ___ Weight: ___ Height:___ BSA: ___. Referral Diagnosis: **ARF. AGH03.1952.** | | | |
| --- | --- | --- | --- |
| **Features** | **Finding** | **Features** | **Finding** |
| **Profile** |  | **Atria** |  |
| Abdominal situs | Solitus | Left atrium | Mildly Dilated |
| Cardiac position | Levocardia | Right atrium | Normal |
| Systemic venous drainage | Normal | **Atrioventricular valves** |  |
| Pulmonary venous drainage | Normal | Mitral valve | Annulus = 18mm. Thickened MVL. |
| Atrioventricular connection | Concordant | Tricuspid valve | Annulus = 20mm.  TAPSE = 21mm |
| Ventriculoarterial connection | Concordant | **Ventricles** |  |
| Ventricular loop | d-Loop | Left ventricle | Dilated |
|  |  | Right ventricle | Normal |
| **Septae** |  | **Coronary arteries** | ----- |
| Interventricular septum | Intact | **Doppler Measurement** |  |
| Interatrial septum | Intact | Mitral | Moderate MR, Holosystolic, Posterior jet with jet velocity of 4m.sec. |
| **Semilunal valves** |  | Aortic | ---- |
| Aortic valve | Annulus = 14mm | Tricuspid | Trivial TR, PPG = 19mmHg |
| Pulmonary valve | Annulus = 15mm | pulmonic |  |
| **Great arteries** | NRGA | **Aortic arch** |  |
| Aorta |  | **PDA** | No |
| Pulmonary artery | Normal MPA and confluent Branch PAs. |  |  |
| **M-Mode:** | | | |
| AO | mm | PWd | mm |
| LA | mm | EDV | ml |
| LVIDd | mm | ESV | ml |
| LVIDs | mm | LVEF | 62% |
| IVSd | mm | FS | 33% |
| **Additional Information**: |  | | |
| No pleural/pericardial effusion | | | |
| **Final Diagnosis:** | | | |
| 1. {S, D, S} Levocardia 2. Thickened Mitral valve leaflet 3. Moderate MR 4. Good Biventricular Function | | | |
| SIGNATURE  Done by: Tesfaye T., Pediatric Cardiologist _______________ 02/04/2012Eth.C | | | |

| Patient Name: **Bethlehem Yaregal**. Patient ID: ADINAS H. SEX/ Age: F/ 1 7/12 Date of Report: 02**/04/2012_**.  BP: ___ Weight: ___ Height:___ BSA: ___. Referral Diagnosis: **RD + CHF. AGH03.1953.** | | | |
| --- | --- | --- | --- |
| **Features** | **Finding** | **Features** | **Finding** |
| **Profile** |  | **Atria** |  |
| Abdominal situs | Solitus | Left atrium | Normal |
| Cardiac position | Levocardia | Right atrium | Dilated |
| Systemic venous drainage | Normal | **Atrioventricular valves** |  |
| Pulmonary venous drainage | Normal | Mitral valve | Annulus = 14mm |
| Atrioventricular connection | Concordant | Tricuspid valve | Annulus = 22 mm.  TAPSE = 21mm |
| Ventriculoarterial connection | Concordant | **Ventricles** |  |
| Ventricular loop | d-Loop | Left ventricle | Normal |
|  |  | Right ventricle | Dilated |
| **Septae** |  | **Coronary arteries** | ----- |
| Interventricular septum | Intact | **Doppler Measurement** |  |
| Interatrial septum | 8 X 10mm OS ASD, L – R Shunt | Mitral | ---- |
| **Semilunal valves** |  | Aortic | ---- |
| Aortic valve | Annulus = 12mm | Tricuspid | Mild TR, PPG = 51mmHg |
| Pulmonary valve | Annulus = 14mm | pulmonic | ---- |
| **Great arteries** | NRGA | **Aortic arch** |  |
| Aorta |  | **PDA** | 4mm, BD Shunt |
| Pulmonary artery | MPA = 19mm. Confluent Branch PAs. |  |  |
| **M-Mode:** | | | |
| AO | mm | PWd | mm |
| LA | mm | EDV | ml |
| LVIDd | mm | ESV | ml |
| LVIDs | mm | LVEF | 63% |
| IVSd | mm | FS | 31% |
| **Additional Information**: |  | | |
| No pleural/pericardial effusion | | | |
| **Final Diagnosis:** | | | |
| 1. {S, D, S} Levocardia 2. Moderate OS ASD, L – R Shunt 3. Large PDA, BD Shunt 4. Moderate to Severe Pulmonary Hypertension 5. RA/RV Dilated 6. Good Function | | | |
| SIGNATURE  Done by: Tesfaye T., Pediatric Cardiologist _______________ 02/04/2012Eth.C | | | |

| Patient Name: **Masresha Ayalew**. Patient ID: FHRH. SEX/ Age: M/ 3Years Date of Report: 06**/04/2012_**.  BP: ___ Weight: ___ Height:___ BSA: ___. Referral Diagnosis: **Incidental Murmur. AGH03.1954.** | | | |
| --- | --- | --- | --- |
| **Features** | **Finding** | **Features** | **Finding** |
| **Profile** |  | **Atria** |  |
| Abdominal situs | Solitus | Left atrium | Normal |
| Cardiac position | Levocardia | Right atrium | Normal |
| Systemic venous drainage | Normal | **Atrioventricular valves** |  |
| Pulmonary venous drainage | Normal | Mitral valve | Annulus = 18mm |
| Atrioventricular connection | Concordant | Tricuspid valve | Annulus = 21mm. |
| Ventriculoarterial connection | Concordant | **Ventricles** |  |
| Ventricular loop | d-Loop | Left ventricle | Normal |
|  |  | Right ventricle | Normal |
| **Septae** |  | **Coronary arteries** | ----- |
| Interventricular septum | 4mm PM VSD, L – R Shunt | **Doppler Measurement** |  |
| Interatrial septum | Intact | Mitral | ---- |
| **Semilunal valves** |  | Aortic | ---- |
| Aortic valve | Annulus = 14mm | Tricuspid | ---- |
| Pulmonary valve | Annulus = 14mm | pulmonic | ---- |
| **Great arteries** | NRGA | **Aortic arch** |  |
| Aorta |  | **PDA** | No |
| Pulmonary artery | Normal MPA and confluent Branch PAs. |  |  |
| **M-Mode:** | | | |
| AO | mm | PWd | mm |
| LA | mm | EDV | ml |
| LVIDd | mm | ESV | ml |
| LVIDs | mm | LVEF | 63% |
| IVSd | mm | FS | 32% |
| **Additional Information**: |  | | |
| No pleural/pericardial effusion | | | |
| **Final Diagnosis:** | | | |
| 1. {S, D, S} Levocardia 2. Small Perimembranous VSD, L – R Shunt 3. Good Function | | | |
| SIGNATURE  Done by: Tesfaye T., Pediatric Cardiologist _______________ 06/04/2012Eth.C | | | |

| Patient Name: **Sewnet Gizachew**. Patient ID: FHRH. SEX/ Age: F/ 6Years Date of Report: 06**/04/2012_**.  BP: ___ Weight: ___ Height:___ BSA: ___. Referral Diagnosis: **Incidental Murmur. AGH03.1955.** | | | |
| --- | --- | --- | --- |
| **Features** | **Finding** | **Features** | **Finding** |
| **Profile** |  | **Atria** |  |
| Abdominal situs | Solitus | Left atrium | Normal |
| Cardiac position | Levocardia | Right atrium | Normal |
| Systemic venous drainage | Normal | **Atrioventricular valves** |  |
| Pulmonary venous drainage | Normal | Mitral valve | Annulus = 19mm |
| Atrioventricular connection | Concordant | Tricuspid valve | Annulus = 22mm.  TAPSE = 20mm |
| Ventriculoarterial connection | Concordant | **Ventricles** |  |
| Ventricular loop | d-Loop | Left ventricle | Normal |
|  |  | Right ventricle | Normal |
| **Septae** |  | **Coronary arteries** | ----- |
| Interventricular septum | Intact | **Doppler Measurement** |  |
| Interatrial septum | Intact | Mitral | ---- |
| **Semilunal valves** |  | Aortic | ---- |
| Aortic valve | Annulus = 17mm | Tricuspid | ---- |
| Pulmonary valve | Annulus = 19mm. Doming PV | pulmonic | Moderate Valvar PS, PPG = 43mmHg |
| **Great arteries** | NRGA | **Aortic arch** | Left |
| Aorta | ---- | **PDA** | No |
| Pulmonary artery | Normal MPA and confluent Branch PAs. |  | No Coarctation of Aorta. |
| **M-Mode:** | | | |
| AO | mm | PWd | mm |
| LA | mm | EDV | ml |
| LVIDd | mm | ESV | ml |
| LVIDs | mm | LVEF | 64% |
| IVSd | mm | FS | 34% |
| **Additional Information**: |  | | |
| No pleural/pericardial effusion | | | |
| **Final Diagnosis:** | | | |
| 1. {S, D, S} Levocardia 2. Doming Pulmonary Valve 3. Moderate Valvar PS 4. Good Biventricular Function | | | |
| SIGNATURE  Done by: Tesfaye T., Pediatric Cardiologist _______________ 06/04/2012Eth.C | | | |

| Patient Name: **Baby Tsegaye Kassahun**. Patient ID: FHRH. SEX/ Age: F/ 37Days Date of Report: 06**/04/2012_**.  BP: ___ Weight: ___ Height:___ BSA: ___. Referral Diagnosis: **DS. AGH03.1956.** | | | |
| --- | --- | --- | --- |
| **Features** | **Finding** | **Features** | **Finding** |
| **Profile** |  | **Atria** |  |
| Abdominal situs | Solitus | Left atrium | Normal |
| Cardiac position | Levocardia | Right atrium | Normal |
| Systemic venous drainage | Normal | **Atrioventricular valves** |  |
| Pulmonary venous drainage | Normal | Mitral valve | Annulus = 11mm |
| Atrioventricular connection | Concordant | Tricuspid valve | Annulus = 13mm.  TAPSE = 12mm |
| Ventriculoarterial connection | Concordant | **Ventricles** |  |
| Ventricular loop | d-Loop | Left ventricle | Normal |
|  |  | Right ventricle | Normal |
| **Septae** |  | **Coronary arteries** | ----- |
| Interventricular septum | Intact | **Doppler Measurement** |  |
| Interatrial septum | PFO, L – R Shunt | Mitral | ---- |
| **Semilunal valves** |  | Aortic | ---- |
| Aortic valve | Annulus = 10mm | Tricuspid | ---- |
| Pulmonary valve | Annulus = 10mm | pulmonic |  |
| **Great arteries** | NRGA | **Aortic arch** |  |
| Aorta |  | **PDA** | No |
| Pulmonary artery | Normal MPA and confluent Branch PAs. |  |  |
| **M-Mode:** | | | |
| AO | mm | PWd | mm |
| LA | mm | EDV | ml |
| LVIDd | mm | ESV | ml |
| LVIDs | mm | LVEF | 60% |
| IVSd | mm | FS | 30% |
| **Additional Information**: |  | | |
| No pleural/pericardial effusion | | | |
| **Final Diagnosis:** | | | |
| 1. {S, D, S} Levocardia 2. PFO, L – R Shunt 3. Good Biventricular Function | | | |
| SIGNATURE  Done by: Tesfaye T., Pediatric Cardiologist _______________ 06/04/2012Eth.C | | | |

| Patient Name: **Sinknesh Ayalew**. Patient ID: FHRH. SEX/ Age: F/ 2Years Date of Report: 06**/04/2012_**.  BP: ___ Weight: ___ Height:___ BSA: ___. Referral Diagnosis: **Incidental Murmur. AGH03.1957.** | | | |
| --- | --- | --- | --- |
| **Features** | **Finding** | **Features** | **Finding** |
| **Profile** |  | **Atria** |  |
| Abdominal situs | Solitus | Left atrium | Normal |
| Cardiac position | Levocardia | Right atrium | Normal |
| Systemic venous drainage | Normal | **Atrioventricular valves** |  |
| Pulmonary venous drainage | Normal | Mitral valve | Annulus = 16mm |
| Atrioventricular connection | Concordant | Tricuspid valve | Annulus = 18mm.  TAPSE = 17mm |
| Ventriculoarterial connection | Concordant | **Ventricles** |  |
| Ventricular loop | d-Loop | Left ventricle | Normal |
|  |  | Right ventricle | Normal |
| **Septae** |  | **Coronary arteries** | ----- |
| Interventricular septum | 5mm PM VSD, L – R Shunt | **Doppler Measurement** |  |
| Interatrial septum | Intact | Mitral | ---- |
| **Semilunal valves** |  | Aortic | ---- |
| Aortic valve | Annulus = 11mm | Tricuspid | ---- |
| Pulmonary valve | Annulus = 13mm | pulmonic | ---- |
| **Great arteries** | NRGA | **Aortic arch** |  |
| Aorta | ----- | **PDA** | No |
| Pulmonary artery | Normal MPA and confluent Branch PAs. |  |  |
| **M-Mode:** | | | |
| AO | mm | PWd | mm |
| LA | mm | EDV | ml |
| LVIDd | mm | ESV | ml |
| LVIDs | mm | LVEF | 59% |
| IVSd | mm | FS | 30% |
| **Additional Information**: |  | | |
| No pleural/pericardial effusion | | | |
| **Final Diagnosis:** | | | |
| 1. {S, D, S} Levocardia 2. Small PM VSD, L – R Shunt 3. Good Biventricular Function. | | | |
| SIGNATURE  Done by: Tesfaye T., Pediatric Cardiologist _______________ 06/04/2012Eth.C | | | |

| Patient Name: **Abebe Mirtu** . Patient ID: FHRH. SEX/ Age: M/ 6Monthss Date of Report: 09**/04/2012_**.  BP: ___ Weight: ___ Height:___ BSA: ___. Referral Diagnosis: **DS. AGH03.1958.** | | | |
| --- | --- | --- | --- |
| **Features** | **Finding** | **Features** | **Finding** |
| **Profile** |  | **Atria** |  |
| Abdominal situs | Solitus | Left atrium | Normal |
| Cardiac position | Levocardia | Right atrium | Normal |
| Systemic venous drainage | Normal | **Atrioventricular valves** |  |
| Pulmonary venous drainage | Normal | Mitral valve | Annulus = 13mm |
| Atrioventricular connection | Concordant | Tricuspid valve | Annulus = 14mm. |
| Ventriculoarterial connection | Concordant | **Ventricles** |  |
| Ventricular loop | d-Loop | Left ventricle | Normal |
|  |  | Right ventricle | Normal |
| **Septae** |  | **Coronary arteries** | ----- |
| Interventricular septum | Intact | **Doppler Measurement** |  |
| Interatrial septum | PFO, L – R Shunt | Mitral | ---- |
| **Semilunal valves** |  | Aortic | ---- |
| Aortic valve | Annulus = 10mm | Tricuspid | ---- |
| Pulmonary valve | Annulus = 13mm | pulmonic | ---- |
| **Great arteries** | NRGA | **Aortic arch** |  |
| Aorta |  | **PDA** | No |
| Pulmonary artery | Normal MPA and confluent Branch PAs. |  |  |
| **M-Mode:** | | | |
| AO | mm | PWd | mm |
| LA | mm | EDV | ml |
| LVIDd | mm | ESV | ml |
| LVIDs | mm | LVEF | 55% |
| IVSd | mm | FS | 29% |
| **Additional Information**: |  | | |
| No pleural/pericardial effusion | | | |
| **Final Diagnosis:** | | | |
| 1. {S, D, S} Levocardia 2. PFO, L – R Shunt | | | |
| SIGNATURE  Done by: Tesfaye T., Pediatric Cardiologist _______________ 09/04/2012Eth.C | | | |

| Patient Name: **Senayit Agegnehu**. Patient ID: SBC. SEX/ Age: F/ 11Years Date of Report: 09**/04/2012_**.  BP: ___ Weight: ___ Height:___ BSA: ___. Referral Diagnosis: **Sydenham’s Chorea. AGH03.1959.** | | | |
| --- | --- | --- | --- |
| **Features** | **Finding** | **Features** | **Finding** |
| **Profile** |  | **Atria** |  |
| Abdominal situs | Solitus | Left atrium | Normal |
| Cardiac position | Levocardia | Right atrium | Normal |
| Systemic venous drainage | Normal | **Atrioventricular valves** |  |
| Pulmonary venous drainage | Normal | Mitral valve | Annulus = 18mm. Thickened MVL |
| Atrioventricular connection | Concordant | Tricuspid valve | Annulus = 21mm.  TAPSE = 22mm |
| Ventriculoarterial connection | Concordant | **Ventricles** |  |
| Ventricular loop | d-Loop | Left ventricle | Mildly Dilated |
|  |  | Right ventricle | Normal |
| **Septae** |  | **Coronary arteries** | ----- |
| Interventricular septum | Intact | **Doppler Measurement** |  |
| Interatrial septum | Intact | Mitral | Moderate MR, Holosystolic, with posterior projection and jet velocity of 3.8m/sec |
| **Semilunal valves** |  | Aortic | ---- |
| Aortic valve | Annulus = 17mm | Tricuspid | ---- |
| Pulmonary valve | Annulus = 18mm | pulmonic |  |
| **Great arteries** | NRGA | **Aortic arch** |  |
| Aorta | ---- | **PDA** | No |
| Pulmonary artery | Normal MPA and confluent Branch PAs. |  |  |
| **M-Mode:** | | | |
| AO | mm | PWd | mm |
| LA | mm | EDV | ml |
| LVIDd | mm | ESV | ml |
| LVIDs | mm | LVEF | 55% |
| IVSd | mm | FS | 28% |
| **Additional Information**: |  | | |
| No pleural/pericardial effusion | | | |
| **Final Diagnosis:** | | | |
| 1. {S, D, S} Levocardia 2. Moderate MR 3. Thickened MVL | | | |
| Remark: the Echocardiographic finding goes with rheumatic valve disease. Correlate with clinical findings | | | |
| SIGNATURE  Done by: Tesfaye T., Pediatric Cardiologist _______________ 09/04/2012Eth.C | | | |

| Patient Name: **Fikrte Desalegn**. Patient ID: FHRH. SEX/ Age: F/ 7Years Date of Report: 10**/04/2012_**.  BP: ___ Weight: ___ Height:___ BSA: ___. Referral Diagnosis: **Syncope. AGH03.1960.** | | | |
| --- | --- | --- | --- |
| **Features** | **Finding** | **Features** | **Finding** |
| **Profile** |  | **Atria** |  |
| Abdominal situs | Solitus | Left atrium | Normal |
| Cardiac position | Levocardia | Right atrium | Normal |
| Systemic venous drainage | Normal | **Atrioventricular valves** |  |
| Pulmonary venous drainage | Normal | Mitral valve | Annulus =17mm |
| Atrioventricular connection | Concordant | Tricuspid valve | Annulus = 19mm.  TAPSE = 18mm |
| Ventriculoarterial connection | Concordant | **Ventricles** |  |
| Ventricular loop | d-Loop | Left ventricle | Normal |
|  |  | Right ventricle | Normal |
| **Septae** |  | **Coronary arteries** | ----- |
| Interventricular septum | Intact | **Doppler Measurement** |  |
| Interatrial septum | Intact | Mitral | ---- |
| **Semilunal valves** |  | Aortic | ---- |
| Aortic valve | Annulus = 14mm | Tricuspid | ---- |
| Pulmonary valve | Annulus = 17mm | pulmonic | Trivial PR |
| **Great arteries** | NRGA | **Aortic arch** | Left |
| Aorta |  | **PDA** | No |
| Pulmonary artery | Normal MPA and confluent Branch PAs. |  |  |
| **M-Mode:** | | | |
| AO | mm | PWd | mm |
| LA | mm | EDV | ml |
| LVIDd | mm | ESV | ml |
| LVIDs | mm | LVEF | 68% |
| IVSd | mm | FS | 38% |
| **Additional Information**: |  | | |
| No pleural/pericardial effusion | | | |
| **Final Diagnosis:** | | | |
| 1. Normal Echocardiography Study. | | | |
| SIGNATURE  Done by: Tesfaye T., Pediatric Cardiologist _______________ 10/04/2012Eth.C | | | |

| Patient Name: **Mahilet Werkie**. Patient ID: Finote Selam H. SEX/ Age: F/ 7Years Date of Report: 10**/04/2012_**.  BP: ___ Weight: ___ Height:___ BSA: ___. Referral Diagnosis: **Easy Fatigability. AGH03.1961.** | | | |
| --- | --- | --- | --- |
| **Features** | **Finding** | **Features** | **Finding** |
| **Profile** |  | **Atria** |  |
| Abdominal situs | Solitus | Left atrium | Normal |
| Cardiac position | Levocardia | Right atrium | Normal |
| Systemic venous drainage | Normal | **Atrioventricular valves** |  |
| Pulmonary venous drainage | Normal | Mitral valve | Annulus = 16mm |
| Atrioventricular connection | Concordant | Tricuspid valve | Annulus = 20mm. |
| Ventriculoarterial connection | Concordant | **Ventricles** |  |
| Ventricular loop | d-Loop | Left ventricle | Normal |
|  |  | Right ventricle | Normal |
| **Septae** |  | **Coronary arteries** | ----- |
| Interventricular septum | Intact | **Doppler Measurement** |  |
| Interatrial septum | Intact | Mitral | ---- |
| **Semilunal valves** |  | Aortic | ---- |
| Aortic valve | Annulus = 12mm | Tricuspid | ---- |
| Pulmonary valve | Annulus = 14mm | pulmonic | ---- |
| **Great arteries** | NRGA | **Aortic arch** | Left |
| Aorta |  | **PDA** | No |
| Pulmonary artery | Normal MPA and confluent Branch PAs. |  |  |
| **M-Mode:** | | | |
| AO | mm | PWd | mm |
| LA | mm | EDV | ml |
| LVIDd | mm | ESV | ml |
| LVIDs | mm | LVEF | 61% |
| IVSd | mm | FS | 31% |
| **Additional Information**: |  | | |
| No pleural/pericardial effusion | | | |
| **Final Diagnosis:** | | | |
| 1. Normal Echocardiography Study | | | |
| SIGNATURE  Done by: Tesfaye T., Pediatric Cardiologist _______________ 10/04/2012Eth.C | | | |

| Patient Name: **Zinegnaw Gasha**. Patient ID: FHRH. SEX/ Age: M/ 5 Months Date of Report: 11**/04/2012_**.  BP: ___ Weight: ___ Height:___ BSA: ___. Referral Diagnosis: **Incidental Murmur. AGH03.1962.** | | | |
| --- | --- | --- | --- |
| **Features** | **Finding** | **Features** | **Finding** |
| **Profile** |  | **Atria** |  |
| Abdominal situs | Solitus | Left atrium | Dilated |
| Cardiac position | Levocardia | Right atrium | Normal |
| Systemic venous drainage | Normal | **Atrioventricular valves** |  |
| Pulmonary venous drainage | Impressive | Mitral valve | Annulus = 12mm |
| Atrioventricular connection | Concordant | Tricuspid valve | Annulus = 12mm.  TAPSE = 14mm |
| Ventriculoarterial connection | Concordant | **Ventricles** |  |
| Ventricular loop | d-Loop | Left ventricle | Dilated |
|  |  | Right ventricle | Normal |
| **Septae** |  | **Coronary arteries** | ----- |
| Interventricular septum | Intact | **Doppler Measurement** |  |
| Interatrial septum | Intact | Mitral | ---- |
| **Semilunal valves** |  | Aortic | ---- |
| Aortic valve | Annulus = 10mm | Tricuspid | ---- |
| Pulmonary valve | Annulus = 10mm | pulmonic | ---- |
| **Great arteries** | NRGA | **Aortic arch** |  |
| Aorta | ------ | **PDA** | 2.5mm PDA, L – R Shunt |
| Pulmonary artery | Normal MPA and confluent Branch PAs. |  |  |
| **M-Mode:** | | | |
| AO | mm | PWd | mm |
| LA | mm | EDV | ml |
| LVIDd | mm | ESV | ml |
| LVIDs | mm | LVEF | 68% |
| IVSd | mm | FS | 36% |
| **Additional Information**: |  | | |
| No pleural/pericardial effusion | | | |
| **Final Diagnosis:** | | | |
| 1. {S, D, S} Levocardia 2. Impressive Pulmonary Venous return 3. Moderate PDA, L – R Shunt 4. Good Biventricular Function | | | |
| SIGNATURE  Done by: Tesfaye T., Pediatric Cardiologist _______________ 11/04/2012Eth.C | | | |

| Patient Name: **Achefer Wendimagegn**. Patient ID: FHRH. SEX/ Age: F/ 6Years Date of Report: 11**/04/2012_**.  BP: ___ Weight: ___ Height:___ BSA: ___. Referral Diagnosis: **FTT. AGH03.1963.** | | | |
| --- | --- | --- | --- |
| **Features** | **Finding** | **Features** | **Finding** |
| **Profile** |  | **Atria** |  |
| Abdominal situs | Solitus | Left atrium | Normal |
| Cardiac position | Levocardia | Right atrium | Normal |
| Systemic venous drainage | Normal | **Atrioventricular valves** |  |
| Pulmonary venous drainage | Normal | Mitral valve | Annulus = 18mm |
| Atrioventricular connection | Concordant | Tricuspid valve | Annulus = 20mm.  TAPSE = 16mm |
| Ventriculoarterial connection | Concordant | **Ventricles** |  |
| Ventricular loop | d-Loop | Left ventricle | Normal |
|  |  | Right ventricle | Normal |
| **Septae** |  | **Coronary arteries** | ----- |
| Interventricular septum | Intact | **Doppler Measurement** |  |
| Interatrial septum | Intact | Mitral | ---- |
| **Semilunal valves** |  | Aortic | ---- |
| Aortic valve | Annulus = 14mm | Tricuspid | ---- |
| Pulmonary valve | Annulus = 17mm | pulmonic | ---- |
| **Great arteries** | NRGA | **Aortic arch** | Left |
| Aorta | ----- | **PDA** | No |
| Pulmonary artery | Normal MPA and confluent Branch PAs. |  |  |
| **M-Mode:** | | | |
| AO | mm | PWd | mm |
| LA | mm | EDV | ml |
| LVIDd | mm | ESV | ml |
| LVIDs | mm | LVEF | 69% |
| IVSd | mm | FS | 38% |
| **Additional Information**: |  | | |
| No pleural/pericardial effusion | | | |
| **Final Diagnosis:** | | | |
| 1. Normal Echocardiography Study | | | |
| SIGNATURE  Done by: Tesfaye T., Pediatric Cardiologist _______________ 11/04/2012Eth.C | | | |

| Patient Name: **Bizunesh Shiferaw**. Patient ID: FHRH. SEX/ Age: F/ 14Years Date of Report: 13**/04/2012_**.  BP: __ Weight: __ Height:__ BSA: __. Referral Diagnosis: **Palpitation + DOE + Rheumatic Recurrence. AGH03.1964.** | | | | | | |
| --- | --- | --- | --- | --- | --- | --- |
| **Features** | **Finding** | | **Features** | | **Finding** | |
| **Profile** |  | | **Atria** | |  | |
| Abdominal situs | Solitus | | Left atrium | | Dilated | |
| Cardiac position | Levocardia | | Right atrium | | Dilated | |
| Systemic venous drainage | Normal | | **AV valves** | |  | |
| Pulmonary venous drainage | Normal | | Mitral valve | | Annulus = 23mm. thickened MVL. MVA = 0.9cm**2**. | |
| Atrioventricular connection | Concordant | | TV | | Annulus = 22mm.  TAPSE = 26mm | |
| Ventriculoarterial connection | Concordant | | **Ventricles** | |  | |
| Ventricular loop | d-Loop | | LV | | Dilated | |
|  |  | | RV | | Dilated | |
| **Septae** |  | | **Coronary arteries** | | ----- | |
| Interventricular septum | Intact | | **Doppler** | |  | |
| Interatrial septum | Intact | | Mitral | | Severe MR with jet velocity of 3.5m/sec, Holosystolic. Severe MS with PPG/MPG = 18/13mmHg | |
| **Semilunal valves** |  | | Aortic | | Mild AR, PHT = 664ms. | |
| Aortic valve | Annulus = 16mm | | Tricuspid | | Mild TR, PPG = 49mmHg | |
| Pulmonary valve | Annulus = 18mm | | pulmonic | |  | |
| **Great arteries** | NRGA | | **Aortic arch** | |  | |
| Aorta | ----- | | **PDA** | | No | |
| Pulmonary artery | Normal MPA and confluent Branch PAs. | |  | |  | |
| **M-Mode:** | | | | | | |
| AO | | mm | | PWd | | mm |
| LA | | mm | | EDV | | ml |
| LVIDd | | mm | | ESV | | ml |
| LVIDs | | mm | | LVEF | | 66% |
| IVSd | | mm | | FS | | 37% |
| **Additional Information**: | |  | | | | |
| 12mm pericardial effusion on RA side and 10mm on RV side | | | | | | |
| **Final Diagnosis:** | | | | | | |
| 1. {S, D, S} Levocardia 2. LA/LV Dilated 3. Severe MR 4. Severe MS 5. Mild TR 6. Moderate Pulmonary Hypertension 7. Moderate Pericardial effusion on RA/RV Side 8. Good Biventricular Function | | | | | | |
| SIGNATURE  Done by: Tesfaye T., Pediatric Cardiologist _______________ 13/04/2012Eth.C | | | | | | |

| Patient Name: **Muluhabit Pawlos**. Patient ID: ADINAS H. SEX/ Age: F/ 4Years Date of Report: 13**/04/2012_**.  BP: ___ Weight: ___ Height:___ BSA: ___. Referral Diagnosis: **Incidental Murmur. AGH03.1965.** | | | |
| --- | --- | --- | --- |
| **Features** | **Finding** | **Features** | **Finding** |
| **Profile** |  | **Atria** |  |
| Abdominal situs | Solitus | Left atrium | Normal |
| Cardiac position | Levocardia | Right atrium | Normal |
| Systemic venous drainage | Normal | **Atrioventricular valves** |  |
| Pulmonary venous drainage | Normal | Mitral valve | Annulus = 17mm |
| Atrioventricular connection | Concordant | Tricuspid valve | Annulus = 23mm. |
| Ventriculoarterial connection | Concordant | **Ventricles** |  |
| Ventricular loop | d-Loop | Left ventricle | Normal |
|  |  | Right ventricle | Normal |
| **Septae** |  | **Coronary arteries** | ----- |
| Interventricular septum | 3mm PM VSD, L – R Shunt. | **Doppler Measurement** |  |
| Interatrial septum | Intact | Mitral | ---- |
| **Semilunal valves** |  | Aortic | ---- |
| Aortic valve | Annulus = 15mm | Tricuspid | ---- |
| Pulmonary valve | Annulus = 17mm | pulmonic |  |
| **Great arteries** | NRGA | **Aortic arch** |  |
| Aorta |  | **PDA** | No |
| Pulmonary artery | Normal MPA and confluent Branch PAs. |  |  |
| **M-Mode:** | | | |
| AO | mm | PWd | mm |
| LA | mm | EDV | ml |
| LVIDd | mm | ESV | ml |
| LVIDs | mm | LVEF | 69% |
| IVSd | mm | FS | 38% |
| **Additional Information**: |  | | |
| No pleural/pericardial effusion | | | |
| **Final Diagnosis:** | | | |
| 1. {S, D, S} Levocardia 2. Small Perimembranous VSD, L – R Shunt 3. Good Biventricular Function. | | | |
| SIGNATURE  Done by: Tesfaye T., Pediatric Cardiologist _______________ 13/04/2012Eth.C | | | |

| Patient Name: **Meaza Gashaw**. Patient ID: ADINAS H. SEX/ Age: F/ 11Years Date of Report: 13**/04/2012_**.  BP: ___ Weight: ___ Height:___ BSA: ___. Referral Diagnosis: **Easy Fatigability. AGH03.1966.** | | | |
| --- | --- | --- | --- |
| **Features** | **Finding** | **Features** | **Finding** |
| **Profile** |  | **Atria** |  |
| Abdominal situs | Solitus | Left atrium | Normal |
| Cardiac position | Levocardia | Right atrium | Normal |
| Systemic venous drainage | Normal | **Atrioventricular valves** |  |
| Pulmonary venous drainage | Normal | Mitral valve | Annulus = 18mm |
| Atrioventricular connection | Concordant | Tricuspid valve | Annulus = 23mm. |
| Ventriculoarterial connection | Concordant | **Ventricles** |  |
| Ventricular loop | d-Loop | Left ventricle | Normal |
|  |  | Right ventricle | Normal |
| **Septae** |  | **Coronary arteries** | ----- |
| Interventricular septum | Intact | **Doppler Measurement** |  |
| Interatrial septum | Intact | Mitral | ---- |
| **Semilunal valves** |  | Aortic | ---- |
| Aortic valve | Annulus = 17mm | Tricuspid | ---- |
| Pulmonary valve | Annulus = 21mm | pulmonic | Trivial PR |
| **Great arteries** | NRGA | **Aortic arch** | Left |
| Aorta | ----- | **PDA** | No |
| Pulmonary artery | Normal MPA and confluent Branch PAs. |  |  |
| **M-Mode:** | | | |
| AO | mm | PWd | mm |
| LA | mm | EDV | ml |
| LVIDd | mm | ESV | ml |
| LVIDs | mm | LVEF | 58% |
| IVSd | mm | FS | 30% |
| **Additional Information**: |  | | |
| No pleural/pericardial effusion | | | |
| **Final Diagnosis:** | | | |
| 1. Normal Echocardiography Study | | | |
| SIGNATURE  Done by: Tesfaye T., Pediatric Cardiologist _______________ 13/04/2012Eth.C | | | |

| Patient Name: **Me’eraf Tewodros**. Patient ID: FHRH. SEX/ Age: F/ 2 5/12Years Date of Report: 14**/04/2012_**.  BP: ___ Weight: ___ Height:___ BSA: ___. Referral Diagnosis: **Recurrent Chest Infection. AGH03.1967.** | | | |
| --- | --- | --- | --- |
| **Features** | **Finding** | **Features** | **Finding** |
| **Profile** |  | **Atria** |  |
| Abdominal situs | Solitus | Left atrium | Normal |
| Cardiac position | Levocardia | Right atrium | Normal |
| Systemic venous drainage | Normal | **Atrioventricular valves** |  |
| Pulmonary venous drainage | Normal | Mitral valve | Annulus = 14mm |
| Atrioventricular connection | Concordant | Tricuspid valve | Annulus =18 mm.  TAPSE = 15mm |
| Ventriculoarterial connection | Concordant | **Ventricles** |  |
| Ventricular loop | d-Loop | Left ventricle | Normal |
|  |  | Right ventricle | Normal |
| **Septae** |  | **Coronary arteries** | ----- |
| Interventricular septum | Intact | **Doppler Measurement** |  |
| Interatrial septum | Intact | Mitral | ---- |
| **Semilunal valves** |  | Aortic | ---- |
| Aortic valve | Annulus = 11mm | Tricuspid | Trivial TR, PPG = 27mmHg |
| Pulmonary valve | Annulus = 13mm | pulmonic | ---- |
| **Great arteries** | NRGA | **Aortic arch** | Left |
| Aorta | ----- | **PDA** | No |
| Pulmonary artery | Normal MPA and confluent Branch PAs. |  |  |
| **M-Mode:** | | | |
| AO | mm | PWd | mm |
| LA | mm | EDV | ml |
| LVIDd | mm | ESV | ml |
| LVIDs | mm | LVEF | 64% |
| IVSd | mm | FS | 33% |
| **Additional Information**: |  | | |
| No pleural/pericardial effusion | | | |
| **Final Diagnosis:** | | | |
| 1. Normal Echocardiography Study. | | | |
| SIGNATURE  Done by: Tesfaye T., Pediatric Cardiologist _______________ 14/04/2012Eth.C | | | |

| Patient Name: **Yitayal Shumet**. Patient ID: FHRH. SEX/ Age: M/ 3 Months Date of Report: 14**/04/2012_**.  BP: ___ Weight: ___ Height:___ BSA: ___. Referral Diagnosis: **DS. AGH03.1968.** | | | |
| --- | --- | --- | --- |
| **Features** | **Finding** | **Features** | **Finding** |
| **Profile** |  | **Atria** |  |
| Abdominal situs | Solitus | Left atrium | Normal |
| Cardiac position | Levocardia | Right atrium | Normal |
| Systemic venous drainage | Normal | **Atrioventricular valves** |  |
| Pulmonary venous drainage | Normal | Mitral valve | Annulus = 10mm |
| Atrioventricular connection | Concordant | Tricuspid valve | Annulus = 14mm.  TAPSE = 14mm |
| Ventriculoarterial connection | Concordant | **Ventricles** |  |
| Ventricular loop | d-Loop | Left ventricle | Normal |
|  |  | Right ventricle | Normal |
| **Septae** |  | **Coronary arteries** | ----- |
| Interventricular septum | Intact | **Doppler Measurement** |  |
| Interatrial septum | Intact | Mitral | ---- |
| **Semilunal valves** |  | Aortic | ---- |
| Aortic valve | Annulus = 11mm | Tricuspid | ---- |
| Pulmonary valve | Annulus = 11mm | pulmonic | ---- |
| **Great arteries** | NRGA | **Aortic arch** | Left |
| Aorta | ----- | **PDA** | No |
| Pulmonary artery | Normal MPA and confluent Branch PAs. |  |  |
| **M-Mode:** | | | |
| AO | mm | PWd | mm |
| LA | mm | EDV | ml |
| LVIDd | mm | ESV | ml |
| LVIDs | mm | LVEF | 59% |
| IVSd | mm | FS | 29% |
| **Additional Information**: |  | | |
| No pleural/pericardial effusion | | | |
| **Final Diagnosis:** | | | |
| 1. Normal Echocardiographic Study. | | | |
| SIGNATURE  Done by: Tesfaye T., Pediatric Cardiologist _______________ 14/04/2012Eth.C | | | |

| Patient Name: **Desalew Mossie**. Patient ID: FHRH. SEX/ Age: M/ 7Years Date of Report: 14**/04/2012_**.  BP: ___ Weight: ___ Height:___ BSA: ___. Referral Diagnosis: **Recurrent Chest Infection. AGH03.1969.** | | | |
| --- | --- | --- | --- |
| **Features** | **Finding** | **Features** | **Finding** |
| **Profile** |  | **Atria** |  |
| Abdominal situs | Solitus | Left atrium | Normal |
| Cardiac position | Levocardia | Right atrium | Normal |
| Systemic venous drainage | Normal | **Atrioventricular valves** |  |
| Pulmonary venous drainage | Normal | Mitral valve | Annulus = 17mm |
| Atrioventricular connection | Concordant | Tricuspid valve | Annulus = 17mm.  TAPSE = 21mm |
| Ventriculoarterial connection | Concordant | **Ventricles** |  |
| Ventricular loop | d-Loop | Left ventricle | Normal |
|  |  | Right ventricle | Normal |
| **Septae** |  | **Coronary arteries** | ----- |
| Interventricular septum | Intact | **Doppler Measurement** |  |
| Interatrial septum | Intact | Mitral | ---- |
| **Semilunal valves** |  | Aortic | ---- |
| Aortic valve | Annulus = 14mm | Tricuspid | ---- |
| Pulmonary valve | Annulus = 16mm | pulmonic | Trivial PR, PPG = 10mmHg |
| **Great arteries** | NRGA | **Aortic arch** | Left |
| Aorta | ----- | **PDA** | No |
| Pulmonary artery | Normal MPA and confluent Branch PAs. |  |  |
| **M-Mode:** | | | |
| AO | mm | PWd | mm |
| LA | mm | EDV | ml |
| LVIDd | mm | ESV | ml |
| LVIDs | mm | LVEF | 65% |
| IVSd | mm | FS | 34% |
| **Additional Information**: |  | | |
| No pleural/pericardial effusion | | | |
| **Final Diagnosis:** | | | |
| 1. Normal Echocardiography Study | | | |
| SIGNATURE  Done by: Tesfaye T., Pediatric Cardiologist _______________ 14/04/2012Eth.C | | | |

| Patient Name: **Yitayish Alemayehu**. Patient ID: FHRH. SEX/ Age: F/ 14Years Date of Report: 14**/04/2012_**.  BP: ___ Weight: ___ Height:___ BSA: ___. Referral Diagnosis: **Palpitation. AGH03.1970.** | | | |
| --- | --- | --- | --- |
| **Features** | **Finding** | **Features** | **Finding** |
| **Profile** |  | **Atria** |  |
| Abdominal situs | Solitus | Left atrium | Normal |
| Cardiac position | Levocardia | Right atrium | Normal |
| Systemic venous drainage | Normal | **Atrioventricular valves** |  |
| Pulmonary venous drainage | Normal | Mitral valve | Annulus = 18mm |
| Atrioventricular connection | Concordant | Tricuspid valve | Annulus = 24mm.  TAPSE = 25mm |
| Ventriculoarterial connection | Concordant | **Ventricles** |  |
| Ventricular loop | d-Loop | Left ventricle | Normal |
|  |  | Right ventricle | Normal |
| **Septae** |  | **Coronary arteries** | ----- |
| Interventricular septum | Intact | **Doppler Measurement** |  |
| Interatrial septum | Intact | Mitral | ---- |
| **Semilunal valves** |  | Aortic | ---- |
| Aortic valve | Annulus = 19mm | Tricuspid | ---- |
| Pulmonary valve | Annulus = 19mm | pulmonic | ---- |
| **Great arteries** | NRGA | **Aortic arch** | Left |
| Aorta | ----- | **PDA** | No |
| Pulmonary artery | Normal MPA and confluent Branch PAs. |  |  |
| **M-Mode:** | | | |
| AO | mm | PWd | mm |
| LA | mm | EDV | ml |
| LVIDd | mm | ESV | ml |
| LVIDs | mm | LVEF | 60% |
| IVSd | mm | FS | 32% |
| **Additional Information**: |  | | |
| No pleural/pericardial effusion | | | |
| **Final Diagnosis:** | | | |
| 1. Normal Echocardiography Study. | | | |
| SIGNATURE  Done by: Tesfaye T., Pediatric Cardiologist _______________ 14/04/2012Eth.C | | | |

| Patient Name: **Maritu Azimeraw**. Patient ID: LIBEN H. SEX/ Age: F/ 12Years Date of Report: 15**/04/2012_**.  BP: ___ Weight: ___ Height:___ BSA: ___. Referral Diagnosis: **Sepsis (Galloping Infection). AGH03.1971.** | | | |
| --- | --- | --- | --- |
| **Features** | **Finding** | **Features** | **Finding** |
| **Profile** |  | **Atria** |  |
| Abdominal situs | Solitus | Left atrium | Normal |
| Cardiac position | Levocardia | Right atrium | Normal |
| Systemic venous drainage | Normal | **Atrioventricular valves** |  |
| Pulmonary venous drainage | Normal | Mitral valve | Annulus = 20mm |
| Atrioventricular connection | Concordant | Tricuspid valve | Annulus = 20mm.  TAPSE = 16mm |
| Ventriculoarterial connection | Concordant | **Ventricles** |  |
| Ventricular loop | d-Loop | Left ventricle | Normal |
|  |  | Right ventricle | Normal |
| **Septae** |  | **Coronary arteries** | ----- |
| Interventricular septum | Intact | **Doppler Measurement** |  |
| Interatrial septum | Intact | Mitral | ---- |
| **Semilunal valves** |  | Aortic | ---- |
| Aortic valve | Annulus = 17mm | Tricuspid | ---- |
| Pulmonary valve | Annulus = 18mm | pulmonic | ---- |
| **Great arteries** | NRGA | **Aortic arch** | Left |
| Aorta | ----- | **PDA** | No |
| Pulmonary artery | Normal MPA and confluent Branch PAs. |  |  |
| **M-Mode:** | | | |
| AO | mm | PWd | mm |
| LA | mm | EDV | ml |
| LVIDd | mm | ESV | ml |
| LVIDs | mm | LVEF | 60% |
| IVSd | mm | FS | 32% |
| **Additional Information**: |  | | |
| No pleural/pericardial effusion | | | |
| **Final Diagnosis:** | | | |
| 1. Normal Echocardiography Study. | | | |
| SIGNATURE  Done by: Tesfaye T., Pediatric Cardiologist _______________ 15/04/2012Eth.C | | | |

| Patient Name: **Eyerus Ademe**. Patient ID: TGSH. SEX/ Age: F/ 14Years Date of Report: 16**/04/2012_**.  BP: ___ Weight: ___ Height:___ BSA: ___. Referral Diagnosis: **Easy Fatigability. AGH03.1972.** | | | |
| --- | --- | --- | --- |
| **Features** | **Finding** | **Features** | **Finding** |
| **Profile** |  | **Atria** |  |
| Abdominal situs | Solitus | Left atrium | Normal |
| Cardiac position | Levocardia | Right atrium | Normal |
| Systemic venous drainage | Normal | **Atrioventricular valves** |  |
| Pulmonary venous drainage | Normal | Mitral valve | Annulus = 20mm |
| Atrioventricular connection | Concordant | Tricuspid valve | Annulus = 20mm. |
| Ventriculoarterial connection | Concordant | **Ventricles** |  |
| Ventricular loop | d-Loop | Left ventricle | Normal |
|  |  | Right ventricle | Normal |
| **Septae** |  | **Coronary arteries** | ----- |
| Interventricular septum | Intact | **Doppler Measurement** |  |
| Interatrial septum | Intact | Mitral | ---- |
| **Semilunal valves** |  | Aortic | ---- |
| Aortic valve | Annulus = 18mm | Tricuspid | ---- |
| Pulmonary valve | Annulus = 17mm | pulmonic | ---- |
| **Great arteries** | NRGA | **Aortic arch** | Left |
| Aorta | ----- | **PDA** | No |
| Pulmonary artery | Normal MPA and confluent Branch PAs. |  | No Coarctation of Aorta. |
| **M-Mode:** | | | |
| AO | mm | PWd | mm |
| LA | mm | EDV | ml |
| LVIDd | mm | ESV | ml |
| LVIDs | mm | LVEF | 63% |
| IVSd | mm | FS | 33% |
| **Additional Information**: |  | | |
| No pleural/pericardial effusion | | | |
| **Final Diagnosis:** | | | |
| 1. Normal Echocardiography Study. | | | |
| SIGNATURE  Done by: Tesfaye T., Pediatric Cardiologist _______________ 16/04/2012Eth.C | | | |

| Patient Name: **Yeshambel Emwedew**. Patient ID: FHRH. SEX/ Age: M/ 3Years Date of Report: 16**/04/2012_**.  BP: ___ Weight: ___ Height:___ BSA: ___. Referral Diagnosis: **CHF. AGH03.1973.** | | | | |
| --- | --- | --- | --- | --- |
| **Features** | **Finding** | **Features** | | **Finding** |
| **Profile** |  | **Atria** | |  |
| Abdominal situs | Solitus | Left atrium | | Normal |
| Cardiac position | Levocardia | Right atrium | | **Dilated, 34 X 37mm** |
| Systemic venous drainage | Normal | **Atrioventricular valves** | |  |
| Pulmonary venous drainage | Normal | Mitral valve | | Annulus = 14mm |
| Atrioventricular connection | Concordant | Tricuspid valve | | Annulus = 21mm.  **TAPSE = 8mm** |
| Ventriculoarterial connection | Concordant | **Ventricles** | |  |
| Ventricular loop | d-Loop | Left ventricle | | Normal |
|  |  | Right ventricle | | Dilated, Dysfunctional |
| **Septae** |  | **Coronary arteries** | | ----- |
| Interventricular septum | Intact | **Doppler Measurement** | |  |
| Interatrial septum | PFO, R – L Shunt | Mitral | | ---- |
| **Semilunal valves** |  | Aortic | | ---- |
| Aortic valve | Annulus = 13mm | Tricuspid | | **Severe TR, PPG = 60mmHg** |
| Pulmonary valve | Annulus = 16mm. thickened Pulmonary valve leaflets. | pulmonic | | Moderate PR, PPG = 70mmHg. Mild PS, PPG = 35mmHg(Underestimated) |
| **Great arteries** | NRGA | **Aortic arch** | | Left |
| Aorta | ----- | **PDA** | | No |
| Pulmonary artery | MPA = **15mm**. Confluent Branch PAs. |  | |  |
| **M-Mode:** | | | | |
| AO | mm | PWd | mm | |
| LA | mm | EDV | ml | |
| LVIDd | mm | ESV | ml | |
| LVIDs | mm | LVEF | 68% | |
| IVSd | mm | FS | 36% | |
| **Additional Information**: | No pleural/pericardial effusion | | | |
| **Final Diagnosis:** | | | | |
| 1. {S, D, S} Levocardia 2. PFO, R – L Shunt 3. RA Dilated 4. Severe TR 5. Moderate PR 6. PS (Under estimated gradient because of RV Failure) 7. Thickened Pulmonary valve leaflet 8. Dilated, Dysfunctional RV 9. Good LV Function | | | | |
| SIGNATURE  Done by: Tesfaye T., Pediatric Cardiologist _______________ 16/04/2012Eth.C | | | | |

| Patient Name: **Hulu – Ager Amara**. Patient ID: FHRH. SEX/ Age: F/ 1 8/12 Date of Report: 17**/04/2012_**.  BP: ___ Weight: ___ Height:___ BSA: ___. Referral Diagnosis: **CHF. AGH03.1974.** | | | |
| --- | --- | --- | --- |
| **Features** | **Finding** | **Features** | **Finding** |
| **Profile** |  | **Atria** |  |
| Abdominal situs | Solitus | Left atrium | Normal |
| Cardiac position | Levocardia | Right atrium | Normal |
| Systemic venous drainage | Normal | **Atrioventricular valves** |  |
| Pulmonary venous drainage | Normal | Mitral valve | Annulus = 15mm |
| Atrioventricular connection | Concordant | Tricuspid valve | Annulus = 13mm. |
| Ventriculoarterial connection | Concordant | **Ventricles** |  |
| Ventricular loop | d-Loop | Left ventricle | Mildly Dilated |
|  |  | Right ventricle | Normal |
| **Septae** |  | **Coronary arteries** | ----- |
| Interventricular septum | 12mm Perimembranous VSD, L – R Shunt | **Doppler Measurement** |  |
| Interatrial septum | Intact | Mitral | Mild MR |
| **Semilunal valves** |  | Aortic | ---- |
| Aortic valve | Annulus = 12mm | Tricuspid | ---- |
| Pulmonary valve | Annulus = 15mm | pulmonic | ---- |
| **Great arteries** | NRGA | **Aortic arch** | Left |
| Aorta | ----- | **PDA** | No |
| Pulmonary artery | Normal MPA and confluent Branch PAs. |  |  |
| **M-Mode:** | | | |
| AO | mm | PWd | mm |
| LA | mm | EDV | ml |
| LVIDd | mm | ESV | ml |
| LVIDs | mm | LVEF | 62% |
| IVSd | mm | FS | 32% |
| **Additional Information**: |  | | |
| No pleural/pericardial effusion | | | |
| **Final Diagnosis:** | | | |
| 1. {S, D, S} Levocardia 2. Mild MR 3. Large Perimembranous VSD, L – R Shunt 4. Good Function | | | |
| SIGNATURE  Done by: Tesfaye T., Pediatric Cardiologist _______________ 17/04/2012Eth.C | | | |

| Patient Name: **Nigussu Getahun**. Patient ID: FHRH. SEX/ Age: M/ 1 3/12 Date of Report: 18**/04/2012**.  BP: ___ Weight: ___ Height:___ BSA: ___. Referral Diagnosis: **RD + DS. AGH03.1975.** | | | |
| --- | --- | --- | --- |
| **Features** | **Finding** | **Features** | **Finding** |
| **Profile** |  | **Atria** |  |
| Abdominal situs | Solitus | Left atrium | Normal |
| Cardiac position | Levocardia | Right atrium | Normal |
| Systemic venous drainage | Normal | **Atrioventricular valves** |  |
| Pulmonary venous drainage | Normal | Mitral valve | Annulus = 14mm |
| Atrioventricular connection | Concordant | Tricuspid valve | Annulus = 16mm.  TAPSE = 14mm |
| Ventriculoarterial connection | Concordant | **Ventricles** |  |
| Ventricular loop | d-Loop | Left ventricle | Normal |
|  |  | Right ventricle | Normal |
| **Septae** |  | **Coronary arteries** | ----- |
| Interventricular septum | Intact | **Doppler Measurement** |  |
| Interatrial septum | Intact | Mitral | ---- |
| **Semilunal valves** |  | Aortic | ---- |
| Aortic valve | Annulus = 11mm | Tricuspid | ---- |
| Pulmonary valve | Annulus = 12mm | pulmonic | ---- |
| **Great arteries** | NRGA | **Aortic arch** | Left |
| Aorta | ----- | **PDA** | No |
| Pulmonary artery | Normal MPA and confluent Branch PAs. |  |  |
| **M-Mode: Normal LV Function on eye balling** | | | |
| AO | mm | PWd | mm |
| LA | mm | EDV | ml |
| LVIDd | mm | ESV | ml |
| LVIDs | mm | LVEF | % |
| IVSd | mm | FS | % |
| **Additional Information**: |  | | |
| No pleural/pericardial effusion | | | |
| **Final Diagnosis:** | | | |
| 1. Normal Echocardiography Study. | | | |
| SIGNATURE  Done by: Tesfaye T., Pediatric Cardiologist _______________ 18/04/2012Eth.C | | | |

| Patient Name: **Tsigereda Mulu**. Patient ID: FHRH. SEX/ Age: F/ 4 7/12 Date of Report: 21**/04/2012_**.  BP: ___ Weight: ___ Height:___ BSA: ___. Referral Diagnosis: **IE. AGH03.1976.** | | | |
| --- | --- | --- | --- |
| **Features** | **Finding** | **Features** | **Finding** |
| **Profile** |  | **Atria** |  |
| Abdominal situs | Solitus | Left atrium | Mildly dilated |
| Cardiac position | Levocardia | Right atrium | Normal |
| Systemic venous drainage | Normal | **Atrioventricular valves** |  |
| Pulmonary venous drainage | Normal | Mitral valve | Annulus = 19mm |
| Atrioventricular connection | Concordant | Tricuspid valve | Annulus = 18mm.  TAPSE = 14mm |
| Ventriculoarterial connection | Concordant | **Ventricles** |  |
| Ventricular loop | d-Loop | Left ventricle | Mildly Dilated |
|  |  | Right ventricle | Normal |
| **Septae** |  | **Coronary arteries** | ----- |
| Interventricular septum | 9mm PM VSD, L – R Shunt | **Doppler Measurement** |  |
| Interatrial septum | Intact | Mitral | ---- |
| **Semilunal valves** |  | Aortic | ---- |
| Aortic valve | Annulus = 14mm | Tricuspid | ---- |
| Pulmonary valve | Annulus = 18mm | pulmonic | ---- |
| **Great arteries** | NRGA | **Aortic arch** | Left |
| Aorta | ----- | **PDA** | No |
| Pulmonary artery | Normal MPA and confluent Branch PAs. |  |  |
| **M-Mode:** | | | |
| AO | mm | PWd | mm |
| LA | mm | EDV | ml |
| LVIDd | mm | ESV | ml |
| LVIDs | mm | LVEF | 54% |
| IVSd | mm | FS | 27% |
| **Additional Information**: | - No evidence of vegetation so far | | |
| No pleural/pericardial effusion | | | |
| **Final Diagnosis:** | | | |
| 1. {S, D, S} Levocardia 2. Moderate Perimembranous VSD, L – R Shunt 3. Good Biventricular Function. | | | |
| SIGNATURE  Done by: Tesfaye T., Pediatric Cardiologist _______________ 21/04/2012Eth.C | | | |

| Patient Name: **Alefech Yismaw**. Patient ID: FHRH. SEX/ Age: F/ 6Monthss Date of Report: 21**/04/2012_**.  BP: ___ Weight: ___ Height:___ BSA: ___. Referral Diagnosis: **CHF + DS. AGH03.1977.** | | | |
| --- | --- | --- | --- |
| **Features** | **Finding** | **Features** | **Finding** |
| **Profile** |  | **Atria** |  |
| Abdominal situs | Solitus | Left atrium | Dilated |
| Cardiac position | Levocardia | Right atrium | Dilated |
| Systemic venous drainage | Normal | **Atrioventricular valves** |  |
| Pulmonary venous drainage | Normal | Mitral valve | Annulus = 11mm |
| Atrioventricular connection | Concordant | Tricuspid valve | Annulus = 15mm.  TAPSE = 12mm |
| Ventriculoarterial connection | Concordant | **Ventricles** |  |
| Ventricular loop | d-Loop | Left ventricle | Dilated |
|  |  | Right ventricle | Dilated |
| **Septae** |  | **Coronary arteries** | ----- |
| Interventricular septum | 10mm Inlet VSD, L – R Shunt | **Doppler Measurement** |  |
| Interatrial septum | Intact | Mitral | Moderate MR |
| **Semilunal valves** |  | Aortic | ---- |
| Aortic valve | Annulus = 9mm | Tricuspid | Moderate TR |
| Pulmonary valve | Annulus = 10mm | pulmonic | ---- |
| **Great arteries** | NRGA | **Aortic arch** | Left |
| Aorta | ----- | **PDA** | No |
| Pulmonary artery | **MPA = 14mm**. Good sized confluent Branch PAs. |  |  |
| **M-Mode:** | | | |
| AO | mm | PWd | mm |
| LA | mm | EDV | ml |
| LVIDd | mm | ESV | ml |
| LVIDs | mm | LVEF | 64% |
| IVSd | mm | FS | 33% |
| **Additional Information**: |  | | |
| No pleural/pericardial effusion | | | |
| **Final Diagnosis:** | | | |
| 1. {S, D, S} Levocardia 2. Large Inlet VSD, L – R Shunt 3. Severe Pulmonary Hypertension 4. Normal Biventricular Function | | | |
| SIGNATURE  Done by: Tesfaye T., Pediatric Cardiologist _______________ 21/04/2012Eth.C | | | |

| Patient Name: **Silenat Anim**. Patient ID: FHRH. SEX/ Age: F/ 10Years Date of Report: 22**/04/2012_**.  BP: ___ Weight: ___ Height:___ BSA: ___. Referral Diagnosis: **Rheumatic Recurrence. AGH03.1978.** | | | |
| --- | --- | --- | --- |
| **Features** | **Finding** | **Features** | **Finding** |
| **Profile** |  | **Atria** |  |
| Abdominal situs | Solitus | Left atrium | Dilated |
| Cardiac position | Levocardia | Right atrium | Normal |
| Systemic venous drainage | Normal | **Atrioventricular valves** |  |
| Pulmonary venous drainage | Normal | Mitral valve | Annulus =23mm. Thickened MVL |
| Atrioventricular connection | Concordant | Tricuspid valve | Annulus = 19mm.  TAPSE = 21mm |
| Ventriculoarterial connection | Concordant | **Ventricles** |  |
| Ventricular loop | d-Loop | Left ventricle | Dilated |
|  |  | Right ventricle | Normal |
| **Septae** |  | **Coronary arteries** | ----- |
| Interventricular septum | Intact | **Doppler Measurement** |  |
| Interatrial septum | Intact | Mitral | Severe MR with posterior projection and velocity of 4m/sec. Holosystolic |
| **Semilunal valves** |  | Aortic | Mild AR, PHT = 592ms |
| Aortic valve | Annulus = 22mm | Tricuspid | Trivial TR, PPG = 27mmHg |
| Pulmonary valve | Annulus = 21mm | pulmonic | ---- |
| **Great arteries** | NRGA | **Aortic arch** | Left |
| Aorta | ----- | **PDA** | No |
| Pulmonary artery | Normal MPA and confluent Branch PAs. |  |  |
| **M-Mode:** | | | |
| AO | mm | PWd | mm |
| LA | mm | EDV | ml |
| LVIDd | mm | ESV | ml |
| LVIDs | mm | LVEF | 55% |
| IVSd | mm | FS | 28% |
| **Additional Information**: | No pleural/pericardial effusion | | |
| **Final Diagnosis:** | | | |
| 1. {S, D, S} Levocardia 2. Severe MR 3. Mild AR 4. Dilated LA/LV 5. Thickened Mitral Valve leaflet 6. Good Biventricular Function. | | | |
| SIGNATURE  Done by: Tesfaye T., Pediatric Cardiologist _______________ 22/04/2012Eth.C | | | |

| Patient Name: **Kalkidan Asmamaw**. Patient ID: FHRH. SEX/ Age: F/ 10Years Date of Report: 23**/04/2012_**.  BP: ___ Weight: ___ Height:___ BSA: ___. Referral Diagnosis: **Rheumatic Recurrence. AGH03.1979.** | | | |
| --- | --- | --- | --- |
| **Features** | **Finding** | **Features** | **Finding** |
| **Profile** |  | **Atria** |  |
| Abdominal situs | Solitus | Left atrium | Dilated. 37mm X 59mm |
| Cardiac position | Levocardia | Right atrium | Normal |
| Systemic venous drainage | Normal | **Atrioventricular valves** |  |
| Pulmonary venous drainage | Normal | Mitral valve | Annulus = 30mm. Thickened MVL. MVA = 3.5cm2. |
| Atrioventricular connection | Concordant | Tricuspid valve | Annulus = 23mm.  TAPSE = 14mm |
| Ventriculoarterial connection | Concordant | **Ventricles** |  |
| Ventricular loop | d-Loop | Left ventricle | DILATED |
|  |  | Right ventricle | Normal |
| **Septae** |  | **Coronary arteries** | ----- |
| Interventricular septum | Intact | **Doppler Measurement** |  |
| Interatrial septum | Intact | Mitral | Severe MR, Holosystolic, posterior projection. Jet velocity 4.4m/sec. inflow gradient = 9/4mmHg |
| **Semilunal valves** |  | Aortic | ---- |
| Aortic valve | Annulus = 18mm | Tricuspid | Trivial TR, PPG = 30mmHg |
| Pulmonary valve | Annulus = 19mm | pulmonic | ---- |
| **Great arteries** | NRGA | **Aortic arch** | Left |
| Aorta | ----- | **PDA** | No |
| Pulmonary artery | Normal MPA and Branch PAs. |  |  |
| **M-Mode:** | | | |
| AO | mm | PWd | mm |
| LA | mm | EDV | ml |
| LVIDd | mm | ESV | ml |
| LVIDs | mm | LVEF | 43% |
| IVSd | mm | FS | 21% |
| **Additional Information**: | 9mm pericardial effusion on RA/RV Side | | |
| **Final Diagnosis:** | | | |
| 1. {S, D, S} Levocardia 2. Dilated LA/LV 3. Thickened MVL 4. Severe MR 5. Pericardial effusion 6. Reduced LV Function. | | | |
| SIGNATURE  Done by: Tesfaye T., Pediatric Cardiologist _______________ 23/04/2012Eth.C | | | |

| Patient Name: **Henock Nigus**. Patient ID: ADINAS H. SEX/ Age: M/ 9Years Date of Report: 23**/04/2012_**.  BP: ___ Weight: ___ Height:___ BSA: ___. Referral Diagnosis: **Chest Pain. AGH03.1980.** | | | |
| --- | --- | --- | --- |
| **Features** | **Finding** | **Features** | **Finding** |
| **Profile** |  | **Atria** |  |
| Abdominal situs | Solitus | Left atrium | Normal |
| Cardiac position | Levocardia | Right atrium | Normal |
| Systemic venous drainage | Normal | **Atrioventricular valves** |  |
| Pulmonary venous drainage | Normal | Mitral valve | Annulus = 15mm |
| Atrioventricular connection | Concordant | Tricuspid valve | Annulus = 19mm.  TAPSE = 19mm |
| Ventriculoarterial connection | Concordant | **Ventricles** |  |
| Ventricular loop | d-Loop | Left ventricle | Normal |
|  |  | Right ventricle | Normal |
| **Septae** |  | **Coronary arteries** | ----- |
| Interventricular septum | Intact | **Doppler Measurement** |  |
| Interatrial septum | Intact | Mitral | ---- |
| **Semilunal valves** |  | Aortic | ---- |
| Aortic valve | Annulus = 19mm | Tricuspid | ---- |
| Pulmonary valve | Annulus = 21mm | pulmonic | ---- |
| **Great arteries** | NRGA | **Aortic arch** | Left |
| Aorta | ----- | **PDA** | No |
| Pulmonary artery | Normal MPA and confluent Branch PAs. |  |  |
| **M-Mode:** | | | |
| AO | mm | PWd | mm |
| LA | mm | EDV | ml |
| LVIDd | mm | ESV | ml |
| LVIDs | mm | LVEF | 63% |
| IVSd | mm | FS | 33% |
| **Additional Information**: |  | | |
| No pleural/pericardial effusion | | | |
| **Final Diagnosis:** | | | |
| 1. Normal Echocardiography Study | | | |
| SIGNATURE  Done by: Tesfaye T., Pediatric Cardiologist _______________ 23/04/2012Eth.C | | | |

| Patient Name: **Kalkidan Yenewa**. Patient ID: FHRH. SEX/ Age: F/ 1 3/12 Date of Report: 23**/04/2012_**.  BP: ___ Weight: ___ Height:___ BSA: ___. Referral Diagnosis: **Incidental Murmur. AGH03.1981.** | | | |
| --- | --- | --- | --- |
| **Features** | **Finding** | **Features** | **Finding** |
| **Profile** |  | **Atria** |  |
| Abdominal situs | Solitus | Left atrium | Normal |
| Cardiac position | Levocardia | Right atrium | Normal |
| Systemic venous drainage | Normal | **Atrioventricular valves** |  |
| Pulmonary venous drainage | Normal | Mitral valve | Annulus = 13mm |
| Atrioventricular connection | Concordant | Tricuspid valve | Annulus = 13mm. |
| Ventriculoarterial connection | Concordant | **Ventricles** |  |
| Ventricular loop | d-Loop | Left ventricle | Normal |
|  |  | Right ventricle | Normal |
| **Septae** |  | **Coronary arteries** | ----- |
| Interventricular septum | Intact | **Doppler Measurement** |  |
| Interatrial septum | PFO, L – R Shunt | Mitral | ---- |
| **Semilunal valves** |  | Aortic | ---- |
| Aortic valve | Annulus = 9mm | Tricuspid | ---- |
| Pulmonary valve | Annulus = 9mm | pulmonic | Flow acceleration across pulmonary valve with peak gradient of 19mmHg |
| **Great arteries** | NRGA | **Aortic arch** | Left |
| Aorta | ----- | **PDA** | No |
| Pulmonary artery | Normal MPA and confluent Branch PAs. |  |  |
| **M-Mode: Good LV Function on eye balling** | | | |
| AO | mm | PWd | mm |
| LA | mm | EDV | ml |
| LVIDd | mm | ESV | ml |
| LVIDs | mm | LVEF | % |
| IVSd | mm | FS | % |
| **Additional Information**: | No pleural/pericardial effusion | | |
| Remark: Baby was mobile. Needs Follow up echo. | | | |
| **Final Diagnosis:** | | | |
| 1. {S, D, S} Levocardia 2. PFO, L – R Shunt 3. Flow acceleration across Pulmonary Valve with no significant obstruction. 4. Good LV Function | | | |
| SIGNATURE  Done by: Tesfaye T., Pediatric Cardiologist _______________ 23/04/2012Eth.C | | | |

| Patient Name: **Mekdes Tsegaye**. Patient ID: ADINAS H. SEX/ Age: F/ 7Years Date of Report: 23**/04/2012_**.  BP: ___ Weight: ___ Height:___ BSA: ___. Referral Diagnosis: **Incidental Murmur. AGH03.1982.** | | | |
| --- | --- | --- | --- |
| **Features** | **Finding** | **Features** | **Finding** |
| **Profile** |  | **Atria** |  |
| Abdominal situs | Solitus | Left atrium | Normal |
| Cardiac position | Levocardia | Right atrium | Normal |
| Systemic venous drainage | Normal | **Atrioventricular valves** |  |
| Pulmonary venous drainage | Normal | Mitral valve | Annulus = 17mm |
| Atrioventricular connection | Concordant | Tricuspid valve | Annulus = 17mm.  TAPSE = 21mm |
| Ventriculoarterial connection | Concordant | **Ventricles** |  |
| Ventricular loop | d-Loop | Left ventricle | Normal |
|  |  | Right ventricle | Normal |
| **Septae** |  | **Coronary arteries** | ----- |
| Interventricular septum | 4mm PM VSD, L – R Shunt. Restrictive with gradient of 61mmHg. Partially covered by tricuspid tissue | **Doppler Measurement** |  |
| Interatrial septum | Intact | Mitral | ---- |
| **Semilunal valves** |  | Aortic | ---- |
| Aortic valve | Annulus = 16mm | Tricuspid | ---- |
| Pulmonary valve | Annulus = 19mm | pulmonic | ---- |
| **Great arteries** | NRGA | **Aortic arch** | Left |
| Aorta | ----- | **PDA** | No |
| Pulmonary artery | Normal MPA and confluent Branch PAs. |  |  |
| **M-Mode:** | | | |
| AO | mm | PWd | mm |
| LA | mm | EDV | ml |
| LVIDd | mm | ESV | ml |
| LVIDs | mm | LVEF | 68% |
| IVSd | mm | FS | 37% |
| **Additional Information**: |  | | |
| No pleural/pericardial effusion | | | |
| **Final Diagnosis:** | | | |
| 1. {S, D, S} Levocardia 2. Small restrictive PM VSD, L – R Shunt 3. Good Biventricular Function | | | |
| SIGNATURE  Done by: Tesfaye T., Pediatric Cardiologist _______________ 23/04/2012Eth.C | | | |

| Patient Name: **Baby of Yidenek Wallelign**. Patient ID: TGSH. SEX/ Age: F/ 13Days Date of Report: 27**/04/2012_**.  BP: ___ Weight: ___ Height:___ BSA: ___. Referral Diagnosis: **RD + DS. AGH03.1983.** | | | |
| --- | --- | --- | --- |
| **Features** | **Finding** | **Features** | **Finding** |
| **Profile** |  | **Atria** |  |
| Abdominal situs | Solitus | Left atrium | Normal |
| Cardiac position | Levocardia | Right atrium | Normal |
| Systemic venous drainage | Normal | **Atrioventricular valves** |  |
| Pulmonary venous drainage | Normal | Mitral valve | Annulus = 12mm |
| Atrioventricular connection | Concordant | Tricuspid valve | Annulus = 13mm.  TAPSE = 10mm |
| Ventriculoarterial connection | Concordant | **Ventricles** |  |
| Ventricular loop | d-Loop | Left ventricle | Normal |
|  |  | Right ventricle | Normal |
| **Septae** |  | **Coronary arteries** | ----- |
| Interventricular septum | Intact | **Doppler Measurement** |  |
| Interatrial septum | Intact | Mitral | ---- |
| **Semilunal valves** |  | Aortic | ---- |
| Aortic valve | Annulus = 9mm | Tricuspid | ---- |
| Pulmonary valve | Annulus = 8mm | pulmonic | ---- |
| **Great arteries** | NRGA | **Aortic arch** | Left |
| Aorta | ----- | **PDA** | No |
| Pulmonary artery | Normal MPA and confluent Branch PAs. |  |  |
| **M-Mode:** | | | |
| AO | mm | PWd | mm |
| LA | mm | EDV | ml |
| LVIDd | mm | ESV | ml |
| LVIDs | mm | LVEF | 75% |
| IVSd | mm | FS | 41% |
| **Additional Information**: |  | | |
| No pleural/pericardial effusion | | | |
| **Final Diagnosis:** | | | |
| 1. Normal Echocardiography Study. | | | |
| SIGNATURE  Done by: Tesfaye T., Pediatric Cardiologist _______________ 27/04/2012Eth.C | | | |

| Patient Name: **Bewuket Enyew**. Patient ID: SBC. SEX/ Age: M/ 6Monthss Date of Report: 29**/04/2012_**.  BP: ___ Weight: ___ Height:___ BSA: ___. Referral Diagnosis: **CHF. AGH03.1984**. | | | |
| --- | --- | --- | --- |
| **Features** | **Finding** | **Features** | **Finding** |
| **Profile** |  | **Atria** |  |
| Abdominal situs | Solitus | Left atrium | Dilated |
| Cardiac position | Levocardia | Right atrium | Dilated |
| Systemic venous drainage | Normal | **Atrioventricular valves** |  |
| Pulmonary venous drainage | Normal | Mitral valve | Annulus = 13mm |
| Atrioventricular connection | Concordant | Tricuspid valve | Annulus = 13mm.  TAPSE = 14mm |
| Ventriculoarterial connection | DORV | **Ventricles** |  |
| Ventricular loop | d-Loop | Left ventricle | Dilated |
|  |  | Right ventricle | Dilated |
| **Septae** |  | **Coronary arteries** | ----- |
| Interventricular septum | 11mm Inlet VSD, Predominantly L – R Shunt | **Doppler Measurement** |  |
| Interatrial septum | Intact | Mitral | ---- |
| **Semilunal valves** |  | Aortic | ---- |
| Aortic valve | Annulus = 14mm | Tricuspid | Mild TR |
| Pulmonary valve | Annulus = 10mm | pulmonic | ---- |
| **Great arteries** | NRGA | **Aortic arch** | Left |
| Aorta | ----- | **PDA** | No |
[truncated: 747,368 more chars]
