# Supplementary material for: Two–Dimensional and Doppler trans-thoracic echocardiographic patterns of suspected pediatric heart diseases at Tibebe-—Ghion specialized Teaching Hospital and Adinas General Hospital, Bahir Dar, North-west Ethiopia:–An experience from an LMIC
Source: PLoS One. 2024 Mar 11;19(3):e0292694. doi: 10.1371/journal.pone.0292694 (PMC10927071; doi:10.1371/journal.pone.0292694)
Supplement: S1 File — (ZIP) [file pone.0292694.s002.zip › TGSH1 Pediatric Transthoracic Echocardiography Report August 2021 SPSS FILLED.docx]

| **Patient Name:** Yitayal Yenew. **Sex/Age**: M/3 4/12. **Date of Report**: 06/12/13Eth.C.  **Clinical Diagnosis: ?CHF + Murmur + Diaphoresis. TGSH1.2381** | | | |
| --- | --- | --- | --- |
| **Features:** | **Findings** | **Features** | **Findings** |
| **Profile** | | **Atria** | |
| Abdominal Situs | Solitus | Left Atrium | Dilated |
| Cardiac Position | Levocardia | Right Atrium | Dilated |
| Systemic Venous Drainage | To RA | **Atrio-Ventricular Valves** | |
| Pulmonary Venous Drainage | To LA | Mitral Valve | Annulus = 24mm. thickened MVL. |
| Atrio-ventricular Connection | Concordant | Tricuspid Valve | Annulus = 15mm |
| Ventriculo-Arterial Connection | Concordant | **Ventricle** | |
| Ventricular Loop | d-Loop | Left Ventricle | Dilated |
| **Septae** |  | Right Ventricle | Dilated |
| Interatrial Septum | Intact | **Doppler Measurement** | |
| Interventricular Septum | Intact | Mitral | Moderate MR, Holosystolic, posterior projection, seen in two planes with jet velocity = 4.3m/sec |
| **Semilunar Valves** |  | Aortic | Valvular AS, PPG/MPG = 21mmHg |
| Aortic Valve | Annulus = 12mm. Trileaflet, thickened | Tricuspid | Mild TR, PPG = 66mmHg |
| Pulmonary Valve | Annulus = 16MM | Pulmonic | ------ |
| **Great Arteries** | NRGA | **Coronary Arteries** |  |
| Aorta |  | **Aortic Arch** |  |
| Pulmonary Arteries | **MPA = 22mm** | **PDA** | 2.5mm PDA, L – R Shunt |
| **M-Mode**: Normal LV Function on eye balling | | | |
| Ao | mm | PWd | mm |
| LA | mm | EDV | ml |
| LVIDd | mm | ESV | ml |
| LVIDs | mm | FS | % |
| IVSd | mm | LVEF | % |
| **Additional Information:** | | | |
| **Conclusion:** | | | |
| 1. {S, D, S} Levocardia 2. All chambers Dilated 3. Thickened MVL and AVL 4. Moderate MR 5. Mild AS 6. Mild TR 7. Large PDA 8. Severe Pulmonary Hypertension 9. Normal LV Function | | | |
| **Remark** | Deformed chest, limited echo window | | |
| **Recommendation** |  | | |
| **Done By** | **Signature** | **Date** | **Remark** |
| Tesfaye T., Pediatric Cardiologist |  | 06/12/13Eth.C |  |

| **Patient Name:** Alemu Getinet. **Sex/Age**: M/14years. **Date of Report**: 06/12/13Eth.C.  **Clinical Diagnosis: CHF + Rheumatic Recurrence + DOE + Easy Fatigability + RD + Palpitation. TGSH1.2382**. | | | |
| --- | --- | --- | --- |
| **Features:** | **Findings** | **Features** | **Findings** |
| **Profile** | | **Atria** | |
| Abdominal Situs | Solitus | Left Atrium | More Dilated. 70 x78mm |
| Cardiac Position | Levocardia | Right Atrium | Dilated |
| Systemic Venous Drainage | To RA. IVC Dilated | **Atrio-Ventricular Valves** | |
| Pulmonary Venous Drainage | To LA | Mitral Valve | Annulus = 34mm. thickened MVL. Non coapting |
| Atrio-ventricular Connection | Concordant | Tricuspid Valve | Annulus = 29mm. Non coapting  TAPSE = 20mm |
| Ventriculo-Arterial Connection | Concordant | **Ventricle** | |
| Ventricular Loop | d-Loop | Left Ventricle | More dilated |
| **Septae** |  | Right Ventricle | Dilated |
| Interatrial Septum | Intact | **Doppler Measurement** |  |
| Interventricular Septum | Intact | Mitral | Severe MR, Holosystolic, posterior projection, seen in two planes with jet velocity = 4.1m/sec. |
| **Semilunar Valves** |  | Aortic | Moderate AR, PHT = 293ms |
| Aortic Valve | Annulus = 20mm | Tricuspid | Severe TR, PPG = 56mmHg |
| Pulmonary Valve | Annulus = 24mm | Pulmonic |  |
| **Great Arteries** | NRGA | **Coronary Arteries** |  |
| Aorta | Posterior & to right | **Aortic Arch** | Left |
| Pulmonary Arteries | Anterior & to left | **PDA** |  |
| **M-Mode**: | | | |
| Ao | mm | PWd | mm |
| LA | mm | EDV | ml |
| LVIDd | mm | ESV | ml |
| LVIDs | mm | FS | 34% |
| IVSd | mm | LVEF | 61% |
| **Additional Information:** Trace Pericardial effusion on RA/RV Side.7mm right Pleural effusion. | | | |
| **Conclusion:** | | | |
| 1. {S, D, S} Levocardia 2. All chambers dilated 3. Thickened MVL 4. Non Coapting Mitral and Tricuspid valve leaflets 5. Severe MR 6. Severe TR 7. Moderate AR 8. Severe Pulmonary Hypertension 9. Trace Pericardial effusion 10. Small Right Pleural effusion 11. Normal Biventricular Function | | | |
| **Recommendation** |  | | |
| **Done By** | **Signature** | **Date** | **Remark** |
| Tesfaye T., Pediatric Cardiologist |  | 06/12/13Eth.C |  |

| **Patient Name:** Baby of Medina Ibrahim. **Sex/Age**: F/4months. **Date of Report**: 06/12/13Eth.C.  **Clinical Diagnosis: ?PPHTN. TGSH1.2383**. | | | |
| --- | --- | --- | --- |
| **Features:** | **Findings** | **Features** | **Findings** |
| **Profile** | | **Atria** | |
| Abdominal Situs | Solitus | Left Atrium | Normal |
| Cardiac Position | Levocardia | Right Atrium | Normal |
| Systemic Venous Drainage | To RA | **Atrio-Ventricular Valves** | |
| Pulmonary Venous Drainage | To LA | Mitral Valve | Annulus = 12mm |
| Atrio-ventricular Connection | Concordant | Tricuspid Valve | Annulus = 12mm  TAPSE = 13mm |
| Ventriculo-Arterial Connection | Concordant | **Ventricle** | |
| Ventricular Loop | d-Loop | Left Ventricle | Normal |
| **Septae** |  | Right Ventricle | Normal |
| Interatrial Septum | Intact | **Doppler Measurement** |  |
| Interventricular Septum | Intact | Mitral |  |
| **Semilunar Valves** |  | Aortic |  |
| Aortic Valve | Annulus = 10mm | Tricuspid |  |
| Pulmonary Valve | Annulus = 11mm | Pulmonic |  |
| **Great Arteries** | NRGA | **Coronary Arteries** |  |
| Aorta | Posterior and to the right | **Aortic Arch** | Left |
| Pulmonary Arteries | Anterior and to the left | **PDA** |  |
| **M-Mode**: Normal LV Function on eye balling. | | | |
| Ao | mm | PWd | mm |
| LA | mm | EDV | ml |
| LVIDd | mm | ESV | ml |
| LVIDs | mm | FS | % |
| IVSd | mm | LVEF | % |
| **Additional Information:** | | | |
| **Conclusion:** | | | |
| 1. Normal Echocardiography Study. | | | |
| **Remark** |  | | |
| **Recommendation** |  | | |
| **Done By** | **Signature** | **Date** | **Remark** |
| Tesfaye T., Pediatric Cardiologist |  | 06/12/13Eth.C. |  |

| **Patient Name:** Baby of Hulgizie Getinet. **Sex/Age**: M/8days. **Date of Report**: 11/12/13Eth.C.  **Clinical Diagnosis: RD. TGSH1.2384**. | | | |
| --- | --- | --- | --- |
| **Features:** | **Findings** | **Features** | **Findings** |
| **Profile** | | **Atria** | |
| Abdominal Situs | Solitus | Left Atrium | Normal |
| Cardiac Position | Levocardia | Right Atrium | Normal |
| Systemic Venous Drainage | To RA | **Atrio-Ventricular Valves** | |
| Pulmonary Venous Drainage | To LA | Mitral Valve | Annulus = 10mm |
| Atrio-ventricular Connection | Concordant | Tricuspid Valve | Annulus = 12mm |
| Ventriculo-Arterial Connection | Concordant | **Ventricle** | |
| Ventricular Loop | d-Loop | Left Ventricle | Normal |
| **Septae** |  | Right Ventricle | Normal |
| Interatrial Septum | Intact | **Doppler Measurement** |  |
| Interventricular Septum | Intact | Mitral |  |
| **Semilunar Valves** |  | Aortic |  |
| Aortic Valve | Annulus = 8mm | Tricuspid |  |
| Pulmonary Valve | Annulus = 10mm | Pulmonic |  |
| **Great Arteries** | NRGA | **Coronary Arteries** |  |
| Aorta | Posterior and to the right | **Aortic Arch** | Left |
| Pulmonary Arteries | Anterior and to the left | **PDA** |  |
| **M-Mode**: | | | |
| Ao | mm | PWd | mm |
| LA | mm | EDV | ml |
| LVIDd | mm | ESV | ml |
| LVIDs | mm | FS | % |
| IVSd | mm | LVEF | % |
| **Additional Information:** | | | |
| **Conclusion:** | | | |
| 1. Normal Echocardiography Study | | | |
| **Remark** |  | | |
| **Recommendation** |  | | |
| **Done By** | **Signature** | **Date** | **Remark** |
| Tesfaye T., Pediatric Cardiologist |  | 11/12/13Eth.C |  |

| **Patient Name:** Mebe’a Ashenafi. **Sex/Age**: F/3years. **Date of Report**: 13/12/13Eth.C.  Clinical Diagnosis: Follow up echo for Pulmonary hypertension sec.to OSA/H (Currently operated).  **Clinical Diagnosis: ______. TGSH1.2385**. (TGSH4) | | | |
| --- | --- | --- | --- |
| **Features:** | **Findings** | **Features** | **Findings** |
| **Profile** | | **Atria** | |
| Abdominal Situs | Solitus | Left Atrium | Normal |
| Cardiac Position | Levocardia | Right Atrium | Normal |
| Systemic Venous Drainage | To RA | **Atrio-Ventricular Valves** | |
| Pulmonary Venous Drainage | To LA | Mitral Valve | Annulus = 14mm |
| Atrio-ventricular Connection | Concordant | Tricuspid Valve | Annulus = 15mm  TAPSE = 18mm |
| Ventriculo-Arterial Connection | Concordant | **Ventricle** | |
| Ventricular Loop | d-Loop | Left Ventricle | Normal |
| **Septae** |  | Right Ventricle | Normal. RV TDI S wave = 12cm/sec |
| Interatrial Septum | Intact | **Doppler Measurement** |  |
| Interventricular Septum | Intact | Mitral |  |
| **Semilunar Valves** |  | Aortic |  |
| Aortic Valve | Annulus = 12mm | Tricuspid |  |
| Pulmonary Valve | Annulus = 16mm | Pulmonic |  |
| **Great Arteries** | NRGA | **Coronary Arteries** |  |
| Aorta | Posterior and to the right | **Aortic Arch** | Left |
| Pulmonary Arteries | Anterior and to the left | **PDA** |  |
| **M-Mode**: | | | |
| Ao | mm | PWd | mm |
| LA | mm | EDV | ml |
| LVIDd | mm | ESV | ml |
| LVIDs | mm | FS | 40% |
| IVSd | mm | LVEF | 72% |
| **Additional Information:** | | | |
| **Conclusion:** | | | |
| 1. Normal Echocardiography Study | | | |
| **Remark** | Resolved | | |
| **Recommendation** | No need of cardiac follow up | | |
| **Done By** | **Signature** | **Date** | **Remark** |
| Tesfaye T., Pediatric Cardiologist |  | 13/12/13Eth.C. |  |

| **Patient Name:** Samuel Bahiru. **Sex/Age**: M/4 1/12. **Date of Report**: 13/12/13Eth.C.  **Clinical Diagnosis: Incidental Murmur. TGSH1.2386**. | | | |
| --- | --- | --- | --- |
| **Features:** | **Findings** | **Features** | **Findings** |
| **Profile** | | **Atria** | |
| Abdominal Situs | Solitus | Left Atrium | Normal |
| Cardiac Position | Levocardia | Right Atrium | Normal |
| Systemic Venous Drainage | To RA | **Atrio-Ventricular Valves** | |
| Pulmonary Venous Drainage | To LA | Mitral Valve | Annulus = 16mm |
| Atrio-ventricular Connection | Concordant | Tricuspid Valve | Annulus = 16mm |
| Ventriculo-Arterial Connection | Concordant | **Ventricle** | |
| Ventricular Loop | d-Loop | Left Ventricle | Normal |
| **Septae** |  | Right Ventricle | Normal |
| Interatrial Septum | Intact | **Doppler Measurement** |  |
| Interventricular Septum | Intact | Mitral |  |
| **Semilunar Valves** |  | Aortic |  |
| Aortic Valve | Annulus = 14mm | Tricuspid |  |
| Pulmonary Valve | Annulus = 17mm | Pulmonic |  |
| **Great Arteries** | NRGA | **Coronary Arteries** |  |
| Aorta | Posterior and to the right | **Aortic Arch** | Left |
| Pulmonary Arteries | Anterior and to the left | **PDA** | 1mm PDA, L – R Shunt |
| **M-Mode**: Normal LV Function | | | |
| Ao | mm | PWd | mm |
| LA | mm | EDV | ml |
| LVIDd | mm | ESV | ml |
| LVIDs | mm | FS | % |
| IVSd | mm | LVEF | % |
| **Additional Information:** | | | |
| **Conclusion:** | | | |
| 1. {S, D, S} Levocardia 2. Small PDA, L – R Shunt 3. Normal LV Function | | | |
| **Remark** |  | | |
| **Recommendation** | Yearly Echocardiography follow up. | | |
| **Done By** | **Signature** | **Date** | **Remark** |
| Tesfaye T., Pediatric Cardiologist |  | 13/12/13Eth.C. |  |

| **Patient Name:** Bete Mequanint. **Sex/Age**: F/3years. **Date of Report**: 17/12/13Eth.C.  **Clinical Diagnosis: DOE + CHF + RD. TGSH1.2387**. | | | |
| --- | --- | --- | --- |
| **Features:** | **Findings** | **Features** | **Findings** |
| **Profile** | | **Atria** | |
| Abdominal Situs | Solitus | Left Atrium | Normal |
| Cardiac Position | Levocardia | Right Atrium | Dilated |
| Systemic Venous Drainage | To RA | **Atrio-Ventricular Valves** | |
| Pulmonary Venous Drainage | To LA | Mitral Valve | Annulus = 18mm |
| Atrio-ventricular Connection | Concordant | Tricuspid Valve | Annulus = 23mm  TAPSE = 12mm |
| Ventriculo-Arterial Connection | Concordant | **Ventricle** | |
| Ventricular Loop | d-Loop | Left Ventricle | Normal |
| **Septae** |  | Right Ventricle | Dilated, Hypertrophied |
| Interatrial Septum | PFO, R – L Shunt | **Doppler Measurement** |  |
| Interventricular Septum | Intact | Mitral |  |
| **Semilunar Valves** |  | Aortic |  |
| Aortic Valve | Annulus = 15mm | Tricuspid | Mild TR, PPG = 60mmHg |
| Pulmonary Valve | Annulus = 19mm | Pulmonic |  |
| **Great Arteries** | NRGA | **Coronary Arteries** |  |
| Aorta | -- | **Aortic Arch** | Left |
| Pulmonary Arteries | -- | **PDA** |  |
| **M-Mode**: Normal LV Function on eye balling | | | |
| Ao | mm | PWd | mm |
| LA | mm | EDV | ml |
| LVIDd | mm | ESV | ml |
| LVIDs | mm | FS | % |
| IVSd | mm | LVEF | % |
| **Additional Information: Trace pericardial effusion on RA/RV Side** | | | |
| **Conclusion:** | | | |
| 1. {S, D, S} Levocardia 2. RA/RV Dilated 3. PFO, R – L Shunt 4. Severe Pulmonary Hypertension 5. RV Dysfunctional 6. Trace pericardial effusion | | | |
| **Done By** | **Signature** | **Date** | **Remark** |
| Tesfaye T., Pediatric Cardiologist |  | 17/12/13Eth.C. |  |

| **Patient Name:** Chilot Dessie. **Sex/Age**: M/ 3 8/12. **Date of Report**: 18/12/13Eth.C.  **Clinical Diagnosis:** ?TB Perimyocarditis ? Pyogenic**. TGSH1.2388**. | | | |
| --- | --- | --- | --- |
| **Features:** | **Findings** | **Features** | **Findings** |
| **Profile** | | **Atria** | |
| Abdominal Situs | Solitus | Left Atrium | Normal |
| Cardiac Position | Levocardia | Right Atrium | Normal |
| Systemic Venous Drainage | To RA | **Atrio-Ventricular Valves** | |
| Pulmonary Venous Drainage | To LA | Mitral Valve | Annulus = 15mm |
| Atrio-ventricular Connection | Concordant | Tricuspid Valve | Annulus = 18mm  TAPSE = 16mm |
| Ventriculo-Arterial Connection | Concordant | **Ventricle** | |
| Ventricular Loop | d-Loop | Left Ventricle | Normal |
| **Septae** |  | Right Ventricle | Normal |
| Interatrial Septum | Intact | **Doppler Measurement** | E/A = 2.58 |
| Interventricular Septum | Intact | Mitral |  |
| **Semilunar Valves** |  | Aortic |  |
| Aortic Valve | Annulus = 13mm | Tricuspid |  |
| Pulmonary Valve | Annulus = 15mm | Pulmonic |  |
| **Great Arteries** | NRGA | **Coronary Arteries** |  |
| Aorta | Posterior & to the right | **Aortic Arch** | Left |
| Pulmonary Arteries | Anterior and to the left | **PDA** |  |
| **M-Mode**: Normal LV Function on eye balling | | | |
| Ao | mm | PWd | mm |
| LA | mm | EDV | ml |
| LVIDd | mm | ESV | ml |
| LVIDs | mm | FS | % |
| IVSd | mm | LVEF | % |
| **Additional Information: Pericardial thickening on LV Side** | | | |
| **Conclusion:** | | | |
| 1. {S, D, S} Levocardia 2. Pericardial thickening 3. Normal Systolic and Diastolic LV Function 4. Normal RV Function | | | |
| **Remark** | Improving | | |
| **Recommendation** | Complete treatment | | |
| **Done By** | **Signature** | **Date** | **Remark** |
| Tesfaye T., Pediatric Cardiologist |  | 18/12/13Eth.C. |  |

| **Patient Name:** Baby of Mender Twin B. **Sex/Age**: F/22days. **Date of Report**: 18/12/13Eth.C.  **Clinical Diagnosis: Incidental Murmur. TGSH1.2389**. | | | |
| --- | --- | --- | --- |
| **Features:** | **Findings** | **Features** | **Findings** |
| **Profile** | | **Atria** | |
| Abdominal Situs | Solitus | Left Atrium | Normal |
| Cardiac Position | Levocardia | Right Atrium | Normal |
| Systemic Venous Drainage | To RA | **Atrio-Ventricular Valves** | |
| Pulmonary Venous Drainage | To LA | Mitral Valve | Annulus = 9mm |
| Atrio-ventricular Connection | Concordant | Tricuspid Valve | Annulus = 9mm |
| Ventriculo-Arterial Connection | Concordant | **Ventricle** | |
| Ventricular Loop | d-Loop | Left Ventricle | Normal |
| **Septae** |  | Right Ventricle | Normal |
| Interatrial Septum | PFO, L – R Shunt | **Doppler Measurement** |  |
| Interventricular Septum | Intact | Mitral |  |
| **Semilunar Valves** |  | Aortic |  |
| Aortic Valve | Annulus = 6mm | Tricuspid |  |
| Pulmonary Valve | Annulus = 8mm. doming PV | Pulmonic | Mild Valvular PS, PPG = 20mmHg |
| **Great Arteries** | NRGA | **Coronary Arteries** |  |
| Aorta | Posterior and to the right | **Aortic Arch** | Left |
| Pulmonary Arteries | Anterior and to the left | **PDA** |  |
| **M-Mode**: Normal LV Function on eye balling | | | |
| Ao | mm | PWd | mm |
| LA | mm | EDV | ml |
| LVIDd | mm | ESV | ml |
| LVIDs | mm | FS | % |
| IVSd | mm | LVEF | % |
| **Additional Information:** | | | |
| **Conclusion:** | | | |
| 1. {S, D, S} Levocardia 2. PFO, L – R Shunt 3. Mild Valvular PS 4. Doming Pulmonary Valve | | | |
| **Done By** | **Signature** | **Date** | **Remark** |
| Tesfaye T., Pediatric Cardiologist |  | 18/12/13Eth.C. |  |

| **Patient Name:** Tesfahun Asabneh. **Sex/Age**: M/3 9/12. **Date of Report**: 18/12/13Eth.C.  **Clinical Diagnosis: Recurrent Chest Infection. TGSH1.2390**. | | | |
| --- | --- | --- | --- |
| **Features:** | **Findings** | **Features** | **Findings** |
| **Profile** | | **Atria** | |
| Abdominal Situs | Solitus | Left Atrium | Normal |
| Cardiac Position | Levocardia | Right Atrium | Normal |
| Systemic Venous Drainage | To RA | **Atrio-Ventricular Valves** | |
| Pulmonary Venous Drainage | To LA | Mitral Valve | Annulus = 14mm |
| Atrio-ventricular Connection | Concordant | Tricuspid Valve | Annulus = 15mm |
| Ventriculo-Arterial Connection | Concordant | **Ventricle** | |
| Ventricular Loop | d-Loop | Left Ventricle | Normal |
| **Septae** |  | Right Ventricle | Normal |
| Interatrial Septum | Intact | **Doppler Measurement** |  |
| Interventricular Septum | Intact | Mitral |  |
| **Semilunar Valves** |  | Aortic |  |
| Aortic Valve | Annulus = 13mm | Tricuspid |  |
| Pulmonary Valve | Annulus = 14mm | Pulmonic |  |
| **Great Arteries** | NRGA | **Coronary Arteries** |  |
| Aorta | Posterior and to the right | **Aortic Arch** | Left |
| Pulmonary Arteries | Anterior and to the left | **PDA** |  |
| **M-Mode**: Normal LV Function on eye balling. | | | |
| Ao | mm | PWd | mm |
| LA | mm | EDV | ml |
| LVIDd | mm | ESV | ml |
| LVIDs | mm | FS | % |
| IVSd | mm | LVEF | % |
| **Additional Information:** No Pericardial/Pleural Effusion. | | | |
| **Conclusion:** | | | |
| 1. Normal Echocardiography Study | | | |
| **Remark** |  | | |
| **Recommendation** |  | | |
| **Done By** | **Signature** | **Date** | **Remark** |
| Tesfaye T., Pediatric Cardiologist |  | 18/12/13Eth.C. |  |

| **Patient Name:** Amanu’el Esubalew. **Sex/Age**: M/5 months. **Date of Report**: 20/12/13Eth.C.  **Clinical Diagnosis: CHF. TGSH1.2391**. | | | |
| --- | --- | --- | --- |
| **Features:** | **Findings** | **Features** | **Findings** |
| **Profile** | | **Atria** | |
| Abdominal Situs | Solitus | Left Atrium | Mildly Dilated |
| Cardiac Position | Levocardia | Right Atrium | Normal |
| Systemic Venous Drainage | To RA | **Atrio-Ventricular Valves** | |
| Pulmonary Venous Drainage | To LA | Mitral Valve | Annulus = 14mm |
| Atrio-ventricular Connection | Concordant | Tricuspid Valve | Annulus = 13mm |
| Ventriculo-Arterial Connection | Concordant | **Ventricle** | |
| Ventricular Loop | d-Loop | Left Ventricle | Mildly Dilated |
| **Septae** |  | Right Ventricle | Normal |
| Interatrial Septum | PFO, L – R Shunt | **Doppler Measurement** |  |
| Interventricular Septum | 6mm subaortic VSD, L – R Shunt | Mitral |  |
| **Semilunar Valves** |  | Aortic |  |
| Aortic Valve | Annulus = 11mm | Tricuspid |  |
| Pulmonary Valve | Annulus = 14mm | Pulmonic |  |
| **Great Arteries** | NRGA | **Coronary Arteries** |  |
| Aorta | Posterior and to the right | **Aortic Arch** | Left |
| Pulmonary Arteries | Anterior and to the left | **PDA** |  |
| **M-Mode**: Normal LV Function on eye balling. | | | |
| Ao | mm | PWd | mm |
| LA | mm | EDV | ml |
| LVIDd | mm | ESV | ml |
| LVIDs | mm | FS | % |
| IVSd | mm | LVEF | % |
| **Additional Information:** | | | |
| **Conclusion:**   1. {S, D, S} Levocardia 2. PFO, L – R Shunt 3. Moderate Subaortic VSD, L – R Shunt 4. Normal LV Function | | | |
| **Remark** |  | | |
| **Recommendation** |  | | |
| **Done By** | **Signature** | **Date** | **Remark** |
| Tesfaye T., Pediatric Cardiologist |  | 20/12/13Eth.C. |  |

| **Patient Name:** Gizenew Belay. **Sex/Age**: M/12years. **Date of Report**: 24/12/13Eth.C.  **Dx: ?Constrictive pericarditis with BV Systolic Dysfunction and RV Dysfunction + DOE + CHF. TGSH1.2392**. | | | |
| --- | --- | --- | --- |
| **Features:** | **Findings** | **Features** | **Findings** |
| **Profile** | | **Atria** | |
| Abdominal Situs | Solitus | Left Atrium | Dilated |
| Cardiac Position | Levocardia | Right Atrium | Dilated |
| Systemic Venous Drainage | To RA | **Atrio-Ventricular Valves** | |
| Pulmonary Venous Drainage | To LA | Mitral Valve | Annulus = 16mm |
| Atrio-ventricular Connection | Concordant | Tricuspid Valve | Annulus = 20mm  TAPSE = 7mm |
| Ventriculo-Arterial Connection | Concordant | **Ventricle** | |
| Ventricular Loop | d-Loop | Left Ventricle | Normal |
| **Septae** | Bowing to LV | Right Ventricle | Normal |
| Interatrial Septum | Intact | **Doppler Measurement** |  |
| Interventricular Septum | Intact | Mitral | E/A = 1.7 |
| **Semilunar Valves** |  | Aortic |  |
| Aortic Valve | Annulus = 18mm | Tricuspid |  |
| Pulmonary Valve | Annulus = 19mm | Pulmonic |  |
| **Great Arteries** | NRGA | **Coronary Arteries** |  |
| Aorta | Posterior and to the right. From LV. | **Aortic Arch** | Left |
| Pulmonary Arteries | Anterior and to the left. From RV. | **PDA** |  |
| **M-Mode**: | | | |
| Ao | mm | PWd | mm |
| LA | mm | EDV | ml |
| LVIDd | mm | ESV | ml |
| LVIDs | mm | FS | 19% |
| IVSd | mm | LVEF | 38% |
| **Additional Information:** Hyper-echoic pericardial thickening | | | |
| **Conclusion:**   1. {S, D, S} Levocardia 2. Pericardial Thickening 3. LV Systolic and Diastolic Dysfunction 4. Dysfunctional RV | | | |
| **Remark:** |  | | |
| **Recommendation:** |  | | |
| **Done By:** | **Signature** | **Date** | **Remark** |
| Tesfaye T., Pediatric Cardiologist |  | 20/12/13Eth.C. |  |

| **Patient Name:** Tensaynesh **Sex/Age**F/12yr. **Date of Report**: 25/12/13Eth.C.  **Clinical Diagnosis: CHF + DOE + Palpitation + RD + Murmur. TGSH1.2393**. | | | |
| --- | --- | --- | --- |
| **Features:** | **Findings** | **Features** | **Findings** |
| **Profile** | | **Atria** | |
| Abdominal Situs | Solitus | Left Atrium | Dilated |
| Cardiac Position | Levocardia | Right Atrium | More dilated |
| Systemic Venous Drainage | To RA | **Atrio-Ventricular Valves** | |
| Pulmonary Venous Drainage | To LA | Mitral Valve | Annulus = 21mm |
| Atrio-ventricular Connection | Concordant | Tricuspid Valve | Annulus = 36mm  TAPSE = 21mm |
| Ventriculo-Arterial Connection | Concordant | **Ventricle** | |
| Ventricular Loop | d-Loop | Left Ventricle | Dilated |
| **Septae** |  | Right Ventricle | More dilated |
| Interatrial Septum | 29mm Primum defect, L – R Shunt | **Doppler Measurement** |  |
| Interventricular Septum | 7mm Aneurysmal Distension of the Inlet septum to RV | Mitral | Moderate MR |
| **Semilunar Valves** |  | Aortic |  |
| Aortic Valve | Annulus = 19mm | Tricuspid | Moderate TR |
| Pulmonary Valve | Annulus = 25mm | Pulmonic |  |
| **Great Arteries** | NRGA | **Coronary Arteries** |  |
| Aorta | Posterior and to the right. From LV. | **Aortic Arch** | Left |
| Pulmonary Arteries | Anterior and to the left. From RV. | **PDA** |  |
| **M-Mode**: | | | |
| Ao | mm | PWd | mm |
| LA | mm | EDV | ml |
| LVIDd | mm | ESV | ml |
| LVIDs | mm | FS | 24% |
| IVSd | mm | LVEF | 47% |
| **Additional Information:** circumferential Pericardial effusion with Max. depth of 13mm on RA/RV Side. | | | |
| **Conclusion:**   1. {S, D, S} Levocardia 2. Transitional AVSD, L – R Shunt 3. Severe Pulmonary Hypertension 4. Mildly reduced LV Function 5. Moderate Pericardial effusion | | | |
| **Done By:** | **Signature** | **Date** | **Remark** |
| Tesfaye T., Pediatric Cardiologist |  | 25/12/13Eth.C. |  |

| **Patient Name:** Baby of Dejiynsu Demelash. **Sex/Age**: F/16days. **Date of Report**: 25/12/13Eth.C.  **Clinical Diagnosis: Incidental Murmur. TGSH1.2394**. | | | |
| --- | --- | --- | --- |
| **Features:** | **Findings** | **Features** | **Findings** |
| **Profile** | | **Atria** | |
| Abdominal Situs | Solitus | Left Atrium | Normal |
| Cardiac Position | Levocardia | Right Atrium | Normal |
| Systemic Venous Drainage | To RA | **Atrio-Ventricular Valves** | |
| Pulmonary Venous Drainage | To LA | Mitral Valve | Annulus = 8mm |
| Atrio-ventricular Connection | Concordant | Tricuspid Valve | Annulus = 8mm |
| Ventriculo-Arterial Connection | Concordant | **Ventricle** | |
| Ventricular Loop | d-Loop | Left Ventricle | Normal |
| **Septae** |  | Right Ventricle | Normal |
| Interatrial Septum | PFO, L – R Shunt | **Doppler Measurement** |  |
| Interventricular Septum | Intact | Mitral |  |
| **Semilunar Valves** |  | Aortic |  |
| Aortic Valve | Annulus = 6mm | Tricuspid |  |
| Pulmonary Valve | Annulus = 7mm | Pulmonic |  |
| **Great Arteries** | NRGA | **Coronary Arteries** |  |
| Aorta | Posterior and to the right. From LV. | **Aortic Arch** | Left |
| Pulmonary Arteries | Anterior and to the left. From RV. | **PDA** | 1mm PDA, L – R Shunt |
| **M-Mode**: Normal LV Function on eye balling | | | |
| Ao | mm | PWd | mm |
| LA | mm | EDV | ml |
| LVIDd | mm | ESV | ml |
| LVIDs | mm | FS | % |
| IVSd | mm | LVEF | % |
| **Additional Information:** | | | |
| **Conclusion:**   1. {S, D, S} Levocardia 2. PFO, L – R Shunt 3. Small PDA, L – R Shunt | | | |
| **Remark:** |  | | |
| **Recommendation:** |  | | |
| **Done By:** | **Signature** | **Date** | **Remark** |
| Tesfaye T., Pediatric Cardiologist |  | 25/12/13Eth.C. |  |

| **Patient Name:** Mar Guadie. **Sex/Age**: F/9years. **Date of Report**: 25/12/13Eth.C.  **Clinical Diagnosis: CHF + IE + ?Rheumatic Recurrence + RD + DOE + Murmur + Palpitation. TGSH1.2395**. | | | |
| --- | --- | --- | --- |
| **Features:** | **Findings** | **Features** | **Findings** |
| **Profile** | | **Atria** | |
| Abdominal Situs | Solitus | Left Atrium | Dilated |
| Cardiac Position | Levocardia | Right Atrium | Normal |
| Systemic Venous Drainage | To RA | **Atrio-Ventricular Valves** | |
| Pulmonary Venous Drainage | To LA | Mitral Valve | Annulus = 25mm. Thickened MVL |
| Atrio-ventricular Connection | Concordant | Tricuspid Valve | Annulus = 29mm  TAPSE = 16mm |
| Ventriculo-Arterial Connection | Concordant | **Ventricle** | |
| Ventricular Loop | d-Loop | Left Ventricle | Dilated |
| **Septae** |  | Right Ventricle | Normal. RV TDI S wave = 12cm/sec |
| Interatrial Septum | Intact | **Doppler Measurement** |  |
| Interventricular Septum | Intact | Mitral | Mild MR, Holosystolic, posterior projection, seen in two planes with jet velocity = 3.9m/sec |
| **Semilunar Valves** |  | Aortic | Severe AR, PHT = 118ms |
| Aortic Valve | Annulus = 14mm. Oscillating echogenic mass on the LV side of aortic annulus. Thickened AVL | Tricuspid | Mild TR, PPG = 39mmHg |
| Pulmonary Valve | Annulus = 18mm | Pulmonic |  |
| **Great Arteries** | NRGA | **Coronary Arteries** |  |
| Aorta | -- | **Aortic Arch** | Left |
| Pulmonary Arteries | -- | **PDA** |  |
| **M-Mode**: | | | |
| Ao | mm | PWd | mm |
| LA | mm | EDV | ml |
| LVIDd | mm | ESV | ml |
| LVIDs | mm | FS | 17% |
| IVSd | mm | LVEF | 35% |
| **Additional Information:** 15mm Pericardial effusion on RA/RV Side. 20mm Left Pleural effusion. 23mm Right Pleural effusion. | | | |
| **Conclusion:**   1. {S, D, S} Levocardia 2. Thickened MVL and AVL 3. Mild MR, Mild TR 4. Severe AR 5. Mild Pulmonary Hypertension 6. Reduced LV Function 7. Echogenic Oscillating mass over the LVOT adjacent to the AVL Secondary to ?IE 8. Large Pericardial and Bilateral Pleural Effusion | | | |
| **Done By:** | **Signature** | **Date** | **Remark** |
| Tesfaye T., Pediatric Cardiologist |  | 25/12/13Eth.C. |  |

| **Patient Name:** Nardos Addisu. **Sex/Age**: F/3 9/12. **Date of Report**: 25/12/13Eth.C.  **Clinical Diagnosis: Echo for DCM with reduced EF (done before a year). TGSH1.2396**. (@2years age) | | | |
| --- | --- | --- | --- |
| **Features:** | **Findings** | **Features** | **Findings** |
| **Profile** | | **Atria** | |
| Abdominal Situs | Solitus | Left Atrium | Normal |
| Cardiac Position | Levocardia | Right Atrium | Normal |
| Systemic Venous Drainage | To RA | **Atrio-Ventricular Valves** | |
| Pulmonary Venous Drainage | To LA | Mitral Valve | Annulus = 16mm |
| Atrio-ventricular Connection | Concordant | Tricuspid Valve | Annulus = 17mm |
| Ventriculo-Arterial Connection | Concordant | **Ventricle** | |
| Ventricular Loop | d-Loop | Left Ventricle | Normal |
| **Septae** |  | Right Ventricle | Normal |
| Interatrial Septum | Intact | **Doppler Measurement** |  |
| Interventricular Septum | Intact | Mitral |  |
| **Semilunar Valves** |  | Aortic |  |
| Aortic Valve | Annulus = 14mm | Tricuspid |  |
| Pulmonary Valve | Annulus = 14mm | Pulmonic |  |
| **Great Arteries** | NRGA | **Coronary Arteries** |  |
| Aorta | Posterior and to the right. From LV. | **Aortic Arch** | Left |
| Pulmonary Arteries | Anterior and to the left. From RV. | **PDA** |  |
| **M-Mode**: | | | |
| Ao | mm | PWd | mm |
| LA | mm | EDV | ml |
| LVIDd | mm | ESV | ml |
| LVIDs | mm | FS | 30% |
| IVSd | mm | LVEF | 58% |
| **Additional Information:** | | | |
| **Conclusion:**   1. {S, D, S} Levocardia 2. Normal LV Function | | | |
| **Remark:** | Improving | | |
| **Recommendation:** | Reduce Diuretics  Echo after 6 months | | |
| **Done By:** | **Signature** | **Date** | **Remark** |
| Tesfaye T., Pediatric Cardiologist |  | 25/12/13Eth.C. |  |

| **Patient Name:** Yimegn Amare. **Sex/Age**: F/13years. **Date of Report**: 27/12/13Eth.C.  **Clinical Diagnosis: CHF + Rheumatic Recurrence. TGSH1.2397** | | | |
| --- | --- | --- | --- |
| **Features:** | **Findings** | **Features** | **Findings** |
| **Profile** | | **Atria** | |
| Abdominal Situs | Solitus | Left Atrium | Dilated |
| Cardiac Position | Levocardia | Right Atrium | Normal |
| Systemic Venous Drainage | To RA | **Atrio-Ventricular Valves** | |
| Pulmonary Venous Drainage | To LA | Mitral Valve | Annulus = 26mm. Thickened, clubbed MVL. MVA = 0.5cm2. |
| Atrio-ventricular Connection | Concordant | Tricuspid Valve | Annulus = 24mm TAPSE = 21mm |
| Ventriculo-Arterial Connection | Concordant | **Ventricle** | |
| Ventricular Loop | d-Loop | Left Ventricle | Dilated |
| **Septae** |  | Right Ventricle | Normal |
| Interatrial Septum | Intact | **Doppler Measurement** |  |
| Interventricular Septum | Intact | Mitral | Mild MR with jet velocity = 3.6m/sec. Severe MS, PPG/MPG = 19/13mmHg |
| **Semilunar Valves** |  | Aortic | Mild AR, PHT = 509ms |
| Aortic Valve | Annulus = 15mm | Tricuspid | Moderate TR, PPG = 48mmHg |
| Pulmonary Valve | Annulus = 20mm | Pulmonic |  |
| **Great Arteries** | NRGA | **Coronary Arteries** |  |
| Aorta | Posterior and to the right. From LV. | **Aortic Arch** | Left |
| Pulmonary Arteries | Anterior and to the left. From RV. | **PDA** |  |
| **M-Mode**: | | | |
| Ao | mm | PWd | mm |
| LA | mm | EDV | ml |
| LVIDd | mm | ESV | ml |
| LVIDs | mm | FS | 36% |
| IVSd | mm | LVEF | 67% |
| **Additional Information:** 6mm Pericardial effusion on RA/RV Side. | | | |
| **Conclusion:**   1. {S, D, S} Levocardia 2. LA/LV Dilated 3. Thickened, clubbed MVL 4. Mild MR 5. Severe MS 6. Moderate TR 7. Mild AR 8. Moderate Pulmonary Hypertension 9. Normal Biventricular Function 10. Small Pericardial effusion | | | |
| **Remark:** |  | | |
| **Recommendation:** |  | | |
| **Done By:** | **Signature** | **Date** | **Remark** |
| Tesfaye T., Pediatric Cardiologist |  | 27/12/13Eth.C. |  |

| **Patient Name:** Fekadu Birhanu. **Sex/Age**: M/6months. **Date of Report**: 27/12/13Eth.C.  **Clinical Diagnosis: DS. TGSH1.2398.** | | | |
| --- | --- | --- | --- |
| **Features:** | **Findings** | **Features** | **Findings** |
| **Profile** | | **Atria** | |
| Abdominal Situs | Solitus | Left Atrium | Normal |
| Cardiac Position | Levocardia | Right Atrium | Normal |
| Systemic Venous Drainage | To RA | **Atrio-Ventricular Valves** | |
| Pulmonary Venous Drainage | To LA | Mitral Valve | Annulus = 13mm |
| Atrio-ventricular Connection | Concordant | Tricuspid Valve | Annulus = 13mm  TAPSE = 17mm |
| Ventriculo-Arterial Connection | Concordant | **Ventricle** | |
| Ventricular Loop | d-Loop | Left Ventricle | Normal |
| **Septae** |  | Right Ventricle | Normal |
| Interatrial Septum | Intact | **Doppler Measurement** |  |
| Interventricular Septum | Intact | Mitral |  |
| **Semilunar Valves** |  | Aortic |  |
| Aortic Valve | Annulus = 12mm | Tricuspid |  |
| Pulmonary Valve | Annulus = 12mm | Pulmonic |  |
| **Great Arteries** | NRGA | **Coronary Arteries** |  |
| Aorta | Posterior and to the right. From LV. | **Aortic Arch** | Left |
| Pulmonary Arteries | Anterior and to the left. From RV. | **PDA** |  |
| **M-Mode**: | | | |
| Ao | mm | PWd | mm |
| LA | mm | EDV | ml |
| LVIDd | mm | ESV | ml |
| LVIDs | mm | FS | 38% |
| IVSd | mm | LVEF | 71% |
| **Additional Information:** 3mm Pericardial effusion on RV Side. | | | |
| **Conclusion:**   1. {S, D, S} Levocardia 2. Trace Pericardial effusion 3. Normal Biventricular Function | | | |
| **Remark:** |  | | |
| **Recommendation:** |  | | |
| **Done By:** | **Signature** | **Date** | **Remark** |
| Tesfaye T., Pediatric Cardiologist |  | 27/12/13Eth.C. |  |

| **Patient Name:** Sifelig Tebikew. **Sex/Age**: F/7years. **Date of Report**: 04/13/13Eth.C.  **Clinical Diagnosis: CHF + Pulmonary Hypertension. TGSH1.2399.** | | | |
| --- | --- | --- | --- |
| **Features:** | **Findings** | **Features** | **Findings** |
| **Profile** | | **Atria** | |
| Abdominal Situs | Solitus | Left Atrium | Dilated |
| Cardiac Position | Levocardia | Right Atrium | Normal |
| Systemic Venous Drainage | To RA | **Atrio-Ventricular Valves** | |
| Pulmonary Venous Drainage | To LA | Mitral Valve | Annulus = 27mm. Thickened MVL |
| Atrio-ventricular Connection | Concordant | Tricuspid Valve | Annulus = 24mm |
| Ventriculo-Arterial Connection | Concordant | **Ventricle** | |
| Ventricular Loop | d-Loop | Left Ventricle | Dilated |
| **Septae** |  | Right Ventricle | Normal |
| Interatrial Septum | Intact | **Doppler Measurement** |  |
| Interventricular Septum | Intact | Mitral | Severe MR, Holosystolic, posterior projection, seen in two planes with jet velocity = 4.7m/sec |
| **Semilunar Valves** |  | Aortic | Moderate AR, PHT = 280ms. |
| Aortic Valve | Annulus = 16mm | Tricuspid | Moderate TR, PPG = 41mmHg. |
| Pulmonary Valve | Annulus = 20mm | Pulmonic | Mild PR, PPG = 24mmHg |
| **Great Arteries** | NRGA | **Coronary Arteries** |  |
| Aorta |  | **Aortic Arch** | Left |
| Pulmonary Arteries |  | **PDA** |  |
| **M-Mode**: | | | |
| Ao | mm | PWd | mm |
| LA | mm | EDV | ml |
| LVIDd | mm | ESV | ml |
| LVIDs | mm | FS | 32% |
| IVSd | mm | LVEF | 59% |
| **Additional Information:** circumferential pericardial effusion with max. depth of 26mm on RA/RV Junction. | | | |
| **Conclusion:**   1. {S, D, S} Levocardia 2. LA/LV Dilated 3. Thickened MVL 4. Severe MR 5. Moderate AR 6. Moderate TR 7. Mild PR 8. Normal LV Function | | | |
| **Remark:** |  | | |
| **Recommendation:** |  | | |
| **Done By:** | **Signature** | **Date** | **Remark** |
| Tesfaye T., Pediatric Cardiologist |  | 04/13/13Eth.C. |  |

| **Patient Name:** Siraye Bikis. **Sex/Age**: F/9years. **Date of Report**: 04/01/14Eth.C.  **Clinical Diagnosis: Sydenham’s Chorea. TGSH1.2400** | | | |
| --- | --- | --- | --- |
| **Features:** | **Findings** | **Features** | **Findings** |
| **Profile** | | **Atria** | |
| Abdominal Situs | Solitus | Left Atrium | Normal |
| Cardiac Position | Levocardia | Right Atrium | Normal |
| Systemic Venous Drainage | To RA | **Atrio-Ventricular Valves** | |
| Pulmonary Venous Drainage | To LA | Mitral Valve | Annulus = 21mm |
| Atrio-ventricular Connection | Concordant | Tricuspid Valve | Annulus = 25mm |
| Ventriculo-Arterial Connection | Concordant | **Ventricle** | |
| Ventricular Loop | d-Loop | Left Ventricle | Normal |
| **Septae** |  | Right Ventricle | Normal |
| Interatrial Septum | Intact | **Doppler Measurement** |  |
| Interventricular Septum | Intact | Mitral | Trivial MR, Incomplete signal, jet velocity = 2m/sec |
| **Semilunar Valves** |  | Aortic |  |
| Aortic Valve | Annulus = 17mm | Tricuspid | Trivial TR, PPG = 15mmHg, incomplete signal |
| Pulmonary Valve | Annulus = 19mm | Pulmonic |  |
| **Great Arteries** | NRGA | **Coronary Arteries** |  |
| Aorta |  | **Aortic Arch** | Left |
| Pulmonary Arteries |  | **PDA** |  |
| **M-Mode**: | | | |
| Ao | mm | PWd | mm |
| LA | mm | EDV | ml |
| LVIDd | mm | ESV | ml |
| LVIDs | mm | FS | 28% |
| IVSd | mm | LVEF | 55% |
| **Additional Information:** | | | |
| **Conclusion:**   1. Normal Echocardiography Study | | | |
| **Remark:** |  | | |
| **Recommendation:** |  | | |
| **Done By:** | **Signature** | **Date** | **Remark** |
| Tesfaye T., Pediatric Cardiologist |  | 04/01/14Eth.C. |  |

| **Patient Name:** Keralem Zewudu. **Sex/Age**: F/10months. **Date of Report**: 06/01/14Eth.C.  **Clinical Diagnosis: Incidental Murmur. TGSH1.2401** | | | |
| --- | --- | --- | --- |
| **Features:** | **Findings** | **Features** | **Findings** |
| **Profile** | | **Atria** | |
| Abdominal Situs | Solitus | Left Atrium | Normal |
| Cardiac Position | Levocardia | Right Atrium | Normal |
| Systemic Venous Drainage | To RA | **Atrio-Ventricular Valves** | |
| Pulmonary Venous Drainage | To LA | Mitral Valve | Annulus = 12mm |
| Atrio-ventricular Connection | Concordant | Tricuspid Valve | Annulus = 12mm |
| Ventriculo-Arterial Connection | Concordant | **Ventricle** | |
| Ventricular Loop | d-Loop | Left Ventricle | Normal |
| **Septae** |  | Right Ventricle | Normal |
| Interatrial Septum | Intact | **Doppler Measurement** |  |
| Interventricular Septum | 3mm PM VSD, L – R Shunt, Restrictive with PPG = 58mmHg. | Mitral |  |
| **Semilunar Valves** |  | Aortic |  |
| Aortic Valve | Annulus = 10mm | Tricuspid |  |
| Pulmonary Valve | Annulus = 10mm | Pulmonic |  |
| **Great Arteries** | NRGA | **Coronary Arteries** |  |
| Aorta |  | **Aortic Arch** | Left |
| Pulmonary Arteries |  | **PDA** |  |
| **M-Mode**: Normal LV Function on eye balling. | | | |
| Ao | mm | PWd | mm |
| LA | mm | EDV | ml |
| LVIDd | mm | ESV | ml |
| LVIDs | mm | FS | % |
| IVSd | mm | LVEF | % |
| **Additional Information:** | | | |
| **Conclusion:**   1. {S, D, S} Levocardia 2. Small PM VSD, L – R Shunt 3. Normal LV Function | | | |
| **Done By:** | **Signature** | **Date** | **Remark** |
| Tesfaye T., Pediatric Cardiologist |  | 06/01/14Eth.C. |  |

| **Patient Name:** Agegnew Belachew. **Sex/Age**: M/6months. **Date of Report**: 06/01/14Eth.C.  **Clinical Diagnosis: Recurrent Chest Infection. TGSH1.2402** | | | |
| --- | --- | --- | --- |
| **Features:** | **Findings** | **Features** | **Findings** |
| **Profile** | | **Atria** | |
| Abdominal Situs | Solitus | Left Atrium | Normal |
| Cardiac Position | Levocardia | Right Atrium | Normal |
| Systemic Venous Drainage | To RA | **Atrio-Ventricular Valves** | |
| Pulmonary Venous Drainage | To LA | Mitral Valve | Annulus = 10mm |
| Atrio-ventricular Connection | Concordant | Tricuspid Valve | Annulus = 13mm |
| Ventriculo-Arterial Connection | Concordant | **Ventricle** | |
| Ventricular Loop | d-Loop | Left Ventricle | Normal |
| **Septae** |  | Right Ventricle | Normal |
| Interatrial Septum | Intact | **Doppler Measurement** |  |
| Interventricular Septum | Intact | Mitral |  |
| **Semilunar Valves** |  | Aortic |  |
| Aortic Valve | Annulus = 10mm | Tricuspid |  |
| Pulmonary Valve | Annulus = 11mm | Pulmonic |  |
| **Great Arteries** | NRGA | **Coronary Arteries** |  |
| Aorta |  | **Aortic Arch** | Left |
| Pulmonary Arteries |  | **PDA** | No |
| **M-Mode**: Normal LV Function on eye balling. | | | |
| Ao | mm | PWd | mm |
| LA | mm | EDV | ml |
| LVIDd | mm | ESV | ml |
| LVIDs | mm | FS | % |
| IVSd | mm | LVEF | % |
| **Additional Information:** | | | |
| **Conclusion:**   1. Normal Echocardiography Study | | | |
| **Remark:** |  | | |
| **Recommendation:** |  | | |
| **Done By:** | **Signature** | **Date** | **Remark** |
| Tesfaye T., Pediatric Cardiologist |  | 06/01/14Eth.C. |  |

| **Patient Name:** Eyerusalem Shimelis. **Sex/Age**: F/12years. **Date of Report**: 13/01/14Eth.C.  **Clinical Diagnosis: DOE + CHF + Murmur. TGSH1.2403.** | | | |
| --- | --- | --- | --- |
| **Features:** | **Findings** | **Features** | **Findings** |
| **Profile** | | **Atria** | |
| Abdominal Situs | Solitus | Left Atrium | Dilated |
| Cardiac Position | Levocardia | Right Atrium | Normal |
| Systemic Venous Drainage | To RA | **Atrio-Ventricular Valves** | |
| Pulmonary Venous Drainage | To LA | Mitral Valve | Annulus = 32mm |
| Atrio-ventricular Connection | Concordant | Tricuspid Valve | Annulus = 23mm |
| Ventriculo-Arterial Connection | Concordant | **Ventricle** | |
| Ventricular Loop | d-Loop | Left Ventricle | Dilated |
| **Septae** |  | Right Ventricle | Normal |
| Interatrial Septum | Intact | **Doppler Measurement** |  |
| Interventricular Septum | Intact | Mitral | Mild MR, Holosystolic |
| **Semilunar Valves** |  | Aortic |  |
| Aortic Valve | Annulus = 21mm | Tricuspid | Mild TR, PPG = 47mmHg |
| Pulmonary Valve | Annulus = 24mm | Pulmonic |  |
| **Great Arteries** | NRGA | **Coronary Arteries** |  |
| Aorta | ---- | **Aortic Arch** | Left |
| Pulmonary Arteries | ------ | **PDA** | 3.5mm PDA, L – R Shunt |
| **M-Mode**: | | | |
| Ao | mm | PWd | mm |
| LA | mm | EDV | ml |
| LVIDd | mm | ESV | ml |
| LVIDs | mm | FS | 30% |
| IVSd | mm | LVEF | 56% |
| **Additional Information:** | | | |
| **Conclusion:**   1. {S, D, S} Levocardia 2. Large PDA, L – R Shunt 3. Mild MR 4. Mild TR 5. Moderate Pulmonary Hypertension 6. Normal LV Function | | | |
| **Remark:** |  | | |
| **Recommendation:** | Needs closure | | |
| **Done By:** | **Signature** | **Date** | **Remark** |
| Tesfaye T., Pediatric Cardiologist |  | 13/01/14Eth.C. |  |

| **Patient Name:** Minwuye Ayal. **Sex/Age**: M/14years. **Date of Report**: 13/01/14Eth.C. MRN: 100094.  **Clinical Diagnosis: CHF + Murmur + Rheumatic Recurrence + DOE + Plpitation. TGSH1.2404.** | | | |
| --- | --- | --- | --- |
| **Features:** | **Findings** | **Features** | **Findings** |
| **Profile** | | **Atria** | |
| Abdominal Situs | Solitus | Left Atrium | Dilated |
| Cardiac Position | Levocardia | Right Atrium | Dilated |
| Systemic Venous Drainage | To RA | **Atrio-Ventricular Valves** | |
| Pulmonary Venous Drainage | To LA | Mitral Valve | Annulus = 34mm. Thickened, clubbed MVL. Shortened PMVL. MVA = 0.5cm2. |
| Atrio-ventricular Connection | Concordant | Tricuspid Valve | Annulus = 28mm  TAPSE = 24mm |
| Ventriculo-Arterial Connection | Concordant | **Ventricle** | |
| Ventricular Loop | d-Loop | Left Ventricle | Dilated |
| **Septae** |  | Right Ventricl | Dilated |
| Interatrial Septum | Intact | **Doppler Measurement** |  |
| Interventricular Septum | Intact | Mitral | Severe MS, PPG/MPG = 22/14mmHg |
| **Semilunar Valves** |  | Aortic | Mild AR, PHT = 505ms. |
| Aortic Valve | Annulus = 19mm | Tricuspid | Severe TR, PPG = 100mmHg |
| Pulmonary Valve | Annulus = 27mm | Pulmonic |  |
| **Great Arteries** | NRGA | **Coronary Arteries** |  |
| Aorta | ---- | **Aortic Arch** | Left |
| Pulmonary Arteries | ---- | **PDA** | No |
| **M-Mode**: | | | |
| Ao | mm | PWd | mm |
| LA | mm | EDV | ml |
| LVIDd | mm | ESV | ml |
| LVIDs | mm | FS | 37% |
| IVSd | mm | LVEF | 67% |
| **Additional Information:** | | | |
| **Conclusion:**   1. {S, D, S} Levocardia 2. All chambers Dilated 3. Thickened, clubbed MVL. Shortened PMVL 4. Severe MS 5. Mild AR 6. Severe TR 7. Severe Pulmonary Hypertension 8. Normal Biventricular Function | | | |
| **Remark:** |  | | |
| **Recommendation:** | Needs intervention | | |
| **Done By:** | **Signature** | **Date** | **Remark** |
| Tesfaye T., Pediatric Cardiologist |  | 13/01/14Eth.C. |  |

| **Patient Name:** Anguach Habtie. **Sex/Age**: F/6years. **Date of Report**: 13/01/14Eth.C. MRN: 100167.  **Clinical Diagnosis: Rheumatic Recurrence + CHF + Murmur + RD. TGSH1.2405.** | | | |
| --- | --- | --- | --- |
| **Features:** | **Findings** | **Features** | **Findings** |
| **Profile** | | **Atria** | |
| Abdominal Situs | Solitus | Left Atrium | Dilated |
| Cardiac Position | Levocardia | Right Atrium | Normal |
| Systemic Venous Drainage | To RA | **Atrio-Ventricular Valves** | |
| Pulmonary Venous Drainage | To LA | Mitral Valve | Annulus = 22mm. Thickened MVL. |
| Atrio-ventricular Connection | Concordant | TV | Annulus = 22mm. TAPSE = 17mm |
| Ventriculo-Arterial Connection | Concordant | **Ventricle** | |
| Ventricular Loop | d-Loop | Left Ventricle | Dilated |
| **Septae** |  | Right Ventricl | Normal |
| Interatrial Septum | Intact | **Doppler Measurement** | |
| Interventricular Septum | Intact | Mitral | Severe MR, Holosystolic, posterior projection, seen in two planes, jet velocity = 4m/sec. |
| **Semilunar Valves** |  | Aortic | Moderate AR, PHT = 315ms |
| Aortic Valve | Annulus = 12mm | Tricuspid | Mild TR, PPG = 48mmHg. |
| Pulmonary Valve | Annulus = 15mm | Pulmonic | Moderate PR, PPG = 47mmHg |
| **Great Arteries** | NRGA | **Coronary Arteries** |  |
| Aorta | ----- | **Aortic Arch** | Left |
| Pulmonary Arteries | ----- | **PDA** | No |
| **M-Mode**: | | | |
| Ao | mm | PWd | mm |
| LA | mm | EDV | ml |
| LVIDd | mm | ESV | ml |
| LVIDs | mm | FS | 30% |
| IVSd | mm | LVEF | 57% |
| **Additional Information:** Trace Pericardial effusion. | | | |
| **Conclusion:**   1. {S, D, S} Levocardia 2. LA/LV Dilated 3. Thickened MVL 4. Severe MR, Moderate AR, Mild TR, Moderate PR 5. Moderate Pulmonary Hypertension 6. Trace Pericardial effusion | | | |
| **Done By:** | **Signature** | **Date** | **Remark** |
| Tesfaye T., Pediatric Cardiologist |  | 13/01/14Eth.C. |  |

| **Patient Name:** Meskerem Abebaw. **Sex/Age**: F/8years. **Date of Report**: 20/01/14Eth.C. MRN: 100843.  **Clinical Diagnosis: DOE + Murmur + palpitation + Easy Fatigability + CHF. TGSH1.2406.** | | | |
| --- | --- | --- | --- |
| **Features:** | **Findings** | **Features** | **Findings** |
| **Profile** | | **Atria** | |
| Abdominal Situs | Solitus | Left Atrium | Dilated |
| Cardiac Position | Levocardia | Right Atrium | Normal |
| Systemic Venous Drainage | To RA | **Atrio-Ventricular Valves** | |
| Pulmonary Venous Drainage | To LA | Mitral Valve | Annulus = 23mm |
| Atrio-ventricular Connection | Concordant | Tricuspid Valve | Annulus = 21mm |
| Ventriculo-Arterial Connection | Concordant | **Ventricle** | |
| Ventricular Loop | d-Loop | Left Ventricle | Dilated |
| **Septae** |  | Right Ventricle | Normal |
| Interatrial Septum | 14mm OS ASD, L – R Shunt | **Doppler Measurement** |  |
| Interventricular Septum | 7mm PM VSD, L – R Shunt | Mitral |  |
| **Semilunar Valves** |  | Aortic |  |
| Aortic Valve | Annulus = 13mm | Tricuspid |  |
| Pulmonary Valve | Annulus = 15mm | Pulmonic | Moderate PR, PPG = 52mmHg. Mild PS, PPG = 21mmHg |
| **Great Arteries** | NRGA | **Coronary Arteries** |  |
| Aorta | ---- | **Aortic Arch** | Left |
| Pulmonary Arteries | ---- | **PDA** | No |
| **M-Mode**: Normal LV Function on eye balling | | | |
| Ao | mm | PWd | mm |
| LA | mm | EDV | ml |
| LVIDd | mm | ESV | ml |
| LVIDs | mm | FS | % |
| IVSd | mm | LVEF | % |
| **Additional Information:** circumferential Pericardial effusion with MX. Depth on RA Side measuring 15mm | | | |
| **Conclusion:**   1. {S, D, S} Levocardia 2. Large OS ASD, L – R Shunt 3. Moderate PM VSD, L – R Shunt 4. Moderate PR 5. Mild PS 6. Moderate Pulmonary Hypertension 7. Normal LV Function 8. Moderate Pericardial effusion | | | |
| **Done By:** | **Signature** | **Date** | **Remark** |
| Tesfaye T., Pediatric Cardiologist |  | 20/01/14Eth.C. |  |

| **Patient Name:** Baby of Jemila Abdi. **Sex/Age**: M/11days. **Date of Report**: 20/01/14Eth.C. MRN: 100925.  **Clinical Diagnosis: DS. TGSH1.2407.** | | | |
| --- | --- | --- | --- |
| **Features:** | **Findings** | **Features** | **Findings** |
| **Profile** | | **Atria** | |
| Abdominal Situs | Solitus | Left Atrium | Normal |
| Cardiac Position | Levocardia | Right Atrium | Normal |
| Systemic Venous Drainage | To RA | **Atrio-Ventricular Valves** | |
| Pulmonary Venous Drainage | To LA | Mitral Valve | Annulus = 11mm |
| Atrio-ventricular Connection | Concordant | Tricuspid Valve | Annulus = 11mm  TAPSE = 14mm |
| Ventriculo-Arterial Connection | Concordant | **Ventricle** | |
| Ventricular Loop | d-Loop | Left Ventricle | Normal |
| **Septae** |  | Right Ventricle | Normal |
| Interatrial Septum | PFO, L – R Shunt | **Doppler Measurement** |  |
| Interventricular Septum | Intact | Mitral |  |
| **Semilunar Valves** |  | Aortic |  |
| Aortic Valve | Annulus = 8mm | Tricuspid |  |
| Pulmonary Valve | Annulus = 9mm | Pulmonic |  |
| **Great Arteries** | NRGA | **Coronary Arteries** |  |
| Aorta |  | **Aortic Arch** | Left |
| Pulmonary Arteries |  | **PDA** | No |
| **M-Mode**: Normal LV Function on eye balling | | | |
| Ao | mm | PWd | mm |
| LA | mm | EDV | ml |
| LVIDd | mm | ESV | ml |
| LVIDs | mm | FS | % |
| IVSd | mm | LVEF | % |
| **Additional Information:** | | | |
| **Conclusion:**   1. {S, D, S} Levocardia 2. PFO, L – R Shunt | | | |
| **Remark:** |  | | |
| **Recommendation:** |  | | |
| **Done By:** | **Signature** | **Date** | **Remark** |
| Tesfaye T., Pediatric Cardiologist |  | 20/01/14Eth.C. |  |

| **Patient Name:** Baby of Enkutatash Melaku. **Sex/Age**: F/ 1 1/12. **Date of Report**: 20/01/14Eth.C. MRN: 053495.  Clinical Diagnosis: Follow up Echocardiography for small Muscular VSD (Done before a year).  **Clinical Diagnosis: Incidental Murmur. TGSH1.2408.** | | | |
| --- | --- | --- | --- |
| **Features:** | **Findings** | **Features** | **Findings** |
| **Profile** | | **Atria** | |
| Abdominal Situs | Solitus | Left Atrium | Normal |
| Cardiac Position | Levocardia | Right Atrium | Normal |
| Systemic Venous Drainage | To RA | **Atrio-Ventricular Valves** | |
| Pulmonary Venous Drainage | To LA | Mitral Valve | Annulus = 13mm |
| Atrio-ventricular Connection | Concordant | Tricuspid Valve | Annulus = 15mm |
| Ventriculo-Arterial Connection | Concordant | **Ventricle** | |
| Ventricular Loop | d-Loop | Left Ventricle | Normal |
| **Septae** |  | Right Ventricle | Normal |
| Interatrial Septum | Intact | **Doppler Measurement** |  |
| Interventricular Septum | Intact | Mitral |  |
| **Semilunar Valves** |  | Aortic |  |
| Aortic Valve | Annulus = 12mm | Tricuspid |  |
| Pulmonary Valve | Annulus = 13mm | Pulmonic |  |
| **Great Arteries** | NRGA | **Coronary Arteries** |  |
| Aorta |  | **Aortic Arch** | Left |
| Pulmonary Arteries |  | **PDA** | No |
| **M-Mode**: Normal LV Function on eye balling | | | |
| Ao | mm | PWd | mm |
| LA | mm | EDV | ml |
| LVIDd | mm | ESV | ml |
| LVIDs | mm | FS | % |
| IVSd | mm | LVEF | % |
| **Additional Information:** | | | |
| **Conclusion:**   1. Normal Echocardiography Study | | | |
| **Remark:** | The Muscular VSD has closed. | | |
| **Recommendation:** |  | | |
| **Done By:** | **Signature** | **Date** | **Remark** |
| Tesfaye T., Pediatric Cardiologist |  | 20/01/14Eth.C. |  |

| **Patient Name:** Abrham Haile=Mariam. **Sex/Age**: M/5years. **Date of Report**: 20/01/14Eth.C. MRN: 002717.  **Clinical Diagnosis: Incidental Murmur. TGSH1.2409.** | | | |
| --- | --- | --- | --- |
| **Features:** | **Findings** | **Features** | **Findings** |
| **Profile** | | **Atria** | |
| Abdominal Situs | Solitus | Left Atrium | Normal |
| Cardiac Position | Levocardia | Right Atrium | Normal |
| Systemic Venous Drainage | To RA | **Atrio-Ventricular Valves** | |
| Pulmonary Venous Drainage | To LA | Mitral Valve | Annulus = 22mm |
| Atrio-ventricular Connection | Concordant | Tricuspid Valve | Annulus = 23mm  TAPSE = 22mm |
| Ventriculo-Arterial Connection | Concordant | **Ventricle** | |
| Ventricular Loop | d-Loop | Left Ventricle | Normal |
| **Septae** |  | Right Ventricle | Normal |
| Interatrial Septum | Intact | **Doppler Measurement** |  |
| Interventricular Septum | 5mm PM VSD, L – R Shunt | Mitral |  |
| **Semilunar Valves** |  | Aortic |  |
| Aortic Valve | Annulus = 15mm | Tricuspid |  |
| Pulmonary Valve | Annulus = 16mm | Pulmonic |  |
| **Great Arteries** | NRGA | **Coronary Arteries** |  |
| Aorta | ----- | **Aortic Arch** | Left |
| Pulmonary Arteries | ------ | **PDA** | No |
| **M-Mode**: | | | |
| Ao | mm | PWd | mm |
| LA | mm | EDV | ml |
| LVIDd | mm | ESV | ml |
| LVIDs | mm | FS | 38% |
| IVSd | mm | LVEF | 69% |
| **Additional Information:** | | | |
| **Conclusion:**   1. {S, D, S} Levocardia 2. Small PM VSD, L – R Shunt 3. Normal Biventricular Function | | | |
| **Remark:** |  | | |
| **Recommendation:** | 1. No need to put on any form of medication 2. No restriction from sporting activities 3. Echocardiography follow up yearly | | |
| **Done By:** | **Signature** | **Date** | **Remark** |
| Tesfaye T., Pediatric Cardiologist |  | 20/01/14Eth.C. |  |

| **Patient Name:** Seble-Werk Dires. **Sex/Age**: F/8years. **Date of Report**: 25/01/14Eth.C. MRN: 021774.  **Clinical Diagnosis: CHF + Rheumatic Recurrence + DOE + Palpitation + Easy Fatigability + RD. TGSH1.2410.** | | | |
| --- | --- | --- | --- |
| **Features:** | **Findings** | **Features** | **Findings** |
| **Profile** | | **Atria** | |
| Abdominal Situs | Solitus | Left Atrium | Markedly Dilated. SEC+ |
| Cardiac Position | Levocardia | Right Atrium | Dilated |
| Systemic Venous Drainage | To RA | **Atrio-Ventricular Valves** | |
| Pulmonary Venous Drainage | To LA | Mitral Valve | Annulus = 30mm. Thickened MVL |
| Atrio-ventricular Connection | Concordant | Tricuspid Valve | Annulus = 21mm |
| Ventriculo-Arterial Connection | Concordant | **Ventricle** | |
| Ventricular Loop | d-Loop | Left Ventricle | Markedly Dilated. SEC + |
| **Septae** |  | Right Ventricle | Dilated |
| Interatrial Septum | Intact | **Doppler Measurement** |  |
| Interventricular Septum | Intact | Mitral | Severe MR, Holosystolic, Posterior projection, seen in two planes with jet velocity = 4m/sec |
| **Semilunar Valves** |  | Aortic | Mild AR, PHT = 505ms |
| Aortic Valve | Annulus = 17mm | Tricuspid |  |
| Pulmonary Valve | Annulus = 27mm | Pulmonic | Moderate PR, PPG = 61mmHg |
| **Great Arteries** | NRGA | **Coronary Arteries** |  |
| Aorta |  | **Aortic Arch** | Left |
| Pulmonary Arteries | MPA = 28mm. | **PDA** | No |
| **M-Mode**: | | | |
| Ao | mm | PWd | mm |
| LA | mm | EDV | ml |
| LVIDd | mm | ESV | ml |
| LVIDs | mm | FS | 35% |
| IVSd | mm | LVEF | 63% |
| **Additional Information:** Trace pericardial effusion. | | | |
| **Conclusion:**   1. {S, D, S} Levocardia 2. All chambers Dilated 3. SEC+ 4. Thickened MVL 5. Moderate to severe MR 6. Mild AR 7. Mild PR 8. Severe Pulmonary Hypertension 9. Normal LV Function 10. Trace pericardial effusion | | | |
| **Remark:** |  | | |
| **Recommendation:** |  | | |
| **Done By:** | **Signature** | **Date** | **Remark** |
| Tesfaye T., Pediatric Cardiologist |  | 25/01/14Eth.C. |  |

| **Patient Name:** Habtamu Maru. **Sex/Age**: M/14years. **Date of Report**: 25/01/14Eth.C. MRN: 101615.  **Clinical Diagnosis: Incidental Murmur + Rheumatic Recurrence. TGSH1.2411.** | | | |
| --- | --- | --- | --- |
| **Features:** | **Findings** | **Features** | **Findings** |
| **Profile** | | **Atria** | |
| Abdominal Situs | Solitus | Left Atrium | Normal |
| Cardiac Position | Levocardia | Right Atrium | Normal |
| Systemic Venous Drainage | To RA | **Atrio-Ventricular Valves** | |
| Pulmonary Venous Drainage | To LA | Mitral Valve | Annulus = 23mm. Thickened, Clubbed MVL. MVA = 1.1cm**2**. |
| Atrio-ventricular Connection | Concordant | Tricuspid Valve | Annulus = 23mm  TAPSE = 21mm |
| Ventriculo-Arterial Connection | Concordant | **Ventricle** | |
| Ventricular Loop | d-Loop | Left Ventricle | Normal |
| **Septae** |  | Right Ventricle | Normal |
| Interatrial Septum | Intact | **Doppler Measurement** |  |
| Interventricular Septum | Intact | Mitral | Moderate MS, PPG/MPG = 14/9mmHg |
| **Semilunar Valves** |  | Aortic |  |
| Aortic Valve | Annulus = 18mm | Tricuspid | Trivial TR, PPG = 22mmHg |
| Pulmonary Valve | Annulus = 26mm | Pulmonic |  |
| **Great Arteries** | NRGA | **Coronary Arteries** |  |
| Aorta |  | **Aortic Arch** | Left |
| Pulmonary Arteries |  | **PDA** | No |
| **M-Mode**: Normal LV Function on eye balling. | | | |
| Ao | mm | PWd | mm |
| LA | mm | EDV | ml |
| LVIDd | mm | ESV | ml |
| LVIDs | mm | FS | % |
| IVSd | mm | LVEF | % |
| **Additional Information:** | | | |
| **Conclusion:**   1. {S, D, S} Levocardia 2. Thickened, Clubbed MVL 3. Moderate MS 4. Normal Biventricular Function | | | |
| **Done By:** | **Signature** | **Date** | **Remark** |
| Tesfaye T., Pediatric Cardiologist |  | 25/01/14Eth.C. |  |

| **Patient Name:** Werkneh Abebe. **Sex/Age**: M/9months. **Date of Report**: 02/02/14Eth.C. MRN: 102088.  **Clinical Diagnosis: Incidental Murmur + DS. TGSH1.2412.** | | | |
| --- | --- | --- | --- |
| **Features:** | **Findings** | **Features** | **Findings** |
| **Profile** | | **Atria** | |
| Abdominal Situs | Solitus | Left Atrium | Normal |
| Cardiac Position | Levocardia | Right Atrium | Normal |
| Systemic Venous Drainage | To RA | **Atrio-Ventricular Valves** | |
| Pulmonary Venous Drainage | To LA | Mitral Valve | Annulus = 12mm |
| Atrio-ventricular Connection | Concordant | Tricuspid Valve | Annulus = 14mm |
| Ventriculo-Arterial Connection | Concordant | **Ventricle** | |
| Ventricular Loop | d-Loop | Left Ventricle | Normal |
| **Septae** |  | Right Ventricle | Normal |
| Interatrial Septum | PFO, L – R Shunt | **Doppler Measurement** |  |
| Interventricular Septum | 5mm PM VSD, L – R Shunt | Mitral |  |
| **Semilunar Valves** |  | Aortic |  |
| Aortic Valve | Annulus = 12mm | Tricuspid |  |
| Pulmonary Valve | Annulus = 13mm | Pulmonic |  |
| **Great Arteries** | NRGA | **Coronary Arteries** |  |
| Aorta |  | **Aortic Arch** | Left |
| Pulmonary Arteries |  | **PDA** | No |
| **M-Mode**: Normal LV Function on eye balling. | | | |
| Ao | mm | PWd | mm |
| LA | mm | EDV | ml |
| LVIDd | mm | ESV | ml |
| LVIDs | mm | FS | % |
| IVSd | mm | LVEF | % |
| **Additional Information:** | | | |
| **Conclusion:**   1. {S, D, S} Levocardia 2. PFO, L – R Shunt 3. Small PM VSD, L – R Shunt 4. Normal LV Function | | | |
| **Remark:** | Limited Echo window (Sub costal and apical view only). | | |
| **Recommendation:** | Follow up echocardiography yearly  No need of cardiac medicine currently | | |
| **Done By:** | **Signature** | **Date** | **Remark** |
| Tesfaye T., Pediatric Cardiologist |  | 02/02/14Eth.C. |  |

| **Patient Name:** Werkitu Wetet. **Sex/Age**: F/13 years. **Date of Report**: 04/02/14Eth.C. MRN: 107687.  **Clinical Diagnosis: Rheumatic Recurrence + Murmur. TGSH1.2413.** | | | |
| --- | --- | --- | --- |
| **Features:** | **Findings** | **Features** | **Findings** |
| **Profile** | | **Atria** | |
| Abdominal Situs | Solitus | Left Atrium | Dilated |
| Cardiac Position | Levocardia | Right Atrium | Normal |
| Systemic Venous Drainage | To RA | **Atrio-Ventricular Valves** | |
| Pulmonary Venous Drainage | To LA | Mitral Valve | Annulus = 27mm. Thickened MVL. Shortened PMVL. |
| Atrio-ventricular Connection | Concordant | Tricuspid Valve | Annulus = 30mm  TAPSE = 23mm |
| Ventriculo-Arterial Connection | Concordant | **Ventricle** | |
| Ventricular Loop | d-Loop | Left Ventricle | Dilated |
| **Septae** |  | Right Ventricle | Normal |
| Interatrial Septum | Intact | **Doppler Measurement** |  |
| Interventricular Septum | Intact | Mitral | Moderate MR, Holosystolic, posterior projection, seen in two planes with jet velocity = 4.7m/sec. |
| **Semilunar Valves** |  | Aortic | Mild AR, PHT = 506ms. |
| Aortic Valve | Annulus = 19mm | Tricuspid | Mild TR, PPG = 36mmHg |
| Pulmonary Valve | Annulus = 23mm | Pulmonic |  |
| **Great Arteries** | NRGA | **Coronary Arteries** |  |
| Aorta |  | **Aortic Arch** | Left |
| Pulmonary Arteries |  | **PDA** | No |
| **M-Mode**: Normal LV Function on eye balling. | | | |
| Ao | mm | PWd | mm |
| LA | mm | EDV | ml |
| LVIDd | mm | ESV | ml |
| LVIDs | mm | FS | % |
| IVSd | mm | LVEF | % |
| **Additional Information:** 4mm pericardial effusion on RV Side. | | | |
| **Conclusion:**   1. {S, D, S} Levocardia 2. Thickened MVL 3. LA/LV Dilated 4. Moderate MR 5. Mild TR 6. Mild AR 7. Normal Function | | | |
| **Remark:** |  | | |
| **Recommendation:** |  | | |
| **Done By:** | **Signature** | **Date** | **Remark** |
| Tesfaye T., Pediatric Cardiologist |  | 04/02/14Eth.C. |  |

| **Patient Name:** Mastewal Belete. **Sex/Age**: F/1year. **Date of Report**: 04/02/14Eth.C. MRN: 102155.  **Clinical Diagnosis: CHF + RD + Diaphoresis + Murmur. TGSH1.2414.** | | | |
| --- | --- | --- | --- |
| **Features:** | **Findings** | **Features** | **Findings** |
| **Profile** | | **Atria** | |
| Abdominal Situs | Solitus | Left Atrium | Dilated |
| Cardiac Position | Levocardia | Right Atrium | Dilated |
| Systemic Venous Drainage | To RA | **Atrio-Ventricular Valves** | |
| Pulmonary Venous Drainage | To LA | Mitral Valve | Annulus = 14mm |
| Atrio-ventricular Connection | Concordant | Tricuspid Valve | Annulus = 17mm |
| Ventriculo-Arterial Connection | Concordant | **Ventricle** | |
| Ventricular Loop | d-Loop | Left Ventricle | Dilated |
| **Septae** |  | Right Ventricle | Dilated |
| Interatrial Septum | 4 x 7mm OS ASD, L – R Shunt | **Doppler Measurement** |  |
| Interventricular Septum | 10 mm Upper Muscular VSD, BD Shunt. Additional 4mm Apical Muscular VSD, BD Shunt | Mitral |  |
| **Semilunar Valves** |  | Aortic |  |
| Aortic Valve | Annulus = 9mm | Tricuspid | Mild TR |
| Pulmonary Valve | Annulus = 16mm | Pulmonic | Moderate PR, PPG = 64mmHg |
| **Great Arteries** | NRGA | **Coronary Arteries** |  |
| Aorta |  | **Aortic Arch** | Left |
| Pulmonary Arteries | MPA = 16mm | **PDA** | No |
| **M-Mode**: | | | |
| Ao | mm | PWd | mm |
| LA | mm | EDV | ml |
| LVIDd | mm | ESV | ml |
| LVIDs | mm | FS | 24% |
| IVSd | mm | LVEF | 51% |
| **Conclusion:**   1. {S, D, S} Levocardia 2. All chambers dilated 3. Moderate OS ASD, L – R Shunt 4. Large Upper Muscular & Small Apical Muscular VSD (Swiss cheese), BD Shunt 5. Moderate PR 6. Severe Pulmonary Hypertension 7. Reduced LV Function | | | |
| **Done By:** | **Signature** | **Date** | **Remark** |
| Tesfaye T., Pediatric Cardiologist |  | 04/02/14Eth.C. |  |

| **Patient Name:** Migbaru Gizachew. **Sex/Age**: M/1 4/12. **Date of Report**: 10/02/14Eth.C. MRN: 103357.  **Clinical Diagnosis: Cyanosis + RD . TGSH1.2415.** | | | |
| --- | --- | --- | --- |
| **Features:** | **Findings** | **Features** | **Findings** |
| **Profile** | | **Atria** | |
| Abdominal Situs | Solitus | Left Atrium | Normal |
| Cardiac Position | Levocardia | Right Atrium | Dilated |
| Systemic Venous Drainage | To RA | **Atrio-Ventricular Valves** | |
| Pulmonary Venous Drainage | To LA | Mitral Valve | Atretic |
| Atrio-ventricular Connection | Concordant | Tricuspid Valve | Annulus = 22mm |
| Ventriculo-Arterial Connection | Concordant | **Ventricle** | |
| Ventricular Loop | d-Loop | Left Ventricle | Smallish LV |
| **Septae** |  | Right Ventricle | Dilated |
| Interatrial Septum | 6mm OP ASD, L – R Shunt | **Doppler Measurement** |  |
| Interventricular Septum | 10mm Inlet VSD, R – L Shunt | Mitral | Atretic |
| **Semilunar Valves** |  | Aortic |  |
| Aortic Valve | Annulus = 13mm | Tricuspid | Moderate TR |
| Pulmonary Valve | Annulus = 14mm | Pulmonic |  |
| **Great Arteries** | NRGA | **Coronary Arteries** |  |
| Aorta |  | **Aortic Arch** | Left |
| Pulmonary Arteries |  | **PDA** | No |
| **M-Mode**: | | | |
| Ao | mm | PWd | mm |
| LA | mm | EDV | ml |
| LVIDd | mm | ESV | ml |
| LVIDs | mm | FS | % |
| IVSd | mm | LVEF | % |
| **Additional Information:** | | | |
| **Conclusion:**   1. {S, D, S} Levocardia 2. RA/RV Dilated 3. Small Primum defect, L – R Shunt 4. Moderate Inlet VSD, R – L Shunt 5. Moderate TR 6. Mitral Atresia 7. Smallish LV | | | |
| **Done By:** | **Signature** | **Date** | **Remark** |
| Tesfaye T., Pediatric Cardiologist |  | 10/02/14Eth.C. |  |

| **Patient Name:** Dinberu Yirdaw. **Sex/Age**: M/9years. **Date of Report**: 11/02/14Eth.C. MRN: ________.  **Clinical Diagnosis: Palpitation. TGSH1.2416.** | | | |
| --- | --- | --- | --- |
| **Features:** | **Findings** | **Features** | **Findings** |
| **Profile** | | **Atria** | |
| Abdominal Situs | Solitus | Left Atrium | Normal |
| Cardiac Position | Levocardia | Right Atrium | Normal |
| Systemic Venous Drainage | To RA | **Atrio-Ventricular Valves** | |
| Pulmonary Venous Drainage | To LA | Mitral Valve | Annulus = 19mm |
| Atrio-ventricular Connection | Concordant | Tricuspid Valve | Annulus = 20mm  TAPSE = 21mm |
| Ventriculo-Arterial Connection | Concordant | **Ventricle** | |
| Ventricular Loop | d-Loop | Left Ventricle | Normal |
| **Septae** |  | Right Ventricle | Normal |
| Interatrial Septum | Intact | **Doppler Measurement** |  |
| Interventricular Septum | Intact | Mitral |  |
| **Semilunar Valves** |  | Aortic |  |
| Aortic Valve | Annulus = 17mm | Tricuspid |  |
| Pulmonary Valve | Annulus = 19mm | Pulmonic | Trivial PR, PPG = 10mmHg |
| **Great Arteries** | NRGA | **Coronary Arteries** |  |
| Aorta |  | **Aortic Arch** | Left |
| Pulmonary Arteries |  | **PDA** | No |
| **M-Mode**: | | | |
| Ao | mm | PWd | mm |
| LA | mm | EDV | ml |
| LVIDd | mm | ESV | ml |
| LVIDs | mm | FS | 30% |
| IVSd | mm | LVEF | 59% |
| **Additional Information:** | | | |
| **Conclusion:**   1. Normal Echocardiography Study | | | |
| **Done By:** | **Signature** | **Date** | **Remark** |
| Tesfaye T., Pediatric Cardiologist |  | 11/02/14Eth.C. |  |

| **Patient Name:** Zemenay Gashaw. **Sex/Age**: F/2 7/12. **Date of Report**: 11/02/14Eth.C. MRN: 034105.  **Clinical Diagnosis: Recurrent Chest Infection. TGSH1.2417.** | | | |
| --- | --- | --- | --- |
| **Features:** | **Findings** | **Features** | **Findings** |
| **Profile** | | **Atria** | |
| Abdominal Situs | Solitus | Left Atrium | Normal |
| Cardiac Position | Levocardia | Right Atrium | Normal |
| Systemic Venous Drainage | To RA | **Atrio-Ventricular Valves** | |
| Pulmonary Venous Drainage | To LA | Mitral Valve | Annulus = 14mm |
| Atrio-ventricular Connection | Concordant | Tricuspid Valve | Annulus = 15mm |
| Ventriculo-Arterial Connection | Concordant | **Ventricle** | |
| Ventricular Loop | d-Loop | Left Ventricle | Normal |
| **Septae** |  | Right Ventricle | Normal |
| Interatrial Septum | Intact | **Doppler Measurement** |  |
| Interventricular Septum | Intact | Mitral |  |
| **Semilunar Valves** |  | Aortic |  |
| Aortic Valve | Annulus = 12mm | Tricuspid |  |
| Pulmonary Valve | Annulus = 13mm | Pulmonic |  |
| **Great Arteries** | NRGA | **Coronary Arteries** |  |
| Aorta |  | **Aortic Arch** | Left |
| Pulmonary Arteries |  | **PDA** | No |
| **M-Mode**: Normal LV Function on eye balling | | | |
| Ao | mm | PWd | mm |
| LA | mm | EDV | ml |
| LVIDd | mm | ESV | ml |
| LVIDs | mm | FS | % |
| IVSd | mm | LVEF | % |
| **Additional Information:** pericardial effusion measuring 3mm on RV side and 7mm on RA/RV Junction. | | | |
| **Conclusion:**   1. {S, D, S} Levocardia 2. Small Pericardial effusion | | | |
| **Remark:** | No PDA on Current study | | |
| **Done By:** | **Signature** | **Date** | **Remark** |
| Tesfaye T., Pediatric Cardiologist |  | 11/02/14Eth.C. |  |

| **Patient Name:** Sefinew Eskezia. **Sex/Age**: M/6months. **Date of Report**: 11/02/14Eth.C. MRN: 103546.  **Clinical Diagnosis: DS. TGSH1.2418.** | | | |
| --- | --- | --- | --- |
| **Features:** | **Findings** | **Features** | **Findings** |
| **Profile** | | **Atria** | |
| Abdominal Situs | Solitus | Left Atrium | Normal |
| Cardiac Position | Levocardia | Right Atrium | Normal |
| Systemic Venous Drainage | To RA | **Atrio-Ventricular Valves** | |
| Pulmonary Venous Drainage | To LA | Mitral Valve | Annulus = 12mm |
| Atrio-ventricular Connection | Concordant | Tricuspid Valve | Annulus = 15mm |
| Ventriculo-Arterial Connection | Concordant | **Ventricle** | |
| Ventricular Loop | d-Loop | Left Ventricle | Normal |
| **Septae** |  | Right Ventricle | Normal |
| Interatrial Septum | Intact | **Doppler Measurement** |  |
| Interventricular Septum | Intact | Mitral |  |
| **Semilunar Valves** |  | Aortic |  |
| Aortic Valve | Annulus = 12mm | Tricuspid |  |
| Pulmonary Valve | Annulus = 14mm | Pulmonic |  |
| **Great Arteries** | NRGA | **Coronary Arteries** |  |
| Aorta |  | **Aortic Arch** | Left |
| Pulmonary Arteries |  | **PDA** | No |
| **M-Mode**: Normal LV Function on eye balling | | | |
| Ao | mm | PWd | mm |
| LA | mm | EDV | ml |
| LVIDd | mm | ESV | ml |
| LVIDs | mm | FS | % |
| IVSd | mm | LVEF | % |
| **Additional Information:** Pericardial effusion with max depth on RA/RV Junction measuring 6mm. | | | |
| **Conclusion:**   1. {S, D, S} Levocardia 2. Small Pericardial effusion | | | |
| **Recommendation:** |  | | |
| **Done By:** | **Signature** | **Date** | **Remark** |
| Tesfaye T., Pediatric Cardiologist |  | 11/02/14Eth.C. |  |

| **Patient Name:** Bewuketu Simegnew. **Sex/Age**: M/13years. **Date of Report**: 23/02/14Eth.C. MRN: 104766.  **Clinical Diagnosis: CHF + Rheumatic Recurrence + DOE + Palpitation + Easy Fatigability. TGSH1.2419.** | | | |
| --- | --- | --- | --- |
| **Features:** | **Findings** | **Features** | **Findings** |
| **Profile** | | **Atria** | |
| Abdominal Situs | Solitus | Left Atrium | Markedly dilated |
| Cardiac Position | Levocardia | Right Atrium | Dilated |
| Systemic Venous Drainage | To RA | **Atrio-Ventricular Valves** | |
| Pulmonary Venous Drainage | To LA | Mitral Valve | Annulus = 29mm. thickened MVL. Shortened PMVL. MVA = 2.6cm**2**. |
| Atrio-ventricular Connection | Concordant | Tricuspid Valve | Annulus = 26mm  TAPSE = 20mm |
| Ventriculo-Arterial Connection | Concordant | **Ventricle** | |
| Ventricular Loop | d-Loop | Left Ventricle | Markedly dilated |
| **Septae** |  | Right Ventricle | Dilated |
| Interatrial Septum | Intact | **Doppler Measurement** |  |
| Interventricular Septum | Intact | Mitral | Severe MR, Holosystolic, posterior projection, seen in two planes with jet velocity = 4m/sec. |
| **Semilunar Valves** |  | Aortic | Mild AR, PHT = 585ms. |
| Aortic Valve | Annulus = 15mm | Tricuspid | Severe TR, PPG = 60mmHg |
| Pulmonary Valve | Annulus = 20mm | Pulmonic | Moderate PR |
| **Great Arteries** | NRGA | **Coronary Arteries** |  |
| Aorta |  | **Aortic Arch** | Left |
| Pulmonary Arteries |  | **PDA** | No |
| **M-Mode**: | | | |
| Ao | mm | PWd | mm |
| LA | mm | EDV | ml |
| LVIDd | mm | ESV | ml |
| LVIDs | mm | FS | % |
| IVSd | mm | LVEF | % |
| **Additional Information:** | | | |
| **Conclusion:**   1. {S, D, S} Levocardia 2. All chambers dilated 3. Thickened MVL. Shortened PMVL 4. Severe MR 5. Severe TR 6. Mild AR 7. Moderate PR 8. Severe Pulmonary Hypertension 9. Normal Biventricular Function | | | |
| **Done By:** | **Signature** | **Date** | **Remark** |
| Tesfaye T., Pediatric Cardiologist |  | 23/02/14Eth.C. |  |

| **Patient Name:** Nafkot Fekadu. **Sex/Age**: F/8months. **Date of Report**: 23/02/14Eth.C. MRN: ________.  **Clinical Diagnosis: CHF + RD + Pulmonary Hypertension. TGSH1.2420.** | | | |
| --- | --- | --- | --- |
| **Features:** | **Findings** | **Features** | **Findings** |
| **Profile** | | **Atria** | |
| Abdominal Situs | Solitus | Left Atrium | Normal |
| Cardiac Position | Levocardia | Right Atrium | Hugely dilated |
| Systemic Venous Drainage | To RA | **Atrio-Ventricular Valves** | |
| Pulmonary Venous Drainage | To LA | Mitral Valve | Annulus = 11mm |
| Atrio-ventricular Connection | Concordant | Tricuspid Valve | Annulus = 22mm  TAPSE = 6mm |
| Ventriculo-Arterial Connection | Concordant | **Ventricle** | |
| Ventricular Loop | d-Loop | Left Ventricle | Banana shaped |
| **Septae** |  | Right Ventricle | Hugely dilated, hypertrophied & dysfunctional |
| Interatrial Septum | Probe patent PFO | **Doppler Measurement** |  |
| Interventricular Septum | Intact, Bowed to LV. | Mitral |  |
| **Semilunar Valves** |  | Aortic |  |
| Aortic Valve | Annulus = 10mm | Tricuspid | Mild TR |
| Pulmonary Valve | Annulus = 13mm | Pulmonic | Moderate PR, PPG = 57mmHg. |
| **Great Arteries** | NRGA | **Coronary Arteries** |  |
| Aorta |  | **Aortic Arch** | Left |
| Pulmonary Arteries |  | **PDA** | No |
| **M-Mode**: LV Dysfunctional on eye balling | | | |
| Ao | mm | PWd | mm |
| LA | mm | EDV | ml |
| LVIDd | mm | ESV | ml |
| LVIDs | mm | FS | % |
| IVSd | mm | LVEF | % |
| **Additional Information:** | | | |
| **Conclusion:**   1. {S, D, S} Levocardia 2. Probe Patent PFO 3. RA/RV Dilated 4. Hypertrophied, dilated and Dysfunctional RV 5. Severe Pulmonary Hypertension | | | |
| **Remark:** | Infant was crying during study | | |
| **Done By:** | **Signature** | **Date** | **Remark** |
| Tesfaye T., Pediatric Cardiologist |  | 23/02/14Eth.C. |  |

| **Patient Name:** Baby Enkuahonech Gebrie. **Sex/Age**: **M/13days**. **Date of Report**: 30/02/14Eth.C. MRN: 106219.  **Clinical Diagnosis: DS. TGSH1.2421.** | | | |
| --- | --- | --- | --- |
| **Features:** | **Findings** | **Features** | **Findings** |
| **Profile** | | **Atria** | |
| Abdominal Situs | Solitus | Left Atrium | Normal |
| Cardiac Position | Levocardia | Right Atrium | Normal |
| Systemic Venous Drainage | To RA | **Atrio-Ventricular Valves** | |
| Pulmonary Venous Drainage | To LA | Mitral Valve | Annulus = 9mm |
| Atrio-ventricular Connection | Concordant | Tricuspid Valve | Annulus = 10mm |
| Ventriculo-Arterial Connection | Concordant | **Ventricle** | |
| Ventricular Loop | d-Loop | Left Ventricle | Normal |
| **Septae** | Tongue of tissue in b/n | Right Ventricle | Normal |
| Interatrial Septum | PFO, L – R Shunt. Additional 5mm Primum defect, L – R Shunt | **Doppler Measurement** |  |
| Interventricular Septum | 2.5mm Inlet VSD, L – R Shunt | Mitral |  |
| **Semilunar Valves** |  | Aortic |  |
| Aortic Valve | Annulus = 9mm | Tricuspid |  |
| Pulmonary Valve | Annulus = 9mm | Pulmonic |  |
| **Great Arteries** | NRGA | **Coronary Arteries** |  |
| Aorta |  | **Aortic Arch** | Left |
| Pulmonary Arteries |  | **PDA** | No |
| **M-Mode**: Normal LV Function on eye balling. | | | |
| Ao | mm | PWd | mm |
| LA | mm | EDV | ml |
| LVIDd | mm | ESV | ml |
| LVIDs | mm | FS | % |
| IVSd | mm | LVEF | % |
| **Additional Information:** | | | |
| **Conclusion:**   1. {S, D, S} Levocardia 2. PFO, L – R Shunt 3. Transitional AVSD, L – R Shunt | | | |
| **Remark:** |  | | |
| **Recommendation:** |  | | |
| **Done By:** | **Signature** | **Date** | **Remark** |
| Tesfaye T., Pediatric Cardiologist |  | 30/02/14Eth.C. |  |

| **Patient Name:** Bekalu Teketay. **Sex/Age**: M/2months. **Date of Report**: 30/02/14Eth.C. MRN: 103712.  **Clinical Diagnosis: Incidental Murmur. TGSH1.2422.** | | | |
| --- | --- | --- | --- |
| **Features:** | **Findings** | **Features** | **Findings** |
| **Profile** | | **Atria** | |
| Abdominal Situs | Solitus | Left Atrium | Normal |
| Cardiac Position | Levocardia | Right Atrium | Normal |
| Systemic Venous Drainage | To RA | **Atrio-Ventricular Valves** | |
| Pulmonary Venous Drainage | To LA | Mitral Valve | Annulus = 11mm |
| Atrio-ventricular Connection | Concordant | Tricuspid Valve | Annulus = 11mm |
| Ventriculo-Arterial Connection | Concordant | **Ventricle** | |
| Ventricular Loop | d-Loop | Left Ventricle | Normal |
| **Septae** |  | Right Ventricle | Normal |
| Interatrial Septum | Intact | **Doppler Measurement** |  |
| Interventricular Septum | 2.5mm PM VSD, L – R Shunt | Mitral |  |
| **Semilunar Valves** |  | Aortic |  |
| Aortic Valve | Annulus = 9mm | Tricuspid |  |
| Pulmonary Valve | Annulus = 11mm | Pulmonic |  |
| **Great Arteries** | NRGA | **Coronary Arteries** |  |
| Aorta |  | **Aortic Arch** | Left |
| Pulmonary Arteries |  | **PDA** | No |
| **M-Mode**: Normal LV Function on eye balling | | | |
| Ao | mm | PWd | mm |
| LA | mm | EDV | ml |
| LVIDd | mm | ESV | ml |
| LVIDs | mm | FS | % |
| IVSd | mm | LVEF | % |
| **Additional Information:** | | | |
| **Conclusion:**   1. {S, D, S} Levocardia 2. Small PM VSD, L – R Shunt 3. Normal Function | | | |
| **Remark:** |  | | |
| **Recommendation:** |  | | |
| **Done By:** | **Signature** | **Date** | **Remark** |
| Tesfaye T., Pediatric Cardiologist |  | 30/02/14Eth.C. |  |

| **Patient Name:** Baby of Tiru Yigzaw. **Sex/Age**: M/13days. **Date of Report**: 30/02/14Eth.C. MRN: 104104.  **Clinical Diagnosis: DS. TGSH1.2423.** | | | |
| --- | --- | --- | --- |
| **Features:** | **Findings** | **Features** | **Findings** |
| **Profile** | | **Atria** | |
| Abdominal Situs | Solitus | Left Atrium | Normal |
| Cardiac Position | Levocardia | Right Atrium | Normal |
| Systemic Venous Drainage | To RA | **Atrio-Ventricular Valves** | |
| Pulmonary Venous Drainage | To LA | Mitral Valve | Annulus = 10mm |
| Atrio-ventricular Connection | Concordant | Tricuspid Valve | Annulus = 10mm |
| Ventriculo-Arterial Connection | Concordant | **Ventricle** | |
| Ventricular Loop | d-Loop | Left Ventricle | Normal |
| **Septae** |  | Right Ventricle | Normal |
| Interatrial Septum | PFO, L – R Shunt | **Doppler Measurement** |  |
| Interventricular Septum | Intact | Mitral |  |
| **Semilunar Valves** |  | Aortic |  |
| Aortic Valve | Annulus = 8mm | Tricuspid |  |
| Pulmonary Valve | Annulus = 9mm | Pulmonic |  |
| **Great Arteries** | NRGA | **Coronary Arteries** |  |
| Aorta | ---- | **Aortic Arch** | Left |
| Pulmonary Arteries | ------ | **PDA** | No |
| **M-Mode**: Normal LV Function on eye balling. | | | |
| Ao | mm | PWd | mm |
| LA | mm | EDV | ml |
| LVIDd | mm | ESV | ml |
| LVIDs | mm | FS | % |
| IVSd | mm | LVEF | % |
| **Additional Information:** | | | |
| **Conclusion:**   1. {S, D, S} Levocardia 2. PFO, L – R Shunt | | | |
| **Remark:** |  | | |
| **Recommendation:** |  | | |
| **Done By:** | **Signature** | **Date** | **Remark** |
| Tesfaye T., Pediatric Cardiologist |  | 30/02/14Eth.C. |  |

| **Patient Name:** Bethelihem Demeke. **Sex/Age**: F/5months. **Date of Report**: 30/02/14Eth.C. MRN: 106673.  **Clinical Diagnosis: Cyanosis. TGSH1.2424.** | | | |
| --- | --- | --- | --- |
| **Features:** | **Findings** | **Features** | **Findings** |
| **Profile** | | **Atria** | |
| Abdominal Situs | Solitus | Left Atrium | Normal |
| Cardiac Position | Levocardia | Right Atrium | Normal |
| Systemic Venous Drainage | To RA | **Atrio-Ventricular Valves** | |
| Pulmonary Venous Drainage | To LA | Mitral Valve | Annulus = 11mm |
| Atrio-ventricular Connection | Concordant | Tricuspid Valve | Annulus = 13mm |
| Ventriculo-Arterial Connection | Concordant | **Ventricle** | |
| Ventricular Loop | d-Loop | Left Ventricle | Normal |
| **Septae** |  | Right Ventricle | Hypertrophied |
| Interatrial Septum | Intact | **Doppler Measurement** |  |
| Interventricular Septum | Non-restrictive subaortic VSD, R – L Shunt | Mitral |  |
| **Semilunar Valves** |  | Aortic |  |
| Aortic Valve | Annulus = 12mm | Tricuspid |  |
| Pulmonary Valve | Annulus = 6mm | Pulmonic | Infundibular, Valvular and supra valvular PS, PPG = 60mmHg. |
| **Great Arteries** | NRGA | **Coronary Arteries** |  |
| Aorta | Over-riding aorta | **Aortic Arch** | Left |
| Pulmonary Arteries | smallish | **PDA** | No |
| **M-Mode**: Normal LV Function on eye balling | | | |
| Ao | mm | PWd | mm |
| LA | mm | EDV | ml |
| LVIDd | mm | ESV | ml |
| LVIDs | mm | FS | % |
| IVSd | mm | LVEF | % |
| **Additional Information:** | | | |
| **Conclusion:**   1. {S, D, S} Levocardia 2. TOF | | | |
| **Remark:** |  | | |
| **Recommendation:** |  | | |
| **Done By:** | **Signature** | **Date** | **Remark** |
| Tesfaye T., Pediatric Cardiologist |  | 30/02/14Eth.C. |  |

| **Patient Name:** Birtukan Tesfaye. **Sex/Age**: F/4years. **Date of Report**: 02/03/14Eth.C. MRN: 106971.  **Clinical Diagnosis: Easy Fatigability. TGSH1.2425.** | | | |
| --- | --- | --- | --- |
| **Features:** | **Findings** | **Features** | **Findings** |
| **Profile** | | **Atria** | |
| Abdominal Situs | Solitus | Left Atrium | Dilated |
| Cardiac Position | Levocardia | Right Atrium | Normal |
| Systemic Venous Drainage | To RA | **Atrio-Ventricular Valves** | |
| Pulmonary Venous Drainage | To LA | Mitral Valve | Annulus = 18mm |
| Atrio-ventricular Connection | Concordant | Tricuspid Valve | Annulus = 22mm. Failed delamination of the STL. |
| Ventriculo-Arterial Connection | Concordant | **Ventricle** | |
| Ventricular Loop | d-Loop | Left Ventricle | Dilated |
| **Septae** |  | Right Ventricle | Atrialized RV |
| Interatrial Septum | Intact | **Doppler Measurement** |  |
| Interventricular Septum | 9mm Sub-arterial VSD, BD Shunt | Mitral |  |
| **Semilunar Valves** |  | Aortic |  |
| Aortic Valve | Annulus = 16mm | Tricuspid |  |
| Pulmonary Valve | Annulus = 18mm | Pulmonic |  |
| **Great Arteries** | NRGA | **Coronary Arteries** |  |
| Aorta |  | **Aortic Arch** | Left |
| Pulmonary Arteries |  | **PDA** | No |
| **M-Mode**: Normal LV Function on eye balling | | | |
| Ao | mm | PWd | mm |
| LA | mm | EDV | ml |
| LVIDd | mm | ESV | ml |
| LVIDs | mm | FS | % |
| IVSd | mm | LVEF | % |
| **Additional Information:** 4mm pericardial effusion on RV Side | | | |
| **Conclusion:**   1. {S, D, S} Levocardia 2. Ebstein anomaly of the Tricuspid valve 3. Moderate Sub-arterial VSD, BD Shunt 4. Normal LV Function 5. Trace Pericardial effusion | | | |
| **Recommendation:** |  | | |
| **Done By:** | **Signature** | **Date** | **Remark** |
| Tesfaye T., Pediatric Cardiologist |  | 02/03/14Eth.C. |  |

| **Patient Name:** Baby of Birtukan Yihun. **Sex/Age**: M/4months. **Date of Report**: 02/03/14Eth.C. MRN: 090871.  **Clinical Diagnosis: RD + PPHTN. TGSH1.2426. (TGSH4)** | | | |
| --- | --- | --- | --- |
| **Features:** | **Findings** | **Features** | **Findings** |
| **Profile** | | **Atria** | |
| Abdominal Situs | Solitus | Left Atrium | Normal |
| Cardiac Position | Levocardia | Right Atrium | Normal |
| Systemic Venous Drainage | To RA | **Atrio-Ventricular Valves** | |
| Pulmonary Venous Drainage | To LA | Mitral Valve | Annulus = 15mm |
| Atrio-ventricular Connection | Concordant | Tricuspid Valve | Annulus = 18mm  TAPSE = 17mm |
| Ventriculo-Arterial Connection | Concordant | **Ventricle** | |
| Ventricular Loop | d-Loop | Left Ventricle | Normal |
| **Septae** |  | Right Ventricle | Normal |
| Interatrial Septum | Intact | **Doppler Measurement** |  |
| Interventricular Septum | Intact | Mitral |  |
| **Semilunar Valves** |  | Aortic |  |
| Aortic Valve | Annulus = 12mm | Tricuspid | Mild TR, PPG = 38mmHg |
| Pulmonary Valve | Annulus = 13mm | Pulmonic |  |
| **Great Arteries** | NRGA | **Coronary Arteries** |  |
| Aorta |  | **Aortic Arch** | Left |
| Pulmonary Arteries |  | **PDA** | No |
| **M-Mode**: Normal LV Function on eye balling | | | |
| Ao | mm | PWd | mm |
| LA | mm | EDV | ml |
| LVIDd | mm | ESV | ml |
| LVIDs | mm | FS | % |
| IVSd | mm | LVEF | % |
| **Additional Information:** | | | |
| **Conclusion:**   1. {S, D, S} Levocardia 2. Mild TR 3. Mild Pulmonary Hypertension 4. Normal Biventricular Function | | | |
| **Remark:** | Good clinical and echocardiographic progress from the previous finding | | |
| **Recommendation:** |  | | |
| **Done By:** | **Signature** | **Date** | **Remark** |
| Tesfaye T., Pediatric Cardiologist |  | 02/03/14Eth.C. |  |

| **Patient Name:** Tirualem Tenesa. **Sex/Age**: F/11years. **Date of Report**: 02/03/14Eth.C. MRN: 106989.  **Clinical Diagnosis: Sydenham’s Chorea. TGSH1.2427.** | | | |
| --- | --- | --- | --- |
| **Features:** | **Findings** | **Features** | **Findings** |
| **Profile** | | **Atria** | |
| Abdominal Situs | Solitus | Left Atrium | Dilated |
| Cardiac Position | Levocardia | Right Atrium | Normal |
| Systemic Venous Drainage | To RA | **Atrio-Ventricular Valves** | |
| Pulmonary Venous Drainage | To LA | Mitral Valve | Annulus = 27mm |
| Atrio-ventricular Connection | Concordant | Tricuspid Valve | Annulus = 25mm |
| Ventriculo-Arterial Connection | Concordant | **Ventricle** | |
| Ventricular Loop | d-Loop | Left Ventricle | Dilated |
| **Septae** |  | Right Ventricle | Normal |
| Interatrial Septum | Intact | **Doppler Measurement** |  |
| Interventricular Septum | Intact | Mitral | Severe MR, Holosystolic, posterior projection, seen in two planes with jet velocity = 4.5m/sec |
| **Semilunar Valves** |  | Aortic |  |
| Aortic Valve | Annulus = 18mm | Tricuspid | Trivial TR, PPG = 19mmHg |
| Pulmonary Valve | Annulus = 19mm | Pulmonic |  |
| **Great Arteries** | NRGA | **Coronary Arteries** |  |
| Aorta |  | **Aortic Arch** | Left |
| Pulmonary Arteries |  | **PDA** | No |
| **M-Mode**: | | | |
| Ao | mm | PWd | mm |
| LA | mm | EDV | ml |
| LVIDd | mm | ESV | ml |
| LVIDs | mm | FS | % |
| IVSd | mm | LVEF | % |
| **Additional Information:** | | | |
| **Conclusion:**   1. {S, D, S} Levocardia 2. LA/LV Dilated 3. Patulous, thickened MVL 4. Severe MR 5. Trivial TR 6. Normal Biventricular Function | | | |
| **Done By:** | **Signature** | **Date** | **Remark** |
| Tesfaye T., Pediatric Cardiologist |  | 02/03/14Eth.C. |  |

| **Patient Name:** Gashaw Guadie. **Sex/Age**: M/9 6/12. **Date of Report**: 02/03/14Eth.C. MRN: 106863.  **Clinical Diagnosis: Cyanosis + Clubbing. TGSH1.2428.** | | | |
| --- | --- | --- | --- |
| **Features:** | **Findings** | **Features** | **Findings** |
| **Profile** | | **Atria** | |
| Abdominal Situs | Solitus | Left Atrium | Normal |
| Cardiac Position | Levocardia | Right Atrium | Dilated |
| Systemic Venous Drainage | To RA | **Atrio-Ventricular Valves** | |
| Pulmonary Venous Drainage | To LA | Mitral Valve | Annulus = 16mm |
| Atrio-ventricular Connection | Concordant | Tricuspid Valve | Annulus = 22mm |
| Ventriculo-Arterial Connection | Concordant | **Ventricle** | |
| Ventricular Loop | d-Loop | Left Ventricle | Normal |
| **Septae** |  | Right Ventricle | Dilated & Hypertrophied |
| Interatrial Septum | Intact | **Doppler Measurement** |  |
| Interventricular Septum | Malaligned non-restrictive sub-aortic VSD, R – L Shunt | Mitral |  |
| **Semilunar Valves** |  | Aortic |  |
| Aortic Valve | Annulus = 21mm | Tricuspid |  |
| Pulmonary Valve | Annulus = 14mm | Pulmonic | Severe PS, PPG = 66mmHg |
| **Great Arteries** | NRGA | **Coronary Arteries** |  |
| Aorta | Over-riding aorta | **Aortic Arch** |  |
| Pulmonary Arteries |  | **PDA** | No |
| **M-Mode**: | | | |
| Ao | mm | PWd | mm |
| LA | mm | EDV | ml |
| LVIDd | mm | ESV | ml |
| LVIDs | mm | FS | % |
| IVSd | mm | LVEF | % |
| **Additional Information:** | | | |
| **Conclusion:**   1. {S, D, S} Levocardia 2. TOF | | | |
| **Remark:** |  | | |
| **Recommendation:** |  | | |
| **Done By:** | **Signature** | **Date** | **Remark** |
| Tesfaye T., Pediatric Cardiologist |  | 02/03/14Eth.C. |  |

| **Patient Name:** Kabaw Sultan. **Sex/Age**: M/1 9/12. **Date of Report**: 02/03/14Eth.C. MRN: 07343.  **Clinical Diagnosis: RD + Murmur + CHF. TGSH1.2429.** | | | |
| --- | --- | --- | --- |
| **Features:** | **Findings** | **Features** | **Findings** |
| **Profile** | | **Atria** | |
| Abdominal Situs | Solitus | Left Atrium | More dilated |
| Cardiac Position | Levocardia | Right Atrium | Dilated |
| Systemic Venous Drainage | To RA | **Atrio-Ventricular Valves** | |
| Pulmonary Venous Drainage | To LA | Mitral Valve | Annulus = 27mm |
| Atrio-ventricular Connection | Concordant | Tricuspid Valve | Annulus = 21mm |
| Ventriculo-Arterial Connection | Concordant | **Ventricle** | |
| Ventricular Loop | d-Loop | Left Ventricle | More dilated |
| **Septae** |  | Right Ventricle | Dilated |
| Interatrial Septum | 14mm anterior OS ASD, L – R Shunt | **Doppler Measurement** |  |
| Interventricular Septum | 12mm Inlet VSD, L – R Shunt | Mitral |  |
| **Semilunar Valves** |  | Aortic |  |
| Aortic Valve | Annulus = 16mm | Tricuspid |  |
| Pulmonary Valve | Annulus = 20mm | Pulmonic |  |
| **Great Arteries** | Side by side | **Coronary Arteries** |  |
| Aorta |  | **Aortic Arch** | Left |
| Pulmonary Arteries | MPA = 22mm | **PDA** | No |
| **M-Mode**: Reduced LV Function | | | |
| Ao | mm | PWd | mm |
| LA | mm | EDV | ml |
| LVIDd | mm | ESV | ml |
| LVIDs | mm | FS | % |
| IVSd | mm | LVEF | % |
| **Additional Information:** 5mm Pericardial effusion on RV Side | | | |
| **Conclusion:**   1. {S, D, Side by side} Levocardia 2. All chambers dilated 3. Large OS ASD, L – R Shunt 4. Large inlet VSD, L – R Shunt 5. Severe Pulmonary Hypertension 6. Reduced LV Function 7. Small Pericardial effusion | | | |
| **Done By:** | **Signature** | **Date** | **Remark** |
| Tesfaye T., Pediatric Cardiologist |  | 02/03/14Eth.C. |  |

| **Patient Name:** Dersolign Yazie. **Sex/Age**: M/13years. **Date of Report**: 03/03/14Eth.C. MRN: ________.  **Clinical Diagnosis: CHF + DOE. TGSH1.2430.** | | | |
| --- | --- | --- | --- |
| **Features:** | **Findings** | **Features** | **Findings** |
| **Profile** | | **Atria** | |
| Abdominal Situs | Solitus | Left Atrium | Dilated |
| Cardiac Position | Levocardia | Right Atrium | Dilated |
| Systemic Venous Drainage | To RA. IVC Dilated | **Atrio-Ventricular Valves** | |
| Pulmonary Venous Drainage | To LA | Mitral Valve | Annulus = 20mm. E/A = 2.35. DT = 114ms |
| Atrio-ventricular Connection | Concordant | Tricuspid Valve | Annulus = 22mm  TAPSE = 8mm |
| Ventriculo-Arterial Connection | Concordant | **Ventricle:** thickened pericardium | |
| Ventricular Loop | d-Loop | Left Ventricle | Normal |
| **Septae** | Bouncing | Right Ventricle | Normal |
| Interatrial Septum | Intact | **Doppler Measurement** |  |
| Interventricular Septum | Intact | Mitral | Trivial MR |
| **Semilunar Valves** |  | Aortic |  |
| Aortic Valve | Annulus = 16mm | Tricuspid | Severe TR, PPG = 60mmHg |
| Pulmonary Valve | Annulus = 23mm | Pulmonic |  |
| **Great Arteries** | NRGA | **Coronary Arteries** |  |
| Aorta | ---- | **Aortic Arch** | Left |
| Pulmonary Arteries | ---- | **PDA** | No |
| **M-Mode**: Reduced LV Function on eye balling | | | |
| Ao | mm | PWd | mm |
| LA | mm | EDV | ml |
| LVIDd | mm | ESV | ml |
| LVIDs | mm | FS | % |
| IVSd | mm | LVEF | % |
| **Additional Information:** thickened Pericardium. | | | |
| **Conclusion:**   1. {S, D, S} Levocardia 2. LA/RA Dilated (Dwarf sign) 3. Severe TR 4. Severe Pulmonary Hypertension 5. Diastolic Dysfunction 6. Biventricular Systolic Dysfunction 7. Pericardial thickening | | | |
| **Done By:** | **Signature** | **Date** | **Remark** |
| Tesfaye T., Pediatric Cardiologist |  | 03/03/14Eth.C. |  |
| **Patient Name:** Zimam Habtam. **Sex/Age**: F/6months. **Date of Report**: 07/03/14Eth.C. MRN: 107369.  **Clinical Diagnosis: Cyanosis. TGSH1.2431.** | | | |
| **Features:** | **Findings** | **Features** | **Findings** |
| **Profile** | | **Atria** | |
| Abdominal Situs | Solitus | Left Atrium | Normal |
| Cardiac Position | Levocardia | Right Atrium | Normal |
| Systemic Venous Drainage | To RA | **Atrio-Ventricular Valves** | |
| Pulmonary Venous Drainage | To LA | Mitral Valve | Annulus = 18mm |
| Atrio-ventricular Connection | Concordant | Tricuspid Valve | Annulus = 18mm |
| Ventriculo-Arterial Connection | Discordant | **Ventricle** | |
| Ventricular Loop | d-Loop | Left Ventricle | Normal |
| **Septae** |  | Right Ventricle | Normal |
| Interatrial Septum | 15mm OS ASD, L – R Shunt. | **Doppler Measurement** |  |
| Interventricular Septum | Intact | Mitral |  |
| **Semilunar Valves** |  | Aortic |  |
| Aortic Valve | Annulus = 12mm | Tricuspid | Severe TR |
| Pulmonary Valve | Annulus = 16mm | Pulmonic |  |
| **Great Arteries** | d-TGA | **Coronary Arteries** |  |
| Aorta | anterior and to the right | **Aortic Arch** | Left |
| Pulmonary Arteries | Posterior and to the left. **MPA = 16mm** | **PDA** | No |
| **M-Mode**: Normal LV Function | | | |
| Ao | mm | PWd | mm |
| LA | mm | EDV | ml |
| LVIDd | mm | ESV | ml |
| LVIDs | mm | FS | % |
| IVSd | mm | LVEF | % |
| **Additional Information:** | | | |
| **Conclusion:**   1. {S, D, D} Levocardia 2. Large OS ASD, L – R Shunt 3. d-TGA 4. Severe Pulmonary Hypertension | | | |
| **Remark:** |  | | |
| **Recommendation:** |  | | |
| **Done By:** | **Signature** | **Date** | **Remark** |
| Tesfaye T., Pediatric Cardiologist |  | 07/03/14Eth.C. |  |

| **Patient Name:** Baby of Yeshareg Andualem. **Sex/Age**: M/17days. **Date of Report**: 07/03/14Eth.C. MRN: 105495.  **Clinical Diagnosis: DS. TGSH1.2432.** | | | |
| --- | --- | --- | --- |
| **Features:** | **Findings** | **Features** | **Findings** |
| **Profile** | | **Atria** | |
| Abdominal Situs | Solitus | Left Atrium | Normal |
| Cardiac Position | Levocardia | Right Atrium | Normal |
| Systemic Venous Drainage | To RA | **Atrio-Ventricular Valves** | |
| Pulmonary Venous Drainage | To LA | Mitral Valve | Annulus = 8mm |
| Atrio-ventricular Connection | Concordant | Tricuspid Valve | Annulus = 8mm |
| Ventriculo-Arterial Connection | Concordant | **Ventricle** | |
| Ventricular Loop | d-Loop | Left Ventricle | Normal |
| **Septae** |  | Right Ventricle | Normal |
| Interatrial Septum | PFO, Probe patent | **Doppler Measurement** |  |
| Interventricular Septum | Intact | Mitral |  |
| **Semilunar Valves** |  | Aortic |  |
| Aortic Valve | Annulus = 7mm | Tricuspid | Trivial TR, PPG = 22mmHg |
| Pulmonary Valve | Annulus = 8mm | Pulmonic |  |
| **Great Arteries** | NRGA | **Coronary Arteries** |  |
| Aorta |  | **Aortic Arch** | Left |
| Pulmonary Arteries |  | **PDA** | No |
| **M-Mode**: Normal LV Function on eye balling | | | |
| Ao | mm | PWd | mm |
| LA | mm | EDV | ml |
| LVIDd | mm | ESV | ml |
| LVIDs | mm | FS | % |
| IVSd | mm | LVEF | % |
| **Additional Information:** | | | |
| **Conclusion:**   1. {S, D, S} Levocardia 2. Probe Patent PFO | | | |
| **Remark:** |  | | |
| **Recommendation:** |  | | |
| **Done By:** | **Signature** | **Date** | **Remark** |
| Tesfaye T., Pediatric Cardiologist |  | 07/03/14Eth.C. |  |

| **Patient Name:** Hanna-Mariam Gashaw. **Sex/Age**: F/7months. **Date of Report**: 07/03/14Eth.C. MRN: 106210.  **Clinical Diagnosis: DS. TGSH1.2433.** | | | |
| --- | --- | --- | --- |
| **Features:** | **Findings** | **Features** | **Findings** |
| **Profile** | | **Atria** | |
| Abdominal Situs | Solitus | Left Atrium | Normal |
| Cardiac Position | Levocardia | Right Atrium | Normal |
| Systemic Venous Drainage | To RA | **Atrio-Ventricular Valves** | |
| Pulmonary Venous Drainage | To LA | Mitral Valve | Complete AVSD |
| Atrio-ventricular Connection | AVSD | Tricuspid Valve |  |
| Ventriculo-Arterial Connection | Concordant | **Ventricle** | |
| Ventricular Loop | d-Loop | Left Ventricle | Normal |
| **Septae** |  | Right Ventricle | Normal |
| Interatrial Septum | Complete AVSD, L – R Shunt | **Doppler Measurement** |  |
| Interventricular Septum |  | Mitral | Mild Left AVVR |
| **Semilunar Valves** |  | Aortic |  |
| Aortic Valve | Annulus = 12mm | Tricuspid | Moderate Right AVVR |
| Pulmonary Valve | Annulus = 13mm | Pulmonic |  |
| **Great Arteries** | NRGA | **Coronary Arteries** |  |
| Aorta |  | **Aortic Arch** | Left |
| Pulmonary Arteries |  | **PDA** | No |
| **M-Mode**: Normal LV Function on eye balling | | | |
| Ao | mm | PWd | mm |
| LA | mm | EDV | ml |
| LVIDd | mm | ESV | ml |
| LVIDs | mm | FS | % |
| IVSd | mm | LVEF | % |
| **Additional Information:** 5mm pericardial effusion seen on RV Side. | | | |
| **Conclusion:**   1. {S, D, S} Levocardia 2. Common Complete Balanced AVSD, L – R Shunt 3. Small Pericardial effusion | | | |
| **Remark:** |  | | |
| **Recommendation:** |  | | |
| **Done By:** | **Signature** | **Date** | **Remark** |
| Tesfaye T., Pediatric Cardiologist |  | 07/03/14Eth.C. |  |

| **Patient Name:** Ermias Getu. **Sex/Age**: M/2years. **Date of Report**: 09/03/14Eth.C. MRN: 040470.  **Clinical Diagnosis: Diaphoresis + Murmur + FTT. TGSH1.2434.** | | | |
| --- | --- | --- | --- |
| **Features:** | **Findings** | **Features** | **Findings** |
| **Profile** | | **Atria** | |
| Abdominal Situs | Solitus | Left Atrium | Dilated |
| Cardiac Position | Levocardia | Right Atrium | Normal |
| Systemic Venous Drainage | To RA | **Atrio-Ventricular Valves** | |
| Pulmonary Venous Drainage | To LA | Mitral Valve | Annulus = 22mm |
| Atrio-ventricular Connection | Concordant | Tricuspid Valve | Annulus = 18mm |
| Ventriculo-Arterial Connection | Concordant | **Ventricle** | |
| Ventricular Loop | d-Loop | Left Ventricle | Dilated |
| **Septae** |  | Right Ventricle | Normal |
| Interatrial Septum | Intact | **Doppler Measurement** |  |
| Interventricular Septum | 8mm PM VSD, L – R Shunt | Mitral |  |
| **Semilunar Valves** |  | Aortic |  |
| Aortic Valve | Annulus = 11mm | Tricuspid |  |
| Pulmonary Valve | Annulus = 20mm | Pulmonic |  |
| **Great Arteries** | NRGA | **Coronary Arteries** |  |
| Aorta |  | **Aortic Arch** | Left |
| Pulmonary Arteries | **MPA = 26mm** | **PDA** | No |
| **M-Mode**: Normal LV Function on eye balling | | | |
| Ao | mm | PWd | mm |
| LA | mm | EDV | ml |
| LVIDd | mm | ESV | ml |
| LVIDs | mm | FS | % |
| IVSd | mm | LVEF | % |
| **Additional Information:** | | | |
| **Conclusion:**   1. {S, D, S} Levocardia 2. LA/LV Dilated 3. Large PM VSD, L – R Shunt 4. Severe Pulmonary Hypertension 5. Normal LV Function | | | |
| **Remark:** | Child was crying during study | | |
| **Recommendation:** |  | | |
| **Done By:** | **Signature** | **Date** | **Remark** |
| Tesfaye T., Pediatric Cardiologist |  | 09/03/14Eth.C. |  |

| **Patient Name:** Baby of Habiba. **Sex/Age**: M/15days. **Date of Report**: 09/03/14Eth.C. MRN: 103641.  **Clinical Diagnosis: DS + RD. TGSH1.2435.** | | | |
| --- | --- | --- | --- |
| **Features:** | **Findings** | **Features** | **Findings** |
| **Profile** | | **Atria** | |
| Abdominal Situs | Solitus | Left Atrium | Normal |
| Cardiac Position | Levocardia | Right Atrium | Dilated |
| Systemic Venous Drainage | To RA | **Atrio-Ventricular Valves** | |
| Pulmonary Venous Drainage | To LA | Mitral Valve | Annulus = 9mm |
| Atrio-ventricular Connection | Concordant | Tricuspid Valve | Annulus = 9mm |
| Ventriculo-Arterial Connection | Concordant | **Ventricle** | |
| Ventricular Loop | d-Loop | Left Ventricle | Normal |
| **Septae** |  | Right Ventricle | Dilated |
| Interatrial Septum | PFO, L – R Shunt | **Doppler Measurement** |  |
| Interventricular Septum | Intact | Mitral |  |
| **Semilunar Valves** |  | Aortic |  |
| Aortic Valve | Annulus = 8mm | Tricuspid | Moderate TR, PPG = 51mmHg |
| Pulmonary Valve | Annulus = 9mm | Pulmonic |  |
| **Great Arteries** | NRGA | **Coronary Arteries** |  |
| Aorta |  | **Aortic Arch** | Left |
| Pulmonary Arteries |  | **PDA** | No |
| **M-Mode**: Normal LV Function on eye balling | | | |
| Ao | mm | PWd | mm |
| LA | mm | EDV | ml |
| LVIDd | mm | ESV | ml |
| LVIDs | mm | FS | % |
| IVSd | mm | LVEF | % |
| **Additional Information:** | | | |
| **Conclusion:**   1. {S, D, S} Levocardia 2. PFO, L – R Shunt 3. Moderate Pulmonary Hypertension secondary to ? | | | |
| **Remark:** |  | | |
| **Recommendation:** |  | | |
| **Done By:** | **Signature** | **Date** | **Remark** |
| Tesfaye T., Pediatric Cardiologist |  | 09/03/14Eth.C. |  |

| **Patient Name:** Samuel Alemu. **Sex/Age**: M/5months. **Date of Report**: 14/03/14Eth.C. MRN: 091464.  **Clinical Diagnosis: Incidental Murmur. TGSH1.2436.** | | | |
| --- | --- | --- | --- |
| **Features:** | **Findings** | **Features** | **Findings** |
| **Profile** | | **Atria** | |
| Abdominal Situs | Solitus | Left Atrium | Normal |
| Cardiac Position | Levocardia | Right Atrium | Normal |
| Systemic Venous Drainage | To RA | **Atrio-Ventricular Valves** | |
| Pulmonary Venous Drainage | To LA | Mitral Valve | Annulus = 12mm |
| Atrio-ventricular Connection | Concordant | Tricuspid Valve | Annulus = 12mm |
| Ventriculo-Arterial Connection | Concordant | **Ventricle** | |
| Ventricular Loop | d-Loop | Left Ventricle | Normal |
| **Septae** |  | Right Ventricle | Normal |
| Interatrial Septum | Intact | **Doppler Measurement** |  |
| Interventricular Septum | Intact | Mitral |  |
| **Semilunar Valves** |  | Aortic |  |
| Aortic Valve | Annulus = 11mm | Tricuspid |  |
| Pulmonary Valve | Annulus = 11mm | Pulmonic |  |
| **Great Arteries** | NRGA | **Coronary Arteries** |  |
| Aorta |  | **Aortic Arch** | Left |
| Pulmonary Arteries |  | **PDA** | 1mm PDA, L – R Shunt |
| **M-Mode**: Normal LV Function on eye balling | | | |
| Ao | mm | PWd | mm |
| LA | mm | EDV | ml |
| LVIDd | mm | ESV | ml |
| LVIDs | mm | FS | % |
| IVSd | mm | LVEF | % |
| **Additional Information:** | | | |
| **Conclusion:**   1. {S, D, S} Levocardia 2. Small PDA, L – R Shunt 3. Normal Function | | | |
| **Remark:** |  | | |
| **Recommendation:** |  | | |
| **Done By:** | **Signature** | **Date** | **Remark** |
| Tesfaye T., Pediatric Cardiologist |  | 14/03/14Eth.C. |  |

| **Patient Name:** Getinet Munaw. **Sex/Age**: M/11years. **Date of Report**: 14/03/14Eth.C. MRN: 108264.  **Clinical Diagnosis: ARF + Murmur. TGSH1.2437.** | | | |
| --- | --- | --- | --- |
| **Features:** | **Findings** | **Features** | **Findings** |
| **Profile** | | **Atria** | |
| Abdominal Situs | Solitus | Left Atrium | Normal |
| Cardiac Position | Levocardia | Right Atrium | Normal |
| Systemic Venous Drainage | To RA | **Atrio-Ventricular Valves** | |
| Pulmonary Venous Drainage | To LA | Mitral Valve | Annulus = 24mm. patulous, elongated and mildly thickened MVL |
| Atrio-ventricular Connection | Concordant | Tricuspid Valve | Annulus = 25mm |
| Ventriculo-Arterial Connection | Concordant | **Ventricle** | |
| Ventricular Loop | d-Loop | Left Ventricle | Normal |
| **Septae** |  | Right Ventricle | Normal |
| Interatrial Septum | Intact | **Doppler Measurement** |  |
| Interventricular Septum | Intact | Mitral | Mild MR, Holosystolic, posterior projection, seen in two planes with jet velocity = 3.5m/sec. |
| **Semilunar Valves** |  | Aortic |  |
| Aortic Valve | Annulus = 19mm | Tricuspid |  |
| Pulmonary Valve | Annulus = 21mm | Pulmonic |  |
| **Great Arteries** | NRGA | **Coronary Arteries** |  |
| Aorta |  | **Aortic Arch** | Left |
| Pulmonary Arteries |  | **PDA** | No |
| **M-Mode**: Normal LV Function on eye balling | | | |
| Ao | mm | PWd | mm |
| LA | mm | EDV | ml |
| LVIDd | mm | ESV | ml |
| LVIDs | mm | FS | % |
| IVSd | mm | LVEF | % |
| **Additional Information:** | | | |
| **Conclusion:**   1. {S, D, S} Levocardia 2. Patulous, elongated and mildly thickened MVL 3. Mild MR 4. Normal LV Function | | | |
| **Done By:** | **Signature** | **Date** | **Remark** |
| Tesfaye T., Pediatric Cardiologist |  | 14/03/14Eth.C. |  |

| **Patient Name:** Kalkidan Shitahun. **Sex/Age**: F/12years. **Date of Report**: 16/03/14Eth.C. MRN: 079312.  **Clinical Diagnosis: _______. Inconclusive report.** | | | |
| --- | --- | --- | --- |
| **Features:** | **Findings** | **Features** | **Findings** |
| **Profile** | | **Atria** | |
| Abdominal Situs | Solitus | Left Atrium | Normal |
| Cardiac Position | Levocardia | Right Atrium | Normal |
| Systemic Venous Drainage | Flow acceleration at the IVC – RA Junction with gradient of 12mmHg | **Atrio-Ventricular Valves** | |
| Pulmonary Venous Drainage | To LA | Mitral Valve | Annulus = 19mm |
| Atrio-ventricular Connection | Concordant | Tricuspid Valve | Annulus = 20mm |
| Ventriculo-Arterial Connection | Concordant | **Ventricle** | |
| Ventricular Loop | d-Loop | Left Ventricle | Normal |
| **Septae** |  | Right Ventricle | Normal |
| Interatrial Septum | Intact | **Doppler Measurement** |  |
| Interventricular Septum | Intact | Mitral |  |
| **Semilunar Valves** |  | Aortic |  |
| Aortic Valve | Annulus = 20mm | Tricuspid |  |
| Pulmonary Valve | Annulus = mm (not visusalized, poor echo window) | Pulmonic |  |
| **Great Arteries** | NRGA | **Coronary Arteries** |  |
| Aorta |  | **Aortic Arch** | Left |
| Pulmonary Arteries |  | **PDA** | No |
| **M-Mode**: | | | |
| Ao | mm | PWd | mm |
| LA | mm | EDV | ml |
| LVIDd | mm | ESV | ml |
| LVIDs | mm | FS | % |
| IVSd | mm | LVEF | % |
| **Additional Information:** Circumferential Pericardial effusion with a maximum depth of 6mm on RV Side. | | | |
| **Conclusion:**   1. {S, D, S} Levocardia 2. Flow acceleration at the IVC – RA Junction with a gradient of 12mmHg 3. Small circumferential Pericardial effusion | | | |
| **Remark:** | 1. Poor echo window (hyper-inflated chest) 2. Conduction abnormality detected during study 3. Heart is swinging | | |
| **Recommendation:** | Do ECG | | |
| **Done By:** | **Signature** | **Date** | **Remark** |
| Tesfaye T., Pediatric Cardiologist |  | 16/03/14Eth.C. |  |

| **Patient Name:** Eleni Fekede. **Sex/Age**: F/9months. **Date of Report**: 21/03/14Eth.C. MRN: 109043.  **Clinical Diagnosis: DS + RD + Murmur. TGSH1.2438.** | | | |
| --- | --- | --- | --- |
| **Features:** | **Findings** | **Features** | **Findings** |
| **Profile** | | **Atria** | |
| Abdominal Situs | Solitus | Left Atrium | Dilated |
| Cardiac Position | Levocardia | Right Atrium | Dilated |
| Systemic Venous Drainage | To RA | **Atrio-Ventricular Valves** | |
| Pulmonary Venous Drainage | To LA | Mitral Valve | Common Complete AVVR. |
| Atrio-ventricular Connection | Concordant | Tricuspid Valve |  |
| Ventriculo-Arterial Connection | Concordant | **Ventricle** | |
| Ventricular Loop | d-Loop | Left Ventricle | Dilated |
| **Septae** |  | Right Ventricle | Dilated |
| Interatrial Septum | Common Complete AVVR, L – R Shunt | **Doppler Measurement** |  |
| Interventricular Septum |  | Mitral |  |
| **Semilunar Valves** |  | Aortic |  |
| Aortic Valve | Annulus = 9mm | Tricuspid |  |
| Pulmonary Valve | Annulus = 14mm | Pulmonic |  |
| **Great Arteries** | NRGA | **Coronary Arteries** |  |
| Aorta |  | **Aortic Arch** | Left |
| Pulmonary Arteries | **MPA = 17mm** | **PDA** | No |
| **M-Mode**: Normal LV Function on eye balling. | | | |
| Ao | mm | PWd | mm |
| LA | mm | EDV | ml |
| LVIDd | mm | ESV | ml |
| LVIDs | mm | FS | % |
| IVSd | mm | LVEF | % |
| **Additional Information:** Circumferential Pericardial effusion with maximum depth of 12mm on RA/RV Side. | | | |
| **Conclusion:**   1. {S, D, S} Levocardia 2. Common Complete AVSD, L – R Shunt 3. Severe Pulmonary Hypertension 4. Normal LV Function 5. Moderate circumferential Pericardial effusion | | | |
| **Remark:** |  | | |
| **Recommendation:** |  | | |
| **Done By:** | **Signature** | **Date** | **Remark** |
| Tesfaye T., Pediatric Cardiologist |  | 21/03/14Eth.C. |  |

| **Patient Name:** Baby of Zinaye Getahun. **Sex/Age**: F/18days. **Date of Report**: 21/03/14Eth.C. MRN: 107342.  **Clinical Diagnosis: Pre-Op Screening. TGSH1.2439.** | | | |
| --- | --- | --- | --- |
| **Features:** | **Findings** | **Features** | **Findings** |
| **Profile** | | **Atria** | |
| Abdominal Situs | Solitus | Left Atrium | Normal |
| Cardiac Position | Levocardia | Right Atrium | Normal |
| Systemic Venous Drainage | To RA | **Atrio-Ventricular Valves** | |
| Pulmonary Venous Drainage | To LA | Mitral Valve | Annulus = 11mm |
| Atrio-ventricular Connection | Concordant | Tricuspid Valve | Annulus = 12mm |
| Ventriculo-Arterial Connection | Concordant | **Ventricle** | |
| Ventricular Loop | d-Loop | Left Ventricle | Normal |
| **Septae** |  | Right Ventricle | Normal |
| Interatrial Septum | Intact | **Doppler Measurement** |  |
| Interventricular Septum | Intact | Mitral |  |
| **Semilunar Valves** |  | Aortic |  |
| Aortic Valve | Annulus = 9mm | Tricuspid |  |
| Pulmonary Valve | Annulus = 8mm | Pulmonic |  |
| **Great Arteries** | NRGA | **Coronary Arteries** |  |
| Aorta |  | **Aortic Arch** | Left |
| Pulmonary Arteries |  | **PDA** | No |
| **M-Mode**: Normal LV Function on eye balling. | | | |
| Ao | mm | PWd | mm |
| LA | mm | EDV | ml |
| LVIDd | mm | ESV | ml |
| LVIDs | mm | FS | % |
| IVSd | mm | LVEF | % |
| **Additional Information:** | | | |
| **Conclusion:**   1. Normal Echocardiography Study | | | |
| **Remark:** |  | | |
| **Recommendation:** |  | | |
| **Done By:** | **Signature** | **Date** | **Remark** |
| Tesfaye T., Pediatric Cardiologist |  | 21/03/14Eth.C. |  |

| **Patient Name:** Masresha Teshome. **Sex/Age**: M/8years. **Date of Report**: 21/03/14Eth.C. MRN: 109045.  **Clinical Diagnosis: Rheumatic Recurrence + Murmur + DOE + Plpitation. TGSH1.2440.** | | | |
| --- | --- | --- | --- |
| **Features:** | **Findings** | **Features** | **Findings** |
| **Profile** | | **Atria** | |
| Abdominal Situs | Solitus | Left Atrium | Dilated |
| Cardiac Position | Levocardia | Right Atrium | Normal |
| Systemic Venous Drainage | To RA. IVC Dilated | **Atrio-Ventricular Valves** | |
| Pulmonary Venous Drainage | To LA | Mitral Valve | Annulus = 28mm. Thickened MVL |
| Atrio-ventricular Connection | Concordant | Tricuspid Valve | Annulus = 18mm |
| Ventriculo-Arterial Connection | Concordant | **Ventricle** | |
| Ventricular Loop | d-Loop | Left Ventricle | Dilated |
| **Septae** |  | Right Ventricle | Normal |
| Interatrial Septum | Intact | **Doppler Measurement** |  |
| Interventricular Septum | Intact | Mitral | Mild MR, Holosystolic, posterior projection, seen in two planes with jet velocity = 4.5m/sec. |
| **Semilunar Valves** |  | Aortic | Moderate AR, PHT = 243ms |
| Aortic Valve | Annulus = 23mm. Thickened AVL | Tricuspid | Trivial TR, PPG = 30mmHg |
| Pulmonary Valve | Annulus = 20mm | Pulmonic |  |
| **Great Arteries** | NRGA | **Coronary Arteries** |  |
| Aorta |  | **Aortic Arch** | Left |
| Pulmonary Arteries |  | **PDA** | No |
| **M-Mode**: | | | |
| Ao | mm | PWd | mm |
| LA | mm | EDV | ml |
| LVIDd | mm | ESV | ml |
| LVIDs | mm | FS | 38% |
| IVSd | mm | LVEF | 68% |
| **Additional Information:** | | | |
| **Conclusion:**   1. {S, D, S} Levocardia 2. LA/LV Dilated 3. Thickened MVL and AVL 4. Mild MR 5. Moderate AR 6. Normal LV Function | | | |
| **Done By:** | **Signature** | **Date** | **Remark** |
| Tesfaye T., Pediatric Cardiologist |  | 21/03/14Eth.C. |  |

| **Patient Name:** Mirtzer Tegegne. **Sex/Age**: F/11years. **Date of Report**: 21/03/14Eth.C. MRN: 108974.  **Clinical Diagnosis: Rheumatic Recurrence + DOE + Murmur + CHF. TGSH1.2441.** | | | |
| --- | --- | --- | --- |
| **Features:** | **Findings** | **Features** | **Findings** |
| **Profile** | | **Atria** | |
| Abdominal Situs | Solitus | Left Atrium | Dilated. 53 X 65mm. |
| Cardiac Position | Levocardia | Right Atrium | Dilated |
| Systemic Venous Drainage | To RA | **Atrio-Ventricular Valves** | |
| Pulmonary Venous Drainage | To LA | Mitral Valve | Annulus = 32mm. Thickened, clubbed MV. MVA = 0.3cm2. |
| Atrio-ventricular Connection | Concordant | Tricuspid Valve | Annulus = 25mm |
| Ventriculo-Arterial Connection | Concordant | **Ventricle** | |
| Ventricular Loop | d-Loop | Left Ventricle | Dilated |
| **Septae** | Bowed to left | Right Ventricle | Dilated |
| Interatrial Septum | Intact | **Doppler Measurement** |  |
| Interventricular Septum | Intact | Mitral | Moderate MR, Holosystolic, posterior projection, seen in two planes with jet velocity = 3.5cm/sec. severe MS with PPG/MPG = 24/17mmHg. |
| **Semilunar Valves** |  | Aortic |  |
| Aortic Valve | Annulus = 13mm | Tricuspid | Mild TR, PPG = 65mmHg |
| Pulmonary Valve | Annulus = 21mm | Pulmonic |  |
| **Great Arteries** | NRGA | **Coronary Arteries** |  |
| Aorta |  | **Aortic Arch** | Left |
| Pulmonary Arteries |  | **PDA** | No |
| **M-Mode**: Normal LV Function on eye balling. | | | |
| Ao | mm | PWd | mm |
| LA | mm | EDV | ml |
| LVIDd | mm | ESV | ml |
| LVIDs | mm | FS | % |
| IVSd | mm | LVEF | % |
| **Conclusion:**   1. {S, D, S} Levocardia 2. All chambers dilated 3. Moderate MR 4. Severe MS 5. Mild TR 6. Severe Pulmonary Hypertension | | | |
| **Done By:** | **Signature** | **Date** | **Remark** |
| Tesfaye T., Pediatric Cardiologist |  | 21/03/14Eth.C. |  |

| **Patient Name:** Baby of Maralem Assema. **Sex/Age**: F/32days. **Date of Report**: 22/03/14Eth.C. MRN: 108703.  **Clinical Diagnosis: RD. TGSH1.2442.** | | | |
| --- | --- | --- | --- |
| **Features:** | **Findings** | **Features** | **Findings** |
| **Profile** | | **Atria** | |
| Abdominal Situs | Solitus | Left Atrium | Normal |
| Cardiac Position | Levocardia | Right Atrium | Normal |
| Systemic Venous Drainage | To RA | **Atrio-Ventricular Valves** | |
| Pulmonary Venous Drainage | To LA | Mitral Valve | Annulus = 8mm |
| Atrio-ventricular Connection | Concordant | Tricuspid Valve | Annulus = 9mm |
| Ventriculo-Arterial Connection | Concordant | **Ventricle** | |
| Ventricular Loop | d-Loop | Left Ventricle | Normal |
| **Septae** |  | Right Ventricle | Normal |
| Interatrial Septum | Intact | **Doppler Measurement** |  |
| Interventricular Septum | Intact | Mitral |  |
| **Semilunar Valves** |  | Aortic |  |
| Aortic Valve | Annulus = 9mm | Tricuspid |  |
| Pulmonary Valve | Annulus = 10mm | Pulmonic |  |
| **Great Arteries** | NRGA | **Coronary Arteries** |  |
| Aorta |  | **Aortic Arch** | Left |
| Pulmonary Arteries |  | **PDA** | No |
| **M-Mode**: Normal LV Function on eye balling | | | |
| Ao | mm | PWd | mm |
| LA | mm | EDV | ml |
| LVIDd | mm | ESV | ml |
| LVIDs | mm | FS | % |
| IVSd | mm | LVEF | % |
| **Additional Information:** | | | |
| **Conclusion:**   1. Normal Echocardiography Study | | | |
| **Remark:** |  | | |
| **Recommendation:** |  | | |
| **Done By:** | **Signature** | **Date** | **Remark** |
| Tesfaye T., Pediatric Cardiologist |  | 21/03/14Eth.C. |  |

| **Patient Name:** Enat-Tihun Getachew. **Sex/Age**: F/13years. **Date of Report**: 21/03/14Eth.C. MRN: 055687.  **Clinical Diagnosis: HTN + Pulseless upper extrimity. TGSH1.2443.** | | | |
| --- | --- | --- | --- |
| **Features:** | **Findings** | **Features** | **Findings** |
| **Profile** | | **Atria** | |
| Abdominal Situs | Solitus | Left Atrium | Mildly dilated |
| Cardiac Position | Levocardia | Right Atrium | Normal |
| Systemic Venous Drainage | To RA | **Atrio-Ventricular Valves** | |
| Pulmonary Venous Drainage | To LA | Mitral Valve | Annulus = 23mm |
| Atrio-ventricular Connection | Concordant | Tricuspid Valve | Annulus = 21mm |
| Ventriculo-Arterial Connection | Concordant | **Ventricle** | |
| Ventricular Loop | d-Loop | Left Ventricle | Mildly dilated. LVH |
| **Septae** |  | Right Ventricle | Normal |
| Interatrial Septum | Intact | **Doppler Measurement** |  |
| Interventricular Septum | Intact | Mitral |  |
| **Semilunar Valves** |  | Aortic |  |
| Aortic Valve | Annulus = 17mm | Tricuspid |  |
| Pulmonary Valve | Annulus = 21mm | Pulmonic |  |
| **Great Arteries** | NRGA | **Coronary Arteries** |  |
| Aorta |  | **Aortic Arch** | Left. Flow acceleration over the branching neck vessels. |
| Pulmonary Arteries |  | **PDA** | No |
| **M-Mode**: | | | |
|  |  | IVSs | 18mm |
| Ao | mm | PWd | 12mm |
| LA | mm | EDV | ml |
| LVIDd | 38mm | ESV | ml |
| LVIDs | 23mm | FS | 40% |
| IVSd | 15mm | LVEF | 70% |
| **Additional Information:** trace pericardial effusion measuring maximum of 4mm on RV Side. | | | |
| **Conclusion:**   1. {S, D, S} Levocardia 2. Mild LA/LV Dilated 3. LVH 4. Flow acceleration over the branching neck vessels 5. Trace pericardial effusion | | | |
| **Done By:** | **Signature** | **Date** | **Remark** |
| Tesfaye T., Pediatric Cardiologist |  | 21/03/14Eth.C. |  |

| **Patient Name:** Tiru-Edil Atalay. **Sex/Age**: F/1 5/12. **Date of Report**: 23/03/14Eth.C. MRN: 106319.  **Clinical Diagnosis: Diaphoresis during BF. TGSH1.2444.** | | | |
| --- | --- | --- | --- |
| **Features:** | **Findings** | **Features** | **Findings** |
| **Profile** | | **Atria** | |
| Abdominal Situs | Solitus | Left Atrium | Normal |
| Cardiac Position | Levocardia | Right Atrium | Normal |
| Systemic Venous Drainage | To RA | **Atrio-Ventricular Valves** | |
| Pulmonary Venous Drainage | To LA | Mitral Valve | Annulus = 9mm |
| Atrio-ventricular Connection | Concordant | Tricuspid Valve | Annulus = 10mm |
| Ventriculo-Arterial Connection | Concordant | **Ventricle** | |
| Ventricular Loop | d-Loop | Left Ventricle | Normal |
| **Septae** |  | Right Ventricle | Normal |
| Interatrial Septum | Intact | **Doppler Measurement** |  |
| Interventricular Septum | Intact | Mitral |  |
| **Semilunar Valves** |  | Aortic |  |
| Aortic Valve | Annulus = 10mm | Tricuspid |  |
| Pulmonary Valve | Annulus = 10mm | Pulmonic |  |
| **Great Arteries** | NRGA | **Coronary Arteries** |  |
| Aorta |  | **Aortic Arch** | Left |
| Pulmonary Arteries |  | **PDA** | No |
| **M-Mode**: Normal LV Function on eye balling | | | |
| Ao | mm | PWd | mm |
| LA | mm | EDV | ml |
| LVIDd | mm | ESV | ml |
| LVIDs | mm | FS | % |
| IVSd | mm | LVEF | % |
| **Additional Information:** | | | |
| **Conclusion:**   1. Normal Echocardiography Study | | | |
| **Remark:** |  | | |
| **Recommendation:** |  | | |
| **Done By:** | **Signature** | **Date** | **Remark** |
| Tesfaye T., Pediatric Cardiologist |  | 23/03/14Eth.C. |  |

| **Patient Name:** Belay Alemnew. **Sex/Age**: M/ 1 6/12. **Date of Report**: 28/03/14Eth.C. MRN: 109957.  **Clinical Diagnosis: Cyanosis + Clubbing + FTT + Murmur. TGSH1.2445.** | | | |
| --- | --- | --- | --- |
| **Features:** | **Findings** | **Features** | **Findings** |
| **Profile** | | **Atria** | |
| Abdominal Situs | Solitus | Left Atrium | Normal |
| Cardiac Position | Levocardia | Right Atrium | Dilated |
| Systemic Venous Drainage | To RA | **Atrio-Ventricular Valves** | |
| Pulmonary Venous Drainage | To LA | Mitral Valve | Annulus = 12mm |
| Atrio-ventricular Connection | Concordant | Tricuspid Valve | Annulus = 20mm |
| Ventriculo-Arterial Connection | Discordant | **Ventricle** | |
| Ventricular Loop | d-Loop | Left Ventricle | Dilated |
| **Septae** |  | Right Ventricle | Normal |
| Interatrial Septum | 8mm OS ASD, BD Shunt | **Doppler Measurement** |  |
| Interventricular Septum | 8mm upper muscular VSD, BD Shunt | Mitral |  |
| **Semilunar Valves** |  | Aortic |  |
| Aortic Valve | Annulus = 13mm | Tricuspid |  |
| Pulmonary Valve | Annulus = 12mm | Pulmonic |  |
| **Great Arteries** | d-TGA | **Coronary Arteries** |  |
| Aorta |  | **Aortic Arch** | Left |
| Pulmonary Arteries |  | **PDA** | 2mm PDA, aorta to Pul. artery |
| **M-Mode**: | | | |
| Ao | mm | PWd | mm |
| LA | mm | EDV | ml |
| LVIDd | mm | ESV | ml |
| LVIDs | mm | FS | % |
| IVSd | mm | LVEF | % |
| **Additional Information:** | | | |
| **Conclusion:**   1. {S, D, D} Levocardia 2. d-TGA 3. Moderate ASD, BD Shunt 4. Moderate Upper Muscular VSD, BD Shunt 5. Moderate PDA, from aorta to Pulmonary artery | | | |
| **Done By:** | **Signature** | **Date** | **Remark** |
| Tesfaye T., Pediatric Cardiologist |  | 28/03/14Eth.C. |  |

| **Patient Name:** Baby of Nitsuh. **Sex/Age**: F/10days. **Date of Report**: 28/03/14Eth.C. MRN: 108959.  **Clinical Diagnosis: Incidental Murmur. TGSH1.2446.** | | | |
| --- | --- | --- | --- |
| **Features:** | **Findings** | **Features** | **Findings** |
| **Profile** | | **Atria** | |
| Abdominal Situs | Solitus | Left Atrium | Normal |
| Cardiac Position | Levocardia | Right Atrium | Normal |
| Systemic Venous Drainage | To RA | **Atrio-Ventricular Valves** | |
| Pulmonary Venous Drainage | To LA | Mitral Valve | Annulus = 10mm |
| Atrio-ventricular Connection | Concordant | Tricuspid Valve | Annulus = 9mm |
| Ventriculo-Arterial Connection | Concordant | **Ventricle** | |
| Ventricular Loop | d-Loop | Left Ventricle | Normal |
| **Septae** |  | Right Ventricle | Normal |
| Interatrial Septum | PFO, L – R Shunt | **Doppler Measurement** |  |
| Interventricular Septum | 2mm PM VSD, L – R Shunt | Mitral |  |
| **Semilunar Valves** |  | Aortic |  |
| Aortic Valve | Annulus = 8mm | Tricuspid |  |
| Pulmonary Valve | Annulus = 10mm | Pulmonic |  |
| **Great Arteries** | NRGA | **Coronary Arteries** |  |
| Aorta |  | **Aortic Arch** | Left |
| Pulmonary Arteries |  | **PDA** | No |
| **M-Mode**: Normal LV Function on eye balling. | | | |
| Ao | mm | PWd | mm |
| LA | mm | EDV | ml |
| LVIDd | mm | ESV | ml |
| LVIDs | mm | FS | % |
| IVSd | mm | LVEF | % |
| **Additional Information:** | | | |
| **Conclusion:**   1. {S, D, S} Levocardia 2. PFO, L – R Shunt 3. Small PM VSD, L – R Shunt 4. Normal LV Function | | | |
| **Remark:** |  | | |
| **Recommendation:** |  | | |
| **Done By:** | **Signature** | **Date** | **Remark** |
| Tesfaye T., Pediatric Cardiologist |  | 28/03/14Eth.C. |  |

| **Patient Name:** Temechew Kefale. **Sex/Age**: M/15years. **Date of Report**: 28/03/14Eth.C. MRN: 017241.  **Clinical Diagnosis: Incidental Murmur. TGSH1.2447.** | | | |
| --- | --- | --- | --- |
| **Features:** | **Findings** | **Features** | **Findings** |
| **Profile** | | **Atria** | |
| Abdominal Situs | Solitus | Left Atrium | Normal |
| Cardiac Position | Levocardia | Right Atrium | Normal |
| Systemic Venous Drainage | To RA | **Atrio-Ventricular Valves** | |
| Pulmonary Venous Drainage | To LA | Mitral Valve | Annulus = 24mm. Mildly thickened, patulous MVL |
| Atrio-ventricular Connection | Concordant | Tricuspid Valve | Annulus = 24mm |
| Ventriculo-Arterial Connection | Concordant | **Ventricle** | |
| Ventricular Loop | d-Loop | Left Ventricle | Normal |
| **Septae** |  | Right Ventricle | Normal |
| Interatrial Septum | Intact | **Doppler Measurement** |  |
| Interventricular Septum | Intact | Mitral | Mild MR, Incomplete signal, seen in two planes with jet velocity = 3.6m/sec |
| **Semilunar Valves** |  | Aortic |  |
| Aortic Valve | Annulus = 21mm | Tricuspid | Trivial TR, PPG = 21mmHg |
| Pulmonary Valve | Annulus = 21mm | Pulmonic | Trivial PR, PPG = 10mmHg |
| **Great Arteries** | NRGA | **Coronary Arteries** |  |
| Aorta |  | **Aortic Arch** | Left |
| Pulmonary Arteries |  | **PDA** | No |
| **M-Mode**: | | | |
| Ao | mm | PWd | mm |
| LA | mm | EDV | ml |
| LVIDd | mm | ESV | ml |
| LVIDs | mm | FS | % |
| IVSd | mm | LVEF | % |
| **Additional Information:** | | | |
| **Conclusion:**   1. {S, D, S} Levocardia 2. Mild MR 3. Mildly thickened, Patulous MVL (Borderline RHD) | | | |
| **Done By:** | **Signature** | **Date** | **Remark** |
| Tesfaye T., Pediatric Cardiologist |  | 28/03/14Eth.C. |  |
|  |  |  |  |
| **Patient Name:** Baby of Emuye Acham. **Sex/Age**: M/20days. **Date of Report**: 28/03/14Eth.C. MRN: 107581.  **Clinical Diagnosis: Incidental Murmur. TGSH1.2448.** | | | |
| **Features:** | **Findings** | **Features** | **Findings** |
| **Profile** | | **Atria** | |
| Abdominal Situs | Solitus | Left Atrium | Normal |
| Cardiac Position | Levocardia | Right Atrium | Dilated |
| Systemic Venous Drainage | To RA | **Atrio-Ventricular Valves** | |
| Pulmonary Venous Drainage | To LA | Mitral Valve | Annulus = 7mm. |
| Atrio-ventricular Connection | Concordant | Tricuspid Valve | Annulus = 8mm |
| Ventriculo-Arterial Connection | Concordant | **Ventricle** | |
| Ventricular Loop | d-Loop | Left Ventricle | Normal |
| **Septae** |  | Right Ventricle | Dilated, Hypertrophied |
| Interatrial Septum | 5mm OS ASD, L – R Shunt | **Doppler Measurement** |  |
| Interventricular Septum | Intact | Mitral |  |
| **Semilunar Valves** |  | Aortic |  |
| Aortic Valve | Annulus = 6mm | Tricuspid |  |
| Pulmonary Valve | Annulus = 7mm | Pulmonic | Severe Valvular PS, PPG = 60mmHg |
| **Great Arteries** | NRGA | **Coronary Arteries** |  |
| Aorta |  | **Aortic Arch** | Left |
| Pulmonary Arteries |  | **PDA** | No |
| **M-Mode**: Normal LV Function on eye balling | | | |
| Ao | mm | PWd | mm |
| LA | mm | EDV | ml |
| LVIDd | mm | ESV | ml |
| LVIDs | mm | FS | % |
| IVSd | mm | LVEF | % |
| **Additional Information:** | | | |
| **Conclusion:**   1. {S, D, S} Levocardia 2. Small OS ASD, L – R Shunt 3. RA/RV Dilated 4. Severe Pulmonary Stenosis 5. Normal LV Function | | | |
| **Done By:** | **Signature** | **Date** | **Remark** |
| Tesfaye T., Pediatric Cardiologist |  | 28/03/14Eth.C. |  |

| **Patient Name:** Degisew Delie. **Sex/Age**: M/11years. **Date of Report**: 28/03/14Eth.C. MRN: __________.  **Clinical Diagnosis: Rheumatic Recurrence + Murmur + DOE + Palpitation. TGSH1.2449.** | | | |
| --- | --- | --- | --- |
| **Features:** | **Findings** | **Features** | **Findings** |
| **Profile** | | **Atria** | |
| Abdominal Situs | Solitus | Left Atrium | Dilated |
| Cardiac Position | Levocardia | Right Atrium | Normal |
| Systemic Venous Drainage | To RA | **Atrio-Ventricular Valves** | |
| Pulmonary Venous Drainage | To LA | Mitral Valve | Annulus = 24mm. thickened, clubbed MVL |
| Atrio-ventricular Connection | Concordant | Tricuspid Valve | Annulus = 20mm |
| Ventriculo-Arterial Connection | Concordant | **Ventricle** | |
| Ventricular Loop | d-Loop | Left Ventricle | Dilated |
| **Septae** |  | Right Ventricle | Normal |
| Interatrial Septum | Intact | **Doppler Measurement** |  |
| Interventricular Septum | Intact | Mitral | Severe MR, Holosystolic, posterior projection, seen in two planes with jet velocity = 3.7m/sec |
| **Semilunar Valves** |  | Aortic | Moderate AR, PHT = 317ms |
| Aortic Valve | Annulus = 18mm | Tricuspid |  |
| Pulmonary Valve | Annulus = 20mm | Pulmonic |  |
| **Great Arteries** | NRGA | **Coronary Arteries** |  |
| Aorta |  | **Aortic Arch** | Left |
| Pulmonary Arteries |  | **PDA** | No |
| **M-Mode**: Normal LV Function on eye balling | | | |
| Ao | mm | PWd | mm |
| LA | mm | EDV | ml |
| LVIDd | mm | ESV | ml |
| LVIDs | mm | FS | % |
| IVSd | mm | LVEF | % |
| **Additional Information:** | | | |
| **Conclusion:**   1. {S, D, S} Levocardia 2. LA/LV Dilated 3. Thickened MVL 4. Severe MR 5. Moderate AR 6. Normal LV Function | | | |
| **Done By:** | **Signature** | **Date** | **Remark** |
| Tesfaye T., Pediatric Cardiologist |  | 28/03/14Eth.C. |  |

| **Patient Name:** Banchigizie Haile. **Sex/Age**: F/1 2/12. **Date of Report**: 28/03/14Eth.C. MRN: 110139.  **Clinical Diagnosis: Incidental Murmur + Diaphoresis during BF. TGSH1.2450.** | | | |
| --- | --- | --- | --- |
| **Features:** | **Findings** | **Features** | **Findings** |
| **Profile** | | **Atria** | |
| Abdominal Situs | Solitus | Left Atrium | Normal |
| Cardiac Position | Dextroposition | Right Atrium | Dilated |
| Systemic Venous Drainage | To RA | **Atrio-Ventricular Valves** | |
| Pulmonary Venous Drainage | To LA | Mitral Valve | Annulus = 12mm. |
| Atrio-ventricular Connection | Concordant | Tricuspid Valve | Annulus = 20mm |
| Ventriculo-Arterial Connection | Concordant | **Ventricle** | |
| Ventricular Loop | d-Loop | Left Ventricle | Normal |
| **Septae** |  | Right Ventricle | Dilated |
| Interatrial Septum | 9mm OS ASD, L – R Shunt | **Doppler Measurement** |  |
| Interventricular Septum | 6mm Trabecular VSD, L – R Shunt | Mitral |  |
| **Semilunar Valves** |  | Aortic |  |
| Aortic Valve | Annulus = 12mm | Tricuspid |  |
| Pulmonary Valve | Annulus = mm | Pulmonic |  |
| **Great Arteries** | NRGA | **Coronary Arteries** |  |
| Aorta |  | **Aortic Arch** | Left |
| Pulmonary Arteries |  | **PDA** | No |
| **M-Mode**: Normal LV Function on eye balling | | | |
| Ao | mm | PWd | mm |
| LA | mm | EDV | ml |
| LVIDd | mm | ESV | ml |
| LVIDs | mm | FS | % |
| IVSd | mm | LVEF | % |
| **Additional Information:** | | | |
| **Conclusion:**   1. {S, D, S} Dextroposition 2. RA/RV Dilated 3. Moderate OS ASD, L – R Shunt 4. Small Trabecular VSD, L – R Shunt | | | |
| **Done By:** | **Signature** | **Date** | **Remark** |
| Tesfaye T., Pediatric Cardiologist |  | 28/03/14Eth.C. |  |

| **Patient Name:** Abrham Enchalew. **Sex/Age**: M/34days. **Date of Report**: 28/03/14Eth.C. MRN: 110142.  **Clinical Diagnosis: Cyanosis + Murmur. TGSH1.2451.** | | | |
| --- | --- | --- | --- |
| **Features:** | **Findings** | **Features** | **Findings** |
| **Profile** | | **Atria** | |
| Abdominal Situs | Solitus | Left Atrium | Normal |
| Cardiac Position | Levocardia | Right Atrium | Normal |
| Systemic Venous Drainage | To RA | **Atrio-Ventricular Valves** | |
| Pulmonary Venous Drainage | To LA | Mitral Valve | Annulus = 12mm. |
| Atrio-ventricular Connection | Concordant | Tricuspid Valve | Annulus = 11mm |
| Ventriculo-Arterial Connection | DORV, Discordant | **Ventricle** | |
| Ventricular Loop | d-Loop | Left Ventricle | Normal |
| **Septae** |  | Right Ventricle | Normal |
| Interatrial Septum | PFO, L – R Shunt | **Doppler Measurement** |  |
| Interventricular Septum | Malaligned Sub Pulmonic VSD, BD Shunt. >50% RV overriding | Mitral | TRIVIAL MR |
| **Semilunar Valves** |  | Aortic |  |
| Aortic Valve | Annulus = 13mm | Tricuspid | Trivial TR |
| Pulmonary Valve | Annulus = 10mm | Pulmonic |  |
| **Great Arteries** | d-TGA | **Coronary Arteries** |  |
| Aorta | Anterior and from RV | **Aortic Arch** | Left |
| Pulmonary Arteries | MPA = 18mm. posterior and from LV | **PDA** | No |
| **M-Mode**: | | | |
| Ao | mm | PWd | mm |
| LA | mm | EDV | ml |
| LVIDd | mm | ESV | ml |
| LVIDs | mm | FS | % |
| IVSd | mm | LVEF | % |
| **Additional Information:** | | | |
| **Conclusion:**   1. {S, D, D} Levocardia 2. PFO, L – R Shunt 3. DORV (Taussig-Bing anomaly) 4. Malaligned Sub Pulmonic VSD, BD Shunt 5. Severe Pulmonary Hypertension | | | |
| **Done By:** | **Signature** | **Date** | **Remark** |
| Tesfaye T., Pediatric Cardiologist |  | 28/03/14Eth.C. |  |

| **Patient Name:** Kemelew Ibrahim. **Sex/Age**: M/14years. **Date of Report**: 30/03/14Eth.C. MRN: 110210.  **Clinical Diagnosis: DOE + Rheumatic Recurrence + palpitation + easy fatigability. TGSH1.2452.** | | | |
| --- | --- | --- | --- |
| **Features:** | **Findings** | **Features** | **Findings** |
| **Profile** | | **Atria** | |
| Abdominal Situs | Solitus | Left Atrium | Dilated |
| Cardiac Position | Levocardia | Right Atrium | Normal |
| Systemic Venous Drainage | To RA | **Atrio-Ventricular Valves** | |
| Pulmonary Venous Drainage | To LA | Mitral Valve | Annulus = 28mm. Thickened, clubbed MVL. MVA = 0.9cm2. |
| Atrio-ventricular Connection | Concordant | Tricuspid Valve | Annulus = 24mm |
| Ventriculo-Arterial Connection | Concordant | **Ventricle** | |
| Ventricular Loop | d-Loop | Left Ventricle | Normal |
| **Septae** |  | Right Ventricle | Normal |
| Interatrial Septum | Intact | **Doppler Measurement** |  |
| Interventricular Septum | Intact | Mitral | Moderate MR, Holosystolic, central projection, seen in two planes with jet velocity = 4.6m/sec. severe MS, PPG/MPG = 28/11mmHg. |
| **Semilunar Valves** |  | Aortic | Moderate AR, PHT = 331ms. |
| Aortic Valve | Annulus = 18mm. Trileaflet, thickened AVL | Tricuspid | Mild TR, PPG = 24mmHg |
| Pulmonary Valve | Annulus = 21mm | Pulmonic |  |
| **Great Arteries** | NRGA | **Coronary Arteries** |  |
| Aorta |  | **Aortic Arch** | Left |
| Pulmonary Arteries |  | **PDA** | No |
| **M-Mode**: Normal LV Function on eye balling | | | |
| Ao | mm | PWd | mm |
| LA | mm | EDV | ml |
| LVIDd | mm | ESV | ml |
| LVIDs | mm | FS | % |
| IVSd | mm | LVEF | % |
| **Additional Information:** | | | |
| **Conclusion:**   1. {S, D, S} Levocardia 2. LA Dilated 3. Moderate MR 4. Severe MS 5. Moderate AR 6. Mild TR 7. Normal LV Function | | | |
| **Done By:** | **Signature** | **Date** | **Remark** |
| Tesfaye T., Pediatric Cardiologist |  | 30/03/14Eth.C. |  |

| **Patient Name:** _Tihitina Manayew. **Sex F /Age**: 10months. **Date of Report**: 30/03/14Eth.C. MRN: 110137.  **Clinical Diagnosis: Diaphoresis during BF + Murmur + CHF. TGSH1.2453.** | | | |
| --- | --- | --- | --- |
| **Features:** | **Findings** | **Features** | **Findings** |
| **Profile** | | **Atria** | |
| Abdominal Situs | Solitus | Left Atrium | Dilated |
| Cardiac Position | Levocardia | Right Atrium | Dilated |
| Systemic Venous Drainage | To RA | **Atrio-Ventricular Valves** | |
| Pulmonary Venous Drainage | To LA | Mitral Valve | Annulus = 16mm. |
| Atrio-ventricular Connection | Concordant | Tricuspid Valve | Annulus = 15mm |
| Ventriculo-Arterial Connection | Concordant | **Ventricle** | |
| Ventricular Loop | d-Loop | Left Ventricle | Dilated |
| **Septae** |  | Right Ventricle | Dilated |
| Interatrial Septum | 12mm OS ASD, L – R Shunt. | **Doppler Measurement** |  |
| Interventricular Septum | 11mm subaortic VSD, L – R Shunt | Mitral |  |
| **Semilunar Valves** |  | Aortic |  |
| Aortic Valve | Annulus = 14mm | Tricuspid |  |
| Pulmonary Valve | Annulus = 21mm | Pulmonic | Mild PR, PPG = 63mmHg |
| **Great Arteries** | NRGA | **Coronary Arteries** |  |
| Aorta |  | **Aortic Arch** | Left |
| Pulmonary Arteries | MPA = 23mm | **PDA** | No |
| **M-Mode**: Normal LV Function on eye balling | | | |
| Ao | mm | PWd | mm |
| LA | mm | EDV | ml |
| LVIDd | mm | ESV | ml |
| LVIDs | mm | FS | % |
| IVSd | mm | LVEF | % |
| **Additional Information:** Pericardial effusion with max depth of 6mm on RV Side. | | | |
| **Conclusion:**   1. {S, D, S} Levocardia 2. RA/RV Dilated 3. Large OS ASD, L – R Shunt 4. Large Subaortic VSD, L – R Shunt 5. Severe Pulmonary Hypertension 6. Small Pericardial effusion 7. Normal LV Function | | | |
| **Done By:** | **Signature** | **Date** | **Remark** |
| Tesfaye T., Pediatric Cardiologist |  | 30/03/14Eth.C. |  |
| **Patient Name:** Shega Birie. **Sex/Age**: F/8years. **Date of Report**: 30/03/14Eth.C. MRN: 109872.  **Clinical Diagnosis: CHF + ARF + RD + Murmur. TGSH1.2454.** | | | |
| **Features:** | **Findings** | **Features** | **Findings** |
| **Profile** | | **Atria** | |
| Abdominal Situs | Solitus | Left Atrium | Dilated |
| Cardiac Position | Levocardia | Right Atrium | Dilated |
| Systemic Venous Drainage | To RA | **Atrio-Ventricular Valves** | |
| Pulmonary Venous Drainage | To LA | Mitral Valve | Annulus = 30mm. |
| Atrio-ventricular Connection | Concordant | Tricuspid Valve | Annulus = 30mm |
| Ventriculo-Arterial Connection | Concordant | **Ventricle** | |
| Ventricular Loop | d-Loop | Left Ventricle | Dilated |
| **Septae** |  | Right Ventricle | Dilated |
| Interatrial Septum |  | **Doppler Measurement** |  |
| Interventricular Septum |  | Mitral | Severe MR, Holosystolic, posterior projection, seen in two planes with jet velocity = 3.2m/sec |
| **Semilunar Valves** |  | Aortic | Severe AR, PHT = 133ms |
| Aortic Valve | Annulus = 21mm | Tricuspid | Moderate TR, PPG = 45mmHg |
| Pulmonary Valve | Annulus = 22mm | Pulmonic | Mild PR, PPG = 51mmHg |
| **Great Arteries** | NRGA | **Coronary Arteries** |  |
| Aorta |  | **Aortic Arch** | Left |
| Pulmonary Arteries |  | **PDA** | No |
| **M-Mode**: Normal LV Function on eye balling | | | |
| Ao | mm | PWd | mm |
| LA | mm | EDV | ml |
| LVIDd | mm | ESV | ml |
| LVIDs | mm | FS | % |
| IVSd | mm | LVEF | % |
| **Additional Information:** | | | |
| **Conclusion:**   1. {S, D, S} Levocardia 2. All chambers dilated 3. Severe MR 4. Severe AR 5. Moderate TR 6. Mild PR 7. Moderate Pulmonary Hypertension 8. Normal LV Function | | | |
| **Done By:** | **Signature** | **Date** | **Remark** |
| Tesfaye T., Pediatric Cardiologist |  | 30/03/14Eth.C. |  |

| **Patient Name:** Baby of Emebet Ketema. **Sex/Age**: M/13days. **Date of Report**: 30/03/14Eth.C. MRN: 105512.  **Clinical Diagnosis: DS. TGSH1.2455.** | | | |
| --- | --- | --- | --- |
| **Features:** | **Findings** | **Features** | **Findings** |
| **Profile** | | **Atria** | |
| Abdominal Situs | Solitus | Left Atrium | Normal |
| Cardiac Position | Dextroposition | Right Atrium | Normal |
| Systemic Venous Drainage | To RA | **Atrio-Ventricular Valves** | |
| Pulmonary Venous Drainage | To LA | Mitral Valve | Annulus = 11mm. |
| Atrio-ventricular Connection | Concordant | Tricuspid Valve | Annulus = 9mm |
| Ventriculo-Arterial Connection | Concordant | **Ventricle** | |
| Ventricular Loop | d-Loop | Left Ventricle | Normal |
| **Septae** |  | Right Ventricle | Normal |
| Interatrial Septum | 4mm OS ASD, L – R Shunt | **Doppler Measurement** |  |
| Interventricular Septum | Intact | Mitral |  |
| **Semilunar Valves** |  | Aortic |  |
| Aortic Valve | Annulus = 10mm | Tricuspid |  |
| Pulmonary Valve | Annulus = 9mm | Pulmonic |  |
| **Great Arteries** | NRGA | **Coronary Arteries** |  |
| Aorta |  | **Aortic Arch** | Left |
| Pulmonary Arteries |  | **PDA** | No |
| **M-Mode**: Normal LV Function on eye balling | | | |
| Ao | mm | PWd | mm |
| LA | mm | EDV | ml |
| LVIDd | mm | ESV | ml |
| LVIDs | mm | FS | % |
| IVSd | mm | LVEF | % |
| **Additional Information:** | | | |
| **Conclusion:**   1. {S, D, S} Dextroposition 2. Small OS ASD, L – R Shunt | | | |
| **Remark:** Sternal defect(main) | | | |
| **Recommendation:** Pentalogy of Cantrell can be considered as DDx. | | | |
| **Done By:** | **Signature** | **Date** | **Remark** |
| Tesfaye T., Pediatric Cardiologist |  | 30/03/14Eth.C. |  |

| **Patient Name:** Atsede Tsegaye. **Sex/Age**: F/12years. **Date of Report**: 30/03/14Eth.C. MRN: 110351.  **Clinical Diagnosis: Chest Pain + Friction ru. TGSH1.2456.** | | | |
| --- | --- | --- | --- |
| **Features:** | **Findings** | **Features** | **Findings** |
| **Profile** | | **Atria** | |
| Abdominal Situs | Solitus | Left Atrium | Normal |
| Cardiac Position | Levocardia | Right Atrium | Normal |
| Systemic Venous Drainage | To RA | **Atrio-Ventricular Valves** | |
| Pulmonary Venous Drainage | To LA | Mitral Valve | Annulus = 16mm. |
| Atrio-ventricular Connection | Concordant | Tricuspid Valve | Annulus = 18mm |
| Ventriculo-Arterial Connection | Concordant | **Ventricle** | |
| Ventricular Loop | d-Loop | Left Ventricle | Normal |
| **Septae** |  | Right Ventricle | Normal |
| Interatrial Septum |  | **Doppler Measurement** |  |
| Interventricular Septum |  | Mitral |  |
| **Semilunar Valves** |  | Aortic |  |
| Aortic Valve | Annulus = 15mm | Tricuspid |  |
| Pulmonary Valve | Annulus = 20mm | Pulmonic |  |
| **Great Arteries** | NRGA | **Coronary Arteries** |  |
| Aorta |  | **Aortic Arch** | Left |
| Pulmonary Arteries |  | **PDA** | No |
| **M-Mode**: | | | |
| Ao | mm | PWd | mm |
| LA | mm | EDV | ml |
| LVIDd | mm | ESV | ml |
| LVIDs | mm | FS | % |
| IVSd | mm | LVEF | % |
| **Additional Information:** Pericardial thickening with 4mm Pericardial effusion | | | |
| **Conclusion:**   1. {S, D, S} Levocardia 2. Pericardial thickening with trace effusion secondary to ? | | | |
| **Done By:** | **Signature** | **Date** | **Remark** |
| Tesfaye T., Pediatric Cardiologist |  | 30/03/14Eth.C. |  |

| **Patient Name:** Sitra Nuru. **Sex/Age**: F/4years. **Date of Report**: 07/03/14Eth.C. MRN: 109980.  **Clinical Diagnosis: Incidental Murmur. TGSH1.2457.** | | | |
| --- | --- | --- | --- |
| **Features:** | **Findings** | **Features** | **Findings** |
| **Profile** | | **Atria** | |
| Abdominal Situs | Solitus | Left Atrium | Normal |
| Cardiac Position | Levocardia | Right Atrium | Normal |
| Systemic Venous Drainage | To RA | **Atrio-Ventricular Valves** | |
| Pulmonary Venous Drainage | To LA | Mitral Valve | Annulus = 19mm. Patulous MVL |
| Atrio-ventricular Connection | Concordant | Tricuspid Valve | Annulus = 21mm |
| Ventriculo-Arterial Connection | Concordant | **Ventricle** | |
| Ventricular Loop | d-Loop | Left Ventricle | Normal |
| **Septae** |  | Right Ventricle | Normal |
| Interatrial Septum |  | **Doppler Measurement** |  |
| Interventricular Septum |  | Mitral | Moderate MR, Central Projection, seen in two planes with jet velocity of 4.3m/sec |
| **Semilunar Valves** |  | Aortic | Mild AR |
| Aortic Valve | Annulus = 14mm | Tricuspid | Mild TR, PPG = 34mmHg |
| Pulmonary Valve | Annulus = 17mm | Pulmonic |  |
| **Great Arteries** | NRGA | **Coronary Arteries** |  |
| Aorta |  | **Aortic Arch** | Left |
| Pulmonary Arteries |  | **PDA** | No |
| **M-Mode**: | | | |
| Ao | mm | PWd | mm |
| LA | mm | EDV | ml |
| LVIDd | mm | ESV | ml |
| LVIDs | mm | FS | % |
| IVSd | mm | LVEF | % |
| **Additional Information:** Circumferential Pericardial effusion with maximum depth of 6mm on RA/RV Side. | | | |
| **Conclusion:**   1. {S, D, S} Levocardia 2. Moderate MR 3. Mild AR 4. Mild TR 5. Small Pericardial effusion | | | |
| **Done By:** | **Signature** | **Date** | **Remark** |
| Tesfaye T., Pediatric Cardiologist |  | 07/03/14Eth.C. |  |

| **Patient Name:** Baby of Hirut Walelign. **Sex/Age**: M/37days. **Date of Report**: 07/03/14Eth.C. MRN: 110379.  **Clinical Diagnosis: DS. TGSH1.2458.** | | | |
| --- | --- | --- | --- |
| **Features:** | **Findings** | **Features** | **Findings** |
| **Profile** | | **Atria** | |
| Abdominal Situs | Solitus | Left Atrium | Normal |
| Cardiac Position | Levocardia | Right Atrium | Normal |
| Systemic Venous Drainage | To RA | **Atrio-Ventricular Valves** | |
| Pulmonary Venous Drainage | To LA | Mitral Valve | Annulus = 9mm. |
| Atrio-ventricular Connection | Concordant | Tricuspid Valve | Annulus = 10mm |
| Ventriculo-Arterial Connection | Concordant | **Ventricle** | |
| Ventricular Loop | d-Loop | Left Ventricle | Normal |
| **Septae** |  | Right Ventricle | Normal |
| Interatrial Septum |  | **Doppler Measurement** |  |
| Interventricular Septum |  | Mitral |  |
| **Semilunar Valves** |  | Aortic |  |
| Aortic Valve | Annulus = 7mm | Tricuspid |  |
| Pulmonary Valve | Annulus = 9mm | Pulmonic |  |
| **Great Arteries** | NRGA | **Coronary Arteries** |  |
| Aorta |  | **Aortic Arch** | Left |
| Pulmonary Arteries |  | **PDA** | No |
| **M-Mode**: Normal LV Function on eye balling | | | |
| Ao | mm | PWd | mm |
| LA | mm | EDV | ml |
| LVIDd | mm | ESV | ml |
| LVIDs | mm | FS | % |
| IVSd | mm | LVEF | % |
| **Additional Information:** | | | |
| **Conclusion:**   1. Normal Echocardiography Study | | | |
| **Done By:** | **Signature** | **Date** | **Remark** |
| Tesfaye T., Pediatric Cardiologist |  | 07/03/14Eth.C. |  |

| **Patient Name:** Adugnaw Kassie. **Sex/Age**: M/14years. **Date of Report**: 07/03/14Eth.C. MRN: 088461.  **Clinical Diagnosis: Rheumatic Recurrence + DOE + Murmur + Palpitation. TGSH1.2459.** | | | |
| --- | --- | --- | --- |
| **Features:** | **Findings** | **Features** | **Findings** |
| **Profile** | | **Atria** | |
| Abdominal Situs | Solitus | Left Atrium | Dilated |
| Cardiac Position | Levocardia | Right Atrium | Dilated |
| Systemic Venous Drainage | To RA | **Atrio-Ventricular Valves** | |
| Pulmonary Venous Drainage | To LA | Mitral Valve | Annulus = 32mm. clubbed, thickened MVL. Shortened PMVL |
| Atrio-ventricular Connection | Concordant | Tricuspid Valve | Annulus = 33mm  TAPSE = 25mm |
| Ventriculo-Arterial Connection | Concordant | **Ventricle** | |
| Ventricular Loop | d-Loop | Left Ventricle | Dilated |
| **Septae** |  | Right Ventricle | Dilated |
| Interatrial Septum |  | **Doppler Measurement** |  |
| Interventricular Septum |  | Mitral | Moderate MR, Holosystolic, posterior projection, seen in two planes with jet velocity = 3.2m/sec. |
| **Semilunar Valves** |  | Aortic |  |
| Aortic Valve | Annulus = 15mm | Tricuspid | Moderate TR, PPG = 50mmHg |
| Pulmonary Valve | Annulus = 25mm | Pulmonic |  |
| **Great Arteries** | NRGA | **Coronary Arteries** |  |
| Aorta |  | **Aortic Arch** | Left |
| Pulmonary Arteries | MPA = 26mm | **PDA** | No |
| **M-Mode**: | | | |
| Ao | mm | PWd | 9mm |
| LA | mm | EDV | 148ml |
| LVIDd | 55mm | ESV | 65ml |
| LVIDs | 39mm | FS | 30% |
| IVSd | 8mm | LVEF | 56% |
| **Additional Information:** pericardial effusion with maximum depth of 10mm on RA/RV Side. | | | |
| **Conclusion:**   1. {S, D, S} Levocardia 2. All chambers dilated 3. Thickened, Clubbed MVL 4. Moderate MR 5. Moderate TR 6. Moderate Pulmonary Hypertension 7. Normal Function | | | |
| **Done By:** | **Signature** | **Date** | **Remark** |
| Tesfaye T., Pediatric Cardiologist |  | 07/03/14Eth.C. |  |

| **Patient Name:** Gardachew Yaregal. **Sex/Age**: M/13years. **Date of Report**: 12/04/14Eth.C. MRN: 109944.  **Clinical Diagnosis: Sepsis + Chest Pain + Friction rub. TGSH1.2460.** | | | |
| --- | --- | --- | --- |
| **Features:** | **Findings** | **Features** | **Findings** |
| **Profile** | | **Atria** | |
| Abdominal Situs | Solitus | Left Atrium | Normal |
| Cardiac Position | Levocardia | Right Atrium | Normal |
| Systemic Venous Drainage | To RA | **Atrio-Ventricular Valves** | |
| Pulmonary Venous Drainage | To LA | Mitral Valve | Annulus = 21mm. |
| Atrio-ventricular Connection | Concordant | Tricuspid Valve | Annulus = 20mm  TAPSE =19mm |
| Ventriculo-Arterial Connection | Concordant | **Ventricle** | |
| Ventricular Loop | d-Loop | Left Ventricle | Normal |
| **Septae** |  | Right Ventricle | Normal |
| Interatrial Septum |  | **Doppler Measurement** |  |
| Interventricular Septum |  | Mitral |  |
| **Semilunar Valves** |  | Aortic |  |
| Aortic Valve | Annulus = 16mm | Tricuspid | Mild TR, PPG = 41mmHg |
| Pulmonary Valve | Annulus = 19mm | Pulmonic | Mild PR, PPG = 36mmHg |
| **Great Arteries** | NRGA | **Coronary Arteries** |  |
| Aorta |  | **Aortic Arch** | Left |
| Pulmonary Arteries |  | **PDA** | No |
| **M-Mode**: | | | |
| Ao | mm | PWd | mm |
| LA | mm | EDV | ml |
| LVIDd | mm | ESV | ml |
| LVIDs | mm | FS | 36% |
| IVSd | mm | LVEF | 67% |
| **Additional Information:**  5mm Circumferential pericardial effusion with thickening and echo debris. 30mm Right Pleural effusion | | | |
| **Conclusion:**   1. {S, D, S} Levocardia 2. Small Circumferential Pericardial effusion 3. Large Right Pleural Effusion 4. Mild TR 5. Mild PR 6. Mild Pulmonary Hypertension 7. Normal Biventricular Systolic Function | | | |
| **Done By:** | **Signature** | **Date** | **Remark** |
| Tesfaye T., Pediatric Cardiologist |  | 12/04/14Eth.C. |  |

| **Patient Name:** Abubeker Ahmed. **Sex/Age**: M/1 1/12. **Date of Report**: 18/04/14Eth.C. MRN: __________.  **Clinical Diagnosis: Diaphoresis + RD. TGSH1.2461.** | | | |
| --- | --- | --- | --- |
| **Features:** | **Findings** | **Features** | **Findings** |
| **Profile** | | **Atria** | |
| Abdominal Situs | Solitus | Left Atrium | Normal |
| Cardiac Position | Levocardia | Right Atrium | Normal |
| Systemic Venous Drainage | To RA | **Atrio-Ventricular Valves** | |
| Pulmonary Venous Drainage | To LA | Mitral Valve | Annulus = mm. |
| Atrio-ventricular Connection | Concordant | Tricuspid Valve | Annulus = mm |
| Ventriculo-Arterial Connection | Concordant | **Ventricle** | |
| Ventricular Loop | d-Loop | Left Ventricle | Normal |
| **Septae** |  | Right Ventricle | Normal |
| Interatrial Septum |  | **Doppler Measurement** |  |
| Interventricular Septum |  | Mitral |  |
| **Semilunar Valves** |  | Aortic |  |
| Aortic Valve | Annulus = mm | Tricuspid |  |
| Pulmonary Valve | Annulus = mm | Pulmonic |  |
| **Great Arteries** | NRGA | **Coronary Arteries** |  |
| Aorta |  | **Aortic Arch** | Left |
| Pulmonary Arteries |  | **PDA** | No |
| **M-Mode**: | | | |
| Ao | mm | PWd | mm |
| LA | mm | EDV | ml |
| LVIDd | mm | ESV | ml |
| LVIDs | mm | FS | % |
| IVSd | mm | LVEF | % |
| **Additional Information:** | | | |
| **Conclusion:**   1. {S, D, S} Levocardia | | | |
| **Done By:** | **Signature** | **Date** | **Remark** |
| Tesfaye T., Pediatric Cardiologist |  | 18/04/14Eth.C. |  |

| **Patient Name:** Atinkut Tewachew. **Sex/Age**: M/7months. **Date of Report**: 18/04/14Eth.C. MRN: 112001.  **Clinical Diagnosis: DS + RD. TGSH1.2462.** | | | |
| --- | --- | --- | --- |
| **Features:** | **Findings** | **Features** | **Findings** |
| **Profile** | | **Atria** | |
| Abdominal Situs | Solitus | Left Atrium | Dilated |
| Cardiac Position | Levocardia | Right Atrium | Dilated |
| Systemic Venous Drainage | To RA | **Atrio-Ventricular Valves** | |
| Pulmonary Venous Drainage | To LA | Mitral Valve | Common Complete AVSD, L – R Shunt |
| Atrio-ventricular Connection | Concordant | Tricuspid Valve |
| Ventriculo-Arterial Connection | Concordant | **Ventricle** | |
| Ventricular Loop | d-Loop | Left Ventricle | Dilated |
| **Septae** |  | Right Ventricle | Dilated |
| Interatrial Septum |  | **Doppler Measurement** |  |
| Interventricular Septum |  | Mitral |  |
| **Semilunar Valves** |  | Aortic |  |
| Aortic Valve | Annulus = 16mm | Tricuspid | Moderate Right AVVR |
| Pulmonary Valve | Annulus = 16mm | Pulmonic |  |
| **Great Arteries** | NRGA | **Coronary Arteries** |  |
| Aorta |  | **Aortic Arch** | Left |
| Pulmonary Arteries | MPA = 16mm | **PDA** | No |
| **M-Mode**: Normal LV Function on eye balling | | | |
| Ao | mm | PWd | mm |
| LA | mm | EDV | ml |
| LVIDd | mm | ESV | ml |
| LVIDs | mm | FS | % |
| IVSd | mm | LVEF | % |
| **Additional Information:** | | | |
| **Conclusion:**   1. {S, D, S} Levocardia 2. Common Complete Balanced AVSD, L – R Shunt 3. Severe Pulmonary Hypertension 4. Normal LV Function | | | |
| **Done By:** | **Signature** | **Date** | **Remark** |
| Tesfaye T., Pediatric Cardiologist |  | 18/04/14Eth.C. |  |

| **Patient Name:** Temeliket Shumet. **Sex/Age**: M/4years. **Date of Report**: 26/04/14Eth.C. MRN: 005194.  **Clinical Diagnosis: Incidental Murmu. TGSH1.2463.** | | | |
| --- | --- | --- | --- |
| **Features:** | **Findings** | **Features** | **Findings** |
| **Profile** | | **Atria** | |
| Abdominal Situs | Solitus | Left Atrium | Normal |
| Cardiac Position | Levocardia | Right Atrium | Normal |
| Systemic Venous Drainage | To RA | **Atrio-Ventricular Valves** | |
| Pulmonary Venous Drainage | To LA | Mitral Valve | Annulus = 18mm. |
| Atrio-ventricular Connection | Concordant | Tricuspid Valve | Annulus = 18mm |
| Ventriculo-Arterial Connection | Concordant | **Ventricle** | |
| Ventricular Loop | d-Loop | Left Ventricle | Normal |
| **Septae** |  | Right Ventricle | Normal |
| Interatrial Septum | PFO, L – R Shunt | **Doppler Measurement** |  |
| Interventricular Septum |  | Mitral |  |
| **Semilunar Valves** |  | Aortic |  |
| Aortic Valve | Annulus = 19mm | Tricuspid |  |
| Pulmonary Valve | Annulus = 20mm | Pulmonic |  |
| **Great Arteries** | NRGA | **Coronary Arteries** |  |
| Aorta |  | **Aortic Arch** | Left |
| Pulmonary Arteries |  | **PDA** | 1.5mm PDA, L – R Shunt |
| **M-Mode**: Normal LV Function on eye balling | | | |
| Ao | mm | PWd | mm |
| LA | mm | EDV | ml |
| LVIDd | mm | ESV | ml |
| LVIDs | mm | FS | % |
| IVSd | mm | LVEF | % |
| **Additional Information:** | | | |
| **Conclusion:**   1. {S, D, S} Levocardia 2. PFO, L – R Shunt 3. Small PDA, L – R Shunt 4. Normal LV Function | | | |
| **Done By:** | **Signature** | **Date** | **Remark** |
| Tesfaye T., Pediatric Cardiologist |  | 26/04/14Eth.C. |  |

| **Patient Name:** Baby of Endashash wendie. **Sex/Age**: M/11days. **Date of Report**: 26/04/14Eth.C. MRN: 113037.  **Clinical Diagnosis: Incidental Murmur. TGSH1.2464.** | | | |
| --- | --- | --- | --- |
| **Features:** | **Findings** | **Features** | **Findings** |
| **Profile** | | **Atria** | |
| Abdominal Situs | Solitus | Left Atrium | Normal |
| Cardiac Position | Levocardia | Right Atrium | Normal |
| Systemic Venous Drainage | To RA | **Atrio-Ventricular Valves** | |
| Pulmonary Venous Drainage | To LA | Mitral Valve | Annulus = 10mm. |
| Atrio-ventricular Connection | Concordant | Tricuspid Valve | Annulus = 13mm |
| Ventriculo-Arterial Connection | Concordant | **Ventricle** | |
| Ventricular Loop | d-Loop | Left Ventricle | Normal |
| **Septae** |  | Right Ventricle | Mildly Hypertrophied |
| Interatrial Septum | 9mm OS ASD, L – R Shunt | **Doppler Measurement** |  |
| Interventricular Septum | Non-Restrictive Mal-aligned Sub-Aortic VSD, BD Shunt | Mitral |  |
| **Semilunar Valves** |  | Aortic | Mild AR |
| Aortic Valve | Annulus = 9mm | Tricuspid |  |
| Pulmonary Valve | Annulus = 6mm | Pulmonic | Mild to moderate PS, PPG = 37 – 46mmHg |
| **Great Arteries** | NRGA | **Coronary Arteries** |  |
| Aorta | Over-riding VSD | **Aortic Arch** | Left |
| Pulmonary Arteries |  | **PDA** | No |
| **M-Mode**: Normal LV Function on eye balling | | | |
| Ao | mm | PWd | mm |
| LA | mm | EDV | ml |
| LVIDd | mm | ESV | ml |
| LVIDs | mm | FS | % |
| IVSd | mm | LVEF | % |
| **Additional Information:** | | | |
| **Conclusion:**   1. {S, D, S} Levocardia 2. Moderate OS ASD, L – R Shunt 3. TOF | | | |
| **Done By:** | **Signature** | **Date** | **Remark** |
| Tesfaye T., Pediatric Cardiologist |  | 26/04/14Eth.C. |  |

| **Patient Name:** Abdurahizaq Hamid. **Sex/Age**: M/2 4/12. **Date of Report**: 26/04/14Eth.C. MRN: 113686.  **Clinical Diagnosis: CHF + Murmur + rd. TGSH1.2465.** | | | |
| --- | --- | --- | --- |
| **Features:** | **Findings** | **Features** | **Findings** |
| **Profile** | | **Atria** | |
| Abdominal Situs | Solitus | Left Atrium | Dilated |
| Cardiac Position | Levocardia | Right Atrium | Dilated |
| Systemic Venous Drainage | To RA | **Atrio-Ventricular Valves** | |
| Pulmonary Venous Drainage | To LA | Mitral Valve | Annulus = 10mm. thickened MVL. MVA = 0.2cm2. |
| Atrio-ventricular Connection | Concordant | Tricuspid Valve | Annulus = 20mm. TAPSE = 14mm |
| Ventriculo-Arterial Connection | Concordant | **Ventricle** | |
| Ventricular Loop | d-Loop | Left Ventricle | Normal |
| **Septae** |  | Right Ventricle | Dilated |
| Interatrial Septum |  | **Doppler Measurement** |  |
| Interventricular Septum |  | Mitral | Severe MS, PPG/MPG = 21/14mmHg |
| **Semilunar Valves** |  | Aortic |  |
| Aortic Valve | Annulus = 11mm | Tricuspid | Moderate TR, PPG = 94mmHg |
| Pulmonary Valve | Annulus = 17mm | Pulmonic | Moderate PR, PPG = 54mmHg |
| **Great Arteries** | NRGA | **Coronary Arteries** |  |
| Aorta |  | **Aortic Arch** | Left |
| Pulmonary Arteries | MPA = 19mm | **PDA** | No |
| **M-Mode**: Normal LV Function | | | |
| Ao | mm | PWd | mm |
| LA | mm | EDV | ml |
| LVIDd | mm | ESV | ml |
| LVIDs | mm | FS | % |
| IVSd | mm | LVEF | % |
| **Additional Information:** | | | |
| **Conclusion:**   1. {S, D, S} Levocardia 2. LA/RA/RV Dilated 3. Severe MS, ?Dysplastic MVL 4. Moderate TR 5. Moderate PR 6. Severe Pulmonary Hypertension | | | |
| **Done By:** | **Signature** | **Date** | **Remark** |
| Tesfaye T., Pediatric Cardiologist |  | 26/04/14Eth.C. |  |

| **Patient Name:** Tihitina Endaweke. **Sex/Age**: F/9years. **Date of Report**: 26/04/14Eth.C. MRN: 106988.  **Clinical Diagnosis: Rheumatic Recurrence + Murmur. TGSH1.2466.** | | | |
| --- | --- | --- | --- |
| **Features:** | **Findings** | **Features** | **Findings** |
| **Profile** | | **Atria** | |
| Abdominal Situs | Solitus | Left Atrium | Mildly dilated |
| Cardiac Position | Levocardia | Right Atrium | Normal |
| Systemic Venous Drainage | To RA | **Atrio-Ventricular Valves** | |
| Pulmonary Venous Drainage | To LA | Mitral Valve | Annulus = 22mm. |
| Atrio-ventricular Connection | Concordant | Tricuspid Valve | Annulus = 19mm |
| Ventriculo-Arterial Connection | Concordant | **Ventricle** | |
| Ventricular Loop | d-Loop | Left Ventricle | Mildly dilated |
| **Septae** |  | Right Ventricle | Normal |
| Interatrial Septum |  | **Doppler Measurement** |  |
| Interventricular Septum |  | Mitral | Moderate MR, Holosystolic, posterior projection, with jet velocity = 4m/sec |
| **Semilunar Valves** |  | Aortic | Moderate AR, PHT = 244ms |
| Aortic Valve | Annulus = 23mm | Tricuspid |  |
| Pulmonary Valve | Annulus = 23mm | Pulmonic |  |
| **Great Arteries** | NRGA | **Coronary Arteries** |  |
| Aorta |  | **Aortic Arch** | Left |
| Pulmonary Arteries |  | **PDA** | No |
| **M-Mode**: | | | |
| Ao | mm | PWd | mm |
| LA | mm | EDV | ml |
| LVIDd | mm | ESV | ml |
| LVIDs | mm | FS | 32% |
| IVSd | mm | LVEF | 61% |
| **Additional Information:** 2mm circumferential Pericardial effusion. | | | |
| **Conclusion:**   1. {S, D, S} Levocardia 2. Moderate MR 3. Moderate AR 4. Normal LV Function 5. Trace Pericardial perfusion | | | |
| **Done By:** | **Signature** | **Date** | **Remark** |
| Tesfaye T., Pediatric Cardiologist |  | 26/04/14Eth.C. |  |

| **Patient Name:** Baby of Genet Tilahun. **Sex/Age**: F/7months. **Date of Report**: 26/04/14Eth.C. MRN: 087611.  **Clinical Diagnosis: DS. TGSH1.2467.** | | | |
| --- | --- | --- | --- |
| **Features:** | **Findings** | **Features** | **Findings** |
| **Profile** | | **Atria** | |
| Abdominal Situs | Solitus | Left Atrium | Normal |
| Cardiac Position | Levocardia | Right Atrium | Normal |
| Systemic Venous Drainage | To RA | **Atrio-Ventricular Valves** | |
| Pulmonary Venous Drainage | To LA | Mitral Valve | Annulus = 13mm. |
| Atrio-ventricular Connection | Concordant | Tricuspid Valve | Annulus = 13mm |
| Ventriculo-Arterial Connection | Concordant | **Ventricle** | |
| Ventricular Loop | d-Loop | Left Ventricle | Normal |
| **Septae** |  | Right Ventricle | Normal |
| Interatrial Septum | 4mm OS ASD, L – R Shunt | **Doppler Measurement** |  |
| Interventricular Septum |  | Mitral |  |
| **Semilunar Valves** |  | Aortic |  |
| Aortic Valve | Annulus = 10mm | Tricuspid |  |
| Pulmonary Valve | Annulus = 12mm | Pulmonic |  |
| **Great Arteries** | NRGA | **Coronary Arteries** |  |
| Aorta |  | **Aortic Arch** | Left |
| Pulmonary Arteries |  | **PDA** | No |
| **M-Mode**: Normal LV Function on eye balling | | | |
| Ao | mm | PWd | mm |
| LA | mm | EDV | ml |
| LVIDd | mm | ESV | ml |
| LVIDs | mm | FS | % |
| IVSd | mm | LVEF | % |
| **Additional Information:** | | | |
| **Conclusion:**   1. {S, D, S} Levocardia 2. Small OS ASD, L – R Shunt | | | |
| **Done By:** | **Signature** | **Date** | **Remark** |
| Tesfaye T., Pediatric Cardiologist |  | 26/04/14Eth.C. |  |

| **Patient Name:** Solomie Getachew. **Sex/Age**: F/2 8/12. **Date of Report**: 26/04/14Eth.C. MRN: 608216.  **Clinical Diagnosis: Incidental Murmur. TGSH1.2468. (TGSH4)** | | | |
| --- | --- | --- | --- |
| **Features:** | **Findings** | **Features** | **Findings** |
| **Profile** | | **Atria** | |
| Abdominal Situs | Solitus | Left Atrium | Normal |
| Cardiac Position | Levocardia | Right Atrium | Normal |
| Systemic Venous Drainage | To RA | **Atrio-Ventricular Valves** | |
| Pulmonary Venous Drainage | To LA | Mitral Valve | Annulus = 17mm. |
| Atrio-ventricular Connection | Concordant | Tricuspid Valve | Annulus = 17mm |
| Ventriculo-Arterial Connection | Concordant | **Ventricle** | |
| Ventricular Loop | d-Loop | Left Ventricle | Normal |
| **Septae** |  | Right Ventricle | Normal |
| Interatrial Septum |  | **Doppler Measurement** |  |
| Interventricular Septum | 2 – 2.5mm PM VSD, L – R Shunt | Mitral |  |
| **Semilunar Valves** |  | Aortic |  |
| Aortic Valve | Annulus = 13mm | Tricuspid |  |
| Pulmonary Valve | Annulus = 15mm | Pulmonic |  |
| **Great Arteries** | NRGA | **Coronary Arteries** |  |
| Aorta |  | **Aortic Arch** | Left |
| Pulmonary Arteries |  | **PDA** | No |
| **M-Mode**: Normal LV Function on eye balling | | | |
| Ao | mm | PWd | mm |
| LA | mm | EDV | ml |
| LVIDd | mm | ESV | ml |
| LVIDs | mm | FS | % |
| IVSd | mm | LVEF | % |
| **Additional Information:** | | | |
| **Conclusion:**   1. {S, D, S} Levocardia 2. Small PM VSD, L – R Shunt | | | |
| **Done By:** | **Signature** | **Date** | **Remark** |
| Tesfaye T., Pediatric Cardiologist |  | 26/04/14Eth.C. |  |

| **Patient Name:** Likie Gebeyehu. **Sex/Age**: F/12years. **Date of Report**: 28/04/14Eth.C. MRN: 113543.  **Clinical Diagnosis: DOE + RD + CHF + Murmur + Rheumatic Recurrence. TGSH1.2469.** | | | |
| --- | --- | --- | --- |
| **Features:** | **Findings** | **Features** | **Findings** |
| **Profile** | | **Atria** | |
| Abdominal Situs | Solitus | Left Atrium | Dilated |
| Cardiac Position | Levocardia | Right Atrium | Dilated |
| Systemic Venous Drainage | To RA | **Atrio-Ventricular Valves** | |
| Pulmonary Venous Drainage | To LA | Mitral Valve | Annulus = 28mm. Thickened, clubbed, drum stick appearance MVL. MVA = 0.4cm2. |
| Atrio-ventricular Connection | Concordant | Tricuspid Valve | Annulus = 26mm. thickened TVL |
| Ventriculo-Arterial Connection | Concordant | **Ventricle** | |
| Ventricular Loop | d-Loop | Left Ventricle | Dilated |
| **Septae** |  | Right Ventricle | Dilated |
| Interatrial Septum |  | **Doppler Measurement** |  |
| Interventricular Septum |  | Mitral | Mild MR, Holosystolic, seen in two planes with jet velocity = 3.8m/sec. Severe MS, PPG/MPG = 13/10mmHg. |
| **Semilunar Valves** |  | Aortic | Moderate AR, PHT = 227ms. |
| Aortic Valve | Annulus = 17mm. Thickened AVL, Trileaflet | Tricuspid | Moderate TR, PPG = 86mmHg. |
| Pulmonary Valve | Annulus = 21mm | Pulmonic |  |
| **Great Arteries** | NRGA | **Coronary Arteries** |  |
| Aorta |  | **Aortic Arch** | Left |
| Pulmonary Arteries | MPA = 32cm | **PDA** | No |
| **M-Mode**: | | | |
| Ao | mm | PWd | mm |
| LA | mm | EDV | ml |
| LVIDd | mm | ESV | ml |
| LVIDs | mm | FS | 33% |
| IVSd | mm | LVEF | 61% |
| **Conclusion:**   1. {S, D, S} Levocardia 2. All chambers dilated 3. Thickened, clubbed, drum stick appearance MVL 4. Thickened AVL and TVL 5. Mild MR 6. Severe MS 7. Moderate TR 8. Severe Pulmonary Hypertension 9. Normal LV Function | | | |
| **Done By:** | **Signature** | **Date** | **Remark** |
| Tesfaye T., Pediatric Cardiologist |  | 28/04/14Eth.C. |  |

| **Patient Name:** Baby of Degitu Debeb. **Sex/Age**: M/11days. **Date of Report**: 28/04/14Eth.C. MRN: 112669.  **Clinical Diagnosis: RD. TGSH1.2470.** | | | |
| --- | --- | --- | --- |
| **Features:** | **Findings** | **Features** | **Findings** |
| **Profile** | | **Atria** | |
| Abdominal Situs | Solitus | Left Atrium | Normal |
| Cardiac Position | Levocardia | Right Atrium | Normal |
| Systemic Venous Drainage | To RA | **Atrio-Ventricular Valves** | |
| Pulmonary Venous Drainage | To LA | Mitral Valve | Annulus = 9mm. |
| Atrio-ventricular Connection | Concordant | Tricuspid Valve | Annulus = 9mm |
| Ventriculo-Arterial Connection | Concordant | **Ventricle** | |
| Ventricular Loop | d-Loop | Left Ventricle | Normal |
| **Septae** |  | Right Ventricle | Normal |
| Interatrial Septum | PFO, L – R Shunt | **Doppler Measurement** |  |
| Interventricular Septum |  | Mitral |  |
| **Semilunar Valves** |  | Aortic |  |
| Aortic Valve | Annulus = 9mm | Tricuspid |  |
| Pulmonary Valve | Annulus = 8mm | Pulmonic |  |
| **Great Arteries** | NRGA | **Coronary Arteries** |  |
| Aorta |  | **Aortic Arch** | Left |
| Pulmonary Arteries |  | **PDA** | No |
| **M-Mode**: Normal LV Function | | | |
| Ao | mm | PWd | mm |
| LA | mm | EDV | ml |
| LVIDd | mm | ESV | ml |
| LVIDs | mm | FS | % |
| IVSd | mm | LVEF | % |
| **Additional Information:** | | | |
| **Conclusion:**   1. {S, D, S} Levocardia 2. PFO, L – R Shunt | | | |
| **Done By:** | **Signature** | **Date** | **Remark** |
| Tesfaye T., Pediatric Cardiologist |  | 28/04/14Eth.C. |  |

| **Patient Name:** Mulugeta Emiwedew. **Sex/Age**: M/10years. **Date of Report**: 28/04/14Eth.C. MRN: 113711.  **Clinical Diagnosis: DOE + Murmur. TGSH1.2471.** | | | |
| --- | --- | --- | --- |
| **Features:** | **Findings** | **Features** | **Findings** |
| **Profile** | | **Atria** | |
| Abdominal Situs | Solitus | Left Atrium | Mildly dilated |
| Cardiac Position | Levocardia | Right Atrium | Normal |
| Systemic Venous Drainage | To RA | **Atrio-Ventricular Valves** | |
| Pulmonary Venous Drainage | To LA | Mitral Valve | Annulus = 24mm. |
| Atrio-ventricular Connection | Concordant | Tricuspid Valve | Annulus = 26mm  TAPSE = 23mm |
| Ventriculo-Arterial Connection | Concordant | **Ventricle** | |
| Ventricular Loop | d-Loop | Left Ventricle | Dilated |
| **Septae** |  | Right Ventricle | Normal |
| Interatrial Septum |  | **Doppler Measurement** |  |
| Interventricular Septum |  | Mitral |  |
| **Semilunar Valves** |  | Aortic | Mild AR, PHT = 523ms. AS, PPG/MPG = 59/30mmHg |
| Aortic Valve | Annulus = 17mm. Trileaflet. | Tricuspid |  |
| Pulmonary Valve | Annulus = 25mm | Pulmonic |  |
| **Great Arteries** | NRGA | **Coronary Arteries** |  |
| Aorta |  | **Aortic Arch** | Left |
| Pulmonary Arteries |  | **PDA** | 4mm PDA, L – R Shunt |
| **M-Mode**: | | | |
| Ao | mm | PWd | mm |
| LA | mm | EDV | ml |
| LVIDd | mm | ESV | ml |
| LVIDs | mm | FS | 34% |
| IVSd | mm | LVEF | 62% |
| **Additional Information:** | | | |
| **Conclusion:**   1. {S, D, S} Levocardia 2. LA/LV Dilated 3. Mild AR 4. Moderate AS 5. Large PDA, L – R Shunt 6. Normal Biventricular Systolic Function | | | |
| **Done By:** | **Signature** | **Date** | **Remark** |
| Tesfaye T., Pediatric Cardiologist |  | 28/04/14Eth.C. |  |

| **Patient Name:** Eyob Enyew. **Sex/Age**: M/57days. **Date of Report**: 28/04/14Eth.C. MRN: 113715.  **Clinical Diagnosis: RD. TGSH1.2472.** | | | |
| --- | --- | --- | --- |
| **Features:** | **Findings** | **Features** | **Findings** |
| **Profile** | | **Atria** | |
| Abdominal Situs | Solitus | Left Atrium | Normal |
| Cardiac Position | Dextrocardia | Right Atrium | Normal |
| Systemic Venous Drainage | To RA | **Atrio-Ventricular Valves** | |
| Pulmonary Venous Drainage | To LA | Mitral Valve | Annulus = 10mm. |
| Atrio-ventricular Connection | Concordant | Tricuspid Valve | Annulus = 11mm |
| Ventriculo-Arterial Connection | Concordant | **Ventricle** | |
| Ventricular Loop | d-Loop | Left Ventricle | Normal |
| **Septae** |  | Right Ventricle | Normal |
| Interatrial Septum |  | **Doppler Measurement** |  |
| Interventricular Septum |  | Mitral |  |
| **Semilunar Valves** |  | Aortic |  |
| Aortic Valve | Annulus = 6mm | Tricuspid |  |
| Pulmonary Valve | Annulus = 9mm | Pulmonic |  |
| **Great Arteries** | NRGA | **Coronary Arteries** |  |
| Aorta |  | **Aortic Arch** | Left |
| Pulmonary Arteries |  | **PDA** | No |
| **M-Mode**: | | | |
| Ao | mm | PWd | mm |
| LA | mm | EDV | ml |
| LVIDd | mm | ESV | ml |
| LVIDs | mm | FS | % |
| IVSd | mm | LVEF | % |
| **Additional Information:** | | | |
| **Conclusion:**   1. {S, D, S} Dextrocardia | | | |
| **Remark:** infant was irritable and restless during study | | | |
| **Done By:** | **Signature** | **Date** | **Remark** |
| Tesfaye T., Pediatric Cardiologist |  | 28/04/14Eth.C. |  |

| **Patient Name:** Baby of Nitsuh Asfaw. **Sex/Age**: F/11days. **Date of Report**: 05/05/14Eth.C. MRN: 113333.  **Clinical Diagnosis: RD. TGSH1.2473.** | | | |
| --- | --- | --- | --- |
| **Features:** | **Findings** | **Features** | **Findings** |
| **Profile** | | **Atria** | |
| Abdominal Situs | Solitus | Left Atrium | Normal |
| Cardiac Position | Levocardia | Right Atrium | Normal |
| Systemic Venous Drainage | To RA | **Atrio-Ventricular Valves** | |
| Pulmonary Venous Drainage | To LA | Mitral Valve | Annulus = 8mm. |
| Atrio-ventricular Connection | Concordant | Tricuspid Valve | Annulus = 9mm |
| Ventriculo-Arterial Connection | Concordant | **Ventricle** | |
| Ventricular Loop | d-Loop | Left Ventricle | Normal |
| **Septae** |  | Right Ventricle | Normal |
| Interatrial Septum |  | **Doppler Measurement** |  |
| Interventricular Septum |  | Mitral |  |
| **Semilunar Valves** |  | Aortic |  |
| Aortic Valve | Annulus = 7mm | Tricuspid |  |
| Pulmonary Valve | Annulus = 8mm | Pulmonic |  |
| **Great Arteries** | NRGA | **Coronary Arteries** |  |
| Aorta |  | **Aortic Arch** | Left |
| Pulmonary Arteries |  | **PDA** | No |
| **M-Mode**: Normal LV Function on eye balling | | | |
| Ao | mm | PWd | mm |
| LA | mm | EDV | ml |
| LVIDd | mm | ESV | ml |
| LVIDs | mm | FS | % |
| IVSd | mm | LVEF | % |
| **Additional Information:** | | | |
| **Conclusion:**   1. Normal Echocardiography Study | | | |
| **Done By:** | **Signature** | **Date** | **Remark** |
| Tesfaye T., Pediatric Cardiologist |  | 05/05/14Eth.C. |  |

| **Patient Name:** Daniel Tadele. **Sex/Age**: M/6months. **Date of Report**: 05/05/14Eth.C. MRN: 114166.  **Clinical Diagnosis: CHF + RD + Murmur. TGSH1.2474.** | | | |
| --- | --- | --- | --- |
| **Features:** | **Findings** | **Features** | **Findings** |
| **Profile** | | **Atria** | |
| Abdominal Situs | Solitus | Left Atrium | Dilated |
| Cardiac Position | Levocardia | Right Atrium | Dilated |
| Systemic Venous Drainage | To RA | **Atrio-Ventricular Valves** | |
| Pulmonary Venous Drainage | To LA | Mitral Valve | Annulus = 13mm. |
| Atrio-ventricular Connection | Concordant | Tricuspid Valve | Annulus = 15mm |
| Ventriculo-Arterial Connection | Concordant | **Ventricle** | |
| Ventricular Loop | d-Loop | Left Ventricle | Dilated |
| **Septae** |  | Right Ventricle | Dilated |
| Interatrial Septum |  | **Doppler Measurement** |  |
| Interventricular Septum | Sub aortic nonrestrictive VSD, BD Shunt | Mitral |  |
| **Semilunar Valves** |  | Aortic |  |
| Aortic Valve | Truncal Annulus = 15mm | Tricuspid | Moderate TR |
| Pulmonary Valve | Arising from the truncus as MPA and branch then after | Pulmonic |  |
| **Great Arteries** | Truncus | **Coronary Arteries** |  |
| Aorta |  | **Aortic Arch** | Left |
| Pulmonary Arteries |  | **PDA** | No |
| **M-Mode**: | | | |
| Ao | mm | PWd | mm |
| LA | mm | EDV | ml |
| LVIDd | mm | ESV | ml |
| LVIDs | mm | FS | % |
| IVSd | mm | LVEF | % |
| **Additional Information:** | | | |
| **Conclusion:**   1. {S, D, S} Levocardia 2. Moderate TR 3. Truncus Arteriosus Type I | | | |
| **Done By:** | **Signature** | **Date** | **Remark** |
| Tesfaye T., Pediatric Cardiologist |  | 05/05/14Eth.C. |  |

| **Patient Name:** Demeke Alemneh. **Sex/Age**: M/13years. **Date of Report**: 05/05/14Eth.C. MRN: 077780.  **Clinical Diagnosis: Rheumatic Recurrence + Easy Fatigability + Murmur. TGSH1.2475.** | | | |
| --- | --- | --- | --- |
| **Features:** | **Findings** | **Features** | **Findings** |
| **Profile** | | **Atria** | |
| Abdominal Situs | Solitus | Left Atrium | Normal |
| Cardiac Position | Levocardia | Right Atrium | Normal |
| Systemic Venous Drainage | To RA | **Atrio-Ventricular Valves** | |
| Pulmonary Venous Drainage | To LA | Mitral Valve | Annulus = 26mm. Thickened, clubbed MVL. Shortened PMVL. MVA = 1.8cm2. |
| Atrio-ventricular Connection | Concordant | Tricuspid Valve | Annulus = 28mm |
| Ventriculo-Arterial Connection | Concordant | **Ventricle** | |
| Ventricular Loop | d-Loop | Left Ventricle | Normal |
| **Septae** |  | Right Ventricle | Normal |
| Interatrial Septum |  | **Doppler Measurement** |  |
| Interventricular Septum |  | Mitral | Mild MR, Jet velocity = 4.1m/sec. Mild MS, PPG/MPG = 18/6mmHg. |
| **Semilunar Valves** |  | Aortic | Mild AR |
| Aortic Valve | Annulus = 18mm. thickened AVL, Trileaflet. | Tricuspid | Moderate TR, PPG = 23mmHg |
| Pulmonary Valve | Annulus = 21mm | Pulmonic |  |
| **Great Arteries** | NRGA | **Coronary Arteries** |  |
| Aorta |  | **Aortic Arch** | Left |
| Pulmonary Arteries |  | **PDA** | No |
| **M-Mode**: Normal LV Function on eye balling. | | | |
| Ao | mm | PWd | mm |
| LA | mm | EDV | ml |
| LVIDd | mm | ESV | ml |
| LVIDs | mm | FS | % |
| IVSd | mm | LVEF | % |
| **Additional Information:** | | | |
| **Conclusion:**   1. {S, D, S} Levocardia 2. Thickened MVL and AVL 3. Mild MR 4. Mild MS 5. Mild AR 6. Moderate TR 7. Normal Function | | | |
| **Done By:** | **Signature** | **Date** | **Remark** |
| Tesfaye T., Pediatric Cardiologist |  | 05/05/14Eth.C. |  |

| **Patient Name:** Birhanu Mengie. **Sex/Age**: M/12years. **Date of Report**: 10/05/14Eth.C. MRN: 114018.  **Clinical Diagnosis: DOE + Murmur. TGSH1.2476.** | | | |
| --- | --- | --- | --- |
| **Features:** | **Findings** | **Features** | **Findings** |
| **Profile** | | **Atria** | |
| Abdominal Situs | Solitus | Left Atrium | Normal |
| Cardiac Position | Levocardia | Right Atrium | Mildly Dilated |
| Systemic Venous Drainage | To RA | **Atrio-Ventricular Valves** | |
| Pulmonary Venous Drainage | To LA | Mitral Valve | Annulus = 22mm. |
| Atrio-ventricular Connection | Concordant | Tricuspid Valve | Annulus = 24mm |
| Ventriculo-Arterial Connection | Concordant | **Ventricle** | |
| Ventricular Loop | d-Loop | Left Ventricle | Normal |
| **Septae** |  | Right Ventricle | Mildly Dilated |
| Interatrial Septum | 8mm High Secundum ASD, BD Shunt. | **Doppler Measurement** |  |
| Interventricular Septum |  | Mitral |  |
| **Semilunar Valves** |  | Aortic |  |
| Aortic Valve | Annulus = 20mm | Tricuspid |  |
| Pulmonary Valve | Annulus = 13mm | Pulmonic | Moderate to severe Valvular and Supra valvular PS, PPG = 49 – 56mmHg. |
| **Great Arteries** | NRGA | **Coronary Arteries** |  |
| Aorta |  | **Aortic Arch** | Left |
| Pulmonary Arteries | Smallish MPA and Branch PAs. | **PDA** | No |
| **M-Mode**: Normal LV Function on eye balling | | | |
| Ao | mm | PWd | mm |
| LA | mm | EDV | ml |
| LVIDd | mm | ESV | ml |
| LVIDs | mm | FS | % |
| IVSd | mm | LVEF | % |
| **Additional Information:** | | | |
| **Conclusion:**   1. {S, D, S} Levocardia 2. Moderate High Secundum ASD, BD Shunt 3. Moderate to severe Valvular and supra valvular PS 4. Smallish MPA and Branch PAs. 5. Normal LV Function | | | |
| **Done By:** | **Signature** | **Date** | **Remark** |
| Tesfaye T., Pediatric Cardiologist |  | 10/05/14Eth.C. |  |

| **Patient Name:** Baby of Genet Teshome. **Sex/Age**: F/10days. **Date of Report**: 10/05/14Eth.C. MRN: 113897.  **Clinical Diagnosis: Incidental Murmur. TGSH1.2477.** | | | |
| --- | --- | --- | --- |
| **Features:** | **Findings** | **Features** | **Findings** |
| **Profile** | | **Atria** | |
| Abdominal Situs | Solitus | Left Atrium | Normal |
| Cardiac Position | Levocardia | Right Atrium | Normal |
| Systemic Venous Drainage | To RA | **Atrio-Ventricular Valves** | |
| Pulmonary Venous Drainage | To LA | Mitral Valve | Annulus = 9mm. |
| Atrio-ventricular Connection | Concordant | Tricuspid Valve | Annulus = 10mm |
| Ventriculo-Arterial Connection | Concordant | **Ventricle** | |
| Ventricular Loop | d-Loop | Left Ventricle | Normal |
| **Septae** |  | Right Ventricle | Normal |
| Interatrial Septum |  | **Doppler Measurement** |  |
| Interventricular Septum |  | Mitral |  |
| **Semilunar Valves** |  | Aortic |  |
| Aortic Valve | Annulus = 7mm | Tricuspid | Trivial TR, Incomplete Signal, PPG = 13mmHg. |
| Pulmonary Valve | Annulus = 8mm | Pulmonic |  |
| **Great Arteries** | NRGA | **Coronary Arteries** |  |
| Aorta |  | **Aortic Arch** | Left |
| Pulmonary Arteries |  | **PDA** | 1mm PDA, L – R Shunt |
| **M-Mode**: Normal LV Function on eye balling | | | |
| Ao | mm | PWd | mm |
| LA | mm | EDV | ml |
| LVIDd | mm | ESV | ml |
| LVIDs | mm | FS | % |
| IVSd | mm | LVEF | % |
| **Additional Information:** | | | |
| **Conclusion:**   1. {S, D, S} Levocardia 2. Small PDA, L – R Shunt | | | |
| **Remark:** Preterm Baby. | | | |
| **Recommendation:** Echocardiographic evaluation after 3months. | | | |
| **Done By:** | **Signature** | **Date** | **Remark** |
| Tesfaye T., Pediatric Cardiologist |  | 10/05/14Eth.C. |  |

| **Patient Name:** Tesfahun Yitayal. **Sex/Age**: M/14years. **Date of Report**: 12/05/14Eth.C. MRN: 112688.  **Clinical Diagnosis: Sydenham’s Chorea. TGSH1.2478.** | | | |
| --- | --- | --- | --- |
| **Features:** | **Findings** | **Features** | **Findings** |
| **Profile** | | **Atria** | |
| Abdominal Situs | Solitus | Left Atrium | Normal |
| Cardiac Position | Levocardia | Right Atrium | Normal |
| Systemic Venous Drainage | To RA | **Atrio-Ventricular Valves** | |
| Pulmonary Venous Drainage | To LA | Mitral Valve | Annulus = 24mm. |
| Atrio-ventricular Connection | Concordant | Tricuspid Valve | Annulus = 23mm  TAPSE = 17mm |
| Ventriculo-Arterial Connection | Concordant | **Ventricle** | |
| Ventricular Loop | d-Loop | Left Ventricle | Normal |
| **Septae** |  | Right Ventricle | Normal |
| Interatrial Septum | Intact | **Doppler Measurement** |  |
| Interventricular Septum | Intact | Mitral |  |
| **Semilunar Valves** |  | Aortic |  |
| Aortic Valve | Annulus = 19mm | Tricuspid |  |
| Pulmonary Valve | Annulus = 24mm | Pulmonic |  |
| **Great Arteries** | NRGA | **Coronary Arteries** |  |
| Aorta |  | **Aortic Arch** | Left |
| Pulmonary Arteries |  | **PDA** | No |
| **M-Mode**: | | | |
| Ao | mm | PWd | mm |
| LA | mm | EDV | ml |
| LVIDd | mm | ESV | ml |
| LVIDs | mm | FS | 30% |
| IVSd | mm | LVEF | 57% |
| **Additional Information:** | | | |
| **Conclusion:**   1. Normal Echocardiography Study | | | |
| **Done By:** | **Signature** | **Date** | **Remark** |
| Tesfaye T., Pediatric Cardiologist |  | 12/05/14Eth.C. |  |

| **Patient Name:** Tenagne Masresha. **Sex/Age**: M/18days. **Date of Report**: 12/05/14Eth.C. MRN: 115084.  **Clinical Diagnosis: Incidental Murmur. TGSH1.2479.** | | | |
| --- | --- | --- | --- |
| **Features:** | **Findings** | **Features** | **Findings** |
| **Profile** | | **Atria** | |
| Abdominal Situs | Solitus | Left Atrium | Normal |
| Cardiac Position | Levocardia | Right Atrium | Dilated |
| Systemic Venous Drainage | To RA | **Atrio-Ventricular Valves** | |
| Pulmonary Venous Drainage | To LA | Mitral Valve | Annulus = 8mm. |
| Atrio-ventricular Connection | Concordant | Tricuspid Valve | Annulus = 14mm |
| Ventriculo-Arterial Connection | Concordant | **Ventricle** | |
| Ventricular Loop | d-Loop | Left Ventricle | Normal |
| **Septae** |  | Right Ventricle | Dilated |
| Interatrial Septum | 9mm Fenestrated ASD, L – R Shunt | **Doppler Measurement** |  |
| Interventricular Septum | 1mm Upper Muscular VSD, L – R Shunt | Mitral |  |
| **Semilunar Valves** |  | Aortic |  |
| Aortic Valve | Annulus = 9mm | Tricuspid |  |
| Pulmonary Valve | Annulus = 11mm | Pulmonic | Moderate Valvular PS , PPG = 50mmHg |
| **Great Arteries** | NRGA | **Coronary Arteries** |  |
| Aorta |  | **Aortic Arch** | Left |
| Pulmonary Arteries |  | **PDA** | 1mm PDA, L – R Shunt |
| **M-Mode**: Normal LV Function on eye balling | | | |
| Ao | mm | PWd | mm |
| LA | mm | EDV | ml |
| LVIDd | mm | ESV | ml |
| LVIDs | mm | FS | % |
| IVSd | mm | LVEF | % |
| **Additional Information:** | | | |
| **Conclusion:**   1. {S, D, S} Levocardia 2. RA/RV Dilated 3. Moderate Fenestrated ASD, L – R Shunt 4. Tiny Upper Muscular VSD, L – R Shunt 5. Small PDA, L – R Shunt 6. Moderate PS 7. Normal LV Function | | | |
| **Done By:** | **Signature** | **Date** | **Remark** |
| Tesfaye T., Pediatric Cardiologist |  | 12/05/14Eth.C. |  |

| **Patient Name:** Biruk Teferi. **Sex/Age**: M/33days. **Date of Report**: 12/05/14Eth.C. MRN: 115108.  **Clinical Diagnosis: RD + PPHTN. TGSH1.2480.** | | | |
| --- | --- | --- | --- |
| **Features:** | **Findings** | **Features** | **Findings** |
| **Profile** | | **Atria** | |
| Abdominal Situs | Solitus | Left Atrium | Normal |
| Cardiac Position | Levocardia | Right Atrium | Dilated |
| Systemic Venous Drainage | To RA | **Atrio-Ventricular Valves** | |
| Pulmonary Venous Drainage | To LA | Mitral Valve | Annulus = 10mm. |
| Atrio-ventricular Connection | Concordant | Tricuspid Valve | Annulus = 14mm  **TAPSE = 6mm** |
| Ventriculo-Arterial Connection | Concordant | **Ventricle** | |
| Ventricular Loop | d-Loop | Left Ventricle | Normal |
| **Septae** |  | Right Ventricle | Dilated |
| Interatrial Septum | PFO, R – L Shunt | **Doppler Measurement** |  |
| Interventricular Septum | Intact | Mitral |  |
| **Semilunar Valves** |  | Aortic |  |
| Aortic Valve | Annulus = 7mm | Tricuspid | Moderate TR, PPG = 65mmHg |
| Pulmonary Valve | Annulus = 9mm | Pulmonic |  |
| **Great Arteries** | NRGA | **Coronary Arteries** |  |
| Aorta |  | **Aortic Arch** | Left |
| Pulmonary Arteries |  | **PDA** | PDA, < 1mm, R – L Shunt. |
| **M-Mode**: | | | |
| Ao | mm | PWd | mm |
| LA | mm | EDV | ml |
| LVIDd | mm | ESV | ml |
| LVIDs | mm | FS | % |
| IVSd | mm | LVEF | % |
| **Additional Information:** | | | |
| **Conclusion:**   1. {S, D, S} Levocardia 2. RA/RV Dilated 3. PFO, R – L Shunt 4. Moderate TR 5. Small PDA, R – L Shunt 6. RV Dysfunctional 7. Severe Pulmonary Hypertension ? PPHN. | | | |
| **Recommendation:** secondary causes shall be ruled out. | | | |
| **Done By:** | **Signature** | **Date** | **Remark** |
| Tesfaye T., Pediatric Cardiologist |  | 12/05/14Eth.C. |  |

| **Patient Name:** Yibeltal Desalegn. **Sex/Age**: M/8months. **Date of Report**: 19/05/14Eth.C. MRN: 125121.  **Clinical Diagnosis: CHF + Diaphoresis during BF + DS + RD. TGSH1.2481.** | | | |
| --- | --- | --- | --- |
| **Features:** | **Findings** | **Features** | **Findings** |
| **Profile** | | **Atria** | |
| Abdominal Situs | Solitus | Left Atrium | Dilated |
| Cardiac Position | Levocardia | Right Atrium | Dilated |
| Systemic Venous Drainage | To RA | **Atrio-Ventricular Valves** | |
| Pulmonary Venous Drainage | To LA | Mitral Valve | Annulus = 16mm. |
| Atrio-ventricular Connection | Concordant | Tricuspid Valve | Annulus = 16mm |
| Ventriculo-Arterial Connection | Concordant | **Ventricle** | |
| Ventricular Loop | d-Loop | Left Ventricle | Dilated |
| **Septae** |  | Right Ventricle | Dilated |
| Interatrial Septum |  | **Doppler Measurement** |  |
| Interventricular Septum | 17mm Inlet VSD with PM extension, L – R Shunt | Mitral | Mild MR |
| **Semilunar Valves** |  | Aortic |  |
| Aortic Valve | Annulus = 13mm | Tricuspid | Moderate TR |
| Pulmonary Valve | Annulus = 20mm | Pulmonic | Moderate PR, PPG = 58mmHg |
| **Great Arteries** | NRGA | **Coronary Arteries** |  |
| Aorta |  | **Aortic Arch** | Left |
| Pulmonary Arteries | MPD = 26mm | **PDA** | No |
| **M-Mode**: Normal LV Function | | | |
| Ao | mm | PWd | mm |
| LA | mm | EDV | ml |
| LVIDd | mm | ESV | ml |
| LVIDs | mm | FS | % |
| IVSd | mm | LVEF | % |
| **Additional Information:** | | | |
| **Conclusion:**   1. {S, D, S} Levocardia 2. All chambers dilated 3. Large Inlet VSD with PM extension, L – R Shunt 4. Severe Pulmonary Hypertension 5. Normal LV Function | | | |
| **Done By:** | **Signature** | **Date** | **Remark** |
| Tesfaye T., Pediatric Cardiologist |  | 19/05/14Eth.C. |  |

| **Patient Name:** Simegn Ameshe. **Sex/Age**: F/4years. **Date of Report**: 19/05/14Eth.C. MRN: _115629.  **Clinical Diagnosis: RD + CHF. TGSH1.2482.** | | | |
| --- | --- | --- | --- |
| **Features:** | **Findings** | **Features** | **Findings** |
| **Profile** | | **Atria** | |
| Abdominal Situs | Solitus | Left Atrium | Markedly dilated |
| Cardiac Position | Levocardia | Right Atrium | Normal |
| Systemic Venous Drainage | To RA | **Atrio-Ventricular Valves** | |
| Pulmonary Venous Drainage | To LA | Mitral Valve | Annulus = 21mm. |
| Atrio-ventricular Connection | Concordant | Tricuspid Valve | Annulus = 18mm |
| Ventriculo-Arterial Connection | Concordant | **Ventricle** | |
| Ventricular Loop | d-Loop | Left Ventricle | Markedly dilated & Dysfunctional |
| **Septae** |  | Right Ventricle | Normal |
| Interatrial Septum | Intact | **Doppler Measurement** |  |
| Interventricular Septum | Intact | Mitral | Mild MR |
| **Semilunar Valves** |  | Aortic | Mild AR |
| Aortic Valve | Annulus = 13mm | Tricuspid | Moderate TR |
| Pulmonary Valve | Annulus = 19mm | Pulmonic | Moderate PR, PPG = 65mmHg |
| **Great Arteries** | NRGA | **Coronary Arteries** | No ALCAPA |
| Aorta |  | **Aortic Arch** | Left. No CoA. |
| Pulmonary Arteries |  | **PDA** | 1mm PDA, L – R Shunt |
| **M-Mode**: | | | |
| Ao | mm | PWd | mm |
| LA | mm | EDV | ml |
| LVIDd | mm | ESV | ml |
| LVIDs | mm | FS | 8% |
| IVSd | mm | LVEF | 14% |
| **Additional Information:** Pericardial effusion with maximum depth of 20mm on RA/RV Side. | | | |
| **Conclusion:**   1. {S, D, S} Levocardia 2. LA/LV Markedly Dilated 3. Mild MR 4. Mild AR 5. Moderate TR 6. Moderate PR 7. Severe Pulmonary Hypertension 8. Severe LV Dysfunction 9. Large Pericardial effusion | | | |
| **Recommendation:** | Consider DCM as a DDx. | | |
| **Done By:** | **Signature** | **Date** | **Remark** |
| Tesfaye T., Pediatric Cardiologist |  | 19/05/14Eth.C. |  |

| **Patient Name: Temesgen Wondmnew**. **Sex/Age. M/23 months**. **Date of Report**: 19/05/14Eth.C. MRN:**063139**  **Clinical Diagnosis: Incidental Murmur + Recurrent Chest Infection. TGSH1.2483.** | | | |
| --- | --- | --- | --- |
| **Features:** | **Findings** | **Features** | **Findings** |
| **Profile** | | **Atria** | |
| Abdominal Situs | Solitus | Left Atrium | Normal |
| Cardiac Position | Levocardia | Right Atrium | Mildly dilated |
| Systemic Venous Drainage | To RA | **Atrio-Ventricular Valves** | |
| Pulmonary Venous Drainage | To LA | Mitral Valve | Annulus = 14mm. |
| Atrio-ventricular Connection | Concordant | Tricuspid Valve | Annulus = 19mm  TAPSE = 17mm |
| Ventriculo-Arterial Connection | Concordant | **Ventricle** | |
| Ventricular Loop | d-Loop | Left Ventricle | Normal |
| **Septae** |  | Right Ventricle | Mildly dilated & hypertrophied |
| Interatrial Septum | 9mm OS ASD, L – R Shunt. | **Doppler Measurement** |  |
| Interventricular Septum | 7mm Subaortic VSD, L – R Shunt | Mitral |  |
| **Semilunar Valves** |  | Aortic |  |
| Aortic Valve | Annulus = 14mm | Tricuspid |  |
| Pulmonary Valve | Annulus = 15mm | Pulmonic | Mild PS, PPG = 23mmHg |
| **Great Arteries** | NRGA | **Coronary Arteries** |  |
| Aorta |  | **Aortic Arch** | Left |
| Pulmonary Arteries |  | **PDA** | No |
| **M-Mode**: | | | |
| Ao | mm | PWd | mm |
| LA | mm | EDV | ml |
| LVIDd | mm | ESV | ml |
| LVIDs | mm | FS | % |
| IVSd | mm | LVEF | % |
| **Additional Information:** | | | |
| **Conclusion:**   1. {S, D, S} Levocardia 2. RA/RV Dilated 3. Moderate OS ASD, L – R Shunt 4. TOF (Pink type) 5. Normal Biventricular Function | | | |
| **Done By:** | **Signature** | **Date** | **Remark** |
| Tesfaye T., Pediatric Cardiologist |  | 19/05/14Eth.C. |  |

| **Patient Name: Selam Tewachew. Sex Age: F/ 6 month Date of Report: 19/05/14Eth.C. MRN:115369**.  **Clinical Diagnosis: Diaphoresis during BF + Incidental Murmur. TGSH1.2484.** | | | |
| --- | --- | --- | --- |
| **Features:** | **Findings** | **Features** | **Findings** |
| **Profile** | | **Atria** | |
| Abdominal Situs | Solitus | Left Atrium | Mildly Dilated |
| Cardiac Position | Levocardia | Right Atrium | Normal |
| Systemic Venous Drainage | To RA | **Atrio-Ventricular Valves** | |
| Pulmonary Venous Drainage | To LA | Mitral Valve | Annulus = 14mm. |
| Atrio-ventricular Connection | Concordant | Tricuspid Valve | Annulus = 14mm |
| Ventriculo-Arterial Connection | Concordant | **Ventricle** | |
| Ventricular Loop | d-Loop | Left Ventricle | Mildly Dilated |
| **Septae** |  | Right Ventricle | Normal |
| Interatrial Septum | Intact | **Doppler Measurement** |  |
| Interventricular Septum | 7mm Subaortic VSD, L – R Shunt | Mitral |  |
| **Semilunar Valves** |  | Aortic |  |
| Aortic Valve | Annulus = 12mm | Tricuspid |  |
| Pulmonary Valve | Annulus = 13mm | Pulmonic | Mild PS, PPG = 27mmHg |
| **Great Arteries** | NRGA | **Coronary Arteries** |  |
| Aorta |  | **Aortic Arch** | Left |
| Pulmonary Arteries |  | **PDA** | 2.5mm PDA, L – R Shunt. |
| **M-Mode**: | | | |
| Ao | mm | PWd | mm |
| LA | mm | EDV | ml |
| LVIDd | mm | ESV | ml |
| LVIDs | mm | FS | % |
| IVSd | mm | LVEF | % |
| **Additional Information:** 3mm Pericardial effusion. | | | |
| **Conclusion:**   1. {S, D, S} Levocardia 2. Moderate Subaortic VSD, L – R Shunt 3. Moderate PDA, L – R Shunt 4. Mild Valvular PS 5. Normal LV Function | | | |
| **Done By:** | **Signature** | **Date** | **Remark** |
| Tesfaye T., Pediatric Cardiologist |  | 19/05/14Eth.C. |  |

| **Patient Name: Yabibal Walle. Sex M Age: M/9month. Date of Report: 19/05/14Eth.C. MRN:115428.**  **Clinical Diagnosis: Incidental Murmur + Diaphoresis during BF. TGSH1.2485.** | | | |
| --- | --- | --- | --- |
| **Features:** | **Findings** | **Features** | **Findings** |
| **Profile** | | **Atria** | |
| Abdominal Situs | Solitus | Left Atrium | Mildly dilated |
| Cardiac Position | Levocardia | Right Atrium | Normal |
| Systemic Venous Drainage | To RA | **Atrio-Ventricular Valves** | |
| Pulmonary Venous Drainage | To LA | Mitral Valve | Annulus = 15mm. |
| Atrio-ventricular Connection | Concordant | Tricuspid Valve | Annulus = 14mm |
| Ventriculo-Arterial Connection | Concordant | **Ventricle** | |
| Ventricular Loop | d-Loop | Left Ventricle | Mildly Dilated |
| **Septae** |  | Right Ventricle | Normal |
| Interatrial Septum | Intact | **Doppler Measurement** |  |
| Interventricular Septum | Intact | Mitral |  |
| **Semilunar Valves** |  | Aortic |  |
| Aortic Valve | Annulus = 10mm | Tricuspid |  |
| Pulmonary Valve | Annulus = 13mm | Pulmonic |  |
| **Great Arteries** | NRGA | **Coronary Arteries** |  |
| Aorta |  | **Aortic Arch** | Left |
| Pulmonary Arteries |  | **PDA** | 2mm PDA, L – R Shunt |
| **M-Mode**: Normal LV Function on eye balling | | | |
| Ao | mm | PWd | mm |
| LA | mm | EDV | ml |
| LVIDd | mm | ESV | ml |
| LVIDs | mm | FS | % |
| IVSd | mm | LVEF | % |
| **Additional Information:** | | | |
| **Conclusion:**   1. {S, D, S} Levocardia 2. Moderate PDA, L – R Shunt 3. Normal LV Function | | | |
| **Done By:** | **Signature** | **Date** | **Remark** |
| Tesfaye T., Pediatric Cardiologist |  | 19/05/14Eth.C. |  |

| **Patient Name: Amanuel Birhanu. Sex/Age: M/3years Date of Report: 19/05/14Eth.C. MRN:113091.**  **Clinical Diagnosis: DS. TGSH1.2486.** | | | |
| --- | --- | --- | --- |
| **Features:** | **Findings** | **Features** | **Findings** |
| **Profile** | | **Atria** | |
| Abdominal Situs | Solitus | Left Atrium | Normal |
| Cardiac Position | Levocardia | Right Atrium | Normal |
| Systemic Venous Drainage | To RA | **Atrio-Ventricular Valves** | |
| Pulmonary Venous Drainage | To LA | Mitral Valve | Annulus = 16mm. |
| Atrio-ventricular Connection | Concordant | Tricuspid Valve | Annulus = 17mm |
| Ventriculo-Arterial Connection | Concordant | **Ventricle** | |
| Ventricular Loop | d-Loop | Left Ventricle | Normal |
| **Septae** |  | Right Ventricle | Normal |
| Interatrial Septum | Intact | **Doppler Measurement** |  |
| Interventricular Septum | Intact | Mitral |  |
| **Semilunar Valves** |  | Aortic |  |
| Aortic Valve | Annulus = 14mm | Tricuspid |  |
| Pulmonary Valve | Annulus = 16mm | Pulmonic |  |
| **Great Arteries** | NRGA | **Coronary Arteries** |  |
| Aorta |  | **Aortic Arch** | Left |
| Pulmonary Arteries |  | **PDA** | No |
| **M-Mode**: Normal LV Function on eye balling | | | |
| Ao | mm | PWd | mm |
| LA | mm | EDV | ml |
| LVIDd | mm | ESV | ml |
| LVIDs | mm | FS | % |
| IVSd | mm | LVEF | % |
| **Additional Information:** | | | |
| **Conclusion:**   1. Normal Echocardiography. | | | |
| **Done By:** | **Signature** | **Date** | **Remark** |
| Tesfaye T., Pediatric Cardiologist |  | 19/05/14Eth.C. |  |

| **Patient Name: Weineshet Atinkut. Sex/Age: F/1year. Date of Report: 19/05/14Eth.C. MRN:115296.**  **Clinical Diagnosis: Diaphoresis + RD + CHF + Murmur. TGSH1.2487.** | | | |
| --- | --- | --- | --- |
| **Features:** | **Findings** | **Features** | **Findings** |
| **Profile** | | **Atria** | |
| Abdominal Situs | Solitus | Left Atrium | Dilated |
| Cardiac Position | Levocardia | Right Atrium | Dilated |
| Systemic Venous Drainage | To RA | **Atrio-Ventricular Valves** | |
| Pulmonary Venous Drainage | To LA | Mitral Valve | Annulus = 16mm. |
| Atrio-ventricular Connection | Concordant | Tricuspid Valve | Annulus = 14mm |
| Ventriculo-Arterial Connection | Concordant | **Ventricle** | |
| Ventricular Loop | d-Loop | Left Ventricle | Dilated |
| **Septae** |  | Right Ventricle | Dilated |
| Interatrial Septum | Intact | **Doppler Measurement** |  |
| Interventricular Septum | Intact | Mitral | Mild TR |
| **Semilunar Valves** |  | Aortic |  |
| Aortic Valve | Annulus = 11mm | Tricuspid | Mild TR, PPG = 65mmHg |
| Pulmonary Valve | Annulus = 15mm | Pulmonic |  |
| **Great Arteries** | NRGA | **Coronary Arteries** |  |
| Aorta |  | **Aortic Arch** | Left |
| Pulmonary Arteries | **MPA = 21mm** | **PDA** | 3mm PDA, L – R Shunt |
| **M-Mode**: Normal LV Function | | | |
| Ao | mm | PWd | mm |
| LA | mm | EDV | ml |
| LVIDd | mm | ESV | ml |
| LVIDs | mm | FS | % |
| IVSd | mm | LVEF | % |
| **Additional Information:** | | | |
| **Conclusion:**   1. {S, D, S} Levocardia 2. LA/LV Dilated 3. Mild TR 4. Large PDA, L – R Shunt 5. Severe Pulmonary Hypertension 6. Normal LV Function | | | |
| **Done By:** | **Signature** | **Date** | **Remark** |
| Tesfaye T., Pediatric Cardiologist |  | 19/05/14Eth.C. |  |

| **Patient Name: Yohannes Derso. Sex/Age: M/83days. Date of Report: 23/05/14Eth.C. MRN:115911.**  **Clinical Diagnosis: Cyanosis + RD. TGSH1.2488.** | | | |
| --- | --- | --- | --- |
| **Features:** | **Findings** | **Features** | **Findings** |
| **Profile** | | **Atria** | |
| Abdominal Situs | Solitus | Left Atrium | Normal |
| Cardiac Position | Levocardia | Right Atrium | Dilated |
| Systemic Venous Drainage | To RA | **Atrio-Ventricular Valves** | |
| Pulmonary Venous Drainage | To LA | Mitral Valve | Annulus = 14mm. |
| Atrio-ventricular Connection | Concordant | Tricuspid Valve | Annulus = 15mm |
| Ventriculo-Arterial Connection | Discordant | **Ventricle** | |
| Ventricular Loop | d-Loop | Left Ventricle | Normal |
| **Septae** |  | Right Ventricle | Dilated |
| Interatrial Septum | PFO, LA - RA | **Doppler Measurement** |  |
| Interventricular Septum | Intact | Mitral |  |
| **Semilunar Valves** |  | Aortic |  |
| Aortic Valve | Annulus = 10mm | Tricuspid |  |
| Pulmonary Valve | Annulus = 9mm | Pulmonic |  |
| **Great Arteries** | d-TGA | **Coronary Arteries** |  |
| Aorta | Anterior and to the right. From RV | **Aortic Arch** | Left |
| Pulmonary Arteries | Posterior and to the left. From LV | **PDA** | 1.5mm PDA, PA – Ao. |
| **M-Mode**: | | | |
| Ao | mm | PWd | mm |
| LA | mm | EDV | ml |
| LVIDd | mm | ESV | ml |
| LVIDs | mm | FS | % |
| IVSd | mm | LVEF | % |
| **Additional Information:** | | | |
| **Conclusion:**   1. {S, D, D} Levocardia 2. PFO, LA – RA Shunt 3. d-TGA with Intact IVS 4. Small PDA, PA – Ao Shunt | | | |
| **Done By:** | **Signature** | **Date** | **Remark** |
| Tesfaye T., Pediatric Cardiologist |  | 23/05/14Eth.C. |  |

| **Patient Name: Senayit Habtamu. Sex/Age: F/6years. Date of Report: 23/05/14Eth.C. MRN:116162.**  **Clinical Diagnosis: ARF + CHF + Murmur + RD. TGSH1.2489.** | | | |
| --- | --- | --- | --- |
| **Features:** | **Findings** | **Features** | **Findings** |
| **Profile** | | **Atria** | |
| Abdominal Situs | Solitus | Left Atrium | Markedly Dilated |
| Cardiac Position | Levocardia | Right Atrium | Normal |
| Systemic Venous Drainage | To RA | **Atrio-Ventricular Valves** | |
| Pulmonary Venous Drainage | To LA | Mitral Valve | Annulus = 22mm. Thickened MVL. Shortened PMVL. |
| Atrio-ventricular Connection | Concordant | Tricuspid Valve | Annulus = 21mm |
| Ventriculo-Arterial Connection | concordant | **Ventricle** | |
| Ventricular Loop | d-Loop | Left Ventricle | Markedly Dilated |
| **Septae** |  | Right Ventricle | Normal |
| Interatrial Septum | Intact | **Doppler Measurement** |  |
| Interventricular Septum | Intact | Mitral | Severe MR, Holosystolic, posterior projection, seen in two planes with jet velocity = 4.2m/sec. Mild MS, PPG/MPG = 10/5mmHg. |
| **Semilunar Valves** |  | Aortic | Mild AR, PHT = 521ms. |
| Aortic Valve | Annulus = 14mm | Tricuspid | Mild TR, PPG = 40mmHg. |
| Pulmonary Valve | Annulus = 17mm | Pulmonic |  |
| **Great Arteries** | NRGA | **Coronary Arteries** |  |
| Aorta |  | **Aortic Arch** | Left |
| Pulmonary Arteries |  | **PDA** | No |
| **M-Mode**: | | | |
| Ao | mm | PWd | mm |
| LA | mm | EDV | ml |
| LVIDd | mm | ESV | ml |
| LVIDs | mm | FS | 25% |
| IVSd | mm | LVEF | 49% |
| **Additional Information:** Pericardial effusion with maximum depth of 5mm on RV Side. | | | |
| **Conclusion:**   1. {S, D, S} Levocardia 2. LA/LV Markedly Dilated 3. Thickened MVL, Shortened PMVL 4. Severe MR 5. Mild MS 6. Mild AR 7. Mild TR 8. Reduced LV Function 9. Small Pericardial effusion. | | | |
| **Done By:** | **Signature** | **Date** | **Remark** |
| Tesfaye T., Pediatric Cardiologist |  | 23/05/14Eth.C. |  |

| **Patient Name: Baby of Emebet Abera. Sex/Age: M/4months. Date of Report: 23/05/14Eth.C. MRN:102737.**  **Clinical Diagnosis: DS. TGSH1.2490.** | | | |
| --- | --- | --- | --- |
| **Features:** | **Findings** | **Features** | **Findings** |
| **Profile** | | **Atria** | |
| Abdominal Situs | Solitus | Left Atrium | Normal |
| Cardiac Position | Levocardia | Right Atrium | Mildly Dilated |
| Systemic Venous Drainage | To RA | **Atrio-Ventricular Valves** | |
| Pulmonary Venous Drainage | To LA | Mitral Valve | Annulus = 11mm. |
| Atrio-ventricular Connection | Concordant | Tricuspid Valve | Annulus = 12mm |
| Ventriculo-Arterial Connection | concordant | **Ventricle** | |
| Ventricular Loop | d-Loop | Left Ventricle | Normal |
| **Septae** |  | Right Ventricle | Mildly Dilated |
| Interatrial Septum | 8 X 9mm OS ASD, L – R Shunt | **Doppler Measurement** |  |
| Interventricular Septum | Intact | Mitral |  |
| **Semilunar Valves** |  | Aortic |  |
| Aortic Valve | Annulus = 11mm | Tricuspid |  |
| Pulmonary Valve | Annulus = 11mm | Pulmonic |  |
| **Great Arteries** | NRGA | **Coronary Arteries** |  |
| Aorta |  | **Aortic Arch** | Left |
| Pulmonary Arteries |  | **PDA** | No |
| **M-Mode**: Normal LV Function on eye balling | | | |
| Ao | mm | PWd | mm |
| LA | mm | EDV | ml |
| LVIDd | mm | ESV | ml |
| LVIDs | mm | FS | % |
| IVSd | mm | LVEF | % |
| **Additional Information:** | | | |
| **Conclusion:**   1. {S, D, S} Levocardia 2. RA/RV Dilated 3. Moderate OS ASD, L – R Shunt 4. Normal LV Function | | | |
| **Done By:** | **Signature** | **Date** | **Remark** |
| Tesfaye T., Pediatric Cardiologist |  | 23/05/14Eth.C. |  |

| **Patient Name: Nardos Shitu. Sex/Age: M/2 6/12. Date of Report: 24/05/14Eth.C. MRN:023026.**  **Clinical Diagnosis: Easy Fatigability + Murmur. TGSH1.2491. (TGSH4)** | | | |
| --- | --- | --- | --- |
| **Features:** | **Findings** | **Features** | **Findings** |
| **Profile** | | **Atria** | |
| Abdominal Situs | Solitus | Left Atrium | Normal |
| Cardiac Position | Levocardia | Right Atrium | Normal |
| Systemic Venous Drainage | To RA | **Atrio-Ventricular Valves** | |
| Pulmonary Venous Drainage | To LA | Mitral Valve | Annulus = 17mm. |
| Atrio-ventricular Connection | Concordant | Tricuspid Valve | Annulus = 20mm |
| Ventriculo-Arterial Connection | concordant | **Ventricle** | |
| Ventricular Loop | d-Loop | Left Ventricle | Normal |
| **Septae** |  | Right Ventricle | Normal |
| Interatrial Septum | Intact | **Doppler Measurement** |  |
| Interventricular Septum | 5mm Subaortic VSD, L – R Shunt | Mitral |  |
| **Semilunar Valves** |  | Aortic |  |
| Aortic Valve | Annulus = 18mm | Tricuspid |  |
| Pulmonary Valve | Annulus = 18mm | Pulmonic |  |
| **Great Arteries** | NRGA | **Coronary Arteries** |  |
| Aorta |  | **Aortic Arch** | Left |
| Pulmonary Arteries |  | **PDA** | No |
| **M-Mode**: Normal LV Function on eye balling | | | |
| Ao | mm | PWd | mm |
| LA | mm | EDV | ml |
| LVIDd | mm | ESV | ml |
| LVIDs | mm | FS | % |
| IVSd | mm | LVEF | % |
| **Additional Information:** | | | |
| **Conclusion:**   1. {S, D, S} Levocardia 2. Small Subaortic VSD, L – R Shunt 3. Normal LV Function | | | |
| **Done By:** | **Signature** | **Date** | **Remark** |
| Tesfaye T., Pediatric Cardiologist |  | 24/05/14Eth.C. |  |

| **Patient Name: Atsede Kelemwork. Sex /Age: F/5 6/12. Date of Report: 24/05/14Eth.C. MRN:_116349.**  **Clinical Diagnosis: ARF + Murmur. TGSH1.2492.** | | | |
| --- | --- | --- | --- |
| **Features:** | **Findings** | **Features** | **Findings** |
| **Profile** | | **Atria** | |
| Abdominal Situs | Solitus | Left Atrium | Dilated |
| Cardiac Position | Levocardia | Right Atrium | Normal |
| Systemic Venous Drainage | To RA | **Atrio-Ventricular Valves** | |
| Pulmonary Venous Drainage | To LA | Mitral Valve | Annulus = 24mm. Thickened MVL. MVA = 1cm2. |
| Atrio-ventricular Connection | Concordant | Tricuspid Valve | Annulus = 21mm |
| Ventriculo-Arterial Connection | concordant | **Ventricle** | |
| Ventricular Loop | d-Loop | Left Ventricle | Dilated |
| **Septae** |  | Right Ventricle | Normal |
| Interatrial Septum | Intact | **Doppler Measurement** |  |
| Interventricular Septum | Intact | Mitral | Moderate MR, Holosystolic, posterior projection, seen in two planes with jet velocity = 4.7m/sec. Moderate MS, PPG/MPG = 12/7mmHg. |
| **Semilunar Valves** |  | Aortic | Mild AR, PHT = 520ms |
| Aortic Valve | Annulus = 15mm. | Tricuspid | Trivial TR, PPG = 26mmHg |
| Pulmonary Valve | Annulus = 19mm | Pulmonic |  |
| **Great Arteries** | NRGA | **Coronary Arteries** |  |
| Aorta |  | **Aortic Arch** | Left |
| Pulmonary Arteries |  | **PDA** | No |
| **M-Mode**: Normal LV Function on eye balling. | | | |
| Ao | mm | PWd | mm |
| LA | mm | EDV | ml |
| LVIDd | mm | ESV | ml |
| LVIDs | mm | FS | % |
| IVSd | mm | LVEF | % |
| **Additional Information:** 4mm Circumferential Pericardial effusion. 22mm Right Pleural effusion. | | | |
| **Conclusion:**   1. {S, D, S} Levocardia 2. LA/LV Dilated 3. Thickened MVL 4. Moderate MR 5. Moderate MS 6. Mild AR 7. Trivial TR 8. Trace Circumferential pericardial effusion 9. Large Right Pleural effusion 10. Normal Systolic LV Function | | | |
| **Done By:** | **Signature** | **Date** | **Remark** |
| Tesfaye T., Pediatric Cardiologist |  | 24/05/14Eth.C. |  |

| **Patient Name: Bereket Melkamu. Sex/Age: M/3 month. Date of Report: 24/05/14Eth.C. MRN:116370.**  **Clinical Diagnosis: Incidental Murmur + Diaphoresis. TGSH1.2493.** | | | |
| --- | --- | --- | --- |
| **Features:** | **Findings** | **Features** | **Findings** |
| **Profile** | | **Atria** | |
| Abdominal Situs | Solitus | Left Atrium | Normal |
| Cardiac Position | Levocardia | Right Atrium | Normal |
| Systemic Venous Drainage | To RA | **Atrio-Ventricular Valves** | |
| Pulmonary Venous Drainage | To LA | Mitral Valve | Annulus = 14mm. |
| Atrio-ventricular Connection | Concordant | Tricuspid Valve | Annulus = 16mm |
| Ventriculo-Arterial Connection | concordant | **Ventricle** | |
| Ventricular Loop | d-Loop | Left Ventricle | Normal |
| **Septae** |  | Right Ventricle | Normal |
| Interatrial Septum | Intact | **Doppler Measurement** |  |
| Interventricular Septum | 5mm Sub-arterial VSD, Nonrestrictive, L – R Shunt | Mitral |  |
| **Semilunar Valves** |  | Aortic |  |
| Aortic Valve | Annulus = 11mm | Tricuspid |  |
| Pulmonary Valve | Annulus = 11mm | Pulmonic |  |
| **Great Arteries** | NRGA | **Coronary Arteries** |  |
| Aorta |  | **Aortic Arch** | Left |
| Pulmonary Arteries |  | **PDA** | No |
| **M-Mode**: Normal LV Function on eye balling. | | | |
| Ao | mm | PWd | mm |
| LA | mm | EDV | ml |
| LVIDd | mm | ESV | ml |
| LVIDs | mm | FS | % |
| IVSd | mm | LVEF | % |
| **Additional Information:** | | | |
| **Conclusion:**   1. {S, D, S} Levocardia 2. Moderate Sub – Arterial Non – Restrictive VSD, L – R Shunt 3. Normal LV Function | | | |
| **Done By:** | **Signature** | **Date** | **Remark** |
| Tesfaye T., Pediatric Cardiologist |  | 24/05/14Eth.C. |  |

| **Patient Name: Baby Tiringo Tamiru. Sex/Age: F/4 days. Date of Report: 24/05/14Eth.C. MRN:116208.**  **Clinical Diagnosis: Incidental Murmur. TGSH1.2494.** | | | |
| --- | --- | --- | --- |
| **Features:** | **Findings** | **Features** | **Findings** |
| **Profile** | | **Atria** | |
| Abdominal Situs | Solitus | Left Atrium | Normal |
| Cardiac Position | Levocardia | Right Atrium | Normal |
| Systemic Venous Drainage | To RA | **Atrio-Ventricular Valves** | |
| Pulmonary Venous Drainage | To LA | Mitral Valve | Annulus = 9mm. aorto-mitral Discontinuity |
| Atrio-ventricular Connection | Concordant | Tricuspid Valve | Annulus = 11mm |
| Ventriculo-Arterial Connection | DORV | **Ventricle** | |
| Ventricular Loop | d-Loop | Left Ventricle | Normal |
| **Septae** |  | Right Ventricle | Normal |
| Interatrial Septum | PFO, L – R Shunt | **Doppler Measurement** |  |
| Interventricular Septum | 11mm Inlet VSD with PM extension, L – R Shunt | Mitral |  |
| **Semilunar Valves** |  | Aortic |  |
| Aortic Valve | Annulus = 12mm | Tricuspid |  |
| Pulmonary Valve | Annulus = 9mm | Pulmonic |  |
| **Great Arteries** | NRGA | **Coronary Arteries** |  |
| Aorta | Posterior and to the right. From RV | **Aortic Arch** | Left |
| Pulmonary Arteries | Anterior and to the left. From RV | **PDA** | 1mm PDA, L – R Shunt |
| **M-Mode**: Normal LV Function on eye balling | | | |
| Ao | mm | PWd | mm |
| LA | mm | EDV | ml |
| LVIDd | mm | ESV | ml |
| LVIDs | mm | FS | % |
| IVSd | mm | LVEF | % |
| **Additional Information:** | | | |
| **Conclusion:**   1. {S, D, D} Levocardia 2. DORV 3. PFO, L – R Shunt 4. Large Inlet VSD with PM extension, L – R Shunt (Doubly committed) 5. Small PDA, L – R Shunt | | | |
| **Done By:** | **Signature** | **Date** | **Remark** |
| Tesfaye T., Pediatric Cardiologist |  | 24/05/14Eth.C. |  |

| **Patient Name: Nathan Ayana. Sex/Age: M/7 Month. Date of Report: 24/05/14Eth.C. MRN:116344.**  **Clinical Diagnosis: Incidental Murmur. TGSH1.2495.** | | | |
| --- | --- | --- | --- |
| **Features:** | **Findings** | **Features** | **Findings** |
| **Profile** | | **Atria** | |
| Abdominal Situs | Solitus | Left Atrium | Normal |
| Cardiac Position | Levocardia | Right Atrium | Markedly Dilated |
| Systemic Venous Drainage | To RA | **Atrio-Ventricular Valves** | |
| Pulmonary Venous Drainage | To LA | Mitral Valve | Annulus = 13mm. |
| Atrio-ventricular Connection | Concordant | Tricuspid Valve | Annulus = 18mm  TAPSE = 11mm |
| Ventriculo-Arterial Connection | concordant | **Ventricle** | |
| Ventricular Loop | d-Loop | Left Ventricle | Normal |
| **Septae** |  | Right Ventricle | Markedly Dilated & Hypertrophied |
| Interatrial Septum | 10 X 6mm OS ASD, R – L Shunt | **Doppler Measurement** |  |
| Interventricular Septum | Intact | Mitral |  |
| **Semilunar Valves** |  | Aortic |  |
| Aortic Valve | Annulus = 12mm | Tricuspid |  |
| Pulmonary Valve | Annulus = 10mm. doming PV | Pulmonic | Severe Valvular PS, PPG = 125mmHg. |
| **Great Arteries** | NRGA | **Coronary Arteries** |  |
| Aorta |  | **Aortic Arch** | Left |
| Pulmonary Arteries |  | **PDA** | No |
| **M-Mode**: Normal LV Function on eye balling | | | |
| Ao | mm | PWd | mm |
| LA | mm | EDV | ml |
| LVIDd | mm | ESV | ml |
| LVIDs | mm | FS | % |
| IVSd | mm | LVEF | % |
| **Additional Information:** | | | |
| **Conclusion:**   1. {S, D, S} Levocardia 2. RA/RV Markedly Dilated, RV Hypertrophied 3. Moderate to large OS ASD, R – L Shunt 4. Severe Valvular PS 5. Normal Biventricular Function | | | |
| **Done By:** | **Signature** | **Date** | **Remark** |
| Tesfaye T., Pediatric Cardiologist |  | 24/05/14Eth.C. |  |

| **Patient Name: Bemnet Bamlaku . Sex/Age: F/5 month. Date of Report: 24/05/14Eth.C. MRN:106840.**  **Clinical Diagnosis: DS. TGSH1.2496.** | | | |
| --- | --- | --- | --- |
| **Features:** | **Findings** | **Features** | **Findings** |
| **Profile** | | **Atria** | |
| Abdominal Situs | Solitus | Left Atrium | Normal |
| Cardiac Position | Levocardia | Right Atrium | Normal |
| Systemic Venous Drainage | To RA | **Atrio-Ventricular Valves** | |
| Pulmonary Venous Drainage | To LA | Mitral Valve | Annulus = mm. |
| Atrio-ventricular Connection | Concordant | Tricuspid Valve | Annulus = mm |
| Ventriculo-Arterial Connection | concordant | **Ventricle** | |
| Ventricular Loop | d-Loop | Left Ventricle | Normal |
| **Septae** |  | Right Ventricle | Normal |
| Interatrial Septum | Intact | **Doppler Measurement** |  |
| Interventricular Septum | Intact | Mitral |  |
| **Semilunar Valves** |  | Aortic |  |
| Aortic Valve | Annulus = mm | Tricuspid |  |
| Pulmonary Valve | Annulus = mm | Pulmonic |  |
| **Great Arteries** | NRGA | **Coronary Arteries** |  |
| Aorta |  | **Aortic Arch** | Left |
| Pulmonary Arteries |  | **PDA** | No |
| **M-Mode**: | | | |
| Ao | mm | PWd | mm |
| LA | mm | EDV | ml |
| LVIDd | mm | ESV | ml |
| LVIDs | mm | FS | % |
| IVSd | mm | LVEF | % |
| **Additional Information:** | | | |
| **Conclusion:**   1. {S, D, S} Levocardia | | | |
| **Done By:** | **Signature** | **Date** | **Remark** |
| Tesfaye T., Pediatric Cardiologist |  | 24/05/14Eth.C. |  |

| **Patient Name: Nardos Addisu. Sex/Age: F/4 years. Date of Report: 26/05/14Eth.C. MRN:008004.**  **Follow up Echocardiography for Myocarditis.** | | | |
| --- | --- | --- | --- |
| **Features:** | **Findings** | **Features** | **Findings** |
| **Profile** | | **Atria** | |
| Abdominal Situs | Solitus | Left Atrium | Normal |
| Cardiac Position | Levocardia | Right Atrium | Normal |
| Systemic Venous Drainage | To RA | **Atrio-Ventricular Valves** | |
| Pulmonary Venous Drainage | To LA | Mitral Valve | Annulus = 19mm. |
| Atrio-ventricular Connection | Concordant | Tricuspid Valve | Annulus = 21mm  TAPSE = 18mm |
| Ventriculo-Arterial Connection | concordant | **Ventricle** | |
| Ventricular Loop | d-Loop | Left Ventricle | Normal |
| **Septae** |  | Right Ventricle | Normal |
| Interatrial Septum | Intact | **Doppler Measurement** |  |
| Interventricular Septum | Intact | Mitral |  |
| **Semilunar Valves** |  | Aortic |  |
| Aortic Valve | Annulus = 14mm | Tricuspid |  |
| Pulmonary Valve | Annulus = 16mm | Pulmonic |  |
| **Great Arteries** | NRGA | **Coronary Arteries** |  |
| Aorta |  | **Aortic Arch** | Left |
| Pulmonary Arteries |  | **PDA** | No |
| **M-Mode**: | | | |
| Ao | mm | PWd | mm |
| LA | mm | EDV | ml |
| LVIDd | mm | ESV | ml |
| LVIDs | mm | FS | 30% |
| IVSd | mm | LVEF | 59% |
| **Additional Information:** the Features of Myocarditis has resolved. | | | |
| **Conclusion:**   1. Normal Echocardiography Study | | | |
| **Done By:** | **Signature** | **Date** | **Remark** |
| Tesfaye T., Pediatric Cardiologist |  | 24/05/14Eth.C. |  |

| **Patient Name: Baby shega Dessie. Sex/Age: F/11 Days. Date of Report: 26/05/14Eth.C. MRN:115412.**  **Clinical Diagnosis: Incidental Murmur. TGSH1.2497.** | | | |
| --- | --- | --- | --- |
| **Features:** | **Findings** | **Features** | **Findings** |
| **Profile** | | **Atria** | |
| Abdominal Situs | Solitus | Left Atrium | Normal |
| Cardiac Position | Levocardia | Right Atrium | Normal |
| Systemic Venous Drainage | To RA | **Atrio-Ventricular Valves** | |
| Pulmonary Venous Drainage | To LA | Mitral Valve | Annulus = 8mm. |
| Atrio-ventricular Connection | Concordant | Tricuspid Valve | Annulus = 8mm |
| Ventriculo-Arterial Connection | concordant | **Ventricle** | |
| Ventricular Loop | d-Loop | Left Ventricle | Normal |
| **Septae** |  | Right Ventricle | Normal |
| Interatrial Septum | PFO, L – R Shunt | **Doppler Measurement** |  |
| Interventricular Septum | Intact | Mitral |  |
| **Semilunar Valves** |  | Aortic |  |
| Aortic Valve | Annulus = 7mm | Tricuspid |  |
| Pulmonary Valve | Annulus = 7mm | Pulmonic |  |
| **Great Arteries** | NRGA | **Coronary Arteries** |  |
| Aorta |  | **Aortic Arch** | Left |
| Pulmonary Arteries |  | **PDA** | 1.5mm PDA, L – R Shunt. |
| **M-Mode**: Normal LV Function on eye balling | | | |
| Ao | mm | PWd | mm |
| LA | mm | EDV | ml |
| LVIDd | mm | ESV | ml |
| LVIDs | mm | FS | % |
| IVSd | mm | LVEF | % |
| **Additional Information:** | | | |
| **Conclusion:**   1. {S, D, S} Levocardia 2. PFO, L – R Shunt 3. Small PDA, L – R Shunt 4. Normal LV Function on eye balling | | | |
| **Done By:** | **Signature** | **Date** | **Remark** |
| Tesfaye T., Pediatric Cardiologist |  | 24/05/14Eth.C. |  |

| **Patient Name: B/Habtam Twin A. Sex/Age: F/10 Days. Date of Report: 26/05/14Eth.C. MRN: 115548**  **Clinical Diagnosis: incidental Murmur + DS. TGSH1.2498.** | | | |
| --- | --- | --- | --- |
| **Features:** | **Findings** | **Features** | **Findings** |
| **Profile** | | **Atria** | |
| Abdominal Situs | Solitus | Left Atrium | Dilated |
| Cardiac Position | Levocardia | Right Atrium | Normal |
| Systemic Venous Drainage | To RA | **Atrio-Ventricular Valves** | |
| Pulmonary Venous Drainage | To LA | Mitral Valve | Annulus = 10mm. |
| Atrio-ventricular Connection | Concordant | Tricuspid Valve | Annulus = 8mm |
| Ventriculo-Arterial Connection | concordant | **Ventricle** | |
| Ventricular Loop | d-Loop | Left Ventricle | Dilated |
| **Septae** |  | Right Ventricle | Normal |
| Interatrial Septum | 5mm OS ASD, BD Shunt | **Doppler Measurement** |  |
| Interventricular Septum | Intact | Mitral | Trivial MR |
| **Semilunar Valves** |  | Aortic |  |
| Aortic Valve | Annulus = 7mm | Tricuspid | Mild TR |
| Pulmonary Valve | Annulus = 7mm | Pulmonic |  |
| **Great Arteries** | NRGA | **Coronary Arteries** |  |
| Aorta |  | **Aortic Arch** | Left |
| Pulmonary Arteries |  | **PDA** | 2mm PDA, L – R Shunt |
| **M-Mode**: | | | |
| Ao | mm | PWd | mm |
| LA | mm | EDV | ml |
| LVIDd | mm | ESV | ml |
| LVIDs | mm | FS | 32% |
| IVSd | mm | LVEF | 65% |
| **Additional Information:** | | | |
| **Conclusion:**   1. {S, D, S} Levocardia 2. All Chambers Dilated 3. Small OS ASD, BD Shunt 4. Trivial MR 5. Mild TR 6. Moderate PDA, L – R Shunt | | | |
| **Done By:** | **Signature** | **Date** | **Remark** |
| Tesfaye T., Pediatric Cardiologist |  | 26/05/14Eth.C. |  |

| **Patient Name: Yikeber Yibel Sex/Age: M/11 years. Date of Report: 01/06/14Eth.C. MRN:026680.**  **Clinical Diagnosis: CHF + DOE. TGSH1.2499.** | | | |
| --- | --- | --- | --- |
| **Features:** | **Findings** | **Features** | **Findings** |
| **Profile** | | **Atria** | |
| Abdominal Situs | Solitus | Left Atrium | Dilated |
| Cardiac Position | Levocardia | Right Atrium | Dilated |
| Systemic Venous Drainage | To RA | **Atrio-Ventricular Valves** | |
| Pulmonary Venous Drainage | To LA | Mitral Valve | Annulus = 20mm. E/A = 1.6. MV DT = 123ms. |
| Atrio-ventricular Connection | Concordant | Tricuspid Valve | Annulus = 20mm  TAPSE = 17mm |
| Ventriculo-Arterial Connection | concordant | **Ventricle** | |
| Ventricular Loop | d-Loop | Left Ventricle | Normal |
| **Septae** |  | Right Ventricle | Normal |
| Interatrial Septum | Intact | **Doppler Measurement** |  |
| Interventricular Septum | Intact | Mitral |  |
| **Semilunar Valves** |  | Aortic |  |
| Aortic Valve | Annulus = 15mm | Tricuspid |  |
| Pulmonary Valve | Annulus = 18mm | Pulmonic |  |
| **Great Arteries** | NRGA | **Coronary Arteries** |  |
| Aorta |  | **Aortic Arch** | Left |
| Pulmonary Arteries |  | **PDA** | No |
| **M-Mode**: | | | |
| Ao | mm | PWd | mm |
| LA | mm | EDV | ml |
| LVIDd | mm | ESV | ml |
| LVIDs | mm | FS | 27% |
| IVSd | mm | LVEF | 54% |
| **Additional Information:** Hyper-echoic Pericardium. | | | |
| **Conclusion:**   1. {S, D, S} Levocardia 2. Severe LV Diastolic Dysfunction 3. Mild LV Systolic Dysfunction 4. Hyper-echoic Pericardium secondary to ? | | | |
| **Done By:** | **Signature** | **Date** | **Remark** |
| Tesfaye T., Pediatric Cardiologist |  | 01/06/14Eth.C. |  |

| **Patient Name: Habitemariam Tadesse. Sex/Age: M/1years. Date of Report:01/06/14Eth.C. MRN:116714**  **Clinical Diagnosis: DS + CHF + RD. TGSH1.2500.** | | | |
| --- | --- | --- | --- |
| **Features:** | **Findings** | **Features** | **Findings** |
| **Profile** | | **Atria** | |
| Abdominal Situs | Solitus | Left Atrium | Dilated |
| Cardiac Position | Levocardia | Right Atrium | Dilated |
| Systemic Venous Drainage | To RA | **Atrio-Ventricular Valves** | |
| Pulmonary Venous Drainage | To LA | Mitral Valve | Common Complete AVSD |
| Atrio-ventricular Connection | Common Complete AVSD | Tricuspid Valve |  |
| Ventriculo-Arterial Connection | concordant | **Ventricle** | |
| Ventricular Loop | d-Loop | Left Ventricle | Dilated |
| **Septae** |  | Right Ventricle | Dilated |
| Interatrial Septum | Common Complete AVSD, L – R Shunt | **Doppler Measurement** |  |
| Interventricular Septum |  | Mitral | Moderate Left AVVR |
| **Semilunar Valves** |  | Aortic |  |
| Aortic Valve | Annulus = 9mm | Tricuspid |  |
| Pulmonary Valve | Annulus = 13mm | Pulmonic |  |
| **Great Arteries** | NRGA | **Coronary Arteries** |  |
| Aorta |  | **Aortic Arch** | Left |
| Pulmonary Arteries | MPA = 20mm | **PDA** | No |
| **M-Mode**: Normal LV Function on eye balling | | | |
| Ao | mm | PWd | mm |
| LA | mm | EDV | ml |
| LVIDd | mm | ESV | ml |
| LVIDs | mm | FS | % |
| IVSd | mm | LVEF | % |
| **Additional Information:** | | | |
| **Conclusion:**   1. {S, D, S} Levocardia 2. Common Complete Balanced AVSD, L – R Shunt 3. Severe Pulmonary Hypertension | | | |
| **Done By:** | **Signature** | **Date** | **Remark** |
| Tesfaye T., Pediatric Cardiologist |  | 01/06/14Eth.C. |  |

| **Patient Name: Abeba Gebiya. Sex/Age: F/8years. Date of Report: 01/06/14Eth.C. MRN:117214.**  **Clinical Diagnosis: Acute Rheumatic Fever + DOE + Palpitation + Murmur. TGSH1.2501.** | | | |
| --- | --- | --- | --- |
| **Features:** | **Findings** | **Features** | **Findings** |
| **Profile** | | **Atria** | |
| Abdominal Situs | Solitus | Left Atrium | Markedly Dilated. 58 X 68mm. |
| Cardiac Position | Levocardia | Right Atrium | Normal |
| Systemic Venous Drainage | To RA | **Atrio-Ventricular Valves** | |
| Pulmonary Venous Drainage | To LA | Mitral Valve | Annulus = 28mm. Thickened MVL. Shortened PMVL. |
| Atrio-ventricular Connection | Concordant | Tricuspid Valve | Annulus = 23mm. TAPSE = 17mm |
| Ventriculo-Arterial Connection | concordant | **Ventricle** | |
| Ventricular Loop | d-Loop | Left Ventricle | Dilated |
| **Septae** |  | Right Ventricle | Normal |
| Interatrial Septum | Intact | **Doppler Measurement** |  |
| Interventricular Septum | Intact | Mitral | Severe MR, Holosystolic, posterior projection, seen in two planes with jet velocity = 5m/sec. |
| **Semilunar Valves** |  | Aortic | Moderate AR, PHT = 220ms. |
| Aortic Valve | Annulus = 15mm | Tricuspid | Mild TR, PPG = 26mmHg. |
| Pulmonary Valve | Annulus = 19mm | Pulmonic |  |
| **Great Arteries** | NRGA | **Coronary Arteries** |  |
| Aorta |  | **Aortic Arch** | Left |
| Pulmonary Arteries |  | **PDA** | No |
| **M-Mode**: | | | |
| Ao | mm | PWd | mm |
| LA | mm | EDV | ml |
| LVIDd | mm | ESV | ml |
| LVIDs | mm | FS | 27% |
| IVSd | mm | LVEF | 53% |
| **Additional Information:** Circumferential Pericardial effusion with max. depth of 18mm on RA/RV Junction. | | | |
| **Conclusion:**   1. {S, D, S} Levocardia 2. LA/LV Dilated 3. Thickened MVL, Shortened PMVL 4. Severe MR 5. Moderate AR 6. Mild TR 7. Mildly Reduced LV Function 8. Moderate Pericardial effusion | | | |
| **Done By:** | **Signature** | **Date** | **Remark** |
| Tesfaye T., Pediatric Cardiologist |  | 01/06/14Eth.C. |  |

| **Patient Name: Theophlos Yhenew Sex/Age: M/1 years. Date of Report: 01/06/14Eth.C. MRN:067212.**  **Clinical Diagnosis: FTT + Diaphoresis + Murmur. TGSH1.2502.** | | | |
| --- | --- | --- | --- |
| **Features:** | **Findings** | **Features** | **Findings** |
| **Profile** | | **Atria** | |
| Abdominal Situs | Solitus | Left Atrium | Dilated |
| Cardiac Position | Levocardia | Right Atrium | Dilated |
| Systemic Venous Drainage | To RA | **Atrio-Ventricular Valves** | |
| Pulmonary Venous Drainage | To LA | Mitral Valve | Annulus = 13mm. |
| Atrio-ventricular Connection | Concordant | Tricuspid Valve | Annulus = 15mm |
| Ventriculo-Arterial Connection | concordant | **Ventricle** | |
| Ventricular Loop | d-Loop | Left Ventricle | Dilated |
| **Septae** |  | Right Ventricle | Dilated |
| Interatrial Septum | Intact | **Doppler Measurement** |  |
| Interventricular Septum | 11mm PM VSD, L – R Shunt. | Mitral |  |
| **Semilunar Valves** |  | Aortic |  |
| Aortic Valve | Annulus = 13mm | Tricuspid |  |
| Pulmonary Valve | Annulus = 14mm. Dysplastic | Pulmonic | Severe Valvular PS, PPG = 72mmHg. Moderate PR, PPG = 48mmHg |
| **Great Arteries** | NRGA | **Coronary Arteries** |  |
| Aorta |  | **Aortic Arch** | Left |
| Pulmonary Arteries | MPA = 25mm | **PDA** | 1.5mm PDA |
| **M-Mode**: Normal LV Function on eye balling | | | |
| Ao | mm | PWd | mm |
| LA | mm | EDV | ml |
| LVIDd | mm | ESV | ml |
| LVIDs | mm | FS | % |
| IVSd | mm | LVEF | % |
| **Additional Information:** | | | |
| **Conclusion:**   1. {S, D, S} Levocardia 2. Large PM VSD, L – R Shunt 3. Dysplastic Pulmonary Valve 4. Severe PS 5. Moderate PR 6. Small PDA, L – R Shunt | | | |
| **Done By:** | **Signature** | **Date** | **Remark** |
| Tesfaye T., Pediatric Cardiologist |  | 01/06/14Eth.C. |  |

| **Patient Name: Yostina Demewez. Sex/Age: F/1 9/12 years. Date of Report: 01/06/14Eth.C. MRN:108137.**  **Clinical Diagnosis: IE + RD. TGSH1.2503.** | | | |
| --- | --- | --- | --- |
| **Features:** | **Findings** | **Features** | **Findings** |
| **Profile** | | **Atria** | |
| Abdominal Situs | Solitus | Left Atrium | Normal |
| Cardiac Position | Levocardia | Right Atrium | Normal |
| Systemic Venous Drainage | To RA | **Atrio-Ventricular Valves** | |
| Pulmonary Venous Drainage | To LA | Mitral Valve | Annulus = 15mm. |
| Atrio-ventricular Connection | Concordant | Tricuspid Valve | Annulus = 17mm  TAPSE = 15mm |
| Ventriculo-Arterial Connection | concordant | **Ventricle** | |
| Ventricular Loop | d-Loop | Left Ventricle | Normal |
| **Septae** |  | Right Ventricle | Normal |
| Interatrial Septum | 10mm Primum defect, L – R Shunt | **Doppler Measurement** |  |
| Interventricular Septum | 2mm Restrictive Inlet VSD, L – R Shunt | Mitral | Mild MR |
| **Semilunar Valves** |  | Aortic |  |
| Aortic Valve | Annulus = 10mm | Tricuspid | Trivial TR |
| Pulmonary Valve | Annulus = 14mm | Pulmonic |  |
| **Great Arteries** | NRGA | **Coronary Arteries** |  |
| Aorta |  | **Aortic Arch** | Left |
| Pulmonary Arteries |  | **PDA** | No |
| **M-Mode**: | | | |
| Ao | mm | PWd | mm |
| LA | mm | EDV | ml |
| LVIDd | mm | ESV | ml |
| LVIDs | mm | FS | 34% |
| IVSd | mm | LVEF | 65% |
| **Additional Information:** | | | |
| **Conclusion:**   1. {S, D, S} Levocardia 2. Transitional AVSD, L – R Shunt 3. Normal LV Systolic Function | | | |
| **Remark:** | No echocardiographic Clue suggestive of IE. | | |
| **Recommendation:** | Correlate with your clinical finding. | | |
| **Done By:** | **Signature** | **Date** | **Remark** |
| Tesfaye T., Pediatric Cardiologist |  | 01/06/14Eth.C. |  |

| **Patient Name: Sera Lakew. Sex/Age: F/4 years. Date of Report: 03/06/14Eth.C. MRN:117574.**  **Clinical Diagnosis: Pre-op Screening. TGSH1.2504.** | | | |
| --- | --- | --- | --- |
| **Features:** | **Findings** | **Features** | **Findings** |
| **Profile** | | **Atria** | |
| Abdominal Situs | Solitus | Left Atrium | Normal |
| Cardiac Position | Levocardia | Right Atrium | Normal |
| Systemic Venous Drainage | To RA | **Atrio-Ventricular Valves** | |
| Pulmonary Venous Drainage | To LA | Mitral Valve | Annulus = 17mm. |
| Atrio-ventricular Connection | Concordant | Tricuspid Valve | Annulus = 19mm  TAPSE = 16mm |
| Ventriculo-Arterial Connection | concordant | **Ventricle** | |
| Ventricular Loop | d-Loop | Left Ventricle | Normal |
| **Septae** |  | Right Ventricle | Normal |
| Interatrial Septum | Intact | **Doppler Measurement** |  |
| Interventricular Septum | Intact | Mitral |  |
| **Semilunar Valves** |  | Aortic |  |
| Aortic Valve | Annulus = 12mm | Tricuspid | Trivial TR, PPG = 25mmHg |
| Pulmonary Valve | Annulus = 16mm | Pulmonic |  |
| **Great Arteries** | NRGA | **Coronary Arteries** |  |
| Aorta |  | **Aortic Arch** | Left |
| Pulmonary Arteries |  | **PDA** | No |
| **M-Mode**: | | | |
| Ao | mm | PWd | mm |
| LA | mm | EDV | ml |
| LVIDd | mm | ESV | ml |
| LVIDs | mm | FS | 34% |
| IVSd | mm | LVEF | 65% |
| **Additional Information:** | | | |
| **Conclusion:**   1. Normal Echocardiography Study | | | |
| **Done By:** | **Signature** | **Date** | **Remark** |
| Tesfaye T., Pediatric Cardiologist |  | 03/06/14Eth.C. |  |

| **Patient Name: Rahel Abebe Sex/Age: F/8 month. Date of Report: 03/06/14Eth.C. MRN:176118.**  **Clinical Diagnosis: DS + RD + Murmur. TGSH1.2505.** | | | |
| --- | --- | --- | --- |
| **Features:** | **Findings** | **Features** | **Findings** |
| **Profile** | | **Atria** | |
| Abdominal Situs | Solitus | Left Atrium | Dilated |
| Cardiac Position | Levocardia | Right Atrium | Dilated |
| Systemic Venous Drainage | To RA | **Atrio-Ventricular Valves** | |
| Pulmonary Venous Drainage | To LA | Mitral Valve | Annulus = 16mm. |
| Atrio-ventricular Connection | Concordant | Tricuspid Valve | Annulus = 15mm  TAPSE = 15mm |
| Ventriculo-Arterial Connection | concordant | **Ventricle** | |
| Ventricular Loop | d-Loop | Left Ventricle | Dilated |
| **Septae** |  | Right Ventricle | Dilated |
| Interatrial Septum | PFO, L – R Shunt | **Doppler Measurement** |  |
| Interventricular Septum | 9mm Inlet VSD, L – R Shunt | Mitral |  |
| **Semilunar Valves** |  | Aortic |  |
| Aortic Valve | Annulus = 12mm | Tricuspid |  |
| Pulmonary Valve | Annulus = 18mm | Pulmonic |  |
| **Great Arteries** | NRGA | **Coronary Arteries** |  |
| Aorta |  | **Aortic Arch** | Left |
| Pulmonary Arteries | **MPA = 19mm** | **PDA** | No |
| **M-Mode**: | | | |
| Ao | mm | PWd | mm |
| LA | mm | EDV | ml |
| LVIDd | mm | ESV | ml |
| LVIDs | mm | FS | 39% |
| IVSd | mm | LVEF | 72% |
| **Additional Information:** | | | |
| **Conclusion:**   1. {S, D, S} Levocardia 2. All chambers Dilated 3. PFO, L – R Shunt 4. Large Inlet VSD, L – R Shunt 5. Severe Pulmonary Hypertension (Left Heart Failure) 6. Normal Biventricular Systolic Function | | | |
| **Done By:** | **Signature** | **Date** | **Remark** |
| Tesfaye T., Pediatric Cardiologist |  | 03/06/14Eth.C. |  |

| **Patient Name: Baby of Abeba Sitota. Sex/Age: F/3days. Date of Report: 04/06/14Eth.C. MRN:_______.**  **Clinical Diagnosis: RD. TGSH1.2506.** | | | |
| --- | --- | --- | --- |
| **Features:** | **Findings** | **Features** | **Findings** |
| **Profile** | | **Atria** | |
| Abdominal Situs | Solitus | Left Atrium | Normal |
| Cardiac Position | Levocardia | Right Atrium | Normal |
| Systemic Venous Drainage | To RA | **Atrio-Ventricular Valves** | |
| Pulmonary Venous Drainage | To LA | Mitral Valve | Annulus = 9mm. |
| Atrio-ventricular Connection | Concordant | Tricuspid Valve | Annulus = 9mm |
| Ventriculo-Arterial Connection | concordant | **Ventricle** | |
| Ventricular Loop | d-Loop | Left Ventricle | Normal |
| **Septae** |  | Right Ventricle | Normal |
| Interatrial Septum | PFO, L – R Shunt | **Doppler Measurement** |  |
| Interventricular Septum | Intact | Mitral |  |
| **Semilunar Valves** |  | Aortic |  |
| Aortic Valve | Annulus = 9mm | Tricuspid |  |
| Pulmonary Valve | Annulus = 9mm | Pulmonic |  |
| **Great Arteries** | NRGA | **Coronary Arteries** |  |
| Aorta |  | **Aortic Arch** | Left |
| Pulmonary Arteries | Normal MPA & BPAs. | **PDA** | No |
| **M-Mode**: Normal LV Function on eye balling | | | |
| Ao | mm | PWd | mm |
| LA | mm | EDV | ml |
| LVIDd | mm | ESV | ml |
| LVIDs | mm | FS | % |
| IVSd | mm | LVEF | % |
| **Additional Information:** | | | |
| **Conclusion:**   1. {S, D, S} Levocardia 2. PFO, L – R Shunt 3. Normal Systolic LV Function | | | |
| **Done By:** | **Signature** | **Date** | **Remark** |
| Tesfaye T., Pediatric Cardiologist |  | 04/06/14Eth.C. |  |

| **Patient Name: Siraye Ebabu Sex/Age: F / 1 6/12 years. Date of Report: 08/06/14Eth.C. MRN:117904.**  **Clinical Diagnosis: DS. TGSH1.2507.** | | | |
| --- | --- | --- | --- |
| **Features:** | **Findings** | **Features** | **Findings** |
| **Profile** | | **Atria** | |
| Abdominal Situs | Solitus | Left Atrium | Normal |
| Cardiac Position | Levocardia | Right Atrium | Normal |
| Systemic Venous Drainage | To RA | **Atrio-Ventricular Valves** | |
| Pulmonary Venous Drainage | To LA | Mitral Valve | Annulus = 12mm. |
| Atrio-ventricular Connection | Concordant | Tricuspid Valve | Annulus = 15mm |
| Ventriculo-Arterial Connection | concordant | **Ventricle** | |
| Ventricular Loop | d-Loop | Left Ventricle | Normal |
| **Septae** |  | Right Ventricle | Normal |
| Interatrial Septum | Intact | **Doppler Measurement** |  |
| Interventricular Septum | Intact | Mitral |  |
| **Semilunar Valves** |  | Aortic |  |
| Aortic Valve | Annulus = 12mm | Tricuspid |  |
| Pulmonary Valve | Annulus = 14mm | Pulmonic |  |
| **Great Arteries** | NRGA | **Coronary Arteries** |  |
| Aorta |  | **Aortic Arch** | Left |
| Pulmonary Arteries |  | **PDA** | <1mm PDA, L – R Shunt. |
| **M-Mode**: | | | |
| Ao | mm | PWd | mm |
| LA | mm | EDV | ml |
| LVIDd | mm | ESV | ml |
| LVIDs | mm | FS | % |
| IVSd | mm | LVEF | % |
| **Additional Information:** Pericardial effusion with maximum depth of 3mm on RV Side. | | | |
| **Conclusion:**   1. {S, D, S} Levocardia 2. Small PDA (Silent PDA), L – R Shunt 3. Trace Pericardial effusion | | | |
| **Remark:** on Clinical evaluation No Murmur. | | | |
| **Done By:** | **Signature** | **Date** | **Remark** |
| Tesfaye T., Pediatric Cardiologist |  | 08/06/14Eth.C. |  |

| **Patient Name: Kalkidan Tamiru . Sex/Age: F/10 months. Date of Report: 08/06/14Eth.C. MRN:117707.**  **Clinical Diagnosis: DS. TGSH1.2508.** | | | |
| --- | --- | --- | --- |
|  | | | |
| **Features:** | **Findings** | **Features** | **Findings** |
| **Profile** | | **Atria** | |
| Abdominal Situs | Solitus | Left Atrium | Normal |
| Cardiac Position | Levocardia | Right Atrium | Normal |
| Systemic Venous Drainage | To RA | **Atrio-Ventricular Valves** | |
| Pulmonary Venous Drainage | To LA | Mitral Valve | Annulus = 11mm. |
| Atrio-ventricular Connection | Concordant | Tricuspid Valve | Annulus = 13mm  TAPSE = 15mm |
| Ventriculo-Arterial Connection | concordant | **Ventricle** | |
| Ventricular Loop | d-Loop | Left Ventricle | Normal |
| **Septae** |  | Right Ventricle | Normal |
| Interatrial Septum | 7mm OS ASD, L – R Shunt. 9mm protrusion of fossa ovalis to left and 6mm protrusion to RA. | **Doppler Measurement** |  |
| Interventricular Septum | Intact | Mitral |  |
| **Semilunar Valves** |  | Aortic |  |
| Aortic Valve | Annulus = 10mm | Tricuspid |  |
| Pulmonary Valve | Annulus = 10mm | Pulmonic |  |
| **Great Arteries** | NRGA | **Coronary Arteries** |  |
| Aorta |  | **Aortic Arch** | Left |
| Pulmonary Arteries |  | **PDA** | No |
| **M-Mode**: Normal LV Function on eye balling | | | |
| Ao | mm | PWd | mm |
| LA | mm | EDV | ml |
| LVIDd | mm | ESV | ml |
| LVIDs | mm | FS | % |
| IVSd | mm | LVEF | % |
| **Additional Information:** | | | |
| **Conclusion:**   1. {S, D, S} Levocardia 2. Moderate OS ASD, L – R Shunt 3. Type 2 Fossa Ovalis Atrial Septal Aneurysm 4. Normal Biventricular Systolic Function | | | |
| **Done By:** | **Signature** | **Date** | **Remark** |
| Tesfaye T., Pediatric Cardiologist |  | 04/06/14Eth.C. |  |

| **Patient Name: Abaynew Mulatie. Sex/Age: M/2 2/12 years. Date of Report: 08/06/14Eth.C. MRN: 118104.**  **Clinical Diagnosis: CHF + Murmur. TGSH1.2509.** | | | |
| --- | --- | --- | --- |
| **Features:** | **Findings** | **Features** | **Findings** |
| **Profile** | | **Atria** | |
| Abdominal Situs | Solitus | Left Atrium | Dilated |
| Cardiac Position | Levocardia | Right Atrium | Normal |
| Systemic Venous Drainage | To RA | **Atrio-Ventricular Valves** | |
| Pulmonary Venous Drainage | To LA | Mitral Valve | Annulus = 18mm. |
| Atrio-ventricular Connection | Concordant | Tricuspid Valve | Annulus = 18mm  TAPSE = 15mm |
| Ventriculo-Arterial Connection | concordant | **Ventricle** | |
| Ventricular Loop | d-Loop | Left Ventricle | Dilated |
| **Septae** |  | Right Ventricle | Normal |
| Interatrial Septum | Intact | **Doppler Measurement** |  |
| Interventricular Septum | Intact | Mitral | Moderate MR, Jet velocity = 4m/sec, Holosystolic, Central Projection |
| **Semilunar Valves** |  | Aortic |  |
| Aortic Valve | Annulus = 13mm | Tricuspid | Mild TR |
| Pulmonary Valve | Annulus = 15mm | Pulmonic |  |
| **Great Arteries** | NRGA | **Coronary Arteries** | Arising from Aortic Sinus. |
| Aorta |  | **Aortic Arch** | Left. No CoA |
| Pulmonary Arteries |  | **PDA** | No |
| **M-Mode**: | | | |
| Ao | mm | PWd | 6mm |
| LA | mm | EDV | 63ml |
| LVIDd | 38mm | ESV | 40ml |
| LVIDs | 32mm | FS | 17% |
| IVSd | 5mm | LVEF | 36% |
| **Additional Information:** | | | |
| **Conclusion:**   1. {S, D, S} Levocardia 2. LA/LV Dilated 3. Moderate MR 4. Mild TR 5. Moderately reduced LV Function | | | |
| **Remark:** No ALCAPA, No AS, No CoA. | | | |
| **Done By:** | **Signature** | **Date** | **Remark** |
| Tesfaye T., Pediatric Cardiologist |  | 04/06/14Eth.C. |  |

| **Patient Name: Yewagnesh Agegnehu Sex/Age: F/7 years. Date of Report: 08/06/14Eth.C. MRN:118135.**  **Clinical Diagnosis: Rheumatic Recurrence + CHF + Murmur. TGSH1.2510.** | | | | | | | | |
| --- | --- | --- | --- | --- | --- | --- | --- | --- |
| **Features:** | | **Findings** | | **Features** | | **Findings** | | |
| **Profile** | | | | **Atria** | | | | |
| Abdominal Situs | | Solitus | | Left Atrium | | Dilated | | |
| Cardiac Position | | Levocardia | | Right Atrium | | Normal | | |
| Systemic Venous Drainage | | To RA | | **Atrio-Ventricular Valves** | | | | |
| Pulmonary Venous Drainage | | To LA | | Mitral Valve | | Annulus = 26mm. Thickened, clubbed MVL. MVA = 1.1cm2. | | |
| Atrio-ventricular Connection | | Concordant | | Tricuspid Valve | | Annulus = 19mm  TAPSE = 18mm | | |
| Ventriculo-Arterial Connection | | concordant | | **Ventricle** | | | | |
| Ventricular Loop | | d-Loop | | Left Ventricle | | Dilated | | |
| **Septae** | |  | | Right Ventricle | | Normal | | |
| Interatrial Septum | | Intact | | **Doppler Measurement** | |  | | |
| Interventricular Septum | | Intact | | Mitral | | Severe MR, Holosystolic, posterior projection, seen in two planes with jet velocity = 4m/sec. Moderate MS, PPG/MPG = 16/8mmHg. | | |
| **Semilunar Valves** | |  | | Aortic | |  | | |
| Aortic Valve | | Annulus = 14mm | | Tricuspid | | Moderate TR, PPG = 44mmHg. | | |
| Pulmonary Valve | | Annulus = 17mm | | Pulmonic | |  | | |
| **Great Arteries** | | NRGA | | **Coronary Arteries** | |  | | |
| Aorta | |  | | **Aortic Arch** | | Left | | |
| Pulmonary Arteries | |  | | **PDA** | | No | | |
| **M-Mode**: | | | | | | | | |
| Ao | | mm | | PWd | | mm | | |
| LA | | mm | | EDV | | ml | | |
| LVIDd | | mm | | ESV | | ml | | |
| LVIDs | | mm | | FS | | 27% | | |
| IVSd | | mm | | LVEF | | 53% | | |
| **Additional Information:** Pericardial effusion with maximum depth of 5mm on RA/RV Junction. | | | | | | | | |
| **Conclusion:**   1. {S, D, S} Levocardia 2. LA/LV Dilated 3. Thickened, clubbed MVL 4. Severe MR 5. Moderate MS 6. Moderate TR 7. Small Pericardial effusion 8. Mildly Reduced LV Systolic Function | | | | | | | | |
| **Done By:** | | **Signature** | | **Date** | | **Remark** | | |
| Tesfaye T., Pediatric Cardiologist | |  | | 04/06/14Eth.C. | |  | | |
| **Patient Name: B/ Kebebushe Tilaye Sex/Age: M/24 days Date of Report: 08/06/14Eth.C. MRN: 118280**  **Clinical Diagnosis: Cyanosis + Murmur. TGSH1.2511.** | | | | | | |
| **Features:** | | **Findings** | | **Features** | | **Findings** |
| **Profile** | | | | **Atria** | | |
| Abdominal Situs | | Solitus | | Left Atrium | | Normal |
| Cardiac Position | | Levocardia | | Right Atrium | | Normal |
| Systemic Venous Drainage | | To RA | | **Atrio-Ventricular Valves** | | |
| Pulmonary Venous Drainage | | To LA | | Mitral Valve | | Annulus = 13mm. |
| Atrio-ventricular Connection | | Concordant | | Tricuspid Valve | | Annulus = 11mm |
| Ventriculo-Arterial Connection | | Discordant | | **Ventricle** | | |
| Ventricular Loop | | d-Loop | | Left Ventricle | | Normal |
| **Septae** | |  | | Right Ventricle | | Normal |
| Interatrial Septum | | 6mm OS ASD, L – R Shunt | | **Doppler Measurement** | |  |
| Interventricular Septum | | 4mm Mid Muscular VSD, only on 2D. No color flow | | Mitral | |  |
| **Semilunar Valves** | |  | | Aortic | |  |
| Aortic Valve | | Annulus = 8mm | | Tricuspid | |  |
| Pulmonary Valve | | Annulus = 13mm | | Pulmonic | |  |
| **Great Arteries** | | d-TGA | | **Coronary Arteries** | |  |
| Aorta | | Anterior and to the right. From RV | | **Aortic Arch** | | Left |
| Pulmonary Arteries | | Posterior and to the left. From LV | | **PDA** | | No |
| **M-Mode**: Normal LV Function on eye balling | | | | | | |
| Ao | | mm | | PWd | | mm |
| LA | | mm | | EDV | | ml |
| LVIDd | | mm | | ESV | | ml |
| LVIDs | | mm | | FS | | % |
| IVSd | | mm | | LVEF | | % |
| **Additional Information:** | | | | | | |
| **Conclusion:**   1. {S, D, D} Levocardia 2. Small OS ASD, L – R Shunt 3. d-TGA 4. Small Mid Muscular VSD (2D Defect) | | | | | | |
| **Done By:** | | **Signature** | | **Date** | | **Remark** |
| Tesfaye T., Pediatric Cardiologist | |  | | 04/06/14Eth.C. | |  |

| **Patient Name: Yabibal Enchalew. Sex/Age: M/6 years. Date of Report: 08/06/14Eth.C. MRN:117730.**  **Clinical Diagnosis: Pre-Op Screening. TGSH1.2512.** | | | |
| --- | --- | --- | --- |
| **Features:** | **Findings** | **Features** | **Findings** |
| **Profile** | | **Atria** | |
| Abdominal Situs | Solitus | Left Atrium | Normal |
| Cardiac Position | Levocardia | Right Atrium | Normal |
| Systemic Venous Drainage | To RA | **Atrio-Ventricular Valves** | |
| Pulmonary Venous Drainage | To LA | Mitral Valve | Annulus = 19mm. |
| Atrio-ventricular Connection | Concordant | Tricuspid Valve | Annulus = 20mm  TAPSE = 20mm |
| Ventriculo-Arterial Connection | concordant | **Ventricle** | |
| Ventricular Loop | d-Loop | Left Ventricle | Normal |
| **Septae** |  | Right Ventricle | Normal |
| Interatrial Septum | Intact | **Doppler Measurement** |  |
| Interventricular Septum | Intact | Mitral |  |
| **Semilunar Valves** |  | Aortic |  |
| Aortic Valve | Annulus = 16mm | Tricuspid |  |
| Pulmonary Valve | Annulus = 20mm | Pulmonic |  |
| **Great Arteries** | NRGA | **Coronary Arteries** |  |
| Aorta |  | **Aortic Arch** | Left |
| Pulmonary Arteries |  | **PDA** | No |
| **M-Mode**: Normal LV Function on eye balling. | | | |
| Ao | mm | PWd | mm |
| LA | mm | EDV | ml |
| LVIDd | mm | ESV | ml |
| LVIDs | mm | FS | % |
| IVSd | mm | LVEF | % |
| **Additional Information:** | | | |
| **Conclusion:**   1. Normal Echocardiography Study | | | |
| **Done By:** | **Signature** | **Date** | **Remark** |
| Tesfaye T., Pediatric Cardiologist |  | 08/06/14Eth.C. |  |

| **Patient Name: B/Aniley Sex/Age: F/7days . Date of Report: 08/06/14Eth.C. MRN: .**  **Clinical Diagnosis: RD. TGSH1.2513.** | | | |
| --- | --- | --- | --- |
|  | | | |
| **Features:** | **Findings** | **Features** | **Findings** |
| **Profile** | | **Atria** | |
| Abdominal Situs | Solitus | Left Atrium | Normal |
| Cardiac Position | Levocardia | Right Atrium | Normal |
| Systemic Venous Drainage | To RA | **Atrio-Ventricular Valves** | |
| Pulmonary Venous Drainage | To LA | Mitral Valve | Annulus = mm. |
| Atrio-ventricular Connection | Concordant | Tricuspid Valve | Annulus = mm |
| Ventriculo-Arterial Connection | concordant | **Ventricle** | |
| Ventricular Loop | d-Loop | Left Ventricle | Normal |
| **Septae** |  | Right Ventricle | Normal |
| Interatrial Septum | Intact | **Doppler Measurement** |  |
| Interventricular Septum | Intact | Mitral |  |
| **Semilunar Valves** |  | Aortic |  |
| Aortic Valve | Annulus = mm | Tricuspid |  |
| Pulmonary Valve | Annulus = mm | Pulmonic |  |
| **Great Arteries** | NRGA | **Coronary Arteries** |  |
| Aorta |  | **Aortic Arch** | Left |
| Pulmonary Arteries |  | **PDA** | No |
| **M-Mode**: | | | |
| Ao | mm | PWd | mm |
| LA | mm | EDV | ml |
| LVIDd | mm | ESV | ml |
| LVIDs | mm | FS | % |
| IVSd | mm | LVEF | % |
| **Additional Information:** | | | |
| **Conclusion:**   1. {S, D, S} Levocardia | | | |
| **Done By:** | **Signature** | **Date** | **Remark** |
| Tesfaye T., Pediatric Cardiologist |  | 08/06/14Eth.C. |  |

| **Patient Name: Bezawit Derbew Sex/Age: F/40days. Date of Report: 10/06/14Eth.C. MRN: 118110 .**  **Clinical Diagnosis: Incidental Murmur. TGSH1.2514.** | | | |
| --- | --- | --- | --- |
|  | | | |
| **Features:** | **Findings** | **Features** | **Findings** |
| **Profile** | | **Atria** | |
| Abdominal Situs | Solitus | Left Atrium | Normal |
| Cardiac Position | Levocardia | Right Atrium | Normal |
| Systemic Venous Drainage | To RA | **Atrio-Ventricular Valves** | |
| Pulmonary Venous Drainage | To LA | Mitral Valve | Annulus = 11mm. |
| Atrio-ventricular Connection | Concordant | Tricuspid Valve | Annulus = 12mm |
| Ventriculo-Arterial Connection | concordant | **Ventricle** | |
| Ventricular Loop | d-Loop | Left Ventricle | Normal |
| **Septae** |  | Right Ventricle | Normal |
| Interatrial Septum | PFO, L – R Shunt | **Doppler Measurement** |  |
| Interventricular Septum | Intact | Mitral |  |
| **Semilunar Valves** |  | Aortic |  |
| Aortic Valve | Annulus = 8mm | Tricuspid |  |
| Pulmonary Valve | Annulus = 8mm | Pulmonic | Mild Valvular PS, PPG = 26mmHg |
| **Great Arteries** | NRGA | **Coronary Arteries** |  |
| Aorta |  | **Aortic Arch** | Left |
| Pulmonary Arteries |  | **PDA** | No |
| **M-Mode**: Normal LV Function on eye balling | | | |
| Ao | mm | PWd | mm |
| LA | mm | EDV | ml |
| LVIDd | mm | ESV | ml |
| LVIDs | mm | FS | % |
| IVSd | mm | LVEF | % |
| **Additional Information:** | | | |
| **Conclusion:**   1. {S, D, S} Levocardia 2. PFO, L – R Shunt 3. Mild Valvular PS | | | |
| **Done By:** | **Signature** | **Date** | **Remark** |
| Tesfaye T., Pediatric Cardiologist |  | 10/06/14Eth.C. |  |

| **Patient Name: Agegnehush Jember Sex/Age: F /2years. Date of Report: 10/06/14Eth.C. MRN: 118435**  **Clinical Diagnosis: DS + RD + Recurrent Chest Infection. TGSH1.2515.** | | | |
| --- | --- | --- | --- |
| **Features:** | **Findings** | **Features** | **Findings** |
| **Profile** | | **Atria** | |
| Abdominal Situs | Solitus | Left Atrium | Dilated |
| Cardiac Position | Levocardia | Right Atrium | Dilated |
| Systemic Venous Drainage | To RA | **Atrio-Ventricular Valves** | |
| Pulmonary Venous Drainage | To LA | Mitral Valve | Annulus = 14mm. |
| Atrio-ventricular Connection | Concordant | Tricuspid Valve | Annulus = 19mm |
| Ventriculo-Arterial Connection | concordant | **Ventricle** | |
| Ventricular Loop | d-Loop | Left Ventricle | Dilated |
| **Septae** | Tongue of tissue in b/n | Right Ventricle | Dilated |
| Interatrial Septum | 14mm Primum defect, L – R Shunt. Additional PFO, L – R Shunt | **Doppler Measurement** |  |
| Interventricular Septum | 9mm Inlet VSD, L – R Shunt | Mitral | Moderate to severe MR |
| **Semilunar Valves** |  | Aortic |  |
| Aortic Valve | Annulus = 11mm | Tricuspid | Moderate to severe TR |
| Pulmonary Valve | Annulus = 13mm | Pulmonic |  |
| **Great Arteries** | NRGA | **Coronary Arteries** |  |
| Aorta |  | **Aortic Arch** | Left |
| Pulmonary Arteries | MPA = 16mm | **PDA** | No |
| **M-Mode**: Normal LV Function | | | |
| Ao | mm | PWd | mm |
| LA | mm | EDV | ml |
| LVIDd | mm | ESV | ml |
| LVIDs | mm | FS | % |
| IVSd | mm | LVEF | % |
| **Additional Information:** | | | |
| **Conclusion:**   1. {S, D, S} Levocardia 2. RA/RV Dilated 3. PFO, L – R Shunt 4. Intermediate AVSD, L – R Shunt 5. Moderate to severe MR 6. Moderate to severe TR 7. Pulmonary Hypertension 8. Normal LV Function | | | |
| **Done By:** | **Signature** | **Date** | **Remark** |
| Tesfaye T., Pediatric Cardiologist |  | 10/06/14Eth.C. |  |

| **Patient Name: B/ Lubaba Nuru Sex/Age: M/17 days Date of Report: 10/06/14Eth.C. MRN: 116528**  **Clinical Diagnosis: DS. TGSH1.2516.** | | | |
| --- | --- | --- | --- |
|  | | | |
| **Features:** | **Findings** | **Features** | **Findings** |
| **Profile** | | **Atria** | |
| Abdominal Situs | Solitus | Left Atrium | Normal |
| Cardiac Position | Levocardia | Right Atrium | Normal |
| Systemic Venous Drainage | To RA | **Atrio-Ventricular Valves** | |
| Pulmonary Venous Drainage | To LA | Mitral Valve | Annulus = 10mm. |
| Atrio-ventricular Connection | Concordant | Tricuspid Valve | Annulus = 11mm |
| Ventriculo-Arterial Connection | concordant | **Ventricle** | |
| Ventricular Loop | d-Loop | Left Ventricle | Normal |
| **Septae** |  | Right Ventricle | Normal |
| Interatrial Septum | PFO, L – R Shunt | **Doppler Measurement** |  |
| Interventricular Septum | Intact | Mitral |  |
| **Semilunar Valves** |  | Aortic |  |
| Aortic Valve | Annulus = 9mm | Tricuspid |  |
| Pulmonary Valve | Annulus = 9mm | Pulmonic |  |
| **Great Arteries** | NRGA | **Coronary Arteries** |  |
| Aorta |  | **Aortic Arch** | Left |
| Pulmonary Arteries |  | **PDA** | No |
| **M-Mode**: | | | |
| Ao | mm | PWd | mm |
| LA | mm | EDV | ml |
| LVIDd | mm | ESV | ml |
| LVIDs | mm | FS | % |
| IVSd | mm | LVEF | % |
| **Additional Information:** | | | |
| **Conclusion:**   1. {S, D, S} Levocardia 2. PFO, L – R Shunt | | | |
| **Done By:** | **Signature** | **Date** | **Remark** |
| Tesfaye T., Pediatric Cardiologist |  | 10/06/14Eth.C. |  |

| **Patient Name: Toweled Moges S ex/Age: M/5Months. Date of Report:10/06/14Eth.C. MRN: 118436.**  **Clinical Diagnosis: Recurrent Chest Infection + DS + RD. TGSH1.2517.** | | | |
| --- | --- | --- | --- |
|  | | | |
| **Features:** | **Findings** | **Features** | **Findings** |
| **Profile** | | **Atria** | |
| Abdominal Situs | Solitus | Left Atrium | Normal |
| Cardiac Position | Levocardia | Right Atrium | Normal |
| Systemic Venous Drainage | To RA | **Atrio-Ventricular Valves** | |
| Pulmonary Venous Drainage | To LA | Mitral Valve | Annulus = 8mm. |
| Atrio-ventricular Connection | Concordant | Tricuspid Valve | Annulus = 11mm |
| Ventriculo-Arterial Connection | concordant | **Ventricle** | |
| Ventricular Loop | d-Loop | Left Ventricle | Normal |
| **Septae** | Tongue of tissue in b/n | Right Ventricle | Normal |
| Interatrial Septum | 7mm Primum defect, L – R Shunt | **Doppler Measurement** |  |
| Interventricular Septum | 6mm Inlet VSD, L – R Shunt | Mitral |  |
| **Semilunar Valves** |  | Aortic |  |
| Aortic Valve | Annulus = 9mm | Tricuspid |  |
| Pulmonary Valve | Annulus = 9mm | Pulmonic |  |
| **Great Arteries** | NRGA | **Coronary Arteries** |  |
| Aorta |  | **Aortic Arch** | Left |
| Pulmonary Arteries |  | **PDA** | No |
| **M-Mode**: | | | |
| Ao | mm | PWd | mm |
| LA | mm | EDV | ml |
| LVIDd | mm | ESV | ml |
| LVIDs | mm | FS | % |
| IVSd | mm | LVEF | % |
| **Additional Information:** | | | |
| **Conclusion:**   1. {S, D, S} Levocardia 2. Intermediate AVSD, L – R Shunt | | | |
| **Done By:** | **Signature** | **Date** | **Remark** |
| Tesfaye T., Pediatric Cardiologist |  | 10/06/14Eth.C. |  |

| **Patient Name: Demeke Zewdie Sex/Age: M/1, 1/12. Date of Report 10/06/14Eth.C. MRN: 118484 .**  **Clinical Diagnosis: Recurrent Chest Infection. TGSH1.2518.** | | | |
| --- | --- | --- | --- |
|  | | | |
| **Features:** | **Findings** | **Features** | **Findings** |
| **Profile** | | **Atria** | |
| Abdominal Situs | Solitus | Left Atrium | Normal |
| Cardiac Position | Levocardia | Right Atrium | Normal |
| Systemic Venous Drainage | To RA | **Atrio-Ventricular Valves** | |
| Pulmonary Venous Drainage | To LA | Mitral Valve | Annulus = 15mm. |
| Atrio-ventricular Connection | Concordant | Tricuspid Valve | Annulus = 16mm  TAPSE = 13mm |
| Ventriculo-Arterial Connection | concordant | **Ventricle** | |
| Ventricular Loop | d-Loop | Left Ventricle | Normal |
| **Septae** |  | Right Ventricle | Normal |
| Interatrial Septum | Intact | **Doppler Measurement** |  |
| Interventricular Septum | Intact | Mitral |  |
| **Semilunar Valves** |  | Aortic |  |
| Aortic Valve | Annulus = 12mm | Tricuspid |  |
| Pulmonary Valve | Annulus = 14mm | Pulmonic |  |
| **Great Arteries** | NRGA | **Coronary Arteries** |  |
| Aorta |  | **Aortic Arch** | Left |
| Pulmonary Arteries |  | **PDA** | No |
| **M-Mode**: Normal LV Function on eye balling | | | |
| Ao | mm | PWd | mm |
| LA | mm | EDV | ml |
| LVIDd | mm | ESV | ml |
| LVIDs | mm | FS | % |
| IVSd | mm | LVEF | % |
| **Additional Information:** | | | |
| **Conclusion:**   1. Normal Echocardiography Study | | | |
| **Done By:** | **Signature** | **Date** | **Remark** |
| Tesfaye T., Pediatric Cardiologist |  | 10/06/14Eth.C. |  |

| **Patient Name: B/Teje Alemenew Sex/Age: F/33days . Date of Report: 10/06/14Eth.C. MRN: 113036.**  **Clinical Diagnosis: RD + DS. TGSH1.2519.** | | | |
| --- | --- | --- | --- |
|  | | | |
| **Features:** | **Findings** | **Features** | **Findings** |
| **Profile** | | **Atria** | |
| Abdominal Situs | Solitus | Left Atrium | Normal |
| Cardiac Position | Levocardia | Right Atrium | Mildly dilated |
| Systemic Venous Drainage | To RA | **Atrio-Ventricular Valves** | |
| Pulmonary Venous Drainage | To LA | Mitral Valve | Annulus = 9mm. |
| Atrio-ventricular Connection | Concordant | Tricuspid Valve | Annulus = 10mm  TAPSE = 11mm |
| Ventriculo-Arterial Connection | concordant | **Ventricle** | |
| Ventricular Loop | d-Loop | Left Ventricle | Normal |
| **Septae** |  | Right Ventricle | Mildly dilated |
| Interatrial Septum | 6mm OS ASD, L – R Shunt | **Doppler Measurement** |  |
| Interventricular Septum | Intact | Mitral |  |
| **Semilunar Valves** |  | Aortic |  |
| Aortic Valve | Annulus = mm | Tricuspid | Mild TR, PPG = 52mmHg |
| Pulmonary Valve | Annulus = 8mm | Pulmonic | Moderate PR, PPG = 54mmHg |
| **Great Arteries** | NRGA | **Coronary Arteries** |  |
| Aorta |  | **Aortic Arch** | Left |
| Pulmonary Arteries | MPA = 13mm | **PDA** | No |
| **M-Mode**: Normal LV Function on eye balling | | | |
| Ao | mm | PWd | mm |
| LA | mm | EDV | ml |
| LVIDd | mm | ESV | ml |
| LVIDs | mm | FS | % |
| IVSd | mm | LVEF | % |
| **Additional Information:** | | | |
| **Conclusion:**   1. {S, D, S} Levocardia 2. Small OS ASD, L – R Shunt 3. Mild TR 4. Moderate PR 5. Moderate Pulmonary Hypertension | | | |
| **Done By:** | **Signature** | **Date** | **Remark** |
| Tesfaye T., Pediatric Cardiologist |  | 10/06/14Eth.C. |  |

| **Patient Name: Abebe Worket. Sex/Age: M/13yrs . Date of Report: 15/06/14Eth.C. MRN: 118883.**  **Clinical Diagnosis: IE + Rheumatic Recurrence + CHF + Murmur + DOE + Palpitation. TGSH1.2520.** | | | | |
| --- | --- | --- | --- | --- |
| **Features:** | **Findings** | **Features** | | **Findings** |
| **Profile** | | **Atria** | | |
| Abdominal Situs | Solitus | Left Atrium | | Dilated. Oscillating mass attached to the Anterior MVL. |
| Cardiac Position | Levocardia | Right Atrium | | Dilated |
| Systemic Venous Drainage | To RA | **Atrio-Ventricular Valves** | | |
| Pulmonary Venous Drainage | To LA | Mitral Valve | | Annulus = 21mm. Thickened MVL. Shortened PMVL. Oscillating mass arising from AMVL. MVA = 0.9cm2. |
| Atrio-ventricular Connection | Concordant | Tricuspid Valve | | Annulus = 23mm. TAPSE = 24mm |
| Ventriculo-Arterial Connection | concordant | **Ventricle** | | |
| Ventricular Loop | d-Loop | Left Ventricle | | Dilated |
| **Septae** |  | Right Ventricle | | Dilated |
| Interatrial Septum | Intact | **Doppler Measurement** | |  |
| Interventricular Septum | Intact | Mitral | | Severe MR, Holosystolic, posterior projection, seen in two planes with jet velocity = 4.2m/sec. Severe MS, PPG/MPG = 26/16mmHg. |
| **Semilunar Valves** |  | Aortic | | Moderate AR, PHT = 428ms. |
| Aortic Valve | Annulus = 17mm. | Tricuspid | | Moderate TR, PPG = 65mmHg |
| Pulmonary Valve | Annulus = 22mm | Pulmonic | | Mild PR, PPG = 60mmHg |
| **Great Arteries** | NRGA | **Coronary Arteries** | |  |
| Aorta |  | **Aortic Arch** | | Left |
| Pulmonary Arteries | MPA = 24mm | **PDA** | | No |
| **M-Mode**: | | | | |
| Ao | mm | PWd | | mm |
| LA | mm | EDV | | ml |
| LVIDd | mm | ESV | | ml |
| LVIDs | mm | FS | | 38% |
| IVSd | mm | LVEF | | 67% |
| **Additional Information:** | | | | |
| **Conclusion:**   1. {S, D, S} Levocardia 2. All chambers dilated 3. Thickened MVL and Aortic valve leaflet. Shortened PMVL 4. Hyperechoic Oscillating mass in the LA attached to the AMVL 5. Severe MR | | | 1. Severe MS 2. Moderate TR 3. Moderate AR 4. Severe Pulmonary Hypertension 5. Normal Biventricular Systolic Function | |
| **Done By:** | **Signature** | **Date** | | **Remark** |
| Tesfaye T., Pediatric Cardiologist |  | 15/06/14Eth.C. | |  |

| **Patient Name: Dagim Sigermegne Sex/Age: M/7 month. Date of Report: 15/06/14Eth.C. MRN: 100006**  **Clinical Diagnosis: DS + Murmur. TGSH1.2522.** | | | |
| --- | --- | --- | --- |
|  | | | |
| **Features:** | **Findings** | **Features** | **Findings** |
| **Profile** | | **Atria** | |
| Abdominal Situs | Solitus | Left Atrium | Normal |
| Cardiac Position | Levocardia | Right Atrium | Dilated |
| Systemic Venous Drainage | To RA | **Atrio-Ventricular Valves** | |
| Pulmonary Venous Drainage | To LA | Mitral Valve | Annulus = 9mm |
| Atrio-ventricular Connection | Concordant | Tricuspid Valve | Annulus = 13mm |
| Ventriculo-Arterial Connection | concordant | **Ventricle** | |
| Ventricular Loop | d-Loop | Left Ventricle | Normal |
| **Septae** | Tongue of tissue in b/n | Right Ventricle | Dilated |
| Interatrial Septum | 16mm Primum defect, L – R Shunt | **Doppler Measurement** |  |
| Interventricular Septum | 2mm Inlet VSD, L – R Shunt | Mitral |  |
| **Semilunar Valves** |  | Aortic |  |
| Aortic Valve | Annulus = 12mm | Tricuspid | Trivial TR |
| Pulmonary Valve | Annulus = 12mm | Pulmonic |  |
| **Great Arteries** | NRGA | **Coronary Arteries** |  |
| Aorta |  | **Aortic Arch** | Left |
| Pulmonary Arteries |  | **PDA** | No |
| **M-Mode**: Normal LV Function on eye balling | | | |
| Ao | mm | PWd | mm |
| LA | mm | EDV | ml |
| LVIDd | mm | ESV | ml |
| LVIDs | mm | FS | % |
| IVSd | mm | LVEF | % |
| **Additional Information:** | | | |
| **Conclusion:**   1. {S, D, S} Levocardia 2. Transitional AVSD, L – R Shunt | | | |
| **Done By:** | **Signature** | **Date** | **Remark** |
| Tesfaye T., Pediatric Cardiologist |  | 15/06/14Eth.C. |  |

| **Patient Name: Tseganesh Workie. Sex/Age: F/2 6/12years. Date of Report: 15/06/14Eth.C. MRN: 017334 .**  **Clinical Diagnosis: Incidental Murmur. TGSH1.2522.** | | | |
| --- | --- | --- | --- |
|  | | | |
| **Features:** | **Findings** | **Features** | **Findings** |
| **Profile** | | **Atria** | |
| Abdominal Situs | Solitus | Left Atrium | Mildly Dilated |
| Cardiac Position | Levocardia | Right Atrium | Normal |
| Systemic Venous Drainage | To RA | **Atrio-Ventricular Valves** | |
| Pulmonary Venous Drainage | To LA | Mitral Valve | Annulus = 17mm |
| Atrio-ventricular Connection | Concordant | Tricuspid Valve | Annulus = 16mm  TAPSE = 16mm |
| Ventriculo-Arterial Connection | concordant | **Ventricle** | |
| Ventricular Loop | d-Loop | Left Ventricle | Mildly dilated |
| **Septae** |  | Right Ventricle | Normal |
| Interatrial Septum | Intact | **Doppler Measurement** |  |
| Interventricular Septum | Intact | Mitral |  |
| **Semilunar Valves** |  | Aortic |  |
| Aortic Valve | Annulus = 14mm | Tricuspid |  |
| Pulmonary Valve | Annulus = 16mm | Pulmonic |  |
| **Great Arteries** | NRGA | **Coronary Arteries** |  |
| Aorta |  | **Aortic Arch** | Left |
| Pulmonary Arteries |  | **PDA** | 2mm PDA, L – R Shunt |
| **M-Mode**: Normal LV Function on eye balling | | | |
| Ao | mm | PWd | mm |
| LA | mm | EDV | ml |
| LVIDd | mm | ESV | ml |
| LVIDs | mm | FS | % |
| IVSd | mm | LVEF | % |
| **Additional Information:** | | | |
| **Conclusion:**   1. {S, D, S} Levocardia 2. Moderate PDA, L – R Shunt | | | |
| **Done By:** | **Signature** | **Date** | **Remark** |
| Tesfaye T., Pediatric Cardiologist |  | 15/06/14Eth.C. |  |

| **Patient Name: Felegush Endshaw. Sex/Age: F/8years. Date of Report: 15/06/14Eth.C. MRN: 118682.**  **Clinical Diagnosis: Rheumatic Recurrence + DOE + Murmur. TGSH1.2523.** | | | |
| --- | --- | --- | --- |
|  | | | |
| **Features:** | **Findings** | **Features** | **Findings** |
| **Profile** | | **Atria** | |
| Abdominal Situs | Solitus | Left Atrium | Dilated |
| Cardiac Position | Levocardia | Right Atrium | Normal |
| Systemic Venous Drainage | To RA | **Atrio-Ventricular Valves** | |
| Pulmonary Venous Drainage | To LA | Mitral Valve | Annulus = 29mm. thickened MVL. |
| Atrio-ventricular Connection | Concordant | Tricuspid Valve | Annulus = 20mm. TAPSE = 19mm |
| Ventriculo-Arterial Connection | concordant | **Ventricle** | |
| Ventricular Loop | d-Loop | Left Ventricle | Dilated |
| **Septae** |  | Right Ventricle | Normal |
| Interatrial Septum | Intact | **Doppler Measurement** |  |
| Interventricular Septum | Intact | Mitral | Severe MR, Holosystolic, posterior projection, seen in two planes with jet velocity = 4.3m/sec. |
| **Semilunar Valves** |  | Aortic | Trivial AR |
| Aortic Valve | Annulus = 18mm | Tricuspid | Mild TR, PPG = 34mmHg |
| Pulmonary Valve | Annulus = 20mm | Pulmonic | Mild TR, PPG = 23mmHg |
| **Great Arteries** | NRGA | **Coronary Arteries** |  |
| Aorta |  | **Aortic Arch** | Left |
| Pulmonary Arteries |  | **PDA** | No |
| **M-Mode**: | | | |
| Ao | mm | PWd | mm |
| LA | mm | EDV | ml |
| LVIDd | mm | ESV | ml |
| LVIDs | mm | FS | 30% |
| IVSd | mm | LVEF | 57% |
| **Additional Information:** | | | |
| **Conclusion:**   1. {S, D, S} Levocardia 2. LA/LV Dilated 3. Thickened MVL 4. Severe MR 5. Mild TR 6. Trivial AR 7. Normal Biventricular Systolic Function | | | |
| **Done By:** | **Signature** | **Date** | **Remark** |
| Tesfaye T., Pediatric Cardiologist |  | 15/06/14Eth.C. |  |

| **Patient Name: Tigist Tadele. Sex/Age: F/12years. Date of Report: 17/06/14Eth.C. MRN: 114372.**  **Clinical Diagnosis: _Rheumatic Fever + Murmur. TGSH1.2524.** | | | |
| --- | --- | --- | --- |
| **Features:** | **Findings** | **Features** | **Findings** |
| **Profile** | | **Atria** | |
| Abdominal Situs | Solitus | Left Atrium | Dilated |
| Cardiac Position | Levocardia | Right Atrium | Normal |
| Systemic Venous Drainage | To RA | **Atrio-Ventricular Valves** | |
| Pulmonary Venous Drainage | To LA | Mitral Valve | Annulus = 28mm. Patulous MVL. |
| Atrio-ventricular Connection | Concordant | Tricuspid Valve | Annulus = 21mm. TAPSE = 20mm |
| Ventriculo-Arterial Connection | concordant | **Ventricle** | |
| Ventricular Loop | d-Loop | Left Ventricle | Dilated |
| **Septae** |  | Right Ventricle | Normal |
| Interatrial Septum | Intact | **Doppler Measurement** |  |
| Interventricular Septum | Intact | Mitral | Severe MR, Holosystolic, Posterior Projection, seen in two planes, jet velocity = 4.8m/sec |
| **Semilunar Valves** |  | Aortic | Trivial AR |
| Aortic Valve | Annulus = 17mm | Tricuspid | Trivial TR, PPG = 24mmHg |
| Pulmonary Valve | Annulus = 23mm | Pulmonic |  |
| **Great Arteries** | NRGA | **Coronary Arteries** |  |
| Aorta |  | **Aortic Arch** | Left |
| Pulmonary Arteries | MPA = 19mm | **PDA** | No |
| **M-Mode**: | | | |
| Ao | mm | PWd | mm |
| LA | mm | EDV | ml |
| LVIDd | mm | ESV | ml |
| LVIDs | mm | FS | 32% |
| IVSd | mm | LVEF | 60% |
| **Additional Information:** | | | |
| **Conclusion:**   1. {S, D, S} Levocardia 2. LA/LV Dilated 3. Patulous MVL 4. Severe MR 5. Trivial AR 6. Trivial TR 7. Normal Biventricular Systolic Function | | | |
| **Done By:** | **Signature** | **Date** | **Remark** |
| Tesfaye T., Pediatric Cardiologist |  | 17/06/14Eth.C. |  |

| **Patient Name: Habtamu Feten. Sex/Age: M/10 years. Date of Report: 17/06/14Eth.C. MRN: 119419.**  **Clinical Diagnosis: Sydenham’s Chorea. TGSH1.2525.** | | | |
| --- | --- | --- | --- |
|  | | | |
| **Features:** | **Findings** | **Features** | **Findings** |
| **Profile** | | **Atria** | |
| Abdominal Situs | Solitus | Left Atrium | Normal |
| Cardiac Position | Levocardia | Right Atrium | Normal |
| Systemic Venous Drainage | To RA | **Atrio-Ventricular Valves** | |
| Pulmonary Venous Drainage | To LA | Mitral Valve | Annulus = 22mm |
| Atrio-ventricular Connection | Concordant | Tricuspid Valve | Annulus = 25mm  TAPSE = 21mm |
| Ventriculo-Arterial Connection | concordant | **Ventricle** | |
| Ventricular Loop | d-Loop | Left Ventricle | Normal |
| **Septae** |  | Right Ventricle | Normal |
| Interatrial Septum | Intact | **Doppler Measurement** |  |
| Interventricular Septum | Intact | Mitral |  |
| **Semilunar Valves** |  | Aortic |  |
| Aortic Valve | Annulus = 15mm | Tricuspid | Trivial TR, PPG = 28mmHg |
| Pulmonary Valve | Annulus = 19mm | Pulmonic |  |
| **Great Arteries** | NRGA | **Coronary Arteries** |  |
| Aorta |  | **Aortic Arch** | Left |
| Pulmonary Arteries | MPA = 19mm | **PDA** | No |
| **M-Mode**: | | | |
| Ao | mm | PWd | mm |
| LA | mm | EDV | ml |
| LVIDd | mm | ESV | ml |
| LVIDs | mm | FS | 37% |
| IVSd | mm | LVEF | 68% |
| **Additional Information:** pericardial effusion measuring maximum depth of 8mm on RV Side. | | | |
| **Conclusion:**   1. {S, D, S} Levocardia 2. Small Pericardial effusion 3. Normal Biventricular Systolic Function | | | |
| **Done By:** | **Signature** | **Date** | **Remark** |
| Tesfaye T., Pediatric Cardiologist |  | 17/06/14Eth.C. |  |

| **Patient Name: Kalkidan Desalegn. Sex/Age: F/1 9/12. Date of Report: 18/06/14Eth.C. MRN: 119047.**  **Clinical Diagnosis: DS + Incidental Murmur. TGSH1.2526.** | | | |
| --- | --- | --- | --- |
| **Features:** | **Findings** | **Features** | **Findings** |
| **Profile** | | **Atria** | |
| Abdominal Situs | Solitus | Left Atrium | Normal |
| Cardiac Position | Levocardia | Right Atrium | Normal |
| Systemic Venous Drainage | To RA | **Atrio-Ventricular Valves** | |
| Pulmonary Venous Drainage | To LA | Mitral Valve | Annulus = 11mm |
| Atrio-ventricular Connection | Concordant | Tricuspid Valve | Annulus = 12mm |
| Ventriculo-Arterial Connection | concordant | **Ventricle** | |
| Ventricular Loop | d-Loop | Left Ventricle | Normal |
| **Septae** |  | Right Ventricle | Normal |
| Interatrial Septum | Intact | **Doppler Measurement** |  |
| Interventricular Septum | 4.5mm PM VSD, L – R Shunt | Mitral |  |
| **Semilunar Valves** |  | Aortic |  |
| Aortic Valve | Annulus = 14mm | Tricuspid |  |
| Pulmonary Valve | Annulus = 15mm | Pulmonic |  |
| **Great Arteries** | NRGA | **Coronary Arteries** |  |
| Aorta |  | **Aortic Arch** | Left |
| Pulmonary Arteries |  | **PDA** | No |
| **M-Mode**: Normal LV Function on eye balling | | | |
| Ao | mm | PWd | mm |
| LA | mm | EDV | ml |
| LVIDd | mm | ESV | ml |
| LVIDs | mm | FS | % |
| IVSd | mm | LVEF | % |
| **Additional Information:** Circumferential Pericardial effusion measuring maximum depth of 5mm. | | | |
| **Conclusion:**   1. {S, D, S} Levocardia 2. Small PM VSD, L – R Shunt 3. Small Circumferential pericardial effusion 4. Normal LV Systolic Function | | | |
| **Remark:** Tachycardia during study | | | |
| **Recommendation:** Link to cardiac follow up clinic | | | |
| **Done By:** | **Signature** | **Date** | **Remark** |
| Tesfaye T., Pediatric Cardiologist |  | 18/06/14Eth.C. |  |

| **Patient Name: yetagesu Abeje . Sex/Age: M/6years. Date of Report: 20/07/14Eth.C. MRN: 123873.**  **Clinical Diagnosis: ARF + Murmur. TGSH1.2527.** | | | |
| --- | --- | --- | --- |
|  | | | |
| **Features:** | **Findings** | **Features** | **Findings** |
| **Profile** | | **Atria** | |
| Abdominal Situs | Solitus | Left Atrium | Dilated |
| Cardiac Position | Levocardia | Right Atrium | Normal |
| Systemic Venous Drainage | To RA | **Atrio-Ventricular Valves** | |
| Pulmonary Venous Drainage | To LA | Mitral Valve | Annulus = 24mm. thickened, patulous MVL. |
| Atrio-ventricular Connection | Concordant | Tricuspid Valve | Annulus = 19mm  TAPSE = 16mm |
| Ventriculo-Arterial Connection | concordant | **Ventricle** | |
| Ventricular Loop | d-Loop | Left Ventricle | Dilated |
| **Septae** |  | Right Ventricle | Normal |
| Interatrial Septum | Intact | **Doppler Measurement** |  |
| Interventricular Septum | Intact | Mitral | Severe MR, Holosystolic, posterior projection, seen in two planes with jet velocity = 3.8m/sec. |
| **Semilunar Valves** |  | Aortic | Mild AR, PHT = 534ms |
| Aortic Valve | Annulus = 16mm | Tricuspid |  |
| Pulmonary Valve | Annulus = 19mm | Pulmonic |  |
| **Great Arteries** | NRGA | **Coronary Arteries** |  |
| Aorta |  | **Aortic Arch** | Left |
| Pulmonary Arteries | MPA = 20mm | **PDA** | No |
| **M-Mode**: | | | |
| Ao | mm | PWd | mm |
| LA | mm | EDV | ml |
| LVIDd | mm | ESV | ml |
| LVIDs | mm | FS | 28% |
| IVSd | mm | LVEF | 55% |
| **Additional Information:** Pericardial effusion with a maximum depth of 12mm on RV Side. | | | |
| **Conclusion:**   1. {S, D, S} Levocardia 2. LA/LV Dilated 3. Thickened, patulous MVL 4. Severe MR 5. Mild AR 6. Moderate Pericardial effusion | | | |
| **Done By:** | **Signature** | **Date** | **Remark** |
| Tesfaye T., Pediatric Cardiologist |  | 18/06/14Eth.C. |  |

| **Patient Name: B/Fentaye Gedefaw. Sex/Age: F/13 days. Date of Report: 20/07/14Eth.C. MRN: 123908.**  **Clinical Diagnosis: Incidental Murmur. TGSH1.2528.** | | | |
| --- | --- | --- | --- |
| **Features:** | **Findings** | **Features** | **Findings** |
| **Profile** | | **Atria** | |
| Abdominal Situs | Solitus | Left Atrium | Smallish (? Compressed) |
| Cardiac Position | Dextroposition | Right Atrium | Normal |
| Systemic Venous Drainage | To RA | **Atrio-Ventricular Valves** | |
| Pulmonary Venous Drainage | To LA. Flow acceleration from LUPV to LA with a gradient of 8/6mmHg. | Mitral Valve | Annulus = 9mm |
| Atrio-ventricular Connection | Concordant | Tricuspid Valve | Annulus = 11mm |
| Ventriculo-Arterial Connection | concordant | **Ventricle** | |
| Ventricular Loop | d-Loop | Left Ventricle | Normal |
| **Septae** |  | Right Ventricle | Normal |
| Interatrial Septum | 4mm OS ASD, L – R Shunt | **Doppler Measurement** |  |
| Interventricular Septum | Intact | Mitral |  |
| **Semilunar Valves** |  | Aortic |  |
| Aortic Valve | Annulus = 5mm | Tricuspid |  |
| Pulmonary Valve | Annulus = 8mm | Pulmonic |  |
| **Great Arteries** | NRGA | **Coronary Arteries** |  |
| Aorta |  | **Aortic Arch** | Left |
| Pulmonary Arteries |  | **PDA** | No |
| **M-Mode**: Normal LV Function on eye balling | | | |
| Ao | mm | PWd | mm |
| LA | mm | EDV | ml |
| LVIDd | mm | ESV | ml |
| LVIDs | mm | FS | % |
| IVSd | mm | LVEF | % |
| **Additional Information:** | | | |
| **Conclusion:**   1. {S, D, S} Dextroposition 2. Small OS ASD, L – R Shunt 3. Flow acceleration across LUPV to LA Secondary to ? 4. Smallish LA (?Compressed) | | | |
| **Remark:** Needs further imaging | | | |
| **Done By:** | **Signature** | **Date** | **Remark** |
| Tesfaye T., Pediatric Cardiologist |  | 20/07/14Eth.C. |  |

| **Patient Name: Ayinadis Simachew. Sex/Age: F/10/12months. Date of Report: 21/07/14Eth.C. MRN: 121076.**  **Clinical Diagnosis: Incidental Murmur + DS. TGSH1.2529.** | | | |
| --- | --- | --- | --- |
| **Features:** | **Findings** | **Features** | **Findings** |
| **Profile** | | **Atria** | |
| Abdominal Situs | Solitus | Left Atrium | Dilated |
| Cardiac Position | Levocardia | Right Atrium | Normal |
| Systemic Venous Drainage | To RA | **Atrio-Ventricular Valves** | |
| Pulmonary Venous Drainage | To LA | Mitral Valve | Annulus = 15mm |
| Atrio-ventricular Connection | Concordant | Tricuspid Valve | Annulus = 11mm |
| Ventriculo-Arterial Connection | concordant | **Ventricle** | |
| Ventricular Loop | d-Loop | Left Ventricle | Dilated |
| **Septae** |  | Right Ventricle | Normal |
| Interatrial Septum | Intact | **Doppler Measurement** |  |
| Interventricular Septum | Intact | Mitral |  |
| **Semilunar Valves** |  | Aortic |  |
| Aortic Valve | Annulus = 11mm | Tricuspid |  |
| Pulmonary Valve | Annulus = 11mm | Pulmonic | Mild PR, PPG = 46mmHg |
| **Great Arteries** | NRGA | **Coronary Arteries** |  |
| Aorta |  | **Aortic Arch** | Left |
| Pulmonary Arteries |  | **PDA** | 2.5mm PDA, L – R Shunt |
| **M-Mode**: | | | |
| Ao | mm | PWd | mm |
| LA | mm | EDV | ml |
| LVIDd | mm | ESV | ml |
| LVIDs | mm | FS | 38% |
| IVSd | mm | LVEF | 70% |
| **Additional Information:** pericardial effusion measuring maximum depth of 3mm on RV Side. | | | |
| **Conclusion:**   1. {S, D, S} Levocardia 2. LA/LV Dilated 3. Moderate PDA, L – R Shunt 4. Mild Pul HTN 5. Normal LV Systolic Function 6. Trace Pericardial effusion | | | |
| **Done By:** | **Signature** | **Date** | **Remark** |
| Tesfaye T., Pediatric Cardiologist |  | 21/07/14Eth.C. |  |

| **Patient Name: B/Tigist Birara . Sex/Age: M/9 days . Date of Report: 21/07/14Eth.C. MRN: 123343.** | | | |
| --- | --- | --- | --- |
| **Clinical Diagnosis: RD + Incidental Murmur. TGSH1.2530.** | | | |
| **Features:** | **Findings** | **Features** | **Findings** |
| **Profile** | | **Atria** | |
| Abdominal Situs | Solitus | Left Atrium | Dilated |
| Cardiac Position | Levocardia | Right Atrium | Normal |
| Systemic Venous Drainage | To RA | **Atrio-Ventricular Valves** | |
| Pulmonary Venous Drainage | To LA | Mitral Valve | Annulus = 15mm |
| Atrio-ventricular Connection | Concordant | Tricuspid Valve | Annulus = 13mm  TAPSE = 17mm |
| Ventriculo-Arterial Connection | concordant | **Ventricle** | |
| Ventricular Loop | d-Loop | Left Ventricle | Dilated |
| **Septae** |  | Right Ventricle | Normal |
| Interatrial Septum | Intact | **Doppler Measurement** |  |
| Interventricular Septum | Intact | Mitral | Moderate MR, Holosystolic, posterior projection, seen in two planes with jet velocity = 3.9m/sec |
| **Semilunar Valves** |  | Aortic |  |
| Aortic Valve | Annulus = 11mm | Tricuspid |  |
| Pulmonary Valve | Annulus = 12mm | Pulmonic |  |
| **Great Arteries** | NRGA | **Coronary Arteries** |  |
| Aorta |  | **Aortic Arch** | Left |
| Pulmonary Arteries |  | **PDA** | 3.2mm PDA, L – R Shunt |
| **M-Mode**: | | | |
| Ao | mm | PWd | mm |
| LA | mm | EDV | ml |
| LVIDd | mm | ESV | ml |
| LVIDs | mm | FS | 32% |
| IVSd | mm | LVEF | 61% |
| **Additional Information:** | | | |
| **Conclusion:**   1. {S, D, S} Levocardia 2. LA/LV Dilated 3. Large PDA, L – R Shunt 4. Normal Biventricular Systolic Function | | | |
| **Done By:** | **Signature** | **Date** | **Remark** |
| Tesfaye T., Pediatric Cardiologist |  | 20/07/14Eth.C. |  |

| **Patient Name: Awuraris Kefyalew. Sex/Age: M/6 7/12. Date of Report: 21/07/2014Eth.C. MRN: 124023.**  **Clinical Diagnosis: CHF + DOE + Palpitation + RD + Murmur. TGSH1.2531.** | | | |
| --- | --- | --- | --- |
| **Features:** | **Findings** | **Features** | **Findings** |
| **Profile** | | **Atria** | |
| Abdominal Situs | Solitus | Left Atrium | Dilated |
| Cardiac Position | Levocardia | Right Atrium | More dilated |
| Systemic Venous Drainage | To RA | **Atrio-Ventricular Valves** | |
| Pulmonary Venous Drainage | To LA | Mitral Valve | Annulus = 25mm |
| Atrio-ventricular Connection | Concordant | Tricuspid Valve | Annulus = 29mm. TAPSE = 24mm |
| Ventriculo-Arterial Connection | concordant | **Ventricle** | |
| Ventricular Loop | d-Loop | Left Ventricle | Dilated |
| **Septae** |  | Right Ventricle | More dilated |
| Interatrial Septum | 19mm Fenestrated OS ASD, L – R Shunt | **Doppler Measurement** |  |
| Interventricular Septum | 14mm PM VSD, L – R Shunt | Mitral | Mild MR, Holosystolic, posterior projection, seen in two planes with jet velocity = 4.5m/sec |
| **Semilunar Valves** |  | Aortic |  |
| Aortic Valve | Annulus = 17mm | Tricuspid | Moderate TR |
| Pulmonary Valve | Annulus = 23mm | Pulmonic | Mild PR, PPG = 58mmHg |
| **Great Arteries** | NRGA | **Coronary Arteries** |  |
| Aorta |  | **Aortic Arch** | Left |
| Pulmonary Arteries | MPA = 26mm | **PDA** | No PDA |
| **M-Mode**: | | | |
| Ao | mm | PWd | mm |
| LA | mm | EDV | ml |
| LVIDd | mm | ESV | ml |
| LVIDs | mm | FS | 41% |
| IVSd | mm | LVEF | 73% |
| **Additional Information:** Pericardial effusion measuring with maximum depth of 5mm on RV Side. | | | |
| **Conclusion:**   1. {S, D, S} Levocardia 2. All chambers dilated 3. Large Fenestrated OS ASD, L – R Shunt 4. Large PM VSD, L – R Shunt 5. Moderate TR 6. Mild MR 7. Severe Pulmonary Hypertension 8. Normal Biventricular Systolic Function 9. Small Pericardial effusion | | | |
| **Done By:** | **Signature** | **Date** | **Remark** |
| Tesfaye T., Pediatric Cardiologist |  | 21/07/14Eth.C. |  |

| **Patient Name: Belgie Agmas . Sex/Age: F/13years. Date of Report: 21/07/2014Eth.C. MRN: 121567.** | | | |
| --- | --- | --- | --- |
| **Clinical Diagnosis: Rheumatic Recurrence + CHF + Murmur. TGSH1.2532.** | | | |
| **Features:** | **Findings** | **Features** | **Findings** |
| **Profile** | | **Atria** | |
| Abdominal Situs | Solitus | Left Atrium | Markedly Dilated |
| Cardiac Position | Levocardia | Right Atrium | Normal |
| Systemic Venous Drainage | To RA | **Atrio-Ventricular Valves** | |
| Pulmonary Venous Drainage | To LA | Mitral Valve | Annulus = 34mm. thickened, clubbed MVL. MVA = 1.64cm2. |
| Atrio-ventricular Connection | Concordant | Tricuspid Valve | Annulus = 19mm |
| Ventriculo-Arterial Connection | concordant | **Ventricle** | |
| Ventricular Loop | d-Loop | Left Ventricle | Markedly Dilated |
| **Septae** |  | Right Ventricle | Normal |
| Interatrial Septum | Intact | **Doppler Measurement** |  |
| Interventricular Septum | Intact | Mitral | Severe MR, Holosystolic, posterior projection, seen in two planes with jet velocity = 4.9m/sec. MS, PPG/MPG = 15/8mmHg. |
| **Semilunar Valves** |  | Aortic | Moderate AR, PHT = 460ms |
| Aortic Valve | Annulus = 16mm | Tricuspid |  |
| Pulmonary Valve | Annulus = 19mm | Pulmonic |  |
| **Great Arteries** | NRGA | **Coronary Arteries** |  |
| Aorta |  | **Aortic Arch** | Left |
| Pulmonary Arteries |  | **PDA** | No PDA |
| **M-Mode**: | | | |
| Ao | mm | PWd | mm |
| LA | mm | EDV | ml |
| LVIDd | mm | ESV | ml |
| LVIDs | mm | FS | 31% |
| IVSd | mm | LVEF | 57% |
| **Additional Information:** | | | |
| **Conclusion:**   1. {S, D, S} Levocardia 2. LA/LV Markedly Dilated 3. Thickened, clubbed MVL 4. Severe MR 5. Mild MS 6. Moderate AR 7. Normal LV Systolic Function | | | |
| **Done By:** | **Signature** | **Date** | **Remark** |
| Tesfaye T., Pediatric Cardiologist |  | 21/07/14Eth.C. |  |

| **Patient Name: Fekadu Shegaw . Sex/Age: M/1 3/12 years. Date of Report: 22/07/2014Eth.C. MRN: -121844.** | | | |
| --- | --- | --- | --- |
| **Clinical Diagnosis: Incidental Murmur + Diaphoresis on BF. TGSH1.2533.** | | | |
| **Features:** | **Findings** | **Features** | **Findings** |
| **Profile** | | **Atria** | |
| Abdominal Situs | Solitus | Left Atrium | Normal |
| Cardiac Position | Levocardia | Right Atrium | Normal |
| Systemic Venous Drainage | To RA | **Atrio-Ventricular Valves** | |
| Pulmonary Venous Drainage | To LA | Mitral Valve | Annulus = 13mm. ? Parachute MV |
| Atrio-ventricular Connection | Concordant | Tricuspid Valve | Annulus = 18mm  TAPSE = mm |
| Ventriculo-Arterial Connection | concordant | **Ventricle** | |
| Ventricular Loop | d-Loop | Left Ventricle | LVH |
| **Septae** |  | Right Ventricle | Normal |
| Interatrial Septum | Intact | **Doppler Measurement** |  |
| Interventricular Septum | Intact | Mitral | Mild MS, PPG/MPG = 11.5mmHg. |
| **Semilunar Valves** |  | Aortic | Valvular AS, PPG/MPG = 46/25mmHg |
| Aortic Valve | Annulus = 11mm | Tricuspid |  |
| Pulmonary Valve | Annulus = 16mm | Pulmonic |  |
| **Great Arteries** | NRGA | **Coronary Arteries** |  |
| Aorta |  | **Aortic Arch** | Left. CoA with PPG = 34mmHg |
| Pulmonary Arteries |  | **PDA** | 2mm PDA, L – R Shunt |
| **M-Mode**: | | | |
| Ao | mm | PWd | 6mm |
| LA | mm | EDV | 41ml |
| LVIDd | 32mm | ESV | 16ml |
| LVIDs | 22mm | FS | 32% |
| IVSd | 6mm | LVEF | 61% |
| **Additional Information:** | | | |
| **Conclusion:**   1. {S, D, S} Levocardia 2. ?Parachute MV 3. Mild MS 4. Mild Valvular AS 5. Moderate PDA, L – R Shunt 6. Mild CoA | | | |
| **Remark: Shone’s Complex** | | | |
| **Done By:** | **Signature** | **Date** | **Remark** |
| Tesfaye T., Pediatric Cardiologist |  | 22/07/14Eth.C. |  |

| **Patient Name: -Eyerus Dimetse Sex/Age: F/13years. Date of Report: 22/07/2014Eth.C. MRN: 124103.**  **Clinical Diagnosis: Rheumatic Recurrence + CHF + DOE + Palpitation + Murmur. TGSH1.2534.** | | | |
| --- | --- | --- | --- |
| **Features:** | **Findings** | **Features** | **Findings** |
| **Profile** | | **Atria** | |
| Abdominal Situs | Solitus | Left Atrium | More dilated |
| Cardiac Position | Levocardia | Right Atrium | Dilated |
| Systemic Venous Drainage | To RA | **Atrio-Ventricular Valves** | |
| Pulmonary Venous Drainage | To LA | Mitral Valve | Annulus = 21mm. Thickened MVL |
| Atrio-ventricular Connection | Concordant | Tricuspid Valve | Annulus = 21mm  TAPSE = 9mm |
| Ventriculo-Arterial Connection | concordant | **Ventricle** | |
| Ventricular Loop | d-Loop | Left Ventricle | More dilated |
| **Septae** |  | Right Ventricle | Dilated |
| Interatrial Septum | Intact | **Doppler Measurement** |  |
| Interventricular Septum | Intact | Mitral | Severe MR, Holosystolic, PPG = 3.3m/sec. Mild MS, PPG/MPG = 12/6mmHg |
| **Semilunar Valves** |  | Aortic |  |
| Aortic Valve | Annulus = 19mm | Tricuspid | Moderate TR, PPG = 39mmHg |
| Pulmonary Valve | Annulus = 21mm | Pulmonic | Mild PR, PPG = 37mmHg |
| **Great Arteries** | NRGA | **Coronary Arteries** |  |
| Aorta |  | **Aortic Arch** | Left |
| Pulmonary Arteries |  | **PDA** | No PDA |
| **M-Mode**: | | | |
| Ao | mm | PWd | mm |
| LA | mm | EDV | ml |
| LVIDd | mm | ESV | ml |
| LVIDs | mm | FS | 34% |
| IVSd | mm | LVEF | 66% |
| **Additional Information:** | | | |
| **Conclusion:**   1. {S, D, S} Levocardia 2. All chambers dilated 3. Thickened MVL tips 4. Severe MR 5. Mild MS 6. Moderate TR 7. Mild PR 8. Mild Pulmonary Hypertension 9. Normal Biventricular Systolic Function | | | |
| **Done By:** | **Signature** | **Date** | **Remark** |
| Tesfaye T., Pediatric Cardiologist |  | 22/07/14Eth.C. |  |

| **Patient Name: Yeshiwork Amare. Sex/Age: F/3years. Date of Report: 22/07/2014Eth.C. MRN: 123740.** | | | |
| --- | --- | --- | --- |
| **Clinical Diagnosis: Recurrent Chest Infection. TGSH1.2535.** | | | |
| **Features:** | **Findings** | **Features** | **Findings** |
| **Profile** | | **Atria** | |
| Abdominal Situs | Solitus | Left Atrium | Normal |
| Cardiac Position | Levocardia | Right Atrium | Normal |
| Systemic Venous Drainage | To RA | **Atrio-Ventricular Valves** | |
| Pulmonary Venous Drainage | To LA | Mitral Valve | Annulus = 18mm |
| Atrio-ventricular Connection | Concordant | Tricuspid Valve | Annulus = 19mm  TAPSE = 18mm |
| Ventriculo-Arterial Connection | concordant | **Ventricle** | |
| Ventricular Loop | d-Loop | Left Ventricle | Normal |
| **Septae** |  | Right Ventricle | Normal |
| Interatrial Septum | Intact | **Doppler Measurement** |  |
| Interventricular Septum | Intact | Mitral |  |
| **Semilunar Valves** |  | Aortic |  |
| Aortic Valve | Annulus = 13mm | Tricuspid |  |
| Pulmonary Valve | Annulus = 14mm | Pulmonic |  |
| **Great Arteries** | NRGA | **Coronary Arteries** |  |
| Aorta |  | **Aortic Arch** | Left |
| Pulmonary Arteries |  | **PDA** | No PDA |
| **M-Mode**: | | | |
| Ao | mm | PWd | mm |
| LA | mm | EDV | ml |
| LVIDd | mm | ESV | ml |
| LVIDs | mm | FS | 38% |
| IVSd | mm | LVEF | 69% |
| **Additional Information:** | | | |
| **Conclusion:**   1. Normal Echocardiography Study | | | |
| **Done By:** | **Signature** | **Date** | **Remark** |
| Tesfaye T., Pediatric Cardiologist |  | 22/07/14Eth.C. |  |

| **Patient Name: B/Tigist Birara . Sex/Age: M/9days. Date of Report: 22/07/2014Eth.C. MRN: 122752.** | | | |
| --- | --- | --- | --- |
| **Clinical Diagnosis: RD. TGSH1.2536.** | | | |
| **Features:** | **Findings** | **Features** | **Findings** |
| **Profile** | | **Atria** | |
| Abdominal Situs | Solitus | Left Atrium | Normal |
| Cardiac Position | Levocardia | Right Atrium | Normal |
| Systemic Venous Drainage | To RA | **Atrio-Ventricular Valves** | |
| Pulmonary Venous Drainage | To LA | Mitral Valve | Annulus = 10mm |
| Atrio-ventricular Connection | Concordant | Tricuspid Valve | Annulus = 11mm |
| Ventriculo-Arterial Connection | concordant | **Ventricle** | |
| Ventricular Loop | d-Loop | Left Ventricle | Normal |
| **Septae** |  | Right Ventricle | Normal |
| Interatrial Septum | Intact | **Doppler Measurement** |  |
| Interventricular Septum | Intact | Mitral |  |
| **Semilunar Valves** |  | Aortic |  |
| Aortic Valve | Annulus = 8mm | Tricuspid |  |
| Pulmonary Valve | Annulus = 10mm | Pulmonic |  |
| **Great Arteries** | NRGA | **Coronary Arteries** |  |
| Aorta |  | **Aortic Arch** | Left |
| Pulmonary Arteries |  | **PDA** | No PDA |
| **M-Mode**: Normal LV Function on eye balling | | | |
| Ao | mm | PWd | mm |
| LA | mm | EDV | ml |
| LVIDd | mm | ESV | ml |
| LVIDs | mm | FS | % |
| IVSd | mm | LVEF | % |
| **Additional Information:** | | | |
| **Conclusion:**   1. Normal Echocardiography Study | | | |
| **Done By:** | **Signature** | **Date** | **Remark** |
| Tesfaye T., Pediatric Cardiologist |  | 22/07/14Eth.C. |  |

| **Patient Name: B/Rekeya Yesuf. Sex/Age: F/2/12. Date of Report: 22/07/2014Eth.C. MRN: 112418.** | | | |
| --- | --- | --- | --- |
| **Clinical Diagnosis: DS. TGSH1.2537.** | | | |
| **Features:** | **Findings** | **Features** | **Findings** |
| **Profile** | | **Atria** | |
| Abdominal Situs | Solitus | Left Atrium | Normal |
| Cardiac Position | Levocardia | Right Atrium | Normal |
| Systemic Venous Drainage | To RA | **Atrio-Ventricular Valves** | |
| Pulmonary Venous Drainage | To LA | Mitral Valve | Annulus = 9mm |
| Atrio-ventricular Connection | Concordant | Tricuspid Valve | Annulus = 9mm |
| Ventriculo-Arterial Connection | concordant | **Ventricle** | |
| Ventricular Loop | d-Loop | Left Ventricle | Normal |
| **Septae** |  | Right Ventricle | Normal |
| Interatrial Septum | PFO, L – R Shunt | **Doppler Measurement** |  |
| Interventricular Septum | Intact | Mitral |  |
| **Semilunar Valves** |  | Aortic |  |
| Aortic Valve | Annulus = 8mm | Tricuspid |  |
| Pulmonary Valve | Annulus = 7mm | Pulmonic |  |
| **Great Arteries** | NRGA | **Coronary Arteries** |  |
| Aorta |  | **Aortic Arch** | Left |
| Pulmonary Arteries |  | **PDA** | No PDA |
| **M-Mode**: Normal LV Function on eye balling | | | |
| Ao | mm | PWd | mm |
| LA | mm | EDV | ml |
| LVIDd | mm | ESV | ml |
| LVIDs | mm | FS | % |
| IVSd | mm | LVEF | % |
| **Additional Information:** | | | |
| **Conclusion:**   1. {S, D, S} Levocardia 2. PFO, L – R Shunt | | | |
| **Done By:** | **Signature** | **Date** | **Remark** |
| Tesfaye T., Pediatric Cardiologist |  | 22/07/14Eth.C. |  |

| **Patient Name: Yohannes Belayneh. Sex/Age: M/5 4/12years. Date of Report: 27/07/2014Eth.C. MRN:056621.** | | | |
| --- | --- | --- | --- |
| **Clinical Diagnosis: RD + Recurrent Chest Infection. TGSH1.2538.** | | | |
| **Features:** | **Findings** | **Features** | **Findings** |
| **Profile** | | **Atria** | |
| Abdominal Situs | Solitus | Left Atrium | Normal |
| Cardiac Position | Levocardia | Right Atrium | Normal |
| Systemic Venous Drainage | To RA | **Atrio-Ventricular Valves** | |
| Pulmonary Venous Drainage | To LA | Mitral Valve | Annulus = 19mm |
| Atrio-ventricular Connection | Concordant | Tricuspid Valve | Annulus = 19mm  TAPSE = 17mm |
| Ventriculo-Arterial Connection | concordant | **Ventricle** | |
| Ventricular Loop | d-Loop | Left Ventricle | Normal |
| **Septae** |  | Right Ventricle | Normal |
| Interatrial Septum | Intact | **Doppler Measurement** |  |
| Interventricular Septum | Intact | Mitral |  |
| **Semilunar Valves** |  | Aortic |  |
| Aortic Valve | Annulus = 15mm | Tricuspid |  |
| Pulmonary Valve | Annulus = 18mm | Pulmonic |  |
| **Great Arteries** | NRGA | **Coronary Arteries** |  |
| Aorta |  | **Aortic Arch** | Left |
| Pulmonary Arteries |  | **PDA** | No PDA |
| **M-Mode**: | | | |
| Ao | mm | PWd | mm |
| LA | mm | EDV | ml |
| LVIDd | mm | ESV | ml |
| LVIDs | mm | FS | 33% |
| IVSd | mm | LVEF | 62% |
| **Additional Information:** | | | |
| **Conclusion:**   1. Normal Echocardiography | | | |
| **Done By:** | **Signature** | **Date** | **Remark** |
| Tesfaye T., Pediatric Cardiologist |  | 27/07/14Eth.C. |  |

| **Patient Name: Endalew Mamoye. Sex/Age: M/12years. Date of Report:27/07/2014Eth.C. MRN:125102.**  **Clinical Diagnosis: Rheumatic Recurrence + DOE + Palpitation + Murmur. TGSH1.2539.** | | | |
| --- | --- | --- | --- |
| **Features:** | **Findings** | **Features** | **Findings** |
| **Profile** | | **Atria** | |
| Abdominal Situs | Solitus | Left Atrium | Dilated |
| Cardiac Position | Levocardia | Right Atrium | Normal |
| Systemic Venous Drainage | To RA | **Atrio-Ventricular Valves** | |
| Pulmonary Venous Drainage | To LA | Mitral Valve | Annulus = 22mm. thickened, clubbed MVL. Short PMVL. MVA = 1.5cm**2**. |
| Atrio-ventricular Connection | Concordant | Tricuspid Valve | Annulus = 19mm. TAPSE = 19mm |
| Ventriculo-Arterial Connection | concordant | **Ventricle** | |
| Ventricular Loop | d-Loop | Left Ventricle | Dilated |
| **Septae** |  | Right Ventricle | Normal |
| Interatrial Septum | Intact | **Doppler Measurement** | |
| Interventricular Septum | Intact | Mitral | Severe MR, Holosystolic, posterior projection, seen in two planes with jet velocity = 4.7m/sec. Mild MS, PPG/MPG = 8/6mmHg. |
| **Semilunar Valves** |  | Aortic | Mild AR, PHT = 540ms. |
| Aortic Valve | Annulus = 17mm. thickened AVL. Trileaflet | Tricuspid |  |
| Pulmonary Valve | Annulus = 19mm | Pulmonic |  |
| **Great Arteries** | NRGA | **Coronary Arteries** |  |
| Aorta |  | **Aortic Arch** | Left |
| Pulmonary Arteries |  | **PDA** | No PDA |
| **M-Mode**: | | | |
| Ao | mm | PWd | mm |
| LA | mm | EDV | ml |
| LVIDd | mm | ESV | ml |
| LVIDs | mm | FS | 35% |
| IVSd | mm | LVEF | 63% |
| **Conclusion:**   1. {S, D, S} Levocardia 2. LA/LV Dilated 3. Thickened, clubbed MVL. Shortened PMVL 4. Thickened AVL 5. Severe MR 6. Mild MS 7. Mild AR 8. Normal Biventricular Systolic Function | | | |
| **Done By:** | **Signature** | **Date** | **Remark** |
| Tesfaye T., Pediatric Cardiologist |  | 27/07/14Eth.C. |  |

| **Patient Name: Mubarak Tesema. Sex/Age: 1 5/12. Date of Report: 27/07/2014Eth.C. MRN:124717.** | | | |
| --- | --- | --- | --- |
| **Clinical Diagnosis: Sepsis + RD + Friction rub + Cardiomegaly on CXR. TGSH1.2540.** | | | |
| **Features:** | **Findings** | **Features** | **Findings** |
| **Profile** | | **Atria** | |
| Abdominal Situs | Solitus | Left Atrium | Normal |
| Cardiac Position | Levocardia | Right Atrium | Normal |
| Systemic Venous Drainage | To RA | **Atrio-Ventricular Valves** | |
| Pulmonary Venous Drainage | To LA | Mitral Valve | Annulus = 14mm |
| Atrio-ventricular Connection | Concordant | Tricuspid Valve | Annulus = 15mm. TAPSE = 15mm |
| Ventriculo-Arterial Connection | concordant | **Ventricle** | |
| Ventricular Loop | d-Loop | Left Ventricle | Normal |
| **Septae** |  | Right Ventricle | Normal |
| Interatrial Septum | Intact | **Doppler Measurement** |  |
| Interventricular Septum | Intact | Mitral |  |
| **Semilunar Valves** |  | Aortic |  |
| Aortic Valve | Annulus = mm | Tricuspid |  |
| Pulmonary Valve | Annulus = mm | Pulmonic |  |
| **Great Arteries** | NRGA | **Coronary Arteries** |  |
| Aorta |  | **Aortic Arch** | Left |
| Pulmonary Arteries |  | **PDA** | No PDA |
| **M-Mode**: Normal LV Function on eye balling | | | |
| Ao | mm | PWd | mm |
| LA | mm | EDV | ml |
| LVIDd | mm | ESV | ml |
| LVIDs | mm | FS | % |
| IVSd | mm | LVEF | % |
| **Additional Information:** Circumferential Pericardial effusion, maximum depth of 5mm on LV Side with pericardial thickening.15mm Left Pleural effusion with echodebris seen. | | | |
| **Conclusion:**   1. {S, D, S} Levocardia 2. Small Circumferential Pericardial effusion with pericardial thickening 3. Moderate Left Pleural effusion with echodebris. | | | |
| **Done By:** | **Signature** | **Date** | **Remark** |
| Tesfaye T., Pediatric Cardiologist |  | 27/07/14Eth.C. |  |

| **Patient Name: B/ Mucheye Metaled. Sex/Age: F/9days. Date of Report: 21/07/2014Eth.C. MRN:123902.** | | | |
| --- | --- | --- | --- |
| **Clinical Diagnosis: DS. TGSH1.2541.** | | | |
| **Features:** | **Findings** | **Features** | **Findings** |
| **Profile** | | **Atria** | |
| Abdominal Situs | Solitus | Left Atrium | Normal |
| Cardiac Position | Levocardia | Right Atrium | Normal |
| Systemic Venous Drainage | To RA | **Atrio-Ventricular Valves** | |
| Pulmonary Venous Drainage | To LA | Mitral Valve | Annulus = 11mm |
| Atrio-ventricular Connection | Concordant | Tricuspid Valve | Annulus = 13mm |
| Ventriculo-Arterial Connection | concordant | **Ventricle** | |
| Ventricular Loop | d-Loop | Left Ventricle | Normal |
| **Septae** |  | Right Ventricle | Normal |
| Interatrial Septum | 6 X 8mm High Secundum ASD, L – R Shunt | **Doppler Measurement** |  |
| Interventricular Septum | Intact | Mitral |  |
| **Semilunar Valves** |  | Aortic |  |
| Aortic Valve | Annulus = 8mm | Tricuspid |  |
| Pulmonary Valve | Annulus = 8mm | Pulmonic |  |
| **Great Arteries** | NRGA | **Coronary Arteries** |  |
| Aorta |  | **Aortic Arch** | Left |
| Pulmonary Arteries |  | **PDA** | No PDA |
| **M-Mode**: Normal LV Function on eye balling | | | |
| Ao | mm | PWd | mm |
| LA | mm | EDV | ml |
| LVIDd | mm | ESV | ml |
| LVIDs | mm | FS | % |
| IVSd | mm | LVEF | % |
| **Additional Information:** | | | |
| **Conclusion:**   1. {S, D, S} Levocardia 2. Moderate OS ASD, L – R Shunt | | | |
| **Done By:** | **Signature** | **Date** | **Remark** |
| Tesfaye T., Pediatric Cardiologist |  | 27/07/14Eth.C. |  |

| **Patient Name: B/Tete Negus . Sex/Age: M/ 23days . Date of Report: 27/07/2014Eth.C. MRN: 122274.** | | | |
| --- | --- | --- | --- |
| **Clinical Diagnosis: Incidental Murmur. TGSH1.2542.** | | | |
| **Features:** | **Findings** | **Features** | **Findings** |
| **Profile** | | **Atria** | |
| Abdominal Situs | Solitus | Left Atrium | Normal |
| Cardiac Position | Levocardia | Right Atrium | Normal |
| Systemic Venous Drainage | To RA | **Atrio-Ventricular Valves** | |
| Pulmonary Venous Drainage | To LA | Mitral Valve | Annulus = 10mm |
| Atrio-ventricular Connection | Concordant | Tricuspid Valve | Annulus = 12mm  TAPSE = 12mm |
| Ventriculo-Arterial Connection | concordant | **Ventricle** | |
| Ventricular Loop | d-Loop | Left Ventricle | Normal |
| **Septae** |  | Right Ventricle | Normal |
| Interatrial Septum | PFO, L – R Shunt | **Doppler Measurement** |  |
| Interventricular Septum | Intact | Mitral |  |
| **Semilunar Valves** |  | Aortic |  |
| Aortic Valve | Annulus = 9mm | Tricuspid | Mild TR, PPG = 37mmHg |
| Pulmonary Valve | Annulus = 10mm | Pulmonic |  |
| **Great Arteries** | NRGA | **Coronary Arteries** |  |
| Aorta |  | **Aortic Arch** | Left |
| Pulmonary Arteries |  | **PDA** | No PDA |
| **M-Mode**: Normal LV Function on eye balling | | | |
| Ao | mm | PWd | mm |
| LA | mm | EDV | ml |
| LVIDd | mm | ESV | ml |
| LVIDs | mm | FS | % |
| IVSd | mm | LVEF | % |
| **Additional Information:** 2.5mm defect from RCS to RA adjacent to TV, L – R Shunt. | | | |
| **Conclusion:**   1. {S, D, S} Levocardia 2. ?RSOV Type IIIa. 3. Mild Pulmonary Hypertension | | | |
| **Done By:** | **Signature** | **Date** | **Remark** |
| Tesfaye T., Pediatric Cardiologist |  | 27/07/14Eth.C. |  |

| **Patient Name: Kidusie Wassie. Sex/Age: M/2months. Date of Report: 27/07/2014Eth.C. MRN:124782.**  **Clinical Diagnosis: Cyanosis. TGSH1.2543.** | | | |
| --- | --- | --- | --- |
| **Features:** | **Findings** | **Features** | **Findings** |
| **Profile** | | **Atria** | |
| Abdominal Situs | Solitus | Left Atrium | Normal |
| Cardiac Position | Levocardia | Right Atrium | Normal |
| Systemic Venous Drainage | To RA | **Atrio-Ventricular Valves** | |
| Pulmonary Venous Drainage | To LA | Mitral Valve | Annulus = 13mm |
| Atrio-ventricular Connection | Concordant | Tricuspid Valve | Annulus = 12mm |
| Ventriculo-Arterial Connection | Discordant | **Ventricle** | |
| Ventricular Loop | d-Loop | Left Ventricle | Normal |
| **Septae** |  | Right Ventricle | Normal |
| Interatrial Septum | 10mm OS ASD, L – R Shunt | **Doppler Measurement** |  |
| Interventricular Septum | 10mm Inlet VSD, R – L Shunt | Mitral |  |
| **Semilunar Valves** |  | Aortic |  |
| Aortic Valve | Annulus = 9mm | Tricuspid |  |
| Pulmonary Valve | Annulus = 12mm | Pulmonic | Moderate LVOTO(PS) with PPG = 50mmHg |
| **Great Arteries** | Side by side, d | **Coronary Arteries** |  |
| Aorta | From RV | **Aortic Arch** | Left |
| Pulmonary Arteries | From LV | **PDA** | 1.5mm PDA |
| **M-Mode**: | | | |
| Ao | mm | PWd | mm |
| LA | mm | EDV | ml |
| LVIDd | mm | ESV | ml |
| LVIDs | mm | FS | % |
| IVSd | mm | LVEF | % |
| **Additional Information:** | | | |
| **Conclusion:**   1. {S, D, Side by Side} Levocardia 2. Moderate OS ASD, L – R Shunt 3. Large Inlet VSD, R – L Shunt 4. d-TGA 5. Severe LVOTO(PS) 6. Small PDA, PA to Aorta | | | |
| **Done By:** | **Signature** | **Date** | **Remark** |
| Tesfaye T., Pediatric Cardiologist |  | 27/07/14Eth.C. |  |

| **Patient Name: Addise Shita. Sex/Age: F/8 years. Date of Report: 29/07/2014Eth.C. MRN:125265** | | | |
| --- | --- | --- | --- |
| **Clinical Diagnosis: Sydenham’s Chorea. TGSH1.2544.** | | | |
| **Features:** | **Findings** | **Features** | **Findings** |
| **Profile** | | **Atria** | |
| Abdominal Situs | Solitus | Left Atrium | Normal |
| Cardiac Position | Levocardia | Right Atrium | Normal |
| Systemic Venous Drainage | To RA | **Atrio-Ventricular Valves** | |
| Pulmonary Venous Drainage | To LA | Mitral Valve | Annulus = 20mm. thickened MVL |
| Atrio-ventricular Connection | Concordant | Tricuspid Valve | Annulus = 20mm |
| Ventriculo-Arterial Connection | concordant | **Ventricle** | |
| Ventricular Loop | d-Loop | Left Ventricle | Normal |
| **Septae** |  | Right Ventricle | Normal |
| Interatrial Septum | Intact | **Doppler Measurement** |  |
| Interventricular Septum | Intact | Mitral | Mild MR, Incomplete signal, seen in two planes with jet velocity = 4.3m/sec |
| **Semilunar Valves** |  | Aortic |  |
| Aortic Valve | Annulus = 14mm | Tricuspid |  |
| Pulmonary Valve | Annulus = 17mm | Pulmonic |  |
| **Great Arteries** | NRGA | **Coronary Arteries** |  |
| Aorta |  | **Aortic Arch** | Left |
| Pulmonary Arteries |  | **PDA** | No PDA |
| **M-Mode**: | | | |
| Ao | mm | PWd | mm |
| LA | mm | EDV | ml |
| LVIDd | mm | ESV | ml |
| LVIDs | mm | FS | 40% |
| IVSd | mm | LVEF | 71% |
| **Additional Information:** | | | |
| **Conclusion:**   1. {S, D, S} Levocardia 2. Thickened MVL 3. Mild MR 4. Normal Systolic LV Function | | | |
| **Done By:** | **Signature** | **Date** | **Remark** |
| Tesfaye T., Pediatric Cardiologist |  | 29/07/14Eth.C. |  |

| **Patient Name: Hilina Teramaje . Sex/Age: F/ 2 3/12. Date of Report: 29/07/2014Eth.C. MRN: 124774.** | | | |
| --- | --- | --- | --- |
| **Clinical Diagnosis: RD + Recurrent Chest Infection. TGSH1.2545.** | | | |
| **Features:** | **Findings** | **Features** | **Findings** |
| **Profile** | | **Atria** | |
| Abdominal Situs | Solitus | Left Atrium | Normal |
| Cardiac Position | Levocardia | Right Atrium | Normal |
| Systemic Venous Drainage | To RA | **Atrio-Ventricular Valves** | |
| Pulmonary Venous Drainage | To LA | Mitral Valve | Annulus = mm |
| Atrio-ventricular Connection | Concordant | Tricuspid Valve | Annulus = mm  TAPSE = mm |
| Ventriculo-Arterial Connection | concordant | **Ventricle** | |
| Ventricular Loop | d-Loop | Left Ventricle | Normal |
| **Septae** |  | Right Ventricle | Normal |
| Interatrial Septum | Intact | **Doppler Measurement** |  |
| Interventricular Septum | Intact | Mitral |  |
| **Semilunar Valves** |  | Aortic |  |
| Aortic Valve | Annulus = mm | Tricuspid |  |
| Pulmonary Valve | Annulus = mm | Pulmonic |  |
| **Great Arteries** | NRGA | **Coronary Arteries** |  |
| Aorta |  | **Aortic Arch** | Left |
| Pulmonary Arteries |  | **PDA** | No PDA |
| **M-Mode**: | | | |
| Ao | mm | PWd | mm |
| LA | mm | EDV | ml |
| LVIDd | mm | ESV | ml |
| LVIDs | mm | FS | % |
| IVSd | mm | LVEF | % |
| **Additional Information:** | | | |
| **Conclusion:**   1. {S, D, S} Levocardia 2. Normal | | | |
| **Done By:** | **Signature** | **Date** | **Remark** |
| Tesfaye T., Pediatric Cardiologist |  | 29/07/14Eth.C. |  |

| **Patient Name: B/Yezbalem Wale . Sex/Age: F/ 7 days. Date of Report: 04/08/2014Eth.C. MRN: 125912.** | | | |
| --- | --- | --- | --- |
| **Clinical Diagnosis: Incidental Murmur. TGSH1.2546.** | | | |
| **Features:** | **Findings** | **Features** | **Findings** |
| **Profile** | | **Atria** | |
| Abdominal Situs | Solitus | Left Atrium | Normal |
| Cardiac Position | Levocardia | Right Atrium | Normal |
| Systemic Venous Drainage | To RA | **Atrio-Ventricular Valves** | |
| Pulmonary Venous Drainage | To LA | Mitral Valve | Annulus = 8mm |
| Atrio-ventricular Connection | Concordant | Tricuspid Valve | Annulus = 9mm |
| Ventriculo-Arterial Connection | concordant | **Ventricle** | |
| Ventricular Loop | d-Loop | Left Ventricle | Normal |
| **Septae** |  | Right Ventricle | Normal |
| Interatrial Septum | Intact | **Doppler Measurement** |  |
| Interventricular Septum | Intact | Mitral |  |
| **Semilunar Valves** |  | Aortic |  |
| Aortic Valve | Annulus = 7mm | Tricuspid |  |
| Pulmonary Valve | Annulus = 7mm. PV Dysplastic | Pulmonic | Mild Valvular PS, PPG = 22mmHg |
| **Great Arteries** | NRGA | **Coronary Arteries** |  |
| Aorta |  | **Aortic Arch** | Left |
| Pulmonary Arteries |  | **PDA** | No PDA |
| **M-Mode**: Normal LV Function on eye balling | | | |
| Ao | mm | PWd | mm |
| LA | mm | EDV | ml |
| LVIDd | mm | ESV | ml |
| LVIDs | mm | FS | % |
| IVSd | mm | LVEF | % |
| **Additional Information:** | | | |
| **Conclusion:**   1. {S, D, S} Levocardia 2. Dysplastic PV 3. Mild Valvular PS | | | |
| **Done By:** | **Signature** | **Date** | **Remark** |
| Tesfaye T., Pediatric Cardiologist |  | 04/08/14Eth.C. |  |

| **Patient Name: Kidest Ayenew. Sex/Age: F/1 3/12. Date of Report: 04/08/2014Eth.C. MRN: 125963.** | | | |
| --- | --- | --- | --- |
| **Clinical Diagnosis: Cyanosis + CHF + RD + Murmur. TGSH1.2547.** | | | |
| **Features:** | **Findings** | **Features** | **Findings** |
| **Profile** | | **Atria** | |
| Abdominal Situs | Solitus | Left Atrium | Dilated |
| Cardiac Position | Levocardia | Right Atrium | Dilated |
| Systemic Venous Drainage | To RA | **Atrio-Ventricular Valves** | |
| Pulmonary Venous Drainage | To LA | Mitral Valve | Annulus = 14mm |
| Atrio-ventricular Connection | Concordant | Tricuspid Valve | Annulus = 17mm  **TAPSE = 12mm** |
| Ventriculo-Arterial Connection | DORV | **Ventricle** | |
| Ventricular Loop | d-Loop | Left Ventricle | Dilated |
| **Septae** |  | Right Ventricle | Dilated |
| Interatrial Septum | Intact | **Doppler Measurement** |  |
| Interventricular Septum | 12mm Sub-aortic VSD, BD Shunt | Mitral |  |
| **Semilunar Valves** |  | Aortic |  |
| Aortic Valve | Annulus = 11mm. aorto-mitral valve fibrous discontinuity | Tricuspid | Mild TR |
| Pulmonary Valve | Annulus = 17mm | Pulmonic | Moderate PR, PPG = 59mmHg |
| **Great Arteries** | NRGA | **Coronary Arteries** |  |
| Aorta | >50% over-ride to RV | **Aortic Arch** | Left |
| Pulmonary Arteries | **MPA = 17mm** | **PDA** | 1mm PDA, L – R Shunt |
| **M-Mode**: Normal LV Function on eye balling | | | |
| Ao | mm | PWd | mm |
| LA | mm | EDV | ml |
| LVIDd | mm | ESV | ml |
| LVIDs | mm | FS | % |
| IVSd | mm | LVEF | % |
| **Additional Information:** pericardial effusion with maximum depth of 5mm on RV Side | | | |
| **Conclusion:**   1. {S, D, D} Levocardia 2. RA/RV Dilated 3. DORV 4. Large Sub-aortic VSD, BD Shunt 5. Dysfunctional RV 6. Severe Pulmonary Hypertension 7. Small Pericardial effusion | | | |
| **Done By:** | **Signature** | **Date** | **Remark** |
| Tesfaye T., Pediatric Cardiologist |  | 04/08/14Eth.C. |  |

| **Patient Name: Belay Ayalew. Sex/Age: M/ 36 days. Date of Report: 04/08/2014Eth.C. MRN: 125909.** | | | |
| --- | --- | --- | --- |
| **Clinical Diagnosis: RD + DS. TGSH1.2548.** | | | |
| **Features:** | **Findings** | **Features** | **Findings** |
| **Profile** | | **Atria** | |
| Abdominal Situs | Solitus | Left Atrium | Normal |
| Cardiac Position | Levocardia | Right Atrium | Normal |
| Systemic Venous Drainage | To RA | **Atrio-Ventricular Valves** | |
| Pulmonary Venous Drainage | To LA | Mitral Valve | Annulus = 11mm |
| Atrio-ventricular Connection | Concordant | Tricuspid Valve | Annulus = 11mm |
| Ventriculo-Arterial Connection | concordant | **Ventricle** | |
| Ventricular Loop | d-Loop | Left Ventricle | Normal |
| **Septae** |  | Right Ventricle | Normal |
| Interatrial Septum | PFO, L – R Shunt | **Doppler Measurement** |  |
| Interventricular Septum | Intact | Mitral |  |
| **Semilunar Valves** |  | Aortic |  |
| Aortic Valve | Annulus = 9mm | Tricuspid |  |
| Pulmonary Valve | Annulus = 10mm | Pulmonic |  |
| **Great Arteries** | NRGA | **Coronary Arteries** |  |
| Aorta |  | **Aortic Arch** | Left |
| Pulmonary Arteries |  | **PDA** | No PDA |
| **M-Mode**: Normal LV Function on eye balling | | | |
| Ao | mm | PWd | mm |
| LA | mm | EDV | ml |
| LVIDd | mm | ESV | ml |
| LVIDs | mm | FS | % |
| IVSd | mm | LVEF | % |
| **Additional Information:** | | | |
| **Conclusion:**   1. {S, D, S} Levocardia 2. PFO, L – R Shunt | | | |
| **Done By:** | **Signature** | **Date** | **Remark** |
| Tesfaye T., Pediatric Cardiologist |  | 04/08/14Eth.C. |  |
| **Remark** | Can do the surgery safely. | | |

| **Patient Name: B/ Yemegn Getie Sex/Age: M/8 days. Date of Report: 04/08/2014Eth.C. MRN:126584.** | | | |
| --- | --- | --- | --- |
| **Clinical Diagnosis: DS + RD + Murmur. TGSH1.2549.** | | | |
| **Features:** | **Findings** | **Features** | **Findings** |
| **Profile** | | **Atria** | |
| Abdominal Situs | Solitus | Left Atrium | Normal |
| Cardiac Position | Levocardia | Right Atrium | Normal |
| Systemic Venous Drainage | To RA | **Atrio-Ventricular Valves** | |
| Pulmonary Venous Drainage | To LA | Mitral Valve | Annulus = 11mm |
| Atrio-ventricular Connection | Concordant | Tricuspid Valve | Annulus = 12mm |
| Ventriculo-Arterial Connection | concordant | **Ventricle** | |
| Ventricular Loop | d-Loop | Left Ventricle | Normal |
| **Septae** | Tongue of tissue in b/n | Right Ventricle | Normal |
| Interatrial Septum | 5 X8mm OS ASD, L – R Shunt. Additional 5mm Primum ASD, L – R Shunt | **Doppler Measurement** |  |
| Interventricular Septum | 4mm Inlet VSD, L – R Shunt | Mitral |  |
| **Semilunar Valves** |  | Aortic |  |
| Aortic Valve | Annulus = 9mm | Tricuspid |  |
| Pulmonary Valve | Annulus = 8mm | Pulmonic |  |
| **Great Arteries** | NRGA | **Coronary Arteries** |  |
| Aorta |  | **Aortic Arch** | Left |
| Pulmonary Arteries |  | **PDA** | No PDA |
| **M-Mode**: Normal LV Function on eye balling | | | |
| Ao | mm | PWd | mm |
| LA | mm | EDV | ml |
| LVIDd | mm | ESV | ml |
| LVIDs | mm | FS | % |
| IVSd | mm | LVEF | % |
| **Additional Information:** | | | |
| **Conclusion:**   1. {S, D, S} Levocardia 2. Transitional AVSD, L – R Shunt 3. Additional Moderate OS ASD, L – R Shunt 4. Normal LV Function | | | |
| **Done By:** | **Signature** | **Date** | **Remark** |
| Tesfaye T., Pediatric Cardiologist |  | 04/08/14Eth.C. |  |

| **Patient Name: Getaneh Bahiru. Sex/Age: Male/6months. Date of Report: 04/08/2014Eth.C. MRN: 126255.**  **Clinical Diagnosis: RD + Murmur. TGSH1.2550.** | | | |
| --- | --- | --- | --- |
| **Features:** | **Findings** | **Features** | **Findings** |
| **Profile** | | **Atria** | |
| Abdominal Situs | Solitus | Left Atrium | There is an oblique septum crossing from Left upper to right lower end of LA. |
| Cardiac Position | Levocardia | Right Atrium | Dilated |
| Systemic Venous Drainage | To RA | **Atrio-Ventricular Valves** | |
| Pulmonary Venous Drainage | To LA | Mitral Valve | Annulus = 14mm |
| Atrio-ventricular Connection | Concordant | Tricuspid Valve | Annulus = 19mm |
| Ventriculo-Arterial Connection | concordant | **Ventricle** | |
| Ventricular Loop | d-Loop | Left Ventricle | Normal |
| **Septae** |  | Right Ventricle | Dilated |
| Interatrial Septum | 5mm High Secundum ASD, L – R Shunt | **Doppler Measurement** |  |
| Interventricular Septum | Intact | Mitral |  |
| **Semilunar Valves** |  | Aortic |  |
| Aortic Valve | Annulus = 9mm | Tricuspid | Moderate TR, PPG = 70mmHg |
| Pulmonary Valve | Annulus = mm | Pulmonic | Moderate PR, PPG = 56mmHg |
| **Great Arteries** | NRGA | **Coronary Arteries** |  |
| Aorta |  | **Aortic Arch** | Left |
| Pulmonary Arteries |  | **PDA** | No PDA |
| **M-Mode**: | | | |
| Ao | mm | PWd | mm |
| LA | mm | EDV | ml |
| LVIDd | mm | ESV | ml |
| LVIDs | mm | FS | 35% |
| IVSd | mm | LVEF | 67% |
| **Additional Information:** Pericardial effusion with maximum depth of 5mm on RA/RV Junction area. | | | |
| **Conclusion:**   1. {S, D, S} Levocardia 2. Small High secundum ASD, L – R Shunt 3. ? Cortriatriatum 4. Moderate TR 5. Moderate PR 6. Severe Pulmonary Hypertension 7. Small Pericardial effusion | | | |
| **Done By:** | **Signature** | **Date** | **Remark** |
| Tesfaye T., Pediatric Cardiologist |  | 04/08/14Eth.C. |  |

| **Patient Name: Sifelig Tebikew. Sex/Age: F/8years. Date of Report:06/08/2014Eth.C. MRN: 098088.**  **Clinical Diagnosis: CHF + Rheumatic Recurrence + Murmur. TGSH1.2551.** | | | |
| --- | --- | --- | --- |
| **Features:** | **Findings** | **Features** | **Findings** |
| **Profile** | | **Atria** | |
| Abdominal Situs | Solitus | Left Atrium | More dilated |
| Cardiac Position | Levocardia | Right Atrium | Dilated |
| Systemic Venous Drainage | To RA. IVC Dilated | **Atrio-Ventricular Valves** | |
| Pulmonary Venous Drainage | To LA | Mitral Valve | Annulus = 30mm. thickened MVL |
| Atrio-ventricular Connection | Concordant | Tricuspid Valve | Annulus = 26mm  TAPSE = 21mm |
| Ventriculo-Arterial Connection | concordant | **Ventricle** | |
| Ventricular Loop | d-Loop | Left Ventricle | More dilated |
| **Septae** |  | Right Ventricle | Dilated |
| Interatrial Septum | Intact | **Doppler Measurement** |  |
| Interventricular Septum | Intact | Mitral | Severe MR, Holosystolic, posterior projection, seen in two planes with jet velocity = 5.4m/sec |
| **Semilunar Valves** |  | Aortic | Moderate AR, PHT = 380ms |
| Aortic Valve | Annulus = 14mm. | Tricuspid | Severe TR, PPG = 71mmHg |
| Pulmonary Valve | Annulus = 20mm | Pulmonic |  |
| **Great Arteries** | NRGA | **Coronary Arteries** |  |
| Aorta |  | **Aortic Arch** | Left |
| Pulmonary Arteries | MPA Dilated | **PDA** | No PDA |
| **M-Mode**: | | | |
| Ao | mm | PWd | mm |
| LA | mm | EDV | ml |
| LVIDd | mm | ESV | ml |
| LVIDs | mm | FS | 40% |
| IVSd | mm | LVEF | 70% |
| **Additional Information:** circumferential Pericardial effusion with maximum depth of 17mm in RA Side. | | | |
| **Conclusion:**   1. {S, D, S} Levocardia 2. All chambers dilated 3. Thickened MVL 4. Severe MR 5. Severe TR 6. Moderate AR 7. Severe Pulmonary Hypertension 8. Normal Biventricular Systolic Function | | | |
| **Done By:** | **Signature** | **Date** | **Remark** |
| Tesfaye T., Pediatric Cardiologist |  | 06/08/14Eth.C. |  |

| **Patient Name: B/Tesfaye Abeje. Sex/Age: F/9days. Date of Report: 06/08/2014Eth.C. MRN: 125501.** | | | |
| --- | --- | --- | --- |
| **Clinical Diagnosis: DS + RD. TGSH1.2552.** | | | |
| **Features:** | **Findings** | **Features** | **Findings** |
| **Profile** | | **Atria** | |
| Abdominal Situs | Solitus | Left Atrium | Normal |
| Cardiac Position | Levocardia | Right Atrium | Normal |
| Systemic Venous Drainage | To RA | **Atrio-Ventricular Valves** | |
| Pulmonary Venous Drainage | To LA | Mitral Valve | Annulus = 11mm |
| Atrio-ventricular Connection | Concordant | Tricuspid Valve | Annulus = 10mm  TAPSE = 12mm |
| Ventriculo-Arterial Connection | concordant | **Ventricle** | |
| Ventricular Loop | d-Loop | Left Ventricle | Normal |
| **Septae** |  | Right Ventricle | Normal |
| Interatrial Septum | 8mm OS ASD, L – R Shunt. | **Doppler Measurement** |  |
| Interventricular Septum | Intact | Mitral |  |
| **Semilunar Valves** |  | Aortic |  |
| Aortic Valve | Annulus = 10mm | Tricuspid |  |
| Pulmonary Valve | Annulus = 9mm | Pulmonic |  |
| **Great Arteries** | NRGA | **Coronary Arteries** |  |
| Aorta |  | **Aortic Arch** | Left |
| Pulmonary Arteries |  | **PDA** | No PDA |
| **M-Mode**: Normal LV Function on eye balling | | | |
| Ao | mm | PWd | mm |
| LA | mm | EDV | ml |
| LVIDd | mm | ESV | ml |
| LVIDs | mm | FS | % |
| IVSd | mm | LVEF | % |
| **Additional Information:** | | | |
| **Conclusion:**   1. {S, D, S} Levocardia 2. Moderate OS ASD, L – R Shunt 3. Normal Biventricular Systolic Function | | | |
| **Done By:** | **Signature** | **Date** | **Remark** |
| Tesfaye T., Pediatric Cardiologist |  | 06/08/14Eth.C. |  |

| **Patient Name: Kishen Tegegne. Sex/Age: F/9years. Date of Report:06/08/2014Eth.C. MRN: 126911.** | | | |
| --- | --- | --- | --- |
| **Clinical Diagnosis: Connective tissue disorder + easy fatigability. TGSH1.2553.** | | | |
| **Features:** | **Findings** | **Features** | **Findings** |
| **Profile** | | **Atria** | |
| Abdominal Situs | Solitus | Left Atrium | Normal |
| Cardiac Position | Levocardia | Right Atrium | Normal |
| Systemic Venous Drainage | To RA | **Atrio-Ventricular Valves** | |
| Pulmonary Venous Drainage | To LA | Mitral Valve | Annulus = 16mm |
| Atrio-ventricular Connection | Concordant | Tricuspid Valve | Annulus = 17mm  TAPSE = 17mm |
| Ventriculo-Arterial Connection | concordant | **Ventricle** | |
| Ventricular Loop | d-Loop | Left Ventricle | Normal |
| **Septae** |  | Right Ventricle | Normal |
| Interatrial Septum | Intact | **Doppler Measurement** |  |
| Interventricular Septum | Intact | Mitral |  |
| **Semilunar Valves** |  | Aortic |  |
| Aortic Valve | Annulus = 15mm | Tricuspid |  |
| Pulmonary Valve | Annulus = 20mm | Pulmonic |  |
| **Great Arteries** | NRGA | **Coronary Arteries** |  |
| Aorta |  | **Aortic Arch** | Left |
| Pulmonary Arteries |  | **PDA** | No PDA |
| **M-Mode**: | | | |
| Ao | mm | PWd | mm |
| LA | mm | EDV | ml |
| LVIDd | mm | ESV | ml |
| LVIDs | mm | FS | 31% |
| IVSd | mm | LVEF | 60% |
| **Additional Information:** | | | |
| **Conclusion:**   1. Normal Echocardiography Study | | | |
| **Remark:** consider Micronutrient replacement, work up for the clubbing and secondary causes of malnutrition. | | | |
| **Done By:** | **Signature** | **Date** | **Remark** |
| Tesfaye T., Pediatric Cardiologist |  | 06/08/14Eth.C. |  |

| **Patient Name: Birhanu Awoke. Sex/Age: M/5 8/12 Date of Report:11/08/2014Eth.C. MRN:123227** | | | |
| --- | --- | --- | --- |
| **Clinical Diagnosis: Pre-Op Screening. TGSH1.2554.** | | | |
| **Features:** | **Findings** | **Features** | **Findings** |
| **Profile** | | **Atria** | |
| Abdominal Situs | Solitus | Left Atrium | Normal |
| Cardiac Position | Levocardia | Right Atrium | Normal |
| Systemic Venous Drainage | To RA | **Atrio-Ventricular Valves** | |
| Pulmonary Venous Drainage | To LA | Mitral Valve | Annulus = 16mm |
| Atrio-ventricular Connection | Concordant | Tricuspid Valve | Annulus = 17mm  TAPSE = 17mm |
| Ventriculo-Arterial Connection | concordant | **Ventricle** | |
| Ventricular Loop | d-Loop | Left Ventricle | Normal |
| **Septae** |  | Right Ventricle | Normal |
| Interatrial Septum | Intact | **Doppler Measurement** |  |
| Interventricular Septum | Intact | Mitral |  |
| **Semilunar Valves** |  | Aortic |  |
| Aortic Valve | Annulus = 15mm | Tricuspid |  |
| Pulmonary Valve | Annulus = 20mm | Pulmonic |  |
| **Great Arteries** | NRGA | **Coronary Arteries** |  |
| Aorta |  | **Aortic Arch** | Left |
| Pulmonary Arteries |  | **PDA** | No PDA |
| **M-Mode**: | | | |
| Ao | mm | PWd | mm |
| LA | mm | EDV | ml |
| LVIDd | mm | ESV | ml |
| LVIDs | mm | FS | 31% |
| IVSd | mm | LVEF | 60% |
| **Additional Information:** | | | |
| **Conclusion:**   1. Normal Echocardiography Study | | | |
| **Done By:** | **Signature** | **Date** | **Remark** |
| Tesfaye T., Pediatric Cardiologist |  | 11/08/14Eth.C. |  |

| **Patient Name: Birhanu Le’alem. Sex/Age: M/8/12. Date of Report:11/08/2014Eth.C. MRN: 123224.** | | | |
| --- | --- | --- | --- |
| **Clinical Diagnosis: Incidental Murmur + Diaphoresis during BF. TGSH1.2555.** | | | |
| **Features:** | **Findings** | **Features** | **Findings** |
| **Profile** | | **Atria** | |
| Abdominal Situs | Solitus | Left Atrium | Normal |
| Cardiac Position | Levocardia | Right Atrium | Dilated |
| Systemic Venous Drainage | To RA | **Atrio-Ventricular Valves** | |
| Pulmonary Venous Drainage | To LA | Mitral Valve | Annulus = 15mm |
| Atrio-ventricular Connection | Concordant | Tricuspid Valve | Annulus = 24mm. TAPSE = 20mm |
| Ventriculo-Arterial Connection | concordant | **Ventricle** | |
| Ventricular Loop | d-Loop | Left Ventricle | Normal |
| **Septae** |  | Right Ventricle | Dilated |
| Interatrial Septum | 15mm X 25mm OS ASD, L – R Shunt | **Doppler Measurement** |  |
| Interventricular Septum | Intact | Mitral |  |
| **Semilunar Valves** |  | Aortic |  |
| Aortic Valve | Annulus = 11mm | Tricuspid | Moderate TR |
| Pulmonary Valve | Annulus = 19mm. Doming PV | Pulmonic | Moderate PS, PPG = 45 – 55 mmHg |
| **Great Arteries** | NRGA | **Coronary Arteries** |  |
| Aorta |  | **Aortic Arch** | Left |
| Pulmonary Arteries |  | **PDA** | No PDA |
| **M-Mode**: | | | |
| Ao | mm | PWd | mm |
| LA | mm | EDV | ml |
| LVIDd | mm | ESV | ml |
| LVIDs | mm | FS | 31% |
| IVSd | mm | LVEF | 60% |
| **Additional Information:** | | | |
| **Conclusion:**   1. {S, D, S} Levocardia 2. RA/RV Dilated 3. Large OS ASD, L – R Shunt 4. Moderate TR 5. Doming Pulmonary Valve 6. Moderate Pulmonary Stenosis 7. Normal Biventricular Systolic Function | | | |
| **Done By:** | **Signature** | **Date** | **Remark** |
| Tesfaye T., Pediatric Cardiologist |  | ___/08/14Eth.C. |  |

| **Patient Name: Tamere Teshale. Sex/Age: F /1 3/12yr. Date of Report:13/08/2014Eth.C. MRN:127798.** | | | |
| --- | --- | --- | --- |
| **Clinical Diagnosis: DS. TGSH1.2556.** | | | |
| **Features:** | **Findings** | **Features** | **Findings** |
| **Profile** | | **Atria** | |
| Abdominal Situs | Solitus | Left Atrium | Normal |
| Cardiac Position | Levocardia | Right Atrium | Normal |
| Systemic Venous Drainage | To RA | **Atrio-Ventricular Valves** | |
| Pulmonary Venous Drainage | To LA | Mitral Valve | Annulus = 14mm |
| Atrio-ventricular Connection | Concordant | Tricuspid Valve | Annulus = 17mm |
| Ventriculo-Arterial Connection | concordant | **Ventricle** | |
| Ventricular Loop | d-Loop | Left Ventricle | Normal |
| **Septae** |  | Right Ventricle | Normal |
| Interatrial Septum | Intact | **Doppler Measurement** |  |
| Interventricular Septum | Intact | Mitral |  |
| **Semilunar Valves** |  | Aortic |  |
| Aortic Valve | Annulus = 13mm | Tricuspid |  |
| Pulmonary Valve | Annulus = 17mm | Pulmonic |  |
| **Great Arteries** | NRGA | **Coronary Arteries** |  |
| Aorta |  | **Aortic Arch** | Left |
| Pulmonary Arteries |  | **PDA** | No PDA |
| **M-Mode**: | | | |
| Ao | mm | PWd | mm |
| LA | mm | EDV | ml |
| LVIDd | mm | ESV | ml |
| LVIDs | mm | FS | 36% |
| IVSd | mm | LVEF | 66% |
| **Additional Information:** Pericardial effusion with maximum depth of 5mm measurement**.** | | | |
| **Conclusion:**   1. {S, D, S} Levocardia 2. Small Pericardial effusion | | | |
| **Done By:** | **Signature** | **Date** | **Remark** |
| Tesfaye T., Pediatric Cardiologist |  | 13/08/14Eth.C. |  |

| **Patient Name: Asmamaw Tesfahun. Sex/Age: M/ 5/12. Date of Report:13/8/2014Eth.C. MRN: 127777.**  **Clinical Diagnosis: Diaphoresis + RD + Murmur. TGSH1.2557.** | | | |
| --- | --- | --- | --- |
| **Features:** | **Findings** | **Features** | **Findings** |
| **Profile** | | **Atria** | |
| Abdominal Situs | Solitus | Left Atrium | Markedly Dilated |
| Cardiac Position | Levocardia | Right Atrium | Normal |
| Systemic Venous Drainage | To RA | **Atrio-Ventricular Valves** | |
| Pulmonary Venous Drainage | To LA | Mitral Valve | Annulus = mm |
| Atrio-ventricular Connection | Concordant | Tricuspid Valve | Annulus = mm |
| Ventriculo-Arterial Connection | concordant | **Ventricle** | |
| Ventricular Loop | d-Loop | Left Ventricle | Markedly Dilated, Hypertrophied |
| **Septae** |  | Right Ventricle | Normal |
| Interatrial Septum | Intact | **Doppler Measurement** |  |
| Interventricular Septum | Intact | Mitral | Moderate MR, Holosystolic |
| **Semilunar Valves** |  | Aortic | Moderate Valvular AS, PPG/MPG = 67/31mmHg |
| Aortic Valve | Annulus = 8mm. | Tricuspid | Trial TR, PPG = 20mmHg |
| Pulmonary Valve | Annulus = 11mm | Pulmonic |  |
| **Great Arteries** | NRGA | **Coronary Arteries** |  |
| Aorta |  | **Aortic Arch** | Left |
| Pulmonary Arteries |  | **PDA** | 3mm PDA, L – R Shunt |
| **M-Mode**: Normal LV Function on eye balling | | | |
| Ao | mm | PWd | mm |
| LA | mm | EDV | ml |
| LVIDd | mm | ESV | ml |
| LVIDs | mm | FS | % |
| IVSd | mm | LVEF | % |
| **Additional Information:** | | | |
| **Conclusion:**   1. {S, D, S} Levocardia 2. LA/LV Dilated 3. Moderate MR 4. Moderate Valvular AS 5. Moderate PDA, L – R Shunt 6. LV Hypertrophied 7. Normal LV Systolic Function | | | |
| **Done By:** | **Signature** | **Date** | **Remark** |
| Tesfaye T., Pediatric Cardiologist |  | 13/08/14Eth.C. |  |

| **Patient Name: Kidane Behailu . Sex/Age: M/8 yrs. Date of Report: 13/08/2014Eth.C. MRN: 127599.** | | | |
| --- | --- | --- | --- |
| **Clinical Diagnosis: ARF + Murmur. TGSH1.2558.** | | | |
| **Features:** | **Findings** | **Features** | **Findings** |
| **Profile** | | **Atria** | |
| Abdominal Situs | Solitus | Left Atrium | Mildly Dilated |
| Cardiac Position | Levocardia | Right Atrium | Normal |
| Systemic Venous Drainage | To RA | **Atrio-Ventricular Valves** | |
| Pulmonary Venous Drainage | To LA | Mitral Valve | Annulus = 23mm. Thickened MVL |
| Atrio-ventricular Connection | Concordant | Tricuspid Valve | Annulus = 22mm  TAPSE = 21mm |
| Ventriculo-Arterial Connection | concordant | **Ventricle** | |
| Ventricular Loop | d-Loop | Left Ventricle | Mildly Dilated |
| **Septae** |  | Right Ventricle | Normal |
| Interatrial Septum | Intact | **Doppler Measurement** |  |
| Interventricular Septum | Intact | Mitral | Moderate MR, Holosystolic, posterior projection, seen in two planes with jet velocity = 4.7m/sec. |
| **Semilunar Valves** |  | Aortic |  |
| Aortic Valve | Annulus = 18mm | Tricuspid |  |
| Pulmonary Valve | Annulus = 22mm | Pulmonic |  |
| **Great Arteries** | NRGA | **Coronary Arteries** |  |
| Aorta |  | **Aortic Arch** | Left |
| Pulmonary Arteries |  | **PDA** | No PDA |
| **M-Mode**: | | | |
| Ao | mm | PWd | mm |
| LA | mm | EDV | ml |
| LVIDd | mm | ESV | ml |
| LVIDs | mm | FS | 38% |
| IVSd | mm | LVEF | 68% |
| **Additional Information:** | | | |
| **Conclusion:**   1. {S, D, S} Levocardia 2. Mildly Dilated LA/LV 3. Thickened MVL 4. Moderate MR 5. Normal Biventricular Systolic Function | | | |
| **Done By:** | **Signature** | **Date** | **Remark** |
| Tesfaye T., Pediatric Cardiologist |  | 13/08/14Eth.C. |  |

| **Patient Name: Eyob Andualem. Sex/Age: M/41days. Date of Report:19/08/2014Eth.C. MRN: 127965.** | | | |
| --- | --- | --- | --- |
| **Clinical Diagnosis: Incidental Murmur. TGSH1.2559.** | | | |
| **Features:** | **Findings** | **Features** | **Findings** |
| **Profile** | | **Atria** | |
| Abdominal Situs | Solitus | Left Atrium | Normal |
| Cardiac Position | Levocardia | Right Atrium | Normal |
| Systemic Venous Drainage | To RA | **Atrio-Ventricular Valves** | |
| Pulmonary Venous Drainage | To LA | Mitral Valve | Annulus = 13mm |
| Atrio-ventricular Connection | Concordant | Tricuspid Valve | Annulus = 14mm |
| Ventriculo-Arterial Connection | Concordant | **Ventricle** | |
| Ventricular Loop | d-Loop | Left Ventricle | Normal |
| **Septae** |  | Right Ventricle | Normal |
| Interatrial Septum | PFO, L – R Shunt | **Doppler Measurement** |  |
| Interventricular Septum | 4mm PM VSD, L – R Shunt with PPG = 52mmHg | Mitral |  |
| **Semilunar Valves** |  | Aortic |  |
| Aortic Valve | Annulus = 11mm | Tricuspid |  |
| Pulmonary Valve | Annulus = 11mm | Pulmonic |  |
| **Great Arteries** | NRGA | **Coronary Arteries** |  |
| Aorta |  | **Aortic Arch** | Left |
| Pulmonary Arteries |  | **PDA** | No PDA |
| **M-Mode**: Normal LV Function on eye balling | | | |
| Ao | mm | PWd | mm |
| LA | mm | EDV | ml |
| LVIDd | mm | ESV | ml |
| LVIDs | mm | FS | % |
| IVSd | mm | LVEF | % |
| **Additional Information:** | | | |
| **Conclusion:**   1. {S, D, S} Levocardia 2. PFO, L – R Shunt 3. Small Restrictive PM VSD, L – R Shunt 4. Normal LV Systolic Function | | | |
| **Done By:** | **Signature** | **Date** | **Remark** |
| Tesfaye T., Pediatric Cardiologist |  | 19/08/14Eth.C. |  |

| **Patient Name: Eyosias Meseret. Sex/Age: M/11yrs. Date of Report:19/08/2014Eth.C. MRN: __________.** | | | |
| --- | --- | --- | --- |
| **Clinical Diagnosis: Easy Fatigability. TGSH1.2560.** | | | |
| **Features:** | **Findings** | **Features** | **Findings** |
| **Profile** | | **Atria** | |
| Abdominal Situs | Solitus | Left Atrium | Normal |
| Cardiac Position | Levocardia | Right Atrium | Normal |
| Systemic Venous Drainage | To RA | **Atrio-Ventricular Valves** | |
| Pulmonary Venous Drainage | To LA | Mitral Valve | Annulus = 22mm |
| Atrio-ventricular Connection | Concordant | Tricuspid Valve | Annulus = 21mm |
| Ventriculo-Arterial Connection | concordant | **Ventricle** | |
| Ventricular Loop | d-Loop | Left Ventricle | Normal |
| **Septae** |  | Right Ventricle | Normal |
| Interatrial Septum | Intact | **Doppler Measurement** |  |
| Interventricular Septum | Intact | Mitral |  |
| **Semilunar Valves** |  | Aortic |  |
| Aortic Valve | Annulus = 19mm | Tricuspid |  |
| Pulmonary Valve | Annulus = 19mm | Pulmonic |  |
| **Great Arteries** | NRGA | **Coronary Arteries** |  |
| Aorta |  | **Aortic Arch** | Left |
| Pulmonary Arteries |  | **PDA** | No PDA |
| **M-Mode**: Normal LV Function on eye balling | | | |
| Ao | mm | PWd | mm |
| LA | mm | EDV | ml |
| LVIDd | mm | ESV | ml |
| LVIDs | mm | FS | % |
| IVSd | mm | LVEF | % |
| **Additional Information:** | | | |
| **Conclusion:**   1. Normal Echocardiography Study | | | |
| **Done By:** | **Signature** | **Date** | **Remark** |
| Tesfaye T., Pediatric Cardiologist |  | 19/08/14Eth.C. |  |

| **Patient Name: Ferehiwot Tigabu Sex/Age: F/4yrs. Date of Report: 25/08/2014Eth.C. MRN: 128680**  **Clinical Diagnosis: CHF + Murmur + Diaphoresis. TGSH1.2561.** | | | |
| --- | --- | --- | --- |
| **Features:** | **Findings** | **Features** | **Findings** |
| **Profile** | | **Atria** | |
| Abdominal Situs | Solitus | Left Atrium | Dilated |
| Cardiac Position | Levocardia | Right Atrium | More Dilated |
| Systemic Venous Drainage | To RA | **Atrio-Ventricular Valves** | |
| Pulmonary Venous Drainage | To LA | Mitral Valve | Annulus = 17mm |
| Atrio-ventricular Connection | Concordant | Tricuspid Valve | Annulus = 21mm  TAPSE = 19mm |
| Ventriculo-Arterial Connection | concordant | **Ventricle** | |
| Ventricular Loop | d-Loop | Left Ventricle | Dilated |
| **Septae** |  | Right Ventricle | More Dilated |
| Interatrial Septum | Intact | **Doppler Measurement** |  |
| Interventricular Septum | 11mm Mid-Muscular VSD, L – R Shunt | Mitral |  |
| **Semilunar Valves** |  | Aortic |  |
| Aortic Valve | Annulus = 14mm | Tricuspid | Moderate TR |
| Pulmonary Valve | Annulus = 21mm | Pulmonic |  |
| **Great Arteries** | NRGA | **Coronary Arteries** |  |
| Aorta |  | **Aortic Arch** | Left |
| Pulmonary Arteries | MPA = 21mm. confluent Branch PAs. | **PDA** | No PDA |
| **M-Mode**: | | | |
| Ao | mm | PWd | mm |
| LA | mm | EDV | ml |
| LVIDd | mm | ESV | ml |
| LVIDs | mm | FS | 36% |
| IVSd | mm | LVEF | 67% |
| **Additional Information:** | | | |
| **Conclusion:**   1. {S, D, S} Levocardia 2. All chambers dilated 3. Moderate TR 4. Large Mid-Muscular VSD, L – R Shunt 5. Severe Pulmonary Hypertension 6. Normal Biventricular Systolic Function | | | |
| **Done By:** | **Signature** | **Date** | **Remark** |
| Tesfaye T., Pediatric Cardiologist |  | 25/08/14Eth.C. |  |

| **Patient Name: Kalkidan Atalo. Sex/Age: F/3/12. Date of Report: 25/08/2014Eth.C. MRN:128472** | | | |
| --- | --- | --- | --- |
| **Clinical Diagnosis: Incidental Murmur. TGSH1.2562.** | | | |
| **Features:** | **Findings** | **Features** | **Findings** |
| **Profile** | | **Atria** | |
| Abdominal Situs | Solitus | Left Atrium | Normal |
| Cardiac Position | Levocardia | Right Atrium | Normal |
| Systemic Venous Drainage | To RA | **Atrio-Ventricular Valves** | |
| Pulmonary Venous Drainage | To LA | Mitral Valve | Annulus = 11mm |
| Atrio-ventricular Connection | Concordant | Tricuspid Valve | Annulus = 11mm |
| Ventriculo-Arterial Connection | concordant | **Ventricle** | |
| Ventricular Loop | d-Loop | Left Ventricle | Normal |
| **Septae** |  | Right Ventricle | RVH |
| Interatrial Septum | PFO, L – R Shunt | **Doppler Measurement** |  |
| Interventricular Septum | 2mm Supracristal VSD, L – R Shunt | Mitral |  |
| **Semilunar Valves** |  | Aortic |  |
| Aortic Valve | Annulus = 11mm | Tricuspid |  |
| Pulmonary Valve | Annulus = 7mm. dysplastic PV | Pulmonic | Severe Valvular PS, PPG = 67mmHg |
| **Great Arteries** | NRGA | **Coronary Arteries** |  |
| Aorta |  | **Aortic Arch** | Left |
| Pulmonary Arteries | Confluent BPAs. Post stenotic PA Dilatation | **PDA** | No PDA |
| **M-Mode**: Normal LV Function on eye balling | | | |
| Ao | mm | PWd | mm |
| LA | mm | EDV | ml |
| LVIDd | mm | ESV | ml |
| LVIDs | mm | FS | % |
| IVSd | mm | LVEF | % |
| **Additional Information:** | | | |
| **Conclusion:**   1. {S, D, S} Levocardia 2. PFO, L – R Shunt 3. Small Supracristal VSD, L – R Shunt 4. Severe Pulmonary Stenosis 5. Dysplastic Pulmonary Valve 6. RVH 7. Normal LV Systolic Function | | | |
| **Done By:** | **Signature** | **Date** | **Remark** |
| Tesfaye T., Pediatric Cardiologist |  | 25/08/14Eth.C. |  |

| **Patient Name: Bemnet Bamlaku. Sex/Age: F/8months. Date of Report:25/08/2014Eth.C. MRN: 106840.**  **Clinical Diagnosis: Cyanosis. TGSH1.2563.** | | | |
| --- | --- | --- | --- |
| **Features:** | **Findings** | **Features** | **Findings** |
| **Profile** | | **Atria** | |
| Abdominal Situs | Solitus | Left Atrium | Normal |
| Cardiac Position | Levocardia | Right Atrium | Normal |
| Systemic Venous Drainage | To RA | **Atrio-Ventricular Valves** | |
| Pulmonary Venous Drainage | To LA | Mitral Valve | Annulus = 13mm |
| Atrio-ventricular Connection | Concordant | Tricuspid Valve | Annulus = 12mm |
| Ventriculo-Arterial Connection | concordant | **Ventricle** | |
| Ventricular Loop | d-Loop | Left Ventricle | Normal |
| **Septae** |  | Right Ventricle | RVH |
| Interatrial Septum | Intact | **Doppler Measurement** |  |
| Interventricular Septum | Non-Restrictive Malaligned Subaortic VSD, R – L Shunt | Mitral |  |
| **Semilunar Valves** |  | Aortic |  |
| Aortic Valve | Annulus = 11mm | Tricuspid |  |
| Pulmonary Valve | Atretic | Pulmonic |  |
| **Great Arteries** | NRGA | **Coronary Arteries** |  |
| Aorta | Overriding aorta | **Aortic Arch** | Left |
| Pulmonary Arteries |  | **PDA** | No PDA |
| **M-Mode**: Normal LV Function on eye balling | | | |
| Ao | mm | PWd | mm |
| LA | mm | EDV | ml |
| LVIDd | mm | ESV | ml |
| LVIDs | mm | FS | % |
| IVSd | mm | LVEF | % |
| **Additional Information:** | | | |
| **Conclusion:**   1. {S, D, S} Levocardia 2. Non-Restrictive Malaligned Sub aortic VSD, R – L Shunt 3. Pulmonary Atresia 4. RVH (Extreme variant of TOF) | | | |
| **Done By:** | **Signature** | **Date** | **Remark** |
| Tesfaye T., Pediatric Cardiologist |  | 25/08/14Eth.C. |  |

| **Patient Name: Hiwote Getnet. Sex/Age: F/1yr. Date of Report:25/08/2014Eth.C. MRN: 128732.** | | | |
| --- | --- | --- | --- |
| **Clinical Diagnosis: Incidental Murmur. TGSH1.2564.** | | | |
| **Features:** | **Findings** | **Features** | **Findings** |
| **Profile** | | **Atria** | |
| Abdominal Situs | Solitus | Left Atrium | Normal |
| Cardiac Position | Levocardia | Right Atrium | Normal |
| Systemic Venous Drainage | To RA | **Atrio-Ventricular Valves** | |
| Pulmonary Venous Drainage | To LA | Mitral Valve | Annulus = 13mm |
| Atrio-ventricular Connection | Concordant | Tricuspid Valve | Annulus = 14mm  TAPSE = 16mm |
| Ventriculo-Arterial Connection | concordant | **Ventricle** | |
| Ventricular Loop | d-Loop | Left Ventricle | Normal |
| **Septae** |  | Right Ventricle | Normal |
| Interatrial Septum | Intact | **Doppler Measurement** |  |
| Interventricular Septum | Intact | Mitral |  |
| **Semilunar Valves** |  | Aortic |  |
| Aortic Valve | Annulus = 10mm | Tricuspid |  |
| Pulmonary Valve | Annulus = 12mm | Pulmonic |  |
| **Great Arteries** | NRGA | **Coronary Arteries** |  |
| Aorta |  | **Aortic Arch** | Left |
| Pulmonary Arteries |  | **PDA** | 1mm PDA, L – R Shunt |
| **M-Mode**: Normal LV Function on eye balling | | | |
| Ao | mm | PWd | mm |
| LA | mm | EDV | ml |
| LVIDd | mm | ESV | ml |
| LVIDs | mm | FS | % |
| IVSd | mm | LVEF | % |
| **Additional Information:** | | | |
| **Conclusion:**   1. {S, D, S} Levocardia 2. Small PDA, L – R Shunt | | | |
| **Recommendation:**   1. Follow up echocardiography study annually. 2. No need to start medicine. | | | |
| **Done By:** | **Signature** | **Date** | **Remark** |
| Tesfaye T., Pediatric Cardiologist |  | 25/08/14Eth.C. |  |

| **Patient Name: Natnael Desalegn. Sex/Age: M/40days. Date of Report: 28/08/2014Eth.C. MRN: 129177.**  **Clinical Diagnosis: RD + CHF. TGSH1.2565.** | | | |
| --- | --- | --- | --- |
| **Features:** | **Findings** | **Features** | **Findings** |
| **Profile** | | **Atria** | |
| Abdominal Situs | Solitus | Left Atrium | Normal |
| Cardiac Position | Levocardia | Right Atrium | Dilated |
| Systemic Venous Drainage | To RA | **Atrio-Ventricular Valves** | |
| Pulmonary Venous Drainage | To LA | Mitral Valve | Annulus = 11mm |
| Atrio-ventricular Connection | Concordant | Tricuspid Valve | Annulus = 12mm. **TAPSE = 5mm** |
| Ventriculo-Arterial Connection | concordant | **Ventricle** | |
| Ventricular Loop | d-Loop | Left Ventricle | Normal |
| **Septae** |  | Right Ventricle | Dilated. Two layered RV wall with thinner compacted epicardial layer and inner non compacted layer with prominent trabeculations associated with deep inter trabecular recesses. |
| Interatrial Septum | PFO, L – R Shunt | **Doppler Measurement** |  |
| Interventricular Septum | Intact | Mitral |  |
| **Semilunar Valves** |  | Aortic |  |
| Aortic Valve | Annulus = 7mm | Tricuspid | Trivial TR |
| Pulmonary Valve | Annulus = 12mm | Pulmonic | Trivial PR |
| **Great Arteries** | NRGA | **Coronary Arteries** |  |
| Aorta |  | **Aortic Arch** | Left |
| Pulmonary Arteries | **MPA = 15mm** | **PDA** | No PDA |
| **M-Mode**: | | | |
| Ao | mm | PWd | mm |
| LA | mm | EDV | ml |
| LVIDd | mm | ESV | ml |
| LVIDs | mm | FS | 34% |
| IVSd | mm | LVEF | 65% |
| **Additional Information:** Pericardial effusion on RA Side with maximum depth of 8mm. | | | |
| **Conclusion:**   1. {S, D, S} Levocardia 2. PFO, L – R Shunt 3. RA/RV Dilated 4. RV NCM 5. Dysfunctional RV 6. Severe pulmonary Hypertension 7. Normal LV Systolic Function 8. Small Pericardial effusion | | | |
| **Done By:** | **Signature** | **Date** | **Remark** |
| Tesfaye T., Pediatric Cardiologist |  | 28/08/14Eth.C. |  |

| **Patient Name: Meron Asmamaw. Sex/Age: F/13years. Date of Report: 01/09/2014Eth.C. MRN: 129655.** | | | |
| --- | --- | --- | --- |
| **Clinical Diagnosis: RD + ACCENTUATED P2 + Murmur. TGSH1.2566.** | | | |
| **Features:** | **Findings** | **Features** | **Findings** |
| **Profile** | | **Atria** | |
| Abdominal Situs | Solitus | Left Atrium | Normal |
| Cardiac Position | Levocardia | Right Atrium | Dilated |
| Systemic Venous Drainage | To RA | **Atrio-Ventricular Valves** | |
| Pulmonary Venous Drainage | To LA | Mitral Valve | Annulus = 19mm |
| Atrio-ventricular Connection | Concordant | Tricuspid Valve | Annulus = 33mm  TAPSE = 22mm |
| Ventriculo-Arterial Connection | concordant | **Ventricle** | |
| Ventricular Loop | d-Loop | Left Ventricle | Normal |
| **Septae** |  | Right Ventricle | Dilated |
| Interatrial Septum | Intact | **Doppler Measurement** |  |
| Interventricular Septum | Intact | Mitral |  |
| **Semilunar Valves** |  | Aortic |  |
| Aortic Valve | Annulus = 19mm | Tricuspid | Moderate TR, PPG = 98mmHg |
| Pulmonary Valve | Annulus = 19mm | Pulmonic |  |
| **Great Arteries** | NRGA | **Coronary Arteries** |  |
| Aorta |  | **Aortic Arch** | Left |
| Pulmonary Arteries |  | **PDA** | No PDA |
| **M-Mode**: | | | |
| Ao | mm | PWd | mm |
| LA | mm | EDV | ml |
| LVIDd | mm | ESV | ml |
| LVIDs | mm | FS | 40% |
| IVSd | mm | LVEF | 71% |
| **Additional Information:** | | | |
| **Conclusion:**   1. {S, D, S} Levocardia 2. RA/RV Dilated 3. Moderate TR 4. Severe Pulmonary Hypertension 5. Normal Biventricular Systolic Function | | | |
| **Remark:** extreme tachycardia during study. | | | |
| **Done By:** | **Signature** | **Date** | **Remark** |
| Tesfaye T., Pediatric Cardiologist |  | ___/08/14Eth.C. |  |

| **Patient Name: B/Alembanche Abie. Sex/Age: F/31days . Date of Report: 01/09/2014Eth.C. MRN: 129144.** | | | |
| --- | --- | --- | --- |
| **Clinical Diagnosis: DS + Incidental Murmur. TGSH1.2567.** | | | |
| **Features:** | **Findings** | **Features** | **Findings** |
| **Profile** | | **Atria** | |
| Abdominal Situs | Solitus | Left Atrium | Normal |
| Cardiac Position | Levocardia | Right Atrium | Normal |
| Systemic Venous Drainage | To RA | **Atrio-Ventricular Valves** | |
| Pulmonary Venous Drainage | To LA | Mitral Valve | Annulus = 10mm |
| Atrio-ventricular Connection | Concordant | Tricuspid Valve | Annulus = 10mm |
| Ventriculo-Arterial Connection | concordant | **Ventricle** | |
| Ventricular Loop | d-Loop | Left Ventricle | Normal |
| **Septae** |  | Right Ventricle | Normal |
| Interatrial Septum | 7mm OS ASD, L – R Shunt | **Doppler Measurement** |  |
| Interventricular Septum | Intact | Mitral |  |
| **Semilunar Valves** |  | Aortic |  |
| Aortic Valve | Annulus = 7mm | Tricuspid |  |
| Pulmonary Valve | Annulus = 7mm | Pulmonic |  |
| **Great Arteries** | NRGA | **Coronary Arteries** |  |
| Aorta |  | **Aortic Arch** | Left |
| Pulmonary Arteries |  | **PDA** | No PDA |
| **M-Mode**: Normal LV Function on eye balling | | | |
| Ao | mm | PWd | mm |
| LA | mm | EDV | ml |
| LVIDd | mm | ESV | ml |
| LVIDs | mm | FS | % |
| IVSd | mm | LVEF | % |
| **Additional Information:** | | | |
| **Conclusion:**   1. {S, D, S} Levocardia 2. Moderate OS ASD, L – R Shunt | | | |
| **Done By:** | **Signature** | **Date** | **Remark** |
| Tesfaye T., Pediatric Cardiologist |  | 01/09/14Eth.C. |  |

| **Patient Name: Cheru Kendie. Sex/Age: M/13 yrs. Date of Report: 02/09/2014Eth.C. MRN: __________.**  **Clinical Diagnosis: ARF + Murmur. TGSH1.2568.** | | | |
| --- | --- | --- | --- |
| **Features:** | **Findings** | **Features** | **Findings** |
| **Profile** | | **Atria** | |
| Abdominal Situs | Solitus | Left Atrium | Normal |
| Cardiac Position | Levocardia | Right Atrium | Normal |
| Systemic Venous Drainage | To RA | **Atrio-Ventricular Valves** | |
| Pulmonary Venous Drainage | To LA | Mitral Valve | Annulus = 22mm. thickened MVL |
| Atrio-ventricular Connection | Concordant | Tricuspid Valve | Annulus = 23mm  TAPSE = 21mm |
| Ventriculo-Arterial Connection | concordant | **Ventricle** | |
| Ventricular Loop | d-Loop | Left Ventricle | Normal |
| **Septae** |  | Right Ventricle | Normal |
| Interatrial Septum | Intact | **Doppler Measurement** |  |
| Interventricular Septum | Intact | Mitral | Mild MR, Holosystolic, posterior projection, seen in two planes with jet velocity = 4.7m/sec |
| **Semilunar Valves** |  | Aortic | Mild AR, PHT = 527ms |
| Aortic Valve | Annulus = 20mm | Tricuspid |  |
| Pulmonary Valve | Annulus = 22mm | Pulmonic |  |
| **Great Arteries** | NRGA | **Coronary Arteries** |  |
| Aorta |  | **Aortic Arch** | Left |
| Pulmonary Arteries |  | **PDA** | No PDA |
| **M-Mode**: | | | |
| Ao | mm | PWd | mm |
| LA | mm | EDV | ml |
| LVIDd | mm | ESV | ml |
| LVIDs | mm | FS | 32% |
| IVSd | mm | LVEF | 60% |
| **Additional Information:** | | | |
| **Conclusion:**   1. {S, D, S} Levocardia 2. Thickened MVL 3. Mild MR 4. Mild AR 5. Normal Biventricular Systolic Function | | | |
| **Done By:** | **Signature** | **Date** | **Remark** |
| Tesfaye T., Pediatric Cardiologist |  | 02/09/14Eth.C. |  |

| **Patient Name: B/Muluken Genet. Sex/Age: F/21 Days. Date of Report:02/09/2014Eth.C. MRN: 129161.** | | | |
| --- | --- | --- | --- |
| **Clinical Diagnosis: Incidental Murmur. TGSH1.2569.** | | | |
| **Features:** | **Findings** | **Features** | **Findings** |
| **Profile** | | **Atria** | |
| Abdominal Situs | Solitus | Left Atrium | Normal |
| Cardiac Position | Levocardia | Right Atrium | Normal |
| Systemic Venous Drainage | To RA | **Atrio-Ventricular Valves** | |
| Pulmonary Venous Drainage | To LA | Mitral Valve | Annulus = 10mm |
| Atrio-ventricular Connection | Concordant | Tricuspid Valve | Annulus = 9mm |
| Ventriculo-Arterial Connection | concordant | **Ventricle** | |
| Ventricular Loop | d-Loop | Left Ventricle | Normal |
| **Septae** |  | Right Ventricle | Normal |
| Interatrial Septum | PFO, L – R Shunt | **Doppler Measurement** |  |
| Interventricular Septum | 2.5mm PM VSD, L – R Shunt | Mitral |  |
| **Semilunar Valves** |  | Aortic |  |
| Aortic Valve | Annulus = 8mm | Tricuspid |  |
| Pulmonary Valve | Annulus = 8mm | Pulmonic |  |
| **Great Arteries** | NRGA | **Coronary Arteries** |  |
| Aorta |  | **Aortic Arch** | Left |
| Pulmonary Arteries |  | **PDA** | 1mm PDA, L – R Shunt |
| **M-Mode**: Normal LV Function on eye balling | | | |
| Ao | mm | PWd | mm |
| LA | mm | EDV | ml |
| LVIDd | mm | ESV | ml |
| LVIDs | mm | FS | % |
| IVSd | mm | LVEF | % |
| **Additional Information:** | | | |
| **Conclusion:**   1. {S, D, S} Levocardia 2. PFO, L – R Shunt 3. Small PM VSD, L – R Shunt 4. Small PDA, L – R Shunt 5. Normal LV Systolic Function | | | |
| **Done By:** | **Signature** | **Date** | **Remark** |
| Tesfaye T., Pediatric Cardiologist |  | 02/09/14Eth.C. |  |

| **Patient Name: Yechale Belay. Sex/Age: M/14. Date of Report:_02/09/2014Eth.C. MRN: 128485.** | | | |
| --- | --- | --- | --- |
| **Clinical Diagnosis: Systemic Hypertension 20 to AGN. TGSH1.2570.** | | | |
| **Features:** | **Findings** | **Features** | **Findings** |
| **Profile** | | **Atria** | |
| Abdominal Situs | Solitus | Left Atrium | Normal |
| Cardiac Position | Levocardia | Right Atrium | Normal |
| Systemic Venous Drainage | To RA | **Atrio-Ventricular Valves** | |
| Pulmonary Venous Drainage | To LA | Mitral Valve | Annulus = 19mm |
| Atrio-ventricular Connection | Concordant | Tricuspid Valve | Annulus = 23mm |
| Ventriculo-Arterial Connection | concordant | **Ventricle** | |
| Ventricular Loop | d-Loop | Left Ventricle | Normal |
| **Septae** |  | Right Ventricle | Normal |
| Interatrial Septum | Intact | **Doppler Measurement** |  |
| Interventricular Septum | Intact | Mitral |  |
| **Semilunar Valves** |  | Aortic |  |
| Aortic Valve | Annulus = 19mm | Tricuspid |  |
| Pulmonary Valve | Annulus = 23mm | Pulmonic |  |
| **Great Arteries** | NRGA | **Coronary Arteries** |  |
| Aorta | Ascending aorta = 20mm. sinus = 34mm. SAJ = 23mm | **Aortic Arch** | Left. No CoA. |
| Pulmonary Arteries |  | **PDA** | No PDA |
| **M-Mode**: | | | |
| Ao | mm | PWd | 12mm |
| LA | mm | EDV | 69ml |
| LVIDd | 40mm | ESV | 21ml |
| LVIDs | 24mm | FS | 39% |
| IVSd | 13mm | LVEF | 70% |
| **Additional Information:** | | | |
| **Conclusion:**   1. {S, D, S} Levocardia 2. LVH | | | |
| **Done By:** | **Signature** | **Date** | **Remark** |
| Tesfaye T., Pediatric Cardiologist |  | 02/09/14Eth.C. |  |

| **Patient Name: Rediet Masresha. Sex/Age: F/11monthes. Date of Report:04/09/2014Eth.C. MRN: 129775.**  **Clinical Diagnosis: Cyanosis + CHF + RD + DS. TGSH1.2571.** | | | |
| --- | --- | --- | --- |
| **Features:** | **Findings** | **Features** | **Findings** |
| **Profile** | | **Atria** | |
| Abdominal Situs | Solitus | Left Atrium | Dilated |
| Cardiac Position | Levocardia | Right Atrium | Dilated |
| Systemic Venous Drainage | To RA | **Atrio-Ventricular Valves** | |
| Pulmonary Venous Drainage | To LA | Mitral Valve | Common Complete AVSD |
| Atrio-ventricular Connection | Common Complete AVSD | Tricuspid Valve |  |
| Ventriculo-Arterial Connection | DORV | **Ventricle** | |
| Ventricular Loop | d-Loop | Left Ventricle | Smallish |
| **Septae** |  | Right Ventricle | Dilated |
| Interatrial Septum | Common Complete AVSD, L – R Shunt | **Doppler Measurement** |  |
| Interventricular Septum |  | Mitral |  |
| **Semilunar Valves** |  | Aortic |  |
| Aortic Valve | Annulus = 11mm | Tricuspid | Moderate Right AVVR |
| Pulmonary Valve | Annulus = 9mm. Doming PV | Pulmonic | Mild PS, PPG = 30mmHg |
| **Great Arteries** | d-TGA(Side by side) | **Coronary Arteries** |  |
| Aorta | Anterior and to the right. From RV | **Aortic Arch** | Left |
| Pulmonary Arteries | Posterior and to the left. From RV | **PDA** | 1mm PDA, Aorta to PA |
| **M-Mode**: | | | |
| Ao | mm | PWd | mm |
| LA | mm | EDV | ml |
| LVIDd | mm | ESV | ml |
| LVIDs | mm | FS | % |
| IVSd | mm | LVEF | % |
| **Additional Information:** | | | |
| **Conclusion:**   1. {S, D, Side by side} Levocardia 2. DORV 3. Common Complete Unbalanced AVSD, L – R Shunt 4. Moderate Right AVVR 5. Doming Pulmonary Valve 6. Mild Valvular PS 7. Smallish LV | | | |
| **Done By:** | **Signature** | **Date** | **Remark** |
| Tesfaye T., Pediatric Cardiologist |  | 04/09/14Eth.C. |  |

| **Patient Name: B/Tete Nigus. Sex/Age: F/27days. Date of Report: 04/09/2014Eth.C. MRN: 122774.** | | | |
| --- | --- | --- | --- |
| **Clinical Diagnosis: Incidental Murmur. TGSH1.2572.** | | | |
| **Features:** | **Findings** | **Features** | **Findings** |
| **Profile** | | **Atria** | |
| Abdominal Situs | Solitus | Left Atrium | Normal |
| Cardiac Position | Levocardia | Right Atrium | Normal |
| Systemic Venous Drainage | To RA | **Atrio-Ventricular Valves** | |
| Pulmonary Venous Drainage | To LA | Mitral Valve | Annulus = 13mm |
| Atrio-ventricular Connection | Concordant | Tricuspid Valve | Annulus = 12mm |
| Ventriculo-Arterial Connection | concordant | **Ventricle** | |
| Ventricular Loop | d-Loop | Left Ventricle | Normal |
| **Septae** |  | Right Ventricle | Normal |
| Interatrial Septum | 5mm OS ASD, L – R Shunt | **Doppler Measurement** |  |
| Interventricular Septum | Intact | Mitral |  |
| **Semilunar Valves** |  | Aortic |  |
| Aortic Valve | Annulus = 11mm | Tricuspid |  |
| Pulmonary Valve | Annulus = 11mm | Pulmonic |  |
| **Great Arteries** | NRGA | **Coronary Arteries** |  |
| Aorta |  | **Aortic Arch** | Left |
| Pulmonary Arteries |  | **PDA** | No PDA |
| **M-Mode**: | | | |
| Ao | mm | PWd | mm |
| LA | mm | EDV | ml |
| LVIDd | mm | ESV | ml |
| LVIDs | mm | FS | % |
| IVSd | mm | LVEF | % |
| **Additional Information:** 3mm defect from RCS to RA adjacent to TV, L – R Shunt. | | | |
| **Conclusion:**   1. {S, D, S} Levocardia 2. Small OS ASD, L – R Shunt 3. ?RSOV Type IIIa. | | | |
| **Done By:** | **Signature** | **Date** | **Remark** |
| Tesfaye T., Pediatric Cardiologist |  | 04/09/14Eth.C. |  |

| **Patient Name: B/Hiwot Bayih. Sex/Age: F/15days. Date of Report: 04/09/2014Eth.C. MRN: 128629.** | | | |
| --- | --- | --- | --- |
| **Clinical Diagnosis: RD. TGSH1.2573.** | | | |
| **Features:** | **Findings** | **Features** | **Findings** |
| **Profile** | | **Atria** | |
| Abdominal Situs | Solitus | Left Atrium | Normal |
| Cardiac Position | Levocardia | Right Atrium | Normal |
| Systemic Venous Drainage | To RA | **Atrio-Ventricular Valves** | |
| Pulmonary Venous Drainage | To LA | Mitral Valve | Annulus = 9mm |
| Atrio-ventricular Connection | Concordant | Tricuspid Valve | Annulus = 10mm |
| Ventriculo-Arterial Connection | concordant | **Ventricle** | |
| Ventricular Loop | d-Loop | Left Ventricle | Normal |
| **Septae** |  | Right Ventricle | Normal |
| Interatrial Septum | PFO, L – R Shunt | **Doppler Measurement** |  |
| Interventricular Septum | Intact | Mitral |  |
| **Semilunar Valves** |  | Aortic |  |
| Aortic Valve | Annulus = 9mm | Tricuspid |  |
| Pulmonary Valve | Annulus = 9mm | Pulmonic |  |
| **Great Arteries** | NRGA | **Coronary Arteries** |  |
| Aorta |  | **Aortic Arch** | Left |
| Pulmonary Arteries |  | **PDA** | No PDA |
| **M-Mode**: Normal LV Function on eye balling | | | |
| Ao | mm | PWd | mm |
| LA | mm | EDV | ml |
| LVIDd | mm | ESV | ml |
| LVIDs | mm | FS | % |
| IVSd | mm | LVEF | % |
| **Additional Information:** | | | |
| **Conclusion:**   1. {S, D, S} Levocardia 2. PFO, L – R Shunt | | | |
| **Done By:** | **Signature** | **Date** | **Remark** |
| Tesfaye T., Pediatric Cardiologist |  | 04/09/14Eth.C. |  |

| **Patient Name: Baby of mister Adamu. Sex/Age: M/ 13months. Date of Report: 04/09/2014Eth.C. MRN: __________.** | | | |
| --- | --- | --- | --- |
| **Clinical Diagnosis: Cyanosis + Murmur. TGSH1.2574.** | | | |
| **Features:** | **Findings** | **Features** | **Findings** |
| **Profile** | | **Atria** | |
| Abdominal Situs | Solitus | Left Atrium | Normal |
| Cardiac Position | Levocardia | Right Atrium | Normal |
| Systemic Venous Drainage | To RA | **Atrio-Ventricular Valves** | |
| Pulmonary Venous Drainage | To LA | Mitral Valve | Annulus = 14mm |
| Atrio-ventricular Connection | Concordant | Tricuspid Valve | Annulus = 14mm  TAPSE = 15mm |
| Ventriculo-Arterial Connection | concordant | **Ventricle** | |
| Ventricular Loop | d-Loop | Left Ventricle | Normal |
| **Septae** |  | Right Ventricle | RVH |
| Interatrial Septum | 8mm X 9mm OS ASD, L – R Shunt | **Doppler Measurement** |  |
| Interventricular Septum | Non- restrictive Malaligned subaortic VSD, R – L Shunt | Mitral |  |
| **Semilunar Valves** |  | Aortic |  |
| Aortic Valve | Annulus = 13mm | Tricuspid |  |
| Pulmonary Valve | Annulus = 13mm | Pulmonic | Severe RVOTO, PPG = 87mmHg |
| **Great Arteries** | NRGA | **Coronary Arteries** |  |
| Aorta | Overriding aorta | **Aortic Arch** | Left |
| Pulmonary Arteries |  | **PDA** | No PDA |
| **M-Mode**: | | | |
| Ao | mm | PWd | mm |
| LA | mm | EDV | ml |
| LVIDd | mm | ESV | ml |
| LVIDs | mm | FS | % |
| IVSd | mm | LVEF | % |
| **Additional Information:** | | | |
| **Conclusion:**   1. {S, D, S} Levocardia 2. Moderate OS ASD, L – R Shunt 3. TOF | | | |
| **Done By:** | **Signature** | **Date** | **Remark** |
| Tesfaye T., Pediatric Cardiologist |  | 04/09/14Eth.C. |  |

| **Patient Name: Amanuel Alem-work . Sex/Age: M/3 9/12 yrs. Date of Report: 09/09/2014Eth.C. MRN.044586** | | | |
| --- | --- | --- | --- |
| **Clinical Diagnosis: Pre-Op Screening. TGSH1.2575.** | | | |
| **Features:** | **Findings** | **Features** | **Findings** |
| **Profile** | | **Atria** | |
| Abdominal Situs | Solitus | Left Atrium | Normal |
| Cardiac Position | Levocardia | Right Atrium | Normal |
| Systemic Venous Drainage | To RA | **Atrio-Ventricular Valves** | |
| Pulmonary Venous Drainage | To LA | Mitral Valve | Annulus = 17mm |
| Atrio-ventricular Connection | Concordant | Tricuspid Valve | Annulus = 18mm  TAPSE = 19mm |
| Ventriculo-Arterial Connection | concordant | **Ventricle** | |
| Ventricular Loop | d-Loop | Left Ventricle | Normal |
| **Septae** |  | Right Ventricle | Normal |
| Interatrial Septum | Intact | **Doppler Measurement** |  |
| Interventricular Septum | Intact | Mitral |  |
| **Semilunar Valves** |  | Aortic |  |
| Aortic Valve | Annulus = 14mm | Tricuspid | Trivial TR, PPG = 16mmHg |
| Pulmonary Valve | Annulus = 16mm | Pulmonic |  |
| **Great Arteries** | NRGA | **Coronary Arteries** |  |
| Aorta |  | **Aortic Arch** | Left |
| Pulmonary Arteries |  | **PDA** | No PDA |
| **M-Mode**: | | | |
| Ao | mm | PWd | mm |
| LA | mm | EDV | ml |
| LVIDd | mm | ESV | ml |
| LVIDs | mm | FS | 42% |
| IVSd | mm | LVEF | 75% |
| **Additional Information:** | | | |
| **Conclusion:**   1. Normal Echocardiography Study | | | |
| **Done By:** | **Signature** | **Date** | **Remark** |
| Tesfaye T., Pediatric Cardiologist |  | 09/09/14Eth.C. |  |

| **Patient Name: Lukas Asefaw. Sex/Age: M/11yrs. Date of Report: 09/09/2014Eth.C. MRN: 002985.** | | | |
| --- | --- | --- | --- |
| **Clinical Diagnosis: Murmrur. TGSH1.2576.** | | | |
| **Features:** | **Findings** | **Features** | **Findings** |
| **Profile** | | **Atria** | |
| Abdominal Situs | Solitus | Left Atrium | Normal |
| Cardiac Position | Levocardia | Right Atrium | Mildly dilated |
| Systemic Venous Drainage | To RA | **Atrio-Ventricular Valves** | |
| Pulmonary Venous Drainage | To LA | Mitral Valve | Annulus = 27mm |
| Atrio-ventricular Connection | Concordant | Tricuspid Valve | Annulus = 31mm  TAPSE = mm |
| Ventriculo-Arterial Connection | concordant | **Ventricle** | |
| Ventricular Loop | d-Loop | Left Ventricle | Normal |
| **Septae** |  | Right Ventricle | Mildly dilated |
| Interatrial Septum | Intact | **Doppler Measurement** |  |
| Interventricular Septum | Intact | Mitral |  |
| **Semilunar Valves** |  | Aortic |  |
| Aortic Valve | Annulus = 22mm | Tricuspid |  |
| Pulmonary Valve | Annulus = 29mm | Pulmonic |  |
| **Great Arteries** | NRGA | **Coronary Arteries** |  |
| Aorta |  | **Aortic Arch** | Left |
| Pulmonary Arteries |  | **PDA** | No PDA |
| **M-Mode**: | | | |
| Ao | mm | PWd | mm |
| LA | mm | EDV | ml |
| LVIDd | mm | ESV | ml |
| LVIDs | mm | FS | % |
| IVSd | mm | LVEF | % |
| **Additional Information:** Dilated Coronary Sinus with diameter 11.5mm. Coronary Sinus ostium stenosis measuring 4.5mm with PPG = 45mmHg | | | |
| **Conclusion:**   1. {S, D, S} Levocardia 2. RA/RV Dilated 3. Moderate Coronary Sinus Ostium Stenosis 4. Coronary Sinus dilatation 5. Normal LV Systolic Function | | | |
| **Remark:** Consider diuretics if the right chamber dilatation increases. | | | |
| **Done By:** | **Signature** | **Date** | **Remark** |
| Tesfaye T., Pediatric Cardiologist |  | 09/09/14Eth.C. |  |

| **Patient Name: Ephrem Mulat . Sex/Age: M/10 yr. Date of Report: 09/09/2014Eth.C. MRN: 130325.**  **Clinical Diagnosis: DOE + CHF + Rheumatic Recurrence + Murmur. TGSH1.2577.** | | | |
| --- | --- | --- | --- |
| **Features:** | **Findings** | **Features** | **Findings** |
| **Profile** | | **Atria** | |
| Abdominal Situs | Solitus | Left Atrium | Dilated |
| Cardiac Position | Levocardia | Right Atrium | Dilated |
| Systemic Venous Drainage | To RA | **Atrio-Ventricular Valves** | |
| Pulmonary Venous Drainage | To LA | Mitral Valve | Annulus = 31mm. Thickened MVL. |
| Atrio-ventricular Connection | Concordant | Tricuspid Valve | Annulus = 29mm  TAPSE = 20mm |
| Ventriculo-Arterial Connection | concordant | **Ventricle** | |
| Ventricular Loop | d-Loop | Left Ventricle | Dilated |
| **Septae** |  | Right Ventricle | Dilated |
| Interatrial Septum | Intact | **Doppler Measurement** |  |
| Interventricular Septum | Intact | Mitral | Severe MR, Holosystolic, posterior projection, seen in two planes with jet velocity = 4.9m/sec. |
| **Semilunar Valves** |  | Aortic | Severe AR, PHT = 135ms |
| Aortic Valve | Annulus = 14mm. | Tricuspid | Moderate TR, PPG = 63mmHg. |
| Pulmonary Valve | Annulus = 22mm | Pulmonic | Severe PR, PPG = 48mmHg |
| **Great Arteries** | NRGA | **Coronary Arteries** |  |
| Aorta |  | **Aortic Arch** | Left |
| Pulmonary Arteries | MPA = 21mm | **PDA** | No PDA |
| **M-Mode**: | | | |
| Ao | mm | PWd | mm |
| LA | mm | EDV | ml |
| LVIDd | mm | ESV | ml |
| LVIDs | mm | FS | 37% |
| IVSd | mm | LVEF | 66% |
| **Conclusion:**   1. {S, D, S} Levocardia 2. All chambers dilated 3. Thickened MVL and AVL, Shortened PMVL 4. Severe MR 5. Moderate TR 6. Severe AR 7. Severe PR 8. Severe Pulmonary Hypertension 9. Normal Biventricular Systolic Function | | | |
| **Done By:** | **Signature** | **Date** | **Remark** |
| Tesfaye T., Pediatric Cardiologist |  | 09/09/14Eth.C. |  |

| **Patient Name: Elsabeth Getaneh .Sex/Age: F/9 month. Date of Report: 09/09/2014Eth.C. MRN:130551.** | | | |
| --- | --- | --- | --- |
| **Clinical Diagnosis: DS. TGSH1.2578.** | | | |
| **Features:** | **Findings** | **Features** | **Findings** |
| **Profile** | | **Atria** | |
| Abdominal Situs | Solitus | Left Atrium | Normal |
| Cardiac Position | Levocardia | Right Atrium | Mild dilatation |
| Systemic Venous Drainage | To RA | **Atrio-Ventricular Valves** | |
| Pulmonary Venous Drainage | To LA | Mitral Valve | Annulus = 14mm |
| Atrio-ventricular Connection | Concordant | Tricuspid Valve | Annulus = 16mm  TAPSE = 16mm |
| Ventriculo-Arterial Connection | concordant | **Ventricle** | |
| Ventricular Loop | d-Loop | Left Ventricle | Normal |
| **Septae** |  | Right Ventricle | Mild dilatation |
| Interatrial Septum | PFO, L – R Shunt | **Doppler Measurement** |  |
| Interventricular Septum | Intact | Mitral |  |
| **Semilunar Valves** |  | Aortic |  |
| Aortic Valve | Annulus = 11mm | Tricuspid |  |
| Pulmonary Valve | Annulus = 16mm | Pulmonic | Moderate PR, PPG = 44mmHg |
| **Great Arteries** | NRGA | **Coronary Arteries** |  |
| Aorta |  | **Aortic Arch** | Left |
| Pulmonary Arteries |  | **PDA** | No PDA |
| **M-Mode**: | | | |
| Ao | mm | PWd | mm |
| LA | mm | EDV | ml |
| LVIDd | mm | ESV | ml |
| LVIDs | mm | FS | 34% |
| IVSd | mm | LVEF | 66% |
| **Additional Information:** | | | |
| **Conclusion:**   1. {S, D, S} Levocardia 2. PFO, L – R Shunt 3. Mild RA/RV Dilatation 4. Moderate PR 5. Mild Pulmonary Hypertension 6. Normal Biventricular Systolic Function | | | |
| **Done By:** | **Signature** | **Date** | **Remark** |
| Tesfaye T., Pediatric Cardiologist |  | 09/09/14Eth.C. |  |

| **Patient Name: B/Selam Awoke. Sex/Age: M/9days. Date of Report: 11/09/2014Eth.C. MRN: 130029.**  **Referral Diagnosis: LBO + Post op after colostomy (for cardiac screening). TGSH1.2579.** | | | |
| --- | --- | --- | --- |
|  | | | |
| **Features:** | **Findings** | **Features** | **Findings** |
| **Profile** | | **Atria** | |
| Abdominal Situs | Solitus | Left Atrium | Normal |
| Cardiac Position | Levocardia | Right Atrium | Normal |
| Systemic Venous Drainage | To RA | **Atrio-Ventricular Valves** | |
| Pulmonary Venous Drainage | To LA | Mitral Valve | Annulus = 10mm |
| Atrio-ventricular Connection | Concordant | Tricuspid Valve | Annulus = 11mm |
| Ventriculo-Arterial Connection | concordant | **Ventricle** | |
| Ventricular Loop | d-Loop | Left Ventricle | Normal |
| **Septae** |  | Right Ventricle | Normal |
| Interatrial Septum | PFO, L – R Shunt | **Doppler Measurement** |  |
| Interventricular Septum | Intact | Mitral |  |
| **Semilunar Valves** |  | Aortic |  |
| Aortic Valve | Annulus = 7mm | Tricuspid |  |
| Pulmonary Valve | Annulus = 10mm | Pulmonic |  |
| **Great Arteries** | NRGA | **Coronary Arteries** |  |
| Aorta |  | **Aortic Arch** | Left |
| Pulmonary Arteries |  | **PDA** | No PDA |
| **M-Mode**: Normal LV Function on eye balling | | | |
| Ao | mm | PWd | mm |
| LA | mm | EDV | ml |
| LVIDd | mm | ESV | ml |
| LVIDs | mm | FS | % |
| IVSd | mm | LVEF | % |
| **Additional Information:** | | | |
| **Conclusion:**   1. {S, D, S} Levocardia 2. PFO, L – R Shunt | | | |
| **Done By:** | **Signature** | **Date** | **Remark** |
| Tesfaye T., Pediatric Cardiologist |  | 11/09/14Eth.C. |  |

| **Patient Name: Sisay Ashagerie. Sex/Age: M/2yrs. Date of Report: 11/09/2014Eth.C. MRN: 130774.**  **Clinical Diagnosis: RD + CHF + Accentuated p2. TGSH1.2580. (TGSH3)** | | | |
| --- | --- | --- | --- |
| **Features:** | **Findings** | **Features** | **Findings** |
| **Profile** | | **Atria** | |
| Abdominal Situs | Solitus | Left Atrium | Normal |
| Cardiac Position | Levocardia | Right Atrium | Dilated |
| Systemic Venous Drainage | To RA | **Atrio-Ventricular Valves** | |
| Pulmonary Venous Drainage | To LA | Mitral Valve | Annulus = 15mm |
| Atrio-ventricular Connection | Concordant | Tricuspid Valve | Annulus = 21mm  TAPSE = 12mm |
| Ventriculo-Arterial Connection | concordant | **Ventricle** | |
| Ventricular Loop | d-Loop | Left Ventricle | Normal |
| **Septae** |  | Right Ventricle | Dilated, Hypertrophied & Dysfunctional. RV TDI S wave = 9cm/sec. |
| Interatrial Septum | PFO, BD Shunt | **Doppler Measurement** |  |
| Interventricular Septum | Intact | Mitral |  |
| **Semilunar Valves** |  | Aortic |  |
| Aortic Valve | Annulus = 12mm | Tricuspid | Mild TR, PPG = 67mmHg |
| Pulmonary Valve | Annulus = 17mm | Pulmonic | Mild to moderate PR, PPG = 63mmHg |
| **Great Arteries** | NRGA | **Coronary Arteries** |  |
| Aorta |  | **Aortic Arch** | Left |
| Pulmonary Arteries | MPA = 20mm. | **PDA** | No PDA |
| **M-Mode**: | | | |
| Ao | mm | PWd | mm |
| LA | mm | EDV | ml |
| LVIDd | mm | ESV | ml |
| LVIDs | mm | FS | 35% |
| IVSd | mm | LVEF | 66% |
| **Additional Information:** | | | |
| **Conclusion:**   1. {S, D, S} Levocardia 2. RA/RV Dilated 3. PFO, BD Shunt 4. Mild TR 5. Mild to moderate PR 6. Severe Pulmonary Hypertension 7. RV Hypertrophied, Dilated and Dysfunctional 8. Normal LV Systolic Function | | | |
| **Done By:** | **Signature** | **Date** | **Remark** |
| Tesfaye T., Pediatric Cardiologist |  | 11/09/14Eth.C. |  |

| **Patient Name: Mollalign Tamene. Sex/Age: M/9yrs. Date of Report: 11/09/2014Eth.C. MRN: 020978.** | | | |
| --- | --- | --- | --- |
|  | | | |
| **Features:** | **Findings** | **Features** | **Findings** |
| **Profile** | | **Atria** | |
| Abdominal Situs | Solitus | Left Atrium | Dilated |
| Cardiac Position | Levocardia | Right Atrium | Normal |
| Systemic Venous Drainage | To RA | **Atrio-Ventricular Valves** | |
| Pulmonary Venous Drainage | To LA | Mitral Valve | Annulus = 29mm. Thickened MVL. |
| Atrio-ventricular Connection | Concordant | Tricuspid Valve | Annulus = 24mm  TAPSE = 22mm |
| Ventriculo-Arterial Connection | concordant | **Ventricle** | |
| Ventricular Loop | d-Loop | Left Ventricle | Dilated |
| **Septae** |  | Right Ventricle | Normal |
| Interatrial Septum | Intact | **Doppler Measurement** |  |
| Interventricular Septum | Intact | Mitral | Mild MR, Holosystolic, posterior projection, seen in two planes with jet velocity = 4.3m/sec. |
| **Semilunar Valves** |  | Aortic | Moderate AR, PHT = 493ms. |
| Aortic Valve | Annulus = 20mm. Thickened AVL | Tricuspid |  |
| Pulmonary Valve | Annulus = 22mm | Pulmonic |  |
| **Great Arteries** | NRGA | **Coronary Arteries** |  |
| Aorta |  | **Aortic Arch** | Left |
| Pulmonary Arteries |  | **PDA** | No PDA |
| **M-Mode**: | | | |
| Ao | mm | PWd | mm |
| LA | mm | EDV | ml |
| LVIDd | mm | ESV | ml |
| LVIDs | mm | FS | 32% |
| IVSd | mm | LVEF | 60% |
| **Additional Information:** | | | |
| **Conclusion:**   1. {S, D, S} Levocardia 2. LA/LV Dilated 3. Thickened MVL, AVL 4. Mild MR 5. Moderate AR 6. Normal Biventricular Systolic Function | | | |
| **Done By:** | **Signature** | **Date** | **Remark** |
| Tesfaye T., Pediatric Cardiologist |  | 11/09/14Eth.C. |  |

| **Patient Name: B/Rihanna Beshir. Sex/Age: M/2 7/12yrs. Date of Report: 11/09/2014Eth.C. MRN: 002277.** | | | |
| --- | --- | --- | --- |
| **Clinical Diagnosis: Recurrent Chest Infection. TGSH1.2581.** | | | |
| **Features:** | **Findings** | **Features** | **Findings** |
| **Profile** | | **Atria** | |
| Abdominal Situs | Solitus | Left Atrium | Normal |
| Cardiac Position | Levocardia | Right Atrium | Normal |
| Systemic Venous Drainage | To RA | **Atrio-Ventricular Valves** | |
| Pulmonary Venous Drainage | To LA | Mitral Valve | Annulus = mm |
| Atrio-ventricular Connection | Concordant | Tricuspid Valve | Annulus = mm  TAPSE = mm |
| Ventriculo-Arterial Connection | concordant | **Ventricle** | |
| Ventricular Loop | d-Loop | Left Ventricle | Normal |
| **Septae** |  | Right Ventricle | Normal |
| Interatrial Septum | Intact | **Doppler Measurement** |  |
| Interventricular Septum | Intact | Mitral |  |
| **Semilunar Valves** |  | Aortic |  |
| Aortic Valve | Annulus = mm | Tricuspid |  |
| Pulmonary Valve | Annulus = mm | Pulmonic |  |
| **Great Arteries** | NRGA | **Coronary Arteries** |  |
| Aorta |  | **Aortic Arch** | Left |
| Pulmonary Arteries |  | **PDA** | No PDA |
| **M-Mode**: | | | |
| Ao | mm | PWd | mm |
| LA | mm | EDV | ml |
| LVIDd | mm | ESV | ml |
| LVIDs | mm | FS | % |
| IVSd | mm | LVEF | % |
| **Additional Information:** | | | |
| **Conclusion:**   1. {S, D, S} Levocardia | | | |
| **Done By:** | **Signature** | **Date** | **Remark** |
| Tesfaye T., Pediatric Cardiologist |  | 11/09/14Eth.C. |  |
|  |  |  |  |
| **Patient Name: Meaza K/Muluye. Sex/Age F/12 yrs. . Date of Report: 11/09/2014Eth.C. MRN:131306.**  **Clinical Diagnosis: ARF + Murmur. TGSH1.2582.** | | | |
| **Features:** | **Findings** | **Features** | **Findings** |
| **Profile** | | **Atria** | |
| Abdominal Situs | Solitus | Left Atrium | Dilated |
| Cardiac Position | Levocardia | Right Atrium | Normal |
| Systemic Venous Drainage | To RA | **Atrio-Ventricular Valves** | |
| Pulmonary Venous Drainage | To LA | Mitral Valve | Annulus = 26mm. thickened MVL. Shortened PMVL. |
| Atrio-ventricular Connection | Concordant | Tricuspid Valve | Annulus = 20mm  TAPSE = 16mm |
| Ventriculo-Arterial Connection | concordant | **Ventricle** | |
| Ventricular Loop | d-Loop | Left Ventricle | Dilated |
| **Septae** |  | Right Ventricle | Normal |
| Interatrial Septum | Intact | **Doppler Measurement** |  |
| Interventricular Septum | Intact | Mitral | Moderate MR, Holosystolic, posterior projection, seen in two planes with jet velocity = 4.6m/sec. |
| **Semilunar Valves** |  | Aortic | Moderate AR, PHT = 419ms |
| Aortic Valve | Annulus = 19mm | Tricuspid |  |
| Pulmonary Valve | Annulus = 20mm | Pulmonic |  |
| **Great Arteries** | NRGA | **Coronary Arteries** |  |
| Aorta |  | **Aortic Arch** | Left |
| Pulmonary Arteries |  | **PDA** | No PDA |
| **M-Mode**: | | | |
| Ao | mm | PWd | mm |
| LA | mm | EDV | ml |
| LVIDd | mm | ESV | ml |
| LVIDs | mm | FS | 29% |
| IVSd | mm | LVEF | 56% |
| **Additional Information:** | | | |
| **Conclusion:**   1. {S, D, S} Levocardia 2. LA/LV Dilated 3. Thickened MVL, Shortened PMVL 4. Moderate MR 5. Moderate AR 6. Normal Biventricular Systolic Function | | | |
| **Done By:** | **Signature** | **Date** | **Remark** |
| Tesfaye T., Pediatric Cardiologist |  | 11/09/14Eth.C. |  |

| **Patient Name: Tibebu Getahun. Sex/Age: M/84days. Date of Report: 16/09/2014Eth.C. MRN: 130761.**  **Clinical Diagnosis: RD. TGSH1.2583.** | | | |
| --- | --- | --- | --- |
| **Features:** | **Findings** | **Features** | **Findings** |
| **Profile** | | **Atria** | |
| Abdominal Situs | Solitus | Left Atrium | Normal |
| Cardiac Position | Levocardia | Right Atrium | Normal |
| Systemic Venous Drainage | To RA | **Atrio-Ventricular Valves** | |
| Pulmonary Venous Drainage | To LA | Mitral Valve | Annulus = 11mm |
| Atrio-ventricular Connection | Concordant | Tricuspid Valve | Annulus = 11mm |
| Ventriculo-Arterial Connection | concordant | **Ventricle** | |
| Ventricular Loop | d-Loop | Left Ventricle | Normal |
| **Septae** |  | Right Ventricle | Normal |
| Interatrial Septum | PFO, L – R Shunt | **Doppler Measurement** |  |
| Interventricular Septum | Intact | Mitral |  |
| **Semilunar Valves** |  | Aortic |  |
| Aortic Valve | Annulus = 9mm | Tricuspid |  |
| Pulmonary Valve | Annulus = 10mm | Pulmonic |  |
| **Great Arteries** | NRGA | **Coronary Arteries** |  |
| Aorta |  | **Aortic Arch** | Left |
| Pulmonary Arteries |  | **PDA** | No PDA |
| **M-Mode**: Normal LV Function on eye balling | | | |
| Ao | mm | PWd | mm |
| LA | mm | EDV | ml |
| LVIDd | mm | ESV | ml |
| LVIDs | mm | FS | % |
| IVSd | mm | LVEF | % |
| **Additional Information:** | | | |
| **Conclusion:**   1. {S, D, S} Levocardia 2. PFO, L – R Shunt | | | |
| **Done By:** | **Signature** | **Date** | **Remark** |
| Tesfaye T., Pediatric Cardiologist |  | 16/09/14Eth.C. |  |

| **Patient Name: B/Firnussie Bitew. Sex/Age: F/55days. Date of Report: 16/09/2014Eth.C. MRN: 127123.** | | | |
| --- | --- | --- | --- |
| **Clinical Diagnosis: RD. TGSH1.2584.** | | | |
| **Features:** | **Findings** | **Features** | **Findings** |
| **Profile** | | **Atria** | |
| Abdominal Situs | Solitus | Left Atrium | Normal |
| Cardiac Position | Levocardia | Right Atrium | Normal |
| Systemic Venous Drainage | To RA | **Atrio-Ventricular Valves** | |
| Pulmonary Venous Drainage | To LA | Mitral Valve | Annulus = 10mm |
| Atrio-ventricular Connection | Concordant | Tricuspid Valve | Annulus = 11mm |
| Ventriculo-Arterial Connection | concordant | **Ventricle** | |
| Ventricular Loop | d-Loop | Left Ventricle | Normal |
| **Septae** |  | Right Ventricle | Normal |
| Interatrial Septum | PFO, L – R Shunt | **Doppler Measurement** |  |
| Interventricular Septum | Intact | Mitral |  |
| **Semilunar Valves** |  | Aortic |  |
| Aortic Valve | Annulus = 8mm | Tricuspid |  |
| Pulmonary Valve | Annulus = 9mm | Pulmonic |  |
| **Great Arteries** | NRGA | **Coronary Arteries** |  |
| Aorta |  | **Aortic Arch** | Left |
| Pulmonary Arteries |  | **PDA** | No PDA |
| **M-Mode**: Normal LV Function on eye balling | | | |
| Ao | mm | PWd | mm |
| LA | mm | EDV | ml |
| LVIDd | mm | ESV | ml |
| LVIDs | mm | FS | % |
| IVSd | mm | LVEF | % |
| **Additional Information:** | | | |
| **Conclusion:**   1. {S, D, S} Levocardia 2. PFO, L – R Shunt | | | |
| **Done By:** | **Signature** | **Date** | **Remark** |
| Tesfaye T., Pediatric Cardiologist |  | 16/09/14Eth.C. |  |

| **Patient Name: Aregash Melkamu. Sex/Age: F/14years. Date of Report: 16/09/2014Eth.C. MRN: 131810.**  **Clinical Diagnosis: Rheumatic Recurrence + CHF + Murmur. TGSH1.2585.** | | | |
| --- | --- | --- | --- |
| **Features:** | **Findings** | **Features** | **Findings** |
| **Profile** | | **Atria** | |
| Abdominal Situs | Solitus | Left Atrium | Dilated. 54mm X 77mm |
| Cardiac Position | Levocardia | Right Atrium | Normal |
| Systemic Venous Drainage | To RA | **Atrio-Ventricular Valves** | |
| Pulmonary Venous Drainage | To LA. IVC Dilated | Mitral Valve | Annulus = 30mm. thickened MVL. Shortened PMVL. MVA = 2.8cm2. |
| Atrio-ventricular Connection | Concordant | Tricuspid Valve | Annulus = 30mm. TAPSE = 23mm |
| Ventriculo-Arterial Connection | concordant | **Ventricle** | |
| Ventricular Loop | d-Loop | Left Ventricle | Dilated |
| **Septae** |  | Right Ventricle | Normal |
| Interatrial Septum | Intact | **Doppler Measurement** |  |
| Interventricular Septum | Intact | Mitral | Severe MR, Holosystolic, posterior projection, seen in two planes with jet velocity = 4.4m/sec. MIV PPG/MPG -= 12/6mmHg. |
| **Semilunar Valves** |  | Aortic | Moderate AR, PHT = 360ms. |
| Aortic Valve | Annulus = 17mm | Tricuspid | Moderate TR, PPG = 44mmHg |
| Pulmonary Valve | Annulus = 27mm | Pulmonic |  |
| **Great Arteries** | NRGA | **Coronary Arteries** |  |
| Aorta |  | **Aortic Arch** | Left |
| Pulmonary Arteries | MPA = 28mm. | **PDA** | No PDA |
| **M-Mode**: | | | |
| Ao | mm | PWd | mm |
| LA | mm | EDV | ml |
| LVIDd | mm | ESV | ml |
| LVIDs | mm | FS | 34% |
| IVSd | mm | LVEF | 62% |
| **Additional Information:** Circumferential Pericardial effusion with Maximum depth of 11mm. 29mm Right Pleural effusion. | | | |
| **Conclusion:**   1. {S, D, S} Levocardia 2. LA/LV Dilated 3. Thickened MVL. Shortened PMVL 4. Severe MR 5. Moderate AR 6. Moderate TR 7. Mild Pulmonary Hypertension 8. Moderate Pericardial effusion 9. Large Right Pleural effusion 10. Normal Biventricular Systolic Function | | | |
| **Done By:** | **Signature** | **Date** | **Remark** |
| Tesfaye T., Pediatric Cardiologist |  | 16/09/14Eth.C. |  |

| **Patient Name: Bancheayimelu Yingu. Sex/Age: F/8yrs. Date of Report: 16/09/2014Eth.C. MRN: 104977.** | | | |
| --- | --- | --- | --- |
| **Clinical Diagnosis: ARF + CHF + Murmur. TGSH1.2586.** | | | |
| **Features:** | **Findings** | **Features** | **Findings** |
| **Profile** | | **Atria** | |
| Abdominal Situs | Solitus | Left Atrium | More Dilated. 58mm X 74mm |
| Cardiac Position | Levocardia | Right Atrium | Dilated |
| Systemic Venous Drainage | To RA | **Atrio-Ventricular Valves** | |
| Pulmonary Venous Drainage | To LA | Mitral Valve | Annulus = 27mm. thickened MVL |
| Atrio-ventricular Connection | Concordant | Tricuspid Valve | Annulus = 26mm  TAPSE = 21mm |
| Ventriculo-Arterial Connection | concordant | **Ventricle** | |
| Ventricular Loop | d-Loop | Left Ventricle | More Dilated |
| **Septae** |  | Right Ventricle | Dilated |
| Interatrial Septum | Intact | **Doppler Measurement** |  |
| Interventricular Septum | Intact | Mitral | Severe MR, Holosystolic, posterior projection, seen in two planes with jet velocity = 4.6m/sec. |
| **Semilunar Valves** |  | Aortic | Mild AR, PHT = 520ms |
| Aortic Valve | Annulus = 14mm | Tricuspid | Severe TR, PPG = 65mmHg |
| Pulmonary Valve | Annulus = 18mm | Pulmonic |  |
| **Great Arteries** | NRGA | **Coronary Arteries** |  |
| Aorta |  | **Aortic Arch** | Left |
| Pulmonary Arteries |  | **PDA** | No PDA |
| **M-Mode**: | | | |
| Ao | mm | PWd | mm |
| LA | mm | EDV | ml |
| LVIDd | mm | ESV | ml |
| LVIDs | mm | FS | 32% |
| IVSd | mm | LVEF | 60% |
| **Additional Information:** | | | |
| **Conclusion:**   1. {S, D, S} Levocardia 2. All chambers Dilated 3. Thickened MVL 4. Severe MR 5. Severe TR 6. Mild AR 7. Severe Pulmonary Hypertension 8. Normal Biventricular Systolic Function | | | |
| **Done By:** | **Signature** | **Date** | **Remark** |
| Tesfaye T., Pediatric Cardiologist |  | 16/09/14Eth.C. |  |

| **Patient Name: Amnen Haile-Mariam. Sex/Age: F/1 7/12. Date of Report: 16/09/2014Eth.C. MRN: __________.** | | | |
| --- | --- | --- | --- |
| **Clinical Diagnosis: CHB. TGSH1.2587.** | | | |
| **Features:** | **Findings** | **Features** | **Findings** |
| **Profile** | | **Atria** | |
| Abdominal Situs | Solitus | Left Atrium | Normal |
| Cardiac Position | Levocardia | Right Atrium | Normal |
| Systemic Venous Drainage | To RA | **Atrio-Ventricular Valves** | |
| Pulmonary Venous Drainage | To LA | Mitral Valve | Annulus = 16mm |
| Atrio-ventricular Connection | Concordant | Tricuspid Valve | Annulus = 17mm |
| Ventriculo-Arterial Connection | concordant | **Ventricle** | |
| Ventricular Loop | d-Loop | Left Ventricle | Normal |
| **Septae** |  | Right Ventricle | Normal |
| Interatrial Septum | Intact | **Doppler Measurement** |  |
| Interventricular Septum | Intact | Mitral |  |
| **Semilunar Valves** |  | Aortic |  |
| Aortic Valve | Annulus = 13mm | Tricuspid |  |
| Pulmonary Valve | Annulus = 14mm | Pulmonic | Trivial PR, PPG = 12mmHg |
| **Great Arteries** | NRGA | **Coronary Arteries** |  |
| Aorta |  | **Aortic Arch** | Left |
| Pulmonary Arteries |  | **PDA** | No PDA |
| **M-Mode**: | | | |
| Ao | mm | PWd | mm |
| LA | mm | EDV | ml |
| LVIDd | mm | ESV | ml |
| LVIDs | mm | FS | 31% |
| IVSd | mm | LVEF | 60% |
| **Additional Information:** | | | |
| **Conclusion:**   1. {S, D, S} Levocardia 2. Normal Echocardiography study | | | |
| **Remark:** Brady Cardia detected during study. | | | |
| **Done By:** | **Signature** | **Date** | **Remark** |
| Tesfaye T., Pediatric Cardiologist |  | 16/09/14Eth.C. |  |

| **Patient Name: Semere Getu. Sex/Age: M/11yrs. Date of Report:18/09/2014Eth. MRN:132242.**  **Clinical Diagnosis: Rheumatic Recurrence + CHF + DOE + Murmur. TGSH1.2588.** | | | |
| --- | --- | --- | --- |
| **Features:** | **Findings** | **Features** | **Findings** |
| **Profile** | | **Atria** | |
| Abdominal Situs | Solitus | Left Atrium | More dilated |
| Cardiac Position | Levocardia | Right Atrium | Dilated |
| Systemic Venous Drainage | To RA | **Atrio-Ventricular Valves** | |
| Pulmonary Venous Drainage | To LA | Mitral Valve | Annulus = 21mm. thickened, clubbed MVL. Shortened PMVL. MVA = 0.6cm2. |
| Atrio-ventricular Connection | Concordant | Tricuspid Valve | Annulus = 21mm  TAPSE = 19mm |
| Ventriculo-Arterial Connection | concordant | **Ventricle** | |
| Ventricular Loop | d-Loop | Left Ventricle | Dilated |
| **Septae** |  | Right Ventricle | Dilated |
| Interatrial Septum | Intact | **Doppler Measurement** |  |
| Interventricular Septum | Intact | Mitral | Severe MS, PPG/MPG = 20/13mmHg |
| **Semilunar Valves** |  | Aortic | Mild AR, PHT = 684ms. |
| Aortic Valve | Annulus = 17mm | Tricuspid | Moderate TR, PPG = 70mmHg |
| Pulmonary Valve | Annulus = 21mm | Pulmonic |  |
| **Great Arteries** | NRGA | **Coronary Arteries** |  |
| Aorta |  | **Aortic Arch** | Left |
| Pulmonary Arteries |  | **PDA** | No PDA |
| **M-Mode**: | | | |
| Ao | mm | PWd | mm |
| LA | mm | EDV | ml |
| LVIDd | mm | ESV | ml |
| LVIDs | mm | FS | 38% |
| IVSd | mm | LVEF | 67% |
| **Additional Information:** circumferential pericardial effusion with maximum depth of 13mm on RV Side. | | | |
| **Conclusion:**   1. {S, D, S} Levocardia 2. All chambers dilated 3. Severe MS 4. Mild AR 5. Moderate TR 6. Severe Pulmonary Hypertension 7. Normal LV Systolic Function 8. Moderate Circumferential Pericardial effusion | | | |
| **Done By:** | **Signature** | **Date** | **Remark** |
| Tesfaye T., Pediatric Cardiologist |  | 18/09/14Eth.C. |  |

| **Patient Name: Kalkidan Shishigu. Sex/Age: F/3months. Date of Report: 18/09/2014Eth.C. MRN: 132247.** | | | |
| --- | --- | --- | --- |
| **Clinical Diagnosis: Cyanosis + Murmur. TGSH1.2589.** | | | |
| **Features:** | **Findings** | **Features** | **Findings** |
| **Profile** | | **Atria** | |
| Abdominal Situs | Solitus | Left Atrium | Normal |
| Cardiac Position | Levocardia | Right Atrium | Normal |
| Systemic Venous Drainage | To RA | **Atrio-Ventricular Valves** | |
| Pulmonary Venous Drainage | To LA | Mitral Valve | Annulus = 18mm |
| Atrio-ventricular Connection | Concordant | Tricuspid Valve | Annulus = 18mm |
| Ventriculo-Arterial Connection | Discordant | **Ventricle** | |
| Ventricular Loop | d-Loop | Left Ventricle | Normal |
| **Septae** |  | Right Ventricle | Normal |
| Interatrial Septum | 8mm OS ASD, BD Shunt | **Doppler Measurement** |  |
| Interventricular Septum | 16mm posteriorly Malaligned septum VSD, BD Shunt | Mitral |  |
| **Semilunar Valves** |  | Aortic |  |
| Aortic Valve | Annulus = 16mm | Tricuspid |  |
| Pulmonary Valve | Annulus = 10mm | Pulmonic | Severe LVOTO, PPG = 71mmHg |
| **Great Arteries** | d-TGA | **Coronary Arteries** |  |
| Aorta | Anterior and to the right. From RV | **Aortic Arch** | Left |
| Pulmonary Arteries | Posterior and to the left. From LV | **PDA** | No PDA |
| **M-Mode**: | | | |
| Ao | mm | PWd | mm |
| LA | mm | EDV | ml |
| LVIDd | mm | ESV | ml |
| LVIDs | mm | FS | % |
| IVSd | mm | LVEF | % |
| **Additional Information:** | | | |
| **Conclusion:**   1. {S, D, S} Levocardia 2. Moderate OS ASD, BD Shunt 3. d-TGA with posteriorly Malaligned VSD, BD Shunt 4. Severe LVOTO(PS) | | | |
| **Done By:** | **Signature** | **Date** | **Remark** |
| Tesfaye T., Pediatric Cardiologist |  | 18/09/14Eth.C. |  |

| **Patient Name: Fentanesh Tsegaw. Sex/Age: F/ 8months Date of Report: 03/10/2014Eth.C. MRN: 134354.** | | | |
| --- | --- | --- | --- |
| **Clinical Diagnosis: RD. TGSH1.2590.** | | | |
| **Features:** | **Findings** | **Features** | **Findings** |
| **Profile** | | **Atria** | |
| Abdominal Situs | Solitus | Left Atrium | Normal |
| Cardiac Position | Levocardia | Right Atrium | Normal |
| Systemic Venous Drainage | To RA | **Atrio-Ventricular Valves** | |
| Pulmonary Venous Drainage | To LA | Mitral Valve | Annulus = mm |
| Atrio-ventricular Connection | Concordant | Tricuspid Valve | Annulus = mm  TAPSE = mm |
| Ventriculo-Arterial Connection | concordant | **Ventricle** | |
| Ventricular Loop | d-Loop | Left Ventricle | Normal |
| **Septae** |  | Right Ventricle | Normal |
| Interatrial Septum | Intact | **Doppler Measurement** |  |
| Interventricular Septum | Intact | Mitral |  |
| **Semilunar Valves** |  | Aortic |  |
| Aortic Valve | Annulus = mm | Tricuspid |  |
| Pulmonary Valve | Annulus = mm | Pulmonic |  |
| **Great Arteries** | NRGA | **Coronary Arteries** |  |
| Aorta |  | **Aortic Arch** | Left |
| Pulmonary Arteries |  | **PDA** | No PDA |
| **M-Mode**: | | | |
| Ao | mm | PWd | mm |
| LA | mm | EDV | ml |
| LVIDd | mm | ESV | ml |
| LVIDs | mm | FS | % |
| IVSd | mm | LVEF | % |
| **Additional Information:** | | | |
| **Conclusion:**   1. Normal Echocardiography Study. | | | |
| **Done By:** | **Signature** | **Date** | **Remark** |
| Tesfaye T., Pediatric Cardiologist |  | 03/10/14Eth.C. |  |

| **Patient Name: Gubayinesh Abebaw. Sex/Age: F/1yr. Date of Report:23/09/2014Eth.C. MRN: 132725.**  **Clinical Diagnosis: CHF + DS. TGSH1.2591.** | | | |
| --- | --- | --- | --- |
| **Features:** | **Findings** | **Features** | **Findings** |
| **Profile** | | **Atria** | |
| Abdominal Situs | Solitus | Left Atrium | Dilated |
| Cardiac Position | Levocardia | Right Atrium | Dilated |
| Systemic Venous Drainage | To RA | **Atrio-Ventricular Valves** | |
| Pulmonary Venous Drainage | To LA | Mitral Valve | Common Complete AVSD  TAPSE = 14mm |
| Atrio-ventricular Connection | Common Complete AVSD | Tricuspid Valve |  |
| Ventriculo-Arterial Connection | concordant | **Ventricle** | |
| Ventricular Loop | d-Loop | Left Ventricle | Dilated |
| **Septae** |  | Right Ventricle | Dilated |
| Interatrial Septum | Complete AVSD, L – R Shunt. Additional 8mm Fenestrated OS ASD, L – R Shunt | **Doppler Measurement** |  |
| Interventricular Septum |  | Mitral | Mild Left AVVR |
| **Semilunar Valves** |  | Aortic |  |
| Aortic Valve | Annulus = 11mm | Tricuspid | Moderate Right AVVR |
| Pulmonary Valve | Annulus = 15mm | Pulmonic |  |
| **Great Arteries** | NRGA | **Coronary Arteries** |  |
| Aorta |  | **Aortic Arch** | Left |
| Pulmonary Arteries | MPA = 17mm. Confluent Branch PAs. | **PDA** | 1mm PDA, predominantly L – R Shunt |
| **M-Mode**: | | | |
| Ao | Mm | PWd | mm |
| LA | Mm | EDV | ml |
| LVIDd | mm | ESV | ml |
| LVIDs | mm | FS | 40% |
| IVSd | mm | LVEF | 74% |
| **Additional Information:** Pericardial effusion on RV Side with maximum depth of 6mm. | | | |
| **Conclusion:**   1. {S, D, S} Levocardia 2. All chambers dilated 3. Common Complete AVSD, L – R Shunt 4. Moderate Fenestrated OS ASD, L – R Shunt 5. Small PDA, L – R Shunt 6. Mild Left AVVR 7. Moderate Right AVVR 8. Severe Pulmonary Hypertension 9. Small Pericardial effusion | | | |
| **Done By:** | **Signature** | **Date** | **Remark** |
| Tesfaye T., Pediatric Cardiologist |  | 23/09/14Eth.C. |  |

| **Patient Name: Mahlet Kifle Sex/Age: F/4 7/12. Date of Report: 23/09/2014Eth.C. MRN: 064802.** | | | |
| --- | --- | --- | --- |
| **Clinical Diagnosis: Cyanosis + Clubbing + Murmur. TGSH1.2592.** | | | |
| **Features:** | **Findings** | **Features** | **Findings** |
| **Profile** | | **Atria** | |
| Abdominal Situs | Solitus | Left Atrium | Normal |
| Cardiac Position | Levocardia | Right Atrium | Dilated |
| Systemic Venous Drainage | To RA | **Atrio-Ventricular Valves** | |
| Pulmonary Venous Drainage | To LA | Mitral Valve | Atretic |
| Atrio-ventricular Connection | Concordant | Tricuspid Valve | Annulus = 31mm |
| Ventriculo-Arterial Connection | concordant | **Ventricle** | |
| Ventricular Loop | d-Loop | Left Ventricle | Smallish |
| **Septae** |  | Right Ventricle | Dilated |
| Interatrial Septum | 6mm OS ASD, L – R Shunt | **Doppler Measurement** |  |
| Interventricular Septum | 17mm anterior muscular VSD, R – L Shunt | Mitral | Atretic |
| **Semilunar Valves** |  | Aortic |  |
| Aortic Valve | Annulus = 16mm | Tricuspid |  |
| Pulmonary Valve | Annulus = 14mm | Pulmonic |  |
| **Great Arteries** | NRGA | **Coronary Arteries** |  |
| Aorta |  | **Aortic Arch** | Left |
| Pulmonary Arteries |  | **PDA** | No PDA |
| **M-Mode**: | | | |
| Ao | mm | PWd | mm |
| LA | mm | EDV | ml |
| LVIDd | mm | ESV | ml |
| LVIDs | mm | FS | % |
| IVSd | mm | LVEF | % |
| **Additional Information:** | | | |
| **Conclusion:**   1. {S, D, S} Levocardia 2. RA/RV Dilated 3. Small OS ASD, L – R Shunt 4. Large anterior Muscular VSD, R – L Shunt 5. Smallish LV 6. Mitral Atresia | | | |
| **Done By:** | **Signature** | **Date** | **Remark** |
| Tesfaye T., Pediatric Cardiologist |  | 23/09/14Eth.C. |  |

| **Patient Name: Alemu Getinet. Sex/Age: M/16 yrs. Date of Report: 23/09/2014Eth.C. MRN:053223.**  **Clinical Diagnosis: Rheumatic Recurrence + CHF + DOE + Palpitation + Murmur. TGSH1.2593.** | | | |
| --- | --- | --- | --- |
| **Features:** | **Findings** | **Features** | **Findings** |
| **Profile** | | **Atria** | |
| Abdominal Situs | Solitus | Left Atrium | Markedly dilated |
| Cardiac Position | Levocardia | Right Atrium | Markedly dilated |
| Systemic Venous Drainage | To RA | **Atrio-Ventricular Valves** | |
| Pulmonary Venous Drainage | To LA | Mitral Valve | Annulus = 34mm. Thickened MVL. Shortened PMVL. |
| Atrio-ventricular Connection | Concordant | Tricuspid Valve | Annulus = 37mm. TAPSE = 17mm |
| Ventriculo-Arterial Connection | concordant | **Ventricle** | |
| Ventricular Loop | d-Loop | Left Ventricle | Markedly dilated |
| **Septae** |  | Right Ventricle | Markedly dilated |
| Interatrial Septum | Intact | **Doppler Measurement** |  |
| Interventricular Septum | Intact | Mitral | Severe MR, Holosystolic, posterior projection, seen in two planes with jet velocity = 3.7m/sec. |
| **Semilunar Valves** |  | Aortic | Moderate AR, PHT = 303ms |
| Aortic Valve | Annulus = 17mm | Tricuspid | Severe TR, PPG = 61mmHg |
| Pulmonary Valve | Annulus = 25mm | Pulmonic |  |
| **Great Arteries** | NRGA | **Coronary Arteries** |  |
| Aorta |  | **Aortic Arch** | Left |
| Pulmonary Arteries |  | **PDA** | No PDA |
| **M-Mode**: | | | |
| Ao | Mm | PWd | Mm |
| LA | Mm | EDV | Ml |
| LVIDd | Mm | ESV | Ml |
| LVIDs | Mm | FS | 35% |
| IVSd | Mm | LVEF | 64% |
| **Additional Information:** Pericardial effusion with 4mm Size on RV Side | | | |
| **Conclusion:**   1. {S, D, S} Levocardia 2. All chambers dilated 3. Thickened MVL, Shortened PMVL. 4. Severe MR 5. Severe TR 6. Moderate AR 7. Severe Pulmonary Hypertension 8. Trace Pericardial effusion 9. Normal Biventricular Systolic Function | | | |
| **Done By:** | **Signature** | **Date** | **Remark** |
| Tesfaye T., Pediatric Cardiologist |  | 23/09/14Eth.C. |  |

| **Patient Name: B/Abez Tobiaw . Sex/Age: F/3 days Date of Report: 25/09/2014Eth.C. MRN: 133151.** | | | |
| --- | --- | --- | --- |
| **Clinical Diagnosis: RD. TGSH1.2594.** | | | |
| **Features:** | **Findings** | **Features** | **Findings** |
| **Profile** | | **Atria** | |
| Abdominal Situs | Solitus | Left Atrium | Normal |
| Cardiac Position | Levocardia | Right Atrium | Normal |
| Systemic Venous Drainage | To RA | **Atrio-Ventricular Valves** | |
| Pulmonary Venous Drainage | To LA | Mitral Valve | Annulus = mm |
| Atrio-ventricular Connection | Concordant | Tricuspid Valve | Annulus = mm  TAPSE = mm |
| Ventriculo-Arterial Connection | concordant | **Ventricle** | |
| Ventricular Loop | d-Loop | Left Ventricle | Normal |
| **Septae** |  | Right Ventricle | Normal |
| Interatrial Septum | Intact | **Doppler Measurement** |  |
| Interventricular Septum | Intact | Mitral |  |
| **Semilunar Valves** |  | Aortic |  |
| Aortic Valve | Annulus = mm | Tricuspid |  |
| Pulmonary Valve | Annulus = mm | Pulmonic |  |
| **Great Arteries** | NRGA | **Coronary Arteries** |  |
| Aorta |  | **Aortic Arch** | Left |
| Pulmonary Arteries |  | **PDA** | No PDA |
| **M-Mode**: | | | |
| Ao | mm | PWd | mm |
| LA | mm | EDV | ml |
| LVIDd | mm | ESV | ml |
| LVIDs | mm | FS | % |
| IVSd | mm | LVEF | % |
| **Additional Information:** | | | |
| **Conclusion:**   1. Normal Echocardiography Study | | | |
| **Done By:** | **Signature** | **Date** | **Remark** |
| Tesfaye T., Pediatric Cardiologist |  | 25/09/14Eth.C. |  |

| **Patient Name: Kidus Azmeraw. Sex/Age: M/7 months. Date of Report: 25/09/2014Eth.C. MRN:132837.** | | | |
| --- | --- | --- | --- |
| **Clinical Diagnosis: DS. TGSH1.2595.** | | | |
| **Features:** | **Findings** | **Features** | **Findings** |
| **Profile** | | **Atria** | |
| Abdominal Situs | Solitus | Left Atrium | Normal |
| Cardiac Position | Levocardia | Right Atrium | Normal |
| Systemic Venous Drainage | To RA | **Atrio-Ventricular Valves** | |
| Pulmonary Venous Drainage | To LA | Mitral Valve | Annulus = mm |
| Atrio-ventricular Connection | Concordant | Tricuspid Valve | Annulus = mm  TAPSE = mm |
| Ventriculo-Arterial Connection | concordant | **Ventricle** | |
| Ventricular Loop | d-Loop | Left Ventricle | Normal |
| **Septae** |  | Right Ventricle | Normal |
| Interatrial Septum | Intact | **Doppler Measurement** |  |
| Interventricular Septum | Intact | Mitral |  |
| **Semilunar Valves** |  | Aortic |  |
| Aortic Valve | Annulus = mm | Tricuspid |  |
| Pulmonary Valve | Annulus = mm | Pulmonic |  |
| **Great Arteries** | NRGA | **Coronary Arteries** |  |
| Aorta |  | **Aortic Arch** | Left |
| Pulmonary Arteries |  | **PDA** | No PDA |
| **M-Mode**: | | | |
| Ao | mm | PWd | mm |
| LA | mm | EDV | ml |
| LVIDd | mm | ESV | ml |
| LVIDs | mm | FS | % |
| IVSd | mm | LVEF | % |
| **Additional Information:** | | | |
| **Conclusion:**   1. Normal Echocardiography Study | | | |
| **Done By:** | **Signature** | **Date** | **Remark** |
| Tesfaye T., Pediatric Cardiologist |  | 25/09/14Eth.C. |  |

| **Patient Name: Hayat Ebrahim. Sex/Age: F/3yrs. Date of Report:30/09/2014Eth.C. MRN: 064421.**  **Clinical Diagnosis: IE + RD + DS. TGSH1.2596.** | | | |
| --- | --- | --- | --- |
| **Features:** | **Findings** | **Features** | **Findings** |
| **Profile** | | **Atria** | |
| Abdominal Situs | Solitus | Left Atrium | Dilated |
| Cardiac Position | Levocardia | Right Atrium | Dilated |
| Systemic Venous Drainage | To RA | **Atrio-Ventricular Valves** | |
| Pulmonary Venous Drainage | To LA | Mitral Valve | Common Complete AVSD, L – R Shunt |
| Atrio-ventricular Connection | Common Complete AVSD | Tricuspid Valve |  |
| Ventriculo-Arterial Connection | concordant | **Ventricle** | |
| Ventricular Loop | d-Loop | Left Ventricle | Dilated |
| **Septae** |  | Right Ventricle | Dilated |
| Interatrial Septum | Common Complete AVSD, L – R Shunt.  Additional 5mm OS ASD, L – R Shunt | **Doppler Measurement** |  |
| Interventricular Septum |  | Mitral | Mild Left AVVR |
| **Semilunar Valves** |  | Aortic |  |
| Aortic Valve | Annulus = 13mm | Tricuspid | Moderate Right AVVR |
| Pulmonary Valve | Annulus = 17mm | Pulmonic |  |
| **Great Arteries** | NRGA | **Coronary Arteries** |  |
| Aorta |  | **Aortic Arch** | Left |
| Pulmonary Arteries | MPA Dilated | **PDA** | No PDA |
| **M-Mode**: Normal LV Function on eye balling | | | |
| Ao | mm | PWd | mm |
| LA | mm | EDV | ml |
| LVIDd | mm | ESV | ml |
| LVIDs | mm | FS | % |
| IVSd | mm | LVEF | % |
| **Additional Information:** No evidences of IE at the current moment. | | | |
| **Conclusion:**   1. {S, D, S} Levocardia 2. All chambers dilated 3. Common Complete AVSD, L – R Shunt 4. Additional Small OS ASD, L – R Shunt 5. Mild Left AVVR 6. Moderate Right AVVR | | | |
| **Remark:** Clinical assessment for IE is of paramount importance. | | | |
| **Done By:** | **Signature** | **Date** | **Remark** |
| Tesfaye T., Pediatric Cardiologist |  | 30/09/14Eth.C. |  |

| **Patient Name: Amen Tagel. Sex/Age: F/3 7/12yrs. Date of Report:30/09/2014Eth.C. MRN:133420_.** | | | |
| --- | --- | --- | --- |
| **Clinical Diagnosis: Cyanosis + Clubbing + Murmur. TGSH1.2597.** | | | |
| **Features:** | **Findings** | **Features** | **Findings** |
| **Profile** | | **Atria** | |
| Abdominal Situs | Solitus | Left Atrium | Normal |
| Cardiac Position | Levocardia | Right Atrium | Dilated |
| Systemic Venous Drainage | To RA | **Atrio-Ventricular Valves** | |
| Pulmonary Venous Drainage | To LA | Mitral Valve | Annulus = 13mm |
| Atrio-ventricular Connection | Concordant | Tricuspid Valve | Annulus = 17mm  TAPSE = 15mm |
| Ventriculo-Arterial Connection | concordant | **Ventricle** | |
| Ventricular Loop | d-Loop | Left Ventricle | Normal |
| **Septae** |  | Right Ventricle | Dilated, RVH |
| Interatrial Septum | Intact | **Doppler Measurement** |  |
| Interventricular Septum | Non-Restrictive Malaligned Sub – aortic VSD, R – L Shunt | Mitral |  |
| **Semilunar Valves** |  | Aortic |  |
| Aortic Valve | Annulus = 16mm | Tricuspid |  |
| Pulmonary Valve | Annulus = 6mm | Pulmonic | Severe PS, PPG = 86mmHg |
| **Great Arteries** | NRGA | **Coronary Arteries** |  |
| Aorta | Aortic over-ride to the VSD. | **Aortic Arch** | Right |
| Pulmonary Arteries | Smallish MPA and Confluent Branch PAs. | **PDA** | No PDA |
| **M-Mode**: | | | |
| Ao | mm | PWd | mm |
| LA | mm | EDV | ml |
| LVIDd | mm | ESV | ml |
| LVIDs | mm | FS | 30% |
| IVSd | mm | LVEF | 60% |
| **Additional Information:** | | | |
| **Conclusion:**   1. {S, D, S} Levocardia 2. TOF 3. Smallish MPA and Confluent Branch PAs. 4. Right Aortic Arch | | | |
| **Done By:** | **Signature** | **Date** | **Remark** |
| Tesfaye T., Pediatric Cardiologist |  | 30/09/14Eth.C. |  |

| **Patient Name: Baby Tirualem Yeshi. Sex/Age M/1 Day: Date of Report:01/10/2014Eth. MRN:134001.**  **Clinical Diagnosis: Pre-Op Screening. TGSH1.2598.** | | | |
| --- | --- | --- | --- |
| **Features:** | **Findings** | **Features** | **Findings** |
| **Profile** | | **Atria** | |
| Abdominal Situs | Solitus | Left Atrium | Normal |
| Cardiac Position | Levocardia | Right Atrium | Normal |
| Systemic Venous Drainage | To RA | **Atrio-Ventricular Valves** | |
| Pulmonary Venous Drainage | To LA | Mitral Valve | Annulus = 9mm |
| Atrio-ventricular Connection | Concordant | Tricuspid Valve | Annulus = 10mm  TAPSE = 9mm |
| Ventriculo-Arterial Connection | concordant | **Ventricle** | |
| Ventricular Loop | d-Loop | Left Ventricle | Normal |
| **Septae** |  | Right Ventricle | Normal |
| Interatrial Septum | PFO, L – R Shunt | **Doppler Measurement** |  |
| Interventricular Septum | Intact | Mitral |  |
| **Semilunar Valves** |  | Aortic |  |
| Aortic Valve | Annulus = 10mm | Tricuspid |  |
| Pulmonary Valve | Annulus = 10mm | Pulmonic |  |
| **Great Arteries** | NRGA | **Coronary Arteries** |  |
| Aorta |  | **Aortic Arch** | Left |
| Pulmonary Arteries |  | **PDA** | <1mm PDA, L – R Shunt |
| **M-Mode**: | | | |
| Ao | mm | PWd | mm |
| LA | mm | EDV | ml |
| LVIDd | mm | ESV | ml |
| LVIDs | mm | FS | % |
| IVSd | mm | LVEF | % |
| **Additional Information:** | | | |
| **Conclusion:**   1. {S, D, S} Levocardia 2. PFO, L – R Shunt 3. Silent PDA, L – R Shunt | | | |
| **Remark:** No Hemodynamic Impact. Can undergo General Anesthesia and Major Surgery from cardiac evaluation point. | | | |
| **Done By:** | **Signature** | **Date** | **Remark** |
| Tesfaye T., Pediatric Cardiologist |  | 25/09/14Eth.C. |  |

| **Patient Name: Enatnesh Wasu. Sex/Age: F/5months. Date of Report: 02/10/2014Eth.C. MRN:113100.** | | | |
| --- | --- | --- | --- |
| **Clinical Diagnosis: Recurrent Chest Infection + Murmur. TGSH1.2599.** | | | |
| **Features:** | **Findings** | **Features** | **Findings** |
| **Profile** | | **Atria** | |
| Abdominal Situs | Solitus | Left Atrium | Normal |
| Cardiac Position | Levocardia | Right Atrium | Normal |
| Systemic Venous Drainage | To RA | **Atrio-Ventricular Valves** | |
| Pulmonary Venous Drainage | To LA | Mitral Valve | Annulus = 9mm |
| Atrio-ventricular Connection | Concordant | Tricuspid Valve | Annulus = 9mm |
| Ventriculo-Arterial Connection | concordant | **Ventricle** | |
| Ventricular Loop | d-Loop | Left Ventricle | Normal |
| **Septae** |  | Right Ventricle | Normal |
| Interatrial Septum | Intact | **Doppler Measurement** |  |
| Interventricular Septum | Intact | Mitral |  |
| **Semilunar Valves** |  | Aortic |  |
| Aortic Valve | Annulus = 9mm | Tricuspid |  |
| Pulmonary Valve | Annulus = 10mm | Pulmonic |  |
| **Great Arteries** | NRGA | **Coronary Arteries** |  |
| Aorta |  | **Aortic Arch** | Left |
| Pulmonary Arteries |  | **PDA** | No PDA |
| **M-Mode**: Normal LV Function on eye balling | | | |
| Ao | mm | PWd | mm |
| LA | mm | EDV | ml |
| LVIDd | mm | ESV | ml |
| LVIDs | mm | FS | % |
| IVSd | mm | LVEF | % |
| **Additional Information:** | | | |
| **Conclusion:**   1. Normal Echocardiography Study | | | |
| **Done By:** | **Signature** | **Date** | **Remark** |
| Tesfaye T., Pediatric Cardiologist |  | 02/10/14Eth.C. |  |

| **Patient Name: Fasikaw Demeke. Sex/Age: M/8yrs. Date of Report:02/10/2014Eth.C. MRN:134167.**  **Clinical Diagnosis: ARF + Murmur. TGSH1.2600.** | | | |
| --- | --- | --- | --- |
| **Features:** | **Findings** | **Features** | **Findings** |
| **Profile** | | **Atria** | |
| Abdominal Situs | Solitus | Left Atrium | Dilated |
| Cardiac Position | Levocardia | Right Atrium | Normal |
| Systemic Venous Drainage | To RA | **Atrio-Ventricular Valves** | |
| Pulmonary Venous Drainage | To LA | Mitral Valve | Annulus = 31mm. thickened MVL. Shortened PMVL |
| Atrio-ventricular Connection | Concordant | Tricuspid Valve | Annulus = 28mm  TAPSE = 22mm |
| Ventriculo-Arterial Connection | concordant | **Ventricle** | |
| Ventricular Loop | d-Loop | Left Ventricle | Dilated |
| **Septae** |  | Right Ventricle | Normal |
| Interatrial Septum | Intact | **Doppler Measurement** |  |
| Interventricular Septum | Intact | Mitral | Severe MR, Holosystolic, Posterior projection, seen in two planes with jet velocity = 4.3m/sec. |
| **Semilunar Valves** |  | Aortic | Trivial AR |
| Aortic Valve | Annulus = 16mm | Tricuspid | Moderate TR, PPG = 29mmHg |
| Pulmonary Valve | Annulus = 22mm | Pulmonic | Trivial PR, PPG = 20mmHg |
| **Great Arteries** | NRGA | **Coronary Arteries** |  |
| Aorta |  | **Aortic Arch** | Left |
| Pulmonary Arteries |  | **PDA** | No PDA |
| **M-Mode**: | | | |
| Ao | mm | PWd | mm |
| LA | mm | EDV | ml |
| LVIDd | mm | ESV | ml |
| LVIDs | mm | FS | 33% |
| IVSd | mm | LVEF | 61% |
| **Additional Information:** | | | |
| **Conclusion:**   1. {S, D, S} Levocardia 2. LA/LV Dilated 3. Thickened MVL, Shortened PMVL 4. Severe MR 5. Moderate TR 6. Trivial AR 7. Normal Biventricular Systolic Function | | | |
| **Done By:** | **Signature** | **Date** | **Remark** |
| Tesfaye T., Pediatric Cardiologist |  | 02/10/14Eth.C |  |

| **Patient Name: Fentanesh Tsegaw. Sex/Age: F/8months. Date of Report:03/10/2014Eth.C. MRN: 134354.**  **Clinical Diagnosis: CHF + RD. TGSH1.2601.** | | | |
| --- | --- | --- | --- |
| **Features:** | **Findings** | **Features** | **Findings** |
| **Profile** | | **Atria** | |
| Abdominal Situs | Solitus | Left Atrium | Dilated |
| Cardiac Position | Levocardia | Right Atrium | Normal |
| Systemic Venous Drainage | To RA | **Atrio-Ventricular Valves** | |
| Pulmonary Venous Drainage | To LA | Mitral Valve | Annulus = 15mm |
| Atrio-ventricular Connection | Concordant | Tricuspid Valve | Annulus = 15mm |
| Ventriculo-Arterial Connection | concordant | **Ventricle** | |
| Ventricular Loop | d-Loop | Left Ventricle | Globularly Dilated & Dysfunctional |
| **Septae** |  | Right Ventricle | Normal |
| Interatrial Septum | Intact | **Doppler Measurement** |  |
| Interventricular Septum | Intact | Mitral | Moderate MR, Holosystolic, posterior projection, seen in two planes with jet velocity = 3.9m/sec. |
| **Semilunar Valves** |  | Aortic |  |
| Aortic Valve | Annulus = 10mm | Tricuspid | Trivial TR, PPG = 18mmHg |
| Pulmonary Valve | Annulus = 11mm | Pulmonic |  |
| **Great Arteries** | NRGA | **Coronary Arteries** | No ALCAPA |
| Aorta |  | **Aortic Arch** | Left. No arch abnormality |
| Pulmonary Arteries |  | **PDA** | No PDA |
| **M-Mode**: | | | |
| Ao | mm | PWd | mm |
| LA | mm | EDV | ml |
| LVIDd | mm | ESV | ml |
| LVIDs | mm | FS | 10% |
| IVSd | mm | LVEF | 22% |
| **Additional Information:** pericardial effusion measuring a maximum of 3mm on RA/RV Side. | | | |
| **Conclusion:**   1. {S, D, S} Levocardia 2. Moderate MR 3. Trivial TR 4. Globularly Dilated & Severely Dysfunctional LV | | | |
| **Remark:** work up in the line of DCM | | | |
| **Done By:** | **Signature** | **Date** | **Remark** |
| Tesfaye T., Pediatric Cardiologist |  | 23/09/14Eth.C. |  |

| **Patient Name: Mirtzer Belete . Sex/Age: F/10 yrs. Date of Report:07/10/2014Eth.C. MRN: 133825.** | | | |
| --- | --- | --- | --- |
| **Clinical Diagnosis: Palpitation. TGSH1.2602.** | | | |
| **Features:** | **Findings** | **Features** | **Findings** |
| **Profile** | | **Atria** | |
| Abdominal Situs | Solitus | Left Atrium | Normal |
| Cardiac Position | Levocardia | Right Atrium | Normal |
| Systemic Venous Drainage | To RA | **Atrio-Ventricular Valves** | |
| Pulmonary Venous Drainage | To LA | Mitral Valve | Annulus = mm |
| Atrio-ventricular Connection | Concordant | Tricuspid Valve | Annulus = mm  TAPSE = mm |
| Ventriculo-Arterial Connection | concordant | **Ventricle** | |
| Ventricular Loop | d-Loop | Left Ventricle | Normal |
| **Septae** |  | Right Ventricle | Normal |
| Interatrial Septum | Intact | **Doppler Measurement** |  |
| Interventricular Septum | Intact | Mitral |  |
| **Semilunar Valves** |  | Aortic |  |
| Aortic Valve | Annulus = mm | Tricuspid |  |
| Pulmonary Valve | Annulus = mm | Pulmonic |  |
| **Great Arteries** | NRGA | **Coronary Arteries** |  |
| Aorta |  | **Aortic Arch** | Left |
| Pulmonary Arteries |  | **PDA** | No PDA |
| **M-Mode**: | | | |
| Ao | mm | PWd | mm |
| LA | mm | EDV | ml |
| LVIDd | mm | ESV | ml |
| LVIDs | mm | FS | % |
| IVSd | mm | LVEF | % |
| **Additional Information:** | | | |
| **Conclusion:**   1. Normal Echocardiography Study | | | |
| **Done By:** | **Signature** | **Date** | **Remark** |
| Tesfaye T., Pediatric Cardiologist |  | 23/09/14Eth.C. |  |

| **Patient Name: Kalkidan Mengistu. Sex/Age: F/12. Date of Report:09/10/2014Eth.C. MRN: 135256.**  **Clinical Diagnosis: DOE + Murmur + Palpitation + CHF. TGSH1.2603.** | | | |
| --- | --- | --- | --- |
| **Features:** | **Findings** | **Features** | **Findings** |
| **Profile** | | **Atria** | |
| Abdominal Situs | Solitus | Left Atrium | Dilated |
| Cardiac Position | Levocardia | Right Atrium | Normal |
| Systemic Venous Drainage | To RA | **Atrio-Ventricular Valves** | |
| Pulmonary Venous Drainage | To LA | Mitral Valve | Annulus = 32mm |
| Atrio-ventricular Connection | Concordant | Tricuspid Valve | Annulus = 18mm  TAPSE = 21mm |
| Ventriculo-Arterial Connection | concordant | **Ventricle** | |
| Ventricular Loop | d-Loop | Left Ventricle | Dilated |
| **Septae** |  | Right Ventricle | Normal. RV TDI S wave = 11cm/sec. |
| Interatrial Septum | Intact | **Doppler Measurement** |  |
| Interventricular Septum | Intact | Mitral | Mild MR, Holosystolic, posterior projection, seen in two planes with jet velocity = 4.2m/sec. |
| **Semilunar Valves** |  | Aortic |  |
| Aortic Valve | Annulus = 18mm | Tricuspid |  |
| Pulmonary Valve | Annulus = 25mm | Pulmonic |  |
| **Great Arteries** | NRGA | **Coronary Arteries** |  |
| Aorta |  | **Aortic Arch** | Left |
| Pulmonary Arteries | **MPA = 25mm.** Confluent Branches | **PDA** | 4mm PDA, L – R Shunt with SPG/DPG = 66/42mmHg |
| **M-Mode**: | | | |
| Ao | mm | PWd | mm |
| LA | mm | EDV | ml |
| LVIDd | mm | ESV | ml |
| LVIDs | mm | FS | 31% |
| IVSd | mm | LVEF | 58% |
| **Additional Information:** | | | |
| **Conclusion:**   1. {S, D, S} Levocardia 2. LA/LV Dilated 3. Mild MR 4. Large PDA, L – R Shunt 5. Pulmonary Hypertension 6. Normal Biventricular Systolic Function | | | |
| **Done By:** | **Signature** | **Date** | **Remark** |
| Tesfaye T., Pediatric Cardiologist |  | 09/10/14Eth.C. |  |

| **Patient Name: B/Simegn Bassie. Sex/Age: M/19days. Date of Report:09109/2014Eth.C. MRN: 135053.**  **Clinical Diagnosis: Incidental Murmur. TGSH1.2604.** | | | |
| --- | --- | --- | --- |
|  | | | |
| **Features:** | **Findings** | **Features** | **Findings** |
| **Profile** | | **Atria** | |
| Abdominal Situs | Solitus | Left Atrium | Normal |
| Cardiac Position | Levocardia | Right Atrium | Normal |
| Systemic Venous Drainage | To RA | **Atrio-Ventricular Valves** | |
| Pulmonary Venous Drainage | To LA | Mitral Valve | Annulus = 11mm |
| Atrio-ventricular Connection | Concordant | Tricuspid Valve | Annulus = 12mm |
| Ventriculo-Arterial Connection | concordant | **Ventricle** | |
| Ventricular Loop | d-Loop | Left Ventricle | Normal |
| **Septae** |  | Right Ventricle | Normal |
| Interatrial Septum | PFO, L – R Shunt | **Doppler Measurement** |  |
| Interventricular Septum | 4mm PM VSD, L – R Shunt | Mitral | ---- |
| **Semilunar Valves** |  | Aortic | ---- |
| Aortic Valve | Annulus = 9mm | Tricuspid | ----- |
| Pulmonary Valve | Annulus = 9mm. Doming | Pulmonic | Mild Valvular PS, PPG = 29mmHg |
| **Great Arteries** | NRGA | **Coronary Arteries** |  |
| Aorta |  | **Aortic Arch** | Left |
| Pulmonary Arteries |  | **PDA** | No PDA |
[truncated: 249,024 more chars]
