# Supplementary material for: Two–Dimensional and Doppler trans-thoracic echocardiographic patterns of suspected pediatric heart diseases at Tibebe-—Ghion specialized Teaching Hospital and Adinas General Hospital, Bahir Dar, North-west Ethiopia:–An experience from an LMIC
Source: PLoS One. 2024 Mar 11;19(3):e0292694. doi: 10.1371/journal.pone.0292694 (PMC10927071; doi:10.1371/journal.pone.0292694)
Supplement: S1 File — (ZIP) [file pone.0292694.s002.zip › TGSH2 Pediatric Transthoracic Echocardiography Report August 2021 SPSS FILLED.docx]

| **Patient Name: Baby Haymanot Birhanu. Sex/Age: M/2days.Date of Report:04/11/2014Eth.C. MRN:135467.** | | | |
| --- | --- | --- | --- |
| **Clinical Diagnosis: Cyanosis + RD. TGSH2.2837** | | | |
| **Features:** | **Findings** | **Features** | **Findings** |
| **Profile** | | **Atria** | |
| Abdominal Situs | Solitus | Left Atrium | Normal |
| Cardiac Position | Levocardia | Right Atrium | Normal |
| Systemic Venous Drainage | To RA | **Atrio-Ventricular Valves** | |
| Pulmonary Venous Drainage | To LA | Mitral Valve | Annulus = 11mm |
| Atrio-ventricular Connection | Concordant | Tricuspid Valve | Atretic |
| Ventriculo-Arterial Connection | concordant | **Ventricle** | |
| Ventricular Loop | d-Loop | Left Ventricle | Normal |
| **Septae** |  | Right Ventricle | Smallish/Hypoplastic |
| Interatrial Septum | PFO, R – L Shunt | **Doppler Measurement** |  |
| Interventricular Septum | Intact | Mitral |  |
| **Semilunar Valves** |  | Aortic |  |
| Aortic Valve | Annulus = 11mm | Tricuspid |  |
| Pulmonary Valve | Annulus = 6mm | Pulmonic | Atretic |
| **Great Arteries** | NRGA | **Coronary Arteries** |  |
| Aorta |  | **Aortic Arch** | Left |
| Pulmonary Arteries | Smallish MPA and Branch PAs/Hypoplastic | **PDA** | 1mm PDA, L – R Shunt |
| **M-Mode**: | | | |
| Ao | mm | PWd | mm |
| LA | mm | EDV | ml |
| LVIDd | mm | ESV | ml |
| LVIDs | mm | FS | % |
| IVSd | mm | LVEF | % |
| **Additional Information:** | | | |
| **Conclusion:**   1. {S, D, S} Levocardia 2. PFO, R – L Shunt 3. Small PDA, L – R Shunt 4. Smallish MPA and Branch PAs 5. Tricuspid Atresia Type IA | | | |
| **Done By:** | **Signature** | **Date** | **Remark** |
| Tesfaye T., Pediatric Cardiologist |  | 04/11/14Eth.C. |  |

| **Patient Name: Kalkidan Atalay. Sex/Age: F/5/12. Date of Report:05/11/2014Eth.C. MRN:128472.** | | | |
| --- | --- | --- | --- |
| **Clinical Diagnosis: Incidental Murmur. TGSH2.2838.** | | | |
| **Features:** | **Findings** | **Features** | **Findings** |
| **Profile** | | **Atria** | |
| Abdominal Situs | Solitus | Left Atrium | Normal |
| Cardiac Position | Levocardia | Right Atrium | Mildly Dilated |
| Systemic Venous Drainage | To RA | **Atrio-Ventricular Valves** | |
| Pulmonary Venous Drainage | To LA | Mitral Valve | Annulus = 12mm |
| Atrio-ventricular Connection | Concordant | Tricuspid Valve | Annulus = 13mm |
| Ventriculo-Arterial Connection | concordant | **Ventricle** | |
| Ventricular Loop | d-Loop | Left Ventricle | Normal |
| **Septae** |  | Right Ventricle | Mildly Dilated |
| Interatrial Septum | PFO, L – R Shunt | **Doppler Measurement** |  |
| Interventricular Septum | 2mm Supracristal VSD, Predominantly L – R Shunt | Mitral |  |
| **Semilunar Valves** |  | Aortic |  |
| Aortic Valve | Annulus = 12mm | Tricuspid |  |
| Pulmonary Valve | Annulus = 12mm. Dysplastic | Pulmonic | Severe Valvular PS, PPG = 60mmHg |
| **Great Arteries** | NRGA | **Coronary Arteries** |  |
| Aorta |  | **Aortic Arch** | Left |
| Pulmonary Arteries |  | **PDA** | No PDA |
| **M-Mode**: Normal LV Function on eye balling | | | |
| Ao | mm | PWd | mm |
| LA | mm | EDV | ml |
| LVIDd | mm | ESV | ml |
| LVIDs | mm | FS | % |
| IVSd | mm | LVEF | % |
| **Additional Information:** | | | |
| **Conclusion:**   1. {S, D, S} Levocardia 2. PFO, L – R Shunt 3. Small Supracristal VSD, Predominantly L – R Shunt 4. Dysplastic PV 5. Severe PS | | | |
| **Done By:** | **Signature** | **Date** | **Remark** |
| Tesfaye T., Pediatric Cardiologist |  | 05/11/14Eth.C. |  |

| **Patient Name: Baby Emebet Adugna. Sex/Age: M/14days. Date of Report:05/11/2014Eth.C. MRN: 138339.** | | | |
| --- | --- | --- | --- |
| **Clinical Diagnosis : Incidental Murmur. TGSH2.2839.** | | | |
| **Features:** | **Findings** | **Features** | **Findings** |
| **Profile** | | **Atria** | |
| Abdominal Situs | Solitus | Left Atrium | Normal |
| Cardiac Position | Levocardia | Right Atrium | Normal |
| Systemic Venous Drainage | To RA | **Atrio-Ventricular Valves** | |
| Pulmonary Venous Drainage | To LA | Mitral Valve | Annulus = 11mm |
| Atrio-ventricular Connection | Concordant | Tricuspid Valve | Annulus = 12mm  TAPSE = 12mm |
| Ventriculo-Arterial Connection | concordant | **Ventricle** | |
| Ventricular Loop | d-Loop | Left Ventricle | Normal |
| **Septae** |  | Right Ventricle | Normal |
| Interatrial Septum | PFO, L – R Shunt | **Doppler Measurement** |  |
| Interventricular Septum | 4mm PM VSD, L – R Shunt | Mitral |  |
| **Semilunar Valves** |  | Aortic |  |
| Aortic Valve | Annulus = 7mm | Tricuspid |  |
| Pulmonary Valve | Annulus = 10mm | Pulmonic |  |
| **Great Arteries** | NRGA | **Coronary Arteries** |  |
| Aorta |  | **Aortic Arch** | Left |
| Pulmonary Arteries |  | **PDA** | No PDA |
| **M-Mode**: Normal LV Function on eye balling | | | |
| Ao | mm | PWd | mm |
| LA | mm | EDV | ml |
| LVIDd | mm | ESV | ml |
| LVIDs | mm | FS | % |
| IVSd | mm | LVEF | % |
| **Additional Information:** | | | |
| **Conclusion:**   1. {S, D, S} Levocardia 2. PFO, L – R Shunt 3. Moderate PM VSD, L – R Shunt | | | |
| **Done By:** | **Signature** | **Date** | **Remark** |
| Tesfaye T., Pediatric Cardiologist |  | 05/11/14Eth.C. |  |

| **Patient Name: Baby of Fikir Adugnaw. Sex/Age: F/15days. Date of Report:05/11/2014Eth.C. MRN: 137893.** | | | |
| --- | --- | --- | --- |
| **Clinical Diagnosis : RD. TGSH2.2840.** | | | |
| **Features:** | **Findings** | **Features** | **Findings** |
| **Profile** | | **Atria** | |
| Abdominal Situs | Solitus | Left Atrium | Normal |
| Cardiac Position | Levocardia | Right Atrium | Normal |
| Systemic Venous Drainage | To RA | **Atrio-Ventricular Valves** | |
| Pulmonary Venous Drainage | To LA | Mitral Valve | Annulus = 9mm |
| Atrio-ventricular Connection | Concordant | Tricuspid Valve | Annulus = 9mm |
| Ventriculo-Arterial Connection | concordant | **Ventricle** | |
| Ventricular Loop | d-Loop | Left Ventricle | Normal |
| **Septae** |  | Right Ventricle | Normal |
| Interatrial Septum | Intact | **Doppler Measurement** |  |
| Interventricular Septum | Intact | Mitral |  |
| **Semilunar Valves** |  | Aortic |  |
| Aortic Valve | Annulus = 7mm | Tricuspid |  |
| Pulmonary Valve | Annulus = 8mm | Pulmonic |  |
| **Great Arteries** | NRGA | **Coronary Arteries** |  |
| Aorta |  | **Aortic Arch** | Left |
| Pulmonary Arteries |  | **PDA** | No PDA |
| **M-Mode**: Normal LV Function on eye balling | | | |
| Ao | mm | PWd | mm |
| LA | mm | EDV | ml |
| LVIDd | mm | ESV | ml |
| LVIDs | mm | FS | % |
| IVSd | mm | LVEF | % |
| **Additional Information:** | | | |
| **Conclusion:**   1. Normal Echocardiography Study. | | | |
| **Done By:** | **Signature** | **Date** | **Remark** |
| Tesfaye T., Pediatric Cardiologist |  | 05/11/2014Eth.C. |  |

| **Patient Name: Mekedes Lingerew. Sex/Age: F/7months. Date of Report:12/11/2014Eth.C. MRN: 138879.** | | | |
| --- | --- | --- | --- |
| **Clinical Diagnosis : DS + Murmur + RD. TGSH2.2841.** | | | |
| **Features:** | **Findings** | **Features** | **Findings** |
| **Profile** | | **Atria** | |
| Abdominal Situs | Solitus | Left Atrium | Dilated |
| Cardiac Position | Levocardia | Right Atrium | Dilated |
| Systemic Venous Drainage | To RA | **Atrio-Ventricular Valves** | |
| Pulmonary Venous Drainage | To LA | Mitral Valve | Common Complete AVSD |
| Atrio-ventricular Connection | Common Complete AVSD | Tricuspid Valve |  |
| Ventriculo-Arterial Connection | concordant | **Ventricle** | |
| Ventricular Loop | d-Loop | Left Ventricle | Dilated |
| **Septae** |  | Right Ventricle | Dilated |
| Interatrial Septum | Common Complete AVSD, L – R Shunt | **Doppler Measurement** |  |
| Interventricular Septum |  | Mitral | Moderate Left AVVR |
| **Semilunar Valves** |  | Aortic |  |
| Aortic Valve | Annulus = 10mm | Tricuspid | Mild Right AVVR |
| Pulmonary Valve | Annulus = 16mm | Pulmonic | Mild PR, PPG = 57mmHg |
| **Great Arteries** | NRGA | **Coronary Arteries** |  |
| Aorta |  | **Aortic Arch** | Left |
| Pulmonary Arteries | MPA = 18mm | **PDA** | No PDA |
| **M-Mode**: Normal LV Function on eye balling | | | |
| Ao | mm | PWd | mm |
| LA | mm | EDV | ml |
| LVIDd | mm | ESV | ml |
| LVIDs | mm | FS | % |
| IVSd | mm | LVEF | % |
| **Additional Information:** | | | |
| **Conclusion:**   1. {S, D, S} Levocardia 2. RA/RV Dilated 3. Common Complete Balanced AVSD, L – R Shunt 4. Mild Right AVVR 5. Moderate Left AVVR 6. Mild PR 7. Moderate to severe Pulmonary Hypertension 8. Normal LV Systolic Function | | | |
| **Done By:** | **Signature** | **Date** | **Remark** |
| Tesfaye T., Pediatric Cardiologist |  | 12/11/14Eth.C. |  |

| **Patient Name: Yalemwork Aschale. Sex/Age: F/5 2/12. Date of Report:12/11/2014Eth.C. MRN:119263.** | | | |
| --- | --- | --- | --- |
| **Clinical Diagnosis : ?Duchenne Dystrophy. TGSH2.2842.** | | | |
| **Features:** | **Findings** | **Features** | **Findings** |
| **Profile** | | **Atria** | |
| Abdominal Situs | Solitus | Left Atrium | Normal |
| Cardiac Position | Levocardia | Right Atrium | Normal |
| Systemic Venous Drainage | To RA | **Atrio-Ventricular Valves** | |
| Pulmonary Venous Drainage | To LA | Mitral Valve | Annulus = 17mm |
| Atrio-ventricular Connection | Concordant | Tricuspid Valve | Annulus = 21mm  TAPSE = 18mm |
| Ventriculo-Arterial Connection | concordant | **Ventricle** | |
| Ventricular Loop | d-Loop | Left Ventricle | LVH |
| **Septae** |  | Right Ventricle | RVH |
| Interatrial Septum | Intact | **Doppler Measurement** |  |
| Interventricular Septum | Intact | Mitral |  |
| **Semilunar Valves** |  | Aortic | Mild AR, PHT = 586ms |
| Aortic Valve | Annulus = 15mm | Tricuspid | Trivial TR, PPG = 38mmHg |
| Pulmonary Valve | Annulus = 19mm | Pulmonic | Trivial PR, PPG = 22mmHg |
| **Great Arteries** | NRGA | **Coronary Arteries** |  |
| Aorta |  | **Aortic Arch** | Left |
| Pulmonary Arteries |  | **PDA** | No PDA |
| **M-Mode**: | | | |
| Ao | mm | PWd | 11mm |
| LA | mm | PWs | 15mm |
| LVIDd | 30mm | EDV | 35ml |
| LVIDs | 16mm | ESV | 7ml |
| IVSd | 15mm | FS | 48% |
| IVSs | 17mm | LVEF | 81% |
| **Additional Information:** | | | |
| **Conclusion:**   1. {S, D, S} Levocardia 2. Trivial TR 3. Trivial PR 4. Mild AR 5. Biventricular Ventricular Hypertrophy 6. Mild Pulmonary Hypertension | | | |
| **Done By:** | **Signature** | **Date** | **Remark** |
| Tesfaye T., Pediatric Cardiologist |  | 12/11/14Eth.C. |  |

| **Patient Name: Baby Gojjam Dereje. Sex/Age: M/22 days. Date of Report:12/11/2014Eth.C. MRN:138774.** | | | |
| --- | --- | --- | --- |
| **Clinical Diagnosis : RD. TGSH2.2843.** | | | |
| **Features:** | **Findings** | **Features** | **Findings** |
| **Profile** | | **Atria** | |
| Abdominal Situs | Solitus | Left Atrium | Normal |
| Cardiac Position | Levocardia | Right Atrium | Normal |
| Systemic Venous Drainage | To RA | **Atrio-Ventricular Valves** | |
| Pulmonary Venous Drainage | To LA | Mitral Valve | Annulus = 8mm |
| Atrio-ventricular Connection | Concordant | Tricuspid Valve | Annulus = 9mm |
| Ventriculo-Arterial Connection | concordant | **Ventricle** | |
| Ventricular Loop | d-Loop | Left Ventricle | Normal |
| **Septae** |  | Right Ventricle | Normal |
| Interatrial Septum | Intact | **Doppler Measurement** |  |
| Interventricular Septum | Intact | Mitral |  |
| **Semilunar Valves** |  | Aortic |  |
| Aortic Valve | Annulus = 7mm | Tricuspid |  |
| Pulmonary Valve | Annulus = 9mm | Pulmonic |  |
| **Great Arteries** | NRGA | **Coronary Arteries** |  |
| Aorta |  | **Aortic Arch** | Left |
| Pulmonary Arteries |  | **PDA** | No PDA |
| **M-Mode**: Normal LV Function on eye balling | | | |
| Ao | mm | PWd | mm |
| LA | mm | EDV | ml |
| LVIDd | mm | ESV | ml |
| LVIDs | mm | FS | % |
| IVSd | mm | LVEF | % |
| **Additional Information:** | | | |
| **Conclusion:**   1. Normal Echocardiography Study | | | |
| **Done By:** | **Signature** | **Date** | **Remark** |
| Tesfaye T., Pediatric Cardiologist |  | 12/11/14Eth.C. |  |

| **Patient Name: Dawit Kibatie. Sex/Age: M/3years. Date of Report:14/11/2014Eth.C. MRN:139724.** | | | |
| --- | --- | --- | --- |
| **Clinical Diagnosis : CHF + Murmur + Palpitation + easy fatigability. TGSH2.2844.** | | | |
| **Features:** | **Findings** | **Features** | **Findings** |
| **Profile** | | **Atria** | |
| Abdominal Situs | Solitus | Left Atrium | Dilated |
| Cardiac Position | Levocardia | Right Atrium | Dilated |
| Systemic Venous Drainage | To RA | **Atrio-Ventricular Valves** | |
| Pulmonary Venous Drainage | To LA | Mitral Valve | Annulus = 17mm |
| Atrio-ventricular Connection | Concordant | Tricuspid Valve | Annulus = 17mm |
| Ventriculo-Arterial Connection | concordant | **Ventricle** | |
| Ventricular Loop | d-Loop | Left Ventricle | Dilated |
| **Septae** |  | Right Ventricle | Dilated |
| Interatrial Septum | Intact | **Doppler Measurement** |  |
| Interventricular Septum | Intact | Mitral |  |
| **Semilunar Valves** |  | Aortic |  |
| Aortic Valve | Annulus = 14mm | Tricuspid |  |
| Pulmonary Valve | Annulus = 16mm | Pulmonic | Moderate PR, PPG = 63mmHg |
| **Great Arteries** | NRGA | **Coronary Arteries** |  |
| Aorta |  | **Aortic Arch** | Left |
| Pulmonary Arteries |  | **PDA** | 15mm Aorto-Pulmonary Window, L – R Shunt |
| **M-Mode**: | | | |
| Ao | mm | PWd | mm |
| LA | mm | EDV | ml |
| LVIDd | mm | ESV | ml |
| LVIDs | mm | FS | 40% |
| IVSd | mm | LVEF | 71% |
| **Additional Information:** | | | |
| **Conclusion:**   1. {S, D, S} Levocardia 2. All chambers dilated 3. Large Aorto-pulmonary Window, Predominantly L – R Shunt 4. Severe Pulmonary Hypertension 5. Normal LV Systolic Function | | | |
| **Done By:** | **Signature** | **Date** | **Remark** |
| Tesfaye T., Pediatric Cardiologist |  | 14/11/14Eth.C. |  |

| **Patient Name: Baby Azagne Setegn . Sex/Age: F/25 days . Date of Report:14/11/2014Eth.C. MRN:136734.** | | | |
| --- | --- | --- | --- |
| **Clinical Diagnosis : RD + PPHTN. TGSH2.2845.** | | | |
| **Features:** | **Findings** | **Features** | **Findings** |
| **Profile** | | **Atria** | |
| Abdominal Situs | Solitus | Left Atrium | Normal |
| Cardiac Position | Levocardia | Right Atrium | Normal |
| Systemic Venous Drainage | To RA | **Atrio-Ventricular Valves** | |
| Pulmonary Venous Drainage | To LA | Mitral Valve | Annulus = 8mm |
| Atrio-ventricular Connection | Concordant | Tricuspid Valve | Annulus = 8mm |
| Ventriculo-Arterial Connection | concordant | **Ventricle** | |
| Ventricular Loop | d-Loop | Left Ventricle | Normal |
| **Septae** |  | Right Ventricle | Normal |
| Interatrial Septum | PFO, L – R Shunt | **Doppler Measurement** |  |
| Interventricular Septum | Intact | Mitral |  |
| **Semilunar Valves** |  | Aortic |  |
| Aortic Valve | Annulus = 7mm | Tricuspid |  |
| Pulmonary Valve | Annulus = 7mm | Pulmonic |  |
| **Great Arteries** | NRGA | **Coronary Arteries** |  |
| Aorta |  | **Aortic Arch** | Left |
| Pulmonary Arteries |  | **PDA** | No PDA |
| **M-Mode**: Normal LV Systolic Function | | | |
| Ao | mm | PWd | mm |
| LA | mm | EDV | ml |
| LVIDd | mm | ESV | ml |
| LVIDs | mm | FS | % |
| IVSd | mm | LVEF | % |
| **Additional Information:** | | | |
| **Conclusion:**   1. {S, D, S} Levocardia 2. PFO, L – R Shunt | | | |
| **Done By:** | **Signature** | **Date** | **Remark** |
| Tesfaye T., Pediatric Cardiologist |  | 14/11/14Eth.C. |  |

| **Patient Name: Baby of Wuditu Taddesse. Sex/Age: M/3years. Date of Report:14/11/2014Eth.C. MRN: 139724.** | | | |
| --- | --- | --- | --- |
| **Clinical Diagnosis : Incidental Murmur + DS. TGSH2.2846.** | | | |
| **Features:** | **Findings** | **Features** | **Findings** |
| **Profile** | | **Atria** | |
| Abdominal Situs | Solitus | Left Atrium | Normal |
| Cardiac Position | Levocardia | Right Atrium | Normal |
| Systemic Venous Drainage | To RA | **Atrio-Ventricular Valves** | |
| Pulmonary Venous Drainage | To LA | Mitral Valve | Annulus = 6mm |
| Atrio-ventricular Connection | Concordant | Tricuspid Valve | Annulus = 7mm |
| Ventriculo-Arterial Connection | concordant | **Ventricle** | |
| Ventricular Loop | d-Loop | Left Ventricle | Normal |
| **Septae** |  | Right Ventricle | Normal |
| Interatrial Septum | 4mm OS ASD, L – R Shunt | **Doppler Measurement** |  |
| Interventricular Septum | Intact | Mitral |  |
| **Semilunar Valves** |  | Aortic |  |
| Aortic Valve | Annulus = 6mm | Tricuspid |  |
| Pulmonary Valve | Annulus = 7mm | Pulmonic |  |
| **Great Arteries** | NRGA | **Coronary Arteries** |  |
| Aorta |  | **Aortic Arch** | Left |
| Pulmonary Arteries |  | **PDA** | 1mm PDA, PDA, L – R Shunt |
| **M-Mode**: Normal LV Function on eye balling | | | |
| Ao | mm | PWd | mm |
| LA | mm | EDV | ml |
| LVIDd | mm | ESV | ml |
| LVIDs | mm | FS | % |
| IVSd | mm | LVEF | % |
| **Additional Information:** | | | |
| **Conclusion:**   1. {S, D, S} Levocardia 2. Small OS ASD, L – R Shunt 3. Small PDA, L – R Shunt | | | |
| **Done By:** | **Signature** | **Date** | **Remark** |
| Tesfaye T., Pediatric Cardiologist |  | 14/11/14Eth.C. |  |

| **Patient Name: Birhanu Mulat . Sex/Age:M/2 8/12. Date of Report:14/11/2014Eth.C. MRN: 045397.** | | | |
| --- | --- | --- | --- |
| **Follow up echocardiography (TGSH7 (age 7months). Clinical Diagnosis : Incidental Murmur. TGSH2.2847.** | | | |
| **Features:** | **Findings** | **Features** | **Findings** |
| **Profile** | | **Atria** | |
| Abdominal Situs | Solitus | Left Atrium | Normal |
| Cardiac Position | Levocardia | Right Atrium | Normal |
| Systemic Venous Drainage | To RA | **Atrio-Ventricular Valves** | |
| Pulmonary Venous Drainage | To LA | Mitral Valve | Annulus = 15mm |
| Atrio-ventricular Connection | Concordant | Tricuspid Valve | Annulus = 13mm |
| Ventriculo-Arterial Connection | concordant | **Ventricle** | |
| Ventricular Loop | d-Loop | Left Ventricle | Normal |
| **Septae** |  | Right Ventricle | Normal |
| Interatrial Septum | 8mm OS ASD, L – R Shunt. The septum bow to RA 10mm. | **Doppler Measurement** |  |
| Interventricular Septum | 6mm PM VSD almost closed by STL with an effective defect of 1mm, L – R Shunt | Mitral |  |
| **Semilunar Valves** |  | Aortic |  |
| Aortic Valve | Annulus = 13mm | Tricuspid |  |
| Pulmonary Valve | Annulus = 13mm | Pulmonic |  |
| **Great Arteries** | NRGA | **Coronary Arteries** |  |
| Aorta |  | **Aortic Arch** | Left |
| Pulmonary Arteries |  | **PDA** | No PDA |
| **M-Mode**: Normal LV Function on eye balling | | | |
| Ao | mm | PWd | mm |
| LA | mm | EDV | ml |
| LVIDd | mm | ESV | ml |
| LVIDs | mm | FS | % |
| IVSd | mm | LVEF | % |
| **Additional Information:** | | | |
| **Conclusion:**   1. {S, D, S} Levocardia 2. Moderate OS ASD, L – R Shunt 3. Moderate PM VSD, Almost closed by STL with an effective defect of 1mm, L – R Shunt | | | |
| **Done By:** | **Signature** | **Date** | **Remark** |
| Tesfaye T., Pediatric Cardiologist |  | 14/11/14Eth.C. |  |

| **Patient Name: Baby of Yeshiwerk Mequanint. Sex/Age: F/18days. Date of Report:14/11/2014Eth.C. MRN: 139660.** | | | |
| --- | --- | --- | --- |
| **Clinical Diagnosis: RD TGSH2.2848.** | | | |
| **Features:** | **Findings** | **Features** | **Findings** |
| **Profile** | | **Atria** | |
| Abdominal Situs | Solitus | Left Atrium | Normal |
| Cardiac Position | Levocardia | Right Atrium | Normal |
| Systemic Venous Drainage | To RA | **Atrio-Ventricular Valves** | |
| Pulmonary Venous Drainage | To LA | Mitral Valve | Annulus = 8mm |
| Atrio-ventricular Connection | Concordant | Tricuspid Valve | Annulus = 9mm |
| Ventriculo-Arterial Connection | concordant | **Ventricle** | |
| Ventricular Loop | d-Loop | Left Ventricle | Normal |
| **Septae** |  | Right Ventricle | Normal |
| Interatrial Septum | Intact | **Doppler Measurement** |  |
| Interventricular Septum | Intact | Mitral |  |
| **Semilunar Valves** |  | Aortic |  |
| Aortic Valve | Annulus = 8mm | Tricuspid |  |
| Pulmonary Valve | Annulus = 9mm | Pulmonic |  |
| **Great Arteries** | NRGA | **Coronary Arteries** |  |
| Aorta |  | **Aortic Arch** | Left |
| Pulmonary Arteries |  | **PDA** | No PDA |
| **M-Mode**: | | | |
| Ao | mm | PWd | mm |
| LA | mm | EDV | ml |
| LVIDd | mm | ESV | ml |
| LVIDs | mm | FS | % |
| IVSd | mm | LVEF | % |
| **Additional Information:** | | | |
| **Conclusion:**   1. Normal Echocardiography Study | | | |
| **Done By:** | **Signature** | **Date** | **Remark** |
| Tesfaye T., Pediatric Cardiologist |  | 23/09/14Eth.C. |  |

| **Patient Name: Biruk Nahusenay. Sex/Age: M /10 6/12. Date of Report:14/11/2014Eth.C. MRN: 139995.** | | | |
| --- | --- | --- | --- |
| **Clinical Diagnosis: Bradycardia. TGSH2.2849.** | | | |
| **Features:** | **Findings** | **Features** | **Findings** |
| **Profile** | | **Atria** | |
| Abdominal Situs | Solitus | Left Atrium | Normal |
| Cardiac Position | Levocardia | Right Atrium | Normal |
| Systemic Venous Drainage | To RA | **Atrio-Ventricular Valves** | |
| Pulmonary Venous Drainage | To LA | Mitral Valve | Annulus = 20mm |
| Atrio-ventricular Connection | Concordant | Tricuspid Valve | Annulus = 20mm |
| Ventriculo-Arterial Connection | concordant | **Ventricle** | |
| Ventricular Loop | d-Loop | Left Ventricle | Normal |
| **Septae** |  | Right Ventricle | Normal |
| Interatrial Septum | Intact | **Doppler Measurement** |  |
| Interventricular Septum | Intact | Mitral |  |
| **Semilunar Valves** |  | Aortic |  |
| Aortic Valve | Annulus = 16mm | Tricuspid |  |
| Pulmonary Valve | Annulus = 18mm | Pulmonic |  |
| **Great Arteries** | NRGA | **Coronary Arteries** |  |
| Aorta |  | **Aortic Arch** | Left |
| Pulmonary Arteries |  | **PDA** | No PDA |
| **M-Mode**: | | | |
| Ao | mm | PWd | mm |
| LA | mm | EDV | ml |
| LVIDd | mm | ESV | ml |
| LVIDs | mm | FS | 32% |
| IVSd | mm | LVEF | 60% |
| **Additional Information:** | | | |
| **Conclusion:**   1. Normal Echocardiography Study | | | |
| **Remark:** Bradycardia during study | | | |
| **ECG:** Sinus bradycardia, Normal Axis, LV Forces | | | |
| **Recommendation:** Follow up | | | |
| **Done By:** | **Signature** | **Date** | **Remark** |
| Tesfaye T., Pediatric Cardiologist |  | 14/11/14Eth.C. |  |

| **Patient Name: Tiruneh Tesfaye. Sex/Age: M/6years. Date of Report:19/12/2014Eth.C. MRN: 143909.**  **Clinical Diagnosis: DOE + CHF + Murmur. TGSH2.2850.** | | | | | |
| --- | --- | --- | --- | --- | --- |
| **Features:** | **Findings** | | **Features** | **Findings** | |
| **Profile** | | | **Atria** | | |
| Abdominal Situs | Solitus | | Left Atrium | Mildly Dilated | |
| Cardiac Position | Levocardia | | Right Atrium | Mildly Dilated | |
| Systemic Venous Drainage | To RA | | **Atrio-Ventricular Valves** | | |
| Pulmonary Venous Drainage | To LA | | Mitral Valve | Annulus = 18mm | |
| Atrio-ventricular Connection | Concordant | | Tricuspid Valve | Annulus = 19mm  TAPSE = 20mm | |
| Ventriculo-Arterial Connection | concordant | | **Ventricle** | | |
| Ventricular Loop | d-Loop | | Left Ventricle | | Mildly Dilated |
| **Septae** |  | | Right Ventricle | | Mildly Dilated |
| Interatrial Septum | 14mm Fenestrated ASD, L – R Shunt | | **Doppler Measurement** | |  |
| Interventricular Septum | 11mm Inlet VSD with PM extension, L – R Shunt | | Mitral | |  |
| **Semilunar Valves** |  | | Aortic | |  |
| Aortic Valve | Annulus = 15mm | | Tricuspid | |  |
| Pulmonary Valve | Annulus = 20mm | | Pulmonic | | Mild PR, PPG = 53mmHg |
| **Great Arteries** | NRGA | | **Coronary Arteries** | |  |
| Aorta |  | | **Aortic Arch** | | Left |
| Pulmonary Arteries |  | | **PDA** | | No PDA |
| **M-Mode**: | | | | | |
| Ao | mm | | PWd | | mm |
| LA | mm | | EDV | | ml |
| LVIDd | mm | | ESV | | ml |
| LVIDs | mm | | FS | | 31% |
| IVSd | mm | | LVEF | | 60% |
| **Additional Information:** pericardial effusion with a maximum depth of 4mm on RA side. | | | | | |
| **Conclusion:**   1. {S, D, S} Levocardia 2. All chambers mildly dilated 3. Large Fenestratesd ASD, L – R Shunt 4. Large Inlet VSD with PM extension, L – R Shunt 5. Moderate Pulmonary Hypertension 6. Normal Biventricular Systolic Function | | | | | |
| **Done By:** | **Signature** | **Date** | | **Remark** | |
| Tesfaye T., Pediatric Cardiologist |  | 19/12/14Eth.C. | |  | |

| **Patient Name: Sofiya Bekele. Sex/Age: F/85days. Date of Report:19/12/2014Eth.C. MRN: . 143412**  **Clinical Diagnosis: CHF + RD. TGSH2.2851.** | | | |
| --- | --- | --- | --- |
|  | | | |
| **Features:** | **Findings** | **Features** | **Findings** |
| **Profile** | | **Atria** | |
| Abdominal Situs | Solitus | Left Atrium | Dilated |
| Cardiac Position | Levocardia | Right Atrium | Dilated |
| Systemic Venous Drainage | To RA | **Atrio-Ventricular Valves** | |
| Pulmonary Venous Drainage | To LA | Mitral Valve | Annulus = 10mm |
| Atrio-ventricular Connection | Concordant | Tricuspid Valve | Annulus = 12mm  **TAPSE = 6mm** |
| Ventriculo-Arterial Connection | concordant | **Ventricle** | |
| Ventricular Loop | d-Loop | Left Ventricle | Dilated |
| **Septae** |  | Right Ventricle | Dilated and Dysfunctional |
| Interatrial Septum | Intact | **Doppler Measurement** |  |
| Interventricular Septum | Intact | Mitral |  |
| **Semilunar Valves** |  | Aortic |  |
| Aortic Valve | Annulus = 8mm | Tricuspid |  |
| Pulmonary Valve | Annulus = 11mm | Pulmonic |  |
| **Great Arteries** | NRGA | **Coronary Arteries** |  |
| Aorta |  | **Aortic Arch** | Left |
| Pulmonary Arteries |  | **PDA** | No PDA |
| **M-Mode**: | | | |
| Ao | mm | PWd | 6mm |
| LA | mm | EDV | 8ml |
| LVIDd | 17mm | ESV | 2ml |
| LVIDs | 10mm | FS | 26% |
| IVSd | **8mm** | LVEF | 50% |
| **Additional Information:** | | | |
| **Conclusion:**   1. {S, D, S} Levocardia 2. All chambers Dilated 3. Thick IVS 4. Dysfunctional LV and RV | | | |
| **Remark:** Myocarditis shall be considered as a DDx. | | | |
| **Done By:** | **Signature** | **Date** | **Remark** |
| Tesfaye T., Pediatric Cardiologist |  | 19/12/14Eth.C. |  |

| **Patient Name: Kibre-Werk Yismaw. Sex/Age: F/10years. Date of Report:19/12/2014Eth.C. MRN: 144012.**  **Clinical Diagnosis: CHF + DOE + Murmur + Palpitation + RD. TGSH2.2852.** | | | |
| --- | --- | --- | --- |
| **Features:** | **Findings** | **Features** | **Findings** |
| **Profile** | | **Atria** | |
| Abdominal Situs | Solitus | Left Atrium | Markedly dilated |
| Cardiac Position | Levocardia | Right Atrium | Dilated |
| Systemic Venous Drainage | To RA | **Atrio-Ventricular Valves** | |
| Pulmonary Venous Drainage | To LA | Mitral Valve | Annulus = 21mm. Thickened Clubbed MVL. .6 X 8mm echogenic mass at the tip of the AMVL on LA side |
| Atrio-ventricular Connection | Concordant | Tricuspid Valve | Annulus = 20mm. TAPSE = 23mm |
| Ventriculo-Arterial Connection | concordant | **Ventricle** | |
| Ventricular Loop | d-Loop | Left Ventricle | Markedly dilated. 9 X 14mm echogenic mass, oscillating in the LV attached to Posterior wall. |
| **Septae** |  | Right Ventricle | Dilated |
| Interatrial Septum | Intact | **Doppler Measurement** |  |
| Interventricular Septum | Intact | Mitral | Severe MR, Holosystolic, posterior projection, seen in two planes with jet velocity = 3.4m/sec. |
| **Semilunar Valves** |  | Aortic | Mild AR |
| Aortic Valve | Annulus = 15mm | Tricuspid | Moderate TR, PPG = 59mmHg |
| Pulmonary Valve | Annulus = 19mm | Pulmonic |  |
| **Great Arteries** | NRGA | **Coronary Arteries** |  |
| Aorta |  | **Aortic Arch** | Left |
| Pulmonary Arteries |  | **PDA** | No PDA |
| **M-Mode**: | | | |
| Ao | mm | PWd | mm |
| LA | mm | EDV | ml |
| LVIDd | mm | ESV | ml |
| LVIDs | mm | FS | 42% |
| IVSd | mm | LVEF | 72% |
| **Additional Information:** Pericardial effusion with maximum depth of 10mm on RA Side | | | |
| **Conclusion:**   1. {S, D, S} Levocardia 2. All chambers dilated 3. Thickened, clubbed MVL. 4. Severe MR 5. Moderate TR 6. Mild AR 7. Echogenic mass attached on the LA Side of the AMVL 8. Echogenic mass attached to the Posterior wall of the LV, Oscillating 9. Severe Pulmonary Hypertension 10. Normal Biventricular Systolic Function 11. Moderate Pericardial effusion | | | |
| **Done By:** | **Signature** | **Date** | **Remark** |
| Tesfaye T., Pediatric Cardiologist |  | 19/12/14Eth.C. |  |

| **Patient Name: Kishin Tegegne. Sex/Age: F/9 6/12. Date of Report:19/12/2014Eth.C. MRN: 126911. (AGH)** | | | |
| --- | --- | --- | --- |
|  | | | |
| **Features:** | **Findings** | **Features** | **Findings** |
| **Profile** | | **Atria** | |
| Abdominal Situs | Solitus | Left Atrium | Normal |
| Cardiac Position | Levocardia | Right Atrium | Dilated |
| Systemic Venous Drainage | To RA | **Atrio-Ventricular Valves** | |
| Pulmonary Venous Drainage | To LA | Mitral Valve | Annulus = 17mm. E/A = 2.2 |
| Atrio-ventricular Connection | Concordant | Tricuspid Valve | Annulus = 20mm  **TAPSE = 13mm** |
| Ventriculo-Arterial Connection | concordant | **Ventricle** | |
| Ventricular Loop | d-Loop | Left Ventricle | Normal |
| **Septae** |  | Right Ventricle | Dilated |
| Interatrial Septum | Intact | **Doppler Measurement** |  |
| Interventricular Septum | Intact | Mitral | ----- |
| **Semilunar Valves** |  | Aortic | ------ |
| Aortic Valve | Annulus = 15mm | Tricuspid | ------ |
| Pulmonary Valve | Annulus = 21mm | Pulmonic | ------ |
| **Great Arteries** | NRGA | **Coronary Arteries** |  |
| Aorta |  | **Aortic Arch** | Left |
| Pulmonary Arteries |  | **PDA** | No PDA |
| **M-Mode**: | | | |
| Ao | mm | PWd | mm |
| LA | mm | EDV | ml |
| LVIDd | mm | ESV | ml |
| LVIDs | mm | FS | **25%** |
| IVSd | mm | LVEF | **51%** |
| **Additional Information:** | | | |
| **Conclusion:**   1. {S, D, S} Levocardia 2. Biventricular Systolic Dysfunction secondary to ? | | | |
| **Remark:**   1. Work up for systemic disease is recommended. 2. Cardiac MRI would have been beneficiary (if available in the country) | | | |
| **Done By:** | **Signature** | **Date** | **Remark** |
| Tesfaye T., Pediatric Cardiologist |  | 19/12/14Eth.C. |  |

| **Patient Name: Haymanot Enawgaw. Sex/Age: M/6years. Date of Report:19/12/2014Eth.C. MRN: 144160.** | | | |
| --- | --- | --- | --- |
| **Clinical Diagnosis: ARF + Murmur. TGSH2.2853.** | | | |
| **Features:** | **Findings** | **Features** | **Findings** |
| **Profile** | | **Atria** | |
| Abdominal Situs | Solitus | Left Atrium | Dilated |
| Cardiac Position | Levocardia | Right Atrium | Normal |
| Systemic Venous Drainage | To RA | **Atrio-Ventricular Valves** | |
| Pulmonary Venous Drainage | To LA | Mitral Valve | Annulus = 29mm. thickened MVL |
| Atrio-ventricular Connection | Concordant | Tricuspid Valve | Annulus = 21mm. TAPSE = 20mm |
| Ventriculo-Arterial Connection | concordant | **Ventricle** | |
| Ventricular Loop | d-Loop | Left Ventricle | Dilated |
| **Septae** |  | Right Ventricle | Normal |
| Interatrial Septum | Intact | **Doppler Measurement** |  |
| Interventricular Septum | Intact | Mitral | Moderate MR, Holosystolic, posterior projection, seen in two planes with jet velocity = 4.6m/sec. |
| **Semilunar Valves** |  | Aortic | Moderate AR, PHT = 462ms |
| Aortic Valve | Annulus = 18mm | Tricuspid | Trivial TR, PPG = 21mmHg |
| Pulmonary Valve | Annulus = 21mm | Pulmonic |  |
| **Great Arteries** | NRGA | **Coronary Arteries** |  |
| Aorta |  | **Aortic Arch** | Left |
| Pulmonary Arteries |  | **PDA** | No PDA |
| **M-Mode**: | | | |
| Ao | mm | PWd | mm |
| LA | mm | EDV | ml |
| LVIDd | mm | ESV | ml |
| LVIDs | mm | FS | 31% |
| IVSd | mm | LVEF | 58% |
| **Additional Information:** | | | |
| **Conclusion:**   1. {S, D, S} Levocardia 2. LA/LV Dilated 3. Thickened MVL 4. Moderate MR 5. Moderate AR 6. Normal Biventricular Systolic Function | | | |
| **Done By:** | **Signature** | **Date** | **Remark** |
| Tesfaye T., Pediatric Cardiologist |  | 19/12/14Eth.C. |  |

| **Patient Name: Bekalu Bilelegn. Sex/Age: M/1 3/12. Date of Report:20/12/2014Eth.C. MRN: .144131** | | | |
| --- | --- | --- | --- |
| **Clinical Diagnosis: DS + RD + Murmur. TGSH2.2854.** | | | |
| **Features:** | **Findings** | **Features** | **Findings** |
| **Profile** | | **Atria** | |
| Abdominal Situs | Solitus | Left Atrium | Normal |
| Cardiac Position | Levocardia | Right Atrium | Dilated |
| Systemic Venous Dr  ainage | To RA | **Atrio-Ventricular Valves** | |
| Pulmonary Venous Drainage | To LA | Mitral Valve | Annulus = 12mm |
| Atrio-ventricular Connection | Concordant | Tricuspid Valve | Annulus = 18mm  TAPSE = 14mm |
| Ventriculo-Arterial Connection | concordant | **Ventricle** | |
| Ventricular Loop | d-Loop | Left Ventricle | Normal |
| **Septae** |  | Right Ventricle | Dilated |
| Interatrial Septum | Intact | **Doppler Measurement** |  |
| Interventricular Septum | Intact | Mitral |  |
| **Semilunar Valves** |  | Aortic |  |
| Aortic Valve | Annulus = 10mm | Tricuspid | Mild TR, PPG = 72mmHg |
| Pulmonary Valve | Annulus = 15mm | Pulmonic | Moderate PR, PPG = 59mmHg |
| **Great Arteries** | NRGA | **Coronary Arteries** |  |
| Aorta |  | **Aortic Arch** | Left |
| Pulmonary Arteries |  | **PDA** | No PDA |
| **M-Mode**: | | | |
| Ao | mm | PWd | mm |
| LA | mm | EDV | ml |
| LVIDd | mm | ESV | ml |
| LVIDs | mm | FS | % |
| IVSd | mm | LVEF | % |
| **Additional Information:** | | | |
| **Conclusion:**   1. {S, D, S} Levocardia 2. RA/RV Dilated 3. Mild TR 4. Moderate PR 5. Severe Pulmonary Hypertension 6. Normal Biventricular Systolic Function | | | |
| **Done By:** | **Signature** | **Date** | **Remark** |
| Tesfaye T., Pediatric Cardiologist |  | 20/12/14Eth.C. |  |

| **Patient Name: Metadel Geremew. Sex/Age: M/12 years. Date of Report:20/12/2014Eth.C. MRN: 144161.**  **Clinical Diagnosis: CHF + RD + Rheumatic Recurrence + Murmur. TGSH2.2855.** | | | |
| --- | --- | --- | --- |
| **Features:** | **Findings** | **Features** | **Findings** |
| **Profile** | | **Atria** | |
| Abdominal Situs | Solitus | Left Atrium | Markedly Dilated |
| Cardiac Position | Levocardia | Right Atrium | Dilated |
| Systemic Venous Drainage | To RA | **Atrio-Ventricular Valves** | |
| Pulmonary Venous Drainage | To LA | Mitral Valve | Annulus = 27mm. thickened MVL. |
| Atrio-ventricular Connection | Concordant | Tricuspid Valve | Annulus = 27mm. TAPSE = 17mm |
| Ventriculo-Arterial Connection | concordant | **Ventricle** | |
| Ventricular Loop | d-Loop | Left Ventricle | Markedly Dilated |
| **Septae** |  | Right Ventricle | Dilated. RV TDI S wave = 10cm/sec. |
| Interatrial Septum | Intact | **Doppler Measurement** |  |
| Interventricular Septum | Intact | Mitral | Severe MR, Holosystolic, posterior projection, seen in two planes with jet velocity = 3.8m/sec. |
| **Semilunar Valves** |  | Aortic | Mild AR |
| Aortic Valve | Annulus = 14mm | Tricuspid | Moderate TR, PPG = 63mmHg |
| Pulmonary Valve | Annulus = 18mm | Pulmonic |  |
| **Great Arteries** | NRGA | **Coronary Arteries** |  |
| Aorta |  | **Aortic Arch** | Left |
| Pulmonary Arteries |  | **PDA** | No PDA |
| **M-Mode**: | | | |
| Ao | mm | PWd | mm |
| LA | mm | EDV | ml |
| LVIDd | mm | ESV | ml |
| LVIDs | mm | FS | 11% |
| IVSd | mm | LVEF | 23% |
| **Additional Information:** 31mm Left Pleural effusion. 16mm Right Pleural effusion. Pericardial effusion measuring maximum depth of 5mm on RV Side. | | | |
| **Conclusion:**   1. {S, D, S} Levocardia 2. All chambers dilated 3. Thickened MVL. 4. Severe MR 5. Moderate TR 6. Trivial AR 7. Markedly Reduced LV Systolic Function 8. Small Pericardial effusion 9. Large Bilateral Pleural effusion | | | |
| **Done By:** | **Signature** | **Date** | **Remark** |
| Tesfaye T., Pediatric Cardiologist |  | 20/12/14Eth.C. |  |

| **Patient Name: Habtamu Awoke. Sex/Age: M/4 /12. Date of Report:26/12/2014Eth.C. MRN: 144799.**  **Clinical Diagnosis: DS + Diaphoresis. TGSH2.2856.** | | | |
| --- | --- | --- | --- |
| **Features:** | **Findings** | **Features** | **Findings** |
| **Profile** | | **Atria** | |
| Abdominal Situs | Solitus | Left Atrium | Dilated |
| Cardiac Position | Levocardia | Right Atrium | Dilated |
| Systemic Venous Drainage | To RA | **Atrio-Ventricular Valves** | |
| Pulmonary Venous Drainage | To LA | Mitral Valve | Annulus = 10mm |
| Atrio-ventricular Connection | Concordant | Tricuspid Valve | Annulus = 13mm  TAPSE = 14mm |
| Ventriculo-Arterial Connection | concordant | **Ventricle** | |
| Ventricular Loop | d-Loop | Left Ventricle | Dilated |
| **Septae** |  | Right Ventricle | Dilated |
| Interatrial Septum | 8mm OS ASD, L – R Shunt | **Doppler Measurement** |  |
| Interventricular Septum | 6mm PM VSD, L – R Shunt | Mitral |  |
| **Semilunar Valves** |  | Aortic |  |
| Aortic Valve | Annulus = 11mm | Tricuspid |  |
| Pulmonary Valve | Annulus = 12mm | Pulmonic | Mild PR, PPG = 41mmHg |
| **Great Arteries** | NRGA | **Coronary Arteries** |  |
| Aorta |  | **Aortic Arch** | Left |
| Pulmonary Arteries |  | **PDA** | No PDA |
| **M-Mode**: Normal LV Function on eye balling | | | |
| Ao | mm | PWd | mm |
| LA | mm | EDV | ml |
| LVIDd | mm | ESV | ml |
| LVIDs | mm | FS | % |
| IVSd | mm | LVEF | % |
| **Additional Information:** | | | |
| **Conclusion:**   1. {S, D, S} Levocardia 2. RA/RV Dilated 3. Moderate OS ASD, L – R Shunt 4. Moderate PM VSD, L – R Shunt 5. Mild PR 6. Mild Pulmonary Hypertension 7. Normal Biventricular Systolic Function | | | |
| **Done By:** | **Signature** | **Date** | **Remark** |
| Tesfaye T., Pediatric Cardiologist |  | 26/12/14Eth.C. |  |

| **Patient Name: B/Fasika Asefa. Sex/Age: M/15daye. Date of Report:26/12/2014Eth.C. MRN: 144953.** | | | |
| --- | --- | --- | --- |
| **Clinical Diagnosis: Cyanosis + Murmur. TGSH2.2857.** | | | |
| **Features:** | **Findings** | **Features** | **Findings** |
| **Profile** | | **Atria** | |
| Abdominal Situs | Solitus | Left Atrium | Normal |
| Cardiac Position | Levocardia | Right Atrium | Normal |
| Systemic Venous Drainage | To RA | **Atrio-Ventricular Valves** | |
| Pulmonary Venous Drainage | To LA | Mitral Valve | Annulus = 10mm |
| Atrio-ventricular Connection | DIRV | Tricuspid Valve | Annulus = 10mm |
| Ventriculo-Arterial Connection | Concordant | **Ventricle** | |
| Ventricular Loop | d-Loop | Left Ventricle | Smallish |
| **Septae** |  | Right Ventricle | Normal |
| Interatrial Septum | 6mm OS ASD, L – R Shunt | **Doppler Measurement** |  |
| Interventricular Septum | 8mm Muscular VSD, R – L Shunt. | Mitral |  |
| **Semilunar Valves** |  | Aortic |  |
| Aortic Valve | Annulus = 9mm | Tricuspid |  |
| Pulmonary Valve | Annulus = 7mm | Pulmonic |  |
| **Great Arteries** | L-Posed Great Arteries | **Coronary Arteries** |  |
| Aorta |  | **Aortic Arch** | Left |
| Pulmonary Arteries |  | **PDA** | No PDA |
| **M-Mode**: | | | |
| Ao | mm | PWd | mm |
| LA | mm | EDV | ml |
| LVIDd | mm | ESV | ml |
| LVIDs | mm | FS | % |
| IVSd | mm | LVEF | % |
| **Additional Information:** | | | |
| **Conclusion:**   1. {S, D, L} Levocardia 2. DIRV 3. Small OS ASD, L – R Shunt 4. Large Muscular VSD, R – L Shunt 5. L – Posed Great Arteries 6. Smallish LV | | | |
| **Done By:** | **Signature** | **Date** | **Remark** |
| Tesfaye T., Pediatric Cardiologist |  | 26/12/14Eth.C. |  |

| **Patient Name: Amar Mohammed. Sex/Age:M/47Days. Date of Report:30/12/2014Eth.C. MRN: 144742.** | | | |
| --- | --- | --- | --- |
| **Clinical Diagnosis: Incidentam Murmur. TGSH2.2858.** | | | |
| **Features:** | **Findings** | **Features** | **Findings** |
| **Profile** | | **Atria** | |
| Abdominal Situs | Solitus | Left Atrium | Normal |
| Cardiac Position | Levocardia | Right Atrium | Normal |
| Systemic Venous Drainage | To RA | **Atrio-Ventricular Valves** | |
| Pulmonary Venous Drainage | To LA | Mitral Valve | Annulus = 12mm |
| Atrio-ventricular Connection | Concordant | Tricuspid Valve | Annulus = 12mm |
| Ventriculo-Arterial Connection | concordant | **Ventricle** | |
| Ventricular Loop | d-Loop | Left Ventricle | Normal |
| **Septae** |  | Right Ventricle | Normal |
| Interatrial Septum | PFO, L – R Shunt | **Doppler Measurement** |  |
| Interventricular Septum | 8mm PM VSD, Predominantly L – R Shunt | Mitral |  |
| **Semilunar Valves** |  | Aortic |  |
| Aortic Valve | Annulus = 11mm | Tricuspid |  |
| Pulmonary Valve | Annulus = 11mm | Pulmonic |  |
| **Great Arteries** | NRGA | **Coronary Arteries** |  |
| Aorta |  | **Aortic Arch** | Left |
| Pulmonary Arteries |  | **PDA** | No PDA |
| **M-Mode**: Normal LV Function on eye balling | | | |
| Ao | mm | PWd | mm |
| LA | mm | EDV | ml |
| LVIDd | mm | ESV | ml |
| LVIDs | mm | FS | % |
| IVSd | mm | LVEF | % |
| **Additional Information:** | | | |
| **Conclusion:**   1. {S, D, S} Levocardia 2. PFO, L – R Shunt 3. Moderate to Large PM VSD, L – R Shunt 4. Normal LV Systolic Function | | | |
| **Done By:** | **Signature** | **Date** | **Remark** |
| Tesfaye T., Pediatric Cardiologist |  | 27/12/14Eth.C. |  |

| **Patient Name: Mekdes Derje. Sex/Age:F/3/12. Date of Report:30/12/2014Eth.C. MRN: 144137.** | | | |
| --- | --- | --- | --- |
| **Clinical Diagnosis: DR. TGSH2.2859.** | | | |
| **Features:** | **Findings** | **Features** | **Findings** |
| **Profile** | | **Atria** | |
| Abdominal Situs | Solitus | Left Atrium | Normal |
| Cardiac Position | Levocardia | Right Atrium | Normal |
| Systemic Venous Drainage | To RA | **Atrio-Ventricular Valves** | |
| Pulmonary Venous Drainage | To LA | Mitral Valve | Annulus = 11mm |
| Atrio-ventricular Connection | Concordant | Tricuspid Valve | Annulus = 11mm |
| Ventriculo-Arterial Connection | concordant | **Ventricle** | |
| Ventricular Loop | d-Loop | Left Ventricle | Normal |
| **Septae** |  | Right Ventricle | Normal |
| Interatrial Septum | Probe Patent PFO. | **Doppler Measurement** |  |
| Interventricular Septum | Intact | Mitral |  |
| **Semilunar Valves** |  | Aortic |  |
| Aortic Valve | Annulus = 10mm | Tricuspid |  |
| Pulmonary Valve | Annulus = 12mm | Pulmonic |  |
| **Great Arteries** | NRGA | **Coronary Arteries** |  |
| Aorta |  | **Aortic Arch** | Left |
| Pulmonary Arteries |  | **PDA** | No PDA |
| **M-Mode**: Normal LV Function on eye balling | | | |
| Ao | mm | PWd | mm |
| LA | mm | EDV | ml |
| LVIDd | mm | ESV | ml |
| LVIDs | mm | FS | % |
| IVSd | mm | LVEF | % |
| **Additional Information:** | | | |
| **Conclusion:**   1. {S, D, S} Levocardia 2. Probe Patent PFO | | | |
| **Done By:** | **Signature** | **Date** | **Remark** |
| Tesfaye T., Pediatric Cardiologist |  | 30/12/14Eth.C. |  |

| **Patient Name: HKibre – Werk Yismaw. Sex/Age: F/10years. Date of Report:01/13/2014Eth.C. MRN: 144012.** | | | |
| --- | --- | --- | --- |
| **Follow up Echocardiography for Mural IE (tip of MV & Mural endocardium) TGSH10** | | | |
| **Features:** | **Findings** | **Features** | **Findings** |
| **Profile** | | **Atria** | |
| Abdominal Situs | Solitus | Left Atrium | Dilated |
| Cardiac Position | Levocardia | Right Atrium | Normal |
| Systemic Venous Drainage | To RA | **Atrio-Ventricular Valves** | |
| Pulmonary Venous Drainage | To LA | Mitral Valve | Annulus = 22mm |
| Atrio-ventricular Connection | Concordant | Tricuspid Valve | Annulus = 20mm. TAPSE = 18mm |
| Ventriculo-Arterial Connection | concordant | **Ventricle** | |
| Ventricular Loop | d-Loop | Left Ventricle | Dilated. 8 X 8mm vegetation attached to the PW endocardium of LV |
| **Septae** |  | Right Ventricle | Normal |
| Interatrial Septum | Intact | **Doppler Measurement** |  |
| Interventricular Septum | Intact | Mitral | Moderate MR, Holosystolic, posterior projection, seen in two planes with jet velocity = 3.6m/sec. |
| **Semilunar Valves** |  | Aortic | Trivial AR |
| Aortic Valve | Annulus = 16mm | Tricuspid | Mild TR, PPG = 41mmHg |
| Pulmonary Valve | Annulus = 21mm | Pulmonic |  |
| **Great Arteries** | NRGA | **Coronary Arteries** |  |
| Aorta |  | **Aortic Arch** | Left |
| Pulmonary Arteries |  | **PDA** | No PDA |
| **M-Mode**: | | | |
| Ao | mm | PWd | mm |
| LA | mm | EDV | ml |
| LVIDd | mm | ESV | ml |
| LVIDs | mm | FS | 32% |
| IVSd | mm | LVEF | 59% |
| **Conclusion:**   1. {S, D, S} Levocardia 2. LA/LV Dilated 3. Thickened MVL 4. Moderate MR 5. Mild TR 6. Echogenic mass attached to mural endocardium on the PW of LV 7. Mild Pulmonary Hypertension 8. Normal Biventricular Systolic Function | | | |
| **Remark:** the vegetation at the tip of MV has disappeared. The mural mass has decreased in size. | | | |
| **Done By:** | **Signature** | **Date** | **Remark** |
| Tesfaye T., Pediatric Cardiologist |  | 01/13/14Eth.C. |  |

| **Patient Name: G/Hiwot wubetu. Sex/Age:.M/2 6/12 Date of Report:01/13/2014Eth.C. MRN: 092974.** | | | |
| --- | --- | --- | --- |
| **Clinical Diagnosis: RD + Murmur + CHF. TGSH2.2860.** | | | |
| **Features:** | **Findings** | **Features** | **Findings** |
| **Profile** | | **Atria** | |
| Abdominal Situs | Solitus | Left Atrium | Dilated |
| Cardiac Position | Levocardia | Right Atrium | Normal |
| Systemic Venous Drainage | To RA | **Atrio-Ventricular Valves** | |
| Pulmonary Venous Drainage | To LA | Mitral Valve | Annulus = 19mm |
| Atrio-ventricular Connection | Concordant | Tricuspid Valve | Annulus = 16mm |
| Ventriculo-Arterial Connection | concordant | **Ventricle** | |
| Ventricular Loop | d-Loop | Left Ventricle | Dilated |
| **Septae** |  | Right Ventricle | Hypertrophied |
| Interatrial Septum | Intact | **Doppler Measurement** |  |
| Interventricular Septum | Non – Restrictive Sub aortic VSD, L – R Shunt | Mitral |  |
| **Semilunar Valves** |  | Aortic | Mild Truncal Regurgitation.  Mild Truncal Stenosis, |
| Truncus Valve | Annulus = 17mm | Tricuspid |  |
|  |  |  |  |
| **Great Arteries** | Truncus Arteriosus | **Coronary Arteries** |  |
| Aorta |  | **Aortic Arch** | Left |
| Pulmonary Arteries | Arises from Truncus from the left side with an opening of 7mm. | **PDA** | No PDA |
| **M-Mode**: Normal LV Function on eye balling | | | |
| Ao | mm | PWd | mm |
| LA | mm | EDV | ml |
| LVIDd | mm | ESV | ml |
| LVIDs | mm | FS | % |
| IVSd | mm | LVEF | % |
| **Additional Information:** | | | |
| **Conclusion:**   1. {S, D, S} Levocardia 2. Truncus Arteriosus 3. Mild Truncal Regurgitation 4. Mild Truncal Stenosis | | | |
| **Done By:** | **Signature** | **Date** | **Remark** |
| Tesfaye T., Pediatric Cardiologist |  | 01/13/14Eth.C. |  |

| **Patient Name: Bekalu Molla. Sex/Age:M/1 4/12. Date of Report:01/13/2014Eth.C. MRN: 142500.** | | | |
| --- | --- | --- | --- |
| **Follow up echo for Perimyocarditis (06/12/14) ( AGH8.502)** | | | |
| **Features:** | **Findings** | **Features** | **Findings** |
| **Profile** | | **Atria** | |
| Abdominal Situs | Solitus | Left Atrium | Normal |
| Cardiac Position | Levocardia | Right Atrium | Mildly Dilated |
| Systemic Venous Drainage | To RA | **Atrio-Ventricular Valves** | |
| Pulmonary Venous Drainage | To LA | Mitral Valve | Annulus = 18mm |
| Atrio-ventricular Connection | Concordant | Tricuspid Valve | Annulus = 18mm  TAPSE = 19mm |
| Ventriculo-Arterial Connection | concordant | **Ventricle** | |
| Ventricular Loop | d-Loop | Left Ventricle | Normal |
| **Septae** |  | Right Ventricle | Mildly Dilated |
| Interatrial Septum | Intact | **Doppler Measurement** |  |
| Interventricular Septum | Intact | Mitral |  |
| **Semilunar Valves** |  | Aortic |  |
| Aortic Valve | Annulus = 15mm | Tricuspid | Mild TR, PPG = 40mmHg |
| Pulmonary Valve | Annulus = 16mm | Pulmonic |  |
| **Great Arteries** | NRGA | **Coronary Arteries** |  |
| Aorta |  | **Aortic Arch** | Left |
| Pulmonary Arteries |  | **PDA** | <1mm PDA, L – R Shunt |
| **M-Mode**: | | | |
| Ao | mm | PWd | 7mm |
| LA | mm | EDV | 34ml |
| LVIDd | 30mm | ESV | 13ml |
| LVIDs | 20mm | FS | 32% |
| IVSd | **7mm** | LVEF | 62% |
| **Additional Information:** 4mm Circumferential Pericardial effusion. | | | |
| **Conclusion:**   1. {S, D, S} Levocardia. 2. Silent PDA, L – R Shunt 3. Mild Pulmonary Hypertension 4. Trace Pericardial effusion 5. Normal Biventricular Function | | | |
| **Remark:** Improvement from previous report | | | |
| **Done By:** | **Signature** | **Date** | **Remark** |
| Tesfaye T., Pediatric Cardiologist |  | 01/13/14Eth.C. |  |

| **Patient Name: Mebre Abay. Sex/Age: M/14 years. Date of Report:01/13/2014Eth.C. MRN: 145623.** | | | |
| --- | --- | --- | --- |
| **Clinical Diagnosis: Easy Fatigability. TGSH2.2861.** | | | |
| **Features:** | **Findings** | **Features** | **Findings** |
| **Profile** | | **Atria** | |
| Abdominal Situs | Solitus | Left Atrium | Normal |
| Cardiac Position | Levocardia | Right Atrium | Normal |
| Systemic Venous Drainage | To RA | **Atrio-Ventricular Valves** | |
| Pulmonary Venous Drainage | To LA | Mitral Valve | Annulus = 23mm |
| Atrio-ventricular Connection | Concordant | Tricuspid Valve | Annulus = 25mm  TAPSE = 25mm |
| Ventriculo-Arterial Connection | concordant | **Ventricle** | |
| Ventricular Loop | d-Loop | Left Ventricle | Normal |
| **Septae** |  | Right Ventricle | Normal |
| Interatrial Septum | Intact | **Doppler Measurement** |  |
| Interventricular Septum | Intact | Mitral | ------ |
| **Semilunar Valves** |  | Aortic | ------- |
| Aortic Valve | Annulus = 17mm | Tricuspid | Trivial TR, PPG = 27mmHg |
| Pulmonary Valve | Annulus = 21mm | Pulmonic | -------- |
| **Great Arteries** | NRGA | **Coronary Arteries** |  |
| Aorta | ------- | **Aortic Arch** | Left. No CoA. Normal Branching neck vessels. |
| Pulmonary Arteries | -------- | **PDA** | No PDA |
| **M-Mode**: | | | |
| Ao | mm | PWd | mm |
| LA | mm | EDV | ml |
| LVIDd | mm | ESV | ml |
| LVIDs | mm | FS | 39% |
| IVSd | mm | LVEF | 70% |
| **Additional Information:** | | | |
| **Conclusion:**   1. Normal Echocardiography Study. | | | |
| **Done By:** | **Signature** | **Date** | **Remark** |
| Tesfaye T., Pediatric Cardiologist |  | 01/13/14Eth.C. |  |

| **Patient Name: Tigistu Belay. Sex/Age: M/11months. Date of Report:04/13/2014Eth.C. MRN: 145857.** | | | |
| --- | --- | --- | --- |
| **Clinical Diagnosis: Incidental Murmur. TGSH2.2862.** | | | |
| **Features:** | **Findings** | **Features** | **Findings** |
| **Profile** | | **Atria** | |
| Abdominal Situs | Solitus | Left Atrium | Normal |
| Cardiac Position | Levocardia | Right Atrium | Dilated |
| Systemic Venous Drainage | To RA | **Atrio-Ventricular Valves** | |
| Pulmonary Venous Drainage | To LA | Mitral Valve | Annulus = 14mm |
| Atrio-ventricular Connection | Concordant | Tricuspid Valve | Annulus = 19mm  TAPSE = 18mm |
| Ventriculo-Arterial Connection | concordant | **Ventricle** | |
| Ventricular Loop | d-Loop | Left Ventricle | Normal |
| **Septae** |  | Right Ventricle | Dilated |
| Interatrial Septum | 14mm X 16mm Fenestrated ASD, L – R Shunt | **Doppler Measurement** |  |
| Interventricular Septum | Intact | Mitral |  |
| **Semilunar Valves** |  | Aortic |  |
| Aortic Valve | Annulus = 13mm | Tricuspid |  |
| Pulmonary Valve | Annulus = 14mm | Pulmonic | Pulmonary Stenosis, ppg = 28mmHg |
| **Great Arteries** | NRGA | **Coronary Arteries** |  |
| Aorta |  | **Aortic Arch** | Left |
| Pulmonary Arteries |  | **PDA** | No PDA |
| **M-Mode**: | | | |
| Ao | mm | PWd | mm |
| LA | mm | EDV | ml |
| LVIDd | mm | ESV | ml |
| LVIDs | mm | FS | 36% |
| IVSd | mm | LVEF | 69% |
| **Additional Information:** | | | |
| **Conclusion:**   1. {S, D, S} Levocardia 2. RA/RV Dilated 3. Large Fenestrated ASD, L – R Shunt 4. Mild Pulmonary Stenosis (?Physiologic) 5. Normal Biventricular Systolic Function | | | |
| **Done By:** | **Signature** | **Date** | **Remark** |
| Tesfaye T., Pediatric Cardiologist |  | 04/13/14Eth.C. |  |

| **Patient Name: Tazebew Bekele. Sex/Age: M/ 1 6/12. Date of Report:01/13/2014Eth.C. MRN: 145933.**  **Clinical Diagnosis: Murmur. TGSH3.2763** | | | | | | |
| --- | --- | --- | --- | --- | --- | --- |
| **Features:** | **Findings** | | **Features** | | | **Findings** |
| **Profile** | | | **Atria** | | | |
| Abdominal Situs | Solitus | | Left Atrium | | | Normal |
| Cardiac Position | Levocardia | | Right Atrium | | | Normal |
| Systemic Venous Drainage | To RA | | **Atrio-Ventricular Valves** | | | |
| Pulmonary Venous Drainage | To LA | | Mitral Valve | | | Annulus = 20mm (Right Side |
| Atrio-ventricular Connection | Discordant | | Tricuspid Valve | | | Annulus = 17mm(Left Side) |
| Ventriculo-Arterial Connection | Discordant | | **Ventricle** | | | |
| Ventricular Loop | l-Loop | | Left Ventricle | | | Right side |
| **Septae** |  | | Right Ventricle | | | Left side |
| Interatrial Septum | Intact | | **Doppler Measurement** | | |  |
| Interventricular Septum | 12mm Inlet VSD, L – R (From Morphologically RV to Morphologically LV) | | Mitral | | |  |
| **Semilunar Valves** |  | | Aortic | | |  |
| Aortic Valve | Annulus = 16mm | | Tricuspid | | | Moderate TR to LA. 11mm Downward STL Displacement |
| Pulmonary Valve | Annulus = 19mm | | Pulmonic | | |  |
| **Great Arteries** | l-TGA | | **Coronary Arteries** | | |  |
| Aorta | From left side Morphologically RV | | **Aortic Arch** | | | Left |
| Pulmonary Arteries | From Right side Morphologically LV | | **PDA** | | | No PDA |
| **M-Mode**: | | | | | | |
| Ao | mm | | PWd | | mm | |
| LA | mm | | EDV | | ml | |
| LVIDd | mm | | ESV | | ml | |
| LVIDs | mm | | FS | | % | |
| IVSd | mm | | LVEF | | % | |
| **Additional Information:** | | | | | | |
| **Conclusion:**   1. {S, L, L} Levocardia 2. ccTGA 3. Large VSD, L – R Shunt 4. Ebstein anomaly of the tricuspid valve 5. Moderate TR | | | | | | |
| **Done By:** | **Signature** | **Date** | | **Remark** | | |
| Tesfaye T., Pediatric Cardiologist |  | 01/13/14Eth.C. | |  | | |

| **Patient Name: Abdulaziz Ibrahim. Sex/Age: M/4years. Date of Report:04/01/2015Eth.C. MRN: 146277.** | | | |
| --- | --- | --- | --- |
| **Clinical Diagnosis: Cyanosis + DOE + Clubbing + Murmur. TGSH2.2863.** | | | |
| **Features:** | **Findings** | **Features** | **Findings** |
| **Profile** | | **Atria** | |
| Abdominal Situs | Solitus | Left Atrium | Normal |
| Cardiac Position | Levocardia | Right Atrium | Normal |
| Systemic Venous Drainage | To RA | **Atrio-Ventricular Valves** | |
| Pulmonary Venous Drainage | To LA | Mitral Valve | Annulus = 16mm |
| Atrio-ventricular Connection | Concordant | Tricuspid Valve | Annulus = 18mm  TAPSE = 15mm |
| Ventriculo-Arterial Connection | concordant | **Ventricle** | |
| Ventricular Loop | d-Loop | Left Ventricle | Normal |
| **Septae** |  | Right Ventricle | RVH |
| Interatrial Septum | Intact | **Doppler Measurement** |  |
| Interventricular Septum | Non –Restrictive Malaligned Subaortic VSD, R – L Shunt | Mitral |  |
| **Semilunar Valves** |  | Aortic |  |
| Aortic Valve | Annulus = 14mm | Tricuspid |  |
| Pulmonary Valve | Annulus = 10mm | Pulmonic | Severe PS, PPG = 64mmHg |
| **Great Arteries** | NRGA | **Coronary Arteries** |  |
| Aorta | Over – riding aorta | **Aortic Arch** |  |
| Pulmonary Arteries |  | **PDA** | No PDA |
| **M-Mode**: | | | |
| Ao | mm | PWd | mm |
| LA | mm | EDV | ml |
| LVIDd | mm | ESV | ml |
| LVIDs | mm | FS | 33% |
| IVSd | mm | LVEF | 63% |
| **Additional Information:** | | | |
| **Conclusion:**   1. {S, D, S} Levocardia 2. TOF | | | |
| **Done By:** | **Signature** | **Date** | **Remark** |
| Tesfaye T., Pediatric Cardiologist |  | 04/01/15Eth.C. |  |

| **Patient Name: Baby of wuberist Antehun. Sex/Age: M/6days. Date of Report:05/01/2015Eth.C. MRN: 146517.** | | | |
| --- | --- | --- | --- |
| **Clinical Diagnosis : _Incidental Murmur. TGSH2.2864.** | | | |
| **Features:** | **Findings** | **Features** | **Findings** |
| **Profile** | | **Atria** | |
| Abdominal Situs | Solitus | Left Atrium | Normal |
| Cardiac Position | Levocardia | Right Atrium | Normal |
| Systemic Venous Drainage | To RA | **Atrio-Ventricular Valves** | |
| Pulmonary Venous Drainage | To LA | Mitral Valve | Annulus = 11mm |
| Atrio-ventricular Connection | Concordant | Tricuspid Valve | Annulus = 11mm |
| Ventriculo-Arterial Connection | concordant | **Ventricle** | |
| Ventricular Loop | d-Loop | Left Ventricle | Normal |
| **Septae** |  | Right Ventricle | Normal |
| Interatrial Septum | Intact | **Doppler Measurement** |  |
| Interventricular Septum | 12mm OS ASD, L – R Shunt | Mitral |  |
| **Semilunar Valves** |  | Aortic |  |
| Aortic Valve | Annulus = 8mm | Tricuspid |  |
| Pulmonary Valve | Annulus = 9mm | Pulmonic |  |
| **Great Arteries** | NRGA | **Coronary Arteries** |  |
| Aorta |  | **Aortic Arch** | Left |
| Pulmonary Arteries |  | **PDA** | <1mm PDA, PDA, L – R Shunt |
| **M-Mode**: Normal LV Function on eye balling | | | |
| Ao | mm | PWd | mm |
| LA | mm | EDV | ml |
| LVIDd | mm | ESV | ml |
| LVIDs | mm | FS | % |
| IVSd | mm | LVEF | % |
| **Additional Information:** | | | |
| **Conclusion:**   1. {S, D, S} Levocardia 2. Large OS ASD, L – R Shunt 3. Small PDA, L – R Shunt 4. Normal Systolic LV Function | | | |
| **Done By:** | **Signature** | **Date** | **Remark** |
| Tesfaye T., Pediatric Cardiologist |  | 05/01/15Eth.C. |  |

| **Patient Name: Michael Girmaw. Sex/Age: M/1 4/12. Date of Report:09/01/2015Eth.C. MRN: 087559.** | | | |
| --- | --- | --- | --- |
| **Clinical Diagnosis : Cyanosis + Clubbing + Murmur. TGSH2.2865.** | | | |
| **Features:** | **Findings** | **Features** | **Findings** |
| **Profile** | | **Atria** | |
| Abdominal Situs | Solitus | Left Atrium | Normal |
| Cardiac Position | Levocardia | Right Atrium | Dilated |
| Systemic Venous Drainage | To RA | **Atrio-Ventricular Valves** | |
| Pulmonary Venous Drainage | To LA | Mitral Valve | Annulus = 11mm |
| Atrio-ventricular Connection | Concordant | Tricuspid Valve | Annulus = 16mm  TAPSE = 14mm |
| Ventriculo-Arterial Connection | Discordant | **Ventricle** | |
| Ventricular Loop | d-Loop | Left Ventricle | Normal |
| **Septae** |  | Right Ventricle | Dilated |
| Interatrial Septum | Intact | **Doppler Measurement** |  |
| Interventricular Septum | 8mm Sub arterial (Sub pulmonic) VSD, L – R Shunt | Mitral |  |
| **Semilunar Valves** |  | Aortic |  |
| Aortic Valve | Annulus = 13mm | Tricuspid |  |
| Pulmonary Valve | Annulus = 6mm | Pulmonic | Severe PS, PPG = 66mmHg. |
| **Great Arteries** | d-TGA | **Coronary Arteries** |  |
| Aorta | Anterior and to the right. From RV | **Aortic Arch** | Left |
| Pulmonary Arteries | Posterior and to the left. From LV | **PDA** | No PDA |
| **M-Mode**: | | | |
| Ao | mm | PWd | mm |
| LA | mm | EDV | ml |
| LVIDd | mm | ESV | ml |
| LVIDs | mm | FS | 39% |
| IVSd | mm | LVEF | 73% |
| **Additional Information:** | | | |
| **Conclusion:**   1. {S, D, D} Levocardia 2. RA/RV Dilated 3. d-TGA 4. Large Sub arterial VSD, L – R Shunt 5. Severe PS 6. Normal Biventricular Systolic Function | | | |
| **Done By:** | **Signature** | **Date** | **Remark** |
| Tesfaye T., Pediatric Cardiologist |  | 09/01/15Eth.C. |  |

| **Patient Name: Hilina Wuletaw. Sex/Age: F/9monthsyears. Date of Report:09/01/2015Eth.C. MRN: 142033.** | | | |
| --- | --- | --- | --- |
| **Follow up echo for Moderate PDA + ASD + Atrial septal aneurysm type I + IE ( AGH8.429)** | | | |
| **Features:** | **Findings** | **Features** | **Findings** |
| **Profile** | | **Atria** | |
| Abdominal Situs | Solitus | Left Atrium | Normal |
| Cardiac Position | Levocardia | Right Atrium | Normal |
| Systemic Venous Drainage | To RA | **Atrio-Ventricular Valves** | |
| Pulmonary Venous Drainage | To LA | Mitral Valve | Annulus = 11mm |
| Atrio-ventricular Connection | Concordant | Tricuspid Valve | Annulus = 14mm |
| Ventriculo-Arterial Connection | concordant | **Ventricle** | |
| Ventricular Loop | d-Loop | Left Ventricle | Normal |
| **Septae** |  | Right Ventricle | Normal |
| Interatrial Septum | 9 X 8mm OS ASD, L – R Shunt. Atrial Septal Aneurysm. | **Doppler Measurement** |  |
| Interventricular Septum | Intact | Mitral |  |
| **Semilunar Valves** |  | Aortic |  |
| Aortic Valve | Annulus = 9mm | Tricuspid |  |
| Pulmonary Valve | Annulus = 10mm | Pulmonic |  |
| **Great Arteries** | NRGA | **Coronary Arteries** |  |
| Aorta |  | **Aortic Arch** | Left |
| Pulmonary Arteries |  | **PDA** | 2mm PDA, L – R Shunt |
| **M-Mode**: Normal LV Function on eye balling | | | |
| Ao | mm | PWd | mm |
| LA | mm | EDV | ml |
| LVIDd | mm | ESV | ml |
| LVIDs | mm | FS | % |
| IVSd | mm | LVEF | % |
| **Additional Information:** Circumferential Pericardial effusion with maximum depth of 9mm on RV Side. | | | |
| **Conclusion:**   1. {S, D, S} Levocardia 2. Moderate OS ASD, L – R Shunt 3. Atrial Septal Anuerysm 4. Moderate PDA, L – R Shunt 5. Normal LV Systolic Function 6. Small Circumferential Pericardial effusion | | | |
| **Done By:** | **Signature** | **Date** | **Remark** |
| Tesfaye T., Pediatric Cardiologist |  | 09/01/15Eth.C. |  |
