# Supplementary material for: Two–Dimensional and Doppler trans-thoracic echocardiographic patterns of suspected pediatric heart diseases at Tibebe-—Ghion specialized Teaching Hospital and Adinas General Hospital, Bahir Dar, North-west Ethiopia:–An experience from an LMIC
Source: PLoS One. 2024 Mar 11;19(3):e0292694. doi: 10.1371/journal.pone.0292694 (PMC10927071; doi:10.1371/journal.pone.0292694)
Supplement: S1 File — (ZIP) [file pone.0292694.s002.zip › TGSH3 Pediatric Echo Report Format 2023 SPSS FILLED.docx]

| **Tibebe – Ghion Specialized Teaching Hospital, Bahir Dar University,**  **Bahir Dar, Ethiopia** | | | | |
| --- | --- | --- | --- | --- |
| **Name: Baby of Eyerus Yeshanew. Sex/Age: M/2M22D. MRN: 148685. Date of Report: 13/04/15Eth.C.**  **Clinical Diagnosis: Diaphoresis + Incidental Murmur. TGSH3.2737.** | | | | |
| **Features:** | **Findings** | | **Features** | **Findings** |
| **Profile** | | | **Atria** | |
| Abdominal Situs | Solitus | | Left Atrium | Normal |
| Cardiac Position | Levocardia | | Right Atrium | Normal |
| Systemic Venous Drainage | To RA | | **Atrio-Ventricular Valves** | |
| Pulmonary Venous Drainage | To LA | | Mitral Valve | Annulus = 11mm |
| Atrio-ventricular Connection | Concordant | | Tricuspid Valve | Annulus = 13mm |
| Ventriculo-Arterial Connection | concordant | | **Ventricle** | |
| Ventricular Loop | d-Loop | | Left Ventricle | Normal |
| **Septae** |  | | Right Ventricle | Normal |
| Interatrial Septum | Intact | | **Doppler Measurement** |  |
| Interventricular Septum | Intact | | Mitral | ------------- |
| **Semilunar Valves** |  | | Aortic | ------------- |
| Aortic Valve | Annulus = 9mm | | Tricuspid | ------------- |
| Pulmonary Valve | Annulus = 12mm | | Pulmonic | ------------- |
| **Great Arteries** | NRGA | | **Coronary Arteries** |  |
| Aorta |  | | **Aortic Arch** | Left. No CoA |
| Pulmonary Arteries | Normal MPA & Branch PAs. | | **PDA** | 2.5mm PDA, L – R Shunt. |
| **M-Mode**: Normal LV Function on eye balling | | | | |
| Ao | mm | | PWd | mm |
| LA | mm | | EDV | ml |
| LVIDd | mm | | ESV | ml |
| LVIDs | mm | | FS | % |
| IVSd | mm | | LVEF | % |
| **Additional Information:** ?Echogenic mass at the pulmonary end of the PDA. Correlate with clinical finding. | | | | |
| **Conclusion:**   1. {S, D, S} Levocardia 2. Moderate PDA, L – R Shunt 3. Echogenic mass at the PA end of the PDA Secondary to ? 4. Normal LV Systolic Function | | | | |
| **Done By:** | | **Signature** | **Date** | **Remark** |
| Tesfaye T., Paediatrician, Paediatric Cardiologist | |  | 13/04/15Eth.C. |  |

| **Tibebe – Ghion Specialized Teaching Hospital, Bahir Dar University,** | | | | |
| --- | --- | --- | --- | --- |
| **Name: Dawit Yihenew. Sex/Age: M/6years. MRN: 154816. Date of Report: 13/04/15Eth.C.**  **Follow up echo for MR+ Vegetation on the LA side of MV + Vegetation occluding PDA. TGSH1.2708.** | | | | |
| **Features:** | **Findings** | | **Features** | **Findings** |
| **Profile** | | | **Atria** | |
| Abdominal Situs | Solitus | | Left Atrium | Normal |
| Cardiac Position | Levocardia | | Right Atrium | Normal |
| Systemic Venous Drainage | To RA | | **Atrio-Ventricular Valves** | |
| Pulmonary Venous Drainage | To LA | | Mitral Valve | Annulus = 20mm |
| Atrio-ventricular Connection | Concordant | | Tricuspid Valve | Annulus = 23mm  TAPSE = mm |
| Ventriculo-Arterial Connection | concordant | | **Ventricle** | |
| Ventricular Loop | d-Loop | | Left Ventricle | Normal |
| **Septae** |  | | Right Ventricle | Normal |
| Interatrial Septum | Intact | | **Doppler Measurement** |  |
| Interventricular Septum | Intact | | Mitral | Mild MR, Holosystolic, posterior projection, seen in two planes with jet velocity = 4.5m/sec. |
| **Semilunar Valves** |  | | Aortic | ------------- |
| Aortic Valve | Annulus = 13mm | | Tricuspid | ------------- |
| Pulmonary Valve | Annulus = 18mm | | Pulmonic | ------------- |
| **Great Arteries** | NRGA | | **Coronary Arteries** |  |
| Aorta |  | | **Aortic Arch** | Left. No CoA |
| Pulmonary Arteries | Normal MPA & BPAs. | | **PDA** | PDA closed by hyper-echoic mass. |
| **M-Mode**: Normal LV Function on eye balling. | | | | |
| Ao | mm | | PWd | mm |
| LA | mm | | EDV | ml |
| LVIDd | mm | | ESV | ml |
| LVIDs | mm | | FS | % |
| IVSd | mm | | LVEF | % |
| **Additional Information:** | | | | |
| **Conclusion:**   1. {S, D, S} Levocardia 2. Mild MR 3. No vegetation seen | | | | |
| **Recommendation:** Complete anti – infective treatment for 6weeks. | | | | |
| **Done By:** | | **Signature** | **Date** | **Remark** |
| Tesfaye T., Paediatrician, Paediatric Cardiologist | |  | 13/04/15Eth.C. |  |

| **Tibebe – Ghion Specialized Teaching Hospital, Bahir Dar University,**  **Bahir Dar, Ethiopia** | | | | |
| --- | --- | --- | --- | --- |
| **Name: Baby of Marie Kenaw. Sex/Age: F/8days. MRN: 157935. Date of Report: 13/04/15Eth.C.**  **Clinical Diagnosis: DS + Murmur. TGSH3.2738.** | | | | |
| **Features:** | **Findings** | | **Features** | **Findings** |
| **Profile** | | | **Atria** | |
| Abdominal Situs | Solitus | | Left Atrium | Normal |
| Cardiac Position | Levocardia | | Right Atrium | Normal |
| Systemic Venous Drainage | To RA | | **Atrio-Ventricular Valves** | |
| Pulmonary Venous Drainage | To LA | | Mitral Valve | Annulus = 10mm |
| Atrio-ventricular Connection | Concordant | | Tricuspid Valve | Annulus = 10mm |
| Ventriculo-Arterial Connection | concordant | | **Ventricle** | |
| Ventricular Loop | d-Loop | | Left Ventricle | Normal |
| **Septae** |  | | Right Ventricle | Normal |
| Interatrial Septum | 5mm OS ASD, L – R Shunt | | **Doppler Measurement** |  |
| Interventricular Septum | Intact | | Mitral | ------------- |
| **Semilunar Valves** |  | | Aortic | ------------- |
| Aortic Valve | Annulus = 9mm | | Tricuspid | ------------- |
| Pulmonary Valve | Annulus = 10mm | | Pulmonic | ------------- |
| **Great Arteries** | NRGA | | **Coronary Arteries** |  |
| Aorta |  | | **Aortic Arch** | Left. No CoA |
| Pulmonary Arteries | Normal MPA & Branch PAs. | | **PDA** | 1mm PDA, PDA, L – R Shunt |
| **M-Mode**: Normal LV Function on eye balling. | | | | |
| Ao | mm | | PWd | mm |
| LA | mm | | EDV | ml |
| LVIDd | mm | | ESV | ml |
| LVIDs | mm | | FS | % |
| IVSd | mm | | LVEF | % |
| **Additional Information:** | | | | |
| **Conclusion:**   1. {S, D, S} Levocardia 2. Small OS ASD, L – R Shunt 3. Small PDA, L – R Shunt | | | | |
| **Recommendation:** | | | | |
| **Done By:** | | **Signature** | **Date** | **Remark** |
| Tesfaye T., Paediatrician, Paediatric Cardiologist | |  | 13/04/15Eth.C. |  |

| **Tibebe – Ghion Specialized Teaching Hospital, Bahir Dar University,** | | | | |
| --- | --- | --- | --- | --- |
| **Name: Yared Werkneh. Sex/Age: M/9months. MRN: 154045. Date of Report: 13/04/15Eth.C.**  **Clinical Diagnosis: Diaphoresis + RD + Murmur + CHF. TGSH3.2739.** | | | | |
| **Features:** | **Findings** | | **Features** | **Findings** |
| **Profile** | | | **Atria** | |
| Abdominal Situs | Solitus | | Left Atrium | Dilated |
| Cardiac Position | Levocardia | | Right Atrium | Dilated |
| Systemic Venous Drainage | To RA | | **Atrio-Ventricular Valves** | |
| Pulmonary Venous Drainage | To LA | | Mitral Valve | Annulus = 15mm |
| Atrio-ventricular Connection | Concordant | | Tricuspid Valve | Annulus = 17mm. TAPSE = 17mm |
| Ventriculo-Arterial Connection | concordant | | **Ventricle** | |
| Ventricular Loop | d-Loop | | Left Ventricle | Dilated |
| **Septae** |  | | Right Ventricle | Dilated |
| Interatrial Septum | Intact | | **Doppler Measurement** |  |
| Interventricular Septum | 9mm Non-Restrictive Sub-aortic VSD, partially covered by STL, L – R Shunt | | Mitral | ------------- |
| **Semilunar Valves** |  | | Aortic | ------------- |
| Aortic Valve | Annulus = 11mm | | Tricuspid | ------------- |
| Pulmonary Valve | Annulus = 17mm | | Pulmonic | ------------- |
| **Great Arteries** | NRGA | | **Coronary Arteries** |  |
| Aorta |  | | **Aortic Arch** | Left. No CoA |
| Pulmonary Arteries | **MPA = 21mm.** | | **PDA** | No PDA |
| **M-Mode**: Normal LV Function on eye balling. | | | | |
| Ao | mm | | PWd | mm |
| LA | mm | | EDV | ml |
| LVIDd | mm | | ESV | ml |
| LVIDs | mm | | FS | % |
| IVSd | mm | | LVEF | % |
| **Additional Information:** | | | | |
| **Conclusion:**   1. {S, D, S} Levocardia 2. All chambers dilated 3. Large Non-Restrictive Sub –aortic VSD, Partially covered by STL, L – R Shunt 4. Pulmonary Hypertension | | | | |
| **Recommendation:** the initial report was not correct. | | | | |
| **Done By:** | | **Signature** | **Date** | **Remark** |
| Tesfaye T., Paediatrician, Paediatric Cardiologist | |  | 13/04/15Eth.C. |  |

| **Tibebe – Ghion Specialized Teaching Hospital, Bahir Dar University,**  **Bahir Dar, Ethiopia** | | | | |
| --- | --- | --- | --- | --- |
| **Name: Bezawit Senay. Sex/Age: F/2 3/12. MRN: 158376. Date of Report: 17/04/15Eth.C.**  **Clinical Diagnosis: CHF + RD + Diaphoresis. TGSH3.2740.** | | | | |
| **Features:** | **Findings** | | **Features** | **Findings** |
| **Profile** | | | **Atria** | |
| Abdominal Situs | Solitus | | Left Atrium | Markedly Dilated |
| Cardiac Position | Levocardia | | Right Atrium | Normal |
| Systemic Venous Drainage | To RA | | **Atrio-Ventricular Valves** | |
| Pulmonary Venous Drainage | To LA | | Mitral Valve | Annulus = 17mm |
| Atrio-ventricular Connection | Concordant | | Tricuspid Valve | Annulus = 17mm. TAPSE = 15mm |
| Ventriculo-Arterial Connection | concordant | | **Ventricle** | |
| Ventricular Loop | d-Loop | | Left Ventricle | Markedly Dilated. 9 X 9mm Thrombus attached to LVPW, Mobile. |
| **Septae** |  | | Right Ventricle | Normal |
| Interatrial Septum | Intact | | **Doppler Measurement** |  |
| Interventricular Septum | Intact | | Mitral | ------------- |
| **Semilunar Valves** |  | | Aortic | ------------- |
| Aortic Valve | Annulus = 14mm | | Tricuspid | ------------- |
| Pulmonary Valve | Annulus = 16mm | | Pulmonic | ------------- |
| **Great Arteries** | NRGA | | **Coronary Arteries** |  |
| Aorta |  | | **Aortic Arch** | Left. No CoA |
| Pulmonary Arteries | Normal | | **PDA** | No PDA |
| **M-Mode**: | | | | |
| Ao | mm | | PWd | mm |
| LA | mm | | EDV | ml |
| LVIDd | mm | | ESV | ml |
| LVIDs | mm | | FS | 14% |
| IVSd | mm | | LVEF | 29% |
| **Conclusion:**   1. {S, D, S} Levocardia 2. Markedly Dilated LA/LV 3. Thrombus in LV attached to Posterior wall 4. Markedly Reduced LV Systolic Function | | | | |
| **Recommendation:** | | | | |
| **Done By:** | | **Signature** | **Date** | **Remark** |
| Tesfaye T., Paediatrician, Paediatric Cardiologist | |  | 17/04/15Eth.C. |  |

| **Tibebe – Ghion Specialized Teaching Hospital, Bahir Dar University,**  **Bahir Dar, Ethiopia** | | | | |
| --- | --- | --- | --- | --- |
| **Name: Babey Asefa. Sex/Age: M/7years. MRN: 158905__. Date of Report: 17/04/15Eth.C.**  **Clinical Diagnosis: ARF + Rhythm abnormality. TGSH3.2741.** | | | | |
| **Features:** | **Findings** | | **Features** | **Findings** |
| **Profile** | | | **Atria** | |
| Abdominal Situs | Solitus | | Left Atrium | Markedly Dilated |
| Cardiac Position | Levocardia | | Right Atrium | Normal |
| Systemic Venous Drainage | To RA | | **Atrio-Ventricular Valves** | |
| Pulmonary Venous Drainage | To LA | | Mitral Valve | Annulus = 18mm. thickened MVL. Shortened PMVL. |
| Atrio-ventricular Connection | Concordant | | Tricuspid Valve | Annulus = 17mm |
| Ventriculo-Arterial Connection | concordant | | **Ventricle** | |
| Ventricular Loop | d-Loop | | Left Ventricle | Markedly Dilated & Dysfunctional |
| **Septae** |  | | Right Ventricle | Normal |
| Interatrial Septum | Intact | | **Doppler Measurement** |  |
| Interventricular Septum | Intact | | Mitral | Mild MR |
| **Semilunar Valves** |  | | Aortic | Mild AR, Severe AS, PPG/MPG = 131/76mmHg |
| Aortic Valve | Annulus = 12mm | | Tricuspid | Mild TR, PPG = 48mmHg |
| Pulmonary Valve | Annulus = 17mm | | Pulmonic | ------------- |
| **Great Arteries** | NRGA | | **Coronary Arteries** |  |
| Aorta |  | | **Aortic Arch** | Left. No CoA |
| Pulmonary Arteries | Normal MPA & BPAs. | | **PDA** | No PDA |
| **M-Mode**: Reduced LV Function on eye balling | | | | |
| Ao | mm | | PWd | mm |
| LA | mm | | EDV | ml |
| LVIDd | mm | | ESV | ml |
| LVIDs | mm | | FS | % |
| IVSd | mm | | LVEF | % |
| **Additional Information:** Rhythm abnormality detected during study. | | | | |
| **Conclusion:**   1. {S, D, S} Levocardia 2. LA/LV Markedly Dilated 3. Thickened MVL, Shortened PMVL 4. Severe AS 5. Mild AR 6. Mild MR 7. Mild TR 8. Reduced LV Systolic Function | | | | |
| **Done By:** | | **Signature** | **Date** | **Remark** |
| Tesfaye T., Paediatrician, Paediatric Cardiologist | |  | 17/04/15Eth.C. |  |

| **Tibebe – Ghion Specialized Teaching Hospital, Bahir Dar University,**  **Bahir Dar, Ethiopia** | | | | |
| --- | --- | --- | --- | --- |
| **Name: Abebaye Misganaw . Sex/Age: _F/10months. MRN: 159485. Date of Report: 20/04/15Eth.C.**  **Clinical Diagnosis: DS + Murmur. TGSH3.2742.** | | | | |
| **Features:** | **Findings** | | **Features** | **Findings** |
| **Profile** | | | **Atria** | |
| Abdominal Situs | Solitus | | Left Atrium | Dilated |
| Cardiac Position | Levocardia | | Right Atrium | Dilated |
| Systemic Venous Drainage | To RA | | **Atrio-Ventricular Valves** | |
| Pulmonary Venous Drainage | To LA | | Mitral Valve | Common Complete AVSD, L – R Shunt |
| Atrio-ventricular Connection | Common Complete AVSD | | Tricuspid Valve |  |
| Ventriculo-Arterial Connection | concordant | | **Ventricle** | |
| Ventricular Loop | d-Loop | | Left Ventricle | Dilated |
| **Septae** |  | | Right Ventricle | Dilated |
| Interatrial Septum | Common Complete AVSD, L – R Shunt | | **Doppler Measurement** |  |
| Interventricular Septum |  |  | Mitral | ------------- |
| **Semilunar Valves** |  | | Aortic | ------------- |
| Aortic Valve | Annulus = 11mm | | Tricuspid | ------------- |
| Pulmonary Valve | Annulus = 14mm | | Pulmonic | Mild PR, PPG = 53mmHg. Mild Valvular PS, PPG = 23mmHg (?Physiologic) |
| **Great Arteries** | NRGA | | **Coronary Arteries** |  |
| Aorta |  | | **Aortic Arch** | Left. No CoA |
| Pulmonary Arteries | Normal MPA & BPAs. | | **PDA** | 1mm PDA, L – R Shunt |
| **M-Mode**: Normal LV Function on eye balling | | | | |
| Ao | mm | | PWd | mm |
| LA | mm | | EDV | ml |
| LVIDd | mm | | ESV | ml |
| LVIDs | mm | | FS | % |
| IVSd | mm | | LVEF | % |
| **Additional Information:** Pericardial effusion measuring 9mm on RV Side | | | | |
| **Conclusion:**   1. {S, D, S} Levocardia 2. Common Complete AVSD, L – R Shunt 3. Mild PR 4. Small PDA, L – R Shunt 5. Moderate Pulmonary Hypertension 6. Normal LV Systolic Function 7. Small Pericardial effusion | | | | |
| **Done By:** | | **Signature** | **Date** | **Remark** |
| Tesfaye T., Paediatrician, Paediatric Cardiologist | |  | 20/04/15Eth.C. |  |
| **Tibebe – Ghion Specialized Teaching Hospital, Bahir Dar University,**  **Bahir Dar, Ethiopia** | | | | |
| **Name: Amanuel Getaw. Sex/Age: M/ 1years. MRN: 159210_. Date of Report: 20/04/15Eth.C.**  **Clinical Diagnosis: Murmur + Diaphoresis + FTT. TGSH3.2743.** | | | | |
| **Features:** | **Findings** | | **Features** | **Findings** |
| **Profile** | | | **Atria** | |
| Abdominal Situs | Solitus | | Left Atrium | Normal |
| Cardiac Position | Mesocardia | | Right Atrium | Dilated |
| Systemic Venous Drainage | To RA | | **Atrio-Ventricular Valves** | |
| Pulmonary Venous Drainage | RUPV to RA through 8mm High Secundum ASD. | | Mitral Valve | Annulus = 10mm |
| Atrio-ventricular Connection | Concordant | | Tricuspid Valve | Annulus = 11mm |
| Ventriculo-Arterial Connection | concordant | | **Ventricle** | |
| Ventricular Loop | d-Loop | | Left Ventricle | Normal |
| **Septae** |  | | Right Ventricle | Dilated |
| Interatrial Septum | 8mm High Secundum ASD, L – R Shunt | | **Doppler Measurement** |  |
| Interventricular Septum | Intact | | Mitral | ------------- |
| **Semilunar Valves** |  | | Aortic | ------------- |
| Aortic Valve | Annulus = 9mm | | Tricuspid | ------------- |
| Pulmonary Valve | Annulus = 9mm | | Pulmonic | ------------- |
| **Great Arteries** | NRGA | | **Coronary Arteries** |  |
| Aorta |  | | **Aortic Arch** | Left. No CoA |
| Pulmonary Arteries | Normal MPA & BPAs. | | **PDA** | No PDA |
| **M-Mode**: Normal LV Function on eye balling. | | | | |
| Ao | mm | | PWd | mm |
| LA | mm | | EDV | ml |
| LVIDd | mm | | ESV | ml |
| LVIDs | mm | | FS | % |
| IVSd | mm | | LVEF | % |
| **Additional Information:** | | | | |
| **Conclusion:**   1. {S, D, S} Mesocardia 2. RA/RV Dilated 3. Moderate High Secundum ASD, L – R Shunt 4. PAPVC of the RUPV to RA. 5. Normal LV Systolic Function | | | | |
| **Done By:** | | **Signature** | **Date** | **Remark** |
| Tesfaye T., Paediatrician, Paediatric Cardiologist | |  | 20/04/15Eth.C. |  |

| **Tibebe – Ghion Specialized Teaching Hospital, Bahir Dar University,**  **Bahir Dar, Ethiopia** | | | | |
| --- | --- | --- | --- | --- |
| **Name: Amare Fentie . Sex/Age: M/10years. MRN: 158194 _. Date of Report: 20/04/15Eth.C.**  **Clinical Diagnosis: RD + Disseminated Staph. Infection? + Distant Heart sound. TGSH3.2744.** | | | | |
| **Features:** | **Findings** | | **Features** | **Findings** |
| **Profile** | | | **Atria** | |
| Abdominal Situs | Solitus | | Left Atrium | Normal |
| Cardiac Position | Levocardia | | Right Atrium | Normal |
| Systemic Venous Drainage | To RA | | **Atrio-Ventricular Valves** | |
| Pulmonary Venous Drainage | To LA | | Mitral Valve | Annulus = 20mm |
| Atrio-ventricular Connection | Concordant | | Tricuspid Valve | Annulus = 23mm. TAPSE = 17mm |
| Ventriculo-Arterial Connection | concordant | | **Ventricle** | |
| Ventricular Loop | d-Loop | | Left Ventricle | Normal |
| **Septae** |  | | Right Ventricle | Normal |
| Interatrial Septum | Intact | | **Doppler Measurement** |  |
| Interventricular Septum | Intact | | Mitral | Trivial MR, Incomplete Signal, with jet velocity = 2.9m/sec. |
| **Semilunar Valves** |  | | Aortic | ------------- |
| Aortic Valve | Annulus = 18mm | | Tricuspid | ------------- |
| Pulmonary Valve | Annulus = 20mm | | Pulmonic | ------------- |
| **Great Arteries** | NRGA | | **Coronary Arteries** |  |
| Aorta |  | | **Aortic Arch** | Left. No CoA |
| Pulmonary Arteries | Normal MPA & BPAs. | | **PDA** | No PDA |
| **M-Mode**: | | | | |
| Ao | mm | | PWd | mm |
| LA | mm | | EDV | ml |
| LVIDd | mm | | ESV | ml |
| LVIDs | mm | | FS | 33% |
| IVSd | mm | | LVEF | 62% |
| **Additional Information:** Circumferential Pericardial effusion with maximum depth of 9mm on RV Side. 14mm Right Pleural effusion. | | | | |
| **Conclusion:**   1. {S, D, S} Levocardia 2. Trivial MR 3. Small Circumferential Pericardial effusion 4. Moderate Right Pleural effusion 5. Normal Biventricular Systolic Function | | | | |
| **Recommendation:** | | | | |
| **Done By:** | | **Signature** | **Date** | **Remark** |
| Tesfaye T., Paediatrician, Paediatric Cardiologist | |  | 20/04/15Eth.C. |  |

| **Tibebe – Ghion Specialized Teaching Hospital, Bahir Dar University,**  **Bahir Dar, Ethiopia** | | | | |
| --- | --- | --- | --- | --- |
| **Name: Soliana Manyazewal. Sex/Age: F /7years. MRN: 069121_. Date of Report: 25/04/15Eth.C.**  **Follow up Echo for d-TGA + Inlet VSD,**  **TGSH1.2719** | | | | |
| **Features:** | **Findings** | | **Features** | **Findings** |
| **Profile** | | | **Atria** | |
| Abdominal Situs | Solitus | | Left Atrium | Normal |
| Cardiac Position | Levocardia | | Right Atrium | Normal |
| Systemic Venous Drainage | To RA | | **Atrio-Ventricular Valves** | |
| Pulmonary Venous Drainage | To LA | | Mitral Valve | Annulus = 25mm |
| Atrio-ventricular Connection | Concordant | | Tricuspid Valve | Annulus = 27mm |
| Ventriculo-Arterial Connection | Discordant | | **Ventricle** | |
| Ventricular Loop | d-Loop | | Left Ventricle | Normal |
| **Septae** |  | | Right Ventricle | Normal |
| Interatrial Septum | Intact | | **Doppler Measurement** |  |
| Interventricular Septum | 11mm Inlet VSD, BD Shunt | | Mitral | Mild MR |
| **Semilunar Valves** |  | | Aortic | ------------- |
| Aortic Valve | Annulus = 18mm | | Tricuspid | Trivial TR |
| Pulmonary Valve | Annulus = 23mm | | Pulmonic | Mild PR, PPG = 53mmHg |
| **Great Arteries** | d-TGA | | **Coronary Arteries** |  |
| Aorta |  | | **Aortic Arch** | Left. No CoA |
| Pulmonary Arteries | MPA = 31mm. | | **PDA** | No PDA |
| **M-Mode**: Normal LV Function on eye balling. | | | | |
| Ao | mm | | PWd | mm |
| LA | mm | | EDV | ml |
| LVIDd | mm | | ESV | ml |
| LVIDs | mm | | FS | % |
| IVSd | mm | | LVEF | % |
| **Additional Information:** | | | | |
| **Conclusion:**   1. {S, D, D} Levocardia 2. d-TGA with Large VSD 3. Mild PR 4. Moderate Pulmonary Hypertension 5. Normal LV Systolic Function | | | | |
| **Recommendation:** | | | | |
| **Done By:** | | **Signature** | **Date** | **Remark** |
| Tesfaye T., Paediatrician, Paediatric Cardiologist | |  | 25/04/15Eth.C. |  |
| **Tibebe – Ghion Specialized Teaching Hospital, Bahir Dar University,**  **Bahir Dar, Ethiopia** | | | | |
| **Name: Belestie Teshager. Sex/Age: M/13years. MRN: 159065. Date of Report: 25/04/15Eth.C.**  **Clinical Diagnosis: Easy Fatigability. TGSH3.2745.** | | | | |
| **Features:** | **Findings** | | **Features** | **Findings** |
| **Profile** | | | **Atria** | |
| Abdominal Situs | Solitus | | Left Atrium | Normal |
| Cardiac Position | Levocardia | | Right Atrium | Normal |
| Systemic Venous Drainage | To RA | | **Atrio-Ventricular Valves** | |
| Pulmonary Venous Drainage | To LA | | Mitral Valve | Annulus = 23mm |
| Atrio-ventricular Connection | Concordant | | Tricuspid Valve | Annulus = 26mm  TAPSE = 20mm |
| Ventriculo-Arterial Connection | concordant | | **Ventricle** | |
| Ventricular Loop | d-Loop | | Left Ventricle | Normal |
| **Septae** |  | | Right Ventricle | Normal |
| Interatrial Septum | Intact | | **Doppler Measurement** |  |
| Interventricular Septum | Intact | | Mitral | ------------- |
| **Semilunar Valves** |  | | Aortic | ------------- |
| Aortic Valve | Annulus = 17mm | | Tricuspid | Trivial TR, PPG = 26mmHg |
| Pulmonary Valve | Annulus = 23mm | | Pulmonic | ------------- |
| **Great Arteries** | NRGA | | **Coronary Arteries** |  |
| Aorta |  | | **Aortic Arch** | Left. No CoA |
| Pulmonary Arteries | Normal MPA & BPAs. | | **PDA** | No PDA |
| **M-Mode**: | | | | |
| Ao | mm | | PWd | mm |
| LA | mm | | EDV | ml |
| LVIDd | mm | | ESV | ml |
| LVIDs | mm | | FS | 33% |
| IVSd | mm | | LVEF | 63% |
| **Additional Information:** | | | | |
| **Conclusion:**   1. {S, D, S} Levocardia 2. Trivial TR 3. Normal Biventricular Systolic Function. | | | | |
| **Done By:** | | **Signature** | **Date** | **Remark** |
| Tesfaye T., Paediatrician, Paediatric Cardiologist | |  | 25/04/15Eth.C. |  |

| **Tibebe – Ghion Specialized Teaching Hospital, Bahir Dar University,**  **Bahir Dar, Ethiopia** | | | | |
| --- | --- | --- | --- | --- |
| **Name: Baby of Kidija Nure. Sex/Age: M /8 Days. MRN: 159495. Date of Report: 25/04/15Eth.C.**  **Clinical Diagnosis: RD. TGSH3.2746.** | | | | |
| **Features:** | **Findings** | | **Features** | **Findings** |
| **Profile** | | | **Atria** | |
| Abdominal Situs | Solitus | | Left Atrium | Normal |
| Cardiac Position | Levocardia | | Right Atrium | Normal |
| Systemic Venous Drainage | To RA | | **Atrio-Ventricular Valves** | |
| Pulmonary Venous Drainage | To LA | | Mitral Valve | Annulus = 10mm |
| Atrio-ventricular Connection | Concordant | | Tricuspid Valve | Annulus = 11mm |
| Ventriculo-Arterial Connection | concordant | | **Ventricle** | |
| Ventricular Loop | d-Loop | | Left Ventricle | Normal |
| **Septae** |  | | Right Ventricle | Normal |
| Interatrial Septum | PFO, L – R Shunt | | **Doppler Measurement** |  |
| Interventricular Septum | Intact | | Mitral | ------------- |
| **Semilunar Valves** |  | | Aortic | ------------- |
| Aortic Valve | Annulus = 10mm | | Tricuspid | ------------- |
| Pulmonary Valve | Annulus = 10mm | | Pulmonic | ------------- |
| **Great Arteries** | NRGA | | **Coronary Arteries** |  |
| Aorta |  | | **Aortic Arch** | Left. No CoA |
| Pulmonary Arteries | Normal MPA & BPAs. | | **PDA** | No PDA |
| **M-Mode**: Normal LV Function on eye balling. | | | | |
| Ao | mm | | PWd | mm |
| LA | mm | | EDV | ml |
| LVIDd | mm | | ESV | ml |
| LVIDs | mm | | FS | % |
| IVSd | mm | | LVEF | % |
| **Additional Information:** | | | | |
| **Conclusion:**   1. {S, D, S} Levocardia 2. .PFO, L – R Shunt | | | | |
| **Recommendation:** | | | | |
| **Done By:** | | **Signature** | **Date** | **Remark** |
| Tesfaye T., Paediatrician, Paediatric Cardiologist | |  | 25/04/15Eth.C. |  |

| **Tibebe – Ghion Specialized Teaching Hospital, Bahir Dar University,**  **Bahir Dar, Ethiopia** | | | | |
| --- | --- | --- | --- | --- |
| **Name: Mamen Alemu. Sex/Age: F/12years. MRN: 053224_. Date of Report: 27/04/15Eth.C.**  **Clinical Diagnosis: CHF + DOE + Murmur + Rheumatic Recurrence. TGSH3.2747.** | | | | |
| **Features:** | **Findings** | | **Features** | **Findings** |
| **Profile** | | | **Atria** | |
| Abdominal Situs | Solitus | | Left Atrium | Markedly Dilated |
| Cardiac Position | Levocardia | | Right Atrium | Normal |
| Systemic Venous Drainage | To RA | | **Atrio-Ventricular Valves** | |
| Pulmonary Venous Drainage | To LA | | Mitral Valve | Annulus = 42mm. Thickened, clubbed MVL. MVA = 0.5cm**^2^**. |
| Atrio-ventricular Connection | Concordant | | Tricuspid Valve | Annulus = 28mm |
| Ventriculo-Arterial Connection | concordant | | **Ventricle** | |
| Ventricular Loop | d-Loop | | Left Ventricle | Markedly Dilated & Dysfunctional |
| **Septae** |  | | Right Ventricle | Normal |
| Interatrial Septum | Intact | | **Doppler Measurement** |  |
| Interventricular Septum | Intact | | Mitral | Moderate MR, Holosystolic, posterior projection, seen in two planes with jet velocity = 4m/sec. Severe MS, PPG/MPG = 21/12mmHg. |
| **Semilunar Valves** |  | | Aortic | Mild AR, PHT = 513ms |
| Aortic Valve | Annulus = 15mm | | Tricuspid | Moderate TR, PPG = 42mmHg |
| Pulmonary Valve | Annulus = 21mm | | Pulmonic | ------------ |
| **Great Arteries** | NRGA | | **Coronary Arteries** |  |
| Aorta |  | | **Aortic Arch** | Left. No CoA |
| Pulmonary Arteries | Normal MPA & BPAs. | | **PDA** | No PDA |
| **M-Mode**: | | | | |
| Ao | mm | | PWd | mm |
| LA | mm | | EDV | ml |
| LVIDd | mm | | ESV | ml |
| LVIDs | mm | | FS | 24% |
| IVSd | mm | | LVEF | 47% |
| **Additional Information:** Circumferential Pericardial effusion with maximum depth of 13mm on RA Side. | | | | |
| **Conclusion:**   1. {S, D, S} Levocardia 2. .LA/LV Markedly Dilated 3. Moderate MR 4. Severe MS 5. Moderate TR 6. Mild AR 7. Moderate Pericardial effusion 8. Mild Pulmonary Hypertension 9. Reduced LV Systolic Function | | | | |
| **Remark:** Rhythm abnormality during study. | | | | |
| **Done By:** | | **Signature** | **Date** | **Remark** |
| Tesfaye T., Paediatrician, Paediatric Cardiologist | |  | 27/04/15Eth.C. |  |

| **Tibebe – Ghion Specialized Teaching Hospital, Bahir Dar University,**  **Bahir Dar, Ethiopia** | | | | |
| --- | --- | --- | --- | --- |
| **Name: Yalemsira Dessie . Sex/Age: F/6/12 . MRN: 159815. Date of Report: 27/04/15Eth.C.**  **Follow up Echo for Cardiac Tamponade + Large ASD + Severe Pulmonary Hypertension (After pericardial drainage) (AGH)** | | | | |
| **Features:** | **Findings** | | **Features** | **Findings** |
| **Profile** | | | **Atria** | |
| Abdominal Situs | Solitus | | Left Atrium | Normal |
| Cardiac Position | Levocardia | | Right Atrium | Dilated |
| Systemic Venous Drainage | To RA | | **Atrio-Ventricular Valves** | |
| Pulmonary Venous Drainage | To LA | | Mitral Valve | Annulus = 13mm |
| Atrio-ventricular Connection | Concordant | | Tricuspid Valve | Annulus = 16mm |
| Ventriculo-Arterial Connection | concordant | | **Ventricle** | |
| Ventricular Loop | d-Loop | | Left Ventricle | Normal |
| **Septae** |  | | Right Ventricle | Dilated |
| Interatrial Septum | 13mm OS ASD, L – R Shunt | | **Doppler Measurement** |  |
| Interventricular Septum | Intact | | Mitral | Mild MR |
| **Semilunar Valves** |  | | Aortic | ------------- |
| Aortic Valve | Annulus = 12mm | | Tricuspid | Moderate TR, PPG = 34mmHg |
| Pulmonary Valve | Annulus = 12mm | | Pulmonic | ------------- |
| **Great Arteries** | NRGA | | **Coronary Arteries** |  |
| Aorta |  | | **Aortic Arch** | Left. No CoA |
| Pulmonary Arteries | Normal MPA & BPAs. | | **PDA** | No PDA |
| **M-Mode**: Normal LV Function on eye balling. | | | | |
| Ao | mm | | PWd | mm |
| LA | mm | | EDV | ml |
| LVIDd | mm | | ESV | ml |
| LVIDs | mm | | FS | % |
| IVSd | mm | | LVEF | % |
| **Additional Information:** Pericardial effusion measuring maximum of 3mm on RV Side | | | | |
| **Conclusion:**   1. {S, D, S} Levocardia 2. .RA/RV Dilated 3. Large OS ASD, L – R Shunt 4. Mild MR 5. Moderate TR 6. Normal LV Systolic Function | | | | |
| **Recommendation:** | | | | |
| **Done By:** | | **Signature** | **Date** | **Remark** |
| Tesfaye T., Paediatrician, Paediatric Cardiologist | |  | 27/04/15Eth.C. |  |

| **Tibebe – Ghion Specialized Teaching Hospital, Bahir Dar University,**  **Bahir Dar, Ethiopia** | | | | |
| --- | --- | --- | --- | --- |
| **Name: Tigist Tigabu. Sex/Age: F/3 months. MRN: 158031. Date of Report: 27/04/15Eth.C.**  **Clinical Diagnosis: Incidental Murmur. TGSH3.2748.** | | | | |
| **Features:** | **Findings** | | **Features** | **Findings** |
| **Profile** | | | **Atria** | |
| Abdominal Situs | Solitus | | Left Atrium | Normal |
| Cardiac Position | Levocardia | | Right Atrium | Normal |
| Systemic Venous Drainage | To RA | | **Atrio-Ventricular Valves** | |
| Pulmonary Venous Drainage | To LA | | Mitral Valve | Annulus = 14mm |
| Atrio-ventricular Connection | Concordant | | Tricuspid Valve | Annulus = 13mm |
| Ventriculo-Arterial Connection | concordant | | **Ventricle** | |
| Ventricular Loop | d-Loop | | Left Ventricle | Normal |
| **Septae** |  | | Right Ventricle | Normal |
| Interatrial Septum | Intact | | **Doppler Measurement** |  |
| Interventricular Septum | 3mm PM VSD, L – R Shunt | | Mitral | ------------- |
| **Semilunar Valves** |  | | Aortic | ------------- |
| Aortic Valve | Annulus = 11mm | | Tricuspid | ------------- |
| Pulmonary Valve | Annulus = 12mm | | Pulmonic | ------------- |
| **Great Arteries** | NRGA | | **Coronary Arteries** |  |
| Aorta |  | | **Aortic Arch** | Left. No CoA |
| Pulmonary Arteries | Normal MPA & BPAs. | | **PDA** | No PDA |
| **M-Mode**: Normal LV Function on eye balling. | | | | |
| Ao | mm | | PWd | mm |
| LA | mm | | EDV | ml |
| LVIDd | mm | | ESV | ml |
| LVIDs | mm | | FS | % |
| IVSd | mm | | LVEF | % |
| **Additional Information:** | | | | |
| **Conclusion:**   1. {S, D, S} Levocardia 2. .Small PM VSD, L – R Shunt | | | | |
| **Recommendation:** | | | | |
| **Done By:** | | **Signature** | **Date** | **Remark** |
| Tesfaye T., Paediatrician, Paediatric Cardiologist | |  | 27/04/15Eth.C. |  |

| **Tibebe – Ghion Specialized Teaching Hospital, Bahir Dar University,**  **Bahir Dar, Ethiopia** | | | | |
| --- | --- | --- | --- | --- |
| **Name: Sefinew Eyilet. Sex/Age: M/7years. MRN: 018276. Date of Report: 02/05/15Eth.C.**  **Clinical Diagnosis: CHF + Murmur + easy fatigability + FTT. TGSH3.2749.** | | | | |
| **Features:** | **Findings** | | **Features** | **Findings** |
| **Profile** | | | **Atria** | |
| Abdominal Situs | Solitus | | Left Atrium | More Dilated |
| Cardiac Position | Levocardia | | Right Atrium | Dilated |
| Systemic Venous Drainage | To RA | | **Atrio-Ventricular Valves** | |
| Pulmonary Venous Drainage | To LA | | Mitral Valve | Annulus = 32mm |
| Atrio-ventricular Connection | Concordant | | Tricuspid Valve | Annulus = 27mm. TAPSE = 19mm |
| Ventriculo-Arterial Connection | concordant | | **Ventricle** | |
| Ventricular Loop | d-Loop | | Left Ventricle | More Dilated |
| **Septae** |  | | Right Ventricle | Dilated |
| Interatrial Septum | Intact | | **Doppler Measurement** |  |
| Interventricular Septum | 19mm PM VSD, L – R Shunt | | Mitral | ------------- |
| **Semilunar Valves** |  | | Aortic | ------------- |
| Aortic Valve | Annulus = 16mm | | Tricuspid | ------------- |
| Pulmonary Valve | Annulus = 25mm | | Pulmonic | Mild PR, PPG = 63mmHg |
| **Great Arteries** | NRGA | | **Coronary Arteries** |  |
| Aorta |  | | **Aortic Arch** | Left. No CoA |
| Pulmonary Arteries | MPA =32mm. | | **PDA** | 7mm PDA, L – R Shunt |
| **M-Mode**: Normal LV Function on eye balling. | | | | |
| Ao | mm | | PWd | mm |
| LA | mm | | EDV | ml |
| LVIDd | mm | | ESV | ml |
| LVIDs | mm | | FS | % |
| IVSd | mm | | LVEF | % |
| **Additional Information:** | | | | |
| **Conclusion:**   1. {S, D, S} Levocardia 2. All Chambers Dilated 3. Large PM VSD, L – R Shunt 4. Large PDA, L – R Shunt 5. .Severe Pulmonary Hypertension 6. Normal Biventricular Systolic Function | | | | |
| **Recommendation:** | | | | |
| **Done By:** | | **Signature** | **Date** | **Remark** |
| Tesfaye T., Paediatrician, Paediatric Cardiologist | |  | 02/05/15Eth.C. |  |

| **Tibebe – Ghion Specialized Teaching Hospital, Bahir Dar University,**  **Bahir Dar, Ethiopia** | | | | |
| --- | --- | --- | --- | --- |
| **Name: Sefinew Eyelet. Sex/Age: M/7years. MRN: 018276. Date of Report: 02/05/15Eth.C.**  **Clinical Diagnosis: Easy Fatigability. TGSH3.2750.** | | | | |
| **Features:** | **Findings** | | **Features** | **Findings** |
| **Profile** | | | **Atria** | |
| Abdominal Situs | Solitus | | Left Atrium | Normal |
| Cardiac Position | Levocardia | | Right Atrium | Normal |
| Systemic Venous Drainage | To RA | | **Atrio-Ventricular Valves** | |
| Pulmonary Venous Drainage | To LA | | Mitral Valve | Annulus = mm |
| Atrio-ventricular Connection | Concordant | | Tricuspid Valve | Annulus = mm  TAPSE = mm |
| Ventriculo-Arterial Connection | concordant | | **Ventricle** | |
| Ventricular Loop | d-Loop | | Left Ventricle | Normal |
| **Septae** |  | | Right Ventricle | Normal |
| Interatrial Septum | Intact | | **Doppler Measurement** |  |
| Interventricular Septum | Intact | | Mitral | ------------- |
| **Semilunar Valves** |  | | Aortic | ------------- |
| Aortic Valve | Annulus = mm | | Tricuspid | ------------- |
| Pulmonary Valve | Annulus = mm | | Pulmonic | ------------- |
| **Great Arteries** | NRGA | | **Coronary Arteries** |  |
| Aorta |  | | **Aortic Arch** | Left. No CoA |
| Pulmonary Arteries | Normal MPA & BPAs. | | **PDA** | No PDA |
| **M-Mode**: | | | | |
| Ao | mm | | PWd | mm |
| LA | mm | | EDV | ml |
| LVIDd | mm | | ESV | ml |
| LVIDs | mm | | FS | % |
| IVSd | mm | | LVEF | % |
| **Additional Information:** | | | | |
| **Conclusion:**   1. Normal Echocardiography Study 2. . | | | | |
| **Recommendation:** | | | | |
| **Done By:** | | **Signature** | **Date** | **Remark** |
| Tesfaye T., Paediatrician, Paediatric Cardiologist | |  | 02/05/15Eth.C. |  |

| **Tibebe – Ghion Specialized Teaching Hospital, Bahir Dar University,**  **Bahir Dar, Ethiopia** | | | | |
| --- | --- | --- | --- | --- |
| **Name: Muluken Dessie . Sex/Age: M/1 Month. MRN: 160531 . Date of Report: 02/05/15Eth.C.**  **Clinical Diagnosis: RD. TGSH3.2751.** | | | | |
| **Features:** | **Findings** | | **Features** | **Findings** |
| **Profile** | | | **Atria** | |
| Abdominal Situs | Solitus | | Left Atrium | Normal |
| Cardiac Position | Levocardia | | Right Atrium | Normal |
| Systemic Venous Drainage | To RA | | **Atrio-Ventricular Valves** | |
| Pulmonary Venous Drainage | To LA | | Mitral Valve | Annulus = 10mm |
| Atrio-ventricular Connection | Concordant | | Tricuspid Valve | Annulus = 9mm |
| Ventriculo-Arterial Connection | concordant | | **Ventricle** | |
| Ventricular Loop | d-Loop | | Left Ventricle | Normal |
| **Septae** |  | | Right Ventricle | Normal |
| Interatrial Septum | Intact | | **Doppler Measurement** |  |
| Interventricular Septum | Intact | | Mitral | ------------- |
| **Semilunar Valves** |  | | Aortic | ------------- |
| Aortic Valve | Annulus = 8mm | | Tricuspid | ------------- |
| Pulmonary Valve | Annulus = 9mm | | Pulmonic | ------------- |
| **Great Arteries** | NRGA | | **Coronary Arteries** |  |
| Aorta |  | | **Aortic Arch** | Left. No CoA |
| Pulmonary Arteries | Normal MPA & BPAs. | | **PDA** | No PDA |
| **M-Mode**: Normal LV Function on eye balling. | | | | |
| Ao | mm | | PWd | mm |
| LA | mm | | EDV | ml |
| LVIDd | mm | | ESV | ml |
| LVIDs | mm | | FS | % |
| IVSd | mm | | LVEF | % |
| **Additional Information:** | | | | |
| **Conclusion:**   1. Normal Echocardiography Study | | | | |
| **Recommendation:** | | | | |
| **Done By:** | | **Signature** | **Date** | **Remark** |
| Tesfaye T., Paediatrician, Paediatric Cardiologist | |  | 02/05/15Eth.C. |  |

| **Tibebe – Ghion Specialized Teaching Hospital, Bahir Dar University,**  **Bahir Dar, Ethiopia** | | | | | |
| --- | --- | --- | --- | --- | --- |
| **Name: Dejitinu Akalu . Sex/Age: F/11years. MRN: 074031. Date of Report: 02/05/15Eth.C.**  **Clinical Diagnosis: Cyanosis + clubbing. TGSH3.2752.** | | | | | |
| **Features:** | | **Findings** | | **Features** | **Findings** |
| **Profile** | | | | **Atria** | |
| Abdominal Situs | Solitus | | | Left Atrium | Normal |
| Cardiac Position | Levocardia | | | Right Atrium | Dilated |
| Systemic Venous Drainage | To RA | | | **Atrio-Ventricular Valves** | |
| Pulmonary Venous Drainage | To LA | | | Mitral Valve | Annulus = 15mm |
| Atrio-ventricular Connection | Concordant | | | Tricuspid Valve | Annulus = 22mm  TAPSE = 10mm |
| Ventriculo-Arterial Connection | concordant | | | **Ventricle** | |
| Ventricular Loop | d-Loop | | | Left Ventricle | Normal |
| **Septae** |  | | | Right Ventricle | Dilated, Hypertrophied & Dysfunctional. |
| Interatrial Septum | Intact | | | **Doppler Measurement** |  |
| Interventricular Septum | Mal-aligned Sub-aortic Non-Restrictive VSD, R – L Shunt | | | Mitral | ------------- |
| **Semilunar Valves** |  | | | Aortic | ------------- |
| Aortic Valve | Annulus = 24mm | | | Tricuspid | ------------- |
| Pulmonary Valve | Annulus = 8mm | | | Pulmonic | Severe PS, PPG = 64mmHg |
| **Great Arteries** | NRGA | | | **Coronary Arteries** |  |
| Aorta | Over-riding aorta | | | **Aortic Arch** | No CoA |
| Pulmonary Arteries | Smallish MPA & BPAs. | | | **PDA** | No PDA |
| **M-Mode**: Normal LV Systolic Function on eye balling. | | | | | |
| Ao | mm | | | PWd | mm |
| LA | mm | | | EDV | ml |
| LVIDd | mm | | | ESV | ml |
| LVIDs | mm | | | FS | % |
| IVSd | mm | | | LVEF | % |
| **Additional Information:** | | | | | |
| **Conclusion:**   1. {S, D, S} Levocardia 2. RA/RV Dilated 3. TOF 4. Smallish MPA and Branch Pas. 5. RV Hypertrophic and Dysfunctional | | | | | |
| **Done By:** | | | **Signature** | **Date** | **Remark** |
| Tesfaye T., Paediatrician, Paediatric Cardiologist | | |  | 02/05/15Eth.C. |  |

| **Tibebe – Ghion Specialized Teaching Hospital, Bahir Dar University,**  **Bahir Dar, Ethiopia** | | | | |
| --- | --- | --- | --- | --- |
| **Name: Sofia Ebabu. Sex/Age: F /12years. MRN: 157646_. Date of Report: 04/05/15Eth.C.**  **Follow up echocardiography for Large Purulent pericardial effusion (AGH)** | | | | |
| **Features:** | **Findings** | | **Features** | **Findings** |
| **Profile** | | | **Atria** | |
| Abdominal Situs | Solitus | | Left Atrium | Normal |
| Cardiac Position | Levocardia | | Right Atrium | Normal |
| Systemic Venous Drainage | To RA | | **Atrio-Ventricular Valves** | |
| Pulmonary Venous Drainage | To LA | | Mitral Valve | Annulus = 22mm |
| Atrio-ventricular Connection | Concordant | | Tricuspid Valve | Annulus = 22mm  TAPSE = 17mm |
| Ventriculo-Arterial Connection | concordant | | **Ventricle** | |
| Ventricular Loop | d-Loop | | Left Ventricle | Normal |
| **Septae** |  | | Right Ventricle | Normal |
| Interatrial Septum | Intact | | **Doppler Measurement** |  |
| Interventricular Septum | Intact | | Mitral | ------------- |
| **Semilunar Valves** |  | | Aortic | ------------- |
| Aortic Valve | Annulus = 17mm | | Tricuspid | ------------- |
| Pulmonary Valve | Annulus = 20mm | | Pulmonic | ------------- |
| **Great Arteries** | NRGA | | **Coronary Arteries** |  |
| Aorta |  | | **Aortic Arch** | Left. No CoA |
| Pulmonary Arteries | Normal MPA & BPAs. | | **PDA** | No PDA |
| **M-Mode**: | | | | |
| Ao | mm | | PWd | mm |
| LA | mm | | EDV | ml |
| LVIDd | mm | | ESV | ml |
| LVIDs | mm | | FS | 31% |
| IVSd | mm | | LVEF | 60% |
| **Additional Information:** No pericardial effusion | | | | |
| **Conclusion:**   1. Normal Echocardiography Study | | | | |
| **Recommendation:** | | | | |
| **Done By:** | | **Signature** | **Date** | **Remark** |
| Tesfaye T., Paediatrician, Paediatric Cardiologist | |  | 04/05/15Eth.C. |  |

| **Tibebe – Ghion Specialized Teaching Hospital, Bahir Dar University,**  **Bahir Dar, Ethiopia** | | | | |
| --- | --- | --- | --- | --- |
| **Name: Hiwot Endeshaw. Sex/Age: F/1 month. MRN: 161031. Date of Report: 04/05/15Eth.C.**  **Clinical Diagnosis: Cyanosis + Cardiogenic Shock + RD. TGSH3.2753.** | | | | |
| **Features:** | **Findings** | | **Features** | **Findings** |
| **Profile** | | | **Atria** | |
| Abdominal Situs | Solitus | | Left Atrium | Normal |
| Cardiac Position | Levocardia | | Right Atrium | Dilated |
| Systemic Venous Drainage | To RA | | **Atrio-Ventricular Valves** | |
| Pulmonary Venous Drainage | To LA | | Mitral Valve | Annulus = 11mm |
| Atrio-ventricular Connection | Concordant | | Tricuspid Valve | Annulus = 12mm |
| Ventriculo-Arterial Connection | Discordant | | **Ventricle** | |
| Ventricular Loop | d-Loop | | Left Ventricle | Regressed |
| **Septae** |  | | Right Ventricle | Dilated & Hypertrophied |
| Interatrial Septum | PFO, L – R Shunt, Restrictive | | **Doppler Measurement** |  |
| Interventricular Septum | Intact | | Mitral | ------------- |
| **Semilunar Valves** |  | | Aortic | ------------- |
| Aortic Valve | Annulus = 8mm | | Tricuspid | ------------- |
| Pulmonary Valve | Annulus = 6mm | | Pulmonic | Mild PS (LVOTO), PPG = 37mmHg |
| **Great Arteries** | d-TGA | | **Coronary Arteries** |  |
| Aorta | Anterior & from RV | | **Aortic Arch** | Left. No CoA |
| Pulmonary Arteries | Posterior & from LV | | **PDA** | No PDA |
| **M-Mode**: | | | | |
| Ao | mm | | PWd | mm |
| LA | mm | | EDV | ml |
| LVIDd | mm | | ESV | ml |
| LVIDs | mm | | FS | % |
| IVSd | mm | | LVEF | % |
| **Additional Information:** | | | | |
| **Conclusion:**   1. {S, D, D} Levocardia 2. .Restrictive PFO, L – R Shunt 3. d-TGA with Intact IVS 4. Mild PS(LVOTO) | | | | |
| **Recommendation:** | | | | |
| **Done By:** | | **Signature** | **Date** | **Remark** |
| Tesfaye T., Paediatrician, Paediatric Cardiologist | |  | 04/05/15Eth.C. |  |

| **Tibebe – Ghion Specialized Teaching Hospital, Bahir Dar University,**  **Bahir Dar, Ethiopia** | | | | |
| --- | --- | --- | --- | --- |
| **Name: Balew Nibret. Sex/Age: M/8months. MRN: 160955. Date of Report: 04/05/15Eth.C.**  **Clinical Diagnosis: RD + Cardiogenic Shock. TGSH3.2754. TGSH10** | | | | |
| **Features:** | **Findings** | | **Features** | **Findings** |
| **Profile** | | | **Atria** | |
| Abdominal Situs | Solitus | | Left Atrium | Normal |
| Cardiac Position | Levocardia | | Right Atrium | Normal. Early diastolic RA Collapse |
| Systemic Venous Drainage | To RA | | **Atrio-Ventricular Valves** | |
| Pulmonary Venous Drainage | To LA | | Mitral Valve | Annulus = 12mm |
| Atrio-ventricular Connection | Concordant | | Tricuspid Valve | Annulus = 12mm |
| Ventriculo-Arterial Connection | concordant | | **Ventricle** | |
| Ventricular Loop | d-Loop | | Left Ventricle | Normal |
| **Septae** |  | | Right Ventricle | Normal |
| Interatrial Septum | Intact | | **Doppler Measurement** |  |
| Interventricular Septum | Intact | | Mitral | >25% respiratory variability of mitral inflow velocity. |
| **Semilunar Valves** |  | | Aortic | ------------- |
| Aortic Valve | Annulus = 9mm | | Tricuspid | ------------- |
| Pulmonary Valve | Annulus = 10mm | | Pulmonic | ------------- |
| **Great Arteries** | NRGA | | **Coronary Arteries** |  |
| Aorta |  | | **Aortic Arch** | Left. No CoA |
| Pulmonary Arteries | Normal MPA & BPAs. | | **PDA** | No PDA |
| **M-Mode**: | | | | |
| Ao | mm | | PWd | mm |
| LA | mm | | EDV | ml |
| LVIDd | mm | | ESV | ml |
| LVIDs | mm | | FS | 38% |
| IVSd | mm | | LVEF | 70% |
| **Additional Information:** Circumferential Pericardial effusion with maximum depth of 26mm on LV Side. Echo-debris inside. Swinging Heart | | | | |
| **Conclusion:**   1. {S, D, S} Levocardia 2. Large Circumferential pericardial effusion with features of cardiac tamponade (? Purulent) 3. Normal LV Systolic Function | | | | |
| **Recommendation:** | | | | |
| **Done By:** | | **Signature** | **Date** | **Remark** |
| Tesfaye T., Paediatrician, Paediatric Cardiologist | |  | 04/05/15Eth.C. |  |

| **Tibebe – Ghion Specialized Teaching Hospital, Bahir Dar University,**  **Bahir Dar, Ethiopia** | | | | |
| --- | --- | --- | --- | --- |
| **Name: Kidest Abebaw . Sex/Age: F/2 3/12 years. MRN: 160853. Date of Report: 09/05/15Eth.C.**  **Clinical Diagnosis: Recurrent Chest Infection. TGSH3.2755.** | | | | |
| **Features:** | **Findings** | | **Features** | **Findings** |
| **Profile** | | | **Atria** | |
| Abdominal Situs | Solitus | | Left Atrium | Normal |
| Cardiac Position | Levocardia | | Right Atrium | Normal |
| Systemic Venous Drainage | To RA | | **Atrio-Ventricular Valves** | |
| Pulmonary Venous Drainage | To LA | | Mitral Valve | Annulus = mm |
| Atrio-ventricular Connection | Concordant | | Tricuspid Valve | Annulus = mm  TAPSE = mm |
| Ventriculo-Arterial Connection | concordant | | **Ventricle** | |
| Ventricular Loop | d-Loop | | Left Ventricle | Normal |
| **Septae** |  | | Right Ventricle | Normal |
| Interatrial Septum | Intact | | **Doppler Measurement** |  |
| Interventricular Septum | Intact | | Mitral | ------------- |
| **Semilunar Valves** |  | | Aortic | ------------- |
| Aortic Valve | Annulus = mm | | Tricuspid | ------------- |
| Pulmonary Valve | Annulus = mm | | Pulmonic | ------------- |
| **Great Arteries** | NRGA | | **Coronary Arteries** |  |
| Aorta |  | | **Aortic Arch** | Left. No CoA |
| Pulmonary Arteries | Normal MPA & BPAs. | | **PDA** | No PDA |
| **M-Mode**: | | | | |
| Ao | mm | | PWd | mm |
| LA | mm | | EDV | ml |
| LVIDd | mm | | ESV | ml |
| LVIDs | mm | | FS | % |
| IVSd | mm | | LVEF | % |
| **Additional Information:** | | | | |
| **Conclusion:**   1. {S, D, S} Levocardia 2. . | | | | |
| **Recommendation:** | | | | |
| **Done By:** | | **Signature** | **Date** | **Remark** |
| Tesfaye T., Paediatrician, Paediatric Cardiologist | |  | 09/05/15Eth.C. |  |

| **Tibebe – Ghion Specialized Teaching Hospital, Bahir Dar University,**  **Bahir Dar, Ethiopia** | | | | |
| --- | --- | --- | --- | --- |
| **Name: Nardos Adissu. Sex/Age: F/5years. MRN: 008004. Date of Report: 09/05/15Eth.C.**  **Follow up echocardiography for ?Healed Myocarditis (AGH)** | | | | |
| **Features:** | **Findings** | | **Features** | **Findings** |
| **Profile** | | | **Atria** | |
| Abdominal Situs | Solitus | | Left Atrium | Normal |
| Cardiac Position | Levocardia | | Right Atrium | Normal |
| Systemic Venous Drainage | To RA | | **Atrio-Ventricular Valves** | |
| Pulmonary Venous Drainage | To LA | | Mitral Valve | Annulus = 19mm |
| Atrio-ventricular Connection | Concordant | | Tricuspid Valve | Annulus = 19mm  TAPSE = 18mm |
| Ventriculo-Arterial Connection | concordant | | **Ventricle** | |
| Ventricular Loop | d-Loop | | Left Ventricle | Normal |
| **Septae** |  | | Right Ventricle | Normal |
| Interatrial Septum | Intact | | **Doppler Measurement** |  |
| Interventricular Septum | Intact | | Mitral | ------------- |
| **Semilunar Valves** |  | | Aortic | ------------- |
| Aortic Valve | Annulus = 13mm | | Tricuspid | ------------- |
| Pulmonary Valve | Annulus = 17mm | | Pulmonic | ------------- |
| **Great Arteries** | NRGA | | **Coronary Arteries** |  |
| Aorta |  | | **Aortic Arch** | Left. No CoA |
| Pulmonary Arteries | Normal MPA & BPAs. | | **PDA** | No PDA |
| **M-Mode**: | | | | |
| Ao | mm | | PWd | mm |
| LA | mm | | EDV | ml |
| LVIDd | mm | | ESV | ml |
| LVIDs | mm | | FS | 33% |
| IVSd | mm | | LVEF | 63% |
| **Additional Information:** | | | | |
| **Conclusion:**   1. Normal Echocardiography Study | | | | |
| **Recommendation:** Do echo after a year | | | | |
| **Done By:** | | **Signature** | **Date** | **Remark** |
| Tesfaye T., Paediatrician, Paediatric Cardiologist | |  | 09/05/15Eth.C. |  |

| **Tibebe – Ghion Specialized Teaching Hospital, Bahir Dar University,**  **Bahir Dar, Ethiopia** | | | | |
| --- | --- | --- | --- | --- |
| **Name: Kidest Yaregal. Sex/Age: F/1 5/12years. MRN: 160788. Date of Report: 09/05/15Eth.C.**  **Clinical Diagnosis: Diaphoresis + Murmur. TGSH3.2756.** | | | | |
| **Features:** | **Findings** | | **Features** | **Findings** |
| **Profile** | | | **Atria** | |
| Abdominal Situs | Solitus | | Left Atrium | Dilated |
| Cardiac Position | Levocardia | | Right Atrium | Normal |
| Systemic Venous Drainage | To RA | | **Atrio-Ventricular Valves** | |
| Pulmonary Venous Drainage | To LA. Impressive. | | Mitral Valve | Annulus = 14mm |
| Atrio-ventricular Connection | Concordant | | Tricuspid Valve | Annulus = 15mm |
| Ventriculo-Arterial Connection | concordant | | **Ventricle** | |
| Ventricular Loop | d-Loop | | Left Ventricle | Dilated |
| **Septae** |  | | Right Ventricle | Normal |
| Interatrial Septum | PFO, L – R Shunt | | **Doppler Measurement** |  |
| Interventricular Septum | Non-Restrictive Sub-aortic VSD, L – R Shunt | | Mitral | ------------- |
| **Semilunar Valves** |  | | Aortic | ------------- |
| Aortic Valve | Annulus = 12mm | | Tricuspid | ------------- |
| Pulmonary Valve | Annulus = 14mm | | Pulmonic | ------------- |
| **Great Arteries** | NRGA | | **Coronary Arteries** |  |
| Aorta |  | | **Aortic Arch** | Left. No CoA |
| Pulmonary Arteries | Normal MPA & BPAs. | | **PDA** | No PDA |
| **M-Mode**: Normal LV Function on eye balling. | | | | |
| Ao | mm | | PWd | mm |
| LA | mm | | EDV | ml |
| LVIDd | mm | | ESV | ml |
| LVIDs | mm | | FS | % |
| IVSd | mm | | LVEF | % |
| **Additional Information:** | | | | |
| **Conclusion:**   1. {S, D, S} Levocardia 2. LA/LV Dilated 3. PFO, L – R Shunt 4. Non –Restrictive Sub-Aortic VSD, L – R Shunt 5. Normal LV Systolic Function | | | | |
| **Recommendation:** | | | | |
| **Done By:** | | **Signature** | **Date** | **Remark** |
| Tesfaye T., Paediatrician, Paediatric Cardiologist | |  | 09/05/15Eth.C. |  |

| **Tibebe – Ghion Specialized Teaching Hospital, Bahir Dar University,**  **Bahir Dar, Ethiopia** | | | | |
| --- | --- | --- | --- | --- |
| **Name: Nataniem Assefa. Sex/Age: M/2years. MRN: 081799. Date of Report: 09/05/15Eth.C.**  **Clinical Diagnosis: ATH with OSA/H + ?Pulmonary Hypertension + Pre-Op screening. TGSH3.2757.** | | | | |
| **Features:** | **Findings** | | **Features** | **Findings** |
| **Profile** | | | **Atria** | |
| Abdominal Situs | Solitus | | Left Atrium | Normal |
| Cardiac Position | Levocardia | | Right Atrium | Dilated |
| Systemic Venous Drainage | To RA | | **Atrio-Ventricular Valves** | |
| Pulmonary Venous Drainage | To LA | | Mitral Valve | Annulus = 12mm |
| Atrio-ventricular Connection | Concordant | | Tricuspid Valve | Annulus = 18mm |
| Ventriculo-Arterial Connection | concordant | | **Ventricle** | |
| Ventricular Loop | d-Loop | | Left Ventricle | Normal |
| **Septae** |  | | Right Ventricle | Dilated |
| Interatrial Septum | Intact | | **Doppler Measurement** |  |
| Interventricular Septum | Intact | | Mitral | ------------- |
| **Semilunar Valves** |  | | Aortic | ------------- |
| Aortic Valve | Annulus = 11mm | | Tricuspid | Moderate TR, PPG = 55mmHg |
| Pulmonary Valve | Annulus = 14mm | | Pulmonic | Mild PR, PPG = 50mmHg |
| **Great Arteries** | NRGA | | **Coronary Arteries** |  |
| Aorta |  | | **Aortic Arch** | Left. No CoA |
| Pulmonary Arteries | Normal MPA & BPAs. | | **PDA** | No PDA |
| **M-Mode**: Normal LV Function on eye balling. | | | | |
| Ao | mm | | PWd | mm |
| LA | mm | | EDV | ml |
| LVIDd | mm | | ESV | ml |
| LVIDs | mm | | FS | % |
| IVSd | mm | | LVEF | % |
| **Additional Information:** | | | | |
| **Conclusion:**   1. {S, D, S} Levocardia 2. RA/RV Dilated 3. Moderate TR 4. Mild PR 5. Moderate Pulmonary Hypertension | | | | |
| **Recommendation:** | | | | |
| **Done By:** | | **Signature** | **Date** | **Remark** |
| Tesfaye T., Paediatrician, Paediatric Cardiologist | |  | 09/05/15Eth.C. |  |

| **Tibebe – Ghion Specialized Teaching Hospital, Bahir Dar University,**  **Bahir Dar, Ethiopia** | | | | |
| --- | --- | --- | --- | --- |
| **Name: Mekdes Sibhat. Sex/Age: F/5years. MRN: 161409. Date of Report: 09/05/15Eth.C.**  **Clinical Diagnosis: RD + Sepsis + r/o Pericardial involvement. TGSH3.2758.** | | | | |
| **Features:** | **Findings** | | **Features** | **Findings** |
| **Profile** | | | **Atria** | |
| Abdominal Situs | Solitus | | Left Atrium | Normal |
| Cardiac Position | Levocardia | | Right Atrium | Normal |
| Systemic Venous Drainage | To RA | | **Atrio-Ventricular Valves** | |
| Pulmonary Venous Drainage | To LA | | Mitral Valve | Annulus = 22mm |
| Atrio-ventricular Connection | Concordant | | Tricuspid Valve | Annulus = 20mm  TAPSE = 15mm |
| Ventriculo-Arterial Connection | concordant | | **Ventricle** | |
| Ventricular Loop | d-Loop | | Left Ventricle | Normal |
| **Septae** |  | | Right Ventricle | Normal |
| Interatrial Septum | Intact | | **Doppler Measurement** |  |
| Interventricular Septum | Intact | | Mitral | ------------- |
| **Semilunar Valves** |  | | Aortic | ------------- |
| Aortic Valve | Annulus = 16mm | | Tricuspid | ------------- |
| Pulmonary Valve | Annulus = 16mm | | Pulmonic | ------------- |
| **Great Arteries** | NRGA | | **Coronary Arteries** |  |
| Aorta |  | | **Aortic Arch** | Left. No CoA |
| Pulmonary Arteries | Normal MPA & BPAs. | | **PDA** | No PDA |
| **M-Mode**: | | | | |
| Ao | mm | | PWd | mm |
| LA | mm | | EDV | ml |
| LVIDd | mm | | ESV | ml |
| LVIDs | mm | | FS | % |
| IVSd | mm | | LVEF | % |
| **Additional Information:** 17mm Right Pleural effusion with echo debris. | | | | |
| **Conclusion:**   1. {S, D, S} Levocardia 2. Moderate Right Pleural effusion. | | | | |
| **Recommendation:** | | | | |
| **Done By:** | | **Signature** | **Date** | **Remark** |
| Tesfaye T., Paediatrician, Paediatric Cardiologist | |  | 09/05/15Eth.C. |  |

| **Tibebe – Ghion Specialized Teaching Hospital, Bahir Dar University,**  **Bahir Dar, Ethiopia** | | | | |
| --- | --- | --- | --- | --- |
| **Name: Baby Anguach Tigabu. Sex/Age: M/2 5 /12years. MRN: 065765. Date of Report: 09/05/15Eth.C.**  **Clinical Diagnosis: RD + Murmur. TGSH3.2759. (TGSH4) (13days)** | | | | |
| **Features:** | **Findings** | | **Features** | **Findings** |
| **Profile** | | | **Atria** | |
| Abdominal Situs | Solitus | | Left Atrium | Dilated |
| Cardiac Position | Levocardia | | Right Atrium | Normal |
| Systemic Venous Drainage | To RA | | **Atrio-Ventricular Valves** | |
| Pulmonary Venous Drainage | To LA | | Mitral Valve | Annulus = 18mm |
| Atrio-ventricular Connection | Concordant | | Tricuspid Valve | Annulus = 18mm |
| Ventriculo-Arterial Connection | concordant | | **Ventricle** | |
| Ventricular Loop | d-Loop | | Left Ventricle | Dilated |
| **Septae** |  | | Right Ventricle | Normal |
| Interatrial Septum | Intact | | **Doppler Measurement** |  |
| Interventricular Septum | Large Sub aortic VSD, L – R Shunt | | Mitral | ------------- |
| **Semilunar Valves** |  | | Aortic | ------------- |
| Aortic Valve | Annulus = 17mm | | Tricuspid | ------------- |
| Pulmonary Valve | Annulus = 20mm | | Pulmonic | Moderate PR, PPG = 55mmHg |
| **Great Arteries** | NRGA | | **Coronary Arteries** |  |
| Aorta |  | | **Aortic Arch** | Left. No CoA |
| Pulmonary Arteries | Normal MPA & BPAs. | | **PDA** | No PDA |
| **M-Mode**: | | | | |
| Ao | mm | | PWd | mm |
| LA | mm | | EDV | ml |
| LVIDd | mm | | ESV | ml |
| LVIDs | mm | | FS | % |
| IVSd | mm | | LVEF | % |
| **Additional Information:** | | | | |
| **Conclusion:**   1. {S, D, S} Levocardia 2. LA/LV Dilated 3. Non-Restrictive Sub-aortic VSD, L – R Shunt 4. Moderate PR 5. Moderate Pulmonary Hypertension | | | | |
| **Recommendation:** | | | | |
| **Done By:** | | **Signature** | **Date** | **Remark** |
| Tesfaye T., Paediatrician, Paediatric Cardiologist | |  | 09/05/15Eth.C. |  |

| **Tibebe – Ghion Specialized Teaching Hospital, Bahir Dar University,**  **Bahir Dar, Ethiopia** | | | | |
| --- | --- | --- | --- | --- |
| **Name: Nabil Ahmed. Sex/Age: M_/39days. MRN: 162343. Date of Report: 16/05/15Eth.C.**  **Clinical Diagnosis: DS. TGSH3.2760.** | | | | |
| **Features:** | **Findings** | | **Features** | **Findings** |
| **Profile** | | | **Atria** | |
| Abdominal Situs | Solitus | | Left Atrium | Dilated |
| Cardiac Position | Levocardia | | Right Atrium | Dilated |
| Systemic Venous Drainage | To RA | | **Atrio-Ventricular Valves** | |
| Pulmonary Venous Drainage | To LA | | Mitral Valve | Annulus = 8mm |
| Atrio-ventricular Connection | Concordant | | Tricuspid Valve | Annulus = 16mm |
| Ventriculo-Arterial Connection | concordant | | **Ventricle** | |
| Ventricular Loop | d-Loop | | Left Ventricle | Small |
| **Septae** |  | | Right Ventricle | Dilated and Hypertrophied |
| Interatrial Septum | 30mm Primum defect amounting to single atrium | | **Doppler Measurement** |  |
| Interventricular Septum | Intact | | Mitral | Moderate MR |
| **Semilunar Valves** |  | | Aortic | ------------- |
| Aortic Valve | Annulus = 8mm | | Tricuspid | Severe TR |
| Pulmonary Valve | Annulus = 11mm | | Pulmonic | ------------- |
| **Great Arteries** | NRGA | | **Coronary Arteries** |  |
| Aorta |  | | **Aortic Arch** | Left. No CoA |
| Pulmonary Arteries | Normal MPA & BPAs. | | **PDA** | No PDA |
| **M-Mode**: | | | | |
| Ao | mm | | PWd | mm |
| LA | mm | | EDV | ml |
| LVIDd | mm | | ESV | ml |
| LVIDs | mm | | FS | % |
| IVSd | mm | | LVEF | % |
| **Additional Information:** | | | | |
| **Conclusion:**   1. {S, D, S} Levocardia 2. Partial AVSD amounting to single atrium 3. Moderate MR 4. Severe TR. | | | | |
| **Recommendation:** | | | | |
| **Done By:** | | **Signature** | **Date** | **Remark** |
| Tesfaye T., Paediatrician, Paediatric Cardiologist | |  | 16/05/15Eth.C. |  |

| **Tibebe – Ghion Specialized Teaching Hospital, Bahir Dar University,**  **Bahir Dar, Ethiopia** | | | | |
| --- | --- | --- | --- | --- |
| **Name: Amanuel Endeg. Sex/Age: M /81days. MRN:162494. Date of Report: 16/05/15Eth.C.**  **Clinical Diagnosis: Cyanosis + RD + Cardiogenic Shock. TGSH3.2761.** | | | | |
| **Features:** | **Findings** | | **Features** | **Findings** |
| **Profile** | | | **Atria** | |
| Abdominal Situs | Solitus | | Left Atrium | Normal |
| Cardiac Position | Levocardia | | Right Atrium | Normal |
| Systemic Venous Drainage | To RA | | **Atrio-Ventricular Valves** | |
| Pulmonary Venous Drainage | To LA | | Mitral Valve | Annulus = 12mm |
| Atrio-ventricular Connection | Concordant | | Tricuspid Valve | Annulus = 11mm |
| Ventriculo-Arterial Connection | Discordant | | **Ventricle** | |
| Ventricular Loop | d-Loop | | Left Ventricle | Normal |
| **Septae** |  | | Right Ventricle | Normal |
| Interatrial Septum | PFO, L – R Shunt | | **Doppler Measurement** |  |
| Interventricular Septum | 3.5mm Apical Muscular VSD, R – L Shunt | | Mitral | ------------- |
| **Semilunar Valves** |  | | Aortic | ------------- |
| Aortic Valve | Annulus = 13mm | | Tricuspid | ------------- |
| Pulmonary Valve | Annulus = 10mm | | Pulmonic | Mild Valvular PS, PPG, 23mmHg |
| **Great Arteries** | d-TGA | | **Coronary Arteries** |  |
| Aorta | From RV & anterior and to the right | | **Aortic Arch** | Left. No CoA |
| Pulmonary Arteries | From LV & Posterior & to the left | | **PDA** | No PDA |
| **M-Mode**: | | | | |
| Ao | mm | | PWd | mm |
| LA | mm | | EDV | ml |
| LVIDd | mm | | ESV | ml |
| LVIDs | mm | | FS | % |
| IVSd | mm | | LVEF | % |
| **Additional Information:** | | | | |
| **Conclusion:**   1. {S, D, D} Levocardia 2. d-TGA 3. PFO, L – R Shunt 4. Small Apical Muscular VSD, R – L Shunt 5. Mild Valvular PS. | | | | |
| **Recommendation:** | | | | |
| **Done By:** | | **Signature** | **Date** | **Remark** |
| Tesfaye T., Paediatrician, Paediatric Cardiologist | |  | 16/05/15Eth.C. |  |

| **Tibebe – Ghion Specialized Teaching Hospital, Bahir Dar University,**  **Bahir Dar, Ethiopia** | | | | |
| --- | --- | --- | --- | --- |
| **Name: Surafel Me’adu. Sex/Age: M/14years. MRN: 162491. Date of Report: 16/05/15Eth.C.**  **Clinical Diagnosis: Incidental Murmur. TGSH3.2762.** | | | | |
| **Features:** | **Findings** | | **Features** | **Findings** |
| **Profile** | | | **Atria** | |
| Abdominal Situs | Solitus | | Left Atrium | Normal |
| Cardiac Position | Levocardia | | Right Atrium | Normal |
| Systemic Venous Drainage | To RA | | **Atrio-Ventricular Valves** | |
| Pulmonary Venous Drainage | To LA | | Mitral Valve | Annulus = 27mm |
| Atrio-ventricular Connection | Concordant | | Tricuspid Valve | Annulus = 27mm  TAPSE = 18mm |
| Ventriculo-Arterial Connection | concordant | | **Ventricle** | |
| Ventricular Loop | d-Loop | | Left Ventricle | Normal |
| **Septae** |  | | Right Ventricle | Normal |
| Interatrial Septum | Intact | | **Doppler Measurement** |  |
| Interventricular Septum | Intact | | Mitral | Trivial MR, Incomplete Signal, seen in two planes with jet velocity = 3.7m/sec. |
| **Semilunar Valves** |  | | Aortic | ------------- |
| Aortic Valve | Annulus = 20mm | | Tricuspid | ------------- |
| Pulmonary Valve | Annulus = 24mm | | Pulmonic | ------------- |
| **Great Arteries** | NRGA | | **Coronary Arteries** |  |
| Aorta |  | | **Aortic Arch** | Left. No CoA |
| Pulmonary Arteries | Normal MPA & BPAs. | | **PDA** | No PDA |
| **M-Mode**: | | | | |
| Ao | mm | | PWd | mm |
| LA | mm | | EDV | ml |
| LVIDd | mm | | ESV | ml |
| LVIDs | mm | | FS | 32% |
| IVSd | mm | | LVEF | 60% |
| **Additional Information:** | | | | |
| **Conclusion:**   1. {S, D, S} Levocardia 2. Trivial MR. | | | | |
| **Recommendation:** Consider Borderline RHD. | | | | |
| **Done By:** | | **Signature** | **Date** | **Remark** |
| Tesfaye T., Paediatrician, Paediatric Cardiologist | |  | 16/05/15Eth.C. |  |

| **Tibebe – Ghion Specialized Teaching Hospital, Bahir Dar University,**  **Bahir Dar, Ethiopia** | | | | |
| --- | --- | --- | --- | --- |
| **Name: Sisay Ashagrie. Sex/Age: M/2 7/12. MRN: 130174. Date of Report: 16/05/15Eth.C.**  **Follow up echo: Previous Echocardiography severe Pul.HTN + Moderate PR + Mild TR + Dysfunctional RV. (TGSH1.2580)** | | | | |
| **Features:** | **Findings** | | **Features** | **Findings** |
| **Profile** | | | **Atria** | |
| Abdominal Situs | Solitus | | Left Atrium | Normal |
| Cardiac Position | Levocardia | | Right Atrium | Normal |
| Systemic Venous Drainage | To RA | | **Atrio-Ventricular Valves** | |
| Pulmonary Venous Drainage | To LA | | Mitral Valve | Annulus = 18mm |
| Atrio-ventricular Connection | Concordant | | Tricuspid Valve | Annulus = 20mm  TAPSE = 16mm |
| Ventriculo-Arterial Connection | concordant | | **Ventricle** | |
| Ventricular Loop | d-Loop | | Left Ventricle | Normal |
| **Septae** |  | | Right Ventricle | Normal |
| Interatrial Septum | Intact | | **Doppler Measurement** |  |
| Interventricular Septum | Intact | | Mitral | ------------- |
| **Semilunar Valves** |  | | Aortic | ------------- |
| Aortic Valve | Annulus = 14mm | | Tricuspid | Trivial TR, PPG = 27mmHg |
| Pulmonary Valve | Annulus = 19mm | | Pulmonic | ------------- |
| **Great Arteries** | NRGA | | **Coronary Arteries** |  |
| Aorta |  | | **Aortic Arch** | Left. No CoA |
| Pulmonary Arteries | Normal MPA & BPAs. | | **PDA** | No PDA |
| **M-Mode**: Normal LV Function on eye balling | | | | |
| Ao | mm | | PWd | mm |
| LA | mm | | EDV | ml |
| LVIDd | mm | | ESV | ml |
| LVIDs | mm | | FS | % |
| IVSd | mm | | LVEF | % |
| **Additional Information:** | | | | |
| **Conclusion:**   1. {S, D, S} Levocardia 2. Trivial TR. 3. Normal Biventricular Function | | | | |
| **Remark:** The Pulmonary Hypertension and RV Dysfunction has resolved | | | | |
| **Recommendation:** Can Undergo GA and Surgery. | | | | |
| **Done By:** | | **Signature** | **Date** | **Remark** |
| Tesfaye T., Paediatrician, Paediatric Cardiologist | |  | 16/05/15Eth.C. |  |

| **Tibebe – Ghion Specialized Teaching Hospital, Bahir Dar University,**  **Bahir Dar, Ethiopia** | | | | |
| --- | --- | --- | --- | --- |
| **Name: Tazebew Bekele . Sex/Age: M /2years. MRN: 145933. Date of Report: 16/05/15Eth.C.**  **Follow up Echo for: ccTGA + Ebstein anomaly of the TV + Large VSD + Clinical Dx: Murmur. TGSH3.2763. (TGSH2)** | | | | |
| **Features:** | **Findings** | | **Features** | **Findings** |
| **Profile** | | | **Atria** | |
| Abdominal Situs | Solitus | | Left Atrium | Normal |
| Cardiac Position | Levocardia | | Right Atrium | Normal |
| Systemic Venous Drainage | To RA | | **Atrio-Ventricular Valves** | |
| Pulmonary Venous Drainage | To LA | | Mitral Valve | Annulus = 20mm. Right Side, basophilic |
| Atrio-ventricular Connection | Discordant | | Tricuspid Valve | Annulus = mm. left side, apicophilic. 12mm Down ward STL Displacement. |
| Ventriculo-Arterial Connection | Discordant | | **Ventricle** | |
| Ventricular Loop | l-Loop | | Left Ventricle | Normal |
| **Septae** |  | | Right Ventricle | Normal |
| Interatrial Septum | Intact | | **Doppler Measurement** |  |
| Interventricular Septum | 11mm Inlet VSD, L – R Shunt. | | Mitral | ------------- |
| **Semilunar Valves** |  | | Aortic | Moderate AR |
| Aortic Valve | Annulus = 18mm | | Tricuspid | Moderate TR |
| Pulmonary Valve | Annulus = 21mm | | Pulmonic | ------------- |
| **Great Arteries** | L-TGA | | **Coronary Arteries** |  |
| Aorta | From Left side RV, anterior & to the right | | **Aortic Arch** | Left. No CoA |
| Pulmonary Arteries | From Right Side LV, Posterior & to the left | | **PDA** | No PDA |
| **M-Mode**: | | | | |
| Ao | mm | | PWd | mm |
| LA | mm | | EDV | ml |
| LVIDd | mm | | ESV | ml |
| LVIDs | mm | | FS | % |
| IVSd | mm | | LVEF | % |
| **Additional Information:** | | | | |
| **Conclusion:**   1. {S, L, L} Levocardia 2. ccTGA 3. Ebstein anomaly of the Left sided TV. 4. Large inlet VSD, L – R Shunt 5. Moderate AR 6. Moderate TR 7. Normal Function | | | | |
| **Done By:** | | **Signature** | **Date** | **Remark** |
| Tesfaye T., Paediatrician, Paediatric Cardiologist | |  | 16/05/15Eth.C. |  |

| **Tibebe – Ghion Specialized Teaching Hospital, Bahir Dar University,**  **Bahir Dar, Ethiopia** | | | | |
| --- | --- | --- | --- | --- |
| **Name: Birhan Awoke. Sex/Age: M /6years. MRN: 123224. Date of Report: 18/05/15Eth.C.**  **Follow up echo. Clinical Diagnosis: Easy Fatigability + Murmur. TGSH3.2764. (TGSH2)** | | | | |
| **Features:** | **Findings** | | **Features** | **Findings** |
| **Profile** | | | **Atria** | |
| Abdominal Situs | Solitus | | Left Atrium | Normal |
| Cardiac Position | Levocardia | | Right Atrium | Dilated |
| Systemic Venous Drainage | To RA | | **Atrio-Ventricular Valves** | |
| Pulmonary Venous Drainage | To LA | | Mitral Valve | Annulus = 18mm |
| Atrio-ventricular Connection | Concordant | | Tricuspid Valve | Annulus = 24mm. TAPSE = 20mm |
| Ventriculo-Arterial Connection | concordant | | **Ventricle** | |
| Ventricular Loop | d-Loop | | Left Ventricle | Normal |
| **Septae** |  | | Right Ventricle | Dilated |
| Interatrial Septum | 18 X 23mm OS ASD, L – R Shunt. | | **Doppler Measurement** |  |
| Interventricular Septum | Intact | | Mitral | ------------- |
| **Semilunar Valves** |  | | Aortic | ------------- |
| Aortic Valve | Annulus = 12mm | | Tricuspid | Mild TR |
| Pulmonary Valve | Annulus = 17mm. Doming PV. | | Pulmonic | Moderate Valvular PS, PPG = 48mmHg. |
| **Great Arteries** | NRGA | | **Coronary Arteries** |  |
| Aorta |  | | **Aortic Arch** | Left. No CoA |
| Pulmonary Arteries | Normal MPA & BPAs. | | **PDA** | No PDA |
| **M-Mode**: | | | | |
| Ao | mm | | PWd | mm |
| LA | mm | | EDV | ml |
| LVIDd | mm | | ESV | ml |
| LVIDs | mm | | FS | 34% |
| IVSd | mm | | LVEF | 65% |
| **Additional Information:** | | | | |
| **Conclusion:**   1. {S, D, S} Levocardia 2. RA/RV Dilated 3. Large OS ASD, L – R Shunt 4. Moderate Valvular PS. 5. Doming PV 6. Normal Biventricular Systolic Function | | | | |
| **Recommendation:** | | | | |
| **Done By:** | | **Signature** | **Date** | **Remark** |
| Tesfaye T., Paediatrician, Paediatric Cardiologist | |  | 18/05/15Eth.C. |  |

| **Tibebe – Ghion Specialized Teaching Hospital, Bahir Dar University,**  **Bahir Dar, Ethiopia** | | | | |
| --- | --- | --- | --- | --- |
| **Name: Baby Yesharge Addis . Sex/Age: F/48hrs. MRN: ______. Date of Report: 18/05/15Eth.C.**  **Clinical Diagnosis: RD + PPHTN. TGSH3.2765.** | | | | |
| **Features:** | **Findings** | | **Features** | **Findings** |
| **Profile** | | | **Atria** | |
| Abdominal Situs | Solitus | | Left Atrium | Normal |
| Cardiac Position | Levocardia | | Right Atrium | Normal |
| Systemic Venous Drainage | To RA | | **Atrio-Ventricular Valves** | |
| Pulmonary Venous Drainage | To LA | | Mitral Valve | Annulus = 10mm |
| Atrio-ventricular Connection | Concordant | | Tricuspid Valve | Annulus = 10mm |
| Ventriculo-Arterial Connection | concordant | | **Ventricle** | |
| Ventricular Loop | d-Loop | | Left Ventricle | Normal |
| **Septae** |  | | Right Ventricle | Normal |
| Interatrial Septum | PFO, L – R Shunt | | **Doppler Measurement** |  |
| Interventricular Septum | Intact | | Mitral | ------------- |
| **Semilunar Valves** |  | | Aortic | ------------- |
| Aortic Valve | Annulus = 10mm | | Tricuspid | ------------- |
| Pulmonary Valve | Annulus = 9mm | | Pulmonic | ------------- |
| **Great Arteries** | NRGA | | **Coronary Arteries** |  |
| Aorta |  | | **Aortic Arch** | Left. No CoA |
| Pulmonary Arteries | Normal MPA & BPAs. | | **PDA** | No PDA |
| **M-Mode**: Normal LV Function on eye balling. | | | | |
| Ao | mm | | PWd | mm |
| LA | mm | | EDV | ml |
| LVIDd | mm | | ESV | ml |
| LVIDs | mm | | FS | % |
| IVSd | mm | | LVEF | % |
| **Additional Information:** | | | | |
| **Conclusion:**   1. {S, D, S} Levocardia 2. PFO, L – R Shunt. | | | | |
| **Remark:** Newborn was crying during study. | | | | |
| **Recommendation:** | | | | |
| **Done By:** | | **Signature** | **Date** | **Remark** |
| Tesfaye T., Paediatrician, Paediatric Cardiologist | |  | 18/05/15Eth.C. |  |

| **Tibebe – Ghion Specialized Teaching Hospital, Bahir Dar University,**  **Bahir Dar, Ethiopia** | | | | |
| --- | --- | --- | --- | --- |
| **Name: Baby of Bosena Mebratu. Sex/Age: F/5days. MRN: 163029. Date of Report: 19/05/15Eth.C.**  **Clinical Diagnosis: RD + ?PPHTN. TGSH3.2766.** | | | | |
| **Features:** | **Findings** | | **Features** | **Findings** |
| **Profile** | | | **Atria** | |
| Abdominal Situs | Solitus | | Left Atrium | Normal |
| Cardiac Position | Levocardia | | Right Atrium | Normal |
| Systemic Venous Drainage | To RA | | **Atrio-Ventricular Valves** | |
| Pulmonary Venous Drainage | To LA | | Mitral Valve | Annulus = 10mm |
| Atrio-ventricular Connection | Concordant | | Tricuspid Valve | Annulus = 10mm |
| Ventriculo-Arterial Connection | concordant | | **Ventricle** | |
| Ventricular Loop | d-Loop | | Left Ventricle | Normal |
| **Septae** |  | | Right Ventricle | Normal |
| Interatrial Septum | PFO, L – R Shunt | | **Doppler Measurement** |  |
| Interventricular Septum | Intact | | Mitral | ------------- |
| **Semilunar Valves** |  | | Aortic | ------------- |
| Aortic Valve | Annulus = 8mm | | Tricuspid | ------------- |
| Pulmonary Valve | Annulus = 9mm | | Pulmonic | ------------- |
| **Great Arteries** | NRGA | | **Coronary Arteries** |  |
| Aorta |  | | **Aortic Arch** | Left. No CoA |
| Pulmonary Arteries | Normal MPA & BPAs. | | **PDA** | No PDA |
| **M-Mode**: Normal LV Function on eye balling. | | | | |
| Ao | mm | | PWd | mm |
| LA | mm | | EDV | ml |
| LVIDd | mm | | ESV | ml |
| LVIDs | mm | | FS | % |
| IVSd | mm | | LVEF | % |
| **Additional Information:** | | | | |
| **Conclusion:**   1. {S, D, S} Levocardia 2. PFO, L – R Shunt. | | | | |
| **Recommendation:** | | | | |
| **Done By:** | | **Signature** | **Date** | **Remark** |
| Tesfaye T., Paediatrician, Paediatric Cardiologist | |  | 19/05/15Eth.C. |  |

| **Tibebe – Ghion Specialized Teaching Hospital, Bahir Dar University,**  **Bahir Dar, Ethiopia** | | | | |
| --- | --- | --- | --- | --- |
| **Name: Abenezer Dereje. Sex/Age: M /4months. MRN: 163384. Date of Report: 23/05/15Eth.C.**  **Clinical Diagnosis: RD + Diaphoresis + Cyanosis + Murmur. TGSH3.2767.** | | | | |
| **Features:** | **Findings** | | **Features** | **Findings** |
| **Profile** | | | **Atria** | |
| Abdominal Situs | Solitus | | Left Atrium | Normal |
| Cardiac Position | Levocardia | | Right Atrium | Normal |
| Systemic Venous Drainage | To RA | | **Atrio-Ventricular Valves** | |
| Pulmonary Venous Drainage | To LA | | Mitral Valve | Annulus = 11mm. Aorto-Mitral Fibrous Discontinuity. |
| Atrio-ventricular Connection | Concordant | | Tricuspid Valve | Annulus = 13mm |
| Ventriculo-Arterial Connection | DORV | | **Ventricle** | |
| Ventricular Loop | d-Loop | | Left Ventricle | Normal |
| **Septae** |  | | Right Ventricle | Normal |
| Interatrial Septum | Intact | | **Doppler Measurement** |  |
| Interventricular Septum | Non-Restrictive Sub-aortic VSD, L – R Shunt | | Mitral | ------------- |
| **Semilunar Valves** |  | | Aortic | ------------- |
| Aortic Valve | Annulus = 11mm | | Tricuspid | ------------- |
| Pulmonary Valve | Annulus = 11mm | | Pulmonic | Mild Valvular PS, PPG = 36mmHg |
| **Great Arteries** | NRGA | | **Coronary Arteries** |  |
| Aorta | >50% arising from RV | | **Aortic Arch** | Left. No CoA |
| Pulmonary Arteries | Normal MPA & BPAs. | | **PDA** | 1.5mm PDA, L – R Shunt |
| **M-Mode**: Normal LV Function on eye balling. | | | | |
| Ao | mm | | PWd | mm |
| LA | mm | | EDV | ml |
| LVIDd | mm | | ESV | ml |
| LVIDs | mm | | FS | % |
| IVSd | mm | | LVEF | % |
| **Additional Information:** | | | | |
| **Conclusion:**   1. {S, D, S} Levocardia 2. DORV 3. Non-Restrictive Sub-Aortic VSD, L – R Shunt 4. Small PDA, L – R Shunt 5. Mild Valvular PS. 6. Normal LV Systolic Function | | | | |
| **Recommendation:** | | | | |
| **Done By:** | | **Signature** | **Date** | **Remark** |
| Tesfaye T., Paediatrician, Paediatric Cardiologist | |  | 23/05/15Eth.C. |  |

| **Tibebe – Ghion Specialized Teaching Hospital, Bahir Dar University,**  **Bahir Dar, Ethiopia** | | | | |
| --- | --- | --- | --- | --- |
| **Name: Kalkidan Lealem . Sex/Age: F /7 months. MRN: 161408. Date of Report: 23/05/15Eth.C.**  **Clinical Diagnosis: Diaphoresis + Recurrent chest Infection + RD. TGSH3.2768.** | | | | |
| **Features:** | **Findings** | | **Features** | **Findings** |
| **Profile** | | | **Atria** | |
| Abdominal Situs | Solitus | | Left Atrium | Normal |
| Cardiac Position | Levocardia | | Right Atrium | Normal |
| Systemic Venous Drainage | To RA | | **Atrio-Ventricular Valves** | |
| Pulmonary Venous Drainage | To LA | | Mitral Valve | Annulus = 11mm |
| Atrio-ventricular Connection | Concordant | | Tricuspid Valve | Annulus = 11mm |
| Ventriculo-Arterial Connection | concordant | | **Ventricle** | |
| Ventricular Loop | d-Loop | | Left Ventricle | Normal |
| **Septae** |  | | Right Ventricle | Normal |
| Interatrial Septum | Intact | | **Doppler Measurement** |  |
| Interventricular Septum | Intact | | Mitral | ------------- |
| **Semilunar Valves** |  | | Aortic | ------------- |
| Aortic Valve | Annulus = 9mm | | Tricuspid | ------------- |
| Pulmonary Valve | Annulus = 10mm | | Pulmonic | ------------- |
| **Great Arteries** | NRGA | | **Coronary Arteries** |  |
| Aorta |  | | **Aortic Arch** | Left. No CoA |
| Pulmonary Arteries | Normal MPA & BPAs. | | **PDA** | No PDA |
| **M-Mode**: Normal LV Function on eye balling. | | | | |
| Ao | mm | | PWd | mm |
| LA | mm | | EDV | ml |
| LVIDd | mm | | ESV | ml |
| LVIDs | mm | | FS | % |
| IVSd | mm | | LVEF | % |
| **Additional Information:** | | | | |
| **Conclusion:**   1. Normal Echocardiography Study | | | | |
| **Recommendation:** | | | | |
| **Done By:** | | **Signature** | **Date** | **Remark** |
| Tesfaye T., Paediatrician, Paediatric Cardiologist | |  | 23/05/15Eth.C. |  |

| **Tibebe – Ghion Specialized Teaching Hospital, Bahir Dar University,**  **Bahir Dar, Ethiopia** | | | | |
| --- | --- | --- | --- | --- |
| **Name: Redeat Bayeleygn. Sex/Age: F/3/8years. MRN:163531. Date of Report: 23/05/15Eth.C.**  **INCOMPLETE DATA (AGE NOT CLEAR)** | | | | |
| **Features:** | **Findings** | | **Features** | **Findings** |
| **Profile** | | | **Atria** | |
| Abdominal Situs | Solitus | | Left Atrium | Normal |
| Cardiac Position | Levocardia | | Right Atrium | Normal |
| Systemic Venous Drainage | To RA | | **Atrio-Ventricular Valves** | |
| Pulmonary Venous Drainage | To LA | | Mitral Valve | Annulus = 15mm. Thickened MVL. |
| Atrio-ventricular Connection | Concordant | | Tricuspid Valve | Annulus = 15mm  TAPSE = 17mm |
| Ventriculo-Arterial Connection | concordant | | **Ventricle** | |
| Ventricular Loop | d-Loop | | Left Ventricle | Normal |
| **Septae** |  | | Right Ventricle | Normal |
| Interatrial Septum | Intact | | **Doppler Measurement** |  |
| Interventricular Septum | Intact | | Mitral | Mild MR, Holosystolic, central projection, seen in two planes with jet velocity = 4.6m/sec |
| **Semilunar Valves** |  | | Aortic | ------------- |
| Aortic Valve | Annulus = 13mm | | Tricuspid | ------------- |
| Pulmonary Valve | Annulus = 16mm | | Pulmonic | ------------- |
| **Great Arteries** | NRGA | | **Coronary Arteries** |  |
| Aorta |  | | **Aortic Arch** | Left. No CoA |
| Pulmonary Arteries | Normal MPA & BPAs. | | **PDA** | No PDA |
| **M-Mode**: Normal LV Function on eye balling. | | | | |
| Ao | mm | | PWd | mm |
| LA | mm | | EDV | ml |
| LVIDd | mm | | ESV | ml |
| LVIDs | mm | | FS | % |
| IVSd | mm | | LVEF | % |
| **Additional Information:** | | | | |
| **Conclusion:**   1. {S, D, S} Levocardia 2. Thickened MVL 3. Mild MR 4. Normal LV Systolic Function. | | | | |
| **Recommendation:** | | | | |
| **Done By:** | | **Signature** | **Date** | **Remark** |
| Tesfaye T., Paediatrician, Paediatric Cardiologist | |  | 23/05/15Eth.C. |  |

| **Tibebe – Ghion Specialized Teaching Hospital, Bahir Dar University,**  **Bahir Dar, Ethiopia** | | | | |
| --- | --- | --- | --- | --- |
| **Name: Yalemsira Dessie. Sex/Age: F/6/12years. MRN: 159815. Date of Report: 23/05/15Eth.C.**  **Follow up echo for Large ASD + Large Pericardial effusion with Tamponade + Severe Pulmonary HTN + SVT. (AGH2.140)** | | | | |
| **Features:** | **Findings** | | **Features** | **Findings** |
| **Profile** | | | **Atria** | |
| Abdominal Situs | Solitus | | Left Atrium | Dilated |
| Cardiac Position | Levocardia | | Right Atrium | Markedly Dilated |
| Systemic Venous Drainage | To RA | | **Atrio-Ventricular Valves** | |
| Pulmonary Venous Drainage | To LA | | Mitral Valve | Annulus = 19mm |
| Atrio-ventricular Connection | Concordant | | Tricuspid Valve | Annulus = 20mm |
| Ventriculo-Arterial Connection | concordant | | **Ventricle** | |
| Ventricular Loop | d-Loop | | Left Ventricle | Dilated |
| **Septae** |  | | Right Ventricle | Markedly Dilated |
| Interatrial Septum | 14mm OS ASD, BD Shunt | | **Doppler Measurement** |  |
| Interventricular Septum | Intact | | Mitral | ------------- |
| **Semilunar Valves** |  | | Aortic | ------------- |
| Aortic Valve | Annulus = 9mm | | Tricuspid | Moderate TR, PPG = 55mmHg |
| Pulmonary Valve | Annulus = 11mm | | Pulmonic | Mild PR, PPG = 50mmHg |
| **Great Arteries** | NRGA | | **Coronary Arteries** |  |
| Aorta |  | | **Aortic Arch** | Left. No CoA |
| Pulmonary Arteries | Normal MPA & BPAs. | | **PDA** | No PDA |
| **M-Mode**: | | | | |
| Ao | mm | | PWd | mm |
| LA | mm | | EDV | ml |
| LVIDd | mm | | ESV | ml |
| LVIDs | mm | | FS | % |
| IVSd | mm | | LVEF | % |
| **Additional Information: No Pericardial effusion** | | | | |
| **Conclusion:**   1. {S, D, S} Levocardia 2. All chambers dilated. 3. Large OS ASD, BD Shunt 4. Moderate TR 5. Mild PR 6. Moderate Pulmonary Hypertension | | | | |
| **Remark:** Rhythm Abnormality and tachycardia detected during study | | | | |
| **Recommendation:** Do ECG | | | | |
| **Done By:** | | **Signature** | **Date** | **Remark** |
| Tesfaye T., Paediatrician, Paediatric Cardiologist | |  | 23/05/15Eth.C. |  |
| **Tibebe – Ghion Specialized Teaching Hospital, Bahir Dar University,**  **Bahir Dar, Ethiopia** | | | | |
| **Name: B/Eldana Kelem. Sex/Age: M/8days. MRN: 163033. Date of Report: 29/05/15Eth.C.**  **Follow up echo for PPHTNN. (AGH)** | | | | |
| **Features:** | **Findings** | | **Features** | **Findings** |
| **Profile** | | | **Atria** | |
| Abdominal Situs | Solitus | | Left Atrium | Normal |
| Cardiac Position | Levocardia | | Right Atrium | Normal |
| Systemic Venous Drainage | To RA | | **Atrio-Ventricular Valves** | |
| Pulmonary Venous Drainage | To LA | | Mitral Valve | Annulus = 11mm |
| Atrio-ventricular Connection | Concordant | | Tricuspid Valve | Annulus = 10mm  TAPSE = 10mm |
| Ventriculo-Arterial Connection | concordant | | **Ventricle** | |
| Ventricular Loop | d-Loop | | Left Ventricle | Normal |
| **Septae** |  | | Right Ventricle | Normal |
| Interatrial Septum | Intact | | **Doppler Measurement** |  |
| Interventricular Septum | Intact | | Mitral | ------------- |
| **Semilunar Valves** |  | | Aortic | ------------- |
| Aortic Valve | Annulus = 10mm | | Tricuspid | Trivial TR, PPG = 27mmHg |
| Pulmonary Valve | Annulus = 9mm | | Pulmonic | ------------- |
| **Great Arteries** | NRGA | | **Coronary Arteries** |  |
| Aorta |  | | **Aortic Arch** | Left. No CoA |
| Pulmonary Arteries | Normal MPA & BPAs. | | **PDA** | No PDA |
| **M-Mode**: Normal LV Function on eye balling. | | | | |
| Ao | mm | | PWd | mm |
| LA | mm | | EDV | ml |
| LVIDd | mm | | ESV | ml |
| LVIDs | mm | | FS | % |
| IVSd | mm | | LVEF | % |
| **Additional Information:** | | | | |
| **Conclusion:**   1. Normal Echocardiography Study | | | | |
| **Recommendation:** Resolved PPHTNN. | | | | |
| **Done By:** | | **Signature** | **Date** | **Remark** |
| Tesfaye T., Paediatrician, Paediatric Cardiologist | |  | 23/05/15Eth.C. |  |

| **Tibebe – Ghion Specialized Teaching Hospital, Bahir Dar University,**  **Bahir Dar, Ethiopia** | | | | | | |
| --- | --- | --- | --- | --- | --- | --- |
| **Name: B/ Yirgedu Fentahun. Sex/Age: M/15days. MRN: 164506. Date of Report: 29/05/15Eth.C.**  **Clinical Diagnosis: Cyanosis + Murmur. TGSH3.2769.** | | | | | | |
| **Features:** | **Findings** | | | **Features** | | **Findings** |
| **Profile** | | | | **Atria** | | |
| Abdominal Situs | Solitus | | | Left Atrium | | Normal |
| Cardiac Position | Mesocardia | | | Right Atrium | | Normal |
| Systemic Venous Drainage | To RA | | | **Atrio-Ventricular Valves** | | |
| Pulmonary Venous Drainage | To LA | | | Mitral Valve | | Annulus = 7mm |
| Atrio-ventricular Connection | Discordant/Criss-Cross | | | Tricuspid Valve | | Annulus = 12mm |
| Ventriculo-Arterial Connection | DOLV | | | **Ventricle** | | |
| Ventricular Loop | l-Loop | | | Left Ventricle | | Hypoplastic |
| **Septae** |  | | | Right Ventricle | | Normal |
| Interatrial Septum | 10mm OS ASD, amounting to single atrium, BD Shunt | | | **Doppler Measurement** | |  |
| Interventricular Septum | 8mm Inlet VSD, R – L Shunt | | | Mitral | | ------------- |
| **Semilunar Valves** |  | | | Aortic | | ------------- |
| Aortic Valve | Annulus = 10mm | | | Tricuspid | | Trivial TR |
| Pulmonary Valve | Annulus = 3mm | | | Pulmonic | | Severe Valvular & Supra Valvular PS, PPG = 63mmHg |
| **Great Arteries** | l-TGA | | | **Coronary Arteries** | |  |
| Aorta | From LV, anterior & to the left | | | **Aortic Arch** | | Left. No CoA |
| Pulmonary Arteries | Hypoplastic MPA & BPAs. From LV, Posterior & to the right. | | | **PDA** | | No PDA |
| **M-Mode**: | | | | | | |
| Ao | mm | | | PWd | | mm |
| LA | mm | | | EDV | | ml |
| LVIDd | mm | | | ESV | | ml |
| LVIDs | mm | | | FS | | % |
| IVSd | mm | | | LVEF | | % |
| **Additional Information:** | | | | | | |
| **Conclusion:**   1. {S, L, L} Mesocardia 2. Criss-Cross AV Connection/Discordant 3. DOLV 4. Large OS ASD, amounting to single atrium, BD Shunt. 5. Large Inlet VSD, R – L Shunt 6. Hypoplastic LV 7. Hypoplastic MPA and Branch PAs. 8. Severe Valvular and Supra-Valvular PS 9. Trivial TR | | | | | | |
| **Done By:** | | **Signature** | **Date** | | **Remark** | |
| Tesfaye T., Paediatrician, Paediatric Cardiologist | |  | 23/05/15Eth.C. | |  | |

| **Tibebe – Ghion Specialized Teaching Hospital, Bahir Dar University,**  **Bahir Dar, Ethiopia** | | | | |
| --- | --- | --- | --- | --- |
| **Name: Baby Hawa Dawid. Sex/Age: F/ 6days. MRN: 164195. Date of Report: 30/05/15Eth.C.**  **Clinical Diagnosis: Incidental Murmur. TGSH3.2770.** | | | | |
| **Features:** | **Findings** | | **Features** | **Findings** |
| **Profile** | | | **Atria** | |
| Abdominal Situs | Solitus | | Left Atrium | Normal |
| Cardiac Position | Levocardia | | Right Atrium | Dilated |
| Systemic Venous Drainage | To RA | | **Atrio-Ventricular Valves** | |
| Pulmonary Venous Drainage | To LA | | Mitral Valve | Annulus = 10mm |
| Atrio-ventricular Connection | Concordant | | Tricuspid Valve | Annulus = 12mm. 8.5mm Downward displacement of STL attached to the septum. |
| Ventriculo-Arterial Connection | concordant | | **Ventricle** | |
| Ventricular Loop | d-Loop | | Left Ventricle | Normal |
| **Septae** |  | | Right Ventricle | Smallish. |
| Interatrial Septum | 6mm High Secundum ASD, BD Shunt | | **Doppler Measurement** |  |
| Interventricular Septum | Intact | | Mitral | ------------- |
| **Semilunar Valves** |  | | Aortic | ------------- |
| Aortic Valve | Annulus = 7mm | | Tricuspid | Moderate TR, PPG = 20mmHg |
| Pulmonary Valve | Annulus = 6mm | | Pulmonic | ------------- |
| **Great Arteries** | NRGA | | **Coronary Arteries** |  |
| Aorta |  | | **Aortic Arch** | Left. No CoA |
| Pulmonary Arteries | Normal MPA & BPAs. | | **PDA** | <1mm PDA, L – R Shunt |
| **M-Mode**: Normal LV Function on eye balling. | | | | |
| Ao | mm | | PWd | mm |
| LA | mm | | EDV | ml |
| LVIDd | mm | | ESV | ml |
| LVIDs | mm | | FS | % |
| IVSd | mm | | LVEF | % |
| **Additional Information:** | | | | |
| **Conclusion:**   1. {S, D, S} Levocardia 2. Small OS High ASD, BD Shunt 3. Ebstein anomaly of the tricuspid Valve 4. Moderate TR 5. Small PDA, L – R Shunt. 6. Normal LV Systolic Function | | | | |
| **Recommendation:** | | | | |
| **Done By:** | | **Signature** | **Date** | **Remark** |
| Tesfaye T., Paediatrician, Paediatric Cardiologist | |  | 30/05/15Eth.C. |  |

| **Tibebe – Ghion Specialized Teaching Hospital, Bahir Dar University,**  **Bahir Dar, Ethiopia** | | | | |
| --- | --- | --- | --- | --- |
| **Name: H/ Giorgis Endawok. Sex/Age: M /7months. MRN: 164007. Date of Report: 30/05/15Eth.C.**  **Clinical Diagnosis: Incidental Murmur. TGSH3.2771.** | | | | |
| **Features:** | **Findings** | | **Features** | **Findings** |
| **Profile** | | | **Atria** | |
| Abdominal Situs | Solitus | | Left Atrium | Normal |
| Cardiac Position | Levocardia | | Right Atrium | Normal |
| Systemic Venous Drainage | To RA | | **Atrio-Ventricular Valves** | |
| Pulmonary Venous Drainage | To LA | | Mitral Valve | Annulus = 18mm |
| Atrio-ventricular Connection | Concordant | | Tricuspid Valve | Annulus = 19mm  TAPSE = 19mm |
| Ventriculo-Arterial Connection | concordant | | **Ventricle** | |
| Ventricular Loop | d-Loop | | Left Ventricle | Normal |
| **Septae** |  | | Right Ventricle | Normal |
| Interatrial Septum | PFO, L – R Shunt | | **Doppler Measurement** |  |
| Interventricular Septum | Intact | | Mitral | ------------- |
| **Semilunar Valves** |  | | Aortic | ------------- |
| Aortic Valve | Annulus = 15mm | | Tricuspid | ------------- |
| Pulmonary Valve | Annulus = 15mm. Doming PV | | Pulmonic | Mild Valvular PS, PPG = 35mmHg |
| **Great Arteries** | NRGA | | **Coronary Arteries** |  |
| Aorta |  | | **Aortic Arch** | Left. No CoA |
| Pulmonary Arteries | Normal MPA & BPAs. | | **PDA** | No PDA |
| **M-Mode**: Normal LV Function on eye balling | | | | |
| Ao | mm | | PWd | mm |
| LA | mm | | EDV | ml |
| LVIDd | mm | | ESV | ml |
| LVIDs | mm | | FS | % |
| IVSd | mm | | LVEF | % |
| **Additional Information:** | | | | |
| **Conclusion:**   1. {S, D, S} Levocardia 2. Doming Pulmonary Valve 3. Mild Valvular PS. 4. Normal Biventricular Systolic Function | | | | |
| **Recommendation:** | | | | |
| **Done By:** | | **Signature** | **Date** | **Remark** |
| Tesfaye T., Paediatrician, Paediatric Cardiologist | |  | 30/05/15Eth.C. |  |

| **Tibebe – Ghion Specialized Teaching Hospital, Bahir Dar University,**  **Bahir Dar, Ethiopia** | | | | |
| --- | --- | --- | --- | --- |
| **Name: Leul Saleamlak. Sex/Age: M/2months. MRN: 164864. Date of Report: 30/05/15Eth.C.**  **Clinical Diagnosis: Incidental Murmur. TGSH3.2772.** | | | | |
| **Features:** | **Findings** | | **Features** | **Findings** |
| **Profile** | | | **Atria** | |
| Abdominal Situs | Solitus | | Left Atrium | Normal |
| Cardiac Position | Levocardia | | Right Atrium | Normal |
| Systemic Venous Drainage | To RA | | **Atrio-Ventricular Valves** | |
| Pulmonary Venous Drainage | To LA | | Mitral Valve | Annulus = 13mm |
| Atrio-ventricular Connection | Concordant | | Tricuspid Valve | Annulus = 13mm  TAPSE = 15mm |
| Ventriculo-Arterial Connection | concordant | | **Ventricle** | |
| Ventricular Loop | d-Loop | | Left Ventricle | Normal |
| **Septae** |  | | Right Ventricle | Normal |
| Interatrial Septum | 4mm OS ASD, L – R Shunt | | **Doppler Measurement** |  |
| Interventricular Septum | 3mm PM VSD, L – R Shunt, with a gradient of 63mmHg. | | Mitral | ------------- |
| **Semilunar Valves** |  | | Aortic | ------------- |
| Aortic Valve | Annulus = 9mm | | Tricuspid | ------------- |
| Pulmonary Valve | Annulus = 11mm | | Pulmonic | Mild Valvular PS, PPG = 26mmHg |
| **Great Arteries** | NRGA | | **Coronary Arteries** |  |
| Aorta |  | | **Aortic Arch** | Left. No CoA |
| Pulmonary Arteries | Normal MPA & BPAs. | | **PDA** | No PDA |
| **M-Mode**: Normal LV Function on eye balling. | | | | |
| Ao | mm | | PWd | mm |
| LA | mm | | EDV | ml |
| LVIDd | mm | | ESV | ml |
| LVIDs | mm | | FS | % |
| IVSd | mm | | LVEF | % |
| **Additional Information:** | | | | |
| **Conclusion:**   1. {S, D, S} Levocardia 2. Small OS ASD, L – R Shunt 3. Restrictive PM VSD, L – R Shunt 4. Mild Valvular PS 5. Normal Biventricular Systolic Function. | | | | |
| **Recommendation:** | | | | |
| **Done By:** | | **Signature** | **Date** | **Remark** |
| Tesfaye T., Paediatrician, Paediatric Cardiologist | |  | 30/05/15Eth.C. |  |

| **Tibebe – Ghion Specialized Teaching Hospital, Bahir Dar University,**  **Bahir Dar, Ethiopia** | | | | |
| --- | --- | --- | --- | --- |
| **Name: Hirut Berhanu. Sex/Age: F/4years. MRN: 164670. Date of Report: 30/05/15Eth.C.**  **Clinical Diagnosis: Cyanosis + Clubbing + Murmur. TGSH3.2773.** | | | | |
| **Features:** | **Findings** | | **Features** | **Findings** |
| **Profile** | | | **Atria** | |
| Abdominal Situs | Solitus | | Left Atrium | Normal |
| Cardiac Position | Levocardia | | Right Atrium | Normal |
| Systemic Venous Drainage | To RA | | **Atrio-Ventricular Valves** | |
| Pulmonary Venous Drainage | To LA | | Mitral Valve | Annulus = 17mm |
| Atrio-ventricular Connection | Concordant | | Tricuspid Valve | Annulus = 19mm  TAPSE = 16mm |
| Ventriculo-Arterial Connection | concordant | | **Ventricle** | |
| Ventricular Loop | d-Loop | | Left Ventricle | Normal |
| **Septae** |  | | Right Ventricle | Dilated & Hypertrophied |
| Interatrial Septum | Intact | | **Doppler Measurement** |  |
| Interventricular Septum | Non – Restrictive Sub-Aortic VSD, R – L Shunt | | Mitral | ------------- |
| **Semilunar Valves** |  | | Aortic | ------------- |
| Aortic Valve | Annulus = 16mm | | Tricuspid | ------------- |
| Pulmonary Valve | Annulus = 18mm | | Pulmonic | Severe PS, PPG = 68mmHg |
| **Great Arteries** | NRGA | | **Coronary Arteries** |  |
| Aorta | Over-riding aorta | | **Aortic Arch** | Left. No CoA |
| Pulmonary Arteries | Normal MPA & BPAs. | | **PDA** | No PDA |
| **M-Mode**: Normal LV Function on eye balling. | | | | |
| Ao | mm | | PWd | mm |
| LA | mm | | EDV | ml |
| LVIDd | mm | | ESV | ml |
| LVIDs | mm | | FS | % |
| IVSd | mm | | LVEF | % |
| **Additional Information:** | | | | |
| **Conclusion:**   1. {S, D, S} Levocardia 2. RA/RV Dilated, RV Hypertrophied 3. TOF. | | | | |
| **Recommendation:** | | | | |
| **Done By:** | | **Signature** | **Date** | **Remark** |
| Tesfaye T., Paediatrician, Paediatric Cardiologist | |  | 30/05/15Eth.C. |  |

| **Tibebe – Ghion Specialized Teaching Hospital, Bahir Dar University,**  **Bahir Dar, Ethiopia** | | | | |
| --- | --- | --- | --- | --- |
| **Name: B/Hawa Getu. Sex/Age: F/11days. MRN: 164882. Date of Report: 30/05/15Eth.C.**  **Clinical Diagnosis: DS. TGSH3.2774.** | | | | |
| **Features:** | **Findings** | | **Features** | **Findings** |
| **Profile** | | | **Atria** | |
| Abdominal Situs | Solitus | | Left Atrium | Normal |
| Cardiac Position | Levocardia | | Right Atrium | Mildly dilated |
| Systemic Venous Drainage | To RA | | **Atrio-Ventricular Valves** | |
| Pulmonary Venous Drainage | To LA | | Mitral Valve | Annulus = 8mm |
| Atrio-ventricular Connection | Concordant | | Tricuspid Valve | Annulus = 9mm. TAPSE = 12mm |
| Ventriculo-Arterial Connection | concordant | | **Ventricle** | |
| Ventricular Loop | d-Loop | | Left Ventricle | Normal |
| **Septae** |  | | Right Ventricle | Mildly Dilated |
| Interatrial Septum | 8mm High OS ASD, L – R Shunt | | **Doppler Measurement** |  |
| Interventricular Septum | Intact | | Mitral | ------------- |
| **Semilunar Valves** |  | | Aortic | ------------- |
| Aortic Valve | Annulus = 8mm | | Tricuspid | Trivial TR, PPG = 33mmHg |
| Pulmonary Valve | Annulus = 10mm | | Pulmonic | ------------- |
| **Great Arteries** | NRGA | | **Coronary Arteries** |  |
| Aorta |  | | **Aortic Arch** | Left. No CoA |
| Pulmonary Arteries | Normal MPA & BPAs. | | **PDA** | No PDA |
| **M-Mode**: Normal LV Function on eye balling. | | | | |
| Ao | mm | | PWd | mm |
| LA | mm | | EDV | ml |
| LVIDd | mm | | ESV | ml |
| LVIDs | mm | | FS | % |
| IVSd | mm | | LVEF | % |
| **Additional Information:** | | | | |
| **Conclusion:**   1. {S, D, S} Levocardia 2. RA/RV Dilated 3. Moderate High OS ASD, L – R Shunt. 4. Trivial TR 5. Normal Biventricular Systolic Function | | | | |
| **Recommendation:** | | | | |
| **Done By:** | | **Signature** | **Date** | **Remark** |
| Tesfaye T., Paediatrician, Paediatric Cardiologist | |  | 30/05/15Eth.C. |  |

| **Tibebe – Ghion Specialized Teaching Hospital, Bahir Dar University,**  **Bahir Dar, Ethiopia** | | | | |
| --- | --- | --- | --- | --- |
| **Name: Sewhareg mongo. Sex/Age: F/_2 3/12 years. MRN: 164180. Date of Report: 30/05/15Eth.C.**  **Clinical Diagnosis: Diaphoresis + RD + CHF + Murmur. TGSH3.2775.** | | | | |
| **Features:** | **Findings** | | **Features** | **Findings** |
| **Profile** | | | **Atria** | |
| Abdominal Situs | Solitus | | Left Atrium | Dilated |
| Cardiac Position | Levocardia | | Right Atrium | Dilated |
| Systemic Venous Drainage | To RA | | **Atrio-Ventricular Valves** | |
| Pulmonary Venous Drainage | To LA | | Mitral Valve | Annulus = 13mm |
| Atrio-ventricular Connection | Concordant | | Tricuspid Valve | Annulus = 20mm. **TAPSE = 13mm** |
| Ventriculo-Arterial Connection | concordant | | **Ventricle** | |
| Ventricular Loop | d-Loop | | Left Ventricle | Dilated |
| **Septae** |  | | Right Ventricle | Dilated |
| Interatrial Septum | Intact | | **Doppler Measurement** |  |
| Interventricular Septum | Non-Restrictive Sub-Aortic VSD, R – L Shunt | | Mitral | ------------- |
| **Semilunar Valves** |  | | Aortic | ------------- |
| Aortic Valve | Annulus = 14mm | | Tricuspid | Mild TR |
| Pulmonary Valve | Annulus = 15mm | | Pulmonic | Moderate PR, PPG = 66mmHg |
| **Great Arteries** | NRGA | | **Coronary Arteries** |  |
| Aorta |  | | **Aortic Arch** | Left. No CoA |
| Pulmonary Arteries | **MPA =20mm.** | | **PDA** | No PDA |
| **M-Mode**: | | | | |
| Ao | mm | | PWd | mm |
| LA | mm | | EDV | ml |
| LVIDd | mm | | ESV | ml |
| LVIDs | mm | | FS | 25% |
| IVSd | mm | | LVEF | 52% |
| **Additional Information:** | | | | |
| **Conclusion:**   1. {S, D, S} Levocardia 2. RA/RV Dilated 3. Non-Restrictive Sub-Aortic VSD, R – L Shunt 4. Mild TR 5. Moderate PR. 6. Severe Pulmonary Hypertension 7. Reduced Biventricular Systolic Function | | | | |
| **Recommendation:** | | | | |
| **Done By:** | | **Signature** | **Date** | **Remark** |
| Tesfaye T., Paediatrician, Paediatric Cardiologist | |  | 30/05/15Eth.C. |  |

| **Tibebe – Ghion Specialized Teaching Hospital, Bahir Dar University,**  **Bahir Dar, Ethiopia** | | | | |
| --- | --- | --- | --- | --- |
| **Name: Baby of Tagegn Asnake. Sex/Age: M/4days. MRN: 164818. Date of Report: 01/06/15Eth.C.**  **Clinical Diagnosis: RD + DS. TGSH3.2776.** | | | | |
| **Features:** | **Findings** | | **Features** | **Findings** |
| **Profile** | | | **Atria** | |
| Abdominal Situs | Solitus | | Left Atrium | Normal |
| Cardiac Position | Levocardia | | Right Atrium | Normal |
| Systemic Venous Drainage | To RA | | **Atrio-Ventricular Valves** | |
| Pulmonary Venous Drainage | To LA | | Mitral Valve | Annulus = 10mm |
| Atrio-ventricular Connection | Concordant | | Tricuspid Valve | Annulus = 11mm |
| Ventriculo-Arterial Connection | concordant | | **Ventricle** | |
| Ventricular Loop | d-Loop | | Left Ventricle | Normal |
| **Septae** |  | | Right Ventricle | Normal |
| Interatrial Septum | Intact | | **Doppler Measurement** |  |
| Interventricular Septum | Intact | | Mitral | Mild MR |
| **Semilunar Valves** |  | | Aortic | ------------- |
| Aortic Valve | Annulus = 8mm | | Tricuspid | Mild TR, PPG = 38mmHg |
| Pulmonary Valve | Annulus = 9mm | | Pulmonic | ------------- |
| **Great Arteries** | NRGA | | **Coronary Arteries** |  |
| Aorta |  | | **Aortic Arch** | Left. No CoA |
| Pulmonary Arteries | Normal MPA & BPAs. | | **PDA** | No PDA |
| **M-Mode**: Normal LV Systolic Function | | | | |
| Ao | mm | | PWd | mm |
| LA | mm | | EDV | ml |
| LVIDd | mm | | ESV | ml |
| LVIDs | mm | | FS | % |
| IVSd | mm | | LVEF | % |
| **Additional Information:** | | | | |
| **Conclusion:**   1. {S, D, S} Levocardia 2. Mild MR 3. Mild TR 4. Mild Pulmonary Hypertension 5. Normal LV Systolic Function. | | | | |
| **Recommendation:** | | | | |
| **Done By:** | | **Signature** | **Date** | **Remark** |
| Tesfaye T., Paediatrician, Paediatric Cardiologist | |  | 01/06/15Eth.C. |  |

| **Tibebe – Ghion Specialized Teaching Hospital, Bahir Dar University,**  **Bahir Dar, Ethiopia** | | | | |
| --- | --- | --- | --- | --- |
| **Name: Muluken Wubayhu . Sex/Age: F/1 6/12. MRN: 166296. Date of Report: 21/06/15Eth.C.**  **Clinical Diagnosis: RD + CHF + Murmur. TGSH3.2777.** | | | | |
| **Features:** | **Findings** | | **Features** | **Findings** |
| **Profile** | | | **Atria** | |
| Abdominal Situs | Solitus | | Left Atrium | Dilated |
| Cardiac Position | Levocardia | | Right Atrium | Normal |
| Systemic Venous Drainage | To RA | | **Atrio-Ventricular Valves** | |
| Pulmonary Venous Drainage | To LA | | Mitral Valve | Annulus = 19mm |
| Atrio-ventricular Connection | Concordant | | Tricuspid Valve | Annulus = 14mm. TAPSE = 15mm |
| Ventriculo-Arterial Connection | concordant | | **Ventricle** | |
| Ventricular Loop | d-Loop | | Left Ventricle | Globularly Dilated |
| **Septae** |  | | Right Ventricle | Normal |
| Interatrial Septum | Intact | | **Doppler Measurement** |  |
| Interventricular Septum | Intact | | Mitral | Moderate MR, Holosystolic, posterior projection, seen in two planes with jet velocity = 3.9m/sec. |
| **Semilunar Valves** |  | | Aortic | ------------- |
| Aortic Valve | Annulus = 20mm | | Tricuspid | ------------- |
| Pulmonary Valve | Annulus = 12mm | | Pulmonic | ------------- |
| **Great Arteries** | NRGA | | **Coronary Arteries** | No ALCAPA |
| Aorta |  | | **Aortic Arch** | Left. No CoA |
| Pulmonary Arteries | Normal MPA & BPAs. | | **PDA** | No PDA |
| **M-Mode**: | | | | |
| Ao | mm | | PWd | mm |
| LA | mm | | EDV | ml |
| LVIDd | mm | | ESV | ml |
| LVIDs | mm | | FS | 16% |
| IVSd | mm | | LVEF | 34% |
| **Additional Information:** Pericardial effusion with a maximum depth of 5mm on RA Side | | | | |
| **Conclusion:**   1. {S, D, S} Levocardia 2. LA/LV Dilated 3. Moderate MR 4. LV Systolic Dysfunction 5. Small Pericardial effusion | | | | |
| **Recommendation:** DCM can be considered as DDx | | | | |
| **Done By:** | | **Signature** | **Date** | **Remark** |
| Tesfaye T., Paediatrician, Paediatric Cardiologist | |  | 21/06/15Eth.C. |  |

| **Tibebe – Ghion Specialized Teaching Hospital, Bahir Dar University,**  **Bahir Dar, Ethiopia** | | | | | |
| --- | --- | --- | --- | --- | --- |
| **Name: Ahlam Kindu. Sex/Age: F/7/12. MRN: 165734. Date of Report: 21/06/15Eth.C.**  **Clinical Diagnosis: DS + Murmur. TGSH3.2778.** | | | | | |
| **Features:** | | **Findings** | | **Features** | **Findings** |
| **Profile** | | | | **Atria** | |
| Abdominal Situs | Solitus | | | Left Atrium | Mildly dilated |
| Cardiac Position | Levocardia | | | Right Atrium | Mildly dilated |
| Systemic Venous Drainage | To RA | | | **Atrio-Ventricular Valves** | |
| Pulmonary Venous Drainage | To LA | | | Mitral Valve | Annulus = 13mm |
| Atrio-ventricular Connection | Concordant | | | Tricuspid Valve | Annulus = 15mm. TAPSE = 17mm |
| Ventriculo-Arterial Connection | concordant | | | **Ventricle** | |
| Ventricular Loop | d-Loop | | | Left Ventricle | Mildly dilated |
| **Septae** |  | | | Right Ventricle | Mildly dilated |
| Interatrial Septum | 8mm OS ASD, L – R Shunt. | | | **Doppler Measurement** |  |
| Interventricular Septum | 7mm PM VSD, L – R Shunt. | | | Mitral | Trivial MR, VELOCITY = 4.3m/sec. |
| **Semilunar Valves** |  | | | Aortic | ------------- |
| Aortic Valve | Annulus = 13mm | | | Tricuspid | ------------- |
| Pulmonary Valve | Annulus = 16mm | | | Pulmonic | Moderate PR, PPG = 46mmHg |
| **Great Arteries** | NRGA | | | **Coronary Arteries** |  |
| Aorta |  | | | **Aortic Arch** | Left. No CoA |
| Pulmonary Arteries | Normal MPA & BPAs. | | | **PDA** | No PDA |
| **M-Mode**: | | | | | |
| Ao | | mm | | PWd | mm |
| LA | | mm | | EDV | ml |
| LVIDd | | mm | | ESV | ml |
| LVIDs | | mm | | FS | 33% |
| IVSd | | mm | | LVEF | 64% |
| **Additional Information:** Pericardial effusion with maximum depth of 4mm on RA/RV Side | | | | | |
| **Conclusion:**   1. {S, D, S} Levocardia 2. All chambers mildly dilated. 3. Moderate OS ASD, L – R Shunt 4. Moderate PM VSD, L – R Shunt 5. Moderate PR 6. Mild Pulmonary Hypertension 7. Normal Biventricular Systolic Function 8. Trace Pericardial effusion | | | | | |
| **Recommendation:** | | | | | |
| **Done By:** | | | **Signature** | **Date** | **Remark** |
| Tesfaye T., Paediatrician, Paediatric Cardiologist | | |  | 21/06/15Eth.C. |  |

| **Tibebe – Ghion Specialized Teaching Hospital, Bahir Dar University,**  **Bahir Dar, Ethiopia** | | | | |
| --- | --- | --- | --- | --- |
| **Name: Netsanet Getachew. Sex/Age: F/10/12 months. MRN: 167038. Date of Report: 21/06/15Eth.C.**  **Clinical Diagnosis: Incidental Murmur. TGSH3.2779.** | | | | |
| **Features:** | **Findings** | | **Features** | **Findings** |
| **Profile** | | | **Atria** | |
| Abdominal Situs | Solitus | | Left Atrium | Dilated |
| Cardiac Position | Levocardia | | Right Atrium | Normal |
| Systemic Venous Drainage | To RA | | **Atrio-Ventricular Valves** | |
| Pulmonary Venous Drainage | To LA | | Mitral Valve | Annulus = 20mm |
| Atrio-ventricular Connection | Concordant | | Tricuspid Valve | Annulus = 14mm |
| Ventriculo-Arterial Connection | concordant | | **Ventricle** | |
| Ventricular Loop | d-Loop | | Left Ventricle | Dilated |
| **Septae** |  | | Right Ventricle | Normal |
| Interatrial Septum | 8mm OS ASD, L – R Shunt | | **Doppler Measurement** |  |
| Interventricular Septum | 8mm Sub-Pulmonic VSD, L – R Shunt | | Mitral | ------------- |
| **Semilunar Valves** |  | | Aortic | ------------- |
| Aortic Valve | Annulus = 12mm | | Tricuspid | ------------- |
| Pulmonary Valve | Annulus = 12mm | | Pulmonic | ------------- |
| **Great Arteries** | NRGA | | **Coronary Arteries** |  |
| Aorta |  | | **Aortic Arch** | Left. No CoA |
| Pulmonary Arteries | Normal MPA & BPAs. | | **PDA** | No PDA |
| **M-Mode**: | | | | |
| Ao | mm | | PWd | mm |
| LA | mm | | EDV | ml |
| LVIDd | mm | | ESV | ml |
| LVIDs | mm | | FS | 38% |
| IVSd | mm | | LVEF | 69% |
| **Additional Information:** | | | | |
| **Conclusion:**   1. {S, D, S} Levocardia 2. Moderate OS ASD, L – R Shunt 3. Moderate Sub-Pulmonic VSD, L – R Shunt 4. Normal Systolic LV Function. | | | | |
| **Recommendation:** | | | | |
| **Done By:** | | **Signature** | **Date** | **Remark** |
| Tesfaye T., Paediatrician, Paediatric Cardiologist | |  | 21/06/15Eth.C. |  |

| **Tibebe – Ghion Specialized Teaching Hospital, Bahir Dar University,**  **Bahir Dar, Ethiopia** | | | | |
| --- | --- | --- | --- | --- |
| **Name: Baby of Felegush Ertiban. Sex/Age: M/ 23 days. MRN: 167540. Date of Report: 21/06/15Eth.C.**  **Clinical Diagnosis: Cyanosis + Cardiogenic Shock + RD. TGSH3.2780.** | | | | |
| **Features:** | **Findings** | | **Features** | **Findings** |
| **Profile** | | | **Atria** | |
| Abdominal Situs | Solitus | | Left Atrium | Normal |
| Cardiac Position | Levocardia | | Right Atrium | Mildly dilated |
| Systemic Venous Drainage | To RA | | **Atrio-Ventricular Valves** | |
| Pulmonary Venous Drainage | To LA | | Mitral Valve | Annulus = mm |
| Atrio-ventricular Connection | Concordant | | Tricuspid Valve | Annulus = mm  TAPSE = mm |
| Ventriculo-Arterial Connection | Discordant | | **Ventricle** | |
| Ventricular Loop | d-Loop | | Left Ventricle | Normal |
| **Septae** |  | | Right Ventricle | Mildly dilated |
| Interatrial Septum | Restrictive PFO, LA to RA. | | **Doppler Measurement** |  |
| Interventricular Septum | Intact | | Mitral | ------------- |
| **Semilunar Valves** |  | | Aortic | ------------- |
| Aortic Valve | Annulus = 11mm | | Tricuspid | ------------- |
| Pulmonary Valve | Annulus = 8mm | | Pulmonic | Mild PS(LVOTO), PPG = 40mmHg. |
| **Great Arteries** | d-TGA | | **Coronary Arteries** |  |
| Aorta | Anterior and from RV | | **Aortic Arch** | Left. No CoA |
| Pulmonary Arteries | Posterior & from LV | | **PDA** | 1mm PDA, from Aorta to MPA |
| **M-Mode**: | | | | |
| Ao | mm | | PWd | mm |
| LA | mm | | EDV | ml |
| LVIDd | mm | | ESV | ml |
| LVIDs | mm | | FS | % |
| IVSd | mm | | LVEF | % |
| **Additional Information:** | | | | |
| **Conclusion:**   1. {S, D, S} Levocardia 2. Mildly dilated RA/RV 3. Restrictive PFO, LA to RA 4. d-TGA with Intact IVS. 5. Mild PS (LVOTO) 6. Small PDA, Aorta to MPA | | | | |
| **Recommendation:** | | | | |
| **Done By:** | | **Signature** | **Date** | **Remark** |
| Tesfaye T., Paediatrician, Paediatric Cardiologist | |  | 21/06/15Eth.C. |  |

| **Tibebe – Ghion Specialized Teaching Hospital, Bahir Dar University,**  **Bahir Dar, Ethiopia** | | | | |
| --- | --- | --- | --- | --- |
| **Name: Amsalu Dagnaw . Sex/Age: M /1 7/12years. MRN: 167960. Date of Report: 30/06/15Eth.C.**  **Clinical Diagnosis: RD + Diaphoresis + Murmur + CHF. TGSH3.2781.** | | | | |
| **Features:** | **Findings** | | **Features** | **Findings** |
| **Profile** | | | **Atria** | |
| Abdominal Situs | Solitus | | Left Atrium | Dilated |
| Cardiac Position | Levocardia | | Right Atrium | Dilated |
| Systemic Venous Drainage | To RA | | **Atrio-Ventricular Valves** | |
| Pulmonary Venous Drainage | To LA | | Mitral Valve | Annulus = 10mm |
| Atrio-ventricular Connection | Concordant | | Tricuspid Valve | Annulus = 20mm  TAPSE = 18mm |
| Ventriculo-Arterial Connection | concordant | | **Ventricle** | |
| Ventricular Loop | d-Loop | | Left Ventricle | Dilated |
| **Septae** |  | | Right Ventricle | Dilated |
| Interatrial Septum | 9mm OS ASD, L – R Shunt | | **Doppler Measurement** |  |
| Interventricular Septum | Intact | | Mitral | ------------- |
| **Semilunar Valves** |  | | Aortic | ------------- |
| Aortic Valve | Annulus = 12mm | | Tricuspid | Mild TR, PPG = 63mmHg |
| Pulmonary Valve | Annulus = 15mm | | Pulmonic | ------------- |
| **Great Arteries** | NRGA | | **Coronary Arteries** |  |
| Aorta |  | | **Aortic Arch** | Left. No CoA |
| Pulmonary Arteries | Normal MPA & BPAs. | | **PDA** | 2.5mm PDA, L – R Shunt |
| **M-Mode**: Normal LV Function on eye balling. | | | | |
| Ao | mm | | PWd | mm |
| LA | mm | | EDV | ml |
| LVIDd | mm | | ESV | ml |
| LVIDs | mm | | FS | % |
| IVSd | mm | | LVEF | % |
| **Additional Information:** Pericardial effusion, circumferential with maximum depth of 7mm on RV Side. | | | | |
| **Conclusion:**   1. {S, D, S} Levocardia 2. Moderate OS ASD, L – R Shunt 3. Large PDA, L – R Shunt 4. Severe Pulmonary Hypertension 5. Normal Biventricular Systolic Function | | | | |
| **Recommendation:** | | | | |
| **Done By:** | | **Signature** | **Date** | **Remark** |
| Tesfaye T., Paediatrician, Paediatric Cardiologist | |  | 30/06/15Eth.C. |  |

| **Tibebe – Ghion Specialized Teaching Hospital, Bahir Dar University,**  **Bahir Dar, Ethiopia** | | | | |
| --- | --- | --- | --- | --- |
| **Name: B/Haymanot W/Gebreal. Sex/Age: M/17 Days. MRN: 166806. Date of Report: 30/06/15Eth.C.**  **Clinical Diagnosis: RD. TGSH3.2782.** | | | | |
| **Features:** | **Findings** | | **Features** | **Findings** |
| **Profile** | | | **Atria** | |
| Abdominal Situs | Solitus | | Left Atrium | Normal |
| Cardiac Position | Levocardia | | Right Atrium | Normal |
| Systemic Venous Drainage | To RA | | **Atrio-Ventricular Valves** | |
| Pulmonary Venous Drainage | To LA | | Mitral Valve | Annulus = 10mm |
| Atrio-ventricular Connection | Concordant | | Tricuspid Valve | Annulus = 9mm |
| Ventriculo-Arterial Connection | concordant | | **Ventricle** | |
| Ventricular Loop | d-Loop | | Left Ventricle | Normal |
| **Septae** |  | | Right Ventricle | Normal |
| Interatrial Septum | PFO, L – R Shunt | | **Doppler Measurement** |  |
| Interventricular Septum | Intact | | Mitral | ------------- |
| **Semilunar Valves** |  | | Aortic | ------------- |
| Aortic Valve | Annulus = 9mm | | Tricuspid | ------------- |
| Pulmonary Valve | Annulus = 8mm | | Pulmonic | ------------- |
| **Great Arteries** | NRGA | | **Coronary Arteries** |  |
| Aorta |  | | **Aortic Arch** | Left. No CoA |
| Pulmonary Arteries | Normal MPA & BPAs. | | **PDA** | No PDA |
| **M-Mode**: Normal LV Function on eye balling. | | | | |
| Ao | mm | | PWd | mm |
| LA | mm | | EDV | ml |
| LVIDd | mm | | ESV | ml |
| LVIDs | mm | | FS | % |
| IVSd | mm | | LVEF | % |
| **Additional Information:** | | | | |
| **Conclusion:**   1. {S, D, S} Levocardia 2. PFO, L – R Shunt. | | | | |
| **Recommendation:** | | | | |
| **Done By:** | | **Signature** | **Date** | **Remark** |
| Tesfaye T., Paediatrician, Paediatric Cardiologist | |  | 30/06/15Eth.C. |  |

| **Tibebe – Ghion Specialized Teaching Hospital, Bahir Dar University,**  **Bahir Dar, Ethiopia** | | | | |
| --- | --- | --- | --- | --- |
| **Name: Baby of Teshalu Berie . Sex/Age: M/13days. MRN: 167824. Date of Report: 30/06/15Eth.C.**  **Clinical Diagnosis: Incidental Murmur. TGSH3.2783.** | | | | |
| **Features:** | **Findings** | | **Features** | **Findings** |
| **Profile** | | | **Atria** | |
| Abdominal Situs | Solitus | | Left Atrium | Normal |
| Cardiac Position | Levocardia | | Right Atrium | Normal |
| Systemic Venous Drainage | To RA | | **Atrio-Ventricular Valves** | |
| Pulmonary Venous Drainage | To LA | | Mitral Valve | Annulus = 10mm |
| Atrio-ventricular Connection | Concordant | | Tricuspid Valve | Annulus = 11mm |
| Ventriculo-Arterial Connection | concordant | | **Ventricle** | |
| Ventricular Loop | d-Loop | | Left Ventricle | Normal |
| **Septae** |  | | Right Ventricle | Normal |
| Interatrial Septum | 6mm High Secundum ASD, L – R Shunt. | | **Doppler Measurement** |  |
| Interventricular Septum | Non-restrictive Sub-aortic VSD, L – R Shunt. | | Mitral | ------------- |
| **Semilunar Valves** |  | | Aortic | ------------- |
| Aortic Valve | Annulus = 10mm | | Tricuspid | Trivial TR, PPG = 31mmHg |
| Pulmonary Valve | Annulus = mm | | Pulmonic | ------------- |
| **Great Arteries** | NRGA | | **Coronary Arteries** |  |
| Aorta |  | | **Aortic Arch** | Left. No CoA |
| Pulmonary Arteries | Normal MPA & BPAs. | | **PDA** | 1mm PDA, L – R Shunt |
| **M-Mode**: Normal LV Function on eye balling. | | | | |
| Ao | mm | | PWd | mm |
| LA | mm | | EDV | ml |
| LVIDd | mm | | ESV | ml |
| LVIDs | mm | | FS | % |
| IVSd | mm | | LVEF | % |
| **Additional Information:** Pericardial effusion with maximum depth of 4mm on RV Side. | | | | |
| **Conclusion:**   1. {S, D, S} Levocardia 2. Small High Secundum ASD, L – R Shunt. 3. Non-Restrictive Sub-aortic VSD, L – R Shunt 4. Small PDA, L – R Shunt 5. Trace Pericardial effusion | | | | |
| **Recommendation:** | | | | |
| **Done By:** | | **Signature** | **Date** | **Remark** |
| Tesfaye T., Paediatrician, Paediatric Cardiologist | |  | 30/06/15Eth.C. |  |

| **Tibebe – Ghion Specialized Teaching Hospital, Bahir Dar University,**  **Bahir Dar, Ethiopia** | | | | |
| --- | --- | --- | --- | --- |
| **Name:_____________. Sex/Age: ____ /_____years. MRN: ___________. Date of Report: 20/06/15Eth.C.** | | | | |
| **Features:** | **Findings** | | **Features** | **Findings** |
| **Profile** | | | **Atria** | |
| Abdominal Situs | Solitus | | Left Atrium | Normal |
| Cardiac Position | Levocardia | | Right Atrium | Normal |
| Systemic Venous Drainage | To RA | | **Atrio-Ventricular Valves** | |
| Pulmonary Venous Drainage | To LA | | Mitral Valve | Annulus = mm |
| Atrio-ventricular Connection | Concordant | | Tricuspid Valve | Annulus = mm  TAPSE = mm |
| Ventriculo-Arterial Connection | concordant | | **Ventricle** | |
| Ventricular Loop | d-Loop | | Left Ventricle | Normal |
| **Septae** |  | | Right Ventricle | Normal |
| Interatrial Septum | Intact | | **Doppler Measurement** |  |
| Interventricular Septum | Intact | | Mitral | ------------- |
| **Semilunar Valves** |  | | Aortic | ------------- |
| Aortic Valve | Annulus = mm | | Tricuspid | ------------- |
| Pulmonary Valve | Annulus = mm | | Pulmonic | ------------- |
| **Great Arteries** | NRGA | | **Coronary Arteries** |  |
| Aorta |  | | **Aortic Arch** | Left. No CoA |
| Pulmonary Arteries | Normal MPA & BPAs. | | **PDA** | No PDA |
| **M-Mode**: | | | | |
| Ao | mm | | PWd | Mm |
| LA | mm | | EDV | Ml |
| LVIDd | mm | | ESV | Ml |
| LVIDs | mm | | FS | % |
| IVSd | mm | | LVEF | % |
| **Additional Information:** | | | | |
| **Conclusion:**   1. {S, D, S} Levocardia 2. . | | | | |
| **Recommendation:** | | | | |
| **Done By:** | | **Signature** | **Date** | **Remark** |
| Tesfaye T., Paediatrician, Paediatric Cardiologist | |  | 20/06/15Eth.C. |  |

| **Tibebe – Ghion Specialized Teaching Hospital, Bahir Dar University,**  **Bahir Dar, Ethiopia** | | | | |
| --- | --- | --- | --- | --- |
| **Name:_____________. Sex/Age: ____ /_____years. MRN: ___________. Date of Report: 20/06/15Eth.C.** | | | | |
| **Features:** | **Findings** | | **Features** | **Findings** |
| **Profile** | | | **Atria** | |
| Abdominal Situs | Solitus | | Left Atrium | Normal |
| Cardiac Position | Levocardia | | Right Atrium | Normal |
| Systemic Venous Drainage | To RA | | **Atrio-Ventricular Valves** | |
| Pulmonary Venous Drainage | To LA | | Mitral Valve | Annulus = mm |
| Atrio-ventricular Connection | Concordant | | Tricuspid Valve | Annulus = mm  TAPSE = mm |
| Ventriculo-Arterial Connection | concordant | | **Ventricle** | |
| Ventricular Loop | d-Loop | | Left Ventricle | Normal |
| **Septae** |  | | Right Ventricle | Normal |
| Interatrial Septum | Intact | | **Doppler Measurement** |  |
| Interventricular Septum | Intact | | Mitral | ------------- |
| **Semilunar Valves** |  | | Aortic | ------------- |
| Aortic Valve | Annulus = mm | | Tricuspid | ------------- |
| Pulmonary Valve | Annulus = mm | | Pulmonic | ------------- |
| **Great Arteries** | NRGA | | **Coronary Arteries** |  |
| Aorta |  | | **Aortic Arch** | Left. No CoA |
| Pulmonary Arteries | Normal MPA & BPAs. | | **PDA** | No PDA |
| **M-Mode**: | | | | |
| Ao | mm | | PWd | Mm |
| LA | mm | | EDV | Ml |
| LVIDd | mm | | ESV | Ml |
| LVIDs | mm | | FS | % |
| IVSd | mm | | LVEF | % |
| **Additional Information:** | | | | |
| **Conclusion:**   1. {S, D, S} Levocardia 2. . | | | | |
| **Recommendation:** | | | | |
| **Done By:** | | **Signature** | **Date** | **Remark** |
| Tesfaye T., Paediatrician, Paediatric Cardiologist | |  | 20/06/15Eth.C. |  |

| **Tibebe – Ghion Specialized Teaching Hospital, Bahir Dar University,**  **Bahir Dar, Ethiopia** | | | | |
| --- | --- | --- | --- | --- |
| **Name:_____________. Sex/Age: ____ /_____years. MRN: ___________. Date of Report: 20/06/15Eth.C.** | | | | |
| **Features:** | **Findings** | | **Features** | **Findings** |
| **Profile** | | | **Atria** | |
| Abdominal Situs | Solitus | | Left Atrium | Normal |
| Cardiac Position | Levocardia | | Right Atrium | Normal |
| Systemic Venous Drainage | To RA | | **Atrio-Ventricular Valves** | |
| Pulmonary Venous Drainage | To LA | | Mitral Valve | Annulus = mm |
| Atrio-ventricular Connection | Concordant | | Tricuspid Valve | Annulus = mm  TAPSE = mm |
| Ventriculo-Arterial Connection | concordant | | **Ventricle** | |
| Ventricular Loop | d-Loop | | Left Ventricle | Normal |
| **Septae** |  | | Right Ventricle | Normal |
| Interatrial Septum | Intact | | **Doppler Measurement** |  |
| Interventricular Septum | Intact | | Mitral | ------------- |
| **Semilunar Valves** |  | | Aortic | ------------- |
| Aortic Valve | Annulus = mm | | Tricuspid | ------------- |
| Pulmonary Valve | Annulus = mm | | Pulmonic | ------------- |
| **Great Arteries** | NRGA | | **Coronary Arteries** |  |
| Aorta |  | | **Aortic Arch** | Left. No CoA |
| Pulmonary Arteries | Normal MPA & BPAs. | | **PDA** | No PDA |
| **M-Mode**: | | | | |
| Ao | mm | | PWd | Mm |
| LA | mm | | EDV | Ml |
| LVIDd | mm | | ESV | Ml |
| LVIDs | mm | | FS | % |
| IVSd | mm | | LVEF | % |
| **Additional Information:** | | | | |
| **Conclusion:**   1. {S, D, S} Levocardia 2. . | | | | |
| **Recommendation:** | | | | |
| **Done By:** | | **Signature** | **Date** | **Remark** |
| Tesfaye T., Paediatrician, Paediatric Cardiologist | |  | 20/06/15Eth.C. |  |

| **Tibebe – Ghion Specialized Teaching Hospital, Bahir Dar University,**  **Bahir Dar, Ethiopia** | | | | |
| --- | --- | --- | --- | --- |
| **Name:_____________. Sex/Age: ____ /_____years. MRN: ___________. Date of Report: 20/06/15Eth.C.** | | | | |
| **Features:** | **Findings** | | **Features** | **Findings** |
| **Profile** | | | **Atria** | |
| Abdominal Situs | Solitus | | Left Atrium | Normal |
| Cardiac Position | Levocardia | | Right Atrium | Normal |
| Systemic Venous Drainage | To RA | | **Atrio-Ventricular Valves** | |
| Pulmonary Venous Drainage | To LA | | Mitral Valve | Annulus = mm |
| Atrio-ventricular Connection | Concordant | | Tricuspid Valve | Annulus = mm  TAPSE = mm |
| Ventriculo-Arterial Connection | concordant | | **Ventricle** | |
| Ventricular Loop | d-Loop | | Left Ventricle | Normal |
| **Septae** |  | | Right Ventricle | Normal |
| Interatrial Septum | Intact | | **Doppler Measurement** |  |
| Interventricular Septum | Intact | | Mitral | ------------- |
| **Semilunar Valves** |  | | Aortic | ------------- |
| Aortic Valve | Annulus = mm | | Tricuspid | ------------- |
| Pulmonary Valve | Annulus = mm | | Pulmonic | ------------- |
| **Great Arteries** | NRGA | | **Coronary Arteries** |  |
| Aorta |  | | **Aortic Arch** | Left. No CoA |
| Pulmonary Arteries | Normal MPA & BPAs. | | **PDA** | No PDA |
| **M-Mode**: | | | | |
| Ao | mm | | PWd | Mm |
| LA | mm | | EDV | Ml |
| LVIDd | mm | | ESV | Ml |
| LVIDs | mm | | FS | % |
| IVSd | mm | | LVEF | % |
| **Additional Information:** | | | | |
| **Conclusion:**   1. {S, D, S} Levocardia 2. . | | | | |
| **Recommendation:** | | | | |
| **Done By:** | | **Signature** | **Date** | **Remark** |
| Tesfaye T., Paediatrician, Paediatric Cardiologist | |  | 20/06/15Eth.C. |  |

| **Tibebe – Ghion Specialized Teaching Hospital, Bahir Dar University,**  **Bahir Dar, Ethiopia** | | | | |
| --- | --- | --- | --- | --- |
| **Name:_____________. Sex/Age: ____ /_____years. MRN: ___________. Date of Report: 20/06/15Eth.C.** | | | | |
| **Features:** | **Findings** | | **Features** | **Findings** |
| **Profile** | | | **Atria** | |
| Abdominal Situs | Solitus | | Left Atrium | Normal |
| Cardiac Position | Levocardia | | Right Atrium | Normal |
| Systemic Venous Drainage | To RA | | **Atrio-Ventricular Valves** | |
| Pulmonary Venous Drainage | To LA | | Mitral Valve | Annulus = mm |
| Atrio-ventricular Connection | Concordant | | Tricuspid Valve | Annulus = mm  TAPSE = mm |
| Ventriculo-Arterial Connection | concordant | | **Ventricle** | |
| Ventricular Loop | d-Loop | | Left Ventricle | Normal |
| **Septae** |  | | Right Ventricle | Normal |
| Interatrial Septum | Intact | | **Doppler Measurement** |  |
| Interventricular Septum | Intact | | Mitral | ------------- |
| **Semilunar Valves** |  | | Aortic | ------------- |
| Aortic Valve | Annulus = mm | | Tricuspid | ------------- |
| Pulmonary Valve | Annulus = mm | | Pulmonic | ------------- |
| **Great Arteries** | NRGA | | **Coronary Arteries** |  |
| Aorta |  | | **Aortic Arch** | Left. No CoA |
| Pulmonary Arteries | Normal MPA & BPAs. | | **PDA** | No PDA |
| **M-Mode**: | | | | |
| Ao | mm | | PWd | Mm |
| LA | mm | | EDV | Ml |
| LVIDd | mm | | ESV | Ml |
| LVIDs | mm | | FS | % |
| IVSd | mm | | LVEF | % |
| **Additional Information:** | | | | |
| **Conclusion:**   1. {S, D, S} Levocardia 2. . | | | | |
| **Recommendation:** | | | | |
| **Done By:** | | **Signature** | **Date** | **Remark** |
| Tesfaye T., Paediatrician, Paediatric Cardiologist | |  | 20/06/15Eth.C. |  |
