# Supplementary material for: Two–Dimensional and Doppler trans-thoracic echocardiographic patterns of suspected pediatric heart diseases at Tibebe-—Ghion specialized Teaching Hospital and Adinas General Hospital, Bahir Dar, North-west Ethiopia:–An experience from an LMIC
Source: PLoS One. 2024 Mar 11;19(3):e0292694. doi: 10.1371/journal.pone.0292694 (PMC10927071; doi:10.1371/journal.pone.0292694)
Supplement: S1 File — (ZIP) [file pone.0292694.s002.zip › TGSH4 Pediatric Echocardiography report 2021 SPSS FILLED.docx]

| Pediatric Echocardiography report  Patient Name: baby of Atsede - Wein. Patient ID: 061845. Gender: Female. Age: 31days.  Date of Report : 24/03/2013Eth.C Clinical Diagnosis: _DS. TGSH4.2866. | | | | |
| --- | --- | --- | --- | --- |
| Features | **Finding** | **Features** | | **Finding** |
| Profile | | **Atria** | | |
| Abdominal situs | Solitus | Left atrium | | Normal |
| Cardiac position | Levocardia | Right atrium | | Normal |
| Systemic venous drainage | to RA | **Atrioventricular valves** | | |
| Pulmonary venous drainage | to LA | Mitral valve | | Annulus = 9mm. |
| Atrioventricular connection | Concordant | Tricuspid valve | | Annulus = 10mm. |
| Ventriculoarterial connection | Concordant | **Ventricles** | | |
| Ventricular loop | d-Loop | Left ventricle | | Normal |
| Septae | | Right ventricle | | Normal |
| Interventricular septum | Intact | **M-Mode:** | | |
| Interatrial septum | Intact | AO | |  |
| Great arteries | NRGA | LA | |  |
| Aorta | ----- | LVIDd | | mm |
| Pulmonary artery | Normal MPA & Branch PAs. | LVIDs | | mm |
| Semilunal valves |  | IVSd | | mm |
| Aortic valve | Annulus = 10mm. | IVSs | | mm |
| Pulmonary valve | Annulus = 9mm | LVPWd | | mm |
| Doppler Measurement | | LVPWs | | mm |
| Mitral | ---- | EDV | | ml |
| Aortic | ------ | ESV | | ml |
| Tricuspid | Trivial TR, PPG = 30mmHg | FS | | % |
| pulmonic | --------- | LVEF | | % |
| Aortic arch | ------- | **Coronary arteries** | |  |
| PDA | No |  | |  |
| Pericardium |  | | | |
| Pleura |  | | | |
| Final Diagnosis | 1. Normal Echocardiography Study. | | | |
| Done By: | **Signature** | | **Date of Reporting** | |
| Dr. Tesfaye Taye,  Pediatrician, Pediatric Cardiologist |  | | **24/03/2013Eth.C** | |

| Pediatric Echocardiography report  Patient Name: Destaw Dimamu. Patient ID: 062734. Gender: M. Age: 5months.  Clinical Diagnosis: _Incidental Murmur + Diaphoresis. TGSH4.2867. | | | | |
| --- | --- | --- | --- | --- |
| Features | **Finding** | **Features** | | **Finding** |
| Profile | | **Atria** | | |
| Abdominal situs | Solitus | Left atrium | | Normal |
| Cardiac position | Levocardia | Right atrium | | Normal |
| Systemic venous drainage | to RA | **Atrioventricular valves** | | |
| Pulmonary venous drainage | to LA | Mitral valve | | Annulus = 12mm. |
| Atrioventricular connection | Concordant | Tricuspid valve | | Annulus = 12mm. |
| Ventriculoarterial connection | Concordant | **Ventricles** | | |
| Ventricular loop | d-Loop | Left ventricle | | Normal |
| Septae | | Right ventricle | | Normal |
| Interventricular septum | 6mm PM VSD, L – R Shunt | **M-Mode:** | | |
| Interatrial septum | PFO, L – R Shunt | AO | |  |
| Great arteries | NRGA | LA | |  |
| Aorta | ----- | LVIDd | | mm |
| Pulmonary artery | Normal MPA normal Branch PAs. | LVIDs | | mm |
| Semilunal valves |  | IVSd | | mm |
| Aortic valve | Annulus = 11mm. | IVSs | | mm |
| Pulmonary valve | Annulus = 11mm | LVPWd | | mm |
| Doppler Measurement | | LVPWs | | mm |
| Mitral | ---- | EDV | | ml |
| Aortic | ------ | ESV | | ml |
| Tricuspid | ------- | FS | | 33% |
| pulmonic | --------- | LVEF | | 63% |
| Aortic arch | ------- | **Coronary arteries** | |  |
| PDA | No |  | |  |
| Pericardium/Pleura |  | | | |
| Final Diagnosis | 1. {S, D, S} Levocardia. 2. PFO, L – R Shunt 3. Moderate PM VSD, L – R Shunt 4. Normal LV Function | | | |
| Done By: | **Signature** | | **Date of Reporting** | |
| Dr. Tesfaye Taye,  Pediatrician, Pediatric Cardiologist |  | | **29/03/2013Eth.C** | |

| Pediatric Echocardiography report  Patient Name: Netsanet Habitamu. Patient ID: 062733. Gender: F. Age: 7months.  Clinical Diagnosis: DS + RD + Murmur + Diaphoresis. TGSH4.2868. | | | | |
| --- | --- | --- | --- | --- |
| Features | **Finding** | **Features** | | **Finding** |
| Profile | | **Atria** | | |
| Abdominal situs | Solitus | Left atrium | | Dilated |
| Cardiac position | Levocardia | Right atrium | | Dilated |
| Systemic venous drainage | to RA | **Atrioventricular valves** | | |
| Pulmonary venous drainage | to LA | Mitral valve | | Common Complete AVSD |
| Atrioventricular connection | Concordant | Tricuspid valve | | Common Complete AVSD |
| Ventriculoarterial connection | Concordant | **Ventricles** | | |
| Ventricular loop | d-Loop | Left ventricle | | Dilated |
| Septae | | Right ventricle | | Dilated |
| Interventricular septum | Common Complete AVSD, L – R Shunt | **M-Mode:** | | |
| Interatrial septum | Common Complete AVSD, L – R Shunt. Additional PFO, L – R Shunt. | AO | |  |
| Great arteries | NRGA | LA | |  |
| Aorta | ----- | LVIDd | | mm |
| Pulmonary artery | Normal MPA normal Branch PAs. | LVIDs | | mm |
| Semilunal valves |  | IVSd | | mm |
| Aortic valve | Annulus = 12mm. | IVSs | | mm |
| Pulmonary valve | Annulus = 12mm | LVPWd | | mm |
| Doppler Measurement | | LVPWs | | mm |
| Mitral | Mild Left AVVR | EDV | | ml |
| Aortic | ------ | ESV | | ml |
| Tricuspid | Mild Right AVVR | FS | | % |
| pulmonic | --------- | LVEF | | % |
| Aortic arch | ------- | **Coronary arteries** | |  |
| PDA | No |  | |  |
| Pericardium/Pleura |  | | | |
| Final Diagnosis | 1. {S, D, S} Levocardia. 2. PFO, L – R Shunt 3. Common Complete AVSD, L – R Shunt 4. Normal LV Function | | | |
| Done By: | **Signature** | | **Date of Reporting** | |
| Dr. Tesfaye Taye,  Pediatrician, Pediatric Cardiologist |  | | **29/03/2013Eth.C** | |

| Pediatric Echocardiography report  Patient Name: Negese Lijalem. Patient ID: 053076_. Gender: Male. Age: 9months. | | | | |
| --- | --- | --- | --- | --- |
| Clinical Diagnosis: RD + Diaphoresis + Recurrent Chest Infection. TGSH4.2869. (TGSH7) | | | | |
| Features | **Finding** | **Features** | | **Finding** |
| Profile | | **Atria** | | |
| Abdominal situs | Solitus | Left atrium | | Normal |
| Cardiac position | Levocardia | Right atrium | | Dilated |
| Systemic venous drainage | to RA | **Atrioventricular valves** | | |
| Pulmonary venous drainage | to LA | Mitral valve | | Annulus = 11mm. |
| Atrioventricular connection | Concordant | Tricuspid valve | | Annulus = 14mm.  TAPSE = 11mm |
| Ventriculoarterial connection | Concordant | **Ventricles** | | |
| Ventricular loop | d-Loop | Left ventricle | | Normal |
| Septae | | Right ventricle | | Dilated  RV TDI S wave = 9cm/sec |
| Interventricular septum | Intact | **M-Mode:** | | |
| Interatrial septum | 14mm OS ASD, L – R Shunt | AO | |  |
| Great arteries | NRGA | LA | |  |
| Aorta | ----- | LVIDd | | mm |
| Pulmonary artery | Normal MPA normal Branch PAs. | LVIDs | | mm |
| Semilunal valves |  | IVSd | | mm |
| Aortic valve | Annulus = 11mm. | IVSs | | mm |
| Pulmonary valve | Annulus = 12mm | LVPWd | | mm |
| Doppler Measurement | | LVPWs | | mm |
| Mitral | ---- | EDV | | ml |
| Aortic | ------ | ESV | | ml |
| Tricuspid | Mild TR, PPG = 47mmHg | FS | | % |
| pulmonic | --------- | LVEF | | % |
| Aortic arch | ------- | **Coronary arteries** | |  |
| PDA | No |  | |  |
| Pericardium/Pleura |  | | | |
| Final Diagnosis | 1. {S, D, S} Levocardia 2. RA/RV Dilated 3. Large OS ASD, L – R Shunt 4. Mild Pulmonary Hypertension 5. Normal LV Function | | | |
| Done By: | **Signature** | | **Date of Reporting** | |
| Dr. Tesfaye Taye,  Pediatrician, Pediatric Cardiologist |  | | **06/04/2013Eth.C** | |

| Pediatric Echocardiography report  Patient Name: B/Nigist Molla. Patient ID: 063318. Gender: Male. Age: 9 Days. | | | | |
| --- | --- | --- | --- | --- |
| Clinical Diagnosis: Incidental Murmur. TGSH4.2870. | | | | |
| Features | **Finding** | **Features** | | **Finding** |
| Profile | | **Atria** | | |
| Abdominal situs | Solitus | Left atrium | | Normal |
| Cardiac position | Levocardia | Right atrium | | Normal |
| Systemic venous drainage | to RA | **Atrioventricular valves** | | |
| Pulmonary venous drainage | to LA | Mitral valve | | Annulus = 11mm. |
| Atrioventricular connection | Concordant | Tricuspid valve | | Annulus = 12mm. |
| Ventriculoarterial connection | Concordant | **Ventricles** | | |
| Ventricular loop | d-Loop | Left ventricle | | Normal |
| Septae | | Right ventricle | | Normal |
| Interventricular septum | 4mm Muscular VSD, L – R Shunt. | **M-Mode:** | | |
| Interatrial septum | PFO, L – R Shunt | AO | |  |
| Great arteries | NRGA | LA | |  |
| Aorta | ----- | LVIDd | | mm |
| Pulmonary artery | Normal MPA normal Branch PAs. | LVIDs | | mm |
| Semilunal valves |  | IVSd | | mm |
| Aortic valve | Annulus = 9mm. | IVSs | | mm |
| Pulmonary valve | Annulus = 10mm | LVPWd | | mm |
| Doppler Measurement | | LVPWs | | mm |
| Mitral | ---- | EDV | | ml |
| Aortic | ------ | ESV | | ml |
| Tricuspid | ------- | FS | | % |
| pulmonic | --------- | LVEF | | % |
| Aortic arch | ------- | **Coronary arteries** | |  |
| PDA | No |  | |  |
| Pericardium/Pleura |  | | | |
| Final Diagnosis | 1. {S, D, S} Levocardia 2. PFO, L – R Shunt 3. Small Muscular VSD, L – R Shunt | | | |
| Done By: | **Signature** | | **Date of Reporting** | |
| Dr. Tesfaye Taye,  Pediatrician, Pediatric Cardiologist |  | | **06/04/2013Eth.C** | |

| Pediatric Echocardiography report  Patient Name: Haile – Gebriel Amare. Patient ID: 063607. Gender: Male. Age: 5/12.  Clinical Diagnosis: Incidental Murmur. TGSH4.2871. | | | | |
| --- | --- | --- | --- | --- |
| Features | **Finding** | **Features** | | **Finding** |
| Profile | | **Atria** | | |
| Abdominal situs | Solitus | Left atrium | | Dilated |
| Cardiac position | Levocardia | Right atrium | | Normal |
| Systemic venous drainage | to RA | **Atrioventricular valves** | | |
| Pulmonary venous drainage | to LA | Mitral valve | | Annulus = 15mm. |
| Atrioventricular connection | Concordant | Tricuspid valve | | Annulus = 9mm. |
| Ventriculoarterial connection | Concordant | **Ventricles** | | |
| Ventricular loop | d-Loop | Left ventricle | | Dilated |
| Septae | | Right ventricle | | Normal |
| Interventricular septum | 11mm Subaortic VSD, BD Shunt predominantly L – R. | **M-Mode:** | | |
| Interatrial septum | Intact | AO | |  |
| Great arteries | NRGA | LA | |  |
| Aorta | ----- | LVIDd | | mm |
| Pulmonary artery | MPA =13mm. Normal Branch PAs. | LVIDs | | mm |
| Semilunal valves |  | IVSd | | mm |
| Aortic valve | Annulus = 13mm. | IVSs | | mm |
| Pulmonary valve | Annulus = 14mm | LVPWd | | mm |
| Doppler Measurement | | LVPWs | | mm |
| Mitral | ---- | EDV | | ml |
| Aortic | ------ | ESV | | ml |
| Tricuspid | ------- | FS | | 32% |
| pulmonic | Mild Valvar PS, PPG = 23mmHg | LVEF | | 62% |
| Aortic arch | ------- | **Coronary arteries** | |  |
| PDA | No |  | |  |
| Pericardium/Pleura |  | | | |
| Final Diagnosis | 1. {S, D, S} Levocardia 2. LA/LV Dilated 3. Large Subaortic VSD 4. Mild Valvar PS 5. Normal LV Function | | | |
| Done By: | **Signature** | | **Date of Reporting** | |
| Dr. Tesfaye Taye,  Pediatrician, Pediatric Cardiologist |  | | **08/04/2013Eth.C** | |

| Pediatric Echocardiography report  Patient Name: Belmaley Demelash. Patient ID: 063541. Gender: Female. Age: 2 Years.  Clinical Diagnosis: CHF + RD. TGSH4.2872. | | | | |
| --- | --- | --- | --- | --- |
| Features | **Finding** | **Features** | | **Finding** |
| Profile | | **Atria** | | |
| Abdominal situs | Solitus | Left atrium | | Hugely Dilated |
| Cardiac position | Levocardia | Right atrium | | Normal |
| Systemic venous drainage | to RA | **Atrioventricular valves** | | |
| Pulmonary venous drainage | to LA | Mitral valve | | Annulus = 19mm. |
| Atrioventricular connection | Concordant | Tricuspid valve | | Annulus = 16mm. |
| Ventriculoarterial connection | Concordant | **Ventricles** | | |
| Ventricular loop | d-Loop | Left ventricle | | Markedly Dilated |
| Septae | | Right ventricle | | Normal |
| Interventricular septum | Intact | **M-Mode:** | | |
| Interatrial septum | Intact | AO | |  |
| Great arteries | NRGA | LA | |  |
| Aorta | ----- | LVIDd | | mm |
| Pulmonary artery | Normal MPA & Branch PAs. | LVIDs | | mm |
| Semilunal valves |  | IVSd | | mm |
| Aortic valve | Annulus = 14mm. | IVSs | | mm |
| Pulmonary valve | Annulus = 14mm | LVPWd | | mm |
| Doppler Measurement | | LVPWs | | mm |
| Mitral | Moderate to severe MR, Holosystolic | EDV | | ml |
| Aortic | ------ | ESV | | ml |
| Tricuspid | Mild TR, PPG = 55mmHg | FS | | 23% |
| pulmonic | --------- | LVEF | | 40% |
| Aortic arch | ------- | **Coronary arteries** | |  |
| PDA | No |  | |  |
| Pericardium/Pleura | 4mm pericardial effusion on RA Side. | | | |
| Final Diagnosis | 1. {S, D, S} Levocardia. 2. LA/LV Dilated 3. Moderate to severe MR, Mild TR 4. Moderate Pulmonary Hypertension 5. Trace Pericardial Effusion | | | |
| Done By: | **Signature** | | **Date of Reporting** | |
| Dr. Tesfaye Taye, Pediatrician, Pediatric Cardiologist | | | **08/04/2013Eth.C** | |

| Pediatric Echocardiography report  Patient Name: Bethlehem Zelalem. Patient ID: 064211. Gender: Female. Age: 7 months.  Clinical Diagnosis: RD + Recurrent Chest Infection + Murmur. TGSH4.2873. | | | | |
| --- | --- | --- | --- | --- |
| Features | **Finding** | **Features** | | **Finding** |
| Profile | | **Atria** | | |
| Abdominal situs | Solitus | Left atrium | | Dilated |
| Cardiac position | Levocardia | Right atrium | | Dilated |
| Systemic venous drainage | to RA | **Atrioventricular valves** | | |
| Pulmonary venous drainage | to LA | Mitral valve | | Annulus = 11mm. |
| Atrioventricular connection | Concordant | Tricuspid valve | | Annulus = 13mm.  TAPSE = 15mm. |
| Ventriculoarterial connection | DORV | **Ventricles** | | |
| Ventricular loop | d-Loop | Left ventricle | | Dilated. |
| Septae | | RV | | Dilated |
| Interventricular septum | 7mm subaortic VSD, L – R Shunt | **M-Mode:** | | |
| Interatrial septum | PFO, L – R Shunt | AO | |  |
| Great arteries | NRGA | LA | |  |
| Aorta | ----- | LVIDd | | mm |
| Pulmonary artery | MPA = 14mm. Normal Branch PAs. | LVIDs | | mm |
| Semilunal valves |  | IVSd | | mm |
| Aortic valve | Annulus = 11mm. | IVSs | | mm |
| Pulmonary valve | Annulus = 14mm | LVPWd | | mm |
| Doppler Measurement | | LVPWs | | mm |
| Mitral | Moderate MR | EDV | | ml |
| Aortic | ------ | ESV | | ml |
| Tricuspid | ------- | FS | | 37% |
| pulmonic | --------- | LVEF | | 69% |
| Aortic arch | Left | **Coronary arteries** | |  |
| PDA | No |  | |  |
| Pericardium/Pleura | 4mm Pericardial effusion on RA/RV Side | | | |
| Final Diagnosis | 1. {S, D, S} Levocardia. 2. DORV 3. All Chambers dilated 4. Moderate Subaortic VSD, L – R Shunt 5. Trace Pericardial effusion 6. Severe Pulmonary Hypertension 7. Normal Biventricular Function | | | |
| Done By: | **Signature** | | **Date of Reporting** | |
| Dr. Tesfaye Taye,  Pediatrician, Pediatric Cardiologist |  | | **12/04/2013Eth.C** | |

| Pediatric Echocardiography report  Patient Name: Yeabsira Mogninet. Patient ID: 064368. Gender: Male. Age: 53days.  Clinical Diagnosis: Cyanosis + RD + Shock. TGSH4.2874. | | | | |
| --- | --- | --- | --- | --- |
| Features | **Finding** | **Features** | | **Finding** |
| Profile | | **Atria** | | |
| Abdominal situs | Solitus | Left atrium | | Normal |
| Cardiac position | Levocardia | Right atrium | | Dilated |
| Systemic venous drainage | to RA | **Atrioventricular valves** | | |
| Pulmonary venous drainage | to LA | Mitral valve | | Atretic |
| Atrioventricular connection | Concordant | Tricuspid valve | | Annulus = 19mm.  TAPSE = 9mm |
| Ventriculoarterial connection | Concordant | **Ventricles** | | |
| Ventricular loop | d-Loop | Left ventricle | | Smallish. |
| Septae | | Right ventricle | | Hypertrophied and dilated. RV TDI S wave = 11cm/sec |
| Interventricular septum | Intact | **M-Mode:** | | |
| Interatrial septum | 10mm OS ASD, BD, Predominantly L – R Shunt | AO | |  |
| Great arteries | NRGA | LA | |  |
| Aorta | ----- | LVIDd | | mm |
| Pulmonary artery | MPA = 12mm. Normal PAs. | LVIDs | | mm |
| Semilunal valves |  | IVSd | | mm |
| Aortic valve | Annulus = 4mm. Hypoplastic | IVSs | | mm |
| Pulmonary valve | Annulus = 12mm | LVPWd | | mm |
| Doppler Measurement | | LVPWs | | mm |
| Mitral | Atretic | EDV | | ml |
| Aortic | ------ | ESV | | ml |
| Tricuspid | Severe TR, PPG = 142mmHg | FS | | % |
| pulmonic | --------- | LVEF | | % |
| Aortic arch | Hypoplastic | **Coronary arteries** | |  |
| PDA | 1mm PDA, R – L Shunt |  | |  |
| Pericardium/Pleura |  | | | |
| Final Diagnosis | 1. {S, D, S} Levocardia. 2. HLHS 3. Large OS ASD, BD Shunt, predominantly L – R Shunt 4. Small PDA, R – L Shunt 5. Severe Pulmonary Hypertension | | | |
| Done By: | **Signature** | | **Date of Reporting** | |
| Dr. Tesfaye Taye, Pediatrician, Pediatric Cardiologist | | | **13/04/2013Eth.C** | |

| Pediatric Echocardiography report  Patient Name: Baby of Yenenesh Enkuahone. Patient ID: 062596. Gender: Male. Age: 17days.  Clinical Diagnosis: RD + Sepsis. TGSH4.2875. | | | | |
| --- | --- | --- | --- | --- |
| Features | **Finding** | **Features** | | **Finding** |
| Profile | | **Atria** | | |
| Abdominal situs | Solitus | Left atrium | | Normal |
| Cardiac position | Levocardia | Right atrium | | Normal |
| Systemic venous drainage | to RA | **Atrioventricular valves** | | |
| Pulmonary venous drainage | to LA | Mitral valve | | Annulus = 9mm. |
| Atrioventricular connection | Concordant | Tricuspid valve | | Annulus = 10mm. |
| Ventriculoarterial connection | Concordant | **Ventricles** | | |
| Ventricular loop | d-Loop | Left ventricle | | Normal |
| Septae | | Right ventricle | | Normal |
| Interventricular septum | Intact | **M-Mode:** | | |
| Interatrial septum | PFO, L – R Shunt | AO | |  |
| Great arteries | NRGA | LA | |  |
| Aorta | ----- | LVIDd | | mm |
| Pulmonary artery | Normal MPA & Branch PAs. | LVIDs | | mm |
| Semilunal valves |  | IVSd | | mm |
| Aortic valve | Annulus = 7mm. | IVSs | | mm |
| Pulmonary valve | Annulus = 7mm | LVPWd | | mm |
| Doppler Measurement | | LVPWs | | mm |
| Mitral | ---- | EDV | | ml |
| Aortic | ------ | ESV | | ml |
| Tricuspid | Trivial TR, PPG = 23mmHg | FS | | % |
| pulmonic | --------- | LVEF | | % |
| Aortic arch | Left. No CoA | **Coronary arteries** | |  |
| PDA | No |  | |  |
| Pericardium/Pleura |  | | | |
| Final Diagnosis | 1. {S, D, S} Levocardia. 2. PFO, L – R Shunt | | | |
| Done By: | **Signature** | | **Date of Reporting** | |
| Dr. Tesfaye Taye, Pediatrician, Pediatric Cardiologist | | | **13/04/2013Eth.C** | |

| Pediatric Echocardiography report  Patient Name: Adbaru Tewubo. Patient ID: _____________. Gender: Male. Age: 2years.  Clinical Diagnosis: Diaphoresis + palpitation. TGSH4.2876. | | | | |
| --- | --- | --- | --- | --- |
| Features | **Finding** | **Features** | | **Finding** |
| Profile | | **Atria** | | |
| Abdominal situs | Solitus | Left atrium | | Normal |
| Cardiac position | Levocardia | Right atrium | | Dilated |
| Systemic venous drainage | to RA | **Atrioventricular valves** | | |
| Pulmonary venous drainage | to LA | Mitral valve | | Annulus = 19mm. |
| Atrioventricular connection | Concordant | Tricuspid valve | | Annulus = 9mm. |
| Ventriculoarterial connection | Concordant | **Ventricles** | | |
| Ventricular loop | d-Loop | Left ventricle | | Normal |
| Septae | | Right ventricle | | Dilated |
| Interventricular septum | ___ | **M-Mode:** | | |
| Interatrial septum | Large OS ASD amounting to single atrium. | AO | |  |
| Great arteries | NRGA | LA | |  |
| Aorta | ----- | LVIDd | | mm |
| Pulmonary artery | Normal MPA & Branch PAs. | LVIDs | | mm |
| Semilunal valves |  | IVSd | | mm |
| Aortic valve | Annulus = 12mm. | IVSs | | mm |
| Pulmonary valve | Annulus = 13mm | LVPWd | | mm |
| Doppler Measurement | | LVPWs | | mm |
| Mitral | ---- | EDV | | ml |
| Aortic | ------ | ESV | | ml |
| Tricuspid | ------- | FS | | % |
| pulmonic | --------- | LVEF | | % |
| Aortic arch | ------- | **Coronary arteries** | |  |
| PDA | No |  | |  |
| Pericardium/Pleura |  | | | |
| Final Diagnosis | 1. {S, D, S} Levocardia. 2. Large OS ASD amounting to Single Atrium | | | |
| Done By: | **Signature** | | **Date of Reporting** | |
| Dr. Tesfaye Taye, Pediatrician, Pediatric Cardiologist | | | **13/04/2013Eth.C** | |

| Pediatric Echocardiography report  Patient Name: Hayat Ibrahim. Patient ID: 064421. Gender: F. Age: 1 8/12.  Clinical Diagnosis: Diaphoresis + DS + RD. TGSH4.2877. | | | | |
| --- | --- | --- | --- | --- |
| Features | **Finding** | **Features** | | **Finding** |
| Profile | | **Atria** | | |
| Abdominal situs | Solitus | Left atrium | | Dilated |
| Cardiac position | Levocardia | Right atrium | | DILATED |
| Systemic venous drainage | to RA | **Atrioventricular valves** | | |
| Pulmonary venous drainage | to LA | Mitral valve | | AVSD |
| Atrioventricular connection | AVSD | Tricuspid valve | | AVSD |
| Ventriculoarterial connection | Concordant | **Ventricles** | | |
| Ventricular loop | d-Loop | Left ventricle | | Dilated |
| Septae : No tongue of tissue in b/n | | Right ventricle | | Dilated |
| Interventricular septum | AVSD, L – R Shunt | **M-Mode:** | | |
| Interatrial septum | AVSD, L – R Shunt. Additional 5mm OS ASD, L – R Shunt | AO | |  |
| Great arteries | NRGA | LA | |  |
| Aorta | ----- | LVIDd | | mm |
| Pulmonary artery | Normal MPA & Branch PAs. | LVIDs | | mm |
| Semilunal valves |  | IVSd | | mm |
| Aortic valve | Annulus = 12mm. | IVSs | | mm |
| Pulmonary valve | Annulus = 16mm | LVPWd | | mm |
| Doppler Measurement | | LVPWs | | mm |
| Mitral | Mild Left AVVR | EDV | | ml |
| Aortic | ------ | ESV | | ml |
| Tricuspid | Trivial Right AVVR | FS | | % |
| pulmonic | --------- | LVEF | | % |
| Aortic arch | ------- | **Coronary arteries** | |  |
| PDA | No |  | |  |
| Pericardium/Pleura |  | | | |
| Final Diagnosis | 1. {S, D, S} Levocardia. 2. Common Complete Balanced AVSD, L – R Shunt 3. Additional OS ASD, L – R Shunt 4. Trivial Right AVVR 5. Mild Left AVVR | | | |
| Done By: | **Signature** | | **Date of Reporting** | |
| Dr. Tesfaye Taye, Pediatrician, Pediatric Cardiologist | | | **13/04/2013Eth.C** | |

| Pediatric Echocardiography report  Patient Name: Simegnew Aynalem. Patient ID: 064518. Gender: M. Age: 4 years.  Clinical Diagnosis: CHF + RD + Murmur. TGSH4.2878. | | | | |
| --- | --- | --- | --- | --- |
| Features | **Finding** | **Features** | | **Finding** |
| Profile | | **Atria** | | |
| Abdominal situs | Solitus | Left atrium | | Normal |
| Cardiac position | Levocardia | Right atrium | | Markedly Dilated |
| Systemic venous drainage | to RA | **Atrioventricular valves** | | |
| Pulmonary venous drainage | to LA | Mitral valve | | Annulus = 14mm. |
| Atrioventricular connection | Concordant | Tricuspid valve | | Annulus = 31mm.  TAPSE = 6mm |
| Ventriculoarterial connection | Concordant | **Ventricles** | | |
| Ventricular loop | d-Loop | Left ventricle | | Normal |
| Septae | | Right ventricle | | Markedly Dilated, Hypertrophied and Dysfunctional |
| Interventricular septum | Intact | **M-Mode:** Normal LV Function on eye balling. | | |
| Interatrial septum | Intact | AO | |  |
| Great arteries | NRGA | LA | |  |
| Aorta | ----- | LVIDd | | mm |
| Pulmonary artery | Normal MPA & Branch PAs. | LVIDs | | mm |
| Semilunal valves |  | IVSd | | mm |
| Aortic valve | Annulus = 16mm. | IVSs | | mm |
| Pulmonary valve | Annulus = 8mm | LVPWd | | mm |
| Doppler Measurement | | LVPWs | | mm |
| Mitral | ---- | EDV | | ml |
| Aortic | ------ | ESV | | ml |
| Tricuspid | Severe TR | FS | | % |
| pulmonic | Severe PS, PPG = 106mmHg | LVEF | | % |
| Aortic arch | ------- | **Coronary arteries** | |  |
| PDA | No |  | |  |
| Pericardium/Pleura | 9mm Pericardial effusion on RA Side. 8mm Right Pleural effusion. | | | |
| Final Diagnosis | 1. {S, D, S} Levocardia. 2. RA/RV Dilated 3. Severe TR 4. Severe Valvar PS 5. Dilated, Hypertrophied and Dysfunctional RV | | | |
| Recommendation | Candidate for BPV | | | |
| Done By: | **Signature** | | **Date of Reporting** | |
| Dr. Tesfaye Taye, Pediatrician, Pediatric Cardiologist | | | **15/04/2013Eth.C** | |

| Pediatric Echocardiography report  Patient Name: Fekadu Sintie. Patient ID: 064093. Gender: M. Age: 1 5/12.  Clinical Diagnosis: Incidental Murmur. TGSH4.2879. | | | | |
| --- | --- | --- | --- | --- |
| Features | **Finding** | **Features** | | **Finding** |
| Profile | | **Atria** | | |
| Abdominal situs | Solitus | Left atrium | | Normal |
| Cardiac position | Levocardia | Right atrium | | Normal |
| Systemic venous drainage | to RA | **Atrioventricular valves** | | |
| Pulmonary venous drainage | to LA | Mitral valve | | Annulus = 13mm. |
| Atrioventricular connection | Concordant | Tricuspid valve | | Annulus = 13mm. |
| Ventriculoarterial connection | Concordant | **Ventricles** | | |
| Ventricular loop | d-Loop | Left ventricle | | Normal |
| Septae | | Right ventricle | | Normal |
| Interventricular septum | Intact | **M-Mode:** Normal LV Function on eye balling. | | |
| Interatrial septum | Intact | AO | |  |
| Great arteries | NRGA | LA | |  |
| Aorta | ----- | LVIDd | | mm |
| Pulmonary artery | Normal MPA & Branch PAs. | LVIDs | | mm |
| Semilunal valves |  | IVSd | | mm |
| Aortic valve | Annulus = 11mm. | IVSs | | mm |
| Pulmonary valve | Annulus = 13mm | LVPWd | | mm |
| Doppler Measurement | | LVPWs | | mm |
| Mitral | ---- | EDV | | ml |
| Aortic | ------ | ESV | | ml |
| Tricuspid | ------- | FS | | % |
| pulmonic | Mild PS, PPG = 35mmHg | LVEF | | % |
| Aortic arch | ------- | **Coronary arteries** | |  |
| PDA | No |  | |  |
| Pericardium/Pleura |  | | | |
| Final Diagnosis | 1. {S, D, S} Levocardia. 2. Mild PS | | | |
| Remark | Only subcostal and apical window. Child was restless | | | |
| Done By: | **Signature** | | **Date of Reporting** | |
| Dr. Tesfaye Taye, Pediatrician, Pediatric Cardiologist | | | **15/04/2013Eth.C** | |

| Pediatric Echocardiography report  Patient Name: Amen Habtamu. Patient ID: 012710. Gender: F. Age: 4 10/12. | | | | |
| --- | --- | --- | --- | --- |
| Clinical Diagnosis | Supraventricular Tachycardia **TGSH1.2629** | | | |
| Features | **Finding** | **Features** | | **Finding** |
| Profile | | **Atria** | | |
| Abdominal situs | Solitus | Left atrium | | Normal |
| Cardiac position | Levocardia | Right atrium | | Normal |
| Systemic venous drainage | to RA | **Atrioventricular valves** | | |
| Pulmonary venous drainage | to LA | Mitral valve | | Annulus = 18mm. |
| Atrioventricular connection | Concordant | Tricuspid valve | | Annulus = 21mm.  TAPSE = 15mm. |
| Ventriculoarterial connection | Concordant | **Ventricles** | | |
| Ventricular loop | d-Loop | Left ventricle | | Normal |
| Septae | | Right ventricle | | Normal |
| Interventricular septum | Intact | **M-Mode:** | | |
| Interatrial septum | Intact | AO | |  |
| Great arteries | NRGA | LA | |  |
| Aorta | ----- | LVIDd | | mm |
| Pulmonary artery | Normal MPA normal Branch PAs. | LVIDs | | mm |
| Semilunal valves |  | IVSd | | mm |
| Aortic valve | Annulus = 14mm. | IVSs | | mm |
| Pulmonary valve | Annulus = 18mm | LVPWd | | mm |
| Doppler Measurement | | LVPWs | | mm |
| Mitral | ---- | EDV | | ml |
| Aortic | ------ | ESV | | ml |
| Tricuspid | ------- | FS | | 32% |
| pulmonic | --------- | LVEF | | 62% |
| Aortic arch | ------- | **Coronary arteries** | |  |
| PDA | No |  | |  |
| Pericardium/Pleura |  | | | |
| Final Diagnosis | 1. Normal Echocardiography Study. | | | |
| Done By: | **Signature** | | **Date of Reporting** | |
| Dr. Tesfaye Taye, Pediatrician, Pediatric Cardiologist | | | **20/04/2013Eth.C** | |

| Pediatric Echocardiography report  Patient Name: Silenat Antehunegn. Patient ID: 064730. Gender: F. Age: 1 10/12.  Clinical Diagnosis: Cyanosis + Murmur. TGSH4.2880. | | | | |
| --- | --- | --- | --- | --- |
| Features | **Finding** | **Features** | | **Finding** |
| Profile | | **Atria** | | |
| Abdominal situs | Solitus | Left atrium | | Normal |
| Cardiac position | Levocardia | Right atrium | | Dilated |
| Systemic venous drainage | to RA | **Atrioventricular valves** | | |
| Pulmonary venous drainage | to LA | Mitral valve | | Annulus = 14mm. |
| Atrioventricular connection | Concordant | Tricuspid valve | | Annulus = 16mm. |
| Ventriculoarterial connection | Concordant | **Ventricles** | | |
| Ventricular loop | d-Loop | Left ventricle | | Normal |
| Septae | | Right ventricle | | Hypertrophied |
| Interventricular septum | 13mm Malaligned Non restrictive sub aortic VSD, Predominantly R – L Shunt | **M-Mode:** | | |
| Interatrial septum | Intact | AO | |  |
| Great arteries | NRGA | LA | |  |
| Aorta | ----- | LVIDd | | mm |
| Pulmonary artery | MPA = 23mm. Branch PAs. | LVIDs | | mm |
| Semilunal valves |  | IVSd | | mm |
| Aortic valve | Annulus = mm. | IVSs | | mm |
| Pulmonary valve | Annulus = 15mm. thickened Pulmonary valve leaflets. | LVPWd | | mm |
| Doppler Measurement | | LVPWs | | mm |
| Mitral | ---- | EDV | | ml |
| Aortic | ------ | ESV | | ml |
| Tricuspid | ------- | FS | | 40% |
| pulmonic | Severe PS, PPG = 63mmHg, Moderate PR, PPG = 38mmHg. | LVEF | | 73% |
| Aortic arch | Left | **Coronary arteries** | |  |
| PDA | No |  | |  |
| Pericardium/Pleura |  | | | |
| Final Diagnosis | 1. {S, D, S} Levocardia. 2. TOF 3. ?Dysplastic Pulmonary Valve | | | |
| Done By: | **Signature** | | **Date of Reporting** | |
| Dr. Tesfaye Taye, Pediatrician, Pediatric Cardiologist | | | **20/04/2013Eth.C** | |

| Pediatric Echocardiography report  Patient Name: Tamralech Walle. Patient ID: 064606. Gender: F. Age: 13years.  Clinical Diagnosis: Hypertension. TGSH4.2881. | | | | |
| --- | --- | --- | --- | --- |
| Features | **Finding** | **Features** | | **Finding** |
| Profile | | **Atria** | | |
| Abdominal situs | Solitus | Left atrium | | Normal |
| Cardiac position | Levocardia | Right atrium | | Normal |
| Systemic venous drainage | to RA | **Atrioventricular valves** | | |
| Pulmonary venous drainage | to LA | Mitral valve | | Annulus = 24mm. |
| Atrioventricular connection | Concordant | Tricuspid valve | | Annulus = 21mm. |
| Ventriculoarterial connection | Concordant | **Ventricles** | | |
| Ventricular loop | d-Loop | Left ventricle | | Dilated and Hypertrophied |
| Septae | | Right ventricle | | Normal |
| Interventricular septum | Intact | **M-Mode:** | | |
| Interatrial septum | Intact | AO | |  |
| Great arteries | NRGA | LA | |  |
| Aorta | ----- | LVIDd | | 44mm |
| Pulmonary artery | Normal MPA & Branch PAs. | LVIDs | | 31mm |
| Semilunal valves |  | IVSd | | 8mm |
| Aortic valve | Annulus = 17mm. | IVSs | | 10mm |
| Pulmonary valve | Annulus = 20mm | LVPWd | | 8mm |
| Doppler Measurement | | LVPWs | | 9mm |
| Mitral | Trivial MR | EDV | | 86ml |
| Aortic | ------ | ESV | | 38ml |
| Tricuspid | ------- | FS | | 29% |
| pulmonic | Mild PR, PPG = 10mmHg | LVEF | | 56% |
| Aortic arch | No CoA | **Coronary arteries** | |  |
| PDA | No |  | |  |
| Pericardium/Pleura | Trace pericardial effusion on RV Side (2mm) | | | |
| Final Diagnosis | 1. {S, D, S} Levocardia. 2. LV Dilated 3. LVH 4. Normal LV Function | | | |
| Done By: | **Signature** | | **Date of Reporting** | |
| Dr. Tesfaye Taye, Pediatrician, Pediatric Cardiologist | | | **20/04/2013Eth.C** | |

| Pediatric Echocardiography report  Patient Name: Baby of Melkam Addis (twin B). Patient ID: 063524. Gender: F. Age: 19days.  Clinical Diagnosis: Incidental Murmur. TGSH4.2882. | | | | |
| --- | --- | --- | --- | --- |
| Features | **Finding** | **Features** | | **Finding** |
| Profile | | **Atria** | | |
| Abdominal situs | Solitus | Left atrium | | Normal |
| Cardiac position | Levocardia | Right atrium | | Normal |
| Systemic venous drainage | to RA | **Atrioventricular valves** | | |
| Pulmonary venous drainage | to LA | Mitral valve | | Annulus = 9mm. |
| Atrioventricular connection | Concordant | Tricuspid valve | | Annulus = 9mm. |
| Ventriculoarterial connection | Concordant | **Ventricles** | | |
| Ventricular loop | d-Loop | Left ventricle | | Normal |
| Septae | | Right ventricle | | Normal |
| Interventricular septum | Intact | **M-Mode: Normal LV Function** | | |
| Interatrial septum | 4mm OS ASD, L – R Shunt | AO | |  |
| Great arteries | NRGA | LA | |  |
| Aorta | ----- | LVIDd | | mm |
| Pulmonary artery | Normal MPA normal Branch PAs. | LVIDs | | mm |
| Semilunal valves |  | IVSd | | mm |
| Aortic valve | Annulus = 8mm. | IVSs | | mm |
| Pulmonary valve | Annulus = 9mm | LVPWd | | mm |
| Doppler Measurement | | LVPWs | | mm |
| Mitral | ---- | EDV | | ml |
| Aortic | ------ | ESV | | ml |
| Tricuspid | ------- | FS | | % |
| pulmonic | --------- | LVEF | | % |
| Aortic arch | ------- | **Coronary arteries** | |  |
| PDA | 1mm PDA, L – R Shunt |  | |  |
| Pericardium/Pleura |  | | | |
| Final Diagnosis | 1. {S, D, S} Levocardia. 2. Small OS ASD, L – R Shunt 3. Small PDA, L – R Shunt 4. Normal LV Function | | | |
| Done By: | **Signature** | | **Date of Reporting** | |
| Dr. Tesfaye Taye, Pediatrician, Pediatric Cardiologist | | | **20/04/2013Eth.C** | |

| Pediatric Echocardiography report  Patient Name: Solomie Getachew. Patient ID: 008216. Gender: F. Age: 1 7/12. | | | | |
| --- | --- | --- | --- | --- |
| Follow up Echo | Small PM VSD (3.5mm) done before a year. (TGSH7 & **TGSH1.2468.**) | | | |
| Features | **Finding** | **Features** | | **Finding** |
| Profile | | **Atria** | | |
| Abdominal situs | Solitus | Left atrium | | Normal |
| Cardiac position | Levocardia | Right atrium | | Normal |
| Systemic venous drainage | to RA | **Atrioventricular valves** | | |
| Pulmonary venous drainage | to LA | Mitral valve | | Annulus = 14mm. |
| Atrioventricular connection | Concordant | Tricuspid valve | | Annulus = 14mm. |
| Ventriculoarterial connection | Concordant | **Ventricles** | | |
| Ventricular loop | d-Loop | Left ventricle | | Normal |
| Septae | | Right ventricle | | Normal |
| Interventricular septum | 2mm PM VSD, L – R Shunt | **M-Mode:** | | |
| Interatrial septum | Intact | AO | |  |
| Great arteries | NRGA | LA | |  |
| Aorta | ----- | LVIDd | | mm |
| Pulmonary artery | Normal MPA normal Branch PAs. | LVIDs | | mm |
| Semilunal valves |  | IVSd | | mm |
| Aortic valve | Annulus = 11mm. | IVSs | | mm |
| Pulmonary valve | Annulus = 12mm | LVPWd | | mm |
| Doppler Measurement | | LVPWs | | mm |
| Mitral | ---- | EDV | | ml |
| Aortic | ------ | ESV | | ml |
| Tricuspid | Trivial TR | FS | | 36% |
| pulmonic | --------- | LVEF | | 68% |
| Aortic arch | ------- | **Coronary arteries** | |  |
| PDA | No |  | |  |
| Pericardium/Pleura |  | | | |
| Final Diagnosis | 1. {S, D, S} Levocardia. 2. Small Perimembranous VSD, L – R Shunt 3. Normal LV Function | | | |
| Done By: | **Signature** | | **Date of Reporting** | |
| Dr. Tesfaye Taye, Pediatrician, Pediatric Cardiologist | | | **20/04/2013Eth.C** | |

| Pediatric Echocardiography report  Patient Name: Sofonias Desalegn. Patient ID: 066024. Gender: M. Age: 80days.  AGH2.070 | | | | |
| --- | --- | --- | --- | --- |
| Features | **Finding** | **Features** | | **Finding** |
| Profile | | **Atria** | | |
| Abdominal situs | Solitus | Left atrium | | Normal |
| Cardiac position | Levocardia | Right atrium | | Normal |
| Systemic venous drainage | to RA | **Atrioventricular valves** | | |
| Pulmonary venous drainage | to LA | Mitral valve | | Annulus = 12mm. |
| Atrioventricular connection | Concordant | Tricuspid valve | | Annulus = 13mm. |
| Ventriculoarterial connection | Discordant | **Ventricles** | | |
| Ventricular loop | d-Loop | Left ventricle | | Normal |
| Septae | | Right ventricle | | Normal |
| Interventricular septum | 7mm Inlet VSD with PM extension, BD Shunt. | **M-Mode: Normal LV Function** | | |
| Interatrial septum | 5mm OS ASD, L – R Shunt | AO | |  |
| Great arteries | d-TGA | LA | |  |
| Aorta | From RV, anterior and to the right | LVIDd | | mm |
| Pulmonary artery | From LV, Posterior and to the left | LVIDs | | mm |
| Semilunal valves |  | IVSd | | mm |
| Aortic valve | Annulus = 13mm. | IVSs | | mm |
| Pulmonary valve | Annulus = 10mm | LVPWd | | mm |
| Doppler Measurement | | LVPWs | | mm |
| Mitral | ---- | EDV | | ml |
| Aortic | ------ | ESV | | ml |
| Tricuspid | ------- | FS | | % |
| pulmonic | --------- | LVEF | | % |
| Aortic arch | ------- | **Coronary arteries** | |  |
| PDA | No |  | |  |
| Pericardium/Pleura |  | | | |
| Final Diagnosis | 1. {S, D, D} Levocardia 2. Small OS ASD, L – R Shunt 3. Moderate Inlet VSD with PM extension, BD Shunt 4. d-TGA. 5. Normal LV Function | | | |
| Done By: | **Signature** | | **Date of Reporting** | |
| Dr. Tesfaye Taye, Pediatrician, Pediatric Cardiologist | | | **27/04/2013Eth.C** | |

| Pediatric Echocardiography report  Patient Name: Gashanew Birhan. Patient ID: 066084. Gender: M. Age: 13years.  Clinical Diagnosis: Rheumatic Recurrence + DOE + Palpitation + CHF + Murmur. TGSH4.2883. | | | | |
| --- | --- | --- | --- | --- |
| Features | **Finding** | **Features** | | **Finding** |
| Profile | | **Atria** | | |
| Abdominal situs | Solitus | Left atrium | | Dilated. 85mm X 56mm |
| Cardiac position | Levocardia | Right atrium | | Dilated |
| Systemic venous drainage | to RA | **Atrioventricular valves** | | |
| Pulmonary venous drainage | to LA | Mitral valve | | Annulus = 24mm. Non coapting MVL, Thickened, Shortened PML. Mildly clubbed MVL. MVA = 1.02cm^2^. |
| Atrioventricular connection | Concordant | Tricuspid valve | | Annulus = 23mm.  TAPSE = 19mm. |
| Ventriculoarterial connection | Concordant | **Ventricles** | | |
| Ventricular loop | d-Loop | Left ventricle | | Dilated |
| Septae | | Right ventricle | | Dilated |
| Interventricular septum | Intact | **M-Mode:** | | |
| Interatrial septum | Intact | AO | |  |
| Great arteries | NRGA | LA | |  |
| Aorta | ----- | LVIDd | | mm |
| Pulmonary artery | MPA =28mm. | LVIDs | | mm |
| Semilunal valves |  | IVSd | | mm |
| Aortic valve | Annulus = 14mm. | IVSs | | mm |
| Pulmonary valve | Annulus = 26mm | LVPWd | | mm |
| Doppler Measurement | | LVPWs | | mm |
| Mitral | Severe MR, Holosystolic, posterior projection with Jet velocity = 3.7m/sec. Moderate MS, PPG/MPG = 16/8mmHg. | EDV | | ml |
| Aortic | Moderate AR, PHT = 283ms. | ESV | | ml |
| Tricuspid | Moderate TR, PPG = 56 | FS | | 30% |
| pulmonic | Mild PR, PPG = 42mmHg | LVEF | | 56% |
| Aortic arch | ------- | **Coronary arteries** | |  |
| PDA | No |  | |  |
| Pericardium/Pleura | 21mm Right Pleural Effusion. 8mm Left Pleural Effusion. 8mm Pericardial Effusion on RV Side. | | | |
| Final Diagnosis | 1. {S, D, S} Levocardia. 2. All chambers dilated 3. Thickened, Clubbed MVL 4. Severe MR 5. Moderate MS 6. Moderate AR 7. Moderate TR 8. Mild PR 9. Moderate Pulmonary Hypertension 10. Mild Pericardial Effusion 11. Large Pleural Effusion | | | |
| Done By: | **Signature** | | **Date of Reporting** | |
| Dr. Tesfaye Taye, Pediatrician, Pediatric Cardiologist | | | **27/04/2013Eth.C** | |

| Pediatric Echocardiography report  Patient Name: Gizenew Belay. Patient ID: 0521238. Gender: M. Age: 11years.  Clinical Diagnosis: CHF + RD + DOE + Easy Fatigability. TGSH4.2884. | | | | |
| --- | --- | --- | --- | --- |
| Features | **Finding** | **Features** | | **Finding** |
| Profile | | **Atria** | | |
| Abdominal situs | Solitus | Left atrium | | Dilated |
| Cardiac position | Levocardia | Right atrium | | Dilated |
| Systemic venous drainage | to RA. IVC Dilated | **Atrioventricular valves** | | |
| Pulmonary venous drainage | to LA | Mitral valve | | Annulus = 16mm. E/A = 1.8 |
| Atrioventricular connection | Concordant | Tricuspid valve | | Annulus = 20mm.  TAPSE = 10mm. |
| Ventriculoarterial connection | Concordant | **Ventricles** | | |
| Ventricular loop | d-Loop | Left ventricle | | N & Dysfunctional |
| Septae : abnormal Septal Motion | | Right ventricle | | Normal |
| Interventricular septum | Intact | **M-Mode:** | | |
| Interatrial septum | Intact | AO | |  |
| Great arteries | NRGA | LA | |  |
| Aorta | ----- | LVIDd | | mm |
| Pulmonary artery | Normal MPA & Branch PAs. | LVIDs | | mm |
| Semilunal valves |  | IVSd | | mm |
| Aortic valve | Annulus = 13mm. | IVSs | | mm |
| Pulmonary valve | Annulus = 18mm | LVPWd | | mm |
| Doppler Measurement | | LVPWs | | mm |
| Mitral | ---- | EDV | | ml |
| Aortic | ------ | ESV | | ml |
| Tricuspid | ------- | FS | | 23% |
| pulmonic | --------- | LVEF | | 48% |
| Aortic arch | ------- | **Coronary arteries** | |  |
| PDA | No |  | |  |
| Pericardium/Pleura | Thickened Pericardium with trace pericardial effusion | | | |
| Final Diagnosis | 1. {S, D, S} Levocardia. 2. Biventricular Systolic Dysfunction 3. Severe Diastolic Dysfunction 4. Thickened Pericardium with trace pericardial effusion | | | |
| Remark | Constrictive pericarditis is highly likely | | | |
| Recommendation | Would Benefit from pericardiectomy | | | |
| Done By: | **Signature** | | **Date of Reporting** | |
| Dr. Tesfaye Taye, Pediatrician, Pediatric Cardiologist | | | **20/04/2013Eth.C** | |

| Pediatric Echocardiography report  Patient Name: Mengistu Aleka. Patient ID: 066051. Gender: M. Age: 14years.  Clinical Diagnosis: RD + CHF + DOE. TGSH4.2885. | | | | |
| --- | --- | --- | --- | --- |
| Features | **Finding** | **Features** | | **Finding** |
| Profile: Swinging Heart. | | **Atria** | | |
| Abdominal situs | Solitus | Left atrium | | Normal |
| Cardiac position | Levocardia | Right atrium | | Normal. Late Diastolic collapse |
| Systemic venous drainage | to RA. IVC Dilated. | **Atrioventricular valves** | | |
| Pulmonary venous drainage | to LA | Mitral valve | | Annulus = 24mm. |
| Atrioventricular connection | Concordant | Tricuspid valve | | Annulus = 21mm.  TAPSE = 19mm. |
| Ventriculoarterial connection | Concordant | **Ventricles** | | |
| Ventricular loop | d-Loop | Left ventricle | | Normal |
| Septae | | Right ventricle | | Normal. Early Diastolic collapse |
| Interventricular septum | Intact | **M-Mode:** | | |
| Interatrial septum | Intact | AO | |  |
| Great arteries | NRGA | LA | |  |
| Aorta | ----- | LVIDd | | mm |
| Pulmonary artery | Normal MPA normal Branch PAs. | LVIDs | | mm |
| Semilunal valves |  | IVSd | | mm |
| Aortic valve | Annulus = 26mm. | IVSs | | mm |
| Pulmonary valve | Annulus = 26mm | LVPWd | | mm |
| Doppler Measurement | | LVPWs | | mm |
| Mitral | ---- | EDV | | ml |
| Aortic | ------ | ESV | | ml |
| Tricuspid | Trivial TR, PPG = 34mmHg | FS | | 41% |
| pulmonic | --------- | LVEF | | 71% |
| Aortic arch | ------- | **Coronary arteries** | |  |
| PDA | No |  | |  |
| Pericardium/Pleura | Circumferential Pericardial Effusion; 22mm on LV Side, 27mm on RA Side & 28mm on RV Side. Echo debris inside. | | | |
| Final Diagnosis | 1. {S, D, S} Levocardia. 2. Large Pericardial effusion with echo debris inside | | | |
|  | Cardiac Tamponade | | | |
| Done By: | **Signature** | | **Date of Reporting** | |
| Dr. Tesfaye Taye, Pediatrician, Pediatric Cardiologist | | | **27/04/2013Eth.C** | |

| Pediatric Echocardiography report  Patient Name: Baby of Haymanot Andargie . Patient ID: 063705. Gender: F. Age: 16days.  Clinical Diagnosis: RD. TGSH4.2886. | | | | |
| --- | --- | --- | --- | --- |
| Features | **Finding** | **Features** | | **Finding** |
| Profile | | **Atria** | | |
| Abdominal situs | Solitus | Left atrium | | Normal |
| Cardiac position | Levocardia | Right atrium | | Normal |
| Systemic venous drainage | to RA | **Atrioventricular valves** | | |
| Pulmonary venous drainage | to LA | Mitral valve | | Annulus = 12mm. |
| Atrioventricular connection | Concordant | Tricuspid valve | | Annulus = 11mm. |
| Ventriculoarterial connection | Concordant | **Ventricles** | | |
| Ventricular loop | d-Loop | Left ventricle | | Normal |
| Septae | | Right ventricle | | Normal |
| Interventricular septum | Intact | **M-Mode:** | | |
| Interatrial septum | PFO, L – R Shunt | AO | |  |
| Great arteries | NRGA | LA | |  |
| Aorta | ----- | LVIDd | | mm |
| Pulmonary artery | Normal MPA & Branch PAs. | LVIDs | | mm |
| Semilunal valves |  | IVSd | | mm |
| Aortic valve | Annulus = 9mm. | IVSs | | mm |
| Pulmonary valve | Annulus = 9mm | LVPWd | | mm |
| Doppler Measurement | | LVPWs | | mm |
| Mitral | ---- | EDV | | ml |
| Aortic | ------ | ESV | | ml |
| Tricuspid | ------- | FS | | % |
| pulmonic | --------- | LVEF | | % |
| Aortic arch | ------- | **Coronary arteries** | |  |
| PDA | No |  | |  |
| Pericardium/Pleura |  | | | |
| Final Diagnosis | 1. {S, D, S} Levocardia. 2. PFO, L – R Shunt | | | |
| Done By: | **Signature** | | **Date of Reporting** | |
| Dr. Tesfaye Taye, Pediatrician, Pediatric Cardiologist | | | **27/04/2013Eth.C** | |

| Pediatric Echocardiography report  Patient Name: Lulit Gebrye. Patient ID: 066711. Gender: F. Age: 9years.  Clinical Diagnosis: CHF + Rheumatic Recurrence + IE + Easy Fatigability + Palpitation. TGSH4.2887. | | | | |
| --- | --- | --- | --- | --- |
| Features | **Finding** | **Features** | | **Finding** |
| Profile | | **Atria** | | |
| Abdominal situs | Solitus | Left atrium | | Dilated. Echogenic mass above the posterior MVL. |
| Cardiac position | Levocardia | Right atrium | | Normal |
| Systemic venous drainage | to RA | **Atrioventricular valves** | | |
| Pulmonary venous drainage | to LA | MV | | Annulus = 33mm. Freely moving echogenic mass at the tips of the MVLs. |
| Atrioventricular connection | Concordant | TV | | Annulus = 21mm. TAPSE = 20mm. |
| Ventriculoarterial connection | Concordant | **Ventricles** | | |
| Ventricular loop | d-Loop | Left ventricle | | Dilated |
| Septae | | Right ventricle | | Normal |
| Interventricular septum | Intact | **M-Mode:** | | |
| Interatrial septum | Intact | AO | |  |
| Great arteries | NRGA | LA | |  |
| Aorta | ----- | LVIDd | | mm |
| Pulmonary artery | Normal MPA & Branch PAs. | LVIDs | | mm |
| Semilunal valves |  | IVSd | | mm |
| Aortic valve | Annulus = 16mm. | IVSs | | mm |
| Pulmonary valve | Annulus = 23mm | LVPWd | | mm |
| Doppler Measurement | | LVPWs | | mm |
| Mitral | Severe MR, Holosystolic, posterior projection seen in two planes with jet velocity of 5.1m/sec. | EDV | | ml |
| Aortic | ------ | ESV | | ml |
| Tricuspid | Trivial TR | FS | | 39% |
| pulmonic | --------- | LVEF | | 69% |
| Aortic arch | ------- | **Coronary arteries** | |  |
| PDA | No |  | |  |
| Pericardium/Pleura | 10mm pericardial effusion on RV Side | | | |
| Final Diagnosis | 1. {S, D, S} Levocardia. 2. LA/LV Dilated 3. Severe MR 4. ? Infective Endocarditis 5. Normal Biventricular Function | | | |
| Done By: | **Signature** | | **Date of Reporting** | |
| Dr. Tesfaye Taye, Pediatrician, Pediatric Cardiologist | | | **04/05/2013Eth.C** | |

| Pediatric Echocardiography report  Patient Name: _Baby of Anguach Tigabu. Patient ID: 065765. Gender: M. Age: 13days. (TGSH3.2759) RD + Murmur | | | | |
| --- | --- | --- | --- | --- |
| Features | **Finding** | **Features** | | **Finding** |
| Profile | | **Atria** | | |
| Abdominal situs | Solitus | Left atrium | | Normal |
| Cardiac position | Levocardia | Right atrium | | Normal |
| Systemic venous drainage | to RA | **Atrioventricular valves** | | |
| Pulmonary venous drainage | to LA | Mitral valve | | Annulus = 11mm. |
| Atrioventricular connection | Concordant | Tricuspid valve | | Annulus = 13mm. |
| Ventriculoarterial connection | Concordant | **Ventricles** | | |
| Ventricular loop | d-Loop | Left ventricle | | Normal |
| Septae | | Right ventricle | | Normal |
| Interventricular septum | 9mm Subaortic VSD, L – R Shunt | **M-Mode:** | | |
| Interatrial septum | PFO, L – R Shunt | AO | |  |
| Great arteries | NRGA | LA | |  |
| Aorta | ----- | LVIDd | | mm |
| Pulmonary artery | Normal MPA normal Branch PAs. | LVIDs | | mm |
| Semilunal valves |  | IVSd | | mm |
| Aortic valve | Annulus = 11mm. | IVSs | | mm |
| Pulmonary valve | Annulus = 12mm | LVPWd | | mm |
| Doppler Measurement | | LVPWs | | mm |
| Mitral | ---- | EDV | | ml |
| Aortic | ------ | ESV | | ml |
| Tricuspid | ------- | FS | | 36% |
| pulmonic | --------- | LVEF | | 68% |
| Aortic arch | ------- | **Coronary arteries** | |  |
| PDA | 1mm PDA, L – R Shunt |  | |  |
| Pericardium/Pleura |  | | | |
| Final Diagnosis | 1. {S, D, S} Levocardia. 2. PFO, L – R Shunt 3. Large Subaortic VSD, L – R Shunt 4. Small PDA, L – R Shunt (Silent) 5. Normal LV Function | | | |
| Done By: | **Signature** | | **Date of Reporting** | |
| Dr. Tesfaye Taye, Pediatrician, Pediatric Cardiologist | | | **04/05/2013Eth.C** | |

| Pediatric Echocardiography report  Patient Name: Amanuel Yirdaw. Patient ID: 064728. Gender: M. Age: 4years.  Clinical Diagnosis: Easy Fatigability + Murmur + FTT. TGSH4.2888. | | | | |
| --- | --- | --- | --- | --- |
| Features | **Finding** | **Features** | | **Finding** |
| Profile | | **Atria** | | |
| Abdominal situs | Solitus | Left atrium | | Normal |
| Cardiac position | Levocardia | Right atrium | | Dilated |
| Systemic venous drainage | to RA | **Atrioventricular valves** | | |
| Pulmonary venous drainage | to LA | Mitral valve | | Annulus = 13mm. |
| Atrioventricular connection | Concordant | Tricuspid valve | | Annulus = 20mm. |
| Ventriculoarterial connection | Concordant | **Ventricles** | | |
| Ventricular loop | d-Loop | Left ventricle | | Normal |
| Septae | | Right ventricle | | Dilated |
| Interventricular septum | Intact | **M-Mode:** Normal LV Function on eye balling. | | |
| Interatrial septum | 10mm Fenestrated OS ASD, L – R Shunt | AO | |  |
| Great arteries | NRGA | LA | |  |
| Aorta | ----- | LVIDd | | mm |
| Pulmonary artery | Normal MPA normal Branch PAs. | LVIDs | | mm |
| Semilunal valves |  | IVSd | | mm |
| Aortic valve | Annulus = 16mm. | IVSs | | mm |
| Pulmonary valve | Annulus = 15mm | LVPWd | | mm |
| Doppler Measurement | | LVPWs | | mm |
| Mitral | ---- | EDV | | ml |
| Aortic | ------ | ESV | | ml |
| Tricuspid | ------- | FS | | % |
| pulmonic | --------- | LVEF | | % |
| Aortic arch | ------- | **Coronary arteries** | |  |
| PDA | No |  | |  |
| Pericardium/Pleura |  | | | |
| Final Diagnosis | 1. {S, D, S} Levocardia. 2. RA/RV Dilated 3. Moderate Fenestrated OS ASD, L – R Shunt 4. Normal Function | | | |
| Done By: | **Signature** | | **Date of Reporting** | |
| Dr. Tesfaye Taye, Pediatrician, Pediatric Cardiologist | | | **04/05/2013Eth.C** | |

| Pediatric Echocardiography report  Patient Name: Baby of Mastewal Werku. Patient ID: 062024. Gender: M. Age: 44days.  Clinical Diagnosis: RD. TGSH4.2889. | | | | |
| --- | --- | --- | --- | --- |
| Features | **Finding** | **Features** | | **Finding** |
| Profile | | **Atria** | | |
| Abdominal situs | Solitus | Left atrium | | Normal |
| Cardiac position | Levocardia | Right atrium | | Normal |
| Systemic venous drainage | to RA | **Atrioventricular valves** | | |
| Pulmonary venous drainage | to LA | Mitral valve | | Annulus = 9mm. |
| Atrioventricular connection | Concordant | Tricuspid valve | | Annulus = 12mm. |
| Ventriculoarterial connection | Concordant | **Ventricles** | | |
| Ventricular loop | d-Loop | Left ventricle | | Normal |
| Septae | | Right ventricle | | Normal |
| Interventricular septum | Intact | **M-Mode:** Normal LV Function on eye balling. | | |
| Interatrial septum | PFO, L – R Shunt | AO | |  |
| Great arteries | NRGA | LA | |  |
| Aorta | ----- | LVIDd | | mm |
| Pulmonary artery | Normal MPA & Branch PAs. | LVIDs | | mm |
| Semilunal valves |  | IVSd | | mm |
| Aortic valve | Annulus = 9mm. | IVSs | | mm |
| Pulmonary valve | Annulus = 9mm | LVPWd | | mm |
| Doppler Measurement | | LVPWs | | mm |
| Mitral | ---- | EDV | | ml |
| Aortic | ------ | ESV | | ml |
| Tricuspid | ------- | FS | | % |
| pulmonic | --------- | LVEF | | % |
| Aortic arch | ------- | **Coronary arteries** | |  |
| PDA | No |  | |  |
| Pericardium/Pleura |  | | | |
| Final Diagnosis | 1. {S, D, S} Levocardia. 2. PFO, L – R Shunt | | | |
| Done By: | **Signature** | | **Date of Reporting** | |
| Dr. Tesfaye Taye, Pediatrician, Pediatric Cardiologist | | | **04/05/2013Eth.C** | |

| Pediatric Echocardiography report  Patient Name: Baby of Birknesh Tibebu. Patient ID: 066444. Gender: F. Age: 23days.  Clinical Diagnosis: Incidental Murmur. TGSH4.2890. | | | | |
| --- | --- | --- | --- | --- |
| Features | **Finding** | **Features** | | **Finding** |
| Profile | | **Atria** | | |
| Abdominal situs | Solitus | Left atrium | | Normal |
| Cardiac position | Levocardia | Right atrium | | Normal |
| Systemic venous drainage | to RA | **Atrioventricular valves** | | |
| Pulmonary venous drainage | to LA | Mitral valve | | Annulus = 11mm. |
| Atrioventricular connection | Concordant | Tricuspid valve | | Annulus = 12mm. |
| Ventriculoarterial connection | Concordant | **Ventricles** | | |
| Ventricular loop | d-Loop | Left ventricle | | Normal |
| Septae | | Right ventricle | | Normal |
| Interventricular septum | 6mm Inlet VSD, L – R Shunt | **M-Mode:** Normal LV Function on eye balling. | | |
| Interatrial septum | PFO, L – R Shunt | AO | |  |
| Great arteries | NRGA | LA | |  |
| Aorta | ----- | LVIDd | | mm |
| Pulmonary artery | Normal MPA normal Branch PAs. | LVIDs | | mm |
| Semilunal valves |  | IVSd | | mm |
| Aortic valve | Annulus = 10mm. | IVSs | | mm |
| Pulmonary valve | Annulus = 10mm | LVPWd | | mm |
| Doppler Measurement | | LVPWs | | mm |
| Mitral | ---- | EDV | | ml |
| Aortic | ------ | ESV | | ml |
| Tricuspid | ------- | FS | | % |
| pulmonic | --------- | LVEF | | % |
| Aortic arch | ------- | **Coronary arteries** | |  |
| PDA | No |  | |  |
| Pericardium/Pleura |  | | | |
| Final Diagnosis | 1. {S, D, S} Levocardia. 2. PFO, L – R Shunt 3. Inlet VSD, L – R Shunt 4. Normal LV Function | | | |
| Done By: | **Signature** | | **Date of Reporting** | |
| Dr. Tesfaye Taye, Pediatrician, Pediatric Cardiologist | | | **04/05/2013Eth.C** | |

| Pediatric Echocardiography report  Patient Name: Yezina Tadie. Patient ID: 065178. Gender: F. Age: 8years.  Clinical Diagnosis: ARF. TGSH4.2891. | | | | |
| --- | --- | --- | --- | --- |
| Features | **Finding** | **Features** | | **Finding** |
| Profile | | **Atria** | | |
| Abdominal situs | Solitus | Left atrium | | Dilated |
| Cardiac position | Levocardia | Right atrium | | Normal |
| Systemic venous drainage | to RA | **Atrioventricular valves** | | |
| Pulmonary venous drainage | to LA | Mitral valve | | Annulus = 20mm. Thickened MVL. |
| Atrioventricular connection | Concordant | Tricuspid valve | | Annulus = 21mm.  TAPSE = 17mm. |
| Ventriculoarterial connection | Concordant | **Ventricles** | | |
| Ventricular loop | d-Loop | Left ventricle | | Dilated |
| Septae | | Right ventricle | | Normal |
| Interventricular septum | Intact | **M-Mode:** | | |
| Interatrial septum | Intact | AO | |  |
| Great arteries | NRGA | LA | |  |
| Aorta | ----- | LVIDd | | mm |
| Pulmonary artery | Normal MPA & Branch PAs. | LVIDs | | mm |
| Semilunal valves |  | IVSd | | mm |
| Aortic valve | Annulus = 14mm. | IVSs | | mm |
| Pulmonary valve | Annulus = 18mm | LVPWd | | mm |
| Doppler Measurement | | LVPWs | | mm |
| Mitral | Moderate MR, Holosystolic, posterior projection seen in two planes with jet velocity = 4.4m/sec. | EDV | | ml |
| Aortic | ------ | ESV | | ml |
| Tricuspid | Mild TR, PPG = 31mmHg | FS | | 33% |
| pulmonic | Trivial PR, PPG = 15mmHg | LVEF | | 62% |
| Aortic arch | ------- | **Coronary arteries** | |  |
| PDA | No |  | |  |
| Pericardium/Pleura |  | | | |
| Final Diagnosis | 1. {S, D, S} Levocardia. 2. LA/LV Dilated 3. Thickened MVL 4. Moderate MR 5. Mild TR 6. Normal Biventricular Function | | | |
| Done By: | **Signature** | | **Date of Reporting** | |
| Dr. Tesfaye Taye, Pediatrician, Pediatric Cardiologist | | | **06/05/2013Eth.C** | |

| Pediatric Echocardiography report  Patient Name: Tamralech Yadam. Patient ID: 066717. Gender: F. Age: 2 6/12.  Clinical Diagnosis: DS + Diaphoresis + RD + Murmur. TGSH4.2892. | | | | |
| --- | --- | --- | --- | --- |
| Features | **Finding** | **Features** | | **Finding** |
| Profile | | **Atria** | | |
| Abdominal situs | Solitus | Left atrium | | Normal |
| Cardiac position | Levocardia | Right atrium | | Normal |
| Systemic venous drainage | to RA | **Atrioventricular valves** | | |
| Pulmonary venous drainage | to LA | Mitral valve | | Annulus = mm. |
| Atrioventricular connection | Concordant | Tricuspid valve | | Annulus = mm. |
| Ventriculoarterial connection | Concordant | **Ventricles** | | |
| Ventricular loop | d-Loop | Left ventricle | | Normal |
| Septae | | Right ventricle | | Normal |
| Interventricular septum | Small Inlet VSD | **M-Mode:** Normal LV Function on eye balling. | | |
| Interatrial septum | 15mm Primum ASD, L – R Shunt | AO | |  |
| Great arteries | NRGA | LA | |  |
| Aorta | ----- | LVIDd | | mm |
| Pulmonary artery | Normal MPA normal Branch PAs. | LVIDs | | mm |
| Semilunal valves |  | IVSd | | mm |
| Aortic valve | Annulus = 11mm. | IVSs | | mm |
| Pulmonary valve | Annulus = 17mm | LVPWd | | mm |
| Doppler Measurement | | LVPWs | | mm |
| Mitral | Moderate MR | EDV | | ml |
| Aortic | ------ | ESV | | ml |
| Tricuspid | Moderate TR | FS | | % |
| pulmonic | --------- | LVEF | | % |
| Aortic arch | ------- | **Coronary arteries** | |  |
| PDA | No |  | |  |
| Pericardium/Pleura |  | | | |
| Final Diagnosis | 1. {S, D, S} Levocardia. 2. Transitional AVSD, L – R Shunt 3. Moderate MR 4. Moderate TR 5. Normal LV Function | | | |
| Done By: | **Signature** | | **Date of Reporting** | |
| Dr. Tesfaye Taye, Pediatrician, Pediatric Cardiologist | | | **06/05/2013Eth.C** | |

| Pediatric Echocardiography report  Patient Name: Seada Mussa. Patient ID: ___________. Gender: F. Age: 12Years.  Clinical Diagnosis: DOE + Murmur + CHF + Rheumatic Recurrence + Palpitation. TGSH4.2893. | | | | |
| --- | --- | --- | --- | --- |
| Features | **Finding** | **Features** | | **Finding** |
| Profile | | **Atria** | | |
| Abdominal situs | Solitus | Left atrium | | More Dilated |
| Cardiac position | Levocardia | Right atrium | | Dilated |
| Systemic venous drainage | to RA | **Atrioventricular valves** | | |
| Pulmonary venous drainage | to LA | Mitral valve | | Annulus = 26mm. thickened MVL. |
| Atrioventricular connection | Concordant | Tricuspid valve | | Annulus = 23mm.TAPSE = 14mm. |
| Ventriculoarterial connection | Concordant | **Ventricles** | | |
| Ventricular loop | d-Loop | Left ventricle | | More Dilated |
| Septae | | Right ventricle | | Dilated. RV TDI S Wave = 9cm/sec. |
| Interventricular septum | Intact | **M-Mode:**. | | |
| Interatrial septum | Intact | AO | |  |
| Great arteries | NRGA | LA | |  |
| Aorta | ----- | LVIDd | | mm |
| Pulmonary artery | MPA = 26mm. | LVIDs | | mm |
| Semilunal valves |  | IVSd | | mm |
| Aortic valve | Annulus = 14mm. | IVSs | | mm |
| Pulmonary valve | Annulus = 25mm | LVPWd | | mm |
| Doppler Measurement | | LVPWs | | mm |
| Mitral | Severe MR, Holosystolic, posterior projection, seen in two planes with jet velocity = 5.2m/sec. | EDV | | ml |
| Aortic | Mild AR | ESV | | ml |
| Tricuspid | Severe TR, PPG = 60mmHg | FS | | 20% |
| pulmonic | --------- | LVEF | | 41% |
| PDA | No |  | |  |
| Pericardium/Pleura | Trace pericardial effusion on RA/RV Side | | | |
| Final Diagnosis | 1. {S, D, S} Levocardia. 2. All chambers dilated 3. Thickened MVL 4. Severe MR 5. Severe TR 6. Severe Pulmonary Hypertension 7.Trace Pericardial effusion 7. Reduced Biventricular Function | | | |
| Done By: | **Signature** | | **Date of Reporting** | |
| Dr. Tesfaye Taye, Pediatrician, Pediatric Cardiologist | | | **06/05/2013Eth.C** | |

| Pediatric Echocardiography report  Patient Name: Habtam Tadesse. Patient ID: 023476. Gender: F. Age: 12Years.  Clinical Diagnosis: ARF + Murmur. TGSH4.2894. | | | | |
| --- | --- | --- | --- | --- |
| Features | **Finding** | **Features** | | **Finding** |
| Profile | | **Atria** | | |
| Abdominal situs | Solitus | Left atrium | | Normal |
| Cardiac position | Levocardia | Right atrium | | Normal |
| Systemic venous drainage | to RA | **Atrioventricular valves** | | |
| Pulmonary venous drainage | to LA | Mitral valve | | Annulus = 23mm. Mildly thickened MVL |
| Atrioventricular connection | Concordant | Tricuspid valve | | Annulus = 26mm.  TAPSE = 16mm. |
| Ventriculoarterial connection | Concordant | **Ventricles** | | |
| Ventricular loop | d-Loop | Left ventricle | | Normal |
| Septae | | Right ventricle | | Normal |
| Interventricular septum | Intact | **M-Mode:**. | | |
| Interatrial septum | Intact | AO | |  |
| Great arteries | NRGA | LA | |  |
| Aorta | ----- | LVIDd | | mm |
| Pulmonary artery | Normal MPA & Branch PAs. | LVIDs | | mm |
| Semilunal valves |  | IVSd | | mm |
| Aortic valve | Annulus = 17mm. | IVSs | | mm |
| Pulmonary valve | Annulus = 19mm | LVPWd | | mm |
| Doppler Measurement | | LVPWs | | mm |
| Mitral | Mild MR, Holosystolic, Posterior Projection, Seen In Two Planes With Jet Velocity = 4.7m/sec | EDV | | ml |
| Aortic | ------ | ESV | | ml |
| Tricuspid | Trivial TR, PPG = 26mmHg | FS | | % |
| pulmonic | --------- | LVEF | | % |
| Aortic arch | ------- | **Coronary arteries** | |  |
| PDA | No |  | |  |
| Pericardium/Pleura |  | | | |
| Final Diagnosis | 1. {S, D, S} Levocardia. 2. Mildly thickened MVL 3. Mild MR 4. Normal Function | | | |
| Done By: | **Signature** | | **Date of Reporting** | |
| Dr. Tesfaye Taye, Pediatrician, Pediatric Cardiologist | | | **13/05/2013Eth.C** | |

| Pediatric Echocardiography report  Patient Name: Mengistu Aleka. Patient ID: 066051. Gender: _M. Age: 14Years.  Clinical Diagnosis: Sepsis + pericardial effusion. TGSH4.2895. | | | | |
| --- | --- | --- | --- | --- |
|  | | | | |
| Features | **Finding** | **Features** | | **Finding** |
| Profile | | **Atria** | | |
| Abdominal situs | Solitus | Left atrium | | Normal |
| Cardiac position | Levocardia | Right atrium | | Normal |
| Systemic venous drainage | to RA | **Atrioventricular valves** | | |
| Pulmonary venous drainage | to LA | Mitral valve | | Annulus = 23mm. |
| Atrioventricular connection | Concordant | Tricuspid valve | | Annulus = 29mm. |
| Ventriculoarterial connection | Concordant | **Ventricles** | | |
| Ventricular loop | d-Loop | Left ventricle | | Normal |
| Septae | | Right ventricle | | Normal |
| Interventricular septum | Intact | **M-Mode:**. | | |
| Interatrial septum | Intact | AO | |  |
| Great arteries | NRGA | LA | |  |
| Aorta | ----- | LVIDd | | mm |
| Pulmonary artery | Normal MPA & Branch PAs. | LVIDs | | mm |
| Semilunal valves |  | IVSd | | mm |
| Aortic valve | Annulus = 22mm. | IVSs | | mm |
| Pulmonary valve | Annulus = 25mm | LVPWd | | mm |
| Doppler Measurement | | LVPWs | | mm |
| Mitral | ------- | EDV | | ml |
| Aortic | ------ | ESV | | ml |
| Tricuspid | -------- | FS | | 21% |
| pulmonic | --------- | LVEF | | 42% |
| Aortic arch | ------- | **Coronary arteries** | |  |
| PDA | 15mm Large communication b/n MPA and Descending aorta, L – R Shunt | | | |
| Pericardium/Pleura | Large Septated circumferential Pericardial effusion (22mm on LV Side, 22mm on RA Side & 24mm on RV Side) | | | |
| Final Diagnosis | 1. {S, D, S} Levocardia. 2. Large Septated Pericardial effusion (? Pyogenic) 3. Large MPA to Descending Aorta fistula, L – R Shunt 2^0^ to ? + communication with the pericardium 4. Reduced LV Function | | | |
| Recommendation | **Refer for surgical evaluation and management.** | | | |
| Done By: | **Signature** | | **Date of Reporting** | |
| Dr. Tesfaye Taye, Pediatrician, Pediatric Cardiologist | | | **06/05/2013Eth.C** | |

| Pediatric Echocardiography report  Patient Name: Baby of Jemila Siraj. Patient ID: 060795. Gender: F. Age: 65days.  Clinical Diagnosis: Incidental Murmur. TGSH4.2896 | | | | |
| --- | --- | --- | --- | --- |
| Features | **Finding** | **Features** | | **Finding** |
| Profile | | **Atria** | | |
| Abdominal situs | Solitus | Left atrium | | Normal |
| Cardiac position | Levocardia | Right atrium | | Normal |
| Systemic venous drainage | to RA | **Atrioventricular valves** | | |
| Pulmonary venous drainage | to LA | Mitral valve | | Annulus = 10mm. |
| Atrioventricular connection | Concordant | Tricuspid valve | | Annulus = 9mm. |
| Ventriculoarterial connection | Concordant | **Ventricles** | | |
| Ventricular loop | d-Loop | Left ventricle | | Normal |
| Septae | | Right ventricle | | Normal |
| Interventricular septum | Intact | **M-Mode:**. | | |
| Interatrial septum | Intact | AO | |  |
| Great arteries | NRGA | LA | |  |
| Aorta | ----- | LVIDd | | mm |
| Pulmonary artery | Normal MPA & Branch PAs. | LVIDs | | mm |
| Semilunal valves |  | IVSd | | mm |
| Aortic valve | Annulus = 10mm. | IVSs | | mm |
| Pulmonary valve | Annulus = 11mm | LVPWd | | mm |
| Doppler Measurement | | LVPWs | | mm |
| Mitral | ------- | EDV | | ml |
| Aortic | ------ | ESV | | ml |
| Tricuspid | -------- | FS | | % |
| pulmonic | --------- | LVEF | | % |
| Aortic arch | ------- | **Coronary arteries** | |  |
| PDA | 1mm PDA, L – R Shunt |  | |  |
| Pericardium/Pleura |  | | | |
| Final Diagnosis | 1. {S, D, S} Levocardia. 2. Small PDA, L – R Shunt 3. Normal Function | | | |
| Done By: | **Signature** | | **Date of Reporting** | |
| Dr. Tesfaye Taye, Pediatrician, Pediatric Cardiologist | | | **13/05/2013Eth.C** | |

| Pediatric Echocardiography report  Patient Name: Hiwet Astewul. Patient ID: 066404. Gender: F. Age: 15days.  Clinical Diagnosis: Incidental Murmur. TGSH4.2897. | | | | |
| --- | --- | --- | --- | --- |
| Features | **Finding** | **Features** | | **Finding** |
| Profile | | **Atria** | | |
| Abdominal situs | Solitus | Left atrium | | Normal |
| Cardiac position | Levocardia | Right atrium | | Normal |
| Systemic venous drainage | to RA | **Atrioventricular valves** | | |
| Pulmonary venous drainage | to LA | Mitral valve | | Annulus = mm. |
| Atrioventricular connection | Concordant | Tricuspid valve | | Annulus = mm. |
| Ventriculoarterial connection | Concordant | **Ventricles** | | |
| Ventricular loop | d-Loop | Left ventricle | | Normal |
| Septae | | Right ventricle | | Normal |
| Interventricular septum | Intact | **M-Mode:**. | | |
| Interatrial septum | Intact | AO | |  |
| Great arteries | NRGA | LA | |  |
| Aorta | ----- | LVIDd | | mm |
| Pulmonary artery | Normal MPA & Branch PAs. | LVIDs | | mm |
| Semilunal valves |  | IVSd | | mm |
| Aortic valve | Annulus = mm. | IVSs | | mm |
| Pulmonary valve | Annulus = mm | LVPWd | | mm |
| Doppler Measurement | | LVPWs | | mm |
| Mitral | ------- | EDV | | ml |
| Aortic | ------ | ESV | | ml |
| Tricuspid | -------- | FS | | % |
| pulmonic | --------- | LVEF | | % |
| Aortic arch | ------- | **Coronary arteries** | |  |
| PDA | 1.5mm PDA, L – R Shunt |  | |  |
| Pericardium/Pleura |  | | | |
| Final Diagnosis | 1. {. 2. Small PDA, L – R Shunt 3. Good Function | | | |
| Remark | Only subcoastal and parasternal short axis view | | | |
| Done By: | **Signature** | | **Date of Reporting** | |
| Dr. Tesfaye Taye, Pediatrician, Pediatric Cardiologist | | | **13/05/2013Eth.C** | |

| Pediatric Echocardiography report  Patient Name: Amet Bal Kindu. Patient ID: 067462. Gender: F. Age: 1Year.  Clinical Diagnosis: DS + RD + Murmur + CHF. TGSH4.2898. | | | | |
| --- | --- | --- | --- | --- |
| Features | **Finding** | **Features** | | **Finding** |
| Profile | | **Atria** | | |
| Abdominal situs | Solitus | Left atrium | | Normal |
| Cardiac position | Levocardia | Right atrium | | Dilated |
| Systemic venous drainage | to RA | **Atrioventricular valves** | | |
| Pulmonary venous drainage | to LA | Mitral valve | | Annulus = 13mm. |
| Atrioventricular connection | Concordant | Tricuspid valve | | Annulus = 20mm.  TAPSE = 15mm. |
| Ventriculoarterial connection | Concordant | **Ventricles** | | |
| Ventricular loop | d-Loop | Left ventricle | | Normal |
| Septae | | Right ventricle | | Dilated |
| Interventricular septum | 6mm Inlet VSD, L – R Shunt | **M-Mode:**. | | |
| Interatrial septum | 16mm Primum ASD, L – R Shunt. Additional 9mm OS ASD, L – R Shunt | AO | |  |
| Great arteries | NRGA | LA | |  |
| Aorta | ----- | LVIDd | | mm |
| Pulmonary artery | Normal MPA & Branch PAs. | LVIDs | | mm |
| Semilunal valves |  | IVSd | | mm |
| Aortic valve | Annulus = 10mm. | IVSs | | mm |
| Pulmonary valve | Annulus = 13mm | LVPWd | | mm |
| Doppler Measurement | | LVPWs | | mm |
| Mitral | ------- | EDV | | ml |
| Aortic | ------ | ESV | | ml |
| Tricuspid | Severe TR | FS | | % |
| pulmonic | --------- | LVEF | | % |
| Aortic arch | ------- | **Coronary arteries** | |  |
| PDA | 2.5mm PDA, L – R Shunt |  | |  |
| Pericardium/Pleura | Circumferential Pericardial effusion (Max. 10mm on RA/RV Side) | | | |
| Final Diagnosis | 1. {S, D, S} Levocardia. 2. Intermediate AVSD, L – R Shunt 3. Additional Moderate OS ASD, L – R Shunt 4. Large PDA, L – R Shunt 5. Moderate Pericardial effusion 6. Pulmonary Hypertension | | | |
| Done By: | **Signature** | | **Date of Reporting** | |
| Dr. Tesfaye Taye, Pediatrician, Pediatric Cardiologist | | | **13/05/2013Eth.C** | |

| Pediatric Echocardiography report  Patient Name: Mekdes Animut. Patient ID: 049561. Gender: F. Age: 9 6/12.  Clinical Diagnosis: Rheumatic Recurrence + Murmur + Palpitation. TGSH4.2899. | | | | |
| --- | --- | --- | --- | --- |
|  | | | | |
| Features | **Finding** | **Features** | | **Finding** |
| Profile | | **Atria** | | |
| Abdominal situs | Solitus | Left atrium | | Dilated |
| Cardiac position | Levocardia | Right atrium | | Normal |
| Systemic venous drainage | to RA | **Atrioventricular valves** | | |
| Pulmonary venous drainage | to LA | Mitral valve | | Annulus = 28mm. Mildly thickened MVL |
| Atrioventricular connection | Concordant | Tricuspid valve | | Annulus = 19mm.  TAPSE = 18mm. |
| Ventriculoarterial connection | Concordant | **Ventricles** | | |
| Ventricular loop | d-Loop | Left ventricle | | Dilated |
| Septae | | Right ventricle | | Normal |
| Interventricular septum | Intact | **M-Mode:**. | | |
| Interatrial septum | Intact | AO | |  |
| Great arteries | NRGA | LA | |  |
| Aorta | ----- | LVIDd | | mm |
| Pulmonary artery | Normal MPA & Branch PAs. | LVIDs | | mm |
| Semilunal valves |  | IVSd | | mm |
| Aortic valve | Annulus = 20mm. | IVSs | | mm |
| Pulmonary valve | Annulus = 22mm | LVPWd | | mm |
| Doppler Measurement | | LVPWs | | mm |
| Mitral | Severe MR, Holosystolic, jet velocity = 5m/sec | EDV | | ml |
| Aortic | ------ | ESV | | ml |
| Tricuspid | -------- | FS | | 43% |
| pulmonic | Trivial PR, PPG = 22mmHg. | LVEF | | 74% |
| Aortic arch | ------- | **Coronary arteries** | |  |
| PDA | No |  | |  |
| Pericardium/Pleura |  | | | |
| Final Diagnosis | 1. {S, D, S} Levocardia. 2. LA/LV Dilated 3. Severe MR 4. Mildly thickened MVL 5. Normal Function | | | |
| Done By: | **Signature** | | **Date of Reporting** | |
| Dr. Tesfaye Taye, Pediatrician, Pediatric Cardiologist | | | **18/05/2013Eth.C** | |

| Pediatric Echocardiography report  Patient Name: Werkneh Alene. Patient ID: 067094. Gender: F. Age: 5 6/12.  Clinical Diagnosis: ARF + Murmur. TGSH4.2900. | | | | |
| --- | --- | --- | --- | --- |
| Features | **Finding** | **Features** | | **Finding** |
| Profile | | **Atria** | | |
| Abdominal situs | Solitus | Left atrium | | Dilated |
| Cardiac position | Levocardia | Right atrium | | Normal |
| Systemic venous drainage | to RA | **Atrioventricular valves** | | |
| Pulmonary venous drainage | to LA | Mitral valve | | Annulus = 25mm. Mildly thickened |
| Atrioventricular connection | Concordant | Tricuspid valve | | Annulus = 22mm.  TAPSE = 19mm. |
| Ventriculoarterial connection | Concordant | **Ventricles** | | |
| Ventricular loop | d-Loop | Left ventricle | | Dilated |
| Septae | | Right ventricle | | Normal |
| Interventricular septum | Intact | **M-Mode:**. | | |
| Interatrial septum | Intact | AO | |  |
| Great arteries | NRGA | LA | |  |
| Aorta | ----- | LVIDd | | mm |
| Pulmonary artery | Normal MPA & Branch PAs. | LVIDs | | mm |
| Semilunal valves |  | IVSd | | mm |
| Aortic valve | Annulus = 19mm. | IVSs | | mm |
| Pulmonary valve | Annulus = 18mm | LVPWd | | mm |
| Doppler Measurement | | LVPWs | | mm |
| Mitral | Moderate MR, Holosystolic seen in two planes with jet velocity = 4.3m/sec | EDV | | ml |
| Aortic | ------ | ESV | | ml |
| Tricuspid | -------- | FS | | 30% |
| pulmonic | --------- | LVEF | | 58% |
| Aortic arch | ------- | **Coronary arteries** | |  |
| PDA | No |  | |  |
| Pericardium/Pleura |  | | | |
| Final Diagnosis | 1. {S, D, D} Levocardia. 2. LA/LV Dilated 3. Mildly thickened MVL 4. Moderate MR 5. Normal Function | | | |
| Done By: | **Signature** | | **Date of Reporting** | |
| Dr. Tesfaye Taye, Pediatrician, Pediatric Cardiologist | | | **18/05/2013Eth.C** | |

| Pediatric Echocardiography report  Patient Name: Baby of Yekitie Dagnaw. Patient ID: 067874. Gender: F. Age: 6days.  Clinical Diagnosis: Cyanosis + Murmur + DS. TGSH4.2901. | | | | |
| --- | --- | --- | --- | --- |
| Features | **Finding** | **Features** | | **Finding** |
| Profile | | **Atria** | | |
| Abdominal situs | Solitus | Left atrium | | Normal |
| Cardiac position | Levocardia | Right atrium | | Dilated |
| Systemic venous drainage | to RA | **Atrioventricular valves** | | |
| Pulmonary venous drainage | to LA | Mitral valve | | Common Complete AVSD, predominantly , L – R shunt |
| Atrioventricular connection | Concordant | Tricuspid valve | |  |
| Ventriculoarterial connection | DORV | **Ventricles** | | |
| Ventricular loop | d-Loop | Left ventricle | | Smallish |
| Septae | | Right ventricle | | Dilated |
| Interventricular septum | Common Complete AVSD, predominantly , L – R shunt | **M-Mode:**. | | |
| Interatrial septum |  | AO | |  |
| Great arteries | NRGA | LA | |  |
| Aorta | ----- | LVIDd | | mm |
| Pulmonary artery | Normal MPA & Branch PAs. | LVIDs | | mm |
| Semilunal valves |  | IVSd | | mm |
| Aortic valve | Annulus = 10mm. | IVSs | | mm |
| Pulmonary valve | Annulus = 11mm | LVPWd | | mm |
| Doppler Measurement | | LVPWs | | mm |
| Mitral | ------- | EDV | | ml |
| Aortic | ------ | ESV | | ml |
| Tricuspid | -------- | FS | | % |
| pulmonic | --------- | LVEF | | % |
| Aortic arch | ------- | **Coronary arteries** | |  |
| PDA | No |  | |  |
| Pericardium/Pleura |  | | | |
| Final Diagnosis | 1. {S, D, S} Levocardia. 2. DORV 3. RA/RV Dilated 4. Common Complete Unbalanced AVSD, L – R Shunt | | | |
| Done By: | **Signature** | | **Date of Reporting** | |
| Dr. Tesfaye Taye, Pediatrician, Pediatric Cardiologist | | | **18/05/2013Eth.C** | |

| Pediatric Echocardiography report  Patient Name: Baby of Birtukan Ayisheshim. Patient ID: 067212. Gender: M. Age: 10days.  Clinical Diagnosis: Incidental Murmur. TGSH4.2902. | | | | |
| --- | --- | --- | --- | --- |
| Features | **Finding** | **Features** | | **Finding** |
| Profile | | **Atria** | | |
| Abdominal situs | Solitus | Left atrium | | Normal |
| Cardiac position | Levocardia | Right atrium | | Dilated |
| Systemic venous drainage | to RA | **Atrioventricular valves** | | |
| Pulmonary venous drainage | to LA | Mitral valve | | Annulus = 9mm. |
| Atrioventricular connection | Concordant | Tricuspid valve | | Annulus = 13mm. |
| Ventriculoarterial connection | Concordant | **Ventricles** | | |
| Ventricular loop | d-Loop | Left ventricle | | Normal |
| Septae | | Right ventricle | | Dilated, Hypertrophied |
| Interventricular septum | 7mm Inlet VSD, Predominantly L- R Shunt | **M-Mode:**. | | |
| Interatrial septum | 5mm OS ASD, L – R Shunt | AO | |  |
| Great arteries | NRGA | LA | |  |
| Aorta | ----- | LVIDd | | mm |
| Pulmonary artery | Normal MPA & Branch PAs. | LVIDs | | mm |
| Semilunal valves |  | IVSd | | mm |
| Aortic valve | Annulus = 10mm. | IVSs | | mm |
| Pulmonary valve | Annulus = 8mm. Dysplastic. Post stenotic dilatation | LVPWd | | mm |
| Doppler Measurement | | LVPWs | | mm |
| Mitral | ------- | EDV | | ml |
| Aortic | ------ | ESV | | ml |
| Tricuspid | -------- | FS | | % |
| pulmonic | Severe Valvar PS, PPG = 65mmHg. Mild to moderate PR | LVEF | | % |
| Aortic arch | ------- | **Coronary arteries** | |  |
| PDA | 2mm PDA, L – R Shunt |  | |  |
| Final Diagnosis | 1. {S, D, S} Levocardia. 2. RA/RV Dilated 3. Small OS ASD, L – R Shunt 4. Moderate Inlet VSD, L – R Shunt 5. Moderate PDA, L – R Shunt 6. Severe Valvar PS 7. ? Dysplastic Pulmonary Valve | | | |
| Done By: | **Signature** | | **Date of Reporting** | |
| Dr. Tesfaye Taye, Pediatrician, Pediatric Cardiologist | | | **18/05/2013Eth.C** | |

| Pediatric Echocardiography report  Patient Name: Baby of Genet. Patient ID: 067928. Gender: M. Age: 5days.  Clinical Diagnosis: Incidental Murmur + RD. TGSH4.2903. | | | | |
| --- | --- | --- | --- | --- |
| Features | **Finding** | **Features** | | **Finding** |
| Profile | | **Atria** | | |
| Abdominal situs | Solitus | Left atrium | | Normal |
| Cardiac position | Levocardia | Right atrium | | Dilated |
| Systemic venous drainage | to RA | **Atrioventricular valves** | | |
| Pulmonary venous drainage | to LA | Mitral valve | | Annulus = 9mm. |
| Atrioventricular connection | Concordant | Tricuspid valve | | Annulus = 9mm. |
| Ventriculoarterial connection | Concordant | **Ventricles** | | |
| Ventricular loop | d-Loop | Left ventricle | | Normal |
| Septae | | Right ventricle | | Dilated |
| Interventricular septum | Intact | **M-Mode:**. | | |
| Interatrial septum | 6mm OS ASD, R – L Shunt | AO | |  |
| Great arteries | NRGA | LA | |  |
| Aorta | ----- | LVIDd | | mm |
| Pulmonary artery | Normal MPA & Branch PAs. | LVIDs | | mm |
| Semilunal valves |  | IVSd | | mm |
| Aortic valve | Annulus = 6mm. | IVSs | | mm |
| Pulmonary valve | Annulus = 8mm | LVPWd | | mm |
| Doppler Measurement | | LVPWs | | mm |
| Mitral | ------- | EDV | | ml |
| Aortic | ------ | ESV | | ml |
| Tricuspid | -------- | FS | | % |
| pulmonic | --------- | LVEF | | % |
| Aortic arch | ------- | **Coronary arteries** | |  |
| PDA | 1.5mm PDA, Predominantly R – L Shunt | | | |
| Pericardium/Pleura |  | | | |
| Final Diagnosis | 1. {S, D, S} Levocardia. 2. Small OS ASD, R – L Shunt 3. Small PDA, Predominantly R – L Shunt | | | |
| Remark | Consider in the line of PPHN. | | | |
| Done By: | **Signature** | | **Date of Reporting** | |
| Dr. Tesfaye Taye, Pediatrician, Pediatric Cardiologist | | | **20/05/2013Eth.C** | |

| Pediatric Echocardiography report  Patient Name: Dawit Tegegne. Patient ID: 011492. Gender: M. Age: 4 3/12Years.  Clinical Diagnosis: Murmur + RD + ARF. TGSH4.2904. (TGSH11) | | | | |
| --- | --- | --- | --- | --- |
| Features | **Finding** | **Features** | | **Finding** |
| Profile | | **Atria** | | |
| Abdominal situs | Solitus | Left atrium | | Dilated |
| Cardiac position | Levocardia | Right atrium | | Normal |
| Systemic venous drainage | to RA | **Atrioventricular valves** | | |
| Pulmonary venous drainage | to LA | Mitral valve | | Annulus = 26mm. Thickened MVL. |
| Atrioventricular connection | Concordant | Tricuspid valve | | Annulus = 24mm.  TAPSE = 15mm |
| Ventriculoarterial connection | Concordant | **Ventricles** | | |
| Ventricular loop | d-Loop | Left ventricle | | Dilated |
| Septae | | Right ventricle | | Normal |
| Interventricular septum | Intact | **M-Mode:**. | | |
| Interatrial septum | Intact | AO | |  |
| Great arteries | NRGA | LA | |  |
| Aorta | ----- | LVIDd | | mm |
| Pulmonary artery | Normal MPA & Branch PAs. | LVIDs | | mm |
| Semilunal valves |  | IVSd | | mm |
| Aortic valve | Annulus = 15mm. | IVSs | | mm |
| Pulmonary valve | Annulus = 19mm | LVPWd | | mm |
| Doppler Measurement | | LVPWs | | mm |
| Mitral | Moderate MR, Holosystolic, Posterior projection, seen in two planes with jet velocity = 5.3m/sec | EDV | | ml |
| Aortic | Mild AR | ESV | | ml |
| Tricuspid | Trivial TR, PPG = 23mmHg | FS | | 32% |
| pulmonic | --------- | LVEF | | 60% |
| Aortic arch | ------- | **Coronary arteries** | |  |
| PDA | No |  | |  |
| Final Diagnosis | 1. {S, D, S} Levocardia. 2. LA/LV Dilated 3. Moderate MR 4. Mild AR 5. Normal Biventricular Function | | | |
| Remark | Significant improvement | | | |
| Done By: | **Signature** | | **Date of Reporting** | |
| Dr. Tesfaye Taye, Pediatrician, Pediatric Cardiologist | | | **27/05/2013Eth.C** | |

| Pediatric Echocardiography report  Patient Name: Surafel Getachew. Patient ID: 069169. Gender: M. Age: 2 5/12.  Clinical Diagnosis: Incidental Murmur. TGSH4.2905 | | | | |
| --- | --- | --- | --- | --- |
| Features | **Finding** | **Features** | | **Finding** |
| Profile | | **Atria** | | |
| Abdominal situs | Solitus | Left atrium | | Normal |
| Cardiac position | Levocardia | Right atrium | | Dilated |
| Systemic venous drainage | to RA | **Atrioventricular valves** | | |
| Pulmonary venous drainage | to LA | Mitral valve | | Annulus = 15mm. |
| Atrioventricular connection | Concordant | Tricuspid valve | | Annulus = 19mm.  TAPSE = 14mm |
| Ventriculoarterial connection | Concordant | **Ventricles** | | |
| Ventricular loop | d-Loop | Left ventricle | | Normal |
| Septae | | Right ventricle | | Dilated and Hypertrophied. RV TDI S Wave = 10cm/sec |
| Interventricular septum | Intact | **M-Mode:**. | | |
| Interatrial septum | Intact | AO | |  |
| Great arteries | NRGA | LA | |  |
| Aorta | ----- | LVIDd | | mm |
| Pulmonary artery | Normal MPA & BPAs. | LVIDs | | mm |
| Semilunal valves |  | IVSd | | mm |
| Aortic valve | Annulus = 11mm. | IVSs | | mm |
| Pulmonary valve | Annulus = 16mm. Doming PV. | LVPWd | | mm |
| Doppler Measurement | | LVPWs | | mm |
| Mitral | ------- | EDV | | ml |
| Aortic | ------ | ESV | | ml |
| Tricuspid | -------- | FS | | 35% |
| pulmonic | Severe Valvar PS, PPG = 100mmHg. | LVEF | | 68% |
| Aortic arch | ------- | **Coronary arteries** | |  |
| PDA | No |  | |  |
| Pericardium/Pleura | Trace Pericardial effusion/ Apical Area | | | |
| Final Diagnosis | 1. {S, D, S} Levocardia. 2. RA/RV Dilated 3. Severe Valvar PS 4. Doming Pulmonary Valve 5. Normal Biventricular Function | | | |
| Done By: | **Signature** | | **Date of Reporting** | |
| Dr. Tesfaye Taye, Pediatrician, Pediatric Cardiologist | | | **27/05/2013Eth.C** | |

| Pediatric Echocardiography report  Patient Name: Yeabsira Embiale. Patient ID: ___________. Gender: F. Age: 2 8/12.  Clinical Diagnosis: RD + Distant heart sound + Cardiomegaly on CXR. TGSH4.2906. | | | | |
| --- | --- | --- | --- | --- |
| Features | **Finding** | **Features** | | **Finding** |
| Profile | | **Atria** | | |
| Abdominal situs | Solitus | Left atrium | | Normal |
| Cardiac position | Levocardia | Right atrium | | Late Diastolic RV Collapse |
| Systemic venous drainage | to RA | **Atrioventricular valves** | | |
| Pulmonary venous drainage | to LA | Mitral valve | | Annulus = 13mm. |
| Atrioventricular connection | Concordant | Tricuspid valve | | Annulus = 14mm. |
| Ventriculoarterial connection | Concordant | **Ventricles** | | |
| Ventricular loop | d-Loop | Left ventricle | | Normal |
| Septae : Abnormal septal motion | | Right ventricle | | No early Diastolic RV Collapse |
| Interventricular septum | Intact | **M-Mode:**. | | |
| Interatrial septum | Intact | AO | |  |
| Great arteries | NRGA | LA | |  |
| Aorta | ----- | LVIDd | | mm |
| Pulmonary artery | Normal MPA normal Branch PAs. | LVIDs | | mm |
| Semilunal valves |  | IVSd | | mm |
| Aortic valve | Annulus = 12mm. | IVSs | | mm |
| Pulmonary valve | Annulus = 13mm | LVPWd | | mm |
| Doppler Measurement | | LVPWs | | mm |
| Mitral | Mitral inflow velocity variability with respiration > 40% | EDV | | ml |
| Aortic | No features of Pulsus paradoxus | ESV | | ml |
| Tricuspid | -------- | FS | | 35% |
| pulmonic | --------- | LVEF | | 67% |
| Aortic arch | ------- | **Coronary arteries** | |  |
| PDA | No |  | |  |
| Pericardium/Pleura | Pericardial effusion 20mm on LV Side, 16mm on RA Side and 13m on RV side, clear.  Swinging Heart | | | |
| Final Diagnosis | 1. {S, D, S} Levocardia. 2. Large Pericardial effusion with some features of Cardiac Tamponade. 3. Normal LV Function | | | |
| Done By: | **Signature** | | **Date of Reporting** | |
| Dr. Tesfaye Taye, Pediatrician, Pediatric Cardiologist | | | **28/05/2013Eth.C** | |

| Pediatric Echocardiography report  Patient Name: Kalkidan Tazeb. Patient ID: 069053. Gender: F. Age: 7Years.  Clinical Diagnosis: Rheumatic Recurrence + CHF + RD + Murmur. TGSH4.2907. | | | | |
| --- | --- | --- | --- | --- |
| Features | **Finding** | **Features** | | **Finding** |
| Profile | | **Atria** | | |
| Abdominal situs | Solitus | Left atrium | | Very Dilated |
| Cardiac position | Levocardia | Right atrium | | Dilated |
| Systemic venous drainage | to RA | **Atrioventricular valves** | | |
| Pulmonary venous drainage | to LA | Mitral valve | | Annulus = 25mm. Thickened MVL. Shortened PMVL, Non coapting MVL |
| Atrioventricular connection | Concordant | Tricuspid valve | | Annulus = 23mm. |
| Ventriculoarterial connection | Concordant | **Ventricles** | | |
| Ventricular loop | d-Loop | Left ventricle | | Very Dilated |
| Septae | | Right ventricle | | Dilated |
| Interventricular septum | Intact | **M-Mode:**. | | |
| Interatrial septum | Intact | AO | |  |
| Great arteries | NRGA | LA | |  |
| Aorta | ----- | LVIDd | | mm |
| Pulmonary artery | Normal MPA normal Branch PAs. | LVIDs | | mm |
| Semilunal valves |  | IVSd | | mm |
| Aortic valve | Annulus = 15mm. | IVSs | | mm |
| Pulmonary valve | Annulus = 19mm | LVPWd | | mm |
| Doppler Measurement | | LVPWs | | mm |
| Mitral | Severe MR, Holosystolic, posterior projection, seen in two planes with jet velocity = 4.6m/sec. | EDV | | ml |
| Aortic | Moderate AR, PHT = 366ms. | ESV | | ml |
| Tricuspid | Severe TR, PPG = 66mmHg | FS | | 29% |
| pulmonic | Moderate PR, PPG = 58mmHg | LVEF | | 56% |
| Pericardium/Pleura | 4mm Pericardial effusion on RA Side. | | | |
| Final Diagnosis | 1. {S, D, S} Levocardia. 2. All Chambers 3. Thickened, Non Coapting MVL. Shortened PMVL 4. Severe MR 5. Severe TR 6. Moderate AR 7. Moderate PR 8. Severe Pulmonary Hypertension 9. Trace Pericardial Effusion 10. Normal LV Function | | | |
| Done By: | **Signature** | | **Date of Reporting** | |
| Dr. Tesfaye Taye, Pediatrician, Pediatric Cardiologist | | | **02/06/2013Eth.C** | |

| Pediatric Echocardiography report  Patient Name: Mebe’a Ashenafi. Patient ID: ___________. Gender: F. Age: 2 7/12. TGSH1.2385 (Cor Pulmonale) | | | | |
| --- | --- | --- | --- | --- |
| Features | **Finding** | **Features** | | **Finding** |
| Profile | | **Atria** | | |
| Abdominal situs | Solitus | Left atrium | | Normal |
| Cardiac position | Levocardia | Right atrium | | Mildly Dilated |
| Systemic venous drainage | to RA | **Atrioventricular valves** | | |
| Pulmonary venous drainage | to LA | Mitral valve | | Annulus = 14mm. |
| Atrioventricular connection | Concordant | Tricuspid valve | | Annulus = 20mm.  TAPSE = 18mm |
| Ventriculoarterial connection | Concordant | **Ventricles** | | |
| Ventricular loop | d-Loop | Left ventricle | | Normal |
| Septae | | Right ventricle | | Mildly Dilated. RV TDI S wave = 12cm/sec |
| Interventricular septum | Intact | **M-Mode:**. | | |
| Interatrial septum | Intact | AO | |  |
| Great arteries | NRGA | LA | |  |
| Aorta | ----- | LVIDd | | 28mm |
| Pulmonary artery | Normal MPA normal Branch PAs. | LVIDs | | 16mm |
| Semilunal valves |  | IVSd | | 7mm |
| Aortic valve | Annulus = 13mm. | IVSs | | 7mm |
| Pulmonary valve | Annulus = 15mm | LVPWd | | 7mm |
| Doppler Measurement | | LVPWs | | 6mm |
| Mitral | ------- | EDV | | 29ml |
| Aortic | ------ | ESV | | 7ml |
| Tricuspid | Mild TR, PPG = 42 - 46mmHg | FS | | 42% |
| pulmonic | --------- | LVEF | | 74% |
| Aortic arch | ------- | **Coronary arteries** | |  |
| PDA | No |  | |  |
| Pericardium/Pleura |  | | | |
| Final Diagnosis | 1. {S, D, S} Levocardia. 2. RA/RV Dilated 3. Mild to moderate Pulmonary Hypertension | | | |
| Recommendation | Investigate the underlying cause. | | | |
| Done By: | **Signature** | | **Date of Reporting** | |
| Dr. Tesfaye Taye, Pediatrician, Pediatric Cardiologist | | | **03/06/2013Eth.C** | |

| Pediatric Echocardiography report  Patient Name: Dersolign Yazie. Patient ID: 068259. Gender: M. Age: 13Years.  Clinical Diagnosis: CHF + DOE + Syncope + easy fatigability. TGSH4.2908. | | | | |
| --- | --- | --- | --- | --- |
| Features | **Finding** | **Features** | | **Finding** |
| Profile | | **Atria** | | |
| Abdominal situs | Solitus | Left atrium | | Normal |
| Cardiac position | Levocardia | Right atrium | | Normal |
| Systemic venous drainage | to RA. IVC Dilated | **Atrioventricular valves** | | |
| Pulmonary venous drainage | to LA | Mitral valve | | Annulus = 23mm. MV E/A = 1.9 |
| Atrioventricular connection | Concordant | Tricuspid valve | | Annulus = 22mm. |
| Ventriculoarterial connection | Concordant | **Ventricles** | | |
| Ventricular loop | d-Loop | Left ventricle | | Normal |
| Septae | | Right ventricle | | Normal |
| Interventricular septum | Intact | **M-Mode:**. | | |
| Interatrial septum | Intact | AO | |  |
| Great arteries | NRGA | LA | |  |
| Aorta | ----- | LVIDd | | mm |
| Pulmonary artery | Normal MPA & Branch PAs. | LVIDs | | mm |
| Semilunal valves |  | IVSd | | mm |
| Aortic valve | Annulus = 15mm. | IVSs | | mm |
| Pulmonary valve | Annulus = 22mm | LVPWd | | mm |
| Doppler Measurement | | LVPWs | | mm |
| Mitral | Trivial MR, Incomplete signal, jet velocity = 3.7m/sec | EDV | | ml |
| Aortic | ------ | ESV | | ml |
| Tricuspid | Mild TR, PPG = 60mmHg | FS | | 23% |
| pulmonic | --------- | LVEF | | 48% |
| Aortic arch | ------- | **Coronary arteries** | |  |
| PDA | No |  | |  |
| Pericardium/Pleura | 7mm Pericardial effusion. Thickened Pericardium | | | |
| Final Diagnosis | 1. {S, D, S} Levocardia. 2. Mild TR 3. Moderate to Severe Pulmonary Hypertension 4. Mild Systolic LV Dysfunction 5. Severe Diastolic Dysfunction | | | |
| DDx | Constrictive Pericarditis | | | |
| Done By: | **Signature** | | **Date of Reporting** | |
| Dr. Tesfaye Taye, Pediatrician, Pediatric Cardiologist | | | **04/06/2013Eth.C** | |

| Pediatric Echocardiography report  Patient Name: Baby of Tirualem Aweke. Patient ID: 069430. Gender: M. Age: 31days.  Clinical Diagnosis: RD. TGSH4.2909. | | | | |
| --- | --- | --- | --- | --- |
| Features | **Finding** | **Features** | | **Finding** |
| Profile | | **Atria** | | |
| Abdominal situs | Solitus | Left atrium | | Normal |
| Cardiac position | Levocardia | Right atrium | | Normal |
| Systemic venous drainage | to RA | **Atrioventricular valves** | | |
| Pulmonary venous drainage | to LA | Mitral valve | | Annulus = 10mm. |
| Atrioventricular connection | Concordant | Tricuspid valve | | Annulus = 9mm. |
| Ventriculoarterial connection | Concordant | **Ventricles** | | |
| Ventricular loop | d-Loop | Left ventricle | | Normal |
| Septae | | Right ventricle | | Normal |
| Interventricular septum | Intact | **M-Mode:** Normal LV Function on eye balling. | | |
| Interatrial septum | Intact | AO | |  |
| Great arteries | NRGA | LA | |  |
| Aorta | ----- | LVIDd | | mm |
| Pulmonary artery | Normal MPA & Branch PAs. | LVIDs | | mm |
| Semilunal valves |  | IVSd | | mm |
| Aortic valve | Annulus = 8mm. | IVSs | | mm |
| Pulmonary valve | Annulus = 9mm | LVPWd | | mm |
| Doppler Measurement | | LVPWs | | mm |
| Mitral | ------- | EDV | | ml |
| Aortic | ------ | ESV | | ml |
| Tricuspid | -------- | FS | | % |
| pulmonic | --------- | LVEF | | % |
| Aortic arch | ------- | **Coronary arteries** | |  |
| PDA | No |  | |  |
| Pericardium/Pleura |  | | | |
| Final Diagnosis | 1. Normal Echocardiography Study | | | |
| Done By: | **Signature** | | **Date of Reporting** | |
| Dr. Tesfaye Taye, Pediatrician, Pediatric Cardiologist | | | **09/06/2013Eth.C** | |

| Pediatric Echocardiography report  Patient Name: Baby of Emebet Bamlaku Patient ID: 069618. Gender: M. Age: 23days.  Clinical Diagnosis: RD. TGSH4.2910. | | | | |
| --- | --- | --- | --- | --- |
| Features | **Finding** | **Features** | | **Finding** |
| Profile | | **Atria** | | |
| Abdominal situs | Solitus | Left atrium | | Normal |
| Cardiac position | Levocardia | Right atrium | | Normal |
| Systemic venous drainage | to RA | **Atrioventricular valves** | | |
| Pulmonary venous drainage | to LA | Mitral valve | | Annulus = 11mm. |
| Atrioventricular connection | Concordant | Tricuspid valve | | Annulus = 13mm. |
| Ventriculoarterial connection | Concordant | **Ventricles** | | |
| Ventricular loop | d-Loop | Left ventricle | | Normal |
| Septae | | Right ventricle | | Normal |
| Interventricular septum | Intact | **M-Mode:** Normal LV Function on eye balling. | | |
| Interatrial septum | PFO, L – R Shunt | AO | |  |
| Great arteries | NRGA | LA | |  |
| Aorta | ----- | LVIDd | | mm |
| Pulmonary artery | Normal MPA & Branch PAs. | LVIDs | | mm |
| Semilunal valves |  | IVSd | | mm |
| Aortic valve | Annulus = 10mm. | IVSs | | mm |
| Pulmonary valve | Annulus = 10mm | LVPWd | | mm |
| Doppler Measurement | | LVPWs | | mm |
| Mitral | ------- | EDV | | ml |
| Aortic | ------ | ESV | | ml |
| Tricuspid | -------- | FS | | % |
| pulmonic | --------- | LVEF | | % |
| Aortic arch | ------- | **Coronary arteries** | |  |
| PDA | No |  | |  |
| Pericardium/Pleura |  | | | |
| Final Diagnosis | 1. {S, D, S} Levocardia 2. PFO, L – R Shunt | | | |
| Done By: | **Signature** | | **Date of Reporting** | |
| Dr. Tesfaye Taye, Pediatrician, Pediatric Cardiologist | | | **09/06/2013Eth.C** | |

| Pediatric Echocardiography report  Patient Name: Baby of Eyerus ayele. Patient ID: 066555. Gender: M. Age: 38days.  Clinical Diagnosis: Cyanosis + Murmur. TGSH4.2911. | | | | |
| --- | --- | --- | --- | --- |
| Features | **Finding** | **Features** | | **Finding** |
| Profile | | **Atria** | | |
| Abdominal situs | Solitus | Left atrium | | Normal |
| Cardiac position | Levocardia | Right atrium | | Normal |
| Systemic venous drainage | to RA | **Atrioventricular valves** | | |
| Pulmonary venous drainage | to LA | Mitral valve | | Annulus = 13mm. |
| Atrioventricular connection | Concordant | Tricuspid valve | | Annulus = 10mm. |
| Ventriculoarterial connection | Discordant | **Ventricles** | | |
| Ventricular loop | d-Loop | Left ventricle | | Normal |
| Septae | | Right ventricle | | Normal |
| Interventricular septum | 6mm Sub pulmonic VSD, BD Shunt | **M-Mode:** Normal LV Function on eye balling | | |
| Interatrial septum | PFO, L – R Shunt | AO | |  |
| Great arteries | d-TGA | LA | |  |
| Aorta | Anterior and to the right. Arises from RV | LVIDd | | mm |
| Pulmonary artery | Posterior and to the left. Arises from LV | LVIDs | | mm |
| Semilunal valves |  | IVSd | | mm |
| Aortic valve | Annulus = 11mm. | IVSs | | mm |
| Pulmonary valve | Annulus = 10mm | LVPWd | | mm |
| Doppler Measurement | | LVPWs | | mm |
| Mitral | ------- | EDV | | ml |
| Aortic | ------ | ESV | | ml |
| Tricuspid | -------- | FS | | % |
| pulmonic | --------- | LVEF | | % |
| Aortic arch | ------- | **Coronary arteries** | |  |
| PDA | 2mm PDA, L – R Shunt |  | |  |
| Pericardium/Pleura |  | | | |
| Final Diagnosis | 1. {S, D, D} Levocardia. 2. PFO, L – R Shunt 3. Sub pulmonic VSD, BD Shunt 4. PDA, L – R Shunt 5. d-TGA 6. Normal Function | | | |
| Done By: | **Signature** | | **Date of Reporting** | |
| Dr. Tesfaye Taye, Pediatrician, Pediatric Cardiologist | | | **09/06/2013Eth.C** | |

| Pediatric Echocardiography report  Patient Name: Arsema Sewmehon. Patient ID: 070035 Gender: F. Age: 63days.  Clinical Diagnosis: RD. TGSH4.2912. | | | | |
| --- | --- | --- | --- | --- |
| Features | **Finding** | **Features** | | **Finding** |
| Profile | | **Atria** | | |
| Abdominal situs | Solitus | Left atrium | | Normal |
| Cardiac position | Levocardia | Right atrium | | Normal |
| Systemic venous drainage | to RA | **Atrioventricular valves** | | |
| Pulmonary venous drainage | to LA | Mitral valve | | Annulus = 8mm. |
| Atrioventricular connection | Concordant | Tricuspid valve | | Annulus = 10mm. |
| Ventriculoarterial connection | Concordant | **Ventricles** | | |
| Ventricular loop | d-Loop | Left ventricle | | Normal |
| Septae | | Right ventricle | | Normal |
| Interventricular septum | Intact | **M-Mode:**. | | |
| Interatrial septum | PFO, L – R Shunt | AO | |  |
| Great arteries | NRGA | LA | |  |
| Aorta | ----- | LVIDd | | mm |
| Pulmonary artery | Normal MPA & Branch PAs. | LVIDs | | mm |
| Semilunal valves |  | IVSd | | mm |
| Aortic valve | Annulus = 8mm. | IVSs | | mm |
| Pulmonary valve | Annulus = 8mm | LVPWd | | mm |
| Doppler Measurement | | LVPWs | | mm |
| Mitral | ------- | EDV | | ml |
| Aortic | ------ | ESV | | ml |
| Tricuspid | -------- | FS | | % |
| pulmonic | --------- | LVEF | | % |
| Aortic arch | ------- | **Coronary arteries** | |  |
| PDA | No |  | |  |
| Pericardium/Pleura |  | | | |
| Final Diagnosis | 1. {S, D, S} Levocardia. 2. PFO, L – R Shunt | | | |
| Done By: | **Signature** | | **Date of Reporting** | |
| Dr. Tesfaye Taye, Pediatrician, Pediatric Cardiologist | | | **09/06/2013Eth.C** | |

| Pediatric Echocardiography report  Patient Name: Mulugeta Fekadu. Patient ID: 067901. Gender: M. Age: 5/12.  Clinical Diagnosis: Incidental Murmur. TGSH4.2913. | | | | |
| --- | --- | --- | --- | --- |
| Features | **Finding** | **Features** | | **Finding** |
| Profile | | **Atria** | | |
| Abdominal situs | Solitus | Left atrium | | Normal |
| Cardiac position | Levocardia | Right atrium | | Normal |
| Systemic venous drainage | to RA | **Atrioventricular valves** | | |
| Pulmonary venous drainage | to LA | Mitral valve | | Annulus = 10mm. |
| Atrioventricular connection | Concordant | Tricuspid valve | | Annulus = 13mm. |
| Ventriculoarterial connection | Concordant | **Ventricles** | | |
| Ventricular loop | d-Loop | Left ventricle | | Normal |
| Septae | | Right ventricle | | Normal |
| Interventricular septum | 2.5mm PM VSD, L – R Shunt | **M-Mode:** Normal LV Function on eye balling | | |
| Interatrial septum | Intact | AO | |  |
| Great arteries | NRGA | LA | |  |
| Aorta | ----- | LVIDd | | mm |
| Pulmonary artery | Normal MPA & Branch PAs. | LVIDs | | mm |
| Semilunal valves |  | IVSd | | mm |
| Aortic valve | Annulus = 10mm. | IVSs | | mm |
| Pulmonary valve | Annulus = 11mm | LVPWd | | mm |
| Doppler Measurement | | LVPWs | | mm |
| Mitral | ------- | EDV | | ml |
| Aortic | ------ | ESV | | ml |
| Tricuspid | -------- | FS | | % |
| pulmonic | --------- | LVEF | | % |
| Aortic arch | ------- | **Coronary arteries** | |  |
| PDA | No |  | |  |
| Pericardium/Pleura |  | | | |
| Final Diagnosis | 1. {S, D, S} Levocardia. 2. Small PM VSD, L – R Shunt 3. Normal Function | | | |
| Done By: | **Signature** | | **Date of Reporting** | |
| Dr. Tesfaye Taye, Pediatrician, Pediatric Cardiologist | | | **11/06/2013Eth.C** | |

| Pediatric Echocardiography report  Patient Name: Baby of Mulunesh Werkie Patient ID: 070181 Gender: M. Age: 12days.  Clinical Diagnosis: Incidental Murmur. TGSH4.2914. | | | | |
| --- | --- | --- | --- | --- |
| Features | **Finding** | **Features** | | **Finding** |
| Profile | | **Atria** | | |
| Abdominal situs | Solitus | Left atrium | | Normal |
| Cardiac position | Levocardia | Right atrium | | Normal |
| Systemic venous drainage | to RA | **Atrioventricular valves** | | |
| Pulmonary venous drainage | to LA | Mitral valve | | Annulus = 8mm. |
| Atrioventricular connection | Concordant | Tricuspid valve | | Annulus = 10mm. |
| Ventriculoarterial connection | Concordant | **Ventricles** | | |
| Ventricular loop | d-Loop | Left ventricle | | Normal |
| Septae | | Right ventricle | | Normal |
| Interventricular septum | 2mm Inlet VSD with PM Extension, L – R Shunt | **M-Mode:** Normal Function on eye balling. | | |
| Interatrial septum | PFO, L – R Shunt | AO | |  |
| Great arteries | NRGA | LA | |  |
| Aorta | ----- | LVIDd | | mm |
| Pulmonary artery | Normal MPA & Branch PAs. | LVIDs | | mm |
| Semilunal valves |  | IVSd | | mm |
| Aortic valve | Annulus = 7mm. | IVSs | | mm |
| Pulmonary valve | Annulus = 8mm | LVPWd | | mm |
| Doppler Measurement | | LVPWs | | mm |
| Mitral | ------- | EDV | | ml |
| Aortic | ------ | ESV | | ml |
| Tricuspid | -------- | FS | | % |
| pulmonic | --------- | LVEF | | % |
| Aortic arch | ------- | **Coronary arteries** | |  |
| PDA | No |  | |  |
| Pericardium/Pleura |  | | | |
| Final Diagnosis | 1. {S, D, S} Levocardia. 2. PFO, L – R Shunt 3. Small Inlet VSD with PM extension, L – R Shunt 4. Normal Function | | | |
| Remark | No hemodynamic Significance. | | | |
| Done By: | **Signature** | | **Date of Reporting** | |
| Dr. Tesfaye Taye, Pediatrician, Pediatric Cardiologist | | | **11/06/2013Eth.C** | |

| Pediatric Echocardiography report  Patient Name: Baby of Asiya Isleman. Patient ID: 070284. Gender: M. Age: 11days.  Clinical Diagnosis: Cyanosis + RD + Murmur. TGSH4.2915. | | | | |
| --- | --- | --- | --- | --- |
| Features | **Finding** | **Features** | | **Finding** |
| Profile | | **Atria** | | |
| Abdominal situs | Solitus | Left atrium | | Smallish |
| Cardiac position | Levocardia | Right atrium | | Normal |
| Systemic venous drainage | to RA | **Atrioventricular valves** | | |
| Pulmonary venous drainage | to LA | Mitral valve | | Atretic |
| Atrioventricular connection | Concordant | Tricuspid valve | | Annulus = 12mm. |
| Ventriculoarterial connection | DORV | **Ventricles** | | |
| Ventricular loop | d-Loop | Left ventricle | | Hypoplastic |
| Septae | | Right ventricle | | Dilated |
| Interventricular septum | 6mm Inlet VSD, BD Shunt | **M-Mode:**. | | |
| Interatrial septum | Large OS ASD, R – L Shunt | AO | |  |
| Great arteries | NRGA | LA | |  |
| Aorta | ----- | LVIDd | | mm |
| Pulmonary artery | Normal MPA & Branch PAs. | LVIDs | | mm |
| Semilunal valves |  | IVSd | | mm |
| Aortic valve | Annulus = 11mm. | IVSs | | mm |
| Pulmonary valve | Annulus = 9mm | LVPWd | | mm |
| Doppler Measurement | | LVPWs | | mm |
| Mitral | ------- | EDV | | ml |
| Aortic | ------ | ESV | | ml |
| Tricuspid | Moderate TR | FS | | % |
| pulmonic | --------- | LVEF | | % |
| Aortic arch | ------- | **Coronary arteries** | |  |
| PDA | 2mm PDA, L – R Shunt |  | |  |
| Pericardium/Pleura |  | | | |
| Final Diagnosis | 1. {S, D, D} Levocardia. 2. DORV 3. Large OS ASD,R – L Shunt 4. Moderate Inlet VSD, BD Shunt 5. Mitral Atresia 6. Hypoplastic LV 7. PDA, L – R Shunt | | | |
| Done By: | **Signature** | | **Date of Reporting** | |
| Dr. Tesfaye Taye, Pediatrician, Pediatric Cardiologist | | | **11/06/2013Eth.C** | |

| Pediatric Echocardiography report  Patient Name: Gashaw Eshet. Patient ID: 07898. Gender: M. Age: 13Years.  Clinical Diagnosis: CHF + Murmur + Rheumatic Recurrence + ?IE. TGSH4.2916. | | | | |
| --- | --- | --- | --- | --- |
| Features | **Finding** | **Features** | | **Finding** |
| Profile | | **Atria** | | |
| Abdominal situs | Solitus | Left atrium | | Dilated |
| Cardiac position | Levocardia | Right atrium | | Normal |
| Systemic venous drainage | to RA | **Atrioventricular valves** | | |
| Pulmonary venous drainage | to LA | Mitral valve | | Annulus = 29mm. thickened MVL. Mild clubbing |
| Atrioventricular connection | Concordant | Tricuspid valve | | Annulus = 26mm.TAPSE = 21mmHg |
| Ventriculoarterial connection | Concordant | **Ventricles** | | |
| Ventricular loop | d-Loop | Left ventricle | | Dilated. There is echogenic mass in the LVOT beneath the AV. |
| Septae | | Right ventricle | | Normal |
| Interventricular septum | Intact | **M-Mode:**. | | |
| Interatrial septum | Intact | AO | |  |
| Great arteries | NRGA | LA | |  |
| Aorta | ----- | LVIDd | | mm |
| Pulmonary artery | MPA =25mm. normal Branch PAs. | LVIDs | | mm |
| Semilunal valves |  | IVSd | | mm |
| Aortic valve | Annulus = 18mm. thickened AVL | IVSs | | mm |
| Pulmonary valve | Annulus = 23mm | LVPWd | | mm |
| Doppler Measurement | | LVPWs | | mm |
| Mitral | Severe MR, Holosystolic, Posterior projection, seen in two planes with jet velocity = 4m/sec | EDV | | ml |
| Aortic | Moderate AR, PHT = 332ms. | ESV | | ml |
| Tricuspid | Moderate TR, PPG = 56mmHg | FS | | 35% |
| pulmonic | Moderate PR, PPG = 49mmHg | LVEF | | 65% |
| Aortic arch | ------- | **Coronary arteries** | |  |
| PDA | No |  | |  |
| Pericardium/Pleura | 15mm Right Pleural Effusion. Trace pericardial Effusion more on RA/RV Side. | | | |
| Final Diagnosis | 1. {S, D, S} Levocardia. 2. LA/LV Dilated 3. Thickened MVL and AVL 4. Severe MR 5. Moderate TR 6. Moderate AR 7. Moderate PR 8. Moderate Pulmonary Hypertension 9. Right pleural effusion 10. Trace Pericardial effusion 11. Normal Biventricular Function | | | |
| Remark | **Investigate in the line of IE** | | | |
| Done By: | **Signature** | | **Date of Reporting** | |
| Dr. Tesfaye Taye, Pediatrician, Pediatric Cardiologist | | | **16/06/2013Eth.C** | |

| Pediatric Echocardiography report  Patient Name: Nardos Shitu. Patient ID: 024132. Gender: M. Age: 1 6/12. | | | | |
| --- | --- | --- | --- | --- |
| Follow up Echo for (Pink TOF (Mild PS, Mild RVH, L – R Shunt) & PFO. (TGSH1.2491.) | | | | |
| Features | **Finding** | **Features** | | **Finding** |
| Profile | | **Atria** | | |
| Abdominal situs | Solitus | Left atrium | | Normal |
| Cardiac position | Levocardia | Right atrium | | Normal |
| Systemic venous drainage | to RA | **Atrioventricular valves** | | |
| Pulmonary venous drainage | to LA | Mitral valve | | Annulus = 16mm. |
| Atrioventricular connection | Concordant | Tricuspid valve | | Annulus = 17mm. |
| Ventriculoarterial connection | Concordant | **Ventricles** | | |
| Ventricular loop | d-Loop | Left ventricle | | Normal |
| Septae | | Right ventricle | | Normal (No RVH) |
| Interventricular septum | 5mm Subaortic VSD, L – R Shunt | **M-Mode:**. | | |
| Interatrial septum | Intact (PFO Closed) | AO | |  |
| Great arteries | NRGA | LA | |  |
| Aorta | ----- | LVIDd | | mm |
| Pulmonary artery | Normal MPA normal Branch PAs. | LVIDs | | mm |
| Semilunal valves |  | IVSd | | mm |
| Aortic valve | Annulus = 15mm. | IVSs | | mm |
| Pulmonary valve | Annulus = 19mm | LVPWd | | mm |
| Doppler Measurement | | LVPWs | | mm |
| Mitral | ------- | EDV | | ml |
| Aortic | ------ | ESV | | ml |
| Tricuspid | -------- | FS | | % |
| pulmonic | The mild PS (PPG = 24mmHg) has reduced to 11mmHg | LVEF | | % |
| Aortic arch | ------- | **Coronary arteries** | |  |
| PDA | No |  | |  |
| Pericardium/Pleura | Trace Pericardial effusion on RA side | | | |
| Final Diagnosis | 1. {S, D, S} Levocardia. 2. Small Sub aortic VSD, L – R Shunt 3. Good Function | | | |
| Done By: | **Signature** | | **Date of Reporting** | |
| Dr. Tesfaye Taye, Pediatrician, Pediatric Cardiologist | | | **16/06/2013Eth.C** | |

| Pediatric Echocardiography report  Patient Name: Beza Getie. Patient ID: ___________. Gender: ___________. Age: ______________Years.  Clinical Diagnosis: ______________. INCOMPLETE DATA. | | | | |
| --- | --- | --- | --- | --- |
| Features | **Finding** | **Features** | | **Finding** |
| Profile | | **Atria** | | |
| Abdominal situs | Solitus | Left atrium | | Normal |
| Cardiac position | Dextrocardia | Right atrium | | Normal |
| Systemic venous drainage | to RA | **Atrioventricular valves** | | |
| Pulmonary venous drainage | to LA | Mitral valve | | Common Complete AVSD |
| Atrioventricular connection | AVSD | Tricuspid valve | |  |
| Ventriculoarterial connection | Truncus | **Ventricles** | | |
| Ventricular loop | d-Loop | Left ventricle | | Smallish |
| Septae | | Right ventricle | | Normal |
| Interventricular septum | Common Complete AVSD | **M-Mode:**. | | |
| Interatrial septum | Common Complete AVSD. Additional PFO | AO | |  |
| Great arteries | TRUNCUS | LA | |  |
| Aorta |  | LVIDd | | mm |
| Pulmonary artery | Seems arising from the truncus on the left lateral side | LVIDs | | mm |
| Semilunal valves |  | IVSd | | mm |
| Aortic valve | Annulus = 12mm. | IVSs | | mm |
| Pulmonary valve |  | LVPWd | | mm |
| Doppler Measurement | | LVPWs | | mm |
| Mitral | ------- | EDV | | ml |
| Aortic | ------ | ESV | | ml |
| Tricuspid | -------- | FS | | % |
| pulmonic | --------- | LVEF | | % |
| Aortic arch | ------- | **Coronary arteries** | |  |
| PDA | No |  | |  |
| Pericardium/Pleura |  | | | |
| Final Diagnosis | 1. {S, D, S} Dextrocardia. 2. PFO 3. Common Complete Unbalanced AVSD 4. ?Truncus Arteriosus | | | |
| Done By: | **Signature** | | **Date of Reporting** | |
| Dr. Tesfaye Taye, Pediatrician, Pediatric Cardiologist | | | **16/06/2013Eth.C** | |

| Pediatric Echocardiography report  Patient Name: Baby Alemitu Gizatie. Patient ID: 071205. Gender: F. Age: 11days.  Clinical Diagnosis: Incidental Murmur. TGSH4.2917. | | | | |
| --- | --- | --- | --- | --- |
| Features | **Finding** | **Features** | | **Finding** |
| Profile | | **Atria** | | |
| Abdominal situs | Solitus | Left atrium | | Normal |
| Cardiac position | Levocardia | Right atrium | | Normal |
| Systemic venous drainage | to RA | **Atrioventricular valves** | | |
| Pulmonary venous drainage | to LA | Mitral valve | | Annulus = 7mm |
| Atrioventricular connection | Normal | Tricuspid valve | | Annulus = 7mm |
| Ventriculoarterial connection | Normal | **Ventricles** | | |
| Ventricular loop | d-Loop | Left ventricle | | Normal |
| Septae | | Right ventricle | | Normal |
| Interventricular septum | Intact | **M-Mode:** Normal LV Function on eye balling. | | |
| Interatrial septum | Intact | AO | |  |
| Great arteries | NRGA | LA | |  |
| Aorta | ------- | LVIDd | | mm |
| Pulmonary artery | ------- | LVIDs | | mm |
| Semilunal valves |  | IVSd | | mm |
| Aortic valve | Annulus = 6mm. | IVSs | | mm |
| Pulmonary valve | Annulus = 7mm | LVPWd | | mm |
| Doppler Measurement | | LVPWs | | mm |
| Mitral | ------- | EDV | | ml |
| Aortic | ------ | ESV | | ml |
| Tricuspid | -------- | FS | | % |
| pulmonic | --------- | LVEF | | % |
| Aortic arch | ------- | **Coronary arteries** | |  |
| PDA | 1.5mm PDA, L – R Shunt |  | |  |
| Pericardium/Pleura |  | | | |
| Final Diagnosis | 1. {S, D, S} Levocardia. 2. Small PDA, L – R Shunt 3. Normal LV Function | | | |
| Done By: | **Signature** | | **Date of Reporting** | |
| Dr. Tesfaye Taye, Pediatrician, Pediatric Cardiologist | | | **16/06/2013Eth.C** | |

| Pediatric Echocardiography report  Patient Name: Abrham Asmamaw. Patient ID: 071850. Gender: M. Age: 62days.  Clinical Diagnosis: Diaphoresis + Murmur. TGSH4.2918. | | | | |
| --- | --- | --- | --- | --- |
| Features | **Finding** | **Features** | | **Finding** |
| Profile | | **Atria** | | |
| Abdominal situs | Solitus | Left atrium | | Dilated |
| Cardiac position | Levocardia | Right atrium | | Normal |
| Systemic venous drainage | to RA | **Atrioventricular valves** | | |
| Pulmonary venous drainage | to LA | Mitral valve | | Annulus = 13mm |
| Atrioventricular connection | Normal | Tricuspid valve | | Annulus = 12mm |
| Ventriculoarterial connection | Normal | **Ventricles** | | |
| Ventricular loop | d-Loop | Left ventricle | | Dilated |
| Septae | | Right ventricle | | Normal |
| Interventricular septum | 7mm inlet VSD with PM extension, L – R Shunt | **M-Mode:**. | | |
| Interatrial septum | PFO, L – R Shunt | AO | |  |
| Great arteries | NRGA | LA | |  |
| Aorta | ------- | LVIDd | | mm |
| Pulmonary artery | ------- | LVIDs | | mm |
| Semilunal valves |  | IVSd | | mm |
| Aortic valve | Annulus = 8mm. | IVSs | | mm |
| Pulmonary valve | Annulus = 12mm | LVPWd | | mm |
| Doppler Measurement | | LVPWs | | mm |
| Mitral | ------- | EDV | | ml |
| Aortic | ------ | ESV | | ml |
| Tricuspid | -------- | FS | | % |
| pulmonic | Mild PS, PPG = 31mmHg | LVEF | | % |
| Aortic arch | ------- | **Coronary arteries** | |  |
| PDA | ------------------- |  | |  |
| Pericardium/Pleura |  | | | |
| Final Diagnosis | 1. {S, D, S} Levocardia. 2. PFO, L – R Shunt 3. Large Inlet VSD with PM extension, L – R Shunt 4. Mild Valvar PS 5. Normal Function | | | |
| Done By: | **Signature** | | **Date of Reporting** | |
| Dr. Tesfaye Taye, Pediatrician, Pediatric Cardiologist | | | **18/06/2013Eth.C** | |

| Pediatric Echocardiography report  Patient Name: Behailu Habtamu. Patient ID: 071098. Gender: M. Age: 1 1/12.  Clinical Diagnosis: Diaphoresis + Recurrent Chest Infection. TGSH4.2919. | | | | |
| --- | --- | --- | --- | --- |
| Features | **Finding** | **Features** | | **Finding** |
| Profile | | **Atria** | | |
| Abdominal situs | Solitus | Left atrium | | Normal |
| Cardiac position | Levocardia | Right atrium | | Normal |
| Systemic venous drainage | to RA | **Atrioventricular valves** | | |
| Pulmonary venous drainage | to LA | Mitral valve | | Annulus = 16mm |
| Atrioventricular connection | Normal | Tricuspid valve | | Annulus = 16mm |
| Ventriculoarterial connection | Normal | **Ventricles** | | |
| Ventricular loop | d-Loop | Left ventricle | | Normal |
| Septae | | Right ventricle | | Normal |
| Interventricular septum | Intact | **M-Mode:** Normal LV Function on eye balling. | | |
| Interatrial septum | Intact | AO | |  |
| Great arteries | NRGA | LA | |  |
| Aorta | ------- | LVIDd | | mm |
| Pulmonary artery | ------- | LVIDs | | mm |
| Semilunal valves |  | IVSd | | mm |
| Aortic valve | Annulus = 13mm. | IVSs | | mm |
| Pulmonary valve | Annulus = 14mm | LVPWd | | mm |
| Doppler Measurement | | LVPWs | | mm |
| Mitral | ------- | EDV | | ml |
| Aortic | ------ | ESV | | ml |
| Tricuspid | Trivial TR, PPG = 28mmHg | FS | | % |
| pulmonic | --------- | LVEF | | % |
| Aortic arch | ------- | **Coronary arteries** | |  |
| PDA | ---- |  | |  |
| Pericardium/Pleura |  | | | |
| Final Diagnosis | 1. Normal Echocardiography Study. | | | |
| Done By: | **Signature** | | **Date of Reporting** | |
| Dr. Tesfaye Taye, Pediatrician, Pediatric Cardiologist | | | **18/06/2013Eth.C** | |

| Pediatric Echocardiography report  Patient Name: _Bethelihem Wendim. Patient ID: 061608. Gender: F. Age: 4/12.  Clinical Diagnosis: Incidental Murmur. TGSH4.2920. (TGSH7./ 50days) | | | | |
| --- | --- | --- | --- | --- |
| Follow up echo for PDA done on 22/03/13 | | | | |
| Features | **Finding** | **Features** | | **Finding** |
| Profile | | **Atria** | | |
| Abdominal situs | Solitus | Left atrium | | Normal |
| Cardiac position | Levocardia | Right atrium | | Normal |
| Systemic venous drainage | to RA | **Atrioventricular valves** | | |
| Pulmonary venous drainage | to LA | Mitral valve | | Annulus = 13mm |
| Atrioventricular connection | Normal | Tricuspid valve | | Annulus = 13mm  TAPSE = 13mmHg |
| Ventriculoarterial connection | Normal | **Ventricles** | | |
| Ventricular loop | d-Loop | Left ventricle | | Normal |
| Septae | | Right ventricle | | Normal |
| Interventricular septum | Intact | **M-Mode:** Normal LV Function on eye balling. | | |
| Interatrial septum | PFO, L – R Shunt | AO | |  |
| Great arteries | NRGA | LA | |  |
| Aorta | ------- | LVIDd | | mm |
| Pulmonary artery | ------- | LVIDs | | mm |
| Semilunal valves |  | IVSd | | mm |
| Aortic valve | Annulus = 11mm. | IVSs | | mm |
| Pulmonary valve | Annulus = 13mm | LVPWd | | mm |
| Doppler Measurement | | LVPWs | | mm |
| Mitral | ------- | EDV | | ml |
| Aortic | ------ | ESV | | ml |
| Tricuspid | -------- | FS | | % |
| pulmonic | --------- | LVEF | | % |
| Aortic arch | ------- | **Coronary arteries** | |  |
| PDA | 1.5mm PDA, L – R Shunt with SPG/DPG = 68/36mmHg | | | |
| Pericardium/Pleura |  | | | |
| Final Diagnosis | 1. {S, D, S} Levocardia. 2. PFO, L – R Shunt 3. Small PDA, L – R Shunt 4. Normal Biventricular Function | | | |
| Done By: | **Signature** | | **Date of Reporting** | |
| Dr. Tesfaye Taye, Pediatrician, Pediatric Cardiologist | | | **18/06/2013Eth.C** | |

| Pediatric Echocardiography report  Patient Name: Tilahun Girmie. Patient ID: 072556. Gender: M. Age: 14years.  Clinical Finding: CHF + Rheumatic Recurrence + DOE + Easy Fatigability + Palpitation. TGSH4.2921 | | | | |
| --- | --- | --- | --- | --- |
| Features | **Finding** | **Features** | | **Finding** |
| Profile | | **Atria** | | |
| Abdominal situs | Solitus | Left atrium | | More dilated |
| Cardiac position | Levocardia | Right atrium | | Dilated |
| Systemic venous drainage | to RA | **Atrioventricular valves** | | |
| Pulmonary venous drainage | to LA | Mitral valve | | Annulus = 31mm. Thickened MVL. Shortened PMVL |
| Atrioventricular connection | Normal | Tricuspid valve | | Annulus = 30mm  **TAPSE = 17mm** |
| Ventriculoarterial connection | Normal | **Ventricles** | | |
| Ventricular loop | d-Loop | Left ventricle | | More Dilated |
| Septae | | Right ventricle | | Dilated, |
| Interventricular septum | Intact | **M-Mode:**. | | |
| Interatrial septum | Intact | AO | |  |
| Great arteries | NRGA | LA | |  |
| Aorta | ------- | LVIDd | | mm |
| Pulmonary artery | **MPA = 30mm**. Normal Branch PAs. | LVIDs | | mm |
| Semilunal valves |  | IVSd | | mm |
| Aortic valve | Annulus = mm. | IVSs | | mm |
| Pulmonary valve | **Annulus = 31mm** | LVPWd | | mm |
| Doppler Measurement | | LVPWs | | mm |
| Mitral | Severe MR, Holosystolic, posterior projection, seen in two planes with jet velocity = 4.9m/sec. | EDV | | ml |
| Aortic | Mild AR, PHT = 668ms. | ESV | | ml |
| Tricuspid | Severe TR, PPG = 60mmHg | FS | | 32% |
| pulmonic | Moderate PR, PPG = 54mmHg | LVEF | | 59% |
| Aortic arch | ------- | **Coronary aa** | |  |
| PDA | -------- |  | |  |
| Pericardium/Pleura | 19mm right Pleural effusion | | | |
| Final Diagnosis | 1. {S, D, S} Levocardia. 2. All chambers Dilated 3. Thickened MVL, Shortened PMVL 4. Severe MR 5. Severe TR 6. Moderate PR 7. Mild AR 8. Severe Pulmonary Hypertension 9. Moderate Right Pleural Effusion | | | |
| Done By: | **Signature** | | **Date of Reporting** | |
| Dr. Tesfaye Taye, Pediatrician, Pediatric Cardiologist | | | **22/06/2013Eth.C** | |

| Pediatric Echocardiography report  Patient Name: Hiwet Tichew. Patient ID: 070375. Gender: F. Age: 32days.  Clinical Finding: RD + Sepsis. TGSH4.2922. | | | | |
| --- | --- | --- | --- | --- |
| Features | **Finding** | **Features** | | **Finding** |
| Profile | | **Atria** | | |
| Abdominal situs | Solitus | Left atrium | | Normal |
| Cardiac position | Levocardia | Right atrium | | Normal |
| Systemic venous drainage | to RA | **Atrioventricular valves** | | |
| Pulmonary venous drainage | to LA | Mitral valve | | Annulus = 13mm |
| Atrioventricular connection | Normal | Tricuspid valve | | Annulus = 14mm |
| Ventriculoarterial connection | Normal | **Ventricles** | | |
| Ventricular loop | d-Loop | Left ventricle | | Normal |
| Septae | | Right ventricle | | Normal |
| Interventricular septum | Intact | **M-Mode:** Normal LV Function on eye balling. | | |
| Interatrial septum | Intact | AO | |  |
| Great arteries | NRGA | LA | |  |
| Aorta | ------- | LVIDd | | mm |
| Pulmonary artery | ------- | LVIDs | | mm |
| Semilunal valves |  | IVSd | | mm |
| Aortic valve | Annulus = 10mm. | IVSs | | mm |
| Pulmonary valve | Annulus = 10mm | LVPWd | | mm |
| Doppler Measurement | | LVPWs | | mm |
| Mitral | ------- | EDV | | ml |
| Aortic | ------ | ESV | | ml |
| Tricuspid | -------- | FS | | % |
| pulmonic | --------- | LVEF | | % |
| Aortic arch | ------- | **Coronary arteries** | |  |
| PDA | -------- |  | |  |
| Pericardium/Pleura |  | | | |
| Final Diagnosis | 1. Normal Echocardiography Study. | | | |
| Done By: | **Signature** | | **Date of Reporting** | |
| Dr. Tesfaye Taye, Pediatrician, Pediatric Cardiologist | | | **25/06/2013Eth.C** | |

| Pediatric Echocardiography report  Patient Name: Hiwet Shumye. Patient ID: 072777. Gender: F. Age: 1 1/12.  Clinical Finding: CHF + RD + Murmur + Diaphoresis + Recurrent Chest Infection. TGSH4.2923. | | | | |
| --- | --- | --- | --- | --- |
| Features | **Finding** | **Features** | | **Finding** |
| Profile | | **Atria** | | |
| Abdominal situs | Solitus | Left atrium | | Dilated |
| Cardiac position | Levocardia | Right atrium | | Dilated |
| Systemic venous drainage | to RA | **Atrioventricular valves** | | |
| Pulmonary venous drainage | to LA | Mitral valve | | Annulus = 14mm. |
| Atrioventricular connection | Normal | Tricuspid valve | | Annulus = 19mm. STL Prolapsing into the septal defect.  TAPSE = 13mm |
| Ventriculoarterial connection | Normal | **Ventricles** | | |
| Ventricular loop | d-Loop | Left ventricle | | Dilated |
| Septae | | Right ventricle | | Dilated |
| Interventricular septum | 12mm Inlet VSD, Partially covered by the STL, L – R Shunt | **M-Mode:**. | | |
| Interatrial septum | Intact | AO | |  |
| Great arteries | NRGA | LA | |  |
| Aorta | ------- | LVIDd | | mm |
| Pulmonary artery | ------- | LVIDs | | mm |
| Semilunal valves |  | IVSd | | mm |
| Aortic valve | Annulus = 14mm. | IVSs | | mm |
| Pulmonary valve | Annulus = 18mm | LVPWd | | mm |
| Doppler Measurement | | LVPWs | | mm |
| Mitral | Mild MR | EDV | | ml |
| Aortic | ------ | ESV | | ml |
| Tricuspid | Moderate to severe TR. | FS | | 21% |
| pulmonic | Moderate PR, PPG = 63mmHg | LVEF | | 44% |
| Aortic arch | ------- | **Coronary arteries** | |  |
| PDA | 2mm PDA, L – R Shunt |  | |  |
| Pericardium/Pleura | 3mm Pericardial effusion on RA/RV Side | | | |
| Final Diagnosis | 1. {S, D, S} Levocardia. 2. RA/RV Dilated 3. Large Inlet VSD partially covered by STL, STL Prolapsing to the defect, L – R Shunt 4. Moderate PDA, L – R Shunt 5. Severe TR, Moderate PR, Mild MR 6. LV Dysfunction 7. Severe Pulmonary Hypertension 8. Trace Pericardial effusion | | | |
| Done By: | **Signature** | | **Date of Reporting** | |
| Dr. Tesfaye Taye, Pediatrician, Pediatric Cardiologist | | | **25/06/2013Eth.C** | |

| Pediatric Echocardiography report  Patient Name: Zemenu Bogale. Patient ID: 072792. Gender: M. Age: 46days.  Clinical Finding: CHF + Shock + RD. TGSH4.2924. | | | | |
| --- | --- | --- | --- | --- |
| Features | **Finding** | **Features** | | **Finding** |
| Profile | | **Atria** | | |
| Abdominal situs | Solitus | Left atrium | | Dilated |
| Cardiac position | Levocardia | Right atrium | | Dilated |
| Systemic venous drainage | to RA | **Atrioventricular valves** | | |
| Pulmonary venous drainage | to LA | Mitral valve | | Annulus = 13mm |
| Atrioventricular connection | Normal | Tricuspid valve | | Annulus = 17mm  TAPSE = 8mm. |
| Ventriculoarterial connection | Normal | **Ventricles** | | |
| Ventricular loop | d-Loop | Left ventricle | | Dilated |
| Septae | | Right ventricle | | Dilated |
| Interventricular septum | Intact | **M-Mode:** Severe LV Dysfunction | | |
| Interatrial septum | Intact | AO | |  |
| Great arteries | NRGA | LA | |  |
| Aorta | ------- | LVIDd | | mm |
| Pulmonary artery | ------- | LVIDs | | mm |
| Semilunal valves |  | IVSd | | mm |
| Aortic valve | Annulus = 10mm. | IVSs | | mm |
| Pulmonary valve | Annulus = 13mm | LVPWd | | mm |
| Doppler Measurement | | LVPWs | | mm |
| Mitral | Mild MR | EDV | | ml |
| Aortic | ------ | ESV | | ml |
| Tricuspid | Mild TR | FS | | % |
| pulmonic | Mild PR | LVEF | | % |
| Aortic arch | ------- | **Coronary arteries** | |  |
| PDA | -------- |  | |  |
| Pericardium/Pleura |  | | | |
| Final Diagnosis | 1. {S, D, S} Levocardia. 2. All chambers dilated 3. Mild MR, Mild TR, Mild PR 4. Severe Biventricular Dysfunction | | | |
| Done By: | **Signature** | | **Date of Reporting** | |
| Dr. Tesfaye Taye, Pediatrician, Pediatric Cardiologist | | | **25/06/2013Eth.C** | |

| Pediatric Echocardiography report  Patient Name: Baby Etewa Mulualem. Patient ID: 072932. Gender: M. Age: 27days.  Clinical Finding: Cyanosis + Mumrur. TGSH4.2925. | | | | |
| --- | --- | --- | --- | --- |
| Features | **Finding** | **Features** | | **Finding** |
| Profile | | **Atria** | | |
| Abdominal situs | Solitus | Left atrium | | Normal |
| Cardiac position | Levocardia | Right atrium | | Normal |
| Systemic venous drainage | to RA | **Atrioventricular valves** | | |
| Pulmonary venous drainage | to LA | Mitral valve | | Annulus = 13mm |
| Atrioventricular connection | Normal | Tricuspid valve | | Atretic |
| Ventriculoarterial connection | Normal | **Ventricles** | | |
| Ventricular loop | d-Loop | Left ventricle | | Normal |
| Septae | | Right ventricle | | Smallish |
| Interventricular septum | 4mm Inlet VSD, L – R Shunt | **M-Mode:** Normal Function | | |
| Interatrial septum | 8mm OS ASD, R – L Shunt | AO | |  |
| Great arteries | NRGA | LA | |  |
| Aorta | ------- | LVIDd | | mm |
| Pulmonary artery | ------- | LVIDs | | mm |
| Semilunal valves |  | IVSd | | mm |
| Aortic valve | Annulus = 11mm. | IVSs | | mm |
| Pulmonary valve | Annulus = 9mm | LVPWd | | mm |
| Doppler Measurement | | LVPWs | | mm |
| Mitral | ------- | EDV | | ml |
| Aortic | ------ | ESV | | ml |
| Tricuspid | -------- | FS | | % |
| pulmonic | --------- | LVEF | | % |
| Aortic arch | ------- | **Coronary arteries** | |  |
| PDA | -------- |  | |  |
| Pericardium/Pleura |  | | | |
| Final Diagnosis | 1. {S, D, S} Levocardia. 2. Moderate OS ASD, R – L Shunt 3. Non Restrictive inlet VSD, L – R Shunt 4. Smallish RV 5. Tricuspid Atresia type IC | | | |
| Done By: | **Signature** | | **Date of Reporting** | |
| Dr. Tesfaye Taye, Pediatrician, Pediatric Cardiologist | | | **25/06/2013Eth.C** | |

| Pediatric Echocardiography report  Patient Name: Tigist Selamsew. Patient ID: _073001. Gender: F. Age: 3 8/12.  Clinical Finding: Cyanosis + DS + Murmur. TGSH4.2926. | | | | |
| --- | --- | --- | --- | --- |
| Features | **Finding** | **Features** | | **Finding** |
| Profile | | **Atria** | | |
| Abdominal situs | Solitus | Left atrium | | Normal |
| Cardiac position | Levocardia | Right atrium | | Normal |
| Systemic venous drainage | to RA | **Atrioventricular valves** | | |
| Pulmonary venous drainage | to LA | Mitral valve | | Common Complete AVSD |
| Atrioventricular connection | DIRV, common Complete AVSD | Tricuspid valve | |  |
| Ventriculoarterial connection | DORV | **Ventricles** | | |
| Ventricular loop | d-Loop | Left ventricle | | Smallish |
| Septae | | Right ventricle | | Normal |
| Interventricular septum | Common Complete AVSD, BD Shunt | **M-Mode:**. | | |
| Interatrial septum |  | AO | |  |
| Great arteries | NRGA | LA | |  |
| Aorta | ------- | LVIDd | | mm |
| Pulmonary artery | ------- | LVIDs | | mm |
| Semilunal valves |  | IVSd | | mm |
| Aortic valve | Annulus = 18mm. | IVSs | | mm |
| Pulmonary valve | Annulus = 9mm | LVPWd | | mm |
| Doppler Measurement | | LVPWs | | mm |
| Mitral | Mild Left AVVR | EDV | | ml |
| Aortic | ------ | ESV | | ml |
| Tricuspid | -------- | FS | | % |
| pulmonic | Severe PS, PPG = 65mmHg | LVEF | | % |
| Aortic arch | ------- | **Coronary arteries** | |  |
| PDA | -------- |  | |  |
| Pericardium/Pleura |  | | | |
| Final Diagnosis | 1. {S, D, D} Levocardia. 2. DIRV/DORV 3. Unbalanced Common Complete AVSD 4. Mild Left AVVR 5. Severe PS | | | |
| Done By: | **Signature** | | **Date of Reporting** | |
| Dr. Tesfaye Taye, Pediatrician, Pediatric Cardiologist | | | **25/06/2013Eth.C** | |

| Pediatric Echocardiography report  Patient Name: Hewan Berihun. Patient ID: 073490. Gender: F. Age: 3years.  Clinical Finding: Dextroposition on CXR. TGSH4.2927. | | | | |
| --- | --- | --- | --- | --- |
| Features | **Finding** | **Features** | | **Finding** |
| Profile | | **Atria** | | |
| Abdominal situs | Solitus | Left atrium | | Normal |
| Cardiac position | Dextroposition | Right atrium | | Normal |
| Systemic venous drainage | to RA | **Atrioventricular valves** | | |
| Pulmonary venous drainage | to LA | Mitral valve | | Annulus = 15mm |
| Atrioventricular connection | Normal | Tricuspid valve | | Annulus = 15mm |
| Ventriculoarterial connection | Normal | **Ventricles** | | |
| Ventricular loop | d-Loop | Left ventricle | | Normal |
| Septae | | Right ventricle | | Normal |
| Interventricular septum | Intact | **M-Mode:** Normal LV Function on eye balling. | | |
| Interatrial septum | Intact | AO | |  |
| Great arteries | NRGA | LA | |  |
| Aorta | ------- | LVIDd | | mm |
| Pulmonary artery | ------- | LVIDs | | mm |
| Semilunal valves |  | IVSd | | mm |
| Aortic valve | Annulus = 12mm. | IVSs | | mm |
| Pulmonary valve | Annulus = 14mm | LVPWd | | mm |
| Doppler Measurement | | LVPWs | | mm |
| Mitral | ------- | EDV | | ml |
| Aortic | ------ | ESV | | ml |
| Tricuspid | -------- | FS | | % |
| pulmonic | --------- | LVEF | | % |
| Aortic arch | ------- | **Coronary arteries** | |  |
| PDA | -------- |  | |  |
| Pericardium/Pleura |  | | | |
| Final Diagnosis | 1. {S, D, S} Dextroposition. 2. Otherwise Normal Heart Study | | | |
| Done By: | **Signature** | | **Date of Reporting** | |
| Dr. Tesfaye Taye, Pediatrician, Pediatric Cardiologist | | | **02/07/2013Eth.C** | |

| Pediatric Echocardiography report  Patient Name: Abay Fentaw. Patient ID: 073902. Gender: M. Age: 9/12.  Clinical Finding: Diaphoresis + Murmur. TGSH4.2928. | | | | |
| --- | --- | --- | --- | --- |
| Features | **Finding** | **Features** | | **Finding** |
| Profile | | **Atria** | | |
| Abdominal situs | Solitus | Left atrium | | Dilated |
| Cardiac position | Levocardia | Right atrium | | Normal |
| Systemic venous drainage | to RA | **Atrioventricular valves** | | |
| Pulmonary venous drainage | to LA | Mitral valve | | Annulus = 17mm |
| Atrioventricular connection | Normal | Tricuspid valve | | Annulus = 15mm  TAPSE = 13mm |
| Ventriculoarterial connection | Normal | **Ventricles** | | |
| Ventricular loop | d-Loop | Left ventricle | | Dilated |
| Septae | | Right ventricle | | Normal |
| Interventricular septum | 8mm PM VSD, L – R Shunt with a gradient of 42mmHg | **M-Mode:**. | | |
| Interatrial septum | Intact | AO | |  |
| Great arteries | NRGA | LA | |  |
| Aorta | ------- | LVIDd | | mm |
| Pulmonary artery | ------- | LVIDs | | mm |
| Semilunal valves |  | IVSd | | mm |
| Aortic valve | Annulus = 13mm. | IVSs | | mm |
| Pulmonary valve | Annulus = 16mm | LVPWd | | mm |
| Doppler Measurement | | LVPWs | | mm |
| Mitral | ------- | EDV | | ml |
| Aortic | ------ | ESV | | ml |
| Tricuspid | -------- | FS | | 33% |
| pulmonic | --------- | LVEF | | 63% |
| Aortic arch | ------- | **Coronary arteries** | |  |
| PDA | -------- |  | |  |
| Pericardium/Pleura |  | | | |
| Final Diagnosis | 1. {S, D, S} Levocardia. 2. Moderate PM VSD, L – R Shunt 3. Normal Function | | | |
| Done By: | **Signature** | | **Date of Reporting** | |
| Dr. Tesfaye Taye, Pediatrician, Pediatric Cardiologist | | | **02/07/2013Eth.C** | |

| Pediatric Echocardiography report  Patient Name: Baby of Etihun Belete_. Patient ID: ___________. Gender: M. Age: 6/12.  Clinical Finding: Diaphoresis + Murmur + Recurrent Chest Infection. TGSH4.2929. | | | | |
| --- | --- | --- | --- | --- |
| Features | **Finding** | **Features** | | **Finding** |
| Profile | | **Atria** | | |
| Abdominal situs | Solitus | Left atrium | | Dilated |
| Cardiac position | Levocardia | Right atrium | | Normal |
| Systemic venous drainage | to RA | **Atrioventricular valves** | | |
| Pulmonary venous drainage | to LA | Mitral valve | | Annulus = 12mm |
| Atrioventricular connection | Normal | Tricuspid valve | | Annulus = 11mm  TAPSE = 16mm |
| Ventriculoarterial connection | Normal | **Ventricles** | | |
| Ventricular loop | d-Loop | Left ventricle | | Dilated |
| Septae | | Right ventricle | | Normal |
| Interventricular septum | Intact | **M-Mode:**. | | |
| Interatrial septum | Intact | AO | |  |
| Great arteries | NRGA | LA | |  |
| Aorta | ------- | LVIDd | | mm |
| Pulmonary artery | **MPA = 16mm** | LVIDs | | mm |
| Semilunal valves |  | IVSd | | mm |
| Aortic valve | Annulus = 10mm. | IVSs | | mm |
| Pulmonary valve | Annulus = 12mm | LVPWd | | mm |
| Doppler Measurement | | LVPWs | | mm |
| Mitral | ------- | EDV | | ml |
| Aortic | ------ | ESV | | ml |
| Tricuspid | -------- | FS | | 35% |
| pulmonic | --------- | LVEF | | 66% |
| Aortic arch | ------- | **Coronary arteries** | |  |
| PDA | 2.5mm PDA, PPG/DPG = 64/26mmHg |  | |  |
| Pericardium/Pleura |  | | | |
| Final Diagnosis | 1. {S, D, S} Levocardia. 2. Moderate PDA, L – R Shunt 3. Moderate Pulmonary Hypertension 4. Normal Biventricular Function | | | |
| Done By: | **Signature** | | **Date of Reporting** | |
| Dr. Tesfaye Taye, Pediatrician, Pediatric Cardiologist | | | **02/07/2013Eth.C** | |

| Pediatric Echocardiography report  Patient Name: Baby of Zebua Genanew. Patient ID: 072182 Gender: F. Age: 13days.  Clinical Finding: Incidental Murmur. TGSH4.2930. | | | | |
| --- | --- | --- | --- | --- |
| Features | **Finding** | **Features** | | **Finding** |
| Profile | | **Atria** | | |
| Abdominal situs | Solitus | Left atrium | | Normal |
| Cardiac position | Levocardia | Right atrium | | Normal |
| Systemic venous drainage | to RA | **Atrioventricular valves** | | |
| Pulmonary venous drainage | to LA | Mitral valve | | Annulus = mm |
| Atrioventricular connection | Normal | Tricuspid valve | | Annulus = mm |
| Ventriculoarterial connection | Normal | **Ventricles** | | |
| Ventricular loop | d-Loop | Left ventricle | | Normal |
| Septae | | Right ventricle | | Normal |
| Interventricular septum | Intact | **M-Mode:**. | | |
| Interatrial septum | Intact | AO | |  |
| Great arteries | NRGA | LA | |  |
| Aorta | ------- | LVIDd | | mm |
| Pulmonary artery | ------- | LVIDs | | mm |
| Semilunal valves |  | IVSd | | mm |
| Aortic valve | Annulus = mm. | IVSs | | mm |
| Pulmonary valve | Annulus = mm | LVPWd | | mm |
| Doppler Measurement | | LVPWs | | mm |
| Mitral | ------- | EDV | | ml |
| Aortic | ------ | ESV | | ml |
| Tricuspid | -------- | FS | | % |
| pulmonic | --------- | LVEF | | % |
| Aortic arch | ------- | **Coronary arteries** | |  |
| PDA | <1mm PDA, L – R Shunt |  | |  |
| Pericardium/Pleura |  | | | |
| Final Diagnosis | 1. {S, D, S} Levocardia. 2. Small PDA, L – R Shunt 3. All chambers normal size | | | |
| Remark | 1. **Probe is to big for this tiny creature (preterm 32)** 2. **Repeat echo after 6 months** 3. **No need to put on medication** | | | |
| Done By: | **Signature** | | **Date of Reporting** | |
| Dr. Tesfaye Taye, Pediatrician, Pediatric Cardiologist | | | **02/07/2013Eth.C** | |

| Pediatric Echocardiography report  Patient Name: Tenanesh Debasu. Patient ID: 074074. Gender: F. Age: 10years.  Clinical Finding: DOE + Palpitation + Recurrent Chest Infection + Murmur. TGSH4.2931. | | | | |
| --- | --- | --- | --- | --- |
| Features | **Finding** | **Features** | | **Finding** |
| Profile | | **Atria** | | |
| Abdominal situs | Solitus | Left atrium | | Dilated |
| Cardiac position | Levocardia | Right atrium | | Normal |
| Systemic venous drainage | to RA | **Atrioventricular valves** | | |
| Pulmonary venous drainage | to LA | Mitral valve | | Annulus = 18mm |
| Atrioventricular connection | Normal | Tricuspid valve | | Annulus = 18mm |
| Ventriculoarterial connection | Normal | **Ventricles** | | |
| Ventricular loop | d-Loop | Left ventricle | | Dilated |
| Septae | | Right ventricle | | Normal |
| Interventricular septum | 16mm Inlet VSD, L – R Shunt | **M-Mode:** Normal LV Function on eye balling. | | |
| Interatrial septum | Intact | AO | |  |
| Great arteries | NRGA | LA | |  |
| Aorta | ------- | LVIDd | | mm |
| Pulmonary artery | ------- | LVIDs | | mm |
| Semilunal valves |  | IVSd | | mm |
| Aortic valve | Annulus = 15mm. | IVSs | | mm |
| Pulmonary valve | Annulus = 19mm | LVPWd | | mm |
| Doppler Measurement | | LVPWs | | mm |
| Mitral | Moderate MR | EDV | | ml |
| Aortic | ------ | ESV | | ml |
| Tricuspid | Moderate TR | FS | | % |
| pulmonic | --------- | LVEF | | % |
| Aortic arch | ------- | **Coronary arteries** | |  |
| PDA | -------- |  | |  |
| Pericardium/Pleura |  | | | |
| Final Diagnosis | 1. {S, D, S} Levocardia. 2. Large Inlet VSD 3. Normal Function | | | |
| Done By: | **Signature** | | **Date of Reporting** | |
| Dr. Tesfaye Taye, Pediatrician, Pediatric Cardiologist | | | **02/07/2013Eth.C** | |

| Pediatric Echocardiography report  Patient Name: Lakachew Abita. Patient ID: 073840. Gender: M. Age: 3 9/12.  Clinical Finding: Cyanosis + Murmur. TGSH4.2932. | | | | |
| --- | --- | --- | --- | --- |
| Features | **Finding** | **Features** | | **Finding** |
| Profile | | **Atria** | | |
| Abdominal situs | Solitus | Left atrium | | Normal |
| Cardiac position | Levocardia | Right atrium | | Dilated |
| Systemic venous drainage | to RA | **Atrioventricular valves** | | |
| Pulmonary venous drainage | to LA | Mitral valve | | Annulus = 15mm |
| Atrioventricular connection | Normal | Tricuspid valve | | Annulus = 20mm |
| Ventriculoarterial connection | Normal | **Ventricles** | | |
| Ventricular loop | d-Loop | Left ventricle | | Normal |
| Septae | | Right ventricle | | Dilated and Hypertrophied |
| Interventricular septum | 8mm Malaligned Sub Aortic VSD, R – L Shunt | **M-Mode:**. | | |
| Interatrial septum | PFO, L – R Shunt | AO | |  |
| Great arteries | NRGA | LA | |  |
| Aorta | Overriding aorta | LVIDd | | mm |
| Pulmonary artery | ------- | LVIDs | | mm |
| Semilunal valves |  | IVSd | | mm |
| Aortic valve | Annulus = 18mm. | IVSs | | mm |
| Pulmonary valve | Annulus = 12mm | LVPWd | | mm |
| Doppler Measurement | | LVPWs | | mm |
| Mitral | ------- | EDV | | ml |
| Aortic | ------ | ESV | | ml |
| Tricuspid | -------- | FS | | % |
| pulmonic | Severe Sub pulmonic and Pulmonic PS, PPG = 70mmHg | LVEF | | % |
| Aortic arch | ------- | **Coronary arteries** | |  |
| PDA | -------- |  | |  |
| Pericardium/Pleura |  | | | |
| Final Diagnosis | 1. {S, D, S} Levocardia. 2. PFO, L – R Shunt 3. TOF | | | |
| Done By: | **Signature** | | **Date of Reporting** | |
| Dr. Tesfaye Taye, Pediatrician, Pediatric Cardiologist | | | **02/07/2013Eth.C** | |

| Pediatric Echocardiography report  Patient Name: Tadsual Ma’edu. Patient ID: ___________. Gender: M. Age: 1 7/12.  Clinical Finding: Recurrent Chest Infection. TGSH4.2933. | | | | |
| --- | --- | --- | --- | --- |
| Features | **Finding** | **Features** | | **Finding** |
| Profile | | **Atria** | | |
| Abdominal situs | Solitus | Left atrium | | Normal |
| Cardiac position | Levocardia | Right atrium | | Normal |
| Systemic venous drainage | to RA | **Atrioventricular valves** | | |
| Pulmonary venous drainage | to LA | Mitral valve | | Annulus = 12mm |
| Atrioventricular connection | Normal | Tricuspid valve | | Annulus = 15mm |
| Ventriculoarterial connection | Normal | **Ventricles** | | |
| Ventricular loop | d-Loop | Left ventricle | | Normal |
| Septae | | Right ventricle | | Normal |
| Interventricular septum | Intact | **M-Mode:** Normal LV Function on eye balling. | | |
| Interatrial septum | PFO, L – R Shunt | AO | |  |
| Great arteries | NRGA | LA | |  |
| Aorta | ------- | LVIDd | | mm |
| Pulmonary artery | ------- | LVIDs | | mm |
| Semilunal valves |  | IVSd | | mm |
| Aortic valve | Annulus = 12mm. | IVSs | | mm |
| Pulmonary valve | Annulus = 13mm | LVPWd | | mm |
| Doppler Measurement | | LVPWs | | mm |
| Mitral | ------- | EDV | | ml |
| Aortic | ------ | ESV | | ml |
| Tricuspid | -------- | FS | | % |
| pulmonic | --------- | LVEF | | % |
| Aortic arch | ------- | **Coronary arteries** | |  |
| PDA | -------- |  | |  |
| Pericardium/Pleura |  | | | |
| Final Diagnosis | 1. {S, D, S} Levocardia. 2. PFO, L – R Shunt | | | |
| Done By: | **Signature** | | **Date of Reporting** | |
| Dr. Tesfaye Taye, Pediatrician, Pediatric Cardiologist | | | **07/07/2013Eth.C** | |

| Pediatric Echocardiography report  Patient Name: Kalkidan Seye. Patient ID: 072576. Gender: F. Age: 10years. (PREVIOUS ECHO STUDY NOT FOUND) | | | | |
| --- | --- | --- | --- | --- |
| Follow up Echocardiography for Pericardial Tamponade (After Window Drain) | | | | |
| Features | **Finding** | **Features** | | **Finding** |
| Profile | | **Atria** | | |
| Abdominal situs | Solitus | Left atrium | | Normal |
| Cardiac position | Levocardia | Right atrium | | Normal |
| Systemic venous drainage | to RA | **Atrioventricular valves** | | |
| Pulmonary venous drainage | to LA | Mitral valve | | Annulus = 15mm |
| Atrioventricular connection | Normal | Tricuspid valve | | Annulus = 16mm  TAPSE = 18mm |
| Ventriculoarterial connection | Normal | **Ventricles** | | |
| Ventricular loop | d-Loop | Left ventricle | | Normal |
| Septae | | Right ventricle | | Normal |
| Interventricular septum | Intact | **M-Mode:**. | | |
| Interatrial septum | Intact | AO | |  |
| Great arteries | NRGA | LA | |  |
| Aorta | ------- | LVIDd | | mm |
| Pulmonary artery | ------- | LVIDs | | mm |
| Semilunal valves |  | IVSd | | mm |
| Aortic valve | Annulus = 17mm. | IVSs | | mm |
| Pulmonary valve | Annulus = 22mm | LVPWd | | mm |
| Doppler Measurement | | LVPWs | | mm |
| Mitral | ------- | EDV | | ml |
| Aortic | ------ | ESV | | ml |
| Tricuspid | -------- | FS | | 29% |
| pulmonic | --------- | LVEF | | 57% |
| Aortic arch | ------- | **Coronary arteries** | |  |
| PDA | -------- |  | |  |
| Pericardium/Pleura | 6mm Pericardial effusion on RV Side. 15mm Pericardial effusion on LV Side | | | |
| Final Diagnosis | 1. S/P Pericardial window drainage 2. {S, D, S} Levocardia. 3. Small Pericardial effusion 4. Normal Biventricular Function 5. No tamponade features | | | |
| Done By: | **Signature** | | **Date of Reporting** | |
| Dr. Tesfaye Taye, Pediatrician, Pediatric Cardiologist | | | **07/07/2013Eth.C** | |

| Pediatric Echocardiography report  Patient Name: Nebiyat Abiyot. Patient ID: 074460. Gender: F. Age: 8months.  Clinical Finding: RD + Diaphoresis + Murmur + CHF  . TGSH4. 2934. | | | | |
| --- | --- | --- | --- | --- |
| Features | **Finding** | **Features** | | **Finding** |
| Profile | | **Atria** | | |
| Abdominal situs | Solitus | Left atrium | | Dilated |
| Cardiac position | Levocardia | Right atrium | | Dilated |
| Systemic venous drainage | to RA | **Atrioventricular valves** | | |
| Pulmonary venous drainage | to LA | Mitral valve | | Annulus = 14mm |
| Atrioventricular connection | Normal | Tricuspid valve | | Annulus = 15mm  TAPSE = 13mm |
| Ventriculoarterial connection | Normal | **Ventricles** | | |
| Ventricular loop | d-Loop | Left ventricle | | Dilated |
| Septae | | Right ventricle | | Dilated. RV TDI S wave = 12cm/sec |
| Interventricular septum | Intact | **M-Mode:**. | | |
| Interatrial septum | Intact | AO | |  |
| Great arteries | NRGA | LA | |  |
| Aorta | ------- | LVIDd | | mm |
| Pulmonary artery | MPA = 16mm | LVIDs | | mm |
| Semilunal valves |  | IVSd | | mm |
| Aortic valve | Annulus = 12mm. | IVSs | | mm |
| Pulmonary valve | Annulus = 13mm | LVPWd | | mm |
| Doppler Measurement | | LVPWs | | mm |
| Mitral | ------- | EDV | | ml |
| Aortic | ------ | ESV | | ml |
| Tricuspid | -------- | FS | | 23% |
| pulmonic | Severe PR, PPG = 67mmHg | LVEF | | 48% |
| Aortic arch | ------- | **Coronary arteries** | |  |
| PDA | 3mm PDA, L – R Shunt |  | |  |
| Pericardium/Pleura |  | | | |
| Final Diagnosis | 1. {S, D, S} Levocardia. 2. Mildly dilated RA/RV 3. Large PDA, L – R Shunt 4. Severe PR 5. Severe Pulmonary Hypertension 6. Reduced LV Function | | | |
| Done By: | **Signature** | | **Date of Reporting** | |
| Dr. Tesfaye Taye, Pediatrician, Pediatric Cardiologist | | | **07/07/2013Eth.C** | |

| Pediatric Echocardiography report  Patient Name: Yetimwerk Baye. Patient ID: 074952. Gender: F. Age: 8/`12_.  Clinical Finding: Murmur + Diaphoresis. TGSH4. 2935. | | | | |
| --- | --- | --- | --- | --- |
| Features | **Finding** | **Features** | | **Finding** |
| Profile | | **Atria** | | |
| Abdominal situs | Solitus | Left atrium | | Dilated |
| Cardiac position | Levocardia | Right atrium | | Normal |
| Systemic venous drainage | to RA | **Atrioventricular valves** | | |
| Pulmonary venous drainage | to LA | Mitral valve | | Annulus = 20mm |
| Atrioventricular connection | Normal | Tricuspid valve | | Annulus = 15mm |
| Ventriculoarterial connection | Normal | **Ventricles** | | |
| Ventricular loop | d-Loop | Left ventricle | | Dilated |
| Septae | | Right ventricle | | Normal |
| Interventricular septum | Intact | **M-Mode:** Normal LV Function on eye balling. | | |
| Interatrial septum | Intact | AO | |  |
| Great arteries | NRGA | LA | |  |
| Aorta | ------- | LVIDd | | mm |
| Pulmonary artery | ------- | LVIDs | | mm |
| Semilunal valves |  | IVSd | | mm |
| Aortic valve | Annulus = 12mm. | IVSs | | mm |
| Pulmonary valve | Annulus = 14mm | LVPWd | | mm |
| Doppler Measurement | | LVPWs | | mm |
| Mitral | Moderate MR, Incomplete signal with jet velocity = 4m/sec | EDV | | ml |
| Aortic | ------ | ESV | | ml |
| Tricuspid | -------- | FS | | % |
| pulmonic | --------- | LVEF | | % |
| Aortic arch | ------- | **Coronary arteries** | |  |
| PDA | 2mm PDA, L – R Shunt |  | |  |
| Pericardium/Pleura |  | | | |
| Final Diagnosis | 1. {S, D, S} Levocardia. 2. Moderate PDA, L – R Shunt 3. Normal LV Function | | | |
| Done By: | **Signature** | | **Date of Reporting** | |
| Dr. Tesfaye Taye, Pediatrician, Pediatric Cardiologist | | | **09/07/2013Eth.C** | |

| Pediatric Echocardiography report  Patient Name: Tinsae Marye. Patient ID: _060906. Gender: F. Age: _10/12.  Clinical Finding: Recurrent Chest Infection. TGSH4. 2936. | | | | |
| --- | --- | --- | --- | --- |
| Features | **Finding** | **Features** | | **Finding** |
| Profile | | **Atria** | | |
| Abdominal situs | Solitus | Left atrium | | Normal |
| Cardiac position | Levocardia | Right atrium | | Normal |
| Systemic venous drainage | to RA | **Atrioventricular valves** | | |
| Pulmonary venous drainage | to LA | Mitral valve | | Annulus = mm |
| Atrioventricular connection | Normal | Tricuspid valve | | Annulus = mm |
| Ventriculoarterial connection | Normal | **Ventricles** | | |
| Ventricular loop | d-Loop | Left ventricle | | Normal |
| Septae | | Right ventricle | | Normal |
| Interventricular septum | Intact | **M-Mode:**. | | |
| Interatrial septum | Intact | AO | |  |
| Great arteries | NRGA | LA | |  |
| Aorta | ------- | LVIDd | | mm |
| Pulmonary artery | ------- | LVIDs | | mm |
| Semilunal valves |  | IVSd | | mm |
| Aortic valve | Annulus = mm. | IVSs | | mm |
| Pulmonary valve | Annulus = mm | LVPWd | | mm |
| Doppler Measurement | | LVPWs | | mm |
| Mitral | ------- | EDV | | ml |
| Aortic | ------ | ESV | | ml |
| Tricuspid | -------- | FS | | % |
| pulmonic | --------- | LVEF | | % |
| Aortic arch | ------- | **Coronary arteries** | |  |
| PDA | -------- |  | |  |
| Pericardium/Pleura |  | | | |
| Final Diagnosis | 1. Normal Echocardiography Study. | | | |
| Done By: | **Signature** | | **Date of Reporting** | |
| Dr. Tesfaye Taye, Pediatrician, Pediatric Cardiologist | | | **09/07/2013Eth.C** | |

| Pediatric Echocardiography report  Patient Name: Aman Yihun Alie. Patient ID: 075543. Gender: M. Age: 69days.  Clinical Finding: Incidental Murmur + cyanosis. TGSH4. 2937. | | | | |
| --- | --- | --- | --- | --- |
| Features | **Finding** | **Features** | | **Finding** |
| Profile | | **Atria** | | |
| Abdominal situs | Solitus | Left atrium | | Normal |
| Cardiac position | Levocardia | Right atrium | | Normal |
| Systemic venous drainage | to RA | **Atrioventricular valves** | | |
| Pulmonary venous drainage | to LA | Mitral valve | | Annulus = 14mm |
| Atrioventricular connection | Concordant | Tricuspid valve | | Annulus = 13mm |
| Ventriculoarterial connection | DORV | **Ventricles** | | |
| Ventricular loop | d-Loop | Left ventricle | | Normal |
| Septae | | Right ventricle | | Normal |
| Interventricular septum | 6mm Sub Arterial VSD, L – R Shunt | **M-Mode:**. | | |
| Interatrial septum | PFO, L – R Shunt | AO | |  |
| Great arteries | d-TGA | LA | |  |
| Aorta | From RV, to the right and anterior | LVIDd | | mm |
| Pulmonary artery | From RV, to the left and Posterior | LVIDs | | mm |
| Semilunal valves |  | IVSd | | mm |
| Aortic valve | Annulus = 13mm. | IVSs | | mm |
| Pulmonary valve | Annulus = 9mm | LVPWd | | mm |
| Doppler Measurement | | LVPWs | | mm |
| Mitral | ------- | EDV | | ml |
| Aortic | ------ | ESV | | ml |
| Tricuspid | -------- | FS | | % |
| pulmonic | Mild PS, PPG = 33mmHg | LVEF | | % |
| Aortic arch | ------- | **Coronary arteries** | |  |
| PDA | -------- |  | |  |
| Pericardium/Pleura |  | | | |
| Final Diagnosis | 1. {S, D, D} Levocardia. 2. DORV 3. PFO, L – R Shunt 4. Sub Arterial VSD, L – R Shunt 5. d-TGA 6. Mild PS 7. Normal Function | | | |
| Done By: | **Signature** | | **Date of Reporting** | |
| Dr. Tesfaye Taye, Pediatrician, Pediatric Cardiologist | | | **14/07/2013Eth.C** | |

| Pediatric Echocardiography report  Patient Name: Abebaw Tadele. Patient ID: 075855. Gender: M. Age: 6months.  Clinical Finding: Cyanosis + Murmur. TGSH4. 2938. | | | | |
| --- | --- | --- | --- | --- |
| Features | **Finding** | **Features** | | **Finding** |
| Profile | | **Atria** | | |
| Abdominal situs | Solitus | Left atrium | | Normal |
| Cardiac position | Levocardia | Right atrium | | Normal |
| Systemic venous drainage | to RA | **Atrioventricular valves** | | |
| Pulmonary venous drainage | to LA | Mitral valve | | Annulus = 13mm |
| Atrioventricular connection | Concordant | Tricuspid valve | | Annulus = 12mm |
| Ventriculoarterial connection | Discordant | **Ventricles** | | |
| Ventricular loop | d-Loop | Left ventricle | | Normal |
| Septae | | Right ventricle | | Hypertrophied |
| Interventricular septum | 3mm Upper Muscular VSD, RV to LV | **M-Mode:**. | | |
| Interatrial septum | PFO, LA to RA Shunt | AO | |  |
| Great arteries | d-TGA | LA | |  |
| Aorta | From RV, anterior and to the right | LVIDd | | mm |
| Pulmonary artery | From LV, Posterior and to the left | LVIDs | | mm |
| Semilunal valves |  | IVSd | | mm |
| Aortic valve | Annulus = 11mm. | IVSs | | mm |
| Pulmonary valve | Annulus = 14mm | LVPWd | | mm |
| Doppler Measurement | | LVPWs | | mm |
| Mitral | ------- | EDV | | ml |
| Aortic | ------ | ESV | | ml |
| Tricuspid | -------- | FS | | % |
| pulmonic | --------- | LVEF | | % |
| Aortic arch | ------- | **Coronary arteries** | |  |
| PDA | 1mm PDA, BD Shunt |  | |  |
| Pericardium/Pleura | Trace Pericardial effusion on RA/RV Side. | | | |
| Final Diagnosis | 1. {S, D, D} Levocardia. 2. d-TGA 3. Restrictive PFO 4. Restrictive Upper Muscular VSD, RV to LV 5. Hypertrophied RV 6. Small PDA, BD Shunt 7. Pulmonary Hypertension | | | |
| Done By: | **Signature** | | **Date of Reporting** | |
| Dr. Tesfaye Taye, Pediatrician, Pediatric Cardiologist | | | **14/07/2013Eth.C** | |

| Pediatric Echocardiography report  Patient Name: Baby of Fentanesh Fekadu. Patient ID: 074575. Gender: M. Age: 13days.  Clinical Finding: Incidental Murmur. TGSH4. 2939. | | | | |
| --- | --- | --- | --- | --- |
| Features | **Finding** | **Features** | | **Finding** |
| Profile | | **Atria** | | |
| Abdominal situs | Solitus | Left atrium | | Normal |
| Cardiac position | Levocardia | Right atrium | | Normal |
| Systemic venous drainage | to RA | **Atrioventricular valves** | | |
| Pulmonary venous drainage | to LA | Mitral valve | | Annulus = 9mm |
| Atrioventricular connection | Concordant | Tricuspid valve | | Annulus = 10mm |
| Ventriculoarterial connection | Concordant | **Ventricles** | | |
| Ventricular loop | d-Loop | Left ventricle | | Normal |
| Septae | | Right ventricle | | Normal |
| Interventricular septum | Intact | **M-Mode:**. | | |
| Interatrial septum | PFO, L – R Shunt | AO | |  |
| Great arteries | NRGA | LA | |  |
| Aorta | ------- | LVIDd | | mm |
| Pulmonary artery | ------- | LVIDs | | mm |
| Semilunal valves |  | IVSd | | mm |
| Aortic valve | Annulus = 8mm. | IVSs | | mm |
| Pulmonary valve | Annulus = 8mm | LVPWd | | mm |
| Doppler Measurement | | LVPWs | | mm |
| Mitral | ------- | EDV | | ml |
| Aortic | ------ | ESV | | ml |
| Tricuspid | Trivial TR, PPG = 29mmHg | FS | | % |
| pulmonic | --------- | LVEF | | % |
| Aortic arch | ------- | **Coronary arteries** | |  |
| PDA | 2mm PDA, L – R Shunt |  | |  |
| Pericardium/Pleura |  | | | |
| Final Diagnosis | 1. {S, D, S} Levocardia. 2. PFO, L – R Shunt 3. Moderate PDA, L – R Shunt 4. Normal Biventricular Function | | | |
| Done By: | **Signature** | | **Date of Reporting** | |
| Dr. Tesfaye Taye, Pediatrician, Pediatric Cardiologist | | | **14/07/2013Eth.C** | |

| Pediatric Echocardiography report  Patient Name: Baby of Alemnat Addisie. Patient ID: 074230. Gender: M. Age: 14days.  Clinical Finding: _RD + DS. TGSH4. 2940. | | | | |
| --- | --- | --- | --- | --- |
| Features | **Finding** | **Features** | | **Finding** |
| Profile | | **Atria** | | |
| Abdominal situs | Solitus | Left atrium | | Normal |
| Cardiac position | Levocardia | Right atrium | | Normal |
| Systemic venous drainage | to RA | **Atrioventricular valves** | | |
| Pulmonary venous drainage | to LA | Mitral valve | | Annulus = 9mm |
| Atrioventricular connection | Concordant | Tricuspid valve | | Annulus = 11mm  TAPSE = 13mm |
| Ventriculoarterial connection | Concordant | **Ventricles** | | |
| Ventricular loop | d-Loop | Left ventricle | | Normal |
| Septae | | Right ventricle | | Normal |
| Interventricular septum | Intact | **M-Mode:**. | | |
| Interatrial septum | PFO , L – R Shunt | AO | |  |
| Great arteries | NRGA | LA | |  |
| Aorta | ------- | LVIDd | | mm |
| Pulmonary artery | ------- | LVIDs | | mm |
| Semilunal valves |  | IVSd | | mm |
| Aortic valve | Annulus = 8mm. | IVSs | | mm |
| Pulmonary valve | Annulus = 10mm | LVPWd | | mm |
| Doppler Measurement | | LVPWs | | mm |
| Mitral | ------- | EDV | | ml |
| Aortic | ------ | ESV | | ml |
| Tricuspid | -------- | FS | | 40% |
| pulmonic | --------- | LVEF | | 75% |
| Aortic arch | ------- | **Coronary arteries** | |  |
| PDA | <1mm PDA, L – R Shunt |  | |  |
| Pericardium/Pleura |  | | | |
| Final Diagnosis | 1. {S, D, S} Levocardia. 2. PFO, L – R Shunt 3. Silent PDA, L – R Shunt (No clinical Finding; Murmur) 4. Normal Biventricular Function | | | |
| Recommendation | Echocardiography after a year. | | | |
| Done By: | **Signature** | | **Date of Reporting** | |
| Dr. Tesfaye Taye, Pediatrician, Pediatric Cardiologist | | | **16/07/2013Eth.C** | |

| Pediatric Echocardiography report  Patient Name: Yenie wud Andualem_. Patient ID: 048467. Gender: F. Age: 1 7/12.  Clinical Finding: Incidental Murmur + Recurrent Chest Infection + Diaphoresis. TGSH4. 2941. | | | | |
| --- | --- | --- | --- | --- |
| Features | **Finding** | **Features** | | **Finding** |
| Profile | | **Atria** | | |
| Abdominal situs | Solitus | Left atrium | | Normal |
| Cardiac position | Levocardia | Right atrium | | Dilated |
| Systemic venous drainage | to RA | **Atrioventricular valves** | | |
| Pulmonary venous drainage | to LA | Mitral valve | | Annulus = 13mm |
| Atrioventricular connection | Concordant | Tricuspid valve | | Annulus = 21mm. Non coapting TV.  TAPSE = 22mm |
| Ventriculoarterial connection | Concordant | **Ventricles** | | |
| Ventricular loop | d-Loop | Left ventricle | | Normal |
| Septae | | Right ventricle | | Dilated |
| Interventricular septum | Intact | **M-Mode:** Normal LV Function on eye balling. | | |
| Interatrial septum | 13 X 17mm Fenestrated OS ASD, L –R Shunt | AO | |  |
| Great arteries | NRGA | LA | |  |
| Aorta | ------- | LVIDd | | mm |
| Pulmonary artery | ------- | LVIDs | | mm |
| Semilunal valves |  | IVSd | | mm |
| Aortic valve | Annulus = mm. | IVSs | | mm |
| Pulmonary valve | Annulus = 10mm | LVPWd | | mm |
| Doppler Measurement | | LVPWs | | mm |
| Mitral | ------- | EDV | | ml |
| Aortic | ------ | ESV | | ml |
| Tricuspid | Severe TR, PPG = 40mmHg | FS | | % |
| pulmonic | --------- | LVEF | | % |
| Aortic arch | ------- | **Coronary arteries** | |  |
| PDA | -------- |  | |  |
| Pericardium/Pleura |  | | | |
| Final Diagnosis | 1. {S, D, S} Levocardia. 2. RA/RV Dilated 3. Large Fenestrated OS ASD, L – R Shunt 4. Non coapting TV 5. Severe TR 6. Normal Biventricular Function | | | |
| Done By: | **Signature** | | **Date of Reporting** | |
| Dr. Tesfaye Taye, Pediatrician, Pediatric Cardiologist | | | **16/07/2013Eth.C** | |

| Pediatric Echocardiography report  Patient Name: Shikur Abdulqadir. Patient ID: _075927. Gender: M. Age: 104days.  Clinical Finding: Incidental Murmur. TGSH4. 2942. | | | | |
| --- | --- | --- | --- | --- |
| Features | **Finding** | **Features** | | **Finding** |
| Profile | | **Atria** | | |
| Abdominal situs | Solitus | Left atrium | | Normal |
| Cardiac position | Levocardia | Right atrium | | Normal |
| Systemic venous drainage | to RA | **Atrioventricular valves** | | |
| Pulmonary venous drainage | to LA | Mitral valve | | Annulus = 9mm |
| Atrioventricular connection | Concordant | Tricuspid valve | | Annulus = 9mm  TAPSE = mm. |
| Ventriculoarterial connection | Concordant | **Ventricles** | | |
| Ventricular loop | d-Loop | Left ventricle | | Normal |
| Septae | | Right ventricle | | Normal |
| Interventricular septum | Intact | **M-Mode:** Normal LV Function on eye balling. | | |
| Interatrial septum | Intact | AO | |  |
| Great arteries | NRGA | LA | |  |
| Aorta | ------- | LVIDd | | mm |
| Pulmonary artery | ------- | LVIDs | | mm |
| Semilunal valves |  | IVSd | | mm |
| Aortic valve | Annulus = 7mm. | IVSs | | mm |
| Pulmonary valve | Annulus = 10mm | LVPWd | | mm |
| Doppler Measurement | | LVPWs | | mm |
| Mitral | ------- | EDV | | ml |
| Aortic | ------ | ESV | | ml |
| Tricuspid | -------- | FS | | % |
| pulmonic | Mild to Moderate PS, PPG = 42 – 50mmHg | LVEF | | % |
| Aortic arch | ------- | **Coronary arteries** | |  |
| PDA | -------- |  | |  |
| Pericardium/Pleura |  | | | |
| Final Diagnosis | 1. {S, D, S} Levocardia. 2. Mild to Moderate PS 3. Normal Function | | | |
| Recommendation | Yearly Echocardiographic Assessment | | | |
| Done By: | **Signature** | | **Date of Reporting** | |
| Dr. Tesfaye Taye, Pediatrician, Pediatric Cardiologist | | | **16/07/2013Eth.C** | |

| Pediatric Echocardiography report  Patient Name: Waltengus walle . Patient ID: ____________. Gender: M. Age: 12years_.  Clinical Finding: RD + CHF + Murmur + Palpitation. TGSH4. 2943. | | | | |
| --- | --- | --- | --- | --- |
| Features | **Finding** | **Features** | | **Finding** |
| Profile | | **Atria** | | |
| Abdominal situs | Solitus | Left atrium | | Dilated |
| Cardiac position | Levocardia | Right atrium | | Dilated |
| Systemic venous drainage | to RA | **Atrioventricular valves** | | |
| Pulmonary venous drainage | to LA | Mitral valve | | Annulus = 15mm |
| Atrioventricular connection | Concordant | Tricuspid valve | | Annulus = 31mm  TAPSE = 12mm. |
| Ventriculoarterial connection | Concordant | **Ventricles** | | |
| Ventricular loop | d-Loop | Left ventricle | | Dilated |
| Septae | | Right ventricle | | Dilated and Dysfunctional |
| Interventricular septum | Intact | **M-Mode:**. | | |
| Interatrial septum | 13mm OS ASD, R – L Shunt | AO | |  |
| Great arteries | NRGA | LA | |  |
| Aorta | ------- | LVIDd | | mm |
| Pulmonary artery | ------- | LVIDs | | mm |
| Semilunal valves |  | IVSd | | mm |
| Aortic valve | Annulus = 17mm. | IVSs | | mm |
| Pulmonary valve | Annulus = 22mm | LVPWd | | mm |
| Doppler Measurement | | LVPWs | | mm |
| Mitral | ------- | EDV | | ml |
| Aortic | ------ | ESV | | ml |
| Tricuspid | Moderate TR, PPG = 65mmHg | FS | | 25% |
| pulmonic | Moderate PR, PPG = 66mmHg | LVEF | | 51% |
| Aortic arch | ------- | **Coronary arteries** | |  |
| PDA | -------- |  | |  |
| Pericardium/Pleura |  | | | |
| Final Diagnosis | 1. {S, D, S} Levocardia. 2. Large OS ASD, R – L Shunt 3. All chambers Dilated 4. Moderate TR 5. Moderate PR 6. Severe Pulmonary Hypertension 7. Reduced Biventricular Function | | | |
| Done By: | **Signature** | | **Date of Reporting** | |
| Dr. Tesfaye Taye, Pediatrician, Pediatric Cardiologist | | | **17/07/2013Eth.C** | |

| Pediatric Echocardiography report  Patient Name: Abaynew Defaru. Patient ID: 076519. Gender: M. Age: 8years.  Clinical Finding: CHF + Murmur + Palpitation + DOE. TGSH4. 2944. | | | | |
| --- | --- | --- | --- | --- |
| Features | **Finding** | **Features** | | **Finding** |
| Profile | | **Atria** | | |
| Abdominal situs | Solitus | Left atrium | | Dilated |
| Cardiac position | Levocardia | Right atrium | | Dilated |
| Systemic venous drainage | to RA | **Atrioventricular valves** | | |
| Pulmonary venous drainage | to LA | Mitral valve | | Annulus = mm. Thickened MVL. Echogenic mass on the anterior leaflet of the MV on the atrial side. |
| Atrioventricular connection | Concordant | Tricuspid valve | | Annulus = mm |
| Ventriculoarterial connection | Concordant | **Ventricles** | | |
| Ventricular loop | d-Loop | Left ventricle | | Dilated |
| Septae | | Right ventricle | | Dilated |
| Interventricular septum | 18mm PM VSD, L – R Shunt Partially covered by STL. | **M-Mode:**. | | |
| Interatrial septum | Intact | AO | |  |
| Great arteries | NRGA | LA | |  |
| Aorta | ------- | LVIDd | | mm |
| Pulmonary artery | **MPA = 26mm** | LVIDs | | mm |
| Semilunal valves |  | IVSd | | mm |
| Aortic valve | Annulus = 18mm. | IVSs | | mm |
| Pulmonary valve | Annulus = 26mm | LVPWd | | mm |
| Doppler Measurement | | LVPWs | | mm |
| Mitral | Moderate MR, Holosystolic, posterior projection, seen in two planes with jet velocity = 4.3m/sec | EDV | | ml |
| Aortic | ------ | ESV | | ml |
| Tricuspid | Severe TR | FS | | 33% |
| pulmonic | --------- | LVEF | | 61% |
| Aortic arch | ------- | **Coronary arteries** | |  |
| PDA | -------- |  | |  |
| Pericardium/Pleura | 9mm Pericardial effusion on RA Side | | | |
| Final Diagnosis | 1. {S, D, S} Levocardia. 2. All chambers Dilated 3. Moderate MR 4. Severe TR 5. Large PM VSD, L – R Shunt 6. Echogenic mass on the AML on LA side 7. Severe Pulmonary Hypertension 8. Small Pericardial effusion 9. Normal Function | | | |
| Done By: | **Signature** | | **Date of Reporting** | |
| Dr. Tesfaye Taye, Pediatrician, Pediatric Cardiologist | | | **17/07/2013Eth.C** | |

| Pediatric Echocardiography report  Patient Name: Agernesh Kassa. Patient ID: 076087. Gender: F. Age: 10years.  Clinical Finding: ARF. TGSH4. 2945. | | | | |
| --- | --- | --- | --- | --- |
| Features | **Finding** | **Features** | | **Finding** |
| Profile | | **Atria** | | |
| Abdominal situs | Solitus | Left atrium | | Normal |
| Cardiac position | Levocardia | Right atrium | | Normal |
| Systemic venous drainage | to RA | **Atrioventricular valves** | | |
| Pulmonary venous drainage | to LA | Mitral valve | | Annulus = 18mm. thickened MVL |
| Atrioventricular connection | Concordant | Tricuspid valve | | Annulus = 21mm |
| Ventriculoarterial connection | Concordant | **Ventricles** | | |
| Ventricular loop | d-Loop | Left ventricle | | Normal |
| Septae | | Right ventricle | | Normal |
| Interventricular septum | Intact | **M-Mode:**. | | |
| Interatrial septum | Intact | AO | |  |
| Great arteries | NRGA | LA | |  |
| Aorta | ------- | LVIDd | | mm |
| Pulmonary artery | ------- | LVIDs | | mm |
| Semilunal valves |  | IVSd | | mm |
| Aortic valve | Annulus = 16mm. | IVSs | | mm |
| Pulmonary valve | Annulus = 19mm | LVPWd | | mm |
| Doppler Measurement | | LVPWs | | mm |
| Mitral | Mild MR, Holosystolic, posterior projection, seen in two planes with jet velocity = 4.4m/sec | EDV | | ml |
| Aortic | ------ | ESV | | ml |
| Tricuspid | -------- | FS | | 32% |
| pulmonic | Trivial PR, PPG = 10mmHg | LVEF | | 62% |
| Aortic arch | ------- | **Coronary arteries** | |  |
| PDA | -------- |  | |  |
| Pericardium/Pleura |  | | | |
| Final Diagnosis | 1. {S, D, S} Levocardia. 2. Thickened MVL 3. Mild MR 4. Normal Function | | | |
| Done By: | **Signature** | | **Date of Reporting** | |
| Dr. Tesfaye Taye, Pediatrician, Pediatric Cardiologist | | | **21/07/2013Eth.C** | |

| Pediatric Echocardiography report  Patient Name: Enat Temesgen. Patient ID: 076766. Gender: F. Age: 12Years.  Clinical Finding: Rheumatic Recurrence + Murmur. TGSH4. 2946. | | | | |
| --- | --- | --- | --- | --- |
| Features | **Finding** | **Features** | | **Finding** |
| Profile | | **Atria** | | |
| Abdominal situs | Solitus | Left atrium | | Dilated |
| Cardiac position | Levocardia | Right atrium | | Normal |
| Systemic venous drainage | to RA | **Atrioventricular valves** | | |
| Pulmonary venous drainage | to LA | Mitral valve | | Annulus = 29mm. Thickened, mildly clubbed MVL. |
| Atrioventricular connection | Concordant | Tricuspid valve | | Annulus = 27mm  TAPSE = 17mm. |
| Ventriculoarterial connection | Concordant | **Ventricles** | | |
| Ventricular loop | d-Loop | Left ventricle | | Dilated |
| Septae | | Right ventricle | | Normal |
| Interventricular septum | Intact | **M-Mode:**. | | |
| Interatrial septum | Intact | AO | |  |
| Great arteries | NRGA | LA | |  |
| Aorta | ------- | LVIDd | | mm |
| Pulmonary artery | ------- | LVIDs | | mm |
| Semilunal valves |  | IVSd | | mm |
| Aortic valve | Annulus = 19mm. | IVSs | | mm |
| Pulmonary valve | Annulus = 21mm | LVPWd | | mm |
| Doppler Measurement | | LVPWs | | mm |
| Mitral | Moderate MR, Holosystolic, posterior projection, seen in two planes with jet velocity = 4.4m/sec | EDV | | ml |
| Aortic | Mild AR | ESV | | ml |
| Tricuspid | Moderate TR, PPG = 39mmHg | FS | | 31% |
| pulmonic | Trivial PR, PPG = 18mmHg | LVEF | | 58% |
| Final Diagnosis | 1. {S, D, S} Levocardia. 2. LA/LV Dilated 3. Thickened MVL 4. Moderate MR 5. Moderate TR 6. Mild AR 7. Mild Pulmonary Hypertension 8. Normal LV Function | | | |
| Done By: | **Signature** | | **Date of Reporting** | |
| Dr. Tesfaye Taye, Pediatrician, Pediatric Cardiologist | | | **21/07/2013Eth.C** | |

| Pediatric Echocardiography report  Patient Name: _Amlaku Abebaw. Patient ID: 076920. Gender: M. Age: 1 3/12.  Clinical Finding: DS + Murmur + RD. TGSH4. 2947. | | | | |
| --- | --- | --- | --- | --- |
| Features | **Finding** | **Features** | | **Finding** |
| Profile | | **Atria** | | |
| Abdominal situs | Solitus | Left atrium | | Dilated |
| Cardiac position | Levocardia | Right atrium | | Dilated |
| Systemic venous drainage | to RA | **Atrioventricular valves** | | |
| Pulmonary venous drainage | to LA | Mitral valve | | Annulus = mm |
| Atrioventricular connection | Complete AVSD | Tricuspid valve | | Annulus = mm  TAPSE = 11mm. |
| Ventriculoarterial connection | Concordant | **Ventricles** | | |
| Ventricular loop | d-Loop | Left ventricle | | Dilated |
| Septae | | Right ventricle | | Dilated |
| Interventricular septum | Complete AVSD, L – R Shunt | **M-Mode:** Normal LV Function on eye balling. | | |
| Interatrial septum | 12mm Additional Fenestrated OS ASD, L – R Shunt | AO | |  |
| Great arteries | NRGA | LA | |  |
| Aorta | ------- | LVIDd | | mm |
| Pulmonary artery | ------- | LVIDs | | mm |
| Semilunal valves |  | IVSd | | mm |
| Aortic valve | Annulus = 13mm. | IVSs | | mm |
| Pulmonary valve | Annulus = 14mm | LVPWd | | mm |
| Doppler Measurement | | LVPWs | | mm |
| Mitral | Mild Left AVVR | EDV | | ml |
| Aortic | ------ | ESV | | ml |
| Tricuspid | Mild Right AVVR | FS | | % |
| pulmonic | --------- | LVEF | | % |
| Aortic arch | ------- | **Coronary arteries** | |  |
| PDA | 1mm PDA, L – R Shunt |  | |  |
| Pericardium/Pleura |  | | | |
| Final Diagnosis | 1. {S, D, S} Levocardia. 2. Common Complete AVSD, L – R Shunt 3. Additional Large Fenestrated OS ASD, L – R Shunt 4. Mild Left AVVR 5. Mild Right AVVR 6. Small PDA, L – R Shunt 7. Normal LV Function | | | |
| Done By: | **Signature** | | **Date of Reporting** | |
| Dr. Tesfaye Taye, Pediatrician, Pediatric Cardiologist | | | **21/07/2013Eth.C** | |

| Pediatric Echocardiography report  Patient Name: Abebech Bekalu. Patient ID: 076890. Gender: _F_. Age: 1 3/12_.  Clinical Finding: Incidental Murmur. TGSH4. 2948. | | | | |
| --- | --- | --- | --- | --- |
| Features | **Finding** | **Features** | | **Finding** |
| Profile | | **Atria** | | |
| Abdominal situs | Solitus | Left atrium | | Normal |
| Cardiac position | Levocardia | Right atrium | | Normal |
| Systemic venous drainage | to RA | **Atrioventricular valves** | | |
| Pulmonary venous drainage | to LA | Mitral valve | | Annulus = 15mm |
| Atrioventricular connection | Concordant | Tricuspid valve | | Annulus = 15mm |
| Ventriculoarterial connection | Concordant | **Ventricles** | | |
| Ventricular loop | d-Loop | Left ventricle | | Normal |
| Septae | | Right ventricle | | Normal |
| Interventricular septum | Inlet VSD, Partially covered by STL with Functional Opening = 4mm, PPG = 80mmHg | **M-Mode:** Normal LV Function on eye balling. | | |
| Interatrial septum | Intact | AO | |  |
| Great arteries | NRGA | LA | |  |
| Aorta | ------- | LVIDd | | mm |
| Pulmonary artery | ------- | LVIDs | | mm |
| Semilunal valves |  | IVSd | | mm |
| Aortic valve | Annulus = 15mm. | IVSs | | mm |
| Pulmonary valve | Annulus = 14mm | LVPWd | | mm |
| Doppler Measurement | | LVPWs | | mm |
| Mitral | ------- | EDV | | ml |
| Aortic | ------ | ESV | | ml |
| Tricuspid | -------- | FS | | % |
| pulmonic | --------- | LVEF | | % |
| Aortic arch | ------- | **Coronary arteries** | |  |
| PDA | -------- |  | |  |
| Pericardium/Pleura |  | | | |
| Final Diagnosis | 1. {S, D, S} Levocardia. 2. Restrictive Inlet VSD, L – R Shunt, partially covered by STL 3. Normal Function | | | |
| Done By: | **Signature** | | **Date of Reporting** | |
| Dr. Tesfaye Taye, Pediatrician, Pediatric Cardiologist | | | **21/07/2013Eth.C** | |

| Pediatric Echocardiography report  Patient Name: Mariamawit Pawulos. Patient ID: 077545. Gender: Female. Age: 7/12.  Clinical Finding: RD + Murmur + Diaphoresis. TGSH4. 2949. | | | | |
| --- | --- | --- | --- | --- |
| Features | **Finding** | **Features** | | **Finding** |
| Profile | | **Atria** | | |
| Abdominal situs | Solitus | Left atrium | | Dilated |
| Cardiac position | Levocardia | Right atrium | | Dilated |
| Systemic venous drainage | to RA | **Atrioventricular valves** | | |
| Pulmonary venous drainage | to LA | Mitral valve | | Annulus = 13mm |
| Atrioventricular connection | Concordant | Tricuspid valve | | Annulus = 13mm  TAPSE = 17mm. |
| Ventriculoarterial connection | Truncus Arteriosus | **Ventricles** | | |
| Ventricular loop | d-Loop | Left ventricle | | Dilated |
| Septae | | Right ventricle | | Dilated |
| Interventricular septum | Non Restrictive Sub Aortic VSD | **M-Mode:** Normal LV Function on eye balling. | | |
| Interatrial septum | Intact | AO | |  |
| Great arteries | Truncus Arteriosus | LA | |  |
| Aorta | ------- | LVIDd | | mm |
| Pulmonary artery | A major vessel arising from left side of the Truncus | LVIDs | | mm |
| Semilunal valves |  | IVSd | | mm |
| Aortic valve | Truncal Annulus = 15mm. | IVSs | | mm |
| Pulmonary valve | --------------- | LVPWd | | mm |
| Doppler Measurement | | LVPWs | | mm |
| Mitral | ------- | EDV | | ml |
| Aortic | Moderate Truncal Regurgitation | ESV | | ml |
| Tricuspid | -------- | FS | | % |
| pulmonic | --------- | LVEF | | % |
| Aortic arch | ------- | **Coronary arteries** | |  |
| PDA | -------- |  | |  |
| Pericardium/Pleura | 5mm Pericardial effusion on RA/RV Junction. | | | |
| Final Diagnosis | 1. {S, D, S} Levocardia. 2. Truncus Arteriosus 3. Small Pericardial Effusion 4. Normal Biventricular Function | | | |
| Done By: | **Signature** | | **Date of Reporting** | |
| Dr. Tesfaye Taye, Pediatrician, Pediatric Cardiologist | | | **23/07/2013Eth.C** | |

| Pediatric Echocardiography report  Patient Name: Tirualem Azmeraw. Patient ID: 077117. Gender: Female. Age: 11/12.  Clinical Finding: RD + CHF + Murmur + Diaphoresis + ?IE. TGSH4. 2950. | | | | |
| --- | --- | --- | --- | --- |
| Features | **Finding** | **Features** | | **Finding** |
| Profile | | **Atria** | | |
| Abdominal situs | Solitus | Left atrium | | Dilated |
| Cardiac position | Levocardia | Right atrium | | Normal |
| Systemic venous drainage | to RA | **Atrioventricular valves** | | |
| Pulmonary venous drainage | to LA | Mitral valve | | Annulus = 14mm |
| Atrioventricular connection | Concordant | Tricuspid valve | | Annulus = 14mm |
| Ventriculoarterial connection | Concordant | **Ventricles** | | |
| Ventricular loop | d-Loop | Left ventricle | | Dilated |
| Septae | | Right ventricle | | Normal |
| Interventricular septum | 3mm Sub Pulmonic VSD, L – R Shunt | **M-Mode:** Normal Function on eye balling. | | |
| Interatrial septum | PFO, L – R Shunt | AO | |  |
| Great arteries | NRGA | LA | |  |
| Aorta | ------- | LVIDd | | mm |
| Pulmonary artery | **MPA = 18mm** | LVIDs | | mm |
| Semilunal valves |  | IVSd | | mm |
| Aortic valve | Annulus = 10mm. | IVSs | | mm |
| Pulmonary valve | Annulus = 18mm | LVPWd | | mm |
| Doppler Measurement | | LVPWs | | mm |
| Mitral | Mild MR, Holosystolic, seen in apical view with Jet velocity = 3.5m/sec | EDV | | ml |
| Aortic | ------ | ESV | | ml |
| Tricuspid | -------- | FS | | % |
| pulmonic | --------- | LVEF | | % |
| Aortic arch | ------- | **Coronary arteries** | |  |
| PDA | 3mm PDA, L – R Shunt. Echogenic mass at the Pulmonic side of the PDA, Mobile. | | | |
| Pericardium/Pleura | Trace Pericardial effusion | | | |
| Final Diagnosis | 1. {S, D, S} Levocardia. 2. LA/LV Dilated 3. PFO, L – R Shunt 4. Small Sub pulmonic VSD, L – R Shunt 5. Large PDA, L – R Shunt 6. Echogenic, Mobile mass at the Pulmonic end of the PDA. 7. Severe Pulmonary Hypertension 8. Trace pericardial effusion 9. Normal Function | | | |
| Done By: | **Signature** | | **Date of Reporting** | |
| Dr. Tesfaye Taye, Pediatrician, Pediatric Cardiologist | | | **23/07/2013Eth.C** | |

| Pediatric Echocardiography report  Patient Name: Baby of Zinash Amlak Patient ID: 043922. Gender: Male. Age: 10/12.  Clinical Finding: Recurrent Chest Infection. TGSH4. 2951. | | | | |
| --- | --- | --- | --- | --- |
| Features | **Finding** | **Features** | | **Finding** |
| Profile | | **Atria** | | |
| Abdominal situs | Solitus | Left atrium | | Normal |
| Cardiac position | Levocardia | Right atrium | | Normal |
| Systemic venous drainage | to RA | **Atrioventricular valves** | | |
| Pulmonary venous drainage | to LA | Mitral valve | | Annulus = 13mm |
| Atrioventricular connection | Concordant | Tricuspid valve | | Annulus = 13mm |
| Ventriculoarterial connection | Concordant | **Ventricles** | | |
| Ventricular loop | d-Loop | Left ventricle | | Normal |
| Septae | | Right ventricle | | Normal |
| Interventricular septum | Intact | **M-Mode:** Normal LV Function on eye balling. | | |
| Interatrial septum | PFO, L – R Shunt | AO | |  |
| Great arteries | NRGA | LA | |  |
| Aorta | ------- | LVIDd | | mm |
| Pulmonary artery | ------- | LVIDs | | mm |
| Semilunal valves |  | IVSd | | mm |
| Aortic valve | Annulus = 10mm. | IVSs | | mm |
| Pulmonary valve | Annulus = 11mm | LVPWd | | mm |
| Doppler Measurement | | LVPWs | | mm |
| Mitral | ------- | EDV | | ml |
| Aortic | ------ | ESV | | ml |
| Tricuspid | -------- | FS | | % |
| pulmonic | --------- | LVEF | | % |
| Aortic arch | ------- | **Coronary arteries** | |  |
| PDA | -------- |  | |  |
| Pericardium/Pleura |  | | | |
| Final Diagnosis | 1. {S, D, S} Levocardia. 2. PFO, L – R Shunt | | | |
| Done By: | **Signature** | | **Date of Reporting** | |
| Dr. Tesfaye Taye, Pediatrician, Pediatric Cardiologist | | | **23/07/2013Eth.C** | |

| Pediatric Echocardiography report  Patient Name: Mequanint Challie. Patient ID: 077381. Gender: Male. Age: 13years.  Clinical Finding: Rheumatic Recurrence + Murmur + CHF + Palpitation + DOE. TGSH4. 2952. | | | | |
| --- | --- | --- | --- | --- |
| Features | **Finding** | **Features** | | **Finding** |
| Profile | | **Atria** | | |
| Abdominal situs | Solitus | Left atrium | | Dilated |
| Cardiac position | Levocardia | Right atrium | | Dilated |
| Systemic venous drainage | to RA | **Atrioventricular valves** | | |
| Pulmonary venous drainage | to LA | Mitral valve | | Annulus = 22mm. Thickened MVL. Shortened PMVL |
| Atrioventricular connection | Concordant | Tricuspid valve | | Annulus = 23mm |
| Ventriculoarterial connection | Concordant | **Ventricles** | | |
| Ventricular loop | d-Loop | Left ventricle | | Dilated |
| Septae | | Right ventricle | | Dilated |
| Interventricular septum | Intact | **M-Mode:** Normal LV Function on eye balling. | | |
| Interatrial septum | Intact | AO | |  |
| Great arteries | NRGA | LA | |  |
| Aorta | ------- | LVIDd | | mm |
| Pulmonary artery | ------- | LVIDs | | mm |
| Semilunal valves |  | IVSd | | mm |
| Aortic valve | Annulus = 17mm. | IVSs | | mm |
| Pulmonary valve | Annulus = 24mm | LVPWd | | mm |
| Doppler Measurement | | LVPWs | | mm |
| Mitral | Moderate MR, Holosystolic, posterior projection, seen in two planes with jet velocity = 3.8m/sec | EDV | | ml |
| Aortic | ------ | ESV | | ml |
| Tricuspid | Moderate TR, PPG = 77mmHg | FS | | % |
| pulmonic | --------- | LVEF | | % |
| PDA | -------- |  | |  |
| Pericardium/Pleura |  | | | |
| Final Diagnosis | 1. {S, D, S} Levocardia. 2. Thickened MVL. Shortened PMVL 3. Moderate MR 4. Moderate TR 5. Severe Pulmonary Hypertension 6. Normal LV Function | | | |
| Done By: | **Signature** | | **Date of Reporting** | |
| Dr. Tesfaye Taye, Pediatrician, Pediatric Cardiologist | | | **23/07/2013Eth.C** | |

| Pediatric Echocardiography report  Patient Name: Yihalem Yigrem. Patient ID: 077549. Gender: Male. Age: 5/12.  Clinical Finding: Cyanosis + RD + Diaphoresis. TGSH4. 2953. | | | | |
| --- | --- | --- | --- | --- |
| Features | **Finding** | **Features** | | **Finding** |
| Profile | | **Atria** | | |
| Abdominal situs | Solitus | Left atrium | | Normal |
| Cardiac position | Levocardia | Right atrium | | Normal |
| Systemic venous drainage | to RA | **Atrioventricular valves** | | |
| Pulmonary venous drainage | to LA | Mitral valve | | Common Complete AVSD |
| Atrioventricular connection | AVSD | Tricuspid valve | | Common Complete AVSD |
| Ventriculoarterial connection | Concordant | **Ventricles** | | |
| Ventricular loop | d-Loop | Left ventricle | | Smallish |
| Septae | | Right ventricle | | Normal |
| Interventricular septum | Common Complete AVSD | **M-Mode:**. | | |
| Interatrial septum | Additional 6mm High Secundum ASD, L – R Shunt | AO | |  |
| Great arteries | NRGA | LA | |  |
| Aorta | ------- | LVIDd | | mm |
| Pulmonary artery | ------- | LVIDs | | mm |
| Semilunal valves |  | IVSd | | mm |
| Aortic valve | ?Atretic | IVSs | | mm |
| Pulmonary valve | Annulus = 13mm | LVPWd | | mm |
| Doppler Measurement | | LVPWs | | mm |
| Mitral | ------- | EDV | | ml |
| Aortic | ------ | ESV | | ml |
| Tricuspid | -------- | FS | | % |
| pulmonic | --------- | LVEF | | % |
| Aortic arch | ------- | **Coronary arteries** | |  |
| PDA | PDA from PA to AO | | | |
| Pericardium/Pleura |  | | | |
| Final Diagnosis | 1. {S, D, S} Levocardia. 2. Common Complete Unbalanced AVSD, L – R Shunt 3. Additional High Secundum ASD, L – R Shunt 4. ? Atretic Aortic Valve | | | |
| Recommendation | **Repeat Echo after 3months (HLHS + AVSD + ASD)** | | | |
| Done By: | **Signature** | | **Date of Reporting** | |
| Dr. Tesfaye Taye, Pediatrician, Pediatric Cardiologist | | | **23/07/2013Eth.C** | |

| Pediatric Echocardiography report  Patient Name: Simegn Tigabu. Patient ID: 078361. Gender: Female. Age: 7months.  Clinical Finding: Murmur + Diaphoresis. TGSH4. 2954. | | | | |
| --- | --- | --- | --- | --- |
| Features | **Finding** | **Features** | | **Finding** |
| Profile | | **Atria** | | |
| Abdominal situs | Solitus | Left atrium | | Normal |
| Cardiac position | Levocardia | Right atrium | | Dilated |
| Systemic venous drainage | to RA | **Atrioventricular valves** | | |
| Pulmonary venous drainage | to LA | Mitral valve | | Annulus = 10mm |
| Atrioventricular connection | Concordant | Tricuspid valve | | Annulus = 14mm |
| Ventriculoarterial connection | Concordant | **Ventricles** | | |
| Ventricular loop | d-Loop | Left ventricle | | Normal |
| Septae | | Right ventricle | | Dilated |
| Interventricular septum | 4mm Inlet VSD, L – R Shunt | **M-Mode:**. | | |
| Interatrial septum | 12mm OS ASD, L – R Shunt  Ostium Primum defect, L – R Shunt | AO | |  |
| Great arteries | NRGA | LA | |  |
| Aorta | ------- | LVIDd | | mm |
| Pulmonary artery | ------- | LVIDs | | mm |
| Semilunal valves |  | IVSd | | mm |
| Aortic valve | Annulus = 12mm. | IVSs | | mm |
| Pulmonary valve | Annulus = 16mm | LVPWd | | mm |
| Doppler Measurement | | LVPWs | | mm |
| Mitral | ------- | EDV | | ml |
| Aortic | ------ | ESV | | ml |
| Tricuspid | Mild TR | FS | | % |
| pulmonic | --------- | LVEF | | % |
| Aortic arch | ------- | **Coronary arteries** | |  |
| PDA | <1mm PDA, L – R Shunt |  | |  |
| Pericardium/Pleura |  | | | |
| Final Diagnosis | 1. {S, D, S} Levocardia. 2. RA/RV Dilated 3. Transitional AVSD, L – R Shunt 4. Additional Large OS ASD, L – R Shunt 5. Small (Silent) PDA, L – R Shunt | | | |
| Done By: | **Signature** | | **Date of Reporting** | |
| Dr. Tesfaye Taye, Pediatrician, Pediatric Cardiologist | | | **30/07/2013Eth.C** | |

| Pediatric Echocardiography report  Patient Name: Tekeste Meseret. Patient ID: 077816. Gender: Male. Age: 1 6/12.  Clinical Finding: RD + CHF + DOE + Murmur. TGSH4. 2955. | | | | |
| --- | --- | --- | --- | --- |
| Features | **Finding** | **Features** | | **Finding** |
| Profile | | **Atria** | | |
| Abdominal situs | Solitus | Left atrium | | More dilated |
| Cardiac position | Levocardia | Right atrium | | Dilated |
| Systemic venous drainage | to RA | **Atrioventricular valves** | | |
| Pulmonary venous drainage | to LA | Mitral valve | | Annulus = 20mm. thickened and shortened PMVL. |
| Atrioventricular connection | Concordant | Tricuspid valve | | Annulus = 21mm |
| Ventriculoarterial connection | Concordant | **Ventricles** | | |
| Ventricular loop | d-Loop | Left ventricle | | More dilated. Hypertrophy |
| Septae | | Right ventricle | | Dilated. RV TDI S wave = 11cm/sec |
| Interventricular septum | Intact | **M-Mode:**. | | |
| Interatrial septum | Intact | AO | |  |
| Great arteries | NRGA | LA | |  |
| Aorta | ------- | LVIDd | | mm |
| Pulmonary artery | MPA = 23mm | LVIDs | | mm |
| Semilunal valves |  | IVSd | | mm |
| Aortic valve | Annulus = 11mm. | IVSs | | mm |
| Pulmonary valve | Annulus = 20mm | LVPWd | | mm |
| Doppler Measurement | | LVPWs | | mm |
| Mitral | Moderate MR, Holosystolic, posterior projection, seen in two planes with jet velocity = 3.7m/sec. Mitral inflow gradient = 10/6mmHg. | EDV | | ml |
| Aortic | ------ | ESV | | ml |
| Tricuspid | Mild TR, PPG = 88mmHg | FS | | 33% |
| pulmonic | Mild PR, PPG = 62mmHg | LVEF | | 62% |
| Aortic arch | Left. Gradient across the aortic isthmus= 21mmHg | **Coronary arteries** | |  |
| PDA | -------- |  | |  |
| Pericardium/Pleura | Trace pericardial effusion | | | |
| Final Diagnosis | 1. {S, D, S} Levocardia. 2. All chambers dilated 3. Thickened, shortened PMVL 4. Moderate MR 5. Mild TR 6. Mild MS (Flow related) 7. Coarctation substrate 8. Severe Pulmonary Hypertension 9. Normal Function | | | |
| Done By: | **Signature** | | **Date of Reporting** | |
| Dr. Tesfaye Taye, Pediatrician, Pediatric Cardiologist | | | **30/07/2013Eth.C** | |

| Pediatric Echocardiography report  Patient Name: Amnen Haile Mariam. Patient ID: _074164_. Gender: F_. Age: 6/12_.  Clinical Finding: + CHB. AGH. | | | | |
| --- | --- | --- | --- | --- |
| Features | **Finding** | **Features** | | **Finding** |
| Follow up echo for Congenital Complete Heart Block | | | | |
| Profile | | **Atria** | | |
| Abdominal situs | Solitus | Left atrium | | Normal |
| Cardiac position | Levocardia | Right atrium | | Normal |
| Systemic venous drainage | to RA | **Atrioventricular valves** | | |
| Pulmonary venous drainage | to LA | Mitral valve | | Annulus = 14mm |
| Atrioventricular connection | Concordant | Tricuspid valve | | Annulus = 15mm  TAPSE = 17mm. |
| Ventriculoarterial connection | Concordant | **Ventricles** | | |
| Ventricular loop | d-Loop | Left ventricle | | Normal |
| Septae | | Right ventricle | | Normal |
| Interventricular septum | Intact | **M-Mode:**. | | |
| Interatrial septum | PFO, L – R Shunt | AO | |  |
| Great arteries | NRGA | LA | |  |
| Aorta | ------- | LVIDd | | mm |
| Pulmonary artery | ------- | LVIDs | | mm |
| Semilunal valves |  | IVSd | | mm |
| Aortic valve | Annulus = 12mm. | IVSs | | mm |
| Pulmonary valve | Annulus = 12mm | LVPWd | | mm |
| Doppler Measurement | | LVPWs | | mm |
| Mitral | ------- | EDV | | ml |
| Aortic | ------ | ESV | | ml |
| Tricuspid | Trivial TR, PPG = 25mmHg | FS | | 35% |
| pulmonic | --------- | LVEF | | 66% |
| Aortic arch | ------- | **Coronary arteries** | |  |
| PDA | -------- |  | |  |
| Pericardium/Pleura |  | | | |
| Final Diagnosis | 1. {S, D, S} Levocardia. 2. PFO, L – R Shunt 3. Normal Biventricular Function | | | |
| Done By: | **Signature** | | **Date of Reporting** | |
| Dr. Tesfaye Taye, Pediatrician, Pediatric Cardiologist | | | **05/08/2013Eth.C** | |

| Pediatric Echocardiography report  Patient Name: Bekalu Hunegnaw. Patient ID: 040944. Gender: M. Age: 1 2/12. | | | | |
| --- | --- | --- | --- | --- |
| Clinical Finding: Follow up echo for Mild valvar PS | | | | |
| Features | **Finding** | **Features** | | **Finding** |
| Profile | | **Atria** | | |
| Abdominal situs | Solitus | Left atrium | | Normal |
| Cardiac position | Levocardia | Right atrium | | Normal |
| Systemic venous drainage | to RA | **Atrioventricular valves** | | |
| Pulmonary venous drainage | to LA | Mitral valve | | Annulus = 13mm |
| Atrioventricular connection | Concordant | Tricuspid valve | | Annulus = 14mm |
| Ventriculoarterial connection | Concordant | **Ventricles** | | |
| Ventricular loop | d-Loop | Left ventricle | | Normal |
| Septae | | Right ventricle | | Normal |
| Interventricular septum | Intact | **M-Mode:**. | | |
| Interatrial septum | Intact | AO | |  |
| Great arteries | NRGA | LA | |  |
| Aorta | ------- | LVIDd | | mm |
| Pulmonary artery | ------- | LVIDs | | mm |
| Semilunal valves |  | IVSd | | mm |
| Aortic valve | Annulus = 11mm. | IVSs | | mm |
| Pulmonary valve | Annulus = 11mm | LVPWd | | mm |
| Doppler Measurement | | LVPWs | | mm |
| Mitral | ------- | EDV | | ml |
| Aortic | ------ | ESV | | ml |
| Tricuspid | -------- | FS | | % |
| pulmonic | --------- | LVEF | | % |
| Aortic arch | ------- | **Coronary arteries** | |  |
| PDA | -------- |  | |  |
| Pericardium/Pleura |  | | | |
| Final Diagnosis | 1. Normal Echocardiography Study | | | |
| Remark | Resolved Valvar PS | | | |
| Done By: | **Signature** | | **Date of Reporting** | |
| Dr. Tesfaye Taye, Pediatrician, Pediatric Cardiologist | | | **05/08/2013Eth.C** | |

| Pediatric Echocardiography report  Patient Name: _Melaku Getachew. Patient ID: 078876. Gender: M_. Age: _1 7/12_.  Clinical Finding: RD + Murmur + CHF + Diaphoresis. TGSH4. 2956. | | | | |
| --- | --- | --- | --- | --- |
| Features | **Finding** | **Features** | | **Finding** |
| Profile | | **Atria** | | |
| Abdominal situs | Solitus | Left atrium | | Dilated |
| Cardiac position | Levocardia | Right atrium | | Dilated |
| Systemic venous drainage | to RA | **Atrioventricular valves** | | |
| Pulmonary venous drainage | to LA | Mitral valve | | Annulus = 16mm |
| Atrioventricular connection | Concordant | Tricuspid valve | | Annulus = 16mm |
| Ventriculoarterial connection | DORV. Aorto-Mitral Fibrous Discontinuity. | **Ventricles** | | |
| Ventricular loop | d-Loop | Left ventricle | | Dilated |
| Septae | | Right ventricle | | Dilated |
| Interventricular septum | 15mm Inlet VSD, L – R Shunt | **M-Mode:**. | | |
| Interatrial septum | Intact | AO | |  |
| Great arteries | NRGA | LA | |  |
| Aorta | From RV and posterior & to the right | LVIDd | | mm |
| Pulmonary artery | **MPA = 21mm.** From RV and anterior & to the left. | LVIDs | | mm |
| Semilunal valves |  | IVSd | | mm |
| Aortic valve | Annulus = 14mm. | IVSs | | mm |
| Pulmonary valve | Annulus = 16mm | LVPWd | | mm |
| Doppler Measurement | | LVPWs | | mm |
| Mitral | Trivial MR | EDV | | ml |
| Aortic | ------ | ESV | | ml |
| Tricuspid | Trivial TR | FS | | 36% |
| pulmonic | --------- | LVEF | | 67% |
| Aortic arch | ------- | **Coronary arteries** | |  |
| PDA | -------- |  | |  |
| Pericardium/Pleura |  | | | |
| Final Diagnosis | 1. {S, D, D} Levocardia. 2. DORV 3. All chambers dilated 4. Large Inlet VSD, L – R Shunt 5. Severe Pulmonary Hypertension 6. Normal LV Function | | | |
| Done By: | **Signature** | | **Date of Reporting** | |
| Dr. Tesfaye Taye, Pediatrician, Pediatric Cardiologist | | | **05/08/2013Eth.C** | |

| Pediatric Echocardiography report  Patient Name: Yenealem Selamsew. Patient ID: 078173. Gender: F. Age: 7years.  Clinical Finding: DOE + Murmur + Palpitation. TGSH4. 2957. | | | | |
| --- | --- | --- | --- | --- |
| Features | **Finding** | **Features** | | **Finding** |
| Profile | | **Atria** | | |
| Abdominal situs | Solitus | Left atrium | | Normal |
| Cardiac position | Levocardia | Right atrium | | Dilated |
| Systemic venous drainage | to RA | **Atrioventricular valves** | | |
| Pulmonary venous drainage | to LA | Mitral valve | | Annulus = 16mm |
| Atrioventricular connection | Concordant | Tricuspid valve | | Annulus = 18mm  TAPSE = 17mm. |
| Ventriculoarterial connection | Concordant | **Ventricles** | | |
| Ventricular loop | d-Loop | Left ventricle | | Normal |
| Septae | | Right ventricle | | Dilated, Hypertrophied |
| Interventricular septum | --------- | **M-Mode:**. | | |
| Interatrial septum | 15mm OS ASD, L – R Shunt | AO | |  |
| Great arteries | NRGA | LA | |  |
| Aorta | ------- | LVIDd | | mm |
| Pulmonary artery | MPA Narrow = 9mm mid level | LVIDs | | mm |
| Semilunal valves |  | IVSd | | mm |
| Aortic valve | Annulus = 11mm. | IVSs | | mm |
| Pulmonary valve | Annulus = 15mm. | LVPWd | | mm |
| Doppler Measurement | | LVPWs | | mm |
| Mitral | ------- | EDV | | ml |
| Aortic | ------ | ESV | | ml |
| Tricuspid | -------- | FS | | 37% |
| pulmonic | Severe valvar & Supra valvar PS, PPG = 110mmHg. | LVEF | | 69% |
| Aortic arch | ------- | **Coronary arteries** | |  |
| PDA | -------- |  | |  |
| Pericardium/Pleura |  | | | |
| Final Diagnosis | 1. {S, D, S} Levocardia. 2. Large OS ASD, L – R Shunt 3. Severe Valvar and Supra valvar PS | | | |
| Done By: | **Signature** | | **Date of Reporting** | |
| Dr. Tesfaye Taye, Pediatrician, Pediatric Cardiologist | | | **05/08/2013Eth.C** | |

| Pediatric Echocardiography report  Patient Name: Bante-Gizie Demlie. Patient ID: 079513. Gender: M. Age: 3years.  Clinical Finding: RD + Murmur + Diaphoresis + Cyanosis. TGSH4. 2958. | | | | |
| --- | --- | --- | --- | --- |
| Features | **Finding** | **Features** | | **Finding** |
| Profile | | **Atria** | | |
| Abdominal situs | Solitus | Left atrium | | Dilated |
| Cardiac position | Levocardia | Right atrium | | Dilated |
| Systemic venous drainage | to RA | **Atrioventricular valves** | | |
| Pulmonary venous drainage | to LA | Mitral valve | | Annulus = 18mm |
| Atrioventricular connection | Concordant | Tricuspid valve | | Annulus = 15mm |
| Ventriculoarterial connection | Concordant | **Ventricles** | | |
| Ventricular loop | d-Loop | Left ventricle | | Dilated |
| Septae | | Right ventricle | | Dilated |
| Interventricular septum | 11mm Muscular VSD, R – L Shunt | **M-Mode:**. | | |
| Interatrial septum |  | AO | |  |
| Great arteries | l-TGA | LA | |  |
| Aorta | Anterior and to the left. From LV | LVIDd | | mm |
| Pulmonary artery | Smallish MPA and branch PAs. Posterior and to the right. From RV | LVIDs | | mm |
| Semilunal valves |  | IVSd | | mm |
| Aortic valve | Annulus = 18mm. | IVSs | | mm |
| Pulmonary valve | Atretic | LVPWd | | mm |
| Doppler Measurement | | LVPWs | | mm |
| Mitral | ------- | EDV | | ml |
| Aortic | ------ | ESV | | ml |
| Tricuspid | -------- | FS | | % |
| pulmonic | -------- | LVEF | | % |
| Aortic arch | ------- | **Coronary arteries** | |  |
| PDA | -------- |  | |  |
| Pericardium/Pleura |  | | | |
| Final Diagnosis | 1. {S, D, L} Levocardia. 2. L-TGA 3. Large Muscular VSD, BD Shunt 4. Pulmonary atresia 5. Smallish MPA and Branch PAs. | | | |
| Done By: | **Signature** | | **Date of Reporting** | |
| Dr. Tesfaye Taye, Pediatrician, Pediatric Cardiologist | | | **05/08/2013Eth.C** | |

| Pediatric Echocardiography report  Patient Name: Mita Kasa. Patient ID: 079430. Gender: Female. Age: 14years.  Clinical Finding: Palpitation + DOE + Murmur + easy fatigability. TGSH4. 2959. | | | | |
| --- | --- | --- | --- | --- |
| Features | **Finding** | **Features** | | **Finding** |
| Profile | | **Atria** | | |
| Abdominal situs | Solitus | Left atrium | | Normal |
| Cardiac position | Levocardia | Right atrium | | Normal |
| Systemic venous drainage | to RA | **Atrioventricular valves** | | |
| Pulmonary venous drainage | to LA | Mitral valve | | Annulus = 24mm |
| Atrioventricular connection | Concordant | Tricuspid valve | | Annulus = 23mm  TAPSE = 21mm. |
| Ventriculoarterial connection | Concordant | **Ventricles** | | |
| Ventricular loop | d-Loop | Left ventricle | | Concentric hypertrophy |
| Septae | | Right ventricle | | Normal |
| Interventricular septum |  | **M-Mode:**. | | |
| Interatrial septum |  | AO | |  |
| Great arteries | NRGA | LA | |  |
| Aorta | ------- | LVIDd | | 38mm |
| Pulmonary artery |  | LVIDs | | 17mm |
| Semilunal valves |  | IVSd | | 16mm |
| Aortic valve | Annulus = 15mm. Trileaflet | IVSs | | 17mm |
| Pulmonary valve | Annulus = 19mm. | LVPWd | | 15mm |
| Doppler Measurement | | LVPWs | | 16mm |
| Mitral | ------- | EDV | | 63ml |
| Aortic | Severe AS, PPG/MPG = 103/63mmHg. Moderate AR, PHT = 400ms. | ESV | | 8ml |
| Tricuspid | Trivial TR, PPG = 25mmHg | FS | | 56% |
| pulmonic | -------- | LVEF | | 87% |
| Aortic arch | ------- | **Coronary arteries** | |  |
| PDA | -------- |  | |  |
| Pericardium/Pleura |  | | | |
| Final Diagnosis | 1. {S, D, S} Levocardia. 2. Moderate AR 3. Severe AS 4. LVH 5. Hyperdynamic LV | | | |
| Recommendation | **Needs Urgent intervention.** | | | |
| Done By: | **Signature** | | **Date of Reporting** | |
| Dr. Tesfaye Taye, Pediatrician, Pediatric Cardiologist | | | **07/08/2013Eth.C** | |

| Pediatric Echocardiography report  Patient Name: Nebiyat Abiy. Patient ID: 067143. Gender: _Female. Age: 2years. | | | | |
| --- | --- | --- | --- | --- |
| Follow up echo after PDA Surgical closure. | | | | |
| Features | **Finding** | **Features** | | **Finding** |
| Profile | | **Atria** | | |
| Abdominal situs | Solitus | Left atrium | | Mildly dilated |
| Cardiac position | Levocardia | Right atrium | | Normal |
| Systemic venous drainage | to RA | **Atrioventricular valves** | | |
| Pulmonary venous drainage | to LA | Mitral valve | | Annulus = 18mm |
| Atrioventricular connection | Concordant | Tricuspid valve | | Annulus = 16mm |
| Ventriculoarterial connection | Concordant | **Ventricles** | | |
| Ventricular loop | d-Loop | Left ventricle | | Mildly dilated |
| Septae | | Right ventricle | | Normal |
| Interventricular septum |  | **M-Mode:** Normal LV Function on eye balling | | |
| Interatrial septum |  | AO | |  |
| Great arteries | NRGA | LA | |  |
| Aorta | ------- | LVIDd | | mm |
| Pulmonary artery | No obstruction across MPA, RPA | LVIDs | | mm |
| Semilunal valves |  | IVSd | | mm |
| Aortic valve | Annulus = 12mm. | IVSs | | mm |
| Pulmonary valve | Annulus = 13mm. | LVPWd | | mm |
| Doppler Measurement | | LVPWs | | mm |
| Mitral | Mild MR | EDV | | ml |
| Aortic | Trivial AR | ESV | | ml |
| Tricuspid | -------- | FS | | % |
| pulmonic | -------- | LVEF | | % |
| PDA | No PDA |  | |  |
| Pericardium/Pleura | 4mm pericardial effusion on RV Side | | | |
| Final Diagnosis | 1. S/P PDA Surgical Closure 2. {S, D, S} Levocardia. 3. Mild MR 4. Trivial AR 5. Normal Function | | | |
| Remark | No echocardiographic Evidences for IE | | | |
| Done By: | **Signature** | | **Date of Reporting** | |
| Dr. Tesfaye Taye, Pediatrician, Pediatric Cardiologist | | | **07/08/2013Eth.C** | |

| Pediatric Echocardiography report  Patient Name: _Nigussie Misganaw. Patient ID: 079146. Gender: Male. Age: 12years.  Clinical Finding: Rheumatic Recurrence + DOE + Palpitation + Murmur. TGSH4. 2960. | | | |
| --- | --- | --- | --- |
| Features | **Finding** | **Features** | **Finding** |
| Profile | | **Atria** | |
| Abdominal situs | Solitus | Left atrium | Dilated |
| Cardiac position | Levocardia | Right atrium | Normal |
| Systemic venous drainage | to RA | **Atrioventricular valves** | |
| Pulmonary venous drainage | to LA | Mitral valve | Annulus = 23mm. Thickened, Clubbed MVL. MVA = 1.2cm^2^. |
| Atrioventricular connection | Concordant | TV | Annulus = 24mm |
| Ventriculoarterial connection | Concordant | **Ventricles** | |
| Ventricular loop | d-Loop | Left ventricle | Dilated |
| Septae | | Right ventricle | Normal. RV TDI S Wave = 16cm/sec. |
| Interventricular septum |  | **M-Mode:**. | |
| Interatrial septum |  | AO |  |
| Great arteries | NRGA | LA |  |
| Aorta | ------- | LVIDd | mm |
| Pulmonary artery |  | LVIDs | mm |
| Semilunal valves |  | IVSd | mm |
| Aortic valve | Annulus = 17mm. | IVSs | mm |
| Pulmonary valve | Annulus = 24mm. | LVPWd | mm |
| Doppler Measurement | | LVPWs | mm |
| Mitral | Severe MR, Holosystolic, Posterior projection, seen in two planes, jet velocity = 3.6m/sec. Moderate MS, PPG/MPG = 14/7mmHg | EDV | ml |
| Aortic | Moderate AR, PHT = 319ms. | ESV | ml |
| Tricuspid | Trivial TR, PPG = 21mmHg | FS | 35% |
| pulmonic | -------- | LVEF | 65% |
| Aortic arch | ------- | **Coronary arteries** |  |
| PDA | -------- | **Pericardium/Pleura** |  |
| Final Diagnosis | 1. {S, D, S} Levocardia. 2. LA/LV Dilated, Thickened, clubbed MVL 3. Severe MR 4. Moderate MS 5. Moderate AR 6. Normal Function | | |
| Done By: | **Signature** | **Date of Reporting** | |
| Dr. Tesfaye Taye, Pediatrician, Pediatric Cardiologist | | **07/08/2013Eth.C** | |

| Pediatric Echocardiography report  Patient Name: Habtamnesh Asferaw. Patient ID: PICU. Gender: Female. Age: 8years.  Clinical Finding: Sepsis + ?Cardiogenic Shock + RD. TGSH4. 2961. | | | | |
| --- | --- | --- | --- | --- |
| Features | **Finding** | **Features** | | **Finding** |
| Profile | | **Atria** | | |
| Abdominal situs | Solitus | Left atrium | | Normal |
| Cardiac position | Levocardia | Right atrium | | Normal |
| Systemic venous drainage | to RA | **Atrioventricular valves** | | |
| Pulmonary venous drainage | to LA | Mitral valve | | Annulus = 20mm |
| Atrioventricular connection | Concordant | Tricuspid valve | | Annulus = 23mm |
| Ventriculoarterial connection | Concordant | **Ventricles** | | |
| Ventricular loop | d-Loop | Left ventricle | | Normal |
| Septae | | Right ventricle | | Normal  RV TDI S wave = 14cm/sec |
| Interventricular septum |  | **M-Mode:**. | | |
| Interatrial septum |  | AO | |  |
| Great arteries | NRGA | LA | |  |
| Aorta | ------- | LVIDd | | mm |
| Pulmonary artery |  | LVIDs | | mm |
| Semilunal valves |  | IVSd | | mm |
| Aortic valve | Annulus = 15mm. | IVSs | | mm |
| Pulmonary valve | Annulus = 21mm. | LVPWd | | mm |
| Doppler Measurement | | LVPWs | | mm |
| Mitral | ------- | EDV | | ml |
| Aortic | ------ | ESV | | ml |
| Tricuspid | Trivial TR, PPG = 36mmHg | FS | | 31% |
| pulmonic | -------- | LVEF | | 60% |
| Aortic arch | ------- | **Coronary arteries** | |  |
| PDA | -------- |  | |  |
| Pericardium/Pleura | 33mm Right Pleural Effusion. | | | |
| Final Diagnosis | 1. {S, D, S} Levocardia. 2. Large Right Pleural Effusion | | | |
| Remark | Tachycardia During Study | | | |
| Done By: | **Signature** | | **Date of Reporting** | |
| Dr. Tesfaye Taye, Pediatrician, Pediatric Cardiologist | | | **07/08/2013Eth.C** | |

| Pediatric Echocardiography report  Patient Name: Baby of Birhane Getawey. Patient ID: 080295. Gender: Female. Age: 6days.  Clinical Finding: Incidental Murmur. TGSH4. 2962. | | | | |
| --- | --- | --- | --- | --- |
| Features | **Finding** | **Features** | | **Finding** |
| Profile | | **Atria** | | |
| Abdominal situs | Solitus | Left atrium | | Normal |
| Cardiac position | Levocardia | Right atrium | | Normal |
| Systemic venous drainage | to RA | **Atrioventricular valves** | | |
| Pulmonary venous drainage | to LA | Mitral valve | | Annulus = 10mm |
| Atrioventricular connection | Concordant | Tricuspid valve | | Annulus = 10mm |
| Ventriculoarterial connection | Concordant | **Ventricles** | | |
| Ventricular loop | d-Loop | Left ventricle | | Normal |
| Septae | | Right ventricle | | Normal |
| Interventricular septum |  | **M-Mode:** Normal LV Function on eye balling. | | |
| Interatrial septum |  | AO | |  |
| Great arteries | NRGA | LA | |  |
| Aorta | ------- | LVIDd | | mm |
| Pulmonary artery |  | LVIDs | | mm |
| Semilunal valves |  | IVSd | | mm |
| Aortic valve | Annulus = 8mm. | IVSs | | mm |
| Pulmonary valve | Annulus = 8mm. | LVPWd | | mm |
| Doppler Measurement | | LVPWs | | mm |
| Mitral | ------- | EDV | | ml |
| Aortic | ------ | ESV | | ml |
| Tricuspid | -------- | FS | | % |
| pulmonic | Mild Valvar PS, PPG = 31mmHg | LVEF | | % |
| Aortic arch | ------- | **Coronary arteries** | |  |
| PDA | 1.5mm PDA, L – R Shunt |  | |  |
| Pericardium/Pleura |  | | | |
| Final Diagnosis | 1. {S, D, S} Levocardia. 2. Small PDA, L – R Shunt 3. Mild Valvar PS 4. Normal Function | | | |
| Done By: | **Signature** | | **Date of Reporting** | |
| Dr. Tesfaye Taye, Pediatrician, Pediatric Cardiologist | | | **12/08/2013Eth.C** | |

| Pediatric Echocardiography report  Patient Name: Dinknesh Mulu. Patient ID: 062455. Gender: Female. Age: 5months.  Clinical Finding: Diaphoresis + Murmur. TGSH4. 2963. | | | | |
| --- | --- | --- | --- | --- |
| Features | **Finding** | **Features** | | **Finding** |
| Profile | | **Atria** | | |
| Abdominal situs | Solitus | Left atrium | | Dilated |
| Cardiac position | Levocardia | Right atrium | | Mildly Dilated |
| Systemic venous drainage | to RA | **Atrioventricular valves** | | |
| Pulmonary venous drainage | to LA | Mitral valve | | Annulus = 13mm |
| Atrioventricular connection | Concordant | Tricuspid valve | | Annulus = 13mm |
| Ventriculoarterial connection | Concordant | **Ventricles** | | |
| Ventricular loop | d-Loop | Left ventricle | | Dilated |
| Septae | | Right ventricle | | Mildly Dilated |
| Interventricular septum |  | **M-Mode:** Normal LV Function on eye balling. | | |
| Interatrial septum |  | AO | |  |
| Great arteries | NRGA | LA | |  |
| Aorta | ------- | LVIDd | | mm |
| Pulmonary artery |  | LVIDs | | mm |
| Semilunal valves |  | IVSd | | mm |
| Aortic valve | Annulus = 12mm. | IVSs | | mm |
| Pulmonary valve | Annulus = 14mm. | LVPWd | | mm |
| Doppler Measurement | | LVPWs | | mm |
| Mitral | ------- | EDV | | ml |
| Aortic | ------ | ESV | | ml |
| Tricuspid | Mild TR, PPG = 50mmHg | FS | | % |
| pulmonic | -------- | LVEF | | % |
| Aortic arch | ------- | **Coronary arteries** | |  |
| PDA | 2mm PDA, L – R Shunt |  | |  |
| Pericardium/Pleura |  | | | |
| Final Diagnosis | 1. {S, D, S} Levocardia. 2. Moderate PDA, L – R Shunt 3. Moderate Pulmonary Hypertension 4. Normal LV Function | | | |
| Done By: | **Signature** | | **Date of Reporting** | |
| Dr. Tesfaye Taye, Pediatrician, Pediatric Cardiologist | | | **12/08/2013Eth.C** | |

| Pediatric Echocardiography report  Patient Name: Temesgen Gizat. Patient ID: 080087. Gender: Male. Age: 10months.  Clinical Finding: DS + RD. TGSH4. 2964. | | | | |
| --- | --- | --- | --- | --- |
| Features | **Finding** | **Features** | | **Finding** |
| Profile | | **Atria** | | |
| Abdominal situs | Solitus | Left atrium | | Normal |
|  |  |  | |  |
| Cardiac position | Levocardia | Right atrium | | Normal |
| Systemic venous drainage | to RA | **Atrioventricular valves** | | |
| Pulmonary venous drainage | to LA | Mitral valve | | Annulus = 15mm |
| Atrioventricular connection | Concordant | Tricuspid valve | | Annulus = 16mm  TAPSE = 18mm. |
| Ventriculoarterial connection | Concordant | **Ventricles** | | |
| Ventricular loop | d-Loop | Left ventricle | | Normal |
| Septae | | Right ventricle | | Normal |
| Interventricular septum |  | **M-Mode:**. | | |
| Interatrial septum | PFO, L – R Shunt | AO | |  |
| Great arteries | NRGA | LA | |  |
| Aorta | ------- | LVIDd | | mm |
| Pulmonary artery |  | LVIDs | | mm |
| Semilunal valves |  | IVSd | | mm |
| Aortic valve | Annulus = 13mm. | IVSs | | mm |
| Pulmonary valve | Annulus = 13mm. | LVPWd | | mm |
| Doppler Measurement | | LVPWs | | mm |
| Mitral | ------- | EDV | | ml |
| Aortic | ------ | ESV | | ml |
| Tricuspid | Trivial TR, PPG = 30mmHg | FS | | 38% |
| pulmonic | -------- | LVEF | | 70% |
| Aortic arch | ------- | **Coronary arteries** | |  |
| PDA | -------- |  | |  |
| Pericardium/Pleura | Trace pericardial effusion on RV Side | | | |
| Final Diagnosis | 1. {S, D, S} Levocardia. 2. PFO, L – R Shunt 3. Trace Pericardial Effusion 4. Normal Biventricular Function | | | |
| Done By: | **Signature** | | **Date of Reporting** | |
| Dr. Tesfaye Taye, Pediatrician, Pediatric Cardiologist | | | **12/08/2013Eth.C** | |

| Pediatric Echocardiography report  Patient Name: Lema Abay. Patient ID: 059560. Gender: Male. Age: 6years.  Clinical Finding: CHF + IE + RD + ?Cardio-embolic Stroke + Murmur + DOE. TGSH4. 2965. | | | | |
| --- | --- | --- | --- | --- |
| Features | **Finding** | **Features** | | **Finding** |
| Profile | | **Atria** | | |
| Abdominal situs | Solitus | Left atrium | | Markedly Dilated |
| Cardiac position | Levocardia | Right atrium | | Dilated |
| Systemic venous drainage | to RA | **Atrioventricular valves** | | |
| Pulmonary venous drainage | to LA | Mitral valve | | Annulus = 41mm. thickened, shortened PMVL. Freely mobile echogenic mass attached to the AMVL on the LA Side with protrusion in to LV |
| Atrioventricular connection | Concordant | Tricuspid valve | | Annulus = 32mm  TAPSE = 17mm. |
| Ventriculoarterial connection | Concordant | **Ventricles** | | |
| Ventricular loop | d-Loop | Left ventricle | | Markedly Dilated |
| Septae | | Right ventricle | | Dilated |
| Interventricular septum | 11mm PM VSD, L – R Shunt with PPG = 16mmHg | **M-Mode:**. | | |
| Interatrial septum |  | AO | |  |
| Great arteries | NRGA | LA | |  |
| Aorta | ------- | LVIDd | | mm |
| Pulmonary artery | MPA = 32mm | LVIDs | | mm |
| Semilunal valves |  | IVSd | | mm |
| Aortic valve | Annulus = 14mm. | IVSs | | mm |
| Pulmonary valve | Annulus = 32mm. | LVPWd | | mm |
| Doppler Measurement | | LVPWs | | mm |
| Mitral | Severe MR, Holosystolic, posterior projection, seen in two planes with jet velocity = 4.2m/sec | | | EDV ml |
| Aortic | Mild AR | ESV | | ml |
| Tricuspid | Severe TR | FS | | 32% |
| pulmonic | -------- | LVEF | | 59% |
| Pericardium/Pleura | 7mm Pericardial effusion on RV Side | | | |
| Final Diagnosis | 1. {S, D, S} Levocardia. 2. All chambers dilated 3. Large PM VSD, L – R Shunt 4. Severe MR 5. Severe TR 6. Mild AR 7. Severe Pulmonary Hypertension 8. Echogenic mass attached to the AMVL on LA Side 9. Small Pericardial effusion | | | |
| Done By: | **Signature** | | **Date of Reporting** | |
| Dr. Tesfaye Taye, Pediatrician, Pediatric Cardiologist | | | **12/08/2013Eth.C** | |

| Pediatric Echocardiography report  Patient Name: Abriw Ayele. Patient ID: ___________. Gender: Female. Age: 13years.  Clinical Finding: Chest Pain. TGSH4. 2966. | | | | |
| --- | --- | --- | --- | --- |
| Features | **Finding** | **Features** | | **Finding** |
| Profile | | **Atria** | | |
| Abdominal situs | Solitus | Left atrium | | Normal |
| Cardiac position | Levocardia | Right atrium | | Normal |
| Systemic venous drainage | to RA | **Atrioventricular valves** | | |
| Pulmonary venous drainage | to LA | Mitral valve | | Annulus = 23mm |
| Atrioventricular connection | Concordant | Tricuspid valve | | Annulus = 22mm  TAPSE = 17mm. |
| Ventriculoarterial connection | Concordant | **Ventricles** | | |
| Ventricular loop | d-Loop | Left ventricle | | Normal |
| Septae | | Right ventricle | | Normal  RV TDI S wave = 13cm/sec |
| Interventricular septum |  | **M-Mode:**. | | |
| Interatrial septum |  | AO | |  |
| Great arteries | NRGA | LA | |  |
| Aorta | ------- | LVIDd | | 41mm |
| Pulmonary artery |  | LVIDs | | 27mm |
| Semilunal valves |  | IVSd | | 7.6mm |
| Aortic valve | Annulus = 19mm. | IVSs | | 9mm |
| Pulmonary valve | Annulus = 26mm. | LVPWd | | 7.6mm |
| Doppler Measurement | | LVPWs | | 10mm |
| Mitral | ------- | EDV | | 76ml |
| Aortic | ------ | ESV | | 28ml |
| Tricuspid | Trivial TR, PPG = 25mmHg | FS | | 34% |
| pulmonic | -------- | LVEF | | 64% |
| Aortic arch | Left. No CoA | **Coronary arteries** | |  |
| PDA | -------- |  | |  |
| Pericardium/Pleura | Pericardial effusion, 9mm on LV Side and 8mm on RA/RV Side | | | |
| Final Diagnosis | 1. {S, D, S} Levocardia. 2. Small Pericardial effusion 3. Normal Biventricular Function | | | |
| Done By: | **Signature** | | **Date of Reporting** | |
| Dr. Tesfaye Taye, Pediatrician, Pediatric Cardiologist | | | **15/08/2013Eth.C** | |

| Pediatric Echocardiography report  Patient Name: Birhanu Mengie. Patient ID: 081174. Gender: Male. Age: 13years.  Clinical Finding: Pulseless extrimities + ? Takayasu. TGSH4. 2967. | | | | |
| --- | --- | --- | --- | --- |
| Features | **Finding** | **Features** | | **Finding** |
| Profile | | **Atria** | | |
| Abdominal situs | Solitus | Left atrium | | Normal |
| Cardiac position | Levocardia | Right atrium | | Normal |
| Systemic venous drainage | to RA | **Atrioventricular valves** | | |
| Pulmonary venous drainage | to LA | Mitral valve | | Annulus = 19mm |
| Atrioventricular connection | Concordant | Tricuspid valve | | Annulus = 22mm |
| Ventriculoarterial connection | Concordant | **Ventricles** | | |
| Ventricular loop | d-Loop | Left ventricle | | Normal |
| Septae | | Right ventricle | | Normal |
| Interventricular septum |  | **M-Mode:**. | | |
| Interatrial septum |  | AO | |  |
| Great arteries | NRGA | LA | |  |
| Aorta | ------- | LVIDd | | 32mm |
| Pulmonary artery |  | LVIDs | | 21mm |
| Semilunal valves |  | IVSd | | 11mm |
| Aortic valve | Annulus = 17mm. SoV = 23mm | IVSs | | 13mm |
| Pulmonary valve | Annulus = 20mm. | LVPWd | | 8.6mm |
| Doppler Measurement | | LVPWs | | 10mm |
| Mitral | ------- | EDV | | 41ml |
| Aortic | ------ | ESV | | 15ml |
| Tricuspid | -------- | FS | | 34% |
| pulmonic | -------- | LVEF | | 64% |
| Aortic arch | Flow acceleration across left common carotid, left subclavian and Brachiocephalic arteries. There seems to be luminal narrowing of these vessels. No CoA. | **Coronary arteries** | |  |
| PDA | -------- |  | |  |
| Final Diagnosis | 1. {S, D, S} Levocardia. 2. Flow acceleration over the aortic arch branching vessels | | | |
| Remark | Work on the line of Takayasu arteritis | | | |
| Recommendation | Additional imaging on the abdominal and renal arteries | | | |
| Done By: | **Signature** | | **Date of Reporting** | |
| Dr. Tesfaye Taye, Pediatrician, Pediatric Cardiologist | | | **15/08/2013Eth.C** | |

| Pediatric Echocardiography report  Patient Name: Baby of Bicha-werk . Patient ID: 078102. Gender: Male. Age: 39days.  Clinical Finding : RD. TGSH4.2968. | | | | |
| --- | --- | --- | --- | --- |
| Features | **Finding** | **Features** | | **Finding** |
| Profile | | **Atria** | | |
| Abdominal situs | Solitus | Left atrium | | Normal |
| Cardiac position | Levocardia | Right atrium | | Normal |
| Systemic venous drainage | to RA | **Atrioventricular valves** | | |
| Pulmonary venous drainage | to LA | Mitral valve | | Annulus = 9mm |
| Atrioventricular connection | Concordant | Tricuspid valve | | Annulus = 12mm |
| Ventriculoarterial connection | Concordant | **Ventricles** | | |
| Ventricular loop | d-Loop | Left ventricle | | Normal |
| Septae | | Right ventricle | | Normal |
| Interventricular septum |  | **M-Mode:** Normal LV Function on eye balling. | | |
| Interatrial septum | PFO, L – R Shunt | AO | |  |
| Great arteries | NRGA | LA | |  |
| Aorta | ------- | LVIDd | | mm |
| Pulmonary artery |  | LVIDs | | mm |
| Semilunal valves |  | IVSd | | mm |
| Aortic valve | Annulus = 9mm. | IVSs | | mm |
| Pulmonary valve | Annulus = 9mm. | LVPWd | | mm |
| Doppler Measurement | | LVPWs | | mm |
| Mitral | ------- | EDV | | ml |
| Aortic | ------ | ESV | | ml |
| Tricuspid | -------- | FS | | % |
| pulmonic | -------- | LVEF | | % |
| Aortic arch | ------- | **Coronary arteries** | |  |
| PDA | -------- |  | |  |
| Pericardium/Pleura |  | | | |
| Final Diagnosis | 1. {S, D, S} Levocardia. 2. PFO, L – R Shunt | | | |
| Done By: | **Signature** | | **Date of Reporting** | |
| Dr. Tesfaye Taye, Pediatrician, Pediatric Cardiologist | | | **15/08/2013Eth.C** | |

| Pediatric Echocardiography report  Patient Name: Werknesh Alem. Patient ID: _079902 Gender: Female. Age: 11years.  Clinical Finding : CHF + DOE + Rheumatic Recurrence + Palpitation + . TGSH4.2969. | | | |
| --- | --- | --- | --- |
| Features | **Finding** | **Features** | **Finding** |
| Profile | | **Atria** | |
| Abdominal situs | Solitus | Left atrium | Markedly Dilated |
| Cardiac position | Levocardia | Right atrium | Dilated |
| Systemic venous drainage | to RA | **Atrioventricular valves** | |
| Pulmonary venous drainage | to LA | Mitral valve | Annulus = 28mm. thickened, clubbed MVL. MVA = 0.9cm^2^. |
| Atrioventricular connection | Concordant | Tricuspid valve | Annulus = 20mm. |
| Ventriculoarterial connection | Concordant | **Ventricles** | |
| Ventricular loop | d-Loop | Left ventricle | Markedly Dilated |
| Septae | | Right ventricle | Dilated |
| Interventricular septum |  | **M-Mode:**. | |
| Interatrial septum |  | AO |  |
| Great arteries | NRGA | LA |  |
| Aorta | ------- | LVIDd | mm |
| Pulmonary artery | Normal MPA and Branch PAs | LVIDs | mm |
| Semilunal valves |  | IVSd | mm |
| Aortic valve | Annulus = 13mm. | IVSs | mm |
| Pulmonary valve | Annulus = 17mm. | LVPWd | mm |
| Doppler Measurement | | LVPWs | mm |
| Mitral | Severe MR, Holosystolic, posterior projection, seen in two planes with jet velocity = 4.2m/sec. Severe MS, PPG/MPG = 20/10mmHg | EDV | ml |
| Aortic | Moderate AR, PHT = 281ms. | ESV | ml |
| Tricuspid | Severe TR, PPG = 60mmHg | FS | % |
| pulmonic | -------- | LVEF | % |
| Pericardium/Pleura | 4mm pericardial effusion on RA Side. | | |
| Final Diagnosis | 1. {S, D, S} Levocardia. 2. All chambers dilated 3. Thickened, clubbed MVL 4. Severe MR, Severe MS 5. Severe TR 6. Moderate AR 7. Severe Pulmonary Hypertension 8. Trace pericardial effusion 9. Normal Function | | |
| Done By: | **Signature** | **Date of Reporting** | |
| Dr. Tesfaye Taye, Pediatrician, Pediatric Cardiologist | | **19/08/2013Eth.C** | |

| Pediatric Echocardiography report  Patient Name: Tiruyitu Yihalem. Patient ID: 081048. Gender: Female. Age: 10years.  Clinical Finding : DOE + Murmur. TGSH4.2970. | | | | |
| --- | --- | --- | --- | --- |
| Features | **Finding** | **Features** | | **Finding** |
| Profile | | **Atria** | | |
| Abdominal situs | Solitus | Left atrium | | Normal |
| Cardiac position | Levocardia | Right atrium | | Dilated |
| Systemic venous drainage | to RA | **Atrioventricular valves** | | |
| Pulmonary venous drainage | to LA | Mitral valve | | Annulus = 17mm |
| Atrioventricular connection | Concordant | Tricuspid valve | | Annulus = 21mm  TAPSE = 21mm. |
| Ventriculoarterial connection | Concordant | **Ventricles** | | |
| Ventricular loop | d-Loop | Left ventricle | | Normal |
| Septae | | Right ventricle | | Dilated |
| Interventricular septum | Intact | **M-Mode:**. | | |
| Interatrial septum | 20mm OS ASD, L – R Shunt | AO | |  |
| Great arteries | NRGA | LA | |  |
| Aorta | ------- | LVIDd | | mm |
| Pulmonary artery | Normal MPA and Branch PAs | LVIDs | | mm |
| Semilunal valves |  | IVSd | | mm |
| Aortic valve | Annulus = 16mm. | IVSs | | mm |
| Pulmonary valve | Annulus = 22mm. Doming | LVPWd | | mm |
| Doppler Measurement | | LVPWs | | mm |
| Mitral | ------- | EDV | | ml |
| Aortic | ------ | ESV | | ml |
| Tricuspid | -------- | FS | | 33% |
| pulmonic | Mild PS, PPG = 35mmHg. Trivial PR, PPG = 10mmHg | LVEF | | 63% |
| Aortic arch | ------- | **Coronary arteries** | |  |
| PDA | -------- |  | |  |
| Pericardium/Pleura |  | | | |
| Final Diagnosis | 1. {S, D, S} Levocardia. 2. Large OS ASD, L – R Shunt 3. Doming Pulmonary Valve 4. Mild PS 5. Normal Biventricular Function | | | |
| Done By: | **Signature** | | **Date of Reporting** | |
| Dr. Tesfaye Taye, Pediatrician, Pediatric Cardiologist | | | **19/08/2013Eth.C** | |

| Pediatric Echocardiography report  Patient Name: Bilen Yidnekachew. Patient ID: 044473. Gender: Female. Age: 1year.  (Baby of Bemnet Mekete) (TGSH7) (13days old) | | | | |
| --- | --- | --- | --- | --- |
| Follow up echo for Small PM VSD and small ASD (Incidental Murmur) (TGSH4.2971.) | | | | |
| Features | **Finding** | **Features** | | **Finding** |
| Profile | | **Atria** | | |
| Abdominal situs | Solitus | Left atrium | | Normal |
| Cardiac position | Levocardia | Right atrium | | Normal |
| Systemic venous drainage | to RA | **Atrioventricular valves** | | |
| Pulmonary venous drainage | to LA | Mitral valve | | Annulus = 12mm |
| Atrioventricular connection | Concordant | Tricuspid valve | | Annulus = 12mm |
| Ventriculoarterial connection | Concordant | **Ventricles** | | |
| Ventricular loop | d-Loop | Left ventricle | | Normal |
| Septae | | Right ventricle | | Normal |
| Interventricular septum | 5mm PM VSD, partially covered by septal leaflet of TV, L – R Shunt | **M-Mode:**. | | |
| Interatrial septum | 4mm OS ASD, L – R Shunt | AO | |  |
| Great arteries | NRGA | LA | |  |
| Aorta | ------- | LVIDd | | mm |
| Pulmonary artery | Normal MPA and Branch PAs | LVIDs | | mm |
| Semilunal valves |  | IVSd | | mm |
| Aortic valve | Annulus = 13mm. | IVSs | | mm |
| Pulmonary valve | Annulus = 11mm. | LVPWd | | mm |
| Doppler Measurement | | LVPWs | | mm |
| Mitral | ------- | EDV | | ml |
| Aortic | ------ | ESV | | ml |
| Tricuspid | -------- | FS | | % |
| pulmonic | -------- | LVEF | | % |
| Aortic arch | ------- | **Coronary arteries** | |  |
| PDA | -------- |  | |  |
| Pericardium/Pleura |  | | | |
| Final Diagnosis | 1. {S, D, S} Levocardia. 2. Small OS ASD, L – R Shunt 3. Small PM VSD, L – R Shunt 4. Normal Function | | | |
| Done By: | **Signature** | | **Date of Reporting** | |
| Dr. Tesfaye Taye, Pediatrician, Pediatric Cardiologist | | | **19/08/2013Eth.C** | |

| Pediatric Echocardiography report  Patient Name: Yonas Adela. Patient ID: 082614 Gender: Male. Age: 3years.  Clinical Diagnosis: RD + CHF + Cyanosis + Diaphoresis. TGSH4.2972. | | | | |
| --- | --- | --- | --- | --- |
| Features | **Finding** | **Features** | | **Finding** |
| Profile | | **Atria** | | |
| Abdominal situs | Solitus | Left atrium | | Dilated |
| Cardiac position | Levocardia | Right atrium | | Dilated |
| Systemic venous drainage | to RA | **Atrioventricular valves** | | |
| Pulmonary venous drainage | to LA | Mitral valve | | Annulus = 13mm |
| Atrioventricular connection | Concordant | Tricuspid valve | | Annulus = 19mm  TAPSE = 14mm. |
| Ventriculoarterial connection | DORV | **Ventricles** | | |
| Ventricular loop | d-Loop | Left ventricle | | Dilated |
| Septae | | Right ventricle | | Dilated, Hypertrophied. RV TDI S wave = 8cm/sec |
| Interventricular septum | Malaligned Non-restrictive Sub aortic VSD, R – L Shunt. | **M-Mode:**. | | |
| Interatrial septum |  | AO | |  |
| Great arteries | NRGA | LA | |  |
| Aorta | Overriding aorta with greater than 50% aortic valve attachment to the RV. Posterior and to the right. | LVIDd | | mm |
| Pulmonary artery | Smallish MPA and Branch PAs. Anterior and to the left. From RV | LVIDs | | mm |
| Semilunal valves |  | IVSd | | mm |
| Aortic valve | Annulus = 19mm. Aorto Mitral Discontinuity. | IVSs | | mm |
| Pulmonary valve | Annulus = 9mm. | LVPWd | | mm |
| Doppler Measurement | | LVPWs | | mm |
| Mitral | ------- | EDV | | ml |
| Aortic | ------ | ESV | | ml |
| Tricuspid | -------- | FS | | 21% |
| pulmonic | Severe PS, PPG = 62mmHg | LVEF | | 44% |
| Aortic arch | ------- | **Coronary arteries** | |  |
| Pericardium/Pleura |  | | | |
| Final Diagnosis | 1. {S, D, S} Levocardia. 2. DORV (TOF Type) 3. Large Mal-aligned Sub aortic VSD, R – L Shunt 4. Severe PS 5. Smallish MPA and Branch PAs. 6. Hypertrophied, Dilated and Dysfunctional RV 7. Mildly reduced Systolic LV Function | | | |
| Done By: | **Signature** | | **Date of Reporting** | |
| Dr. Tesfaye Taye, Pediatrician, Pediatric Cardiologist | | | **21/08/2013Eth.C** | |

| Pediatric Echocardiography report  Patient Name: Mamen Alemu. Patient ID: 053224. Gender: Female. Age: 11years.  Clinical Diagnosis: Rheumatic Recurrence + DOE + Easy Fatigability + Murmur. TGSH4.2973. | | | | |
| --- | --- | --- | --- | --- |
| Features | **Finding** | **Features** | | **Finding** |
| Profile | | **Atria** | | |
| Abdominal situs | Solitus | Left atrium | | Dilated |
| Cardiac position | Levocardia | Right atrium | | Normal |
| Systemic venous drainage | to RA | **Atrioventricular valves** | | |
| Pulmonary venous drainage | to LA | Mitral valve | | Annulus = 35mm. MVA = 1.8cm**^2^**. Thickened MVL |
| Atrioventricular connection | Concordant | Tricuspid valve | | Annulus = 29mm |
| Ventriculoarterial connection | Concordant | **Ventricles** | | |
| Ventricular loop | d-Loop | Left ventricle | | Dilated |
| Septae | | Right ventricle | | Normal |
| Interventricular septum |  | **M-Mode:**. | | |
| Interatrial septum |  | AO | |  |
| Great arteries | NRGA | LA | |  |
| Aorta | ------- | LVIDd | | mm |
| Pulmonary artery | Normal MPA and Branch PAs | LVIDs | | mm |
| Semilunal valves |  | IVSd | | mm |
| Aortic valve | Annulus = 15mm. | IVSs | | mm |
| Pulmonary valve | Annulus = 21mm. | LVPWd | | mm |
| Doppler Measurement | | LVPWs | | mm |
| Mitral | Severe MR, Holosystolic, posterior projection, seen in two planes with jet velocity = 5m/sec | EDV | | ml |
| Aortic | Moderate AR, PHT = 303ms | ESV | | ml |
| Tricuspid | Moderate TR, PPG = 55mmHg | FS | | 37% |
| pulmonic | -------- | LVEF | | 66% |
| Aortic arch | ------- | **Coronary arteries** | |  |
| Pericardium/Pleura | 4mm Circumferential pericardial effusion | | | |
| Final Diagnosis | 1. {S, D, S} Levocardia. 2. LA/LV Dilated 3. Thickened MVL 4. Severe MR 5. Mild MS 6. Moderate TR 7. Moderate AR 8. Moderate Pulmonary Hypertension 9. Normal LV Function | | | |
| Done By: | **Signature** | | **Date of Reporting** | |
| Dr. Tesfaye Taye, Pediatrician, Pediatric Cardiologist | | | **03/09/2013Eth.C** | |

| Pediatric Echocardiography report  Patient Name: Temelket Atirsaw. Patient ID: 082860. Gender: male. Age: 13years.  Clinical Diagnosis: Murmur + Rheumatic Fever. TGSH4.2974. | | | | |
| --- | --- | --- | --- | --- |
| Features | **Finding** | **Features** | | **Finding** |
| Profile | | **Atria** | | |
| Abdominal situs | Solitus | Left atrium | | Dilated |
| Cardiac position | Levocardia | Right atrium | | Normal |
| Systemic venous drainage | to RA | **Atrioventricular valves** | | |
| Pulmonary venous drainage | to LA | Mitral valve | | Annulus = 25mm |
| Atrioventricular connection | Concordant | Tricuspid valve | | Annulus = 20mm |
| Ventriculoarterial connection | Concordant | **Ventricles** | | |
| Ventricular loop | d-Loop | Left ventricle | | Dilated |
| Septae | | Right ventricle | | Normal |
| Interventricular septum |  | **M-Mode:**. | | |
| Interatrial septum |  | AO | |  |
| Great arteries | NRGA | LA | |  |
| Aorta | ------- | LVIDd | | mm |
| Pulmonary artery | Normal MPA and Branch PAs | LVIDs | | mm |
| Semilunal valves |  | IVSd | | mm |
| Aortic valve | Annulus = 26mm. thickened AVL | IVSs | | mm |
| Pulmonary valve | Annulus = 21mm. | LVPWd | | mm |
| Doppler Measurement | | LVPWs | | mm |
| Mitral | Mild MR, Incomplete Signal, seen in two planes with jet velocity = 4.6m/sec | EDV | | ml |
| Aortic | Moderate AR, PHT = 260ms | ESV | | ml |
| Tricuspid | -------- | FS | | 38% |
| pulmonic | -------- | LVEF | | 67% |
| Aortic arch | ------- | **Coronary arteries** | |  |
| Pericardium/Pleura | 7mm Right Pleural effusion | | | |
| Final Diagnosis | 1. {S, D, S} Levocardia. 2. LA/LV Dilated 3. Mild MR 4. Moderate AR 5. Small Right Pleural Effusion 6. Normal LV Function | | | |
| Done By: | **Signature** | | **Date of Reporting** | |
| Dr. Tesfaye Taye, Pediatrician, Pediatric Cardiologist | | | **03/09/2013Eth.C** | |

| Pediatric Echocardiography report  Patient Name: Eyerus Minale. Patient ID: 084780. Gender: Female. Age: 64days.  Clinical Diagnosis: Incidental Murmur. TGSH4.2975. | | | | |
| --- | --- | --- | --- | --- |
| Features | **Finding** | **Features** | | **Finding** |
| Profile | | **Atria** | | |
| Abdominal situs | Solitus | Left atrium | | Normal |
| Cardiac position | Levocardia | Right atrium | | Normal |
| Systemic venous drainage | to RA | **Atrioventricular valves** | | |
| Pulmonary venous drainage | to LA | Mitral valve | | Annulus = 13mm |
| Atrioventricular connection | Concordant | Tricuspid valve | | Annulus = 15mm |
| Ventriculoarterial connection | Concordant | **Ventricles** | | |
| Ventricular loop | d-Loop | Left ventricle | | Normal |
| Septae | | Right ventricle | | Normal |
| Interventricular septum | 8mm upper Muscular VSD, L – R Shunt | **M-Mode:** Normal LV Function on eye balling. | | |
| Interatrial septum | PFO, L – R Shunt | AO | |  |
| Great arteries | NRGA | LA | |  |
| Aorta | ------- | LVIDd | | mm |
| Pulmonary artery | Normal MPA and Branch PAs | LVIDs | | mm |
| Semilunal valves |  | IVSd | | mm |
| Aortic valve | Annulus = 7mm. | IVSs | | mm |
| Pulmonary valve | Annulus = 11mm. | LVPWd | | mm |
| Doppler Measurement | | LVPWs | | mm |
| Mitral | ------- | EDV | | ml |
| Aortic | ------ | ESV | | ml |
| Tricuspid | -------- | FS | | % |
| pulmonic | -------- | LVEF | | % |
| Aortic arch | ------- | **Coronary arteries** | |  |
| PDA | -------- |  | |  |
| Pericardium/Pleura |  | | | |
| Final Diagnosis | 1. {S, D, S} Levocardia. 2. PFO, L – R Shunt 3. Large Upper Muscular VSD, L – R Shunt 4. Normal LV Function | | | |
| Done By: | **Signature** | | **Date of Reporting** | |
| Dr. Tesfaye Taye, Pediatrician, Pediatric Cardiologist | | | **17/09/2013Eth.C** | |

| Pediatric Echocardiography report  Patient Name: Bereket Getie. Patient ID: 083370. Gender: male. Age: 7years.  Clinical Diagnosis: Chest Pain + Friction rub. TGSH4.2976. | | | | |
| --- | --- | --- | --- | --- |
| Features | **Finding** | **Features** | | **Finding** |
| Profile | | **Atria** | | |
| Abdominal situs | Solitus | Left atrium | | Normal |
| Cardiac position | Levocardia | Right atrium | | Normal |
| Systemic venous drainage | to RA | **Atrioventricular valves** | | |
| Pulmonary venous drainage | to LA | Mitral valve | | Annulus = 18mm |
| Atrioventricular connection | Concordant | Tricuspid valve | | Annulus = 20mm  TAPSE = 17mm. |
| Ventriculoarterial connection | Concordant | **Ventricles** | | |
| Ventricular loop | d-Loop | Left ventricle | | Normal |
| Septae | | Right ventricle | | Normal |
| Interventricular septum |  | **M-Mode:**. | | |
| Interatrial septum |  | AO | |  |
| Great arteries | NRGA | LA | |  |
| Aorta | ------- | LVIDd | | mm |
| Pulmonary artery | Normal MPA and Branch PAs | LVIDs | | mm |
| Semilunal valves |  | IVSd | | mm |
| Aortic valve | Annulus = 15mm. | IVSs | | mm |
| Pulmonary valve | Annulus = 19mm. | LVPWd | | mm |
| Doppler Measurement | | LVPWs | | mm |
| Mitral | ------- | EDV | | ml |
| Aortic | ------ | ESV | | ml |
| Tricuspid | -------- | FS | | 36% |
| pulmonic | -------- | LVEF | | 67% |
| Aortic arch | ------- | **Coronary arteries** | |  |
| PDA | -------- |  | |  |
| Pericardium/Pleura | 3mm Pericardial effusion | | | |
| Final Diagnosis | 1. {S, D, S} Levocardia. 2. Trace Pericardial effusion | | | |
| Done By: | **Signature** | | **Date of Reporting** | |
| Dr. Tesfaye Taye, Pediatrician, Pediatric Cardiologist | | | **17/09/2013Eth.C** | |

| Pediatric Echocardiography report  Patient Name: Linger Adamu. Patient ID: ___________. Gender: Female. Age: 8Years.  Clinical Diagnosis: Rheumatic Recurrence + Murmur + CHF + DOE + Palpitation. TGSH4.2977. | | | | |
| --- | --- | --- | --- | --- |
| Features | **Finding** | **Features** | | **Finding** |
| Profile | | **Atria** | | |
| Abdominal situs | Solitus | Left atrium | | More Dilated |
| Cardiac position | Levocardia | Right atrium | | Dilated |
| Systemic venous drainage | to RA | **Atrioventricular valves** | | |
| Pulmonary venous drainage | to LA | Mitral valve | | Annulus = 30mm. Thickened, Shortened PMVL. Non coapting MVL |
| Atrioventricular connection | Concordant | Tricuspid valve | | Annulus = 26mm |
| Ventriculoarterial connection | Concordant | **Ventricles** | | |
| Ventricular loop | d-Loop | Left ventricle | | More Dilated |
| Septae | | Right ventricle | | Dilated |
| Interventricular septum |  | **M-Mode:**. | | |
| Interatrial septum |  | AO | |  |
| Great arteries | NRGA | LA | |  |
| Aorta | ------- | LVIDd | | mm |
| Pulmonary artery | Dilated MPA and Branch PAs | LVIDs | | mm |
| Semilunal valves |  | IVSd | | mm |
| Aortic valve | Annulus = 16mm. | IVSs | | mm |
| Pulmonary valve | Annulus = 22mm. | LVPWd | | mm |
| Doppler Measurement | | LVPWs | | mm |
| Mitral | Severe MR, Holosystolic, posterior projection, seen in two planes with jet velocity = 3.9m/sec | EDV | | ml |
| Aortic | Moderate AR, PHT = 401cm/sec | ESV | | ml |
| Tricuspid | Severe TR, PPG = 74mmHg | FS | | 29% |
| pulmonic | Mild PR, PPG = 53mmHg | LVEF | | 55% |
| Aortic arch | ------- | **Coronary arteries** | |  |
| Pericardium/Pleura |  | | | |
| Final Diagnosis | 1. {S, D, S} Levocardia. 2. Thickened, Shortened PMVL, Non coapting MVL 3. Severe MR 4. Severe TR 5. Moderate AR 6. Mild PR 7. Severe Pulmonary Hypertension 8. Normal LV Function | | | |
| Done By: | **Signature** | | **Date of Reporting** | |
| Dr. Tesfaye Taye, Pediatrician, Pediatric Cardiologist | | | **17/09/2013Eth.C** | |

| Pediatric Echocardiography report  Patient Name: Yohannes Misganaw. Patient ID: 083812. Gender: Male. Age: 3/12.  Clinical Diagnosis: CHF + RD + Murmur + Diaphoresis. TGSH4.2978. | | | | |
| --- | --- | --- | --- | --- |
| Features | **Finding** | **Features** | | **Finding** |
| Profile | | **Atria** | | |
| Abdominal situs | Solitus | Left atrium | | Mildly dilated |
| Cardiac position | Levocardia | Right atrium | | Normal |
| Systemic venous drainage | to RA | **Atrioventricular valves** | | |
| Pulmonary venous drainage | to LA | Mitral valve | | Annulus = 16mm |
| Atrioventricular connection | Concordant | Tricuspid valve | | Annulus = 15mm |
| Ventriculoarterial connection | Truncus | **Ventricles** | | |
| Ventricular loop | d-Loop | Left ventricle | | Mildly dilated |
| Septae | | Right ventricle | | Normal |
| Interventricular septum | Non- restrictive Sub arterial VSD | **M-Mode:**. | | |
| Interatrial septum |  | AO | |  |
| Great arteries | Truncus | LA | |  |
| Aorta | ------- | LVIDd | | mm |
| Pulmonary artery | Branching from Truncus on the left side and posteriorly | LVIDs | | mm |
| Semilunal valves |  | IVSd | | mm |
| Aortic valve | Truncal Annulus = 15mm. | IVSs | | mm |
| Pulmonary valve |  | LVPWd | | mm |
| Doppler Measurement | | LVPWs | | mm |
| Mitral | ------- | EDV | | ml |
| Aortic | ------ | ESV | | ml |
| Tricuspid | -------- | FS | | % |
| pulmonic | -------- | LVEF | | % |
| Aortic arch | ------- | **Coronary arteries** | |  |
| PDA | -------- |  | |  |
| Pericardium/Pleura |  | | | |
| Final Diagnosis | 1. {S, D, S} Levocardia. 2. ? Truncus Arteriosus | | | |
| Remark |  | | | |
| Done By: | **Signature** | | **Date of Reporting** | |
| Dr. Tesfaye Taye, Pediatrician, Pediatric Cardiologist | | | **17/09/2013Eth.C** | |

| Pediatric Echocardiography report  Patient Name: Adane Fisseha. Patient ID: 084770. Gender: Male. Age: 5/12.  Clinical Diagnosis: DS + RD + Murmur + Diaphoresis. TGSH4.2979. | | | | |
| --- | --- | --- | --- | --- |
| Features | **Finding** | **Features** | | **Finding** |
| Profile | | **Atria** | | |
| Abdominal situs | Solitus | Left atrium | | Normal |
| Cardiac position | Levocardia | Right atrium | | Dilated |
| Systemic venous drainage | to RA | **Atrioventricular valves** | | |
| Pulmonary venous drainage | to LA | Mitral valve | | AVSD |
| Atrioventricular connection | Concordant | Tricuspid valve | | AVSD |
| Ventriculoarterial connection | Concordant | **Ventricles** | | |
| Ventricular loop | d-Loop | Left ventricle | | Normal |
| Septae | | Right ventricle | | Dilated |
| Interventricular septum | 1mm Inlet VSD, L – R Shunt | **M-Mode:** Normal LV Function on eye balling | | |
| Interatrial septum | 8mm OS ASD, L – R Shunt.  Primum defect, 6mm, L – R Shunt | AO | |  |
| Great arteries | NRGA | LA | |  |
| Aorta | ------- | LVIDd | | mm |
| Pulmonary artery | Normal MPA and Branch PAs | LVIDs | | mm |
| Semilunal valves |  | IVSd | | mm |
| Aortic valve | Annulus = 10mm. | IVSs | | mm |
| Pulmonary valve | Annulus = 12mm. | LVPWd | | mm |
| Doppler Measurement | | LVPWs | | mm |
| Mitral | ------- | EDV | | ml |
| Aortic | ------ | ESV | | ml |
| Tricuspid | Trivial TR | FS | | % |
| pulmonic | -------- | LVEF | | % |
| Aortic arch | ------- | **Coronary arteries** | |  |
| PDA | -------- |  | |  |
| Pericardium/Pleura |  | | | |
| Final Diagnosis | 1. {S, D, S} Levocardia. 2. Moderate OS ASD, L – R Shunt 3. Transitional AVSD, L – R Shunt 4. Normal LV Function | | | |
| Done By: | **Signature** | | **Date of Reporting** | |
| Dr. Tesfaye Taye, Pediatrician, Pediatric Cardiologist | | | **19/09/2013Eth.C** | |

| Pediatric Echocardiography report  Patient Name: Baby of Birhan Muluye. Patient ID: 084546. Gender: Female. Age: 18days.  Clinical Diagnosis: Incidental Murmur. TGSH4.2980. | | | | |
| --- | --- | --- | --- | --- |
| Features | **Finding** | **Features** | | **Finding** |
| Profile | | **Atria** | | |
| Abdominal situs | Solitus | Left atrium | | Normal |
| Cardiac position | Levocardia | Right atrium | | Normal |
| Systemic venous drainage | to RA | **Atrioventricular valves** | | |
| Pulmonary venous drainage | to LA | Mitral valve | | Annulus = 10mm |
| Atrioventricular connection | Concordant | Tricuspid valve | | Annulus = 9mm |
| Ventriculoarterial connection | Concordant | **Ventricles** | | |
| Ventricular loop | d-Loop | Left ventricle | | Normal |
| Septae | | Right ventricle | | Normal |
| Interventricular septum | Intact | **M-Mode:** Normal LV Function on eye balling | | |
| Interatrial septum | PFO, L – R Shunt | AO | |  |
| Great arteries | NRGA | LA | |  |
| Aorta | ------- | LVIDd | | mm |
| Pulmonary artery | Normal MPA and Branch PAs | LVIDs | | mm |
| Semilunal valves |  | IVSd | | mm |
| Aortic valve | Annulus = 7mm. | IVSs | | mm |
| Pulmonary valve | Annulus = 9mm. | LVPWd | | mm |
| Doppler Measurement | | LVPWs | | mm |
| Mitral | ------- | EDV | | ml |
| Aortic | ------ | ESV | | ml |
| Tricuspid | -------- | FS | | % |
| pulmonic | -------- | LVEF | | % |
| Aortic arch | ------- | **Coronary arteries** | |  |
| PDA | 1mm PDA, L – R Shunt |  | |  |
| Pericardium/Pleura |  | | | |
| Final Diagnosis | 1. {S, D, S} Levocardia. 2. PFO, L – R Shunt 3. Small PDA, L – R Shunt 4. Normal LV Function | | | |
| Done By: | **Signature** | | **Date of Reporting** | |
| Dr. Tesfaye Taye, Pediatrician, Pediatric Cardiologist | | | **19/09/2013Eth.C** | |

| Pediatric Echocardiography report  Patient Name: Muluken Melash. Patient ID: 084301. Gender: Male. Age: 13Months.  Clinical Diagnosis: RD + CHF + Murmur + Diaphoresis. TGSH4.2981. | | | | |
| --- | --- | --- | --- | --- |
| Features | **Finding** | **Features** | | **Finding** |
| Profile | | **Atria** | | |
| Abdominal situs | Solitus | Left atrium | | Dilated |
| Cardiac position | Levocardia | Right atrium | | Dilated |
| Systemic venous drainage | to RA | **Atrioventricular valves** | | |
| Pulmonary venous drainage | to LA | Mitral valve | | Annulus = 17mm |
| Atrioventricular connection | Concordant | Tricuspid valve | | Annulus = 19mm |
| Ventriculoarterial connection | TRUCNCUS | **Ventricles** | | |
| Ventricular loop | d-Loop | Left ventricle | | Dilated |
| Septae | | Right ventricle | | Dilated |
| Interventricular septum | Sub arterial VSD | **M-Mode:**. | | |
| Interatrial septum | 4mm High secundum ASD, L – R Shunt | AO | |  |
| Great arteries | Truncus Arteriosus | LA | |  |
| Aorta | ------- | LVIDd | | mm |
| Pulmonary artery | Normal MPA and Branch PAs | LVIDs | | mm |
| Semilunal valves |  | IVSd | | mm |
| Truncus valve | Annulus = 23mm. | IVSs | | mm |
|  |  | LVPWd | | mm |
| Doppler Measurement | | LVPWs | | mm |
| Mitral | ------- | EDV | | ml |
| Aortic | Severe Truncal Regurgitation. Mild Truncal Stenosis | ESV | | ml |
| Tricuspid | -------- | FS | | % |
| pulmonic | -------- | LVEF | | % |
| Aortic arch | ------- | **Coronary arteries** | |  |
| PDA | -------- |  | |  |
| Pericardium/Pleura |  | | | |
| Final Diagnosis | 1. {S, D, S} Levocardia. 2. Small High Secundum ASD, L – R Shunt 3. Truncus Arteriosus | | | |
| Done By: | **Signature** | | **Date of Reporting** | |
| Dr. Tesfaye Taye, Pediatrician, Pediatric Cardiologist | | | **19/09/2013Eth.C** | |

| Pediatric Echocardiography report  Patient Name: Bereket Birku. Patient ID: 084897. Gender: Male. Age: 3 6/12.  Clinical Diagnosis: DS + RD + CHF + Murmur + IE. TGSH4.2982. | | | | |
| --- | --- | --- | --- | --- |
| Features | **Finding** | **Features** | | **Finding** |
| Profile | | **Atria** | | |
| Abdominal situs | Solitus | Left atrium | | Dilated |
| Cardiac position | Levocardia | Right atrium | | Dilated |
| Systemic venous drainage | to RA. IVC Dilated | **Atrioventricular valves** | | |
| Pulmonary venous drainage | to LA | Mitral valve | | Common Complete AVSD, L – R Shunt |
| Atrioventricular connection | Complete AVSD | Tricuspid valve | |  |
| Ventriculoarterial connection | Concordant | **Ventricles** | | |
| Ventricular loop | d-Loop | Left ventricle | | Dilated |
| Septae | | Right ventricle | | Dilated |
| Interventricular septum |  | **M-Mode:** Normal LV Function on eye balling | | |
| Interatrial septum |  | AO | |  |
| Great arteries | NRGA | LA | |  |
| Aorta | ------- | LVIDd | | mm |
| Pulmonary artery | Normal MPA and Branch PAs | LVIDs | | mm |
| Semilunal valves |  | IVSd | | mm |
| Aortic valve | Annulus = 16mm. | IVSs | | mm |
| Pulmonary valve | Annulus = 17mm. | LVPWd | | mm |
| Doppler Measurement | | LVPWs | | mm |
| Mitral | Mild left AVVR | EDV | | ml |
| Aortic | ------ | ESV | | ml |
| Tricuspid | Mild right AVVR | FS | | % |
| pulmonic | Mild PS, PPG = 22mmHg | LVEF | | % |
| Aortic arch | ------- | **Coronary arteries** | |  |
| PDA | 2mm PDA, L – R |  | |  |
| Pericardium/Pleura | 13mm Pericardial effusion on RA/RV Side | | | |
| Final Diagnosis | 1. {S, D, S} Levocardia. 2. Common Complete Balanced AVSD, L – R Shunt 3. Moderate PDA, L – R Shunt 4. Mild PS 5. Normal LV Function | | | |
| Done By: | **Signature** | | **Date of Reporting** | |
| Dr. Tesfaye Taye, Pediatrician, Pediatric Cardiologist | | | **19/09/2013Eth.C** | |

| Pediatric Echocardiography report  Patient Name: Denasew Melak. Patient ID: 069535. Gender: Male. Age: 8Months.  Clinical Diagnosis: Incidental Murmur. TGSH4.2983. | | | | |
| --- | --- | --- | --- | --- |
| Features | **Finding** | **Features** | | **Finding** |
| Profile | | **Atria** | | |
| Abdominal situs | Solitus | Left atrium | | Normal |
| Cardiac position | Levocardia | Right atrium | | Normal |
| Systemic venous drainage | to RA | **Atrioventricular valves** | | |
| Pulmonary venous drainage | to LA | Mitral valve | | Annulus = 13mm |
| Atrioventricular connection | Concordant | Tricuspid valve | | Annulus = 15mm |
| Ventriculoarterial connection | Concordant | **Ventricles** | | |
| Ventricular loop | d-Loop | Left ventricle | | Normal |
| Septae | | Right ventricle | | Normal |
| Interventricular septum |  | **M-Mode:** Normal LV Function on eye balling. | | |
| Interatrial septum |  | AO | |  |
| Great arteries | NRGA | LA | |  |
| Aorta | ------- | LVIDd | | mm |
| Pulmonary artery | Normal MPA and Branch PAs | LVIDs | | mm |
| Semilunal valves |  | IVSd | | mm |
| Aortic valve | Annulus = 10mm. | IVSs | | mm |
| Pulmonary valve | Annulus = 13mm. | LVPWd | | mm |
| Doppler Measurement | | LVPWs | | mm |
| Mitral | ------- | EDV | | ml |
| Aortic | ------ | ESV | | ml |
| Tricuspid | -------- | FS | | % |
| pulmonic | -------- | LVEF | | % |
| Aortic arch | ------- | **Coronary arteries** | |  |
| PDA | 1.5mm PDA, L – R Shunt |  | |  |
| Pericardium/Pleura |  | | | |
| Final Diagnosis | 1. {S, D, S} Levocardia. 2. Small PDA, L – R Shunt 3. Normal LV Function | | | |
| Done By: | **Signature** | | **Date of Reporting** | |
| Dr. Tesfaye Taye, Pediatrician, Pediatric Cardiologist | | | **19/09/2013Eth.C** | |

| Pediatric Echocardiography report  Patient Name: _Tsehaynesh Gashew. Patient ID: 082041. Gender: Female. Age: 14years.  Clinical Diagnosis: ARF + Sydenham’s Chorea. TGSH4.2984. | | | | |
| --- | --- | --- | --- | --- |
| Features | **Finding** | **Features** | | **Finding** |
| Profile | | **Atria** | | |
| Abdominal situs | Solitus | Left atrium | | Normal |
| Cardiac position | Levocardia | Right atrium | | Normal |
| Systemic venous drainage | to RA | **Atrioventricular valves** | | |
| Pulmonary venous drainage | to LA | Mitral valve | | Annulus = 21mm. Mildly Thickened MVL |
| Atrioventricular connection | Concordant | Tricuspid valve | | Annulus = 22mm  TAPSE = 19mm. |
| Ventriculoarterial connection | Concordant | **Ventricles** | | |
| Ventricular loop | d-Loop | Left ventricle | | Normal |
| Septae | | Right ventricle | | Normal |
| Interventricular septum |  | **M-Mode:**. | | |
| Interatrial septum |  | AO | |  |
| Great arteries | NRGA | LA | |  |
| Aorta | ------- | LVIDd | | mm |
| Pulmonary artery | Normal MPA and Branch PAs | LVIDs | | mm |
| Semilunal valves |  | IVSd | | mm |
| Aortic valve | Annulus = 18mm. | IVSs | | mm |
| Pulmonary valve | Annulus = 21mm. | LVPWd | | mm |
| Doppler Measurement | | LVPWs | | mm |
| Mitral | Mild MR, Holosystolic, posterior projection, seen in two planes with jet velocity = 4.7m/sec | EDV | | ml |
| Aortic | ------ | ESV | | ml |
| Tricuspid | -------- | FS | | 30% |
| pulmonic | -------- | LVEF | | 58% |
| Aortic arch | ------- | **Coronary arteries** | |  |
| PDA | -------- |  | |  |
| Pericardium/Pleura |  | | | |
| Final Diagnosis | 1. {S, D, S} Levocardia. 2. Mildly thickened MVL 3. Mild MR 4. Normal Biventricular Function | | | |
| Done By: | **Signature** | | **Date of Reporting** | |
| Dr. Tesfaye Taye, Pediatrician, Pediatric Cardiologist | | | **24/09/2013Eth.C** | |

| Pediatric Echocardiography report  Patient Name: _Emebet Ayalew Patient ID: 084789. Gender: Female. Age: 8/12.  Clinical Diagnosis: DS + Murmur + RD + CHF + Diaphoresi. TGSH4.2985. | | | | |
| --- | --- | --- | --- | --- |
| Features | **Finding** | **Features** | | **Finding** |
| Profile | | **Atria** | | |
| Abdominal situs | Solitus | Left atrium | | Normal |
| Cardiac position | Levocardia | Right atrium | | Dilated |
| Systemic venous drainage | to RA | **Atrioventricular valves** | | |
| Pulmonary venous drainage | to LA | Mitral valve | | Annulus = 11mm |
| Atrioventricular connection | Concordant | Tricuspid valve | | Annulus = 14mm  TAPSE = 5mm. |
| Ventriculoarterial connection | Concordant | **Ventricles** | | |
| Ventricular loop | d-Loop | Left ventricle | | Normal |
| Septae | | Right ventricle | | Dilated, Hypertrophied |
| Interventricular septum |  | **M-Mode:**. | | |
| Interatrial septum | 5mm OS ASD, BD Shunt | AO | |  |
| Great arteries | NRGA | LA | |  |
| Aorta | ------- | LVIDd | | mm |
| Pulmonary artery | Normal MPA and Branch PAs | LVIDs | | mm |
| Semilunal valves |  | IVSd | | mm |
| Aortic valve | Annulus = 9mm. | IVSs | | mm |
| Pulmonary valve | Annulus = 11mm. | LVPWd | | mm |
| Doppler Measurement | | LVPWs | | mm |
| Mitral | ------- | EDV | | ml |
| Aortic | ------ | ESV | | ml |
| Tricuspid | -------- | FS | | 36% |
| pulmonic | Moderate PR, PPG = 61mmHg | LVEF | | 68% |
| Aortic arch | ------- | **Coronary arteries** | |  |
| Pericardium/Pleura | 4mm Pericardial effusion | | | |
| Final Diagnosis | 1. {S, D, S} Levocardia. 2. RA/RV Dilated 3. Small OS ASD, BD Shunt 4. Moderate PR 5. Severe Pulmonary Hypertension 6. Dilated, Hypertrophied and Dysfunctional RV 7. Trace Pericardial effusion | | | |
| Done By: | **Signature** | | **Date of Reporting** | |
| Dr. Tesfaye Taye, Pediatrician, Pediatric Cardiologist | | | **24/09/2013Eth.C** | |

| Pediatric Echocardiography report  Patient Name: Anchinesh Demelash. Patient ID: 085412. Gender: Female. Age: 7/12.  Clinical Diagnosis: DS + RD. TGSH4.2986. | | | | |
| --- | --- | --- | --- | --- |
| Features | **Finding** | **Features** | | **Finding** |
| Profile | | **Atria** | | |
| Abdominal situs | Solitus | Left atrium | | Normal |
| Cardiac position | Levocardia | Right atrium | | Normal |
| Systemic venous drainage | to RA | **Atrioventricular valves** | | |
| Pulmonary venous drainage | to LA | Mitral valve | | Annulus = 10mm |
| Atrioventricular connection | Concordant | Tricuspid valve | | Annulus = 11mm |
| Ventriculoarterial connection | Concordant | **Ventricles** | | |
| Ventricular loop | d-Loop | Left ventricle | | Normal |
| Septae | | Right ventricle | | Normal |
| Interventricular septum |  | **M-Mode:** Normal LV Function on eye balling | | |
| Interatrial septum |  | AO | |  |
| Great arteries | NRGA | LA | |  |
| Aorta | ------- | LVIDd | | mm |
| Pulmonary artery | Normal MPA and Branch PAs | LVIDs | | mm |
| Semilunal valves |  | IVSd | | mm |
| Aortic valve | Annulus = 9mm. | IVSs | | mm |
| Pulmonary valve | Annulus = 10mm. | LVPWd | | mm |
| Doppler Measurement | | LVPWs | | mm |
| Mitral | ------- | EDV | | ml |
| Aortic | ------ | ESV | | ml |
| Tricuspid | -------- | FS | | % |
| pulmonic | -------- | LVEF | | % |
| Aortic arch | ------- | **Coronary arteries** | |  |
| PDA | -------- |  | |  |
| Pericardium/Pleura |  | | | |
| Final Diagnosis | 1. Normal Echocardiography Study. | | | |
| Done By: | **Signature** | | **Date of Reporting** | |
| Dr. Tesfaye Taye, Pediatrician, Pediatric Cardiologist | | | **24/09/2013Eth.C** | |

| Pediatric Echocardiography report  Patient Name: Baby of Wubalech Gashaw. Patient ID: 085444. Gender: Male. Age: 15days.  Clinical Diagnosis: RD. TGSH4.2987. | | | | |
| --- | --- | --- | --- | --- |
| Features | **Finding** | **Features** | | **Finding** |
| Profile | | **Atria** | | |
| Abdominal situs | Solitus | Left atrium | | Normal |
| Cardiac position | Levocardia | Right atrium | | Normal |
| Systemic venous drainage | to RA | **Atrioventricular valves** | | |
| Pulmonary venous drainage | to LA | Mitral valve | | Annulus = 9mm |
| Atrioventricular connection | Concordant | Tricuspid valve | | Annulus = 10mm |
| Ventriculoarterial connection | Concordant | **Ventricles** | | |
| Ventricular loop | d-Loop | Left ventricle | | Normal |
| Septae | | Right ventricle | | Normal |
| Interventricular septum |  | **M-Mode:** Normal LV Function on eye balling | | |
| Interatrial septum |  | AO | |  |
| Great arteries | NRGA | LA | |  |
| Aorta | ------- | LVIDd | | mm |
| Pulmonary artery | Normal MPA and Branch PAs | LVIDs | | mm |
| Semilunal valves |  | IVSd | | mm |
| Aortic valve | Annulus = 7mm. | IVSs | | mm |
| Pulmonary valve | Annulus = 8mm. | LVPWd | | mm |
| Doppler Measurement | | LVPWs | | mm |
| Mitral | ------- | EDV | | ml |
| Aortic | ------ | ESV | | ml |
| Tricuspid | -------- | FS | | % |
| pulmonic | -------- | LVEF | | % |
| Aortic arch | ------- | **Coronary arteries** | |  |
| PDA | -------- |  | |  |
| Pericardium/Pleura |  | | | |
| Final Diagnosis | 1. Normal Echocardiography Study. | | | |
| Done By: | **Signature** | | **Date of Reporting** | |
| Dr. Tesfaye Taye, Pediatrician, Pediatric Cardiologist | | | **24/09/2013Eth.C** | |

| Pediatric Echocardiography report  Patient Name: Agegnehu Misganaw. Patient ID: 082608. Gender: male. Age: 6years.  Clinical Diagnosis: FTT. TGSH4.2988. | | | | |
| --- | --- | --- | --- | --- |
| Features | **Finding** | **Features** | | **Finding** |
| Profile | | **Atria** | | |
| Abdominal situs | Solitus | Left atrium | | Normal |
| Cardiac position | Levocardia | Right atrium | | Normal |
| Systemic venous drainage | to RA | **Atrioventricular valves** | | |
| Pulmonary venous drainage | to LA | Mitral valve | | Annulus = 16mm |
| Atrioventricular connection | Concordant | Tricuspid valve | | Annulus = 21mm |
| Ventriculoarterial connection | Concordant | **Ventricles** | | |
| Ventricular loop | d-Loop | Left ventricle | | Normal |
| Septae | | Right ventricle | | Normal |
| Interventricular septum |  | **M-Mode:**. | | |
| Interatrial septum |  | AO | |  |
| Great arteries | NRGA | LA | |  |
| Aorta | ------- | LVIDd | | mm |
| Pulmonary artery | Normal MPA and Branch PAs | LVIDs | | mm |
| Semilunal valves |  | IVSd | | mm |
| Aortic valve | Annulus = 16mm. | IVSs | | mm |
| Pulmonary valve | Annulus = 19mm. | LVPWd | | mm |
| Doppler Measurement | | LVPWs | | mm |
| Mitral | ------- | EDV | | ml |
| Aortic | ------ | ESV | | ml |
| Tricuspid | -------- | FS | | 31% |
| pulmonic | -------- | LVEF | | 59% |
| Aortic arch | Left, normal branching neck arteries. No CoA | **Coronary arteries** | |  |
| PDA | -------- |  | |  |
| Pericardium/Pleura |  | | | |
| Final Diagnosis | 1. Normal Echocardiography Study. | | | |
| Done By: | **Signature** | | **Date of Reporting** | |
| Dr. Tesfaye Taye, Pediatrician, Pediatric Cardiologist | | | **24/09/2013Eth.C** | |

| Pediatric Echocardiography report  Patient Name: Baby of Ayalnesh Yismaw. Patient ID: ___________. Gender: Male. Age: 12days.  Clinical Diagnosis: Cyanosis + RD. TGSH4.2989. | | | | |
| --- | --- | --- | --- | --- |
| Features | **Finding** | **Features** | | **Finding** |
| Profile | | **Atria** | | |
| Abdominal situs | Solitus | Left atrium | | Normal |
| Cardiac position | Levocardia | Right atrium | | Dilated |
| Systemic venous drainage | to RA | **Atrioventricular valves** | | |
| Pulmonary venous drainage | to LA | Mitral valve | | Annulus = 10mm |
| Atrioventricular connection | Concordant | Tricuspid valve | | Annulus = 13mm |
| Ventriculoarterial connection | Concordant | **Ventricles** | | |
| Ventricular loop | d-Loop | Left ventricle | | Banana shaped |
| Septae : Bowing to LV | | Right ventricle | | Dilated |
| Interventricular septum | Intact | **M-Mode:**. | | |
| Interatrial septum | PFO, L – R Shunt | AO | |  |
| Great arteries | d-TGA | LA | |  |
| Aorta | Anterior and to the right. Arises from RV | LVIDd | | mm |
| Pulmonary artery | Posterior and to the left. Arises from LV | LVIDs | | mm |
| Semilunal valves |  | IVSd | | mm |
| Aortic valve | Annulus = 11mm. | IVSs | | mm |
| Pulmonary valve | Annulus = 9mm. | LVPWd | | mm |
| Doppler Measurement | | LVPWs | | mm |
| Mitral | ------- | EDV | | ml |
| Aortic | ------ | ESV | | ml |
| Tricuspid | Moderate TR | FS | | % |
| pulmonic | -------- | LVEF | | % |
| Aortic arch | ------- | **Coronary arteries** | |  |
| PDA | 1mm PDA, from L –R Shunt |  | |  |
| Pericardium/Pleura |  | | | |
| Final Diagnosis | 1. {S, D, S} Levocardia. 2. PFO, L – R Shunt 3. Small PDA, L – R Shunt 4. d-TGA with Intact Ventricular Septum | | | |
| Done By: | **Signature** | | **Date of Reporting** | |
| Dr. Tesfaye Taye, Pediatrician, Pediatric Cardiologist | | | **27/09/2013Eth.C** | |

| Pediatric Echocardiography report  Patient Name: _Enatihun Getachew. Patient ID: 055687_. Gender: Female. Age: 12years.  Clinical Diagnosis: HTN + Feeble lower extremities pulse. TGSH4.2990. | | | | |
| --- | --- | --- | --- | --- |
| Features | **Finding** | **Features** | | **Finding** |
| Profile | | **Atria** | | |
| Abdominal situs | Solitus | Left atrium | | Normal |
| Cardiac position | Levocardia | Right atrium | | Normal |
| Systemic venous drainage | to RA | **Atrioventricular valves** | | |
| Pulmonary venous drainage | to LA | Mitral valve | | Annulus = 25mm |
| Atrioventricular connection | Concordant | Tricuspid valve | | Annulus = 24mm  TAPSE = 20mm. |
| Ventriculoarterial connection | Concordant | **Ventricles** | | |
| Ventricular loop | d-Loop | Left ventricle | | Concentric Hypertrophy |
| Septae | | Right ventricle | | Normal |
| Interventricular septum |  | **M-Mode:**. | | |
| Interatrial septum |  | AO | |  |
| Great arteries | NRGA | LA | |  |
| Aorta | ------- | LVIDd | | 41mm |
| Pulmonary artery | Normal MPA and Branch PAs | LVIDs | | 28mm |
| Semilunal valves |  | IVSd | | 12mm |
| Aortic valve | Annulus = 17mm. | IVSs | | 13mm |
| Pulmonary valve | Annulus = 20mm. | LVPWd | | 9mm |
| Doppler Measurement | | LVPWs | | 14mm |
| Mitral | Trivial MR, Incomplete signal with jet velocity = 2m/sec | EDV | | 75ml |
| Aortic | ------ | ESV | | 29ml |
| Tricuspid | -------- | FS | | 33% |
| pulmonic | -------- | LVEF | | 62% |
| Aortic arch | Left. Branching neck vessels are Normal. No thoracic aorta coarctation. | **Coronary arteries** | |  |
| PDA | -------- |  | |  |
| Pericardium/Pleura | 3mm pericardial effusion on RV Side. | | | |
| Final Diagnosis | 1. {S, D, S} Levocardia. 2. LVH 3. Normal Function | | | |
| Remark | 1. **Considering the clinical finding, do Abdominal CT.** 2. **R/O Coarctation of Abdominal Aorta** | | | |
| Done By: | **Signature** | | **Date of Reporting** | |
| Dr. Tesfaye Taye, Pediatrician, Pediatric Cardiologist | | | **01/10/2013Eth.C** | |

| Pediatric Echocardiography report  Patient Name: Zufan Abay. Patient ID: o85148. Gender: Female. Age: 8months.  Clinical Finding: DS + RD. TGSH4.2991. | | | | |
| --- | --- | --- | --- | --- |
| Features | **Finding** | **Features** | | **Finding** |
| Profile | | **Atria** | | |
| Abdominal situs | Solitus | Left atrium | | Normal |
| Cardiac position | Levocardia | Right atrium | | Mildly dilayed |
| Systemic venous drainage | to RA | **Atrioventricular valves** | | |
| Pulmonary venous drainage | to LA | Mitral valve | | Common complete AVSD |
| Atrioventricular connection | Common Complete AVSD | Tricuspid valve | |  |
| Ventriculoarterial connection | Concordant | **Ventricles** | | |
| Ventricular loop | d-Loop | Left ventricle | | Normal |
| Septae | | Right ventricle | | Mildly Dilated |
| Interventricular septum | Common Complete AVSD, L – R Shunt | **M-Mode:**. | | |
| Interatrial septum | PFO, L – R Shunt | AO | |  |
| Great arteries | NRGA | LA | |  |
| Aorta | ------- | LVIDd | | mm |
| Pulmonary artery | Normal MPA and Branch PAs | LVIDs | | mm |
| Semilunal valves |  | IVSd | | mm |
| Aortic valve | Annulus = 10mm. | IVSs | | mm |
| Pulmonary valve | Annulus = 10mm. | LVPWd | | mm |
| Doppler Measurement | | LVPWs | | mm |
| Mitral | Mild left AVVR | EDV | | ml |
| Aortic | ------ | ESV | | ml |
| Tricuspid | Mild Right AVVR | FS | | 32% |
| pulmonic | Mild PS, PPG = 36mmHg | LVEF | | 61% |
| Aortic arch | ------- | **Coronary arteries** | |  |
| PDA | -------- |  | |  |
| Pericardium/Pleura |  | | | |
| Final Diagnosis | 1. {S, D, S} Levocardia. 2. PFO, L – R Shunt 3. Common Complete Balanced AVSD 4. Mild Valvular PS 5. Normal LV Function | | | |
| Done By: | **Signature** | | **Date of Reporting** | |
| Dr. Tesfaye Taye, Pediatrician, Pediatric Cardiologist | | | **01/10/2013Eth.C** | |

| Pediatric Echocardiography report  Patient Name: Mirtzer Takele. Patient ID: 084149. Gender: Female. Age: 4months.  Clinical Finding: Incidental Murmur. TGSH4.2992. | | | | | | | |
| --- | --- | --- | --- | --- | --- | --- | --- |
| Features | | | **Finding** | **Features** | | **Finding** | |
| Profile | | | | **Atria** | | | |
| Abdominal situs | | | Solitus | Left atrium | | Normal | |
| Cardiac position | | | Levocardia | Right atrium | | Normal | |
| Systemic venous drainage | | | to RA | **Atrioventricular valves** | | | |
| Pulmonary venous drainage | | | to LA | Mitral valve | | Annulus = 10mm | |
| Atrioventricular connection | | | Concordant | Tricuspid valve | | Annulus = 12mm | |
| Ventriculoarterial connection | | | Concordant | **Ventricles** | | | |
| Ventricular loop | | | d-Loop | Left ventricle | | Normal | |
| Septae | | | | Right ventricle | | Normal | |
| Interventricular septum | | | 2mm PM VSD, L – R Shunt | **M-Mode:** Normal LV Function on eye balling | | | |
| Interatrial septum | | | Intact | AO | |  | |
| Great arteries | | | NRGA | LA | |  | |
| Aorta | | | ------- | LVIDd | | mm | |
| Pulmonary artery | | | Normal MPA and Branch PAs | LVIDs | | mm | |
| Semilunal valves | | |  | IVSd | | mm | |
| Aortic valve | | | Annulus = 9mm. | IVSs | | mm | |
| Pulmonary valve | | | Annulus = 11mm. | LVPWd | | mm | |
| Doppler Measurement | | | | LVPWs | | mm | |
| Mitral | | | ------- | EDV | | ml | |
| Aortic | | | ------ | ESV | | ml | |
| Tricuspid | | | -------- | FS | | % | |
| pulmonic | | | -------- | LVEF | | % | |
| Aortic arch | | | ------- | **Coronary arteries** | |  | |
| PDA | | | -------- |  | |  | |
| Pericardium/Pleura | | |  | | | | |
| Final Diagnosis | | | 1. {S, D, S} Levocardia. 2. Small PM VSD, L – R Shunt | | | | |
| Remark | 1. **Yearly Echocardiography follow up** 2. **No need of medication** | | | | |  |  |
| Done By: | | | **Signature** | | **Date of Reporting** | | |
| Dr. Tesfaye Taye, Pediatrician, Pediatric Cardiologist | | | | | **01/10/2013Eth.C** | | |

| Pediatric Echocardiography report  Patient Name: _Muhammed Kemal. Patient ID: 044668. Gender: male. Age: 1 8/12.  Clinical Finding: RD + CHF. TGSH4.2993. | | | | |
| --- | --- | --- | --- | --- |
| Features | **Finding** | **Features** | | **Finding** |
| Profile | | **Atria** | | |
| Abdominal situs | Solitus | Left atrium | | Dilated |
| Cardiac position | Levocardia | Right atrium | | Normal |
| Systemic venous drainage | to RA | **Atrioventricular valves** | | |
| Pulmonary venous drainage | to LA | Mitral valve | | Annulus = 25mm |
| Atrioventricular connection | Concordant | Tricuspid valve | | Annulus = 15mm |
| Ventriculoarterial connection | Concordant | **Ventricles** | | |
| Ventricular loop | d-Loop | Left ventricle | | Globularly Dilated and Dysfunctional |
| Septae | | Right ventricle | | Normal |
| Interventricular septum | Intact | **M-Mode:**. | | |
| Interatrial septum | Intact | AO | |  |
| Great arteries | NRGA | LA | |  |
| Aorta | ------- | LVIDd | | mm |
| Pulmonary artery | Normal MPA and Branch PAs | LVIDs | | mm |
| Semilunal valves |  | IVSd | | mm |
| Aortic valve | Annulus = 14mm. | IVSs | | mm |
| Pulmonary valve | Annulus = 15mm. | LVPWd | | mm |
| Doppler Measurement | | LVPWs | | mm |
| Mitral | Moderate MR | EDV | | ml |
| Aortic | ------ | ESV | | ml |
| Tricuspid | -------- | FS | | 21% |
| pulmonic | -------- | LVEF | | 43% |
| Aortic arch | Left. No CoA | **Coronary arteries** | | No ALCAPA |
| PDA | -------- |  | |  |
| Pericardium/Pleura |  | | | |
| Final Diagnosis | 1. {S, D, S} Levocardia. 2. LA/LV Dilated 3. Moderate MR 4. Globularly Dilated Dysfunctional LV | | | |
| Remark | 1. **Likely to be DCM** | | | |
| Done By: | **Signature** | | **Date of Reporting** | |
| Dr. Tesfaye Taye, Pediatrician, Pediatric Cardiologist | | | **01/10/2013Eth.C** | |

| Pediatric Echocardiography report  Patient Name: Dawit Mollalign. Patient ID: 021040. Gender: Male. Age: 5years. TGSH1.2628. | | | | |
| --- | --- | --- | --- | --- |
| Features | **Finding** | **Features** | | **Finding** |
| Profile | | **Atria** | | |
| Abdominal situs | Solitus | Left atrium | | Normal |
| Cardiac position | Levocardia | Right atrium | | Normal |
| Systemic venous drainage | to RA | **Atrioventricular valves** | | |
| Pulmonary venous drainage | to LA | Mitral valve | | Annulus = 17mm |
| Atrioventricular connection | Concordant | Tricuspid valve | | Annulus = 20mm |
| Ventriculoarterial connection | Concordant | **Ventricles** | | |
| Ventricular loop | d-Loop | Left ventricle | | Normal |
| Septae | | Right ventricle | | Normal |
| Interventricular septum | Intact | **M-Mode:**. | | |
| Interatrial septum | Intact | AO | |  |
| Great arteries | NRGA | LA | |  |
| Aorta | ------- | LVIDd | | mm |
| Pulmonary artery | Normal MPA and Branch PAs | LVIDs | | mm |
| Semilunal valves |  | IVSd | | mm |
| Aortic valve | Annulus = 15mm. | IVSs | | mm |
| Pulmonary valve | Annulus = 16mm. | LVPWd | | mm |
| Doppler Measurement | | LVPWs | | mm |
| Mitral | Trivial MR, incomplete signal, seen in apical plane only with jet velocity = 3.5m/sec | EDV | | ml |
| Aortic | ------ | ESV | | ml |
| Tricuspid | -------- | FS | | 29% |
| pulmonic | -------- | LVEF | | 56% |
| Aortic arch | ------- | **Coronary arteries** | |  |
| PDA | -------- |  | |  |
| Pericardium/Pleura |  | | | |
| Final Diagnosis | 1. {S, D, S} Levocardia. 2. Trivial MR 3. Normal LV Function | | | |
| Done By: | **Signature** | | **Date of Reporting** | |
| Dr. Tesfaye Taye, Pediatrician, Pediatric Cardiologist | | | **03/10/2013Eth.C** | |

| Pediatric Echocardiography report  Patient Name: Zekarias Mulie. Patient ID: 082245. Gender: Male. Age: 1year.  Clinical Finding: Diaphoresis + Murmur. TGSH4.2994. | | | | |
| --- | --- | --- | --- | --- |
| Features | **Finding** | **Features** | | **Finding** |
| Profile | | **Atria** | | |
| Abdominal situs | Solitus | Left atrium | | Normal |
| Cardiac position | Levocardia | Right atrium | | Normal |
| Systemic venous drainage | to RA | **Atrioventricular valves** | | |
| Pulmonary venous drainage | to LA | Mitral valve | | Annulus = 13mm |
| Atrioventricular connection | Concordant | Tricuspid valve | | Annulus = 16mm |
| Ventriculoarterial connection | Concordant | **Ventricles** | | |
| Ventricular loop | d-Loop | Left ventricle | | Normal |
| Septae | | Right ventricle | | Normal |
| Interventricular septum | 7.5mm PM VSD, L – R Shunt | **M-Mode:** Normal LV Function on eye balling. | | |
| Interatrial septum | Intact | AO | |  |
| Great arteries | NRGA | LA | |  |
| Aorta | ------- | LVIDd | | mm |
| Pulmonary artery | Normal MPA and Branch PAs | LVIDs | | mm |
| Semilunal valves |  | IVSd | | mm |
| Aortic valve | Annulus = 12mm. | IVSs | | mm |
| Pulmonary valve | Annulus = 13mm. | LVPWd | | mm |
| Doppler Measurement | | LVPWs | | mm |
| Mitral | ------- | EDV | | ml |
| Aortic | ------ | ESV | | ml |
| Tricuspid | -------- | FS | | % |
| pulmonic | -------- | LVEF | | % |
| Aortic arch | ------- | **Coronary arteries** | |  |
| PDA | 1.5mm PDA, L – R Shunt |  | |  |
| Pericardium/Pleura |  | | | |
| Final Diagnosis | 1. {S, D, S} Levocardia. 2. Moderate PM VSD, L – R Shunt 3. Small PDA, L – R Shunt 4. Normal LV Function | | | |
| Done By: | **Signature** | | **Date of Reporting** | |
| Dr. Tesfaye Taye, Pediatrician, Pediatric Cardiologist | | | **03/10/2013Eth.C** | |

| Pediatric Echocardiography report  Patient Name: Baby Genet Tilahun. Patient ID: 087611. Gender: Female. Age: 17days.  Clinical Finding: Incidental Murmur. TGSH4.2995. | | | | |
| --- | --- | --- | --- | --- |
| Features | **Finding** | **Features** | | **Finding** |
| Profile | | **Atria** | | |
| Abdominal situs | Solitus | Left atrium | | Normal |
| Cardiac position | Levocardia | Right atrium | | Normal |
| Systemic venous drainage | to RA | **Atrioventricular valves** | | |
| Pulmonary venous drainage | to LA | Mitral valve | | Annulus = 10mm |
| Atrioventricular connection | Concordant | Tricuspid valve | | Annulus = 11mm |
| Ventriculoarterial connection | Concordant | **Ventricles** | | |
| Ventricular loop | d-Loop | Left ventricle | | Normal |
| Septae | | Right ventricle | | Normal |
| Interventricular septum | Intact | **M-Mode:** Normal LV Function on eye balling | | |
| Interatrial septum | PFO, L – R Shunt | AO | |  |
| Great arteries | NRGA | LA | |  |
| Aorta | ------- | LVIDd | | mm |
| Pulmonary artery | Normal MPA and Branch PAs | LVIDs | | mm |
| Semilunal valves |  | IVSd | | mm |
| Aortic valve | Annulus = 9mm. | IVSs | | mm |
| Pulmonary valve | Annulus = 10mm. | LVPWd | | mm |
| Doppler Measurement | | LVPWs | | mm |
| Mitral | ------- | EDV | | ml |
| Aortic | ------ | ESV | | ml |
| Tricuspid | -------- | FS | | % |
| pulmonic | -------- | LVEF | | % |
| Aortic arch | ------- | **Coronary arteries** | |  |
| PDA | <1mm PDA, L – R Shunt |  | |  |
| Pericardium/Pleura |  | | | |
| Final Diagnosis | 1. {S, D, S} Levocardia. 2. PFO, L – R Shunt 3. Small PDA, L – R Shunt | | | |
| Done By: | **Signature** | | **Date of Reporting** | |
| Dr. Tesfaye Taye, Pediatrician, Pediatric Cardiologist | | | **08/10/2013Eth.C** | |

| Pediatric Echocardiography report  Patient Name: Baby of Sewhareg Biadgie. Patient ID: 085831. Gender: Male. Age: 21days.  Clinical Finding: Incidental Murmur. TGSH4.2996. | | | | |
| --- | --- | --- | --- | --- |
| Features | **Finding** | **Features** | | **Finding** |
| Profile | | **Atria** | | |
| Abdominal situs | Solitus | Left atrium | | Normal |
| Cardiac position | Levocardia | Right atrium | | Normal |
| Systemic venous drainage | to RA | **Atrioventricular valves** | | |
| Pulmonary venous drainage | to LA | Mitral valve | | Annulus = 10mm |
| Atrioventricular connection | Concordant | Tricuspid valve | | Annulus = 10mm |
| Ventriculoarterial connection | Concordant | **Ventricles** | | |
| Ventricular loop | d-Loop | Left ventricle | | Normal |
| Septae | | Right ventricle | | Normal |
| Interventricular septum | 2.5mm PM VSD, L – R Shunt | **M-Mode:** Normal LV Function on eye balling | | |
| Interatrial septum | PFO, L – R Shunt | AO | |  |
| Great arteries | NRGA | LA | |  |
| Aorta | ------- | LVIDd | | mm |
| Pulmonary artery | Normal MPA and Branch PAs | LVIDs | | mm |
| Semilunal valves |  | IVSd | | mm |
| Aortic valve | Annulus = 8mm. | IVSs | | mm |
| Pulmonary valve | Annulus = 9mm. | LVPWd | | mm |
| Doppler Measurement | | LVPWs | | mm |
| Mitral | ------- | EDV | | ml |
| Aortic | ------ | ESV | | ml |
| Tricuspid | -------- | FS | | % |
| pulmonic | -------- | LVEF | | % |
| Aortic arch | ------- | **Coronary arteries** | |  |
| PDA | -------- |  | |  |
| Pericardium/Pleura |  | | | |
| Final Diagnosis | 1. {S, D, S} Levocardia. 2. PFO, L – R Shunt 3. Small Perimembranous VSD, L – R Shunt 4. Normal LV Function | | | |
| Done By: | **Signature** | | **Date of Reporting** | |
| Dr. Tesfaye Taye, Pediatrician, Pediatric Cardiologist | | | **08/10/2013Eth.C** | |

| Pediatric Echocardiography report  Patient Name: Habte-Mariam Tenesa. Patient ID: 085637. Gender: Male. Age: 3 8/12.  Clinical Finding: DS. TGSH4.2997. | | | | |
| --- | --- | --- | --- | --- |
| Features | **Finding** | **Features** | | **Finding** |
| Profile | | **Atria** | | |
| Abdominal situs | Solitus | Left atrium | | Normal |
| Cardiac position | Levocardia | Right atrium | | Normal |
| Systemic venous drainage | to RA | **Atrioventricular valves** | | |
| Pulmonary venous drainage | to LA | Mitral valve | | Annulus = 13mm |
| Atrioventricular connection | Concordant | Tricuspid valve | | Annulus = 15mm  TAPSE = 13mm. |
| Ventriculoarterial connection | Concordant | **Ventricles** | | |
| Ventricular loop | d-Loop | Left ventricle | | Normal |
| Septae | | Right ventricle | | Normal |
| Interventricular septum | Intact | **M-Mode:**. | | |
| Interatrial septum | Intact | AO | |  |
| Great arteries | NRGA | LA | |  |
| Aorta | ------- | LVIDd | | mm |
| Pulmonary artery | Normal MPA and Branch PAs | LVIDs | | mm |
| Semilunal valves |  | IVSd | | mm |
| Aortic valve | Annulus = 12mm. | IVSs | | mm |
| Pulmonary valve | Annulus = 16mm. | LVPWd | | mm |
| Doppler Measurement | | LVPWs | | mm |
| Mitral | ------- | EDV | | ml |
| Aortic | ------ | ESV | | ml |
| Tricuspid | Mild TR, PPG = 38mmHg | FS | | 33% |
| pulmonic | -------- | LVEF | | 63% |
| Aortic arch | ------- | **Coronary arteries** | |  |
| PDA | 1mm PDA, L – R Shunt |  | |  |
| Pericardium/Pleura | 4mm pericardial effusion on RV Side. | | | |
| Final Diagnosis | 1. {S, D, S} Levocardia. 2. Mild TR 3. Silent PDA, L – R Shunt 4. Normal Biventricular Function | | | |
| Remark |  | | | |
| Done By: | **Signature** | | **Date of Reporting** | |
| Dr. Tesfaye Taye, Pediatrician, Pediatric Cardiologist | | | **08/10/2013Eth.C** | |

| Pediatric Echocardiography report  Patient Name: Baby of Nigist Molla. Patient ID: 063318. Gender: Male. Age: 6months. | | | | |
| --- | --- | --- | --- | --- |
| Clinical Finding: Incidental Murmur. TGSH4.2998. | | | | |
| Features | **Finding** | **Features** | | **Finding** |
| Profile | | **Atria** | | |
| Abdominal situs | Solitus | Left atrium | | Normal |
| Cardiac position | Levocardia | Right atrium | | Normal |
| Systemic venous drainage | to RA | **Atrioventricular valves** | | |
| Pulmonary venous drainage | to LA | Mitral valve | | Annulus = 12mm |
| Atrioventricular connection | Concordant | Tricuspid valve | | Annulus = 14mm |
| Ventriculoarterial connection | Concordant | **Ventricles** | | |
| Ventricular loop | d-Loop | Left ventricle | | Normal |
| Septae | | Right ventricle | | Normal |
| Interventricular septum | 2.5mm Posterior Muscular VSD, L – R Shunt | **M-Mode:** Normal LV Function on eye balling | | |
| Interatrial septum | Intact | AO | |  |
| Great arteries | NRGA | LA | |  |
| Aorta | ------- | LVIDd | | mm |
| Pulmonary artery | Normal MPA and Branch PAs | LVIDs | | mm |
| Semilunal valves |  | IVSd | | mm |
| Aortic valve | Annulus = 11mm. | IVSs | | mm |
| Pulmonary valve | Annulus = 12mm. | LVPWd | | mm |
| Doppler Measurement | | LVPWs | | mm |
| Mitral | ------- | EDV | | ml |
| Aortic | ------ | ESV | | ml |
| Tricuspid | -------- | FS | | % |
| pulmonic | -------- | LVEF | | % |
| Aortic arch | ------- | **Coronary arteries** | |  |
| PDA | -------- |  | |  |
| Pericardium/Pleura |  | | | |
| Final Diagnosis | 1. {S, D, S} Levocardia. 2. Small Posterior Muscular VSD, L – R Shunt 3. Normal LV Function | | | |
| Remark | 1. Do yearly Echocardiography 2. PFO, CLOSED 3. No need of Cardiac Medicine | | | |
| Done By: | **Signature** | | **Date of Reporting** | |
| Dr. Tesfaye Taye, Pediatrician, Pediatric Cardiologist | | | **08/10/2013Eth.C** | |

| Pediatric Echocardiography report  Patient Name: Eyerus Tadie. Patient ID: 087635. Gender: Female. Age: 7years.  Clinical Finding: Incidental Murmur. TGSH4.2999. | | | | |
| --- | --- | --- | --- | --- |
| Features | **Finding** | **Features** | | **Finding** |
| Profile | | **Atria** | | |
| Abdominal situs | Solitus | Left atrium | | Dilated |
| Cardiac position | Levocardia | Right atrium | | Normal |
| Systemic venous drainage | to RA | **Atrioventricular valves** | | |
| Pulmonary venous drainage | to LA | Mitral valve | | Annulus = 15mm |
| Atrioventricular connection | Concordant | Tricuspid valve | | Annulus = 14mm  TAPSE = 20mm. |
| Ventriculoarterial connection | Concordant | **Ventricles** | | |
| Ventricular loop | d-Loop | Left ventricle | | Dilated |
| Septae | | Right ventricle | | Normal |
| Interventricular septum | Intact | **M-Mode:**. | | |
| Interatrial septum | Intact | AO | |  |
| Great arteries | NRGA | LA | |  |
| Aorta | ------- | LVIDd | | mm |
| Pulmonary artery | Normal MPA and Branch PAs | LVIDs | | mm |
| Semilunal valves |  | IVSd | | mm |
| Aortic valve | Annulus = 13mm. | IVSs | | mm |
| Pulmonary valve | Annulus = 14mm. Doming Pulmonary Valve | LVPWd | | mm |
| Doppler Measurement | | LVPWs | | mm |
| Mitral | ------- | EDV | | ml |
| Aortic | ------ | ESV | | ml |
| Tricuspid | -------- | FS | | 40% |
| pulmonic | Mild PS, PPG = 24mmHg. Moderate PR, PPG = 53mmHg | LVEF | | 72% |
| Aortic arch | ------- | **Coronary arteries** | |  |
| PDA | 2mm PDA, L – R Shunt |  | |  |
| Pericardium/Pleura |  | | | |
| Final Diagnosis | 1. {S, D, S} Levocardia. 2. Moderate PDA, L – R Shunt 3. Moderate PR 4. Mild PS 5. Doming Pulmonary Valve 6. Moderate Pulmonary Hypertension 7. Normal Biventricular Function | | | |
| Done By: | **Signature** | | **Date of Reporting** | |
| Dr. Tesfaye Taye, Pediatrician, Pediatric Cardiologist | | | **08/10/2013Eth.C** | |

| Pediatric Echocardiography report  Patient Name: Biruk Markos. Patient ID: 088279. Gender: Male. Age: 13years.  Clinical Finding: Rheumatic Recurrence + DOE + Murmur + Palpitation. TGSH4.3000. | | | | |
| --- | --- | --- | --- | --- |
| Features | **Finding** | **Features** | | **Finding** |
| Profile | | **Atria** | | |
| Abdominal situs | Solitus | Left atrium | | Markedly dilated |
| Cardiac position | Levocardia | Right atrium | | Normal |
| Systemic venous drainage | to RA | **Atrioventricular valves** | | |
| Pulmonary venous drainage | to LA | Mitral valve | | Annulus = 33mm. thickened, Shortened Posterior MVL |
| Atrioventricular connection | Concordant | Tricuspid valve | | Annulus = 19mm  TAPSE = 18mm. |
| Ventriculoarterial connection | Concordant | **Ventricles** | | |
| Ventricular loop | d-Loop | Left ventricle | | Markedly dilated |
| Septae | | Right ventricle | | Normal |
| Interventricular septum | Intact | **M-Mode:**. | | |
| Interatrial septum | Intact | AO | |  |
| Great arteries | NRGA | LA | |  |
| Aorta | ------- | LVIDd | | mm |
| Pulmonary artery | Normal MPA and Branch PAs | LVIDs | | mm |
| Semilunal valves |  | IVSd | | mm |
| Aortic valve | Annulus = 18mm. | IVSs | | mm |
| Pulmonary valve | Annulus = 25mm. | LVPWd | | mm |
| Doppler Measurement | | LVPWs | | mm |
| Mitral | Severe MR, Holosystolic, posterior projection, seen in two planes with jet velocity = 4.4m/sec | EDV | | ml |
| Aortic | ------ | ESV | | ml |
| Tricuspid | Mild to moderate TR, PPG = 48mmHg | FS | | 24% |
| pulmonic | -------- | LVEF | | 47% |
| Aortic arch | ------- | **Coronary arteries** | |  |
| Pericardium/Pleura |  | | | |
| Final Diagnosis | 1. {S, D, S} Levocardia. 2. LA/LV Dilated 3. Thickened Shortened Posterior MVL 4. Severe MR 5. Mild to Moderate TR 6. Mild Pulmonary Hypertension 7. Moderately reduced LV Function | | | |
| Done By: | **Signature** | | **Date of Reporting** | |
| Dr. Tesfaye Taye, Pediatrician, Pediatric Cardiologist | | | **10/10/2013Eth.C** | |

| Pediatric Echocardiography report  Patient Name: Tatey Abere. Patient ID: 088351. Gender: Female. Age: 8years.  Clinical Finding: Rheumatic Recurrence + DOE + Murmur + CHF + Palpitation + RD. TGSH4.3001. | | | | |
| --- | --- | --- | --- | --- |
| Features | **Finding** | | **Features** | **Finding** |
| Profile | | | **Atria** | |
| Abdominal situs | Solitus | | Left atrium | Dilated |
| Cardiac position | Levocardia | | Right atrium | Normal |
| Systemic venous drainage | to RA | | **Atrioventricular valves** | |
| Pulmonary venous drainage | to LA | | Mitral valve | Annulus = 22mm. Thickened clubbed and shortened MVL |
| Atrioventricular connection | Concordant | | TV | Annulus = 18mm |
| Ventriculoarterial connection | Concordant | | **Ventricles** | |
| Ventricular loop | d-Loop | | Left ventricle | Dilated |
| Septae | | | Right ventricle | Normal |
| Interventricular septum | Intact | | **M-Mode:**. | |
| Interatrial septum | Intact | | AO |  |
| Great arteries | NRGA | | LA |  |
| Aorta | ------- | | LVIDd | mm |
| Pulmonary artery | Normal MPA and Branch PAs | | LVIDs | mm |
| Semilunal valves |  | | IVSd | mm |
| Aortic valve | Annulus = 17mm. | | IVSs | mm |
| Pulmonary valve | Annulus = 18mm. | | LVPWd | mm |
| Doppler Measurement | | | LVPWs | mm |
| Mitral | Severe MR, Holosystolic, posterior projection, seen in two planes with jet velocity = 5.1m/sec. | | EDV | ml |
| Aortic | Mild AR, PHT = 519ms | | ESV | ml |
| Tricuspid | Moderate TR, PPG = 48mmHg | | FS | 30% |
| pulmonic | Mild to moderate PR, PPG = 41mmHg | | LVEF | 57% |
| Pericardium/Pleura |  | | | |
| Final Diagnosis | 1. {S, D, S} Levocardia. 2. LA/LV Dilated 3. Thickened, clubbed and shortened MVL 4. Severe MR 5. Moderate TR 6. Mild AR 7. Mild to Moderate PR 8. Mild Pulmonary Hypertension 9. Normal LV Function | | | |
| Done By: | **Signature** | **Date of Reporting** | | |
| Dr. Tesfaye Taye, Pediatrician, Pediatric Cardiologist | | **10/10/2013Eth.C** | | |

| Pediatric Echocardiography report  Patient Name: Birhanu Tilahun. Patient ID: 086300. Gender: Male. Age: 2 8/12.  Clinical Finding: Recurrent Chest Infection. TGSH4.3002. | | | | |
| --- | --- | --- | --- | --- |
| Features | **Finding** | **Features** | | **Finding** |
| Profile | | **Atria** | | |
| Abdominal situs | Solitus | Left atrium | | Normal |
| Cardiac position | Levocardia | Right atrium | | Normal |
| Systemic venous drainage | to RA | **Atrioventricular valves** | | |
| Pulmonary venous drainage | to LA | Mitral valve | | Annulus = 15mm |
| Atrioventricular connection | Concordant | Tricuspid valve | | Annulus = 17mm  TAPSE = 16mm. |
| Ventriculoarterial connection | Concordant | **Ventricles** | | |
| Ventricular loop | d-Loop | Left ventricle | | Normal |
| Septae | | Right ventricle | | Normal |
| Interventricular septum | Intact | **M-Mode:**. | | |
| Interatrial septum | PFO, L – R Shunt | AO | |  |
| Great arteries | NRGA | LA | |  |
| Aorta | ------- | LVIDd | | mm |
| Pulmonary artery | Normal MPA and Branch PAs | LVIDs | | mm |
| Semilunal valves |  | IVSd | | mm |
| Aortic valve | Annulus = 12mm. | IVSs | | mm |
| Pulmonary valve | Annulus = 15mm. | LVPWd | | mm |
| Doppler Measurement | | LVPWs | | mm |
| Mitral | ------- | EDV | | ml |
| Aortic | ------ | ESV | | ml |
| Tricuspid | -------- | FS | | 38% |
| pulmonic | -------- | LVEF | | 70% |
| Aortic arch | ------- | **Coronary arteries** | |  |
| PDA | -------- |  | |  |
| Pericardium/Pleura |  | | | |
| Final Diagnosis | 1. {S, D, S} Levocardia. 2. PFO, L – R Shunt | | | |
| Done By: | **Signature** | | **Date of Reporting** | |
| Dr. Tesfaye Taye, Pediatrician, Pediatric Cardiologist | | | **10/10/2013Eth.C** | |

| Pediatric Echocardiography report  Patient Name: Adugnaw Kassie. Patient ID: ___________. Gender: Male. Age: 13years.  Clinical Finding: Rheumatic Recurrence + DOE + Murmur + Palpitation + CHF. TGSH4.3003. | | | | |
| --- | --- | --- | --- | --- |
| Features | **Finding** | **Features** | | **Finding** |
| Profile | | **Atria** | | |
| Abdominal situs | Solitus | Left atrium | | Dilated |
| Cardiac position | Levocardia | Right atrium | | Normal |
| Systemic venous drainage | to RA | **Atrioventricular valves** | | |
| Pulmonary venous drainage | to LA | Mitral valve | | Annulus = 28mm. Mildly thickened MVL. |
| Atrioventricular connection | Concordant | Tricuspid valve | | Annulus = 26mm  TAPSE = 22mm. |
| Ventriculoarterial connection | Concordant | **Ventricles** | | |
| Ventricular loop | d-Loop | Left ventricle | | Dilated |
| Septae | | Right ventricle | | Normal |
| Interventricular septum | Intact | **M-Mode:**. | | |
| Interatrial septum | Intact | AO | |  |
| Great arteries | NRGA | LA | |  |
| Aorta | ------- | LVIDd | | mm |
| Pulmonary artery | Normal MPA and Branch PAs | LVIDs | | mm |
| Semilunal valves |  | IVSd | | mm |
| Aortic valve | Annulus = 17mm. | IVSs | | mm |
| Pulmonary valve | Annulus = 23mm. | LVPWd | | mm |
| Doppler Measurement | | LVPWs | | mm |
| Mitral | Severe MR, Holosystolic, posterior projection, seen in two planes with jet velocity = 4.7m/sec | EDV | | ml |
| Aortic | Mild AR, PHT = 552ms. | ESV | | ml |
| Tricuspid | Moderate TR, PPG = 70mmHg | FS | | 41% |
| pulmonic | Mild PR, PPG = 52mmHg | LVEF | | 72% |
| Pericardium/Pleura | 10mm Pericardial effusion on RA/RV Side | | | |
| Final Diagnosis | 1. {S, D, S} Levocardia. 2. LA/LV Dilated 3. Thickened MVL 4. Severe MR 5. Moderate TR 6. Mild AR 7. Mild PR 8. Severe Pulmonary Hypertension 9. Mild Pericardial effusion 10. Normal Biventricular Function | | | |
| Done By: | **Signature** | | **Date of Reporting** | |
| Dr. Tesfaye Taye, Pediatrician, Pediatric Cardiologist | | | **10/10/2013Eth.C** | |

| Pediatric Echocardiography report  Patient Name: Mamush Enchalew. Patient ID: 086300. Gender: Male. Age: 14years.  Clinical Finding: Rheumatic Recurrence + Murmur + DOE + RD + CHF. TGSH4.3004. | | | | |
| --- | --- | --- | --- | --- |
| Features | **Finding** | **Features** | | **Finding** |
| Profile | | **Atria** | | |
| Abdominal situs | Solitus | Left atrium | | Dilated |
| Cardiac position | Levocardia | Right atrium | | Normal |
| Systemic venous drainage | to RA | **Atrioventricular valves** | | |
| Pulmonary venous drainage | to LA | Mitral valve | | Annulus = 30mm. Thickened, clubbed MVL. MVA = 1.3cm^2^. |
| Atrioventricular connection | Concordant | Tricuspid valve | | Annulus = 26mm |
| Ventriculoarterial connection | Concordant | **Ventricles** | | |
| Ventricular loop | d-Loop | Left ventricle | | Dilated |
| Septae | | Right ventricle | | Normal |
| Interventricular septum | Intact | **M-Mode:**. | | |
| Interatrial septum | Intact | AO | |  |
| Great arteries | NRGA | LA | |  |
| Aorta | ------- | LVIDd | | mm |
| Pulmonary artery | Normal MPA and Branch PAs | LVIDs | | mm |
| Semilunal valves |  | IVSd | | mm |
| Aortic valve | Annulus = 18mm. | IVSs | | mm |
| Pulmonary valve | Annulus = 20mm. | LVPWd | | mm |
| Doppler Measurement | | LVPWs | | mm |
| Mitral | Severe MR, Holosystolic, posterior projection, seen in two planes with jet velocity = 4.7m/sec. Moderate MS, PPG/MPG = 15/8mmHg | EDV | | ml |
| Aortic | Moderate AR, PHT = 490ms. | ESV | | ml |
| Tricuspid | Mild TR, PPG = 39mmHg | FS | | 34% |
| pulmonic | -------- | LVEF | | 62% |
| Pericardium/Pleura | 3mm pericardial effusion on RA/RV Side | | | |
| Final Diagnosis | 1. {S, D, S} Levocardia. 2. LA/LV Dilated 3. Thickened, clubbed MVL 4. Severe MR 5. Moderate MS 6. Moderate AR 7. Mild TR 8. Mild Pulmonary Hypertension 9. Trace Pericardial effusion 10. Normal LV Function | | | |
| Done By: | **Signature** | | **Date of Reporting** | |
| Dr. Tesfaye Taye, Pediatrician, Pediatric Cardiologist | | | **10/10/2013Eth.C** | |

| Pediatric Echocardiography report  Patient Name: Yeshiwerk Tesfaw. Patient ID: 088647. Gender: Female. Age: 1 6/12.  Clinical Finding: RD + CHF + Murmur + Pulmonary HTN. TGSH4.3005. | | | | |
| --- | --- | --- | --- | --- |
| Features | **Finding** | **Features** | | **Finding** |
| Profile | | **Atria** | | |
| Abdominal situs | Solitus | Left atrium | | Normal |
| Cardiac position | Levocardia | Right atrium | | Dilated |
| Systemic venous drainage | to RA | **Atrioventricular valves** | | |
| Pulmonary venous drainage | to LA | Mitral valve | | Annulus = 14mm |
| Atrioventricular connection | Concordant | Tricuspid valve | | Annulus = 21mm  TAPSE = 8mm. |
| Ventriculoarterial connection | Concordant | **Ventricles** | | |
| Ventricular loop | d-Loop | Left ventricle | | Normal |
| Septae | | Right ventricle | | Dilated, Hypertrophied and Dysfunctional. RV TDI S wave = 7cm/sec |
| Interventricular septum | Intact | **M-Mode:**. | | |
| Interatrial septum | PFO, BD Shunt | AO | |  |
| Great arteries | NRGA | LA | |  |
| Aorta | ------- | LVIDd | | mm |
| Pulmonary artery | Normal MPA and Branch PAs | LVIDs | | mm |
| Semilunal valves |  | IVSd | | mm |
| Aortic valve | Annulus = 11mm. | IVSs | | mm |
| Pulmonary valve | Annulus = 13mm. | LVPWd | | mm |
| Doppler Measurement | | LVPWs | | mm |
| Mitral | ------- | EDV | | ml |
| Aortic | ------ | ESV | | ml |
| Tricuspid | Moderate TR, PPG = 60mmHg | FS | | 33% |
| pulmonic | -------- | LVEF | | 64% |
| Aortic arch | ------- | **Coronary arteries** | |  |
| Pericardium/Pleura | Trace pericardial effusion | | | |
| Final Diagnosis | 1. {S, D, S} Levocardia. 2. PFO, BD Shunt 3. RA/RV Dilated 4. Moderate TR 5. Severe Pulmonary Hypertension 6. Dilated, Dysfunctional RV 7. Normal LV Function 8. Trace Pericardial effusion | | | |
| Done By: | **Signature** | | **Date of Reporting** | |
| Dr. Tesfaye Taye, Pediatrician, Pediatric Cardiologist | | | **15/10/2013Eth.C** | |

| Pediatric Echocardiography report  Patient Name: Selam Asmamaw. Patient ID: 088756. Gender: Female. Age: 5years.  Clinical Finding: ARF. TGSH4.3006. | | | | |
| --- | --- | --- | --- | --- |
| Features | **Finding** | **Features** | | **Finding** |
| Profile | | **Atria** | | |
| Abdominal situs | Solitus | Left atrium | | Normal |
| Cardiac position | Levocardia | Right atrium | | Normal |
| Systemic venous drainage | to RA | **Atrioventricular valves** | | |
| Pulmonary venous drainage | to LA | Mitral valve | | Annulus = 18mm. Thickened MVL |
| Atrioventricular connection | Concordant | Tricuspid valve | | Annulus = 18mm  TAPSE = 17mm. |
| Ventriculoarterial connection | Concordant | **Ventricles** | | |
| Ventricular loop | d-Loop | Left ventricle | | Normal |
| Septae | | Right ventricle | | Normal |
| Interventricular septum | Intact | **M-Mode:**. | | |
| Interatrial septum | Intact | AO | |  |
| Great arteries | NRGA | LA | |  |
| Aorta | ------- | LVIDd | | mm |
| Pulmonary artery | Normal MPA and Branch PAs | LVIDs | | mm |
| Semilunal valves |  | IVSd | | mm |
| Aortic valve | Annulus = 15mm. | IVSs | | mm |
| Pulmonary valve | Annulus = 16mm. | LVPWd | | mm |
| Doppler Measurement | | LVPWs | | mm |
| Mitral | Mild MR, Holosystolic, posterior projection, seen in two planes with jet velocity = 4.1m/sec | EDV | | ml |
| Aortic | ------ | ESV | | ml |
| Tricuspid | Mild TR, PPG = 15mmHg | FS | | 32% |
| pulmonic | Mild PR, PPG = 9mmHg | LVEF | | 62% |
| Aortic arch | ------- | **Coronary arteries** | |  |
| PDA | -------- |  | |  |
| Pericardium/Pleura |  | | | |
| Final Diagnosis | 1. {S, D, S} Levocardia. 2. Thickened MVL 3. Mild MR 4. Normal Biventricular Function | | | |
| Recommendation | Work in the line of ARF | | | |
| Done By: | **Signature** | | **Date of Reporting** | |
| Dr. Tesfaye Taye, Pediatrician, Pediatric Cardiologist | | | **17/10/2013Eth.C** | |

| Pediatric Echocardiography report  Patient Name: Biruk Gizeaddis. Patient ID: 087673. Gender: Male. Age: 1 3/12.  Clinical Finding: Recurrent Chest Infection. TGSH4.3007. | | | | |
| --- | --- | --- | --- | --- |
| Features | **Finding** | **Features** | | **Finding** |
| Profile | | **Atria** | | |
| Abdominal situs | Solitus | Left atrium | | Normal |
| Cardiac position | Levocardia | Right atrium | | Normal |
| Systemic venous drainage | to RA | **Atrioventricular valves** | | |
| Pulmonary venous drainage | to LA | Mitral valve | | Annulus = 13mm |
| Atrioventricular connection | Concordant | Tricuspid valve | | Annulus = 15mm  TAPSE = 16mm. |
| Ventriculoarterial connection | Concordant | **Ventricles** | | |
| Ventricular loop | d-Loop | Left ventricle | | Normal |
| Septae | | Right ventricle | | Normal |
| Interventricular septum | Intact | **M-Mode:**. | | |
| Interatrial septum | Intact | AO | |  |
| Great arteries | NRGA | LA | |  |
| Aorta | ------- | LVIDd | | mm |
| Pulmonary artery | Normal MPA and Branch PAs | LVIDs | | mm |
| Semilunal valves |  | IVSd | | mm |
| Aortic valve | Annulus = 12mm. | IVSs | | mm |
| Pulmonary valve | Annulus = 13mm. | LVPWd | | mm |
| Doppler Measurement | | LVPWs | | mm |
| Mitral | ------- | EDV | | ml |
| Aortic | ------ | ESV | | ml |
| Tricuspid | -------- | FS | | 41% |
| pulmonic | Mild PR, PPG = 15mmHg | LVEF | | 74% |
| Aortic arch | ------- | **Coronary arteries** | |  |
| PDA | -------- |  | |  |
| Pericardium/Pleura |  | | | |
| Final Diagnosis | 1. {S, D, S} Levocardia. 2. Mild PR 3. Normal Biventricular Function | | | |
| Done By: | **Signature** | | **Date of Reporting** | |
| Dr. Tesfaye Taye, Pediatrician, Pediatric Cardiologist | | | **17/10/2013Eth.C** | |

| Pediatric Echocardiography report  Patient Name: Baby of Enguday Birhanu. Patient ID: 088847. Gender: Male. Age: 72hours.  Clinical Finding: RD. TGSH4.3008. | | | | |
| --- | --- | --- | --- | --- |
| Features | **Finding** | **Features** | | **Finding** |
| Profile | | **Atria** | | |
| Abdominal situs | Solitus | Left atrium | | Normal |
| Cardiac position | Levocardia | Right atrium | | Normal |
| Systemic venous drainage | to RA | **Atrioventricular valves** | | |
| Pulmonary venous drainage | to LA | Mitral valve | | Annulus = 8mm |
| Atrioventricular connection | Concordant | Tricuspid valve | | Annulus = 10mm  TAPSE = 11mm. |
| Ventriculoarterial connection | Concordant | **Ventricles** | | |
| Ventricular loop | d-Loop | Left ventricle | | Normal |
| Septae | | Right ventricle | | Normal |
| Interventricular septum | Intact | **M-Mode:**. | | |
| Interatrial septum | Probe Patent PFO | AO | |  |
| Great arteries | NRGA | LA | |  |
| Aorta | ------- | LVIDd | | mm |
| Pulmonary artery | Normal MPA and Branch PAs | LVIDs | | mm |
| Semilunal valves |  | IVSd | | mm |
| Aortic valve | Annulus = 8mm. | IVSs | | mm |
| Pulmonary valve | Annulus = 8mm. | LVPWd | | mm |
| Doppler Measurement | | LVPWs | | mm |
| Mitral | ------- | EDV | | ml |
| Aortic | ------ | ESV | | ml |
| Tricuspid | Trivial TR, PPG = 40mmHg | FS | | 33% |
| pulmonic | -------- | LVEF | | 65% |
| Aortic arch | ------- | **Coronary arteries** | |  |
| PDA | -------- |  | |  |
| Pericardium/Pleura |  | | | |
| Final Diagnosis | 1. {S, D, S} Levocardia. 2. Probe patent PFO | | | |
| Done By: | **Signature** | | **Date of Reporting** | |
| Dr. Tesfaye Taye, Pediatrician, Pediatric Cardiologist | | | **17/10/2013Eth.C** | |

| Pediatric Echocardiography report  Patient Name: Haymanot Yibeltal. Patient ID: 089160. Gender: Female. Age: 9years.  Clinical Finding: Sydenham’s Chorea. TGSH4.3009. | | | | |
| --- | --- | --- | --- | --- |
| Features | **Finding** | **Features** | | **Finding** |
| Profile | | **Atria** | | |
| Abdominal situs | Solitus | Left atrium | | Normal |
| Cardiac position | Levocardia | Right atrium | | Normal |
| Systemic venous drainage | to RA | **Atrioventricular valves** | | |
| Pulmonary venous drainage | to LA | Mitral valve | | Annulus = 21mm. Mildly thickened MVL |
| Atrioventricular connection | Concordant | Tricuspid valve | | Annulus = 20mm  TAPSE = 18mm. |
| Ventriculoarterial connection | Concordant | **Ventricles** | | |
| Ventricular loop | d-Loop | Left ventricle | | Normal |
| Septae | | Right ventricle | | Normal |
| Interventricular septum | Intact | **M-Mode:**. | | |
| Interatrial septum | Intact | AO | |  |
| Great arteries | NRGA | LA | |  |
| Aorta | ------- | LVIDd | | mm |
| Pulmonary artery | Normal MPA and Branch PAs | LVIDs | | mm |
| Semilunal valves |  | IVSd | | mm |
| Aortic valve | Annulus = 15mm. | IVSs | | mm |
| Pulmonary valve | Annulus = 18mm. | LVPWd | | mm |
| Doppler Measurement | | LVPWs | | mm |
| Mitral | Mild MR, Holosystolic, posterior projection, seen in two planes with jet velocity = 4.5m/sec | EDV | | ml |
| Aortic | ------ | ESV | | ml |
| Tricuspid | -------- | FS | | 31% |
| pulmonic | -------- | LVEF | | 59% |
| Aortic arch | ------- | **Coronary arteries** | |  |
| PDA | -------- |  | |  |
| Pericardium/Pleura |  | | | |
| Final Diagnosis | 1. {S, D, S} Levocardia. 2. Mildly thickened MVL 3. Mild MR 4. Normal Biventricular Function | | | |
| Recommendation | Work up in the line of Rheumatic carditis | | | |
| Done By: | **Signature** | | **Date of Reporting** | |
| Dr. Tesfaye Taye, Pediatrician, Pediatric Cardiologist | | | **17/10/2013Eth.C** | |

| Pediatric Echocardiography report  Patient Name: Hewan Challachew. Patient ID: 089091. Gender: Female. Age: 5/12.  Clinical Finding: CHF + RD. TGSH4.3010. | | | | |
| --- | --- | --- | --- | --- |
| Features | **Finding** | **Features** | | **Finding** |
| Profile | | **Atria** | | |
| Abdominal situs | Solitus | Left atrium | | Dilated |
| Cardiac position | Levocardia | Right atrium | | Normal |
| Systemic venous drainage | to RA | **Atrioventricular valves** | | |
| Pulmonary venous drainage | to LA | Mitral valve | | Annulus = 13mm |
| Atrioventricular connection | Concordant | Tricuspid valve | | Annulus = 11mm  TAPSE = 14mm. |
| Ventriculoarterial connection | Concordant | **Ventricles** | | |
| Ventricular loop | d-Loop | Left ventricle | | Hugely dilated and dysfunctional |
| Septae | | Right ventricle | | Normal |
| Interventricular septum | Intact | **M-Mode:**. | | |
| Interatrial septum | PFO, L – R Shunt | AO | |  |
| Great arteries | NRGA | LA | |  |
| Aorta | ------- | LVIDd | | 4mm |
| Pulmonary artery | Normal MPA and Branch PAs | LVIDs | | 3mm |
| Semilunal valves |  | IVSd | | 4mm |
| Aortic valve | Annulus = 11mm. | IVSs | | 5mm |
| Pulmonary valve | Annulus = 12mm. | LVPWd | | 4mm |
| Doppler Measurement | | LVPWs | | 5mm |
| Mitral | Moderate MR, Holosystolic, central, seen in two planes with jet velocity = 4.6m/sec | EDV | | 65ml |
| Aortic | Trivial AR, PHT = 507ms | ESV | | 45ml |
| Tricuspid | -------- | FS | | 14% |
| pulmonic | -------- | LVEF | | 30% |
| Aortic arch | Left, No CoA. | **Coronary arteries** | | From Aorta |
| Pericardium/Pleura |  | | | |
| Final Diagnosis | 1. {S, D, S} Levocardia. 2. LA/LV Dilated 3. PFO, L – R Shunt 4. Moderate MR 5. Trivial AR 6. Dilated Dysfunctional LV | | | |
| Remark | Consider Dilated Cardiomyopathy as a Differential Diagnosis | | | |
| Done By: | **Signature** | | **Date of Reporting** | |
| Dr. Tesfaye Taye, Pediatrician, Pediatric Cardiologist | | | **22/10/2013Eth.C** | |

| Pediatric Echocardiography report  Patient Name: Temechew Kefale. Patient ID: 017241. Gender: Male. Age: 14 10/12.  Clinical Finding: Incidental Murmur. TGSH4.3011. | | | | |
| --- | --- | --- | --- | --- |
| Features | **Finding** | **Features** | | **Finding** |
| Profile | | **Atria** | | |
| Abdominal situs | Solitus | Left atrium | | Normal |
| Cardiac position | Levocardia | Right atrium | | Normal |
| Systemic venous drainage | to RA | **Atrioventricular valves** | | |
| Pulmonary venous drainage | to LA | Mitral valve | | Annulus = 25mm. Mildly thickened MVL |
| Atrioventricular connection | Concordant | Tricuspid valve | | Annulus = 25mm |
| Ventriculoarterial connection | Concordant | **Ventricles** | | |
| Ventricular loop | d-Loop | Left ventricle | | Normal |
| Septae | | Right ventricle | | Normal |
| Interventricular septum | Intact | **M-Mode:**. | | |
| Interatrial septum | Intact | AO | |  |
| Great arteries | NRGA | LA | |  |
| Aorta | ------- | LVIDd | | mm |
| Pulmonary artery | Normal MPA and Branch PAs | LVIDs | | mm |
| Semilunal valves |  | IVSd | | mm |
| Aortic valve | Annulus = 19mm. | IVSs | | mm |
| Pulmonary valve | Annulus = 23mm. | LVPWd | | mm |
| Doppler Measurement | | LVPWs | | mm |
| Mitral | Trivial MR, Incomplete signal, seen in two planes with jet velocity = 3.5m/sec | EDV | | ml |
| Aortic | ------ | ESV | | ml |
| Tricuspid | Trivial TR, PPG = 18mmHg | FS | | 34% |
| pulmonic | Trivial PR, PPG = 10mmHg | LVEF | | 63% |
| Aortic arch | ------- | **Coronary arteries** | |  |
| PDA | -------- |  | |  |
| Pericardium/Pleura |  | | | |
| Final Diagnosis | 1. {S, D, S} Levocardia. 2. Mildly thickened MVL 3. Trivial MR 4. Normal LV Function | | | |
| Done By: | **Signature** | | **Date of Reporting** | |
| Dr. Tesfaye Taye, Pediatrician, Pediatric Cardiologist | | | **24/10/2013Eth.C** | |

| Pediatric Echocardiography report  Patient Name: Addisu Alie. Patient ID: 089621. Gender: Male. Age: 10years.  Clinical Finding: Incidental Murmur + DOE. TGSH4.3012. | | | | |
| --- | --- | --- | --- | --- |
| Features | **Finding** | **Features** | | **Finding** |
| Profile | | **Atria** | | |
| Abdominal situs | Solitus | Left atrium | | Normal |
| Cardiac position | Levocardia | Right atrium | | Dilated |
| Systemic venous drainage | to RA | **Atrioventricular valves** | | |
| Pulmonary venous drainage | to LA | Mitral valve | | Annulus = 15mm |
| Atrioventricular connection | Concordant | Tricuspid valve | | Annulus = 23mm  TAPSE = 23mm. |
| Ventriculoarterial connection | Concordant | **Ventricles** | | |
| Ventricular loop | d-Loop | Left ventricle | | Normal |
| Septae | | Right ventricle | | Dilated |
| Interventricular septum | Intact | **M-Mode:**. | | |
| Interatrial septum | 18mm OS ASD, L – R Shunt | AO | |  |
| Great arteries | NRGA | LA | |  |
| Aorta | ------- | LVIDd | | mm |
| Pulmonary artery | Normal MPA and Branch PAs | LVIDs | | mm |
| Semilunal valves |  | IVSd | | mm |
| Aortic valve | Annulus = 15mm. | IVSs | | mm |
| Pulmonary valve | Annulus = 21mm. | LVPWd | | mm |
| Doppler Measurement | | LVPWs | | mm |
| Mitral | ------- | EDV | | ml |
| Aortic | ------ | ESV | | ml |
| Tricuspid | Mild TR, PPG = 45mmHg | FS | | 38% |
| pulmonic | Trivial PR, PPG = 19mmHg | LVEF | | 69% |
| Aortic arch | ------- | **Coronary arteries** | |  |
| PDA | -------- |  | |  |
| Pericardium/Pleura |  | | | |
| Final Diagnosis | 1. {S, D, S} Levocardia. 2. Large OS ASD, L – R Shunt 3. Mild Pulmonary Hypertension 4. Normal Biventricular Function | | | |
| Done By: | **Signature** | | **Date of Reporting** | |
| Dr. Tesfaye Taye, Pediatrician, Pediatric Cardiologist | | | **24/10/2013Eth.C** | |

| Pediatric Echocardiography report  Patient Name: Haymanot Shumet. Patient ID: 090102. Gender: Female. Age: 6years.  Clinical Finding: Incidental Murmur. TGSH4.3013. | | | | |
| --- | --- | --- | --- | --- |
| Features | **Finding** | **Features** | | **Finding** |
| Profile | | **Atria** | | |
| Abdominal situs | Solitus | Left atrium | | Normal |
| Cardiac position | Levocardia | Right atrium | | Dilated |
| Systemic venous drainage | to RA | **Atrioventricular valves** | | |
| Pulmonary venous drainage | to LA | Mitral valve | | Annulus = 14mm |
| Atrioventricular connection | Concordant | Tricuspid valve | | Annulus = 27mm  TAPSE = 24mm. |
| Ventriculoarterial connection | Concordant | **Ventricles** | | |
| Ventricular loop | d-Loop | Left ventricle | | Normal |
| Septae | | Right ventricle | | Dilated |
| Interventricular septum | Intact | **M-Mode:**. | | |
| Interatrial septum | 30mm X 34mm OS ASD, L – R Shunt | AO | |  |
| Great arteries | NRGA | LA | |  |
| Aorta | ------- | LVIDd | | mm |
| Pulmonary artery | MPA 26mm | LVIDs | | mm |
| Semilunal valves |  | IVSd | | mm |
| Aortic valve | Annulus = 11mm. | IVSs | | mm |
| Pulmonary valve | Annulus = 20mm. | LVPWd | | mm |
| Doppler Measurement | | LVPWs | | mm |
| Mitral | ------- | EDV | | ml |
| Aortic | ------ | ESV | | ml |
| Tricuspid | -------- | FS | | 38% |
| pulmonic | Mild PS, PPG = 25mmHg | LVEF | | 70% |
| Aortic arch | ------- | **Coronary arteries** | |  |
| PDA | -------- |  | |  |
| Pericardium/Pleura |  | | | |
| Final Diagnosis | 1. {S, D, S} Levocardia. 2. Large OS ASD, L – R Shunt 3. Normal Biventricular Function | | | |
| Done By: | **Signature** | | **Date of Reporting** | |
| Dr. Tesfaye Taye, Pediatrician, Pediatric Cardiologist | | | **24/10/2013Eth.C** | |

| Pediatric Echocardiography report  Patient Name: Haymanot Mihretie. Patient ID: 090186. Gender: Female. Age: 12years.  Clinical Finding: Easy Fatigability + Palpitation. TGSH4.3014. | | | | |
| --- | --- | --- | --- | --- |
| Features | **Finding** | **Features** | | **Finding** |
| Profile | | **Atria** | | |
| Abdominal situs | Solitus | Left atrium | | Normal |
| Cardiac position | Levocardia | Right atrium | | Normal |
| Systemic venous drainage | to RA | **Atrioventricular valves** | | |
| Pulmonary venous drainage | to LA | Mitral valve | | Annulus = 20mm |
| Atrioventricular connection | Concordant | Tricuspid valve | | Annulus = 23mm  TAPSE = 22mm. |
| Ventriculoarterial connection | Concordant | **Ventricles** | | |
| Ventricular loop | d-Loop | Left ventricle | | Normal |
| Septae | | Right ventricle | | Normal |
| Interventricular septum | Intact | **M-Mode:**. | | |
| Interatrial septum | Intact | AO | |  |
| Great arteries | NRGA | LA | |  |
| Aorta | ------- | LVIDd | | mm |
| Pulmonary artery | Normal MPA and Branch PAs | LVIDs | | mm |
| Semilunal valves |  | IVSd | | mm |
| Aortic valve | Annulus = 19mm. | IVSs | | mm |
| Pulmonary valve | Annulus = 21mm. | LVPWd | | mm |
| Doppler Measurement | | LVPWs | | mm |
| Mitral | ------- | EDV | | ml |
| Aortic | ------ | ESV | | ml |
| Tricuspid | -------- | FS | | 35% |
| pulmonic | -------- | LVEF | | 65% |
| Aortic arch | ------- | **Coronary arteries** | |  |
| PDA | -------- |  | |  |
| Pericardium/Pleura |  | | | |
| Final Diagnosis | 1. Normal Echocardiography Study. | | | |
| Done By: | **Signature** | | **Date of Reporting** | |
| Dr. Tesfaye Taye, Pediatrician, Pediatric Cardiologist | | | **24/10/2013Eth.C** | |

| Pediatric Echocardiography report  Patient Name: Baby of Abeba Bogale. Patient ID: 090494. Gender: Male. Age: 31days.  Clinical Finding: Cyanosis + RD. TGSH4.3015. | | | | |
| --- | --- | --- | --- | --- |
| Features | **Finding** | **Features** | | **Finding** |
| Profile | | **Atria** | | |
| Abdominal situs | Solitus | Left atrium | | Normal |
| Cardiac position | Levocardia | Right atrium | | Normal |
| Systemic venous drainage | to RA | **Atrioventricular valves** | | |
| Pulmonary venous drainage | to LA | Mitral valve | | Annulus = 14mm |
| Atrioventricular connection | Concordant | Tricuspid valve | | Atretic |
| Ventriculoarterial connection | DORV | **Ventricles** | | |
| Ventricular loop | d-Loop | Left ventricle | | Normal |
| Septae | | Right ventricle | | Normal |
| Interventricular septum | 5mm Inlet VSD, L – R Shunt | **M-Mode:**. | | |
| Interatrial septum | 10mm Primum defect, R – L Shunt | AO | |  |
| Great arteries | d-TGA | LA | |  |
| Aorta | Anterior and to the right. From RV | LVIDd | | mm |
| Pulmonary artery | Posterior and to the left. From RV | LVIDs | | mm |
| Semilunal valves |  | IVSd | | mm |
| Aortic valve | Annulus = 9mm. | IVSs | | mm |
| Pulmonary valve | Annulus = 10mm. | LVPWd | | mm |
| Doppler Measurement | | LVPWs | | mm |
| Mitral | ------- | EDV | | ml |
| Aortic | ------ | ESV | | ml |
| Tricuspid | Tricuspid atresia | FS | | % |
| pulmonic | Moderate PS, PPG = 46mmHg | LVEF | | % |
| Aortic arch | ------- | **Coronary arteries** | |  |
| PDA | -------- |  | |  |
| Pericardium/Pleura |  | | | |
| Final Diagnosis | 1. {S, D, D} Levocardia. 2. DORV 3. d-TGA 4. Primum defect, R – L Shunt 5. Inlet VSD, L – R Shunt 6. Moderate PS 7. Tricuspid atresia type IIB | | | |
| Done By: | **Signature** | | **Date of Reporting** | |
| Dr. Tesfaye Taye, Pediatrician, Pediatric Cardiologist | | | **29/10/2013Eth.C** | |

| Pediatric Echocardiography report  Patient Name: Tsehayitu Wudu. Patient ID: 090549. Gender: Female. Age: 52days.  Clinical Finding: Incidental Murmur. TGSH4.3016. | | | | |
| --- | --- | --- | --- | --- |
| Features | **Finding** | **Features** | | **Finding** |
| Profile | | **Atria** | | |
| Abdominal situs | Solitus | Left atrium | | Normal |
| Cardiac position | Levocardia | Right atrium | | Normal |
| Systemic venous drainage | to RA | **Atrioventricular valves** | | |
| Pulmonary venous drainage | to LA | Mitral valve | | Annulus = 12mm |
| Atrioventricular connection | Concordant | Tricuspid valve | | Annulus = 12mm |
| Ventriculoarterial connection | Concordant | **Ventricles** | | |
| Ventricular loop | d-Loop | Left ventricle | | Normal |
| Septae | | Right ventricle | | Normal |
| Interventricular septum | Intact | **M-Mode:** Normal LV Function on eye balling | | |
| Interatrial septum | Intact | AO | |  |
| Great arteries | NRGA | LA | |  |
| Aorta | ------- | LVIDd | | mm |
| Pulmonary artery | Normal MPA and Branch PAs | LVIDs | | mm |
| Semilunal valves |  | IVSd | | mm |
| Aortic valve | Annulus = 9mm. | IVSs | | mm |
| Pulmonary valve | Annulus = 11mm. | LVPWd | | mm |
| Doppler Measurement | | LVPWs | | mm |
| Mitral | ------- | EDV | | ml |
| Aortic | ------ | ESV | | ml |
| Tricuspid | -------- | FS | | % |
| pulmonic | -------- | LVEF | | % |
| Aortic arch | ------- | **Coronary arteries** | |  |
| PDA | 1mm PDA, L – R Shunt |  | |  |
| Pericardium/Pleura |  | | | |
| Final Diagnosis | 1. {S, D, S} Levocardia. 2. Small PDA, L – R Shunt 3. Normal LV Function | | | |
| Done By: | **Signature** | | **Date of Reporting** | |
| Dr. Tesfaye Taye, Pediatrician, Pediatric Cardiologist | | | **29/10/2013Eth.C** | |

| Pediatric Echocardiography report  Patient Name: Tirualem Yihunie. Patient ID: 087613. Gender: Female. Age: 13years.  Clinical Finding: DOE + Murmur. TGSH4.3017. | | | |
| --- | --- | --- | --- |
| Features | **Finding** | **Features** | **Finding** |
| Profile | | **Atria** | |
| Abdominal situs | Solitus | Left atrium | Normal |
| Cardiac position | Levocardia | Right atrium | Mildly dilated |
| Systemic venous drainage | to RA. IVC Dilated | **Atrioventricular valves** | |
| Pulmonary venous drainage | to LA | Mitral valve | Annulus = 25mm |
| Atrioventricular connection | Concordant | Tricuspid valve | Annulus = 31mm  TAPSE = 21mm. |
| Ventriculoarterial connection | Concordant | **Ventricles** | |
| Ventricular loop | d-Loop | Left ventricle | Normal |
| Septae | | Right ventricle | Mildly Dilated |
| Interventricular septum | Intact | **M-Mode:**. | |
| Interatrial septum | 20 mm Primum defect, L – R Shunt. 10mm additional Fenestrated OS ASD, L – R Shunt. | AO |  |
| Great arteries | NRGA | LA |  |
| Aorta | ------- | LVIDd | mm |
| Pulmonary artery | Normal MPA and Branch PAs | LVIDs | mm |
| Semilunal valves |  | IVSd | mm |
| Aortic valve | Annulus = 16mm. | IVSs | mm |
| Pulmonary valve | Annulus = 24mm. Doming Pulmonary Valve. | LVPWd | mm |
| Doppler Measurement | | LVPWs | mm |
| Mitral | Moderate MR | EDV | ml |
| Aortic | ------ | ESV | ml |
| Tricuspid | Mild TR | FS | 30% |
| pulmonic | Mild PS, PPG = 40mmHg | LVEF | 58% |
| Aortic arch | ------- | **Coronary arteries** |  |
| PDA | -------- |  |  |
| Pericardium/Pleura | 4mm Pericardial effusion on RA/RV Junction | | |
| Final Diagnosis | 1. {S, D, S} Levocardia. 2. Partial AVSD, L – R Shunt 3. Additional Moderate Fenestrated OS ASD, L – R Shunt 4. Moderate MR 5. Mild TR 6. Mild PS 7. Doming Pulmonary Valve 8. Trace Pericardial effusion 9. Normal LV Function | | |
| Done By: | **Signature** | **Date of Reporting** | |
| Dr. Tesfaye Taye, Pediatrician, Pediatric Cardiologist | | **29/10/2013Eth.C** | |

| Pediatric Echocardiography report  Patient Name: Yesewmar Alehegn. Patient ID: 090727. Gender: Female. Age: 7/12.  INCOMPLETE DOCUMENTATION | | | | |
| --- | --- | --- | --- | --- |
| Features | **Finding** | **Features** | | **Finding** |
| Profile | | **Atria** | | |
| Abdominal situs | Solitus | Left atrium | | Dilated |
| Cardiac position | Levocardia | Right atrium | | Normal |
| Systemic venous drainage | to RA | **Atrioventricular valves** | | |
| Pulmonary venous drainage | to LA | Mitral valve | | Annulus = 15mm |
| Atrioventricular connection | Concordant | Tricuspid valve | | Annulus = 14mm. |
| Ventriculoarterial connection | Concordant | **Ventricles** | | |
| Ventricular loop | d-Loop | Left ventricle | | Dilated |
| Septae | | Right ventricle | | Normal |
| Interventricular septum | Intact | **M-Mode:**. | | |
| Interatrial septum | Intact | AO | |  |
| Great arteries | NRGA | LA | |  |
| Aorta | ------- | LVIDd | | mm |
| Pulmonary artery | Normal MPA and Branch PAs | LVIDs | | mm |
| Semilunal valves |  | IVSd | | mm |
| Aortic valve | Annulus = 10mm. | IVSs | | mm |
| Pulmonary valve | Annulus = 15mm. | LVPWd | | mm |
| Doppler Measurement | | LVPWs | | mm |
| Mitral | Moderate MR, Jet velocity = 5.9m/sec, Holosystolic | EDV | | ml |
| Aortic | ------ | ESV | | ml |
| Tricuspid | Trivial TR, PPG = 51mmHg | FS | | 40% |
| pulmonic | Moderate PR, PPG = 55mmHg | LVEF | | 73% |
| Aortic arch | ------- | **Coronary arteries** | |  |
| PDA | -------- |  | |  |
| Pericardium/Pleura | 6mm Pericardial effusion | | | |
| Final Diagnosis | 1. {S, D, S} Levocardia. 2. LA/LV Dilated 3. Moderate MR 4. Moderate PR 5. Moderate Pulmonary Hypertension 6. Small Pericardial effusion 7. Normal LV Function | | | |
| Done By: | **Signature** | | **Date of Reporting** | |
| Dr. Tesfaye Taye, Pediatrician, Pediatric Cardiologist | | | **01/11/2013Eth.C** | |

| Pediatric Echocardiography report  Patient Name: Baby of Birtukan Yihun. Patient ID: 090871. Gender: Female. Age: 8days.  Clinical Diagnosis: RD + PPHTN. TGSH1.2426. | | | | |
| --- | --- | --- | --- | --- |
| Features | **Finding** | **Features** | | **Finding** |
| Profile | | **Atria** | | |
| Abdominal situs | Solitus | Left atrium | | Normal |
| Cardiac position | Levocardia | Right atrium | | Dilated |
| Systemic venous drainage | to RA | **Atrioventricular valves** | | |
| Pulmonary venous drainage | to LA | Mitral valve | | Annulus = 8mm |
| Atrioventricular connection | Concordant | Tricuspid valve | | Annulus = 13mm  TAPSE = 9mm. |
| Ventriculoarterial connection | Concordant | **Ventricles** | | |
| Ventricular loop | d-Loop | Left ventricle | | Normal |
| Septae | | Right ventricle | | Dilated |
| Interventricular septum | Intact | **M-Mode:** Normal LV Function on eye balling | | |
| Interatrial septum | Intact | AO | |  |
| Great arteries | NRGA | LA | |  |
| Aorta | ------- | LVIDd | | mm |
| Pulmonary artery | Normal MPA and Branch PAs | LVIDs | | mm |
| Semilunal valves |  | IVSd | | mm |
| Aortic valve | Annulus = 9mm. | IVSs | | mm |
| Pulmonary valve | Annulus = 10mm. | LVPWd | | mm |
| Doppler Measurement | | LVPWs | | mm |
| Mitral | ------- | EDV | | ml |
| Aortic | ------ | ESV | | ml |
| Tricuspid | Moderate TR, PPG = 72mmHg | FS | | % |
| pulmonic | -------- | LVEF | | % |
| Aortic arch | ------- | **Coronary arteries** | |  |
| PDA | 1mm PDA, R – L Shunt |  | |  |
| Pericardium/Pleura |  | | | |
| Final Diagnosis | 1. {S, D, S} Levocardia. 2. RA/RV Dilated 3. Moderate TR 4. Severe Pulmonary Hypertension 5. Boarder line RV Function 6. Normal LV Function | | | |
| Done By: | **Signature** | | **Date of Reporting** | |
| Dr. Tesfaye Taye, Pediatrician, Pediatric Cardiologist | | | **01/11/2013Eth.C** | |

| Pediatric Echocardiography report  Patient Name: Baby of Wieneshet Shiferaw. Patient ID: 091354. Gender: Male. Age: 8days.  Date: 06/11/13Eth.C. Weight: _______________Kg. Height: _______________cm. BSA: _____________.  Clinical Finding: RD + Incidental Murmur. TGSH4.3018. | | | | |
| --- | --- | --- | --- | --- |
| Features | **Finding** | **Features** | | **Finding** |
| Profile | | **Atria** | | |
| Abdominal situs | Solitus | Left atrium | | Normal |
| Cardiac position | Levocardia | Right atrium | | Normal |
| Systemic venous drainage | to RA | **Atrioventricular valves** | | |
| Pulmonary venous drainage | to LA | Mitral valve | | Annulus = 13mm |
| Atrioventricular connection | Concordant | Tricuspid valve | | Annulus = 12mm |
| Ventriculoarterial connection | Concordant | **Ventricles** | | |
| Ventricular loop | d-Loop | Left ventricle | | Normal |
| Septae | | Right ventricle | | Normal |
| Interventricular septum | Intact | **M-Mode:**. | | |
| Interatrial septum | 5mm OS ASD, L – R Shunt | AO | |  |
| Great arteries | NRGA | LA | |  |
| Aorta | ------- | LVIDd | | mm |
| Pulmonary artery | Normal MPA and Branch PAs | LVIDs | | mm |
| Semilunal valves |  | IVSd | | mm |
| Aortic valve | Annulus = 8mm. | IVSs | | mm |
| Pulmonary valve | Annulus = 10mm. | LVPWd | | mm |
| Doppler Measurement | | LVPWs | | mm |
| Mitral | ------- | EDV | | ml |
| Aortic | ------ | ESV | | ml |
| Tricuspid | Mild to Moderate TR, PPG = 31mmHg | FS | | 29% |
| pulmonic | -------- | LVEF | | 58% |
| Aortic arch | ------- | **Coronary arteries** | |  |
| PDA | <1mm PDA, L – R Shunt |  | |  |
| Pericardium/Pleura |  | | | |
| Final Diagnosis | 1. {S, D, S} Levocardia. 2. Small OS ASD, L – R Shunt 3. Mild to moderate TR 4. Small PDA, L – R Shunt 5. Normal LV Function | | | |
| Done By: | **Signature** | | **Date of Reporting** | |
| Dr. Tesfaye Taye, Pediatrician, Pediatric Cardiologist | | | **06/11/2013Eth.C** | |

| Transthoracic Pediatric Echocardiography report  Patient Name: Baby of Mister. Patient ID: 090892. Gender: Male. Age: 13days. Date: 06/11/13Eth.C.  INCOMPLETE DOCUMENTATION | | | | |
| --- | --- | --- | --- | --- |
|  |  |  | |  |
| Limited Echocardiography Window  Small OS ASD, L – R Shunt  Moderate Inlet VSD, L – R Shunt  ? Mild PS  Normal LV Function (eye balling) | |  | | |
| Remark | Repeat Echocardiography after 3 months  The windows were limited because of deformed chest and the probe was not appropriate for age. | | | |
| Done By: | **Signature** | | **Date of Reporting** | |
| Dr. Tesfaye Taye, Pediatrician, Pediatric Cardiologist | | | **06/11/2013Eth.C** | |

| Pediatric Echocardiography report  Patient Name: Samuel Alemu. Patient ID: 091464. Gender: Male. Age: 23days. Date: 06/11/13Eth.C.  Clinical Finding: Incidental Murmur. TGSH4.3019. | | | | |
| --- | --- | --- | --- | --- |
| Features | **Finding** | **Features** | | **Finding** |
| Profile | | **Atria** | | |
| Abdominal situs | Solitus | Left atrium | | Normal |
| Cardiac position | Levocardia | Right atrium | | Normal |
| Systemic venous drainage | to RA | **Atrioventricular valves** | | |
| Pulmonary venous drainage | to LA | Mitral valve | | Annulus = 10mm |
| Atrioventricular connection | Concordant | Tricuspid valve | | Annulus = 11mm  TAPSE = 13mm. |
| Ventriculoarterial connection | Concordant | **Ventricles** | | |
| Ventricular loop | d-Loop | Left ventricle | | Normal |
| Septae | | Right ventricle | | Normal |
| Interventricular septum | Intact | **M-Mode:** Normal LV Function on eye balling | | |
| Interatrial septum | PFO, L – R Shunt | AO | |  |
| Great arteries | NRGA | LA | |  |
| Aorta | ------- | LVIDd | | mm |
| Pulmonary artery | Normal MPA and Branch PAs | LVIDs | | mm |
| Semilunal valves |  | IVSd | | mm |
| Aortic valve | Annulus = 9mm. | IVSs | | mm |
| Pulmonary valve | Annulus = 9mm. | LVPWd | | mm |
| Doppler Measurement | | LVPWs | | mm |
| Mitral | ------- | EDV | | ml |
| Aortic | ------ | ESV | | ml |
| Tricuspid | -------- | FS | | % |
| pulmonic | -------- | LVEF | | % |
| Aortic arch | ------- | **Coronary arteries** | |  |
| PDA | 2mm PDA, L – R Shunt, SPG/DPG = 48/36mmHg | | | |
| Pericardium/Pleura |  | | | |
| Final Diagnosis | 1. {S, D, S} Levocardia. 2. PFO, L – R Shunt 3. Moderate PDA, L – R Shunt 4. Normal Biventricular Function | | | |
| Done By: | **Signature** | | **Date of Reporting** | |
| Dr. Tesfaye Taye, Pediatrician, Pediatric Cardiologist | | | **06/11/2013Eth.C** | |

| Pediatric Echocardiography report  Patient Name: Nobel Alemu. Patient ID: 010909. Gender: Male. Age: 5 3/12. Date: 08/11/13Eth.C.  AGH | | | | |
| --- | --- | --- | --- | --- |
| Features | **Finding** | **Features** | | **Finding** |
| Profile | | **Atria** | | |
| Abdominal situs | Solitus | Left atrium | | Normal |
| Cardiac position | Levocardia | Right atrium | | Normal |
| Systemic venous drainage | to RA | **Atrioventricular valves** | | |
| Pulmonary venous drainage | to LA | Mitral valve | | Annulus = 18mm |
| Atrioventricular connection | Concordant | Tricuspid valve | | Annulus = 18mm |
| Ventriculoarterial connection | Concordant | **Ventricles** | | |
| Ventricular loop | d-Loop | Left ventricle | | Normal |
| Septae | | Right ventricle | | Normal |
| Interventricular septum | 4mm PM VSD, L – R Shunt with gradient of 78mmHg | **M-Mode:**. | | |
| Interatrial septum | Intact | AO | |  |
| Great arteries | NRGA | LA | |  |
| Aorta | ------- | LVIDd | | mm |
| Pulmonary artery | Normal MPA and Branch PAs | LVIDs | | mm |
| Semilunal valves |  | IVSd | | mm |
| Aortic valve | Annulus = 15mm. | IVSs | | mm |
| Pulmonary valve | Annulus = 17mm. | LVPWd | | mm |
| Doppler Measurement | | LVPWs | | mm |
| Mitral | ------- | EDV | | ml |
| Aortic | ------ | ESV | | ml |
| Tricuspid | -------- | FS | | 30% |
| pulmonic | -------- | LVEF | | 58% |
| Aortic arch | ------- | **Coronary arteries** | |  |
| PDA | -------- |  | |  |
| Pericardium/Pleura |  | | | |
| Final Diagnosis | 1. {S, D, S} Levocardia. 2. Small Restrictive PM VSD, L – R Shunt 3. Normal LV Function | | | |
| Done By: | **Signature** | | **Date of Reporting** | |
| Dr. Tesfaye Taye, Pediatrician, Pediatric Cardiologist | | | **08/11/2013Eth.C** | |

| Pediatric Echocardiography report  Patient Name: Yitayish Aragaw. Patient ID: 091770. Gender: Female. Age: 1 7/12. Date: 08/11/13Eth.C.  Clinical Finding: _Diaphoresis + RD + Murmur + CHF. TGSH4.3020. | | | | |
| --- | --- | --- | --- | --- |
| Features | **Finding** | **Features** | | **Finding** |
| Profile | | **Atria** | | |
| Abdominal situs | Solitus | Left atrium | | Dilated |
| Cardiac position | Levocardia | Right atrium | | Dilated |
| Systemic venous drainage | to RA | **Atrioventricular valves** | | |
| Pulmonary venous drainage | to LA | Mitral valve | | Annulus = 13mm |
| Atrioventricular connection | Concordant | Tricuspid valve | | Annulus = 16mm |
| Ventriculoarterial connection | Concordant | **Ventricles** | | |
| Ventricular loop | d-Loop | Left ventricle | | Dilated |
| Septae | | Right ventricle | | Dilated |
| Interventricular septum | 7mm PM VSD, L – R Shunt | **M-Mode:**. | | |
| Interatrial septum | Intact | AO | |  |
| Great arteries | NRGA | LA | |  |
| Aorta | ------- | LVIDd | | mm |
| Pulmonary artery | **MPA =18mm** | LVIDs | | mm |
| Semilunal valves |  | IVSd | | mm |
| Aortic valve | Annulus = 10mm. | IVSs | | mm |
| Pulmonary valve | Annulus = 17mm. | LVPWd | | mm |
| Doppler Measurement | | LVPWs | | mm |
| Mitral | ------- | EDV | | ml |
| Aortic | ------ | ESV | | ml |
| Tricuspid | Mild TR | FS | | 35% |
| pulmonic | -------- | LVEF | | 67% |
| Aortic arch | ------- | **Coronary arteries** | |  |
| PDA | -------- |  | |  |
| Pericardium/Pleura | 6mm Pericardial effusion on RA side | | | |
| Final Diagnosis | 1. {S, D, S} Levocardia. 2. Large PM VSD, L – R Shunt 3. Pulmonary Hypertension 4. Small Pericardial effusion 5. Normal LV Function | | | |
| Done By: | **Signature** | | **Date of Reporting** | |
| Dr. Tesfaye Taye, Pediatrician, Pediatric Cardiologist | | | **08/11/2013Eth.C** | |

| Pediatric Echocardiography report  Patient Name: Mitiku Melesse. Patient ID: 081642. Gender: Male. Age: 2 8/12. Date: 08/11/13Eth.C.  Clinical Finding: _RD + CHF + RD + Murmur + Easy Fatigability + Pul.HTN. TGSH4.3021. | | | | |
| --- | --- | --- | --- | --- |
| Features | **Finding** | **Features** | | **Finding** |
| Profile | | **Atria** | | |
| Abdominal situs | Solitus | Left atrium | | Normal |
| Cardiac position | Levocardia | Right atrium | | Dilated |
| Systemic venous drainage | to RA | **Atrioventricular valves** | | |
| Pulmonary venous drainage | to LA | Mitral valve | | Annulus = 14mm |
| Atrioventricular connection | Concordant | Tricuspid valve | | Annulus = 22mm  TAPSE = 12mm. |
| Ventriculoarterial connection | Concordant | **Ventricles** | | |
| Ventricular loop | d-Loop | Left ventricle | | Normal |
| Septae | | Right ventricle | | Dilated, Hypertrophied,  RV TDI S wave = 9.5cm/sec |
| Interventricular septum | Intact | **M-Mode:**. | | |
| Interatrial septum | Intact | AO | |  |
| Great arteries | NRGA | LA | |  |
| Aorta | ------- | LVIDd | | mm |
| Pulmonary artery | Dilated MPA and Branch PAs | LVIDs | | mm |
| Semilunal valves |  | IVSd | | mm |
| Aortic valve | Annulus = 13mm. | IVSs | | mm |
| Pulmonary valve | Annulus = 17mm. | LVPWd | | mm |
| Doppler Measurement | | LVPWs | | mm |
| Mitral | ------- | EDV | | ml |
| Aortic | ------ | ESV | | ml |
| Tricuspid | Moderate TR, PPG = 69mmHg | FS | | 34% |
| pulmonic | Mild PR, PPG = 61mmHg | LVEF | | 65% |
| Aortic arch | ------- | **Coronary arteries** | |  |
| PDA | -------- |  | |  |
| Pericardium/Pleura |  | | | |
| Final Diagnosis | 1. {S, D, S} Levocardia. 2. RA/RV Dilated 3. Moderate TR 4. Mild PR 5. RV Dilated, Hypertrophied and Dysfunctional 6. Severe Pulmonary Hypertension 7. Normal LV Function | | | |
| Done By: | **Signature** | | **Date of Reporting** | |
| Dr. Tesfaye Taye, Pediatrician, Pediatric Cardiologist | | | **08/11/2013Eth.C** | |

| Pediatric Echocardiography report  Patient Name: Simegn Zemene. Patient ID: 092598. Gender: Female. Age: 2 3/12. Date: 16/11/13Eth.C.  Clinical Finding: DS  . TGSH4.3022. | | | | |
| --- | --- | --- | --- | --- |
| Features | **Finding** | **Features** | | **Finding** |
| Profile | | **Atria** | | |
| Abdominal situs | Solitus | Left atrium | | Normal |
| Cardiac position | Levocardia | Right atrium | | Normal |
| Systemic venous drainage | to RA | **Atrioventricular valves** | | |
| Pulmonary venous drainage | to LA | Mitral valve | | Annulus = 14mm |
| Atrioventricular connection | Concordant | Tricuspid valve | | Annulus = 14mm  TAPSE = 15mm. |
| Ventriculoarterial connection | Concordant | **Ventricles** | | |
| Ventricular loop | d-Loop | Left ventricle | | Normal |
| Septae | | Right ventricle | | Normal |
| Interventricular septum | Intact | **M-Mode:**. | | |
| Interatrial septum | Intact | AO | |  |
| Great arteries | NRGA | LA | |  |
| Aorta | ------- | LVIDd | | mm |
| Pulmonary artery | Normal MPA and Branch PAs | LVIDs | | mm |
| Semilunal valves |  | IVSd | | mm |
| Aortic valve | Annulus = 12mm. | IVSs | | mm |
| Pulmonary valve | Annulus = 15mm. | LVPWd | | mm |
| Doppler Measurement | | LVPWs | | mm |
| Mitral | ------- | EDV | | ml |
| Aortic | ------ | ESV | | ml |
| Tricuspid | -------- | FS | | 35% |
| pulmonic | -------- | LVEF | | 66% |
| Aortic arch | ------- | **Coronary arteries** | |  |
| PDA | -------- |  | |  |
| Pericardium/Pleura | 5mm Circumferential pericardial effusion | | | |
| Final Diagnosis | 1. {S, D, S} Levocardia. 2. Small Pericardial effusion | | | |
| Done By: | **Signature** | | **Date of Reporting** | |
| Dr. Tesfaye Taye, Pediatrician, Pediatric Cardiologist | | | **16/11/2013Eth.C** | |

| Pediatric Echocardiography report  Patient Name: Addisu Melesse. Patient ID: 092550. Gender: Male. Age: 11/12. Date: 19/11/13Eth.C.  Clinical Finding: CHF + RD + Murmur + cyanosis + Diaphoresis. TGSH4.3023. | | | | |
| --- | --- | --- | --- | --- |
| Features | **Finding** | **Features** | | **Finding** |
| Profile | | **Atria** | | |
| Abdominal situs | Solitus | Left atrium | | Dilated |
| Cardiac position | Levocardia | Right atrium | | Dilated |
| Systemic venous drainage | to RA | **Atrioventricular valves** | | |
| Pulmonary venous drainage | to LA | Mitral valve | | Annulus = 16mm |
| Atrioventricular connection | Concordant | Tricuspid valve | | Annulus = 15mm  TAPSE = 15mm. |
| Ventriculoarterial connection | DORV | **Ventricles** | | |
| Ventricular loop | d-Loop | Left ventricle | | Dilated |
| Septae | | Right ventricle | | Dilated |
| Interventricular septum | 17mm Sub arterial VSD, BD Shunt | **M-Mode:** Normal LV Function on eye balling. | | |
| Interatrial septum | Intact | AO | |  |
| Great arteries | NRGA | LA | |  |
| Aorta | Posterior and to the right. From RV. | LVIDd | | mm |
| Pulmonary artery | Anterior and to the left. From RV. **MPA = 19mm** | LVIDs | | mm |
| Semilunal valves |  | IVSd | | mm |
| Aortic valve | Annulus = 11mm. | IVSs | | mm |
| Pulmonary valve | Annulus = 15mm. | LVPWd | | mm |
| Doppler Measurement | | LVPWs | | mm |
| Mitral | Aorto-Mitral valve discontinuity | EDV | | ml |
| Aortic | ------ | ESV | | ml |
| Tricuspid | Mild TR | FS | | % |
| pulmonic | Moderate PR, PPG = 51mmHg.  Mild PS, PPG = 31mmHg. | LVEF | | % |
| Aortic arch | ------- | **Coronary arteries** | |  |
| PDA | -------- |  | |  |
| Final Diagnosis | 1. {S, D, S} Levocardia. 2. All chambers dilated 3. DORV 4. Sub – arterial Large VSD, BD Shunt 5. Moderate PR 6. Mild PS 7. Moderate Pulmonary Hypertension 8. Normal Biventricular Function | | | |
| Done By: | **Signature** | | **Date of Reporting** | |
| Dr. Tesfaye Taye, Pediatrician, Pediatric Cardiologist | | | **19/11/2013Eth.C** | |

| Pediatric Echocardiography report  Patient Name: Lijalem Aderaw. Patient ID: 093049. Gender: Male. Age: 1 11/12. Date: 20/11/13Eth.C.  Clinical Finding: Incidental Murmur. TGSH4.3024. | | | | |
| --- | --- | --- | --- | --- |
| Features | **Finding** | **Features** | | **Finding** |
| Profile | | **Atria** | | |
| Abdominal situs | Solitus | Left atrium | | Normal |
| Cardiac position | Levocardia | Right atrium | | Dilated |
| Systemic venous drainage | to RA | **Atrioventricular valves** | | |
| Pulmonary venous drainage | to LA | Mitral valve | | Annulus = 10mm |
| Atrioventricular connection | Concordant | Tricuspid valve | | Annulus = 16mm |
| Ventriculoarterial connection | Concordant | **Ventricles** | | |
| Ventricular loop | d-Loop | Left ventricle | | Normal |
| Septae | | Right ventricle | | Dilated |
| Interventricular septum | Intact | **M-Mode:** Normal LV Function on eye balling | | |
| Interatrial septum | 4mm OS ASD, L – R Shunt | AO | |  |
| Great arteries | NRGA | LA | |  |
| Aorta | ------- | LVIDd | | mm |
| Pulmonary artery | Smallish MPA and Branch PAs | LVIDs | | mm |
| Semilunal valves |  | IVSd | | mm |
| Aortic valve | Annulus = 11mm. | IVSs | | mm |
| Pulmonary valve | Annulus = 6mm. | LVPWd | | mm |
| Doppler Measurement | | LVPWs | | mm |
| Mitral | ------- | EDV | | ml |
| Aortic | ------ | ESV | | ml |
| Tricuspid | -------- | FS | | % |
| pulmonic | Moderate PS, PPG = 56mmHg | LVEF | | % |
| Aortic arch | ------- | **Coronary arteries** | |  |
| PDA | -------- |  | |  |
| Pericardium/Pleura |  | | | |
| Final Diagnosis | 1. {S, D, S} Levocardia. 2. Small OS ASD, L – R Shunt 3. Moderate PS 4. Smallish MPA and Branch PAs | | | |
| Done By: | **Signature** | | **Date of Reporting** | |
| Dr. Tesfaye Taye, Pediatrician, Pediatric Cardiologist | | | **20/11/2013Eth.C** | |

| Pediatric Echocardiography report  Patient Name: Hanna Getaneh. Patient ID: ___________. Gender: Female. Age: 9years. Date: 20/11/13Eth.C.  Clinical Finding: CHF + RD + Murmur + DOE + Easy Fatigability + Palpitation + Recurrence. TGSH4.3025. | | | | |
| --- | --- | --- | --- | --- |
| Features | **Finding** | **Features** | | **Finding** |
| Profile | | **Atria** | | |
| Abdominal situs | Solitus | Left atrium | | Dilated. 56mm X 46mm |
| Cardiac position | Levocardia | Right atrium | | Dilated |
| Systemic venous drainage | to RA. IVC Dilated. | **Atrioventricular valves** | | |
| Pulmonary venous drainage | to LA | Mitral valve | | Annulus = 24mm. Thickened, clubbed MVL |
| Atrioventricular connection | Concordant | Tricuspid valve | | Annulus = 20mm |
| Ventriculoarterial connection | Concordant | **Ventricles** | | |
| Ventricular loop | d-Loop | Left ventricle | | Dilated |
| Septae | | Right ventricle | | Dilated |
| Interventricular septum | Intact | **M-Mode:**. | | |
| Interatrial septum | Intact | AO | |  |
| Great arteries | NRGA | LA | |  |
| Aorta | ------- | LVIDd | | mm |
| Pulmonary artery | MPA = 21mm | LVIDs | | mm |
| Semilunal valves |  | IVSd | | mm |
| Aortic valve | Annulus = 14mm. | IVSs | | mm |
| Pulmonary valve | Annulus = 21mm. | LVPWd | | mm |
| Doppler Measurement | | LVPWs | | mm |
| Mitral | Severe MR, Holosystolic, posterior projection, seen in two planes with jet velocity = 4m/sec | EDV | | ml |
| Aortic | Mild AR, PHT = 527ms | ESV | | ml |
| Tricuspid | Moderate TR, PPG = 65mmHg | FS | | 30% |
| pulmonic | Moderate PR, PPG = 53mmHg | LVEF | | 57% |
| Aortic arch | ------- | **Coronary arteries** | |  |
| Pericardium/Pleura | Circumferential Pericardial effusion with maximum depth of 13mm on RA Side | | | |
| Final Diagnosis | 1. {S, D, S} Levocardia. 2. LA/LV Dilated 3. Thickened, clubbed MVL 4. Severe MR 5. Moderate TR 6. Mild AR 7. Moderate PR 8. Severe Pulmonary Hypertension 9. Normal LV Function 10. Moderate Pericardial effusion | | | |
| Done By: | **Signature** | | **Date of Reporting** | |
| Dr. Tesfaye Taye, Pediatrician, Pediatric Cardiologist | | | **20/11/2013Eth.C** | |

| Pediatric Echocardiography report  Patient Name: _Baby of Birtukan Yihun. Patient ID: 090871. Gender: Female. Age: 27days. Date: 20/11/13Eth.C.  Clinical Diagnosis: _______. TGSH1.2426. | | | | |
| --- | --- | --- | --- | --- |
| Follow up Echocardiography for Pulmonary Hypertension | | | | |
| Features | **Finding** | **Features** | | **Finding** |
| Profile | | **Atria** | | |
| Abdominal situs | Solitus | Left atrium | | Normal |
| Cardiac position | Levocardia | Right atrium | | Dilated |
| Systemic venous drainage | to RA | **Atrioventricular valves** | | |
| Pulmonary venous drainage | to LA | Mitral valve | | Annulus = 13mm |
| Atrioventricular connection | Concordant | Tricuspid valve | | Annulus = 16mm  TAPSE = 12mm. |
| Ventriculoarterial connection | Concordant | **Ventricles** | | |
| Ventricular loop | d-Loop | Left ventricle | | Normal |
| Septae | | Right ventricle | | Dilated |
| Interventricular septum | Intact | **M-Mode:** Normal LV Function on eye balling | | |
| Interatrial septum | PFO, L – R Shunt | AO | |  |
| Great arteries | NRGA | LA | |  |
| Aorta | ------- | LVIDd | | mm |
| Pulmonary artery | Normal MPA and Branch PAs | LVIDs | | mm |
| Semilunal valves |  | IVSd | | mm |
| Aortic valve | Annulus = 9mm. | IVSs | | mm |
| Pulmonary valve | Annulus = 9mm. | LVPWd | | mm |
| Doppler Measurement | | LVPWs | | mm |
| Mitral | ------- | EDV | | ml |
| Aortic | ------ | ESV | | ml |
| Tricuspid | Mild TR, PPG = 48mmHg | FS | | % |
| pulmonic | -------- | LVEF | | % |
| Aortic arch | ------- | **Coronary arteries** | |  |
| PDA | -------- |  | |  |
| Pericardium/Pleura |  | | | |
| Final Diagnosis | 1. {S, D, S} Levocardia. 2. RA/RV Dilated 3. PFO, L – R Shunt 4. Mild TR 5. ? Moderate Pulmonary Hypertension 6. Normal Function | | | |
| Remark | There is improvement from the previous Echo study | | | |
| Done By: | **Signature** | | **Date of Reporting** | |
| Dr. Tesfaye Taye, Pediatrician, Pediatric Cardiologist | | | **20/11/2013Eth.C** | |

| Pediatric Echocardiography report  Patient Name: Yohannes Dagnew. Patient ID: 058661. Gender: Male. Age: _5 10/12. Date: 20/11/13Eth.C.  Clinical Finding: Recurrent Chest Infection + FTT. TGSH4.3026. | | | | |
| --- | --- | --- | --- | --- |
| Features | **Finding** | **Features** | | **Finding** |
| Profile | | **Atria** | | |
| Abdominal situs | Solitus | Left atrium | | Normal |
| Cardiac position | Levocardia | Right atrium | | Normal |
| Systemic venous drainage | to RA | **Atrioventricular valves** | | |
| Pulmonary venous drainage | to LA | Mitral valve | | Annulus = 17mm |
| Atrioventricular connection | Concordant | Tricuspid valve | | Annulus = 19mm |
| Ventriculoarterial connection | Concordant | **Ventricles** | | |
| Ventricular loop | d-Loop | Left ventricle | | Normal |
| Septae | | Right ventricle | | Normal |
| Interventricular septum | Intact | **M-Mode:** Normal LV Function on eye balling | | |
| Interatrial septum | Intact | AO | |  |
| Great arteries | NRGA | LA | |  |
| Aorta | ------- | LVIDd | | mm |
| Pulmonary artery | Normal MPA and Branch PAs | LVIDs | | mm |
| Semilunal valves |  | IVSd | | mm |
| Aortic valve | Annulus = 13mm. | IVSs | | mm |
| Pulmonary valve | Annulus = 13mm. | LVPWd | | mm |
| Doppler Measurement | | LVPWs | | mm |
| Mitral | ------- | EDV | | ml |
| Aortic | ------ | ESV | | ml |
| Tricuspid | -------- | FS | | % |
| pulmonic | -------- | LVEF | | % |
| Aortic arch | ------- | **Coronary arteries** | |  |
| PDA | -------- |  | |  |
| Pericardium/Pleura |  | | | |
| Final Diagnosis | 1. Normal Echocardiography Study. | | | |
| Done By: | **Signature** | | **Date of Reporting** | |
| Dr. Tesfaye Taye, Pediatrician, Pediatric Cardiologist | | | **20/11/2013Eth.C** | |

| Pediatric Echocardiography report  Patient Name: Tibeltalech Anley. Patient ID: 092609. Gender: Female. Age: 13years. Date: 20/11/13Eth.C.  Clinical Finding: Rheumatic Recurrence + Murmur + DOE. TGSH4.3027. | | | | |
| --- | --- | --- | --- | --- |
| Features | **Finding** | **Features** | | **Finding** |
| Profile | | **Atria** | | |
| Abdominal situs | Solitus | Left atrium | | Dilated |
| Cardiac position | Levocardia | Right atrium | | Normal |
| Systemic venous drainage | to RA | **Atrioventricular valves** | | |
| Pulmonary venous drainage | to LA | Mitral valve | | Annulus = 24mm. Mildly Thickened MVL |
| Atrioventricular connection | Concordant | Tricuspid valve | | Annulus = 25mm. TAPSE = 21mm. |
| Ventriculoarterial connection | Concordant | **Ventricles** | | |
| Ventricular loop | d-Loop | Left ventricle | | Dilated |
| Septae | | Right ventricle | | Normal |
| Interventricular septum | Intact | **M-Mode:**. | | |
| Interatrial septum | Intact | AO | |  |
| Great arteries | NRGA | LA | |  |
| Aorta | ------- | LVIDd | | mm |
| Pulmonary artery | Normal MPA and Branch PAs | LVIDs | | mm |
| Semilunal valves |  | IVSd | | mm |
| Aortic valve | Annulus = 19mm. | IVSs | | mm |
| Pulmonary valve | Annulus = 21mm. | LVPWd | | mm |
| Doppler Measurement | | LVPWs | | mm |
| Mitral | Severe MR, Holosystolic, posterior projection, seen in two planes with jet velocity = 4.8m/sec | EDV | | ml |
| Aortic | Mild AR, PHT = 510ms | ESV | | ml |
| Tricuspid | Trivial TR, PPG = 30mmHg | FS | | 42% |
| pulmonic | -------- | LVEF | | 74% |
| Aortic arch | ------- | **Coronary arteries** | |  |
| PDA | -------- |  | |  |
| Pericardium/Pleura |  | | | |
| Final Diagnosis | 1. {S, D, S} Levocardia. 2. Mildly thickened MVL 3. Severe MR 4. Mild AR 5. Normal Biventricular Function | | | |
| Done By: | **Signature** | | **Date of Reporting** | |
| Dr. Tesfaye Taye, Pediatrician, Pediatric Cardiologist | | | **20/11/2013Eth.C** | |

| Pediatric Echocardiography report  Patient Name: Bosena Gashaw. Patient ID: 093053. Gender: Female. Age: 14years. Date: 20/11/13Eth.C.  Clinical Finding: Rheumatic Recurrence + Murmur + Palpitation + Easy Fatigability + DOE. TGSH4.3028. | | | | |
| --- | --- | --- | --- | --- |
| Features | **Finding** | **Features** | | **Finding** |
| Profile | | **Atria** | | |
| Abdominal situs | Solitus | Left atrium | | Dilated |
| Cardiac position | Levocardia | Right atrium | | Normal |
| Systemic venous drainage | to RA | **Atrioventricular valves** | | |
| Pulmonary venous drainage | to LA | Mitral valve | | Annulus = 28mm. Thickened MVL |
| Atrioventricular connection | Concordant | Tricuspid valve | | Annulus = 24mm  TAPSE = 20mm. |
| Ventriculoarterial connection | Concordant | **Ventricles** | | |
| Ventricular loop | d-Loop | Left ventricle | | Dilated |
| Septae | | Right ventricle | | Normal |
| Interventricular septum | Intact | **M-Mode:**. | | |
| Interatrial septum | Intact | AO | |  |
| Great arteries | NRGA | LA | |  |
| Aorta | ------- | LVIDd | | mm |
| Pulmonary artery | Normal MPA and Branch PAs | LVIDs | | mm |
| Semilunal valves |  | IVSd | | mm |
| Aortic valve | Annulus = 21mm. | IVSs | | mm |
| Pulmonary valve | Annulus = 24mm. | LVPWd | | mm |
| Doppler Measurement | | LVPWs | | mm |
| Mitral | Severe MR, Holosystolic, posterior projection, seen in two planes with jet velocity = 4.4m/sec | EDV | | ml |
| Aortic | Moderate AR, PHT = 343ms | ESV | | ml |
| Tricuspid | -------- | FS | | 33% |
| pulmonic | -------- | LVEF | | 60% |
| Aortic arch | ------- | **Coronary arteries** | |  |
| Pericardium/Pleura | Circumferential Pericardial effusion with maximum depth of 6mm On RA/RV Side | | | |
| Final Diagnosis | 1. {S, D, S} Levocardia. 2. LA/LV Dilated 3. Severe MR 4. Moderate AR 5. Normal Biventricular Function 6. Small Pericardial effusion | | | |
| Done By: | **Signature** | | **Date of Reporting** | |
| Dr. Tesfaye Taye, Pediatrician, Pediatric Cardiologist | | | **20/11/2013Eth.C** | |

| Pediatric Echocardiography report  Patient Name: Addisu Ashebir. Patient ID: 092322. Gender: Male. Age: 8/12. Date: 20/11/13Eth.C.  Clinical Finding: DS + Murmur + RD + Diaphoresis. TGSH4.3029. | | | | |
| --- | --- | --- | --- | --- |
| Features | **Finding** | **Features** | | **Finding** |
| Profile | | **Atria** | | |
| Abdominal situs | Solitus | Left atrium | | Dilated |
| Cardiac position | Levocardia | Right atrium | | Normal |
| Systemic venous drainage | to RA | **Atrioventricular valves** | | |
| Pulmonary venous drainage | to LA | Mitral valve | | Annulus = 16mm |
| Atrioventricular connection | Concordant | Tricuspid valve | | Annulus = 13mm |
| Ventriculoarterial connection | Concordant | **Ventricles** | | |
| Ventricular loop | d-Loop | Left ventricle | | Dilated |
| Septae | | Right ventricle | | Normal |
| Interventricular septum | 5mm Inlet VSD , L – R Shunt | **M-Mode:**. | | |
| Interatrial septum | 16mm Primum defect, L – R Shunt. Additional 5mm OS ASD, L – R Shunt | AO | |  |
| Great arteries | NRGA | LA | |  |
| Aorta | ------- | LVIDd | | mm |
| Pulmonary artery | Normal MPA and Branch PAs | LVIDs | | mm |
| Semilunal valves |  | IVSd | | mm |
| Aortic valve | Annulus = 13mm. | IVSs | | mm |
| Pulmonary valve | Annulus = 13mm. | LVPWd | | mm |
| Doppler Measurement | | LVPWs | | mm |
| Mitral | Moderate MR | EDV | | ml |
| Aortic | ------ | ESV | | ml |
| Tricuspid | Mild TR | FS | | % |
| pulmonic | -------- | LVEF | | % |
| Aortic arch | ------- | **Coronary arteries** | |  |
| PDA | 1mm PDA, L – R Shunt |  | |  |
| Pericardium/Pleura |  | | | |
| Final Diagnosis | 1. {S, D, S} Levocardia. 2. LA/LV Dilated 3. Transitional AVSD, L – R Shunt 4. Additional Small OS ASD, L – R Shunt 5. Small PDA, L – R Shunt 6. Moderate MR 7. Mild TR | | | |
| Done By: | **Signature** | | **Date of Reporting** | |
| Dr. Tesfaye Taye, Pediatrician, Pediatric Cardiologist | | | **20/11/2013Eth.C** | |

| Pediatric Echocardiography report  Patient Name: Baby of Etaferaw Melak. Patient ID: 093611. Gender: Male. Age: 14days. Date: 27/11/13Eth.C.  Clinical Finding: Incidental Murmur. TGSH4.3030. | | | | |
| --- | --- | --- | --- | --- |
| Features | **Finding** | **Features** | | **Finding** |
| Profile | | **Atria** | | |
| Abdominal situs | Solitus | Left atrium | | Normal |
| Cardiac position | Levocardia | Right atrium | | Normal |
| Systemic venous drainage | to RA | **Atrioventricular valves** | | |
| Pulmonary venous drainage | to LA | Mitral valve | | Annulus = 11mm |
| Atrioventricular connection | Concordant | Tricuspid valve | | Annulus = 11mm  TAPSE = 10mm. |
| Ventriculoarterial connection | Concordant | **Ventricles** | | |
| Ventricular loop | d-Loop | Left ventricle | | Normal |
| Septae | | Right ventricle | | Normal |
| Interventricular septum | 2mm PM VSD, L – R Shunt | **M-Mode:** Normal LV Function on eye balling | | |
| Interatrial septum | 6mm OS ASD, L – R Shunt | AO | |  |
| Great arteries | NRGA | LA | |  |
| Aorta | ------- | LVIDd | | mm |
| Pulmonary artery | Normal MPA and Branch PAs | LVIDs | | mm |
| Semilunal valves |  | IVSd | | mm |
| Aortic valve | Annulus = 9mm. | IVSs | | mm |
| Pulmonary valve | Annulus = 9mm. | LVPWd | | mm |
| Doppler Measurement | | LVPWs | | mm |
| Mitral | ------- | EDV | | ml |
| Aortic | ------ | ESV | | ml |
| Tricuspid | -------- | FS | | % |
| pulmonic | -------- | LVEF | | % |
| Aortic arch | ------- | **Coronary arteries** | |  |
| PDA | -------- |  | |  |
| Pericardium/Pleura |  | | | |
| Final Diagnosis | 1. {S, D, S} Levocardia. 2. Small OS ASD, L – R Shunt 3. Small PM VSD, L – R Shunt 4. Normal Biventricular Function | | | |
| Done By: | **Signature** | | **Date of Reporting** | |
| Dr. Tesfaye Taye, Pediatrician, Pediatric Cardiologist | | | **27/11/2013Eth.C** | |

| Pediatric Echocardiography report  Patient Name: Abi Bamlaku. Patient ID: 093925. Gender: Male. Age: 10years. Date: 27/11/13Eth.C.  Clinical Finding: RD + DOE + Murmur + CHF + Rheumatic Recurrence + Easy Fatigability + Palpitation. TGSH4.3031. | | | | |
| --- | --- | --- | --- | --- |
| Features | **Finding** | **Features** | | **Finding** |
| Profile | | **Atria** | | |
| Abdominal situs | Solitus | Left atrium | | Dilated |
| Cardiac position | Levocardia | Right atrium | | Dilated |
| Systemic venous drainage | to RA. IVC Dilated. SEC+ | **Atrioventricular valves** | | |
| Pulmonary venous drainage | to LA | Mitral valve | | Annulus = 22mm. thickened, clubbed MVL. Shortened PMVL. |
| Atrioventricular connection | Concordant | Tricuspid valve | | Annulus = 23mm  TAPSE = 20mm. |
| Ventriculoarterial connection | Concordant | **Ventricles** | | |
| Ventricular loop | d-Loop | Left ventricle | | Dilated |
| Septae | | Right ventricle | | Dilated. RV TDI S wave = 12cm/sec |
| Interventricular septum | Intact | **M-Mode:**. | | |
| Interatrial septum | Intact | AO | |  |
| Great arteries | NRGA | LA | |  |
| Aorta | ------- | LVIDd | | mm |
| Pulmonary artery | Dilated MPA and Branch PAs | LVIDs | | mm |
| Semilunal valves |  | IVSd | | mm |
| Aortic valve | Annulus = mm. | IVSs | | mm |
| Pulmonary valve | Annulus = 27mm. | LVPWd | | mm |
| Doppler Measurement | | LVPWs | | mm |
| Mitral | Severe MR, Posterior projection, seen in two planes | EDV | | ml |
| Aortic | Mild AR, PHT = 545ms | ESV | | ml |
| Tricuspid | Severe TR, PPG = 64mmHg | FS | | 24% |
| pulmonic | Moderate PR, PPG = 48mmHg | LVEF | | 49% |
| Pericardium/Pleura | 33mm left pleural effusion. 14mm Right Pleural effusion. Circumferential pericardial effusion with max. depth on RA side measuring = 21mm. | | | |
| Final Diagnosis | 1. {S, D, S} Levocardia. 2. All chambers dilated 3. Thickened, clubbed MVL. Thickened PMVL 4. Severe MR 5. Severe TR 6. Mild AR 7. Moderate PR 8. Severe Pulmonary Hypertension 9. Reduced LV Function 10. Large Pleural effusion 11. Large Circumferential Pericardial effusion | | | |
| Done By: | **Signature** | | **Date of Reporting** | |
| Dr. Tesfaye Taye, Pediatrician, Pediatric Cardiologist | | | **27/11/2013Eth.C** | |

| Pediatric Echocardiography report  Patient Name: Habtamnesh Mekete. Patient ID: 093830. Gender: Female. Age: 8/12. Date: 27/11/13Eth.C.  Clinical Finding: Diaphoresis + Murmur. TGSH4.3032. | | | | |
| --- | --- | --- | --- | --- |
| Features | **Finding** | **Features** | | **Finding** |
| Profile | | **Atria** | | |
| Abdominal situs | Solitus | Left atrium | | Dilated |
| Cardiac position | Levocardia | Right atrium | | Normal |
| Systemic venous drainage | to RA | **Atrioventricular valves** | | |
| Pulmonary venous drainage | to LA | Mitral valve | | Annulus = 13mm |
| Atrioventricular connection | Concordant | Tricuspid valve | | Annulus = 15mm |
| Ventriculoarterial connection | Concordant | **Ventricles** | | |
| Ventricular loop | d-Loop | Left ventricle | | Dilated |
| Septae | | Right ventricle | | Normal |
| Interventricular septum | 7mm PM VSD, L – R Shunt with a gradient of 31mmHg | **M-Mode:**. | | |
| Interatrial septum | Intact | AO | |  |
| Great arteries | NRGA | LA | |  |
| Aorta | ------- | LVIDd | | mm |
| Pulmonary artery | Normal MPA and Branch PAs | LVIDs | | mm |
| Semilunal valves |  | IVSd | | mm |
| Aortic valve | Annulus = 11mm. | IVSs | | mm |
| Pulmonary valve | Annulus = 14mm. | LVPWd | | mm |
| Doppler Measurement | | LVPWs | | mm |
| Mitral | ------- | EDV | | ml |
| Aortic | ------ | ESV | | ml |
| Tricuspid | -------- | FS | | 40% |
| pulmonic | -------- | LVEF | | 72% |
| Aortic arch | ------- | **Coronary arteries** | |  |
| PDA | -------- |  | |  |
| Pericardium/Pleura |  | | | |
| Final Diagnosis | 1. {S, D, S} Levocardia. 2. LA/LV Dilated 3. Moderate PM VSD, L – R Shunt 4. Normal LV Function | | | |
| Done By: | **Signature** | | **Date of Reporting** | |
| Dr. Tesfaye Taye, Pediatrician, Pediatric Cardiologist | | | **27/11/2013Eth.C** | |

| Pediatric Echocardiography report  Patient Name: Baby of Enana Alem. Patient ID: 048840. Gender: Female. Age: 8/12. Date: 27/11/13Eth.C.  Clinical Finding: Incidental Murmur. TGSH4.3033. | | | | |
| --- | --- | --- | --- | --- |
| Features | **Finding** | **Features** | | **Finding** |
| Profile | | **Atria** | | |
| Abdominal situs | Solitus | Left atrium | | Normal |
| Cardiac position | Levocardia | Right atrium | | Normal |
| Systemic venous drainage | to RA | **Atrioventricular valves** | | |
| Pulmonary venous drainage | to LA | Mitral valve | | Annulus = 14mm |
| Atrioventricular connection | Concordant | Tricuspid valve | | Annulus = 16mm |
| Ventriculoarterial connection | Concordant | **Ventricles** | | |
| Ventricular loop | d-Loop | Left ventricle | | Normal |
| Septae | | Right ventricle | | Normal |
| Interventricular septum | <2mm Mid-Muscular VSD, L – R Shunt | **M-Mode:** Normal LV Function on eye balling | | |
| Interatrial septum | Intact | AO | |  |
| Great arteries | NRGA | LA | |  |
| Aorta | ------- | LVIDd | | mm |
| Pulmonary artery | Normal MPA and Branch PAs | LVIDs | | mm |
| Semilunal valves |  | IVSd | | mm |
| Aortic valve | Annulus = 12mm. | IVSs | | mm |
| Pulmonary valve | Annulus = 12mm. | LVPWd | | mm |
| Doppler Measurement | | LVPWs | | mm |
| Mitral | ------- | EDV | | ml |
| Aortic | ------ | ESV | | ml |
| Tricuspid | -------- | FS | | % |
| pulmonic | -------- | LVEF | | % |
| Aortic arch | ------- | **Coronary arteries** | |  |
| PDA | -------- |  | |  |
| Pericardium/Pleura |  | | | |
| Final Diagnosis | 1. {S, D, S} Levocardia. 2. Tiny Mid-Muscular VSD, L – R Shunt | | | |
| Recommendation | Echocardiographic Follow up yearly Until it closes | | | |
| Done By: | **Signature** | | **Date of Reporting** | |
| Dr. Tesfaye Taye, Pediatrician, Pediatric Cardiologist | | | **27/11/2013Eth.C** | |

| Pediatric Echocardiography report  Patient Name: Dagnanesh Dawit. Patient ID: 093548. Gender: Female. Age: 2 1/12. Date: 27/11/13Eth.C.  Clinical Finding: Incidental Murmur. TGSH4.3034. | | | | |
| --- | --- | --- | --- | --- |
| Features | **Finding** | **Features** | | **Finding** |
| Profile | | **Atria** | | |
| Abdominal situs | Solitus | Left atrium | | Normal |
| Cardiac position | Levocardia | Right atrium | | Normal |
| Systemic venous drainage | to RA | **Atrioventricular valves** | | |
| Pulmonary venous drainage | to LA | Mitral valve | | Annulus = 19mm |
| Atrioventricular connection | Concordant | Tricuspid valve | | Annulus = 19mm |
| Ventriculoarterial connection | Concordant | **Ventricles** | | |
| Ventricular loop | d-Loop | Left ventricle | | Normal |
| Septae | | Right ventricle | | Normal |
| Interventricular septum | Intact | **M-Mode:**. | | |
| Interatrial septum | Intact | AO | |  |
| Great arteries | NRGA | LA | |  |
| Aorta | ------- | LVIDd | | mm |
| Pulmonary artery | Normal MPA and Branch PAs | LVIDs | | mm |
| Semilunal valves |  | IVSd | | mm |
| Aortic valve | Annulus = 13mm. | IVSs | | mm |
| Pulmonary valve | Annulus = 15mm. | LVPWd | | mm |
| Doppler Measurement | | LVPWs | | mm |
| Mitral | Trivial MR, Incomplete signal | EDV | | ml |
| Aortic | ------ | ESV | | ml |
| Tricuspid | -------- | FS | | 30% |
| pulmonic | -------- | LVEF | | 60% |
| Aortic arch | ------- | **Coronary arteries** | |  |
| PDA | -------- |  | |  |
| Pericardium/Pleura |  | | | |
| Final Diagnosis | 1. Normal Echocardiography Study | | | |
| Done By: | **Signature** | | **Date of Reporting** | |
| Dr. Tesfaye Taye, Pediatrician, Pediatric Cardiologist | | | **27/11/2013Eth.C** | |

| Pediatric Echocardiography report  Patient Name: Se’alite Mihret. Patient ID: 087134. Gender: Female. Age: 83days. Date: 29/11/13Eth.C.  Clinical Finding: RD. TGSH4.3035. | | | | |
| --- | --- | --- | --- | --- |
| Features | **Finding** | **Features** | | **Finding** |
| Profile | | **Atria** | | |
| Abdominal situs | Solitus | Left atrium | | Normal |
| Cardiac position | Levocardia | Right atrium | | Normal |
| Systemic venous drainage | to RA | **Atrioventricular valves** | | |
| Pulmonary venous drainage | to LA | Mitral valve | | Annulus = 10mm |
| Atrioventricular connection | Concordant | Tricuspid valve | | Annulus = 10mm |
| Ventriculoarterial connection | Concordant | **Ventricles** | | |
| Ventricular loop | d-Loop | Left ventricle | | Normal |
| Septae | | Right ventricle | | Normal |
| Interventricular septum | Intact | **M-Mode:**. | | |
| Interatrial septum | Intact | AO | |  |
| Great arteries | NRGA | LA | |  |
| Aorta | ------- | LVIDd | | mm |
| Pulmonary artery | Normal MPA and Branch PAs | LVIDs | | mm |
| Semilunal valves |  | IVSd | | mm |
| Aortic valve | Annulus = 9mm. | IVSs | | mm |
| Pulmonary valve | Annulus = 9mm. | LVPWd | | mm |
| Doppler Measurement | | LVPWs | | mm |
| Mitral | ------- | EDV | | ml |
| Aortic | ------ | ESV | | ml |
| Tricuspid | -------- | FS | | % |
| pulmonic | -------- | LVEF | | % |
| Aortic arch | ------- | **Coronary arteries** | |  |
| PDA | -------- |  | |  |
| Pericardium/Pleura |  | | | |
| Final Diagnosis | 1. Normal Echocardiography Study. | | | |
| Done By: | **Signature** | | **Date of Reporting** | |
| Dr. Tesfaye Taye, Pediatrician, Pediatric Cardiologist | | | **29/11/2013Eth.C** | |

| Pediatric Echocardiography report  Patient Name: Yesewmar Alehegn. Patient ID: 090727. Gender: Female. Age: 8months. Date: 29/11/13Eth.C.  Clinical Finding: RD + Cardiogenic Shock + Tachycardia. TGSH4.3036. | | | | |
| --- | --- | --- | --- | --- |
| Features | **Finding** | **Features** | | **Finding** |
| Profile | | **Atria** | | |
| Abdominal situs | Solitus | Left atrium | | Dilated |
| Cardiac position | Levocardia | Right atrium | | Dilated |
| Systemic venous drainage | to RA | **Atrioventricular valves** | | |
| Pulmonary venous drainage | to LA | Mitral valve | | Annulus = 15mm. Thickened MVL |
| Atrioventricular connection | Concordant | Tricuspid valve | | Annulus = 14mm  TAPSE = 14mm. |
| Ventriculoarterial connection | Concordant | **Ventricles** | | |
| Ventricular loop | d-Loop | Left ventricle | | Dilated |
| Septae | | Right ventricle | | Dilated |
| Interventricular septum | Intact | **M-Mode:** Reduced LV Function on eye balling | | |
| Interatrial septum | Intact | AO | |  |
| Great arteries | NRGA | LA | |  |
| Aorta | ------- | LVIDd | | mm |
| Pulmonary artery | MPA = 16mm, RPA = 8mm and LPA = 7mm. | LVIDs | | mm |
| Semilunal valves |  | IVSd | | mm |
| Aortic valve | Annulus = 13mm. | IVSs | | mm |
| Pulmonary valve | Annulus = 14mm. | LVPWd | | mm |
| Doppler Measurement | | LVPWs | | mm |
| Mitral | Severe MR, Holosystolic, posterior projection, seen in two planes with jet velocity = 4.3m/sec | EDV | | ml |
| Aortic | ------ | ESV | | ml |
| Tricuspid | -------- | FS | | % |
| pulmonic | Moderate PR, PPG = 62mmHg | LVEF | | % |
| Aortic arch | ------- | **Coronary arteries** | |  |
| Pericardium/Pleura | Circumferential Pericardial effusion with maximum depth in the RA/RV Side measuring 12mm with echo contrast in it. | | | |
| Final Diagnosis | 1. {S, D, S} Levocardia. 2. LA/LV Dilated 3. Severe MR 4. Moderate PR 5. Severe Pulmonary Hypertension 6. Moderate pericardial effusion (Echo contrast seen) | | | |
| Done By: | **Signature** | | **Date of Reporting** | |
| Dr. Tesfaye Taye, Pediatrician, Pediatric Cardiologist | | | **29/11/2013Eth.C** | |

| Pediatric Echocardiography report  Patient Name: Henock Wendale. Patient ID: 066301. Gender: Male. Age: 8/12. Date: 04/12/13Eth.C.  Clinical Finding: Diaphoresis + RD + Murmur + DS. TGSH4.3037. | | | | |
| --- | --- | --- | --- | --- |
| Features | **Finding** | **Features** | | **Finding** |
| Profile | | **Atria** | | |
| Abdominal situs | Solitus | Left atrium | | Dilated |
| Cardiac position | Levocardia | Right atrium | | Dilated |
| Systemic venous drainage | to RA | **Atrioventricular valves** | | |
| Pulmonary venous drainage | to LA | Mitral valve | | Common Complete AVSD |
| Atrioventricular connection | Concordant | Tricuspid valve | |  |
| Ventriculoarterial connection | Concordant | **Ventricles** | | |
| Ventricular loop | d-Loop | Left ventricle | | Dilated |
| Septae | | Right ventricle | | Dilated |
| Interventricular septum | Common Complete AVSD, L – R Shunt.  Additional 8mm OS ASD, L – R Shunt | **M-Mode:**. | | |
| Interatrial septum |  | AO | |  |
| Great arteries | NRGA | LA | |  |
| Aorta | ------- | LVIDd | | mm |
| Pulmonary artery | MPA =14mm. | LVIDs | | mm |
| Semilunal valves |  | IVSd | | mm |
| Aortic valve | Annulus = 12mm. | IVSs | | mm |
| Pulmonary valve | Annulus = 14mm. | LVPWd | | mm |
| Doppler Measurement | | LVPWs | | mm |
| Mitral | Moderate Left AVVR | EDV | | ml |
| Aortic | ------ | ESV | | ml |
| Tricuspid | Mild Right AVVR | FS | | 71% |
| pulmonic | -------- | LVEF | | 38% |
| Aortic arch | ------- | **Coronary arteries** | |  |
| PDA | -------- |  | |  |
| Pericardium/Pleura | 7mm Pericardial effusion on RA/RV Side | | | |
| Final Diagnosis | 1. {S, D, S} Levocardia. 2. Common Complete AVSD, L – R Shunt 3. Additional Moderate OS ASD, L – R Shunt 4. Normal LV Function | | | |
| Done By: | **Signature** | | **Date of Reporting** | |
| Dr. Tesfaye Taye, Pediatrician, Pediatric Cardiologist | | | **04/12/2013Eth.C** | |

| Pediatric Echocardiography report  Patient Name: Abrham Tenaw. Patient ID: 094226. Gender: Male. Age: 1 2/12. Date: 04/12/13Eth.C.  Clinical Finding: Recurrent Chest Infection. TGSH4.3038. | | | | |
| --- | --- | --- | --- | --- |
| Features | **Finding** | **Features** | | **Finding** |
| Profile | | **Atria** | | |
| Abdominal situs | Solitus | Left atrium | | Normal |
| Cardiac position | Levocardia | Right atrium | | Normal |
| Systemic venous drainage | to RA | **Atrioventricular valves** | | |
| Pulmonary venous drainage | to LA | Mitral valve | | Annulus = 14mm |
| Atrioventricular connection | Concordant | Tricuspid valve | | Annulus = 16mm  TAPSE = 15mm. |
| Ventriculoarterial connection | Concordant | **Ventricles** | | |
| Ventricular loop | d-Loop | Left ventricle | | Normal |
| Septae | | Right ventricle | | Normal |
| Interventricular septum | Intact | **M-Mode:**. | | |
| Interatrial septum | Intact | AO | |  |
| Great arteries | NRGA | LA | |  |
| Aorta | ------- | LVIDd | | mm |
| Pulmonary artery | Normal MPA and Branch PAs | LVIDs | | mm |
| Semilunal valves |  | IVSd | | mm |
| Aortic valve | Annulus = 12mm. | IVSs | | mm |
| Pulmonary valve | Annulus = 15mm. | LVPWd | | mm |
| Doppler Measurement | | LVPWs | | mm |
| Mitral | ------- | EDV | | ml |
| Aortic | ------ | ESV | | ml |
| Tricuspid | -------- | FS | | 36% |
| pulmonic | -------- | LVEF | | 67% |
| Aortic arch | ------- | **Coronary arteries** | |  |
| PDA | -------- |  | |  |
| Pericardium/Pleura |  | | | |
| Final Diagnosis | 1. Normal Echocardiography Study. | | | |
| Done By: | **Signature** | | **Date of Reporting** | |
| Dr. Tesfaye Taye, Pediatrician, Pediatric Cardiologist | | | **04/12/2013Eth.C** | |

| Pediatric Echocardiography report  Patient Name: Abrham Esubalew. Patient ID: 094417. Gender: Male. Age: 8/12. Date: 04/12/13Eth.C.  Clinical Finding: Incidental Murmur. TGSH4.3039. | | | | |
| --- | --- | --- | --- | --- |
| Features | **Finding** | **Features** | | **Finding** |
| Profile | | **Atria** | | |
| Abdominal situs | Solitus | Left atrium | | Normal |
| Cardiac position | Levocardia | Right atrium | | Normal |
| Systemic venous drainage | to RA | **Atrioventricular valves** | | |
| Pulmonary venous drainage | to LA | Mitral valve | | Annulus = 13mm |
| Atrioventricular connection | Concordant | Tricuspid valve | | Annulus = 13mm |
| Ventriculoarterial connection | Concordant | **Ventricles** | | |
| Ventricular loop | d-Loop | Left ventricle | | Normal |
| Septae | | Right ventricle | | Normal |
| Interventricular septum | Intact | **M-Mode:**. | | |
| Interatrial septum | PFO, L – R Shunt | AO | |  |
| Great arteries | NRGA | LA | |  |
| Aorta | ------- | LVIDd | | mm |
| Pulmonary artery | Normal MPA and Branch PAs | LVIDs | | mm |
| Semilunal valves |  | IVSd | | mm |
| Aortic valve | Annulus = 10mm. | IVSs | | mm |
| Pulmonary valve | Annulus = 10mm. | LVPWd | | mm |
| Doppler Measurement | | LVPWs | | mm |
| Mitral | ------- | EDV | | ml |
| Aortic | ------ | ESV | | ml |
| Tricuspid | -------- | FS | | 37% |
| pulmonic | -------- | LVEF | | 69% |
| Aortic arch | ------- | **Coronary arteries** | |  |
| PDA | 1mm PDA, L – R Shunt | | | |
| Pericardium/Pleura |  | | | |
| Final Diagnosis | 1. {S, D, S} Levocardia. 2. PFO, L – R Shunt 3. Small PDA, L – R Shunt 4. Normal LV Function | | | |
| Done By: | **Signature** | | **Date of Reporting** | |
| Dr. Tesfaye Taye, Pediatrician, Pediatric Cardiologist | | | **04/12/2013Eth.C** | |

| Pediatric Echocardiography report  Patient Name: Chilotu Dessie. Patient ID: 094957. Gender: Male. Age: 3 8/12. Date: 04/12/13Eth.C.  Clinical Finding: RD + Sepsis + CHF. TGSH4.3040. | | | | |
| --- | --- | --- | --- | --- |
| Features | **Finding** | **Features** | | **Finding** |
| Profile | | **Atria** | | |
| Abdominal situs | Solitus | Left atrium | | Normal |
| Cardiac position | Levocardia | Right atrium | | Normal |
| Systemic venous drainage | to RA | **Atrioventricular valves** | | |
| Pulmonary venous drainage | to LA | Mitral valve | | Annulus = 16mm |
| Atrioventricular connection | Concordant | Tricuspid valve | | Annulus = 16mm  TAPSE = 12mm. |
| Ventriculoarterial connection | Concordant | **Ventricles** | | |
| Ventricular loop | d-Loop | Left ventricle | | Normal |
| Septae | | Right ventricle | | Normal.  RV TDI S wave = 10cm/sec |
| Interventricular septum | Intact | **M-Mode:**. | | |
| Interatrial septum | Intact | AO | |  |
| Great arteries | NRGA | LA | |  |
| Aorta | ------- | LVIDd | | mm |
| Pulmonary artery | Normal MPA and Branch PAs | LVIDs | | mm |
| Semilunal valves |  | IVSd | | mm |
| Aortic valve | Annulus = 12mm. | IVSs | | mm |
| Pulmonary valve | Annulus = 13mm. | LVPWd | | mm |
| Doppler Measurement | | LVPWs | | mm |
| Mitral | ------- | EDV | | ml |
| Aortic | ------ | ESV | | ml |
| Tricuspid | -------- | FS | | 26 - 29% |
| pulmonic | -------- | LVEF | | 52 - 56% |
| Aortic arch | ------- | **Coronary arteries** | |  |
| PDA | -------- |  | |  |
| Pericardium/Pleura | Circumferential Pericardial effusion with pericardial thickening with max. depth of 7mm on RA/RV Side | | | |
| Final Diagnosis | 1. {S, D, S} Levocardia. 2. Small Circumferential Pericardial effusion with thickening 3. Mildly reduced LV Function | | | |
| Remark | Myopericarditis. | | | |
| Done By: | **Signature** | | **Date of Reporting** | |
| Dr. Tesfaye Taye, Pediatrician, Pediatric Cardiologist | | | **04/12/2013Eth.C** | |

| Pediatric Echocardiography report  Patient Name: Godada Atalo. Patient ID: 094973. Gender: Female. Age: 11years. Date: 04/12/13Eth.C.  Clinical Finding: Rheumatic Recurrence + Murmur + CHF + Palpitation + DOE + Easy Fatigability. TGSH4.3041. | | | | |
| --- | --- | --- | --- | --- |
| Features | **Finding** | **Features** | | **Finding** |
| Profile | | **Atria** | | |
| Abdominal situs | Solitus | Left atrium | | Hugely Dilated. 71 X 63mm |
| Cardiac position | Levocardia | Right atrium | | Normal |
| Systemic venous drainage | to RA | **Atrioventricular valves** | | |
| Pulmonary venous drainage | to LA | Mitral valve | | Annulus = 23mm. thickened, clubbed AMVL. Thickened, shortened PMVL |
| Atrioventricular connection | Concordant | Tricuspid valve | | Annulus = 18mm. Thickened TVL. |
| Ventriculoarterial connection | Concordant | **Ventricles** | | |
| Ventricular loop | d-Loop | Left ventricle | | Dilated |
| Septae | | Right ventricle | | Normal |
| Interventricular septum | Intact | **M-Mode:**. | | |
| Interatrial septum | Intact | AO | |  |
| Great arteries | NRGA | LA | |  |
| Aorta | ------- | LVIDd | | mm |
| Pulmonary artery | Normal MPA and Branch PAs | LVIDs | | mm |
| Semilunal valves |  | IVSd | | mm |
| Aortic valve | Annulus = 17mm. thickened AVL. Trileaflet | IVSs | | mm |
| Pulmonary valve | Annulus = 18mm. | LVPWd | | mm |
| Doppler Measurement | | LVPWs | | mm |
| Mitral | Severe MR, Holosystolic, posterior projection, seen in two planes with Jet velocity = 4.5m/sec. | EDV | | ml |
| Aortic | Moderate AR, PHT = 347ms | ESV | | ml |
| Tricuspid | Mild TR, PPG = 27mmHg | FS | | 28% |
| pulmonic | -------- | LVEF | | 54% |
| Aortic arch | ------- | **Coronary arteries** | |  |
| PDA | -------- |  | |  |
| Pericardium/Pleura | Circumferential Pericardial effusion with maximum depth of 6mm on RA Side | | | |
| Final Diagnosis | 1. {S, D, S} Levocardia. 2. Thickened MVL, TVL, AVL 3. LA/LV Dilated 4. Mild TR 5. Severe MR 6. Moderate AR 7. Small Circumferential Pericardial effusion 8. Mildly reduced LV Function | | | |
| Done By: | **Signature** | | **Date of Reporting** | |
| Dr. Tesfaye Taye, Pediatrician, Pediatric Cardiologist | | | **04/12/2013Eth.C** | |

| Pediatric Echocardiography report  Patient Name: Yibeltal Muche. Patient ID: 094362. Gender: Male. Age: 13years. Date: 04/12/13Eth.C.  Clinical Finding: Easy Fatigability. TGSH4.3042. | | | | |
| --- | --- | --- | --- | --- |
| Features | **Finding** | **Features** | | **Finding** |
| Profile | | **Atria** | | |
| Abdominal situs | Solitus | Left atrium | | Normal |
| Cardiac position | Levocardia | Right atrium | | Normal |
| Systemic venous drainage | to RA | **Atrioventricular valves** | | |
| Pulmonary venous drainage | to LA | Mitral valve | | Annulus = 18mm |
| Atrioventricular connection | Concordant | Tricuspid valve | | Annulus = 18mm |
| Ventriculoarterial connection | Concordant | **Ventricles** | | |
| Ventricular loop | d-Loop | Left ventricle | | Normal |
| Septae | | Right ventricle | | Normal |
| Interventricular septum | Intact | **M-Mode:**. | | |
| Interatrial septum | Intact | AO | |  |
| Great arteries | NRGA | LA | |  |
| Aorta | ------- | LVIDd | | mm |
| Pulmonary artery | Normal MPA and Branch PAs | LVIDs | | mm |
| Semilunal valves |  | IVSd | | mm |
| Aortic valve | Annulus = 17mm. | IVSs | | mm |
| Pulmonary valve | Annulus = 24mm. | LVPWd | | mm |
| Doppler Measurement | | LVPWs | | mm |
| Mitral | ------- | EDV | | ml |
| Aortic | ------ | ESV | | ml |
| Tricuspid | -------- | FS | | 32% |
| pulmonic | -------- | LVEF | | 60% |
| Aortic arch | ------- | **Coronary arteries** | |  |
| PDA | -------- |  | |  |
| Pericardium/Pleura |  | | | |
| Final Diagnosis | 1. Normal Echocardiography Study. | | | |
| Done By: | **Signature** | | **Date of Reporting** | |
| Dr. Tesfaye Taye, Pediatrician, Pediatric Cardiologist | | | **04/12/2013Eth.C** | |

| Pediatric Echocardiography report  Patient Name: _____________. Patient ID: ___________. Gender: Female. Age: _________years. Date: 04/12/13Eth.C. | | | | |
| --- | --- | --- | --- | --- |
| Features | **Finding** | **Features** | | **Finding** |
| Profile | | **Atria** | | |
| Abdominal situs | Solitus | Left atrium | | Normal |
| Cardiac position | Levocardia | Right atrium | | Normal |
| Systemic venous drainage | to RA | **Atrioventricular valves** | | |
| Pulmonary venous drainage | to LA | Mitral valve | | Annulus = mm |
| Atrioventricular connection | Concordant | Tricuspid valve | | Annulus = mm  TAPSE = mm. |
| Ventriculoarterial connection | Concordant | **Ventricles** | | |
| Ventricular loop | d-Loop | Left ventricle | | Normal |
| Septae | | Right ventricle | | Normal |
| Interventricular septum | Intact | **M-Mode:**. | | |
| Interatrial septum | Intact | AO | |  |
| Great arteries | NRGA | LA | |  |
| Aorta | ------- | LVIDd | | mm |
| Pulmonary artery | Normal MPA and Branch PAs | LVIDs | | mm |
| Semilunal valves |  | IVSd | | mm |
| Aortic valve | Annulus = mm. | IVSs | | mm |
| Pulmonary valve | Annulus = mm. | LVPWd | | mm |
| Doppler Measurement | | LVPWs | | mm |
| Mitral | ------- | EDV | | ml |
| Aortic | ------ | ESV | | ml |
| Tricuspid | -------- | FS | | % |
| pulmonic | -------- | LVEF | | % |
| Aortic arch | ------- | **Coronary arteries** | |  |
| PDA | -------- |  | |  |
| Pericardium/Pleura |  | | | |
| Final Diagnosis | 1. {S, D, S} Levocardia. | | | |
| Done By: | **Signature** | | **Date of Reporting** | |
| Dr. Tesfaye Taye, Pediatrician, Pediatric Cardiologist | | | **04/12/2013Eth.C** | |
