# Supplementary material for: Two–Dimensional and Doppler trans-thoracic echocardiographic patterns of suspected pediatric heart diseases at Tibebe-—Ghion specialized Teaching Hospital and Adinas General Hospital, Bahir Dar, North-west Ethiopia:–An experience from an LMIC
Source: PLoS One. 2024 Mar 11;19(3):e0292694. doi: 10.1371/journal.pone.0292694 (PMC10927071; doi:10.1371/journal.pone.0292694)
Supplement: S1 File — (ZIP) [file pone.0292694.s002.zip › TGSH7 Pediatric Echocardiography report.docx]

| Pediatric Echocardiography report  Patient Name: Abereham Bireku Patient ID: 030781  Gender: M Age:14 Date of Report: 05/05/2011EC  Clinical Finding: Rheumatic Recurrence + Murmu. TGSH7.3043. | | | |
| --- | --- | --- | --- |
| Features | **Finding** | **Features** | **Finding** |
| Profile | | **Atria** | |
| Abdominal situs | Solitus | Left atrium | Enlarged |
| Cardiac position | Levocardia | Right atrium | Normal |
| Systemic venous drainage | to RA | **Atrioventricular valves** | |
| Pulmonary venous drainage | to LA | Mitral valve | N |
| Atrioventricular connection | Concordant | Tricuspid valve | N |
| Ventriculoarterial connection | Concordant | **Ventricles** | |
| Ventricular loop | d Loop | Left ventricle | Enlarged |
| Septae | | Right ventricle | Normal, TAPSE = 17mm |
| Interventricular septum | Intact, deviated to RV | **Semilunal valves** | |
| Interatrial septum | Intact | Aortic valve | N |
| Great arteries | NRGA | Pulmonary valve | N |
| Aorta |  | **M-Mode** |  |
| Pulmonary artery | Confluent | **AO** | mm |
| Coronary arteries | Normal | **LA** | mm |
| Doppler Measurement | | **LVIDd** | mm |
| Mitral | Moderate MR | **LVIDs** | mm |
| Aortic | Mild AR | **IVSd** | mm |
| Tricuspid | Trivial TR, PPG = 9mmHg | **PWd** | mm |
| pulmonic | Trivial PR | **EDV** | ml |
| Aortic arch |  | **ESV** | ml |
| PDA | No PDA | **LVEF** | 28% |
| Final Diagnosis | 1. Moderate MR 2. Mild AR 3. Dilated and dysfunctional LV (DCM | | |

**Done by sign----------------------**

**Name: Dr. Tesfaye T. Pediatric Cardiologist Date-04/10/2011 E.C**

| 2.Pediatric Echocardiography report  Patient Name. Patient ID: 010288  Gender: M Age:7 years Date of Report: 06/10/2011Eth.C Weight: _____________  INCOMPLETE DATA (PATIENT NAME MISSING) | | | |
| --- | --- | --- | --- |
| Features | **Finding** | **Features** | **Finding** |
| Profile | | **Atria** | |
| Abdominal situs | Solitus | Left atrium | Normal |
| Cardiac position | Levocardia | Right atrium | Normal |
| Systemic venous drainage | To RA | **Atrioventricular valves** | |
| Pulmonary venous drainage | To LA | Mitral valve |  |
| Atrioventricular connection | Concordant | Tricuspid valve |  |
| Ventriculoarterial connection | Concordant | **Ventricles** | |
| Ventricular loop | d Loop | Left ventricle | Normal |
| Septae | | Right ventricle | TAPSE = 19mm, RV TDI S wave= 15cm/sec |
| Interventricular septum | Intact | **Semilunal valves** | |
| Interatrial septum | Intact | Aortic valve |  |
| Great arteries | NRGA | Pulmonary valve |  |
| Aorta |  | **M-Mode** |  |
| Pulmonary artery |  | **AO** | mm |
| Coronary arteries |  | **LA** | mm |
| Doppler Measurement | | **LVIDd** | 49.7mm |
| Mitral |  | **LVIDs** | 30.2mm |
| Aortic |  | **IVSd** | 8.64mm |
| Tricuspid | Mild TR, PPG =25mmHg | **PWd** | 8.64mm |
| pulmonic | Trivial PR, PPG = 15mmHg | **EDV** | 117ml |
| Aortic arch | Left Arch | **ESV** | 35.6ml |
|  |  | **FS** | 39.2% |
| PDA | No PDA | **LVEF** | 69.6% |
| Final Diagnosis | 1. Mild TR 2. Trivial PR | | |

**Done by sign---------------------**

**Name: Dr. Tesfaye T. Pediatrician & Pediatric Cardiologist. Date-06/10/2011 E.C**

| 3.Pediatric Echocardiography report  Patient Name: Be’emnet Getu Patient ID: 009955  Gender: F Age: 1 7/12 years Date of Report: 06/10/2011Eth.C  Clinical Finding: FTT + Murmur + Diaphoresi. TGSH7.3044. | | | |
| --- | --- | --- | --- |
| Features | **Finding** | **Features** | **Finding** |
| Profile | | **Atria** | |
| Abdominal situs | Solitus | Left atrium | Normal |
| Cardiac position | Levocardia | Right atrium | Normal |
| Systemic venous drainage | to RA | **Atrioventricular valves** | |
| Pulmonary venous drainage | to LA | Mitral valve |  |
| Atrioventricular connection | Concordant | Tricuspid valve |  |
| Ventriculoarterial connection | Concordant | **Ventricles** | |
| Ventricular loop | d- Loop | Left ventricle | Normal |
| Septae | | Right ventricle | TAPSE = 20mm |
| Interventricular septum | Intact | **Semilunal valves** | |
| Interatrial septum | 10mm premium septal defect with L-R shunt | Aortic valve | 12mm |
| Great arteries | NRGA | Pulmonary valve | 12mm |
| Aorta |  | **M-Mode** |  |
| Pulmonary artery |  | **AO** | mm |
| Coronary arteries |  | **LA** | mm |
| Doppler Measurement | | **LVIDd** | 20.3mm |
| Mitral | No MR/MS | **LVIDs** | 13.3mm |
| Aortic | No AR/AS | **IVSd** | 6.05mm |
| Tricuspid | No TR/TS | **PWd** | 4.84mm |
| pulmonic | No PR/PS | **EDV** | 13.2ml |
| Aortic arch |  | **ESV** | 4.42ml |
|  |  | **FS** | 34.5% |
| PDA | No PDA | **LVEF** | 66.5% |
| Final Diagnosis | 1. **Moderate Ostium Primum ASD, L – R Shunt** | | |

**Done by Signature**

**Name: Dr. Tesfaye T. Pediatrician & Pediatric Cardiologist Date: 06/10/2011Eth.C**

| 4.Pediatric Echocardiography report  Patient Name: Solome Getachew Patient ID:008216  Gender: F Age: 42days Date of Report: 06/10/2011 E.C. TGSH1.2468. + TGSH4 | | | |
| --- | --- | --- | --- |
| Features | **Finding** | **Features** | **Finding** |
| Profile | | **Atria** | |
| Abdominal situs | Solitus | Left atrium | N |
| Cardiac position | Levocardia | Right atrium | N |
| Systemic venous drainage | to RA | **Atrioventricular valves** | |
| Pulmonary venous drainage | to LA | Mitral valve |  |
| Atrioventricular connection | Concordant | Tricuspid valve |  |
| Ventriculoarterial connection | Concordant | **Ventricles** | |
| Ventricular loop | d- Loop | Left ventricle | N |
| Septae | | Right ventricle | Normal , TAPSE = 15mm |
| Interventricular septum | 3.5mm perimembranous VSD with L-R shunt. | **Semilunal valves** | |
| Interatrial septum | Intact | Aortic valve |  |
| Great arteries | NRGA | Pulmonary valve |  |
| Aorta |  | **M-Mode** |  |
| Pulmonary artery |  | **AO** | mm |
| Coronary arteries |  | **LA** | mm |
| Doppler Measurement | | **LVIDd** | 16.5mm |
| Mitral |  | **LVIDs** | 10.2mm |
| Aortic |  | **IVSd** | 5.5mm |
| Tricuspid | Mild TR, PPG = 28mmHg | **PWd** | 5.5mm |
| pulmonic |  | **EDV** | 7.76ml |
| Aortic arch |  | **ESV** | 2.17ml |
|  |  | **FS** | 38.2% |
| PDA | No PDA | **LVEF** | 72% |
| Final Diagnosis | 1. **Perimembranous VSD** | | |

**Done by Signature**

**Name: Tesfaye T., Pediatrician & Pediatric Cardiologist. Date: 06/10/2011Eth.C**

| 5.Pediatric Echocardiography report  Patient Name: Baby of Bayu Patient ID:010694  Gender: M Age:03day_ Date of Report: 10/10/2011Eth.C  Clinical Finding: RD + MAS. TGSH7.3045. | | | |
| --- | --- | --- | --- |
| Features | **Finding** | **Features** | **Finding** |
| Profile | | **Atria** | |
| Abdominal situs | Solitus | Left atrium | Normal |
| Cardiac position | Levocardia | Right atrium | Dilated |
| Systemic venous drainage | To RA | **Atrioventricular valves** | |
| Pulmonary venous drainage | To LA | Mitral valve | Normal size |
| Atrioventricular connection | Concordant | Tricuspid valve | Normal size |
| Ventriculoarterial connection | Concordant | **Ventricles** | |
| Ventricular loop | d-Loop | Left ventricle | Normal |
| Septae | | Right ventricle | Dilated, TAPSE = 8mm, RVTDI S wave = 8cm/sec |
| Interventricular septum | Intact | **Semilunal valves** | |
| Interatrial septum | Septal bouncing to LA | Aortic valve | Normal size |
| Great arteries | NRGA | Pulmonary valve | Normal size |
| Aorta | Normal size | **M-Mode** |  |
| Pulmonary artery | Confluent branch PAs | **AO** | mm |
| Coronary arteries |  | **LA** | mm |
| Doppler Measurement | | **LVIDd** | mm |
| Mitral | Mild MR | **LVIDs** | mm |
| Aortic | Normal | **IVSd** | mm |
| Tricuspid | Moderate TR, PPG = 60mmHg | **PWd** | mm |
| pulmonic | Mild PR, PPG = 25mmHg | **EDV** | ml |
| Aortic arch |  | **ESV** | ml |
| PDA | 1mm PDA, R – L Shunt | **LVEF** |  |
| Final Diagnosis | 1. Severe Pulmonary Hypertension secondary to ?PPHTN | | |

**Done by sign----------------------**

**Name: Dr. Tesfaye T., Pediatrician, Pediatric Cardiologist Date: 10/10/2011Eth.C**

| 6.Pediatric Echocardiography report  Patient Name: Bekalu Yetayew Patient ID: 010170  Gender: M Age: - 9years_ Date of Report13/01/2012Eth.C  Clinical Finding: Sydenham’s Chorea. TGSH7.3046. | | | |
| --- | --- | --- | --- |
| Features | **Finding** | **Features** | **Finding** |
| Profile | | **Atria** | |
| Abdominal situs | Solitus | Left atrium | Normal |
| Cardiac position | Levocardia | Right atrium | Normal |
| Systemic venous drainage | to RA | **Atrioventricular valves** | |
| Pulmonary venous drainage | to LA | Mitral valve | Annulus = 19mm |
| Atrioventricular connection | Concordant | Tricuspid valve | Annulus = 22mm, TAPSE = 21mm |
| Ventriculoarterial connection | Concordant | **Ventricles** | |
| Ventricular loop | d-Loop | Left ventricle | Normal |
| Septae | | Right ventricle | Normal |
| Interventricular septum | Intact | **Semilunal valves** | |
| Interatrial septum | Intact | Aortic valve | Annulus = 19mm |
| Great arteries | NRGA | Pulmonary valve | Annulus = 18mm |
| Aorta |  | **M-Mode** |  |
| Pulmonary artery |  | **AO** | mm |
| Coronary arteries | ------ | **LA** | mm |
| Doppler Measurement | | **LVIDd** | 32.7mm |
| Mitral | ------- | **LVIDs** | 19.4mm |
| Aortic | ------- | **IVSd** | 6.045mm |
| Tricuspid | Mild TR, PPG = 28mmHg | **PWd** | 9.98mm |
| pulmonic | Trivial PR, PPG = 16mmHg | **EDV** | 43.2ml |
| Aortic arch | ---- | **ESV** | 11.8ml |
| PDA | ------ | **LVEF** | 72% |
| Final Diagnosis | 1. Normal Heart Study | | |

**Done by Signature**

**Name: Dr. Tesfaye T., Pediatric Cardiologist Date: 13/012012Eth.C.**

| 7.Pediatric Echocardiography report  Patient Name: Dawit Baye Patient ID:010750  Gender: M. Age: 5 years_ Date of Report: 11/10/2011Eth.C  Clinical Finding: FTT + Murmur. TGSH7.3047. | | | |
| --- | --- | --- | --- |
| Features | **Finding** | **Features** | **Finding** |
| Profile | | **Atria** | |
| Abdominal situs | Solitus | Left atrium | Normal |
| Cardiac position | Levocardia | Right atrium | Normal |
| Systemic venous drainage | to RA | **Atrioventricular valves** | |
| Pulmonary venous drainage | to LA | Mitral valve | Normal |
| Atrioventricular connection | Concordant | Tricuspid valve | Normal |
| Ventriculoarterial connection | Concordant | **Ventricles** | |
| Ventricular loop | d-Loop | Left ventricle | Normal |
| Septae | | Right ventricle | Normal |
| Interventricular septum | Intact | **Semilunal valves** | |
| Interatrial septum | Intact | Aortic valve | Normal |
| Great arteries | NTGA | Pulmonary valve | Normal |
| Aorta | ---- | **M-Mode** |  |
| Pulmonary artery | ------ | **AO** | mm |
| Coronary arteries | ------ | **LA** | mm |
| Doppler Measurement | | **LVIDd** | 26.2mm |
| Mitral | ------ | **LVIDs** | 19.4mm |
| Aortic | ------ | **IVSd** | 7.06mm |
| Tricuspid | Trivial TR, PPG = 20mmHg | **PWd** | 5.04mm |
| pulmonic | ------ | **EDV** | 25.1ml |
| Aortic arch | --- | **ESV** | 11.8ml |
| PDA | No PDA | **LVEF** | 57% |
| Final Diagnosis | 1. Normal Heart Study | | |

**Done by Signature**

**Name: Dr. Tesfaye T., Pediatric Cardiologist Date: 11/10/2011Eth.C**

| 8.Pediatric Echocardiography report  Patient Name: Dejamach Abere Patient ID:009968  Gender: M. Age: 12years_ Date of Report 11/10/2011Eth.C  Clinical Finding: _RD + DOE + Cardiomegaly on CXR + Sepsis. TGSH7.3048. | | | |
| --- | --- | --- | --- |
| Features | **Finding** | **Features** | **Finding** |
| Profile | | **Atria** | |
| Abdominal situs | Solitus | Left atrium | Normal |
| Cardiac position | Levocardia | Right atrium | Diastolic collapse |
| Systemic venous drainage | to RA | **Atrioventricular valves** | |
| Pulmonary venous drainage | to LA | Mitral valve | ------- |
| Atrioventricular connection | Concordant | Tricuspid valve | ------- |
| Ventriculoarterial connection | Concordant | **Ventricles** | |
| Ventricular loop | d-Loop | Left ventricle | -------- |
| Septae | | Right ventricle | Diastolic collapse |
| Interventricular septum | Intact, paradoxical septal motion | **Semilunal valves** | |
| Interatrial septum | Intact | Aortic valve | ------ |
| Great arteries | NRGA | Pulmonary valve | -------- |
| Aorta | ------- | **M-Mode** |  |
| Pulmonary artery | -------- | **AO** | mm |
| Coronary arteries | -------- | **LA** | mm |
| Doppler Measurement | | **LVIDd** | 47.5mm |
| Mitral | No inspiratory variability in mitral inflow velocity | **LVIDs** | 36.3mm |
| Aortic | No echo pulsus paradoxus | **IVSd** | 12.5mm |
| Tricuspid | ----- | **PWd** | 12.2mm |
| pulmonic | -------- | **EDV** | 105ml |
| Aortic arch | ------ | **ESV** | 55.5ml |
| PDA | ------- | **LVEF** | 47% |
| Additional finding: | 1. **17.4mm Right pleural effusion.** 2. **18.4mm circumferential pericardial effusion with debris inside.** 3. **Swinging heart** 4. **RA/RV Diastolic collapse** 5. **IVC Plethoric** | | |
| Final Diagnosis | 1. Cardiac tamponade secondary to ?pyogenic/tuberculosis 2. Right pleural effusion 3. Slightly reduced LV Function | | |

**Done by sign----------------------**

**Name: Dr. Tesfaye T. Pediatric Cardiologist Date-11/10/2011 E.C**

| 9.Pediatric Echocardiography report  Patient Name: Nobel Alemu. Patient ID: 010909  Gender: M Age: 3 years_ Date of Report: 13/10/2011Eth.C  Clinical Finding: Incidental Murmur. TGSH7.3049. | | | |
| --- | --- | --- | --- |
| Features | **Finding** | **Features** | **Finding** |
| Profile | | **Atria** | |
| Abdominal situs | Solitus | Left atrium | Normal |
| Cardiac position | Levocardia | Right atrium | Normal |
| Systemic venous drainage | to RA | **Atrioventricular valves** | |
| Pulmonary venous drainage | to LA | Mitral valve | ------- |
| Atrioventricular connection | Concordant | Tricuspid valve | -------- |
| Ventriculoarterial connection | Concordant | **Ventricles** | |
| Ventricular loop | d-Loop | Left ventricle | Normal |
| Septae | | Right ventricle | Normal |
| Interventricular septum | 3.4mm PM VSD | **Semilunal valves** | |
| Interatrial septum | Intact | Aortic valve |  |
| Great arteries | NRGA | Pulmonary valve |  |
| Aorta |  | **M-Mode** |  |
| Pulmonary artery |  | **AO** | mm |
| Coronary arteries |  | **LA** | mm |
| Doppler Measurement | | **LVIDd** | 28mm |
| Mitral |  | **LVIDs** | 19.7mm |
| Aortic |  | **IVSd** | 7.56mm |
| Tricuspid |  | **PWd** | 7.18mm |
| pulmonic |  | **EDV** | 29.6ml |
| Aortic arch | Left | **ESV** | 12.2ml |
| PDA | ------- | **LVEF** | 58% |
| Final Diagnosis | 1. PM VSD | | |

**Done by Signature**

**Name: Dr. Tesfaye T. Pediatric Cardiologist Date: 13/10/2011Eth.C**

| Pediatric Echocardiography report  Patient Name: Alazar Getnet Patient ID:011016  Gender: M Age:6 years_ Date of Report: 13/10/2011Eth.C  Clinical Finding: Incidental Murmur/Innocent. TGSH7.3050. | | | |
| --- | --- | --- | --- |
| Features | **Finding** | **Features** | **Finding** |
| Profile | | **Atria** | |
| Abdominal situs | Solitus | Left atrium | Normal |
| Cardiac position | Levocardia | Right atrium | Normal |
| Systemic venous drainage | to RA | **Atrioventricular valves** | |
| Pulmonary venous drainage | to LA | Mitral valve | ---- |
| Atrioventricular connection | Concordant | Tricuspid valve | ------ |
| Ventriculoarterial connection | Concordant | **Ventricles** | |
| Ventricular loop | d-Loop | Left ventricle | Normal |
| Septae | | Right ventricle | Normal |
| Interventricular septum | Intact | **Semilunal valves** | |
| Interatrial septum | Intact | Aortic valve | ----- |
| Great arteries | NRGA | Pulmonary valve | ------ |
| Aorta |  | **M-Mode** |  |
| Pulmonary artery |  | **AO** | mm |
| Coronary arteries |  | **LA** | mm |
| Doppler Measurement | | **LVIDd** | 30.2mm |
| Mitral |  | **LVIDs** | 20mm |
| Aortic |  | **IVSd** | 7.56mm |
| Tricuspid |  | **PWd** | 7.56mm |
| pulmonic | Trivial PR, PPG = 10mmHg | **EDV** | 35.6ml |
| Aortic arch | Left | **ESV** | 12.7ml |
| PDA | ----- | **LVEF** | 64% |
| Final Diagnosis | 1. NHS | | |

**Done by Signature**

**Name: Dr. Tesfaye T. Pediatric Cardiologist Date-13/10/2011 E.C**

| | Pediatric Echocardiography report  Patient Name: Sina Getnet Patient ID:011016  Gender: M Age:4 5/12 years_ Date of Report: 13/10/2011Eth.C  Clinical Finding: _Recurrent Chest Infection. TGSH7.3051. | | | | | --- | --- | --- | --- | | Features | **Finding** | **Features** | **Finding** | | Profile | | **Atria** | | | Abdominal situs | Solitus | Left atrium | Normal | | Cardiac position | Levocardia | Right atrium | Normal | | Systemic venous drainage | to RA | **Atrioventricular valves** | | | Pulmonary venous drainage | to LA | Mitral valve | ---- | | Atrioventricular connection | Concordant | Tricuspid valve | ------ | | Ventriculoarterial connection | Concordant | **Ventricles** | | | Ventricular loop | d-Loop | Left ventricle | Normal | | Septae | | Right ventricle | Normal | | Interventricular septum | Intact | **Semilunal valves** | | | Interatrial septum | Intact | Aortic valve | ----- | | Great arteries | NRGA | Pulmonary valve | ------ | | Aorta |  | **M-Mode** |  | | Pulmonary artery |  | **AO** | mm | | Coronary arteries |  | **LA** | mm | | Doppler Measurement | | **LVIDd** | 22.2mm | | Mitral |  | **LVIDs** | 16.4mm | | Aortic |  | **IVSd** | 7.15mm | | Tricuspid | Trivial TR, PPG = 10mmHg | **PWd** | 7.15mm | | pulmonic | Trivial PR, PPG = 12mmHg | **EDV** | 16.6ml | | Aortic arch | Left | **ESV** | 7.64ml | | PDA | ----- | **LVEF** | 55% | | Final Diagnosis | 1. NHS | | |   Done by Signature  Name: Dr. Tesfaye T. Pediatric Cardiologist Date-13/10/2011 E.C  Pediatric Echocardiography report  Patient Name: Tsinu kal Sisay Patient ID:010892  Gender: F Age:2 years_ Dateof Report: 13/10/2011Eth.C  Clinical Finding: Diaphoresis + RD + Murmur + FTT. TGSH7.3052. | | | |
| --- | --- | --- | --- | --- | --- | --- | --- | --- | --- | --- | --- | --- | --- | --- | --- | --- | --- | --- | --- | --- | --- | --- | --- | --- | --- | --- | --- | --- | --- | --- | --- | --- | --- | --- | --- | --- | --- | --- | --- | --- | --- | --- | --- | --- | --- | --- | --- | --- | --- | --- | --- | --- | --- | --- | --- | --- | --- | --- | --- | --- | --- | --- | --- | --- | --- | --- | --- | --- | --- | --- | --- | --- | --- | --- | --- | --- | --- | --- | --- | --- | --- | --- | --- | --- | --- | --- | --- | --- | --- | --- | --- | --- | --- | --- | --- | --- | --- | --- | --- | --- | --- | --- | --- |
| Features | **Finding** | **Features** | **Finding** |
| Profile | | **Atria** | |
| Abdominal situs | Solitus | Left atrium | Dilated |
| Cardiac position | Levocardia | Right atrium | Normal |
| Systemic venous drainage | to RA | **Atrioventricular valves** | |
| Pulmonary venous drainage | to LA | Mitral valve |  |
| Atrioventricular connection | Concordant | Tricuspid valve |  |
| Ventriculoarterial connection | Concordant | **Ventricles** | |
| Ventricular loop | d-Loop | Left ventricle | Dilated |
| Septae | | Right ventricle | Normal |
| Interventricular septum | Intact | **Semilunal valves** | |
| Interatrial septum | Intact | Aortic valve |  |
| Great arteries | NRGA | Pulmonary valve |  |
| Aorta | ---- | **M-Mode** | Normal Function (Eye balling) |
| Pulmonary artery | ------- | **AO** | mm |
| Coronary arteries |  | **LA** | mm |
| Doppler Measurement | | **LVIDd** | mm |
| Mitral | ----- | **LVIDs** | mm |
| Aortic | ------ | **IVSd** | mm |
| Tricuspid | ------ | **PWd** | mm |
| pulmonic | ------- | **EDV** | ml |
| Aortic arch | Left | **ESV** | ml |
| PDA | 3.5mm PDA, L – R shunt | **LVEF** |  |
| Comment: | Child was crying during study | | |
| Final Diagnosis | 1. Large PDA, L – R Shunt | | |

**Done by Signature**

**Name: Dr. Tesfaye T. Pediatric Cardiologist Date----------------------**

| Pediatric Echocardiography report  Patient Name: Mestayit Abera Patient ID:011180  Gender: F Age: -14 years_ Date of Report: 14/10/2011Eth.C  Clinical Finding: DOE + Murmur + Easy Fatigability + Palpitation. TGSH7.3053. | | | |
| --- | --- | --- | --- |
| Features | **Finding** | **Features** | **Finding** |
| Profile | | **Atria** | |
| Abdominal situs | Solitus | Left atrium | Normal |
| Cardiac position | Levocardia | Right atrium | Dilated 44 X 44mm |
| Systemic venous drainage | to RA | **Atrioventricular valves** | |
| Pulmonary venous drainage | to LA | Mitral valve | ------- |
| Atrioventricular connection | Concordant | Tricuspid valve | Trivial TR, TAPSE = 27mm |
| Ventriculoarterial connection | Concordant | **Ventricles** | |
| Ventricular loop | d-Loop | Left ventricle | Normal size |
| Septae | | Right ventricle | Dilated, RV TDI S wave = 15cm/s |
| Interventricular septum | Intact | **Semilunal valves** | |
| Interatrial septum | 22mm OS ASD, L – R Shunt | Aortic valve | Trileaflet |
| Great arteries | NRGA | Pulmonary valve | Annulus = 33mm |
| Aorta | Normal | **M-Mode** |  |
| Pulmonary artery | Dilated | **AO** | mm |
| Coronary arteries | -------- | **LA** | mm |
| Doppler Measurement | | **LVIDd** | 37.1mm |
| Mitral |  | **LVIDs** | 25.1mm |
| Aortic |  | **IVSd** | 6.62mm |
| Tricuspid |  | **PWd** | 6.62mm |
| pulmonic |  | **EDV** | 58.5ml |
| Aortic arch | Left | **ESV** | 22.5ml |
| PDA | ------ | **LVEF** | 61% |
| Final Diagnosis | 1. Large OS ASD 2. Dilated RA/RV 3. Good Bi-Ventricular function | | |

**Done by signature**

**Dr. Tesfaye T. Pediatric Cardiologist Date-14/10/2011 E.C**

| Pediatric Echocardiography report  Patient Name: Baby of Workie Patient ID: 011168  Gender: M Age:- 16day Date of Report: 17/10/2011Eth.C  Clinical Finding: Incidental Murmur. TGSH7.3054. | | | |
| --- | --- | --- | --- |
| Features | **Finding** | **Features** | **Finding** |
| Profile | | **Atria** | |
| Abdominal situs | Solitus | Left atrium | Normal |
| Cardiac position | Levocardia | Right atrium | Normal |
| Systemic venous drainage | to RA | **Atrioventricular valves** | |
| Pulmonary venous drainage | to LA | Mitral valve | Annulus =15mm |
| Atrioventricular connection | Concordant | Tricuspid valve | Annulus = 14mm |
| Ventriculoarterial connection | Concordant | **Ventricles** | |
| Ventricular loop | d-Loop | Left ventricle | Normal |
| Septae | | Right ventricle | TAPSE = 12mm |
| Interventricular septum | 6mm PM defect, L – R shunt | **Semilunal valves** | |
| Interatrial septum | PFO, L – R shunt | Aortic valve | A = 13mm |
| Great arteries | NRGA | Pulmonary valve | Annulus = 13mm |
| Aorta |  | **M-Mode** |  |
| Pulmonary artery |  | **AO** | mm |
| Coronary arteries | ------- | **LA** | mm |
| Doppler Measurement | | **LVIDd** | 16.3mm |
| Mitral | Moderate MR | **LVIDs** | 10.5mm |
| Aortic | No AR/AS | **IVSd** | 4.58mm |
| Tricuspid | Mild to Moderate TR | **PWd** | 4.58mm |
| pulmonic | No PR/PS | **EDV** | 7.52ml |
| Aortic arch | Left | **ESV** | 2.35ml |
| PDA | ------ | **LVEF** | 68% |
| Final Diagnosis | 1. PFO, L-R Shunt 2. Small Perimembranous VSD, L – R Shunt 3. Moderate MR | | |
| Comment: Needs follow up echo for confirmation as the Neonate was crying & restless | | | |

**Done by signature**

**Dr. Tesfaye T. Pediatric Cardiologist Date-17/10/2011 E.C**

| Pediatric Echocardiography report  Patient Name: Dawit Tegegn Patient ID:011492  Gender: M Age:- 3 years_ Date of Report: 18/10/2011 Eth.C  Clinical Finding: CHF + RD + Murmur. TGSH7.3055. | | | |
| --- | --- | --- | --- |
| Features | **Finding** | **Features** | **Finding** |
| Profile | | **Atria** | |
| Abdominal situs | Solitus | Left atrium | Dilated, 39 x 39mm |
| Cardiac position | Levocardia | Right atrium | Dilated, 33X31mm |
| Systemic venous drainage | to RA | **Atrioventricular valves** | |
| Pulmonary venous drainage | to LA | Mitral valve | A=26mm, Thickened MVL |
| Atrioventricular connection | Concordant | Tricuspid valve | A=19mm |
| Ventriculoarterial connection | Concordant | **Ventricles** | |
| Ventricular loop | d-Loop | Left ventricle | Dilated |
| Septae | | Right ventricle | Dilated. TAPSE = 14mm |
| Interventricular septum | Intact | **Semilunal valves** | |
| Interatrial septum | Intact | Aortic valve | Normal |
| Great arteries | NRGA | Pulmonary valve | Normal |
| Aorta | ----------- | **M-Mode** |  |
| Pulmonary artery | ------------- | **AO** | mm |
| Coronary arteries |  | **LA** | mm |
| Doppler Measurement | | **LVIDd** | 38.9mm |
| Mitral | Severe MR, lateral projection | **LVIDs** | 29.1mm |
| Aortic | Moderate AR, PHT = 325ms | **IVSd** | 7.56mm |
| Tricuspid | Moderate to severe TR, PPG = 67mmHg | **PWd** | 7.56mm |
| pulmonic | Mild PR, PPG = 39mmHg | **EDV** | 65.5ml |
| Aortic arch | Left | **ESV** | 32.5ml |
| PDA | No PDA | **LVEF** | 50% |
| Final Diagnosis | 1. {S, D, S} Levocardia 2. Severe MR 3. Moderate AR 4. Severe TR 5. Mild PR 6. LA/LV Dilated 7. Severe pulmonary hypertension 8. Normal biventricular function | | |

**Done by Signature**

**Dr. Tesfaye T. Pediatric Cardiologist Date-18/10/2011 E.C**

| Pediatric Echocardiography report  Patient Name: Mekides Yeshiwas Patient ID: 011500  Gender: F Age: - 4 years Date of Report: 20/10/2011Eth.C  Clinical Finding: RD + CHF + Murmur. TGSH7.3056. | | | |
| --- | --- | --- | --- |
| Features | **Finding** | **Features** | **Finding** |
| Profile | | **Atria** | |
| Abdominal situs | Solitus | Left atrium | Normal |
| Cardiac position | Levocardia | Right atrium | Dilated |
| Systemic venous drainage | to RA | **Atrioventricular valves** | |
| Pulmonary venous drainage | to LA | Mitral valve | A = 19mm |
| Atrioventricular connection | Concordant | Tricuspid valve | A = 22mm, TAPSE = 6mm |
| Ventriculoarterial connection | Concordant | **Ventricles** | |
| Ventricular loop | d-Loop | Left ventricle | Normal |
| Septae | | Right ventricle | Dilated, RV TDI S wave = 8cm/sec |
| Interventricular septum | Intact | **Semilunal valves** | |
| Interatrial septum | Intact | Aortic valve | A = 15mm |
| Great arteries | NRGA | Pulmonary valve | A = 18mm |
| Aorta | ------- | **M-Mode** |  |
| Pulmonary artery | -------- | **AO** | mm |
| Coronary arteries | --------- | **LA** | mm |
| Doppler Measurement | | **LVIDd** | 27.6mm |
| Mitral | No MR/MS | **LVIDs** | 17.8mm |
| Aortic | No AR/AS | **IVSd** | 9.8mm |
| Tricuspid | Grade II TR, PPG = 65mmHg | **PWd** | 7.56mm |
| pulmonic | Mild PR, PPG = 60mmHg | **EDV** | 28.5ml |
| Aortic arch | ----- | **ESV** | 9.44ml |
| PDA | No PDA | **LVEF** | 65% |
| Final Diagnosis | 1. {S, D, S} Levocardia 2. Grade II TR 3. Mild PR 4. Severe pulmonary Hypertension 5. Dilated and Dysfunctional RV 6. Normal LF Function | | |
| Remark: Search cause for pulmonary hypertension | | | |

**Done by Signature**

**Name: Dr. Tesfaye T., Pediatric Cardiologist Date:- 20/10/2011Eth.C**

| Pediatric Echocardiography report  Patient Name: Sale-Amlak Ayen Patient ID: FHRRH  Gender: M Age:- 14years_ Date of Report: 26/10/2011Eth.C  Clinical Finding: _Cyanosis + Clubbing + Murmur + DOE + Palpitation. TGSH7.3057. | | | |
| --- | --- | --- | --- |
| Features | **Finding** | **Features** | **Finding** |
| Profile | | **Atria** | |
| Abdominal situs | Solitus | Left atrium | Normal |
| Cardiac position | Levocardia | Right atrium | Dilated |
| Systemic venous drainage | to RA | **Atrioventricular valves** | |
| Pulmonary venous drainage | to LA | Mitral valve | ------ |
| Atrioventricular connection | Concordant | Tricuspid valve | Thick valve tips  TAPSE = 15mm |
| Ventriculoarterial connection | Concordant | **Ventricles** | |
| Ventricular loop | d-Loop | Left ventricle | ----- |
| Septae | | Right ventricle | Hypertrophied |
| Interventricular septum | Tiny PM VSD, BD Shunt, Predominantly R-L. | **Semilunal valves** | |
| Interatrial septum | 16mm OS ASD, BD shunt, predominantly R-L. | Aortic valve | Anterior leaflet prolapsing to PM VSD partially obstructing the VSD. |
| Great arteries | NRGA | Pulmonary valve |  |
| Aorta |  | **M-Mode** | Good LV Function (eye balling) |
| Pulmonary artery |  | **AO** | mm |
| Coronary arteries | -------- | **LA** | mm |
| Doppler Measurement | | **LVIDd** | mm |
| Mitral | ------ | **LVIDs** | mm |
| Aortic | Moderate AR, PHT = 287ms | **IVSd** | mm |
| Tricuspid | Trivial TR | **PWd** | mm |
| pulmonic | Mild PR | **EDV** | ml |
| Aortic arch | ----- | **ESV** | ml |
| PDA | No PDA | **LVEF** |  |
| Final Diagnosis | 1. {S, D, S} Levocardia. 2. Large OS ASD, BD Shunt Predominantly R – L. 3. Perimembranous VSD Partially obstructed by prolapsing aortic valve, BD Shunt Predominantly R – L. 4. Moderate AR secondary to prolapsing aortic valve. 5. Good biventricular function. | | |

**Done by signature**

**Name: Dr. Tesfaye T. Pediatric Cardiologist Date-26/10/2011Eth.C**

| Pediatric Echocardiography report  Patient Name: Agumas Yalelet Patient ID: 011602  Gender: M Age: 7 years_ Date of Report: 27/10/2011 E.C  Clinical Finding: Chest Pain. TGSH7.3058. | | | |
| --- | --- | --- | --- |
| Features | **Finding** | **Features** | **Finding** |
| Profile | | **Atria** | |
| Abdominal situs | Solitus | Left atrium | Normal |
| Cardiac position | Levocardia | Right atrium | Normal |
| Systemic venous drainage | to RA | **Atrioventricular valves** | |
| Pulmonary venous drainage | to LA | Mitral valve | Normal |
| Atrioventricular connection | Concordant | Tricuspid valve | TAPSE = 21mm |
| Ventriculoarterial connection | Concordant | **Ventricles** | |
| Ventricular loop | d-Loop | Left ventricle | Normal |
| Septae | | Right ventricle | Normal |
| Interventricular septum | Intact | **Semilunal valves** | |
| Interatrial septum | Intact | Aortic valve | Trileaflet |
| Great arteries | NRGA | Pulmonary valve | ------ |
| Aorta | ----- | **M-Mode** |  |
| Pulmonary artery | Confluent Branch PAs | **AO** | mm |
| Coronary arteries | ----- | **LA** | mm |
| Doppler Measurement | | **LVIDd** | 32.1mm |
| Mitral | Trivial MR | **LVIDs** | 21.2mm |
| Aortic | No AR/AS | **IVSd** | 9.8mm |
| Tricuspid | Trivial TR, PPG = 26mmHg | **PWd** | 9.4mm |
| pulmonic | No PR/PS | **EDV** | 41ml |
| Aortic arch | ---- | **ESV** | 14.8ml |
| PDA | NO PDA | **LVEF** | 64% |
| Final Diagnosis | 1. {S, D, S} Levocardia 2. Trivial TR/Trivial MR 3. Good biventricular Function | | |

**Done by Signature**

**Name: Dr. Tesfaye T. Pediatric Cardiologist Date 27/10/2011 E.C**

| Pediatric Echocardiography report  Patient Name: Felegush Assefa Patient ID: 011038  Gender: Female Age: 11Day Date of Report: 02/11/2011Eth.C  Clinical Finding: RD. TGSH7.3059. | | | |
| --- | --- | --- | --- |
| Features | **Finding** | **Features** | **Finding** |
| Profile | | **Atria** | |
| Abdominal situs | Solitus | Left atrium | Normal |
| Cardiac position | Levocardia | Right atrium | Normal |
| Systemic venous drainage | to RA | **Atrioventricular valves** | |
| Pulmonary venous drainage | to LA | Mitral valve | Normal |
| Atrioventricular connection | Concordant | Tricuspid valve | Normal |
| Ventriculoarterial connection | Concordant | **Ventricles** | |
| Ventricular loop | d-Loop | Left ventricle | Normal |
| Septae | | Right ventricle | TAPSE = 11mm |
| Interventricular septum | Intact | **Semilunal valves** | |
| Interatrial septum | PFO, L–R shunt | Aortic valve | Normal |
| Great arteries | NRGA | Pulmonary valve | Normal |
| Aorta | Posterior & to the right | **M-Mode** |  |
| Pulmonary artery | Anterior & to the left | **AO** | mm |
| Coronary arteries | ------- | **LA** | mm |
| Doppler Measurement | | **LVIDd** | 16.6mm |
| Mitral | No MR/MS | **LVIDs** | 11.8mm |
| Aortic | No AR/AS | **IVSd** | 3.28mm |
| Tricuspid | Trivial TR, PPG = 26mmHg | **PWd** | 6.55mm |
| pulmonic | No PR/PS | **EDV** | 7.89ml |
| Aortic arch | Left | **ESV** | 3.21ml |
| PDA | No PDA | **LVEF** | 59% |
|  |  | **FS** | 28.9% |
| Final Diagnosis | 1. {S, D, S} Levocardia 2. PFO | | |

**Done by Signature**

**Dr. Tesfaye T. Pediatric Cardiologist Date 02/11/2011 E.C**

| Pediatric Echocardiography report  Patient Name: Amen Habtamu Patient ID: 12710  Gender: F Age:3- 6/12 years_ Date of Report: 02/11/2011Eth.C  Clinical Finding: RD + Recurrent Chest Infection. TGSH7.3060. | | | |
| --- | --- | --- | --- |
| Features | **Finding** | **Features** | **Finding** |
| Profile | | **Atria** | |
| Abdominal situs | Solitus | Left atrium | Normal |
| Cardiac position | Levocardia | Right atrium | Normal |
| Systemic venous drainage | to RA | **Atrioventricular valves** | |
| Pulmonary venous drainage | to LA | Mitral valve | Normal |
| Atrioventricular connection | Concordant | Tricuspid valve | TAPSE = 18mm |
| Ventriculoarterial connection | Concordant | **Ventricles** | |
| Ventricular loop | d-Loop | Left ventricle | Normal |
| Septae | | Right ventricle | Normal |
| Interventricular septum | Intact | **M-Mode** |  |
| Interatrial septum | Intact | AO |  |
| Great arteries | NRGA | LA |  |
| Aorta | ---- | LVIDd | 16.6mm |
| Pulmonary artery | ------ | LVIDs | 11.8mm |
| Semilunal valves |  | IVSd | 3.28mm |
| Aortic valve | Normal | IVSs | 2.52mm |
| Pulmonary valve | Normal | LVPWd | 6.55mm |
| Doppler Measurement | | LVPWs | 7.56mm |
| Mitral | No MR/MS | **EDV** | 7.89ml |
| Aortic | No AR/AS | ESV | 3.21ml |
| Tricuspid | Trivial TR | FS | 28.9% |
| pulmonic | Trivial PR | LVEF | 59% |
| Aortic arch | Left | **Coronary arteries** | -------- |
| PDA | ------- |  |  |
| Additional information | ------- | | |
| Final Diagnosis | 1. {S, D, S} Levocardia 2. Normal Heart Study | | |

**Done by Signature**

**Name: Dr. Tesfaye T. Pediatric Cardiologist Date-02/11/2011 E.C**

| Pediatric Echocardiography report  Patient Name: Baby of Kassaye Patient ID: 012624  Gender: Male Age: 20days_ Date of Report: 04/11/2011Eth.C  Clinical Finding: Incidental Murmur + Cyanosis. TGSH7.3061. | | | |
| --- | --- | --- | --- |
| Features | **Finding** | **Features** | **Finding** |
| Profile | | **Atria** | |
| Abdominal situs | Solitus | Left atrium | Normal |
| Cardiac position | Levocardia | Right atrium | Dilated, 17mm X 27mm |
| Systemic venous drainage | to RA | **Atrioventricular valves** | |
| Pulmonary venous drainage | to LA | Mitral valve | Normal |
| Atrioventricular connection | Concordant | Tricuspid valve | 10mm downward displacement of PTL |
| Ventriculoarterial connection | Concordant | **Ventricles** | |
| Ventricular loop | d-Loop | Left ventricle | Normal |
| Septae | | Right ventricle | Small sized, RV TDI S wave = 10cm/sec |
| Interventricular septum | Intact | **M-Mode** |  |
| Interatrial septum | 7.5mm OS ASD, BD shunt | AO |  |
| Great arteries | NRGA | LA |  |
| Aorta | ---- | LVIDd | 15.8mm |
| Pulmonary artery | ------- | LVIDs | 11.5mm |
| Semilunal valves |  | IVSd | 4.58mm |
| Aortic valve | Trileaflet | IVSs | 4.58mm |
| Pulmonary valve | Normal | LVPWd | 2.5mm |
| Doppler Measurement | | LVPWs | 2.75mm |
| Mitral | No MS/MR | **EDV** | 6.94ml |
| Aortic | No AS/AR | ESV | 3ml |
| Tricuspid | Moderate TR | FS | 27.2% |
| pulmonic | No PS/PR. Decreased ante grade flow across the pulmonary valve | LVEF | 56.8% |
| Aortic arch | ----- | **Coronary arteries** |  |
| PDA | ------- |  |  |
| Additional information | Baby was crying and restless during study | | |
| Final Diagnosis | 1. {S, D, S} Levocardia 2. Moderate OS ASD, BD shunt 3. Ebstein Anomaly of the Tricuspid valve 4. Good Biventricular Function | | |

**Done by Signature**

**Name: Dr. Tesfaye T. Pediatric Cardiologist Date-04/11/2011 E.C**

| Pediatric Echocardiography report  Patient Name: Wereket Tayachew Patient ID: 007144  Gender: F Age: 14years_ Date of Report: 04/11/2011Eth.C  Clinical Finding: Rheumatic Recurrence + Murmur + DOE + Plpitation. TGSH7.3062. | | | |
| --- | --- | --- | --- |
| Features | **Finding** | **Features** | **Finding** |
| Profile | | **Atria** | |
| Abdominal situs | Solitus | Left atrium | Dilated |
| Cardiac position | Levocardia | Right atrium | Normal |
| Systemic venous drainage | to RA | **Atrioventricular valves** | |
| Pulmonary venous drainage | to LA | Mitral valve | Thickened, clubbed |
| Atrioventricular connection | Concordant | Tricuspid valve | TAPSE = 22mm |
| Ventriculoarterial connection | Concordant | **Ventricles** | |
| Ventricular loop | d-Loop | Left ventricle | Dilated |
| Septae | | Right ventricle | Normal |
| Interventricular septum | Intact | **M-Mode** |  |
| Interatrial septum | Intact | AO |  |
| Great arteries | NRGA | LA |  |
| Aorta | ------ | LVIDd | 56.7mm |
| Pulmonary artery | ------ | LVIDs | 42.1 |
| Semilunal valves |  | IVSd | 9.69mm |
| Aortic valve | Trileaflet | IVSs | 12.6mm |
| Pulmonary valve | Normal | LVPWd | 9.69mm |
| Doppler Measurement | | LVPWs | 9.69mm |
| Mitral | Moderate MR, Mitral inflow gradient PPG/MPG = 16/6mmHg | **EDV** | 158ml |
| Aortic | Moderate AR, PHT = 316ms | ESV | 79ml |
| Tricuspid | No TR/TS | FS | 28% |
| pulmonic | Trivial PR, PPG = 10mmHg | LVEF | 55% |
| Aortic arch | Left | **Coronary arteries** |  |
| PDA | ----- |  |  |
| Additional information |  | | |
| Final Diagnosis | 1. {S, D, S} Levocardia 2. Moderate MS 3. Moderate MR, Thickened & clubbed Mitral Valve 4. Moderate AR 5. Good Biventricular Function | | |

**Done by Signature**

**Name: Dr. Tesfaye T. Pediatric Cardiologist Date-02/11/2011 E.C**

| Pediatric Echocardiography report  Patient Name: Beletu Waga Patient ID: 010955  Gender: F Age: 11 years_ Date of Report: 04/11/2011Eth.C  Clinical Finding: Incidental Murmur + Palpitation + Easy Fatigability. TGSH7.3063. | | | |
| --- | --- | --- | --- |
| Features | **Finding** | **Features** | **Finding** |
| Profile | | **Atria** | |
| Abdominal situs | Solitus | Left atrium | Dilated |
| Cardiac position | Levocardia | Right atrium | Normal |
| Systemic venous drainage | to RA | **Atrioventricular valves** | |
| Pulmonary venous drainage | to LA | Mitral valve | Thickened MVL |
| Atrioventricular connection | Concordant | Tricuspid valve | TAPSE = 26mm |
| Ventriculoarterial connection | Concordant | **Ventricles** | |
| Ventricular loop | d-Loop | Left ventricle | Dilated |
| Septae | | Right ventricle | Normal |
| Interventricular septum | 7mm PM VSD, L- R shunt. | **M-Mode** |  |
| Interatrial septum | Intact | AO |  |
| Great arteries | NRGA | LA |  |
| Aorta | ---- | LVIDd | 48mm |
| Pulmonary artery | ------ | LVIDs | 34.8mm |
| Semilunal valves |  | IVSd | 7.56mm |
| Aortic valve | A = 23mm, Trileaflet | IVSs | 7.56mm |
| Pulmonary valve | A = 23mm | LVPWd | 7.56mm |
| Doppler Measurement | | LVPWs | 7.18mm |
| Mitral | No MR/MS | **EDV** | 108ml |
| Aortic | No AR/AS | ESV | 50ml |
| Tricuspid | G – II TR | FS | 28% |
| pulmonic | No PR/PS | LVEF | 55% |
| Aortic arch | Left | **Coronary arteries** | ----- |
| PDA | No PDA |  |  |
| Additional information |  | | |
| Final Diagnosis | 1. {S, D, S} Levocardia 2. Moderate PM VSD, L – R shunt 3. Good Biventricular Function | | |

**Done by Signature**

**Name: Dr. Tesfaye T. Pediatric Cardiologist Date-02/11/2011 E.C**

| Pediatric Echocardiography report  Patient Name: Ateka Tadie Patient ID: 011386  Gender: M Age: 6years_ Date of Report: 04/11/2011Eth.C  Clinical Finding: Easy Fatigability. TGSH7.3064. | | | |
| --- | --- | --- | --- |
| Features | **Finding** | **Features** | **Finding** |
| Profile | | **Atria** | |
| Abdominal situs | Solitus | Left atrium | Normal |
| Cardiac position | Levocardia | Right atrium | Normal |
| Systemic venous drainage | to RA | **Atrioventricular valves** | |
| Pulmonary venous drainage | to LA | Mitral valve | Normal |
| Atrioventricular connection | Concordant | Tricuspid valve | TAPSE = 21mm |
| Ventriculoarterial connection | Concordant | **Ventricles** | |
| Ventricular loop | d-Loop | Left ventricle | Normal |
| Septae | | Right ventricle | Normal |
| Interventricular septum | Intact | **M-Mode** |  |
| Interatrial septum | Intact | AO |  |
| Great arteries | NRGA | LA |  |
| Aorta | ------ | LVIDd | 37mm |
| Pulmonary artery | ------ | LVIDs | 22mm |
| Semilunal valves |  | IVSd | 4.9mm |
| Aortic valve | Normal | IVSs | 9.07mm |
| Pulmonary valve | Normal | LVPWd | 7.56mm |
| Doppler Measurement | | LVPWs | 7.56mm |
| Mitral | ----- | **EDV** | 58.5ml |
| Aortic | ------- | ESV | 16.8ml |
| Tricuspid | Trivial TR, PPG = 26mmHg | FS | 39% |
| pulmonic | ------ | LVEF | 71% |
| Aortic arch | Left | **Coronary arteries** | ------ |
| PDA | No PDA |  |  |
| Additional information |  | | |
| Final Diagnosis | 1. Normal Heart Study | | |

**Done by Signature**

**Name: Dr. Tesfaye T. Pediatric Cardiologist Date-06/11/2011 E.C**

| Pediatric Echocardiography report  Patient Name: Dejamach Abere Patient ID: 009968  Gender: M Age:12 years_ Date of Report: 09/11/2011Eth.C (TGSH7.3048) | | | |
| --- | --- | --- | --- |
| Features | **Finding** | **Features** | **Finding** |
| Profile | | **Atria** | |
| Abdominal situs | Solitus | Left atrium | Normal |
| Cardiac position | Levocardia | Right atrium | Normal |
| Systemic venous drainage | to RA | **Atrioventricular valves** | |
| Pulmonary venous drainage | to LA | Mitral valve | Annulus = 23cm |
| Atrioventricular connection | Concordant | Tricuspid valve | Annulus = 24mm, TAPSE = 17mm |
| Ventriculoarterial connection | Concordant | **Ventricles** | |
| Ventricular loop | d-Loop | Left ventricle | Normal |
| Septae | | Right ventricle | RV TDI S wave – 11cm/sec |
| Interventricular septum | Intact | **M-Mode** |  |
| Interatrial septum | Intact | AO |  |
| Great arteries | NRGA | LA |  |
| Aorta |  | LVIDd | 42mm |
| Pulmonary artery |  | LVIDs | 30.6mm |
| Semilunal valves |  | IVSd | 7.56mm |
| Aortic valve | Annulus = 18mm | IVSs | 9.0mm |
| Pulmonary valve | Annulus = 19mm | LVPWd | 7.56mm |
| Doppler Measurement | | LVPWs | 9.07mm |
| Mitral | No MS/MR | **EDV** | 78.6ml |
| Aortic | No AS/AR | ESV | 76.7ml |
| Tricuspid | Trivial TR, PPG = 16mmHg | FS | 27% |
| pulmonic | Trivial PR, PPG = 25mmHg | LVEF | 53% |
| Aortic arch | Left | **Coronary arteries** | ------- |
| PDA | No PDA |  |  |
| Additional information | No pericardial/pleural effusion | | |
| Final Diagnosis | 1. S/P Pericardiocentesis for Cardiac Tamponade (? Pyogenic) 2. Trivial TR 3. Trivial PR 4. Normal Heart Study | | |

**Done by Signature**

**Name: Dr. Tesfaye T. Pediatric Cardiologist Date-09/11/2011 E.C**

| Pediatric Echocardiography report  Patient Name: Tsinu kal Sisay Patient ID: 010892  Gender: F Age:2 years_ Date of Report: 09/11/2011Eth.C (TGSH7.3052.) | | | |
| --- | --- | --- | --- |
| Features | **Finding** | **Features** | **Finding** |
| Profile | | **Atria** | |
| Abdominal situs | Solitus | Left atrium | Normal |
| Cardiac position | Levocardia | Right atrium | Normal |
| Systemic venous drainage | to RA | **Atrioventricular valves** | |
| Pulmonary venous drainage | to LA | Mitral valve | Annulus = 14mm |
| Atrioventricular connection | Concordant | Tricuspid valve | Annulus = 15mm, TAPSE = 13mm |
| Ventriculoarterial connection | Concordant | **Ventricles** | |
| Ventricular loop | d-Loop | Left ventricle |  |
| Septae | | Right ventricle | RV TDI S wave = 12cm/sec |
| Interventricular septum | Intact | **M-Mode** |  |
| Interatrial septum | Intact | AO |  |
| Great arteries | NRGA | LA |  |
| Aorta | --- | LVIDd | 29mm |
| Pulmonary artery | ----- | LVIDs | 21mm |
| Semilunal valves |  | IVSd | 6mm |
| Aortic valve | Annulus = 12mm | IVSs | 6mm |
| Pulmonary valve | Annulus =13mm | LVPWd | 6mm |
| Doppler Measurement | | LVPWs | 6mm |
| Mitral | ------ | **EDV** | 32ml |
| Aortic | ------- | ESV | 14ml |
| Tricuspid | Trivial TR = 26mmHg | FS | 28% |
| pulmonic | ------ | LVEF | 56% |
| Aortic arch | Left | **Coronary arteries** | ------ |
| PDA | 1mm PDA, L – R Shunt with PPG/DPG = 75/40mmHg |  |  |
| Additional information | Follow up & IE prophylaxis | | |
| Final Diagnosis | 1. {S, D, S} Levocardia 2. Trivial TR 3. Small PDA | | |

**Done by Signature**

**Name: Dr. Tesfaye T. Pediatric Cardiologist Date-09/11/2011 E.C**

| Pediatric Echocardiography report  Patient Name: Workinesh Getahun Patient ID: 013026  Gender: F Age: 10years Date of Report: 09/11/2011Eth.C  Clinical Finding: Murmur + ARF. TGSH7.3065. | | | |
| --- | --- | --- | --- |
| Features | **Finding** | **Features** | **Finding** |
| Profile | | **Atria** | |
| Abdominal situs | Solitus | Left atrium | Dilated |
| Cardiac position | Levocardia | Right atrium | Normal |
| Systemic venous drainage | to RA | **Atrioventricular valves** | |
| Pulmonary venous drainage | to LA | Mitral valve | Annulus = 23mm. Patulous MVL |
| Atrioventricular connection | Concordant | Tricuspid valve | Annulus =19mm, TAPSE = 13mm |
| Ventriculoarterial connection | Concordant | **Ventricles** | |
| Ventricular loop | d-Loop | Left ventricle | Dilated |
| Septae | | Right ventricle | RV TDI S wave = 14cm/sec |
| Interventricular septum | Intact | **M-Mode** |  |
| Interatrial septum | Intact | AO |  |
| Great arteries | NRGA | LA |  |
| Aorta | ----- | LVIDd | 37mm |
| Pulmonary artery | ----- | LVIDs | 29mm |
| Semilunal valves |  | IVSd | 6.6mm |
| Aortic valve | Annulus = 18mm | IVSs | 7.6mm |
| Pulmonary valve | Annulus = 18mm | LVPWd | 6.6mm |
| Doppler Measurement | | LVPWs | 10mm |
| Mitral | Mild MR | **EDV** | 59.6ml |
| Aortic | Moderate AR, PHT = 387ms | ESV | 33.3ml |
| Tricuspid | Trivial TR = 23mmHg | FS | 21% |
| pulmonic | ------- | LVEF | 44% |
| Aortic arch | Left | **Coronary arteries** | ------ |
| PDA | No PDA |  |  |
| Additional information | No Coarctation | | |
| Final Diagnosis | 1. {S, D, S} Levocardia 2. Mild MR 3. Moderate AR 4. Normal RV function 5. Mildly Reduced LV Function | | |

**Done by Signature**

**Name: Dr. Tesfaye T. Pediatric Cardiologist**

**Date-09/11/2011 E.C**

| Pediatric Echocardiography report  Patient Name: Yetsedaw Walle Patient ID: 012806  Gender: M Age:5 years Date of Report: 09/11/2011Eth.C  Clinical Finding: CHF + RD + Murmur + Easy Fatigability + DOE. TGSH7.3066. | | | |
| --- | --- | --- | --- |
| Features | **Finding** | **Features** | **Finding** |
| Profile | | **Atria** | |
| Abdominal situs | Solitus | Left atrium | Normal |
| Cardiac position | Levocardia | Right atrium | Dilated, 81mm X 90mm |
| Systemic venous drainage | to RA | **Atrioventricular valves** | |
| Pulmonary venous drainage | to LA | Mitral valve | Annulus = 21mm |
| Atrioventricular connection | Concordant | Tricuspid valve | Annulus = 32mm, TAPSE = 9mm |
| Ventriculoarterial connection | Concordant | **Ventricles** | |
| Ventricular loop | d-Loop | Left ventricle | Dilated |
| Septae | | Right ventricle | Dilated, Dysfunctional, RV TDI S wave = 8cm/sec |
| Interventricular septum | Intact | **M-Mode** |  |
| Interatrial septum | Intact | AO |  |
| Great arteries | NRGA | LA |  |
| Aorta | ----- | LVIDd | 19mm |
| Pulmonary artery | ----- | LVIDs | 15mm |
| Semilunal valves |  | IVSd | 9.7mm |
| Aortic valve | Annulus = 15mm | IVSs | 8.7mm |
| Pulmonary valve | Annulus = 11mm, dysplastic. No post stenotic dilatation | LVPWd | 9.69mm |
| Doppler Measurement | | LVPWs | 9.69mm |
| Mitral | Mild to Moderate MR | **EDV** | 11ml |
| Aortic | No AR/AS | ESV | 6.6ml |
| Tricuspid | Severe TR, PPG = 116mmHg | FS | 18% |
| pulmonic | Mild PR, Severe PS, PPG/MPG = 92/58mmHg | LVEF | 40% |
| Aortic arch | Left | **Coronary arteries** | ------ |
| PDA | No PDA |  |  |
| Additional information | MV E/A ration = 0.7 | | |
| Final Diagnosis | 1. {S, D, S} Levocardia 2. Severe TR 3. Mild to Moderate MR 4. Mild PR 5. Severe PS 6. Hugely dilated RA 7. RV Dilated and Dysfunctional 8. LV Dysfunction (both systolic and diastolic) | | |

**Done by Signature**

**Name: Dr. Tesfaye T. Pediatric Cardiologist Date-09/11/2011 E.C.**

| Pediatric Echocardiography report  Patient Name: Le’elena Haile Patient ID: 013356  Gender: F Age: 7/12 Date of Report: 11/11/11Eth.C  Clinical Finding: RD. TGSH7.3067. | | | |
| --- | --- | --- | --- |
| Features | **Finding** | **Features** | **Finding** |
| Profile | | **Atria** | |
| Abdominal situs | Solitus | Left atrium | Normal |
| Cardiac position | Levocardia | Right atrium | Normal |
| Systemic venous drainage | to RA | **Atrioventricular valves** | |
| Pulmonary venous drainage | to LA | Mitral valve | Normal |
| Atrioventricular connection | Concordant | Tricuspid valve | TAPSE = 13mm |
| Ventriculoarterial connection | Concordant | **Ventricles** | |
| Ventricular loop | d-Loop | Left ventricle | Normal |
| Septae | | Right ventricle | Normal |
| Interventricular septum | Intact | **M-Mode** | Normal LV Function (eye balling) |
| Interatrial septum | Intact | AO |  |
| Great arteries | NRGA | LA |  |
| Aorta | ----- | LVIDd | mm |
| Pulmonary artery | Confluent branch PAs, MPA = 10mm, RPA = 8mm, LPA = 8mm | LVIDs | mm |
| Semilunal valves |  | IVSd | mm |
| Aortic valve | Normal | IVSs | mm |
| Pulmonary valve | Normal | LVPWd | mm |
| Doppler Measurement | | LVPWs | mm |
| Mitral | ----- | **EDV** | ml |
| Aortic | ----- | ESV | ml |
| Tricuspid | Trivial TR, PPG = 27mmHg | FS |  |
| pulmonic | ------- | LVEF |  |
| Aortic arch | Left | **Coronary arteries** | ------ |
| PDA | No |  |  |
| Additional information |  | | |
| Final Diagnosis | 1. Normal Heart Study | | |

**Done by Signature**

**Name: Dr. Tesfaye T. Pediatric Cardiologist Date-11/11/11 E.C**

| Pediatric Echocardiography report  Patient Name:B/ Kalkidan Nigussie Patient ID 034570  Gender: M Age: 40hrs Date of Report 15/05/12 E. C  Clinical Finding: RD. TGSH7.3068. | | | |
| --- | --- | --- | --- |
| Features | **Finding** | **Features** | **Finding** |
| Profile | | **Atria** | |
| Abdominal situs | Solitus | Left atrium | Normal |
| Cardiac position | Levocardia | Right atrium | Normal |
| Systemic venous drainage | to RA | **Atrioventricular valves** | |
| Pulmonary venous drainage | to LA | Mitral valve | Annulus = 25mm |
| Atrioventricular connection | Concordant | Tricuspid valve | Annulus =26mm , TAPSE = 23mm |
| Ventriculoarterial connection | Concordant | **Ventricles** | |
| Ventricular loop | d-Loop | Left ventricle | Mildly dilated |
| Septae | | Right ventricle | Normal |
| Interventricular septum | Intact | **M-Mode** |  |
| Interatrial septum | Intact | AO |  |
| Great arteries | NRGA | LA |  |
| Aorta | ----- | LVIDd | 42.1mm |
| Pulmonary artery | Confluent branch PAs | LVIDs | **32mm** |
| Semilunal valves |  | IVSd | **9.69mm** |
| Aortic valve | Annulus = 18mm | IVSs | 9.69mm |
| Pulmonary valve | Annulus = 24mm | LVPWd | **9.69mm** |
| Doppler Measurement | | LVPWs | 9.69mm |
| Mitral | Trivial MR | **EDV** | 79ml |
| Aortic | Mild AR, PHT = | ESV | 41ml |
| Tricuspid | Trivial TR, PPG= 27mmHg | FS | **24%** |
| pulmonic | ------- | LVEF | **48%** |
| Aortic arch | Left | **Coronary arteries** | ------ |
| PDA | No |  |  |
| Additional information | No coarctation | | |
| Final Diagnosis | 1. {S, D, S} Levocardia 2. Mild AR 3. Trivial MR 4. Trivial TR 5. Mildly Reduced LV Function | | |

**Done by Signature**

**Name: Dr. Tesfaye T. Pediatric Cardiologist Date-15/05/12 E.C**

| Pediatric Echocardiography report  Patient Name: Getasew Yayew Patient ID: 013736  Gender: M Age: 12years Date of Report: 11/11/11Eth.C  Clinical Finding: CHF + Murmur + RD + DOE + Easy Fatigability + Palpitation + Recurrence. TGSH7.3069. | | | |
| --- | --- | --- | --- |
| Features | **Finding** | **Features** | **Finding** |
| Profile | | **Atria** | |
| Abdominal situs | Solitus | Left atrium | Dilated, 53mm X 60mm |
| Cardiac position | Levocardia | Right atrium | 44mm X 39mm |
| Systemic venous drainage | to RA, IVC dilated | **Atrioventricular valves** | |
| Pulmonary venous drainage | to LA | Mitral valve | Annulus = 32mm, Thickened MVL |
| Atrioventricular connection | Concordant | Tricuspid valve | Annulus = 28mm, TAPSE = 19mm |
| Ventriculoarterial connection | Concordant | **Ventricles** | |
| Ventricular loop | d-Loop | Left ventricle | Dilated |
| Septae | | Right ventricle | Dilated |
| Interventricular septum | Intact | **M-Mode** |  |
| Interatrial septum | Intact | AO |  |
| Great arteries | NRGA | LA |  |
| Aorta | ----- | LVIDd | 55.5mm |
| Pulmonary artery | ----- | LVIDs | 41mm |
| Semilunal valves |  | IVSd | 10mm |
| Aortic valve | Trileaflet | IVSs | 10mm |
| Pulmonary valve | ------ | LVPWd | 10mm |
| Doppler Measurement | | LVPWs | 10mm |
| Mitral | Severe MR | **EDV** | 151ml |
| Aortic | Mild AR, PHT = 614ms | ESV | 75.5ml |
| Tricuspid | Severe TR, PPG = 113mmHg | FS | 25.6% |
| pulmonic | Mild PR, | LVEF | 50% |
| Aortic arch |  | **Coronary arteries** | ------ |
| PDA | No |  |  |
| Additional information |  | | |
| Final Diagnosis | 1. {S, D, S} Levocardia 2. Severe MR, Prolapsing PML 3. Severe TR 4. Mild AR 5. Mild PR 6. Severe Pulmonary Hypertension 7. Reduced LV Systolic Function | | |

**Done by Signature**

**Name: Dr. Tesfaye T. Pediatric Cardiologist**

**Date-11/11/11 E.C**

| Pediatric Echocardiography report  Patient Name: Asnakew Tarekegn Patient ID: 014115  Gender: M Age: 12yrs Date of Report: 16/11/11Eth.C  Clinical Finding: Rheumatic Recurrence + Murmur. TGSH7.3070. | | | |
| --- | --- | --- | --- |
| Features | **Finding** | **Features** | **Finding** |
| Profile | | **Atria** | |
| Abdominal situs | Solitus | Left atrium | Dilated |
| Cardiac position | Levocardia | Right atrium | Normal |
| Systemic venous drainage | to RA | **Atrioventricular valves** | |
| Pulmonary venous drainage | to LA | Mitral valve | Mildly thickened MVL |
| Atrioventricular connection | Concordant | Tricuspid valve | TAPSE = 23mm |
| Ventriculoarterial connection | Concordant | **Ventricles** | |
| Ventricular loop | d-Loop | Left ventricle | Dilated |
| Septae | | Right ventricle | Normal |
| Interventricular septum | Deviated to right | **M-Mode** |  |
| Interatrial septum | Intact | AO |  |
| Great arteries | NRGA | LA |  |
| Aorta | ----- | LVIDd | 49.7mm |
| Pulmonary artery | ----- | LVIDs | 35.4mm |
| Semilunal valves |  | IVSd | 8.64mm |
| Aortic valve | ---- | IVSs | 8.64mm |
| Pulmonary valve | ----- | LVPWd | 9.94mm |
| Doppler Measurement | | LVPWs | 10.8mm |
| Mitral | Mild to Moderate MR. Mild MS with PPG/MPG = 12/5mmHg | **EDV** | 117ml |
| Aortic | ----- | ESV | 52.3ml |
| Tricuspid | Trivial TR , PPG = 23mmHg | FS | 29% |
| pulmonic | ------- | LVEF | 55% |
| Aortic arch | Left | **Coronary arteries** | ------ |
| PDA | No |  |  |
| Additional information |  | | |
| Final Diagnosis | 1. {S, D, S} Levocardia 2. Mild to Moderate MR 3. Mild MS (? Functional) 4. Mildly thickened MVL 5. Good Biventricular Function | | |

**Done by Signature**

**Name: Dr. Tesfaye T. Pediatric Cardiologist Date-16/11/11 E.C**

| Pediatric Echocardiography report  Patient Name: Dagmawi Anmut Patient ID: 014216  Gender: M Age: 9months Date of Report: 16/11/2011Eth.C  Clinical Finding: CHF + RD + Tachycardia. TGSH7.3071. | | | |
| --- | --- | --- | --- |
| Features | **Finding** | **Features** | **Finding** |
| Profile | | **Atria** | |
| Abdominal situs | Solitus | Left atrium | Normal |
| Cardiac position | Levocardia | Right atrium | Normal |
| Systemic venous drainage | to RA | **Atrioventricular valves** | |
| Pulmonary venous drainage | to LA | Mitral valve | Normal |
| Atrioventricular connection | Concordant | Tricuspid valve | TAPSE = 15mm |
| Ventriculoarterial connection | Concordant | **Ventricles** | |
| Ventricular loop | d-Loop | Left ventricle | Dilated |
| Septae | | Right ventricle | Normal |
| Interventricular septum | Intact | **M-Mode** |  |
| Interatrial septum | Intact | AO |  |
| Great arteries | NRGA | LA |  |
| Aorta | ----- | LVIDd | **30mm** |
| Pulmonary artery | ----- | LVIDs | **23.6mm** |
| Semilunal valves |  | IVSd | **5.5mm** |
| Aortic valve | ----- | IVSs | 5.5mm |
| Pulmonary valve | --------- | LVPWd | 5.22mm |
| Doppler Measurement | | LVPWs | 5.5mm |
| Mitral | Mild MR | **EDV** | 35.6ml |
| Aortic | -------- | ESV | 19.3ml |
| Tricuspid | Mild TR, PPG = 48mmHg | FS | 22% |
| pulmonic | Trivial PR | LVEF | 46% |
| Aortic arch | LEFT | **Coronary arteries** | ------ |
| PDA | No |  |  |
| Additional information |  | | |
| Final Diagnosis | 1. {S, D, S} Levocardia 2. Mild MR 3. Mild TR 4. Moderate Pulmonary Hypertension (increased circulation) 5. Reduced LV Function | | |

**Done by Signature**

**Name: Dr. Tesfaye T. Pediatric Cardiologist Date-16/11/2011 E.C**

| Pediatric Echocardiography report  Patient Name: Zekarias Abiyot Patient ID: 006401  Gender: M Age: 3months Date of Report: 16/11/2011Eth.C  Clinical Finding: Incidental Murmur. TGSH7.3072. | | | |
| --- | --- | --- | --- |
| Features | **Finding** | **Features** | **Finding** |
| Profile | | **Atria** | |
| Abdominal situs | Solitus | Left atrium | Normal |
| Cardiac position | Levocardia | Right atrium | Normal |
| Systemic venous drainage | to RA | **Atrioventricular valves** | |
| Pulmonary venous drainage | to LA | Mitral valve | -------------- |
| Atrioventricular connection | Concordant | Tricuspid valve | -------------- |
| Ventriculoarterial connection | Concordant | **Ventricles** | |
| Ventricular loop | d-Loop | Left ventricle | Normal |
| Septae | | Right ventricle | Normal |
| Interventricular septum | 4mm PM VSD | **M-Mode** |  |
| Interatrial septum | Intact | AO |  |
| Great arteries | NRGA | LA |  |
| Aorta | ----- | LVIDd | 24.7mm |
| Pulmonary artery | ----- | LVIDs | 18.1mm |
| Semilunal valves |  | IVSd | 3.56mm |
| Aortic valve | ---------- | IVSs | 3.56mm |
| Pulmonary valve | --------------- | LVPWd | 3.56mm |
| Doppler Measurement | | LVPWs | 3.56mm |
| Mitral | ----- | **EDV** | 21.7ml |
| Aortic | ----- | ESV | 9.88ml |
| Tricuspid | ------ | FS | 27% |
| pulmonic | ------- | LVEF | 55% |
| Aortic arch | Left | **Coronary arteries** | ------ |
| PDA | No |  |  |
| Additional information |  | | |
| Final Diagnosis | 1. {S, D, S} Levocardia 2. Small Perimembranous VSD 3. Good Function | | |

**Done by Signature**

**Name: Dr. Tesfaye T. Pediatric Cardiologist Date: 16/11/2011 E.C**

| Pediatric Echocardiography report  Patient Name: Baby of Fentanesh Patient ID:013711  Gender: M Age: 3months Date of Report: 18/11/2011Eth.C  Clinical Finding: _DS + RD. TGSH7.3073. | | | |
| --- | --- | --- | --- |
| Features | **Finding** | **Features** | **Finding** |
| Profile | | **Atria** | |
| Abdominal situs | Solitus | Left atrium | Normal |
| Cardiac position | Levocardia | Right atrium | Normal |
| Systemic venous drainage | to RA | **Atrioventricular valves** | |
| Pulmonary venous drainage | to LA | Mitral valve | Normal |
| Atrioventricular connection | Concordant | Tricuspid valve | Normal |
| Ventriculoarterial connection | Concordant | **Ventricles** | |
| Ventricular loop | d-Loop | Left ventricle | Normal |
| Septae | | Right ventricle | Normal |
| Interventricular septum | Intact | **M-Mode : Normal LV Function on eye balling** | |
| Interatrial septum | Intact | AO |  |
| Great arteries | NRGA | LA |  |
| Aorta | ----- | LVIDd | mm |
| Pulmonary artery | Confluent branch PAs | LVIDs | mm |
| Semilunal valves |  | IVSd | mm |
| Aortic valve | Normal | IVSs | mm |
| Pulmonary valve | Normal | LVPWd | mm |
| Doppler Measurement | | LVPWs | mm |
| Mitral | -------- | **EDV** | ml |
| Aortic | --------- | ESV | ml |
| Tricuspid | -------- | FS |  |
| pulmonic | ------- | LVEF |  |
| Aortic arch | Left | **Coronary arteries** | ------ |
| PDA | No |  |  |
| Additional information | No coarctation of Aorta | | |
| Final Diagnosis | 1. NHS | | |

**Done by Signature**

**Name: Dr. Tesfaye T. Pediatric Cardiologist**

**Date-18/11/2011 E.C**

| Pediatric Echocardiography report  Patient Name: Addisse Gedif Patient ID: 014613  Gender: F Age:11years Date of Report: 23/11/2011Eth.C  Clinical Finding: Murmur + DOE + Easy Fatigability + Palpitation. TGSH7.3074. | | | |
| --- | --- | --- | --- |
| Features | **Finding** | **Features** | **Finding** |
| Profile | | **Atria** | |
| Abdominal situs | Solitus | Left atrium | Normal |
| Cardiac position | Dextrocardia | Right atrium | Normal |
| Systemic venous drainage | to RA | **Atrioventricular valves** | |
| Pulmonary venous drainage | to LA | Mitral valve | Annulus = 23mm |
| Atrioventricular connection | Concordant | Tricuspid valve | Annulus = 27mm, TAPSE= 22mm |
| Ventriculoarterial connection | Concordant | **Ventricles** | |
| Ventricular loop | d-Loop | Left ventricle | Normal |
| Septae | | Right ventricle | Normal |
| Interventricular septum | 7mm infra crista defect, L – R Shunt | **M-Mode** |  |
| Interatrial septum | Intact | AO |  |
| Great arteries | NRGA | LA |  |
| Aorta | ----- | LVIDd | 33.8mm |
| Pulmonary artery | ----- | LVIDs | 21.9mm |
| Semilunal valves |  | IVSd | 11.3mm |
| Aortic valve | Annulus = 25mm | IVSs | 11.3mm |
| Pulmonary valve | Annulus = 26mm | LVPWd | 11.3mm |
| Doppler Measurement | | LVPWs | 11.3mm |
| Mitral |  | **EDV** | 46.8ml |
| Aortic |  | ESV | 16ml |
| Tricuspid | Mild TR, PPG = 53mmHg | FS | 35% |
| pulmonic | Mild PR, PPG = 55mmHg  Mild PS, PPG/MPG = 23/9mmHg | LVEF | 65% |
| Aortic arch | ------ | **Coronary arteries** | ------ |
| PDA | No |  |  |
| Additional information |  | | |
| Final Diagnosis | 1. {S, D, S} Dextrocardia 2. Small Infra crista VSD, L – R Shunt 3. Mild PS 4. Mild PR 5. Good biventricular Function | | |

**Done by Signature**

**Name: Dr. Tesfaye T. Pediatric Cardiologist**

**Date-23/11/2011 E.C**

| Pediatric Echocardiography report  Patient Name: Ayenew Semaw Patient ID:014644  Gender: M Age: 14years Date of Report: 23/11/2011Eth.C  Clinical Finding: DOE. TGSH7.3075. | | | |
| --- | --- | --- | --- |
| Features | **Finding** | **Features** | **Finding** |
| Profile | | **Atria** | |
| Abdominal situs | Solitus | Left atrium | Normal |
| Cardiac position | Levocardia | Right atrium | Normal |
| Systemic venous drainage | to RA | **Atrioventricular valves** | |
| Pulmonary venous drainage | to LA | Mitral valve | Annulus = 26mm |
| Atrioventricular connection | Concordant | Tricuspid valve | Annulus = 25mm  TAPSE = 20mm |
| Ventriculoarterial connection | Concordant | **Ventricles** | |
| Ventricular loop | d-Loop | Left ventricle | Symmetric Hypertrophy |
| Septae | | Right ventricle | RV TDI S Wave = 10cm/sec |
| Interventricular septum | Intact | **M-Mode** |  |
| Interatrial septum | Intact | AO |  |
| Great arteries | NRGA | LA |  |
| Aorta | ----- | LVIDd | 48.4mm |
| Pulmonary artery | ----- | LVIDs | 30.8mm |
| Semilunal valves |  | IVSd | **16.1mm** |
| Aortic valve | Annulus = 19mm | IVSs | **18.1mm** |
| Pulmonary valve | Annulus = 22 | LVPWd | **12.6mm** |
| Doppler Measurement | | LVPWs | **20.7mm** |
| Mitral | ------ | **EDV** | 110ml |
| Aortic | ---- | ESV | 37.3ml |
| Tricuspid | ----- | FS | 36.4% |
| pulmonic | Trivial PR, PPG = 14mmHg | LVEF | 66% |
| Aortic arch | Left | **Coronary arteries** | ------ |
| PDA | No |  |  |
| Additional information | No coarctation of aorta | | |
| Final Diagnosis | 1. {S, D, S} Levocardia 2. Symmetric left ventricular hypertrophy 3. Good Biventricular Function | | |

**Done by Signature**

**Name: Dr. Tesfaye T. Pediatric Cardiologist Date-23/11/2011 E.C**

| Pediatric Echocardiography report  Patient Name: Emebet Tesfahun Patient ID: 14400  Gender: F Age: 14years Date of Report: 23/11/2011Eth.C  Clinical Finding: DOE + Rheumatic Recurrence + Palpitation. TGSH7.3076. | | | |
| --- | --- | --- | --- |
| Features | **Finding** | **Features** | **Finding** |
| Profile | | **Atria** | |
| Abdominal situs | Solitus | Left atrium | Dilated, 52 X 58mm |
| Cardiac position | Levocardia | Right atrium | Normal |
| Systemic venous drainage | to RA | **Atrioventricular valves** | |
| Pulmonary venous drainage | to LA | Mitral valve | Thickened, clubbed & deformed AMVL & Shortened and thickened PMVL. Annulus = 0.6cm2 |
| Atrioventricular connection | Concordant | Tricuspid valve | ------- |
| Ventriculoarterial connection | Concordant | **Ventricles** | |
| Ventricular loop | d-Loop | Left ventricle | Normal |
| Septae | | Right ventricle | Normal |
| Interventricular septum | Intact | **M-Mode** |  |
| Interatrial septum | Intact | AO |  |
| Great arteries | NRGA | LA |  |
| Aorta | ----- | LVIDd | 37.3mm |
| Pulmonary artery | ----- | LVIDs | 24.9mm |
| Semilunal valves |  | IVSd | 6.72mm |
| Aortic valve | ----- | IVSs | 8.07mm |
| Pulmonary valve | ------- | LVPWd | 6.72mm |
| Doppler Measurement | | LVPWs | 10.8mm |
| Mitral | Severe MS, PPG/MPG = 30/19mmHg. Mild MR | **EDV** | 59.3ml |
| Aortic | ----- | ESV | 22.1ml |
| Tricuspid | Trivial TR, PPG = 26mmHg | FS | 33% |
| pulmonic | ------- | LVEF | 62% |
| Aortic arch | Left | **Coronary arteries** | ------ |
| PDA | No |  |  |
| Additional information |  | | |
| Final Diagnosis | 1. {S, D, S} Levocardia 2. Severe MS 3. Mild MR 4. Trivial TR 5. Good Function | | |

**Done by Signature**

**Name: Dr. Tesfaye T. Pediatric Cardiologist Date-23/11/2011 E.C**

| Pediatric Echocardiography report  Patient Name: Emebet Mignonette Patient ID: 014873  Gender: F Age: 13years Date of Report: 23/11/2011Eth.C  Clinical Finding: _Sydenham’s Chorea. TGSH7.3077. | | | |
| --- | --- | --- | --- |
| Features | **Finding** | **Features** | **Finding** |
| Profile | | **Atria** | |
| Abdominal situs | Solitus | Left atrium | Normal |
| Cardiac position | Levocardia | Right atrium | Normal |
| Systemic venous drainage | to RA | **Atrioventricular valves** | |
| Pulmonary venous drainage | to LA | Mitral valve | Normal |
| Atrioventricular connection | Concordant | Tricuspid valve | TAPSE = 25mm |
| Ventriculoarterial connection | Concordant | **Ventricles** | |
| Ventricular loop | d-Loop | Left ventricle |  |
| Septae | | Right ventricle | RV TDI S wave = 12cm/sec |
| Interventricular septum |  | **M-Mode** |  |
| Interatrial septum |  | AO |  |
| Great arteries |  | LA |  |
| Aorta | ----- | LVIDd | 39.6mm |
| Pulmonary artery | ----- | LVIDs | 28.6mm |
| Semilunal valves |  | IVSd | 8.17mm |
| Aortic valve | Annulus = 20mm | IVSs | 12.3mm |
| Pulmonary valve | Annulus = 22mm | LVPWd | 8.17mm |
| Doppler Measurement | | LVPWs | 8.17mm |
| Mitral | ---- | **EDV** | 68.3ml |
| Aortic | ---- | ESV | 31.1ml |
| Tricuspid | ------ | FS | 28% |
| pulmonic | ------- | LVEF | 55% |
| Aortic arch | Left | **Coronary arteries** | ------ |
| PDA | No |  |  |
| Additional information |  | | |
| Final Diagnosis | 1. NHS (Normal Heart Study) | | |

**Done by Signature**

**Name: Dr. Tesfaye T. Pediatric Cardiologist**

**Date-23/11/2011 E.C**

| Pediatric Echocardiography report  Patient Name: Yirega Dagne Patient ID: 014799  Gender: M Age:14years Date of Report: 23/11/2011Eth.C  Clinical Finding: Easy Fatigability + sepsis. TGSH7.3078. | | | |
| --- | --- | --- | --- |
| Features | **Finding** | **Features** | **Finding** |
| Profile | | **Atria** | |
| Abdominal situs | Solitus | Left atrium | Normal |
| Cardiac position | Levocardia | Right atrium | Normal |
| Systemic venous drainage | to RA | **Atrioventricular valves** | |
| Pulmonary venous drainage | to LA | Mitral valve | Annulus = 27mm |
| Atrioventricular connection | Concordant | Tricuspid valve | Annulus = 32mm  TAPSE = 27mm |
| Ventriculoarterial connection | Concordant | **Ventricles** | |
| Ventricular loop | d-Loop | Left ventricle | Normal |
| Septae | | Right ventricle | Normal |
| Interventricular septum |  | **M-Mode** |  |
| Interatrial septum |  | AO |  |
| Great arteries |  | LA |  |
| Aorta | ----- | LVIDd | **55.3mm** |
| Pulmonary artery | ----- | LVIDs | **39.4mm** |
| Semilunal valves |  | IVSd | 8.82mm |
| Aortic valve | Normal | IVSs | 11.8mm |
| Pulmonary valve | Annulus = 22mm | LVPWd | 10mm |
| Doppler Measurement | | LVPWs | 14.1mm |
| Mitral | --------- | **EDV** | 149ml |
| Aortic | Trivial AR | ESV | 67.5ml |
| Tricuspid | Trivial TR, PPG = 18mmHg | FS | 28.8% |
| pulmonic | ------- | LVEF | 57% |
| Aortic arch | Left | **Coronary arteries** | ------ |
| PDA | No |  |  |
| Additional information | No Coarctation of Aorta | | |
| Final Diagnosis | 1. {S, D, S} Levocardia 2. Trivial AR 3. Trivial TR 4. Good Biventricular Function | | |

**Done by Signature**

**Name: Dr. Tesfaye T. Pediatric Cardiologist**

**Date-23/11/2011 E.C**

| Pediatric Echocardiography report  Patient Name: Bamlaku Asferaw Patient ID: 013789  Gender: M Age: 5months Date of Report: 25/11/2011Eth.C  Clinical Finding: _RD. TGSH7.3079. | | | |
| --- | --- | --- | --- |
| Features | **Finding** | **Features** | **Finding** |
| Profile | | **Atria** | |
| Abdominal situs | Solitus | Left atrium | Normal |
| Cardiac position | Levocardia | Right atrium | Normal |
| Systemic venous drainage | to RA | **Atrioventricular valves** | |
| Pulmonary venous drainage | to LA | Mitral valve | Annulus = 13mm |
| Atrioventricular connection | Concordant | Tricuspid valve | Annulus = 16mm  TAPSE = 17mm |
| Ventriculoarterial connection | Concordant | **Ventricles** | |
| Ventricular loop | d-Loop | Left ventricle | Normal |
| Septae | | Right ventricle | Normal |
| Interventricular septum | Intact | **M-Mode** |  |
| Interatrial septum | Intact | AO |  |
| Great arteries | NRGA | LA |  |
| Aorta | ----- | LVIDd | 22.3mm |
| Pulmonary artery | ----- | LVIDs | 17mm |
| Semilunal valves |  | IVSd | 7.56mm |
| Aortic valve | Annulus = 10mm | IVSs | 7.56mm |
| Pulmonary valve | Annulus = 11mm | LVPWd | 7.56mm |
| Doppler Measurement | | LVPWs | 7.56mm |
| Mitral | ----- | **EDV** | 16.8ml |
| Aortic | ------- | ESV | 8.39ml |
| Tricuspid | Trivial TR, PPG = 27mmHg | FS | 28% |
| pulmonic | ------- | LVEF | 55% |
| Aortic arch | Left | **Coronary arteries** | ------ |
| PDA | No |  |  |
| Additional information |  | | |
| Final Diagnosis | 1. Normal Heart Study | | |

**Done by Signature**

**Name: Dr. Tesfaye T. Pediatric Cardiologist**

**Date-25/11/2011 E.C**

| Pediatric Echocardiography report  Patient Name: Samrawit Yilkal Patient ID: 015595  Gender: F Age: 11months Date of Report: 02/12/2011Eth.C  Clinical Finding: _Cyanosis + RD. TGSH7.3080. | | | |
| --- | --- | --- | --- |
| Features | **Finding** | **Features** | **Finding** |
| Profile | | **Atria** | |
| Abdominal situs | Solitus | Left atrium |  |
| Cardiac position | Levocardia | Right atrium |  |
| Systemic venous drainage | to RA | **Atrioventricular valves** | |
| Pulmonary venous drainage | to LA | Mitral valve | Annulus = 22mm |
| Atrioventricular connection | Concordant | Tricuspid valve | Atretic |
| Ventriculoarterial connection | Concordant | **Ventricles** | |
| Ventricular loop | d-Loop | Left ventricle | Normal |
| Septae | | Right ventricle | Smallish |
| Interventricular septum | 8mm inlet VSD with L – R Shunt | **M-Mode** |  |
| Interatrial septum | 12mm OS ASD with R – L Shunt | AO |  |
| Great arteries | NRGA | LA |  |
| Aorta |  | LVIDd | 41.5mm |
| Pulmonary artery |  | LVIDs | 28.9mm |
| Semilunal valves |  | IVSd | 6.6mm |
| Aortic valve | Annulus = 14mm | IVSs | 6mm |
| Pulmonary valve | Annulus = 18mm | LVPWd | 5.5mm |
| Doppler Measurement | | LVPWs | 6mm |
| Mitral | ------ | **EDV** | 76ml |
| Aortic | ------ | ESV | 32ml |
| Tricuspid | No flow across the valve | FS | 30 |
| pulmonic | Flow acceleration across the pulmonary valve without outflow obstruction. | LVEF | 58 |
| Aortic arch | Left | **Coronary arteries** | ------ |
| PDA | No |  |  |
| Final Diagnosis | 1. {S, D, S} Levocardia 2. Ostium Secundum ASD, R – L Shunt 3. Inlet VSD, L- R Shunt 4. Tricuspid Atresia Type IC 5. Good LV Function | | |

**Done by Signature**

**Name: Dr. Tesfaye T. Pediatric Cardiologist Date-02/12/2011 E.C**

| Pediatric Echocardiography report  Patient Name: Mahlet Balew Patient ID: 015874  Gender: F Age: 3yrs Date of Report: 02/12/2011Eth.C  Clinical Finding: Diaphoresis + Murmur. TGSH7.3081. | | | |
| --- | --- | --- | --- |
| Features | **Finding** | **Features** | **Finding** |
| Profile | | **Atria** | |
| Abdominal situs | Solitus | Left atrium | Dilated |
| Cardiac position | Levocardia | Right atrium | Normal |
| Systemic venous drainage | to RA | **Atrioventricular valves** | |
| Pulmonary venous drainage | to LA | Mitral valve | Annulus = 17mm |
| Atrioventricular connection | Concordant | Tricuspid valve | Annulus = 17mm |
| Ventriculoarterial connection | Concordant | **Ventricles** | |
| Ventricular loop | d-Loop | Left ventricle | Dilated |
| Septae | | Right ventricle | Normal |
| Interventricular septum | Intact | **M-Mode** |  |
| Interatrial septum | Intact | AO |  |
| Great arteries | NRGA | LA |  |
| Aorta |  | LVIDd | 37.4mm |
| Pulmonary artery |  | LVIDs | 26.8mm |
| Semilunal valves |  | IVSd | 6.6mm |
| Aortic valve | Annulus = | IVSs | 6.6mm |
| Pulmonary valve | Annulus = 17mm | LVPWd | 6.3mm |
| Doppler Measurement | | LVPWs | 6.6mm |
| Mitral | ----- | **EDV** | 59.6ml |
| Aortic | ------ | ESV | 26.5ml |
| Tricuspid | Trivial TR, PPG = 20mmHg | FS | 28% |
| pulmonic | ------- | LVEF | 55% |
| Aortic arch | Left | **Coronary arteries** |  |
| PDA | 3mm PDA, L – R Shunt |  |  |
| Additional information | No coarctation of Aorta | | |
| Final Diagnosis | 1. {S, D, S} Levocardia 2. Large PDA, L – R Shunt | | |

**Done by Signature**

**Name: Dr. Tesfaye T. Pediatric Cardiologist**

**Date-02/12/2011 E.C**

| Pediatric Echocardiography report  Patient Name: Dimetros Ediget Patient ID: 015616  Gender: M Age: 68days Date of Report: 02/12/2011Eth.C  Clinical Finding: Incidental Murmur + RD. TGSH7.3082. | | | |
| --- | --- | --- | --- |
| Features | **Finding** | **Features** | **Finding** |
| Profile | | **Atria** | |
| Abdominal situs | Solitus | Left atrium | Dilated |
| Cardiac position | Levocardia | Right atrium | Normal |
| Systemic venous drainage | to RA | **Atrioventricular valves** | |
| Pulmonary venous drainage | to LA | Mitral valve | Annulus = 11mm |
| Atrioventricular connection | Concordant | Tricuspid valve | Annulus = 14mm  TAPSE = 12mm |
| Ventriculoarterial connection | Concordant | **Ventricles** | |
| Ventricular loop | d-Loop | Left ventricle | Dilated |
| Septae | | Right ventricle | Normal |
| Interventricular septum | Intact | **M-Mode** |  |
| Interatrial septum | 10mm OS ASD, L – R Shunt | AO |  |
| Great arteries | NRGA | LA |  |
| Aorta |  | LVIDd | 19.5mm |
| Pulmonary artery |  | LVIDs | 14mm |
| Semilunal valves |  | IVSd | 5.5mm |
| Aortic valve |  | IVSs | 5.2mm |
| Pulmonary valve |  | LVPWd | 5.5mm |
| Doppler Measurement | | LVPWs | 8mm |
| Mitral | --- | **EDV** | 12ml |
| Aortic | ----- | ESV | 5ml |
| Tricuspid | Trivial TR, PPG = 26mmHg | FS | 28% |
| pulmonic | Mild PS, PPG 46mmHg | LVEF | 57% |
| Aortic arch | Left | **Coronary arteries** |  |
| PDA | 2.5mm PDA, L – R Shunt |  |  |
| Additional information |  | | |
| Final Diagnosis | 1. {S, D, S} Levocardia 2. Moderate OS ASD, L – R Shunt 3. Mild PS 4. Moderate PDA, L – R Shunt | | |

**Done by Signature**

**Name: Dr. Tesfaye T. Pediatric Cardiologist**

**Date-02/12/2011 E.C**

| Pediatric Echocardiography report  Patient Name: Abriham hailemariam Patient ID: 002717  Gender: M Age: 3yrs Date of Report: 02/12/2011Eth.C  Clinical Finding: _Incidental Murmur. TGSH7.3083. | | | |
| --- | --- | --- | --- |
| Features | **Finding** | **Features** | **Finding** |
| Profile | | **Atria** | |
| Abdominal situs | Solitus | Left atrium | Normal |
| Cardiac position | Levocardia | Right atrium | Normal |
| Systemic venous drainage | to RA | **Atrioventricular valves** | |
| Pulmonary venous drainage | to LA | Mitral valve | Annulus = 16mm |
| Atrioventricular connection | Concordant | Tricuspid valve | Annulus = 19mm  TAPSE = 21mm |
| Ventriculoarterial connection | Concordant | **Ventricles** | |
| Ventricular loop | d-Loop | Left ventricle | Normal |
| Septae | | Right ventricle | Normal |
| Interventricular septum | 4mm Perimembranous VSD, L – R Shunt | **M-Mode** |  |
| Interatrial septum | Intact | AO |  |
| Great arteries | NRGA | LA |  |
| Aorta | --- | LVIDd | 33.3mm |
| Pulmonary artery | ------ | LVIDs | 23.1mm |
| Semilunal valves |  | IVSd | 7.56mm |
| Aortic valve | Annulus = 14mm | IVSs | 8.3mm |
| Pulmonary valve | Annulus = 17mm | LVPWd | 10.6mm |
| Doppler Measurement | | LVPWs | 9.45mm |
| Mitral | ----- | **EDV** | 45.1ml |
| Aortic | ----- | ESV | 18.3ml |
| Tricuspid | ------ | FS | 30% |
| pulmonic | ----------- | LVEF | 59% |
| Aortic arch | Left | **Coronary arteries** |  |
| PDA | No |  |  |
| Additional information |  | | |
| Final Diagnosis | 1. {S, D, S} Levocardia 2. Small Perimembranous VSD, L – R Shunt 3. Good Biventricular Function | | |

**Done by Signature**

**Name: Dr. Tesfaye T. Pediatric Cardiologist Date-02/12/2011 E.C**

| Pediatric Echocardiography report  Patient Name: Kalkidan Mulugeta Patient ID: 015938  Gender: F Age: 3months Date of Report: 09/12/2011Eth.C  Clinical Finding: RD. TGSH7.3084. | | | |
| --- | --- | --- | --- |
| Features | **Finding** | **Features** | **Finding** |
| Profile | | **Atria** | |
| Abdominal situs | Solitus | Left atrium | Normal |
| Cardiac position | Levocardia | Right atrium | Normal |
| Systemic venous drainage | to RA | **Atrioventricular valves** | |
| Pulmonary venous drainage | to LA | Mitral valve | Normal |
| Atrioventricular connection | Concordant | Tricuspid valve | Normal |
| Ventriculoarterial connection | Concordant | **Ventricles** | |
| Ventricular loop | d-Loop | Left ventricle |  |
| Septae | | Right ventricle |  |
| Interventricular septum | Intact | **M-Mode** |  |
| Interatrial septum | Intact | AO |  |
| Great arteries | NRGA | LA |  |
| Aorta | ------- | LVIDd | mm |
| Pulmonary artery | Normal sized confluent Branch PAs | LVIDs | mm |
| Semilunal valves |  | IVSd | mm |
| Aortic valve | Normal | IVSs | mm |
| Pulmonary valve | Normal | LVPWd | mm |
| Doppler Measurement | | LVPWs | mm |
| Mitral | ------ | **EDV** | ml |
| Aortic | ----- | ESV | ml |
| Tricuspid | Trivial TR | FS |  |
| pulmonic | ----- | LVEF |  |
| Aortic arch | Left | **Coronary arteries** |  |
| PDA | No | Normal LV Function (eye balling) | |
| Additional information |  | | |
| Final Diagnosis | Normal Heart Study | | |

**Done by Signature**

**Name: Dr. Tesfaye T. Pediatric Cardiologist Date-09/12/2011 E.C**

| Pediatric Echocardiography report  Patient Name: Zemenu Misganaw Patient ID: 016361  Gender: M Age: 76days Date of Report: 09/12/2011Eth.C  Clinical Finding: _Incidental Murmur. TGSH7.3085. | | | |
| --- | --- | --- | --- |
| Features | **Finding** | **Features** | **Finding** |
| Profile | | **Atria** | |
| Abdominal situs | Solitus | Left atrium | Normal |
| Cardiac position | Levocardia | Right atrium | Normal |
| Systemic venous drainage | to RA | **Atrioventricular valves** | |
| Pulmonary venous drainage | to LA | Mitral valve | Annulus =14mm |
| Atrioventricular connection | Concordant | Tricuspid valve | Annulus = 16mm |
| Ventriculoarterial connection | Concordant | **Ventricles** | |
| Ventricular loop | d-Loop | Left ventricle | Normal |
| Septae | | Right ventricle | Normal |
| Interventricular septum | 3mm PM VSD, L- R Shunt | **M-Mode** |  |
| Interatrial septum | PFO, L- R Shunt | AO |  |
| Great arteries | NRGA | LA |  |
| Aorta | --- | LVIDd | 25.5mm |
| Pulmonary artery | Dilated | LVIDs | 17.4mm |
| Semilunal valves |  | IVSd | 5.04mm |
| Aortic valve | Annulus =11mm | IVSs | 5.04mm |
| Pulmonary valve | Annulus = 13mm | LVPWd | 5.04mm |
| Doppler Measurement | | LVPWs | 5.04mm |
| Mitral | ---- | **EDV** | 23.4ml |
| Aortic | ----- | ESV | 8.91ml |
| Tricuspid | ----- | FS | 31.8% |
| pulmonic | ---- | LVEF | 62% |
| Aortic arch | Left | **Coronary arteries** |  |
| PDA | No |  |  |
| Additional information | Baby was crying | | |
| Final Diagnosis | 1. {S, D, S} Levocardia 2. PFO, L – R Shunt 3. Small Perimembranous VSD, L – R Shunt 4. Normal LV Function | | |

**Done by Signature**

**Name: Dr. Tesfaye T. Pediatric Cardiologist Date-09/12/2011 E.C**

| Pediatric Echocardiography report  Patient Name: Baby of Etenat Patient ID: 015936  Gender: F Age: 9days Date of Report: 09/12/2011Eth.C  Clinical Finding: _RD. TGSH7.3086. | | | |
| --- | --- | --- | --- |
| Features | **Finding** | **Features** | **Finding** |
| Profile | | **Atria** | |
| Abdominal situs | Solitus | Left atrium | Normal |
| Cardiac position | Levocardia | Right atrium | Normal |
| Systemic venous drainage | to RA | **Atrioventricular valves** | |
| Pulmonary venous drainage | to LA | Mitral valve | Annulus 10mm |
| Atrioventricular connection | Concordant | Tricuspid valve | Annulus = 10mm |
| Ventriculoarterial connection | Concordant | **Ventricles** | |
| Ventricular loop | d-Loop | Left ventricle | Normal |
| Septae | | Right ventricle | Normal |
| Interventricular septum | Intact | **M-Mode** |  |
| Interatrial septum | Intact | AO |  |
| Great arteries | NRGA | LA |  |
| Aorta |  | LVIDd | mm |
| Pulmonary artery |  | LVIDs | mm |
| Semilunal valves |  | IVSd | mm |
| Aortic valve | Annulus = 10mm | IVSs | mm |
| Pulmonary valve | Annulus = 12mm | LVPWd | mm |
| Doppler Measurement | | LVPWs | mm |
| Mitral | ---- | **EDV** | ml |
| Aortic | ---- | ESV | ml |
| Tricuspid | Trivial TR, PPG = 25mmHg | FS | 28% |
| pulmonic | ------- | LVEF | 57% |
| Aortic arch | Left | **Coronary arteries** |  |
| PDA | No |  |  |
| Additional information |  | | |
| Final Diagnosis | Normal Echocardiography | | |

**Done by Signature**

**Name: Dr. Tesfaye T. Pediatric Cardiologist Date-09/12/2011 E.C**

| Pediatric Echocardiography report  Patient Name: Temesgen Simachew Patient ID: 016050  Gender: M Age: 1yrs Date of Report: 09/12/2011Eth.C  Clinical Finding: RD + CHF + Accentuated P2 + Murmur. TGSH7.3087. | | | |
| --- | --- | --- | --- |
| Features | **Finding** | **Features** | **Finding** |
| Profile | | **Atria** | |
| Abdominal situs | Solitus | Left atrium | Normal |
| Cardiac position | Levocardia | Right atrium | Dilated, 44 X 35mm |
| Systemic venous drainage | to RA | **Atrioventricular valves** | |
| Pulmonary venous drainage | to LA | Mitral valve | Annulus = 13mm |
| Atrioventricular connection | Concordant | Tricuspid valve | Annulus = 18mm  TAPSE = 5mm |
| Ventriculoarterial connection | Concordant | **Ventricles** | |
| Ventricular loop | d-Loop | Left ventricle | Normal |
| Septae | | Right ventricle | Dilated, Hypertrophied & Dysfunctional |
| Interventricular septum | Intact | **M-Mode** |  |
| Interatrial septum | Intact | AO |  |
| Great arteries | NRGA | LA |  |
| Aorta |  | LVIDd | 25.5mm |
| Pulmonary artery | MPA Dilated | LVIDs | 17.5mm |
| Semilunal valves |  | IVSd | 7.61mm |
| Aortic valve | Normal | IVSs | 7.94mm |
| Pulmonary valve | Annulus = 16mm | LVPWd | 6.62mm |
| Doppler Measurement | | LVPWs | 6.62mm |
| Mitral | ---- | **EDV** | 23.4ml |
| Aortic | --- | ESV | 9.04ml |
| Tricuspid | Mild TR, PPG = 78mmHg | FS | 31% |
| pulmonic | Moderate PR, PPG = 58mmHg | LVEF | 61% |
| Aortic arch | Left | **Coronary arteries** |  |
| PDA | No |  |  |
| Additional information | 24mm Right Pleural effusion | | |
| Final Diagnosis | 1. {S, D, S} Levocardia 2. Mild TR 3. Moderate PR 4. Severe Pulmonary Hypertension 5. Hypertrophied, dilated, Dysfunctional RV 6. Good LV Function | | |

**Done by Signature**

**Name: Dr. Tesfaye T. Pediatric Cardiologist Date-09/12/2011 E.C**

| Pediatric Echocardiography report  Patient Name: baby of Hadja Patient ID:  Gender: M Age: 11days Date of Report: 09/12/2011Eth.C  Clinical Finding: RD + Incidental Murmur + DS. TGSH7.3088. | | | |
| --- | --- | --- | --- |
| Features | **Finding** | **Features** | **Finding** |
| Profile | | **Atria** | |
| Abdominal situs | Solitus | Left atrium | Dilated |
| Cardiac position | Levocardia | Right atrium | Normal |
| Systemic venous drainage | to RA | **Atrioventricular valves** | |
| Pulmonary venous drainage | to LA | Mitral valve | Annulus = 11mm |
| Atrioventricular connection | Concordant | Tricuspid valve | Annulus =13mm  TAPSE = 8mm |
| Ventriculoarterial connection | Concordant | **Ventricles** | |
| Ventricular loop | d-Loop | Left ventricle | Dilated |
| Septae | | Right ventricle | Normal |
| Interventricular septum | Intact | **M-Mode** |  |
| Interatrial septum | Intact | AO |  |
| Great arteries | NRGA | LA |  |
| Aorta |  | LVIDd | 15.1mm |
| Pulmonary artery |  | LVIDs | 13.1mm |
| Semilunal valves |  | IVSd | 6.05mm |
| Aortic valve | Annulus = 7mm | IVSs | 5.04mm |
| Pulmonary valve | Annulus = 12mm | LVPWd | 5.04mm |
| Doppler Measurement | | LVPWs | 5.04mm |
| Mitral | Trivial MR | **EDV** | 6.16ml |
| Aortic | Mild AR | ESV | 4.24ml |
| Tricuspid | Trivial TR, PPG = 19mmHg | FS | 13.2% |
| pulmonic | Mild PR, PPG = 15mmHg | LVEF | 31.2% |
| Aortic arch | Left | **Coronary arteries** |  |
| PDA | 1.5mm PDA, L- R Shunt |  |  |
| Additional information | Turbulent flow at the CS opening with gradient of PPG/MPG = 9/5mmHg | | |
| Final Diagnosis | 1. {S, D, S} Levocardia 2. Coronary Ostium stenosis 3. Mild AR 4. Mild PR 5. Trivial TR 6. Trivial MR 7. Reduced LV Function | | |

**Done by Signature**

**Name: Dr. Tesfaye T. Pediatric Cardiologist Date-09/12/2011 E.C**

| Pediatric Echocardiography report  Patient Name: Kidest Yazzie Patient ID: 012716  Gender: F Age: 8yrs Date of Report: 09/12/2011Eth.C  Clinical Finding: Palpitation + arrhythmia. TGSH7.3089. | | | |
| --- | --- | --- | --- |
| Features | **Finding** | **Features** | **Finding** |
| Profile | | **Atria** | |
| Abdominal situs | Solitus | Left atrium | Normal |
| Cardiac position | Levocardia | Right atrium | Normal |
| Systemic venous drainage | to RA | **Atrioventricular valves** | |
| Pulmonary venous drainage | to LA | Mitral valve | Annulus =21mm |
| Atrioventricular connection | Concordant | Tricuspid valve | Annulus = 21mm |
| Ventriculoarterial connection | Concordant | **Ventricles** | |
| Ventricular loop | d-Loop | Left ventricle | Normal |
| Septae | | Right ventricle | Normal |
| Interventricular septum | Intact | **M-Mode** |  |
| Interatrial septum | Intact | AO |  |
| Great arteries | NRGA | LA |  |
| Aorta |  | LVIDd | 31mm |
| Pulmonary artery |  | LVIDs | 21.2mm |
| Semilunal valves |  | IVSd | 7.56mm |
| Aortic valve | Annulus = 17mm | IVSs | 7.56mm |
| Pulmonary valve | Annulus = 17mm | LVPWd | 6.05mm |
| Doppler Measurement | | LVPWs | 8.32mm |
| Mitral | ---- | **EDV** | 37.9ml |
| Aortic | ---- | ESV | 14.8ml |
| Tricuspid | Trivial TR, PPG = 18mmHg | FS | 31% |
| pulmonic | ----- | LVEF | 60% |
| Aortic arch | Left | **Coronary arteries** |  |
| PDA | No |  |  |
| Additional information |  | | |
| Final Diagnosis | Normal Echocardiographic Study | | |

**Done by Signature**

**Name: Dr. Tesfaye T. Pediatric Cardiologist Date-09/12/2011 E.C**

| Pediatric Echocardiography report  Patient Name: Dagim Kassie Patient ID: 016791  Gender: M Age: 14yrs Date of Report: 16/12/2011Eth.C  Clinical Finding: Easy Fatigability. TGSH7.3090. | | | |
| --- | --- | --- | --- |
| Features | **Finding** | **Features** | **Finding** |
| Profile | | **Atria** | |
| Abdominal situs | Solitus | Left atrium | Normal |
| Cardiac position | Levocardia | Right atrium | Normal |
| Systemic venous drainage | to RA | **Atrioventricular valves** | |
| Pulmonary venous drainage | to LA | Mitral valve | Annulus = 20mm |
| Atrioventricular connection | Concordant | Tricuspid valve | Annulus = 22mm  TAPSE = 20mm |
| Ventriculoarterial connection | Concordant | **Ventricles** | |
| Ventricular loop | d-Loop | Left ventricle | Normal |
| Septae | | Right ventricle | Normal |
| Interventricular septum | intact | **M-Mode** |  |
| Interatrial septum | Intact | AO |  |
| Great arteries | NRGA | LA |  |
| Aorta | ---- | LVIDd | 32.5mm |
| Pulmonary artery | ---- | LVIDs | 23.7mm |
| Semilunal valves |  | IVSd | 9.69mm |
| Aortic valve | Annulus = 20mm | IVSs | 10.7mm |
| Pulmonary valve | Annulus = 24mm | LVPWd | 11.6mm |
| Doppler Measurement | | LVPWs | 11.6mm |
| Mitral | ---- | **EDV** | 42.5ml |
| Aortic | ---- | ESV | 19.5ml |
| Tricuspid | ---- | FS | 29% |
| pulmonic | ---- | LVEF | 59% |
| Aortic arch | Left | **Coronary arteries** |  |
| PDA | No |  |  |
| Additional information |  | | |
| Final Diagnosis | 1. Normal Echocardiographic Study | | |

**Done by Signature**

**Name: Dr. Tesfaye T. Pediatric Cardiologist Date-16/12/2011 E.C**

| Pediatric Echocardiography report  Patient Name: Nardos adisu Patient ID: 008004  Gender: F Age: 1- 6/12. Date of Report: 16/12/2011Eth.C  Clinical Finding: _RD + CHF + Shock. TGSH7.3091. (TGSH7 DOWN) | | | |
| --- | --- | --- | --- |
| Features | **Finding** | **Features** | **Finding** |
| Profile | | **Atria** | |
| Abdominal situs | Solitus | Left atrium | Dilated |
| Cardiac position | Levocardia | Right atrium | Normal |
| Systemic venous drainage | to RA | **Atrioventricular valves** | |
| Pulmonary venous drainage | to LA | Mitral valve | Annulus = 21mm |
| Atrioventricular connection | Concordant | Tricuspid valve | Annulus =13mm  TAPSE = 10mm |
| Ventriculoarterial connection | Concordant | **Ventricles** | |
| Ventricular loop | d-Loop | Left ventricle | Dilated, Dysfunctional |
| Septae | | Right ventricle | Normal |
| Interventricular septum | Intact | **M-Mode** |  |
| Interatrial septum | Intact | AO |  |
| Great arteries | NRGA | LA |  |
| Aorta |  | LVIDd | 45mm |
| Pulmonary artery | Normal sized confluent Branch PAs | LVIDs | 38.2mm |
| Semilunal valves |  | IVSd | 6.05mm |
| Aortic valve | Annulus = 12mm, Trileaflet | IVSs | 7.56mm |
| Pulmonary valve | Annulus = 15mm | LVPWd | 7.56mm |
| Doppler Measurement | | LVPWs | 7.56mm |
| Mitral | Mild MR | **EDV** | 92.4ml |
| Aortic | ----- | ESV | 62.7ml |
| Tricuspid | Trivial TR, PPG = 26mmHg | FS | 15% |
| pulmonic | ---- | LVEF | 32% |
| Aortic arch | Left | **Coronary arteries** | No ALCAPA |
| PDA | No |  |  |
| Additional information | No pleural/pericardial effusion | | |
| Final Diagnosis | 1. {S, D, S} Levocardia 2. Mild MR 3. Trivial TR 4. Dilated LA 5. Dilated Dysfunction LV with reduced EF | | |

**Done by Signature**

**Name: Dr. Tesfaye T. Pediatric Cardiologist Date-16/12/2011 E.C**

| Pediatric Echocardiography report  Patient Name: Yeshizerf Chaklie Patient ID: 16972  Gender: Female Age: 6years Date of Report: 16/12/2011Eth.C  Clinical Finding: Easy Fatigability. TGSH7.3092. | | | |
| --- | --- | --- | --- |
| Features | **Finding** | **Features** | **Finding** |
| Profile | | **Atria** | |
| Abdominal situs | Solitus | Left atrium | Normal |
| Cardiac position | Levocardia | Right atrium | Normal |
| Systemic venous drainage | to RA | **Atrioventricular valves** | |
| Pulmonary venous drainage | to LA | Mitral valve | Annulus = 17mm |
| Atrioventricular connection | Concordant | Tricuspid valve | Annulus =17mm |
| Ventriculoarterial connection | Concordant | **Ventricles** | |
| Ventricular loop | d-Loop | Left ventricle |  |
| Septae | | Right ventricle |  |
| Interventricular septum | Intact | **M-Mode** |  |
| Interatrial septum | Intact | AO |  |
| Great arteries | NRGA | LA |  |
| Aorta | --- | LVIDd | 32.9mm |
| Pulmonary artery | --- | LVIDs | 23.4mm |
| Semilunal valves |  | IVSd | 6.81mm |
| Aortic valve | Annulus = 16mm, Trileaflet | IVSs | 7.56mm |
| Pulmonary valve | Annulus = 19mm | LVPWd | 4.92mm |
| Doppler Measurement | | LVPWs | 7.56mm |
| Mitral | --- | **EDV** | 43.8ml |
| Aortic | ---- | ESV | 18.9ml |
| Tricuspid | Trivial TR, PPG = 21mmHg | FS | 29% |
| pulmonic |  | LVEF | 56% |
| Aortic arch | Left | **Coronary arteries** |  |
| PDA | No |  |  |
| Additional information |  | | |
| Final Diagnosis | 1. Normal Echocardiographic Study | | |

**Done by Signature**

**Name: Dr. Tesfaye T. Pediatric Cardiologist Date-16/12/2011 E.C**

| Pediatric Echocardiography report  Patient Name: Kassahun Chekole Patient ID: 016503  Gender: M Age: 9months Date of Report: 16/12/2011Eth.C  Clinical Finding: DS + RD. TGSH7.3093. | | | |
| --- | --- | --- | --- |
| Features | **Finding** | **Features** | **Finding** |
| Profile | | **Atria** | |
| Abdominal situs | Solitus | Left atrium | Normal |
| Cardiac position | Levocardia | Right atrium | Dilated |
| Systemic venous drainage | to RA | **Atrioventricular valves** | |
| Pulmonary venous drainage | to LA | Mitral valve | Annulus = 10mm |
| Atrioventricular connection | Concordant | Tricuspid valve | Annulus = 11mm  TAPSE = 13mm |
| Ventriculoarterial connection | Concordant | **Ventricles** | |
| Ventricular loop | d-Loop | Left ventricle | Normal |
| Septae | | Right ventricle | Dilated |
| Interventricular septum |  | **M-Mode** |  |
| Interatrial septum | Intact | AO |  |
| Great arteries | NRGA | LA |  |
| Aorta |  | LVIDd | 19.7mm |
| Pulmonary artery |  | LVIDs | 13.6mm |
| Semilunal valves |  | IVSd | **7.15mm** |
| Aortic valve | Annulus = 11mm | IVSs | 7.15mm |
| Pulmonary valve | Annulus = 13mm | LVPWd | **7.15mm** |
| Doppler Measurement | | LVPWs | 7.15mm |
| Mitral | ---- | **EDV** | 12.2ml |
| Aortic | ------ | ESV | 4.68ml |
| Tricuspid | Mild to Grade II TR, PPG = 58mmHg | FS | 31% |
| pulmonic | Mild PR, PPG = 52mmHg | LVEF | 61% |
| Aortic arch | Left | **Coronary arteries** |  |
| PDA | No |  |  |
| Additional information |  | | |
| Final Diagnosis | 1. {S, D, S} Levocardia 2. RA/RV Dilated 3. Mild to Grade II TR 4. Mild PR 5. Moderate Pulmonary Hypertension | | |

**Done by Signature**

**Name: Dr. Tesfaye T. Pediatric Cardiologist Date-16/12/2011 E.C**

| Pediatric Echocardiography report  Patient Name: Mubarak Abraham Patient ID: 017336  Gender: M Age: 7yrs Date of Report: 20/12/2011Eth.C  Clinical Finding: Chest Pain + Pericardial friction rub. TGSH7.3094. | | | |
| --- | --- | --- | --- |
| Features | **Finding** | **Features** | **Finding** |
| Profile | | **Atria** | |
| Abdominal situs | Solitus | Left atrium | Normal |
| Cardiac position | Levocardia | Right atrium | Normal |
| Systemic venous drainage | to RA | **Atrioventricular valves** | |
| Pulmonary venous drainage | to LA | Mitral valve | Annulus = 17mm |
| Atrioventricular connection | Concordant | Tricuspid valve | Annulus = 22mm  TAPSE = 13mm |
| Ventriculoarterial connection | Concordant | **Ventricles** | |
| Ventricular loop | d-Loop | Left ventricle | Normal |
| Septae | | Right ventricle | Normal |
| Interventricular septum | Intact | **M-Mode** |  |
| Interatrial septum | Intact | AO |  |
| Great arteries | NRGA | LA |  |
| Aorta |  | LVIDd | 33.5mm |
| Pulmonary artery |  | LVIDs | 22.8mm |
| Semilunal valves |  | IVSd | 5.5mm |
| Aortic valve | Annulus = 15mm, Trileaflet | IVSs | 9.35mm |
| Pulmonary valve | Annulus = 19mm | LVPWd | 8.5mm |
| Doppler Measurement | | LVPWs | 11.5mm |
| Mitral | Trivial MR | **EDV** | 45.8ml |
| Aortic | ---- | ESV | 17.7ml |
| Tricuspid | Mild TR, PPG = 28mmHg | FS | 32% |
| pulmonic | ----- | LVEF | 61% |
| Aortic arch | Left | **Coronary arteries** |  |
| PDA | No |  |  |
| Additional information | 6.5mm pericardial effusion on RV side and 3 mm on LV side  12mm right pleural effusion | | |
| Final Diagnosis | 1. {S, D, S} Levocardia 2. Small Pericardial Effusion 3. Right Pleural Effusion 4. Good Biventricular Function | | |

**Done by Signature**

**Name: Dr. Tesfaye T. Pediatric Cardiologist Date-20/12/2011 E.C**

| Pediatric Echocardiography report  Patient Name: Temechew Kefale Patient ID: 017241  Gender: M Age: 13Years Date of Report: 21/12/2011Eth.C  BP: Weight: Height: BSA: TGSH1.2638 | | | |
| --- | --- | --- | --- |
| Features | **Finding** | **Features** | **Finding** |
| Profile | | **Atria** | |
| Abdominal situs | Solitus | Left atrium | Normal |
| Cardiac position | Levocardia | Right atrium | Normal |
| Systemic venous drainage | to RA | **Atrioventricular valves** | |
| Pulmonary venous drainage | to LA | Mitral valve | Thickened |
| Atrioventricular connection | Concordant | Tricuspid valve | Normal  TAPSE = 22mm |
| Ventriculoarterial connection | Concordant | **Ventricles** | |
| Ventricular loop | d-Loop | Left ventricle |  |
| Septae | | Right ventricle |  |
| Interventricular septum | Intact | **M-Mode** |  |
| Interatrial septum | Intact | AO |  |
| Great arteries | NRGA | LA |  |
| Aorta |  | LVIDd | 44.5mm |
| Pulmonary artery | Normal sized confluent Branch PAs | LVIDs | 32.8mm |
| Semilunal valves |  | IVSd | 8.64mm |
| Aortic valve | Annulus = 16mm, | IVSs | 8.64mm |
| Pulmonary valve | Annulus = 25mm | LVPWd | 8.64mm |
| Doppler Measurement | | LVPWs | 8.64mm |
| Mitral | Mild MR, Posterior jet | **EDV** | 90.1ml |
| Aortic | ---- | ESV | 43.5ml |
| Tricuspid | Mild TR, PPG = 28mmHg | FS | 26% |
| pulmonic | Mild PR, PPG = 10mmHg | LVEF | 51% |
| Aortic arch | Left | **Coronary arteries** |  |
| PDA | No |  |  |
| Additional information |  | | |
| Final Diagnosis | 1. {S, D, S} Levocardia 2. Thickened Mitral valve, Mild MR 3. Mild TR 4. Mild PR 5. Good Biventricular Function | | |

**Done by Signature**

**Name: Dr. Tesfaye T. Pediatric Cardiologist Date-21/12/2011 E.C**

| Pediatric Echocardiography report  Patient Name: Temesgen Abebe Patient ID: 017342  Gender: M Age: 9months Date of Report: 21/12/2011Eth.C  Clinical Finding: DS + CHF + RD + Recurrent Chest Infection + Murmur. TGSH7.3095. | | | |
| --- | --- | --- | --- |
| Features | **Finding** | **Features** | **Finding** |
| Profile | | **Atria** | |
| Abdominal situs | Solitus | Left atrium | Normal |
| Cardiac position | Levocardia | Right atrium | Dilated, 31 X 32mm |
| Systemic venous drainage | to RA | **Atrioventricular valves** | |
| Pulmonary venous drainage | to LA | Mitral valve | Annulus = 11mm |
| Atrioventricular connection | Concordant | Tricuspid valve | Annulus = 19mm  TAPSE = 22mm |
| Ventriculoarterial connection | Concordant | **Ventricles** | |
| Ventricular loop | d-Loop | Left ventricle | Normal |
| Septae | | Right ventricle | Dilated |
| Interventricular septum | Abnormal septal motion | **M-Mode: Normal LV Function on eye balling (Abnormal septal motion)** | |
| Interatrial septum | 7mm OS ASD, R – L Shunt | AO |  |
| Great arteries | NRGA | LA |  |
| Aorta |  | LVIDd | mm |
| Pulmonary artery | Dilated MPA. Confluent Branch PAs | LVIDs | mm |
| Semilunal valves |  | IVSd | mm |
| Aortic valve | Normal | IVSs | mm |
| Pulmonary valve | Annulus = 13mm | LVPWd | mm |
| Doppler Measurement | | LVPWs | mm |
| Mitral | ---- | **EDV** | ml |
| Aortic | ---- | ESV | ml |
| Tricuspid | Severe TR, PPG = 101mmHg | FS |  |
| pulmonic | Mild PR, PPG = 63mmHg | LVEF |  |
| Aortic arch | Left | **Coronary arteries** |  |
| PDA | No |  |  |
| Additional information |  | | |
| Final Diagnosis | 1. {S, D, S} Levocardia 2. Small OS ASD, R – L Shunt 3. RA/RV Dilated, Dilated MPA 4. Severe TR, Mild PR 5. Severe Pulmonary Hypertension secondary to ? | | |

**Done by Signature**

**Name: Dr. Tesfaye T. Pediatric Cardiologist Date-21/12/2011 E.C**

| Pediatric Echocardiography report  Patient Name: Yared Atinkut Patient ID: 017270  Gender: M Age: 9months Date of Report: 21/12/2011Eth.C  Clinical Finding: DS. TGSH7.3096. | | | |
| --- | --- | --- | --- |
| Features | **Finding** | **Features** | **Finding** |
| Profile | | **Atria** | |
| Abdominal situs | Solitus | Left atrium | Normal |
| Cardiac position | Levocardia | Right atrium | Normal |
| Systemic venous drainage | to RA | **Atrioventricular valves** | |
| Pulmonary venous drainage | to LA | Mitral valve | Normal |
| Atrioventricular connection | Concordant | Tricuspid valve | Normal |
| Ventriculoarterial connection | Concordant | **Ventricles** | |
| Ventricular loop | d-Loop | Left ventricle |  |
| Septae | | Right ventricle |  |
| Interventricular septum | Intact | **M-Mode** | Normal LV Function |
| Interatrial septum | PFO, L – R shunt | AO |  |
| Great arteries | NRGA | LA |  |
| Aorta | ---- | LVIDd | mm |
| Pulmonary artery | ---- | LVIDs | mm |
| Semilunal valves |  | IVSd | mm |
| Aortic valve | Normal | IVSs | mm |
| Pulmonary valve | Normal | LVPWd | mm |
| Doppler Measurement | | LVPWs | mm |
| Mitral | ---- | **EDV** | ml |
| Aortic | ---- | ESV | ml |
| Tricuspid | ---- | FS |  |
| pulmonic | ---- | LVEF |  |
| Aortic arch | Left | **Coronary arteries** |  |
| PDA | No |  |  |
| Additional information |  | | |
| Final Diagnosis | PFO | | |

**Done by Signature**

**Name: Dr. Tesfaye T. Pediatric Cardiologist Date-21/12/2011 E.C**

| Pediatric Echocardiography report  Patient Name: Amar Seid Patient ID: 017301  Gender: M Age: 1Yr Date of Report: 28/12/2011Eth.C  Clinical Finding: Diaphoresis + RD + Murmur. TGSH7.3097. | | | |
| --- | --- | --- | --- |
| Features | **Finding** | **Features** | **Finding** |
| Profile | | **Atria** | |
| Abdominal situs | Solitus | Left atrium | Dilated |
| Cardiac position | Levocardia | Right atrium | Dilated |
| Systemic venous drainage | to RA | **Atrioventricular valves** | |
| Pulmonary venous drainage | to LA | Mitral valve | Normal |
| Atrioventricular connection | Concordant | Tricuspid valve | TAPSE = 16mm |
| Ventriculoarterial connection | Concordant | **Ventricles** | |
| Ventricular loop | d-Loop | Left ventricle | Dilated |
| Septae | | Right ventricle | Dilated |
| Interventricular septum | 10mm inlet VSD, L – R Shunt | **M-Mode** |  |
| Interatrial septum | Intact | AO |  |
| Great arteries | NRGA | LA |  |
| Aorta |  | LVIDd | 30.6mm |
| Pulmonary artery | Dilated MPA | LVIDs | 21.9mm |
| Semilunal valves |  | IVSd | 7.56mm |
| Aortic valve | Annulus = 10mm | IVSs | 7.56mm |
| Pulmonary valve | Annulus = 17mm | LVPWd | 7.56mm |
| Doppler Measurement | | LVPWs | 7.56mm |
| Mitral | ---- | **EDV** | 36.7ml |
| Aortic | ---- | ESV | 16ml |
| Tricuspid | Mild TR | FS | 28% |
| pulmonic | Mild PR, PPG = 55mmHg | LVEF | 56% |
| Aortic arch | Left | **Coronary arteries** |  |
| PDA | No |  |  |
| Additional information |  | | |
| Final Diagnosis | 1. {S, D, S} Levocardia 2. Large Inlet VSD, L – R Shunt 3. Moderate pulmonary Hypertension 4. Good Biventricular Function | | |

**Done by Signature**

**Name: Dr. Tesfaye T. Pediatric Cardiologist Date-28/12/2011 E.C**

| Pediatric Echocardiography report  Patient Name: Naod Sewale Patient ID:018175  Gender: M Age: 4yrs Date of Report: 28/12/2011Eth.C  Clinical Finding: Easy Fatigability. TGSH7.3098. | | | |
| --- | --- | --- | --- |
| Features | **Finding** | **Features** | **Finding** |
| Profile | | **Atria** | |
| Abdominal situs | Solitus | Left atrium | Normal |
| Cardiac position | Levocardia | Right atrium | Normal |
| Systemic venous drainage | to RA | **Atrioventricular valves** | |
| Pulmonary venous drainage | to LA | Mitral valve | Annulus = 14mm |
| Atrioventricular connection | Concordant | Tricuspid valve | Annulus = 16mm  TAPSE = 15mm |
| Ventriculoarterial connection | Concordant | **Ventricles** | |
| Ventricular loop | d-Loop | Left ventricle | Normal |
| Septae | | Right ventricle | Normal |
| Interventricular septum | Intact | **M-Mode** |  |
| Interatrial septum | Intact | AO |  |
| Great arteries | NRGA | LA |  |
| Aorta | ---- | LVIDd | 32.5mm |
| Pulmonary artery | Normal sized confluent Branch PAs | LVIDs | 21.9mm |
| Semilunal valves |  | IVSd | 4.92mm |
| Aortic valve | Annulus = 12mm | IVSs | 6.43mm |
| Pulmonary valve | Annulus = 15mm | LVPWd | 4.92mm |
| Doppler Measurement | | LVPWs | 7.56mm |
| Mitral | ---- | **EDV** | 42.5ml |
| Aortic | ---- | ESV | 16ml |
| Tricuspid | Trivial TR, PPG = 25mmHg | FS | 32% |
| pulmonic | ---- | LVEF | 58% |
| Aortic arch | Left | **Coronary arteries** |  |
| PDA | No |  |  |
| Additional information | No Coarctation of Aorta | | |
| Final Diagnosis | 1. {S, D, S} Levocardia 2. Normal Echocardiography Study   N.B: Needs follow up | | |

**Done by Signature**

**Name: Dr. Tesfaye T. Pediatric Cardiologist Date-28/12/2011 E.C**

| Pediatric Echocardiography report  Patient Name: B/Siraye Amare Patient ID: 017334  Gender: F Age: 3days Date of Report: 28/12/2011Eth.C  INCOMPLETE DATA | | | |
| --- | --- | --- | --- |
| Features | **Finding** | **Features** | **Finding** |
| Profile | | **Atria** | |
| Abdominal situs | Solitus | Left atrium | Normal |
| Cardiac position | Levocardia | Right atrium | Normal |
| Systemic venous drainage | to RA | **Atrioventricular valves** | |
| Pulmonary venous drainage | to LA | Mitral valve | Normal |
| Atrioventricular connection | Concordant | Tricuspid valve | Normal |
| Ventriculoarterial connection | Concordant | **Ventricles** | |
| Ventricular loop | d-Loop | Left ventricle | Normal |
| Septae | | Right ventricle | Normal |
| Interventricular septum | Intact | **M-Mode** |  |
| Interatrial septum | PFO, L – R Shunt | AO |  |
| Great arteries | NRGA | LA |  |
| Aorta | ---- | LVIDd | mm |
| Pulmonary artery | ----- | LVIDs | mm |
| Semilunal valves |  | IVSd | mm |
| Aortic valve | Normal | IVSs | mm |
| Pulmonary valve | Normal | LVPWd | mm |
| Doppler Measurement | | LVPWs | mm |
| Mitral | ---- | **EDV** | ml |
| Aortic | ---- | ESV | ml |
| Tricuspid | ---- | FS |  |
| pulmonic | ---- | LVEF |  |
| Aortic arch | Left | **Coronary arteries** |  |
| PDA | Suspicious of PDA |  |  |
| Additional information |  | | |
|  |  | | |
| Final Diagnosis |  | | |

**Done by Signature**

**Name: Dr. Tesfaye T. Pediatric Cardiologist Date-28/12/2011 E.C**

| Pediatric Echocardiography report  Patient Name: Gashaw Birara Patient ID:017686  Gender: M Age: 13yrs Date of Report: 28/12/2011Eth.C  BP: Weight:21.6kg Height: 125cm BSA:  Clinical Finding: DOE + Murmur. TGSH7.3099. | | | |
| --- | --- | --- | --- |
| Features | **Finding** | **Features** | **Finding** |
| Profile | | **Atria** | |
| Abdominal situs | Solitus | Left atrium | Normal |
| Cardiac position | Levocardia | Right atrium | Dilated, 42 X 58mm |
| Systemic venous drainage | to RA | **Atrioventricular valves** | |
| Pulmonary venous drainage | to LA, impressing pulmonary venous return | Mitral valve | Annulus = 18mm |
| Atrioventricular connection | Concordant | Tricuspid valve | Annulus = 32mm  TAPSE = 27mm |
| Ventriculoarterial connection | Concordant | **Ventricles** | |
| Ventricular loop | d-Loop | Left ventricle | Normal |
| Septae | | Right ventricle | Dilated |
| Interventricular septum | Intact | **M-Mode** |  |
| Interatrial septum | 25mm OS ASD, L – R Shunt | AO |  |
| Great arteries | NRGA | LA |  |
| Aorta |  | LVIDd | 32.9mm |
| Pulmonary artery | MPA = **25mm** | LVIDs | 23.1mm |
| Semilunal valves |  | IVSd | 7.56mm |
| Aortic valve | Annulus = 19mm | IVSs | 7.56mm |
| Pulmonary valve | Annulus = 26mm | LVPWd | 7.56mm |
| Doppler Measurement | | LVPWs | 7.56mm |
| Mitral |  | **EDV** | 43.8ml |
| Aortic |  | ESV | 18.3ml |
| Tricuspid | Trivial TR, PPG = 28mmHg | FS | 29% |
| pulmonic | Trivial PR, PPG = 25mmHg | LVEF | 58% |
| Aortic arch | Left | **Coronary arteries** |  |
| PDA | No |  |  |
| Additional information |  | | |
| Final Diagnosis | 1. {S, D, S} Levocardia 2. Large OS ASD, L – R Shunt 3. RA/RV Dilated 4. Impressing pulmonary venous return 5. Good Biventricular Function | | |

**Done by Signature**

**Name: Dr. Tesfaye T. Pediatric Cardiologist Date-28/12/2011 E.C**

| Pediatric Echocardiography report  Patient Name: Zigju Yeneneh Patient ID:015886  Gender:F Age:8yrs Date of Report: 28/12/2011Eth.C  Clinical Finding: ARF + Murmur + Sydenham’s Chorea. TGSH7.3100. | | | |
| --- | --- | --- | --- |
| Features | **Finding** | **Features** | **Finding** |
| Profile | | **Atria** | |
| Abdominal situs | Solitus | Left atrium | Normal |
| Cardiac position | Levocardia | Right atrium | Normal |
| Systemic venous drainage | to RA | **Atrioventricular valves** | |
| Pulmonary venous drainage | to LA | Mitral valve | Annulus = 21mm, thickened valve leaflet |
| Atrioventricular connection | Concordant | Tricuspid valve | Annulus = 22mm |
| Ventriculoarterial connection | Concordant | **Ventricles** | |
| Ventricular loop | d-Loop | Left ventricle | Normal |
| Septae | | Right ventricle | Normal |
| Interventricular septum | Intact | **M-Mode** |  |
| Interatrial septum | Intact | AO |  |
| Great arteries | NRGA | LA |  |
| Aorta | ---- | LVIDd | 38.2mm |
| Pulmonary artery | ---- | LVIDs | 28.4mm |
| Semilunal valves |  | IVSd | 7.56mm |
| Aortic valve | Annulus = 20mm | IVSs | 9.45mm |
| Pulmonary valve | Annulus = 21mm | LVPWd | 7.56mm |
| Doppler Measurement | | LVPWs | 7.56mm |
| Mitral | Mild MR, lateral projection | **EDV** | 62.7ml |
| Aortic | Moderate AR, PHT = 434ms | ESV | 30.6ml |
| Tricuspid | ---- | FS | 31% |
| pulmonic | ---- | LVEF | 59% |
| Aortic arch | Left | **Coronary arteries** |  |
| PDA | No |  |  |
| Additional information |  | | |
|  |  | | |
| Final Diagnosis | 1. {S, D, S} Levocardia 2. Mild MR 3. Moderate AR 4. Good Biventricular Function | | |

**Done by Name: Dr. Tesfaye T. Pediatric Cardiologist Pediatric Echocardiography report**

| Pediatric Echocardiography report  Patient Name: Ashagerie Muche Patient ID:011667  Gender: M Age:12 Date of Report:05/13/2011Eth.C  Clinical Finding: bradycardia. TGSH7.3101. | | | |
| --- | --- | --- | --- |
| Features | **Finding** | **Features** | **Finding** |
| Profile | | **Atria** | |
| Abdominal situs | Solitus | Left atrium | Normal |
| Cardiac position | Levocardia | Right atrium | Normal |
| Systemic venous drainage | to RA | **Atrioventricular valves** | |
| Pulmonary venous drainage | to LA | Mitral valve | Normal |
| Atrioventricular connection | Concordant | Tricuspid valve | Normal |
| Ventriculoarterial connection | Concordant | **Ventricles** | |
| Ventricular loop | d-Loop | Left ventricle | Normal |
| Septae | | Right ventricle | Normal |
| Interventricular septum | Intact | **M-Mode** |  |
| Interatrial septum | Intact | AO |  |
| Great arteries | NRGA | LA |  |
| Aorta | ---- | LVIDd | 33.3mm |
| Pulmonary artery | ---- | LVIDs | 24.2mm |
| Semilunal valves |  | IVSd | 8.64mm |
| Aortic valve | Normal | IVSs | 8.64mm |
| Pulmonary valve | Normal | LVPWd | 5.62mm |
| Doppler Measurement | | LVPWs | 8.64mm |
| Mitral | ---- | **EDV** | 45ml |
| Aortic | ---- | ESV | 20.6ml |
| Tricuspid | ---- | FS | 32% |
| pulmonic | ---- | LVEF | 59% |
| Aortic arch | Left | **Coronary arteries** |  |
| PDA | No |  |  |
| Additional information | Bradycardia is noted (Do ECG) | | |
|  |  | | |
| Final Diagnosis | 1. Normal echocardiography Study | | |

| Pediatric Echocardiography report  Patient Name: Habitamu Alamir Patient ID:018832  Gender: M Age: 1 8/12 Date of Report: 05/13/2011Eth.C  Clinical Finding: Recurrent Chest Infection + RD. TGSH7.3102. | | | |
| --- | --- | --- | --- |
| Features | **Finding** | **Features** | **Finding** |
| Profile | | **Atria** | |
| Abdominal situs | Solitus | Left atrium | Normal |
| Cardiac position | Levocardia | Right atrium | Normal |
| Systemic venous drainage | to RA | **Atrioventricular valves** | |
| Pulmonary venous drainage | to LA | Mitral valve | Normal |
| Atrioventricular connection | Concordant | Tricuspid valve | Normal |
| Ventriculoarterial connection | Concordant | **Ventricles** | |
| Ventricular loop | d-Loop | Left ventricle |  |
| Septae | | Right ventricle |  |
| Interventricular septum | Intact | **M-Mode** | Normal LV Function (eye balling) |
| Interatrial septum | Intact | AO |  |
| Great arteries | NRGA | LA |  |
| Aorta |  | LVIDd | mm |
| Pulmonary artery |  | LVIDs | mm |
| Semilunal valves |  | IVSd | mm |
| Aortic valve | Normal | IVSs | mm |
| Pulmonary valve | Normal | LVPWd | mm |
| Doppler Measurement | | LVPWs | mm |
| Mitral |  | **EDV** | ml |
| Aortic |  | ESV | ml |
| Tricuspid |  | FS |  |
| pulmonic |  | LVEF |  |
| Aortic arch | Left | **Coronary arteries** |  |
| PDA | No |  |  |
| Additional information |  | | |
|  |  | | |
| Final Diagnosis | 1. Normal Echocardiographic Study | | |

**Done by Signature**

**Name: Dr. Tesfaye T. Pediatric Cardiologist Date-05/13/2011 E.C**

| Pediatric Echocardiography report  Patient Name: Zelalem Yinges Patient ID:019050  Gender: M Age:14 Date of Report: 05/13/2011Eth.C  Clinical Finding: Palpitation. TGSH7.3103. | | | |
| --- | --- | --- | --- |
| Features | **Finding** | **Features** | **Finding** |
| Profile | | **Atria** | |
| Abdominal situs | Solitus | Left atrium | Normal |
| Cardiac position | Levocardia | Right atrium | Normal |
| Systemic venous drainage | to RA | **Atrioventricular valves** | |
| Pulmonary venous drainage | to LA | Mitral valve | Annulus = 23mm |
| Atrioventricular connection | Concordant | Tricuspid valve | Annulus = 23mm  TAPSE = 18mm |
| Ventriculoarterial connection | Concordant | **Ventricles** | |
| Ventricular loop | d-Loop | Left ventricle | Normal |
| Septae | | Right ventricle | Normal |
| Interventricular septum | Intact | **M-Mode** |  |
| Interatrial septum | Intact | AO |  |
| Great arteries | NRGA | LA |  |
| Aorta | ---- | LVIDd | 48mm |
| Pulmonary artery | ---- | LVIDs | 35.4mm |
| Semilunal valves |  | IVSd | 7.27mm |
| Aortic valve | Annulus = 23mm, Trileaflet | IVSs | 7.75mm |
| Pulmonary valve | Annulus = 23mm | LVPWd | 9.69mm |
| Doppler Measurement | | LVPWs | 9.2mm |
| Mitral | ---- | **EDV** | 108ml |
| Aortic | ---- | ESV | 52ml |
| Tricuspid | ---- | FS | 26% |
| pulmonic | ---- | LVEF | 51% |
| Aortic arch | Left | **Coronary arteries** |  |
| PDA | No |  |  |
| Additional information |  | | |
|  |  | | |
| Final Diagnosis | 1. Normal Echocardiographic Study | | |

**Done by Signature**

**Name: Dr. Tesfaye T. Pediatric Cardiologist Date-05/13/2011 E.C**

| Pediatric Echocardiography report  Patient Name: Molalign Mirtu Patient ID:018513  Gender: M Age:1 yr. Date of Report: 05/13/2011Eth.C  Clinical Finding: Diaphoresis. TGSH7.3104. | | | |
| --- | --- | --- | --- |
| Features | **Finding** | **Features** | **Finding** |
| Profile | | **Atria** | |
| Abdominal situs | Solitus | Left atrium | Normal |
| Cardiac position | Levocardia | Right atrium | Normal |
| Systemic venous drainage | to RA | **Atrioventricular valves** | |
| Pulmonary venous drainage | to LA | Mitral valve | Normal |
| Atrioventricular connection | Concordant | Tricuspid valve | Normal |
| Ventriculoarterial connection | Concordant | **Ventricles** | |
| Ventricular loop | d-Loop | Left ventricle | Normal |
| Septae | | Right ventricle | Normal |
| Interventricular septum | Intact | **M-Mode** | Normal LV Function (eye balling) |
| Interatrial septum | Intact | AO |  |
| Great arteries | NRGA | LA |  |
| Aorta |  | LVIDd | mm |
| Pulmonary artery |  | LVIDs | mm |
| Semilunal valves |  | IVSd | mm |
| Aortic valve | Normal | IVSs | mm |
| Pulmonary valve | Normal | LVPWd | mm |
| Doppler Measurement | | LVPWs | mm |
| Mitral | ---- | **EDV** | ml |
| Aortic | ---- | ESV | ml |
| Tricuspid | ---- | FS |  |
| pulmonic | ---- | LVEF |  |
| Aortic arch | Left | **Coronary arteries** |  |
| PDA | No |  |  |
| Additional information |  | | |
|  |  | | |
| Final Diagnosis | 1. Normal Echocardiography Study | | |

**Done by Signature**

**Name: Dr. Tesfaye T. Pediatric Cardiologist Date: 05 /13/2011 E.C**

| Pediatric Echocardiography report  Patient Name: Yonas Tegenew Patient ID:019148  Gender: M Age:46 days Date of Report: 06/01/2012Eth.C  Clinical Finding: DS + Murmur + RD. TGSH7.3105. | | | |  |
| --- | --- | --- | --- | --- |
| Features | **Finding** | **Features** | **Finding** |  |
| Profile | | **Atria** | |  |
| Abdominal situs | Solitus | Left atrium | Normal |  |
| Cardiac position | Levocardia | Right atrium | Normal |  |
| Systemic venous drainage | to RA | **Atrioventricular valves** | |  |
| Pulmonary venous drainage | to LA | Mitral valve |  |  |
| Atrioventricular connection | Common AV valve | Tricuspid valve |  |  |
| Ventriculoarterial connection | Concordant | **Ventricles** | |  |
| Ventricular loop | d-Loop | Left ventricle | Normal |  |
| Septae : No Tongue of tissue | | Right ventricle | Normal |  |
| Interventricular septum | Common complete AVSD, L – R Shunt | **M-Mode** |  |  |
| Interatrial septum |  | AO |  |  |
| Great arteries | NRGA | LA |  |  |
| Aorta | ----- | LVIDd | 11.8mm |  |
| Pulmonary artery | ------- | LVIDs | 7.56mm |  |
| Semilunal valves |  | IVSd | 6mm |  |
| Aortic valve | Normal | IVSs | 6mm |  |
| Pulmonary valve | Normal | LVPWd | 6mm |  |
| Doppler Measurement | | LVPWs | 6mm |  |
| Mitral | Mild Left AVVR | **EDV** | 3.21ml |  |
| Aortic | ---- | ESV | 1ml |  |
| Tricuspid | Moderate Right AVVR | FS | 35% |  |
| pulmonic | ---- | LVEF | 70% |  |
| Aortic arch | Left | **Coronary arteries** |  |  |
| PDA | No |  |  |  |
| Additional information |  | | |  |
|  |  |  |  |  |
| Final Diagnosis | 1. {S, D, S} Levocardia 2. Common Complete Balanced AVSD 3. Mild Left AVVR 4. Moderate Right AVVR 5. Good LV Function | | |  |

**Done by Signature**

**Name: Dr. Tesfaye T. Pediatric Cardiologist Date-06/01/2012 E.C**

**AVVR = Atrio – Ventricular Valve Regurgitation**

| Pediatric Echocardiography report  Patient Name: Abriham Adamu Patient ID:019272  Gender: M Age: 11 months Date of Report: 0 6/01/2012Eth.C  Clinical Finding: RD + Recurrent Chest Infection. TGSH7.3106. | | | |
| --- | --- | --- | --- |
| Features | **Finding** | **Features** | **Finding** |
| Profile | | **Atria** | |
| Abdominal situs | Solitus | Left atrium | Normal |
| Cardiac position | Levocardia | Right atrium | Normal |
| Systemic venous drainage | to RA | **Atrioventricular valves** | |
| Pulmonary venous drainage | to LA | Mitral valve | Normal |
| Atrioventricular connection | Concordant | Tricuspid valve | Normal |
| Ventriculoarterial connection | Concordant | **Ventricles** | |
| Ventricular loop | d-Loop | Left ventricle | Normal |
| Septae | | Right ventricle | Normal |
| Interventricular septum | Intact | **M-Mode** |  |
| Interatrial septum | Intact | AO |  |
| Great arteries | NRGA | LA |  |
| Aorta | ---- | LVIDd | 23.4mm |
| Pulmonary artery | Good sized MPA & Confluent Branch PAs | LVIDs | 16.6mm |
| Semilunal valves |  | IVSd | 7.56mm |
| Aortic valve | Normal | IVSs | 7.56mm |
| Pulmonary valve | Normal | LVPWd | 7.56mm |
| Doppler Measurement | | LVPWs | 7.56mm |
| Mitral | Trivial MR | **EDV** | 18.9ml |
| Aortic | ---- | ESV | 7.89ml |
| Tricuspid | Trivial TR | FS | 29% |
| pulmonic | ---- | LVEF | 58% |
| Aortic arch | Left | **Coronary arteries** |  |
| PDA | No |  |  |
| Additional information |  | | |
|  |  | | |
| Final Diagnosis | Normal Echocardiographic Study | | |

**Done by Signature**

**Name: Dr. Tesfaye T. Pediatric Cardiologist Date-06/01/2012 E.C**

| Pediatric Echocardiography report  Patient Name: Miftah Ali Patient ID:019395  Gender: M Age:7 months Date of Report: 06/01/2012Eth.C  Clinical Finding: RD + Murmur + CHF + DS + . TGSH7.3107. | | | |
| --- | --- | --- | --- |
| Features | **Finding** | **Features** | **Finding** |
| Profile | | **Atria** | |
| Abdominal situs | Solitus | Left atrium | Dilated |
| Cardiac position | Levocardia | Right atrium | Dilated |
| Systemic venous drainage | to RA | **Atrioventricular valves** | |
| Pulmonary venous drainage | to LA | Mitral valve | Common AV Valve |
| Atrioventricular connection | Common AV Valve | Tricuspid valve | Common AV Valve |
| Ventriculoarterial connection | Concordant | **Ventricles** | |
| Ventricular loop | d-Loop | Left ventricle | Dilated |
| Septae : No tongue of tissue in b/n | | Right ventricle | Dilated |
| Interventricular septum | Large Inlet VSD, L – R Shunt | **M-Mode** |  |
| Interatrial septum | Large Primmum septal defect, L – R Shunt | AO |  |
| Great arteries | NRGA | LA |  |
| Aorta | ---- | LVIDd | mm |
| Pulmonary artery | ---- | LVIDs | mm |
| Semilunal valves | ---- | IVSd | mm |
| Aortic valve | Annulus = 13mm | IVSs | mm |
| Pulmonary valve | Annulus = 15mm | LVPWd | mm |
| Doppler Measurement | | LVPWs | mm |
| Mitral | Mild Left AVVR | **EDV** | ml |
| Aortic |  | ESV | ml |
| Tricuspid | Moderate Right AVVR | FS | 30% |
| pulmonic |  | LVEF | 60% |
| Aortic arch | Left | **Coronary arteries** |  |
| PDA | No |  |  |
| Additional information | 1. 7mm right pleural effusion 2. 5mm left pleural effusion 3. Trace pericardial effusion, circumferential | | |
|  |
| Final Diagnosis | 1. {S, D, S} Levocardia 2. Common complete Balanced AVSD 3. Mild to Moderate Right AVVR 4. Mild Left AVVR 5. Good LV Function | | |

**Done by Signature**

**Name: Dr. Tesfaye T. Pediatric Cardiologist Date-06/01/20112 E.C**

AVVR = Atrio – Ventricular Valve Regurgitation

| Pediatric Echocardiography report  Patient Name: Baby of yeshi Patient ID:019392  Gender M Age:14 Day Date of Report: 06/01/2012Eth.C  Clinical Finding: Incidental Murmur. TGSH7.3108. | | | |
| --- | --- | --- | --- |
| Features | **Finding** | **Features** | **Finding** |
| Profile | | **Atria** | |
| Abdominal situs | Solitus | Left atrium | Normal |
| Cardiac position | Levocardia | Right atrium | Normal |
| Systemic venous drainage | to RA | **Atrioventricular valves** | |
| Pulmonary venous drainage | to LA | Mitral valve | Annulus = 11mm |
| Atrioventricular connection | Concordant | Tricuspid valve | Annulus = 9mm  TAPSE = 9mm |
| Ventriculoarterial connection | Concordant | **Ventricles** | |
| Ventricular loop | d-Loop | Left ventricle | Normal |
| Septae | | Right ventricle | Normal |
| Interventricular septum | Intact | **M-Mode** |  |
| Interatrial septum | 4mm OS ASD, L – R Shunt | AO |  |
| Great arteries | NRGA | LA |  |
| Aorta | ---- | LVIDd | mm |
| Pulmonary artery | ---- | LVIDs | mm |
| Semilunal valves |  | IVSd | mm |
| Aortic valve | Annulus = 9mm | IVSs | mm |
| Pulmonary valve | Annulus = 9mm | LVPWd | mm |
| Doppler Measurement | | LVPWs | mm |
| Mitral | ---- | **EDV** | ml |
| Aortic | ---- | ESV | ml |
| Tricuspid | ---- | FS | 26% |
| pulmonic | ---- | LVEF | 54% |
| Aortic arch | Left | **Coronary arteries** |  |
| PDA | 1.5mm PDA, L – R Shunt |  |  |
| Additional information |  | | |
|  |  | | |
| Final Diagnosis | 1. {S, D, S} Levocardia 2. Small OS ASD, L – R Shunt 3. Small PDA, L – R Shunt 4. Good Biventricular Function | | |

**Done by Signature**

**Name: Dr. Tesfaye T. Pediatric Cardiologist Date-06/01/2012 E.C**

| Pediatric Echocardiography report  Patient Name: Temesigen Werku Patient ID: 018781  Gender: Male Age: 2 6/12 Date of Report 08/01/2012 Eth.C  Clinical Finding: Diaphoresis + Recurrent Chest Infection. TGSH7.3109. | | | |
| --- | --- | --- | --- |
| Features | **Finding** | **Features** | **Finding** |
| Profile | | **Atria** | |
| Abdominal situs | Solitus | Left atrium | Normal |
| Cardiac position | Levocardia | Right atrium | Normal |
| Systemic venous drainage | to RA | **Atrioventricular valves** | |
| Pulmonary venous drainage | to LA | Mitral valve | ---- |
| Atrioventricular connection | Concordant | Tricuspid valve | TAPSE = 12mm |
| Ventriculoarterial connection | Concordant | **Ventricles** | |
| Ventricular loop | d-Loop | Left ventricle | Normal |
| Septae | | Right ventricle | Normal |
| Interventricular septum | Intact | **M-Mode** |  |
| Interatrial septum | Intact | AO |  |
| Great arteries | NRGA | LA |  |
| Aorta | ----- | LVIDd | mm |
| Pulmonary artery | ---- | LVIDs | mm |
| Semilunal valves |  | IVSd | mm |
| Aortic valve | Annulus = 11mm | IVSs | mm |
| Pulmonary valve | Annulus = 14mm | LVPWd | mm |
| Doppler Measurement | | LVPWs | mm |
| Mitral | ---- | **EDV** | ml |
| Aortic | ---- | ESV | ml |
| Tricuspid | Trivial TR | FS | 26% |
| pulmonic | ---- | LVEF | 54% |
| Aortic arch | Left | **Coronary arteries** |  |
| PDA | No |  |  |
| Additional information |  | | |
|  |  | | |
| Final Diagnosis | Normal Echocardiographic Study | | |

**Done by Signature**

**Name: Dr. Tesfaye T. Pediatric Cardiologist Date-08/01/2012 E.C**

| Pediatric Echocardiography report  Patient Name: Mitiku Molla Patient ID:019710  Gender: M Age:11 Date of Report: 08/01/2012Eth.C  Clinical Finding: Easy Fatigability. TGSH7.3110. | | | |
| --- | --- | --- | --- |
| Features | **Finding** | **Features** | **Finding** |
| Profile | | **Atria** | |
| Abdominal situs | Solitus | Left atrium | Normal |
| Cardiac position | Levocardia | Right atrium | Normal |
| Systemic venous drainage | to RA | **Atrioventricular valves** | |
| Pulmonary venous drainage | to LA | Mitral valve | Annulus = 19mm |
| Atrioventricular connection | Concordant | Tricuspid valve | Annulus = 20mm  TAPSE = 16mm |
| Ventriculoarterial connection | Concordant | **Ventricles** | |
| Ventricular loop | d-Loop | Left ventricle | Normal |
| Septae | | Right ventricle | Normal |
| Interventricular septum | Intact | **M-Mode** |  |
| Interatrial septum | Intact | AO |  |
| Great arteries | NRGA | LA |  |
| Aorta | ---- | LVIDd | 40mm |
| Pulmonary artery | ---- | LVIDs | 26.5mm |
| Semilunal valves |  | IVSd | 7.6mm |
| Aortic valve | Annulus = 13mm | IVSs | 7.6mm |
| Pulmonary valve | Annulus = 17mm | LVPWd | 7.5mm |
| Doppler Measurement | | LVPWs | 7.5mm |
| Mitral | ---- | **EDV** | 70ml |
| Aortic | ---- | ESV | 25.8ml |
| Tricuspid | Trivial TR, PPG = 28mmHg | FS | 34% |
| pulmonic | ---- | LVEF | 63% |
| Aortic arch | Left | **Coronary arteries** |  |
| PDA | No |  |  |
| Additional information | No coarctation of aorta | | |
|  |  | | |
| Final Diagnosis | Normal Echocardiographic Study | | |

**Done by Signature**

**Name: Dr. Tesfaye T. Pediatric Cardiologist Date-08/01/2012 E.C**

| Pediatric Echocardiography report  Patient Name: Hilina Yayeh Patient ID: 019701  Gender: F Age:2 yrs. &9 months Date of Report: 08/01/2012 Eth.C  Clinical Finding: RD + Murmur + CHF + Easy Fatigability. TGSH7.3111. | | | |
| --- | --- | --- | --- |
| Features | **Finding** | **Features** | **Finding** |
| Profile | | **Atria** | |
| Abdominal situs | Solitus | Left atrium | Dilated, 33 X 31mm |
| Cardiac position | Levocardia | Right atrium | Dilated |
| Systemic venous drainage | to RA | **Atrioventricular valves** | |
| Pulmonary venous drainage | to LA. There is turbulence across entry of LLPV in to LA with PPG/MPG = 10mmHg/5mmHg | Mitral valve | Annulus = 22mm |
| Atrioventricular connection | Concordant | Tricuspid valve | Annulus = 18mm |
| Ventriculoarterial connection | Concordant | **Ventricles** | |
| Ventricular loop | d-Loop | Left ventricle | Dilated |
| Septae | | Right ventricle | Dilated |
| Interventricular septum | Intact | **M-Mode** |  |
| Interatrial septum | Intact | AO |  |
| Great arteries | NRGA | LA |  |
| Aorta | ---- | LVIDd | 48.4mm |
| Pulmonary artery | ---- | LVIDs | 30.2mm |
| Semilunal valves |  | IVSd | 7.5mm |
| Aortic valve | Annulus = 11mm, Trileaflet | IVSs | 9.4mm |
| Pulmonary valve | Annulus = 18mm | LVPWd | 6mm |
| Doppler Measurement | | LVPWs | 7.5mm |
| Mitral | Mild MR | **EDV** | 110ml |
| Aortic | ---- | ESV | 35.6ml |
| Tricuspid | Mild TR, PPG = 62mmHg | FS | 37% |
| pulmonic | Mild PR, PPG = 56mmHg | LVEF | 67% |
| Aortic arch | Left | **Coronary arteries** |  |
| PDA | 5mm PDA L – R Shunt, SPG/EDG = 67/32mmHg |  |  |
| Additional information | 3mm pericardial effusion on RA/RV Side | | |
|  |  | | |
| Final Diagnosis | 1. {S, D, S} Levocardia 2. Large PDA, L – R Shunt 3. LLPV Stenosis 4. Mild MR 5. Mild TR 6. Mild PR 7. Severe pulmonary Hypertension 8. Small pericardial effusion 9. Good LV Function | | |

**Done by Signature**

**Name: Dr. Tesfaye T. Pediatric Cardiologist Date-08/01/2012 E.C**

| Pediatric Echocardiography report  Patient Name: Haile Getasew Patient ID:019304  Gender: M Age:14 Date of Report: 08/01/2012Eth.C  Clinical Finding: Rheumatic Recurrence + DOE + Murmur + CHF + Palpitation + RD. TGSH7.3112. | | | |
| --- | --- | --- | --- |
| Features | **Finding** | **Features** | **Finding** |
| Profile | | **Atria** | |
| Abdominal situs | Solitus | Left atrium | Mildly dilated |
| Cardiac position | Levocardia | Right atrium | Dilated, 64 X 70mm |
| Systemic venous drainage | to RA | **Atrioventricular valves** | |
| Pulmonary venous drainage | to LA | Mitral valve | Annulus = 24mm, thickened, clubbed & calcified. Valve area = 0.6cm**2** |
| Atrioventricular connection | Concordant | Tricuspid valve | Annulus = 35mm, non coapting valves. Calcified valve leaflets.  TAPSE = 19mm |
| Ventriculoarterial connection | Concordant | **Ventricles** | |
| Ventricular loop | d-Loop | Left ventricle | Normal |
| Septae | | Right ventricle | Dilated |
| Interventricular septum | Intact | **M-Mode** | Good LV Function on eye balling |
| Interatrial septum | Intact | AO |  |
| Great arteries | NRGA | LA |  |
| Aorta | ---- | LVIDd | mm |
| Pulmonary artery | ---- | LVIDs | mm |
| Semilunal valves |  | IVSd | mm |
| Aortic valve | Annulus = 17mm | IVSs | mm |
| Pulmonary valve | Annulus = 18mm | LVPWd | mm |
| Doppler Measurement | | LVPWs | mm |
| Mitral | Severe MS, PPG/MPG = 44/22mmHg | **EDV** | ml |
| Aortic | Mild AR, PHT = 547ms | ESV | ml |
| Tricuspid | Severe TR, PPG = 170mmHg | FS |  |
| pulmonic | ---- | LVEF |  |
| Aortic arch | Left | **Coronary arteries** |  |
| PDA | No |  |  |
| Additional information | 6mm Right Pleural effusion | | |
|  |  | | |
| Final Diagnosis | 1. {S, D, S} Levocardia 2. RA/RV Dilated 3. Severe MS 4. Thickened calcified Mitral Valve 5. Thickened calcified, non coapting Tricuspid valve 6. Severe Pulmonary Hypertension 7. Right Pleural effusion 8. Good biventricular Function | | |

**Done by Signature**

**Name: Dr. Tesfaye T. Pediatric Cardiologist Date-08/01/2012 E.C**

| Pediatric Echocardiography report  Patient Name: Weredaw Mogninet Patient ID:  Gender: M Age: 3/12 Date of Report: 09/01/2012Eth.C  Clinical Finding: Cyanosis + Murmur + Diaphoresis. TGSH7.3113. | | | |
| --- | --- | --- | --- |
| Features | **Finding** | **Features** | **Finding** |
| Profile | | **Atria** | |
| Abdominal situs | Solitus | Left atrium | Normal |
| Cardiac position | Levocardia | Right atrium | Normal |
| Systemic venous drainage | to RA | **Atrioventricular valves** | |
| Pulmonary venous drainage | to LA | Mitral valve | Normal |
| Atrioventricular connection | Concordant | Tricuspid valve | Atretic |
| Ventriculoarterial connection | DORV | **Ventricles** | |
| Ventricular loop | d-Loop | Left ventricle | Normal |
| Septae | | Right ventricle | Echogenic mass in the RV beneath the Atretic valve on the lateral side |
| Interventricular septum | 10mm inlet VSD, L – R Shunt | **M-Mode** |  |
| Interatrial septum | 8mm OS ASD, R – L Shunt | AO |  |
| Great arteries | NRGA | LA |  |
| Aorta | From RV | LVIDd | 17mm |
| Pulmonary artery | From RV. | LVIDs | 11mm |
| Semilunal valves |  | IVSd | 4mm |
| Aortic valve | Annulus = 8cm | IVSs | 4mm |
| Pulmonary valve | Annulus = 8cm | LVPWd | 4mm |
| Doppler Measurement | | LVPWs | 4mm |
| Mitral | ---- | **EDV** | 9ml |
| Aortic | ---- | ESV | 2.6ml |
| Tricuspid | Atretic | FS | 37% |
| pulmonic | ---- | LVEF | 70% |
| Aortic arch | ----- | **Coronary arteries** |  |
| PDA | No |  |  |
| Additional information | Aorto – Mitral valve fibrous discontinuity | | |
|  |  | | |
| Final Diagnosis | 1. {S, D, S} Levocardia 2. Moderate OS ASD, R – L Shunt 3. Large Inlet VSD, L – R Shunt 4. DORV 5. Tricuspid Atresia Type IIIC 6. ? Echogenic mass in the RV secondary to ? 7. Good LV Function | | |

**Done by Signature**

**Name: Dr. Tesfaye T. Pediatric Cardiologist Date-09/01/2012 E.C**

| Pediatric Echocardiography report  Patient Name: Rebeka Sisay Patient ID: 020325  Gender: F Age: 1 2/12 Date of Report: 13/01/2012Eth.C  Clinical Finding: Murmur + Diaphoresis + Cyanosis. TGSH7.3114. | | | |
| --- | --- | --- | --- |
| Features | **Finding** | **Features** | **Finding** |
| Profile | | **Atria** | |
| Abdominal situs | Solitus | Left atrium | Dilated |
| Cardiac position | Dextrocardia | Right atrium | Dilated |
| Systemic venous drainage | to RA | **Atrioventricular valves** | |
| Pulmonary venous drainage | to LA | Mitral valve | ---- |
| Atrioventricular connection | Concordant | Tricuspid valve | ----- |
| Ventriculoarterial connection | DORV | **Ventricles** | |
| Ventricular loop | d-Loop | Left ventricle | Dilated |
| Septae | | Right ventricle | Hypertrophied |
| Interventricular septum | 8mm Sub aortic VSD, L – R Shunt | **M-Mode** |  |
| Interatrial septum | 13mm OS ASD, L – R Shunt | AO |  |
| Great arteries | Side by side | LA |  |
| Aorta | Smallish ascending Aorta | LVIDd | mm |
| Pulmonary artery |  | LVIDs | mm |
| Semilunal valves |  | IVSd | mm |
| Aortic valve | Annulus = 5mm | IVSs | mm |
| Pulmonary valve | Annulus = 14mm | LVPWd | mm |
| Doppler Measurement | | LVPWs | mm |
| Mitral | ---- | **EDV** | ml |
| Aortic | Severe valvar & supra valvar AS, PPG/MPG = 100/67mmHg | ESV | ml |
| Tricuspid | Trivial TR, PPG = 27mmHg | FS |  |
| pulmonic | ---- | LVEF |  |
| Aortic arch | Left | **Coronary arteries** |  |
| PDA | No |  |  |
| Additional information |  | | |
|  |  | | |
| Final Diagnosis | 1. {S, D, D} Dextrocardia 2. DORV 3. Large OS ASD, L – R Shunt 4. 8mm subaortic VSD, L – R Shunt 5. Smallish ascending aorta and aortic valve 6. Severe valvar and supra valvar AS | | |

**Done by Signature**

**Name: Dr. Tesfaye T. Pediatric Cardiologist Date-13/01/2012E.C**

| Pediatric Echocardiography report  Patient Name: Yechale Dejen Patient ID:022101  Gender:M Age:1 Date of Report: 06/02/2012Eth.C  Clinical Finding: Cyanosis + Clubbing + Murmur. TGSH7.3115. | | | |
| --- | --- | --- | --- |
| Features | **Finding** | **Features** | **Finding** |
| Profile | | **Atria** | |
| Abdominal situs | Solitus | Left atrium | Normal |
| Cardiac position | Levocardia | Right atrium | Normal |
| Systemic venous drainage | to RA | **Atrioventricular valves** | |
| Pulmonary venous drainage | to LA | Mitral valve | Annulus= 13mm |
| Atrioventricular connection | Concordant | Tricuspid valve | Annulus =17mm  TAPSE = 12mm |
| Ventriculoarterial connection | Over – riding aorta | **Ventricles** | |
| Ventricular loop | d-Loop | Left ventricle | Normal |
| Septae | | Right ventricle | hypertrophied |
| Interventricular septum | 6mm mal – aligned Sub Aortic VSD, R – L Shunt | **M-Mode** |  |
| Interatrial septum | Intact | AO |  |
| Great arteries | NRGA | LA |  |
| Aorta | Over-riding aorta | LVIDd | 17mm |
| Pulmonary artery | Smallish MPA (8mm), RPA (4mm) and LPA(4mm) | LVIDs | 11mm |
| Semilunal valves |  | IVSd | 6mm |
| Aortic valve | Annulus =14 | IVSs | 7mm |
| Pulmonary valve | Annulus = 6 | LVPWd | 7mm |
| Doppler Measurement | | LVPWs | 7mm |
| Mitral | ---- | **EDV** | 9ml |
| Aortic | ---- | ESV | 3ml |
| Tricuspid | ----- | FS | 35% |
| pulmonic | Valvar and supra valvar PS, PPG = 81mmHg | LVEF | 68% |
| Aortic arch | Left | **Coronary arteries** |  |
| PDA | No |  |  |
| Additional information |  | | |
|  | Child was irritable and restless during study | | |
| Final Diagnosis | 1. {S, D, S} Levocardia 2. TOF 3. Smallish MPA and Branch PAs | | |

**Done by Signature**

**Name: Dr. Tesfaye T. Pediatric Cardiologist Date-06/02/2012 E.C**

| Pediatric Echocardiography report  Patient Name: Desalegn Sintayehu Patient ID:023161  Gender: F Age:4/12 months Date of Report: 07/02/2012Eth.C  Clinical Finding: Cyanosis + Shock TGSH7.3116. | | | |
| --- | --- | --- | --- |
| Features | **Finding** | **Features** | **Finding** |
| Profile | | **Atria** | |
| Abdominal situs | Solitus | Left atrium | Smallish |
| Cardiac position | Levocardia | Right atrium | dilated |
| Systemic venous drainage | to RA | **Atrioventricular valves** | |
| Pulmonary venous drainage | to LA | Mitral valve | Annulus= 6mm |
| Atrioventricular connection | Concordant | Tricuspid valve | Annulus = 11mm  TAPSE = |
| Ventriculoarterial connection | Concordant | **Ventricles** | |
| Ventricular loop | d-Loop | Left ventricle | Hypoplastic |
| Septae | | Right ventricle | Dilated |
| Interventricular septum | 5mm Inlet VSD, BD Shunt | **M-Mode** |  |
| Interatrial septum | Intact | AO |  |
| Great arteries | NRGA | LA |  |
| Aorta | Hypoplastic Ascending aorta and arch | LVIDd | mm |
| Pulmonary artery | ----- | LVIDs | mm |
| Semilunal valves |  | IVSd | mm |
| Aortic valve | Annulus = Atretic, 2mm | IVSs | mm |
| Pulmonary valve | Annulus = 9mm | LVPWd | mm |
| Doppler Measurement | | LVPWs | mm |
| Mitral | ---- | **EDV** | ml |
| Aortic | ---- | ESV | ml |
| Tricuspid | Trivial TR | FS |  |
| pulmonic | ------ | LVEF |  |
| Aortic arch | Left | **Coronary arteries** |  |
| PDA |  |  |  |
| Additional information |  | | |
|  |  | | |
| Final Diagnosis | 1. {S, D, S} Levocardia 2. Hypoplastic LV Syndrome | | |

**Done by Signature**

**Name: Dr. Tesfaye T. Pediatric Cardiologist Date-07/02/2012 E.C**

| Pediatric Echocardiography report  Patient Name: Abrham Tizazu Patient ID:023270  Gender: F Age:2 Date of Report: 07/02/2012Eth.C  Clinical Finding: Incidental Murmur/Innocent. TGSH7.3117. | | | |
| --- | --- | --- | --- |
| Features | **Finding** | **Features** | **Finding** |
| Profile | | **Atria** | |
| Abdominal situs | Solitus | Left atrium | Normal |
| Cardiac position | Levocardia | Right atrium | Normal |
| Systemic venous drainage | to RA | **Atrioventricular valves** | |
| Pulmonary venous drainage | to LA | Mitral valve | Annulus = 18mm |
| Atrioventricular connection | Concordant | Tricuspid valve | Annulus = 23mm  TAPSE = 17mm |
| Ventriculoarterial connection | Concordant | **Ventricles** | |
| Ventricular loop | d-Loop | Left ventricle | Normal |
| Septae | | Right ventricle | Normal |
| Interventricular septum | Intact | **M-Mode** |  |
| Interatrial septum | Intact | AO |  |
| Great arteries | NRGA | LA |  |
| Aorta | ---- | LVIDd | mm |
| Pulmonary artery | Good sized MPA and Confluent Branch Pas. | LVIDs | mm |
| Semilunal valves |  | IVSd | mm |
| Aortic valve | Annulus = 19mm | IVSs | mm |
| Pulmonary valve | Annulus = 22mm | LVPWd | mm |
| Doppler Measurement | | LVPWs | mm |
| Mitral | ---- | **EDV** | ml |
| Aortic | ---- | ESV | ml |
| Tricuspid | ---- | FS | 34% |
| pulmonic | ---- | LVEF | 63% |
| Aortic arch | Left | **Coronary arteries** |  |
| PDA | No |  |  |
| Additional information | NO Coarctation | | |
|  |  | | |
| Final Diagnosis | 1. Normal Echocardiographic Study | | |

**Done by Signature**

**Name: Dr. Tesfaye T. Pediatric Cardiologist Date-07/02/2012 E.C**

| Pediatric Echocardiography report  Patient Name: Biwota Yirsew Patient ID:023267  Gender:M Age:10yrs Date of Report: 11/02/2012Eth.C  Clinical Finding: Easy Fatigability + Arrhthmia. TGSH7.3118. | | | |
| --- | --- | --- | --- |
| Features | **Finding** | **Features** | **Finding** |
| Profile | | **Atria** | |
| Abdominal situs | Solitus | Left atrium | Normal |
| Cardiac position | Levocardia | Right atrium | Normal |
| Systemic venous drainage | to RA | **Atrioventricular valves** | |
| Pulmonary venous drainage | to LA | Mitral valve | Annulus = 21mm |
| Atrioventricular connection | Concordant | Tricuspid valve | Annulus = 123mm |
| Ventriculoarterial connection | Concordant | **Ventricles** | |
| Ventricular loop | d-Loop | Left ventricle | Normal |
| Septae | | Right ventricle | Normal |
| Interventricular septum | Intact | **M-Mode** |  |
| Interatrial septum | Intact | AO |  |
| Great arteries | NRGA | LA |  |
| Aorta |  | LVIDd | 37mm |
| Pulmonary artery | Good sized MPA and confluent Branch PAs. | LVIDs | 25mm |
| Semilunal valves |  | IVSd | 6mm |
| Aortic valve | Annulus = 17mm, Trileaflet | IVSs | 6mm |
| Pulmonary valve | Annulus = 20mm | LVPWd | 7mm |
| Doppler Measurement | | LVPWs | 7mm |
| Mitral | ---- | **EDV** | 57ml |
| Aortic | ---- | ESV | 24ml |
| Tricuspid | ---- | FS | 30% |
| pulmonic | Trivial PR, PPG = 12mmHg | LVEF | 58% |
| Aortic arch | Left | **Coronary arteries** |  |
| PDA | No |  |  |
| Additional information | No Coarctation of aorta | | |
|  |  | | |
| Final Diagnosis | 1. Normal Echocardiography Study. | | |

**Done by Signature**

**Name: Dr. Tesfaye T. Pediatric Cardiologist Date-11/01/2012 E.C**

| Pediatric Echocardiography report  Patient Name: biyadegelegn Melese Patient ID: 020602  Gender:M Age: 14 days Date of Report: 11/01/2012Eth.C  Clinical Finding: RD. TGSH7.3119. | | | |
| --- | --- | --- | --- |
| Features | **Finding** | **Features** | **Finding** |
| Profile | | **Atria** | |
| Abdominal situs | Solitus | Left atrium | Normal |
| Cardiac position | Levocardia | Right atrium | Normal |
| Systemic venous drainage | to RA | **Atrioventricular valves** | |
| Pulmonary venous drainage | to LA | Mitral valve | Annulus = 11mm |
| Atrioventricular connection | Concordant | Tricuspid valve | Annulus = 12mm  TAPSE = 10mm |
| Ventriculoarterial connection | Concordant | **Ventricles** | |
| Ventricular loop | d-Loop | Left ventricle | Normal |
| Septae | | Right ventricle | Normal |
| Interventricular septum | Intact | **M-Mode** |  |
| Interatrial septum | Intact | AO |  |
| Great arteries | NRGA | LA |  |
| Aorta |  | LVIDd | 18.5mm |
| Pulmonary artery | Good sized MPA and Confluent Branch PAs. | LVIDs | 11mm |
| Semilunal valves |  | IVSd | 4mm |
| Aortic valve | Annulus = 9mm | IVSs | 5mm |
| Pulmonary valve | Annulus = 11mm | LVPWd | 6mm |
| Doppler Measurement | | LVPWs | 6mm |
| Mitral | ---- | **EDV** | 10ml |
| Aortic | ---- | ESV | 3ml |
| Tricuspid | ---- | FS | 39% |
| pulmonic | ----- | LVEF | 73% |
| Aortic arch | Left | **Coronary arteries** |  |
| PDA | No |  |  |
| Additional information | No Coarctation of aorta | | |
|  |  | | |
| Final Diagnosis | 1. Normal Echocardiography Study | | |

**Done by Signature**

**Name: Dr. Tesfaye T. Pediatric Cardiologist Date-11/01/2012 E.C**

| Pediatric Echocardiography report  Patient Name: Nardos Shitu Patient ID: 023076  Gender:M Age:83 days Date of Report: 11/02/2012Eth.C  Clinical Finding: Incidental Murmur. TGSH7.3120. | | | |
| --- | --- | --- | --- |
| Features | **Finding** | **Features** | **Finding** |
| Profile | | **Atria** | |
| Abdominal situs | Solitus | Left atrium | Normal |
| Cardiac position | Levocardia | Right atrium | Normal |
| Systemic venous drainage | to RA | **Atrioventricular valves** | |
| Pulmonary venous drainage | to LA | Mitral valve | Annulus = 10mm |
| Atrioventricular connection | Concordant | Tricuspid valve | Annulus = 12mm  TAPSE = 12mm |
| Ventriculoarterial connection | Concordant | **Ventricles** | |
| Ventricular loop | d-Loop | Left ventricle | Normal |
| Septae | | Right ventricle | Mildly Hypertrophied  RV TDI S wave = 12cm/sec |
| Interventricular septum | 8mm Mal aligned Sub aortic VSD, L – R Shunt | **M-Mode** |  |
| Interatrial septum | PFO, L – R Shunt | AO |  |
| Great arteries | NRGA | LA |  |
| Aorta | ---- | LVIDd | 25mm |
| Pulmonary artery | Good sized MPA and Confluent Branch PAs. | LVIDs | 14.6mm |
| Semilunal valves |  | IVSd | 3.85mm |
| Aortic valve | Annulus = 9mm | IVSs | 5.5mm |
| Pulmonary valve | Annulus = 12mm, doming pulmonary Valve | LVPWd | 5.5mm |
| Doppler Measurement | | LVPWs | 5.5mm |
| Mitral | ---- | **EDV** | 22ml |
| Aortic | ------ | ESV | 5.64ml |
| Tricuspid | ------ | FS | 41% |
| pulmonic | Mild Valvar PS with PPG = 24mmHg | LVEF | 74% |
| Aortic arch | Left | **Coronary arteries** |  |
| PDA | No |  |  |
| Additional information |  | | |
|  |  | | |
| Final Diagnosis | 1. {S, D, S} Levocardia 2. TOF (Pink) 3. PFO, L – R Shunt 4. Good Biventricular Function | | |

**Done by Signature**

**Name: Dr. Tesfaye T. Pediatric Cardiologist Date-11/01/2012 E.C**

| Pediatric Echocardiography report  Patient Name: Habtam Tadesse Patient ID:023476  Gender: F Age: 12Years Date of Report: 10/02/2012Eth.C  Clinical Finding: Sydenham’s Chorea. TGSH7.3121. | | | |
| --- | --- | --- | --- |
| Features | **Finding** | **Features** | **Finding** |
| Profile | | **Atria** | |
| Abdominal situs | Solitus | Left atrium | Normal |
| Cardiac position | Levocardia | Right atrium | Normal |
| Systemic venous drainage | to RA | **Atrioventricular valves** | |
| Pulmonary venous drainage | to LA | Mitral valve | Annulus = 20mm, mildly Thickened Mitral valve leaflets |
| Atrioventricular connection | Concordant | Tricuspid valve | Annulus = 20mm |
| Ventriculoarterial connection | Concordant | **Ventricles** | |
| Ventricular loop | d-Loop | Left ventricle | Normal |
| Septae | | Right ventricle | Normal  RV TDI S wave = 12cm/sec |
| Interventricular septum | Intact | **M-Mode** |  |
| Interatrial septum | Intact | AO |  |
| Great arteries | NRGA | LA |  |
| Aorta | ---- | LVIDd | 40mm |
| Pulmonary artery | Good sized MPA and confluent Branch PAs. | LVIDs | 28mm |
| Semilunal valves |  | IVSd | 7.5mm |
| Aortic valve | Annulus = 16mm, Trileaflet | IVSs | 7.5mm |
| Pulmonary valve | Annulus = 18mm | LVPWd | 8mm |
| Doppler Measurement | | LVPWs | 8mm |
| Mitral | Mild MR, Posterior projection with velocity of 4m/sec seen on two planes | **EDV** | 70ml |
| Aortic | ---- | ESV | 29ml |
| Tricuspid | ---- | FS | 31% |
| pulmonic | ---- | LVEF | 60% |
| Aortic arch | Left | **Coronary arteries** |  |
| PDA | No |  |  |
| Additional information | No Coarctation of Aorta | | |
|  |  | | |
| Final Diagnosis | 1. {S, D, S} Levocardia 2. Mild MR 3. Thickened mitral valve leaflets 4. Good Biventricular Function | | |

**Done by Signature**

**Name: Dr. Tesfaye T. Pediatric Cardiologist Date-10/02/2012 E.C**

| Pediatric Echocardiography report  Patient Name: Kibur Mekuanint Patient ID:023561  Gender:M Age:10months Date of Report: 11/02/2012Eth.C  Clinical Finding: Cyanosis + Murmur + Diaphoresis. TGSH7.3122. | | | |
| --- | --- | --- | --- |
| Features | **Finding** | **Features** | **Finding** |
| Profile | | **Atria** | |
| Abdominal situs | Solitus | Left atrium | Normal |
| Cardiac position | Levocardia | Right atrium | Dilated |
| Systemic venous drainage | to RA | **Atrioventricular valves** | |
| Pulmonary venous drainage | to LA | Mitral valve | Atretic |
| Atrioventricular connection | Concordant (Atretic MV) | Tricuspid valve | Annulus = 23mm |
| Ventriculoarterial connection | Concordant | **Ventricles** | |
| Ventricular loop | d-Loop | Left ventricle | Smallish |
| Septae | | Right ventricle | Dilated |
| Interventricular septum | Multiple VSD (4mm Inlet and 4mm mid muscular) R – L Shunt | **M-Mode** |  |
| Interatrial septum | 7mm High OS ASD, L – R Shunt | AO |  |
| Great arteries | NRGA | LA |  |
| Aorta |  | LVIDd | mm |
| Pulmonary artery | MPA=**19**mm. Good sized confluent Branch PAs. | LVIDs | mm |
| Semilunal valves |  | IVSd | mm |
| Aortic valve | Annulus = 10mm | IVSs | mm |
| Pulmonary valve | Annulus = 16mm | LVPWd | mm |
| Doppler Measurement | | LVPWs | mm |
| Mitral | Atretic | **EDV** | ml |
| Aortic | ------ | ESV | ml |
| Tricuspid | ----- | FS |  |
| pulmonic | Mild PR, PPG = 22mmHg | LVEF |  |
| Aortic arch | Left | **Coronary arteries** |  |
| PDA | No |  |  |
| Additional information |  | | |
| Final Diagnosis | 1. {S, D, S} Levocardia 2. High OS ASD, L – R Shunt 3. Multiple VSD, R – L Shunt 4. Mitral atresia 5. Smallish LV 6. Severe Pulmonary Hypertension | | |

**Done by Signature**

**Name: Dr. Tesfaye T. Pediatric Cardiologist Date-11/02/2012 E.C**

| Pediatric Echocardiography report  Patient Name: Nahom Addiss Patient ID:022447  Gender:M Age:4months Date of Report: 11/02/2012Eth.C  Clinical Finding: DS. TGSH7.3123. | | | |
| --- | --- | --- | --- |
| Features | **Finding** | **Features** | **Finding** |
| Profile | | **Atria** | |
| Abdominal situs | Solitus | Left atrium | Normal |
| Cardiac position | Levocardia | Right atrium | Normal |
| Systemic venous drainage | to RA | **Atrioventricular valves** | |
| Pulmonary venous drainage | to LA | Mitral valve | Annulus = 9mm |
| Atrioventricular connection | Concordant | Tricuspid valve | Annulus = 12mm  TAPSE = 13mm |
| Ventriculoarterial connection | Concordant | **Ventricles** | |
| Ventricular loop | d-Loop | Left ventricle | Normal |
| Septae | | Right ventricle | Normal |
| Interventricular septum | Intact | **M-Mode** |  |
| Interatrial septum | 5mm OS ASD, L – R Shunt | AO |  |
| Great arteries | NRGA | LA |  |
| Aorta | --- | LVIDd | mm |
| Pulmonary artery | ---- | LVIDs | mm |
| Semilunal valves |  | IVSd | mm |
| Aortic valve | Annulus = 10mm | IVSs | mm |
| Pulmonary valve | Annulus = 10mm | LVPWd | mm |
| Doppler Measurement | | LVPWs | mm |
| Mitral | --- | **EDV** | ml |
| Aortic | ---- | ESV | ml |
| Tricuspid | Trivial TR, PPG = 33mmHg | FS | 31% |
| pulmonic | ---- | LVEF | 64% |
| Aortic arch | Left | **Coronary arteries** |  |
| PDA | No |  |  |
| Additional information |  | | |
|  |  | | |
| Final Diagnosis | 1. {S, D, S} Levocardia 2. Small OS ASD, L – R Shunt 3. Good Biventricular Function | | |

**Done by Signature**

**Name: Dr. Tesfaye T. Pediatric Cardiologist Date-11/02/2012 E.C**

| Pediatric Echocardiography report  Patient Name: Netsanet Tamiru Patient ID:023626  Gender: F Age:9yrs Date of Report: 13/02/2012Eth.C  Clinical Finding: Chest Pain + Friction rub. TGSH7.3124. | | | |
| --- | --- | --- | --- |
| Features | **Finding** | **Features** | **Finding** |
| Profile | | **Atria** | |
| Abdominal situs | Solitus | Left atrium | Normal |
| Cardiac position | Levocardia | Right atrium | Normal |
| Systemic venous drainage | to RA | **Atrioventricular valves** | |
| Pulmonary venous drainage | to LA | Mitral valve | Annulus = 19mm |
| Atrioventricular connection | Concordant | Tricuspid valve | Annulus = 19mm  TAPSE = 22mm |
| Ventriculoarterial connection | Concordant | **Ventricles** | |
| Ventricular loop | d-Loop | Left ventricle | Normal |
| Septae | | Right ventricle | Normal |
| Interventricular septum | Intact | **M-Mode** |  |
| Interatrial septum | Intact | AO |  |
| Great arteries | NRGA | LA |  |
| Aorta | ---- | LVIDd | 31mm |
| Pulmonary artery | ---- | LVIDs | 21mm |
| Semilunal valves |  | IVSd | 7mm |
| Aortic valve | Annulus = 14mm | IVSs | 8mm |
| Pulmonary valve | Annulus = 16mm | LVPWd | 9mm |
| Doppler Measurement | | LVPWs | 10mm |
| Mitral | ---- | **EDV** | 39ml |
| Aortic | ---- | ESV | 15ml |
| Tricuspid | Trivial TR, PPG = 11mmHg | FS | 32% |
| pulmonic | ---- | LVEF | 62% |
| Aortic arch | Left | **Coronary arteries** |  |
| PDA | No |  |  |
| Additional information | 6mm pericardial effusion on RV side | | |
|  |  | | |
| Final Diagnosis | 1. {S, D, S} Levocardia 2. Small Pericardial effusion secondary to ? 3. Good Biventricular Function | | |

**Done by Signature**

**Name: Dr. Tesfaye T. Pediatric Cardiologist Date-13/02/2012 E.C**

| Pediatric Echocardiography report  Patient Name: Tagele Aynayehu Patient ID:023794  Gender: M Age: 13yrs Date of Report: 13/02/2012Eth.C  Clinical Finding: Rheumatic Recurrence + Murmur + CHF + DOE + Palpitation. TGSH7.3125. | | | |
| --- | --- | --- | --- |
| Features | **Finding** | **Features** | **Finding** |
| Profile | | **Atria** | |
| Abdominal situs | Solitus | Left atrium | Dilated, 49 X 49mm |
| Cardiac position | Levocardia | Right atrium | Normal |
| Systemic venous drainage | to RA | **Atrioventricular valves** | |
| Pulmonary venous drainage | to LA | Mitral valve | Annulus = 19mm, thickened MVL. MVA = 2cm**2**. |
| Atrioventricular connection | Concordant | Tricuspid valve | Annulus = 23mm  TAPSE = 19mm |
| Ventriculoarterial connection | Concordant | **Ventricles** | |
| Ventricular loop | d-Loop | Left ventricle | Dilated |
| Septae | | Right ventricle | Normal |
| Interventricular septum | Intact | **M-Mode** |  |
| Interatrial septum | Intact | AO |  |
| Great arteries | NRGA | LA |  |
| Aorta | ---- | LVIDd | 53mm |
| Pulmonary artery | ---- | LVIDs | 36mm |
| Semilunal valves |  | IVSd | 5mm |
| Aortic valve | Annulus = 18mm | IVSs | 7.5mm |
| Pulmonary valve | Annulus = 21mm | LVPWd | 5mm |
| Doppler Measurement | | LVPWs | 7.5mm |
| Mitral | Severe MR, Posterior projection with a velocity of 5m/sec. Mild MS(?Functional PPG/MPG = 13/5mmHg) | **EDV** | 135ml |
| Aortic | ---- | ESV | 54ml |
| Tricuspid | Mild TR, PPG = 46mmHg | FS | 32% |
| pulmonic | Trivial PR | LVEF | 60% |
| Aortic arch | Left | **Coronary arteries** |  |
| PDA | No |  |  |
| Additional information |  | | |
| Final Diagnosis | 1. {S, D, S} Levocardia 2. Mild TR 3. Severe MR/ Mild MS (Functional) 4. Thickened Mitral valve leaflet 5. LA/LV Dilated 6. Good Biventricular Function 7. Mild pulmonary Hypertension | | |

**Done by Signature**

**Name: Dr. Tesfaye T. Pediatric Cardiologist Date-13/02/2012 E.C**

| Pediatric Echocardiography report  Patient Name: B/Tadfe Patient ID:022765  Gender:M Age:6days Date of Report: 13/02/2012Eth.C  Clinical Finding: Pre-op screening. TGSH7.3126. | | | |
| --- | --- | --- | --- |
| Features | **Finding** | **Features** | **Finding** |
| Profile | | **Atria** | |
| Abdominal situs | Solitus | Left atrium | Normal |
| Cardiac position | Levocardia | Right atrium | Normal |
| Systemic venous drainage | to RA | **Atrioventricular valves** | |
| Pulmonary venous drainage | to LA | Mitral valve | Annulus = 10mm |
| Atrioventricular connection | Concordant | Tricuspid valve | Annulus = 11mm  TAPSE = 11mm |
| Ventriculoarterial connection | Concordant | **Ventricles** | |
| Ventricular loop | d-Loop | Left ventricle | Normal |
| Septae | | Right ventricle | Normal |
| Interventricular septum | Intact | **M-Mode** |  |
| Interatrial septum | 4mm OS ASD, L – R Shunt | AO |  |
| Great arteries | NRGA | LA |  |
| Aorta |  | LVIDd | 15mm |
| Pulmonary artery |  | LVIDs | 11mm |
| Semilunal valves |  | IVSd | 5.7mm |
| Aortic valve | Annulus = 10mm | IVSs | 5mm |
| Pulmonary valve | Annulus = 11mm | LVPWd | 3mm |
| Doppler Measurement | | LVPWs | 5mm |
| Mitral | ---- | **EDV** | 6ml |
| Aortic | ---- | ESV | 2.5ml |
| Tricuspid | ---- | FS | 29 |
| Pulmonic` | ----- | LVEF | 60% |
| Aortic arch | Left | **Coronary arteries** |  |
| PDA | No |  |  |
| Additional information | **PROMINENT DILATED CORONARY SINUS** | | |
|  |  | | |
| Final Diagnosis | 1. {S, D, S} Levocardia 2. Small OS ASD, L – R shunt 3. Prominent Coronary sinus secondary to ? 4. Good Biventricular Function | | |

**Done by Signature**

**Name: Dr. Tesfaye T. Pediatric Cardiologist Date-13/02/2012 E.C**

| Pediatric Echocardiography report  Patient Name: Bersabeh Patient ID:  Gender: Age: Date of Report: 13/02/2012Eth.C  INCOMPLETE DOCUMENT | | | |
| --- | --- | --- | --- |
| Features | **Finding** | **Features** | **Finding** |
| Profile | | **Atria** | |
| Abdominal situs | Solitus | Left atrium | Normal |
| Cardiac position | Levocardia | Right atrium | Normal |
| Systemic venous drainage | to RA | **Atrioventricular valves** | |
| Pulmonary venous drainage | to LA | Mitral valve | Annulus = 15mm |
| Atrioventricular connection | Concordant | Tricuspid valve | Annulus = 19mm  TAPSE = 20mm |
| Ventriculoarterial connection | Concordant | **Ventricles** | |
| Ventricular loop | d-Loop | Left ventricle | Normal |
| Septae | | Right ventricle | Normal  RV TDI S wave = 12cm/sec |
| Interventricular septum | Intact | **M-Mode** |  |
| Interatrial septum | Intact | AO |  |
| Great arteries | NRGA | LA |  |
| Aorta | ---- | LVIDd | 30mm |
| Pulmonary artery | ---- | LVIDs | 19mm |
| Semilunal valves |  | IVSd | 4.5mm |
| Aortic valve | Annulus = 14mm | IVSs | 7.5mm |
| Pulmonary valve | Annulus = 16mm | LVPWd | 5mm |
| Doppler Measurement | | LVPWs | 7.5mm |
| Mitral | ------ | **EDV** | 35ml |
| Aortic | ---- | ESV | 11ml |
| Tricuspid | ---- | FS | 36% |
| pulmonic | ---- | LVEF | 68% |
| Aortic arch | Left | **Coronary arteries** |  |
| PDA | No |  |  |
| Additional information | No coarctation of aorta | | |
|  |  | | |
| Final Diagnosis | 1. Normal Echocardiography Study | | |

**Done by Signature**

**Name: Dr. Tesfaye T. Pediatric Cardiologist Date-13/02/2012 E.C**

| Pediatric Echocardiography report  Patient Name: Alemush Wassie Patient ID:024015  Gender: F Age:12 Date of Report: 18/02/2012Eth.C  Clinical Finding: Palpitation. TGSH7.3127. | | | |
| --- | --- | --- | --- |
| Features | **Finding** | **Features** | **Finding** |
| Profile | | **Atria** | |
| Abdominal situs | Solitus | Left atrium | Normal |
| Cardiac position | Levocardia | Right atrium | Normal |
| Systemic venous drainage | to RA | **Atrioventricular valves** | |
| Pulmonary venous drainage | to LA | Mitral valve | Annulus = 16mm |
| Atrioventricular connection | Concordant | Tricuspid valve | Annulus = 19mm  TAPSE = 20mm |
| Ventriculoarterial connection | Concordant | **Ventricles** | |
| Ventricular loop | d-Loop | Left ventricle | Normal |
| Septae | | Right ventricle | Normal |
| Interventricular septum | Intact | **M-Mode** |  |
| Interatrial septum | Intact | AO |  |
| Great arteries | NRGA | LA |  |
| Aorta | ---- | LVIDd | 34mm |
| Pulmonary artery | Good sized MPA and confluent Branch PAs. | LVIDs | 23mm |
| Semilunal valves |  | IVSd | 7mm |
| Aortic valve | Annulus = 18mm, Trileaflet | IVSs | 6mm |
| Pulmonary valve | Annulus = 17mm | LVPWd | 5mm |
| Doppler Measurement | | LVPWs | 7mm |
| Mitral | ---- | **EDV** | 49ml |
| Aortic | ---- | ESV | 19ml |
| Tricuspid | Trivial TR, PPG = 35mmHg | FS | 32% |
| pulmonic | Trivial PR, PPG = 21mmHg | LVEF | 61% |
| Aortic arch | Left | **Coronary arteries** |  |
| PDA | No |  |  |
| Additional information | No coarctation of Aorta | | |
|  | No effusion | | |
| Final Diagnosis | Normal Echocardiography Study | | |

**Done by Signature**

**Name: Dr. Tesfaye T. Pediatric Cardiologist Date-18/02/2012 E.C**

| Pediatric Echocardiography report  Patient Name: Sewnet Dereje Patient ID:024524  Gender: F Age:8yrs Date of Report: 18/02/2012Eth.C  Clinical Finding: ARF + Murmur. TGSH7.3128. | | | |
| --- | --- | --- | --- |
| Features | **Finding** | **Features** | **Finding** |
| Profile | | **Atria** | |
| Abdominal situs | Solitus | Left atrium | Dilated, 57 X 50mm |
| Cardiac position | Levocardia | Right atrium |  |
| Systemic venous drainage | to RA | **Atrioventricular valves** | |
| Pulmonary venous drainage | to LA | Mitral valve | Annulus = 25mm. thickened mitral valve leaflet. MVA = 2.7cm**2**. |
| Atrioventricular connection | Concordant | Tricuspid valve | Annulus = 23mm  TAPSE = 23mm |
| Ventriculoarterial connection | Concordant | **Ventricles** | |
| Ventricular loop | d-Loop | Left ventricle | Dilated |
| Septae | | Right ventricle | RV TDI S wave 11cm/sec |
| Interventricular septum | Intact | **M-Mode** |  |
| Interatrial septum | Intact | AO |  |
| Great arteries | NRGA | LA |  |
| Aorta |  | LVIDd | 5mm |
| Pulmonary artery | MPA = 19mm, RPA = 14mm, LPA = 14mm | LVIDs | 3mm |
| Semilunal valves |  | IVSd | 7mm |
| Aortic valve | Annulus = 21mm, Trileaflet | IVSs | 7mm |
| Pulmonary valve | Annulus = 17mm | LVPWd | 7mm |
| Doppler Measurement | | LVPWs | 9mm |
| Mitral | Severe MR, Posterior projection with velocity of 5m/sec. | **EDV** | 135ml |
| Aortic | ------- | ESV | 44ml |
| Tricuspid | Mild TR, PPG = 32mmHg | FS | 37% |
| pulmonic | Trivial PR, PPG = 30mmHg | LVEF | 67% |
| Aortic arch | Left | **Coronary arteries** |  |
| PDA | No |  |  |
| Additional information |  | | |
|  |  | | |
| Final Diagnosis | 1. {S, D, S} Levocardia 2. LA/LV Dilated 3. Severe MR 4. Mild TR 5. Good Biventricular Function | | |

**Done by Signature**

**Name: Dr. Tesfaye T. Pediatric Cardiologist Date-18/02/2012 E.C**

| Pediatric Echocardiography report  Patient Name: B/Ayal Gashaw Patient ID: 022084 Gender: F Age: 17 days  Date of Report: 18/02/2012Eth.C  Clinical Finding: Cyanosis +RD + Murmur. TGSH7.3129. | | | |
| --- | --- | --- | --- |
| Features | **Finding** | **Features** | **Finding** |
| Profile | | **Atria** | |
| Abdominal situs | Solitus | Left atrium | Normal |
| Cardiac position | Levocardia | Right atrium | Normal |
| Systemic venous drainage | to RA | **Atrioventricular valves** | |
| Pulmonary venous drainage | to LA | Mitral valve | Annulus = 12mm |
| Atrioventricular connection | Concordant | Tricuspid valve | atretic |
| Ventriculoarterial connection | Concordant | **Ventricles** | |
| Ventricular loop | d-Loop | Left ventricle | Normal |
| Septae | | Right ventricle | Smallish |
| Interventricular septum | Intact | **M-Mode** |  |
| Interatrial septum | 6mm Fenestrated ASD, R – L Shunt | AO |  |
| Great arteries | NRGA | LA |  |
| Aorta | ----- | LVIDd | 20mm |
| Pulmonary artery | MPA = 6.8mm, RPA = 3.2mm, LPA = 4.9mm. | LVIDs | 12mm |
| Semilunal valves |  | IVSd | 5.5mm |
| Aortic valve | Annulus = 9mm | IVSs | 5.5mm |
| Pulmonary valve | Annulus = Dysplastic | LVPWd | 5.5mm |
| Doppler Measurement | | LVPWs | 5.5mm |
| Mitral | ----- | **EDV** | 12ml |
| Aortic | ------ | ESV | 3ml |
| Tricuspid | Atretic | FS | 38% |
| pulmonic | Dysplastic | LVEF | 72% |
| Aortic arch | Left | **Coronary arteries** |  |
| PDA | 3mm PDA, L – R Shunt |  |  |
| Additional information | No Coarctation of Aorta | | |
|  |  | | |
| Final Diagnosis | 1. {S, D, S} Levocardia 2. Small Fenestrated ASD, R – L Shunt 3. Smallish RV 4. Tricuspid Atresia type IA 5. Large PDA, L – R Shunt 6. Good LV Function | | |

**Done by Signature**

**Dr. Tesfaye T. Pediatric Cardiologist ________________ Date: 18/02/2012Eth.C**

| Pediatric Echocardiography report  Patient Name Misrak Muhabaw Patient ID: 023804  Gender: f Age: 1 2/12 Date of Report: 20/02/2012Eth.C  Clinical Finding: Syndromic + Murmur + Diaphoresis. TGSH7.3130. | | | |
| --- | --- | --- | --- |
| Features | **Finding** | **Features** | **Finding** |
| Profile | | **Atria** | |
| Abdominal situs | Solitus | Left atrium | Dilated |
| Cardiac position | Levocardia | Right atrium | Normal |
| Systemic venous drainage | to RA | **Atrioventricular valves** | |
| Pulmonary venous drainage | to LA | Mitral valve | Annulus = 15mm |
| Atrioventricular connection | Concordant | Tricuspid valve | Annulus = 14mm  TAPSE = 12mm |
| Ventriculoarterial connection | Concordant | **Ventricles** | |
| Ventricular loop | d-Loop | Left ventricle | Dilated |
| Septae | | Right ventricle | Normal |
| Interventricular septum | Intact | **M-Mode** |  |
| Interatrial septum | Intact | AO |  |
| Great arteries | NRGA | LA |  |
| Aorta | ---- | LVIDd | 38mm |
| Pulmonary artery | ---- | LVIDs | 26mm |
| Semilunal valves |  | IVSd | 4mm |
| Aortic valve | Annulus = 9mm | IVSs | 4mm |
| Pulmonary valve | Annulus = 13mm | LVPWd | 7.5mm |
| Doppler Measurement | | LVPWs | 7.5mm |
| Mitral | ---- | **EDV** | 61ml |
| Aortic | ---- | ESV | 25ml |
| Tricuspid | ---- | FS | 31 |
| pulmonic | ---- | LVEF | 60 |
| Aortic arch | Left | **Coronary arteries** |  |
| PDA | 2.5mm PDA, L – R Shunt |  |  |
| Additional information | Syndromic baby. | | |
|  |  | | |
| Final Diagnosis | 1. {S, D, S} Levocardia 2. Moderate PDA, L – R Shunt 3. Good Biventricular Function | | |

**Done by Signature**

**Name: Dr. Tesfaye T. Pediatric Cardiologist Date-20/02/2012 E.C**

| Pediatric Echocardiography report  Patient Name: Eyerusalm Bayinesagne Patient ID: 024438  Gender: F Age: 5/12 Date of Report: 20/02/2012Eth.C  Clinical Finding: DS + Diaphoresis + Murmur. TGSH7.3131. | | | |
| --- | --- | --- | --- |
| Features | **Finding** | **Features** | **Finding** |
| Profile | | **Atria** | |
| Abdominal situs | Solitus | Left atrium | Dilated |
| Cardiac position | Levocardia | Right atrium | Dilated |
| Systemic venous drainage | to RA | **Atrioventricular valves** | |
| Pulmonary venous drainage | to LA | Mitral valve | ------ |
| Atrioventricular connection | Common AV Valve | Tricuspid valve | ------- |
| Ventriculoarterial connection | Concordant | **Ventricles** | |
| Ventricular loop | d-Loop | Left ventricle | Mildly Dilated |
| Septae : No tongue of tissue in b/n the defects | | Right ventricle | Mildly Dilated |
| Interventricular septum | Large inlet VSD, L – R Shunt | **M-Mode** |  |
| Interatrial septum | Large premium ASD, L – R Shunt | AO |  |
| Great arteries | NRGA | LA |  |
| Aorta |  | LVIDd | 21mm |
| Pulmonary artery | MPA = 11mm | LVIDs | 14mm |
| Semilunal valves |  | IVSd | 5mm |
| Aortic valve | Annulus = 8mm | IVSs | 6mm |
| Pulmonary valve | Annulus = 13mm | LVPWd | 6.5mm |
| Doppler Measurement | | LVPWs | 6.5mm |
| Mitral | Severe left AVVR | **EDV** | 15ml |
| Aortic | ---- | ESV | 5ml |
| Tricuspid | Mild right AVVR | FS | 33% |
| pulmonic | ---- | LVEF | 65% |
| Aortic arch | Left | **Coronary arteries** |  |
| PDA | No |  |  |
| Additional information |  | | |
|  |  | | |
| Final Diagnosis | 1. {S, D, S} Levocardia 2. Common complete AVSD 3. Severe Left AVVR 4. Mild right AVVR 5. Good LV Function | | |

**Done by Signature**

**Name: Dr. Tesfaye T. Pediatric Cardiologist Date-20/02/2012 E.C**

| Pediatric Echocardiography report  Patient Name: Gebeyew Wendyifraw Patient ID:024487  Gender: M Age: 11Months Date of Report: 20/02/2012Eth.C  Clinical Finding: Incidental Murmur + Diaphoresis. TGSH7.3132. | | | |
| --- | --- | --- | --- |
| Features | **Finding** | **Features** | **Finding** |
| Profile | | **Atria** | |
| Abdominal situs | Solitus | Left atrium | Dilated |
| Cardiac position | Levocardia | Right atrium | Normal |
| Systemic venous drainage | to RA | **Atrioventricular valves** | |
| Pulmonary venous drainage | to LA | Mitral valve | Annulus = 16mm |
| Atrioventricular connection | Concordant | Tricuspid valve | Annulus = 13mm  TAPSE = 14mm |
| Ventriculoarterial connection | Concordant | **Ventricles** | |
| Ventricular loop | d-Loop | Left ventricle | Dilated |
| Septae | | Right ventricle | Normal |
| Interventricular septum | Intact, bowed to RV | **M-Mode** |  |
| Interatrial septum | PFO, L – R Shunt. Bowed to RA | AO |  |
| Great arteries | NRGA | LA |  |
| Aorta | ---- | LVIDd | mm |
| Pulmonary artery | ----- | LVIDs | mm |
| Semilunal valves |  | IVSd | mm |
| Aortic valve | Annulus = 12mm | IVSs | mm |
| Pulmonary valve | Annulus = 12mm | LVPWd | mm |
| Doppler Measurement | | LVPWs | mm |
| Mitral | Mild MR | **EDV** | ml |
| Aortic | ---- | ESV | ml |
| Tricuspid | Mild TR, PPG = 43mmHg | FS | 32% |
| pulmonic | Mild PR, PPG = 48mmHg | LVEF | 62% |
| Aortic arch | Left | **Coronary arteries** |  |
| PDA | 4mm PDA, L – R Shunt |  |  |
| Additional information | 12mm Pericardial effusion on the RA, RV Side. | | |
|  |  | | |
| Final Diagnosis | 1. {S, D, S} Levocardia 2. LA/LV Dilated 3. PFO, L – R Shunt 4. Large PDA, L – R Shunt 5. Good Biventricular Function 6. Moderate Pericardial effusion on the RA/RV Side | | |

**Done by Signature**

**Name: Dr. Tesfaye T. Pediatric Cardiologist Date-20 /02/2012 E.C**

| Pediatric Echocardiography report  Patient Name: Demekech Birhanu Patient ID:024277  Gender: F Age:4 Month Date of Report: 20/02/2012Eth.C  Clinical Finding: Cyanosis + Murmur. TGSH7.3133. | | | |
| --- | --- | --- | --- |
| Features | **Finding** | **Features** | **Finding** |
| Profile | | **Atria** | |
| Abdominal situs | Solitus | Left atrium | Normal |
| Cardiac position | Levocardia | Right atrium | Normal |
| Systemic venous drainage | to RA | **Atrioventricular valves** | |
| Pulmonary venous drainage | to LA | Mitral valve | Annulus = 12mm |
| Atrioventricular connection | Concordant | Tricuspid valve | Annulus = 15mm |
| Ventriculoarterial connection | Discordant | **Ventricles** | |
| Ventricular loop | d-Loop | Left ventricle | Normal |
| Septae | | Right ventricle | Normal |
| Interventricular septum | 11mm Sub-aortic VSD | **M-Mode: Normal LV Function on eye balling** | |
| Interatrial septum | Intact | AO |  |
| Great arteries | d-TGA | LA |  |
| Aorta | To the right and anterior | LVIDd | mm |
| Pulmonary artery | To the left and posterior | LVIDs | mm |
| Semilunal valves |  | IVSd | mm |
| Aortic valve | Annulus = 10mm | IVSs | mm |
| Pulmonary valve | Annulus = 14mm | LVPWd | mm |
| Doppler Measurement | | LVPWs | mm |
| Mitral | ---- | **EDV** | ml |
| Aortic | ----- | ESV | ml |
| Tricuspid | ------- | FS |  |
| pulmonic | Mild valvar PS, PPG = 30mmHg | LVEF |  |
| Aortic arch | Left | **Coronary arteries** |  |
| PDA | No |  |  |
| Additional information |  | | |
|  | LIMITTED Echo window | | |
| Final Diagnosis | 1. {S, D, D} Levocardia 2. d-TGA with Sub-aortic VSD 3. Mild Valvar PS (LVOTO) 4. Good Function | | |

**Done by Signature**

**Name: Dr. Tesfaye T. Pediatric Cardiologist Date-20/02/2012 E.C**

| Pediatric Echocardiography report  Patient Name: B/Etatu Molla Patient ID: 022734  Gender: M Age:24days Date of Report: 25/02/2012Eth.C  Clinical Finding: RD. TGSH7.3134. | | | |
| --- | --- | --- | --- |
| Features | **Finding** | **Features** | **Finding** |
| Profile | | **Atria** | |
| Abdominal situs | Solitus | Left atrium | Normal |
| Cardiac position | Levocardia | Right atrium | Normal |
| Systemic venous drainage | to RA | **Atrioventricular valves** | |
| Pulmonary venous drainage | to LA | Mitral valve | Annulus= 8mm |
| Atrioventricular connection | Concordant | Tricuspid valve | Annulus = 9mm  TAPSE = 9mm |
| Ventriculoarterial connection | Concordant | **Ventricles** | |
| Ventricular loop | d-Loop | Left ventricle | Normal |
| Septae | | Right ventricle | Normal |
| Interventricular septum | Intact | **M-Mode** |  |
| Interatrial septum | PFO, L – R Shunt | AO |  |
| Great arteries | NRGA | LA |  |
| Aorta | ---- | LVIDd | 14.5mm |
| Pulmonary artery | ---- | LVIDs | 9mm |
| Semilunal valves |  | IVSd | 3mm |
| Aortic valve | Annulus = 7mm | IVSs | 4mm |
| Pulmonary valve | Annulus = 9mm | LVPWd | 3.5mm |
| Doppler Measurement | | LVPWs | 5mm |
| Mitral | ---- | **EDV** | 5.6ml |
| Aortic | ---- | ESV | 1.7ml |
| Tricuspid | ---- | FS | 36% |
| pulmonic | ---- | LVEF | 69% |
| Aortic arch | Left | **Coronary arteries** |  |
| PDA | No |  |  |
| Additional information |  | | |
|  |  | | |
| Final Diagnosis | 1. {S, D, S} Levocardia 2. PFO, L – R Shunt | | |

**Done by Signature**

**Name: Dr. Tesfaye T. Pediatric Cardiologist Date-25/02/2012 E.C**

| Pediatric Echocardiography report  Patient Name: Aysheshim Muluken Patient ID:025346  Gender: M Age:7months Date of Report: 27/02/2012Eth.C  Clinical Finding: _Incidental Murmur. TGSH7.3135. | | | |
| --- | --- | --- | --- |
| Features | **Finding** | **Features** | **Finding** |
| Profile | | **Atria** | |
| Abdominal situs | Solitus | Left atrium | Normal |
| Cardiac position | Levocardia | Right atrium | Normal |
| Systemic venous drainage | to RA | **Atrioventricular valves** | |
| Pulmonary venous drainage | to LA | Mitral valve | Annulus = 14mm, |
| Atrioventricular connection | Concordant | Tricuspid valve | Annulus = 17mm |
| Ventriculoarterial connection | Concordant | **Ventricles** | |
| Ventricular loop | d-Loop | Left ventricle | Normal |
| Septae | | Right ventricle | Normal |
| Interventricular septum | 4mm sub arterial VSD, L – R Shunt. | **M-Mode** |  |
| Interatrial septum | Intact | AO |  |
| Great arteries | NRGA | LA |  |
| Aorta | Asc. | LVIDd | 29mm |
| Pulmonary artery | Good sized MPA and Branch PAs. | LVIDs | 20mm |
| Semilunal valves |  | IVSd | 5.6mm |
| Aortic valve | **Annulus = 15mm** | IVSs | 7.5mm |
| Pulmonary valve | Annulus = 14mm | LVPWd | 5mm |
| Doppler Measurement | | LVPWs | 5.6mm |
| Mitral |  | **EDV** | 32ml |
| Aortic |  | ESV | 13ml |
| Tricuspid |  | FS | 31% |
| pulmonic |  | LVEF | 61% |
| Aortic arch | Left | **Coronary arteries** |  |
| PDA | No |  |  |
| Additional information |  | | |
|  | | |
| Final Diagnosis | 1. {S, D, S} Levocardia 2. Small Sub-aortic VSD, L – R Shunt 3. Good LV Function | | |

**Done by Signature**

**Name: Dr. Tesfaye T. Pediatric Cardiologist Date-27/02/2012 E.C**

| Pediatric Echocardiography report  Patient Name: Baby of Amaledech Addis Patient ID: 023220  Gender: F Age:20 Daye Date of Report: 25/02/2012Eth.C  Clinical Finding: RD. TGSH7.3136. | | | |
| --- | --- | --- | --- |
| Features | **Finding** | **Features** | **Finding** |
| Profile | | **Atria** | |
| Abdominal situs | Solitus | Left atrium | Normal |
| Cardiac position | Levocardia | Right atrium | Normal |
| Systemic venous drainage | to RA | **Atrioventricular valves** | |
| Pulmonary venous drainage | to LA | Mitral valve | Annulus = 9mm |
| Atrioventricular connection | Concordant | Tricuspid valve | Annulus = 12mm  TAPSE = 10mm |
| Ventriculoarterial connection | Concordant | **Ventricles** | |
| Ventricular loop | d-Loop | Left ventricle | Normal |
| Septae | | Right ventricle | Normal |
| Interventricular septum | Intact | **M-Mode** |  |
| Interatrial septum | PFO, L – R Shunt | AO |  |
| Great arteries | NRGA | LA |  |
| Aorta | ---- | LVIDd | 16.5mm |
| Pulmonary artery | ---- | LVIDs | 11.3mm |
| Semilunal valves |  | IVSd | 5.5mm |
| Aortic valve | Annulus = 8mm | IVSs | 5mm |
| Pulmonary valve | Annulus = 10mm | LVPWd | 4mm |
| Doppler Measurement | | LVPWs | 5mm |
| Mitral | ----- | **EDV** | 7.76ml |
| Aortic | ----- | ESV | 2.86ml |
| Tricuspid | ----- | FS | 31% |
| pulmonic | Trivial PR | LVEF | 63% |
| Aortic arch | Left | **Coronary arteries** |  |
| PDA | No |  |  |
| Additional information |  | | |
|  |  | | |
| Final Diagnosis | 1. {S, D, S} Levocardia 2. PFO, L – R Shunt | | |

**Done by Signature**

**Name: Dr. Tesfaye T. Pediatric Cardiologist Date-25/02/2012 E.C**

| Pediatric Echocardiography report  Patient Name: Yebirgual Lijalem Patient ID: 025247  Gender: F Age: 9yrs Date of Report: 25/02/2012Eth.C  Clinical Finding: _Rheumatic Recurrence + murmur + DOE + Palpitation. TGSH7.3137. | | | |
| --- | --- | --- | --- |
| Features | **Finding** | **Features** | **Finding** |
| Profile | | **Atria** | |
| Abdominal situs | Solitus | Left atrium | Dilated, 40mm X 47mm |
| Cardiac position | Levocardia | Right atrium | Normal |
| Systemic venous drainage | to RA | **Atrioventricular valves** | |
| Pulmonary venous drainage | to LA | Mitral valve | Thickened, clubbed MV leaflet. MVA = 1.15cm2. |
| Atrioventricular connection | Concordant | Tricuspid valve | TAPSE = 20mm |
| Ventriculoarterial connection | Concordant | **Ventricles** | |
| Ventricular loop | d-Loop | Left ventricle | Dilated |
| Septae | | Right ventricle | Normal |
| Interventricular septum |  | **M-Mode** |  |
| Interatrial septum | Intact | AO |  |
| Great arteries | NRGA | LA |  |
| Aorta |  | LVIDd | 42mm |
| Pulmonary artery |  | LVIDs | 28mm |
| Semilunal valves |  | IVSd | 7mm |
| Aortic valve | Annulus = 16mm | IVSs | 7mm |
| Pulmonary valve | Annulus = 16mm | LVPWd | 7mm |
| Doppler Measurement | | LVPWs | 7mm |
| Mitral | Severe MR, VELOCITY OF 4.6m/sec, posteriorly projected, holosystolic. Moderate MS, PPG/MPG = 12/9mmHg. | **EDV** | 80ml |
| Aortic | Moderate AR, PHT = 361ms. | ESV | 30ml |
| Tricuspid | Trivial TR, PPG = 15mmHg | FS | 33% |
| pulmonic | ------- | LVEF | 63% |
| Aortic arch | Left | **Coronary arteries** |  |
| PDA | No |  |  |
| Additional information |  | | |
|  |  | | |
| Final Diagnosis | 1. {S, D, S} Levocardia 2. LA/LV Dilated 3. Thickened Mitral valve leaflet 4. Severe MR 5. Moderate MS 6. Moderate AR 7. Good Biventricular Function | | |

**Done by Signature**

**Name: Dr. Tesfaye T. Pediatric Cardiologist Date-25/02/2012 E.C**

| Pediatric Echocardiography report  Patient Name: Hana Amsalu Patient ID:024899  Gender: F Age:2yrs Date of Report: 25/02/2012Eth.C  Clinical Finding: _Diaphoresis + Murmur. TGSH7.3138. | | | |
| --- | --- | --- | --- |
| Features | **Finding** | **Features** | **Finding** |
| Profile | | **Atria** | |
| Abdominal situs | Solitus | Left atrium | Dilated |
| Cardiac position | Levocardia | Right atrium | Normal |
| Systemic venous drainage | to RA | **Atrioventricular valves** | |
| Pulmonary venous drainage | to LA | Mitral valve | Annulus = 22mm |
| Atrioventricular connection | Concordant | Tricuspid valve | Annulus = 17mm  TAPSE = 20mm |
| Ventriculoarterial connection | Concordant | **Ventricles** | |
| Ventricular loop | d-Loop | Left ventricle | Dilated |
| Septae | | Right ventricle | Normal |
| Interventricular septum | Intact | **M-Mode** |  |
| Interatrial septum | Intact | AO |  |
| Great arteries | NRGA | LA |  |
| Aorta | ---- | LVIDd | 45mm |
| Pulmonary artery | Good sized MPA and Confluent Branch PAs. | LVIDs | 28mm |
| Semilunal valves |  | IVSd | 5mm |
| Aortic valve | Annulus = 15mm | IVSs | 6mm |
| Pulmonary valve | Annulus = 17mm | LVPWd | 5mm |
| Doppler Measurement | | LVPWs | 6mm |
| Mitral | Moderate MR | **EDV** | 92ml |
| Aortic | ---- | ESV | 31ml |
| Tricuspid | ---- | FS | 36% |
| pulmonic | ---- | LVEF | 66% |
| Aortic arch | Left | **Coronary arteries** |  |
| PDA | 2.5mm PDA, L – R Shunt |  |  |
| Additional information |  | | |
|  |  | | |
| Final Diagnosis | 1. {S, D, S} Levocardia 2. LA/LV Dilated 3. Moderate MR 4. Moderate PDA, L – R Shunt 5. Good Biventricular Function | | |

**Done by Signature**

**Name: Dr. Tesfaye T. Pediatric Cardiologist Date-25/02/2012 E.C**

| Pediatric Echocardiography report  Patient Name: Amanuel Andargie Patient ID:025376  Gender: M Age: 39Day Date of Report: 27/02/2012Eth.C  Clinical Finding: DS + RD. TGSH7.3139. | | | |
| --- | --- | --- | --- |
| Features | **Finding** | **Features** | **Finding** |
| Profile | | **Atria** | |
| Abdominal situs | Solitus | Left atrium |  |
| Cardiac position | Levocardia | Right atrium |  |
| Systemic venous drainage | to RA | **Atrioventricular valves** | |
| Pulmonary venous drainage | to LA | Mitral valve | Annulus = 12mm |
| Atrioventricular connection | Concordant | Tricuspid valve | Annulus = 16mm |
| Ventriculoarterial connection | Concordant | **Ventricles** | |
| Ventricular loop | d-Loop | Left ventricle |  |
| Septae | | Right ventricle |  |
| Interventricular septum | Intact | **M-Mode: Normal LV Function (eye balling)** | |
| Interatrial septum | PFO, L – R Shunt | AO |  |
| Great arteries | NRGA | LA |  |
| Aorta |  | LVIDd | mm |
| Pulmonary artery |  | LVIDs | mm |
| Semilunal valves |  | IVSd | mm |
| Aortic valve | Annulus = 9mm | IVSs | mm |
| Pulmonary valve | Annulus = 12mm | LVPWd | mm |
| Doppler Measurement | | LVPWs | mm |
| Mitral |  | **EDV** | ml |
| Aortic |  | ESV | ml |
| Tricuspid | Trivial TR, PPG = 23mmHg | FS |  |
| pulmonic |  | LVEF |  |
| Aortic arch | Left | **Coronary arteries** |  |
| PDA | No |  |  |
| Additional information |  | | |
|  |  | | |
| Final Diagnosis | 1. {S, D, S} Levocardia 2. PFO, L – R Shunt | | |

**Done by Signature**

**Name: Dr. Tesfaye T. Pediatric Cardiologist Date-27/02/2012 E.C**

| Pediatric Echocardiography report  Patient Name: Agenagni Muche Patient ID:0125669  Gender: M Age:5months Date of Report: 27/02/2012Eth.C  Clinical Finding: Recurrent Chest Infection. TGSH7.3140. | | | |
| --- | --- | --- | --- |
| Features | **Finding** | **Features** | **Finding** |
| Profile | | **Atria** | |
| Abdominal situs | Solitus | Left atrium | Normal |
| Cardiac position | Levocardia | Right atrium | Normal |
| Systemic venous drainage | to RA | **Atrioventricular valves** | |
| Pulmonary venous drainage | to LA | Mitral valve | Annulus = 12 |
| Atrioventricular connection | Concordant | Tricuspid valve | Annulus = 16mm  TAPSE = 12mm |
| Ventriculoarterial connection | Concordant | **Ventricles** | |
| Ventricular loop | d-Loop | Left ventricle | Normal |
| Septae | | Right ventricle | Normal |
| Interventricular septum | Intact | **M-Mode** |  |
| Interatrial septum | PFO, L – R Shunt | AO |  |
| Great arteries | NRGA | LA |  |
| Aorta |  | LVIDd | 17.6mm |
| Pulmonary artery |  | LVIDs | 10.2mm |
| Semilunal valves |  | IVSd | 4mm |
| Aortic valve | Annulus = 11mm | IVSs | 4mm |
| Pulmonary valve | Annulus = 13mm | LVPWd | 4mm |
| Doppler Measurement | | LVPWs | 5mm |
| Mitral | ---- | **EDV** | 9ml |
| Aortic | ---- | ESV | 2ml |
| Tricuspid | Trivial TR, PPG = 13mmHg | FS | 40% |
| pulmonic | Trivial PR, PPG = 32mmHg | LVEF | 76% |
| Aortic arch | Left | **Coronary arteries** |  |
| PDA | No |  |  |
| Additional information |  | | |
|  |  | | |
| Final Diagnosis | 1. {S, D, S} Levocardia 2. PFO, L – R Shunt | | |

**Done by Signature**

**Name: Dr. Tesfaye T. Pediatric Cardiologist Date-27/02/2012 E.C**

| Pediatric Echocardiography report  Patient Name: Bethlehem wota Patient ID: 025458  Gender: F Age: 6 Months Date of Report: 27/02/2012Eth.C  Clinical Finding: DS(Screening). TGSH7.3141. | | | |
| --- | --- | --- | --- |
| Features | **Finding** | **Features** | **Finding** |
| Profile | | **Atria** | |
| Abdominal situs | Solitus | Left atrium | Normal |
| Cardiac position | Levocardia | Right atrium | Normal |
| Systemic venous drainage | to RA | **Atrioventricular valves** | |
| Pulmonary venous drainage | to LA | Mitral valve | Annulus = 12mm |
| Atrioventricular connection | Concordant | Tricuspid valve | Annulus = 14mm  TAPSE = 12mm |
| Ventriculoarterial connection | Concordant | **Ventricles** | |
| Ventricular loop | d-Loop | Left ventricle | Normal |
| Septae | | Right ventricle | Normal |
| Interventricular septum | Intact | **M-Mode** |  |
| Interatrial septum | Intact | AO |  |
| Great arteries | NRGA | LA |  |
| Aorta |  | LVIDd | mm |
| Pulmonary artery |  | LVIDs | mm |
| Semilunal valves |  | IVSd | mm |
| Aortic valve | Annulus = 10mm | IVSs | mm |
| Pulmonary valve | Annulus = 12mm | LVPWd | mm |
| Doppler Measurement | | LVPWs | mm |
| Mitral |  | **EDV** | ml |
| Aortic |  | ESV | ml |
| Tricuspid |  | FS | 30% |
| pulmonic |  | LVEF | 61% |
| Aortic arch | Left | **Coronary arteries** |  |
| PDA | No |  |  |
| Additional information |  | | |
|  |  | | |
| Final Diagnosis | 1. Normal Echocardiography Study. | | |

**Done by Signature**

**Name: Dr. Tesfaye T. Pediatric Cardiologist Date-27/02/2012 E.C**

| Pediatric Echocardiography report  Patient Name: Bemnet Firew Patient ID: 003412  Gender: F Age: 5 6/12 Date of Report: 27/02/2012Eth.C  Clinical Finding: _FTT + RD + CHF + Murmur. TGSH7.3142. | | | |
| --- | --- | --- | --- |
| Features | **Finding** | **Features** | **Finding** |
| Profile | | **Atria** | |
| Abdominal situs | Solitus | Left atrium | Dilated |
| Cardiac position | Levocardia | Right atrium | Normal |
| Systemic venous drainage | to RA | **Atrioventricular valves** | |
| Pulmonary venous drainage | to LA | Mitral valve | Annulus = 20mm |
| Atrioventricular connection | Concordant | Tricuspid valve | Annulus = 13mm  TAPSE = 20mm |
| Ventriculoarterial connection | Concordant | **Ventricles** | |
| Ventricular loop | d-Loop | Left ventricle | Dilated |
| Septae | | Right ventricle | Normal |
| Interventricular septum | Intact | **M-Mode** |  |
| Interatrial septum | Intact | AO |  |
| Great arteries | NRGA | LA |  |
| Aorta |  | LVIDd | 45mm |
| Pulmonary artery |  | LVIDs | 32mm |
| Semilunal valves |  | IVSd | 7mm |
| Aortic valve | Annulus = 16mm | IVSs | 7mm |
| Pulmonary valve | Annulus = 19mm | LVPWd | 7mm |
| Doppler Measurement | | LVPWs | 7mm |
| Mitral | Severe MR, Mild MS (Functional), PPG/MPG = 11/4mmHg | **EDV** | 91ml |
| Aortic |  | ESV | 39ml |
| Tricuspid | Moderate TR, PPG = 60mmHg | FS | 29% |
| pulmonic |  | LVEF | 57% |
| Aortic arch | Left | **Coronary arteries** |  |
| PDA | 5mm PDA, L – R Shunt |  |  |
| Additional information | No evidence of vegetation so far. | | |
|  |  | | |
| Final Diagnosis | 1. {S, D, S} Levocardia 2. LA/LV Dilated 3. Severe MR 4. Mild MS (Functional) 5. Large PDA, L – R Shunt 6. Severe pulmonary Hypertension 7. Good Biventricular Function | | |

**Done by Signature**

**Name: Dr. Tesfaye T. Pediatric Cardiologist Date-27/02/2012 E.C**

| Pediatric Echocardiography report  Patient Name: Yihealem Adugna Patient ID: 025022  Gender: F Age: 8 Month Date of Report: 27/02/2012Eth.C  Clinical Finding: RD + Recurrent Chest Infection. TGSH7.3143. | | | |
| --- | --- | --- | --- |
| Features | **Finding** | **Features** | **Finding** |
| Profile | | **Atria** | |
| Abdominal situs | Solitus | Left atrium | Normal |
| Cardiac position | Levocardia | Right atrium | Normal |
| Systemic venous drainage | to RA | **Atrioventricular valves** | |
| Pulmonary venous drainage | to LA | Mitral valve | Annulus = 12mm |
| Atrioventricular connection | Concordant | Tricuspid valve | Annulus = 13mm  TAPSE = 14mm |
| Ventriculoarterial connection | Concordant | **Ventricles** | |
| Ventricular loop | d-Loop | Left ventricle | Normal |
| Septae | | Right ventricle | Normal |
| Interventricular septum | Intact | **M-Mode: Normal LV Function** | |
| Interatrial septum | Intact | AO |  |
| Great arteries | NRGA | LA |  |
| Aorta | --- | LVIDd | mm |
| Pulmonary artery | ---- | LVIDs | mm |
| Semilunal valves |  | IVSd | mm |
| Aortic valve | Annulus = 14mm | IVSs | mm |
| Pulmonary valve | Annulus = 11mm | LVPWd | mm |
| Doppler Measurement | | LVPWs | mm |
| Mitral | ----- | **EDV** | ml |
| Aortic | ---- | ESV | ml |
| Tricuspid | Trivial TR, PPG = 23mmHg | FS |  |
| pulmonic | ----- | LVEF |  |
| Aortic arch | Left | **Coronary arteries** |  |
| PDA | No |  |  |
| Additional information |  | | |
|  |  | | |
| Final Diagnosis | 1. Normal Echocardiography Study. | | |

**Done by Signature**

**Name: Dr. Tesfaye T. Pediatric Cardiologist Date-27/02/2012 E.C**

| Pediatric Echocardiography report  Patient Name: Aragaw Alemye Patient ID: 006728  Gender: M Age:12 Date of Report: 27/02/2012Eth.C  BP: Weight: Height: BSA:  Follow up echocardiography Report | | | |
| --- | --- | --- | --- |
| Features | **Finding** | **Features** | **Finding** |
| Profile | | **Atria** | |
| Abdominal situs | Solitus | Left atrium | Dilated, 40 X 58mm |
| Cardiac position | Levocardia | Right atrium | Normal |
| Systemic venous drainage | to RA | **Atrioventricular valves** | |
| Pulmonary venous drainage | to LA | Mitral valve | Annulus = 28mm |
| Atrioventricular connection | Concordant | Tricuspid valve | Annulus = 19mm  TAPSE = 19mm |
| Ventriculoarterial connection | Concordant | **Ventricles** | |
| Ventricular loop | d-Loop | Left ventricle | Dilated, Dysfunctional |
| Septae | | Right ventricle | Normal |
| Interventricular septum | Aneurysmal | **M-Mode** |  |
| Interatrial septum | Intact | AO |  |
| Great arteries | NRGA | LA |  |
| Aorta | Sinus = 25mm, STJ = 21mm, Asc.Aorta = 19mm. | LVIDd | 57mm |
| Pulmonary artery | MPA = 18mm, LPA = 12mm, RPA = 10mm | LVIDs | 48mm |
| Semilunal valves |  | IVSd | 7mm |
| Aortic valve | Annulus = 17mm, | IVSs | 7mm |
| Pulmonary valve | Annulus = 22mm | LVPWd | 5mm |
| Doppler Measurement | | LVPWs | 7mm |
| Mitral | Mild MR | **EDV** | 161ml |
| Aortic | ---- | ESV | 110ml |
| Tricuspid | Trivial TR, PPG = 18mmHg | FS | 15% |
| pulmonic | ---- | LVEF | 32% |
| Aortic arch | Left | **Coronary arteries** |  |
| PDA | No |  |  |
| Additional information |  | | |
|  |  | | |
| Final Diagnosis | 1. {S, D, S} Levocardia 2. LA/LV Dilated 3. Mild MR 4. Reduced LV Function   N.B: Similar condition with the previous Echocardiography study. | | |

**Done by Signature**

**Name: Dr. Tesfaye T. Pediatric Cardiologist Date-27/02/2012 E.C**

| Pediatric Echocardiography report  Patient Name: B/ Birtukan Patient ID: 025575  Gender: F Age: 06 Day Date of Report: 28/02/2012Eth.C  Clinical Finding: Feeble Pulse lower extrimities. TGSH7.3144. | | | |
| --- | --- | --- | --- |
| Features | **Finding** | **Features** | **Finding** |
| Profile | | **Atria** | |
| Abdominal situs | Solitus | Left atrium | Normal |
| Cardiac position | Levocardia | Right atrium | Normal |
| Systemic venous drainage | to RA | **Atrioventricular valves** | |
| Pulmonary venous drainage | to LA | Mitral valve | Annulus = 10mm |
| Atrioventricular connection | Concordant | Tricuspid valve | Annulus = 11mm  TAPSE = 10mm |
| Ventriculoarterial connection | Concordant | **Ventricles** | |
| Ventricular loop | d-Loop | Left ventricle | Normal |
| Septae | | Right ventricle | Normal |
| Interventricular septum | Intact | **M-Mode** |  |
| Interatrial septum | Intact | AO |  |
| Great arteries | NRGA | LA |  |
| Aorta | ----- | LVIDd | 15.6mm |
| Pulmonary artery | Good sized MPA and Confluent Branch PAs. | LVIDs | 10mm |
| Semilunal valves |  | IVSd | 4.5mm |
| Aortic valve | Annulus = 8mm, Trileaflet | IVSs | 3.4mm |
| Pulmonary valve | Annulus = 10mm | LVPWd | 4.5mm |
| Doppler Measurement | | LVPWs | 4.5mm |
| Mitral | ---- | **EDV** | 6.7ml |
| Aortic | ---- | ESV | 2ml |
| Tricuspid | ---- | FS | 37% |
| pulmonic | ---- | LVEF | 70% |
| Aortic arch | Left | **Coronary arteries** |  |
| PDA | No |  |  |
| Additional information | No Coarctation of Aorta | | |
|  | No pericardial effusion. | | |
| Final Diagnosis | 1. Normal Echocardiography Study. | | |

**Done by Signature**

**Name: Dr. Tesfaye T. Pediatric Cardiologist Date-28/02/2012 E.C**

| Pediatric Echocardiography report  Patient Name: Yinebeb Mezegebu Patient ID: 018252  Gender: M Age: 3 3/12 Date of Report: 02/03/2012Eth.C  Clinical Finding: Cardiomegaly on CXR + Sepsis. TGSH7.3145. | | | |
| --- | --- | --- | --- |
| Features | **Finding** | **Features** | **Finding** |
| Profile | | **Atria** | |
| Abdominal situs | Solitus | Left atrium | Normal |
| Cardiac position | Levocardia | Right atrium | Normal |
| Systemic venous drainage | to RA | **Atrioventricular valves** | |
| Pulmonary venous drainage | to LA | Mitral valve | Annulus = 15mm |
| Atrioventricular connection | Concordant | Tricuspid valve | Annulus = 16mm  TAPSE = 15mm |
| Ventriculoarterial connection | Concordant | **Ventricles** | |
| Ventricular loop | d-Loop | Left ventricle | Normal |
| Septae | | Right ventricle | Normal |
| Interventricular septum | Intact | **M-Mode** |  |
| Interatrial septum | Intact | AO |  |
| Great arteries | NRGA | LA |  |
| Aorta |  | LVIDd | 30mm |
| Pulmonary artery | MPA = 13mm. Good sized confluent Branch PAs. | LVIDs | 20mm |
| Semilunal valves |  | IVSd | 5mm |
| Aortic valve | Annulus = 12mm, Trileaflet. | IVSs | 5.6mm |
| Pulmonary valve | Annulus = 14mm. | LVPWd | 7.5mm |
| Doppler Measurement | | LVPWs | 7.5mm |
| Mitral | ---- | **EDV** | 36ml |
| Aortic | ---- | ESV | 12ml |
| Tricuspid | Mild TR, PPG = 28mmHg | FS | 35% |
| pulmonic |  | LVEF | 66% |
| Aortic arch | Left | **Coronary arteries** |  |
| PDA | No |  |  |
| Additional information | No Coarctation of Aorta | | |
|  | Pericardial effusion 4mm on the RA side, 5mm on the LA side and trace in the RV, LV side | | |
| Final Diagnosis | 1. {S, D, S} Levocardia 2. Small Pericardial effusion 3. Good Biventricular Function. | | |

**Done by Signature**

**Name: Dr. Tesfaye T. Pediatric Cardiologist Date-02/03/2012 E.C**

| Pediatric Echocardiography report  Patient Name: B/Yalganesh Patient ID: 024621  Gender: M Age: 11days Date of Report: 09/03/2012Eth.C  Clinical Finding: RD. TGSH7.3146. | | | |
| --- | --- | --- | --- |
| Features | **Finding** | **Features** | **Finding** |
| Profile | | **Atria** | |
| Abdominal situs | Solitus | Left atrium | Normal |
| Cardiac position | Levocardia | Right atrium | Normal |
| Systemic venous drainage | to RA | **Atrioventricular valves** | |
| Pulmonary venous drainage | to LA | Mitral valve | Annulus= 10mm |
| Atrioventricular connection | Concordant | Tricuspid valve | Annulus = 14mm |
| Ventriculoarterial connection | Concordant | **Ventricles** | |
| Ventricular loop | d-Loop | Left ventricle | Normal |
| Septae | | Right ventricle | Normal |
| Interventricular septum | Intact | **M-Mode** |  |
| Interatrial septum | PFO, L – R Shunt | AO |  |
| Great arteries | NRGA | LA |  |
| Aorta | ---- | LVIDd | 16.7mm |
| Pulmonary artery | Good sized MPA and Confluent Branch PAs. | LVIDs | 11mm |
| Semilunal valves |  | IVSd | 4mm |
| Aortic valve | Annulus = 8mm | IVSs | 4mm |
| Pulmonary valve | Annulus = 9mm | LVPWd | 4mm |
| Doppler Measurement | | LVPWs | 4mm |
| Mitral | Trivial MR | **EDV** | 8ml |
| Aortic | ---- | ESV | 2.6ml |
| Tricuspid | ---- | FS | 35% |
| pulmonic | ---- | LVEF | 67% |
| Aortic arch | Left | **Coronary arteries** |  |
| PDA | No |  |  |
| Additional information |  | | |
|  |  | | |
| Final Diagnosis | 1. {S, D, S} Levocardia 2. PFO, L – R Shunt | | |

**Done by Signature**

**Name: Dr. Tesfaye T. Pediatric Cardiologist Date-09/03/2012 E.C**

| Pediatric Echocardiography report  Patient Name: Yohannes Gebrie Patient ID:026506  Gender:M Age:10month Date of Report: 02/03/2012Eth.C  BP: Weight: 7kg Height: 71cm BSA: TGSH10.2827 | | | |
| --- | --- | --- | --- |
| Features | **Finding** | **Features** | **Finding** |
| Profile | | **Atria** | |
| Abdominal situs | Solitus | Left atrium | Normal |
| Cardiac position | Levocardia | Right atrium | Normal |
| Systemic venous drainage | to RA | **Atrioventricular valves** | |
| Pulmonary venous drainage | to LA | Mitral valve | Annulus =17mm. Right side to the base |
| Atrioventricular connection | Discordant | Tricuspid valve | Annulus = 15mm. to the left and apical |
| Ventriculoarterial connection | DORV, Both from left sided morphologically RV. | **Ventricles** | |
| Ventricular loop | d-Loop | Left ventricle | Morphologically left ventricle and normal |
| Septae | | Right ventricle | Morphologically right ventricle on the left and smallish |
| Interventricular septum | Large inlet VSD amounting to single Ventricle, BD Shunt | **M-Mode: Good Function(Eye balling)** | |
| Interatrial septum | 6mm OS ASD, L – R Shunt | AO |  |
| Great arteries | NRGA | LA |  |
| Aorta | Posterior and to the right of PA arising from Left sided Morphologically RV | LVIDd | mm |
| Pulmonary artery | Anterior and to the left of Aorta, arising from the left sided morphologically RV.  MPA = 20mm | LVIDs | mm |
| Semilunal valves |  | IVSd | mm |
| Aortic valve | Annulus =14mm. Echogenic mass in the sub valvar area. | IVSs | mm |
| Pulmonary valve | Annulus = 19mm | LVPWd | mm |
| Doppler Measurement | | LVPWs | mm |
| Mitral | Mild MR | **EDV** | ml |
| Aortic | Mild LVOTO, PPG = 20mmHg | ESV | ml |
| Tricuspid | Mild TR | FS |  |
| Pulmonic | Moderate PR, PPG = 60mmHg | LVEF |  |
| Aortic arch | Left | **Coronary arteries** |  |
| PDA | No |  |  |
| Additional information |  | | |
| Final Diagnosis | 1. {S, D, S} Levocardia 2. Isolated Ventricular Inversion 3. DORV (Both arising from the left sided Morphologically RV.) 4. Mild AS 5. Large inlet VSD amounting single Ventricle, BD Shunt 6. Moderate PR. 7. Severe Pulmonary Hypertension 8. ? Vegetation (correlate with the clinical scenario) | | |

**Done by Signature**

**Name: Dr. Tesfaye T. Pediatric Cardiologist Date-02/03/2012 E.C**

| Pediatric Echocardiography report  Patient Name: Tewedros Sendekie Patient ID: 025107  Gender: M Age: 9months Date of Report: 02/03/2012Eth.C  Clinical Finding: Recurrent Chest Infection + Innocent Murmur. TGSH7.3147. | | | |
| --- | --- | --- | --- |
| Features | **Finding** | **Features** | **Finding** |
| Profile | | **Atria** | |
| Abdominal situs | Solitus | Left atrium | Normal |
| Cardiac position | Levocardia | Right atrium | Normal |
| Systemic venous drainage | to RA | **Atrioventricular valves** | |
| Pulmonary venous drainage | to LA | Mitral valve | Annulus = 12mm |
| Atrioventricular connection | Concordant | Tricuspid valve | Annulus = 14mm  TAPSE = 15mm |
| Ventriculoarterial connection | Concordant | **Ventricles** | |
| Ventricular loop | d-Loop | Left ventricle | Normal |
| Septae | | Right ventricle | Normal |
| Interventricular septum | Intact | **M-Mode** |  |
| Interatrial septum | Intact | AO |  |
| Great arteries | NRGA | LA |  |
| Aorta | ---- | LVIDd | 20mm |
| Pulmonary artery | Good sized MPA and Confluent Branch PAs. | LVIDs | 14mm |
| Semilunal valves |  | IVSd | 6.6mm |
| Aortic valve | Annulus = 11mm | IVSs | 6.6mm |
| Pulmonary valve | Annulus = 12mm | LVPWd | 6.5mm |
| Doppler Measurement | | LVPWs | 6.5mm |
| Mitral | ---- | **EDV** | 12.6ml |
| Aortic | ---- | ESV | 5ml |
| Tricuspid | ---- | FS | 30% |
| pulmonic | ---- | LVEF | 60% |
| Aortic arch | Left | **Coronary arteries** |  |
| PDA | No |  |  |
| Additional information |  | | |
|  |  | | |
| Final Diagnosis | 1. Normal Echocardiography Study. | | |

**Done by Signature**

**Name: Dr. Tesfaye T. Pediatric Cardiologist Date-02/03/2012Eth.C.**

| Pediatric Echocardiography report  Patient Name: Yikeber Yibel Patient ID: 026680  Gender: M Age: 9yrs Date of Report: 09/03/2012Eth.C  Clinical Finding: CHF + DOE + RD. TGSH7.3148. | | | |
| --- | --- | --- | --- |
| Features | **Finding** | **Features** | **Finding** |
| Profile | | **Atria** | |
| Abdominal situs | Solitus | Left atrium | Dilated |
| Cardiac position | Levocardia | Right atrium | Dilated |
| Systemic venous drainage | to RA. SEC+ | **Atrioventricular valves** | |
| Pulmonary venous drainage | to LA | Mitral valve | Annulus= 22mm. MV E/A Ratio = 2 |
| Atrioventricular connection | Concordant | Tricuspid valve | Annulus = 20mm.  TAPSE = 17mm |
| Ventriculoarterial connection | Concordant | **Ventricles** | |
| Ventricular loop | d-Loop | Left ventricle | Normal |
| Septae | | Right ventricle | Normal |
| Interventricular septum | Intact | **M-Mode** |  |
| Interatrial septum | Intact | AO |  |
| Great arteries | NRGA | LA |  |
| Aorta |  | LVIDd | 31mm |
| Pulmonary artery |  | LVIDs | 23mm |
| Semilunal valves |  | IVSd | 8.6mm |
| Aortic valve | Annulus = 15mm | IVSs | 6.5mm |
| Pulmonary valve | Annulus = 19mm | LVPWd | 7.3mm |
| Doppler Measurement | | LVPWs | 7.3mm |
| Mitral | ---- | **EDV** | 38ml |
| Aortic | ---- | ESV | 19ml |
| Tricuspid | Trivial TR, PPG = 14mmHg. | FS | 25% |
| Pulmonic | Trivial PR | LVEF | 51% |
| Aortic arch | Left | **Coronary arteries** |  |
| PDA | No |  |  |
| Additional information | Trace pericardial effusion on LA side. Thickened Hyper echoic pericardium | | |
| Final Diagnosis | 1. {S, D, S} Levocardia 2. LA/RA Dilated 3. Mildly reduced Systolic LV Function 4. Reduced LV Diastolic Function 5. Thickened Hyperechoic Pericardium 6. Constrictive Pericarditis | | |

**Done by Signature**

**Name: Dr. Tesfaye T. Pediatric Cardiologist Date-09/03/2012**

| Pediatric Echocardiography report  Patient Name: Yordanos Dessie Patient ID: 027167  Gender: F Age: 10 months Date of Report: 09/03/2012Eth.C  Clinical Finding: RD + Murmur + CHF. TGSH7.3149. | | | |
| --- | --- | --- | --- |
| Features | **Finding** | **Features** | **Finding** |
| Profile | | **Atria** | |
| Abdominal situs | Solitus | Left atrium | Mildly dilated |
| Cardiac position | Levocardia | Right atrium | Normal |
| Systemic venous drainage | to RA | **Atrioventricular valves** | |
| Pulmonary venous drainage | to LA | Mitral valve | Annulus = 15mm. |
| Atrioventricular connection | Concordant | Tricuspid valve | Annulus = 12mm.  TAPSE = 13mm. |
| Ventriculoarterial connection | Concordant | **Ventricles** | |
| Ventricular loop | d-Loop | Left ventricle | Dilated |
| Septae | | Right ventricle | Normal |
| Interventricular septum | 10mm inlet VSD, L – R Shunt | **M-Mode** |  |
| Interatrial septum | Intact | AO |  |
| Great arteries | NRGA | LA |  |
| Aorta | ---- | LVIDd | 25mm |
| Pulmonary artery | Good sized MPA and Confluent Branch PAs. | LVIDs | 18mm |
| Semilunal valves |  | IVSd | 6.6mm |
| Aortic valve | Annulus = 10mm | IVSs | 6.6mm |
| Pulmonary valve | Annulus = 12mm | LVPWd | 5mm |
| Doppler Measurement | | LVPWs | 5mm |
| Mitral | Mild MR. | **EDV** | 22.5ml |
| Aortic | ---- | ESV | 9.6ml |
| Tricuspid | ---- | FS | 29% |
| pulmonic | ---- | LVEF | 57% |
| Aortic arch | Left | **Coronary arteries** |  |
| PDA | No |  |  |
| Additional information | Circumferential Pericardial effusion with maximum measurement of 7mm on RV Side. | | |
| Final Diagnosis | 1. {S, D, S} Levocardia 2. Large inlet VSD, L – R Shunt 3. Small Circumferential pericardial effusion 4. Good Biventricular Function. | | |

**Done by Signature**

**Name: Dr. Tesfaye T. Pediatric Cardiologist Date-09/03/2012 E.C**

| Pediatric Echocardiography report  Patient Name: Selewa Yassin Patient ID: 027070  Gender: F Age:3 6/12 Date of Report: 09/03/2012Eth.C  Clinical Finding: _Incidental Murmur. TGSH7.3150. | | | |
| --- | --- | --- | --- |
| Features | **Finding** | **Features** | **Finding** |
| Profile | | **Atria** | |
| Abdominal situs | Solitus | Left atrium | Normal |
| Cardiac position | Levocardia | Right atrium | Normal |
| Systemic venous drainage | to RA | **Atrioventricular valves** | |
| Pulmonary venous drainage | to LA | Mitral valve | Annulus = 19mm |
| Atrioventricular connection | Concordant | Tricuspid valve | Annulus = 20mm |
| Ventriculoarterial connection | Concordant | **Ventricles** | |
| Ventricular loop | d-Loop | Left ventricle | Normal |
| Septae | | Right ventricle | Normal |
| Interventricular septum | 4mm Perimembranous VSD, L – R Shunt. | **M-Mode** |  |
| Interatrial septum | Intact | AO |  |
| Great arteries | NRGA | LA |  |
| Aorta |  | LVIDd | 30mm |
| Pulmonary artery |  | LVIDs | 19mm |
| Semilunal valves |  | IVSd | 5mm |
| Aortic valve | Annulus = 15mm | IVSs | 5mm |
| Pulmonary valve | Annulus = 18mm | LVPWd | 5mm |
| Doppler Measurement | | LVPWs | 5mm |
| Mitral | ---- | **EDV** | 35ml |
| Aortic | ---- | ESV | 11.5ml |
| Tricuspid | ---- | FS | 36% |
| pulmonic | ---- | LVEF | 67% |
| Aortic arch | Left | **Coronary arteries** |  |
| PDA | No |  |  |
| Additional information | No coarctation of aorta | | |
| Final Diagnosis | 1. {S, D, S} Levocardia 2. Small Perimembranous VSD, L – R Shunt 3. Good Biventricular Function | | |

**Done by Signature**

**Name: Dr. Tesfaye T. Pediatric Cardiologist Date-09/03/2012 E.C**

| Pediatric Echocardiography report  Patient Name: Eliana Adebabay Patient ID:027162  Gender: 22Day Age: F Date of Report: 09/03/2012Eth.C  Clinical Finding: CHF + RD + Murmur + . TGSH7.3151. | | | |
| --- | --- | --- | --- |
| Features | **Finding** | **Features** | **Finding** |
| Profile | | **Atria** | |
| Abdominal situs | Solitus | Left atrium | Dilated |
| Cardiac position | Levocardia | Right atrium | Dilated |
| Systemic venous drainage | to RA | **Atrioventricular valves** | |
| Pulmonary venous drainage | to LA | Mitral valve | Annulus = 12mm. Non – Coapting |
| Atrioventricular connection | Concordant | Tricuspid valve | Annulus = 13mm.  TAPSE = 10mm. |
| Ventriculoarterial connection | Concordant | **Ventricles** | |
| Ventricular loop | d-Loop | Left ventricle | Dilated |
| Septae | | Right ventricle | Hypertrabeculated |
| Interventricular septum | Intact | **M-Mode** |  |
| Interatrial septum | OS ASD, 4mm X 6mm, BD Shunt Predominantly R – L. | AO |  |
| Great arteries | NRGA | LA |  |
| Aorta | 6mm aorto – Pulmonary window, BD Shunt | LVIDd | 21mm |
| Pulmonary artery |  | LVIDs | 15mm |
| Semilunal valves |  | IVSd | 5mm |
| Aortic valve | Annulus = 10mm | IVSs | 5mm |
| Pulmonary valve | Annulus = 9mm | LVPWd | 5mm |
| Doppler Measurement | | LVPWs | 5mm |
| Mitral | Severe MR | **EDV** | 14ml |
| Aortic | ---- | ESV | 6ml |
| Tricuspid | Severe TR, PPG= 119mmHg | FS | 29% |
| pulmonic | ---- | LVEF | 58% |
| Aortic arch | Left | **Coronary arteries** |  |
| PDA | No |  |  |
| Additional information |  | | |
|  |  | | |
| Final Diagnosis | 1. {S, D, S} Levocardia 2. RA Dilated 3. OS ASD, BID Shunt, predominantly R – L Shunt 4. Severe MR 5. Severe TR 6. Aorto – Pulmonary Window, BD Shunt 7. Severe Pulmonary Hypertension 8. Good LV Function. | | |

**Done by Signature**

**Name: Dr. Tesfaye T. Pediatric Cardiologist Date-09/03/2012 E.C**

| Pediatric Echocardiography report  Patient Name: Mira Misganaw Patient ID: 027023  Gender: F Age: 3/12 Date of Report: 09/03/2012Eth.C  Clinical Finding: RD + Murmur. TGSH7.3152. | | | |
| --- | --- | --- | --- |
| Features | **Finding** | **Features** | **Finding** |
| Profile | | **Atria** | |
| Abdominal situs | Solitus | Left atrium | Dilated, 33mm X 43mm |
| Cardiac position | Levocardia | Right atrium | Dilated |
| Systemic venous drainage | to RA | **Atrioventricular valves** | |
| Pulmonary venous drainage | to LA | Mitral valve | Annulus = 18mm |
| Atrioventricular connection | Concordant | Tricuspid valve | Annulus = 16mm |
| Ventriculoarterial connection | Concordant | **Ventricles** | |
| Ventricular loop | d-Loop | Left ventricle | Dilated |
| Septae | | Right ventricle | Dilated |
| Interventricular septum | Intact | **M-Mode** |  |
| Interatrial septum | PFO, L – R Shunt. Aneurysmal Septum. | AO |  |
| Great arteries | NRGA | LA |  |
| Aorta |  | LVIDd | 30mm |
| Pulmonary artery | MPA = 10mm. good sized Confluent Branch PAs. | LVIDs | 19mm |
| Semilunal valves |  | IVSd | 5.4mm |
| Aortic valve | Annulus = 9mm | IVSs | 6mm |
| Pulmonary valve | Annulus = 9mm | LVPWd | 6mm |
| Doppler Measurement | | LVPWs | 6mm |
| Mitral | Severe MR. | **EDV** | 34ml |
| Aortic | ---- | ESV | 11ml |
| Tricuspid | Moderate TR, PPG = 66mmHg | FS | 37% |
| pulmonic |  | LVEF | 68% |
| Aortic arch | Left | **Coronary arteries** |  |
| PDA | 4mm PDA |  |  |
| Additional information |  | | |
| Final Diagnosis | 1. {S, D, S} Levocardia 2. PFO, L – R Shunt 3. LA/LV Dilated 4. Severe MR 5. Moderate TR 6. Large PDA 7. Severe Pulmonary Hypertension 8. Good LV Function | | |
| Recommendation: | Needs follow up echo after 3 months. There was limited echo window. | | |

**Done by Signature**

**Name: Dr. Tesfaye T. Pediatric Cardiologist Date-09/03/2012 E.C**

| Pediatric Echocardiography report  Patient Name: Firdows Tesfaye Patient ID: 027249  Gender: F Age: 2 5/12 Date of Report: 11/03/2012Eth.C  Clinical Finding: _Diaphoresis + Murmur. TGSH7.3153. | | | |
| --- | --- | --- | --- |
| Features | **Finding** | **Features** | **Finding** |
| Profile | | **Atria** | |
| Abdominal situs | Solitus | Left atrium | Dilated |
| Cardiac position | Levocardia | Right atrium | Mildly Dilated |
| Systemic venous drainage | to RA | **Atrioventricular valves** | |
| Pulmonary venous drainage | to LA | Mitral valve | Annulus = 17mm |
| Atrioventricular connection | Concordant | Tricuspid valve | Annulus = 23mm  TAPSE = 15mm. |
| Ventriculoarterial connection | Concordant | **Ventricles** | |
| Ventricular loop | d-Loop | Left ventricle | Dilated |
| Septae | | Right ventricle | Mildly Dilated |
| Interventricular septum | Intact | **M-Mode** |  |
| Interatrial septum | Intact | AO |  |
| Great arteries | NRGA | LA |  |
| Aorta |  | LVIDd | 30mm |
| Pulmonary artery | MPA = 21mm, RPA = 11mm, LPA = 11mm. | LVIDs | 19mm |
| Semilunal valves |  | IVSd | 6.6mm |
| Aortic valve | Annulus = 14mm | IVSs | 6.6mm |
| Pulmonary valve | Annulus = 19mm | LVPWd | 6.6mm |
| Doppler Measurement | | LVPWs | 6.6mm |
| Mitral |  | **EDV** | 35ml |
| Aortic |  | ESV | 11.5ml |
| Tricuspid | Trivial TR, PPG = 30mmHg | FS | 36% |
| pulmonic | Moderate PR, PPG = 58mmHg | LVEF | 67% |
| Aortic arch | Left | **Coronary arteries** |  |
| PDA | 2.5mm PDA, L – R Shunt. |  |  |
| Additional information | No coarctation | | |
| Final Diagnosis | 1. {S, D, S} Levocardia 2. Moderate PDA 3. Moderate Pulmonary Hypertension 4. Good Biventricular Function | | |

**Done by Signature Date-11/03/2012 E.C Name: Dr. Tesfaye T. Pediatric Cardiologist**

| Pediatric Echocardiography report  Patient Name: Solomon Walle Patient ID: 027595  Gender: M Age: 6years Date of Report: 11/03/2012Eth.C  Clinical Finding: _CHF + DOE + Murmur + RD. TGSH7.3154. | | | |
| --- | --- | --- | --- |
| Features | **Finding** | **Features** | **Finding** |
| Profile | | **Atria** | |
| Abdominal situs | Solitus | Left atrium | Normal |
| Cardiac position | Levocardia | Right atrium | Dilated |
| Systemic venous drainage | to RA | **Atrioventricular valves** | |
| Pulmonary venous drainage | to LA | Mitral valve | Annulus = 17mm. |
| Atrioventricular connection | Concordant | Tricuspid valve | Annulus = 23mm.  TAPSE = 12mm |
| Ventriculoarterial connection | Concordant | **Ventricles** | |
| Ventricular loop | d-Loop | Left ventricle | Banana shaped LV. hyperdynamic. |
| Septae | | Right ventricle | Dilated, Hypertrabeculated and mildly Dysfunctional |
| Interventricular septum | Thickened IVS and bowed to the RV. | **M-Mode** |  |
| Interatrial septum | Intact | AO |  |
| Great arteries | NRGA | LA |  |
| Aorta | ---- | LVIDd | 17.5mm |
| Pulmonary artery | ---- | LVIDs | 7.6mm |
| Semilunal valves |  | IVSd | 13mm |
| Aortic valve | Annulus = 17mm, Trileaflet. | IVSs | 14mm |
| Pulmonary valve | Annulus = 17mm. Doming Pulmonary Valve. | LVPWd | 6.6mm |
| Doppler Measurement | | LVPWs | 6.6mm |
| Mitral | Trivial MR | **EDV** | 9ml |
| Aortic | ---- | ESV | 9.8ml |
| Tricuspid | Mild to moderate TR. | FS | ---- |
| pulmonic | Severe valvar PS, PPG/MPG = 130/70mmHg. Trivial PR. | LVEF | ---- |
| Aortic arch | Left | **Coronary arteries** |  |
| PDA | No |  |  |
| Additional information | No coarctation of aorta/ no effusion. | | |
| Final Diagnosis | 1. {S, D, S} Levocardia 2. RA Dilated 3. Thickened IVS without LVOTO. 4. Mild to Moderate TR 5. Severe Valvar PS 6. Doming Pulmonary Valve 7. Dilated, Hypertrabeculated and Mildly Dysfunctional RV. | | |
| Recommendation: | **Urgent Referral for Intervention.** | | |

**Done by Signature**

**Name: Dr. Tesfaye T. Pediatric Cardiologist Date-11/03/2012 E.C**

| Pediatric Echocardiography report  Patient Name: Firdows Tesfaye Patient ID: 027249  Gender: F Age: 2 5/12 Date of Report: 11/03/2012Eth.C  Clinical Finding: ____________. INCOMPLETE DOCUMENTATION | | | |
| --- | --- | --- | --- |
| Features | **Finding** | **Features** | **Finding** |
| Profile | | **Atria** | |
| Abdominal situs | Solitus | Left atrium | Normal |
| Cardiac position | Levocardia | Right atrium | Normal |
| Systemic venous drainage | to RA | **Atrioventricular valves** | |
| Pulmonary venous drainage | to LA | Mitral valve | Annulus = 16mm |
| Atrioventricular connection | Concordant | Tricuspid valve | Annulus = 20mm  TAPSE = 16mm. |
| Ventriculoarterial connection | Concordant | **Ventricles** | |
| Ventricular loop | d-Loop | Left ventricle |  |
| Septae | | Right ventricle |  |
| Interventricular septum |  | **M-Mode** |  |
| Interatrial septum | Intact | AO |  |
| Great arteries | NRGA | LA |  |
| Aorta |  | LVIDd | 30mm |
| Pulmonary artery |  | LVIDs | 19mm |
| Semilunal valves |  | IVSd | 6.6mm |
| Aortic valve | Annulus = 14mm | IVSs | 6.6mm |
| Pulmonary valve | Annulus = 17mm | LVPWd | 6.6mm |
| Doppler Measurement | | LVPWs | 6.6mm |
| Mitral |  | **EDV** | 35ml |
| Aortic |  | ESV | 11.5ml |
| Tricuspid | Trivial TR, PPG = 30mmHg | FS | 36% |
| pulmonic | Mild PR, PPG = 45mmHg | LVEF | 67% |
| Aortic arch | Left | **Coronary arteries** |  |
| PDA | No |  |  |
| Additional information |  | | |
|  |  | | |
| Final Diagnosis |  | | |

**Done by Signature Date-11/03/2012 E.C Name: Dr. Tesfaye T. Pediatric Cardiologist**

| Pediatric Echocardiography report  Patient Name: Sefiager Takele Patient ID: 027926  Gender: F Age:6yrs Date of Report: 16/03/2012Eth.C  Clinical Finding: ?ARF + Murmur + CHF + DOE + RD + Shock. TGSH7.3155. | | | |
| --- | --- | --- | --- |
| Features | **Finding** | **Features** | **Finding** |
| Profile | | **Atria** | |
| Abdominal situs | Solitus | Left atrium | Dilated |
| Cardiac position | Levocardia | Right atrium | Normal |
| Systemic venous drainage | to RA | **Atrioventricular valves** | |
| Pulmonary venous drainage | to LA | Mitral valve | Annulus = 19mm. Patulous |
| Atrioventricular connection | Concordant | Tricuspid valve | Annulus = 15mm.  TAPSE = 11mm |
| Ventriculoarterial connection | Concordant | **Ventricles** | |
| Ventricular loop | d-Loop | Left ventricle | Dilated & Dysfunctional |
| Septae | | Right ventricle | Normal |
| Interventricular septum | Intact | **M-Mode** |  |
| Interatrial septum | Intact | AO |  |
| Great arteries | NRGA | LA |  |
| Aorta | ---- | LVIDd | 59mm |
| Pulmonary artery |  | LVIDs | 54mm |
| Semilunal valves |  | IVSd | **9.45mm** |
| Aortic valve | Annulus = 18mm. Trileaflet | IVSs | 9.45mm |
| Pulmonary valve | Annulus = 21mm | LVPWd | 7.56mm |
| Doppler Measurement | | LVPWs | 7.56mm |
| Mitral | Moderate MR | **EDV** | 173ml |
| Aortic | Moderate AR, PHT = 313ms. | ESV | 140ml |
| Tricuspid | Mild TR, PPG = 38mmHg | FS | 9% |
| pulmonic | Trivial PR, PPG = 22mmHg | LVEF | 19% |
| Aortic arch | Left | **Coronary arteries** |  |
| PDA | No |  |  |
| Additional information | No coarctation of Aorta | | |
|  | Pericardial effusion measuring 8mm on RV Side. | | |
| Final Diagnosis | 1. {S, D, S} Levocardia 2. Dilated LA 3. Moderate MR 4. Moderate AR 5. Mild TR 6. Reduced RV Function 7. Dilated, Dysfunctional LV. 8. Minimal Pericardial effusion. | | |

**Done by Signature Date-16/03/2012 E.C Name: Dr. Tesfaye T. Pediatric Cardiologist**

| Pediatric Echocardiography report  Patient Name: Ashenafie Walelign Patient ID: 027922  Gender: M Age: 11yrs Date of Report: 16/03/2012Eth.C  Clinical Finding: _Rheumatic Recurrence + Murmur + DOE + Palpitation + Easy Fatigability. TGSH7.3156. | | | |
| --- | --- | --- | --- |
| Features | **Finding** | **Features** | **Finding** |
| Profile | | **Atria** | |
| 23Abdominal situs | Solitus | Left atrium | Dilated, 62 X 72mm |
| Cardiac position | Levocardia | Right atrium | Dilated |
| Systemic venous drainage | to RA | **Atrioventricular valves** | |
| Pulmonary venous drainage | to LA | Mitral valve | Annulus = 35mm. Thickened, Clubbed, calcified MV. MVA = 0.8cm**2**. |
| Atrioventricular connection | Concordant | Tricuspid valve | Annulus = 29mm.  TAPSE = 14mm. |
| Ventriculoarterial connection | Concordant | **Ventricles** | |
| Ventricular loop | d-Loop | Left ventricle | Dilated |
| Septae | | Right ventricle | Dilated |
| Interventricular septum | Intact | **M-Mode** |  |
| Interatrial septum | Intact | AO |  |
| Great arteries | NRGA | LA |  |
| Aorta |  | LVIDd | mm |
| Pulmonary artery | Good sized MPA and Confluent Branch PAs. | LVIDs | mm |
| Semilunal valves |  | IVSd | mm |
| Aortic valve | Annulus = 18mm. thickened and calcified. | IVSs | mm |
| Pulmonary valve | Annulus = 22mm | LVPWd | mm |
| Doppler Measurement | | LVPWs | mm |
| Mitral | Severe MR. Severe MS. | **EDV** | ml |
| Aortic | Moderate AR | ESV | ml |
| Tricuspid | Moderate TR, PPG = 38mmHg | FS | 29% |
| pulmonic | ---- | LVEF | 55% |
| Aortic arch | Left | **Coronary arteries** |  |
| PDA | No |  |  |
| Additional information |  | | |
|  | Trace pericardial effusion | | |
| Final Diagnosis | 1. {S, D, S} Levocardia 2. Severe MR 3. Severe MS 4. Moderate AR 5. Moderate AS 6. Moderate TR. 7. Thickened, clubbed, calcified Mitral Valve 8. Thickened, calcified Aortic Valve 9. Good Function | | |

**Done by Signature Date-16/03/2012 E.C Name: Dr. Tesfaye T. Pediatric Cardiologist**

| Pediatric Echocardiography report  Patient Name: Abaynew Dinberu Patient ID: 279163  Gender: M Age: 9 months Date of Report: 16/03/2012Eth.C  Clinical Finding: RD + CHF + DS. TGSH7.3157. | | | |
| --- | --- | --- | --- |
| Features | **Finding** | **Features** | **Finding** |
| Profile | | **Atria** | |
| Abdominal situs | Solitus | Left atrium | Normal |
| Cardiac position | Levocardia | Right atrium | Dilated |
| Systemic venous drainage | to RA | **Atrioventricular valves** | |
| Pulmonary venous drainage | to LA | Mitral valve | Annulus = 10mm |
| Atrioventricular connection | Concordant | Tricuspid valve | Annulus = 19mm.  TAPSE = 10mm |
| Ventriculoarterial connection | Concordant | **Ventricles** | |
| Ventricular loop | d-Loop | Left ventricle |  |
| Septae | | Right ventricle | Dilated, Hypertrabeculated |
| Interventricular septum | 2mm Apical Muscular VSD | **M-Mode: Good Function on eye balling.** | |
| Interatrial septum | Intact | AO |  |
| Great arteries | NRGA | LA |  |
| Aorta | ---- | LVIDd | mm |
| Pulmonary artery | Good Sized MPA and Confluent Branch PAs | LVIDs | mm |
| Semilunal valves |  | IVSd | mm |
| Aortic valve | Annulus = 12mm | IVSs | mm |
| Pulmonary valve | Annulus = 16mm | LVPWd | mm |
| Doppler Measurement | | LVPWs | mm |
| Mitral | ---- | **EDV** | ml |
| Aortic | ---- | ESV | ml |
| Tricuspid | Mild TR, PPG = 61mmHg | FS |  |
| pulmonic | Moderate PR, PPG = 60mmHg | LVEF |  |
| Aortic arch | Left | **Coronary arteries** |  |
| PDA | No |  |  |
| Additional information | No coarctation of aorta | | |
|  | 6mm pericardial effusion on RA/RV Side | | |
| Final Diagnosis | 1. {S, D, S} Levocardia 2. Dilated RA/RV 3. Mild TR 4. Moderate PR 5. Small Apical Muscular VSD 6. Severe Pulmonary Hypertension 7. Right Ventricular Dysfunction | | |
| Recommendation: Search Pulmonary Cause for the pulmonary Hypertension and RV Dysfunction. | | | |

**Done by Signature Date-16/03/2012 E.C Name: Dr. Tesfaye T. Pediatric Cardiologist**

| Pediatric Echocardiography report  Patient Name: Bantegize Asnake Patient ID: 024339  Gender: M Age: 14yrs Date of Report: 16/03/2012Eth.C  Clinical Finding: Rheumatic Recurrence + DOE + Palpitation + Murmur + CHF + Easy Fatigability. TGSH7.3158. | | | |
| --- | --- | --- | --- |
| Features | **Finding** | **Features** | **Finding** |
| Profile | | **Atria** | |
| Abdominal situs | Solitus | Left atrium | Dilated, 81mm X 106mm |
| Cardiac position | Levocardia | Right atrium | Dilated |
| Systemic venous drainage | to RA | **Atrioventricular valves** | |
| Pulmonary venous drainage | to LA | Mitral valve | Annulus = 19mm. Thickened, clubbed MVL. |
| Atrioventricular connection | Concordant | Tricuspid valve | Annulus = 21mm. |
| Ventriculoarterial connection | Concordant | **Ventricles** | |
| Ventricular loop | d-Loop | Left ventricle | Dilated |
| Septae | | Right ventricle | Dilated |
| Interventricular septum |  | **M-Mode** |  |
| Interatrial septum | Intact | AO |  |
| Great arteries | NRGA | LA |  |
| Aorta | ---- | LVIDd | mm |
| Pulmonary artery | ---- | LVIDs | mm |
| Semilunal valves |  | IVSd | mm |
| Aortic valve | Annulus = 18mm. | IVSs | mm |
| Pulmonary valve | Annulus = 25mm. | LVPWd | mm |
| Doppler Measurement | | LVPWs | mm |
| Mitral | Severe MR, Severe MS | **EDV** | ml |
| Aortic | Moderate AR | ESV | ml |
| Tricuspid | Mild TR, PPG = 65mmHg | FS | 29% |
| pulmonic | ---- | LVEF | 54% |
| Aortic arch | Left | **Coronary arteries** |  |
| PDA | No |  |  |
| Additional information |  | | |
|  |  | | |
| Final Diagnosis | 1. {S, D, S} Levocardia 2. Severe MR 3. Severe MS 4. Moderate AR 5. Severe Pulmonary Hypertension 6. Reduced LV Systolic Function | | |

**Done by Signature Date-16/03/2012 E.C Name: Dr. Tesfaye T. Pediatric Cardiologist**

| Pediatric Echocardiography report  Patient Name: Nardos adisu Patient ID: 008004  Gender: F Age: 2yrs Date of Report: 18/03/2012Eth.C  Clinical Finding: ____. TGSH7.3091. | | | | | | |
| --- | --- | --- | --- | --- | --- | --- |
| Follow up Echo |  | | | | | |
| Features | **Finding** | **Features** | | | **Finding** | |
| Profile | | **Atria** | | | | |
| Abdominal situs | Solitus | Left atrium | | | Dilated | |
| Cardiac position | Levocardia | Right atrium | | | Normal | |
| Systemic venous drainage | to RA | **Atrioventricular valves** | | | | |
| Pulmonary venous drainage | to LA | Mitral valve | | | Annulus = 16mm | |
| Atrioventricular connection | Concordant | Tricuspid valve | | | Annulus =17mm  TAPSE = 15mm | |
| Ventriculoarterial connection | Concordant | **Ventricles** | | | | |
| Ventricular loop | d-Loop | Left ventricle | | | Dilated, Dysfunctional | |
| Septae | | Right ventricle | | | Normal | |
| Interventricular septum | Intact | **M-Mode** | | |  | |
| Interatrial septum | Intact | AO | | |  | |
| Great arteries | NRGA | LA | | |  | |
| Aorta |  | LVIDd | | | 45mm | |
| Pulmonary artery | MPA = 14mm, LPA = 10mm, RPA= 8mm. | LVIDs | | | 37mm | |
| Semilunal valves |  | IVSd | | | 6mm | |
| Aortic valve | Annulus = 12mm, Trileaflet | IVSs | | | 7.5mm | |
| Pulmonary valve | Annulus = 14mm | LVPWd | | | 7.5mm | |
| Doppler Measurement | | LVPWs | | | 7.5mm | |
| Mitral | Trivial MR | **EDV** | | | 90.5ml | |
| Aortic | ----- | ESV | | | 58.5ml | |
| Tricuspid | Trivial TR, PPG = 12mmHg | FS | | | 17% | |
| pulmonic | ---- | LVEF | | | 36% | |
| Aortic arch | Left | **Coronary arteries** | | | No ALCAPA | |
| PDA | No |  | | |  | |
| Additional information | No pleural/pericardial effusion | | | | | |
| Final Diagnosis | 1. {S, D, S} Levocardia 2. Trivial MR 3. Trivial TR 4. Dilated LA 5. Dilated Dysfunction LV with reduced EF. | | | | | |
| Conclusion: | Slight improvement in Function (see after 6 months) | | | | | |
| Done by | **Signature** | | | **Date Echo Performed** | | |
| Dr. Tesfaye T., Pediatric Cardiologist |  | | | 18/03/2012Eth.C | | |
| Pediatric Echocardiography report  Patient Name: Tesera Zewudu Patient ID:028135  Gender:M Age: 3 yrs Date of Report: 18/03/2012Eth.C  BP: Weight:9.8kg Height: 90cm BSA: | | | | | | |
| Features | **Finding** | | | **Features** | | **Finding** |
| Profile | | | | **Atria** | | |
| Abdominal situs | Solitus | | | Left atrium | | Dilated |
| Cardiac position | Levocardia | | | Right atrium | | Dilated |
| Systemic venous drainage | to RA | | | **Atrioventricular valves** | | |
| Pulmonary venous drainage | to LA | | | Mitral valve | | Annulus = 17mm |
| Atrioventricular connection | Concordant | | | Tricuspid valve | | Annulus =16mm  TAPSE = mm |
| Ventriculoarterial connection | Concordant | | | **Ventricles** | | |
| Ventricular loop | d-Loop | | | Left ventricle | | Mildly dilated |
| Septae | | | | Right ventricle | | More dilated |
| Interventricular septum | Intact | | | **M-Mode** | |  |
| Interatrial septum | 26mm X 18mm OS ASD, L – R Shunt amounting to single atrium | | | AO | |  |
| Great arteries | NRGA | | | LA | |  |
| Aorta |  | | | LVIDd | | mm |
| Pulmonary artery | Normal sized MPA and confluent Branch PAs | | | LVIDs | | mm |
| Semilunal valves |  | | | IVSd | | mm |
| Aortic valve | Annulus = 16mm, | | | IVSs | | mm |
| Pulmonary valve | Annulus = 17mm | | | LVPWd | | mm |
| Doppler Measurement | | | | LVPWs | | mm |
| Mitral | Mild MR | | | **EDV** | | ml |
| Aortic | ----- | | | ESV | | ml |
| Tricuspid | Moderate TR, PPG = 53mmHg | | | FS | | 34% |
| pulmonic | Moderate PR, PPG = 51mmHg | | | LVEF | | 64% |
| Aortic arch | Left | | | **Coronary arteries** | |  |
| PDA | 4mm PDA, L – R Shunt | | |  | |  |
| Additional information | Trace Pericardial effusion, circumferential. | | | | | |
| Final Diagnosis | 1. {S, D, S} Levocardia 2. Large OS ASD amounting to single atrium, L – R Shunt. 3. Large PDA, L – R Shunt 4. Moderate to severe Pulmonary Hypertension | | | | | |
| Done by | **Signature** | | | **Date Echo Performed** | | |
| Dr. Tesfaye T., Pediatric Cardiologist |  | | | 18/03/2012Eth.C | | |
| Pediatric Echocardiography report  Patient Name: Rozina Yeshiwas Patient ID: 028153  Gender: F Age: 11Months Date of Report: 18/03/2012Eth.C  BP: Weight: 6kg Height: 65cm BSA: | | | | | | |
| Features | **Finding** | | **Features** | | **Finding** | |
| Profile | | | **Atria** | | | |
| Abdominal situs | Solitus | | Left atrium | | Normal | |
| Cardiac position | Levocardia | | Right atrium | | Dilated | |
| Systemic venous drainage | to RA | | **Atrioventricular valves** | | | |
| Pulmonary venous drainage | to LA | | Mitral valve | | Normal | |
| Atrioventricular connection | Concordant | | Tricuspid valve | | TAPSE = 12mm | |
| Ventriculoarterial connection | Concordant | | **Ventricles** | | | |
| Ventricular loop | d-Loop | | Left ventricle | | Normal | |
| Septae | | | Right ventricle | | Dilated  RV TDI S Wave = 10cm/sec | |
| Interventricular septum | Intact | | **M-Mode** | |  | |
| Interatrial septum | PFO, BD Shunt | | AO | |  | |
| Great arteries | NRGA | | LA | |  | |
| Aorta |  | | LVIDd | | mm | |
| Pulmonary artery | Good sized MPA and confluent Branch PAs | | LVIDs | | mm | |
| Semilunal valves |  | | IVSd | | mm | |
| Aortic valve | Annulus = 8mm, Trileaflet | | IVSs | | mm | |
| Pulmonary valve | Doming Pulmonary valve | | LVPWd | | mm | |
| Doppler Measurement | | | LVPWs | | mm | |
| Mitral | ---- | | **EDV** | | ml | |
| Aortic | ----- | | ESV | | ml | |
| Tricuspid | Moderate TR, PPG = 66mmHg | | FS | | 32% | |
| pulmonic | Severe Valvar PS, PPG = 61mmHg | | LVEF | | 63% | |
| Aortic arch | Left | | **Coronary arteries** | |  | |
| PDA | No | |  | |  | |
| Additional information | No pleural/pericardial effusion | | | | | |
| Final Diagnosis | 1. {S, D, S} Levocardia 2. PFO, BD Shunt. 3. Severe Valvar PS 4. Good Biventricular Function. | | | | | |
| Recommendation | Candidate for BPV. | | | | | |
| Done by | **Signature** | | | **Date Echo Performed** | | |
| Dr. Tesfaye T., Pediatric Cardiologist |  | | | 18/03/2012Eth.C | | |

| Pediatric Echocardiography report | | | | |
| --- | --- | --- | --- | --- |
| Patient Name: Samuel Alebachew  Patient ID: 028457 | **Age: 8months**  Gender: Male | **BP: ___.**  **Weight: 5.7 kg** | | **Height: 63cm**  **BSA: ____m2.** |
| Clinical Finding: Incidental Murmur. TGSH7.3159. | | | | |
| Features | **Finding** | **Features** | | **Finding** |
| Profile | | **Atria** | | |
| Abdominal situs | Solitus | Left atrium | | Normal |
| Cardiac position | Levocardia | Right atrium | | Dilated |
| Systemic venous drainage | to RA | **Atrioventricular valves** | | |
| Pulmonary venous drainage | to LA | Mitral valve | | Annulus = 12mm |
| Atrioventricular connection | Concordant | Tricuspid valve | | Annulus = 17mm  TAPSE = 14mm |
| Ventriculoarterial connection | Concordant | **Ventricles** | | |
| Ventricular loop | d-Loop | Left ventricle | | Normal |
| Septae | | Right ventricle | | Dilated |
| Interventricular septum | Intact | **M-Mode** | |  |
| Interatrial septum | Intact | AO | |  |
| Great arteries | NRGA | LA | |  |
| Aorta |  | LVIDd | | mm |
| Pulmonary artery | Good sized MPA and confluent Branch PAs | LVIDs | | mm |
| Semilunal valves |  | IVSd | | mm |
| Aortic valve | Annulus = 10mm, Trileaflet | IVSs | | mm |
| Pulmonary valve | Annulus = 12mm. Doming. | LVPWd | | mm |
| Doppler Measurement | | LVPWs | | mm |
| Mitral | ---- | **EDV** | | ml |
| Aortic | ----- | ESV | | ml |
| Tricuspid | ---- | FS | | 27% |
| pulmonic | Severe Valvar PS, PPG = 60mmHg. | LVEF | | 55% |
| Aortic arch | Left | **Coronary arteries** | |  |
| PDA | No |  | |  |
| Additional information |  | | | |
| Final Diagnosis | 1. {S, D, S} Levocardia 2. Severe Valvar PS 3. Doming Pulmonary Valve 4. Good Biventricular Function. | | | |
| Recommendation: | BPV | | | |
| Done by | **Signature** | | **Date Echo Performed** | |
| Dr. Tesfaye T., Pediatric Cardiologist |  | | 18/03/2012Eth.C | |

| Pediatric Echocardiography report  Patient Name: Amen Getasew Patient ID: 021801  Gender: F Age: 1 3/12 Date of Report: 23/03/2012Eth.C  Clinical Finding: ?Pulmonary Hypertension. TGSH7.3160. | | | |
| --- | --- | --- | --- |
| Features | **Finding** | **Features** | **Finding** |
| Profile | | **Atria** | |
| Abdominal situs | Solitus | Left atrium | Normal |
| Cardiac position | Levocardia | Right atrium | Normal |
| Systemic venous drainage | to RA | **Atrioventricular valves** | |
| Pulmonary venous drainage | to LA | Mitral valve | Annulus = 14mm |
| Atrioventricular connection | Concordant | Tricuspid valve | Annulus = 18mm.  TAPSE = 14mm |
| Ventriculoarterial connection | Concordant | **Ventricles** | |
| Ventricular loop | d-Loop | Left ventricle | Normal |
| Septae | | Right ventricle | Normal |
| Interventricular septum | Intact | **M-Mode** |  |
| Interatrial septum | Intact | AO |  |
| Great arteries | NRGA | LA |  |
| Aorta | ---- | LVIDd | 24.6mm |
| Pulmonary artery | Good sized MPA and Confluent Branch PAs. | LVIDs | 17mm |
| Semilunal valves |  | IVSd | 4.5mm |
| Aortic valve | Annulus = 13mm. Trileaflet | IVSs | 5.6mm |
| Pulmonary valve | Annulus = 14mm | LVPWd | 5mm |
| Doppler Measurement | | LVPWs | 5.6mm |
| Mitral | ---- | **EDV** | 21ml |
| Aortic | ---- | ESV | 8.4ml |
| Tricuspid | Trivial TR, PPG = 31mmHg | FS | 31% |
| pulmonic | Trivial PR | LVEF | 61% |
| Aortic arch | Left | **Coronary arteries** |  |
| PDA | No |  |  |
| Additional information |  | | |
| Final Diagnosis | 1. {S, D, S} Levocardia 2. Normal Echocardiographic Study.   No pulmonary Hypertension. | | |

**Done by Signature Date-23/03/2012 E.C Name: Dr. Tesfaye T. Pediatric Cardiologist**

| Pediatric Echocardiography report  Patient Name: Melkam Tazebew Patient ID: 028674  Gender: F Age:14 Date of Report: 23/03/2012Eth.C  Clinical Finding: Rheumatic Recurrence + Murmur + DOE + CHF + Easy Fatigability + Palpitation. TGSH7.3161. | | | | |
| --- | --- | --- | --- | --- |
| Features | Finding | | Features | Finding |
| Profile | | | Atria | |
| Abdominal situs | Solitus | | Left atrium | Dilated, 68 X 73mm |
| Cardiac position | Levocardia | | Right atrium | Dilated |
| Systemic venous drainage | to RA | | Atrioventricular valves | |
| Pulmonary venous drainage | to LA | | Mitral valve | Annulus = 33mm. Thickened, clubbed and calcified Mitral valve leaflet. MVA = 0.9cm2. |
| Atrioventricular connection | Concordant | | Tricuspid valve | Annulus = 24mm. thickened. |
| VA connection | Concordant | | Ventricles | |
| Ventricular loop | d-Loop | | Left ventricle | Dilated |
| Septae | | | Right ventricle | Dilated |
| IVS |  | | M-Mode |  |
| Interatrial septum | Intact | | AO |  |
| Great arteries | NRGA | | LA |  |
| Aorta |  | | LVIDd | 53mm |
| Pulmonary artery | MPA = 21mm, RPA = 15mm, LPA = 15mm. | | LVIDs | 39mm |
| Semilunal valves |  | | IVSd | 9.5mm |
| Aortic valve | Annulus = 13mm, Trileaflet, thickened | | IVSs | 8.6mm |
| Pulmonary valve | Annulus = 24mm | | LVPWd | 8.6mm |
| Doppler Measurement | | | LVPWs | 8.6mm |
| Mitral | Severe MR with velocity of 4.7m/s, holosystolic and posterior jet seen in two planes. Severe MS, PPG/MPG = 21/14mmHg. | | EDV | 137ml |
| Aortic | Moderate AR, PHT = 366ms. | | ESV | 65.5ml |
| Tricuspid | Severe TR, PPG = 75mmHg | | FS | 27% |
| pulmonic | Trivial PR, PPG = 45mmHg | | LVEF | 52% |
| Aortic arch | Left | | Coronary arteries |  |
| PDA | No | |  |  |
| Additional information | |  | | | |
|  | |  | | | |
| Final Diagnosis | 1. {S, D, S} Levocardia 2. Severe MR 3. Severe MS 4. Severe TR 5. Moderate AR 6. Trivial PR 7. Calcified, thickened, clubbed Mitral Valve 8. Thickened Tricuspid valve and aortic valve 9. Severe Pulmonary Hypertension   Good LV Function | | | |

Done by Signature Date-23/03/2012 E.C

Name: Dr. Tesfaye T. Pediatric Cardiologist

| Pediatric Echocardiography report  Patient Name: Temechew Kefale Patient ID: 017241  Gender: M Age: 13Years Date of Report: 21/12/2011Eth.C  BP: Weight: Height: BSA: TGSH1.2638 | | | |
| --- | --- | --- | --- |
| Features | **Finding** | **Features** | **Finding** |
| Profile | | **Atria** | |
| Abdominal situs | Solitus | Left atrium | Normal |
| Cardiac position | Levocardia | Right atrium | Normal |
| Systemic venous drainage | to RA | **Atrioventricular valves** | |
| Pulmonary venous drainage | to LA | Mitral valve | Thickened |
| Atrioventricular connection | Concordant | Tricuspid valve | Normal  TAPSE = 22mm |
| Ventriculoarterial connection | Concordant | **Ventricles** | |
| Ventricular loop | d-Loop | Left ventricle |  |
| Septae | | Right ventricle |  |
| Interventricular septum | Intact | **M-Mode** |  |
| Interatrial septum | Intact | AO |  |
| Great arteries | NRGA | LA |  |
| Aorta |  | LVIDd | 44.5mm |
| Pulmonary artery | Normal sized confluent Branch PAs | LVIDs | 32.8mm |
| Semilunal valves |  | IVSd | 8.64mm |
| Aortic valve | Annulus = 16mm, | IVSs | 8.64mm |
| Pulmonary valve | Annulus = 25mm | LVPWd | 8.64mm |
| Doppler Measurement | | LVPWs | 8.64mm |
| Mitral | Mild MR, Posterior jet | **EDV** | 90.1ml |
| Aortic | ---- | ESV | 43.5ml |
| Tricuspid | Mild TR, PPG = 28mmHg | FS | 26% |
| pulmonic | Mild PR, PPG = 10mmHg | LVEF | 51% |
| Aortic arch | Left | **Coronary arteries** |  |
| PDA | No |  |  |
| Additional information |  | | |
| Final Diagnosis | 1. {S, D, S} Levocardia 2. Thickened Mitral valve, Mild MR 3. Mild TR 4. Mild PR 5. Good Biventricular Function | | |
| Pediatric Echocardiography report  Patient Name: Temechew Kefale. Patient ID: 017241. Gender: M Age: 14  Date of Report: 02/04/2012Eth.C BP: Weight: Height: BSA: TGSH1.2638 | | | |
| Features | **Finding** | **Features** | **Finding** |
| Profile | | **Atria** | |
| Abdominal situs | Solitus | Left atrium | Normal |
| Cardiac position | Levocardia | Right atrium | Normal |
| Systemic venous drainage | to RA | **Atrioventricular valves** | |
| Pulmonary venous drainage | to LA | Mitral valve | Annulus = 25mm. thickened MVL. |
| Atrioventricular connection | Concordant | Tricuspid valve | Annulus = 24mm  TAPSE = 18mm |
| Ventriculoarterial connection | Concordant | **Ventricles** | |
| Ventricular loop | d-Loop | Left ventricle | Normal |
| Septae | | Right ventricle | Normal |
| Interventricular septum |  | **M-Mode** |  |
| Interatrial septum | Intact | AO |  |
| Great arteries | NRGA | LA |  |
| Aorta |  | LVIDd | 48mm |
| Pulmonary artery |  | LVIDs | 28mm |
| Semilunal valves |  | IVSd | 6mm |
| Aortic valve | Annulus = 13mm | IVSs | 7mm |
| Pulmonary valve | Annulus = 15mm | LVPWd | 5mm |
| Doppler Measurement | | LVPWs | 5mm |
| Mitral | Trivial MR | **EDV** | 108ml |
| Aortic | ---- | ESV | 30ml |
| Tricuspid | ---- | FS | 42% |
| pulmonic | Trivial PR, PPG = 10mmHg | LVEF | 72% |
| Aortic arch | Left | **Coronary arteries** |  |
| PDA | No |  |  |
| Additional information |  | | |
|  |  | | |
| Final Diagnosis | 1. {S, D, S} Levocardia 2. Trivial MR 3. Thickened Mitral Valve Leaflet 4. Good Biventricular Function | | |

**Done by Signature Date-02/04/2012 E.C Name: Dr. Tesfaye T. Pediatric Cardiologist**

| Pediatric Echocardiography report  Patient Name: Surafel Tadesse Patient ID: 027857  Gender: M Age: 1 5/12 Date of Report: 02/04/2012Eth.C  Clinical Finding: Recurrent Chest Infection. TGSH7.3162. | | | |
| --- | --- | --- | --- |
| Features | **Finding** | **Features** | **Finding** |
| Profile | | **Atria** | |
| Abdominal situs | Solitus | Left atrium | Normal |
| Cardiac position | Levocardia | Right atrium | Normal |
| Systemic venous drainage | to RA | **Atrioventricular valves** | |
| Pulmonary venous drainage | to LA | Mitral valve | Annulus = 10mm |
| Atrioventricular connection | Concordant | Tricuspid valve | Annulus = 12mm |
| Ventriculoarterial connection | Concordant | **Ventricles** | |
| Ventricular loop | d-Loop | Left ventricle | Normal |
| Septae | | Right ventricle | Normal |
| Interventricular septum | Intact | **M-Mode** |  |
| Interatrial septum | Intact | AO |  |
| Great arteries | NRGA | LA |  |
| Aorta | ---- | LVIDd | mm |
| Pulmonary artery |  | LVIDs | mm |
| Semilunal valves |  | IVSd | mm |
| Aortic valve | Annulus = 11mm | IVSs | mm |
| Pulmonary valve | Annulus = 10mm | LVPWd | mm |
| Doppler Measurement | | LVPWs | mm |
| Mitral | ---- | **EDV** | ml |
| Aortic | ---- | ESV | ml |
| Tricuspid | Trivial TR, PPG = 29mmHg | FS |  |
| pulmonic | ---- | LVEF |  |
| Aortic arch | Left | **Coronary arteries** |  |
| PDA | No |  |  |
| Additional information |  | | |
| Final Diagnosis | 1. Normal Echocardiographic Study. | | |

**Done by Signature Date-02/04/2012 E.C Name: Dr. Tesfaye T. Pediatric Cardiologist**

| Pediatric Echocardiography report  Patient Name: B/Azagne Patient ID: 028941 Gender: F Age: 35 days  Date of Report: 02/04/2012Eth.C BP: Weight: 3.1kg Height: 48cms BSA:  Clinical Finding: Incidental Murmur. TGSH7.3163. | | | |
| --- | --- | --- | --- |
| Features | **Finding** | **Features** | **Finding** |
| Profile | | **Atria** | |
| Abdominal situs | Solitus | Left atrium | Normal |
| Cardiac position | Levocardia | Right atrium | Normal |
| Systemic venous drainage | to RA | **Atrioventricular valves** | |
| Pulmonary venous drainage | to LA | Mitral valve | Annulus = 9mm |
| Atrioventricular connection | Concordant | Tricuspid valve | Annulus = 11mm  TAPSE = 8mm |
| Ventriculoarterial connection | Concordant | **Ventricles** | |
| Ventricular loop | d-Loop | Left ventricle | Normal |
| Septae | | Right ventricle | Normal |
| Interventricular septum | Intact | **M-Mode** |  |
| Interatrial septum | PFO, L – R Shunt | AO |  |
| Great arteries | NRGA | LA |  |
| Aorta | ------ | LVIDd | mm |
| Pulmonary artery | Good sized MPA and Confluent Branch PAs. | LVIDs | mm |
| Semilunal valves |  | IVSd | mm |
| Aortic valve | Annulus = 8mm | IVSs | mm |
| Pulmonary valve | Annulus = 6mm | LVPWd | mm |
| Doppler Measurement | | LVPWs | mm |
| Mitral |  | **EDV** | ml |
| Aortic |  | ESV | ml |
| Tricuspid |  | FS | % |
| pulmonic | Mild PS, PPG = 30mmHg | LVEF | % |
| Aortic arch | Left | **Coronary arteries** |  |
| PDA | No |  |  |
| Additional information |  | | |
| Final Diagnosis | 1. {S, D, S} Levocardia 2. PFO, L – R Shunt 3. Mild PS 4. Good Biventricular Function | | |

**Done by Signature Date-02/04/2012 E.C Name: Dr. Tesfaye T. Pediatric Cardiologist**

| Pediatric Echocardiography report  Patient Name: Bemnet Aragaw Patient ID: 028889 Gender: F Age: 2  Date of Report: 02/04/2012Eth.C BP: Weight: 8.4kg Height: 80cmsrdd BSA:  Clinical Finding: __________. INCOMPLETE DATA | | | |
| --- | --- | --- | --- |
| Features | **Finding** | **Features** | **Finding** |
| Profile | | **Atria** | |
| Abdominal situs | Solitus | Left atrium | Normal |
| Cardiac position | Levocardia | Right atrium | Normal |
| Systemic venous drainage | to RA | **Atrioventricular valves** | |
| Pulmonary venous drainage | to LA | Mitral valve | Annulus = mm |
| Atrioventricular connection | Concordant | Tricuspid valve | Annulus = mm |
| Ventriculoarterial connection | Concordant | **Ventricles** | |
| Ventricular loop | d-Loop | Left ventricle | Normal |
| Septae | | Right ventricle | Normal |
| Interventricular septum | Intact | **M-Mode** |  |
| Interatrial septum | Intact | AO |  |
| Great arteries | NRGA | LA |  |
| Aorta | ------ | LVIDd | mm |
| Pulmonary artery | Good sized MPA and Confluent Branch PAs. | LVIDs | mm |
| Semilunal valves |  | IVSd | mm |
| Aortic valve | Annulus = mm | IVSs | mm |
| Pulmonary valve | Annulus = mm | LVPWd | mm |
| Doppler Measurement | | LVPWs | mm |
| Mitral |  | **EDV** | ml |
| Aortic |  | ESV | ml |
| Tricuspid |  | FS | % |
| pulmonic |  | LVEF | % |
| Aortic arch | Left | **Coronary arteries** |  |
| PDA | No |  |  |
| Additional information |  | | |
|  |  | | |
| Final Diagnosis |  | | |

**Done by Signature Date-02/04/2012 E.C Name: Dr. Tesfaye T. Pediatric Cardiologist**

| Pediatric Echocardiography report  Patient Name: Wude Demeke Patient ID: 029206 Gender: F Age: 13  Date of Report: 02/04/2012Eth.C BP: Weight: Height: BSA:  Clinical Finding: Cyanosis + Clubbing + Murmur_. TGSH7.3164. | | | |
| --- | --- | --- | --- |
| Features | **Finding** | **Features** | **Finding** |
| Profile | | **Atria** | |
| Abdominal situs | Solitus | Left atrium | Normal |
| Cardiac position | Levocardia | Right atrium | Normal |
| Systemic venous drainage | to RA | **Atrioventricular valves** | |
| Pulmonary venous drainage | to LA | Mitral valve | Annulus = mm |
| Atrioventricular connection | Concordant | Tricuspid valve | Annulus = 20mm  TAPSE = 18mm |
| Ventriculoarterial connection | Concordant | **Ventricles** | |
| Ventricular loop | d-Loop | Left ventricle | Normal |
| Septae | | Right ventricle | Hypertrophied |
| Interventricular septum | 13mm Mal aligned sub aortic VSD, L – R Shunt. Not restrictive. | **M-Mode** |  |
| Interatrial septum | Intact | AO |  |
| Great arteries | NRGA | LA |  |
| Aorta | Aortic over riding. | LVIDd | mm |
| Pulmonary artery | Small sized MPA and Confluent Branch PAs. | LVIDs | mm |
| Semilunal valves |  | IVSd | mm |
| Aortic valve | Annulus = 20mm. Aorto – mitral fibrous continuity present. | IVSs | mm |
| Pulmonary valve | Annulus = 11mm | LVPWd | mm |
| Doppler Measurement | | LVPWs | mm |
| Mitral | ---- | **EDV** | ml |
| Aortic | ---- | ESV | ml |
| Tricuspid | Mild TR | FS | 37% |
| pulmonic | Severe PS, PPG = 112mmHg | LVEF | 69% |
| Aortic arch | Left | **Coronary arteries** |  |
| PDA | No |  |  |
| Additional information |  | | |
|  |  | | |
| Final Diagnosis | 1. {S, D, S} Levocardia 2. TOF 3. Good Biventricular Function | | |

**Done by Signature Date-02/04/2012 E.C Name: Dr. Tesfaye T. Pediatric Cardiologist**

| Pediatric Echocardiography report  Patient Name: Solomie Getachew Patient ID: 00846 Gender: F Age: 7 months  Date of Report: 02/04/2012Eth.C BP: Weight: 11kg Height: BSA: | | | |
| --- | --- | --- | --- |
| Features | **Finding** | **Features** | **Finding** |
| Profile | | **Atria** | |
| Abdominal situs | Solitus | Left atrium | Normal |
| Cardiac position | Levocardia | Right atrium | Normal |
| Systemic venous drainage | to RA | **Atrioventricular valves** | |
| Pulmonary venous drainage | to LA | Mitral valve | Normal |
| Atrioventricular connection | Concordant | Tricuspid valve | Normal |
| Ventriculoarterial connection | Concordant | **Ventricles** | |
| Ventricular loop | d-Loop | Left ventricle | Normal |
| Septae | | Right ventricle | Normal |
| Interventricular septum | 3mm PM VSD, L – R Shunt with PPG= 66mmHg. | **M-Mode: Normal LV Function on eye balling.** | |
| Interatrial septum | Intact | AO |  |
| Great arteries | NRGA | LA |  |
| Aorta | ------ | LVIDd | mm |
| Pulmonary artery | Good sized MPA and Confluent Branch PAs. | LVIDs | mm |
| Semilunal valves |  | IVSd | mm |
| Aortic valve | Normal | IVSs | mm |
| Pulmonary valve | Normal | LVPWd | mm |
| Doppler Measurement | | LVPWs | mm |
| Mitral | ---- | **EDV** | ml |
| Aortic | ----- | ESV | ml |
| Tricuspid | ----- | FS | % |
| pulmonic | ----- | LVEF | % |
| Aortic arch | Left | **Coronary arteries** |  |
| PDA | No |  |  |
| Additional information |  | | |
|  |  | | |
| Final Diagnosis | 1. {S, D, S} Levocardia 2. Small PM VSD, L – R Shunt 3. Good Function. | | |

**Done by Signature Date: 02/04/2012 E.C**

**Name: Dr. Tesfaye T. Pediatric Cardiologist**

| Pediatric Echocardiography report  Patient Name: Minalush Asirat Patient ID: 028290 Gender: F Age: 19days  Date of Report: 07/04/2012Eth.C BP: Weight: Height: BSA:  Clinical Finding: RD. TGSH7.3165. | | | |
| --- | --- | --- | --- |
| Features | **Finding** | **Features** | **Finding** |
| Profile | | **Atria** | |
| Abdominal situs | Solitus | Left atrium | Normal |
| Cardiac position | Levocardia | Right atrium | Normal |
| Systemic venous drainage | to RA | **Atrioventricular valves** | |
| Pulmonary venous drainage | to LA | Mitral valve | Annulus = 12mm |
| Atrioventricular connection | Concordant | Tricuspid valve | Annulus = 10mm  TAPSE = 12mm |
| Ventriculoarterial connection | Concordant | **Ventricles** | |
| Ventricular loop | d-Loop | Left ventricle | Normal |
| Septae | | Right ventricle | Normal |
| Interventricular septum | Intact | **M-Mode** |  |
| Interatrial septum | PFO, L – R Shunt | AO |  |
| Great arteries | NRGA | LA |  |
| Aorta | ------ | LVIDd | mm |
| Pulmonary artery | Good sized MPA and Confluent Branch PAs. | LVIDs | mm |
| Semilunal valves |  | IVSd | mm |
| Aortic valve | Annulus = 10mm | IVSs | mm |
| Pulmonary valve | Annulus = 10mm | LVPWd | mm |
| Doppler Measurement | | LVPWs | mm |
| Mitral | ---- | **EDV** | ml |
| Aortic | ----- | ESV | ml |
| Tricuspid | ---- | FS | 33% |
| pulmonic | ---- | LVEF | 64% |
| Aortic arch | Left | **Coronary arteries** |  |
| PDA | No |  |  |
| Additional information |  | | |
|  |  | | |
| Final Diagnosis | 1. {S, D, S} Levocardia 2. PFO, L – R Shunt | | |

**Done by Signature Date-07/04/2012 E.C Name: Dr. Tesfaye T. Pediatric Cardiologist**

| Pediatric Echocardiography report  Patient Name: Leulseged Admaw Patient ID: 029862 Gender: M Age: 2 3/12  Date of Report: 07/04/2012Eth.C BP: Weight: 12kg Height: 80cms BSA:  Clinical Finding: DS + RD + CHF + Murmur + Diaphoresis. TGSH7.3166. | | | |
| --- | --- | --- | --- |
| Features | **Finding** | **Features** | **Finding** |
| Profile | | **Atria** | |
| Abdominal situs | Solitus | Left atrium | Dilated |
| Cardiac position | Levocardia | Right atrium | Dilated |
| Systemic venous drainage | to RA | **Atrioventricular valves** | |
| Pulmonary venous drainage | to LA | Mitral valve | Common AV Valve |
| Atrioventricular connection | Common AV Valve | Tricuspid valve | Common AV Valve |
| Ventriculoarterial connection | Concordant | **Ventricles** | |
| Ventricular loop | d-Loop | Left ventricle | Dilated |
| Septae | | Right ventricle | Dilated |
| Interventricular septum | Large Inlet VSD, BD Shunt | **M-Mode** |  |
| Interatrial septum | Large Primmum ASD, BD Shunt. Additional 7mm OS ASD, BD Shunt. | AO |  |
| Great arteries | NRGA | LA |  |
| Aorta | ------ | LVIDd | mm |
| Pulmonary artery | MPA DILATED. Good sized Confluent Branch PAs. | LVIDs | mm |
| Semilunal valves |  | IVSd | mm |
| Aortic valve | Annulus = 16mm | IVSs | mm |
| Pulmonary valve | Annulus = 16mm | LVPWd | mm |
| Doppler Measurement | | LVPWs | mm |
| Mitral | ---- | **EDV** | ml |
| Aortic | ---- | ESV | ml |
| Tricuspid | Mild right AVVR | FS | 34% |
| pulmonic | Moderate PR | LVEF | 65% |
| Aortic arch | Left | **Coronary arteries** |  |
| PDA | No |  |  |
| Additional information | No effusion | | |
| Final Diagnosis | 1. {S, D, S} Levocardia 2. Moderate OS ASD, BD Shunt 3. Common Complete AVSD, BD Shunt 4. Mild Right AVVR 5. Severe Pulmonary Hypertension 6. Good Function | | |
| Remark: Child was restless and crying. | | | |

**Done by Signature Date-07/04/2012 E.C Name: Dr. Tesfaye T. Pediatric Cardiologist**

| Pediatric Echocardiography report  Patient Name: Nibret Melash Patient ID: 1 3/12 Gender: F Age: 029746  Date of Report: 07/04/2012Eth.C BP: Weight: 5.1 kg Height: 72cms BSA:  Clinical Finding: CHF + RD + Murmur. TGSH7.3167. | | | |
| --- | --- | --- | --- |
| Features | **Finding** | **Features** | **Finding** |
| Profile | | **Atria** | |
| Abdominal situs | Solitus | Left atrium | Dilated |
| Cardiac position | Levocardia | Right atrium | Dilated |
| Systemic venous drainage | to RA | **Atrioventricular valves** | |
| Pulmonary venous drainage | to LA | Mitral valve | Annulus = 16mm. thickened MVL. |
| Atrioventricular connection | Concordant | Tricuspid valve | Annulus = 17mm |
| Ventriculoarterial connection | Concordant | **Ventricles** | |
| Ventricular loop | d-Loop | Left ventricle | Dilated |
| Septae | | Right ventricle | Dilated |
| Interventricular septum | Intact | **M-Mode** |  |
| Interatrial septum | Intact | AO |  |
| Great arteries | NRGA | LA |  |
| Aorta | ------ | LVIDd | 34mm |
| Pulmonary artery | Good sized MPA and Confluent Branch PAs. | LVIDs | 24mm |
| Semilunal valves |  | IVSd | 8.7mm |
| Aortic valve | Annulus = 10mm | IVSs | 9.8mm |
| Pulmonary valve | Annulus = 13mm | LVPWd | 7.6mm |
| Doppler Measurement | | LVPWs | 7.6mm |
| Mitral | Moderate MR | **EDV** | 49ml |
| Aortic | ---- | ESV | 21ml |
| Tricuspid | Mild TR, PPG = 50mmHg | FS | 30% |
| pulmonic | ---- | LVEF | 58% |
| Aortic arch | Left | **Coronary arteries** |  |
| PDA | No |  |  |
| Additional information | 6mm Aorto Pulmonary Window, L – R Shunt | | |
| Final Diagnosis | 1. {S, D, S} Levocardia 2. Aorto Pulmonary Window, L – R Shunt 3. Moderate MR 4. Moderate Pulmonary Hypertension 5. Good LV Function | | |
| Remark: the MR doesn’t go with characteristics of Rheumatic. | | | |

**Done by Signature Date-07/04/2012 E.C Name: Dr. Tesfaye T. Pediatric Cardiologist**

| Pediatric Echocardiography report  Patient Name: Hiwet Mekonnen Patient ID: 026506 Gender: F Age: 6 10/12  Date of Report: 09/04/2012Eth.C BP: Weight: Height: cms BSA:  Clinical Finding: Incidental Murmur. TGSH7.3168. | | | |
| --- | --- | --- | --- |
| Features | **Finding** | **Features** | **Finding** |
| Profile | | **Atria** | |
| Abdominal situs | Solitus | Left atrium | Normal |
| Cardiac position | Levocardia | Right atrium | Normal |
| Systemic venous drainage | to RA | **Atrioventricular valves** | |
| Pulmonary venous drainage | to LA | Mitral valve | Annulus = 17mm |
| Atrioventricular connection | Concordant | Tricuspid valve | Annulus = 16mm |
| Ventriculoarterial connection | Concordant | **Ventricles** | |
| Ventricular loop | d-Loop | Left ventricle | Normal |
| Septae | | Right ventricle | Normal |
| Interventricular septum | 5mm PM VSD, L – R Shunt | **M-Mode** |  |
| Interatrial septum | PFO, L – R Shunt | AO |  |
| Great arteries | NRGA | LA |  |
| Aorta | ------ | LVIDd | mm |
| Pulmonary artery | Good sized MPA and Confluent Branch PAs. | LVIDs | mm |
| Semilunal valves |  | IVSd | mm |
| Aortic valve | Annulus = 18mm | IVSs | mm |
| Pulmonary valve | Annulus = 21mm | LVPWd | mm |
| Doppler Measurement | | LVPWs | mm |
| Mitral | ---- | **EDV** | ml |
| Aortic | ---- | ESV | ml |
| Tricuspid | ---- | FS | 29% |
| pulmonic | Trivial PR | LVEF | 58% |
| Aortic arch | Left | **Coronary arteries** |  |
| PDA | No |  |  |
| Additional information |  | | |
|  |  | | |
| Final Diagnosis | 1. {S, D, S} Levocardia 2. PFO, L – R Shunt 3. Small PM VSD, L – R Shunt 4. Good Function | | |

**Done by Signature Date-09/04/2012 E.C Name: Dr. Tesfaye T. Pediatric Cardiologist**

| Pediatric Echocardiography report  Patient Name: Baby of Belaynesh Zelalem Patient ID: 022016 Gender: M Age: 3/12  Date of Report: 09/04/2012Eth.C BP: Weight: Height: BSA:  Clinical Finding: RD. TGSH7.3169. | | | |
| --- | --- | --- | --- |
| Features | **Finding** | **Features** | **Finding** |
| Profile | | **Atria** | |
| Abdominal situs | Solitus | Left atrium | Normal |
| Cardiac position | Levocardia | Right atrium | Normal |
| Systemic venous drainage | to RA | **Atrioventricular valves** | |
| Pulmonary venous drainage | to LA | Mitral valve | Normal |
| Atrioventricular connection | Concordant | Tricuspid valve | Normal |
| Ventriculoarterial connection | Concordant | **Ventricles** | |
| Ventricular loop | d-Loop | Left ventricle | Normal |
| Septae | | Right ventricle | Normal |
| Interventricular septum | Intact | **M-Mode** |  |
| Interatrial septum | PFO, L – R Shunt | AO |  |
| Great arteries | NRGA | LA |  |
| Aorta | ------ | LVIDd | mm |
| Pulmonary artery | Good sized MPA and Confluent Branch PAs. | LVIDs | mm |
| Semilunal valves |  | IVSd | mm |
| Aortic valve | Normal | IVSs | mm |
| Pulmonary valve | Normal | LVPWd | mm |
| Doppler Measurement | | LVPWs | mm |
| Mitral | ---- | **EDV** | ml |
| Aortic | ---- | ESV | ml |
| Tricuspid | ---- | FS | % |
| pulmonic | ---- | LVEF | % |
| Aortic arch | Left | **Coronary arteries** |  |
| PDA | No |  |  |
| Additional information |  | | |
|  |  | | |
| Final Diagnosis | 1. {S, D, S} Levocardia 2. PFO, L – R Shunt. | | |

**Done by Signature Date-09/04/2012 E.C Name: Dr. Tesfaye T. Pediatric Cardiologist**

| Pediatric Echocardiography report  Patient Name: Solomon Asmare Patient ID: 029918 Gender: M Age: 9 years  Date of Report: 07/04/2012Eth.C BP: Weight: Height: BSA:  Clinical Finding: ARF + Murmur. TGSH7.3170. | | | |
| --- | --- | --- | --- |
| Features | **Finding** | **Features** | **Finding** |
| Profile | | **Atria** | |
| Abdominal situs | Solitus | Left atrium | Normal |
| Cardiac position | Levocardia | Right atrium | Normal |
| Systemic venous drainage | to RA | **Atrioventricular valves** | |
| Pulmonary venous drainage | to LA | Mitral valve | Annulus = 18mm. thickened MVL. Shortened posterior leaflet |
| Atrioventricular connection | Concordant | Tricuspid valve | Annulus = 17mm  TAPSE = 20mm |
| Ventriculoarterial connection | Concordant | **Ventricles** | |
| Ventricular loop | d-Loop | Left ventricle | Normal |
| Septae | | Right ventricle | Normal |
| Interventricular septum | Intact | **M-Mode** |  |
| Interatrial septum | Intact | AO |  |
| Great arteries | NRGA | LA |  |
| Aorta | ------ | LVIDd | mm |
| Pulmonary artery | Good sized MPA and Confluent Branch PAs. | LVIDs | mm |
| Semilunal valves |  | IVSd | mm |
| Aortic valve | Annulus = 16mm | IVSs | mm |
| Pulmonary valve | Annulus = 17mm | LVPWd | mm |
| Doppler Measurement | | LVPWs | mm |
| Mitral | Mild MR, Holosystolic, posteriorly projected with jet velocity of 5m/sec | **EDV** | ml |
| Aortic | Mid AR, PHT = 500ms | ESV | ml |
| Tricuspid | Trivial TR, PPG = 30mmHg | FS | 35% |
| pulmonic | ---- | LVEF | 65% |
| Aortic arch | Left | **Coronary arteries** |  |
| PDA | No |  |  |
| Additional information |  | | |
|  |  | | |
| Final Diagnosis | 1. {S, D, S} Levocardia 2. Thickened MVL. Shortened Posterior MVL 3. Mild MR 4. Mild AR 5. Good Biventricular Function | | |
| Remark: The Echocardiographic Finding goes for Rheumatic Valve disease. Please correlate with clinical findings. | | | |

**Done by Signature Date-07/04/2012 E.C Name: Dr. Tesfaye T. Pediatric Cardiologist**

| Pediatric Echocardiography report  Patient Name: Yohannes Addisu Patient ID: 030369 Gender: M Age: 1 3/12  Date of Report: 09/04/2012Eth.C BP: Weight: Height: BSA:  Clinical Finding: Cyanosis + Clubbing + Murmur. TGSH7.3171. | | | | |
| --- | --- | --- | --- | --- |
| Features | **Finding** | **Features** | | **Finding** |
| Profile | | **Atria** | | |
| Abdominal situs | Solitus | Left atrium | | Normal |
| Cardiac position | Levocardia | Right atrium | | Normal |
| Systemic venous drainage | to RA | **Atrioventricular valves** | | |
| Pulmonary venous drainage | to LA | Mitral valve | | Annulus = 12mm |
| Atrioventricular connection | Concordant | Tricuspid valve | | Annulus = 14mm |
| Ventriculoarterial connection | Concordant | **Ventricles** | | |
| Ventricular loop | d-Loop | Left ventricle | | Normal |
| Septae | | Right ventricle | | Hypertrophied |
| Interventricular septum | Mal-aligned Subaortic VSD, Non-restrictive, R – L Shunt | **M-Mode** | |  |
| Interatrial septum | PFO, R – L Shunt | AO | |  |
| Great arteries | NRGA | LA | |  |
| Aorta | Aortic over riding | LVIDd | | mm |
| Pulmonary artery | Small sized MPA and Branch PAs. | LVIDs | | mm |
| Semilunal valves |  | IVSd | | mm |
| Aortic valve | Annulus = 17mm | IVSs | | mm |
| Pulmonary valve | Annulus = 4mm | LVPWd | | mm |
| Doppler Measurement | | LVPWs | | mm |
| Mitral | ---- | **EDV** | | ml |
| Aortic | ---- | ESV | | ml |
| Tricuspid | ---- | FS | | 30% |
| pulmonic | Severe PS, PPG = 90mmHg | LVEF | | 60% |
| Aortic arch | Left | **Coronary arteries** | |  |
| PDA | No |  | |  |
| Additional information |  | | | |
|  |  | | | |
| Final Diagnosis | 1. {S, D, S} Levocardia 2. PFO, R – L Shunt 3. TOF 4. Smallish MPA and Branch PAs. | | | |
| Done By: | **Signature** | | **Date of Reporting** | |
| Dr. Tesfaye Taye, Pediatric Cardiologist |  | | 09/04/2012Eth.C | |

| Pediatric Echocardiography report  Patient Name: Sefinew Semayneh Patient ID: 030121 Gender: M Age: 1 6/12  Date of Report: 14/04/2012Eth.C BP: Weight: Height: BSA:  Clinical Finding: Recurrent Chest Infection. TGSH7.3172. | | | | |
| --- | --- | --- | --- | --- |
| Features | **Finding** | **Features** | | **Finding** |
| Profile | | **Atria** | | |
| Abdominal situs | Solitus | Left atrium | | Normal |
| Cardiac position | Levocardia | Right atrium | | Normal |
| Systemic venous drainage | to RA | **Atrioventricular valves** | | |
| Pulmonary venous drainage | to LA | Mitral valve | | Annulus = 12mm |
| Atrioventricular connection | Concordant | Tricuspid valve | | Annulus = 15mm  TAPSE = 15mm |
| Ventriculoarterial connection | Concordant | **Ventricles** | | |
| Ventricular loop | d-Loop | Left ventricle | | Normal |
| Septae | | Right ventricle | | Normal |
| Interventricular septum | Intact | **M-Mode** | |  |
| Interatrial septum | Intact | AO | |  |
| Great arteries | NRGA | LA | |  |
| Aorta |  | LVIDd | | mm |
| Pulmonary artery |  | LVIDs | | mm |
| Semilunal valves |  | IVSd | | mm |
| Aortic valve | Annulus = 11mm | IVSs | | mm |
| Pulmonary valve | Annulus = 13mm | LVPWd | | mm |
| Doppler Measurement | | LVPWs | | mm |
| Mitral | ---- | **EDV** | | ml |
| Aortic | ---- | ESV | | ml |
| Tricuspid | Trivial PR, PPG = 31mmHg | FS | | 31% |
| pulmonic | ----- | LVEF | | 60% |
| Aortic arch | Left | **Coronary arteries** | |  |
| PDA | No |  | |  |
| Additional information |  | | | |
| Final Diagnosis | 1. Normal Echocardiography Study | | | |
| Done By: | **Signature** | | **Date of Reporting** | |
| Dr. Tesfaye Taye,  Pediatric Cardiologist |  | | 14/04/2012Eth.C | |

| Pediatric Echocardiography report  Patient Name: Semeredin Ahimed Patient ID: 030970 Gender: M Age: 7  Date of Report: 14/04/2012Eth.C BP: Weight: Height: BSA:  Clinical Finding: Chest Pain. TGSH7.3173. | | | | |
| --- | --- | --- | --- | --- |
| Features | **Finding** | **Features** | | **Finding** |
| Profile | | **Atria** | | |
| Abdominal situs | Solitus | Left atrium | | Normal |
| Cardiac position | Levocardia | Right atrium | | Normal |
| Systemic venous drainage | to RA | **Atrioventricular valves** | | |
| Pulmonary venous drainage | to LA | Mitral valve | | Annulus = 14mm |
| Atrioventricular connection | Concordant | Tricuspid valve | | Annulus = 18mm  TAPSE = 15mm |
| Ventriculoarterial connection | Concordant | **Ventricles** | | |
| Ventricular loop | d-Loop | Left ventricle | | Normal |
| Septae | | Right ventricle | | Normal |
| Interventricular septum | Intact | **M-Mode** | |  |
| Interatrial septum | Intact | AO | |  |
| Great arteries | NRGA | LA | |  |
| Aorta | ------ | LVIDd | | mm |
| Pulmonary artery | Good sized MPA & Branch PAs. | LVIDs | | mm |
| Semilunal valves |  | IVSd | | mm |
| Aortic valve | Annulus = 14mm | IVSs | | mm |
| Pulmonary valve | Annulus = 16mm | LVPWd | | mm |
| Doppler Measurement | | LVPWs | | mm |
| Mitral | ---- | **EDV** | | ml |
| Aortic | ---- | ESV | | ml |
| Tricuspid | Trivial TR, PPG = 15mmHg | FS | | 35% |
| pulmonic | Trivial PR, PPG = 14mmHg | LVEF | | 65% |
| Aortic arch | Left | **Coronary arteries** | |  |
| PDA | No |  | |  |
| Additional information | Trace pericardial effusion on RV side | | | |
| Final Diagnosis | 1. {S, D, S} Levocardia 2. Trace Pericardial Effusion Secondary to ? | | | |
| Done By: | **Signature** | | **Date of Reporting** | |
| Dr. Tesfaye Taye,  Pediatric Cardiologist |  | | 14/04/2012Eth.C | |

| Pediatric Echocardiography report  Patient Name: B/Abeba Yehuala Patient ID: 028735 Gender: M Age: 25days  Date of Report: 14/04/2012Eth.C BP: Weight: 1.3kg Height: 37cms BSA:  INCOMPLETE DOCUMENTATION | | |
| --- | --- | --- |
| Limited Echo window  Only subcostal and suprasternal window evaluation.  Situs Solitus  Levocardia  Intact interventricular septum  PFO, L – R Shunt  No TR/MR  No ventricular inflow obstruction  No LVOTO/RVOTO  Good sized MPA and Branch PAs.  No pulmonary Hypertension  Good Biventricular Function | | |
| Final Diagnosis | 1. {S, D, S} Levocardia 2. PFO, L – R Shunt 3. No pulmonary Hypertension | |
| Done By: | **Signature** | **Date of Reporting** |
| Dr. Tesfaye Taye,  Pediatric Cardiologist |  | 14/04/2012Eth.C |

| Pediatric Echocardiography report  Patient Name: Chalachew Mengiste Patient ID: Gender: M Age: 12  Date of Report: 14/04/2012Eth.C BP: Weight: Height: BSA:  Clinical Finding: Easy Fatigability. TGSH7.3174. | | | | |
| --- | --- | --- | --- | --- |
| Features | **Finding** | **Features** | | **Finding** |
| Profile | | **Atria** | | |
| Abdominal situs | Solitus | Left atrium | | Normal |
| Cardiac position | Levocardia | Right atrium | | Normal |
| Systemic venous drainage | to RA | **Atrioventricular valves** | | |
| Pulmonary venous drainage | to LA | Mitral valve | | Annulus = 22mm |
| Atrioventricular connection | Concordant | Tricuspid valve | | Annulus = 22mm |
| Ventriculoarterial connection | Concordant | **Ventricles** | | |
| Ventricular loop | d-Loop | Left ventricle | | Normal |
| Septae | | Right ventricle | | Normal |
| Interventricular septum | Intact | **M-Mode** | |  |
| Interatrial septum | Intact | AO | |  |
| Great arteries | NRGA | LA | |  |
| Aorta | ----- | LVIDd | | mm |
| Pulmonary artery | Good sized MPA & Branch PAs. | LVIDs | | mm |
| Semilunal valves |  | IVSd | | mm |
| Aortic valve | Annulus = 18mm | IVSs | | mm |
| Pulmonary valve | Annulus = 25mm | LVPWd | | mm |
| Doppler Measurement | | LVPWs | | mm |
| Mitral | ---- | **EDV** | | ml |
| Aortic | ---- | ESV | | ml |
| Tricuspid | Trivial TR, PPG = 27mmHg | FS | | 33% |
| pulmonic | Trivial PR, PPG = 15mmHg | LVEF | | 62% |
| Aortic arch | Left | **Coronary arteries** | |  |
| PDA | No |  | |  |
| Additional information |  | | | |
| Final Diagnosis | 1. Normal Echocardiography Study | | | |
| Done By: | **Signature** | | **Date of Reporting** | |
| Dr. Tesfaye Taye,  Pediatric Cardiologist |  | | 14/04/2012Eth.C | |

| Pediatric Echocardiography report  Patient Name: Nibret Yhenew Patient ID: 032029 Gender: M Age:3months  Date of Report: 20/04/2012Eth.C BP: Weight: Height: BSA:  Clinical Finding: Incidental Murmur. TGSH7.3175. | | | | |
| --- | --- | --- | --- | --- |
| Features | **Finding** | **Features** | | **Finding** |
| Profile | | **Atria** | | |
| Abdominal situs | Solitus | Left atrium | | Normal |
| Cardiac position | Levocardia | Right atrium | | Normal |
| Systemic venous drainage | to RA | Atrioventricular valves | | |
| Pulmonary venous drainage | to LA | Mitral valve | | Annulus = 10mm |
| Atrioventricular connection | Concordant | Tricuspid valve | | Annulus = 11mm  TAPSE = 12mm |
| Ventriculoarterial connection | Concordant | Ventricles | | |
| Ventricular loop | d-Loop | Left ventricle | | Normal |
| Septae | | Right ventricle | | Normal |
| Interventricular septum | Intact | M-Mode: **Normal LV Function on eye balling** | | |
| Interatrial septum | Intact | AO | |  |
| Great arteries | NRGA | LA | |  |
| Aorta | ----- | LVIDd | | mm |
| Pulmonary artery | Good sized MPA & Branch PAs. | LVIDs | | mm |
| Semilunal valves |  | IVSd | | mm |
| Aortic valve | Annulus = 8mm | IVSs | | mm |
| Pulmonary valve | Annulus = 8mm | LVPWd | | mm |
| Doppler Measurement | | LVPWs | | mm |
| Mitral | ---- | EDV | | ml |
| Aortic | ---- | ESV | | ml |
| Tricuspid | Trivial TR | FS | | % |
| pulmonic | Mild Valvar PS, PPG = 28mmHg. | LVEF | | % |
| Aortic arch | Left | **Coronary arteries** | |  |
| PDA | No |  | |  |
| Additional information |  | | | |
|  |  | | | |
| Final Diagnosis | 1. {S, D, S} Levocardia 2. Mild Valvar PS 3. Good Biventricular Function | | | |
| Done By: | **Signature:** | | **Date of Reporting** | |
| Dr. Tesfaye Taye,  Pediatric Cardiologist |  | | 20/04/2012Eth.C | |

| Pediatric Echocardiography report  Patient Name: Alebel Yazzie Patient ID: 022934 Gender: M Age: 13Yrs  Date of Report: 21/04/2012Eth.C BP: Weight: Height: BSA:  Clinical Finding: Easy Fatigability. TGSH7.3176. | | | | |
| --- | --- | --- | --- | --- |
| Features | Finding | Features | | Finding |
| Profile | | Atria | | |
| Abdominal situs | Solitus | Left atrium | | Normal |
| Cardiac position | Levocardia | Right atrium | | Normal |
| Systemic venous drainage | to RA | Atrioventricular valves | | |
| Pulmonary venous drainage | to LA | Mitral valve | | Annulus = 23mm |
| Atrioventricular connection | Concordant | Tricuspid valve | | Annulus = 20mm |
| Ventriculoarterial connection | Concordant | Ventricles | | |
| Ventricular loop | d-Loop | Left ventricle | | Normal |
| Septae | | Right ventricle | | Normal |
| Interventricular septum | Intact | M-Mode | |  |
| Interatrial septum | Intact | AO | |  |
| Great arteries | NRGA | LA | |  |
| Aorta | ----- | LVIDd | | mm |
| Pulmonary artery | Good sized MPA & Confluent Branch PAs. | LVIDs | | mm |
| Semilunal valves |  | IVSd | | mm |
| Aortic valve | Annulus = 18mm | IVSs | | mm |
| Pulmonary valve | Annulus = 24mm | LVPWd | | mm |
| Doppler Measurement | | LVPWs | | mm |
| Mitral | ---- | EDV | | ml |
| Aortic | ---- | ESV | | ml |
| Tricuspid | ---- | FS | | 26% |
| pulmonic | Trivial PR, PPG = 11mmHg | LVEF | | 52% |
| Aortic arch | Left | Coronary arteries | |  |
| PDA | No |  | |  |
| Additional information | No Coarctation of Aorta | | | |
|  | No pericardial effusion. | | | |
| Final Diagnosis | 1. Normal Echocardiography Study. | | | |
| Done By: | Signature | | Date of Reporting | |
| Dr. Tesfaye Taye,  Pediatric Cardiologist |  | | 21/04/2012Eth.C | |

| Pediatric Echocardiography report  Patient Name: Desalew Yenealem Patient ID: 032232 Gender: M Age: 2Yrs  Date of Report: 21/04/2012Eth.C BP: Weight: Height: BSA:  Clinical Finding: Incidental Murmur. TGSH7.3177. | | | | |
| --- | --- | --- | --- | --- |
| Features | Finding | Features | | Finding |
| Profile | | Atria | | |
| Abdominal situs | Solitus | Left atrium | | Normal |
| Cardiac position | Levocardia | Right atrium | | Normal |
| Systemic venous drainage | to RA | Atrioventricular valves | | |
| Pulmonary venous drainage | to LA | Mitral valve | | Annulus = 15mm |
| Atrioventricular connection | Concordant | Tricuspid valve | | Annulus = 17mm  TAPSE = 11mm |
| Ventriculoarterial connection | Concordant | Ventricles | | |
| Ventricular loop | d-Loop | Left ventricle | | Normal |
| Septae | | Right ventricle | | Normal |
| Interventricular septum | 4.5mm PM VSD, L – R Shunt | M-Mode | |  |
| Interatrial septum | Intact | AO | |  |
| Great arteries | NRGA | LA | |  |
| Aorta | ----- | LVIDd | | mm |
| Pulmonary artery | Good sized MPA & Branch PAs. | LVIDs | | mm |
| Semilunal valves |  | IVSd | | mm |
| Aortic valve | Annulus = 15mm | IVSs | | mm |
| Pulmonary valve | Annulus = 17mm | LVPWd | | mm |
| Doppler Measurement | | LVPWs | | mm |
| Mitral | Mild to Moderate MR | EDV | | ml |
| Aortic | ---- | ESV | | ml |
| Tricuspid | Trivial TR | FS | | 26% |
| pulmonic | ---- | LVEF | | 52% |
| Aortic arch | Left | Coronary arteries | |  |
| PDA | 1.5mm PDA, L – R Shunt |  | |  |
| Additional information |  | | | |
|  |  | | | |
| Final Diagnosis | 1. {S, D, S} Levocardia 2. Small Perimembranous VSD, L – R Shunt 3. Small PDA, L – R Shunt 4. Good Function | | | |
| Done By: | Signature | | Date of Reporting | |
| Dr. Tesfaye Taye,  Pediatric Cardiologist |  | | 21/04/2012Eth.C | |

| Pediatric Echocardiography report  Patient Name: Temesgen Berihun Patient ID:032290 Gender: M Age: 13  Date of Report: 21/04/2012Eth.C BP: Weight: Height: BSA:  Clinical Finding: DOE + CHF + Murmur. TGSH7.3178. | | | | |
| --- | --- | --- | --- | --- |
| Features | Finding | Features | | Finding |
| Profile | | Atria | | |
| Abdominal situs | Solitus | Left atrium | | Normal |
| Cardiac position | Levocardia | Right atrium | | Normal |
| Systemic venous drainage | to RA | Atrioventricular valves | | |
| Pulmonary venous drainage | to LA | Mitral valve | | Annulus = 20mm |
| Atrioventricular connection | Concordant | Tricuspid valve | | Annulus = 23mm  TAPSE = 16mm. |
| Ventriculoarterial connection | Concordant | Ventricles | | |
| Ventricular loop | d-Loop | Left ventricle | | Normal |
| Septae | | Right ventricle | | Hypertrophied RV |
| Interventricular septum | Intact | M-Mode | |  |
| Interatrial septum | Intact | AO | |  |
| Great arteries | NRGA | LA | |  |
| Aorta | ----- | LVIDd | | mm |
| Pulmonary artery | Smallish MPA & Branch PAs. | LVIDs | | mm |
| Semilunal valves |  | IVSd | | mm |
| Aortic valve | Annulus = 13mm | IVSs | | mm |
| Pulmonary valve | Annulus = mm | LVPWd | | mm |
| Doppler Measurement | | LVPWs | | mm |
| Mitral | ---- | EDV | | ml |
| Aortic | Mild AR | ESV | | ml |
| Tricuspid | Severe TR, PPG = 116mmHg | FS | | 46% |
| pulmonic | Severe Valvar and Supra valvar PS, PPG = 85mmHg. Mild PR, PPG = 12mmHg | LVEF | | 79% |
| Aortic arch | Left | Coronary arteries | |  |
| PDA | No |  | |  |
| Additional information |  | | | |
|  |  | | | |
| Final Diagnosis | 1. {S, D, S} Levocardia 2. Severe Valvar and supra valvar PS 3. Severe TR 4. RVH + RV Dysfunction | | | |
| Done By: | Signature | | Date of Reporting | |
| Dr. Tesfaye Taye,  Pediatric Cardiologist |  | | 21/04/2012Eth.C | |

| Pediatric Echocardiography report  Patient Name: B/Bizu Azo Patient ID: 031766 Gender: F Age: 28day  Date of Report: 21/04/2012Eth.C BP: Weight: 27kg Height:56cm BSA:  Clinical Finding: DS. TGSH7.3179. | | | | |
| --- | --- | --- | --- | --- |
| Features | Finding | Features | | Finding |
| Profile | | Atria | | |
| Abdominal situs | Solitus | Left atrium | | Normal |
| Cardiac position | Levocardia | Right atrium | | Normal |
| Systemic venous drainage | to RA | Atrioventricular valves | | |
| Pulmonary venous drainage | to LA | Mitral valve | | Annulus = 8mm |
| Atrioventricular connection | Concordant | Tricuspid valve | | Annulus = 11mm |
| Ventriculoarterial connection | Concordant | Ventricles | | |
| Ventricular loop | d-Loop | Left ventricle | | Normal |
| Septae | | Right ventricle | | Normal |
| Interventricular septum | Intact | M-Mode | |  |
| Interatrial septum | 6mm OS ASD, L – R Shunt | AO | |  |
| Great arteries | NRGA | LA | |  |
| Aorta | ----- | LVIDd | | mm |
| Pulmonary artery | Good sized MPA & Branch PAs. | LVIDs | | mm |
| Semilunal valves |  | IVSd | | mm |
| Aortic valve | Annulus = 9mm | IVSs | | mm |
| Pulmonary valve | Annulus = 10mm | LVPWd | | mm |
| Doppler Measurement | | LVPWs | | mm |
| Mitral | ---- | EDV | | ml |
| Aortic | ---- | ESV | | ml |
| Tricuspid | ---- | FS | | 40% |
| pulmonic | ---- | LVEF | | 75% |
| Aortic arch | Left | Coronary arteries | |  |
| PDA | No |  | |  |
| Additional information | Limited Echo window (Only sub costal and partially apical were used) | | | |
|  |  | | | |
| Final Diagnosis | 1. {S, D, S} Levocardia 2. Small Ostium Secundum ASD, L – R Shunt 3. Good Function | | | |
| Done By: | Signature | | Date of Reporting | |
| Dr. Tesfaye Taye,  Pediatric Cardiologist |  | | 21/04/2012Eth.C | |

| Pediatric Echocardiography report  Patient Name: Hana Melak Patient ID: 032076 Gender: F Age: 3yrs  Date of Report: 23/04/2012Eth.C BP: Weight:8kg Height: 78cms BSA:  Clinical Finding: CHF + RD. TGSH7.3180. | | | | |
| --- | --- | --- | --- | --- |
| Features | Finding | Features | | Finding |
| Profile | | Atria | | |
| Abdominal situs | Solitus | Left atrium | | Normal |
| Cardiac position | Levocardia | Right atrium | | Normal |
| Systemic venous drainage | to RA | Atrioventricular valves | | |
| Pulmonary venous drainage | to LA | Mitral valve | | Annulus = 16mm |
| Atrioventricular connection | Concordant | Tricuspid valve | | Annulus = 18mm  TAPSE = 14mm |
| Ventriculoarterial connection | Concordant | Ventricles | | |
| Ventricular loop | d-Loop | Left ventricle | | Normal |
| Septae | | Right ventricle | | Normal |
| Interventricular septum | Intact | M-Mode | |  |
| Interatrial septum | Intact | AO | |  |
| Great arteries | NRGA | LA | |  |
| Aorta | ----- | LVIDd | | 27.6mm |
| Pulmonary artery | Good sized MPA & Branch PAs. | LVIDs | | 20.8mm |
| Semilunal valves |  | IVSd | | **9mm** |
| Aortic valve | Annulus = 12mm | IVSs | | 9mm |
| Pulmonary valve | Annulus = 13mm | LVPWd | | 5mm |
| Doppler Measurement | | LVPWs | | 6mm |
| Mitral | Mild MR | EDV | | 28.5ml |
| Aortic | ---- | ESV | | 14ml |
| Tricuspid | Trivial TR, PPG = 27mmHg | FS | | 24% |
| pulmonic | ---- | LVEF | | 50% |
| Aortic arch | Left | Coronary arteries | |  |
| PDA | No |  | |  |
| Additional information | Trace pericardial effusion, circumferential. | | | |
|  |  | | | |
| Final Diagnosis | 1. {S, D, S} Levocardia 2. Mild MR 3. Trivial TR 4. Thickened IVS 5. Mild LV Dysfunction | | | |
| Suggestion: | Echocardiography Feature goes for Perimyocarditis. | | | |
| Done By: | Signature | | Date of Reporting | |
| Dr. Tesfaye Taye,  Pediatric Cardiologist |  | | 23/04/2012Eth.C | |

| Pediatric Echocardiography report  Patient Name: Abrham Birku. Patient ID: 030781 Gender: Male. Age: 14years.  Date of Report: 05/05/2012Eth.C BP: Weight: Height: BSA:  Clinical Finding: Easy Fatigability. TGSH7.3181. | | | | |
| --- | --- | --- | --- | --- |
| Features | Finding | Features | | Finding |
| Profile | | Atria | | |
| Abdominal situs | Solitus | Left atrium | | Normal |
| Cardiac position | Levocardia | Right atrium | | Normal |
| Systemic venous drainage | to RA | Atrioventricular valves | | |
| Pulmonary venous drainage | to LA | Mitral valve | | Annulus = 25mm |
| Atrioventricular connection | Concordant | Tricuspid valve | | Annulus = 28mm |
| Ventriculoarterial connection | Concordant | Ventricles | | |
| Ventricular loop | d-Loop | Left ventricle | | Normal |
| Septae | | Right ventricle | | Normal |
| Interventricular septum | Intact | M-Mode | |  |
| Interatrial septum | Intact | AO | |  |
| Great arteries | NRGA | LA | |  |
| Aorta | ----- | LVIDd | | mm |
| Pulmonary artery | Good sized MPA & Branch PAs. | LVIDs | | mm |
| Semilunal valves |  | IVSd | | mm |
| Aortic valve | Annulus = 17mm | IVSs | | mm |
| Pulmonary valve | Annulus = 19mm | LVPWd | | mm |
| Doppler Measurement | | LVPWs | | mm |
| Mitral | ---- | EDV | | ml |
| Aortic | ---- | ESV | | ml |
| Tricuspid | Trivial TR, PPG = 25mmHg | FS | | 36% |
| pulmonic | Trivial PR, PPG = 16mmHg | LVEF | | 66% |
| Aortic arch | Left | Coronary arteries | |  |
| PDA | No |  | |  |
| Additional information |  | | | |
|  |  | | | |
| Final Diagnosis | 1. Normal Echocardiography. | | | |
| Done By: | Signature | | Date of Reporting | |
| Dr. Tesfaye Taye,  Pediatric Cardiologist |  | | 05/05/2012Eth.C | |

| Pediatric Echocardiography report  Patient Name: Amar Seid Patient ID: 017301  Gender: M Age: 1 4/12 Date of Report: 05/05/2012Eth.C  Clinical Finding: DS + RD + CHF + Murmur. TGSH7.3182. | | | | |
| --- | --- | --- | --- | --- |
| Features | **Finding** | **Features** | | **Finding** |
| Profile | | **Atria** | | |
| Abdominal situs | Solitus | Left atrium | | Dilated |
| Cardiac position | Levocardia | Right atrium | | Dilated |
| Systemic venous drainage | to RA | **Atrioventricular valves** | | |
| Pulmonary venous drainage | to LA | Mitral valve | | Annulus = 17mm |
| Atrioventricular connection | Concordant | Tricuspid valve | | Annulus = 17mm |
| Ventriculoarterial connection | Concordant | **Ventricles** | | |
| Ventricular loop | d-Loop | Left ventricle | | Dilated |
| Septae | | Right ventricle | | Dilated |
| Interventricular septum | 10mm inlet VSD with PM Extension, L – R Shunt | **M-Mode** | |  |
| Interatrial septum | Intact | AO | |  |
| Great arteries | NRGA | LA | |  |
| Aorta |  | LVIDd | | mm |
| Pulmonary artery | MPA = 21mm. Good sized confluent Branch PAs | LVIDs | | mm |
| Semilunal valves |  | IVSd | | mm |
| Aortic valve | Annulus = 13mm | IVSs | | mm |
| Pulmonary valve | Annulus = 17mm | LVPWd | | mm |
| Doppler Measurement | | LVPWs | | mm |
| Mitral | ---- | **EDV** | | ml |
| Aortic | ---- | ESV | | ml |
| Tricuspid | Mild TR | FS | | 29% |
| pulmonic | Mild PR, PPG = 55mmHg | LVEF | | 57% |
| Aortic arch | Left | **Coronary arteries** | |  |
| PDA | No |  | |  |
| Additional information | 5mm Pericardial effusion on the RA/RV Side | | | |
| Final Diagnosis | 1. {S, D, S} Levocardia 2. Large Inlet VSD with PM extension, L – R Shunt 3. Moderate pulmonary Hypertension 4. Trace pericardial effusion on RA/RV Side 5. Good Biventricular Function | | | |
| Done By: | Signature | | Date of Reporting | |
| Dr. Tesfaye Taye, Pediatric Cardiologist |  | | 05/05/2012Eth.C. | |

| Pediatric Echocardiography report  Patient Name: Agumas Metalign. Patient ID: 032709 Gender: Male. Age: 6 9/12.  Date of Report: 05/05/2012Eth.C BP: Weight: Height: BSA:  Clinical Finding: Easy Fatigability. TGSH7.3183. | | | | |
| --- | --- | --- | --- | --- |
| Features | Finding | Features | | Finding |
| Profile | | Atria | | |
| Abdominal situs | Solitus | Left atrium | | Normal |
| Cardiac position | Levocardia | Right atrium | | Normal |
| Systemic venous drainage | to RA | Atrioventricular valves | | |
| Pulmonary venous drainage | to LA | Mitral valve | | Annulus = 17mm |
| Atrioventricular connection | Concordant | Tricuspid valve | | Annulus = 20mm  TAPSE = 15mm. |
| Ventriculoarterial connection | Concordant | Ventricles | | |
| Ventricular loop | d-Loop | Left ventricle | | Normal |
| Septae | | Right ventricle | | Normal |
| Interventricular septum | Intact | M-Mode | |  |
| Interatrial septum | Intact | AO | |  |
| Great arteries | NRGA | LA | |  |
| Aorta | ----- | LVIDd | | mm |
| Pulmonary artery | Good sized MPA & Branch PAs. | LVIDs | | mm |
| Semilunal valves |  | IVSd | | mm |
| Aortic valve | Annulus = 14mm | IVSs | | mm |
| Pulmonary valve | Annulus = 18mm | LVPWd | | mm |
| Doppler Measurement | | LVPWs | | mm |
| Mitral | ---- | EDV | | ml |
| Aortic | ---- | ESV | | ml |
| Tricuspid | ---- | FS | | 32% |
| pulmonic | Trivial PR, PPG = 12mmHg | LVEF | | 62% |
| Aortic arch | Left | Coronary arteries | |  |
| PDA | No |  | |  |
| Additional information |  | | | |
|  |  | | | |
| Final Diagnosis | 1. Normal Echocardiography Study. | | | |
| Done By: | Signature | | Date of Reporting | |
| Dr. Tesfaye Taye,  Pediatric Cardiologist |  | | 05/05/2012Eth.C | |

| Pediatric Echocardiography report  Patient Name: Samuel Habtamu. Patient ID: 033358. Gender: Male . Age: 7years .  Date of Report: 05/05/2012Eth.C BP: Weight: Height: BSA:  Clinical Finding: Easy Fatigability. TGSH7.3184. | | | | |
| --- | --- | --- | --- | --- |
| Features | Finding | Features | | Finding |
| Profile | | Atria | | |
| Abdominal situs | Solitus | Left atrium | | Normal |
| Cardiac position | Levocardia | Right atrium | | Normal |
| Systemic venous drainage | to RA | Atrioventricular valves | | |
| Pulmonary venous drainage | to LA | Mitral valve | | Annulus = 21mm |
| Atrioventricular connection | Concordant | Tricuspid valve | | Annulus = 23mm  TAPSE = 22mm. |
| Ventriculoarterial connection | Concordant | Ventricles | | |
| Ventricular loop | d-Loop | Left ventricle | | Normal |
| Septae | | Right ventricle | | Normal |
| Interventricular septum | Intact | M-Mode | |  |
| Interatrial septum | Intact | AO | |  |
| Great arteries | NRGA | LA | |  |
| Aorta | ----- | LVIDd | | mm |
| Pulmonary artery | Good sized MPA & Branch PAs. | LVIDs | | mm |
| Semilunal valves |  | IVSd | | mm |
| Aortic valve | Annulus = 16mm | IVSs | | mm |
| Pulmonary valve | Annulus = 20mm | LVPWd | | mm |
| Doppler Measurement | | LVPWs | | mm |
| Mitral | ---- | EDV | | ml |
| Aortic | ---- | ESV | | ml |
| Tricuspid | Trivial PR, PPG = 20mmHg | FS | | 35% |
| pulmonic | Trivial PR, PPG = 20mmHg | LVEF | | 65% |
| Aortic arch | Left | Coronary arteries | |  |
| PDA | No |  | |  |
| Additional information |  | | | |
|  |  | | | |
| Final Diagnosis | 1. Normal Echocardiography Study. | | | |
| Done By: | Signature | | Date of Reporting | |
| Dr. Tesfaye Taye,  Pediatric Cardiologist |  | | 05/05/2012Eth.C | |

| Pediatric Echocardiography report  Patient Name: baby Melkam Gashaw. Patient ID 033117: Gender: male Age: 6days  Date of Report:05 /05/2012 ETC BP: Weight:3kg Height: 48 cm BSA:  Clinical Finding: Incidental Murmur. TGSH7.3185. | | | | |
| --- | --- | --- | --- | --- |
| Features | Finding | Features | | Finding |
| Profile | | Atria | | |
| Abdominal situs | Solitus | Left atrium | | Normal |
| Cardiac position | Levocardia | Right atrium | | Normal |
| Systemic venous drainage | to RA | Atrioventricular valves | | |
| Pulmonary venous drainage | to LA | Mitral valve | | Normal |
| Atrioventricular connection | Concordant | Tricuspid valve | | Normal |
| Ventriculoarterial connection | Concordant | Ventricles | | |
| Ventricular loop | d-Loop | Left ventricle | | Normal |
| Septae | | Right ventricle | | Normal |
| Interventricular septum | 4mm anterior mid muscular VSD, L – R Shunt, restrictive | M-Mode | |  |
| Interatrial septum | PFO, L – R Shunt | AO | |  |
| Great arteries | NRGA | LA | |  |
| Aorta | ----- | LVIDd | | mm |
| Pulmonary artery | Good sized MPA & Confluent Branch PAs. | LVIDs | | mm |
| Semilunal valves |  | IVSd | | mm |
| Aortic valve | Annulus = 6mm | IVSs | | mm |
| Pulmonary valve | Annulus = 7mm | LVPWd | | mm |
| Doppler Measurement | | LVPWs | | mm |
| Mitral | ---- | EDV | | ml |
| Aortic | ---- | ESV | | ml |
| Tricuspid | ---- | FS | | 38% |
| pulmonic | ---- | LVEF | | 73% |
| Aortic arch | Left | Coronary arteries | |  |
| PDA | No |  | |  |
| Additional information |  | | | |
|  |  | | | |
| Final Diagnosis | 1. {S, D, S} Levocardia 2. PFO, L – R Shunt 3. Restrictive anterior mid muscular VSD, L – R Shunt 4. Good Biventricular Function. | | | |
| Done By: | Signature | | Date of Reporting | |
| Dr. Tesfaye Taye,  Pediatric Cardiologist |  | | 05/05/2012Eth.C | |

| Pediatric Echocardiography report  Patient Name: Yitayal Misganaw. Patient ID: 034038 Gender: Male. Age: 11Years.  Date of Report: 12/05/2012Eth.C BP: Weight: Height: BSA:  Clinical Finding: Sydenham’s Chorea. TGSH7.3186. | | | | |
| --- | --- | --- | --- | --- |
| Features | Finding | Features | | Finding |
| Profile | | Atria | | |
| Abdominal situs | Solitus | Left atrium | | Normal |
| Cardiac position | Levocardia | Right atrium | | Normal |
| Systemic venous drainage | to RA | Atrioventricular valves | | |
| Pulmonary venous drainage | to LA | Mitral valve | | Annulus = 20mm |
| Atrioventricular connection | Concordant | Tricuspid valve | | Annulus = 25mm.  TAPSE = 25mm. |
| Ventriculoarterial connection | Concordant | Ventricles | | |
| Ventricular loop | d-Loop | Left ventricle | | Normal |
| Septae | | Right ventricle | | Normal |
| Interventricular septum | Intact | M-Mode | |  |
| Interatrial septum | Intact | AO | |  |
| Great arteries | NRGA | LA | |  |
| Aorta | ----- | LVIDd | | mm |
| Pulmonary artery | Good sized MPA & Branch PAs. | LVIDs | | mm |
| Semilunal valves |  | IVSd | | mm |
| Aortic valve | Annulus = 16mm | IVSs | | mm |
| Pulmonary valve | Annulus = 22mm | LVPWd | | mm |
| Doppler Measurement | | LVPWs | | mm |
| Mitral | ---- | EDV | | ml |
| Aortic | ---- | ESV | | ml |
| Tricuspid | ---- | FS | | 37% |
| pulmonic | ---- | LVEF | | 68% |
| Aortic arch | Left | Coronary arteries | |  |
| PDA | No |  | |  |
| Additional information |  | | | |
|  |  | | | |
| Final Diagnosis | 1. Normal Echocardiography Study. | | | |
| Done By: | Signature | | Date of Reporting | |
| Dr. Tesfaye Taye,  Pediatric Cardiologist |  | | 12/05/2012Eth.C | |

| Pediatric Echocardiography report  Patient Name: Bethlehem Ayana Patient ID:034278 Gender: F Age: 3yr 6/12  Date of Report: 12/05/2012Eth.C BP: Weight: Height: BSA:  Clinical Finding: DS + CHF + Murmur + RD + Diaphoresis. TGSH7.3187. | | | | |
| --- | --- | --- | --- | --- |
| Features | Finding | Features | | Finding |
| Profile | | Atria | | |
| Abdominal situs | Solitus | Left atrium | | Dilated |
| Cardiac position | Levocardia | Right atrium | | More dilated |
| Systemic venous drainage | to RA | Atrioventricular valves | | |
| Pulmonary venous drainage | to LA | Mitral valve | | Common AV Valve. |
| Atrioventricular connection | Common valve | Tricuspid valve | | Common AV Valve.  TAPSE = 16mm |
| Ventriculoarterial connection | Concordant | Ventricles | | |
| Ventricular loop | d-Loop | Left ventricle | | Dilated |
| Septae : No tongue of tissue in b/n | | Right ventricle | | More dilated |
| Interventricular septum | Nonrestrictive Inlet VSD, L – R Shunt | M-Mode | |  |
| Interatrial septum | Large primum defect, L – R Shunt. | AO | |  |
| Great arteries | NRGA | LA | |  |
| Aorta | ----- | LVIDd | | mm |
| Pulmonary artery | **MPA = 20mm**. Confluent Branch PAs. | LVIDs | | mm |
| Semilunal valves |  | IVSd | | mm |
| Aortic valve | Annulus = mm | IVSs | | mm |
| Pulmonary valve | Annulus = 20mm | LVPWd | | mm |
| Doppler Measurement | | LVPWs | | mm |
| Mitral | Severe Left AVVR | EDV | | ml |
| Aortic | ---- | ESV | | ml |
| Tricuspid | Severe right AVVR | FS | | 23% |
| pulmonic | ---- | LVEF | | 47% |
| Aortic arch | Left | Coronary arteries | |  |
| PDA | No |  | |  |
| Additional information |  | | | |
|  | 3mm pericardial effusion on RA/RV Side | | | |
| Final Diagnosis | 1. {S, D, S} Levocardia 2. Common Complete Balanced AVSD 3. Severe Left AVVR 4. Severe Right AVVR 5. Trace pericardial effusion (RA/RV Side) 6. Severe Pulmonary Hypertension 7. Mildly Reduced LV Function | | | |
| Done By: | Signature | | Date of Reporting | |
| Dr. Tesfaye Taye, Pediatric Cardiologist |  | | 12/05/2012Eth.C | |

| Pediatric Echocardiography report  Patient Name: Tigist Sendekie. Patient ID: 033603 Gender: F Age: 8 Years  Date of Report: 12/05/2012Eth.C BP: Weight: Height: BSA:  Clinical Finding: Incidental Murmur + Easy Fatigability. TGSH7.3188. | | | | |
| --- | --- | --- | --- | --- |
| Features | Finding | Features | | Finding |
| Profile | | Atria | | |
| Abdominal situs | Solitus | Left atrium | | Dilated |
| Cardiac position | Levocardia | Right atrium | | Normal |
| Systemic venous drainage | to RA | Atrioventricular valves | | |
| Pulmonary venous drainage | to LA | Mitral valve | | Annulus = 25mm |
| Atrioventricular connection | Concordant | Tricuspid valve | | Annulus = 13mm |
| Ventriculoarterial connection | Concordant | Ventricles | | |
| Ventricular loop | d-Loop | Left ventricle | | Dilated |
| Septae | | Right ventricle | | Normal |
| Interventricular septum | Intact | M-Mode | |  |
| Interatrial septum | Intact | AO | |  |
| Great arteries | NRGA | LA | |  |
| Aorta | ----- | LVIDd | | mm |
| Pulmonary artery | Good sized MPA & Branch PAs. | LVIDs | | mm |
| Semilunal valves |  | IVSd | | mm |
| Aortic valve | Annulus = 19mm | IVSs | | mm |
| Pulmonary valve | Annulus = 20mm | LVPWd | | mm |
| Doppler Measurement | | LVPWs | | mm |
| Mitral | Mild MR | EDV | | ml |
| Aortic | Trivial AR | ESV | | ml |
| Tricuspid | Trivial TR, PPG = 31mmHg | FS | | 36% |
| pulmonic | ---- | LVEF | | 65% |
| Aortic arch | Left | Coronary arteries | |  |
| PDA | 2.5mm PDA, L – R Shunt. |  | |  |
| Additional information |  | | | |
|  |  | | | |
| Final Diagnosis | 1. {S, D, S} Levocardia 2. Moderate PDA, L – R Shunt 3. Good Function | | | |
| Done By: | Signature | | Date of Reporting | |
| Dr. Tesfaye Taye,  Pediatric Cardiologist |  | | 12/05/2012Eth.C | |

| Pediatric Echocardiography report  Patient Name: Yechale Wubet Patient ID: 034263 Gender: M Age: 4 YEARS 4/12  Date of Report: 12/05/2012Eth.C BP: Weight: Height: BSA:  Clinical Finding: CHF + Murmur. TGSH7.3189. | | | | |
| --- | --- | --- | --- | --- |
| Features | Finding | Features | | Finding |
| Profile | | Atria | | |
| Abdominal situs | Solitus | Left atrium | | Dilated |
| Cardiac position | Levocardia | Right atrium | | Normal |
| Systemic venous drainage | to RA | Atrioventricular valves | | |
| Pulmonary venous drainage | to LA | Mitral valve | | Annulus = 22mm. Thickened MVL. |
| Atrioventricular connection | Concordant | Tricuspid valve | | Annulus = 22mm.  TAPSE = 16mm. |
| Ventriculoarterial connection | Concordant | Ventricles | | |
| Ventricular loop | d-Loop | Left ventricle | | Dilated |
| Septae | | Right ventricle | | Normal |
| Interventricular septum | Intact | M-Mode | |  |
| Interatrial septum | Intact | AO | |  |
| Great arteries | NRGA | LA | |  |
| Aorta | ----- | LVIDd | | mm |
| Pulmonary artery | Good sized MPA & Branch PAs. | LVIDs | | mm |
| Semilunal valves |  | IVSd | | mm |
| Aortic valve | Annulus = 15mm | IVSs | | mm |
| Pulmonary valve | Annulus = 18mm | LVPWd | | mm |
| Doppler Measurement | | LVPWs | | mm |
| Mitral | Severe MR, Holosystolic, posterior projection, seen in two planes with velocity of 4m/sec. | EDV | | ml |
| Aortic | ---- | ESV | | ml |
| Tricuspid | Severe TR, PPG = 52mmHg | FS | | 35% |
| pulmonic | ---- | LVEF | | 66% |
| Aortic arch | Left | PDA | | No |
| Additional information | 6mm pericardial effusion on LV Side. 13.6mm pericardial effusion on the RA/RV Side. | | | |
|  | Trace right pleural effusion. | | | |
| Final Diagnosis | 1. {S, D, S} Levocardia 2. Severe TR 3. Severe MR 4. Thickened Mitral valve leaflet 5. Moderate Pericardial effusion 6. Trace right pleural effusion. | | | |
| Remark: correlate with clinical scenario. The echocardiography finding goes with Rheumatic carditis. | | | | |
| Done By: | Signature | | Date of Reporting | |
| Dr. Tesfaye Taye, Pediatric Cardiologist |  | | 12/05/2012Eth.C | |

| Pediatric Echocardiography report  Patient Name: B. Kalkidan Nigussie. Patient ID: 034570 Gender: F Age: 46hrs  Date of Report: 15/05/2012Eth.C BP: Weight: Height: BSA:  Clinical Finding: PNA. TGSH7.3190. | | | | |
| --- | --- | --- | --- | --- |
| Features | Finding | Features | | Finding |
| Profile | | Atria | | |
| Abdominal situs | Solitus | Left atrium | | Normal |
| Cardiac position | Levocardia | Right atrium | | Normal |
| Systemic venous drainage | to RA | Atrioventricular valves | | |
| Pulmonary venous drainage | to LA | Mitral valve | | Annulus = 8mm |
| Atrioventricular connection | Concordant | Tricuspid valve | | Annulus = 8mm  TAPSE = 8mm |
| Ventriculoarterial connection | Concordant | Ventricles | | |
| Ventricular loop | d-Loop | Left ventricle | | Normal |
| Septae | | Right ventricle | | Hypertrophied.  RV TDI S Wave =8cm/sec |
| Interventricular septum | Intact | M-Mode | |  |
| Interatrial septum | Intact | AO | |  |
| Great arteries | NRGA | LA | |  |
| Aorta | ----- | LVIDd | | mm |
| Pulmonary artery | Good sized MPA & Branch PAs. | LVIDs | | mm |
| Semilunal valves |  | IVSd | | 5mm |
| Aortic valve | Annulus = 8mm | IVSs | | 4mm |
| Pulmonary valve | Annulus = 8mm | LVPWd | | mm |
| Doppler Measurement | | LVPWs | | mm |
| Mitral | Trivial MR | EDV | | ml |
| Aortic | ---- | ESV | | ml |
| Tricuspid | Mild TR, PPG = 37mmHg | FS | | 24% |
| pulmonic | ---- | LVEF | | 52% |
| Aortic arch | Left | Coronary arteries | |  |
| PDA | No |  | |  |
| Additional information | 4mm Pericardial effusion on RA/RV Side. Trace circumferential Pericardial effusion | | | |
|  |  | | | |
| Final Diagnosis | 1. {S, D, S} Levocardia 2. Mild TR 3. Trivial MR 4. Reduced Biventricular Function 5. Trace Pericardial Effusion. 6. ?PNA Associated Myocardial Dysfunction | | | |
| Done By: | Signature | | Date of Reporting | |
| Dr. Tesfaye Taye,  Pediatric Cardiologist |  | | 15/05/2012Eth.C | |

| Pediatric Echocardiography report  Patient Name: Eskeziaw Bizengaw. patient ID: 034733 Gender: F Age: 5  Date of Report: 19/05/2012Eth.C BP: Weight: 13.6kg Height: 96cm BSA:  Clinical Finding: Incidental Murmur. TGSH7.3191. | | | | |
| --- | --- | --- | --- | --- |
| Features | Finding | Features | | Finding |
| Profile | | Atria | | |
| Abdominal situs | Solitus | Left atrium | | Dilated |
| Cardiac position | Levocardia | Right atrium | | Normal |
| Systemic venous drainage | to RA | Atrioventricular valves | | |
| Pulmonary venous drainage | to LA | Mitral valve | | Annulus = 21mm |
| Atrioventricular connection | Concordant | Tricuspid valve | | Annulus = 20mm |
| Ventriculoarterial connection | Concordant | Ventricles | | |
| Ventricular loop | d-Loop | Left ventricle | | Dilated |
| Septae | | Right ventricle | | Normal |
| Interventricular septum | 7mm supra Cristal VSD, L – R Shunt | M-Mode | |  |
| Interatrial septum | PFO, L – R Shunt | AO | |  |
| Great arteries | NRGA | LA | |  |
| Aorta | ----- | LVIDd | | mm |
| Pulmonary artery | **MPA = 23mm**. Confluent Branch PAs. | LVIDs | | mm |
| Semilunal valves |  | IVSd | | mm |
| Aortic valve | Annulus = 16mm | IVSs | | mm |
| Pulmonary valve | Annulus = 19mm | LVPWd | | mm |
| Doppler Measurement | | LVPWs | | mm |
| Mitral | ---- | EDV | | ml |
| Aortic | Mild AR, PPG = 726ms | ESV | | ml |
| Tricuspid | Trivial TR | FS | | 30% |
| pulmonic | ---- | LVEF | | 58% |
| Aortic arch | Left | Coronary arteries | |  |
| PDA | No |  | |  |
| Additional information |  | | | |
|  |  | | | |
| Final Diagnosis | 1. {S, D, S} Levocardia 2. PFO, L – R Shunt 3. Moderate Supra Cristal VSD, L – R Shunt 4. Mild AR 5. Pulmonary Hypertension 6. Good Function | | | |
| Recommendation: | Surgical Closure | | | |
| Done By: | Signature | | Date of Reporting | |
| Dr. Tesfaye Taye,  Pediatric Cardiologist |  | | 19/04/2012Eth.C | |

| Pediatric Echocardiography report  Patient Name: Mastewal Shibabaw. Patient ID: 035837 Gender: F Age: 2 years 6/12  Date of Report: 19/05/2012Eth.C BP: Weight: Height: BSA:  Clinical Finding: Cyanosis + Clubbing + RD + CHF + Diaphoresis. TGSH7.3192. | | | | |
| --- | --- | --- | --- | --- |
|  | | | | |
| Features | Finding | Features | | Finding |
| Profile | | Atria | | |
| Abdominal situs | Solitus | Left atrium | | Dilated |
| Cardiac position | Levocardia | Right atrium | | Dilated |
| Systemic venous drainage | to RA. Prominent CS. | Atrioventricular valves | | |
| Pulmonary venous drainage | to LA | Mitral valve | | Annulus = 10mm |
| Atrioventricular connection | Concordant | Tricuspid valve | | Annulus = 12mm |
| Ventriculoarterial connection | Discordant | Ventricles | | |
| Ventricular loop | d-Loop | Left ventricle | | Dilated |
| Septae | | Right ventricle | | Dilated |
| Interventricular septum | Large Sub-Aortic VSD, BD Shunt | **M-Mode: Good Function on eye balling** | | |
| Interatrial septum | PFO, L – R Shunt | AO | |  |
| Great arteries | d-TGA | LA | |  |
| Aorta | Anterior and to the right | LVIDd | | mm |
| Pulmonary artery | Posterior and to the left | LVIDs | | mm |
| Semilunal valves |  | IVSd | | mm |
| Aortic valve | Annulus = 8mm | IVSs | | mm |
| Pulmonary valve | Annulus = 10mm | LVPWd | | mm |
| Doppler Measurement | | LVPWs | | mm |
| Mitral | ---- | EDV | | ml |
| Aortic | ---- | ESV | | ml |
| Tricuspid | ---- | FS | | % |
| pulmonic | ---- | LVEF | | % |
| Aortic arch | Left | Coronary arteries | |  |
| PDA | No |  | |  |
| Additional information |  | | | |
| Final Diagnosis | 1. {S, D, S} Levocardia 2. d-TGA 3. Large non - restrictive Sub-Aortic VSD, BD Shunt 4. Good Function | | | |
| Done By: | Signature | | Date of Reporting | |
| Dr. Tesfaye Taye,  Pediatric Cardiologist |  | | 19/04/2012Eth.C | |
| REMARK: DILV = Double Inlet Left Ventricle. | | | | |

| Pediatric Echocardiography report  Patient Name: Amen Getasew Patient ID: 021801 Gender: F Age: 1 year 4/12  Date of Report: 19/05/2012Eth.C BP: Weight: Height: BSA:  Clinical Finding: ________. INCOMPLETE DOCUMENTATION | | | | |
| --- | --- | --- | --- | --- |
| Features | Finding | Features | | Finding |
| Profile | | Atria | | |
| Abdominal situs | Solitus | Left atrium | | Normal |
| Cardiac position | Levocardia | Right atrium | | Normal |
| Systemic venous drainage | to RA | Atrioventricular valves | | |
| Pulmonary venous drainage | to LA | Mitral valve | | Annulus = mm |
| Atrioventricular connection | Concordant | Tricuspid valve | | Annulus = mm |
| Ventriculoarterial connection | Concordant | Ventricles | | |
| Ventricular loop | d-Loop | Left ventricle | | Normal |
| Septae | | Right ventricle | | Normal |
| Interventricular septum | Intact | M-Mode | |  |
| Interatrial septum | Intact | AO | |  |
| Great arteries | NRGA | LA | |  |
| Aorta | ----- | LVIDd | | mm |
| Pulmonary artery | Good sized MPA & Branch PAs. | LVIDs | | mm |
| Semilunal valves |  | IVSd | | mm |
| Aortic valve | Annulus = mm | IVSs | | mm |
| Pulmonary valve | Annulus = mm | LVPWd | | mm |
| Doppler Measurement | | LVPWs | | mm |
| Mitral | ---- | EDV | | ml |
| Aortic | ---- | ESV | | ml |
| Tricuspid | ---- | FS | | % |
| pulmonic | ---- | LVEF | | % |
| Aortic arch | Left | Coronary arteries | |  |
| PDA | No |  | |  |
| Additional information |  | | | |
|  |  | | | |
| Final Diagnosis | 1. {S, D, S} Levocardia | | | |
| Done By: | Signature | | Date of Reporting | |
| Dr. Tesfaye Taye,  Pediatric Cardiologist |  | | 19/05/2012Eth.C | |

| Pediatric Echocardiography report  Patient Name: Samuel Bahiru , patient ID: 031400 , Gender: M Age: 2 YEARS 5/12  Date of Report: 26/05/2012Eth.C BP: Weight: Height: BSA:  Clinical Finding: Incidental Murmur. TGSH7.3193. | | | | |
| --- | --- | --- | --- | --- |
| Features | Finding | Features | | Finding |
| Profile | | Atria | | |
| Abdominal situs | Solitus | Left atrium | | Normal |
| Cardiac position | Levocardia | Right atrium | | Normal |
| Systemic venous drainage | to RA | Atrioventricular valves | | |
| Pulmonary venous drainage | to LA | Mitral valve | | Annulus = 16mm |
| Atrioventricular connection | Concordant | Tricuspid valve | | Annulus = 16mm  TAPSE = 14mm. |
| Ventriculoarterial connection | Concordant | Ventricles | | |
| Ventricular loop | d-Loop | Left ventricle | | Normal |
| Septae | | Right ventricle | | Normal |
| Interventricular septum | Intact | M-Mode | |  |
| Interatrial septum | Intact | AO | |  |
| Great arteries | NRGA | LA | |  |
| Aorta | ----- | LVIDd | | mm |
| Pulmonary artery | Good sized MPA & Branch PAs. | LVIDs | | mm |
| Semilunal valves |  | IVSd | | mm |
| Aortic valve | Annulus = 13mm | IVSs | | mm |
| Pulmonary valve | Annulus = 15mm | LVPWd | | mm |
| Doppler Measurement | | LVPWs | | mm |
| Mitral | ---- | EDV | | ml |
| Aortic | ---- | ESV | | ml |
| Tricuspid | ---- | FS | | 32% |
| pulmonic | ---- | LVEF | | 62% |
| Aortic arch | Left | Coronary arteries | |  |
| PDA | 1.5mm PDA, L – R Shunt with SPG/DPG = 74/20mmHg | | | |
| Additional information |  | | | |
|  |  | | | |
| Final Diagnosis | 1. {S, D, S} Levocardia 2. Small PDA, L – R Shunt 3. Good Biventricular Function | | | |
| Done By: | Signature | | Date of Reporting | |
| Dr. Tesfaye Taye, Pediatric Cardiologist |  | | 26/05/2012Eth.C | |

| Pediatric Echocardiography report  Patient Name: Nobel Alemu Patient ID: 010909 Gender: M Age: 3 years 8/12  Date of Report: 26/05/2012Eth.C BP: Weight: Height: BSA:  Clinical Finding: Incidental Murmur. TGSH7.3194. | | | | |
| --- | --- | --- | --- | --- |
| Features | **Finding** | **Features** | | **Finding** |
| Profile | | **Atria** | | |
| Abdominal situs | Solitus | Left atrium | | Normal |
| Cardiac position | Levocardia | Right atrium | | Normal |
| Systemic venous drainage | to RA | **Atrioventricular valves** | | |
| Pulmonary venous drainage | to LA | Mitral valve | | Annulus = 18mm |
| Atrioventricular connection | Concordant | Tricuspid valve | | Annulus = 18mm |
| Ventriculoarterial connection | Concordant | **Ventricles** | | |
| Ventricular loop | d-Loop | Left ventricle | | Normal |
| Septae | | Right ventricle | | Normal |
| Interventricular septum | 3.5mm PM VSD, L – R Shunt with peak pressure gradient of 75mmHg. | **M-Mode** | | |
| Interatrial septum | Intact | AO | |  |
| Great arteries | NRGA | LA | |  |
| Aorta | ----- | LVIDd | | mm |
| Pulmonary artery | Good sized MPA & Branch PAs. | LVIDs | | mm |
| Semilunal valves |  | IVSd | | mm |
| Aortic valve | Annulus = 15mm | IVSs | | mm |
| Pulmonary valve | Annulus = 17mm | LVPWd | | mm |
| Doppler Measurement | | LVPWs | | mm |
| Mitral | ---- | EDV | | ml |
| Aortic | ---- | ESV | | ml |
| Tricuspid | ---- | FS | | % |
| pulmonic | ---- | LVEF | | % |
| Aortic arch | Left | **Coronary arteries** | |  |
| PDA | No |  | |  |
| Additional information |  | | | |
|  |  | | | |
| Final Diagnosis | 1. {S, D, S} Levocardia 2. Small Restrictive Perimembranous VSD, L – R Shunt 3. Good Function. | | | |
| Done By: | **Signature** | | **Date of Reporting** | |
| Dr. Tesfaye Taye, Pediatric Cardiologist |  | | 26/05/2012Eth.C | |

| Pediatric Echocardiography report  Patient Name: Abubeker Wendye Patient ID: 035352 Gender: M Age: 1 years 7/12  Date of Report: 26/05/2012Eth.C BP: Weight: Height: BSA:  Clinical Finding: Diaphoresis. TGSH7.3195. | | | | |
| --- | --- | --- | --- | --- |
| Features | **Finding** | **Features** | | **Finding** |
| Profile | | **Atria** | | |
| Abdominal situs | Solitus | Left atrium | | Normal |
| Cardiac position | Levocardia | Right atrium | | Normal |
| Systemic venous drainage | to RA | **Atrioventricular valves** | | |
| Pulmonary venous drainage | to LA | Mitral valve | | Annulus = 15mm |
| Atrioventricular connection | Concordant | Tricuspid valve | | Annulus = 17mm  TAPSE = 16mm. |
| Ventriculoarterial connection | Concordant | **Ventricles** | | |
| Ventricular loop | d-Loop | Left ventricle | | Normal |
| Septae | | Right ventricle | | Normal |
| Interventricular septum | Intact | **M-Mode** | | |
| Interatrial septum | 5mm OS ASD, L – R Shunt. | AO | |  |
| Great arteries | NRGA | LA | |  |
| Aorta | ----- | LVIDd | | mm |
| Pulmonary artery | Good sized MPA & Branch PAs. | LVIDs | | mm |
| Semilunal valves |  | IVSd | | mm |
| Aortic valve | Annulus = 13mm | IVSs | | mm |
| Pulmonary valve | Annulus = 17mm | LVPWd | | mm |
| Doppler Measurement | | LVPWs | | mm |
| Mitral | ---- | EDV | | ml |
| Aortic | ---- | ESV | | ml |
| Tricuspid | ---- | FS | | % |
| pulmonic | Trivial PR, PPG = 21mmHg | LVEF | | % |
| Aortic arch | Left | **Coronary arteries** | |  |
| PDA | No |  | |  |
| Additional information |  | | | |
|  |  | | | |
| Final Diagnosis | 1. {S, D, S} Levocardia 2. Small OS ASD, L – R Shunt 3. Good Biventricular Function. | | | |
| Done By: | **Signature** | | **Date of Reporting** | |
| Dr. Tesfaye Taye, Pediatric Cardiologist |  | | 26/05/2012Eth.C | |

| Pediatric Echocardiography report  Patient Name: Mekdelawit Yalew Patient ID: _018667 Gender: _F Age: 4 YEARS  Date of Report: 26/05/202Eth.C BP: Weight: Height: BSA:  Clinical Finding: Diaphoresis + Murmur. TGSH7.3196. | | | | |
| --- | --- | --- | --- | --- |
| Features | **Finding** | **Features** | | **Finding** |
| Profile | | **Atria** | | |
| Abdominal situs | Solitus | Left atrium | | Normal |
| Cardiac position | Levocardia | Right atrium | | Normal |
| Systemic venous drainage | to RA | **Atrioventricular valves** | | |
| Pulmonary venous drainage | to LA | Mitral valve | | Annulus = 16mm |
| Atrioventricular connection | Concordant | Tricuspid valve | | Annulus = 17mm  TAPSE = 16mm |
| Ventriculoarterial connection | Concordant | **Ventricles** | | |
| Ventricular loop | d-Loop | Left ventricle | | Normal |
| Septae | | Right ventricle | | Normal |
| Interventricular septum | Intact | **M-Mode** | | |
| Interatrial septum | 4mm OS ASD, L – R Shunt. | AO | |  |
| Great arteries | NRGA | LA | |  |
| Aorta | ----- | LVIDd | | mm |
| Pulmonary artery | Good sized MPA & Branch PAs. | LVIDs | | mm |
| Semilunal valves |  | IVSd | | mm |
| Aortic valve | Annulus = 13mm | IVSs | | mm |
| Pulmonary valve | Annulus = 14mm | LVPWd | | mm |
| Doppler Measurement | | LVPWs | | mm |
| Mitral | ---- | EDV | | ml |
| Aortic | ---- | ESV | | ml |
| Tricuspid | Trivial TR, PPG = 34mmHg | FS | | 37% |
| pulmonic | ---- | LVEF | | 68% |
| Aortic arch | Left | **Coronary arteries** | |  |
| PDA | No |  | |  |
| Additional information |  | | | |
|  |  | | | |
| Final Diagnosis | 1. {S, D, S} Levocardia 2. Small OS ASD, L – R Shunt 3. Good Biventricular Function | | | |
| Done By: | **Signature** | | **Date of Reporting** | |
| Dr. Tesfaye Taye, Pediatric Cardiologist |  | | 26/05/2012Eth.C | |

| Pediatric Echocardiography report  Patient Name: Meheretie Dereje Patient ID: 035308 Gender: M Age: 1 year 5/12  Date of Report: 26/05/2012Eth.C BP: Weight: Height: BSA:  Clinical Finding: Incidental Murmur. TGSH7.3197. | | | | |
| --- | --- | --- | --- | --- |
| Features | **Finding** | **Features** | | **Finding** |
| Profile | | **Atria** | | |
| Abdominal situs | Solitus | Left atrium | | Normal |
| Cardiac position | Levocardia | Right atrium | | Normal |
| Systemic venous drainage | to RA | **Atrioventricular valves** | | |
| Pulmonary venous drainage | to LA | Mitral valve | | Annulus = 11mm |
| Atrioventricular connection | Concordant | Tricuspid valve | | Annulus = 11mm |
| Ventriculoarterial connection | Concordant | **Ventricles** | | |
| Ventricular loop | d-Loop | Left ventricle | | Normal |
| Septae | | Right ventricle | | Normal |
| Interventricular septum | Intact | **M-Mode** | | |
| Interatrial septum | 4.5mm OS ASD, L – R Shunt | AO | |  |
| Great arteries | NRGA | LA | |  |
| Aorta | ----- | LVIDd | | mm |
| Pulmonary artery | Good sized MPA & Branch PAs. | LVIDs | | mm |
| Semilunal valves |  | IVSd | | mm |
| Aortic valve | Annulus = 10mm | IVSs | | mm |
| Pulmonary valve | Annulus = 12mm | LVPWd | | mm |
| Doppler Measurement | | LVPWs | | mm |
| Mitral | ---- | EDV | | ml |
| Aortic | ---- | ESV | | ml |
| Tricuspid | ---- | FS | | 33% |
| pulmonic | ---- | LVEF | | 63% |
| Aortic arch | Left | **Coronary arteries** | |  |
| PDA | No |  | |  |
| Additional information |  | | | |
| Final Diagnosis | 1. {S, D, S} Levocardia 2. Small OS ASD, L – R Shunt. | | | |
| Recommendation: Follow up echo every year. | | | | |
| Done By: | **Signature** | | **Date of Reporting** | |
| Dr. Tesfaye Taye, Pediatric Cardiologist |  | | 26/05/2012Eth.C | |

| Pediatric Echocardiography report  Patient Name: Zemenay Gashaw Patient ID: 034105 Gender: F Age: 11/12  Date of Report: 26/05/2012Eth.C BP: Weight: Height: BSA:  Clinical Finding: DS. TGSH7.3198. | | | | |
| --- | --- | --- | --- | --- |
| Features | **Finding** | **Features** | | **Finding** |
| Profile | | **Atria** | | |
| Abdominal situs | Solitus | Left atrium | | Normal |
| Cardiac position | Levocardia | Right atrium | | Normal |
| Systemic venous drainage | to RA | **Atrioventricular valves** | | |
| Pulmonary venous drainage | to LA | Mitral valve | | Annulus = 10mm |
| Atrioventricular connection | Concordant | Tricuspid valve | | Annulus = 12mm |
| Ventriculoarterial connection | Concordant | **Ventricles** | | |
| Ventricular loop | d-Loop | Left ventricle | | Normal |
| Septae | | Right ventricle | | Normal |
| Interventricular septum | Intact | **M-Mode:** | | |
| Interatrial septum | Intact | AO | |  |
| Great arteries | NRGA | LA | |  |
| Aorta | ----- | LVIDd | | mm |
| Pulmonary artery | Good sized MPA & Branch PAs. | LVIDs | | mm |
| Semilunal valves |  | IVSd | | mm |
| Aortic valve | Annulus = 10mm | IVSs | | mm |
| Pulmonary valve | Annulus = 13mm | LVPWd | | mm |
| Doppler Measurement | | LVPWs | | mm |
| Mitral | ---- | EDV | | ml |
| Aortic | ---- | ESV | | ml |
| Tricuspid | ---- | FS | | 27% |
| pulmonic | ---- | LVEF | | 58% |
| Aortic arch | Left | **Coronary arteries** | |  |
| PDA | 1mm PDA, L – R Shunt |  | |  |
| Remark: | No murmur appreciated on clinical evaluation. | | | |
| Final Diagnosis | 1. {S, D, S} Levocardia 2. Silent PDA, L – R Shunt. | | | |
| Recommendation: Follow up echo every year. | | | | |
| Done By: | **Signature** | | **Date of Reporting** | |
| Dr. Tesfaye Taye, Pediatric Cardiologist |  | | 26/05/2012Eth.C | |

| Pediatric Echocardiography report  Patient Name: Agenagn Muche Patient ID: 025669 Gender: M Age: 9 month  Date of Report: 28/05/2012Eth.C BP: Weight: 5.6 kg Height: 62 cm BSA:  Clinical Finding: Innocent Murmur. TGSH7.3199. | | | | |
| --- | --- | --- | --- | --- |
| Features | **Finding** | **Features** | | **Finding** |
| Profile | | **Atria** | | |
| Abdominal situs | Solitus | Left atrium | | Normal |
| Cardiac position | Levocardia | Right atrium | | Normal |
| Systemic venous drainage | to RA | **Atrioventricular valves** | | |
| Pulmonary venous drainage | to LA | Mitral valve | | Annulus = 12mm |
| Atrioventricular connection | Concordant | Tricuspid valve | | Annulus = 12mm |
| Ventriculoarterial connection | Concordant | **Ventricles** | | |
| Ventricular loop | d-Loop | Left ventricle | | Normal |
| Septae | | Right ventricle | | Normal |
| Interventricular septum | Intact | **M-Mode: Good LV Function on eye balling** | | |
| Interatrial septum | PFO, L – R Shunt | AO | |  |
| Great arteries | NRGA | LA | |  |
| Aorta | ----- | LVIDd | | mm |
| Pulmonary artery | Good sized MPA & Branch PAs. | LVIDs | | mm |
| Semilunal valves |  | IVSd | | mm |
| Aortic valve | Annulus = 12mm | IVSs | | mm |
| Pulmonary valve | Annulus = 14mm | LVPWd | | mm |
| Doppler Measurement | | LVPWs | | mm |
| Mitral | ---- | EDV | | ml |
| Aortic | ---- | ESV | | ml |
| Tricuspid | Trivial TR, PPG = 37mmHg. | FS | | % |
| pulmonic | Flow acceleration across the MPA with PPG = 10mmHg.  Trivial PR, PPG = 38mmHg. | LVEF | | % |
| Aortic arch | Left | **Coronary arteries** | |  |
| PDA |  |  | |  |
| Remark: | No murmur appreciated on clinical evaluation. | | | |
| Final Diagnosis | 1. {S, D, S} Levocardia 2. PFO, L – R Shunt 3. Flow acceleration across MPA without significant Gradient 4. Good Function | | | |
| Recommendation: Follow up echo every year. | | | | |
| Done By: | **Signature** | | **Date of Reporting** | |
| Dr. Tesfaye Taye, Pediatric Cardiologist |  | | 28/05/2012Eth.C | |

| Pediatric Echocardiography report  Patient Name: Dagmawi Kassa Patient ID:035740 Gender: _M Age:6/12  Date of Report: 28/_05_/2012Eth.C BP: Weight: ________ Height: _________ cm BSA:  Clinical Finding: RD + CHF + Sepsis. TGSH7.3200. | | | | |
| --- | --- | --- | --- | --- |
| Features | **Finding** | **Features** | | **Finding** |
| Profile | | **Atria** | | |
| Abdominal situs | Solitus | Left atrium | | Normal |
| Cardiac position | Levocardia | Right atrium | | Normal |
| Systemic venous drainage | to RA | **Atrioventricular valves** | | |
| Pulmonary venous drainage | to LA | Mitral valve | | Annulus = 14mm |
| Atrioventricular connection | Concordant | Tricuspid valve | | Annulus = 15mm |
| Ventriculoarterial connection | Concordant | **Ventricles** | | |
| Ventricular loop | d-Loop | Left ventricle | | Normal |
| Septae | | Right ventricle | | Normal  RV TDI S wave = 8cm/sec |
| Interventricular septum | Intact | **M-Mode:** | | |
| Interatrial septum | Intact | AO | |  |
| Great arteries | NRGA | LA | |  |
| Aorta | ----- | LVIDd | | mm |
| Pulmonary artery | Good sized MPA & Branch PAs. | LVIDs | | mm |
| Semilunal valves |  | IVSd | | mm |
| Aortic valve | Annulus = 9mm | IVSs | | mm |
| Pulmonary valve | Annulus = 12mm | LVPWd | | mm |
| Doppler Measurement | | LVPWs | | mm |
| Mitral | ---- | EDV | | ml |
| Aortic | ---- | ESV | | ml |
| Tricuspid | ---- | FS | | 22% |
| pulmonic | ---- | LVEF | | 47% |
| Aortic arch | Left | **Coronary arteries** | |  |
| PDA |  |  | |  |
| Pericardium | 7mm Pericardial effusion on RA Side with debris inside. Trace pericardial effusion on LV Side. | | | |
| Final Diagnosis | 1. {S, D, S} Levocardia 2. REDUCED BIVENTRICULAR FUNCTION 3. PERIRDIAL EFFUSION WITH DEBRIS INSIDE | | | |
| Recommendation: Manage the patient in line with Perimyocarditis. | | | | |
| Done By: | **Signature** | | **Date of Reporting** | |
| Dr. Tesfaye Taye, Pediatric Cardiologist |  | | 28/05/2012Eth.C | |

| Pediatric Echocardiography report  Patient Name: Erkihune Amlaku Patient ID:036875 Gender: M Age: 75days.  Date of Report: 05/06/2012Eth.C BP: ____ Weight: 2.4 kg Height: 50cm BSA:  Clinical Finding: Cyanosis + Murmur. TGSH7.3201. | | | | |
| --- | --- | --- | --- | --- |
| Features | **Finding** | **Features** | | **Finding** |
| Profile | | **Atria** | | |
| Abdominal situs | Solitus | Left atrium | | Normal |
| Cardiac position | Levocardia | Right atrium | | Normal |
| Systemic venous drainage | to RA | **Atrioventricular valves** | | |
| Pulmonary venous drainage | to LA | Mitral valve | | Annulus = 14mm |
| Atrioventricular connection | Concordant | Tricuspid valve | | Annulus = 13mm  TAPSE = 13mm |
| Ventriculoarterial connection | Discordant | **Ventricles** | | |
| Ventricular loop | d-Loop | Left ventricle | | Normal |
| Septae | | Right ventricle | | Dilated |
| Interventricular septum | Intact | **M-Mode: Good LV Function on eye balling** | | |
| Interatrial septum | PFO | AO | |  |
| Great arteries | d-TGA | LA | |  |
| Aorta | Anterior and to the right | LVIDd | | mm |
| Pulmonary artery | Posterior and to the left | LVIDs | | mm |
| Semilunal valves |  | IVSd | | mm |
| Aortic valve | Annulus = 12mm | IVSs | | mm |
| Pulmonary valve | Annulus = 10mm | LVPWd | | mm |
| Doppler Measurement | | LVPWs | | mm |
| Mitral | ---- | EDV | | ml |
| Aortic | ---- | ESV | | ml |
| Tricuspid | ---- | FS | | % |
| pulmonic | Mild PS, PPG = 37mmHg (LVOTO) | LVEF | | % |
| Aortic arch | Left | **Coronary arteries** | |  |
| PDA | No |  | |  |
| Remark: |  | | | |
| Final Diagnosis | 1. {S, D, D} Levocardia 2. d-TGA with intact IVS 3. PFO, L – R Shunt 4. Mild LVOTO (PS) | | | |
| Recommendation: Surgical intervention is recommended. | | | | |
| Done By: | **Signature** | | **Date of Reporting** | |
| Dr. Tesfaye Taye, Pediatric Cardiologist |  | | 05/06/2012Eth.C | |

| Pediatric Echocardiography report  Patient Name: Elsabeth Semene Patient ID:035728 Gender: F Age: 58 day  Date of Report: 05/2012Eth.C BP: Weight: 5.4 kg Height: cm BSA:  Clinical Finding: RD. TGSH7.3202. | | | | |
| --- | --- | --- | --- | --- |
| Features | **Finding** | **Features** | | **Finding** |
|  |  |  | |  |
| Profile | | **Atria** | | |
| Abdominal situs | Solitus | Left atrium | | Normal |
|  |  |  | |  |
| Cardiac position | Levocardia | Right atrium | | Normal |
| Systemic venous drainage | to RA | **Atrioventricular valves** | | |
| Pulmonary venous drainage | to LA | Mitral valve | | Annulus = 10mm |
| Atrioventricular connection | Concordant | Tricuspid valve | | Annulus = 11mm |
| Ventriculoarterial connection | Concordant | **Ventricles** | | |
| Ventricular loop | d-Loop | Left ventricle | | Normal |
| Septae | | Right ventricle | | Normal |
| Interventricular septum | Intact | **M-Mode: Normal LV Function on eye balling** | | |
| Interatrial septum | Intact | AO | |  |
| Great arteries | NRGA | LA | |  |
| Aorta | ----- | LVIDd | | mm |
| Pulmonary artery | Good sized MPA & Branch PAs. | LVIDs | | mm |
| Semilunal valves |  | IVSd | | mm |
| Aortic valve | Annulus = 9mm | IVSs | | mm |
| Pulmonary valve | Annulus = 10mm | LVPWd | | mm |
| Doppler Measurement | | LVPWs | | mm |
| Mitral | ---- | EDV | | ml |
| Aortic | ---- | ESV | | ml |
| Tricuspid | ---- | FS | | % |
| pulmonic | ---- | LVEF | | % |
| Aortic arch | Left | **Coronary arteries** | |  |
| PDA | No |  | |  |
| Remark: | No murmur appreciated on clinical evaluation. | | | |
| Final Diagnosis | 1. Normal Echocardiography Study | | | |
| Recommendation: treat the respiratory cause. | | | | |
| Done By: | **Signature** | | **Date of Reporting** | |
| Dr. Tesfaye Taye, Pediatric Cardiologist |  | | 28/05/2012Eth.C | |

| Pediatric Echocardiography report  Patient Name: B/Desta _ Patient ID: __036260 Gender: _F Age: 9 days  Date of Report: _05/06/2012Eth.C BP: Weight: 2.7 kg Height: _________ cm BSA:  Clinical Finding: Pre-op screening. TGSH7.3203. | | | | |
| --- | --- | --- | --- | --- |
| Features | **Finding** | **Features** | | **Finding** |
| Profile | | **Atria** | | |
| Abdominal situs | Solitus | Left atrium | | Normal |
| Cardiac position | Levocardia | Right atrium | | Normal |
| Systemic venous drainage | to RA | **Atrioventricular valves** | | |
| Pulmonary venous drainage | to LA | Mitral valve | | Annulus = 9mm |
| Atrioventricular connection | Concordant | Tricuspid valve | | Annulus = 9mm |
| Ventriculoarterial connection | Concordant | **Ventricles** | | |
| Ventricular loop | d-Loop | Left ventricle | | Normal |
| Septae | | Right ventricle | | Normal |
| Interventricular septum | Intact | **M-Mode:** | | |
| Interatrial septum | PFO, L – R Shunt | AO | |  |
| Great arteries | NRGA | LA | |  |
| Aorta | ----- | LVIDd | | mm |
| Pulmonary artery | Good sized MPA & Branch PAs. | LVIDs | | mm |
| Semilunal valves |  | IVSd | | mm |
| Aortic valve | Annulus = 8mm | IVSs | | mm |
| Pulmonary valve | Annulus = 7mm | LVPWd | | mm |
| Doppler Measurement | | LVPWs | | mm |
| Mitral | ---- | EDV | | ml |
| Aortic | ---- | ESV | | ml |
| Tricuspid | ---- | FS | | % |
| pulmonic | ---- | LVEF | | % |
| Aortic arch | Left | **Coronary arteries** | |  |
| PDA |  |  | |  |
| Remark: | No murmur appreciated on clinical evaluation. | | | |
| Final Diagnosis | 1. {S, D, S} Levocardia 2. PFO, L – R Shunt | | | |
| Recommendation: can undergo surgical intervention without significant risk from the PFO | | | | |
| Done By: | **Signature** | | **Date of Reporting** | |
| Dr. Tesfaye Taye, Pediatric Cardiologist |  | | 06/05/2012Eth.C | |

| Pediatric Echocardiography report  Patient Name: Fasil Gebeyehu Patient ID: 034197 Gender: M_ Age: 13 yrs.  Date of Report: 05/06/2012Eth.C BP: Weight: 34 kg Height: _________ cm BSA:  Clinical Finding: Easy Fatigability. TGSH7.3204. | | | | |
| --- | --- | --- | --- | --- |
| Features | **Finding** | **Features** | | **Finding** |
| Profile | | **Atria** | | |
| Abdominal situs | Solitus | Left atrium | | Normal |
| Cardiac position | Levocardia | Right atrium | | Normal |
| Systemic venous drainage | to RA | **Atrioventricular valves** | | |
| Pulmonary venous drainage | to LA | Mitral valve | | Annulus = 24mm |
| Atrioventricular connection | Concordant | Tricuspid valve | | Annulus = 28mm |
| Ventriculoarterial connection | Concordant | **Ventricles** | | |
| Ventricular loop | d-Loop | Left ventricle | | Normal |
| Septae | | Right ventricle | | Normal |
| Interventricular septum | Intact | **M-Mode:** | | |
| Interatrial septum | Intact | AO | |  |
| Great arteries | NRGA | LA | |  |
| Aorta | ----- | LVIDd | | mm |
| Pulmonary artery | Good sized MPA & Branch PAs. | LVIDs | | mm |
| Semilunal valves |  | IVSd | | mm |
| Aortic valve | Annulus = 24mm | IVSs | | mm |
| Pulmonary valve | Annulus = 26mm | LVPWd | | mm |
| Doppler Measurement | | LVPWs | | mm |
| Mitral | ---- | EDV | | ml |
| Aortic | ---- | ESV | | ml |
| Tricuspid | Trivial TR, PPG = 26mmHg | FS | | 30% |
| pulmonic | Trivial PR, PPG = 22mmHg | LVEF | | 58% |
| Aortic arch | Left | **Coronary arteries** | |  |
| PDA |  |  | |  |
| Remark: |  | | | |
| Final Diagnosis | 1. Normal Echocardiography Study | | | |
| Recommendation: | | | | |
| Done By: | **Signature** | | **Date of Reporting** | |
| Dr. Tesfaye Taye, Pediatric Cardiologist |  | | 05/06/2012Eth.C | |

| Pediatric Echocardiography report  Patient Name: Zemen Simachew Patient ID: 033895 Gender: _M Age: 3 3/12  Date of Report: 10/06/2012Eth.C BP: Weight: ________ kg Height: _________ cm BSA:  Clinical Finding: Sepsis + friction rub + RD. TGSH7.3205. | | | | |
| --- | --- | --- | --- | --- |
| Features | **Finding** | **Features** | | **Finding** |
| Profile | | **Atria** | | |
| Abdominal situs | Solitus | Left atrium | | Normal |
| Cardiac position | Levocardia | Right atrium | | Normal |
| Systemic venous drainage | to RA | **Atrioventricular valves** | | |
| Pulmonary venous drainage | to LA | Mitral valve | | Annulus = 16mm |
| Atrioventricular connection | Concordant | Tricuspid valve | | Annulus = 20mm  TAPSE = 12mm |
| Ventriculoarterial connection | Concordant | **Ventricles** | | |
| Ventricular loop | d-Loop | Left ventricle | | Normal |
| Septae | | Right ventricle | | Normal |
| Interventricular septum | Intact | **M-Mode:** | | |
| Interatrial septum | Intact | AO | |  |
| Great arteries | NRGA | LA | |  |
| Aorta | ----- | LVIDd | | mm |
| Pulmonary artery | Good sized MPA & Branch PAs. | LVIDs | | mm |
| Semilunal valves |  | IVSd | | mm |
| Aortic valve | Annulus = 13mm | IVSs | | mm |
| Pulmonary valve | Annulus = 16mm | LVPWd | | mm |
| Doppler Measurement | | LVPWs | | mm |
| Mitral | Trivial MR | EDV | | ml |
| Aortic | ---- | ESV | | ml |
| Tricuspid | Trivial TR, PPG = 28mmHg | FS | | 30% |
| pulmonic | ---- | LVEF | | 58% |
| Aortic arch | Left | **Coronary arteries** | |  |
| PDA |  |  | |  |
| Pericardium | 10mm pericardial effusion on RV side with echodebris inside. 9mm pericardia effusion on RA side. | | | |
| Final Diagnosis | 1. {S, D, S} Levocardia 2. Small to Moderate Pericardial effusion with echo debris inside 3. Good Biventricular Function | | | |
| Recommendation:. | | | | |
| Done By: | **Signature** | | **Date of Reporting** | |
| Dr. Tesfaye Taye, Pediatric Cardiologist |  | | 10/06/2012Eth.C | |

| Pediatric Echocardiography report  Patient Name: Hailemariam Degu Patient ID: _035446 Gender: __M Age: 15 Days  Date of Report: 10/06_/2012Eth.C BP: Weight: ________ kg Height: _________ cm BSA:  Clinical Finding: RD. TGSH7.3206. | | | | |
| --- | --- | --- | --- | --- |
| Features | **Finding** | **Features** | | **Finding** |
| Profile | | **Atria** | | |
| Abdominal situs | Solitus | Left atrium | | Normal |
| Cardiac position | Levocardia | Right atrium | | Normal |
| Systemic venous drainage | to RA | **Atrioventricular valves** | | |
| Pulmonary venous drainage | to LA | Mitral valve | | Annulus = 9mm |
| Atrioventricular connection | Concordant | Tricuspid valve | | Annulus = 9mm |
| Ventriculoarterial connection | Concordant | **Ventricles** | | |
| Ventricular loop | d-Loop | Left ventricle | | Normal |
| Septae | | Right ventricle | | Normal |
| Interventricular septum | Intact | **M-Mode:** | | |
| Interatrial septum | PFO, L – R Shunt | AO | |  |
| Great arteries | NRGA | LA | |  |
| Aorta | ----- | LVIDd | | mm |
| Pulmonary artery | Good sized MPA & Branch PAs. | LVIDs | | mm |
| Semilunal valves |  | IVSd | | mm |
| Aortic valve | Annulus = 10mm | IVSs | | mm |
| Pulmonary valve | Annulus = 10mm | LVPWd | | mm |
| Doppler Measurement | | LVPWs | | mm |
| Mitral | ---- | EDV | | ml |
| Aortic | ---- | ESV | | ml |
| Tricuspid | ---- | FS | | % |
| pulmonic | ---- | LVEF | | % |
| Aortic arch | Left | **Coronary arteries** | |  |
| PDA |  |  | |  |
| PERICARDIUM | No effusion | | | |
| Final Diagnosis | 1. {S, D, S} Levocardia 2. PFO, L – R Shunt | | | |
| Recommendation:. | | | | |
| Done By: | **Signature** | | **Date of Reporting** | |
| Dr. Tesfaye Taye, Pediatric Cardiologist |  | | 10/06/2012Eth.C | |

| Echocardiography report  Patient Name: Kabew Sultan Patient ID: 037453 Gender: M_ Age: 5 days  Date of Report: 10/06/2012Eth.C BP: Weight: ________ kg Height: _________ cm BSA:  Clinical Finding: Incidental Murmur. TGSH7.3207. | | | | |
| --- | --- | --- | --- | --- |
| Features | **Finding** | **Features** | | **Finding** |
| Profile | | **Atria** | | |
| Abdominal situs | Solitus | Left atrium | | Dilated |
| Cardiac position | Levocardia | Right atrium | | Normal |
| Systemic venous drainage | to RA | **Atrioventricular valves** | | |
| Pulmonary venous drainage | to LA | Mitral valve | | Annulus = 12mm |
| Atrioventricular connection | Concordant | Tricuspid valve | | Annulus = 10mm |
| Ventriculoarterial connection | Concordant | **Ventricles** | | |
| Ventricular loop | d-Loop | Left ventricle | | Dilated |
| Septae | | Right ventricle | | Normal |
| Interventricular septum | 6mm inlet VSD, BD Shunt | **M-Mode:** | | |
| Interatrial septum | 10mm OS ASD, L – R Shunt | AO | |  |
| Great arteries | LTGA | LA | |  |
| Aorta | Anterior and to the left | LVIDd | | mm |
| Pulmonary artery | Posterior and to the right | LVIDs | | mm |
| Semilunal valves |  | IVSd | | mm |
| Aortic valve | Annulus = 10mm | IVSs | | mm |
| Pulmonary valve | Annulus = 11mm | LVPWd | | mm |
| Doppler Measurement | | LVPWs | | mm |
| Mitral | ---- | EDV | | ml |
| Aortic | ---- | ESV | | ml |
| Tricuspid | ---- | FS | | % |
| pulmonic | ---- | LVEF | | % |
| Aortic arch | Left | **Coronary arteries** | |  |
| PDA |  |  | |  |
| Remark: | . | | | |
| Final Diagnosis | 1. {S, D, L} Levocardia 2. Moderate OS ASD, L – R Shunt 3. Moderate Inlet VSD, BD Shunt 4. Good function (eye balling) | | | |
| Recommendation:. | | | | |
| Done By: | **Signature** | | **Date of Reporting** | |
| Dr. Tesfaye Taye, Pediatric Cardiologist |  | | 10/06/2012Eth.C | |

| Echocardiography report  Patient Name: __ Belete Yawukal Patient ID: _037017 Gender: M Age: _6 YEARS  Date of Report: 10 /06/2012Eth.C BP: Weight: ________ kg Height: _________ cm BSA:  Clinical Finding: Incidental Murmur. TGSH7.3208. | | | | |
| --- | --- | --- | --- | --- |
| Features | **Finding** | **Features** | | **Finding** |
| Profile | | **Atria** | | |
| Abdominal situs | Solitus | Left atrium | | Normal |
| Cardiac position | Levocardia | Right atrium | | Normal |
| Systemic venous drainage | to RA | **Atrioventricular valves** | | |
| Pulmonary venous drainage | to LA | Mitral valve | | Annulus = 19mm |
| Atrioventricular connection | Concordant | Tricuspid valve | | Annulus = 19mm |
| Ventriculoarterial connection | Concordant | **Ventricles** | | |
| Ventricular loop | d-Loop | Left ventricle | | Normal |
| Septae | | Right ventricle | | Normal |
| Interventricular septum | Intact | **M-Mode:** | | |
| Interatrial septum | Intact | AO | |  |
| Great arteries | NRGA | LA | |  |
| Aorta | ----- | LVIDd | | mm |
| Pulmonary artery | Good sized MPA & Branch PAs. | LVIDs | | mm |
| Semilunal valves |  | IVSd | | mm |
| Aortic valve | Annulus = 17mm | IVSs | | mm |
| Pulmonary valve | Annulus = 20mm | LVPWd | | mm |
| Doppler Measurement | | LVPWs | | mm |
| Mitral | ---- | EDV | | ml |
| Aortic | ---- | ESV | | ml |
| Tricuspid | ---- | FS | | 35% |
| pulmonic | ---- | LVEF | | 66% |
| Aortic arch | Left | **Coronary arteries** | |  |
| PDA |  |  | |  |
| PERICARDIUM | No effusion | | | |
| Final Diagnosis | 1. Normal Echocardiography Study | | | |
| Recommendation:. | | | | |
| Done By: | **Signature** | | **Date of Reporting** | |
| Dr. Tesfaye Taye, Pediatric Cardiologist |  | | 10/06/2012Eth.C | |

| Echocardiography report  Patient Name: Kabew Sultan Patient ID: 037453 Gender: M_ Age: 5 days  Date of Report: 10/06/2012Eth.C BP: Weight: ________ kg Height: _________ cm BSA:  Clinical Finding: ________. INCOMPLETE DOCUMENTATION | | | | |
| --- | --- | --- | --- | --- |
| Features | **Finding** | **Features** | | **Finding** |
| Profile | | **Atria** | | |
| Abdominal situs | Solitus | Left atrium | | Normal |
| Cardiac position | Levocardia | Right atrium | | Normal |
| Systemic venous drainage | to RA | **Atrioventricular valves** | | |
| Pulmonary venous drainage | to LA | Mitral valve | | Annulus = mm |
| Atrioventricular connection | Concordant | Tricuspid valve | | Annulus = mm |
| Ventriculoarterial connection | Concordant | **Ventricles** | | |
| Ventricular loop | d-Loop | Left ventricle | | Normal |
| Septae | | Right ventricle | | Normal |
| Interventricular septum | Intact | **M-Mode:** | | |
| Interatrial septum | Intact | AO | |  |
| Great arteries | NRGA | LA | |  |
| Aorta | ----- | LVIDd | | mm |
| Pulmonary artery | Good sized MPA & Branch PAs. | LVIDs | | mm |
| Semilunal valves |  | IVSd | | mm |
| Aortic valve | Annulus = mm | IVSs | | mm |
| Pulmonary valve | Annulus = mm | LVPWd | | mm |
| Doppler Measurement | | LVPWs | | mm |
| Mitral | ---- | EDV | | ml |
| Aortic | ---- | ESV | | ml |
| Tricuspid | ---- | FS | | % |
| pulmonic | ---- | LVEF | | % |
| Aortic arch | Left | **Coronary arteries** | |  |
| PDA |  |  | |  |
| Remark: | . | | | |
| Final Diagnosis | 1. {S, D, S} Levocardia | | | |
| Recommendation:. | | | | |
| Done By: | **Signature** | | **Date of Reporting** | |
| Dr. Tesfaye Taye, Pediatric Cardiologist |  | | 10/06/2012Eth.C | |

| Echocardiography report  Patient Name: Erimiyas Getachew Patient ID: 002801 Gender: M Age: 5 years.  Date of Report: 12/06/2012Eth.C BP: Weight: ________ kg Height: _________ cm BSA:  Clinical Finding: Incidental Murmur + Easy Fatigability. TGSH7.3209. | | | | |
| --- | --- | --- | --- | --- |
| Features | **Finding** | **Features** | | **Finding** |
| Profile | | **Atria** | | |
| Abdominal situs | Solitus | Left atrium | | Dilated |
| Cardiac position | Levocardia | Right atrium | | Normal |
| Systemic venous drainage | to RA | **Atrioventricular valves** | | |
| Pulmonary venous drainage | to LA | Mitral valve | | Annulus = 23mm |
| Atrioventricular connection | Concordant | Tricuspid valve | | Annulus = 20mm  TAPSE = 14mm |
| Ventriculoarterial connection | Concordant | **Ventricles** | | |
| Ventricular loop | d-Loop | Left ventricle | | Dilated |
| Septae | | Right ventricle | | Normal |
| Interventricular septum | Intact | **M-Mode:** | | |
| Interatrial septum | Intact | AO | |  |
| Great arteries | NRGA | LA | |  |
| Aorta | ----- | LVIDd | | mm |
| Pulmonary artery | Good sized MPA & Branch PAs. | LVIDs | | mm |
| Semilunal valves |  | IVSd | | mm |
| Aortic valve | Annulus = 19mm | IVSs | | mm |
| Pulmonary valve | Annulus = 21mm | LVPWd | | mm |
| Doppler Measurement | | LVPWs | | mm |
| Mitral | Trivial MR | EDV | | ml |
| Aortic | ---- | ESV | | ml |
| Tricuspid | Trivial TR, PPG = 35mmHg | FS | | 30% |
| pulmonic | ---- | LVEF | | 62% |
| Aortic arch | Left | **Coronary arteries** | |  |
| PDA | 2.5mm PDA, L – R Shunt. |  | |  |
| Remark: | . | | | |
| Final Diagnosis | 1. {S, D, S} Levocardia 2. LA/LV Dilated 3. Moderate PDA, L – R Shunt 4. Good Biventricular Function | | | |
| Recommendation:. | | | | |
| Done By: | **Signature** | | **Date of Reporting** | |
| Dr. Tesfaye Taye, Pediatric Cardiologist |  | | 12/06/2012Eth.C | |

| Echocardiography report  Patient Name: Baby Bogalech Twin B Patient ID: 037542_ Gender: F Age: 14 Days  Date of Report: 12/06/2012Eth.C BP: Weight: 1.4 kg Height: 43 cm BSA:  Clinical Finding: Incidental Murmur. TGSH7.3210. | | | | |
| --- | --- | --- | --- | --- |
| Features | **Finding** | **Features** | | **Finding** |
| Profile | | **Atria** | | |
| Abdominal situs | Solitus | Left atrium | | Normal |
| Cardiac position | Levocardia | Right atrium | | Normal |
| Systemic venous drainage | to RA | **Atrioventricular valves** | | |
| Pulmonary venous drainage | to LA | Mitral valve | | Annulus = 9mm |
| Atrioventricular connection | Concordant | Tricuspid valve | | Annulus = 8mm |
| Ventriculoarterial connection | Concordant | **Ventricles** | | |
| Ventricular loop | d-Loop | Left ventricle | | Normal |
| Septae | | Right ventricle | | Normal |
| Interventricular septum | Intact | **M-Mode: normal LV Function on eye balling** | | |
| Interatrial septum | PFO, L – R Shunt | AO | |  |
| Great arteries | NRGA | LA | |  |
| Aorta | ----- | LVIDd | | mm |
| Pulmonary artery | Good sized MPA & Branch PAs. | LVIDs | | mm |
| Semilunal valves |  | IVSd | | mm |
| Aortic valve | Annulus = 7mm | IVSs | | mm |
| Pulmonary valve | Annulus = 8mm | LVPWd | | mm |
| Doppler Measurement | | LVPWs | | mm |
| Mitral | ---- | EDV | | ml |
| Aortic | ---- | ESV | | ml |
| Tricuspid | ---- | FS | | % |
| pulmonic | ---- | LVEF | | % |
| Aortic arch | Left | **Coronary arteries** | |  |
| PDA | 1.5mm PDA, L – R Shunt |  | |  |
| Remark: | .Limited Echo window (Only subcostal and apical) | | | |
| Final Diagnosis | 1. {S, D, S} Levocardia 2. PFO, L – R Shunt 3. Small PDA, L – R Shunt 4. Good Function | | | |
| Recommendation: Follow up Echocardiography after 3 months | | | | |
| Done By: | **Signature** | | **Date of Reporting** | |
| Dr. Tesfaye Taye, Pediatric Cardiologist |  | | 12/06/2012Eth.C | |

| Echocardiography report  Patient Name: Zemenay Derejaw Patient ID: _037853 Gender: F Age: 8.5Years  Date of Report: 12/06/2012Eth.C BP: Weight: 21kg Height: _118cm BSA:  Clinical Finding: Rheumatic Recurrence + Palpitation + Murmur + Easy Fatigability + DOE. TGSH7.3211. | | | |
| --- | --- | --- | --- |
| Features | **Finding** | **Features** | **Finding** |
| Profile | | **Atria** | |
| Abdominal situs | Solitus | Left atrium | DILATED |
| Cardiac position | Levocardia | Right atrium | Normal |
| Systemic venous drainage | to RA | **Atrioventricular valves** | |
| Pulmonary venous drainage | to LA | Mitral valve | Thickened, Clubbed MVL, MVA = 0.6cm2. |
| Atrioventricular connection | Concordant | Tricuspid valve | TAPSE = 14mm |
| Ventriculoarterial connection | Concordant | **Ventricles** | |
| Ventricular loop | d-Loop | Left ventricle | Dilated |
| Septae | | Right ventricle | Normal |
| Interventricular septum | Intact | **M-Mode:** | |
| Interatrial septum | Intact | AO |  |
| Great arteries | NRGA | LA |  |
| Aorta | ----- | LVIDd | mm |
| Pulmonary artery | Good sized MPA & Branch PAs. | LVIDs | mm |
| Semilunal valves |  | IVSd | mm |
| Aortic valve | Annulus = 17mm | IVSs | mm |
| Pulmonary valve | Annulus = 20mm | LVPWd | mm |
| Doppler Measurement | | LVPWs | mm |
| Mitral | Severe MR, Holosystolic, posterior projection with jet velocity of 4.8m/sec. Severe MS, PPG/MPG = 18/11mmHg | EDV | ml |
| Aortic | ---- | ESV | ml |
| Tricuspid | Moderate to severe TR, PPG = 53mmHg | FS | 28% |
| pulmonic | Trivial PR | LVEF | 53% |
| Aortic arch | Left | **Coronary arteries** |  |
| PDA | No |  |  |
| Final Diagnosis | 1. {S, D, S} Levocardia 2. LA/LV Dilated 3. Thickened MVL 4. Severe MR 5. Severe MS 6. Moderate to severe TR 7. Moderate pulmonary Hypertension 8. Mildly reduced LV Function | | |
| Done By: | **Signature** | **Date of Reporting** | |
| Dr. Tesfaye Taye, Pediatric Cardiologist |  | 12/06/2012Eth.C | |

| Echocardiography report  Patient Name: Agere Bawuke. Patient ID: _037636 Gender: _F Age: 12 Years  Date of Report: 12/06/2012Eth.C BP: Weight: 37 kg Height: 131 cm BSA:  Clinical Finding: Rheumatic Recurrence + Murmur. TGSH7.3212. | | | | |
| --- | --- | --- | --- | --- |
| Features | **Finding** | **Features** | | **Finding** |
| Profile | | **Atria** | | |
| Abdominal situs | Solitus | Left atrium | | Dilated |
| Cardiac position | Levocardia | Right atrium | | Normal |
| Systemic venous drainage | to RA | **Atrioventricular valves** | | |
| Pulmonary venous drainage | to LA | Mitral valve | | Annulus = 24mm. thickened clubbed MVL. MVA = 1.5cm2. |
| Atrioventricular connection | Concordant | Tricuspid valve | | Annulus = 24mm  TAPSE = 17mm |
| Ventriculoarterial connection | Concordant | **Ventricles** | | |
| Ventricular loop | d-Loop | Left ventricle | | Normal |
| Septae | | Right ventricle | | Normal |
| Interventricular septum | Intact | **M-Mode:** | | |
| Interatrial septum | Intact | AO | |  |
| Great arteries | NRGA | LA | |  |
| Aorta | ----- | LVIDd | | mm |
| Pulmonary artery | Good sized MPA & Branch PAs. | LVIDs | | mm |
| Semilunal valves |  | IVSd | | mm |
| Aortic valve | Annulus = 15mm | IVSs | | mm |
| Pulmonary valve | Annulus = 17mm | LVPWd | | mm |
| Doppler Measurement | | LVPWs | | mm |
| Mitral | Moderate MR, Holosystolic, posterior projection with jet velocity of 5m/sec. Mild MS with PPG/MPG = 9/5mmHg | EDV | | ml |
| Aortic | ---- | ESV | | ml |
| Tricuspid | ---- | FS | | 31% |
| pulmonic | ---- | LVEF | | 59% |
| PDA | No PDA |  | |  |
| Final Diagnosis | 1. {S, D, S} Levocardia 2. Thickened MVL 3. Mildly dilated LA 4. Moderate MR 5. Mild MS 6. Good Function | | | |
| Done By: | **Signature** | | **Date of Reporting** | |
| Dr. Tesfaye Taye, Pediatric Cardiologist |  | | 12/06/2012Eth.C | |

| Echocardiography report  Patient Name: B/ Emenesh Ayele Patient ID:037869 Gender: F Age: 12 Days.  Date of Report: 17/06/2012Eth.C BP: Weight: 3 kg Height: 49 cm BSA:  Clinical Finding: Incidental Murmur + RD. TGSH7.3213. | | | | |
| --- | --- | --- | --- | --- |
| Features | **Finding** | **Features** | | **Finding** |
| Profile | | **Atria** | | |
| Abdominal situs | Solitus | Left atrium | | Normal |
| Cardiac position | Levocardia | Right atrium | | Normal |
| Systemic venous drainage | to RA | **Atrioventricular valves** | | |
| Pulmonary venous drainage | All pulmonary veins form a confluence above the LA and drain into ? vertical vein to innominate to SVC to RA. Flow acceleration across the SVC – RA Junction with gradient of 32mmHg. | Mitral valve | | Annulus = 9mm |
| Atrioventricular connection | Concordant | Tricuspid valve | | Annulus = 8mm |
| Ventriculoarterial connection | Concordant | **Ventricles** | | |
| Ventricular loop | d-Loop | Left ventricle | | Normal |
| Septae | | Right ventricle | | Normal |
| Interventricular septum | Intact | **M-Mode:** | | |
| Interatrial septum | 7mm High Secundum ASD, R – L Shunt | AO | |  |
| Great arteries | NRGA | LA | |  |
| Aorta | ----- | LVIDd | | mm |
| Pulmonary artery | MPA = 10mm. LPA = 4mm, RPA = 4mm | LVIDs | | mm |
| Semilunal valves |  | IVSd | | mm |
| Aortic valve | Annulus = 7mm | IVSs | | mm |
| Pulmonary valve | Annulus = 9mm | LVPWd | | mm |
| Doppler Measurement | | LVPWs | | mm |
| Mitral | ---- | EDV | | ml |
| Aortic | ---- | ESV | | ml |
| Tricuspid | ---- | FS | | 32% |
| pulmonic | Mild PS with PPG = 28mmHg. Peripheral PS across Branch PAs with a gradient of 40mmHg across RPA and 38mmHg across LPA. | LVEF | | 65% |
| Aortic arch | Left | **Coronary arteries** | |  |
| Final Diagnosis | 1. {S, D, S} Levocardia 2. Obstructed TAPVC 3. High Secundum ASD, R – L Shunt 4. Mild PS 5. Peripheral PS 6. Good Function | | | |
| Done By: | **Signature** | | **Date of Reporting** | |
| Dr. Tesfaye Taye, Pediatric Cardiologist |  | | 17/06/2012Eth.C | |

| Echocardiography report  Patient Name: B/Yibelu Alemante Patient ID: 037661 Gender: M Age: 30 days  Date of Report: 17/06/2012Eth.C BP: Weight: 3.8 kg Height: 48 cm BSA:  Clinical Finding: Incidental Murmur + Diaphoresis. TGSH7.3214. | | | | |
| --- | --- | --- | --- | --- |
| Features | **Finding** | **Features** | | **Finding** |
| Profile | | **Atria** | | |
| Abdominal situs | Solitus | Left atrium | | Normal |
| Cardiac position | Levocardia | Right atrium | | Normal |
| Systemic venous drainage | to RA | **Atrioventricular valves** | | |
| Pulmonary venous drainage | to LA | Mitral valve | | Annulus = 10mm |
| Atrioventricular connection | Concordant | Tricuspid valve | | Annulus = 10mm |
| Ventriculoarterial connection | Concordant | **Ventricles** | | |
| Ventricular loop | d-Loop | Left ventricle | | Normal |
| Septae | | Right ventricle | | Normal |
| Interventricular septum | Intact | **M-Mode:** | | |
| Interatrial septum | 7mm OS ASD, L – R Shunt | AO | |  |
| Great arteries | NRGA | LA | |  |
| Aorta | ----- | LVIDd | | mm |
| Pulmonary artery | Good sized MPA & Branch PAs. | LVIDs | | mm |
| Semilunal valves |  | IVSd | | mm |
| Aortic valve | Annulus = 8mm | IVSs | | mm |
| Pulmonary valve | Annulus = 9mm | LVPWd | | mm |
| Doppler Measurement | | LVPWs | | mm |
| Mitral | Trivial MR | EDV | | ml |
| Aortic | ---- | ESV | | ml |
| Tricuspid | Mild TR, PPG = 50mmHg | FS | | % |
| pulmonic | Moderate PR, PPG = 45mmHg | LVEF | | % |
| Aortic arch | Left | **Coronary arteries** | |  |
| PDA | 2.5mm PDA, L – R Shunt |  | |  |
| Final Diagnosis | 1. {S, D, S} Levocardia 2. Moderate OS ASD, L – R Shunt 3. Moderate PDA, L – R Shunt 4. Good LV Function (eye balling) | | | |
| Recommendation:. | | | | |
| Done By: | **Signature** | | **Date of Reporting** | |
| Dr. Tesfaye Taye, Pediatric Cardiologist |  | | 17/06/2012Eth.C | |

| Echocardiography report  Patient Name: Tena Birhanu Patient ID: 038321 Gender: F Age: 11 years  Date of Report: 17/06/2012Eth.C BP: Weight: 13.8 kg Height: 117cm BSA:  Clinical Finding: Rheumatic Recurrence + Murmur + DOE + Palpitation + Easy Fatigability + CHF. TGSH7.3215. | | | | |
| --- | --- | --- | --- | --- |
| Features | **Finding** | **Features** | | **Finding** |
| Profile | | **Atria** | | |
| Abdominal situs | Solitus | Left atrium | | Dilated, 55 X 40mm |
| Cardiac position | Levocardia | Right atrium | | Normal |
| Systemic venous drainage | to RA | **Atrioventricular valves** | | |
| Pulmonary venous drainage | to LA | Mitral valve | | Annulus = 23mm. Thickened & calcified MVL. MVA = 2.8cm2. |
| Atrioventricular connection | Concordant | Tricuspid valve | | Annulus = 18mm |
| Ventriculoarterial connection | Concordant | **Ventricles** | | |
| Ventricular loop | d-Loop | Left ventricle | | Dilated |
| Septae | | Right ventricle | | Normal |
| Interventricular septum | Intact | **M-Mode:** | | |
| Interatrial septum | Intact | AO | |  |
| Great arteries | NRGA | LA | |  |
| Aorta | ----- | LVIDd | | mm |
| Pulmonary artery | Good sized MPA & Branch PAs. | LVIDs | | mm |
| Semilunal valves |  | IVSd | | mm |
| Aortic valve | Annulus = 16mm | IVSs | | mm |
| Pulmonary valve | Annulus = 23mm | LVPWd | | mm |
| Doppler Measurement | | LVPWs | | mm |
| Mitral | Moderate to severe MR, Holosystolic with jet velocity of 5m/sec, posterior projection jet. | EDV | | ml |
| Aortic | Mild AR, PHT = 719ms. | ESV | | ml |
| Tricuspid | Moderate TR, PPG = 51mmHg | FS | | 28% |
| pulmonic | Moderate PR, PPG = 50mmHg | LVEF | | 53% |
| Aortic arch | Left | **Coronary arteries** | |  |
| PDA | No |  | |  |
| Final Diagnosis | 1. {S, D, S} Levocardia 2. LA/LV Dilated 3. Thickened, Calcified MVL 4. Mild AR 5. Moderate PR 6. Moderate TR 7. Moderate to severe MR 8. Moderate Pulmonary Hypertension 9. Mildly reduced LV Function | | | |
| Done By: | **Signature** | | **Date of Reporting** | |
| Dr. Tesfaye Taye, Pediatric Cardiologist |  | | 17/06/2012Eth.C | |

| Echocardiography report  Patient Name: B/ Zina Asmare Patient ID:037496 Gender: M Age: 15 days  Date of Report: 19/06/2012Eth.C BP: Weight: 3.5 kg Height: 48 cm BSA:  Clinical Finding: Murmur + RD + CHF. TGSH7.3216. | | | | |
| --- | --- | --- | --- | --- |
| Features | **Finding** | **Features** | | **Finding** |
| Profile | | **Atria** | | |
| Abdominal situs | Solitus | Left atrium | | Normal |
| Cardiac position | Levocardia | Right atrium | | Normal |
| Systemic venous drainage | to RA | **Atrioventricular valves** | | |
| Pulmonary venous drainage | to LA | Mitral valve | | Annulus = 10mm |
| Atrioventricular connection | Concordant | Tricuspid valve | | Annulus = 9mm |
| Ventriculoarterial connection | Concordant | **Ventricles** | | |
| Ventricular loop | d-Loop | Left ventricle | | Normal |
| Septae: Tongue of tissue in b/n | | Right ventricle | | Normal |
| Interventricular septum | 9mm inlet VSD, BD Shunt | **M-Mode:** reduced LV Function (eye balling) | | |
| Interatrial septum | 8mm primum defect, BD Shunt | AO | |  |
| Great arteries | NRGA | LA | |  |
| Aorta | ----- | LVIDd | | mm |
| Pulmonary artery | Good sized MPA & Branch PAs. | LVIDs | | mm |
| Semilunal valves |  | IVSd | | mm |
| Aortic valve | Annulus = 9mm | IVSs | | mm |
| Pulmonary valve | Annulus = 11mm | LVPWd | | mm |
| Doppler Measurement | | LVPWs | | mm |
| Mitral | ---- | EDV | | ml |
| Aortic | ---- | ESV | | ml |
| Tricuspid | Moderate Right AVVR | FS | | % |
| pulmonic | ---- | LVEF | | % |
| Aortic arch | Left | **Coronary arteries** | |  |
| PDA | 1.5mm PDA, L – R Shunt |  | |  |
| Final Diagnosis | 1. {S, D, S} Levocardia 2. Intermediate AVSD 3. Small PDA, L – R shunt 4. Reduced LV Function | | | |
| Done By: | **Signature** | | **Date of Reporting** | |
| Dr. Tesfaye Taye, Pediatric cardiologist |  | | 19/06/2012Eth.C | |

| Echocardiography report  Patient Name: Adisu Ayenew Patient ID: 038320 Gender: M Age: 10 years  Date of Report: 24/06/2012Eth.C BP: Weight: 19 kg Height: 116 cm BSA:  Clinical Finding: Galloping Staph. infection. TGSH7.3217. (TGSH9) | | | | |
| --- | --- | --- | --- | --- |
| Features | **Finding** | **Features** | | **Finding** |
| Profile | | **Atria** | | |
| Abdominal situs | Solitus | Left atrium | | Normal |
| Cardiac position | Levocardia | Right atrium | | Normal |
| Systemic venous drainage | to RA. IVC Dilated | **Atrioventricular valves** | | |
| Pulmonary venous drainage | to LA | Mitral valve | | Annulus = 20mm |
| Atrioventricular connection | Concordant | Tricuspid valve | | Annulus = 20mm |
| Ventriculoarterial connection | Concordant | **Ventricles** | | |
| Ventricular loop | d-Loop | Left ventricle | | Normal |
| Septae | | Right ventricle | | Normal |
| Interventricular septum | Intact | **M-Mode:** | | |
| Interatrial septum | Intact | AO | |  |
| Great arteries | NRGA | LA | |  |
| Aorta | ----- | LVIDd | | mm |
| Pulmonary artery | Good sized MPA & Branch PAs. | LVIDs | | mm |
| Semilunal valves |  | IVSd | | mm |
| Aortic valve | Annulus = 16mm | IVSs | | mm |
| Pulmonary valve | Annulus = 22mm | LVPWd | | mm |
| Doppler Measurement | | LVPWs | | mm |
| Mitral | Moderate MR | EDV | | ml |
| Aortic | ---- | ESV | | ml |
| Tricuspid | Moderate TR, PPG = 55mmHg | FS | | 22% |
| pulmonic | ---- | LVEF | | 44% |
| Aortic arch | Left | **Coronary arteries** | |  |
| PDA |  |  | |  |
| Pericardium: | 12mm Pericardial effusion on RV Side, 23mm Pericardial effusion. Septated and echodebris inside. | | | |
| Final Diagnosis | 1. S/P Pericardiocentesis for cardiac tamponade 2. {S, D, S} Levocardia 3. Moderate MR 4. Moderate TR 5. Moderate Pulmonary Hypertension 6. Reduced LV Function 7. Circumferential Pericardial effusion, Septated with echodebris inside 8. No features of tamponade | | | |
| Recommendation: strict follow up for re-accumulation and features of tamponade. | | | | |
| Done By: | **Signature** | | **Date of Reporting** | |
| Dr. Tesfaye Taye, Pediatric Cardiologist |  | | 24/06/2012Eth.C | |

| Echocardiography report  Patient Name: B/Deberie Patient ID: 038037 Gender: M Age: 27 Days  Date of Report: 24/06/2012Eth.C BP: Weight: 1.3 kg Height: _________ cm BSA:  Clinical Finding: RD + Murmur. TGSH7.3218. | | | | |
| --- | --- | --- | --- | --- |
| Features | **Finding** | **Features** | | **Finding** |
| Profile | | **Atria** | | |
| Abdominal situs | Solitus | Left atrium | | Normal |
| Cardiac position | Levocardia | Right atrium | | Normal |
| Systemic venous drainage | to RA | **Atrioventricular valves** | | |
| Pulmonary venous drainage | to LA | Mitral valve | | Annulus = 8mm |
| Atrioventricular connection | Concordant | Tricuspid valve | | Annulus = 10mm |
| Ventriculoarterial connection | Concordant | **Ventricles** | | |
| Ventricular loop | d-Loop | Left ventricle | | Normal |
| Septae | | Right ventricle | | Normal |
| Interventricular septum | 9mm inlet VSD with perimembranous extension, Predominantly L – R Shunt. | **M-Mode:** | | |
| Interatrial septum | PFO, L – R Shunt | AO | |  |
| Great arteries | NRGA | LA | |  |
| Aorta | ----- | LVIDd | | mm |
| Pulmonary artery | Good sized MPA & Branch PAs. | LVIDs | | mm |
| Semilunal valves |  | IVSd | | mm |
| Aortic valve | Annulus = 8mm | IVSs | | mm |
| Pulmonary valve | Annulus = 9mm | LVPWd | | mm |
| Doppler Measurement | | LVPWs | | mm |
| Mitral | ---- | EDV | | ml |
| Aortic | ---- | ESV | | ml |
| Tricuspid | ---- | FS | | 31% |
| pulmonic | ---- | LVEF | | 63% |
| Aortic arch | Left | **Coronary arteries** | |  |
| PDA |  |  | |  |
| Remark: | . | | | |
| Final Diagnosis | 1. {S, D, S} Levocardia 2. PFO, L – R Shunt 3. Large inlet VSD with perimembranous extension, predominantly L – R Shunt 4. Good LV Function | | | |
| Recommendation:. | | | | |
| Done By: | **Signature** | | **Date of Reporting** | |
| Dr. Tesfaye Taye, Pediatric Cardiologist |  | | 24/06/2012Eth.C | |

| Echocardiography report  Patient Name: B/ Dagim Teshome Patient ID: 038600 Gender: M Age: 17 Days  Date of Report: 24/06/2012Eth.C BP: Weight: 3.1 kg Height: 48 cm BSA:  Clinical Finding: _ ________. INCOMPLETE DOCUMENTATION | | | | |
| --- | --- | --- | --- | --- |
| Features | **Finding** | **Features** | | **Finding** |
| Profile | | **Atria** | | |
| Abdominal situs | Solitus | Left atrium | | Normal |
| Cardiac position | Levocardia | Right atrium | | Normal |
| Systemic venous drainage | to RA | **Atrioventricular valves** | | |
| Pulmonary venous drainage | to LA | Mitral valve | | Annulus = mm |
| Atrioventricular connection | Concordant | Tricuspid valve | | Annulus = mm  TAPSE = mm |
| Ventriculoarterial connection | Concordant | **Ventricles** | | |
| Ventricular loop | d-Loop | Left ventricle | | Normal |
| Septae | | Right ventricle | | Normal |
| Interventricular septum | Intact | **M-Mode:** | | |
| Interatrial septum | Intact | AO | |  |
| Great arteries | NRGA | LA | |  |
| Aorta | ----- | LVIDd | | mm |
| Pulmonary artery | Good sized MPA & Branch PAs. | LVIDs | | mm |
| Semilunal valves |  | IVSd | | mm |
| Aortic valve | Annulus = mm | IVSs | | mm |
| Pulmonary valve | Annulus = mm | LVPWd | | mm |
| Doppler Measurement | | LVPWs | | mm |
| Mitral | ---- | EDV | | ml |
| Aortic | ---- | ESV | | ml |
| Tricuspid | ---- | FS | | % |
| pulmonic | ---- | LVEF | | % |
| Aortic arch | Left | **Coronary arteries** | |  |
| PDA |  |  | |  |
| Remark: | . | | | |
| Final Diagnosis | 1. {S, D, S} Levocardia | | | |
| Recommendation:. | | | | |
| Done By: | **Signature** | | **Date of Reporting** | |
| Dr. Tesfaye Taye, Pediatric Cardiologist |  | | 24/06/2012Eth.C | |

| Echocardiography report  Patient Name: Ephrata Motbayenor Patient ID: 039502 Gender: F Age: 6 Years  Date of Report: 26/06/2012Eth.C BP: Weight: 17 kg Height: 87 BSA:  Clinical Finding: RD + Murmur + DOE + Diaphoresis + CHF + Recurrent Chest Infection. TGSH7.3219. | | | | |
| --- | --- | --- | --- | --- |
| Features | **Finding** | **Features** | | **Finding** |
| Profile | | **Atria** | | |
| Abdominal situs | Solitus | Left atrium | | Dilated |
| Cardiac position | Levocardia | Right atrium | | Dilated |
| Systemic venous drainage | to RA | **Atrioventricular valves** | | |
| Pulmonary venous drainage | to LA | Mitral valve | | Common Complete AVSD |
| Atrioventricular connection | Common complete AVSD | Tricuspid valve | |
| Ventriculoarterial connection | Concordant | **Ventricles** | | |
| Ventricular loop | d-Loop | Left ventricle | | Dilated |
| Septae | | Right ventricle | | Smallish and hypertrophied |
| Interventricular septum | Non-restrictive large inlet VSD, L – R Shunt | **M-Mode:** | | |
| Interatrial septum | Non-restrictive Primum defect, L – R Shunt | AO | |  |
| Great arteries | NRGA | LA | |  |
| Aorta | ----- | LVIDd | | mm |
| Pulmonary artery | Good sized MPA & Branch PAs. | LVIDs | | mm |
| Semilunal valves |  | IVSd | | mm |
| Aortic valve | Annulus = 11mm | IVSs | | mm |
| Pulmonary valve | Annulus = 16mm | LVPWd | | mm |
| Doppler Measurement | | LVPWs | | mm |
| Mitral | Moderate Left AVVR | EDV | | ml |
| Aortic | ---- | ESV | | ml |
| Tricuspid | Moderate Right AVVR | FS | | 38% |
| pulmonic | Moderate PR, PPG = 62mmHg | LVEF | | 70% |
| Aortic arch | Left | **Coronary arteries** | |  |
| PDA | 2.5mm PDA, L – R Shunt |  | |  |
| Remark: | . | | | |
| Final Diagnosis | 1. {S, D, S} Levocardia 2. Common Complete Unbalanced AVSD 3. Moderate PDA, L – R 4. Severe Pulmonary Hypertension 5. Good LV Function | | | |
| Recommendation:. | | | | |
| Done By: | **Signature** | | **Date of Reporting** | |
| Dr. Tesfaye Taye, Pediatric Cardiologist |  | | /07/2012Eth.C | |

| Echocardiography report  Patient Name: Mengistu Zelalem Patient ID: 039787 Gender: M Age: 2/12  Date of Report: 01/07/2012Eth.C BP: Weight: Height: BSA:  Clinical Finding: Incidental Murmur + Diaphoresis. TGSH7.3220. | | | | |
| --- | --- | --- | --- | --- |
| Features | **Finding** | **Features** | | **Finding** |
| Profile | | **Atria** | | |
| Abdominal situs | Solitus | Left atrium | | Dilated |
| Cardiac position | Levocardia | Right atrium | | Normal |
| Systemic venous drainage | to RA | **Atrioventricular valves** | | |
| Pulmonary venous drainage | to LA | Mitral valve | | Annulus = 12mm |
| Atrioventricular connection | Concordant | Tricuspid valve | | Annulus = 10mm |
| Ventriculoarterial connection | Concordant | **Ventricles** | | |
| Ventricular loop | d-Loop | Left ventricle | | Dilated |
| Septae | | Right ventricle | | Normal |
| Interventricular septum | Intact | **M-Mode:** | | |
| Interatrial septum | Intact | AO | |  |
| Great arteries | NRGA | LA | |  |
| Aorta | ----- | LVIDd | | mm |
| Pulmonary artery | Good sized MPA & Branch PAs. | LVIDs | | mm |
| Semilunal valves |  | IVSd | | mm |
| Aortic valve | Annulus = 11mm | IVSs | | mm |
| Pulmonary valve | Annulus = 10mm | LVPWd | | mm |
| Doppler Measurement | | LVPWs | | mm |
| Mitral | --- | EDV | | ml |
| Aortic | ---- | ESV | | ml |
| Tricuspid | ----- | FS | | 44% |
| pulmonic | ----- | LVEF | | 78% |
| Aortic arch |  | **Coronary arteries** | |  |
| PDA | 2.5mm PDA, L – R Shunt |  | |  |
| Remark: | . | | | |
| Final Diagnosis | 1. {S, D, S} Levocardia 2. Moderate PDA, L – R Shunt 3. Good Function | | | |
| Recommendation:. | | | | |
| Done By: | **Signature** | | **Date of Reporting** | |
| Dr. Tesfaye Taye, Pediatric Cardiologist |  | | 01/07/2012Eth.C | |

| Echocardiography report  Patient Name: Sindie Enchalew Patient ID: 039747 Gender: M Age: 13  Date of Report: 03/07/2012Eth.C BP: Weight: 29 Height: BSA:  Clinical Finding: Palpitation. TGSH7.3221. | | | | |
| --- | --- | --- | --- | --- |
| Features | **Finding** | **Features** | | **Finding** |
| Profile | | **Atria** | | |
| Abdominal situs | Solitus | Left atrium | | Normal |
| Cardiac position | Levocardia | Right atrium | | Normal |
| Systemic venous drainage | to RA | **Atrioventricular valves** | | |
| Pulmonary venous drainage | to LA | Mitral valve | | Annulus = 19mm |
| Atrioventricular connection | Concordant | Tricuspid valve | | Annulus = 22mm  TAPSE = 21mm |
| Ventriculoarterial connection | Concordant | **Ventricles** | | |
| Ventricular loop | d-Loop | Left ventricle | | Normal |
| Septae | | Right ventricle | | Normal |
| Interventricular septum | Intact | **M-Mode:** | | |
| Interatrial septum | Intact | AO | |  |
| Great arteries | NRGA | LA | |  |
| Aorta | ----- | LVIDd | | mm |
| Pulmonary artery | Good sized MPA & Branch PAs. | LVIDs | | mm |
| Semilunal valves |  | IVSd | | mm |
| Aortic valve | Annulus = 18mm | IVSs | | mm |
| Pulmonary valve | Annulus = 20mm | LVPWd | | mm |
| Doppler Measurement | | LVPWs | | mm |
| Mitral |  | EDV | | ml |
| Aortic |  | ESV | | ml |
| Tricuspid |  | FS | | 36% |
| pulmonic |  | LVEF | | 66% |
| Aortic arch |  | **Coronary arteries** | |  |
| PDA |  |  | |  |
| Remark: | . | | | |
| Final Diagnosis | 1. Normal Echocardiography Study | | | |
| Recommendation:. | | | | |
| Done By: | **Signature** | | **Date of Reporting** | |
| Dr. Tesfaye Taye, Pediatric Cardiologist |  | | 03/07/2012Eth.C | |

| Echocardiography report  Patient Name : Tadsual shegaye Patient ID: 040267 Gender: M Age: 8  Date of Report: 03/07/2012 ETC BP: Weight: 20kg Height: 114cm. BSA: 0.8m2.  Clinical Finding: DOE + Murmur + easy fatigability + Palpitation + Recurrent Chest Infection. TGSH7.3222. | | | | |
| --- | --- | --- | --- | --- |
| Features | **Finding** | **Features** | | **Finding** |
| Profile | | **Atria** | | |
| Abdominal situs | Solitus | Left atrium | | Normal |
| Cardiac position | Levocardia | Right atrium | | Dilated |
| Systemic venous drainage | to RA | **Atrioventricular valves** | | |
| Pulmonary venous drainage | to LA | Mitral valve | | Annulus = 16mm |
| Atrioventricular connection | Concordant | Tricuspid valve | | Annulus = 22mm |
| Ventriculoarterial connection | Concordant | **Ventricles** | | |
| Ventricular loop | d-Loop | Left ventricle | | Normal |
| Septae | | Right ventricle | | Dilated |
| Interventricular septum | Intact | **M-Mode:** | | |
| Interatrial septum | 14mm Primum defect, L – R Shunt | AO | |  |
| Great arteries | NRGA | LA | |  |
| Aorta | ----- | LVIDd | | mm |
| Pulmonary artery | MPA = 20mm. Good sized Branch PAs. | LVIDs | | mm |
| Semilunal valves |  | IVSd | | mm |
| Aortic valve | Annulus = 14mm | IVSs | | mm |
| Pulmonary valve | Annulus = **24mm** | LVPWd | | mm |
| Doppler Measurement | | LVPWs | | mm |
| Mitral | Moderate to severe MR | EDV | | ml |
| Aortic | ---- | ESV | | ml |
| Tricuspid | Moderate to severe TR | FS | | 32% |
| pulmonic | Moderate PR, PPG = 49mmHg. Mild PS, PPG = 33mmHg | LVEF | | 62% |
| Aortic arch | ---- | **Coronary arteries** | | ---- |
| PDA | No |  | |  |
| Final Diagnosis | 1. {S, D, S} Levocardia 2. Partial AVSD 3. Moderate to severe MR/TR 4. Mild PS 5. Moderate PR 6. Moderate Pulmonary Hypertension 7. Good Function | | | |
| Done By: | **Signature** | | **Date of Reporting** | |
| Dr. Tesfaye Taye, Pediatric Cardiologist |  | | 01/07/2012Eth.C | |

| Echocardiography report  Patient Name: Mekides Melese Patient ID: 040500 Gender: F Age: 8/12  Date of Report: 03/07/2012Eth.C BP: Weight: 4.5 kg Height: 66 cm BSA:  Clinical Finding: Incidental Murmur. TGSH7.3223. | | | | |
| --- | --- | --- | --- | --- |
| Features | **Finding** | **Features** | | **Finding** |
| Profile | | **Atria** | | |
| Abdominal situs | Solitus | Left atrium | | Normal |
| Cardiac position | Levocardia | Right atrium | | Normal |
| Systemic venous drainage | to RA | **Atrioventricular valves** | | |
| Pulmonary venous drainage | to LA | Mitral valve | | Annulus = 13mm |
| Atrioventricular connection | Concordant | Tricuspid valve | | Annulus = 18mm  TAPSE = 13mm |
| Ventriculoarterial connection | Concordant | **Ventricles** | | |
| Ventricular loop | d-Loop | Left ventricle | | Normal |
| Septae | | Right ventricle | | Normal |
| Interventricular septum | 4mm Supra Cristal VSD, L – R Shunt | **M-Mode:** | | |
| Interatrial septum | Intact | AO | |  |
| Great arteries | NRGA | LA | |  |
| Aorta | ----- | LVIDd | | mm |
| Pulmonary artery | Good sized MPA & Branch PAs. | LVIDs | | mm |
| Semilunal valves |  | IVSd | | mm |
| Aortic valve | Annulus = 9mm | IVSs | | mm |
| Pulmonary valve | Annulus = 12mm | LVPWd | | mm |
| Doppler Measurement | | LVPWs | | mm |
| Mitral | Trivial MR | EDV | | ml |
| Aortic | ------ | ESV | | ml |
| Tricuspid | Trivial TR, Trivial TR, PPG = 31mmHg | FS | | % |
| pulmonic | ----- | LVEF | | % |
| Aortic arch |  | **Coronary arteries** | |  |
| PDA |  |  | |  |
| Final Diagnosis | 1. {S, D, S} Levocardia 2. Small Supra Cristal VSD, L – R Shunt 3. Good Biventricular Function | | | |
| Done By: | **Signature** | | **Date of Reporting** | |
| Dr. Tesfaye Taye, Pediatric Cardiologist |  | | 03/07/2012Eth.C | |

| Echocardiography report  Patient Name: Baby Belaynesh Taye Patient ID: 013439 Gender: M Age: 1 years 1 /12  Date of Report: 03/07/2012Eth.C BP: Weight: 9 kg Height: 43 cm BSA:  Clinical Finding: DS. TGSH7.3224. | | | | |
| --- | --- | --- | --- | --- |
| Features | **Finding** | **Features** | | **Finding** |
| Profile | | **Atria** | | |
| Abdominal situs | Solitus | Left atrium | | Normal |
| Cardiac position | Levocardia | Right atrium | | Normal |
| Systemic venous drainage | to RA | **Atrioventricular valves** | | |
| Pulmonary venous drainage | to LA | Mitral valve | | Annulus = 15mm |
| Atrioventricular connection | Concordant | Tricuspid valve | | Annulus = 16mm. |
| Ventriculoarterial connection | Concordant | **Ventricles** | | |
| Ventricular loop | d-Loop | Left ventricle | | Normal |
| Septae | | Right ventricle | | Normal |
| Interventricular septum | Intact | **M-Mode:** | | |
| Interatrial septum | Intact | AO | |  |
| Great arteries | NRGA | LA | |  |
| Aorta | ----- | LVIDd | | mm |
| Pulmonary artery | Good sized MPA & Branch PAs. | LVIDs | | mm |
| Semilunal valves |  | IVSd | | mm |
| Aortic valve | Annulus = 15mm | IVSs | | mm |
| Pulmonary valve | Annulus = 15mm | LVPWd | | mm |
| Doppler Measurement | | LVPWs | | mm |
| Mitral |  | EDV | | ml |
| Aortic |  | ESV | | ml |
| Tricuspid | Trivial TR, PPG = 16mmHg | FS | | 38% |
| pulmonic | Trivial PR, PPG = 33mmHg | LVEF | | 70% |
| Aortic arch | Left | **Coronary arteries** | |  |
| PDA | No |  | |  |
| Final Diagnosis | 1. Normal Echocardiography Study. | | | |
| Done By: | **Signature** | | **Date of Reporting** | |
| Dr. Tesfaye Taye, Pediatric Cardiologist |  | | 03/07/2012Eth.C | |

| Echocardiography report  Patient Name: Ermias Getu Patient ID: 040470 Gender: M Age: 6/12  Date of Report: 03/07/2012Eth.C BP: Weight: 5kg. Height: 59cn. BSA: 0.27m2.  Clinical Finding: RD + CHF + Murmur + Recurrent Chest Infection + Diaphoresis. TGSH7.3225. | | | | |
| --- | --- | --- | --- | --- |
| Features | **Finding** | **Features** | | **Finding** |
| Profile | | **Atria** | | |
| Abdominal situs | Solitus | Left atrium | | Dilated |
| Cardiac position | Levocardia | Right atrium | | Dilated |
| Systemic venous drainage | to RA | **Atrioventricular valves** | | |
| Pulmonary venous drainage | to LA | Mitral valve | | **Annulus = 20mm** |
| Atrioventricular connection | Concordant | Tricuspid valve | | Annulus = 15mm |
| Ventriculoarterial connection | Concordant | **Ventricles** | | |
| Ventricular loop | d-Loop | Left ventricle | | Dilated |
| Septae | | Right ventricle | | Dilated |
| Interventricular septum | 12mm PM VSD, L – R Shunt | **M-Mode:** | | |
| Interatrial septum | Intact | AO | |  |
| Great arteries | NRGA | LA | |  |
| Aorta | ----- | LVIDd | | mm |
| Pulmonary artery | **MPA = 18mm**. Normal Branch PAs. | LVIDs | | mm |
| Semilunal valves |  | IVSd | | mm |
| Aortic valve | Annulus = 10mm | IVSs | | mm |
| Pulmonary valve | **Annulus = 18mm** | LVPWd | | mm |
| Doppler Measurement | | LVPWs | | mm |
| Mitral |  | EDV | | ml |
| Aortic |  | ESV | | ml |
| Tricuspid |  | FS | | 29% |
| pulmonic | Moderate PR, PPG = 76mmHg | LVEF | | 56% |
| Aortic arch |  | **Coronary arteries** | |  |
| PDA |  |  | |  |
| Final Diagnosis | 1. {S, D, S} Levocardia 2. Large Perimembranous VSD, L – R Shunt 3. Severe Pulmonary Hypertension 4. Good Function | | | |
| Done By: | **Signature** | | **Date of Reporting** | |
| Dr. Tesfaye Taye, Pediatric Cardiologist |  | | 03/07/2012Eth.C | |

| Echocardiography report  Patient Name: Chalachew Senechaw Patient ID: 040539 Gender: M Age: 2/12  Date of Report: 03/07/2012Eth.C BP: Weight: 5 kg Height: 58 cm BSA:  Clinical Finding: RD + Murmur + Diaphoresis. TGSH7.3226. | | | | |
| --- | --- | --- | --- | --- |
| Features | **Finding** | **Features** | | **Finding** |
| Profile | | **Atria** | | |
| Abdominal situs | Solitus | Left atrium | | Dilated |
| Cardiac position | Levocardia | Right atrium | | Normal |
| Systemic venous drainage | to RA | **Atrioventricular valves** | | |
| Pulmonary venous drainage | to LA | Mitral valve | | Common complete AVSD |
| Atrioventricular connection | Complete AVSD | Tricuspid valve | | Common complete AVSD |
| Ventriculoarterial connection | Concordant | **Ventricles** | | |
| Ventricular loop | d-Loop | Left ventricle | | Dilated |
| Septae | | Right ventricle | | Normal |
| Interventricular septum | Intact | **M-Mode:** | | |
| Interatrial septum | Intact | AO | |  |
| Great arteries | NRGA | LA | |  |
| Aorta | ----- | LVIDd | | mm |
| Pulmonary artery | MPA = 13mm. Normal Branch PAs. | LVIDs | | mm |
| Semilunal valves |  | IVSd | | mm |
| Aortic valve | Annulus = mm | IVSs | | mm |
| Pulmonary valve | Annulus = 11mm | LVPWd | | mm |
| Doppler Measurement | | LVPWs | | mm |
| Mitral | Mild Left AVVR | EDV | | ml |
| Aortic | ------- | ESV | | ml |
| Tricuspid | Mild right AVVR | FS | | 31% |
| pulmonic | --------- | LVEF | | 62% |
| Aortic arch | ----- | **Coronary arteries** | |  |
| PDA | No |  | |  |
| Final Diagnosis | 1. {S, D, S} Levocardia 2. Common Complete Balanced AVSD 3. Mild Right AVVR 4. Mild left AVVR 5. Good Function | | | |
| Done By: | **Signature** | | **Date of Reporting** | |
| Dr. Tesfaye Taye, Pediatric Cardiologist |  | | 03/07/2012Eth.C | |

| Echocardiography report  Patient Name: Yohannes Tadele Patient ID 040911 Gender: M Age: 57days  Date of Report: 08/07/2012Eth.C BP: Weight: 3.1kg Height: 53 cm BSA:  Clinical Finding: Cyanosis + RD. TGSH7.3227. | | | | |
| --- | --- | --- | --- | --- |
| Features | **Finding** | **Features** | | **Finding** |
| Profile | | **Atria** | | |
| Abdominal situs | Solitus | Left atrium | | Normal |
| Cardiac position | Levocardia | Right atrium | | Mildly dilated |
| Systemic venous drainage | to RA | **Atrioventricular valves** | | |
| Pulmonary venous drainage | to LA | Mitral valve | | Annulus = 12mm |
| Atrioventricular connection | Concordant | Tricuspid valve | | Annulus = 15mm |
| Ventriculoarterial connection | Discordant | **Ventricles** | | |
| Ventricular loop | d-Loop | Left ventricle | | Banana Shaped |
| Septae | | Right ventricle | | Mildly dilated |
| Interventricular septum | Intact | **M-Mode: Good Function** | | |
| Interatrial septum | PFO, L – R Shunt | AO | |  |
| Great arteries | d-TGA | LA | |  |
| Aorta | Posterior and to the right | LVIDd | | mm |
| Pulmonary artery | Anterior and to the left | LVIDs | | mm |
| Semilunal valves |  | IVSd | | mm |
| Aortic valve | Annulus = 11mm | IVSs | | mm |
| Pulmonary valve | Annulus = 10mm | LVPWd | | mm |
| Doppler Measurement | | LVPWs | | mm |
| Mitral | Trivial MR | EDV | | ml |
| Aortic | ------- | ESV | | ml |
| Tricuspid | ------ | FS | | % |
| pulmonic | --------- | LVEF | | % |
| Aortic arch | ----- | **Coronary arteries** | |  |
| PDA | No |  | |  |
| Final Diagnosis | 1. {S, D, D} Levocardia 2. PFO, L – R Shunt, restrictive 3. Intact IVS 4. d-TGA | | | |
| Done By: | **Signature** | | **Date of Reporting** | |
| Dr. Tesfaye Taye, Pediatric Cardiologist |  | | 08/07/2012Eth.C | |

| Echocardiography report  Patient Name: Temechew Kefale Patient ID 027241 Gender: M Age 14  Date of Report: 08/07/2012Eth.C BP: Weight: 37 Height: 1.47 cm BSA: TGSH1.2638 | | | | |
| --- | --- | --- | --- | --- |
| Follow up echocardiography Study | | | | |
|  | **Finding** | **Features** | | **Finding** |
| Profile | | **Atria** | | |
| Abdominal situs | Solitus | Left atrium | | Normal |
| Cardiac position | Levocardia | Right atrium | | Normal |
| Systemic venous drainage | to RA | **Atrioventricular valves** | | |
| Pulmonary venous drainage | to LA | Mitral valve | | Annulus = 23mm. thickened MVL. |
| Atrioventricular connection | Concordant | Tricuspid valve | | Annulus = 31mm |
| Ventriculoarterial connection | Concordant | **Ventricles** | | |
| Ventricular loop | d-Loop | Left ventricle | | Normal |
| Septae | | Right ventricle | | Normal |
| Interventricular septum | Intact | **M-Mode:** | | |
| Interatrial septum | Intact | AO | |  |
| Great arteries | NRGA | LA | |  |
| Aorta | ----- | LVIDd | | mm |
| Pulmonary artery | Normal MPA normal Branch PAs. | LVIDs | | mm |
| Semilunal valves |  | IVSd | | mm |
| Aortic valve | Annulus = 19mm | IVSs | | mm |
| Pulmonary valve | Annulus = 20mm | LVPWd | | mm |
| Doppler Measurement | | LVPWs | | mm |
| Mitral | Trivial MR, Holosystolic, posterior projection, seen in 2 planes with jet velocity of 3m/sec | EDV | | ml |
| Aortic | ------- | ESV | | ml |
| Tricuspid | Trivial TR, PPG = 18mmHg | FS | | 29% |
| pulmonic | Trivial PR, PPG = 12mmHg | LVEF | | 56% |
| Aortic arch | ----- | **Coronary arteries** | |  |
| PDA | No |  | |  |
| Final Diagnosis | 1. {S, D, S} Levocardia 2. Trivial MR 3. Trivial TR 4. Good Function | | | |
| Conclusion | Improving. Continue Benzathin penicillin | | | |
| Done By: | **Signature** | | **Date of Reporting** | |
| Dr. Tesfaye Taye, Pediatric Cardiologist |  | | 08/07/2012Eth.C | |

| Echocadiography report  Patient Name: Eleni Geremew Patient ID 038731 Gender: F Age 9/12  Date of Report: 08/07/2012Eth.C BP: Weight: 4.6 kg Height: 57 cm BSA:  Clinical Finding: Incidental Murmur. TGSH7.3228. | | | | |
| --- | --- | --- | --- | --- |
| Features | **Finding** | **Features** | | **Finding** |
| Profile | | **Atria** | | |
| Abdominal situs | Solitus | Left atrium | | Normal |
| Cardiac position | Levocardia | Right atrium | | Normal |
| Systemic venous drainage | to RA | **Atrioventricular valves** | | |
| Pulmonary venous drainage | to LA | Mitral valve | | Annulus = 14mm |
| Atrioventricular connection | Concordant | Tricuspid valve | | Annulus = 16mm |
| Ventriculoarterial connection | Concordant | **Ventricles** | | |
| Ventricular loop | d-Loop | Left ventricle | | Normal |
| Septae | | Right ventricle | | Normal |
| Interventricular septum | Intact | **M-Mode:** | | |
| Interatrial septum | 10mm OS ASD, L – R Shunt | AO | |  |
| Great arteries | NRGA | LA | |  |
| Aorta | ----- | LVIDd | | mm |
| Pulmonary artery | Normal MPA normal Branch PAs. | LVIDs | | mm |
| Semilunal valves |  | IVSd | | mm |
| Aortic valve | Annulus = 10mm | IVSs | | mm |
| Pulmonary valve | Annulus = 10mm | LVPWd | | mm |
| Doppler Measurement | | LVPWs | | mm |
| Mitral | ------ | EDV | | ml |
| Aortic | ------- | ESV | | ml |
| Tricuspid | ------- | FS | | 35% |
| pulmonic | Mild valvar PS, PPG = 25mmHg | LVEF | | 68% |
| Aortic arch | ----- | **Coronary arteries** | |  |
| PDA | No |  | |  |
| Final Diagnosis | 1. {S, D, S} Levocardia 2. Moderate OS ASD, L – R Shunt 3. Mild Valvar PS 4. Good Biventricular Function. | | | |
| Done By: | **Signature** | | **Date of Reporting** | |
| Dr. Tesfaye Taye, Pediatric Cardiologist |  | | 08/07/2012Eth.C | |

| Echocardiography report  Patient Name: Alemenesh Belay Patient ID 039988 Gender: F Age 25 Days  Date of Report: 08/07/2012Eth.C BP: Weight: 3.6 kg Height: 96 cm BSA:  Clinical Finding: Incidental Murmur. TGSH7.3229. | | | | |
| --- | --- | --- | --- | --- |
| Features | **Finding** | **Features** | | **Finding** |
| Profile | | **Atria** | | |
| Abdominal situs | Solitus | Left atrium | | Normal |
| Cardiac position | Levocardia | Right atrium | | Normal |
| Systemic venous drainage | to RA | **Atrioventricular valves** | | |
| Pulmonary venous drainage | to LA | Mitral valve | | Annulus = 11mm |
| Atrioventricular connection | Concordant | Tricuspid valve | | Annulus = 10mm |
| Ventriculoarterial connection | Concordant | **Ventricles** | | |
| Ventricular loop | d-Loop | Left ventricle | | Normal |
| Septae | | Right ventricle | | Normal |
| Interventricular septum | Small Apical Muscular VSD, L – R Shunt, restrictive | **M-Mode: Good Function** | | |
| Interatrial septum | PFO, L – R Shunt | AO | |  |
| Great arteries | NRGA | LA | |  |
| Aorta | ----- | LVIDd | | mm |
| Pulmonary artery | Normal MPA normal Branch PAs. | LVIDs | | mm |
| Semilunal valves |  | IVSd | | mm |
| Aortic valve | Annulus = 9mm | IVSs | | mm |
| Pulmonary valve | Annulus = 9mm | LVPWd | | mm |
| Doppler Measurement | | LVPWs | | mm |
| Mitral | ------ | EDV | | ml |
| Aortic | ------- | ESV | | ml |
| Tricuspid | ------- | FS | | % |
| pulmonic | Mild Valvar PS, PPG = 20mmHg | LVEF | | % |
| Aortic arch | ----- | **Coronary arteries** | |  |
| PDA | No |  | |  |
| Final Diagnosis | 1. {S, D, S} Levocardia 2. PFO, L – R Shunt 3. Small Apical Muscular Restrictive VSD, L – R Shunt 4. Mild Valvar PS 5. Good Function | | | |
| Done By: | **Signature** | | **Date of Reporting** | |
| Dr. Tesfaye Taye, Pediatric Cardiologist |  | | 08/07/2012Eth.C | |

| Echocardiography report  Patient Name: Meseret Menber Patient ID: Gender: F Age: 1 years 1/12  Date of Report: 17/07 /2012Eth.C BP: Weight: 5.6 kg Height: 63 cm BSA:  Clinical Finding: Diaphoresis + Murmur. TGSH7.3230. | | | | |
| --- | --- | --- | --- | --- |
| Features | **Finding** | **Features** | | **Finding** |
| Profile | | **Atria** | | |
| Abdominal situs | Solitus | Left atrium | | Normal |
| Cardiac position | Levocardia | Right atrium | | Dilated |
| Systemic venous drainage | to RA | **Atrioventricular valves** | | |
| Pulmonary venous drainage | to LA | Mitral valve | | Annulus = 11mm |
| Atrioventricular connection | Concordant | Tricuspid valve | | Annulus = 16mm |
| Ventriculoarterial connection | Concordant | **Ventricles** | | |
| Ventricular loop | d-Loop | Left ventricle | | Normal |
| Septae | | Right ventricle | | Dilated |
| Interventricular septum | Intact | **M-Mode:** | | |
| Interatrial septum | 13mm OS ASD, L – R Shunt | AO | |  |
| Great arteries | NRGA | LA | |  |
| Aorta | ----- | LVIDd | | mm |
| Pulmonary artery | Normal MPA normal Branch PAs. | LVIDs | | mm |
| Semilunal valves |  | IVSd | | mm |
| Aortic valve | Annulus = 12mm | IVSs | | mm |
| Pulmonary valve | Annulus = 15mm | LVPWd | | mm |
| Doppler Measurement | | LVPWs | | mm |
| Mitral | ------ | EDV | | ml |
| Aortic | ------- | ESV | | ml |
| Tricuspid | ------- | FS | | 39% |
| pulmonic | Trivial PR, PPG = 13mmHg | LVEF | | 73% |
| Aortic arch | ----- | **Coronary arteries** | |  |
| PDA | No |  | |  |
| Final Diagnosis | 1. {S, D, S} Levocardia 2. RA/RV Dilated 3. Large OS ASD, L – R Shunt 4. Good Biventricular Function. | | | |
| Done By: | **Signature** | | **Date of Reporting** | |
| Dr. Tesfaye Taye, Pediatric Cardiologist |  | | 17/07/2012Eth.C | |

| Echocardiography report  Patient Name: Chalachew Degalem Patient ID :042071 Gender: M Age :7 years  Date of Report: 17/07/2012Eth.C BP: Weight: 13 kg Height: 104 cm BSA:  Clinical Finding: IE. TGSH7.3231. | | | | |
| --- | --- | --- | --- | --- |
| Features | **Finding** | **Features** | | **Finding** |
| Profile | | **Atria** | | |
| Abdominal situs | Solitus | Left atrium | | Normal |
| Cardiac position | Levocardia | Right atrium | | Dilated |
| Systemic venous drainage | to RA | **Atrioventricular valves** | | |
| Pulmonary venous drainage | to LA | Mitral valve | | Annulus = 22mm |
| Atrioventricular connection | Concordant | Tricuspid valve | | Annulus = 24mm. Non coapting TV Leaflets with calcification ?old vegetation  TAPSE = 23mm |
| Ventriculoarterial connection | Concordant | **Ventricles** | | |
| Ventricular loop | d-Loop | Left ventricle | | Normal |
| Septae | | Right ventricle | | Dilated.  RV TDI S wave = 16cm/sec |
| Interventricular septum | 6mm PM VSD partially covered by septal tricuspid leaflet, L – R Shunt With gradient = 62mmHg. | **M-Mode:** | | |
| Interatrial septum | Intact | AO | |  |
| Great arteries | NRGA | LA | |  |
| Aorta | ----- | LVIDd | | mm |
| Pulmonary artery | MPA = 20mm. normal Branch PAs. | LVIDs | | mm |
| Semilunal valves |  | IVSd | | mm |
| Aortic valve | Annulus = 18mm | IVSs | | mm |
| Pulmonary valve | Annulus = 22mm | LVPWd | | mm |
| Doppler Measurement | | LVPWs | | mm |
| Mitral | ------ | EDV | | ml |
| Aortic | ------- | ESV | | ml |
| Tricuspid | Severe TR. | FS | | 33% |
| pulmonic | Trivial PR | LVEF | | 63% |
| Aortic arch | ----- | **Coronary arteries** | |  |
| PDA | No |  | |  |
| Final Diagnosis | 1. {S, D, S} Levocardia 2. Calcified, non coapting tricuspid valve leaflet ?old vegetation 3. RA/RV Dilated 4. Severe TR 5. Moderate PM VSD, PARTIALLY covered by septal leaflet of tricuspid valve. 6. Good Biventricular Function | | | |
| Done By: | **Signature** | | **Date of Reporting** | |
| Dr. Tesfaye Taye, Pediatric Cardiologist |  | | 17/07/2012Eth.C | |

| Echocardiography report  Patient Name: Mubarak Dires Patient ID :041534 Gender: M Age :7/12  Date of Report: 17/07/2012Eth.C BP: Weight: 6.1 kg Height: 63 cm B SA:  Clinical Finding: RD + Murmur + CHF. TGSH7.3232. | | | | |
| --- | --- | --- | --- | --- |
| Features | **Finding** | **Features** | | **Finding** |
| Profile | | **Atria** | | |
| Abdominal situs | Solitus | Left atrium | | Dilated |
| Cardiac position | Levocardia | Right atrium | | Dilated |
| Systemic venous drainage | to RA | **Atrioventricular valves** | | |
| Pulmonary venous drainage | to LA | Mitral valve | | Annulus = 14mm |
| Atrioventricular connection | Concordant | Tricuspid valve | | Annulus = 22mm |
| Ventriculoarterial connection | Concordant | **Ventricles** | | |
| Ventricular loop | d-Loop | Left ventricle | | Dilated. Banana shaped LV |
| Septae | | Right ventricle | | Dilated. RV TDI S wave = 9cm/sec |
| Interventricular septum | Intact | **M-Mode:** | | |
| Interatrial septum | 4mm OS ASD, R – L Shunt | AO | |  |
| Great arteries | NRGA | LA | |  |
| Aorta | ----- | LVIDd | | mm |
| Pulmonary artery | Normal MPA normal Branch PAs. | LVIDs | | mm |
| Semilunal valves |  | IVSd | | mm |
| Aortic valve | Annulus = 13mm | IVSs | | mm |
| Pulmonary valve | Annulus = 16mm | LVPWd | | mm |
| Doppler Measurement | | LVPWs | | mm |
| Mitral | ------ | EDV | | ml |
| Aortic | ------- | ESV | | ml |
| Tricuspid | Severe TR, PPG = 66mmHg | FS | | 35% |
| pulmonic | Moderate to severe PR, PPG = 60mmHg | LVEF | | 67% |
| Aortic arch | ----- | **Coronary arteries** | |  |
| PDA | 4mm PDA, BD shunt |  | |  |
| Final Diagnosis | 1. {S, D, S} Levocardia 2. Small OS ASD, R – L Shunt 3. RA/RV Dilated 4. Severe TR 5. Moderate to severe PR 6. Large PDA 7. RV Dilated and dysfunctional 8. Banana Shaped LV 9. Severe Pulmonary Hypertension | | | |
| Done By: | **Signature** | | **Date of Reporting** | |
| Dr. Tesfaye Taye, Pediatric Cardiologist |  | | 17/07/2012Eth.C | |

| Echocardiography report  Patient Name: Bekalu Hunegnaw Patient ID : 040944 Gender: M Age : 35 days  Date of Report: 17/07/2012Eth.C BP: Weight: 3.3 kg Height: 52 cm BSA:  Clinical Finding: Incidental Murmur. TGSH7.3233. | | | | |
| --- | --- | --- | --- | --- |
| Features | **Finding** | **Features** | | **Finding** |
| Profile | | **Atria** | | |
| Abdominal situs | Solitus | Left atrium | | Normal |
| Cardiac position | Levocardia | Right atrium | | Normal |
| Systemic venous drainage | to RA | **Atrioventricular valves** | | |
| Pulmonary venous drainage | to LA | Mitral valve | | Annulus = 12mm |
| Atrioventricular connection | Concordant | Tricuspid valve | | Annulus = 12mm |
| Ventriculoarterial connection | Concordant | **Ventricles** | | |
| Ventricular loop | d-Loop | Left ventricle | | Normal |
| Septae | | Right ventricle | | Normal |
| Interventricular septum | Intact | **M-Mode:** | | |
| Interatrial septum | Intact | AO | |  |
| Great arteries | NRGA | LA | |  |
| Aorta | ----- | LVIDd | | mm |
| Pulmonary artery | Normal MPA normal Branch PAs. | LVIDs | | mm |
| Semilunal valves |  | IVSd | | mm |
| Aortic valve | Annulus = 9mm | IVSs | | mm |
| Pulmonary valve | Annulus = 8mm. Doming Valve | LVPWd | | mm |
| Doppler Measurement | | LVPWs | | mm |
| Mitral | ------ | EDV | | ml |
| Aortic | ------- | ESV | | ml |
| Tricuspid | ------- | FS | | % |
| pulmonic | Mild valvar PS, PPG = 27mmHg. | LVEF | | % |
| Aortic arch | ----- | **Coronary arteries** | |  |
| PDA | No |  | |  |
| Final Diagnosis | 1. {S, D, S} Levocardia 2. Mild Valvar PS 3. Doming Pulmonary Valve 4. Good Biventricular Function | | | |
| Done By: | **Signature** | | **Date of Reporting** | |
| Dr. Tesfaye Taye, Pediatric Cardiologist |  | | 17/07/2012Eth.C | |

| Echocardiography report  Patient Name: Bereket Abebaw Patient ID :041504 Gender: M Age:8 /12  Date of Report: 17/07/2012Eth.C BP: Weight: 6.14 kg Height: 62 cm BSA:  Clinical Finding: RD + Murmur + accentuated P2. TGSH7.3234. | | | | |
| --- | --- | --- | --- | --- |
| Features | **Finding** | **Features** | | **Finding** |
| Profile | | **Atria** | | |
| Abdominal situs | Solitus | Left atrium | | Normal |
| Cardiac position | Levocardia | Right atrium | | Dilated |
| Systemic venous drainage | to RA | **Atrioventricular valves** | | |
| Pulmonary venous drainage | to LA | Mitral valve | | Annulus = 13mm |
| Atrioventricular connection | Concordant | Tricuspid valve | | Annulus = 16mm. Non coapting TVL.  TAPSE = 12mm. |
| Ventriculoarterial connection | Concordant | **Ventricles** | | |
| Ventricular loop | d-Loop | Left ventricle | | Normal |
| Septae | | Right ventricle | | Dilated |
| Interventricular septum | Intact | **M-Mode:** | | |
| Interatrial septum | Intact | AO | |  |
| Great arteries | NRGA | LA | |  |
| Aorta | ----- | LVIDd | | mm |
| Pulmonary artery | MPA = 8mm. normal Branch PAs. | LVIDs | | mm |
| Semilunal valves |  | IVSd | | mm |
| Aortic valve | Annulus = 9mm | IVSs | | mm |
| Pulmonary valve | Annulus = 9mm | LVPWd | | mm |
| Doppler Measurement | | LVPWs | | mm |
| Mitral | ------ | EDV | | ml |
| Aortic | ------- | ESV | | ml |
| Tricuspid | Severe TR, PPG = 62mmHg | FS | | 31% |
| pulmonic | --------- | LVEF | | 61% |
| Aortic arch | ----- | **Coronary arteries** | |  |
| PDA | No |  | |  |
| Final Diagnosis | 1. {S, D, S} Levocardia 2. RA/RV Dilated 3. Non Coapting TVL 4. Severe TR 5. ?Severe Pulmonary Hypertension | | | |
| Recommendation: | Please correlate with the clinical feature and other imaging findings | | | |
| Done By: | **Signature** | | **Date of Reporting** | |
| Dr. Tesfaye Taye, Pediatric Cardiologist |  | | 17/07/2012Eth.C | |

| Echocardiography report  Patient Name: Gizachew Dessie Patient ID: 042539 Gender: M Age : 1 year 1/12  Date of Report: 01/08/2012Eth.C BP: Weight: 8 kg Height: 71 cm BSA:  Clinical Finding: Cardiomegaly on CXR + Sepsis + RD. TGSH7.3235. | | | | |
| --- | --- | --- | --- | --- |
| Features | **Finding** | **Features** | | **Finding** |
| Profile | | **Atria** | | |
| Abdominal situs | Solitus | Left atrium | | Normal |
| Cardiac position | Levocardia | Right atrium | | Normal |
| Systemic venous drainage | to RA | **Atrioventricular valves** | | |
| Pulmonary venous drainage | to LA | Mitral valve | | ---- |
| Atrioventricular connection | Concordant | Tricuspid valve | | ---- |
| Ventriculoarterial connection | Concordant | **Ventricles** | | |
| Ventricular loop | d-Loop | Left ventricle | | Normal |
| Septae | | Right ventricle | | Normal |
| Interventricular septum | Intact | **M-Mode:** | | |
| Interatrial septum | Intact | AO | |  |
| Great arteries | NRGA | LA | |  |
| Aorta | ----- | LVIDd | | mm |
| Pulmonary artery | Normal MPA normal Branch PAs. | LVIDs | | mm |
| Semilunal valves |  | IVSd | | mm |
| Aortic valve | Normal valves. No echo pulsus paradoxus. | IVSs | | mm |
| Pulmonary valve | Normal | LVPWd | | mm |
| Doppler Measurement | | LVPWs | | mm |
| Mitral | ------ | EDV | | ml |
| Aortic | ------- | ESV | | ml |
| Tricuspid | ------- | FS | | 39% |
| pulmonic | --------- | LVEF | | 71% |
| Aortic arch | ----- | **Coronary arteries** | |  |
| PDA | No |  | |  |
| Pericardium: | 13mm pericardial effusion on LV Side. 12mm pericardial effusion on RA side. Echodebris inside. | | | |
| Final Diagnosis | 1. {S, D, S} Levocardia 2. Moderate pericardial effusion with echodebris inside 3. No features of Tamponade. 4. Good Function | | | |
| Done By: | **Signature** | | **Date of Reporting** | |
| Dr. Tesfaye Taye, Pediatric Cardiologist |  | | 01/08/2012Eth.C | |

| Echocardiography report/ Follow up Echo  Patient Name: Gizachew Dessie Patient ID: 042539 Gender: M Age : 1 year 1/12  Date of Report: 15/08/2012Eth.C BP: Weight: 8 kg Height: 71 cm BSA:  Clinical Finding: _ ________. FOLLOW UP ECHO FOR TGSH7.3235. | | | | |
| --- | --- | --- | --- | --- |
| Features | **Finding** | **Features** | | **Finding** |
| Profile | | **Atria** | | |
| Abdominal situs | Solitus | Left atrium | | Normal |
| Cardiac position | Levocardia | Right atrium | | Normal |
| Systemic venous drainage | to RA | **Atrioventricular valves** | | |
| Pulmonary venous drainage | to LA | Mitral valve | | ANNULUS = 15mm |
| Atrioventricular connection | Concordant | Tricuspid valve | | Annulus = 16mm |
| Ventriculoarterial connection | Concordant | **Ventricles** | | |
| Ventricular loop | d-Loop | Left ventricle | | Normal |
| Septae | | Right ventricle | | Normal |
| Interventricular septum | Intact | **M-Mode:** | | |
| Interatrial septum | Intact | AO | |  |
| Great arteries | NRGA | LA | |  |
| Aorta | ----- | LVIDd | | mm |
| Pulmonary artery | Normal MPA normal Branch PAs. | LVIDs | | mm |
| Semilunal valves |  | IVSd | | mm |
| Aortic valve | Annulus = 10mm | IVSs | | mm |
| Pulmonary valve | Annulus = 14mm | LVPWd | | mm |
| Doppler Measurement | | LVPWs | | mm |
| Mitral | ------ | EDV | | ml |
| Aortic | ------- | ESV | | ml |
| Tricuspid | ------- | FS | | 45% |
| pulmonic | --------- | LVEF | | 78% |
| Aortic arch | ----- | **Coronary arteries** | |  |
| PDA | No |  | |  |
| Pericardium: | 6mm pericardial effusion on LV Side. 2mm pericardial effusion on RV side. | | | |
| Final Diagnosis | 1. {S, D, S} Levocardia 2. Small pericardial effusion 3. Good Function | | | |
| Conclusion: | Improving | | | |
| Done By: | **Signature** | | **Date of Reporting** | |
| Dr. Tesfaye Taye, Pediatric Cardiologist |  | | 15/08/2012Eth.C | |

| Echocardiography report  Patient Name: Welde – Senbet Demelash Patient ID Gender: M Age: 43days.  Date of Report: 15/08/2012Eth.C BP: Weight: Height: cm BSA:  Clinical Finding: Incidental Murmur. TGSH7.3236. | | | | |
| --- | --- | --- | --- | --- |
| Features | **Finding** | **Features** | | **Finding** |
| Profile | | **Atria** | | |
| Abdominal situs | Solitus | Left atrium | | Normal |
| Cardiac position | Levocardia | Right atrium | | Normal |
| Systemic venous drainage | to RA | **Atrioventricular valves** | | |
| Pulmonary venous drainage | to LA | Mitral valve | | Normal |
| Atrioventricular connection | Concordant | Tricuspid valve | | Normal |
| Ventriculoarterial connection | Concordant | **Ventricles** | | |
| Ventricular loop | d-Loop | Left ventricle | | Normal |
| Septae | | Right ventricle | | Normal |
| Interventricular septum | Intact | **M-Mode:** | | |
| Interatrial septum | Intact | AO | |  |
| Great arteries | NRGA | LA | |  |
| Aorta | ----- | LVIDd | | mm |
| Pulmonary artery | Normal MPA normal Branch PAs. | LVIDs | | mm |
| Semilunal valves |  | IVSd | | mm |
| Aortic valve | Normal | IVSs | | mm |
| Pulmonary valve | Normal | LVPWd | | mm |
| Doppler Measurement | | LVPWs | | mm |
| Mitral | ------ | EDV | | ml |
| Aortic | ------- | ESV | | ml |
| Tricuspid | ------- | FS | | % |
| pulmonic | --------- | LVEF | | % |
| Aortic arch | ----- | **Coronary arteries** | |  |
| PDA | 1.5mm PDA, L – R Shunt |  | |  |
| Final Diagnosis | 1. {S, D, S} Levocardia 2. Small PDA, L – R Shunt 3. Good Function | | | |
| Done By: | **Signature** | | **Date of Reporting** | |
| Dr. Tesfaye Taye, Pediatric Cardiologist |  | | 15/08/2012Eth.C. | |

| Echocardiography report  Patient Name: Zemenu Estibel Patient ID: 043180 Gender: M Age : 3 month 24 days  Date of Report: 20/08/2012Eth.C BP: Weight: 3.3 kg Height: 50 cm BSA:  Clinical Finding: Incidental Murmur. TGSH7.3237. | | | | |
| --- | --- | --- | --- | --- |
| Features | **Finding** | **Features** | | **Finding** |
| Profile | | **Atria** | | |
| Abdominal situs | Solitus | Left atrium | | Normal |
| Cardiac position | Levocardia | Right atrium | | Normal |
| Systemic venous drainage | to RA | **Atrioventricular valves** | | |
| Pulmonary venous drainage | to LA | Mitral valve | | Annulus = 13mm |
| Atrioventricular connection | Concordant | Tricuspid valve | | Annulus = 13mm |
| Ventriculoarterial connection | Concordant | **Ventricles** | | |
| Ventricular loop | d-Loop | Left ventricle | | Normal |
| Septae | | Right ventricle | | Normal |
| Interventricular septum | 4mm PM VSD, L – R Shunt | **M-Mode:** | | |
| Interatrial septum | PFO, L – R Shunt | AO | |  |
| Great arteries | NRGA | LA | |  |
| Aorta | ----- | LVIDd | | mm |
| Pulmonary artery | Normal MPA normal Branch PAs. | LVIDs | | mm |
| Semilunal valves |  | IVSd | | mm |
| Aortic valve | Annulus = 12mm | IVSs | | mm |
| Pulmonary valve | Annulus = 11mm | LVPWd | | mm |
| Doppler Measurement | | LVPWs | | mm |
| Mitral | ------ | EDV | | ml |
| Aortic | ------- | ESV | | ml |
| Tricuspid | ------- | FS | | 35% |
| pulmonic | Trivial PR | LVEF | | 66% |
| Aortic arch | ----- | **Coronary arteries** | |  |
| PDA | No |  | |  |
| Final Diagnosis | 1. {S, D, S} Levocardia 2. PFO, L – R Shunt 3. Small Perimembranous VSD, L – R Shunt 4. Good LV Function | | | |
| Done By: | **Signature** | | **Date of Reporting** | |
| Dr. Tesfaye Taye, Pediatric Cardiologist |  | | 20/08/2012Eth.C | |

| Echocardiography report  Patient Name: Abel Anteneh Patient ID: 043275 Gender: M Age :33 Days  Date of Report: 29/08/2012Eth.C BP: Weight: 2.8 kg Height: 48 cm BSA:  Clinical Finding: RD. TGSH7.3238. | | | | |
| --- | --- | --- | --- | --- |
| Features | **Finding** | **Features** | | **Finding** |
| Profile | | **Atria** | | |
| Abdominal situs | Solitus | Left atrium | | Normal |
| Cardiac position | Levocardia | Right atrium | | Normal |
| Systemic venous drainage | to RA | **Atrioventricular valves** | | |
| Pulmonary venous drainage | to LA | Mitral valve | | Annulus = 10mm |
| Atrioventricular connection | Concordant | Tricuspid valve | | Annulus = 12mm |
| Ventriculoarterial connection | Concordant | **Ventricles** | | |
| Ventricular loop | d-Loop | Left ventricle | | Normal |
| Septae | | Right ventricle | | Normal |
| Interventricular septum | Intact | **M-Mode: Normal LV Function.** | | |
| Interatrial septum | Intact | AO | |  |
| Great arteries | NRGA | LA | |  |
| Aorta | ----- | LVIDd | | mm |
| Pulmonary artery | Normal MPA normal Branch PAs. | LVIDs | | mm |
| Semilunal valves |  | IVSd | | mm |
| Aortic valve | Annulus = 8mm | IVSs | | mm |
| Pulmonary valve | Annulus = 8mm | LVPWd | | mm |
| Doppler Measurement | | LVPWs | | mm |
| Mitral | ------ | EDV | | ml |
| Aortic | ------- | ESV | | ml |
| Tricuspid | ------- | FS | | % |
| pulmonic | --------- | LVEF | | % |
| Aortic arch | ----- | **Coronary arteries** | |  |
| PDA | No |  | |  |
| Final Diagnosis | 1. {S, D, S} Levocardia 2. Normal Echocardiography Study | | | |
| Done By: | **Signature** | | **Date of Reporting** | |
| Dr. Tesfaye Taye, Pediatric Cardiologist |  | | 29/08/2012Eth.C | |

| Echocardiography report  Patient Name: Kelem Abebe Patient ID : 044116 Gender: F Age :11 Years  Date of Report: 06/09/2012Eth.C BP: Weight: 23 kg Height: 139 cm BSA:  Clinical Finding: Rheumatic Recurrence + easy fatigability + Murmur + DOE + CHF + RD. TGSH7.3239. | | | |
| --- | --- | --- | --- |
| Features | **Finding** | **Features** | **Finding** |
| Profile | | **Atria** | |
| Abdominal situs | Solitus | Left atrium | Hugely dilated |
| Cardiac position | Levocardia | Right atrium | Dilated |
| Systemic venous drainage | to RA | **Atrioventricular valves** | |
| Pulmonary venous drainage | to LA | Mitral valve | Annulus = 31mm. Thickened, clubbed and PML is shortened. |
| Atrioventricular connection | Concordant | Tricuspid valve | Annulus = mm  TAPSE = 20mm. |
| Ventriculoarterial connection | Concordant | **Ventricles** | |
| Ventricular loop | d-Loop | Left ventricle | Hugely dilated |
| Septae | | RV | Dilated |
| Interventricular septum | Intact | **M-Mode:** | |
| Interatrial septum | Intact | AO |  |
| Great arteries | NRGA | LA |  |
| Aorta | ----- | LVIDd | mm |
| Pulmonary artery | MPA =29mmHg. | LVIDs | mm |
| Semilunal valves |  | IVSd | mm |
| Aortic valve | Annulus = 17mm | IVSs | mm |
| Pulmonary valve | Annulus = 24mm | LVPWd | mm |
| Doppler Measurement | | LVPWs | mm |
| Mitral | Severe MR, Holosystolic, Posterior projection with Jet velocity = 5m/sec. Severe MS, PPG/MPG = 35/17mmHg. | EDV | ml |
| Aortic | Moderate AR, PHT = 308cm/sec | ESV | ml |
| Tricuspid | Severe TR, PPG = 45mmHg. ?TS | FS | 25% |
| pulmonic | --------- | LVEF | 48% |
| Additional information | 12mm pericardial effusion on LA/LV Side, clear | | |
| Final Diagnosis | 1. {S, D, S} Levocardia 2. All chambers Dilated 3. Severe MR 4. Severe MS 5. Severe TR 6. Moderate AR 7. Severe pulmonary Hypertension 8. Reduced LV Function 9. Pericardial effusion | | |
| Done By: | **Signature** | **Date of Reporting** | |
| Dr. Tesfaye Taye, Pediatric Cardiologist |  | 06/09/2012Eth.C | |

| Echocardiography report  Patient Name: Mekides Melkamu Patient ID: 043757 Gender: F Age : 5 Years  Date of Report: 06/09/2012Eth.C BP: Weight: 15 kg Height: 89 cm BSA:  Clinical Finding: DOE + Murmur + CHF + Easy Fatigability. TGSH7.3240. | | | | |
| --- | --- | --- | --- | --- |
| Features | **Finding** | **Features** | | **Finding** |
| Profile | | **Atria** | | |
| Abdominal situs | Solitus | Left atrium | | Normal |
| Cardiac position | Levocardia | Right atrium | | Hugely Dilated |
| Systemic venous drainage | to RA | **Atrioventricular valves** | | |
| Pulmonary venous drainage | to LA | Mitral valve | | Annulus = 15mm |
| Atrioventricular connection | Concordant | Tricuspid valve | | Annulus = 28mm |
| Ventriculoarterial connection | Concordant | **Ventricles** | | |
| Ventricular loop | d-Loop | Left ventricle | | Normal |
| Septae | | Right ventricle | | Hugely Dilated and Dysfunctional |
| Interventricular septum | Intact | **M-Mode:** | | |
| Interatrial septum | Intact | AO | |  |
| Great arteries | NRGA | LA | |  |
| Aorta | ----- | LVIDd | | mm |
| Pulmonary artery | Normal MPA normal Branch PAs. | LVIDs | | mm |
| Semilunal valves |  | IVSd | | mm |
| Aortic valve | Annulus = 13mm | IVSs | | mm |
| Pulmonary valve | Annulus = 11mm | LVPWd | | mm |
| Doppler Measurement | | LVPWs | | mm |
| Mitral | ------ | EDV | | ml |
| Aortic | ------- | ESV | | ml |
| Tricuspid | Severe TR | FS | | 30% |
| pulmonic | Severe PS, PPG = 120mmHg | LVEF | | 61% |
| Aortic arch | ----- | **Coronary arteries** | |  |
| PDA | No |  | |  |
| Final Diagnosis | 1. {S, D, S} Levocardia 2. RA/RV Dilated 3. Severe TR 4. Severe PS 5. Dysfunctional RV | | | |
| Done By: | **Signature** | | **Date of Reporting** | |
| Dr. Tesfaye Taye, Pediatric Cardiologist |  | | 06/09/2012Eth.C | |

| Echocardiography report  Patient Name: B/ Be’emnet Mekete Patient ID:044473 Gender: M Age :13 Days  Date of Report: 11/09/2012Eth.C BP: Weight: 3.4 kg Height:36 cm BSA: (TGSH4.2971) | | | | |
| --- | --- | --- | --- | --- |
| Features | **Finding** | **Features** | | **Finding** |
| Profile | | **Atria** | | |
| Abdominal situs | Solitus | Left atrium | | Normal |
| Cardiac position | Levocardia | Right atrium | | Normal |
| Systemic venous drainage | to RA | **Atrioventricular valves** | | |
| Pulmonary venous drainage | to LA | Mitral valve | | Annulus = 9mm |
| Atrioventricular connection | Concordant | Tricuspid valve | | Annulus = 11mm  TAPSE = 11mm. |
| Ventriculoarterial connection | Concordant | **Ventricles** | | |
| Ventricular loop | d-Loop | Left ventricle | | Normal |
| Septae | | Right ventricle | | Normal |
| Interventricular septum | 5mm PM VSD, L – R Shunt | **M-Mode:** | | |
| Interatrial septum | 4mm OS ASD, L – R Shunt | AO | |  |
| Great arteries | NRGA | LA | |  |
| Aorta | ----- | LVIDd | | mm |
| Pulmonary artery | Normal MPA normal Branch PAs. | LVIDs | | mm |
| Semilunal valves |  | IVSd | | mm |
| Aortic valve | Annulus = 9mm | IVSs | | mm |
| Pulmonary valve | Annulus = 10mm | LVPWd | | mm |
| Doppler Measurement | | LVPWs | | mm |
| Mitral | ------ | EDV | | ml |
| Aortic | ------- | ESV | | ml |
| Tricuspid | ------- | FS | | 29% |
| pulmonic | --------- | LVEF | | 58% |
| Aortic arch | ----- | **Coronary arteries** | |  |
| PDA | No |  | |  |
| Final Diagnosis | 1. {S, D, S} Levocardia 2. Small OS ASD, L – R Shunt 3. Small Perimembranous VSD, L – R Shunt 4. Good Biventricular Function | | | |
| Done By: | **Signature** | | **Date of Reporting** | |
| Dr. Tesfaye Taye, Pediatric Cardiologist |  | | 11/09/2012Eth.C | |

| Echocardiography report  Patient Name: B/ Yirbeb Lingerew Patient ID: 044253 Gender: F Age 7day  Date of Report: 11/09/2012Eth.C BP: Weight: 1.2kg Height: 40 cm BSA:  Clinical Finding: Incidental Murmur. TGSH7.3241. | | | | |
| --- | --- | --- | --- | --- |
| Features | **Finding** | **Features** | | **Finding** |
| Profile | | **Atria** | | |
| Abdominal situs | Solitus | Left atrium | | Normal |
| Cardiac position | Levocardia | Right atrium | | Normal |
| Systemic venous drainage | to RA | **Atrioventricular valves** | | |
| Pulmonary venous drainage | to LA | Mitral valve | | Annulus = 9mm |
| Atrioventricular connection | Concordant | Tricuspid valve | | Annulus = 11mm |
| Ventriculoarterial connection | Concordant | **Ventricles** | | |
| Ventricular loop | d-Loop | Left ventricle | | Normal |
| Septae | | Right ventricle | | Normal |
| Interventricular septum | 3mm PM VSD, L – R Shunt | **M-Mode:** | | |
| Interatrial septum | 5mm OS ASD,L – R Shunt | AO | |  |
| Great arteries | NRGA | LA | |  |
| Aorta | ----- | LVIDd | | mm |
| Pulmonary artery | Normal MPA normal Branch PAs. | LVIDs | | mm |
| Semilunal valves |  | IVSd | | mm |
| Aortic valve | Annulus = 8mm | IVSs | | mm |
| Pulmonary valve | Annulus = 7mm | LVPWd | | mm |
| Doppler Measurement | | LVPWs | | mm |
| Mitral | ------ | EDV | | ml |
| Aortic | ------- | ESV | | ml |
| Tricuspid | ------- | FS | | 30% |
| pulmonic | --------- | LVEF | | 62% |
| Aortic arch | ----- | **Coronary arteries** | |  |
| PDA |  | | | |
| Final Diagnosis | 1. {S, D, S} Levocardia 2. Small OS ASD, L – R Shunt 3. Small Perimembranous VSD, L – R Shunt 4. Good ventricular Function | | | |
| Remark: |  | | | |
| Recommendation: |  | | | |
| Done By: | **Signature** | | **Date of Reporting** | |
| Dr. Tesfaye Taye, Pediatric Cardiologist |  | | 11/09/2012Eth.C | |

| Echocardiography report  Patient Name: Dawit Molalign Patient ID:021040 Gender: M Age :4 Years  Date of Report: 13/09/2012Eth.C BP: Weight: 18 kg Height: 101 cm BSA:  TGSH1.2628. AND TGSH4 | | | | |
| --- | --- | --- | --- | --- |
| Follow up Echocardiography | | | | |
| Features | **Finding** | **Features** | | **Finding** |
| Profile | | **Atria** | | |
| Abdominal situs | Solitus | Left atrium | | Normal |
| Cardiac position | Levocardia | Right atrium | | Normal |
| Systemic venous drainage | to RA | **Atrioventricular valves** | | |
| Pulmonary venous drainage | to LA | Mitral valve | | Annulus = 21mm. Mildly thickened MVL |
| Atrioventricular connection | Concordant | Tricuspid valve | | Annulus = 20mm.  TAPSE = 23mm |
| Ventriculoarterial connection | Concordant | **Ventricles** | | |
| Ventricular loop | d-Loop | Left ventricle | | Normal |
| Septae | | Right ventricle | | Normal |
| Interventricular septum | Intact | **M-Mode:** | | |
| Interatrial septum | Intact | AO | |  |
| Great arteries | NRGA | LA | |  |
| Aorta | ----- | LVIDd | | mm |
| Pulmonary artery | Normal MPA normal Branch PAs. | LVIDs | | mm |
| Semilunal valves |  | IVSd | | mm |
| Aortic valve | Annulus = 16mm | IVSs | | mm |
| Pulmonary valve | Annulus = 20mm | LVPWd | | mm |
| Doppler Measurement | | LVPWs | | mm |
| Mitral | Trivial to Mild MR, Posterior projection, velocity = 3.5m/sec | EDV | | ml |
| Aortic | ------- | ESV | | ml |
| Tricuspid | ------- | FS | | 34% |
| pulmonic | --------- | LVEF | | 63% |
| Aortic arch | ----- | **Coronary arteries** | |  |
| PDA | No |  | |  |
| Final Diagnosis | 1. {S, D, S} Levocardia 2. Trivial to Mild MR 3. Mildly thickened MVL 4. Good Biventricular Function | | | |
| Done By: | **Signature** | | **Date of Reporting** | |
| Dr. Tesfaye Taye, Pediatric Cardiologist |  | | 13/09/2012Eth.C | |

| Echocardiography report  Patient Name: Mohammed Kemal Patient ID: 044668 Gender: M Age : 7 month  Date of Report: 13/09/2012Eth.C BP: Weight: 7.5 kg Height: 65 cm BSA:  Clinical Finding: _CHF + RD. TGSH7.3242. | | | | |
| --- | --- | --- | --- | --- |
| Features | **Finding** | **Features** | | **Finding** |
| Profile | | **Atria** | | |
| Abdominal situs | Solitus | Left atrium | | Dilated |
| Cardiac position | Levocardia | Right atrium | | Normal |
| Systemic venous drainage | to RA | **Atrioventricular valves** | | |
| Pulmonary venous drainage | to LA | Mitral valve | | Annulus = 16mm |
| Atrioventricular connection | Concordant | Tricuspid valve | | Annulus = 12mm  TAPSE = 13mm. |
| Ventriculoarterial connection | Concordant | **Ventricles** | | |
| Ventricular loop | d-Loop | Left ventricle | | Globularly dilated & dysfunctional |
| Septae | | Right ventricle | | Normal |
| Interventricular septum | Intact | **M-Mode:** | | |
| Interatrial septum | Intact | AO | |  |
| Great arteries | NRGA | LA | |  |
| Aorta | ----- | LVIDd | | mm |
| Pulmonary artery | Normal MPA normal Branch PAs. | LVIDs | | mm |
| Semilunal valves |  | IVSd | | mm |
| Aortic valve | Annulus = 13mm | IVSs | | mm |
| Pulmonary valve | Annulus = 15mm | LVPWd | | mm |
| Doppler Measurement | | LVPWs | | mm |
| Mitral | Moderate MR | EDV | | ml |
| Aortic | ------- | ESV | | ml |
| Tricuspid | ------- | FS | | 14% |
| pulmonic | --------- | LVEF | | 29% |
| Aortic arch | ----- | **Coronary arteries** | |  |
| PDA | No |  | |  |
| Final Diagnosis | 1. {S, D, S} Levocardia 2. Dilated LA 3. Moderate MR 4. Dilated, Dysfunctional LV | | | |
| Done By: | **Signature** | | **Date of Reporting** | |
| Dr. Tesfaye Taye, Pediatric Cardiologist |  | | 13/09/2012Eth.C | |

| Echocardiography report  Patient Name: Bizayehu Belete Patient ID: 044724 Gender: F Age: 37 Days  Date of Report: 13/09/2012Eth.C BP: Weight: 3 kg Height: 51 cm BSA:  Clinical Finding: Cyanosis + Murmur. TGSH7.3243. | | | | |
| --- | --- | --- | --- | --- |
| Features | **Finding** | **Features** | | **Finding** |
| Profile | | **Atria** | | |
| Abdominal situs | Solitus | Left atrium | | Normal |
| Cardiac position | Levocardia | Right atrium | | Dilated |
| Systemic venous drainage | to RA | **Atrioventricular valves** | | |
| Pulmonary venous drainage | to LA | Mitral valve | | Annulus = 7mm. small valve opening measuring = 3mm |
| Atrioventricular connection | Concordant | Tricuspid valve | | Annulus = 11mm.  TAPSE = 6mm |
| Ventriculoarterial connection | DORV | **Ventricles** | | |
| Ventricular loop | d-Loop | Left ventricle | | Smallish |
| Septae | | Right ventricle | | Dilated, Dysfunctional and Hypertrophied |
| Interventricular septum | 7mm Sub-arterial VSD, BD Shunt | **M-Mode:** Normal LV Function on eye balling | | |
| Interatrial septum | 14mm OS ASD, L – R Shunt | AO | |  |
| Great arteries | NRGA | LA | |  |
| Aorta | Anterior & arising from RV. | LVIDd | | mm |
| Pulmonary artery | Normal MPA normal Branch PAs. Posterior & arising from RV | LVIDs | | mm |
| Semilunal valves |  | IVSd | | mm |
| Aortic valve | Annulus = 9mm. Aorto Mitral Discontinuity. | IVSs | | mm |
| Pulmonary valve | Annulus = 10mm | LVPWd | | mm |
| Doppler Measurement | | LVPWs | | mm |
| Mitral | ------ | EDV | | ml |
| Aortic | ------- | ESV | | ml |
| Tricuspid | ------- | FS | | % |
| pulmonic | Mild PS, PPG = 21mmHg. Mild PR, PPG = 27mmHg. | LVEF | | % |
[truncated: 211,819 more chars]
