# Supplementary material for: Two–Dimensional and Doppler trans-thoracic echocardiographic patterns of suspected pediatric heart diseases at Tibebe-—Ghion specialized Teaching Hospital and Adinas General Hospital, Bahir Dar, North-west Ethiopia:–An experience from an LMIC
Source: PLoS One. 2024 Mar 11;19(3):e0292694. doi: 10.1371/journal.pone.0292694 (PMC10927071; doi:10.1371/journal.pone.0292694)
Supplement: S1 File — (ZIP) [file pone.0292694.s002.zip › TGSH10 Pediatric Echo Report 2023 - March - May 30 2015 SPSS FILLED.docx]

| **Tibebe – Ghion Specialized Teaching Hospital, Bahir Dar University,**  **Bahir Dar, Ethiopia** | | | | |
| --- | --- | --- | --- | --- |
| **Name: Yalem-Sira Dessie. Sex/Age: f/9months. MRN: 159815. Date of Report: 21/07/15Eth.C.**  **Referral Diagnosis: Follow up for Large ASD + Cardiac tamponade + SVT. (AGH)** | | | | |
| **Features:** | **Findings** | | **Features** | **Findings** |
| **Profile** | | | **Atria** | |
| Abdominal Situs | Solitus | | Left Atrium | Normal |
| Atrial Situs | Solitus | | Right Atrium | Dilated |
| Cardiac Position | Levocardia | | **Atrio-Ventricular Valves** | |
| Systemic Venous Drainage | To RA | | Mitral Valve | Annulus = 16mm |
| Pulmonary Venous Drainage | To LA | | Tricuspid Valve | Annulus = 22mm |
| Atrio-ventricular Connection | Concordant | |  | TAPSE = 11mm |
| Ventriculo-Arterial Connection | concordant | | **Ventricle** | |
| Ventricular Loop | d-Loop | | Left Ventricle | Normal |
| **Septae** |  | | Right Ventricle | Dilated |
| Interatrial Septum | 14mm OS ASD, BD Shunt | | **Doppler Measurement** |  |
| Interventricular Septum | Intact | | Mitral | ------------- |
| **Semilunar Valves** |  | | Aortic | ------------- |
| Aortic Valve | Annulus = 11mm | | Tricuspid | ------------- |
| Pulmonary Valve | Annulus = 13mm | | Pulmonic | ------------- |
| **Great Arteries** | NRGA | | **Coronary Arteries** |  |
| Aorta |  | | **Aortic Arch** | Left. No CoA |
| Pulmonary Arteries | Normal MPA & BPAs. | | **PDA** | No PDA |
| **M-Mode**: Normal LV Function | | | | |
| Ao | mm | | PWd | mm |
| LA | mm | | EDV | ml |
| LVIDd | mm | | ESV | ml |
| LVIDs | mm | | FS | % |
| IVSd | mm | | LVEF | % |
| **Additional Information:** | | | | |
| **Conclusion:**   1. {S, D, S} Levocardia 2. Large OS ASD, BD Shunt | | | | |
| **Done By:** | | **Signature** | **Date** | **Remark** |
| Tesfaye T., Paediatrician, Paediatric Cardiologist | |  | 21/07/15Eth.C. |  |

| **Tibebe – Ghion Specialized Teaching Hospital, Bahir Dar University,**  **Bahir Dar, Ethiopia** | | | | |
| --- | --- | --- | --- | --- |
| **Name: Brihanu Dejenie. Sex/Age: M/2 6/12. MRN: 110657. Date of Report: 21/07/15Eth.C.**  **Referral Diagnosis: Incidental Murmur. TGSH10.2784** | | | | |
| **Features:** | **Findings** | | **Features** | **Findings** |
| **Profile** | | | **Atria** | |
| Abdominal Situs | Solitus | | Left Atrium | Normal |
| Atrial Situs | Solitus | | Right Atrium | Normal |
| Cardiac Position | Levocardia | | **Atrio-Ventricular Valves** | |
| Systemic Venous Drainage | To RA | | Mitral Valve | Annulus = 19mm |
| Pulmonary Venous Drainage | To LA | | Tricuspid Valve | Annulus = 18mm |
| Atrio-ventricular Connection | Concordant | |  | TAPSE = 19mm |
| Ventriculo-Arterial Connection | concordant | | **Ventricle** | |
| Ventricular Loop | d-Loop | | Left Ventricle | Normal |
| **Septae** |  | | Right Ventricle | Normal |
| Interatrial Septum | Intact | | **Doppler Measurement** |  |
| Interventricular Septum | Intact | | Mitral | ------------- |
| **Semilunar Valves** |  | | Aortic | ------------- |
| Aortic Valve | Annulus = 15mm | | Tricuspid | ------------- |
| Pulmonary Valve | Annulus = 15mm | | Pulmonic | ------------- |
| **Great Arteries** | NRGA | | **Coronary Arteries** |  |
| Aorta |  | | **Aortic Arch** | Left. No CoA |
| Pulmonary Arteries | Normal MPA & BPAs. | | **PDA** | 2mm PDA, L – R Shunt |
| **M-Mode**: | | | | |
| Ao | mm | | PWd | mm |
| LA | mm | | EDV | ml |
| LVIDd | mm | | ESV | ml |
| LVIDs | mm | | FS | 34% |
| IVSd | mm | | LVEF | 63% |
| **Additional Information:** | | | | |
| **Conclusion:**   1. {S, D, S} Levocardia 2. Moderate PDA, L – R Shunt 3. Normal Biventricular Systolic Function | | | | |
| **Done By:** | | **Signature** | **Date** | **Remark** |
| Tesfaye T., Paediatrician, Paediatric Cardiologist | |  | 21/07/15Eth.C. |  |

| **Tibebe – Ghion Specialized Teaching Hospital, Bahir Dar University,**  **Bahir Dar, Ethiopia** | | | | |
| --- | --- | --- | --- | --- |
| **Name: Baby of Tirusew Tadesse. Sex/Age: M/15days. MRN: 170976. Date of Report: 21/07/15Eth.C.**  **Referral Diagnosis: PPHTN. TGSH10.2785** | | | | |
| **Features:** | **Findings** | | **Features** | **Findings** |
| **Profile** | | | **Atria** | |
| Abdominal Situs | Solitus | | Left Atrium | Normal |
| Atrial Situs | Solitus | | Right Atrium | Normal |
| Cardiac Position | Levocardia | | **Atrio-Ventricular Valves** | |
| Systemic Venous Drainage | To RA | | Mitral Valve | Annulus = 10mm |
| Pulmonary Venous Drainage | To LA | | Tricuspid Valve | Annulus = 10mm |
| Atrio-ventricular Connection | Concordant | |  | TAPSE = 11mm |
| Ventriculo-Arterial Connection | concordant | | **Ventricle** | |
| Ventricular Loop | d-Loop | | Left Ventricle | Normal |
| **Septae** |  | | Right Ventricle | Normal |
| Interatrial Septum | Intact | | **Doppler Measurement** |  |
| Interventricular Septum | Intact | | Mitral | ------------- |
| **Semilunar Valves** |  | | Aortic | ------------- |
| Aortic Valve | Annulus = 8mm | | Tricuspid | Trivial TR, PPG = 30mmHg |
| Pulmonary Valve | Annulus = 9mm | | Pulmonic | ------------- |
| **Great Arteries** | NRGA | | **Coronary Arteries** |  |
| Aorta |  | | **Aortic Arch** | Left. No CoA |
| Pulmonary Arteries | Normal MPA & BPAs. | | **PDA** | No PDA |
| **M-Mode**: | | | | |
| Ao | mm | | PWd | mm |
| LA | mm | | EDV | ml |
| LVIDd | mm | | ESV | ml |
| LVIDs | mm | | FS | % |
| IVSd | mm | | LVEF | % |
| **Additional Information:** | | | | |
| **Conclusion:**   1. Normal Echocardiography Study | | | | |
| **Remark:** No features of PPHTN | | | | |
| **Recommendation:** | | | | |
| **Done By:** | | **Signature** | **Date** | **Remark** |
| Tesfaye T., Paediatrician, Paediatric Cardiologist | |  | 21/07/15Eth.C. |  |

| **Tibebe – Ghion Specialized Teaching Hospital, Bahir Dar University,**  **Bahir Dar, Ethiopia** | | | | |
| --- | --- | --- | --- | --- |
| **Name: Tesfahun Amare. Sex/Age: M/10years. MRN: 173328. Date of Report: 21/07/15 Eth.C.**  **Referral Diagnosis: Sydenham’s Chorea. TGSH10.2786** | | | | |
| **Features:** | **Findings** | | **Features** | **Findings** |
| **Profile** | | | **Atria** | |
| Abdominal Situs | Solitus | | Left Atrium | Normal |
| Atrial Situs | Solitus | | Right Atrium | Normal |
| Cardiac Position | Levocardia | | **Atrio-Ventricular Valves** | |
| Systemic Venous Drainage | To RA | | Mitral Valve | Annulus = 25mm. Thickened MVL |
| Pulmonary Venous Drainage | To LA | | Tricuspid Valve | Annulus = 24mm |
| Atrio-ventricular Connection | Concordant | |  | TAPSE = 19mm |
| Ventriculo-Arterial Connection | concordant | | **Ventricle** | |
| Ventricular Loop | d-Loop | | Left Ventricle | Normal |
| **Septae** |  | | Right Ventricle | Normal |
| Interatrial Septum | Intact | | **Doppler Measurement** |  |
| Interventricular Septum | Intact | | Mitral | Mild MR, Holosystolic, posterior projection, seen in two planes with jet velocity = 4.4m/sec |
| **Semilunar Valves** |  | | Aortic | ------------- |
| Aortic Valve | Annulus = 18mm | | Tricuspid | ------------- |
| Pulmonary Valve | Annulus = 21mm | | Pulmonic | ------------- |
| **Great Arteries** | NRGA | | **Coronary Arteries** |  |
| Aorta |  | | **Aortic Arch** | Left. No CoA |
| Pulmonary Arteries | Normal MPA & BPAs. | | **PDA** | No PDA |
| **M-Mode**: | | | | |
| Ao | mm | | PWd | mm |
| LA | mm | | EDV | ml |
| LVIDd | mm | | ESV | ml |
| LVIDs | mm | | FS | 31% |
| IVSd | mm | | LVEF | 58% |
| **Additional Information:** | | | | |
| **Conclusion:**   1. {S, D, S} Levocardia 2. Thickened MVL 3. Mild MR 4. Normal Biventricular Systolic Function | | | | |
| **Done By:** | | **Signature** | **Date** | **Remark** |
| Tesfaye T., Paediatrician, Paediatric Cardiologist | |  | 21/­­­­­­­­­­­­­­07/15 Eth.C. |  |

| **Tibebe – Ghion Specialized Teaching Hospital, Bahir Dar University,**  **Bahir Dar, Ethiopia** | | | | |
| --- | --- | --- | --- | --- |
| **Name: Yeshwas Andualem. Sex/Age: M/8years. MRN: 173642. Date of Report: 21/07/15Eth.C.**  **Referral Diagnosis: CHF + Rheumatic Recurrence. TGSH10.2787** | | | | |
| **Features:** | **Findings** | | **Features** | **Findings** |
| **Profile** | | | **Atria** | |
| Abdominal Situs | Solitus | | Left Atrium | Markedly Dilated |
| Atrial Situs | Solitus | | Right Atrium | Normal |
| Cardiac Position | Levocardia | | **Atrio-Ventricular Valves** | |
| Systemic Venous Drainage | To RA | | Mitral Valve | Annulus = 27mm. Thickened MVL |
| Pulmonary Venous Drainage | To LA | | Tricuspid Valve | Annulus = 18mm |
| Atrio-ventricular Connection | Concordant | |  | TAPSE = 19mm |
| Ventriculo-Arterial Connection | concordant | | **Ventricle** | |
| Ventricular Loop | d-Loop | | Left Ventricle | Markedly Dilated |
| **Septae** |  | | Right Ventricle | Normal |
| Interatrial Septum | Intact | | **Doppler Measurement** |  |
| Interventricular Septum | Intact | | Mitral | Severe MR. Holosystolic, Posterior projection, seen in two planes with jet velocity = 4.7m/sec |
| **Semilunar Valves** |  | | Aortic | ------------- |
| Aortic Valve | Annulus = 16mm | | Tricuspid | Trivial TR, PPG = 26mmHg |
| Pulmonary Valve | Annulus = 19mm | | Pulmonic | ------------- |
| **Great Arteries** | NRGA | | **Coronary Arteries** |  |
| Aorta |  | | **Aortic Arch** | Left. No CoA |
| Pulmonary Arteries | Normal MPA & BPAs. | | **PDA** | No PDA |
| **M-Mode**: | | | | |
| Ao | mm | | PWd | mm |
| LA | mm | | EDV | ml |
| LVIDd | mm | | ESV | ml |
| LVIDs | mm | | FS | 30% |
| IVSd | mm | | LVEF | 56% |
| **Additional Information:** Pericardial effusion with maximum depth of 6mm on RA side. | | | | |
| **Conclusion:**   1. {S, D, S} Levocardia 2. LA/LV Markedly Dilated 3. Thickened MVL 4. Severe MR 5. Normal Biventricular Systolic Function | | | | |
| **Recommendation:** | | | | |
| **Done By:** | | **Signature** | **Date** | **Remark** |
| Tesfaye T., Paediatrician, Paediatric Cardiologist | |  | 21/ ­­­­­­­­­­­­­­­07/15Eth.C. |  |

| **Tibebe – Ghion Specialized Teaching Hospital, Bahir Dar University,**  **Bahir Dar, Ethiopia** | | | | |
| --- | --- | --- | --- | --- |
| **Name: Baby of Mekdes Asefaw. Sex/Age: F/80hrs. MRN: 172931. Date of Report: 21/07/15Eth.C.**  **Referral Diagnosis: Cardiomegaly on CXR. TGSH10.2788** | | | | |
| **Features:** | **Findings** | | **Features** | **Findings** |
| **Profile** | | | **Atria** | |
| Abdominal Situs | Solitus | | Left Atrium | Normal |
| Atrial Situs | Solitus | | Right Atrium | Normal |
| Cardiac Position | Levocardia | | **Atrio-Ventricular Valves** | |
| Systemic Venous Drainage | To RA | | Mitral Valve | Annulus = 11mm |
| Pulmonary Venous Drainage | To LA | | Tricuspid Valve | Annulus = 11mm |
| Atrio-ventricular Connection | Concordant | |  |  |
| Ventriculo-Arterial Connection | concordant | | **Ventricle** | |
| Ventricular Loop | d-Loop | | Left Ventricle | Normal |
| **Septae** |  | | Right Ventricle | Normal |
| Interatrial Septum | 6 X 8mm OS ASD, L – R Shunt | | **Doppler Measurement** |  |
| Interventricular Septum | Intact | | Mitral | ------------- |
| **Semilunar Valves** |  | | Aortic | ------------- |
| Aortic Valve | Annulus = 9mm | | Tricuspid | ------------- |
| Pulmonary Valve | Annulus = 8mm | | Pulmonic | ------------- |
| **Great Arteries** | NRGA | | **Coronary Arteries** |  |
| Aorta |  | | **Aortic Arch** | Left. No CoA |
| Pulmonary Arteries | Normal MPA & BPAs. | | **PDA** | No PDA |
| **M-Mode**: Normal LV Function on eye balling | | | | |
| Ao | mm | | PWd | mm |
| LA | mm | | EDV | ml |
| LVIDd | mm | | ESV | ml |
| LVIDs | mm | | FS | % |
| IVSd | mm | | LVEF | % |
| **Additional Information:** | | | | |
| **Conclusion:**   1. {S, D, S} Levocardia 2. Moderate OS ASD, L – R Shunt | | | | |
| **Done By:** | | **Signature** | **Date** | **Remark** |
| Tesfaye T., Paediatrician, Paediatric Cardiologist | |  | 21/ ­­07­­­­­­­­­­­­­­/15Eth.C. |  |

| **Tibebe – Ghion Specialized Teaching Hospital, Bahir Dar University,**  **Bahir Dar, Ethiopia** | | | | | |
| --- | --- | --- | --- | --- | --- |
| **Name: Baby of Etenat Demele . Sex/Age: M/9days. MRN: 173893. Date of Report: 26 / 07 /15 Eth.C.**  **Referral Diagnosis: Incidental Murmur. TGSH10.2789** | | | | | |
| **Features:** | | **Findings** | | **Features** | **Findings** |
| **Profile** | | | | **Atria** | |
| Abdominal Situs | Solitus | | | Left Atrium | Normal |
| Atrial Situs | Solitus | | | Right Atrium | Normal |
| Cardiac Position | Levocardia | | | **Atrio-Ventricular Valves** | |
| Systemic Venous Drainage | To RA | | | Mitral Valve | Annulus = 12mm |
| Pulmonary Venous Drainage | To LA | | | Tricuspid Valve | Annulus = 12mm |
| Atrio-ventricular Connection | Concordant | | |  | TAPSE = 10mm |
| Ventriculo-Arterial Connection | concordant | | | **Ventricle** | |
| Ventricular Loop | d-Loop | | | Left Ventricle | Normal |
| **Septae** |  | | | Right Ventricle | Hypertrophied. |
| Interatrial Septum | PFO, R – L Shunt. | | | **Doppler Measurement** |  |
| Interventricular Septum | Intact | | | Mitral | ------------- |
| **Semilunar Valves** |  | | | Aortic | ------------- |
| Aortic Valve | Annulus = 8mm | | | Tricuspid | ------------- |
| Pulmonary Valve | Annulus = 8mm. Dysplastic PVL | | | Pulmonic | Severe Valvular PS, PPG = 89mmHg |
| **Great Arteries** | NRGA | | | **Coronary Arteries** |  |
| Aorta |  | | | **Aortic Arch** | Left. No CoA |
| Pulmonary Arteries | Normal MPA & BPAs. | | | **PDA** | No PDA |
| **M-Mode**: | | | | | |
| Ao | | mm | | PWd | mm |
| LA | | mm | | EDV | ml |
| LVIDd | | mm | | ESV | ml |
| LVIDs | | mm | | FS | % |
| IVSd | | mm | | LVEF | % |
| **Additional Information:** | | | | | |
| **Conclusion:**   1. {S, D, S} Levocardia 2. PFO, R – L Shunt 3. Hypertrophied RV 4. Dysplastic Pulmonary Valve Leaflet (PVL) 5. Severe PS | | | | | |
| **Recommendation:** Needs Urgent RVOTO Relief. | | | | | |
| **Done By:** | | | **Signature** | **Date** | **Remark** |
| Tesfaye T., Paediatrician, Paediatric Cardiologist | | |  | 26 / ­­­­­­­­­­­­­­­07/15Eth.C. |  |

| **Tibebe – Ghion Specialized Teaching Hospital, Bahir Dar University,**  **Bahir Dar, Ethiopia** | | | | |
| --- | --- | --- | --- | --- |
| **Name: Tirumar Mitiku Sex/Age: F/10years. MRN: 174371. Date of Report: 26/ 07 / 15Eth.C.**  **Referral Diagnosis: ARF + Sydenhams Chorea. TGSH10.2790** | | | | |
| **Features:** | **Findings** | | **Features** | **Findings** |
| **Profile** | | | **Atria** | |
| Abdominal Situs | Solitus | | Left Atrium | Normal |
| Atrial Situs | Solitus | | Right Atrium | Normal |
| Cardiac Position | Levocardia | | **Atrio-Ventricular Valves** | |
| Systemic Venous Drainage | To RA | | Mitral Valve | Annulus = 19mm. Patulous MVL. |
| Pulmonary Venous Drainage | To LA | | Tricuspid Valve | Annulus = 20mm |
| Atrio-ventricular Connection | Concordant | |  | TAPSE = 16mm |
| Ventriculo-Arterial Connection | concordant | | **Ventricle** | |
| Ventricular Loop | d-Loop | | Left Ventricle | Normal |
| **Septae** |  | | Right Ventricle | Normal |
| Interatrial Septum | Intact | | **Doppler Measurement** |  |
| Interventricular Septum | Intact | | Mitral | Mild MR, Incomplete Signal, seen in two planes with jet velocity = 4,4m/sec. |
| **Semilunar Valves** |  | | Aortic | ------------- |
| Aortic Valve | Annulus = 16mm | | Tricuspid | ------------- |
| Pulmonary Valve | Annulus = 18mm | | Pulmonic | ------------- |
| **Great Arteries** | NRGA | | **Coronary Arteries** |  |
| Aorta |  | | **Aortic Arch** | Left. No CoA |
| Pulmonary Arteries | Normal MPA & BPAs. | | **PDA** | No PDA |
| **M-Mode**: | | | | |
| Ao | mm | | PWd | mm |
| LA | mm | | EDV | ml |
| LVIDd | mm | | ESV | ml |
| LVIDs | mm | | FS | % |
| IVSd | mm | | LVEF | % |
| **Additional Information:** | | | | |
| **Conclusion:**   1. {S, D, S} Levocardia 2. Patulous MVL 3. Mild MR 4. Normal Biventricular Systolic Function | | | | |
| **Done By:** | | **Signature** | **Date** | **Remark** |
| Tesfaye T., Paediatrician, Paediatric Cardiologist | |  | 26 /­­­­­­­­­­­­­­­07 / 15 Eth.C. |  |

| **Tibebe – Ghion Specialized Teaching Hospital, Bahir Dar University,**  **Bahir Dar, Ethiopia** | | | | |
| --- | --- | --- | --- | --- |
| **Name: Sefelg Muche . Sex/Age: F /1. 9/12years. MRN: 173606. Date of Report: 26/07 / 15Eth.C.**  **Referral Diagnosis: Down Syndrome + Recurrent Chest Infection. TGSH10.2791** | | | | |
| **Features:** | **Findings** | | **Features** | **Findings** |
| **Profile** | | | **Atria** | |
| Abdominal Situs | Solitus | | Left Atrium | Normal |
| Atrial Situs | Solitus | | Right Atrium | Normal |
| Cardiac Position | Levocardia | | **Atrio-Ventricular Valves** | |
| Systemic Venous Drainage | To RA | | Mitral Valve | Annulus = 15mm |
| Pulmonary Venous Drainage | To LA | | Tricuspid Valve | Annulus = 15mm |
| Atrio-ventricular Connection | Concordant | |  | TAPSE = 16mm |
| Ventriculo-Arterial Connection | concordant | | **Ventricle** | |
| Ventricular Loop | d-Loop | | Left Ventricle | Normal |
| **Septae** |  | | Right Ventricle | Normal |
| Interatrial Septum | Intact | | **Doppler Measurement** |  |
| Interventricular Septum | 6mm PM VSD, L – R Shunt | | Mitral | ------------- |
| **Semilunar Valves** |  | | Aortic | ------------- |
| Aortic Valve | Annulus = 13mm | | Tricuspid | ------------- |
| Pulmonary Valve | Annulus = 14mm | | Pulmonic | ------------- |
| **Great Arteries** | NRGA | | **Coronary Arteries** |  |
| Aorta |  | | **Aortic Arch** | Left. No CoA |
| Pulmonary Arteries | Normal MPA & BPAs. | | **PDA** | No PDA |
| **M-Mode**: Normal LV Function (On eye balling) | | | | |
| Ao | mm | | PWd | mm |
| LA | mm | | EDV | ml |
| LVIDd | mm | | ESV | ml |
| LVIDs | mm | | FS | % |
| IVSd | mm | | LVEF | % |
| **Additional Information:** | | | | |
| **Conclusion:**   1. {S, D, S} Levocardia 2. Small PM VSD, L – R Shunt 3. Normal Biventricular Systolic Function | | | | |
| **Remark:** Child was crying during study | | | | |
| **Done By:** | | **Signature** | **Date** | **Remark** |
| Tesfaye T., Paediatrician, Paediatric Cardiologist | |  | 26 / 07/2015 Eth.C. |  |

| **Tibebe – Ghion Specialized Teaching Hospital, Bahir Dar University,**  **Bahir Dar, Ethiopia** | | | | |
| --- | --- | --- | --- | --- |
| **Name: B/ Yeshmebet Semeneh _. Sex/Age: M/12 Days. MRN: 173415_. Date of Report: 26/ 07/15 Eth.C.**  **Referral Diagnosis: ?Syndromic + incidental Murmur. TGSH10.2792** | | | | |
| **Features:** | **Findings** | | **Features** | **Findings** |
| **Profile** | | | **Atria** | |
| Abdominal Situs | Solitus | | Left Atrium | Normal |
| Atrial Situs | Solitus | | Right Atrium | Normal |
| Cardiac Position | Levocardia | | **Atrio-Ventricular Valves** | |
| Systemic Venous Drainage | To RA | | Mitral Valve | Annulus = 9mm |
| Pulmonary Venous Drainage | To LA | | Tricuspid Valve | Annulus = 9mm |
| Atrio-ventricular Connection | Concordant | |  | TAPSE = 14mm |
| Ventriculo-Arterial Connection | concordant | | **Ventricle** | |
| Ventricular Loop | d-Loop | | Left Ventricle | Normal |
| **Septae** |  | | Right Ventricle | Normal |
| Interatrial Septum | 6mm OS ASD, L – R Shunt | | **Doppler Measurement** |  |
| Interventricular Septum | Intact | | Mitral | ------------- |
| **Semilunar Valves** |  | | Aortic | ------------- |
| Aortic Valve | Annulus = 7mm | | Tricuspid | ------------- |
| Pulmonary Valve | Annulus = 9mm | | Pulmonic | ------------- |
| **Great Arteries** | NRGA | | **Coronary Arteries** |  |
| Aorta |  | | **Aortic Arch** | Left. No CoA |
| Pulmonary Arteries | Normal MPA & BPAs. | | **PDA** | No PDA |
| **M-Mode**: | | | | |
| Ao | mm | | PWd | mm |
| LA | mm | | EDV | ml |
| LVIDd | mm | | ESV | ml |
| LVIDs | mm | | FS | % |
| IVSd | mm | | LVEF | % |
| **Additional Information:** | | | | |
| **Conclusion:**   1. {S, D, S} Levocardia 2. Small OS ASD, L – R Shunt | | | | |
| **Remark:** | | | | |
| **Recommendation:** | | | | |
| **Done By:** | | **Signature** | **Date** | **Remark** |
| Tesfaye T., Paediatrician, Paediatric Cardiologist | |  | 26/07­­­­­­­­­­­­­/15Eth.C. |  |

| **Tibebe – Ghion Specialized Teaching Hospital, Bahir Dar University,**  **Bahir Dar, Ethiopia** | | | | |
| --- | --- | --- | --- | --- |
| **Name: Baby of Adelash Wale. Sex/Age: M/26days. MRN: 172641. Date of Report: 26/07/15Eth.C.**  **Referral Diagnosis: Incidental Murmur Finding + Down Syndrome. TGSH10.2793** | | | | |
| **Features:** | **Findings** | | **Features** | **Findings** |
| **Profile** | | | **Atria** | |
| Abdominal Situs | Solitus | | Left Atrium | Normal |
| Atrial Situs | Solitus | | Right Atrium | Normal |
| Cardiac Position | Levocardia | | **Atrio-Ventricular Valves** | |
| Systemic Venous Drainage | To RA | | Mitral Valve | Annulus = 11mm |
| Pulmonary Venous Drainage | To LA | | Tricuspid Valve | Annulus = 10mm |
| Atrio-ventricular Connection | Concordant | |  |  |
| Ventriculo-Arterial Connection | concordant | | **Ventricle** | |
| Ventricular Loop | d-Loop | | Left Ventricle | Normal |
| **Septae** |  | | Right Ventricle | Normal |
| Interatrial Septum | 5mm OS ASD, L – R Shunt | | **Doppler Measurement** |  |
| Interventricular Septum | Intact | | Mitral | Trivial MR, |
| **Semilunar Valves** |  | | Aortic | ------------- |
| Aortic Valve | Annulus = 10mm | | Tricuspid | ------------- |
| Pulmonary Valve | Annulus = 10mm | | Pulmonic | ------------- |
| **Great Arteries** | NRGA | | **Coronary Arteries** |  |
| Aorta |  | | **Aortic Arch** | Left. No CoA |
| Pulmonary Arteries | Normal MPA & BPAs. | | **PDA** | 1mm PDA, L – R Shunt |
| **M-Mode**: Normal LV Function on eye balling. | | | | |
| Ao | mm | | PWd | mm |
| LA | mm | | EDV | ml |
| LVIDd | mm | | ESV | ml |
| LVIDs | mm | | FS | % |
| IVSd | mm | | LVEF | % |
| **Additional Information:** | | | | |
| **Conclusion:**   1. {S, D, S} Levocardia 2. Small OS ASD, L – R Shunt 3. Small PDA, L – R Shunt | | | | |
| **Remark:** | | | | |
| **Recommendation:** | | | | |
| **Done By:** | | **Signature** | **Date** | **Remark** |
| Tesfaye T., Paediatrician, Paediatric Cardiologist | |  | 26/07/15Eth.C. |  |

| **Tibebe – Ghion Specialized Teaching Hospital, Bahir Dar University,**  **Bahir Dar, Ethiopia** | | | | | |
| --- | --- | --- | --- | --- | --- |
| **Name: Robel Aragie . Sex/Age: M/9 Months. MRN: 159437. Date of Report: 03 /08 /15 Eth.C.**  **Referral Diagnosis: Follow up echo for TOF + Smallish MPA and Branch PAs. AGH2.121** | | | | | |
| **Features:** | | **Findings** | | **Features** | **Findings** |
| **Profile** | | | | **Atria** | |
| Abdominal Situs | Solitus | | | Left Atrium | Normal |
| Atrial Situs | Solitus | | | Right Atrium | Dilated |
| Cardiac Position | Levocardia | | | **Atrio-Ventricular Valves** | |
| Systemic Venous Drainage | To RA | | | Mitral Valve | Annulus = 10mm |
| Pulmonary Venous Drainage | To LA | | | Tricuspid Valve | Annulus = 16mm |
| Atrio-ventricular Connection | Concordant | | |  | TAPSE = 16mm |
| Ventriculo-Arterial Connection | concordant | | | **Ventricle** | |
| Ventricular Loop | d-Loop | | | Left Ventricle | Normal |
| **Septae** |  | | | Right Ventricle | Dilated & Hypertrophied |
| Interatrial Septum | Intact | | | **Doppler Measurement** |  |
| Interventricular Septum | Non-Restrictive Mal-aligned Sub-Aortic VSD, R – L Shunt | | | Mitral | ------------- |
| **Semilunar Valves** |  | | | Aortic | ------------- |
| Aortic Valve | Annulus = 11mm | | | Tricuspid | ------------- |
| Pulmonary Valve | Annulus = 6mm | | | Pulmonic | Severe PS, PPG = 100mmHg |
| **Great Arteries** | NRGA | | | **Coronary Arteries** |  |
| Aorta | Over-riding aorta | | | **Aortic Arch** | Left. No CoA |
| Pulmonary Arteries | Smallish MPA & BPAs. | | | **PDA** | No PDA |
| **M-Mode**: | | | | | |
| Ao | | mm | | PWd | mm |
| LA | | mm | | EDV | ml |
| LVIDd | | mm | | ESV | ml |
| LVIDs | | mm | | FS | % |
| IVSd | | mm | | LVEF | % |
| **Additional Information:** | | | | | |
| **Conclusion:**   1. {S, D, S} Levocardia 2. TOF 3. Smallish MPA and Branch Pas. | | | | | |
| **Recommendation:** | | | | | |
| **Done By:** | | | **Signature** | **Date** | **Remark** |
| Tesfaye T., Paediatrician, Paediatric Cardiologist | | |  | 03/ ­­­­­­­­­­­­­­­­08/15Eth.C. |  |

| **Tibebe – Ghion Specialized Teaching Hospital, Bahir Dar University,**  **Bahir Dar, Ethiopia** | | | | |
| --- | --- | --- | --- | --- |
| **Name: Gebeyanesh Misganaw. Sex/Age: F/14years. MRN: 164903. Date of Report: 03/ 08/15Eth.C.**  **Referral Diagnosis: Follow up echo for HTN + AGN (Reduced LV Systolic Function). AGH2.217** | | | | |
| **Features:** | **Findings** | | **Features** | **Findings** |
| **Profile** | | | **Atria** | |
| Abdominal Situs | Solitus | | Left Atrium | Normal |
| Atrial Situs | Solitus | | Right Atrium | Normal |
| Cardiac Position | Levocardia | | **Atrio-Ventricular Valves** | |
| Systemic Venous Drainage | To RA | | Mitral Valve | Annulus = 21mm |
| Pulmonary Venous Drainage | To LA | | Tricuspid Valve | Annulus = 24mm |
| Atrio-ventricular Connection | Concordant | |  | TAPSE = mm |
| Ventriculo-Arterial Connection | concordant | | **Ventricle** | |
| Ventricular Loop | d-Loop | | Left Ventricle | Normal |
| **Septae** |  | | Right Ventricle | Normal |
| Interatrial Septum | Intact | | **Doppler Measurement** |  |
| Interventricular Septum | Intact | | Mitral | ------------- |
| **Semilunar Valves** |  | | Aortic | ------------- |
| Aortic Valve | Annulus = 20mm | | Tricuspid | ------------- |
| Pulmonary Valve | Annulus = 21mm | | Pulmonic | ------------- |
| **Great Arteries** | NRGA | | **Coronary Arteries** |  |
| Aorta |  | | **Aortic Arch** | Left. No CoA |
| Pulmonary Arteries | Normal MPA & BPAs. | | **PDA** | No PDA |
| **M-Mode**: | | | | |
| Ao | mm | | PWd | mm |
| LA | mm | | EDV | ml |
| LVIDd | mm | | ESV | ml |
| LVIDs | mm | | FS | 31% |
| IVSd | mm | | LVEF | 60% |
| **Additional Information:** | | | | |
| **Conclusion:**   1. Normal Echocardiography Study | | | | |
| **Remark:** | | | | |
| **Recommendation:** | | | | |
| **Done By:** | | **Signature** | **Date** | **Remark** |
| Tesfaye T., Paediatrician, Paediatric Cardiologist | |  | 03/ ­­­­­­­­­­­­­­­­08 /15Eth.C. |  |

| **Tibebe – Ghion Specialized Teaching Hospital, Bahir Dar University,**  **Bahir Dar, Ethiopia** | | | | |
| --- | --- | --- | --- | --- |
| **Name: Hirut Shumet . Sex/Age: F/4years. MRN: 174927. Date of Report: 03/08 /15Eth.C.**  **Referral Diagnosis: R/O Diss.tbc to pericardium. TGSH10.2794** | | | | |
| **Features:** | **Findings** | | **Features** | **Findings** |
| **Profile** | | | **Atria** | |
| Abdominal Situs | Solitus | | Left Atrium | Normal |
| Atrial Situs | Solitus | | Right Atrium | Normal |
| Cardiac Position | Levocardia | | **Atrio-Ventricular Valves** | |
| Systemic Venous Drainage | To RA | | Mitral Valve | Annulus = 17mm |
| Pulmonary Venous Drainage | To LA | | Tricuspid Valve | Annulus = 17mm |
| Atrio-ventricular Connection | Concordant | |  | TAPSE = 20mm |
| Ventriculo-Arterial Connection | concordant | | **Ventricle** | |
| Ventricular Loop | d-Loop | | Left Ventricle | Normal |
| **Septae** |  | | Right Ventricle | Normal |
| Interatrial Septum | Intact | | **Doppler Measurement** |  |
| Interventricular Septum | Intact | | Mitral | ------------- |
| **Semilunar Valves** |  | | Aortic | ------------- |
| Aortic Valve | Annulus = 15mm | | Tricuspid | ------------- |
| Pulmonary Valve | Annulus = 16mm | | Pulmonic | ------------- |
| **Great Arteries** | NRGA | | **Coronary Arteries** |  |
| Aorta |  | | **Aortic Arch** | Left. No CoA |
| Pulmonary Arteries | Normal MPA & BPAs. | | **PDA** | No PDA |
| **M-Mode**: | | | | |
| Ao | mm | | PWd | mm |
| LA | mm | | EDV | ml |
| LVIDd | mm | | ESV | ml |
| LVIDs | mm | | FS | 33% |
| IVSd | mm | | LVEF | 63% |
| **Additional Information:** | | | | |
| **Conclusion:**   1. Normal Echocardiography Study | | | | |
| **Remark:** | | | | |
| **Recommendation:** | | | | |
| **Done By:** | | **Signature** | **Date** | **Remark** |
| Tesfaye T., Paediatrician, Paediatric Cardiologist | |  | 03/ ­­­­­­­­­­­­­­­08 /15 Eth.C. |  |

| **Tibebe – Ghion Specialized Teaching Hospital, Bahir Dar University,**  **Bahir Dar, Ethiopia** | | | | |
| --- | --- | --- | --- | --- |
| **Name: Baby of Banchalem Tesema Sex/Age: F/15days. MRN: 170268. Date of Report: 03/08/15Eth.C.**  **Referral Diagnosis: ?PPHTN. TGSH10.2795** | | | | |
| **Features:** | **Findings** | | **Features** | **Findings** |
| **Profile** | | | **Atria** | |
| Abdominal Situs | Solitus | | Left Atrium | Normal |
| Atrial Situs | Solitus | | Right Atrium | Normal |
| Cardiac Position | Levocardia | | **Atrio-Ventricular Valves** | |
| Systemic Venous Drainage | To RA | | Mitral Valve | Annulus = 10mm |
| Pulmonary Venous Drainage | To LA | | Tricuspid Valve | Annulus = 10mm |
| Atrio-ventricular Connection | Concordant | |  | TAPSE = mm |
| Ventriculo-Arterial Connection | concordant | | **Ventricle** | |
| Ventricular Loop | d-Loop | | Left Ventricle | Normal |
| **Septae** |  | | Right Ventricle | Normal |
| Interatrial Septum | PFO, L – R Shunt | | **Doppler Measurement** |  |
| Interventricular Septum | Intact | | Mitral | ------------- |
| **Semilunar Valves** |  | | Aortic | ------------- |
| Aortic Valve | Annulus = 9mm | | Tricuspid | ------------- |
| Pulmonary Valve | Annulus = 9mm | | Pulmonic | ------------- |
| **Great Arteries** | NRGA | | **Coronary Arteries** |  |
| Aorta |  | | **Aortic Arch** | Left. No CoA |
| Pulmonary Arteries | Normal MPA & BPAs. | | **PDA** | No PDA |
| **M-Mode**: | | | | |
| Ao | mm | | PWd | mm |
| LA | mm | | EDV | ml |
| LVIDd | mm | | ESV | ml |
| LVIDs | mm | | FS | % |
| IVSd | mm | | LVEF | % |
| **Additional Information:** | | | | |
| **Conclusion:**   1. Normal Echocardiography Study | | | | |
| **Remark:** | | | | |
| **Recommendation:** | | | | |
| **Done By:** | | **Signature** | **Date** | **Remark** |
| Tesfaye T., Paediatrician, Paediatric Cardiologist | |  | 03/­­­­­­­­­­­­­­­­­08/15Eth.C. |  |

| **Tibebe – Ghion Specialized Teaching Hospital, Bahir Dar University,**  **Bahir Dar, Ethiopia** | | | | |
| --- | --- | --- | --- | --- |
| **Name: B/Mastewal Getahun . Sex/Age: F/24days. MRN: 175063. Date of Report: 03/08/15Eth.C.**  **Referral Diagnosis: ?Edward Syndrome + RD + Murmur. TGSH10.2796** | | | | |
| **Features:** | **Findings** | | **Features** | **Findings** |
| **Profile** | | | **Atria** | |
| Abdominal Situs | Solitus | | Left Atrium | Normal |
| Atrial Situs | Solitus | | Right Atrium | Normal |
| Cardiac Position | Levocardia | | **Atrio-Ventricular Valves** | |
| Systemic Venous Drainage | To RA | | Mitral Valve | Annulus = 10mm |
| Pulmonary Venous Drainage | To LA | | Tricuspid Valve | Annulus = 11mm |
| Atrio-ventricular Connection | Concordant | |  | TAPSE = mm |
| Ventriculo-Arterial Connection | concordant | | **Ventricle** | |
| Ventricular Loop | d-Loop | | Left Ventricle | Normal |
| **Septae** |  | | Right Ventricle | Normal |
| Interatrial Septum | 6mm OS ASD, L – R Shunt | | **Doppler Measurement** |  |
| Interventricular Septum | 2mm Sub Aortic VSD, L – R Shunt | | Mitral | ------------- |
| **Semilunar Valves** |  | | Aortic | ------------- |
| Aortic Valve | Annulus = 8mm | | Tricuspid | ------------- |
| Pulmonary Valve | Annulus = 8mm | | Pulmonic | ------------- |
| **Great Arteries** | NRGA | | **Coronary Arteries** |  |
| Aorta |  | | **Aortic Arch** | Left. No CoA |
| Pulmonary Arteries | Normal MPA & BPAs. | | **PDA** | No PDA |
| **M-Mode**: | | | | |
| Ao | mm | | PWd | mm |
| LA | mm | | EDV | ml |
| LVIDd | mm | | ESV | ml |
| LVIDs | mm | | FS | % |
| IVSd | mm | | LVEF | % |
| **Additional Information:** | | | | |
| **Conclusion:**   1. {S, D, S} Levocardia 2. Small OS ASD, L – R Shunt 3. Small Sub-aortic VSD, L – R Shunt | | | | |
| **Remark:** | | | | |
| **Recommendation:** | | | | |
| **Done By:** | | **Signature** | **Date** | **Remark** |
| Tesfaye T., Paediatrician, Paediatric Cardiologist | |  | 03/ ­­­­­­­­­­­­­­­08/15Eth.C. |  |

| **Tibebe – Ghion Specialized Teaching Hospital, Bahir Dar University,**  **Bahir Dar, Ethiopia** | | | | |
| --- | --- | --- | --- | --- |
| **Name: Berket Melaku . Sex/Age: M/ 1 6/12 years. MRN: 116370. Date of Report: 010/08 /15 Eth.C.**  **Referral Diagnosis: Murmur + DS. TGSH10.2797** | | | | |
| **Features:** | **Findings** | | **Features** | **Findings** |
| **Profile** | | | **Atria** | |
| Abdominal Situs | Solitus | | Left Atrium | Normal |
| Atrial Situs | Solitus | | Right Atrium | Normal |
| Cardiac Position | Levocardia | | **Atrio-Ventricular Valves** | |
| Systemic Venous Drainage | To RA | | Mitral Valve | Annulus = 13mm |
| Pulmonary Venous Drainage | To LA | | Tricuspid Valve | Annulus = 14mm |
| Atrio-ventricular Connection | Concordant | |  | TAPSE = 16mm |
| Ventriculo-Arterial Connection | concordant | | **Ventricle** | |
| Ventricular Loop | d-Loop | | Left Ventricle | Normal |
| **Septae** |  | | Right Ventricle | Normal |
| Interatrial Septum | Intact | | **Doppler Measurement** |  |
| Interventricular Septum | 8mm Non-Restrictive Sub-aortic VSD, L – R Shunt, PPG = 26mmHg | | Mitral | ------------- |
| **Semilunar Valves** |  | | Aortic | ------------- |
| Aortic Valve | Annulus = 13mm | | Tricuspid | ------------- |
| Pulmonary Valve | Annulus = 13mm | | Pulmonic | ------------- |
| **Great Arteries** | NRGA | | **Coronary Arteries** |  |
| Aorta |  | | **Aortic Arch** | Left. No CoA |
| Pulmonary Arteries | Normal MPA & BPAs. | | **PDA** | No PDA |
| **M-Mode**: | | | | |
| Ao | mm | | PWd | mm |
| LA | mm | | EDV | ml |
| LVIDd | mm | | ESV | ml |
| LVIDs | mm | | FS | % |
| IVSd | mm | | LVEF | % |
| **Additional Information:** | | | | |
| **Conclusion:**   1. {S, D, S} Levocardia 2. Moderate Non-Restrictive Sub-Aortic VSD, L – R Shunt 3. Normal Biventricular Systolic Function | | | | |
| **Recommendation:** Optimize Medical treatment | | | | |
| **Done By:** | | **Signature** | **Date** | **Remark** |
| Tesfaye T., Paediatrician, Paediatric Cardiologist | |  | 10 /08 ­­­­­­­­­­­­­­­­­/15 Eth.C. |  |
| **Tibebe – Ghion Specialized Teaching Hospital, Bahir Dar University,**  **Bahir Dar, Ethiopia** | | | | |
| **Name: Lijalem Ayenew . Sex/Age: M/ 9years. MRN: 174959. Date of Report: 010/08 /15 Eth.C.**  **Referral Diagnosis: Rheumatic Recurrence.TGSH10.2798** | | | | |
| **Features:** | **Findings** | | **Features** | **Findings** |
| **Profile** | | | **Atria** | |
| Abdominal Situs | Solitus | | Left Atrium | Normal |
| Atrial Situs | Solitus | | Right Atrium | Normal |
| Cardiac Position | Levocardia | | **Atrio-Ventricular Valves** | |
| Systemic Venous Drainage | To RA | | Mitral Valve | Annulus = 20mm. thickened MVL |
| Pulmonary Venous Drainage | To LA | | Tricuspid Valve | Annulus = 20mm |
| Atrio-ventricular Connection | Concordant | |  | TAPSE = mm |
| Ventriculo-Arterial Connection | concordant | | **Ventricle** | |
| Ventricular Loop | d-Loop | | Left Ventricle | Normal |
| **Septae** |  | | Right Ventricle | Normal |
| Interatrial Septum | Intact | | **Doppler Measurement** |  |
| Interventricular Septum | Intact | | Mitral | Mild MR, Holosystolic, posterior projection, seen in two planes with jet velocity = 5m/sec. |
| **Semilunar Valves** |  | | Aortic | ------------- |
| Aortic Valve | Annulus = 16mm | | Tricuspid | ------------- |
| Pulmonary Valve | Annulus = 15mm | | Pulmonic | ------------- |
| **Great Arteries** | NRGA | | **Coronary Arteries** |  |
| Aorta |  | | **Aortic Arch** | Left. No CoA |
| Pulmonary Arteries | Normal MPA & BPAs. | | **PDA** | No PDA |
| **M-Mode**: | | | | |
| Ao | mm | | PWd | mm |
| LA | mm | | EDV | ml |
| LVIDd | mm | | ESV | ml |
| LVIDs | mm | | FS | 36% |
| IVSd | mm | | LVEF | 65% |
| **Additional Information:** | | | | |
| **Conclusion:**   1. {S, D, S} Levocardia 2. Thickened MVL 3. Mild MR 4. Normal Biventricular Systolic Function | | | | |
| **Done By:** | | **Signature** | **Date** | **Remark** |
| Tesfaye T., Paediatrician, Paediatric Cardiologist | |  | 10 /08 ­­­­­­­­­­­­­­­­­/15 Eth.C. |  |

| **Tibebe – Ghion Specialized Teaching Hospital, Bahir Dar University,**  **Bahir Dar, Ethiopia** | | | | | | | |
| --- | --- | --- | --- | --- | --- | --- | --- |
| **Name: Addisie Gerie. Sex/Age: F /14years. MRN: 124294 . Date: 010/08 /15 Eth.C.**  **R.Dx: CHF + Rheumatic Recurrence. TGSH10.2799** | | | | | | | |
| **Features:** | | **Findings** | | **Features** | | **Findings** | |
| **Profile** | | | | **Atria** | | | |
| Abdominal Situs | Solitus | | | Left Atrium | | Markedly Dilated | |
| Atrial Situs | Solitus | | | Right Atrium | | Dilated | |
| Cardiac Position | Levocardia | | | **Atrio-Ventricular Valves** | | | |
| Systemic Venous Drainage | To RA | | | Mitral Valve | | Annulus = 34mm. thickened, clubbed MVL. | |
| Pulmonary Venous Drainage | To LA | | | Tricuspid Valve | | Annulus = 28mm | |
| Atrio-ventricular Connection | Concordant | | |  | | TAPSE = 19mm | |
| Ventriculo-Arterial Connection | concordant | | | **Ventricle** | | | |
| Ventricular Loop | d-Loop | | | Left Ventricle | | Markedly Dilated & Dysfunctional | |
| **Septae** |  | | | Right Ventricle | | Dilated | |
| Interatrial Septum | Intact | | | **Doppler Measurement** | |  | |
| Interventricular Septum | Intact | | | Mitral | | Trivial MR, Holosystolic, Posterior projection, seen in two planes with jet velocity = 3.3m/sec. Severe MS, PPG/MPG = 34/25mmHg | |
| **Semilunar Valves** |  | | | Aortic | | Moderate AR, PHT = 474ms | |
| Aortic Valve | Annulus = 15mm | | | Tricuspid | | ------------- | |
| Pulmonary Valve | Annulus = 25mm | | | Pulmonic | | ------------- | |
| **Great Arteries** | NRGA | | | **Coronary Arteries** | |  | |
| Aorta |  | | | **Aortic Arch** | | Left. No CoA | |
| Pulmonary Arteries | MPA = 28mm. | | | **PDA** | | No PDA | |
| **M-Mode**: | | | | | | | |
| Ao | | mm | | | PWd | | mm |
| LA | | mm | | | EDV | | ml |
| LVIDd | | mm | | | ESV | | ml |
| LVIDs | | mm | | | FS | | 19% |
| IVSd | | mm | | | LVEF | | 39% |
| **Additional Information:** 3mm Pericardial effusion | | | | | | | |
| **Conclusion:**   1. {S, D, S} Levocardia 2. All chambers dilated 3. Thickened, clubbed MVL 4. Trivial MR 5. Severe MS 6. Moderate AR 7. Mild TR 8. Severe Pulmonary Hypertension 9. Trace pericardial effusion 10. Reduced LVB Systolic Function | | | | | | | |
| **Done By:** | | | **Signature** | | **Date** | | **Remark** |
| Tesfaye T., Paediatrician, Paediatric Cardiologist | | |  | | 10 /08 ­­­­­­­­­­­­­­­­­/15 Eth.C. | |  |

| **Tibebe – Ghion Specialized Teaching Hospital, Bahir Dar University,**  **Bahir Dar, Ethiopia** | | | | |
| --- | --- | --- | --- | --- |
| **Name: Syoum Belete. Sex/Age: M/12years. MRN: 176165. Date of Report: 12/08 /15 Eth.C.**  **Referral Diagnosis: Pericarditis (tuberculosis). TGSH10.27800** | | | | |
| **Features:** | **Findings** | | **Features** | **Findings** |
| **Profile** | | | **Atria** | |
| Abdominal Situs | Solitus | | Left Atrium | Normal |
| Atrial Situs | Solitus | | Right Atrium | Normal |
| Cardiac Position | Levocardia | | **Atrio-Ventricular Valves** | |
| Systemic Venous Drainage | To RA. IVC Dilated | | Mitral Valve | Annulus = 20mm |
| Pulmonary Venous Drainage | To LA | | Tricuspid Valve | Annulus = 20mm |
| Atrio-ventricular Connection | Concordant | |  | TAPSE = mm |
| Ventriculo-Arterial Connection | concordant | | **Ventricle** | |
| Ventricular Loop | d-Loop | | Left Ventricle | Normal |
| **Septae** |  | | Right Ventricle | Normal |
| Interatrial Septum | Intact | | **Doppler Measurement** |  |
| Interventricular Septum | Intact | | Mitral | ------------- |
| **Semilunar Valves** |  | | Aortic | ------------- |
| Aortic Valve | Annulus = 15mm | | Tricuspid | ------------- |
| Pulmonary Valve | Annulus = 18mm | | Pulmonic | ------------- |
| **Great Arteries** | NRGA | | **Coronary Arteries** |  |
| Aorta |  | | **Aortic Arch** | Left. No CoA |
| Pulmonary Arteries | Normal MPA & BPAs. | | **PDA** | No PDA |
| **M-Mode**: | | | | |
| Ao | mm | | PWd | mm |
| LA | mm | | EDV | ml |
| LVIDd | mm | | ESV | ml |
| LVIDs | mm | | FS | % |
| IVSd | mm | | LVEF | % |
| **Additional Information:** 28mm Circumferential Pericardial effusion. 26mm Right Pleural effusion. Echodebris seen. Swinging Heart. | | | | |
| **Conclusion:**   1. {S, D, S} Levocardia 2. Large Circumferential Pericardial effusion 3. Large Right Pleural effusion | | | | |
| **Remark:** | | | | |
| **Recommendation:** | | | | |
| **Done By:** | | **Signature** | **Date** | **Remark** |
| Tesfaye T., Paediatrician, Paediatric Cardiologist | |  | 12 /08 ­­­­­­­­­­­­­­­­­/15 Eth.C. |  |

| **Tibebe – Ghion Specialized Teaching Hospital, Bahir Dar University,**  **Bahir Dar, Ethiopia** | | | | | | | |
| --- | --- | --- | --- | --- | --- | --- | --- |
| **Name: Dereje Tirualem. Sex/Age: M /4 5/12. MRN:176193. Date of Report: 12/08 /15 Eth.C.**  **Referral Diagnosis: CHF. TGSH10.2801** | | | | | | | |
| **Features:** | | **Findings** | | | **Features** | | **Findings** |
| **Profile** | | | | | **Atria** | | |
| Abdominal Situs | Solitus | | | | Left Atrium | | Dilated |
| Atrial Situs | Solitus | | | | Right Atrium | | Dilated |
| Cardiac Position | Levocardia | | | | **Atrio-Ventricular Valves** | | |
| Systemic Venous Drainage | To RA | | | | Mitral Valve | | Annulus = 17mm |
| Pulmonary Venous Drainage | To LA | | | | Tricuspid Valve | | Annulus = 23mm |
| Atrio-ventricular Connection | Concordant | | | |  | | TAPSE = 18mm |
| Ventriculo-Arterial Connection | concordant | | | | **Ventricle** | | |
| Ventricular Loop | d-Loop | | | | Left Ventricle | | Dilated |
| **Septae** | Tongue of tissue in b/n the ASD + VSD | | | | Right Ventricle | | Dilated |
| Interatrial Septum | 20mm Primum ASD, L – R Shunt | | | | **Doppler Measurement** | |  |
| Interventricular Septum | 11mm Inlet VSD, L – R Shunt | | | | Mitral | | Moderate MR |
| **Semilunar Valves** |  | | | | Aortic | | ------------- |
| Aortic Valve | Annulus = 16mm | | | | Tricuspid | | Moderate TR |
| Pulmonary Valve | Annulus = 21mm | | | | Pulmonic | | Mild PR, PPG = 56mmHg |
| **Great Arteries** | NRGA | | | | **Coronary Arteries** | |  |
| Aorta |  | | | | **Aortic Arch** | | Left. No CoA |
| Pulmonary Arteries | Normal MPA & BPAs. | | | | **PDA** | | No PDA |
| **M-Mode**: Normal LV Function on eye balling | | | | | | | |
| Ao | | mm | | PWd | | mm | |
| LA | | mm | | EDV | | ml | |
| LVIDd | | mm | | ESV | | ml | |
| LVIDs | | mm | | FS | | % | |
| IVSd | | mm | | LVEF | | % | |
| **Additional Information:** | | | | | | | |
| **Conclusion:**   1. {S, D, S} Levocardia 2. All chambers dilated 3. Intermediate AVSD 4. Moderate MR 5. Moderate TR 6. Mild PR 7. Moderate to severe Pulmonary Hypertension 8. Normal Biventricular Systolic Function | | | | | | | |
| **Done By:** | | | **Signature** | **Date** | | **Remark** | |
| Tesfaye T., Paediatrician, Paediatric Cardiologist | | |  | 12/08 ­­­­­­­­­­­­­­­­­/15 Eth.C. | |  | |
| **Tibebe – Ghion Specialized Teaching Hospital, Bahir Dar University,**  **Bahir Dar, Ethiopia** | | | | | | | |
| **Name: Balew Nibret. Sex/Age: M/1 years. MRN: 161365. Date of Report: 26/08/15 Eth.C.**  **Referral Diagnosis: Follow up echo for large pericardial effusion with cardiac tamponade. TGSH3.2754.** | | | | | | | |
| **Features:** | | **Findings** | | **Features** | | **Findings** | |
| **Profile** | | | | **Atria** | | | |
| Abdominal Situs | | Solitus | | Left Atrium | | Normal | |
| Atrial Situs | | Solitus | | Right Atrium | | Normal | |
| Cardiac Position | | Levocardia | | **Atrio-Ventricular Valves** | | | |
| Systemic Venous Drainage | | To RA | | Mitral Valve | | Annulus = 16mm. E/A = 1.7 | |
| Pulmonary Venous Drainage | | To LA | | Tricuspid Valve | | Annulus = 15mm | |
| Atrio-ventricular Connection | | Concordant | |  | | TAPSE = mm | |
| Ventriculo-Arterial Connection | | concordant | | **Ventricle** | | | |
| Ventricular Loop | | d-Loop | | Left Ventricle | | Normal | |
| **Septae** | |  | | Right Ventricle | | Normal | |
| Interatrial Septum | | Intact | | **Doppler Measurement** | |  | |
| Interventricular Septum | | Intact | | Mitral | | ------------- | |
| **Semilunar Valves** | |  | | Aortic | | ------------- | |
| Aortic Valve | | Annulus = 11mm | | Tricuspid | | ------------- | |
| Pulmonary Valve | | Annulus = 12mm | | Pulmonic | | ------------- | |
| **Great Arteries** | | NRGA | | **Coronary Arteries** | |  | |
| Aorta | |  | | **Aortic Arch** | | Left. No CoA | |
| Pulmonary Arteries | | Normal MPA & BPAs. | | **PDA** | | No PDA | |
| **M-Mode**: | | | | | | | |
| Ao | | mm | | PWd | | mm | |
| LA | | mm | | EDV | | ml | |
| LVIDd | | mm | | ESV | | ml | |
| LVIDs | | mm | | FS | | 31% | |
| IVSd | | mm | | LVEF | | 61% | |
| **Additional Information: No pericardial effusion** | | | | | | | |
| **Conclusion:**   1. Normal Echocardiography Study | | | | | | | |
| **Remark:** | | | | | | | |
| **Recommendation:** Echo after a year | | | | | | | |
| **Done By:** | | | **Signature** | **Date** | | **Remark** | |
| Tesfaye T., Paediatrician, Paediatric Cardiologist | | |  | 26/08 ­­­­­­­­­­­­­­­­­/15 Eth.C. | |  | |

| **Tibebe – Ghion Specialized Teaching Hospital, Bahir Dar University,**  **Bahir Dar, Ethiopia** | | | | |
| --- | --- | --- | --- | --- |
| **Name: Yibeltal Mulat. Sex/Age: M/5years. MRN: 173649. Date of Report: 26/08 /15 Eth.C.**  **Referral Diagnosis: DOE + CHF + Sepsis. TGSH10.2802.** | | | | |
| **Features:** | **Findings** | | **Features** | **Findings** |
| **Profile** | | | **Atria** | |
| Abdominal Situs | Solitus | | Left Atrium | Normal |
| Atrial Situs | Solitus | | Right Atrium | Normal |
| Cardiac Position | Levocardia | | **Atrio-Ventricular Valves** | |
| Systemic Venous Drainage | To RA | | Mitral Valve | Annulus = mm |
| Pulmonary Venous Drainage | To LA | | Tricuspid Valve | Annulus = mm |
| Atrio-ventricular Connection | Concordant | |  | TAPSE = mm |
| Ventriculo-Arterial Connection | concordant | | **Ventricle** | |
| Ventricular Loop | d-Loop | | Left Ventricle | 2mm fistulous communication with pericardium |
| **Septae** |  | | Right Ventricle | Normal |
| Interatrial Septum | Intact | | **Doppler Measurement** |  |
| Interventricular Septum | Intact | | Mitral | ------------- |
| **Semilunar Valves** |  | | Aortic | ------------- |
| Aortic Valve | Annulus = mm | | Tricuspid | ------------- |
| Pulmonary Valve | Annulus = mm | | Pulmonic | ------------- |
| **Great Arteries** | NRGA | | **Coronary Arteries** |  |
| Aorta |  | | **Aortic Arch** | Left. No CoA |
| Pulmonary Arteries | Normal MPA & BPAs. | | **PDA** | No PDA |
| **M-Mode**: | | | | |
| Ao | mm | | PWd | mm |
| LA | mm | | EDV | ml |
| LVIDd | mm | | ESV | ml |
| LVIDs | mm | | FS | % |
| IVSd | mm | | LVEF | % |
| **Additional Information: LV Defect has decreased to 2mm** | | | | |
| **Conclusion:**   1. {S, D, S} Levocardia 2. LV Pseudoaneurysm | | | | |
| **Remark:** | | | | |
| **Recommendation:** | | | | |
| **Done By:** | | **Signature** | **Date** | **Remark** |
| Tesfaye T., Paediatrician, Paediatric Cardiologist | |  | 26/08 ­­­­­­­­­­­­­­­­­/15 Eth.C. |  |

| **Tibebe – Ghion Specialized Teaching Hospital, Bahir Dar University,**  **Bahir Dar, Ethiopia** | | | | |
| --- | --- | --- | --- | --- |
| **Name: Dawit Belayneh. Sex/Age: M/5years. MRN: 164180. Date of Report: 26/08 /15 Eth.C.**  **Referral Diagnosis: Incidental Murmur. TGSH10.2803.** | | | | |
| **Features:** | **Findings** | | **Features** | **Findings** |
| **Profile** | | | **Atria** | |
| Abdominal Situs | Solitus | | Left Atrium | Normal |
| Atrial Situs | Solitus | | Right Atrium | Normal |
| Cardiac Position | Levocardia | | **Atrio-Ventricular Valves** | |
| Systemic Venous Drainage | To RA | | Mitral Valve | Annulus = 17mm |
| Pulmonary Venous Drainage | To LA | | Tricuspid Valve | Annulus = 18mm |
| Atrio-ventricular Connection | Concordant | |  | TAPSE = 21mm |
| Ventriculo-Arterial Connection | concordant | | **Ventricle** | |
| Ventricular Loop | d-Loop | | Left Ventricle | Normal |
| **Septae** |  | | Right Ventricle | Normal |
| Interatrial Septum | Intact | | **Doppler Measurement** |  |
| Interventricular Septum | Intact | | Mitral | ------------- |
| **Semilunar Valves** |  | | Aortic | ------------- |
| Aortic Valve | Annulus = 14mm | | Tricuspid | ------------- |
| Pulmonary Valve | Annulus = 16mm | | Pulmonic | ------------- |
| **Great Arteries** | NRGA | | **Coronary Arteries** |  |
| Aorta |  | | **Aortic Arch** | Left. No CoA |
| Pulmonary Arteries | Normal MPA & BPAs. | | **PDA** | No PDA |
| **M-Mode**: | | | | |
| Ao | mm | | PWd | mm |
| LA | mm | | EDV | ml |
| LVIDd | mm | | ESV | ml |
| LVIDs | mm | | FS | % |
| IVSd | mm | | LVEF | % |
| **Additional Information:** | | | | |
| **Conclusion:**   1. Normal Echocardiography Study | | | | |
| **Remark:** | | | | |
| **Recommendation:** | | | | |
| **Done By:** | | **Signature** | **Date** | **Remark** |
| Tesfaye T., Paediatrician, Paediatric Cardiologist | |  | 26/08 ­­­­­­­­­­­­­­­­­/15 Eth.C. |  |

| **Tibebe – Ghion Specialized Teaching Hospital, Bahir Dar University,**  **Bahir Dar, Ethiopia** | | | | |
| --- | --- | --- | --- | --- |
| **Name: Hana Asaye. Sex/Age: F/2 6/12. MRN:176594. Date of Report: 26/08 /15 Eth.C.**  **Referral Diagnosis: RD + Cyanosis. TGSH10.2804.** | | | | |
| **Features:** | **Findings** | | **Features** | **Findings** |
| **Profile** | | | **Atria** | |
| Abdominal Situs | Solitus | | Left Atrium | Normal |
| Atrial Situs | Solitus | | Right Atrium | Dilated |
| Cardiac Position | Levocardia | | **Atrio-Ventricular Valves** | |
| Systemic Venous Drainage | To RA | | Mitral Valve | Annulus = 16mm |
| Pulmonary Venous Drainage | To LA | | Tricuspid Valve | Annulus = 17mm |
| Atrio-ventricular Connection | Concordant | |  | TAPSE = mm |
| Ventriculo-Arterial Connection | Discordant | | **Ventricle** | |
| Ventricular Loop | d-Loop | | Left Ventricle | Normal |
| **Septae** |  | | Right Ventricle | Dilated & Hypertrophied |
| Interatrial Septum | 7mm OS ASD, L – R Shunt | | **Doppler Measurement** |  |
| Interventricular Septum | Intact | | Mitral | ------------- |
| **Semilunar Valves** |  | | Aortic | ------------- |
| Aortic Valve | Annulus = 16mm | | Tricuspid | ------------- |
| Pulmonary Valve | Annulus = 12mm | | Pulmonic | ------------- |
| **Great Arteries** | d-TGA | | **Coronary Arteries** |  |
| Aorta | Anterior, to the right & from RV | | **Aortic Arch** | Left. No CoA |
| Pulmonary Arteries | Posterior, to the left and from LV | | **PDA** | No PDA |
| **M-Mode**: | | | | |
| Ao | mm | | PWd | mm |
| LA | mm | | EDV | ml |
| LVIDd | mm | | ESV | ml |
| LVIDs | mm | | FS | % |
| IVSd | mm | | LVEF | % |
| **Additional Information:** | | | | |
| **Conclusion:**   1. {S, D, D} Levocardia 2. d-TGA with Moderate OS ASD 3. RA/RV Dilated, RV Hypertrophied | | | | |
| **Remark:** | | | | |
| **Recommendation:** | | | | |
| **Done By:** | | **Signature** | **Date** | **Remark** |
| Tesfaye T., Paediatrician, Paediatric Cardiologist | |  | 26/08 ­­­­­­­­­­­­­­­­­/15 Eth.C. |  |

| **Tibebe – Ghion Specialized Teaching Hospital, Bahir Dar University,**  **Bahir Dar, Ethiopia** | | | | |
| --- | --- | --- | --- | --- |
| **Name: Abel Mekuriya . Sex/Age: M/8 months. MRN: 177160. Date of Report: 26/08 /15 Eth.C.**  **Referral Diagnosis: Pulmonary Hypertension R/O Ebstein anomaly. TGSH10.2805.** | | | | |
| **Features:** | **Findings** | | **Features** | **Findings** |
| **Profile** | | | **Atria** | |
| Abdominal Situs | Solitus | | Left Atrium | Normal |
| Atrial Situs | Solitus | | Right Atrium | Dilated |
| Cardiac Position | Levocardia | | **Atrio-Ventricular Valves** | |
| Systemic Venous Drainage | To RA | | Mitral Valve | Annulus = 14mm |
| Pulmonary Venous Drainage | To LA | | Tricuspid Valve | Annulus = 19mm |
| Atrio-ventricular Connection | Concordant | |  | TAPSE = 10mm |
| Ventriculo-Arterial Connection | concordant | | **Ventricle** | |
| Ventricular Loop | d-Loop | | Left Ventricle | Normal |
| **Septae** |  | | Right Ventricle | Dilated, Hypertrophied and Dysfunctional |
| Interatrial Septum | 5mm OS ASD, R – L Shunt | | **Doppler Measurement** |  |
| Interventricular Septum | Intact | | Mitral | ------------- |
| **Semilunar Valves** |  | | Aortic | ------------- |
| Aortic Valve | Annulus = 12mm | | Tricuspid | Severe TR, PPG = 94mmHg |
| Pulmonary Valve | Annulus = 13mm | | Pulmonic | ------------- |
| **Great Arteries** | NRGA | | **Coronary Arteries** |  |
| Aorta |  | | **Aortic Arch** | Left. No CoA |
| Pulmonary Arteries | Normal MPA & BPAs. | | **PDA** | No PDA |
| **M-Mode**: Normal LV Function on eye balling | | | | |
| Ao | mm | | PWd | mm |
| LA | mm | | EDV | ml |
| LVIDd | mm | | ESV | ml |
| LVIDs | mm | | FS | % |
| IVSd | mm | | LVEF | % |
| **Additional Information:** | | | | |
| **Conclusion:**   1. {S, D, S} Levocardia 2. RA/RV Dilated, RV Dilated and Dysfunctional 3. Moderate OS ASD, R – L Shunt 4. Severe TR 5. Severe Pulmonary Hypertension | | | | |
| **Recommendation:** | | | | |
| **Done By:** | | **Signature** | **Date** | **Remark** |
| Tesfaye T., Paediatrician, Paediatric Cardiologist | |  | 26/08 ­­­­­­­­­­­­­­­­­/15 Eth.C. |  |

| **Tibebe – Ghion Specialized Teaching Hospital, Bahir Dar University,**  **Bahir Dar, Ethiopia** | | | | |
| --- | --- | --- | --- | --- |
| **Name: Sentayehu Genet. Sex/Age: F/1 7/12years. MRN: 178742. Date of Report: 26/08 /15 Eth.C.**  **Referral Diagnosis: CHF + PHTN. TGSH10.2806.** | | | | |
| **Features:** | **Findings** | | **Features** | **Findings** |
| **Profile** | | | **Atria** | |
| Abdominal Situs | Solitus | | Left Atrium | Dilated |
| Atrial Situs | Solitus | | Right Atrium | Dilated |
| Cardiac Position | Levocardia | | **Atrio-Ventricular Valves** | |
| Systemic Venous Drainage | To RA | | Mitral Valve | Annulus = 15mm |
| Pulmonary Venous Drainage | To LA | | Tricuspid Valve | Annulus = 20mm |
| Atrio-ventricular Connection | Concordant | |  | TAPSE = 10mm |
| Ventriculo-Arterial Connection | concordant | | **Ventricle** | |
| Ventricular Loop | d-Loop | | Left Ventricle | Dilated |
| **Septae** |  | | Right Ventricle | Dilated, Hypertrophied & Dysfunctional |
| Interatrial Septum | Intact | | **Doppler Measurement** |  |
| Interventricular Septum | Non-Restrictive Sub-aortic VSD, R – L Shunt | | Mitral | ------------- |
| **Semilunar Valves** |  | | Aortic | ------------- |
| Aortic Valve | Annulus = 12mm | | Tricuspid | ------------- |
| Pulmonary Valve | Annulus = 18mm | | Pulmonic | ------------- |
| **Great Arteries** | NRGA | | **Coronary Arteries** |  |
| Aorta |  | | **Aortic Arch** | Left. No CoA |
| Pulmonary Arteries | Dilated MPA & BPAs. | | **PDA** | No PDA |
| **M-Mode**: | | | | |
| Ao | mm | | PWd | mm |
| LA | mm | | EDV | ml |
| LVIDd | mm | | ESV | ml |
| LVIDs | mm | | FS | 18% |
| IVSd | mm | | LVEF | 38% |
| **Additional Information: 6mm Pericardial effusion on RV Side** | | | | |
| **Conclusion:**   1. {S, D, S} Levocardia 2. All chambers Dilated 3. Non-Restrictive Sub-aortic VSD, R – L Shunt 4. Severe Pulmonary Hypertension 5. Biventricular Systolic Dysfunction 6. Small Pericardial effusion | | | | |
| **Done By:** | | **Signature** | **Date** | **Remark** |
| Tesfaye T., Paediatrician, Paediatric Cardiologist | |  | 26/08 ­­­­­­­­­­­­­­­­­/15 Eth.C. |  |

| **Tibebe – Ghion Specialized Teaching Hospital, Bahir Dar University,**  **Bahir Dar, Ethiopia** | | | | |
| --- | --- | --- | --- | --- |
| **Name: Adanech Guadu . Sex/Age: F/34days. MRN: 178451 . Date of Report: 30/08 /15 Eth.C.**  **Referral Diagnosis: CHF + RD. TGSH10.2807.** | | | | |
| **Features:** | **Findings** | | **Features** | **Findings** |
| **Profile** | | | **Atria** | |
| Abdominal Situs | Solitus | | Left Atrium | Dilated |
| Atrial Situs | Solitus | | Right Atrium | Dilated |
| Cardiac Position | Levocardia | | **Atrio-Ventricular Valves** | |
| Systemic Venous Drainage | To RA | | Mitral Valve | Annulus = 13mm |
| Pulmonary Venous Drainage | To LA | | Tricuspid Valve | Annulus = 14mm |
| Atrio-ventricular Connection | Concordant | |  | **TAPSE = 7mm** |
| Ventriculo-Arterial Connection | concordant | | **Ventricle** | |
| Ventricular Loop | d-Loop | | Left Ventricle | Dilated |
| **Septae** |  | | Right Ventricle | **Hypertrophied, Dysfunctional & DCRV** |
| Interatrial Septum | **PFO, L – R Shunt** | | **Doppler Measurement** |  |
| Interventricular Septum | **2mm PM VSD, L – R Shunt. 9mm Lower Muscular VSD, L – R Shunt** | | Mitral | Mild MR, PPG = 3.3mmHg |
| **Semilunar Valves** |  | | Aortic | ------------- |
| Aortic Valve | Annulus = 7mm | | Tricuspid | ------------- |
| Pulmonary Valve | Annulus = 13mm | | Pulmonic | ------------- |
| **Great Arteries** | NRGA | | **Coronary Arteries** |  |
| Aorta |  | | **Aortic Arch** | Left. No CoA |
| Pulmonary Arteries | Normal MPA & BPAs. | | **PDA** | No PDA |
| **M-Mode**: | | | | |
| Ao | mm | | PWd | mm |
| LA | mm | | EDV | ml |
| LVIDd | mm | | ESV | ml |
| LVIDs | mm | | FS | **13%** |
| IVSd | mm | | LVEF | **30%** |
| **Additional Information:** | | | | |
| **Conclusion:**   1. {S, D, S} Levocardia 2. PFO, L – R Shunt 3. Small PM VSD, L – R Shunt 4. Large Lower Muscular VSD, L – R Shunt 5. DCRV 6. Reduced Biventricular Systolic Function | | | | |
| **Done By:** | | **Signature** | **Date** | **Remark** |
| Tesfaye T., Paediatrician, Paediatric Cardiologist | |  | 19/08 ­­­­­­­­­­­­­­­­­/15 Eth.C. |  |

| **Tibebe – Ghion Specialized Teaching Hospital, Bahir Dar University,**  **Bahir Dar, Ethiopia** | | | | |
| --- | --- | --- | --- | --- |
| **Name: Baby of Sewarege Necho. Sex/Age: M/11days . MRN: 177516. Date of Report: 01/09 /15 Eth.C.**  **Referral Diagnosis: Incidental Murmur. TGSH10.2808.** | | | | |
| **Features:** | **Findings** | | **Features** | **Findings** |
| **Profile** | | | **Atria** | |
| Abdominal Situs | Solitus | | Left Atrium | Normal |
| Atrial Situs | Solitus | | Right Atrium | Normal |
| Cardiac Position | Levocardia | | **Atrio-Ventricular Valves** | |
| Systemic Venous Drainage | To RA | | Mitral Valve | Annulus = mm |
| Pulmonary Venous Drainage | To LA | | Tricuspid Valve | Annulus = mm |
| Atrio-ventricular Connection | Concordant | |  | TAPSE = mm |
| Ventriculo-Arterial Connection | concordant | | **Ventricle** | |
| Ventricular Loop | d-Loop | | Left Ventricle | Normal |
| **Septae** |  | | Right Ventricle | Normal |
| Interatrial Septum | Intact | | **Doppler Measurement** |  |
| Interventricular Septum | Intact | | Mitral | ------------- |
| **Semilunar Valves** |  | | Aortic | ------------- |
| Aortic Valve | Annulus = mm | | Tricuspid | ------------- |
| Pulmonary Valve | Annulus = mm | | Pulmonic | ------------- |
| **Great Arteries** | NRGA | | **Coronary Arteries** |  |
| Aorta |  | | **Aortic Arch** | Left. No CoA |
| Pulmonary Arteries | Normal MPA & BPAs. | | **PDA** | No PDA |
| **M-Mode**: | | | | |
| Ao | mm | | PWd | mm |
| LA | mm | | EDV | ml |
| LVIDd | mm | | ESV | ml |
| LVIDs | mm | | FS | % |
| IVSd | mm | | LVEF | % |
| **Additional Information:** | | | | |
| **Conclusion:**   1. Normal Echocardiography Study | | | | |
| **Remark:** | | | | |
| **Recommendation:** | | | | |
| **Done By:** | | **Signature** | **Date** | **Remark** |
| Tesfaye T., Paediatrician, Paediatric Cardiologist | |  | 01/09 ­­­­­­­­­­­­­­­­­/15 Eth.C. |  |

| **Tibebe – Ghion Specialized Teaching Hospital, Bahir Dar University,**  **Bahir Dar, Ethiopia** | | | | |
| --- | --- | --- | --- | --- |
| **Name: Meseret Bitew . Sex/Age: F/10years. MRN: 176999. Date of Report: 03/09 /15 Eth.C.**  **Referral Diagnosis: Sydenham’s Chorea. TGSH10.2809.** | | | | |
| **Features:** | **Findings** | | **Features** | **Findings** |
| **Profile** | | | **Atria** | |
| Abdominal Situs | Solitus | | Left Atrium | Normal |
| Atrial Situs | Solitus | | Right Atrium | Normal |
| Cardiac Position | Levocardia | | **Atrio-Ventricular Valves** | |
| Systemic Venous Drainage | To RA | | Mitral Valve | Annulus = 21mm. Thickened MVL |
| Pulmonary Venous Drainage | To LA | | Tricuspid Valve | Annulus = 19mm |
| Atrio-ventricular Connection | Concordant | |  | TAPSE = mm |
| Ventriculo-Arterial Connection | concordant | | **Ventricle** | |
| Ventricular Loop | d-Loop | | Left Ventricle | Normal |
| **Septae** |  | | Right Ventricle | Normal |
| Interatrial Septum | Intact | | **Doppler Measurement** |  |
| Interventricular Septum | Intact | | Mitral | Mild MR, Holosystolic, Posterior projection, seen in two planes with jet velocity = 4.5m/sec. |
| **Semilunar Valves** |  | | Aortic | Moderate AR, PHT = 313ms |
| Aortic Valve | Annulus = 16mm | | Tricuspid | ------------- |
| Pulmonary Valve | Annulus = 18mm | | Pulmonic | ------------- |
| **Great Arteries** | NRGA | | **Coronary Arteries** |  |
| Aorta |  | | **Aortic Arch** | Left. No CoA |
| Pulmonary Arteries | Normal MPA & BPAs. | | **PDA** | No PDA |
| **M-Mode**: Normal LV Function on eye balling. | | | | |
| Ao | mm | | PWd | mm |
| LA | mm | | EDV | ml |
| LVIDd | mm | | ESV | ml |
| LVIDs | mm | | FS | % |
| IVSd | mm | | LVEF | % |
| **Additional Information:** | | | | |
| **Conclusion:**   1. {S, D, S} Levocardia 2. Thickened MVL 3. Mild MR 4. Moderate AR 5. Normal LV Systolic Function | | | | |
| **Done By:** | | **Signature** | **Date** | **Remark** |
| Tesfaye T., Paediatrician, Paediatric Cardiologist | |  | 03/09­­­­­­­­­­­­­­­­­/15 Eth.C. |  |

| **Tibebe – Ghion Specialized Teaching Hospital, Bahir Dar University,**  **Bahir Dar, Ethiopia** | | | | |
| --- | --- | --- | --- | --- |
| **Name: Amare Tilahun. Sex/Age: M /3 6/12years. MRN: 142690. Date of Report: 03/09 /15Eth.C.**  **Referral Diagnosis: Follow up echo for RV Dysfunction secondary to ?. AGH8.506** | | | | |
| **Features:** | **Findings** | | **Features** | **Findings** |
| **Profile** | | | **Atria** | |
| Abdominal Situs | Solitus | | Left Atrium | Normal |
| Atrial Situs | Solitus | | Right Atrium | Normal |
| Cardiac Position | Levocardia | | **Atrio-Ventricular Valves** | |
| Systemic Venous Drainage | To RA | | Mitral Valve | Annulus = 13mm |
| Pulmonary Venous Drainage | To LA | | Tricuspid Valve | Annulus = 15mm |
| Atrio-ventricular Connection | Concordant | |  | TAPSE = 15mm |
| Ventriculo-Arterial Connection | concordant | | **Ventricle** | |
| Ventricular Loop | d-Loop | | Left Ventricle | Normal |
| **Septae** |  | | Right Ventricle | Normal |
| Interatrial Septum | Intact | | **Doppler Measurement** |  |
| Interventricular Septum | Intact | | Mitral | ------------- |
| **Semilunar Valves** |  | | Aortic | ------------- |
| Aortic Valve | Annulus = 12mm | | Tricuspid | ------------- |
| Pulmonary Valve | Annulus = 12mm | | Pulmonic | ------------- |
| **Great Arteries** | NRGA | | **Coronary Arteries** |  |
| Aorta |  | | **Aortic Arch** | Left. No CoA |
| Pulmonary Arteries | Normal MPA & BPAs. | | **PDA** | No PDA |
| **M-Mode**: | | | | |
| Ao | mm | | PWd | mm |
| LA | mm | | EDV | ml |
| LVIDd | mm | | ESV | ml |
| LVIDs | mm | | FS | 41% |
| IVSd | mm | | LVEF | 75% |
| **Additional Information:** | | | | |
| **Conclusion:**   1. Normal Echocardiography Study | | | | |
| **Remark:** Work up for the polycythemia, if it is there. | | | | |
| **Recommendation:** | | | | |
| **Done By:** | | **Signature** | **Date** | **Remark** |
| Tesfaye T., Paediatrician, Paediatric Cardiologist | |  | 03/09 ­­­­­­­­­­­­­­­­­/15 Eth.C. |  |

| **Tibebe – Ghion Specialized Teaching Hospital, Bahir Dar University,**  **Bahir Dar, Ethiopia** | | | | |
| --- | --- | --- | --- | --- |
| **Name: Baby Sewareg Necho . Sex/Age: M/ 11days. MRN: . Date of Report: 03/09/15 Eth.C.**  **Referral Diagnosis: CHF + Incidental Murmur. TGSH10.2810.** | | | | |
| **Features:** | **Findings** | | **Features** | **Findings** |
| **Profile** | | | **Atria** | |
| Abdominal Situs | Solitus | | Left Atrium | Normal |
| Atrial Situs | Solitus | | Right Atrium | Normal |
| Cardiac Position | Levocardia | | **Atrio-Ventricular Valves** | |
| Systemic Venous Drainage | To RA | | Mitral Valve | Annulus = 10mm |
| Pulmonary Venous Drainage | To LA | | Tricuspid Valve | Annulus = 9mm |
| Atrio-ventricular Connection | Concordant | |  | TAPSE = mm |
| Ventriculo-Arterial Connection | concordant | | **Ventricle** | |
| Ventricular Loop | d-Loop | | Left Ventricle | Normal |
| **Septae** |  | | Right Ventricle | Normal |
| Interatrial Septum | Intact | | **Doppler Measurement** |  |
| Interventricular Septum | 7mm Inlet VSD, L – R Shunt | | Mitral | ------------- |
| **Semilunar Valves** |  | | Aortic | ------------- |
| Aortic Valve | Annulus = 9mm | | Tricuspid | ------------- |
| Pulmonary Valve | Annulus = 11mm | | Pulmonic | ------------- |
| **Great Arteries** | NRGA | | **Coronary Arteries** |  |
| Aorta |  | | **Aortic Arch** | Left. No CoA |
| Pulmonary Arteries | Normal MPA & BPAs. | | **PDA** | No PDA |
| **M-Mode**: | | | | |
| Ao | mm | | PWd | mm |
| LA | mm | | EDV | ml |
| LVIDd | mm | | ESV | ml |
| LVIDs | mm | | FS | % |
| IVSd | mm | | LVEF | % |
| **Additional Information:** | | | | |
| **Conclusion:**   1. {S, D, S} Levocardia 2. Large inlet VSD, L – R Shunt | | | | |
| **Remark:** | | | | |
| **Recommendation:** | | | | |
| **Done By:** | | **Signature** | **Date** | **Remark** |
| Tesfaye T., Paediatrician, Paediatric Cardiologist | |  | 03/09/15 Eth.C. |  |

| **Tibebe – Ghion Specialized Teaching Hospital, Bahir Dar University,**  **Bahir Dar, Ethiopia** | | | | |
| --- | --- | --- | --- | --- |
| **Name: Baye Getaye. Sex/Age: M/5 months. MRN: 179136. Date of Report: 03/09 /15 Eth.C.**  **Referral Diagnosis: Diaphoresis + Feeding interruption. TGSH10.2811.** | | | | |
| **Features:** | **Findings** | | **Features** | **Findings** |
| **Profile** | | | **Atria** | |
| Abdominal Situs | Inversus | | Left Atrium | Normal |
| Atrial Situs | Inversus | | Right Atrium | Normal |
| Cardiac Position | Dextrocardia | | **Atrio-Ventricular Valves** | |
| Systemic Venous Drainage | To RA | | Mitral Valve | Annulus = 17mm |
| Pulmonary Venous Drainage | To LA | | Tricuspid Valve | Annulus = 16mm |
| Atrio-ventricular Connection | Concordant | |  | TAPSE = mm |
| Ventriculo-Arterial Connection | DORV | | **Ventricle** | |
| Ventricular Loop | L-Loop | | Left Ventricle | Normal |
| **Septae** |  | | Right Ventricle | Normal |
| Interatrial Septum | PFO, L – R Shunt | | **Doppler Measurement** |  |
| Interventricular Septum | 11mm Inlet VSD, Right Side LV to Left Side RV. Non-Committed | | Mitral | ------------- |
| **Semilunar Valves** | Side by side | | Aortic | ------------- |
| Aortic Valve | Annulus = 10mm | | Tricuspid | ------------- |
| Pulmonary Valve | Annulus = 10mm | | Pulmonic | ------------- |
| **Great Arteries** | L-TGA | | **Coronary Arteries** |  |
| Aorta | From RV, left side | | **Aortic Arch** | Left. No CoA |
| Pulmonary Arteries | From RV, right side | | **PDA** | No PDA |
| **M-Mode**: | | | | |
| Ao | mm | | PWd | mm |
| LA | mm | | EDV | ml |
| LVIDd | mm | | ESV | ml |
| LVIDs | mm | | FS | % |
| IVSd | mm | | LVEF | % |
| **Additional Information:** | | | | |
| **Conclusion:**   1. {I, L, L} Dextrocardia 2. Situs Inversus Abdominalis 3. PFO, L – R Shunt 4. DORV With Non-Committed Large Inlet VSD, L – R Shunt | | | | |
| **Recommendation:** | | | | |
| **Done By:** | | **Signature** | **Date** | **Remark** |
| Tesfaye T., Paediatrician, Paediatric Cardiologist | |  | 03/09 ­­­­­­­­­­­­­­­­­/15 Eth.C. |  |

| **Tibebe – Ghion Specialized Teaching Hospital, Bahir Dar University,**  **Bahir Dar, Ethiopia** | | | | |
| --- | --- | --- | --- | --- |
| **Name: Gedefaw Birhanu. Sex/Age: M/11 months. MRN: 178479. Date of Report: 03/09 /15 Eth.C.**  **Referral Diagnosis: Feeding interruption. TGSH10.2812.** | | | | |
| **Features:** | **Findings** | | **Features** | **Findings** |
| **Profile** | | | **Atria** | |
| Abdominal Situs | Solitus | | Left Atrium | Normal |
| Atrial Situs | Solitus | | Right Atrium | Normal |
| Cardiac Position | Levocardia | | **Atrio-Ventricular Valves** | |
| Systemic Venous Drainage | To RA | | Mitral Valve | Annulus = 13mm |
| Pulmonary Venous Drainage | To LA | | Tricuspid Valve | Annulus = 14mm |
| Atrio-ventricular Connection | Concordant | |  | TAPSE = mm |
| Ventriculo-Arterial Connection | concordant | | **Ventricle** | |
| Ventricular Loop | d-Loop | | Left Ventricle | Normal |
| **Septae** |  | | Right Ventricle | Normal |
| Interatrial Septum | Intact | | **Doppler Measurement** |  |
| Interventricular Septum | 1.5mm PM VSD,,L – R Shunt with a gradient of 73mmHg | | Mitral | ------------- |
| **Semilunar Valves** |  | | Aortic | ------------- |
| Aortic Valve | Annulus = 12mm | | Tricuspid | ------------- |
| Pulmonary Valve | Annulus = 13mm | | Pulmonic | ------------- |
| **Great Arteries** | NRGA | | **Coronary Arteries** |  |
| Aorta |  | | **Aortic Arch** | Left. No CoA |
| Pulmonary Arteries | Normal MPA & BPAs. | | **PDA** | No PDA |
| **M-Mode**: | | | | |
| Ao | mm | | PWd | mm |
| LA | mm | | EDV | ml |
| LVIDd | mm | | ESV | ml |
| LVIDs | mm | | FS | % |
| IVSd | mm | | LVEF | % |
| **Additional Information:** | | | | |
| **Conclusion:**   1. {S, D, S} Levocardia 2. Restrictive PM VSD, L – R Shunt | | | | |
| **Remark:** | | | | |
| **Recommendation:** | | | | |
| **Done By:** | | **Signature** | **Date** | **Remark** |
| Tesfaye T., Paediatrician, Paediatric Cardiologist | |  | 03/09 ­­­­­­­­­­­­­­­­­/15 Eth.C. |  |

| **Tibebe – Ghion Specialized Teaching Hospital, Bahir Dar University,**  **Bahir Dar, Ethiopia** | | | | | | | |
| --- | --- | --- | --- | --- | --- | --- | --- |
| **Name: Ananya Abrham. Sex/Age: M/1 4/12. MRN: 113307. Date of Report:08/09 /15 Eth.C.**  **Referral Diagnosis: ?Cardio-embolic Stroke. TGSH10.2813.** | | | | | | | |
| **Features:** | | **Findings** | | | **Features** | | **Findings** |
| **Profile** | | | | | **Atria** | | |
| Abdominal Situs | Solitus | | | | Left Atrium | | Normal |
| Atrial Situs | Solitus | | | | Right Atrium | | Dilated |
| Cardiac Position | Levocardia | | | | **Atrio-Ventricular Valves** | | |
| Systemic Venous Drainage | To RA | | | | Mitral Valve | | Annulus = 10mm |
| Pulmonary Venous Drainage | To LA | | | | Tricuspid Valve | | Annulus = 16mm |
| Atrio-ventricular Connection | Concordant | | | |  | | TAPSE = 16mm |
| Ventriculo-Arterial Connection | concordant | | | | **Ventricle** | | |
| Ventricular Loop | d-Loop | | | | Left Ventricle | | Normal |
| **Septae** |  | | | | Right Ventricle | | Dilated |
| Interatrial Septum | 11 X 12mm OS ASD, L – R Shunt | | | | **Doppler Measurement** | |  |
| Interventricular Septum | Intact | | | | Mitral | | ------------- |
| **Semilunar Valves** |  | | | | Aortic | | ------------- |
| Aortic Valve | Annulus = 13mm | | | | Tricuspid | | Mild TR, PPG = 50mmHg |
| Pulmonary Valve | Annulus = 14mm | | | | Pulmonic | | ------------- |
| **Great Arteries** | NRGA | | | | **Coronary Arteries** | |  |
| Aorta |  | | | | **Aortic Arch** | | Left. No CoA |
| Pulmonary Arteries | Normal MPA & BPAs. | | | | **PDA** | | No PDA |
| **M-Mode**: Normal LV Function on eye balling. | | | | | | | |
| Ao | mm | | | | PWd | | mm |
| LA | mm | | | | EDV | | ml |
| LVIDd | mm | | | | ESV | | ml |
| LVIDs | mm | | | | FS | | % |
| IVSd | mm | | | | LVEF | | % |
| **Additional Information:** | | | | | | | |
| **Conclusion:**   1. {S, D, S} Levocardia 2. RA/RV Dilated 3. Moderate OS ASD, L – R Shunt 4. Moderate Pulmonary Hypertension 5. Normal Biventricular Systolic Function | | | | | | | |
| **Remark:** | | | | | | | |
| **Recommendation:** | | | | | | | |
| **Done By:** | | | **Signature** | **Date** | | **Remark** | |
| Tesfaye T., Paediatrician, Paediatric Cardiologist | | |  | 08/09 ­­­­­­­­­­­­­­­­­/15 Eth.C. | |  | |

| **Tibebe – Ghion Specialized Teaching Hospital, Bahir Dar University,**  **Bahir Dar, Ethiopia** | | | | | |
| --- | --- | --- | --- | --- | --- |
| **Name: Me’eraf Nega. Sex/Age: F/1 11/12years. MRN: 116688. Date of Report: 08/09 /15 Eth.C.**  **Referral Diagnosis: RD + Diaphoresis + CHF. TGSH10.2814.** | | | | | |
| **Features:** | | **Findings** | | **Features** | **Findings** |
| **Profile** | | | | **Atria** | |
| Abdominal Situs | Solitus | | | Left Atrium | Dilated |
| Atrial Situs | Solitus | | | Right Atrium | Dilated |
| Cardiac Position | Levocardia | | | **Atrio-Ventricular Valves** | |
| Systemic Venous Drainage | To RA | | | Mitral Valve | Annulus = 19mm |
| Pulmonary Venous Drainage | To LA | | | Tricuspid Valve | Annulus = 20mm |
| Atrio-ventricular Connection | Concordant | | |  | TAPSE = mm |
| Ventriculo-Arterial Connection | concordant | | | **Ventricle** | |
| Ventricular Loop | d-Loop | | | Left Ventricle | Dilated |
| **Septae** |  | | | Right Ventricle | Dilated |
| Interatrial Septum | Intact | | | **Doppler Measurement** |  |
| Interventricular Septum | 10mm PM VSD, L – R Shunt | | | Mitral | ------------- |
| **Semilunar Valves** |  | | | Aortic | ------------- |
| Aortic Valve | Annulus = 16mm | | | Tricuspid | ------------- |
| Pulmonary Valve | Annulus = 21mm | | | Pulmonic | ------------- |
| **Great Arteries** | NRGA | | | **Coronary Arteries** |  |
| Aorta |  | | | **Aortic Arch** | Left. No CoA |
| Pulmonary Arteries | MPA = 22mm. | | | **PDA** | No PDA |
| **M-Mode**: Normal LV Function on eye balling. | | | | | |
| Ao | mm | | | PWd | mm |
| LA | mm | | | EDV | ml |
| LVIDd | mm | | | ESV | ml |
| LVIDs | mm | | | FS | % |
| IVSd | mm | | | LVEF | % |
| **Additional Information:** | | | | | |
| **Conclusion:**   1. {S, D, S} Levocardia 2. All chambers dilated 3. Large PM VSD, L – R Shunt 4. Pulmonary Hypertension 5. Normal LV Function on eye balling | | | | | |
| **Recommendation:** | | | | | |
| **Done By:** | | | **Signature** | **Date** | **Remark** |
| Tesfaye T., Paediatrician, Paediatric Cardiologist | | |  | 08/09 ­­­­­­­­­­­­­­­­­/15 Eth.C. |  |

| **Tibebe – Ghion Specialized Teaching Hospital, Bahir Dar University,**  **Bahir Dar, Ethiopia** | | | | |
| --- | --- | --- | --- | --- |
| **Name: Baby of Sindu Zerie . Sex/Age: F/24days. MRN: . Date of Report: 08/09 /15 Eth.C.**  **Referral Diagnosis: Incidental Murmur. (No Conclusion)** | | | | |
| **Features:** | **Findings** | | **Features** | **Findings** |
| **Profile** | | | **Atria** | |
| Abdominal Situs | Solitus | | Left Atrium | Dilated |
| Atrial Situs | Solitus | | Right Atrium | Normal |
| Cardiac Position | Levocardia | | **Atrio-Ventricular Valves** | |
| Systemic Venous Drainage | To RA | | Mitral Valve | Annulus = 12mm. Thickened MVL. |
| Pulmonary Venous Drainage | To LA | | Tricuspid Valve | Annulus = 12mm |
| Atrio-ventricular Connection | Concordant | |  | TAPSE = mm |
| Ventriculo-Arterial Connection | concordant | | **Ventricle** | |
| Ventricular Loop | d-Loop | | Left Ventricle | Dilated |
| **Septae** |  | | Right Ventricle | Normal |
| Interatrial Septum | PFO, L – R Shunt | | **Doppler Measurement** |  |
| Interventricular Septum | Intact | | Mitral | Moderate MR, Posterior projection, seen in two planes with jet velocity = 3.6m/sec. |
| **Semilunar Valves** |  | | Aortic | ------------- |
| Aortic Valve | Annulus = 8mm | | Tricuspid | ------------- |
| Pulmonary Valve | Annulus = 9mm | | Pulmonic | ------------- |
| **Great Arteries** | NRGA | | **Coronary Arteries** |  |
| Aorta |  | | **Aortic Arch** | Left. No CoA |
| Pulmonary Arteries | Normal MPA & BPAs. | | **PDA** | No PDA |
| **M-Mode**: Normal LV Function on eye balling. | | | | |
| Ao | mm | | PWd | mm |
| LA | mm | | EDV | ml |
| LVIDd | mm | | ESV | ml |
| LVIDs | mm | | FS | % |
| IVSd | mm | | LVEF | % |
| **Additional Information:** | | | | |
| **Conclusion:**   1. {S, D, S} Levocardia 2. LA/LV Dilated 3. PFO, L – R Shunt 4. Thickened MVL 5. Moderate MR 6. Normal LV Systolic Function | | | | |
| **Done By:** | | **Signature** | **Date** | **Remark** |
| Tesfaye T., Paediatrician, Paediatric Cardiologist | |  | 08/09 ­­­­­­­­­­­­­­­­­/15 Eth.C. |  |

| **Tibebe – Ghion Specialized Teaching Hospital, Bahir Dar University,**  **Bahir Dar, Ethiopia** | | | | |
| --- | --- | --- | --- | --- |
| **Name: Sisay Ashagrie. Sex/Age: M/3years. MRN: 130774. Date of Report: 08/09 /15 Eth.C.**  **Referral Diagnosis: Follow up echo for after OSA/H surgery + Pulmonary Hypertension.** | | | | |
| **Features:** | **Findings** | | **Features** | **Findings** |
| **Profile** | | | **Atria** | |
| Abdominal Situs | Solitus | | Left Atrium | Normal |
| Atrial Situs | Solitus | | Right Atrium | Normal |
| Cardiac Position | Levocardia | | **Atrio-Ventricular Valves** | |
| Systemic Venous Drainage | To RA | | Mitral Valve | Annulus = 18mm |
| Pulmonary Venous Drainage | To LA | | Tricuspid Valve | Annulus = 20mm |
| Atrio-ventricular Connection | Concordant | |  | TAPSE = 19mm |
| Ventriculo-Arterial Connection | concordant | | **Ventricle** | |
| Ventricular Loop | d-Loop | | Left Ventricle | Normal |
| **Septae** |  | | Right Ventricle | Normal |
| Interatrial Septum | Intact | | **Doppler Measurement** |  |
| Interventricular Septum | Intact | | Mitral | ------------- |
| **Semilunar Valves** |  | | Aortic | ------------- |
| Aortic Valve | Annulus = 15mm | | Tricuspid | ------------- |
| Pulmonary Valve | Annulus = 18mm | | Pulmonic | ------------- |
| **Great Arteries** | NRGA | | **Coronary Arteries** |  |
| Aorta |  | | **Aortic Arch** | Left. No CoA |
| Pulmonary Arteries | Normal MPA & BPAs. | | **PDA** | No PDA |
| **M-Mode**: | | | | |
| Ao | mm | | PWd | mm |
| LA | mm | | EDV | ml |
| LVIDd | mm | | ESV | ml |
| LVIDs | mm | | FS | 33% |
| IVSd | mm | | LVEF | 63% |
| **Additional Information:** | | | | |
| **Conclusion:**   1. Normal Echocardiography Study | | | | |
| **Remark:** | | | | |
| **Recommendation:** | | | | |
| **Done By:** | | **Signature** | **Date** | **Remark** |
| Tesfaye T., Paediatrician, Paediatric Cardiologist | |  | 12/08 ­­­­­­­­­­­­­­­­­/15 Eth.C. |  |

| **Tibebe – Ghion Specialized Teaching Hospital, Bahir Dar University,**  **Bahir Dar, Ethiopia** | | | | |
| --- | --- | --- | --- | --- |
| **Name: Shikur Endris. Sex/Age: M/14years. MRN: 075288. Date of Report: 10/09 /15 Eth.C.**  **Referral Diagnosis: Follow up echo for RHD (Moderate MR). SEE AGH06.1510** | | | | |
| **Features:** | **Findings** | | **Features** | **Findings** |
| **Profile** | | | **Atria** | |
| Abdominal Situs | Solitus | | Left Atrium | Normal |
| Atrial Situs | Solitus | | Right Atrium | Normal |
| Cardiac Position | Levocardia | | **Atrio-Ventricular Valves** | |
| Systemic Venous Drainage | To RA | | Mitral Valve | Annulus = 20mm. Thickened MVL |
| Pulmonary Venous Drainage | To LA | | Tricuspid Valve | Annulus = 20mm |
| Atrio-ventricular Connection | Concordant | |  | TAPSE = 17mm |
| Ventriculo-Arterial Connection | concordant | | **Ventricle** | |
| Ventricular Loop | d-Loop | | Left Ventricle | Normal |
| **Septae** |  | | Right Ventricle | Normal |
| Interatrial Septum | Intact | | **Doppler Measurement** |  |
| Interventricular Septum | Intact | | Mitral | Mild MR, Holosystolic, posterior projection, seen in two planes with jet velocity = 5.2m/sec. |
| **Semilunar Valves** |  | | Aortic | ------------- |
| Aortic Valve | Annulus = 19mm | | Tricuspid | ------------- |
| Pulmonary Valve | Annulus = 21mm | | Pulmonic | ------------- |
| **Great Arteries** | NRGA | | **Coronary Arteries** |  |
| Aorta |  | | **Aortic Arch** | Left. No CoA |
| Pulmonary Arteries | Normal MPA & BPAs. | | **PDA** | No PDA |
| **M-Mode**: | | | | |
| Ao | mm | | PWd | mm |
| LA | mm | | EDV | ml |
| LVIDd | mm | | ESV | ml |
| LVIDs | mm | | FS | 31% |
| IVSd | mm | | LVEF | 58% |
| **Additional Information:** | | | | |
| **Conclusion:**   1. {S, D, S} Levocardia 2. Thickened MVL 3. Mild MR 4. Normal Biventricular Systolic Function | | | | |
| **Recommendation:** | | | | |
| **Done By:** | | **Signature** | **Date** | **Remark** |
| Tesfaye T., Paediatrician, Paediatric Cardiologist | |  | 10/09 ­­­­­­­­­­­­­­­­­/15 Eth.C. |  |

| **Tibebe – Ghion Specialized Teaching Hospital, Bahir Dar University,**  **Bahir Dar, Ethiopia** | | | | |
| --- | --- | --- | --- | --- |
| **Name: Fasika Getachew. Sex/Age: F/12years. MRN: 180246. Date of Report: 10/09 /15 Eth.C.**  **Referral Diagnosis: ARF with Carditis. TGSH10.2815.** | | | | |
| **Features:** | **Findings** | | **Features** | **Findings** |
| **Profile** | | | **Atria** | |
| Abdominal Situs | Solitus | | Left Atrium | Normal |
| Atrial Situs | Solitus | | Right Atrium | Normal |
| Cardiac Position | Levocardia | | **Atrio-Ventricular Valves** | |
| Systemic Venous Drainage | To RA | | Mitral Valve | Annulus = 20mm. Thickened MVL |
| Pulmonary Venous Drainage | To LA | | Tricuspid Valve | Annulus = 19mm |
| Atrio-ventricular Connection | Concordant | |  | TAPSE = 22mm |
| Ventriculo-Arterial Connection | concordant | | **Ventricle** | |
| Ventricular Loop | d-Loop | | Left Ventricle | Normal |
| **Septae** |  | | Right Ventricle | Normal |
| Interatrial Septum | Intact | | **Doppler Measurement** |  |
| Interventricular Septum | Intact | | Mitral | Moderate MR, Holosystolic, posterior projection, seen in two planes with jet velocity = 4.4m/sec |
| **Semilunar Valves** |  | | Aortic | ------------- |
| Aortic Valve | Annulus = 17mm | | Tricuspid | ------------- |
| Pulmonary Valve | Annulus = 19mm | | Pulmonic | ------------- |
| **Great Arteries** | NRGA | | **Coronary Arteries** |  |
| Aorta |  | | **Aortic Arch** | Left. No CoA |
| Pulmonary Arteries | Normal MPA & BPAs. | | **PDA** | No PDA |
| **M-Mode**: | | | | |
| Ao | mm | | PWd | mm |
| LA | mm | | EDV | ml |
| LVIDd | mm | | ESV | ml |
| LVIDs | mm | | FS | 36% |
| IVSd | mm | | LVEF | 67% |
| **Additional Information:** | | | | |
| **Conclusion:**   1. {S, D, S} Levocardia 2. Thickened MVL 3. Moderate MR 4. Normal Biventricular Systolic Function | | | | |
| **Recommendation:** | | | | |
| **Done By:** | | **Signature** | **Date** | **Remark** |
| Tesfaye T., Paediatrician, Paediatric Cardiologist | |  | 10/09 ­­­­­­­­­­­­­­­­­/15 Eth.C. |  |

| **Tibebe – Ghion Specialized Teaching Hospital, Bahir Dar University,**  **Bahir Dar, Ethiopia** | | | | |
| --- | --- | --- | --- | --- |
| **Name: Haymanot Tafere. Sex/Age: M/3years. MRN: 180259 . Date of Report: 10/09 /15 Eth.C.**  **Referral Diagnosis: FTT + Incidental Murmur. TGSH10.2816.** | | | | |
| **Features:** | **Findings** | | **Features** | **Findings** |
| **Profile** | | | **Atria** | |
| Abdominal Situs | Solitus | | Left Atrium | Mildly Dilated |
| Atrial Situs | Solitus | | Right Atrium | Normal |
| Cardiac Position | Levocardia | | **Atrio-Ventricular Valves** | |
| Systemic Venous Drainage | To RA | | Mitral Valve | Annulus = 18mm |
| Pulmonary Venous Drainage | To LA | | Tricuspid Valve | Annulus = 15mm |
| Atrio-ventricular Connection | Concordant | |  | TAPSE = mm |
| Ventriculo-Arterial Connection | concordant | | **Ventricle** | |
| Ventricular Loop | d-Loop | | Left Ventricle | Mildly Dilated |
| **Septae** |  | | Right Ventricle | Normal |
| Interatrial Septum | Intact | | **Doppler Measurement** |  |
| Interventricular Septum | 6mm Upper Muscular VSD, L – R Shunt | | Mitral | ------------- |
| **Semilunar Valves** |  | | Aortic | ------------- |
| Aortic Valve | Annulus = 15mm | | Tricuspid | ------------- |
| Pulmonary Valve | Annulus = 17mm | | Pulmonic | ------------- |
| **Great Arteries** | NRGA | | **Coronary Arteries** |  |
| Aorta |  | | **Aortic Arch** | Left. No CoA |
| Pulmonary Arteries | Normal MPA & BPAs. | | **PDA** | 1mm PDA, L – R Shunt |
| **M-Mode**: | | | | |
| Ao | mm | | PWd | mm |
| LA | mm | | EDV | ml |
| LVIDd | mm | | ESV | ml |
| LVIDs | mm | | FS | 35% |
| IVSd | mm | | LVEF | 64% |
| **Additional Information:** Echogenic Mass Measuring 0.2 X 0.5mm On The RV Side Of The VSD. | | | | |
| **Conclusion:**   1. {S, D, S} Levocardia 2. Small Upper Muscular VSD, L – R Shunt 3. Small PDA, L – R Shunt 4. ?Infective Endocarditis 5. Normal LV Systolic Function | | | | |
| **Recommendation:** Work up in the line of IE | | | | |
| **Done By:** | | **Signature** | **Date** | **Remark** |
| Tesfaye T., Paediatrician, Paediatric Cardiologist | |  | 10/09 ­­­­­­­­­­­­­­­­­/15 Eth.C. |  |

| **Tibebe – Ghion Specialized Teaching Hospital, Bahir Dar University,**  **Bahir Dar, Ethiopia** | | | | |
| --- | --- | --- | --- | --- |
| **Name: Simegn Engedaw. Sex/Age: F/6years. MRN: 180289. Date of Report: 10/09 /15 Eth.C.**  **Referral Diagnosis: CRVHD + Clubbing. TGSH10.2817.** | | | | |
| **Features:** | **Findings** | | **Features** | **Findings** |
| **Profile** | | | **Atria** | |
| Abdominal Situs | Solitus | | Left Atrium | Normal |
| Atrial Situs | Solitus | | Right Atrium | Dilated |
| Cardiac Position | Levocardia | | **Atrio-Ventricular Valves** | |
| Systemic Venous Drainage | To RA | | Mitral Valve | Annulus = 18mm |
| Pulmonary Venous Drainage | To LA | | Tricuspid Valve | Annulus = 21mm |
| Atrio-ventricular Connection | Concordant | |  | TAPSE = mm |
| Ventriculo-Arterial Connection | concordant | | **Ventricle** | |
| Ventricular Loop | d-Loop | | Left Ventricle | Normal |
| **Septae** |  | | Right Ventricle | Dilated & Hypertrophied |
| Interatrial Septum | Intact | | **Doppler Measurement** |  |
| Interventricular Septum | Mal-aligned Non-Restrictive sub-aortic VSD, R – L Shunt | | Mitral | ------------- |
| **Semilunar Valves** |  | | Aortic | ------------- |
| Aortic Valve | Annulus = 23mm | | Tricuspid | ------------- |
| Pulmonary Valve | Annulus = 12mm | | Pulmonic | Severe PS, PPG = 67mmHg |
| **Great Arteries** | NRGA | | **Coronary Arteries** |  |
| Aorta | Over-riding aorta | | **Aortic Arch** | Left. No CoA |
| Pulmonary Arteries | Smallish MPA | | **PDA** | No PDA |
| **M-Mode**: Normal LV Function on eye balling | | | | |
| Ao | mm | | PWd | mm |
| LA | mm | | EDV | ml |
| LVIDd | mm | | ESV | ml |
| LVIDs | mm | | FS | % |
| IVSd | mm | | LVEF | % |
| **Additional Information: echogenic mass in the MPA measuring 5 X 8mm** | | | | |
| **Conclusion:**   1. {S, D, S} Levocardia 2. TOF 3. Echogenic mass in the MPA (?IE) | | | | |
| **Remark:** | | | | |
| **Recommendation:** | | | | |
| **Done By:** | | **Signature** | **Date** | **Remark** |
| Tesfaye T., Paediatrician, Paediatric Cardiologist | |  | 10/09 ­­­­­­­­­­­­­­­­­/15 Eth.C. |  |

| **Tibebe – Ghion Specialized Teaching Hospital, Bahir Dar University,**  **Bahir Dar, Ethiopia** | | | | |
| --- | --- | --- | --- | --- |
| **Name: Alemu Demelashe. Sex/Age: M/79days. MRN: 180251. Date of Report: 10/09 /15 Eth.C.**  **Referral Diagnosis: Cardiomegaly on CXR + DS. TGSH10.2818.** | | | | |
| **Features:** | **Findings** | | **Features** | **Findings** |
| **Profile** | | | **Atria** | |
| Abdominal Situs | Solitus | | Left Atrium | Normal |
| Atrial Situs | Solitus | | Right Atrium | Dilated |
| Cardiac Position | Levocardia | | **Atrio-Ventricular Valves** | |
| Systemic Venous Drainage | To RA | | Mitral Valve | Annulus = 10mm |
| Pulmonary Venous Drainage | To LA | | Tricuspid Valve | Annulus = 14mm |
| Atrio-ventricular Connection | Concordant | |  | TAPSE = 13mm |
| Ventriculo-Arterial Connection | concordant | | **Ventricle** | |
| Ventricular Loop | d-Loop | | Left Ventricle | Normal |
| **Septae** | Tongue of tissue in b/n | | Right Ventricle | Dilated |
| Interatrial Septum | 11mm Primum defect, L- R Shunt | | **Doppler Measurement** |  |
| Interventricular Septum | 3mm Inlet VSD, L – R Shunt | | Mitral | ------------- |
| **Semilunar Valves** |  | | Aortic | ------------- |
| Aortic Valve | Annulus = 8mm | | Tricuspid | ------------- |
| Pulmonary Valve | Annulus = 10mm | | Pulmonic | ------------- |
| **Great Arteries** | NRGA | | **Coronary Arteries** |  |
| Aorta |  | | **Aortic Arch** | Left. No CoA |
| Pulmonary Arteries | Normal MPA & BPAs. | | **PDA** | No PDA |
| **M-Mode**: Normal LV Function on eye balling. | | | | |
| Ao | mm | | PWd | mm |
| LA | mm | | EDV | ml |
| LVIDd | mm | | ESV | ml |
| LVIDs | mm | | FS | % |
| IVSd | mm | | LVEF | % |
| **Additional Information:** | | | | |
| **Conclusion:**   1. {S, D, S} Levocardia 2. Transitional AVSD, L – R Shunt | | | | |
| **Remark:** | | | | |
| **Recommendation:** | | | | |
| **Done By:** | | **Signature** | **Date** | **Remark** |
| Tesfaye T., Paediatrician, Paediatric Cardiologist | |  | 10/09 ­­­­­­­­­­­­­­­­­/15 Eth.C. |  |

| **Tibebe – Ghion Specialized Teaching Hospital, Bahir Dar University,**  **Bahir Dar, Ethiopia** | | | | |
| --- | --- | --- | --- | --- |
| **Name: Baby of Alemnesh Getie . Sex/Age: F/9days. MRN:179650 . Date of Report: 03/08 /15 Eth.C.**  **Referral Diagnosis: DS + Incidental Murmur. TGSH10.2819.** | | | | |
| **Features:** | **Findings** | | **Features** | **Findings** |
| **Profile** | | | **Atria** | |
| Abdominal Situs | Solitus | | Left Atrium | Normal |
| Atrial Situs | Solitus | | Right Atrium | Normal |
| Cardiac Position | Levocardia | | **Atrio-Ventricular Valves** | |
| Systemic Venous Drainage | To RA | | Mitral Valve | Annulus = 9mm |
| Pulmonary Venous Drainage | To LA | | Tricuspid Valve | Annulus = 10mm |
| Atrio-ventricular Connection | Concordant | |  | TAPSE = mm |
| Ventriculo-Arterial Connection | concordant | | **Ventricle** | |
| Ventricular Loop | d-Loop | | Left Ventricle | Normal |
| **Septae** |  | | Right Ventricle | Normal |
| Interatrial Septum | 4mm OS ASD, L – R Shunt | | **Doppler Measurement** |  |
| Interventricular Septum | 1mm PM VSD, L – R Shunt | | Mitral | ------------- |
| **Semilunar Valves** |  | | Aortic | ------------- |
| Aortic Valve | Annulus = 8mm | | Tricuspid | ------------- |
| Pulmonary Valve | Annulus = 9mm | | Pulmonic | ------------- |
| **Great Arteries** | NRGA | | **Coronary Arteries** |  |
| Aorta |  | | **Aortic Arch** | Left. No CoA |
| Pulmonary Arteries | Normal MPA & BPAs. | | **PDA** | 1.5mm PDA, L – R Shunt |
| **M-Mode**: Normal LV Function on eye balling | | | | |
| Ao | mm | | PWd | mm |
| LA | mm | | EDV | ml |
| LVIDd | mm | | ESV | ml |
| LVIDs | mm | | FS | % |
| IVSd | mm | | LVEF | % |
| **Additional Information:** | | | | |
| **Conclusion:**   1. {S, D, S} Levocardia 2. Small OS ASD, L – R Shunt 3. Tiny PM VSD, L – R Shunt 4. Small PDA, L – R Shunt 5. Normal LV Systolic Function | | | | |
| **Remark:** | | | | |
| **Recommendation:** | | | | |
| **Done By:** | | **Signature** | **Date** | **Remark** |
| Tesfaye T., Paediatrician, Paediatric Cardiologist | |  | 12/08 ­­­­­­­­­­­­­­­­­/15 Eth.C. |  |

| **Tibebe – Ghion Specialized Teaching Hospital, Bahir Dar University,**  **Bahir Dar, Ethiopia** | | | | |
| --- | --- | --- | --- | --- |
| **Name: Abrham Molla . Sex/Age: M / 8 years. MRN: 179756 . Date of Report: 03/08 /15 Eth.C.**  **Referral Diagnosis: Sepsis + ? vascular disorder + gangrene(wet). TGSH10.2820.** | | | | |
| **Features:** | **Findings** | | **Features** | **Findings** |
| **Profile** | | | **Atria** | |
| Abdominal Situs | Solitus | | Left Atrium | Normal |
| Atrial Situs | Solitus | | Right Atrium | Normal |
| Cardiac Position | Levocardia | | **Atrio-Ventricular Valves** | |
| Systemic Venous Drainage | To RA | | Mitral Valve | Annulus = 21mm |
| Pulmonary Venous Drainage | To LA | | Tricuspid Valve | Annulus = 20mm |
| Atrio-ventricular Connection | Concordant | |  | TAPSE = 22mm |
| Ventriculo-Arterial Connection | concordant | | **Ventricle** | |
| Ventricular Loop | d-Loop | | Left Ventricle | Normal |
| **Septae** |  | | Right Ventricle | Normal |
| Interatrial Septum | Intact | | **Doppler Measurement** |  |
| Interventricular Septum | Intact | | Mitral | ------------- |
| **Semilunar Valves** |  | | Aortic | ------------- |
| Aortic Valve | Annulus = 17mm | | Tricuspid | ------------- |
| Pulmonary Valve | Annulus = 20mm | | Pulmonic | ------------- |
| **Great Arteries** | NRGA | | **Coronary Arteries** |  |
| Aorta |  | | **Aortic Arch** | Left. No CoA |
| Pulmonary Arteries | Normal MPA & BPAs. | | **PDA** | No PDA |
| **M-Mode**: | | | | |
| Ao | mm | | PWd | mm |
| LA | mm | | EDV | ml |
| LVIDd | mm | | ESV | ml |
| LVIDs | mm | | FS | 41% |
| IVSd | mm | | LVEF | 73% |
| **Additional Information:** | | | | |
| **Conclusion:**   1. Normal Echocardiography Study | | | | |
| **Remark:** | | | | |
| **Recommendation:** | | | | |
| **Done By:** | | **Signature** | **Date** | **Remark** |
| Tesfaye T., Paediatrician, Paediatric Cardiologist | |  | 12/08 ­­­­­­­­­­­­­­­­­/15 Eth.C. |  |

| **Tibebe – Ghion Specialized Teaching Hospital, Bahir Dar University,**  **Bahir Dar, Ethiopia** | | | | |
| --- | --- | --- | --- | --- |
| **Name: Tadla Esubalew. Sex/Age: F/7years. MRN:179824. Date of Report: 14/08 /15 Eth.C.**  **Referral Diagnosis: Sydenham’s Chorea. TGSH10.2821.** | | | | |
| **Features:** | **Findings** | | **Features** | **Findings** |
| **Profile** | | | **Atria** | |
| Abdominal Situs | Solitus | | Left Atrium | Normal |
| Atrial Situs | Solitus | | Right Atrium | Normal |
| Cardiac Position | Levocardia | | **Atrio-Ventricular Valves** | |
| Systemic Venous Drainage | To RA | | Mitral Valve | Annulus = 22mm. Thickened MVL |
| Pulmonary Venous Drainage | To LA | | Tricuspid Valve | Annulus = 25mm |
| Atrio-ventricular Connection | Concordant | |  | TAPSE = mm |
| Ventriculo-Arterial Connection | concordant | | **Ventricle** | |
| Ventricular Loop | d-Loop | | Left Ventricle | Normal |
| **Septae** |  | | Right Ventricle | Normal |
| Interatrial Septum | Intact | | **Doppler Measurement** |  |
| Interventricular Septum | Intact | | Mitral | Mild MR, Holosystolic, posterior projection, seen in two planes with jet velocity = 4.6m/sec. |
| **Semilunar Valves** |  | | Aortic | ------------- |
| Aortic Valve | Annulus = 18mm | | Tricuspid | Trivial TR, PPG = 22mmHg |
| Pulmonary Valve | Annulus = 20mm | | Pulmonic | ------------- |
| **Great Arteries** | NRGA | | **Coronary Arteries** |  |
| Aorta |  | | **Aortic Arch** | Left. No CoA |
| Pulmonary Arteries | Normal MPA & BPAs. | | **PDA** | No PDA |
| **M-Mode**: | | | | |
| Ao | mm | | PWd | mm |
| LA | mm | | EDV | ml |
| LVIDd | mm | | ESV | ml |
| LVIDs | mm | | FS | 39% |
| IVSd | mm | | LVEF | 71% |
| **Additional Information:** | | | | |
| **Conclusion:**   1. {S, D, S} Levocardia 2. Thickened MVL 3. Mild MR | | | | |
| **Done By:** | | **Signature** | **Date** | **Remark** |
| Tesfaye T., Paediatrician, Paediatric Cardiologist | |  | 14/08 ­­­­­­­­­­­­­­­­­/15 Eth.C. |  |

| **Tibebe – Ghion Specialized Teaching Hospital, Bahir Dar University,**  **Bahir Dar, Ethiopia** | | | | |
| --- | --- | --- | --- | --- |
| **Name: Abebe Eyilet. Sex/Age: M/ 7yrs. MRN: 181699. Date of Report: 17/09 /15 E.C.**  **R.Dx: CHF + Rheumatic Recurrence. TGSH10.2822.** | | | | |
| **Features:** | **Findings** | | **Features** | **Findings** |
| **Profile** | | | **Atria** | |
| Abdominal Situs | Solitus | | Left Atrium | Dilated |
| Atrial Situs | Solitus | | Right Atrium | Dilated |
| Cardiac Position | Levocardia | | **Atrio-Ventricular Valves** | |
| Systemic Venous Drainage | To RA | | Mitral Valve | Annulus = 24mm. Thickened MVL |
| Pulmonary Venous Drainage | To LA | | Tricuspid Valve | Annulus = 23mm |
| Atrio-ventricular Connection | Concordant | |  | TAPSE = 17mm |
| Ventriculo-Arterial Connection | concordant | | **Ventricle** | |
| Ventricular Loop | d-Loop | | Left Ventricle | Dilated |
| **Septae** |  | | Right Ventricle | Dilated |
| Interatrial Septum | Intact | | **Doppler Measurement** |  |
| Interventricular Septum | Intact | | Mitral | Severe MR, Holosystolic, Posterior projection, seen in two planes with jet velocity = 4.7m/sec. |
| **Semilunar Valves** |  | | Aortic | Mild AR, PHT = 644ms.Moderate AS, PPG/MPG = 56/33mmHg |
| Aortic Valve | Annulus = 16mm. Trileaflet | | Tricuspid | Mild TR, PPG = 55mmHg |
| Pulmonary Valve | Annulus = 23mm | | Pulmonic | Mild PR, PPG = 52mmHg |
| **Great Arteries** | NRGA | | **Coronary Arteries** |  |
| Aorta |  | | **Aortic Arch** | Left. No CoA |
| Pulmonary Arteries | Normal MPA & BPAs. | | **PDA** | No PDA |
| **M-Mode**: | | | | |
| Ao | mm | | PWd | mm |
| LA | mm | | EDV | ml |
| LVIDd | mm | | ESV | ml |
| LVIDs | mm | | FS | 16% |
| IVSd | mm | | LVEF | 34% |
| **Additional Information:** 11mm Right Pleural effusion. Circumferential Pericardial effusion with Maximum depth of 9mm on RA/RV Side. | | | | |
| **Conclusion:**   1. {S, D, S} Levocardia 2. LA/LV Dilated 3. Thickened MVL and AVL 4. Severe MR 5. Moderate AS 6. Mild AR 7. Mild TR 8. Mild PR 9. Moderate Pulmonary Hypertension 10. Moderate Right Pleural effusion 11. Small Circumferential Pericardial effusion 12. Moderately reduced LV Systolic Function | | | | |
| **Done By:** | | **Signature** | **Date** | **Remark** |
| Tesfaye T., Paediatrician, Paediatric Cardiologist | |  | 17/09 ­­­­­­­­­­­­­­­­­/15 Eth.C. |  |

| **Tibebe – Ghion Specialized Teaching Hospital, Bahir Dar University,**  **Bahir Dar, Ethiopia** | | | | |
| --- | --- | --- | --- | --- |
| **Name: Zebura Ibrahim. Sex/Age: F/ 42 Days. MRN: 180661. Date of Report: 17/09 /15 Eth.C.**  **Referral Diagnosis: Diaphoresis + RD + Murmur. TGSH10.2823.** | | | | |
| **Features:** | **Findings** | | **Features** | **Findings** |
| **Profile** | | | **Atria** | |
| Abdominal Situs | Solitus | | Left Atrium | Normal |
| Atrial Situs | Solitus | | Right Atrium | Dilated |
| Cardiac Position | Levocardia | | **Atrio-Ventricular Valves** | |
| Systemic Venous Drainage | To RA | | Mitral Valve | Annulus = 9mm |
| Pulmonary Venous Drainage | To LA | | Tricuspid Valve | Annulus = 11mm |
| Atrio-ventricular Connection | Concordant | |  | TAPSE = mm |
| Ventriculo-Arterial Connection | concordant | | **Ventricle** | |
| Ventricular Loop | d-Loop | | Left Ventricle | Normal |
| **Septae** |  | | Right Ventricle | Dilated |
| Interatrial Septum | 8mm Ostium Primum Defect, L – R Shunt | | **Doppler Measurement** |  |
| Interventricular Septum | 8mm Sub-aortic VSD, L – R Shunt | | Mitral | ------------- |
| **Semilunar Valves** |  | | Aortic | ------------- |
| Aortic Valve | Annulus = 7mm | | Tricuspid | ------------- |
| Pulmonary Valve | Annulus = 13mm | | Pulmonic | ------------- |
| **Great Arteries** | NRGA | | **Coronary Arteries** |  |
| Aorta |  | | **Aortic Arch** | Left. No CoA |
| Pulmonary Arteries | Normal MPA & BPAs. | | **PDA** | No PDA |
| **M-Mode**: Normal LV Function on eye balling | | | | |
| Ao | mm | | PWd | mm |
| LA | mm | | EDV | ml |
| LVIDd | mm | | ESV | ml |
| LVIDs | mm | | FS | % |
| IVSd | mm | | LVEF | % |
| **Additional Information:** | | | | |
| **Conclusion:**   1. {S, D, S} Levocardia 2. Moderate Ostium Primum Defect, L – R Shunt 3. Large Sub-aortic VSD, L – R Shunt 4. Normal LV Systolic Function | | | | |
| **Done By:** | | **Signature** | **Date** | **Remark** |
| Tesfaye T., Paediatrician, Paediatric Cardiologist | |  | 17/09 ­­­­­­­­­­­­­­­­­/15 Eth.C. |  |

| **Tibebe – Ghion Specialized Teaching Hospital, Bahir Dar University,**  **Bahir Dar, Ethiopia** | | | | | | |
| --- | --- | --- | --- | --- | --- | --- |
| **Name: Baby Belaynesh Gedef . Sex/Age: F/ 13days. MRN: 179794 . Date of Report: 17/09 /15Eth.C.**  **Referral Diagnosis: Dextrocardia + Cyanosis. TGSH10.2824.** | | | | | | |
| **Features:** | **Findings** | | | **Features** | | **Findings** |
| **Profile** | | | | **Atria** | | |
| Abdominal Situs | Inversus | | | Left Atrium | | Normal |
| Atrial Situs | Inversus | | | Right Atrium | | Normal |
| Cardiac Position | Dextrocardia | | | **Atrio-Ventricular Valves** | | |
| Systemic Venous Drainage | To RA | | | Mitral Valve | | Annulus = 8mm |
| Pulmonary Venous Drainage | To LA | | | Tricuspid Valve | | Annulus = 10mm |
| Atrio-ventricular Connection | Concordant | | |  | | TAPSE = mm |
| Ventriculo-Arterial Connection | concordant | | | **Ventricle** | | |
| Ventricular Loop | l-Loop | | | Left Ventricle | | Normal |
| **Septae** |  | | | Right Ventricle | | Normal |
| Interatrial Septum | 10mm Ostium Primum Defect, L – R Shunt  5mm OS ASD, L – R Shunt | | | **Doppler Measurement** | |  |
| Interventricular Septum | 4mm Inlet VSD, L – R Shunt | | | Mitral | | ------------- |
| **Semilunar Valves** |  | | | Aortic | | ------------- |
| Aortic Valve | Annulus = 11mm | | | Tricuspid | | ------------- |
| Pulmonary Valve | Annulus = 9mm | | | Pulmonic | | ------------- |
| **Great Arteries** | I-TGA | | | **Coronary Arteries** | |  |
| Aorta |  | | | **Aortic Arch** | | Left. No CoA |
| Pulmonary Arteries | Normal MPA & BPAs. | | | **PDA** | | No PDA |
| **M-Mode**: | | | | | | |
| Ao | mm | | | PWd | | mm |
| LA | mm | | | EDV | | ml |
| LVIDd | mm | | | ESV | | ml |
| LVIDs | mm | | | FS | | % |
| IVSd | mm | | | LVEF | | % |
| **Additional Information:** | | | | | | |
| **Conclusion:**   1. Abdominal Situs Inversus totalis 2. {I, L, I} Dextrocardia 3. Transitional AVSD, L – R Shunt 4. Small OS ASD, L – R Shunt | | | | | | |
| **Remark:** | | | | | | |
| **Recommendation:** | | | | | | |
| **Done By:** | | **Signature** | **Date** | | **Remark** | |
| Tesfaye T., Paediatrician, Paediatric Cardiologist | |  | 17/09 ­­­­­­­­­­­­­­­­­/15 Eth.C. | |  | |

| **Tibebe – Ghion Specialized Teaching Hospital, Bahir Dar University,**  **Bahir Dar, Ethiopia** | | | | |
| --- | --- | --- | --- | --- |
| **Name: Baby Kenu Tiru. Sex/Age: M/6 days. MRN: 181540. Date of Report: 17/09 /15 Eth.C.**  **Referral Diagnosis: Incidental Murmur. TGSH10.2825.** | | | | |
| **Features:** | **Findings** | | **Features** | **Findings** |
| **Profile** | | | **Atria** | |
| Abdominal Situs | Solitus | | Left Atrium | Normal |
| Atrial Situs | Solitus | | Right Atrium | Dilated |
| Cardiac Position | Levocardia | | **Atrio-Ventricular Valves** | |
| Systemic Venous Drainage | To RA | | Mitral Valve | Annulus = 12mm |
| Pulmonary Venous Drainage | To LA | | Tricuspid Valve | Annulus = 15mm |
| Atrio-ventricular Connection | Concordant | |  | TAPSE = 13mm |
| Ventriculo-Arterial Connection | concordant | | **Ventricle** | |
| Ventricular Loop | d-Loop | | Left Ventricle | Normal |
| **Septae** |  | | Right Ventricle | Dilated |
| Interatrial Septum | Intact | | **Doppler Measurement** |  |
| Interventricular Septum | 5mm Inlet VSD, L – R Shunt | | Mitral | Mild MR |
| **Semilunar Valves** |  | | Aortic | ------------- |
| Aortic Valve | Annulus = 10mm | | Tricuspid | Moderate TR |
| Pulmonary Valve | Annulus = 11mm | | Pulmonic | ------------- |
| **Great Arteries** | NRGA | | **Coronary Arteries** |  |
| Aorta |  | | **Aortic Arch** | Left. No CoA |
| Pulmonary Arteries | Normal MPA & BPAs. | | **PDA** | No PDA |
| **M-Mode**: Normal LV Function on eye balling | | | | |
| Ao | mm | | PWd | mm |
| LA | mm | | EDV | ml |
| LVIDd | mm | | ESV | ml |
| LVIDs | mm | | FS | % |
| IVSd | mm | | LVEF | % |
| **Additional Information:** | | | | |
| **Conclusion:**   1. {S, D, S} Levocardia 2. Small Inlet VSD, L – R Shunt 3. Moderate TR 4. Mild MR | | | | |
| **Remark:** | | | | |
| **Recommendation:** | | | | |
| **Done By:** | | **Signature** | **Date** | **Remark** |
| Tesfaye T., Paediatrician, Paediatric Cardiologist | |  | 17/09 ­­­­­­­­­­­­­­­­­/15 Eth.C. |  |

| **Tibebe – Ghion Specialized Teaching Hospital, Bahir Dar University,**  **Bahir Dar, Ethiopia** | | | | |
| --- | --- | --- | --- | --- |
| **Name: Yihenew Getu. Sex/Age: M/ 4years. MRN: 180914. Date of Report: 17/09/15 Eth.C.**  **Referral Diagnosis: DS + Recurrent Chest Infection. TGSH10.2826.** | | | | |
| **Features:** | **Findings** | | **Features** | **Findings** |
| **Profile** | | | **Atria** | |
| Abdominal Situs | Solitus | | Left Atrium | Normal |
| Atrial Situs | Solitus | | Right Atrium | Normal |
| Cardiac Position | Levocardia | | **Atrio-Ventricular Valves** | |
| Systemic Venous Drainage | To RA | | Mitral Valve | Annulus = 15mm |
| Pulmonary Venous Drainage | To LA | | Tricuspid Valve | Annulus = 18mm |
| Atrio-ventricular Connection | Concordant | |  | TAPSE = 17mm |
| Ventriculo-Arterial Connection | concordant | | **Ventricle** | |
| Ventricular Loop | d-Loop | | Left Ventricle | Normal |
| **Septae** |  | | Right Ventricle | Normal |
| Interatrial Septum | Intact | | **Doppler Measurement** |  |
| Interventricular Septum | Intact | | Mitral | ------------- |
| **Semilunar Valves** |  | | Aortic | ------------- |
| Aortic Valve | Annulus = 15mm | | Tricuspid | ------------- |
| Pulmonary Valve | Annulus = 16mm | | Pulmonic | ------------- |
| **Great Arteries** | NRGA | | **Coronary Arteries** |  |
| Aorta |  | | **Aortic Arch** | Left. No CoA |
| Pulmonary Arteries | Normal MPA & BPAs. | | **PDA** | 1.5mm PDA, L – R Shunt |
| **M-Mode**: Normal LV Function on eye balling | | | | |
| Ao | mm | | PWd | mm |
| LA | mm | | EDV | ml |
| LVIDd | mm | | ESV | ml |
| LVIDs | mm | | FS | % |
| IVSd | mm | | LVEF | % |
| **Additional Information:** | | | | |
| **Conclusion:**   1. {S, D, S} Levocardia 2. Small PDA, L – R Shunt | | | | |
| **Remark:** | | | | |
| **Recommendation:** | | | | |
| **Done By:** | | **Signature** | **Date** | **Remark** |
| Tesfaye T., Paediatrician, Paediatric Cardiologist | |  | 17/09 ­­­­­­­­­­­­­­­­­/15 Eth.C. |  |

| **Tibebe – Ghion Specialized Teaching Hospital, Bahir Dar University,**  **Bahir Dar, Ethiopia** | | | | |
| --- | --- | --- | --- | --- |
| **Name: Melona Emiamrew. Sex/Age: F/5Months. MRN: 181722. Date of Report: 18/09 /15 Eth.C.**  **Referral Diagnosis: Follow up echo for ASD + PDA + PM VSD (All small) + RD. AGH2.120** | | | | |
| **Features:** | **Findings** | | **Features** | **Findings** |
| **Profile** | | | **Atria** | |
| Abdominal Situs | Solitus | | Left Atrium | Normal |
| Atrial Situs | Solitus | | Right Atrium | Normal |
| Cardiac Position | Levocardia | | **Atrio-Ventricular Valves** | |
| Systemic Venous Drainage | To RA | | Mitral Valve | Annulus = 12mm |
| Pulmonary Venous Drainage | To LA | | Tricuspid Valve | Annulus = 12mm |
| Atrio-ventricular Connection | Concordant | |  | TAPSE = mm |
| Ventriculo-Arterial Connection | concordant | | **Ventricle** | |
| Ventricular Loop | d-Loop | | Left Ventricle | Normal |
| **Septae** |  | | Right Ventricle | Normal |
| Interatrial Septum | 4mm OS ASD, L – R Shunt | | **Doppler Measurement** |  |
| Interventricular Septum | 5mm PM VSD, L – R Shunt | | Mitral | ------------- |
| **Semilunar Valves** |  | | Aortic | ------------- |
| Aortic Valve | Annulus = 11mm | | Tricuspid | ------------- |
| Pulmonary Valve | Annulus = 12mm | | Pulmonic | ------------- |
| **Great Arteries** | NRGA | | **Coronary Arteries** |  |
| Aorta |  | | **Aortic Arch** | Left. No CoA |
| Pulmonary Arteries | Normal MPA & BPAs. | | **PDA** | 1mm PDA, L – R Shunt |
| **M-Mode**: Normal LV Function on eye balling. | | | | |
| Ao | mm | | PWd | mm |
| LA | mm | | EDV | ml |
| LVIDd | mm | | ESV | ml |
| LVIDs | mm | | FS | % |
| IVSd | mm | | LVEF | % |
| **Additional Information:** trace pericardial effusion | | | | |
| **Conclusion:**   1. {S, D, S} Levocardia 2. Small OS ASD, L – R Shunt 3. Small PM VSD, L – R Shunt 4. Small PDA, L – R Shunt 5. Trace pericardial effusion 6. Normal LV Systolic Function | | | | |
| **Done By:** | | **Signature** | **Date** | **Remark** |
| Tesfaye T., Paediatrician, Paediatric Cardiologist | |  | 18/09 ­­­­­­­­­­­­­­­­­/15 Eth.C. |  |

| **Tibebe – Ghion Specialized Teaching Hospital, Bahir Dar University,**  **Bahir Dar, Ethiopia** | | | | | |
| --- | --- | --- | --- | --- | --- |
| **Name: Yohanes Gebrie . Sex/Age: M/4 5/12years. MRN: 026506. Date of Report: 18/09 /15 Eth.C.**  **Referral Diagnosis: Follow up echo for Double inlet Indeterminate single ventricle + Mild AS. TGSH10.2827 (TGSH7)** | | | | | |
| **Features:** | | **Findings** | | **Features** | **Findings** |
| **Profile** | | | | **Atria** | |
| Abdominal Situs | Solitus | | | Left Atrium | Dilated |
| Atrial Situs | Solitus | | | Right Atrium | Dilated |
| Cardiac Position | Levocardia | | | **Atrio-Ventricular Valves** | |
| Systemic Venous Drainage | To RA | | | Mitral Valve | Annulus = 26mm. 28mm Apical displacement of the left AVV |
| Pulmonary Venous Drainage | To LA | | | Tricuspid Valve | Annulus = 29mm |
| Atrio-ventricular Connection | Indeterminate Double inlet Single Ventricle | | |  | TAPSE = mm |
| Ventriculo-Arterial Connection | concordant | | | **Ventricle** | |
| Ventricular Loop | x-Loop | | | Left Ventricle | Indeterminate single ventricle |
| **Septae** |  | | | Right Ventricle |  |
| Interatrial Septum | Intact | | | **Doppler Measurement** |  |
| Interventricular Septum | Intact | | | Mitral | Moderate Left AVVR |
| **Semilunar Valves** |  | | | Aortic | ------------- |
| Aortic Valve | Annulus = 17mm | | | Tricuspid | Mild Right AVVR |
| Pulmonary Valve | Annulus = 24mm | | | Pulmonic | Mild PR |
| **Great Arteries** | NRGA | | | **Coronary Arteries** |  |
| Aorta |  | | | **Aortic Arch** | Left. No CoA |
| Pulmonary Arteries | MPA = 30mm. | | | **PDA** | No PDA |
| **M-Mode**: | | | | | |
| Ao | mm | | | PWd | mm |
| LA | mm | | | EDV | ml |
| LVIDd | mm | | | ESV | ml |
| LVIDs | mm | | | FS | % |
| IVSd | mm | | | LVEF | % |
| **Additional Information:** Echogenic mass at the ventricular side of LVOT measuring 6 X 3mm | | | | | |
| **Conclusion:**   1. {S, X, D} Levocardia 2. Indeterminate Double Inlet Single Ventricle 3. Ebstein like anomaly of the Left AV Valve 4. Severe Pulmonary Hypertension | | | | | |
| **Recommendation:** | | | | | |
| **Done By:** | | | **Signature** | **Date** | **Remark** |
| Tesfaye T., Paediatrician, Paediatric Cardiologist | | |  | 18/09­­­­­­­­­­­­­­­­­/15 Eth.C. |  |

| **Tibebe – Ghion Specialized Teaching Hospital, Bahir Dar University,**  **Bahir Dar, Ethiopia** | | | | |
| --- | --- | --- | --- | --- |
| **Name: Tsegaye Zelalem. Sex/Age: M/1year. MRN: 181620. Date of Report: 24/09 /15 Eth.C.**  **Referral Diagnosis: FTT + G-III HSM + ?Syndromic (DS). TGSH10.2828.** | | | | |
| **Features:** | **Findings** | | **Features** | **Findings** |
| **Profile** | | | **Atria** | |
| Abdominal Situs | Solitus | | Left Atrium | Normal |
| Atrial Situs | Solitus | | Right Atrium | Normal |
| Cardiac Position | Levocardia | | **Atrio-Ventricular Valves** | |
| Systemic Venous Drainage | To RA | | Mitral Valve | Common Complete AVSD |
| Pulmonary Venous Drainage | To LA | | Tricuspid Valve |  |
| Atrio-ventricular Connection | Concordant | |  | TAPSE = mm |
| Ventriculo-Arterial Connection | concordant | | **Ventricle** | |
| Ventricular Loop | d-Loop | | Left Ventricle | Normal |
| **Septae** |  | | Right Ventricle | Normal |
| Interatrial Septum | Common Complete AVSD, L – R Shunt | | **Doppler Measurement** |  |
| Interventricular Septum |  |  | Mitral | ------------- |
| **Semilunar Valves** |  | | Aortic | ------------- |
| Aortic Valve | Annulus = 13mm | | Tricuspid | Mild Right AVVR |
| Pulmonary Valve | Annulus = 14mm | | Pulmonic | ------------- |
| **Great Arteries** | NRGA | | **Coronary Arteries** |  |
| Aorta |  | | **Aortic Arch** | Left. No CoA |
| Pulmonary Arteries | Normal MPA & BPAs. | | **PDA** | No PDA |
| **M-Mode**: Normal LV Function on eye balling. | | | | |
| Ao | mm | | PWd | mm |
| LA | mm | | EDV | ml |
| LVIDd | mm | | ESV | ml |
| LVIDs | mm | | FS | % |
| IVSd | mm | | LVEF | % |
| **Additional Information:** | | | | |
| **Conclusion:**   1. {S, D, S} Levocardia 2. Common Complete Balanced AVSD, L – R Shunt 3. Mild Right AVVR | | | | |
| **Remark:** | | | | |
| **Recommendation:** | | | | |
| **Done By:** | | **Signature** | **Date** | **Remark** |
| Tesfaye T., Paediatrician, Paediatric Cardiologist | |  | 24/09­­­­­­­­­­­­­­­­­/15 Eth.C. |  |

| **Tibebe – Ghion Specialized Teaching Hospital, Bahir Dar University,**  **Bahir Dar, Ethiopia** | | | | |
| --- | --- | --- | --- | --- |
| **Name: Baby of Yekitie Mengestu. Sex/Age: F/27days. MRN: 179232. Date of Report: 24/09 /15 Eth.C.**  **Referral Diagnosis: Sepsis + Incidental Murmur + PNA. TGSH10.2829.** | | | | |
| **Features:** | **Findings** | | **Features** | **Findings** |
| **Profile** | | | **Atria** | |
| Abdominal Situs | Solitus | | Left Atrium | Normal |
| Atrial Situs | Solitus | | Right Atrium | Normal |
| Cardiac Position | Levocardia | | **Atrio-Ventricular Valves** | |
| Systemic Venous Drainage | To RA | | Mitral Valve | Annulus = 12mm |
| Pulmonary Venous Drainage | To LA | | Tricuspid Valve | Annulus = 12mm |
| Atrio-ventricular Connection | Concordant | |  | TAPSE = 9mm |
| Ventriculo-Arterial Connection | concordant | | **Ventricle** | |
| Ventricular Loop | d-Loop | | Left Ventricle | Normal |
| **Septae** |  | | Right Ventricle | Normal |
| Interatrial Septum | PFO, L – R Shunt | | **Doppler Measurement** |  |
| Interventricular Septum | Intact | | Mitral | ------------- |
| **Semilunar Valves** |  | | Aortic | ------------- |
| Aortic Valve | Annulus = 8mm | | Tricuspid | Mild TR, PPG = 41mmHg |
| Pulmonary Valve | Annulus = 10mm | | Pulmonic | ------------- |
| **Great Arteries** | NRGA | | **Coronary Arteries** |  |
| Aorta |  | | **Aortic Arch** | Left. No CoA |
| Pulmonary Arteries | Normal MPA & BPAs. | | **PDA** | 1mm PDA, L – R Shunt |
| **M-Mode**: | | | | |
| Ao | mm | | PWd | mm |
| LA | mm | | EDV | ml |
| LVIDd | mm | | ESV | ml |
| LVIDs | mm | | FS | 33% |
| IVSd | mm | | LVEF | 65% |
| **Additional Information:** | | | | |
| **Conclusion:**   1. {S, D, S} Levocardia 2. PFO, L – R Shunt 3. Mild TR 4. Small PDA, L – R Shunt 5. Mild Pulmonary Hypertension | | | | |
| **Recommendation:** | | | | |
| **Done By:** | | **Signature** | **Date** | **Remark** |
| Tesfaye T., Paediatrician, Paediatric Cardiologist | |  | 24/09­­­­­­­­­­­­­­­­­/15 Eth.C. |  |

| **Tibebe – Ghion Specialized Teaching Hospital, Bahir Dar University,**  **Bahir Dar, Ethiopia** | | | | |
| --- | --- | --- | --- | --- |
| **Name: Baby of Manamnosh Zelalem. Sex/Age: M/15days. MRN: 179903. Date of Report: 24/09 /15 Eth.C.**  **Referral Diagnosis: Cardiomegaly on CXR + G-III HSM. TGSH10.2830.** | | | | |
| **Features:** | **Findings** | | **Features** | **Findings** |
| **Profile** | | | **Atria** | |
| Abdominal Situs | Solitus | | Left Atrium | Normal |
| Atrial Situs | Solitus | | Right Atrium | Dilated |
| Cardiac Position | Levocardia | | **Atrio-Ventricular Valves** | |
| Systemic Venous Drainage | To RA | | Mitral Valve | Annulus = 8mm |
| Pulmonary Venous Drainage | To LA | | Tricuspid Valve | Annulus = 11mm |
| Atrio-ventricular Connection | Concordant | |  | TAPSE = 9mm |
| Ventriculo-Arterial Connection | concordant | | **Ventricle** | |
| Ventricular Loop | d-Loop | | Left Ventricle | Normal |
| **Septae** |  | | Right Ventricle | Dilated |
| Interatrial Septum | 7mm OS ASD, L – R Shunt | | **Doppler Measurement** |  |
| Interventricular Septum | Intact | | Mitral | ------------- |
| **Semilunar Valves** |  | | Aortic | ------------- |
| Aortic Valve | Annulus = 7mm | | Tricuspid | Moderate TR, PPG = 45mmHg |
| Pulmonary Valve | Annulus = 9mm | | Pulmonic | ------------- |
| **Great Arteries** | NRGA | | **Coronary Arteries** |  |
| Aorta |  | | **Aortic Arch** | Left. No CoA |
| Pulmonary Arteries | Normal MPA & BPAs. | | **PDA** | <1mm PDA, L – R Shunt |
| **M-Mode**: | | | | |
| Ao | mm | | PWd | mm |
| LA | mm | | EDV | ml |
| LVIDd | mm | | ESV | ml |
| LVIDs | mm | | FS | % |
| IVSd | mm | | LVEF | % |
| **Additional Information:** | | | | |
| **Conclusion:**   1. {S, D, S} Levocardia 2. RA/RV Dilated 3. Moderate OS ASD, L – R Shunt 4. Small PDA, L – R Shunt 5. Moderate TR 6. Normal Biventricular Systolic Function | | | | |
| **Recommendation:** | | | | |
| **Done By:** | | **Signature** | **Date** | **Remark** |
| Tesfaye T., Paediatrician, Paediatric Cardiologist | |  | 24/09­­­­­­­­­­­­­­­­­/15 Eth.C. |  |

| **Tibebe – Ghion Specialized Teaching Hospital, Bahir Dar University,**  **Bahir Dar, Ethiopia** | | | | |
| --- | --- | --- | --- | --- |
| **Name: Barkot Mulualem. Sex/Age: M/6monthes. MRN: 182801. Date of Report: 24/09 /15 Eth.C.**  **Referral Diagnosis: Incidental Murmur. TGSH10.2831.** | | | | |
| **Features:** | **Findings** | | **Features** | **Findings** |
| **Profile** | | | **Atria** | |
| Abdominal Situs | Solitus | | Left Atrium | Normal |
| Atrial Situs | Solitus | | Right Atrium | Dilated |
| Cardiac Position | Levocardia | | **Atrio-Ventricular Valves** | |
| Systemic Venous Drainage | To RA | | Mitral Valve | Annulus = 12mm |
| Pulmonary Venous Drainage | To LA | | Tricuspid Valve | Annulus = 16mm |
| Atrio-ventricular Connection | Concordant | |  | TAPSE = 16mm |
| Ventriculo-Arterial Connection | concordant | | **Ventricle** | |
| Ventricular Loop | d-Loop | | Left Ventricle | Normal |
| **Septae** |  | | Right Ventricle | Dilated and Hypertrophied |
| Interatrial Septum | Intact | | **Doppler Measurement** |  |
| Interventricular Septum | Intact | | Mitral | ------------- |
| **Semilunar Valves** |  | | Aortic | ------------- |
| Aortic Valve | Annulus = 11mm | | Tricuspid | ------------- |
| Pulmonary Valve | Annulus = 9mm | | Pulmonic | Severe PS, PPG = 65mmHg |
| **Great Arteries** | NRGA | | **Coronary Arteries** |  |
| Aorta |  | | **Aortic Arch** | Left. No CoA |
| Pulmonary Arteries | Adequate sized MPA. BPAs not well visualized (Hyper-inflated Chest). | | **PDA** | No PDA |
| **M-Mode**: Normal LV Function on eye balling. | | | | |
| Ao | mm | | PWd | mm |
| LA | mm | | EDV | ml |
| LVIDd | mm | | ESV | ml |
| LVIDs | mm | | FS | % |
| IVSd | mm | | LVEF | % |
| **Additional Information:** | | | | |
| **Conclusion:**   1. {S, D, S} Levocardia 2. RA/RV Dilated, RV Hypertrophied 3. Severe PS | | | | |
| **Recommendation:** | | | | |
| **Done By:** | | **Signature** | **Date** | **Remark** |
| Tesfaye T., Paediatrician, Paediatric Cardiologist | |  | 24/09­­­­­­­­­­­­­­­­­/15 Eth.C. |  |

| **Tibebe – Ghion Specialized Teaching Hospital, Bahir Dar University,**  **Bahir Dar, Ethiopia** | | | | |
| --- | --- | --- | --- | --- |
| **Name: Dawit Abeje. Sex/Age: M/4years. MRN: 182539. Date of Report: 24/09 /15Eth.C.**  **Referral Diagnosis: RD + G-III HSM + CHF + IE. TGSH10.2832.** | | | | |
| **Features:** | **Findings** | | **Features** | **Findings** |
| **Profile** | | | **Atria** | |
| Abdominal Situs | Solitus | | Left Atrium | Dilated |
| Atrial Situs | Solitus | | Right Atrium | Dilated |
| Cardiac Position | Levocardia | | **Atrio-Ventricular Valves** | |
| Systemic Venous Drainage | To RA | | Mitral Valve | Annulus = 27mm. Patulous MVL |
| Pulmonary Venous Drainage | To LA | | Tricuspid Valve | Annulus = 28mm |
| Atrio-ventricular Connection | Concordant | |  | TAPSE = 17mm |
| Ventriculo-Arterial Connection | concordant | | **Ventricle** | |
| Ventricular Loop | d-Loop | | Left Ventricle | Dilated |
| **Septae** |  | | Right Ventricle | Dilated |
| Interatrial Septum | Intact | | **Doppler Measurement** |  |
| Interventricular Septum | Intact | | Mitral | Severe MR, Holosystolic, Posterior projection, seen in two planes with jet velocity = 5.2m/sec |
| **Semilunar Valves** |  | | Aortic | Mild AR, PHT = 507ms |
| Aortic Valve | Annulus = 16mm | | Tricuspid | Severe TR, PPG = 61mmHg |
| Pulmonary Valve | Annulus = 19mm | | Pulmonic | ------------- |
| **Great Arteries** | NRGA | | **Coronary Arteries** |  |
| Aorta |  | | **Aortic Arch** | Left. No CoA |
| Pulmonary Arteries | Normal MPA & BPAs. | | **PDA** | No PDA |
| **M-Mode**: | | | | |
| Ao | mm | | PWd | mm |
| LA | mm | | EDV | ml |
| LVIDd | mm | | ESV | ml |
| LVIDs | mm | | FS | 34% |
| IVSd | mm | | LVEF | 63% |
| **Conclusion:**   1. {S, D, S} Levocardia 2. All chambers Dilated 3. Patulous MVL 4. Severe MR 5. Severe TR 6. Normal Biventricular Systolic Function | | | | |
| **Done By:** | | **Signature** | **Date** | **Remark** |
| Tesfaye T., Paediatrician, Paediatric Cardiologist | |  | 24/09­­­­­­­­­­­­­­­­­/15 Eth.C. |  |

| **Tibebe – Ghion Specialized Teaching Hospital, Bahir Dar University,**  **Bahir Dar, Ethiopia** | | | | |
| --- | --- | --- | --- | --- |
| **Name: Rediet Sinamaw. Sex/Age: F/2 5/12. MRN: 182531. Date of Report: 24/09 /15 Eth.C.**  **Referral Diagnosis: FTT + Murmur. TGSH10.2833.** | | | | |
| **Features:** | **Findings** | | **Features** | **Findings** |
| **Profile** | | | **Atria** | |
| Abdominal Situs | Solitus | | Left Atrium | Dilated |
| Atrial Situs | Solitus | | Right Atrium | Dilated |
| Cardiac Position | Levocardia | | **Atrio-Ventricular Valves** | |
| Systemic Venous Drainage | To RA | | Mitral Valve | Annulus = 19mm |
| Pulmonary Venous Drainage | To LA | | Tricuspid Valve | Annulus = 18mm |
| Atrio-ventricular Connection | Concordant | |  | TAPSE = 17mm |
| Ventriculo-Arterial Connection | concordant | | **Ventricle** | |
| Ventricular Loop | d-Loop | | Left Ventricle | Dilated |
| **Septae** |  | | Right Ventricle | Dilated |
| Interatrial Septum | Intact | | **Doppler Measurement** |  |
| Interventricular Septum | Intact | | Mitral | Mild MR, Posterior projection, seen in two planes with jet velocity = 3.9m/sec |
| **Semilunar Valves** |  | | Aortic | ------------- |
| Aortic Valve | Annulus = 16mm | | Tricuspid | Moderate TR, PPG = 57mmHg |
| Pulmonary Valve | Annulus = 19mm | | Pulmonic | ------------- |
| **Great Arteries** | NRGA | | **Coronary Arteries** |  |
| Aorta |  | | **Aortic Arch** | Left. No CoA |
| Pulmonary Arteries | Normal MPA & BPAs. | | **PDA** | 4mm PDA, L – R Shunt |
| **M-Mode**: | | | | |
| Ao | mm | | PWd | mm |
| LA | mm | | EDV | ml |
| LVIDd | mm | | ESV | ml |
| LVIDs | mm | | FS | 29% |
| IVSd | mm | | LVEF | 56% |
| **Additional Information:** | | | | |
| **Conclusion:**   1. {S, D, S} Levocardia 2. All chambers dilated 3. Mild MR 4. Moderate TR 5. Large PDA, L – R Shunt 6. Normal Biventricular Systolic Function | | | | |
| **Done By:** | | **Signature** | **Date** | **Remark** |
| Tesfaye T., Paediatrician, Paediatric Cardiologist | |  | 24/09­­­­­­­­­­­­­­­­­/15 Eth.C. |  |

| **Tibebe – Ghion Specialized Teaching Hospital, Bahir Dar University,**  **Bahir Dar, Ethiopia** | | | | |
| --- | --- | --- | --- | --- |
| **Name: Rediet Menber. Sex/Age: F/2years. MRN: 183363. Date of Report: 29/09 /15 Eth.C.**  **Referral Diagnosis: CHF 2^0^ VSD. Being worked up for Malignancy metastasis TGSH10.2834.** | | | | |
| **Features:** | **Findings** | | **Features** | **Findings** |
| **Profile** | | | **Atria** | |
| Abdominal Situs | Solitus | | Left Atrium | Hypertrophied |
| Atrial Situs | Solitus | | Right Atrium | Hypertrophied |
| Cardiac Position | Levocardia | | **Atrio-Ventricular Valves** | |
| Systemic Venous Drainage | To RA | | Mitral Valve | Annulus = 15mm |
| Pulmonary Venous Drainage | To LA | | Tricuspid Valve | Annulus = 12mm |
| Atrio-ventricular Connection | Concordant | |  | TAPSE = 7mm |
| Ventriculo-Arterial Connection | concordant | | **Ventricle** | |
| Ventricular Loop | d-Loop | | Left Ventricle | Hypertrophied |
| **Septae** |  | | Right Ventricle | Hypertrophied |
| Interatrial Septum | Intact | | **Doppler Measurement** |  |
| Interventricular Septum | Intact | | Mitral | Trivial MR |
| **Semilunar Valves** |  | | Aortic | ------------- |
| Aortic Valve | Annulus = 7mm | | Tricuspid | Trivial TR |
| Pulmonary Valve | Annulus = 9mm | | Pulmonic | ------------- |
| **Great Arteries** | NRGA | | **Coronary Arteries** |  |
| Aorta |  | | **Aortic Arch** | Left. No CoA |
| Pulmonary Arteries | Normal MPA & BPAs. | | **PDA** | No PDA |
| **M-Mode**: Reduced LV Systolic Function. Markedly thickened Septum and chamber walls | | | | |
| Ao | mm | | PWd | mm |
| LA | mm | | EDV | ml |
| LVIDd | mm | | ESV | ml |
| LVIDs | mm | | FS | % |
| IVSd | mm | | LVEF | % |
| **Additional Information:** Circumferential Pericardial effusion with maximum depth of 11mm. | | | | |
| **Conclusion:**   1. {S, D, S} Levocardia 2. Biventricular Systolic Dysfunction 3. Biventricular Hypertrophy 4. Moderate Circumferential Pericardial effusion | | | | |
| **Remark:** Secondary Cardiomyopathies are top in the list of the DDx. | | | | |
| **Recommendation:** | | | | |
| **Done By:** | | **Signature** | **Date** | **Remark** |
| Tesfaye T., Paediatrician, Paediatric Cardiologist | |  | 29/09­­­­­­­­­­­­­­­­­/15 Eth.C. |  |

| **Tibebe – Ghion Specialized Teaching Hospital, Bahir Dar University,**  **Bahir Dar, Ethiopia** | | | | |
| --- | --- | --- | --- | --- |
| **Name: Kibrework Yesmaw. Sex/Age: F/11years. MRN: 144012. Date of Report: 29/09 /15Eth.C.**  **Referral Diagnosis: Cardio-embolic Stroke + R/O IE + DS. TGSH10.2835.** | | | | |
| **Features:** | **Findings** | | **Features** | **Findings** |
| **Profile** | | | **Atria** | |
| Abdominal Situs | Solitus | | Left Atrium | Dilated |
| Atrial Situs | Solitus | | Right Atrium | Normal |
| Cardiac Position | Levocardia | | **Atrio-Ventricular Valves** | |
| Systemic Venous Drainage | To RA | | Mitral Valve | Annulus = 24mm. Thickened MVL |
| Pulmonary Venous Drainage | To LA | | Tricuspid Valve | Annulus = 22mm |
| Atrio-ventricular Connection | Concordant | |  | TAPSE = 23mm |
| Ventriculo-Arterial Connection | concordant | | **Ventricle** | |
| Ventricular Loop | d-Loop | | Left Ventricle | Dilated. Echogenic mass attached to the MVL + Chordae tendenae |
| **Septae** |  | | Right Ventricle | Normal |
| Interatrial Septum | Intact | | **Doppler Measurement** |  |
| Interventricular Septum | Intact | | Mitral | Severe MR, Holosystolic, Posterior projection, seen in two planes with jet velocity = 4.3m/sec. |
| **Semilunar Valves** |  | | Aortic | ------------- |
| Aortic Valve | Annulus = 16mm | | Tricuspid | Mild TR, PPG = 41mmHg |
| Pulmonary Valve | Annulus = 17mm | | Pulmonic | ------------- |
| **Great Arteries** | NRGA | | **Coronary Arteries** |  |
| Aorta |  | | **Aortic Arch** | Left. No CoA |
| Pulmonary Arteries | Normal MPA & BPAs. | | **PDA** | No PDA |
| **M-Mode**: Normal LV Function on eye balling. | | | | |
| Ao | mm | | PWd | mm |
| LA | mm | | EDV | ml |
| LVIDd | mm | | ESV | ml |
| LVIDs | mm | | FS | % |
| IVSd | mm | | LVEF | % |
| **Additional Information:** | | | | |
| **Conclusion:**   1. {S, D, S} Levocardia 2. LA/LV Dilated 3. Thickened MVL 4. Echogenic mass attached to the MVL and Chordae Tendenae 5. Mild Pulmonary Hypertension 6. Normal Biventricular Systolic Function | | | | |
| **Done By:** | | **Signature** | **Date** | **Remark** |
| Tesfaye T., Paediatrician, Paediatric Cardiologist | |  | 29/09­­­­­­­­­­­­­­­­­/15 Eth.C. |  |

| **Tibebe – Ghion Specialized Teaching Hospital, Bahir Dar University,**  **Bahir Dar, Ethiopia** | | | | |
| --- | --- | --- | --- | --- |
| **Name: Misgana Sisay. Sex/Age: F/9monthes. MRN: 183252. Date of Report: 29/09 /15 Eth.C.**  **Referral Diagnosis: Incidental Murmur. TGSH10.2836.** | | | | |
| **Features:** | **Findings** | | **Features** | **Findings** |
| **Profile** | | | **Atria** | |
| Abdominal Situs | Solitus | | Left Atrium | Normal |
| Atrial Situs | Solitus | | Right Atrium | Normal |
| Cardiac Position | Levocardia | | **Atrio-Ventricular Valves** | |
| Systemic Venous Drainage | To RA | | Mitral Valve | Annulus = 15mm |
| Pulmonary Venous Drainage | To LA | | Tricuspid Valve | Annulus = 14mm |
| Atrio-ventricular Connection | Concordant | |  | TAPSE = 17mm |
| Ventriculo-Arterial Connection | concordant | | **Ventricle** | |
| Ventricular Loop | d-Loop | | Left Ventricle | Normal |
| **Septae** |  | | Right Ventricle | Hypertrophied |
| Interatrial Septum | Intact | | **Doppler Measurement** |  |
| Interventricular Septum | Non – Restrictive Mal-aligned Sub-aortic VSD, L – R Shunt | | Mitral | ------------- |
| **Semilunar Valves** |  | | Aortic | ------------- |
| Aortic Valve | Annulus = 13mm | | Tricuspid | ------------- |
| Pulmonary Valve | Annulus = 6mm | | Pulmonic | Severe PS, PPG = 94mmHg |
| **Great Arteries** | NRGA | | **Coronary Arteries** |  |
| Aorta | Over-riding aorta | | **Aortic Arch** | Left. No CoA |
| Pulmonary Arteries | Smallish MPA & BPAs. | | **PDA** | No PDA |
| **M-Mode**: | | | | |
| Ao | mm | | PWd | mm |
| LA | mm | | EDV | ml |
| LVIDd | mm | | ESV | ml |
| LVIDs | mm | | FS | % |
| IVSd | mm | | LVEF | % |
| **Additional Information:** | | | | |
| **Conclusion:**   1. {S, D, S} Levocardia 2. TOF 3. Smallish MPA and Branch Pas. | | | | |
| **Remark:** | | | | |
| **Recommendation:** | | | | |
| **Done By:** | | **Signature** | **Date** | **Remark** |
| Tesfaye T., Paediatrician, Paediatric Cardiologist | |  | 29/09­­­­­­­­­­­­­­­­­/15 Eth.C. |  |

| **Tibebe – Ghion Specialized Teaching Hospital, Bahir Dar University,**  **Bahir Dar, Ethiopia** | | | | |
| --- | --- | --- | --- | --- |
| **Name: . Sex/Age: / years. MRN: . Date of Report: 29/09 /15 Eth.C.**  **Referral Diagnosis: ___________.** | | | | |
| **Features:** | **Findings** | | **Features** | **Findings** |
| **Profile** | | | **Atria** | |
| Abdominal Situs | Solitus | | Left Atrium | Normal |
| Atrial Situs | Solitus | | Right Atrium | Normal |
| Cardiac Position | Levocardia | | **Atrio-Ventricular Valves** | |
| Systemic Venous Drainage | To RA | | Mitral Valve | Annulus = mm |
| Pulmonary Venous Drainage | To LA | | Tricuspid Valve | Annulus = mm |
| Atrio-ventricular Connection | Concordant | |  | TAPSE = mm |
| Ventriculo-Arterial Connection | concordant | | **Ventricle** | |
| Ventricular Loop | d-Loop | | Left Ventricle | Normal |
| **Septae** |  | | Right Ventricle | Normal |
| Interatrial Septum | Intact | | **Doppler Measurement** |  |
| Interventricular Septum | Intact | | Mitral | ------------- |
| **Semilunar Valves** |  | | Aortic | ------------- |
| Aortic Valve | Annulus = mm | | Tricuspid | ------------- |
| Pulmonary Valve | Annulus = mm | | Pulmonic | ------------- |
| **Great Arteries** | NRGA | | **Coronary Arteries** |  |
| Aorta |  | | **Aortic Arch** | Left. No CoA |
| Pulmonary Arteries | Normal MPA & BPAs. | | **PDA** | No PDA |
| **M-Mode**: | | | | |
| Ao | mm | | PWd | mm |
| LA | mm | | EDV | ml |
| LVIDd | mm | | ESV | ml |
| LVIDs | mm | | FS | % |
| IVSd | mm | | LVEF | % |
| **Additional Information:** | | | | |
| **Conclusion:**   1. {S, D, S} Levocardia | | | | |
| **Remark:** | | | | |
| **Recommendation:** | | | | |
| **Done By:** | | **Signature** | **Date** | **Remark** |
| Tesfaye T., Paediatrician, Paediatric Cardiologist | |  | 29/09­­­­­­­­­­­­­­­­­/15 Eth.C. |  |
