# Supplementary material for: Two–Dimensional and Doppler trans-thoracic echocardiographic patterns of suspected pediatric heart diseases at Tibebe-—Ghion specialized Teaching Hospital and Adinas General Hospital, Bahir Dar, North-west Ethiopia:–An experience from an LMIC
Source: PLoS One. 2024 Mar 11;19(3):e0292694. doi: 10.1371/journal.pone.0292694 (PMC10927071; doi:10.1371/journal.pone.0292694)
Supplement: S2 File — (ZIP) [file pone.0292694.s003.zip › AGH2 Paediatric Echocardiography Report Novemeber 18, 2022 SPSS Filled.docx]

| Patient Name: **Baby of Adanech Kassahun**. Referring Institute: **TGSH**. SEX/ Age: **M/22days**.  Date of Report: **09/03/15**. Referral Diagnosis: **RD. AGH2.001** | | | |
| --- | --- | --- | --- |
| **Features** | **Finding** | **Features** | **Finding** |
| **Profile** |  | **Atria** |  |
| Abdominal situs | Solitus | Left atrium | Normal |
| Cardiac position | Levocardia | Right atrium | Normal |
| Systemic venous drainage | Normal. | **Atrioventricular valves** |  |
| Pulmonary venous drainage | Normal | Mitral valve | Annulus = 9mm |
| Atrioventricular connection | Concordant | Tricuspid valve | Annulus = 9mm |
| Ventriculoarterial connection | Concordant | **Ventricles** |  |
| Ventricular loop | d-Loop | Left ventricle | Normal |
|  |  | Right ventricle | Normal |
| **Septae** |  | **Coronary arteries** | ----- |
| Interventricular septum | Intact | **Doppler Measurement** |  |
| Interatrial septum | Intact | Mitral | ----- |
| **Semilunar valves** |  | Aortic | ------- |
| Aortic valve | Annulus = 7mm | Tricuspid | ------- |
| Pulmonary valve | Annulus = 7mm | pulmonic | -------- |
| **Great arteries** | NRGA | **Aortic arch** | Left. No CoA. |
| Aorta | ----- | **PDA** | No |
| Pulmonary artery | Normal MPA and Branch PAs. |  |  |
| **M-Mode:**  Normal LV Function on eye balling | | | |
| AO | mm | PWd | mm |
| LA | mm | PWs | mm |
| LVIDd | mm | EDV | ml |
| LVIDs | mm | ESV | ml |
| IVSs | mm | LVEF | % |
| IVSd | mm | FS | % |
| **Additional Information**: |  | | |
| No pericardial/Pleural effusion. | | | |
| **Final Diagnosis:** | | | |
| 1. Normal Echocardiography Study. | | | |
| **Remark**: | | | |
| **Recommendation**: | | | |
| SIGNATURE  Done by: Tesfaye T., Pediatrician, Pediatric Cardiologist _______________ 09/03/2015Eth.C | | | |

| Patient Name: **Kidist Melesse**. Referring Institute: **Dr. Addisu PSC**. SEX/ Age: **F/2 3/12**. Date of Report: **09/03/15**.  Referral Diagnosis: **Incidental Murmur**.AGH2.002 | | | |
| --- | --- | --- | --- |
| **Features** | **Finding** | **Features** | **Finding** |
| **Profile** |  | **Atria** |  |
| Abdominal situs | Solitus | Left atrium | Normal |
| Cardiac position | Levocardia | Right atrium | Normal |
| Systemic venous drainage | Normal. | **Atrioventricular valves** |  |
| Pulmonary venous drainage | Normal | Mitral valve | Annulus = 15mm |
| Atrioventricular connection | Concordant | Tricuspid valve | Annulus = 16mm  TAPSE = 18mm |
| Ventriculoarterial connection | Concordant | **Ventricles** |  |
| Ventricular loop | d-Loop | Left ventricle | Normal |
|  |  | Right ventricle | Normal |
| **Septae** |  | **Coronary arteries** | ----- |
| Interventricular septum | 2mm Lower Muscular VSD, L – R Shunt | **Doppler Measurement** |  |
| Interatrial septum | Intact | Mitral | ----- |
| **Semilunar valves** |  | Aortic | ------- |
| Aortic valve | Annulus = 12mm | Tricuspid | ------- |
| Pulmonary valve | Annulus = 14mm | pulmonic | -------- |
| **Great arteries** | NRGA | **Aortic arch** | Left. No CoA. |
| Aorta | ----- | **PDA** | No |
| Pulmonary artery | Normal MPA & Branch PAs. |  |  |
| **M-Mode:**  Normal LV Function on eye balling | | | |
| AO | mm | PWd | mm |
| LA | mm | PWs | mm |
| LVIDd | mm | EDV | ml |
| LVIDs | mm | ESV | ml |
| IVSs | mm | LVEF | % |
| IVSd | mm | FS | % |
| **Additional Information**: |  | | |
| No pericardial/Pleural effusion. | | | |
| **Final Diagnosis:** | | | |
| 1. {S, D, S} Levocardia. 2. Small Lower Muscular VSD, L – R Shunt 3. Normal Biventricular Systolic Function | | | |
| **Remark**: | | | |
| **Recommendation**: | | | |
| SIGNATURE  Done by: Tesfaye T., Pediatrician, Pediatric Cardiologist _______________ 09/03/2015Eth.C | | | |

| Patient Name: **Baby of Agerie Gedamu**. Referring Institute: **FHRH**. SEX/ Age: **F/19days**. Date of Report: **09/03/15**.  Referral Diagnosis: **DS**. AGH2.003 | | | |
| --- | --- | --- | --- |
| **Features** | **Finding** | **Features** | **Finding** |
| **Profile** |  | **Atria** |  |
| Abdominal situs | Solitus | Left atrium | Normal |
| Cardiac position | Levocardia | Right atrium | Normal |
| Systemic venous drainage | Normal. | **Atrioventricular valves** |  |
| Pulmonary venous drainage | Normal | Mitral valve | Annulus = 10mm |
| Atrioventricular connection | Concordant | Tricuspid valve | Annulus = 10mm  TAPSE = 12mm |
| Ventriculoarterial connection | Concordant | **Ventricles** |  |
| Ventricular loop | d-Loop | Left ventricle | Normal |
|  |  | Right ventricle | Normal |
| **Septae** |  | **Coronary arteries** | ----- |
| Interventricular septum | Intact | **Doppler Measurement** |  |
| Interatrial septum | 5X6 Fenestrated OS ASD, L – R Shunt | Mitral | ----- |
| **Semilunar valves** |  | Aortic | ------- |
| Aortic valve | Annulus = 7mm | Tricuspid | ------- |
| Pulmonary valve | Annulus = 8mm | pulmonic | -------- |
| **Great arteries** | NRGA | **Aortic arch** | Left. No CoA. |
| Aorta | ----- | **PDA** | No |
| Pulmonary artery | Normal MPA and Branch PAs. |  |  |
| **M-Mode:** | | | |
| AO | mm | PWd | mm |
| LA | mm | PWs | mm |
| LVIDd | mm | EDV | ml |
| LVIDs | mm | ESV | ml |
| IVSs | mm | LVEF | 69% |
| IVSd | mm | FS | 35% |
| **Additional Information**: |  | | |
| No pericardial/Pleural effusion. | | | |
| **Final Diagnosis:** | | | |
| 1. {S, D, S} Levocardia. 2. Small Fenestrated OS ASD, L – R Shunt 3. Normal Biventricular Systolic Function | | | |
| **Remark**: | | | |
| **Recommendation**: | | | |
| SIGNATURE  Done by: Tesfaye T., Pediatrician, Pediatric Cardiologist _______________ 09/03/2015Eth.C | | | |

| Patient Name: **Amanuel Tiruneh**. Referring Institute: **FHRH**. SEX/ Age: **M/6years**. Date of Report: **09/03/15**.  Referral Diagnosis: **Rheumatic Heart Disease**. AGH2.004 | | | |
| --- | --- | --- | --- |
| **Features** | **Finding** | **Features** | **Finding** |
| **Profile** |  | **Atria** |  |
| Abdominal situs | Solitus | Left atrium | Markedly Dilated |
| Cardiac position | Levocardia | Right atrium | Normal |
| Systemic venous drainage | Normal. | **Atrioventricular valves** |  |
| Pulmonary venous drainage | Normal | Mitral valve | Annulus = 27mm. Thickened MVL |
| Atrioventricular connection | Concordant | Tricuspid valve | Annulus = 22mm  TAPSE = 20mm |
| Ventriculoarterial connection | Concordant | **Ventricles** |  |
| Ventricular loop | d-Loop | Left ventricle | Markedly Dilated |
|  |  | Right ventricle | Normal |
| **Septae** |  | **Coronary arteries** | ----- |
| Interventricular septum | Intact | **Doppler Measurement** |  |
| Interatrial septum | Intact | Mitral | Moderate MR, Holosystolic, posterior projection, seen in two planes with jet velocity = 4m/sec. |
| **Semilunar valves** |  | Aortic | ------- |
| Aortic valve | Annulus = 14mm | Tricuspid | ------- |
| Pulmonary valve | Annulus = 19mm | pulmonic | -------- |
| **Great arteries** | NRGA | **Aortic arch** | Left. No CoA. |
| Aorta | ----- | **PDA** | 4mm PDA, L – R Shunt |
| Pulmonary artery | Normal MPA & BPA |  |  |
| **M-Mode:** | | | |
| AO | mm | PWd | mm |
| LA | mm | PWs | mm |
| LVIDd | mm | EDV | ml |
| LVIDs | mm | ESV | ml |
| IVSs | mm | LVEF | 56% |
| IVSd | mm | FS | 30% |
| **Additional Information**: |  | | |
| No pericardial/Pleural effusion. | | | |
| **Final Diagnosis:** | | | |
| 1. {S, D, S} Levocardia. 2. LA/LV Dilated Moderate MR 3. Thickened MVL 4. Large PDA, L – R Shunt 5. Normal Biventricular Systolic Function | | | |
| SIGNATURE  Done by: Tesfaye T., Pediatrician, Pediatric Cardiologist _______________ 09/03/2015Eth.C | | | |

| Patient Name: **Fanuel Tamirat**. Referring Institute: **Adinas GH**. SEX/ Age: **M/1 11/12**. Date of Report: **09/03/15**.  Referral Diagnosis: Failure to thrive. **AGH2.005**. | | | |
| --- | --- | --- | --- |
| **Features** | **Finding** | **Features** | **Finding** |
| **Profile** |  | **Atria** |  |
| Abdominal situs | Solitus | Left atrium | Normal |
| Cardiac position | Levocardia | Right atrium | Dilated |
| Systemic venous drainage | Normal. | **Atrioventricular valves** |  |
| Pulmonary venous drainage | Normal | Mitral valve | Annulus = 15mm |
| Atrioventricular connection | Concordant | Tricuspid valve | Annulus = 22mm  TAPSE = 15mm |
| Ventriculoarterial connection | Concordant | **Ventricles** |  |
| Ventricular loop | d-Loop | Left ventricle | Normal |
|  |  | Right ventricle | Dilated |
| **Septae** |  | **Coronary arteries** | ----- |
| Interventricular septum | 5mm Sub-Aortic VSD, L – R Shunt | **Doppler Measurement** |  |
| Interatrial septum | 14mm OS ASD, L – R Shunt | Mitral | ----- |
| **Semilunar valves** |  | Aortic | ------- |
| Aortic valve | Annulus = 13mm | Tricuspid | ------- |
| Pulmonary valve | Annulus = 16mm | pulmonic | -------- |
| **Great arteries** | NRGA | **Aortic arch** | Left. No CoA. |
| Aorta | ----- | **PDA** | No |
| Pulmonary artery | Normal MPA & BPAs. |  |  |
| **M-Mode:** | | | |
| AO | mm | PWd | mm |
| LA | mm | PWs | mm |
| LVIDd | mm | EDV | ml |
| LVIDs | mm | ESV | ml |
| IVSs | mm | LVEF | 67% |
| IVSd | mm | FS | 35% |
| **Additional Information**: |  | | |
| No pericardial/Pleural effusion. | | | |
| **Final Diagnosis:** | | | |
| 1. {S, D, S} Levocardia. 2. RA/RV Dilated 3. Large OS ASD, L – R Shunt 4. Small Sub-Aortic VSD, L – R Shunt 5. Normal LV Systolic Function | | | |
| **Recommendation**: | | | |
| SIGNATURE  Done by: Tesfaye T., Pediatrician, Pediatric Cardiologist _______________ 09/03/2015Eth.C | | | |

| Patient Name: **Rafael Adane**. Referring Institute: **Adinas GH**. SEX/ Age: **M/10 10/12**. Date of Report: **10/03/15**.  Referral Diagnosis: easy fatigability. AGH2.006 | | | |
| --- | --- | --- | --- |
| **Features** | **Finding** | **Features** | **Finding** |
| **Profile** |  | **Atria** |  |
| Abdominal situs | Solitus | Left atrium | Normal |
| Cardiac position | Levocardia | Right atrium | Normal |
| Systemic venous drainage | Normal. | **Atrioventricular valves** |  |
| Pulmonary venous drainage | Normal | Mitral valve | Annulus = 22mm |
| Atrioventricular connection | Concordant | Tricuspid valve | Annulus = 22mm  TAPSE = 21mm |
| Ventriculoarterial connection | Concordant | **Ventricles** |  |
| Ventricular loop | d-Loop | Left ventricle | Normal |
|  |  | Right ventricle | Normal |
| **Septae** |  | **Coronary arteries** | ----- |
| Interventricular septum | Intact | **Doppler Measurement** |  |
| Interatrial septum | Intact | Mitral | ----- |
| **Semilunar valves** |  | Aortic | ------- |
| Aortic valve | Annulus = 17mm | Tricuspid | ------- |
| Pulmonary valve | Annulus = 19mm | pulmonic | -------- |
| **Great arteries** | NRGA | **Aortic arch** | Left. No CoA. |
| Aorta | ----- | **PDA** | No |
| Pulmonary artery | Normal MPA and Branch PAs. |  |  |
| **M-Mode:** | | | |
| AO | mm | PWd | mm |
| LA | mm | PWs | mm |
| LVIDd | mm | EDV | ml |
| LVIDs | mm | ESV | ml |
| IVSs | mm | LVEF | 65% |
| IVSd | mm | FS | 35% |
| **Additional Information**: |  | | |
| No pericardial/Pleural effusion. | | | |
| **Final Diagnosis:** | | | |
| 1. Normal Echocardiography Study. | | | |
| **Remark**: | | | |
| **Recommendation**: | | | |
| SIGNATURE  Done by: Tesfaye T., Pediatrician, Pediatric Cardiologist _______________ 10/03/2015Eth.C | | | |

| Patient Name: **Ze-mariam Andargie**. Referring Institute: **Adinas GH**. SEX/ Age: **M/3 1/12**. Date of Report: **11/03/15**. Referral Diagnosis: Down Syndrome (**Follow up Echo for Small ASD + Mild Pul.HTN.) AGH2.007.** | | | |
| --- | --- | --- | --- |
| **Features** | **Finding** | **Features** | **Finding** |
| **Profile** |  | **Atria** |  |
| Abdominal situs | Solitus | Left atrium | Normal |
| Cardiac position | Levocardia | Right atrium | Normal |
| Systemic venous drainage | Normal. | **Atrioventricular valves** |  |
| Pulmonary venous drainage | Normal | Mitral valve | Annulus = 15mm |
| Atrioventricular connection | Concordant | Tricuspid valve | Annulus = 16m |
| Ventriculoarterial connection | Concordant | **Ventricles** |  |
| Ventricular loop | d-Loop | Left ventricle | Normal |
|  |  | Right ventricle | Normal |
| **Septae** |  | **Coronary arteries** | ----- |
| Interventricular septum | Intact | **Doppler Measurement** |  |
| Interatrial septum | 4mm OS ASD, L – R Shunt | Mitral | ----- |
| **Semilunar valves** |  | Aortic | ------- |
| Aortic valve | Annulus = 13mm | Tricuspid | ------- |
| Pulmonary valve | Annulus = 15mm | pulmonic | -------- |
| **Great arteries** | NRGA | **Aortic arch** | Left. No CoA. |
| Aorta | ----- | **PDA** | No |
| Pulmonary artery | Normal MPA and Branch PAs. |  |  |
| **M-Mode:**  Normal LV Function on eye balling | | | |
| AO | mm | PWd | mm |
| LA | mm | PWs | mm |
| LVIDd | mm | EDV | ml |
| LVIDs | mm | ESV | ml |
| IVSs | mm | LVEF | % |
| IVSd | mm | FS | % |
| **Additional Information**: |  | | |
| No pericardial/Pleural effusion. | | | |
| **Final Diagnosis:** | | | |
| 1. {S, D, S} Levocardia. 2. Small OS ASD, L – R Shunt 3. Normal LV Systolic Function | | | |
| **Remark**: | | | |
| **Recommendation**: | | | |
| SIGNATURE  Done by: Tesfaye T., Pediatrician, Pediatric Cardiologist _______________ 11/03/2015Eth.C | | | |

| Patient Name: **Solomon Mengistu**. Referring Institute: **Mekane – Selam Hosp**. SEX/ Age: **M/1 8/12**.  Date of Report: **12/03/15**. Referral Diagnosis: **RD + CHF.AGH2.008** | | | |
| --- | --- | --- | --- |
| **Features** | **Finding** | **Features** | **Finding** |
| **Profile** |  | **Atria** |  |
| Abdominal situs | Solitus | Left atrium | Dilated |
| Cardiac position | Levocardia | Right atrium | Dilated |
| Systemic venous drainage | Normal. | **Atrioventricular valves** |  |
| Pulmonary venous drainage | Normal | Mitral valve | Annulus = 22mm |
| Atrioventricular connection | Concordant | Tricuspid valve | Annulus = 26mm |
| Ventriculoarterial connection | Concordant | **Ventricles** |  |
| Ventricular loop | d-Loop | Left ventricle | Dilated |
|  |  | Right ventricle | Dilated |
| **Septae** |  | **Coronary arteries** | ----- |
| Interventricular septum | 10mm PM VSD, BD Shunt. | **Doppler Measurement** |  |
| Interatrial septum | Intact | Mitral | Moderate MR |
| **Semilunar valves** |  | Aortic | ------- |
| Aortic valve | Annulus = 14mm | Tricuspid | Mild TR |
| Pulmonary valve | Annulus = 19mm | pulmonic | -------- |
| **Great arteries** | NRGA | **Aortic arch** | Left. No CoA. |
| Aorta | ----- | **PDA** | No |
| Pulmonary artery | MPA =20mm. |  |  |
| **M-Mode:**  Normal LV Function on eye balling | | | |
| AO | mm | PWd | mm |
| LA | mm | PWs | mm |
| LVIDd | mm | EDV | ml |
| LVIDs | mm | ESV | ml |
| IVSs | mm | LVEF | % |
| IVSd | mm | FS | % |
| **Additional Information**: |  | | |
| No pericardial/Pleural effusion. | | | |
| **Final Diagnosis:** | | | |
| 1. {S, D, S} Levocardia. 2. All chambers Dilated 3. Large PM VSD, BD Shunt 4. Severe Pulmonary Hypertension 5. Normal LV Systolic Function | | | |
| **Remark**: Toddler was crying throughout the study. | | | |
| **Recommendation**: | | | |
| SIGNATURE  Done by: Tesfaye T., Pediatrician, Pediatric Cardiologist _______________ 12/03/2015Eth.C | | | |

| Patient Name: **Hablye Wendie_**. Referring Institute: **Mekane-Selam Hosp**. SEX/ Age: **F/2 7/12**.  Date of Report: **13/03/15**. Referral Diagnosis: **Recurrent Chest infection. AGH2.009** | | | |
| --- | --- | --- | --- |
| **Features** | **Finding** | **Features** | **Finding** |
| **Profile** |  | **Atria** |  |
| Abdominal situs | Solitus | Left atrium | Normal |
| Cardiac position | Levocardia | Right atrium | Normal |
| Systemic venous drainage | Normal. | **Atrioventricular valves** |  |
| Pulmonary venous drainage | Normal | Mitral valve | Annulus = 15mm |
| Atrioventricular connection | Concordant | Tricuspid valve | Annulus = 15mm |
| Ventriculoarterial connection | Concordant | **Ventricles** |  |
| Ventricular loop | d-Loop | Left ventricle | Normal |
|  |  | Right ventricle | Normal |
| **Septae** |  | **Coronary arteries** | ----- |
| Interventricular septum | Intact | **Doppler Measurement** |  |
| Interatrial septum | Intact | Mitral | ----- |
| **Semilunar valves** |  | Aortic | ------- |
| Aortic valve | Annulus = 11mm | Tricuspid | ------- |
| Pulmonary valve | Annulus = 13mm | pulmonic | -------- |
| **Great arteries** | NRGA | **Aortic arch** | Left. No CoA. |
| Aorta | ----- | **PDA** | No |
| Pulmonary artery | Normal MPA and Branch PAs. |  |  |
| **M-Mode:**  Normal LV Function on eye balling | | | |
| AO | mm | PWd | mm |
| LA | mm | PWs | mm |
| LVIDd | mm | EDV | ml |
| LVIDs | mm | ESV | ml |
| IVSs | mm | LVEF | % |
| IVSd | mm | FS | % |
| **Additional Information**: |  | | |
| No pericardial/Pleural effusion. | | | |
| **Final Diagnosis:** | | | |
| 1. Normal Echocardiography Study. | | | |
| **Remark**: | | | |
| **Recommendation**: | | | |
| SIGNATURE  Done by: Tesfaye T., Pediatrician, Pediatric Cardiologist _______________ 13/03/2015Eth.C | | | |

| Patient Name: **Sewareg Geremew**. Referring Institute: **Pawe GH**. SEX/ Age: **F/4months**.  Date of Report: **13/03/15**. Referral Diagnosis: **RD. AGH2.010** | | | |
| --- | --- | --- | --- |
| **Features** | **Finding** | **Features** | **Finding** |
| **Profile** |  | **Atria** |  |
| Abdominal situs | Solitus | Left atrium | Dilated |
| Cardiac position | Levocardia | Right atrium | Normal |
| Systemic venous drainage | Normal. | **Atrioventricular valves** |  |
| Pulmonary venous drainage | Normal | Mitral valve | Annulus = 14mm |
| Atrioventricular connection | Concordant | Tricuspid valve | Annulus = 12mm |
| Ventriculoarterial connection | Concordant | **Ventricles** |  |
| Ventricular loop | d-Loop | Left ventricle | Dilated |
|  |  | Right ventricle | Normal |
| **Septae** |  | **Coronary arteries** | ----- |
| Interventricular septum | 7mm PM VSD, L – R Shunt | **Doppler Measurement** |  |
| Interatrial septum | PFO, L – R Shunt | Mitral | ----- |
| **Semilunar valves** |  | Aortic | ------- |
| Aortic valve | Annulus = 9mm | Tricuspid | ------- |
| Pulmonary valve | Annulus = 13mm | pulmonic | Moderate PR, PPG = 53 – 58mmHg |
| **Great arteries** | NRGA | **Aortic arch** | Left. No CoA. |
| Aorta | ----- | **PDA** | No |
| Pulmonary artery | MPA = 18mm |  |  |
| **M-Mode:** | | | |
| AO | mm | PWd | mm |
| LA | mm | PWs | mm |
| LVIDd | mm | EDV | ml |
| LVIDs | mm | ESV | ml |
| IVSs | mm | LVEF | 70% |
| IVSd | mm | FS | 38% |
| **Additional Information**: |  | | |
| No pericardial/Pleural effusion. | | | |
| **Final Diagnosis:** | | | |
| 1. {S, D, S} Levocardia. 2. LA/LV Dilated 3. PFO, L – R Shunt 4. Large PM VSD, L – R Shunt 5. Moderate PR 6. Moderate Pulmonary Hypertension 7. Normal LV Systolic Function | | | |
| **Remark**: | | | |
| **Recommendation**: | | | |
| SIGNATURE  Done by: Tesfaye T., Pediatrician, Pediatric Cardiologist _______________ 13/03/2015Eth.C | | | |

| Patient Name: **Ristie Abazxi**. Referring Institute: **Adinas GH**. SEX/ Age: **F/45days**.  Date of Report: **13/03/15**. Referral Diagnosis: **DS**. **AGH2.011.** | | | |
| --- | --- | --- | --- |
| **Features** | **Finding** | **Features** | **Finding** |
| **Profile** |  | **Atria** |  |
| Abdominal situs | Solitus | Left atrium | Normal |
| Cardiac position | Levocardia | Right atrium | Normal |
| Systemic venous drainage | Normal. | **Atrioventricular valves** |  |
| Pulmonary venous drainage | Normal | Mitral valve | Annulus = 12mm |
| Atrioventricular connection | Concordant | Tricuspid valve | Annulus = 12mm |
| Ventriculoarterial connection | Concordant | **Ventricles** |  |
| Ventricular loop | d-Loop | Left ventricle | Normal |
|  |  | Right ventricle | Normal |
| **Septae** |  | **Coronary arteries** | ----- |
| Interventricular septum | 4mm PM VSD, L – R Shunt | **Doppler Measurement** |  |
| Interatrial septum | Intact | Mitral | ----- |
| **Semilunar valves** |  | Aortic | ------- |
| Aortic valve | Annulus = 10mm | Tricuspid | ------- |
| Pulmonary valve | Annulus = 11mm | pulmonic | -------- |
| **Great arteries** | NRGA | **Aortic arch** | Left. No CoA. |
| Aorta | ----- | **PDA** | No |
| Pulmonary artery | Normal MPA and Branch PAs. |  |  |
| **M-Mode:** | | | |
| AO | mm | PWd | mm |
| LA | mm | PWs | mm |
| LVIDd | mm | EDV | ml |
| LVIDs | mm | ESV | ml |
| IVSs | mm | LVEF | 65% |
| IVSd | mm | FS | 33% |
| **Additional Information**: |  | | |
| No pericardial/Pleural effusion. | | | |
| **Final Diagnosis:** | | | |
| 1. {S, D, S} Levocardia. 2. Small PM VSD, L – R Shunt 3. Normal LV Systolic Function | | | |
| **Remark**: | | | |
| **Recommendation**: | | | |
| SIGNATURE  Done by: Tesfaye T., Pediatrician, Pediatric Cardiologist _______________ 13/03/2015Eth.C | | | |

| Patient Name: **Rahel Berihun**. Referring Institute: **FHRH**. SEX/ Age: **F/5months**.  Date of Report: **13/03/15**. Referral Diagnosis: **Incidental Murmur Detection. AGH2.012** | | | |
| --- | --- | --- | --- |
| **Features** | **Finding** | **Features** | **Finding** |
| **Profile** |  | **Atria** |  |
| Abdominal situs | Solitus | Left atrium | Normal |
| Cardiac position | Levocardia | Right atrium | Dilated |
| Systemic venous drainage | Normal. | **Atrioventricular valves** |  |
| Pulmonary venous drainage | Normal | Mitral valve | Annulus = 11mm |
| Atrioventricular connection | Concordant | Tricuspid valve | Annulus = 14mm |
| Ventriculoarterial connection | Concordant | **Ventricles** |  |
| Ventricular loop | d-Loop | Left ventricle | Normal |
|  |  | Right ventricle | Dilated |
| **Septae** |  | **Coronary arteries** | ----- |
| Interventricular septum | Intact | **Doppler Measurement** |  |
| Interatrial septum | 6mm OS ASD, L – R Shunt | Mitral | ----- |
| **Semilunar valves** |  | Aortic | ------- |
| Aortic valve | Annulus = 8mm | Tricuspid | ------- |
| Pulmonary valve | Annulus = 12mm | pulmonic | Mild PR, PPG = 41mmHg |
| **Great arteries** | NRGA | **Aortic arch** | Left. No CoA. |
| Aorta | ----- | **PDA** | 2mm PDA, L – R Shunt |
| Pulmonary artery | Normal MPA and Branch PAs. |  |  |
| **M-Mode:** | | | |
| AO | mm | PWd | mm |
| LA | mm | PWs | mm |
| LVIDd | mm | EDV | ml |
| LVIDs | mm | ESV | ml |
| IVSs | mm | LVEF | % |
| IVSd | mm | FS | % |
| **Additional Information**: |  | | |
| No pericardial/Pleural effusion. | | | |
| **Final Diagnosis:** | | | |
| 1. {S, D, S} Levocardia. 2. RA/RV Dilated 3. Moderate OS ASD, L – R Shunt 4. Moderate PDA, L – R Shunt 5. Mild Pulmonary Hypertension 6. Normal LV Systolic Function | | | |
| **Remark**: | | | |
| **Recommendation**: | | | |
| SIGNATURE  Done by: Tesfaye T., Pediatrician, Pediatric Cardiologist _______________ 13/03/2015Eth.C | | | |

| Patient Name: **Beselam Tihitinaw**. Referring Institute: **Debre-Markos RH**. SEX/ Age: **F/5years**.  Date of Report: **13/03/15**. Referral Diagnosis: **Incidental Murmur. AGH2.013** | | | |
| --- | --- | --- | --- |
| **Features** | **Finding** | **Features** | **Finding** |
| **Profile** |  | **Atria** |  |
| Abdominal situs | Solitus | Left atrium | Normal |
| Cardiac position | Levocardia | Right atrium | Normal |
| Systemic venous drainage | Normal. | **Atrioventricular valves** |  |
| Pulmonary venous drainage | Normal | Mitral valve | Annulus = 18mm |
| Atrioventricular connection | Concordant | Tricuspid valve | Annulus = 19mm |
| Ventriculoarterial connection | Concordant | **Ventricles** |  |
| Ventricular loop | d-Loop | Left ventricle | Normal |
|  |  | Right ventricle | Normal |
| **Septae** |  | **Coronary arteries** | ----- |
| Interventricular septum | 3mm PM VSD, L – R Shunt with PPG = 57mmHg. | **Doppler Measurement** |  |
| Interatrial septum | PFO, L – R Shunt | Mitral | ----- |
| **Semilunar valves** |  | Aortic | ------- |
| Aortic valve | Annulus = 15mm | Tricuspid | ------- |
| Pulmonary valve | Annulus = 16mm | pulmonic | -------- |
| **Great arteries** | NRGA | **Aortic arch** | Left. No CoA. |
| Aorta | ----- | **PDA** | No |
| Pulmonary artery | Normal MPA and Branch PAs. |  |  |
| **M-Mode:**  Normal LV Systolic Function on eye balling | | | |
| AO | mm | PWd | mm |
| LA | mm | PWs | mm |
| LVIDd | mm | EDV | ml |
| LVIDs | mm | ESV | ml |
| IVSs | mm | LVEF | % |
| IVSd | mm | FS | % |
| **Additional Information**: |  | | |
| No pericardial/Pleural effusion. | | | |
| **Final Diagnosis:** | | | |
| 1. {S, D, S} Levocardia. 2. PFO, L – R Shunt 3. Small Restrictive PM VSD, L – R Shunt 4. Normal LV Systolic Function | | | |
| **Remark**: | | | |
| **Recommendation**: | | | |
| SIGNATURE  Done by: Tesfaye T., Pediatrician, Pediatric Cardiologist _______________ 13/03/2015Eth.C | | | |

| Patient Name: **Fikre-Mariam Dagim**. Referring Institute: **TGSH**. SEX/ Age: **M/1year**.  Date of Report: **13/03/15**. Referral Diagnosis: **Pre-operative Screening. AGH2.014** | | | |
| --- | --- | --- | --- |
| **Features** | **Finding** | **Features** | **Finding** |
| **Profile** |  | **Atria** |  |
| Abdominal situs | Solitus | Left atrium | Normal |
| Cardiac position | Levocardia | Right atrium | Normal |
| Systemic venous drainage | Normal. | **Atrioventricular valves** |  |
| Pulmonary venous drainage | Normal | Mitral valve | Annulus = 13mm |
| Atrioventricular connection | Concordant | Tricuspid valve | Annulus = 13mm |
| Ventriculoarterial connection | Concordant | **Ventricles** |  |
| Ventricular loop | d-Loop | Left ventricle | Normal |
|  |  | Right ventricle | Normal |
| **Septae** |  | **Coronary arteries** | ----- |
| Interventricular septum | Intact | **Doppler Measurement** |  |
| Interatrial septum | Intact | Mitral | ----- |
| **Semilunar valves** |  | Aortic | ------- |
| Aortic valve | Annulus = 12mm | Tricuspid | ------- |
| Pulmonary valve | Annulus = 13mm | pulmonic | -------- |
| **Great arteries** | NRGA | **Aortic arch** | Left. No CoA. |
| Aorta | ----- | **PDA** | No |
| Pulmonary artery | Normal MPA and Branch PAs. |  |  |
| **M-Mode:**  Normal LV Function on eye balling | | | |
| AO | mm | PWd | mm |
| LA | mm | PWs | mm |
| LVIDd | mm | EDV | ml |
| LVIDs | mm | ESV | ml |
| IVSs | mm | LVEF | % |
| IVSd | mm | FS | % |
| **Additional Information**: |  | | |
| No pericardial/Pleural effusion. | | | |
| **Final Diagnosis:** | | | |
| 1. Normal Echocardiography Study. | | | |
| **Remark**: | | | |
| **Recommendation**: | | | |
| SIGNATURE  Done by: Tesfaye T., Pediatrician, Pediatric Cardiologist _______________ 13/03/2015Eth.C | | | |

| Patient Name: **Betselot Getachew**. Referring Institute: **Amaris PSC**. SEX/ Age: **F/4 4/12**.  Date of Report: **13/03/15**. Referral Diagnosis: **Recurrent chest infection. AGH2.015.** | | | |
| --- | --- | --- | --- |
| **Features** | **Finding** | **Features** | **Finding** |
| **Profile** |  | **Atria** |  |
| Abdominal situs | Solitus | Left atrium | Mildly Dilated |
| Cardiac position | Levocardia | Right atrium | Normal |
| Systemic venous drainage | Normal. | **Atrioventricular valves** |  |
| Pulmonary venous drainage | Normal | Mitral valve | Annulus = 15mm |
| Atrioventricular connection | Concordant | Tricuspid valve | Annulus = 16mm |
| Ventriculoarterial connection | Concordant | **Ventricles** |  |
| Ventricular loop | d-Loop | Left ventricle | Mildly Dilated |
|  |  | Right ventricle | Normal |
| **Septae** |  | **Coronary arteries** | ----- |
| Interventricular septum | 7mm PM VSD, L – R Shunt | **Doppler Measurement** |  |
| Interatrial septum | Intact | Mitral | ----- |
| **Semilunar valves** |  | Aortic | ------- |
| Aortic valve | Annulus = 12mm | Tricuspid | ------- |
| Pulmonary valve | Annulus = 12mm | pulmonic | -------- |
| **Great arteries** | NRGA | **Aortic arch** | Left. No CoA. |
| Aorta | ----- | **PDA** | No |
| Pulmonary artery | Normal MPA and Branch PAs. |  |  |
| **M-Mode:**  Normal LV Function on eye balling | | | |
| AO | mm | PWd | mm |
| LA | mm | PWs | mm |
| LVIDd | mm | EDV | ml |
| LVIDs | mm | ESV | ml |
| IVSs | mm | LVEF | % |
| IVSd | mm | FS | % |
| **Additional Information**: |  | | |
| No pericardial/Pleural effusion. | | | |
| **Final Diagnosis:** | | | |
| 1. {S, D, S} Levocardia. 2. Mildly dilated LA/LV 3. Moderate PM VSD, L – R Shunt | | | |
| **Remark**: Child was restless during study with limited echo window | | | |
| **Recommendation**: | | | |
| SIGNATURE  Done by: Tesfaye T., Pediatrician, Pediatric Cardiologist _______________ 13/03/2015Eth.C | | | |

| Patient Name: **Dawit Belachew**. Referring Institute: **FHRH**. SEX/ Age: **M/11months**.  Date of Report: **13/03/15**. Referral Diagnosis: **DS. AGH2.016** | | | |
| --- | --- | --- | --- |
| **Features** | **Finding** | **Features** | **Finding** |
| **Profile** |  | **Atria** |  |
| Abdominal situs | Solitus | Left atrium | Normal |
| Cardiac position | Levocardia | Right atrium | Normal |
| Systemic venous drainage | Normal. | **Atrioventricular valves** |  |
| Pulmonary venous drainage | Normal | Mitral valve | Annulus = 14mm |
| Atrioventricular connection | Concordant | Tricuspid valve | Annulus = 15mm  TAPSE = 18mm |
| Ventriculoarterial connection | Concordant | **Ventricles** |  |
| Ventricular loop | d-Loop | Left ventricle | Normal |
|  |  | Right ventricle | Normal |
| **Septae** |  | **Coronary arteries** | ----- |
| Interventricular septum | Intact | **Doppler Measurement** |  |
| Interatrial septum | Intact | Mitral | ----- |
| **Semilunar valves** |  | Aortic | ------- |
| Aortic valve | Annulus = 11mm | Tricuspid | ------- |
| Pulmonary valve | Annulus = 12mm | pulmonic | -------- |
| **Great arteries** | NRGA | **Aortic arch** | Left. No CoA. |
| Aorta | ----- | **PDA** | No |
| Pulmonary artery | Normal MPA and Branch PAs. |  |  |
| **M-Mode:**  Normal LV Function on eye balling | | | |
| AO | mm | PWd | mm |
| LA | mm | PWs | mm |
| LVIDd | mm | EDV | ml |
| LVIDs | mm | ESV | ml |
| IVSs | mm | LVEF | % |
| IVSd | mm | FS | % |
| **Additional Information**: |  | | |
| No pericardial/Pleural effusion. | | | |
| **Final Diagnosis:** | | | |
| 1. Normal Echocardiography Study. | | | |
| **Remark**: | | | |
| **Recommendation**: | | | |
| SIGNATURE  Done by: Tesfaye T., Pediatrician, Pediatric Cardiologist _______________ 13/03/2015Eth.C | | | |

| Patient Name: **Tadlo Nigussie**. Referring Institute: **FHRH**. SEX/ Age: **M/8months**.  Date of Report: **13/03/15**. Referral Diagnosis: **Recurrent Chest Infection. AGH2.017** | | | |
| --- | --- | --- | --- |
| **Features** | **Finding** | **Features** | **Finding** |
| **Profile** |  | **Atria** |  |
| Abdominal situs | Solitus | Left atrium | Normal |
| Cardiac position | Levocardia | Right atrium | Normal |
| Systemic venous drainage | Normal. | **Atrioventricular valves** |  |
| Pulmonary venous drainage | Normal | Mitral valve | Annulus = 14mm |
| Atrioventricular connection | Concordant | Tricuspid valve | Annulus = 15mm |
| Ventriculoarterial connection | Concordant | **Ventricles** |  |
| Ventricular loop | d-Loop | Left ventricle | Normal |
|  |  | Right ventricle | Normal |
| **Septae** |  | **Coronary arteries** | ----- |
| Interventricular septum | Intact | **Doppler Measurement** |  |
| Interatrial septum | PFO, L – R Shunt | Mitral | ----- |
| **Semilunar valves** |  | Aortic | ------- |
| Aortic valve | Annulus = 11mm | Tricuspid | ------- |
| Pulmonary valve | Annulus = 13mm | pulmonic | -------- |
| **Great arteries** | NRGA | **Aortic arch** | Left. No CoA. |
| Aorta | ----- | **PDA** | No |
| Pulmonary artery | Normal MPA and Branch PAs. |  |  |
| **M-Mode:**  Normal LV Function on eye balling | | | |
| AO | mm | PWd | mm |
| LA | mm | PWs | mm |
| LVIDd | mm | EDV | ml |
| LVIDs | mm | ESV | ml |
| IVSs | mm | LVEF | % |
| IVSd | mm | FS | % |
| **Additional Information**: |  | | |
| No pericardial/Pleural effusion. | | | |
| **Final Diagnosis:** | | | |
| 1. {S, D, S} Levocardia. 2. PFO, L – R Shunt | | | |
| **Remark**: Poor echo window (Hyper-Inflated chest) | | | |
| **Recommendation**: | | | |
| SIGNATURE  Done by: Tesfaye T., Pediatrician, Pediatric Cardiologist _______________ 13/03/2015Eth.C | | | |

| Patient Name: **Soliana Yemataw**. Referring Institute: **Efrata PSC**. SEX/ Age: **F/4 3/12**.  Date of Report: **13/03/15**. Referral Diagnosis: **Recurrent Chest Infection. AGH2.018.** | | | |
| --- | --- | --- | --- |
| **Features** | **Finding** | **Features** | **Finding** |
| **Profile** |  | **Atria** |  |
| Abdominal situs | Solitus | Left atrium | Normal |
| Cardiac position | Levocardia | Right atrium | Normal |
| Systemic venous drainage | Normal. | **Atrioventricular valves** |  |
| Pulmonary venous drainage | Normal | Mitral valve | Annulus = 16mm |
| Atrioventricular connection | Concordant | Tricuspid valve | Annulus = 16mm  TAPSE = 18mm |
| Ventriculoarterial connection | Concordant | **Ventricles** |  |
| Ventricular loop | d-Loop | Left ventricle | Normal |
|  |  | Right ventricle | Normal |
| **Septae** |  | **Coronary arteries** | ----- |
| Interventricular septum | Intact | **Doppler Measurement** |  |
| Interatrial septum | Intact | Mitral | ----- |
| **Semilunar valves** |  | Aortic | ------- |
| Aortic valve | Annulus = 15mm | Tricuspid | ------- |
| Pulmonary valve | Annulus = 16mm | pulmonic | -------- |
| **Great arteries** | NRGA | **Aortic arch** | Left. No CoA. |
| Aorta | ----- | **PDA** | No |
| Pulmonary artery | Normal MPA and Branch PAs. |  |  |
| **M-Mode:** | | | |
| AO | mm | PWd | mm |
| LA | mm | PWs | mm |
| LVIDd | mm | EDV | ml |
| LVIDs | mm | ESV | ml |
| IVSs | mm | LVEF | 66% |
| IVSd | mm | FS | 35% |
| **Additional Information**: |  | | |
| No pericardial/Pleural effusion. | | | |
| **Final Diagnosis:** | | | |
| 1. Normal Echocardiography Study. | | | |
| **Remark**: | | | |
| **Recommendation**: | | | |
| SIGNATURE  Done by: Tesfaye T., Pediatrician, Pediatric Cardiologist _______________ 13/03/2015Eth.C | | | |

| Patient Name: **Mathias Habtamu**. Referring Institute: **TGSH**. SEX/ Age: **M/6months**.  Date of Report: **13/03/15**. Referral Diagnosis: **RD. AGH2.019.** | | | |
| --- | --- | --- | --- |
| **Features** | **Finding** | **Features** | **Finding** |
| **Profile** |  | **Atria** |  |
| Abdominal situs | Solitus | Left atrium | Dilated |
| Cardiac position | Levocardia | Right atrium | Normal |
| Systemic venous drainage | Normal. | **Atrioventricular valves** |  |
| Pulmonary venous drainage | Normal | Mitral valve | Annulus = 15mm |
| Atrioventricular connection | Concordant | Tricuspid valve | Annulus = 13mm |
| Ventriculoarterial connection | Concordant | **Ventricles** |  |
| Ventricular loop | d-Loop | Left ventricle | Dilated |
|  |  | Right ventricle | Normal |
| **Septae** |  | **Coronary arteries** | ----- |
| Interventricular septum | 8mm PM VSD, L – R Shunt | **Doppler Measurement** |  |
| Interatrial septum | Intact | Mitral | ----- |
| **Semilunar valves** |  | Aortic | ------- |
| Aortic valve | Annulus = 10mm | Tricuspid | ------- |
| Pulmonary valve | Annulus = 12mm | pulmonic | -------- |
| **Great arteries** | NRGA | **Aortic arch** | Left. No CoA. |
| Aorta | ----- | **PDA** | No |
| Pulmonary artery | MPA = 19mm. |  |  |
| **M-Mode:** | | | |
| AO | mm | PWd | mm |
| LA | mm | PWs | mm |
| LVIDd | mm | EDV | ml |
| LVIDs | mm | ESV | ml |
| IVSs | mm | LVEF | 73% |
| IVSd | mm | FS | 40% |
| **Additional Information**: |  | | |
| No pericardial/Pleural effusion. | | | |
| **Final Diagnosis:** | | | |
| 1. {S, D, S} Levocardia. 2. LA/LV Dilated 3. Large PM VSD, L – R Shunt 4. Pulmonary Hypertension 5. Normal LV Systolic Function | | | |
| **Remark**: | | | |
| **Recommendation**: | | | |
| SIGNATURE  Done by: Tesfaye T., Pediatrician, Pediatric Cardiologist _______________ 13/03/2015Eth.C | | | |

| Patient Name: **Betre-Mariam Atrsaw**. Referring Institute: **TGSH**. SEX/ Age: **M/10years**.  Date of Report: **13/03/15**. Referral Diagnosis: **Easy fatigability. AGH2.020** | | | |
| --- | --- | --- | --- |
| **Features** | **Finding** | **Features** | **Finding** |
| **Profile** |  | **Atria** |  |
| Abdominal situs | Solitus | Left atrium | Normal |
| Cardiac position | Levocardia | Right atrium | Normal |
| Systemic venous drainage | Normal. | **Atrioventricular valves** |  |
| Pulmonary venous drainage | Normal | Mitral valve | Annulus = 20mm |
| Atrioventricular connection | Concordant | Tricuspid valve | Annulus = 20mm  TAPSE = 19mm |
| Ventriculoarterial connection | Concordant | **Ventricles** |  |
| Ventricular loop | d-Loop | Left ventricle | Normal |
|  |  | Right ventricle | Normal |
| **Septae** |  | **Coronary arteries** | ----- |
| Interventricular septum | Intact | **Doppler Measurement** |  |
| Interatrial septum | Intact | Mitral | ----- |
| **Semilunar valves** |  | Aortic | ------- |
| Aortic valve | Annulus = 15mm | Tricuspid | ------- |
| Pulmonary valve | Annulus = 18mm | pulmonic | Trivial PR, PPG = 15mmHg |
| **Great arteries** | NRGA | **Aortic arch** | Left. No CoA. |
| Aorta | ----- | **PDA** | No |
| Pulmonary artery | Normal MPA and Branch PAs. |  |  |
| **M-Mode:** | | | |
| AO | mm | PWd | mm |
| LA | mm | PWs | mm |
| LVIDd | mm | EDV | ml |
| LVIDs | mm | ESV | ml |
| IVSs | mm | LVEF | 70% |
| IVSd | mm | FS | 38% |
| **Additional Information**: |  | | |
| No pericardial/Pleural effusion. | | | |
| **Final Diagnosis:** | | | |
| 1. Normal Echocardiography Study. | | | |
| **Remark**: | | | |
| **Recommendation**: | | | |
| SIGNATURE  Done by: Tesfaye T., Pediatrician, Pediatric Cardiologist _______________ 13/03/2015Eth.C | | | |

| Patient Name: **Kidus Abera**. Referring Institute: **TGSH**. SEX/ Age: **M/4 4/12**. Date of Report: **14/03/15**.  Referral Diagnosis: **Pre-op screening. AGH2.021** | | | |
| --- | --- | --- | --- |
| **Features** | **Finding** | **Features** | **Finding** |
| **Profile** |  | **Atria** |  |
| Abdominal situs | Solitus | Left atrium | Normal |
| Cardiac position | Levocardia | Right atrium | Normal |
| Systemic venous drainage | Normal. | **Atrioventricular valves** |  |
| Pulmonary venous drainage | Normal | Mitral valve | Annulus = 16mm |
| Atrioventricular connection | Concordant | Tricuspid valve | Annulus = 18mm  TAPSE = 18mm |
| Ventriculoarterial connection | Concordant | **Ventricles** |  |
| Ventricular loop | d-Loop | Left ventricle | Normal |
|  |  | Right ventricle | Normal |
| **Septae** |  | **Coronary arteries** | ----- |
| Interventricular septum | Intact | **Doppler Measurement** |  |
| Interatrial septum | Intact | Mitral | ----- |
| **Semilunar valves** |  | Aortic | ------- |
| Aortic valve | Annulus = 15mm | Tricuspid | ------- |
| Pulmonary valve | Annulus = 17mm | pulmonic | -------- |
| **Great arteries** | NRGA | **Aortic arch** | Left. No CoA. |
| Aorta | ----- | **PDA** | No |
| Pulmonary artery | Normal MPA and Branch PAs. |  |  |
| **M-Mode:** | | | |
| AO | mm | PWd | mm |
| LA | mm | PWs | mm |
| LVIDd | mm | EDV | ml |
| LVIDs | mm | ESV | ml |
| IVSs | mm | LVEF | 58% |
| IVSd | mm | FS | 29% |
| **Additional Information**: |  | | |
| No pericardial/Pleural effusion. | | | |
| **Final Diagnosis:** | | | |
| 1. Normal Echocardiography Study. | | | |
| **Remark**: | | | |
| **Recommendation**: | | | |
| SIGNATURE  Done by: Tesfaye T., Pediatrician, Pediatric Cardiologist _______________ 14/03/2015Eth.C | | | |

| Patient Name: **Nadab Desalew**. Referring Institute: **GAMBY GH**. SEX/ Age: **M/2months**.  Date of Report: **14/03/15**. Referral Diagnosis: **DS AGH2.022** | | | |
| --- | --- | --- | --- |
| **Features** | **Finding** | **Features** | **Finding** |
| **Profile** |  | **Atria** |  |
| Abdominal situs | Solitus | Left atrium | Normal |
| Cardiac position | Levocardia | Right atrium | Normal |
| Systemic venous drainage | Normal. | **Atrioventricular valves** |  |
| Pulmonary venous drainage | Normal | Mitral valve | Annulus = 11mm |
| Atrioventricular connection | Concordant | Tricuspid valve | Annulus = 12mm |
| Ventriculoarterial connection | Concordant | **Ventricles** |  |
| Ventricular loop | d-Loop | Left ventricle | Normal |
|  |  | Right ventricle | Normal |
| **Septae** |  | **Coronary arteries** | ----- |
| Interventricular septum | Intact | **Doppler Measurement** |  |
| Interatrial septum | PFO, L – R Shunt | Mitral | ----- |
| **Semilunar valves** |  | Aortic | ------- |
| Aortic valve | Annulus = 10mm | Tricuspid | ------- |
| Pulmonary valve | Annulus = 10mm | pulmonic | -------- |
| **Great arteries** | NRGA | **Aortic arch** | Left. No CoA. |
| Aorta | ----- | **PDA** | No |
| Pulmonary artery | Normal MPA and Branch PAs. |  |  |
| **M-Mode:**  Normal LV Function on eye balling | | | |
| AO | mm | PWd | mm |
| LA | mm | PWs | mm |
| LVIDd | mm | EDV | ml |
| LVIDs | mm | ESV | ml |
| IVSs | mm | LVEF | % |
| IVSd | mm | FS | % |
| **Additional Information**: |  | | |
| No pericardial/Pleural effusion. | | | |
| **Final Diagnosis:** | | | |
| 1. {S, D, S} Levocardia. 2. PFO, L – R Shunt | | | |
| **Remark**: | | | |
| **Recommendation**: | | | |
| SIGNATURE  Done by: Tesfaye T., Pediatrician, Pediatric Cardiologist _______________ 14/03/2015Eth.C | | | |

| Patient Name: **Desta Tilahun**. Referring Institute: **TGSH**. SEX/ Age: **F/4 6/12**. Date of Report: **14/03/15**.  Referral Diagnosis: **Incidental Murmur. AGH2.023** | | | |
| --- | --- | --- | --- |
| **Features** | **Finding** | **Features** | **Finding** |
| **Profile** |  | **Atria** |  |
| Abdominal situs | Solitus | Left atrium | Normal |
| Cardiac position | Levocardia | Right atrium | Normal |
| Systemic venous drainage | Normal. | **Atrioventricular valves** |  |
| Pulmonary venous drainage | Normal | Mitral valve | Annulus = 19mm |
| Atrioventricular connection | Concordant | Tricuspid valve | Annulus = 22mm  TAPSE = 20mm |
| Ventriculoarterial connection | Concordant | **Ventricles** |  |
| Ventricular loop | d-Loop | Left ventricle | Normal |
|  |  | Right ventricle | Normal |
| **Septae** |  | **Coronary arteries** | ----- |
| Interventricular septum | Intact | **Doppler Measurement** |  |
| Interatrial septum | PFO, L – R Shunt | Mitral | ----- |
| **Semilunar valves** |  | Aortic | ------- |
| Aortic valve | Annulus = 15mm | Tricuspid | Moderate TR, PPG = 18mmHg |
| Pulmonary valve | Annulus = 16mm | pulmonic | -------- |
| **Great arteries** | NRGA | **Aortic arch** | Left. No CoA. |
| Aorta | ----- | **PDA** | No |
| Pulmonary artery | Normal MPA and Branch PAs. |  |  |
| **M-Mode:** | | | |
| AO | mm | PWd | mm |
| LA | mm | PWs | mm |
| LVIDd | mm | EDV | ml |
| LVIDs | mm | ESV | ml |
| IVSs | mm | LVEF | 57% |
| IVSd | mm | FS | 29% |
| **Additional Information**: |  | | |
| No pericardial/Pleural effusion. | | | |
| **Final Diagnosis:** | | | |
| 1. {S, D, S} Levocardia. 2. PFO, L – R Shunt 3. Moderate TR 4. Normal Biventricular Systolic Function | | | |
| **Remark**: | | | |
| **Recommendation**: | | | |
| SIGNATURE  Done by: Tesfaye T., Pediatrician, Pediatric Cardiologist _______________ 14/03/2015Eth.C | | | |

| Patient Name: **Baby of Endayehu Aweke**. Referring Institute: **FHRH**. SEX/ Age: **M/14days**. Date of Report: **15/03/15**. Referral Diagnosis: **RD. AGH2.024.** | | | |
| --- | --- | --- | --- |
| **Features** | **Finding** | **Features** | **Finding** |
| **Profile** |  | **Atria** |  |
| Abdominal situs | Solitus | Left atrium | Normal |
| Cardiac position | Levocardia | Right atrium | Normal |
| Systemic venous drainage | Normal. | **Atrioventricular valves** |  |
| Pulmonary venous drainage | Normal | Mitral valve | Annulus = 8 |
| Atrioventricular connection | Concordant | Tricuspid valve | Annulus = 10mm |
| Ventriculoarterial connection | Concordant | **Ventricles** |  |
| Ventricular loop | d-Loop | Left ventricle | Normal |
|  |  | Right ventricle | Normal |
| **Septae** |  | **Coronary arteries** | ----- |
| Interventricular septum | Intact | **Doppler Measurement** |  |
| Interatrial septum | 5mm Fenestrated OS ASD, L – R Shunt | Mitral | ----- |
| **Semilunar valves** |  | Aortic | ------- |
| Aortic valve | Annulus = 7mm | Tricuspid | ------- |
| Pulmonary valve | Annulus = 8mm | pulmonic | -------- |
| **Great arteries** | NRGA | **Aortic arch** | Left. No CoA. |
| Aorta | ----- | **PDA** | No |
| Pulmonary artery | Normal MPA and Branch PAs. |  |  |
| **M-Mode:**  Normal LV Function on eye balling. | | | |
| AO | mm | PWd | mm |
| LA | mm | PWs | mm |
| LVIDd | mm | EDV | ml |
| LVIDs | mm | ESV | ml |
| IVSs | mm | LVEF | % |
| IVSd | mm | FS | % |
| **Additional Information**: |  | | |
| No pericardial/Pleural effusion. | | | |
| **Final Diagnosis:** | | | |
| 1. {S, D, S} Levocardia. 2. Small Fenestrated OS ASD, L – R Shunt 3. Normal LV Systolic Function | | | |
| **Remark**: | | | |
| **Recommendation**: | | | |
| SIGNATURE  Done by: Tesfaye T., Pediatrician, Pediatric Cardiologist _______________ 15/03/2015Eth.C | | | |

| Patient Name: **Haset Yiretal**. Referring Institute: **Amaris PSC**. SEX/ Age: **F/1 2/12**. Date of Report: **15/03/15**.  Referral Diagnosis: Incidental Murmur (**Follow up echocardiography for Small PM VSD, L – R Shunt). AGH2.025** | | | |
| --- | --- | --- | --- |
| **Features** | **Finding** | **Features** | **Finding** |
| **Profile** |  | **Atria** |  |
| Abdominal situs | Solitus | Left atrium | Normal |
| Cardiac position | Levocardia | Right atrium | Normal |
| Systemic venous drainage | Normal. | **Atrioventricular valves** |  |
| Pulmonary venous drainage | Normal | Mitral valve | Annulus = 14mm |
| Atrioventricular connection | Concordant | Tricuspid valve | Annulus = 15mm  TAPSE = 17mm |
| Ventriculoarterial connection | Concordant | **Ventricles** |  |
| Ventricular loop | d-Loop | Left ventricle | Normal |
|  |  | Right ventricle | Normal |
| **Septae** |  | **Coronary arteries** | ----- |
| Interventricular septum | 5mm PM VSD, L – R Shunt, Partially closed by STL. | **Doppler Measurement** |  |
| Interatrial septum | Intact | Mitral | ----- |
| **Semilunar valves** |  | Aortic | ------- |
| Aortic valve | Annulus = 12mm | Tricuspid | ------- |
| Pulmonary valve | Annulus = 14mm | pulmonic | -------- |
| **Great arteries** | NRGA | **Aortic arch** | Left. No CoA. |
| Aorta | ----- | **PDA** | No |
| Pulmonary artery | Normal MPA and Branch PAs. |  |  |
| **M-Mode:**  Normal LV Function on eye balling. | | | |
| AO | mm | PWd | mm |
| LA | mm | PWs | mm |
| LVIDd | mm | EDV | ml |
| LVIDs | mm | ESV | ml |
| IVSs | mm | LVEF | % |
| IVSd | mm | FS | % |
| **Additional Information**: |  | | |
| No pericardial/Pleural effusion. | | | |
| **Final Diagnosis:** | | | |
| 1. {S, D, S} Levocardia. 2. Small PM VSD, Partially closed by STL, L – R Shunt 3. Normal Biventricular Systolic Function | | | |
| **Remark**: | | | |
| **Recommendation**: | | | |
| SIGNATURE  Done by: Tesfaye T., Pediatrician, Pediatric Cardiologist _______________ 15/03/2015Eth.C | | | |

| Patient Name: **Yeab-sira Werku**. Referring Institute: **FHRH**. SEX/ Age: **F/3months**.  Date of Report: **15/03/15**. Referral Diagnosis: **DS. AGH2.026** | | | |
| --- | --- | --- | --- |
| **Features** | **Finding** | **Features** | **Finding** |
| **Profile** |  | **Atria** |  |
| Abdominal situs | Solitus | Left atrium | Normal |
| Cardiac position | Levocardia | Right atrium | Normal |
| Systemic venous drainage | Normal. | **Atrioventricular valves** |  |
| Pulmonary venous drainage | Normal | Mitral valve | Annulus = 9mm |
| Atrioventricular connection | Concordant | Tricuspid valve | Annulus = 10mm |
| Ventriculoarterial connection | Concordant | **Ventricles** |  |
| Ventricular loop | d-Loop | Left ventricle | Normal |
|  |  | Right ventricle | Normal |
| **Septae** |  | **Coronary arteries** | ----- |
| Interventricular septum | Intact | **Doppler Measurement** |  |
| Interatrial septum | PFO, L – R Shunt | Mitral | ----- |
| **Semilunar valves** |  | Aortic | ------- |
| Aortic valve | Annulus = 9mm | Tricuspid | ------- |
| Pulmonary valve | Annulus = 11mm | pulmonic | -------- |
| **Great arteries** | NRGA | **Aortic arch** | Left. No CoA. |
| Aorta | ----- | **PDA** | No |
| Pulmonary artery | Normal MPA and Branch PAs. |  |  |
| **M-Mode:**  Normal VL Function on eye balling. | | | |
| AO | mm | PWd | mm |
| LA | mm | PWs | mm |
| LVIDd | mm | EDV | ml |
| LVIDs | mm | ESV | ml |
| IVSs | mm | LVEF | % |
| IVSd | mm | FS | % |
| **Additional Information**: |  | | |
| No pericardial/Pleural effusion. | | | |
| **Final Diagnosis:** | | | |
| 1. {S, D, S} Levocardia. 2. PFO, L – R Shunt 3. Normal LV Systolic Function | | | |
| **Remark**: | | | |
| **Recommendation**: | | | |
| SIGNATURE  Done by: Tesfaye T., Pediatrician, Pediatric Cardiologist _______________ 15/03/2015Eth.C | | | |

| Patient Name: **Habtamu Birhanu**. Referring Institute: **TGSH**. SEX/ Age: **M/2years**. Date of Report: **15/03/15**.  Referral Diagnosis: **Incidental Murmur. AGH2.027** | | | |
| --- | --- | --- | --- |
| **Features** | **Finding** | **Features** | **Finding** |
| **Profile** |  | **Atria** |  |
| Abdominal situs | Solitus | Left atrium | Normal |
| Cardiac position | Levocardia | Right atrium | Normal |
| Systemic venous drainage | Normal. | **Atrioventricular valves** |  |
| Pulmonary venous drainage | Normal | Mitral valve | Annulus = 15mm |
| Atrioventricular connection | Concordant | Tricuspid valve | Annulus = 17mm  TAPSE = 17mm |
| Ventriculoarterial connection | Concordant | **Ventricles** |  |
| Ventricular loop | d-Loop | Left ventricle | Normal |
|  |  | Right ventricle | Normal |
| **Septae** |  | **Coronary arteries** | ----- |
| Interventricular septum | Intact | **Doppler Measurement** |  |
| Interatrial septum | PFO, L – R Shunt | Mitral | ----- |
| **Semilunar valves** |  | Aortic | ------- |
| Aortic valve | Annulus = 14mm | Tricuspid | ------- |
| Pulmonary valve | Annulus =15 mm | pulmonic | -------- |
| **Great arteries** | NRGA | **Aortic arch** | Left. No CoA. |
| Aorta | ----- | **PDA** | 1.5mm PDA, L – R Shunt |
| Pulmonary artery | Normal MPA and Branch PAs. |  |  |
| **M-Mode:**  Normal LV Function on eye balling | | | |
| AO | mm | PWd | mm |
| LA | mm | PWs | mm |
| LVIDd | mm | EDV | ml |
| LVIDs | mm | ESV | ml |
| IVSs | mm | LVEF | % |
| IVSd | mm | FS | % |
| **Additional Information**: |  | | |
| No pericardial/Pleural effusion. | | | |
| **Final Diagnosis:** | | | |
| 1. {S, D, S} Levocardia. 2. PFO, L – R Shunt 3. Small PDA, L – R Shunt 4. Normal Biventricular Systolic Function | | | |
| **Remark**: | | | |
| **Recommendation**: | | | |
| SIGNATURE  Done by: Tesfaye T., Pediatrician, Pediatric Cardiologist _______________ 15/03/2015Eth.C | | | |

| Patient Name: **Naod Netsanet**. Referring Institute: **Amaris PSC.**  SEX/ Age: **M/46days**.  Date of Report: **16/03/15**. Referral Diagnosis: **DS. AGH2.028** | | | |
| --- | --- | --- | --- |
| **Features** | **Finding** | **Features** | **Finding** |
| **Profile** |  | **Atria** |  |
| Abdominal situs | Solitus | Left atrium | Normal |
| Cardiac position | Levocardia | Right atrium | Normal |
| Systemic venous drainage | Normal. | **Atrioventricular valves** |  |
| Pulmonary venous drainage | Normal | Mitral valve | Annulus = 12mm |
| Atrioventricular connection | Concordant | Tricuspid valve | Annulus = 13mm |
| Ventriculoarterial connection | Concordant | **Ventricles** |  |
| Ventricular loop | d-Loop | Left ventricle | Normal |
|  |  | Right ventricle | Normal |
| **Septae** |  | **Coronary arteries** | ----- |
| Interventricular septum | Intact | **Doppler Measurement** |  |
| Interatrial septum | PFO, L – R Shunt | Mitral | ----- |
| **Semilunar valves** |  | Aortic | ------- |
| Aortic valve | Annulus = 10mm | Tricuspid | ------- |
| Pulmonary valve | Annulus = 11mm | pulmonic | -------- |
| **Great arteries** | NRGA | **Aortic arch** | Left. No CoA. |
| Aorta | ----- | **PDA** | No |
| Pulmonary artery | Normal MPA and Branch PAs. |  |  |
| **M-Mode:**  Normal LV Function on eye balling | | | |
| AO | mm | PWd | mm |
| LA | mm | PWs | mm |
| LVIDd | mm | EDV | ml |
| LVIDs | mm | ESV | ml |
| IVSs | mm | LVEF | % |
| IVSd | mm | FS | % |
| **Additional Information**: |  | | |
| No pericardial/Pleural effusion. | | | |
| **Final Diagnosis:** | | | |
| 1. {S, D, S} Levocardia. 2. PFO,L – R Shunt | | | |
| **Remark**: | | | |
| **Recommendation**: | | | |
| SIGNATURE  Done by: Tesfaye T., Pediatrician, Pediatric Cardiologist _______________ 16/03/2015Eth.C | | | |

| Patient Name: **Alemneh Derso**. Referring Institute: **FHRH**. SEX/ Age: **M/3years**. Date of Report: **16/03/15**.  Referral Diagnosis: **Recurrent Chest Infection. AGH2.029** | | | |
| --- | --- | --- | --- |
| **Features** | **Finding** | **Features** | **Finding** |
| **Profile** |  | **Atria** |  |
| Abdominal situs | Solitus | Left atrium | Normal |
| Cardiac position | Levocardia | Right atrium | Normal |
| Systemic venous drainage | Normal. | **Atrioventricular valves** |  |
| Pulmonary venous drainage | Normal | Mitral valve | Annulus = 15mm |
| Atrioventricular connection | Concordant | Tricuspid valve | Annulus = 15mm |
| Ventriculoarterial connection | Concordant | **Ventricles** |  |
| Ventricular loop | d-Loop | Left ventricle | Normal |
|  |  | Right ventricle | Normal |
| **Septae** |  | **Coronary arteries** | ----- |
| Interventricular septum | Intact | **Doppler Measurement** |  |
| Interatrial septum | Intact | Mitral | ----- |
| **Semilunar valves** |  | Aortic | ------- |
| Aortic valve | Annulus = 12mm | Tricuspid | ------- |
| Pulmonary valve | Annulus = 11mm | pulmonic | -------- |
| **Great arteries** | NRGA | **Aortic arch** | Left. No CoA. |
| Aorta | ----- | **PDA** | No |
| Pulmonary artery | Normal MPA and Branch PAs. |  |  |
| **M-Mode:**  Normal LV Function on eye balling | | | |
| AO | mm | PWd | mm |
| LA | mm | PWs | mm |
| LVIDd | mm | EDV | ml |
| LVIDs | mm | ESV | ml |
| IVSs | mm | LVEF | % |
| IVSd | mm | FS | % |
| **Additional Information**: |  | | |
| No pericardial/Pleural effusion. | | | |
| **Final Diagnosis:** | | | |
| 1. Normal Echocardiography Study. | | | |
| **Remark**: Limited Echocardiography window (only subcostal) | | | |
| **Recommendation**: | | | |
| SIGNATURE  Done by: Tesfaye T., Pediatrician, Pediatric Cardiologist _______________ 16/03/2015Eth.C | | | |

| Patient Name: **Baby of Hedija Mohammed**. Referring Institute: **FHRH**. SEX/ Age: **M/53days**. Date of Report: **16/03/15**.  Referral Diagnosis: **DS. AGH2.030** | | | |
| --- | --- | --- | --- |
| **Features** | **Finding** | **Features** | **Finding** |
| **Profile** |  | **Atria** |  |
| Abdominal situs | Solitus | Left atrium | Normal |
| Cardiac position | Levocardia | Right atrium | Normal |
| Systemic venous drainage | Normal. | **Atrioventricular valves** |  |
| Pulmonary venous drainage | Normal | Mitral valve | Annulus = 10mm |
| Atrioventricular connection | Concordant | Tricuspid valve | Annulus = 10mm |
| Ventriculoarterial connection | Concordant | **Ventricles** |  |
| Ventricular loop | d-Loop | Left ventricle | Normal |
|  |  | Right ventricle | Normal |
| **Septae** |  | **Coronary arteries** | ----- |
| Interventricular septum | Intact | **Doppler Measurement** |  |
| Interatrial septum | Intact | Mitral | ----- |
| **Semilunar valves** |  | Aortic | ------- |
| Aortic valve | Annulus = 9mm | Tricuspid | ------- |
| Pulmonary valve | Annulus = 11mm | pulmonic | -------- |
| **Great arteries** | NRGA | **Aortic arch** | Left. No CoA. |
| Aorta | ----- | **PDA** | No |
| Pulmonary artery | Normal MPA and Branch PAs. |  |  |
| **M-Mode:**  Normal LV Function on eye balling | | | |
| AO | mm | PWd | mm |
| LA | mm | PWs | mm |
| LVIDd | mm | EDV | ml |
| LVIDs | mm | ESV | ml |
| IVSs | mm | LVEF | % |
| IVSd | mm | FS | % |
| **Additional Information**: |  | | |
| No pericardial/Pleural effusion. | | | |
| **Final Diagnosis:** | | | |
| 1. Normal Echocardiography Study. | | | |
| **Remark**: Limited Echocardiography Window (Only Subcostal). | | | |
| **Recommendation**: | | | |
| SIGNATURE  Done by: Tesfaye T., Pediatrician, Pediatric Cardiologist _______________ 16/03/2015Eth.C | | | |

| Patient Name: **Mahlet Nega**. Referring Institute: **TGSH**. SEX/ Age: **F/4 6/12**. Date of Report: **16/03/15**.  Referral Diagnosis: **FTT. AGH2.031** | | | |
| --- | --- | --- | --- |
| **Features** | **Finding** | **Features** | **Finding** |
| **Profile** |  | **Atria** |  |
| Abdominal situs | Solitus | Left atrium | Normal |
| Cardiac position | Levocardia | Right atrium | Dilated |
| Systemic venous drainage | Normal. | **Atrioventricular valves** |  |
| Pulmonary venous drainage | Normal | Mitral valve | Annulus = 16mm |
| Atrioventricular connection | Concordant | Tricuspid valve | Annulus = 22mm  TAPSE = 20mm |
| Ventriculoarterial connection | Concordant | **Ventricles** |  |
| Ventricular loop | d-Loop | Left ventricle | Normal |
|  |  | Right ventricle | Dilated |
| **Septae** |  | **Coronary arteries** | ----- |
| Interventricular septum | Intact | **Doppler Measurement** |  |
| Interatrial septum | 11 X 15mm OS ASD, L – R Shunt | Mitral | ----- |
| **Semilunar valves** |  | Aortic | ------- |
| Aortic valve | Annulus = 13mm | Tricuspid | ------- |
| Pulmonary valve | Annulus = 16mm | pulmonic | Trivial PR, PPG = 10mmHg |
| **Great arteries** | NRGA | **Aortic arch** | Left. No CoA. |
| Aorta | ----- | **PDA** | No |
| Pulmonary artery | Normal MPA and Branch PAs. |  |  |
| **M-Mode:** | | | |
| AO | mm | PWd | mm |
| LA | mm | PWs | mm |
| LVIDd | mm | EDV | ml |
| LVIDs | mm | ESV | ml |
| IVSs | mm | LVEF | 64% |
| IVSd | mm | FS | 33% |
| **Additional Information**: |  | | |
| No pericardial/Pleural effusion. | | | |
| **Final Diagnosis:** | | | |
| 1. {S, D, S} Levocardia. 2. RA/RV Dilated 3. Large OS ASD, L – R Shunt 4. Normal Biventricular Systolic Function | | | |
| **Remark**: | | | |
| **Recommendation**: | | | |
| SIGNATURE  Done by: Tesfaye T., Pediatrician, Pediatric Cardiologist _______________ 16/03/2015Eth.C | | | |

| Patient Name: **Amen Mastewal**. Referring Institute: **Adinas GH**. SEX/ Age: **M/8years**. Date of Report: **16/03/15**.  Referral Diagnosis: **easy fatigability. AGH2.032.** | | | |
| --- | --- | --- | --- |
| **Features** | **Finding** | **Features** | **Finding** |
| **Profile** |  | **Atria** |  |
| Abdominal situs | Solitus | Left atrium | Normal |
| Cardiac position | Levocardia | Right atrium | Normal |
| Systemic venous drainage | Normal. | **Atrioventricular valves** |  |
| Pulmonary venous drainage | Normal | Mitral valve | Annulus = 18mm |
| Atrioventricular connection | Concordant | Tricuspid valve | Annulus = 18mm |
| Ventriculoarterial connection | Concordant | **Ventricles** |  |
| Ventricular loop | d-Loop | Left ventricle | Normal |
|  |  | Right ventricle | Normal |
| **Septae** |  | **Coronary arteries** | ----- |
| Interventricular septum | Intact | **Doppler Measurement** |  |
| Interatrial septum | Intact | Mitral | ----- |
| **Semilunar valves** |  | Aortic | ------- |
| Aortic valve | Annulus = 16mm | Tricuspid | ------- |
| Pulmonary valve | Annulus = 17mm | pulmonic | -------- |
| **Great arteries** | NRGA | **Aortic arch** | Left. No CoA. |
| Aorta | ----- | **PDA** | No |
| Pulmonary artery | Normal MPA and Branch PAs. |  |  |
| **M-Mode:** | | | |
| AO | mm | PWd | mm |
| LA | mm | PWs | mm |
| LVIDd | mm | EDV | ml |
| LVIDs | mm | ESV | 31ml |
| IVSs | mm | LVEF | 61% |
| IVSd | mm | FS | % |
| **Additional Information**: |  | | |
| No pericardial/Pleural effusion. | | | |
| **Final Diagnosis:** | | | |
| 1. Normal Echocardiography Study. | | | |
| **Remark**: | | | |
| **Recommendation**: | | | |
| SIGNATURE  Done by: Tesfaye T., Pediatrician, Pediatric Cardiologist _______________ 16/03/2015Eth.C | | | |

| Patient Name: **Fetiha Mohammed**. Referring Institute: **TGSH**. SEX/ Age: **F/33days**.  Date of Report: **17/03/15**. Referral Diagnosis: **RD.AGH2.033** | | | |
| --- | --- | --- | --- |
| **Features** | **Finding** | **Features** | **Finding** |
| **Profile** |  | **Atria** |  |
| Abdominal situs | Solitus | Left atrium | Normal |
| Cardiac position | Levocardia | Right atrium | Normal |
| Systemic venous drainage | Normal. | **Atrioventricular valves** |  |
| Pulmonary venous drainage | Normal | Mitral valve | Annulus = 13mm |
| Atrioventricular connection | Concordant | Tricuspid valve | Annulus = 13mm  TAPSE = 14mm |
| Ventriculoarterial connection | Concordant | **Ventricles** |  |
| Ventricular loop | d-Loop | Left ventricle | Normal |
|  |  | Right ventricle | Normal |
| **Septae** |  | **Coronary arteries** | ----- |
| Interventricular septum | 6mm Sub-Aortic VSD, L – R Shunt | **Doppler Measurement** |  |
| Interatrial septum | Intact | Mitral | ----- |
| **Semilunar valves** |  | Aortic | ------- |
| Aortic valve | Annulus = 10mm | Tricuspid | ------- |
| Pulmonary valve | Annulus = 13mm | pulmonic | -------- |
| **Great arteries** | NRGA | **Aortic arch** | Left. No CoA. |
| Aorta | ----- | **PDA** | No |
| Pulmonary artery | Normal MPA and Branch PAs. |  |  |
| **M-Mode:** | | | |
| AO | mm | PWd | mm |
| LA | mm | PWs | mm |
| LVIDd | mm | EDV | ml |
| LVIDs | mm | ESV | ml |
| IVSs | mm | LVEF | 68% |
| IVSd | mm | FS | 35% |
| **Additional Information**: |  | | |
| No pericardial/Pleural effusion. | | | |
| **Final Diagnosis:** | | | |
| 1. {S, D, S} Levocardia. 2. Moderate Sub-Aortic VSD, L – R Shunt 3. Normal Biventricular Systolic Function | | | |
| **Remark**: | | | |
| **Recommendation**: | | | |
| SIGNATURE  Done by: Tesfaye T., Pediatrician, Pediatric Cardiologist _______________ 17/03/2015Eth.C | | | |

| Patient Name: **Belete Dessie**. Referring Institute: **FHRH**. SEX/ Age: **M/8years**. Date of Report: **17/03/15**.  Referral Diagnosis: **Easy fatigability. AGH2.034** | | | |
| --- | --- | --- | --- |
| **Features** | **Finding** | **Features** | **Finding** |
| **Profile** |  | **Atria** |  |
| Abdominal situs | Solitus | Left atrium | Normal |
| Cardiac position | Levocardia | Right atrium | Normal |
| Systemic venous drainage | Normal. | **Atrioventricular valves** |  |
| Pulmonary venous drainage | Normal | Mitral valve | Annulus = 18mm |
| Atrioventricular connection | Concordant | Tricuspid valve | Annulus = 22mm  TAPSE = 19mm |
| Ventriculoarterial connection | Concordant | **Ventricles** |  |
| Ventricular loop | d-Loop | Left ventricle | Normal |
|  |  | Right ventricle | Normal |
| **Septae** |  | **Coronary arteries** | ----- |
| Interventricular septum | Intact | **Doppler Measurement** |  |
| Interatrial septum | Intact | Mitral | ----- |
| **Semilunar valves** |  | Aortic | ------- |
| Aortic valve | Annulus = 15mm | Tricuspid | ------- |
| Pulmonary valve | Annulus = 17mm | pulmonic | -------- |
| **Great arteries** | NRGA | **Aortic arch** | Left. No CoA. |
| Aorta | ----- | **PDA** | No |
| Pulmonary artery | Normal MPA and Branch PAs. |  |  |
| **M-Mode:** | | | |
| AO | mm | PWd | mm |
| LA | mm | PWs | mm |
| LVIDd | mm | EDV | ml |
| LVIDs | mm | ESV | ml |
| IVSs | mm | LVEF | 56% |
| IVSd | mm | FS | 29% |
| **Additional Information**: |  | | |
| No pericardial/Pleural effusion. | | | |
| **Final Diagnosis:** | | | |
| 1. Normal Echocardiography Study. | | | |
| **Remark**: | | | |
| **Recommendation**: | | | |
| SIGNATURE  Done by: Tesfaye T., Pediatrician, Pediatric Cardiologist _______________ 17/03/2015Eth.C | | | |

| Patient Name: **Mellon Anteneh**. Referring Institute: **Adinas GH**. SEX/ Age: **F/5 3/12**. Date of Report: **17/03/15**.  Referral Diagnosis: **Sydenham’s Chorea. AGH2.035** | | | |
| --- | --- | --- | --- |
| **Features** | **Finding** | **Features** | **Finding** |
| **Profile** |  | **Atria** |  |
| Abdominal situs | Solitus | Left atrium | Normal |
| Cardiac position | Levocardia | Right atrium | Normal |
| Systemic venous drainage | Normal. | **Atrioventricular valves** |  |
| Pulmonary venous drainage | Normal | Mitral valve | Annulus = 18mm |
| Atrioventricular connection | Concordant | Tricuspid valve | Annulus = 18mm  TAPSE = 21mm |
| Ventriculoarterial connection | Concordant | **Ventricles** |  |
| Ventricular loop | d-Loop | Left ventricle | Normal |
|  |  | Right ventricle | Normal |
| **Septae** |  | **Coronary arteries** | ----- |
| Interventricular septum | Intact | **Doppler Measurement** |  |
| Interatrial septum | Intact | Mitral | Trivial MR, Incomplete Signal, seen in apical view with jet velocity = 1.6m/sec. |
| **Semilunar valves** |  | Aortic | ------- |
| Aortic valve | Annulus = 13mm | Tricuspid | ------- |
| Pulmonary valve | Annulus = 16mm | pulmonic | -------- |
| **Great arteries** | NRGA | **Aortic arch** | Left. No CoA. |
| Aorta | ----- | **PDA** | No |
| Pulmonary artery | Normal MPA and Branch PAs. |  |  |
| **M-Mode:** | | | |
| AO | mm | PWd | mm |
| LA | mm | PWs | mm |
| LVIDd | mm | EDV | ml |
| LVIDs | mm | ESV | ml |
| IVSs | mm | LVEF | 66% |
| IVSd | mm | FS | 35% |
| **Additional Information**: |  | | |
| No pericardial/Pleural effusion. | | | |
| **Final Diagnosis:** | | | |
| 1. {S, D, S} Levocardia. 2. Trivial MR 3. Normal Echocardiography Study | | | |
| **Remark**: | | | |
| **Recommendation**: | | | |
| SIGNATURE  Done by: Tesfaye T., Pediatrician, Pediatric Cardiologist _______________ 17/03/2015Eth.C | | | |

| Patient Name: **Rafael Anteneh**. Referring Institute: **Adinas GH**. SEX/ Age: **M/3 2/12**. Date of Report: **17/03/15**.  Referral Diagnosis: **DS.AGH2.036** | | | |
| --- | --- | --- | --- |
| **Features** | **Finding** | **Features** | **Finding** |
| **Profile** |  | **Atria** |  |
| Abdominal situs | Solitus | Left atrium | Normal |
| Cardiac position | Levocardia | Right atrium | Normal |
| Systemic venous drainage | Normal. | **Atrioventricular valves** |  |
| Pulmonary venous drainage | Normal | Mitral valve | Annulus = 14mm |
| Atrioventricular connection | Concordant | Tricuspid valve | Annulus = 16mm  TAPSE = 19mm |
| Ventriculoarterial connection | Concordant | **Ventricles** |  |
| Ventricular loop | d-Loop | Left ventricle | Normal |
|  |  | Right ventricle | Normal |
| **Septae** |  | **Coronary arteries** | ----- |
| Interventricular septum | Intact | **Doppler Measurement** |  |
| Interatrial septum | Intact | Mitral | ----- |
| **Semilunar valves** |  | Aortic | ------- |
| Aortic valve | Annulus = 13mm | Tricuspid | Trivial TR, PPG = 13mmHg. Jet velocity= 1.8m/sec. |
| Pulmonary valve | Annulus = 14mm | pulmonic | -------- |
| **Great arteries** | NRGA | **Aortic arch** | Left. No CoA. |
| Aorta | ----- | **PDA** | No |
| Pulmonary artery | Normal MPA and Branch PAs. |  |  |
| **M-Mode:** | | | |
| AO | mm | PWd | mm |
| LA | mm | PWs | mm |
| LVIDd | mm | EDV | ml |
| LVIDs | mm | ESV | ml |
| IVSs | mm | LVEF | 60% |
| IVSd | mm | FS | 31% |
| **Additional Information**: |  | | |
| No pericardial/Pleural effusion. | | | |
| **Final Diagnosis:** | | | |
| 1. Normal Echocardiography Study. | | | |
| **Remark**: | | | |
| **Recommendation**: | | | |
| SIGNATURE  Done by: Tesfaye T., Pediatrician, Pediatric Cardiologist _______________ 17/03/2015Eth.C | | | |

| Patient Name: **Alem Selam**. Referring Institute: **Addis Zemen PH**. SEX/ Age: **M/6months**.  Date of Report: **19/03/15**. Referral Diagnosis: **Recurrent Chest Infection. AGH2.037** | | | |
| --- | --- | --- | --- |
| **Features** | **Finding** | **Features** | **Finding** |
| **Profile** |  | **Atria** |  |
| Abdominal situs | Solitus | Left atrium | Normal |
| Cardiac position | Levocardia | Right atrium | Normal |
| Systemic venous drainage | Normal. | **Atrioventricular valves** |  |
| Pulmonary venous drainage | Normal | Mitral valve | Annulus = 12mm |
| Atrioventricular connection | Concordant | Tricuspid valve | Annulus = 14mm |
| Ventriculoarterial connection | Concordant | **Ventricles** |  |
| Ventricular loop | d-Loop | Left ventricle | Normal |
|  |  | Right ventricle | Normal |
| **Septae** |  | **Coronary arteries** | ----- |
| Interventricular septum | Intact | **Doppler Measurement** |  |
| Interatrial septum | Intact | Mitral | ----- |
| **Semilunar valves** |  | Aortic | ------- |
| Aortic valve | Annulus = 12mm | Tricuspid | ------- |
| Pulmonary valve | Annulus = 13mm | pulmonic | -------- |
| **Great arteries** | NRGA | **Aortic arch** | Left. No CoA. |
| Aorta | ----- | **PDA** | No |
| Pulmonary artery | Normal MPA and Branch PAs. |  |  |
| **M-Mode:**  Normal LV Function on eye balling | | | |
| AO | mm | PWd | mm |
| LA | mm | PWs | mm |
| LVIDd | mm | EDV | ml |
| LVIDs | mm | ESV | ml |
| IVSs | mm | LVEF | % |
| IVSd | mm | FS | % |
| **Additional Information**: |  | | |
| No pericardial/Pleural effusion. | | | |
| **Final Diagnosis:** | | | |
| 1. Normal Echocardiography Study. | | | |
| **Remark**: | | | |
| **Recommendation**: | | | |
| SIGNATURE  Done by: Tesfaye T., Pediatrician, Pediatric Cardiologist _______________ 19/03/2015Eth.C | | | |

| Patient Name: **Amanuel Gashaw**. Referring Institute: **Addis Alem PH**. SEX/ Age: **M/8months**.  Date of Report: **19/03/15**. Referral Diagnosis: **DS. AGH2.038** | | | |
| --- | --- | --- | --- |
| **Features** | **Finding** | **Features** | **Finding** |
| **Profile** |  | **Atria** |  |
| Abdominal situs | Solitus | Left atrium | Normal |
| Cardiac position | Levocardia | Right atrium | Normal |
| Systemic venous drainage | Normal. | **Atrioventricular valves** |  |
| Pulmonary venous drainage | Normal | Mitral valve | Common Complete AVSD |
| Atrioventricular connection | Common Complete AVSD | Tricuspid valve |  |
| Ventriculoarterial connection | Concordant | **Ventricles** |  |
| Ventricular loop | d-Loop | Left ventricle | Normal |
|  |  | Right ventricle | Normal |
| **Septae** |  | **Coronary arteries** | ----- |
| Interventricular septum | Common Complete AVSD, L – R Shunt | **Doppler Measurement** |  |
| Interatrial septum |  | Mitral | ----- |
| **Semilunar valves** |  | Aortic | ------- |
| Aortic valve | Annulus = 11mm | Tricuspid | ------- |
| Pulmonary valve | Annulus = 9mm | pulmonic | Severe Valvular, supra-valvular and sub-valvular PS, PPG = 62mmHg. |
| **Great arteries** | NRGA | **Aortic arch** | Left. No CoA. |
| Aorta | ----- | **PDA** | No |
| Pulmonary artery | Smallish MPA and Branch PAs. |  |  |
| **M-Mode:**  Normal LV Function on eye balling | | | |
| AO | mm | PWd | mm |
| LA | mm | PWs | mm |
| LVIDd | mm | EDV | ml |
| LVIDs | mm | ESV | ml |
| IVSs | mm | LVEF | % |
| IVSd | mm | FS | % |
| **Additional Information**: |  | | |
| No pericardial/Pleural effusion. | | | |
| **Final Diagnosis:** | | | |
| 1. {S, D, S} Levocardia. 2. Common Complete Balanced AVSD, L – R Shunt 3. Severe PS 4. Smallish MPA and Branch PAs 5. Normal LV Systolic Function | | | |
| **Remark**: | | | |
| **Recommendation**: | | | |
| SIGNATURE  Done by: Tesfaye T., Pediatrician, Pediatric Cardiologist _______________ 19/03/2015Eth.C | | | |

| Patient Name: **Baby of Medina Ahmed**. Referring Institute: **MSI-Ethiopia, Bahir Dar**. SEX/ Age: **M/45days**.  Date of Report: **19/03/15**. Referral Diagnosis: **Infant of Diabetic Mother. AGH2.039.** | | | |
| --- | --- | --- | --- |
| **Features** | **Finding** | **Features** | **Finding** |
| **Profile** |  | **Atria** |  |
| Abdominal situs | Solitus | Left atrium | Normal |
| Cardiac position | Levocardia | Right atrium | Normal |
| Systemic venous drainage | Normal. | **Atrioventricular valves** |  |
| Pulmonary venous drainage | Normal | Mitral valve | Annulus = 10mm |
| Atrioventricular connection | Concordant | Tricuspid valve | Annulus = 13mm |
| Ventriculoarterial connection | Concordant | **Ventricles** |  |
| Ventricular loop | d-Loop | Left ventricle | Normal |
|  |  | Right ventricle | Normal |
| **Septae** |  | **Coronary arteries** | ----- |
| Interventricular septum | Intact | **Doppler Measurement** |  |
| Interatrial septum | Intact | Mitral | ----- |
| **Semilunar valves** |  | Aortic | ------- |
| Aortic valve | Annulus = 11mm | Tricuspid | ------- |
| Pulmonary valve | Annulus = 10mm | pulmonic | -------- |
| **Great arteries** | NRGA | **Aortic arch** | Left. No CoA. |
| Aorta | ----- | **PDA** | No |
| Pulmonary artery | Normal MPA and Branch PAs. |  |  |
| **M-Mode:**  Normal Echocardiography Study | | | |
| AO | mm | PWd | mm |
| LA | mm | PWs | mm |
| LVIDd | mm | EDV | ml |
| LVIDs | mm | ESV | ml |
| IVSs | mm | LVEF | % |
| IVSd | mm | FS | % |
| **Additional Information**: |  | | |
| No pericardial/Pleural effusion. | | | |
| **Final Diagnosis:** | | | |
| 1. Normal Echocardiography Study. | | | |
| **Remark**: | | | |
| **Recommendation**: | | | |
| SIGNATURE  Done by: Tesfaye T., Pediatrician, Pediatric Cardiologist _______________ 19/03/2015Eth.C | | | |

| Patient Name: **Sale-Amlak Kassaye**. Referring Institute: **FHRH**. SEX/ Age: **M/5months**. Date of Report: **19/03/15**.  Referral Diagnosis: **Cyanosis. AGH2.040.** | | | |
| --- | --- | --- | --- |
| **Features** | **Finding** | **Features** | **Finding** |
| **Profile** |  | **Atria** |  |
| Abdominal situs | Solitus | Left atrium | Normal |
| Cardiac position | Levocardia | Right atrium | Dilated |
| Systemic venous drainage | Normal. | **Atrioventricular valves** |  |
| Pulmonary venous drainage | Normal | Mitral valve | Atretic |
| Atrioventricular connection | DIRV | Tricuspid valve | Annulus = 21mm |
| Ventriculoarterial connection | Concordant | **Ventricles** |  |
| Ventricular loop | d-Loop | Left ventricle | Smallish |
|  |  | Right ventricle | Dilated & Hypertrophied |
| **Septae** |  | **Coronary arteries** | ----- |
| Interventricular septum | 3mm Inlet VSD, R – L Shunt | **Doppler Measurement** |  |
| Interatrial septum | Large Primum defect, L – R Shunt. Additional 4mm OS ASD, L – R Shunt | Mitral | ----- |
| **Semilunar valves** |  | Aortic | ------- |
| Aortic valve | Annulus = 6mm | Tricuspid | Moderate TR |
| Pulmonary valve | Annulus = 12mm | pulmonic | Mild PR, PPG = 50mmHg |
| **Great arteries** | NRGA | **Aortic arch** | Left. No CoA. |
| Aorta | Smallish | **PDA** | No |
| Pulmonary artery | Normal MPA and Branch PAs. |  |  |
| **M-Mode:** | | | |
| AO | mm | PWd | mm |
| LA | mm | PWs | mm |
| LVIDd | mm | EDV | ml |
| LVIDs | mm | ESV | ml |
| IVSs | mm | LVEF | % |
| IVSd | mm | FS | % |
| **Additional Information**: |  | | |
| **Final Diagnosis:** | | | |
| 1. {S, D, S} Levocardia. 2. RA/RV Dilated. RV Hypertrophied 3. DIRV 4. Small OS ASD, L – R Shunt 5. Transitional AVSD, Unbalanced 6. Mitral Atresia 7. Smallish LV 8. Smallish Aorta 9. Moderate TR 10. Mild PR 11. Moderate Pulmonary Hypertension HLHS + Transitional AVSD + DIRV | | | |
| SIGNATURE  Done by: Tesfaye T., Pediatrician, Pediatric Cardiologist _______________ 19/03/2015Eth.C | | | |

| Patient Name: **Getachew Tadele**. Referring Institute: **FHRH**. SEX/ Age: **M/1 4/12**. Date of Report: **19/03/15**.  Referral Diagnosis: **RD. AGH2.042** | | | |
| --- | --- | --- | --- |
| **Features** | **Finding** | **Features** | **Finding** |
| **Profile** |  | **Atria** |  |
| Abdominal situs | Solitus | Left atrium | Normal |
| Cardiac position | Levocardia | Right atrium | Dilated |
| Systemic venous drainage | Normal. | **Atrioventricular valves** |  |
| Pulmonary venous drainage | Normal | Mitral valve | Annulus = 11mm |
| Atrioventricular connection | Concordant | Tricuspid valve | Annulus = 17mm  TAPSE = 18mm |
| Ventriculoarterial connection | Concordant | **Ventricles** |  |
| Ventricular loop | d-Loop | Left ventricle | Normal |
|  |  | Right ventricle | Dilated |
| **Septae** |  | **Coronary arteries** | ----- |
| Interventricular septum | Intact | **Doppler Measurement** |  |
| Interatrial septum | 8mm OS ASD, L – R Shunt.  Atrial Septal Aneurysm bowing to RA 19mm and to LA 4mm. | Mitral | ----- |
| **Semilunar valves** |  | Aortic | ------- |
| Aortic valve | Annulus = 13mm | Tricuspid | Mild TR, PPG = 47mmHg |
| Pulmonary valve | Annulus = 14mm | pulmonic | -------- |
| **Great arteries** | NRGA | **Aortic arch** | Left. No CoA. |
| Aorta | ----- | **PDA** | 2.5mm PDA, L – R Shunt |
| Pulmonary artery | Normal MPA and Branch PAs. |  |  |
| **M-Mode:**  Normal LV Function on eye balling | | | |
| AO | mm | PWd | mm |
| LA | mm | PWs | mm |
| LVIDd | mm | EDV | ml |
| LVIDs | mm | ESV | ml |
| IVSs | mm | LVEF | % |
| IVSd | mm | FS | % |
| **Additional Information**: |  | | |
| No pericardial/Pleural effusion. | | | |
| **Final Diagnosis:** | | | |
| 1. {S, D, S} Levocardia. 2. RA/RV Dilated 3. Atrial Septal Aneurysm(ASA) Type 3RL 4. Moderate OS ASD, L – R Shunt 5. Moderate PDA, L – R Shunt 6. Moderate Pulmonary Hypertension 7. Normal Biventricular Systolic Function | | | |
| SIGNATURE  Done by: Tesfaye T., Pediatrician, Pediatric Cardiologist _______________ 19/03/2015Eth.C | | | |

| Patient Name: **Abate Werkie**. Referring Institute: **FHRH**. SEX/ Age: **M/8years**. Date of Report: **19/03/15**.  Referral Diagnosis: **Palpitation. AGH2.042** | | | |
| --- | --- | --- | --- |
| **Features** | **Finding** | **Features** | **Finding** |
| **Profile** |  | **Atria** |  |
| Abdominal situs | Solitus | Left atrium | Normal |
| Cardiac position | Levocardia | Right atrium | Normal |
| Systemic venous drainage | Normal. | **Atrioventricular valves** |  |
| Pulmonary venous drainage | Normal | Mitral valve | Annulus = 15mm |
| Atrioventricular connection | Concordant | Tricuspid valve | Annulus = 43mm. 38mm downward displacement of STL.  TAPSE = mm |
| Ventriculoarterial connection | Concordant | **Ventricles** |  |
| Ventricular loop | d-Loop | Left ventricle | Normal |
|  |  | Right ventricle | Normal |
| **Septae** |  | **Coronary arteries** | ----- |
| Interventricular septum | Intact | **Doppler Measurement** |  |
| Interatrial septum | 8mm High Secundum ASD, L – R Shunt | Mitral | ----- |
| **Semilunar valves** |  | Aortic | ------- |
| Aortic valve | Annulus = 13mm | Tricuspid | Mild TR, PPG = 28mmHg |
| Pulmonary valve | Annulus = 16mm | pulmonic | -------- |
| **Great arteries** | NRGA | **Aortic arch** | Left. No CoA. |
| Aorta | ----- | **PDA** | No |
| Pulmonary artery | Normal MPA and Branch PAs. |  |  |
| **M-Mode:** | | | |
| AO | mm | PWd | mm |
| LA | mm | PWs | mm |
| LVIDd | mm | EDV | ml |
| LVIDs | mm | ESV | ml |
| IVSs | mm | LVEF | % |
| IVSd | mm | FS | % |
| **Additional Information**: |  | | |
| No pericardial/Pleural effusion. | | | |
| **Final Diagnosis:** | | | |
| 1. {S, D, S} Levocardia. 2. Moderate High Secundum ASD, L – R Shunt 3. Ebstein anomaly of the tricuspid valve Type C 4. Mild TR | | | |
| **Remark**: | | | |
| **Recommendation**: | | | |
| SIGNATURE  Done by: Tesfaye T., Pediatrician, Pediatric Cardiologist _______________ 19/03/2015Eth.C | | | |

| Patient Name: **Rahel Yilak**. Referring Institute: **Adinas GH**. SEX/ Age: **F/1 8/12**. Date of Report: **19/03/15**.  Referral Diagnosis: **Incidental Murmur. AGH2. 043** | | | |
| --- | --- | --- | --- |
| **Features** | **Finding** | **Features** | **Finding** |
| **Profile** |  | **Atria** |  |
| Abdominal situs | Solitus | Left atrium | Normal |
| Cardiac position | Levocardia | Right atrium | Mildly Dilated |
| Systemic venous drainage | Normal. | **Atrioventricular valves** |  |
| Pulmonary venous drainage | Normal | Mitral valve | Annulus = 16mm |
| Atrioventricular connection | Concordant | Tricuspid valve | Annulus = 17mm  TAPSE = 19mm |
| Ventriculoarterial connection | Concordant | **Ventricles** |  |
| Ventricular loop | d-Loop | Left ventricle | Normal |
|  |  | Right ventricle | Mildly Dilated |
| **Septae** |  | **Coronary arteries** | ----- |
| Interventricular septum | Intact | **Doppler Measurement** |  |
| Interatrial septum | 12mm High Secundum ASD, L – R Shunt | Mitral | ----- |
| **Semilunar valves** |  | Aortic | ------- |
| Aortic valve | Annulus = 11mm | Tricuspid | Trivial TR, PPG = 29mmHg |
| Pulmonary valve | Annulus = 13mm. Doming PV | pulmonic | Mild Valvular PS, PPG = 20mmHg |
| **Great arteries** | NRGA | **Aortic arch** | Left. No CoA. |
| Aorta | ----- | **PDA** | No |
| Pulmonary artery | Normal MPA and Branch PAs. |  |  |
| **M-Mode:** | | | |
| AO | mm | PWd | mm |
| LA | mm | PWs | mm |
| LVIDd | mm | EDV | ml |
| LVIDs | mm | ESV | ml |
| IVSs | mm | LVEF | % |
| IVSd | mm | FS | % |
| **Additional Information**: |  | | |
| No pericardial/Pleural effusion. | | | |
| **Final Diagnosis:** | | | |
| 1. {S, D, S} Levocardia. 2. RA/RV Mildly Dilated 3. Large High Secundum ASD, L – R Shunt 4. Mild Valvular PS 5. Doming Pulmonary Valve 6. Normal Biventricular Systolic Function | | | |
| **Remark**: | | | |
| **Recommendation**: | | | |
| SIGNATURE  Done by: Tesfaye T., Pediatrician, Pediatric Cardiologist _______________ 19/03/2015Eth.C | | | |

| Patient Name: **Habtamu Kassaw**. Referring Institute: **FHRH**. SEX/ Age: **M/7months**. Date of Report: **20/03/15**.  Referral Diagnosis: **Incidental Murmur Detection.AGH2.044** | | | |
| --- | --- | --- | --- |
| **Features** | **Finding** | **Features** | **Finding** |
| **Profile** |  | **Atria** |  |
| Abdominal situs | Solitus | Left atrium | Normal |
| Cardiac position | Levocardia | Right atrium | Normal |
| Systemic venous drainage | Normal. | **Atrioventricular valves** |  |
| Pulmonary venous drainage | Normal | Mitral valve | Annulus = 11mm |
| Atrioventricular connection | Concordant | Tricuspid valve | Annulus = 12mm  TAPSE = 17mm |
| Ventriculoarterial connection | Concordant | **Ventricles** |  |
| Ventricular loop | d-Loop | Left ventricle | Normal |
|  |  | Right ventricle | Normal |
| **Septae** |  | **Coronary arteries** | ----- |
| Interventricular septum | Intact | **Doppler Measurement** |  |
| Interatrial septum | Intact | Mitral | ----- |
| **Semilunar valves** |  | Aortic | ------- |
| Aortic valve | Annulus = 10mm | Tricuspid | ------- |
| Pulmonary valve | Annulus = 12mm | pulmonic | -------- |
| **Great arteries** | NRGA | **Aortic arch** | Left. No CoA. |
| Aorta | ----- | **PDA** | <1mm PDA, L – R Shunt |
| Pulmonary artery | Normal MPA and Branch PAs. |  |  |
| **M-Mode:** | | | |
| AO | mm | PWd | mm |
| LA | mm | PWs | mm |
| LVIDd | mm | EDV | ml |
| LVIDs | mm | ESV | ml |
| IVSs | mm | LVEF | 70% |
| IVSd | mm | FS | 37% |
| **Additional Information**: |  | | |
| No pericardial/Pleural effusion. | | | |
| **Final Diagnosis:** | | | |
| 1. {S, D, S} Levocardia. 2. Silent PDA, L – R Shunt 3. Normal Biventricular Systolic Function | | | |
| SIGNATURE  Done by: Tesfaye T., Pediatrician, Pediatric Cardiologist _______________ 20/03/2015Eth.C | | | |

| Patient Name: **Sale-Amlak Adane**. Referring Institute: **TGSH**. SEX/ Age: **M/12 8/12**.  Date of Report: **20/03/15**. Referral Diagnosis: **Sydenham’s Chorea.AGH2.045** | | | |
| --- | --- | --- | --- |
| **Features** | **Finding** | **Features** | **Finding** |
| **Profile** |  | **Atria** |  |
| Abdominal situs | Solitus | Left atrium | Normal |
| Cardiac position | Levocardia | Right atrium | Normal |
| Systemic venous drainage | Normal. | **Atrioventricular valves** |  |
| Pulmonary venous drainage | Normal | Mitral valve | Annulus = 20mm |
| Atrioventricular connection | Concordant | Tricuspid valve | Annulus = 23mm  TAPSE = 19mm |
| Ventriculoarterial connection | Concordant | **Ventricles** |  |
| Ventricular loop | d-Loop | Left ventricle | Normal |
|  |  | Right ventricle | Normal. RV TDI S wave = 15cm/sec. |
| **Septae** |  | **Coronary arteries** | ----- |
| Interventricular septum | Intact | **Doppler Measurement** |  |
| Interatrial septum | Intact | Mitral | ----- |
| **Semilunar valves** |  | Aortic | ------- |
| Aortic valve | Annulus = 20mm | Tricuspid | Trivial TR, PPG = 21mmHg |
| Pulmonary valve | Annulus = 24mm | pulmonic | -------- |
| **Great arteries** | NRGA | **Aortic arch** | Left. No CoA. |
| Aorta | ----- | **PDA** | No |
| Pulmonary artery | Normal MPA and Branch PAs. |  |  |
| **M-Mode:** | | | |
| AO | mm | PWd | mm |
| LA | mm | PWs | mm |
| LVIDd | mm | EDV | ml |
| LVIDs | mm | ESV | ml |
| IVSs | mm | LVEF | 61% |
| IVSd | mm | FS | 32% |
| **Additional Information**: |  | | |
| No pericardial/Pleural effusion. | | | |
| **Final Diagnosis:** | | | |
| 1. Normal Echocardiography Study. | | | |
| **Remark**: | | | |
| **Recommendation**: | | | |
| SIGNATURE  Done by: Tesfaye T., Pediatrician, Pediatric Cardiologist _______________ 20/03/2015Eth.C | | | |

| Patient Name: **Mekdes Esuyawkal**. Referring Institute: **TGSH**. SEX/ Age: **F/13years**. Date of Report: **20/03/15**.  Referral Diagnosis: **Easy fatigability.AGH2.046** | | | |
| --- | --- | --- | --- |
| **Features** | **Finding** | **Features** | **Finding** |
| **Profile** |  | **Atria** |  |
| Abdominal situs | Solitus | Left atrium | Normal |
| Cardiac position | Levocardia | Right atrium | Normal |
| Systemic venous drainage | Normal. | **Atrioventricular valves** |  |
| Pulmonary venous drainage | Normal | Mitral valve | Annulus = 23mm |
| Atrioventricular connection | Concordant | Tricuspid valve | Annulus = 24mm |
| Ventriculoarterial connection | Concordant | **Ventricles** |  |
| Ventricular loop | d-Loop | Left ventricle | Normal |
|  |  | Right ventricle | Normal |
| **Septae** |  | **Coronary arteries** | ----- |
| Interventricular septum | Intact | **Doppler Measurement** |  |
| Interatrial septum | Intact | Mitral | ----- |
| **Semilunar valves** |  | Aortic | ------- |
| Aortic valve | Annulus = 18mm | Tricuspid | ------- |
| Pulmonary valve | Annulus = 22mm | pulmonic | -------- |
| **Great arteries** | NRGA | **Aortic arch** | Left. No CoA. |
| Aorta | ----- | **PDA** | No |
| Pulmonary artery | Normal MPA and Branch PAs. |  |  |
| **M-Mode:** | | | |
| AO | mm | PWd | mm |
| LA | mm | PWs | mm |
| LVIDd | mm | EDV | ml |
| LVIDs | mm | ESV | ml |
| IVSs | mm | LVEF | 65% |
| IVSd | mm | FS | 35% |
| **Additional Information**: |  | | |
| No pericardial/Pleural effusion. | | | |
| **Final Diagnosis:** | | | |
| 1. Normal Echocardiography Study. | | | |
| **Remark**: | | | |
| **Recommendation**: | | | |
| SIGNATURE  Done by: Tesfaye T., Pediatrician, Pediatric Cardiologist _______________ 20/03/2015Eth.C | | | |

| Patient Name: **Baby of Dehninet Tesfaw**. Referring Institute: **TGSH**. SEX/ Age: **M/23days**. Date of Report: **21/03/15**.  Referral Diagnosis: **RD.AGH2.047** | | | |
| --- | --- | --- | --- |
| **Features** | **Finding** | **Features** | **Finding** |
| **Profile** |  | **Atria** |  |
| Abdominal situs | Solitus | Left atrium | Normal |
| Cardiac position | Levocardia | Right atrium | Normal |
| Systemic venous drainage | Normal. | **Atrioventricular valves** |  |
| Pulmonary venous drainage | Normal | Mitral valve | Annulus = 12mm |
| Atrioventricular connection | Concordant | Tricuspid valve | Annulus = 12mm |
| Ventriculoarterial connection | Concordant | **Ventricles** |  |
| Ventricular loop | d-Loop | Left ventricle | Normal |
|  |  | Right ventricle | Normal |
| **Septae** |  | **Coronary arteries** | ----- |
| Interventricular septum | Intact | **Doppler Measurement** |  |
| Interatrial septum | 4mm High secundum ASD, L – R Shunt | Mitral | ----- |
| **Semilunar valves** |  | Aortic | ------- |
| Aortic valve | Annulus = 10mm | Tricuspid | ------- |
| Pulmonary valve | Annulus = 10mm | pulmonic | -------- |
| **Great arteries** | NRGA | **Aortic arch** | Left. No CoA. |
| Aorta | ----- | **PDA** | No |
| Pulmonary artery | Normal MPA and Branch PAs. |  |  |
| **M-Mode:**  Normal LV Function on eye balling | | | |
| AO | mm | PWd | mm |
| LA | mm | PWs | mm |
| LVIDd | mm | EDV | ml |
| LVIDs | mm | ESV | ml |
| IVSs | mm | LVEF | % |
| IVSd | mm | FS | % |
| **Additional Information**: |  | | |
| No pericardial/Pleural effusion. | | | |
| **Final Diagnosis:** | | | |
| 1. {S, D, S} Levocardia. 2. Small High secundum ASD, L – R Shunt | | | |
| **Remark**: | | | |
| **Recommendation**: | | | |
| SIGNATURE  Done by: Tesfaye T., Pediatrician, Pediatric Cardiologist _______________ 21/03/2015Eth.C | | | |

| Patient Name: **Brook Solomon**. Referring Institute: **FHRH**. SEX/ Age: **M/11months**.  Date of Report: **21/03/15**. Referral Diagnosis: **RD.AGH2.048** | | | |
| --- | --- | --- | --- |
| **Features** | **Finding** | **Features** | **Finding** |
| **Profile** |  | **Atria** |  |
| Abdominal situs | Solitus | Left atrium | Normal |
| Cardiac position | Levocardia | Right atrium | Normal |
| Systemic venous drainage | Normal. | **Atrioventricular valves** |  |
| Pulmonary venous drainage | Normal | Mitral valve | Annulus = 14mm |
| Atrioventricular connection | Concordant | Tricuspid valve | Annulus = 14mm  TAPSE = 15mm |
| Ventriculoarterial connection | Concordant | **Ventricles** |  |
| Ventricular loop | d-Loop | Left ventricle | Normal |
|  |  | Right ventricle | Normal |
| **Septae** |  | **Coronary arteries** | ----- |
| Interventricular septum | Intact | **Doppler Measurement** |  |
| Interatrial septum | Intact | Mitral | ----- |
| **Semilunar valves** |  | Aortic | ------- |
| Aortic valve | Annulus = 11mm | Tricuspid | ------- |
| Pulmonary valve | Annulus = 12mm | pulmonic | -------- |
| **Great arteries** | NRGA | **Aortic arch** | Left. No CoA. |
| Aorta | ----- | **PDA** | No |
| Pulmonary artery | Normal MPA and Branch PAs. |  |  |
| **M-Mode:** | | | |
| AO | mm | PWd | mm |
| LA | mm | PWs | mm |
| LVIDd | mm | EDV | ml |
| LVIDs | mm | ESV | ml |
| IVSs | mm | LVEF | 69% |
| IVSd | mm | FS | 37% |
| **Additional Information**: |  | | |
| No pericardial/Pleural effusion. | | | |
| **Final Diagnosis:** | | | |
| 1. Normal Echocardiography Study. | | | |
| **Remark**: | | | |
| **Recommendation**: | | | |
| SIGNATURE  Done by: Tesfaye T., Pediatrician, Pediatric Cardiologist _______________ 21/03/2015Eth.C | | | |

| Patient Name: **Kewser Sualih**. Referring Institute: **Dream Care GH**. SEX/ Age: **F/3years**. Date of Report: **21/03/15**.  Referral Diagnosis: **Incidental Murmur. AGH2.049** | | | |
| --- | --- | --- | --- |
| **Features** | **Finding** | **Features** | **Finding** |
| **Profile** |  | **Atria** |  |
| Abdominal situs | Solitus | Left atrium | Normal |
| Cardiac position | Levocardia | Right atrium | Normal |
| Systemic venous drainage | Normal. | **Atrioventricular valves** |  |
| Pulmonary venous drainage | Normal | Mitral valve | Annulus = 16mm |
| Atrioventricular connection | Concordant | Tricuspid valve | Annulus = 18mm  TAPSE = 18mm |
| Ventriculoarterial connection | Concordant | **Ventricles** |  |
| Ventricular loop | d-Loop | Left ventricle | Normal |
|  |  | Right ventricle | Normal. RV TDI S wave = 16cm/sec. |
| **Septae** |  | **Coronary arteries** | ----- |
| Interventricular septum | 1.5mm Supra-Cristal VSD, L – R Shunt | **Doppler Measurement** |  |
| Interatrial septum | Intact | Mitral | ----- |
| **Semilunar valves** |  | Aortic | ------- |
| Aortic valve | Annulus = 13mm | Tricuspid | ------- |
| Pulmonary valve | Annulus = 15mm | pulmonic | Mild Valvular PS, PPG = 20mmHg |
| **Great arteries** | NRGA | **Aortic arch** | Left. No CoA. |
| Aorta | ----- | **PDA** | No |
| Pulmonary artery | Normal MPA and Branch PAs. |  |  |
| **M-Mode:**  Normal LV Function on eye balling. | | | |
| AO | mm | PWd | mm |
| LA | mm | PWs | mm |
| LVIDd | mm | EDV | ml |
| LVIDs | mm | ESV | ml |
| IVSs | mm | LVEF | % |
| IVSd | mm | FS | % |
| **Additional Information**: |  | | |
| No pericardial/Pleural effusion. | | | |
| **Final Diagnosis:** | | | |
| 1. {S, D, S} Levocardia. 2. Small Restrictive Supra-Cristal VSD, L – R Shunt 3. Mild Valvular PS 4. Normal Biventricular Systolic Function | | | |
| **Remark**: | | | |
| **Recommendation**: | | | |
| SIGNATURE  Done by: Tesfaye T., Pediatrician, Pediatric Cardiologist _______________ 21/03/2015Eth.C | | | |

| Patient Name: **Baby of Zina Kindie**. Referring Institute: **TGSH**. SEX/ Age: **F/1 3/12**. Date of Report: **21/03/15**.  Referral Diagnosis: **Follow up echocardiography for Small PM VSD(2mm) diagnosed before a year. (Incidental) AGH2.050** | | | |
| --- | --- | --- | --- |
| **Features** | **Finding** | **Features** | **Finding** |
| **Profile** |  | **Atria** |  |
| Abdominal situs | Solitus | Left atrium | Normal |
| Cardiac position | Levocardia | Right atrium | Normal |
| Systemic venous drainage | Normal. | **Atrioventricular valves** |  |
| Pulmonary venous drainage | Normal | Mitral valve | Annulus = 16mm |
| Atrioventricular connection | Concordant | Tricuspid valve | Annulus = 16mm |
| Ventriculoarterial connection | Concordant | **Ventricles** |  |
| Ventricular loop | d-Loop | Left ventricle | Normal |
|  |  | Right ventricle | Normal |
| **Septae** |  | **Coronary arteries** | ----- |
| Interventricular septum | 2mm PM VSD, L – R Shunt with PPG = 54mmHg. | **Doppler Measurement** |  |
| Interatrial septum | Intact | Mitral | ----- |
| **Semilunar valves** |  | Aortic | ------- |
| Aortic valve | Annulus = 13mm | Tricuspid | ------- |
| Pulmonary valve | Annulus = 14mm | pulmonic | -------- |
| **Great arteries** | NRGA | **Aortic arch** | Left. No CoA. |
| Aorta | ----- | **PDA** | No |
| Pulmonary artery | Normal MPA and Branch PAs. |  |  |
| **M-Mode:**  Normal LV Systolic function on eye balling | | | |
| AO | mm | PWd | mm |
| LA | mm | PWs | mm |
| LVIDd | mm | EDV | ml |
| LVIDs | mm | ESV | ml |
| IVSs | mm | LVEF | % |
| IVSd | mm | FS | % |
| **Additional Information**: |  | | |
| No pericardial/Pleural effusion. | | | |
| **Final Diagnosis:** | | | |
| 1. {S, D, S} Levocardia. 2. Small Restrictive PM VSD, L – R Shunt | | | |
| **Remark**: | | | |
| **Recommendation**: | | | |
| SIGNATURE  Done by: Tesfaye T., Pediatrician, Pediatric Cardiologist _______________ 21/03/2015Eth.C | | | |

| Patient Name: **Baby of Hawa Hassen**. Referring Institute: **TGSH**. SEX/ Age: **M/7days**. Date of Report: **21/03/15**.  Referral Diagnosis: **Cyanosis. AGH2.051** | | | |
| --- | --- | --- | --- |
| **Features** | **Finding** | **Features** | **Finding** |
| **Profile** |  | **Atria** |  |
| Abdominal situs | Solitus | Left atrium | Normal |
| Cardiac position | Levocardia | Right atrium | Dilated |
| Systemic venous drainage | Normal. | **Atrioventricular valves** |  |
| Pulmonary venous drainage | Normal | Mitral valve | Annulus = 9mm |
| Atrioventricular connection | Concordant | Tricuspid valve | Annulus = 12mm |
| Ventriculoarterial connection | Concordant | **Ventricles** |  |
| Ventricular loop | d-Loop | Left ventricle | Normal |
|  |  | Right ventricle | Dilated & Hypertrophied |
| **Septae** |  | **Coronary arteries** | ----- |
| Interventricular septum | Non-Restrictive Sub-Aortic VSD, BD Shunt | **Doppler Measurement** |  |
| Interatrial septum | 4mm OS ASD, L – R Shunt | Mitral | ----- |
| **Semilunar valves** |  | Aortic | Trivial AR |
| Aortic valve | Annulus = 9mm | Tricuspid | Mild TR |
| Pulmonary valve | Annulus = 7mm | pulmonic | Moderate Valvular & supra-valvular PS, PPG = 50mmHg |
| **Great arteries** | NRGA | **Aortic arch** | Left. No CoA. |
| Aorta | Over-riding aorta | **PDA** | No |
| Pulmonary artery | Smallish MPA and Branch PAs. |  |  |
| **M-Mode:**  Normal LV Function on eye balling | | | |
| AO | mm | PWd | mm |
| LA | mm | PWs | mm |
| LVIDd | mm | EDV | ml |
| LVIDs | mm | ESV | ml |
| IVSs | mm | LVEF | % |
| IVSd | mm | FS | % |
| **Additional Information**: |  | | |
| No pericardial/Pleural effusion. | | | |
| **Final Diagnosis:** | | | |
| 1. {S, D, S} Levocardia. 2. Small OS ASD, L - R Shunt 3. TOF 4. Smallish MPA and Branch PAs. | | | |
| **Remark**: | | | |
| **Recommendation**: | | | |
| SIGNATURE  Done by: Tesfaye T., Pediatrician, Pediatric Cardiologist _______________ 21/03/2015Eth.C | | | |

| Patient Name: **Meskerem Mamaru**. Referring Institute: **TGSH**. SEX/ Age: **F/8years**. Date of Report: **21/03/15**.  Referral Diagnosis: **Palpitation. AGH2.052** | | | |
| --- | --- | --- | --- |
| **Features** | **Finding** | **Features** | **Finding** |
| **Profile** |  | **Atria** |  |
| Abdominal situs | Solitus | Left atrium | Normal |
| Cardiac position | Levocardia | Right atrium | Normal |
| Systemic venous drainage | Normal. | **Atrioventricular valves** |  |
| Pulmonary venous drainage | Normal | Mitral valve | Annulus = 19mm |
| Atrioventricular connection | Concordant | Tricuspid valve | Annulus = 22mm |
| Ventriculoarterial connection | Concordant | **Ventricles** |  |
| Ventricular loop | d-Loop | Left ventricle | Normal |
|  |  | Right ventricle | Normal |
| **Septae** |  | **Coronary arteries** | ----- |
| Interventricular septum | Intact | **Doppler Measurement** |  |
| Interatrial septum | Intact | Mitral | ----- |
| **Semilunar valves** |  | Aortic | ------- |
| Aortic valve | Annulus = 16mm | Tricuspid | ------- |
| Pulmonary valve | Annulus = 17mm | pulmonic | -------- |
| **Great arteries** | NRGA | **Aortic arch** | Left. No CoA. |
| Aorta | ----- | **PDA** | No |
| Pulmonary artery | Normal MPA and Branch PAs. |  |  |
| **M-Mode:** | | | |
| AO | mm | PWd | mm |
| LA | mm | PWs | mm |
| LVIDd | mm | EDV | ml |
| LVIDs | mm | ESV | ml |
| IVSs | mm | LVEF | 62% |
| IVSd | mm | FS | 33% |
| **Additional Information**: |  | | |
| No pericardial/Pleural effusion. | | | |
| **Final Diagnosis:** | | | |
| 1. Normal Echocardiography Study. | | | |
| **Remark**: | | | |
| **Recommendation**: | | | |
| SIGNATURE  Done by: Tesfaye T., Pediatrician, Pediatric Cardiologist _______________ 21/03/2015Eth.C | | | |

| Patient Name: **Halid Hussien**. Referring Institute: **Amaris PSC**. SEX/ Age: **M/7 6/12**. Date of Report: **22/03/15**.  Referral Diagnosis: **DS. AGH2.053**. | | | |
| --- | --- | --- | --- |
| **Features** | **Finding** | **Features** | **Finding** |
| **Profile** |  | **Atria** |  |
| Abdominal situs | Solitus | Left atrium | Dilated |
| Cardiac position | Levocardia | Right atrium | Dilated |
| Systemic venous drainage | Normal. | **Atrioventricular valves** |  |
| Pulmonary venous drainage | Normal | Mitral valve | Common Complete AVSD.  TAPSE = 20mm |
| Atrioventricular connection | Common Complete AVSD | Tricuspid valve |  |
| Ventriculoarterial connection | Concordant | **Ventricles** |  |
| Ventricular loop | d-Loop | Left ventricle | Dilated |
|  |  | Right ventricle | Dilated. RV TDI S wave = 14cm/sec. |
| **Septae** |  | **Coronary arteries** | ----- |
| Interventricular septum | Common Complete AVSD, L – R Shunt | **Doppler Measurement** |  |
| Interatrial septum |  | Mitral | Moderate Left AVVR |
| **Semilunar valves** |  | Aortic | ------- |
| Aortic valve | Annulus = 19mm | Tricuspid | Mild Right AVVR |
| Pulmonary valve | Annulus = 21mm | pulmonic | Moderate PR, PPG = 65mmHg. |
| **Great arteries** | NRGA | **Aortic arch** | Left. No CoA. |
| Aorta | ----- | **PDA** | No |
| Pulmonary artery | MPA = 24mm. |  |  |
| **M-Mode:** | | | |
| AO | mm | PWd | mm |
| LA | mm | PWs | mm |
| LVIDd | mm | EDV | ml |
| LVIDs | mm | ESV | ml |
| IVSs | mm | LVEF | 65% |
| IVSd | mm | FS | 35% |
| **Additional Information**: |  | | |
| No pericardial/Pleural effusion. | | | |
| **Final Diagnosis:** | | | |
| 1. {S, D, S} Levocardia. 2. All chambers dilated 3. Common Complete Balanced AVSD, L – R Shunt 4. Moderate Left AVVR 5. Mild Right AVVR 6. Moderate PR 7. Severe Pulmonary Hypertension 8. Normal Biventricular Systolic Function | | | |
| **Remark**: | | | |
| **Recommendation**: | | | |
| SIGNATURE  Done by: Tesfaye T., Pediatrician, Pediatric Cardiologist _______________ 22/03/2015Eth.C | | | |

| Patient Name: **Bezawit Senay**. Referring Institute: **FHRH**. SEX/ Age: **F/2 3/12**. Date of Report: **22/03/15**.  Referral Diagnosis: **CHF. AGH2.054.** | | | |
| --- | --- | --- | --- |
| **Features** | **Finding** | **Features** | **Finding** |
| **Profile** |  | **Atria** |  |
| Abdominal situs | Solitus | Left atrium | Markedly Dilated |
| Cardiac position | Levocardia | Right atrium | Normal |
| Systemic venous drainage | Normal. | **Atrioventricular valves** |  |
| Pulmonary venous drainage | Normal | Mitral valve | Annulus = 18mm |
| Atrioventricular connection | Concordant | Tricuspid valve | Annulus = 16mm  TAPSE = 15mm |
| Ventriculoarterial connection | Concordant | **Ventricles** |  |
| Ventricular loop | d-Loop | Left ventricle | Markedly Dilated & Dysfunctional. |
|  |  | Right ventricle | Normal |
| **Septae** |  | **Coronary arteries** | ----- |
| Interventricular septum | Intact | **Doppler Measurement** |  |
| Interatrial septum | Intact | Mitral | Mild MR, Holosystolic, seen in two planes with jet velocity = 2.9m/sec. |
| **Semilunar valves** |  | Aortic | ------- |
| Aortic valve | Annulus = 12mm | Tricuspid | Trivial TR, PPG = 7mmHg |
| Pulmonary valve | Annulus = 14mm | pulmonic | -------- |
| **Great arteries** | NRGA | **Aortic arch** | Left. No CoA. |
| Aorta | ----- | **PDA** | No |
| Pulmonary artery | Normal MPA and Branch PAs. | **Coronaries** | No ALCAPA |
| **M-Mode:** | | | |
| AO | mm | PWd | mm |
| LA | mm | PWs | mm |
| LVIDd | mm | EDV | ml |
| LVIDs | mm | ESV | ml |
| IVSs | mm | LVEF | 24% |
| IVSd | mm | FS | 11% |
| **Additional Information**: |  | | |
| No pericardial/Pleural effusion. | | | |
| **Final Diagnosis:** | | | |
| 1. {S, D, S} Levocardia. 2. LA/LV Dilated 3. Mild MR 4. Trivial TR 5. LV Markedly Dilated and Dysfunctional | | | |
| **Remark**: | | | |
| **Recommendation**: | | | |
| SIGNATURE  Done by: Tesfaye T., Pediatrician, Pediatric Cardiologist _______________ 22/03/2015Eth.C | | | |

| Patient Name: **Abrham Desalegn**. Referring Institute: **FHRH**. SEX/ Age: **M/1year**. Date of Report: **22/03/15**.  Referral Diagnosis: **Incidental Murmur. AGH2.055.** | | | |
| --- | --- | --- | --- |
| **Features** | **Finding** | **Features** | **Finding** |
| **Profile** |  | **Atria** |  |
| Abdominal situs | Solitus | Left atrium | Normal |
| Cardiac position | Levocardia | Right atrium | Normal |
| Systemic venous drainage | Normal. | **Atrioventricular valves** |  |
| Pulmonary venous drainage | Normal | Mitral valve | Annulus = 14mm |
| Atrioventricular connection | Concordant | Tricuspid valve | Annulus = 17mm |
| Ventriculoarterial connection | Concordant | **Ventricles** |  |
| Ventricular loop | d-Loop | Left ventricle | Normal |
|  |  | Right ventricle | Normal |
| **Septae** |  | **Coronary arteries** | ----- |
| Interventricular septum | 1.5mm PM VSD, L – R Shunt | **Doppler Measurement** |  |
| Interatrial septum | Intact | Mitral | ----- |
| **Semilunar valves** |  | Aortic | ------- |
| Aortic valve | Annulus = 14mm | Tricuspid | ------- |
| Pulmonary valve | Annulus = 13mm | pulmonic | -------- |
| **Great arteries** | NRGA | **Aortic arch** | Left. No CoA. |
| Aorta | ----- | **PDA** | No |
| Pulmonary artery | Normal MPA and Branch PAs. |  |  |
| **M-Mode:**  Normal LV Function on eye balling | | | |
| AO | mm | PWd | mm |
| LA | mm | PWs | mm |
| LVIDd | mm | EDV | ml |
| LVIDs | mm | ESV | ml |
| IVSs | mm | LVEF | % |
| IVSd | mm | FS | % |
| **Additional Information**: |  | | |
| No pericardial/Pleural effusion. | | | |
| **Final Diagnosis:** | | | |
| 1. {S, D, S} Levocardia. 2. Tiny PM VSD, L – R Shunt | | | |
| **Remark**: | | | |
| **Recommendation**: | | | |
| SIGNATURE  Done by: Tesfaye T., Pediatrician, Pediatric Cardiologist _______________ 22/03/2015Eth.C | | | |

| Patient Name: **Misbah Hamid**. Referring Institute: **Mekane-Selam Hospital**. SEX/ Age: **M/3months**.  Date of Report: **22/03/15**. Referral Diagnosis: **Cyanosis. AGH2.056.** | | | |
| --- | --- | --- | --- |
| **Features** | **Finding** | **Features** | **Finding** |
| **Profile** |  | **Atria** |  |
| Abdominal situs | Solitus | Left atrium | Normal |
| Cardiac position | Levocardia | Right atrium | Dilated |
| Systemic venous drainage | Normal. | **Atrioventricular valves** |  |
| Pulmonary venous drainage | Normal | Mitral valve | Annulus = 13mm |
| Atrioventricular connection | Concordant | Tricuspid valve | Annulus = 14mm  TAPSE = 12mm |
| Ventriculoarterial connection | Concordant | **Ventricles** |  |
| Ventricular loop | d-Loop | Left ventricle | Normal |
|  |  | Right ventricle | Dilated |
| **Septae** |  | **Coronary arteries** | ----- |
| Interventricular septum | Intact | **Doppler Measurement** |  |
| Interatrial septum | 7mm OS ASD, BD Shunt | Mitral | ----- |
| **Semilunar valves** |  | Aortic | ------- |
| Aortic valve | Annulus = 11mm | Tricuspid | ------- |
| Pulmonary valve | Annulus = 6mm | pulmonic | Severe PS, PPG = 125mmHg. |
| **Great arteries** | NRGA | **Aortic arch** | Left. No CoA. |
| Aorta | ----- | **PDA** | No |
| Pulmonary artery | Smallish MPA and Branch PAs. |  |  |
| **M-Mode:**  Normal LV Function on eye balling | | | |
| AO | mm | PWd | mm |
| LA | mm | PWs | mm |
| LVIDd | mm | EDV | ml |
| LVIDs | mm | ESV | ml |
| IVSs | mm | LVEF | % |
| IVSd | mm | FS | % |
| **Additional Information**: |  | | |
| No pericardial/Pleural effusion. | | | |
| **Final Diagnosis:** | | | |
| 1. {S, D, S} Levocardia. 2. RA/RV Dilated 3. Moderate OS ASD, BD Shunt 4. Severe Pulmonary stenosis 5. Smallish MPA 6. Normal Biventricular Systolic Function | | | |
| **Remark**: | | | |
| **Recommendation**: | | | |
| SIGNATURE  Done by: Tesfaye T., Pediatrician, Pediatric Cardiologist _______________ 22/03/2015Eth.C | | | |

| Patient Name: **Dawit Adane**. Referring Institute: **FHRH**. SEX/ Age: **M/4 10/12**. Date of Report: **22/03/15**.  Referral Diagnosis: **FTT. AGH2.057.** | | | |
| --- | --- | --- | --- |
| **Features** | **Finding** | **Features** | **Finding** |
| **Profile** |  | **Atria** |  |
| Abdominal situs | Solitus | Left atrium | Normal |
| Cardiac position | Levocardia | Right atrium | Normal |
| Systemic venous drainage | Normal. | **Atrioventricular valves** |  |
| Pulmonary venous drainage | Normal | Mitral valve | Annulus = 18mm |
| Atrioventricular connection | Concordant | Tricuspid valve | Annulus = 18mm  TAPSE = 16mm |
| Ventriculoarterial connection | Concordant | **Ventricles** |  |
| Ventricular loop | d-Loop | Left ventricle | Normal |
|  |  | Right ventricle | Normal |
| **Septae** |  | **Coronary arteries** | ----- |
| Interventricular septum | Intact | **Doppler Measurement** |  |
| Interatrial septum | Intact | Mitral | ----- |
| **Semilunar valves** |  | Aortic | ------- |
| Aortic valve | Annulus = 15mm | Tricuspid | ------- |
| Pulmonary valve | Annulus = 18mm | pulmonic | -------- |
| **Great arteries** | NRGA | **Aortic arch** | Left. No CoA. |
| Aorta | ----- | **PDA** | No |
| Pulmonary artery | Normal MPA and Branch PAs. |  |  |
| **M-Mode:** | | | |
| AO | mm | PWd | mm |
| LA | mm | PWs | mm |
| LVIDd | mm | EDV | ml |
| LVIDs | mm | ESV | ml |
| IVSs | mm | LVEF | 59% |
| IVSd | mm | FS | 31% |
| **Additional Information**: |  | | |
| No pericardial/Pleural effusion. | | | |
| **Final Diagnosis:** | | | |
| 1. Normal Echocardiography Study. | | | |
| **Remark**: | | | |
| **Recommendation**: | | | |
| SIGNATURE  Done by: Tesfaye T., Pediatrician, Pediatric Cardiologist _______________ 22/03/2015Eth.C | | | |

| Patient Name: **Yeab-Sira Gelaw**. Referring Institute: **TGSH**. SEX/ Age: **F/1 9/12**. Date of Report: **22/03/15**.  Referral Diagnosis: **CHF. AGH2.58** | | | |
| --- | --- | --- | --- |
| **Features** | **Finding** | **Features** | **Finding** |
| **Profile** |  | **Atria** |  |
| Abdominal situs | Solitus | Left atrium | Markedly Dilated |
| Cardiac position | Levocardia | Right atrium | Dilated |
| Systemic venous drainage | Normal. | **Atrioventricular valves** |  |
| Pulmonary venous drainage | Normal | Mitral valve | Annulus = 21mm |
| Atrioventricular connection | Concordant | Tricuspid valve | Annulus = 19mm  **TAPSE = 12mm** |
| Ventriculoarterial connection | Concordant | **Ventricles** |  |
| Ventricular loop | d-Loop | Left ventricle | Markedly Dilated & Dysfunctional |
|  |  | Right ventricle | Dilated |
| **Septae** |  | **Coronary arteries** | ----- |
| Interventricular septum | Intact | **Doppler Measurement** |  |
| Interatrial septum | Intact | Mitral | Trivial MR |
| **Semilunar valves** |  | Aortic | ------- |
| Aortic valve | Annulus = 12mm | Tricuspid | Trivial TR |
| Pulmonary valve | Annulus = 12mm | pulmonic | -------- |
| **Great arteries** | NRGA | **Aortic arch** | Left. No CoA. |
| Aorta | ----- | **PDA** | No |
| Pulmonary artery | Normal MPA and Branch PAs. | **Coronaries** | No ALCAPA |
| **M-Mode:** | | | |
| AO | mm | PWd | mm |
| LA | mm | PWs | mm |
| LVIDd | mm | EDV | ml |
| LVIDs | mm | ESV | ml |
| IVSs | mm | LVEF | 30% |
| IVSd | mm | FS | 14% |
| **Additional Information**: |  | | |
| No pericardial/Pleural effusion. | | | |
| **Final Diagnosis:** | | | |
| 1. {S, D, S} Levocardia. 2. Markedly Dilated LA/LV 3. Biventricular Systolic Dysfunction | | | |
| **Remark**: | | | |
| **Recommendation**: | | | |
| SIGNATURE  Done by: Tesfaye T., Pediatrician, Pediatric Cardiologist _______________ 22/03/2015Eth.C | | | |

| Patient Name: **Baby of Tigist Mulualem**. Referring Institute: **TGSH**. SEX/ Age: **M/28hours**. Date of Report: **22/03/15**.  Referral Diagnosis: **DS. AGH2.059.** | | | |
| --- | --- | --- | --- |
| **Features** | **Finding** | **Features** | **Finding** |
| **Profile** |  | **Atria** |  |
| Abdominal situs | Solitus | Left atrium | Normal |
| Cardiac position | Levocardia | Right atrium | Normal |
| Systemic venous drainage | Normal. | **Atrioventricular valves** |  |
| Pulmonary venous drainage | Normal | Mitral valve | Annulus = 9mm |
| Atrioventricular connection | Concordant | Tricuspid valve | Annulus = 9mm |
| Ventriculoarterial connection | Concordant | **Ventricles** |  |
| Ventricular loop | d-Loop | Left ventricle | Normal |
|  |  | Right ventricle | Normal |
| **Septae** |  | **Coronary arteries** | ----- |
| Interventricular septum | Intact | **Doppler Measurement** |  |
| Interatrial septum | 4mm OS ASD, L - R Shunt | Mitral | ----- |
| **Semilunar valves** |  | Aortic | ------- |
| Aortic valve | Annulus = 8mm | Tricuspid | ------- |
| Pulmonary valve | Annulus = 8mm | pulmonic | -------- |
| **Great arteries** | NRGA | **Aortic arch** | Left. No CoA. |
| Aorta | ----- | **PDA** | No |
| Pulmonary artery | Normal MPA and Branch PAs. |  |  |
| **M-Mode:**  Normal LV Function on eye balling | | | |
| AO | mm | PWd | mm |
| LA | mm | PWs | mm |
| LVIDd | mm | EDV | ml |
| LVIDs | mm | ESV | ml |
| IVSs | mm | LVEF | % |
| IVSd | mm | FS | % |
| **Additional Information**: |  | | |
| No pericardial/Pleural effusion. | | | |
| **Final Diagnosis:** | | | |
| 1. {S, D, S} Levocardia. 2. Small OS ASD, L – R Shunt | | | |
| **Remark**: | | | |
| **Recommendation**: | | | |
| SIGNATURE  Done by: Tesfaye T., Pediatrician, Pediatric Cardiologist _______________ 22/03/2015Eth.C | | | |

| Patient Name: **Zekarias Berihun**. Referring Institute: **Dr. Addisu PSC**. SEX/ Age: **M/10days**. Date of Report: **23/03/15**.  Referral Diagnosis: **DS. AGH2.060** | | | |
| --- | --- | --- | --- |
| **Features** | **Finding** | **Features** | **Finding** |
| **Profile** |  | **Atria** |  |
| Abdominal situs | Solitus | Left atrium | Normal |
| Cardiac position | Levocardia | Right atrium | Normal |
| Systemic venous drainage | Normal. | **Atrioventricular valves** |  |
| Pulmonary venous drainage | Normal | Mitral valve | Annulus = 8mm |
| Atrioventricular connection | Concordant | Tricuspid valve | Annulus = 11mm |
| Ventriculoarterial connection | Concordant | **Ventricles** |  |
| Ventricular loop | d-Loop | Left ventricle | Normal |
|  |  | Right ventricle | Normal |
| **Septae** |  | **Coronary arteries** | ----- |
| Interventricular septum | Intact | **Doppler Measurement** |  |
| Interatrial septum | 4mm OS ASD, L – R Shunt | Mitral | ----- |
| **Semilunar valves** |  | Aortic | ------- |
| Aortic valve | Annulus = 9mm | Tricuspid | ------- |
| Pulmonary valve | Annulus = 9mm | pulmonic | -------- |
| **Great arteries** | NRGA | **Aortic arch** | Left. No CoA. |
| Aorta | ----- | **PDA** | No |
| Pulmonary artery | Normal MPA and Branch PAs. |  |  |
| **M-Mode:**  Normal LV Function on eye balling | | | |
| AO | mm | PWd | mm |
| LA | mm | PWs | mm |
| LVIDd | mm | EDV | ml |
| LVIDs | mm | ESV | ml |
| IVSs | mm | LVEF | % |
| IVSd | mm | FS | % |
| **Additional Information**: |  | | |
| No pericardial/Pleural effusion. | | | |
| **Final Diagnosis:** | | | |
| 1. {S, D, S} Levocardia. 2. Small OS ASD, L – R Shunt | | | |
| **Remark**: Neonate was crying throughout study. | | | |
| **Recommendation**: | | | |
| SIGNATURE  Done by: Tesfaye T., Pediatrician, Pediatric Cardiologist _______________ 23/03/2015Eth.C | | | |

| Patient Name: **Baby of Fantaye Tebikew**. Referring Institute: **TGSH**. SEX/ Age: **M/26days**.  Date of Report: **24/03/15**. Referral Diagnosis: **Cardiomegaly. AGH2.061.** | | | |
| --- | --- | --- | --- |
| **Features** | **Finding** | **Features** | **Finding** |
| **Profile** |  | **Atria** |  |
| Abdominal situs | Solitus | Left atrium | Normal |
| Cardiac position | Levocardia | Right atrium | Normal |
| Systemic venous drainage | Normal. | **Atrioventricular valves** |  |
| Pulmonary venous drainage | Normal | Mitral valve | Annulus = 11mm |
| Atrioventricular connection | Concordant | Tricuspid valve | Annulus = 11mm |
| Ventriculoarterial connection | Concordant | **Ventricles** |  |
| Ventricular loop | d-Loop | Left ventricle | Normal |
|  |  | Right ventricle | Normal |
| **Septae** |  | **Coronary arteries** | ----- |
| Interventricular septum | Intact | **Doppler Measurement** |  |
| Interatrial septum | PFO, L – R Shunt | Mitral | ----- |
| **Semilunar valves** |  | Aortic | ------- |
| Aortic valve | Annulus = 9mm | Tricuspid | ------- |
| Pulmonary valve | Annulus = 11mm | pulmonic | -------- |
| **Great arteries** | NRGA | **Aortic arch** | Left. No CoA. |
| Aorta | ----- | **PDA** | No |
| Pulmonary artery | Normal MPA and Branch PAs. |  |  |
| **M-Mode:**  Normal LV Function on eye balling. | | | |
| AO | mm | PWd | mm |
| LA | mm | PWs | mm |
| LVIDd | mm | EDV | ml |
| LVIDs | mm | ESV | ml |
| IVSs | mm | LVEF | % |
| IVSd | mm | FS | % |
| **Additional Information**: |  | | |
| No pericardial/Pleural effusion. | | | |
| **Final Diagnosis:** | | | |
| 1. {S, D, S} Levocardia. 2. PFO, L – R Shunt | | | |
| **Remark**: | | | |
| **Recommendation**: | | | |
| SIGNATURE  Done by: Tesfaye T., Pediatrician, Pediatric Cardiologist _______________ 24/03/2015Eth.C | | | |

| Patient Name: **Biruktawit Getachew**. Referring Institute: **Addis Alem PH**. SEX/ Age: **F/5 10/12**. Date of Report: **24/03/15**.  Referral Diagnosis: **Recurrent Chest Infection. AGH2.062.** | | | |
| --- | --- | --- | --- |
| **Features** | **Finding** | **Features** | **Finding** |
| **Profile** |  | **Atria** |  |
| Abdominal situs | Solitus | Left atrium | Normal |
| Cardiac position | Levocardia | Right atrium | Normal |
| Systemic venous drainage | Normal. | **Atrioventricular valves** |  |
| Pulmonary venous drainage | Normal | Mitral valve | Annulus = 19mm |
| Atrioventricular connection | Concordant | Tricuspid valve | Annulus = 20mm  TAPSE = 19mm |
| Ventriculoarterial connection | Concordant | **Ventricles** |  |
| Ventricular loop | d-Loop | Left ventricle | Normal |
|  |  | Right ventricle | Normal. RV TDI S wave = 16cm/sec. |
| **Septae** |  | **Coronary arteries** | ----- |
| Interventricular septum | Intact | **Doppler Measurement** |  |
| Interatrial septum | Intact | Mitral | ----- |
| **Semilunar valves** |  | Aortic | ------- |
| Aortic valve | Annulus = 15mm | Tricuspid | ------- |
| Pulmonary valve | Annulus = 15mm | pulmonic | -------- |
| **Great arteries** | NRGA | **Aortic arch** | Left. No CoA. |
| Aorta | ----- | **PDA** | No |
| Pulmonary artery | Normal MPA and Branch PAs. |  |  |
| **M-Mode:** | | | |
| AO | mm | PWd | mm |
| LA | mm | PWs | mm |
| LVIDd | mm | EDV | ml |
| LVIDs | mm | ESV | ml |
| IVSs | mm | LVEF | 63% |
| IVSd | mm | FS | 33% |
| **Additional Information**: |  | | |
| No pericardial/Pleural effusion. | | | |
| **Final Diagnosis:** | | | |
| 1. Normal Echocardiography Study. | | | |
| **Remark**: | | | |
| **Recommendation**: | | | |
| SIGNATURE  Done by: Tesfaye T., Pediatrician, Pediatric Cardiologist _______________ 24/03/2015Eth.C | | | |

| Patient Name: **Endeshaw Zemen**. Referring Institute: **Adinas GH**. SEX/ Age: **M/14years**. Date of Report: **24/03/15**.  Referral Diagnosis: **Sydenham’s Chorea. AGH2.063.** | | | |
| --- | --- | --- | --- |
| **Features** | **Finding** | **Features** | **Finding** |
| **Profile** |  | **Atria** |  |
| Abdominal situs | Solitus | Left atrium | Normal |
| Cardiac position | Levocardia | Right atrium | Normal |
| Systemic venous drainage | Normal. | **Atrioventricular valves** |  |
| Pulmonary venous drainage | Normal | Mitral valve | Annulus = 27mm. Thickened MVL. |
| Atrioventricular connection | Concordant | Tricuspid valve | Annulus = 25mm  TAPSE = 26mm |
| Ventriculoarterial connection | Concordant | **Ventricles** |  |
| Ventricular loop | d-Loop | Left ventricle | Normal |
|  |  | Right ventricle | Normal. RV TDI S wave = 17cm/sec. |
| **Septae** |  | **Coronary arteries** | ----- |
| Interventricular septum | Intact | **Doppler Measurement** |  |
| Interatrial septum | Intact | Mitral | Trivial MR, Incomplete Signal, jet velocity = 3.6m/sec, seen in apical view |
| **Semilunar valves** |  | Aortic | ------- |
| Aortic valve | Annulus = 18mm | Tricuspid | Trivial TR, PPG = 12mmHg |
| Pulmonary valve | Annulus = 20mm | pulmonic | -------- |
| **Great arteries** | NRGA | **Aortic arch** | Left. No CoA. |
| Aorta | ----- | **PDA** | No |
| Pulmonary artery | Normal MPA and Branch PAs. |  |  |
| **M-Mode:** | | | |
| AO | mm | PWd | mm |
| LA | mm | PWs | mm |
| LVIDd | mm | EDV | ml |
| LVIDs | mm | ESV | ml |
| IVSs | mm | LVEF | 66% |
| IVSd | mm | FS | 36% |
| **Additional Information**: |  | | |
| No pericardial/Pleural effusion. | | | |
| **Final Diagnosis:** | | | |
| 1. {S, D, S} Levocardia. 2. Thickened MVL 3. Trivial MR | | | |
| **Remark**: See description above | | | |
| **Recommendation**: Better to manage as borderline RHD. | | | |
| SIGNATURE  Done by: Tesfaye T., Pediatrician, Pediatric Cardiologist _______________ 24/03/2015Eth.C | | | |

| Patient Name: **Baby of Mitikie Gashaw**. Referring Institute: **FHRH**. SEX/ Age: **F/14days**.  Date of Report: **24/03/15**. Referral Diagnosis: **Palpitation. AGH2.064.** | | | |
| --- | --- | --- | --- |
| **Features** | **Finding** | **Features** | **Finding** |
| **Profile** |  | **Atria** |  |
| Abdominal situs | Solitus | Left atrium | Normal |
| Cardiac position | Levocardia | Right atrium | Normal |
| Systemic venous drainage | Normal. | **Atrioventricular valves** |  |
| Pulmonary venous drainage | Normal | Mitral valve | Annulus = 10mm |
| Atrioventricular connection | Concordant | Tricuspid valve | Annulus = 10mm |
| Ventriculoarterial connection | Concordant | **Ventricles** |  |
| Ventricular loop | d-Loop | Left ventricle | Normal |
|  |  | Right ventricle | Normal |
| **Septae** |  | **Coronary arteries** | ----- |
| Interventricular septum | Intact | **Doppler Measurement** |  |
| Interatrial septum | Intact | Mitral | ----- |
| **Semilunar valves** |  | Aortic | ------- |
| Aortic valve | Annulus = 10mm | Tricuspid | Trivial TR, PPG = 20mmHg |
| Pulmonary valve | Annulus = 10mm | pulmonic | -------- |
| **Great arteries** | NRGA | **Aortic arch** | Left. No CoA. |
| Aorta | ----- | **PDA** | No |
| Pulmonary artery | Normal MPA and Branch PAs. |  |  |
| **M-Mode:**  Normal LV Function on eye balling | | | |
| AO | mm | PWd | mm |
| LA | mm | PWs | mm |
| LVIDd | mm | EDV | ml |
| LVIDs | mm | ESV | ml |
| IVSs | mm | LVEF | % |
| IVSd | mm | FS | % |
| **Additional Information**: |  | | |
| No pericardial/Pleural effusion. | | | |
| **Final Diagnosis:** | | | |
| 1. Normal Echocardiography Study. | | | |
| **Remark**: | | | |
| **Recommendation**: | | | |
| SIGNATURE  Done by: Tesfaye T., Pediatrician, Pediatric Cardiologist _______________ 24/03/2015Eth.C | | | |

| Patient Name: **Nathan Zewdu**. Referring Institute: **Adinas GH**. SEX/ Age: **M/11 6/12**. Date of Report: **24/03/15**.  Referral Diagnosis: **Easy fatigability. AGH2. 065.** | | | |
| --- | --- | --- | --- |
| **Features** | **Finding** | **Features** | **Finding** |
| **Profile** |  | **Atria** |  |
| Abdominal situs | Solitus | Left atrium | Normal |
| Cardiac position | Levocardia | Right atrium | Normal |
| Systemic venous drainage | Normal. | **Atrioventricular valves** |  |
| Pulmonary venous drainage | Normal | Mitral valve | Annulus = 25mm |
| Atrioventricular connection | Concordant | Tricuspid valve | Annulus = 26mm  TAPSE = 24mm |
| Ventriculoarterial connection | Concordant | **Ventricles** |  |
| Ventricular loop | d-Loop | Left ventricle | Normal |
|  |  | Right ventricle | Normal |
| **Septae** |  | **Coronary arteries** | ----- |
| Interventricular septum | Intact | **Doppler Measurement** |  |
| Interatrial septum | Intact | Mitral | ----- |
| **Semilunar valves** |  | Aortic | ------- |
| Aortic valve | Annulus = 19mm | Tricuspid | ------- |
| Pulmonary valve | Annulus = 21mm | pulmonic | -------- |
| **Great arteries** | NRGA | **Aortic arch** | Left. No CoA. |
| Aorta | ----- | **PDA** | No |
| Pulmonary artery | Normal MPA and Branch PAs. |  |  |
| **M-Mode:** | | | |
| AO | mm | PWd | mm |
| LA | mm | PWs | mm |
| LVIDd | mm | EDV | ml |
| LVIDs | mm | ESV | ml |
| IVSs | mm | LVEF | 67% |
| IVSd | mm | FS | 36% |
| **Additional Information**: |  | | |
| No pericardial/Pleural effusion. | | | |
| **Final Diagnosis:** | | | |
| 1. Normal Echocardiography Study. | | | |
| **Remark**: | | | |
| **Recommendation**: | | | |
| SIGNATURE  Done by: Tesfaye T., Pediatrician, Pediatric Cardiologist _______________ 24/03/2015Eth.C | | | |

| Patient Name: **Bereket Abebe**. Referring Institute: **Adinas GH**. SEX/ Age: **M/3 6/12**. Date of Report: **24/03/15**.  Referral Diagnosis: **Cyanosis. AGH2.066.** | | | |
| --- | --- | --- | --- |
| **Features** | **Finding** | **Features** | **Finding** |
| **Profile** |  | **Atria** |  |
| Abdominal situs | Solitus | Left atrium | Normal |
| Cardiac position | Levocardia | Right atrium | Dilated |
| Systemic venous drainage | Normal. | **Atrioventricular valves** |  |
| Pulmonary venous drainage | Normal | Mitral valve | Annulus = 16mm |
| Atrioventricular connection | Concordant | Tricuspid valve | Annulus = 18mm  TAPSE = 20mm |
| Ventriculoarterial connection | Concordant | **Ventricles** |  |
| Ventricular loop | d-Loop | Left ventricle | Normal |
|  |  | Right ventricle | Dilated and Hypertrophied. RV TDI S wave = 17cm/sec. |
| **Septae** |  | **Coronary arteries** | ----- |
| Interventricular septum | Non Restrictive mal-aligned Sub-Aortic VSD, R – L Shunt. | **Doppler Measurement** |  |
| Interatrial septum | 8mm OS ASD, R – L Shunt. | Mitral | ----- |
| **Semilunar valves** |  | Aortic | ------- |
| Aortic valve | Annulus = 15mm | Tricuspid | ------- |
| Pulmonary valve | Atretic | pulmonic | Severe PS |
| **Great arteries** | NRGA | **Aortic arch** | Left. No CoA. |
| Aorta | Over-riding aorta | **PDA** | No |
| Pulmonary artery | Smallish MPA (8mm) and Branch PAs. |  |  |
| **M-Mode:**  Normal LV Function on eye balling. | | | |
| AO | mm | PWd | mm |
| LA | mm | PWs | mm |
| LVIDd | mm | EDV | ml |
| LVIDs | mm | ESV | ml |
| IVSs | mm | LVEF | % |
| IVSd | mm | FS | % |
| **Additional Information**: |  | | |
| No pericardial/Pleural effusion. | | | |
| **Final Diagnosis:** | | | |
| 1. {S, D, S} Levocardia. 2. Moderate OS ASD, R – L Shunt 3. TOF 4. Smallish MPA and Branch PA | | | |
| **Recommendation**: | | | |
| SIGNATURE  Done by: Tesfaye T., Pediatrician, Pediatric Cardiologist _______________ 24/03/2015Eth.C | | | |

| Patient Name: **Tekle –Ab Yilma**. Referring Institute: **FHRH**. SEX/ Age: **M/4months**.  Date of Report: **26/03/15**. Referral Diagnosis: **Incidental Murmur. AGH2.067** | | | |
| --- | --- | --- | --- |
| **Features** | **Finding** | **Features** | **Finding** |
| **Profile** |  | **Atria** |  |
| Abdominal situs | Solitus | Left atrium | Normal |
| Cardiac position | Levocardia | Right atrium | Normal |
| Systemic venous drainage | Normal. | **Atrioventricular valves** |  |
| Pulmonary venous drainage | Normal | Mitral valve | Annulus = 12mm |
| Atrioventricular connection | Concordant | Tricuspid valve | Annulus = 13mm |
| Ventriculoarterial connection | Concordant | **Ventricles** |  |
| Ventricular loop | d-Loop | Left ventricle | Normal |
|  |  | Right ventricle | Normal |
| **Septae** |  | **Coronary arteries** | ----- |
| Interventricular septum | Intact | **Doppler Measurement** |  |
| Interatrial septum | PFO, L – R Shunt | Mitral | ----- |
| **Semilunar valves** |  | Aortic | ------- |
| Aortic valve | Annulus = 10mm | Tricuspid | ------- |
| Pulmonary valve | Annulus = 11mm | pulmonic | -------- |
| **Great arteries** | NRGA | **Aortic arch** | Left. No CoA. |
| Aorta | ----- | **PDA** | 1mm PDA, L – R Shunt |
| Pulmonary artery | Normal MPA and Branch PAs. |  |  |
| **M-Mode:**  Normal LV Function on eye balling | | | |
| AO | mm | PWd | mm |
| LA | mm | PWs | mm |
| LVIDd | mm | EDV | ml |
| LVIDs | mm | ESV | ml |
| IVSs | mm | LVEF | % |
| IVSd | mm | FS | % |
| **Additional Information**: |  | | |
| No pericardial/Pleural effusion. | | | |
| **Final Diagnosis:** | | | |
| 1. {S, D, S} Levocardia. 2. PFO, L – R Shunt 3. Small PDA, L – R Shunt | | | |
| **Remark**: | | | |
| **Recommendation**: | | | |
| SIGNATURE  Done by: Tesfaye T., Pediatrician, Pediatric Cardiologist _______________ 26/03/2015Eth.C | | | |

| Patient Name: **Eyerus Kefyalew**. Referring Institute: **Amaris PSC**. SEX/ Age: **F/6 11/12**. Date of Report: **26/03/15**.  Referral Diagnosis: **______________. AGH2.068.** | | | |
| --- | --- | --- | --- |
| **Features** | **Finding** | **Features** | **Finding** |
| **Profile** |  | **Atria** |  |
| Abdominal situs | Solitus | Left atrium | Dilated |
| Cardiac position | Levocardia | Right atrium | Normal |
| Systemic venous drainage | Normal. | **Atrioventricular valves** |  |
| Pulmonary venous drainage | Normal | Mitral valve | Annulus = 26mm. The anterior MVL bows to the LA more than 8mm. Thickened AMVL = 7mm. |
| Atrioventricular connection | Concordant | Tricuspid valve | Annulus = 20mm  TAPSE = 21mm |
| Ventriculoarterial connection | Concordant | **Ventricles** |  |
| Ventricular loop | d-Loop | Left ventricle | Dilated |
|  |  | Right ventricle | Normal |
| **Septae** |  | **Coronary arteries** | ----- |
| Interventricular septum | Intact | **Doppler Measurement** |  |
| Interatrial septum | 8mm OS ASD, L – R Shunt | Mitral | Moderate MR, Posterior projection, seen in two planes with jet velocity = 4.5m/sec. |
| **Semilunar valves** |  | Aortic | ------- |
| Aortic valve | Annulus = 14mm | Tricuspid | Trivial TR, PPG = 19mmHg |
| Pulmonary valve | Annulus = 20mm | pulmonic | -------- |
| **Great arteries** | NRGA | **Aortic arch** | Left. No CoA. |
| Aorta | ----- | **PDA** | No |
| Pulmonary artery | Normal MPA and Branch PAs. |  |  |
| **M-Mode:** | | | |
| AO | mm | PWd | mm |
| LA | mm | PWs | mm |
| LVIDd | mm | EDV | ml |
| LVIDs | mm | ESV | ml |
| IVSs | mm | LVEF | 62% |
| IVSd | mm | FS | 33% |
| **Additional Information**: |  | | |
| No pericardial/Pleural effusion. | | | |
| **Final Diagnosis:** | | | |
| 1. {S, D, S} Levocardia. 2. LA/LV Dilated 3. Moderate OS ASD, L – R Shunt 4. Moderate MR 5. Thickened MVL 6. Mitral Valve Prolapse 7. Normal Biventricular Systolic Function | | | |
| **Recommendation**: | | | |
| SIGNATURE  Done by: Tesfaye T., Pediatrician, Pediatric Cardiologist _______________ 26/03/2015Eth.C | | | |

| Patient Name: **Meklit Wassie**. Referring Institute: **Adinas GH**. SEX/ Age: **F/1year**. Date of Report: **26/03/15**.  Referral Diagnosis: **IE + CHF. AGH2.069.** | | | |
| --- | --- | --- | --- |
| **Features** | **Finding** | **Features** | **Finding** |
| **Profile** |  | **Atria** |  |
| Abdominal situs | Solitus | Left atrium | Markedly Dilated |
| Cardiac position | Levocardia | Right atrium | Dilated |
| Systemic venous drainage | Normal. | **Atrioventricular valves** |  |
| Pulmonary venous drainage | Normal | Mitral valve | Annulus = 17mm |
| Atrioventricular connection | Concordant | Tricuspid valve | Annulus = 16mm  TAPSE = 15mm |
| Ventriculoarterial connection | Concordant | **Ventricles** |  |
| Ventricular loop | d-Loop | Left ventricle | Markedly Dilated |
|  |  | Right ventricle | Dilated |
| **Septae** |  | **Coronary arteries** | ----- |
| Interventricular septum | Intact | **Doppler Measurement** |  |
| Interatrial septum | Intact | Mitral | Moderate MR, JET VELOCITY = 4.2m/sec. |
| **Semilunar valves** |  | Aortic | ------- |
| Aortic valve | Annulus = 11mm | Tricuspid | Mild TR, PPG = 65mmHg |
| Pulmonary valve | Annulus = 15mm | pulmonic | -------- |
| **Great arteries** | NRGA | **Aortic arch** | Left. No CoA. |
| Aorta | ----- | **PDA** | 4mm PDA, L – R Shunt. Echogenic mass at the pulmonary end of PDA. |
| Pulmonary artery | MPA = 21mm. |  |  |
| **M-Mode:** | | | |
| AO | mm | PWd | mm |
| LA | mm | PWs | mm |
| LVIDd | mm | EDV | ml |
| LVIDs | mm | ESV | ml |
| IVSs | mm | LVEF | 69% |
| IVSd | mm | FS | 38% |
| **Additional Information**: |  | | |
| No pericardial/Pleural effusion. | | | |
| **Final Diagnosis:** | | | |
| 1. {S, D, S} Levocardia. 2. All chambers Dilated 3. Moderate MR 4. Mild TR 5. Large PDA, L – R Shunt 6. Severe Pulmonary Hypertension 7. Echogenic mass @ the Pulmonary end of the PDA 8. Normal Biventricular Systolic Function | | | |
| SIGNATURE  Done by: Tesfaye T., Pediatrician, Pediatric Cardiologist _______________ 26/03/2015Eth.C | | | |

| Patient Name: **Sofonias Desalegn**. Referring Institute: **Adinas GH**. SEX/ Age: **M/2years**. Date of Report: **26/03/15**.  Referral Diagnosis: **S/P ASO + VSD Closure + PDA Ligation. AGH2.070** | | | |
| --- | --- | --- | --- |
| **Features** | **Finding** | **Features** | **Finding** |
| **Profile** |  | **Atria** |  |
| Abdominal situs | Solitus | Left atrium | Normal |
| Cardiac position | Levocardia | Right atrium | Normal |
| Systemic venous drainage | Normal. | **Atrioventricular valves** |  |
| Pulmonary venous drainage | Normal | Mitral valve | Annulus = 15mm |
| Atrioventricular connection | Concordant | Tricuspid valve | Annulus = 16mm  TAPSE = 16mm |
| Ventriculoarterial connection | Concordant | **Ventricles** |  |
| Ventricular loop | d-Loop | Left ventricle | Normal |
|  |  | Right ventricle | Normal |
| **Septae** |  | **Coronary arteries** | ----- |
| Interventricular septum | Tiny (1mm) Residual VSD, L – R Shunt | **Doppler Measurement** |  |
| Interatrial septum | Intact | Mitral | ----- |
| **Semilunar valves** |  | Aortic | ------- |
| Aortic valve | Annulus = 14mm | Tricuspid | ------- |
| Pulmonary valve | Annulus = 15mm | pulmonic | -------- |
| **Great arteries** | NRGA | **Aortic arch** | Left. No CoA. |
| Aorta | ----- | **PDA** | No |
| Pulmonary artery | Normal MPA and Branch PAs. |  |  |
| **M-Mode:**  Normal LV Function on eye balling | | | |
| AO | mm | PWd | mm |
| LA | mm | PWs | mm |
| LVIDd | mm | EDV | ml |
| LVIDs | mm | ESV | ml |
| IVSs | mm | LVEF | % |
| IVSd | mm | FS | % |
| **Additional Information**: |  | | |
| No pericardial/Pleural effusion. | | | |
| **Final Diagnosis:** | | | |
| 1. S/P ASO for d-TGA + VSD Closure + PDA Ligation 2. {S, D, S} Levocardia. 3. VSD Patch is intact 4. Residual tiny VSD , L – R Shunt 5. No residual PDA 6. Good Biventricular Systolic Function | | | |
| **Remark**: | | | |
| **Recommendation**: | | | |
| SIGNATURE  Done by: Tesfaye T., Pediatrician, Pediatric Cardiologist _______________ 26/03/2015Eth.C | | | |

| Patient Name: **Yordanos Gebre-Silassie**. Referring Institute: **Adinas GH**. SEX/ Age: **F/2years**. Date of Report: **26/03/15**.  Referral Diagnosis: **Follow up echo for Large VSD, Dextroposition.FTT. AGH2.071** | | | |
| --- | --- | --- | --- |
| **Features** | **Finding** | **Features** | **Finding** |
| **Profile** |  | **Atria** |  |
| Abdominal situs | Solitus | Left atrium | Dilated |
| Cardiac position | Dextroposition | Right atrium | Dilated |
| Systemic venous drainage | Normal. | **Atrioventricular valves** |  |
| Pulmonary venous drainage | RUPV draining to RA through 8mm ASD | Mitral valve | Annulus = 20mm |
| Atrioventricular connection | Concordant | Tricuspid valve | Annulus = 20mm |
| Ventriculoarterial connection | Concordant | **Ventricles** |  |
| Ventricular loop | d-Loop | Left ventricle | Dilated |
|  |  | Right ventricle | Dilated |
| **Septae** |  | **Coronary arteries** | ----- |
| Interventricular septum | 8mm PM VSD, L – R Shunt. Additional 18mm Muscular VSD, L – R Shunt | **Doppler Measurement** |  |
| Interatrial septum | 8mm High secundum ASD, L – R Shunt | Mitral | ----- |
| **Semilunar valves** |  | Aortic | ------- |
| Aortic valve | Annulus = 13mm | Tricuspid | ------- |
| Pulmonary valve | Annulus = 19mm. Doming PV | pulmonic | Moderate PR, PPG = 55mmHg. Mild PS, PPG = 21mmHg. |
| **Great arteries** | NRGA | **Aortic arch** | Left. No CoA. |
| Aorta | ----- | **PDA** | No |
| Pulmonary artery | Normal MPA and Branch PAs. |  |  |
| **M-Mode:** | | | |
| AO | mm | PWd | mm |
| LA | mm | PWs | mm |
| LVIDd | mm | EDV | ml |
| LVIDs | mm | ESV | ml |
| IVSs | mm | LVEF | % |
| IVSd | mm | FS | % |
| **Additional Information**: |  | | |
| No pericardial/Pleural effusion. | | | |
| **Final Diagnosis:** | | | |
| 1. {S, D, S} Dextroposition 2. PAPVC of the RUPV 3. Moderate PM VSD, L – R Shunt 4. Large Muscular VSD, L – R Shunt 5. Doming PV 6. Mild Valvular PS 7. Moderate PR 8. Moderate to severe Pulmonary Hypertension. | | | |
| SIGNATURE  Done by: Tesfaye T., Pediatrician, Pediatric Cardiologist _______________ 26/03/2015Eth.C | | | |

| Patient Name: **Dagim Sigermegn**. Referring Institute: **TGSH**. SEX/ Age: **M/1 4/12**. Date of Report: **27/03/15**.  Referral Diagnosis: **Follow up Echo for Transitional AVSD. DS. AGH2.072.** | | | |
| --- | --- | --- | --- |
| **Features** | **Finding** | **Features** | **Finding** |
| **Profile** |  | **Atria** |  |
| Abdominal situs | Solitus | Left atrium | Normal |
| Cardiac position | Levocardia | Right atrium | Normal |
| Systemic venous drainage | Normal. | **Atrioventricular valves** |  |
| Pulmonary venous drainage | Normal | Mitral valve | Annulus = 14mm |
| Atrioventricular connection | Concordant | Tricuspid valve | Annulus = 15mm |
| Ventriculoarterial connection | Concordant | **Ventricles** |  |
| Ventricular loop | d-Loop | Left ventricle | Normal |
|  |  | Right ventricle | Normal |
| **Septae** |  | **Coronary arteries** | ----- |
| Interventricular septum | Transitional AVSD, L – R Shunt | **Doppler Measurement** |  |
| Interatrial septum |  | Mitral | ----- |
| **Semilunar valves** |  | Aortic | ------- |
| Aortic valve | Annulus = 13mm | Tricuspid | ------- |
| Pulmonary valve | Annulus = 14mm | pulmonic | -------- |
| **Great arteries** | NRGA | **Aortic arch** | Left. No CoA. |
| Aorta | ----- | **PDA** | No |
| Pulmonary artery | Normal MPA and Branch PAs. |  |  |
| **M-Mode:**  Normal LV Function on eye balling | | | |
| AO | mm | PWd | mm |
| LA | mm | PWs | mm |
| LVIDd | mm | EDV | ml |
| LVIDs | mm | ESV | ml |
| IVSs | mm | LVEF | % |
| IVSd | mm | FS | % |
| **Additional Information**: |  | | |
| No pericardial/Pleural effusion. | | | |
| **Final Diagnosis:** | | | |
| 1. {S, D, S} Levocardia. 2. Transitional AVSD, L – R Shunt | | | |
| **Remark**: Very poor echo window (only sub costal) | | | |
| **Recommendation**: | | | |
| SIGNATURE  Done by: Tesfaye T., Pediatrician, Pediatric Cardiologist _______________ 27/03/2015Eth.C | | | |

| Patient Name: **Eyorika Getaneh**. Referring Institute: **Dr. Mirtzer MC**. SEX/ Age: **M/4 7/12**. Date of Report: **27/03/15**.  Referral Diagnosis: **Recurrent chest infection.AGH2.073** | | | |
| --- | --- | --- | --- |
| **Features** | **Finding** | **Features** | **Finding** |
| **Profile** |  | **Atria** |  |
| Abdominal situs | Solitus | Left atrium | Normal |
| Cardiac position | Levocardia | Right atrium | Normal |
| Systemic venous drainage | Normal. | **Atrioventricular valves** |  |
| Pulmonary venous drainage | Normal | Mitral valve | Annulus = 16mm |
| Atrioventricular connection | Concordant | Tricuspid valve | Annulus = 18mm  TAPSE = 20mm |
| Ventriculoarterial connection | Concordant | **Ventricles** |  |
| Ventricular loop | d-Loop | Left ventricle | Normal |
|  |  | Right ventricle | Normal |
| **Septae** |  | **Coronary arteries** | ----- |
| Interventricular septum | Intact | **Doppler Measurement** |  |
| Interatrial septum | Intact | Mitral | ----- |
| **Semilunar valves** |  | Aortic | ------- |
| Aortic valve | Annulus = 14mm | Tricuspid | Trivial TR, PPG = 20mmHg |
| Pulmonary valve | Annulus = 14mm | pulmonic | -------- |
| **Great arteries** | NRGA | **Aortic arch** | Left. No CoA. |
| Aorta | ----- | **PDA** | No |
| Pulmonary artery | Normal MPA and Branch PAs. |  |  |
| **M-Mode:** | | | |
| AO | mm | PWd | mm |
| LA | mm | PWs | mm |
| LVIDd | mm | EDV | ml |
| LVIDs | mm | ESV | ml |
| IVSs | mm | LVEF | 63% |
| IVSd | mm | FS | 33% |
| **Additional Information**: |  | | |
| No pericardial/Pleural effusion. | | | |
| **Final Diagnosis:** | | | |
| 1. Normal Echocardiography Study. | | | |
| **Remark**: | | | |
| **Recommendation**: | | | |
| SIGNATURE  Done by: Tesfaye T., Pediatrician, Pediatric Cardiologist _______________ 27/03/2015Eth.C | | | |

| Patient Name: **Hiwet Getinet**. Referring Institute: **TGSH**. SEX/ Age: **F/1 7/12**. Date of Report: **27/03/15**.  Referral Diagnosis: **Incidental Murmur.AGH2. 074.** | | | |
| --- | --- | --- | --- |
| **Features** | **Finding** | **Features** | **Finding** |
| **Profile** |  | **Atria** |  |
| Abdominal situs | Solitus | Left atrium | Normal |
| Cardiac position | Levocardia | Right atrium | Normal |
| Systemic venous drainage | Normal. | **Atrioventricular valves** |  |
| Pulmonary venous drainage | Normal | Mitral valve | Annulus = 15mm |
| Atrioventricular connection | Concordant | Tricuspid valve | Annulus = 16mm  TAPSE = 15mm |
| Ventriculoarterial connection | Concordant | **Ventricles** |  |
| Ventricular loop | d-Loop | Left ventricle | Normal |
|  |  | Right ventricle | Normal |
| **Septae** |  | **Coronary arteries** | ----- |
| Interventricular septum | Intact | **Doppler Measurement** |  |
| Interatrial septum | Intact | Mitral | ----- |
| **Semilunar valves** |  | Aortic | ------- |
| Aortic valve | Annulus = 12mm | Tricuspid | ------- |
| Pulmonary valve | Annulus = 13mm | pulmonic | -------- |
| **Great arteries** | NRGA | **Aortic arch** | Left. No CoA. |
| Aorta | ----- | **PDA** | 1mm PDA, L – R Shunt |
| Pulmonary artery | Normal MPA and Branch PAs. |  |  |
| **M-Mode:**  Normal LV Function on eye balling | | | |
| AO | mm | PWd | mm |
| LA | mm | PWs | mm |
| LVIDd | mm | EDV | ml |
| LVIDs | mm | ESV | ml |
| IVSs | mm | LVEF | % |
| IVSd | mm | FS | % |
| **Additional Information**: |  | | |
| No pericardial/Pleural effusion. | | | |
| **Final Diagnosis:** | | | |
| 1. {S, D, S} Levocardia. 2. Small PDA, L – R Shunt | | | |
| **Remark**: | | | |
| **Recommendation**: | | | |
| SIGNATURE  Done by: Tesfaye T., Pediatrician, Pediatric Cardiologist _______________ 27/03/2015Eth.C | | | |

| Patient Name: **Baby of Mayet Achenef**. Referring Institute: **FHRH**. SEX/ Age: **M/30days**. Date of Report: **28/03/15**.  Referral Diagnosis: **Incidental Murmur finding. AGH2.075** | | | |
| --- | --- | --- | --- |
| **Features** | **Finding** | **Features** | **Finding** |
| **Profile** |  | **Atria** |  |
| Abdominal situs | Solitus | Left atrium | Normal |
| Cardiac position | Levocardia | Right atrium | Normal |
| Systemic venous drainage | Normal. | **Atrioventricular valves** |  |
| Pulmonary venous drainage | Normal | Mitral valve | Annulus = 10mm |
| Atrioventricular connection | Concordant | Tricuspid valve | Annulus = 10mm |
| Ventriculoarterial connection | Concordant | **Ventricles** |  |
| Ventricular loop | d-Loop | Left ventricle | Normal |
|  |  | Right ventricle | Normal |
| **Septae** |  | **Coronary arteries** | ----- |
| Interventricular septum | Intact | **Doppler Measurement** |  |
| Interatrial septum | 4mm OS ASD, L – R Shunt | Mitral | ----- |
| **Semilunar valves** |  | Aortic | ------- |
| Aortic valve | Annulus = 8mm | Tricuspid | ------- |
| Pulmonary valve | Annulus = 9mm | pulmonic | -------- |
| **Great arteries** | NRGA | **Aortic arch** | Left. No CoA. |
| Aorta | ----- | **PDA** | 1mm PDA, L – R Shunt |
| Pulmonary artery | Normal MPA and Branch PAs. |  |  |
| **M-Mode:**  Normal LV Function on eye balling | | | |
| AO | mm | PWd | mm |
| LA | mm | PWs | mm |
| LVIDd | mm | EDV | ml |
| LVIDs | mm | ESV | ml |
| IVSs | mm | LVEF | % |
| IVSd | mm | FS | % |
| **Additional Information**: |  | | |
| No pericardial/Pleural effusion. | | | |
| **Final Diagnosis:** | | | |
| 1. {S, D, S} Levocardia. 2. Small OS ASD, L – R Shunt 3. Small PDA, L – R Shunt | | | |
| **Remark**: | | | |
| **Recommendation**: | | | |
| SIGNATURE  Done by: Tesfaye T., Pediatrician, Pediatric Cardiologist _______________ 28/03/2015Eth.C | | | |

| Patient Name: **Befekadu Asaye**. Referring Institute: **Adinas GH**. SEX/ Age: **M/5years**. Date of Report: **28/03/15**.  Referral Diagnosis: **Follow up echo for RHD (Severe MR + Moderate Pericardial effusion + Mod. Pul.HTN). CHF. AGH2.076.** | | | |
| --- | --- | --- | --- |
| **Features** | **Finding** | **Features** | **Finding** |
| **Profile** |  | **Atria** |  |
| Abdominal situs | Solitus | Left atrium | Mildly Dilated |
| Cardiac position | Levocardia | Right atrium | Normal |
| Systemic venous drainage | Normal. | **Atrioventricular valves** |  |
| Pulmonary venous drainage | Normal | Mitral valve | Annulus = 23mm. Thickened MVL |
| Atrioventricular connection | Concordant | Tricuspid valve | Annulus = 19mm  TAPSE = 19mm |
| Ventriculoarterial connection | Concordant | **Ventricles** |  |
| Ventricular loop | d-Loop | Left ventricle | Mildly Dilated |
|  |  | Right ventricle | Normal |
| **Septae** |  | **Coronary arteries** | ----- |
| Interventricular septum | Intact | **Doppler Measurement** |  |
| Interatrial septum | Intact | Mitral | Moderate MR, Holosystolic, posterior projection, seen in two planes with jet velocity = 4.6m/sec. |
| **Semilunar valves** |  | Aortic | ------- |
| Aortic valve | Annulus = 14mm | Tricuspid | Trivial TR, PPG = 16mmHg |
| Pulmonary valve | Annulus = 16mm | pulmonic | -------- |
| **Great arteries** | NRGA | **Aortic arch** | Left. No CoA. |
| Aorta | ----- | **PDA** | No |
| Pulmonary artery | Normal MPA and Branch PAs. |  |  |
| **M-Mode:** | | | |
| AO | mm | PWd | mm |
| LA | mm | PWs | mm |
| LVIDd | mm | EDV | ml |
| LVIDs | mm | ESV | ml |
| IVSs | mm | LVEF | 69% |
| IVSd | mm | FS | 38% |
| **Additional Information**: |  | | |
| No pericardial/Pleural effusion. | | | |
| **Final Diagnosis:** | | | |
| 1. {S, D, S} Levocardia. 2. LA/LV Mildly Dilated 3. Mildly thickened MVL 4. Moderate MR 5. Normal Biventricular Systolic Function | | | |
| **Remark**: | | | |
| **Recommendation**: | | | |
| SIGNATURE  Done by: Tesfaye T., Pediatrician, Pediatric Cardiologist _______________ 28/03/2015Eth.C | | | |

| Patient Name: **Absalat Mekonnen**. Referring Institute: **TGSH**. SEX/ Age: **F/1 1/12**. Date of Report: **28/03/15**.  Referral Diagnosis: **RD + Murmur. AGH2.077** | | | |
| --- | --- | --- | --- |
| **Features** | **Finding** | **Features** | **Finding** |
| **Profile** |  | **Atria** |  |
| Abdominal situs | Solitus | Left atrium | Normal |
| Cardiac position | Levocardia | Right atrium | Dilated |
| Systemic venous drainage | Normal. | **Atrioventricular valves** |  |
| Pulmonary venous drainage | Normal | Mitral valve | Annulus = 14mm |
| Atrioventricular connection | Concordant | Tricuspid valve | Annulus = 17mm  TAPSE = 18mm |
| Ventriculoarterial connection | Concordant | **Ventricles** |  |
| Ventricular loop | d-Loop | Left ventricle | Normal |
|  |  | Right ventricle | Dilated |
| **Septae** |  | **Coronary arteries** | ----- |
| Interventricular septum | Non-Restrictive Sub aortic VSD, L – R Shunt | **Doppler Measurement** |  |
| Interatrial septum | 10mm OS ASD, L – R Shunt | Mitral | ----- |
| **Semilunar valves** |  | Aortic | ------- |
| Aortic valve | Annulus = 12mm | Tricuspid | ------- |
| Pulmonary valve | Annulus = 13mm | pulmonic | -------- |
| **Great arteries** | NRGA | **Aortic arch** | Left. No CoA. |
| Aorta | ----- | **PDA** | No |
| Pulmonary artery | Normal MPA and Branch PAs. |  |  |
| **M-Mode:**  Normal LV Function on eye balling | | | |
| AO | mm | PWd | mm |
| LA | mm | PWs | mm |
| LVIDd | mm | EDV | ml |
| LVIDs | mm | ESV | ml |
| IVSs | mm | LVEF | % |
| IVSd | mm | FS | % |
| **Additional Information**: |  | | |
| No pericardial/Pleural effusion. | | | |
| **Final Diagnosis:** | | | |
| 1. {S, D, S} Levocardia. 2. RA/RV Dilated 3. Moderate OS ASD, L – R Shunt 4. Non-Restrictive Sub-Aortic VSD, L – R Shunt | | | |
| **Remark**: | | | |
| **Recommendation**: | | | |
| SIGNATURE  Done by: Tesfaye T., Pediatrician, Pediatric Cardiologist _______________ 28/03/2015Eth.C | | | |

| Patient Name: **Yohannes Simegnew**. Referring Institute: **FHRH**. SEX/ Age: **M/3 5/12**. Date of Report: **28/03/15**.  Referral Diagnosis: **SOB. AGH2.078** | | | |
| --- | --- | --- | --- |
| **Features** | **Finding** | **Features** | **Finding** |
| **Profile** |  | **Atria** |  |
| Abdominal situs | Solitus | Left atrium | Normal |
| Cardiac position | Levocardia | Right atrium | Normal |
| Systemic venous drainage | Normal. | **Atrioventricular valves** |  |
| Pulmonary venous drainage | Normal | Mitral valve | Annulus = 16mm |
| Atrioventricular connection | Concordant | Tricuspid valve | Annulus = 18mm |
| Ventriculoarterial connection | Concordant | **Ventricles** |  |
| Ventricular loop | d-Loop | Left ventricle | Normal |
|  |  | Right ventricle | Normal |
| **Septae** |  | **Coronary arteries** | ----- |
| Interventricular septum | Intact | **Doppler Measurement** |  |
| Interatrial septum | Intact | Mitral | ----- |
| **Semilunar valves** |  | Aortic | ------- |
| Aortic valve | Annulus = 13mm | Tricuspid | ------- |
| Pulmonary valve | Annulus = 16mm | pulmonic | -------- |
| **Great arteries** | NRGA | **Aortic arch** | Left. No CoA. |
| Aorta | ----- | **PDA** | No |
| Pulmonary artery | Normal MPA and Branch PAs. |  |  |
| **M-Mode:**  Normal LV Function on eye balling | | | |
| AO | mm | PWd | mm |
| LA | mm | PWs | mm |
| LVIDd | mm | EDV | ml |
| LVIDs | mm | ESV | ml |
| IVSs | mm | LVEF | % |
| IVSd | mm | FS | % |
| **Additional Information**: |  | | |
| No pericardial/Pleural effusion. | | | |
| **Final Diagnosis:** | | | |
| 1. Normal Echocardiography Study. | | | |
| **Remark**: | | | |
| **Recommendation**: | | | |
| SIGNATURE  Done by: Tesfaye T., Pediatrician, Pediatric Cardiologist _______________ 28/03/2015Eth.C | | | |

| Patient Name: **Fasika Techilo**. Referring Institute: **FHRH**. SEX/ Age: **F/12years**. Date of Report: **28/03/15**.  R.Dx: **CHF. AGH2.079**. | | | |
| --- | --- | --- | --- |
| **Features** | **Finding** | **Features** | **Finding** |
| **Profile** |  | **Atria** |  |
| Abdominal situs | Solitus | Left atrium | Dilated |
| Cardiac position | Levocardia | Right atrium | Partitioned in to two from Right anterior to left posterior with an opening = 12mm draining from the septal side to the IVC Side. |
| Systemic venous drainage | Normal. | **Atrioventricular valves** |  |
| Pulmonary venous drainage | Normal | Mitral valve | Annulus = 20mm |
| Atrioventricular connection | Concordant | Tricuspid valve | Annulus = 22mm.TAPSE = 26mm |
| Ventriculoarterial connection | Concordant | **Ventricles** |  |
| Ventricular loop | d-Loop | Left ventricle | Dilated |
|  |  | Right ventricle | Dilated & Hypertrophied |
| **Septae** |  | **Coronary arteries** | ----- |
| Interventricular septum | 19mm Sub aortic VSD, BD Shunt, predominantly L – R | **Doppler Measurement** |  |
| Interatrial septum | 16mm OS ASD, L – R Shunt | Mitral | ----- |
| **Semilunar valves** | Aorto-Mitral Discontinuity | Aortic | ------- |
| Aortic valve | Annulus = 19mm | Tricuspid | ------- |
| Pulmonary valve | Annulus = 11mm | pulmonic | Severe PS, PPG = 90mmHg. Moderate PR, PPG = 33mmHg |
| **Great arteries** | NRGA | **Aortic arch** | Left. No CoA. |
| Aorta | Posterior & from RV | **PDA** | No |
| Pulmonary artery | Anterior & from RV. Smallish MPA and BPAs. |  |  |
| **M-Mode:** | | | |
| AO | mm | PWd | mm |
| LA | mm | PWs | mm |
| LVIDd | mm | EDV | ml |
| LVIDs | mm | ESV | ml |
| IVSs | mm | LVEF | 45% |
| IVSd | mm | FS | 22% |
| **Additional Information**: | Trace Pericardial effusion | | |
| **Final Diagnosis:** | | | |
| 1. {S, D, S} Levocardia. 2. All chambers dilated 3. DORV (TOF Variant) 4. Large OS ASD, L – R Shunt 5. Cor triatriatum Dextrum 6. Large Sub-aortic VSD, BD Shunt predominantly L – R Shunt 7. Severe PS 8. Moderate PR 9. Reduced LV Systolic Function 10. Hypertrophied RV 11. Smallish MPA and Branch PAs | | | |
| SIGNATURE  Done by: Tesfaye T., Pediatrician, Pediatric Cardiologist _______________ 28/03/2015Eth.C | | | |

| Patient Name: **Haile-Yesus Destaw**. Referring Institute: **Debre-Tabour GH**. SEX/ Age: **M/5months**. Date of Report: **29/03/15**.  Referral Diagnosis: **Incidental Murmur finding.AGH2.080.** | | | |
| --- | --- | --- | --- |
| **Features** | **Finding** | **Features** | **Finding** |
| **Profile** |  | **Atria** |  |
| Abdominal situs | Solitus | Left atrium | Normal |
| Cardiac position | Levocardia | Right atrium | Normal |
| Systemic venous drainage | Normal. | **Atrioventricular valves** |  |
| Pulmonary venous drainage | Normal | Mitral valve | Annulus = 11mm |
| Atrioventricular connection | Concordant | Tricuspid valve | Annulus = 13mm |
| Ventriculoarterial connection | Concordant | **Ventricles** |  |
| Ventricular loop | d-Loop | Left ventricle | Normal |
|  |  | Right ventricle | Normal |
| **Septae** |  | **Coronary arteries** | ----- |
| Interventricular septum | 2mm Mid Muscular VSD, L – R Shunt | **Doppler Measurement** |  |
| Interatrial septum | PFO, L – R Shunt | Mitral | ----- |
| **Semilunar valves** |  | Aortic | ------- |
| Aortic valve | Annulus = 11mm | Tricuspid | ------- |
| Pulmonary valve | Annulus = 11mm | pulmonic | -------- |
| **Great arteries** | NRGA | **Aortic arch** | Left. No CoA. |
| Aorta | ----- | **PDA** | 1.5mm PDA, L – R Shunt |
| Pulmonary artery | Normal MPA and Branch PAs. |  |  |
| **M-Mode:**  Normal LV Function on eye balling | | | |
| AO | mm | PWd | mm |
| LA | mm | PWs | mm |
| LVIDd | mm | EDV | ml |
| LVIDs | mm | ESV | ml |
| IVSs | mm | LVEF | % |
| IVSd | mm | FS | % |
| **Additional Information**: |  | | |
| No pericardial/Pleural effusion. | | | |
| **Final Diagnosis:** | | | |
| 1. {S, D, S} Levocardia. 2. PFO, L – R Shunt 3. Small Mid Muscular VSD, L – R Shunt 4. Small PDA, L – R Shunt | | | |
| **Remark**: | | | |
| **Recommendation**: | | | |
| SIGNATURE  Done by: Tesfaye T., Pediatrician, Pediatric Cardiologist _______________ 29/03/2015Eth.C | | | |

| Patient Name: **Chernet Moges**. Referring Institute: **TGSH**. SEX/ Age: **F/27days**. Date of Report: **30/03/15**.  Referral Diagnosis: **DS. AGH2.081** | | | |
| --- | --- | --- | --- |
| **Features** | **Finding** | **Features** | **Finding** |
| **Profile** |  | **Atria** |  |
| Abdominal situs | Solitus | Left atrium | Normal |
| Cardiac position | Levocardia | Right atrium | Normal |
| Systemic venous drainage | Normal. | **Atrioventricular valves** |  |
| Pulmonary venous drainage | Normal | Mitral valve | Annulus = 9mm |
| Atrioventricular connection | Concordant | Tricuspid valve | Annulus = 11mm |
| Ventriculoarterial connection | Concordant | **Ventricles** |  |
| Ventricular loop | d-Loop | Left ventricle | Normal |
|  |  | Right ventricle | Normal |
| **Septae** | Tongue of conal tissue in b/n the two AV Valves | **Coronary arteries** | ----- |
| Interventricular septum | 5mm Inlet VSD, L – R Shunt | **Doppler Measurement** |  |
| Interatrial septum | 6mm Primum Defect, L – R Shunt | Mitral | ----- |
| **Semilunar valves** |  | Aortic | ------- |
| Aortic valve | Annulus = 9mm | Tricuspid | ------- |
| Pulmonary valve | Annulus = 11mm | pulmonic | Mild PR, PPG = 40mmHg |
| **Great arteries** | NRGA | **Aortic arch** | Left. No CoA. |
| Aorta | ----- | **PDA** | No |
| Pulmonary artery | Normal MPA and Branch PAs. |  |  |
| **M-Mode:**  Normal LV Function on eye balling | | | |
| AO | mm | PWd | mm |
| LA | mm | PWs | mm |
| LVIDd | mm | EDV | ml |
| LVIDs | mm | ESV | ml |
| IVSs | mm | LVEF | % |
| IVSd | mm | FS | % |
| **Additional Information**: |  | | |
| No pericardial/Pleural effusion. | | | |
| **Final Diagnosis:** | | | |
| 1. {S, D, S} Levocardia. 2. Transitional AVSD 3. Mild PR 4. Mild Pulmonary Hypertension 5. Normal LV Systolic Function | | | |
| **Remark**: | | | |
| **Recommendation**: | | | |
| SIGNATURE  Done by: Tesfaye T., Pediatrician, Pediatric Cardiologist _______________ 30/03/2015Eth.C | | | |

| Patient Name: **Yordanos Abiyot**. Referring Institute: **Injibara GH**. SEX/ Age: **M/9months**. Date of Report: **30/03/15**.  Referral Diagnosis: **Incidental Finding. AGH2.082** | | | |
| --- | --- | --- | --- |
| **Features** | **Finding** | **Features** | **Finding** |
| **Profile** |  | **Atria** |  |
| Abdominal situs | Solitus | Left atrium | Normal |
| Cardiac position | Levocardia | Right atrium | Normal |
| Systemic venous drainage | Normal. | **Atrioventricular valves** |  |
| Pulmonary venous drainage | Normal | Mitral valve | Annulus = 11mm |
| Atrioventricular connection | Concordant | Tricuspid valve | Annulus = 14mm  TAPSE = mm |
| Ventriculoarterial connection | Concordant | **Ventricles** |  |
| Ventricular loop | d-Loop | Left ventricle | Normal |
|  |  | Right ventricle | Normal |
| **Septae** |  | **Coronary arteries** | ----- |
| Interventricular septum | 2.5mm Sub-Pulmonic VSD, L – R Shunt | **Doppler Measurement** |  |
| Interatrial septum | 5mm High Secundum ASD, L – R Shunt | Mitral | ----- |
| **Semilunar valves** |  | Aortic | ------- |
| Aortic valve | Annulus = 11mm | Tricuspid | ------- |
| Pulmonary valve | Annulus = 13mm | pulmonic | -------- |
| **Great arteries** | NRGA | **Aortic arch** | Left. No CoA. |
| Aorta | ----- | **PDA** | No |
| Pulmonary artery | Normal MPA and Branch PAs. |  |  |
| **M-Mode:**  Normal LV Function on eye balling | | | |
| AO | mm | PWd | mm |
| LA | mm | PWs | mm |
| LVIDd | mm | EDV | ml |
| LVIDs | mm | ESV | ml |
| IVSs | mm | LVEF | % |
| IVSd | mm | FS | % |
| **Additional Information**: |  | | |
| No pericardial/Pleural effusion. | | | |
| **Final Diagnosis:** | | | |
| 1. {S, D, S} Levocardia. 2. Small High Secundum ASD, L – R Shunt 3. Small Sub-Pulmonic VSD, L – R Shunt | | | |
| **Remark**: | | | |
| **Recommendation**: | | | |
| SIGNATURE  Done by: Tesfaye T., Pediatrician, Pediatric Cardiologist _______________ 30/03/2015Eth.C | | | |

| Patient Name: **Baby of Eyerus Debie**. Referring Institute: **TGSH**. SEX/ Age: **F/18hours**. Date of Report: **30/03/15**.  Referral Diagnosis: **Incidental Murmur finding. AGH2.083** | | | |
| --- | --- | --- | --- |
| **Features** | **Finding** | **Features** | **Finding** |
| **Profile** |  | **Atria** |  |
| Abdominal situs | Solitus | Left atrium | Normal |
| Cardiac position | Levocardia | Right atrium | Normal |
| Systemic venous drainage | Normal. | **Atrioventricular valves** |  |
| Pulmonary venous drainage | Normal | Mitral valve | Annulus = 11mm |
| Atrioventricular connection | Concordant | Tricuspid valve | Annulus = 11mm |
| Ventriculoarterial connection | Concordant | **Ventricles** |  |
| Ventricular loop | d-Loop | Left ventricle | Normal |
|  |  | Right ventricle | Normal |
| **Septae** |  | **Coronary arteries** | ----- |
| Interventricular septum | Intact | **Doppler Measurement** |  |
| Interatrial septum | 9 X 10mm OS ASD, L – R Shunt | Mitral | ----- |
| **Semilunar valves** |  | Aortic | ------- |
| Aortic valve | Annulus = 9mm | Tricuspid | ------- |
| Pulmonary valve | Annulus = 9mm | pulmonic | Severe Valvular PS, PPG = 70mmHg. |
| **Great arteries** | NRGA | **Aortic arch** | Left. No CoA. |
| Aorta | ----- | **PDA** | 1.5mm PDA, L – R Shunt |
| Pulmonary artery | Normal MPA and Branch PAs. |  |  |
| **M-Mode:**  Normal LV Function on eye balling | | | |
| AO | mm | PWd | mm |
| LA | mm | PWs | mm |
| LVIDd | mm | EDV | ml |
| LVIDs | mm | ESV | ml |
| IVSs | mm | LVEF | % |
| IVSd | mm | FS | % |
| **Additional Information**: |  | | |
| No pericardial/Pleural effusion. | | | |
| **Final Diagnosis:** | | | |
| 1. {S, D, S} Levocardia. 2. Moderate OS ASD, L – R Shunt 3. Small PDA, L – R Shunt 4. Severe Valvular PS 5. Normal LV Systolic Function | | | |
| **Remark**: | | | |
| **Recommendation**: | | | |
| SIGNATURE  Done by: Tesfaye T., Pediatrician, Pediatric Cardiologist _______________ 30/03/2015Eth.C | | | |

| Patient Name: **Banchamlak Ebabu**. Referring Institute: **FHRH**. SEX/ Age: **F/8years**. Date of Report: **30/03/15**.  Referral Diagnosis: **Easy Fatigability. AGH2.084.** | | | |
| --- | --- | --- | --- |
| **Features** | **Finding** | **Features** | **Finding** |
| **Profile** |  | **Atria** |  |
| Abdominal situs | Solitus | Left atrium | Normal |
| Cardiac position | Levocardia | Right atrium | Normal |
| Systemic venous drainage | Normal. | **Atrioventricular valves** |  |
| Pulmonary venous drainage | Normal | Mitral valve | Annulus = 18mm |
| Atrioventricular connection | Concordant | Tricuspid valve | Annulus = 18mm |
| Ventriculoarterial connection | Concordant | **Ventricles** |  |
| Ventricular loop | d-Loop | Left ventricle | Normal |
|  |  | Right ventricle | Normal |
| **Septae** |  | **Coronary arteries** | ----- |
| Interventricular septum | Intact | **Doppler Measurement** |  |
| Interatrial septum | Intact | Mitral | ----- |
| **Semilunar valves** |  | Aortic | ------- |
| Aortic valve | Annulus = 15mm | Tricuspid | Trivial TR, PPG = 14mmHg |
| Pulmonary valve | Annulus = 17mm | pulmonic | -------- |
| **Great arteries** | NRGA | **Aortic arch** | Left. No CoA. |
| Aorta | ----- | **PDA** | No |
| Pulmonary artery | Normal MPA and Branch PAs. |  |  |
| **M-Mode:** | | | |
| AO | mm | PWd | mm |
| LA | mm | PWs | mm |
| LVIDd | mm | EDV | ml |
| LVIDs | mm | ESV | ml |
| IVSs | mm | LVEF | 64% |
| IVSd | mm | FS | 34% |
| **Additional Information**: |  | | |
| No pericardial/Pleural effusion. | | | |
| **Final Diagnosis:** | | | |
| 1. Normal Echocardiography Study. | | | |
| **Remark**: | | | |
| **Recommendation**: | | | |
| SIGNATURE  Done by: Tesfaye T., Pediatrician, Pediatric Cardiologist _______________ 30/03/2015Eth.C | | | |

| Patient Name: **Bethelihem Desalegn**. Referring Institute: **Adinas GH**. SEX/ Age: **F/5 4/12**. Date of Report: **30/03/15**.  Referral Diagnosis: **S/P PDA Ligation (a month back). AGH2.085.** | | | |
| --- | --- | --- | --- |
| **Features** | **Finding** | **Features** | **Finding** |
| **Profile** |  | **Atria** |  |
| Abdominal situs | Solitus | Left atrium | Normal |
| Cardiac position | Levocardia | Right atrium | Normal |
| Systemic venous drainage | Normal. | **Atrioventricular valves** |  |
| Pulmonary venous drainage | Normal | Mitral valve | Annulus = 17mm |
| Atrioventricular connection | Concordant | Tricuspid valve | Annulus = 18mm  TAPSE = 22mm |
| Ventriculoarterial connection | Concordant | **Ventricles** |  |
| Ventricular loop | d-Loop | Left ventricle | Normal |
|  |  | Right ventricle | Normal |
| **Septae** |  | **Coronary arteries** | ----- |
| Interventricular septum | Intact | **Doppler Measurement** |  |
| Interatrial septum | Intact | Mitral | ----- |
| **Semilunar valves** |  | Aortic | ------- |
| Aortic valve | Annulus = 15mm | Tricuspid | ------- |
| Pulmonary valve | Annulus = 18mm | pulmonic | -------- |
| **Great arteries** | NRGA | **Aortic arch** | Left. No CoA. |
| Aorta | ----- | **PDA** | No |
| Pulmonary artery | Unobstructed Blood flow across MPA and Branch PAs |  |  |
| **M-Mode:** | | | |
| AO | mm | PWd | mm |
| LA | mm | PWs | mm |
| LVIDd | mm | EDV | ml |
| LVIDs | mm | ESV | ml |
| IVSs | mm | LVEF | 61% |
| IVSd | mm | FS | 32% |
| **Additional Information**: |  | | |
| No pericardial/Pleural effusion. | | | |
| **Final Diagnosis:** | | | |
| 1. {S, D, S} Levocardia. 2. S/P PDA Surgical Ligation (a month back) 3. No Residual PDA 4. Unobstructed Blood Flow across MPA & Branch PAs. 5. Normal Biventricular Systolic Function | | | |
| **Remark**: | | | |
| **Recommendation**: | | | |
| SIGNATURE  Done by: Tesfaye T., Pediatrician, Pediatric Cardiologist _______________ 30/03/2015Eth.C | | | |

| Patient Name: **Haile-Yesus Dawit**. Referring Institute: **FHRH**. SEX/ Age: **M/8years**. Date of Report: **01/04/15**.  Referral Diagnosis: **Palpitation. AGH2.086.** | | | |
| --- | --- | --- | --- |
| **Features** | **Finding** | **Features** | **Finding** |
| **Profile** |  | **Atria** |  |
| Abdominal situs | Solitus | Left atrium | Normal |
| Cardiac position | Levocardia | Right atrium | Normal |
| Systemic venous drainage | Normal. | **Atrioventricular valves** |  |
| Pulmonary venous drainage | Normal | Mitral valve | Annulus = mm |
| Atrioventricular connection | Concordant | Tricuspid valve | Annulus = mm  TAPSE = mm |
| Ventriculoarterial connection | Concordant | **Ventricles** |  |
| Ventricular loop | d-Loop | Left ventricle | Normal |
|  |  | Right ventricle | Normal |
| **Septae** |  | **Coronary arteries** | ----- |
| Interventricular septum | Intact | **Doppler Measurement** |  |
| Interatrial septum | Intact | Mitral | ----- |
| **Semilunar valves** |  | Aortic | ------- |
| Aortic valve | Annulus = 15mm | Tricuspid | ------- |
| Pulmonary valve | Annulus = 16mm | pulmonic | Trivial PR, PPG = 7mmHg |
| **Great arteries** | NRGA | **Aortic arch** | Left. No CoA. |
| Aorta | ----- | **PDA** | No |
| Pulmonary artery | Normal MPA and Branch PAs. |  |  |
| **M-Mode:** | | | |
| AO | mm | PWd | 6mm |
| LA | mm | PWs | 8mm |
| LVIDd | 40mm | EDV | 72ml |
| LVIDs | 28mm | ESV | 28ml |
| IVSs | 8mm | LVEF | 61% |
| IVSd | 7mm | FS | 32% |
| **Additional Information**: |  | | |
| No pericardial/Pleural effusion. | | | |
| **Final Diagnosis:** | | | |
| 1. Normal Echocardiography Study. | | | |
| **Remark**: | | | |
| **Recommendation**: Needs serial echocardiography Follow up. | | | |
| SIGNATURE  Done by: Tesfaye T., Pediatrician, Pediatric Cardiologist _______________ 01/04/2015Eth.C | | | |

| Patient Name: **Mulusew Tazebew**. Referring Institute: **Amaris PSC**. SEX/ Age: **M/4months**. Date of Report: **03/04/15**.  Referral Diagnosis: **DS. AGH2.087.** | | | |
| --- | --- | --- | --- |
| **Features** | **Finding** | **Features** | **Finding** |
| **Profile** |  | **Atria** |  |
| Abdominal situs | Solitus | Left atrium | Normal |
| Cardiac position | Levocardia | Right atrium | Normal |
| Systemic venous drainage | Normal. | **Atrioventricular valves** |  |
| Pulmonary venous drainage | Normal | Mitral valve | Annulus = 12mm |
| Atrioventricular connection | Concordant | Tricuspid valve | Annulus = 13mm |
| Ventriculoarterial connection | Concordant | **Ventricles** |  |
| Ventricular loop | d-Loop | Left ventricle | Normal |
|  |  | Right ventricle | Normal |
| **Septae** |  | **Coronary arteries** | ----- |
| Interventricular septum | Intact | **Doppler Measurement** |  |
| Interatrial septum | Intact | Mitral | ----- |
| **Semilunar valves** |  | Aortic | ------- |
| Aortic valve | Annulus = 10mm | Tricuspid | ------- |
| Pulmonary valve | Annulus = 10mm | pulmonic | -------- |
| **Great arteries** | NRGA | **Aortic arch** | Left. No CoA. |
| Aorta | ----- | **PDA** | No |
| Pulmonary artery | Normal MPA and Branch PAs. |  |  |
| **M-Mode:**  Normal LV Function on eye balling | | | |
| AO | mm | PWd | mm |
| LA | mm | PWs | mm |
| LVIDd | mm | EDV | ml |
| LVIDs | mm | ESV | ml |
| IVSs | mm | LVEF | % |
| IVSd | mm | FS | % |
| **Additional Information**: |  | | |
| No pericardial/Pleural effusion. | | | |
| **Final Diagnosis:** | | | |
| 1. Normal Echocardiography Study. | | | |
| **Remark**: | | | |
| **Recommendation**: | | | |
| SIGNATURE  Done by: Tesfaye T., Pediatrician, Pediatric Cardiologist _______________ 03/04/2015Eth.C | | | |

| Patient Name: **Abeselom Temesgen**. Referring Institute: **Adinas GH**. SEX/ Age: **M/5 5/12**. Date of Report: **03/04/15**.  Referral Diagnosis: **_______. AGH2.088.** | | | |
| --- | --- | --- | --- |
| **Features** | **Finding** | **Features** | **Finding** |
| **Profile** |  | **Atria** |  |
| Abdominal situs | Solitus | Left atrium | Normal |
| Cardiac position | Levocardia | Right atrium | Dilated |
| Systemic venous drainage | Normal. | **Atrioventricular valves** |  |
| Pulmonary venous drainage | Normal | Mitral valve | Annulus = 14mm |
| Atrioventricular connection | Concordant | Tricuspid valve | Annulus = 21mm |
| Ventriculoarterial connection | Concordant | **Ventricles** |  |
| Ventricular loop | d-Loop | Left ventricle | Normal |
|  |  | Right ventricle | Dilated & Hypertrophied |
| **Septae** |  | **Coronary arteries** | ----- |
| Interventricular septum | Non-Restrictive Mal-aligned Sub-Aortic VSD, R – L Shunt. | **Doppler Measurement** |  |
| Interatrial septum | Intact | Mitral | ----- |
| **Semilunar valves** |  | Aortic | Mild AR |
| Aortic valve | Annulus = 17mm | Tricuspid | ------- |
| Pulmonary valve | Annulus = 9mm | pulmonic | Severe Valvular, Sub-Valvular & Supra-Valvular PS, PPG = 73mmHg. |
| **Great arteries** | NRGA | **Aortic arch** | No CoA. |
| Aorta | Over-riding aorta | **PDA** | No |
| Pulmonary artery | Smallish MPA and Branch PAs. |  |  |
| **M-Mode:**  Normal LV Function on eye balling | | | |
| AO | mm | PWd | mm |
| LA | mm | PWs | mm |
| LVIDd | mm | EDV | ml |
| LVIDs | mm | ESV | ml |
| IVSs | mm | LVEF | % |
| IVSd | mm | FS | % |
| **Additional Information**: |  | | |
| No pericardial/Pleural effusion. | | | |
| **Final Diagnosis:** | | | |
| 1. {S, D, S} Levocardia. 2. TOF 3. Smallish MPA | | | |
| **Remark**: | | | |
| **Recommendation**: | | | |
| SIGNATURE  Done by: Tesfaye T., Pediatrician, Pediatric Cardiologist _______________ 03/04/2015Eth.C | | | |

| Patient Name: **Mehammed Degarege**. Referring Institute: **TGSH**. SEX/ Age: **M/2months**. Date of Report: **03/04/15**.  Referral Diagnosis: **DS. AGH2.089** | | | |
| --- | --- | --- | --- |
| **Features** | **Finding** | **Features** | **Finding** |
| **Profile** |  | **Atria** |  |
| Abdominal situs | Solitus | Left atrium | Normal |
| Cardiac position | Levocardia | Right atrium | Normal |
| Systemic venous drainage | Normal. | **Atrioventricular valves** |  |
| Pulmonary venous drainage | Normal | Mitral valve | Annulus = 12mm |
| Atrioventricular connection | Concordant | Tricuspid valve | Annulus = 12mm |
| Ventriculoarterial connection | Concordant | **Ventricles** |  |
| Ventricular loop | d-Loop | Left ventricle | Normal |
|  |  | Right ventricle | Normal |
| **Septae** |  | **Coronary arteries** | ----- |
| Interventricular septum | Intact | **Doppler Measurement** |  |
| Interatrial septum | PFO, L – R Shunt | Mitral | ----- |
| **Semilunar valves** |  | Aortic | ------- |
| Aortic valve | Annulus = 11mm | Tricuspid | ------- |
| Pulmonary valve | Annulus = 13mm | pulmonic | -------- |
| **Great arteries** | NRGA | **Aortic arch** | Left. No CoA. |
| Aorta | ----- | **PDA** | <1mm PDA, L – R Shunt |
| Pulmonary artery | Normal MPA and Branch PAs. |  |  |
| **M-Mode:**  Normal LV Function on eye balling | | | |
| AO | mm | PWd | mm |
| LA | mm | PWs | mm |
| LVIDd | mm | EDV | ml |
| LVIDs | mm | ESV | ml |
| IVSs | mm | LVEF | % |
| IVSd | mm | FS | % |
| **Additional Information**: |  | | |
| No pericardial/Pleural effusion. | | | |
| **Final Diagnosis:** | | | |
| 1. {S, D, S} Levocardia. 2. PFO, L – R Shunt 3. Silent PDA, L – R Shunt | | | |
| **Remark**: | | | |
| **Recommendation**: | | | |
| SIGNATURE  Done by: Tesfaye T., Pediatrician, Pediatric Cardiologist _______________ 03/04/2015Eth.C | | | |

| Patient Name: **Yemengist Werku**. Referring Institute: **Addis Alem PH**. SEX/ Age: **F/8years**. Date of Report: **04/04/15**.  Referral Diagnosis: **ARF. AGH2.090** | | | |
| --- | --- | --- | --- |
| **Features** | **Finding** | **Features** | **Finding** |
| **Profile** |  | **Atria** |  |
| Abdominal situs | Solitus | Left atrium | Mildly Dilated |
| Cardiac position | Levocardia | Right atrium | Normal |
| Systemic venous drainage | Normal. | **Atrioventricular valves** |  |
| Pulmonary venous drainage | Normal | Mitral valve | Annulus = 25mm. Patulous, elongated MVL. |
| Atrioventricular connection | Concordant | Tricuspid valve | Annulus = 21mm. TAPSE = 20mm |
| Ventriculoarterial connection | Concordant | **Ventricles** |  |
| Ventricular loop | d-Loop | Left ventricle | Mildly Dilated |
|  |  | Right ventricle | Normal. RV TDI S wave = 16cm/sec. |
| **Septae** |  | **Coronary arteries** | ----- |
| Interventricular septum | Intact | **Doppler Measurement** |  |
| Interatrial septum | Intact | Mitral | Moderate MR, Holosystolic, posterior projection, seen in two planes with jet velocity = 4.2m/sec. |
| **Semilunar valves** |  | Aortic | ------- |
| Aortic valve | Annulus = 16mm | Tricuspid | Trivial TR, PPG = 18mmHg |
| Pulmonary valve | Annulus = 18mm | pulmonic | -------- |
| **Great arteries** | NRGA | **Aortic arch** | Left. No CoA. |
| Aorta | ----- | **PDA** | No |
| Pulmonary artery | Normal MPA and Branch PAs. |  |  |
| **M-Mode:** | | | |
| AO | mm | PWd | mm |
| LA | mm | PWs | mm |
| LVIDd | mm | EDV | ml |
| LVIDs | mm | ESV | ml |
| IVSs | mm | LVEF | 63% |
| IVSd | mm | FS | 34% |
| **Additional Information**: |  | | |
| No pericardial/Pleural effusion. | | | |
| **Final Diagnosis:** | | | |
| 1. {S, D, S} Levocardia. 2. LA/LV Mildly Dilated 3. Patulous, elongated MVL 4. Moderate MR 5. Trivial TR 6. Normal Biventricular Systolic Function | | | |
| **Remark**: | | | |
| **Recommendation**: | | | |
| SIGNATURE  Done by: Tesfaye T., Pediatrician, Pediatric Cardiologist _______________ 04/04/2015Eth.C | | | |

| Patient Name: **Fanuel Solomon**. Referring Institute: **Adinas GH**. SEX/ Age: **M/9 4/12**. Date of Report: **04/04/15**.  Referral Diagnosis: **S/P Partial AVSD Surgical Closure. AGH2.091** | | | |
| --- | --- | --- | --- |
| **Features** | **Finding** | **Features** | **Finding** |
| **Profile** |  | **Atria** |  |
| Abdominal situs | Solitus | Left atrium | Normal |
| Cardiac position | Levocardia | Right atrium | Normal |
| Systemic venous drainage | Normal. | **Atrioventricular valves** |  |
| Pulmonary venous drainage | Normal | Mitral valve | Annulus = 20mm |
| Atrioventricular connection | Concordant | Tricuspid valve | Annulus = 20mm |
| Ventriculoarterial connection | Concordant | **Ventricles** |  |
| Ventricular loop | d-Loop | Left ventricle | Normal |
|  |  | Right ventricle | Normal. RV TDI S wave = 19cm/sec. |
| **Septae** |  | **Coronary arteries** | ----- |
| Interventricular septum | Intact | **Doppler Measurement** |  |
| Interatrial septum | Patch Intact. No Residual ASD | Mitral | ----- |
| **Semilunar valves** |  | Aortic | ------- |
| Aortic valve | Annulus = 15mm | Tricuspid | ------- |
| Pulmonary valve | Annulus = 18mm | pulmonic | -------- |
| **Great arteries** | NRGA | **Aortic arch** | Left. No CoA. |
| Aorta | ----- | **PDA** | No |
| Pulmonary artery | Normal MPA and Branch PAs. |  |  |
| **M-Mode:** | | | |
| AO | mm | PWd | mm |
| LA | mm | PWs | mm |
| LVIDd | mm | EDV | ml |
| LVIDs | mm | ESV | ml |
| IVSs | mm | LVEF | 64% |
| IVSd | mm | FS | 34% |
| **Additional Information**: |  | | |
| No pericardial/Pleural effusion. | | | |
| **Final Diagnosis:** | | | |
| 1. S/P Partial AVSD Surgical Closure 2. {S, D, S} Levocardia. 3. ASD patch Intact 4. No residual ASD 5. Normal Biventricular Systolic Function | | | |
| **Remark**: | | | |
| **Recommendation**: | | | |
| SIGNATURE  Done by: Tesfaye T., Pediatrician, Pediatric Cardiologist _______________ 04/04/2015Eth.C | | | |

| Patient Name: **Oumer Ali**. Referring Institute: **Adinas GH**. SEX/ Age: **M/4years**. Date of Report: **04/04/15**.  Referral Diagnosis: **Recurrent Chest Infection. AGH2.092** | | | |
| --- | --- | --- | --- |
| **Features** | **Finding** | **Features** | **Finding** |
| **Profile** |  | **Atria** |  |
| Abdominal situs | Solitus | Left atrium | Normal |
| Cardiac position | Levocardia | Right atrium | Normal |
| Systemic venous drainage | Normal. | **Atrioventricular valves** |  |
| Pulmonary venous drainage | Normal | Mitral valve | Annulus = 15mm |
| Atrioventricular connection | Concordant | Tricuspid valve | Annulus = 16mm  TAPSE = 18mm |
| Ventriculoarterial connection | Concordant | **Ventricles** |  |
| Ventricular loop | d-Loop | Left ventricle | Normal |
|  |  | Right ventricle | Normal |
| **Septae** |  | **Coronary arteries** | ----- |
| Interventricular septum | Intact | **Doppler Measurement** |  |
| Interatrial septum | Intact | Mitral | ----- |
| **Semilunar valves** |  | Aortic | ------- |
| Aortic valve | Annulus = 15mm | Tricuspid | ------- |
| Pulmonary valve | Annulus = 16mm | pulmonic | -------- |
| **Great arteries** | NRGA | **Aortic arch** | Left. No CoA. |
| Aorta | ----- | **PDA** | No |
| Pulmonary artery | Normal MPA and Branch PAs. |  |  |
| **M-Mode:** | | | |
| AO | mm | PWd | mm |
| LA | mm | PWs | mm |
| LVIDd | mm | EDV | ml |
| LVIDs | mm | ESV | ml |
| IVSs | mm | LVEF | 68% |
| IVSd | mm | FS | 37% |
| **Additional Information**: |  | | |
| No pericardial/Pleural effusion. | | | |
| **Final Diagnosis:** | | | |
| 1. Normal Echocardiography Study. | | | |
| **Remark**: | | | |
| **Recommendation**: | | | |
| SIGNATURE  Done by: Tesfaye T., Pediatrician, Pediatric Cardiologist _______________ 04/04/2015Eth.C | | | |

| Patient Name: **Ananya Tesfahun**. Referring Institute: **Adinas GH**. SEX/ Age: **M/8years**. Date of Report: **04/04/15**.  Referral Diagnosis: **Easy fatigability. AGH2.093** | | | |
| --- | --- | --- | --- |
| **Features** | **Finding** | **Features** | **Finding** |
| **Profile** |  | **Atria** |  |
| Abdominal situs | Solitus | Left atrium | Normal |
| Cardiac position | Levocardia | Right atrium | Normal |
| Systemic venous drainage | Normal. | **Atrioventricular valves** |  |
| Pulmonary venous drainage | Normal | Mitral valve | Annulus = 18mm |
| Atrioventricular connection | Concordant | Tricuspid valve | Annulus = 19mm  TAPSE = 18mm |
| Ventriculoarterial connection | Concordant | **Ventricles** |  |
| Ventricular loop | d-Loop | Left ventricle | Normal |
|  |  | Right ventricle | Normal |
| **Septae** |  | **Coronary arteries** | ----- |
| Interventricular septum | Intact | **Doppler Measurement** |  |
| Interatrial septum | Intact | Mitral | ----- |
| **Semilunar valves** |  | Aortic | ------- |
| Aortic valve | Annulus = 14mm | Tricuspid | ------- |
| Pulmonary valve | Annulus = 16mm | pulmonic | -------- |
| **Great arteries** | NRGA | **Aortic arch** | Left. No CoA. |
| Aorta | ----- | **PDA** | No |
| Pulmonary artery | Normal MPA and Branch PAs. |  |  |
| **M-Mode:** | | | |
| AO | mm | PWd | mm |
| LA | mm | PWs | mm |
| LVIDd | mm | EDV | ml |
| LVIDs | mm | ESV | ml |
| IVSs | mm | LVEF | 67% |
| IVSd | mm | FS | 36% |
| **Additional Information**: |  | | |
| No pericardial/Pleural effusion. | | | |
| **Final Diagnosis:** | | | |
| 1. Normal Echocardiography Study. | | | |
| **Remark**: | | | |
| **Recommendation**: | | | |
| SIGNATURE  Done by: Tesfaye T., Pediatrician, Pediatric Cardiologist _______________ 04/04/2015Eth.C | | | |

| Patient Name: **Mekdelawit Temesgen**. Referring Institute: **Adinas GH**. SEX/ Age: **F/8 9/12**. Date of Report: **05/04/15**.  Referral Diagnosis: **Palpitation. AGH2.094.** | | | |
| --- | --- | --- | --- |
| **Features** | **Finding** | **Features** | **Finding** |
| **Profile** |  | **Atria** |  |
| Abdominal situs | Solitus | Left atrium | Normal |
| Cardiac position | Levocardia | Right atrium | Dilated |
| Systemic venous drainage | Normal. | **Atrioventricular valves** |  |
| Pulmonary venous drainage | Normal | Mitral valve | Annulus = 19mm |
| Atrioventricular connection | Concordant | Tricuspid valve | Annulus = 24mm  TAPSE = 23mm |
| Ventriculoarterial connection | Concordant | **Ventricles** |  |
| Ventricular loop | d-Loop | Left ventricle | Normal |
|  |  | Right ventricle | Dilated. RV TDI S wave = 17cm/sec. |
| **Septae** |  | **Coronary arteries** | ----- |
| Interventricular septum | Intact | **Doppler Measurement** |  |
| Interatrial septum | 19mm X 18mm OS ASD, L – R Shunt | Mitral | ----- |
| **Semilunar valves** |  | Aortic | ------- |
| Aortic valve | Annulus = 15mm | Tricuspid | ------- |
| Pulmonary valve | Annulus = 23mm. Doming PV | pulmonic | Mild Valvular PS, PPG = 22mmHg. Trivial PR, PPG = 11mmHg |
| **Great arteries** | NRGA | **Aortic arch** | Left. No CoA. |
| Aorta | ----- | **PDA** | No |
| Pulmonary artery | Normal MPA and Branch PAs. |  |  |
| **M-Mode:** | | | |
| AO | mm | PWd | mm |
| LA | mm | PWs | mm |
| LVIDd | mm | EDV | ml |
| LVIDs | mm | ESV | ml |
| IVSs | mm | LVEF | 69% |
| IVSd | mm | FS | 37% |
| **Additional Information**: |  | | |
| No pericardial/Pleural effusion. | | | |
| **Final Diagnosis:** | | | |
| 1. {S, D, S} Levocardia. 2. RA/RV Dilated 3. Large OS ASD, L – R Shunt 4. Mild Valvular PS 5. Doming Pulmonary Valve 6. Normal Biventricular Systolic Function | | | |
| **Remark**: | | | |
| **Recommendation**: | | | |
| SIGNATURE  Done by: Tesfaye T., Pediatrician, Pediatric Cardiologist _______________ 05/04/2015Eth.C | | | |

| Patient Name: **Tigist Tigab**. Referring Institute: **Ghion Medium Clinic**. SEX/ Age: **F/3months**. Date of Report: **06/04/15**.  Referral Diagnosis: **Incidental Murmur Finding. AGH2.095** | | | |
| --- | --- | --- | --- |
| **Features** | **Finding** | **Features** | **Finding** |
| **Profile** |  | **Atria** |  |
| Abdominal situs | Solitus | Left atrium | Normal |
| Cardiac position | Levocardia | Right atrium | Normal |
| Systemic venous drainage | Normal. | **Atrioventricular valves** |  |
| Pulmonary venous drainage | Normal | Mitral valve | Annulus = 14mm |
| Atrioventricular connection | Concordant | Tricuspid valve | Annulus = 13mm |
| Ventriculoarterial connection | Concordant | **Ventricles** |  |
| Ventricular loop | d-Loop | Left ventricle | Normal |
|  |  | Right ventricle | Normal |
| **Septae** |  | **Coronary arteries** | ----- |
| Interventricular septum | 3mm PM VSD, L – R Shunt with PPG = 57mmHg | **Doppler Measurement** |  |
| Interatrial septum | PFO, L – R Shunt | Mitral | ----- |
| **Semilunar valves** |  | Aortic | ------- |
| Aortic valve | Annulus = 11mm | Tricuspid | ------- |
| Pulmonary valve | Annulus = 12mm | pulmonic | -------- |
| **Great arteries** | NRGA | **Aortic arch** | Left. No CoA. |
| Aorta | ----- | **PDA** | No |
| Pulmonary artery | Normal MPA and Branch PAs. |  |  |
| **M-Mode:**  Normal LV Function on eye balling | | | |
| AO | mm | PWd | mm |
| LA | mm | PWs | mm |
| LVIDd | mm | EDV | ml |
| LVIDs | mm | ESV | ml |
| IVSs | mm | LVEF | % |
| IVSd | mm | FS | % |
| **Additional Information**: |  | | |
| No pericardial/Pleural effusion. | | | |
| **Final Diagnosis:** | | | |
| 1. {S, D, S} Levocardia. 2. PFO,L – R Shunt 3. Small PM VSD, L – R Shunt | | | |
| **Remark**: | | | |
| **Recommendation**: | | | |
| SIGNATURE  Done by: Tesfaye T., Pediatrician, Pediatric Cardiologist _______________ 06/04/2015Eth.C | | | |

| Patient Name: **Sofia Ebabu**. Referring Institute: **Eyasta MS plc.**. SEX/ Age: **F/12years**. Date of Report: **06/04/15**.  Referral Diagnosis: **Chest pain. AGH2.096** | | | |
| --- | --- | --- | --- |
| **Features** | **Finding** | **Features** | **Finding** |
| **Profile** |  | **Atria** |  |
| Abdominal situs | Solitus | Left atrium | Normal |
| Cardiac position | Levocardia | Right atrium | Normal |
| Systemic venous drainage | Normal. | **Atrioventricular valves** |  |
| Pulmonary venous drainage | Normal | Mitral valve | Annulus = 24mm |
| Atrioventricular connection | Concordant | Tricuspid valve | Annulus = 23mm |
| Ventriculoarterial connection | Concordant | **Ventricles** |  |
| Ventricular loop | d-Loop | Left ventricle | Normal |
|  |  | Right ventricle | Normal |
| **Septae** |  | **Coronary arteries** | ----- |
| Interventricular septum | Intact | **Doppler Measurement** |  |
| Interatrial septum | Intact | Mitral | ----- |
| **Semilunar valves** |  | Aortic | ------- |
| Aortic valve | Annulus = 19mm | Tricuspid | ------- |
| Pulmonary valve | Annulus = 21mm | pulmonic | -------- |
| **Great arteries** | NRGA | **Aortic arch** | Left. No CoA. |
| Aorta | ----- | **PDA** | No |
| Pulmonary artery | Normal MPA and Branch PAs. |  |  |
| **M-Mode:** | | | |
| AO | mm | PWd | mm |
| LA | mm | PWs | mm |
| LVIDd | mm | EDV | ml |
| LVIDs | mm | ESV | ml |
| IVSs | mm | LVEF | 52% |
| IVSd | mm | FS | 26% |
| **Additional Information**: |  | | |
| Circumferential pericardial effusion with echo-debris and multiple septation measuring maximum depth of 27mm on LV Side.. | | | |
| **Final Diagnosis:** | | | |
| 1. {S, D, S} Levocardia. 2. Large Septated Circumferential pericardial effusion 3. Reduced LV Systolic Function | | | |
| **Remark**: | | | |
| **Recommendation**: Needs drainage | | | |
| SIGNATURE  Done by: Tesfaye T., Pediatrician, Pediatric Cardiologist _______________ 06/04/2015Eth.C | | | |

| Patient Name: **Abi Getachew**. Referring Institute: **Debre-Markos RH**. SEX/ Age: **M/5months**. Date of Report: **06/04/15**.  Referral Diagnosis: **Cyanosis. AGH2.097.** | | | |
| --- | --- | --- | --- |
| **Features** | **Finding** | **Features** | **Finding** |
| **Profile** |  | **Atria** |  |
| Abdominal situs | Solitus | Left atrium | Normal |
| Cardiac position | Levocardia | Right atrium | Normal |
| Systemic venous drainage | Normal. | **Atrioventricular valves** |  |
| Pulmonary venous drainage | Normal | Mitral valve | Annulus = 13mm |
| Atrioventricular connection | Concordant | Tricuspid valve | Annulus = 14mm |
| Ventriculoarterial connection | Discordant | **Ventricles** |  |
| Ventricular loop | d-Loop | Left ventricle | Normal |
|  |  | Right ventricle | Normal. RV TDI S wave = 15cm/sec |
| **Septae** |  | **Coronary arteries** | ----- |
| Interventricular septum | 5mm Sub-pulmonic VSD, BD Shunt | **Doppler Measurement** |  |
| Interatrial septum | Intact | Mitral | ----- |
| **Semilunar valves** |  | Aortic | ------- |
| Aortic valve | Annulus = 12mm | Tricuspid | ------- |
| Pulmonary valve | Annulus = 10mm | pulmonic | -------- |
| **Great arteries** | d-TGA | **Aortic arch** | Left. No CoA. |
| Aorta | Anterior & from RV | **PDA** | No |
| Pulmonary artery | Posterior & from LV |  |  |
| **M-Mode:** | | | |
| AO | mm | PWd | mm |
| LA | mm | PWs | mm |
| LVIDd | mm | EDV | ml |
| LVIDs | mm | ESV | ml |
| IVSs | mm | LVEF | % |
| IVSd | mm | FS | % |
| **Additional Information**: |  | | |
| No pericardial/Pleural effusion. | | | |
| **Final Diagnosis:** | | | |
| 1. {S, D, D} Levocardia. 2. d-TGA with Non-Restrictive VSD, BD Shunt | | | |
| **Remark**: | | | |
| **Recommendation**: | | | |
| SIGNATURE  Done by: Tesfaye T., Pediatrician, Pediatric Cardiologist _______________ 06/04/2015Eth.C | | | |

| Patient Name: **Esubalew Tadele**. Referring Institute: **FHRH**. SEX/ Age: **M/10years**. Date of Report: **06/04/15**.  Referral Diagnosis: **Cyanosis. AGH2.098** | | | |
| --- | --- | --- | --- |
| **Features** | **Finding** | **Features** | **Finding** |
| **Profile** |  | **Atria** |  |
| Abdominal situs | Solitus | Left atrium | Normal |
| Cardiac position | Levocardia | Right atrium | Dilated |
| Systemic venous drainage | Normal. | **Atrioventricular valves** |  |
| Pulmonary venous drainage | Normal | Mitral valve | Annulus = 16mm |
| Atrioventricular connection | Concordant | Tricuspid valve | Annulus = 21mm  TAPSE = 17mm |
| Ventriculoarterial connection | Concordant | **Ventricles** |  |
| Ventricular loop | d-Loop | Left ventricle | Normal |
|  |  | Right ventricle | Dilated & Hypertrophied |
| **Septae** |  | **Coronary arteries** | ----- |
| Interventricular septum | Mal-Aligned Sub-aortic Non-Restrictive VSD, R – L Shunt. | **Doppler Measurement** |  |
| Interatrial septum | Intact | Mitral | ----- |
| **Semilunar valves** |  | Aortic | ------- |
| Aortic valve | Annulus = 15mm | Tricuspid | ------- |
| Pulmonary valve | Annulus = 7mm | pulmonic | -------- |
| **Great arteries** | NRGA | **Aortic arch** | Left. No CoA. |
| Aorta | Over-riding aorta | **PDA** | No |
| Pulmonary artery | Smallish MPA (5mm). Branch PAs not visualized. |  |  |
| **M-Mode:** | | | |
| AO | mm | PWd | mm |
| LA | mm | PWs | mm |
| LVIDd | mm | EDV | ml |
| LVIDs | mm | ESV | ml |
| IVSs | mm | LVEF | % |
| IVSd | mm | FS | % |
| **Additional Information**: |  | | |
| No pericardial/Pleural effusion. | | | |
| **Final Diagnosis:** | | | |
| 1. {S, D, S} Levocardia. 2. TOF 3. Smallish MPA | | | |
| **Remark**: | | | |
| **Recommendation**: | | | |
| SIGNATURE  Done by: Tesfaye T., Pediatrician, Pediatric Cardiologist _______________ 06/04/2015Eth.C | | | |

| Patient Name: **Mehariw Anteneh**. Referring Institute: **TGSH**. SEX/ Age: **M/49days**. Date of Report: **07/04/15**.  Referral Diagnosis: **DS. AGH2.099** | | | |
| --- | --- | --- | --- |
| **Features** | **Finding** | **Features** | **Finding** |
| **Profile** |  | **Atria** |  |
| Abdominal situs | Solitus | Left atrium | Normal |
| Cardiac position | Levocardia | Right atrium | Normal |
| Systemic venous drainage | Normal. | **Atrioventricular valves** |  |
| Pulmonary venous drainage | Normal | Mitral valve | Annulus = 10mm |
| Atrioventricular connection | Concordant | Tricuspid valve | Annulus = 9mm |
| Ventriculoarterial connection | Concordant | **Ventricles** |  |
| Ventricular loop | d-Loop | Left ventricle | Normal |
|  |  | Right ventricle | Normal |
| **Septae** |  | **Coronary arteries** | ----- |
| Interventricular septum | Intact | **Doppler Measurement** |  |
| Interatrial septum | 5mm OS ASD, L – R Shunt | Mitral | ----- |
| **Semilunar valves** |  | Aortic | ------- |
| Aortic valve | Annulus = 9mm | Tricuspid | ------- |
| Pulmonary valve | Annulus = 9mm | pulmonic | -------- |
| **Great arteries** | NRGA | **Aortic arch** | Left. No CoA. |
| Aorta | ----- | **PDA** | No |
| Pulmonary artery | Normal MPA and Branch PAs. |  |  |
| **M-Mode:**  Normal LV Function on eye balling | | | |
| AO | mm | PWd | mm |
| LA | mm | PWs | mm |
| LVIDd | mm | EDV | ml |
| LVIDs | mm | ESV | ml |
| IVSs | mm | LVEF | % |
| IVSd | mm | FS | % |
| **Additional Information**: |  | | |
| No pericardial/Pleural effusion. | | | |
| **Final Diagnosis:** | | | |
| 1. {S, D, S} Levocardia. 2. Small OS ASD, L – R Shunt | | | |
| **Remark**: | | | |
| **Recommendation**: | | | |
| SIGNATURE  Done by: Tesfaye T., Pediatrician, Pediatric Cardiologist _______________ 07/04/2015Eth.C | | | |

| Patient Name: **Yehualaw Abebe**. Referring Institute: **Adinas GH**. SEX/ Age: **M/8 9/12**. Date of Report: **07/04/15**.  Referral Diagnosis: **Easy fatigability. AGH2.100** | | | |
| --- | --- | --- | --- |
| **Features** | **Finding** | **Features** | **Finding** |
| **Profile** |  | **Atria** |  |
| Abdominal situs | Solitus | Left atrium | Normal |
| Cardiac position | Levocardia | Right atrium | Normal |
| Systemic venous drainage | Normal. | **Atrioventricular valves** |  |
| Pulmonary venous drainage | Normal | Mitral valve | Annulus = 22mm |
| Atrioventricular connection | Concordant | Tricuspid valve | Annulus = 24mm  TAPSE = 20mm |
| Ventriculoarterial connection | Concordant | **Ventricles** |  |
| Ventricular loop | d-Loop | Left ventricle | Normal |
|  |  | Right ventricle | Normal |
| **Septae** |  | **Coronary arteries** | ----- |
| Interventricular septum | Intact | **Doppler Measurement** |  |
| Interatrial septum | Intact | Mitral | ----- |
| **Semilunar valves** |  | Aortic | ------- |
| Aortic valve | Annulus = 16mm | Tricuspid | ------- |
| Pulmonary valve | Annulus = 18mm | pulmonic | -------- |
| **Great arteries** | NRGA | **Aortic arch** | Left. No CoA. |
| Aorta | ----- | **PDA** | No |
| Pulmonary artery | Normal MPA and Branch PAs. |  |  |
| **M-Mode:** | | | |
| AO | mm | PWd | mm |
| LA | mm | PWs | mm |
| LVIDd | mm | EDV | ml |
| LVIDs | mm | ESV | ml |
| IVSs | mm | LVEF | 66% |
| IVSd | mm | FS | 36% |
| **Additional Information**: |  | | |
| No pericardial/Pleural effusion. | | | |
| **Final Diagnosis:** | | | |
| 1. Normal Echocardiography Study. | | | |
| **Remark**: | | | |
| **Recommendation**: | | | |
| SIGNATURE  Done by: Tesfaye T., Pediatrician, Pediatric Cardiologist _______________ 07/04/2015Eth.C | | | |

| Patient Name: **Alemu Dessie**. Referring Institute: **TGSH**. SEX/ Age: **M/4 6/12**. Date of Report: **07/04/15**.  Referral Diagnosis: **DS. AGH2.101** | | | |
| --- | --- | --- | --- |
| **Features** | **Finding** | **Features** | **Finding** |
| **Profile** |  | **Atria** |  |
| Abdominal situs | Solitus | Left atrium | Normal |
| Cardiac position | Levocardia | Right atrium | Normal |
| Systemic venous drainage | Normal. | **Atrioventricular valves** |  |
| Pulmonary venous drainage | Normal | Mitral valve | Annulus = 16mm |
| Atrioventricular connection | Concordant | Tricuspid valve | Annulus = 18mm  TAPSE = 18mm |
| Ventriculoarterial connection | Concordant | **Ventricles** |  |
| Ventricular loop | d-Loop | Left ventricle | Normal |
|  |  | Right ventricle | Normal |
| **Septae** |  | **Coronary arteries** | ----- |
| Interventricular septum | Intact | **Doppler Measurement** |  |
| Interatrial septum | Intact | Mitral | ----- |
| **Semilunar valves** |  | Aortic | ------- |
| Aortic valve | Annulus = 14mm | Tricuspid | Trivial TR, PPG = 25mmHg |
| Pulmonary valve | Annulus = 15mm | pulmonic | -------- |
| **Great arteries** | NRGA | **Aortic arch** | Left. No CoA. |
| Aorta | ----- | **PDA** | No |
| Pulmonary artery | Normal MPA and Branch PAs. |  |  |
| **M-Mode:** | | | |
| AO | mm | PWd | mm |
| LA | mm | PWs | mm |
| LVIDd | mm | EDV | ml |
| LVIDs | mm | ESV | ml |
| IVSs | mm | LVEF | 70% |
| IVSd | mm | FS | 38% |
| **Additional Information**: |  | | |
| No pericardial/Pleural effusion. | | | |
| **Final Diagnosis:** | | | |
| 1. Normal Echocardiography Study. | | | |
| **Remark**: | | | |
| **Recommendation**: | | | |
| SIGNATURE  Done by: Tesfaye T., Pediatrician, Pediatric Cardiologist _______________ 07/04/2015Eth.C | | | |

| Patient Name: **Baby of Mekdes Yohannes**. Referring Institute: **TGSH**. SEX/ Age: **F/3months**. Date of Report: **08/04/15**.  Referral Diagnosis: **RD. AGH2.102** | | | |
| --- | --- | --- | --- |
| **Features** | **Finding** | **Features** | **Finding** |
| **Profile** |  | **Atria** |  |
| Abdominal situs | Solitus | Left atrium | Normal |
| Cardiac position | Levocardia | Right atrium | Normal |
| Systemic venous drainage | Normal. | **Atrioventricular valves** |  |
| Pulmonary venous drainage | Normal | Mitral valve | Annulus = 11mm |
| Atrioventricular connection | Concordant | Tricuspid valve | Annulus = 12mm |
| Ventriculoarterial connection | Concordant | **Ventricles** |  |
| Ventricular loop | d-Loop | Left ventricle | Normal |
|  |  | Right ventricle | Normal |
| **Septae** |  | **Coronary arteries** | ----- |
| Interventricular septum | Intact | **Doppler Measurement** |  |
| Interatrial septum | Intact | Mitral | ----- |
| **Semilunar valves** |  | Aortic | ------- |
| Aortic valve | Annulus = 11mm | Tricuspid | ------- |
| Pulmonary valve | Annulus = 11mm | pulmonic | -------- |
| **Great arteries** | NRGA | **Aortic arch** | Left. No CoA. |
| Aorta | ----- | **PDA** | No |
| Pulmonary artery | Normal MPA and Branch PAs. |  |  |
| **M-Mode:**  Normal LV Function on eye balling. | | | |
| AO | mm | PWd | mm |
| LA | mm | PWs | mm |
| LVIDd | mm | EDV | ml |
| LVIDs | mm | ESV | ml |
| IVSs | mm | LVEF | % |
| IVSd | mm | FS | % |
| **Additional Information**: |  | | |
| No pericardial/Pleural effusion. | | | |
| **Final Diagnosis:** | | | |
| 1. Normal Echocardiography Study. | | | |
| **Remark**: | | | |
| **Recommendation**: | | | |
| SIGNATURE  Done by: Tesfaye T., Pediatrician, Pediatric Cardiologist _______________ 08/04/2015Eth.C | | | |

| Patient Name: **Hablie Kenaw**. Referring Institute: **FHRH**. SEX/ Age: **F/7years**. Date of Report: **12/04/15**.  Referral Diagnosis: **ARF/Recurrence. AGH2.103** | | | |
| --- | --- | --- | --- |
| **Features** | **Finding** | **Features** | **Finding** |
| **Profile** |  | **Atria** |  |
| Abdominal situs | Solitus | Left atrium | Dilated |
| Cardiac position | Levocardia | Right atrium | Normal |
| Systemic venous drainage | Normal. | **Atrioventricular valves** |  |
| Pulmonary venous drainage | Normal | Mitral valve | Annulus = 23mm. Thickened MVL |
| Atrioventricular connection | Concordant | Tricuspid valve | Annulus = 17mm  TAPSE = 20mm |
| Ventriculoarterial connection | Concordant | **Ventricles** |  |
| Ventricular loop | d-Loop | Left ventricle | Dilated |
|  |  | Right ventricle | Normal |
| **Septae** |  | **Coronary arteries** | ----- |
| Interventricular septum | Intact | **Doppler Measurement** |  |
| Interatrial septum | PFO, L – R Shunt | Mitral | Moderate MR, Holosystolic, posterior projection, seen in two planes with jet velocity = 4m/sec. |
| **Semilunar valves** |  | Aortic | Moderate AR |
| Aortic valve | Annulus = 16mm | Tricuspid | ------- |
| Pulmonary valve | Annulus = 16mm | pulmonic | -------- |
| **Great arteries** | NRGA | **Aortic arch** | Left. No CoA. |
| Aorta | ----- | **PDA** | No |
| Pulmonary artery | Normal MPA and Branch PAs. |  |  |
| **M-Mode:** | | | |
| AO | mm | PWd | mm |
| LA | mm | PWs | mm |
| LVIDd | mm | EDV | ml |
| LVIDs | mm | ESV | ml |
| IVSs | mm | LVEF | 66% |
| IVSd | mm | FS | 36% |
| **Additional Information**: |  | | |
| No pericardial/Pleural effusion. | | | |
| **Final Diagnosis:** | | | |
| 1. {S, D, S} Levocardia. 2. PFO, L – R Shunt 3. LA/LV Dilated 4. Thickened MVL 5. Moderate MR 6. Moderate AR 7. Normal Biventricular Systolic Function | | | |
| **Remark**: | | | |
| **Recommendation**: | | | |
| SIGNATURE  Done by: Tesfaye T., Pediatrician, Pediatric Cardiologist _______________ 12/04/2015Eth.C | | | |

| Patient Name: **Cheru-Amlak Wassie**. Referring Institute: **FHRH**. SEX/ Age: **M/14years**. Date of Report: **12/04/15**.  Referral Diagnosis: **Palpitation. AGH2.104** | | | |
| --- | --- | --- | --- |
| **Features** | **Finding** | **Features** | **Finding** |
| **Profile** |  | **Atria** |  |
| Abdominal situs | Solitus | Left atrium | Normal |
| Cardiac position | Levocardia | Right atrium | Normal |
| Systemic venous drainage | Normal. | **Atrioventricular valves** |  |
| Pulmonary venous drainage | Normal | Mitral valve | Annulus = 19mm |
| Atrioventricular connection | Concordant | Tricuspid valve | Annulus = 20mm  TAPSE = 20mm |
| Ventriculoarterial connection | Concordant | **Ventricles** |  |
| Ventricular loop | d-Loop | Left ventricle | Normal |
|  |  | Right ventricle | Normal |
| **Septae** |  | **Coronary arteries** | ----- |
| Interventricular septum | Intact | **Doppler Measurement** |  |
| Interatrial septum | Intact | Mitral | ----- |
| **Semilunar valves** |  | Aortic | ------- |
| Aortic valve | Annulus = 16mm | Tricuspid | Trivial TR, Incomplete signal, PPG = 13mmHg |
| Pulmonary valve | Annulus = 19mm | pulmonic | Trivial PR, PPG = 5mmHg |
| **Great arteries** | NRGA | **Aortic arch** | Left. No CoA. |
| Aorta | ----- | **PDA** | No |
| Pulmonary artery | Normal MPA and Branch PAs. |  |  |
| **M-Mode:** | | | |
| AO | mm | PWd | mm |
| LA | mm | PWs | mm |
| LVIDd | mm | EDV | ml |
| LVIDs | mm | ESV | ml |
| IVSs | mm | LVEF | 71% |
| IVSd | mm | FS | 39% |
| **Additional Information**: |  | | |
| No pericardial/Pleural effusion. | | | |
| **Final Diagnosis:** | | | |
| 1. Normal Echocardiography Study. | | | |
| **Remark**: | | | |
| **Recommendation**: | | | |
| SIGNATURE  Done by: Tesfaye T., Pediatrician, Pediatric Cardiologist _______________ 12/04/2015Eth.C | | | |

| Patient Name: **Sintayehu Abebe**. Referring Institute: **Pawe Hospital**. SEX/ Age: **F/7years**. Date of Report: **12/04/15**.  Referral Diagnosis: **FTT. AGH2.105** | | | |
| --- | --- | --- | --- |
| **Features** | **Finding** | **Features** | **Finding** |
| **Profile** |  | **Atria** |  |
| Abdominal situs | Solitus | Left atrium | Normal |
| Cardiac position | Levocardia | Right atrium | Normal |
| Systemic venous drainage | Normal. | **Atrioventricular valves** |  |
| Pulmonary venous drainage | Normal | Mitral valve | Annulus = 19mm |
| Atrioventricular connection | Concordant | Tricuspid valve | Annulus = 20mm  TAPSE = 24mm |
| Ventriculoarterial connection | Concordant | **Ventricles** |  |
| Ventricular loop | d-Loop | Left ventricle | Normal |
|  |  | Right ventricle | Normal |
| **Septae** |  | **Coronary arteries** | ----- |
| Interventricular septum | Intact | **Doppler Measurement** |  |
| Interatrial septum | Intact | Mitral | ----- |
| **Semilunar valves** |  | Aortic | ------- |
| Aortic valve | Annulus = 15mm | Tricuspid | ------- |
| Pulmonary valve | Annulus = 18mm | pulmonic | Trivial PR, PPG = 10mmHg |
| **Great arteries** | NRGA | **Aortic arch** | Left. No CoA. |
| Aorta | ----- | **PDA** | No |
| Pulmonary artery | Normal MPA and Branch PAs. |  |  |
| **M-Mode:** | | | |
| AO | mm | PWd | mm |
| LA | mm | PWs | mm |
| LVIDd | mm | EDV | ml |
| LVIDs | mm | ESV | ml |
| IVSs | mm | LVEF | 63% |
| IVSd | mm | FS | 34% |
| **Additional Information**: |  | | |
| No pericardial/Pleural effusion. | | | |
| **Final Diagnosis:** | | | |
| 1. Normal Echocardiography Study. | | | |
| **Remark**: | | | |
| **Recommendation**: | | | |
| SIGNATURE  Done by: Tesfaye T., Pediatrician, Pediatric Cardiologist _______________ 12/04/2015Eth.C | | | |

| Patient Name: **Hawulet Mizanu**. Referring Institute: **Amaris PSC**. SEX/ Age: **F/6years**. Date of Report: **12/04/15**.  Referral Diagnosis: **ARF. AGH2.106** | | | |
| --- | --- | --- | --- |
| **Features** | **Finding** | **Features** | **Finding** |
| **Profile** |  | **Atria** |  |
| Abdominal situs | Solitus | Left atrium | Normal |
| Cardiac position | Levocardia | Right atrium | Normal |
| Systemic venous drainage | Normal. | **Atrioventricular valves** |  |
| Pulmonary venous drainage | Normal | Mitral valve | Annulus = 16mm |
| Atrioventricular connection | Concordant | Tricuspid valve | Annulus = 18mm  TAPSE = 19mm |
| Ventriculoarterial connection | Concordant | **Ventricles** |  |
| Ventricular loop | d-Loop | Left ventricle | Normal |
|  |  | Right ventricle | Normal |
| **Septae** |  | **Coronary arteries** | ----- |
| Interventricular septum | Intact | **Doppler Measurement** |  |
| Interatrial septum | Intact | Mitral | Trivial MR, Incomplete signal, seen in two planes with jet velocity = 1.9m/sec |
| **Semilunar valves** |  | Aortic | ------- |
| Aortic valve | Annulus = 13mm | Tricuspid | ------- |
| Pulmonary valve | Annulus = 16mm | pulmonic | -------- |
| **Great arteries** | NRGA | **Aortic arch** | Left. No CoA. |
| Aorta | ----- | **PDA** | No |
| Pulmonary artery | Normal MPA and Branch PAs. |  |  |
| **M-Mode:** | | | |
| AO | mm | PWd | mm |
| LA | mm | PWs | mm |
| LVIDd | mm | EDV | ml |
| LVIDs | mm | ESV | ml |
| IVSs | mm | LVEF | 66% |
| IVSd | mm | FS | 35% |
| **Additional Information**: |  | | |
| No pericardial/Pleural effusion. | | | |
| **Final Diagnosis:** | | | |
| 1. {S, D, S} Levocardia. 2. Trivial MR | | | |
| **Remark**: | | | |
| **Recommendation**: | | | |
| SIGNATURE  Done by: Tesfaye T., Pediatrician, Pediatric Cardiologist _______________ 12/04/2015Eth.C | | | |

| Patient Name: **Aynalem Getasew**. Referring Institute: **FHRH**. SEX/ Age: **F/7years**. Date of Report: **12/04/15**.  Referral Diagnosis: **Easy fatigability. AGH2.107.** | | | |
| --- | --- | --- | --- |
| **Features** | **Finding** | **Features** | **Finding** |
| **Profile** |  | **Atria** |  |
| Abdominal situs | Solitus | Left atrium | Normal |
| Cardiac position | Levocardia | Right atrium | Normal |
| Systemic venous drainage | Normal. | **Atrioventricular valves** |  |
| Pulmonary venous drainage | Normal | Mitral valve | Annulus = 19mm |
| Atrioventricular connection | Concordant | Tricuspid valve | Annulus = 20mm  TAPSE = 16mm |
| Ventriculoarterial connection | Concordant | **Ventricles** |  |
| Ventricular loop | d-Loop | Left ventricle | Normal |
|  |  | Right ventricle | Normal |
| **Septae** |  | **Coronary arteries** | ----- |
| Interventricular septum | Intact | **Doppler Measurement** |  |
| Interatrial septum | Intact | Mitral | ----- |
| **Semilunar valves** |  | Aortic | ------- |
| Aortic valve | Annulus = 14mm | Tricuspid | Trivial TR, PPG = 13mmHg |
| Pulmonary valve | Annulus = 16mm | pulmonic | Trivial PR, PPG = 7mmHg |
| **Great arteries** | NRGA | **Aortic arch** | Left. No CoA. |
| Aorta | ----- | **PDA** | No |
| Pulmonary artery | Normal MPA and Branch PAs. |  |  |
| **M-Mode:** | | | |
| AO | mm | PWd | mm |
| LA | mm | PWs | mm |
| LVIDd | mm | EDV | ml |
| LVIDs | mm | ESV | ml |
| IVSs | mm | LVEF | 65% |
| IVSd | mm | FS | 35% |
| **Additional Information**: |  | | |
| No pericardial/Pleural effusion. | | | |
| **Final Diagnosis:** | | | |
| 1. {S, D, S} Levocardia. 2. Trivial TR 3. Trivial PR | | | |
| **Remark**: | | | |
| **Recommendation**: | | | |
| SIGNATURE  Done by: Tesfaye T., Pediatrician, Pediatric Cardiologist _______________ 12/04/2015Eth.C | | | |

| Patient Name: **Barkot Esubalew**. Referring Institute: **Adinas GH**. SEX/ Age: **M/5years**. Date of Report: **12/04/15**.  Referral Diagnosis: **Easy fatigability. AGH2.108** | | | |
| --- | --- | --- | --- |
| **Features** | **Finding** | **Features** | **Finding** |
| **Profile** |  | **Atria** |  |
| Abdominal situs | Solitus | Left atrium | Normal |
| Cardiac position | Levocardia | Right atrium | Normal |
| Systemic venous drainage | Normal. | **Atrioventricular valves** |  |
| Pulmonary venous drainage | Normal | Mitral valve | Annulus = 18mm |
| Atrioventricular connection | Concordant | Tricuspid valve | Annulus = 19mm  TAPSE = 20mm |
| Ventriculoarterial connection | Concordant | **Ventricles** |  |
| Ventricular loop | d-Loop | Left ventricle | Normal |
|  |  | Right ventricle | Normal |
| **Septae** |  | **Coronary arteries** | ----- |
| Interventricular septum | Intact | **Doppler Measurement** |  |
| Interatrial septum | Intact | Mitral | ----- |
| **Semilunar valves** |  | Aortic | ------- |
| Aortic valve | Annulus = 15mm | Tricuspid | ------- |
| Pulmonary valve | Annulus = 20mm | pulmonic | -------- |
| **Great arteries** | NRGA | **Aortic arch** | Left. No CoA. |
| Aorta | ----- | **PDA** | No |
| Pulmonary artery | Normal MPA and Branch PAs. |  |  |
| **M-Mode:** | | | |
| AO | mm | PWd | mm |
| LA | mm | PWs | mm |
| LVIDd | mm | EDV | ml |
| LVIDs | mm | ESV | ml |
| IVSs | mm | LVEF | 72% |
| IVSd | mm | FS | 41% |
| **Additional Information**: |  | | |
| No pericardial/Pleural effusion. | | | |
| **Final Diagnosis:** | | | |
| 1. Normal Echocardiography Study. | | | |
| **Remark**: | | | |
| **Recommendation**: | | | |
| SIGNATURE  Done by: Tesfaye T., Pediatrician, Pediatric Cardiologist _______________ 12/04/2015Eth.C | | | |

| Patient Name: **Baby of Bethelihem Hailu**. Referring Institute: **FHRH**. SEX/ Age: **M/10days**. Date of Report: **12/04/15**.  Referral Diagnosis: **RD. AGH2.109** | | | |
| --- | --- | --- | --- |
| **Features** | **Finding** | **Features** | **Finding** |
| **Profile** |  | **Atria** |  |
| Abdominal situs | Solitus | Left atrium | Normal |
| Cardiac position | Levocardia | Right atrium | Normal |
| Systemic venous drainage | Normal. | **Atrioventricular valves** |  |
| Pulmonary venous drainage | Normal | Mitral valve | Annulus = 10mm |
| Atrioventricular connection | Concordant | Tricuspid valve | Annulus = 11mm  TAPSE = 14mm |
| Ventriculoarterial connection | Concordant | **Ventricles** |  |
| Ventricular loop | d-Loop | Left ventricle | Normal |
|  |  | Right ventricle | Normal |
| **Septae** |  | **Coronary arteries** | ----- |
| Interventricular septum | Intact | **Doppler Measurement** |  |
| Interatrial septum | PFO, L – R Shunt | Mitral | ----- |
| **Semilunar valves** |  | Aortic | ------- |
| Aortic valve | Annulus = 9mm | Tricuspid | ------- |
| Pulmonary valve | Annulus = 9mm | pulmonic | -------- |
| **Great arteries** | NRGA | **Aortic arch** | Left. No CoA. |
| Aorta | ----- | **PDA** | No |
| Pulmonary artery | Normal MPA and Branch PAs. |  |  |
| **M-Mode:**  Normal LV Function on eye balling | | | |
| AO | mm | PWd | mm |
| LA | mm | PWs | mm |
| LVIDd | mm | EDV | ml |
| LVIDs | mm | ESV | ml |
| IVSs | mm | LVEF | % |
| IVSd | mm | FS | % |
| **Additional Information**: |  | | |
| No pericardial/Pleural effusion. | | | |
| **Final Diagnosis:** | | | |
| 1. {S, D, S} Levocardia. 2. PFO, L – R Shunt | | | |
| **Remark**: | | | |
| **Recommendation**: | | | |
| SIGNATURE  Done by: Tesfaye T., Pediatrician, Pediatric Cardiologist _______________ 12/04/2015Eth.C | | | |

| Patient Name: **Muluken Absew**. Referring Institute: **Adinas GH**. SEX/ Age: **F/1 5/12**. Date of Report: **12/04/15**.  Referral Diagnosis: **Recurrent chest infction.AGH2.110** | | | |
| --- | --- | --- | --- |
| **Features** | **Finding** | **Features** | **Finding** |
| **Profile** |  | **Atria** |  |
| Abdominal situs | Solitus | Left atrium | Normal |
| Cardiac position | Levocardia | Right atrium | Normal |
| Systemic venous drainage | Normal. | **Atrioventricular valves** |  |
| Pulmonary venous drainage | Normal | Mitral valve | Annulus = 12mm |
| Atrioventricular connection | Concordant | Tricuspid valve | Annulus = 13mm |
| Ventriculoarterial connection | Concordant | **Ventricles** |  |
| Ventricular loop | d-Loop | Left ventricle | Normal |
|  |  | Right ventricle | Normal |
| **Septae** |  | **Coronary arteries** | ----- |
| Interventricular septum | Intact | **Doppler Measurement** |  |
| Interatrial septum | Intact | Mitral | ----- |
| **Semilunar valves** |  | Aortic | ------- |
| Aortic valve | Annulus = 12mm | Tricuspid | ------- |
| Pulmonary valve | Annulus = 14mm | pulmonic | -------- |
| **Great arteries** | NRGA | **Aortic arch** | Left. No CoA. |
| Aorta | ----- | **PDA** | No |
| Pulmonary artery | Normal MPA and Branch PAs. |  |  |
| **M-Mode:**  Normal LV Function on eye balling | | | |
| AO | mm | PWd | mm |
| LA | mm | PWs | mm |
| LVIDd | mm | EDV | ml |
| LVIDs | mm | ESV | ml |
| IVSs | mm | LVEF | % |
| IVSd | mm | FS | % |
| **Additional Information**: |  | | |
| No pericardial/Pleural effusion. | | | |
| **Final Diagnosis:** | | | |
| 1. Normal Echocardiography Study. | | | |
| **Remark**: | | | |
| **Recommendation**: | | | |
| SIGNATURE  Done by: Tesfaye T., Pediatrician, Pediatric Cardiologist _______________ 12/04/2015Eth.C | | | |

| Patient Name: **Tsigereda Birhanu**. Referring Institute: **FHRH**. SEX/ Age: **F/5years**. Date of Report: **12/04/15**.  Referral Diagnosis: **CHF. AGH2.111** | | | |
| --- | --- | --- | --- |
| **Features** | **Finding** | **Features** | **Finding** |
| **Profile** |  | **Atria** |  |
| Abdominal situs | Solitus | Left atrium | Markedly Dilated |
| Cardiac position | Levocardia | Right atrium | Dilated |
| Systemic venous drainage | Normal. | **Atrioventricular valves** |  |
| Pulmonary venous drainage | Normal | Mitral valve | Annulus = 22mm |
| Atrioventricular connection | Concordant | Tricuspid valve | Annulus = 18mm  TAPSE = 20mm |
| Ventriculoarterial connection | Concordant | **Ventricles** |  |
| Ventricular loop | d-Loop | Left ventricle | Markedly Dilated |
|  |  | Right ventricle | Dilated |
| **Septae** |  | **Coronary arteries** | ----- |
| Interventricular septum | Intact | **Doppler Measurement** |  |
| Interatrial septum | Intact | Mitral | Mild MR, Incomplete signal, seen in two planes with jet velocity = 3.3m/sec. |
| **Semilunar valves** |  | Aortic | ------- |
| Aortic valve | Annulus = 16mm | Tricuspid | ------- |
| Pulmonary valve | Annulus = 20mm | pulmonic | Mild PR, PPG = 57mmHg |
| **Great arteries** | NRGA | **Aortic arch** | Left. No CoA. |
| Aorta | ----- | **PDA** | 5mm PDA, L – R Shunt |
| Pulmonary artery | MPA = 24mm. |  |  |
| **M-Mode:** | | | |
| AO | mm | PWd | mm |
| LA | mm | PWs | mm |
| LVIDd | mm | EDV | ml |
| LVIDs | mm | ESV | ml |
| IVSs | mm | LVEF | 64% |
| IVSd | mm | FS | 35% |
| **Additional Information**: |  | | |
| No pericardial/Pleural effusion. | | | |
| **Final Diagnosis:** | | | |
| 1. {S, D, S} Levocardia. 2. All chambers dilated 3. Mild MR 4. Mild PR 5. Large PDA, L – R Shunt 6. Moderate Pulmonary Hypertension 7. Normal Biventricular Systolic Function | | | |
| **Remark**: | | | |
| **Recommendation**: | | | |
| SIGNATURE  Done by: Tesfaye T., Pediatrician, Pediatric Cardiologist _______________ 12/04/2015Eth.C | | | |

| Patient Name: **Yeshiwerk Desalegn**. Referring Institute: **TGSH**. SEX/ Age: **F/6years**. Date of Report: **13/04/15**.  Referral Diagnosis: **CHF. AGH2.112** | | | |
| --- | --- | --- | --- |
| **Features** | **Finding** | **Features** | **Finding** |
| **Profile** |  | **Atria** |  |
| Abdominal situs | Solitus | Left atrium | Dilated |
| Cardiac position | Levocardia | Right atrium | Dilated |
| Systemic venous drainage | Normal. | **Atrioventricular valves** |  |
| Pulmonary venous drainage | Normal | Mitral valve | Annulus = 21mm |
| Atrioventricular connection | Concordant | Tricuspid valve | Annulus = 23mm  TAPSE = 16mm |
| Ventriculoarterial connection | Concordant | **Ventricles** |  |
| Ventricular loop | d-Loop | Left ventricle | Dilated |
|  |  | Right ventricle | Dilated |
| **Septae** |  | **Coronary arteries** | ----- |
| Interventricular septum | Intact | **Doppler Measurement** |  |
| Interatrial septum | Intact | Mitral | Mild MR, Jet velocity = 1.8m/sec. |
| **Semilunar valves** |  | Aortic | ------- |
| Aortic valve | Annulus = 14mm | Tricuspid | ------- |
| Pulmonary valve | Annulus = 21mm | pulmonic | -------- |
| **Great arteries** | NRGA | **Aortic arch** | Left. No CoA. |
| Aorta | 9mm Aorto-pulmonary window, L – R Shunt | **PDA** | No |
| Pulmonary artery |  |  |
| **M-Mode:** | | | |
| AO | mm | PWd | mm |
| LA | mm | PWs | mm |
| LVIDd | mm | EDV | ml |
| LVIDs | mm | ESV | ml |
| IVSs | mm | LVEF | 61% |
| IVSd | mm | FS | 32% |
| **Additional Information**: |  | | |
| No pericardial/Pleural effusion. | | | |
| **Final Diagnosis:** | | | |
| 1. {S, D, S} Levocardia. 2. All chambers dilated 3. Mild MR 4. Aorto-Pulmonary Window, L – R Shunt 5. Normal Biventricular Systolic Function | | | |
| **Remark**: | | | |
| **Recommendation**: | | | |
| SIGNATURE  Done by: Tesfaye T., Pediatrician, Pediatric Cardiologist _______________ 13/04/2015Eth.C | | | |

| Patient Name: **Bereket Adane**. Referring Institute: **Adinas GH**. SEX/ Age: **M/10months**. Date of Report: **13/04/15**.  Referral Diagnosis: **CHF. AGH2.113** | | | |
| --- | --- | --- | --- |
| **Features** | **Finding** | **Features** | **Finding** |
| **Profile** |  | **Atria** |  |
| Abdominal situs | Solitus | Left atrium | Dilated |
| Cardiac position | Levocardia | Right atrium | Dilated |
| Systemic venous drainage | Normal. | **Atrioventricular valves** |  |
| Pulmonary venous drainage | Normal | Mitral valve | Annulus = 16mm |
| Atrioventricular connection | Concordant | Tricuspid valve | Annulus = 15mm  TAPSE = mm |
| Ventriculoarterial connection | Concordant | **Ventricles** |  |
| Ventricular loop | d-Loop | Left ventricle | Dilated |
|  |  | Right ventricle | Dilated |
| **Septae** |  | **Coronary arteries** | ----- |
| Interventricular septum | 10mm Inlet VSD with PM extension, L – R Shunt | **Doppler Measurement** |  |
| Interatrial septum | Intact | Mitral | Trivial MR |
| **Semilunar valves** |  | Aortic | ------- |
| Aortic valve | Annulus = 8mm | Tricuspid | ------- |
| Pulmonary valve | Annulus = 13mm | pulmonic | Moderate PR, PPG = 55mmHg |
| **Great arteries** | NRGA | **Aortic arch** | Left. No CoA. |
| Aorta | ----- | **PDA** | No |
| Pulmonary artery | MPA = 20m. |  |  |
| **M-Mode:** | | | |
| AO | mm | PWd | mm |
| LA | mm | PWs | mm |
| LVIDd | mm | EDV | ml |
| LVIDs | mm | ESV | ml |
| IVSs | mm | LVEF | 71% |
| IVSd | mm | FS | 38% |
| **Additional Information**: |  | | |
| No pericardial/Pleural effusion. | | | |
| **Final Diagnosis:** | | | |
| 1. {S, D, S} Levocardia. 2. All chambers Dilated 3. Large Inlet VSD with PM extension, L – R Shunt 4. Moderate PR 5. Moderate Pulmonary Hypertension 6. Normal Biventricular Systolic Function | | | |
| **Remark**: | | | |
| **Recommendation**: | | | |
| SIGNATURE  Done by: Tesfaye T., Pediatrician, Pediatric Cardiologist _______________ 13/04/2015Eth.C | | | |

| Patient Name: **Ahmed Hassen**. Referring Institute: **Adinas GH**. SEX/ Age: **M/3years**. Date of Report: **13/04/15**.  Referral Diagnosis: **DS + CHF. AGH2.114** | | | |
| --- | --- | --- | --- |
| **Features** | **Finding** | **Features** | **Finding** |
| **Profile** |  | **Atria** |  |
| Abdominal situs | Solitus | Left atrium | Dilated |
| Cardiac position | Levocardia | Right atrium | More Dilated |
| Systemic venous drainage | Normal. | **Atrioventricular valves** |  |
| Pulmonary venous drainage | Normal | Mitral valve | Annulus = 13mm |
| Atrioventricular connection | Concordant | Tricuspid valve | Annulus = 19mm  TAPSE = 19mm |
| Ventriculoarterial connection | Concordant | **Ventricles** |  |
| Ventricular loop | d-Loop | Left ventricle | Dilated |
|  |  | Right ventricle | More Dilated |
| **Septae** |  | **Coronary arteries** | ----- |
| Interventricular septum | 10mm Inlet VSD, L – R Shunt | **Doppler Measurement** |  |
| Interatrial septum | 14mm Primum defect, L – R Shunt | Mitral | Moderate MR |
| **Semilunar valves** |  | Aortic | ------- |
| Aortic valve | Annulus = 14mm | Tricuspid | Mild TR |
| Pulmonary valve | Annulus = 16mm | pulmonic | -------- |
| **Great arteries** | NRGA | **Aortic arch** | Left. No CoA. |
| Aorta | ----- | **PDA** | No |
| Pulmonary artery | Normal MPA and Branch PAs. |  |  |
| **M-Mode:**  Normal LV Function on eye balling | | | |
| AO | mm | PWd | mm |
| LA | mm | PWs | mm |
| LVIDd | mm | EDV | ml |
| LVIDs | mm | ESV | ml |
| IVSs | mm | LVEF | % |
| IVSd | mm | FS | % |
| **Additional Information**: |  | | |
| No pericardial/Pleural effusion. | | | |
| **Final Diagnosis:** | | | |
| 1. {S, D, S} Levocardia. 2. All chambers dilated 3. Intermediate AVSD, L – R Shunt 4. Moderate MR 5. Mild TR 6. Pulmonary Hypertension 7. Normal Biventricular Systolic Function | | | |
| **Remark**: | | | |
| **Recommendation**: | | | |
| SIGNATURE  Done by: Tesfaye T., Pediatrician, Pediatric Cardiologist _______________ 13/04/2015Eth.C | | | |

| Patient Name: **Animut Maru**. Referring Institute: **Addis Alem PH**. SEX/ Age: **M/5years**. Date of Report: **14/04/15**.  Referral Diagnosis: **Recurrent Chest Infection. AGH2.115.** | | | |
| --- | --- | --- | --- |
| **Features** | **Finding** | **Features** | **Finding** |
| **Profile** |  | **Atria** |  |
| Abdominal situs | Solitus | Left atrium | Normal |
| Cardiac position | Levocardia | Right atrium | Normal |
| Systemic venous drainage | Normal. | **Atrioventricular valves** |  |
| Pulmonary venous drainage | Normal | Mitral valve | Annulus = 16mm |
| Atrioventricular connection | Concordant | Tricuspid valve | Annulus = 17mm  TAPSE = 16mm |
| Ventriculoarterial connection | Concordant | **Ventricles** |  |
| Ventricular loop | d-Loop | Left ventricle | Normal |
|  |  | Right ventricle | Normal |
| **Septae** |  | **Coronary arteries** | ----- |
| Interventricular septum | Intact | **Doppler Measurement** |  |
| Interatrial septum | Intact | Mitral | ----- |
| **Semilunar valves** |  | Aortic | ------- |
| Aortic valve | Annulus = 13mm | Tricuspid | Trivial TR, PPG = 29mmHg |
| Pulmonary valve | Annulus = 17mm | pulmonic | -------- |
| **Great arteries** | NRGA | **Aortic arch** | Left. No CoA. |
| Aorta | ----- | **PDA** | No |
| Pulmonary artery | Normal MPA and Branch PAs. |  |  |
| **M-Mode:** | | | |
| AO | mm | PWd | mm |
| LA | mm | PWs | mm |
| LVIDd | mm | EDV | ml |
| LVIDs | mm | ESV | ml |
| IVSs | mm | LVEF | 67% |
| IVSd | mm | FS | 35% |
| **Additional Information**: |  | | |
| No pericardial/Pleural effusion. | | | |
| **Final Diagnosis:** | | | |
| 1. Normal Echocardiography Study. | | | |
| **Remark**: | | | |
| **Recommendation**: | | | |
| SIGNATURE  Done by: Tesfaye T., Pediatrician, Pediatric Cardiologist _______________ 14/04/2015Eth.C | | | |

| Patient Name: **Biniam Wedaje**. Referring Institute: **TGSH**. SEX/ Age: **M/10years**. Date of Report: **14/04/15**.  Referral Diagnosis: **Sydenham’s Chorea. AGH2.116** | | | |
| --- | --- | --- | --- |
| **Features** | **Finding** | **Features** | **Finding** |
| **Profile** |  | **Atria** |  |
| Abdominal situs | Solitus | Left atrium | Normal |
| Cardiac position | Levocardia | Right atrium | Normal |
| Systemic venous drainage | Normal. | **Atrioventricular valves** |  |
| Pulmonary venous drainage | Normal | Mitral valve | Annulus = 19mm. Patulous MVL. |
| Atrioventricular connection | Concordant | Tricuspid valve | Annulus = 22mm  TAPSE = 22mm |
| Ventriculoarterial connection | Concordant | **Ventricles** |  |
| Ventricular loop | d-Loop | Left ventricle | Normal |
|  |  | Right ventricle | Normal |
| **Septae** |  | **Coronary arteries** | ----- |
| Interventricular septum | Intact | **Doppler Measurement** |  |
| Interatrial septum | Intact | Mitral | Mild MR, Holosystolic, Central Projection, seen in two planes with jet velocity = 2.9m/sec. |
| **Semilunar valves** |  | Aortic | ------- |
| Aortic valve | Annulus = 17mm | Tricuspid | ------- |
| Pulmonary valve | Annulus = 19mm | pulmonic | -------- |
| **Great arteries** | NRGA | **Aortic arch** | Left. No CoA. |
| Aorta | ----- | **PDA** | No |
| Pulmonary artery | Normal MPA and Branch PAs. |  |  |
| **M-Mode:** | | | |
| AO | mm | PWd | mm |
| LA | mm | PWs | mm |
| LVIDd | mm | EDV | ml |
| LVIDs | mm | ESV | ml |
| IVSs | mm | LVEF | 57% |
| IVSd | mm | FS | 29% |
| **Additional Information**: |  | | |
| No pericardial/Pleural effusion. | | | |
| **Final Diagnosis:** | | | |
| 1. {S, D, S} Levocardia. 2. Patulous MVL 3. Mild MR 4. Normal Biventricular Systolic Function | | | |
| **Remark**: | | | |
| **Recommendation**: | | | |
| SIGNATURE  Done by: Tesfaye T., Pediatrician, Pediatric Cardiologist _______________ 14/04/2015Eth.C | | | |

| Patient Name: **Atsede-Mariam Habtamu**. Referring Institute: **Adinas GH**. SEX/ Age: **F/2years**. Date of Report: **14/04/15**.  Referral Diagnosis: **Recurrent chest infection. AGH2.117** | | | |
| --- | --- | --- | --- |
| **Features** | **Finding** | **Features** | **Finding** |
| **Profile** |  | **Atria** |  |
| Abdominal situs | Solitus | Left atrium | Normal |
| Cardiac position | Levocardia | Right atrium | Normal |
| Systemic venous drainage | Normal. | **Atrioventricular valves** |  |
| Pulmonary venous drainage | Normal | Mitral valve | Annulus = 12mm |
| Atrioventricular connection | Concordant | Tricuspid valve | Annulus = 13mm |
| Ventriculoarterial connection | Concordant | **Ventricles** |  |
| Ventricular loop | d-Loop | Left ventricle | Normal |
|  |  | Right ventricle | Normal |
| **Septae** |  | **Coronary arteries** | ----- |
| Interventricular septum | Intact | **Doppler Measurement** |  |
| Interatrial septum | Intact | Mitral | ----- |
| **Semilunar valves** |  | Aortic | ------- |
| Aortic valve | Annulus = 12mm | Tricuspid | ------- |
| Pulmonary valve | Annulus = 14mm | pulmonic | -------- |
| **Great arteries** | NRGA | **Aortic arch** | Left. No CoA. |
| Aorta | ----- | **PDA** | No |
| Pulmonary artery | Normal MPA and Branch PAs. |  |  |
| **M-Mode:**  Normal LV Function on eye balling | | | |
| AO | mm | PWd | mm |
| LA | mm | PWs | mm |
| LVIDd | mm | EDV | ml |
| LVIDs | mm | ESV | ml |
| IVSs | mm | LVEF | % |
| IVSd | mm | FS | % |
| **Additional Information**: |  | | |
| No pericardial/Pleural effusion. | | | |
| **Final Diagnosis:** | | | |
| 1. Normal Echocardiography Study. | | | |
| **Remark**: | | | |
| **Recommendation**: | | | |
| SIGNATURE  Done by: Tesfaye T., Pediatrician, Pediatric Cardiologist _______________ 14/04/2015Eth.C | | | |

| Patient Name: **Meklit Wassie**. Referring Institute: **FHRH**. SEX/ Age: **F/1year**. Date of Report: **15/04/15**.  Referral Diagnosis: **Follow up echo for Infective endarteritis after Rx completion. AGH2.118** | | | |
| --- | --- | --- | --- |
| **Features** | **Finding** | **Features** | **Finding** |
| **Profile** |  | **Atria** |  |
| Abdominal situs | Solitus | Left atrium | Dilated |
| Cardiac position | Levocardia | Right atrium | Dilated |
| Systemic venous drainage | Normal. | **Atrioventricular valves** |  |
| Pulmonary venous drainage | Normal | Mitral valve | Annulus = 17mm |
| Atrioventricular connection | Concordant | Tricuspid valve | Annulus = 16mm |
| Ventriculoarterial connection | Concordant | **Ventricles** |  |
| Ventricular loop | d-Loop | Left ventricle | Dilated |
|  |  | Right ventricle | Dilated |
| **Septae** |  | **Coronary arteries** | ----- |
| Interventricular septum | Intact | **Doppler Measurement** |  |
| Interatrial septum | Intact | Mitral | Moderate MR, Holosystolic, posterior projection, seen in two planes with jet velocity = 3.7m/sec. |
| **Semilunar valves** |  | Aortic | ------- |
| Aortic valve | Annulus = 14mm | Tricuspid | Mild TR, PPG = 60mmHg |
| Pulmonary valve | Annulus = 15mm | pulmonic | -------- |
| **Great arteries** | NRGA | **Aortic arch** | Left. No CoA. |
| Aorta | ----- | **PDA** | 4mm PDA, L – R Shunt. Echogenic mass on the PA end of the PDA |
| Pulmonary artery | Normal MPA and Branch PAs. |  |  |
| **M-Mode:** | | | |
| AO | mm | PWd | mm |
| LA | mm | PWs | mm |
| LVIDd | mm | EDV | ml |
| LVIDs | mm | ESV | ml |
| IVSs | mm | LVEF | % |
| IVSd | mm | FS | % |
| **Additional Information**: |  | | |
| No pericardial/Pleural effusion. | | | |
| **Final Diagnosis:** | | | |
| 1. {S, D, S} Levocardia. 2. All chambers dilated 3. Large PDA, L – R Shunt 4. Severe Pulmonary Hypertension 5. Echogenic mass at the PA end of PDA (on Rx for IE) | | | |
| **Remark**: | | | |
| **Recommendation**: | | | |
| SIGNATURE  Done by: Tesfaye T., Pediatrician, Pediatric Cardiologist _______________ 15/04/2015Eth.C | | | |

| Patient Name: **Baby of Atsede-Mariam Alebachew**. Referring Institute: **MSI – E**. SEX/ Age: **M/5days**.  Date of Report: **15/04/15**. Referral Diagnosis: **IDM. AGH2.119** | | | |
| --- | --- | --- | --- |
| **Features** | **Finding** | **Features** | **Finding** |
| **Profile** |  | **Atria** |  |
| Abdominal situs | Solitus | Left atrium | Normal |
| Cardiac position | Levocardia | Right atrium | Normal |
| Systemic venous drainage | Normal. | **Atrioventricular valves** |  |
| Pulmonary venous drainage | Normal | Mitral valve | Annulus = 11mm |
| Atrioventricular connection | Concordant | Tricuspid valve | Annulus = 12mm  TAPSE = 12mm |
| Ventriculoarterial connection | Concordant | **Ventricles** |  |
| Ventricular loop | d-Loop | Left ventricle | Normal |
|  |  | Right ventricle | Normal |
| **Septae** |  | **Coronary arteries** | ----- |
| Interventricular septum | Intact | **Doppler Measurement** |  |
| Interatrial septum | PFO, L – R Shunt | Mitral | ----- |
| **Semilunar valves** |  | Aortic | ------- |
| Aortic valve | Annulus = 9mm | Tricuspid | ------- |
| Pulmonary valve | Annulus = 10mm | pulmonic | -------- |
| **Great arteries** | NRGA | **Aortic arch** | Left. No CoA. |
| Aorta | ----- | **PDA** | No |
| Pulmonary artery | Normal MPA and Branch PAs. |  |  |
| **M-Mode:**  Normal LV Function on eye balling | | | |
| AO | mm | PWd | mm |
| LA | mm | PWs | mm |
| LVIDd | mm | EDV | ml |
| LVIDs | mm | ESV | ml |
| IVSs | mm | LVEF | % |
| IVSd | mm | FS | % |
| **Additional Information**: |  | | |
| No pericardial/Pleural effusion. | | | |
| **Final Diagnosis:** | | | |
| 1. {S, D, S} Levocardia. 2. PFO, L – R Shunt | | | |
| **Remark**: | | | |
| **Recommendation**: | | | |
| SIGNATURE  Done by: Tesfaye T., Pediatrician, Pediatric Cardiologist _______________ 15/04/2015Eth.C | | | |

| Patient Name: **Melona Emiamrew**. Referring Institute: **Afilas GH**. SEX/ Age: **F/13days**. Date of Report: **15/04/15**.  Referral Diagnosis: **Patau Syndrome. AGH2.120 (TGSH10)** | | | |
| --- | --- | --- | --- |
| **Features** | **Finding** | **Features** | **Finding** |
| **Profile** |  | **Atria** |  |
| Abdominal situs | Solitus | Left atrium | Normal |
| Cardiac position | Levocardia | Right atrium | Normal |
| Systemic venous drainage | Normal. | **Atrioventricular valves** |  |
| Pulmonary venous drainage | Normal | Mitral valve | Annulus = 9mm |
| Atrioventricular connection | Concordant | Tricuspid valve | Annulus = 10mm |
| Ventriculoarterial connection | Concordant | **Ventricles** |  |
| Ventricular loop | d-Loop | Left ventricle | Normal |
|  |  | Right ventricle | Normal |
| **Septae** |  | **Coronary arteries** | ----- |
| Interventricular septum | 4mm PM VSD, L – R Shunt | **Doppler Measurement** |  |
| Interatrial septum | 5mm OS ASD, L – R Shunt | Mitral | ----- |
| **Semilunar valves** |  | Aortic | ------- |
| Aortic valve | Annulus = 10mm | Tricuspid | ------- |
| Pulmonary valve | Annulus = 12mm | pulmonic | -------- |
| **Great arteries** | NRGA | **Aortic arch** | Left. No CoA. |
| Aorta | ----- | **PDA** | 1.5mm PDA, L – R Shunt |
| Pulmonary artery | Normal MPA and Branch PAs. |  |  |
| **M-Mode:** | | | |
| AO | mm | PWd | mm |
| LA | mm | PWs | mm |
| LVIDd | mm | EDV | ml |
| LVIDs | mm | ESV | ml |
| IVSs | mm | LVEF | 67% |
| IVSd | mm | FS | 33% |
| **Additional Information**: |  | | |
| No pericardial/Pleural effusion. | | | |
| **Final Diagnosis:** | | | |
| 1. {S, D, S} Levocardia. 2. Small OS ASD, L – R Shunt 3. Small PM VSD, L – R Shunt 4. Small PDA, L – R Shunt 5. Normal LV Systolic Function | | | |
| **Remark**: | | | |
| **Recommendation**: | | | |
| SIGNATURE  Done by: Tesfaye T., Pediatrician, Pediatric Cardiologist _______________ 15/04/2015Eth.C | | | |

| Patient Name: **Robel Aragie**. Referring Institute: **Amaris PSC**. SEX/ Age: **M/5months**. Date of Report: **17/04/15**.  Referral Diagnosis: **Cyanosis. AGH2.121 (TGSH10)** | | | |
| --- | --- | --- | --- |
| **Features** | **Finding** | **Features** | **Finding** |
| **Profile** |  | **Atria** |  |
| Abdominal situs | Solitus | Left atrium | Normal |
| Cardiac position | Levocardia | Right atrium | Dilated |
| Systemic venous drainage | Normal. | **Atrioventricular valves** |  |
| Pulmonary venous drainage | Normal | Mitral valve | Annulus = 12mm |
| Atrioventricular connection | Concordant | Tricuspid valve | Annulus = 14mm |
| Ventriculoarterial connection | Concordant | **Ventricles** |  |
| Ventricular loop | d-Loop | Left ventricle | Normal |
|  |  | Right ventricle | Dilated & Hypertrophied |
| **Septae** |  | **Coronary arteries** | ----- |
| Interventricular septum | Non-Restrictive Mal-aligned Sub-aortic VSD, R – L Shunt. | **Doppler Measurement** |  |
| Interatrial septum | Intact | Mitral | ----- |
| **Semilunar valves** |  | Aortic | ------- |
| Aortic valve | Annulus = 13mm | Tricuspid | ------- |
| Pulmonary valve | Annulus = 6mm | pulmonic | Severe PS, PPG = 101mmHg. |
| **Great arteries** | NRGA | **Aortic arch** | No CoA. |
| Aorta | Over-riding aorta | **PDA** | No |
| Pulmonary artery | MPA = 5mm and Smallish Branch PAs. |  |  |
| **M-Mode:**  Normal LV Function on eye balling | | | |
| AO | mm | PWd | mm |
| LA | mm | PWs | mm |
| LVIDd | mm | EDV | ml |
| LVIDs | mm | ESV | ml |
| IVSs | mm | LVEF | % |
| IVSd | mm | FS | % |
| **Additional Information**: |  | | |
| No pericardial/Pleural effusion. | | | |
| **Final Diagnosis:** | | | |
| 1. {S, D, S} Levocardia. 2. RA/RV Dilated 3. TOF 4. Smallish MPA and Branch PAs. | | | |
| **Remark**: | | | |
| **Recommendation**: | | | |
| SIGNATURE  Done by: Tesfaye T., Pediatrician, Pediatric Cardiologist _______________ 17/04/2015Eth.C | | | |

| Patient Name: **Abel Zemenu**. Referring Institute: **TGSH**. SEX/ Age: **M/9months**. Date of Report: **17/04/15**.  Referral Diagnosis: **RD. AGH2.122** | | | |
| --- | --- | --- | --- |
| **Features** | **Finding** | **Features** | **Finding** |
| **Profile** |  | **Atria** |  |
| Abdominal situs | Solitus | Left atrium | Normal |
| Cardiac position | Levocardia | Right atrium | Normal |
| Systemic venous drainage | Normal. | **Atrioventricular valves** |  |
| Pulmonary venous drainage | Normal | Mitral valve | Annulus = 12mm |
| Atrioventricular connection | Concordant | Tricuspid valve | Annulus = 13mm |
| Ventriculoarterial connection | Concordant | **Ventricles** |  |
| Ventricular loop | d-Loop | Left ventricle | Normal |
|  |  | Right ventricle | Normal |
| **Septae** |  | **Coronary arteries** | ----- |
| Interventricular septum | Intact | **Doppler Measurement** |  |
| Interatrial septum | Intact | Mitral | ----- |
| **Semilunar valves** |  | Aortic | ------- |
| Aortic valve | Annulus = 11mm | Tricuspid | ------- |
| Pulmonary valve | Annulus = 12mm | pulmonic | -------- |
| **Great arteries** | NRGA | **Aortic arch** | Left. No CoA. |
| Aorta | ----- | **PDA** | No |
| Pulmonary artery | Normal MPA and Branch PAs. |  |  |
| **M-Mode:**  Normal LV Function on eye balling | | | |
| AO | mm | PWd | mm |
| LA | mm | PWs | mm |
| LVIDd | mm | EDV | ml |
| LVIDs | mm | ESV | ml |
| IVSs | mm | LVEF | % |
| IVSd | mm | FS | % |
| **Additional Information**: |  | | |
| No pericardial/Pleural effusion. | | | |
| **Final Diagnosis:** | | | |
| 1. Normal Echocardiography Study. | | | |
| **Remark**: | | | |
| **Recommendation**: | | | |
| SIGNATURE  Done by: Tesfaye T., Pediatrician, Pediatric Cardiologist _______________ 17/04/2015Eth.C | | | |

| Patient Name: **Liku Mihretu**. Referring Institute: **FHRH**. SEX/ Age: **M/9years**. Date of Report: **17/04/15**.  Referral Diagnosis: **Palpitation. AGH2.123** | | | |
| --- | --- | --- | --- |
| **Features** | **Finding** | **Features** | **Finding** |
| **Profile** |  | **Atria** |  |
| Abdominal situs | Solitus | Left atrium | Normal |
| Cardiac position | Levocardia | Right atrium | Normal |
| Systemic venous drainage | Normal. | **Atrioventricular valves** |  |
| Pulmonary venous drainage | Normal | Mitral valve | Annulus = 20mm |
| Atrioventricular connection | Concordant | Tricuspid valve | Annulus = 20mm  TAPSE = 23mm |
| Ventriculoarterial connection | Concordant | **Ventricles** |  |
| Ventricular loop | d-Loop | Left ventricle | Normal |
|  |  | Right ventricle | Normal |
| **Septae** |  | **Coronary arteries** | ----- |
| Interventricular septum | Intact | **Doppler Measurement** |  |
| Interatrial septum | Intact | Mitral | ----- |
| **Semilunar valves** |  | Aortic | ------- |
| Aortic valve | Annulus = 17mm | Tricuspid | ------- |
| Pulmonary valve | Annulus = 18mm | pulmonic | Trivial PR, PPG = 8mmHg. |
| **Great arteries** | NRGA | **Aortic arch** | Left. No CoA. |
| Aorta | ----- | **PDA** | No |
| Pulmonary artery | Normal MPA and Branch PAs. |  |  |
| **M-Mode:** | | | |
| AO | mm | PWd | mm |
| LA | mm | PWs | mm |
| LVIDd | mm | EDV | ml |
| LVIDs | mm | ESV | ml |
| IVSs | mm | LVEF | 67% |
| IVSd | mm | FS | 36% |
| **Additional Information**: |  | | |
| No pericardial/Pleural effusion. | | | |
| **Final Diagnosis:** | | | |
| 1. Normal Echocardiography Study. | | | |
| **Remark**: | | | |
| **Recommendation**: | | | |
| SIGNATURE  Done by: Tesfaye T., Pediatrician, Pediatric Cardiologist _______________ 17/04/2015Eth.C | | | |

| Patient Name: **Nura Ismael**. Referring Institute: **FHRH**. SEX/ Age: **F/3years**. Date of Report: **17/04/15**.  Referral Diagnosis: **FTT. AGH2.124** | | | |
| --- | --- | --- | --- |
| **Features** | **Finding** | **Features** | **Finding** |
| **Profile** |  | **Atria** |  |
| Abdominal situs | Solitus | Left atrium | Normal |
| Cardiac position | Levocardia | Right atrium | Normal |
| Systemic venous drainage | Normal. | **Atrioventricular valves** |  |
| Pulmonary venous drainage | Normal | Mitral valve | Annulus = 13mm |
| Atrioventricular connection | Concordant | Tricuspid valve | Annulus = 15mm  TAPSE = 17mm |
| Ventriculoarterial connection | Concordant | **Ventricles** |  |
| Ventricular loop | d-Loop | Left ventricle | Normal |
|  |  | Right ventricle | Normal |
| **Septae** |  | **Coronary arteries** | ----- |
| Interventricular septum | Intact | **Doppler Measurement** |  |
| Interatrial septum | Intact | Mitral | ----- |
| **Semilunar valves** |  | Aortic | ------- |
| Aortic valve | Annulus = 13mm | Tricuspid | ------- |
| Pulmonary valve | Annulus = 15mm | pulmonic | -------- |
| **Great arteries** | NRGA | **Aortic arch** | Left. No CoA. |
| Aorta | ----- | **PDA** | No |
| Pulmonary artery | Normal MPA and Branch PAs. |  |  |
| **M-Mode:**  Normal LV Function on eye balling | | | |
| AO | mm | PWd | mm |
| LA | mm | PWs | mm |
| LVIDd | mm | EDV | ml |
| LVIDs | mm | ESV | ml |
| IVSs | mm | LVEF | % |
| IVSd | mm | FS | % |
| **Additional Information**: |  | | |
| No pericardial/Pleural effusion. | | | |
| **Final Diagnosis:** | | | |
| 1. Normal Echocardiography Study. | | | |
| **Remark**: | | | |
| **Recommendation**: | | | |
| SIGNATURE  Done by: Tesfaye T., Pediatrician, Pediatric Cardiologist _______________ 17/04/2015Eth.C | | | |

| Patient Name: **Tarik Tadesse**. Referring Institute: **FHRH**. SEX/ Age: **M/1year**. Date of Report: **18/04/15**.  Referral Diagnosis: **Recurrent Chest Infection. AGH2.125** | | | |
| --- | --- | --- | --- |
| **Features** | **Finding** | **Features** | **Finding** |
| **Profile** |  | **Atria** |  |
| Abdominal situs | Solitus | Left atrium | Normal |
| Cardiac position | Levocardia | Right atrium | Normal |
| Systemic venous drainage | Normal. | **Atrioventricular valves** |  |
| Pulmonary venous drainage | Normal | Mitral valve | Annulus = 15mm |
| Atrioventricular connection | Concordant | Tricuspid valve | Annulus = 16mm  TAPSE = 15mm |
| Ventriculoarterial connection | Concordant | **Ventricles** |  |
| Ventricular loop | d-Loop | Left ventricle | Normal |
|  |  | Right ventricle | Normal |
| **Septae** |  | **Coronary arteries** | ----- |
| Interventricular septum | Intact | **Doppler Measurement** |  |
| Interatrial septum | PFO, L – R Shunt | Mitral | ----- |
| **Semilunar valves** |  | Aortic | ------- |
| Aortic valve | Annulus = 12mm | Tricuspid | ------- |
| Pulmonary valve | Annulus = 13mm | pulmonic | -------- |
| **Great arteries** | NRGA | **Aortic arch** | Left. No CoA. |
| Aorta | ----- | **PDA** | No |
| Pulmonary artery | Normal MPA and Branch PAs. |  |  |
| **M-Mode:**  Normal LV Function on eye balling. | | | |
| AO | mm | PWd | mm |
| LA | mm | PWs | mm |
| LVIDd | mm | EDV | ml |
| LVIDs | mm | ESV | ml |
| IVSs | mm | LVEF | % |
| IVSd | mm | FS | % |
| **Additional Information**: |  | | |
| No pericardial/Pleural effusion. | | | |
| **Final Diagnosis:** | | | |
| 1. {S, D, S} Levocardia. 2. PFO, L – R Shunt 3. Normal Biventricular Systolic Function | | | |
| **Remark**: | | | |
| **Recommendation**: | | | |
| SIGNATURE  Done by: Tesfaye T., Pediatrician, Pediatric Cardiologist _______________ 18/04/2015Eth.C | | | |

| Patient Name: **Estifanos Mesfin**. Referring Institute: **Debre-Tabour Hospital**. SEX/ Age: **M/2years**. Date of Report: **18/04/15**.  Referral Diagnosis: **Follow up Echo for Moderate PM VSD + Small Mid-Muscular VSD + Mild PS.(FTT + RD). AGH2.126** | | | |
| --- | --- | --- | --- |
| **Features** | **Finding** | **Features** | **Finding** |
| **Profile** |  | **Atria** |  |
| Abdominal situs | Solitus | Left atrium | Normal |
| Cardiac position | Levocardia | Right atrium | Normal |
| Systemic venous drainage | Normal. | **Atrioventricular valves** |  |
| Pulmonary venous drainage | Normal | Mitral valve | Annulus = 18mm |
| Atrioventricular connection | Concordant | Tricuspid valve | Annulus = 19mm  TAPSE = 17mm |
| Ventriculoarterial connection | Concordant | **Ventricles** |  |
| Ventricular loop | d-Loop | Left ventricle | Normal |
|  |  | Right ventricle | Normal |
| **Septae** |  | **Coronary arteries** | ----- |
| Interventricular septum | 9mm PM VSD, Partially closed by STL with effective opening of 3mm, PPG = 56mmHg, L – R Shunt | **Doppler Measurement** |  |
| Interatrial septum | Intact | Mitral | ----- |
| **Semilunar valves** |  | Aortic | ------- |
| Aortic valve | Annulus = 16mm | Tricuspid | ------- |
| Pulmonary valve | Annulus = 16mm | pulmonic | Flow acceleration across PV with PPG = 18mmHg. |
| **Great arteries** | NRGA | **Aortic arch** | Left. No CoA. |
| Aorta | ----- | **PDA** | No |
| Pulmonary artery | Normal MPA and Branch PAs. |  |  |
| **M-Mode:** | | | |
| AO | mm | PWd | mm |
| LA | mm | PWs | mm |
| LVIDd | mm | EDV | ml |
| LVIDs | mm | ESV | ml |
| IVSs | mm | LVEF | 61% |
| IVSd | mm | FS | 32% |
| **Additional Information**: |  | | |
| No pericardial/Pleural effusion. | | | |
| **Final Diagnosis:** | | | |
| 1. {S, D, S} Levocardia. 2. Moderate PM VSD, Partially closed by STL with effective opening of 3mm. 3. Normal Biventricular Systolic Function | | | |
| **Remark**: The Small Mid-Muscular VSD has closed | | | |
| **Recommendation**: No need to start cardiac medicine | | | |
| SIGNATURE  Done by: Tesfaye T., Pediatrician, Pediatric Cardiologist _______________ 18/04/2015Eth.C | | | |

| Patient Name: **Biruk Endalamaw**. Referring Institute: **FHRH**. SEX/ Age: **M/4years**. Date of Report: **18/04/15**.  Referral Diagnosis: **?Marfan Syndrome + screening. AGH2.127** | | | |
| --- | --- | --- | --- |
| **Features** | **Finding** | **Features** | **Finding** |
| **Profile** |  | **Atria** |  |
| Abdominal situs | Solitus | Left atrium | Dilated |
| Cardiac position | Levocardia | Right atrium | Normal |
| Systemic venous drainage | Normal. | **Atrioventricular valves** |  |
| Pulmonary venous drainage | Normal | Mitral valve | Annulus = 23mm. Thick and Redundant MVL. 7mm MVL displacement to LA. |
| Atrioventricular connection | Concordant | Tricuspid valve | Annulus = 21mm. Redundant TVL.  TAPSE = 24mm |
| Ventriculoarterial connection | Concordant | **Ventricles** |  |
| Ventricular loop | d-Loop | Left ventricle | Dilated |
|  |  | Right ventricle | Normal |
| **Septae** |  | **Coronary arteries** | ----- |
| Interventricular septum | Intact | **Doppler Measurement** |  |
| Interatrial septum | Intact | Mitral | Moderate MR, Central projection, seen in two planes with jet velocity = 4m/sec. |
| **Semilunar valves** |  | Aortic | ------- |
| Aortic valve | Annulus = 24mm | Tricuspid | Trivial TR, PPG = 16mmHg |
| Pulmonary valve | Annulus = 26mm | pulmonic | Trivial PR, PPG = 10mHg |
| **Great arteries** | NRGA | **Aortic arch** | Left. No CoA. |
| Aorta | Sinus = 23mm, STJ = 16mm, Ascending aorta = 15mm. | **PDA** | No |
| Pulmonary artery | MPA root = 26mm. |  |  |
| **M-Mode:** | | | |
| AO | mm | PWd | mm |
| LA | mm | PWs | mm |
| LVIDd | mm | EDV | ml |
| LVIDs | mm | ESV | ml |
| IVSs | mm | LVEF | 65% |
| IVSd | mm | FS | 35% |
| **Additional Information**: |  | | |
| No pericardial/Pleural effusion. | | | |
| **Final Diagnosis:** | | | |
| 1. {S, D, S} Levocardia. 2. LA/LV Dilated 3. Redundant, Thickened AV Valve leaflets 4. Dilated aortic sinus and Pulmonary Artery base 5. Moderate MR 6. MVP 7. Normal Biventricular Systolic Function | | | |
| **Remark**: Marfan Syndrome can be considered as differential Diagnosis. | | | |
| **Recommendation**: | | | |
| SIGNATURE  Done by: Tesfaye T., Pediatrician, Pediatric Cardiologist _______________ 18/04/2015Eth.C | | | |

| Patient Name: **Baby of Tena Girum**. Referring Institute: **FHRH**. SEX/ Age: **M/10days**. Date of Report: **18/04/15**.  Referral Diagnosis: **RD. AGH2.128** | | | |
| --- | --- | --- | --- |
| **Features** | **Finding** | **Features** | **Finding** |
| **Profile** |  | **Atria** |  |
| Abdominal situs | Solitus | Left atrium | Normal |
| Cardiac position | Levocardia | Right atrium | Normal |
| Systemic venous drainage | Normal. | **Atrioventricular valves** |  |
| Pulmonary venous drainage | Normal | Mitral valve | Annulus = 11mm |
| Atrioventricular connection | Concordant | Tricuspid valve | Annulus = 12mm |
| Ventriculoarterial connection | Concordant | **Ventricles** |  |
| Ventricular loop | d-Loop | Left ventricle | Normal |
|  |  | Right ventricle | Normal |
| **Septae** |  | **Coronary arteries** | ----- |
| Interventricular septum | Intact | **Doppler Measurement** |  |
| Interatrial septum | PFO, L – R Shunt | Mitral | ----- |
| **Semilunar valves** |  | Aortic | ------- |
| Aortic valve | Annulus = 8mm | Tricuspid | Trivial TR, PPG = 15mmHg |
| Pulmonary valve | Annulus = 9mm | pulmonic | -------- |
| **Great arteries** | NRGA | **Aortic arch** | Left. No CoA. |
| Aorta | ----- | **PDA** | No |
| Pulmonary artery | Normal MPA and Branch PAs. |  |  |
| **M-Mode:**  Normal LV Function on eye balling | | | |
| AO | mm | PWd | mm |
| LA | mm | PWs | mm |
| LVIDd | mm | EDV | ml |
| LVIDs | mm | ESV | ml |
| IVSs | mm | LVEF | % |
| IVSd | mm | FS | % |
| **Additional Information**: |  | | |
| No pericardial/Pleural effusion. | | | |
| **Final Diagnosis:** | | | |
| 1. {S, D, S} Levocardia. 2. PFO, L – R Shunt | | | |
| **Remark**: | | | |
| **Recommendation**: | | | |
| SIGNATURE  Done by: Tesfaye T., Pediatrician, Pediatric Cardiologist _______________ 18/04/2015Eth.C | | | |

| Patient Name: **Baby of Yeshi Ejigu**. Referring Institute: **FHRH**. SEX/ Age: **M/11days**. Date of Report: **18/04/15**.  Referral Diagnosis: **Incidental Murmur Finding. AGH2.129** | | | |
| --- | --- | --- | --- |
| **Features** | **Finding** | **Features** | **Finding** |
| **Profile** |  | **Atria** |  |
| Abdominal situs | Solitus | Left atrium | Normal |
| Cardiac position | Levocardia | Right atrium | Normal |
| Systemic venous drainage | Normal. | **Atrioventricular valves** |  |
| Pulmonary venous drainage | Normal | Mitral valve | Annulus = 9mm |
| Atrioventricular connection | Concordant | Tricuspid valve | Annulus = 9mm |
| Ventriculoarterial connection | Concordant | **Ventricles** |  |
| Ventricular loop | d-Loop | Left ventricle | Normal |
|  |  | Right ventricle | Normal |
| **Septae** |  | **Coronary arteries** | ----- |
| Interventricular septum | Intact | **Doppler Measurement** |  |
| Interatrial septum | PFO, L – R Shunt | Mitral | ----- |
| **Semilunar valves** |  | Aortic | ------- |
| Aortic valve | Annulus = 9mm | Tricuspid | ------- |
| Pulmonary valve | Annulus = 9mm | pulmonic | -------- |
| **Great arteries** | NRGA | **Aortic arch** | Left. No CoA. |
| Aorta | ----- | **PDA** | 1.5mm PDA, L – R Shunt |
| Pulmonary artery | Normal MPA and Branch PAs. |  |  |
| **M-Mode:**  Normal LV Function on eye balling | | | |
| AO | mm | PWd | mm |
| LA | mm | PWs | mm |
| LVIDd | mm | EDV | ml |
| LVIDs | mm | ESV | ml |
| IVSs | mm | LVEF | % |
| IVSd | mm | FS | % |
| **Additional Information**: |  | | |
| No pericardial/Pleural effusion. | | | |
| **Final Diagnosis:** | | | |
| 1. {S, D, S} Levocardia. 2. PFO, L – R Shunt 3. Moderate PDA, L – R Shunt 4. Normal LV Systolic Function | | | |
| **Remark**: | | | |
| **Recommendation**: | | | |
| SIGNATURE  Done by: Tesfaye T., Pediatrician, Pediatric Cardiologist _______________ 18/04/2015Eth.C | | | |

| Patient Name: **Baby of Niwaye-werk Tamene**. Referring Institute: **TGSH**. SEX/ Age: **M/32days**. Date of Report: **18/04/15**.  Referral Diagnosis: **RD. AGH2.130** | | | |
| --- | --- | --- | --- |
| **Features** | **Finding** | **Features** | **Finding** |
| **Profile** |  | **Atria** |  |
| Abdominal situs | Solitus | Left atrium | Normal |
| Cardiac position | Levocardia | Right atrium | Normal |
| Systemic venous drainage | Normal. | **Atrioventricular valves** |  |
| Pulmonary venous drainage | Normal | Mitral valve | Annulus = 12mm |
| Atrioventricular connection | Concordant | Tricuspid valve | Annulus = 11mm |
| Ventriculoarterial connection | Concordant | **Ventricles** |  |
| Ventricular loop | d-Loop | Left ventricle | Normal |
|  |  | Right ventricle | Normal |
| **Septae** |  | **Coronary arteries** | ----- |
| Interventricular septum | Intact | **Doppler Measurement** |  |
| Interatrial septum | PFO, L – R Shunt | Mitral | ----- |
| **Semilunar valves** |  | Aortic | ------- |
| Aortic valve | Annulus = 10mm | Tricuspid | ------- |
| Pulmonary valve | Annulus = 12mm | pulmonic | -------- |
| **Great arteries** | NRGA | **Aortic arch** | Left. No CoA. |
| Aorta | ----- | **PDA** | No |
| Pulmonary artery | Normal MPA and Branch PAs. |  |  |
| **M-Mode:**  Normal LV Function on eye balling | | | |
| AO | mm | PWd | mm |
| LA | mm | PWs | mm |
| LVIDd | mm | EDV | ml |
| LVIDs | mm | ESV | ml |
| IVSs | mm | LVEF | % |
| IVSd | mm | FS | % |
| **Additional Information**: |  | | |
| No pericardial/Pleural effusion. | | | |
| **Final Diagnosis:** | | | |
| 1. {S, D, S} Levocardia. 2. PFO, L – R Shunt | | | |
| **Remark**: | | | |
| **Recommendation**: | | | |
| SIGNATURE  Done by: Tesfaye T., Pediatrician, Pediatric Cardiologist _______________ 18/04/2015Eth.C | | | |

| Patient Name: **Selam Siyamir**. Referring Institute: **FHRH**. SEX/ Age: **F/3years**. Date of Report: **19/04/15**.  Referral Diagnosis: **Incidental Murmur Finding. AGH2.131** | | | |
| --- | --- | --- | --- |
| **Features** | **Finding** | **Features** | **Finding** |
| **Profile** |  | **Atria** |  |
| Abdominal situs | Solitus | Left atrium | Normal |
| Cardiac position | Levocardia | Right atrium | Normal |
| Systemic venous drainage | Normal. | **Atrioventricular valves** |  |
| Pulmonary venous drainage | Normal | Mitral valve | Annulus = 15mm |
| Atrioventricular connection | Concordant | Tricuspid valve | Annulus = 15mm |
| Ventriculoarterial connection | Concordant | **Ventricles** |  |
| Ventricular loop | d-Loop | Left ventricle | Normal |
|  |  | Right ventricle | Normal |
| **Septae** |  | **Coronary arteries** | ----- |
| Interventricular septum | Intact | **Doppler Measurement** |  |
| Interatrial septum | Intact | Mitral | ----- |
| **Semilunar valves** |  | Aortic | ------- |
| Aortic valve | Annulus = 14mm | Tricuspid | ------- |
| Pulmonary valve | Annulus = 15mm | pulmonic | -------- |
| **Great arteries** | NRGA | **Aortic arch** | Left. No CoA. |
| Aorta | ----- | **PDA** | 2mm PDA, L – R Shunt |
| Pulmonary artery | Normal MPA and Branch PAs. |  |  |
| **M-Mode:**  Normal LV Function on eye balling | | | |
| AO | mm | PWd | mm |
| LA | mm | PWs | mm |
| LVIDd | mm | EDV | ml |
| LVIDs | mm | ESV | ml |
| IVSs | mm | LVEF | % |
| IVSd | mm | FS | % |
| **Additional Information**: |  | | |
| No pericardial/Pleural effusion. | | | |
| **Final Diagnosis:** | | | |
| 1. {S, D, S} Levocardia. 2. Moderate PDA, L – R Shunt 3. Normal LV Systolic Function | | | |
| **Remark**: | | | |
| **Recommendation**: | | | |
| SIGNATURE  Done by: Tesfaye T., Pediatrician, Pediatric Cardiologist _______________ 19/04/2015Eth.C | | | |

| Patient Name: **Mulugeta Gebrie**. Referring Institute: **TGSH**. SEX/ Age: **M/61days**. Date of Report: **20/04/15**.  Referral Diagnosis: **Incidental Murmur Finding. AGH2.132** | | | |
| --- | --- | --- | --- |
| **Features** | **Finding** | **Features** | **Finding** |
| **Profile** |  | **Atria** |  |
| Abdominal situs | Solitus | Left atrium | Normal |
| Cardiac position | Levocardia | Right atrium | Normal |
| Systemic venous drainage | Normal. | **Atrioventricular valves** |  |
| Pulmonary venous drainage | Normal | Mitral valve | Annulus = 12mm |
| Atrioventricular connection | Concordant | Tricuspid valve | Annulus = 11mm |
| Ventriculoarterial connection | Concordant | **Ventricles** |  |
| Ventricular loop | d-Loop | Left ventricle | Normal |
|  |  | Right ventricle | Normal |
| **Septae** |  | **Coronary arteries** | ----- |
| Interventricular septum | Intact | **Doppler Measurement** |  |
| Interatrial septum | PFO, L – R Shunt | Mitral | ----- |
| **Semilunar valves** |  | Aortic | ------- |
| Aortic valve | Annulus = 8mm | Tricuspid | ------- |
| Pulmonary valve | Annulus = 10mm | pulmonic | -------- |
| **Great arteries** | NRGA | **Aortic arch** | Left. No CoA. |
| Aorta | ----- | **PDA** | <1mm PDA, L – R Shunt |
| Pulmonary artery | Normal MPA and Branch PAs. |  |  |
| **M-Mode:**  Normal LV Function on eye balling. | | | |
| AO | mm | PWd | mm |
| LA | mm | PWs | mm |
| LVIDd | mm | EDV | ml |
| LVIDs | mm | ESV | ml |
| IVSs | mm | LVEF | % |
| IVSd | mm | FS | % |
| **Additional Information**: |  | | |
| No pericardial/Pleural effusion. | | | |
| **Final Diagnosis:** | | | |
| 1. {S, D, S} Levocardia. 2. PFO, L – R Shunt 3. Small PDA, L – R Shunt 4. Normal LV Systolic Function | | | |
| **Remark**: | | | |
| **Recommendation**: | | | |
| SIGNATURE  Done by: Tesfaye T., Pediatrician, Pediatric Cardiologist _______________ 20/04/2015Eth.C | | | |

| Patient Name: **Dawit Genetu**. Referring Institute: **TGSH**. SEX/ Age: **M/13years**. Date of Report: **20/04/15**.  Referral Diagnosis: **__________________. AGH2.133** | | | |
| --- | --- | --- | --- |
| **Features** | **Finding** | **Features** | **Finding** |
| **Profile** |  | **Atria** |  |
| Abdominal situs | Solitus | Left atrium | Normal |
| Cardiac position | Levocardia | Right atrium | Dilated |
| Systemic venous drainage | Normal. | **Atrioventricular valves** |  |
| Pulmonary venous drainage | Normal | Mitral valve | Annulus = 16mm |
| Atrioventricular connection | Concordant | Tricuspid valve | Annulus = 27mm  TAPSE = 19mm |
| Ventriculoarterial connection | DOLV | **Ventricles** |  |
| Ventricular loop | d-Loop | Left ventricle | Smallish |
|  |  | Right ventricle | Dilated |
| **Septae** |  | **Coronary arteries** | ----- |
| Interventricular septum | 20mm Inlet VSD, R – L Shunt | **Doppler Measurement** |  |
| Interatrial septum | 11mm OS ASD, L – R Shunt | Mitral | ----- |
| **Semilunar valves** |  | Aortic | ------- |
| Aortic valve | **Annulus = 28mm** | Tricuspid | ------- |
| Pulmonary valve | Annulus = 16mm. Hyper-echoic mass at the tip of the PV on the PA side | pulmonic | Moderate PS, PPG = 50mmHg |
| **Great arteries** | d-TGA | **Aortic arch** | Left. No CoA. |
| Aorta | Anterior & from LV. Asc.aorta = 18mm. STJ = 18mm, **Sinus = 32mm.** | **PDA** | No |
| Pulmonary artery | Posterior & from LV |  |  |
| **M-Mode:** | | | |
| AO | mm | PWd | mm |
| LA | mm | PWs | mm |
| LVIDd | mm | EDV | ml |
| LVIDs | mm | ESV | ml |
| IVSs | mm | LVEF | % |
| IVSd | mm | FS | % |
| **Additional Information**: |  | | |
| No pericardial/Pleural effusion. | | | |
| **Final Diagnosis:** | | | |
| 1. {S, D, D} Levocardia. 2. RA/RV Dilated 3. DOLV 4. d-TGA 5. Large OS ASD, L – R Shunt 6. Large Inlet VSD, R – L Shunt 7. Smallish LV 8. Moderate PS (LVOTO) 9. Dilated SoV & Aortic annulus 10. Hyper-echoic mass on the tip of the PV on PA Side | | | |
| **Recommendation**: | | | |
| SIGNATURE  Done by: Tesfaye T., Pediatrician, Pediatric Cardiologist _______________ 20/04/2015Eth.C | | | |

| Patient Name: **Hafiza Birhan**. Referring Institute: **FHRH**. SEX/ Age: **F/9years**. Date of Report: **20/04/15**.  Referral Diagnosis: **Easy fatigability. AGH2.134** | | | |
| --- | --- | --- | --- |
| **Features** | **Finding** | **Features** | **Finding** |
| **Profile** |  | **Atria** |  |
| Abdominal situs | Solitus | Left atrium | Normal |
| Cardiac position | Levocardia | Right atrium | Normal |
| Systemic venous drainage | Normal. | **Atrioventricular valves** |  |
| Pulmonary venous drainage | Normal | Mitral valve | Annulus = 16mm |
| Atrioventricular connection | Concordant | Tricuspid valve | Annulus = 18mm  TAPSE = 19mm |
| Ventriculoarterial connection | Concordant | **Ventricles** |  |
| Ventricular loop | d-Loop | Left ventricle | Normal |
|  |  | Right ventricle | Normal |
| **Septae** |  | **Coronary arteries** | ----- |
| Interventricular septum | Intact | **Doppler Measurement** |  |
| Interatrial septum | Intact | Mitral | ----- |
| **Semilunar valves** |  | Aortic | ------- |
| Aortic valve | Annulus = 16mm | Tricuspid | ------- |
| Pulmonary valve | Annulus = 19mm | pulmonic | Trivial PR, PPG = 8mmHg |
| **Great arteries** | NRGA | **Aortic arch** | Left. No CoA. |
| Aorta | ----- | **PDA** | No |
| Pulmonary artery | Normal MPA and Branch PAs. |  |  |
| **M-Mode:** | | | |
| AO | mm | PWd | mm |
| LA | mm | PWs | mm |
| LVIDd | mm | EDV | ml |
| LVIDs | mm | ESV | ml |
| IVSs | mm | LVEF | 59% |
| IVSd | mm | FS | 30% |
| **Additional Information**: |  | | |
| No pericardial/Pleural effusion. | | | |
| **Final Diagnosis:** | | | |
| 1. Normal Echocardiography Study. | | | |
| **Remark**: | | | |
| **Recommendation**: | | | |
| SIGNATURE  Done by: Tesfaye T., Pediatrician, Pediatric Cardiologist _______________ 20/04/2015Eth.C | | | |

| Patient Name: **Yosef Gizachew**. Referring Institute: **Adinas GH**. SEX/ Age: **M/8 9/12**. Date of Report: **20/04/15**.  Referral Diagnosis: **Chest Pain. AGH2.135** | | | |
| --- | --- | --- | --- |
| **Features** | **Finding** | **Features** | **Finding** |
| **Profile** |  | **Atria** |  |
| Abdominal situs | Solitus | Left atrium | Normal |
| Cardiac position | Levocardia | Right atrium | Normal |
| Systemic venous drainage | Normal. | **Atrioventricular valves** |  |
| Pulmonary venous drainage | Normal | Mitral valve | Annulus = 19mm |
| Atrioventricular connection | Concordant | Tricuspid valve | Annulus = 20mm  TAPSE = 20mm |
| Ventriculoarterial connection | Concordant | **Ventricles** |  |
| Ventricular loop | d-Loop | Left ventricle | Normal |
|  |  | Right ventricle | Normal |
| **Septae** |  | **Coronary arteries** | ----- |
| Interventricular septum | Intact | **Doppler Measurement** |  |
| Interatrial septum | Intact | Mitral | ----- |
| **Semilunar valves** |  | Aortic | ------- |
| Aortic valve | Annulus = 16mm | Tricuspid | Trivial TR, PPG = 16mmHg |
| Pulmonary valve | Annulus = 16mm | pulmonic | -------- |
| **Great arteries** | NRGA | **Aortic arch** | Left. No CoA. |
| Aorta | ----- | **PDA** | No |
| Pulmonary artery | Normal MPA and Branch PAs. |  |  |
| **M-Mode:** | | | |
| AO | mm | PWd | mm |
| LA | mm | PWs | mm |
| LVIDd | mm | EDV | ml |
| LVIDs | mm | ESV | ml |
| IVSs | mm | LVEF | 64% |
| IVSd | mm | FS | 34% |
| **Additional Information**: |  | | |
| No pericardial/Pleural effusion. | | | |
| **Final Diagnosis:** | | | |
| 1. Normal Echocardiography Study. | | | |
| **Remark**: | | | |
| **Recommendation**: | | | |
| SIGNATURE  Done by: Tesfaye T., Pediatrician, Pediatric Cardiologist _______________ 20/04/2015Eth.C | | | |

| Patient Name: **Baby of Mekdes Tegegne**. Referring Institute: **TGSH**. SEX/ Age: **F/5days**. Date of Report: **21/04/15**.  Referral Diagnosis: **DS. AGH2.136** | | | |
| --- | --- | --- | --- |
| **Features** | **Finding** | **Features** | **Finding** |
| **Profile** |  | **Atria** |  |
| Abdominal situs | Solitus | Left atrium | Normal |
| Cardiac position | Levocardia | Right atrium | Normal |
| Systemic venous drainage | Normal. | **Atrioventricular valves** |  |
| Pulmonary venous drainage | Normal | Mitral valve | Annulus = 9mm |
| Atrioventricular connection | Concordant | Tricuspid valve | Annulus = 9mm |
| Ventriculoarterial connection | Concordant | **Ventricles** |  |
| Ventricular loop | d-Loop | Left ventricle | Normal |
|  |  | Right ventricle | Normal |
| **Septae** |  | **Coronary arteries** | ----- |
| Interventricular septum | Intact | **Doppler Measurement** |  |
| Interatrial septum | PFO, L – R Shunt | Mitral | ----- |
| **Semilunar valves** |  | Aortic | ------- |
| Aortic valve | Annulus = 9mm | Tricuspid | ------- |
| Pulmonary valve | Annulus = 8mm | pulmonic | -------- |
| **Great arteries** | NRGA | **Aortic arch** | Left. No CoA. |
| Aorta | ----- | **PDA** | No |
| Pulmonary artery | Normal MPA and Branch PAs. |  |  |
| **M-Mode:**  Normal LV Function on eye balling | | | |
| AO | mm | PWd | mm |
| LA | mm | PWs | mm |
| LVIDd | mm | EDV | ml |
| LVIDs | mm | ESV | ml |
| IVSs | mm | LVEF | % |
| IVSd | mm | FS | % |
| **Additional Information**: |  | | |
| No pericardial/Pleural effusion. | | | |
| **Final Diagnosis:** | | | |
| 1. {S, D, S} Levocardia. 2. PFO, L – R Shunt | | | |
| **Remark**: | | | |
| **Recommendation**: | | | |
| SIGNATURE  Done by: Tesfaye T., Pediatrician, Pediatric Cardiologist _______________ 21/04/2015Eth.C | | | |

| Patient Name: **Gojam Mitiku**. Referring Institute: **TGSH**. SEX/ Age: **F/3months**. Date of Report: **21/04/15**.  Referral Diagnosis: **Incidental Murmur Finding. AGH2.137** | | | |
| --- | --- | --- | --- |
| **Features** | **Finding** | **Features** | **Finding** |
| **Profile** |  | **Atria** |  |
| Abdominal situs | Solitus | Left atrium | Normal |
| Cardiac position | Levocardia | Right atrium | Normal |
| Systemic venous drainage | Normal. | **Atrioventricular valves** |  |
| Pulmonary venous drainage | Normal | Mitral valve | Annulus = 11mm |
| Atrioventricular connection | Concordant | Tricuspid valve | Annulus = 11mm  TAPSE = 16mm |
| Ventriculoarterial connection | Concordant | **Ventricles** |  |
| Ventricular loop | d-Loop | Left ventricle | Normal |
|  |  | Right ventricle | Normal |
| **Septae** |  | **Coronary arteries** | ----- |
| Interventricular septum | 5mm PM VSD, Partially covered by STL, L – R Shunt | **Doppler Measurement** |  |
| Interatrial septum | Intact | Mitral | ----- |
| **Semilunar valves** |  | Aortic | ------- |
| Aortic valve | Annulus = 11mm | Tricuspid | ------- |
| Pulmonary valve | Annulus = 10mm | pulmonic | -------- |
| **Great arteries** | NRGA | **Aortic arch** | Left. No CoA. |
| Aorta | ----- | **PDA** | <1mm PDA, L – R Shunt |
| Pulmonary artery | Normal MPA and Branch PAs. |  |  |
| **M-Mode:**  Normal LV Function on eye balling. | | | |
| AO | mm | PWd | mm |
| LA | mm | PWs | mm |
| LVIDd | mm | EDV | ml |
| LVIDs | mm | ESV | ml |
| IVSs | mm | LVEF | % |
| IVSd | mm | FS | % |
| **Additional Information**: |  | | |
| No pericardial/Pleural effusion. | | | |
| **Final Diagnosis:** | | | |
| 1. {S, D, S} Levocardia. 2. Moderate PM VSD, Partially closed by STL, L – R Shunt 3. Small PDA, L – R Shunt 4. Normal Biventricular Systolic Function | | | |
| **Remark**: | | | |
| **Recommendation**: | | | |
| SIGNATURE  Done by: Tesfaye T., Pediatrician, Pediatric Cardiologist _______________ 21/04/2015Eth.C | | | |

| Patient Name: **Baby of Nigistie Chalie**. Referring Institute: **TGSH**. SEX/ Age: **F/1day**. Date of Report: **21/04/15**.  Referral Diagnosis: **DS. AGH2.138** | | | |
| --- | --- | --- | --- |
| **Features** | **Finding** | **Features** | **Finding** |
| **Profile** |  | **Atria** |  |
| Abdominal situs | Solitus | Left atrium | Normal |
| Cardiac position | Levocardia | Right atrium | Dilated |
| Systemic venous drainage | Normal. | **Atrioventricular valves** |  |
| Pulmonary venous drainage | RUPV to RA through High Secundum. | Mitral valve | Annulus = 6mm |
| Atrioventricular connection | Concordant | Tricuspid valve | Annulus = 10mm  TAPSE = 9mm |
| Ventriculoarterial connection | Concordant | **Ventricles** |  |
| Ventricular loop | d-Loop | Left ventricle | Normal |
|  |  | Right ventricle | Dilated |
| **Septae** |  | **Coronary arteries** | ----- |
| Interventricular septum | Intact | **Doppler Measurement** |  |
| Interatrial septum | 8mm High Secundum ASD, L – R Shunt | Mitral | ----- |
| **Semilunar valves** |  | Aortic | ------- |
| Aortic valve | Annulus = 8mm | Tricuspid | ------- |
| Pulmonary valve | Annulus = 9mm | pulmonic | -------- |
| **Great arteries** | NRGA | **Aortic arch** | Left. No CoA. |
| Aorta | ----- | **PDA** | 1.5mm PDA, L – R Shunt |
| Pulmonary artery | Normal MPA and Branch PAs. |  |  |
| **M-Mode:**  Normal LV Function on eye balling | | | |
| AO | mm | PWd | mm |
| LA | mm | PWs | mm |
| LVIDd | mm | EDV | ml |
| LVIDs | mm | ESV | ml |
| IVSs | mm | LVEF | % |
| IVSd | mm | FS | % |
| **Additional Information**: |  | | |
| No pericardial/Pleural effusion. | | | |
| **Final Diagnosis:** | | | |
| 1. {S, D, S} Levocardia. 2. RA/RV Dilated 3. PAPVC of RUPV to RA 4. Moderate High Secundum ASD, L – R Shunt 5. Moderate PDA, L – R Shunt 6. Normal Biventricular Systolic Function | | | |
| **Remark**: | | | |
| **Recommendation**: | | | |
| SIGNATURE  Done by: Tesfaye T., Pediatrician, Pediatric Cardiologist _______________ 21/04/2015Eth.C | | | |

| Patient Name: **Dawit Mulugeta**. Referring Institute: **FHRH**. SEX/ Age: **M/1year**. Date of Report: **22/04/15**.  Referral Diagnosis: **Recurrent chest Infection. AGH2.139** | | | |
| --- | --- | --- | --- |
| **Features** | **Finding** | **Features** | **Finding** |
| **Profile** |  | **Atria** |  |
| Abdominal situs | Solitus | Left atrium | Normal |
| Cardiac position | Levocardia | Right atrium | Normal |
| Systemic venous drainage | Normal. | **Atrioventricular valves** |  |
| Pulmonary venous drainage | Normal | Mitral valve | Annulus = 14mm |
| Atrioventricular connection | Concordant | Tricuspid valve | Annulus = 15mm  TAPSE = 16mm |
| Ventriculoarterial connection | Concordant | **Ventricles** |  |
| Ventricular loop | d-Loop | Left ventricle | Normal |
|  |  | Right ventricle | Normal |
| **Septae** |  | **Coronary arteries** | ----- |
| Interventricular septum | Intact | **Doppler Measurement** |  |
| Interatrial septum | Intact | Mitral | ----- |
| **Semilunar valves** |  | Aortic | ------- |
| Aortic valve | Annulus = 13mm | Tricuspid | ------- |
| Pulmonary valve | Annulus = 14mm | pulmonic | -------- |
| **Great arteries** | NRGA | **Aortic arch** | Left. No CoA. |
| Aorta | ----- | **PDA** | No |
| Pulmonary artery | Normal MPA and Branch PAs. |  |  |
| **M-Mode:**  Normal LV Function on eye balling | | | |
| AO | mm | PWd | mm |
| LA | mm | PWs | mm |
| LVIDd | mm | EDV | ml |
| LVIDs | mm | ESV | ml |
| IVSs | mm | LVEF | % |
| IVSd | mm | FS | % |
| **Additional Information**: |  | | |
| No pericardial/Pleural effusion. | | | |
| **Final Diagnosis:** | | | |
| 1. Normal Echocardiography Study. | | | |
| **Remark**: | | | |
| **Recommendation**: | | | |
| SIGNATURE  Done by: Tesfaye T., Pediatrician, Pediatric Cardiologist _______________ 22/04/2015Eth.C | | | |

| Patient Name: **Yalem-Sira Dessie**. Referring Institute: **FHRH**. SEX/ Age: **F/5months**. Date of Report: **22/04/15**.  Referral Diagnosis: **SVT + CHF + Shock+ RD. AGH2.140 (TGSH3)** | | | |
| --- | --- | --- | --- |
| **Features** | **Finding** | **Features** | **Finding** |
| **Profile** |  | **Atria** |  |
| Abdominal situs | Solitus | Left atrium | Normal |
| Cardiac position | Levocardia | Right atrium | Normal |
| Systemic venous drainage | Normal. | **Atrioventricular valves** |  |
| Pulmonary venous drainage | Normal | Mitral valve | Annulus = 15mm |
| Atrioventricular connection | Concordant | Tricuspid valve | Annulus = 16mm |
| Ventriculoarterial connection | Concordant | **Ventricles** |  |
| Ventricular loop | d-Loop | Left ventricle | Normal |
|  |  | Right ventricle | Normal |
| **Septae** | Abnormal Septal Motion | **Coronary arteries** | ----- |
| Interventricular septum | Intact | **Doppler Measurement** |  |
| Interatrial septum | 13mm OS ASD, BD Shunt | Mitral | Mitral Inflow respiratory Variability > 25% |
| **Semilunar valves** |  | Aortic | ------- |
| Aortic valve | Annulus = 9mm | Tricuspid | Moderate TR, PPG = 60mmHg |
| Pulmonary valve | Annulus = 11mm | pulmonic | -------- |
| **Great arteries** | NRGA | **Aortic arch** | Left. No CoA. |
| Aorta | ----- | **PDA** | No |
| Pulmonary artery | Normal MPA and Branch PAs. |  |  |
| **M-Mode:** | | | |
| AO | mm | PWd | mm |
| LA | mm | PWs | mm |
| LVIDd | mm | EDV | ml |
| LVIDs | mm | ESV | ml |
| IVSs | mm | LVEF | % |
| IVSd | mm | FS | % |
| **Additional Information**: |  | | |
| Circumferential pericardial effusion with a maximum depth of 24mm on LV Side. Echo debris in the effusion with shaggy borders on both the parietal and visceral side. Swinging Heart. | | | |
| **Final Diagnosis:** | | | |
| 1. {S, D, S} Levocardia. 2. Large OS ASD, BD Shunt 3. Moderate TR 4. Severe Pulmonary Hypertension 5. Large Circumferential Pericardial Effusion (?Purulent) 6. Feature of Cardiac Tamponade | | | |
| **Remark**: extreme Tachycardia during study | | | |
| **Recommendation**: Emergency Decompression is recommended. | | | |
| SIGNATURE  Done by: Tesfaye T., Pediatrician, Pediatric Cardiologist _______________ 22/04/2015Eth.C | | | |

| Patient Name: **Tadla Chekole**. Referring Institute: **Addis Alem PH**. SEX/ Age: **F/5years**. Date of Report: **22/04/15**.  Referral Diagnosis: **CHF. AGH2.141** | | | |
| --- | --- | --- | --- |
| **Features** | **Finding** | **Features** | **Finding** |
| **Profile** |  | **Atria** |  |
| Abdominal situs | Solitus | Left atrium | Dilated |
| Cardiac position | Levocardia | Right atrium | Dilated |
| Systemic venous drainage | Normal. | **Atrioventricular valves** |  |
| Pulmonary venous drainage | Normal | Mitral valve | Annulus = 19mm. Aorto-Mitral Discontinuity |
| Atrioventricular connection | Concordant | Tricuspid valve | Annulus = 21mm  TAPSE = 16mm |
| Ventriculoarterial connection | DORV | **Ventricles** |  |
| Ventricular loop | d-Loop | Left ventricle | Dilated |
|  |  | Right ventricle | Dilated & Hypertrophied |
| **Septae** |  | **Coronary arteries** | ----- |
| Interventricular septum | 13mm Sub-Arterial VSD, L – R Shunt | **Doppler Measurement** |  |
| Interatrial septum | Intact | Mitral | ----- |
| **Semilunar valves** |  | Aortic | Mild AR. |
| Aortic valve | Annulus = 20mm | Tricuspid | Mild TR |
| Pulmonary valve | Annulus = 23mm | pulmonic | -------- |
| **Great arteries** | AP | **Aortic arch** | Left. No CoA. |
| Aorta | Posterior and from RV | **PDA** | No |
| Pulmonary artery | Anterior and from RV. MPA = 30mm. |  |  |
| **M-Mode:**  Normal LV Systolic Function on eye balling | | | |
| AO | mm | PWd | mm |
| LA | mm | PWs | mm |
| LVIDd | mm | EDV | ml |
| LVIDs | mm | ESV | ml |
| IVSs | mm | LVEF | % |
| IVSd | mm | FS | % |
| **Additional Information**: |  | | |
| No pericardial/Pleural effusion. | | | |
| **Final Diagnosis:** | | | |
| 1. {S, D, Antero-posterior} Levocardia. 2. All chambers dilated, RV Hypertrophied 3. DORV 4. Large Sub-arterial VSD, L – R Shunt 5. Severe Pulmonary Hypertension 6. Normal Biventricular Systolic Function | | | |
| **Remark**: | | | |
| **Recommendation**: Needs additional imaging | | | |
| SIGNATURE  Done by: Tesfaye T., Pediatrician, Pediatric Cardiologist _______________ 22/04/2015Eth.C | | | |

| Patient Name: **Baby of Sintayehu Demissie**. Referring Institute: **FHRH**. SEX/ Age: **M/13days**. Date of Report: **22/04/15**.  Referral Diagnosis: **DS. AGH2.142** | | | |
| --- | --- | --- | --- |
| **Features** | **Finding** | **Features** | **Finding** |
| **Profile** |  | **Atria** |  |
| Abdominal situs | Solitus | Left atrium | Normal |
| Cardiac position | Levocardia | Right atrium | Normal |
| Systemic venous drainage | Normal. | **Atrioventricular valves** |  |
| Pulmonary venous drainage | Normal | Mitral valve | Annulus = 10mm |
| Atrioventricular connection | Concordant | Tricuspid valve | Annulus = 11mm |
| Ventriculoarterial connection | Concordant | **Ventricles** |  |
| Ventricular loop | d-Loop | Left ventricle | Normal |
|  |  | Right ventricle | Normal |
| **Septae** |  | **Coronary arteries** | ----- |
| Interventricular septum | Intact | **Doppler Measurement** |  |
| Interatrial septum | PFO, L – R Shunt | Mitral | ----- |
| **Semilunar valves** |  | Aortic | ------- |
| Aortic valve | Annulus = 9mm | Tricuspid | ------- |
| Pulmonary valve | Annulus = 9mm | pulmonic | -------- |
| **Great arteries** | NRGA | **Aortic arch** | Left. No CoA. |
| Aorta | ----- | **PDA** | No |
| Pulmonary artery | Normal MPA and Branch PAs. |  |  |
| **M-Mode:**  Normal LV Function on eye balling | | | |
| AO | mm | PWd | mm |
| LA | mm | PWs | mm |
| LVIDd | mm | EDV | ml |
| LVIDs | mm | ESV | ml |
| IVSs | mm | LVEF | % |
| IVSd | mm | FS | % |
| **Additional Information**: |  | | |
| No pericardial/Pleural effusion. | | | |
| **Final Diagnosis:** | | | |
| 1. {S, D, S} Levocardia. 2. PFO, L – R Shunt | | | |
| **Remark**: | | | |
| **Recommendation**: | | | |
| SIGNATURE  Done by: Tesfaye T., Pediatrician, Pediatric Cardiologist _______________ 22/04/2015Eth.C | | | |

| Patient Name: **Baby of Nardos Afe-werk**. Referring Institute: **FHRH**. SEX/ Age: **M/21days**. Date of Report: **24/04/15**.  Referral Diagnosis: **RD R/o PPHN. AGH2.143** | | | |
| --- | --- | --- | --- |
| **Features** | **Finding** | **Features** | **Finding** |
| **Profile** |  | **Atria** |  |
| Abdominal situs | Solitus | Left atrium | Normal |
| Cardiac position | Levocardia | Right atrium | Normal |
| Systemic venous drainage | Normal. | **Atrioventricular valves** |  |
| Pulmonary venous drainage | Normal | Mitral valve | Annulus = 12mm |
| Atrioventricular connection | Concordant | Tricuspid valve | Annulus = 11mm  TAPSE = 12mm |
| Ventriculoarterial connection | Concordant | **Ventricles** |  |
| Ventricular loop | d-Loop | Left ventricle | Normal |
|  |  | Right ventricle | Normal |
| **Septae** |  | **Coronary arteries** | ----- |
| Interventricular septum | Intact | **Doppler Measurement** |  |
| Interatrial septum | PFO, L – R Shunt | Mitral | ----- |
| **Semilunar valves** |  | Aortic | ------- |
| Aortic valve | Annulus = 10mm | Tricuspid | ------- |
| Pulmonary valve | Annulus = 11mm | pulmonic | -------- |
| **Great arteries** | NRGA | **Aortic arch** | Left. No CoA. |
| Aorta | ----- | **PDA** | No |
| Pulmonary artery | Normal MPA and Branch PAs. |  |  |
| **M-Mode:**  Normal LV Function on eye balling | | | |
| AO | mm | PWd | mm |
| LA | mm | PWs | mm |
| LVIDd | mm | EDV | ml |
| LVIDs | mm | ESV | ml |
| IVSs | mm | LVEF | % |
| IVSd | mm | FS | % |
| **Additional Information**: |  | | |
| No pericardial/Pleural effusion. | | | |
| **Final Diagnosis:** | | | |
| 1. {S, D, S} Levocardia. 2. PFO, L – R Shunt 3. Normal Biventricular Systolic Function | | | |
| **Remark**: No features suggestive of PPHN | | | |
| **Recommendation**: | | | |
| SIGNATURE  Done by: Tesfaye T., Pediatrician, Pediatric Cardiologist _______________ 24/04/2015Eth.C | | | |

| Patient Name: **Werku Dessie**. Referring Institute: **FHRH**. SEX/ Age: **M/12years**. Date of Report: **24/04/15**.  Referral Diagnosis: **CHF + Rheumatic recurrence. AGH2.144** | | | |
| --- | --- | --- | --- |
| **Features** | **Finding** | **Features** | **Finding** |
| **Profile** |  | **Atria** |  |
| Abdominal situs | Solitus | Left atrium | Dilated |
| Cardiac position | Levocardia | Right atrium | Normal |
| Systemic venous drainage | Normal. | **Atrioventricular valves** |  |
| Pulmonary venous drainage | Normal | Mitral valve | Annulus = 27mm. Thickened MVL. Shortened PMVL. MVA =3cm**2**. |
| Atrioventricular connection | Concordant | Tricuspid valve | Annulus = 27mm  TAPSE = 21mm |
| Ventriculoarterial connection | Concordant | **Ventricles** |  |
| Ventricular loop | d-Loop | Left ventricle | Dilated |
|  |  | Right ventricle | Normal |
| **Septae** |  | **Coronary arteries** | ----- |
| Interventricular septum | Intact | **Doppler Measurement** |  |
| Interatrial septum | Intact | Mitral | Severe MR, Holosystolic, posterior projection, seen in two planes with jet velocity = 3.8m/sec. Mitral Inflow gradient, PPG/MPG = 12/6mmHg. |
| **Semilunar valves** |  | Aortic | Moderate AR |
| Aortic valve | Annulus = 17mm | Tricuspid | Moderate TR, PPG = 38mmHg. |
| Pulmonary valve | Annulus = 21mm | pulmonic | -------- |
| **Great arteries** | NRGA | **Aortic arch** | Left. No CoA. |
| Aorta | ----- | **PDA** | No |
| Pulmonary artery | Normal MPA and Branch PAs. |  |  |
| **M-Mode:** | | | |
| AO | mm | PWd | mm |
| LA | mm | PWs | mm |
| LVIDd | mm | EDV | ml |
| LVIDs | mm | ESV | ml |
| IVSs | mm | LVEF | 62% |
| IVSd | mm | FS | 34% |
| **Additional Information**: |  | | |
| No pericardial/Pleural effusion. | | | |
| **Final Diagnosis:** | | | |
| 1. {S, D, S} Levocardia. 2. LA/LV Dilated 3. Thickened MVL, Shortened PMVL 4. Severe MR 5. Moderate AR 6. Moderate TR 7. Mild Pulmonary Hypertension 8. Normal Biventricular Systolic Function | | | |
| SIGNATURE  Done by: Tesfaye T., Pediatrician, Pediatric Cardiologist _______________ 24/04/2015Eth.C | | | |

| Patient Name: **Kalkidan Setegn**. Referring Institute: **FHRH**. SEX/ Age: **F/6years**. Date of Report: **24/04/15**.  Referral Diagnosis: **RHD + CHF + RD. AGH2.145** | | | |
| --- | --- | --- | --- |
| **Features** | **Finding** | **Features** | **Finding** |
| **Profile** |  | **Atria** |  |
| Abdominal situs | Solitus | Left atrium | Markedly Dilated |
| Cardiac position | Levocardia | Right atrium | Normal |
| Systemic venous drainage | Normal. | **Atrioventricular valves** |  |
| Pulmonary venous drainage | Normal | Mitral valve | Annulus = 32mm. Thickened MVL. Shortened PMVL. |
| Atrioventricular connection | Concordant | Tricuspid valve | Annulus = 20mm. TAPSE = mm |
| Ventriculoarterial connection | Concordant | **Ventricles** |  |
| Ventricular loop | d-Loop | Left ventricle | Markedly Dilated |
|  |  | Right ventricle | Normal |
| **Septae** |  | **Coronary arteries** | ----- |
| Interventricular septum | Intact | **Doppler Measurement** |  |
| Interatrial septum | Intact | Mitral | Severe MR, Holosystolic, posterior projection, seen in two planes with jet velocity = 5mmHg. |
| **Semilunar valves** |  | Aortic | ------- |
| Aortic valve | Annulus = 14mm | Tricuspid | Moderate TR, PPG = 41mmHg |
| Pulmonary valve | Annulus = 16mm | pulmonic | -------- |
| **Great arteries** | NRGA | **Aortic arch** | Left. No CoA. |
| Aorta | ----- | **PDA** | No |
| Pulmonary artery | Normal MPA and Branch PAs. |  |  |
| **M-Mode:** | | | |
| AO | mm | PWd | mm |
| LA | mm | PWs | mm |
| LVIDd | mm | EDV | ml |
| LVIDs | mm | ESV | 33ml |
| IVSs | mm | LVEF | 61% |
| IVSd | mm | FS | % |
| **Additional Information**: |  | | |
| Circumferential pericardial effusion with maximum depth of 9mm on RA/RV Side. | | | |
| **Final Diagnosis:** | | | |
| 1. {S, D, S} Levocardia. 2. LA/LV Dilated 3. Thickened MVL, Shortened PMVL 4. Severe MR 5. Moderate TR 6. Mild Pulmonary Hypertension 7. Small Circumferential Pericardial effusion 8. Normal Biventricular Systolic Function | | | |
| SIGNATURE  Done by: Tesfaye T., Pediatrician, Pediatric Cardiologist _______________ 24/04/2015Eth.C | | | |

| Patient Name: **Birtukan Temesgen**. Referring Institute: **Addis Alem PH**. SEX/ Age: **F/13days**. Date of Report: **25/04/15**.  Referral Diagnosis: **DS. AGH2.146** | | | |
| --- | --- | --- | --- |
| **Features** | **Finding** | **Features** | **Finding** |
| **Profile** |  | **Atria** |  |
| Abdominal situs | Solitus | Left atrium | Normal |
| Cardiac position | Levocardia | Right atrium | Normal |
| Systemic venous drainage | Normal. | **Atrioventricular valves** |  |
| Pulmonary venous drainage | Normal | Mitral valve | Annulus = 11mm |
| Atrioventricular connection | Concordant | Tricuspid valve | Annulus = 11mm |
| Ventriculoarterial connection | Concordant | **Ventricles** |  |
| Ventricular loop | d-Loop | Left ventricle | Normal |
|  |  | Right ventricle | Normal |
| **Septae** |  | **Coronary arteries** | ----- |
| Interventricular septum | Intact | **Doppler Measurement** |  |
| Interatrial septum | PFO, L – R Shunt | Mitral | ----- |
| **Semilunar valves** |  | Aortic | ------- |
| Aortic valve | Annulus = 9mm | Tricuspid | ------- |
| Pulmonary valve | Annulus = 10mm | pulmonic | -------- |
| **Great arteries** | NRGA | **Aortic arch** | Left. No CoA. |
| Aorta | ----- | **PDA** | No |
| Pulmonary artery | Normal MPA and Branch PAs. |  |  |
| **M-Mode:**  Normal LV Function on eye balliing | | | |
| AO | mm | PWd | mm |
| LA | mm | PWs | mm |
| LVIDd | mm | EDV | ml |
| LVIDs | mm | ESV | ml |
| IVSs | mm | LVEF | % |
| IVSd | mm | FS | % |
| **Additional Information**: |  | | |
| No pericardial/Pleural effusion. | | | |
| **Final Diagnosis:** | | | |
| 1. {S, D, S} Levocardia. 2. PFO, L – R Shunt | | | |
| **Remark**: | | | |
| **Recommendation**: | | | |
| SIGNATURE  Done by: Tesfaye T., Pediatrician, Pediatric Cardiologist _______________ 25/04/2015Eth.C | | | |

| Patient Name: **Amele-werk Mengistu**. Referring Institute: **FHRH**. SEX/ Age: **F/11years**. Date of Report: **25/04/15**.  Referral Diagnosis: **Palpitation. AGH2.147** | | | |
| --- | --- | --- | --- |
| **Features** | **Finding** | **Features** | **Finding** |
| **Profile** |  | **Atria** |  |
| Abdominal situs | Solitus | Left atrium | Normal |
| Cardiac position | Levocardia | Right atrium | Normal |
| Systemic venous drainage | Normal. | **Atrioventricular valves** |  |
| Pulmonary venous drainage | Normal | Mitral valve | Annulus = 19mm |
| Atrioventricular connection | Concordant | Tricuspid valve | Annulus = 20mm  TAPSE = 19mm |
| Ventriculoarterial connection | Concordant | **Ventricles** |  |
| Ventricular loop | d-Loop | Left ventricle | Normal |
|  |  | Right ventricle | Normal |
| **Septae** |  | **Coronary arteries** | ----- |
| Interventricular septum | Intact | **Doppler Measurement** |  |
| Interatrial septum | Intact | Mitral | ----- |
| **Semilunar valves** |  | Aortic | ------- |
| Aortic valve | Annulus = 18mm | Tricuspid | ------- |
| Pulmonary valve | Annulus = 19mm | pulmonic | -------- |
| **Great arteries** | NRGA | **Aortic arch** | Left. No CoA. |
| Aorta | ----- | **PDA** | No |
| Pulmonary artery | Normal MPA and Branch PAs. |  |  |
| **M-Mode:** | | | |
| AO | mm | PWd | mm |
| LA | mm | PWs | mm |
| LVIDd | mm | EDV | ml |
| LVIDs | mm | ESV | ml |
| IVSs | mm | LVEF | 68% |
| IVSd | mm | FS | 38% |
| **Additional Information**: |  | | |
| No pericardial/Pleural effusion. | | | |
| **Final Diagnosis:** | | | |
| 1. Normal Echocardiography Study. | | | |
| **Remark**: | | | |
| **Recommendation**: | | | |
| SIGNATURE  Done by: Tesfaye T., Pediatrician, Pediatric Cardiologist _______________ 25/04/2015Eth.C | | | |

| Patient Name: **Amare Melak**. Referring Institute: **TGSH**. SEX/ Age: **M/8years**. Date of Report: **25/04/15**.  Referral Diagnosis: **Sydenham’s Chorea. AGH2.148** | | | |
| --- | --- | --- | --- |
| **Features** | **Finding** | **Features** | **Finding** |
| **Profile** |  | **Atria** |  |
| Abdominal situs | Solitus | Left atrium | Normal |
| Cardiac position | Levocardia | Right atrium | Normal |
| Systemic venous drainage | Normal. | **Atrioventricular valves** |  |
| Pulmonary venous drainage | Normal | Mitral valve | Annulus = 18mm. Patulous MVL. |
| Atrioventricular connection | Concordant | Tricuspid valve | Annulus = 18mmሸ TAPSE = 22mm |
| Ventriculoarterial connection | Concordant | **Ventricles** |  |
| Ventricular loop | d-Loop | Left ventricle | Normal |
|  |  | Right ventricle | Normal |
| **Septae** |  | **Coronary arteries** | ----- |
| Interventricular septum | Intact | **Doppler Measurement** |  |
| Interatrial septum | Intact | Mitral | Mild MR, Holosystolic, posterior projection, seen in two planes with jet velocity = 3.1m/sec. |
| **Semilunar valves** |  | Aortic | Trivial AR |
| Aortic valve | Annulus = 15mm | Tricuspid | ------- |
| Pulmonary valve | Annulus = 18mm | pulmonic | -------- |
| **Great arteries** | NRGA | **Aortic arch** | Left. No CoA. |
| Aorta | ----- | **PDA** | No |
| Pulmonary artery | Normal MPA and Branch PAs. |  |  |
| **M-Mode:** | | | |
| AO | mm | PWd | mm |
| LA | mm | PWs | mm |
| LVIDd | mm | EDV | ml |
| LVIDs | mm | ESV | ml |
| IVSs | mm | LVEF | 71% |
| IVSd | mm | FS | 39% |
| **Additional Information**: |  | | |
| No pericardial/Pleural effusion. | | | |
| **Final Diagnosis:** | | | |
| 1. {S, D, S} Levocardia. 2. Patulous MVL 3. Mild MR 4. Trivial AR 5. Normal Biventricular Systolic Function | | | |
| **Remark**: | | | |
| **Recommendation**: | | | |
| SIGNATURE  Done by: Tesfaye T., Pediatrician, Pediatric Cardiologist _______________ 25/04/2015Eth.C | | | |

| Patient Name: **Banchigizie Adugna**. Referring Institute: **TGSH**. SEX/ Age: **F/1 1/12**. Date of Report: **25/04/15**.  Referral Diagnosis: **CHF + RD. AGH2.149.** | | | |
| --- | --- | --- | --- |
| **Features** | **Finding** | **Features** | **Finding** |
| **Profile** |  | **Atria** |  |
| Abdominal situs | Solitus | Left atrium | Dilated |
| Cardiac position | Levocardia | Right atrium | Dilated |
| Systemic venous drainage | Normal. | **Atrioventricular valves** |  |
| Pulmonary venous drainage | Normal | Mitral valve | Annulus = 11mm |
| Atrioventricular connection | Concordant | Tricuspid valve | Annulus = 12mm |
| Ventriculoarterial connection | Concordant | **Ventricles** |  |
| Ventricular loop | d-Loop | Left ventricle | Dilated |
|  |  | Right ventricle | Smallish RV |
| **Septae** |  | **Coronary arteries** | ----- |
| Interventricular septum | 10mm Inlet VSD, L – R Shunt. Tongue of tissue in b/n Primum & ventricular defect. | **Doppler Measurement** |  |
| Interatrial septum | 9mm primum defect, L – R Shunt. Additional 8mm Fenestrated OS ASD, L – R Shunt | Mitral | ----- |
| **Semilunar valves** |  | Aortic | ------- |
| Aortic valve | Annulus = 11mm | Tricuspid | Mild TR |
| Pulmonary valve | Annulus = 15mm | pulmonic | -------- |
| **Great arteries** | NRGA | **Aortic arch** | Left. No CoA. |
| Aorta | ----- | **PDA** | No |
| Pulmonary artery | **MPA = 16mm.** |  |  |
| **M-Mode:**  Normal LV Function on eye balling | | | |
| AO | mm | PWd | mm |
| LA | mm | PWs | mm |
| LVIDd | mm | EDV | ml |
| LVIDs | mm | ESV | ml |
| IVSs | mm | LVEF | % |
| IVSd | mm | FS | % |
| **Additional Information**: |  | | |
| Pleural effusion measuring maximum depth of 4mm on RA Side. | | | |
| **Final Diagnosis:** | | | |
| 1. {S, D, S} Levocardia. 2. Moderate Fenestrated OS ASD, L – R Shunt 3. Intermediate Unbalanced AVSD, L – R Shunt 4. Smallish RV 5. Mild TR 6. Moderate Pulmonary Hypertension 7. Normal LV Systolic Function 8. Trace pericardial effusion | | | |
| SIGNATURE  Done by: Tesfaye T., Pediatrician, Pediatric Cardiologist _______________ 25/04/2015Eth.C | | | |

| Patient Name: **Me’aza Teninet**. Referring Institute: **TGSH**. SEX/ Age: **F/5 6/12**. Date of Report: **26/04/15**.  Referral Diagnosis: **Palpitation. AGH2.150** | | | |
| --- | --- | --- | --- |
| **Features** | **Finding** | **Features** | **Finding** |
| **Profile** |  | **Atria** |  |
| Abdominal situs | Solitus | Left atrium | Normal |
| Cardiac position | Levocardia | Right atrium | Normal |
| Systemic venous drainage | Normal. | **Atrioventricular valves** |  |
| Pulmonary venous drainage | Normal | Mitral valve | Annulus = 18mm. Patulous MV. |
| Atrioventricular connection | Concordant | Tricuspid valve | Annulus = 19mm  TAPSE = 18mm |
| Ventriculoarterial connection | Concordant | **Ventricles** |  |
| Ventricular loop | d-Loop | Left ventricle | Normal |
|  |  | Right ventricle | Normal |
| **Septae** |  | **Coronary arteries** | ----- |
| Interventricular septum | Intact | **Doppler Measurement** |  |
| Interatrial septum | Intact | Mitral | Trivial MR, Incomplete Signal, seen in two planes with jet velocity = 2.5m/sec. |
| **Semilunar valves** |  | Aortic | ------- |
| Aortic valve | Annulus = 14mm | Tricuspid | Trivial TR, PPG = 17mmHg |
| Pulmonary valve | Annulus = 18mm | pulmonic | -------- |
| **Great arteries** | NRGA | **Aortic arch** | Left. No CoA. |
| Aorta | ----- | **PDA** | No |
| Pulmonary artery | Normal MPA and Branch PAs. |  |  |
| **M-Mode:** | | | |
| AO | mm | PWd | mm |
| LA | mm | PWs | mm |
| LVIDd | mm | EDV | ml |
| LVIDs | mm | ESV | ml |
| IVSs | mm | LVEF | 60% |
| IVSd | mm | FS | 31% |
| **Additional Information**: |  | | |
| No pericardial/Pleural effusion. | | | |
| **Final Diagnosis:** | | | |
| 1. {S, D, S} Levocardia. 2. Trivial MR 3. Trivial TR 4. Normal Biventricular systolic function | | | |
| **Remark**: | | | |
| **Recommendation**: | | | |
| SIGNATURE  Done by: Tesfaye T., Pediatrician, Pediatric Cardiologist _______________ 26/04/2015Eth.C | | | |

| Patient Name: **Banchigizie Molla**. Referring Institute: **TGSH**. SEX/ Age: **F/6 6/12**. Date of Report: **26/04/15**.  Referral Diagnosis: **Easy fatigability. AGH2.151** | | | |
| --- | --- | --- | --- |
| **Features** | **Finding** | **Features** | **Finding** |
| **Profile** |  | **Atria** |  |
| Abdominal situs | Solitus | Left atrium | Normal |
| Cardiac position | Levocardia | Right atrium | Normal |
| Systemic venous drainage | Normal. | **Atrioventricular valves** |  |
| Pulmonary venous drainage | Normal | Mitral valve | Annulus = 18mm |
| Atrioventricular connection | Concordant | Tricuspid valve | Annulus = 20mm  TAPSE = 17mm |
| Ventriculoarterial connection | Concordant | **Ventricles** |  |
| Ventricular loop | d-Loop | Left ventricle | Normal |
|  |  | Right ventricle | Normal |
| **Septae** |  | **Coronary arteries** | ----- |
| Interventricular septum | Intact | **Doppler Measurement** |  |
| Interatrial septum | Intact | Mitral | ----- |
| **Semilunar valves** |  | Aortic | ------- |
| Aortic valve | Annulus = 15mm | Tricuspid | Trivial TR, PPG = 23mmHg |
| Pulmonary valve | Annulus = 17mm | pulmonic | Trivial PR, PPG = 10mmHg |
| **Great arteries** | NRGA | **Aortic arch** | Left. No CoA. |
| Aorta | ----- | **PDA** | No |
| Pulmonary artery | Normal MPA and Branch PAs. |  |  |
| **M-Mode:** | | | |
| AO | mm | PWd | mm |
| LA | mm | PWs | mm |
| LVIDd | mm | EDV | ml |
| LVIDs | mm | ESV | ml |
| IVSs | mm | LVEF | 61% |
| IVSd | mm | FS | 32% |
| **Additional Information**: |  | | |
| No pericardial/Pleural effusion. | | | |
| **Final Diagnosis:** | | | |
| 1. Normal Echocardiography Study. | | | |
| **Remark**: | | | |
| **Recommendation**: | | | |
| SIGNATURE  Done by: Tesfaye T., Pediatrician, Pediatric Cardiologist _______________ 26/04/2015Eth.C | | | |

| Patient Name: **Arsema Desalegn**. Referring Institute: **Black Lion Hospital**. SEX/ Age: **F/2 6/12**. Date of Report: **26/04/15**.  Referral Diagnosis: **Recurrent chest infection + RD. AGH2.152** | | | |
| --- | --- | --- | --- |
| **Features** | **Finding** | **Features** | **Finding** |
| **Profile** |  | **Atria** |  |
| Abdominal situs | Solitus | Left atrium | Dilated |
| Cardiac position | Levocardia | Right atrium | Dilated |
| Systemic venous drainage | Normal. | **Atrioventricular valves** |  |
| Pulmonary venous drainage | Normal | Mitral valve | Annulus = 20mm |
| Atrioventricular connection | Concordant | Tricuspid valve | Annulus = 23mm |
| Ventriculoarterial connection | Concordant | **Ventricles** |  |
| Ventricular loop | d-Loop | Left ventricle | Dilated |
|  |  | Right ventricle | Dilated |
| **Septae** |  | **Coronary arteries** | ----- |
| Interventricular septum | 15mm Inlet VSD, L – R Shunt.  10mm Primum defect, L – R Shunt.  Separated by tongue of tissue. | **Doppler Measurement** |  |
| Interatrial septum |  | Mitral | Mild MR |
| **Semilunar valves** |  | Aortic | ------- |
| Aortic valve | Annulus = 17mm | Tricuspid | Moderate TR |
| Pulmonary valve | Annulus = 19mm | pulmonic | Mild PR, PPG = 50mmHg |
| **Great arteries** | NRGA | **Aortic arch** | Left. No CoA. |
| Aorta | ----- | **PDA** | 1.5mm PDA, L – R Shunt |
| Pulmonary artery | MPA = 34mm. |  |  |
| **M-Mode:**  Normal LV Function on eye balling. | | | |
| AO | mm | PWd | mm |
| LA | mm | PWs | mm |
| LVIDd | mm | EDV | ml |
| LVIDs | mm | ESV | ml |
| IVSs | mm | LVEF | % |
| IVSd | mm | FS | % |
| **Additional Information**: |  | | |
| No pericardial/Pleural effusion. | | | |
| **Final Diagnosis:** | | | |
| 1. {S, D, S} Levocardia. 2. All chambers Dilated 3. Intermediate AVSD, L – R Shunt 4. Moderate TR 5. Mild MR 6. Mild PR 7. Small PDA, L – R Shunt 8. Moderate Pulmonary Hypertension 9. Normal LV Systolic Function | | | |
| **Remark**: Previous Echocardiography was reported as Complete AVSD | | | |
| **Recommendation**: | | | |
| SIGNATURE  Done by: Tesfaye T., Pediatrician, Pediatric Cardiologist _______________ 26/04/2015Eth.C | | | |

| Patient Name: **Bethelihem Tesfaye**. Referring Institute: **Amaris PSC**. SEX/ Age: **F/3 4/12**. Date of Report: **27/04/15**.  Referral Diagnosis: **Recurrent Chest Infection. AGH2.153** | | | |
| --- | --- | --- | --- |
| **Features** | **Finding** | **Features** | **Finding** |
| **Profile** |  | **Atria** |  |
| Abdominal situs | Solitus | Left atrium | Normal |
| Cardiac position | Levocardia | Right atrium | Normal |
| Systemic venous drainage | Normal. | **Atrioventricular valves** |  |
| Pulmonary venous drainage | Normal | Mitral valve | Annulus = 17mm |
| Atrioventricular connection | Concordant | Tricuspid valve | Annulus = 18mm  TAPSE = 20mm |
| Ventriculoarterial connection | Concordant | **Ventricles** |  |
| Ventricular loop | d-Loop | Left ventricle | Normal |
|  |  | Right ventricle | Normal |
| **Septae** |  | **Coronary arteries** | ----- |
| Interventricular septum | Intact | **Doppler Measurement** |  |
| Interatrial septum | Intact | Mitral | ----- |
| **Semilunar valves** |  | Aortic | ------- |
| Aortic valve | Annulus = 15mm | Tricuspid | ------- |
| Pulmonary valve | Annulus = 17mm | pulmonic | -------- |
| **Great arteries** | NRGA | **Aortic arch** | Left. No CoA. |
| Aorta | ----- | **PDA** | No |
| Pulmonary artery | Normal MPA and Branch PAs. |  |  |
| **M-Mode:** | | | |
| AO | mm | PWd | mm |
| LA | mm | PWs | mm |
| LVIDd | mm | EDV | ml |
| LVIDs | mm | ESV | ml |
| IVSs | mm | LVEF | 71% |
| IVSd | mm | FS | 39% |
| **Additional Information**: |  | | |
| No pericardial/Pleural effusion. | | | |
| **Final Diagnosis:** | | | |
| 1. Normal Echocardiography Study. | | | |
| **Remark**: | | | |
| **Recommendation**: | | | |
| SIGNATURE  Done by: Tesfaye T., Pediatrician, Pediatric Cardiologist _______________ 27/04/2015Eth.C | | | |

| Patient Name: **Ousman Arega**. Referring Institute: **Gamby GH**. SEX/ Age: **M/3years**. Date of Report: **27/04/15**.  Referral Diagnosis: **Incidental Murmur Finding. AGH2.154** | | | |
| --- | --- | --- | --- |
| **Features** | **Finding** | **Features** | **Finding** |
| **Profile** |  | **Atria** |  |
| Abdominal situs | Solitus | Left atrium | Mildly Dilated |
| Cardiac position | Levocardia | Right atrium | Normal |
| Systemic venous drainage | Normal. | **Atrioventricular valves** |  |
| Pulmonary venous drainage | Normal | Mitral valve | Annulus = 20mm |
| Atrioventricular connection | Concordant | Tricuspid valve | Annulus = 17mm  TAPSE = 20mm |
| Ventriculoarterial connection | Concordant | **Ventricles** |  |
| Ventricular loop | d-Loop | Left ventricle | Mildly Dilated |
|  |  | Right ventricle | Normal |
| **Septae** |  | **Coronary arteries** | ----- |
| Interventricular septum | 5mm Sub-pulmonic VSD, L – R Shunt | **Doppler Measurement** |  |
| Interatrial septum | Intact | Mitral | ----- |
| **Semilunar valves** |  | Aortic | ------- |
| Aortic valve | Annulus = 13mm | Tricuspid | ------- |
| Pulmonary valve | Annulus = 18mm | pulmonic | Mild PS, PPG = 23mmHg |
| **Great arteries** | NRGA | **Aortic arch** | Left. No CoA. |
| Aorta | ----- | **PDA** | No |
| Pulmonary artery | Normal MPA and Branch PAs. |  |  |
| **M-Mode:** | | | |
| AO | mm | PWd | mm |
| LA | mm | PWs | mm |
| LVIDd | mm | EDV | ml |
| LVIDs | mm | ESV | ml |
| IVSs | mm | LVEF | 64% |
| IVSd | mm | FS | 35% |
| **Additional Information**: |  | | |
| No pericardial/Pleural effusion. | | | |
| **Final Diagnosis:** | | | |
| 1. {S, D, S} Levocardia. 2. LA/LV Mildly Dilated 3. Moderate Sub-Pulmonic VSD, L – R Shunt 4. Mild PS 5. Normal Biventricular Systolic Function | | | |
| **Remark**: | | | |
| **Recommendation**: Needs referral for possible surgical evaluation | | | |
| SIGNATURE  Done by: Tesfaye T., Pediatrician, Pediatric Cardiologist _______________ 27/04/2015Eth.C | | | |

| Patient Name: **Kidus Haile-yesus**. Referring Institute: **Adinas GH**. SEX/ Age: **M/2 5/12**. Date of Report: **27/04/15**.  Referral Diagnosis: **Recurrent Chest Infection. AGH2.155** | | | |
| --- | --- | --- | --- |
| **Features** | **Finding** | **Features** | **Finding** |
| **Profile** |  | **Atria** |  |
| Abdominal situs | Solitus | Left atrium | Normal |
| Cardiac position | Levocardia | Right atrium | Normal |
| Systemic venous drainage | Normal. | **Atrioventricular valves** |  |
| Pulmonary venous drainage | Normal | Mitral valve | Annulus = 15mm |
| Atrioventricular connection | Concordant | Tricuspid valve | Annulus = 15mm |
| Ventriculoarterial connection | Concordant | **Ventricles** |  |
| Ventricular loop | d-Loop | Left ventricle | Normal |
|  |  | Right ventricle | Normal |
| **Septae** |  | **Coronary arteries** | ----- |
| Interventricular septum | Intact | **Doppler Measurement** |  |
| Interatrial septum | Intact | Mitral | ----- |
| **Semilunar valves** |  | Aortic | ------- |
| Aortic valve | Annulus = 14mm | Tricuspid | ------- |
| Pulmonary valve | Annulus = 14mm | pulmonic | -------- |
| **Great arteries** | NRGA | **Aortic arch** | Left. No CoA. |
| Aorta | ----- | **PDA** | No |
| Pulmonary artery | Normal MPA and Branch PAs. |  |  |
| **M-Mode:**  Normal LV Function on eye balling | | | |
| AO | mm | PWd | mm |
| LA | mm | PWs | mm |
| LVIDd | mm | EDV | ml |
| LVIDs | mm | ESV | ml |
| IVSs | mm | LVEF | % |
| IVSd | mm | FS | % |
| **Additional Information**: |  | | |
| No pericardial/Pleural effusion. | | | |
| **Final Diagnosis:** | | | |
| 1. Normal Echocardiography Study. | | | |
| **Remark**: | | | |
| **Recommendation**: | | | |
| SIGNATURE  Done by: Tesfaye T., Pediatrician, Pediatric Cardiologist _______________ 27/04/2015Eth.C | | | |

| Patient Name: **Zewditu Andualem**. Referring Institute: **FHRH**. SEX/ Age: **F/10years**. Date of Report: **28/04/15**.  Referral Diagnosis: **Easy fatigability. AGH2.156** | | | |
| --- | --- | --- | --- |
| **Features** | **Finding** | **Features** | **Finding** |
| **Profile** |  | **Atria** |  |
| Abdominal situs | Solitus | Left atrium | Normal |
| Cardiac position | Levocardia | Right atrium | Dilated |
| Systemic venous drainage | Normal. | **Atrioventricular valves** |  |
| Pulmonary venous drainage | Normal | Mitral valve | Annulus = 29mm. Right |
| Atrioventricular connection | Discordant | Tricuspid valve | Annulus = 22mm |
| Ventriculoarterial connection | Discordant | **Ventricles** |  |
| Ventricular loop | l-Loop | Left ventricle | Dilated. Right side. |
|  |  | Right ventricle | Normal. Left Side. |
| **Septae** |  | **Coronary arteries** | ----- |
| Interventricular septum | Intact | **Doppler Measurement** |  |
| Interatrial septum | 12mm OS ASD, Predominantly L – R Shunt | Mitral | Moderate MR |
| **Semilunar valves** |  | Aortic | ------- |
| Aortic valve | Annulus = 19mm | Tricuspid | Mild TR |
| Pulmonary valve | Annulus = 23mm | pulmonic | Mild PS, PPG = 27mmHg. |
| **Great arteries** | l-TGA | **Aortic arch** | Left. No CoA. |
| Aorta | Anterior & to the left | **PDA** | No |
| Pulmonary artery | Posterior & to the right |  |  |
| **M-Mode:** | | | |
| AO | mm | PWd | mm |
| LA | mm | PWs | mm |
| LVIDd | mm | EDV | ml |
| LVIDs | mm | ESV | ml |
| IVSs | mm | LVEF | % |
| IVSd | mm | FS | % |
| **Additional Information**: |  | | |
| No pericardial/Pleural effusion. | | | |
| **Final Diagnosis:** | | | |
| 1. {S, l, l} Levocardia. 2. Large OS ASD, Predominantly L – R Shunt 3. ccTGA 4. Moderate MR 5. Mild TR 6. Mild PS | | | |
| **Remark**: | | | |
| **Recommendation**: | | | |
| SIGNATURE  Done by: Tesfaye T., Pediatrician, Pediatric Cardiologist _______________ 28/04/2015Eth.C | | | |

| Patient Name: **Emawayish Yalew**. Referring Institute: **TGSH**. SEX/ Age: **F/9months**. Date of Report: **28/04/15**.  Referral Diagnosis: **Cardiomegaly on CXR. AGH2.157** | | | |
| --- | --- | --- | --- |
| **Features** | **Finding** | **Features** | **Finding** |
| **Profile** |  | **Atria** |  |
| Abdominal situs | Solitus | Left atrium | Normal |
| Cardiac position | Levocardia | Right atrium | Dilated |
| Systemic venous drainage | Normal. | **Atrioventricular valves** |  |
| Pulmonary venous drainage | Normal | Mitral valve | Annulus = 12mm |
| Atrioventricular connection | Concordant | Tricuspid valve | Annulus = 16mm  TAPSE = 17mm |
| Ventriculoarterial connection | Concordant | **Ventricles** |  |
| Ventricular loop | d-Loop | Left ventricle | Normal |
|  |  | Right ventricle | Dilated & Hypertrophied |
| **Septae** |  | **Coronary arteries** | ----- |
| Interventricular septum | Intact | **Doppler Measurement** |  |
| Interatrial septum | PFO, L – R Shunt | Mitral | ----- |
| **Semilunar valves** |  | Aortic | ------- |
| Aortic valve | Annulus = 12mm | Tricuspid | Trivial TR, PPG = 31mmHg |
| Pulmonary valve | Annulus = 16mm | pulmonic | -------- |
| **Great arteries** | NRGA | **Aortic arch** | Left. No CoA. |
| Aorta | ----- | **PDA** | No |
| Pulmonary artery | Normal MPA and Branch PAs. |  |  |
| **M-Mode:** | | | |
| AO | mm | PWd | mm |
| LA | mm | PWs | mm |
| LVIDd | mm | EDV | ml |
| LVIDs | mm | ESV | ml |
| IVSs | mm | LVEF | 71% |
| IVSd | mm | FS | 38% |
| **Additional Information**: |  | | |
| No pericardial/Pleural effusion. | | | |
| **Final Diagnosis:** | | | |
| 1. {S, D, S} Levocardia. 2. RA/RV Dilated, RV Hypertrophied 3. PFO, L – R Shunt 4. Normal Biventricular Systolic Function | | | |
| **Remark**: | | | |
| **Recommendation**: | | | |
| SIGNATURE  Done by: Tesfaye T., Pediatrician, Pediatric Cardiologist _______________ 28/04/2015Eth.C | | | |

| Patient Name: **Dagnanew Sefiw**. Referring Institute: **FHRH**. SEX/ Age: **M/8years**. Date of Report: **01/05/15**.  Referral Diagnosis: **Incidental Murmur Finding. AGH2.158** | | | |
| --- | --- | --- | --- |
| **Features** | **Finding** | **Features** | **Finding** |
| **Profile** |  | **Atria** |  |
| Abdominal situs | Solitus | Left atrium | Normal |
| Cardiac position | Levocardia | Right atrium | Normal |
| Systemic venous drainage | Normal. | **Atrioventricular valves** |  |
| Pulmonary venous drainage | Normal | Mitral valve | Annulus = 20mm |
| Atrioventricular connection | Concordant | Tricuspid valve | Annulus = 20mm  TAPSE = 19mm |
| Ventriculoarterial connection | Concordant | **Ventricles** |  |
| Ventricular loop | d-Loop | Left ventricle | Normal |
|  |  | Right ventricle | Normal |
| **Septae** |  | **Coronary arteries** | ----- |
| Interventricular septum | Intact | **Doppler Measurement** |  |
| Interatrial septum | Intact | Mitral | ----- |
| **Semilunar valves** |  | Aortic | ------- |
| Aortic valve | Annulus = 15mm | Tricuspid | Mild TR, PPG = 18mmHg |
| Pulmonary valve | Annulus = 18mm | pulmonic | Trivial PR, PPG = 16mmHg |
| **Great arteries** | NRGA | **Aortic arch** | Left. No CoA. |
| Aorta | ----- | **PDA** | No |
| Pulmonary artery | Normal MPA and Branch PAs. |  |  |
| **M-Mode:** | | | |
| AO | mm | PWd | mm |
| LA | mm | PWs | mm |
| LVIDd | mm | EDV | ml |
| LVIDs | mm | ESV | ml |
| IVSs | mm | LVEF | 61% |
| IVSd | mm | FS | 32% |
| **Additional Information**: |  | | |
| No pericardial/Pleural effusion. | | | |
| **Final Diagnosis:** | | | |
| 1. {S, D, S} Levocardia. 2. Mild TR 3. Normal Biventricular Systolic Function | | | |
| **Remark**: | | | |
| **Recommendation**: | | | |
| SIGNATURE  Done by: Tesfaye T., Pediatrician, Pediatric Cardiologist _______________ 01/05/2015Eth.C | | | |

| Patient Name: **Nuhamin Kifle-Mariam**. Referring Institute: **Addis Alem PH**. SEX/ Age: **F/9months**. Date of Report: **01/05/15**.  Referral Diagnosis: **Incidental Murmur Finding. AGH2.159** | | | |
| --- | --- | --- | --- |
| **Features** | **Finding** | **Features** | **Finding** |
| **Profile** |  | **Atria** |  |
| Abdominal situs | Solitus | Left atrium | Dilated |
| Cardiac position | Levocardia | Right atrium | Normal |
| Systemic venous drainage | Normal. | **Atrioventricular valves** |  |
| Pulmonary venous drainage | Normal | Mitral valve | Annulus = 15mm |
| Atrioventricular connection | Concordant | Tricuspid valve | Annulus = 13mm  TAPSE = mm |
| Ventriculoarterial connection | Concordant | **Ventricles** |  |
| Ventricular loop | d-Loop | Left ventricle | Dilated |
|  |  | Right ventricle | Normal |
| **Septae** |  | **Coronary arteries** | ----- |
| Interventricular septum | Intact | **Doppler Measurement** |  |
| Interatrial septum | Intact | Mitral | ----- |
| **Semilunar valves** |  | Aortic | ------- |
| Aortic valve | Annulus = 8mm | Tricuspid | ------- |
| Pulmonary valve | Annulus = 10mm | pulmonic | Mild PS, PPG = 30mmHg |
| **Great arteries** | NRGA | **Aortic arch** | Left. No CoA. |
| Aorta | ----- | **PDA** | 2mm PDA, L – R Shunt |
| Pulmonary artery | Normal MPA and Branch PAs. |  |  |
| **M-Mode:** | | | |
| AO | mm | PWd | mm |
| LA | mm | PWs | mm |
| LVIDd | mm | EDV | ml |
| LVIDs | mm | ESV | ml |
| IVSs | mm | LVEF | % |
| IVSd | mm | FS | % |
| **Additional Information**: |  | | |
| Pericardial effusion measuring maximum depth of 8mm on RA Side. | | | |
| **Final Diagnosis:** | | | |
| 1. {S, D, S} Levocardia. 2. LA/LV Dilated 3. Mild PS 4. Moderate PDA, L – R Shunt 5. Small Pericardial effusion | | | |
| **Remark**: Infant was crying throughout the study. | | | |
| **Recommendation**: | | | |
| SIGNATURE  Done by: Tesfaye T., Pediatrician, Pediatric Cardiologist _______________ 01/05/2015Eth.C | | | |

| Patient Name: **Osueymin Ismael**. Referring Institute: **Adinas GH**. SEX/ Age: **M/11years**. Date of Report: **01/05/15**.  Referral Diagnosis: **Chest Pain. AGH2.160** | | | |
| --- | --- | --- | --- |
| **Features** | **Finding** | **Features** | **Finding** |
| **Profile** |  | **Atria** |  |
| Abdominal situs | Solitus | Left atrium | Normal |
| Cardiac position | Levocardia | Right atrium | Normal |
| Systemic venous drainage | Normal. | **Atrioventricular valves** |  |
| Pulmonary venous drainage | Normal | Mitral valve | Annulus = 20mm |
| Atrioventricular connection | Concordant | Tricuspid valve | Annulus = 23mm  TAPSE = 22mm |
| Ventriculoarterial connection | Concordant | **Ventricles** |  |
| Ventricular loop | d-Loop | Left ventricle | Normal |
|  |  | Right ventricle | Normal |
| **Septae** |  | **Coronary arteries** | ----- |
| Interventricular septum | Intact | **Doppler Measurement** |  |
| Interatrial septum | Intact | Mitral | ----- |
| **Semilunar valves** |  | Aortic | ------- |
| Aortic valve | Annulus = 16mm | Tricuspid | Trivial TR, PPG = 31mmHg |
| Pulmonary valve | Annulus = 19mm | pulmonic | -------- |
| **Great arteries** | NRGA | **Aortic arch** | Left. No CoA. |
| Aorta | ----- | **PDA** | No |
| Pulmonary artery | Normal MPA and Branch PAs. |  |  |
| **M-Mode:** | | | |
| AO | mm | PWd | mm |
| LA | mm | PWs | mm |
| LVIDd | mm | EDV | ml |
| LVIDs | mm | ESV | ml |
| IVSs | mm | LVEF | 60% |
| IVSd | mm | FS | 31% |
| **Additional Information**: |  | | |
| No pericardial/Pleural effusion. | | | |
| **Final Diagnosis:** | | | |
| 1. Normal Echocardiography Study. | | | |
| **Remark**: | | | |
| **Recommendation**: | | | |
| SIGNATURE  Done by: Tesfaye T., Pediatrician, Pediatric Cardiologist _______________ 01/05/2015Eth.C | | | |

| Patient Name: **Fikir Zelalem**. Referring Institute: **FHRH**. SEX/ Age: **M/2 5/12**. Date of Report: **01/05/15**.  Referral Diagnosis: **Incidental Murmur Finding. AGH2.161** | | | |
| --- | --- | --- | --- |
| **Features** | **Finding** | **Features** | **Finding** |
| **Profile** |  | **Atria** |  |
| Abdominal situs | Solitus | Left atrium | Dilated |
| Cardiac position | Levocardia | Right atrium | Normal |
| Systemic venous drainage | Normal. | **Atrioventricular valves** |  |
| Pulmonary venous drainage | Normal | Mitral valve | Annulus = 18mm |
| Atrioventricular connection | Concordant | Tricuspid valve | Annulus = 18mm  TAPSE = 21mm |
| Ventriculoarterial connection | Concordant | **Ventricles** |  |
| Ventricular loop | d-Loop | Left ventricle | Dilated |
|  |  | Right ventricle | Normal |
| **Septae** |  | **Coronary arteries** | ----- |
| Interventricular septum | Intact | **Doppler Measurement** |  |
| Interatrial septum | Intact | Mitral | ----- |
| **Semilunar valves** |  | Aortic | ------- |
| Aortic valve | Annulus = 15mm | Tricuspid | ------- |
| Pulmonary valve | Annulus = 19mm | pulmonic | -------- |
| **Great arteries** | NRGA | **Aortic arch** | Left. No CoA. |
| Aorta | ----- | **PDA** | 4mm PDA, L – R Shunt |
| Pulmonary artery | Normal MPA and Branch PAs. |  |  |
| **M-Mode:** | | | |
| AO | mm | PWd | mm |
| LA | mm | PWs | mm |
| LVIDd | mm | EDV | ml |
| LVIDs | mm | ESV | ml |
| IVSs | mm | LVEF | 72% |
| IVSd | mm | FS | 40% |
| **Additional Information**: |  | | |
| No pericardial/Pleural effusion. | | | |
| **Final Diagnosis:** | | | |
| 1. {S, D, S} Levocardia. 2. LA/LV Dilated 3. Large PDA, L – R Shunt 4. Normal Biventricular SYSTOLIC Function | | | |
| **Remark**: | | | |
| **Recommendation**: | | | |
| SIGNATURE  Done by: Tesfaye T., Pediatrician, Pediatric Cardiologist _______________ 01/05/2015Eth.C | | | |

| Patient Name: **Mandefro Gebrie**. Referring Institute: **FHRH**. SEX/ Age: **M/6months**. Date of Report: **01/05/15**.  Referral Diagnosis: **CHF + RD + DS. AGH2.162** | | | |
| --- | --- | --- | --- |
| **Features** | **Finding** | **Features** | **Finding** |
| **Profile** |  | **Atria** |  |
| Abdominal situs | Solitus | Left atrium | Normal |
| Cardiac position | Levocardia | Right atrium | Dilated |
| Systemic venous drainage | Normal. | **Atrioventricular valves** |  |
| Pulmonary venous drainage | Normal | Mitral valve | Common Complete AVSD |
| Atrioventricular connection | Common Complete AVSD, DIRV | Tricuspid valve |  |
| Ventriculoarterial connection | DORV | **Ventricles** |  |
| Ventricular loop | d-Loop | Left ventricle | Smallish |
|  |  | Right ventricle | Dilated |
| **Septae** |  | **Coronary arteries** | ----- |
| Interventricular septum | Common complete AVSD, L – R Shunt | **Doppler Measurement** |  |
| Interatrial septum |  | Mitral | ----- |
| **Semilunar valves** |  | Aortic | ------- |
| Aortic valve | Annulus = 12mm | Tricuspid | Mild Right AVVR |
| Pulmonary valve | Annulus = 15mm | pulmonic | -------- |
| **Great arteries** | NRGA | **Aortic arch** | Left. No CoA. |
| Aorta | From RV | **PDA** | No |
| Pulmonary artery | From RV. Dilated |  |  |
| **M-Mode:** | | | |
| AO | mm | PWd | mm |
| LA | mm | PWs | mm |
| LVIDd | mm | EDV | ml |
| LVIDs | mm | ESV | ml |
| IVSs | mm | LVEF | % |
| IVSd | mm | FS | % |
| **Additional Information**: |  | | |
| No pericardial/Pleural effusion. | | | |
| **Final Diagnosis:** | | | |
| 1. {S, D, D} Levocardia. 2. DIRV 3. DORV 4. Common Complete Unbalanced AVSD, L – R Shunt 5. Smallish LV 6. Severe Pulmonary Hypertension | | | |
| **Remark**: | | | |
| **Recommendation**: | | | |
| SIGNATURE  Done by: Tesfaye T., Pediatrician, Pediatric Cardiologist _______________ 01/05/2015Eth.C | | | |

| Patient Name: **Kidist Abebaw**. Referring Institute: **TGSH**. SEX/ Age: **F/2 3/12**. Date of Report: **02/05/15**.  Referral Diagnosis: **DS + Right Side CHF. AGH2.163** | | | |
| --- | --- | --- | --- |
| **Features** | **Finding** | **Features** | **Finding** |
| **Profile** |  | **Atria** |  |
| Abdominal situs | Solitus | Left atrium | Normal |
| Cardiac position | Levocardia | Right atrium | Dilated |
| Systemic venous drainage | Normal. | **Atrioventricular valves** |  |
| Pulmonary venous drainage | Normal | Mitral valve | Annulus = 13mm |
| Atrioventricular connection | Concordant | Tricuspid valve | Annulus = 19mm  **TAPSE = 12mm** |
| Ventriculoarterial connection | Concordant | **Ventricles** |  |
| Ventricular loop | d-Loop | Left ventricle | Normal |
|  |  | Right ventricle | Dilated |
| **Septae** |  | **Coronary arteries** | ----- |
| Interventricular septum | Intact | **Doppler Measurement** |  |
| Interatrial septum | 11mm OS ASD, BD Shunt | Mitral | ----- |
| **Semilunar valves** |  | Aortic | ------- |
| Aortic valve | Annulus = 10mm | Tricuspid | Mild TR, PPG = 65mmHg |
| Pulmonary valve | Annulus = 17mm | pulmonic | Moderate PR, PPG = 60mmHg |
| **Great arteries** | NRGA | **Aortic arch** | Left. No CoA. |
| Aorta | ----- | **PDA** | No |
| Pulmonary artery | Normal MPA and Branch PAs. |  |  |
| **M-Mode:** | | | |
| AO | mm | PWd | mm |
| LA | mm | PWs | mm |
| LVIDd | mm | EDV | ml |
| LVIDs | mm | ESV | ml |
| IVSs | mm | LVEF | 69% |
| IVSd | mm | FS | 36% |
| **Additional Information**: |  | | |
| No pericardial/Pleural effusion. | | | |
| **Final Diagnosis:** | | | |
| 1. {S, D, S} Levocardia. 2. RA/RV Dilated 3. Large OS ASD, BD Shunt 4. Mild TR 5. Moderate PR 6. Reduced RV Function 7. Severe Pulmonary Hypertension 8. Normal LV Systolic Function | | | |
| **Remark**: | | | |
| **Recommendation**: | | | |
| SIGNATURE  Done by: Tesfaye T., Pediatrician, Pediatric Cardiologist _______________ 02/05/2015Eth.C | | | |

| Patient Name: **Baby of Emeye Ristie**. Referring Institute: **FHRH**. SEX/ Age: **M/7days**. Date of Report: **02/05/15**.  Referral Diagnosis: **Incidental Murmur Finding. AGH2.164** | | | |
| --- | --- | --- | --- |
| **Features** | **Finding** | **Features** | **Finding** |
| **Profile** |  | **Atria** |  |
| Abdominal situs | Solitus | Left atrium | Normal |
| Cardiac position | Levocardia | Right atrium | Normal |
| Systemic venous drainage | Normal. | **Atrioventricular valves** |  |
| Pulmonary venous drainage | Normal | Mitral valve | Annulus = 10mm |
| Atrioventricular connection | Concordant | Tricuspid valve | Annulus = 10mm |
| Ventriculoarterial connection | Concordant | **Ventricles** |  |
| Ventricular loop | d-Loop | Left ventricle | Normal |
|  |  | Right ventricle | Normal |
| **Septae** |  | **Coronary arteries** | ----- |
| Interventricular septum | Intact | **Doppler Measurement** |  |
| Interatrial septum | PFO, L – R Shunt | Mitral | ----- |
| **Semilunar valves** |  | Aortic | ------- |
| Aortic valve | Annulus = 8mm | Tricuspid | ------- |
| Pulmonary valve | Annulus = 9mm | pulmonic | -------- |
| **Great arteries** | NRGA | **Aortic arch** | Left. No CoA. |
| Aorta | ----- | **PDA** | 1mm PDA,L – R Shunt |
| Pulmonary artery | Normal MPA and Branch PAs. |  |  |
| **M-Mode:**  Normal LV Function on eye balling | | | |
| AO | mm | PWd | mm |
| LA | mm | PWs | mm |
| LVIDd | mm | EDV | ml |
| LVIDs | mm | ESV | ml |
| IVSs | mm | LVEF | % |
| IVSd | mm | FS | % |
| **Additional Information**: |  | | |
| No pericardial/Pleural effusion. | | | |
| **Final Diagnosis:** | | | |
| 1. {S, D, S} Levocardia. 2. PFO, L – R Shunt 3. Small PDA, L – R Shunt 4. Normal LV Function | | | |
| **Remark**: | | | |
| **Recommendation**: | | | |
| SIGNATURE  Done by: Tesfaye T., Pediatrician, Pediatric Cardiologist _______________ 02/05/2015Eth.C | | | |

| Patient Name: **Werknesh Belete**. Referring Institute: **Addis Alem PH**. SEX/ Age: **F/12years**. Date of Report: **02/05/15**.  Referral Diagnosis: **Palpitation. AGH2.165** | | | |
| --- | --- | --- | --- |
| **Features** | **Finding** | **Features** | **Finding** |
| **Profile** |  | **Atria** |  |
| Abdominal situs | Solitus | Left atrium | Normal |
| Cardiac position | Levocardia | Right atrium | Normal |
| Systemic venous drainage | Normal. | **Atrioventricular valves** |  |
| Pulmonary venous drainage | Normal | Mitral valve | Annulus = 23mm |
| Atrioventricular connection | Concordant | Tricuspid valve | Annulus = 24mm  TAPSE = 22mm |
| Ventriculoarterial connection | Concordant | **Ventricles** |  |
| Ventricular loop | d-Loop | Left ventricle | Normal |
|  |  | Right ventricle | Normal |
| **Septae** |  | **Coronary arteries** | ----- |
| Interventricular septum | Intact | **Doppler Measurement** |  |
| Interatrial septum | Intact | Mitral | ----- |
| **Semilunar valves** |  | Aortic | ------- |
| Aortic valve | Annulus = 17mm | Tricuspid | Trivial TR, PPG = 17mmHg |
| Pulmonary valve | Annulus = 20mm | pulmonic | -------- |
| **Great arteries** | NRGA | **Aortic arch** | Left. No CoA. |
| Aorta | ----- | **PDA** | No |
| Pulmonary artery | Normal MPA and Branch PAs. |  |  |
| **M-Mode:** | | | |
| AO | mm | PWd | mm |
| LA | mm | PWs | mm |
| LVIDd | mm | EDV | ml |
| LVIDs | mm | ESV | ml |
| IVSs | mm | LVEF | 66% |
| IVSd | mm | FS | 36% |
| **Additional Information**: |  | | |
| No pericardial/Pleural effusion. | | | |
| **Final Diagnosis:** | | | |
| 1. Normal Echocardiography Study. | | | |
| **Remark**: | | | |
| **Recommendation**: | | | |
| SIGNATURE  Done by: Tesfaye T., Pediatrician, Pediatric Cardiologist _______________ 02/05/2015Eth.C | | | |

| Patient Name: **Hewan Ayalneh**. Referring Institute: **FHRH**. SEX/ Age: **F/1 11/12**. Date of Report: **03/05/15**.  Referral Diagnosis: **Incidental Murmur Finding. AGH2.166** | | | |
| --- | --- | --- | --- |
| **Features** | **Finding** | **Features** | **Finding** |
| **Profile** |  | **Atria** |  |
| Abdominal situs | Solitus | Left atrium | Normal |
| Cardiac position | Levocardia | Right atrium | Normal |
| Systemic venous drainage | Normal. | **Atrioventricular valves** |  |
| Pulmonary venous drainage | Normal | Mitral valve | Annulus = 14mm |
| Atrioventricular connection | Concordant | Tricuspid valve | Annulus = 14mm |
| Ventriculoarterial connection | Concordant | **Ventricles** |  |
| Ventricular loop | d-Loop | Left ventricle | Normal |
|  |  | Right ventricle | Normal |
| **Septae** |  | **Coronary arteries** | ----- |
| Interventricular septum | Intact | **Doppler Measurement** |  |
| Interatrial septum | Intact | Mitral | ----- |
| **Semilunar valves** |  | Aortic | ------- |
| Aortic valve | Annulus = 12mm | Tricuspid | ------- |
| Pulmonary valve | Annulus = 14mm | pulmonic | -------- |
| **Great arteries** | NRGA | **Aortic arch** | Left. No CoA. |
| Aorta | ----- | **PDA** | 1.5mm PDA, L – R Shunt |
| Pulmonary artery | Normal MPA and Branch PAs. |  |  |
| **M-Mode:**  Normal LV Function on eye balling. | | | |
| AO | mm | PWd | mm |
| LA | mm | PWs | mm |
| LVIDd | mm | EDV | ml |
| LVIDs | mm | ESV | ml |
| IVSs | mm | LVEF | % |
| IVSd | mm | FS | % |
| **Additional Information**: |  | | |
| No pericardial/Pleural effusion. | | | |
| **Final Diagnosis:** | | | |
| 1. {S, D, S} Levocardia. 2. Small PDA, L – R Shunt | | | |
| **Remark**: | | | |
| **Recommendation**: | | | |
| SIGNATURE  Done by: Tesfaye T., Pediatrician, Pediatric Cardiologist _______________ 03/05/2015Eth.C | | | |

| Patient Name: **Abinet Seto**. Referring Institute: **Adinas GH**. SEX/ Age: **M/10years**. Date of Report: **04/05/15**.  Referral Diagnosis: **Chest Pain. AGH2.167** | | | |
| --- | --- | --- | --- |
| **Features** | **Finding** | **Features** | **Finding** |
| **Profile** |  | **Atria** |  |
| Abdominal situs | Solitus | Left atrium | Normal |
| Cardiac position | Levocardia | Right atrium | Normal |
| Systemic venous drainage | Normal. | **Atrioventricular valves** |  |
| Pulmonary venous drainage | Normal | Mitral valve | Annulus = 19mm |
| Atrioventricular connection | Concordant | Tricuspid valve | Annulus = 22mm  TAPSE = 19mm |
| Ventriculoarterial connection | Concordant | **Ventricles** |  |
| Ventricular loop | d-Loop | Left ventricle | Normal |
|  |  | Right ventricle | Normal |
| **Septae** |  | **Coronary arteries** | ----- |
| Interventricular septum | Intact | **Doppler Measurement** |  |
| Interatrial septum | Intact | Mitral | ----- |
| **Semilunar valves** |  | Aortic | ------- |
| Aortic valve | Annulus = 18mm | Tricuspid | Trivial TR, PPG = 13mmHg |
| Pulmonary valve | Annulus = 20mm | pulmonic | -------- |
| **Great arteries** | NRGA | **Aortic arch** | Left. No CoA. |
| Aorta | ----- | **PDA** | No |
| Pulmonary artery | Normal MPA and Branch PAs. |  |  |
| **M-Mode:** | | | |
| AO | mm | PWd | mm |
| LA | mm | PWs | mm |
| LVIDd | mm | EDV | ml |
| LVIDs | mm | ESV | ml |
| IVSs | mm | LVEF | 58% |
| IVSd | mm | FS | 30% |
| **Additional Information**: |  | | |
| No pericardial/Pleural effusion. | | | |
| **Final Diagnosis:** | | | |
| 1. Normal Echocardiography Study. | | | |
| **Remark**: | | | |
| **Recommendation**: | | | |
| SIGNATURE  Done by: Tesfaye T., Pediatrician, Pediatric Cardiologist _______________ 04/05/2015Eth.C | | | |

| Patient Name: **Baby of Emebet Werku**. Referring Institute: **FHRH**. SEX/ Age: **M/14days**. Date of Report: **04/05/15**.  Referral Diagnosis: **DS. AGH2.168** | | | |
| --- | --- | --- | --- |
| **Features** | **Finding** | **Features** | **Finding** |
| **Profile** |  | **Atria** |  |
| Abdominal situs | Solitus | Left atrium | Normal |
| Cardiac position | Levocardia | Right atrium | Normal |
| Systemic venous drainage | Normal. | **Atrioventricular valves** |  |
| Pulmonary venous drainage | Normal | Mitral valve | Annulus = 8mm |
| Atrioventricular connection | Concordant | Tricuspid valve | Annulus = 9mm |
| Ventriculoarterial connection | Concordant | **Ventricles** |  |
| Ventricular loop | d-Loop | Left ventricle | Normal |
|  |  | Right ventricle | Normal |
| **Septae** |  | **Coronary arteries** | ----- |
| Interventricular septum | Intact | **Doppler Measurement** |  |
| Interatrial septum | PFO, L – R Shunt | Mitral | ----- |
| **Semilunar valves** |  | Aortic | ------- |
| Aortic valve | Annulus = 9mm | Tricuspid | ------- |
| Pulmonary valve | Annulus = 9mm | pulmonic | -------- |
| **Great arteries** | NRGA | **Aortic arch** | Left. No CoA. |
| Aorta | ----- | **PDA** | No |
| Pulmonary artery | Normal MPA and Branch PAs. |  |  |
| **M-Mode:**  Normal LV Function on eye balling | | | |
| AO | mm | PWd | mm |
| LA | mm | PWs | mm |
| LVIDd | mm | EDV | ml |
| LVIDs | mm | ESV | ml |
| IVSs | mm | LVEF | % |
| IVSd | mm | FS | % |
| **Additional Information**: |  | | |
| No pericardial/Pleural effusion. | | | |
| **Final Diagnosis:** | | | |
| 1. {S, D, S} Levocardia. 2. PFO, L – R Shunt | | | |
| **Remark**: | | | |
| **Recommendation**: | | | |
| SIGNATURE  Done by: Tesfaye T., Pediatrician, Pediatric Cardiologist _______________ 04/05/2015Eth.C | | | |

| Patient Name: **Samuel Abel**. Referring Institute: **Durbetie PH**. SEX/ Age: **M/20days**. Date of Report: **05/05/15**.  Referral Diagnosis: **DS. AGH2.169** | | | |
| --- | --- | --- | --- |
| **Features** | **Finding** | **Features** | **Finding** |
| **Profile** |  | **Atria** |  |
| Abdominal situs | Solitus | Left atrium | Normal |
| Cardiac position | Levocardia | Right atrium | Normal |
| Systemic venous drainage | Normal. | **Atrioventricular valves** |  |
| Pulmonary venous drainage | Normal | Mitral valve | Annulus = 11mm |
| Atrioventricular connection | Concordant | Tricuspid valve | Annulus = 12mm |
| Ventriculoarterial connection | Concordant | **Ventricles** |  |
| Ventricular loop | d-Loop | Left ventricle | Normal |
|  |  | Right ventricle | Normal |
| **Septae** |  | **Coronary arteries** | ----- |
| Interventricular septum | Intact | **Doppler Measurement** |  |
| Interatrial septum | 5mm OS ASD, L – R Shunt | Mitral | ----- |
| **Semilunar valves** |  | Aortic | ------- |
| Aortic valve | Annulus = 9mm | Tricuspid | ------- |
| Pulmonary valve | Annulus = 10mm | pulmonic | -------- |
| **Great arteries** | NRGA | **Aortic arch** | Left. No CoA. |
| Aorta | ----- | **PDA** | No |
| Pulmonary artery | Normal MPA and Branch PAs. |  |  |
| **M-Mode:**  Normal LV Function on eye balling | | | |
| AO | mm | PWd | mm |
| LA | mm | PWs | mm |
| LVIDd | mm | EDV | ml |
| LVIDs | mm | ESV | ml |
| IVSs | mm | LVEF | % |
| IVSd | mm | FS | % |
| **Additional Information**: |  | | |
| No pericardial/Pleural effusion. | | | |
| **Final Diagnosis:** | | | |
| 1. {S, D, S} Levocardia. 2. Small OS ASD, L – R Shunt | | | |
| **Remark**: | | | |
| **Recommendation**: | | | |
| SIGNATURE  Done by: Tesfaye T., Pediatrician, Pediatric Cardiologist _______________ 05/05/2015Eth.C | | | |

| Patient Name: **Nuhamin Wudu**. Referring Institute: **Amaris PSC**. SEX/ Age: **F/11years**. Date of Report: **05/05/15**.  Referral Diagnosis: **Easy fatigability. AGH2.170** | | | |
| --- | --- | --- | --- |
| **Features** | **Finding** | **Features** | **Finding** |
| **Profile** |  | **Atria** |  |
| Abdominal situs | Solitus | Left atrium | Normal |
| Cardiac position | Levocardia | Right atrium | Normal |
| Systemic venous drainage | Normal. | **Atrioventricular valves** |  |
| Pulmonary venous drainage | Normal | Mitral valve | Annulus = 22mm |
| Atrioventricular connection | Concordant | Tricuspid valve | Annulus = 22mm  TAPSE = 20mm |
| Ventriculoarterial connection | Concordant | **Ventricles** |  |
| Ventricular loop | d-Loop | Left ventricle | Normal |
|  |  | Right ventricle | Normal |
| **Septae** |  | **Coronary arteries** | ----- |
| Interventricular septum | Intact | **Doppler Measurement** |  |
| Interatrial septum | Intact | Mitral | ----- |
| **Semilunar valves** |  | Aortic | ------- |
| Aortic valve | Annulus = 17mm | Tricuspid | Trivial TR, PPG = 15mmHg |
| Pulmonary valve | Annulus = 19mm | pulmonic | -------- |
| **Great arteries** | NRGA | **Aortic arch** | Left. No CoA. |
| Aorta | ----- | **PDA** | No |
| Pulmonary artery | Normal MPA and Branch PAs. |  |  |
| **M-Mode:** | | | |
| AO | mm | PWd | mm |
| LA | mm | PWs | mm |
| LVIDd | mm | EDV | ml |
| LVIDs | mm | ESV | ml |
| IVSs | mm | LVEF | 66% |
| IVSd | mm | FS | 35% |
| **Additional Information**: |  | | |
| No pericardial/Pleural effusion. | | | |
| **Final Diagnosis:** | | | |
| 1. Normal Echocardiography Study. | | | |
| **Remark**: | | | |
| **Recommendation**: | | | |
| SIGNATURE  Done by: Tesfaye T., Pediatrician, Pediatric Cardiologist _______________ 05/05/2015Eth.C | | | |

| Patient Name: **Shegalem Belay**. Referring Institute: **FHRH**. SEX/ Age: **M/13years**. Date of Report: **05/05/15**.  Referral Diagnosis: **?Sydenham’s Chorea. AGH2.171** | | | |
| --- | --- | --- | --- |
| **Features** | **Finding** | **Features** | **Finding** |
| **Profile** |  | **Atria** |  |
| Abdominal situs | Solitus | Left atrium | Normal |
| Cardiac position | Levocardia | Right atrium | Normal |
| Systemic venous drainage | Normal. | **Atrioventricular valves** |  |
| Pulmonary venous drainage | Normal | Mitral valve | Annulus = 25mm |
| Atrioventricular connection | Concordant | Tricuspid valve | Annulus = 26mm  TAPSE = 19mm |
| Ventriculoarterial connection | Concordant | **Ventricles** |  |
| Ventricular loop | d-Loop | Left ventricle | Normal |
|  |  | Right ventricle | Normal |
| **Septae** |  | **Coronary arteries** | ----- |
| Interventricular septum | Intact | **Doppler Measurement** |  |
| Interatrial septum | Intact | Mitral | ----- |
| **Semilunar valves** |  | Aortic | ------- |
| Aortic valve | Annulus = 18mm | Tricuspid | Trivial TR, Incomplete Signal, PPG = 18mmHg |
| Pulmonary valve | Annulus = 21mm | pulmonic | Trivial PR, PPG = 13mmHg |
| **Great arteries** | NRGA | **Aortic arch** | Left. No CoA. |
| Aorta | ----- | **PDA** | No |
| Pulmonary artery | Normal MPA and Branch PAs. |  |  |
| **M-Mode:** | | | |
| AO | mm | PWd | mm |
| LA | mm | PWs | mm |
| LVIDd | mm | EDV | ml |
| LVIDs | mm | ESV | ml |
| IVSs | mm | LVEF | 59% |
| IVSd | mm | FS | 31% |
| **Additional Information**: |  | | |
| No pericardial/Pleural effusion. | | | |
| **Final Diagnosis:** | | | |
| 1. Normal Echocardiography Study. | | | |
| **Remark**: | | | |
| **Recommendation**: | | | |
| SIGNATURE  Done by: Tesfaye T., Pediatrician, Pediatric Cardiologist _______________ 05/05/2015Eth.C | | | |

| Patient Name: **Senayit Eshete**. Referring Institute: **FHRH**. SEX/ Age: **F/2years**. Date of Report: **05/05/15**.  Referral Diagnosis: **Incidental Murmur Finding. AGH2.172** | | | |
| --- | --- | --- | --- |
| **Features** | **Finding** | **Features** | **Finding** |
| **Profile** |  | **Atria** |  |
| Abdominal situs | Inversus | Left atrium | Right side & dilated |
| Cardiac position | Dextrocardia | Right atrium | Left side |
| Systemic venous drainage | To Left side RA | **Atrioventricular valves** |  |
| Pulmonary venous drainage | To right side LA | Mitral valve | Annulus = 13mm |
| Atrioventricular connection | Concordant | Tricuspid valve | Annulus = 13mm |
| Ventriculoarterial connection | Concordant | **Ventricles** |  |
| Ventricular loop | l-Loop | Left ventricle | Right side and apex to right |
|  |  | Right ventricle | Left Side |
| **Septae** |  | **Coronary arteries** | ----- |
| Interventricular septum | Intact | **Doppler Measurement** |  |
| Interatrial septum | Intact | Mitral | ----- |
| **Semilunar valves** |  | Aortic | ------- |
| Aortic valve | Annulus = mm | Tricuspid | ------- |
| Pulmonary valve | Annulus = mm | pulmonic | -------- |
| **Great arteries** | i-TGA | **Aortic arch** | Left. No CoA. |
| Aorta | ----- | **PDA** | 2mm PDA, L – R Shunt |
| Pulmonary artery | Normal MPA and Branch PAs. |  |  |
| **M-Mode:** | | | |
| AO | mm | PWd | mm |
| LA | mm | PWs | mm |
| LVIDd | mm | EDV | ml |
| LVIDs | mm | ESV | ml |
| IVSs | mm | LVEF | % |
| IVSd | mm | FS | % |
| **Additional Information**: |  | | |
| No pericardial/Pleural effusion. | | | |
| **Final Diagnosis:** | | | |
| 1. Situs Inversus abdominalis 2. {I, L, I} Dextrocardia. 3. LA/LV Dilated 4. Moderate PDA, L – R Shunt (Physiologic) | | | |
| **Remark**: | | | |
| **Recommendation**: | | | |
| SIGNATURE  Done by: Tesfaye T., Pediatrician, Pediatric Cardiologist _______________ 05/05/2015Eth.C | | | |

| Patient Name: **Baby of Asasie Muche**. Referring Institute: **TGSH**. SEX/ Age: **M/20days**. Date of Report: **05/05/15**.  Referral Diagnosis: **DS. AGH2.173** | | | |
| --- | --- | --- | --- |
| **Features** | **Finding** | **Features** | **Finding** |
| **Profile** |  | **Atria** |  |
| Abdominal situs | Solitus | Left atrium | Normal |
| Cardiac position | Levocardia | Right atrium | Normal |
| Systemic venous drainage | Normal. | **Atrioventricular valves** |  |
| Pulmonary venous drainage | Normal | Mitral valve | Annulus = 9mm |
| Atrioventricular connection | Concordant | Tricuspid valve | Annulus = 11mm |
| Ventriculoarterial connection | Concordant | **Ventricles** |  |
| Ventricular loop | d-Loop | Left ventricle | Normal |
|  |  | Right ventricle | Normal |
| **Septae** |  | **Coronary arteries** | ----- |
| Interventricular septum | 3mm Inlet VSD, L – R Shunt | **Doppler Measurement** |  |
| Interatrial septum | 5mm Primum ASD, L – R Shunt. Additional PFO, L – R Shunt | Mitral | ----- |
| **Semilunar valves** |  | Aortic | ------- |
| Aortic valve | Annulus = 9mm | Tricuspid | Mild TR |
| Pulmonary valve | Annulus = 10mm | pulmonic | -------- |
| **Great arteries** | NRGA | **Aortic arch** | Left. No CoA. |
| Aorta | ----- | **PDA** | No |
| Pulmonary artery | Normal MPA and Branch PAs. |  |  |
| **M-Mode:**  Normal LV Function on eye balling | | | |
| AO | mm | PWd | mm |
| LA | mm | PWs | mm |
| LVIDd | mm | EDV | ml |
| LVIDs | mm | ESV | ml |
| IVSs | mm | LVEF | % |
| IVSd | mm | FS | % |
| **Additional Information**: |  | | |
| No pericardial/Pleural effusion. | | | |
| **Final Diagnosis:** | | | |
| 1. {S, D, S} Levocardia. 2. PFO, L – R Shunt 3. Transitional AVSD, L – R Shunt 4. Mild TR 5. Normal LV Function | | | |
| **Remark**: | | | |
| **Recommendation**: | | | |
| SIGNATURE  Done by: Tesfaye T., Pediatrician, Pediatric Cardiologist _______________ 05/05/2015Eth.C | | | |

| Patient Name: **Meklit Wassie**. Referring Institute: **FHRH**. SEX/ Age: **F/1year**. Date of Report: **08/05/15**.  Referral Diagnosis: **Follow up echo for IE + Large PDA + Severe Pul.HTN.** |
| --- |
|  |
| **Conclusion:** |
| 1. {S, D, S} Levocardia. 2. All chambers Dilated 3. Moderate MR 4. Mild TR 5. Large PDA, L – R Shunt 6. Severe Pulmonary Hypertension 7. Normal Biventricular Systolic Function |
| **Remark**: No vegetation seen |
| **Recommendation**: |
| SIGNATURE  Done by: Tesfaye T., Pediatrician, Pediatric Cardiologist _______________ 08/05/2015Eth.C |

| Patient Name: **Birhan Gashaw**. Referring Institute: **FHRH**. SEX/ Age: **F/14years**. Date of Report: **08/05/15**.  Referral Diagnosis: **ARF. AGH2.174** | | | |
| --- | --- | --- | --- |
| **Features** | **Finding** | **Features** | **Finding** |
| **Profile** |  | **Atria** |  |
| Abdominal situs | Solitus | Left atrium | Normal |
| Cardiac position | Levocardia | Right atrium | Normal |
| Systemic venous drainage | Normal. | **Atrioventricular valves** |  |
| Pulmonary venous drainage | Normal | Mitral valve | Annulus = 20mm |
| Atrioventricular connection | Concordant | Tricuspid valve | Annulus = 19mm  TAPSE = 20mm |
| Ventriculoarterial connection | Concordant | **Ventricles** |  |
| Ventricular loop | d-Loop | Left ventricle | Normal |
|  |  | Right ventricle | Normal |
| **Septae** |  | **Coronary arteries** | ----- |
| Interventricular septum | Intact | **Doppler Measurement** |  |
| Interatrial septum | Intact | Mitral | ----- |
| **Semilunar valves** |  | Aortic | ------- |
| Aortic valve | Annulus = 18mm | Tricuspid | ------- |
| Pulmonary valve | Annulus = 19mm | pulmonic | -------- |
| **Great arteries** | NRGA | **Aortic arch** | Left. No CoA. |
| Aorta | ----- | **PDA** | No |
| Pulmonary artery | Normal MPA and Branch PAs. |  |  |
| **M-Mode:** | | | |
| AO | mm | PWd | mm |
| LA | mm | PWs | mm |
| LVIDd | mm | EDV | ml |
| LVIDs | mm | ESV | ml |
| IVSs | mm | LVEF | 68% |
| IVSd | mm | FS | 37% |
| **Additional Information**: |  | | |
| No pericardial/Pleural effusion. | | | |
| **Final Diagnosis:** | | | |
| 1. Normal Echocardiography Study. | | | |
| **Remark**: | | | |
| **Recommendation**: | | | |
| SIGNATURE  Done by: Tesfaye T., Pediatrician, Pediatric Cardiologist _______________ 08/05/2015Eth.C | | | |

| Patient Name: **Hikma Muhammed**. Referring Institute: **Adinas GH**. SEX/ Age: **F/6years**. Date of Report: **08/05/15**.  Referral Diagnosis: **FTT + Murmur . AGH2.175** | | | |
| --- | --- | --- | --- |
| **Features** | **Finding** | **Features** | **Finding** |
| **Profile** |  | **Atria** |  |
| Abdominal situs | Solitus | Left atrium | Dilated |
| Cardiac position | Levocardia | Right atrium | Normal |
| Systemic venous drainage | Normal. | **Atrioventricular valves** |  |
| Pulmonary venous drainage | Normal | Mitral valve | Annulus = 22mm |
| Atrioventricular connection | Concordant | Tricuspid valve | Annulus = 15mm  TAPSE = 20mm |
| Ventriculoarterial connection | Concordant | **Ventricles** |  |
| Ventricular loop | d-Loop | Left ventricle | Dilated |
|  |  | Right ventricle | Normal |
| **Septae** |  | **Coronary arteries** | ----- |
| Interventricular septum | Intact | **Doppler Measurement** |  |
| Interatrial septum | Intact | Mitral | ----- |
| **Semilunar valves** |  | Aortic | ------- |
| Aortic valve | Annulus = 17mm | Tricuspid | ------- |
| Pulmonary valve | Annulus = 21mm | pulmonic | -------- |
| **Great arteries** | NRGA | **Aortic arch** | Left. No CoA. |
| Aorta | ----- | **PDA** | 5mm PDA, L – R Shunt |
| Pulmonary artery | Normal MPA and Branch PAs. |  |  |
| **M-Mode:** | | | |
| AO | mm | PWd | mm |
| LA | mm | PWs | mm |
| LVIDd | mm | EDV | ml |
| LVIDs | mm | ESV | ml |
| IVSs | mm | LVEF | 65% |
| IVSd | mm | FS | 35% |
| **Additional Information**: |  | | |
| No pericardial/Pleural effusion. | | | |
| **Final Diagnosis:** | | | |
| 1. {S, D, S} Levocardia. 2. LA/LV Dilated 3. Large PDA, L – R Shunt 4. Normal Biventricular Systolic Function | | | |
| **Remark**: | | | |
| **Recommendation**: | | | |
| SIGNATURE  Done by: Tesfaye T., Pediatrician, Pediatric Cardiologist _______________ 08/05/2015Eth.C | | | |

| Patient Name: **Tiruwerk Agumas**. Referring Institute: **FHRH**. SEX/ Age: **F/10years**. Date of Report: **09/05/15**.  Referral Diagnosis: **Easy Fatigability. AGH2.176** | | | |
| --- | --- | --- | --- |
| **Features** | **Finding** | **Features** | **Finding** |
| **Profile** |  | **Atria** |  |
| Abdominal situs | Solitus | Left atrium | Normal |
| Cardiac position | Levocardia | Right atrium | Normal |
| Systemic venous drainage | Normal. | **Atrioventricular valves** |  |
| Pulmonary venous drainage | Normal | Mitral valve | Annulus = 21mm |
| Atrioventricular connection | Concordant | Tricuspid valve | Annulus = 21mm  TAPSE = 20mm |
| Ventriculoarterial connection | Concordant | **Ventricles** |  |
| Ventricular loop | d-Loop | Left ventricle | Normal |
|  |  | Right ventricle | Normal |
| **Septae** |  | **Coronary arteries** | ----- |
| Interventricular septum | Intact | **Doppler Measurement** |  |
| Interatrial septum | 6mm OS ASD, L – R Shunt | Mitral | ----- |
| **Semilunar valves** |  | Aortic | ------- |
| Aortic valve | Annulus = 16mm | Tricuspid | ------- |
| Pulmonary valve | Annulus = 18mm | pulmonic | -------- |
| **Great arteries** | NRGA | **Aortic arch** | Left. No CoA. |
| Aorta | ----- | **PDA** | No |
| Pulmonary artery | Normal MPA and Branch PAs. |  |  |
| **M-Mode:** | | | |
| AO | mm | PWd | mm |
| LA | mm | PWs | mm |
| LVIDd | mm | EDV | ml |
| LVIDs | mm | ESV | ml |
| IVSs | mm | LVEF | 67% |
| IVSd | mm | FS | 36% |
| **Additional Information**: |  | | |
| No pericardial/Pleural effusion. | | | |
| **Final Diagnosis:** | | | |
| 1. {S, D, S} Levocardia. 2. Small OS ASD, L – R Shunt | | | |
| **Remark**: | | | |
| **Recommendation**: | | | |
| SIGNATURE  Done by: Tesfaye T., Pediatrician, Pediatric Cardiologist _______________ 09/05/2015Eth.C | | | |

| Patient Name: **Mamen Assefa**. Referring Institute: **TGSH**. SEX/ Age: **M/15years**. Date of Report: **09/05/15**.  Referral Diagnosis: **Easy Fatigability. AGH2.177** | | | |
| --- | --- | --- | --- |
| **Features** | **Finding** | **Features** | **Finding** |
| **Profile** |  | **Atria** |  |
| Abdominal situs | Solitus | Left atrium | Normal |
| Cardiac position | Levocardia | Right atrium | Normal |
| Systemic venous drainage | Normal. | **Atrioventricular valves** |  |
| Pulmonary venous drainage | Normal | Mitral valve | Annulus = 20mm |
| Atrioventricular connection | Concordant | Tricuspid valve | Annulus = 20mm  TAPSE = 21mm |
| Ventriculoarterial connection | Concordant | **Ventricles** |  |
| Ventricular loop | d-Loop | Left ventricle | Normal |
|  |  | Right ventricle | Mildly Hypertrophied. |
| **Septae** |  | **Coronary arteries** | ----- |
| Interventricular septum | Intact | **Doppler Measurement** |  |
| Interatrial septum | Intact | Mitral | ----- |
| **Semilunar valves** |  | Aortic | ------- |
| Aortic valve | Annulus = 16mm | Tricuspid | ------- |
| Pulmonary valve | Annulus = 17mm. Doming PV | pulmonic | Moderate PR, PPG = 22mmHg. Moderate Valvular PS, PPG = 52mmHg. |
| **Great arteries** | NRGA | **Aortic arch** | Left. No CoA. |
| Aorta | ----- | **PDA** | No |
| Pulmonary artery | Normal MPA and Branch PAs. |  |  |
| **M-Mode:** | | | |
| AO | mm | PWd | mm |
| LA | mm | PWs | mm |
| LVIDd | mm | EDV | ml |
| LVIDs | mm | ESV | ml |
| IVSs | mm | LVEF | 69% |
| IVSd | mm | FS | 38% |
| **Additional Information**: |  | | |
| No pericardial/Pleural effusion. | | | |
| **Final Diagnosis:** | | | |
| 1. {S, D, S} Levocardia. 2. Moderate Valvular PS 3. Moderate PR 4. Doming Pulmonary Valve 5. Normal Biventricular Systolic Function | | | |
| **Remark**: | | | |
| **Recommendation**: | | | |
| SIGNATURE  Done by: Tesfaye T., Pediatrician, Pediatric Cardiologist _______________ 09/05/2015Eth.C | | | |

| Patient Name: **Baby of Yemikir Nibret**. Referring Institute: **FHRH**. SEX/ Age: **F/1month**. Date of Report: **09/05/15**.  Referral Diagnosis: **RD. AGH2.178** | | | |
| --- | --- | --- | --- |
| **Features** | **Finding** | **Features** | **Finding** |
| **Profile** |  | **Atria** |  |
| Abdominal situs | Solitus | Left atrium | Normal |
| Cardiac position | Levocardia | Right atrium | Normal |
| Systemic venous drainage | Normal. | **Atrioventricular valves** |  |
| Pulmonary venous drainage | Normal | Mitral valve | Annulus = 13mm |
| Atrioventricular connection | Concordant | Tricuspid valve | Annulus = 13mm |
| Ventriculoarterial connection | Concordant | **Ventricles** |  |
| Ventricular loop | d-Loop | Left ventricle | Normal |
|  |  | Right ventricle | Normal |
| **Septae** |  | **Coronary arteries** | ----- |
| Interventricular septum | Intact | **Doppler Measurement** |  |
| Interatrial septum | PFO, L – R Shunt | Mitral | ----- |
| **Semilunar valves** |  | Aortic | ------- |
| Aortic valve | Annulus = 11mm | Tricuspid | ------- |
| Pulmonary valve | Annulus = 10mm | pulmonic | -------- |
| **Great arteries** | NRGA | **Aortic arch** | Left. No CoA. |
| Aorta | ----- | **PDA** | No |
| Pulmonary artery | Normal MPA and Branch PAs. |  |  |
| **M-Mode:**  Normal LV Function on eye balling | | | |
| AO | mm | PWd | mm |
| LA | mm | PWs | mm |
| LVIDd | mm | EDV | ml |
| LVIDs | mm | ESV | ml |
| IVSs | mm | LVEF | % |
| IVSd | mm | FS | % |
| **Additional Information**: |  | | |
| No pericardial/Pleural effusion. | | | |
| **Final Diagnosis:** | | | |
| 1. {S, D, S} Levocardia. 2. PFO, L – R Shunt | | | |
| **Remark**: | | | |
| **Recommendation**: | | | |
| SIGNATURE  Done by: Tesfaye T., Pediatrician, Pediatric Cardiologist _______________ 09/05/2015Eth.C | | | |

| Patient Name: **Baby of Askal Gebre-Ab**. Referring Institute: **MSI- Ethiopia**. SEX/ Age: **M/4days**. Date of Report: **09/05/15**.  Referral Diagnosis: **IDM. AGH2.179** | | | |
| --- | --- | --- | --- |
| **Features** | **Finding** | **Features** | **Finding** |
| **Profile** |  | **Atria** |  |
| Abdominal situs | Solitus | Left atrium | Normal |
| Cardiac position | Levocardia | Right atrium | Normal |
| Systemic venous drainage | Normal. | **Atrioventricular valves** |  |
| Pulmonary venous drainage | Normal | Mitral valve | Annulus = 12mm |
| Atrioventricular connection | Concordant | Tricuspid valve | Annulus = 12mm |
| Ventriculoarterial connection | Concordant | **Ventricles** |  |
| Ventricular loop | d-Loop | Left ventricle | Normal |
|  |  | Right ventricle | Normal |
| **Septae** |  | **Coronary arteries** | ----- |
| Interventricular septum | Intact | **Doppler Measurement** |  |
| Interatrial septum | 4mm OS ASD, L – R Shunt | Mitral | ----- |
| **Semilunar valves** |  | Aortic | ------- |
| Aortic valve | Annulus = 9mm | Tricuspid | ------- |
| Pulmonary valve | Annulus = 10mm | pulmonic | -------- |
| **Great arteries** | NRGA | **Aortic arch** | Left. No CoA. |
| Aorta | ----- | **PDA** | No |
| Pulmonary artery | Normal MPA and Branch PAs. |  |  |
| **M-Mode:**  Normal LV Function on eye balling | | | |
| AO | mm | PWd | mm |
| LA | mm | PWs | mm |
| LVIDd | mm | EDV | ml |
| LVIDs | mm | ESV | ml |
| IVSs | mm | LVEF | % |
| IVSd | mm | FS | % |
| **Additional Information**: |  | | |
| No pericardial/Pleural effusion. | | | |
| **Final Diagnosis:** | | | |
| 1. {S, D, S} Levocardia. 2. Small OS ASD, L – R Shunt | | | |
| **Remark**: | | | |
| **Recommendation**: | | | |
| SIGNATURE  Done by: Tesfaye T., Pediatrician, Pediatric Cardiologist _______________ 09/05/2015Eth.C | | | |

| Patient Name: **Haile – Michael Mengesha**. Referring Institute: **FHRH**. SEX/ Age: **M/6years**. Date of Report: **09/05/15**.  Referral Diagnosis: **CHF + RD. AGH2.180** | | | |
| --- | --- | --- | --- |
| **Features** | **Finding** | **Features** | **Finding** |
| **Profile** |  | **Atria** |  |
| Abdominal situs | Solitus | Left atrium | Markedly Dilated |
| Cardiac position | Levocardia | Right atrium | Dilated |
| Systemic venous drainage | Normal. IVC Plethora | **Atrioventricular valves** |  |
| Pulmonary venous drainage | Normal | Mitral valve | Annulus = 26mm. Thickened MVL |
| Atrioventricular connection | Concordant | Tricuspid valve | Annulus = 19mm  TAPSE = 16mm |
| Ventriculoarterial connection | Concordant | **Ventricles** |  |
| Ventricular loop | d-Loop | Left ventricle | Markedly Dilated |
|  |  | Right ventricle | Dilated |
| **Septae** |  | **Coronary arteries** | ----- |
| Interventricular septum | Intact | **Doppler Measurement** |  |
| Interatrial septum | Intact | Mitral | Severe MR, Holosystolic, posterior projection, seen in two planes with jet velocity = 3.5m/sec. |
| **Semilunar valves** |  | Aortic | Mild AR |
| Aortic valve | Annulus = 14mm | Tricuspid | Severe TR, PPG = 34mmHg |
| Pulmonary valve | Annulus = 19mm | pulmonic | -------- |
| **Great arteries** | NRGA | **Aortic arch** | Left. No CoA. |
| Aorta | ----- | **PDA** | No |
| Pulmonary artery | Normal MPA and Branch PAs. |  |  |
| **M-Mode:** | | | |
| AO | mm | PWd | mm |
| LA | mm | PWs | mm |
| LVIDd | mm | EDV | ml |
| LVIDs | mm | ESV | ml |
| IVSs | mm | LVEF | 53% |
| IVSd | mm | FS | 26% |
| **Additional Information**: |  | | |
| No pericardial/Pleural effusion. | | | |
| **Final Diagnosis:** | | | |
| 1. {S, D, S} Levocardia. 2. All chambers dilated 3. Thickened MVL 4. Severe MR 5. Severe TR 6. Mild AR 7. Mildly Reduced LV Systolic Function | | | |
| **Remark**: | | | |
| **Recommendation**: | | | |
| SIGNATURE  Done by: Tesfaye T., Pediatrician, Pediatric Cardiologist _______________ 09/05/2015Eth.C | | | |

| Patient Name: **Baby of Asresach Chekol**. Referring Institute: **FHRH**. SEX/ Age: **M/10days**. Date of Report: **09/05/15**.  Referral Diagnosis: **RD. AGH2.181** | | | |
| --- | --- | --- | --- |
| **Features** | **Finding** | **Features** | **Finding** |
| **Profile** |  | **Atria** |  |
| Abdominal situs | Solitus | Left atrium | Normal |
| Cardiac position | Levocardia | Right atrium | Normal |
| Systemic venous drainage | Normal. | **Atrioventricular valves** |  |
| Pulmonary venous drainage | Normal | Mitral valve | Annulus = 10mm |
| Atrioventricular connection | Concordant | Tricuspid valve | Annulus = 10mm |
| Ventriculoarterial connection | Concordant | **Ventricles** |  |
| Ventricular loop | d-Loop | Left ventricle | Normal |
|  |  | Right ventricle | Normal |
| **Septae** |  | **Coronary arteries** | ----- |
| Interventricular septum | Intact | **Doppler Measurement** |  |
| Interatrial septum | PFO, L – R Shunt | Mitral | ----- |
| **Semilunar valves** |  | Aortic | ------- |
| Aortic valve | Annulus = 8mm | Tricuspid | ------- |
| Pulmonary valve | Annulus = 8mm | pulmonic | -------- |
| **Great arteries** | NRGA | **Aortic arch** | Left. No CoA. |
| Aorta | ----- | **PDA** | No |
| Pulmonary artery | Normal MPA and Branch PAs. |  |  |
| **M-Mode:**  Normal LV Function on eye balling | | | |
| AO | mm | PWd | mm |
| LA | mm | PWs | mm |
| LVIDd | mm | EDV | ml |
| LVIDs | mm | ESV | ml |
| IVSs | mm | LVEF | % |
| IVSd | mm | FS | % |
| **Additional Information**: |  | | |
| No pericardial/Pleural effusion. | | | |
| **Final Diagnosis:** | | | |
| 1. {S, D, S} Levocardia. 2. PFO, L – R Shunt | | | |
| **Remark**: | | | |
| **Recommendation**: | | | |
| SIGNATURE  Done by: Tesfaye T., Pediatrician, Pediatric Cardiologist _______________ 09/05/2015Eth.C | | | |

| Patient Name: **Amanuel Abebe**. Referring Institute: **TGSH**. SEX/ Age: **M/8 9/12**. Date of Report: **12/05/15**.  Referral Diagnosis: **RD + CHF. AGH2.182** | | | |
| --- | --- | --- | --- |
| **Features** | **Finding** | **Features** | **Finding** |
| **Profile** |  | **Atria** |  |
| Abdominal situs | Inversus. Liver on the Left side. Aorta – Right. IVC - Left | Left atrium | Dilated. Right Side |
| Cardiac position | Dextrocardia | Right atrium | Normal. Left Side |
| Systemic venous drainage | to Left side RA | **Atrioventricular valves** |  |
| Pulmonary venous drainage | to Right side LA | Mitral valve | Annulus = 26mm |
| Atrioventricular connection | Concordant | Tricuspid valve | Annulus = 25mm |
| Ventriculoarterial connection | Concordant | **Ventricles** |  |
| Ventricular loop | l-Loop | Left ventricle | Dilated. Right Side |
|  |  | Right ventricle | Normal. Left Side |
| **Septae** |  | **Coronary arteries** | ----- |
| Interventricular septum | Non-Restrictive Sub aortic VSD, from Right side LV to Left Side RV. | **Doppler Measurement** |  |
| Interatrial septum | Intact | Mitral | ----- |
| **Semilunar valves** |  | Aortic | Mild AR |
| Aortic valve | Annulus = 20mm | Tricuspid | ------- |
| Pulmonary valve | Annulus = 21mm | pulmonic | -------- |
| **Great arteries** | NRGA (i-TGA) | **Aortic arch** | Left. No CoA. |
| Aorta | ----- | **PDA** | No |
| Pulmonary artery | Normal MPA and Branch PAs. |  |  |
| **M-Mode:** | | | |
| AO | mm | PWd | mm |
| LA | mm | PWs | mm |
| LVIDd | mm | EDV | ml |
| LVIDs | mm | ESV | ml |
| IVSs | mm | LVEF | % |
| IVSd | mm | FS | % |
| **Additional Information**: |  | | |
| No pericardial/Pleural effusion. | | | |
| **Final Diagnosis:** | | | |
| 1. Abdominal Situs Inversus totalis 2. {I, L, I} Dextrocardia. 3. LA/LV Dilated 4. Non-Restrictive Sub-Aortic VSD, L – R shunt 5. Mild AR (Secondary to ?AVP) | | | |
| **Remark**: | | | |
| **Recommendation**: | | | |
| SIGNATURE  Done by: Tesfaye T., Pediatrician, Pediatric Cardiologist _______________ 12/05/2015Eth.C | | | |

| Patient Name: **Baby of Etayehu Abate**. Referring Institute: **TGSH**. SEX/ Age: **F/11days**. Date of Report: **13/05/15**.  Referral Diagnosis: **DS. AGH2.183** | | | |
| --- | --- | --- | --- |
| **Features** | **Finding** | **Features** | **Finding** |
| **Profile** |  | **Atria** |  |
| Abdominal situs | Solitus | Left atrium | Normal |
| Cardiac position | Levocardia | Right atrium | Normal |
| Systemic venous drainage | Normal. | **Atrioventricular valves** |  |
| Pulmonary venous drainage | Normal | Mitral valve | Annulus = 8mm |
| Atrioventricular connection | Concordant | Tricuspid valve | Annulus = 8mm |
| Ventriculoarterial connection | Concordant | **Ventricles** |  |
| Ventricular loop | d-Loop | Left ventricle | Normal |
|  |  | Right ventricle | Normal |
| **Septae** |  | **Coronary arteries** | ----- |
| Interventricular septum | Intact | **Doppler Measurement** |  |
| Interatrial septum | 4mm OS ASD, L – R Shunt | Mitral | ----- |
| **Semilunar valves** |  | Aortic | ------- |
| Aortic valve | Annulus = 8mm | Tricuspid | ------- |
| Pulmonary valve | Annulus = 9mm | pulmonic | -------- |
| **Great arteries** | NRGA | **Aortic arch** | Left. No CoA. |
| Aorta | ----- | **PDA** | 1mm PDA, L – R Shunt |
| Pulmonary artery | Normal MPA and Branch PAs. |  |  |
| **M-Mode:**  Normal LV Function on eye balling. | | | |
| AO | mm | PWd | mm |
| LA | mm | PWs | mm |
| LVIDd | mm | EDV | ml |
| LVIDs | mm | ESV | ml |
| IVSs | mm | LVEF | % |
| IVSd | mm | FS | % |
| **Additional Information**: |  | | |
| No pericardial/Pleural effusion. | | | |
| **Final Diagnosis:** | | | |
| 1. {S, D, S} Levocardia. 2. Small OS ASD, L – R Shunt 3. Small PDA, L – R Shunt | | | |
| **Remark**: | | | |
| **Recommendation**: | | | |
| SIGNATURE  Done by: Tesfaye T., Pediatrician, Pediatric Cardiologist _______________ 13/05/2015Eth.C | | | |

| Patient Name: **Kidist Ayele**. Referring Institute: **TGSH**. SEX/ Age: **F/3years**. Date of Report: **15/05/15**.  Referral Diagnosis: **DS. AGH2.184** | | | |
| --- | --- | --- | --- |
| **Features** | **Finding** | **Features** | **Finding** |
| **Profile** |  | **Atria** |  |
| Abdominal situs | Solitus | Left atrium | Normal |
| Cardiac position | Levocardia | Right atrium | Normal |
| Systemic venous drainage | Normal. | **Atrioventricular valves** |  |
| Pulmonary venous drainage | Normal | Mitral valve | Annulus = 16mm |
| Atrioventricular connection | Concordant | Tricuspid valve | Annulus = 18mm |
| Ventriculoarterial connection | Concordant | **Ventricles** |  |
| Ventricular loop | d-Loop | Left ventricle | Normal |
|  |  | Right ventricle | Normal |
| **Septae** |  | **Coronary arteries** | ----- |
| Interventricular septum | Intact | **Doppler Measurement** |  |
| Interatrial septum | 4mm OS ASD, L – R Shunt. | Mitral | ----- |
| **Semilunar valves** |  | Aortic | ------- |
| Aortic valve | Annulus = 14mm | Tricuspid | ------- |
| Pulmonary valve | Annulus = 17mm | pulmonic | -------- |
| **Great arteries** | NRGA | **Aortic arch** | Left. No CoA. |
| Aorta | ----- | **PDA** | No |
| Pulmonary artery | Normal MPA and Branch PAs. |  |  |
| **M-Mode:**  Normal LV Function on eye balling | | | |
| AO | mm | PWd | mm |
| LA | mm | PWs | mm |
| LVIDd | mm | EDV | ml |
| LVIDs | mm | ESV | ml |
| IVSs | mm | LVEF | % |
| IVSd | mm | FS | % |
| **Additional Information**: |  | | |
| No pericardial/Pleural effusion. | | | |
| **Final Diagnosis:** | | | |
| 1. {S, D, S} Levocardia. 2. Small OS ASD, L – R Shunt | | | |
| **Remark**: | | | |
| **Recommendation**: | | | |
| SIGNATURE  Done by: Tesfaye T., Pediatrician, Pediatric Cardiologist _______________ 15/05/2015Eth.C | | | |

| Patient Name: **Baby of Tsega Mengist**. Referring Institute: **Addis Alem PH**. SEX/ Age: **M/30days**. Date of Report: **15/05/15**.  Referral Diagnosis: **Incidental Murmur Finding. AGH2.185** | | | |
| --- | --- | --- | --- |
| **Features** | **Finding** | **Features** | **Finding** |
| **Profile** |  | **Atria** |  |
| Abdominal situs | Solitus | Left atrium | Normal |
| Cardiac position | Levocardia | Right atrium | Normal |
| Systemic venous drainage | Normal. | **Atrioventricular valves** |  |
| Pulmonary venous drainage | Normal | Mitral valve | Annulus = 13mm |
| Atrioventricular connection | Concordant | Tricuspid valve | Annulus = 13mm |
| Ventriculoarterial connection | Concordant | **Ventricles** |  |
| Ventricular loop | d-Loop | Left ventricle | Normal |
|  |  | Right ventricle | Normal |
| **Septae** |  | **Coronary arteries** | ----- |
| Interventricular septum | 2mm PM VSD, L – R Shunt | **Doppler Measurement** |  |
| Interatrial septum | PFO, L – R Shunt | Mitral | ----- |
| **Semilunar valves** |  | Aortic | ------- |
| Aortic valve | Annulus = 8mm | Tricuspid | ------- |
| Pulmonary valve | Annulus = 11mm | pulmonic | -------- |
| **Great arteries** | NRGA | **Aortic arch** | Left. No CoA. |
| Aorta | ----- | **PDA** | 1mm PDA, L – R Shunt |
| Pulmonary artery | Normal MPA and Branch PAs. |  |  |
| **M-Mode:**  Normal LV Function on eye balling | | | |
| AO | mm | PWd | mm |
| LA | mm | PWs | mm |
| LVIDd | mm | EDV | ml |
| LVIDs | mm | ESV | ml |
| IVSs | mm | LVEF | % |
| IVSd | mm | FS | % |
| **Additional Information**: |  | | |
| No pericardial/Pleural effusion. | | | |
| **Final Diagnosis:** | | | |
| 1. {S, D, S} Levocardia. 2. PFO, L – R Shunt 3. Small PM VSD, L – R Shunt 4. Small PDA, L – R Shunt | | | |
| **Remark**: | | | |
| **Recommendation**: Follow up echo annually, No need of Cardiac Medicine currently. | | | |
| SIGNATURE  Done by: Tesfaye T., Pediatrician, Pediatric Cardiologist _______________ 15/05/2015Eth.C | | | |

| Patient Name: **Tekle – Mariam Getachew**. Referring Institute: **FHRH**. SEX/ Age: **M/10months**. Date of Report: **16/05/15**.  Referral Diagnosis: **Recurrent Chest Infection. AGH2.186** | | | |
| --- | --- | --- | --- |
| **Features** | **Finding** | **Features** | **Finding** |
| **Profile** |  | **Atria** |  |
| Abdominal situs | Solitus | Left atrium | Normal |
| Cardiac position | Levocardia | Right atrium | Normal |
| Systemic venous drainage | Normal. | **Atrioventricular valves** |  |
| Pulmonary venous drainage | Normal | Mitral valve | Annulus = 12mm |
| Atrioventricular connection | Concordant | Tricuspid valve | Annulus = 12mm |
| Ventriculoarterial connection | Concordant | **Ventricles** |  |
| Ventricular loop | d-Loop | Left ventricle | Normal |
|  |  | Right ventricle | Normal |
| **Septae** |  | **Coronary arteries** | ----- |
| Interventricular septum | Intact | **Doppler Measurement** |  |
| Interatrial septum | Intact | Mitral | ----- |
| **Semilunar valves** |  | Aortic | ------- |
| Aortic valve | Annulus = 11mm | Tricuspid | ------- |
| Pulmonary valve | Annulus = 13mm | pulmonic | -------- |
| **Great arteries** | NRGA | **Aortic arch** | Left. No CoA. |
| Aorta | ----- | **PDA** | No |
| Pulmonary artery | Normal MPA and Branch PAs. |  |  |
| **M-Mode:**  Normal LV Function on eye balling. | | | |
| AO | mm | PWd | mm |
| LA | mm | PWs | mm |
| LVIDd | mm | EDV | ml |
| LVIDs | mm | ESV | ml |
| IVSs | mm | LVEF | % |
| IVSd | mm | FS | % |
| **Additional Information**: |  | | |
| No pericardial/Pleural effusion. | | | |
| **Final Diagnosis:** | | | |
| 1. Normal Echocardiography Study. | | | |
| **Remark**: Limited Echo window (Only sub costal) | | | |
| **Recommendation**: | | | |
| SIGNATURE  Done by: Tesfaye T., Pediatrician, Pediatric Cardiologist _______________ 16/05/2015Eth.C | | | |

| Patient Name: **Eldana Fekadu**. Referring Institute: **TGSH**. SEX/ Age: **F/1 8/12**. Date of Report: **16/05/15**.  Referral Diagnosis: **Incidental Murmur Finding. AGH2.187** | | | |
| --- | --- | --- | --- |
| **Features** | **Finding** | **Features** | **Finding** |
| **Profile** |  | **Atria** |  |
| Abdominal situs | Solitus | Left atrium | Normal |
| Cardiac position | Levocardia | Right atrium | Normal |
| Systemic venous drainage | Normal. | **Atrioventricular valves** |  |
| Pulmonary venous drainage | Normal | Mitral valve | Annulus = 15mm |
| Atrioventricular connection | Concordant | Tricuspid valve | Annulus = 16mm |
| Ventriculoarterial connection | Concordant | **Ventricles** |  |
| Ventricular loop | d-Loop | Left ventricle | Normal |
|  |  | Right ventricle | Normal |
| **Septae** |  | **Coronary arteries** | ----- |
| Interventricular septum | 3mm Sub-Pulmonic VSD (Supra-cristal) VSD, L – R Shunt | **Doppler Measurement** |  |
| Interatrial septum | Intact | Mitral | ----- |
| **Semilunar valves** |  | Aortic | ------- |
| Aortic valve | Annulus = 12mm | Tricuspid | ------- |
| Pulmonary valve | Annulus = 14mm | pulmonic | -------- |
| **Great arteries** | NRGA | **Aortic arch** | Left. No CoA. |
| Aorta | ----- | **PDA** | No |
| Pulmonary artery | Normal MPA and Branch PAs. |  |  |
| **M-Mode:**  Normal LV Function on eye balling | | | |
| AO | mm | PWd | mm |
| LA | mm | PWs | mm |
| LVIDd | mm | EDV | ml |
| LVIDs | mm | ESV | ml |
| IVSs | mm | LVEF | % |
| IVSd | mm | FS | % |
| **Additional Information**: |  | | |
| No pericardial/Pleural effusion. | | | |
| **Final Diagnosis:** | | | |
| 1. {S, D, S} Levocardia. 2. Small Sub-Pulmonic (Supra-Cristal) VSD, L – R Shunt 3. Normal LV Systolic LV Function | | | |
| **Remark**: | | | |
| **Recommendation**: | | | |
| SIGNATURE  Done by: Tesfaye T., Pediatrician, Pediatric Cardiologist _______________ 16/05/2015Eth.C | | | |

| Patient Name: **Baby of Eldana Kelemu**. Referring Institute: **MSI-Ethiopia**. SEX/ Age: **M/2days**. Date of Report: **16/05/15**.  Referral Diagnosis: **RD. AGH2.188** | | | |
| --- | --- | --- | --- |
| **Features** | **Finding** | **Features** | **Finding** |
| **Profile** |  | **Atria** |  |
| Abdominal situs | Solitus | Left atrium | Normal |
| Cardiac position | Levocardia | Right atrium | Dilated |
| Systemic venous drainage | Normal. | **Atrioventricular valves** |  |
| Pulmonary venous drainage | Normal | Mitral valve | Annulus = 12mm |
| Atrioventricular connection | Concordant | Tricuspid valve | Annulus = 13mm  TAPSE = 6mm |
| Ventriculoarterial connection | Concordant | **Ventricles** |  |
| Ventricular loop | d-Loop | Left ventricle | Normal |
|  |  | Right ventricle | Dilated |
| **Septae** |  | **Coronary arteries** | ----- |
| Interventricular septum | Intact | **Doppler Measurement** |  |
| Interatrial septum | Intact | Mitral | ----- |
| **Semilunar valves** |  | Aortic | ------- |
| Aortic valve | Annulus = 10mm | Tricuspid | Moderate TR, PPG = 50mmHg. |
| Pulmonary valve | Annulus = 11mm | pulmonic | -------- |
| **Great arteries** | NRGA | **Aortic arch** | Left. No CoA. |
| Aorta | ----- | **PDA** | 1mm PDA, R – L Shunt |
| Pulmonary artery | Normal MPA and Branch PAs. |  |  |
| **M-Mode:**  Normal LV Function on eye balling | | | |
| AO | mm | PWd | mm |
| LA | mm | PWs | mm |
| LVIDd | mm | EDV | ml |
| LVIDs | mm | ESV | ml |
| IVSs | mm | LVEF | % |
| IVSd | mm | FS | % |
| **Additional Information**: |  | | |
| No pericardial/Pleural effusion. | | | |
| **Final Diagnosis:** | | | |
| 1. {S, D, S} Levocardia. 2. RA/RV Dilated 3. Moderate TR 4. Small PDA, R – L Shunt 5. Moderate Pulmonary Hypertension | | | |
| **Remark**: PPHTN can be considered as a differential. | | | |
| **Recommendation**: | | | |
| SIGNATURE  Done by: Tesfaye T., Pediatrician, Pediatric Cardiologist _______________ 16/05/2015Eth.C | | | |

| Patient Name: **Baby of Asmera Melie**. Referring Institute: **FHRH**. SEX/ Age: **M/21days**. Date of Report: **16/05/15**.  Referral Diagnosis: **RD. AGH2.189** | | | |
| --- | --- | --- | --- |
| **Features** | **Finding** | **Features** | **Finding** |
| **Profile** |  | **Atria** |  |
| Abdominal situs | Solitus | Left atrium | Normal |
| Cardiac position | Levocardia | Right atrium | Normal |
| Systemic venous drainage | Normal. | **Atrioventricular valves** |  |
| Pulmonary venous drainage | Normal | Mitral valve | Annulus = 11mm |
| Atrioventricular connection | Concordant | Tricuspid valve | Annulus = 11mm |
| Ventriculoarterial connection | Concordant | **Ventricles** |  |
| Ventricular loop | d-Loop | Left ventricle | Normal |
|  |  | Right ventricle | Normal |
| **Septae** |  | **Coronary arteries** | ----- |
| Interventricular septum | Intact | **Doppler Measurement** |  |
| Interatrial septum | PFO, L – R Shunt | Mitral | ----- |
| **Semilunar valves** |  | Aortic | ------- |
| Aortic valve | Annulus = 8mm | Tricuspid | ------- |
| Pulmonary valve | Annulus = 10mm | pulmonic | -------- |
| **Great arteries** | NRGA | **Aortic arch** | Left. No CoA. |
| Aorta | ----- | **PDA** | No |
| Pulmonary artery | Normal MPA and Branch PAs. |  |  |
| **M-Mode:**  Normal LV Function on eye balling | | | |
| AO | mm | PWd | mm |
| LA | mm | PWs | mm |
| LVIDd | mm | EDV | ml |
| LVIDs | mm | ESV | ml |
| IVSs | mm | LVEF | % |
| IVSd | mm | FS | % |
| **Additional Information**: |  | | |
| No pericardial/Pleural effusion. | | | |
| **Final Diagnosis:** | | | |
| 1. {S, D, S} Levocardia. 2. PFO, L – R Shunt | | | |
| **Remark**: | | | |
| **Recommendation**: | | | |
| SIGNATURE  Done by: Tesfaye T., Pediatrician, Pediatric Cardiologist _______________ 16/05/2015Eth.C | | | |

| Patient Name: **Enaya Nurhusien**. Referring Institute: **Amen SC**. SEX/ Age: **F/6months**. Date of Report: **17/05/15**.  Referral Diagnosis: **DS. AGH2.190** | | | |
| --- | --- | --- | --- |
| **Features** | **Finding** | **Features** | **Finding** |
| **Profile** |  | **Atria** |  |
| Abdominal situs | Solitus | Left atrium | Normal |
| Cardiac position | Levocardia | Right atrium | Normal |
| Systemic venous drainage | Normal. | **Atrioventricular valves** |  |
| Pulmonary venous drainage | Normal | Mitral valve | Annulus = 13mm |
| Atrioventricular connection | Concordant | Tricuspid valve | Annulus = 13mm |
| Ventriculoarterial connection | Concordant | **Ventricles** |  |
| Ventricular loop | d-Loop | Left ventricle | Normal |
|  |  | Right ventricle | Normal |
| **Septae** |  | **Coronary arteries** | ----- |
| Interventricular septum | Intact | **Doppler Measurement** |  |
| Interatrial septum | 7mm Fenestrated OS ASD, L – R Shunt | Mitral | ----- |
| **Semilunar valves** |  | Aortic | ------- |
| Aortic valve | Annulus = 11mm | Tricuspid | ------- |
| Pulmonary valve | Annulus = 11mm | pulmonic | -------- |
| **Great arteries** | NRGA | **Aortic arch** | Left. No CoA. |
| Aorta | ----- | **PDA** | No |
| Pulmonary artery | Normal MPA and Branch PAs. |  |  |
| **M-Mode:**  Normal LV Function on eye balling | | | |
| AO | mm | PWd | mm |
| LA | mm | PWs | mm |
| LVIDd | mm | EDV | ml |
| LVIDs | mm | ESV | ml |
| IVSs | mm | LVEF | % |
| IVSd | mm | FS | % |
| **Additional Information**: |  | | |
| No pericardial/Pleural effusion. | | | |
| **Final Diagnosis:** | | | |
| 1. {S, D, S} Levocardia. 2. Moderate Fenestrated OS ASD, L – R Shunt | | | |
| **Remark**: | | | |
| **Recommendation**: | | | |
| SIGNATURE  Done by: Tesfaye T., Pediatrician, Pediatric Cardiologist _______________ 17/05/2015Eth.C | | | |

| Patient Name: **Biruk Getaneh**. Referring Institute: **Debre – Tabour GH**. SEX/ Age: **M/1 10/12**. Date of Report: **17/05/15**.  Referral Diagnosis: **Incidental Murmur Finding. AGH2.191** | | | |
| --- | --- | --- | --- |
| **Features** | **Finding** | **Features** | **Finding** |
| **Profile** |  | **Atria** |  |
| Abdominal situs | Solitus | Left atrium | Normal |
| Cardiac position | Levocardia | Right atrium | Dilated |
| Systemic venous drainage | Normal. | **Atrioventricular valves** |  |
| Pulmonary venous drainage | Normal | Mitral valve | Annulus = 14mm |
| Atrioventricular connection | Concordant | Tricuspid valve | Annulus = 18mm  TAPSE = 18mm |
| Ventriculoarterial connection | Concordant | **Ventricles** |  |
| Ventricular loop | d-Loop | Left ventricle | Normal |
|  |  | Right ventricle | Dilated |
| **Septae** |  | **Coronary arteries** | ----- |
| Interventricular septum | 2mm Sub-pulmonic VSD, L – R Shunt | **Doppler Measurement** |  |
| Interatrial septum | 4mm OS ASD, L – R Shunt. Additional OS ASD, 4mm, L – R Shunt. | Mitral | ----- |
| **Semilunar valves** |  | Aortic | Trivial AR |
| Aortic valve | Annulus = 12mm | Tricuspid | ------- |
| Pulmonary valve | Annulus = 14mm | pulmonic | -------- |
| **Great arteries** | NRGA | **Aortic arch** | Left. No CoA. |
| Aorta | ----- | **PDA** | No |
| Pulmonary artery | Normal MPA and Branch PAs. |  |  |
| **M-Mode:**  Normal LV Function on eye balling | | | |
| AO | mm | PWd | mm |
| LA | mm | PWs | mm |
| LVIDd | mm | EDV | ml |
| LVIDs | mm | ESV | ml |
| IVSs | mm | LVEF | % |
| IVSd | mm | FS | % |
| **Additional Information**: |  | | |
| No pericardial/Pleural effusion. | | | |
| **Final Diagnosis:** | | | |
| 1. {S, D, S} Levocardia. 2. RA/RV Dilated 3. Moderate Fenestrated OS ASD, L – R Shunt 4. Small Sub-Pulmonic VSD, L – R Shunt 5. Trivial AR 6. Normal Biventricular Systolic Function | | | |
| **Remark**: | | | |
| **Recommendation**: | | | |
| SIGNATURE  Done by: Tesfaye T., Pediatrician, Pediatric Cardiologist _______________ 17/05/2015Eth.C | | | |

| Patient Name: **Lidya Getnet**. Referring Institute: **Debre-Tabour GH**. SEX/ Age: **F/9years**. Date of Report: **17/05/15**.  Referral Diagnosis: **Incidental Murmur Finding. AGH2.192** | | | |
| --- | --- | --- | --- |
| **Features** | **Finding** | **Features** | **Finding** |
| **Profile** |  | **Atria** |  |
| Abdominal situs | Solitus | Left atrium | Normal |
| Cardiac position | Levocardia | Right atrium | Normal |
| Systemic venous drainage | Normal. | **Atrioventricular valves** |  |
| Pulmonary venous drainage | Normal | Mitral valve | Annulus = 18mm |
| Atrioventricular connection | Concordant | Tricuspid valve | Annulus = 19mm |
| Ventriculoarterial connection | Concordant | **Ventricles** |  |
| Ventricular loop | d-Loop | Left ventricle | Normal |
|  |  | Right ventricle | Normal |
| **Septae** |  | **Coronary arteries** | ----- |
| Interventricular septum | 5mm PM VSD, Partially closed by STL, L – R Shunt with a Gradient of 58mmHg. | **Doppler Measurement** |  |
| Interatrial septum | Intact | Mitral | ----- |
| **Semilunar valves** |  | Aortic | ------- |
| Aortic valve | Annulus = 16mm | Tricuspid | ------- |
| Pulmonary valve | Annulus = 19mm | pulmonic | -------- |
| **Great arteries** | NRGA | **Aortic arch** | Left. No CoA. |
| Aorta | ----- | **PDA** | No |
| Pulmonary artery | Normal MPA and Branch PAs. |  |  |
| **M-Mode:**  Normal LV Function on eye balling | | | |
| AO | mm | PWd | mm |
| LA | mm | PWs | mm |
| LVIDd | mm | EDV | ml |
| LVIDs | mm | ESV | ml |
| IVSs | mm | LVEF | % |
| IVSd | mm | FS | % |
| **Additional Information**: |  | | |
| No pericardial/Pleural effusion. | | | |
| **Final Diagnosis:** | | | |
| 1. {S, D, S} Levocardia. 2. Restrictive PM VSD, Partially closed by STL, L – R Shunt 3. Normal LV Systolic Function | | | |
| **Remark**: | | | |
| **Recommendation**: | | | |
| SIGNATURE  Done by: Tesfaye T., Pediatrician, Pediatric Cardiologist _______________ 17/05/2015Eth.C | | | |

| Patient Name: **Nataniem Seife**. Referring Institute: **Adinas GH**. SEX/ Age: **M/13years**. Date of Report: **17/05/15**.  Referral Diagnosis: **Easy fatigability. AGH2.193** | | | |
| --- | --- | --- | --- |
| **Features** | **Finding** | **Features** | **Finding** |
| **Profile** |  | **Atria** |  |
| Abdominal situs | Solitus | Left atrium | Normal |
| Cardiac position | Levocardia | Right atrium | Normal |
| Systemic venous drainage | Normal. | **Atrioventricular valves** |  |
| Pulmonary venous drainage | Normal | Mitral valve | Annulus = 21mm |
| Atrioventricular connection | Concordant | Tricuspid valve | Annulus = 22mm |
| Ventriculoarterial connection | Concordant | **Ventricles** |  |
| Ventricular loop | d-Loop | Left ventricle | Normal |
|  |  | Right ventricle | Normal |
| **Septae** |  | **Coronary arteries** | ----- |
| Interventricular septum | Intact | **Doppler Measurement** |  |
| Interatrial septum | Intact | Mitral | ----- |
| **Semilunar valves** |  | Aortic | ------- |
| Aortic valve | Annulus = 20mm | Tricuspid | ------- |
| Pulmonary valve | Annulus = 21mm | pulmonic | -------- |
| **Great arteries** | NRGA | **Aortic arch** | Left. No CoA. |
| Aorta | ----- | **PDA** | No |
| Pulmonary artery | Normal MPA and Branch PAs. |  |  |
| **M-Mode:**  Normal LV Function on eye balling | | | |
| AO | mm | PWd | mm |
| LA | mm | PWs | mm |
| LVIDd | mm | EDV | ml |
| LVIDs | mm | ESV | ml |
| IVSs | mm | LVEF | % |
| IVSd | mm | FS | % |
| **Additional Information**: |  | | |
| No pericardial/Pleural effusion. | | | |
| **Final Diagnosis:** | | | |
| 1. Normal Echocardiography Study. | | | |
| **Remark**: | | | |
| **Recommendation**: | | | |
| SIGNATURE  Done by: Tesfaye T., Pediatrician, Pediatric Cardiologist _______________ 17/05/2015Eth.C | | | |

| Patient Name: **Amen Menbere**. Referring Institute: **Adinas GH**. SEX/ Age: **M/1 7/12**. Date of Report: **18/05/15**.  Referral Diagnosis: **RD + CHF. AGH2.194** | | | |
| --- | --- | --- | --- |
| **Features** | **Finding** | **Features** | **Finding** |
| **Profile** |  | **Atria** |  |
| Abdominal situs | Solitus | Left atrium | Dilated |
| Cardiac position | Levocardia | Right atrium | Normal |
| Systemic venous drainage | Normal. | **Atrioventricular valves** |  |
| Pulmonary venous drainage | Normal | Mitral valve | Annulus = 14mm |
| Atrioventricular connection | Concordant | Tricuspid valve | Annulus = 13mm |
| Ventriculoarterial connection | Concordant | **Ventricles** |  |
| Ventricular loop | d-Loop | Left ventricle | Dilated |
|  |  | Right ventricle | Normal |
| **Septae** |  | **Coronary arteries** | ----- |
| Interventricular septum | 9mm PM VSD, Partially covered by STL, L – R Shunt | **Doppler Measurement** |  |
| Interatrial septum | Intact | Mitral | ----- |
| **Semilunar valves** |  | Aortic | ------- |
| Aortic valve | Annulus = 11mm | Tricuspid | ------- |
| Pulmonary valve | Annulus = 14mm | pulmonic | -------- |
| **Great arteries** | NRGA | **Aortic arch** | Left. No CoA. |
| Aorta | ----- | **PDA** | 2mm PDA, L – R Shunt |
| Pulmonary artery | Normal MPA and Branch PAs. |  |  |
| **M-Mode:**  Normal LV Function on eye balling | | | |
| AO | mm | PWd | mm |
| LA | mm | PWs | mm |
| LVIDd | mm | EDV | ml |
| LVIDs | mm | ESV | ml |
| IVSs | mm | LVEF | % |
| IVSd | mm | FS | % |
| **Additional Information**: |  | | |
| No pericardial/Pleural effusion. | | | |
| **Final Diagnosis:** | | | |
| 1. {S, D, S} Levocardia. 2. LA/LV Dilated 3. Large PM VSD, Partially covered by STL, L – R Shunt 4. Moderate PDA, L – R Shunt 5. Normal LV Systolic Function | | | |
| **Remark**: | | | |
| **Recommendation**: | | | |
| SIGNATURE  Done by: Tesfaye T., Pediatrician, Pediatric Cardiologist _______________ 18/05/2015Eth.C | | | |

| Patient Name: **Liya Getachew**. Referring Institute: **FHRH**. SEX/ Age: **F/3 3/12**. Date of Report: **18/05/15**.  Referral Diagnosis: **FTT. AGH2.195** | | | |
| --- | --- | --- | --- |
| **Features** | **Finding** | **Features** | **Finding** |
| **Profile** |  | **Atria** |  |
| Abdominal situs | Solitus | Left atrium | Normal |
| Cardiac position | Levocardia | Right atrium | Normal |
| Systemic venous drainage | Normal. | **Atrioventricular valves** |  |
| Pulmonary venous drainage | Normal | Mitral valve | Annulus = 15mm |
| Atrioventricular connection | Concordant | Tricuspid valve | Annulus = 15mm  TAPSE = 19mm |
| Ventriculoarterial connection | Concordant | **Ventricles** |  |
| Ventricular loop | d-Loop | Left ventricle | Normal |
|  |  | Right ventricle | Normal |
| **Septae** |  | **Coronary arteries** | ----- |
| Interventricular septum | Intact | **Doppler Measurement** |  |
| Interatrial septum | Intact | Mitral | ----- |
| **Semilunar valves** |  | Aortic | ------- |
| Aortic valve | Annulus = 13mm | Tricuspid | ------- |
| Pulmonary valve | Annulus = 14mm | pulmonic | -------- |
| **Great arteries** | NRGA | **Aortic arch** | Left. No CoA. |
| Aorta | ----- | **PDA** | No |
| Pulmonary artery | Normal MPA and Branch PAs. |  |  |
| **M-Mode:** | | | |
| AO | mm | PWd | mm |
| LA | mm | PWs | mm |
| LVIDd | mm | EDV | ml |
| LVIDs | mm | ESV | ml |
| IVSs | mm | LVEF | 57% |
| IVSd | mm | FS | 29% |
| **Additional Information**: |  | | |
| No pericardial/Pleural effusion. | | | |
| **Final Diagnosis:** | | | |
| 1. Normal Echocardiography Study. | | | |
| **Remark**: | | | |
| **Recommendation**: | | | |
| SIGNATURE  Done by: Tesfaye T., Pediatrician, Pediatric Cardiologist _______________ 18/05/2015Eth.C | | | |

| Patient Name: **Alemitu Abiye**. Referring Institute: **TGSH**. SEX/ Age: **F/8months**. Date of Report: **19/05/15**.  Referral Diagnosis: **Incidental Murmur Finding. AGH2.196** | | | |
| --- | --- | --- | --- |
| **Features** | **Finding** | **Features** | **Finding** |
| **Profile** |  | **Atria** |  |
| Abdominal situs | Solitus | Left atrium | Normal |
| Cardiac position | Levocardia | Right atrium | Normal |
| Systemic venous drainage | Normal. | **Atrioventricular valves** |  |
| Pulmonary venous drainage | Normal | Mitral valve | Annulus = 11mm |
| Atrioventricular connection | Concordant | Tricuspid valve | Annulus = 12mm |
| Ventriculoarterial connection | Concordant | **Ventricles** |  |
| Ventricular loop | d-Loop | Left ventricle | Normal |
|  |  | Right ventricle | Normal |
| **Septae** |  | **Coronary arteries** | ----- |
| Interventricular septum | Intact | **Doppler Measurement** |  |
| Interatrial septum | PFO, L – R Shunt | Mitral | ----- |
| **Semilunar valves** |  | Aortic | ------- |
| Aortic valve | Annulus = 12mm | Tricuspid | ------- |
| Pulmonary valve | Annulus = 14mm | pulmonic | -------- |
| **Great arteries** | NRGA | **Aortic arch** | Left. No CoA. |
| Aorta | ----- | **PDA** | 1mm PDA, L – R Shunt |
| Pulmonary artery | Normal MPA and Branch PAs. |  |  |
| **M-Mode:**  Normal LV Function on eye balling | | | |
| AO | mm | PWd | mm |
| LA | mm | PWs | mm |
| LVIDd | mm | EDV | ml |
| LVIDs | mm | ESV | ml |
| IVSs | mm | LVEF | % |
| IVSd | mm | FS | % |
| **Additional Information**: |  | | |
| No pericardial/Pleural effusion. | | | |
| **Final Diagnosis:** | | | |
| 1. {S, D, S} Levocardia. 2. PFO, L – R Shunt 3. Small PDA, L – R Shunt 4. Normal LV Systolic Function | | | |
| **Remark**: | | | |
| **Recommendation**: | | | |
| SIGNATURE  Done by: Tesfaye T., Pediatrician, Pediatric Cardiologist _______________ 19/05/2015Eth.C | | | |

| Patient Name: **Baby of Aynalem Kassahun**. Referring Institute: **FHRH**. SEX/ Age: **M/5days**. Date of Report: **19/05/15**.  Referral Diagnosis: **RD. AGH2.197** | | | |
| --- | --- | --- | --- |
| **Features** | **Finding** | **Features** | **Finding** |
| **Profile** |  | **Atria** |  |
| Abdominal situs | Solitus | Left atrium | Normal |
| Cardiac position | Levocardia | Right atrium | Dilated |
| Systemic venous drainage | Normal. | **Atrioventricular valves** |  |
| Pulmonary venous drainage | Normal | Mitral valve | Annulus = 9mm |
| Atrioventricular connection | Concordant | Tricuspid valve | Annulus = 15mm  TAPSE = 7mm |
| Ventriculoarterial connection | Concordant | **Ventricles** |  |
| Ventricular loop | d-Loop | Left ventricle | Banana shaped |
|  |  | Right ventricle | Dilated & Hypertrophied |
| **Septae** |  | **Coronary arteries** | ----- |
| Interventricular septum | Intact | **Doppler Measurement** |  |
| Interatrial septum | 7mm OS ASD, R – L Shunt | Mitral | ----- |
| **Semilunar valves** |  | Aortic | ------- |
| Aortic valve | Annulus = 9mm | Tricuspid | Mild TR, PPG = 54mmHg (?Under estimated) |
| Pulmonary valve | Annulus = 12mm | pulmonic | -------- |
| **Great arteries** | NRGA | **Aortic arch** | Left. No CoA. |
| Aorta | ----- | **PDA** | 1mm PDA, R – L Shunt |
| Pulmonary artery | Normal MPA and Branch PAs. |  |  |
| **M-Mode:** | | | |
| AO | mm | PWd | mm |
| LA | mm | PWs | mm |
| LVIDd | mm | EDV | ml |
| LVIDs | mm | ESV | ml |
| IVSs | mm | LVEF | 75% |
| IVSd | mm | FS | 42% |
| **Additional Information**: |  | | |
| No pericardial/Pleural effusion. | | | |
| **Final Diagnosis:** | | | |
| 1. {S, D, S} Levocardia. 2. RA/RV Dilated 3. Moderate OS ASD, R – L Shunt 4. Small PDA, R – L Shunt 5. Hypertrophied and Dysfunctional RV 6. Normal LV Systolic Function | | | |
| **Remark**: Persistent Pulmonary Hypertension of the Newborn is Highly likely. | | | |
| **Recommendation**: Search for Underlying cause. | | | |
| SIGNATURE  Done by: Tesfaye T., Pediatrician, Pediatric Cardiologist _______________ 19/05/2015Eth.C | | | |

| Patient Name: **Tigist Wubet**. Referring Institute: **Adinas GH**. SEX/ Age: **F/10years**. Date of Report: **19/05/15**.  Referral Diagnosis: **Sydenham’s Chorea. AGH2.198** | | | |
| --- | --- | --- | --- |
| **Features** | **Finding** | **Features** | **Finding** |
| **Profile** |  | **Atria** |  |
| Abdominal situs | Solitus | Left atrium | Normal |
| Cardiac position | Levocardia | Right atrium | Normal |
| Systemic venous drainage | Normal. | **Atrioventricular valves** |  |
| Pulmonary venous drainage | Normal | Mitral valve | Annulus = 18mm. Thickened MVL. |
| Atrioventricular connection | Concordant | Tricuspid valve | Annulus = 18mm  TAPSE = 18mm |
| Ventriculoarterial connection | Concordant | **Ventricles** |  |
| Ventricular loop | d-Loop | Left ventricle | Normal |
|  |  | Right ventricle | Normal |
| **Septae** |  | **Coronary arteries** | ----- |
| Interventricular septum | Intact | **Doppler Measurement** |  |
| Interatrial septum | Intact | Mitral | Mild MR, Holosystolic, posterior projection, seen in two planes with jet velocity = 3.5m/sec |
| **Semilunar valves** |  | Aortic | ------- |
| Aortic valve | Annulus = 14mm | Tricuspid | Mild TR, PPG = 23mmHg. |
| Pulmonary valve | Annulus = 17mm | pulmonic | -------- |
| **Great arteries** | NRGA | **Aortic arch** | Left. No CoA. |
| Aorta | ----- | **PDA** | No |
| Pulmonary artery | Normal MPA and Branch PAs. |  |  |
| **M-Mode:** | | | |
| AO | mm | PWd | mm |
| LA | mm | PWs | mm |
| LVIDd | mm | EDV | ml |
| LVIDs | mm | ESV | ml |
| IVSs | mm | LVEF | 71% |
| IVSd | mm | FS | 40% |
| **Additional Information**: |  | | |
| No pericardial/Pleural effusion. | | | |
| **Final Diagnosis:** | | | |
| 1. {S, D, S} Levocardia. 2. Thickened MVL 3. Mild MR 4. Mild TR 5. Normal Biventricular Systolic Function | | | |
| **Remark**: | | | |
| **Recommendation**: | | | |
| SIGNATURE  Done by: Tesfaye T., Pediatrician, Pediatric Cardiologist _______________ 19/05/2015Eth.C | | | |

| Patient Name: **Yahe Yesuf**. Referring Institute: **Adinas GH**. SEX/ Age: **M/15years**. Date of Report: **19/05/15**.  Referral Diagnosis: **Cough + DOE. AGH2.199** | | | |
| --- | --- | --- | --- |
| **Features** | **Finding** | **Features** | **Finding** |
| **Profile** |  | **Atria** |  |
| Abdominal situs | Solitus | Left atrium | Dilated |
| Cardiac position | Levocardia | Right atrium | Normal |
| Systemic venous drainage | Normal. | **Atrioventricular valves** |  |
| Pulmonary venous drainage | Normal | Mitral valve | Annulus = 27mm. Thickened, clubbed MVL. MVA = 1cm**2**. |
| Atrioventricular connection | Concordant | Tricuspid valve | Annulus = 28mm  TAPSE = 25mm |
| Ventriculoarterial connection | Concordant | **Ventricles** |  |
| Ventricular loop | d-Loop | Left ventricle | Normal |
|  |  | Right ventricle | Normal |
| **Septae** |  | **Coronary arteries** | ----- |
| Interventricular septum | Intact | **Doppler Measurement** |  |
| Interatrial septum | Intact | Mitral | Mild MR. Moderate MS, PPG/MPG = 14/6mmHg |
| **Semilunar valves** |  | Aortic | ------- |
| Aortic valve | Annulus = 20mm | Tricuspid | Mild TR, PPG = 38mmHg |
| Pulmonary valve | Annulus = 26mm | pulmonic | -------- |
| **Great arteries** | NRGA | **Aortic arch** | Left. No CoA. |
| Aorta | ----- | **PDA** | No |
| Pulmonary artery | Normal MPA and Branch PAs. |  |  |
| **M-Mode:** | | | |
| AO | mm | PWd | mm |
| LA | mm | PWs | mm |
| LVIDd | mm | EDV | ml |
| LVIDs | mm | ESV | ml |
| IVSs | mm | LVEF | 46% |
| IVSd | mm | FS | 23% |
| **Additional Information**: |  | | |
| No pericardial/Pleural effusion. | | | |
| **Final Diagnosis:** | | | |
| 1. {S, D, S} Levocardia. 2. LA Dilated 3. Thickened, clubbed MVL 4. Mild MR 5. Moderate MS 6. Mild TR 7. Mild Pulmonary Hypertension 8. Reduced LV Systolic Function | | | |
| **Remark**: | | | |
| **Recommendation**: | | | |
| SIGNATURE  Done by: Tesfaye T., Pediatrician, Pediatric Cardiologist _______________ 19/05/2015Eth.C | | | |

| Patient Name: **Fisseha Zelalem**. Referring Institute: **Guzara SC**. SEX/ Age: **M/3months**. Date of Report: **20/05/15**.  Referral Diagnosis: **DS. AGH2.200** | | | |
| --- | --- | --- | --- |
| **Features** | **Finding** | **Features** | **Finding** |
| **Profile** |  | **Atria** |  |
| Abdominal situs | Solitus | Left atrium | Normal |
| Cardiac position | Levocardia | Right atrium | Normal |
| Systemic venous drainage | Normal. | **Atrioventricular valves** |  |
| Pulmonary venous drainage | Normal | Mitral valve | Annulus = 11mm |
| Atrioventricular connection | Concordant | Tricuspid valve | Annulus = 13mm |
| Ventriculoarterial connection | Concordant | **Ventricles** |  |
| Ventricular loop | d-Loop | Left ventricle | Normal |
|  |  | Right ventricle | Normal |
| **Septae** |  | **Coronary arteries** | ----- |
| Interventricular septum | Intact | **Doppler Measurement** |  |
| Interatrial septum | Intact | Mitral | ----- |
| **Semilunar valves** |  | Aortic | ------- |
| Aortic valve | Annulus = 9mm | Tricuspid | ------- |
| Pulmonary valve | Annulus = 10mm | pulmonic | -------- |
| **Great arteries** | NRGA | **Aortic arch** | Left. No CoA. |
| Aorta | ----- | **PDA** | No |
| Pulmonary artery | Normal MPA and Branch PAs. |  |  |
| **M-Mode:**  Normal LV Function on eye balling. | | | |
| AO | mm | PWd | mm |
| LA | mm | PWs | mm |
| LVIDd | mm | EDV | ml |
| LVIDs | mm | ESV | ml |
| IVSs | mm | LVEF | % |
| IVSd | mm | FS | % |
| **Additional Information**: |  | | |
| No pericardial/Pleural effusion. | | | |
| **Final Diagnosis:** | | | |
| 1. Normal Echocardiography Study. | | | |
| **Remark**: | | | |
| **Recommendation**: | | | |
| SIGNATURE  Done by: Tesfaye T., Pediatrician, Pediatric Cardiologist _______________ 20/05/2015Eth.C | | | |

| Patient Name: **Abinet Setual**. Referring Institute: **TGSH**. SEX/ Age: **M/12years**. Date of Report: **20/05/15**.  Referral Diagnosis: **Sydenham’s Chorea. AGH2.201** | | | |
| --- | --- | --- | --- |
| **Features** | **Finding** | **Features** | **Finding** |
| **Profile** |  | **Atria** |  |
| Abdominal situs | Solitus | Left atrium | Normal |
| Cardiac position | Levocardia | Right atrium | Normal |
| Systemic venous drainage | Normal. | **Atrioventricular valves** |  |
| Pulmonary venous drainage | Normal | Mitral valve | Annulus = 20mm |
| Atrioventricular connection | Concordant | Tricuspid valve | Annulus = 21mm  TAPSE = 21mm |
| Ventriculoarterial connection | Concordant | **Ventricles** |  |
| Ventricular loop | d-Loop | Left ventricle | Normal |
|  |  | Right ventricle | Normal |
| **Septae** |  | **Coronary arteries** | ----- |
| Interventricular septum | Intact | **Doppler Measurement** |  |
| Interatrial septum | Intact | Mitral | ----- |
| **Semilunar valves** |  | Aortic | ------- |
| Aortic valve | Annulus = 19mm | Tricuspid | Trivial TR, PPG = 23mmHg |
| Pulmonary valve | Annulus = 22mm | pulmonic | -------- |
| **Great arteries** | NRGA | **Aortic arch** | Left. No CoA. |
| Aorta | ----- | **PDA** | No |
| Pulmonary artery | Normal MPA and Branch PAs. |  |  |
| **M-Mode:** | | | |
| AO | mm | PWd | mm |
| LA | mm | PWs | mm |
| LVIDd | mm | EDV | ml |
| LVIDs | mm | ESV | ml |
| IVSs | mm | LVEF | 66% |
| IVSd | mm | FS | 36% |
| **Additional Information**: |  | | |
| No pericardial/Pleural effusion. | | | |
| **Final Diagnosis:** | | | |
| 1. Normal Echocardiography Study. | | | |
| **Remark**: | | | |
| **Recommendation**: | | | |
| SIGNATURE  Done by: Tesfaye T., Pediatrician, Pediatric Cardiologist _______________ 20/05/2015Eth.C | | | |

| Patient Name: **Emran Nesredin**. Referring Institute: **MSI-Ethiopia**. SEX/ Age: **M/10years**. Date of Report: **20/05/15**.  Referral Diagnosis: **Palpitation. AGH2.202** | | | |
| --- | --- | --- | --- |
| **Features** | **Finding** | **Features** | **Finding** |
| **Profile** |  | **Atria** |  |
| Abdominal situs | Solitus | Left atrium | Normal |
| Cardiac position | Levocardia | Right atrium | Normal |
| Systemic venous drainage | Normal. | **Atrioventricular valves** |  |
| Pulmonary venous drainage | Normal | Mitral valve | Annulus = 19mm |
| Atrioventricular connection | Concordant | Tricuspid valve | Annulus = 21mm  TAPSE = 20mm |
| Ventriculoarterial connection | Concordant | **Ventricles** |  |
| Ventricular loop | d-Loop | Left ventricle | Normal |
|  |  | Right ventricle | Normal |
| **Septae** |  | **Coronary arteries** | ----- |
| Interventricular septum | Intact | **Doppler Measurement** |  |
| Interatrial septum | Intact | Mitral | ----- |
| **Semilunar valves** |  | Aortic | ------- |
| Aortic valve | Annulus = 16mm | Tricuspid | Trivial TR, PPG = 12mmHg |
| Pulmonary valve | Annulus = 20mm | pulmonic | -------- |
| **Great arteries** | NRGA | **Aortic arch** | Left. No CoA. |
| Aorta | ----- | **PDA** | No |
| Pulmonary artery | Normal MPA and Branch PAs. |  |  |
| **M-Mode:** | | | |
| AO | mm | PWd | mm |
| LA | mm | PWs | mm |
| LVIDd | mm | EDV | ml |
| LVIDs | mm | ESV | ml |
| IVSs | mm | LVEF | 68% |
| IVSd | mm | FS | 37% |
| **Additional Information**: |  | | |
| No pericardial/Pleural effusion. | | | |
| **Final Diagnosis:** | | | |
| 1. Normal Echocardiography Study. | | | |
| **Remark**: | | | |
| **Recommendation**: | | | |
| SIGNATURE  Done by: Tesfaye T., Pediatrician, Pediatric Cardiologist _______________ 20/05/2015Eth.C | | | |

| Patient Name: **Habtie Yitayew**. Referring Institute: **FHRH**. SEX/ Age: **F/12years**. Date of Report: **20/05/15**.  Referral Diagnosis: **CHF + IE. AGH2.203** | | | |
| --- | --- | --- | --- |
| **Features** | **Finding** | **Features** | **Finding** |
| **Profile** |  | **Atria** |  |
| Abdominal situs | Solitus | Left atrium | Normal |
| Cardiac position | Levocardia | Right atrium | Dilated. 24mm X 9mm echogenic mass attached to the RA Side of Anterior TVL, Oscillating & prolapsing to RV. |
| Systemic venous drainage | Normal. | **Atrioventricular valves** |  |
| Pulmonary venous drainage | Normal | Mitral valve | Annulus = 22mm |
| Atrioventricular connection | Concordant | Tricuspid valve | Annulus = 25mm  TAPSE = 24mm |
| Ventriculoarterial connection | Concordant | **Ventricles** |  |
| Ventricular loop | d-Loop | Left ventricle | Normal |
|  |  | Right ventricle | Dilated |
| **Septae** |  | **Coronary arteries** | ----- |
| Interventricular septum | Intact | **Doppler Measurement** |  |
| Interatrial septum | 14mm OS ASD, L – R Shunt | Mitral | ----- |
| **Semilunar valves** |  | Aortic | ------- |
| Aortic valve | Annulus = 18mm | Tricuspid | Moderate TR, PPG = 27mmHg. |
| Pulmonary valve | Annulus = 22mm | pulmonic | -------- |
| **Great arteries** | NRGA | **Aortic arch** | Left. No CoA. |
| Aorta | ----- | **PDA** | No |
| Pulmonary artery | Normal MPA & Branch PAs. |  |  |
| **M-Mode:** | | | |
| AO | mm | PWd | mm |
| LA | mm | PWs | mm |
| LVIDd | mm | EDV | ml |
| LVIDs | mm | ESV | ml |
| IVSs | mm | LVEF | 48% |
| IVSd | mm | FS | 24% |
| **Additional Information**: |  | | |
| No pericardial/Pleural effusion. | | | |
| **Final Diagnosis:** | | | |
| 1. {S, D, S} Levocardia. 2. RA/RV Dilated 3. Large OS ASD, L – R Shunt 4. Moderate TR 5. Large echogenic mass attached to the RA Side of the Anterior TVL, Oscillating and prolapsing to RV 6. Reduced LV Systolic Function | | | |
| **Remark**: Infective Vegetation is highly likely. | | | |
| **Recommendation**: Correlate with the clinical scenario. | | | |
| SIGNATURE  Done by: Tesfaye T., Pediatrician, Pediatric Cardiologist _______________ 20/05/2015Eth.C | | | |

| Patient Name: **Yohannes Molla**. Referring Institute: **Lideta MC**. SEX/ Age: **M/3 3/12**. Date of Report: **22/05/15**.  Referral Diagnosis: **CHF. AGH2.204** | | | |
| --- | --- | --- | --- |
| **Features** | **Finding** | **Features** | **Finding** |
| **Profile** |  | **Atria** |  |
| Abdominal situs | Solitus | Left atrium | Normal |
| Cardiac position | Levocardia | Right atrium | Markedly Dilated |
| Systemic venous drainage | Normal. | **Atrioventricular valves** |  |
| Pulmonary venous drainage | Normal | Mitral valve | Annulus = 15mm |
| Atrioventricular connection | Concordant | Tricuspid valve | Annulus = 26mm  TAPSE = 9mm |
| Ventriculoarterial connection | Concordant | **Ventricles** |  |
| Ventricular loop | d-Loop | Left ventricle | Normal |
|  |  | Right ventricle | Dilated |
| **Septae** |  | **Coronary arteries** | ----- |
| Interventricular septum | Intact | **Doppler Measurement** |  |
| Interatrial septum | Intact | Mitral | ----- |
| **Semilunar valves** |  | Aortic | ------- |
| Aortic valve | Annulus = 13mm | Tricuspid | Moderate TR |
| Pulmonary valve | Annulus = 15mm | pulmonic | Severe Valvular PS, PPG = 82mmHg. |
| **Great arteries** | NRGA | **Aortic arch** | Left. No CoA. |
| Aorta | ----- | **PDA** | No |
| Pulmonary artery | Normal MPA and Branch PAs. |  |  |
| **M-Mode:**  Normal LV Function on eye balling | | | |
| AO | mm | PWd | mm |
| LA | mm | PWs | mm |
| LVIDd | mm | EDV | ml |
| LVIDs | mm | ESV | ml |
| IVSs | mm | LVEF | % |
| IVSd | mm | FS | % |
| **Additional Information**: |  | | |
| Pericardial effusion measuring maximum depth of 11mm on RA/RV Side. | | | |
| **Final Diagnosis:** | | | |
| 1. {S, D, S} Levocardia. 2. RA/RV Dilated 3. Moderate TR 4. Severe Valvular Pulmonary Stenosis 5. Dysfunctional RV 6. Moderate Pericardial effusion 7. Norma LV Systolic Function | | | |
| **Remark**: | | | |
| **Recommendation**: | | | |
| SIGNATURE  Done by: Tesfaye T., Pediatrician, Pediatric Cardiologist _______________ 22/05/2015Eth.C | | | |

| Patient Name: **Meskele Hailu**. Referring Institute: **MSI-Ethiopia**. SEX/ Age: **F/4 7/12**. Date of Report: **22/05/15**.  Referral Diagnosis: **Recurrent Chest Infection. AGH2.205** | | | |
| --- | --- | --- | --- |
| **Features** | **Finding** | **Features** | **Finding** |
| **Profile** |  | **Atria** |  |
| Abdominal situs | Solitus | Left atrium | Normal |
| Cardiac position | Levocardia | Right atrium | Normal |
| Systemic venous drainage | Normal. | **Atrioventricular valves** |  |
| Pulmonary venous drainage | Normal | Mitral valve | Annulus = 17mm |
| Atrioventricular connection | Concordant | Tricuspid valve | Annulus = 17mm  TAPSE = 19mm |
| Ventriculoarterial connection | Concordant | **Ventricles** |  |
| Ventricular loop | d-Loop | Left ventricle | Normal |
|  |  | Right ventricle | Normal |
| **Septae** |  | **Coronary arteries** | ----- |
| Interventricular septum | Intact | **Doppler Measurement** |  |
| Interatrial septum | Intact | Mitral | ----- |
| **Semilunar valves** |  | Aortic | ------- |
| Aortic valve | Annulus = 15mm | Tricuspid | ------- |
| Pulmonary valve | Annulus = 17mm | pulmonic | -------- |
| **Great arteries** | NRGA | **Aortic arch** | Left. No CoA. |
| Aorta | ----- | **PDA** | No |
| Pulmonary artery | Normal MPA and Branch PAs. |  |  |
| **M-Mode:**  Normal LV Function on eye balling | | | |
| AO | mm | PWd | mm |
| LA | mm | PWs | mm |
| LVIDd | mm | EDV | ml |
| LVIDs | mm | ESV | ml |
| IVSs | mm | LVEF | % |
| IVSd | mm | FS | % |
| **Additional Information**: |  | | |
| No pericardial/Pleural effusion. | | | |
| **Final Diagnosis:** | | | |
| 1. Normal Echocardiography Study. | | | |
| **Remark**: | | | |
| **Recommendation**: | | | |
| SIGNATURE  Done by: Tesfaye T., Pediatrician, Pediatric Cardiologist _______________ 22/05/2015Eth.C | | | |

| Patient Name: **Arafat Muhammed**. Referring Institute: **Adinas GH**. SEX/ Age: **M/3 6/12**. Date of Report: **22/05/15**.  Referral Diagnosis: **Follow up echo for Small PM VSD. Family History. AGH2.206** | | | |
| --- | --- | --- | --- |
| **Features** | **Finding** | **Features** | **Finding** |
| **Profile** |  | **Atria** |  |
| Abdominal situs | Solitus | Left atrium | Normal |
| Cardiac position | Levocardia | Right atrium | Normal |
| Systemic venous drainage | Normal. | **Atrioventricular valves** |  |
| Pulmonary venous drainage | Normal | Mitral valve | Annulus = 16mm |
| Atrioventricular connection | Concordant | Tricuspid valve | Annulus = 16mm  TAPSE = 16mm |
| Ventriculoarterial connection | Concordant | **Ventricles** |  |
| Ventricular loop | d-Loop | Left ventricle | Normal |
|  |  | Right ventricle | Normal |
| **Septae** |  | **Coronary arteries** | ----- |
| Interventricular septum | 1.5mm PM VSD, L – R Shunt | **Doppler Measurement** |  |
| Interatrial septum | Intact | Mitral | ----- |
| **Semilunar valves** |  | Aortic | ------- |
| Aortic valve | Annulus = 13mm | Tricuspid | ------- |
| Pulmonary valve | Annulus = 16mm | pulmonic | -------- |
| **Great arteries** | NRGA | **Aortic arch** | Left. No CoA. |
| Aorta | ----- | **PDA** | No |
| Pulmonary artery | Normal MPA and Branch PAs. |  |  |
| **M-Mode:**  Normal LV Function on eye balling | | | |
| AO | mm | PWd | mm |
| LA | mm | PWs | mm |
| LVIDd | mm | EDV | ml |
| LVIDs | mm | ESV | ml |
| IVSs | mm | LVEF | % |
| IVSd | mm | FS | % |
| **Additional Information**: |  | | |
| No pericardial/Pleural effusion. | | | |
| **Final Diagnosis:** | | | |
| 1. {S, D, S} Levocardia. 2. Small PM VSD, L – R Shunt | | | |
| **Remark**: Size decreasing. | | | |
| **Recommendation**: | | | |
| SIGNATURE  Done by: Tesfaye T., Pediatrician, Pediatric Cardiologist _______________ 22/05/2015Eth.C | | | |

| Patient Name: **Yared Tibebu**. Referring Institute: **Dr. Addisu PSC**. SEX/ Age: **M/1 7/12**. Date of Report: **22/05/15**.  Referral Diagnosis: **DS. AGH2.207** | | | |
| --- | --- | --- | --- |
| **Features** | **Finding** | **Features** | **Finding** |
| **Profile** |  | **Atria** |  |
| Abdominal situs | Solitus | Left atrium | Normal |
| Cardiac position | Levocardia | Right atrium | Normal |
| Systemic venous drainage | Normal. | **Atrioventricular valves** |  |
| Pulmonary venous drainage | Normal | Mitral valve | Annulus = 13mm |
| Atrioventricular connection | Concordant | Tricuspid valve | Annulus = 13mm |
| Ventriculoarterial connection | Concordant | **Ventricles** |  |
| Ventricular loop | d-Loop | Left ventricle | Normal |
|  |  | Right ventricle | Normal |
| **Septae** |  | **Coronary arteries** | ----- |
| Interventricular septum | Intact | **Doppler Measurement** |  |
| Interatrial septum | 6mm OS ASD, L – R Shunt | Mitral | ----- |
| **Semilunar valves** |  | Aortic | ------- |
| Aortic valve | Annulus = 12mm | Tricuspid | ------- |
| Pulmonary valve | Annulus = 13mm | pulmonic | -------- |
| **Great arteries** | NRGA | **Aortic arch** | Left. No CoA. |
| Aorta | ----- | **PDA** | No |
| Pulmonary artery | Normal MPA and Branch PAs. |  |  |
| **M-Mode:**  Normal LV Function on eye balling | | | |
| AO | mm | PWd | mm |
| LA | mm | PWs | mm |
| LVIDd | mm | EDV | ml |
| LVIDs | mm | ESV | ml |
| IVSs | mm | LVEF | % |
| IVSd | mm | FS | % |
| **Additional Information**: |  | | |
| No pericardial/Pleural effusion. | | | |
| **Final Diagnosis:** | | | |
| 1. {S, D, S} Levocardia. 2. Small OS ASD, L – R Shunt | | | |
| **Remark**: | | | |
| **Recommendation**: | | | |
| SIGNATURE  Done by: Tesfaye T., Pediatrician, Pediatric Cardiologist _______________ 22/05/2015Eth.C | | | |

| Patient Name: **Bayelign Geremew**. Referring Institute: **Addis Alem PH**. SEX/ Age: **M/6years**. Date of Report: **29/05/15**.  Referral Diagnosis: **CHF.AGH2.208** | | | |
| --- | --- | --- | --- |
| **Features** | **Finding** | **Features** | **Finding** |
| **Profile** |  | **Atria** |  |
| Abdominal situs | Solitus | Left atrium | Dilated |
| Cardiac position | Levocardia | Right atrium | Normal |
| Systemic venous drainage | Normal. | **Atrioventricular valves** |  |
| Pulmonary venous drainage | Normal | Mitral valve | Annulus = 21mm |
| Atrioventricular connection | Concordant | Tricuspid valve | Annulus = 20mm  TAPSE = 22mm |
| Ventriculoarterial connection | Concordant | **Ventricles** |  |
| Ventricular loop | d-Loop | Left ventricle | Dilated |
|  |  | Right ventricle | Normal |
| **Septae** |  | **Coronary arteries** | ----- |
| Interventricular septum | Intact | **Doppler Measurement** |  |
| Interatrial septum | Intact | Mitral | Mild MR, Jet velocity = 3.6m/sec. |
| **Semilunar valves** |  | Aortic | Mild AR |
| Aortic valve | Annulus = 15mm | Tricuspid | ------- |
| Pulmonary valve | Annulus = 21mm | pulmonic | -------- |
| **Great arteries** | NRGA | **Aortic arch** | Left. No CoA. |
| Aorta | ----- | **PDA** | 5mm PDA, L – R Shunt |
| Pulmonary artery | MPA =25mm. |  |  |
| **M-Mode:** | | | |
| AO | mm | PWd | mm |
| LA | mm | PWs | mm |
| LVIDd | mm | EDV | ml |
| LVIDs | mm | ESV | ml |
| IVSs | mm | LVEF | 66% |
| IVSd | mm | FS | 37% |
| **Additional Information**: |  | | |
| No pericardial/Pleural effusion. | | | |
| **Final Diagnosis:** | | | |
| 1. {S, D, S} Levocardia. 2. LA/LV Dilated 3. Mild MR 4. Mild AR 5. Large PDA, L – R Shunt 6. Normal Biventricular Systolic Function 7. Mod. Pulmonary Hypertension | | | |
| **Remark**: | | | |
| **Recommendation**: | | | |
| SIGNATURE  Done by: Tesfaye T., Pediatrician, Pediatric Cardiologist _______________ 29/05/2015Eth.C | | | |

| Patient Name: **Yemata Abitew**. Referring Institute: **FHRH**. SEX/ Age: **F/4 2/12**. Date of Report: **29/05/15**.  Referral Diagnosis: **CHF. AGH2.209** | | | |
| --- | --- | --- | --- |
| **Features** | **Finding** | **Features** | **Finding** |
| **Profile** |  | **Atria** |  |
| Abdominal situs | Solitus | Left atrium | Normal |
| Cardiac position | Levocardia | Right atrium | Normal |
| Systemic venous drainage | Normal. | **Atrioventricular valves** |  |
| Pulmonary venous drainage | Normal | Mitral valve | Annulus = 16mm |
| Atrioventricular connection | Concordant | Tricuspid valve | Annulus = 16mm  TAPSE = 17mm |
| Ventriculoarterial connection | Concordant | **Ventricles** |  |
| Ventricular loop | d-Loop | Left ventricle | Normal. 13mm X 8mm Echogenic mass with smooth surface floating in the apical area. |
|  |  | Right ventricle | Normal |
| **Septae** |  | **Coronary arteries** | ----- |
| Interventricular septum | Intact | **Doppler Measurement** |  |
| Interatrial septum | Intact | Mitral | ----- |
| **Semilunar valves** |  | Aortic | ------- |
| Aortic valve | Annulus = 12mm | Tricuspid | ------- |
| Pulmonary valve | Annulus = 12mm | pulmonic | -------- |
| **Great arteries** | NRGA | **Aortic arch** | Left. No CoA. |
| Aorta | ----- | **PDA** | No |
| Pulmonary artery | Normal MPA and Branch PAs. |  |  |
| **M-Mode:** | | | |
| AO | mm | PWd | mm |
| LA | mm | PWs | mm |
| LVIDd | mm | EDV | ml |
| LVIDs | mm | ESV | ml |
| IVSs | mm | LVEF | 67% |
| IVSd | mm | FS | 35% |
| **Additional Information**: |  | | |
| No pericardial/Pleural effusion. | | | |
| **Final Diagnosis:** | | | |
| 1. {S, D, S} Levocardia. 2. Echogenic mass with smooth surface at the apical area of LV 3. Normal Biventricular Systolic Function | | | |
| **Remark**: Intra-cardiac thrombus is the top differential Diagnosis | | | |
| **Recommendation**: Further work up of the coagulation profile is recommended | | | |
| SIGNATURE  Done by: Tesfaye T., Pediatrician, Pediatric Cardiologist _______________ 29/05/2015Eth.C | | | |

| Patient Name: **Baby of Selamawit Tadesse**. Referring Institute: **TGSH**. SEX/ Age: **M/48days**. Date of Report: **29/05/15**.  Referral Diagnosis: **DS. AGH2.210** | | | |
| --- | --- | --- | --- |
| **Features** | **Finding** | **Features** | **Finding** |
| **Profile** |  | **Atria** |  |
| Abdominal situs | Solitus | Left atrium | Normal |
| Cardiac position | Levocardia | Right atrium | Normal |
| Systemic venous drainage | Normal. | **Atrioventricular valves** |  |
| Pulmonary venous drainage | Normal | Mitral valve | Annulus = 9mm |
| Atrioventricular connection | Concordant | Tricuspid valve | Annulus = 9mm |
| Ventriculoarterial connection | Concordant | **Ventricles** |  |
| Ventricular loop | d-Loop | Left ventricle | Normal |
|  |  | Right ventricle | Normal |
| **Septae** |  | **Coronary arteries** | ----- |
| Interventricular septum | Intact | **Doppler Measurement** |  |
| Interatrial septum | 4mm OS ASD, L – R Shunt | Mitral | ----- |
| **Semilunar valves** |  | Aortic | ------- |
| Aortic valve | Annulus = 9mm | Tricuspid | ------- |
| Pulmonary valve | Annulus = 10mm | pulmonic | -------- |
| **Great arteries** | NRGA | **Aortic arch** | Left. No CoA. |
| Aorta | ----- | **PDA** | No |
| Pulmonary artery | Normal MPA and Branch PAs. |  |  |
| **M-Mode:**  Normal LV Function on eye balling | | | |
| AO | mm | PWd | mm |
| LA | mm | PWs | mm |
| LVIDd | mm | EDV | ml |
| LVIDs | mm | ESV | ml |
| IVSs | mm | LVEF | % |
| IVSd | mm | FS | % |
| **Additional Information**: |  | | |
| No pericardial/Pleural effusion. | | | |
| **Final Diagnosis:** | | | |
| 1. {S, D, S} Levocardia. 2. Small OS ASD, L – R Shunt | | | |
| **Remark**: | | | |
| **Recommendation**: | | | |
| SIGNATURE  Done by: Tesfaye T., Pediatrician, Pediatric Cardiologist _______________ 29/05/2015Eth.C | | | |

| Patient Name: **Medina Muhammed**. Referring Institute: **Adinas GH**. SEX/ Age: **F/8days**. Date of Report: **29/05/15**.  Referral Diagnosis: **DS. AGH2.211** | | | |
[truncated: 119,454 more chars]
