# Supplementary material for: Two–Dimensional and Doppler trans-thoracic echocardiographic patterns of suspected pediatric heart diseases at Tibebe-—Ghion specialized Teaching Hospital and Adinas General Hospital, Bahir Dar, North-west Ethiopia:–An experience from an LMIC
Source: PLoS One. 2024 Mar 11;19(3):e0292694. doi: 10.1371/journal.pone.0292694 (PMC10927071; doi:10.1371/journal.pone.0292694)
Supplement: S2 File — (ZIP) [file pone.0292694.s003.zip › AGH6 Pediatric Echocardiography Report 2013 extra.docx]

| Patient Name: **Alemu Anteneh**. Patient ID: Enjibara GH. SEX/ Age: M/13years. Date of Report: 18**/02/2013**.  BP: ___ Weight: ___ Height:___ BSA: ___. Referral Diagnosis: **ARF + Murmur. AGH06.2381.** | | | |
| --- | --- | --- | --- |
| **Features** | **Finding** | **Features** | **Finding** |
| **Profile** |  | **Atria** |  |
| Abdominal situs | Solitus | Left atrium | Normal |
| Cardiac position | Levocardia | Right atrium | Normal |
| Systemic venous drainage | Normal | **Atrioventricular valves** |  |
| Pulmonary venous drainage | Normal | Mitral valve | Annulus = 26mm. Thickened MVL |
| Atrioventricular connection | Concordant | Tricuspid valve | Annulus = 29mm  TAPSE = 24mm |
| Ventriculoarterial connection | Concordant | **Ventricles** |  |
| Ventricular loop | d-Loop | Left ventricle | Normal |
|  |  | Right ventricle | Normal |
| **Septae** |  | **Coronary arteries** | ----- |
| Interventricular septum | Intact | **Doppler Measurement** |  |
| Interatrial septum | Intact | Mitral | Mild MR, JET VELOCITY = 3.4m/sec, posterior projection, seen in two planes |
| **Semilunal valves** |  | Aortic | ---- |
| Aortic valve | Annulus = 19mm | Tricuspid | Mild TR, PPG = 27mmHg |
| Pulmonary valve | Annulus = 21mm | pulmonic | Trivial PR, PPG = 17mmHg |
| **Great arteries** | NRGA | **Aortic arch** | Left |
| Aorta | ----- | **PDA** | No |
| Pulmonary artery | Normal MPA and Branch PAs. |  |  |
| **M-Mode:** | | | |
| AO | mm | PWd | mm |
| LA | mm | EDV | ml |
| LVIDd | mm | ESV | ml |
| LVIDs | mm | LVEF | 61% |
| IVSd | mm | FS | 33% |
| **Additional Information**: |  | | |
| No pleural/pericardial effusion | | | |
| **Final Diagnosis:** | | | |
| 1. {S, D, S} Levocardia. 2. Thickened MVL 3. Mild MR 4. Mild TR 5. Normal Biventricular Function | | | |
| Remark: | | | |
| SIGNATURE  Done by: Tesfaye T., Pediatric Cardiologist _______________ 18/02/2013Eth.C | | | |

| Patient Name: **Baby of Tigist Ayenew**. Patient ID: TGSH. SEX/ Age: F/11days. Date of Report: 18**/02/2013**.  BP: ___ Weight: ___ Height:___ BSA: ___. Referral Diagnosis: **RD. AGH06.2382.** | | | |
| --- | --- | --- | --- |
| **Features** | **Finding** | **Features** | **Finding** |
| **Profile** |  | **Atria** |  |
| Abdominal situs | Solitus | Left atrium | Normal |
| Cardiac position | Levocardia | Right atrium | Normal |
| Systemic venous drainage | Normal | **Atrioventricular valves** |  |
| Pulmonary venous drainage | Normal | Mitral valve | Annulus = 9mm |
| Atrioventricular connection | Concordant | Tricuspid valve | Annulus = 11mm |
| Ventriculoarterial connection | Concordant | **Ventricles** |  |
| Ventricular loop | d-Loop | Left ventricle | Normal |
|  |  | Right ventricle | Normal |
| **Septae** |  | **Coronary arteries** | ----- |
| Interventricular septum | Intact | **Doppler Measurement** |  |
| Interatrial septum | Intact | Mitral | ----- |
| **Semilunal valves** |  | Aortic | ---- |
| Aortic valve | Annulus = 9mm | Tricuspid | ---- |
| Pulmonary valve | Annulus = 9mm | pulmonic | ---- |
| **Great arteries** | NRGA | **Aortic arch** | Left |
| Aorta | ----- | **PDA** | No |
| Pulmonary artery | Normal MPA and Branch PAs. |  |  |
| **M-Mode:** Normal LV Function on eye balling. | | | |
| AO | mm | PWd | mm |
| LA | mm | EDV | ml |
| LVIDd | mm | ESV | ml |
| LVIDs | mm | LVEF | % |
| IVSd | mm | FS | % |
| **Additional Information**: |  | | |
| No pleural/pericardial effusion | | | |
| **Final Diagnosis:** | | | |
| 1. Normal Echocardiography Study. | | | |
| Remark: | | | |
| SIGNATURE  Done by: Tesfaye T., Pediatric Cardiologist _______________ 18/02/2013Eth.C | | | |

| Patient Name: **Wintana Yeniesew**. Patient ID: FHRH. SEX/ Age: F/8years. Date of Report: 18**/02/2013**.  BP: ___ Weight: __ Height:___ BSA: ___. Referral Diagnosis: **DOE + Murmur + Palpitation. AGH06.2383.** | | | |
| --- | --- | --- | --- |
| **Features** | **Finding** | **Features** | **Finding** |
| **Profile** |  | **Atria** |  |
| Abdominal situs | Solitus | Left atrium | Dilated |
| Cardiac position | Levocardia | Right atrium | Normal |
| Systemic venous drainage | Normal | **Atrioventricular valves** |  |
| Pulmonary venous drainage | Normal | Mitral valve | Annulus = 27mm. Thickened, Hyperechoic MVL |
| Atrioventricular connection | Concordant | Tricuspid valve | Annulus = 20mm |
| Ventriculoarterial connection | Concordant | **Ventricles** |  |
| Ventricular loop | d-Loop | Left ventricle | Dilated |
|  |  | Right ventricle | Normal |
| **Septae** |  | **Coronary arteries** | ----- |
| Interventricular septum | Intact | **Doppler Measurement** |  |
| Interatrial septum | Intact | Mitral | Moderate MR, Holosystolic, posterior projection, seen in two planes with jet velocity = 5.4m/sec |
| **Semilunal valves** |  | Aortic | ---- |
| Aortic valve | Annulus = 17mm | Tricuspid | ---- |
| Pulmonary valve | Annulus = 17mm | pulmonic | Mild PR, PPG = 40mmHg |
| **Great arteries** | NRGA | **Aortic arch** | Left |
| Aorta | ----- | **PDA** | 4mm PDA, L – R Shunt with SPG/DPG = 59/15mmHg |
| Pulmonary artery | Normal MPA and Branch PAs. |  |  |
| **M-Mode:** | | | |
| AO | mm | PWd | mm |
| LA | mm | EDV | ml |
| LVIDd | mm | ESV | ml |
| LVIDs | mm | LVEF | 56% |
| IVSd | mm | FS | 30% |
| **Additional Information**: |  | | |
| No pleural/pericardial effusion | | | |
| **Final Diagnosis:** | | | |
| 1. {S, D, S} Levocardia. 2. LA/LV Dilated 3. Thickened Hyperechoic MVL 4. Moderate MR 5. Large PDA, L – R Shunt 6. Good LV Function | | | |
| SIGNATURE  Done by: Tesfaye T., Pediatric Cardiologist _______________ 18/02/2013Eth.C | | | |

| Patient Name: **Baby of Keleb Mengistie**. Patient ID: FHRH. SEX/ Age: F/4days. Date of Report: 18**/02/2013**.  BP: ___ Weight: ___ Height:___ BSA: ___. Referral Diagnosis: **Incidental Murmur. AGH06.2384.** | | | |
| --- | --- | --- | --- |
| **Features** | **Finding** | **Features** | **Finding** |
| **Profile** |  | **Atria** |  |
| Abdominal situs | Solitus | Left atrium | Normal |
| Cardiac position | Levocardia | Right atrium | Normal |
| Systemic venous drainage | Normal | **Atrioventricular valves** |  |
| Pulmonary venous drainage | Normal | Mitral valve | Annulus = 7mm |
| Atrioventricular connection | Concordant | Tricuspid valve | Annulus = 10mm |
| Ventriculoarterial connection | Concordant | **Ventricles** |  |
| Ventricular loop | d-Loop | Left ventricle | Normal |
|  |  | Right ventricle | Normal |
| **Septae** |  | **Coronary arteries** | ----- |
| Interventricular septum | 3mm PM VSD, L – R Shunt | **Doppler Measurement** |  |
| Interatrial septum | 7mm OS ASD, L – R Shunt | Mitral | ----- |
| **Semilunal valves** |  | Aortic | ---- |
| Aortic valve | Annulus = 9mm | Tricuspid | ---- |
| Pulmonary valve | Annulus = 8mm | pulmonic | ---- |
| **Great arteries** | NRGA | **Aortic arch** | Left |
| Aorta | ----- | **PDA** | 1mm PDA, L – R Shunt |
| Pulmonary artery | Normal MPA and Branch PAs. |  |  |
| **M-Mode:** Normal LV Function on eye balling. | | | |
| AO | mm | PWd | mm |
| LA | mm | EDV | ml |
| LVIDd | mm | ESV | ml |
| LVIDs | mm | LVEF | % |
| IVSd | mm | FS | % |
| **Additional Information**: |  | | |
| No pleural/pericardial effusion | | | |
| **Final Diagnosis:** | | | |
| 1. {S, D, S} Levocardia. 2. RA/RV Dilated 3. Moderate OS ASD, L – R Shunt 4. Small PM VSD, L – R Shunt 5. Small PDA, L – R Shunt 6. Normal LV Function | | | |
| SIGNATURE  Done by: Tesfaye T., Pediatric Cardiologist _______________ 18/02/2013Eth.C | | | |

| Patient Name: **Bezawit Muluye**. Patient ID: Adinas GH. SEX/ Age: F/11years. Date of Report: 19**/02/2013**.  BP: ___ Weight: ___ Height:___ BSA: ___. Referral Diagnosis: **Rhythm Abnormality. AGH06.2385.** | | | |
| --- | --- | --- | --- |
| **Features** | **Finding** | **Features** | **Finding** |
| **Profile** |  | **Atria** |  |
| Abdominal situs | Solitus | Left atrium | Normal |
| Cardiac position | Levocardia | Right atrium | Normal |
| Systemic venous drainage | Normal | **Atrioventricular valves** |  |
| Pulmonary venous drainage | Normal | Mitral valve | Annulus = 20mm |
| Atrioventricular connection | Concordant | Tricuspid valve | Annulus = 21mm |
| Ventriculoarterial connection | Concordant | **Ventricles** |  |
| Ventricular loop | d-Loop | Left ventricle | Normal |
|  |  | Right ventricle | Normal |
| **Septae** |  | **Coronary arteries** | ----- |
| Interventricular septum | Intact | **Doppler Measurement** |  |
| Interatrial septum | Intact | Mitral | ----- |
| **Semilunal valves** |  | Aortic | ---- |
| Aortic valve | Annulus = 16mm | Tricuspid | ---- |
| Pulmonary valve | Annulus = 17mm | pulmonic | ---- |
| **Great arteries** | NRGA | **Aortic arch** | Left |
| Aorta | ----- | **PDA** | No |
| Pulmonary artery | Normal MPA and Branch PAs. |  |  |
| **M-Mode:** | | | |
| AO | mm | PWd | mm |
| LA | mm | EDV | ml |
| LVIDd | mm | ESV | ml |
| LVIDs | mm | LVEF | 67% |
| IVSd | mm | FS | 36% |
| **Additional Information**: |  | | |
| No pleural/pericardial effusion | | | |
| **Final Diagnosis:** | | | |
| 1. Normal Echocardiography Study. | | | |
| Remark: | | | |
| SIGNATURE  Done by: Tesfaye T., Pediatric Cardiologist _______________ 19/02/2013Eth.C | | | |

| Patient Name: **Mendere Metalign**. Patient ID: FHRH. SEX/ Age: F/10days. Date of Report: 19**/02/2013**.  BP: ___ Weight: ___ Height:___ BSA: ___. Referral Diagnosis: **RD. AGH06.2386.** | | | |
| --- | --- | --- | --- |
| **Features** | **Finding** | **Features** | **Finding** |
| **Profile** |  | **Atria** |  |
| Abdominal situs | Solitus | Left atrium | Normal |
| Cardiac position | Levocardia | Right atrium | Normal |
| Systemic venous drainage | Normal | **Atrioventricular valves** |  |
| Pulmonary venous drainage | Normal | Mitral valve | Annulus = 10mm |
| Atrioventricular connection | Concordant | Tricuspid valve | Annulus = 12mm |
| Ventriculoarterial connection | Concordant | **Ventricles** |  |
| Ventricular loop | d-Loop | Left ventricle | Normal |
|  |  | Right ventricle | Normal |
| **Septae** |  | **Coronary arteries** | ----- |
| Interventricular septum | Intact | **Doppler Measurement** |  |
| Interatrial septum | Intact | Mitral | ----- |
| **Semilunal valves** |  | Aortic | ---- |
| Aortic valve | Annulus = 9mm | Tricuspid | ---- |
| Pulmonary valve | Annulus = 8mm | pulmonic | ---- |
| **Great arteries** | NRGA | **Aortic arch** | Left |
| Aorta | ----- | **PDA** | No |
| Pulmonary artery | Normal MPA and Branch PAs. |  |  |
| **M-Mode:** | | | |
| AO | mm | PWd | mm |
| LA | mm | EDV | ml |
| LVIDd | mm | ESV | ml |
| LVIDs | mm | LVEF | % |
| IVSd | mm | FS | % |
| **Additional Information**: |  | | |
| No pleural/pericardial effusion | | | |
| **Final Diagnosis:** | | | |
| 1. Normal Echocardiography Study. | | | |
| Remark: | | | |
| SIGNATURE  Done by: Tesfaye T., Pediatric Cardiologist _______________ 19/02/2013Eth.C | | | |

| Patient Name: **Hanna Getaneh**. Patient ID: Adet PH. SEX/ Age: F/8years. Date of Report: 20**/02/2013**.  Referral Diagnosis: **CHF + Rheumatic Recurrence + DOE + Murmur. AGH06.2387.** | | | |
| --- | --- | --- | --- |
| **Features** | **Finding** | **Features** | **Finding** |
| **Profile** |  | **Atria** |  |
| Abdominal situs | Solitus | Left atrium | Dilated |
| Cardiac position | Levocardia | Right atrium | Dilated |
| Systemic venous drainage | Normal | **Atrioventricular valves** |  |
| Pulmonary venous drainage | Normal | Mitral valve | Annulus = 21mm. Thickened, mildly clubbed MVL with MVA = 1.2cm2. |
| Atrioventricular connection | Concordant | Tricuspid valve | Annulus = 23mm  TAPSE = 20mm |
| Ventriculoarterial connection | Concordant | **Ventricles** |  |
| Ventricular loop | d-Loop | Left ventricle | Dilated |
|  |  | Right ventricle | Dilated |
| **Septae** |  | **Coronary arteries** | ----- |
| Interventricular septum | Intact | **Doppler Measurement** |  |
| Interatrial septum | Intact | Mitral | Moderate MR, Holosystolic, posterior projection, seen in two planes with jet velocity = 4.4m/sec. Moderate MS, PPG/MPG = 14/8mmHg |
| **Semilunal valves** |  | Aortic | Mild AR |
| Aortic valve | Annulus = 15mm | Tricuspid | Mild TR, PPG = 50mmHg |
| Pulmonary valve | Annulus = 16mm | pulmonic | Mild PR, PPG = 50mmHg |
| **Great arteries** | NRGA | **Aortic arch** | Left |
| Aorta | ----- | **PDA** | No |
| Pulmonary artery | Normal MPA and Branch PAs. |  |  |
| **M-Mode:** | | | |
| AO | mm | PWd | mm |
| LA | mm | EDV | ml |
| LVIDd | mm | ESV | ml |
| LVIDs | mm | LVEF | 64% |
| IVSd | mm | FS | 35% |
| **Additional Information**: |  | | |
| 13mm pericardial effusion on RA/RV Side and 9mm on LV Side | | | |
| **Final Diagnosis:** | | | |
| 1. {S, D, S} Levocardia. 2. LA/LV Dilated 3. Thickened Clubbed MVL 4. Moderate MR 5. Moderate MS 6. Mild AR 7. Mild PR 8. Mild TR 9. Moderate Pulmonary Hypertension 10. Normal Biventricular Function | | | |
| SIGNATURE  Done by: Tesfaye T., Pediatric Cardiologist _______________ 20/02/2013Eth.C | | | |

| Patient Name: **Baby of Fentanesh Alelign.** Patient ID: FHRH. SEX/ Age: F/12days. Date of Report: 20**/02/2013**.  BP: ___ Weight: ___ Height:___ BSA: ___. Referral Diagnosis: **RD. AGH06.2388.** | | | |
| --- | --- | --- | --- |
| **Features** | **Finding** | **Features** | **Finding** |
| **Profile** |  | **Atria** |  |
| Abdominal situs | Solitus | Left atrium | Normal |
| Cardiac position | Levocardia | Right atrium | Normal |
| Systemic venous drainage | Normal | **Atrioventricular valves** |  |
| Pulmonary venous drainage | Normal | Mitral valve | Annulus = 8mm |
| Atrioventricular connection | Concordant | Tricuspid valve | Annulus = 11mm |
| Ventriculoarterial connection | Concordant | **Ventricles** |  |
| Ventricular loop | d-Loop | Left ventricle | Normal |
|  |  | Right ventricle | Normal |
| **Septae** |  | **Coronary arteries** | ----- |
| Interventricular septum | Intact | **Doppler Measurement** |  |
| Interatrial septum | Intact | Mitral | ----- |
| **Semilunal valves** |  | Aortic | ---- |
| Aortic valve | Annulus = 8mm | Tricuspid | ---- |
| Pulmonary valve | Annulus = 9mm | pulmonic | ---- |
| **Great arteries** | NRGA | **Aortic arch** | Left |
| Aorta | ----- | **PDA** | No |
| Pulmonary artery | Normal MPA and Branch PAs. |  |  |
| **M-Mode:** | | | |
| AO | mm | PWd | mm |
| LA | mm | EDV | ml |
| LVIDd | mm | ESV | ml |
| LVIDs | mm | LVEF | 65% |
| IVSd | mm | FS | 33% |
| **Additional Information**: |  | | |
| No pleural/pericardial effusion | | | |
| **Final Diagnosis:** | | | |
| 1. Normal Echocardiography Study. | | | |
| Remark: | | | |
| SIGNATURE  Done by: Tesfaye T., Pediatric Cardiologist _______________ 20/02/2013Eth.C | | | |

| Patient Name: **Baby of Birtukan Cheklie**. Patient ID: FHRH. SEX/ Age: M/8days. Date of Report: 20**/02/2013**.  BP: ___ Weight: ___ Height:___ BSA: ___. Referral Diagnosis: **DS. AGH06.2389.** | | | |
| --- | --- | --- | --- |
| **Features** | **Finding** | **Features** | **Finding** |
| **Profile** |  | **Atria** |  |
| Abdominal situs | Solitus | Left atrium | Normal |
| Cardiac position | Levocardia | Right atrium | Normal |
| Systemic venous drainage | Normal | **Atrioventricular valves** |  |
| Pulmonary venous drainage | Normal | Mitral valve | Annulus = 11mm |
| Atrioventricular connection | Concordant | Tricuspid valve | Annulus = 11mm |
| Ventriculoarterial connection | Concordant | **Ventricles** |  |
| Ventricular loop | d-Loop | Left ventricle | Normal |
|  |  | Right ventricle | Normal |
| **Septae** |  | **Coronary arteries** | ----- |
| Interventricular septum | Intact | **Doppler Measurement** |  |
| Interatrial septum | 4mm OS ASD, L – R Shunt | Mitral | ----- |
| **Semilunal valves** |  | Aortic | ---- |
| Aortic valve | Annulus = 9mm | Tricuspid | ---- |
| Pulmonary valve | Annulus = 10mm | pulmonic | ---- |
| **Great arteries** | NRGA | **Aortic arch** | Left |
| Aorta | ----- | **PDA** | No |
| Pulmonary artery | Normal MPA and Branch PAs. |  |  |
| **M-Mode:** Normal LV Function on eye balling. | | | |
| AO | mm | PWd | mm |
| LA | mm | EDV | ml |
| LVIDd | mm | ESV | ml |
| LVIDs | mm | LVEF | % |
| IVSd | mm | FS | % |
| **Additional Information**: |  | | |
| No pleural/pericardial effusion | | | |
| **Final Diagnosis:** | | | |
| 1. {S, D, S} Levocardia. 2. Small OS ASD, L – R Shunt 3. Normal LV Function | | | |
| Remark: | | | |
| SIGNATURE  Done by: Tesfaye T., Pediatric Cardiologist _______________ 20/02/2013Eth.C | | | |

| Patient Name: **Yared Gedamu**. Patient ID: FHRH. SEX/ Age: M/6/12. Date of Report: 20**/02/2013**.  BP: ___ Weight: ___ Height:___ BSA: ___. Referral Diagnosis: **DS. AGH06.2390.** | | | |
| --- | --- | --- | --- |
| **Features** | **Finding** | **Features** | **Finding** |
| **Profile** |  | **Atria** |  |
| Abdominal situs | Solitus | Left atrium | Normal |
| Cardiac position | Levocardia | Right atrium | Normal |
| Systemic venous drainage | Normal | **Atrioventricular valves** |  |
| Pulmonary venous drainage | Normal | Mitral valve | Annulus = 12mm |
| Atrioventricular connection | Concordant | Tricuspid valve | Annulus = 13mm |
| Ventriculoarterial connection | Concordant | **Ventricles** |  |
| Ventricular loop | d-Loop | Left ventricle | Normal |
|  |  | Right ventricle | Normal |
| **Septae** |  | **Coronary arteries** | ----- |
| Interventricular septum | Intact | **Doppler Measurement** |  |
| Interatrial septum | 6mm OS ASD, L – R Shunt | Mitral | ----- |
| **Semilunal valves** |  | Aortic | ---- |
| Aortic valve | Annulus = 10mm | Tricuspid | ---- |
| Pulmonary valve | Annulus = 12mm | pulmonic | ---- |
| **Great arteries** | NRGA | **Aortic arch** | Left |
| Aorta | ----- | **PDA** | No |
| Pulmonary artery | Normal MPA and Branch PAs. |  |  |
| **M-Mode:** Normal LV Function on eye balling. | | | |
| AO | mm | PWd | mm |
| LA | mm | EDV | ml |
| LVIDd | mm | ESV | ml |
| LVIDs | mm | LVEF | % |
| IVSd | mm | FS | % |
| **Additional Information**: |  | | |
| No pleural/pericardial effusion | | | |
| **Final Diagnosis:** | | | |
| 1. {S, D, S} Levocardia. 2. Small OS ASD. L – R Shunt 3. Normal LV Function | | | |
| Remark: | | | |
| SIGNATURE  Done by: Tesfaye T., Pediatric Cardiologist _______________ 20/02/2013Eth.C | | | |

| Patient Name: **Zemenay Werkie**. Patient ID: FHRH. SEX/ Age: F/9months. Date of Report: 20**/02/2013**.  BP: ___ Weight: ___ Height:___ BSA: ___. Referral Diagnosis: **Diaphoresis + Murmur. AGH06.2391.** | | | |
| --- | --- | --- | --- |
| **Features** | **Finding** | **Features** | **Finding** |
| **Profile** |  | **Atria** |  |
| Abdominal situs | Solitus | Left atrium | Dilated |
| Cardiac position | Levocardia | Right atrium | Normal |
| Systemic venous drainage | Normal | **Atrioventricular valves** |  |
| Pulmonary venous drainage | Normal | Mitral valve | Annulus = 13mm |
| Atrioventricular connection | Concordant | Tricuspid valve | Annulus = 9mm |
| Ventriculoarterial connection | Concordant | **Ventricles** |  |
| Ventricular loop | d-Loop | Left ventricle | Dilated |
|  |  | Right ventricle | Normal |
| **Septae** |  | **Coronary arteries** | ----- |
| Interventricular septum | 8mm non -restrictive PM VSD, L – R Shunt | **Doppler Measurement** |  |
| Interatrial septum | Intact | Mitral | ----- |
| **Semilunal valves** |  | Aortic | ---- |
| Aortic valve | Annulus = 11mm | Tricuspid | ---- |
| Pulmonary valve | Annulus = 10mm | pulmonic | Moderate PR, PPG = 52mmHg |
| **Great arteries** | NRGA | **Aortic arch** | Left |
| Aorta | ----- | **PDA** | No |
| Pulmonary artery | MPA = 13mm. |  |  |
| **M-Mode:** | | | |
| AO | mm | PWd | mm |
| LA | mm | EDV | ml |
| LVIDd | mm | ESV | ml |
| LVIDs | mm | LVEF | 69% |
| IVSd | mm | FS | 36% |
| **Additional Information**: |  | | |
| 5mm pericardial effusion on RA/RV Side. | | | |
| **Final Diagnosis:** | | | |
| 1. {S, D, S} Levocardia. 2. LA/LV Dilated 3. Large Non – Restrictive PM VSD, L – R Shunt 4. Small PERICARDIAL EFFUSION 5. Normal LV Function 6. Moderate Pulmonary Hypertension | | | |
| Remark: Infant was restless during Echocardiographic evaluation. | | | |
| SIGNATURE  Done by: Tesfaye T., Pediatric Cardiologist _______________ 20/02/2013Eth.C | | | |

| Patient Name: **Baby of Muluwerk Kassaye**. Patient ID: FHRH. SEX/ Age: F/7days. Date of Report: 21**/02/2013**.  BP: ___ Weight: ___ Height:___ BSA: ___. Referral Diagnosis: **RD. AGH06.2392.** | | | |
| --- | --- | --- | --- |
| **Features** | **Finding** | **Features** | **Finding** |
| **Profile** |  | **Atria** |  |
| Abdominal situs | Solitus | Left atrium | Normal |
| Cardiac position | Levocardia | Right atrium | Normal |
| Systemic venous drainage | Normal | **Atrioventricular valves** |  |
| Pulmonary venous drainage | Normal | Mitral valve | Annulus = 9mm |
| Atrioventricular connection | Concordant | Tricuspid valve | Annulus = 9mm |
| Ventriculoarterial connection | Concordant | **Ventricles** |  |
| Ventricular loop | d-Loop | Left ventricle | Normal |
|  |  | Right ventricle | Normal |
| **Septae** |  | **Coronary arteries** | ----- |
| Interventricular septum | Intact | **Doppler Measurement** |  |
| Interatrial septum | 4mm OS ASD, L – R Shunt. | Mitral | ----- |
| **Semilunal valves** |  | Aortic | ---- |
| Aortic valve | Annulus = 9mm | Tricuspid | ---- |
| Pulmonary valve | Annulus = 9mm | pulmonic | ---- |
| **Great arteries** | NRGA | **Aortic arch** | Left |
| Aorta | ----- | **PDA** | No |
| Pulmonary artery | Normal MPA and Branch PAs. |  |  |
| **M-Mode:** Normal LV Function on eye balling. | | | |
| AO | mm | PWd | mm |
| LA | mm | EDV | ml |
| LVIDd | mm | ESV | ml |
| LVIDs | mm | LVEF | % |
| IVSd | mm | FS | % |
| **Additional Information**: |  | | |
| No pleural/pericardial effusion | | | |
| **Final Diagnosis:** | | | |
| 1. {S, D, S} Levocardia. 2. Small OS ASD, L – R Shunt 3. Normal Ventricular Function | | | |
| Remark: | | | |
| SIGNATURE  Done by: Tesfaye T., Pediatric Cardiologist _______________ 21/02/2013Eth.C | | | |

| Patient Name: **Yared Birhanu**. Patient ID: SBC. SEX/ Age: M/5months. Date of Report: 23**/02/2013**.  BP: ___ Weight: ___ Height:___ BSA: ___. Referral Diagnosis: **Cyanosis + RD. AGH06.2393.** | | | |
| --- | --- | --- | --- |
| **Features** | **Finding** | **Features** | **Finding** |
| **Profile** |  | **Atria** |  |
| Abdominal situs | Solitus | Left atrium | Normal |
| Cardiac position | Levocardia | Right atrium | Dilated |
| Systemic venous drainage | Normal | **Atrioventricular valves** |  |
| Pulmonary venous drainage | Normal | Mitral valve | Annulus = 9mm |
| Atrioventricular connection | Concordant | Tricuspid valve | Annulus = 16mm |
| Ventriculoarterial connection | Discordant | **Ventricles** |  |
| Ventricular loop | d-Loop | Left ventricle | Normal |
|  |  | Right ventricle | Dilated |
| **Septae** |  | **Coronary arteries** | ----- |
| Interventricular septum | 5mm Sub pulmonic VSD, L – R Shunt | **Doppler Measurement** |  |
| Interatrial septum | 7mm OS ASD, L – R Shunt | Mitral | ----- |
| **Semilunal valves** |  | Aortic | ---- |
| Aortic valve | Annulus = 11mm | Tricuspid | ---- |
| Pulmonary valve | Annulus = 12mm | pulmonic | ---- |
| **Great arteries** | d-TGA | **Aortic arch** | Left |
| Aorta | Anterior and to the right | **PDA** | No |
| Pulmonary artery | Posterior and to the left. |  |  |
| **M-Mode:** | | | |
| AO | mm | PWd | mm |
| LA | mm | EDV | ml |
| LVIDd | mm | ESV | ml |
| LVIDs | mm | LVEF | % |
| IVSd | mm | FS | % |
| **Additional Information**: |  | | |
| No pleural/pericardial effusion | | | |
| **Final Diagnosis:** | | | |
| 1. {S, D, D} Levocardia. 2. Moderate OS ASD, L – R Shunt 3. Small Sub pulmonic VSD 4. d-TGA | | | |
| Remark: Child was restless. | | | |
| SIGNATURE  Done by: Tesfaye T., Pediatric Cardiologist _______________ 23/02/2013Eth.C | | | |

| Patient Name: **Yeshihasab Shita**. Patient ID: FHRH. SEX/ Age: F/5months. Date of Report: 24**/02/2013**.  BP: ___ Weight: ___ Height:___ BSA: ___. Referral Diagnosis: **Incidental Murmur. AGH06.2394.** | | | |
| --- | --- | --- | --- |
| **Features** | **Finding** | **Features** | **Finding** |
| **Profile** |  | **Atria** |  |
| Abdominal situs | Solitus | Left atrium | Mildly Dilated |
| Cardiac position | Levocardia | Right atrium | Normal |
| Systemic venous drainage | Normal | **Atrioventricular valves** |  |
| Pulmonary venous drainage | Normal | Mitral valve | Annulus = 12mm |
| Atrioventricular connection | Concordant | Tricuspid valve | Annulus = 13mm |
| Ventriculoarterial connection | Concordant | **Ventricles** |  |
| Ventricular loop | d-Loop | Left ventricle | Mildly Dilated |
|  |  | Right ventricle | Normal |
| **Septae** |  | **Coronary arteries** | ----- |
| Interventricular septum | 5mm PM VSD, L – R Shunt, covered by septal leaflet of the TV. | **Doppler Measurement** |  |
| Interatrial septum | 4mm OS ASD, L – R Shunt | Mitral | ----- |
| **Semilunal valves** |  | Aortic | ---- |
| Aortic valve | Annulus = 11mm | Tricuspid | ---- |
| Pulmonary valve | Annulus = 11mm | pulmonic | ---- |
| **Great arteries** | NRGA | **Aortic arch** | Left |
| Aorta | ----- | **PDA** | No |
| Pulmonary artery | Normal MPA and Branch PAs. |  |  |
| **M-Mode:** | | | |
| AO | mm | PWd | mm |
| LA | mm | EDV | ml |
| LVIDd | mm | ESV | ml |
| LVIDs | mm | LVEF | 67% |
| IVSd | mm | FS | 35% |
| **Additional Information**: |  | | |
| No pleural/pericardial effusion | | | |
| **Final Diagnosis:** | | | |
| 1. {S, D, S} Levocardia. 2. Small OS ASD, L – R Shunt 3. Small PM VSD, L – R Shunt, covered by septal leaflet of the TV 4. Normal LV Function | | | |
| Remark: | | | |
| SIGNATURE  Done by: Tesfaye T., Pediatric Cardiologist _______________ 24/02/2013Eth.C | | | |

| Patient Name: **Baby of Fana Werkneh**. Patient ID: FHRH. SEX/ Age: M/5/12. Date of Report: 24**/02/2013**.  **AGH07.1540** | | | |
| --- | --- | --- | --- |
| **Follow up Echocardiography** for small restrictive apical VSD (2.5mm) | | | |
| **Features** | **Finding** | **Features** | **Finding** |
| **Profile** |  | **Atria** |  |
| Abdominal situs | Solitus | Left atrium | Normal |
| Cardiac position | Levocardia | Right atrium | Normal |
| Systemic venous drainage | Normal | **Atrioventricular valves** |  |
| Pulmonary venous drainage | Normal | Mitral valve | Annulus = 12mm |
| Atrioventricular connection | Concordant | Tricuspid valve | Annulus = 12mm |
| Ventriculoarterial connection | Concordant | **Ventricles** |  |
| Ventricular loop | d-Loop | Left ventricle | Normal |
|  |  | Right ventricle | Normal |
| **Septae** |  | **Coronary arteries** | ----- |
| Interventricular septum | Intact | **Doppler Measurement** |  |
| Interatrial septum | Intact | Mitral | ----- |
| **Semilunal valves** |  | Aortic | ---- |
| Aortic valve | Annulus = 11mm | Tricuspid | ---- |
| Pulmonary valve | Annulus = 11mm | pulmonic | ---- |
| **Great arteries** | NRGA | **Aortic arch** | Left |
| Aorta | ----- | **PDA** | No |
| Pulmonary artery | Normal MPA and Branch PAs. |  |  |
| **M-Mode:** | | | |
| AO | mm | PWd | mm |
| LA | mm | EDV | ml |
| LVIDd | mm | ESV | ml |
| LVIDs | mm | LVEF | % |
| IVSd | mm | FS | % |
| **Additional Information**: |  | | |
| No pleural/pericardial effusion | | | |
| **Final Diagnosis:** | | | |
| 1. {S, D, S} Levocardia. 2. No VSD (Closed Small Apical Muscular VSD (2.5mm)) 3. Normal LV Function | | | |
| Remark: | | | |
| SIGNATURE  Done by: Tesfaye T., Pediatric Cardiologist _______________ 24/02/2013Eth.C | | | |

| Patient Name: **Baby of Ayal Jibril**. Patient ID: TGSH. SEX/ Age: F/17days. Date of Report: 24**/02/2013**.  BP: ___ Weight: ___ Height:___ BSA: ___. Referral Diagnosis: **Cardiomegaly on CXR. AGH06.2395.** | | | |
| --- | --- | --- | --- |
| **Features** | **Finding** | **Features** | **Finding** |
| **Profile** |  | **Atria** |  |
| Abdominal situs | Solitus | Left atrium | Normal |
| Cardiac position | Levocardia | Right atrium | Normal |
| Systemic venous drainage | Normal | **Atrioventricular valves** |  |
| Pulmonary venous drainage | Normal | Mitral valve | Annulus = 11mm |
| Atrioventricular connection | Concordant | Tricuspid valve | Annulus = 10mm |
| Ventriculoarterial connection | Concordant | **Ventricles** |  |
| Ventricular loop | d-Loop | Left ventricle | Normal |
|  |  | Right ventricle | Normal |
| **Septae** |  | **Coronary arteries** | ----- |
| Interventricular septum | Intact | **Doppler Measurement** |  |
| Interatrial septum | Intact | Mitral | ----- |
| **Semilunal valves** |  | Aortic | ---- |
| Aortic valve | Annulus = 9mm | Tricuspid | ---- |
| Pulmonary valve | Annulus = 9mm | pulmonic | ---- |
| **Great arteries** | NRGA | **Aortic arch** | Left |
| Aorta | ----- | **PDA** | No |
| Pulmonary artery | Normal MPA and Branch PAs. |  |  |
| **M-Mode:** | | | |
| AO | mm | PWd | mm |
| LA | mm | EDV | ml |
| LVIDd | mm | ESV | ml |
| LVIDs | mm | LVEF | % |
| IVSd | mm | FS | % |
| **Additional Information**: |  | | |
| No pleural/pericardial effusion | | | |
| **Final Diagnosis:** | | | |
| 1. Normal Echocardiography Study01. | | | |
| Remark: | | | |
| SIGNATURE  Done by: Tesfaye T., Pediatric Cardiologist _______________ 24/02/2013Eth.C | | | |

| Patient Name: **Tigabie Habtamu**. Patient ID: FHRH. SEX/ Age: F/5months. Date of Report: 24**/02/2013**.  BP: ___ Weight: ___ Height:___ BSA: ___. Referral Diagnosis: **DS. AGH06.2396.** | | | |
| --- | --- | --- | --- |
| **Features** | **Finding** | **Features** | **Finding** |
| **Profile** |  | **Atria** |  |
| Abdominal situs | Solitus | Left atrium | Dilated |
| Cardiac position | Levocardia | Right atrium | Dilated |
| Systemic venous drainage | Normal | **Atrioventricular valves** |  |
| Pulmonary venous drainage | Normal | Mitral valve | Common Complete AVSD |
| Atrioventricular connection | Common Complete AVSD | Tricuspid valve |  |
| Ventriculoarterial connection | Concordant | **Ventricles** |  |
| Ventricular loop | d-Loop | Left ventricle | Dilated |
|  |  | Right ventricle | Dilated |
| **Septae** |  | **Coronary arteries** | ----- |
| Interventricular septum | Common Complete AVSD, L – R Shunt | **Doppler Measurement** |  |
| Interatrial septum |  | Mitral | Mild Left AVVR |
| **Semilunal valves** |  | Aortic | ---- |
| Aortic valve | Annulus = 10mm | Tricuspid | Moderate Right AVVR |
| Pulmonary valve | Annulus = 12mm | pulmonic | ---- |
| **Great arteries** | NRGA | **Aortic arch** | Left |
| Aorta | ----- | **PDA** | No |
| Pulmonary artery | MPA = 14mm and Branch PAs. |  |  |
| **M-Mode:** | | | |
| AO | mm | PWd | mm |
| LA | mm | EDV | ml |
| LVIDd | mm | ESV | ml |
| LVIDs | mm | LVEF | 50% |
| IVSd | mm | FS | 23% |
| **Additional Information**: |  | | |
| No pleural/pericardial effusion | | | |
| **Final Diagnosis:** | | | |
| 1. {S, D, S} Levocardia. 2. Moderate Right AVVR 3. Mild Left AVVR 4. Common Complete Balanced AVSD 5. Moderate Pulmonary Hypertension 6. Mildly reduced LV Function | | | |
| Remark: | | | |
| SIGNATURE  Done by: Tesfaye T., Pediatric Cardiologist _______________ 24/02/2013Eth.C | | | |

| Patient Name: **Baby of Habtam Kassahun**. Patient ID: FHRH. SEX/ Age: F/12days. Date of Report: 24**/02/2013**.  BP: ___ Weight: ___ Height:___ BSA: ___. Referral Diagnosis: **RD. AGH06.2397.** | | | |
| --- | --- | --- | --- |
| **Features** | **Finding** | **Features** | **Finding** |
| **Profile** |  | **Atria** |  |
| Abdominal situs | Solitus | Left atrium | Normal |
| Cardiac position | Levocardia | Right atrium | Normal |
| Systemic venous drainage | Normal | **Atrioventricular valves** |  |
| Pulmonary venous drainage | Normal | Mitral valve | Annulus = 9mm |
| Atrioventricular connection | Concordant | Tricuspid valve | Annulus = 11mm |
| Ventriculoarterial connection | Concordant | **Ventricles** |  |
| Ventricular loop | d-Loop | Left ventricle | Normal |
|  |  | Right ventricle | Normal |
| **Septae** |  | **Coronary arteries** | ----- |
| Interventricular septum | Intact | **Doppler Measurement** |  |
| Interatrial septum | 4mm OS ASD, L – R Shunt | Mitral | ----- |
| **Semilunal valves** |  | Aortic | ---- |
| Aortic valve | Annulus = 9mm | Tricuspid | ---- |
| Pulmonary valve | Annulus = 10mm | pulmonic | ---- |
| **Great arteries** | NRGA | **Aortic arch** | Left |
| Aorta | ----- | **PDA** | No |
| Pulmonary artery | Normal MPA and Branch PAs. |  |  |
| **M-Mode:** Normal LV Function on eye balling. | | | |
| AO | mm | PWd | mm |
| LA | mm | EDV | ml |
| LVIDd | mm | ESV | ml |
| LVIDs | mm | LVEF | % |
| IVSd | mm | FS | % |
| **Additional Information**: |  | | |
| No pleural/pericardial effusion | | | |
| **Final Diagnosis:** | | | |
| 1. {S, D, S} Levocardia. 2. Small OS ASD, L – R Shunt 3. Normal LV Function | | | |
| Remark: Neonate was crying. | | | |
| SIGNATURE  Done by: Tesfaye T., Pediatric Cardiologist _______________ 24/02/2013Eth.C | | | |

| Patient Name: **Darik Yihenew**. Patient ID: Adinas GH. SEX/ Age: F/3/12. Date of Report: 25**/02/2013**.  BP: ___ Weight: ___ Height:___ BSA: ___. Referral Diagnosis: **RD. AGH06.2398.** | | | |
| --- | --- | --- | --- |
| **Features** | **Finding** | **Features** | **Finding** |
| **Profile** |  | **Atria** |  |
| Abdominal situs | Solitus | Left atrium | Normal |
| Cardiac position | Levocardia | Right atrium | Normal |
| Systemic venous drainage | Normal | **Atrioventricular valves** |  |
| Pulmonary venous drainage | Normal | Mitral valve | Annulus = 10mm |
| Atrioventricular connection | Concordant | Tricuspid valve | Annulus = 11mm |
| Ventriculoarterial connection | Concordant | **Ventricles** |  |
| Ventricular loop | d-Loop | Left ventricle | Normal |
|  |  | Right ventricle | Normal |
| **Septae** |  | **Coronary arteries** | ----- |
| Interventricular septum | Intact | **Doppler Measurement** |  |
| Interatrial septum | PFO, L – R Shunt | Mitral | ----- |
| **Semilunal valves** |  | Aortic | ---- |
| Aortic valve | Annulus = 7mm | Tricuspid | ---- |
| Pulmonary valve | Annulus = 8mm | pulmonic | ---- |
| **Great arteries** | NRGA | **Aortic arch** | Left |
| Aorta | ----- | **PDA** | No |
| Pulmonary artery | Normal MPA and Branch PAs. |  |  |
| **M-Mode:** Normal LV Function on eye balling. | | | |
| AO | mm | PWd | mm |
| LA | mm | EDV | ml |
| LVIDd | mm | ESV | ml |
| LVIDs | mm | LVEF | % |
| IVSd | mm | FS | % |
| **Additional Information**: |  | | |
| No pleural/pericardial effusion | | | |
| **Final Diagnosis:** | | | |
| 1. {S, D, S} Levocardia. 2. PFO, L – R Shunt | | | |
| Remark: | | | |
| SIGNATURE  Done by: Tesfaye T., Pediatric Cardiologist _______________ 25/02/2013Eth.C | | | |

| Patient Name: **Tirsit Girma**. Patient ID: Adinas GH. SEX/ Age: F/5years. Date of Report: 25**/02/2013**.  BP: ___ Weight: ___ Height:___ BSA: ___. Referral Diagnosis: **FTT + Incidental Murmur. AGH06.2399.** | | | |
| --- | --- | --- | --- |
| **Features** | **Finding** | **Features** | **Finding** |
| **Profile** |  | **Atria** |  |
| Abdominal situs | Solitus | Left atrium | Normal |
| Cardiac position | Levocardia | Right atrium | Normal |
| Systemic venous drainage | Normal | **Atrioventricular valves** |  |
| Pulmonary venous drainage | Normal | Mitral valve | Annulus = 16mm |
| Atrioventricular connection | Concordant | Tricuspid valve | Annulus = 17mm |
| Ventriculoarterial connection | Concordant | **Ventricles** |  |
| Ventricular loop | d-Loop | Left ventricle | Normal |
|  |  | Right ventricle | Normal |
| **Septae** |  | **Coronary arteries** | ----- |
| Interventricular septum | Intact | **Doppler Measurement** |  |
| Interatrial septum | Intact | Mitral | ----- |
| **Semilunal valves** |  | Aortic | ---- |
| Aortic valve | Annulus = 13mm | Tricuspid | ---- |
| Pulmonary valve | Annulus = 16mm | pulmonic | ---- |
| **Great arteries** | NRGA | **Aortic arch** | Left |
| Aorta | ----- | **PDA** | No |
| Pulmonary artery | Normal MPA and Branch PAs. |  |  |
| **M-Mode:** | | | |
| AO | mm | PWd | mm |
| LA | mm | EDV | ml |
| LVIDd | mm | ESV | ml |
| LVIDs | mm | LVEF | 58% |
| IVSd | mm | FS | 29% |
| **Additional Information**: |  | | |
| No pleural/pericardial effusion | | | |
| **Final Diagnosis:** | | | |
| 1. Normal Echocardiography Study. | | | |
| Remark: | | | |
| SIGNATURE  Done by: Tesfaye T., Pediatric Cardiologist _______________ 25/02/2013Eth.C | | | |

| Patient Name: **Haymanot Ambachew**. Patient ID: FHRH. SEX/ Age: F/8years. Date of Report: 26**/01/2013**.  BP: ___ Weight: ___ Height:___ BSA: ___. Referral Diagnosis: **Chest Pain. AGH06.2400.** | | | |
| --- | --- | --- | --- |
| **Features** | **Finding** | **Features** | **Finding** |
| **Profile** |  | **Atria** |  |
| Abdominal situs | Solitus | Left atrium | Normal |
| Cardiac position | Levocardia | Right atrium | Normal |
| Systemic venous drainage | Normal | **Atrioventricular valves** |  |
| Pulmonary venous drainage | Normal | Mitral valve | Annulus = 17mm |
| Atrioventricular connection | Concordant | Tricuspid valve | Annulus = 20mm |
| Ventriculoarterial connection | Concordant | **Ventricles** |  |
| Ventricular loop | d-Loop | Left ventricle | Normal |
|  |  | Right ventricle | Normal |
| **Septae** |  | **Coronary arteries** | ----- |
| Interventricular septum | Intact | **Doppler Measurement** |  |
| Interatrial septum | Intact | Mitral | ----- |
| **Semilunal valves** |  | Aortic | ---- |
| Aortic valve | Annulus = 15mm | Tricuspid | ---- |
| Pulmonary valve | Annulus = 17mm | pulmonic | ---- |
| **Great arteries** | NRGA | **Aortic arch** | Left |
| Aorta | ----- | **PDA** | No |
| Pulmonary artery | Normal MPA and Branch PAs. |  |  |
| **M-Mode:** | | | |
| AO | mm | PWd | mm |
| LA | mm | EDV | ml |
| LVIDd | mm | ESV | ml |
| LVIDs | mm | LVEF | 58% |
| IVSd | mm | FS | 30% |
| **Additional Information**: |  | | |
| No pleural/pericardial effusion | | | |
| **Final Diagnosis:** | | | |
| 1. Normal Echocardiography Study. | | | |
| Remark: | | | |
| SIGNATURE  Done by: Tesfaye T., Pediatric Cardiologist _______________ 26/01/2013Eth.C | | | |

| Patient Name: **Dagmawi Yenieguad**. Patient ID: Amaris SPC. SEX/ Age: M/6 7/12. Date of Report: 26**/02/2013**.  BP: ___ Weight: ___ Height:___ BSA: ___. Referral Diagnosis: **?ARF. AGH06.2401.** | | | |
| --- | --- | --- | --- |
| **Features** | **Finding** | **Features** | **Finding** |
| **Profile** |  | **Atria** |  |
| Abdominal situs | Solitus | Left atrium | Normal |
| Cardiac position | Levocardia | Right atrium | Normal |
| Systemic venous drainage | Normal | **Atrioventricular valves** |  |
| Pulmonary venous drainage | Normal | Mitral valve | Annulus = 17mm |
| Atrioventricular connection | Concordant | Tricuspid valve | Annulus = 17mm  TAPSE = 19mm. |
| Ventriculoarterial connection | Concordant | **Ventricles** |  |
| Ventricular loop | d-Loop | Left ventricle | Normal |
|  |  | Right ventricle | Normal |
| **Septae** |  | **Coronary arteries** | ----- |
| Interventricular septum | Intact | **Doppler Measurement** |  |
| Interatrial septum | Intact | Mitral | ----- |
| **Semilunal valves** |  | Aortic | ---- |
| Aortic valve | Annulus = 15mm | Tricuspid | Trivial TR, PPG = 11mmHg. |
| Pulmonary valve | Annulus = 18mm | pulmonic | ---- |
| **Great arteries** | NRGA | **Aortic arch** | Left |
| Aorta | ----- | **PDA** | No |
| Pulmonary artery | Normal MPA and Branch PAs. |  |  |
| **M-Mode:** | | | |
| AO | mm | PWd | mm |
| LA | mm | EDV | ml |
| LVIDd | mm | ESV | ml |
| LVIDs | mm | LVEF | 56% |
| IVSd | mm | FS | 29% |
| **Additional Information**: |  | | |
| No pleural/pericardial effusion | | | |
| **Final Diagnosis:** | | | |
| 1. Normal Echocardiography Study. | | | |
| Remark: | | | |
| SIGNATURE  Done by: Tesfaye T., Pediatric Cardiologist _______________ 26/02/2013Eth.C | | | |

| Patient Name: **Desta Tsegaye**. Patient ID: FHRH. SEX/ Age: F/1 7/12. Date of Report: 26**/02/2013**.  BP: ___ Weight: ___ Height:___ BSA: ___. Referral Diagnosis: **Sepsis with Pericarditis. AGH06.2402.** | | | |
| --- | --- | --- | --- |
| **Features** | **Finding** | **Features** | **Finding** |
| **Profile** |  | **Atria** |  |
| Abdominal situs | Solitus | Left atrium | Normal |
| Cardiac position | Levocardia | Right atrium | Normal |
| Systemic venous drainage | Normal | **Atrioventricular valves** |  |
| Pulmonary venous drainage | Normal | Mitral valve | Annulus = 14mm |
| Atrioventricular connection | Concordant | Tricuspid valve | Annulus = 13mm |
| Ventriculoarterial connection | Concordant | **Ventricles** |  |
| Ventricular loop | d-Loop | Left ventricle | Normal |
|  |  | Right ventricle | Normal |
| **Septae** |  | **Coronary arteries** | ----- |
| Interventricular septum | Intact | **Doppler Measurement** |  |
| Interatrial septum | Intact | Mitral | ----- |
| **Semilunal valves** |  | Aortic | ---- |
| Aortic valve | Annulus = 12mm | Tricuspid | ---- |
| Pulmonary valve | Annulus = 13mm | pulmonic | ---- |
| **Great arteries** | NRGA | **Aortic arch** | Left |
| Aorta | ----- | **PDA** | No |
| Pulmonary artery | Normal MPA and Branch PAs. |  |  |
| **M-Mode:** | | | |
| AO | mm | PWd | mm |
| LA | mm | EDV | ml |
| LVIDd | mm | ESV | ml |
| LVIDs | mm | LVEF | 59% |
| IVSd | mm | FS | 31% |
| **Additional Information**: |  | | |
| Pericardial effusion 10mm on RA Side, 3mm on RV Side and 9mm on Apical side with pericardial thickening | | | |
| **Final Diagnosis:** | | | |
| 1. {S, D, S} Levocardia. 2. Mild Pericardial effusion with pericardial thickening secondary to ??? 3. Normal LV Function | | | |
| Remark: | | | |
| SIGNATURE  Done by: Tesfaye T., Pediatric Cardiologist _______________ 26/02/2013Eth.C | | | |

| Patient Name: **Markon Solomon**. Patient ID: FHRH. SEX/ Age: M/10months. Date of Report: 27**/02/2013**.  BP: ___ Weight: ___ Height:___ BSA: ___. Referral Diagnosis: **Diaphoresis. AGH06.2403.** | | | |
| --- | --- | --- | --- |
| **Features** | **Finding** | **Features** | **Finding** |
| **Profile** |  | **Atria** |  |
| Abdominal situs | Solitus | Left atrium | Normal |
| Cardiac position | Levocardia | Right atrium | Normal |
| Systemic venous drainage | Normal | **Atrioventricular valves** |  |
| Pulmonary venous drainage | Normal | Mitral valve | Annulus = 13mm |
| Atrioventricular connection | Concordant | Tricuspid valve | Annulus = 14mm |
| Ventriculoarterial connection | Concordant | **Ventricles** |  |
| Ventricular loop | d-Loop | Left ventricle | Normal |
|  |  | Right ventricle | Normal |
| **Septae** |  | **Coronary arteries** | ----- |
| Interventricular septum | Intact | **Doppler Measurement** |  |
| Interatrial septum | Intact | Mitral | ----- |
| **Semilunal valves** |  | Aortic | ---- |
| Aortic valve | Annulus = 12mm | Tricuspid | ---- |
| Pulmonary valve | Annulus = 12mm | pulmonic | ---- |
| **Great arteries** | NRGA | **Aortic arch** | Left |
| Aorta | ----- | **PDA** | No |
| Pulmonary artery | Normal MPA and Branch PAs. |  |  |
| **M-Mode:** Normal LV Function on eye balling. | | | |
| AO | mm | PWd | mm |
| LA | mm | EDV | ml |
| LVIDd | mm | ESV | ml |
| LVIDs | mm | LVEF | % |
| IVSd | mm | FS | % |
| **Additional Information**: |  | | |
| No pleural/pericardial effusion | | | |
| **Final Diagnosis:** | | | |
| 1. Normal Echocardiography Study. | | | |
| Remark: | | | |
| SIGNATURE  Done by: Tesfaye T., Pediatric Cardiologist _______________ 27/02/2013Eth.C | | | |

| Patient Name: **Baby of Meseret Shiferaw**. Patient ID: FHRH. SEX/ Age: F/6/12. Date of Report: 30**/02/2013**.  **AGH07.1540** | | | |
| --- | --- | --- | --- |
| **Follow up echo** | PFO + Flow acceleration across PV | | |
| **Features** | **Finding** | **Features** | **Finding** |
| **Profile** |  | **Atria** |  |
| Abdominal situs | Solitus | Left atrium | Normal |
| Cardiac position | Levocardia | Right atrium | Normal |
| Systemic venous drainage | Normal | **Atrioventricular valves** |  |
| Pulmonary venous drainage | Normal | Mitral valve | Annulus = 11mm |
| Atrioventricular connection | Concordant | Tricuspid valve | Annulus = 13mm |
| Ventriculoarterial connection | Concordant | **Ventricles** |  |
| Ventricular loop | d-Loop | Left ventricle | Normal |
|  |  | Right ventricle | Normal |
| **Septae** |  | **Coronary arteries** | ----- |
| Interventricular septum | Intact | **Doppler Measurement** |  |
| Interatrial septum | Intact | Mitral | ----- |
| **Semilunal valves** |  | Aortic | ---- |
| Aortic valve | Annulus = 11mm | Tricuspid | ---- |
| Pulmonary valve | Annulus = 12mm | pulmonic | No flow acceleration across the PV |
| **Great arteries** | NRGA | **Aortic arch** | Left |
| Aorta | ----- | **PDA** | No |
| Pulmonary artery | Normal MPA and Branch PAs. |  |  |
| **M-Mode:** | | | |
| AO | mm | PWd | mm |
| LA | mm | EDV | ml |
| LVIDd | mm | ESV | ml |
| LVIDs | mm | LVEF | % |
| IVSd | mm | FS | % |
| **Additional Information**: |  | | |
| No pleural/pericardial effusion | | | |
| **Final Diagnosis:** | | | |
| 1. {S, D, S} Levocardia. 2. PFO Closed 3. No flow acceleration across the PV. | | | |
| Remark: Only from subcostal window. | | | |
| SIGNATURE  Done by: Tesfaye T., Pediatric Cardiologist _______________ 30/02/2013Eth.C | | | |

| Patient Name: **Danawit Tessema**. Patient ID: SBSPC. SEX/ Age: F/2years. Date of Report: 30**/02/2013**.  BP: ___ Weight: ___ Height:___ BSA: ___. Referral Diagnosis: **CHF + Diaphoresis + RD. AGH06.2404.** | | | |
| --- | --- | --- | --- |
| **Features** | **Finding** | **Features** | **Finding** |
| **Profile** |  | **Atria** |  |
| Abdominal situs | Solitus | Left atrium | Dilated |
| Cardiac position | Levocardia | Right atrium | Dilated |
| Systemic venous drainage | Normal | **Atrioventricular valves** |  |
| Pulmonary venous drainage | Normal | Mitral valve | Annulus = 16mm |
| Atrioventricular connection | Concordant | Tricuspid valve | Annulus = 18mm |
| Ventriculoarterial connection | Concordant | **Ventricles** |  |
| Ventricular loop | d-Loop | Left ventricle | Dilated |
|  |  | Right ventricle | Dilated |
| **Septae** |  | **Coronary arteries** | ----- |
| Interventricular septum | 11mm Peri Membranous VSD, L – R Shunt | **Doppler Measurement** |  |
| Interatrial septum | 8mm OS ASD, L – R Shunt. | Mitral | ----- |
| **Semilunal valves** |  | Aortic | ---- |
| Aortic valve | Annulus = 13mm | Tricuspid | ---- |
| Pulmonary valve | Annulus = 16mm | pulmonic | Moderate PR, PPG = 66mmHg. |
| **Great arteries** | NRGA | **Aortic arch** | Left |
| Aorta | ----- | **PDA** | No |
| Pulmonary artery | **MPA = 21mm.** |  |  |
| **M-Mode:** Normal LV Function on eye balling. | | | |
| AO | mm | PWd | mm |
| LA | mm | EDV | ml |
| LVIDd | mm | ESV | ml |
| LVIDs | mm | LVEF | % |
| IVSd | mm | FS | % |
| **Additional Information**: |  | | |
| No pleural/pericardial effusion | | | |
| **Final Diagnosis:** | | | |
| 1. {S, D, S} Levocardia. 2. Moderate OS ASD, L – R Shunt 3. Large Peri-membranous VSD, L – R Shunt 4. Severe Pulmonary Hypertension 5. Normal LV Function | | | |
| Remark: | | | |
| SIGNATURE  Done by: Tesfaye T., Pediatric Cardiologist _______________ 30/02/2013Eth.C | | | |

| Patient Name: **Addisu Hunegnaw**. Patient ID: FHRH. SEX/ Age: M/43days. Date of Report: 30**/02/2013**.  BP: ___ Weight: ___ Height:___ BSA: ___. Referral Diagnosis: **Cyanosis + RD. AGH06.2405.** | | | |
| --- | --- | --- | --- |
| **Features** | **Finding** | **Features** | **Finding** |
| **Profile** |  | **Atria** |  |
| Abdominal situs | Solitus | Left atrium | Normal |
| Cardiac position | Levocardia | Right atrium | Dilated |
| Systemic venous drainage | Normal | **Atrioventricular valves** |  |
| Pulmonary venous drainage | Normal | Mitral valve | Annulus = 11mm |
| Atrioventricular connection | Concordant | Tricuspid valve | Annulus = 13mm |
| Ventriculoarterial connection | Discordant | **Ventricles** |  |
| Ventricular loop | d-Loop | Left ventricle | Banana shaped |
|  |  | Right ventricle | Dilated |
| **Septae** |  | **Coronary arteries** | ----- |
| Interventricular septum | 3mm sub pulmonic VSD | **Doppler Measurement** |  |
| Interatrial septum | Intact | Mitral | ----- |
| **Semilunal valves** |  | Aortic | ---- |
| Aortic valve | Annulus = 9mm | Tricuspid | ---- |
| Pulmonary valve | Annulus = 10mm | pulmonic | ---- |
| **Great arteries** | d-TGA | **Aortic arch** | Left |
| Aorta | Anterior and to the right | **PDA** | No |
| Pulmonary artery | Posterior and to the left |  |  |
| **M-Mode:** | | | |
| AO | mm | PWd | mm |
| LA | mm | EDV | ml |
| LVIDd | mm | ESV | ml |
| LVIDs | mm | LVEF | % |
| IVSd | mm | FS | % |
| **Additional Information**: |  | | |
| 3mm pericardial effusion on RA/RV Side. | | | |
| **Final Diagnosis:** | | | |
| 1. {S, D, D} Levocardia. 2. d-TGA 3. Small Restrictive SUBPULMONIC VSD 4. Trace Pericardial Effusion. | | | |
| Remark: | | | |
| SIGNATURE  Done by: Tesfaye T., Pediatric Cardiologist _______________ 30/02/2013Eth.C | | | |

| Patient Name: **Dagim Destaw**. Patient ID: Adinas GH. SEX/ Age: M/11/12. Date of Report: 01**/03/2013**.  BP: ___ Weight: ___ Height:___ BSA: ___. Referral Diagnosis: **Diaphoresis. AGH06.2406.** | | | |
| --- | --- | --- | --- |
| **Features** | **Finding** | **Features** | **Finding** |
| **Profile** |  | **Atria** |  |
| Abdominal situs | Solitus | Left atrium | Normal |
| Cardiac position | Levocardia | Right atrium | Normal |
| Systemic venous drainage | Normal | **Atrioventricular valves** |  |
| Pulmonary venous drainage | Normal | Mitral valve | Annulus = 11mm |
| Atrioventricular connection | Concordant | Tricuspid valve | Annulus = 13mm |
| Ventriculoarterial connection | Concordant | **Ventricles** |  |
| Ventricular loop | d-Loop | Left ventricle | Normal |
|  |  | Right ventricle | Normal |
| **Septae** |  | **Coronary arteries** | ----- |
| Interventricular septum | Intact | **Doppler Measurement** |  |
| Interatrial septum | PFO, L – R Shunt | Mitral | ----- |
| **Semilunal valves** |  | Aortic | ---- |
| Aortic valve | Annulus = 11mm | Tricuspid | ---- |
| Pulmonary valve | Annulus = 10mm | pulmonic | ---- |
| **Great arteries** | NRGA | **Aortic arch** | Left |
| Aorta | ----- | **PDA** | No |
| Pulmonary artery | Normal MPA and Branch PAs. |  |  |
| **M-Mode:** Normal LV Function on eye balling. | | | |
| AO | mm | PWd | mm |
| LA | mm | EDV | ml |
| LVIDd | mm | ESV | ml |
| LVIDs | mm | LVEF | % |
| IVSd | mm | FS | % |
| **Additional Information**: |  | | |
| No pleural/pericardial effusion | | | |
| **Final Diagnosis:** | | | |
| 1. {S, D, S} Levocardia. 2. PFO, L – R Shunt 3. Normal LV Function. | | | |
| Remark: | | | |
| SIGNATURE  Done by: Tesfaye T., Pediatric Cardiologist _______________ 01/03/2013Eth.C | | | |

| Patient Name: **Befikir Takilo**. Patient ID: Addis Alem PH. SEX/ Age: M/2 6/12. Date of Report: 02**/03/2013**.  BP: ___ Weight: ___ Height:___ BSA: ___. Referral Diagnosis: **Recurrent Chest Infection. AGH06.2407.** | | | |
| --- | --- | --- | --- |
| **Features** | **Finding** | **Features** | **Finding** |
| **Profile** |  | **Atria** |  |
| Abdominal situs | Solitus | Left atrium | Normal |
| Cardiac position | Levocardia | Right atrium | Normal |
| Systemic venous drainage | Normal | **Atrioventricular valves** |  |
| Pulmonary venous drainage | Normal | Mitral valve | Annulus = 13mm |
| Atrioventricular connection | Concordant | Tricuspid valve | Annulus = 15mm |
| Ventriculoarterial connection | Concordant | **Ventricles** |  |
| Ventricular loop | d-Loop | Left ventricle | Normal |
|  |  | Right ventricle | Normal |
| **Septae** |  | **Coronary arteries** | ----- |
| Interventricular septum | Intact | **Doppler Measurement** |  |
| Interatrial septum | Intact | Mitral | ----- |
| **Semilunal valves** |  | Aortic | ---- |
| Aortic valve | Annulus = 12mm | Tricuspid | ---- |
| Pulmonary valve | Annulus = 13mm | pulmonic | ---- |
| **Great arteries** | NRGA | **Aortic arch** | Left |
| Aorta | ----- | **PDA** | No |
| Pulmonary artery | Normal MPA and Branch PAs. |  |  |
| **M-Mode:** | | | |
| AO | mm | PWd | mm |
| LA | mm | EDV | ml |
| LVIDd | mm | ESV | ml |
| LVIDs | mm | LVEF | 66% |
| IVSd | mm | FS | 35% |
| **Additional Information**: |  | | |
| No pleural/pericardial effusion | | | |
| **Final Diagnosis:** | | | |
| 1. Normal Echocardiography Study. | | | |
| Remark: | | | |
| SIGNATURE  Done by: Tesfaye T., Pediatric Cardiologist _______________ 02/03/2013Eth.C | | | |

| Patient Name: **Ermias Abiyot**. Patient ID: TGSH. SEX/ Age: M/3 4/12. Date of Report: 02**/03/2013**.  BP: ___ Weight: ___ Height:___ BSA: ___. Referral Diagnosis: **DS. AGH06.2408.** | | | |
| --- | --- | --- | --- |
| **Features** | **Finding** | **Features** | **Finding** |
| **Profile** |  | **Atria** |  |
| Abdominal situs | Solitus | Left atrium | Normal |
| Cardiac position | Levocardia | Right atrium | Normal |
| Systemic venous drainage | Normal | **Atrioventricular valves** |  |
| Pulmonary venous drainage | Normal | Mitral valve | Annulus = 15mm |
| Atrioventricular connection | Concordant | Tricuspid valve | Annulus = 17mm |
| Ventriculoarterial connection | Concordant | **Ventricles** |  |
| Ventricular loop | d-Loop | Left ventricle | Normal |
|  |  | Right ventricle | Normal |
| **Septae** |  | **Coronary arteries** | ----- |
| Interventricular septum | Intact | **Doppler Measurement** |  |
| Interatrial septum | Intact | Mitral | ----- |
| **Semilunal valves** |  | Aortic | ---- |
| Aortic valve | Annulus = 13mm | Tricuspid | ---- |
| Pulmonary valve | Annulus = 15mm | pulmonic | ---- |
| **Great arteries** | NRGA | **Aortic arch** | Left |
| Aorta | ----- | **PDA** | No |
| Pulmonary artery | Normal MPA and Branch PAs. |  |  |
| **M-Mode:** | | | |
| AO | mm | PWd | mm |
| LA | mm | EDV | ml |
| LVIDd | mm | ESV | ml |
| LVIDs | mm | LVEF | 70% |
| IVSd | mm | FS | 38% |
| **Additional Information**: |  | | |
| No pleural/pericardial effusion | | | |
| **Final Diagnosis:** | | | |
| 1. Normal Echocardiography Study. | | | |
| Remark: | | | |
| SIGNATURE  Done by: Tesfaye T., Pediatric Cardiologist _______________ 02/03/2013Eth.C | | | |

| Patient Name: **Yosef Kura**. Patient ID: Addis Alem PH. SEX/ Age: M/1 8/12. Date of Report: 02**/03/2013**.  BP: _______ Weight: ______ Height:____________ BSA: ________ **AGH07. 1556** | | | |
| --- | --- | --- | --- |
| **Follow up Echo** | Myocarditis + DS + Hypothyroidism | | |
| **Features** | **Finding** | **Features** | **Finding** |
| **Profile** |  | **Atria** |  |
| Abdominal situs | Solitus | Left atrium | Normal |
| Cardiac position | Levocardia | Right atrium | Normal |
| Systemic venous drainage | Normal | **Atrioventricular valves** |  |
| Pulmonary venous drainage | Normal | Mitral valve | Annulus = 16mm |
| Atrioventricular connection | Concordant | Tricuspid valve | Annulus = 19mm |
| Ventriculoarterial connection | Concordant | **Ventricles** |  |
| Ventricular loop | d-Loop | Left ventricle | Normal |
|  |  | Right ventricle | Normal |
| **Septae** |  | **Coronary arteries** | ----- |
| Interventricular septum | Intact | **Doppler Measurement** |  |
| Interatrial septum | Intact | Mitral | ----- |
| **Semilunal valves** |  | Aortic | ---- |
| Aortic valve | Annulus = 14mm | Tricuspid | ---- |
| Pulmonary valve | Annulus = 15mm | pulmonic | ---- |
| **Great arteries** | NRGA | **Aortic arch** | Left |
| Aorta | ----- | **PDA** | No |
| Pulmonary artery | Normal MPA and Branch PAs. |  |  |
| **M-Mode:** | | | |
| AO | mm | PWd | mm |
| LA | mm | EDV | ml |
| LVIDd | mm | ESV | ml |
| LVIDs | mm | LVEF | 66% |
| IVSd | mm | FS | 35% |
| **Additional Information**: |  | | |
| No pleural/pericardial effusion | | | |
| **Final Diagnosis:** | | | |
| 1. Normal Echocardiography Study. | | | |
| Remark: Resolved LV Dysfunction | | | |
| SIGNATURE  Done by: Tesfaye T., Pediatric Cardiologist _______________ 02/03/2013Eth.C | | | |

| Patient Name: **Baby of Fantaye Tesfaye**. Patient ID: TGSH. SEX/ Age: M/7days. Date of Report: 02**/03/2013**.  BP: ___ Weight: ___ Height:___ BSA: ___. Referral Diagnosis: **DS. AGH06.2409.** | | | |
| --- | --- | --- | --- |
| **Features** | **Finding** | **Features** | **Finding** |
| **Profile** |  | **Atria** |  |
| Abdominal situs | Solitus | Left atrium | Normal |
| Cardiac position | Levocardia | Right atrium | Normal |
| Systemic venous drainage | Normal | **Atrioventricular valves** |  |
| Pulmonary venous drainage | Normal | Mitral valve | Annulus = 9mm |
| Atrioventricular connection | Concordant | Tricuspid valve | Annulus = 10mm |
| Ventriculoarterial connection | Concordant | **Ventricles** |  |
| Ventricular loop | d-Loop | Left ventricle | Normal |
|  |  | Right ventricle | Normal |
| **Septae** |  | **Coronary arteries** | ----- |
| Interventricular septum | Intact | **Doppler Measurement** |  |
| Interatrial septum | Intact | Mitral | ----- |
| **Semilunal valves** |  | Aortic | ---- |
| Aortic valve | Annulus = 9mm | Tricuspid | ---- |
| Pulmonary valve | Annulus = 9mm | pulmonic | ---- |
| **Great arteries** | NRGA | **Aortic arch** | Left |
| Aorta | ----- | **PDA** | No |
| Pulmonary artery | Normal MPA and Branch PAs. |  |  |
| **M-Mode:** Normal LV Function on eye balling. | | | |
| AO | mm | PWd | mm |
| LA | mm | EDV | ml |
| LVIDd | mm | ESV | ml |
| LVIDs | mm | LVEF | % |
| IVSd | mm | FS | % |
| **Additional Information**: |  | | |
| No pleural/pericardial effusion | | | |
| **Final Diagnosis:** | | | |
| 1. Normal Echocardiography Study. | | | |
| Remark: Neonate was restless. Limited Echo window | | | |
| SIGNATURE  Done by: Tesfaye T., Pediatric Cardiologist _______________ 02/03/2013Eth.C | | | |

| Patient Name: **Abel Yohannes**. Patient ID: SBPSC. SEX/ Age: M/2years. Date of Report: 03**/03/2013**.  BP: ___ Weight: ___ Height:___ BSA: ___. Referral Diagnosis: **Recurrent Chest Infection. AGH06.2410.** | | | |
| --- | --- | --- | --- |
| **Features** | **Finding** | **Features** | **Finding** |
| **Profile** |  | **Atria** |  |
| Abdominal situs | Solitus | Left atrium | Normal |
| Cardiac position | Levocardia | Right atrium | Normal |
| Systemic venous drainage | Normal | **Atrioventricular valves** |  |
| Pulmonary venous drainage | Normal | Mitral valve | Annulus = 14mm |
| Atrioventricular connection | Concordant | Tricuspid valve | Annulus = 16mm |
| Ventriculoarterial connection | Concordant | **Ventricles** |  |
| Ventricular loop | d-Loop | Left ventricle | Normal |
|  |  | Right ventricle | Normal |
| **Septae** |  | **Coronary arteries** | ----- |
| Interventricular septum | Intact | **Doppler Measurement** |  |
| Interatrial septum | Intact | Mitral | ----- |
| **Semilunal valves** |  | Aortic | ---- |
| Aortic valve | Annulus = 13mm | Tricuspid | ---- |
| Pulmonary valve | Annulus = 13mm | pulmonic | ---- |
| **Great arteries** | NRGA | **Aortic arch** | Left |
| Aorta | ----- | **PDA** | No |
| Pulmonary artery | Normal MPA and Branch PAs. |  |  |
| **M-Mode:** Normal LV Function on eye balling. | | | |
| AO | mm | PWd | mm |
| LA | mm | EDV | ml |
| LVIDd | mm | ESV | ml |
| LVIDs | mm | LVEF | % |
| IVSd | mm | FS | % |
| **Additional Information**: |  | | |
| No pleural/pericardial effusion | | | |
| **Final Diagnosis:** | | | |
| 1. Normal Echocardiography Study. | | | |
| Remark: | | | |
| SIGNATURE  Done by: Tesfaye T., Pediatric Cardiologist _______________ 03/03/2013Eth.C | | | |

| Patient Name: **Yohana Belachew**. Patient ID: FHRH. SEX/ Age: F/5/12. Date of Report: 03**/03/2013**.  BP: ___ Weight: ___ Height:___ BSA: ___. Referral Diagnosis: **CHF. AGH06.2411.** | | | |
| --- | --- | --- | --- |
| **Features** | **Finding** | **Features** | **Finding** |
| **Profile** |  | **Atria** |  |
| Abdominal situs | Solitus | Left atrium | Dilated |
| Cardiac position | Levocardia | Right atrium | Dilated |
| Systemic venous drainage | Normal | **Atrioventricular valves** |  |
| Pulmonary venous drainage | Normal | Mitral valve | Annulus = 12mm |
| Atrioventricular connection | Concordant | Tricuspid valve | Annulus = 14mm |
| Ventriculoarterial connection | Concordant | **Ventricles** |  |
| Ventricular loop | d-Loop | Left ventricle | Dilated |
|  |  | Right ventricle | Dilated |
| **Septae** |  | **Coronary arteries** | ----- |
| Interventricular septum | 7mm PM VSD with Inlet Extension, L – R Shunt, PPG = 27mmHg | **Doppler Measurement** |  |
| Interatrial septum | Intact | Mitral | ----- |
| **Semilunal valves** |  | Aortic | ---- |
| Aortic valve | Annulus = 12mm | Tricuspid | ---- |
| Pulmonary valve | Annulus = 13mm. thickened MVL. | pulmonic | Moderate PR, PPG = 63mmHg |
| **Great arteries** | NRGA | **Aortic arch** | Left |
| Aorta | ----- | **PDA** | 2.5mm PDA, L – R Shunt |
| Pulmonary artery | MPA dilated and Branch PAs. |  |  |
| **M-Mode:** | | | |
| AO | mm | PWd | mm |
| LA | mm | EDV | ml |
| LVIDd | mm | ESV | ml |
| LVIDs | mm | LVEF | 66% |
| IVSd | mm | FS | 35% |
| **Additional Information**: |  | | |
| No pleural/pericardial effusion | | | |
| **Final Diagnosis:** | | | |
| 1. {S, D, S} Levocardia. 2. Moderate PM VSD with Inlet Extension, L – R Shunt 3. Large PDA, L – R Shunt 4. Moderate PR 5. Severe Pulmonary Hypertension 6. Normal LV Function | | | |
| Remark: | | | |
| SIGNATURE  Done by: Tesfaye T., Pediatric Cardiologist _______________ 03/03/2013Eth.C | | | |

| Patient Name: **Alemnesh Molla**. Patient ID: FHRH. SEX/ Age: F/4/12. Date of Report: 04**/03/2013**.  BP: ___ Weight: ___ Height:___ BSA: ___. Referral Diagnosis: **_______. AGH06.2412.** | | | |
| --- | --- | --- | --- |
| **Features** | **Finding** | **Features** | **Finding** |
| **Profile** |  | **Atria** |  |
| Abdominal situs | Solitus | Left atrium | Normal |
| Cardiac position | Levocardia | Right atrium | Normal |
| Systemic venous drainage | Normal | **Atrioventricular valves** |  |
| Pulmonary venous drainage | Normal | Mitral valve | Annulus = 11mm |
| Atrioventricular connection | Concordant | Tricuspid valve | Annulus = 12mm |
| Ventriculoarterial connection | Concordant | **Ventricles** |  |
| Ventricular loop | d-Loop | Left ventricle | Normal |
|  |  | Right ventricle | Normal |
| **Septae** |  | **Coronary arteries** | ----- |
| Interventricular septum | Intact | **Doppler Measurement** |  |
| Interatrial septum | Intact | Mitral | ----- |
| **Semilunal valves** |  | Aortic | ---- |
| Aortic valve | Annulus = 10mm | Tricuspid | ---- |
| Pulmonary valve | Annulus = 8mm | pulmonic | ---- |
| **Great arteries** | NRGA | **Aortic arch** | Left |
| Aorta | ----- | **PDA** | No |
| Pulmonary artery | Normal MPA and Branch PAs. |  |  |
| **M-Mode:** | | | |
| AO | mm | PWd | mm |
| LA | mm | EDV | ml |
| LVIDd | mm | ESV | ml |
| LVIDs | mm | LVEF | 68% |
| IVSd | mm | FS | 35% |
| **Additional Information**: |  | | |
| No pleural/pericardial effusion | | | |
| **Final Diagnosis:** | | | |
| 1. Normal Echocardiography Study | | | |
| Remark: | | | |
| SIGNATURE  Done by: Tesfaye T., Pediatric Cardiologist _______________ 04/03/2013Eth.C | | | |

| Patient Name: **Aytenew Mihret**. Patient ID: FHRH. SEX/ Age: M/10years. Date of Report: 06**/03/2013**.  BP: _______ Weight: ______ Height:____________ BSA: ________ | | | |
| --- | --- | --- | --- |
| **Features** | **Finding** | **Features** | **Finding** |
| **Profile** |  | **Atria** |  |
| Abdominal situs | Solitus | Left atrium | Normal |
| Cardiac position | Levocardia | Right atrium | Normal |
| Systemic venous drainage | Normal | **Atrioventricular valves** |  |
| Pulmonary venous drainage | Normal | Mitral valve | Annulus = 21mm |
| Atrioventricular connection | Concordant | Tricuspid valve | Annulus = 23mm  TAPSE = 23mm. |
| Ventriculoarterial connection | Concordant | **Ventricles** |  |
| Ventricular loop | d-Loop | Left ventricle | Normal |
|  |  | Right ventricle | Normal |
| **Septae** |  | **Coronary arteries** | ----- |
| Interventricular septum | Intact | **Doppler Measurement** |  |
| Interatrial septum | Intact | Mitral | ----- |
| **Semilunal valves** |  | Aortic | ---- |
| Aortic valve | Annulus = 17mm | Tricuspid | ---- |
| Pulmonary valve | Annulus = 19mm | pulmonic | ---- |
| **Great arteries** | NRGA | **Aortic arch** | Left |
| Aorta | ----- | **PDA** | No |
| Pulmonary artery | Normal MPA and Branch PAs. |  |  |
| **M-Mode:** | | | |
| AO | mm | PWd | mm |
| LA | mm | EDV | ml |
| LVIDd | mm | ESV | ml |
| LVIDs | mm | LVEF | 68% |
| IVSd | mm | FS | 38% |
| **Additional Information**: |  | | |
| 7mm pericardial effusion on RA/RV Side. No septation. No echo debris. | | | |
| **Final Diagnosis:** | | | |
| 1. Mild Pericardial effusion. | | | |
| Remark: | | | |
| SIGNATURE  Done by: Tesfaye T., Pediatric Cardiologist _______________ 06/03/2013Eth.C | | | |

| Patient Name: **Mekdes Ayana**. Patient ID: Enjibara GH. SEX/ Age: F/1 9/12. Date of Report: 07**/03/2013**.  BP: _______ Weight: ______ Height:____________ BSA: ________ | | | |
| --- | --- | --- | --- |
| **Features** | **Finding** | **Features** | **Finding** |
| **Profile** |  | **Atria** |  |
| Abdominal situs | Solitus | Left atrium | Normal |
| Cardiac position | Levocardia | Right atrium | Normal |
| Systemic venous drainage | Normal | **Atrioventricular valves** |  |
| Pulmonary venous drainage | Normal | Mitral valve | Annulus = 15mm |
| Atrioventricular connection | Concordant | Tricuspid valve | Annulus = 16mm |
| Ventriculoarterial connection | Concordant | **Ventricles** |  |
| Ventricular loop | d-Loop | Left ventricle | Normal |
|  |  | Right ventricle | Normal |
| **Septae** |  | **Coronary arteries** | ----- |
| Interventricular septum | Intact | **Doppler Measurement** |  |
| Interatrial septum | Intact | Mitral | ----- |
| **Semilunal valves** |  | Aortic | ---- |
| Aortic valve | Annulus = 11mm | Tricuspid | ---- |
| Pulmonary valve | Annulus = 11mm | pulmonic | Mild PS, PPG = 25mmHg |
| **Great arteries** | NRGA | **Aortic arch** | Left |
| Aorta | ----- | **PDA** | No |
| Pulmonary artery | Normal MPA and Branch PAs. |  |  |
| **M-Mode:** | | | |
| AO | mm | PWd | mm |
| LA | mm | EDV | ml |
| LVIDd | mm | ESV | ml |
| LVIDs | mm | LVEF | 63% |
| IVSd | mm | FS | 32% |
| **Additional Information**: |  | | |
| No pleural/pericardial effusion | | | |
| **Final Diagnosis:** | | | |
| 1. {S, D, S} Levocardia. 2. Mild PS 3. Normal LV Function | | | |
| Remark: | | | |
| SIGNATURE  Done by: Tesfaye T., Pediatric Cardiologist _______________ 07/03/2013Eth.C | | | |

| Patient Name: **Aman Hunachew**. Patient ID: Addis Alem PH. SEX/ Age: M/25days. Date of Report: 07**/03/2013**.  BP: _______ Weight: ______ Height:____________ BSA: ________ | | | |
| --- | --- | --- | --- |
| **Features** | **Finding** | **Features** | **Finding** |
| **Profile** |  | **Atria** |  |
| Abdominal situs | Solitus | Left atrium | Normal |
| Cardiac position | Levocardia | Right atrium | Normal |
| Systemic venous drainage | Normal | **Atrioventricular valves** |  |
| Pulmonary venous drainage | Normal | Mitral valve | Annulus = 9mm |
| Atrioventricular connection | Concordant | Tricuspid valve | Annulus = 10mm |
| Ventriculoarterial connection | Concordant | **Ventricles** |  |
| Ventricular loop | d-Loop | Left ventricle | Normal |
|  |  | Right ventricle | Normal |
| **Septae** |  | **Coronary arteries** | ----- |
| Interventricular septum | Intact | **Doppler Measurement** |  |
| Interatrial septum | Intact | Mitral | ----- |
| **Semilunal valves** |  | Aortic | ---- |
| Aortic valve | Annulus = 8mm | Tricuspid | ---- |
| Pulmonary valve | Annulus = 8mm | pulmonic | ---- |
| **Great arteries** | NRGA | **Aortic arch** | Left |
| Aorta | ----- | **PDA** | No |
| Pulmonary artery | Normal MPA and Branch PAs. |  |  |
| **M-Mode:** Normal LV Function on eye balling. | | | |
| AO | mm | PWd | mm |
| LA | mm | EDV | ml |
| LVIDd | mm | ESV | ml |
| LVIDs | mm | LVEF | % |
| IVSd | mm | FS | % |
| **Additional Information**: |  | | |
| No pleural/pericardial effusion | | | |
| **Final Diagnosis:** | | | |
| 1. Normal Echocardiography Study. | | | |
| Remark: | | | |
| SIGNATURE  Done by: Tesfaye T., Pediatric Cardiologist _______________ 07/03/2013Eth.C | | | |

| Patient Name: **Bereket Getachew**. Patient ID: FHRH. SEX/ Age: M/4 7/12. Date of Report: 07**/03/2013**.  BP: _______ Weight: ______ Height:____________ BSA: ________ | | | |
| --- | --- | --- | --- |
| **Features** | **Finding** | **Features** | **Finding** |
| **Profile** |  | **Atria** |  |
| Abdominal situs | Solitus | Left atrium | Normal |
| Cardiac position | Levocardia | Right atrium | Normal |
| Systemic venous drainage | Normal | **Atrioventricular valves** |  |
| Pulmonary venous drainage | Normal | Mitral valve | Annulus = 15mm |
| Atrioventricular connection | Concordant | Tricuspid valve | Annulus = 16mm  TAPSE = 20mm |
| Ventriculoarterial connection | Concordant | **Ventricles** |  |
| Ventricular loop | d-Loop | Left ventricle | Normal |
|  |  | Right ventricle | Normal |
| **Septae** |  | **Coronary arteries** | ----- |
| Interventricular septum | Intact | **Doppler Measurement** |  |
| Interatrial septum | Intact | Mitral | ----- |
| **Semilunal valves** |  | Aortic | ---- |
| Aortic valve | Annulus = 13mm | Tricuspid | ---- |
| Pulmonary valve | Annulus = 14mm | pulmonic | ---- |
| **Great arteries** | NRGA | **Aortic arch** | Left |
| Aorta | ----- | **PDA** | No |
| Pulmonary artery | Normal MPA and Branch PAs. |  |  |
| **M-Mode:** | | | |
| AO | mm | PWd | mm |
| LA | mm | EDV | ml |
| LVIDd | mm | ESV | ml |
| LVIDs | mm | LVEF | 67% |
| IVSd | mm | FS | 36% |
| **Additional Information**: |  | | |
| No pleural/pericardial effusion | | | |
| **Final Diagnosis:** | | | |
| 1. Normal Echocardiography Study. | | | |
| **Remark**: Rhythm disturbance detected during echocardiography Study. | | | |
| **Recommendation**: **Do ECG test**. | | | |
| SIGNATURE  Done by: Tesfaye T., Pediatric Cardiologist _______________ 07/03/2013Eth.C | | | |

| Patient Name: **Temesgen Jenberu**. Patient ID: FHRH. SEX/ Age: M/3 3/12years. Date of Report: 08**/03/2013**.  BP: _______ Weight: ______ Height:____________ BSA: ________ | | | |
| --- | --- | --- | --- |
| **Features** | **Finding** | **Features** | **Finding** |
| **Profile** |  | **Atria** |  |
| Abdominal situs | Solitus | Left atrium | Normal |
| Cardiac position | Levocardia | Right atrium | Normal |
| Systemic venous drainage | Normal | **Atrioventricular valves** |  |
| Pulmonary venous drainage | Normal | Mitral valve | Annulus = 15mm |
| Atrioventricular connection | Concordant | Tricuspid valve | Annulus = 19mm  TAPSE = 20mm |
| Ventriculoarterial connection | Concordant | **Ventricles** |  |
| Ventricular loop | d-Loop | Left ventricle | Normal |
|  |  | Right ventricle | Normal |
| **Septae** |  | **Coronary arteries** | ----- |
| Interventricular septum | Intact | **Doppler Measurement** |  |
| Interatrial septum | Intact | Mitral | ----- |
| **Semilunal valves** |  | Aortic | ---- |
| Aortic valve | Annulus = 13mm | Tricuspid | ---- |
| Pulmonary valve | Annulus = 14mm | pulmonic | ---- |
| **Great arteries** | NRGA | **Aortic arch** | Left |
| Aorta | ----- | **PDA** | No |
| Pulmonary artery | Normal MPA and Branch PAs. |  |  |
| **M-Mode:** | | | |
| AO | mm | PWd | mm |
| LA | mm | EDV | ml |
| LVIDd | mm | ESV | ml |
| LVIDs | mm | LVEF | 69% |
| IVSd | mm | FS | 37% |
| **Additional Information**: |  | | |
| No pleural/pericardial effusion | | | |
| **Final Diagnosis:** | | | |
| 1. Normal Echocardiography Study. | | | |
| **Remark**: | | | |
| **Recommendation**: | | | |
| SIGNATURE  Done by: Tesfaye T., Pediatric Cardiologist _______________ 08/03/2013Eth.C | | | |

| Patient Name: **Baby of Hibist Tadesse**. Patient ID: TGSH. SEX/ Age: M/3months. Date of Report: 08**/03/2013**.  BP: _______ Weight: ______ Height:____________ BSA: ________ | | | |
| --- | --- | --- | --- |
| **Features** | **Finding** | **Features** | **Finding** |
| **Profile** |  | **Atria** |  |
| Abdominal situs | Solitus | Left atrium | Normal |
| Cardiac position | Levocardia | Right atrium | Normal |
| Systemic venous drainage | Normal | **Atrioventricular valves** |  |
| Pulmonary venous drainage | Normal | Mitral valve | Annulus = 12mm |
| Atrioventricular connection | Concordant | Tricuspid valve | Annulus = 11mm |
| Ventriculoarterial connection | Concordant | **Ventricles** |  |
| Ventricular loop | d-Loop | Left ventricle | Normal |
|  |  | Right ventricle | Normal |
| **Septae** |  | **Coronary arteries** | ----- |
| Interventricular septum | Intact | **Doppler Measurement** |  |
| Interatrial septum | Intact | Mitral | ----- |
| **Semilunal valves** |  | Aortic | ---- |
| Aortic valve | Annulus = 9mm | Tricuspid | ---- |
| Pulmonary valve | Annulus = 10mm | pulmonic | ---- |
| **Great arteries** | NRGA | **Aortic arch** | Left |
| Aorta | ----- | **PDA** | No |
| Pulmonary artery | Normal MPA and Branch PAs. |  |  |
| **M-Mode:** | | | |
| AO | mm | PWd | mm |
| LA | mm | EDV | ml |
| LVIDd | mm | ESV | ml |
| LVIDs | mm | LVEF | 61% |
| IVSd | mm | FS | 30% |
| **Additional Information**: |  | | |
| No pleural/pericardial effusion | | | |
| **Final Diagnosis:** | | | |
| 1. Normal Echocardiography Study. | | | |
| **Remark**: | | | |
| **Recommendation**: | | | |
| SIGNATURE  Done by: Tesfaye T., Pediatric Cardiologist _______________ 08/03/2013Eth.C | | | |

| Patient Name: **Zeleke Fantahun**. Patient ID: FHRH. SEX/ Age: M/10years. Date of Report: 10**/03/2013**.  BP: _______ Weight: ______ Height:____________ BSA: ________ | | | |
| --- | --- | --- | --- |
| **Features** | **Finding** | **Features** | **Finding** |
| **Profile** |  | **Atria** |  |
| Abdominal situs | Solitus | Left atrium | Normal |
| Cardiac position | Levocardia | Right atrium | Normal |
| Systemic venous drainage | Normal | **Atrioventricular valves** |  |
| Pulmonary venous drainage | Normal | Mitral valve | Annulus = 18mm |
| Atrioventricular connection | Concordant | Tricuspid valve | Annulus = 19mm  TAPSE = 21mm |
| Ventriculoarterial connection | Concordant | **Ventricles** |  |
| Ventricular loop | d-Loop | Left ventricle | Normal |
|  |  | Right ventricle | Normal |
| **Septae** |  | **Coronary arteries** | ----- |
| Interventricular septum | Intact | **Doppler Measurement** |  |
| Interatrial septum | Intact | Mitral | ----- |
| **Semilunal valves** |  | Aortic | ---- |
| Aortic valve | Annulus = 15mm | Tricuspid | ---- |
| Pulmonary valve | Annulus = 18mm | pulmonic | Trivial PR, PPG = 12mmHg. |
| **Great arteries** | NRGA | **Aortic arch** | Left |
| Aorta | ----- | **PDA** | No |
| Pulmonary artery | Normal MPA and Branch PAs. | Aortic Isthmus | No CoA |
| **M-Mode:** | | | |
| AO | mm | PWd | mm |
| LA | mm | EDV | ml |
| LVIDd | mm | ESV | ml |
| LVIDs | mm | LVEF | 67% |
| IVSd | mm | FS | 37% |
| **Additional Information**: |  | | |
| No pleural/pericardial effusion | | | |
| **Final Diagnosis:** | | | |
| 1. Normal Echocardiography Study. | | | |
| **Remark**: | | | |
| **Recommendation**: | | | |
| SIGNATURE  Done by: Tesfaye T., Pediatric Cardiologist _______________ 10/03/2013Eth.C | | | |

| Patient Name: **Yezab Gebeyehu**. Patient ID: FHRH. SEX/ Age: F/11years. Date of Report: 10**/03/2013**.  BP: _______ Weight: ______ Height:____________ BSA: ________ | | | |
| --- | --- | --- | --- |
| **Features** | **Finding** | **Features** | **Finding** |
| **Profile** |  | **Atria** |  |
| Abdominal situs | Solitus | Left atrium | Normal |
| Cardiac position | Levocardia | Right atrium | Normal |
| Systemic venous drainage | Normal | **Atrioventricular valves** |  |
| Pulmonary venous drainage | Normal | Mitral valve | Annulus = 16mm |
| Atrioventricular connection | Concordant | Tricuspid valve | Annulus = 19mm  TAPSE = 19mm |
| Ventriculoarterial connection | Concordant | **Ventricles** |  |
| Ventricular loop | d-Loop | Left ventricle | Normal |
|  |  | Right ventricle | Normal |
| **Septae** |  | **Coronary arteries** | ----- |
| Interventricular septum | Intact | **Doppler Measurement** |  |
| Interatrial septum | Intact | Mitral | ----- |
| **Semilunal valves** |  | Aortic | ---- |
| Aortic valve | Annulus = 15mm | Tricuspid | ---- |
| Pulmonary valve | Annulus = 18mm | pulmonic | ---- |
| **Great arteries** | NRGA | **Aortic arch** | Left |
| Aorta | ----- | **PDA** | No |
| Pulmonary artery | Normal MPA and Branch PAs. |  |  |
| **M-Mode:** | | | |
| AO | mm | PWd | mm |
| LA | mm | EDV | ml |
| LVIDd | mm | ESV | ml |
| LVIDs | mm | LVEF | 62% |
| IVSd | mm | FS | 33% |
| **Additional Information**: |  | | |
| No pleural/pericardial effusion | | | |
| **Final Diagnosis:** | | | |
| 1. Normal Echocardiography Study. | | | |
| **Remark**: | | | |
| **Recommendation**: | | | |
| SIGNATURE  Done by: Tesfaye T., Pediatric Cardiologist _______________ 10/03/2013Eth.C | | | |

| Patient Name: **Sale-Amlakie Estibel**. Patient ID: TGSH. SEX/ Age: M/9years. Date of Report: 10**/03/2013**.  BP: _______ Weight: ______ Height:____________ BSA: ________ | | | |
| --- | --- | --- | --- |
| **Features** | **Finding** | **Features** | **Finding** |
| **Profile** |  | **Atria** |  |
| Abdominal situs | Solitus | Left atrium | Normal |
| Cardiac position | Levocardia | Right atrium | Normal |
| Systemic venous drainage | Normal | **Atrioventricular valves** |  |
| Pulmonary venous drainage | Normal | Mitral valve | Annulus = 21mm |
| Atrioventricular connection | Concordant | Tricuspid valve | Annulus = 21mm |
| Ventriculoarterial connection | Concordant | **Ventricles** |  |
| Ventricular loop | d-Loop | Left ventricle | Normal |
|  |  | Right ventricle | Normal |
| **Septae** |  | **Coronary arteries** | ----- |
| Interventricular septum | Intact | **Doppler Measurement** |  |
| Interatrial septum | Intact | Mitral | ----- |
| **Semilunal valves** |  | Aortic | ---- |
| Aortic valve | Annulus = 15mm | Tricuspid | Trivial TR, PPG = 16mmHg. |
| Pulmonary valve | Annulus = 17mm | pulmonic | ---- |
| **Great arteries** | NRGA | **Aortic arch** | Left |
| Aorta | ----- | **PDA** | No |
| Pulmonary artery | Normal MPA and Branch PAs. |  |  |
| **M-Mode:** | | | |
| AO | mm | PWd | mm |
| LA | mm | EDV | ml |
| LVIDd | mm | ESV | ml |
| LVIDs | mm | LVEF | 64% |
| IVSd | mm | FS | 34% |
| **Additional Information**: |  | | |
| No pleural/pericardial effusion | | | |
| **Final Diagnosis:** | | | |
| 1. Normal Echocardiography Study. | | | |
| **Remark**: | | | |
| **Recommendation**: | | | |
| SIGNATURE  Done by: Tesfaye T., Pediatric Cardiologist _______________ 10/03/2013Eth.C | | | |

| Patient Name: **Abenezer Girma**. Patient ID: Adinas GH. SEX/ Age: M/11 10/12 Date of Report: 10**/03/2013**.  BP: _______ Weight: ______ Height:____________ BSA: ________ | | | |
| --- | --- | --- | --- |
| **Features** | **Finding** | **Features** | **Finding** |
| **Profile** |  | **Atria** |  |
| Abdominal situs | Solitus | Left atrium | Normal |
| Cardiac position | Levocardia | Right atrium | Normal |
| Systemic venous drainage | Normal | **Atrioventricular valves** |  |
| Pulmonary venous drainage | Normal | Mitral valve | Annulus = 22mm |
| Atrioventricular connection | Concordant | Tricuspid valve | Annulus = 24mm  TAPSE = 25mm |
| Ventriculoarterial connection | Concordant | **Ventricles** |  |
| Ventricular loop | d-Loop | Left ventricle | Normal |
|  |  | Right ventricle | Normal |
| **Septae** |  | **Coronary arteries** | ----- |
| Interventricular septum | Intact | **Doppler Measurement** |  |
| Interatrial septum | Intact | Mitral | ----- |
| **Semilunal valves** |  | Aortic | ---- |
| Aortic valve | Annulus = 18mm | Tricuspid | ---- |
| Pulmonary valve | Annulus = 18mm | pulmonic | ---- |
| **Great arteries** | NRGA | **Aortic arch** | Left |
| Aorta | ----- | **PDA** | No |
| Pulmonary artery | Normal MPA and Branch PAs. |  |  |
| **M-Mode:** | | | |
| AO | mm | PWd | mm |
| LA | mm | EDV | ml |
| LVIDd | mm | ESV | ml |
| LVIDs | mm | LVEF | 64% |
| IVSd | mm | FS | 35% |
| **Additional Information**: |  | | |
| No pleural/pericardial effusion | | | |
| **Final Diagnosis:** | | | |
| 1. Normal Echocardiography Study. | | | |
| **Remark**: | | | |
| **Recommendation**: | | | |
| SIGNATURE  Done by: Tesfaye T., Pediatric Cardiologist _______________ 10/03/2013Eth.C | | | |

| Patient Name: **Mekibib Me’areg**. Patient ID: FHRH. SEX/ Age: M/1year. Date of Report: 11**/03/2013**.  BP: _______ Weight: ______ Height:____________ BSA: ________ | | | |
| --- | --- | --- | --- |
| **Features** | **Finding** | **Features** | **Finding** |
| **Profile** |  | **Atria** |  |
| Abdominal situs | Solitus | Left atrium | Normal |
| Cardiac position | Levocardia | Right atrium | Normal |
| Systemic venous drainage | Normal | **Atrioventricular valves** |  |
| Pulmonary venous drainage | Normal | Mitral valve | Annulus = 15mm |
| Atrioventricular connection | Concordant | Tricuspid valve | Annulus = 16mm  TAPSE = 17mm |
| Ventriculoarterial connection | Concordant | **Ventricles** |  |
| Ventricular loop | d-Loop | Left ventricle | Normal |
|  |  | Right ventricle | Normal |
| **Septae** |  | **Coronary arteries** | ----- |
| Interventricular septum | 7mm PM VSD partially covered by STL, L – R Shunt with PPG = 50mmHg | **Doppler Measurement** |  |
| Interatrial septum | Intact | Mitral | ----- |
| **Semilunal valves** |  | Aortic | ---- |
| Aortic valve | Annulus = 14mm | Tricuspid | ---- |
| Pulmonary valve | Annulus = 15mm | pulmonic | ---- |
| **Great arteries** | NRGA | **Aortic arch** | Left |
| Aorta | ----- | **PDA** | No |
| Pulmonary artery | Normal MPA and Branch PAs. |  |  |
| **M-Mode:** | | | |
| AO | mm | PWd | mm |
| LA | mm | EDV | ml |
| LVIDd | mm | ESV | ml |
| LVIDs | mm | LVEF | 55% |
| IVSd | mm | FS | 28% |
| **Additional Information**: |  | | |
| No pleural/pericardial effusion | | | |
| **Final Diagnosis:** | | | |
| 1. {S, D, S} Levocardia. 2. Moderate PM VSD, L – R Shunt 3. Normal Biventricular Function | | | |
| **Remark**: | | | |
| **Recommendation**: | | | |
| SIGNATURE  Done by: Tesfaye T., Pediatric Cardiologist _______________ 11/03/2013Eth.C | | | |

| Patient Name: **Habitamu Girma**. Patient ID: FHRH. SEX/ Age: M/7months. Date of Report: 11**/03/2013**.  BP: _______ Weight: ______ Height:____________ BSA: ________ | | | |
| --- | --- | --- | --- |
| **Features** | **Finding** | **Features** | **Finding** |
| **Profile** |  | **Atria** |  |
| Abdominal situs | Solitus | Left atrium | Normal |
| Cardiac position | Levocardia | Right atrium | Dilated |
| Systemic venous drainage | Normal | **Atrioventricular valves** |  |
| Pulmonary venous drainage | Normal | Mitral valve | Annulus = 11mm |
| Atrioventricular connection | Concordant | Tricuspid valve | Annulus = 15mm  TAPSE = 8mm |
| Ventriculoarterial connection | Concordant | **Ventricles** |  |
| Ventricular loop | d-Loop | Left ventricle | Normal. Banana Shaped. |
|  |  | Right ventricle | Dilated, Dysfunctional |
| **Septae** |  | **Coronary arteries** | ----- |
| Interventricular septum | Intact | **Doppler Measurement** |  |
| Interatrial septum | PFO, R – L Shunt | Mitral | ----- |
| **Semilunal valves** |  | Aortic | ---- |
| Aortic valve | Annulus = 11mm | Tricuspid | Moderate PR, PPG = 88mmHg |
| Pulmonary valve | Annulus = 8mm | pulmonic | Severe Valvar PS, PPG = 96mmHg |
| **Great arteries** | NRGA | **Aortic arch** | Left |
| Aorta | ----- | **PDA** | No |
| Pulmonary artery | Normal MPA and Branch PAs. |  |  |
| **M-Mode: Normal LV Function on eye balling.** | | | |
| AO | mm | PWd | mm |
| LA | mm | EDV | ml |
| LVIDd | mm | ESV | ml |
| LVIDs | mm | LVEF | % |
| IVSd | mm | FS | % |
| **Additional Information**: |  | | |
| No pleural/pericardial effusion | | | |
| **Final Diagnosis:** | | | |
| 1. {S, D, S} Levocardia. 2. RA/RV Dilated 3. PFO, R – L Shunt (Restrictive) 4. Severe Valvar PS 5. RV Dilated, Hypertrophied and Dysfunctional | | | |
| **Remark**: Baby has severe hypoxemia with acidotic breathing while echocardiography was being done. | | | |
| **Recommendation**: | | | |
| SIGNATURE  Done by: Tesfaye T., Pediatric Cardiologist _______________ 11/03/2013Eth.C | | | |

| Patient Name: **Medhanit Addis**. Patient ID: Adinas GH. SEX/ Age: M/2/12. Date of Report: 11**/03/2013**.  BP: _______ Weight: ______ Height:____________ BSA: ________ | | | |
| --- | --- | --- | --- |
| **Features** | **Finding** | **Features** | **Finding** |
| **Profile** |  | **Atria** |  |
| Abdominal situs | Solitus | Left atrium | Normal |
| Cardiac position | Levocardia | Right atrium | Markedly Dilated |
| Systemic venous drainage | Normal | **Atrioventricular valves** |  |
| Pulmonary venous drainage | Normal | Mitral valve | Annulus = 8mm |
| Atrioventricular connection | Concordant | Tricuspid valve | Annulus = 15mm  TAPSE = 7mm |
| Ventriculoarterial connection | Concordant | **Ventricles** |  |
| Ventricular loop | d-Loop | Left ventricle | Normal |
|  |  | Right ventricle | Marked Dilated and Dysfunctional |
| **Septae** |  | **Coronary arteries** | ----- |
| Interventricular septum | Intact | **Doppler Measurement** |  |
| Interatrial septum | 15mm Primmum Defect, BD Shunt | Mitral | ----- |
| **Semilunal valves** |  | Aortic | ---- |
| Aortic valve | Annulus = mm | Tricuspid | Severe TR, PPG = 80mmHg. |
| Pulmonary valve | Annulus = 9mm | pulmonic | Moderate PR |
| **Great arteries** | NRGA | **Aortic arch** | Left |
| Aorta | ----- | **PDA** | No |
| Pulmonary artery | MPA = 13mm. Confluent Branch PAs. |  |  |
| **M-Mode:** | | | |
| AO | mm | PWd | mm |
| LA | mm | EDV | ml |
| LVIDd | mm | ESV | ml |
| LVIDs | mm | LVEF | % |
| IVSd | mm | FS | % |
| **Additional Information**: |  | | |
| No pleural/pericardial effusion | | | |
| **Final Diagnosis:** | | | |
| 1. {S, D, S} Levocardia. 2. RA/RV Dilated 3. Partial AVSD, BD Shunt 4. Severe TR 5. Moderate PR 6. RV Dilated and Dysfunctional | | | |
| **Remark**: | | | |
| **Recommendation**: | | | |
| SIGNATURE  Done by: Tesfaye T., Pediatric Cardiologist _______________ 11/03/2013Eth.C | | | |

| Patient Name: **TEMESGEN wUBYE**. Patient ID: Adinas GH. SEX/ Age: M/12years. Date of Report: 11**/03/2013**.  BP: _______ Weight: ______ Height:____________ BSA: ________ | | | |
| --- | --- | --- | --- |
| **Features** | **Finding** | **Features** | **Finding** |
| **Profile** |  | **Atria** |  |
| Abdominal situs | Solitus | Left atrium | Normal |
| Cardiac position | Levocardia | Right atrium | Normal |
| Systemic venous drainage | Normal | **Atrioventricular valves** |  |
| Pulmonary venous drainage | Normal | Mitral valve | Annulus = 19mm |
| Atrioventricular connection | Concordant | Tricuspid valve | Annulus = 21mm |
| Ventriculoarterial connection | Concordant | **Ventricles** |  |
| Ventricular loop | d-Loop | Left ventricle | Normal |
|  |  | Right ventricle | Normal |
| **Septae** |  | **Coronary arteries** | ----- |
| Interventricular septum | Intact | **Doppler Measurement** |  |
| Interatrial septum | Intact | Mitral | ----- |
| **Semilunal valves** |  | Aortic | ---- |
| Aortic valve | Annulus = 18mm | Tricuspid | ---- |
| Pulmonary valve | Annulus = 19mm | pulmonic | ---- |
| **Great arteries** | NRGA | **Aortic arch** | Left |
| Aorta | ----- | **PDA** | No |
| Pulmonary artery | Normal MPA and Branch PAs. |  |  |
| **M-Mode:** | | | |
| AO | mm | PWd | mm |
| LA | mm | EDV | ml |
| LVIDd | mm | ESV | ml |
| LVIDs | mm | LVEF | 69% |
| IVSd | mm | FS | 38% |
| **Additional Information**: |  | | |
| No pleural/pericardial effusion | | | |
| **Final Diagnosis:** | | | |
| 1. Normal Echocardiography Study. | | | |
| **Remark**: | | | |
| **Recommendation**: | | | |
| SIGNATURE  Done by: Tesfaye T., Pediatric Cardiologist _______________ 11/03/2013Eth.C | | | |

| Patient Name: **Tiruwerk Kefale**. Patient ID: FHRH. SEX/ Age: F/8months. Date of Report: 14**/03/2013**.  BP: _______ Weight: ______ Height:____________ BSA: ________ | | | |
| --- | --- | --- | --- |
| **Follow up Echocardiography for Small PDA.** | | | |
| **Features** | **Finding** | **Features** | **Finding** |
| **Profile** |  | **Atria** |  |
| Abdominal situs | Solitus | Left atrium | Normal |
| Cardiac position | Levocardia | Right atrium | Normal |
| Systemic venous drainage | Normal | **Atrioventricular valves** |  |
| Pulmonary venous drainage | Normal | Mitral valve | Annulus = 12mm |
| Atrioventricular connection | Concordant | Tricuspid valve | Annulus = 13mm |
| Ventriculoarterial connection | Concordant | **Ventricles** |  |
| Ventricular loop | d-Loop | Left ventricle | Normal |
|  |  | Right ventricle | Normal |
| **Septae** |  | **Coronary arteries** | ----- |
| Interventricular septum | Intact | **Doppler Measurement** |  |
| Interatrial septum | Intact | Mitral | ----- |
| **Semilunal valves** |  | Aortic | ---- |
| Aortic valve | Annulus = 11mm | Tricuspid | ---- |
| Pulmonary valve | Annulus = 12mm | pulmonic | ---- |
| **Great arteries** | NRGA | **Aortic arch** | Left |
| Aorta | ----- | **PDA** | No |
| Pulmonary artery | Normal MPA and Branch PAs. |  |  |
| **M-Mode:** | | | |
| AO | mm | PWd | mm |
| LA | mm | EDV | ml |
| LVIDd | mm | ESV | ml |
| LVIDs | mm | LVEF | 59% |
| IVSd | mm | FS | 29% |
| **Additional Information**: |  | | |
| No pleural/pericardial effusion | | | |
| **Final Diagnosis:** | | | |
| 1. {S, D, S} Levocardia. 2. Normal Echocardiography Study (Closed small VSD and PDA) | | | |
| **Remark**: both the small VSD (<3mm and PDA) are Closed. | | | |
| **Recommendation**: No More echocardiography Study is recommended. | | | |
| SIGNATURE  Done by: Tesfaye T., Pediatric Cardiologist _______________ 14/03/2013Eth.C | | | |

| Patient Name: **Baby of Ethiopia Mengist**. Patient ID: FHRH. SEX/ Age: M/3days. Date of Report: 14**/03/2013**.  BP: _______ Weight: ______ Height:____________ BSA: ________ | | | |
| --- | --- | --- | --- |
| **Features** | **Finding** | **Features** | **Finding** |
| **Profile** |  | **Atria** |  |
| Abdominal situs | Solitus | Left atrium | Normal |
| Cardiac position | Levocardia | Right atrium | Normal |
| Systemic venous drainage | Normal | **Atrioventricular valves** |  |
| Pulmonary venous drainage | Normal | Mitral valve | Annulus = 8mm |
| Atrioventricular connection | Concordant | Tricuspid valve | Annulus = 10mm |
| Ventriculoarterial connection | Concordant | **Ventricles** |  |
| Ventricular loop | d-Loop | Left ventricle | Normal |
|  |  | Right ventricle | Normal |
| **Septae** |  | **Coronary arteries** | ----- |
| Interventricular septum | Intact | **Doppler Measurement** |  |
| Interatrial septum | PFO, L – R Shunt | Mitral | ----- |
| **Semilunal valves** |  | Aortic | ---- |
| Aortic valve | Annulus = 9mm | Tricuspid | ---- |
| Pulmonary valve | Annulus = 9mm | pulmonic | ---- |
| **Great arteries** | NRGA | **Aortic arch** | Left |
| Aorta | ----- | **PDA** | 1.5mm PDA, L – R Shunt |
| Pulmonary artery | Normal MPA and Branch PAs. |  |  |
| **M-Mode:** Normal LV Function on eye balling. | | | |
| AO | mm | PWd | mm |
| LA | mm | EDV | ml |
| LVIDd | mm | ESV | ml |
| LVIDs | mm | LVEF | % |
| IVSd | mm | FS | % |
| **Additional Information**: |  | | |
| No pleural/pericardial effusion | | | |
| **Final Diagnosis:** | | | |
| 1. {S, D, S} Levocardia. 2. PFO, L – R Shunt 3. Moderate PDA, L – R Shunt 4. Normal LV Function | | | |
| **Remark**: | | | |
| **Recommendation**: | | | |
| SIGNATURE  Done by: Tesfaye T., Pediatric Cardiologist _______________ 14/03/2013Eth.C | | | |

| Patient Name: **Baby of Birtukan Tenagne**. Patient ID: TGSH. SEX/ Age: M/4months. Date of Report: 14**/03/2013**.  BP: _______ Weight: ______ Height:____________ BSA: ________ | | | |
| --- | --- | --- | --- |
| **Features** | **Finding** | **Features** | **Finding** |
| **Profile** |  | **Atria** |  |
| Abdominal situs | Solitus | Left atrium | Normal |
| Cardiac position | Levocardia | Right atrium | Normal |
| Systemic venous drainage | Normal | **Atrioventricular valves** |  |
| Pulmonary venous drainage | Normal | Mitral valve | Annulus = 11mm |
| Atrioventricular connection | Concordant | Tricuspid valve | Annulus = 11mm |
| Ventriculoarterial connection | Concordant | **Ventricles** |  |
| Ventricular loop | d-Loop | Left ventricle | Normal |
|  |  | Right ventricle | Normal |
| **Septae** |  | **Coronary arteries** | ----- |
| Interventricular septum | Intact | **Doppler Measurement** |  |
| Interatrial septum | Intact | Mitral | ----- |
| **Semilunal valves** |  | Aortic | ---- |
| Aortic valve | Annulus = 9mm | Tricuspid | ---- |
| Pulmonary valve | Annulus = 9mm | pulmonic | ---- |
| **Great arteries** | NRGA | **Aortic arch** | Left |
| Aorta | ----- | **PDA** | No |
| Pulmonary artery | Normal MPA and Branch PAs. |  |  |
| **M-Mode:** Normal LV Function on eye balling. | | | |
| AO | mm | PWd | mm |
| LA | mm | EDV | ml |
| LVIDd | mm | ESV | ml |
| LVIDs | mm | LVEF | % |
| IVSd | mm | FS | % |
| **Additional Information**: |  | | |
| No pleural/pericardial effusion | | | |
| **Final Diagnosis:** | | | |
| 1. Normal Echocardiography Study. | | | |
| **Remark**: | | | |
| **Recommendation**: | | | |
| SIGNATURE  Done by: Tesfaye T., Pediatric Cardiologist _______________ 14/03/2013Eth.C | | | |

| Patient Name: **Biruk Melkie**. Patient ID: Enjibara GH. SEX/ Age: M/7months. Date of Report: 15**/03/2013**.  BP: _______ Weight: ______ Height:____________ BSA: ________ | | | |
| --- | --- | --- | --- |
| **Features** | **Finding** | **Features** | **Finding** |
| **Profile** |  | **Atria** |  |
| Abdominal situs | Solitus | Left atrium | Dilated |
| Cardiac position | Levocardia | Right atrium | More Dilated |
| Systemic venous drainage | Normal | **Atrioventricular valves** |  |
| Pulmonary venous drainage | Normal | Mitral valve | Common Complete AVSD |
| Atrioventricular connection | Concordant | Tricuspid valve | Common Complete AVSD  TAPSE = 18mm |
| Ventriculoarterial connection | Concordant | **Ventricles** |  |
| Ventricular loop | d-Loop | Left ventricle | Dilated |
|  |  | Right ventricle | More Dilated |
| **Septae** |  | **Coronary arteries** | ----- |
| Interventricular septum | Common Complete AVSD | **Doppler Measurement** |  |
| Interatrial septum | Common Complete AVSD | Mitral | Moderate Left AVVR |
| **Semilunal valves** |  | Aortic | ---- |
| Aortic valve | Annulus = 11mm | Tricuspid | Moderate Right AVVR |
| Pulmonary valve | Annulus = 15mm | pulmonic | ---- |
| **Great arteries** | NRGA | **Aortic arch** | Left |
| Aorta | ----- | **PDA** | No |
| Pulmonary artery | MPA dilated and Confluent Branch PAs. |  |  |
| **M-Mode:** Normal LV Function on eye balling. | | | |
| AO | mm | PWd | mm |
| LA | mm | EDV | ml |
| LVIDd | mm | ESV | ml |
| LVIDs | mm | LVEF | % |
| IVSd | mm | FS | % |
| **Additional Information**: |  | | |
| No pleural/pericardial effusion | | | |
| **Final Diagnosis:** | | | |
| 1. {S, D, S} Levocardia. 2. All Chambers Dilated 3. Moderate Right AVVR 4. Moderate Left AVVR 5. Common Complete Balanced AVSD, L – R Shunt | | | |
| **Remark**: | | | |
| **Recommendation**: | | | |
| SIGNATURE  Done by: Tesfaye T., Pediatric Cardiologist _______________ 15/03/2013Eth.C | | | |

| Patient Name: **Ermias Abebe**. Patient ID: SBCH. SEX/ Age: M/1 4/12. Date of Report: 15**/03/2013**.  BP: _______ Weight: ______ Height:____________ BSA: ________ | | | |
| --- | --- | --- | --- |
| **Features** | **Finding** | **Features** | **Finding** |
| **Profile** |  | **Atria** |  |
| Abdominal situs | Solitus | Left atrium | Mildly dilated |
| Cardiac position | Levocardia | Right atrium | Normal |
| Systemic venous drainage | Normal | **Atrioventricular valves** |  |
| Pulmonary venous drainage | Normal | Mitral valve | Annulus = 14mm |
| Atrioventricular connection | Concordant | Tricuspid valve | Annulus = 15mm |
| Ventriculoarterial connection | Concordant | **Ventricles** |  |
| Ventricular loop | d-Loop | Left ventricle | Mildly dilated |
|  |  | Right ventricle | Normal |
| **Septae** |  | **Coronary arteries** | ----- |
| Interventricular septum | Intact | **Doppler Measurement** |  |
| Interatrial septum | Intact | Mitral | Trivial MR |
| **Semilunal valves** |  | Aortic | ---- |
| Aortic valve | Annulus = 12mm | Tricuspid | ---- |
| Pulmonary valve | Annulus = 13mm | pulmonic | ---- |
| **Great arteries** | NRGA | **Aortic arch** | Left |
| Aorta | ----- | **PDA** | 2mm PDA, L – R Shunt. |
| Pulmonary artery | Normal MPA and Branch PAs. |  |  |
| **M-Mode:** Normal LV Function on eye balling. | | | |
| AO | mm | PWd | mm |
| LA | mm | EDV | ml |
| LVIDd | mm | ESV | ml |
| LVIDs | mm | LVEF | % |
| IVSd | mm | FS | % |
| **Additional Information**: |  | | |
| No pleural/pericardial effusion | | | |
| **Final Diagnosis:** | | | |
| 1. {S, D, S} Levocardia. 2. Mildly dilated LA/LV 3. Moderate PDA, L – R Shunt 4. Normal LV Function | | | |
| **Remark**: | | | |
| **Recommendation**: | | | |
| SIGNATURE  Done by: Tesfaye T., Pediatric Cardiologist _______________ 15/03/2013Eth.C | | | |

| Patient Name: **Mengistu Tazebew**. Patient ID: TGSH. SEX/ Age: M/14years. Date of Report: 15**/03/2013**.  BP: _______ Weight: ______ Height:____________ BSA: ________ | | | |
| --- | --- | --- | --- |
| **Features** | **Finding** | **Features** | **Finding** |
| **Profile** |  | **Atria** |  |
| Abdominal situs | Solitus | Left atrium | Normal |
| Cardiac position | Levocardia | Right atrium | Normal |
| Systemic venous drainage | Normal | **Atrioventricular valves** |  |
| Pulmonary venous drainage | Normal | Mitral valve | Annulus = 17mm |
| Atrioventricular connection | Concordant | Tricuspid valve | Annulus = 20mm |
| Ventriculoarterial connection | Concordant | **Ventricles** |  |
| Ventricular loop | d-Loop | Left ventricle | Normal |
|  |  | Right ventricle | Normal |
| **Septae** |  | **Coronary arteries** | ----- |
| Interventricular septum | Intact | **Doppler Measurement** |  |
| Interatrial septum | Intact | Mitral | ----- |
| **Semilunal valves** |  | Aortic | ---- |
| Aortic valve | Annulus = 15mm | Tricuspid | ---- |
| Pulmonary valve | Annulus = 16mm | pulmonic | ---- |
| **Great arteries** | NRGA | **Aortic arch** | Left |
| Aorta | ----- | **PDA** | No |
| Pulmonary artery | Normal MPA and Branch PAs. |  |  |
| **M-Mode:** | | | |
| AO | mm | PWd | 6.8mm |
| LA | mm | EDV | 29ml |
| LVIDd | 27.8mm | ESV | 9ml |
| LVIDs | 17.6mm | LVEF | 68% |
| IVSd | 6mm | FS | 36% |
| **Additional Information**: |  | | |
| No pleural/pericardial effusion | | | |
| **Final Diagnosis:** | | | |
| 1. Normal Echocardiography Study. | | | |
| **Remark**: | | | |
| **Recommendation**: | | | |
| SIGNATURE  Done by: Tesfaye T., Pediatric Cardiologist _______________ 15/03/2013Eth.C | | | |

| Patient Name: **Samrawit Fantahun**. Patient ID: Afilas GH. SEX/ Age: F/15years. Date of Report: 15**/03/2013**.  BP: _______ Weight: ______ Height:____________ BSA: ________ | | | |
| --- | --- | --- | --- |
| **Features** | **Finding** | **Features** | **Finding** |
| **Profile** |  | **Atria** |  |
| Abdominal situs | Solitus | Left atrium | Normal |
| Cardiac position | Levocardia | Right atrium | Normal |
| Systemic venous drainage | Normal | **Atrioventricular valves** |  |
| Pulmonary venous drainage | Normal | Mitral valve | Annulus = 19mm |
| Atrioventricular connection | Concordant | Tricuspid valve | Annulus = 20mm |
| Ventriculoarterial connection | Concordant | **Ventricles** |  |
| Ventricular loop | d-Loop | Left ventricle | Normal |
|  |  | Right ventricle | Normal |
| **Septae** |  | **Coronary arteries** | ----- |
| Interventricular septum | Intact | **Doppler Measurement** |  |
| Interatrial septum | Intact | Mitral | ----- |
| **Semilunal valves** |  | Aortic | ---- |
| Aortic valve | Annulus = 17mm | Tricuspid | ---- |
| Pulmonary valve | Annulus = 21mm | pulmonic | ---- |
| **Great arteries** | NRGA | **Aortic arch** | Left |
| Aorta | ----- | **PDA** | No |
| Pulmonary artery | Normal MPA and Branch PAs. |  |  |
| **M-Mode:** | | | |
| AO | mm | PWd | 6mm |
| LA | mm | EDV | 89ml |
| LVIDd | 44mm | ESV | 25ml |
| LVIDs | 26mm | LVEF | 71% |
| IVSd | 7mm | FS | 40% |
| **Additional Information**: |  | | |
| No pleural/pericardial effusion | | | |
| **Final Diagnosis:** | | | |
| 1. Normal Echocardiography Study. | | | |
| **Remark**: | | | |
| **Recommendation**: | | | |
| SIGNATURE  Done by: Tesfaye T., Pediatric Cardiologist _______________ 15/03/2013Eth.C | | | |

| Patient Name: **Mengistu Tesfa**. Patient ID: FHRH. SEX/ Age: M/9years. Date of Report: 16**/03/2013**.  BP: _______ Weight: ______ Height:____________ BSA: ________ | | | |
| --- | --- | --- | --- |
| **Features** | **Finding** | **Features** | **Finding** |
| **Profile** |  | **Atria** |  |
| Abdominal situs | Solitus | Left atrium | Normal |
| Cardiac position | Levocardia | Right atrium | Markedly dilated |
| Systemic venous drainage | Normal | **Atrioventricular valves** |  |
| Pulmonary venous drainage | Normal | Mitral valve | Annulus = 20mm |
| Atrioventricular connection | Concordant | Tricuspid valve | Annulus = 35mm. non coapting TVL.  TAPSE = 22mm |
| Ventriculoarterial connection | Concordant | **Ventricles** |  |
| Ventricular loop | d-Loop | Left ventricle | Normal |
|  |  | Right ventricle | Markedly dilated |
| **Septae** |  | **Coronary arteries** | ----- |
| Interventricular septum | 7mm PM VSD, L – R Shunt with PPG = 85mmHg. Partially covered by STL. | **Doppler Measurement** |  |
| Interatrial septum | Intact | Mitral | ----- |
| **Semilunal valves** |  | Aortic | ---- |
| Aortic valve | Annulus = 19mm | Tricuspid | Severe TR, PPG = 19mmHg |
| Pulmonary valve | Annulus = 22mm | pulmonic | Trivial PR, PPG = 19mmHg |
| **Great arteries** | NRGA | **Aortic arch** | Left |
| Aorta | ----- | **PDA** | No |
| Pulmonary artery | Normal MPA and Branch PAs. |  |  |
| **M-Mode:** | | | |
| AO | mm | PWd | mm |
| LA | mm | EDV | ml |
| LVIDd | mm | ESV | ml |
| LVIDs | mm | LVEF | % |
| IVSd | mm | FS | % |
| **Additional Information**: |  | | |
| No pleural/pericardial effusion | | | |
| **Final Diagnosis:** | | | |
| 1. {S, D, S} Levocardia. 2. RA/RV Dilated 3. Severe TR 4. Non Coapting TVL 5. Small Restrictive PM VSD Partially covered by STL | | | |
| **Remark**: The dilated RA/RV cannot be explained by the PM VSD. | | | |
| **Recommendation**: | | | |
| SIGNATURE  Done by: Tesfaye T., Pediatrician, Pediatric Cardiologist _______________ 16/03/2013Eth.C | | | |

| Patient Name: **Atirsaw Birhanu**. Patient ID: FHRH. SEX/ Age: M/2/12. Date of Report: 16**/03/2013**.  BP: _______ Weight: ______ Height:____________ BSA: ________ | | | |
| --- | --- | --- | --- |
| **Features** | **Finding** | **Features** | **Finding** |
| **Profile** |  | **Atria** |  |
| Abdominal situs | Solitus | Left atrium | Normal |
| Cardiac position | Levocardia | Right atrium | Dilated |
| Systemic venous drainage | Normal | **Atrioventricular valves** |  |
| Pulmonary venous drainage | Normal | Mitral valve | Annulus = 10mm |
| Atrioventricular connection | Concordant | Tricuspid valve | Annulus = 16mm |
| Ventriculoarterial connection | Discordant | **Ventricles** |  |
| Ventricular loop | d-Loop | Left ventricle | Normal |
|  |  | Right ventricle | Dilated |
| **Septae** |  | **Coronary arteries** | ----- |
| Interventricular septum | 12mm Large Inlet VSD with Muscular Extension | **Doppler Measurement** |  |
| Interatrial septum | PFO, L – R Shunt | Mitral | ----- |
| **Semilunal valves** |  | Aortic | ---- |
| Aortic valve | Annulus = 9mm | Tricuspid | ---- |
| Pulmonary valve | Annulus = 11mm | pulmonic | Mild LVOTO, PPG = 25mmHg. |
| **Great arteries** | d-TGA | **Aortic arch** | Left |
| Aorta | Anterior and to the right | **PDA** | No |
| Pulmonary artery | Posterior and to the left |  |  |
| **M-Mode:** | | | |
| AO | mm | PWd | mm |
| LA | mm | EDV | ml |
| LVIDd | mm | ESV | ml |
| LVIDs | mm | LVEF | % |
| IVSd | mm | FS | % |
| **Additional Information**: |  | | |
| No pleural/pericardial effusion | | | |
| **Final Diagnosis:** | | | |
| 1. {S, D, S} Levocardia. 2. d-TGA 3. PFO, L – R Shunt 4. Large Inlet VSD with Muscular Extension 5. Mild LVOTO (PS) | | | |
| **Remark**: | | | |
| **Recommendation**: | | | |
| SIGNATURE  Done by: Tesfaye T., Pediatrician, Pediatric Cardiologist _______________ 16/03/2013Eth.C | | | |

| Patient Name: **Abduselam Yusuf**. Patient ID: Enjibara GH. SEX/ Age: M/2months. Date of Report: 18**/03/2013**.  BP: _______ Weight: ______ Height:____________ BSA: ________ | | | |
| --- | --- | --- | --- |
| **Features** | **Finding** | **Features** | **Finding** |
| **Profile** |  | **Atria** |  |
| Abdominal situs | Solitus | Left atrium | Normal |
| Cardiac position | Levocardia | Right atrium | Normal |
| Systemic venous drainage | Normal | **Atrioventricular valves** |  |
| Pulmonary venous drainage | Normal | Mitral valve | Annulus = 11mm |
| Atrioventricular connection | Concordant | Tricuspid valve | Annulus = 12mm |
| Ventriculoarterial connection | Concordant | **Ventricles** |  |
| Ventricular loop | d-Loop | Left ventricle | Normal |
|  |  | Right ventricle | Normal |
| **Septae** |  | **Coronary arteries** | ----- |
| Interventricular septum | 4mm PM VSD, L – R Shunt with PPG = 48mmHg | **Doppler Measurement** |  |
| Interatrial septum | Intact | Mitral | ----- |
| **Semilunal valves** |  | Aortic | ---- |
| Aortic valve | Annulus = 10mm | Tricuspid | ---- |
| Pulmonary valve | Annulus = 10mm | pulmonic | ---- |
| **Great arteries** | NRGA | **Aortic arch** | Left |
| Aorta | ----- | **PDA** | No |
| Pulmonary artery | Normal MPA and Branch PAs. |  |  |
| **M-Mode:** Normal LV Function on eye balling. | | | |
| AO | mm | PWd | mm |
| LA | mm | EDV | ml |
| LVIDd | mm | ESV | ml |
| LVIDs | mm | LVEF | % |
| IVSd | mm | FS | % |
| **Additional Information**: |  | | |
| No pleural/pericardial effusion | | | |
| **Final Diagnosis:** | | | |
| 1. {S, D, S} Levocardia. 2. Small PM VSD, L – R Shunt 3. Normal LV Function | | | |
| **Remark**: | | | |
| **Recommendation**: | | | |
| SIGNATURE  Done by: Tesfaye T., Pediatrician, Pediatric Cardiologist _______________ 18/03/2013Eth.C | | | |

| Patient Name: **Adane Mihret**. Patient ID: FHRH. SEX/ Age: M/2months. Date of Report: 18**/03/2013**.  BP: _______ Weight: ______ Height:____________ BSA: ________ | | | |
| --- | --- | --- | --- |
| **Features** | **Finding** | **Features** | **Finding** |
| **Profile** |  | **Atria** |  |
| Abdominal situs | Solitus | Left atrium | Normal |
| Cardiac position | Levocardia | Right atrium | Normal |
| Systemic venous drainage | Normal | **Atrioventricular valves** |  |
| Pulmonary venous drainage | Normal | Mitral valve | Annulus = 10mm |
| Atrioventricular connection | Concordant | Tricuspid valve | Annulus = 11mm |
| Ventriculoarterial connection | Concordant | **Ventricles** |  |
| Ventricular loop | d-Loop | Left ventricle | Normal |
|  |  | Right ventricle | Normal |
| **Septae** |  | **Coronary arteries** | ----- |
| Interventricular septum | Intact | **Doppler Measurement** |  |
| Interatrial septum | 4mm High Secundum ASD, L – R Shunt | Mitral | ----- |
| **Semilunal valves** |  | Aortic | ---- |
| Aortic valve | Annulus = 9mm | Tricuspid | ---- |
| Pulmonary valve | Annulus = 9mm | pulmonic | ---- |
| **Great arteries** | NRGA | **Aortic arch** | Left |
| Aorta | ----- | **PDA** | No |
| Pulmonary artery | Normal MPA and Branch PAs. |  |  |
| **M-Mode:** Normal LV Function on eye balling. | | | |
| AO | mm | PWd | mm |
| LA | mm | EDV | ml |
| LVIDd | mm | ESV | ml |
| LVIDs | mm | LVEF | % |
| IVSd | mm | FS | % |
| **Additional Information**: |  | | |
| No pleural/pericardial effusion | | | |
| **Final Diagnosis:** | | | |
| 1. {S, D, S} Levocardia. 2. Small High Secundum ASD, L – R Shunt 3. Normal LV Function | | | |
| **Remark**: | | | |
| **Recommendation**: | | | |
| SIGNATURE  Done by: Tesfaye T., Pediatrician, Pediatric Cardiologist _______________ 18/03/2013Eth.C | | | |

| Patient Name: **Lisane – werk Melisie**. Patient ID: FHRH. SEX/ Age: F/9months. Date of Report: 18**/03/2013**.  BP: _______ Weight: ______ Height:____________ BSA: ________ | | | |
| --- | --- | --- | --- |
| **Features** | **Finding** | **Features** | **Finding** |
| **Profile** |  | **Atria** |  |
| Abdominal situs | Solitus | Left atrium | Normal |
| Cardiac position | Levocardia | Right atrium | Normal |
| Systemic venous drainage | Normal | **Atrioventricular valves** |  |
| Pulmonary venous drainage | Normal | Mitral valve | Annulus = 11mm |
| Atrioventricular connection | Concordant | Tricuspid valve | Annulus = 13mm |
| Ventriculoarterial connection | Concordant | **Ventricles** |  |
| Ventricular loop | d-Loop | Left ventricle | Normal |
|  |  | Right ventricle | Normal |
| **Septae** |  | **Coronary arteries** | ----- |
| Interventricular septum | Intact | **Doppler Measurement** |  |
| Interatrial septum | Intact | Mitral | ----- |
| **Semilunal valves** |  | Aortic | ---- |
| Aortic valve | Annulus = 12mm | Tricuspid | Trivial TR, PPG = 19mmHg. |
| Pulmonary valve | Annulus = 13mm | pulmonic | ---- |
| **Great arteries** | NRGA | **Aortic arch** | Left |
| Aorta | ----- | **PDA** | No |
| Pulmonary artery | Normal MPA and Branch PAs. |  |  |
| **M-Mode:** | | | |
| AO | mm | PWd | mm |
| LA | mm | EDV | ml |
| LVIDd | mm | ESV | ml |
| LVIDs | mm | LVEF | % |
| IVSd | mm | FS | % |
| **Additional Information**: |  | | |
| No pleural/pericardial effusion | | | |
| **Final Diagnosis:** | | | |
| 1. Normal Echocardiography Study. | | | |
| **Remark**: | | | |
| **Recommendation**: | | | |
| SIGNATURE  Done by: Tesfaye T., Pediatrician, Pediatric Cardiologist _______________ 18/03/2013Eth.C | | | |

| Patient Name: **Haset Mulualem**. Patient ID: Adinas GH. SEX/ Age: F/6/12. Date of Report: 19**/03/2013**.  BP: _______ Weight: ______ Height:____________ BSA: ________ | | | |
| --- | --- | --- | --- |
| **Features** | **Finding** | **Features** | **Finding** |
| **Profile** |  | **Atria** |  |
| Abdominal situs | Solitus | Left atrium | Normal |
| Cardiac position | Levocardia | Right atrium | Normal |
| Systemic venous drainage | Normal | **Atrioventricular valves** |  |
| Pulmonary venous drainage | Normal | Mitral valve | Annulus = 13mm |
| Atrioventricular connection | Concordant | Tricuspid valve | Annulus = 14mm |
| Ventriculoarterial connection | Concordant | **Ventricles** |  |
| Ventricular loop | d-Loop | Left ventricle | Normal |
|  |  | Right ventricle | Mildly Hypertrophied |
| **Septae** |  | **Coronary arteries** | ----- |
| Interventricular septum | 8mm Subaortic Mal-aligned VSD, BD Shunt. | **Doppler Measurement** |  |
| Interatrial septum | Intact | Mitral | ----- |
| **Semilunal valves** |  | Aortic | ---- |
| Aortic valve | Annulus = 12mm | Tricuspid | ---- |
| Pulmonary valve | Annulus = 7mm. Thickened Valves | pulmonic | Severe Valvar & sub – valvar PS, PPG = 65mmHg. Mild PR, PPG = 27mmHg |
| **Great arteries** | NRGA | **Aortic arch** | Left |
| Aorta | Overriding aorta | **PDA** | No |
| Pulmonary artery | Normal MPA and Branch PAs. |  |  |
| **M-Mode: Normal LV Function on eye balling.** | | | |
| AO | mm | PWd | mm |
| LA | mm | EDV | ml |
| LVIDd | mm | ESV | ml |
| LVIDs | mm | LVEF | % |
| IVSd | mm | FS | % |
| **Additional Information**: |  | | |
| No pleural/pericardial effusion | | | |
| **Final Diagnosis:** | | | |
| 1. {S, D, S} Levocardia. 2. TOF | | | |
| **Remark**: | | | |
| **Recommendation**: | | | |
| SIGNATURE  Done by: Tesfaye T., Pediatrician, Pediatric Cardiologist _______________ 19/03/2013Eth.C | | | |

| Patient Name: **Daniel Habitamu**. Patient ID: Adinas GH. SEX/ Age: M/12years. Date of Report: 22**/03/2013**.  BP: _______ Weight: ______ Height:____________ BSA: ________ | | | |
| --- | --- | --- | --- |
| **Features** | **Finding** | **Features** | **Finding** |
| **Profile** |  | **Atria** |  |
| Abdo32minal situs | Solitus | Left atrium | Normal |
| Cardiac position | Levocardia | Right atrium | Normal |
| Systemic venous drainage | Normal | **Atrioventricular valves** |  |
| Pulmonary venous drainage | Normal | Mitral valve | Annulus = 21mm. Mildly thickened MVL |
| Atrioventricular connection | Concordant | Tricuspid valve | Annulus = 24mm |
| Ventriculoarterial connection | Concordant | **Ventricles** |  |
| Ventricular loop | d-Loop | Left ventricle | Normal |
|  |  | Right ventricle | Normal |
| **Septae** |  | **Coronary arteries** | ----- |
| Interventricular septum | Intact | **Doppler Measurement** |  |
| Interatrial septum | Intact | Mitral | Mild MR, Holosystolic, posterior projection, seen in two planes with jet velocity = 4m/sec. |
| **Semilunal valves** |  | Aortic | Mild AR, PHT = 620ms. |
| Aortic valve | Annulus = 18mm | Tricuspid | ---- |
| Pulmonary valve | Annulus = 22mm | pulmonic | ---- |
| **Great arteries** | NRGA | **Aortic arch** | Left |
| Aorta | ----- | **PDA** | No |
| Pulmonary artery | Normal MPA and Branch PAs. |  |  |
| **M-Mode:** | | | |
| AO | mm | PWd | mm |
| LA | mm | EDV | ml |
| LVIDd | mm | ESV | ml |
| LVIDs | mm | LVEF | 69% |
| IVSd | mm | FS | 38% |
| **Additional Information**: |  | | |
| No pleural/pericardial effusion | | | |
| **Final Diagnosis:** | | | |
| 1. {S, D, S} Levocardia. 2. Mildly thickened MVL 3. Mild MR 4. Mild AR 5. Normal LV Function | | | |
| **Remark**: | | | |
| **Recommendation**: | | | |
| SIGNATURE  Done by: Tesfaye T., Pediatrician, Pediatric Cardiologist _______________ 22/03/2013Eth.C | | | |

| Patient Name: **Kassaye Yagegnal**. Patient ID: FHRH. SEX/ Age: F/7months. Date of Report: 22**/03/2013**.  BP: _______ Weight: ______ Height:____________ BSA: ________ | | | |
| --- | --- | --- | --- |
| **Features** | **Finding** | **Features** | **Finding** |
| **Profile** |  | **Atria** |  |
| Abdominal situs | Solitus | Left atrium | Normal |
| Cardiac position | Levocardia | Right atrium | Normal |
| Systemic venous drainage | Normal | **Atrioventricular valves** |  |
| Pulmonary venous drainage | Normal | Mitral valve | Annulus = 10mm |
| Atrioventricular connection | Concordant | Tricuspid valve | Annulus = 12mm |
| Ventriculoarterial connection | Concordant | **Ventricles** |  |
| Ventricular loop | d-Loop | Left ventricle | Normal |
|  |  | Right ventricle | Normal |
| **Septae** |  | **Coronary arteries** | ----- |
| Interventricular septum | Intact | **Doppler Measurement** |  |
| Interatrial septum | Intact | Mitral | ----- |
| **Semilunal valves** |  | Aortic | ---- |
| Aortic valve | Annulus = 11mm | Tricuspid | ---- |
| Pulmonary valve | Annulus = 7mm. Doming Pulmonary Valve. | pulmonic | Mild Valvar PS, PPG = 40mmHg |
| **Great arteries** | NRGA | **Aortic arch** | Left |
| Aorta | ----- | **PDA** | No |
| Pulmonary artery | Normal MPA and Branch PAs. |  |  |
| **M-Mode:** Normal LV Function on eye balling. | | | |
| AO | mm | PWd | mm |
| LA | mm | EDV | ml |
| LVIDd | mm | ESV | ml |
| LVIDs | mm | LVEF | % |
| IVSd | mm | FS | % |
| **Additional Information**: |  | | |
| No pleural/pericardial effusion | | | |
| **Final Diagnosis:** | | | |
| 1. {S, D, S} Levocardia. 2. Mild Valvar PS 3. Doming Pulmonary Valve | | | |
| **Remark**: | | | |
| **Recommendation**: Follow up only. | | | |
| SIGNATURE  Done by: Tesfaye T., Pediatrician, Pediatric Cardiologist _______________ 22/03/2013Eth.C | | | |

| Patient Name: **Hawulet Oumer**. Patient ID: FHRH. SEX/ Age: F/8years. Date of Report: 23**/03/2013**.  BP: _______ Weight: ______ Height:____________ BSA: ________ | | | |
| --- | --- | --- | --- |
| **Features** | **Finding** | **Features** | **Finding** |
| **Profile** |  | **Atria** |  |
| Abdominal situs | Solitus | Left atrium | Normal |
| Cardiac position | Levocardia | Right atrium | Normal |
| Systemic venous drainage | Normal | **Atrioventricular valves** |  |
| Pulmonary venous drainage | Normal | Mitral valve | Annulus = 21mm. Mildly thickened MVL. |
| Atrioventricular connection | Concordant | Tricuspid valve | Annulus = 22mm. |
| Ventriculoarterial connection | Concordant | **Ventricles** |  |
| Ventricular loop | d-Loop | Left ventricle | Normal |
|  |  | Right ventricle | Normal |
| **Septae** |  | **Coronary arteries** | ----- |
| Interventricular septum | Intact | **Doppler Measurement** |  |
| Interatrial septum | Intact | Mitral | Mild MR, Holosystolic, posterior projection, seen in two planes with jet velocity = 3m/sec. |
| **Semilunal valves** |  | Aortic | Trivial AR |
| Aortic valve | Annulus = 15mm | Tricuspid | Moderate TR, PPG = 22mmHg. |
| Pulmonary valve | Annulus = 18mm | pulmonic | Trivial PR, PPG = 14mmHg |
| **Great arteries** | NRGA | **Aortic arch** | Left |
| Aorta | ----- | **PDA** | No |
| Pulmonary artery | Normal MPA and Branch PAs. |  |  |
| **M-Mode:** | | | |
| AO | mm | PWd | mm |
| LA | mm | EDV | ml |
| LVIDd | mm | ESV | ml |
| LVIDs | mm | LVEF | 58% |
| IVSd | mm | FS | 30% |
| **Additional Information**: |  | | |
| No pleural/pericardial effusion | | | |
| **Final Diagnosis:** | | | |
| 1. {S, D, S} Levocardia. 2. Mildly thickened MVL 3. Mild MR 4. Moderate TR 5. Normal LV Function | | | |
| **Remark**: | | | |
| **Recommendation**: | | | |
| SIGNATURE  Done by: Tesfaye T., Pediatrician, Pediatric Cardiologist _______________ 23/03/2013Eth.C | | | |

| Patient Name: **Abebaw Tadesse**. Patient ID: FHRH. SEX/ Age: M/2/12. Date of Report: 23**/03/2013**.  BP: _______ Weight: ______ Height:____________ BSA: ________ | | | |
| --- | --- | --- | --- |
| **Features** | **Finding** | **Features** | **Finding** |
| **Profile** |  | **Atria** |  |
| Abdominal situs | Solitus | Left atrium | Dilated |
| Cardiac position | Levocardia | Right atrium | Normal |
| Systemic venous drainage | Normal | **Atrioventricular valves** |  |
| Pulmonary venous drainage | Normal | Mitral valve | Annulus = 11mm |
| Atrioventricular connection | Concordant | Tricuspid valve | Annulus = 10mm |
| Ventriculoarterial connection | Discordant | **Ventricles** |  |
| Ventricular loop | d-Loop | Left ventricle | Dilated |
|  |  | Right ventricle | Normal |
| **Septae** |  | **Coronary arteries** | ----- |
| Interventricular septum | 5mm Muscular VSD, BD Shunt | **Doppler Measurement** |  |
| Interatrial septum | PFO, L – R Shunt | Mitral | ----- |
| **Semilunal valves** |  | Aortic | ---- |
| Aortic valve | Annulus = 11mm | Tricuspid | ---- |
| Pulmonary valve | Annulus = 10mm | pulmonic | Mild PS, PPG = 23mmHg |
| **Great arteries** | d-TGA | **Aortic arch** | Left |
| Aorta | Anterior and to the right. Arises from RV | **PDA** | No |
| Pulmonary artery | Posterior and to the left. Arises from LV |  |  |
| **M-Mode:** Normal LV Function | | | |
| AO | mm | PWd | mm |
| LA | mm | EDV | ml |
| LVIDd | mm | ESV | ml |
| LVIDs | mm | LVEF | % |
| IVSd | mm | FS | % |
| **Additional Information**: |  | | |
| No pleural/pericardial effusion | | | |
| **Final Diagnosis:** | | | |
| 1. {S, D, D} Levocardia. 2. d-TGA 3. PFO, L – R Shunt 4. Muscular VSD, BD Shunt 5. Mild PS (LVOTO) | | | |
| **Remark**: | | | |
| **Recommendation**: | | | |
| SIGNATURE  Done by: Tesfaye T., Pediatrician, Pediatric Cardiologist _______________ 23/03/2013Eth.C | | | |

| Patient Name: **Selamawit Meseret**. Patient ID: FHRH. SEX/ Age: F/ 3 9/12. Date of Report: 24**/03/2013**.  BP: _______ Weight: ______ Height:____________ BSA: ________ | | | |
| --- | --- | --- | --- |
| **Features** | **Finding** | **Features** | **Finding** |
| **Profile** |  | **Atria** |  |
| Abdominal situs | Solitus | Left atrium | Normal |
| Cardiac position | Levocardia | Right atrium | Normal |
| Systemic venous drainage | Normal | **Atrioventricular valves** |  |
| Pulmonary venous drainage | Normal | Mitral valve | Annulus = 14mm |
| Atrioventricular connection | Concordant | Tricuspid valve | Annulus = 16mm |
| Ventriculoarterial connection | Concordant | **Ventricles** |  |
| Ventricular loop | d-Loop | Left ventricle | Normal |
|  |  | Right ventricle | Normal |
| **Septae** |  | **Coronary arteries** | ----- |
| Interventricular septum | Intact | **Doppler Measurement** |  |
| Interatrial septum | Intact | Mitral | ----- |
| **Semilunal valves** |  | Aortic | ---- |
| Aortic valve | Annulus = 14mm | Tricuspid | ---- |
| Pulmonary valve | Annulus = 14mm | pulmonic | ---- |
| **Great arteries** | NRGA | **Aortic arch** | Left |
| Aorta | ----- | **PDA** | No |
| Pulmonary artery | Normal MPA and Branch PAs. |  |  |
| **M-Mode:** Normal LV Function on eye balling. | | | |
| AO | mm | PWd | mm |
| LA | mm | EDV | ml |
| LVIDd | mm | ESV | ml |
| LVIDs | mm | LVEF | % |
| IVSd | mm | FS | % |
| **Additional Information**: |  | | |
| No pleural/pericardial effusion | | | |
| **Final Diagnosis:** | | | |
| 1. Normal Echocardiography Study. | | | |
| **Remark**: | | | |
| **Recommendation**: | | | |
| SIGNATURE  Done by: Tesfaye T., Pediatrician, Pediatric Cardiologist _______________ 24/03/2013Eth.C | | | |

| Patient Name: **Masrsha Eshetu**. Patient ID: **Nolot Specialty Clinic**. SEX/ Age: M/9yrs. Date of Report: 24**/03/2013**.  BP: _______ Weight: ______ Height:____________ BSA: ________ | | | |
| --- | --- | --- | --- |
| **Features** | **Finding** | **Features** | **Finding** |
| **Profile** |  | **Atria** |  |
| Abdominal situs | Solitus | Left atrium | Dilated |
| Cardiac position | Levocardia | Right atrium | Dilated |
| Systemic venous drainage | Normal | **Atrioventricular valves** |  |
| Pulmonary venous drainage | Normal | Mitral valve | Annulus = 23mm. Thickened MVL. |
| Atrioventricular connection | Concordant | Tricuspid valve | Annulus = 25mm  TAPSE = 9mm |
| Ventriculoarterial connection | Concordant | **Ventricles** |  |
| Ventricular loop | d-Loop | Left ventricle | Dilated |
|  |  | Right ventricle | Dilated |
| **Septae** |  | **Coronary arteries** | ----- |
| Interventricular septum | Intact | **Doppler Measurement** |  |
| Interatrial septum | Intact | Mitral | Moderate MR, Holosystolic Posterior projection, seen in two planes with Jet velocity = 3.5m/sec |
| **Semilunal valves** |  | Aortic | ---- |
| Aortic valve | Annulus = 15mm | Tricuspid | Trivial TR, PPG = 31mmHg. |
| Pulmonary valve | Annulus = 21mm | pulmonic | Moderate PR, PPG = 34mmHg (? Underestimated) |
| **Great arteries** | NRGA | **Aortic arch** | Left |
| Aorta | ----- | **PDA** | No |
| Pulmonary artery | Normal MPA and Branch PAs. |  |  |
| **M-Mode:** | | | |
| AO | mm | PWd | 8mm |
| LA | mm | EDV | 108ml |
| LVIDd | 48mm | ESV | 63ml |
| LVIDs | 38mm | LVEF | 41% |
| IVSd | 8mm | FS | 20% |
| **Additional Information**: |  | | |
| 7mm Pericardial effusion on LV Side  14mm Pericardial effusion on RA/RV Side  22mm Right Pleural Effusion | | | |
| **Final Diagnosis:** | | | |
| 1. {S, D, S} Levocardia. 2. All Chambers Dilated 3. Thickened MVL 4. Moderate MR 5. Moderate PR 6. Biventricular Dysfunction 7. Moderate Pericardial Effusion 8. Right Pleural Effusion | | | |
| **Remark**: Pancarditis | | | |
| **Recommendation**: Rheumatic Carditis and Myopericarditis are to be considered as DDx. | | | |
| SIGNATURE  Done by: Tesfaye T., Pediatrician, Pediatric Cardiologist _______________ 24/03/2013Eth.C | | | |

| Patient Name: **Baby of Yemisirach Alemu**. Patient ID: **MSI - Ethiopia**. SEX/ Age: M/14d. Date of Report: 24**/3/2013**.  BP: _______ Weight: ______ Height:____________ BSA: ________ | | | |
| --- | --- | --- | --- |
| **Features** | **Finding** | **Features** | **Finding** |
| **Profile** |  | **Atria** |  |
| Abdominal situs | Solitus | Left atrium | Normal |
| Cardiac position | Levocardia | Right atrium | Normal |
| Systemic venous drainage | Normal | **Atrioventricular valves** |  |
| Pulmonary venous drainage | Normal | Mitral valve | Annulus = 8mm |
| Atrioventricular connection | Concordant | Tricuspid valve | Annulus = 9mm |
| Ventriculoarterial connection | Concordant | **Ventricles** |  |
| Ventricular loop | d-Loop | Left ventricle | Normal |
|  |  | Right ventricle | Normal |
| **Septae** |  | **Coronary arteries** | ----- |
| Interventricular septum | Intact | **Doppler Measurement** |  |
| Interatrial septum | Probe Patent PFO. | Mitral | ----- |
| **Semilunal valves** |  | Aortic | ---- |
| Aortic valve | Annulus = 9mm | Tricuspid | ---- |
| Pulmonary valve | Annulus = 9mm | pulmonic | ---- |
| **Great arteries** | NRGA | **Aortic arch** | Left |
| Aorta | ----- | **PDA** | No |
| Pulmonary artery | Normal MPA and Branch PAs. |  |  |
| **M-Mode:** Normal LV Function on eye balling. | | | |
| AO | mm | PWd | mm |
| LA | mm | EDV | ml |
| LVIDd | mm | ESV | ml |
| LVIDs | mm | LVEF | % |
| IVSd | mm | FS | % |
| **Additional Information**: |  | | |
| No pleural/pericardial effusion | | | |
| **Final Diagnosis:** | | | |
| 1. {S, D, S} Levocardia. 2. Probe Patent PFO | | | |
| **Remark**: | | | |
| **Recommendation**: | | | |
| SIGNATURE  Done by: Tesfaye T., Pediatrician, Pediatric Cardiologist _______________ 24/03/2013Eth.C | | | |

| Patient Name: **Yared Getinet**. Patient ID: TGSH. SEX/ Age: M/6/12. Date of Report: 24**/03/2013**.  BP: _______ Weight: ______ Height:____________ BSA: ________ | | | |
| --- | --- | --- | --- |
| **Features** | **Finding** | **Features** | **Finding** |
| **Profile** |  | **Atria** |  |
| Abdominal situs | Solitus | Left atrium | Normal |
| Cardiac position | Levocardia | Right atrium | Normal |
| Systemic venous drainage | Normal | **Atrioventricular valves** |  |
| Pulmonary venous drainage | Normal | Mitral valve | Annulus = 10mm |
| Atrioventricular connection | Concordant | Tricuspid valve | Annulus = 12mm |
| Ventriculoarterial connection | Concordant | **Ventricles** |  |
| Ventricular loop | d-Loop | Left ventricle | Normal |
|  |  | Right ventricle | Normal |
| **Septae** |  | **Coronary arteries** | ----- |
| Interventricular septum | Intact | **Doppler Measurement** |  |
| Interatrial septum | PFO, L – R Shunt | Mitral | ----- |
| **Semilunal valves** |  | Aortic | ---- |
| Aortic valve | Annulus = 11mm | Tricuspid | ---- |
| Pulmonary valve | Annulus = 12mm | pulmonic | ---- |
| **Great arteries** | NRGA | **Aortic arch** | Left |
| Aorta | ----- | **PDA** | 1mm PDA, L – R Shunt |
| Pulmonary artery | Normal MPA and Branch PAs. |  |  |
| **M-Mode:** | | | |
| AO | mm | PWd | mm |
| LA | mm | EDV | ml |
| LVIDd | mm | ESV | ml |
| LVIDs | mm | LVEF | 55% |
| IVSd | mm | FS | 28% |
| **Additional Information**: |  | | |
| No pleural/pericardial effusion | | | |
| **Final Diagnosis:** | | | |
| 1. {S, D, S} Levocardia. 2. PFO, L – R Shunt 3. Silent PDA, L – R Shunt 4. Normal Function. | | | |
| **Remark**: No murmur Clinically | | | |
| **Recommendation**: | | | |
| SIGNATURE  Done by: Tesfaye T., Pediatrician, Pediatric Cardiologist _______________ 24/03/2013Eth.C | | | |

| Patient Name: **Yibeltal Dereje**. Patient ID: FHRH. SEX/ Age: M/9/12. Date of Report: 25**/03/2013**.  BP: _______ Weight: ______ Height:____________ BSA: ________ | | | |
| --- | --- | --- | --- |
| **Features** | **Finding** | **Features** | **Finding** |
| **Profile** |  | **Atria** |  |
| Abdominal situs | Solitus | Left atrium | Dilated |
| Cardiac position | Levocardia | Right atrium | Normal |
| Systemic venous drainage | Normal | **Atrioventricular valves** |  |
| Pulmonary venous drainage | Normal | Mitral valve | Annulus = 10mm |
| Atrioventricular connection | Concordant | Tricuspid valve | Annulus = 11mm |
| Ventriculoarterial connection | Concordant | **Ventricles** |  |
| Ventricular loop | d-Loop | Left ventricle | Dilated |
|  |  | Right ventricle | Normal |
| **Septae** |  | **Coronary arteries** | ----- |
| Interventricular septum | 11mm Inlet VSD, L – R Shunt | **Doppler Measurement** |  |
| Interatrial septum | Intact | Mitral | ----- |
| **Semilunal valves** |  | Aortic | ---- |
| Aortic valve | Annulus = 10mm | Tricuspid | Trivial TR, PPG = 52mmHg. |
| Pulmonary valve | Annulus = 13mm | pulmonic | ---- |
| **Great arteries** | NRGA | **Aortic arch** | Left |
| Aorta | ----- | **PDA** | 1mm PDA, L – R Shunt |
| Pulmonary artery | MPA = 16mm. Confluent Branch PAs. |  |  |
| **M-Mode:** Normal LV Function on eye balling. | | | |
| AO | mm | PWd | mm |
| LA | mm | EDV | ml |
| LVIDd | mm | ESV | ml |
| LVIDs | mm | LVEF | % |
| IVSd | mm | FS | % |
| **Additional Information**: |  | | |
| No pleural/pericardial effusion | | | |
| **Final Diagnosis:** | | | |
| 1. {S, D, S} Levocardia. 2. Large Inlet VSD, L – R Shunt 3. Small PDA, L – R Shunt 4. Moderate Pulmonary Hypertension | | | |
| **Remark**: | | | |
| **Recommendation**: | | | |
| SIGNATURE  Done by: Tesfaye T., Pediatrician, Pediatric Cardiologist _______________ 25/03/2013Eth.C | | | |

| Patient Name: **Baby of Alemnesh Alehegn**. Patient ID: FHRH. SEX/ Age: F/8days. Date of Report: 25**/03/2013**.  BP: ____ Weight: ___ Height:___ BSA: ____ R.Dx: **DS**. **AGH10.752** | | | |
| --- | --- | --- | --- |
| **Features** | **Finding** | **Features** | **Finding** |
| **Profile** |  | **Atria** |  |
| Abdominal situs | Solitus | Left atrium | Normal |
| Cardiac position | Levocardia | Right atrium | Normal |
| Systemic venous drainage | Normal | **Atrioventricular valves** |  |
| Pulmonary venous drainage | Normal | Mitral valve | Annulus = 9mm |
| Atrioventricular connection | Concordant | Tricuspid valve | Annulus = 9mm |
| Ventriculoarterial connection | Concordant | **Ventricles** |  |
| Ventricular loop | d-Loop | Left ventricle | Normal |
|  |  | Right ventricle | Normal |
| **Septae** |  | **Coronary arteries** | ----- |
| Interventricular septum | Intact | **Doppler Measurement** |  |
| Interatrial septum | 4mm OS ASD, L – R Shunt | Mitral | ----- |
| **Semilunal valves** |  | Aortic | ---- |
| Aortic valve | Annulus = 9mm | Tricuspid | ---- |
| Pulmonary valve | Annulus = 8mm | pulmonic | ---- |
| **Great arteries** | NRGA | **Aortic arch** | Left |
| Aorta | ----- | **PDA** | 2mm PDA, L – R Shunt |
| Pulmonary artery | Normal MPA and Branch PAs. |  |  |
| **M-Mode:** Normal LV Function on eye balling. | | | |
| AO | mm | PWd | mm |
| LA | mm | EDV | ml |
| LVIDd | mm | ESV | ml |
| LVIDs | mm | LVEF | % |
| IVSd | mm | FS | % |
| **Additional Information**: |  | | |
| No pleural/pericardial effusion | | | |
| **Final Diagnosis:** | | | |
| 1. {S, D, S} Levocardia. 2. Small OS ASD. L – R Shunt 3. Small PDA, L – R Shunt | | | |
| **Remark**: | | | |
| **Recommendation**: | | | |
| SIGNATURE  Done by: Tesfaye T., Pediatrician, Pediatric Cardiologist _______________ 25/03/2013Eth.C | | | |

| Patient Name: **Gebre – Meskel Simeneh**. Patient ID: Enjibara GH. SEX/ Age: M/2/12. Date of Report: 26**/03/2013**.  BP: _______ Weight: ______ Height:____________ BSA: ________ | | | |
| --- | --- | --- | --- |
| **Features** | **Finding** | **Features** | **Finding** |
| **Profile** |  | **Atria** |  |
| Abdominal situs | Solitus | Left atrium | Normal |
| Cardiac position | Levocardia | Right atrium | Normal |
| Systemic venous drainage | Normal | **Atrioventricular valves** |  |
| Pulmonary venous drainage | Normal | Mitral valve | Annulus = 10mm |
| Atrioventricular connection | Concordant | Tricuspid valve | Annulus = 10mm |
| Ventriculoarterial connection | Concordant | **Ventricles** |  |
| Ventricular loop | d-Loop | Left ventricle | Normal |
|  |  | Right ventricle | Normal |
| **Septae** |  | **Coronary arteries** | ----- |
| Interventricular septum | Intact | **Doppler Measurement** |  |
| Interatrial septum | Intact | Mitral | ----- |
| **Semilunal valves** |  | Aortic | ---- |
| Aortic valve | Annulus = 9mm | Tricuspid | ---- |
| Pulmonary valve | Annulus = 10mm | pulmonic | ---- |
| **Great arteries** | NRGA | **Aortic arch** | Left |
| Aorta | ----- | **PDA** | 1mm PDA, L – R Shunt |
| Pulmonary artery | Normal MPA and Branch PAs. |  |  |
| **M-Mode:** Normal LV Function on eye balling. | | | |
| AO | mm | PWd | mm |
| LA | mm | EDV | ml |
| LVIDd | mm | ESV | ml |
| LVIDs | mm | LVEF | % |
| IVSd | mm | FS | % |
| **Additional Information**: |  | | |
| No pleural/pericardial effusion | | | |
| **Final Diagnosis:** | | | |
| 1. {S, D, S} Levocardia. 2. Small PDA, L – R Shunt | | | |
| **Remark**: | | | |
| **Recommendation**: | | | |
| SIGNATURE  Done by: Tesfaye T., Pediatrician, Pediatric Cardiologist _______________ 26/03/2013Eth.C | | | |

| Patient Name: **Baby of Shegaye Asmamaw**. Patient ID: FHRH. SEX/ Age: M10days. Date of Report: 28**/03/2013**.  BP: _______ Weight: ______ Height:____________ BSA: ________ | | | |
| --- | --- | --- | --- |
| **Features** | **Finding** | **Features** | **Finding** |
| **Profile** |  | **Atria** |  |
| Abdominal situs | Solitus | Left atrium | Normal |
| Cardiac position | Levocardia | Right atrium | Normal |
| Systemic venous drainage | Normal | **Atrioventricular valves** |  |
| Pulmonary venous drainage | Normal | Mitral valve | Annulus = 9mm |
| Atrioventricular connection | Concordant | Tricuspid valve | Annulus = 7mm |
| Ventriculoarterial connection | Concordant | **Ventricles** |  |
| Ventricular loop | d-Loop | Left ventricle | Normal |
|  |  | Right ventricle | Normal |
| **Septae** |  | **Coronary arteries** | ----- |
| Interventricular septum | Intact | **Doppler Measurement** |  |
| Interatrial septum | PFO, L – R Shunt | Mitral | Mild MR |
| **Semilunal valves** |  | Aortic | ---- |
| Aortic valve | Annulus = 8mm | Tricuspid | ---- |
| Pulmonary valve | Annulus = 8mm | pulmonic | ---- |
| **Great arteries** | NRGA | **Aortic arch** | Left |
| Aorta | ----- | **PDA** | 2mm PDA, L – R Shunt |
| Pulmonary artery | Normal MPA and Branch PAs. |  |  |
| **M-Mode:** | | | |
| AO | mm | PWd | mm |
| LA | mm | EDV | ml |
| LVIDd | mm | ESV | ml |
| LVIDs | mm | LVEF | 72% |
| IVSd | mm | FS | 38% |
| **Additional Information**: |  | | |
| No pleural/pericardial effusion | | | |
| **Final Diagnosis:** | | | |
| 1. {S, D, S} Levocardia. 2. PFO, L – R Shunt 3. Small to Moderate PDA, L – R Shunt 4. Normal LV Function | | | |
| **Remark**: | | | |
| **Recommendation**: | | | |
| SIGNATURE  Done by: Tesfaye T., Pediatrician, Pediatric Cardiologist _______________ 28/03/2013Eth.C | | | |

| Patient Name: **Alem – Tsehay Nega**. Patient ID: FHRH. SEX/ Age: M/10days. Date of Report: 28**/03/2013**.  BP: _______ Weight: ______ Height:____________ BSA: ________ | | | |
| --- | --- | --- | --- |
| **Features** | **Finding** | **Features** | **Finding** |
| **Profile** |  | **Atria** |  |
| Abdominal situs | Solitus | Left atrium | Normal |
| Cardiac position | Levocardia | Right atrium | Normal |
| Systemic venous drainage | Normal | **Atrioventricular valves** |  |
| Pulmonary venous drainage | Normal | Mitral valve | Annulus = 10mm |
| Atrioventricular connection | Concordant | Tricuspid valve | Annulus = 9mm |
| Ventriculoarterial connection | Concordant | **Ventricles** |  |
| Ventricular loop | d-Loop | Left ventricle | Normal |
|  |  | Right ventricle | Normal |
| **Septae** |  | **Coronary arteries** | ----- |
| Interventricular septum | Intact | **Doppler Measurement** |  |
| Interatrial septum | Intact | Mitral | ----- |
| **Semilunal valves** |  | Aortic | ---- |
| Aortic valve | Annulus = 8mm | Tricuspid | ---- |
| Pulmonary valve | Annulus = 9mm | pulmonic | ---- |
| **Great arteries** | NRGA | **Aortic arch** | Left |
| Aorta | ----- | **PDA** | No |
| Pulmonary artery | Normal MPA and Branch PAs. |  |  |
| **M-Mode:** Normal LV Function on eye balling. | | | |
| AO | mm | PWd | mm |
| LA | mm | EDV | ml |
| LVIDd | mm | ESV | ml |
| LVIDs | mm | LVEF | % |
| IVSd | mm | FS | % |
| **Additional Information**: |  | | |
| No pleural/pericardial effusion | | | |
| **Final Diagnosis:** | | | |
| 1. Normal Echocardiography Study. | | | |
| **Remark**: | | | |
| **Recommendation**: | | | |
| SIGNATURE  Done by: Tesfaye T., Pediatrician, Pediatric Cardiologist _______________ 28/03/2013Eth.C | | | |

| Patient Name: Baby of **Mastie Birhan**. Patient ID: FHRH. SEX/ Age: F/30days. Date of Report: 28**/03/2013**.  BP: _______ Weight: ______ Height:____________ BSA: ________ | | | |
| --- | --- | --- | --- |
| **Features** | **Finding** | **Features** | **Finding** |
| **Profile** |  | **Atria** |  |
| Abdominal situs | Solitus | Left atrium | Normal |
| Cardiac position | Levocardia | Right atrium | Normal |
| Systemic venous drainage | Normal | **Atrioventricular valves** |  |
| Pulmonary venous drainage | Normal | Mitral valve | Annulus = 8mm |
| Atrioventricular connection | Concordant | Tricuspid valve | Annulus = 8mm |
| Ventriculoarterial connection | Concordant | **Ventricles** |  |
| Ventricular loop | d-Loop | Left ventricle | Normal |
|  |  | Right ventricle | Normal |
| **Septae** |  | **Coronary arteries** | ----- |
| Interventricular septum | Intact | **Doppler Measurement** |  |
| Interatrial septum | Intact | Mitral | ----- |
| **Semilunal valves** |  | Aortic | ---- |
| Aortic valve | Annulus = 7.5mm | Tricuspid | ---- |
| Pulmonary valve | Annulus = 8mm | pulmonic | ---- |
| **Great arteries** | NRGA | **Aortic arch** | Left |
| Aorta | ----- | **PDA** | No |
| Pulmonary artery | Normal MPA and Branch PAs. |  |  |
| **M-Mode:** Normal LV Function on eye balling. | | | |
| AO | mm | PWd | mm |
| LA | mm | EDV | ml |
| LVIDd | mm | ESV | ml |
| LVIDs | mm | LVEF | % |
| IVSd | mm | FS | % |
| **Additional Information**: |  | | |
| No pleural/pericardial effusion | | | |
| **Final Diagnosis:** | | | |
| 1. Normal Echocardiography Study. | | | |
| **Remark**: | | | |
| **Recommendation**: | | | |
| SIGNATURE  Done by: Tesfaye T., Pediatrician, Pediatric Cardiologist _______________ 28/03/2013Eth.C | | | |

| Patient Name: **Ramla Mohamed**. Patient ID: Shegaw Motta H. SEX/ Age: F/4/12. Date of Report: 28**/03/2013**.  BP: _______ Weight: ______ Height:____________ BSA: ________ | | | |
| --- | --- | --- | --- |
| **Features** | **Finding** | **Features** | **Finding** |
| **Profile** |  | **Atria** |  |
| Abdominal situs | Solitus | Left atrium | Normal |
| Cardiac position | Levocardia | Right atrium | Normal |
| Systemic venous drainage | Normal | **Atrioventricular valves** |  |
| Pulmonary venous drainage | Normal | Mitral valve | Annulus = 11mm |
| Atrioventricular connection | Concordant | Tricuspid valve | Annulus = 12mm |
| Ventriculoarterial connection | Concordant | **Ventricles** |  |
| Ventricular loop | d-Loop | Left ventricle | Normal |
|  |  | Right ventricle | Normal |
| **Septae** |  | **Coronary arteries** | ----- |
| Interventricular septum | 3mm PM VSD Partially covered by septal leaflet of the TV. | **Doppler Measurement** |  |
| Interatrial septum | Intact | Mitral | ----- |
| **Semilunal valves** |  | Aortic | ---- |
| Aortic valve | Annulus = 9mm | Tricuspid | ---- |
| Pulmonary valve | Annulus = 11mm | pulmonic | ---- |
| **Great arteries** | NRGA | **Aortic arch** | Left |
| Aorta | ----- | **PDA** | No |
| Pulmonary artery | Normal MPA and Branch PAs. |  |  |
| **M-Mode:** Normal LV Function on eye balling. | | | |
| AO | mm | PWd | mm |
| LA | mm | EDV | ml |
| LVIDd | mm | ESV | ml |
| LVIDs | mm | LVEF | % |
| IVSd | mm | FS | % |
| **Additional Information**: |  | | |
| No pleural/pericardial effusion | | | |
| **Final Diagnosis:** | | | |
| 1. {S, D, S} Levocardia. 2. Small PM VSD, partially covered by STL 3. Normal LV Function. | | | |
| **Remark**: | | | |
| **Recommendation**: | | | |
| SIGNATURE  Done by: Tesfaye T., Pediatrician, Pediatric Cardiologist _______________ 28/03/2013Eth.C | | | |

| Patient Name: **Lozan Alebel**. Patient ID: Debre Tabour GH. SEX/ Age: F/2/12. Date of Report: 28**/03/2013**.  BP: _______ Weight: ______ Height:____________ BSA: ________ | | | |
| --- | --- | --- | --- |
| **Features** | **Finding** | **Features** | **Finding** |
| **Profile** |  | **Atria** |  |
| Abdominal situs | Solitus | Left atrium | Normal |
| Cardiac position | Levocardia | Right atrium | Normal |
| Systemic venous drainage | Normal | **Atrioventricular valves** |  |
| Pulmonary venous drainage | Normal | Mitral valve | Annulus = 10mm |
| Atrioventricular connection | Concordant | Tricuspid valve | Annulus = 11mm |
| Ventriculoarterial connection | Concordant | **Ventricles** |  |
| Ventricular loop | d-Loop | Left ventricle | Normal |
|  |  | Right ventricle | Normal |
| **Septae** |  | **Coronary arteries** | ----- |
| Interventricular septum | 3mm PM VSD, L – R Shunt, Restrictive with PPG = 60mmHg. | **Doppler Measurement** |  |
| Interatrial septum | Intact | Mitral | ----- |
| **Semilunal valves** |  | Aortic | ---- |
| Aortic valve | Annulus = 10mm | Tricuspid | ---- |
| Pulmonary valve | Annulus = 11mm | pulmonic | ---- |
| **Great arteries** | NRGA | **Aortic arch** | Left |
| Aorta | ----- | **PDA** | No |
| Pulmonary artery | Normal MPA and Branch PAs. |  |  |
| **M-Mode:** | | | |
| AO | mm | PWd | mm |
| LA | mm | EDV | ml |
| LVIDd | mm | ESV | ml |
| LVIDs | mm | LVEF | % |
| IVSd | mm | FS | % |
| **Additional Information**: |  | | |
| No pleural/pericardial effusion | | | |
| **Final Diagnosis:** | | | |
| 1. {S, D, S} Levocardia. 2. Small Restrictive PM VSD, L – R Shunt 3. Normal LV Function | | | |
| **Remark**: | | | |
| **Recommendation**: Echocardiography after a year | | | |
| SIGNATURE  Done by: Tesfaye T., Pediatrician, Pediatric Cardiologist _______________ 28/03/2013Eth.C | | | |

| Patient Name: **Eyob Walle**. Patient ID: **Amaris SPC**. SEX/ Age: M/3 2/12. Date of Report: 29**/03/2013**.  BP: _______ Weight: ______ Height:____________ BSA: ________ | | | |
| --- | --- | --- | --- |
| **Features** | **Finding** | **Features** | **Finding** |
| **Profile** |  | **Atria** |  |
| Abdominal situs | Solitus | Left atrium | Normal |
| Cardiac position | Levocardia | Right atrium | Normal |
| Systemic venous drainage | Normal | **Atrioventricular valves** |  |
| Pulmonary venous drainage | Normal | Mitral valve | Annulus = 14mm |
| Atrioventricular connection | Concordant | Tricuspid valve | Annulus = 16mm |
| Ventriculoarterial connection | Concordant | **Ventricles** |  |
| Ventricular loop | d-Loop | Left ventricle | Normal |
|  |  | Right ventricle | Normal |
| **Septae** |  | **Coronary arteries** | ----- |
| Interventricular septum | Intact | **Doppler Measurement** |  |
| Interatrial septum | Intact | Mitral | ----- |
| **Semilunal valves** |  | Aortic | ---- |
| Aortic valve | Annulus = 12mm | Tricuspid | ---- |
| Pulmonary valve | Annulus = 15mm | pulmonic | ---- |
| **Great arteries** | NRGA | **Aortic arch** | Left |
| Aorta | ----- | **PDA** | No |
| Pulmonary artery | Normal MPA and Branch PAs. |  |  |
| **M-Mode:** | | | |
| AO | mm | PWd | mm |
| LA | mm | EDV | ml |
| LVIDd | mm | ESV | ml |
| LVIDs | mm | LVEF | 73% |
| IVSd | mm | FS | 40% |
| **Additional Information**: |  | | |
| No pleural/pericardial effusion | | | |
| **Final Diagnosis:** | | | |
| 1. Normal Echocardiography Study. | | | |
| **Remark**: | | | |
| **Recommendation**: | | | |
| SIGNATURE  Done by: Tesfaye T., Pediatrician, Pediatric Cardiologist _______________ 29/03/2013Eth.C | | | |

| Patient Name: **Getaneh Werkie.** Patient ID: **FHRH**. SEX/ Age: M/8/12. Date of Report: 30**/03/2013**.  BP: _______ Weight: ______ Height:____________ BSA: ________ | | | |
| --- | --- | --- | --- |
| **Features** | **Finding** | **Features** | **Finding** |
| **Profile** |  | **Atria** |  |
| Abdominal situs | Solitus | Left atrium | Normal |
| Cardiac position | Levocardia | Right atrium | Normal |
| Systemic venous drainage | Normal | **Atrioventricular valves** |  |
| Pulmonary venous drainage | Normal | Mitral valve | Annulus = 12mm |
| Atrioventricular connection | Concordant | Tricuspid valve | Annulus = 12mm |
| Ventriculoarterial connection | Concordant | **Ventricles** |  |
| Ventricular loop | d-Loop | Left ventricle | Normal |
|  |  | Right ventricle | Normal |
| **Septae** |  | **Coronary arteries** | ----- |
| Interventricular septum | Intact | **Doppler Measurement** |  |
| Interatrial septum | Intact | Mitral | ----- |
| **Semilunal valves** |  | Aortic | ---- |
| Aortic valve | Annulus = 11mm | Tricuspid | ---- |
| Pulmonary valve | Annulus = 13mm | pulmonic | ---- |
| **Great arteries** | NRGA | **Aortic arch** | Left |
| Aorta | ----- | **PDA** | No |
| Pulmonary artery | Normal MPA and Branch PAs. |  |  |
| **M-Mode:** | | | |
| AO | mm | PWd | 6mm |
| LA | mm | EDV | 16ml |
| LVIDd | 22mm | ESV | 5ml |
| LVIDs | 14mm | LVEF | 69% |
| IVSd | 8mm | FS | 36% |
| **Additional Information**: |  | | |
| No pleural/pericardial effusion | | | |
| **Final Diagnosis:** | | | |
| 1. Normal Echocardiography Study. | | | |
| **Remark**: | | | |
| **Recommendation**: | | | |
| SIGNATURE  Done by: Tesfaye T., Pediatrician, Pediatric Cardiologist _______________ 30/03/2013Eth.C | | | |

| Patient Name: **Welde – Senbet Demelash**. Patient ID: **Enjibara GH**. SEX/ Age: M/8/12. Date of Report: 30**/03/2013**.  BP: _______ Weight: ______ Height:____________ BSA: ________ | | | |
| --- | --- | --- | --- |
| **Features** | **Finding** | **Features** | **Finding** |
| **Profile** |  | **Atria** |  |
| Abdominal situs | Solitus | Left atrium | Normal |
| Cardiac position | Levocardia | Right atrium | Normal |
| Systemic venous drainage | Normal | **Atrioventricular valves** |  |
| Pulmonary venous drainage | Normal | Mitral valve | Annulus = 14mm |
| Atrioventricular connection | Concordant | Tricuspid valve | Annulus = 14mm |
| Ventriculoarterial connection | Concordant | **Ventricles** |  |
| Ventricular loop | d-Loop | Left ventricle | Normal |
|  |  | Right ventricle | Normal |
| **Septae** |  | **Coronary arteries** | ----- |
| Interventricular septum | Intact | **Doppler Measurement** |  |
| Interatrial septum | Intact | Mitral | ----- |
| **Semilunal valves** |  | Aortic | ---- |
| Aortic valve | Annulus = 12mm | Tricuspid | ---- |
| Pulmonary valve | Annulus = 14mm | pulmonic | ---- |
| **Great arteries** | NRGA | **Aortic arch** | Left |
| Aorta | ----- | **PDA** | 2mm PDA, L – R Shunt |
| Pulmonary artery | Normal MPA and Branch PAs. |  |  |
| **M-Mode:** Normal LV Function on eye balling. | | | |
| AO | mm | PWd | mm |
| LA | mm | EDV | ml |
| LVIDd | mm | ESV | ml |
| LVIDs | mm | LVEF | % |
| IVSd | mm | FS | % |
| **Additional Information**: |  | | |
| No pleural/pericardial effusion | | | |
| **Final Diagnosis:** | | | |
| 1. {S, D, S} Levocardia. 2. Small PDA, L – R Shunt 3. Normal LV Function | | | |
| **Remark**: | | | |
| **Recommendation**: | | | |
| SIGNATURE  Done by: Tesfaye T., Pediatrician, Pediatric Cardiologist _______________ 30/03/2013Eth.C | | | |

| Patient Name: **Leul Wubete**. Patient ID: **FHRH**. SEX/ Age: M/8years. Date of Report: 30**/03/2013**.  BP: _______ Weight: ______ Height:____________ BSA: ________ | | | |
| --- | --- | --- | --- |
| **Features** | **Finding** | **Features** | **Finding** |
| **Profile** |  | **Atria** |  |
| Abdominal situs | Solitus | Left atrium | Normal |
| Cardiac position | Levocardia | Right atrium | Normal |
| Systemic venous drainage | Normal | **Atrioventricular valves** |  |
| Pulmonary venous drainage | Normal | Mitral valve | Annulus = 16mm |
| Atrioventricular connection | Concordant | Tricuspid valve | Annulus = 16mm |
| Ventriculoarterial connection | Concordant | **Ventricles** |  |
| Ventricular loop | d-Loop | Left ventricle | Asymmetric Hypertrophy |
|  |  | Right ventricle | Hypertrophied |
| **Septae** |  | **Coronary arteries** | ----- |
| Interventricular septum | Intact | **Doppler Measurement** |  |
| Interatrial septum | Intact | Mitral | ----- |
| **Semilunal valves** |  | Aortic | Mild Subaortic LVOTO, PPG/MPG = 35/25mmHg |
| Aortic valve | Annulus = 11mm | Tricuspid | ---- |
| Pulmonary valve | Annulus = 13mm | pulmonic | Mild Sub pulmonic RVOTO, PPG = 25mmHg |
| **Great arteries** | NRGA | **Aortic arch** | Left |
| Aorta | ----- | **PDA** | No |
| Pulmonary artery | Normal MPA and Branch PAs. |  |  |
| **M-Mode:** | | | |
| AO | mm | PWd | 9mm |
| LA | mm | EDV | 12ml |
| LVIDd | 19mm | ESV | 5ml |
| LVIDs | 14mm | LVEF | 58% |
| IVSd | 7mm | FS | 28% |
| **Additional Information**: |  | | |
| No pleural/pericardial effusion | | | |
| **Final Diagnosis:** | | | |
| 1. {S, D, S} Levocardia. 2. Asymmetric Ventricular Hypertrophy secondary to ? 3. Mild LVOTO 4. Mild RVOTO 5. Normal LV Function | | | |
| **Remark**: HCMP shall be considered as DDx. The LVOTO/RVOTO is in relation to the Hypertrophy. | | | |
| **Recommendation**: Try to Correlate with the underlying Clinical condition of the Child. | | | |
| SIGNATURE  Done by: Tesfaye T., Pediatrician, Pediatric Cardiologist _______________ 30/03/2013Eth.C | | | |

| Patient Name: **Abibo Alene**. Patient ID: **FHRH**. SEX/ Age: M/10years. Date of Report: 30**/03/2013**. | | | |
| --- | --- | --- | --- |
| **Features** | **Finding** | **Features** | **Finding** |
| **Profile** |  | **Atria** |  |
| Abdominal situs | Solitus | Left atrium | Normal |
| Cardiac position | Levocardia | Right atrium | Normal |
| Systemic venous drainage | Normal | **Atrioventricular valves** |  |
| Pulmonary venous drainage | Normal | Mitral valve | Annulus = 20mm. Mildly thickened MVL. |
| Atrioventricular connection | Concordant | Tricuspid valve | Annulus = 22mm  TAPSE = 22mm |
| Ventriculoarterial connection | Concordant | **Ventricles** |  |
| Ventricular loop | d-Loop | Left ventricle | Normal |
|  |  | Right ventricle | Normal |
| **Septae** |  | **Coronary arteries** | ----- |
| Interventricular septum | Intact | **Doppler Measurement** |  |
| Interatrial septum | Intact | Mitral | Moderate MR, Holosystolic, posterior projection, seen in two planes with jet velocity = 4.7m/sec |
| **Semilunal valves** |  | Aortic | ------- |
| Aortic valve | Annulus = 17mm | Tricuspid | Moderate TR, PPG = 49mmHg |
| Pulmonary valve | Annulus = 20mm | pulmonic | -------- |
| **Great arteries** | NRGA | **Aortic arch** | Left |
| Aorta | ----- | **PDA** | No |
| Pulmonary artery | Normal MPA and Branch PAs. |  |  |
| **M-Mode:** | | | |
| AO | mm | PWd | mm |
| LA | mm | EDV | ml |
| LVIDd | mm | ESV | ml |
| LVIDs | mm | LVEF | 58% |
| IVSd | mm | FS | 30% |
| **Additional Information**: |  | | |
| Trace pericardial effusion | | | |
| **Final Diagnosis:** | | | |
| 1. {S, D, S} Levocardia. 2. Thickened MVL 3. Moderate MR 4. Moderate TR 5. Moderate Pulmonary Hypertension 6. Trace Pericardial Effusion 7. Normal Biventricular Function | | | |
| SIGNATURE  Done by: Tesfaye T., Pediatrician, Pediatric Cardiologist _______________ 30/03/2013Eth.C | | | |

| Patient Name: **Yiketel Nibretu**. Patient ID: **FHRH**. SEX/ Age: M/9/12. Date of Report: 01**/04/2013**.  BP: _______ Weight: ______ Height:____________ BSA: ________ | | | |
| --- | --- | --- | --- |
| **Features** | **Finding** | **Features** | **Finding** |
| **Profile** |  | **Atria** |  |
| Abdominal situs | Solitus | Left atrium | Normal |
| Cardiac position | Levocardia | Right atrium | Normal |
| Systemic venous drainage | Normal | **Atrioventricular valves** |  |
| Pulmonary venous drainage | Normal | Mitral valve | Annulus = 11mm |
| Atrioventricular connection | Concordant | Tricuspid valve | Annulus = 11mm |
| Ventriculoarterial connection | Concordant | **Ventricles** |  |
| Ventricular loop | d-Loop | Left ventricle | Normal |
|  |  | Right ventricle | Normal |
| **Septae** |  | **Coronary arteries** | ----- |
| Interventricular septum | Intact | **Doppler Measurement** |  |
| Interatrial septum | Intact | Mitral | ----- |
| **Semilunal valves** |  | Aortic | ------- |
| Aortic valve | Annulus = 11mm | Tricuspid | ---- |
| Pulmonary valve | Annulus = 12mm | pulmonic | -------- |
| **Great arteries** | NRGA | **Aortic arch** | Left |
| Aorta | ----- | **PDA** | 2mm PDA, L – R Shunt |
| Pulmonary artery | Normal MPA and Branch PAs. |  |  |
| **M-Mode:** | | | |
| AO | mm | PWd | mm |
| LA | mm | EDV | ml |
| LVIDd | mm | ESV | ml |
| LVIDs | mm | LVEF | 67% |
| IVSd | mm | FS | 35% |
| **Additional Information**: |  | | |
| 4mm pericardial effusion on RA/RV Side. | | | |
| **Final Diagnosis:** | | | |
| 1. {S, D, S} Levocardia. 2. Small PDA, L – R Shunt 3. Small Pericardial Effusion 4. Normal LV Function | | | |
| **Remark**: | | | |
| **Recommendation**: | | | |
| SIGNATURE  Done by: Tesfaye T., Pediatrician, Pediatric Cardiologist _______________ 01/04/2013Eth.C | | | |

| Patient Name: **Haile – Yesus Dawit**. Patient ID: **Guzara MC**. SEX/ Age: M/5years. Date of Report: 02**/04/2013**.  BP: _______ Weight: ______ Height:____________ BSA: ________ | | | |
| --- | --- | --- | --- |
| **Features** | **Finding** | **Features** | **Finding** |
| **Profile** |  | **Atria** |  |
| Abdominal situs | Solitus | Left atrium | Normal |
| Cardiac position | Levocardia | Right atrium | Normal |
| Systemic venous drainage | Normal | **Atrioventricular valves** |  |
| Pulmonary venous drainage | Normal | Mitral valve | Annulus = 17mm |
| Atrioventricular connection | Concordant | Tricuspid valve | Annulus = 17mm |
| Ventriculoarterial connection | Concordant | **Ventricles** |  |
| Ventricular loop | d-Loop | Left ventricle | Normal |
|  |  | Right ventricle | Normal |
| **Septae** |  | **Coronary arteries** | ----- |
| Interventricular septum | Intact | **Doppler Measurement** |  |
| Interatrial septum | Intact | Mitral | ----- |
| **Semilunal valves** |  | Aortic | ------- |
| Aortic valve | Annulus = 14mm | Tricuspid | ---- |
| Pulmonary valve | Annulus = 16mm | pulmonic | -------- |
| **Great arteries** | NRGA | **Aortic arch** | Left |
| Aorta | ----- | **PDA** | No |
| Pulmonary artery | Normal MPA and Branch PAs. |  |  |
| **M-Mode:** | | | |
| AO | mm | PWd | mm |
| LA | mm | EDV | ml |
| LVIDd | mm | ESV | ml |
| LVIDs | mm | LVEF | 64% |
| IVSd | mm | FS | 34% |
| **Additional Information**: |  | | |
| No pleural/pericardial effusion | | | |
| **Final Diagnosis:** | | | |
| 1. Normal Echocardiography Study. | | | |
| **Remark**: | | | |
| **Recommendation**: | | | |
| SIGNATURE  Done by: Tesfaye T., Pediatrician, Pediatric Cardiologist _______________ 02/04/2013Eth.C | | | |

| Patient Name: **Baby of Mekdes Ayalneh**. Patient ID: **FHRH**. SEX/ Age: F/7days. Date of Report: 02**/04/2013**.  BP: _______ Weight: ______ Height:____________ BSA: ________ | | | |
| --- | --- | --- | --- |
| **Features** | **Finding** | **Features** | **Finding** |
| **Profile** |  | **Atria** |  |
| Abdominal situs | Solitus | Left atrium | Normal |
| Cardiac position | Levocardia | Right atrium | Normal |
| Systemic venous drainage | Normal | **Atrioventricular valves** |  |
| Pulmonary venous drainage | Normal | Mitral valve | Annulus = 10mm |
| Atrioventricular connection | Concordant | Tricuspid valve | Annulus = 10mm |
| Ventriculoarterial connection | Concordant | **Ventricles** |  |
| Ventricular loop | d-Loop | Left ventricle | Normal |
|  |  | Right ventricle | Normal |
| **Septae** |  | **Coronary arteries** | ----- |
| Interventricular septum | Intact | **Doppler Measurement** |  |
| Interatrial septum | Probe Patent PFO. No color flow across the defect. | Mitral | ----- |
| **Semilunal valves** |  | Aortic | ------- |
| Aortic valve | Annulus = 8mm | Tricuspid | ---- |
| Pulmonary valve | Annulus = 9mm | pulmonic | -------- |
| **Great arteries** | NRGA | **Aortic arch** | Left |
| Aorta | ----- | **PDA** | No |
| Pulmonary artery | Normal MPA and Branch PAs. |  |  |
| **M-Mode:** | | | |
| AO | mm | PWd | mm |
| LA | mm | EDV | ml |
| LVIDd | mm | ESV | ml |
| LVIDs | mm | LVEF | 63% |
| IVSd | mm | FS | 31% |
| **Additional Information**: |  | | |
| No pleural/pericardial effusion | | | |
| **Final Diagnosis:** | | | |
| 1. {S, D, S} Levocardia. 2. Probe Patent PFO | | | |
| **Remark**: | | | |
| **Recommendation**: | | | |
| SIGNATURE  Done by: Tesfaye T., Pediatrician, Pediatric Cardiologist _______________ 02/04/2013Eth.C | | | |

| Patient Name: **Fikru Andinet**. Patient ID: **SBSPC**. SEX/ Age: M/11/12. Date of Report: 03**/04/2013**.  BP: _______ Weight: ______ Height:____________ BSA: ________ | | | |
| --- | --- | --- | --- |
| **Features** | **Finding** | **Features** | **Finding** |
| **Profile** |  | **Atria** |  |
| Abdominal situs | Solitus | Left atrium | Normal |
| Cardiac position | Levocardia | Right atrium | Mildly dilated |
| Systemic venous drainage | Normal | **Atrioventricular valves** |  |
| Pulmonary venous drainage | Normal | Mitral valve | Annulus = 13mm |
| Atrioventricular connection | Concordant | Tricuspid valve | Annulus = 13mm |
| Ventriculoarterial connection | Concordant | **Ventricles** |  |
| Ventricular loop | d-Loop | Left ventricle | Normal |
|  |  | Right ventricle | Hypertrophied |
| **Septae** |  | **Coronary arteries** | Not clear |
| Interventricular septum | Non Restrictive Subaortic Malaligned VSD, R – L Shunt | **Doppler Measurement** |  |
| Interatrial septum | Intact | Mitral | ----- |
| **Semilunal valves** |  | Aortic | ------- |
| Aortic valve | Annulus = 13mm | Tricuspid | ---- |
| Pulmonary valve | Annulus = 9mm | pulmonic | Severe PS, PPG = 64mmHg. |
| **Great arteries** | NRGA | **Aortic arch** | - |
| Aorta | Overriding aorta | **PDA** | No |
| Pulmonary artery | Small MPA. |  |  |
| **M-Mode:** Normal LV Function on eye balling. | | | |
| AO | mm | PWd | mm |
| LA | mm | EDV | ml |
| LVIDd | mm | ESV | ml |
| LVIDs | mm | LVEF | % |
| IVSd | mm | FS | % |
| **Additional Information**: |  | | |
| No pleural/pericardial effusion | | | |
| **Final Diagnosis:** | | | |
| 1. {S, D, S} Levocardia. 2. TOF | | | |
| **Remark**: Limited view (apical and subcostal) | | | |
| **Recommendation**: | | | |
| SIGNATURE  Done by: Tesfaye T., Pediatrician, Pediatric Cardiologist _______________ 03/04/2013Eth.C | | | |

| Patient Name: **Baby of Tihun**. Patient ID: **FHRH**. SEX/ Age: M/3/12. Date of Report: 05**/04/2013**.  BP: _______ Weight: ______ Height:____________ BSA: ________ | | | |
| --- | --- | --- | --- |
| **Features** | **Finding** | **Features** | **Finding** |
| **Profile** |  | **Atria** |  |
| Abdominal situs | Solitus | Left atrium | Dilated |
| Cardiac position | Levocardia | Right atrium | Normal |
| Systemic venous drainage | Normal | **Atrioventricular valves** |  |
| Pulmonary venous drainage | Normal | Mitral valve | Annulus = 11mm |
| Atrioventricular connection | Concordant | Tricuspid valve | Annulus = 12mm |
| Ventriculoarterial connection | Concordant | **Ventricles** |  |
| Ventricular loop | d-Loop | Left ventricle | Dilated |
|  |  | Right ventricle | Normal |
| **Septae** |  | **Coronary arteries** | ----- |
| Interventricular septum | Intact | **Doppler Measurement** |  |
| Interatrial septum | Intact | Mitral | ----- |
| **Semilunal valves** |  | Aortic | ------- |
| Aortic valve | Annulus = 8mm | Tricuspid | ---- |
| Pulmonary valve | Annulus = 12mm | pulmonic | -------- |
| **Great arteries** | NRGA | **Aortic arch** | Left |
| Aorta | ----- | **PDA** | 3mm PDA, L – R Shunt |
| Pulmonary artery | MPA = 13mm. Confluent Branch PAs. |  |  |
| **M-Mode:** Normal LV Function on eye balling. | | | |
| AO | mm | PWd | mm |
| LA | mm | EDV | ml |
| LVIDd | mm | ESV | ml |
| LVIDs | mm | LVEF | % |
| IVSd | mm | FS | % |
| **Additional Information**: |  | | |
| No pleural/pericardial effusion | | | |
| **Final Diagnosis:** | | | |
| 1. {S, D, S} Levocardia. 2. Moderate to Large PDA, L – R Shunt 3. Normal LV Function. | | | |
| **Remark**: Baby was crying. Not able to measure Pressure gradients | | | |
| **Recommendation**: | | | |
| SIGNATURE  Done by: Tesfaye T., Pediatrician, Pediatric Cardiologist _______________ 05/04/2013Eth.C | | | |

| Patient Name: **Fasika Melesse**. Patient ID: **FHRH**. SEX/ Age: **F/6years**. Date of Report: 06**/04/2013**.  BP: _______ Weight: ______ Height:____________ BSA: ________ | | | |
| --- | --- | --- | --- |
| **Features** | **Finding** | **Features** | **Finding** |
| **Profile** |  | **Atria** |  |
| Abdominal situs | Solitus | Left atrium | Dilated |
| Cardiac position | Levocardia | Right atrium | Normal |
| Systemic venous drainage | Normal | **Atrioventricular valves** |  |
| Pulmonary venous drainage | PPG = 18mmHg across the LLPV | Mitral valve | Annulus = 19mm |
| Atrioventricular connection | Concordant | Tricuspid valve | Annulus = 15mm  TAPSE = 9mm |
| Ventriculoarterial connection | Concordant | **Ventricles** |  |
| Ventricular loop | d-Loop | Left ventricle | Dilated |
|  |  | Right ventricle | Normal |
| **Septae** |  | **Coronary arteries** | ----- |
| Interventricular septum | Intact | **Doppler Measurement** |  |
| Interatrial septum | Intact | Mitral | Mild MR, Jet Velocity = 2.4m/sec |
| **Semilunal valves** |  | Aortic | ------- |
| Aortic valve | Annulus = 15mm. trileaflet. | Tricuspid | ---- |
| Pulmonary valve | Annulus = 17mm | pulmonic | -------- |
| **Great arteries** | NRGA | **Aortic arch** | Left. No CoA. |
| Aorta | ----- | **PDA** | No |
| Pulmonary artery | Normal MPA and Branch PAs. |  |  |
| **M-Mode:** | | | |
| AO | mm | PWd | 6mm |
| LA | mm | EDV | 95ml |
| LVIDd | 46mm | ESV | 60ml |
| LVIDs | 38mm | LVEF | 37% |
| IVSd | 6mm | FS | 18% |
| **Additional Information**: |  | | |
| No pleural/pericardial effusion | | | |
| **Final Diagnosis:** | | | |
| 1. {S, D, S} Levocardia. 2. LA/LV Dilated 3. RV Dysfunctional 4. Moderate LV Dysfunction | | | |
| **Remark**: | | | |
| **Recommendation**: 1. Work on the Line of DCM. Standardize the M-Mode with Weight and Height. | | | |
| SIGNATURE  Done by: Tesfaye T., Pediatrician, Pediatric Cardiologist _______________ 06/04/2013Eth.C | | | |

| Patient Name: **Minale Destaw**. Patient ID: **FHRH**. SEX/ Age: M/10years. Date of Report: 06**/04/2013**.  BP: _______ Weight: ______ Height:____________ BSA: ________ | | | |
| --- | --- | --- | --- |
| **Features** | **Finding** | **Features** | **Finding** |
| **Profile** |  | **Atria** |  |
| Abdominal situs | Solitus | Left atrium | Normal |
| Cardiac position | Levocardia | Right atrium | Normal |
| Systemic venous drainage | Normal | **Atrioventricular valves** |  |
| Pulmonary venous drainage | Normal | Mitral valve | Annulus = 21mm. Mildly Thickened MVL. |
| Atrioventricular connection | Concordant | Tricuspid valve | Annulus = 23mm.  TAPSE = 18mm |
| Ventriculoarterial connection | Concordant | **Ventricles** |  |
| Ventricular loop | d-Loop | Left ventricle | Normal |
|  |  | Right ventricle | Normal |
| **Septae** |  | **Coronary arteries** | ----- |
| Interventricular septum | Intact | **Doppler Measurement** |  |
| Interatrial septum | Intact | Mitral | Moderate MR, Holosystolic, posterior projection seen in two planes with jet velocity = 4m/sec. |
| **Semilunal valves** |  | Aortic | ------- |
| Aortic valve | Annulus = 16mm | Tricuspid | Mild TR, PPG = 25mmHg |
| Pulmonary valve | Annulus = 19mm | pulmonic | Mild PR, PPG = 17mmHg |
| **Great arteries** | NRGA | **Aortic arch** | Left |
| Aorta | ----- | **PDA** | No |
| Pulmonary artery | Normal MPA and Branch PAs. |  |  |
| **M-Mode:** | | | |
| AO | mm | PWd | mm |
| LA | mm | EDV | ml |
| LVIDd | mm | ESV | ml |
| LVIDs | mm | LVEF | 55% |
| IVSd | mm | FS | 28% |
| **Additional Information**: |  | | |
| No pleural/pericardial effusion | | | |
| **Final Diagnosis:** | | | |
| 1. {S, D, S} Levocardia. 2. Mildly thickened MVL 3. Moderate MR 4. Mild TR 5. Mild PR 6. Mildly reduced LV Function. | | | |
| SIGNATURE  Done by: Tesfaye T., Pediatrician, Pediatric Cardiologist _______________ 06/04/2013Eth.C | | | |

| Patient Name: **Mirtie Gedifie**. Patient ID: **FHRH**. SEX/ Age: **F/9years**. Date of Report: 06**/04/2013**.  BP: _______ Weight: ______ Height:____________ BSA: ________ | | | |
| --- | --- | --- | --- |
| **Features** | **Finding** | **Features** | **Finding** |
| **Profile** |  | **Atria** |  |
| Abdominal situs | Solitus | Left atrium | Normal |
| Cardiac position | Levocardia | Right atrium | Normal |
| Systemic venous drainage | Normal | **Atrioventricular valves** |  |
| Pulmonary venous drainage | Normal | Mitral valve | Annulus = 23mm |
| Atrioventricular connection | Concordant | Tricuspid valve | Annulus = 23mm  TAPSE = 25mm |
| Ventriculoarterial connection | Concordant | **Ventricles** |  |
| Ventricular loop | d-Loop | Left ventricle | Normal |
|  |  | Right ventricle | Obstructive Muscular ridge creating DCRV |
| **Septae** |  | **Coronary arteries** | ----- |
| Interventricular septum | 3mm PM VSD, L – R Shunt. | **Doppler Measurement** |  |
| Interatrial septum | Intact | Mitral | ----- |
| **Semilunal valves** |  | Aortic | ------- |
| Aortic valve | Annulus = 18mm | Tricuspid | ---- |
| Pulmonary valve | Annulus = 21mm | pulmonic | Sub pulmonic RVOTO, PPG = 66mmHg |
| **Great arteries** | NRGA | **Aortic arch** | Left |
| Aorta | ----- | **PDA** | No |
| Pulmonary artery | Normal MPA and Branch PAs. |  |  |
| **M-Mode:** Normal LV Function on eye balling. | | | |
| AO | mm | PWd | mm |
| LA | mm | EDV | ml |
| LVIDd | mm | ESV | ml |
| LVIDs | mm | LVEF | % |
| IVSd | mm | FS | % |
| **Additional Information**: |  | | |
| No pleural/pericardial effusion | | | |
| **Final Diagnosis:** | | | |
| 1. {S, D, S} Levocardia. 2. Small PM VSD, L – R Shunt 3. DCRV 4. Severe RVOTO (Sub Pulmonic) | | | |
| **Remark**: CoA PPG = 66mmHg (Severe CoA) + LVH (13/04/13) | | | |
| **Recommendation**: | | | |
| SIGNATURE  Done by: Tesfaye T., Pediatrician, Pediatric Cardiologist _______________ 06/04/2013Eth.C | | | |

| Patient Name: **Remla Muhammed**. Patient ID: **Adinas GH**. SEX/ Age: F/10/12. Date of Report: 06**/04/2013**.  BP: _______ Weight: ______ Height:____________ BSA: ________ | | | |
| --- | --- | --- | --- |
| **Features** | **Finding** | **Features** | **Finding** |
| **Profile** |  | **Atria** |  |
| Abdominal situs | Solitus | Left atrium | Normal |
| Cardiac position | Levocardia | Right atrium | Normal |
| Systemic venous drainage | Normal | **Atrioventricular valves** |  |
| Pulmonary venous drainage | Normal | Mitral valve | Annulus = 11mm. Prolapsing MVL |
| Atrioventricular connection | Concordant | Tricuspid valve | Annulus = 12mm |
| Ventriculoarterial connection | Concordant | **Ventricles** |  |
| Ventricular loop | d-Loop | Left ventricle | Normal |
|  |  | Right ventricle | Normal |
| **Septae** |  | **Coronary arteries** | ----- |
| Interventricular septum | Intact | **Doppler Measurement** |  |
| Interatrial septum | Intact | Mitral | Mild MR, Jet velocity = 4.5m/sec |
| **Semilunal valves** |  | Aortic | ------- |
| Aortic valve | Annulus = 11mm | Tricuspid | ---- |
| Pulmonary valve | Annulus = 12mm | pulmonic | -------- |
| **Great arteries** | NRGA | **Aortic arch** | Left |
| Aorta | ----- | **PDA** | No |
| Pulmonary artery | Normal MPA and Branch PAs. |  |  |
| **M-Mode:** Normal LV Function on eye balling. | | | |
| AO | mm | PWd | mm |
| LA | mm | EDV | ml |
| LVIDd | mm | ESV | ml |
| LVIDs | mm | LVEF | % |
| IVSd | mm | FS | % |
| **Additional Information**: |  | | |
| No pleural/pericardial effusion | | | |
| **Final Diagnosis:** | | | |
| 1. {S, D, S} Levocardia. 2. Mild MR 3. MVP 4. Normal LV Function | | | |
| **Remark**: | | | |
| **Recommendation**: | | | |
| SIGNATURE  Done by: Tesfaye T., Pediatrician, Pediatric Cardiologist _______________ 06/04/2013Eth.C | | | |

| Patient Name: **Habtamnesh Nega**. Patient ID: **DTRH**. SEX/ Age: F/1 2/12. Date of Report: 06**/04/2013**.  BP: _______ Weight: ______ Height:____________ BSA: ________ | | | |
| --- | --- | --- | --- |
| **Features** | **Finding** | **Features** | **Finding** |
| **Profile** |  | **Atria** |  |
| Abdominal situs | Solitus | Left atrium | Normal |
| Cardiac position | Levocardia | Right atrium | Normal |
| Systemic venous drainage | Normal | **Atrioventricular valves** |  |
| Pulmonary venous drainage | Normal | Mitral valve | Annulus = 13mm |
| Atrioventricular connection | Concordant | Tricuspid valve | Annulus = 14mm |
| Ventriculoarterial connection | Concordant | **Ventricles** |  |
| Ventricular loop | d-Loop | Left ventricle | Normal |
|  |  | Right ventricle | Normal |
| **Septae** |  | **Coronary arteries** | ----- |
| Interventricular septum | Intact | **Doppler Measurement** |  |
| Interatrial septum | Intact | Mitral | ----- |
| **Semilunal valves** |  | Aortic | ------- |
| Aortic valve | Annulus = 12mm | Tricuspid | ---- |
| Pulmonary valve | Annulus = 13mm | pulmonic | -------- |
| **Great arteries** | NRGA | **Aortic arch** | Left |
| Aorta | ----- | **PDA** | 2.5mm PDA, L – R Shunt |
| Pulmonary artery | Normal MPA and Branch PAs. |  |  |
| **M-Mode:** Normal LV Function on eye balling. | | | |
| AO | mm | PWd | mm |
| LA | mm | EDV | ml |
| LVIDd | mm | ESV | ml |
| LVIDs | mm | LVEF | % |
| IVSd | mm | FS | % |
| **Additional Information**: |  | | |
| No pleural/pericardial effusion | | | |
| **Final Diagnosis:** | | | |
| 1. {S, D, S} Levocardia. 2. Moderate PDA, L – R Shunt 3. Normal LV Function | | | |
| **Remark**: | | | |
| **Recommendation**: | | | |
| SIGNATURE  Done by: Tesfaye T., Pediatrician, Pediatric Cardiologist _______________ 06/04/2013Eth.C | | | |

| Patient Name: **Betselot Yeshambel**. Patient ID: **SBPSC**. SEX/ Age: F/9/12. Date of Report: 07**/04/2013**.  BP: _______ Weight: ______ Height:____________ BSA: ________ | | | |
| --- | --- | --- | --- |
| **Features** | **Finding** | **Features** | **Finding** |
| **Profile** |  | **Atria** |  |
| Abdominal situs | Solitus | Left atrium | Normal |
| Cardiac position | Levocardia | Right atrium | Dilated |
| Systemic venous drainage | Normal | **Atrioventricular valves** |  |
| Pulmonary venous drainage | Normal | Mitral valve | Annulus = mm |
| Atrioventricular connection | Concordant | Tricuspid valve | Annulus = mm |
| Ventriculoarterial connection | Concordant | **Ventricles** |  |
| Ventricular loop | d-Loop | Left ventricle | Normal |
|  |  | Right ventricle | Dilated |
| **Septae** |  | **Coronary arteries** | ----- |
| Interventricular septum | Intact | **Doppler Measurement** |  |
| Interatrial septum | 9mm OS ASD, L – R Shunt | Mitral | ----- |
| **Semilunal valves** |  | Aortic | ------- |
| Aortic valve | Annulus = 9mm | Tricuspid | ---- |
| Pulmonary valve | Annulus = 9mm. Doming PV. | pulmonic | Mild Valvar PS, PPG = 22mmHg |
| **Great arteries** | NRGA | **Aortic arch** | Left |
| Aorta | ----- | **PDA** | No |
| Pulmonary artery | Normal MPA and Branch PAs. |  |  |
| **M-Mode:** | | | |
| AO | mm | PWd | mm |
| LA | mm | EDV | ml |
| LVIDd | mm | ESV | ml |
| LVIDs | mm | LVEF | 66% |
| IVSd | mm | FS | 34% |
| **Additional Information**: |  | | |
| No pleural/pericardial effusion | | | |
| **Final Diagnosis:** | | | |
| 1. {S, D, S} Levocardia. 2. RA/RV mildly dilated 3. Moderate OS ASD, L – R Shunt 4. Mild Valvar PS 5. Doming Pulmonary Valve 6. Normal LV Function | | | |
| **Remark**: | | | |
| **Recommendation**: | | | |
| SIGNATURE  Done by: Tesfaye T., Pediatrician, Pediatric Cardiologist _______________ 07/04/2013Eth.C | | | |

| Patient Name: **Dagim Zekaryas**. Patient ID: **FHRH**. SEX/ Age: M/2 6/12. Date of Report: 08**/04/2013**.  BP: _______ Weight: ______ Height:____________ BSA: ________ | | | |
| --- | --- | --- | --- |
| **Features** | **Finding** | **Features** | **Finding** |
| **Profile** |  | **Atria** |  |
| Abdominal situs | Solitus | Left atrium | Normal |
| Cardiac position | Levocardia | Right atrium | Dilated |
| Systemic venous drainage | Normal | **Atrioventricular valves** |  |
| Pulmonary venous drainage | Normal | Mitral valve | Annulus = Atretic |
| Atrioventricular connection | Concordant | Tricuspid valve | Annulus = 21mm |
| Ventriculoarterial connection | Concordant | **Ventricles** |  |
| Ventricular loop | d-Loop | Left ventricle | Smallish |
|  |  | Right ventricle | Dilated |
| **Septae** |  | **Coronary arteries** | ----- |
| Interventricular septum | 8mm Muscular VSD, R – L Shunt | **Doppler Measurement** |  |
| Interatrial septum | 11mm OS ASD, L – R Shunt | Mitral | Atretic |
| **Semilunal valves** |  | Aortic | ------- |
| Aortic valve | Annulus = 10mm | Tricuspid | Moderate TR |
| Pulmonary valve | Annulus = 15mm | pulmonic | -------- |
| **Great arteries** | NRGA | **Aortic arch** | Left |
| Aorta | ----- | **PDA** | No |
| Pulmonary artery | Normal MPA and Branch PAs. |  |  |
| **M-Mode:** | | | |
| AO | mm | PWd | mm |
| LA | mm | EDV | ml |
| LVIDd | mm | ESV | ml |
| LVIDs | mm | LVEF | % |
| IVSd | mm | FS | % |
| **Additional Information**: |  | | |
| 7mm pericardial effusion on RA Side | | | |
| **Final Diagnosis:** | | | |
| 1. {S, D, S} Levocardia. 2. Large OS ASD, L – R Shunt 3. Moderate TR 4. Mitral Atresia 5. Large Muscular VSD, R – L Shunt 6. Smallish LV 7. Small Pericardial effusion | | | |
| **Remark**: | | | |
| **Recommendation**: | | | |
| SIGNATURE  Done by: Tesfaye T., Pediatrician, Pediatric Cardiologist _______________ 08/04/2013Eth.C | | | |

| Patient Name: **Mengistu Geremew**. Patient ID: **FHRH**. SEX/ Age: M/12years. Date of Report: 08**/04/2013**.  BP: _______ Weight: ______ Height:____________ BSA: ________ | | | |
| --- | --- | --- | --- |
| **Features** | **Finding** | **Features** | **Finding** |
| **Profile** |  | **Atria** |  |
| Abdominal situs | Solitus | Left atrium | Dilated |
| Cardiac position | Levocardia | Right atrium | Dilated |
| Systemic venous drainage | Normal | **Atrioventricular valves** | |
| Pulmonary venous drainage | Normal | Mitral valve | Annulus = 16mm. MVA = 1cm2. Thickened, Clubbed, calcified MVL. Shortened PMVL. |
| Atrioventricular connection | Concordant | Tricuspid valve | Annulus = 23mm  TAPSE = 25mm. |
| Ventriculoarterial connection | Concordant | **Ventricles** |  |
| Ventricular loop | d-Loop | Left ventricle | Dilated |
|  |  | Right ventricle | Dilated |
| **Septae** |  | **Coronary arteries** | ----- |
| Interventricular septum | Intact | **Doppler Measurement** | |
| Interatrial septum | Intact | Mitral | Severe MR, Holosystolic, posterior projection, seen in 2 planes with jet velocity = 4.6m/sec. Severe MS, PPG/MPG = 25/13mmHg. |
| **Semilunal valves** |  | Aortic | Moderate AR, PHT = 229ms. |
| Aortic valve | Annulus = 16mm | Tricuspid | Severe TR, PPG = 99mmHg |
| Pulmonary valve | Annulus = 24mm | pulmonic | Moderate PR, PPG = 69mmHg. |
| **Great arteries** | NRGA | **Aortic arch** | Left |
| Aorta | ----- | **PDA** | No |
| Pulmonary artery | Normal MPA and Branch PAs. |  |  |
| **M-Mode:** | | | |
| AO | mm | PWd | mm |
| LA | mm | EDV | ml |
| LVIDd | mm | ESV | ml |
| LVIDs | mm | LVEF | 59% |
| IVSd | mm | FS | 31% |
| **Additional Information**: |  | | |
| 4mm pericardial effusion on RA side | | | |
| **Final Diagnosis:** | | | |
| 1. {S, D, S} Levocardia. 2. All Chambers Dilated 3. Severe TR 4. Severe MR 5. Severe MS 6. Moderate AR 7. Severe Pulmonary Hypertension 8. Normal Biventricular Function | | | |
| SIGNATURE  Done by: Tesfaye T., Pediatrician, Pediatric Cardiologist _______________ 08/04/2013Eth.C | | | |

| Patient Name: **Baby of Bosena Addis**. Patient ID: **FHRH**. SEX/ Age: M/20days. Date of Report: 08**/04/2013**.  BP: _______ Weight: ______ Height:____________ BSA: ________ | | | |
| --- | --- | --- | --- |
| **Features** | **Finding** | **Features** | **Finding** |
| **Profile** |  | **Atria** |  |
| Abdominal situs | Solitus | Left atrium | Normal |
| Cardiac position | Levocardia | Right atrium | Normal |
| Systemic venous drainage | Normal | **Atrioventricular valves** |  |
| Pulmonary venous drainage | Normal | Mitral valve | Annulus = 11mm |
| Atrioventricular connection | Concordant | Tricuspid valve | Annulus = 11mm |
| Ventriculoarterial connection | Concordant | **Ventricles** |  |
| Ventricular loop | d-Loop | Left ventricle | Normal |
|  |  | Right ventricle | Normal |
| **Septae** |  | **Coronary arteries** | ----- |
| Interventricular septum | Intact | **Doppler Measurement** |  |
| Interatrial septum | Probe patent PFO. | Mitral | ----- |
| **Semilunal valves** |  | Aortic | ------- |
| Aortic valve | Annulus = 10mm | Tricuspid | ---- |
| Pulmonary valve | Annulus = 11mm | pulmonic | -------- |
| **Great arteries** | NRGA | **Aortic arch** | Left |
| Aorta | ----- | **PDA** | No |
| Pulmonary artery | Normal MPA and Branch PAs. |  |  |
| **M-Mode:** Normal LV Function on eye balling. | | | |
| AO | mm | PWd | mm |
| LA | mm | EDV | ml |
| LVIDd | mm | ESV | ml |
| LVIDs | mm | LVEF | % |
| IVSd | mm | FS | % |
| **Additional Information**: |  | | |
| No pleural/pericardial effusion | | | |
| **Final Diagnosis:** | | | |
| 1. {S, D, S} Levocardia. 2. Probe Patent PFO. | | | |
| **Remark**: | | | |
| **Recommendation**: | | | |
| SIGNATURE  Done by: Tesfaye T., Pediatrician, Pediatric Cardiologist _______________ 08/04/2013Eth.C | | | |

| Patient Name: **Baby of Emebet Getaneh**. Patient ID: **FHRH**. SEX/ Age: M/4days. Date of Report: 08**/04/2013**.  BP: _______ Weight: ______ Height:____________ BSA: ________ | | | |
| --- | --- | --- | --- |
| **Features** | **Finding** | **Features** | **Finding** |
| **Profile** |  | **Atria** |  |
| Abdominal situs | ?Inversus | Left atrium | On the right side |
| Cardiac position | Dextrocardia | Right atrium | On the left side. Dilated |
| Systemic venous drainage | To the Left sided RA | **Atrioventricular valves** |  |
| Pulmonary venous drainage | To the right sided LA | Mitral valve | Annulus = atretic |
| Atrioventricular connection | Concordant | Tricuspid valve | Annulus = 12mm |
| Ventriculoarterial connection | Discordant | **Ventricles** |  |
| Ventricular loop | l-Loop | Left ventricle | On the right side. Smallish |
|  |  | Right ventricle | On the left side |
| **Septae** |  | **Coronary arteries** | ----- |
| Interventricular septum | 6mm inlet VSD, from left side RV to right side LV | **Doppler Measurement** |  |
| Interatrial septum | Large ASD amounting to single atrium | Mitral | ----- |
| **Semilunal valves** |  | Aortic | ------- |
| Aortic valve | Annulus = 7mm | Tricuspid | ---- |
| Pulmonary valve | Annulus = 6mm | pulmonic | -------- |
| **Great arteries** | NRGA | **Aortic arch** | Left |
| Aorta | From left side RV | **PDA** | No |
| Pulmonary artery | From right side LV |  |  |
| **M-Mode:** | | | |
| AO | mm | PWd | mm |
| LA | mm | EDV | ml |
| LVIDd | mm | ESV | ml |
| LVIDs | mm | LVEF | % |
| IVSd | mm | FS | % |
| **Additional Information**: |  | | |
| No pleural/pericardial effusion | | | |
| **Final Diagnosis:** | | | |
| 1. {I, L, D} Dextrocardia. 2. Large ASD amounting to single ventricle 3. Large Inlet VSD, from Morphologic RV (left side) to Morphologic LV (right side) 4. Hypoplastic LV 5. TGA | | | |
| **Conclusion: Complex Congenital Heart Disease** | | | |
| **Remark**: Baby was crying during Echocardiography Study | | | |
| **Recommendation**: Repeat Echo after 3 months | | | |
| SIGNATURE  Done by: Tesfaye T., Pediatrician, Pediatric Cardiologist _______________ 08/04/2013Eth.C | | | |

| Patient Name: **Kalkidan Nigussu**. Patient ID: **Adinas GH**. SEX/ Age: M/6/12. Date of Report: 09**/04/2013**.  BP: _______ Weight: ______ Height:____________ BSA: ________ | | | |
| --- | --- | --- | --- |
| **Features** | **Finding** | **Features** | **Finding** |
| **Profile** |  | **Atria** |  |
| Abdominal situs | Solitus | Left atrium | Normal |
| Cardiac position | Levocardia | Right atrium | Normal |
| Systemic venous drainage | Normal | **Atrioventricular valves** |  |
| Pulmonary venous drainage | Normal | Mitral valve | Annulus = 14mm |
| Atrioventricular connection | Concordant | Tricuspid valve | Annulus = 15mm  TAPSE = 22mm |
| Ventriculoarterial connection | Concordant | **Ventricles** |  |
| Ventricular loop | d-Loop | Left ventricle | Normal |
|  |  | Right ventricle | Normal |
| **Septae** |  | **Coronary arteries** | ----- |
| Interventricular septum | 4mm PM VSD with PPG = 60mmHg L – R Shunt. | **Doppler Measurement** |  |
| Interatrial septum | Intact | Mitral | ----- |
| **Semilunal valves** |  | Aortic | ------- |
| Aortic valve | Annulus = 11mm | Tricuspid | ---- |
| Pulmonary valve | Annulus = 12mm | pulmonic | -------- |
| **Great arteries** | NRGA | **Aortic arch** | Left |
| Aorta | ----- | **PDA** | No |
| Pulmonary artery | Normal MPA and Branch PAs. |  |  |
| **M-Mode:** | | | |
| AO | mm | PWd | mm |
| LA | mm | EDV | ml |
| LVIDd | mm | ESV | ml |
| LVIDs | mm | LVEF | 67% |
| IVSd | mm | FS | 36% |
| **Additional Information**: |  | | |
| No pleural/pericardial effusion | | | |
| **Final Diagnosis:** | | | |
| 1. {S, D, S} Levocardia. 2. Small Restrictive PM VSD, L – R Shunt 3. Normal Biventricular Function250 | | | |
| **Remark**: | | | |
| **Recommendation**: | | | |
| SIGNATURE  Done by: Tesfaye T., Pediatrician, Pediatric Cardiologist _______________ 09/04/2013Eth.C | | | |

| Patient Name: **Mulugeta Fekadu**. Patient ID: **FHRH**. SEX/ Age: M/3/12. Date of Report: 10**/04/2013**.  BP: _______ Weight: ______ Height:____________ BSA: ________ | | | |
| --- | --- | --- | --- |
| **Features** | **Finding** | **Features** | **Finding** |
| **Profile** |  | **Atria** |  |
| Abdominal situs | Solitus | Left atrium | Normal |
| Cardiac position | Levocardia | Right atrium | Normal |
| Systemic venous drainage | Normal | **Atrioventricular valves** |  |
| Pulmonary venous drainage | Normal | Mitral valve | Annulus = 10mm |
| Atrioventricular connection | Concordant | Tricuspid valve | Annulus = 10mm |
| Ventriculoarterial connection | Concordant | **Ventricles** |  |
| Ventricular loop | d-Loop | Left ventricle | Normal |
|  |  | Right ventricle | Normal |
| **Septae** |  | **Coronary arteries** | ----- |
| Interventricular septum | Intact | **Doppler Measurement** |  |
| Interatrial septum | Intact | Mitral | ----- |
| **Semilunal valves** |  | Aortic | ------- |
| Aortic valve | Annulus = 10mm | Tricuspid | ---- |
| Pulmonary valve | Annulus = 11mm | pulmonic | -------- |
| **Great arteries** | NRGA | **Aortic arch** | Left |
| Aorta | ----- | **PDA** | No |
| Pulmonary artery | Normal MPA and Branch PAs. |  |  |
| **M-Mode:** Normal LV Function on eye balling. | | | |
| AO | mm | PWd | mm |
| LA | mm | EDV | ml |
| LVIDd | mm | ESV | ml |
| LVIDs | mm | LVEF | % |
| IVSd | mm | FS | % |
| **Additional Information**: |  | | |
| No pleural/pericardial effusion | | | |
| **Final Diagnosis:** | | | |
| 1. Normal Echocardiography Study | | | |
| **Remark**: Very limited Echo window (Only Subcostal Window) | | | |
| **Recommendation**: | | | |
| SIGNATURE  Done by: Tesfaye T., Pediatrician, Pediatric Cardiologist _______________ 10/04/2013Eth.C | | | |

| Patient Name: **Fanuel Solomon**. Patient ID: **Adinas GH**. SEX/ Age: M/7years. Date of Report: 10**/04/2013**.  BP: _______ Weight: ______ Height:____________ BSA: ________ **AGH10.1148** | | | |
| --- | --- | --- | --- |
| **Features** | **Finding** | **Features** | **Finding** |
| **Profile** |  | **Atria** |  |
| Abdominal situs | Solitus | Left atrium | Normal |
| Cardiac position | Levocardia | Right atrium | Normal |
| Systemic venous drainage | Normal | **Atrioventricular valves** |  |
| Pulmonary venous drainage | Normal | Mitral valve | Annulus = 15mm |
| Atrioventricular connection | Concordant | Tricuspid valve | Annulus = 17mm |
| Ventriculoarterial connection | Concordant | **Ventricles** |  |
| Ventricular loop | d-Loop | Left ventricle | Normal |
|  |  | Right ventricle | Normal |
| **Septae** |  | **Coronary arteries** | ----- |
| Interventricular septum | Intact | **Doppler Measurement** |  |
| Interatrial septum | Intact | Mitral | ----- |
| **Semilunal valves** |  | Aortic | ------- |
| Aortic valve | Annulus = 13mm | Tricuspid | ---- |
| Pulmonary valve | Annulus = 17mm | pulmonic | -------- |
| **Great arteries** | NRGA | **Aortic arch** | Left |
| Aorta | ----- | **PDA** | No |
| Pulmonary artery | Normal MPA and Branch PAs. |  |  |
| **M-Mode:** | | | |
| AO | mm | PWd | mm |
| LA | mm | EDV | ml |
| LVIDd | mm | ESV | ml |
| LVIDs | mm | LVEF | 58% |
| IVSd | mm | FS | 30% |
| **Additional Information**: |  | | |
| No pleural/pericardial effusion | | | |
| **Final Diagnosis:** | | | |
| 1. S/P Primmum ASD Surgical Closure 2. {S, D, S} Levocardia. 3. No Residual ASD 4. Normal LV Function | | | |
| **Remark**: | | | |
| **Recommendation**: | | | |
| SIGNATURE  Done by: Tesfaye T., Pediatrician, Pediatric Cardiologist _______________ 10/04/2013Eth.C | | | |

| Patient Name: **Metadel Dessie**. Patient ID: **TGSH**. SEX/ Age: F/1 3/12. Date of Report: 10**/04/2013**.  BP: _______ Weight: ______ Height:____________ BSA: ________ | | | |
| --- | --- | --- | --- |
| **Features** | **Finding** | **Features** | **Finding** |
| **Profile** |  | **Atria** |  |
| Abdominal situs | Solitus | Left atrium | Normal |
| Cardiac position | Levocardia | Right atrium | Normal |
| Systemic venous drainage | Normal | **Atrioventricular valves** |  |
| Pulmonary venous drainage | Normal | Mitral valve | Annulus = 10mm |
| Atrioventricular connection | Concordant | Tricuspid valve | Annulus = 12mm |
| Ventriculoarterial connection | Concordant | **Ventricles** |  |
| Ventricular loop | d-Loop | Left ventricle | Normal |
|  |  | Right ventricle | Normal |
| **Septae** |  | **Coronary arteries** | ----- |
| Interventricular septum | Intact | **Doppler Measurement** |  |
| Interatrial septum | Intact | Mitral | ----- |
| **Semilunal valves** |  | Aortic | ------- |
| Aortic valve | Annulus = 10mm | Tricuspid | ---- |
| Pulmonary valve | Annulus = 11mm | pulmonic | -------- |
| **Great arteries** | NRGA | **Aortic arch** | Left |
| Aorta | ----- | **PDA** | No |
| Pulmonary artery | Normal MPA and Branch PAs. |  |  |
| **M-Mode:** | | | |
| AO | mm | PWd | mm |
| LA | mm | EDV | ml |
| LVIDd | mm | ESV | ml |
| LVIDs | mm | LVEF | % |
| IVSd | mm | FS | % |
| **Additional Information**: |  | | |
| No pleural/pericardial effusion | | | |
| **Final Diagnosis:** | | | |
| 1. Normal Echocardiography Study. | | | |
| **Remark**: | | | |
| **Recommendation**: | | | |
| SIGNATURE  Done by: Tesfaye T., Pediatrician, Pediatric Cardiologist _______________ 10/04/2013Eth.C | | | |

| Patient Name: **Bilen Shumet**. Patient ID: **Adinas GH**. SEX/ Age: F/8 8/12. Date of Report: 10**/04/2013**.  BP: _______ Weight: ______ Height:____________ BSA: ________ | | | |
| --- | --- | --- | --- |
| **Features** | **Finding** | **Features** | **Finding** |
| **Profile** |  | **Atria** |  |
| Abdominal situs | Solitus | Left atrium | Normal |
| Cardiac position | Levocardia | Right atrium | Normal |
| Systemic venous drainage | Normal | **Atrioventricular valves** |  |
| Pulmonary venous drainage | Normal | Mitral valve | Annulus = 18mm |
| Atrioventricular connection | Concordant | Tricuspid valve | Annulus = 20mm  TAPSE = 21mmHg |
| Ventriculoarterial connection | Concordant | **Ventricles** |  |
| Ventricular loop | d-Loop | Left ventricle | Normal |
|  |  | Right ventricle | Normal |
| **Septae** |  | **Coronary arteries** | ----- |
| Interventricular septum | Intact | **Doppler Measurement** |  |
| Interatrial septum | Intact | Mitral | ----- |
| **Semilunal valves** |  | Aortic | ------- |
| Aortic valve | Annulus = 16mm | Tricuspid | ---- |
| Pulmonary valve | Annulus = 17mm | pulmonic | Trivial PR, PPG = 10mmHg. |
| **Great arteries** | NRGA | **Aortic arch** | Left |
| Aorta | ----- | **PDA** | No |
| Pulmonary artery | Normal MPA and Branch PAs. |  |  |
| **M-Mode:** | | | |
| AO | mm | PWd | mm |
| LA | mm | EDV | ml |
| LVIDd | mm | ESV | ml |
| LVIDs | mm | LVEF | 66% |
| IVSd | mm | FS | 35% |
| **Additional Information**: |  | | |
| No pleural/pericardial effusion | | | |
| **Final Diagnosis:** | | | |
| 1. Normal Echocardiography Study. | | | |
| **Remark**: | | | |
| **Recommendation**: | | | |
| SIGNATURE  Done by: Tesfaye T., Pediatrician, Pediatric Cardiologist _______________ 10/04/2013Eth.C | | | |

| Patient Name: **Baby of Tigist Genet**. Patient ID: **FHRH**. SEX/ Age: M/24days. Date of Report: 12**/04/2013**.  BP: _______ Weight: ______ Height:____________ BSA: ________ | | | |
| --- | --- | --- | --- |
| **Features** | **Finding** | **Features** | **Finding** |
| **Profile** |  | **Atria** |  |
| Abdominal situs | Solitus | Left atrium | Normal |
| Cardiac position | Levocardia | Right atrium | Normal |
| Systemic venous drainage | Normal | **Atrioventricular valves** |  |
| Pulmonary venous drainage | Normal | Mitral valve | Annulus = 9mm |
| Atrioventricular connection | Concordant | Tricuspid valve | Annulus = 11mm |
| Ventriculoarterial connection | Concordant | **Ventricles** |  |
| Ventricular loop | d-Loop | Left ventricle | Normal |
|  |  | Right ventricle | Normal |
| **Septae** |  | **Coronary arteries** | ----- |
| Interventricular septum | Intact | **Doppler Measurement** |  |
| Interatrial septum | Intact | Mitral | ----- |
| **Semilunal valves0** |  | Aortic | ------- |
| Aortic valve | Annulus = 9mm | Tricuspid | ---- |
| Pulmonary valve | Annulus = 10mm | pulmonic | -------- |
| **Great arteries** | NRGA | **Aortic arch** | Left |
| Aorta | ----- | **PDA** | No |
| Pulmonary artery | Normal MPA and Branch PAs. |  |  |
| **M-Mode:** Normal LV Function on eye balling. | | | |
| AO | mm | PWd | mm |
| LA | mm | EDV | ml |
| LVIDd | mm | ESV | ml |
| LVIDs | mm | LVEF | % |
| IVSd | mm | FS | % |
| **Additional Information**: |  | | |
| No pleural/pericardial effusion | | | |
| **Final Diagnosis:** | | | |
| 1. Normal Echocardiography Study. | | | |
| **Remark**: | | | |
| **Recommendation**: | | | |
| SIGNATURE  Done by: Tesfaye T., Pediatrician, Pediatric Cardiologist _______________ 12/04/2013Eth.C | | | |

| Patient Name: **Tsigereda Ayenew**. Patient ID: **Adinas GH**. SEX/ Age: **F/2 10/12**. Date of Report: 12**/04/2013**.  BP: _______ Weight: ______ Height:____________ BSA: ________ | | | |
| --- | --- | --- | --- |
| **Features** | **Finding** | **Features** | **Finding** |
| **Profile** |  | **Atria** |  |
| Abdominal situs | Solitus | Left atrium | Normal |
| Cardiac position | Levocardia | Right atrium | Normal |
| Systemic venous drainage | Normal | **Atrioventricular valves** |  |
| Pulmonary venous drainage | Normal | Mitral valve | Annulus = 15mm |
| Atrioventricular connection | Concordant | Tricuspid valve | Annulus = 16mm  TAPSE = 12mm |
| Ventriculoarterial connection | Concordant | **Ventricles** |  |
| Ventricular loop | d-Loop | Left ventricle | Normal |
|  |  | Right ventricle | Normal |
| **Septae** |  | **Coronary arteries** | ----- |
| Interventricular septum | Intact | **Doppler Measurement** |  |
| Interatrial septum | Intact | Mitral | Mild MR |
| **Semilunal valves** |  | Aortic | ------- |
| Aortic valve | Annulus = 13mm | Tricuspid | ---- |
| Pulmonary valve | Annulus = 14mm | pulmonic | -------- |
| **Great arteries** | NRGA | **Aortic arch** | Left |
| Aorta | ----- | **PDA** | No |
| Pulmonary artery | Normal MPA and Branch PAs. |  |  |
| **M-Mode:** | | | |
| AO | mm | PWd | 6mm |
| LA | mm | EDV | 34ml |
| LVIDd | 30mm | ESV | 21ml |
| LVIDs | 24mm | LVEF | 38% |
| IVSd | 6mm | FS | 17% |
| **Additional Information**: |  | | |
| 5mm pericardial effusion on RA/RV Side. | | | |
| **Final Diagnosis:** | | | |
| 1. {S, D, S} Levocardia. 2. Mild MR 3. Mild Pericardial Effusion 4. Moderately Reduced LV Function | | | |
| **Remark**: Consider Myopericarditis | | | |
| **Recommendation**: | | | |
| SIGNATURE  Done by: Tesfaye T., Pediatrician, Pediatric Cardiologist _______________ 12/04/2013Eth.C | | | |

| Patient Name: **Bethelihem Takele**. Patient ID: **SBPSC**. SEX/ Age: F8/12. Date of Report: 13**/04/2013**.  BP: _______ Weight: ______ Height:____________ BSA: ________ | | | |
| --- | --- | --- | --- |
| **Features** | **Finding** | **Features** | **Finding** |
| **Profile** |  | **Atria** |  |
| Abdominal situs | Solitus | Left atrium | Normal |
| Cardiac position | Levocardia | Right atrium | Normal |
| Systemic venous drainage | Normal | **Atrioventricular valves** |  |
| Pulmonary venous drainage | Normal | Mitral valve | Annulus = 15mm |
| Atrioventricular connection | Concordant | Tricuspid valve | Annulus = 15mm |
| Ventriculoarterial connection | Concordant | **Ventricles** |  |
| Ventricular loop | d-Loop | Left ventricle | Normal |
|  |  | Right ventricle | Normal |
| **Septae** |  | **Coronary arteries** | ----- |
| Interventricular septum | Intact | **Doppler Measurement** |  |
| Interatrial septum | Intact | Mitral | ----- |
| **Semilunal valves** |  | Aortic | ------- |
| Aortic valve | Annulus = 12mm | Tricuspid | ---- |
| Pulmonary valve | Annulus = 9mm | pulmonic | Mild Valvar and Supra Valvar PS, PPG = 42mmHg |
| **Great arteries** | NRGA | **Aortic arch** | Left |
| Aorta | ----- | **PDA** | No |
| Pulmonary artery | MPA =5mm; RPA = 4mm; LPA = 5mm. |  |  |
| **M-Mode:** | | | |
| AO | mm | PWd | mm |
| LA | mm | EDV | ml |
| LVIDd | mm | ESV | ml |
| LVIDs | mm | LVEF | 67% |
| IVSd | mm | FS | 35% |
| **Additional Information**: |  | | |
| No pleural/pericardial effusion | | | |
| **Final Diagnosis:** | | | |
| 1. {S, D, S} Levocardia. 2. Mild PS 3. Smallish MPA and Branch PAs. | | | |
| **Remark**: | | | |
| **Recommendation**: Follow up | | | |
| SIGNATURE  Done by: Tesfaye T., Pediatrician, Pediatric Cardiologist _______________ 13/04/2013Eth.C | | | |

| Patient Name: **Herya Maruf**. Patient ID: **Adinas GH**. SEX/ Age: F/14years. Date of Report: 13**/04/2013**.  BP: _______ Weight: ______ Height:____________ BSA: ________ | | | |
| --- | --- | --- | --- |
| **Features** | **Finding** | **Features** | **Finding** |
| **Profile** |  | **Atria** |  |
| Abdominal situs | Solitus | Left atrium | Normal |
| Cardiac position | Levocardia | Right atrium | Normal |
| Systemic venous drainage | Normal | **Atrioventricular valves** |  |
| Pulmonary venous drainage | Normal | Mitral valve | Annulus = 22mm |
| Atrioventricular connection | Concordant | Tricuspid valve | Annulus = 18mm |
| Ventriculoarterial connection | DORV | **Ventricles** |  |
| Ventricular loop | d-Loop | Left ventricle | Normal |
|  |  | Right ventricle | Normal |
| **Septae** |  | **Coronary arteries** | ----- |
| Interventricular septum | 22mm Inlet VSD, L – R Shunt | **Doppler Measurement** |  |
| Interatrial septum | Intact | Mitral | Mild MR |
| **Semilunal valves** |  | Aortic | ------- |
| Aortic valve | Annulus = 18mm | Tricuspid | ---- |
| Pulmonary valve | Annulus = 22mm | pulmonic | -------- |
| **Great arteries** | NRGA (Side by Side) | **Aortic arch** | Left |
| Aorta | ----- | **PDA** | No |
| Pulmonary artery | Normal MPA and Branch PAs. |  |  |
| **M-Mode:** | | | |
| AO | mm | PWd | mm |
| LA | mm | EDV | ml |
| LVIDd | mm | ESV | ml |
| LVIDs | mm | LVEF | % |
| IVSd | mm | FS | % |
| **Additional Information**: |  | | |
| No pleural/pericardial effusion | | | |
| **Final Diagnosis:** | | | |
| 1. {S, D, Side by Side} Levocardia. 2. DORV 3. Large Inlet VSD, L – R Shunt | | | |
| **Remark**: | | | |
| **Recommendation**: | | | |
| SIGNATURE  Done by: Tesfaye T., Pediatrician, Pediatric Cardiologist _______________ 13/04/2013Eth.C | | | |

| Patient Name: **Banchi – Gizae Endale**. Patient ID: **Adinas GH**. SEX/ Age: F/15years. Date of Report: 15**/04/2013**.  BP: _______ Weight: ______ Height:____________ BSA: ________ | | | |
| --- | --- | --- | --- |
| **Features** | **Finding** | **Features** | **Finding** |
| **Profile** |  | **Atria** |  |
| Abdominal situs | Solitus | Left atrium | Normal |
| Cardiac position | Levocardia | Right atrium | Normal |
| Systemic venous drainage | Normal | **Atrioventricular valves** |  |
| Pulmonary venous drainage | Normal | Mitral valve | Annulus = 18mm |
| Atrioventricular connection | Concordant | Tricuspid valve | Annulus = 24mm  TAPSE = 26mm. |
| Ventriculoarterial connection | Concordant | **Ventricles** |  |
| Ventricular loop | d-Loop | Left ventricle | Normal |
|  |  | Right ventricle | Normal |
| **Septae** |  | **Coronary arteries** | ----- |
| Interventricular septum | Intact | **Doppler Measurement** |  |
| Interatrial septum | Intact | Mitral | Mild MR |
| **Semilunal valves** |  | Aortic | ------- |
| Aortic valve | Annulus = 17mm | Tricuspid | Moderate TR, PPG = 27mmHg |
| Pulmonary valve | Annulus = 24mm | pulmonic | -------- |
| **Great arteries** | NRGA | **Aortic arch** | Left |
| Aorta | ----- | **PDA** | No |
| Pulmonary artery | Normal MPA and Branch PAs. |  |  |
| **M-Mode:** | | | |
| AO | mm | PWd | mm |
| LA | mm | EDV | ml |
| LVIDd | mm | ESV | ml |
| LVIDs | mm | LVEF | 64% |
| IVSd | mm | FS | 34% |
| **Additional Information**: |  | | |
| pericardial effusion 11mm on LV Side and 17mm on RV Side. No echodebris inside. No evidence of tamponade | | | |
| **Final Diagnosis:** | | | |
| 1. {S, D, S} Levocardia. 2. Mild MR 3. Moderate TR 4. Moderate Pericardial Effusion. 5. Normal Biventricular Function | | | |
| **Remark**: | | | |
| **Recommendation**: | | | |
| SIGNATURE  Done by: Tesfaye T., Pediatrician, Pediatric Cardiologist _______________ 15/04/2013Eth.C | | | |

| Patient Name: **Yordanos Enchalew**. Patient ID: **FHRH**. SEX/ Age: M/5/12. Date of Report: 15**/04/2013**.  BP: _______ Weight: ______ Height:____________ BSA: ________ | | | |
| --- | --- | --- | --- |
| **Features** | **Finding** | **Features** | **Finding** |
| **Profile** |  | **Atria** |  |
| Abdominal situs | Solitus | Left atrium | Normal |
| Cardiac position | Levocardia | Right atrium | Normal |
| Systemic venous drainage | Normal | **Atrioventricular valves** |  |
| Pulmonary venous drainage | Normal | Mitral valve | Annulus = 14mm |
| Atrioventricular connection | Concordant | Tricuspid valve | Annulus = 15mm  TAPSE = 12mm |
| Ventriculoarterial connection | Concordant | **Ventricles** |  |
| Ventricular loop | d-Loop | Left ventricle | Normal |
|  |  | Right ventricle | Mildly Dilated |
| **Septae** |  | **Coronary arteries** | ----- |
| Interventricular septum | Intact | **Doppler Measurement** |  |
| Interatrial septum | 8mm OS ASD, L – R Shunt | Mitral | Trivial MR |
| **Semilunal valves** |  | Aortic | ------- |
| Aortic valve | Annulus = 10mm | Tricuspid | ---- |
| Pulmonary valve | Annulus = 7mm | pulmonic | Moderate PS, PPG = 56mmHg |
| **Great arteries** | NRGA | **Aortic arch** | Left |
| Aorta | ----- | **PDA** | No |
| Pulmonary artery | MPA = 8mm. |  |  |
| **M-Mode:** Normal LV Function on eye balling. | | | |
| AO | mm | PWd | mm |
| LA | mm | EDV | ml |
| LVIDd | mm | ESV | ml |
| LVIDs | mm | LVEF | % |
| IVSd | mm | FS | % |
| **Additional Information**: |  | | |
| No pleural/pericardial effusion | | | |
| **Final Diagnosis:** | | | |
| 1. {S, D, S} Levocardia. 2. Moderate OS ASD, L – R Shunt 3. Moderate PS | | | |
| **Remark**: | | | |
| **Recommendation**: | | | |
| SIGNATURE  Done by: Tesfaye T., Pediatrician, Pediatric Cardiologist _______________ 15/04/2013Eth.C | | | |

| Patient Name: **Mahlet Kifle**. Patient ID: **TGSH**. SEX/ Age: F/3 2/12. Date of Report: 15**/04/2013**.  BP: _______ Weight: ______ Height:____________ BSA: ________ | | | |
| --- | --- | --- | --- |
| **Features** | **Finding** | **Features** | **Finding** |
| **Profile** |  | **Atria** |  |
| Abdominal situs | Solitus | Left atrium | Normal |
| Cardiac position | Levocardia | Right atrium | Dilated |
| Systemic venous drainage | Normal | **Atrioventricular valves** |  |
| Pulmonary venous drainage | Normal | Mitral valve | Atretic |
| Atrioventricular connection | Concordant | Tricuspid valve | Annulus = 23mm |
| Ventriculoarterial connection | Concordant | **Ventricles** |  |
| Ventricular loop | d-Loop | Left ventricle | Smallish |
|  |  | Right ventricle | Dilated |
| **Septae** |  | **Coronary arteries** | ----- |
| Interventricular septum | 13mm Muscular VSD, R – L Shunt | **Doppler Measurement** |  |
| Interatrial septum | 5mm OS ASD, L – R Shunt | Mitral | Atretic |
| **Semilunal valves** |  | Aortic | ------- |
| Aortic valve | Annulus = 13mm | Tricuspid | Severe TR |
| Pulmonary valve | Annulus = 11mm | pulmonic | -------- |
| **Great arteries** | NRGA | **Aortic arch** | Left |
| Aorta | ----- | **PDA** | No |
| Pulmonary artery | Normal MPA and Branch PAs. |  |  |
| **M-Mode:** | | | |
| AO | mm | PWd | mm |
| LA | mm | EDV | ml |
| LVIDd | mm | ESV | ml |
| LVIDs | mm | LVEF | % |
| IVSd | mm | FS | % |
| **Additional Information**: |  | | |
| 8mm pericardial effusion on RA/RV Side. | | | |
| **Final Diagnosis:** | | | |
| 1. {S, D, Side by Side} Levocardia. 2. RA/RV Dilated 3. Large Muscular VSD, R – L Shunt 4. Small OS ASD, L – R Shunt 5. Mitral Atresia | | | |
| **Remark**: | | | |
| **Recommendation**: | | | |
| SIGNATURE  Done by: Tesfaye T., Pediatrician, Pediatric Cardiologist _______________ 15/04/2013Eth.C | | | |

| Patient Name: **Mahlet Bayelign**. Patient ID: **Adinas GH**. SEX/ Age: F/6years. Date of Report: 15**/04/2013**.  BP: _______ Weight: ______ Height:____________ BSA: ________ | | | |
| --- | --- | --- | --- |
| **Features** | **Finding** | **Features** | **Finding** |
| **Profile** |  | **Atria** |  |
| Abdominal situs | Solitus | Left atrium | Normal |
| Cardiac position | Levocardia | Right atrium | Normal |
| Systemic venous drainage | Normal | **Atrioventricular valves** |  |
| Pulmonary venous drainage | Normal | Mitral valve | Annulus = 16mm |
| Atrioventricular connection | Concordant | Tricuspid valve | Annulus = 18mm  TAPSE = 20mm |
| Ventriculoarterial connection | Concordant | **Ventricles** |  |
| Ventricular loop | d-Loop | Left ventricle | Normal |
|  |  | Right ventricle | Normal |
| **Septae** |  | **Coronary arteries** | ----- |
| Interventricular septum | Intact | **Doppler Measurement** |  |
| Interatrial septum | Intact | Mitral | ----- |
| **Semilunal valves** |  | Aortic | ------- |
| Aortic valve | Annulus = 14mm | Tricuspid | ---- |
| Pulmonary valve | Annulus = 16mm | pulmonic | -------- |
| **Great arteries** | NRGA | **Aortic arch** | Left |
| Aorta | ----- | **PDA** | No |
| Pulmonary artery | Normal MPA and Branch PAs. |  |  |
| **M-Mode:** | | | |
| AO | mm | PWd | mm |
| LA | mm | EDV | ml |
| LVIDd | mm | ESV | ml |
| LVIDs | mm | LVEF | 61% |
| IVSd | mm | FS | 32% |
| **Additional Information**: |  | | |
| No pleural/pericardial effusion | | | |
| **Final Diagnosis:** | | | |
| 1. Normal Echocardiography Study. | | | |
| **Remark**: | | | |
| **Recommendation**: | | | |
| SIGNATURE  Done by: Tesfaye T., Pediatrician, Pediatric Cardiologist _______________ 15/04/2013Eth.C | | | |

| Patient Name: **Baby of Wubalem**. Patient ID: **FHRH**. SEX/ Age: F/89days. Date of Report: 16**/04/2013**.  BP: _______ Weight: ______ Height:____________ BSA: ________ | | | |
| --- | --- | --- | --- |
| **Features** | **Finding** | **Features** | **Finding** |
| **Profile** |  | **Atria** |  |
| Abdominal situs | Solitus | Left atrium | Normal |
| Cardiac position | Levocardia | Right atrium | Normal |
| Systemic venous drainage | Normal | **Atrioventricular valves** |  |
| Pulmonary venous drainage | Normal | Mitral valve | Annulus = 8mm |
| Atrioventricular connection | Concordant | Tricuspid valve | Annulus = 8mm |
| Ventriculoarterial connection | Concordant | **Ventricles** |  |
| Ventricular loop | d-Loop | Left ventricle | Normal |
|  |  | Right ventricle | Normal |
| **Septae** |  | **Coronary arteries** | ----- |
| Interventricular septum | Intact | **Doppler Measurement** |  |
| Interatrial septum | Intact | Mitral | ----- |
| **Semilunal valves** |  | Aortic | ------- |
| Aortic valve | Annulus = 7mm | Tricuspid | ---- |
| Pulmonary valve | Annulus = 7mm | pulmonic | -------- |
| **Great arteries** | NRGA | **Aortic arch** | Left |
| Aorta | ----- | **PDA** | No window to visualize |
| Pulmonary artery | Normal MPA and Branch PAs. |  |  |
| **M-Mode:** | | | |
| AO | mm | PWd | mm |
| LA | mm | EDV | ml |
| LVIDd | mm | ESV | ml |
| LVIDs | mm | LVEF | % |
| IVSd | mm | FS | % |
| **Additional Information**: |  | | |
| No pleural/pericardial effusion | | | |
| **Final Diagnosis:** | | | |
| 1. Normal Echocardiography Study. | | | |
| **Remark**: Only Subcostal window is accessible. Infant was irritable during study. | | | |
| **Recommendation**: Repeat Echo after 3months for better evaluation. | | | |
| SIGNATURE  Done by: Tesfaye T., Pediatrician, Pediatric Cardiologist _______________ 16/04/2013Eth.C | | | |

| Patient Name: **Misganaw Mulat**. Patient ID: **Adinas GH**. SEX/ Age: M/3 7/12. Date of Report: 18**/04/2013**.  BP: _______ Weight: ______ Height:____________ BSA: ________ | | | |
| --- | --- | --- | --- |
| **Features** | **Finding** | **Features** | **Finding** |
| **Profile** |  | **Atria** |  |
| Abdominal situs | Solitus | Left atrium | Normal |
| Cardiac position | Levocardia | Right atrium | Normal |
| Systemic venous drainage | Normal | **Atrioventricular valves** |  |
| Pulmonary venous drainage | Normal | Mitral valve | Annulus = 15mm |
| Atrioventricular connection | Concordant | Tricuspid valve | Annulus = 15mm |
| Ventriculoarterial connection | Concordant | **Ventricles** |  |
| Ventricular loop | d-Loop | Left ventricle | Normal |
|  |  | Right ventricle | Normal |
| **Septae** |  | **Coronary arteries** | ----- |
| Interventricular septum | Intact | **Doppler Measurement** |  |
| Interatrial septum | Intact | Mitral | ----- |
| **Semilunal valves** |  | Aortic | ------- |
| Aortic valve | Annulus = 13mm | Tricuspid | ---- |
| Pulmonary valve | Annulus = 14mm | pulmonic | -------- |
| **Great arteries** | NRGA | **Aortic arch** | Left |
| Aorta | ----- | **PDA** | No |
| Pulmonary artery | Normal MPA and Branch PAs. |  |  |
| **M-Mode:** | | | |
| AO | mm | PWd | mm |
| LA | mm | EDV | ml |
| LVIDd | mm | ESV | ml |
| LVIDs | mm | LVEF | 61% |
| IVSd | mm | FS | 31% |
| **Additional Information**: |  | | |
| No pleural/pericardial effusion | | | |
| **Final Diagnosis:** | | | |
| 1. Normal Echocardiography Study. | | | |
| **Remark**: | | | |
| **Recommendation**: | | | |
| SIGNATURE  Done by: Tesfaye T., Pediatrician, Pediatric Cardiologist _______________ 18/04/2013Eth.C | | | |

| Patient Name: **Bethelihem Aklog**. Patient ID: **FHRH**. SEX/ Age: F/8/12. Date of Report: 18**/04/2013**.  BP: _______ Weight: ______ Height:____________ BSA: ________ | | | |
| --- | --- | --- | --- |
| **Features** | **Finding** | **Features** | **Finding** |
| **Profile** |  | **Atria** |  |
| Abdominal situs | Solitus | Left atrium | Normal |
| Cardiac position | Levocardia | Right atrium | Dilated |
| Systemic venous drainage | Normal | **Atrioventricular valves** |  |
| Pulmonary venous drainage | Normal | Mitral valve | Complete AVSD |
| Atrioventricular connection | Complete AVSD | Tricuspid valve | Complete AVSD.  TAPSE = 8mm |
| Ventriculoarterial connection | Concordant | **Ventricles** |  |
| Ventricular loop | d-Loop | Left ventricle | Normal |
|  |  | Right ventricle | Dilated |
| **Septae** |  | **Coronary arteries** | ----- |
| Interventricular septum | Complete AVSD, L – R Shunt | **Doppler Measurement** |  |
| Interatrial septum | Complete AVSD, L – R Shunt. Additional OS ASD 11mm X 11mm, L – R Shunt | Mitral | Moderate MR |
| **Semilunal valves** |  | Aortic | ------- |
| Aortic valve | Annulus = 11mm | Tricuspid | ---- |
| Pulmonary valve | Annulus = 12mm | pulmonic | -------- |
| **Great arteries** | NRGA | **Aortic arch** | Left |
| Aorta | ----- | **PDA** | No |
| Pulmonary artery | Normal MPA and Branch PAs. |  |  |
| **M-Mode:** | | | |
| AO | mm | PWd | mm |
| LA | mm | EDV | ml |
| LVIDd | mm | ESV | ml |
| LVIDs | mm | LVEF | 39% |
| IVSd | mm | FS | 18% |
| **Additional Information**: |  | | |
| 5mm Circumferential pericardial effusion. | | | |
| **Final Diagnosis:** | | | |
| 1. {S, D, S} Levocardia. 2. Common Complete AVSD (Balanced), L – R Shunt 3. Additional Large OS ASD, L – R Shunt 4. Moderate MR 5. Biventricular Dysfunction 6. Pulmonary Hypertension 7. Mild Pericardial Effusion | | | |
| **Remark**: | | | |
| **Recommendation**: | | | |
| SIGNATURE  Done by: Tesfaye T., Pediatrician, Pediatric Cardiologist _______________ 18/04/2013Eth.C | | | |

| Patient Name: **Eyob Tibebu**. Patient ID: **FHRH**. SEX/ Age: M/8/12. Date of Report: 19**/04/2013**.  BP: _______ Weight: ______ Height:____________ BSA: ________ | | | |
| --- | --- | --- | --- |
| **Features** | **Finding** | **Features** | **Finding** |
| **Profile** |  | **Atria** |  |
| Abdominal situs | Solitus | Left atrium | Normal |
| Cardiac position | Levocardia | Right atrium | Normal |
| Systemic venous drainage | Normal | **Atrioventricular valves** |  |
| Pulmonary venous drainage | Normal | Mitral valve | Annulus = 12mm |
| Atrioventricular connection | Concordant | Tricuspid valve | Annulus = 12mm  TAPSE = 10mm. |
| Ventriculoarterial connection | Concordant | **Ventricles** |  |
| Ventricular loop | d-Loop | Left ventricle | Normal |
|  |  | Right ventricle | Normal |
| **Septae** |  | **Coronary arteries** | ----- |
| Interventricular septum | Intact | **Doppler Measurement** |  |
| Interatrial septum | Intact | Mitral | ----- |
| **Semilunal valves** |  | Aortic | ------- |
| Aortic valve | Annulus = 9mm | Tricuspid | ---- |
| Pulmonary valve | Annulus = 11mm | pulmonic | -------- |
| **Great arteries** | NRGA | **Aortic arch** | Left |
| Aorta | ----- | **PDA** | No |
| Pulmonary artery | Normal MPA and Branch PAs. |  |  |
| **M-Mode:** | | | |
| AO | mm | PWd | mm |
| LA | mm | EDV | ml |
| LVIDd | mm | ESV | ml |
| LVIDs | mm | LVEF | 63% |
| IVSd | mm | FS | 32% |
| **Additional Information**: |  | | |
| No pleural/pericardial effusion | | | |
| **Final Diagnosis:** | | | |
| 1. Normal Echocardiography Study. | | | |
| **Remark**: | | | |
| **Recommendation**: | | | |
| SIGNATURE  Done by: Tesfaye T., Pediatrician, Pediatric Cardiologist _______________ 19/04/2013Eth.C | | | |

| Patient Name: **Mastewal Asmamaw**. Patient ID: **FHRH**. SEX/ Age: F/14years. Date of Report: 19**/04/2013**.  BP: _______ Weight: ______ Height:____________ BSA: ________ | | | |
| --- | --- | --- | --- |
| **Features** | **Finding** | **Features** | **Finding** |
| **Profile** |  | **Atria** |  |
| Abdominal situs | Solitus | Left atrium | Normal |
| Cardiac position | Levocardia | Right atrium | Normal |
| Systemic venous drainage | Normal | **Atrioventricular valves** |  |
| Pulmonary venous drainage | Normal | Mitral valve | Annulus = 22mm. Thickened MVL. |
| Atrioventricular connection | Concordant | Tricuspid valve | Annulus = 22mm  TAPSE = 20mm. |
| Ventriculoarterial connection | Concordant | **Ventricles** |  |
| Ventricular loop | d-Loop | Left ventricle | Normal |
|  |  | Right ventricle | Normal |
| **Septae** |  | **Coronary arteries** | ----- |
| Interventricular septum | Intact | **Doppler Measurement** |  |
| Interatrial septum | Intact | Mitral | Moderate MR, Holosystolic, seen in two planes, Posterior projection with Jet Velocity = 4.6m/sec |
| **Semilunal valves** |  | Aortic | Mild AR, PHT = 531ms. |
| Aortic valve | Annulus = 19mm | Tricuspid | Mild TR, PPG = 51mmHg |
| Pulmonary valve | Annulus = 20mm | pulmonic | -------- |
| **Great arteries** | NRGA | **Aortic arch** | Left |
| Aorta | ----- | **PDA** | No |
| Pulmonary artery | Normal MPA and Branch PAs. |  |  |
| **M-Mode:** | | | |
| AO | mm | PWd | mm |
| LA | mm | EDV | ml |
| LVIDd | mm | ESV | ml |
| LVIDs | mm | LVEF | 67% |
| IVSd | mm | FS | 37% |
| **Additional Information**: |  | | |
| No pleural/pericardial effusion | | | |
| **Final Diagnosis:** | | | |
| 1. {S, D, S} Levocardia. 2. Thickened MVL 3. Moderate MR 4. Mild TR 5. Mild AR 6. Moderate Pulmonary Hypertension 7. Normal Biventricular Function | | | |
| **Recommendation**: Consider RHD. | | | |
| SIGNATURE  Done by: Tesfaye T., Pediatrician, Pediatric Cardiologist _______________ 19/04/2013Eth.C | | | |

| Patient Name: **Haile – Michael Nigatu**. Patient ID: **TGSH**. SEX/ Age: M/6/12. Date of Report: 20**/04/2013**.  BP: _______ Weight: ______ Height:____________ BSA: ________ | | | |
| --- | --- | --- | --- |
| **Features** | **Finding** | **Features** | **Finding** |
| **Profile** |  | **Atria** |  |
| Abdominal situs | Solitus | Left atrium | Normal |
| Cardiac position | Levocardia | Right atrium | Normal |
| Systemic venous drainage | Normal | **Atrioventricular valves** |  |
| Pulmonary venous drainage | Normal | Mitral valve | Annulus = 13mm |
| Atrioventricular connection | Concordant | Tricuspid valve | Annulus = 14mm  TAPSE = 15mm. |
| Ventriculoarterial connection | Concordant | **Ventricles** |  |
| Ventricular loop | d-Loop | Left ventricle | Normal |
|  |  | Right ventricle | Normal |
| **Septae** |  | **Coronary arteries** | ----- |
| Interventricular septum | Intact | **Doppler Measurement** |  |
| Interatrial septum | Intact | Mitral | ----- |
| **Semilunal valves** |  | Aortic | ------- |
| Aortic valve | Annulus = 10mm | Tricuspid | ---- |
| Pulmonary valve | Annulus = 12mm | pulmonic | -------- |
| **Great arteries** | NRGA | **Aortic arch** | Left |
| Aorta | ----- | **PDA** | No |
| Pulmonary artery | Normal MPA and Branch PAs. |  |  |
| **M-Mode:** | | | |
| AO | mm | PWd | mm |
| LA | mm | EDV | ml |
| LVIDd | mm | ESV | ml |
| LVIDs | mm | LVEF | 58% |
| IVSd | mm | FS | 29% |
| **Additional Information**: |  | | |
| No pleural/pericardial effusion | | | |
| **Final Diagnosis:** | | | |
| 1. Normal Echocardiography Study. | | | |
| **Remark**: | | | |
| **Recommendation**: | | | |
| SIGNATURE  Done by: Tesfaye T., Pediatrician, Pediatric Cardiologist _______________ 20/04/2013Eth.C | | | |

| Patient Name: **Tinsae Wessenie**. Patient ID: **FHRH**. SEX/ Age: M/48days. Date of Report: 20**/04/2013**.  BP: _______ Weight: ______ Height:____________ BSA: ________ | | | |
| --- | --- | --- | --- |
| **Features** | **Finding** | **Features** | **Finding** |
| **Profile** |  | **Atria** |  |
| Abdominal situs | Solitus | Left atrium | Normal |
| Cardiac position | Levocardia | Right atrium | Normal |
| Systemic venous drainage | Normal | **Atrioventricular valves** |  |
| Pulmonary venous drainage | Normal | Mitral valve | Annulus = 11mm |
| Atrioventricular connection | Concordant | Tricuspid valve | Annulus = 11mm |
| Ventriculoarterial connection | Concordant | **Ventricles** |  |
| Ventricular loop | d-Loop | Left ventricle | Normal |
|  |  | Right ventricle | Normal |
| **Septae** |  | **Coronary arteries** | ----- |
| Interventricular septum | Intact | **Doppler Measurement** |  |
| Interatrial septum | PFO, L – R Shunt | Mitral | ----- |
| **Semilunal valves** |  | Aortic | ------- |
| Aortic valve | Annulus = 10mm | Tricuspid | ---- |
| Pulmonary valve | Annulus = 9mm | pulmonic | -------- |
| **Great arteries** | NRGA | **Aortic arch** | Left |
| Aorta | ----- | **PDA** | No |
| Pulmonary artery | Normal MPA and Branch PAs. |  |  |
| **M-Mode:** Normal LV Function on eye balling. | | | |
| AO | mm | PWd | mm |
| LA | mm | EDV | ml |
| LVIDd | mm | ESV | ml |
| LVIDs | mm | LVEF | % |
| IVSd | mm | FS | % |
| **Additional Information**: |  | | |
| No pleural/pericardial effusion | | | |
| **Final Diagnosis:** | | | |
| 1. {S, D, S} Levocardia. 2. PFO, L – R Shunt | | | |
| **Remark**: | | | |
| **Recommendation**: | | | |
| SIGNATURE  Done by: Tesfaye T., Pediatrician, Pediatric Cardiologist _______________ 20/04/2013Eth.C | | | |

| Patient Name: **Tsion Melkamu**. Patient ID: **TGSH**. SEX/ Age: F/70days. Date of Report: 20**/04/2013**.  BP: _______ Weight: ______ Height:____________ BSA: ________ | | | |
| --- | --- | --- | --- |
| **Features** | **Finding** | **Features** | **Finding** |
| **Profile** |  | **Atria** |  |
| Abdominal situs | Solitus | Left atrium | Normal |
| Cardiac position | Mesocardia | Right atrium | Normal |
| Systemic venous drainage | Normal | **Atrioventricular valves** |  |
| Pulmonary venous drainage | Normal | Mitral valve | Annulus = 11mm |
| Atrioventricular connection | Concordant | Tricuspid valve | Annulus = 10mm |
| Ventriculoarterial connection | Concordant | **Ventricles** |  |
| Ventricular loop | d-Loop | Left ventricle | Normal |
|  |  | Right ventricle | Normal |
| **Septae** |  | **Coronary arteries** | ----- |
| Interventricular septum | Intact | **Doppler Measurement** |  |
| Interatrial septum | Intact | Mitral | ----- |
| **Semilunal valves** |  | Aortic | ------- |
| Aortic valve | Annulus = 10mm | Tricuspid | ---- |
| Pulmonary valve | Annulus = 11mm | pulmonic | -------- |
| **Great arteries** | NRGA | **Aortic arch** | Left |
| Aorta | ----- | **PDA** | No |
| Pulmonary artery | Normal MPA and Branch PAs. |  |  |
| **M-Mode:** Normal LV Function on eye balling. | | | |
| AO | mm | PWd | mm |
| LA | mm | EDV | ml |
| LVIDd | mm | ESV | ml |
| LVIDs | mm | LVEF | % |
| IVSd | mm | FS | % |
| **Additional Information**: |  | | |
| No pleural/pericardial effusion | | | |
| **Final Diagnosis:** | | | |
| 1. {S, D, S} Mesocardia. | | | |
| **Remark**: Clinical evaluation finding, weight and height are important for interpretation | | | |
| **Recommendation**: CXR.0 | | | |
| SIGNATURE  Done by: Tesfaye T., Pediatrician, Pediatric Cardiologist _______________ 20/04/2013Eth.C | | | |

| Patient Name: **Amanuel Mekonen**. Patient ID: **Adinas GH**. SEX/ Age: M/11years. Date of Report: 21**/04/2013**.  BP: _______ Weight: ______ Height:____________ BSA: ________ | | | |
| --- | --- | --- | --- |
| **Features** | **Finding** | **Features** | **Finding** |
| **Profile** |  | **Atria** |  |
| Abdominal situs | Solitus | Left atrium | Normal |
| Cardiac position | Levocardia | Right atrium | Dilated |
| Systemic venous drainage | Normal | **Atrioventricular valves** |  |
| Pulmonary venous drainage | Normal | Mitral valve | Common Complete AVSD. |
| Atrioventricular connection | Common Complete AVSD | Tricuspid valve | Common Complete AVSD.  TAPSE = 30mm. |
| Ventriculoarterial connection | Concordant | **Ventricles** |  |
| Ventricular loop | d-Loop | Left ventricle | Normal |
|  |  | Right ventricle | Dilated |
| **Septae** |  | **Coronary arteries** | ----- |
| Interventricular septum | Intact | **Doppler Measurement** |  |
| Interatrial septum | Intact | Mitral | ----- |
| **Semilunal valves** |  | Aortic | ------- |
| Aortic valve | Annulus = 16mm | Tricuspid | Mild Right AVVR |
| Pulmonary valve | Annulus = 25mm | pulmonic | Mild PR, PPG = 48mmHg |
| **Great arteries** | NRGA | **Aortic arch** | Left |
| Aorta | ----- | **PDA** | No |
| Pulmonary artery | MPA =30mm. Normal Branch PAs. |  |  |
| **M-Mode:** | | | |
| AO | mm | PWd | mm |
| LA | mm | EDV | ml |
| LVIDd | mm | ESV | ml |
| LVIDs | mm | LVEF | % |
| IVSd | mm | FS | % |
| **Additional Information**: |  | | |
| No pleural/pericardial effusion | | | |
| **Final Diagnosis:** | | | |
| 1. {S, D, S} Levocardia. 2. Common Complete AVSD, L – R Shunt 3. Moderate Pulmonary Hypertension | | | |
| **Remark**: | | | |
| **Recommendation**: | | | |
| SIGNATURE  Done by: Tesfaye T., Pediatrician, Pediatric Cardiologist _______________ 21/04/2013Eth.C | | | |

| Patient Name: **Yonatan Wendim**. Patient ID: **Adinas GH**. SEX/ Age: M/9years. Date of Report: 21**/04/2013**.  BP: _______ Weight: ______ Height:____________ BSA: ________ | | | |
| --- | --- | --- | --- |
| **Features** | **Finding** | **Features** | **Finding** |
| **Profile** |  | **Atria** |  |
| Abdominal situs | Solitus | Left atrium | Dilated |
| Cardiac position | Levocardia | Right atrium | Dilated |
| Systemic venous drainage | To RA. IVC Plethoric | **Atrioventricular valves** |  |
| Pulmonary venous drainage | Normal | Mitral valve | Annulus = 23mm. thickened MVL, Non coapting. Echogenic mass attached to the atrial side of the Septal MVL. |
| Atrioventricular connection | Concordant | Tricuspid valve | Annulus = 26mm.  TAPSE = 25mm |
| Ventriculoarterial connection | Concordant | **Ventricles** |  |
| Ventricular loop | d-Loop | Left ventricle | Dilated |
|  |  | Right ventricle | Dilated |
| **Septae** |  | **Coronary arteries** | ----- |
| Interventricular septum | Intact | **Doppler Measurement** |  |
| Interatrial septum | Intact | Mitral | Severe MR, Holosystolic, posterior projection seen in two planes with jet velocity = 4.2m/sec. Mild MS, PPG/MPG = 14/5mmHg |
| **Semilunal valves** |  | Aortic | Mild AR, PHT = 739ms. |
| Aortic valve | Annulus = 14mm | Tricuspid | Severe TR, PPG = 54mmHg. |
| Pulmonary valve | Annulus = 22mm | pulmonic | Moderate PR, PPG = 40mmHg |
| **Great arteries** | NRGA | **Aortic arch** | Left |
| Aorta | ----- | **PDA** | No |
| Pulmonary artery | MPA = 22mm. Normal Br. PAs. |  |  |
| **M-Mode:** | | | |
| AO | mm | PWd | mm |
| LA | mm | EDV | ml |
| LVIDd | mm | ESV | ml |
| LVIDs | mm | LVEF | 65% |
| IVSd | mm | FS | 36% |
| **Additional Information**: |  | | |
| 11mm pericardial effusion, circumferential | | | |
| **Final Diagnosis:** | | | |
| 1. {S, D, S} Levocardia. 2. All chambers dilated 3. Thickened, non coapting MVL 4. Severe MR 5. Mild MS 6. Severe TR 7. Mild AR 8. Moderate PR 9. ? Infective endocarditis 10. Moderate Pericardial Effusion 11. Normal Biventricular Function | | | |
| **Remark**: | | | |
| **Recommendation**: | | | |
| SIGNATURE  Done by: Tesfaye T., Pediatrician, Pediatric Cardiologist _______________ 21/04/2013Eth.C | | | |

| Patient Name: **Dejitnu Setie**. Patient ID: **TGSH**. SEX/ Age: F/11/12. Date of Report: 21**/04/2013**.  BP: _______ Weight: ______ Height:____________ BSA: ________ | | | |
| --- | --- | --- | --- |
| **Features** | **Finding** | **Features** | **Finding** |
| **Profile** |  | **Atria** |  |
| Abdominal situs | Solitus | Left atrium | Normal |
| Cardiac position | Levocardia | Right atrium | Normal |
| Systemic venous drainage | Normal | **Atrioventricular valves** |  |
| Pulmonary venous drainage | Normal | Mitral valve | Annulus = 11mm |
| Atrioventricular connection | Concordant | Tricuspid valve | Annulus = 12mm  TAPSE = 13mm |
| Ventriculoarterial connection | Concordant | **Ventricles** |  |
| Ventricular loop | d-Loop | Left ventricle | Normal |
|  |  | Right ventricle | Normal |
| **Septae** |  | **Coronary arteries** | ----- |
| Interventricular septum | Intact | **Doppler Measurement** |  |
| Interatrial septum | PFO, L – R Shunt | Mitral | ----- |
| **Semilunal valves** |  | Aortic | ------- |
| Aortic valve | Annulus = 10mm | Tricuspid | ---- |
| Pulmonary valve | Annulus = 12mm | pulmonic | -------- |
| **Great arteries** | NRGA | **Aortic arch** | Left |
| Aorta | ----- | **PDA** | No |
| Pulmonary artery | Normal MPA and Branch PAs. |  |  |
| **M-Mode:** | | | |
| AO | mm | PWd | mm |
| LA | mm | EDV | ml |
| LVIDd | mm | ESV | ml |
| LVIDs | mm | LVEF | 65% |
| IVSd | mm | FS | 34% |
| **Additional Information**: |  | | |
| No pleural/pericardial effusion | | | |
| **Final Diagnosis:** | | | |
| 1. {S, D, S} Levocardia. 2. PFO, L – R Shunt | | | |
| **Remark**: | | | |
| **Recommendation**: | | | |
| SIGNATURE  Done by: Tesfaye T., Pediatrician, Pediatric Cardiologist _______________ 21/04/2013Eth.C | | | |

| Patient Name: **Baby of Tewabech Alem**. Patient ID: **FHRH**. SEX/ Age: F/2/12. Date of Report: 22**/04/2013**.  BP: _______ Weight: ______ Height:____________ BSA: ________ | | | |
| --- | --- | --- | --- |
| **Features** | **Finding** | **Features** | **Finding** |
| **Profile** |  | **Atria** |  |
| Abdominal situs | Solitus | Left atrium | Normal |
| Cardiac position | Levocardia | Right atrium | Normal |
| Systemic venous drainage | Normal | **Atrioventricular valves** |  |
| Pulmonary venous drainage | Normal | Mitral valve | Annulus = 9mm |
| Atrioventricular connection | Concordant | Tricuspid valve | Annulus = 9mm  TAPSE = 10mm |
| Ventriculoarterial connection | Concordant | **Ventricles** |  |
| Ventricular loop | d-Loop | Left ventricle | Normal |
|  |  | Right ventricle | Normal |
| **Septae** |  | **Coronary arteries** | ----- |
| Interventricular septum | Intact | **Doppler Measurement** |  |
| Interatrial septum | 5mm OS ASD, L – R Shunt | Mitral | ----- |
| **Semilunal valves** |  | Aortic | ------- |
| Aortic valve | Annulus = 8mm | Tricuspid | ---- |
| Pulmonary valve | Annulus = 9mm | pulmonic | -------- |
| **Great arteries** | NRGA | **Aortic arch** | Left |
| Aorta | ----- | **PDA** | No |
| Pulmonary artery | Normal MPA and Branch PAs. |  |  |
| **M-Mode:** Normal LV Function on eye balling. | | | |
| AO | mm | PWd | mm |
| LA | mm | EDV | ml |
| LVIDd | mm | ESV | ml |
| LVIDs | mm | LVEF | % |
| IVSd | mm | FS | % |
| **Additional Information**: |  | | |
| No pleural/pericardial effusion | | | |
| **Final Diagnosis:** | | | |
| 1. {S, D, S} Levocardia. 2. PFO, L – R Shunt | | | |
| **Remark**: | | | |
| **Recommendation**: | | | |
| SIGNATURE  Done by: Tesfaye T., Pediatrician, Pediatric Cardiologist _______________ 22/04/2013Eth.C | | | |

| Patient Name: **Efrata Haymanot**. Patient ID: **TGSH**. SEX/ Age: F/20days. Date of Report: 22**/04/2013**.  BP: _______ Weight: ______ Height:____________ BSA: ________ | | | |
| --- | --- | --- | --- |
| **Features** | **Finding** | **Features** | **Finding** |
| **Profile** |  | **Atria** |  |
| Abdominal situs | Solitus | Left atrium | Normal |
| Cardiac position | Levocardia | Right atrium | Normal |
| Systemic venous drainage | Normal | **Atrioventricular valves** |  |
| Pulmonary venous drainage | Normal | Mitral valve | Annulus = 9mm |
| Atrioventricular connection | Concordant | Tricuspid valve | Annulus = 9mm |
| Ventriculoarterial connection | Concordant | **Ventricles** |  |
| Ventricular loop | d-Loop | Left ventricle | Normal |
|  |  | Right ventricle | Normal |
| **Septae** |  | **Coronary arteries** | ----- |
| Interventricular septum | Intact | **Doppler Measurement** |  |
| Interatrial septum | Intact | Mitral | ----- |
| **Semilunal valves** |  | Aortic | ------- |
| Aortic valve | Annulus = 8mm | Tricuspid | ---- |
| Pulmonary valve | Annulus = 10mm | pulmonic | -------- |
| **Great arteries** | NRGA | **Aortic arch** | Left |
| Aorta | ----- | **PDA** | 1mm PDA, L – R Shunt |
| Pulmonary artery | Normal MPA and Branch PAs. |  |  |
| **M-Mode:** | | | |
| AO | mm | PWd | mm |
| LA | mm | EDV | ml |
| LVIDd | mm | ESV | ml |
| LVIDs | mm | LVEF | % |
| IVSd | mm | FS | % |
| **Additional Information**: |  | | |
| No pleural/pericardial effusion | | | |
| **Final Diagnosis:** | | | |
| 1. {S, D, S} Levocardia. 2. Silent PDA, L – R Shunt | | | |
| **Remark**: Clinical evaluation report says no murmur | | | |
| **Recommendation**: Can undergo General anesthesia and Surgery (No Significant Hemodynamic effect) | | | |
| SIGNATURE  Done by: Tesfaye T., Pediatrician, Pediatric Cardiologist _______________ 22/04/2013Eth.C | | | |

| Patient Name: **Bethelihem Solomon**. Patient ID: **Adinas GH**. SEX/ Age: F/14years. Date of Report: 23**/04/2013**.  BP: _______ Weight: ______ Height:____________ BSA: ________ | | | |
| --- | --- | --- | --- |
| **Features** | **Finding** | **Features** | **Finding** |
| **Profile** |  | **Atria** |  |
| Abdominal situs | Solitus | Left atrium | Normal |
| Cardiac position | Levocardia | Right atrium | Normal |
| Systemic venous drainage | Normal | **Atrioventricular valves** |  |
| Pulmonary venous drainage | Normal | Mitral valve | Annulus = 20mm |
| Atrioventricular connection | Concordant | Tricuspid valve | Annulus = 21mm  TAPSE = 17mm. |
| Ventriculoarterial connection | Concordant | **Ventricles** |  |
| Ventricular loop | d-Loop | Left ventricle | Normal |
|  |  | Right ventricle | Normal |
| **Septae** |  | **Coronary arteries** | ----- |
| Interventricular septum | Intact | **Doppler Measurement** |  |
| Interatrial septum | Intact | Mitral | ----- |
| **Semilunal valves** |  | Aortic | ------- |
| Aortic valve | Annulus = 17mm | Tricuspid | ---- |
| Pulmonary valve | Annulus = 18mm | pulmonic | -------- |
| **Great arteries** | NRGA | **Aortic arch** | Left |
| Aorta | ----- | **PDA** | No |
| Pulmonary artery | Normal MPA and Branch PAs. |  |  |
| **M-Mode:** | | | |
| AO | mm | PWd | mm |
| LA | mm | EDV | ml |
| LVIDd | mm | ESV | ml |
| LVIDs | mm | LVEF | 57% |
| IVSd | mm | FS | 29% |
| **Additional Information**: |  | | |
| No pleural/pericardial effusion | | | |
| **Final Diagnosis:** | | | |
| 1. Normal Echocardiography Study. | | | |
| **Remark**: | | | |
| **Recommendation**: | | | |
| SIGNATURE  Done by: Tesfaye T., Pediatrician, Pediatric Cardiologist _______________ 23/04/2013Eth.C | | | |

| Patient Name: **Mebratu Birhanu**. Patient ID: **THMH(Sekota)**. SEX/ Age: M/13years. Date of Report: 24**/04/2013**.  BP: _______ Weight: ______ Height:____________ BSA: ________ | | | |
| --- | --- | --- | --- |
| **Features** | **Finding** | **Features** | **Finding** |
| **Profile** |  | **Atria** |  |
| Abdominal situs | Solitus | Left atrium | Dilated |
| Cardiac position | Levocardia | Right atrium | Normal |
| Systemic venous drainage | Normal | **Atrioventricular valves** |  |
| Pulmonary venous drainage | Normal | Mitral valve | Annulus = 21mm. Thickened MVL |
| Atrioventricular connection | Concordant | Tricuspid valve | Annulus = 16mm |
| Ventriculoarterial connection | Concordant | **Ventricles** |  |
| Ventricular loop | d-Loop | Left ventricle | More Dilated |
|  |  | Right ventricle | Normal |
| **Septae** |  | **Coronary arteries** | ----- |
| Interventricular septum | Intact | **Doppler Measurement** |  |
| Interatrial septum | Intact | Mitral | Mild MR, Jet velocity = 3.8m/sec. |
| **Semilunal valves** |  | Aortic | Moderate AR, PHT = 262ms. |
| Aortic valve | Annulus = 24mm. Trileaflet, thickened Valve. | Tricuspid | ---- |
| Pulmonary valve | Annulus = 20mm | pulmonic | -------- |
| **Great arteries** | NRGA | **Aortic arch** | Left |
| Aorta | ----- | **PDA** | No |
| Pulmonary artery | Normal MPA and Branch PAs. |  |  |
| **M-Mode:** | | | |
| AO | mm | PWd | mm |
| LA | mm | EDV | ml |
| LVIDd | mm | ESV | ml |
| LVIDs | mm | LVEF | 56% |
| IVSd | mm | FS | 30% |
| **Additional Information**: |  | | |
| No pleural/pericardial effusion | | | |
| **Final Diagnosis:** | | | |
| 1. {S, D, S} Levocardia. 2. Thickened MVL, 3. Mild MR 4. Moderate AR 5. Normal LV Function | | | |
| **Remark**: | | | |
| **Recommendation**: | | | |
| SIGNATURE  Done by: Tesfaye T., Pediatrician, Pediatric Cardiologist _______________ 24/04/2013Eth.C | | | |

| Patient Name: **Meklit Abebe**. Patient ID: **Dangila Hosp**. SEX/ Age: F/6/12. Date of Report: 25**/04/2013**.  BP: _______ Weight: ______ Height:____________ BSA: ________ | | | |
| --- | --- | --- | --- |
| **Features** | **Finding** | **Features** | **Finding** |
| **Profile** |  | **Atria** |  |
| Abdominal situs | Solitus | Left atrium | Normal |
| Cardiac position | Levocardia | Right atrium | Normal |
| Systemic venous drainage | Normal | **Atrioventricular valves** |  |
| Pulmonary venous drainage | Normal | Mitral valve | Annulus = 11mm |
| Atrioventricular connection | Concordant | Tricuspid valve | Annulus = 12mm  TAPSE = 15mm |
| Ventriculoarterial connection | Concordant | **Ventricles** |  |
| Ventricular loop | d-Loop | Left ventricle | Normal |
|  |  | Right ventricle | Normal |
| **Septae** |  | **Coronary arteries** | ----- |
| Interventricular septum | Intact | **Doppler Measurement** |  |
| Interatrial septum | Intact | Mitral | ----- |
| **Semilunal valves** |  | Aortic | ------- |
| Aortic valve | Annulus = 10mm | Tricuspid | ---- |
| Pulmonary valve | Annulus = 11mm | pulmonic | -------- |
| **Great arteries** | NRGA | **Aortic arch** | Left |
| Aorta | ----- | **PDA** | No |
| Pulmonary artery | Normal MPA and Branch PAs. |  |  |
| **M-Mode: Normal LV Function on eye balling** | | | |
| AO | mm | PWd | mm |
| LA | mm | EDV | ml |
| LVIDd | mm | ESV | ml |
| LVIDs | mm | LVEF | % |
| IVSd | mm | FS | % |
| **Additional Information**: |  | | |
| No pleural/pericardial effusion | | | |
| **Final Diagnosis:** | | | |
| 1. Normal Echocardiography Study. | | | |
| **Remark**: | | | |
| **Recommendation**: | | | |
| SIGNATURE  Done by: Tesfaye T., Pediatrician, Pediatric Cardiologist _______________ 25/04/2013Eth.C | | | |

| Patient Name: **Eskedar Tiruneh**. Patient ID: **Enjibara GH**. SEX/ Age: F/1 3/12. Date of Report: 26**/04/2013**.  BP: _______ Weight: ______ Height:____________ BSA: ________ | | | |
| --- | --- | --- | --- |
| **Features** | **Finding** | **Features** | **Finding** |
| **Profile** |  | **Atria** |  |
| Abdominal situs | Solitus | Left atrium | Normal |
| Cardiac position | Levocardia | Right atrium | Normal |
| Systemic venous drainage | Normal | **Atrioventricular valves** |  |
| Pulmonary venous drainage | Normal | Mitral valve | Annulus = 12mm |
| Atrioventricular connection | Concordant | Tricuspid valve | Annulus = 13mm  TAPSE = 11mm. |
| Ventriculoarterial connection | Concordant | **Ventricles** |  |
| Ventricular loop | d-Loop | Left ventricle | Normal |
|  |  | Right ventricle | Normal |
| **Septae** |  | **Coronary arteries** | ----- |
| Interventricular septum | Intact | **Doppler Measurement** |  |
| Interatrial septum | 4mm OS ASD, L – R Shunt | Mitral | ----- |
| **Semilunal valves** |  | Aortic | ------- |
| Aortic valve | Annulus = 11mm | Tricuspid | ---- |
| Pulmonary valve | Annulus = 11mm | pulmonic | -------- |
| **Great arteries** | NRGA | **Aortic arch** | Left |
| Aorta | ----- | **PDA** | No |
| Pulmonary artery | Normal MPA and Branch PAs. |  |  |
| **M-Mode:** Normal LV Function on eye balling. | | | |
| AO | mm | PWd | mm |
| LA | mm | EDV | ml |
| LVIDd | mm | ESV | ml |
| LVIDs | mm | LVEF | % |
| IVSd | mm | FS | % |
| **Additional Information**: |  | | |
| No pleural/pericardial effusion | | | |
| **Final Diagnosis:** | | | |
| 1. {S, D, S} Levocardia. 2. Small OS ASD, L – R Shunt | | | |
| **Remark**: | | | |
| **Recommendation**: | | | |
| SIGNATURE  Done by: Tesfaye T., Pediatrician, Pediatric Cardiologist _______________ 26/04/2013Eth.C | | | |

| Patient Name: **Amanuel Melesse**. Patient ID: **FHRH**. SEX/ Age: M/11/12. Date of Report: 26**/04/2013**.  BP: _______ Weight: ______ Height:____________ BSA: ________ | | | |
| --- | --- | --- | --- |
| **Features** | **Finding** | **Features** | **Finding** |
| **Profile** |  | **Atria** |  |
| Abdominal situs | Solitus | Left atrium | Normal |
| Cardiac position | Levocardia | Right atrium | Normal |
| Systemic venous drainage | Normal | **Atrioventricular valves** |  |
| Pulmonary venous drainage | Normal | Mitral valve | Annulus = 12mm |
| Atrioventricular connection | Concordant | Tricuspid valve | Annulus = 14mm  TAPSE = 15mm |
| Ventriculoarterial connection | Concordant | **Ventricles** |  |
| Ventricular loop | d-Loop | Left ventricle | Normal |
|  |  | Right ventricle | Normal |
| **Septae** |  | **Coronary arteries** | ----- |
| Interventricular septum | Intact | **Doppler Measurement** |  |
| Interatrial septum | PFO, L – R Shunt | Mitral | ----- |
| **Semilunal valves** |  | Aortic | ------- |
| Aortic valve | Annulus = 12mm | Tricuspid | ---- |
| Pulmonary valve | Annulus = 15mm | pulmonic | Mild PR, PPG = 40mmHg |
| **Great arteries** | NRGA | **Aortic arch** | Left |
| Aorta | ----- | **PDA** | <1mm PDA, L – R Shunt |
| Pulmonary artery | Normal MPA and Branch PAs. |  |  |
| **M-Mode:** Normal LV Function on eye balling. | | | |
| AO | mm | PWd | mm |
| LA | mm | EDV | ml |
| LVIDd | mm | ESV | ml |
| LVIDs | mm | LVEF | % |
| IVSd | mm | FS | % |
| **Additional Information**: |  | | |
| No pleural/pericardial effusion | | | |
| **Final Diagnosis:** | | | |
| 1. {S, D, S} Levocardia. 2. PFO, L – R Shunt 3. Small PDA, L – R Shunt 4. Mild Pulmonary Hypertension 5. Normal Biventricular Function | | | |
| **Remark**: Correlate with Clinical Finding, if no murmur, can be leveled as Silent PDA. | | | |
| **Recommendation**: No need of putting the baby on any form of cardiac medication. Just follow up only. | | | |
| SIGNATURE  Done by: Tesfaye T., Pediatrician, Pediatric Cardiologist _______________ 26/04/2013Eth.C | | | |

| Patient Name: **Temesgen Aynalem**. Patient ID: **TGSH**. SEX/ Age: M/12years. Date of Report: 27**/04/2013**.  BP: _______ Weight: ______ Height:____________ BSA: ________ | | | |
| --- | --- | --- | --- |
| **Features** | **Finding** | **Features** | **Finding** |
| **Profile** |  | **Atria** |  |
| Abdominal situs | Solitus | Left atrium | Normal |
| Cardiac position | Levocardia | Right atrium | Normal |
| Systemic venous drainage | Normal | **Atrioventricular valves** |  |
| Pulmonary venous drainage | Normal | Mitral valve | Annulus = 20mm |
| Atrioventricular connection | Concordant | Tricuspid valve | Annulus = 22mm |
| Ventriculoarterial connection | Concordant | **Ventricles** |  |
| Ventricular loop | d-Loop | Left ventricle | Normal |
|  |  | Right ventricle | Normal |
| **Septae** |  | **Coronary arteries** | ----- |
| Interventricular septum | Intact | **Doppler Measurement** |  |
| Interatrial septum | Intact | Mitral | ----- |
| **Semilunal valves** |  | Aortic | ------- |
| Aortic valve | Annulus = 16mm | Tricuspid | ---- |
| Pulmonary valve | Annulus = 17mm | pulmonic | -------- |
| **Great arteries** | NRGA | **Aortic arch** | Left |
| Aorta | ----- | **PDA** | No |
| Pulmonary artery | Normal MPA and Branch PAs. |  |  |
| **M-Mode:** | | | |
| AO | mm | PWd | mm |
| LA | mm | EDV | ml |
| LVIDd | mm | ESV | ml |
| LVIDs | mm | LVEF | 70% |
| IVSd | mm | FS | 39% |
| **Additional Information**: |  | | |
| No pleural/pericardial effusion | | | |
| **Final Diagnosis:** | | | |
| 1. Normal Echocardiography Study. | | | |
| **Remark**: | | | |
| **Recommendation**: | | | |
| SIGNATURE  Done by: Tesfaye T., Pediatrician, Pediatric Cardiologist _______________ 27/04/2013Eth.C | | | |

| Patient Name: **Meskerem Belay**. Patient ID: **FHRH**. SEX/ Age: F/2 3/12. Date of Report: 27**/04/2013**.  BP: _______ Weight: ______ Height:____________ BSA: ________ | | | |
| --- | --- | --- | --- |
| **Features** | **Finding** | **Features** | **Finding** |
| **Profile** |  | **Atria** |  |
| Abdominal situs | Solitus | Left atrium | Normal |
| Cardiac position | Levocardia | Right atrium | Normal |
| Systemic venous drainage | Normal | **Atrioventricular valves** |  |
| Pulmonary venous drainage | Normal | Mitral valve | Common Complete AVSD |
| Atrioventricular connection | Common Complete AVSD | Tricuspid valve |  |
| Ventriculoarterial connection | Concordant | **Ventricles** |  |
| Ventricular loop | d-Loop | Left ventricle | Normal |
|  |  | Right ventricle | Normal |
| **Septae** |  | **Coronary arteries** | ----- |
| Interventricular septum | Common Complete AVSD, L – R Shunt | **Doppler Measurement** |  |
| Interatrial septum |  | Mitral | Mild Left AVVR |
| **Semilunal valves** |  | Aortic | ------- |
| Aortic valve | Annulus = 13mm | Tricuspid | Moderate Right AVVR |
| Pulmonary valve | Annulus = 13mm | pulmonic | -------- |
| **Great arteries** | NRGA | **Aortic arch** | Left |
| Aorta | ----- | **PDA** | No |
| Pulmonary artery | Normal MPA and Branch PAs. |  |  |
| **M-Mode:** Normal LV Function on eye balling. | | | |
| AO | mm | PWd | mm |
| LA | mm | EDV | ml |
| LVIDd | mm | ESV | ml |
| LVIDs | mm | LVEF | % |
| IVSd | mm | FS | % |
| **Additional Information**: |  | | |
| No pleural/pericardial effusion | | | |
| **Final Diagnosis:** | | | |
| 1. {S, D, S} Levocardia. 2. Mild Left AVVR 3. Moderate Right AVVR 4. Common Complete Balanced AVSD | | | |
| **Remark**: Child was irritable throughout the study. | | | |
| **Recommendation**: | | | |
| SIGNATURE  Done by: Tesfaye T., Pediatrician, Pediatric Cardiologist _______________ 27/04/2013Eth.C | | | |

| Patient Name: **Banchialem Bahiru**. Patient ID: **FHRH**. SEX/ Age: F/85days. Date of Report: 28**/04/2013**.  BP: _______ Weight: ______ Height:____________ BSA: ________ | | | |
| --- | --- | --- | --- |
| **Features** | **Finding** | **Features** | **Finding** |
| **Profile** |  | **Atria** |  |
| Abdominal situs | Solitus | Left atrium | Normal |
| Cardiac position | Levocardia | Right atrium | Dilated |
| Systemic venous drainage | Normal | **Atrioventricular valves** |  |
| Pulmonary venous drainage | Normal | Mitral valve | Annulus = 9mm |
| Atrioventricular connection | Concordant | Tricuspid valve | Annulus = 10mm  TAPSE = 11mm. |
| Ventriculoarterial connection | Concordant | **Ventricles** |  |
| Ventricular loop | d-Loop | Left ventricle | Normal |
|  |  | Right ventricle | Dilated |
| **Septae** |  | **Coronary arteries** | ----- |
| Interventricular septum | 7mm LV – RA defect, LV to RA Shunt. | **Doppler Measurement** |  |
| Interatrial septum | PFO, L – R Shunt | Mitral | ----- |
| **Semilunal valves** |  | Aortic | ------- |
| Aortic valve | Annulus = 8mm | Tricuspid | ---- |
| Pulmonary valve | Annulus = 9mm | pulmonic | -------- |
| **Great arteries** | NRGA | **Aortic arch** | Left |
| Aorta | ----- | **PDA** | No |
| Pulmonary artery | Normal MPA and Branch PAs. |  |  |
| **M-Mode:** Normal LV Function on eye balling. | | | |
| AO | mm | PWd | mm |
| LA | mm | EDV | ml |
| LVIDd | mm | ESV | ml |
| LVIDs | mm | LVEF | % |
| IVSd | mm | FS | % |
| **Additional Information**: |  | | |
| No pleural/pericardial effusion | | | |
| **Final Diagnosis:** | | | |
| 1. {S, D, S} Levocardia. 2. PFO, L – R Shunt 3. Gerbode Defect, L – R Shunt 4. Normal Biventricular Function. | | | |
| **Remark**: | | | |
| **Recommendation**: | | | |
| SIGNATURE  Done by: Tesfaye T., Pediatrician, Pediatric Cardiologist _______________ 28/04/2013Eth.C | | | |

| Patient Name: **Nebiat Abiy**. Patient ID: **FHRH**. SEX/ Age: F/1 10/12. Date of Report: 01**/05/2013**.  BP: _______ Weight: ______ Height:____________ BSA: ________ | | | |
| --- | --- | --- | --- |
| **Features** | **Finding** | **Features** | **Finding** |
| **Profile** |  | **Atria** | |
| Abdominal situs | Solitus | Left atrium | Dilated |
| Cardiac position | Levocardia | Right atrium | Normal |
| Systemic venous drainage | Normal | **Atrioventricular valves** | |
| Pulmonary venous drainage | Normal | Mitral valve | Annulus = 19mm |
| Atrioventricular connection | Concordant | Tricuspid valve | Annulus = 13mm  TAPSE = 15mm. |
| Ventriculoarterial connection | Concordant | **Ventricles** | |
| Ventricular loop | d-Loop | Left ventricle | Dilated |
|  |  | Right ventricle | Normal |
| **Septae** |  | **Coronary arteries** | ----- |
| Interventricular septum | Intact | **Doppler Measurement** | |
| Interatrial septum | Intact | Mitral | Moderate to severe MR, Holosystolic, Posterior projection, seen in two planes with jet velocity = 5.2m/sec |
| **Semilunal valves** |  | Aortic | Mild AS, PPG/MPG = 44/26mmHg. Ill-defined hyper-echoic structure at the LVOT adjacent to the Aortic valve |
| Aortic valve | Annulus = 11mm | Tricuspid | ---- |
| Pulmonary valve | Annulus = 15mm | pulmonic | -------- |
| **Great arteries** | NRGA | **Aortic arch** | Left |
| Aorta | ----- | **PDA** | 5mm PDA, L – R Shunt. |
| Pulmonary artery | ------ |  |  |
| **M-Mode:** | | | |
| AO | mm | PWd | mm |
| LA | mm | EDV | ml |
| LVIDd | mm | ESV | ml |
| LVIDs | mm | LVEF | 53% |
| IVSd | mm | FS | 27% |
| **Additional Information**: |  | | |
| 4mm pericardial effusion on RV Side. | | | |
| **Final Diagnosis:** | | | |
| 1. {S, D, S} Levocardia. 2. Moderate to severe MR 3. Mild AS 4. Large PDA, L – R Shunt 5. ? IE 6. Small pericardial Effusion 7. Mildly Reduced LV Function. | | | |
| SIGNATURE  Done by: Tesfaye T., Pediatrician, Pediatric Cardiologist _______________ 01/05/2013Eth.C | | | |

| Patient Name: **Sobrina Adem.** Patient ID: **Pawi H**. SEX/ Age: F/11/12. Date of Report: 03**/05/2013**.  BP: _______ Weight: ______ Height:____________ BSA: ________ | | | |
| --- | --- | --- | --- |
| **Features** | **Finding** | **Features** | **Finding** |
| **Profile** |  | **Atria** |  |
| Abdominal situs | Solitus | Left atrium | Normal |
| Cardiac position | Levocardia | Right atrium | Dilated |
| Systemic venous drainage | Normal | **Atrioventricular valves** |  |
| Pulmonary venous drainage | Normal | Mitral valve | Annulus = 13mm |
| Atrioventricular connection | Concordant | Tricuspid valve | Annulus = 16mm |
| Ventriculoarterial connection | Concordant | **Ventricles** |  |
| Ventricular loop | d-Loop | Left ventricle | Normal |
|  |  | Right ventricle | Dilated |
| **Septae** |  | **Coronary arteries** | ----- |
| Interventricular septum | 11mm Inlet VSD, L – R Shunt | **Doppler Measurement** |  |
| Interatrial septum | 9mm OS ASD, L – R Shunt | Mitral | ----- |
| **Semilunal valves** |  | Aortic | ------- |
| Aortic valve | Annulus = 13mm | Tricuspid | ---- |
| Pulmonary valve | Annulus = 16mm | pulmonic | -------- |
| **Great arteries** | NRGA | **Aortic arch** | Left |
| Aorta | ----- | **PDA** | No |
| Pulmonary artery | MPA = 14mm. |  |  |
| **M-Mode: Normal LV Function on eye balling.** | | | |
| AO | mm | PWd | mm |
| LA | mm | EDV | ml |
| LVIDd | mm | ESV | ml |
| LVIDs | mm | LVEF | % |
| IVSd | mm | FS | % |
| **Additional Information**: |  | | |
| No pleural/pericardial effusion | | | |
| **Final Diagnosis:** | | | |
| 1. {S, D, S} Levocardia. 2. Moderate OS ASD, L – R Shunt 3. Large Inlet VSD, L – R Shunt 4. Normal LV Function | | | |
| **Remark**: Infant was crying during study (Doppler not done) | | | |
| **Recommendation**: | | | |
| SIGNATURE  Done by: Tesfaye T., Pediatrician, Pediatric Cardiologist _______________ 03/05/2013Eth.C | | | |

| Patient Name: **Getnet Wudie**. Patient ID: **Adet PH**. SEX/ Age: M/12Years. Date of Report: 04**/05/2013**.  BP: _______ Weight: ______ Height:____________ BSA: ________ | | | |
| --- | --- | --- | --- |
| **Features** | **Finding** | **Features** | **Finding** |
| **Profile** |  | **Atria** |  |
| Abdominal situs | Solitus | Left atrium | Normal |
| Cardiac position | Levocardia | Right atrium | Normal |
| Systemic venous drainage | Normal | **Atrioventricular valves** |  |
| Pulmonary venous drainage | Normal | Mitral valve | Annulus = 19mm. MV E/A = 3.4. DT = 482ms. |
| Atrioventricular connection | Concordant | Tricuspid valve | Annulus = 17mm  TAPSE = 18mm. |
| Ventriculoarterial connection | Concordant | **Ventricles** |  |
| Ventricular loop | d-Loop | Left ventricle | Normal |
|  |  | Right ventricle | Normal |
| **Septae** |  | **Coronary arteries** | ----- |
| Interventricular septum | Intact | **Doppler Measurement** |  |
| Interatrial septum | Intact | Mitral | ----- |
| **Semilunal valves** |  | Aortic | ------- |
| Aortic valve | Annulus = 16mm | Tricuspid | ---- |
| Pulmonary valve | Annulus = 19mm | pulmonic | -------- |
| **Great arteries** | NRGA | **Aortic arch** | Left |
| Aorta | ----- | **PDA** | No |
| Pulmonary artery | Normal MPA and Branch PAs. |  |  |
| **M-Mode:** | | | |
| AO | mm | PWd | mm |
| LA | mm | EDV | ml |
| LVIDd | mm | ESV | ml |
| LVIDs | mm | LVEF | 54% |
| IVSd | mm | FS | 27% |
| **Additional Information**: |  | | |
| 11mm Right pleural effusion. Pericardial effusion 11mm on RV Side, 12mm at apex and 7mm on LV Side with pericardial thickening and echo debris inside. | | | |
| **Final Diagnosis:** | | | |
| 1. {S, D, S} Levocardia. 2. Mildly Reduced LV Systolic Function 3. Severe Diastolic LV Dysfunction 4. Mild Right Pleural Effusion 5. Moderate Pericardial effusion with pericardial thickening and echo debris inside. | | | |
| **Remark**: | | | |
| **Recommendation**: | | | |
| SIGNATURE  Done by: Tesfaye T., Pediatrician, Pediatric Cardiologist _______________ 04/05/2013Eth.C | | | |

| Patient Name: **Mekdes Zemenu**. Patient ID: **TGSH**. SEX/ Age: F/1 8/12. Date of Report: 04**/05/2013**.  BP: _______ Weight: ______ Height:____________ BSA: ________ | | | |
| --- | --- | --- | --- |
| **Features** | **Finding** | **Features** | **Finding** |
| **Profile** |  | **Atria** |  |
| Abdominal situs | Solitus | Left atrium | Normal |
| Cardiac position | Levocardia | Right atrium | Normal |
| Systemic venous drainage | Normal | **Atrioventricular valves** |  |
| Pulmonary venous drainage | Normal | Mitral valve | Annulus = 13mm |
| Atrioventricular connection | Concordant | Tricuspid valve | Annulus = 14mm  TAPSE = 18mm. |
| Ventriculoarterial connection | Concordant | **Ventricles** |  |
| Ventricular loop | d-Loop | Left ventricle | Normal |
|  |  | Right ventricle | Normal |
| **Septae** |  | **Coronary arteries** | ----- |
| Interventricular septum | Intact | **Doppler Measurement** |  |
| Interatrial septum | Intact | Mitral | ----- |
| **Semilunal valves** |  | Aortic | ------- |
| Aortic valve | Annulus = 11mm | Tricuspid | ---- |
| Pulmonary valve | Annulus = 12mm | pulmonic | -------- |
| **Great arteries** | NRGA | **Aortic arch** | Left |
| Aorta | ----- | **PDA** | No |
| Pulmonary artery | Normal MPA and Branch PAs. |  |  |
| **M-Mode:** Normal LV Function on eye balling. | | | |
| AO | mm | PWd | mm |
| LA | mm | EDV | ml |
| LVIDd | mm | ESV | ml |
| LVIDs | mm | LVEF | % |
| IVSd | mm | FS | % |
| **Additional Information**: |  | | |
| No pleural/pericardial effusion | | | |
| **Final Diagnosis:** | | | |
| 1. Normal Echocardiography Study. | | | |
| **Remark**: | | | |
| **Recommendation**: | | | |
| SIGNATURE  Done by: Tesfaye T., Pediatrician, Pediatric Cardiologist _______________ 04/05/2013Eth.C | | | |

| Patient Name: **Yididiya Getaneh**. Patient ID: **TGSH**. SEX/ Age: F/24days. Date of Report: 05**/05/2013**.  BP: _______ Weight: ______ Height:____________ BSA: ________ | | | |
| --- | --- | --- | --- |
| **Features** | **Finding** | **Features** | **Finding** |
| **Profile** |  | **Atria** |  |
| Abdominal situs | Solitus | Left atrium | Normal |
| Cardiac position | Levocardia | Right atrium | Normal |
| Systemic venous drainage | Normal | **Atrioventricular valves** |  |
| Pulmonary venous drainage | Normal | Mitral valve | Annulus = 10mm |
| Atrioventricular connection | Concordant | Tricuspid valve | Annulus = 9mm |
| Ventriculoarterial connection | Concordant | **Ventricles** |  |
| Ventricular loop | d-Loop | Left ventricle | Normal |
|  |  | Right ventricle | Normal |
| **Septae** |  | **Coronary arteries** | ----- |
| Interventricular septum | Intact | **Doppler Measurement** |  |
| Interatrial septum | PFO, L – R Shunt | Mitral | ----- |
| **Semilunal valves** |  | Aortic | ------- |
| Aortic valve | Annulus = 9mm | Tricuspid | ---- |
| Pulmonary valve | Annulus = 8mm | pulmonic | -------- |
| **Great arteries** | NRGA | **Aortic arch** | Left |
| Aorta | ----- | **PDA** | No |
| Pulmonary artery | Normal MPA and Branch PAs. |  |  |
| **M-Mode:** Normal lv Function on LV Function. | | | |
| AO | mm | PWd | mm |
| LA | mm | EDV | ml |
| LVIDd | mm | ESV | ml |
| LVIDs | mm | LVEF | % |
| IVSd | mm | FS | % |
| **Additional Information**: |  | | |
| No pleural/pericardial effusion | | | |
| **Final Diagnosis:** | | | |
| 1. {S, D, S} Levocardia. 2. PFO, L – R Shunt | | | |
| **Remark**: | | | |
| **Recommendation**: | | | |
| SIGNATURE  Done by: Tesfaye T., Pediatrician, Pediatric Cardiologist _______________ 05/05/2013Eth.C | | | |

| Patient Name: **Imran Mohamed**. Patient ID: **TGSH**. SEX/ Age: M/2 6/12. Date of Report: 06**/05/2013**.  BP: _______ Weight: ______ Height:____________ BSA: ________ | | | |
| --- | --- | --- | --- |
| **Features** | **Finding** | **Features** | **Finding** |
| **Profile** |  | **Atria** |  |
| Abdominal situs | Solitus | Left atrium | Normal |
| Cardiac position | Levocardia | Right atrium | Normal |
| Systemic venous drainage | Normal | **Atrioventricular valves** |  |
| Pulmonary venous drainage | Normal | Mitral valve | Annulus = 10mm |
| Atrioventricular connection | Concordant | Tricuspid valve | Annulus = 16mm |
| Ventriculoarterial connection | Concordant | **Ventricles** |  |
| Ventricular loop | d-Loop | Left ventricle | Normal |
|  |  | Right ventricle | Normal |
| **Septae** |  | **Coronary arteries** | ----- |
| Interventricular septum | 8mm Subaortic Nonrestrictive VSD, Predominantly L – R Shunt | **Doppler Measurement** |  |
| Interatrial septum | Intact | Mitral | ----- |
| **Semilunal valves** |  | Aortic | ------- |
| Aortic valve | Annulus = 12mm | Tricuspid | ---- |
| Pulmonary valve | Annulus = 16mm | pulmonic | -------- |
| **Great arteries** | NRGA | **Aortic arch** | Left |
| Aorta | ----- | **PDA** | No |
| Pulmonary artery | Normal MPA and Branch PAs. |  |  |
| **M-Mode:** | | | |
| AO | mm | PWd | mm |
| LA | mm | EDV | ml |
| LVIDd | mm | ESV | ml |
| LVIDs | mm | LVEF | 58% |
| IVSd | mm | FS | 29% |
| **Additional Information**: |  | | |
| No pleural/pericardial effusion | | | |
| **Final Diagnosis:** | | | |
| 1. {S, D, S} Levocardia. 2. Moderate Subaortic Nonrestrictive VSD, Predominantly L – R Shunt 3. Normal LV Function | | | |
| **Remark**: | | | |
| **Recommendation**: | | | |
| SIGNATURE  Done by: Tesfaye T., Pediatrician, Pediatric Cardiologist _______________ 06/05/2013Eth.C | | | |

| Patient Name: **Alemnew Merie**. Patient ID: **TGSH**. SEX/ Age: M/9years. Date of Report: 06**/05/2013**.  BP: _______ Weight: ______ Height:____________ BSA: ________ | | | |
| --- | --- | --- | --- |
| **Features** | **Finding** | **Features** | **Finding** |
| **Profile** |  | **Atria** |  |
| Abdominal situs | Solitus | Left atrium | Dilated |
| Cardiac position | Levocardia | Right atrium | Normal |
| Systemic venous drainage | Normal | **Atrioventricular valves** | |
| Pulmonary venous drainage | Normal | Mitral valve | Annulus = 22mm. Thickened, clubbed MVL. MVA = 1.05cm2. |
| Atrioventricular connection | Concordant | Tricuspid valve | Annulus = 22mm  TAPSE = 18mm. |
| Ventriculoarterial connection | Concordant | **Ventricles** |  |
| Ventricular loop | d-Loop | Left ventricle | Dilated |
|  |  | Right ventricle | Normal |
| **Septae** |  | **Coronary arteries** | ----- |
| Interventricular septum | Intact | **Doppler Measurement** | |
| Interatrial septum | Intact | Mitral | Moderate MR, Holosystolic, posterior projection, seen in two planes with jet velocity = 4.4m/sec. Moderate MS, PPG/MPG = 18/7mmHg. |
| **Semilunal valves** |  | Aortic | Severe AR, PHT = 170ms. |
| Aortic valve | Annulus = 15mm. Trileaflet, thickened | Tricuspid | Mild TR, PPG = 30mmHg. |
| Pulmonary valve | Annulus = 19mm | pulmonic | -------- |
| **Great arteries** | NRGA | **Aortic arch** | Left |
| Aorta | ----- | **PDA** | No |
| Pulmonary artery | Normal MPA. |  |  |
| **M-Mode:** | | | |
| AO | mm | PWd | mm |
| LA | mm | EDV | ml |
| LVIDd | mm | ESV | ml |
| LVIDs | mm | LVEF | 53% |
| IVSd | mm | FS | 28% |
| **Additional Information**: |  | | |
| No pleural/pericardial effusion | | | |
| **Final Diagnosis:** | | | |
| 1. {S, D, S} Levocardia. 2. LA/LV Dilated 3. Thickened, clubbed MVL. Thickened Aortic valve 4. Moderate MR 5. Moderate MS 6. Severe AR 7. Mildly reduced LV Function | | | |
| SIGNATURE  Done by: Tesfaye T., Pediatrician, Pediatric Cardiologist _______________ 06/05/2013Eth.C | | | |

| Patient Name: **Christian Yohannes**. Patient ID: **GAMBY GH**. SEX/ Age: M/4years. Date of Report: 06**/05/2013**.  BP: _______ Weight: ______ Height:____________ BSA: ________ | | | |
| --- | --- | --- | --- |
| **Features** | **Finding** | **Features** | **Finding** |
| **Profile** |  | **Atria** |  |
| Abdominal situs | Solitus | Left atrium | Normal |
| Cardiac position | Levocardia | Right atrium | Normal |
| Systemic venous drainage | Normal | **Atrioventricular valves** |  |
| Pulmonary venous drainage | Normal | Mitral valve | Annulus = 16mm |
| Atrioventricular connection | Concordant | Tricuspid valve | Annulus = 17mm  TAPSE = 21mm |
| Ventriculoarterial connection | Concordant | **Ventricles** |  |
| Ventricular loop | d-Loop | Left ventricle | Normal |
|  |  | Right ventricle | Normal |
| **Septae** |  | **Coronary arteries** | ----- |
| Interventricular septum | Intact | **Doppler Measurement** |  |
| Interatrial septum | Intact | Mitral | ----- |
| **Semilunal valves** |  | Aortic | ------- |
| Aortic valve | Annulus = 16mm | Tricuspid | ---- |
| Pulmonary valve | Annulus = 18mm | pulmonic | Trivial PR, PPG = 12mmHg. |
| **Great arteries** | NRGA | **Aortic arch** | Left |
| Aorta | ----- | **PDA** | No |
| Pulmonary artery | Normal MPA and Branch PAs. |  |  |
| **M-Mode:** | | | |
| AO | mm | PWd | mm |
| LA | mm | EDV | ml |
| LVIDd | mm | ESV | ml |
| LVIDs | mm | LVEF | 67% |
| IVSd | mm | FS | 36% |
| **Additional Information**: |  | | |
| No pleural/pericardial effusion | | | |
| **Final Diagnosis:** | | | |
| 1. Normal Echocardiography Study. | | | |
| **Remark**: | | | |
| **Recommendation**: | | | |
| SIGNATURE  Done by: Tesfaye T., Pediatrician, Pediatric Cardiologist _______________ 06/05/2013Eth.C | | | |

| Patient Name: **Blen Asnake**. Patient ID: **TGSH**. SEX/ Age: F/6/12. Date of Report: 06**/05/2013**.  BP: _______ Weight: ______ Height:____________ BSA: ________ | | | |
| --- | --- | --- | --- |
| **Features** | **Finding** | **Features** | **Finding** |
| **Profile** |  | **Atria** |  |
| Abdominal situs | Solitus | Left atrium | Dilated |
| Cardiac position | Levocardia | Right atrium | Normal |
| Systemic venous drainage | Normal | **Atrioventricular valves** |  |
| Pulmonary venous drainage | Normal | Mitral valve | Annulus = 13mm |
| Atrioventricular connection | Concordant | Tricuspid valve | Annulus = 12mm |
| Ventriculoarterial connection | Concordant | **Ventricles** |  |
| Ventricular loop | d-Loop | Left ventricle | Dilated |
|  |  | Right ventricle | Normal |
| **Septae** |  | **Coronary arteries** | ----- |
| Interventricular septum | 7mm PM VSD, L – R Shunt | **Doppler Measurement** |  |
| Interatrial septum | Intact | Mitral | ----- |
| **Semilunal valves** |  | Aortic | ------- |
| Aortic valve | Annulus = 9mm | Tricuspid | ---- |
| Pulmonary valve | Annulus = 17mm | pulmonic | Moderate PR, PPG = 56mmHg |
| **Great arteries** | NRGA | **Aortic arch** | Left |
| Aorta | ----- | **PDA** | No |
| Pulmonary artery | MPA =19mm. |  |  |
| **M-Mode:** | | | |
| AO | mm | PWd | mm |
| LA | mm | EDV | ml |
| LVIDd | mm | ESV | ml |
| LVIDs | mm | LVEF | 65% |
| IVSd | mm | FS | 34% |
| **Additional Information**: |  | | |
| No pleural/pericardial effusion | | | |
| **Final Diagnosis:** | | | |
| 1. {S, D, S} Levocardia. 2. Moderate PM VSD, L – R Shunt 3. Moderate Pulmonary Hypertension 4. Normal LV Function | | | |
| **Remark**: | | | |
| **Recommendation**: | | | |
| SIGNATURE  Done by: Tesfaye T., Pediatrician, Pediatric Cardiologist _______________ 06/05/2013Eth.C | | | |

| Patient Name: **Mihretu Sisay**. Patient ID: **Adinas GH**. SEX/ Age: M/10years. Date of Report: 06**/05/2013**.  BP: _______ Weight: ______ Height:____________ BSA: ________ | | | |
| --- | --- | --- | --- |
| **Features** | **Finding** | **Features** | **Finding** |
| **Profile** |  | **Atria** |  |
| Abdominal situs | Solitus | Left atrium | Dilated |
| Cardiac position | Levocardia | Right atrium | Dilated |
| Systemic venous drainage | Normal | **Atrioventricular valves** |  |
| Pulmonary venous drainage | Normal | Mitral valve | Annulus = 19mm |
| Atrioventricular connection | Concordant | Tricuspid valve | Annulus = 20mm  TAPSE = 21mm |
| Ventriculoarterial connection | Concordant | **Ventricles** |  |
| Ventricular loop | d-Loop | Left ventricle | Dilated |
|  |  | Right ventricle | Dilated |
| **Septae** |  | **Coronary arteries** | ----- |
| Interventricular septum | 20mm Subaortic nonrestrictive VSD, L – R Shunt | **Doppler Measurement** |  |
| Interatrial septum | Intact | Mitral | ----- |
| **Semilunal valves** |  | Aortic | ------- |
| Aortic valve | Annulus = 25mm | Tricuspid | ---- |
| Pulmonary valve | Annulus = 28mm | pulmonic | Moderate PR, PPG = 53mmHg |
| **Great arteries** | NRGA | **Aortic arch** | Left |
| Aorta | ----- | **PDA** | No |
| Pulmonary artery | MPA = 30mm. |  |  |
| **M-Mode:** | | | |
| AO | mm | PWd | mm |
| LA | mm | EDV | ml |
| LVIDd | mm | ESV | ml |
| LVIDs | mm | LVEF | 59% |
| IVSd | mm | FS | 31% |
| **Additional Information**: |  | | |
| No pleural/pericardial effusion | | | |
| **Final Diagnosis:** | | | |
| 1. {S, D, S} Levocardia. 2. Large Subaortic VSD, L – R Shunt 3. Moderate to severe Pulmonary Hypertension 4. Normal Biventricular Function | | | |
| **Remark**: | | | |
| **Recommendation**: | | | |
| SIGNATURE  Done by: Tesfaye T., Pediatrician, Pediatric Cardiologist _______________ 06/05/2013Eth.C | | | |

| Patient Name: **Matias Endihnew**. Patient ID: **FHRH**. SEX/ Age: M/2 3/12. Date of Report: 07**/05/2013**.  BP: _______ Weight: ______ Height:____________ BSA: ________ | | | |
| --- | --- | --- | --- |
| **Features** | **Finding** | **Features** | **Finding** |
| **Profile** |  | **Atria** |  |
| Abdominal situs | Solitus | Left atrium | Normal |
| Cardiac position | Levocardia | Right atrium | Normal |
| Systemic venous drainage | Normal | **Atrioventricular valves** |  |
| Pulmonary venous drainage | Normal | Mitral valve | Annulus = 16mm |
| Atrioventricular connection | Concordant | Tricuspid valve | Annulus = 18mm |
| Ventriculoarterial connection | Concordant | **Ventricles** |  |
| Ventricular loop | d-Loop | Left ventricle | Normal |
|  |  | Right ventricle | Normal |
| **Septae** |  | **Coronary arteries** | ----- |
| Interventricular septum | 13mm Inlet VSD, L – R Shunt | **Doppler Measurement** |  |
| Interatrial septum | Intact | Mitral | ----- |
| **Semilunal valves** |  | Aortic | ------- |
| Aortic valve | Annulus = 14mm | Tricuspid | ---- |
| Pulmonary valve | Annulus = 18mm | pulmonic | -------- |
| **Great arteries** | NRGA | **Aortic arch** | Left |
| Aorta | ----- | **PDA** | No |
| Pulmonary artery | Normal MPA and Branch PAs. |  |  |
| **M-Mode:** | | | |
| AO | mm | PWd | mm |
| LA | mm | EDV | ml |
| LVIDd | mm | ESV | ml |
| LVIDs | mm | LVEF | 59% |
| IVSd | mm | FS | 31% |
| **Additional Information**: |  | | |
| No pleural/pericardial effusion | | | |
| **Final Diagnosis:** | | | |
| 1. {S, D, S} Levocardia. 2. Large Inlet VSD, L – R Shunt 3. Normal LV Function | | | |
| **Remark**: | | | |
| **Recommendation**: | | | |
| SIGNATURE  Done by: Tesfaye T., Pediatrician, Pediatric Cardiologist _______________ 07/05/2013Eth.C | | | |

| Patient Name: **Netsanet Muluneh**. Patient ID: **Amaris PSC**. SEX/ Age: F/5/12. Date of Report: 09**/05/2013**.  BP: _______ Weight: ______ Height:____________ BSA: ________ | | | |
| --- | --- | --- | --- |
| **Features** | **Finding** | **Features** | **Finding** |
| **Profile** |  | **Atria** |  |
| Abdominal situs | Solitus | Left atrium | Dilated |
| Cardiac position | Levocardia | Right atrium | Normal |
| Systemic venous drainage | Normal | **Atrioventricular valves** |  |
| Pulmonary venous drainage | Normal | Mitral valve | Annulus = 14mm |
| Atrioventricular connection | Concordant | Tricuspid valve | Annulus = 12mm  TAPSE = 13mm. |
| Ventriculoarterial connection | Concordant | **Ventricles** |  |
| Ventricular loop | d-Loop | Left ventricle | Dilated |
|  |  | Right ventricle | Normal |
| **Septae** |  | **Coronary arteries** | ----- |
| Interventricular septum | Intact | **Doppler Measurement** |  |
| Interatrial septum | Intact | Mitral | ----- |
| **Semilunal valves** |  | Aortic | ------- |
| Aortic valve | Annulus = 10mm | Tricuspid | ---- |
| Pulmonary valve | Annulus = 12mm | pulmonic | -------- |
| **Great arteries** | NRGA | **Aortic arch** | Left |
| Aorta | ----- | **PDA** | 2.5mm PDA, L – R Shunt |
| Pulmonary artery | Normal MPA and Branch PAs. |  |  |
| **M-Mode:** | | | |
| AO | mm | PWd | mm |
| LA | mm | EDV | ml |
| LVIDd | mm | ESV | ml |
| LVIDs | mm | LVEF | 70% |
| IVSd | mm | FS | 38% |
| **Additional Information**: |  | | |
| No pleural/pericardial effusion | | | |
| **Final Diagnosis:** | | | |
| 1. {S, D, S} Levocardia. 2. Moderate PDA, L – R Shunt 3. Normal Biventricular Function. | | | |
| **Remark**: | | | |
| **Recommendation**: | | | |
| SIGNATURE  Done by: Tesfaye T., Pediatrician, Pediatric Cardiologist _______________ 09/05/2013Eth.C | | | |

| Patient Name: **Yohannes Tenaw**. Patient ID: **FHRH**. SEX/ Age: M/7/12. Date of Report: 09**/05/2013**.  BP: _______ Weight: ______ Height:____________ BSA: ________ | | | |
| --- | --- | --- | --- |
| **Features** | **Finding** | **Features** | **Finding** |
| **Profile** |  | **Atria** |  |
| Abdominal situs | Solitus | Left atrium | Normal |
| Cardiac position | Levocardia | Right atrium | Normal |
| Systemic venous drainage | Normal | **Atrioventricular valves** |  |
| Pulmonary venous drainage | Normal | Mitral valve | Annulus = 12mm |
| Atrioventricular connection | Concordant | Tricuspid valve | Annulus = 13mm  TAPSE = 14mm. |
| Ventriculoarterial connection | Concordant | **Ventricles** |  |
| Ventricular loop | d-Loop | Left ventricle | Normal |
|  |  | Right ventricle | Normal |
| **Septae** |  | **Coronary arteries** | ----- |
| Interventricular septum | Intact | **Doppler Measurement** |  |
| Interatrial septum | Intact | Mitral | ----- |
| **Semilunal valves** |  | Aortic | ------- |
| Aortic valve | Annulus = 10mm | Tricuspid | ---- |
| Pulmonary valve | Annulus = 12mm | pulmonic | -------- |
| **Great arteries** | NRGA | **Aortic arch** | Left |
| Aorta | ----- | **PDA** | No |
| Pulmonary artery | Normal MPA and Branch PAs. |  |  |
| **M-Mode:** | | | |
| AO | mm | PWd | mm |
| LA | mm | EDV | ml |
| LVIDd | mm | ESV | ml |
| LVIDs | mm | LVEF | 70% |
| IVSd | mm | FS | 37% |
| **Additional Information**: |  | | |
| No pleural/pericardial effusion | | | |
| **Final Diagnosis:** | | | |
| 1. Normal Echocardiography. | | | |
| **Remark**: | | | |
| **Recommendation**: | | | |
| SIGNATURE  Done by: Tesfaye T., Pediatrician, Pediatric Cardiologist _______________ 09/05/2013Eth.C | | | |

| Patient Name: **Baby of Bizuayehu Werkayehu**. Patient ID: **FHRH**. SEX/ Age: F/41days. Date of Report: 10**/05/2013**.  BP: _______ Weight: ______ Height:____________ BSA: ________ | | | |
| --- | --- | --- | --- |
| **Features** | **Finding** | **Features** | **Finding** |
| **Profile** |  | **Atria** |  |
| Abdominal situs | Solitus | Left atrium | Normal |
| Cardiac position | Levocardia | Right atrium | Normal |
| Systemic venous drainage | Normal | **Atrioventricular valves** |  |
| Pulmonary venous drainage | Normal | Mitral valve | Annulus = 8mm |
| Atrioventricular connection | Concordant | Tricuspid valve | Annulus = 8mm |
| Ventriculoarterial connection | Concordant | **Ventricles** |  |
| Ventricular loop | d-Loop | Left ventricle | Normal |
|  |  | Right ventricle | Normal |
| **Septae** |  | **Coronary arteries** | ----- |
| Interventricular septum | Intact | **Doppler Measurement** |  |
| Interatrial septum | PFO, L – R Shunt | Mitral | ----- |
| **Semilunal valves** |  | Aortic | ------- |
| Aortic valve | Annulus = 7mm | Tricuspid | ---- |
| Pulmonary valve | Annulus = 8mm | pulmonic | -------- |
| **Great arteries** | NRGA | **Aortic arch** | Left |
| Aorta | ----- | **PDA** | No |
| Pulmonary artery | Normal MPA and Branch PAs. |  |  |
| **M-Mode:** Normal LV Function. | | | |
| AO | mm | PWd | mm |
| LA | mm | EDV | ml |
| LVIDd | mm | ESV | ml |
| LVIDs | mm | LVEF | % |
| IVSd | mm | FS | % |
| **Additional Information**: |  | | |
| No pleural/pericardial effusion | | | |
| **Final Diagnosis:** | | | |
| 1. {S, D, S} Levocardia. 2. PFO, L – R Shunt | | | |
| **Remark**: | | | |
| **Recommendation**: | | | |
| SIGNATURE  Done by: Tesfaye T., Pediatrician, Pediatric Cardiologist _______________ 10/05/2013Eth.C | | | |

| Patient Name: **Yordanos Molla**. Patient ID: **TGSH**. SEX/ Age: M/5/12. Date of Report: 13**/05/2013**.  BP: _______ Weight: ______ Height:____________ BSA: ________ | | | |
| --- | --- | --- | --- |
| **Features** | **Finding** | **Features** | **Finding** |
| **Profile** |  | **Atria** |  |
| Abdominal situs | Solitus | Left atrium | Normal |
| Cardiac position | Levocardia | Right atrium | Normal |
| Systemic venous drainage | Normal | **Atrioventricular valves** |  |
| Pulmonary venous drainage | Normal | Mitral valve | Annulus = 12mm |
| Atrioventricular connection | Concordant | Tricuspid valve | Annulus = 12mm  TAPSE = 15mm |
| Ventriculoarterial connection | Concordant | **Ventricles** |  |
| Ventricular loop | d-Loop | Left ventricle | Normal |
|  |  | Right ventricle | Normal |
| **Septae** |  | **Coronary arteries** | ----- |
| Interventricular septum | Intact | **Doppler Measurement** |  |
| Interatrial septum | Intact | Mitral | ----- |
| **Semilunal valves** |  | Aortic | ------- |
| Aortic valve | Annulus = 12mm | Tricuspid | ---- |
| Pulmonary valve | Annulus = 12mm | pulmonic | -------- |
| **Great arteries** | NRGA | **Aortic arch** | Left |
| Aorta | ----- | **PDA** | No |
| Pulmonary artery | Normal MPA and Branch PAs. |  |  |
| **M-Mode: Normal LV Function on eye balling.** | | | |
| AO | mm | PWd | mm |
| LA | mm | EDV | ml |
| LVIDd | mm | ESV | ml |
| LVIDs | mm | LVEF | % |
| IVSd | mm | FS | % |
| **Additional Information**: |  | | |
| No pleural/pericardial effusion | | | |
| **Final Diagnosis:** | | | |
| 1. Normal Echocardiography Study. | | | |
| **Remark**: | | | |
| **Recommendation**: | | | |
| SIGNATURE  Done by: Tesfaye T., Pediatrician, Pediatric Cardiologist _______________ 13/05/2013Eth.C | | | |

| Patient Name: **Sofonias Ayalew**. Patient ID: **Enjibara GH**. SEX/ Age: M/15days. Date of Report: 13**/05/2013**.  BP: _______ Weight: ______ Height:____________ BSA: ________ | | | |
| --- | --- | --- | --- |
| **Features** | **Finding** | **Features** | **Finding** |
| **Profile** |  | **Atria** |  |
| Abdominal situs | Solitus | Left atrium | Normal |
| Cardiac position | Levocardia | Right atrium | Normal |
| Systemic venous drainage | Normal | **Atrioventricular valves** |  |
| Pulmonary venous drainage | Normal | Mitral valve | Annulus = 9mm |
| Atrioventricular connection | Concordant | Tricuspid valve | Annulus = 9mm  TAPSE = 10mm |
| Ventriculoarterial connection | Concordant | **Ventricles** |  |
| Ventricular loop | d-Loop | Left ventricle | Normal |
|  |  | Right ventricle | Normal |
| **Septae** |  | **Coronary arteries** | ----- |
| Interventricular septum | 3mm Muscular VSD, L – R Shunt | **Doppler Measurement** |  |
| Interatrial septum | PFO, L – R Shunt | Mitral | ----- |
| **Semilunal valves** |  | Aortic | ------- |
| Aortic valve | Annulus = 9mm | Tricuspid | ---- |
| Pulmonary valve | Annulus = 9mm | pulmonic | -------- |
| **Great arteries** | NRGA | **Aortic arch** | Left |
| Aorta | ----- | **PDA** | No |
| Pulmonary artery | Normal MPA and Branch PAs. |  |  |
| **M-Mode:** Normal LV Function on eye balling. | | | |
| AO | mm | PWd | mm |
| LA | mm | EDV | ml |
| LVIDd | mm | ESV | ml |
| LVIDs | mm | LVEF | % |
| IVSd | mm | FS | % |
| **Additional Information**: |  | | |
| No pleural/pericardial effusion | | | |
| **Final Diagnosis:** | | | |
| 1. {S, D, S} Levocardia. 2. PFO, L – R Shunt 3. Small Muscular VSD, L – R Shunt | | | |
| **Remark**: Likely to close. | | | |
| **Recommendation**: no need to start medication, Yearly follow up echo, Watch for IE | | | |
| SIGNATURE  Done by: Tesfaye T., Pediatrician, Pediatric Cardiologist _______________ 13/05/2013Eth.C | | | |

| Name: **Baby of Yenie – alem Shiferaw**. Patient ID: **FHRH**. SEX/ Age: M/2 6/12. Date of Report: 13**/05/2013**.  BP: _______ Weight: ______ Height:____________ BSA: ________ | | | |
| --- | --- | --- | --- |
| **Features** | **Finding** | **Features** | **Finding** |
| **Profile** |  | **Atria** |  |
| Abdominal situs | Solitus | Left atrium | Normal |
| Cardiac position | Levocardia | Right atrium | Normal |
| Systemic venous drainage | Normal | **Atrioventricular valves** |  |
| Pulmonary venous drainage | Normal | Mitral valve | Annulus = 14mm |
| Atrioventricular connection | Concordant | Tricuspid valve | Annulus = 15mm  TAPSE = 18mm |
| Ventriculoarterial connection | Concordant | **Ventricles** |  |
| Ventricular loop | d-Loop | Left ventricle | Normal |
|  |  | Right ventricle | Normal |
| **Septae** |  | **Coronary arteries** | ----- |
| Interventricular septum | Intact | **Doppler Measurement** |  |
| Interatrial septum | Intact | Mitral | ----- |
| **Semilunal valves** |  | Aortic | ------- |
| Aortic valve | Annulus = 15mm | Tricuspid | ---- |
| Pulmonary valve | Annulus = 15mm | pulmonic | -------- |
| **Great arteries** | NRGA | **Aortic arch** | Left |
| Aorta | ----- | **PDA** | No |
| Pulmonary artery | Normal MPA and Branch PAs. |  |  |
| **M-Mode:** | | | |
| AO | mm | PWd | mm |
| LA | mm | EDV | ml |
| LVIDd | mm | ESV | ml |
| LVIDs | mm | LVEF | 58% |
| IVSd | mm | FS | 29% |
| **Additional Information**: |  | | |
| No pleural/pericardial effusion | | | |
| **Final Diagnosis:** | | | |
| 1. Normal Echocardiography Study. | | | |
| **Remark**: | | | |
| **Recommendation**: | | | |
| SIGNATURE  Done by: Tesfaye T., Pediatrician, Pediatric Cardiologist _______________ 13/05/2013Eth.C | | | |

| Patient Name: **Meklit Abiyot**. Patient ID: **Guzara MC**. SEX/ Age: F/8/12. Date of Report: 15**/05/2013**.  BP: _______ Weight: ______ Height:____________ BSA: ________ | | | |
| --- | --- | --- | --- |
| **Features** | **Finding** | **Features** | **Finding** |
| **Profile** |  | **Atria** |  |
| Abdominal situs | Solitus | Left atrium | Dilated |
| Cardiac position | Levocardia | Right atrium | Normal |
| Systemic venous drainage | Normal | **Atrioventricular valves** |  |
| Pulmonary venous drainage | Normal | Mitral valve | Annulus = 15mm |
| Atrioventricular connection | Concordant | Tricuspid valve | Annulus = 14mm |
| Ventriculoarterial connection | Discordant | **Ventricles** |  |
| Ventricular loop | d-Loop | Left ventricle | Dilated |
|  |  | Right ventricle | Normal |
| **Septae** |  | **Coronary arteries** | ----- |
| Interventricular septum | 28mm Large Inlet VSD amounting to single ventricle, BD Shunt | **Doppler Measurement** |  |
| Interatrial septum | Intact | Mitral | Mild MR |
| **Semilunal valves** |  | Aortic | ------- |
| Aortic valve | Annulus = 14mm | Tricuspid | ---- |
| Pulmonary valve | Annulus = 12mm | pulmonic | Moderate PR, PPG = 66mmHg |
| **Great arteries** | d-TGA (AP) | **Aortic arch** | Left |
| Aorta | Anterior and to the right | **PDA** | No |
| Pulmonary artery | Posterior and to the left . |  |  |
| **M-Mode:** | | | |
| AO | mm | PWd | mm |
| LA | mm | EDV | ml |
| LVIDd | mm | ESV | ml |
| LVIDs | mm | LVEF | % |
| IVSd | mm | FS | % |
| **Additional Information**: |  | | |
| No pleural/pericardial effusion | | | |
| **Final Diagnosis:** | | | |
| 1. {S, D, AP} Levocardia. 2. d-TGA with large inlet VSD, BD Shunt 3. Severe Pulmonary Hypertension | | | |
| **Remark**: | | | |
| **Recommendation**: | | | |
| SIGNATURE  Done by: Tesfaye T., Pediatrician, Pediatric Cardiologist _______________ 15/05/2013Eth.C | | | |

| Patient Name: **Yonathan Girma**. Patient ID: **Zikuala Hosp**. SEX/ Age: M/5/12. Date of Report: 15**/05/2013**.  BP: _______ Weight: ______ Height:____________ BSA: ________ | | | |
| --- | --- | --- | --- |
| **Features** | **Finding** | **Features** | **Finding** |
| **Profile** |  | **Atria** |  |
| Abdominal situs | Solitus | Left atrium | Normal |
| Cardiac position | Levocardia | Right atrium | Normal |
| Systemic venous drainage | Normal | **Atrioventricular valves** |  |
| Pulmonary venous drainage | Normal | Mitral valve | Annulus = 11mm |
| Atrioventricular connection | Concordant | Tricuspid valve | Annulus = 13mm |
| Ventriculoarterial connection | Concordant | **Ventricles** |  |
| Ventricular loop | d-Loop | Left ventricle | Normal |
|  |  | Right ventricle | Normal |
| **Septae** |  | **Coronary arteries** | ----- |
| Interventricular septum | Intact | **Doppler Measurement** |  |
| Interatrial septum | Intact | Mitral | ----- |
| **Semilunal valves** |  | Aortic | ------- |
| Aortic valve | Annulus = 10mm | Tricuspid | ---- |
| Pulmonary valve | Annulus = 11mm | pulmonic | -------- |
| **Great arteries** | NRGA | **Aortic arch** | Left |
| Aorta | ----- | **PDA** | No |
| Pulmonary artery | Normal MPA and Branch PAs. |  |  |
| **M-Mode:** | | | |
| AO | mm | PWd | mm |
| LA | mm | EDV | ml |
| LVIDd | mm | ESV | ml |
| LVIDs | mm | LVEF | 71% |
| IVSd | mm | FS | 38% |
| **Additional Information**: |  | | |
| No pleural/pericardial effusion | | | |
| **Final Diagnosis:** | | | |
| 1. Normal Echocardiography Study. | | | |
| **Remark**: | | | |
| **Recommendation**: | | | |
| SIGNATURE  Done by: Tesfaye T., Pediatrician, Pediatric Cardiologist _______________ 15/05/2013Eth.C | | | |

| Patient Name: **Baby of Amelewerk Eyasu**. Patient ID: **Adinas GH**. SEX/ Age: F/5days. Date of Report: 15**/05/2013**.  BP: _______ Weight: ______ Height:____________ BSA: ________ | | | |
| --- | --- | --- | --- |
| **Features** | **Finding** | **Features** | **Finding** |
| **Profile** |  | **Atria** |  |
| Abdominal situs | Solitus | Left atrium | Normal |
| Cardiac position | Levocardia | Right atrium | Normal |
| Systemic venous drainage | Normal | **Atrioventricular valves** |  |
| Pulmonary venous drainage | Normal | Mitral valve | Common Complete AVSD |
| Atrioventricular connection | Common Complete AVSD | Tricuspid valve |  |
| Ventriculoarterial connection | Concordant | **Ventricles** |  |
| Ventricular loop | d-Loop | Left ventricle | Normal |
|  |  | Right ventricle | Normal |
| **Septae** |  | **Coronary arteries** | ----- |
| Interventricular septum | Common Complete AVSD, L – R Shunt | **Doppler Measurement** |  |
| Interatrial septum |  | Mitral | ----- |
| **Semilunal valves** |  | Aortic | ------- |
| Aortic valve | Annulus = 8mm | Tricuspid | ---- |
| Pulmonary valve | Annulus = 8mm | pulmonic | -------- |
| **Great arteries** | NRGA | **Aortic arch** | Left |
| Aorta | ----- | **PDA** | No |
| Pulmonary artery | Normal MPA and Branch PAs. |  |  |
| **M-Mode:** Normal LV Function on eye balling. | | | |
| AO | mm | PWd | mm |
| LA | mm | EDV | ml |
| LVIDd | mm | ESV | ml |
| LVIDs | mm | LVEF | % |
| IVSd | mm | FS | % |
| **Additional Information**: |  | | |
| No pleural/pericardial effusion | | | |
| **Final Diagnosis:** | | | |
| 1. {S, D, S} Levocardia. 2. Common Complete Balanced AVSD, L – R Shunt 3. Normal Function | | | |
| **Remark**: | | | |
| **Recommendation**: | | | |
| SIGNATURE  Done by: Tesfaye T., Pediatrician, Pediatric Cardiologist _______________ 15/05/2013Eth.C | | | |

| Patient Name: **Baby of Meseret Alemu**. Patient ID: **FHRH**. SEX/ Age: F/10days. Date of Report: 17**/05/2013**.  BP: _______ Weight: ______ Height:____________ BSA: ________ | | | |
| --- | --- | --- | --- |
| **Features** | **Finding** | **Features** | **Finding** |
| **Profile** |  | **Atria** |  |
| Abdominal situs | Solitus | Left atrium | Normal |
| Cardiac position | Levocardia | Right atrium | Normal |
| Systemic venous drainage | Normal | **Atrioventricular valves** |  |
| Pulmonary venous drainage | Normal | Mitral valve | Annulus = 9mm |
| Atrioventricular connection | Concordant | Tricuspid valve | Annulus = 10mm |
| Ventriculoarterial connection | Concordant | **Ventricles** |  |
| Ventricular loop | d-Loop | Left ventricle | Normal |
|  |  | Right ventricle | Normal |
| **Septae** |  | **Coronary arteries** | ----- |
| Interventricular septum | Intact | **Doppler Measurement** |  |
| Interatrial septum | 5mm OS ASD, L – R Shunt | Mitral | ----- |
| **Semilunal valves** |  | Aortic | ------- |
| Aortic valve | Annulus = 8mm | Tricuspid | ---- |
| Pulmonary valve | Annulus = 8mm | pulmonic | -------- |
| **Great arteries** | NRGA | **Aortic arch** | Left |
| Aorta | ----- | **PDA** | No |
| Pulmonary artery | Normal MPA and Branch PAs. |  |  |
| **M-Mode:** | | | |
| AO | mm | PWd | mm |
| LA | mm | EDV | ml |
| LVIDd | mm | ESV | ml |
| LVIDs | mm | LVEF | % |
| IVSd | mm | FS | % |
| **Additional Information**: |  | | |
| No pleural/pericardial effusion | | | |
| **Final Diagnosis:** | | | |
| 1. {S, D, S} Levocardia. 2. Small OS ASD, L – R Shunt 3. Normal Function | | | |
| **Remark**: | | | |
| **Recommendation**: | | | |
| SIGNATURE  Done by: Tesfaye T., Pediatrician, Pediatric Cardiologist _______________ 17/05/2013Eth.C | | | |

| Patient Name: **Abrham Atalay**. Patient ID: **Finote Selam H**. SEX/ Age: M/10years. Date of Report: 17**/05/2013**.  BP: _______ Weight: ______ Height:____________ BSA: ________ | | | |
| --- | --- | --- | --- |
| **Features** | **Finding** | **Features** | **Finding** |
| **Profile** |  | **Atria** |  |
| Abdominal situs | Solitus | Left atrium | Normal |
| Cardiac position | Levocardia | Right atrium | Normal |
| Systemic venous drainage | Normal | **Atrioventricular valves** |  |
| Pulmonary venous drainage | Normal | Mitral valve | Annulus = 27mm |
| Atrioventricular connection | Concordant | Tricuspid valve | Annulus = 19mm |
| Ventriculoarterial connection | Concordant | **Ventricles** |  |
| Ventricular loop | d-Loop | Left ventricle | Dilated |
|  |  | Right ventricle | Normal |
| **Septae** |  | **Coronary arteries** | ----- |
| Interventricular septum | Intact | **Doppler Measurement** |  |
| Interatrial septum | Intact | Mitral | Mild MR, Decrescendo, Jet velocity, 2.3m/sec |
| **Semilunal valves** |  | Aortic | ------- |
| Aortic valve | Annulus = 18mm. Trileaflet | Tricuspid | ---- |
| Pulmonary valve | Annulus = 20mm | pulmonic | Trivial PR, PPG = 6mmHg |
| **Great arteries** | NRGA | **Aortic arch** | Left, No Coarctation. |
| Aorta | ----- | **PDA** | No |
| Pulmonary artery | Normal MPA and Branch PAs. | **Coronaries** | No ALCAPA |
| **M-Mode:** | | | |
| AO | mm | PWd | 7mm |
| LA | mm | EDV | 140ml |
| LVIDd | 54mm | ESV | 88ml |
| LVIDs | 44mm | LVEF | 37% |
| IVSd | 7mm | FS | 18% |
| **Additional Information**: |  | | |
| No pleural/pericardial effusion | | | |
| **Final Diagnosis:** | | | |
| 1. {S, D, S} Levocardia. 2. Mild MR 3. Dilated LV 4. Moderate LV Dysfunction | | | |
| **Remark**: No LVOTO, No CoA, No ALCAPA | | | |
| **Recommendation**: Investigate in the line of DCMP | | | |
| SIGNATURE  Done by: Tesfaye T., Pediatrician, Pediatric Cardiologist _______________ 17/05/2013Eth.C | | | |

| Patient Name: **Bamlak Dawit**. Patient ID: **THMH**. SEX/ Age: M/28days. Date of Report: 19**/05/2013**.  BP: _______ Weight: ______ Height:____________ BSA: ________ | | | |
| --- | --- | --- | --- |
| **Features** | **Finding** | **Features** | **Finding** |
| **Profile** |  | **Atria** |  |
| Abdominal situs | Solitus | Left atrium | Normal |
| Cardiac position | Levocardia | Right atrium | Normal |
| Systemic venous drainage | Normal | **Atrioventricular valves** |  |
| Pulmonary venous drainage | Normal | Mitral valve | Annulus = 9mm |
| Atrioventricular connection | Concordant | Tricuspid valve | Annulus = 9mm |
| Ventriculoarterial connection | Concordant | **Ventricles** |  |
| Ventricular loop | d-Loop | Left ventricle | Normal |
|  |  | Right ventricle | Normal |
| **Septae** |  | **Coronary arteries** | ----- |
| Interventricular septum | 6mm Inlet VSD, Partially covered by septal leaflet of TV, L – R Shunt | **Doppler Measurement** |  |
| Interatrial septum | PFO, L – R Shunt | Mitral | ----- |
| **Semilunal valves** |  | Aortic | ------- |
| Aortic valve | Annulus = 9mm | Tricuspid | ---- |
| Pulmonary valve | Annulus = 10mm | pulmonic | -------- |
| **Great arteries** | NRGA | **Aortic arch** | Left |
| Aorta | ----- | **PDA** | No |
| Pulmonary artery | Normal MPA and Branch PAs. |  |  |
| **M-Mode:** | | | |
| AO | mm | PWd | mm |
| LA | mm | EDV | ml |
| LVIDd | mm | ESV | ml |
| LVIDs | mm | LVEF | 72% |
| IVSd | mm | FS | 39% |
| **Additional Information**: |  | | |
| No pleural/pericardial effusion | | | |
| **Final Diagnosis:** | | | |
| 1. {S, D, S} Levocardia. 2. PFO, L – R Shunt 3. Moderate Inlet VSD, partially covered by septal leaflet of TV, L – R Shunt 4. Normal Function | | | |
| **Remark**: | | | |
| **Recommendation**: | | | |
| SIGNATURE  Done by: Tesfaye T., Pediatrician, Pediatric Cardiologist _______________ 19/05/2013Eth.C | | | |

| Patient Name: **Abatneh Muluneh**. Patient ID: **Agumas MC**. SEX/ Age: M/9years. Date of Report: 19**/05/2013**.  BP: _______ Weight: ______ Height:____________ BSA: ________ | | | |
| --- | --- | --- | --- |
| **Features** | **Finding** | **Features** | **Finding** |
| **Profile** |  | **Atria** |  |
| Abdominal situs | Solitus | Left atrium | Normal |
| Cardiac position | Levocardia | Right atrium | Normal |
| Systemic venous drainage | Normal | **Atrioventricular valves** |  |
| Pulmonary venous drainage | Normal | Mitral valve | Annulus = 20mm |
| Atrioventricular connection | Concordant | Tricuspid valve | Annulus = 19mm  TAPSE = 20mm |
| Ventriculoarterial connection | Concordant | **Ventricles** |  |
| Ventricular loop | d-Loop | Left ventricle | Normal |
|  |  | Right ventricle | Normal |
| **Septae** |  | **Coronary arteries** | ----- |
| Interventricular septum | Intact | **Doppler Measurement** |  |
| Interatrial septum | Intact | Mitral | ----- |
| **Semilunal valves** |  | Aortic | ------- |
| Aortic valve | Annulus = 17mm | Tricuspid | ---- |
| Pulmonary valve | Annulus = 19mm | pulmonic | -------- |
| **Great arteries** | NRGA | **Aortic arch** | Left |
| Aorta | ----- | **PDA** | No |
| Pulmonary artery | Normal MPA and Branch PAs. |  |  |
| **M-Mode:** | | | |
| AO | mm | PWd | mm |
| LA | mm | EDV | ml |
| LVIDd | mm | ESV | ml |
| LVIDs | mm | LVEF | 66% |
| IVSd | mm | FS | 36% |
| **Additional Information**: |  | | |
| pericardial effusion, 13mm on RA/RV Side and 12mm on LA/LV Side with echo debris inside. No Echocardiographic Features of Cardiac tamponade. | | | |
| **Final Diagnosis:** | | | |
| 1. {S, D, S} Levocardia. 2. Moderate Circumferential Pericardial Effusion with echodebris inside. 3. Normal Biventricular Function 4. Mo Echocardiographic Features of Cardiac tamponade | | | |
| **Remark**: | | | |
| **Recommendation**: | | | |
| SIGNATURE  Done by: Tesfaye T., Pediatrician, Pediatric Cardiologist _______________ 19/05/2013Eth.C | | | |

| Patient Name: **Emebet Gebeyehu**. Patient ID: **Addis Alem H**. SEX/ Age: F/10years. Date of Report: 19**/05/2013**.  BP: _______ Weight: ______ Height:____________ BSA: ________. ARF | | | |
| --- | --- | --- | --- |
| **Features** | **Finding** | **Features** | **Finding** |
| **Profile** |  | **Atria** |  |
| Abdominal situs | Solitus | Left atrium | Normal |
| Cardiac position | Levocardia | Right atrium | Normal |
| Systemic venous drainage | Normal | **Atrioventricular valves** |  |
| Pulmonary venous drainage | Normal | Mitral valve | Annulus = 19mm  Patulous MVL |
| Atrioventricular connection | Concordant | Tricuspid valve | Annulus = 19mm  TAPSE = 17mm |
| Ventriculoarterial connection | Concordant | **Ventricles** |  |
| Ventricular loop | d-Loop | Left ventricle | Normal |
|  |  | Right ventricle | Normal |
| **Septae** |  | **Coronary arteries** | ----- |
| Interventricular septum | Intact | **Doppler Measurement** |  |
| Interatrial septum | Intact | Mitral | Trivial MR, Holosystolic, seen only in apical view, jet velocity = 2m/sec |
| **Semilunal valves** |  | Aortic | ------- |
| Aortic valve | Annulus = 15mm | Tricuspid | ---- |
| Pulmonary valve | Annulus = 18mm | pulmonic | Trivial PR, PPG = 6mmHg. |
| **Great arteries** | NRGA | **Aortic arch** | Left |
| Aorta | ----- | **PDA** | No |
| Pulmonary artery | Normal MPA and Branch PAs. |  |  |
| **M-Mode:** | | | |
| AO | mm | PWd | mm |
| LA | mm | EDV | ml |
| LVIDd | mm | ESV | ml |
| LVIDs | mm | LVEF | 58% |
| IVSd | mm | FS | 30% |
| **Additional Information**: |  | | |
| No pleural/pericardial effusion | | | |
| **Final Diagnosis:** | | | |
| 1. {S, D, S} Levocardia. 2. Trivial MR | | | |
| **Remark**: Consider it as normal Echocardiography Study. | | | |
| **Recommendation**: Follow up depending on the clinical condition of the patient. | | | |
| SIGNATURE  Done by: Tesfaye T., Pediatrician, Pediatric Cardiologist _______________ 19/05/2013Eth.C | | | |

| Patient Name: **Arsema Dessie**. Patient ID: **Adinas GH**. SEX/ Age: F/11/12. Date of Report: 21**/05/2013**.  BP: _______ Weight: ______ Height:____________ BSA: ________ | | | |
| --- | --- | --- | --- |
| **Features** | **Finding** | **Features** | **Finding** |
| **Profile** |  | **Atria** |  |
| Abdominal situs | Solitus | Left atrium | Normal |
| Cardiac position | Levocardia | Right atrium | Normal |
| Systemic venous drainage | Normal | **Atrioventricular valves** |  |
| Pulmonary venous drainage | Normal | Mitral valve | Annulus = 11mm |
| Atrioventricular connection | Concordant | Tricuspid valve | Annulus = 13mm |
| Ventriculoarterial connection | Concordant | **Ventricles** |  |
| Ventricular loop | d-Loop | Left ventricle | Normal |
|  |  | Right ventricle | Normal |
| **Septae** |  | **Coronary arteries** | ----- |
| Interventricular septum | Intact | **Doppler Measurement** |  |
| Interatrial septum | Intact | Mitral | ----- |
| **Semilunal valves** |  | Aortic | ------- |
| Aortic valve | Annulus = 11mm | Tricuspid | ---- |
| Pulmonary valve | Annulus = 13mm | pulmonic | -------- |
| **Great arteries** | NRGA | **Aortic arch** | Left |
| Aorta | ----- | **PDA** | 2.5mm PDA, L – R Shunt |
| Pulmonary artery | Normal MPA and Branch PAs. |  |  |
| **M-Mode:** Normal LV Function on eye balling. | | | |
| AO | mm | PWd | mm |
| LA | mm | EDV | ml |
| LVIDd | mm | ESV | ml |
| LVIDs | mm | LVEF | % |
| IVSd | mm | FS | % |
| **Additional Information**: |  | | |
| pericardial effusion, 4mm on RA/RV Side, clear | | | |
| **Final Diagnosis:** | | | |
| 1. {S, D, S} Levocardia. 2. Moderate PDA, L – R Shunt 3. Small Pericardial Effusion 4. Normal LV Function | | | |
| **Remark**: Infant was restless during study. Needs sedation to estimate pressure gradient. | | | |
| **Recommendation**: | | | |
| SIGNATURE  Done by: Tesfaye T., Pediatrician, Pediatric Cardiologist _______________ 21/05/2013Eth.C | | | |

| Patient Name: **Ma’edot Yilkal**. Patient ID: **Afilas GH**. SEX/ Age: F/4 6/12. Date of Report: 21**/05/2013**.  BP: _______ Weight: ______ Height:____________ BSA: ________ | | | |
| --- | --- | --- | --- |
| **Features** | **Finding** | **Features** | **Finding** |
| **Profile** |  | **Atria** |  |
| Abdominal situs | Solitus | Left atrium | Normal |
| Cardiac position | Levocardia | Right atrium | Normal |
| Systemic venous drainage | Normal | **Atrioventricular valves** |  |
| Pulmonary venous drainage | Normal | Mitral valve | Annulus = 14mm |
| Atrioventricular connection | Concordant | Tricuspid valve | Annulus = 14mm  TAPSE = 18mm |
| Ventriculoarterial connection | Concordant | **Ventricles** |  |
| Ventricular loop | d-Loop | Left ventricle | Normal |
|  |  | Right ventricle | Normal |
| **Septae** |  | **Coronary arteries** | ----- |
| Interventricular septum | Intact | **Doppler Measurement** |  |
| Interatrial septum | Intact | Mitral | ----- |
| **Semilunal valves** |  | Aortic | ------- |
| Aortic valve | Annulus = 13mm | Tricuspid | ---- |
| Pulmonary valve | Annulus = 15mm | pulmonic | -------- |
| **Great arteries** | NRGA | **Aortic arch** | Left |
| Aorta | ----- | **PDA** | No |
| Pulmonary artery | Normal MPA and Branch PAs. |  |  |
| **M-Mode:** | | | |
| AO | mm | PWd | mm |
| LA | mm | EDV | ml |
| LVIDd | mm | ESV | ml |
| LVIDs | mm | LVEF | 73% |
| IVSd | mm | FS | 41% |
| **Additional Information**: |  | | |
| No pleural/pericardial effusion | | | |
| **Final Diagnosis:** | | | |
| 1. Normal Echocardiography Study. | | | |
| **Remark**: | | | |
| **Recommendation**: | | | |
| SIGNATURE  Done by: Tesfaye T., Pediatrician, Pediatric Cardiologist _______________ 21/05/2013Eth.C | | | |

| Patient Name: **Tiruye Shimelash**. Patient ID: **Adinas GH**. SEX/ Age: F/13years. Date of Report: 21**/05/2013**. | | | |
| --- | --- | --- | --- |
| **Features** | **Finding** | **Features** | **Finding** |
| **Profile** |  | **Atria** |  |
| Abdominal situs | Solitus | Left atrium | Dilated |
| Cardiac position | Levocardia | Right atrium | Normal |
| Systemic venous drainage | Normal. IVC Dilated | **Atrioventricular valves** |  |
| Pulmonary venous drainage | Normal | Mitral valve | Annulus = 19mm. thickened, clubbed MVL. MVA = 0.75cm2. |
| Atrioventricular connection | Concordant | Tricuspid valve | Annulus = 23mm  TAPSE = 18mm |
| Ventriculoarterial connection | Concordant | **Ventricles** |  |
| Ventricular loop | d-Loop | Left ventricle | Dilated |
|  |  | Right ventricle | Normal |
| **Septae** |  | **Coronary arteries** | ----- |
| Interventricular septum | Intact | **Doppler Measurement** |  |
| Interatrial septum | Intact | Mitral | Severe MR, Holosystolic, posterior projection, seen in two planes with jet velocity = 4.8m/sec. Severe MS, PPG/MPG = 35/23mmHg. |
| **Semilunal valves** |  | Aortic | Moderate AR, PHT = 283ms. |
| Aortic valve | Annulus = 17mm. thickened valve, trileaflet. | Tricuspid | ---- |
| Pulmonary valve | Annulus = 25mm | pulmonic | Mild PR, PPG = 35mmHg |
| **Great arteries** | NRGA | **Aortic arch** | Left |
| Aorta | ----- | **PDA** | No |
| Pulmonary artery | Normal MPA and Branch PAs. |  |  |
| **M-Mode:** | | | |
| AO | mm | PWd | mm |
| LA | mm | EDV | ml |
| LVIDd | mm | ESV | ml |
| LVIDs | mm | LVEF | 48% |
| IVSd | mm | FS | 24% |
| **Additional Information**: |  | | |
| 5mm pericardial effusion on RA/RV Side | | | |
| **Final Diagnosis:** | | | |
| 1. {S, D, S} Levocardia. 2. LA/LV Dilated 3. Thickened, Clubbed MVL. Thickened AV 4. Severe MR 5. Severe MS 6. Moderate AR 7. Reduced LV Function 8. Small Pericardial effusion | | | |
| **Remark**: | | | |
| **Recommendation**: | | | |
| SIGNATURE  Done by: Tesfaye T., Pediatrician, Pediatric Cardiologist _______________ 21/05/2013Eth.C | | | |

| Patient Name: **Eyuel Tadele**. Patient ID: **FHRH**. SEX/ Age: M/4/12. Date of Report: 21**/05/2013**.  BP: _______ Weight: ______ Height:____________ BSA: ________ | | | |
| --- | --- | --- | --- |
| **Features** | **Finding** | **Features** | **Finding** |
| **Profile** |  | **Atria** |  |
| Abdominal situs | Solitus | Left atrium | Normal |
| Cardiac position | Levocardia | Right atrium | Normal |
| Systemic venous drainage | Normal | **Atrioventricular valves** |  |
| Pulmonary venous drainage | Normal | Mitral valve | Annulus = 11mm |
| Atrioventricular connection | Concordant | Tricuspid valve | Annulus = 12mm |
| Ventriculoarterial connection | Concordant | **Ventricles** |  |
| Ventricular loop | d-Loop | Left ventricle | Normal |
|  |  | Right ventricle | Normal |
| **Septae** |  | **Coronary arteries** | ----- |
| Interventricular septum | Intact | **Doppler Measurement** |  |
| Interatrial septum | Intact | Mitral | ----- |
| **Semilunal valves** |  | Aortic | ------- |
| Aortic valve | Annulus = 10mm | Tricuspid | ---- |
| Pulmonary valve | Annulus = 10mm | pulmonic | Flow acceleration across the PV with PPG = 15mmHg. |
| **Great arteries** | NRGA | **Aortic arch** | Left |
| Aorta | ----- | **PDA** | No |
| Pulmonary artery | Normal MPA and Branch PAs. |  |  |
| **M-Mode:** | | | |
| AO | mm | PWd | mm |
| LA | mm | EDV | ml |
| LVIDd | mm | ESV | ml |
| LVIDs | mm | LVEF | % |
| IVSd | mm | FS | % |
| **Additional Information**: |  | | |
| No pleural/pericardial effusion | | | |
| **Final Diagnosis:** | | | |
| 1. Normal Echocardiography Study. | | | |
| **Remark**: Follow up echo for monitoring of the flow acceleration across the PV is recommended after a year. | | | |
| **Recommendation**: | | | |
| SIGNATURE  Done by: Tesfaye T., Pediatrician, Pediatric Cardiologist _______________ 21/05/2013Eth.C | | | |

| Patient Name: **Arsema Desalegn**. Patient ID: **FHRH**. SEX/ Age: F/11/12. Date of Report: 21**/05/2013**.  BP: _______ Weight: ______ Height:____________ BSA: ________R.Dx: **DS + CHF. AGH10. 898** | | | |
| --- | --- | --- | --- |
| **Features** | **Finding** | **Features** | **Finding** |
| **Profile** |  | **Atria** |  |
| Abdominal situs | Solitus | Left atrium | Normal |
| Cardiac position | Levocardia | Right atrium | Normal |
| Systemic venous drainage | Normal | **Atrioventricular valves** |  |
| Pulmonary venous drainage | Normal | Mitral valve | Common Complete AVSD, L – R Shunt |
| Atrioventricular connection | Common Complete AVSD | Tricuspid valve |  |
| Ventriculoarterial connection | Concordant | **Ventricles** |  |
| Ventricular loop | d-Loop | Left ventricle | Normal |
|  |  | Right ventricle | Normal |
| **Septae** |  | **Coronary arteries** | ----- |
| Interventricular septum | Common Complete AVSD, L – R Shunt | **Doppler Measurement** |  |
| Interatrial septum |  | Mitral | Mild Left AVVR |
| **Semilunal valves** |  | Aortic | ------- |
| Aortic valve | Annulus = 13mm | Tricuspid | Mild Right AVVR |
| Pulmonary valve | Annulus = 14mm | pulmonic | -------- |
| **Great arteries** | NRGA | **Aortic arch** | Left |
| Aorta | ----- | **PDA** | 1.5mm PDA, L – R Shunt |
| Pulmonary artery | Normal MPA and Branch PAs. |  |  |
| **M-Mode:** | | | |
| AO | mm | PWd | mm |
| LA | mm | EDV | ml |
| LVIDd | mm | ESV | ml |
| LVIDs | mm | LVEF | % |
| IVSd | mm | FS | % |
| **Additional Information**: |  | | |
| pericardial effusion, 11mm on RA Side | | | |
| **Final Diagnosis:** | | | |
| 1. {S, D, S} Levocardia. 2. Common Complete AVSD, L – R Shunt 3. Mild Left AVVR 4. Mild Right AVVR 5. Small PDA, L – R Shunt 6. Moderate Pericardial Effusion 7. Normal LV Function | | | |
| **Remark**: | | | |
| **Recommendation**: | | | |
| SIGNATURE  Done by: Tesfaye T., Pediatrician, Pediatric Cardiologist _______________ 21/05/2013Eth.C | | | |

| Patient Name: **Yonathan Abay**. Patient ID: **MSI - Eth**. SEX/ Age: M/4 5/12. Date of Report: 21**/05/2013**.  BP: _______ Weight: ______ Height:____________ BSA: ________ | | | |
| --- | --- | --- | --- |
| **Features** | **Finding** | **Features** | **Finding** |
| **Profile** |  | **Atria** |  |
| Abdominal situs | Solitus | Left atrium | Normal |
| Cardiac position | Levocardia | Right atrium | Normal |
| Systemic venous drainage | Normal | **Atrioventricular valves** |  |
| Pulmonary venous drainage | Normal | Mitral valve | Annulus = 16mm |
| Atrioventricular connection | Concordant | Tricuspid valve | Annulus = 16mm  TAPSE = 18mm |
| Ventriculoarterial connection | Concordant | **Ventricles** |  |
| Ventricular loop | d-Loop | Left ventricle | Normal |
|  |  | Right ventricle | Normal |
| **Septae** |  | **Coronary arteries** | ----- |
| Interventricular septum | Intact | **Doppler Measurement** |  |
| Interatrial septum | Intact | Mitral | ----- |
| **Semilunal valves** |  | Aortic | ------- |
| Aortic valve | Annulus = 14mm | Tricuspid | ---- |
| Pulmonary valve | Annulus = 17mm | pulmonic | -------- |
| **Great arteries** | NRGA | **Aortic arch** | Left. No CoA |
| Aorta | ----- | **PDA** | No |
| Pulmonary artery | Normal MPA and Branch PAs. |  |  |
| **M-Mode:** | | | |
| AO | mm | PWd | mm |
| LA | mm | EDV | ml |
| LVIDd | mm | ESV | ml |
| LVIDs | mm | LVEF | 65% |
| IVSd | mm | FS | 35% |
| **Additional Information**: |  | | |
| No pleural/pericardial effusion | | | |
| **Final Diagnosis:** | | | |
| 1. Normal Echocardiography Study. | | | |
| **Remark**: | | | |
| **Recommendation**: | | | |
| SIGNATURE  Done by: Tesfaye T., Pediatrician, Pediatric Cardiologist _______________ 21/05/2013Eth.C | | | |

| Patient Name: **Baby of Amarech**. Patient ID: **Adinas GH**. SEX/ Age: M/33hours. Date of Report: 22**/05/2013**.  BP: _______ Weight: ______ Height:____________ BSA: ________ | | | |
| --- | --- | --- | --- |
| **Features** | **Finding** | **Features** | **Finding** |
| **Profile** |  | **Atria** |  |
| Abdominal situs | Solitus | Left atrium | Normal |
| Cardiac position | Levocardia | Right atrium | Normal |
| Systemic venous drainage | Normal | **Atrioventricular valves** |  |
| Pulmonary venous drainage | Normal | Mitral valve | Annulus = 10mm |
| Atrioventricular connection | Concordant | Tricuspid valve | Annulus = 10mm  TAPSE = 10mm |
| Ventriculoarterial connection | Concordant | **Ventricles** |  |
| Ventricular loop | d-Loop | Left ventricle | Normal |
|  |  | Right ventricle | Normal |
| **Septae** |  | **Coronary arteries** | ----- |
| Interventricular septum | Intact | **Doppler Measurement** |  |
| Interatrial septum | PFO, L – R Shunt | Mitral | ----- |
| **Semilunal valves** |  | Aortic | ------- |
| Aortic valve | Annulus = 8mm | Tricuspid | Trivial TR, PPG = 24mmHg. |
| Pulmonary valve | Annulus = 9mm | pulmonic | -------- |
| **Great arteries** | NRGA | **Aortic arch** | Left |
| Aorta | ----- | **PDA** | No |
| Pulmonary artery | Normal MPA and Branch PAs. |  |  |
| **M-Mode:** | | | |
| AO | mm | PWd | mm |
| LA | mm | EDV | ml |
| LVIDd | mm | ESV | ml |
| LVIDs | mm | LVEF | 59% |
| IVSd | mm | FS | 29% |
| **Additional Information**: |  | | |
| No pleural/pericardial effusion | | | |
| **Final Diagnosis:** | | | |
| 1. {S, D, S} Levocardia. 2. PFO, L – R Shunt 3. Normal Biventricular Function | | | |
| **Remark**: | | | |
| **Recommendation**: | | | |
| SIGNATURE  Done by: Tesfaye T., Pediatrician, Pediatric Cardiologist _______________ 22/05/2013Eth.C | | | |

| Patient Name: **Natnael Eshetu**. Patient ID: **Adinas GH**. SEX/ Age: M/8 8/12. Date of Report: 22**/05/2013**.  BP: _______ Weight: ______ Height:____________ BSA: ________ | | | |
| --- | --- | --- | --- |
| **Features** | **Finding** | **Features** | **Finding** |
| **Profile** |  | **Atria** |  |
| Abdominal situs | Solitus | Left atrium | Normal |
| Cardiac position | Levocardia | Right atrium | Normal |
| Systemic venous drainage | Normal | **Atrioventricular valves** |  |
| Pulmonary venous drainage | Normal | Mitral valve | Annulus = 17mm |
| Atrioventricular connection | Concordant | Tricuspid valve | Annulus = 17mm  TAPSE = 17mm |
| Ventriculoarterial connection | Concordant | **Ventricles** |  |
| Ventricular loop | d-Loop | Left ventricle | Normal |
|  |  | Right ventricle | Normal |
| **Septae** |  | **Coronary arteries** | ----- |
| Interventricular septum | Intact | **Doppler Measurement** |  |
| Interatrial septum | Intact | Mitral | ----- |
| **Semilunal valves** |  | Aortic | ------- |
| Aortic valve | Annulus = 17mm | Tricuspid | ---- |
| Pulmonary valve | Annulus = 18mm | pulmonic | -------- |
| **Great arteries** | NRGA | **Aortic arch** | Left |
| Aorta | ----- | **PDA** | No |
| Pulmonary artery | Normal MPA and Branch PAs. |  |  |
| **M-Mode:** | | | |
| AO | mm | PWd | mm |
| LA | mm | EDV | ml |
| LVIDd | mm | ESV | ml |
| LVIDs | mm | LVEF | 61% |
| IVSd | mm | FS | 32% |
| **Additional Information**: |  | | |
| No pleural/pericardial effusion | | | |
| **Final Diagnosis:** | | | |
| 1. Normal Echocardiography Study. | | | |
| **Remark**: | | | |
| **Recommendation**: | | | |
| SIGNATURE  Done by: Tesfaye T., Pediatrician, Pediatric Cardiologist _______________ 22/05/2013Eth.C | | | |

| Patient Name: **Mekdm Ayana**. Patient ID: **Adinas GH**. SEX/ Age: F/9/12. Date of Report: 22**/05/2013**.  BP: _______ Weight: ______ Height:____________ BSA: ________ | | | |
| --- | --- | --- | --- |
| **Features** | **Finding** | **Features** | **Finding** |
| **Profile** |  | **Atria** |  |
| Abdominal situs | Solitus | Left atrium | Normal |
| Cardiac position | Levocardia | Right atrium | Normal |
| Systemic venous drainage | Normal | **Atrioventricular valves** |  |
| Pulmonary venous drainage | Normal | Mitral valve | Annulus = 14mm |
| Atrioventricular connection | Concordant | Tricuspid valve | Annulus = 15mm  TAPSE = 18mm |
| Ventriculoarterial connection | Concordant | **Ventricles** |  |
| Ventricular loop | d-Loop | Left ventricle | Normal |
|  |  | Right ventricle | Normal |
| **Septae** |  | **Coronary arteries** | ----- |
| Interventricular septum | Intact | **Doppler Measurement** |  |
| Interatrial septum | Intact | Mitral | ----- |
| **Semilunal valves** |  | Aortic | ------- |
| Aortic valve | Annulus = 10mm | Tricuspid | ---- |
| Pulmonary valve | Annulus = 8mm | pulmonic | Mild Valvar PS, PPG = 30mmHg. |
| **Great arteries** | NRGA | **Aortic arch** | Left |
| Aorta | ----- | **PDA** | No |
| Pulmonary artery | Normal MPA and Branch PAs. |  |  |
| **M-Mode:** | | | |
| AO | mm | PWd | mm |
| LA | mm | EDV | ml |
| LVIDd | mm | ESV | ml |
| LVIDs | mm | LVEF | 67% |
| IVSd | mm | FS | 35% |
| **Additional Information**: |  | | |
| No pleural/pericardial effusion | | | |
| **Final Diagnosis:** | | | |
| 1. {S, D, S} Levocardia. 2. Mild Valvar PS 3. Normal Biventricular Function | | | |
| **Remark**: | | | |
| **Recommendation**: | | | |
| SIGNATURE  Done by: Tesfaye T., Pediatrician, Pediatric Cardiologist _______________ 22/05/2013Eth.C | | | |

| Patient Name: **Kalkidan Admasu**. Patient ID: **Adinas GH**. SEX/ Age: F/7years. Date of Report: 24**/05/2013**.  BP: _______ Weight: ______ Height:____________ BSA: ________ | | | |
| --- | --- | --- | --- |
| **Features** | **Finding** | **Features** | **Finding** |
| **Profile** |  | **Atria** |  |
| Abdominal situs | Solitus | Left atrium | Normal |
| Cardiac position | Levocardia | Right atrium | Normal |
| Systemic venous drainage | Normal | **Atrioventricular valves** |  |
| Pulmonary venous drainage | Normal | Mitral valve | Annulus = 19mm |
| Atrioventricular connection | Concordant | Tricuspid valve | Annulus = 20mm  TAPSE = 16mm |
| Ventriculoarterial connection | Concordant | **Ventricles** |  |
| Ventricular loop | d-Loop | Left ventricle | Normal |
|  |  | Right ventricle | Normal |
| **Septae** |  | **Coronary arteries** | ----- |
| Interventricular septum | Intact | **Doppler Measurement** |  |
| Interatrial septum | Intact | Mitral | ----- |
| **Semilunal valves** |  | Aortic | ------- |
| Aortic valve | Annulus = 15mm | Tricuspid | ---- |
| Pulmonary valve | Annulus = 17mm | pulmonic | -------- |
| **Great arteries** | NRGA | **Aortic arch** | Left |
| Aorta | ----- | **PDA** | No |
| Pulmonary artery | Normal MPA and Branch PAs. |  |  |
| **M-Mode:** | | | |
| AO | mm | PWd | mm |
| LA | mm | EDV | ml |
| LVIDd | mm | ESV | ml |
| LVIDs | mm | LVEF | 58% |
| IVSd | mm | FS | 30% |
| **Additional Information**: |  | | |
| No pleural/pericardial effusion | | | |
| **Final Diagnosis:** | | | |
| 1. Normal Echocardiography Study. | | | |
| **Remark**: | | | |
| **Recommendation**: | | | |
| SIGNATURE  Done by: Tesfaye T., Pediatrician, Pediatric Cardiologist _______________ 24/05/2013Eth.C | | | |

| Patient Name: **Yordanos Techilo**. Patient ID: **Adinas GH**. SEX/ Age: M/6 7/12. Date of Report: 24**/05/2013**.  BP: _______ Weight: ______ Height:____________ BSA: ________ | | | |
| --- | --- | --- | --- |
| **Features** | **Finding** | **Features** | **Finding** |
| **Profile** |  | **Atria** |  |
| Abdominal situs | Solitus | Left atrium | Normal |
| Cardiac position | Levocardia | Right atrium | Normal |
| Systemic venous drainage | Normal | **Atrioventricular valves** |  |
| Pulmonary venous drainage | Normal | Mitral valve | Annulus = 19mm |
| Atrioventricular connection | Concordant | Tricuspid valve | Annulus = 19mm  TAPSE = 19mm |
| Ventriculoarterial connection | Concordant | **Ventricles** |  |
| Ventricular loop | d-Loop | Left ventricle | Normal |
|  |  | Right ventricle | Normal |
| **Septae** |  | **Coronary arteries** | ----- |
| Interventricular septum | Intact | **Doppler Measurement** |  |
| Interatrial septum | Intact | Mitral | ----- |
| **Semilunal valves** |  | Aortic | ------- |
| Aortic valve | Annulus = 16mm | Tricuspid | ---- |
| Pulmonary valve | Annulus = 18mm | pulmonic | -------- |
| **Great arteries** | NRGA | **Aortic arch** | Left |
| Aorta | ----- | **PDA** | No |
| Pulmonary artery | Normal MPA and Branch PAs. |  |  |
| **M-Mode:** | | | |
| AO | mm | PWd | mm |
| LA | mm | EDV | ml |
| LVIDd | mm | ESV | ml |
| LVIDs | mm | LVEF | 64% |
| IVSd | mm | FS | 34% |
| **Additional Information**: |  | | |
| No pleural/pericardial effusion | | | |
| **Final Diagnosis:** | | | |
| 1. Normal Echocardiography Study. | | | |
| **Remark**: | | | |
| **Recommendation**: | | | |
| SIGNATURE  Done by: Tesfaye T., Pediatrician, Pediatric Cardiologist _______________ 24/05/2013Eth.C | | | |

| Patient Name: **Fetiha Sofiyan**. Patient ID: **MSI - Ethio**. SEX/ Age: F/9/12. Date of Report: 24**/05/2013**.  BP: _______ Weight: ______ Height:____________ BSA: ________ | | | |
| --- | --- | --- | --- |
| **Features** | **Finding** | **Features** | **Finding** |
| **Profile** |  | **Atria** |  |
| Abdominal situs | Solitus | Left atrium | Normal |
| Cardiac position | Levocardia | Right atrium | Normal |
| Systemic venous drainage | Normal | **Atrioventricular valves** |  |
| Pulmonary venous drainage | Normal | Mitral valve | Annulus = 11mm |
| Atrioventricular connection | Concordant | Tricuspid valve | Annulus = 13mm  TAPSE = 15mm |
| Ventriculoarterial connection | Concordant | **Ventricles** |  |
| Ventricular loop | d-Loop | Left ventricle | Normal |
|  |  | Right ventricle | Normal |
| **Septae** |  | **Coronary arteries** | ----- |
| Interventricular septum | Intact | **Doppler Measurement** |  |
| Interatrial septum | Intact | Mitral | ----- |
| **Semilunal valves** |  | Aortic | ------- |
| Aortic valve | Annulus = 11mm | Tricuspid | ---- |
| Pulmonary valve | Annulus = 10mm | pulmonic | -------- |
| **Great arteries** | NRGA | **Aortic arch** | Left |
| Aorta | ----- | **PDA** | No |
| Pulmonary artery | Normal MPA and Branch PAs. |  |  |
| **M-Mode:** Normal LV Function on eye balling. | | | |
| AO | mm | PWd | mm |
| LA | mm | EDV | ml |
| LVIDd | mm | ESV | ml |
| LVIDs | mm | LVEF | % |
| IVSd | mm | FS | % |
| **Additional Information**: |  | | |
| No pleural/pericardial effusion | | | |
| **Final Diagnosis:** | | | |
| 1. Normal Echocardiography Study. | | | |
| **Remark**: | | | |
| **Recommendation**: | | | |
| SIGNATURE  Done by: Tesfaye T., Pediatrician, Pediatric Cardiologist _______________ 24/05/2013Eth.C | | | |

| Patient Name: **Abrham Esubalew**. Patient ID: **FHRH**. SEX/ Age: M/4/12. Date of Report: 25**/05/2013**.  BP: _______ Weight: ______ Height:____________ BSA: ________ | | | |
| --- | --- | --- | --- |
| **Features** | **Finding** | **Features** | **Finding** |
| **Profile** |  | **Atria** |  |
| Abdominal situs | Solitus | Left atrium | Normal |
| Cardiac position | Levocardia | Right atrium | Normal |
| Systemic venous drainage | Normal | **Atrioventricular valves** |  |
| Pulmonary venous drainage | Normal | Mitral valve | AVSD |
| Atrioventricular connection | Concordant | Tricuspid valve | AVSD |
| Ventriculoarterial connection | Concordant | **Ventricles** |  |
| Ventricular loop | d-Loop | Left ventricle | Normal |
|  |  | Right ventricle | Normal |
| **Septae** |  | **Coronary arteries** | ----- |
| Interventricular septum | 6mm Inlet VSD, L – R Shunt | **Doppler Measurement** |  |
| Interatrial septum | 17mm Primmum defect, L – R Shunt | Mitral | ----- |
| **Semilunal valves** |  | Aortic | ------- |
| Aortic valve | Annulus = 10mm | Tricuspid | Mild Right AVVR |
| Pulmonary valve | Annulus = 9mm | pulmonic | -------- |
| **Great arteries** | NRGA | **Aortic arch** | Left |
| Aorta | ----- | **PDA** | No |
| Pulmonary artery | Normal MPA and Branch PAs. |  |  |
| **M-Mode:** Normal LV Function on eye balling. | | | |
| AO | mm | PWd | mm |
| LA | mm | EDV | ml |
| LVIDd | mm | ESV | ml |
| LVIDs | mm | LVEF | % |
| IVSd | mm | FS | % |
| **Additional Information**: |  | | |
| pericardial effusion on RA/RV Side measuring 3mm on RA side. | | | |
| **Final Diagnosis:** | | | |
| 1. {S, D, S} Levocardia. 2. Transitional AVSD, L – R Shunt 3. Mild Right AVVR 4. Mild Pericardial Effusion 5. Normal LV Function | | | |
| **Remark**: | | | |
| **Recommendation**: | | | |
| SIGNATURE  Done by: Tesfaye T., Pediatrician, Pediatric Cardiologist _______________ 25/05/2013Eth.C | | | |

| Patient Name: Baby of **Mitinie Nibretu**. Patient ID: **FHRHH**. SEX/ Age: M/18days. Date of Report: 26**/05/2013**.  BP: _______ Weight: ______ Height:____________ BSA: ________ | | | |
| --- | --- | --- | --- |
| **Features** | **Finding** | **Features** | **Finding** |
| **Profile** |  | **Atria** |  |
| Abdominal situs | Solitus | Left atrium | Normal |
| Cardiac position | Levocardia | Right atrium | Normal |
| Systemic venous drainage | Normal | **Atrioventricular valves** |  |
| Pulmonary venous drainage | Normal | Mitral valve | Annulus = 9mm |
| Atrioventricular connection | Concordant | Tricuspid valve | Annulus = 10mm |
| Ventriculoarterial connection | Concordant | **Ventricles** |  |
| Ventricular loop | d-Loop | Left ventricle | Normal |
|  |  | Right ventricle | Normal |
| **Septae** |  | **Coronary arteries** | ----- |
| Interventricular septum | Intact | **Doppler Measurement** |  |
| Interatrial septum | Intact | Mitral | ----- |
| **Semilunal valves** |  | Aortic | ------- |
| Aortic valve | Annulus = 8mm | Tricuspid | ---- |
| Pulmonary valve | Annulus = 8mm | pulmonic | -------- |
| **Great arteries** | NRGA | **Aortic arch** | Left |
| Aorta | ----- | **PDA** | No |
| Pulmonary artery | Normal MPA and Branch PAs. |  |  |
| **M-Mode:** Normal LV Function on eye balling. | | | |
| AO | mm | PWd | mm |
| LA | mm | EDV | ml |
| LVIDd | mm | ESV | ml |
| LVIDs | mm | LVEF | % |
| IVSd | mm | FS | % |
| **Additional Information**: |  | | |
| No pleural/pericardial effusion | | | |
| **Final Diagnosis:** | | | |
| 1. Normal Echocardiography Study. | | | |
| **Remark**: | | | |
| **Recommendation**: | | | |
| SIGNATURE  Done by: Tesfaye T., Pediatrician, Pediatric Cardiologist _______________ 26/05/2013Eth.C | | | |

| Patient Name: **Besifat Mekuriaw**. Patient ID: **FHRH**. SEX/ Age: M/7years. Date of Report: 26**/05/2013**. | | | |
| --- | --- | --- | --- |
| **Features** | **Finding** | **Features** | **Finding** |
| **Profile** |  | **Atria** |  |
| Abdominal situs | Solitus | Left atrium | Normal |
| Cardiac position | Levocardia | Right atrium | Dilated |
| Systemic venous drainage | To RA. **IVC Dilated. SEC+** | **Atrioventricular valves** |  |
| Pulmonary venous drainage | Normal | Mitral valve | Annulus = 21mm |
| Atrioventricular connection | Concordant | Tricuspid valve | Annulus = 22mm  TAPSE = 10mm |
| Ventriculoarterial connection | Concordant | **Ventricles** |  |
| Ventricular loop | d-Loop | Left ventricle | Normal |
|  |  | Right ventricle | Dilated, Hypertrophied & Dysfunctional. |
| **Septae** |  | **Coronary arteries** | ----- |
| Interventricular septum | Intact | **Doppler Measurement** |  |
| Interatrial septum | PFO, R – L Shunt | Mitral | ----- |
| **Semilunal valves** |  | Aortic | ------- |
| Aortic valve | Annulus = 15mm | Tricuspid | Severe TR |
| Pulmonary valve | Annulus = 9mm. Doming Pulmonary Valve | pulmonic | Severe PS, PPG = 100mmHg. |
| **Great arteries** | NRGA | **Aortic arch** | Left |
| Aorta | ----- | **PDA** | No |
| Pulmonary artery | Normal MPA and Branch PAs. |  |  |
| **M-Mode:** Normal LV Function on eye balling. | | | |
| AO | mm | PWd | mm |
| LA | mm | EDV | ml |
| LVIDd | mm | ESV | ml |
| LVIDs | mm | LVEF | % |
| IVSd | mm | FS | % |
| **Additional Information**: |  | | |
| Trace pericardial effusion | | | |
| **Final Diagnosis:** | | | |
| 1. {S, D, S} Levocardia. 2. RA/RV Dilated 3. PFO, R – L Shunt 4. Severe TR 5. Severe Valvar PS 6. Doming Pulmonary Valve 7. RV Dilated, Hypertrophied and Dysfunctional 8. Normal LV Function 9. Trace Pericardial effusion | | | |
| **Remark**: Candidate for intervention (BPV) | | | |
| **Recommendation**: Please refer him to Children’s Heart Fund - Ethiopia | | | |
| SIGNATURE  Done by: Tesfaye T., Pediatrician, Pediatric Cardiologist _______________ 26/05/2013Eth.C | | | |

| Patient Name: **Soliyana Manyazewal**. Patient ID: **Adinas GH**. SEX/ Age: F/5 8/12. Date of Report: 24**/05/2013**.  BP: _______ Weight: ______ Height:____________ BSA: ________ | | | |
| --- | --- | --- | --- |
| **Features** | **Finding** | **Features** | **Finding** |
| **Profile** |  | **Atria** |  |
| Abdominal situs | Solitus | Left atrium | Dilated |
| Cardiac position | Levocardia | Right atrium | Dilated |
| Systemic venous drainage | Normal | **Atrioventricular valves** |  |
| Pulmonary venous drainage | Normal | Mitral valve | Annulus = 18mm |
| Atrioventricular connection | Concordant | Tricuspid valve | Annulus = 20mm  TAPSE = mm |
| Ventriculoarterial connection | Concordant | **Ventricles** |  |
| Ventricular loop | d-Loop | Left ventricle | Dilated |
|  |  | Right ventricle | Dilated |
| **Septae** |  | **Coronary arteries** | ----- |
| Interventricular septum | 16mm Muscular VSD, L – R Shunt, BD Shunt | **Doppler Measurement** |  |
| Interatrial septum | Intact | Mitral | Severe MR, Holosystolic, posterior projection, seen in two planes, jet velocity= 4.6m/sec |
| **Semilunal valves** |  | Aortic | ------- |
| Aortic valve | Annulus = 14mm | Tricuspid | ---- |
| Pulmonary valve | Annulus = 19mm | pulmonic | Moderate PR, PPG = 50mmHg. |
| **Great arteries** | NRGA | **Aortic arch** | Left |
| Aorta | ----- | **PDA** | 4mm PDA, BD Shunt |
| Pulmonary artery | MPA = 30mm. |  |  |
| **M-Mode:** | | | |
| AO | mm | PWd | mm |
| LA | mm | EDV | ml |
| LVIDd | mm | ESV | ml |
| LVIDs | mm | LVEF | % |
| IVSd | mm | FS | % |
| **Additional Information**: |  | | |
| 5mm pericardial effusion on RA/RV Side. | | | |
| **Final Diagnosis:** | | | |
| 1. {S, D, S} Levocardia. 2. All chambers Dilated 3. Severe MR 4. Large Muscular VSD, BD Shunt 5. Large PDA, BD Shunt 6. Severe Pulmonary Hypertension 7. Small Pericardial Effusion 8. ?Eisenmengerized | | | |
| **Remark**: | | | |
| **Recommendation**: | | | |
| SIGNATURE  Done by: Tesfaye T., Pediatrician, Pediatric Cardiologist _______________ 24/05/2013Eth.C | | | |

| Patient Name: **Bereket Endalew**. Patient ID: **FHRH**. SEX/ Age: M/2years. Date of Report: 27**/05/2013**.  BP: _______ Weight: ______ Height:____________ BSA: ________ | | | |
| --- | --- | --- | --- |
| **Features** | **Finding** | **Features** | **Finding** |
| **Profile** |  | **Atria** |  |
| Abdominal situs | Solitus | Left atrium | Normal |
| Cardiac position | Levocardia | Right atrium | Normal |
| Systemic venous drainage | Normal | **Atrioventricular valves** |  |
| Pulmonary venous drainage | Normal | Mitral valve | Annulus = 14mm |
| Atrioventricular connection | Concordant | Tricuspid valve | Annulus = 15mm  TAPSE = 13mm |
| Ventriculoarterial connection | Concordant | **Ventricles** |  |
| Ventricular loop | d-Loop | Left ventricle | Normal |
|  |  | Right ventricle | Normal |
| **Septae** |  | **Coronary arteries** | ----- |
| Interventricular septum | Intact | **Doppler Measurement** |  |
| Interatrial septum | Intact | Mitral | ----- |
| **Semilunal valves** |  | Aortic | ------- |
| Aortic valve | Annulus = 10mm | Tricuspid | ---- |
| Pulmonary valve | Annulus = 13mm | pulmonic | -------- |
| **Great arteries** | NRGA | **Aortic arch** | Left |
| Aorta | ----- | **PDA** | No |
| Pulmonary artery | Normal MPA and Branch PAs. |  |  |
| **M-Mode:** | | | |
| AO | mm | PWd | mm |
| LA | mm | EDV | ml |
| LVIDd | mm | ESV | ml |
| LVIDs | mm | LVEF | 61% |
| IVSd | mm | FS | 32% |
| **Additional Information**: |  | | |
| No pleural/pericardial effusion | | | |
| **Final Diagnosis:** | | | |
| 1. Normal Echocardiography Study. | | | |
| **Remark**: | | | |
| **Recommendation**: | | | |
| SIGNATURE  Done by: Tesfaye T., Pediatrician, Pediatric Cardiologist _______________ 27/05/2013Eth.C | | | |

| Patient Name: **Mamye Fenta**. Patient ID: **TGSH**. SEX/ Age: F/10years. Date of Report: 27**/05/2013**.  BP: _______ Weight: ______ Height:____________ BSA: ________ | | | |
| --- | --- | --- | --- |
| **Features** | **Finding** | **Features** | **Finding** |
| **Profile** |  | **Atria** |  |
| Abdominal situs | Solitus | Left atrium | Normal |
| Cardiac position | Levocardia | Right atrium | Normal |
| Systemic venous drainage | Normal | **Atrioventricular valves** |  |
| Pulmonary venous drainage | Normal | Mitral valve | Annulus = 18mm |
| Atrioventricular connection | Concordant | Tricuspid valve | Annulus = 20mm  TAPSE = 19mm |
| Ventriculoarterial connection | Concordant | **Ventricles** |  |
| Ventricular loop | d-Loop | Left ventricle | Normal |
|  |  | Right ventricle | Normal |
| **Septae** |  | **Coronary arteries** | ----- |
| Interventricular septum | Intact | **Doppler Measurement** |  |
| Interatrial septum | Intact | Mitral | ----- |
| **Semilunal valves** |  | Aortic | ------- |
| Aortic valve | Annulus = 16mm | Tricuspid | ---- |
| Pulmonary valve | Annulus = 19mm | pulmonic | -------- |
| **Great arteries** | NRGA | **Aortic arch** | Left |
| Aorta | ----- | **PDA** | No |
| Pulmonary artery | Normal MPA and Branch PAs. |  |  |
| **M-Mode:** | | | |
| AO | mm | PWd | mm |
| LA | mm | EDV | ml |
| LVIDd | mm | ESV | ml |
| LVIDs | mm | LVEF | 58% |
| IVSd | mm | FS | 30% |
| **Additional Information**: |  | | |
| No pleural/pericardial effusion | | | |
| **Final Diagnosis:** | | | |
| 1. Normal Echocardiography Study. | | | |
| **Remark**: | | | |
| **Recommendation**: | | | |
| SIGNATURE  Done by: Tesfaye T., Pediatrician, Pediatric Cardiologist _______________ 27/05/2013Eth.C | | | |

| Patient Name: **Kalkidan Mamyew**. Patient ID: **FHRH**. SEX/ Age: F/4 3/12. Date of Report: 27**/05/2013**. | | | |
| --- | --- | --- | --- |
| **Features** | **Finding** | **Features** | **Finding** |
| **Profile** |  | **Atria** |  |
| Abdominal situs | Solitus | Left atrium | Normal |
| Cardiac position | Levocardia | Right atrium | Normal |
| Systemic venous drainage | Normal | **Atrioventricular valves** |  |
| Pulmonary venous drainage | Normal | Mitral valve | Annulus = 21mm |
| Atrioventricular connection | Concordant | Tricuspid valve | Annulus = 18mm  TAPSE = 16mm |
| Ventriculoarterial connection | Concordant | **Ventricles** |  |
| Ventricular loop | d-Loop | Left ventricle | Normal |
|  |  | Right ventricle | Normal |
| **Septae** |  | **Coronary arteries** | ----- |
| Interventricular septum | Large, nonrestrictive subaortic VSD, BD Shunt | **Doppler Measurement** |  |
| Interatrial septum | Intact | Mitral | Mild MR, Holosystolic, posterior projection seen in 2 planes with jet velocity = 4m/sec |
| **Semilunal valves** |  | Aortic | ------- |
| Aortic valve | Annulus = 15mm | Tricuspid | Trivial TR |
| Pulmonary valve | Annulus = 22mm. Doming pulmonary Valve | pulmonic | Mild Valvar PS, PPG = 30mmHg |
| **Great arteries** | NRGA | **Aortic arch** | Left |
| Aorta |  | **PDA** | No |
| Pulmonary artery | MPA = 20mm. Normal Branch PAs. |  |  |
| **M-Mode:** | | | |
| AO | mm | PWd | mm |
| LA | mm | EDV | ml |
| LVIDd | mm | ESV | ml |
| LVIDs | mm | LVEF | 50% |
| IVSd | mm | FS | 25% |
| **Additional Information**: |  | | |
| Trace pericardial effusion | | | |
| **Final Diagnosis:** | | | |
| 1. {S, D, S} Levocardia. 2. Mild MR 3. Trivial TR 4. Large Subaortic Non-restrictive VSD, BD Shunt 5. Mild Valvar PS 6. Mildly reduced LV Function | | | |
| **Remark**: No echocardiographic Evidence of IE | | | |
| SIGNATURE  Done by: Tesfaye T., Pediatrician, Pediatric Cardiologist _______________ 27/05/2013Eth.C | | | |

| Patient Name: **Bereket Mulat**. Patient ID: **Adinas GH**. SEX/ Age: M/45days. Date of Report: 27**/05/2013**.  BP: _______ Weight: ______ Height:____________ BSA: ________ | | | |
| --- | --- | --- | --- |
| **Features** | **Finding** | **Features** | **Finding** |
| **Profile** |  | **Atria** |  |
| Abdominal situs | Solitus | Left atrium | Normal |
| Cardiac position | Levocardia | Right atrium | Normal |
| Systemic venous drainage | Normal | **Atrioventricular valves** |  |
| Pulmonary venous drainage | Normal | Mitral valve | Annulus = 8mm |
| Atrioventricular connection | Concordant | Tricuspid valve | Annulus = 10mm  TAPSE = 13mm |
| Ventriculoarterial connection | Concordant | **Ventricles** |  |
| Ventricular loop | d-Loop | Left ventricle | Normal |
|  |  | Right ventricle | Normal |
| **Septae** |  | **Coronary arteries** | ----- |
| Interventricular septum | Intact | **Doppler Measurement** |  |
| Interatrial septum | PFO, L – R Shunt | Mitral | ----- |
| **Semilunal valves** |  | Aortic | ------- |
| Aortic valve | Annulus = mm | Tricuspid | ---- |
| Pulmonary valve | Annulus = 8mm | pulmonic | Moderate PS, PPG = 54mmHg. In addition, there is a gradient of PPG = 48mmHg across branch PAs |
| **Great arteries** | NRGA | **Aortic arch** | Left |
| Aorta | ----- | **PDA** | No |
| Pulmonary artery | MPA =6mm, LPA = 3mm and RPA = 4mm. |  |  |
| **M-Mode:** | | | |
| AO | mm | PWd | mm |
| LA | mm | EDV | ml |
| LVIDd | mm | ESV | ml |
| LVIDs | mm | LVEF | 71% |
| IVSd | mm | FS | 38% |
| **Additional Information**: |  | | |
| No pleural/pericardial effusion | | | |
| **Final Diagnosis:** | | | |
| 1. {S, D, S} Levocardia. 2. PFO, L – R Shunt 3. Moderate Valvar PS and Peripheral PS 4. Normal Biventricular Function | | | |
| **Remark**: | | | |
| **Recommendation**: | | | |
| SIGNATURE  Done by: Tesfaye T., Pediatrician, Pediatric Cardiologist _______________ 27/05/2013Eth.C | | | |

| Patient Name: **Baby of Hiwet Debebe**. Patient ID: **FHRH**. SEX/ Age: M/2hours. Date of Report: 28**/05/2013**.  BP: _______ Weight: ______ Height:____________ BSA: ________ | | | |
| --- | --- | --- | --- |
| **Features** | **Finding** | **Features** | **Finding** |
| **Profile** |  | **Atria** |  |
| Abdominal situs | Solitus | Left atrium | Normal |
| Cardiac position | Levocardia | Right atrium | Normal |
| Systemic venous drainage | Normal | **Atrioventricular valves** |  |
| Pulmonary venous drainage | Normal | Mitral valve | Common Complete AVSD |
| Atrioventricular connection | Common Complete AVSD | Tricuspid valve |  |
| Ventriculoarterial connection | Concordant | **Ventricles** |  |
| Ventricular loop | d-Loop | Left ventricle | Normal |
|  |  | Right ventricle | Normal |
| **Septae** |  | **Coronary arteries** | ----- |
| Interventricular septum | Common Complete AVSD, L – R Shunt | **Doppler Measurement** |  |
| Interatrial septum |  | Mitral | ----- |
| **Semilunal valves** |  | Aortic | ------- |
| Aortic valve | Annulus = 8mm | Tricuspid | Mild TR |
| Pulmonary valve | Annulus = 8mm | pulmonic | -------- |
| **Great arteries** | NRGA | **Aortic arch** | Left |
| Aorta | ----- | **PDA** | 1mm PDA, L – R Shunt. |
| Pulmonary artery | Normal MPA and Branch PAs. |  |  |
| **M-Mode:** Normal LV Function on eye balling. | | | |
| AO | mm | PWd | mm |
| LA | mm | EDV | ml |
| LVIDd | mm | ESV | ml |
| LVIDs | mm | LVEF | % |
| IVSd | mm | FS | % |
| **Additional Information**: |  | | |
| No pleural/pericardial effusion | | | |
| **Final Diagnosis:** | | | |
| 1. {S, D, S} Levocardia. 2. Common Complete Balanced AVSD, L – R Shunt 3. Small PDA, L – R Shunt 4. Normal LV Function | | | |
| **Remark**: | | | |
| **Recommendation**: | | | |
| SIGNATURE  Done by: Tesfaye T., Pediatrician, Pediatric Cardiologist _______________ 28/05/2013Eth.C | | | |

| Patient Name: **Baby of Shashe matebie**. Patient ID: **Adinas GH**. SEX/ Age: F/7days. Date of Report: 29**/05/2013**.  BP: _______ Weight: ______ Height:____________ BSA: ________ | | | |
| --- | --- | --- | --- |
| **Features** | **Finding** | **Features** | **Finding** |
| **Profile** |  | **Atria** |  |
| Abdominal situs | Solitus | Left atrium | Normal |
| Cardiac position | Levocardia | Right atrium | Normal |
| Systemic venous drainage | Normal | **Atrioventricular valves** |  |
| Pulmonary venous drainage | Normal | Mitral valve | Common Complete AVSD |
| Atrioventricular connection | Common Complete AVSD | Tricuspid valve |  |
| Ventriculoarterial connection | Concordant | **Ventricles** |  |
| Ventricular loop | d-Loop | Left ventricle | Normal |
|  |  | Right ventricle | Normal |
| **Septae** |  | **Coronary arteries** | ----- |
| Interventricular septum | Common Complete AVSD, L – R Shunt | **Doppler Measurement** |  |
| Interatrial septum |  | Mitral | Mild Left AVVR |
| **Semilunal valves** |  | Aortic | ------- |
| Aortic valve | Annulus = mm | Tricuspid | ---- |
| Pulmonary valve | Annulus = mm | pulmonic | -------- |
| **Great arteries** | NRGA | **Aortic arch** | Left |
| Aorta | ----- | **PDA** | No |
| Pulmonary artery | Normal MPA and Branch PAs. |  |  |
| **M-Mode:** Normal LV Function on eye balling. | | | |
| AO | mm | PWd | mm |
| LA | mm | EDV | ml |
| LVIDd | mm | ESV | ml |
| LVIDs | mm | LVEF | % |
| IVSd | mm | FS | % |
| **Additional Information**: |  | | |
| Trace pericardial effusion | | | |
| **Final Diagnosis:** | | | |
| 1. {S, D, S} Levocardia. 2. Common Complete AVSD, L – R Shunt 3. Trace Pericardial Effusion 4. Normal Biventricular Function | | | |
| **Remark**: | | | |
| **Recommendation**: | | | |
| SIGNATURE  Done by: Tesfaye T., Pediatrician, Pediatric Cardiologist _______________ 29/05/2013Eth.C | | | |

| Patient Name: **Arsema Wendimnew**. Patient ID: **Finote Selam H**. SEX/ Age: F/8/12. Date of Report: 29**/05/2013**.  BP: _______ Weight: ______ Height:____________ BSA: ________ | | | |
| --- | --- | --- | --- |
| **Features** | **Finding** | **Features** | **Finding** |
| **Profile** |  | **Atria** |  |
| Abdominal situs | Solitus | Left atrium | Normal |
| Cardiac position | Levocardia | Right atrium | Normal |
| Systemic venous drainage | Normal | **Atrioventricular valves** |  |
| Pulmonary venous drainage | Normal | Mitral valve | Annulus = 13mm |
| Atrioventricular connection | Concordant | Tricuspid valve | Annulus = 15mm |
| Ventriculoarterial connection | Concordant | **Ventricles** |  |
| Ventricular loop | d-Loop | Left ventricle | Normal |
|  |  | Right ventricle | Normal |
| **Septae** |  | **Coronary arteries** | ----- |
| Interventricular septum | 3mm Sub pulmonic VSD, L – R Shunt | **Doppler Measurement** |  |
| Interatrial septum | 5mm OS ASD, L – R Shunt | Mitral | ----- |
| **Semilunal valves** |  | Aortic | ------- |
| Aortic valve | Annulus = 10mm | Tricuspid | Mild TR |
| Pulmonary valve | Annulus = 11mm | pulmonic | -------- |
| **Great arteries** | NRGA | **Aortic arch** | Left |
| Aorta | ----- | **PDA** | No |
| Pulmonary artery | Normal MPA and Branch PAs. |  |  |
| **M-Mode:** | | | |
| AO | mm | PWd | mm |
| LA | mm | EDV | ml |
| LVIDd | mm | ESV | ml |
| LVIDs | mm | LVEF | % |
| IVSd | mm | FS | % |
| **Additional Information**: |  | | |
| No pleural/pericardial effusion | | | |
| **Final Diagnosis:** | | | |
| 1. {S, D, S} Levocardia. 2. Small OS ASD, L – R Shunt 3. Small Sub Pulmonic VSD, L – R Shunt 4. Normal Biventricular Function | | | |
| **Remark**: | | | |
| **Recommendation**: | | | |
| SIGNATURE  Done by: Tesfaye T., Pediatrician, Pediatric Cardiologist _______________ 29/05/2013Eth.C | | | |

| Patient Name: **Asfera Yirga**. Patient ID: **Finote Selam H**. SEX/ Age: M/14years. Date of Report: 29**/05/2013**.  BP: _______ Weight: ______ Height:____________ BSA: ________ | | | |
| --- | --- | --- | --- |
| **Features** | **Finding** | **Features** | **Finding** |
| **Profile** |  | **Atria** |  |
| Abdominal situs | Solitus | Left atrium | Normal |
| Cardiac position | Levocardia | Right atrium | Normal |
| Systemic venous drainage | Normal | **Atrioventricular valves** |  |
| Pulmonary venous drainage | Normal | Mitral valve | Annulus = 25mm |
| Atrioventricular connection | Concordant | Tricuspid valve | Annulus = 19mm  TAPSE = 14mm |
| Ventriculoarterial connection | Concordant | **Ventricles** |  |
| Ventricular loop | d-Loop | Left ventricle | Dilated |
|  |  | Right ventricle | Normal |
| **Septae** |  | **Coronary arteries** | ----- |
| Interventricular septum | Intact | **Doppler Measurement** |  |
| Interatrial septum | Intact | Mitral | Mild MR, Not Holosystolic, with jet velocity = 2.2m/sec |
| **Semilunal valves** |  | Aortic | ------- |
| Aortic valve | Annulus = 22mm | Tricuspid | ---- |
| Pulmonary valve | Annulus = 22mm | pulmonic | -------- |
| **Great arteries** | NRGA | **Aortic arch** | Left. No CoA. |
| Aorta | ----- | **PDA** | No |
| Pulmonary artery | Normal MPA and Branch PAs. |  |  |
| **M-Mode:** | | | |
| AO | mm | PWd | mm |
| LA | mm | EDV | ml |
| LVIDd | mm | ESV | ml |
| LVIDs | mm | LVEF | 49% |
| IVSd | mm | FS | 24% |
| **Additional Information**: |  | | |
| No pleural/pericardial effusion | | | |
| **Final Diagnosis:** | | | |
| 1. {S, D, S} Levocardia. 2. Mild MR 3. Dilated and mildly Dysfunctional LV | | | |
| **Remark**: | | | |
| **Recommendation**: | | | |
| SIGNATURE  Done by: Tesfaye T., Pediatrician, Pediatric Cardiologist _______________ 29/05/2013Eth.C | | | |

| Patient Name: **Yidagn Abtew**. Patient ID: **Addis Alem PH**. SEX/ Age: F/3/12. Date of Report: 01**/06/2013**.  BP: _______ Weight: ______ Height:____________ BSA: ________ | | | |
| --- | --- | --- | --- |
| **Features** | **Finding** | **Features** | **Finding** |
| **Profile** |  | **Atria** |  |
| Abdominal situs | Solitus | Left atrium | Normal |
| Cardiac position | Levocardia | Right atrium | Normal |
| Systemic venous drainage | Normal | **Atrioventricular valves** |  |
| Pulmonary venous drainage | Normal | Mitral valve | Annulus = 11mm |
| Atrioventricular connection | Concordant | Tricuspid valve | Annulus = 12mm  TAPSE = 13mm |
| Ventriculoarterial connection | Concordant | **Ventricles** |  |
| Ventricular loop | d-Loop | Left ventricle | Normal |
|  |  | Right ventricle | Normal |
| **Septae** |  | **Coronary arteries** | ----- |
| Interventricular septum | Intact | **Doppler Measurement** |  |
| Interatrial septum | Intact | Mitral | ----- |
| **Semilunal valves** |  | Aortic | ------- |
| Aortic valve | Annulus = 9mm | Tricuspid | ---- |
| Pulmonary valve | Annulus = 9mm | pulmonic | -------- |
| **Great arteries** | NRGA | **Aortic arch** | Left |
| Aorta | ----- | **PDA** | No |
| Pulmonary artery | Normal MPA and Branch PAs. |  |  |
| **M-Mode:** Normal LV Function on eye balling. | | | |
| AO | mm | PWd | mm |
| LA | mm | EDV | ml |
| LVIDd | mm | ESV | ml |
| LVIDs | mm | LVEF | % |
| IVSd | mm | FS | % |
| **Additional Information**: |  | | |
| No pleural/pericardial effusion | | | |
| **Final Diagnosis:** | | | |
| 1. Normal Echocardiography Study. | | | |
| **Remark**: | | | |
| **Recommendation**: | | | |
| SIGNATURE  Done by: Tesfaye T., Pediatrician, Pediatric Cardiologist _______________ 01/06/2013Eth.C | | | |

| Patient Name: **Simeneh Yalew**. Patient ID: **Adinas GH**. SEX/ Age: M/9years. Date of Report: 01**/06/2013**. | | | |
| --- | --- | --- | --- |
| **Features** | **Finding** | **Features** | **Finding** |
| **Profile** |  | **Atria** |  |
| Abdominal situs | Solitus | Left atrium | Dilated |
| Cardiac position | Levocardia | Right atrium | Normal |
| Systemic venous drainage | Normal | **AV Valves** |  |
| Pulmonary venous drainage | Normal | Mitral valve | Annulus = 25mm. Thickened MVL |
| Atrioventricular connection | Concordant | Tricuspid valve | Annulus = 22mm  TAPSE = 22mm |
| Ventriculoarterial connection | Concordant | **Ventricles** |  |
| Ventricular loop | d-Loop | Left ventricle | Dilated |
|  |  | Right ventricle | Normal |
| **Septae** |  | **Coronary arteries** | ----- |
| Interventricular septum | Intact | **Doppler Measurement** | |
| Interatrial septum | Intact | Mitral | Severe MR, Holosystolic, posterior projection, seen in two planes with jet velocity = 3.3m/sec. |
| **Semilunal valves** |  | Aortic | Moderate AR, PHT = 278ms. |
| Aortic valve | Annulus = 15mm. Thickened MVL | Tricuspid | Moderate TR, PPG = 33mmHg |
| Pulmonary valve | Annulus = 19mm | pulmonic | -------- |
| **Great arteries** | NRGA | **Aortic arch** | Left |
| Aorta | ----- | **PDA** | No |
| Pulmonary artery | Normal MPA and Branch PAs. |  |  |
| **M-Mode:** | | | |
| AO | mm | PWd | mm |
| LA | mm | EDV | ml |
| LVIDd | mm | ESV | ml |
| LVIDs | mm | LVEF | 74% |
| IVSd | mm | FS | 43% |
| **Additional Information**: |  | | |
| 6mm pericardial effusion on RA/RV Side | | | |
| **Final Diagnosis:** | | | |
| 1. {S, D, S} Levocardia. 2. LA/LV Dilated 3. Thickened MVL and AVL 4. Severe MR 5. Moderate AR 6. Moderate TR 7. Small Pericardial Effusion 8. Normal Function | | | |
| SIGNATURE  Done by: Tesfaye T., Pediatrician, Pediatric Cardiologist _______________ 01/06/2013Eth.C | | | |

| Patient Name: **Endaley Atlog**. Patient ID: **FHRH**. SEX/ Age: F/7years. Date of Report: 02**/06/2013**.  BP: _______ Weight: ______ Height:____________ BSA: ________ | | | |
| --- | --- | --- | --- |
| **Features** | **Finding** | **Features** | **Finding** |
| **Profile** |  | **Atria** |  |
| Abdominal situs | Solitus | Left atrium | Normal |
| Cardiac position | Levocardia | Right atrium | Normal |
| Systemic venous drainage | Normal | **Atrioventricular valves** |  |
| Pulmonary venous drainage | LSVC to CS to RA | Mitral valve | Annulus = 18mm |
| Atrioventricular connection | Concordant | Tricuspid valve | Annulus = 20mm |
| Ventriculoarterial connection | Concordant | **Ventricles** |  |
| Ventricular loop | d-Loop | Left ventricle | Normal |
|  |  | Right ventricle | Normal |
| **Septae** |  | **Coronary arteries** | ----- |
| Interventricular septum | Intact | **Doppler Measurement** |  |
| Interatrial septum | Intact | Mitral | ----- |
| **Semilunal valves** |  | Aortic | ------- |
| Aortic valve | Annulus = 13mm | Tricuspid | ---- |
| Pulmonary valve | Annulus = 17mm | pulmonic | -------- |
| **Great arteries** | NRGA | **Aortic arch** | Left |
| Aorta | ----- | **PDA** | No |
| Pulmonary artery | Normal MPA and Branch PAs. |  |  |
| **M-Mode:** | | | |
| AO | mm | PWd | mm |
| LA | mm | EDV | ml |
| LVIDd | mm | ESV | ml |
| LVIDs | mm | LVEF | 57% |
| IVSd | mm | FS | 29% |
| **Additional Information**: |  | | |
| No pleural/pericardial effusion | | | |
| **Final Diagnosis:** | | | |
| 1. {S, D, S} Levocardia. 2. Persistent LSVC Draining to CS to RA 3. Normal Function | | | |
| **Remark**: | | | |
| **Recommendation**: Nothing is needed. | | | |
| SIGNATURE  Done by: Tesfaye T., Pediatrician, Pediatric Cardiologist _______________ 02/06/2013Eth.C | | | |

| Patient Name: **Seble Wengel Adgeh**. Patient ID: **Adinas GH**. SEX/ Age: F/10years. Date of Report: 02**/06/2013**.  BP: _______ Weight: ______ Height:____________ BSA: ________ | | | |
| --- | --- | --- | --- |
| **Features** | **Finding** | **Features** | **Finding** |
| **Profile** |  | **Atria** |  |
| Abdominal situs | Solitus | Left atrium | Normal |
| Cardiac position | Levocardia | Right atrium | Normal |
| Systemic venous drainage | Normal | **Atrioventricular valves** |  |
| Pulmonary venous drainage | Normal | Mitral valve | Annulus = 16mm |
| Atrioventricular connection | Concordant | Tricuspid valve | Annulus = 15mm  TAPSE = 16mm |
| Ventriculoarterial connection | Concordant | **Ventricles** |  |
| Ventricular loop | d-Loop | Left ventricle | Normal |
|  |  | Right ventricle | Normal |
| **Septae** |  | **Coronary arteries** | ----- |
| Interventricular septum | Intact | **Doppler Measurement** |  |
| Interatrial septum | Intact | Mitral | ----- |
| **Semilunal valves** |  | Aortic | ------- |
| Aortic valve | Annulus = 18mm | Tricuspid | ---- |
| Pulmonary valve | Annulus = 21mm | pulmonic | -------- |
| **Great arteries** | NRGA | **Aortic arch** | Left |
| Aorta | ----- | **PDA** | No |
| Pulmonary artery | Normal MPA and Branch PAs. |  |  |
| **M-Mode:** Normal LV Function on eye balling. | | | |
| AO | mm | PWd | mm |
| LA | mm | EDV | ml |
| LVIDd | mm | ESV | ml |
| LVIDs | mm | LVEF | % |
| IVSd | mm | FS | % |
| **Additional Information**: |  | | |
| No pleural/pericardial effusion | | | |
| **Final Diagnosis:** | | | |
| 1. Normal Echocardiography Study. | | | |
| **Remark**: | | | |
| **Recommendation**: | | | |
| SIGNATURE  Done by: Tesfaye T., Pediatrician, Pediatric Cardiologist _______________ 02/06/2013Eth.C | | | |

| Patient Name: **Baby of wellela Tegegne**. Patient ID: **FHRH**. SEX/ Age: M/23days. Date of Report: 02**/06/2013**.  BP: _______ Weight: ______ Height:____________ BSA: ________ | | | |
| --- | --- | --- | --- |
| **Features** | **Finding** | **Features** | **Finding** |
| **Profile** |  | **Atria** |  |
| Abdominal situs | Solitus | Left atrium | Normal |
| Cardiac position | Levocardia | Right atrium | Normal |
| Systemic venous drainage | Normal | **Atrioventricular valves** |  |
| Pulmonary venous drainage | Normal | Mitral valve | Annulus = 9mm |
| Atrioventricular connection | Concordant | Tricuspid valve | Annulus = 9mm |
| Ventriculoarterial connection | Concordant | **Ventricles** |  |
| Ventricular loop | d-Loop | Left ventricle | Normal |
|  |  | Right ventricle | Normal |
| **Septae** |  | **Coronary arteries** | ----- |
| Interventricular septum | Intact | **Doppler Measurement** |  |
| Interatrial septum | Intact | Mitral | ----- |
| **Semilunal valves** |  | Aortic | ------- |
| Aortic valve | Annulus = 8mm | Tricuspid | ---- |
| Pulmonary valve | Annulus = 8mm | pulmonic | -------- |
| **Great arteries** | NRGA | **Aortic arch** | Left |
| Aorta | ----- | **PDA** | No |
| Pulmonary artery | Normal MPA and Branch PAs. |  |  |
| **M-Mode:** | | | |
| AO | mm | PWd | mm |
| LA | mm | EDV | ml |
| LVIDd | mm | ESV | ml |
| LVIDs | mm | LVEF | % |
| IVSd | mm | FS | % |
| **Additional Information**: |  | | |
| No pleural/pericardial effusion | | | |
| **Final Diagnosis:** | | | |
| 1. Normal Echocardiography Study. | | | |
| **Remark**: Baby was crying during study | | | |
| **Recommendation**: | | | |
| SIGNATURE  Done by: Tesfaye T., Pediatrician, Pediatric Cardiologist _______________ 02/06/2013Eth.C | | | |

| Patient Name: **Baby of Zyin Endeshaw**. Patient ID: **FHRH**. SEX/ Age: M/11days. Date of Report: 02**/06/2013**.  BP: _______ Weight: ______ Height:____________ BSA: ________ | | | |
| --- | --- | --- | --- |
| **Features** | **Finding** | **Features** | **Finding** |
| **Profile** |  | **Atria** |  |
| Abdominal situs | Solitus | Left atrium | Normal |
| Cardiac position | Levocardia | Right atrium | Normal |
| Systemic venous drainage | Normal | **Atrioventricular valves** |  |
| Pulmonary venous drainage | Normal | Mitral valve | Annulus = 8mm |
| Atrioventricular connection | Concordant | Tricuspid valve | Annulus = 10mm |
| Ventriculoarterial connection | Concordant | **Ventricles** |  |
| Ventricular loop | d-Loop | Left ventricle | Normal |
|  |  | Right ventricle | Normal |
| **Septae** |  | **Coronary arteries** | ----- |
| Interventricular septum | Intact | **Doppler Measurement** |  |
| Interatrial septum | 5mm OS ASD, L – R Shunt | Mitral | ----- |
| **Semilunal valves** |  | Aortic | ------- |
| Aortic valve | Annulus = mm | Tricuspid | ---- |
| Pulmonary valve | Annulus = mm | pulmonic | -------- |
| **Great arteries** | NRGA | **Aortic arch** | Left |
| Aorta | ----- | **PDA** | 1mm PDA, L – R Shunt |
| Pulmonary artery | Normal MPA and Branch PAs. |  |  |
| **M-Mode:** Normal LV Function on eye balling. | | | |
| AO | mm | PWd | mm |
| LA | mm | EDV | ml |
| LVIDd | mm | ESV | ml |
| LVIDs | mm | LVEF | % |
| IVSd | mm | FS | % |
| **Additional Information**: |  | | |
| No pleural/pericardial effusion | | | |
| **Final Diagnosis:** | | | |
| 1. {S, D, S} Levocardia. 2. Small OS ASD, L – R Shunt 3. Small PDA, L – R Shunt 4. Normal Function | | | |
| **Remark**: Limited Echo window (only subcostal and apical) | | | |
| **Recommendation**: | | | |
| SIGNATURE  Done by: Tesfaye T., Pediatrician, Pediatric Cardiologist _______________ 02/06/2013Eth.C | | | |

| Patient Name: **Eyerus Tilahun**. Patient ID: **Addis Zemen H**. SEX/ Age: F/11years. Date of Report: 04**/06/2013**.  BP: _______ Weight: ______ Height:____________ BSA: ________ | | | |
| --- | --- | --- | --- |
| **Features** | **Finding** | **Features** | **Finding** |
| **Profile** |  | **Atria** |  |
| Abdominal situs | Solitus | Left atrium | Normal |
| Cardiac position | Levocardia | Right atrium | Normal |
| Systemic venous drainage | Normal | **Atrioventricular valves** |  |
| Pulmonary venous drainage | Normal | Mitral valve | Annulus = 21mm |
| Atrioventricular connection | Concordant | Tricuspid valve | Annulus = 21mm  TAPSE = 19mm |
| Ventriculoarterial connection | Concordant | **Ventricles** |  |
| Ventricular loop | d-Loop | Left ventricle | Normal |
|  |  | Right ventricle | Normal |
| **Septae** |  | **Coronary arteries** | ----- |
| Interventricular septum | 4mm Inlet VSD partially covered by Septal Leaflet of TV, L – R Shunt, Restrictive with a gradient of 75mmHg. | **Doppler Measurement** |  |
| Interatrial septum | Intact | Mitral | ----- |
| **Semilunal valves** |  | Aortic | ------- |
| Aortic valve | Annulus = 16mm | Tricuspid | ---- |
| Pulmonary valve | Annulus = 20mm | pulmonic | -------- |
| **Great arteries** | NRGA | **Aortic arch** | Left |
| Aorta | ----- | **PDA** | No |
| Pulmonary artery | Normal MPA and Branch PAs. |  |  |
| **M-Mode:** | | | |
| AO | mm | PWd | mm |
| LA | mm | EDV | ml |
| LVIDd | mm | ESV | ml |
| LVIDs | mm | LVEF | 58% |
| IVSd | mm | FS | 30% |
| **Additional Information**: |  | | |
| No pleural/pericardial effusion | | | |
| **Final Diagnosis:** | | | |
| 1. {S, D, S} Levocardia. 2. Small Restrictive Inlet VSD Partially covered by STL, L – R Shunt 3. Normal Biventricular Function. | | | |
| **Remark**: | | | |
| **Recommendation**: Follow up every year | | | |
| SIGNATURE  Done by: Tesfaye T., Pediatrician, Pediatric Cardiologist _______________ 04/06/2013Eth.C | | | |

| Patient Name: **Abel Gashaw**. Patient ID: **Adinas GH**. SEX/ Age: M/2 10/12. Date of Report: 04**/06/2013**.  BP: _______ Weight: ______ Height:____________ BSA: ________ | | | |
| --- | --- | --- | --- |
| **Features** | **Finding** | **Features** | **Finding** |
| **Profile** |  | **Atria** |  |
| Abdominal situs | Solitus | Left atrium | Normal |
| Cardiac position | Levocardia | Right atrium | Normal |
| Systemic venous drainage | Normal | **Atrioventricular valves** |  |
| Pulmonary venous drainage | Normal | Mitral valve | Annulus = 15mm |
| Atrioventricular connection | Concordant | Tricuspid valve | Annulus = 16mm  TAPSE = 21mm |
| Ventriculoarterial connection | Concordant | **Ventricles** |  |
| Ventricular loop | d-Loop | Left ventricle | Normal |
|  |  | Right ventricle | Normal |
| **Septae** |  | **Coronary arteries** | ----- |
| Interventricular septum | Intact | **Doppler Measurement** |  |
| Interatrial septum | Intact | Mitral | ----- |
| **Semilunal valves** |  | Aortic | ------- |
| Aortic valve | Annulus = 15mm | Tricuspid | ---- |
| Pulmonary valve | Annulus = 16mm | pulmonic | -------- |
| **Great arteries** | NRGA | **Aortic arch** | Left |
| Aorta | ----- | **PDA** | No |
| Pulmonary artery | Normal MPA and Branch PAs. |  |  |
| **M-Mode:** | | | |
| AO | mm | PWd | mm |
| LA | mm | EDV | ml |
| LVIDd | mm | ESV | ml |
| LVIDs | mm | LVEF | 69% |
| IVSd | mm | FS | 37% |
| **Additional Information**: |  | | |
| No pleural/pericardial effusion | | | |
| **Final Diagnosis:** | | | |
| 1. Normal Echocardiography Study. | | | |
| **Remark**: | | | |
| **Recommendation**: | | | |
| SIGNATURE  Done by: Tesfaye T., Pediatrician, Pediatric Cardiologist _______________ 04/06/2013Eth.C | | | |

| Patient Name: **Akiya Abiyot**. Patient ID: **TGSH**. SEX/ Age: F/2 5/12. Date of Report: **05/06/2013**.  BP: _______ Weight: ______ Height:____________ BSA: ________ | | | |
| --- | --- | --- | --- |
| **Features** | **Finding** | **Features** | **Finding** |
| **Profile** |  | **Atria** |  |
| Abdominal situs | Solitus | Left atrium | Mildly Dilated |
| Cardiac position | Levocardia | Right atrium | Normal |
| Systemic venous drainage | Normal | **Atrioventricular valves** |  |
| Pulmonary venous drainage | Normal | Mitral valve | Annulus = 16mm |
| Atrioventricular connection | Concordant | Tricuspid valve | Annulus = 16mm  TAPSE = 17mm |
| Ventriculoarterial connection | Concordant | **Ventricles** |  |
| Ventricular loop | d-Loop | Left ventricle | Mildly Dilated |
|  |  | Right ventricle | Normal |
| **Septae** |  | **Coronary arteries** | ----- |
| Interventricular septum | Intact | **Doppler Measurement** |  |
| Interatrial septum | Intact | Mitral | ----- |
| **Semilunal valves** |  | Aortic | ------- |
| Aortic valve | Annulus = 14mm | Tricuspid | ---- |
| Pulmonary valve | Annulus = 16mm | pulmonic | -------- |
| **Great arteries** | NRGA | **Aortic arch** | Left |
| Aorta | ----- | **PDA** | 2mm PDA, L – R Shunt |
| Pulmonary artery | Normal MPA and Branch PAs. |  |  |
| **M-Mode:** | | | |
| AO | mm | PWd | mm |
| LA | mm | EDV | ml |
| LVIDd | mm | ESV | ml |
| LVIDs | mm | LVEF | 66% |
| IVSd | mm | FS | 35% |
| **Additional Information**: |  | | |
| No pleural/pericardial effusion | | | |
| **Final Diagnosis:** | | | |
| 1. {S, D, S} Levocardia. 2. Moderate PDA, L – R Shunt 3. Normal Biventricular Function | | | |
| **Remark**: | | | |
| **Recommendation**: | | | |
| SIGNATURE  Done by: Tesfaye T., Pediatrician, Pediatric Cardiologist _______________ 05/06/2013Eth.C | | | |

| Patient Name: **Kidist Abrham.** Patient ID: **FHRH**. SEX/ Age: F/9years. Date of Report: **05/06/2013**.  BP: _______ Weight: ______ Height:____________ BSA: ________ | | | |
| --- | --- | --- | --- |
| **Features** | **Finding** | **Features** | **Finding** |
| **Profile** |  | **Atria** |  |
| Abdominal situs | Solitus | Left atrium | Normal |
| Cardiac position | Levocardia | Right atrium | Dilated |
| Systemic venous drainage | Normal | **Atrioventricular valves** |  |
| Pulmonary venous drainage | Normal | Mitral valve | Annulus = 16mm |
| Atrioventricular connection | Concordant | Tricuspid valve | Annulus = 21mm |
| Ventriculoarterial connection | Concordant | **Ventricles** |  |
| Ventricular loop | d-Loop | Left ventricle | Normal |
|  |  | Right ventricle | Dilated, Hypertrophied |
| **Septae** |  | **Coronary arteries** | ----- |
| Interventricular septum | Nonrestrictive Malaligned Sub aortic VSD, R – L S hunt | **Doppler Measurement** |  |
| Interatrial septum | Intact | Mitral | ----- |
| **Semilunal valves** |  | Aortic | ------- |
| Aortic valve | Annulus = 22mm | Tricuspid | ---- |
| Pulmonary valve | Annulus = 12mm | pulmonic | Severe PS, PPG = 69mmHg |
| **Great arteries** | NRGA | **Aortic arch** | Left |
| Aorta | Overriding aorta over the VSD | **PDA** | No |
| Pulmonary artery | Normal MPA and Branch PAs. |  |  |
| **M-Mode:** | | | |
| AO | mm | PWd | mm |
| LA | mm | EDV | ml |
| LVIDd | mm | ESV | ml |
| LVIDs | mm | LVEF | 66% |
| IVSd | mm | FS | 35% |
| **Additional Information**: |  | | |
| No pleural/pericardial effusion | | | |
| **Final Diagnosis:** | | | |
| 1. {S, D, S} Levocardia. 2. TOF | | | |
| **Remark**: | | | |
| **Recommendation**: | | | |
| SIGNATURE  Done by: Tesfaye T., Pediatrician, Pediatric Cardiologist _______________ 05/06/2013Eth.C | | | |

| Patient Name: **Bereket Addisu**. Patient ID: **Adinas GH**. SEX/ Age: M/8 9/12. Date of Report: 06**/06/2013**.  BP: _______ Weight: ______ Height:____________ BSA: ________ | | | |
| --- | --- | --- | --- |
| **Features** | **Finding** | **Features** | **Finding** |
| **Profile** |  | **Atria** |  |
| Abdominal situs | Solitus | Left atrium | Normal |
| Cardiac position | Levocardia | Right atrium | Normal |
| Systemic venous drainage | Normal | **Atrioventricular valves** |  |
| Pulmonary venous drainage | Normal | Mitral valve | Annulus = 22mm. Mildly thickened MVL |
| Atrioventricular connection | Concordant | Tricuspid valve | Annulus = 21mm  TAPSE = mm |
| Ventriculoarterial connection | Concordant | **Ventricles** |  |
| Ventricular loop | d-Loop | Left ventricle | Normal |
|  |  | Right ventricle | Normal |
| **Septae** |  | **Coronary arteries** | ----- |
| Interventricular septum | Intact | **Doppler Measurement** |  |
| Interatrial septum | Intact | Mitral | Trivial MR, Incomplete signal, seen in two planes with jet velocity = 2m/sec, Posterior projection |
| **Semilunal valves** |  | Aortic | ------- |
| Aortic valve | Annulus = 18mm | Tricuspid | ---- |
| Pulmonary valve | Annulus = 24mm | pulmonic | -------- |
| **Great arteries** | NRGA | **Aortic arch** | Left |
| Aorta | ----- | **PDA** | No |
| Pulmonary artery | Normal MPA and Branch PAs. |  |  |
| **M-Mode:** | | | |
| AO | mm | PWd | mm |
| LA | mm | EDV | ml |
| LVIDd | mm | ESV | ml |
| LVIDs | mm | LVEF | 59% |
| IVSd | mm | FS | 31% |
| **Additional Information**: |  | | |
| No pleural/pericardial effusion | | | |
| **Final Diagnosis:** | | | |
| 1. {S, D, S} Levocardia. 2. Mildly Thickened MVL 3. Trivial MR 4. Normal Function | | | |
| **Remark**: | | | |
| **Recommendation**: | | | |
| SIGNATURE  Done by: Tesfaye T., Pediatrician, Pediatric Cardiologist _______________ 06/06/2013Eth.C | | | |

| Patient Name: **Sale-Amlak Adane**. Patient ID: **FHRH**. SEX/ Age: M/12years. Date of Report: 06**/06/2013**.  BP: _______ Weight: ______ Height:____________ BSA: ________ | | | |
| --- | --- | --- | --- |
| **Features** | **Finding** | **Features** | **Finding** |
| **Profile** |  | **Atria** |  |
| Abdominal situs | Solitus | Left atrium | Normal |
| Cardiac position | Levocardia | Right atrium | Normal |
| Systemic venous drainage | Normal | **Atrioventricular valves** |  |
| Pulmonary venous drainage | Normal | Mitral valve | Annulus = 20mm |
| Atrioventricular connection | Concordant | Tricuspid valve | Annulus = 23mm  TAPSE = 24mm |
| Ventriculoarterial connection | Concordant | **Ventricles** |  |
| Ventricular loop | d-Loop | Left ventricle | Normal |
|  |  | Right ventricle | Normal |
| **Septae** |  | **Coronary arteries** | ----- |
| Interventricular septum | Intact | **Doppler Measurement** |  |
| Interatrial septum | Intact | Mitral | ----- |
| **Semilunal valves** |  | Aortic | ------- |
| Aortic valve | Annulus = 17mm | Tricuspid | Trivial TR, PPG = 24mmHg |
| Pulmonary valve | Annulus = 20mm | pulmonic | -------- |
| **Great arteries** | NRGA | **Aortic arch** | Left |
| Aorta | ----- | **PDA** | No |
| Pulmonary artery | Normal MPA and Branch PAs. |  |  |
| **M-Mode:** | | | |
| AO | mm | PWd | mm |
| LA | mm | EDV | ml |
| LVIDd | mm | ESV | ml |
| LVIDs | mm | LVEF | 72% |
| IVSd | mm | FS | 40% |
| **Additional Information**: |  | | |
| No pleural/pericardial effusion | | | |
| **Final Diagnosis:** | | | |
| 1. Normal Echocardiography Study. | | | |
| **Remark**: | | | |
| **Recommendation**: | | | |
| SIGNATURE  Done by: Tesfaye T., Pediatrician, Pediatric Cardiologist _______________ 06/06/2013Eth.C | | | |

| Patient Name: **Firdos Abdulqadir**. Patient ID: **Adinas GH**. SEX/ Age: F/9/12. Date of Report: 07**/06/2013**.  BP: _______ Weight: ______ Height:____________ BSA: ________ | | | |
| --- | --- | --- | --- |
| **Features** | **Finding** | **Features** | **Finding** |
| **Profile** |  | **Atria** |  |
| Abdominal situs | Solitus | Left atrium | Normal |
| Cardiac position | Levocardia | Right atrium | Normal |
| Systemic venous drainage | Normal | **Atrioventricular valves** |  |
| Pulmonary venous drainage | Normal | Mitral valve | Annulus = 14mm |
| Atrioventricular connection | Concordant | Tricuspid valve | Annulus = 14mm  TAPSE = 14mm |
| Ventriculoarterial connection | Concordant | **Ventricles** |  |
| Ventricular loop | d-Loop | Left ventricle | Normal |
|  |  | Right ventricle | Normal |
| **Septae** |  | **Coronary arteries** | ----- |
| Interventricular septum | Intact | **Doppler Measurement** |  |
| Interatrial septum | Intact | Mitral | ----- |
| **Semilunal valves** |  | Aortic | ------- |
| Aortic valve | Annulus = 12mm | Tricuspid | ---- |
| Pulmonary valve | Annulus = 11mm | pulmonic | -------- |
| **Great arteries** | NRGA | **Aortic arch** | Left |
| Aorta | ----- | **PDA** | No |
| Pulmonary artery | Normal MPA and Branch PAs. |  |  |
| **M-Mode:** Normal LV Function on eye balling. | | | |
| AO | mm | PWd | mm |
| LA | mm | EDV | ml |
| LVIDd | mm | ESV | ml |
| LVIDs | mm | LVEF | % |
| IVSd | mm | FS | % |
| **Additional Information**: |  | | |
| No pleural/pericardial effusion | | | |
| **Final Diagnosis:** | | | |
| 1. Normal Echocardiography Study. | | | |
| **Remark**: | | | |
| **Recommendation**: | | | |
| SIGNATURE  Done by: Tesfaye T., Pediatrician, Pediatric Cardiologist _______________ 07/06/2013Eth.C | | | |

| Patient Name: **Telayneh Abew**. Patient ID: **Durbete H**. SEX/ Age: M/1Years. Date of Report: 08/**05/2013**.  BP: _______ Weight: ______ Height:____________ BSA: ________ | | | |
| --- | --- | --- | --- |
| **Features** | **Finding** | **Features** | **Finding** |
| **Profile** |  | **Atria** |  |
| Abdominal situs | Solitus | Left atrium | Normal |
| Cardiac position | Levocardia | Right atrium | Normal |
| Systemic venous drainage | Normal | **Atrioventricular valves** |  |
| Pulmonary venous drainage | Normal | Mitral valve | Annulus = 14mm |
| Atrioventricular connection | Concordant | Tricuspid valve | Annulus = 16mm |
| Ventriculoarterial connection | Concordant | **Ventricles** |  |
| Ventricular loop | d-Loop | Left ventricle | Normal |
|  |  | Right ventricle | Normal |
| **Septae** |  | **Coronary arteries** | ----- |
| Interventricular septum | Intact | **Doppler Measurement** |  |
| Interatrial septum | Intact | Mitral | ----- |
| **Semilunal valves** |  | Aortic | ------- |
| Aortic valve | Annulus = 13mm | Tricuspid | ---- |
| Pulmonary valve | Annulus = 13mm | pulmonic | -------- |
| **Great arteries** | NRGA | **Aortic arch** | Left |
| Aorta | ----- | **PDA** | No |
| Pulmonary artery | Normal MPA and Branch PAs. |  |  |
| **M-Mode:** | | | |
| AO | mm | PWd | mm |
| LA | mm | EDV | ml |
| LVIDd | mm | ESV | ml |
| LVIDs | mm | LVEF | % |
| IVSd | mm | FS | % |
| **Additional Information**: |  | | |
| No pleural/pericardial effusion | | | |
| **Final Diagnosis:** | | | |
| 1. Normal Echocardiography Study. | | | |
| **Remark**: Child was crying during study | | | |
| **Recommendation**: | | | |
| SIGNATURE  Done by: Tesfaye T., Pediatrician, Pediatric Cardiologist _______________ 08/05/2013Eth.C | | | |

| Patient Name: **Asmeshew Nigussie**. Patient ID: **FHRH**. SEX/ Age: M/13Years. Date of Report: 08/**05/2013**.  BP: _______ Weight: ______ Height:____________ BSA: ________ | | | |
| --- | --- | --- | --- |
| **Features** | **Finding** | **Features** | **Finding** |
| **Profile** |  | **Atria** |  |
| Abdominal situs | Solitus | Left atrium | Dilated |
| Cardiac position | Levocardia | Right atrium | Normal |
| Systemic venous drainage | Normal | **Atrioventricular valves** |  |
| Pulmonary venous drainage | Normal | Mitral valve | Annulus = 35mm. thickened MVL. |
| Atrioventricular connection | Concordant | Tricuspid valve | Annulus = 21mm  TAPSE = 26mm |
| Ventriculoarterial connection | Concordant | **Ventricles** |  |
| Ventricular loop | d-Loop | Left ventricle | Dilated |
|  |  | Right ventricle | Normal |
| **Septae** |  | **Coronary arteries** | ----- |
| Interventricular septum | Intact | **Doppler Measurement** |  |
| Interatrial septum | Intact | Mitral | Moderate MR, Holosystolic, posterior projection seen in two planes with jet velocity = 4.5m/sec |
| **Semilunal valves** |  | Aortic | Moderate AR, PHT = 261ms. |
| Aortic valve | Annulus = 21mm. Thickened AVL | Tricuspid | Trivial TR, PPG = 20mmHg |
| Pulmonary valve | Annulus = 24mm | pulmonic | -------- |
| **Great arteries** | NRGA | **Aortic arch** | Left |
| Aorta | ----- | **PDA** | No |
| Pulmonary artery | Normal MPA and Branch PAs. |  |  |
| **M-Mode:** | | | |
| AO | mm | PWd | mm |
| LA | mm | EDV | ml |
| LVIDd | mm | ESV | ml |
| LVIDs | mm | LVEF | 70% |
| IVSd | mm | FS | 40% |
| **Additional Information**: |  | | |
| 10mm pericardial effusion on RA Side | | | |
| **Final Diagnosis:** | | | |
| 1. {S, D, S} Levocardia. 2. LA/LV Dilated 3. Thickened MVL and AVL 4. Moderate MR 5. Moderate AR 6. Mild Pericardial effusion 7. Normal Function | | | |
| SIGNATURE  Done by: Tesfaye T., Pediatrician, Pediatric Cardiologist _______________ 08/05/2013Eth.C | | | |

| Patient Name: **Mebe’a – Tsion Abebaw**. Patient ID: **FHRH**. SEX/ Age: F/2 5/12. Date of Report: 08/**05/2013**.  BP: _______ Weight: ______ Height:____________ BSA: ________ | | | |
| --- | --- | --- | --- |
| **Features** | **Finding** | **Features** | **Finding** |
| **Profile** |  | **Atria** |  |
| Abdominal situs | Solitus | Left atrium | Normal |
| Cardiac position | Levocardia | Right atrium | Normal |
| Systemic venous drainage | Normal | **Atrioventricular valves** |  |
| Pulmonary venous drainage | Normal | Mitral valve | Annulus = 16mm |
| Atrioventricular connection | Concordant | Tricuspid valve | Annulus = 16mm |
| Ventriculoarterial connection | Concordant | **Ventricles** |  |
| Ventricular loop | d-Loop | Left ventricle | Normal |
|  |  | Right ventricle | Normal |
| **Septae** |  | **Coronary arteries** | ----- |
| Interventricular septum | Intact | **Doppler Measurement** |  |
| Interatrial septum | Intact | Mitral | ----- |
| **Semilunal valves** |  | Aortic | ------- |
| Aortic valve | Annulus = 14mm | Tricuspid | Trivial TR, PPG = 20mmHg |
| Pulmonary valve | Annulus = 14mm | pulmonic | -------- |
| **Great arteries** | NRGA | **Aortic arch** | Left |
| Aorta | ----- | **PDA** | No |
| Pulmonary artery | Normal MPA and Branch PAs. |  |  |
| **M-Mode:** Normal LV Function on eye balling. | | | |
| AO | mm | PWd | mm |
| LA | mm | EDV | ml |
| LVIDd | mm | ESV | ml |
| LVIDs | mm | LVEF | % |
| IVSd | mm | FS | % |
| **Additional Information**: |  | | |
| No pleural/pericardial effusion | | | |
| **Final Diagnosis:** | | | |
| 1. Normal Echocardiography Study. | | | |
| **Remark**: | | | |
| **Recommendation**: | | | |
| SIGNATURE  Done by: Tesfaye T., Pediatrician, Pediatric Cardiologist _______________ 08/05/2013Eth.C | | | |

| Patient Name: **Kidist Aychew**. Patient ID: **FHRH**. SEX/ Age: F/3/12. Date of Report: 08/**05/2013**.  BP: _______ Weight: ______ Height:____________ BSA: ________ | | | |
| --- | --- | --- | --- |
| **Features** | **Finding** | **Features** | **Finding** |
| **Profile** |  | **Atria** |  |
| Abdominal situs | Solitus | Left atrium | Normal |
| Cardiac position | Levocardia | Right atrium | Normal |
| Systemic venous drainage | Normal | **Atrioventricular valves** |  |
| Pulmonary venous drainage | Normal | Mitral valve | Annulus = 10mm |
| Atrioventricular connection | Concordant | Tricuspid valve | Annulus = 10mm |
| Ventriculoarterial connection | Concordant | **Ventricles** |  |
| Ventricular loop | d-Loop | Left ventricle | Normal |
|  |  | Right ventricle | Normal |
| **Septae** |  | **Coronary arteries** | ----- |
| Interventricular septum | Intact | **Doppler Measurement** |  |
| Interatrial septum | Intact | Mitral | ----- |
| **Semilunal valves** |  | Aortic | ------- |
| Aortic valve | Annulus = 8mm | Tricuspid | ---- |
| Pulmonary valve | Annulus = 9mm | pulmonic | -------- |
| **Great arteries** | NRGA | **Aortic arch** | Left |
| Aorta | ----- | **PDA** | No |
| Pulmonary artery | Normal MPA and Branch PAs. |  |  |
| **M-Mode:** Normal LV Function on eye balling. | | | |
| AO | mm | PWd | mm |
| LA | mm | EDV | ml |
| LVIDd | mm | ESV | ml |
| LVIDs | mm | LVEF | % |
| IVSd | mm | FS | % |
| **Additional Information**: |  | | |
| No pleural/pericardial effusion | | | |
| **Final Diagnosis:** | | | |
| 1. Normal Echocardiography Study. | | | |
| **Remark**: | | | |
| **Recommendation**: | | | |
| SIGNATURE  Done by: Tesfaye T., Pediatrician, Pediatric Cardiologist _______________ 08/05/2013Eth.C | | | |

| Patient Name: **Ayenew Takele**. Patient ID: **FHRH**. SEX/ Age: M/14Years. Date of Report: 09/**05/2013**.  BP: _______ Weight: ______ Height:____________ BSA: ________ | | | |
| --- | --- | --- | --- |
| **Features** | **Finding** | **Features** | **Finding** |
| **Profile** |  | **Atria** |  |
| Abdominal situs | Solitus | Left atrium | Normal |
| Cardiac position | Levocardia | Right atrium | Normal |
| Systemic venous drainage | Normal | **Atrioventricular valves** |  |
| Pulmonary venous drainage | Normal | Mitral valve | Annulus = 22mm |
| Atrioventricular connection | Concordant | Tricuspid valve | Annulus = 24mm  TAPSE = 24mm |
| Ventriculoarterial connection | Concordant | **Ventricles** |  |
| Ventricular loop | d-Loop | Left ventricle | Normal |
|  |  | Right ventricle | Normal |
| **Septae** |  | **Coronary arteries** | ----- |
| Interventricular septum | Intact | **Doppler Measurement** |  |
| Interatrial septum | Intact | Mitral | ----- |
| **Semilunal valves** |  | Aortic | ------- |
| Aortic valve | Annulus = 18mm | Tricuspid | ---- |
| Pulmonary valve | Annulus = 19mm | pulmonic | -------- |
| **Great arteries** | NRGA | **Aortic arch** | Left |
| Aorta | ----- | **PDA** | No |
| Pulmonary artery | Normal MPA and Branch PAs. |  |  |
| **M-Mode:** | | | |
| AO | mm | PWd | mm |
| LA | mm | EDV | ml |
| LVIDd | mm | ESV | ml |
| LVIDs | mm | LVEF | 72% |
| IVSd | mm | FS | 40% |
| **Additional Information**: |  | | |
| No pleural/pericardial effusion | | | |
| **Final Diagnosis:** | | | |
| 1. Normal Echocardiography Study. | | | |
| **Remark**: | | | |
| **Recommendation**: | | | |
| SIGNATURE  Done by: Tesfaye T., Pediatrician, Pediatric Cardiologist _______________ 09/05/2013Eth.C | | | |

| Patient Name: **Fentaye Melaku**. Patient ID: **Finote Selam GH**. SEX/ Age: F/13Years. Date of Report: 09/**05/2013**.  BP: _______ Weight: ______ Height:____________ BSA: ________ | | | |
| --- | --- | --- | --- |
| **Features** | **Finding** | **Features** | **Finding** |
| **Profile** |  | **Atria** |  |
| Abdominal situs | Solitus | Left atrium | Normal |
| Cardiac position | Levocardia | Right atrium | Normal |
| Systemic venous drainage | Normal | **Atrioventricular valves** |  |
| Pulmonary venous drainage | Normal | Mitral valve | Annulus = 23mm |
| Atrioventricular connection | Concordant | Tricuspid valve | Annulus = 27mm |
| Ventriculoarterial connection | Concordant | **Ventricles** |  |
| Ventricular loop | d-Loop | Left ventricle | Symmetrical Concentric Hypertrophy |
|  |  | Right ventricle | Normal |
| **Septae** |  | **Coronary arteries** | ----- |
| Interventricular septum | Intact | **Doppler Measurement** |  |
| Interatrial septum | Intact | Mitral | ----- |
| **Semilunal valves** |  | Aortic | Severe AS, PPG/MPG = 128/88mmHg. Moderate AR, PHT = 361ms. |
| Aortic valve | Annulus = 17mm. Bicuspid | Tricuspid | ---- |
| Pulmonary valve | Annulus = 21mm | pulmonic | -------- |
| **Great arteries** | NRGA | **Aortic arch** | Left |
| Aorta | ----- | **PDA** | No |
| Pulmonary artery | Normal MPA and Branch PAs. |  |  |
| **M-Mode:** | | | |
| AO | mm | PWd | 12mm |
| LA | mm | EDV | 119ml |
| LVIDd | 50mm | ESV | 44ml |
| LVIDs | 33mm | LVEF | 63% |
| IVSd | 12mm | FS | 34% |
| **Additional Information**: |  | | |
| No pleural/pericardial effusion | | | |
| **Final Diagnosis:** | | | |
| 1. {S, D, S} Levocardia. 2. BAV 3. Severe AS 4. Moderate AR 5. Concentric LVH 6. Normal LV Function | | | |
| **Remark**: | | | |
| **Recommendation**: | | | |
| SIGNATURE  Done by: Tesfaye T., Pediatrician, Pediatric Cardiologist _______________ 09/05/2013Eth.C | | | |

| Patient Name: **Baby of Ajebush Tegena**. Patient ID: **FHRH**. SEX/ Age: F/23days. Date of Report: 09/**05/2013**.  BP: _______ Weight: ______ Height:____________ BSA: ________ | | | |
| --- | --- | --- | --- |
| **Features** | **Finding** | **Features** | **Finding** |
| **Profile** |  | **Atria** |  |
| Abdominal situs | Solitus | Left atrium | Normal |
| Cardiac position | Levocardia | Right atrium | Normal |
| Systemic venous drainage | Normal | **Atrioventricular valves** |  |
| Pulmonary venous drainage | Normal | Mitral valve | Annulus = 10mm |
| Atrioventricular connection | Concordant | Tricuspid valve | Annulus = 10mm |
| Ventriculoarterial connection | Concordant | **Ventricles** |  |
| Ventricular loop | d-Loop | Left ventricle | Normal |
|  |  | Right ventricle | Normal |
| **Septae** |  | **Coronary arteries** | ----- |
| Interventricular septum | Intact | **Doppler Measurement** |  |
| Interatrial septum | 4mm OS ASD, L – R Shunt | Mitral | ----- |
| **Semilunal valves** |  | Aortic | ------- |
| Aortic valve | Annulus = 9mm | Tricuspid | ---- |
| Pulmonary valve | Annulus = 8mm | pulmonic | -------- |
| **Great arteries** | NRGA | **Aortic arch** | Left |
| Aorta | ----- | **PDA** | No |
| Pulmonary artery | Normal MPA and Branch PAs. |  |  |
| **M-Mode:** Normal LV Function on eye balling. | | | |
| AO | mm | PWd | mm |
| LA | mm | EDV | ml |
| LVIDd | mm | ESV | ml |
| LVIDs | mm | LVEF | % |
| IVSd | mm | FS | % |
| **Additional Information**: |  | | |
| No pleural/pericardial effusion | | | |
| **Final Diagnosis:** | | | |
| 1. {S, D, S} Levocardia. 2. Small OS ASD, L – R Shunt 3. Normal Function | | | |
| **Remark**: | | | |
| **Recommendation**: | | | |
| SIGNATURE  Done by: Tesfaye T., Pediatrician, Pediatric Cardiologist _______________ 09/05/2013Eth.C | | | |

| Patient Name: **Baby of Fasika Manalew**. Patient ID: **FHRH**. SEX/ Age: F/27days. Date of Report: 09/**05/2013**.  BP: _______ Weight: ______ Height:____________ BSA: ________ | | | |
| --- | --- | --- | --- |
| **Features** | **Finding** | **Features** | **Finding** |
| **Profile** |  | **Atria** |  |
| Abdominal situs | Solitus | Left atrium | Normal |
| Cardiac position | Levocardia | Right atrium | Normal |
| Systemic venous drainage | Normal | **Atrioventricular valves** |  |
| Pulmonary venous drainage | Normal | Mitral valve | Annulus = 10mm |
| Atrioventricular connection | Concordant | Tricuspid valve | Annulus = 11mm |
| Ventriculoarterial connection | Concordant | **Ventricles** |  |
| Ventricular loop | d-Loop | Left ventricle | Normal |
|  |  | Right ventricle | Normal |
| **Septae** |  | **Coronary arteries** | ----- |
| Interventricular septum | Intact | **Doppler Measurement** |  |
| Interatrial septum | Intact | Mitral | ----- |
| **Semilunal valves** |  | Aortic | ------- |
| Aortic valve | Annulus = 9mm | Tricuspid | ---- |
| Pulmonary valve | Annulus = 7mm | pulmonic | Mild Valvar PS, PPG = 30mmHg |
| **Great arteries** | NRGA | **Aortic arch** | Left |
| Aorta | ----- | **PDA** | No |
| Pulmonary artery | Normal MPA and Branch PAs. |  |  |
| **M-Mode:** Normal LV Function on eye balling. | | | |
| AO | mm | PWd | mm |
| LA | mm | EDV | ml |
| LVIDd | mm | ESV | ml |
| LVIDs | mm | LVEF | % |
| IVSd | mm | FS | % |
| **Additional Information**: |  | | |
| No pleural/pericardial effusion | | | |
| **Final Diagnosis:** | | | |
| 1. {S, D, S} Levocardia. 2. Mild Valvar PS 3. Normal Function | | | |
| **Remark**: | | | |
| **Recommendation**: | | | |
| SIGNATURE  Done by: Tesfaye T., Pediatrician, Pediatric Cardiologist _______________ 09/05/2013Eth.C | | | |

| Patient Name: **Metadel Fantahun**. Patient ID: **Merawi PH**. SEX/ Age: M/7/12. Date of Report: 09/**05/2013**.  BP: _______ Weight: ______ Height:____________ BSA: ________ | | | |
| --- | --- | --- | --- |
| **Features** | **Finding** | **Features** | **Finding** |
| **Profile** |  | **Atria** |  |
| Abdominal situs | Solitus | Left atrium | Dilated |
| Cardiac position | Levocardia | Right atrium | Dilated |
| Systemic venous drainage | Normal | **Atrioventricular valves** |  |
| Pulmonary venous drainage | Normal | Mitral valve | Annulus = 16mm |
| Atrioventricular connection | Concordant | Tricuspid valve | Annulus = 13mm |
| Ventriculoarterial connection | Concordant | **Ventricles** |  |
| Ventricular loop | d-Loop | Left ventricle | Dilated, Hypertrophied |
|  |  | Right ventricle | Dilated, Hypertrophied |
| **Septae** |  | **Coronary arteries** | ----- |
| Interventricular septum | Intact | **Doppler Measurement** |  |
| Interatrial septum | 5mm OS ASD, L – R Shunt | Mitral | Moderate MR, Holosystolic, seen in two planes posterior projection with jet velocity = 4.5m/sec |
| **Semilunal valves** |  | Aortic | ------- |
| Aortic valve | Annulus = 14mm | Tricuspid | ---- |
| Pulmonary valve | Annulus = 13mm | pulmonic | Mild PR, PPG = 33mmHg |
| **Great arteries** | NRGA | **Aortic arch** | Left |
| Aorta | ----- | **PDA** | No |
| Pulmonary artery | Normal MPA and Branch PAs. |  |  |
| **M-Mode:** | | | |
| AO | mm | PWd | 7mm |
| LA | mm | EDV | 62ml |
| LVIDd | 38mm | ESV | 30ml |
| LVIDs | 28mm | LVEF | 51% |
| IVSd | 9mm | FS | 26% |
| **Additional Information**: |  | | |
| 6mm pericardial effusion on RA Side | | | |
| **Final Diagnosis:** | | | |
| 1. {S, D, S} Levocardia. 2. All chambers dilated 3. LV/RV Hypertrophied 4. Moderate MR 5. Mild PR 6. Mild Pericardial effusion 7. Mildly reduced LV Function | | | |
| **Remark**: Consider in the line of Connective tissue disorder and Storage disease | | | |
| **Recommendation**: | | | |
| SIGNATURE  Done by: Tesfaye T., Pediatrician, Pediatric Cardiologist _______________ 09/05/2013Eth.C | | | |

| Patient Name: **Mekdes Bazezew**. Patient ID: **Adinas GH**. SEX/ Age: F/10Years. Date of Report: 09/**05/2013**.  BP: _______ Weight: ______ Height:____________ BSA: ________ | | | |
| --- | --- | --- | --- |
| **Features** | **Finding** | **Features** | **Finding** |
| **Profile** |  | **Atria** |  |
| Abdominal situs | Solitus | Left atrium | Normal |
| Cardiac position | Levocardia | Right atrium | Normal |
| Systemic venous drainage | Normal | **Atrioventricular valves** |  |
| Pulmonary venous drainage | Normal | Mitral valve | Annulus = 18mm. thickened MVL |
| Atrioventricular connection | Concordant | Tricuspid valve | Annulus = 18mm  TAPSE = 22mm |
| Ventriculoarterial connection | Concordant | **Ventricles** |  |
| Ventricular loop | d-Loop | Left ventricle | Normal |
|  |  | Right ventricle | Normal |
| **Septae** |  | **Coronary arteries** | ----- |
| Interventricular septum | Intact | **Doppler Measurement** |  |
| Interatrial septum | Intact | Mitral | Mild MR, Holosystolic, posterior projection, seen in two planes with jet velocity = 4.2m/sec |
| **Semilunal valves** |  | Aortic | Mild AR |
| Aortic valve | Annulus = 17 | Tricuspid | ---- |
| Pulmonary valve | Annulus = 20mm | pulmonic | -------- |
| **Great arteries** | NRGA | **Aortic arch** | Left |
| Aorta | ----- | **PDA** | No |
| Pulmonary artery | Normal MPA and Branch PAs. |  |  |
| **M-Mode:** | | | |
| AO | mm | PWd | mm |
| LA | mm | EDV | ml |
| LVIDd | mm | ESV | ml |
| LVIDs | mm | LVEF | 66% |
| IVSd | mm | FS | 36% |
| **Additional Information**: |  | | |
| No pleural/pericardial effusion | | | |
| **Final Diagnosis:** | | | |
| 1. {S, D, S} Levocardia. 2. Thickened MVL 3. Mild MR 4. Mild AR 5. Normal Biventricular Function | | | |
| **Remark**: | | | |
| **Recommendation**: | | | |
| SIGNATURE  Done by: Tesfaye T., Pediatrician, Pediatric Cardiologist _______________ 09/05/2013Eth.C | | | |

| Patient Name: **Chemere Nega**. Patient ID: **TGSH**. SEX/ Age: M/1 8/12. Date of Report: 10/**05/2013**.  BP: _______ Weight: ______ Height:____________ BSA: ________ | | | |
| --- | --- | --- | --- |
| **Features** | **Finding** | **Features** | **Finding** |
| **Profile** |  | **Atria** |  |
| Abdominal situs | Solitus | Left atrium | Normal |
| Cardiac position | Levocardia | Right atrium | Normal |
| Systemic venous drainage | Normal | **Atrioventricular valves** |  |
| Pulmonary venous drainage | Normal | Mitral valve | Annulus = 13mm |
| Atrioventricular connection | Concordant | Tricuspid valve | Annulus = 15mm |
| Ventriculoarterial connection | Concordant | **Ventricles** |  |
| Ventricular loop | d-Loop | Left ventricle | Normal |
|  |  | Right ventricle | Normal |
| **Septae** |  | **Coronary arteries** | ----- |
| Interventricular septum | Intact | **Doppler Measurement** |  |
| Interatrial septum | Intact | Mitral | ----- |
| **Semilunal valves** |  | Aortic | ------- |
| Aortic valve | Annulus = 12mm | Tricuspid | Trivial TR, PPG = 18mmHg. |
| Pulmonary valve | Annulus = 15mm | pulmonic | -------- |
| **Great arteries** | NRGA | **Aortic arch** | Left |
| Aorta | ----- | **PDA** | No |
| Pulmonary artery | Normal MPA and Branch PAs. |  |  |
| **M-Mode:** | | | |
| AO | mm | PWd | mm |
| LA | mm | EDV | ml |
| LVIDd | mm | ESV | ml |
| LVIDs | mm | LVEF | 70% |
| IVSd | mm | FS | 37% |
| **Additional Information**: |  | | |
| No pleural/pericardial effusion | | | |
| **Final Diagnosis:** | | | |
| 1. Normal Echocardiography Study. | | | |
| **Remark**: | | | |
| **Recommendation**: | | | |
| SIGNATURE  Done by: Tesfaye T., Pediatrician, Pediatric Cardiologist _______________ 10/05/2013Eth.C | | | |

| Patient Name: **Baby of Tirualem Tsegaye**. Patient ID: **FHRH**. SEX/ Age: M/23days. Date of Report: 10/**05/2013**.  BP: _______ Weight: ______ Height:____________ BSA: ________ | | | |
| --- | --- | --- | --- |
| **Features** | **Finding** | **Features** | **Finding** |
| **Profile** |  | **Atria** |  |
| Abdominal situs | Solitus | Left atrium | Normal |
| Cardiac position | Levocardia | Right atrium | Normal |
| Systemic venous drainage | Normal | **Atrioventricular valves** |  |
| Pulmonary venous drainage | Normal | Mitral valve | Annulus = 11mm |
| Atrioventricular connection | Concordant | Tricuspid valve | Annulus = 13mm  TAPSE = 12mm |
| Ventriculoarterial connection | Concordant | **Ventricles** |  |
| Ventricular loop | d-Loop | Left ventricle | Normal |
|  |  | Right ventricle | Normal |
| **Septae** |  | **Coronary arteries** | ----- |
| Interventricular septum | 5mm Perimembranous septal defect from LV to RA. Additional Small PM VSD defect, L – R Shunt | **Doppler Measurement** |  |
| Interatrial septum | 5mm OS ASD, L – R Shunt | Mitral | ----- |
| **Semilunal valves** |  | Aortic | ------- |
| Aortic valve | Annulus = 11mm | Tricuspid | ---- |
| Pulmonary valve | Annulus = 11mm | pulmonic | -------- |
| **Great arteries** | NRGA | **Aortic arch** | Left |
| Aorta | ----- | **PDA** | No |
| Pulmonary artery | Normal MPA and Branch PAs. |  |  |
| **M-Mode:** Normal LV Function on eye balling. | | | |
| AO | mm | PWd | mm |
| LA | mm | EDV | ml |
| LVIDd | mm | ESV | ml |
| LVIDs | mm | LVEF | % |
| IVSd | mm | FS | % |
| **Additional Information**: |  | | |
| No pleural/pericardial effusion | | | |
| **Final Diagnosis:** | | | |
| 1. {S, D, S} Levocardia. 2. Small OS ASD, L – R Shunt 3. Gerbode defect (Perimembranous, LV to RA and LV to RV) 4. Normal Function | | | |
| **Remark**: | | | |
| **Recommendation**: | | | |
| SIGNATURE  Done by: Tesfaye T., Pediatrician, Pediatric Cardiologist _______________ 10/05/2013Eth.C | | | |

| Patient Name: **Baby of Tenanesh Enkuahone**. Patient ID: **FHRH**. SEX/ Age: F/22days. Date of Report: 10/**05/2013**.  BP: _______ Weight: ______ Height:____________ BSA: ________ | | | |
| --- | --- | --- | --- |
| **Features** | **Finding** | **Features** | **Finding** |
| **Profile** |  | **Atria** |  |
| Abdominal situs | Solitus | Left atrium | Normal |
| Cardiac position | Levocardia | Right atrium | Normal |
| Systemic venous drainage | Normal | **Atrioventricular valves** |  |
| Pulmonary venous drainage | Normal | Mitral valve | Annulus = 7mm |
| Atrioventricular connection | Concordant | Tricuspid valve | Annulus = 7mm |
| Ventriculoarterial connection | Concordant | **Ventricles** |  |
| Ventricular loop | d-Loop | Left ventricle | Normal |
|  |  | Right ventricle | Normal |
| **Septae** |  | **Coronary arteries** | ----- |
| Interventricular septum | Intact | **Doppler Measurement** |  |
| Interatrial septum | PFO, L – R Shunt | Mitral | ----- |
| **Semilunal valves** |  | Aortic | ------- |
| Aortic valve | Annulus = 7mm | Tricuspid | ---- |
| Pulmonary valve | Annulus = 6mm | pulmonic | -------- |
| **Great arteries** | NRGA | **Aortic arch** | Left |
| Aorta | ----- | **PDA** | PDA, L – R Shunt |
| Pulmonary artery | Normal MPA and Branch PAs. |  |  |
| **M-Mode:** Normal LV Function on eye balling. | | | |
| AO | mm | PWd | mm |
| LA | mm | EDV | ml |
| LVIDd | mm | ESV | ml |
| LVIDs | mm | LVEF | % |
| IVSd | mm | FS | % |
| **Additional Information**: |  | | |
| No pleural/pericardial effusion | | | |
| **Final Diagnosis:** | | | |
| 1. {S, D, S} Levocardia. 2. PFO, L – R Shunt 3. PDA, L – R Shunt 4. Normal Function | | | |
| **Remark**: Only Subcostal window is accessible. Difficult to measure size of the PDA as the window is poor. | | | |
| **Recommendation**: | | | |
| SIGNATURE  Done by: Tesfaye T., Pediatrician, Pediatric Cardiologist _______________ 10/05/2013Eth.C | | | |

| Patient Name: **Esuyawkal SIlie**. Patient ID: **FHRH**. SEX/ Age: M/11Years. Date of Report: 11/**06/2013**.  BP: _______ Weight: ______ Height:____________ BSA: ________ | | | |
| --- | --- | --- | --- |
| **Features** | **Finding** | **Features** | **Finding** |
| **Profile** |  | **Atria** |  |
| Abdominal situs | Solitus | Left atrium | Dilated |
| Cardiac position | Levocardia | Right atrium | Normal |
| Systemic venous drainage | Normal | **Atrioventricular valves** |  |
| Pulmonary venous drainage | Normal | Mitral valve | Annulus = 25mm. thickened MVL |
| Atrioventricular connection | Concordant | Tricuspid valve | Annulus = 28mm |
| Ventriculoarterial connection | Concordant | **Ventricles** |  |
| Ventricular loop | d-Loop | Left ventricle | Dilated |
|  |  | Right ventricle | Normal |
| **Septae** |  | **Coronary arteries** | ----- |
| Interventricular septum | Intact | **Doppler Measurement** |  |
| Interatrial septum | Intact | Mitral | Severe MR, Holosystolic, posterior projection, seen in two planes with jet velocity = 3m/sec |
| **Semilunal valves** |  | Aortic | Severe AR, PHT = 167ms. |
| Aortic valve | Annulus = 19mm | Tricuspid | Severe TR, PPG = 61mmHg |
| Pulmonary valve | Annulus = 18mm | pulmonic | Moderate PR |
| **Great arteries** | NRGA | **Aortic arch** | Left |
| Aorta | ----- | **PDA** | No |
| Pulmonary artery | Normal MPA and Branch PAs. |  |  |
| **M-Mode:** | | | |
| AO | mm | PWd | mm |
| LA | mm | EDV | ml |
| LVIDd | mm | ESV | ml |
| LVIDs | mm | LVEF | 61% |
| IVSd | mm | FS | 33% |
| **Additional Information**: |  | | |
| No pleural/pericardial effusion | | | |
| **Final Diagnosis:** | | | |
| 1. {S, D, S} Levocardia. 2. LA/LV Dilated 3. Thickened MVL 4. Severe MR 5. Severe AR 6. Severe TR 7. Moderate PR 8. Severe Pulmonary Hypertension | | | |
| **Remark**: | | | |
| **Recommendation**: | | | |
| SIGNATURE  Done by: Tesfaye T., Pediatrician, Pediatric Cardiologist _______________ 11/06/2013Eth.C | | | |

| Patient Name: **Getachew Tiruneh**. Patient ID: **Adiss - Alem PH**. SEX/ Age: M/8Years. Date of Report: 11/**06/2013**.  BP: _______ Weight: ______ Height:____________ BSA: ________ | | | |
| --- | --- | --- | --- |
| **Features** | **Finding** | **Features** | **Finding** |
| **Profile** |  | **Atria** |  |
| Abdominal situs | Solitus | Left atrium | Normal |
| Cardiac position | Levocardia | Right atrium | Normal |
| Systemic venous drainage | Normal | **Atrioventricular valves** |  |
| Pulmonary venous drainage | Normal | Mitral valve | Annulus = 18mm |
| Atrioventricular connection | Concordant | Tricuspid valve | Annulus = 18mm |
| Ventriculoarterial connection | Concordant | **Ventricles** |  |
| Ventricular loop | d-Loop | Left ventricle | Normal |
|  |  | Right ventricle | Normal |
| **Septae** |  | **Coronary arteries** | ----- |
| Interventricular septum | Intact | **Doppler Measurement** |  |
| Interatrial septum | Intact | Mitral | ----- |
| **Semilunal valves** |  | Aortic | ------- |
| Aortic valve | Annulus = 15mm | Tricuspid | ---- |
| Pulmonary valve | Annulus = mm | pulmonic | Trivial PR, PPG = 15mmHg |
| **Great arteries** | NRGA | **Aortic arch** | Left |
| Aorta | ----- | **PDA** | No |
| Pulmonary artery | Normal MPA and Branch PAs. |  |  |
| **M-Mode:** | | | |
| AO | mm | PWd | mm |
| LA | mm | EDV | ml |
| LVIDd | mm | ESV | ml |
| LVIDs | mm | LVEF | 63% |
| IVSd | mm | FS | 33% |
| **Additional Information**: |  | | |
| No pleural/pericardial effusion | | | |
| **Final Diagnosis:** | | | |
| 1. Normal Echocardiography Study. | | | |
| **Remark**: | | | |
| **Recommendation**: | | | |
| SIGNATURE  Done by: Tesfaye T., Pediatrician, Pediatric Cardiologist _______________ 11/06/2013Eth.C | | | |

| Patient Name: **Evador Yohannes**. Patient ID: **Gamby GH**. SEX/ Age: F/1 6/12. Date of Report: 11/**06/2013**.  BP: _______ Weight: ______ Height:____________ BSA: ________ | | | |
| --- | --- | --- | --- |
| **Features** | **Finding** | **Features** | **Finding** |
| **Profile** |  | **Atria** |  |
| Abdominal situs | Solitus | Left atrium | Normal |
| Cardiac position | Levocardia | Right atrium | Normal |
| Systemic venous drainage | Normal | **Atrioventricular valves** |  |
| Pulmonary venous drainage | Normal | Mitral valve | Annulus = 13mm |
| Atrioventricular connection | Concordant | Tricuspid valve | Annulus = 15mm |
| Ventriculoarterial connection | Concordant | **Ventricles** |  |
| Ventricular loop | d-Loop | Left ventricle | Normal |
|  |  | Right ventricle | Normal |
| **Septae** |  | **Coronary arteries** | ----- |
| Interventricular septum | Intact | **Doppler Measurement** |  |
| Interatrial septum | Intact | Mitral | ----- |
| **Semilunal valves** |  | Aortic | ------- |
| Aortic valve | Annulus = 14mm | Tricuspid | ---- |
| Pulmonary valve | Annulus = 14mm | pulmonic | -------- |
| **Great arteries** | NRGA | **Aortic arch** | Left |
| Aorta | ----- | **PDA** | 2mm PDA, L – R Shunt |
| Pulmonary artery | Normal MPA and Branch PAs. |  |  |
| **M-Mode:** Normal LV Function on eye balling. | | | |
| AO | mm | PWd | mm |
| LA | mm | EDV | ml |
| LVIDd | mm | ESV | ml |
| LVIDs | mm | LVEF | % |
| IVSd | mm | FS | % |
| **Additional Information**: |  | | |
| No pleural/pericardial effusion | | | |
| **Final Diagnosis:** | | | |
| 1. {S, D, S} Levocardia. 2. Small PDA, L – R Shunt 3. Normal Function | | | |
| **Remark**: | | | |
| **Recommendation**: | | | |
| SIGNATURE  Done by: Tesfaye T., Pediatrician, Pediatric Cardiologist _______________ 11/06/2013Eth.C | | | |

| Patient Name: **Sintayehu Aemiro**. Patient ID: **Enjibara GH**. SEX/ Age: M/3/12. Date of Report: 12/**06/2013**.  BP: _______ Weight: ______ Height:____________ BSA: ________ | | | |
| --- | --- | --- | --- |
| **Features** | **Finding** | **Features** | **Finding** |
| **Profile** |  | **Atria** |  |
| Abdominal situs | Solitus | Left atrium | Normal |
| Cardiac position | Levocardia | Right atrium | Normal |
| Systemic venous drainage | Normal | **Atrioventricular valves** |  |
| Pulmonary venous drainage | Normal | Mitral valve | Annulus = 10mm |
| Atrioventricular connection | Concordant | Tricuspid valve | Annulus = 11mm |
| Ventriculoarterial connection | Concordant | **Ventricles** |  |
| Ventricular loop | d-Loop | Left ventricle | Normal |
|  |  | Right ventricle | Normal |
| **Septae** |  | **Coronary arteries** | ----- |
| Interventricular septum | Intact | **Doppler Measurement** |  |
| Interatrial septum | PFO, L – R Shunt | Mitral | ----- |
| **Semilunal valves** |  | Aortic | ------- |
| Aortic valve | Annulus = 8mm | Tricuspid | ---- |
| Pulmonary valve | Annulus = 9mm | pulmonic | -------- |
| **Great arteries** | NRGA | **Aortic arch** | Left |
| Aorta | ----- | **PDA** | No |
| Pulmonary artery | Normal MPA and Branch PAs. |  |  |
| **M-Mode:** Normal LV Function on eye balling. | | | |
| AO | mm | PWd | mm |
| LA | mm | EDV | ml |
| LVIDd | mm | ESV | ml |
| LVIDs | mm | LVEF | % |
| IVSd | mm | FS | % |
| **Additional Information**: |  | | |
| No pleural/pericardial effusion | | | |
| **Final Diagnosis:** | | | |
| 1. {S, D, S} Levocardia. 2. PFO, L – R Shunt 3. Normal Function | | | |
| **Remark**: | | | |
| **Recommendation**: | | | |
| SIGNATURE  Done by: Tesfaye T., Pediatrician, Pediatric Cardiologist _______________ 12/06/2013Eth.C | | | |

| Patient Name: **Solomon Negesse**. Patient ID: **FHRH**. SEX/ Age: M/2Years. Date of Report: 12/**06/2013**.  BP: _______ Weight: ______ Height:____________ BSA: ________ | | | |
| --- | --- | --- | --- |
| **Features** | **Finding** | **Features** | **Finding** |
| **Profile** |  | **Atria** |  |
| Abdominal situs | Solitus | Left atrium | Normal |
| Cardiac position | Levocardia | Right atrium | Normal |
| Systemic venous drainage | Normal | **Atrioventricular valves** |  |
| Pulmonary venous drainage | Normal | Mitral valve | Annulus = 13mm |
| Atrioventricular connection | Concordant | Tricuspid valve | Annulus = 13mm  TAPSE = 15mm |
| Ventriculoarterial connection | Concordant | **Ventricles** |  |
| Ventricular loop | d-Loop | Left ventricle | Normal |
|  |  | Right ventricle | Normal |
| **Septae** |  | **Coronary arteries** | ----- |
| Interventricular septum | Intact | **Doppler Measurement** |  |
| Interatrial septum | Intact | Mitral | ----- |
| **Semilunal valves** |  | Aortic | ------- |
| Aortic valve | Annulus = 13mm | Tricuspid | ---- |
| Pulmonary valve | Annulus = 14mm | pulmonic | -------- |
| **Great arteries** | NRGA | **Aortic arch** | Left |
| Aorta | ----- | **PDA** | No |
| Pulmonary artery | Normal MPA and Branch PAs. |  |  |
| **M-Mode:** Normal LV Function on eye balling. | | | |
| AO | mm | PWd | mm |
| LA | mm | EDV | ml |
| LVIDd | mm | ESV | ml |
| LVIDs | mm | LVEF | % |
| IVSd | mm | FS | % |
| **Additional Information**: |  | | |
| No pleural/pericardial effusion | | | |
| **Final Diagnosis:** | | | |
| 1. Normal Echocardiography Study. | | | |
| **Remark**: | | | |
| **Recommendation**: | | | |
| SIGNATURE  Done by: Tesfaye T., Pediatrician, Pediatric Cardiologist _______________ 12/06/2013Eth.C | | | |

| Patient Name: **Baby of Ethiopia Mengist**. Patient ID: **FHRH**. SEX/ Age: M/3/12. Date of Report: 12/**06/2013**.  BP: _______ Weight: ______ Height:____________ BSA: ________ | | | |
| --- | --- | --- | --- |
| **Features** | **Finding** | **Features** | **Finding** |
| **Follow up Echocardiography Study.** | | | |
| **Profile** |  | **Atria** |  |
| Abdominal situs | Solitus | Left atrium | Normal |
| Cardiac position | Levocardia | Right atrium | Normal |
| Systemic venous drainage | Normal | **Atrioventricular valves** |  |
| Pulmonary venous drainage | Normal | Mitral valve | Annulus = 11mm |
| Atrioventricular connection | Concordant | Tricuspid valve | Annulus = 12mm |
| Ventriculoarterial connection | Concordant | **Ventricles** |  |
| Ventricular loop | d-Loop | Left ventricle | Normal |
|  |  | Right ventricle | Normal |
| **Septae** |  | **Coronary arteries** | ----- |
| Interventricular septum | Intact | **Doppler Measurement** |  |
| Interatrial septum | PFO, L – R Shunt | Mitral | ----- |
| **Semilunal valves** |  | Aortic | ------- |
| Aortic valve | Annulus = 10mm | Tricuspid | ---- |
| Pulmonary valve | Annulus = 11mm | pulmonic | Trivial PR, PPG = 19mmHg |
| **Great arteries** | NRGA | **Aortic arch** | Left |
| Aorta | ----- | **PDA** | 1mm PDA, L – R Shunt |
| Pulmonary artery | Normal MPA and Branch PAs. |  |  |
| **M-Mode:** Normal LV Function on eye balling. | | | |
| AO | mm | PWd | mm |
| LA | mm | EDV | ml |
| LVIDd | mm | ESV | ml |
| LVIDs | mm | LVEF | % |
| IVSd | mm | FS | % |
| **Additional Information**: |  | | |
| No pleural/pericardial effusion | | | |
| **Final Diagnosis:** | | | |
| 1. {S, D, S} Levocardia. 2. PFO, L – R Shunt 3. Small PDA, L – R Shunt (If no murmur clinically, can be considered as Silent PDA) 4. Normal LV Function | | | |
| **Remark**: | | | |
| **Recommendation**: | | | |
| SIGNATURE  Done by: Tesfaye T., Pediatrician, Pediatric Cardiologist _______________ 12/06/2013Eth.C | | | |

| Patient Name: **Baby of Azmera Misganaw**. Patient ID: **FHRH**. SEX/ Age: M/22days. Date of Report: 12/**06/2013**.  BP: _______ Weight: ______ Height:____________ BSA: ________ | | | |
| --- | --- | --- | --- |
| **Features** | **Finding** | **Features** | **Finding** |
| **Profile** |  | **Atria** |  |
| Abdominal situs | Solitus | Left atrium | Normal |
| Cardiac position | Levocardia | Right atrium | Normal |
| Systemic venous drainage | Normal | **Atrioventricular valves** |  |
| Pulmonary venous drainage | Normal | Mitral valve | Annulus = 11mm |
| Atrioventricular connection | Concordant | Tricuspid valve | Annulus = 12mm |
| Ventriculoarterial connection | Concordant | **Ventricles** |  |
| Ventricular loop | d-Loop | Left ventricle | Normal |
|  |  | Right ventricle | Normal |
| **Septae** |  | **Coronary arteries** | ----- |
| Interventricular septum | Intact | **Doppler Measurement** |  |
| Interatrial septum | PFO, L – R Shunt | Mitral | ----- |
| **Semilunal valves** |  | Aortic | ------- |
| Aortic valve | Annulus = 9mm | Tricuspid | ---- |
| Pulmonary valve | Annulus = 8mm. Doming. | pulmonic | Mild Valvar PS, PPG = 21mmHg. |
| **Great arteries** | NRGA | **Aortic arch** | Left |
| Aorta | ----- | **PDA** | No |
| Pulmonary artery | Normal MPA and Branch PAs. |  |  |
| **M-Mode:** Normal LV Function on eye balling. | | | |
| AO | mm | PWd | mm |
| LA | mm | EDV | ml |
| LVIDd | mm | ESV | ml |
| LVIDs | mm | LVEF | % |
| IVSd | mm | FS | % |
| **Additional Information**: |  | | |
| No pleural/pericardial effusion | | | |
| **Final Diagnosis:** | | | |
| 1. {S, D, S} Levocardia. 2. PFO, L – R Shunt 3. Mild Valvar PS 4. Doming Pulmonary Valve 5. Normal LV Function | | | |
| **Remark**: | | | |
| **Recommendation**: Echocardiographic evaluation yearly. | | | |
| SIGNATURE  Done by: Tesfaye T., Pediatrician, Pediatric Cardiologist _______________ 12/06/2013Eth.C | | | |

| Patient Name: **Mohammed Mossa**. Patient ID: **SBPSC**. SEX/ Age: M/10/12. Date of Report: 14/**06/2013**.  BP: _______ Weight: ______ Height:____________ BSA: ________ | | | |
| --- | --- | --- | --- |
| **Features** | **Finding** | **Features** | **Finding** |
| **Profile** |  | **Atria** |  |
| Abdominal situs | Solitus | Left atrium | Normal |
| Cardiac position | Levocardia | Right atrium | Normal |
| Systemic venous drainage | Normal | **Atrioventricular valves** |  |
| Pulmonary venous drainage | Normal | Mitral valve | Annulus = 13mm |
| Atrioventricular connection | Concordant | Tricuspid valve | Annulus = 14mm |
| Ventriculoarterial connection | Concordant | **Ventricles** |  |
| Ventricular loop | d-Loop | Left ventricle | Normal |
|  |  | Right ventricle | Normal |
| **Septae** |  | **Coronary arteries** | ----- |
| Interventricular septum | 5mm Inlet VSD, L – R Shunt | **Doppler Measurement** |  |
| Interatrial septum | Intact | Mitral | ----- |
| **Semilunal valves** |  | Aortic | ------- |
| Aortic valve | Annulus = 12mm | Tricuspid | ---- |
| Pulmonary valve | Annulus = 12mm | pulmonic | -------- |
| **Great arteries** | NRGA | **Aortic arch** | Left |
| Aorta | ----- | **PDA** | No |
| Pulmonary artery | Normal MPA and Branch PAs. |  |  |
| **M-Mode:** Normal LV Function on eye balling. | | | |
| AO | mm | PWd | mm |
| LA | mm | EDV | ml |
| LVIDd | mm | ESV | ml |
| LVIDs | mm | LVEF | % |
| IVSd | mm | FS | % |
| **Additional Information**: |  | | |
| No pleural/pericardial effusion | | | |
| **Final Diagnosis:** | | | |
| 1. {S, D, S} Levocardia. 2. Small Inlet VSD, L – R Shunt 3. Normal Function | | | |
| **Remark**: | | | |
| **Recommendation**: | | | |
| SIGNATURE  Done by: Tesfaye T., Pediatrician, Pediatric Cardiologist _______________ 14/06/2013Eth.C | | | |

| Patient Name: **Sale – Egziabher Basazinew**. Patient ID: **Mekane-Selam H**. SEX/ Age: M/3Years. Date of Report: 15/**06/2013**. | | | |
| --- | --- | --- | --- |
| **Features** | **Finding** | **Features** | **Finding** |
| **Profile** |  | **Atria** |  |
| Abdominal situs | Solitus | Left atrium | Normal |
| Cardiac position | Levocardia | Right atrium | Mildly Dilated |
| Systemic venous drainage | Normal | **Atrioventricular valves** |  |
| Pulmonary venous drainage | Normal | Mitral valve | Annulus = 15mm |
| Atrioventricular connection | Concordant | Tricuspid valve | Annulus = 16mm |
| Ventriculoarterial connection | Concordant | **Ventricles** |  |
| Ventricular loop | d-Loop | Left ventricle | Normal |
|  |  | Right ventricle | Mildly Dilated |
| **Septae** |  | **Coronary arteries** | ----- |
| Interventricular septum | 6mm Sub aortic VSD, L – R Shunt | **Doppler Measurement** |  |
| Interatrial septum | Intact | Mitral | ----- |
| **Semilunal valves** |  | Aortic | ------- |
| Aortic valve | Annulus = 16mm | Tricuspid | ---- |
| Pulmonary valve | Annulus = 21mm | pulmonic | -------- |
| **Great arteries** | NRGA | **Aortic arch** | Left |
| Aorta | ----- | **PDA** | 2mm PDA, L – R Shunt |
| Pulmonary artery | MPA = 20mm and Normal Branch PAs. |  |  |
| **M-Mode:** Normal LV Function on eye balling. | | | |
| AO | mm | PWd | mm |
| LA | mm | EDV | ml |
| LVIDd | mm | ESV | ml |
| LVIDs | mm | LVEF | % |
| IVSd | mm | FS | % |
| **Additional Information**: |  | | |
| No pleural/pericardial effusion | | | |
| **Final Diagnosis:** | | | |
| 1. {S, D, S} Levocardia. 2. Small Sub aortic VSD, L – R Shunt 3. Moderate PDA, L – R Shunt 4. Moderate Pulmonary Hypertension 5. Normal Function | | | |
| **Remark**: | | | |
| **Recommendation**: | | | |
| SIGNATURE  Done by: Tesfaye T., Pediatrician, Pediatric Cardiologist _______________ 15/06/2013Eth.C | | | |

| Patient Name: **Eyob Temesgen**. Patient ID: **Afilas GH**. SEX/ Age: M/5Years. Date of Report: 15/**06/2013**.  BP: _______ Weight: ______ Height:____________ BSA: ________ | | | |
| --- | --- | --- | --- |
| **Features** | **Finding** | **Features** | **Finding** |
| **Profile** |  | **Atria** |  |
| Abdominal situs | Solitus | Left atrium | Dilated |
| Cardiac position | Levocardia | Right atrium | Normal |
| Systemic venous drainage | Normal | **Atrioventricular valves** |  |
| Pulmonary venous drainage | Normal | Mitral valve | Annulus = 19mm |
| Atrioventricular connection | Concordant | Tricuspid valve | Annulus = 15mm |
| Ventriculoarterial connection | Concordant | **Ventricles** |  |
| Ventricular loop | d-Loop | Left ventricle | Dilated |
|  |  | Right ventricle | Normal |
| **Septae** |  | **Coronary arteries** | ----- |
| Interventricular septum | Intact | **Doppler Measurement** |  |
| Interatrial septum | Intact | Mitral | ----- |
| **Semilunal valves** |  | Aortic | ------- |
| Aortic valve | Annulus = 15mm | Tricuspid | ---- |
| Pulmonary valve | Annulus = 19mm. Doming | pulmonic | -------- |
| **Great arteries** | NRGA | **Aortic arch** | Left |
| Aorta | ----- | **PDA** | 3mm PDA, L – R Shunt |
| Pulmonary artery | Normal MPA and Branch PAs. |  |  |
| **M-Mode:** | | | |
| AO | mm | PWd | mm |
| LA | mm | EDV | ml |
| LVIDd | mm | ESV | ml |
| LVIDs | mm | LVEF | 55% |
| IVSd | mm | FS | 28% |
| **Additional Information**: |  | | |
| No pleural/pericardial effusion | | | |
| **Final Diagnosis:** | | | |
| 1. {S, D, S} Levocardia. 2. Doming Pulmonary Valve 3. Moderate PDA, L – R Shunt 4. Normal LV Function | | | |
| **Remark**: | | | |
| **Recommendation**: | | | |
| SIGNATURE  Done by: Tesfaye T., Pediatrician, Pediatric Cardiologist _______________ 15/06/2013Eth.C | | | |

| Patient Name: **Dagnew Addisu**. Patient ID: **Enjibara GH**. SEX/ Age: M/3/12. Date of Report: 15/**06/2013**.  BP: _______ Weight: ______ Height:____________ BSA: ________ | | | |
| --- | --- | --- | --- |
| **Features** | **Finding** | **Features** | **Finding** |
| **Profile** |  | **Atria** |  |
| Abdominal situs | Solitus | Left atrium | Normal |
| Cardiac position | Levocardia | Right atrium | Normal |
| Systemic venous drainage | Normal | **Atrioventricular valves** |  |
| Pulmonary venous drainage | Normal | Mitral valve | Annulus = 11mm |
| Atrioventricular connection | Concordant | Tricuspid valve | Annulus = 12mm |
| Ventriculoarterial connection | Concordant | **Ventricles** |  |
[truncated: 110,302 more chars]
