# Supplementary material for: Two–Dimensional and Doppler trans-thoracic echocardiographic patterns of suspected pediatric heart diseases at Tibebe-—Ghion specialized Teaching Hospital and Adinas General Hospital, Bahir Dar, North-west Ethiopia:–An experience from an LMIC
Source: PLoS One. 2024 Mar 11;19(3):e0292694. doi: 10.1371/journal.pone.0292694 (PMC10927071; doi:10.1371/journal.pone.0292694)
Supplement: S2 File — (ZIP) [file pone.0292694.s003.zip › AGH8 Pediatric Transthoracic Echocardiography Report May 2022.docx]

| Patient Name: **Sitotaw Gugsa**. Referring Institute: **FHRH**. SEX/ Age: **M/7years**.  Date of Report: **10/09/08/14**. Referral Diagnosis: **FTT. AGH8.273** | | | |
| --- | --- | --- | --- |
| **Features** | **Finding** | **Features** | **Finding** |
| **Profile** |  | **Atria** |  |
| Abdominal situs | Solitus | Left atrium | Normal |
| Cardiac position | Levocardia | Right atrium | Normal |
| Systemic venous drainage | Normal. | **Atrioventricular valves** |  |
| Pulmonary venous drainage | Normal | Mitral valve | Annulus = 19mm |
| Atrioventricular connection | Concordant | Tricuspid valve | Annulus = 19mm  TAPSE = 21mm |
| Ventriculoarterial connection | Concordant | **Ventricles** |  |
| Ventricular loop | d-Loop | Left ventricle | Normal |
|  |  | Right ventricle | Normal |
| **Septae** |  | **Coronary arteries** | ----- |
| Interventricular septum | Intact | **Doppler Measurement** |  |
| Interatrial septum | Intact | Mitral | ----- |
| **Semilunar valves** |  | Aortic | ------- |
| Aortic valve | Annulus = 16mm | Tricuspid | ------- |
| Pulmonary valve | Annulus = 17mm | pulmonic | -------- |
| **Great arteries** | NRGA | **Aortic arch** | Left. No CoA. |
| Aorta | ----- | **PDA** | No |
| Pulmonary artery | Normal MPA and Branch PAs. |  |  |
| **M-Mode:** | | | |
| AO | mm | PWd | mm |
| LA | mm | PWs | mm |
| LVIDd | mm | EDV | ml |
| LVIDs | mm | ESV | ml |
| IVSs | mm | LVEF | 68% |
| IVSd | mm | FS | 37% |
| **Additional Information**: |  | | |
| No pericardial/Pleural effusion. | | | |
| **Final Diagnosis:** | | | |
| 1. Normal Echocardiography Study. | | | |
| **Remark**: | | | |
| **Recommendation**: | | | |
| SIGNATURE  Done by: Tesfaye T., Pediatrician, Pediatric Cardiologist _______________ 10/09/08/2014Eth.C | | | |

| Patient Name: **Haymanot Gugsa**. Referring Institute: **FHRH**. SEX/ Age: **M/10years**.  Date of Report: **10/09/08/14**. Referral Diagnosis: **FTT. AGH8.274** | | | |
| --- | --- | --- | --- |
| **Features** | **Finding** | **Features** | **Finding** |
| **Profile** |  | **Atria** |  |
| Abdominal situs | Solitus | Left atrium | Normal |
| Cardiac position | Levocardia | Right atrium | Normal |
| Systemic venous drainage | Normal. | **Atrioventricular valves** |  |
| Pulmonary venous drainage | Normal | Mitral valve | Annulus = 19mm |
| Atrioventricular connection | Concordant | Tricuspid valve | Annulus = 22mm  TAPSE = 22mm |
| Ventriculoarterial connection | Concordant | **Ventricles** |  |
| Ventricular loop | d-Loop | Left ventricle | Normal |
|  |  | Right ventricle | Normal |
| **Septae** |  | **Coronary arteries** | ----- |
| Interventricular septum | Intact | **Doppler Measurement** |  |
| Interatrial septum | Intact | Mitral | ----- |
| **Semilunar valves** |  | Aortic | ------- |
| Aortic valve | Annulus = 16mm | Tricuspid | ------- |
| Pulmonary valve | Annulus = 16mm | pulmonic | -------- |
| **Great arteries** | NRGA | **Aortic arch** | Left. No CoA. |
| Aorta | ----- | **PDA** | No |
| Pulmonary artery | Normal MPA and Branch PAs. |  |  |
| **M-Mode:**  Normal LV Function on eye balling | | | |
| AO | mm | PWd | mm |
| LA | mm | PWs | mm |
| LVIDd | mm | EDV | ml |
| LVIDs | mm | ESV | ml |
| IVSs | mm | LVEF | % |
| IVSd | mm | FS | % |
| **Additional Information**: |  | | |
| No pericardial/Pleural effusion. | | | |
| **Final Diagnosis:** | | | |
| 1. Normal Echocardiography Study. | | | |
| **Remark**: | | | |
| **Recommendation**: | | | |
| SIGNATURE  Done by: Tesfaye T., Pediatrician, Pediatric Cardiologist _______________ 10/09/08/2014Eth.C | | | |

| Patient Name: **Baby of Agegnewush Ayinie-aye**. Referring Institute: **FHRH**. SEX/ Age: **F/13days**.  Date of Report: **10/09/08/14**. Referral Diagnosis: **DS. AGH8.275** | | | |
| --- | --- | --- | --- |
| **Features** | **Finding** | **Features** | **Finding** |
| **Profile** |  | **Atria** |  |
| Abdominal situs | Solitus | Left atrium | Normal |
| Cardiac position | Levocardia | Right atrium | Normal |
| Systemic venous drainage | Normal. | **Atrioventricular valves** |  |
| Pulmonary venous drainage | Normal | Mitral valve | Annulus = 9mm |
| Atrioventricular connection | Concordant | Tricuspid valve | Annulus = 9mm |
| Ventriculoarterial connection | Concordant | **Ventricles** |  |
| Ventricular loop | d-Loop | Left ventricle | Normal |
|  |  | Right ventricle | Normal |
| **Septae** |  | **Coronary arteries** | ----- |
| Interventricular septum | Intact | **Doppler Measurement** |  |
| Interatrial septum | 6mm OS ASD, L – R Shunt | Mitral | ----- |
| **Semilunar valves** |  | Aortic | ------- |
| Aortic valve | Annulus = 9mm | Tricuspid | ------- |
| Pulmonary valve | Annulus = 9mm | pulmonic | -------- |
| **Great arteries** | NRGA | **Aortic arch** | Left. No CoA. |
| Aorta | ----- | **PDA** | No |
| Pulmonary artery | Normal MPA and Branch PAs. |  |  |
| **M-Mode:**  Normal LV Function on eye balling | | | |
| AO | mm | PWd | mm |
| LA | mm | PWs | mm |
| LVIDd | mm | EDV | ml |
| LVIDs | mm | ESV | ml |
| IVSs | mm | LVEF | % |
| IVSd | mm | FS | % |
| **Additional Information**: |  | | |
| No pericardial/Pleural effusion. | | | |
| **Final Diagnosis:** | | | |
| 1. {S, D, S} Levocardia. 2. Small OS ASD, L – R Shunt | | | |
| **Remark**: | | | |
| **Recommendation**: | | | |
| SIGNATURE  Done by: Tesfaye T., Pediatrician, Pediatric Cardiologist _______________ 10/09/08/2014Eth.C | | | |

| Patient Name: **Baby of Fasika Tilahun**. Referring Institute: **FHRH**. SEX/ Age: **F/40days**.  Date of Report: **10/09/08/14**. Referral Diagnosis: **RD. AGH8.276** | | | |
| --- | --- | --- | --- |
| **Features** | **Finding** | **Features** | **Finding** |
| **Profile** |  | **Atria** |  |
| Abdominal situs | Solitus | Left atrium | Normal |
| Cardiac position | Levocardia | Right atrium | Normal |
| Systemic venous drainage | Normal. | **Atrioventricular valves** |  |
| Pulmonary venous drainage | Normal | Mitral valve | Annulus = 10mm |
| Atrioventricular connection | Concordant | Tricuspid valve | Annulus = 10mm |
| Ventriculoarterial connection | Concordant | **Ventricles** |  |
| Ventricular loop | d-Loop | Left ventricle | Normal |
|  |  | Right ventricle | Normal |
| **Septae** |  | **Coronary arteries** | ----- |
| Interventricular septum | Intact | **Doppler Measurement** |  |
| Interatrial septum | Intact | Mitral | ----- |
| **Semilunar valves** |  | Aortic | ------- |
| Aortic valve | Annulus = 8mm | Tricuspid | ------- |
| Pulmonary valve | Annulus = 8mm | pulmonic | -------- |
| **Great arteries** | NRGA | **Aortic arch** | Left. No CoA. |
| Aorta | ----- | **PDA** | No |
| Pulmonary artery | Normal MPA and Branch PAs. |  |  |
| **M-Mode:**  Normal LV Function on eye balling | | | |
| AO | mm | PWd | mm |
| LA | mm | PWs | mm |
| LVIDd | mm | EDV | ml |
| LVIDs | mm | ESV | ml |
| IVSs | mm | LVEF | % |
| IVSd | mm | FS | % |
| **Additional Information**: |  | | |
| No pericardial/Pleural effusion. | | | |
| **Final Diagnosis:** | | | |
| 1. Normal Echocardiography Study. | | | |
| **Remark**: | | | |
| **Recommendation**: | | | |
| SIGNATURE  Done by: Tesfaye T., Pediatrician, Pediatric Cardiologist _______________ 10/09/08/2014Eth.C | | | |

| Patient Name: **Tewulign Abera**. Referring Institute: **FHRH**. SEX/ Age: **M/1 2/12**.  Date of Report: **11/09/08/14**. Referral Diagnosis: **DS. AGH8.277** | | | |
| --- | --- | --- | --- |
| **Features** | **Finding** | **Features** | **Finding** |
| **Profile** |  | **Atria** |  |
| Abdominal situs | Solitus | Left atrium | Dilated |
| Cardiac position | Levocardia | Right atrium | More dilated |
| Systemic venous drainage | Normal. | **Atrioventricular valves** |  |
| Pulmonary venous drainage | Normal | Mitral valve | Annulus = 12mm |
| Atrioventricular connection | Concordant | Tricuspid valve | Annulus = 12mm |
| Ventriculoarterial connection | Concordant | **Ventricles** |  |
| Ventricular loop | d-Loop | Left ventricle | Dilated |
|  |  | Right ventricle | Dilated |
| **Septae** |  | **Coronary arteries** | ----- |
| Interventricular septum | 8mm Inlet VSD, L – R Shunt | **Doppler Measurement** |  |
| Interatrial septum | 10mm Primum ASD, L – R Shunt | Mitral | ----- |
| **Semilunar valves** |  | Aortic | ------- |
| Aortic valve | Annulus = 11mm | Tricuspid | Mild TR |
| Pulmonary valve | Annulus = 15mm | pulmonic | -------- |
| **Great arteries** | NRGA | **Aortic arch** | Left. No CoA. |
| Aorta | ----- | **PDA** | No |
| Pulmonary artery | MPA = 16mm. Confluent Branch PAs. |  |  |
| **M-Mode:**  Normal LV Function on eye balling | | | |
| AO | mm | PWd | mm |
| LA | mm | PWs | mm |
| LVIDd | mm | EDV | ml |
| LVIDs | mm | ESV | ml |
| IVSs | mm | LVEF | % |
| IVSd | mm | FS | % |
| **Additional Information**: |  | | |
| No pericardial/Pleural effusion. | | | |
| **Final Diagnosis:** | | | |
| 1. {S, D, S} Levocardia. 2. All chambers dilated 3. Intermediate AVSD, L – R Shunt 4. Mild TR 5. Moderate Pulmonary Hypertension 6. Normal LV Systolic Function | | | |
| **Remark**: | | | |
| **Recommendation**: | | | |
| SIGNATURE  Done by: Tesfaye T., Pediatrician, Pediatric Cardiologist _______________ 11/09/08/2014Eth.C | | | |

| Patient Name: **Hiwet Werku**. Referring Institute: **FHRH**. SEX/ Age: **F/7months**.  Date of Report: **11/09/08/14**. Referral Diagnosis: **RD + CHF + Murmur. AGH8.278** | | | |
| --- | --- | --- | --- |
| **Features** | **Finding** | **Features** | **Finding** |
| **Profile** |  | **Atria** |  |
| Abdominal situs | Solitus | Left atrium | Dilated |
| Cardiac position | Levocardia | Right atrium | Normal |
| Systemic venous drainage | Normal. | **Atrioventricular valves** |  |
| Pulmonary venous drainage | Normal | Mitral valve | Annulus = 16mm |
| Atrioventricular connection | Concordant | Tricuspid valve | Annulus = 14mm  TAPSE = 18mm |
| Ventriculoarterial connection | Concordant | **Ventricles** |  |
| Ventricular loop | d-Loop | Left ventricle | Dilated |
|  |  | Right ventricle | Normal |
| **Septae** |  | **Coronary arteries** | ----- |
| Interventricular septum | 7.5mm PM VSD, L – R Shunt | **Doppler Measurement** |  |
| Interatrial septum | Intact | Mitral | Moderate MR, Holosystolic, posterior projection seen in two planes with jet velocity = 4.4m/sec |
| **Semilunar valves** |  | Aortic | ------- |
| Aortic valve | Annulus = 11mm | Tricuspid | ------- |
| Pulmonary valve | Annulus = 12mm | pulmonic | Mild PR, PPG = 49mmHg |
| **Great arteries** | NRGA | **Aortic arch** | Left. No CoA. |
| Aorta | ----- | **PDA** | No |
| Pulmonary artery | MPA =16mm. Confluent Branch PAs. |  |  |
| **M-Mode:** | | | |
| AO | mm | PWd | mm |
| LA | mm | PWs | mm |
| LVIDd | mm | EDV | ml |
| LVIDs | mm | ESV | ml |
| IVSs | mm | LVEF | 59% |
| IVSd | mm | FS | 31% |
| **Additional Information**: |  | | |
| No pericardial/Pleural effusion. | | | |
| **Final Diagnosis:** | | | |
| 1. {S, D, S} Levocardia. 2. LA/LV Dilated 3. Moderate MR 4. Moderate PM VSD, L – R Shunt 5. Moderate Pulmonary Hypertension 6. Normal Biventricular Systolic Function | | | |
| SIGNATURE  Done by: Tesfaye T., Pediatrician, Pediatric Cardiologist _______________ 11/09/08/2014Eth.C | | | |

| Patient Name: **Baby of Hadra Yesuf**. Referring Institute: **FHRH**. SEX/ Age: **F/75days**. (24days, 19/07/14)  Date of Report: **11/09/08/14**. R. Dx: **Follow up echo for PFO & small PM VSD (2mm).Murmur. AGH8.279** | | | |
| --- | --- | --- | --- |
| **Features** | **Finding** | **Features** | **Finding** |
| **Profile** |  | **Atria** |  |
| Abdominal situs | Solitus | Left atrium | Normal |
| Cardiac position | Levocardia | Right atrium | Normal |
| Systemic venous drainage | Normal. | **Atrioventricular valves** |  |
| Pulmonary venous drainage | Normal | Mitral valve | Annulus = 8mm |
| Atrioventricular connection | Concordant | Tricuspid valve | Annulus = 9mm |
| Ventriculoarterial connection | Concordant | **Ventricles** |  |
| Ventricular loop | d-Loop | Left ventricle | Normal |
|  |  | Right ventricle | Normal |
| **Septae** |  | **Coronary arteries** | ----- |
| Interventricular septum | 2mm PM VSD, L – R Shunt | **Doppler Measurement** |  |
| Interatrial septum | Intact | Mitral | ----- |
| **Semilunar valves** |  | Aortic | ------- |
| Aortic valve | Annulus = 7mm | Tricuspid | ------- |
| Pulmonary valve | Annulus = 8mm | pulmonic | -------- |
| **Great arteries** | NRGA | **Aortic arch** | Left. No CoA. |
| Aorta | ----- | **PDA** | No |
| Pulmonary artery | Normal MPA and Branch PAs. |  |  |
| **M-Mode:**  Normal LV Function on eye balling | | | |
| AO | mm | PWd | mm |
| LA | mm | PWs | mm |
| LVIDd | mm | EDV | ml |
| LVIDs | mm | ESV | ml |
| IVSs | mm | LVEF | % |
| IVSd | mm | FS | % |
| **Additional Information**: |  | | |
| No pericardial/Pleural effusion. | | | |
| **Final Diagnosis:** | | | |
| 1. {S, D, S} Levocardia. 2. Small PM VSD, L – R Shunt 3. Normal LV Systolic Function | | | |
| **Remark**: PFO has closed | | | |
| **Recommendation**:   1. No need to start medicine 2. Counseling on possibility of IE 3. Advice on normal Life style( no physical activity restriction and can have a diet with added salt) 4. Follow up Echocardiography yearly | | | |
| SIGNATURE  Done by: Tesfaye T., Pediatrician, Pediatric Cardiologist _______________ 11/09/08/2014Eth.C | | | |

| Patient Name: **Bamlaku Adera**. Referring Institute: **FHRH**. SEX/ Age: **M/3 8/12**.  Date of Report: **12/09/08/14**. Referral Diagnosis: **Cyanosis. AGH8.280** | | | |
| --- | --- | --- | --- |
| **Features** | **Finding** | **Features** | **Finding** |
| **Profile** |  | **Atria** |  |
| Abdominal situs | Solitus | Left atrium | Normal |
| Cardiac position | Levocardia | Right atrium | Dilated |
| Systemic venous drainage | Normal. | **Atrioventricular valves** |  |
| Pulmonary venous drainage | Normal | Mitral valve | Atretic |
| Atrioventricular connection | Concordant | Tricuspid valve | Annulus = 22mm  TAPSE = 16mm |
| Ventriculoarterial connection | DORV | **Ventricles** |  |
| Ventricular loop | d-Loop | Left ventricle | Hypoplastic |
|  |  | Right ventricle | Dilated & Hypertrophied |
| **Septae** |  | **Coronary arteries** | ----- |
| Interventricular septum | Intact | **Doppler Measurement** |  |
| Interatrial septum | PFO, L – R Shunt | Mitral | ----- |
| **Semilunar valves** |  | Aortic | ------- |
| Aortic valve | Annulus = 14mm | Tricuspid | Mild TR, PPG = 77mmHg |
| Pulmonary valve | Annulus = 11mm | pulmonic | PS, PPG = 25mmHg (?under-estimated) |
| **Great arteries** | NRGA | **Aortic arch** | Left. No CoA. |
| Aorta | ----- | **PDA** | No |
| Pulmonary artery | Normal MPA and Branch PAs. |  |  |
| **M-Mode:** | | | |
| AO | mm | PWd | mm |
| LA | mm | PWs | mm |
| LVIDd | mm | EDV | ml |
| LVIDs | mm | ESV | ml |
| IVSs | mm | LVEF | % |
| IVSd | mm | FS | % |
| **Additional Information**: |  | | |
| No pericardial/Pleural effusion. | | | |
| **Final Diagnosis:** | | | |
| 1. {S, D, D} Levocardia. 2. RA/RV Dilated 3. DORV 4. PFO, L – R Shunt 5. Mitral Atresia 6. Hypoplastic LV 7. PS (Under-estimated) | | | |
| **Remark**: | | | |
| **Recommendation**: | | | |
| SIGNATURE  Done by: Tesfaye T., Pediatrician, Pediatric Cardiologist _______________ 12/09/08/2014Eth.C | | | |

| Patient Name: **Amarech Fekadu**. Referring Institute: **TGSH**. SEX/ Age: **F/13years**.  Date of Report: **12/09/08/14**. Referral Diagnosis: **CHF. AGH8.281** | | | |
| --- | --- | --- | --- |
| **Features** | **Finding** | **Features** | **Finding** |
| **Profile** |  | **Atria** |  |
| Abdominal situs | Solitus | Left atrium | More dilated |
| Cardiac position | Levocardia | Right atrium | Dilated |
| Systemic venous drainage | Normal. IVC Dilated | **Atrioventricular valves** |  |
| Pulmonary venous drainage | Normal | Mitral valve | Annulus = 25mm |
| Atrioventricular connection | Concordant | Tricuspid valve | Annulus = 29mm  TAPSE = 12mm |
| Ventriculoarterial connection | Concordant | **Ventricles** |  |
| Ventricular loop | d-Loop | Left ventricle | More dilated with globular Dysfunction |
|  |  | Right ventricle | Dilated |
| **Septae** |  | **Coronary arteries** | ----- |
| Interventricular septum | Intact | **Doppler Measurement** |  |
| Interatrial septum | Intact | Mitral | Moderate MR, Holosystolic, Posterior projection, seen in two planes with jet velocity = 3.3m/sec. |
| **Semilunar valves** |  | Aortic | ------- |
| Aortic valve | Annulus = 16mm | Tricuspid | Mild TR, PPG = 27mmHg |
| Pulmonary valve | Annulus = 18mm | pulmonic | Trivial PR, PPG = 27mmHg |
| **Great arteries** | NRGA | **Aortic arch** | Left. No CoA. |
| Aorta | ----- | **PDA** | No |
| Pulmonary artery | Normal MPA and Branch PAs. | **Coronaries** | No ALCAPA |
| **M-Mode:** | | | |
| AO | mm | PWd | 9mm |
| LA | mm | PWs | 10mm |
| LVIDd | 62mm | EDV | 192ml |
| LVIDs | 56mm | ESV | 152ml |
| IVSs | 7mm | LVEF | 21% |
| IVSd | 7mm | FS | 10% |
| **Additional Information**: |  | | |
| Right Pleural effusion measuring 19mm. | | | |
| **Final Diagnosis:** | | | |
| 1. {S, D, S} Levocardia. 2. All chambers dilated 3. Mild TR 4. Moderate MR 5. Trivia PR 6. Severe LV Systolic Dysfunction 7. RV Dysfunction 8. Moderate Right Pleural effusion | | | |
| **Remark**: | | | |
| **Recommendation**: Work in the line of DCMP. | | | |
| SIGNATURE  Done by: Tesfaye T., Pediatrician, Pediatric Cardiologist _______________ 12/09/08/2014Eth.C | | | |

| Patient Name: **Yalemtila Yekoye**. Referring Institute: **TGSH**. SEX/ Age: **F/3 9/12**.  Date of Report: **13/09/08/14**. Referral Diagnosis: **Incidental Murmur Finding. AGH8.282** | | | |
| --- | --- | --- | --- |
| **Features** | **Finding** | **Features** | **Finding** |
| **Profile** |  | **Atria** |  |
| Abdominal situs | Solitus | Left atrium | Normal |
| Cardiac position | Levocardia | Right atrium | Normal |
| Systemic venous drainage | Normal. | **Atrioventricular valves** |  |
| Pulmonary venous drainage | Normal | Mitral valve | Annulus = 18mm |
| Atrioventricular connection | Concordant | Tricuspid valve | Annulus = 19mm  TAPSE = 17mm |
| Ventriculoarterial connection | Concordant | **Ventricles** |  |
| Ventricular loop | d-Loop | Left ventricle | Normal |
|  |  | Right ventricle | Normal |
| **Septae** |  | **Coronary arteries** | ----- |
| Interventricular septum | 5mm PM VSD, L – R Shunt | **Doppler Measurement** |  |
| Interatrial septum | Intact | Mitral | ----- |
| **Semilunar valves** |  | Aortic | ------- |
| Aortic valve | Annulus = 14mm | Tricuspid | ------- |
| Pulmonary valve | Annulus = 16mm | pulmonic | -------- |
| **Great arteries** | NRGA | **Aortic arch** | Left. No CoA. |
| Aorta | ----- | **PDA** | No |
| Pulmonary artery | Normal MPA and Branch PAs. |  |  |
| **M-Mode:** | | | |
| AO | mm | PWd | mm |
| LA | mm | PWs | mm |
| LVIDd | mm | EDV | ml |
| LVIDs | mm | ESV | ml |
| IVSs | mm | LVEF | 61% |
| IVSd | mm | FS | 33% |
| **Additional Information**: |  | | |
| No pericardial/Pleural effusion. | | | |
| **Final Diagnosis:** | | | |
| 1. {S, D, S} Levocardia. 2. Small PM VSD, L – R Shunt 3. Normal Biventricular Systolic Function | | | |
| **Remark**: | | | |
| **Recommendation**: | | | |
| SIGNATURE  Done by: Tesfaye T., Pediatrician, Pediatric Cardiologist _______________ 13/09/08/2014Eth.C | | | |

| Patient Name: **Leul Geta**. Referring Institute: **Adinas GH**. SEX/ Age: **M/7years**.  Date of Report: **13/09/08/14**. Referral Diagnosis: **ARF. AGH8.283** | | | |
| --- | --- | --- | --- |
| **Features** | **Finding** | **Features** | **Finding** |
| **Profile** |  | **Atria** |  |
| Abdominal situs | Solitus | Left atrium | Normal |
| Cardiac position | Levocardia | Right atrium | Normal |
| Systemic venous drainage | Normal. | **Atrioventricular valves** |  |
| Pulmonary venous drainage | Normal | Mitral valve | Annulus = 19mm |
| Atrioventricular connection | Concordant | Tricuspid valve | Annulus = 20mm  TAPSE = 24mm |
| Ventriculoarterial connection | Concordant | **Ventricles** |  |
| Ventricular loop | d-Loop | Left ventricle | Normal |
|  |  | Right ventricle | Normal |
| **Septae** |  | **Coronary arteries** | ----- |
| Interventricular septum | Intact | **Doppler Measurement** |  |
| Interatrial septum | Intact | Mitral | ----- |
| **Semilunar valves** |  | Aortic | ------- |
| Aortic valve | Annulus = 14mm | Tricuspid | ------- |
| Pulmonary valve | Annulus = 14mm | pulmonic | -------- |
| **Great arteries** | NRGA | **Aortic arch** | Left. No CoA. |
| Aorta | ----- | **PDA** | No |
| Pulmonary artery | Normal MPA and Branch PAs. |  |  |
| **M-Mode:** | | | |
| AO | mm | PWd | mm |
| LA | mm | PWs | mm |
| LVIDd | mm | EDV | ml |
| LVIDs | mm | ESV | ml |
| IVSs | mm | LVEF | 63% |
| IVSd | mm | FS | 33% |
| **Additional Information**: |  | | |
| No pericardial/Pleural effusion. | | | |
| **Final Diagnosis:** | | | |
| 1. Normal Echocardiography Study. | | | |
| **Remark**: | | | |
| **Recommendation**: | | | |
| SIGNATURE  Done by: Tesfaye T., Pediatrician, Pediatric Cardiologist _______________ 13/09/08/2014Eth.C | | | |

| Patient Name: **Bereket Fantahun**. Referring Institute: **Amen SMSC**. SEX/ Age: **M/5years**.  Date of Report: **13/09/08/14**. Referral Diagnosis: **Incidental Murmur Finding. AGH8.284** | | | | | | |
| --- | --- | --- | --- | --- | --- | --- |
| **Features** | **Finding** | | **Features** | | **Finding** | |
| **Profile** |  | | **Atria** | |  | |
| Abdominal situs | Solitus | | Left atrium | | Dilated | |
| Cardiac position | Levocardia | | Right atrium | | Normal | |
| Systemic venous drainage | Normal. | | **Atrioventricular valves** | |  | |
| Pulmonary venous drainage | Normal | | Mitral valve | | Annulus = 22mm. Thickened MVL. | |
| Atrioventricular connection | Concordant | | Tricuspid valve | | Annulus = 23mm  TAPSE = 20mm | |
| Ventriculoarterial connection | Concordant | | **Ventricles** | |  | |
| Ventricular loop | d-Loop | | Left ventricle | | Dilated | |
|  |  | | Right ventricle | | Normal | |
| **Septae** |  | | **Coronary arteries** | | ----- | |
| Interventricular septum | Intact | | **Doppler Measurement** | |  | |
| Interatrial septum | Intact | | Mitral | | Moderate MR, Holosystolic, Posterior projection, seen in two planes with jet velocity = 4m/sec. | |
| **Semilunar valves** |  | | Aortic | | Moderate AR, PHT = 226ms. | |
| Aortic valve | Annulus = 16mm | | Tricuspid | | Moderate TR, PPG = 44mmHg | |
| Pulmonary valve | Annulus = 19mm | | pulmonic | | Trivial PR, PPG = 15mmHg | |
| **Great arteries** | NRGA | | **Aortic arch** | | Left. No CoA. | |
| Aorta | ----- | | **PDA** | | No | |
| Pulmonary artery | Normal MPA and Branch PAs. | |  | |  | |
| **M-Mode:** | | | | | | |
| AO | | mm | | PWd | | mm |
| LA | | mm | | PWs | | mm |
| LVIDd | | mm | | EDV | | ml |
| LVIDs | | mm | | ESV | | ml |
| IVSs | | mm | | LVEF | | 50% |
| IVSd | | mm | | FS | | 26% |
| **Additional Information**: | |  | | | | |
| Pericardial effusion with maximum depth of 7mm on RA/RV Junction. | | | | | | |
| **Final Diagnosis:** | | | | | | |
| 1. {S, D, S} Levocardia. 2. LA/LV Dilated 3. Thickened MVL 4. Moderate MR 5. Moderate AR 6. Moderate TR 7. Mild Pulmonary Hypertension 8. Reduced LV Systolic Function 9. Small Pericardial effusion | | | | | | |
| **Remark**: | | | | | | |
| **Recommendation**: work up the child in the line of Rheumatic Heart Disease | | | | | | |
| SIGNATURE  Done by: Tesfaye T., Pediatrician, Pediatric Cardiologist _______________ 13/09/08/2014Eth.C | | | | | | |

| Patient Name: **Kalkidan Mengistu**. Referring Institute: **Adinas GH**. SEX/ Age: **F/14years**.  Date of Report: **15/09/08/14**. Referral Diagnosis: **Palpitation. AGH8.285** | | | |
| --- | --- | --- | --- |
| **Features** | **Finding** | **Features** | **Finding** |
| **Profile** |  | **Atria** |  |
| Abdominal situs | Solitus | Left atrium | Dilated |
| Cardiac position | Levocardia | Right atrium | Normal |
| Systemic venous drainage | Normal. | **Atrioventricular valves** |  |
| Pulmonary venous drainage | Normal | Mitral valve | Annulus = 25mm |
| Atrioventricular connection | Concordant | Tricuspid valve | Annulus = 24mm  TAPSE = 25mm |
| Ventriculoarterial connection | Concordant | **Ventricles** |  |
| Ventricular loop | d-Loop | Left ventricle | Dilated |
|  |  | Right ventricle | Normal |
| **Septae** |  | **Coronary arteries** | ----- |
| Interventricular septum | Intact | **Doppler Measurement** |  |
| Interatrial septum | Intact | Mitral | ----- |
| **Semilunar valves** |  | Aortic | ------- |
| Aortic valve | Annulus = 20mm | Tricuspid | ------- |
| Pulmonary valve | Annulus = 22mm | pulmonic | -------- |
| **Great arteries** | NRGA | **Aortic arch** | Left. No CoA. |
| Aorta | ----- | **PDA** | 3mm PDA, L – R Shunt |
| Pulmonary artery | Normal MPA and Confluent Branch PAs. |  |  |
| **M-Mode:** | | | |
| AO | mm | PWd | mm |
| LA | mm | PWs | mm |
| LVIDd | mm | EDV | ml |
| LVIDs | mm | ESV | ml |
| IVSs | mm | LVEF | 70% |
| IVSd | mm | FS | 40% |
| **Additional Information**: |  | | |
| No pericardial/Pleural effusion. | | | |
| **Final Diagnosis:** | | | |
| 1. {S, D, S} Levocardia. 2. Dilated LA/LV 3. Large PDA, L – R Shunt 4. Normal Biventricular Systolic Function | | | |
| **Remark**: | | | |
| **Recommendation**: Needs Closure | | | |
| SIGNATURE  Done by: Tesfaye T., Pediatrician, Pediatric Cardiologist _______________ 15/09/08/2014Eth.C | | | |

| Patient Name: **Baby Aklilu**. Referring Institute: **Dr. Addisu PSC**. SEX/ Age: **M/2 1/12**.  Date of Report: **15/09/08/14**. Referral Diagnosis: **Incidental Murmur. AGH8.286** | | | |
| --- | --- | --- | --- |
| **Features** | **Finding** | **Features** | **Finding** |
| **Profile** |  | **Atria** |  |
| Abdominal situs | Solitus | Left atrium | Normal |
| Cardiac position | Levocardia | Right atrium | Normal |
| Systemic venous drainage | Normal. | **Atrioventricular valves** |  |
| Pulmonary venous drainage | Normal | Mitral valve | Annulus = 14mm |
| Atrioventricular connection | Concordant | Tricuspid valve | Annulus = 16mm  TAPSE = 20mm |
| Ventriculoarterial connection | Concordant | **Ventricles** |  |
| Ventricular loop | d-Loop | Left ventricle | Normal |
|  |  | Right ventricle | Normal |
| **Septae** |  | **Coronary arteries** | ----- |
| Interventricular septum | Intact | **Doppler Measurement** |  |
| Interatrial septum | Intact | Mitral | ----- |
| **Semilunar valves** |  | Aortic | ------- |
| Aortic valve | Annulus = 13mm | Tricuspid | ------- |
| Pulmonary valve | Annulus = mm | pulmonic | -------- |
| **Great arteries** | NRGA | **Aortic arch** | Left. No CoA. |
| Aorta | ----- | **PDA** | 0.8mm PDA, L – R Shunt |
| Pulmonary artery | Normal MPA and Branch PAs. |  |  |
| **M-Mode:** | | | |
| AO | mm | PWd | mm |
| LA | mm | PWs | mm |
| LVIDd | mm | EDV | ml |
| LVIDs | mm | ESV | ml |
| IVSs | mm | LVEF | 62% |
| IVSd | mm | FS | 32% |
| **Additional Information**: |  | | |
| No pericardial/Pleural effusion. | | | |
| **Final Diagnosis:** | | | |
| 1. {S, D, S} Levocardia. 2. Small PDA, L – R Shunt | | | |
| **Remark**: If No murmur, can be named as “Silent PDA) | | | |
| **Recommendation**: | | | |
| SIGNATURE  Done by: Tesfaye T., Pediatrician, Pediatric Cardiologist _______________ 15/09/08/2014Eth.C | | | |

| Patient Name: **Arsema dESALEGN**. Referring Institute: **FHRH**. SEX/ Age: **F/39days**.  Date of Report: **15/09/08/14**. Referral Diagnosis: **Incidental Murmur + Cyanosis. AGH8.287** | | | |
| --- | --- | --- | --- |
| **Features** | **Finding** | **Features** | **Finding** |
| **Profile** |  | **Atria** |  |
| Abdominal situs | Solitus | Left atrium | Normal |
| Cardiac position | Levocardia | Right atrium | Normal |
| Systemic venous drainage | Normal. | **Atrioventricular valves** |  |
| Pulmonary venous drainage | Normal | Mitral valve | Annulus = 13mm |
| Atrioventricular connection | Concordant | Tricuspid valve | Annulus = 12mm  TAPSE = 15mm |
| Ventriculoarterial connection | Discordant | **Ventricles** |  |
| Ventricular loop | d-Loop | Left ventricle | Normal |
|  |  | Right ventricle | Normal |
| **Septae** |  | **Coronary arteries** | ----- |
| Interventricular septum | 2mm Gerbode defect, from LV to RA | **Doppler Measurement** |  |
| Interatrial septum | PFO, LA - RA | Mitral | Trivial MR |
| **Semilunar valves** |  | Aortic | ------- |
| Aortic valve | Annulus = 7mm | Tricuspid | ------- |
| Pulmonary valve | Annulus = 11mm | pulmonic | Mild PS(LVOTO), PPG = 23mmHg |
| **Great arteries** | d-TGA | **Aortic arch** | Left. No CoA. |
| Aorta | Anterior and to the right | **PDA** | 2mm PDA, from Aorta to PA |
| Pulmonary artery | Posterior and to the left |  |  |
| **M-Mode:** | | | |
| AO | mm | PWd | mm |
| LA | mm | PWs | mm |
| LVIDd | mm | EDV | ml |
| LVIDs | mm | ESV | ml |
| IVSs | mm | LVEF | % |
| IVSd | mm | FS | % |
| **Additional Information**: |  | | |
| No pericardial/Pleural effusion. | | | |
| **Final Diagnosis:** | | | |
| 1. {S, D, D} Levocardia. 2. d-TGA 3. PFO, LA to RA 4. Small Gerbode defect, from LV to RA 5. Mild PS(LVOTO) 6. Adequate size PDA, from aorta to MPA | | | |
| **Remark**: | | | |
| **Recommendation**: | | | |
| SIGNATURE  Done by: Tesfaye T., Pediatrician, Pediatric Cardiologist _______________ 15/09/08/2014Eth.C | | | |

| Patient Name: **Misganaw Babey**. Referring Institute: **FHRH**. SEX/ Age: **M/14years**.  Date of Report: **15/09/08/14**. Referral Diagnosis: **Incidental Murmur + easy fatigability + Palpitation .AGH8.288** | | | |
| --- | --- | --- | --- |
| **Features** | **Finding** | **Features** | **Finding** |
| **Profile** |  | **Atria** |  |
| Abdominal situs | Solitus | Left atrium | Dilated |
| Cardiac position | Levocardia | Right atrium | Normal |
| Systemic venous drainage | Normal. | **Atrioventricular valves** |  |
| Pulmonary venous drainage | Normal | Mitral valve | Annulus = 21mm |
| Atrioventricular connection | Concordant | Tricuspid valve | Annulus = 19mm  TAPSE = 18mm |
| Ventriculoarterial connection | Concordant | **Ventricles** |  |
| Ventricular loop | d-Loop | Left ventricle | Dilated |
|  |  | Right ventricle | Normal |
| **Septae** |  | **Coronary arteries** | ----- |
| Interventricular septum | 8mm PM VSD, L – R Shunt | **Doppler Measurement** |  |
| Interatrial septum | Intact | Mitral | ----- |
| **Semilunar valves** |  | Aortic | ------- |
| Aortic valve | Annulus = 16mm | Tricuspid | ------- |
| Pulmonary valve | Annulus = 19mm | pulmonic | -------- |
| **Great arteries** | NRGA | **Aortic arch** | Left. No CoA. |
| Aorta | ----- | **PDA** | No |
| Pulmonary artery | Normal MPA and Branch PAs. |  |  |
| **M-Mode:** | | | |
| AO | mm | PWd | mm |
| LA | mm | PWs | mm |
| LVIDd | mm | EDV | ml |
| LVIDs | mm | ESV | ml |
| IVSs | mm | LVEF | 58% |
| IVSd | mm | FS | 30% |
| **Additional Information**: |  | | |
| No pericardial/Pleural effusion. | | | |
| **Final Diagnosis:** | | | |
| 1. {S, D, S} Levocardia. 2. LA/LV Dilated 3. Moderate PM VSD, L – R Shunt 4. Normal Biventricular Systolic Function | | | |
| **Remark**: | | | |
| **Recommendation**: | | | |
| SIGNATURE  Done by: Tesfaye T., Pediatrician, Pediatric Cardiologist _______________ 15/09/08/2014Eth.C | | | |

| Patient Name: **Meseret Yayesew**. Referring Institute: **TGSH**. SEX/ Age: **F/3years**.  Date of Report: **16/09/08/14**. Referral Diagnosis: **Cyanosis. AGH8.289** | | | |
| --- | --- | --- | --- |
| **Features** | **Finding** | **Features** | **Finding** |
| **Profile** |  | **Atria** |  |
| Abdominal situs | Solitus | Left atrium | Normal |
| Cardiac position | Levocardia | Right atrium | Dilated |
| Systemic venous drainage | Normal. | **Atrioventricular valves** |  |
| Pulmonary venous drainage | Normal | Mitral valve | Annulus = 15mm |
| Atrioventricular connection | Concordant | Tricuspid valve | Annulus = 17mm  TAPSE = 15mm |
| Ventriculoarterial connection | Concordant | **Ventricles** |  |
| Ventricular loop | d-Loop | Left ventricle | Normal |
|  |  | Right ventricle | Dilated & Hypertrophied |
| **Septae** |  | **Coronary arteries** | ----- |
| Interventricular septum | 9mm Malaligned Subaortic, R – L Shunt | **Doppler Measurement** |  |
| Interatrial septum | Intact | Mitral | ----- |
| **Semilunar valves** |  | Aortic | ------- |
| Aortic valve | Annulus = 16mm | Tricuspid | ------- |
| Pulmonary valve | Annulus = 6mm. doming PV | pulmonic | Severe Valvular and supra valvular PS, PPG = 64mmHg. |
| **Great arteries** | NRGA | **Aortic arch** | Left. No CoA. |
| Aorta | Aortic Over-riding | **PDA** | No |
| Pulmonary artery | Smallish MPA and Confluent Branch PAs. |  |  |
| **M-Mode:** | | | |
| AO | mm | PWd | mm |
| LA | mm | PWs | mm |
| LVIDd | mm | EDV | ml |
| LVIDs | mm | ESV | ml |
| IVSs | mm | LVEF | 68% |
| IVSd | mm | FS | 36% |
| **Additional Information**: |  | | |
| No pericardial/Pleural effusion. | | | |
| **Final Diagnosis:** | | | |
| 1. {S, D, S} Levocardia. 2. TOF 3. Smallish MPA and Branch PAs | | | |
| **Remark**: | | | |
| **Recommendation**: | | | |
| SIGNATURE  Done by: Tesfaye T., Pediatrician, Pediatric Cardiologist _______________ 16/09/08/2014Eth.C | | | |

| Patient Name: **Lealem Feleke**. Referring Institute: **Adinas GH**. SEX/ Age: **M/10years**.  Date of Report: **16/09/08/14**. Referral Diagnosis: **Chest Pain. AGH8.290** | | | |
| --- | --- | --- | --- |
| **Features** | **Finding** | **Features** | **Finding** |
| **Profile** |  | **Atria** |  |
| Abdominal situs | Solitus | Left atrium | Normal |
| Cardiac position | Levocardia | Right atrium | Normal |
| Systemic venous drainage | Normal. | **Atrioventricular valves** |  |
| Pulmonary venous drainage | Normal | Mitral valve | Annulus = 19mm |
| Atrioventricular connection | Concordant | Tricuspid valve | Annulus = 20mm  TAPSE = 19mm |
| Ventriculoarterial connection | Concordant | **Ventricles** |  |
| Ventricular loop | d-Loop | Left ventricle | Normal |
|  |  | Right ventricle | Normal |
| **Septae** |  | **Coronary arteries** | ----- |
| Interventricular septum | Intact | **Doppler Measurement** |  |
| Interatrial septum | Intact | Mitral | ----- |
| **Semilunar valves** |  | Aortic | ------- |
| Aortic valve | Annulus = 16mm | Tricuspid | ------- |
| Pulmonary valve | Annulus = 16mm | pulmonic | -------- |
| **Great arteries** | NRGA | **Aortic arch** | Left. No CoA. |
| Aorta | ----- | **PDA** | No |
| Pulmonary artery | Normal MPA and Branch PAs. |  |  |
| **M-Mode:** | | | |
| AO | mm | PWd | mm |
| LA | mm | PWs | mm |
| LVIDd | mm | EDV | ml |
| LVIDs | mm | ESV | ml |
| IVSs | mm | LVEF | 67% |
| IVSd | mm | FS | 37% |
| **Additional Information**: |  | | |
| No pericardial/Pleural effusion. | | | |
| **Final Diagnosis:** | | | |
| 1. Normal Echocardiography Study. | | | |
| **Remark**: | | | |
| **Recommendation**: | | | |
| SIGNATURE  Done by: Tesfaye T., Pediatrician, Pediatric Cardiologist _______________ 16/09/08/2014Eth.C | | | |

| Patient Name: **Baby of Alemnesh Mitikie**. Referring Institute: **FHRH**. SEX/ Age: **M/25days**.  Date of Report: **16/09/08/14**. Referral Diagnosis: **RD + Incidental murmur. AGH8.291** | | | |
| --- | --- | --- | --- |
| **Features** | **Finding** | **Features** | **Finding** |
| **Profile** |  | **Atria** |  |
| Abdominal situs | Solitus | Left atrium | Normal |
| Cardiac position | Levocardia | Right atrium | Normal |
| Systemic venous drainage | Normal. | **Atrioventricular valves** |  |
| Pulmonary venous drainage | Normal | Mitral valve | Annulus = 12mm |
| Atrioventricular connection | Concordant | Tricuspid valve | Annulus = 13mm  TAPSE = 13mm |
| Ventriculoarterial connection | Concordant | **Ventricles** |  |
| Ventricular loop | d-Loop | Left ventricle | Normal |
|  |  | Right ventricle | Normal |
| **Septae** |  | **Coronary arteries** | ----- |
| Interventricular septum | Intact | **Doppler Measurement** |  |
| Interatrial septum | 5mm Fenestrated OS ASD, L – R Shunt | Mitral | ----- |
| **Semilunar valves** |  | Aortic | ------- |
| Aortic valve | Annulus = 8mm | Tricuspid | ------- |
| Pulmonary valve | Annulus = 9mm | pulmonic | -------- |
| **Great arteries** | NRGA | **Aortic arch** | Left. No CoA. |
| Aorta | ----- | **PDA** | No |
| Pulmonary artery | Normal MPA and Branch PAs. |  |  |
| **M-Mode:**  Normal LV Systolic Function | | | |
| AO | Mm | PWd | mm |
| LA | Mm | PWs | mm |
| LVIDd | Mm | EDV | ml |
| LVIDs | Mm | ESV | ml |
| IVSs | Mm | LVEF | % |
| IVSd | Mm | FS | % |
| **Additional Information**: |  | | |
| No pericardial/Pleural effusion. | | | |
| **Final Diagnosis:** | | | |
| 1. {S, D, S} Levocardia. 2. Small Fenestrated OS ASD, L – R Shunt | | | |
| **Remark**: | | | |
| **Recommendation**:   1. Follow up only. 2. No need to start any form of cardiac medicine. | | | |
| SIGNATURE  Done by: Tesfaye T., Pediatrician, Pediatric Cardiologist _______________ 16/09/08/2014Eth.C | | | |

| Patient Name: **Mahlet Nega**. Referring Institute: **Addis Alem PH**. SEX/ Age: **F/4years**.  Date of Report: **17/09/08/14**. Referral Diagnosis: **FTT + Incidental Murmur. AGH8.292** | | | |
| --- | --- | --- | --- |
| **Features** | **Finding** | **Features** | **Finding** |
| **Profile** |  | **Atria** |  |
| Abdominal situs | Solitus | Left atrium | Normal |
| Cardiac position | Levocardia | Right atrium | Dilated |
| Systemic venous drainage | Normal. | **Atrioventricular valves** |  |
| Pulmonary venous drainage | Normal | Mitral valve | Annulus = 17mm |
| Atrioventricular connection | Concordant | Tricuspid valve | Annulus = 18mm  TAPSE = 23mm |
| Ventriculoarterial connection | Concordant | **Ventricles** |  |
| Ventricular loop | d-Loop | Left ventricle | Normal |
|  |  | Right ventricle | Dilated |
| **Septae** |  | **Coronary arteries** | ----- |
| Interventricular septum | Intact | **Doppler Measurement** |  |
| Interatrial septum | 11mm OS ASD, L – R Shunt | Mitral | ----- |
| **Semilunar valves** |  | Aortic | ------- |
| Aortic valve | Annulus = 13mm | Tricuspid | ------- |
| Pulmonary valve | Annulus = 15mm | pulmonic | -------- |
| **Great arteries** | NRGA | **Aortic arch** | Left. No CoA. |
| Aorta | ----- | **PDA** | No |
| Pulmonary artery | Normal MPA and Branch PAs. |  |  |
| **M-Mode:** | | | |
| AO | mm | PWd | mm |
| LA | mm | PWs | mm |
| LVIDd | mm | EDV | ml |
| LVIDs | mm | ESV | ml |
| IVSs | mm | LVEF | 64% |
| IVSd | mm | FS | 33% |
| **Additional Information**: |  | | |
| No pericardial/Pleural effusion. | | | |
| **Final Diagnosis:** | | | |
| 1. {S, D, S} Levocardia. 2. RA/RV Dilated 3. Large OS ASD, L – R Shunt 4. Normal Biventricular Systolic Function | | | |
| **Remark**: | | | |
| **Recommendation**: Needs closure | | | |
| SIGNATURE  Done by: Tesfaye T., Pediatrician, Pediatric Cardiologist _______________ 17/09/08/2014Eth.C | | | |

| Patient Name: **Zemenu Getinet**. Referring Institute: **TGSH**. SEX/ Age: **M/6years**.  Date of Report: **17/09/08/14**. Referral Diagnosis: **FTT. AGH8.293** | | | |
| --- | --- | --- | --- |
| **Features** | **Finding** | **Features** | **Finding** |
| **Profile** |  | **Atria** |  |
| Abdominal situs | Solitus | Left atrium | Normal |
| Cardiac position | Levocardia | Right atrium | Normal |
| Systemic venous drainage | Normal. | **Atrioventricular valves** |  |
| Pulmonary venous drainage | Normal | Mitral valve | Annulus = 17mm |
| Atrioventricular connection | Concordant | Tricuspid valve | Annulus = 19mm |
| Ventriculoarterial connection | Concordant | **Ventricles** |  |
| Ventricular loop | d-Loop | Left ventricle | Normal |
|  |  | Right ventricle | Normal |
| **Septae** |  | **Coronary arteries** | ----- |
| Interventricular septum | Intact | **Doppler Measurement** |  |
| Interatrial septum | Intact | Mitral | ----- |
| **Semilunar valves** |  | Aortic | ------- |
| Aortic valve | Annulus = 15mm | Tricuspid | ------- |
| Pulmonary valve | Annulus = 18mm | pulmonic | -------- |
| **Great arteries** | NRGA | **Aortic arch** | Left. No CoA. |
| Aorta | ----- | **PDA** | No |
| Pulmonary artery | Normal MPA and Branch PAs. |  |  |
| **M-Mode:** | | | |
| AO | mm | PWd | mm |
| LA | mm | PWs | mm |
| LVIDd | mm | EDV | ml |
| LVIDs | mm | ESV | ml |
| IVSs | mm | LVEF | 63% |
| IVSd | mm | FS | 33% |
| **Additional Information**: |  | | |
| No pericardial/Pleural effusion. | | | |
| **Final Diagnosis:** | | | |
| 1. Normal Echocardiography Study. | | | |
| **Remark**: | | | |
| **Recommendation**: | | | |
| SIGNATURE  Done by: Tesfaye T., Pediatrician, Pediatric Cardiologist _______________ 17/09/08/2014Eth.C | | | |

| Patient Name: **Surafel Ambachew**. Referring Institute: **Amaris PSC**. SEX/ Age: **M/1 2/12**.  Date of Report: **18/09/08/14**. Referral Diagnosis: **CHF + RD. AGH8.294** | | | |
| --- | --- | --- | --- |
| **Features** | **Finding** | **Features** | **Finding** |
| **Profile** |  | **Atria** |  |
| Abdominal situs | Solitus | Left atrium | Dilated |
| Cardiac position | Levocardia | Right atrium | Dilated |
| Systemic venous drainage | Normal. | **Atrioventricular valves** |  |
| Pulmonary venous drainage | Normal | Mitral valve | Annulus = 14mm |
| Atrioventricular connection | Concordant | Tricuspid valve | Annulus = 14mm |
| Ventriculoarterial connection | Concordant | **Ventricles** |  |
| Ventricular loop | d-Loop | Left ventricle | Dilated |
|  |  | Right ventricle | Dilated |
| **Septae** |  | **Coronary arteries** | ----- |
| Interventricular septum | 10mm PM VSD, L – R Shunt | **Doppler Measurement** |  |
| Interatrial septum | Intact | Mitral | ----- |
| **Semilunar valves** |  | Aortic | ------- |
| Aortic valve | Annulus = 11mm | Tricuspid | ------- |
| Pulmonary valve | Annulus = 14mm | pulmonic | -------- |
| **Great arteries** | NRGA | **Aortic arch** | Left. No CoA. |
| Aorta | ----- | **PDA** | No |
| Pulmonary artery | MPA = 15mm. Confluent Branch PAs. |  |  |
| **M-Mode:**  Normal LV Function on eye balling | | | |
| AO | mm | PWd | mm |
| LA | mm | PWs | mm |
| LVIDd | mm | EDV | ml |
| LVIDs | mm | ESV | ml |
| IVSs | mm | LVEF | % |
| IVSd | mm | FS | % |
| **Additional Information**: |  | | |
| No pericardial/Pleural effusion. | | | |
| **Final Diagnosis:** | | | |
| 1. {S, D, S} Levocardia. 2. All chambers dilated 3. Large PM VSD, L – R Shunt 4. Mod. Pulmonary Hypertension 5. Normal LV Systolic Function | | | |
| **Remark**: | | | |
| **Recommendation**: | | | |
| SIGNATURE  Done by: Tesfaye T., Pediatrician, Pediatric Cardiologist _______________ 18/09/08/2014Eth.C | | | |

| Patient Name: **Lisane-werk Ayalew**. Referring Institute: **TGSH**. SEX/ Age: **F/10years**.  Date of Report: **18/09/08/14**. Referral Diagnosis: **Rheumatic Recurrence. AGH8.295** | | | |
| --- | --- | --- | --- |
| **Features** | **Finding** | **Features** | **Finding** |
| **Profile** |  | **Atria** |  |
| Abdominal situs | Solitus | Left atrium | Dilated |
| Cardiac position | Levocardia | Right atrium | Normal |
| Systemic venous drainage | Normal. | **Atrioventricular valves** |  |
| Pulmonary venous drainage | Normal | Mitral valve | Annulus = 25mm. Thickened MVL |
| Atrioventricular connection | Concordant | Tricuspid valve | Annulus = 20mm  TAPSE = 20mm |
| Ventriculoarterial connection | Concordant | **Ventricles** |  |
| Ventricular loop | d-Loop | Left ventricle | Dilated |
|  |  | Right ventricle | Normal |
| **Septae** |  | **Coronary arteries** | ----- |
| Interventricular septum | Intact | **Doppler Measurement** |  |
| Interatrial septum | Intact | Mitral | Moderate MR, Holosystolic, posterior projection, seen in two planes with jet velocity = 4.6m/sec. |
| **Semilunar valves** |  | Aortic | ------- |
| Aortic valve | Annulus = 18mm | Tricuspid | ------- |
| Pulmonary valve | Annulus = 19mm | pulmonic | -------- |
| **Great arteries** | NRGA | **Aortic arch** | Left. No CoA. |
| Aorta | ----- | **PDA** | No |
| Pulmonary artery | Normal MPA and Confluent Branch PAs. |  |  |
| **M-Mode:** | | | |
| AO | mm | PWd | mm |
| LA | mm | PWs | mm |
| LVIDd | mm | EDV | ml |
| LVIDs | mm | ESV | ml |
| IVSs | mm | LVEF | 59% |
| IVSd | mm | FS | 31% |
| **Additional Information**: |  | | |
| No pericardial/Pleural effusion. | | | |
| **Final Diagnosis:** | | | |
| 1. {S, D, S} Levocardia. 2. LA/LV Dilated 3. Thickened MVL 4. Moderate MR 5. Normal Biventricular Systolic Function | | | |
| **Remark**: | | | |
| **Recommendation**: | | | |
| SIGNATURE  Done by: Tesfaye T., Pediatrician, Pediatric Cardiologist _______________ 18/09/08/2014Eth.C | | | |

| Patient Name: **Fikir Anbesaw**. Referring Institute: **TGSH**. SEX/ Age: **F/1 6/12**.  Date of Report: **19/09/08/14**. Referral Diagnosis: **FTT + Incidental Murmur. AGH8.296** | | | |
| --- | --- | --- | --- |
| **Features** | **Finding** | **Features** | **Finding** |
| **Profile** |  | **Atria** |  |
| Abdominal situs | Solitus | Left atrium | Dilated |
| Cardiac position | Levocardia | Right atrium | Normal |
| Systemic venous drainage | Normal. | **Atrioventricular valves** |  |
| Pulmonary venous drainage | Normal | Mitral valve | Annulus = 21mm |
| Atrioventricular connection | Concordant | Tricuspid valve | Annulus = 14mm  TAPSE = 18mm |
| Ventriculoarterial connection | Concordant | **Ventricles** |  |
| Ventricular loop | d-Loop | Left ventricle | Dilated |
|  |  | Right ventricle | Normal |
| **Septae** |  | **Coronary arteries** | ----- |
| Interventricular septum | 6mm Sub-pulmonic VSD with AV prolapsing to the defect, L – R Shunt | **Doppler Measurement** |  |
| Interatrial septum | Intact | Mitral | ----- |
| **Semilunar valves** |  | Aortic | Mild AR, |
| Aortic valve | Annulus = 12mm. | Tricuspid | ------- |
| Pulmonary valve | Annulus = 18mm | pulmonic | -------- |
| **Great arteries** | NRGA | **Aortic arch** | Left. No CoA. |
| Aorta | ----- | **PDA** | No |
| Pulmonary artery | MPA = 18mm and Confluent Branch PAs. |  |  |
| **M-Mode:**  Normal LV Function on eye balling | | | |
| AO | mm | PWd | mm |
| LA | mm | PWs | mm |
| LVIDd | mm | EDV | ml |
| LVIDs | mm | ESV | ml |
| IVSs | mm | LVEF | % |
| IVSd | mm | FS | % |
| **Additional Information**: |  | | |
| No pericardial/Pleural effusion. | | | |
| **Final Diagnosis:** | | | |
| 1. {S, D, S} Levocardia. 2. LA/LV Dilated 3. Moderate Sub – Pulmonic VSD with Aortic valve prolapsing to the defect, L – R Shunt 4. Mild AR 5. Mod.Pul.HTN 6. Normal Biventricular Systolic Function | | | |
| **Remark**: | | | |
| **Recommendation**: Needs Surgical Closure | | | |
| SIGNATURE  Done by: Tesfaye T., Pediatrician, Pediatric Cardiologist _______________ 19/09/08/2014Eth.C | | | |

| Patient Name: **Matias Tinfash**. Referring Institute: **Adinas GH**. SEX/ Age: **M/1 11/12**.  Date of Report: **19/09/08/14**. Referral Diagnosis: **RD + FTT. AGH8.297** | | | |
| --- | --- | --- | --- |
| **Features** | **Finding** | **Features** | **Finding** |
| **Profile** |  | **Atria** |  |
| Abdominal situs | Solitus | Left atrium | Normal |
| Cardiac position | Levocardia | Right atrium | Dilated |
| Systemic venous drainage | Normal. | **Atrioventricular valves** |  |
| Pulmonary venous drainage | Normal | Mitral valve | Annulus = 15mm |
| Atrioventricular connection | Concordant | Tricuspid valve | Annulus = 23mm  TAPSE = 15mm |
| Ventriculoarterial connection | Concordant | **Ventricles** |  |
| Ventricular loop | d-Loop | Left ventricle | Normal |
|  |  | Right ventricle | Dilated |
| **Septae** |  | **Coronary arteries** | ----- |
| Interventricular septum | Intact | **Doppler Measurement** |  |
| Interatrial septum | Intact | Mitral | ----- |
| **Semilunar valves** |  | Aortic | ------- |
| Aortic valve | Annulus = 11mm | Tricuspid | Moderate TR, PPG = 67mmHg. |
| Pulmonary valve | Annulus = 16mm | pulmonic | -------- |
| **Great arteries** | NRGA | **Aortic arch** | Left. No CoA. |
| Aorta | ----- | **PDA** | No |
| Pulmonary artery | MPA = 17mm. Confluent Branch PAs. |  |  |
| **M-Mode:** | | | |
| AO | mm | PWd | mm |
| LA | mm | PWs | mm |
| LVIDd | mm | EDV | ml |
| LVIDs | mm | ESV | ml |
| IVSs | mm | LVEF | % |
| IVSd | mm | FS | % |
| **Additional Information**: |  | | |
| No pericardial/Pleural effusion. | | | |
| **Final Diagnosis:** | | | |
| 1. {S, D, S} Levocardia. 2. RA/RV Dilated 3. Moderate TR 4. Severe Pulmonary Hypertension | | | |
| **Remark**: | | | |
| **Recommendation**: | | | |
| SIGNATURE  Done by: Tesfaye T., Pediatrician, Pediatric Cardiologist _______________ 19/09/08/2014Eth.C | | | |

| Patient Name: **Tirsit Tadele**. Referring Institute: **Amaris PSC**. SEX/ Age: **F/11months**.  Date of Report: **20/09/08/14**. Referral Diagnosis: **Incidental Murmur Finding. AGH8.298** | | | |
| --- | --- | --- | --- |
| **Features** | **Finding** | **Features** | **Finding** |
| **Profile** |  | **Atria** |  |
| Abdominal situs | Solitus | Left atrium | Normal |
| Cardiac position | Levocardia | Right atrium | Normal |
| Systemic venous drainage | Normal. | **Atrioventricular valves** |  |
| Pulmonary venous drainage | Normal | Mitral valve | Annulus = 13mm |
| Atrioventricular connection | Concordant | Tricuspid valve | Annulus = 15mm  TAPSE = 15mm |
| Ventriculoarterial connection | Concordant | **Ventricles** |  |
| Ventricular loop | d-Loop | Left ventricle | Normal |
|  |  | Right ventricle | Normal |
| **Septae** |  | **Coronary arteries** | ----- |
| Interventricular septum | Intact | **Doppler Measurement** |  |
| Interatrial septum | 5mm OS ASD, L – R Shunt | Mitral | ----- |
| **Semilunar valves** |  | Aortic | ------- |
| Aortic valve | Annulus = 11mm | Tricuspid | ------- |
| Pulmonary valve | Annulus = 11mm | pulmonic | -------- |
| **Great arteries** | NRGA | **Aortic arch** | Left. No CoA. |
| Aorta | ----- | **PDA** | No |
| Pulmonary artery | Normal MPA and Branch PAs. |  |  |
| **M-Mode:**  Normal LV Systolic Function on eye balling | | | |
| AO | mm | PWd | mm |
| LA | mm | PWs | mm |
| LVIDd | mm | EDV | ml |
| LVIDs | mm | ESV | ml |
| IVSs | mm | LVEF | % |
| IVSd | mm | FS | % |
| **Additional Information**: |  | | |
| No pericardial/Pleural effusion. | | | |
| **Final Diagnosis:** | | | |
| 1. {S, D, S} Levocardia. 2. Small OS ASD, L – R Shunt 3. Normal Biventricular Systolic Function | | | |
| **Remark**: | | | |
| **Recommendation**:   1. Follow up echocardiography yearly. 2. No need to start cardiac medicine | | | |
| SIGNATURE  Done by: Tesfaye T., Pediatrician, Pediatric Cardiologist _______________ 20/09/08/2014Eth.C | | | |

| Patient Name: **Mussie Melkamu**. Referring Institute: **Addis Alem PH**. SEX/ Age: **M/14years**.  Date of Report: **22/09/08/14**. Referral Diagnosis: **Rheumatic Recurrence. AGH8.299** | | | |
| --- | --- | --- | --- |
| **Features** | **Finding** | **Features** | **Finding** |
| **Profile** |  | **Atria** |  |
| Abdominal situs | Solitus | Left atrium | Dilated |
| Cardiac position | Levocardia | Right atrium | Normal |
| Systemic venous drainage | Normal. | **Atrioventricular valves** |  |
| Pulmonary venous drainage | Normal | Mitral valve | Annulus = 28mm. thickened MVL. |
| Atrioventricular connection | Concordant | Tricuspid valve | Annulus = 21mm  TAPSE = 20mm |
| Ventriculoarterial connection | Concordant | **Ventricles** |  |
| Ventricular loop | d-Loop | Left ventricle | Dilated |
|  |  | Right ventricle | Normal |
| **Septae** |  | **Coronary arteries** | ----- |
| Interventricular septum | Intact | **Doppler Measurement** |  |
| Interatrial septum | Intact | Mitral | Moderate MR, Holosystolic, central projection, seen in two planes with jet velocity = 3m/sec. |
| **Semilunar valves** |  | Aortic | Moderate AR, PHT = 343ms |
| Aortic valve | Annulus = 20mm | Tricuspid | Mild TR ppg = 44mmHg |
| Pulmonary valve | Annulus = 23mm | pulmonic | -------- |
| **Great arteries** | NRGA | **Aortic arch** | Left. No CoA. |
| Aorta | ----- | **PDA** | No |
| Pulmonary artery | Normal MPA and Branch PAs. |  |  |
| **M-Mode:** | | | |
| AO | mm | PWd | mm |
| LA | mm | PWs | mm |
| LVIDd | mm | EDV | ml |
| LVIDs | mm | ESV | ml |
| IVSs | mm | LVEF | 59% |
| IVSd | mm | FS | 32% |
| **Additional Information**: |  | | |
| Pericardial effusion with maximum depth of 4mm on RA/RV Junction. | | | |
| **Final Diagnosis:** | | | |
| 1. {S, D, S} Levocardia. 2. LA/LV Dilated 3. Thickened MVL 4. Moderate MR 5. Moderate AR 6. Mild TR 7. Mild Pulmonary Hypertension 8. Tace Pericardial effusion | | | |
| **Remark**: | | | |
| **Recommendation**: | | | |
| SIGNATURE  Done by: Tesfaye T., Pediatrician, Pediatric Cardiologist _______________ 22/09/08/2014Eth.C | | | |

| Patient Name: **Sale-amlak Mequanint**. Referring Institute: **FHRH**. SEX/ Age: **M/1 7/12**.  Date of Report: **22/09/08/14**. Referral Diagnosis: **Recurrent Chest Infection. AGH8.300** | | | |
| --- | --- | --- | --- |
| **Features** | **Finding** | **Features** | **Finding** |
| **Profile** |  | **Atria** |  |
| Abdominal situs | Solitus | Left atrium | Normal |
| Cardiac position | Levocardia | Right atrium | Normal |
| Systemic venous drainage | Normal. | **Atrioventricular valves** |  |
| Pulmonary venous drainage | Normal | Mitral valve | Annulus = 14mm |
| Atrioventricular connection | Concordant | Tricuspid valve | Annulus = 16mm  TAPSE = 14mm |
| Ventriculoarterial connection | Concordant | **Ventricles** |  |
| Ventricular loop | d-Loop | Left ventricle | Normal |
|  |  | Right ventricle | Normal |
| **Septae** |  | **Coronary arteries** | ----- |
| Interventricular septum | Intact | **Doppler Measurement** |  |
| Interatrial septum | Intact | Mitral | ----- |
| **Semilunar valves** |  | Aortic | ------- |
| Aortic valve | Annulus = 12mm | Tricuspid | ------- |
| Pulmonary valve | Annulus = 13mm | pulmonic | -------- |
| **Great arteries** | NRGA | **Aortic arch** | Left. No CoA. |
| Aorta | ----- | **PDA** | No |
| Pulmonary artery | Normal MPA and Branch PAs. |  |  |
| **M-Mode:** | | | |
| AO | mm | PWd | 7mm |
| LA | mm | PWs | 5mm |
| LVIDd | 23.7mm | EDV | 19ml |
| LVIDs | 14.9mm | ESV | 6ml |
| IVSs | 8.8mm | LVEF | 70% |
| IVSd | 7mm | FS | 37% |
| **Additional Information**: |  | | |
| No pericardial/Pleural effusion. | | | |
| **Final Diagnosis:** | | | |
| 1. Normal Echocardiography Study. | | | |
| **Remark**: | | | |
| **Recommendation**: | | | |
| SIGNATURE  Done by: Tesfaye T., Pediatrician, Pediatric Cardiologist _______________ 22/09/08/2014Eth.C | | | |

| Patient Name: **Tangut Alene**. Referring Institute: **FHRH**. SEX/ Age: **F/7years**.  Date of Report: **22/09/08/14**. Referral Diagnosis: **Cyanosis + clubbing. AGH8.301** | | | |
| --- | --- | --- | --- |
| **Features** | **Finding** | **Features** | **Finding** |
| **Profile** |  | **Atria** |  |
| Abdominal situs | Solitus | Left atrium | Normal |
| Cardiac position | Levocardia | Right atrium | Dilated |
| Systemic venous drainage | Normal. | **Atrioventricular valves** |  |
| Pulmonary venous drainage | Normal | Mitral valve | Annulus = 14mm |
| Atrioventricular connection | Concordant | Tricuspid valve | Annulus = 15mm  TAPSE = 17mm |
| Ventriculoarterial connection | Concordant | **Ventricles** |  |
| Ventricular loop | d-Loop | Left ventricle | Normal |
|  |  | Right ventricle | Dilated & Hypertrophied |
| **Septae** |  | **Coronary arteries** | ----- |
| Interventricular septum | Non-restrictive Malaligned sub aortic VSD, R – L Shunt. | **Doppler Measurement** |  |
| Interatrial septum | Intact | Mitral | ----- |
| **Semilunar valves** |  | Aortic | ------- |
| Aortic valve | Annulus = 15mm | Tricuspid | ------- |
| Pulmonary valve | Atretic | pulmonic | -------- |
| **Great arteries** | NRGA | **Aortic arch** | Left. No CoA. |
| Aorta | Aortic Over-Ride | **PDA** | No |
| Pulmonary artery | Atretic |  |  |
| **M-Mode:** | | | |
| AO | mm | PWd | mm |
| LA | mm | PWs | mm |
| LVIDd | mm | EDV | ml |
| LVIDs | mm | ESV | ml |
| IVSs | mm | LVEF | % |
| IVSd | mm | FS | % |
| **Additional Information**: |  | | |
| No pericardial/Pleural effusion. | | | |
| **Final Diagnosis:** | | | |
| 1. {S, D, S} Levocardia. 2. RA/RV Dilated 3. TOF 4. Pulmonary Atresia | | | |
| **Remark**: | | | |
| **Recommendation**: | | | |
| SIGNATURE  Done by: Tesfaye T., Pediatrician, Pediatric Cardiologist _______________ 22/09/08/2014Eth.C | | | |

| Patient Name: **Genezeb Belete**. Referring Institute: **FHRH**. SEX/ Age: **F/11years**.  Date of Report: **22/09/08/14**. Referral Diagnosis: **CRHD. AGH8.302** | | | |
| --- | --- | --- | --- |
| **Features** | **Finding** | **Features** | **Finding** |
| **Profile** |  | **Atria** |  |
| Abdominal situs | Solitus | Left atrium | Normal |
| Cardiac position | Levocardia | Right atrium | Normal |
| Systemic venous drainage | Normal. | **Atrioventricular valves** |  |
| Pulmonary venous drainage | Normal | Mitral valve | Annulus = 28mm. thickened MVL |
| Atrioventricular connection | Concordant | Tricuspid valve | Annulus = 29mm  TAPSE = 19mm |
| Ventriculoarterial connection | Concordant | **Ventricles** |  |
| Ventricular loop | d-Loop | Left ventricle | Normal |
|  |  | Right ventricle | Normal |
| **Septae** |  | **Coronary arteries** | ----- |
| Interventricular septum | Intact | **Doppler Measurement** |  |
| Interatrial septum | Intact | Mitral | Severe MR, Holosystolic, posterior projection, seen In two planes with jet velocity = 4.2m/sec. |
| **Semilunar valves** |  | Aortic | Moderate AR, PHT= 353ms |
| Aortic valve | Annulus = 16mm. thickened, trileaflet | Tricuspid | Mild TR, PPG = 36mmHg |
| Pulmonary valve | Annulus = 20mm | pulmonic | Mild PR, PPG = 35mmHg |
| **Great arteries** | NRGA | **Aortic arch** | Left. No CoA. |
| Aorta | ----- | **PDA** | No |
| Pulmonary artery | Normal MPA and Branch PAs. |  |  |
| **M-Mode:** | | | |
| AO | mm | PWd | mm |
| LA | mm | PWs | mm |
| LVIDd | mm | EDV | ml |
| LVIDs | mm | ESV | ml |
| IVSs | mm | LVEF | 60% |
| IVSd | mm | FS | 32% |
| **Additional Information**: |  | | |
| Pericardial effusion with maximum depth of 7mm on RV Side. | | | |
| **Final Diagnosis:** | | | |
| 1. {S, D, S} Levocardia. 2. LA/LV Dilated 3. Thickened MVL, AVL 4. Severe MR 5. Moderate AR 6. Mild TR 7. Mild PR 8. Mild Pulmonary Hypertension 9. Small Pericardial effusion 10. Normal Biventyricular Systolic Function | | | |
| SIGNATURE  Done by: Tesfaye T., Pediatrician, Pediatric Cardiologist _______________ 22/09/08/2014Eth.C | | | |

| Patient Name: **Birke Matebie**. Referring Institute: **Adinas GH**. SEX/ Age: **F/14years**. Date of Report: **22/09/08/14**.  Referral Diagnosis: **CHF + CRVHD + Arrhythmia + palpitation + easy fatigability. AGH8.303** | | | |
| --- | --- | --- | --- |
| **Features** | **Finding** | **Features** | **Finding** |
| **Profile** |  | **Atria** |  |
| Abdominal situs | Solitus | Left atrium | Markedly Dilated |
| Cardiac position | Levocardia | Right atrium | Dilated |
| Systemic venous drainage | Normal. | **Atrioventricular valves** |  |
| Pulmonary venous drainage | Normal | Mitral valve | Annulus = 31mm. thickened, clubbed, dysfigured, calcified MVL. MVA = 0.5cm2. |
| Atrioventricular connection | Concordant | Tricuspid valve | Annulus = 21mm  TAPSE = 19mm |
| Ventriculoarterial connection | Concordant | **Ventricles** |  |
| Ventricular loop | d-Loop | Left ventricle | Dilated |
|  |  | Right ventricle | Dilated |
| **Septae** |  | **Coronary arteries** | ----- |
| Interventricular septum | Intact | **Doppler Measurement** |  |
| Interatrial septum | Intact | Mitral | Severe MS, PPG/MPG = 32/21mmHg |
| **Semilunar valves** |  | Aortic | Mild AR, PHT = 555ms |
| Aortic valve | Annulus = 17mm | Tricuspid | Moderate TR, PPG = 97mmHg |
| Pulmonary valve | Annulus = 20mm | pulmonic | Mild PR, PPG = 81mmHg |
| **Great arteries** | NRGA | **Aortic arch** | Left. No CoA. |
| Aorta | ----- | **PDA** | No |
| Pulmonary artery | Normal MPA and Branch PAs. |  |  |
| **M-Mode:** | | | |
| AO | mm | PWd | mm |
| LA | mm | PWs | mm |
| LVIDd | mm | EDV | ml |
| LVIDs | mm | ESV | ml |
| IVSs | mm | LVEF | 71% |
| IVSd | mm | FS | 38% |
| **Additional Information**: |  | | |
| No pericardial/Pleural effusion. | | | |
| **Final Diagnosis:** | | | |
| 1. {S, D, S} Levocardia. 2. All chambers dilated 3. Thickened, clubbed, disfigured, calcified MVL 4. Severe MS 5. Mild AR 6. Moderate TR 7. Mild PR 8. Severe Pulmonary Hypertension 9. Normal Biventricular Systolic Function | | | |
| **Recommendation**: Needs surgery | | | |
| SIGNATURE  Done by: Tesfaye T., Pediatrician, Pediatric Cardiologist _______________ 22/09/08/2014Eth.C | | | |

| Patient Name: **Derso Muhabaw**. Referring Institute: **FHRH**. SEX/ Age: **M/14years**.  Date of Report: **23/09/08/14**. Referral Diagnosis: **CRVHD + Palpitation + CHF. AGH8.304** | | | |
| --- | --- | --- | --- |
| **Features** | **Finding** | **Features** | **Finding** |
| **Profile** |  | **Atria** |  |
| Abdominal situs | Solitus | Left atrium | Dilated |
| Cardiac position | Levocardia | Right atrium | Normal |
| Systemic venous drainage | Normal. | **Atrioventricular valves** |  |
| Pulmonary venous drainage | Normal | Mitral valve | Annulus = 35mm. thickened MVL. Shortened PMVL |
| Atrioventricular connection | Concordant | Tricuspid valve | Annulus = 26mm  TAPSE = 29mm |
| Ventriculoarterial connection | Concordant | **Ventricles** |  |
| Ventricular loop | d-Loop | Left ventricle | Globularly Dilated |
|  |  | Right ventricle | Normal |
| **Septae** |  | **Coronary arteries** | ----- |
| Interventricular septum | Intact | **Doppler Measurement** |  |
| Interatrial septum | Intact | Mitral | Severe MR, Holosystolic, posterior projection, seen in two planes with jet velocity = 4.2m/sec. |
| **Semilunar valves** |  | Aortic | Moderate AR, PHT = 307ms |
| Aortic valve | Annulus = 26mm. trileaflet, thickened | Tricuspid | ------- |
| Pulmonary valve | Annulus = 25mm | pulmonic | -------- |
| **Great arteries** | NRGA | **Aortic arch** | Left. No CoA. |
| Aorta | ----- | **PDA** | No |
| Pulmonary artery | Normal MPA and Branch PAs. |  |  |
| **M-Mode:** | | | |
| AO | mm | PWd | mm |
| LA | mm | PWs | mm |
| LVIDd | mm | EDV | ml |
| LVIDs | mm | ESV | ml |
| IVSs | mm | LVEF | 61% |
| IVSd | mm | FS | 34% |
| **Additional Information**: |  | | |
| No pericardial/Pleural effusion. | | | |
| **Final Diagnosis:** | | | |
| 1. {S, D, S} Levocardia. 2. LA/LV Dilated 3. Thickened MVL and AVL, Shortened PMVL 4. Severe MR 5. Moderate to severe AR 6. Normal Biventricular Systolic Function | | | |
| **Remark**: | | | |
| **Recommendation**: | | | |
| SIGNATURE  Done by: Tesfaye T., Pediatrician, Pediatric Cardiologist _______________ 23/09/08/2014Eth.C | | | |

| Patient Name: **Muluken Tesfaye**. Referring Institute: **Injibara GH**. SEX/ Age: **M/8years**.  Date of Report: **23/09/08/14**. Referral Diagnosis: **RHD. AGH8.305** | | | |
| --- | --- | --- | --- |
| **Features** | **Finding** | **Features** | **Finding** |
| **Profile** |  | **Atria** |  |
| Abdominal situs | Solitus | Left atrium | Mildly dilated |
| Cardiac position | Levocardia | Right atrium | Normal |
| Systemic venous drainage | Normal. | **Atrioventricular valves** |  |
| Pulmonary venous drainage | Normal | Mitral valve | Annulus = 24mm. thickened MVL. |
| Atrioventricular connection | Concordant | Tricuspid valve | Annulus = 22mm  TAPSE = 22mm |
| Ventriculoarterial connection | Concordant | **Ventricles** |  |
| Ventricular loop | d-Loop | Left ventricle | Mildly dilated |
|  |  | Right ventricle | Normal |
| **Septae** |  | **Coronary arteries** | ----- |
| Interventricular septum | Intact | **Doppler Measurement** |  |
| Interatrial septum | Intact | Mitral | Mild MR, Holosystolic, posterior projection, seen in two planes with jet velocity = 4.5m/sec. |
| **Semilunar valves** |  | Aortic | Mild AR, PHT = 555ms |
| Aortic valve | Annulus = 18mm | Tricuspid | Trivial TR, PPG = 15mmHg. |
| Pulmonary valve | Annulus = 18mm | pulmonic | -------- |
| **Great arteries** | NRGA | **Aortic arch** | Left. No CoA. |
| Aorta | ----- | **PDA** | No |
| Pulmonary artery | Normal MPA and Branch PAs. |  |  |
| **M-Mode:** | | | |
| AO | mm | PWd | mm |
| LA | mm | PWs | mm |
| LVIDd | mm | EDV | ml |
| LVIDs | mm | ESV | ml |
| IVSs | mm | LVEF | 62% |
| IVSd | mm | FS | 33% |
| **Additional Information**: |  | | |
| No pericardial/Pleural effusion. | | | |
| **Final Diagnosis:** | | | |
| 1. {S, D, S} Levocardia. 2. Mildly dilated LA/LV 3. Thickened MVL 4. Mild MR 5. Mild AR 6. Trivial TR 7. Normal Biventricular Systolic Function | | | |
| **Remark**: | | | |
| **Recommendation**: | | | |
| SIGNATURE  Done by: Tesfaye T., Pediatrician, Pediatric Cardiologist _______________ 23/09/08/2014Eth.C | | | |

| Patient Name: **Samrawit Fantahun**. Referring Institute: **Enat Medium Clinic**. SEX/ Age: **F/4months**.  Date of Report: **23/09/08/14**. Referral Diagnosis: **Incidental Murmur Finding. AGH8.306** | | | |
| --- | --- | --- | --- |
| **Features** | **Finding** | **Features** | **Finding** |
| **Profile** |  | **Atria** |  |
| Abdominal situs | Solitus | Left atrium | Normal |
| Cardiac position | Levocardia | Right atrium | Normal |
| Systemic venous drainage | Normal. | **Atrioventricular valves** |  |
| Pulmonary venous drainage | Normal | Mitral valve | Annulus = 13mm |
| Atrioventricular connection | Concordant | Tricuspid valve | Annulus = 14mm  TAPSE = 14mm |
| Ventriculoarterial connection | Concordant | **Ventricles** |  |
| Ventricular loop | d-Loop | Left ventricle | Normal |
|  |  | Right ventricle | Normal |
| **Septae** |  | **Coronary arteries** | ----- |
| Interventricular septum | 2mm PM VSD, L – R Shunt | **Doppler Measurement** |  |
| Interatrial septum | PFO, L – R Shunt | Mitral | ----- |
| **Semilunar valves** |  | Aortic | ------- |
| Aortic valve | Annulus = 10mm | Tricuspid | ------- |
| Pulmonary valve | Annulus = 11mm | pulmonic | -------- |
| **Great arteries** | NRGA | **Aortic arch** | Left. No CoA. |
| Aorta | ----- | **PDA** | No |
| Pulmonary artery | Normal MPA and Branch PAs. |  |  |
| **M-Mode:**  Normal LV Function on eye balling | | | |
| AO | mm | PWd | mm |
| LA | mm | PWs | mm |
| LVIDd | mm | EDV | ml |
| LVIDs | mm | ESV | ml |
| IVSs | mm | LVEF | % |
| IVSd | mm | FS | % |
| **Additional Information**: |  | | |
| No pericardial/Pleural effusion. | | | |
| **Final Diagnosis:** | | | |
| 1. {S, D, S} Levocardia. 2. PFO, L – R Shunt 3. Small PM VSD, L – R Shunt 4. Normal Biventricular Systolic Function | | | |
| **Remark**: | | | |
| **Recommendation**: | | | |
| SIGNATURE  Done by: Tesfaye T., Pediatrician, Pediatric Cardiologist _______________ 23/09/08/2014Eth.C | | | |

| Patient Name: **Kidus Esuyawkal**. Referring Institute: **Adinas GH**. SEX/ Age: **M/1 3/12**. 3/12, 19/09/2013  Date of Report: **23/09/08/14**. Referral Diagnosis: **Follow up Echo for Mild PS. Incidental Murmur. AGH8.307** | | | |
| --- | --- | --- | --- |
| **Features** | **Finding** | **Features** | **Finding** |
| **Profile** |  | **Atria** |  |
| Abdominal situs | Solitus | Left atrium | Normal |
| Cardiac position | Levocardia | Right atrium | Normal |
| Systemic venous drainage | Normal. | **Atrioventricular valves** |  |
| Pulmonary venous drainage | Normal | Mitral valve | Annulus = 15mm |
| Atrioventricular connection | Concordant | Tricuspid valve | Annulus = 16mm |
| Ventriculoarterial connection | Concordant | **Ventricles** |  |
| Ventricular loop | d-Loop | Left ventricle | Normal |
|  |  | Right ventricle | Normal |
| **Septae** |  | **Coronary arteries** | ----- |
| Interventricular septum | Intact | **Doppler Measurement** |  |
| Interatrial septum | Intact | Mitral | ----- |
| **Semilunar valves** |  | Aortic | ------- |
| Aortic valve | Annulus = 14mm | Tricuspid | ------- |
| Pulmonary valve | Annulus = 15mm | pulmonic | Mild Valvular PS, PPG = 20mmHg |
| **Great arteries** | NRGA | **Aortic arch** | Left. No CoA. |
| Aorta | ----- | **PDA** | No |
| Pulmonary artery | Normal MPA and Branch PAs. |  |  |
| **M-Mode:** | | | |
| AO | mm | PWd | mm |
| LA | mm | PWs | mm |
| LVIDd | mm | EDV | ml |
| LVIDs | mm | ESV | ml |
| IVSs | mm | LVEF | 66% |
| IVSd | mm | FS | 35% |
| **Additional Information**: |  | | |
| No pericardial/Pleural effusion. | | | |
| **Final Diagnosis:** | | | |
| 1. {S, D, S} Levocardia. 2. Mild Valvular PS | | | |
| **Remark**: The Pulmonary Valve is not dysplastic (Previous Report was stating “?Dysplastic” | | | |
| **Recommendation**: | | | |
| SIGNATURE  Done by: Tesfaye T., Pediatrician, Pediatric Cardiologist _______________ 23/09/08/2014Eth.C | | | |

| Patient Name: **Rahel Amare**. Referring Institute: **FHRH**. SEX/ Age: **F/10years**.  Date of Report: **23/09/08/14**. Referral Diagnosis: **Easy fatigability + palpitation. AGH8.308** | | | |
| --- | --- | --- | --- |
| **Features** | **Finding** | **Features** | **Finding** |
| **Profile** |  | **Atria** |  |
| Abdominal situs | Solitus | Left atrium | Normal |
| Cardiac position | Levocardia | Right atrium | Normal |
| Systemic venous drainage | Normal. | **Atrioventricular valves** |  |
| Pulmonary venous drainage | Normal | Mitral valve | Annulus = 21mm |
| Atrioventricular connection | Concordant | Tricuspid valve | Annulus = 23mm |
| Ventriculoarterial connection | Concordant | **Ventricles** |  |
| Ventricular loop | d-Loop | Left ventricle | Normal |
|  |  | Right ventricle | Normal |
| **Septae** |  | **Coronary arteries** | ----- |
| Interventricular septum | Intact | **Doppler Measurement** |  |
| Interatrial septum | Intact | Mitral | ----- |
| **Semilunar valves** |  | Aortic | ------- |
| Aortic valve | Annulus = 18mm | Tricuspid | ------- |
| Pulmonary valve | Annulus = 21mm | pulmonic | -------- |
| **Great arteries** | NRGA | **Aortic arch** | Left. No CoA. |
| Aorta | ----- | **PDA** | No |
| Pulmonary artery | Normal MPA and Branch PAs. |  |  |
| **M-Mode:** | | | |
| AO | mm | PWd | mm |
| LA | mm | PWs | mm |
| LVIDd | mm | EDV | ml |
| LVIDs | mm | ESV | ml |
| IVSs | mm | LVEF | 70% |
| IVSd | mm | FS | 39% |
| **Additional Information**: |  | | |
| No pericardial/Pleural effusion. | | | |
| **Final Diagnosis:** | | | |
| 1. Normal Echocardiography Study. | | | |
| **Remark**: | | | |
| **Recommendation**: | | | |
| SIGNATURE  Done by: Tesfaye T., Pediatrician, Pediatric Cardiologist _______________ 23/09/08/2014Eth.C | | | |

| Patient Name: **Redeat Hulunm**. Referring Institute: **Nolot Speciality Clinic**. SEX/ Age: **F/1 8/12**.  Date of Report: **24/09/08/14**. Referral Diagnosis: **Incidental Murmur Finding. AGH8.309** | | | |
| --- | --- | --- | --- |
| **Features** | **Finding** | **Features** | **Finding** |
| **Profile** |  | **Atria** |  |
| Abdominal situs | Solitus | Left atrium | Mildly Dilated |
| Cardiac position | Levocardia | Right atrium | Normal |
| Systemic venous drainage | Normal. | **Atrioventricular valves** |  |
| Pulmonary venous drainage | Normal | Mitral valve | Annulus = 16mm |
| Atrioventricular connection | Concordant | Tricuspid valve | Annulus = 14mm |
| Ventriculoarterial connection | Concordant | **Ventricles** |  |
| Ventricular loop | d-Loop | Left ventricle | Mildly Dilated |
|  |  | Right ventricle | Normal |
| **Septae** |  | **Coronary arteries** | ----- |
| Interventricular septum | Intact | **Doppler Measurement** |  |
| Interatrial septum | Intact | Mitral | ----- |
| **Semilunar valves** |  | Aortic | ------- |
| Aortic valve | Annulus = 13mm | Tricuspid | ------- |
| Pulmonary valve | Annulus = 15mm | pulmonic | -------- |
| **Great arteries** | NRGA | **Aortic arch** | Left. No CoA. |
| Aorta | ----- | **PDA** | 2mm PDA, L – R Shunt |
| Pulmonary artery | Normal MPA and Branch PAs. |  |  |
| **M-Mode:** | | | |
| AO | mm | PWd | mm |
| LA | mm | PWs | mm |
| LVIDd | mm | EDV | ml |
| LVIDs | mm | ESV | ml |
| IVSs | mm | LVEF | 59% |
| IVSd | mm | FS | 30% |
| **Additional Information**: |  | | |
| No pericardial/Pleural effusion. | | | |
| **Final Diagnosis:** | | | |
| 1. {S, D, S} Levocardia. 2. LA/LV Mildly Dilated 3. Small PDA, L – R Shunt 4. Normal LV Systolic Function | | | |
| **Remark**: | | | |
| **Recommendation**: | | | |
| SIGNATURE  Done by: Tesfaye T., Pediatrician, Pediatric Cardiologist _______________ 24/09/08/2014Eth.C | | | |

| Patient Name: **Tesfahun Habtamu**. Referring Institute: **TGSH**. SEX/ Age: **M/4 10/12**.  Date of Report: **24/09/08/14**. Referral Diagnosis: **Pre op screening. AGH8.310** | | | |
| --- | --- | --- | --- |
| **Features** | **Finding** | **Features** | **Finding** |
| **Profile** |  | **Atria** |  |
| Abdominal situs | Solitus | Left atrium | Normal |
| Cardiac position | Levocardia | Right atrium | Normal |
| Systemic venous drainage | Normal. | **Atrioventricular valves** |  |
| Pulmonary venous drainage | Normal | Mitral valve | Annulus = 19mm |
| Atrioventricular connection | Concordant | Tricuspid valve | Annulus = 20mm  TAPSE = 22mm |
| Ventriculoarterial connection | Concordant | **Ventricles** |  |
| Ventricular loop | d-Loop | Left ventricle | Normal |
|  |  | Right ventricle | Normal |
| **Septae** |  | **Coronary arteries** | ----- |
| Interventricular septum | Intact | **Doppler Measurement** |  |
| Interatrial septum | Intact | Mitral | ----- |
| **Semilunar valves** |  | Aortic | ------- |
| Aortic valve | Annulus = 16mm | Tricuspid | ------- |
| Pulmonary valve | Annulus = 18mm | pulmonic | -------- |
| **Great arteries** | NRGA | **Aortic arch** | Left. No CoA. |
| Aorta | ----- | **PDA** | No |
| Pulmonary artery | Normal MPA and Branch PAs. |  |  |
| **M-Mode:**  Normal LV Function on eye balling | | | |
| AO | mm | PWd | mm |
| LA | mm | PWs | mm |
| LVIDd | mm | EDV | ml |
| LVIDs | mm | ESV | ml |
| IVSs | mm | LVEF | % |
| IVSd | mm | FS | % |
| **Additional Information**: |  | | |
| No pericardial/Pleural effusion. | | | |
| **Final Diagnosis:** | | | |
| 1. Normal Echocardiography Study. | | | |
| **Remark**: | | | |
| **Recommendation**: | | | |
| SIGNATURE  Done by: Tesfaye T., Pediatrician, Pediatric Cardiologist _______________ 24/09/08/2014Eth.C | | | |

| Patient Name: **Baby of Tigist Getnet**. Referring Institute: **FHRH**. SEX/ Age: **M/5days**.  Date of Report: **24/09/08/14**. Referral Diagnosis: **DS. AGH8.311** | | | |
| --- | --- | --- | --- |
| **Features** | **Finding** | **Features** | **Finding** |
| **Profile** |  | **Atria** |  |
| Abdominal situs | Solitus | Left atrium | Normal |
| Cardiac position | Levocardia | Right atrium | Normal |
| Systemic venous drainage | Normal. | **Atrioventricular valves** |  |
| Pulmonary venous drainage | Normal | Mitral valve | Annulus = 10mm |
| Atrioventricular connection | Concordant | Tricuspid valve | Annulus = 11mm |
| Ventriculoarterial connection | Concordant | **Ventricles** |  |
| Ventricular loop | d-Loop | Left ventricle | Normal |
|  |  | Right ventricle | Normal |
| **Septae** |  | **Coronary arteries** | ----- |
| Interventricular septum | Intact | **Doppler Measurement** |  |
| Interatrial septum | 4mm OS ASD, L – R Shunt | Mitral | ----- |
| **Semilunar valves** |  | Aortic | ------- |
| Aortic valve | Annulus = 9mm | Tricuspid | ------- |
| Pulmonary valve | Annulus = 9mm | pulmonic | -------- |
| **Great arteries** | NRGA | **Aortic arch** | Left. No CoA. |
| Aorta | ----- | **PDA** | No |
| Pulmonary artery | Normal MPA and Branch PAs. |  |  |
| **M-Mode:**  Normal LV Systolic function on eye balling | | | |
| AO | mm | PWd | mm |
| LA | mm | PWs | mm |
| LVIDd | mm | EDV | ml |
| LVIDs | mm | ESV | ml |
| IVSs | mm | LVEF | % |
| IVSd | mm | FS | % |
| **Additional Information**: |  | | |
| No pericardial/Pleural effusion. | | | |
| **Final Diagnosis:** | | | |
| 1. {S, D, S} Levocardia. 2. Small OS ASD, L – R Shunt 3. Normal LV Systolic Function | | | |
| **Remark**: | | | |
| **Recommendation**: | | | |
| SIGNATURE  Done by: Tesfaye T., Pediatrician, Pediatric Cardiologist _______________ 24/09/08/2014Eth.C | | | |

| Patient Name: **Yohannes Gochye**. Referring Institute: **TGSH**. SEX/ Age: **M/12years**.  Date of Report: **24/09/08/14**. Referral Diagnosis: **_Sydenham’s Chorea. AGH8.312** | | | |
| --- | --- | --- | --- |
| **Features** | **Finding** | **Features** | **Finding** |
| **Profile** |  | **Atria** |  |
| Abdominal situs | Solitus | Left atrium | Normal |
| Cardiac position | Levocardia | Right atrium | Normal |
| Systemic venous drainage | Normal. | **Atrioventricular valves** |  |
| Pulmonary venous drainage | Normal | Mitral valve | Annulus = 21mm |
| Atrioventricular connection | Concordant | Tricuspid valve | Annulus = 21mm  TAPSE = 23mm |
| Ventriculoarterial connection | Concordant | **Ventricles** |  |
| Ventricular loop | d-Loop | Left ventricle | Normal |
|  |  | Right ventricle | Normal |
| **Septae** |  | **Coronary arteries** | ----- |
| Interventricular septum | Intact | **Doppler Measurement** |  |
| Interatrial septum | Intact | Mitral | ----- |
| **Semilunar valves** |  | Aortic | ------- |
| Aortic valve | Annulus = 16mm | Tricuspid | ------- |
| Pulmonary valve | Annulus = mm | pulmonic | -------- |
| **Great arteries** | NRGA | **Aortic arch** | Left. No CoA. |
| Aorta | ----- | **PDA** | No |
| Pulmonary artery | Normal MPA and Branch PAs. |  |  |
| **M-Mode:** | | | |
| AO | mm | PWd | mm |
| LA | mm | PWs | mm |
| LVIDd | mm | EDV | ml |
| LVIDs | mm | ESV | ml |
| IVSs | mm | LVEF | 68% |
| IVSd | mm | FS | 38% |
| **Additional Information**: |  | | |
| No pericardial/Pleural effusion. | | | |
| **Final Diagnosis:** | | | |
| 1. Normal Echocardiography Study. | | | |
| **Remark**: | | | |
| **Recommendation**: | | | |
| SIGNATURE  Done by: Tesfaye T., Pediatrician, Pediatric Cardiologist _______________ 24/09/08/2014Eth.C | | | |

| Patient Name: **Bereket Minyichil**. Referring Institute: **FHRH**. SEX/ Age: **M/8years**.  Date of Report: **25/09/08/14**. Referral Diagnosis: **Palpitation. AGH8.313** | | | |
| --- | --- | --- | --- |
| **Features** | **Finding** | **Features** | **Finding** |
| **Profile** |  | **Atria** |  |
| Abdominal situs | Solitus | Left atrium | Normal |
| Cardiac position | Levocardia | Right atrium | Normal |
| Systemic venous drainage | Normal. | **Atrioventricular valves** |  |
| Pulmonary venous drainage | Normal | Mitral valve | Annulus = 18mm |
| Atrioventricular connection | Concordant | Tricuspid valve | Annulus = 20mm  TAPSE = mm |
| Ventriculoarterial connection | Concordant | **Ventricles** |  |
| Ventricular loop | d-Loop | Left ventricle | Normal |
|  |  | Right ventricle | Normal |
| **Septae** |  | **Coronary arteries** | ----- |
| Interventricular septum | Intact | **Doppler Measurement** |  |
| Interatrial septum | Intact | Mitral | ----- |
| **Semilunar valves** |  | Aortic | ------- |
| Aortic valve | Annulus = 16mm | Tricuspid | ------- |
| Pulmonary valve | Annulus = 16mm | pulmonic | -------- |
| **Great arteries** | NRGA | **Aortic arch** | Left. No CoA. |
| Aorta | ----- | **PDA** | No |
| Pulmonary artery | Normal MPA and Branch PAs. |  |  |
| **M-Mode:** | | | |
| AO | mm | PWd | mm |
| LA | mm | PWs | mm |
| LVIDd | mm | EDV | ml |
| LVIDs | mm | ESV | ml |
| IVSs | mm | LVEF | 63% |
| IVSd | mm | FS | 33% |
| **Additional Information**: |  | | |
| No pericardial/Pleural effusion. | | | |
| **Final Diagnosis:** | | | |
| 1. Normal Echocardiography Study. | | | |
| **Remark**: | | | |
| **Recommendation**: | | | |
| SIGNATURE  Done by: Tesfaye T., Pediatrician, Pediatric Cardiologist _______________ 25/09/08/2014Eth.C | | | |

| Patient Name: **Desalegn Tadlo**. Referring Institute: **Addis Alem PH**. SEX/ Age: **M/1year**.  Date of Report: **25/09/08/14**. Referral Diagnosis: **RD + CHF. AGH8.314** | | | |
| --- | --- | --- | --- |
| **Features** | **Finding** | **Features** | **Finding** |
| **Profile** |  | **Atria** |  |
| Abdominal situs | Solitus | Left atrium | Dilated |
| Cardiac position | Levocardia | Right atrium | Normal |
| Systemic venous drainage | Normal. | **Atrioventricular valves** |  |
| Pulmonary venous drainage | Normal | Mitral valve | Annulus = 17mm |
| Atrioventricular connection | Concordant | Tricuspid valve | Annulus = 14mm |
| Ventriculoarterial connection | Concordant | **Ventricles** |  |
| Ventricular loop | d-Loop | Left ventricle | Dilated |
|  |  | Right ventricle | Normal |
| **Septae** |  | **Coronary arteries** | ----- |
| Interventricular septum | Intact | **Doppler Measurement** |  |
| Interatrial septum | Intact | Mitral | Mild MR |
| **Semilunar valves** |  | Aortic | ------- |
| Aortic valve | Annulus = 12mm | Tricuspid | ------- |
| Pulmonary valve | Annulus = 13mm | pulmonic | -------- |
| **Great arteries** | NRGA | **Aortic arch** | Left. No CoA. |
| Aorta | ----- | **PDA** | 3mm PDA, L – R Shunt |
| Pulmonary artery | MPA = 15mm. Confluent Branch PAs. |  |  |
| **M-Mode:**  Normal LV Function on eye balling | | | |
| AO | mm | PWd | mm |
| LA | mm | PWs | mm |
| LVIDd | mm | EDV | ml |
| LVIDs | mm | ESV | ml |
| IVSs | mm | LVEF | % |
| IVSd | mm | FS | % |
| **Additional Information**: |  | | |
| No pericardial/Pleural effusion. | | | |
| **Final Diagnosis:** | | | |
| 1. {S, D, S} Levocardia. 2. LA/LV Dilated 3. Mild MR 4. Mod. Pulmonary Hypertension 5. Moderate PDA, L – R Shunt 6. Normal LV Systolic Function | | | |
| **Remark**: | | | |
| **Recommendation**: | | | |
| SIGNATURE  Done by: Tesfaye T., Pediatrician, Pediatric Cardiologist _______________ 25/09/08/2014Eth.C | | | |

| Patient Name: **Mirtzer Belete**. Referring Institute: **Nolot Specility Clinic**. SEX/ Age: **F/10years**. Date of Report: **25/09/08/14**. Referral Diagnosis: **CHF. AGH8.315** | | | |
| --- | --- | --- | --- |
| **Features** | **Finding** | **Features** | **Finding** |
| **Profile** |  | **Atria** |  |
| Abdominal situs | Solitus | Left atrium | Dilated |
| Cardiac position | Levocardia | Right atrium | Normal |
| Systemic venous drainage | Normal. | **Atrioventricular valves** |  |
| Pulmonary venous drainage | Normal | Mitral valve | Annulus = 24mm. Thickened MVL. |
| Atrioventricular connection | Concordant | Tricuspid valve | Annulus = 23mm  TAPSE = 18mm |
| Ventriculoarterial connection | Concordant | **Ventricles** |  |
| Ventricular loop | d-Loop | Left ventricle | Dilated |
|  |  | Right ventricle | Normal |
| **Septae** |  | **Coronary arteries** | ----- |
| Interventricular septum | Intact | **Doppler Measurement** |  |
| Interatrial septum | Intact | Mitral | Moderate MR, Holosystolic with posterior projection, seen in two planes with jet velocity = 4.5m/sec. No Mitral inflow velocity variation with respiration. |
| **Semilunar valves** |  | Aortic | ------- |
| Aortic valve | Annulus = 16mm. No features of Pulsus paradoxus on echo. | Tricuspid | ------- |
| Pulmonary valve | Annulus = 17mm | pulmonic | -------- |
| **Great arteries** | NRGA | **Aortic arch** | Left. No CoA. |
| Aorta | ----- | **PDA** | No |
| Pulmonary artery | Normal MPA and Branch PAs. |  |  |
| **M-Mode:** | | | |
| AO | mm | PWd | mm |
| LA | mm | PWs | mm |
| LVIDd | mm | EDV | ml |
| LVIDs | mm | ESV | ml |
| IVSs | mm | LVEF | 60% |
| IVSd | mm | FS | 32% |
| **Additional Information**: |  | | |
| Circumferential Pericardial effusion with depth of 20mm on RA/RV Side and 15mm on LV Side. Echo-debris inside the effusion. Thickened epicardium. | | | |
| **Final Diagnosis:** | | | |
| 1. {S, D, S} Levocardia. 2. LA/LV Dilated 3. Thickened MVL 4. Moderate MR 5. Large Circumferential Pericardial effusion with echodebris inside and thickened Epicardium 6. Normal Biventricular Systolic Function | | | |
| **Recommendation**: | | | |
| SIGNATURE  Done by: Tesfaye T., Pediatrician, Pediatric Cardiologist _______________ 25/09/08/2014Eth.C | | | |

| Patient Name: **Tinsae Desalegn**. Referring Institute: **_______**. SEX/ Age: **____/___years**. **INCOMPLETE DATA**  Date of Report: **25/09/08/14**. Referral Diagnosis: **_______.** | | | |
| --- | --- | --- | --- |
| **Features** | **Finding** | **Features** | **Finding** |
| **Profile** |  | **Atria** |  |
| Abdominal situs | Solitus | Left atrium | Normal |
| Cardiac position | Levocardia | Right atrium | Normal |
| Systemic venous drainage | Normal. | **Atrioventricular valves** |  |
| Pulmonary venous drainage | Normal | Mitral valve | Annulus = 21mm |
| Atrioventricular connection | Concordant | Tricuspid valve | Annulus = 22mm  TAPSE = 23mm |
| Ventriculoarterial connection | Concordant | **Ventricles** |  |
| Ventricular loop | d-Loop | Left ventricle | Normal |
|  |  | Right ventricle | Normal |
| **Septae** |  | **Coronary arteries** | ----- |
| Interventricular septum | Intact | **Doppler Measurement** |  |
| Interatrial septum | Intact | Mitral | ----- |
| **Semilunar valves** |  | Aortic | ------- |
| Aortic valve | Annulus = 16mm | Tricuspid | ------- |
| Pulmonary valve | Annulus = 20mm | pulmonic | -------- |
| **Great arteries** | NRGA | **Aortic arch** | Left. No CoA. |
| Aorta | ----- | **PDA** | No |
| Pulmonary artery | Normal MPA and Branch PAs. |  |  |
| **M-Mode:** | | | |
| AO | mm | PWd | mm |
| LA | mm | PWs | mm |
| LVIDd | mm | EDV | ml |
| LVIDs | mm | ESV | ml |
| IVSs | mm | LVEF | 64% |
| IVSd | mm | FS | 34% |
| **Additional Information**: |  | | |
| No pericardial/Pleural effusion. | | | |
| **Final Diagnosis:** | | | |
| 1. Normal Echocardiography Study. | | | |
| **Remark**: | | | |
| **Recommendation**: | | | |
| SIGNATURE  Done by: Tesfaye T., Pediatrician, Pediatric Cardiologist _______________ 25/09/08/2014Eth.C | | | |

| Patient Name: **Kalkidan Melak**. Referring Institute: **TGSH**. SEX/ Age: **F/12years**.  Date of Report: **25/09/08/14**. Referral Diagnosis: **Palpitation.**  **AGH8.316** | | | |
| --- | --- | --- | --- |
| **Features** | **Finding** | **Features** | **Finding** |
| **Profile** |  | **Atria** |  |
| Abdominal situs | Solitus | Left atrium | Normal |
| Cardiac position | Levocardia | Right atrium | Normal |
| Systemic venous drainage | Normal. | **Atrioventricular valves** |  |
| Pulmonary venous drainage | Normal | Mitral valve | Annulus = 18mm |
| Atrioventricular connection | Concordant | Tricuspid valve | Annulus = 18mm  TAPSE = 19mm |
| Ventriculoarterial connection | Concordant | **Ventricles** |  |
| Ventricular loop | d-Loop | Left ventricle | Normal |
|  |  | Right ventricle | Normal |
| **Septae** |  | **Coronary arteries** | ----- |
| Interventricular septum | Intact | **Doppler Measurement** |  |
| Interatrial septum | Intact | Mitral | ----- |
| **Semilunar valves** |  | Aortic | ------- |
| Aortic valve | Annulus = 17mm | Tricuspid | ------- |
| Pulmonary valve | Annulus = 18mm | pulmonic | -------- |
| **Great arteries** | NRGA | **Aortic arch** | Left. No CoA. |
| Aorta | ----- | **PDA** | No |
| Pulmonary artery | Normal MPA and Branch PAs. |  |  |
| **M-Mode:** | | | |
| AO | mm | PWd | 7.5mm |
| LA | mm | PWs | 10mm |
| LVIDd | 38mm | EDV | 62ml |
| LVIDs | 25mm | ESV | 23ml |
| IVSs | 7.5mm | LVEF | 64% |
| IVSd | 7.5mm | FS | 34% |
| **Additional Information**: |  | | |
| No pericardial/Pleural effusion. | | | |
| **Final Diagnosis:** | | | |
| 1. Normal Echocardiography Study. | | | |
| **Remark**: | | | |
| **Recommendation**: | | | |
| SIGNATURE  Done by: Tesfaye T., Pediatrician, Pediatric Cardiologist _______________ 25/09/08/2014Eth.C | | | |

| Patient Name: **Akiya Wendye**. Referring Institute: **FHRH**. SEX/ Age: **M/10months**.  Date of Report: **26/09/08/14**. Referral Diagnosis: **Recurrent Chest Infection. AGH8.317** | | | |
| --- | --- | --- | --- |
| **Features** | **Finding** | **Features** | **Finding** |
| **Profile** |  | **Atria** |  |
| Abdominal situs | Solitus | Left atrium | Normal |
| Cardiac position | Levocardia | Right atrium | Normal |
| Systemic venous drainage | Normal. | **Atrioventricular valves** |  |
| Pulmonary venous drainage | Normal | Mitral valve | Annulus = 11mm |
| Atrioventricular connection | Concordant | Tricuspid valve | Annulus = 12mm |
| Ventriculoarterial connection | Concordant | **Ventricles** |  |
| Ventricular loop | d-Loop | Left ventricle | Normal |
|  |  | Right ventricle | Normal |
| **Septae** |  | **Coronary arteries** | ----- |
| Interventricular septum | Intact | **Doppler Measurement** |  |
| Interatrial septum | Intact | Mitral | ----- |
| **Semilunar valves** |  | Aortic | ------- |
| Aortic valve | Annulus = 10mm | Tricuspid | ------- |
| Pulmonary valve | Annulus = 12mm | pulmonic | -------- |
| **Great arteries** | NRGA | **Aortic arch** | Left. No CoA. |
| Aorta | ----- | **PDA** | No |
| Pulmonary artery | Normal MPA and Branch PAs. |  |  |
| **M-Mode:**  Normal LV Function on eye balling | | | |
| AO | mm | PWd | mm |
| LA | mm | PWs | mm |
| LVIDd | mm | EDV | ml |
| LVIDs | mm | ESV | ml |
| IVSs | mm | LVEF | % |
| IVSd | mm | FS | % |
| **Additional Information**: |  | | |
| No pericardial/Pleural effusion. | | | |
| **Final Diagnosis:** | | | |
| 1. Normal Echocardiography Study. | | | |
| **Remark**: Baby was crying during study | | | |
| **Recommendation**: | | | |
| SIGNATURE  Done by: Tesfaye T., Pediatrician, Pediatric Cardiologist _______________ 26/09/08/2014Eth.C | | | |

| Patient Name: **Alemnat Kasye**. Referring Institute: **Addis-Alem PH**. SEX/ Age: **F/12years**.  Date of Report: **26/09/08/14**. Referral Diagnosis: **ARF. AGH8.318** | | | |
| --- | --- | --- | --- |
| **Features** | **Finding** | **Features** | **Finding** |
| **Profile** |  | **Atria** |  |
| Abdominal situs | Solitus | Left atrium | Normal |
| Cardiac position | Levocardia | Right atrium | Normal |
| Systemic venous drainage | Normal. | **Atrioventricular valves** |  |
| Pulmonary venous drainage | Normal | Mitral valve | Annulus = 26mm |
| Atrioventricular connection | Concordant | Tricuspid valve | Annulus = 27mm  TAPSE = 27mm |
| Ventriculoarterial connection | Concordant | **Ventricles** |  |
| Ventricular loop | d-Loop | Left ventricle | Normal |
|  |  | Right ventricle | Normal |
| **Septae** |  | **Coronary arteries** | ----- |
| Interventricular septum | Intact | **Doppler Measurement** |  |
| Interatrial septum | Intact | Mitral | ----- |
| **Semilunar valves** |  | Aortic | ------- |
| Aortic valve | Annulus = 18mm | Tricuspid | Trivial TR, PPG = 28mmHg |
| Pulmonary valve | Annulus = 23mm | pulmonic | Trivial PR, PPG = 21mmHg |
| **Great arteries** | NRGA | **Aortic arch** | Left. No CoA. |
| Aorta | ----- | **PDA** | No |
| Pulmonary artery | Normal MPA and Branch PAs. |  |  |
| **M-Mode:** | | | |
| AO | mm | PWd | mm |
| LA | mm | PWs | mm |
| LVIDd | mm | EDV | ml |
| LVIDs | mm | ESV | ml |
| IVSs | mm | LVEF | 60% |
| IVSd | mm | FS | 32% |
| **Additional Information**: |  | | |
| No pericardial/Pleural effusion. | | | |
| **Final Diagnosis:** | | | |
| 1. Normal Echocardiography Study. | | | |
| **Remark**: | | | |
| **Recommendation**: | | | |
| SIGNATURE  Done by: Tesfaye T., Pediatrician, Pediatric Cardiologist _______________ 26/09/08/2014Eth.C | | | |

| Patient Name: **Selam Abera**. Referring Institute: **FHRH**. SEX/ Age: **F/10 11/12**.  Date of Report: **26/09/08/14**. Referral Diagnosis: **RD. AGH8.319** | | | |
| --- | --- | --- | --- |
| **Features** | **Finding** | **Features** | **Finding** |
| **Profile** |  | **Atria** |  |
| Abdominal situs | Solitus | Left atrium | Normal |
| Cardiac position | Levocardia | Right atrium | Normal |
| Systemic venous drainage | Normal. | **Atrioventricular valves** |  |
| Pulmonary venous drainage | Normal | Mitral valve | Annulus = 20mm |
| Atrioventricular connection | Concordant | Tricuspid valve | Annulus = 21mm  TAPSE = 23mm |
| Ventriculoarterial connection | Concordant | **Ventricles** |  |
| Ventricular loop | d-Loop | Left ventricle | Normal |
|  |  | Right ventricle | Normal |
| **Septae** |  | **Coronary arteries** | ----- |
| Interventricular septum | Intact | **Doppler Measurement** |  |
| Interatrial septum | Intact | Mitral | ----- |
| **Semilunar valves** |  | Aortic | ------- |
| Aortic valve | Annulus = 16mm | Tricuspid | ------- |
| Pulmonary valve | Annulus = 19mm | pulmonic | Trivial PR, PPG = 10mmHg |
| **Great arteries** | NRGA | **Aortic arch** | Left. No CoA. |
| Aorta | ----- | **PDA** | No |
| Pulmonary artery | Normal MPA and Branch PAs. |  |  |
| **M-Mode:** | | | |
| AO | mm | PWd | mm |
| LA | mm | PWs | mm |
| LVIDd | mm | EDV | ml |
| LVIDs | mm | ESV | ml |
| IVSs | mm | LVEF | 61% |
| IVSd | mm | FS | 32% |
| **Additional Information**: |  | | |
| No pericardial/Pleural effusion. | | | |
| **Final Diagnosis:** | | | |
| 1. Normal Echocardiography Study3. | | | |
| **Remark**: | | | |
| **Recommendation**: | | | |
| SIGNATURE  Done by: Tesfaye T., Pediatrician, Pediatric Cardiologist _______________ 26/09/08/2014Eth.C | | | |

| Patient Name: **Bereket Addisu**. Referring Institute: **Adinas GH**. SEX/ Age: **M/10 1/12**.  Date of Report: **27/09/08/14**. Referral Diagnosis: **Rheumatic recurrence. AGH8.320** | | | |
| --- | --- | --- | --- |
| **Features** | **Finding** | **Features** | **Finding** |
| **Profile** |  | **Atria** |  |
| Abdominal situs | Solitus | Left atrium | Normal |
| Cardiac position | Levocardia | Right atrium | Normal |
| Systemic venous drainage | Normal. | **Atrioventricular valves** |  |
| Pulmonary venous drainage | Normal | Mitral valve | Annulus = 24mm. Tickened, patulous MVL |
| Atrioventricular connection | Concordant | Tricuspid valve | Annulus = 26mm  TAPSE = 21mm |
| Ventriculoarterial connection | Concordant | **Ventricles** |  |
| Ventricular loop | d-Loop | Left ventricle | Normal |
|  |  | Right ventricle | Normal |
| **Septae** |  | **Coronary arteries** | ----- |
| Interventricular septum | Intact | **Doppler Measurement** |  |
| Interatrial septum | Intact | Mitral | Mild MR, Hlosystolic, posterior projection, seen in two planes with jet velocity = 4m/sec. |
| **Semilunar valves** |  | Aortic | ------- |
| Aortic valve | Annulus = 20mm | Tricuspid | ------- |
| Pulmonary valve | Annulus = 23mm | pulmonic | -------- |
| **Great arteries** | NRGA | **Aortic arch** | Left. No CoA. |
| Aorta | ----- | **PDA** | No |
| Pulmonary artery | Normal MPA and Branch PAs. |  |  |
| **M-Mode:** | | | |
| AO | mm | PWd | mm |
| LA | mm | PWs | mm |
| LVIDd | mm | EDV | ml |
| LVIDs | mm | ESV | ml |
| IVSs | mm | LVEF | 60% |
| IVSd | mm | FS | 32% |
| **Additional Information**: |  | | |
| No pericardial/Pleural effusion. | | | |
| **Final Diagnosis:** | | | |
| 1. {S, D, S} Levocardia. 2. Thickened, patulous MVL 3. Mild MR 4. Normal Biventricular Systolic Function | | | |
| **Remark**: | | | |
| **Recommendation**: Continue Secondary Prophylaxis | | | |
| SIGNATURE  Done by: Tesfaye T., Pediatrician, Pediatric Cardiologist _______________ 27/09/08/2014Eth.C | | | |

| Patient Name: **Tadfalech Shumet**. Referring Institute: **FHRH**. SEX/ Age: **F/11months**.  Date of Report: **29/09/08/14**. Referral Diagnosis: **Incidental Murmur Finding. AGH8.321** | | | |
| --- | --- | --- | --- |
| **Features** | **Finding** | **Features** | **Finding** |
| **Profile** |  | **Atria** |  |
| Abdominal situs | Solitus | Left atrium | Normal |
| Cardiac position | Levocardia | Right atrium | Normal |
| Systemic venous drainage | Normal. | **Atrioventricular valves** |  |
| Pulmonary venous drainage | Normal | Mitral valve | Annulus = 13mm |
| Atrioventricular connection | Concordant | Tricuspid valve | Annulus = 14mm |
| Ventriculoarterial connection | Concordant | **Ventricles** |  |
| Ventricular loop | d-Loop | Left ventricle | Normal |
|  |  | Right ventricle | Normal |
| **Septae** |  | **Coronary arteries** | ----- |
| Interventricular septum | 2mm PM VSD Closed by STL. NO Shunt | **Doppler Measurement** |  |
| Interatrial septum | PFO, L – R Shunt | Mitral | ----- |
| **Semilunar valves** |  | Aortic | ------- |
| Aortic valve | Annulus = 10mm | Tricuspid | ------- |
| Pulmonary valve | Annulus = 11mm. Doming PV. | pulmonic | Flow acceleration across the PV with PPG = 15mmHg. Trivial PR, PPG = 15mmHg. |
| **Great arteries** | NRGA | **Aortic arch** | Left. No CoA. |
| Aorta | ----- | **PDA** | No |
| Pulmonary artery | Normal MPA and Branch PAs. |  |  |
| **M-Mode:**  Normal LV Function on eye balling | | | |
| AO | mm | PWd | mm |
| LA | mm | PWs | mm |
| LVIDd | mm | EDV | ml |
| LVIDs | mm | ESV | ml |
| IVSs | mm | LVEF | % |
| IVSd | mm | FS | % |
| **Additional Information**: |  | | |
| Pericardial effusion with maximum depth of 7mm on RA/RV Junction. | | | |
| **Final Diagnosis:** | | | |
| 1. {S, D, S} Levocardia. 2. PFO, L – R Shunt 3. Small PM VSD, Closed by STL 4. Doming PV 5. Flow acceleration across the PV with no significant gradient 6. Normal LV Function | | | |
| **Remark**: | | | |
| **Recommendation**: | | | |
| SIGNATURE  Done by: Tesfaye T., Pediatrician, Pediatric Cardiologist _______________ 29/09/08/2014Eth.C | | | |

| Patient Name: **Haset Yenesew**. Referring Institute: **FHRH**. SEX/ Age: **F/10months**.  Date of Report: **29/09/08/14**. Referral Diagnosis: **DS. AGH8.322** | | | |
| --- | --- | --- | --- |
| **Features** | **Finding** | **Features** | **Finding** |
| **Profile** |  | **Atria** |  |
| Abdominal situs | Solitus | Left atrium | Normal |
| Cardiac position | Levocardia | Right atrium | Normal |
| Systemic venous drainage | Normal. | **Atrioventricular valves** |  |
| Pulmonary venous drainage | Normal | Mitral valve | Annulus = 11mm |
| Atrioventricular connection | Concordant | Tricuspid valve | Annulus = 14mm |
| Ventriculoarterial connection | Concordant | **Ventricles** |  |
| Ventricular loop | d-Loop | Left ventricle | Normal |
|  |  | Right ventricle | Normal |
| **Septae** |  | **Coronary arteries** | ----- |
| Interventricular septum | Intact | **Doppler Measurement** |  |
| Interatrial septum | Intact | Mitral | ----- |
| **Semilunar valves** |  | Aortic | ------- |
| Aortic valve | Annulus = 11mm | Tricuspid | ------- |
| Pulmonary valve | Annulus = 13mm | pulmonic | -------- |
| **Great arteries** | NRGA | **Aortic arch** | Left. No CoA. |
| Aorta | ----- | **PDA** | <1mm PDA |
| Pulmonary artery | Normal MPA and Branch PAs. |  |  |
| **M-Mode:**  Normal LV Function (eye balling) | | | |
| AO | mm | PWd | mm |
| LA | mm | PWs | mm |
| LVIDd | mm | EDV | ml |
| LVIDs | mm | ESV | ml |
| IVSs | mm | LVEF | % |
| IVSd | mm | FS | % |
| **Additional Information**: |  | | |
| No pericardial/Pleural effusion. | | | |
| **Final Diagnosis:** | | | |
| 1. {S, D, S} Levocardia. 2. Silent PDA, L – R Shunt | | | |
| **Remark**: | | | |
| **Recommendation**: | | | |
| SIGNATURE  Done by: Tesfaye T., Pediatrician, Pediatric Cardiologist _______________ 29/09/08/2014Eth.C | | | |

| Patient Name: **Eldana Mekuriaw**. Referring Institute: **FHRH**. SEX/ Age: **F/7years**.  Date of Report: **29/09/08/14**. Referral Diagnosis: **Easy fatigability. AGH8.323** | | | |
| --- | --- | --- | --- |
| **Features** | **Finding** | **Features** | **Finding** |
| **Profile** |  | **Atria** |  |
| Abdominal situs | Solitus | Left atrium | Normal |
| Cardiac position | Levocardia | Right atrium | Normal |
| Systemic venous drainage | Normal. | **Atrioventricular valves** |  |
| Pulmonary venous drainage | Normal | Mitral valve | Annulus = 18mm |
| Atrioventricular connection | Concordant | Tricuspid valve | Annulus = 18mm  TAPSE = 21mm |
| Ventriculoarterial connection | Concordant | **Ventricles** |  |
| Ventricular loop | d-Loop | Left ventricle | Normal |
|  |  | Right ventricle | Normal |
| **Septae** |  | **Coronary arteries** | ----- |
| Interventricular septum | Intact | **Doppler Measurement** |  |
| Interatrial septum | Intact | Mitral | ----- |
| **Semilunar valves** |  | Aortic | ------- |
| Aortic valve | Annulus = 15mm | Tricuspid | ------- |
| Pulmonary valve | Annulus = 15mm | pulmonic | -------- |
| **Great arteries** | NRGA | **Aortic arch** | Left. No CoA. |
| Aorta | ----- | **PDA** | No |
| Pulmonary artery | Normal MPA and Branch PAs. |  |  |
| **M-Mode:** | | | |
| AO | mm | PWd | mm |
| LA | mm | PWs | mm |
| LVIDd | mm | EDV | ml |
| LVIDs | mm | ESV | ml |
| IVSs | mm | LVEF | 67% |
| IVSd | mm | FS | 36% |
| **Additional Information**: |  | | |
| No pericardial/Pleural effusion. | | | |
| **Final Diagnosis:** | | | |
| 1. Normal Echocardiography Study. | | | |
| **Remark**: | | | |
| **Recommendation**: | | | |
| SIGNATURE  Done by: Tesfaye T., Pediatrician, Pediatric Cardiologist _______________ 29/09/08/2014Eth.C | | | |

| Patient Name: **Selamawit Addis**. Referring Institute: **Adinas GH**. SEX/ Age: **F/9 4/12**.  Date of Report: **29/09/08/14**. Referral Diagnosis: **Easy fatigability. AGH8.324** | | | |
| --- | --- | --- | --- |
| **Features** | **Finding** | **Features** | **Finding** |
| **Profile** |  | **Atria** |  |
| Abdominal situs | Solitus | Left atrium | Normal |
| Cardiac position | Levocardia | Right atrium | Normal |
| Systemic venous drainage | Normal. | **Atrioventricular valves** |  |
| Pulmonary venous drainage | Normal | Mitral valve | Annulus = 18mm |
| Atrioventricular connection | Concordant | Tricuspid valve | Annulus = 20mm  TAPSE = 20mm |
| Ventriculoarterial connection | Concordant | **Ventricles** |  |
| Ventricular loop | d-Loop | Left ventricle | Normal |
|  |  | Right ventricle | Normal |
| **Septae** |  | **Coronary arteries** | ----- |
| Interventricular septum | Intact | **Doppler Measurement** |  |
| Interatrial septum | Intact | Mitral | ----- |
| **Semilunar valves** |  | Aortic | ------- |
| Aortic valve | Annulus = 16mm | Tricuspid | ------- |
| Pulmonary valve | Annulus = 16mm | pulmonic | -------- |
| **Great arteries** | NRGA | **Aortic arch** | Left. No CoA. |
| Aorta | ----- | **PDA** | No |
| Pulmonary artery | Normal MPA and Branch PAs. |  |  |
| **M-Mode:** | | | |
| AO | mm | PWd | mm |
| LA | mm | PWs | mm |
| LVIDd | mm | EDV | ml |
| LVIDs | mm | ESV | ml |
| IVSs | mm | LVEF | 65% |
| IVSd | mm | FS | 34% |
| **Additional Information**: |  | | |
| No pericardial/Pleural effusion. | | | |
| **Final Diagnosis:** | | | |
| 1. Normal Echocardiography Study. | | | |
| **Remark**: | | | |
| **Recommendation**: | | | |
| SIGNATURE  Done by: Tesfaye T., Pediatrician, Pediatric Cardiologist _______________ 29/09/08/2014Eth.C | | | |

| Patient Name: **Metadel Getnet**. Referring Institute: **Adinas GH**. SEX/ Age: **F/6months**.  Date of Report: **30/09/08/14**. Referral Diagnosis: **RD + Murmur. AGH8.325** | | | |
| --- | --- | --- | --- |
| **Features** | **Finding** | **Features** | **Finding** |
| **Profile** |  | **Atria** |  |
| Abdominal situs | Solitus | Left atrium | Dilated |
| Cardiac position | Levocardia | Right atrium | Normal |
| Systemic venous drainage | Normal. | **Atrioventricular valves** |  |
| Pulmonary venous drainage | Normal | Mitral valve | Annulus = 15mm |
| Atrioventricular connection | Concordant | Tricuspid valve | Annulus = 13mm |
| Ventriculoarterial connection | Concordant | **Ventricles** |  |
| Ventricular loop | d-Loop | Left ventricle | Dilated |
|  |  | Right ventricle | Normal |
| **Septae** |  | **Coronary arteries** | ----- |
| Interventricular septum | Intact | **Doppler Measurement** |  |
| Interatrial septum | Intact | Mitral | Mild MR, Holosystolic, posterior projection, seen in two planes with jet velocity = 2.7m/sec |
| **Semilunar valves** |  | Aortic | ------- |
| Aortic valve | Annulus = 11mm | Tricuspid | ------- |
| Pulmonary valve | Annulus = 12mm | pulmonic | -------- |
| **Great arteries** | NRGA | **Aortic arch** | Left. No CoA. |
| Aorta | ----- | **PDA** | 3mm PDA, L – R Shunt |
| Pulmonary artery | MPA = 14mm. Confluent Branch PAs. |  |  |
| **M-Mode:**  Normal LV Systolic Function | | | |
| AO | mm | PWd | mm |
| LA | mm | PWs | mm |
| LVIDd | mm | EDV | ml |
| LVIDs | mm | ESV | ml |
| IVSs | mm | LVEF | % |
| IVSd | mm | FS | % |
| **Additional Information**: |  | | |
| No pericardial/Pleural effusion. | | | |
| **Final Diagnosis:** | | | |
| 1. {S, D, S} Levocardia. 2. LA/LV Dilated 3. Mild MR 4. Large PDA, L – R Shunt 5. Normal LV Systolic Function | | | |
| **Remark**: | | | |
| **Recommendation**: | | | |
| SIGNATURE  Done by: Tesfaye T., Pediatrician, Pediatric Cardiologist _______________ 30/09/08/2014Eth.C | | | |

| Patient Name: **Bereket Wallelign**. Referring Institute: **FHRH**. SEX/ Age: **M/1 5/12**.  Date of Report: **30/09/08/14**. Referral Diagnosis: **Sepsis. AGH8.326** | | | |
| --- | --- | --- | --- |
| **Features** | **Finding** | **Features** | **Finding** |
| **Profile** |  | **Atria** |  |
| Abdominal situs | Solitus | Left atrium | Normal |
| Cardiac position | Levocardia | Right atrium | Normal |
| Systemic venous drainage | Normal. | **Atrioventricular valves** |  |
| Pulmonary venous drainage | Normal | Mitral valve | Annulus = 16mm |
| Atrioventricular connection | Concordant | Tricuspid valve | Annulus = 17mm  TAPSE = 16mm |
| Ventriculoarterial connection | Concordant | **Ventricles** |  |
| Ventricular loop | d-Loop | Left ventricle | Normal |
|  |  | Right ventricle | Normal |
| **Septae** |  | **Coronary arteries** | ----- |
| Interventricular septum | Intact | **Doppler Measurement** |  |
| Interatrial septum | Intact | Mitral | Trivial MR, Central projection seen in two planes with jet velocity = 2.3m/sec |
| **Semilunar valves** |  | Aortic | ------- |
| Aortic valve | Annulus = 11mm | Tricuspid | ------- |
| Pulmonary valve | Annulus = 13mm | pulmonic | -------- |
| **Great arteries** | NRGA | **Aortic arch** | Left. No CoA. |
| Aorta | ----- | **PDA** | No |
| Pulmonary artery | Normal MPA and Branch PAs. |  |  |
| **M-Mode:** | | | |
| AO | mm | PWd | mm |
| LA | mm | PWs | mm |
| LVIDd | mm | EDV | ml |
| LVIDs | mm | ESV | ml |
| IVSs | mm | LVEF | 65% |
| IVSd | mm | FS | 33% |
| **Additional Information**: |  | | |
| No pericardial/Pleural effusion. | | | |
| **Final Diagnosis:** | | | |
| 1. {S, D, S} Levocardia. 2. Trivial MR | | | |
| **Remark**: | | | |
| **Recommendation**: | | | |
| SIGNATURE  Done by: Tesfaye T., Pediatrician, Pediatric Cardiologist _______________ 30/09/08/2014Eth.C | | | |

| Patient Name: **Amarech Molla**. Referring Institute: **FHRH**. SEX/ Age: **F/10years**.  Date of Report: **30/09/08/14**. Referral Diagnosis: **ARF. AGH8.327** | | | |
| --- | --- | --- | --- |
| **Features** | **Finding** | **Features** | **Finding** |
| **Profile** |  | **Atria** |  |
| Abdominal situs | Solitus | Left atrium | Mildly Dilated |
| Cardiac position | Levocardia | Right atrium | Normal |
| Systemic venous drainage | Normal. | **Atrioventricular valves** |  |
| Pulmonary venous drainage | Normal | Mitral valve | Annulus = mm. Patulous MVL |
| Atrioventricular connection | Concordant | Tricuspid valve | Annulus = 17mm  TAPSE = 20mm |
| Ventriculoarterial connection | Concordant | **Ventricles** |  |
| Ventricular loop | d-Loop | Left ventricle | Midly Dilated |
|  |  | Right ventricle | Normal |
| **Septae** |  | **Coronary arteries** | ----- |
| Interventricular septum | Intact | **Doppler Measurement** |  |
| Interatrial septum | Intact | Mitral | Mild MR, Holosystolic, posterior projection, seen in two planes with jet velocity = 4.9m/sec. |
| **Semilunar valves** |  | Aortic | ------- |
| Aortic valve | Annulus = 13mm | Tricuspid | ------- |
| Pulmonary valve | Annulus = 17mm | pulmonic | -------- |
| **Great arteries** | NRGA | **Aortic arch** | Left. No CoA. |
| Aorta | ----- | **PDA** | No |
| Pulmonary artery | Normal MPA and Branch PAs. |  |  |
| **M-Mode:** | | | |
| AO | mm | PWd | mm |
| LA | mm | PWs | mm |
| LVIDd | mm | EDV | ml |
| LVIDs | mm | ESV | ml |
| IVSs | mm | LVEF | 64% |
| IVSd | mm | FS | 34% |
| **Additional Information**: |  | | |
| No pericardial/Pleural effusion. | | | |
| **Final Diagnosis:** | | | |
| 1. {S, D, S} Levocardia. 2. Mildly Dilated LA/LV 3. Patulous, thickened MVL 4. Mild MR 5. Normal Biventricular Systolic Function | | | |
| **Remark**: | | | |
| **Recommendation**: | | | |
| SIGNATURE  Done by: Tesfaye T., Pediatrician, Pediatric Cardiologist _______________ 30/09/08/2014Eth.C | | | |

| Patient Name: **Robel Abebaw**. Referring Institute: **FHRH**. SEX/ Age: **M/1month**.  Date of Report: **30/09/08/14**. Referral Diagnosis: **Cyanosis. AGH8.328** | | | |
| --- | --- | --- | --- |
| **Features** | **Finding** | **Features** | **Finding** |
| **Profile** |  | **Atria** |  |
| Abdominal situs | Solitus | Left atrium | Normal |
| Cardiac position | Levocardia | Right atrium | Dilated |
| Systemic venous drainage | Normal. | **Atrioventricular valves** |  |
| Pulmonary venous drainage | Normal | Mitral valve | Annulus = 11mm |
| Atrioventricular connection | Concordant | Tricuspid valve | Annulus = 13mm |
| Ventriculoarterial connection | Discordant | **Ventricles** |  |
| Ventricular loop | d-Loop | Left ventricle | Regressed |
|  |  | Right ventricle | Dilated |
| **Septae** |  | **Coronary arteries** | ----- |
| Interventricular septum | Intact | **Doppler Measurement** |  |
| Interatrial septum | 4mm OS ASD, L – R Shunt | Mitral | ----- |
| **Semilunar valves** |  | Aortic | ------- |
| Aortic valve | Annulus = 9mm | Tricuspid | Trivial TR |
| Pulmonary valve | Annulus = 8mm | pulmonic | -------- |
| **Great arteries** | d-TGA | **Aortic arch** | Left. No CoA. |
| Aorta | Anterior & from RV | **PDA** | No |
| Pulmonary artery | Posterior & from LV |  |  |
| **M-Mode:** | | | |
| AO | mm | PWd | mm |
| LA | mm | PWs | mm |
| LVIDd | mm | EDV | ml |
| LVIDs | mm | ESV | ml |
| IVSs | mm | LVEF | % |
| IVSd | mm | FS | % |
| **Additional Information**: |  | | |
| No pericardial/Pleural effusion. | | | |
| **Final Diagnosis:** | | | |
| 1. {S, D, D} Levocardia. 2. Small OS ASD, L – R Shunt 3. d-TGA with IVS (Intact Interventricular Septum) 4. LV Regressed | | | |
| **Remark**: | | | |
| **Recommendation**: | | | |
| SIGNATURE  Done by: Tesfaye T., Pediatrician, Pediatric Cardiologist _______________ 30/09/08/2014Eth.C | | | |

| Patient Name: **Natanem Birhanu**. Referring Institute: **MSI-Ethiopia, Bahir Dar**. SEX/ Age: **M/7days**.  Date of Report: **01/10/08/14**. Referral Diagnosis: **Cyanosis. AGH8.329** | | | |
| --- | --- | --- | --- |
| **Features** | **Finding** | **Features** | **Finding** |
| **Profile** |  | **Atria** |  |
| Abdominal situs | Solitus | Left atrium | Normal |
| Cardiac position | Levocardia | Right atrium | Mildly Dilated |
| Systemic venous drainage | Normal. | **Atrioventricular valves** |  |
| Pulmonary venous drainage | Normal | Mitral valve | Annulus = 9mm |
| Atrioventricular connection | Concordant | Tricuspid valve | Annulus = 10mm |
| Ventriculoarterial connection | Concordant | **Ventricles** |  |
| Ventricular loop | d-Loop | Left ventricle | Normal |
|  |  | Right ventricle | Mildly Dilated & Hypertrophied |
| **Septae** |  | **Coronary arteries** | ----- |
| Interventricular septum | Mal-aligned Non-Restrictive Sub-Aortic VSD, R – L Shunt | **Doppler Measurement** |  |
| Interatrial septum | 4mm OS ASD, L – R Shunt | Mitral | ----- |
| **Semilunar valves** |  | Aortic | ------- |
| Aortic valve | Annulus = 9mm | Tricuspid | ------- |
| Pulmonary valve | Annulus = 7mm. Dysplastic. | pulmonic | Moderate Sub-valvular and Valvular PS, PPG = 51mmHg. Mild PR, PPG = 26mmHg |
| **Great arteries** | NRGA | **Aortic arch** | Left. No CoA. |
| Aorta | Aortic Over-ride | **PDA** | No |
| Pulmonary artery | Normal MPA and Branch PAs. |  |  |
| **M-Mode:**  Normal LV Function on eye balling | | | |
| AO | mm | PWd | mm |
| LA | mm | PWs | mm |
| LVIDd | mm | EDV | ml |
| LVIDs | mm | ESV | ml |
| IVSs | mm | LVEF | % |
| IVSd | mm | FS | % |
| **Additional Information**: |  | | |
| No pericardial/Pleural effusion. | | | |
| **Final Diagnosis:** | | | |
| 1. {S, D, S} Levocardia. 2. Small OS ASD, L – R Shunt 3. TOF | | | |
| **Remark**: | | | |
| **Recommendation**: | | | |
| SIGNATURE  Done by: Tesfaye T., Pediatrician, Pediatric Cardiologist _______________ 01/10/08/2014Eth.C | | | |

| Patient Name: **Fetle-werk Yirga**. Referring Institute: **FHRH**. SEX/ Age: **F/3 6/12**.  Date of Report: **01/10/08/14**. Referral Diagnosis: **Incidental Murmur Finding. AGH8.330** | | | |
| --- | --- | --- | --- |
| **Features** | **Finding** | **Features** | **Finding** |
| **Profile** |  | **Atria** |  |
| Abdominal situs | Solitus | Left atrium | Normal |
| Cardiac position | Levocardia | Right atrium | Normal |
| Systemic venous drainage | Normal. | **Atrioventricular valves** |  |
| Pulmonary venous drainage | Normal | Mitral valve | Annulus = 20mm |
| Atrioventricular connection | Concordant | Tricuspid valve | Annulus = 19mm |
| Ventriculoarterial connection | Concordant | **Ventricles** |  |
| Ventricular loop | d-Loop | Left ventricle | Normal |
|  |  | Right ventricle | Normal |
| **Septae** |  | **Coronary arteries** | ----- |
| Interventricular septum | 5mm PM VSD, Partially closed by STL, L – R Shunt with a gradient of 73mmHg. | **Doppler Measurement** |  |
| Interatrial septum | PFO, L – R Shunt | Mitral | ----- |
| **Semilunar valves** |  | Aortic | ------- |
| Aortic valve | Annulus = 14mm | Tricuspid | ------- |
| Pulmonary valve | Annulus = 16mm | pulmonic | -------- |
| **Great arteries** | NRGA | **Aortic arch** | Left. No CoA. |
| Aorta | ----- | **PDA** | No |
| Pulmonary artery | Normal MPA and Branch PAs. |  |  |
| **M-Mode:** | | | |
| AO | mm | PWd | mm |
| LA | mm | PWs | mm |
| LVIDd | mm | EDV | ml |
| LVIDs | mm | ESV | ml |
| IVSs | mm | LVEF | 62% |
| IVSd | mm | FS | 33% |
| **Additional Information**: |  | | |
| No pericardial/Pleural effusion. | | | |
| **Final Diagnosis:** | | | |
| 1. {S, D, S} Levocardia. 2. PFO, L – R Shunt 3. Small Restrictive PM VSD, L – R Shunt 4. Normal LV Systolic Function | | | |
| **Remark**: | | | |
| **Recommendation**: | | | |
| SIGNATURE  Done by: Tesfaye T., Pediatrician, Pediatric Cardiologist _______________ 01/10/08/2014Eth.C | | | |

| Patient Name: **Me’eraf Mesfin**. Referring Institute: **Amaris PSC**. SEX/ Age: **F/4months**.  Date of Report: **02/10/08/14**. Referral Diagnosis: **DS. AGH8.331** | | | |
| --- | --- | --- | --- |
| **Features** | **Finding** | **Features** | **Finding** |
| **Profile** |  | **Atria** |  |
| Abdominal situs | Solitus | Left atrium | Normal |
| Cardiac position | Levocardia | Right atrium | Normal |
| Systemic venous drainage | Normal. | **Atrioventricular valves** |  |
| Pulmonary venous drainage | Normal | Mitral valve | Annulus = 11mm |
| Atrioventricular connection | Concordant | Tricuspid valve | Annulus = 11mm |
| Ventriculoarterial connection | Concordant | **Ventricles** |  |
| Ventricular loop | d-Loop | Left ventricle | Normal |
|  |  | Right ventricle | Normal |
| **Septae** |  | **Coronary arteries** | ----- |
| Interventricular septum | Intact | **Doppler Measurement** |  |
| Interatrial septum | PFO, L – R Shunt | Mitral | ----- |
| **Semilunar valves** |  | Aortic | ------- |
| Aortic valve | Annulus = 10mm | Tricuspid | ------- |
| Pulmonary valve | Annulus = 10mm | pulmonic | -------- |
| **Great arteries** | NRGA | **Aortic arch** | Left. No CoA. |
| Aorta | ----- | **PDA** | No |
| Pulmonary artery | Normal MPA and Branch PAs. |  |  |
| **M-Mode:**  Normal LV Function on eye balling | | | |
| AO | mm | PWd | mm |
| LA | mm | PWs | mm |
| LVIDd | mm | EDV | ml |
| LVIDs | mm | ESV | ml |
| IVSs | mm | LVEF | % |
| IVSd | mm | FS | % |
| **Additional Information**: |  | | |
| No pericardial/Pleural effusion. | | | |
| **Final Diagnosis:** | | | |
| 1. {S, D, S} Levocardia. 2. PFO, L – R Shunt | | | |
| **Remark**: | | | |
| **Recommendation**: | | | |
| SIGNATURE  Done by: Tesfaye T., Pediatrician, Pediatric Cardiologist _______________ 02/10/08/2014Eth.C | | | |

| Patient Name: **Baby of Yalem-Tsehay Tewachew**. Referring Institute: **MSI- Ethiopia, Bahir Dar**. SEX/ Age: **M/One month.** Date of Report: **02/10/08/14**. Referral Diagnosis: **Incidental Murmur.AGH8.332** | | | |
| --- | --- | --- | --- |
| **Features** | **Finding** | **Features** | **Finding** |
| **Profile** |  | **Atria** |  |
| Abdominal situs | Solitus | Left atrium | Normal |
| Cardiac position | Levocardia | Right atrium | Normal |
| Systemic venous drainage | Normal. | **Atrioventricular valves** |  |
| Pulmonary venous drainage | Normal | Mitral valve | Annulus = 11mm |
| Atrioventricular connection | Concordant | Tricuspid valve | Annulus = 12mm |
| Ventriculoarterial connection | Concordant | **Ventricles** |  |
| Ventricular loop | d-Loop | Left ventricle | Normal |
|  |  | Right ventricle | Normal |
| **Septae** |  | **Coronary arteries** | ----- |
| Interventricular septum | Intact | **Doppler Measurement** |  |
| Interatrial septum | PFO, L – R Shunt | Mitral | ----- |
| **Semilunar valves** |  | Aortic | ------- |
| Aortic valve | Annulus = 8mm | Tricuspid | ------- |
| Pulmonary valve | Annulus = 9mm | pulmonic | -------- |
| **Great arteries** | NRGA | **Aortic arch** | Left. No CoA. |
| Aorta | ----- | **PDA** | 1mm PDA, L – R Shunt |
| Pulmonary artery | Normal MPA and Branch PAs. |  |  |
| **M-Mode:** | | | |
| AO | mm | PWd | mm |
| LA | mm | PWs | mm |
| LVIDd | mm | EDV | ml |
| LVIDs | mm | ESV | ml |
| IVSs | mm | LVEF | 61% |
| IVSd | mm | FS | 31% |
| **Additional Information**: |  | | |
| No pericardial/Pleural effusion. | | | |
| **Final Diagnosis:** | | | |
| 1. {S, D, S} Levocardia. 2. PFO, L – R Shunt 3. Small PDA, L – R Shunt 4. Normal LV Systolic Function | | | |
| **Remark**: | | | |
| **Recommendation**: | | | |
| SIGNATURE  Done by: Tesfaye T., Pediatrician, Pediatric Cardiologist _______________ 02/10/08/2014Eth.C | | | |

| Patient Name: **Desalu Chekol**. Referring Institute: **FHRH**. SEX/ Age: **F/12years**.  Date of Report: **02/10/08/14**. Referral Diagnosis: **Palpitation. AGH8.333** | | | |
| --- | --- | --- | --- |
| **Features** | **Finding** | **Features** | **Finding** |
| **Profile** |  | **Atria** |  |
| Abdominal situs | Solitus | Left atrium | Normal |
| Cardiac position | Levocardia | Right atrium | Normal |
| Systemic venous drainage | Normal. | **Atrioventricular valves** |  |
| Pulmonary venous drainage | Normal | Mitral valve | Annulus = 21mm |
| Atrioventricular connection | Concordant | Tricuspid valve | Annulus = 22mm  TAPSE = 25mm |
| Ventriculoarterial connection | Concordant | **Ventricles** |  |
| Ventricular loop | d-Loop | Left ventricle | Normal |
|  |  | Right ventricle | Normal |
| **Septae** |  | **Coronary arteries** | ----- |
| Interventricular septum | Intact | **Doppler Measurement** |  |
| Interatrial septum | Intact | Mitral | ----- |
| **Semilunar valves** |  | Aortic | ------- |
| Aortic valve | Annulus = 20mm | Tricuspid | ------- |
| Pulmonary valve | Annulus = 20mm | pulmonic | -------- |
| **Great arteries** | NRGA | **Aortic arch** | Left. No CoA. |
| Aorta | ----- | **PDA** | No |
| Pulmonary artery | Normal MPA and Branch PAs. |  |  |
| **M-Mode:** | | | |
| AO | mm | PWd | mm |
| LA | mm | PWs | mm |
| LVIDd | mm | EDV | ml |
| LVIDs | mm | ESV | ml |
| IVSs | mm | LVEF | 65% |
| IVSd | mm | FS | 36% |
| **Additional Information**: |  | | |
| No pericardial/Pleural effusion. | | | |
| **Final Diagnosis:** | | | |
| 1. Normal Echocardiography Study. | | | |
| **Remark**: | | | |
| **Recommendation**: | | | |
| SIGNATURE  Done by: Tesfaye T., Pediatrician, Pediatric Cardiologist _______________ 02/10/08/2014Eth.C | | | |

| Patient Name: **Meriem Hassen**. Referring Institute: **Pawe GH**. SEX/ Age: **F/13years**.  Date of Report: **04/10/08/14**. Referral Diagnosis: **Rheumatic Recurrence + CHF. AGH8.334** | | | | |
| --- | --- | --- | --- | --- |
| **Features** | **Finding** | **Features** | | **Finding** |
| **Profile** |  | **Atria** | |  |
| Abdominal situs | Solitus | Left atrium | | More dilated |
| Cardiac position | Levocardia | Right atrium | | Dilated |
| Systemic venous drainage | Normal. | **Atrioventricular valves** | |  |
| Pulmonary venous drainage | Normal | Mitral valve | | Annulus = 32mm. Thickened, clubbed MVL. Shortened PMVL. MVA = 0.8cm2. |
| Atrioventricular connection | Concordant | Tricuspid valve | | Annulus = 26mm |
| Ventriculoarterial connection | Concordant | **Ventricles** | |  |
| Ventricular loop | d-Loop | Left ventricle | | More Dilated |
|  |  | Right ventricle | | Dilated |
| **Septae** |  | **Coronary arteries** | | ----- |
| Interventricular septum | Intact | **Doppler Measurement** | |  |
| Interatrial septum | Intact | Mitral | | Severe MR, Holosystolic, posterior projection, seen in two planes with jet velocity = 3.9m/sec. Severe MS, PPG/MPG = 22/18mmHg. |
| **Semilunar valves** |  | Aortic | | ------- |
| Aortic valve | Annulus = 14mm | Tricuspid | | Mild TR, PPG = 57 – 61mmHg. |
| Pulmonary valve | Annulus = 24mm | pulmonic | | -------- |
| **Great arteries** | NRGA | **Aortic arch** | | Left. No CoA. |
| Aorta | ----- | **PDA** | | No |
| Pulmonary artery | ----- |  | |  |
| **M-Mode:** | | | | |
| AO | mm | | PWd | mm |
| LA | mm | | PWs | mm |
| LVIDd | mm | | EDV | ml |
| LVIDs | mm | | ESV | ml |
| IVSs | mm | | LVEF | 56% |
| IVSd | mm | | FS | 29% |
| **Additional Information**: |  | | | |
| No pericardial/Pleural effusion. | | | | |
| **Final Diagnosis:** | | | | |
| 1. {S, D, S} Levocardia. 2. All chambers dilated 3. Thickened, clubbed PMVL. Shortened PMVL 4. Severe MR 5. Severe MS 6. Mild TR 7. Moderate to Severe Pulmonary Hypertension 8. Boarderline Systolic LV Function | | | | |
| **Remark**: | | | | |
| **Recommendation**: Needs Intervention | | | | |
| SIGNATURE  Done by: Tesfaye T., Pediatrician, Pediatric Cardiologist _______________ 04/10/08/2014Eth.C | | | | |

| Patient Name: **Kidist Aychew**. Referring Institute: **FHRH**. SEX/ Age: **F/1 8/12**. Date of Report: **05/10/08/14**. Referral Diagnosis: **G-III Systolic Murmur. AGH8.335** | | | |
| --- | --- | --- | --- |
| **Features** | **Finding** | **Features** | **Finding** |
| **Profile** |  | **Atria** |  |
| Abdominal situs | Solitus | Left atrium | Normal |
| Cardiac position | Levocardia | Right atrium | Normal |
| Systemic venous drainage | Normal. | **Atrioventricular valves** |  |
| Pulmonary venous drainage | Normal | Mitral valve | Annulus = 12mm |
| Atrioventricular connection | Concordant | Tricuspid valve | Annulus = 13mm  TAPSE = 16mm |
| Ventriculoarterial connection | Concordant | **Ventricles** |  |
| Ventricular loop | d-Loop | Left ventricle | Normal |
|  |  | Right ventricle | Normal |
| **Septae** |  | **Coronary arteries** | ----- |
| Interventricular septum | Intact | **Doppler Measurement** |  |
| Interatrial septum | Intact | Mitral | ----- |
| **Semilunar valves** |  | Aortic | ------- |
| Aortic valve | Annulus = 11mm | Tricuspid | ------- |
| Pulmonary valve | Annulus = 11mm | pulmonic | Flow acceleration across PV with a gradient of 20mmHg |
| **Great arteries** | NRGA | **Aortic arch** | Left. No CoA. |
| Aorta | ----- | **PDA** | No |
| Pulmonary artery | Normal MPA and Branch PAs. |  |  |
| **M-Mode:**  Normal LV Function on eye balling | | | |
| AO | mm | PWd | mm |
| LA | mm | PWs | mm |
| LVIDd | mm | EDV | ml |
| LVIDs | mm | ESV | ml |
| IVSs | mm | LVEF | % |
| IVSd | mm | FS | % |
| **Additional Information**: |  | | |
| No pericardial/Pleural effusion. | | | |
| **Final Diagnosis:** | | | |
| 1. {S, D, S} Levocardia. 2. Mild Valvular PS 3. Normal Biventricular Systolic Function | | | |
| **Remark**: Previous study doesn’t show RVOTO | | | |
| **Recommendation**: | | | |
| SIGNATURE  Done by: Tesfaye T., Pediatrician, Pediatric Cardiologist _______________ 05/10/08/2014Eth.C | | | |

| Patient Name: **Biruk Chalie**. Referring Institute: **TGSH**. SEX/ Age: **M/1 10/12**. Date of Report: **05/10/08/14**. Referral Diagnosis: **CHF + RD. AGH8.336** | | | |
| --- | --- | --- | --- |
| **Features** | **Finding** | **Features** | **Finding** |
| **Profile** |  | **Atria** |  |
| Abdominal situs | Solitus | Left atrium | Normal |
| Cardiac position | Levocardia | Right atrium | Normal |
| Systemic venous drainage | Normal. | **Atrioventricular valves** |  |
| Pulmonary venous drainage | Normal | Mitral valve | Annulus = 17mm |
| Atrioventricular connection | Concordant | Tricuspid valve | Annulus = 20mm  TAPSE = 13mm |
| Ventriculoarterial connection | Concordant | **Ventricles** |  |
| Ventricular loop | d-Loop | Left ventricle | Dilated |
|  |  | Right ventricle | Normal |
| **Septae** |  | **Coronary arteries** | ----- |
| Interventricular septum | Intact | **Doppler Measurement** |  |
| Interatrial septum | Intact | Mitral | Mild MR, Jet velocity = 3.4m/sec |
| **Semilunar valves** |  | Aortic | ------- |
| Aortic valve | Annulus = 12mm | Tricuspid | Mild TR, PPG = 42mmHg |
| Pulmonary valve | Annulus = 16mm | pulmonic | Mild PR, PPG = 41mmHg |
| **Great arteries** | NRGA | **Aortic arch** | Left. No CoA. |
| Aorta | ----- | **PDA** | No |
| Pulmonary artery | MPA = 14mm |  |  |
| **M-Mode:** | | | |
| AO | mm | PWd | **7mm** |
| LA | mm | PWs | 9mm |
| LVIDd | 26.5mm | EDV | 26ml |
| LVIDs | 18.5mm | ESV | 10ml |
| IVSs | 9mm | LVEF | 50% |
| IVSd | **7mm** | FS | 26% |
| **Additional Information**: |  | | |
| No pericardial/Pleural effusion. | | | |
| **Final Diagnosis:** | | | |
| 1. {S, D, S} Levocardia. 2. Mild MR 3. Mild TR 4. Mild PR 5. Mild Pulmonary Hypertension 6. Thickened IVS (LVH) 7. Reduced LV Function | | | |
| **Remark**: | | | |
| **Recommendation**: | | | |
| SIGNATURE  Done by: Tesfaye T., Pediatrician, Pediatric Cardiologist _______________ 05/10/08/2014Eth.C | | | |

| Patient Name: **Mikyas Endalamaw**. Referring Institute: **FHRH**. SEX/ Age: **M/6years**.  Date of Report: **06/10/08/14**. Referral Diagnosis: **Easy fatigability. AGH8.337** | | | |
| --- | --- | --- | --- |
| **Features** | **Finding** | **Features** | **Finding** |
| **Profile** |  | **Atria** |  |
| Abdominal situs | Solitus | Left atrium | Normal |
| Cardiac position | Levocardia | Right atrium | Normal |
| Systemic venous drainage | Normal. | **Atrioventricular valves** |  |
| Pulmonary venous drainage | Normal | Mitral valve | Annulus = 18mm |
| Atrioventricular connection | Concordant | Tricuspid valve | Annulus = 20mm  TAPSE = 19mm |
| Ventriculoarterial connection | Concordant | **Ventricles** |  |
| Ventricular loop | d-Loop | Left ventricle | Normal |
|  |  | Right ventricle | Normal |
| **Septae** |  | **Coronary arteries** | ----- |
| Interventricular septum | Intact | **Doppler Measurement** |  |
| Interatrial septum | Intact | Mitral | ----- |
| **Semilunar valves** |  | Aortic | ------- |
| Aortic valve | Annulus = 16mm. Trileaflet. | Tricuspid | ------- |
| Pulmonary valve | Annulus = 15mm | pulmonic | -------- |
| **Great arteries** | NRGA | **Aortic arch** | Left. No CoA. |
| Aorta | ----- | **PDA** | No |
| Pulmonary artery | Normal MPA and Branch PAs. |  |  |
| **M-Mode:** | | | |
| AO | mm | PWd | mm |
| LA | mm | PWs | mm |
| LVIDd | mm | EDV | ml |
| LVIDs | mm | ESV | ml |
| IVSs | mm | LVEF | 65% |
| IVSd | mm | FS | 35% |
| **Additional Information**: |  | | |
| No pericardial/Pleural effusion. | | | |
| **Final Diagnosis:** | | | |
| 1. Normal Echocardiography Study. | | | |
| **Remark**: | | | |
| **Recommendation**: | | | |
| SIGNATURE  Done by: Tesfaye T., Pediatrician, Pediatric Cardiologist _______________ 06/10/08/2014Eth.C | | | |

| Patient Name: **Huzeif Umer**. Referring Institute: **Adinas GH**. SEX/ Age: **M/12years**.  Date of Report: **06/10/08/14**. Referral Diagnosis: **Chest pain + easy fatigability. AGH8.338** | | | |
| --- | --- | --- | --- |
| **Features** | **Finding** | **Features** | **Finding** |
| **Profile** |  | **Atria** |  |
| Abdominal situs | Solitus | Left atrium | Normal |
| Cardiac position | Levocardia | Right atrium | Normal |
| Systemic venous drainage | Normal. | **Atrioventricular valves** |  |
| Pulmonary venous drainage | Normal | Mitral valve | Annulus = 17mm |
| Atrioventricular connection | Concordant | Tricuspid valve | Annulus = 19mm  TAPSE = 18mm |
| Ventriculoarterial connection | Concordant | **Ventricles** |  |
| Ventricular loop | d-Loop | Left ventricle | Normal |
|  |  | Right ventricle | Normal |
| **Septae** |  | **Coronary arteries** | ----- |
| Interventricular septum | Intact | **Doppler Measurement** |  |
| Interatrial septum | Intact | Mitral | ----- |
| **Semilunar valves** |  | Aortic | ------- |
| Aortic valve | Annulus = 16mm | Tricuspid | ------- |
| Pulmonary valve | Annulus = 17mm | pulmonic | -------- |
| **Great arteries** | NRGA | **Aortic arch** | Left. No CoA. |
| Aorta | ----- | **PDA** | No |
| Pulmonary artery | Normal MPA and Branch PAs. |  |  |
| **M-Mode:** | | | |
| AO | mm | PWd | mm |
| LA | mm | PWs | mm |
| LVIDd | mm | EDV | ml |
| LVIDs | mm | ESV | ml |
| IVSs | mm | LVEF | 59% |
| IVSd | mm | FS | 30% |
| **Additional Information**: |  | | |
| Circumferential Pericardial effusion with maximum depth of 5mm on RV Side and 8mm on LV Side. | | | |
| **Final Diagnosis:** | | | |
| 1. {S, D, S} Levocardia. 2. Small Pericardial effusion 3. Normal Biventricular Systolic Function | | | |
| **Remark**: Pericarditis | | | |
| **Recommendation**: | | | |
| SIGNATURE  Done by: Tesfaye T., Pediatrician, Pediatric Cardiologist _______________ 06/10/08/2014Eth.C | | | |

| Patient Name: **Desta Melak**. Referring Institute: **FHRH**. SEX/ Age: **F/1 1/12**.  Date of Report: **06/10/08/14**. Referral Diagnosis: **DS. AGH8.339** | | | |
| --- | --- | --- | --- |
| **Features** | **Finding** | **Features** | **Finding** |
| **Profile** |  | **Atria** |  |
| Abdominal situs | Solitus | Left atrium | Normal |
| Cardiac position | Levocardia | Right atrium | Normal |
| Systemic venous drainage | Normal. | **Atrioventricular valves** |  |
| Pulmonary venous drainage | Normal | Mitral valve | Annulus = 13mm |
| Atrioventricular connection | Concordant | Tricuspid valve | Annulus = 15mm  TAPSE = 13mm |
| Ventriculoarterial connection | Concordant | **Ventricles** |  |
| Ventricular loop | d-Loop | Left ventricle | Normal |
|  |  | Right ventricle | Normal |
| **Septae** |  | **Coronary arteries** | ----- |
| Interventricular septum | Intact | **Doppler Measurement** |  |
| Interatrial septum | Intact | Mitral | ----- |
| **Semilunar valves** |  | Aortic | ------- |
| Aortic valve | Annulus = 11mm | Tricuspid | ------- |
| Pulmonary valve | Annulus = 13mm | pulmonic | -------- |
| **Great arteries** | NRGA | **Aortic arch** | Left. No CoA. |
| Aorta | ----- | **PDA** | No |
| Pulmonary artery | Normal MPA and Branch PAs. |  |  |
| **M-Mode:** | | | |
| AO | mm | PWd | mm |
| LA | mm | PWs | mm |
| LVIDd | mm | EDV | ml |
| LVIDs | mm | ESV | ml |
| IVSs | mm | LVEF | 72% |
| IVSd | mm | FS | 39% |
| **Additional Information**: |  | | |
| No pericardial/Pleural effusion. | | | |
| **Final Diagnosis:** | | | |
| 1. Normal Echocardiography Study. | | | |
| **Remark**: | | | |
| **Recommendation**: | | | |
| SIGNATURE  Done by: Tesfaye T., Pediatrician, Pediatric Cardiologist _______________ 06/10/08/2014Eth.C | | | |

| Patient Name: **Eyob Derbew**. Referring Institute: **Eyasta MS Plc.**. SEX/ Age: **M/8years**.  Date of Report: **07/10/08/14**. Referral Diagnosis: **Chest Pain. AGH8.340** | | | |
| --- | --- | --- | --- |
| **Features** | **Finding** | **Features** | **Finding** |
| **Profile** |  | **Atria** |  |
| Abdominal situs | Solitus | Left atrium | Normal |
| Cardiac position | Levocardia | Right atrium | Normal |
| Systemic venous drainage | Normal. | **Atrioventricular valves** |  |
| Pulmonary venous drainage | Normal | Mitral valve | Annulus = 20mm |
| Atrioventricular connection | Concordant | Tricuspid valve | Annulus = 21mm  TAPSE = 24mm |
| Ventriculoarterial connection | Concordant | **Ventricles** |  |
| Ventricular loop | d-Loop | Left ventricle | Normal |
|  |  | Right ventricle | Normal |
| **Septae** |  | **Coronary arteries** | ----- |
| Interventricular septum | Intact | **Doppler Measurement** |  |
| Interatrial septum | Intact | Mitral | ----- |
| **Semilunar valves** |  | Aortic | ------- |
| Aortic valve | Annulus = 17mm | Tricuspid | ------- |
| Pulmonary valve | Annulus = 19mm | pulmonic | Trivial PR, PPG = 12mmHg. |
| **Great arteries** | NRGA | **Aortic arch** | Left. No CoA. |
| Aorta | ----- | **PDA** | No |
| Pulmonary artery | Normal MPA and Branch PAs. |  |  |
| **M-Mode:** | | | |
| AO | mm | PWd | mm |
| LA | mm | PWs | mm |
| LVIDd | mm | EDV | ml |
| LVIDs | mm | ESV | ml |
| IVSs | mm | LVEF | 66% |
| IVSd | mm | FS | 35% |
| **Additional Information**: |  | | |
| No pericardial/Pleural effusion. | | | |
| **Final Diagnosis:** | | | |
| 1. Normal Echocardiography Study. | | | |
| **Remark**: | | | |
| **Recommendation**: | | | |
| SIGNATURE  Done by: Tesfaye T., Pediatrician, Pediatric Cardiologist _______________ 07/10/08/2014Eth.C | | | |

| Patient Name: **Ehitnesh Anmut**. Referring Institute: **FHRH**. SEX/ Age: **F/6years**.  Date of Report: **07/10/08/14**. Referral Diagnosis: **CHF. AGH8.341** | | | |
| --- | --- | --- | --- |
| **Features** | **Finding** | **Features** | **Finding** |
| **Profile** |  | **Atria** |  |
| Abdominal situs | Solitus | Left atrium | Dilated |
| Cardiac position | Levocardia | Right atrium | Dilated |
| Systemic venous drainage | Normal. | **Atrioventricular valves** |  |
| Pulmonary venous drainage | Normal | Mitral valve | Annulus = 27mm. Thickened MVL. |
| Atrioventricular connection | Concordant | Tricuspid valve | Annulus = 20mm  TAPSE = 22mm |
| Ventriculoarterial connection | Concordant | **Ventricles** |  |
| Ventricular loop | d-Loop | Left ventricle | Dilated |
|  |  | Right ventricle | Dilated |
| **Septae** |  | **Coronary arteries** | ----- |
| Interventricular septum | Intact | **Doppler Measurement** |  |
| Interatrial septum | Intact | Mitral | Severe MR, Holosystolic, posterior projection, seen in two planes with jet velocity = 3.7m/sec |
| **Semilunar valves** |  | Aortic | Moderate AR, PHT = 314ms. |
| Aortic valve | Annulus = 15mm. trileaflet | Tricuspid | Severe TR, PPG = 53mmHg |
| Pulmonary valve | Annulus = 16mm | pulmonic | -------- |
| **Great arteries** | NRGA | **Aortic arch** | Left. No CoA. |
| Aorta | ----- | **PDA** | No |
| Pulmonary artery | Normal MPA and Branch PAs. |  |  |
| **M-Mode:** | | | |
| AO | mm | PWd | mm |
| LA | mm | PWs | mm |
| LVIDd | mm | EDV | ml |
| LVIDs | mm | ESV | ml |
| IVSs | mm | LVEF | 60% |
| IVSd | mm | FS | 32% |
| **Additional Information**: |  | | |
| Pericardial effusion with maximum depth of 7mm. | | | |
| **Final Diagnosis:** | | | |
| 1. {S, D, S} Levocardia. 2. All chambers dilated 3. Thickened MVL 4. Severe MR 5. Severe TR 6. Moderate AR 7. Moderate Pulmonary Hypertension 8. Small Pericardial effusion 9. Normal Biventricular Systolic Function | | | |
| **Remark**: | | | |
| **Recommendation**: | | | |
| SIGNATURE  Done by: Tesfaye T., Pediatrician, Pediatric Cardiologist _______________ 07/10/08/2014Eth.C | | | |

| Patient Name: **Yonas Alemayehu**. Referring Institute: **FHRH**. SEX/ Age: **M/1 1/12**.  Date of Report: **07/10/08/14**. Referral Diagnosis: **DS. AGH8.342** | | | |
| --- | --- | --- | --- |
| **Features** | **Finding** | **Features** | **Finding** |
| **Profile** |  | **Atria** |  |
| Abdominal situs | Solitus | Left atrium | Normal |
| Cardiac position | Levocardia | Right atrium | Normal |
| Systemic venous drainage | Normal. | **Atrioventricular valves** |  |
| Pulmonary venous drainage | Normal | Mitral valve | Annulus = 14mm |
| Atrioventricular connection | Concordant | Tricuspid valve | Annulus = 14mm |
| Ventriculoarterial connection | Concordant | **Ventricles** |  |
| Ventricular loop | d-Loop | Left ventricle | Normal |
|  |  | Right ventricle | Normal |
| **Septae** |  | **Coronary arteries** | ----- |
| Interventricular septum | Intact | **Doppler Measurement** |  |
| Interatrial septum | Intact | Mitral | ----- |
| **Semilunar valves** |  | Aortic | ------- |
| Aortic valve | Annulus = 12mm | Tricuspid | ------- |
| Pulmonary valve | Annulus = 13mm | pulmonic | -------- |
| **Great arteries** | NRGA | **Aortic arch** | Left. No CoA. |
| Aorta | ----- | **PDA** | No |
| Pulmonary artery | Normal MPA and Branch PAs. |  |  |
| **M-Mode:**  Normal LV Function on eye balling | | | |
| AO | mm | PWd | mm |
| LA | mm | PWs | mm |
| LVIDd | mm | EDV | ml |
| LVIDs | mm | ESV | ml |
| IVSs | mm | LVEF | % |
| IVSd | mm | FS | % |
| **Additional Information**: |  | | |
| No pericardial/Pleural effusion. | | | |
| **Final Diagnosis:** | | | |
| 1. Normal Echocardiography Study. | | | |
| **Remark**: | | | |
| **Recommendation**: | | | |
| SIGNATURE  Done by: Tesfaye T., Pediatrician, Pediatric Cardiologist _______________ 07/10/08/2014Eth.C | | | |

| Patient Name: **Tsigereda Abebe**. Referring Institute: **FHRH**. SEX/ Age: **F/2years**.  Date of Report: **08/10/08/14**. Referral Diagnosis: **DS + CHF. AGH8.343** | | | |
| --- | --- | --- | --- |
| **Features** | **Finding** | **Features** | **Finding** |
| **Profile** |  | **Atria** |  |
| Abdominal situs | Solitus | Left atrium | Dilated |
| Cardiac position | Levocardia | Right atrium | Dilated |
| Systemic venous drainage | Normal. | **Atrioventricular valves** |  |
| Pulmonary venous drainage | Normal | Mitral valve | Common Complete AVSD, L – R Shunt  TAPSE = 17mm |
| Atrioventricular connection | Common Complete AVSD | Tricuspid valve |  |
| Ventriculoarterial connection | Concordant | **Ventricles** |  |
| Ventricular loop | d-Loop | Left ventricle | Dilated |
|  |  | Right ventricle | Dilated |
| **Septae** |  | **Coronary arteries** | ----- |
| Interventricular septum | Common Complete AVSD, L – R Shunt. Additional 8mm OS ASD, L – R Shunt | **Doppler Measurement** |  |
| Interatrial septum |  | Mitral | Severe Left AVVR |
| **Semilunar valves** |  | Aortic | ------- |
| Aortic valve | Annulus = 12mm | Tricuspid | Mild Right AVVR |
| Pulmonary valve | Annulus = 15mm | pulmonic | -------- |
| **Great arteries** | NRGA | **Aortic arch** | Left. No CoA. |
| Aorta | ----- | **PDA** | No |
| Pulmonary artery | MPA = 16mm. |  |  |
| **M-Mode:**  Normal LV Function on eye balling | | | |
| AO | mm | PWd | mm |
| LA | mm | PWs | mm |
| LVIDd | mm | EDV | ml |
| LVIDs | mm | ESV | ml |
| IVSs | mm | LVEF | % |
| IVSd | mm | FS | % |
| **Additional Information**: |  | | |
| No pericardial/Pleural effusion. | | | |
| **Final Diagnosis:** | | | |
| 1. {S, D, S} Levocardia. 2. Common Completed Balanced AVSD, L – R Shunt 3. Additional Moderate OS ASD, L – R Shunt 4. Severe Left AVVR 5. Mild Right AVVR 6. Mod. Pulmonary Hypertension 7. Normal Biventricular Systolic Function | | | |
| **Remark**: | | | |
| **Recommendation**: | | | |
| SIGNATURE  Done by: Tesfaye T., Pediatrician, Pediatric Cardiologist _______________ 08/10/08/2014Eth.C | | | |

| Patient Name: **Abrham Temesgen**. Referring Institute: **FHRH**. SEX/ Age: **M/2 8/12**.  Date of Report: **08/10/08/14**. Referral Diagnosis: **Incidental Murmur finding. AGH8.344** | | | |
| --- | --- | --- | --- |
| **Features** | **Finding** | **Features** | **Finding** |
| **Profile** |  | **Atria** |  |
| Abdominal situs | Solitus | Left atrium | Normal |
| Cardiac position | Levocardia | Right atrium | Normal |
| Systemic venous drainage | Normal. | **Atrioventricular valves** |  |
| Pulmonary venous drainage | Normal | Mitral valve | Annulus = 17mm |
| Atrioventricular connection | Concordant | Tricuspid valve | Annulus = 17mm  TAPSE = 18mm |
| Ventriculoarterial connection | Concordant | **Ventricles** |  |
| Ventricular loop | d-Loop | Left ventricle | Normal |
|  |  | Right ventricle | Normal |
| **Septae** |  | **Coronary arteries** | ----- |
| Interventricular septum | 5mm PM VSD, Partially covered by STL, L – R Shunt | **Doppler Measurement** |  |
| Interatrial septum | Intact | Mitral | ----- |
| **Semilunar valves** |  | Aortic | ------- |
| Aortic valve | Annulus = 13mm | Tricuspid | ------- |
| Pulmonary valve | Annulus = 14mm | pulmonic | -------- |
| **Great arteries** | NRGA | **Aortic arch** | Left. No CoA. |
| Aorta | ----- | **PDA** | No |
| Pulmonary artery | Normal MPA and Branch PAs. |  |  |
| **M-Mode:** | | | |
| AO | mm | PWd | mm |
| LA | mm | PWs | mm |
| LVIDd | mm | EDV | ml |
| LVIDs | mm | ESV | ml |
| IVSs | mm | LVEF | 59% |
| IVSd | mm | FS | 30% |
| **Additional Information**: |  | | |
| No pericardial/Pleural effusion. | | | |
| **Final Diagnosis:** | | | |
| 1. {S, D, S} Levocardia. 2. Small PM VSD, Partially covered by STL, L – R Shunt 3. Normal Biventricular Systolic Function | | | |
| **Remark**: | | | |
| **Recommendation**: | | | |
| SIGNATURE  Done by: Tesfaye T., Pediatrician, Pediatric Cardiologist _______________ 08/10/08/2014Eth.C | | | |

| Patient Name: **Tiliksew Belay**. Referring Institute: **TGSH**. SEX/ Age: **M/13years**.  Date of Report: **09/10/08/14**. Referral Diagnosis: **Rheumatic Recurrence. AGH8.345** | | | |
| --- | --- | --- | --- |
| **Features** | **Finding** | **Features** | **Finding** |
| **Profile** |  | **Atria** |  |
| Abdominal situs | Solitus | Left atrium | Mildly dilated |
| Cardiac position | Levocardia | Right atrium | Normal |
| Systemic venous drainage | Normal. | **Atrioventricular valves** |  |
| Pulmonary venous drainage | Normal | Mitral valve | Annulus = 28mm. Thickened MVL. |
| Atrioventricular connection | Concordant | Tricuspid valve | Annulus = 23mm  TAPSE = 20mm |
| Ventriculoarterial connection | Concordant | **Ventricles** |  |
| Ventricular loop | d-Loop | Left ventricle | Mildly Dilated |
|  |  | Right ventricle | Normal |
| **Septae** |  | **Coronary arteries** | ----- |
| Interventricular septum | Intact | **Doppler Measurement** |  |
| Interatrial septum | Intact | Mitral | Moderate MR, Holosystolic, posterior projection, seen in two planes with jet velocity = 4.2m/sec. |
| **Semilunar valves** |  | Aortic | Moderate AR, PHT = 374ms. |
| Aortic valve | Annulus = 19mm | Tricuspid | Trivial TR, PPG = 26mmHg |
| Pulmonary valve | Annulus = 20mm | pulmonic | -------- |
| **Great arteries** | NRGA | **Aortic arch** | Left. No CoA. |
| Aorta | ----- | **PDA** | No |
| Pulmonary artery | Normal MPA and Branch PAs. |  |  |
| **M-Mode:** | | | |
| AO | mm | PWd | mm |
| LA | mm | PWs | mm |
| LVIDd | mm | EDV | ml |
| LVIDs | mm | ESV | ml |
| IVSs | mm | LVEF | 63% |
| IVSd | mm | FS | 34% |
| **Additional Information**: |  | | |
| No pericardial/Pleural effusion. | | | |
| **Final Diagnosis:** | | | |
| 1. {S, D, S} Levocardia. 2. LA/LV Mildly Dilated 3. Thickened MVL 4. Moderate MR 5. Moderate AR 6. Normal Biventricular Systolic Function | | | |
| **Remark**: | | | |
| **Recommendation**: | | | |
| SIGNATURE  Done by: Tesfaye T., Pediatrician, Pediatric Cardiologist _______________ 09/10/08/2014Eth.C | | | |

| Patient Name: **Baby of Enyat EMWEDEW**. Referring Institute: **FHRH**. SEX/ Age: **F/31days**.  Date of Report: **09/10/08/14**. Referral Diagnosis: **Incidental Murmur Finding. AGH8.346** | | | |
| --- | --- | --- | --- |
| **Features** | **Finding** | **Features** | **Finding** |
| **Profile** |  | **Atria** |  |
| Abdominal situs | Solitus | Left atrium | Normal |
| Cardiac position | Levocardia | Right atrium | Normal |
| Systemic venous drainage | Normal. | **Atrioventricular valves** |  |
| Pulmonary venous drainage | Normal | Mitral valve | Annulus =7mm |
| Atrioventricular connection | Concordant | Tricuspid valve | Annulus = 7mm |
| Ventriculoarterial connection | Concordant | **Ventricles** |  |
| Ventricular loop | d-Loop | Left ventricle | Normal |
|  |  | Right ventricle | Normal |
| **Septae** |  | **Coronary arteries** | ----- |
| Interventricular septum | Intact | **Doppler Measurement** |  |
| Interatrial septum | PFO, L – R Shunt | Mitral | ----- |
| **Semilunar valves** |  | Aortic | ------- |
| Aortic valve | Annulus = 6mm | Tricuspid | ------- |
| Pulmonary valve | Annulus = 6mm | pulmonic | -------- |
| **Great arteries** | NRGA | **Aortic arch** | Left. No CoA. |
| Aorta | ----- | **PDA** | 1mm PDA, L – R Shunt |
| Pulmonary artery | Normal MPA and Branch PAs. |  |  |
| **M-Mode:**  Normal LV Function on eye balling | | | |
| AO | mm | PWd | mm |
| LA | mm | PWs | mm |
| LVIDd | mm | EDV | ml |
| LVIDs | mm | ESV | ml |
| IVSs | mm | LVEF | % |
| IVSd | mm | FS | % |
| **Additional Information**: |  | | |
| No pericardial/Pleural effusion. | | | |
| **Final Diagnosis:** | | | |
| 1. {S, D, S} Levocardia. 2. PFO, L – R Shunt 3. Small PDA, L – R Shunt 4. Normal LV Systolic Function | | | |
| **Remark**: | | | |
| **Recommendation**: | | | |
| SIGNATURE  Done by: Tesfaye T., Pediatrician, Pediatric Cardiologist _______________ 09/10/08/2014Eth.C | | | |

| Patient Name: **Moges Birhanu**. Referring Institute: **TGSH**. SEX/ Age: **M/9years**.  Date of Report: **10/10/08/14**. Referral Diagnosis: **ARF. AGH8.347** | | | |
| --- | --- | --- | --- |
| **Features** | **Finding** | **Features** | **Finding** |
| **Profile** |  | **Atria** |  |
| Abdominal situs | Solitus | Left atrium | Normal |
| Cardiac position | Levocardia | Right atrium | Normal |
| Systemic venous drainage | Normal. | **Atrioventricular valves** |  |
| Pulmonary venous drainage | Normal | Mitral valve | Annulus = 19mm |
| Atrioventricular connection | Concordant | Tricuspid valve | Annulus = 20mm  TAPSE = 21mm |
| Ventriculoarterial connection | Concordant | **Ventricles** |  |
| Ventricular loop | d-Loop | Left ventricle | Normal |
|  |  | Right ventricle | Normal |
| **Septae** |  | **Coronary arteries** | ----- |
| Interventricular septum | Intact | **Doppler Measurement** |  |
| Interatrial septum | Intact | Mitral | ----- |
| **Semilunar valves** |  | Aortic | ------- |
| Aortic valve | Annulus = 17mm | Tricuspid | ------- |
| Pulmonary valve | Annulus = 19mm | pulmonic | -------- |
| **Great arteries** | NRGA | **Aortic arch** | Left. No CoA. |
| Aorta | ----- | **PDA** | No |
| Pulmonary artery | Normal MPA and Branch PAs. |  |  |
| **M-Mode:**  Normal LV Function on eye balling | | | |
| AO | mm | PWd | mm |
| LA | mm | PWs | mm |
| LVIDd | mm | EDV | ml |
| LVIDs | mm | ESV | ml |
| IVSs | mm | LVEF | % |
| IVSd | mm | FS | % |
| **Additional Information**: |  | | |
| No pericardial/Pleural effusion. | | | |
| **Final Diagnosis:** | | | |
| 1. Normal Echocardiography Study. | | | |
| **Remark**: | | | |
| **Recommendation**: | | | |
| SIGNATURE  Done by: Tesfaye T., Pediatrician, Pediatric Cardiologist _______________ 10/10/08/2014Eth.C | | | |

| Patient Name: **Sobrina Kedir**. Referring Institute: **Nolot Speciality Clinic**. SEX/ Age: **F/6years**.  Date of Report: **10/10/08/14**. Referral Diagnosis: **Incidental Murmur Finding. AGH8.348** | | | |
| --- | --- | --- | --- |
| **Features** | **Finding** | **Features** | **Finding** |
| **Profile** |  | **Atria** |  |
| Abdominal situs | Solitus | Left atrium | Normal |
| Cardiac position | Levocardia | Right atrium | Normal |
| Systemic venous drainage | Normal. | **Atrioventricular valves** |  |
| Pulmonary venous drainage | Normal | Mitral valve | Annulus = 19mm |
| Atrioventricular connection | Concordant | Tricuspid valve | Annulus = 19mm  TAPSE = 23mm |
| Ventriculoarterial connection | Concordant | **Ventricles** |  |
| Ventricular loop | d-Loop | Left ventricle | Normal |
|  |  | Right ventricle | Normal |
| **Septae** |  | **Coronary arteries** | ----- |
| Interventricular septum | 4mm PM VSD, L – R Shunt with PPG= 53mmHg. | **Doppler Measurement** |  |
| Interatrial septum | Intact | Mitral | ----- |
| **Semilunar valves** |  | Aortic | ------- |
| Aortic valve | Annulus = 13mm | Tricuspid | ------- |
| Pulmonary valve | Annulus = 15mm | pulmonic | -------- |
| **Great arteries** | NRGA | **Aortic arch** | Left. No CoA. |
| Aorta | ----- | **PDA** | No |
| Pulmonary artery | Normal MPA and Branch PAs. |  |  |
| **M-Mode:** | | | |
| AO | mm | PWd | mm |
| LA | mm | PWs | mm |
| LVIDd | mm | EDV | ml |
| LVIDs | mm | ESV | ml |
| IVSs | mm | LVEF | 64% |
| IVSd | mm | FS | 34% |
| **Additional Information**: |  | | |
| No pericardial/Pleural effusion. | | | |
| **Final Diagnosis:** | | | |
| 1. {S, D, S} Levocardia. 2. Small Restrictive PM VSD, L – R Shunt 3. Normal Biventricular Systolic Function | | | |
| **Remark**: | | | |
| **Recommendation**: | | | |
| SIGNATURE  Done by: Tesfaye T., Pediatrician, Pediatric Cardiologist _______________ 10/10/08/2014Eth.C | | | |

| Patient Name: **Amanawit Tekle-Mariam**. Referring Institute: **Amaris PSC**. SEX/ Age: **F/7 6/12**.  Date of Report: **10/10/08/14**. Referral Diagnosis: **ARF. AGH8.349** | | | |
| --- | --- | --- | --- |
| **Features** | **Finding** | **Features** | **Finding** |
| **Profile** |  | **Atria** |  |
| Abdominal situs | Solitus | Left atrium | Normal |
| Cardiac position | Levocardia | Right atrium | Normal |
| Systemic venous drainage | Normal. | **Atrioventricular valves** |  |
| Pulmonary venous drainage | Normal | Mitral valve | Annulus = 18mm |
| Atrioventricular connection | Concordant | Tricuspid valve | Annulus = 21mm  TAPSE = 17mm |
| Ventriculoarterial connection | Concordant | **Ventricles** |  |
| Ventricular loop | d-Loop | Left ventricle | Normal |
|  |  | Right ventricle | Normal |
| **Septae** |  | **Coronary arteries** | ----- |
| Interventricular septum | Intact | **Doppler Measurement** |  |
| Interatrial septum | Intact | Mitral | ----- |
| **Semilunar valves** |  | Aortic | ------- |
| Aortic valve | Annulus = 16mm | Tricuspid | ------- |
| Pulmonary valve | Annulus = 18mm | pulmonic | -------- |
| **Great arteries** | NRGA | **Aortic arch** | Left. No CoA. |
| Aorta | ----- | **PDA** | No |
| Pulmonary artery | Normal MPA and Branch PAs. |  |  |
| **M-Mode:** | | | |
| AO | mm | PWd | mm |
| LA | mm | PWs | mm |
| LVIDd | mm | EDV | ml |
| LVIDs | mm | ESV | ml |
| IVSs | mm | LVEF | 69% |
| IVSd | mm | FS | 38% |
| **Additional Information**: |  | | |
| No pericardial/Pleural effusion. | | | |
| **Final Diagnosis:** | | | |
| 1. Normal Echocardiography Study. | | | |
| **Remark**: | | | |
| **Recommendation**: | | | |
| SIGNATURE  Done by: Tesfaye T., Pediatrician, Pediatric Cardiologist _______________ 10/10/08/2014Eth.C | | | |

| Patient Name: **Amanuel Getnet**. Referring Institute: **TGSH**. SEX/ Age: **M/8 2/12**.  Date of Report: **11/10/08/14**. Referral Diagnosis: **Sydenham’s Chorea. AGH8.350** | | | |
| --- | --- | --- | --- |
| **Features** | **Finding** | **Features** | **Finding** |
| **Profile** |  | **Atria** |  |
| Abdominal situs | Solitus | Left atrium | Mildly Dilated |
| Cardiac position | Levocardia | Right atrium | Normal |
| Systemic venous drainage | Normal. | **Atrioventricular valves** |  |
| Pulmonary venous drainage | Normal | Mitral valve | Annulus = 20mm. Thickened MVL |
| Atrioventricular connection | Concordant | Tricuspid valve | Annulus = 16mm  TAPSE = 21mm |
| Ventriculoarterial connection | Concordant | **Ventricles** |  |
| Ventricular loop | d-Loop | Left ventricle | Mildly Dilated |
|  |  | Right ventricle | Normal |
| **Septae** |  | **Coronary arteries** | ----- |
| Interventricular septum | Intact | **Doppler Measurement** |  |
| Interatrial septum | Intact | Mitral | Mild MR, Holosystolic, posterior projection, seen in two planes with jet velocity = 4.3m/sec |
| **Semilunar valves** |  | Aortic | ------- |
| Aortic valve | Annulus = 15mm | Tricuspid | ------- |
| Pulmonary valve | Annulus = 18mm | pulmonic | -------- |
| **Great arteries** | NRGA | **Aortic arch** | Left. No CoA. |
| Aorta | ----- | **PDA** | No |
| Pulmonary artery | Normal MPA and Branch PAs. |  |  |
| **M-Mode:** | | | |
| AO | mm | PWd | mm |
| LA | mm | PWs | mm |
| LVIDd | mm | EDV | ml |
| LVIDs | mm | ESV | ml |
| IVSs | mm | LVEF | 63% |
| IVSd | mm | FS | 33% |
| **Additional Information**: |  | | |
| No pericardial/Pleural effusion. | | | |
| **Final Diagnosis:** | | | |
| 1. {S, D, S} Levocardia. 2. LA/LV Mildly Dilated 3. Thickened MVL 4. Mild MR 5. Normal Biventricular Systolic Function | | | |
| **Remark**: | | | |
| **Recommendation**: | | | |
| SIGNATURE  Done by: Tesfaye T., Pediatrician, Pediatric Cardiologist _______________ 11/10/08/2014Eth.C | | | |

| Patient Name: **Fikir Tigistu**. Referring Institute: **Guzara Speciality Clinic**. SEX/ Age: **F/6months**.  Date of Report: **11/10/08/14**. Referral Diagnosis: **Incidental Murmur Finding. AGH8.351** | | | |
| --- | --- | --- | --- |
| **Features** | **Finding** | **Features** | **Finding** |
| **Profile** |  | **Atria** |  |
| Abdominal situs | Solitus | Left atrium | Normal |
| Cardiac position | Levocardia | Right atrium | Normal |
| Systemic venous drainage | Normal. | **Atrioventricular valves** |  |
| Pulmonary venous drainage | Normal | Mitral valve | Annulus = 14mm |
| Atrioventricular connection | Concordant | Tricuspid valve | Annulus = 15mm  TAPSE = 15mm |
| Ventriculoarterial connection | Concordant | **Ventricles** |  |
| Ventricular loop | d-Loop | Left ventricle | Normal |
|  |  | Right ventricle | Normal |
| **Septae** |  | **Coronary arteries** | ----- |
| Interventricular septum | 2mm PM VSD, Partially covered by STL, PPG = 50mmHg. | **Doppler Measurement** |  |
| Interatrial septum | 4mm OSD, L – R Shunt | Mitral | ----- |
| **Semilunar valves** |  | Aortic | ------- |
| Aortic valve | Annulus = 13mm | Tricuspid | ------- |
| Pulmonary valve | Annulus = 13mm | pulmonic | -------- |
| **Great arteries** | NRGA | **Aortic arch** | Left. No CoA. |
| Aorta | ----- | **PDA** | No |
| Pulmonary artery | Normal MPA and Branch PAs. |  |  |
| **M-Mode:**  Normal LV Function on eye balling. | | | |
| AO | mm | PWd | mm |
| LA | mm | PWs | mm |
| LVIDd | mm | EDV | ml |
| LVIDs | mm | ESV | ml |
| IVSs | mm | LVEF | % |
| IVSd | mm | FS | % |
| **Additional Information**: |  | | |
| No pericardial/Pleural effusion. | | | |
| **Final Diagnosis:** | | | |
| 1. {S, D, S} Levocardia. 2. Small OS ASD, L – R Shunt 3. Small Restrictive PM VSD, L – R Shunt 4. Normal Biventricular Systolic Function | | | |
| **Remark**: | | | |
| **Recommendation**: | | | |
| SIGNATURE  Done by: Tesfaye T., Pediatrician, Pediatric Cardiologist _______________ 11/10/08/2014Eth.C | | | |

| Patient Name: **Haile-Mariam Belete**. Referring Institute: **FHRH**. SEX/ Age: **M/5 10/12**.  Date of Report: **13/10/08/14**. Referral Diagnosis: **Easy fatigability. AGH8.352** | | | |
| --- | --- | --- | --- |
| **Features** | **Finding** | **Features** | **Finding** |
| **Profile** |  | **Atria** |  |
| Abdominal situs | Solitus | Left atrium | Normal |
| Cardiac position | Levocardia | Right atrium | Normal |
| Systemic venous drainage | Normal. | **Atrioventricular valves** |  |
| Pulmonary venous drainage | Normal | Mitral valve | Annulus = 16mm |
| Atrioventricular connection | Concordant | Tricuspid valve | Annulus = 17mm |
| Ventriculoarterial connection | Concordant | **Ventricles** |  |
| Ventricular loop | d-Loop | Left ventricle | Normal |
|  |  | Right ventricle | Normal |
| **Septae** |  | **Coronary arteries** | ----- |
| Interventricular septum | Intact | **Doppler Measurement** |  |
| Interatrial septum | Intact | Mitral | ----- |
| **Semilunar valves** |  | Aortic | ------- |
| Aortic valve | Annulus = 14mm | Tricuspid | ------- |
| Pulmonary valve | Annulus = 14mm | pulmonic | -------- |
| **Great arteries** | NRGA | **Aortic arch** | Left. No CoA. |
| Aorta | ----- | **PDA** | No |
| Pulmonary artery | Normal MPA and Branch PAs. |  |  |
| **M-Mode:** | | | |
| AO | mm | PWd | mm |
| LA | mm | PWs | mm |
| LVIDd | mm | EDV | ml |
| LVIDs | mm | ESV | ml |
| IVSs | mm | LVEF | 68% |
| IVSd | mm | FS | 37% |
| **Additional Information**: |  | | |
| No pericardial/Pleural effusion. | | | |
| **Final Diagnosis:** | | | |
| 1. Normal Echocardiography Study. | | | |
| **Remark**: | | | |
| **Recommendation**: | | | |
| SIGNATURE  Done by: Tesfaye T., Pediatrician, Pediatric Cardiologist _______________ 13/10/08/2014Eth.C | | | |

| Patient Name: **Baby of Wederyelesh Dagne**. Referring Institute: **MSI-Ethiopia, Bahir Dar**. SEX/ Age: **F/3months**.  Date of Report: **13/10/08/14**. Referral Diagnosis: **Follow up Echo for Small PDA(1mm). Incidental Murmur Finding. AGH8.353 17/07/14, 11day** | | | |
| --- | --- | --- | --- |
| **Features** | **Finding** | **Features** | **Finding** |
| **Profile** |  | **Atria** |  |
| Abdominal situs | Solitus | Left atrium | Normal |
| Cardiac position | Levocardia | Right atrium | Normal |
| Systemic venous drainage | Normal. | **Atrioventricular valves** |  |
| Pulmonary venous drainage | Normal | Mitral valve | Annulus = 13mm |
| Atrioventricular connection | Concordant | Tricuspid valve | Annulus = 13mm  TAPSE = 13mm |
| Ventriculoarterial connection | Concordant | **Ventricles** |  |
| Ventricular loop | d-Loop | Left ventricle | Normal |
|  |  | Right ventricle | Normal |
| **Septae** |  | **Coronary arteries** | ----- |
| Interventricular septum | Intact | **Doppler Measurement** |  |
| Interatrial septum | Intact | Mitral | ----- |
| **Semilunar valves** |  | Aortic | ------- |
| Aortic valve | Annulus = 10mm | Tricuspid | ------- |
| Pulmonary valve | Annulus = 10mm | pulmonic | -------- |
| **Great arteries** | NRGA | **Aortic arch** | Left. No CoA. |
| Aorta | ----- | **PDA** | PDA, <1mm, L – R Shunt |
| Pulmonary artery | Normal MPA and Branch PAs. |  |  |
| **M-Mode:**  Normal LV Function on eye balling | | | |
| AO | mm | PWd | mm |
| LA | mm | PWs | mm |
| LVIDd | mm | EDV | ml |
| LVIDs | mm | ESV | ml |
| IVSs | mm | LVEF | % |
| IVSd | mm | FS | % |
| **Additional Information**: |  | | |
| No pericardial/Pleural effusion. | | | |
| **Final Diagnosis:** | | | |
| 1. {S, D, S} Levocardia. 2. Small PDA, L – R Shunt 3. Normal Biventricular Systolic Function | | | |
| **Remark**: | | | |
| **Recommendation**:   1. Yearly follow up echocardiography 2. No need to start any cardiac medicine | | | |
| SIGNATURE  Done by: Tesfaye T., Pediatrician, Pediatric Cardiologist _______________ 13/10/08/2014Eth.C | | | |

| Patient Name: **Hemen Gebrie**. Referring Institute: **Adinas GH**. SEX/ Age: **F/2 4/12**. Date of Report: **13/10/08/14**. Referral Diagnosis: **Incidental Murmur Finding. AGH8.354** | | | |
| --- | --- | --- | --- |
| **Features** | **Finding** | **Features** | **Finding** |
| **Profile** |  | **Atria** |  |
| Abdominal situs | Solitus | Left atrium | Normal |
| Cardiac position | Levocardia | Right atrium | Normal |
| Systemic venous drainage | Normal. | **Atrioventricular valves** |  |
| Pulmonary venous drainage | Normal | Mitral valve | Annulus = 12mm |
| Atrioventricular connection | Concordant | Tricuspid valve | Annulus = 12mm |
| Ventriculoarterial connection | Concordant | **Ventricles** |  |
| Ventricular loop | d-Loop | Left ventricle | Normal |
|  |  | Right ventricle | Normal |
| **Septae** |  | **Coronary arteries** | ----- |
| Interventricular septum | 2mm PM VSD, Partially closed by STL, L – R Shunt | **Doppler Measurement** |  |
| Interatrial septum | Intact | Mitral | ----- |
| **Semilunar valves** |  | Aortic | ------- |
| Aortic valve | Annulus = 12mm | Tricuspid | ------- |
| Pulmonary valve | Annulus = 14mm | pulmonic | -------- |
| **Great arteries** | NRGA | **Aortic arch** | Left. No CoA. |
| Aorta | ----- | **PDA** | No |
| Pulmonary artery | Normal MPA and Branch PAs. |  |  |
| **M-Mode:**  Normal LV Function on eye balling | | | |
| AO | mm | PWd | mm |
| LA | mm | PWs | mm |
| LVIDd | mm | EDV | ml |
| LVIDs | mm | ESV | ml |
| IVSs | mm | LVEF | % |
| IVSd | mm | FS | % |
| **Additional Information**: |  | | |
| No pericardial/Pleural effusion. | | | |
| **Final Diagnosis:** | | | |
| 1. {S, D, S} Levocardia. 2. Small PM VSD, Partially closed by STL, L – R Shunt 3. Normal LV Systolic Function | | | |
| **Remark**: | | | |
| **Recommendation**: | | | |
| SIGNATURE  Done by: Tesfaye T., Pediatrician, Pediatric Cardiologist _______________ 13/10/08/2014Eth.C | | | |

| Patient Name: **Belaynesh Molla**. Referring Institute: **FHRH**. SEX/ Age: **F/14years**.  Date of Report: **14/10/08/14**. Referral Diagnosis: **easy fatigability. AGH8.355** | | | |
| --- | --- | --- | --- |
| **Features** | **Finding** | **Features** | **Finding** |
| **Profile** |  | **Atria** |  |
| Abdominal situs | Solitus | Left atrium | Mildly Dilated |
| Cardiac position | Levocardia | Right atrium | Normal |
| Systemic venous drainage | Normal. | **Atrioventricular valves** |  |
| Pulmonary venous drainage | Normal | Mitral valve | Annulus = 28mm |
| Atrioventricular connection | Concordant | Tricuspid valve | Annulus = 25mm  TAPSE = 25mm |
| Ventriculoarterial connection | Concordant | **Ventricles** |  |
| Ventricular loop | d-Loop | Left ventricle | Mildly Dilated |
|  |  | Right ventricle | Normal |
| **Septae** |  | **Coronary arteries** | ----- |
| Interventricular septum | Intact | **Doppler Measurement** |  |
| Interatrial septum | Intact | Mitral | ----- |
| **Semilunar valves** |  | Aortic | Trivial(Mild) AR, with velocity = 2m/sec, seen on apical view, PHT = 735ms. |
| Aortic valve | Annulus = 21mm | Tricuspid | ------- |
| Pulmonary valve | Annulus = 25mm | pulmonic | -------- |
| **Great arteries** | NRGA | **Aortic arch** | Left. No CoA. |
| Aorta | ----- | **PDA** | No |
| Pulmonary artery | Normal MPA and Branch PAs. | **Coronaries** | No ALCAPA |
| **M-Mode:** | | | |
| AO | mm | PWd | 9mm |
| LA | mm | PWs | 9mm |
| LVIDd | 5mm | EDV | 124ml |
| LVIDs | 4mm | ESV | 67ml |
| IVSs | 10mm | LVEF | 46% |
| IVSd | 9mm | FS | 23% |
| **Additional Information**: |  | | |
| No pericardial/Pleural effusion. | | | |
| **Final Diagnosis:** | | | |
| 1. {S, D, S} Levocardia. 2. LA/LV Mildy Dilated 3. Trivial AR 4. Reduced LV Systolic Function | | | |
| **Remark**: | | | |
| **Recommendation**: | | | |
| SIGNATURE  Done by: Tesfaye T., Pediatrician, Pediatric Cardiologist _______________ 14/10/08/2014Eth.C | | | |

| Patient Name: **Haile-Mariam Fantahun**. Referring Institute: **Adinas GH**. SEX/ Age: **M/14 3/12**.  Date of Report: **14/10/08/14**. Referral Diagnosis: **Chest Pain. AGH8.356** | | | |
| --- | --- | --- | --- |
| **Features** | **Finding** | **Features** | **Finding** |
| **Profile** |  | **Atria** |  |
| Abdominal situs | Solitus | Left atrium | Normal |
| Cardiac position | Levocardia | Right atrium | Normal |
| Systemic venous drainage | Normal. | **Atrioventricular valves** |  |
| Pulmonary venous drainage | Normal | Mitral valve | Annulus = 20mm |
| Atrioventricular connection | Concordant | Tricuspid valve | Annulus = 19mm  TAPSE = 22mm |
| Ventriculoarterial connection | Concordant | **Ventricles** |  |
| Ventricular loop | d-Loop | Left ventricle | Normal |
|  |  | Right ventricle | Normal |
| **Septae** |  | **Coronary arteries** | ----- |
| Interventricular septum | Intact | **Doppler Measurement** |  |
| Interatrial septum | Intact | Mitral | ----- |
| **Semilunar valves** |  | Aortic | ------- |
| Aortic valve | Annulus = 16mm | Tricuspid | ------- |
| Pulmonary valve | Annulus = 20mm | pulmonic | Mild PR, PPG = 10mmHg |
| **Great arteries** | NRGA | **Aortic arch** | Left. No CoA. |
| Aorta | ----- | **PDA** | No |
| Pulmonary artery | Normal MPA and Branch PAs. | **Coronaries** | No ALCAPA |
| **M-Mode:** | | | |
| AO | mm | PWd | mm |
| LA | mm | PWs | mm |
| LVIDd | mm | EDV | ml |
| LVIDs | mm | ESV | ml |
| IVSs | mm | LVEF | 65% |
| IVSd | mm | FS | 35% |
| **Additional Information**: |  | | |
| No pericardial/Pleural effusion. | | | |
| **Final Diagnosis:** | | | |
| 1. Normal Echocardiography Study. | | | |
| **Remark**: | | | |
| **Recommendation**: | | | |
| SIGNATURE  Done by: Tesfaye T., Pediatrician, Pediatric Cardiologist _______________ 14/10/08/2014Eth.C | | | |

| Patient Name: **Tofiq Wudu**. Referring Institute: **FHRH**. SEX/ Age: **M/8years**.  Date of Report: **14/10/08/14**. Referral Diagnosis: **Palpitation. AGH8.357** | | | |
| --- | --- | --- | --- |
| **Features** | **Finding** | **Features** | **Finding** |
| **Profile** |  | **Atria** |  |
| Abdominal situs | Solitus | Left atrium | Normal |
| Cardiac position | Levocardia | Right atrium | Normal |
| Systemic venous drainage | Normal. | **Atrioventricular valves** |  |
| Pulmonary venous drainage | Normal | Mitral valve | Annulus = 17mm |
| Atrioventricular connection | Concordant | Tricuspid valve | Annulus = 22mm  TAPSE = 20mm |
| Ventriculoarterial connection | Concordant | **Ventricles** |  |
| Ventricular loop | d-Loop | Left ventricle | Normal |
|  |  | Right ventricle | Normal |
| **Septae** |  | **Coronary arteries** | ----- |
| Interventricular septum | Intact | **Doppler Measurement** |  |
| Interatrial septum | Intact | Mitral | ----- |
| **Semilunar valves** |  | Aortic | ------- |
| Aortic valve | Annulus = 16mm | Tricuspid | Trivial TR, PPG = 25mmHg |
| Pulmonary valve | Annulus = 17mm | pulmonic | -------- |
| **Great arteries** | NRGA | **Aortic arch** | Left. No CoA. |
| Aorta | ----- | **PDA** | No |
| Pulmonary artery | Normal MPA and Branch PAs. |  |  |
| **M-Mode:** | | | |
| AO | mm | PWd | mm |
| LA | mm | PWs | mm |
| LVIDd | mm | EDV | ml |
| LVIDs | mm | ESV | ml |
| IVSs | mm | LVEF | 68% |
| IVSd | mm | FS | 38% |
| **Additional Information**: |  | | |
| No pericardial/Pleural effusion. | | | |
| **Final Diagnosis:** | | | |
| 1. Normal Echocardiography Study. | | | |
| **Remark**: | | | |
| **Recommendation**: | | | |
| SIGNATURE  Done by: Tesfaye T., Pediatrician, Pediatric Cardiologist _______________ 14/10/08/2014Eth.C | | | |

| Patient Name: **Haymanot Adane**. Referring Institute: **FHRH**. SEX/ Age: **F/8years**.  Date of Report: **15/10/08/14**. Referral Diagnosis: **DOE. AGH8.358** | | | |
| --- | --- | --- | --- |
| **Features** | **Finding** | **Features** | **Finding** |
| **Profile** |  | **Atria** |  |
| Abdominal situs | Solitus | Left atrium | Normal |
| Cardiac position | Levocardia | Right atrium | Dilated |
| Systemic venous drainage | Dilated IVC. To RA | **Atrioventricular valves** |  |
| Pulmonary venous drainage | To LA | Mitral valve | Annulus = 19mm |
| Atrioventricular connection | Concordant | Tricuspid valve | Annulus = 25mm  TAPSE = 22mm |
| Ventriculoarterial connection | Concordant | **Ventricles** |  |
| Ventricular loop | d-Loop | Left ventricle | Normal |
|  |  | Right ventricle | Dilated |
| **Septae** |  | **Coronary arteries** | ----- |
| Interventricular septum | Intact | **Doppler Measurement** |  |
| Interatrial septum | 25mm X 19mm OS ASD, L – R Shunt | Mitral | ----- |
| **Semilunar valves** |  | Aortic | ------- |
| Aortic valve | Annulus = 12mm | Tricuspid | ------- |
| Pulmonary valve | Annulus = 16mm | pulmonic | Mild PS, PPG = 22mmHg (?physiologic) |
| **Great arteries** | NRGA | **Aortic arch** | Left. No CoA. |
| Aorta | ----- | **PDA** | No |
| Pulmonary artery | Normal MPA and Branch PAs. |  |  |
| **M-Mode:**  abnormal septal Motion. Reduced LV Function | | | |
| AO | mm | PWd | mm |
| LA | mm | PWs | mm |
| LVIDd | mm | EDV | ml |
| LVIDs | mm | ESV | ml |
| IVSs | mm | LVEF | % |
| IVSd | mm | FS | % |
| **Additional Information**: |  | | |
| Pericardial Effusion With Maximum Depth Of 5mm on RA side. | | | |
| **Final Diagnosis:** | | | |
| 1. {S, D, S} Levocardia. 2. RA/RV Dilated 3. Large OS ASD, L – R Shunt 4. Mild PS (?Physiologic) 5. Abnormal Septal Motion 6. Reduced LV Systolic Function | | | |
| **Remark**: | | | |
| **Recommendation**: | | | |
| SIGNATURE  Done by: Tesfaye T., Pediatrician, Pediatric Cardiologist _______________ 15/10/08/2014Eth.C | | | |

| Patient Name: **Zulkifel Jemal**. Referring Institute: **Adinas GH**. SEX/ Age: **M/8years**.  Date of Report: **15/10/08/14**. Referral Diagnosis: **Incidental Murmur Finding. AGH8.359** | | | |
| --- | --- | --- | --- |
| **Features** | **Finding** | **Features** | **Finding** |
| **Profile** |  | **Atria** |  |
| Abdominal situs | Solitus | Left atrium | Normal |
| Cardiac position | Levocardia | Right atrium | Normal |
| Systemic venous drainage | Normal. | **Atrioventricular valves** |  |
| Pulmonary venous drainage | Normal | Mitral valve | Annulus = 19mm |
| Atrioventricular connection | Concordant | Tricuspid valve | Annulus = 19mm  TAPSE = 17mm |
| Ventriculoarterial connection | Concordant | **Ventricles** |  |
| Ventricular loop | d-Loop | Left ventricle | Normal |
|  |  | Right ventricle | Normal |
| **Septae** |  | **Coronary arteries** | ----- |
| Interventricular septum | Intact | **Doppler Measurement** |  |
| Interatrial septum | Intact | Mitral | ----- |
| **Semilunar valves** |  | Aortic | ------- |
| Aortic valve | Annulus = 17mm | Tricuspid | ------- |
| Pulmonary valve | Annulus = 20mm | pulmonic | -------- |
| **Great arteries** | NRGA | **Aortic arch** | Left. No CoA. |
| Aorta | ----- | **PDA** | No |
| Pulmonary artery | Normal MPA and Branch PAs. |  |  |
| **M-Mode:** | | | |
| AO | mm | PWd | mm |
| LA | mm | PWs | mm |
| LVIDd | mm | EDV | ml |
| LVIDs | mm | ESV | ml |
| IVSs | mm | LVEF | 70% |
| IVSd | mm | FS | 39% |
| **Additional Information**: |  | | |
| No pericardial/Pleural effusion. | | | |
| **Final Diagnosis:** | | | |
| 1. Normal Echocardiography Study. | | | |
| **Remark**: | | | |
| **Recommendation**: | | | |
| SIGNATURE  Done by: Tesfaye T., Pediatrician, Pediatric Cardiologist _______________ 15/10/08/2014Eth.C | | | |

| Patient Name: **Emebet Yeshambel**. Referring Institute: **FHRH**. SEX/ Age: **F/11years**.  Date of Report: **15/10/08/14**. Referral Diagnosis: **Palpitation. AGH8.360** | | | |
| --- | --- | --- | --- |
| **Features** | **Finding** | **Features** | **Finding** |
| **Profile** |  | **Atria** |  |
| Abdominal situs | Solitus | Left atrium | Normal |
| Cardiac position | Levocardia | Right atrium | Normal |
| Systemic venous drainage | Normal. | **Atrioventricular valves** |  |
| Pulmonary venous drainage | Normal | Mitral valve | Annulus = 21mm |
| Atrioventricular connection | Concordant | Tricuspid valve | Annulus = 21mm  TAPSE = 21mm |
| Ventriculoarterial connection | Concordant | **Ventricles** |  |
| Ventricular loop | d-Loop | Left ventricle | Normal |
|  |  | Right ventricle | Normal |
| **Septae** |  | **Coronary arteries** | ----- |
| Interventricular septum | Intact | **Doppler Measurement** |  |
| Interatrial septum | Intact | Mitral | ----- |
| **Semilunar valves** |  | Aortic | ------- |
| Aortic valve | Annulus = 16mm | Tricuspid | ------- |
| Pulmonary valve | Annulus = 17mm | pulmonic | -------- |
| **Great arteries** | NRGA | **Aortic arch** | Left. No CoA. |
| Aorta | ----- | **PDA** | No |
| Pulmonary artery | Normal MPA and Branch PAs. |  |  |
| **M-Mode:** | | | |
| AO | mm | PWd | mm |
| LA | mm | PWs | mm |
| LVIDd | mm | EDV | ml |
| LVIDs | mm | ESV | ml |
| IVSs | mm | LVEF | 60% |
| IVSd | mm | FS | 32% |
| **Additional Information**: |  | | |
| No pericardial/Pleural effusion. | | | |
| **Final Diagnosis:** | | | |
| 1. Normal Echocardiography Study. | | | |
| **Remark**: | | | |
| **Recommendation**: | | | |
| SIGNATURE  Done by: Tesfaye T., Pediatrician, Pediatric Cardiologist _______________ 15/10/08/2014Eth.C | | | |

| Patient Name: **Getaneh Tesfaye**. Referring Institute: **FHRH**. SEX/ Age: **M/11years**.  Date of Report: **16/10/08/14**. Referral Diagnosis: **Easy fatigability. AGH8.361** | | | |
| --- | --- | --- | --- |
| **Features** | **Finding** | **Features** | **Finding** |
| **Profile** |  | **Atria** |  |
| Abdominal situs | Solitus | Left atrium | Normal |
| Cardiac position | Levocardia | Right atrium | Normal |
| Systemic venous drainage | Normal. | **Atrioventricular valves** |  |
| Pulmonary venous drainage | Normal | Mitral valve | Annulus = 21mm |
| Atrioventricular connection | Concordant | Tricuspid valve | Annulus = 21mm  TAPSE = 23mm |
| Ventriculoarterial connection | Concordant | **Ventricles** |  |
| Ventricular loop | d-Loop | Left ventricle | Normal |
|  |  | Right ventricle | Normal |
| **Septae** |  | **Coronary arteries** | ----- |
| Interventricular septum | Intact | **Doppler Measurement** |  |
| Interatrial septum | Intact | Mitral | ----- |
| **Semilunar valves** |  | Aortic | ------- |
| Aortic valve | Annulus = 16mm | Tricuspid | Trivial TR, PPG = 27mmHg |
| Pulmonary valve | Annulus = 19mm | pulmonic | -------- |
| **Great arteries** | NRGA | **Aortic arch** | Left. No CoA. |
| Aorta | ----- | **PDA** | No |
| Pulmonary artery | Normal MPA and Branch PAs. |  |  |
| **M-Mode:** | | | |
| AO | mm | PWd | mm |
| LA | mm | PWs | mm |
| LVIDd | mm | EDV | ml |
| LVIDs | mm | ESV | ml |
| IVSs | mm | LVEF | 69% |
| IVSd | mm | FS | 38% |
| **Additional Information**: |  | | |
| No pericardial/Pleural effusion. | | | |
| **Final Diagnosis:** | | | |
| 1. Normal Echocardiography Study. | | | |
| **Remark**: | | | |
| **Recommendation**: | | | |
| SIGNATURE  Done by: Tesfaye T., Pediatrician, Pediatric Cardiologist _______________ 16/10/08/2014Eth.C | | | |

| Patient Name: **Miftah Demissie**. Referring Institute: **Mekane-Selam GH**. SEX/ Age: **M/7years**.  Date of Report: **16/10/08/14**. Referral Diagnosis: **FTT. AGH8.362** | | | |
| --- | --- | --- | --- |
| **Features** | **Finding** | **Features** | **Finding** |
| **Profile** |  | **Atria** |  |
| Abdominal situs | Solitus | Left atrium | Normal |
| Cardiac position | Levocardia | Right atrium | Normal |
| Systemic venous drainage | Normal. | **Atrioventricular valves** |  |
| Pulmonary venous drainage | Normal | Mitral valve | Annulus = 18mm |
| Atrioventricular connection | Concordant | Tricuspid valve | Annulus = 18mm |
| Ventriculoarterial connection | Concordant | **Ventricles** |  |
| Ventricular loop | d-Loop | Left ventricle | Normal |
|  |  | Right ventricle | Normal |
| **Septae** |  | **Coronary arteries** | ----- |
| Interventricular septum | Intact | **Doppler Measurement** |  |
| Interatrial septum | Intact | Mitral | ----- |
| **Semilunar valves** |  | Aortic | ------- |
| Aortic valve | Annulus = 14mm | Tricuspid | ------- |
| Pulmonary valve | Annulus = 16mm | pulmonic | -------- |
| **Great arteries** | NRGA | **Aortic arch** | Left. No CoA. |
| Aorta | ----- | **PDA** | No |
| Pulmonary artery | Normal MPA and Branch PAs. |  |  |
| **M-Mode:**  Normal LV Functio on eye balling | | | |
| AO | mm | PWd | mm |
| LA | mm | PWs | mm |
| LVIDd | mm | EDV | ml |
| LVIDs | mm | ESV | ml |
| IVSs | mm | LVEF | % |
| IVSd | mm | FS | % |
| **Additional Information**: |  | | |
| No pericardial/Pleural effusion. | | | |
| **Final Diagnosis:** | | | |
| 1. Normal Echocardiography Study. | | | |
| **Remark**: | | | |
| **Recommendation**: | | | |
| SIGNATURE  Done by: Tesfaye T., Pediatrician, Pediatric Cardiologist _______________ 16/10/08/2014Eth.C | | | |

| Patient Name: **Seble Demeke**. Referring Institute: **Mekane-Selam GH**. SEX/ Age: **F/14years**.  Date of Report: **16/10/08/14**. Referral Diagnosis: **Rheumatic Recurrence. AGH8.363** | | | |
| --- | --- | --- | --- |
| **Features** | **Finding** | **Features** | **Finding** |
| **Profile** |  | **Atria** |  |
| Abdominal situs | Solitus | Left atrium | Mildly Dilated |
| Cardiac position | Levocardia | Right atrium | Normal |
| Systemic venous drainage | Normal. | **Atrioventricular valves** |  |
| Pulmonary venous drainage | Normal | Mitral valve | Annulus = 28mm |
| Atrioventricular connection | Concordant | Tricuspid valve | Annulus = 24mm  TAPSE = mm |
| Ventriculoarterial connection | Concordant | **Ventricles** |  |
| Ventricular loop | d-Loop | Left ventricle | Mildly Dilated |
|  |  | Right ventricle | Normal |
| **Septae** |  | **Coronary arteries** | ----- |
| Interventricular septum | Intact | **Doppler Measurement** |  |
| Interatrial septum | Intact | Mitral | ----- |
| **Semilunar valves** |  | Aortic | Moderate AR, PHT = 395ms. |
| Aortic valve | Annulus = 22mm. trileaflet. Thickened AVL. | Tricuspid | ------- |
| Pulmonary valve | Annulus = 26mm | pulmonic | -------- |
| **Great arteries** | NRGA | **Aortic arch** | Left. No CoA. |
| Aorta | ----- | **PDA** | No |
| Pulmonary artery | Normal MPA and Branch PAs. |  |  |
| **M-Mode:** | | | |
| AO | mm | PWd | mm |
| LA | mm | PWs | mm |
| LVIDd | mm | EDV | ml |
| LVIDs | mm | ESV | ml |
| IVSs | mm | LVEF | 62% |
| IVSd | mm | FS | 34% |
| **Additional Information**: |  | | |
| No pericardial/Pleural effusion. | | | |
| **Final Diagnosis:** | | | |
| 1. {S, D, S} Levocardia. 2. Mildly Dilated LA/LV 3. Thickened AVL 4. Moderate AR 5. Normal LV Systolic Function | | | |
| **Remark**: | | | |
| **Recommendation**: | | | |
| SIGNATURE  Done by: Tesfaye T., Pediatrician, Pediatric Cardiologist _______________ 16/10/08/2014Eth.C | | | |

| Patient Name: **Markon Muluken**. Referring Institute: **Adinas GH**. SEX/ Age: **M/10years**. 9years/26/04/14Eth.C  Date of Report: **16/10/08/14**. Referral Diagnosis: **Follow up** **CRHD( Mild MR) + Sydenham’s Chorea. AGH8.364** | | | |
| --- | --- | --- | --- |
| **Features** | **Finding** | **Features** | **Finding** |
| **Profile** |  | **Atria** |  |
| Abdominal situs | Solitus | Left atrium | Normal |
| Cardiac position | Levocardia | Right atrium | Normal |
| Systemic venous drainage | Normal. | **Atrioventricular valves** |  |
| Pulmonary venous drainage | Normal | Mitral valve | Annulus = 21mm |
| Atrioventricular connection | Concordant | Tricuspid valve | Annulus = 22mm  TAPSE = 19mm |
| Ventriculoarterial connection | Concordant | **Ventricles** |  |
| Ventricular loop | d-Loop | Left ventricle | Normal |
|  |  | Right ventricle | Normal |
| **Septae** |  | **Coronary arteries** | ----- |
| Interventricular septum | Intact | **Doppler Measurement** |  |
| Interatrial septum | Intact | Mitral | Trivial MR, Incomplete Signal, seen in apical view, jet velocity = 1.1m/sec |
| **Semilunar valves** |  | Aortic | ------- |
| Aortic valve | Annulus = 16mm | Tricuspid | ------- |
| Pulmonary valve | Annulus = 19mm | pulmonic | -------- |
| **Great arteries** | NRGA | **Aortic arch** | Left. No CoA. |
| Aorta | ----- | **PDA** | No |
| Pulmonary artery | Normal MPA and Branch PAs. |  |  |
| **M-Mode:** | | | |
| AO | Mm | PWd | mm |
| LA | Mm | PWs | mm |
| LVIDd | mm | EDV | ml |
| LVIDs | mm | ESV | ml |
| IVSs | mm | LVEF | 60% |
| IVSd | mm | FS | 31% |
| **Additional Information**: |  | | |
| No pericardial/Pleural effusion. | | | |
| **Final Diagnosis:** | | | |
| 1. {S, D, S} Levocardia. 2. Trivial MR 3. Normal Biventricular Systolic Function | | | |
| **Remark**: | | | |
| **Recommendation**: | | | |
| SIGNATURE  Done by: Tesfaye T., Pediatrician, Pediatric Cardiologist _______________ 16/10/08/2014Eth.C | | | |

| Patient Name: **Mekides Yimesgen**. Referring Institute: **TGSH**. SEX/ Age: **F/5 6/12**.  Date of Report: **17/10/08/14**. Referral Diagnosis: **CHF + ARF. AGH8.365** | | | |
| --- | --- | --- | --- |
| **Features** | **Finding** | **Features** | **Finding** |
| **Profile** |  | **Atria** |  |
| Abdominal situs | Solitus | Left atrium | Dilated |
| Cardiac position | Levocardia | Right atrium | Normal |
| Systemic venous drainage | Normal. | **Atrioventricular valves** |  |
| Pulmonary venous drainage | Normal | Mitral valve | Annulus = 22mm. Thickened MVL |
| Atrioventricular connection | Concordant | Tricuspid valve | Annulus = 18mm  TAPSE = 19mm |
| Ventriculoarterial connection | Concordant | **Ventricles** |  |
| Ventricular loop | d-Loop | Left ventricle | Dilated |
|  |  | Right ventricle | Normal |
| **Septae** |  | **Coronary arteries** | ----- |
| Interventricular septum | Intact | **Doppler Measurement** |  |
| Interatrial septum | Intact | Mitral | Severe MR, Holosystolic, posterior projection, seen in two planes with jet velocity = 3.8m/sec. |
| **Semilunar valves** |  | Aortic | Mild AR, PHT = 505ms. |
| Aortic valve | Annulus = 14mm | Tricuspid | Mild TR, PPG = 31mmHg |
| Pulmonary valve | Annulus = 16mm | pulmonic | -------- |
| **Great arteries** | NRGA | **Aortic arch** | Left. No CoA. |
| Aorta | ----- | **PDA** | No |
| Pulmonary artery | Normal MPA and Branch PAs. |  |  |
| **M-Mode:** | | | |
| AO | Mm | PWd | mm |
| LA | Mm | PWs | mm |
| LVIDd | Mm | EDV | ml |
| LVIDs | Mm | ESV | ml |
| IVSs | Mm | LVEF | 61% |
| IVSd | Mm | FS | 32% |
| **Additional Information**: |  | | |
| No pericardial/Pleural effusion. | | | |
| **Final Diagnosis:** | | | |
| 1. {S, D, S} Levocardia. 2. LA/LV Dilated 3. Thickened MVL 4. Severe MR 5. Mild AR 6. Mild TR 7. Normal Biventricular Systolic Function | | | |
| **Remark**: | | | |
| **Recommendation**: | | | |
| SIGNATURE  Done by: Tesfaye T., Pediatrician, Pediatric Cardiologist _______________ 17/10/08/2014Eth.C | | | |

| Patient Name: **Baby of Mastewal Abeje**. Referring Institute: **TGSH**. SEX/ Age: **M/3days**.  Date of Report: **17/10/08/14**. Referral Diagnosis: **Cardiomegaly on CXR. AGH8.366** | | | |
| --- | --- | --- | --- |
| **Features** | **Finding** | **Features** | **Finding** |
| **Profile** |  | **Atria** |  |
| Abdominal situs | Solitus | Left atrium | Normal |
| Cardiac position | Levocardia | Right atrium | Normal |
| Systemic venous drainage | Normal. | **Atrioventricular valves** |  |
| Pulmonary venous drainage | Normal | Mitral valve | Annulus = 10mm |
| Atrioventricular connection | Concordant | Tricuspid valve | Annulus = 10mm |
| Ventriculoarterial connection | Concordant | **Ventricles** |  |
| Ventricular loop | d-Loop | Left ventricle | Normal |
|  |  | Right ventricle | Normal |
| **Septae** |  | **Coronary arteries** | ----- |
| Interventricular septum | Intact | **Doppler Measurement** |  |
| Interatrial septum | PFO, L – R Shunt | Mitral | ----- |
| **Semilunar valves** |  | Aortic | ------- |
| Aortic valve | Annulus = 9mm | Tricuspid | ------- |
| Pulmonary valve | Annulus = 9mm | pulmonic | -------- |
| **Great arteries** | NRGA | **Aortic arch** | Left. No CoA. |
| Aorta | ----- | **PDA** | No |
| Pulmonary artery | Normal MPA and Branch PAs. |  |  |
| **M-Mode:**  Normal LV Function on eye balling | | | |
| AO | mm | PWd | mm |
| LA | mm | PWs | mm |
| LVIDd | mm | EDV | ml |
| LVIDs | mm | ESV | ml |
| IVSs | mm | LVEF | % |
| IVSd | mm | FS | % |
| **Additional Information**: |  | | |
| No pericardial/Pleural effusion. | | | |
| **Final Diagnosis:** | | | |
| 1. {S, D, S} Levocardia. 2. PFO, L – R Shunt | | | |
| **Remark**: | | | |
| **Recommendation**: | | | |
| SIGNATURE  Done by: Tesfaye T., Pediatrician, Pediatric Cardiologist _______________ 17/10/08/2014Eth.C | | | |

| Patient Name: **Meaza-werq Abie**. Referring Institute: **Adinas GH**. SEX/ Age: **F/3years**.  Date of Report: **17/10/08/14**. Referral Diagnosis: **_______. INCONCLUSIVE DATA** | | | |
| --- | --- | --- | --- |
| **Features** | **Finding** | **Features** | **Finding** |
| **Profile** |  | **Atria** |  |
| Abdominal situs | Solitus | Left atrium | Normal |
| Cardiac position | Levocardia | Right atrium | Normal |
| Systemic venous drainage | Normal. | **Atrioventricular valves** |  |
| Pulmonary venous drainage | Normal | Mitral valve | Annulus = 17mm |
| Atrioventricular connection | Concordant | Tricuspid valve | Annulus = 17mm  TAPSE = mm |
| Ventriculoarterial connection | Concordant | **Ventricles** |  |
| Ventricular loop | d-Loop | Left ventricle | Normal |
|  |  | Right ventricle | Normal |
| **Septae** |  | **Coronary arteries** | ----- |
| Interventricular septum | Intact | **Doppler Measurement** |  |
| Interatrial septum | Intact | Mitral | Mild MR, Holosystolic, anterior projection, seen in two planes with jet velocity = 3.4m/sec. MR arising from the Anterior MV itself. |
| **Semilunar valves** |  | Aortic | ------- |
| Aortic valve | Annulus = 12mm | Tricuspid | ------- |
| Pulmonary valve | Annulus = 14mm | pulmonic | -------- |
| **Great arteries** | NRGA | **Aortic arch** | Left. No CoA. |
| Aorta | ----- | **PDA** | No |
| Pulmonary artery | Normal MPA and Branch PAs. |  |  |
| **M-Mode:** | | | |
| AO | mm | PWd | mm |
| LA | mm | PWs | mm |
| LVIDd | mm | EDV | ml |
| LVIDs | mm | ESV | ml |
| IVSs | mm | LVEF | 59% |
| IVSd | mm | FS | 31% |
| **Additional Information**: |  | | |
| No pericardial/Pleural effusion. | | | |
| **Final Diagnosis:** | | | |
| 1. {S, D, S} Levocardia. 2. Mild MR 3. ?Mild anterior MV Cleft 4. Normal LV Systoic Function | | | |
| **Remark**: | | | |
| **Recommendation**: | | | |
| SIGNATURE  Done by: Tesfaye T., Pediatrician, Pediatric Cardiologist _______________ 17/10/08/2014Eth.C | | | |

| Patient Name: **Bisrat Abateneh**. Referring Institute: **TGSH**. SEX/ Age: **M/2months**.  Date of Report: **18/10/08/14**. Referral Diagnosis: **DS.** **AGH8.367** | | | |
| --- | --- | --- | --- |
| **Features** | **Finding** | **Features** | **Finding** |
| **Profile** |  | **Atria** |  |
| Abdominal situs | Solitus | Left atrium | Normal |
| Cardiac position | Levocardia | Right atrium | Normal |
| Systemic venous drainage | Normal. | **Atrioventricular valves** |  |
| Pulmonary venous drainage | Normal | Mitral valve | Annulus = 10mm |
| Atrioventricular connection | Concordant | Tricuspid valve | Annulus = 11mm |
| Ventriculoarterial connection | Concordant | **Ventricles** |  |
| Ventricular loop | d-Loop | Left ventricle | Normal |
|  |  | Right ventricle | Normal |
| **Septae** |  | **Coronary arteries** | ----- |
| Interventricular septum | Intact | **Doppler Measurement** |  |
| Interatrial septum | Intact | Mitral | ----- |
| **Semilunar valves** |  | Aortic | ------- |
| Aortic valve | Annulus = 9mm | Tricuspid | ------- |
| Pulmonary valve | Annulus = 10mm | pulmonic | -------- |
| **Great arteries** | NRGA | **Aortic arch** | Left. No CoA. |
| Aorta | ----- | **PDA** | No |
| Pulmonary artery | Normal MPA and Branch PAs. |  |  |
| **M-Mode:**  Normal LV Function on eye balling | | | |
| AO | mm | PWd | mm |
| LA | mm | PWs | mm |
| LVIDd | mm | EDV | ml |
| LVIDs | mm | ESV | ml |
| IVSs | mm | LVEF | % |
| IVSd | mm | FS | % |
| **Additional Information**: |  | | |
| No pericardial/Pleural effusion. | | | |
| **Final Diagnosis:** | | | |
| 1. Normal Echocardiography Study. | | | |
| **Remark**: Infant was crying during study | | | |
| **Recommendation**: | | | |
| SIGNATURE  Done by: Tesfaye T., Pediatrician, Pediatric Cardiologist _______________ 18/10/08/2014Eth.C | | | |

| Patient Name: **Baby of Hamda Abuker**. Referring Institute: **TGSH**. SEX/ Age: **F/17days**.  Date of Report: **18/10/08/14**. Referral Diagnosis: **Cyanosis. AGH8.368** | | | |
| --- | --- | --- | --- |
| **Features** | **Finding** | **Features** | **Finding** |
| **Profile** |  | **Atria** |  |
| Abdominal situs | Solitus | Left atrium | Normal |
| Cardiac position | Levocardia | Right atrium | Normal |
| Systemic venous drainage | Normal. | **Atrioventricular valves** |  |
| Pulmonary venous drainage | Normal | Mitral valve | Annulus = 10mm |
| Atrioventricular connection | Concordant | Tricuspid valve | Annulus = 9mm |
| Ventriculoarterial connection | Concordant | **Ventricles** |  |
| Ventricular loop | d-Loop | Left ventricle | Normal |
|  |  | Right ventricle | RVH |
| **Septae** |  | **Coronary arteries** | ----- |
| Interventricular septum | Non- Restrictive Mal-Aligned Sub-Aortic VSD, R – L Shunt | **Doppler Measurement** |  |
| Interatrial septum | PFO, R – L Shunt | Mitral | ----- |
| **Semilunar valves** |  | Aortic | ------- |
| Aortic valve | Annulus = 8mm | Tricuspid | ------- |
| Pulmonary valve | Atretic | pulmonic | -------- |
| **Great arteries** | NRGA | **Aortic arch** |  |
| Aorta | Aortic Over-ride to the VSD | **PDA** | 1mm PDA, L – R Shunt |
| Pulmonary artery | Atretic MPA and Branch PAs |  |  |
| **M-Mode:** | | | |
| AO | mm | PWd | mm |
| LA | mm | PWs | mm |
| LVIDd | mm | EDV | ml |
| LVIDs | mm | ESV | ml |
| IVSs | mm | LVEF | % |
| IVSd | mm | FS | % |
| **Additional Information**: |  | | |
| No pericardial/Pleural effusion. | | | |
| **Final Diagnosis:** | | | |
| 1. {S, D, S} Levocardia. 2. PFO, R – L Shunt 3. TOF (extreme Variant) 4. Atretic MPA and Branch PAs. 5. PDA, L – R Shunt | | | |
| **Remark**: | | | |
| **Recommendation**: | | | |
| SIGNATURE  Done by: Tesfaye T., Pediatrician, Pediatric Cardiologist _______________ 18/10/08/2014Eth.C | | | |

| Patient Name: **Bereket Semahegn**. Referring Institute: **FHRH**. SEX/ Age: **M/5years**.  Date of Report: **20/10/08/14**. Referral Diagnosis: **FTT. AGH8.369** | | | |
| --- | --- | --- | --- |
| **Features** | **Finding** | **Features** | **Finding** |
| **Profile** |  | **Atria** |  |
| Abdominal situs | Solitus | Left atrium | Normal |
| Cardiac position | Levocardia | Right atrium | Normal |
| Systemic venous drainage | Normal. | **Atrioventricular valves** |  |
| Pulmonary venous drainage | Normal | Mitral valve | Annulus = 16mm |
| Atrioventricular connection | Concordant | Tricuspid valve | Annulus = 16mm  TAPSE = 16mm |
| Ventriculoarterial connection | Concordant | **Ventricles** |  |
| Ventricular loop | d-Loop | Left ventricle | Normal |
|  |  | Right ventricle | Normal |
| **Septae** |  | **Coronary arteries** | ----- |
| Interventricular septum | Intact | **Doppler Measurement** |  |
| Interatrial septum | Intact | Mitral | ----- |
| **Semilunar valves** |  | Aortic | ------- |
| Aortic valve | Annulus = 12mm | Tricuspid | ------- |
| Pulmonary valve | Annulus = 14mm | pulmonic | -------- |
| **Great arteries** | NRGA | **Aortic arch** | Left. No CoA. |
| Aorta | ----- | **PDA** | No |
| Pulmonary artery | Normal MPA and Branch PAs. |  |  |
| **M-Mode:**  Normal LV Function on eye balling | | | |
| AO | mm | PWd | mm |
| LA | mm | PWs | mm |
| LVIDd | mm | EDV | ml |
| LVIDs | mm | ESV | ml |
| IVSs | mm | LVEF | % |
| IVSd | mm | FS | % |
| **Additional Information**: |  | | |
| No pericardial/Pleural effusion. | | | |
| **Final Diagnosis:** | | | |
| 1. Normal Echocardiography study. | | | |
| **Remark**: | | | |
| **Recommendation**: | | | |
| SIGNATURE  Done by: Tesfaye T., Pediatrician, Pediatric Cardiologist _______________ 20/10/08/2014Eth.C | | | |

| Patient Name: **Yichalal Babey**. Referring Institute: **Addis Alem PH**. SEX/ Age: **M/10years**.  Date of Report: **20/10/08/14**. Referral Diagnosis: **Easy fatigability. AGH8.370** | | | |
| --- | --- | --- | --- |
| **Features** | **Finding** | **Features** | **Finding** |
| **Profile** |  | **Atria** |  |
| Abdominal situs | Solitus | Left atrium | Normal |
| Cardiac position | Levocardia | Right atrium | Normal |
| Systemic venous drainage | Normal. | **Atrioventricular valves** |  |
| Pulmonary venous drainage | Normal | Mitral valve | Annulus = 21mm |
| Atrioventricular connection | Concordant | Tricuspid valve | Annulus = 24mm  TAPSE = 24mm |
| Ventriculoarterial connection | Concordant | **Ventricles** |  |
| Ventricular loop | d-Loop | Left ventricle | Normal |
|  |  | Right ventricle | Normal |
| **Septae** |  | **Coronary arteries** | ----- |
| Interventricular septum | Intact | **Doppler Measurement** |  |
| Interatrial septum | Intact | Mitral | ----- |
| **Semilunar valves** |  | Aortic | ------- |
| Aortic valve | Annulus = 16mm | Tricuspid | ------- |
| Pulmonary valve | Annulus = 20mm | pulmonic | Trivial PR, PPG = 10mmHg |
| **Great arteries** | NRGA | **Aortic arch** | Left. No CoA. |
| Aorta | ----- | **PDA** | No |
| Pulmonary artery | Normal MPA and Branch PAs. |  |  |
| **M-Mode:** | | | |
| AO | mm | PWd | mm |
| LA | mm | PWs | mm |
| LVIDd | mm | EDV | ml |
| LVIDs | mm | ESV | ml |
| IVSs | mm | LVEF | 59% |
| IVSd | mm | FS | 31% |
| **Additional Information**: |  | | |
| No pericardial/Pleural effusion. | | | |
| **Final Diagnosis:** | | | |
| 1. Normal Echocardiography Study. | | | |
| **Remark**: | | | |
| **Recommendation**: | | | |
| SIGNATURE  Done by: Tesfaye T., Pediatrician, Pediatric Cardiologist _______________ 20/10/08/2014Eth.C | | | |

| Patient Name: **Eyosias Alganeh**. Referring Institute: **Dr. Addisu Pediatric Speciality Clinic**. SEX/ Age: **M/45days**.  Date of Report: **20/10/08/14**. Referral Diagnosis: **DS. AGH8.371** | | | |
| --- | --- | --- | --- |
| **Features** | **Finding** | **Features** | **Finding** |
| **Profile** |  | **Atria** |  |
| Abdominal situs | Solitus | Left atrium | Normal |
| Cardiac position | Levocardia | Right atrium | Normal |
| Systemic venous drainage | Normal. | **Atrioventricular valves** |  |
| Pulmonary venous drainage | Normal | Mitral valve | Annulus = 13mm |
| Atrioventricular connection | Concordant | Tricuspid valve | Annulus = 15mm  TAPSE = 15mm |
| Ventriculoarterial connection | Concordant | **Ventricles** |  |
| Ventricular loop | d-Loop | Left ventricle | Normal |
|  |  | Right ventricle | Normal |
| **Septae** |  | **Coronary arteries** | ----- |
| Interventricular septum | Intact | **Doppler Measurement** |  |
| Interatrial septum | PFO, L – R Shunt | Mitral | ----- |
| **Semilunar valves** |  | Aortic | ------- |
| Aortic valve | Annulus = 11mm | Tricuspid | Trivial TR, PPG = 32mmHg |
| Pulmonary valve | Annulus = 12mm | pulmonic | Mild PR, PPG = 27mmHg |
| **Great arteries** | NRGA | **Aortic arch** | Left. No CoA. |
| Aorta | ----- | **PDA** | No |
| Pulmonary artery | Normal MPA and Branch PAs. |  |  |
| **M-Mode:**  Normal LV Function on eye balling | | | |
| AO | mm | PWd | mm |
| LA | mm | PWs | mm |
| LVIDd | mm | EDV | ml |
| LVIDs | mm | ESV | ml |
| IVSs | mm | LVEF | % |
| IVSd | mm | FS | % |
| **Additional Information**: |  | | |
| No pericardial/Pleural effusion. | | | |
| **Final Diagnosis:** | | | |
| 1. {S, D, S} Levocardia. 2. PFO, L – R Shunt 3. Mild PR 4. Trivial TR | | | |
| **Remark**: | | | |
| **Recommendation**: | | | |
| SIGNATURE  Done by: Tesfaye T., Pediatrician, Pediatric Cardiologist _______________ 20/10/08/2014Eth.C | | | |

| Patient Name: **Dagne Dessie**. Referring Institute: **FHRH**. SEX/ Age: **M/55days**. Date of Report: **20/10/08/14**. Referral Diagnosis: **Incidental Murmur Finding. AGH8.372** | | | |
| --- | --- | --- | --- |
| **Features** | **Finding** | **Features** | **Finding** |
| **Profile** |  | **Atria** |  |
| Abdominal situs | Solitus | Left atrium | Mild Dilatation |
| Cardiac position | Levocardia | Right atrium | Normal |
| Systemic venous drainage | Normal. | **Atrioventricular valves** |  |
| Pulmonary venous drainage | Normal | Mitral valve | Annulus = 9mm |
| Atrioventricular connection | Concordant | Tricuspid valve | Annulus = 8mm |
| Ventriculoarterial connection | Concordant | **Ventricles** |  |
| Ventricular loop | d-Loop | Left ventricle | Mild Dilatation |
|  |  | Right ventricle | Normal |
| **Septae** |  | **Coronary arteries** | ----- |
| Interventricular septum | Intact | **Doppler Measurement** |  |
| Interatrial septum | PFO, L – R Shunt | Mitral | ----- |
| **Semilunar valves** |  | Aortic | ------- |
| Aortic valve | Annulus = 8mm | Tricuspid | ------- |
| Pulmonary valve | Annulus = mm | pulmonic | -------- |
| **Great arteries** | NRGA | **Aortic arch** | Left. No CoA. |
| Aorta | ----- | **PDA** | 1.5mm PDA, L – R Shunt |
| Pulmonary artery | Normal MPA and Branch PAs. |  |  |
| **M-Mode:**  Normal LV Function on eye balling | | | |
| AO | mm | PWd | mm |
| LA | mm | PWs | mm |
| LVIDd | mm | EDV | ml |
| LVIDs | mm | ESV | ml |
| IVSs | mm | LVEF | % |
| IVSd | mm | FS | % |
| **Additional Information**: |  | | |
| No pericardial/Pleural effusion. | | | |
| **Final Diagnosis:** | | | |
| 1. {S, D, S} Levocardia. 2. PFO, L – R Shunt 3. Small PDA, L – R Shunt 4. Normal LV Systolic Function | | | |
| **Remark**: | | | |
| **Recommendation**: | | | |
| SIGNATURE  Done by: Tesfaye T., Pediatrician, Pediatric Cardiologist _______________ 20/10/08/2014Eth.C | | | |

| Patient Name: **Iman Kassie**. Referring Institute: **Mekane-Selam**. SEX/ Age: **F/1 3/12**.  Date of Report: **20/10/08/14**. Referral Diagnosis: **FTT. AGH8.373** | | | |
| --- | --- | --- | --- |
| **Features** | **Finding** | **Features** | **Finding** |
| **Profile** |  | **Atria** |  |
| Abdominal situs | Solitus | Left atrium | Normal |
| Cardiac position | Levocardia | Right atrium | Normal |
| Systemic venous drainage | Normal. | **Atrioventricular valves** |  |
| Pulmonary venous drainage | Normal | Mitral valve | Annulus = 13mm |
| Atrioventricular connection | Concordant | Tricuspid valve | Annulus = 13mm |
| Ventriculoarterial connection | Concordant | **Ventricles** |  |
| Ventricular loop | d-Loop | Left ventricle | Normal |
|  |  | Right ventricle | Normal |
| **Septae** |  | **Coronary arteries** | ----- |
| Interventricular septum | Intact | **Doppler Measurement** |  |
| Interatrial septum | PFO, L – R Shunt | Mitral | ----- |
| **Semilunar valves** |  | Aortic | ------- |
| Aortic valve | Annulus = 12mm | Tricuspid | ------- |
| Pulmonary valve | Annulus = 12mm | pulmonic | -------- |
| **Great arteries** | NRGA | **Aortic arch** | Left. No CoA. |
| Aorta | ----- | **PDA** | No |
| Pulmonary artery | Normal MPA and Branch PAs. |  |  |
| **M-Mode:**  Normal LV Function on eye balling | | | |
| AO | mm | PWd | mm |
| LA | mm | PWs | mm |
| LVIDd | mm | EDV | ml |
| LVIDs | mm | ESV | ml |
| IVSs | mm | LVEF | % |
| IVSd | mm | FS | % |
| **Additional Information**: |  | | |
| No pericardial/Pleural effusion. | | | |
| **Final Diagnosis:** | | | |
| 1. {S, D, S} Levocardia. 2. PFO, L – R Shunt 3. Normal LV Systolic Function | | | |
| **Remark**: | | | |
| **Recommendation**: | | | |
| SIGNATURE  Done by: Tesfaye T., Pediatrician, Pediatric Cardiologist _______________ 20/10/08/2014Eth.C | | | |

| Patient Name: **Bahiru Lamesgin**. Referring Institute: **TGSH**. SEX/ Age: **M/4 10/12**.  Date of Report: **21/10/08/14**. Referral Diagnosis: **Chest Pain. AGH8.374** | | | |
| --- | --- | --- | --- |
| **Features** | **Finding** | **Features** | **Finding** |
| **Profile** |  | **Atria** |  |
| Abdominal situs | Solitus | Left atrium | Normal |
| Cardiac position | Levocardia | Right atrium | Normal |
| Systemic venous drainage | Normal. | **Atrioventricular valves** |  |
| Pulmonary venous drainage | Normal | Mitral valve | Annulus = 18mm |
| Atrioventricular connection | Concordant | Tricuspid valve | Annulus = 18mm  TAPSE = 20mm |
| Ventriculoarterial connection | Concordant | **Ventricles** |  |
| Ventricular loop | d-Loop | Left ventricle | Normal |
|  |  | Right ventricle | Normal |
| **Septae** |  | **Coronary arteries** | ----- |
| Interventricular septum | Intact | **Doppler Measurement** |  |
| Interatrial septum | Intact | Mitral | ----- |
| **Semilunar valves** |  | Aortic | ------- |
| Aortic valve | Annulus = 13mm | Tricuspid | ------- |
| Pulmonary valve | Annulus = 13mm | pulmonic | -------- |
| **Great arteries** | NRGA | **Aortic arch** | Left. No CoA. |
| Aorta | ----- | **PDA** | No |
| Pulmonary artery | Normal MPA and Branch PAs. |  |  |
| **M-Mode:**  Normal LV Function on eye balling | | | |
| AO | mm | PWd | mm |
| LA | mm | PWs | mm |
| LVIDd | mm | EDV | ml |
| LVIDs | mm | ESV | ml |
| IVSs | mm | LVEF | % |
| IVSd | mm | FS | % |
| **Additional Information**: |  | | |
| Pericardial effusion with maximum depth of 3mm on RV Side. | | | |
| **Final Diagnosis:** | | | |
| 1. {S, D, S} Levocardia. 2. Trace pericardial effusion | | | |
| **Remark**: Pericarditis can be considered | | | |
| **Recommendation**: | | | |
| SIGNATURE  Done by: Tesfaye T., Pediatrician, Pediatric Cardiologist _______________ 21/10/08/2014Eth.C | | | |

| Patient Name: **Baby of Aster Asmamaw**. Referring Institute: **FHRH**. SEX/ Age: **F/16days**.  Date of Report: **21/10/08/14**. Referral Diagnosis: **Incidental Murmur Finding. AGH8.375** | | | |
| --- | --- | --- | --- |
| **Features** | **Finding** | **Features** | **Finding** |
| **Profile** |  | **Atria** |  |
| Abdominal situs | Solitus | Left atrium | Normal |
| Cardiac position | Levocardia | Right atrium | Normal |
| Systemic venous drainage | Normal. | **Atrioventricular valves** |  |
| Pulmonary venous drainage | Normal | Mitral valve | Annulus = 11mm |
| Atrioventricular connection | Concordant | Tricuspid valve | Annulus = 10mm |
| Ventriculoarterial connection | Concordant | **Ventricles** |  |
| Ventricular loop | d-Loop | Left ventricle | Normal |
|  |  | Right ventricle | Normal |
| **Septae** |  | **Coronary arteries** | ----- |
| Interventricular septum | 2mm Upper Muscular VSD, L – R Shunt | **Doppler Measurement** |  |
| Interatrial septum | PFO, L – R Shunt | Mitral | ----- |
| **Semilunar valves** |  | Aortic | ------- |
| Aortic valve | Annulus = 8mm | Tricuspid | ------- |
| Pulmonary valve | Annulus = 10mm | pulmonic | -------- |
| **Great arteries** | NRGA | **Aortic arch** | Left. No CoA. |
| Aorta | ----- | **PDA** | No |
| Pulmonary artery | Normal MPA and Branch PAs. |  |  |
| **M-Mode:** Normal LV Function on eye balling | | | |
| AO | mm | PWd | mm |
| LA | mm | PWs | mm |
| LVIDd | mm | EDV | ml |
| LVIDs | mm | ESV | ml |
| IVSs | mm | LVEF | % |
| IVSd | mm | FS | % |
| **Additional Information**: |  | | |
| No pericardial/Pleural effusion. | | | |
| **Final Diagnosis:** | | | |
| 1. {S, D, S} Levocardia. 2. PFO, L – R Shunt 3. Small Upper Muscular VSD, L – R Shunt 4. Normal LV Systolic Function | | | |
| **Remark**: | | | |
| **Recommendation**: | | | |
| SIGNATURE  Done by: Tesfaye T., Pediatrician, Pediatric Cardiologist _______________ 21/10/08/2014Eth.C | | | |

| Patient Name: **Baby of Amaled Mekonen**. Referring Institute: **FHRH**. SEX/ Age: **F/24days**.  Date of Report: **21/10/08/14**. Referral Diagnosis: **RD. AGH8.376** | | | |
| --- | --- | --- | --- |
| **Features** | **Finding** | **Features** | **Finding** |
| **Profile** |  | **Atria** |  |
| Abdominal situs | Solitus | Left atrium | Normal |
| Cardiac position | Levocardia | Right atrium | Normal |
| Systemic venous drainage | Normal. | **Atrioventricular valves** |  |
| Pulmonary venous drainage | Normal | Mitral valve | Annulus = 10mm |
| Atrioventricular connection | Concordant | Tricuspid valve | Annulus = 10mm  TAPSE = 12mm |
| Ventriculoarterial connection | Concordant | **Ventricles** |  |
| Ventricular loop | d-Loop | Left ventricle | Normal |
|  |  | Right ventricle | Normal |
| **Septae** |  | **Coronary arteries** | ----- |
| Interventricular septum | Intact | **Doppler Measurement** |  |
| Interatrial septum | Intact | Mitral | ----- |
| **Semilunar valves** |  | Aortic | ------- |
| Aortic valve | Annulus = 9mm | Tricuspid | ------- |
| Pulmonary valve | Annulus = 9mm | pulmonic | -------- |
| **Great arteries** | NRGA | **Aortic arch** | Left. No CoA. |
| Aorta | ----- | **PDA** | No |
| Pulmonary artery | Normal MPA and Branch PAs. |  |  |
| **M-Mode:**  Normal LV Function on eye balling | | | |
| AO | mm | PWd | mm |
| LA | mm | PWs | mm |
| LVIDd | mm | EDV | ml |
| LVIDs | mm | ESV | ml |
| IVSs | mm | LVEF | % |
| IVSd | mm | FS | % |
| **Additional Information**: |  | | |
| No pericardial/Pleural effusion. | | | |
| **Final Diagnosis:** | | | |
| 1. Normal Echocardiography study. | | | |
| **Remark**: | | | |
| **Recommendation**: | | | |
| SIGNATURE  Done by: Tesfaye T., Pediatrician, Pediatric Cardiologist _______________ 21/10/08/2014Eth.C | | | |

| Patient Name: **Wasihun Tilahun**. Referring Institute: **FHRH**. SEX/ Age: **M/14years**.  Date of Report: **21/10/08/14**. Referral Diagnosis: **RHD.AGH8.377** | | | |
| --- | --- | --- | --- |
| **Features** | **Finding** | **Features** | **Finding** |
| **Profile** |  | **Atria** |  |
| Abdominal situs | Solitus | Left atrium | Mildly dilated |
| Cardiac position | Levocardia | Right atrium | Normal |
| Systemic venous drainage | Normal. | **Atrioventricular valves** |  |
| Pulmonary venous drainage | Normal | Mitral valve | Annulus = 24mm |
| Atrioventricular connection | Concordant | Tricuspid valve | Annulus = 23mm |
| Ventriculoarterial connection | Concordant | **Ventricles** |  |
| Ventricular loop | d-Loop | Left ventricle | Mildly dilated |
|  |  | Right ventricle | Normal |
| **Septae** |  | **Coronary arteries** | ----- |
| Interventricular septum | Intact | **Doppler Measurement** |  |
| Interatrial septum | Intact | Mitral | ----- |
| **Semilunar valves** |  | Aortic | Moderate AR, PHT = 301ms. |
| Aortic valve | Annulus = 25mm. Trileaflet. Thickened AVL. | Tricuspid | ------- |
| Pulmonary valve | Annulus = 21mm | pulmonic | -------- |
| **Great arteries** | NRGA | **Aortic arch** | Left. No CoA. |
| Aorta | ----- | **PDA** | No |
| Pulmonary artery | Normal MPA and Branch PAs. |  |  |
| **M-Mode:** | | | |
| AO | mm | PWd | mm |
| LA | mm | PWs | mm |
| LVIDd | mm | EDV | ml |
| LVIDs | mm | ESV | ml |
| IVSs | mm | LVEF | 66% |
| IVSd | mm | FS | 37% |
| **Additional Information**: |  | | |
| No pericardial/Pleural effusion. | | | |
| **Final Diagnosis:** | | | |
| 1. {S, D, S} Levocardia. 2. Moderate AR 3. Normal LV Systolic Function | | | |
| **Remark**: | | | |
| **Recommendation**: | | | |
| SIGNATURE  Done by: Tesfaye T., Pediatrician, Pediatric Cardiologist _______________ 21/10/08/2014Eth.C | | | |

| Patient Name: **Natan Mesafint**. Referring Institute: **Adinas GH**. SEX/ Age: **M/11/12**.  Date of Report: **23/10/08/14**. Referral Diagnosis: **Easy fatigability/diaphoresis. AGH8.378** | | | |
| --- | --- | --- | --- |
| **Features** | **Finding** | **Features** | **Finding** |
| **Profile** |  | **Atria** |  |
| Abdominal situs | Solitus | Left atrium | Normal |
| Cardiac position | Levocardia | Right atrium | Normal |
| Systemic venous drainage | Normal. | **Atrioventricular valves** |  |
| Pulmonary venous drainage | Normal | Mitral valve | Annulus = 12mm |
| Atrioventricular connection | Concordant | Tricuspid valve | Annulus = 12mm  TAPSE = 16mm |
| Ventriculoarterial connection | Concordant | **Ventricles** |  |
| Ventricular loop | d-Loop | Left ventricle | Normal |
|  |  | Right ventricle | Normal |
| **Septae** |  | **Coronary arteries** | ----- |
| Interventricular septum | Intact | **Doppler Measurement** |  |
| Interatrial septum | Intact | Mitral | ----- |
| **Semilunar valves** |  | Aortic | ------- |
| Aortic valve | Annulus = 11mm | Tricuspid | ------- |
| Pulmonary valve | Annulus = 11mm | pulmonic | -------- |
| **Great arteries** | NRGA | **Aortic arch** | Left. No CoA. |
| Aorta | ----- | **PDA** | No |
| Pulmonary artery | Normal MPA and Branch PAs. |  |  |
| **M-Mode:**  Normal LV Function on eye balling | | | |
| AO | mm | PWd | mm |
| LA | mm | PWs | mm |
| LVIDd | mm | EDV | ml |
| LVIDs | mm | ESV | ml |
| IVSs | mm | LVEF | % |
| IVSd | mm | FS | % |
| **Additional Information**: |  | | |
| No pericardial/Pleural effusion. | | | |
| **Final Diagnosis:** | | | |
| 1. Normal Echocardiography Study. | | | |
| **Remark**: | | | |
| **Recommendation**: | | | |
| SIGNATURE  Done by: Tesfaye T., Pediatrician, Pediatric Cardiologist _______________ 23/10/08/2014Eth.C | | | |

| Patient Name: **Tamrie Wubet**. Referring Institute: **FHRH**. SEX/ Age: **F/9years**. Date of Report: **24/10/08/14**. Referral Diagnosis: **Sydenham’s Chorea. AGH8.379** | | | |
| --- | --- | --- | --- |
| **Features** | **Finding** | **Features** | **Finding** |
| **Profile** |  | **Atria** |  |
| Abdominal situs | Solitus | Left atrium | Normal |
| Cardiac position | Levocardia | Right atrium | Normal |
| Systemic venous drainage | Normal. | **Atrioventricular valves** |  |
| Pulmonary venous drainage | Normal | Mitral valve | Annulus = 17mm |
| Atrioventricular connection | Concordant | Tricuspid valve | Annulus = 17mm  TAPSE = 23mm |
| Ventriculoarterial connection | Concordant | **Ventricles** |  |
| Ventricular loop | d-Loop | Left ventricle | Normal |
|  |  | Right ventricle | Normal |
| **Septae** |  | **Coronary arteries** | ----- |
| Interventricular septum | Intact | **Doppler Measurement** |  |
| Interatrial septum | Intact | Mitral | ----- |
| **Semilunar valves** |  | Aortic | ------- |
| Aortic valve | Annulus = 16mm | Tricuspid | ------- |
| Pulmonary valve | Annulus = 19mm | pulmonic | -------- |
| **Great arteries** | NRGA | **Aortic arch** | Left. No CoA. |
| Aorta | ----- | **PDA** | No |
| Pulmonary artery | Normal MPA and Branch PAs. |  |  |
| **M-Mode:** | | | |
| AO | mm | PWd | mm |
| LA | mm | PWs | mm |
| LVIDd | mm | EDV | ml |
| LVIDs | mm | ESV | ml |
| IVSs | mm | LVEF | 69% |
| IVSd | mm | FS | 38% |
| **Additional Information**: |  | | |
| No pericardial/Pleural effusion. | | | |
| **Final Diagnosis:** | | | |
| 1. Normal Echocardiography Study. | | | |
| **Remark**: | | | |
| **Recommendation**: | | | |
| SIGNATURE  Done by: Tesfaye T., Pediatrician, Pediatric Cardiologist _______________ 24/10/08/2014Eth.C | | | |

| Patient Name: **Mebe’a-Tsion Wendwessen**. Referring Institute: **Amaris PSC**. SEX/ Age: **F/1 10/12**.  Date of Report: **24/10/08/14**. Referral Diagnosis: **Recurrent Chest Infection. AGH8.380** | | | |
| --- | --- | --- | --- |
| **Features** | **Finding** | **Features** | **Finding** |
| **Profile** |  | **Atria** |  |
| Abdominal situs | Solitus | Left atrium | Normal |
| Cardiac position | Levocardia | Right atrium | Normal |
| Systemic venous drainage | Normal. | **Atrioventricular valves** |  |
| Pulmonary venous drainage | Normal | Mitral valve | Annulus = 14mm |
| Atrioventricular connection | Concordant | Tricuspid valve | Annulus = 16mm  TAPSE = 18mm |
| Ventriculoarterial connection | Concordant | **Ventricles** |  |
| Ventricular loop | d-Loop | Left ventricle | Normal |
|  |  | Right ventricle | Normal |
| **Septae** |  | **Coronary arteries** | ----- |
| Interventricular septum | Intact | **Doppler Measurement** |  |
| Interatrial septum | Intact | Mitral | ----- |
| **Semilunar valves** |  | Aortic | ------- |
| Aortic valve | Annulus = 12mm | Tricuspid | ------- |
| Pulmonary valve | Annulus = 14mm | pulmonic | -------- |
| **Great arteries** | NRGA | **Aortic arch** | Left. No CoA. |
| Aorta | ----- | **PDA** | No |
| Pulmonary artery | Normal MPA and Branch PAs. |  |  |
| **M-Mode:**  Normal LV Function on eye balling | | | |
| AO | mm | PWd | mm |
| LA | mm | PWs | mm |
| LVIDd | mm | EDV | ml |
| LVIDs | mm | ESV | ml |
| IVSs | mm | LVEF | % |
| IVSd | mm | FS | % |
| **Additional Information**: |  | | |
| No pericardial/Pleural effusion. | | | |
| **Final Diagnosis:** | | | |
| 1. Normal Echocardiography Study. | | | |
| **Remark**: | | | |
| **Recommendation**: | | | |
| SIGNATURE  Done by: Tesfaye T., Pediatrician, Pediatric Cardiologist _______________ 24/10/08/2014Eth.C | | | |

| Patient Name: **Baby of Netsanet Fekadu**. Referring Institute: **FHRH**. SEX/ Age: **M/9days**.  Date of Report: **24/10/08/14**. Referral Diagnosis: **RD. AGH8.381** | | | |
| --- | --- | --- | --- |
| **Features** | **Finding** | **Features** | **Finding** |
| **Profile** |  | **Atria** |  |
| Abdominal situs | Solitus | Left atrium | Normal |
| Cardiac position | Levocardia | Right atrium | Normal |
| Systemic venous drainage | Normal. | **Atrioventricular valves** |  |
| Pulmonary venous drainage | Normal | Mitral valve | Annulus = 9mm |
| Atrioventricular connection | Concordant | Tricuspid valve | Annulus = 11mm  TAPSE = 10mm |
| Ventriculoarterial connection | Concordant | **Ventricles** |  |
| Ventricular loop | d-Loop | Left ventricle | Normal |
|  |  | Right ventricle | Normal |
| **Septae** |  | **Coronary arteries** | ----- |
| Interventricular septum | Intact | **Doppler Measurement** |  |
| Interatrial septum | Intact | Mitral | ----- |
| **Semilunar valves** |  | Aortic | ------- |
| Aortic valve | Annulus = 8mm | Tricuspid | ------- |
| Pulmonary valve | Annulus = 8mm | pulmonic | -------- |
| **Great arteries** | NRGA | **Aortic arch** | Left. No CoA. |
| Aorta | ----- | **PDA** | No |
| Pulmonary artery | Normal MPA and Branch PAs. |  |  |
| **M-Mode:**  Normal LV Function on eye balling | | | |
| AO | mm | PWd | mm |
| LA | mm | PWs | mm |
| LVIDd | mm | EDV | ml |
| LVIDs | mm | ESV | ml |
| IVSs | mm | LVEF | % |
| IVSd | mm | FS | % |
| **Additional Information**: |  | | |
| No pericardial/Pleural effusion. | | | |
| **Final Diagnosis:** | | | |
| 1. Normal Echocardiography Study. | | | |
| **Remark**: | | | |
| **Recommendation**: | | | |
| SIGNATURE  Done by: Tesfaye T., Pediatrician, Pediatric Cardiologist _______________ 24/10/08/2014Eth.C | | | |

| Patient Name: **Habtamu Moges**. Referring Institute: **FHRH**. SEX/ Age: **M/1 5/12**. Date of Report: **24/10/08/14**. Referral Diagnosis: **Cardiomegaly on CXR. AGH8.382** | | | |
| --- | --- | --- | --- |
| **Features** | **Finding** | **Features** | **Finding** |
| **Profile** |  | **Atria** |  |
| Abdominal situs | Solitus | Left atrium | Normal |
| Cardiac position | Levocardia | Right atrium | Dilated |
| Systemic venous drainage | Normal. | **Atrioventricular valves** |  |
| Pulmonary venous drainage | Normal | Mitral valve | Annulus = 16mm |
| Atrioventricular connection | Concordant | Tricuspid valve | Annulus = 17mm  TAPSE = 10mm |
| Ventriculoarterial connection | Concordant | **Ventricles** |  |
| Ventricular loop | d-Loop | Left ventricle | Normal |
|  |  | Right ventricle | Dilated |
| **Septae** |  | **Coronary arteries** | ----- |
| Interventricular septum | Intact | **Doppler Measurement** |  |
| Interatrial septum | Intact | Mitral | ----- |
| **Semilunar valves** |  | Aortic | ------- |
| Aortic valve | Annulus = 12mm | Tricuspid | Mild TR, PPG = 61mmHg. |
| Pulmonary valve | Annulus = 13mm | pulmonic | -------- |
| **Great arteries** | NRGA | **Aortic arch** | Left. No CoA. |
| Aorta | ----- | **PDA** | No |
| Pulmonary artery | MPA = 16mm. Confluent Branch PAs. |  |  |
| **M-Mode:** | | | |
| AO | mm | PWd | mm |
| LA | mm | PWs | mm |
| LVIDd | mm | EDV | ml |
| LVIDs | mm | ESV | ml |
| IVSs | mm | LVEF | 71% |
| IVSd | mm | FS | 38% |
| **Additional Information**: |  | | |
| No pericardial/Pleural effusion. | | | |
| **Final Diagnosis:** | | | |
| 1. {S, D, S} Levocardia. 2. RA/RV Dilated 3. Mild TR 4. Severe Pulmonary Hypertension secondary to ? 5. RV Systolic Dysfunction | | | |
| **Remark**: | | | |
| **Recommendation**: | | | |
| SIGNATURE  Done by: Tesfaye T., Pediatrician, Pediatric Cardiologist _______________ 24/10/08/2014Eth.C | | | |

| Patient Name: **Arsema Melesse**. Referring Institute: **Adinas GH**. SEX/ Age: **F/1 4/12**. Date of Report: **24/10/08/14**. Referral Diagnosis: **Follow up echo for Small PM VSD(3mm), L – R Shunt. AGH8.383. F/40D (18/07/2013)** | | | |
| --- | --- | --- | --- |
| **Features** | **Finding** | **Features** | **Finding** |
| **Profile** |  | **Atria** |  |
| Abdominal situs | Solitus | Left atrium | Normal |
| Cardiac position | Levocardia | Right atrium | Normal |
| Systemic venous drainage | Normal. | **Atrioventricular valves** |  |
| Pulmonary venous drainage | Normal | Mitral valve | Annulus = 14mm |
| Atrioventricular connection | Concordant | Tricuspid valve | Annulus = 15mm  TAPSE = 17mm |
| Ventriculoarterial connection | Concordant | **Ventricles** |  |
| Ventricular loop | d-Loop | Left ventricle | Normal |
|  |  | Right ventricle | Normal |
| **Septae** |  | **Coronary arteries** | ----- |
| Interventricular septum | 1.5mm PM VSD, L – R Shunt | **Doppler Measurement** |  |
| Interatrial septum | Intact | Mitral | ----- |
| **Semilunar valves** |  | Aortic | ------- |
| Aortic valve | Annulus = 12mm | Tricuspid | ------- |
| Pulmonary valve | Annulus = 15mm | pulmonic | -------- |
| **Great arteries** | NRGA | **Aortic arch** | Left. No CoA. |
| Aorta | ----- | **PDA** | No |
| Pulmonary artery | Normal MPA and Branch PAs. |  |  |
| **M-Mode:** | | | |
| AO | mm | PWd | mm |
| LA | mm | PWs | mm |
| LVIDd | mm | EDV | ml |
| LVIDs | mm | ESV | ml |
| IVSs | mm | LVEF | % |
| IVSd | mm | FS | % |
| **Additional Information**: |  | | |
| No pericardial/Pleural effusion. | | | |
| **Final Diagnosis:** | | | |
| 1. {S, D, S} Levocardia. 2. Small PM VSD, L – R Shunt | | | |
| **Remark**: Size has decreased | | | |
| **Recommendation**: see after a year | | | |
| SIGNATURE  Done by: Tesfaye T., Pediatrician, Pediatric Cardiologist _______________ 24/10/08/2014Eth.C | | | |

| Patient Name: **Eyob Adugna**. Referring Institute: **TGSH**. SEX/ Age: **M/6months**. Date of Report: **25/10/08/14**. Referral Diagnosis: **CHF + RD. AGH8.384** | | | |
| --- | --- | --- | --- |
| **Features** | **Finding** | **Features** | **Finding** |
| **Profile** |  | **Atria** |  |
| Abdominal situs | Solitus | Left atrium | Markedly Dilated |
| Cardiac position | Levocardia | Right atrium | Normal |
| Systemic venous drainage | Normal. | **Atrioventricular valves** |  |
| Pulmonary venous drainage | Normal | Mitral valve | Annulus = 14mm |
| Atrioventricular connection | Concordant | Tricuspid valve | Annulus = 12mm |
| Ventriculoarterial connection | Concordant | **Ventricles** |  |
| Ventricular loop | d-Loop | Left ventricle | Markedly Dilated |
|  |  | Right ventricle | Normal |
| **Septae** |  | **Coronary arteries** | ----- |
| Interventricular septum | Intact | **Doppler Measurement** |  |
| Interatrial septum | Intact | Mitral | Mild MR, Holosystolic, posterior projection, seen in two planes with jet velocity = 4.6m/sec |
| **Semilunar valves** |  | Aortic | ------- |
| Aortic valve | Annulus = 13mm. trileflet | Tricuspid | Mild TR, PPG = 44mmHg |
| Pulmonary valve | Annulus = 13mm | pulmonic | -------- |
| **Great arteries** | NRGA | **Aortic arch** | Left. No CoA. |
| Aorta | ----- | **PDA** | No |
| Pulmonary artery | Normal MPA and Branch PAs. | **Coronaries** | No ALCAPA |
| **M-Mode:** | | | |
| AO | mm | PWd | 7mm |
| LA | mm | PWs | 7mm |
| LVIDd | 48mm | EDV | 107ml |
| LVIDs | 41mm | ESV | 74ml |
| IVSs | 10mm | LVEF | 31% |
| IVSd | 10mm | FS | 15% |
| **Additional Information**: |  | | |
| Circumferential Pericardial effusion with maximum depth of 22mm on RA Side. | | | |
| **Final Diagnosis:** | | | |
| 1. {S, D, S} Levocardia. 2. LA/LV Markedly Dilated 3. Mild MR 4. Mild TR 5. Severely Dysfunctional LV 6. Large Pericardial effusion | | | |
| **Remark**: | | | |
| **Recommendation**: | | | |
| SIGNATURE  Done by: Tesfaye T., Pediatrician, Pediatric Cardiologist _______________ 25/10/08/2014Eth.C | | | |

| Patient Name: **Fisseha Haile-Mariam**. Referring Institute: **Dr. Aweke Pediatric Sp. Clinic**. SEX/ Age: **M/1 1/12**.  Date of Report: **25/10/08/14**. Referral Diagnosis: **Family Hx. Wt. = 9kg. Ht. = 70cm. AGH8.385** | | | |
| --- | --- | --- | --- |
| **Features** | **Finding** | **Features** | **Finding** |
| **Profile** |  | **Atria** |  |
| Abdominal situs | Solitus | Left atrium | Normal |
| Cardiac position | Levocardia | Right atrium | Normal |
| Systemic venous drainage | Normal. | **Atrioventricular valves** |  |
| Pulmonary venous drainage | Normal | Mitral valve | Annulus = 12mm. Myxomatous MVL with 4mm superior displacement of MVL |
| Atrioventricular connection | Concordant | Tricuspid valve | Annulus = 13mm  TAPSE = mm |
| Ventriculoarterial connection | Concordant | **Ventricles** |  |
| Ventricular loop | d-Loop | Left ventricle | Normal |
|  |  | Right ventricle | Normal |
| **Septae** |  | **Coronary arteries** | ----- |
| Interventricular septum | Intact | **Doppler Measurement** |  |
| Interatrial septum | Intact | Mitral | Mild MR |
| **Semilunar valves** |  | Aortic | ------- |
| Aortic valve | Annulus = 12mm. **SV = 20. STJ = 17mm** | Tricuspid | ------- |
| Pulmonary valve | **Annulus = 16mm** | pulmonic | -------- |
| **Great arteries** | NRGA | **Aortic arch** | Left. No CoA. |
| Aorta | ----- | **PDA** | No |
| Pulmonary artery | **MPA = 16mm**. Confluent Branch PAs. |  |  |
| **M-Mode:**  Normal LV Function on eye balling | | | |
| AO | mm | PWd | mm |
| LA | mm | PWs | mm |
| LVIDd | mm | EDV | ml |
| LVIDs | mm | ESV | ml |
| IVSs | mm | LVEF | % |
| IVSd | mm | FS | % |
| **Additional Information**: |  | | |
| No pericardial/Pleural effusion. | | | |
| **Final Diagnosis:** | | | |
| 1. {S, D, S} Levocardia. 2. Myxomatous MVL 3. Dilated SoV, STJ 4. Dilated MPA, PV | | | |
| **Remark**: Ascending aorta, Aortic arch and Descending aorta are not assessed as the baby was crying excessively. | | | |
| **Recommendation**: Findings go with Marfan | | | |
| SIGNATURE  Done by: Tesfaye T., Pediatrician, Pediatric Cardiologist _______________ 25/10/08/2014Eth.C | | | |

| Patient Name: **Nuhamin Minale**. Referring Institute: **Dr. Aemiro OBGY Sp.C**. SEX/ Age: **F/13years**.  Date of Report: **25/10/08/14**. Referral Diagnosis: **Palpitation. AGH8.386** | | | |
| --- | --- | --- | --- |
| **Features** | **Finding** | **Features** | **Finding** |
| **Profile** |  | **Atria** |  |
| Abdominal situs | Solitus | Left atrium | Normal |
| Cardiac position | Levocardia | Right atrium | Normal |
| Systemic venous drainage | Normal. | **Atrioventricular valves** |  |
| Pulmonary venous drainage | Normal | Mitral valve | Annulus = 18mm |
| Atrioventricular connection | Concordant | Tricuspid valve | Annulus = 19mm  TAPSE = 21mm |
| Ventriculoarterial connection | Concordant | **Ventricles** |  |
| Ventricular loop | d-Loop | Left ventricle | Normal |
|  |  | Right ventricle | Normal |
| **Septae** |  | **Coronary arteries** | ----- |
| Interventricular septum | Intact | **Doppler Measurement** |  |
| Interatrial septum | Intact | Mitral | ----- |
| **Semilunar valves** |  | Aortic | ------- |
| Aortic valve | Annulus = 17mm | Tricuspid | ------- |
| Pulmonary valve | Annulus = 19mm | pulmonic | -------- |
| **Great arteries** | NRGA | **Aortic arch** | Left. No CoA. |
| Aorta | ----- | **PDA** | No |
| Pulmonary artery | Normal MPA and Branch PAs. |  |  |
| **M-Mode:** | | | |
| AO | mm | PWd | mm |
| LA | mm | PWs | mm |
| LVIDd | mm | EDV | ml |
| LVIDs | mm | ESV | ml |
| IVSs | mm | LVEF | 62% |
| IVSd | mm | FS | 33% |
| **Additional Information**: |  | | |
| No pericardial/Pleural effusion. | | | |
| **Final Diagnosis:** | | | |
| 1. Normal Echocardiography Study. | | | |
| **Remark**: | | | |
| **Recommendation**: | | | |
| SIGNATURE  Done by: Tesfaye T., Pediatrician, Pediatric Cardiologist _______________ 25/10/08/2014Eth.C | | | |

| Patient Name: **Hilina Tadele**. Referring Institute: **Adinas GH**. SEX/ Age: **F/5 2/12**. Date of Report: **25/10/08/14**. Referral Diagnosis: **Incidental Murmur Finding (G-II HSM). AGH8.387** | | | |
| --- | --- | --- | --- |
| **Features** | **Finding** | **Features** | **Finding** |
| **Profile** |  | **Atria** |  |
| Abdominal situs | Solitus | Left atrium | Normal |
| Cardiac position | Levocardia | Right atrium | Normal |
| Systemic venous drainage | Normal. | **Atrioventricular valves** |  |
| Pulmonary venous drainage | Normal | Mitral valve | Annulus = 14mm |
| Atrioventricular connection | Concordant | Tricuspid valve | Annulus = 14mm  TAPSE = 17mm |
| Ventriculoarterial connection | Concordant | **Ventricles** |  |
| Ventricular loop | d-Loop | Left ventricle | Normal |
|  |  | Right ventricle | Normal |
| **Septae** |  | **Coronary arteries** | ----- |
| Interventricular septum | Intact | **Doppler Measurement** |  |
| Interatrial septum | Intact | Mitral | ----- |
| **Semilunar valves** |  | Aortic | ------- |
| Aortic valve | Annulus = 13mm | Tricuspid | ------- |
| Pulmonary valve | Annulus = 12mm | pulmonic | -------- |
| **Great arteries** | NRGA | **Aortic arch** | Left. No CoA. |
| Aorta | ----- | **PDA** | No |
| Pulmonary artery | Normal MPA and Branch PAs. |  |  |
| **M-Mode:** | | | |
| AO | mm | PWd | mm |
| LA | mm | PWs | mm |
| LVIDd | mm | EDV | ml |
| LVIDs | mm | ESV | ml |
| IVSs | mm | LVEF | 62% |
| IVSd | mm | FS | 32% |
| **Additional Information**: |  | | |
| No pericardial/Pleural effusion. | | | |
| **Final Diagnosis:** | | | |
| 1. Normal Echocardiography. | | | |
| **Remark**: | | | |
| **Recommendation**: | | | |
| SIGNATURE  Done by: Tesfaye T., Pediatrician, Pediatric Cardiologist _______________ 25/10/08/2014Eth.C | | | |

| Patient Name: **Tsehayneh Mequanint**. Referring Institute: **FHRH**. SEX/ Age: **M/2 3/12**. Date of Report: **27/10/14**. Referral Diagnosis: **Incidental Murmur Finding. AGH8.388** | | | |
| --- | --- | --- | --- |
| **Features** | **Finding** | **Features** | **Finding** |
| **Profile** |  | **Atria** |  |
| Abdominal situs | Solitus | Left atrium | Normal |
| Cardiac position | Levocardia | Right atrium | Normal |
| Systemic venous drainage | Normal. | **Atrioventricular valves** |  |
| Pulmonary venous drainage | Normal | Mitral valve | Annulus = 16mm |
| Atrioventricular connection | Concordant | Tricuspid valve | Annulus = 17mm |
| Ventriculoarterial connection | Concordant | **Ventricles** |  |
| Ventricular loop | d-Loop | Left ventricle | Normal |
|  |  | Right ventricle | Normal |
| **Septae** |  | **Coronary arteries** | ----- |
| Interventricular septum | 4mm PM VSD, L – R Shunt with a gradient of 70mmHg. | **Doppler Measurement** |  |
| Interatrial septum | Intact | Mitral | ----- |
| **Semilunar valves** |  | Aortic | ------- |
| Aortic valve | Annulus = 14mm | Tricuspid | ------- |
| Pulmonary valve | Annulus = 16mm | pulmonic | -------- |
| **Great arteries** | NRGA | **Aortic arch** | Left. No CoA. |
| Aorta | ----- | **PDA** | No |
| Pulmonary artery | Normal MPA and Branch PAs. |  |  |
| **M-Mode:**  Normal LV Function on eye balling | | | |
| AO | mm | PWd | mm |
| LA | mm | PWs | mm |
| LVIDd | mm | EDV | ml |
| LVIDs | mm | ESV | ml |
| IVSs | mm | LVEF | % |
| IVSd | mm | FS | % |
| **Additional Information**: |  | | |
| No pericardial/Pleural effusion. | | | |
| **Final Diagnosis:** | | | |
| 1. {S, D, S} Levocardia. 2. Small Restrictive PM VSD, L – R Shunt 3. Normal LV Systolic Function | | | |
| **Remark**: | | | |
| **Recommendation**:   1. No need to start any cardiac Medicine 2. No need to prohibit activity 3. Encourage to live normal life 4. Yearly echocardiography follow up 5. Education on Risk of IE | | | |
| SIGNATURE  Done by: Tesfaye T., Pediatrician, Pediatric Cardiologist _______________ 27/10/2014Eth.C | | | |

| Patient Name: **Zemenu Fantahun**. Referring Institute: **FHRH**. SEX/ Age: **M/1year**. Date of Report: **27/10/14**. Referral Diagnosis: **Cyanosis. AGH8.389** | | | |
| --- | --- | --- | --- |
| **Features** | **Finding** | **Features** | **Finding** |
| **Profile** |  | **Atria** |  |
| Abdominal situs | Solitus | Left atrium | Dilated |
| Cardiac position | Levocardia | Right atrium | Dilated |
| Systemic venous drainage | Normal. | **Atrioventricular valves** |  |
| Pulmonary venous drainage | Normal | Mitral valve | Annulus = 12mm |
| Atrioventricular connection | Concordant | Tricuspid valve | Annulus = 12mm |
| Ventriculoarterial connection | Discordant | **Ventricles** |  |
| Ventricular loop | d-Loop | Left ventricle | Dilated |
|  |  | Right ventricle | Dilated |
| **Septae** |  | **Coronary arteries** | ----- |
| Interventricular septum | 15mm PM VSD, BD Shunt | **Doppler Measurement** |  |
| Interatrial septum | Intact | Mitral | ----- |
| **Semilunar valves** |  | Aortic | ------- |
| Aortic valve | Annulus = 10mm | Tricuspid | ------- |
| Pulmonary valve | Annulus = 12mm | pulmonic | -------- |
| **Great arteries** | d-TGA | **Aortic arch** | Left. No CoA. |
| Aorta | From RV & anterior | **PDA** | No |
| Pulmonary artery | From LV and Posterior |  |  |
| **M-Mode:** | | | |
| AO | mm | PWd | mm |
| LA | mm | PWs | mm |
| LVIDd | mm | EDV | ml |
| LVIDs | mm | ESV | ml |
| IVSs | mm | LVEF | % |
| IVSd | mm | FS | % |
| **Additional Information**: |  | | |
| No pericardial/Pleural effusion. | | | |
| **Final Diagnosis:** | | | |
| 1. {S, D, D} Levocardia. 2. d-TGA 3. Large VSD 4. Severe Pul.HTN | | | |
| **Remark**: Child was crying during study | | | |
| **Recommendation**: | | | |
| SIGNATURE  Done by: Tesfaye T., Pediatrician, Pediatric Cardiologist _______________ 27/10/2014Eth.C | | | |

| Patient Name: **Mahider Melkamu**. Referring Institute: **Debre – Tabour RH**. SEX/ Age: **F/3 2/12**.  Date of Report: **28/10/14**. Referral Diagnosis: **Incidental Murmur Finding. AGH8.390** | | | |
| --- | --- | --- | --- |
| **Features** | **Finding** | **Features** | **Finding** |
| **Profile** |  | **Atria** |  |
| Abdominal situs | Solitus | Left atrium | Normal |
| Cardiac position | Levocardia | Right atrium | Normal |
| Systemic venous drainage | Normal. | **Atrioventricular valves** |  |
| Pulmonary venous drainage | Normal | Mitral valve | Annulus = 18mm |
| Atrioventricular connection | Concordant | Tricuspid valve | Annulus = 19mm |
| Ventriculoarterial connection | Concordant | **Ventricles** |  |
| Ventricular loop | d-Loop | Left ventricle | Normal |
|  |  | Right ventricle | Normal |
| **Septae** |  | **Coronary arteries** | ----- |
| Interventricular septum | Sacular septum at the inlet ventricular septum | **Doppler Measurement** |  |
| Interatrial septum | 9mm Primum defect, L – R Shunt | Mitral | ----- |
| **Semilunar valves** |  | Aortic | ------- |
| Aortic valve | Annulus = 14mm | Tricuspid | Mild TR |
| Pulmonary valve | Annulus = 15mm | pulmonic | Mild PS, PPG = 24mmHg |
| **Great arteries** | NRGA | **Aortic arch** | Left. No CoA. |
| Aorta | ----- | **PDA** | No |
| Pulmonary artery | Normal MPA and Branch PAs. |  |  |
| **M-Mode:**  Normal LV Function on eye balling | | | |
| AO | mm | PWd | mm |
| LA | mm | PWs | mm |
| LVIDd | mm | EDV | ml |
| LVIDs | mm | ESV | ml |
| IVSs | mm | LVEF | % |
| IVSd | mm | FS | % |
| **Additional Information**: |  | | |
| No pericardial/Pleural effusion. | | | |
| **Final Diagnosis:** | | | |
| 1. {S, D, S} Levocardia. 2. Transitional AVSD, L – R Shunt 3. Mild PS | | | |
| **Remark**: | | | |
| **Recommendation**: | | | |
| SIGNATURE  Done by: Tesfaye T., Pediatrician, Pediatric Cardiologist _______________ 28/10/2014Eth.C | | | |

| Patient Name: **Dawit Yitbarek**. Referring Institute: **Injibara GH**. SEX/ Age: **M/7years**.  Date of Report: **28/10/14**. Referral Diagnosis: **ARF. AGH8.391** | | | |
| --- | --- | --- | --- |
| **Features** | **Finding** | **Features** | **Finding** |
| **Profile** |  | **Atria** |  |
| Abdominal situs | Solitus | Left atrium | Normal |
| Cardiac position | Levocardia | Right atrium | Normal |
| Systemic venous drainage | Normal. | **Atrioventricular valves** |  |
| Pulmonary venous drainage | Normal | Mitral valve | Annulus = 15mm |
| Atrioventricular connection | Concordant | Tricuspid valve | Annulus = 16mm  TAPSE = 19mm |
| Ventriculoarterial connection | Concordant | **Ventricles** |  |
| Ventricular loop | d-Loop | Left ventricle | Normal |
|  |  | Right ventricle | Normal |
| **Septae** |  | **Coronary arteries** | ----- |
| Interventricular septum | Intact | **Doppler Measurement** |  |
| Interatrial septum | Intact | Mitral | ----- |
| **Semilunar valves** |  | Aortic | ------- |
| Aortic valve | Annulus = 14mm | Tricuspid | ------- |
| Pulmonary valve | Annulus = 16mm | pulmonic | -------- |
| **Great arteries** | NRGA | **Aortic arch** | Left. No CoA. |
| Aorta | ----- | **PDA** | No |
| Pulmonary artery | Normal MPA and Branch PAs. |  |  |
| **M-Mode:**  Normal LV Function on eye balling | | | |
| AO | mm | PWd | mm |
| LA | mm | PWs | mm |
| LVIDd | mm | EDV | ml |
| LVIDs | mm | ESV | ml |
| IVSs | mm | LVEF | % |
| IVSd | mm | FS | % |
| **Additional Information**: |  | | |
| No pericardial/Pleural effusion. | | | |
| **Final Diagnosis:** | | | |
| 1. Normal Echocardiography study. | | | |
| **Remark**: | | | |
| **Recommendation**: | | | |
| SIGNATURE  Done by: Tesfaye T., Pediatrician, Pediatric Cardiologist _______________ 28/10/2014Eth.C | | | |

| Patient Name: **Abas Oumer**. Referring Institute: **FHRH**. SEX/ Age: **M/2 4/12**.  Date of Report: **28/10/14**. Referral Diagnosis: **Recurrent Chest Infection. AGH8.392** | | | |
| --- | --- | --- | --- |
| **Features** | **Finding** | **Features** | **Finding** |
| **Profile** |  | **Atria** |  |
| Abdominal situs | Solitus | Left atrium | Normal |
| Cardiac position | Levocardia | Right atrium | Normal |
| Systemic venous drainage | Normal. | **Atrioventricular valves** |  |
| Pulmonary venous drainage | Normal | Mitral valve | Annulus = 15mm |
| Atrioventricular connection | Concordant | Tricuspid valve | Annulus = 17mm |
| Ventriculoarterial connection | Concordant | **Ventricles** |  |
| Ventricular loop | d-Loop | Left ventricle | Normal |
|  |  | Right ventricle | Normal |
| **Septae** |  | **Coronary arteries** | ----- |
| Interventricular septum | Intact | **Doppler Measurement** |  |
| Interatrial septum | Intact | Mitral | ----- |
| **Semilunar valves** |  | Aortic | ------- |
| Aortic valve | Annulus = 13mm | Tricuspid | ------- |
| Pulmonary valve | Annulus = 14mm | pulmonic | -------- |
| **Great arteries** | NRGA | **Aortic arch** | Left. No CoA. |
| Aorta | ----- | **PDA** | No |
| Pulmonary artery | Normal MPA and Branch PAs. |  |  |
| **M-Mode:**  Normal LV Function on eye balling | | | |
| AO | mm | PWd | mm |
| LA | mm | PWs | mm |
| LVIDd | mm | EDV | ml |
| LVIDs | mm | ESV | ml |
| IVSs | mm | LVEF | % |
| IVSd | mm | FS | % |
| **Additional Information**: |  | | |
| No pericardial/Pleural effusion. | | | |
| **Final Diagnosis:** | | | |
| 1. Normal Echocardiography Study. | | | |
| **Remark**: Baby was crying during study. Only subcostal window was accessible | | | |
| **Recommendation**: | | | |
| SIGNATURE  Done by: Tesfaye T., Pediatrician, Pediatric Cardiologist _______________ 28/10/2014Eth.C | | | |

| Patient Name: **Efrata Destaw**. Referring Institute: **_FHRH**. SEX/ Age: **F/8years**.  Date of Report: **28/10/14**. Referral Diagnosis: **Easy fatigability. AGH8.393** | | | |
| --- | --- | --- | --- |
| **Features** | **Finding** | **Features** | **Finding** |
| **Profile** |  | **Atria** |  |
| Abdominal situs | Solitus | Left atrium | Dilated |
| Cardiac position | Levocardia | Right atrium | Normal |
| Systemic venous drainage | Normal. | **Atrioventricular valves** |  |
| Pulmonary venous drainage | Normal | Mitral valve | Annulus = 23mm |
| Atrioventricular connection | Concordant | Tricuspid valve | Annulus = 21mm |
| Ventriculoarterial connection | Concordant | **Ventricles** |  |
| Ventricular loop | d-Loop | Left ventricle | Dilated |
|  |  | Right ventricle | Normal |
| **Septae** |  | **Coronary arteries** | ----- |
| Interventricular septum | 8mm PM VSD, L – R Shunt | **Doppler Measurement** |  |
| Interatrial septum | Intact | Mitral | ----- |
| **Semilunar valves** |  | Aortic | ------- |
| Aortic valve | Annulus = 17mm | Tricuspid | ------- |
| Pulmonary valve | Annulus = 18mm | pulmonic | -------- |
| **Great arteries** | NRGA | **Aortic arch** | Left. No CoA. |
| Aorta | ----- | **PDA** | No |
| Pulmonary artery | Normal MPA and Branch PAs. |  |  |
| **M-Mode:**  Normal LV Function on eye balling | | | |
| AO | mm | PWd | mm |
| LA | mm | PWs | mm |
| LVIDd | mm | EDV | ml |
| LVIDs | mm | ESV | ml |
| IVSs | mm | LVEF | % |
| IVSd | mm | FS | % |
| **Additional Information**: |  | | |
| No pericardial/Pleural effusion. | | | |
| **Final Diagnosis:** | | | |
| 1. {S, D, S} Levocardia. 2. LA/LV Dilated 3. Moderate PM VSD, L – R Shunt 4. Normal LV Systolic Function | | | |
| **Remark**: | | | |
| **Recommendation**: | | | |
| SIGNATURE  Done by: Tesfaye T., Pediatrician, Pediatric Cardiologist _______________ 28/10/2014Eth.C | | | |

| Patient Name: **Gojam Abebile**. Referring Institute: **FHRH**. SEX/ Age: **F/12years**.  Date of Report: **28/10/14**. Referral Diagnosis: **Chest pain. AGH8.394** | | | |
| --- | --- | --- | --- |
| **Features** | **Finding** | **Features** | **Finding** |
| **Profile** |  | **Atria** |  |
| Abdominal situs | Solitus | Left atrium | Normal |
| Cardiac position | Levocardia | Right atrium | Normal |
| Systemic venous drainage | Normal. | **Atrioventricular valves** |  |
| Pulmonary venous drainage | Normal | Mitral valve | Annulus = 21mm |
| Atrioventricular connection | Concordant | Tricuspid valve | Annulus = 25mm  TAPSE = 25mm |
| Ventriculoarterial connection | Concordant | **Ventricles** |  |
| Ventricular loop | d-Loop | Left ventricle | Normal |
|  |  | Right ventricle | Normal |
| **Septae** |  | **Coronary arteries** | ----- |
| Interventricular septum | Intact | **Doppler Measurement** |  |
| Interatrial septum | Intact | Mitral | ----- |
| **Semilunar valves** |  | Aortic | ------- |
| Aortic valve | Annulus = 16mm | Tricuspid | ------- |
| Pulmonary valve | Annulus = 17mm | pulmonic | -------- |
| **Great arteries** | NRGA | **Aortic arch** | Left. No CoA. |
| Aorta | ----- | **PDA** | No |
| Pulmonary artery | Normal MPA and Branch PAs. |  |  |
| **M-Mode:**  Normal LV Function on eye balling | | | |
| AO | mm | PWd | mm |
| LA | mm | PWs | mm |
| LVIDd | mm | EDV | ml |
| LVIDs | mm | ESV | ml |
| IVSs | mm | LVEF | % |
| IVSd | mm | FS | % |
| **Additional Information**: |  | | |
| No pericardial/Pleural effusion. | | | |
| **Final Diagnosis:** | | | |
| 1. Normal Echocardiography study. | | | |
| **Remark**: | | | |
| **Recommendation**: | | | |
| SIGNATURE  Done by: Tesfaye T., Pediatrician, Pediatric Cardiologist _______________ 28/10/2014Eth.C | | | |

| Patient Name: **Be’emnet Abebe**. Referring Institute: **Addis-Alem PH**. SEX/ Age: **F/11years**.  Date of Report: **28/10/14**. Referral Diagnosis: **Arrhythmia. AGH8.395** | | | |
| --- | --- | --- | --- |
| **Features** | **Finding** | **Features** | **Finding** |
| **Profile** |  | **Atria** |  |
| Abdominal situs | Solitus | Left atrium | Normal |
| Cardiac position | Levocardia | Right atrium | Normal |
| Systemic venous drainage | Normal. | **Atrioventricular valves** |  |
| Pulmonary venous drainage | Normal | Mitral valve | Annulus = 18mm |
| Atrioventricular connection | Concordant | Tricuspid valve | Annulus = 19mm  TAPSE = mm |
| Ventriculoarterial connection | Concordant | **Ventricles** |  |
| Ventricular loop | d-Loop | Left ventricle | Normal |
|  |  | Right ventricle | Normal |
| **Septae** |  | **Coronary arteries** | ----- |
| Interventricular septum | Intact | **Doppler Measurement** |  |
| Interatrial septum | Intact | Mitral | ----- |
| **Semilunar valves** |  | Aortic | ------- |
| Aortic valve | Annulus = 15mm | Tricuspid | ------- |
| Pulmonary valve | Annulus = 18mm | pulmonic | -------- |
| **Great arteries** | NRGA | **Aortic arch** | Left. No CoA. |
| Aorta | ----- | **PDA** | No |
| Pulmonary artery | Normal MPA and Branch PAs. |  |  |
| **M-Mode:** | | | |
| AO | mm | PWd | mm |
| LA | mm | PWs | mm |
| LVIDd | mm | EDV | ml |
| LVIDs | mm | ESV | ml |
| IVSs | mm | LVEF | % |
| IVSd | mm | FS | % |
| **Additional Information**: |  | | |
| No pericardial/Pleural effusion. | | | |
| **Final Diagnosis:** | | | |
| 1. Normal Echocardiography study. | | | |
| **Remark**: rhythm abnormality detected during study | | | |
| **Recommendation**: do ECG | | | |
| SIGNATURE  Done by: Tesfaye T., Pediatrician, Pediatric Cardiologist _______________ 28/10/2014Eth.C | | | |

| Patient Name: **Hanna Yohannes**. Referring Institute: **TGSH**. SEX/ Age: **F/1 8/12**.  Date of Report: **28/10/14**. Referral Diagnosis: **Incidental Murmur Finding. AGH8.396** | | | |
| --- | --- | --- | --- |
| **Features** | **Finding** | **Features** | **Finding** |
| **Profile** |  | **Atria** |  |
| Abdominal situs | Solitus | Left atrium | Normal |
| Cardiac position | Levocardia | Right atrium | Normal |
| Systemic venous drainage | Normal. | **Atrioventricular valves** |  |
| Pulmonary venous drainage | Normal | Mitral valve | Annulus = 16mm |
| Atrioventricular connection | Concordant | Tricuspid valve | Annulus = 16mm |
| Ventriculoarterial connection | Concordant | **Ventricles** |  |
| Ventricular loop | d-Loop | Left ventricle | Normal |
|  |  | Right ventricle | Normal |
| **Septae** |  | **Coronary arteries** | ----- |
| Interventricular septum | Intact | **Doppler Measurement** |  |
| Interatrial septum | Intact | Mitral | ----- |
| **Semilunar valves** |  | Aortic | ------- |
| Aortic valve | Annulus = 13mm | Tricuspid | ------- |
| Pulmonary valve | Annulus = 13mm. doming PV | pulmonic | Mild PS, PPG = 27mmHg |
| **Great arteries** | NRGA | **Aortic arch** | Left. No CoA. |
| Aorta | ----- | **PDA** | No |
| Pulmonary artery | Normal MPA and Branch PAs. |  |  |
| **M-Mode:**  Normal LV Function on eye balling | | | |
| AO | mm | PWd | mm |
| LA | mm | PWs | mm |
| LVIDd | mm | EDV | ml |
| LVIDs | mm | ESV | ml |
| IVSs | mm | LVEF | % |
| IVSd | mm | FS | % |
| **Additional Information**: |  | | |
| No pericardial/Pleural effusion. | | | |
| **Final Diagnosis:** | | | |
| 1. {S, D, S} Levocardia. 2. Doming PV 3. Mild Valvular PS | | | |
| **Remark**: | | | |
| **Recommendation**: | | | |
| SIGNATURE  Done by: Tesfaye T., Pediatrician, Pediatric Cardiologist _______________ 28/10/2014Eth.C | | | |

| Patient Name: **Aster Getawey**. Referring Institute: **FHRH**. SEX/ Age: **F/6years**.  Date of Report: **28/10/14**. Referral Diagnosis: **Incidental Murmur Finding. AGH8.397** | | | |
| --- | --- | --- | --- |
| **Features** | **Finding** | **Features** | **Finding** |
| **Profile** |  | **Atria** |  |
| Abdominal situs | Solitus | Left atrium | Normal |
| Cardiac position | Levocardia | Right atrium | Normal |
| Systemic venous drainage | Normal. | **Atrioventricular valves** |  |
| Pulmonary venous drainage | Normal | Mitral valve | Annulus = 16mm |
| Atrioventricular connection | Concordant | Tricuspid valve | Annulus = 17mm  TAPSE = 18mm |
| Ventriculoarterial connection | Concordant | **Ventricles** |  |
| Ventricular loop | d-Loop | Left ventricle | Normal |
|  |  | Right ventricle | Normal |
| **Septae** |  | **Coronary arteries** | ----- |
| Interventricular septum | Intact | **Doppler Measurement** |  |
| Interatrial septum | Intact | Mitral | ----- |
| **Semilunar valves** |  | Aortic | ------- |
| Aortic valve | Annulus = 15mm | Tricuspid | ------- |
| Pulmonary valve | Annulus = 14mm | pulmonic | Moderate PS, PPG = 53mmHg. Mild PR, PPG = 12mmHg |
| **Great arteries** | NRGA | **Aortic arch** | Left. No CoA. |
| Aorta | ----- | **PDA** | No |
| Pulmonary artery | Normal MPA and Branch PAs. |  |  |
| **M-Mode:**  Normal LV Function on eye balling | | | |
| AO | mm | PWd | mm |
| LA | mm | PWs | mm |
| LVIDd | mm | EDV | ml |
| LVIDs | mm | ESV | ml |
| IVSs | mm | LVEF | % |
| IVSd | mm | FS | % |
| **Additional Information**: |  | | |
| No pericardial/Pleural effusion. | | | |
| **Final Diagnosis:** | | | |
| 1. {S, D, S} Levocardia. 2. Moderate Valvular PS | | | |
| **Remark**: | | | |
| **Recommendation**: | | | |
| SIGNATURE  Done by: Tesfaye T., Pediatrician, Pediatric Cardiologist _______________ 28/10/2014Eth.C | | | |

| Patient Name: **Abrham Demilew**. Referring Institute: **FHRH**. SEX/ Age: **M/11years**.  Date of Report: **28/10/14**. Referral Diagnosis: **CHF + Rheumatic Recurrence. AGH8.398** | | | |
| --- | --- | --- | --- |
| **Features** | **Finding** | **Features** | **Finding** |
| **Profile** |  | **Atria** |  |
| Abdominal situs | Solitus | Left atrium | Dilated |
| Cardiac position | Levocardia | Right atrium | Dilated |
| Systemic venous drainage | Normal. | **Atrioventricular valves** |  |
| Pulmonary venous drainage | Normal | Mitral valve | Annulus = 27mm. Mildly thickened MVL |
| Atrioventricular connection | Concordant | Tricuspid valve | Annulus = 26mm  TAPSE = 18mm |
| Ventriculoarterial connection | Concordant | **Ventricles** |  |
| Ventricular loop | d-Loop | Left ventricle | Dilated |
|  |  | Right ventricle | Dilated |
| **Septae** |  | **Coronary arteries** | ----- |
| Interventricular septum | Intact | **Doppler Measurement** |  |
| Interatrial septum | Intact | Mitral | Severe MR, Holosystolic, posterior projection, seen in two planes with jet velocity = 4.2m/sec |
| **Semilunar valves** |  | Aortic | Moderate AR, PHT = 253ms |
| Aortic valve | Annulus = 20mm | Tricuspid | Moderate TR, PPG = 34mmHg |
| Pulmonary valve | Annulus = 22mm | pulmonic | -------- |
| **Great arteries** | NRGA | **Aortic arch** | Left. No CoA. |
| Aorta | ----- | **PDA** | No |
| Pulmonary artery | Normal MPA and Branch PAs. |  |  |
| **M-Mode:**  Reduced LV Function | | | |
| AO | mm | PWd | mm |
| LA | mm | PWs | mm |
| LVIDd | mm | EDV | ml |
| LVIDs | mm | ESV | ml |
| IVSs | mm | LVEF | % |
| IVSd | mm | FS | % |
| **Additional Information**: |  | | |
| No pericardial/Pleural effusion. | | | |
| **Final Diagnosis:** | | | |
| 1. {S, D, S} Levocardia. 2. All chambers dilated 3. Severe MR 4. Moderate AR 5. Moderate TR 6. Reduced LV Systolic Function | | | |
| **Remark**: | | | |
| **Recommendation**: | | | |
| SIGNATURE  Done by: Tesfaye T., Pediatrician, Pediatric Cardiologist _______________ 28/10/2014Eth.C | | | |

| Patient Name: **Tsigie Bamlie**. Referring Institute: **TGSH**. SEX/ Age: **F/1 2/12**.  Date of Report: **28/10/14**. Referral Diagnosis: **DS. AGH8.399** | | | |
| --- | --- | --- | --- |
| **Features** | **Finding** | **Features** | **Finding** |
| **Profile** |  | **Atria** |  |
| Abdominal situs | Solitus | Left atrium | Normal |
| Cardiac position | Levocardia | Right atrium | Normal |
| Systemic venous drainage | Normal. | **Atrioventricular valves** |  |
| Pulmonary venous drainage | Normal | Mitral valve | Annulus = 14mm |
| Atrioventricular connection | Concordant | Tricuspid valve | Annulus = 15mm  TAPSE = 16mm |
| Ventriculoarterial connection | Concordant | **Ventricles** |  |
| Ventricular loop | d-Loop | Left ventricle | Normal |
|  |  | Right ventricle | Normal |
| **Septae** |  | **Coronary arteries** | ----- |
| Interventricular septum | Intact | **Doppler Measurement** |  |
| Interatrial septum | Intact | Mitral | ----- |
| **Semilunar valves** |  | Aortic | ------- |
| Aortic valve | Annulus = 10mm | Tricuspid | ------- |
| Pulmonary valve | Annulus = 10mm | pulmonic | -------- |
| **Great arteries** | NRGA | **Aortic arch** | Left. No CoA. |
| Aorta | ----- | **PDA** | No |
| Pulmonary artery | Normal MPA and Branch PAs. |  |  |
| **M-Mode:**  Normal LV Systolic function | | | |
| AO | mm | PWd | mm |
| LA | mm | PWs | mm |
| LVIDd | mm | EDV | ml |
| LVIDs | mm | ESV | ml |
| IVSs | mm | LVEF | % |
| IVSd | mm | FS | % |
| **Additional Information**: |  | | |
| Circumferential Pericardial effusion measuring 3mm. | | | |
| **Final Diagnosis:** | | | |
| 1. {S, D, S} Levocardia. 2. Trace Circumferential Pericardial effusion 3. Normal Biventricular Systolic Function | | | |
| **Remark**: | | | |
| **Recommendation**: | | | |
| SIGNATURE  Done by: Tesfaye T., Pediatrician, Pediatric Cardiologist _______________ 28/10/2014Eth.C | | | |

| Patient Name: **Amen Belew**. Referring Institute: **Adinas GH**. SEX/ Age: **M/5years**.  Date of Report: **29/10/14**. Referral Diagnosis: **Recurrent Chest Infection. AGH8.400** | | | |
| --- | --- | --- | --- |
| **Features** | **Finding** | **Features** | **Finding** |
| **Profile** |  | **Atria** |  |
| Abdominal situs | Solitus | Left atrium | Normal |
| Cardiac position | Levocardia | Right atrium | Normal |
| Systemic venous drainage | Normal. | **Atrioventricular valves** |  |
| Pulmonary venous drainage | Normal | Mitral valve | Annulus = 15mm |
| Atrioventricular connection | Concordant | Tricuspid valve | Annulus = 16mm  TAPSE = 21mm |
| Ventriculoarterial connection | Concordant | **Ventricles** |  |
| Ventricular loop | d-Loop | Left ventricle | Normal |
|  |  | Right ventricle | Normal |
| **Septae** |  | **Coronary arteries** | ----- |
| Interventricular septum | Intact | **Doppler Measurement** |  |
| Interatrial septum | Intact | Mitral | ----- |
| **Semilunar valves** |  | Aortic | ------- |
| Aortic valve | Annulus = 14mm | Tricuspid | ------- |
| Pulmonary valve | Annulus = 16mm | pulmonic | -------- |
| **Great arteries** | NRGA | **Aortic arch** | Left. No CoA. |
| Aorta | ----- | **PDA** | No |
| Pulmonary artery | Normal MPA and Branch PAs. |  |  |
| **M-Mode:** | | | |
| AO | mm | PWd | mm |
| LA | mm | PWs | mm |
| LVIDd | mm | EDV | ml |
| LVIDs | mm | ESV | ml |
| IVSs | mm | LVEF | % |
| IVSd | mm | FS | % |
| **Additional Information**: |  | | |
| No pericardial/Pleural effusion. | | | |
| **Final Diagnosis:** | | | |
| 1. Normal Echocardiography Study. | | | |
| **Remark**: | | | |
| **Recommendation**: | | | |
| SIGNATURE  Done by: Tesfaye T., Pediatrician, Pediatric Cardiologist _______________ 29/10/2014Eth.C | | | |

| Patient Name: **Baby of Seada Muktar**. Referring Institute: **FHRH**. SEX/ Age: **M/6days**.  Date of Report: **29/10/14**. Referral Diagnosis: **RD. AGH8.401** | | | |
| --- | --- | --- | --- |
| **Features** | **Finding** | **Features** | **Finding** |
| **Profile** |  | **Atria** |  |
| Abdominal situs | Solitus | Left atrium | Normal |
| Cardiac position | Levocardia | Right atrium | Normal |
| Systemic venous drainage | Normal. | **Atrioventricular valves** |  |
| Pulmonary venous drainage | Normal | Mitral valve | Annulus = 7mm |
| Atrioventricular connection | Concordant | Tricuspid valve | Annulus = 9mm |
| Ventriculoarterial connection | Concordant | **Ventricles** |  |
| Ventricular loop | d-Loop | Left ventricle | Normal |
|  |  | Right ventricle | Normal |
| **Septae** |  | **Coronary arteries** | ----- |
| Interventricular septum | Intact | **Doppler Measurement** |  |
| Interatrial septum | PFO, L – R Shunt | Mitral | ----- |
| **Semilunar valves** |  | Aortic | ------- |
| Aortic valve | Annulus = 7mm | Tricuspid | ------- |
| Pulmonary valve | Annulus = 8mm | pulmonic | -------- |
| **Great arteries** | NRGA | **Aortic arch** | Left. No CoA. |
| Aorta | ----- | **PDA** | No |
| Pulmonary artery | Normal MPA and Branch PAs. |  |  |
| **M-Mode:** | | | |
| AO | mm | PWd | mm |
| LA | mm | PWs | mm |
| LVIDd | mm | EDV | ml |
| LVIDs | mm | ESV | ml |
| IVSs | mm | LVEF | % |
| IVSd | mm | FS | % |
| **Additional Information**: |  | | |
| No pericardial/Pleural effusion. | | | |
| **Final Diagnosis:** | | | |
| 1. {S, D, S} Levocardia. 2. PFO, L – R Shunt | | | |
| **Remark**: | | | |
| **Recommendation**: | | | |
| SIGNATURE  Done by: Tesfaye T., Pediatrician, Pediatric Cardiologist _______________ 29/10/2014Eth.C | | | |

| Patient Name: **Yohannes Marachew**. Referring Institute: **FHRH**. SEX/ Age: **M/7months**.  Date of Report: **29/10/14**. Referral Diagnosis: **RD. AGH8.402** | | | |
| --- | --- | --- | --- |
| **Features** | **Finding** | **Features** | **Finding** |
| **Profile** |  | **Atria** |  |
| Abdominal situs | Solitus | Left atrium | Mildly dilated |
| Cardiac position | Levocardia | Right atrium | Normal |
| Systemic venous drainage | Normal. | **Atrioventricular valves** |  |
| Pulmonary venous drainage | Normal | Mitral valve | Annulus = 15mm |
| Atrioventricular connection | Concordant | Tricuspid valve | Annulus = 13mm  TAPSE = 17mm |
| Ventriculoarterial connection | Concordant | **Ventricles** |  |
| Ventricular loop | d-Loop | Left ventricle | Mildly dilated |
|  |  | Right ventricle | Normal |
| **Septae** |  | **Coronary arteries** | ----- |
| Interventricular septum | 6mm PM VSD, L – R Shunt with a gradient of 28mmHg | **Doppler Measurement** |  |
| Interatrial septum | Intact | Mitral | ----- |
| **Semilunar valves** |  | Aortic | ------- |
| Aortic valve | Annulus = 11mm | Tricuspid | ------- |
| Pulmonary valve | Annulus = 11mm | pulmonic | -------- |
| **Great arteries** | NRGA | **Aortic arch** | Left. No CoA. |
| Aorta | ----- | **PDA** | No |
| Pulmonary artery | Normal MPA and Branch PAs. |  |  |
| **M-Mode:**  Normal LV Function on eye balling | | | |
| AO | mm | PWd | mm |
| LA | mm | PWs | mm |
| LVIDd | mm | EDV | ml |
| LVIDs | mm | ESV | ml |
| IVSs | mm | LVEF | % |
| IVSd | mm | FS | % |
| **Additional Information**: |  | | |
| No pericardial/Pleural effusion. | | | |
| **Final Diagnosis:** | | | |
| 1. {S, D, S} Levocardia. 2. LA/LV Dilated 3. Moderate PM VSD, L – R Shunt 4. Normal Biventricular Systolic Function | | | |
| **Remark**: | | | |
| **Recommendation**: | | | |
| SIGNATURE  Done by: Tesfaye T., Pediatrician, Pediatric Cardiologist _______________ 29/10/2014Eth.C | | | |

| Patient Name: **Kedija Adem**. Referring Institute: **TGSH**. SEX/ Age: **F/5years**. Date of Report: **30/10/14**. Referral Diagnosis: **RD + CHF. AGH8.403** | | | |
| --- | --- | --- | --- |
| **Features** | **Finding** | **Features** | **Finding** |
| **Profile** |  | **Atria** |  |
| Abdominal situs | Solitus | Left atrium | Normal |
| Cardiac position | Levocardia | Right atrium | Markedly Dilated |
| Systemic venous drainage | Normal. | **Atrioventricular valves** |  |
| Pulmonary venous drainage | Normal | Mitral valve | Annulus = 14mm |
| Atrioventricular connection | Concordant | Tricuspid valve | Annulus = 21mm  TAPSE = 7mm |
| Ventriculoarterial connection | Concordant | **Ventricles** |  |
| Ventricular loop | d-Loop | Left ventricle | Normal |
|  |  | Right ventricle | Markedly Dilated & Dysfunctional |
| **Septae** |  | **Coronary arteries** | ----- |
| Interventricular septum | Intact | **Doppler Measurement** |  |
| Interatrial septum | Intact | Mitral | ----- |
| **Semilunar valves** |  | Aortic | ------- |
| Aortic valve | Annulus = 13mm | Tricuspid | Mild TR, PPG = 65mmHg |
| Pulmonary valve | Annulus = 16mm | pulmonic | -------- |
| **Great arteries** | NRGA | **Aortic arch** | Left. No CoA. |
| Aorta | ----- | **PDA** | No |
| Pulmonary artery | Normal MPA and Branch PAs. |  |  |
| **M-Mode:**  Normal LV Function on eye balling | | | |
| AO | mm | PWd | mm |
| LA | mm | PWs | mm |
| LVIDd | mm | EDV | ml |
| LVIDs | mm | ESV | ml |
| IVSs | mm | LVEF | % |
| IVSd | mm | FS | % |
| **Additional Information**: |  | | |
| Pericardial effusion with maximum depth of 8mm on RA/RV Side. | | | |
| **Final Diagnosis:** | | | |
| 1. {S, D, S} Levocardia. 2. RA/RV Dilated 3. Mild TR 4. Dilated and Dysfunctional RV 5. Severe Pulmonary Hypertension 20 to ? 6. Small Pericardial effusion | | | |
| **Remark**: | | | |
| **Recommendation**: | | | |
| SIGNATURE  Done by: Tesfaye T., Pediatrician, Pediatric Cardiologist _______________ 30/10/2014Eth.C | | | |

| Patient Name: **Thomas Yiketel**. Referring Institute: **MSI-Ethiopia, Bahir Dar**. SEX/ Age: **M/6months**.  Date of Report: **30/10/14**. Referral Diagnosis: **Recurrent chest infection. AGH8.404** | | | |
| --- | --- | --- | --- |
| **Features** | **Finding** | **Features** | **Finding** |
| **Profile** |  | **Atria** |  |
| Abdominal situs | Solitus | Left atrium | Normal |
| Cardiac position | Levocardia | Right atrium | Normal |
| Systemic venous drainage | Normal. | **Atrioventricular valves** |  |
| Pulmonary venous drainage | Normal | Mitral valve | Annulus = 12mm |
| Atrioventricular connection | Concordant | Tricuspid valve | Annulus = 13mm |
| Ventriculoarterial connection | Concordant | **Ventricles** |  |
| Ventricular loop | d-Loop | Left ventricle | Normal |
|  |  | Right ventricle | Normal |
| **Septae** |  | **Coronary arteries** | ----- |
| Interventricular septum | Intact | **Doppler Measurement** |  |
| Interatrial septum | Intact | Mitral | ----- |
| **Semilunar valves** |  | Aortic | ------- |
| Aortic valve | Annulus = 11mm | Tricuspid | ------- |
| Pulmonary valve | Annulus = 12mm | pulmonic | -------- |
| **Great arteries** | NRGA | **Aortic arch** | Left. No CoA. |
| Aorta | ----- | **PDA** | No |
| Pulmonary artery | Normal MPA and Branch PAs. |  |  |
| **M-Mode:**  Normal LV Function on eye balling | | | |
| AO | mm | PWd | mm |
| LA | mm | PWs | mm |
| LVIDd | mm | EDV | ml |
| LVIDs | mm | ESV | ml |
| IVSs | mm | LVEF | % |
| IVSd | mm | FS | % |
| **Additional Information**: |  | | |
| No pericardial/Pleural effusion. | | | |
| **Final Diagnosis:** | | | |
| 1. Normal Echocardiography Study. | | | |
| **Remark**: Limitted Echo window (only subcostal) | | | |
| **Recommendation**: | | | |
| SIGNATURE  Done by: Tesfaye T., Pediatrician, Pediatric Cardiologist _______________ 30/10/2014Eth.C | | | |

| Patient Name: **Yafet Daniel**. Referring Institute: **FHRH**. SEX/ Age: **M/12years**.  Date of Report: **01/11/14**. Referral Diagnosis: **Sydenham’s Chorea. AGH8.404** | | | |
| --- | --- | --- | --- |
| **Features** | **Finding** | **Features** | **Finding** |
| **Profile** |  | **Atria** |  |
| Abdominal situs | Solitus | Left atrium | Normal |
| Cardiac position | Levocardia | Right atrium | Normal |
| Systemic venous drainage | Normal. | **Atrioventricular valves** |  |
| Pulmonary venous drainage | Normal | Mitral valve | Annulus = 18mm. Thickened MVL |
| Atrioventricular connection | Concordant | Tricuspid valve | Annulus = 18mm  TAPSE = 21mm |
| Ventriculoarterial connection | Concordant | **Ventricles** |  |
| Ventricular loop | d-Loop | Left ventricle | Normal |
|  |  | Right ventricle | Normal |
| **Septae** |  | **Coronary arteries** | ----- |
| Interventricular septum | Intact | **Doppler Measurement** |  |
| Interatrial septum | Intact | Mitral | Trivial MR, Incomplete Signal, Posterior projection, seen in two planes with jet velocity = 2.6m/sec. |
| **Semilunar valves** |  | Aortic | ------- |
| Aortic valve | Annulus = 18mm | Tricuspid | ------- |
| Pulmonary valve | Annulus = 21mm | pulmonic | -------- |
| **Great arteries** | NRGA | **Aortic arch** | Left. No CoA. |
| Aorta | ----- | **PDA** | No |
| Pulmonary artery | Normal MPA and Branch PAs. |  |  |
| **M-Mode:** | | | |
| AO | mm | PWd | mm |
| LA | mm | PWs | mm |
| LVIDd | mm | EDV | ml |
| LVIDs | mm | ESV | ml |
| IVSs | mm | LVEF | 67% |
| IVSd | mm | FS | 36% |
| **Additional Information**: |  | | |
| No pericardial/Pleural effusion. | | | |
| **Final Diagnosis:** | | | |
| 1. {S, D, S} Levocardia. 2. Mildly thickened MVL 3. Trivial MR 4. Normal Biventricular Systolic Function | | | |
| **Remark**: Consider Borderline RHD | | | |
| **Recommendation**: Manage as Mild Rheumatic Carditis | | | |
| SIGNATURE  Done by: Tesfaye T., Pediatrician, Pediatric Cardiologist _______________ 01/11/2014Eth.C | | | |

| Patient Name: **Mahider Aknaw**. Referring Institute: **Debre-Tabour RH**. SEX/ Age: **F/1 1/12**.  Date of Report: **01/11/14**. Referral Diagnosis: **Incidental Murmur Finding. AGH8.406** | | | |
| --- | --- | --- | --- |
| **Features** | **Finding** | **Features** | **Finding** |
| **Profile** |  | **Atria** |  |
| Abdominal situs | Solitus | Left atrium | Normal |
| Cardiac position | Levocardia | Right atrium | Normal |
| Systemic venous drainage | Normal. | **Atrioventricular valves** |  |
| Pulmonary venous drainage | Normal | Mitral valve | Annulus =13 mm |
| Atrioventricular connection | Concordant | Tricuspid valve | Annulus = 15mm |
| Ventriculoarterial connection | Concordant | **Ventricles** |  |
| Ventricular loop | d-Loop | Left ventricle | Normal |
|  |  | Right ventricle | Normal |
| **Septae** |  | **Coronary arteries** | ----- |
| Interventricular septum | 3mm Sub – Pulmonic VSD, L – R Shunt. (supra cristal) | **Doppler Measurement** |  |
| Interatrial septum | Intact | Mitral | ----- |
| **Semilunar valves** |  | Aortic | ------- |
| Aortic valve | Annulus = 13mm | Tricuspid | ------- |
| Pulmonary valve | Annulus = 13mm | pulmonic | -------- |
| **Great arteries** | NRGA | **Aortic arch** | Left. No CoA. |
| Aorta | ----- | **PDA** | No |
| Pulmonary artery | Normal MPA and Branch PAs. |  |  |
| **M-Mode:**  Normal LV Function on eye balling | | | |
| AO | mm | PWd | mm |
| LA | mm | PWs | mm |
| LVIDd | mm | EDV | ml |
| LVIDs | mm | ESV | ml |
| IVSs | mm | LVEF | % |
| IVSd | mm | FS | % |
| **Additional Information**: |  | | |
| No pericardial/Pleural effusion. | | | |
| **Final Diagnosis:** | | | |
| 1. {S, D, S} Levocardia. 2. Small Sub – Pulmonic, Infra cristal VSD, L – R Shunt 3. Normal Systolic LV Function | | | |
| **Remark**: | | | |
| **Recommendation**: | | | |
| SIGNATURE  Done by: Tesfaye T., Pediatrician, Pediatric Cardiologist _______________ 01/11/2014Eth.C | | | |

| Patient Name: **Abraham Haile - Maryam**. Referring Institute: **TGSH**. SEX/ Age: **M/6years**.  Date of Report: **01/11/14**. Referral Diagnosis: **IE + CHF. AGH8.407** | | | |
| --- | --- | --- | --- |
| **Features** | **Finding** | **Features** | **Finding** |
| **Profile** |  | **Atria** |  |
| Abdominal situs | Solitus | Left atrium | Normal |
| Cardiac position | Levocardia | Right atrium | Normal |
| Systemic venous drainage | Normal. | **Atrioventricular valves** |  |
| Pulmonary venous drainage | Normal | Mitral valve | Annulus = 22mm. Mobile vegetation at the tip of the MV on LA side Protruding to LV During Diastole |
| Atrioventricular connection | Concordant | Tricuspid valve | Annulus = 21mm. Mobile vegetation at the tip of the TV on RA side protruding to RV during Diastole |
| Ventriculoarterial connection | Concordant | **Ventricles** |  |
| Ventricular loop | d-Loop | Left ventricle | Normal |
|  |  | Right ventricle | Normal |
| **Septae** |  | **Coronary arteries** | ----- |
| Interventricular septum | 7mm PM VSD, L – R Shunt | **Doppler Measurement** |  |
| Interatrial septum | Intact | Mitral | ----- |
| **Semilunar valves** |  | Aortic | ------- |
| Aortic valve | Annulus = 14mm | Tricuspid | ------- |
| Pulmonary valve | Annulus = 17mm. Mobile vegetation at the tip of the PV on RV Side protruding to the PA during systole. | pulmonic | -------- |
| **Great arteries** | NRGA | **Aortic arch** | Left. No CoA. |
| Aorta | ----- | **PDA** | No |
| Pulmonary artery | Normal MPA and Branch PAs. |  |  |
| **M-Mode:** | | | |
| AO | mm | PWd | mm |
| LA | mm | PWs | mm |
| LVIDd | mm | EDV | ml |
| LVIDs | mm | ESV | ml |
| IVSs | mm | LVEF | 67% |
| IVSd | mm | FS | 37% |
| **Additional Information**: |  | | |
| Circumferential pericardial effusion with maximum depth of 14mm on RA Side. | | | |
| **Final Diagnosis:** | | | |
| 1. {S, D, S} Levocardia. 2. Moderate PM VSD, L – R Shunt 3. Vegetation at MV, TV and PV 4. Normal LV Systolic Function 5. Moderate Circumferential Pericardial effusion | | | |
| **Remark**: | | | |
| **Recommendation**: | | | |
| SIGNATURE  Done by: Tesfaye T., Pediatrician, Pediatric Cardiologist _______________ 01/11/2014Eth.C | | | |

| Patient Name: **Tsion Dabere**. Referring Institute: **Afilas GH**. SEX/ Age: **F/7years**.  Date of Report: **01/11/14**. Referral Diagnosis: **easy fatigability. AGH8.408** | | | |
| --- | --- | --- | --- |
| **Features** | **Finding** | **Features** | **Finding** |
| **Profile** |  | **Atria** |  |
| Abdominal situs | Solitus | Left atrium | Normal |
| Cardiac position | Levocardia | Right atrium | Normal |
| Systemic venous drainage | Normal. | **Atrioventricular valves** |  |
| Pulmonary venous drainage | Normal | Mitral valve | Annulus = 14mm |
| Atrioventricular connection | Concordant | Tricuspid valve | Annulus = 14mm  TAPSE = 17mm |
| Ventriculoarterial connection | Concordant | **Ventricles** |  |
| Ventricular loop | d-Loop | Left ventricle | Normal |
|  |  | Right ventricle | Normal |
| **Septae** |  | **Coronary arteries** | ----- |
| Interventricular septum | Intact | **Doppler Measurement** |  |
| Interatrial septum | Intact | Mitral | ----- |
| **Semilunar valves** |  | Aortic | ------- |
| Aortic valve | Annulus = 12mm | Tricuspid | ------- |
| Pulmonary valve | Annulus = 15mm | pulmonic | -------- |
| **Great arteries** | NRGA | **Aortic arch** | Left. No CoA. |
| Aorta | ----- | **PDA** | No |
| Pulmonary artery | Normal MPA and Branch PAs. |  |  |
| **M-Mode:** | | | |
| AO | mm | PWd | mm |
| LA | mm | PWs | mm |
| LVIDd | mm | EDV | ml |
| LVIDs | mm | ESV | ml |
| IVSs | mm | LVEF | 73% |
| IVSd | mm | FS | 41% |
| **Additional Information**: |  | | |
| No pericardial/Pleural effusion. | | | |
| **Final Diagnosis:** | | | |
| 1. Normal Echocardiography Study. | | | |
| **Remark**: | | | |
| **Recommendation**: | | | |
| SIGNATURE  Done by: Tesfaye T., Pediatrician, Pediatric Cardiologist _______________ 01/11/2014Eth.C | | | |

| Patient Name: **Anteneh Getahun**. Referring Institute: **FHRH**. SEX/ Age: **M/3months**.  Date of Report: **01/11/14**. Referral Diagnosis: **Recurrent chest infection. AGH8.409** | | | |
| --- | --- | --- | --- |
| **Features** | **Finding** | **Features** | **Finding** |
| **Profile** |  | **Atria** |  |
| Abdominal situs | Solitus | Left atrium | Normal |
| Cardiac position | Levocardia | Right atrium | Dilated |
| Systemic venous drainage | Normal. | **Atrioventricular valves** |  |
| Pulmonary venous drainage | Normal | Mitral valve | Annulus = 9mm |
| Atrioventricular connection | Concordant | Tricuspid valve | Annulus = 13mm  TAPSE = 11mm |
| Ventriculoarterial connection | Concordant | **Ventricles** |  |
| Ventricular loop | d-Loop | Left ventricle | Normal |
|  |  | Right ventricle | Dilated |
| **Septae** |  | **Coronary arteries** | ----- |
| Interventricular septum | Intact | **Doppler Measurement** |  |
| Interatrial septum | 9mm OS ASD, L – R Shunt | Mitral | ----- |
| **Semilunar valves** |  | Aortic | ------- |
| Aortic valve | Annulus = 8mm | Tricuspid | ------- |
| Pulmonary valve | Annulus = 9mm | pulmonic | Trivial PR, PPG = 21mmHg |
| **Great arteries** | NRGA | **Aortic arch** | Left. No CoA. |
| Aorta | ----- | **PDA** | No |
| Pulmonary artery | Normal MPA and Branch PAs. |  |  |
| **M-Mode:**  Normal LV Function on eye balling | | | |
| AO | mm | PWd | mm |
| LA | mm | PWs | mm |
| LVIDd | mm | EDV | ml |
| LVIDs | mm | ESV | ml |
| IVSs | mm | LVEF | % |
| IVSd | mm | FS | % |
| **Additional Information**: |  | | |
| No pericardial/Pleural effusion. | | | |
| **Final Diagnosis:** | | | |
| 1. {S, D, S} Levocardia. 2. RA.RV Dilated 3. Moderate to Large OS ASD, L – R Shunt 4. Normal LV Systolic Function | | | |
| **Remark**: | | | |
| **Recommendation**: | | | |
| SIGNATURE  Done by: Tesfaye T., Pediatrician, Pediatric Cardiologist _______________ 01/11/2014Eth.C | | | |

| Patient Name: **Temesgen Birku**. Referring Institute: **FHRH**. SEX/ Age: **M/1 4/12**.  Date of Report: **01/11/14**. Referral Diagnosis: **Murmur + RD. AGH8.410** | | | |
| --- | --- | --- | --- |
| **Features** | **Finding** | **Features** | **Finding** |
| **Profile** |  | **Atria** |  |
| Abdominal situs | Solitus | Left atrium | Normal |
| Cardiac position | Levocardia | Right atrium | Dilated |
| Systemic venous drainage | Normal. | **Atrioventricular valves** |  |
| Pulmonary venous drainage | Normal | Mitral valve | Annulus = 11mm |
| Atrioventricular connection | Concordant | Tricuspid valve | Annulus = 14mm  TAPSE = mm |
| Ventriculoarterial connection | Concordant | **Ventricles** |  |
| Ventricular loop | d-Loop | Left ventricle | Normal |
|  |  | Right ventricle | Dilated |
| **Septae** |  | **Coronary arteries** | ----- |
| Interventricular septum | Non-restrictive Sub-aortic VSD, L – R Shunt | **Doppler Measurement** |  |
| Interatrial septum | 7mm OS ASD, L – R Shunt | Mitral | ----- |
| **Semilunar valves** |  | Aortic | ------- |
| Aortic valve | Annulus = 10mm | Tricuspid | ------- |
| Pulmonary valve | Annulus = 11mm | pulmonic | -------- |
| **Great arteries** | NRGA | **Aortic arch** | Left. No CoA. |
| Aorta | ----- | **PDA** | No |
| Pulmonary artery | Normal MPA and Branch PAs. |  |  |
| **M-Mode:**  Normal LV Systolic Function | | | |
| AO | mm | PWd | mm |
| LA | mm | PWs | mm |
| LVIDd | mm | EDV | ml |
| LVIDs | mm | ESV | ml |
| IVSs | mm | LVEF | % |
| IVSd | mm | FS | % |
| **Additional Information**: |  | | |
| No pericardial/Pleural effusion. | | | |
| **Final Diagnosis:** | | | |
| 1. {S, D, S} Levocardia. 2. RA/RV Dilated 3. Moderate OS ASD, L – R Shunt 4. Non – Restrictive Sub aortic VSD, L – R Shunt 5. Normal LV Systolic Function | | | |
| **Remark**: | | | |
| **Recommendation**: | | | |
| SIGNATURE  Done by: Tesfaye T., Pediatrician, Pediatric Cardiologist _______________ 01/11/2014Eth.C | | | |

| Patient Name: **Fozia Kefyalew**. Referring Institute: **Adinas GH**. SEX/ Age: **F/2years**.  Date of Report: **01/11/14**. Referral Diagnosis: **Cyanosis. AGH8.411** | | | |
| --- | --- | --- | --- |
| **Features** | **Finding** | **Features** | **Finding** |
| **Profile** |  | **Atria** |  |
| Abdominal situs | Solitus | Left atrium | Normal |
| Cardiac position | Levocardia | Right atrium | Normal |
| Systemic venous drainage | Normal. | **Atrioventricular valves** |  |
| Pulmonary venous drainage | Normal | Mitral valve | Annulus = 14mm |
| Atrioventricular connection | Concordant | Tricuspid valve | Atretic |
| Ventriculoarterial connection | DOLV | **Ventricles** |  |
| Ventricular loop | d-Loop | Left ventricle | Normal |
|  |  | Right ventricle | Hypoplastic |
| **Septae** |  | **Coronary arteries** | ----- |
| Interventricular septum | 7mm Inlet VSD, BD Shunt | **Doppler Measurement** |  |
| Interatrial septum | 11mm OS ASD, R – L Shunt | Mitral | ----- |
| **Semilunar valves** |  | Aortic | ------- |
| Aortic valve | Annulus = 12mm | Tricuspid | ------- |
| Pulmonary valve | Annulus = 10mm | pulmonic | -------- |
| **Great arteries** | NRGA | **Aortic arch** | Left. No CoA. |
| Aorta | Posterior & from LV, to the right | **PDA** | No |
| Pulmonary artery | Anterior and from LV, to the left |  |  |
| **M-Mode:** | | | |
| AO | mm | PWd | mm |
| LA | mm | PWs | mm |
| LVIDd | mm | EDV | ml |
| LVIDs | mm | ESV | ml |
| IVSs | mm | LVEF | % |
| IVSd | mm | FS | % |
| **Additional Information**: |  | | |
| No pericardial/Pleural effusion. | | | |
| **Final Diagnosis:** | | | |
| 1. {S, D, D} Levocardia. 2. DOLV 3. Large OS ASD, R – L Shunt 4. Moderate Inlet VSD, BD Shunt 5. Tricuspid atresia type IIIC | | | |
| **Remark**: | | | |
| **Recommendation**: | | | |
| SIGNATURE  Done by: Tesfaye T., Pediatrician, Pediatric Cardiologist _______________ 01/11/2014Eth.C | | | |

| Patient Name: **Evador Yohannes**. Referring Institute: **Dr. Addisu PSC**. SEX/ Age: **F/2 8/12**.  Date of Report: **01/11/14**. Referral Diagnosis: **Follow up Echo for small PDA (2mm). Incidental. AGH8.412** | | | |
| --- | --- | --- | --- |
| **Features** | **Finding** | **Features** | **Finding** |
| **Profile** |  | **Atria** |  |
| Abdominal situs | Solitus | Left atrium | Normal |
| Cardiac position | Levocardia | Right atrium | Normal |
| Systemic venous drainage | Normal. | **Atrioventricular valves** |  |
| Pulmonary venous drainage | Normal | Mitral valve | Annulus = 13mm |
| Atrioventricular connection | Concordant | Tricuspid valve | Annulus = 15mm  TAPSE = 16mm |
| Ventriculoarterial connection | Concordant | **Ventricles** |  |
| Ventricular loop | d-Loop | Left ventricle | Normal |
|  |  | Right ventricle | Normal |
| **Septae** |  | **Coronary arteries** | ----- |
| Interventricular septum | Intact | **Doppler Measurement** |  |
| Interatrial septum | Intact | Mitral | ----- |
| **Semilunar valves** |  | Aortic | ------- |
| Aortic valve | Annulus = 13mm | Tricuspid | ------- |
| Pulmonary valve | Annulus = 13mm | pulmonic | -------- |
| **Great arteries** | NRGA | **Aortic arch** | Left. No CoA. |
| Aorta | ----- | **PDA** | 1mm PDA, L – R Shunt |
| Pulmonary artery | Normal MPA and Branch PAs. |  |  |
| **M-Mode:**  Normal LV Function on eye balling | | | |
| AO | mm | PWd | mm |
| LA | mm | PWs | mm |
| LVIDd | mm | EDV | ml |
| LVIDs | mm | ESV | ml |
| IVSs | mm | LVEF | % |
| IVSd | mm | FS | % |
| **Additional Information**: |  | | |
| No pericardial/Pleural effusion. | | | |
| **Final Diagnosis:** | | | |
| 1. {S, D, S} Levocardia. 2. Small PDA, L – R Shunt 3. Normal Biventricular Systolic Function | | | |
| **Remark**: | | | |
| **Recommendation**: | | | |
| SIGNATURE  Done by: Tesfaye T., Pediatrician, Pediatric Cardiologist _______________ 01/11/2014Eth.C | | | |

| Patient Name: **Eptisam Hamid**. Referring Institute: **Dr. Addisu PSC**. SEX/ Age: **F/1 3/12**.  Date of Report: **04/11/14**. Referral Diagnosis: **Cyanosis. AGH8.413** | | | |
| --- | --- | --- | --- |
| **Features** | **Finding** | **Features** | **Finding** |
| **Profile** |  | **Atria** |  |
| Abdominal situs | Solitus | Left atrium | Normal |
| Cardiac position | Levocardia | Right atrium | Mildly dilated |
| Systemic venous drainage | Normal. | **Atrioventricular valves** |  |
| Pulmonary venous drainage | Normal | Mitral valve | Annulus = 12mm |
| Atrioventricular connection | Concordant | Tricuspid valve | Annulus = 13mm  TAPSE = 13mm |
| Ventriculoarterial connection | Concordant | **Ventricles** |  |
| Ventricular loop | d-Loop | Left ventricle | Normal |
|  |  | Right ventricle | Mildly dilated and Hypertrophied |
| **Septae** |  | **Coronary arteries** | ----- |
| Interventricular septum | Non – Restrictive Mal-aligned Sub aortic VSD, R – L Shunt | **Doppler Measurement** |  |
| Interatrial septum | Intact | Mitral | ----- |
| **Semilunar valves** |  | Aortic | ------- |
| Aortic valve | Annulus = 11mm | Tricuspid | ------- |
| Pulmonary valve | Annulus = 11mm | pulmonic | Valvular and Sub-Valvular PS, PPG = 63mmHg |
| **Great arteries** | NRGA | **Aortic arch** | Left. No CoA. |
| Aorta | Aorta over-riding the VSD | **PDA** | No |
| Pulmonary artery | Normal MPA and Branch PAs. |  |  |
| **M-Mode:**  Normal LV Function on eye balling | | | |
| AO | mm | PWd | mm |
| LA | mm | PWs | mm |
| LVIDd | mm | EDV | ml |
| LVIDs | mm | ESV | ml |
| IVSs | mm | LVEF | % |
| IVSd | mm | FS | % |
| **Additional Information**: |  | | |
| No pericardial/Pleural effusion. | | | |
| **Final Diagnosis:** | | | |
| 1. {S, D, S} Levocardia. 2. TOF | | | |
| **Remark**: | | | |
| **Recommendation**: | | | |
| SIGNATURE  Done by: Tesfaye T., Pediatrician, Pediatric Cardiologist _______________ 04/11/2014Eth.C | | | |

| Patient Name: **Baby of Tigist Tigabu**. Referring Institute: **MSI – Ethiopia, Bahir Dar**. SEX/ Age: **M/4months**.  Date of Report: **04/11/14**. Referral Diagnosis: **Cyanosis. AGH8.414** | | | |
| --- | --- | --- | --- |
| **Features** | **Finding** | **Features** | **Finding** |
| **Profile** |  | **Atria** |  |
| Abdominal situs | Solitus | Left atrium | Normal |
| Cardiac position | Levocardia | Right atrium | Normal |
| Systemic venous drainage | Normal. | **Atrioventricular valves** |  |
| Pulmonary venous drainage | Normal | Mitral valve | Annulus = 14mm |
| Atrioventricular connection | Concordant | Tricuspid valve | Annulus = 14mm |
| Ventriculoarterial connection | DORV | **Ventricles** |  |
| Ventricular loop | d-Loop | Left ventricle | Normal |
|  |  | Right ventricle | Normal |
| **Septae** |  | **Coronary arteries** | ----- |
| Interventricular septum | 8mm Sub – aortic VSD, L – R Shunt | **Doppler Measurement** |  |
| Interatrial septum | PFO, L – R Shunt | Mitral | ----- |
| **Semilunar valves** |  | Aortic | ------- |
| Aortic valve | Annulus = 10mm | Tricuspid | ------- |
| Pulmonary valve | Annulus = 13mm | pulmonic | Mild PS, PPG = 21mmHg |
| **Great arteries** | NRGA | **Aortic arch** | Left. No CoA. |
| Aorta | Right, Posterior and from RV | **PDA** | No |
| Pulmonary artery | Left, Anterior and from RV |  |  |
| **M-Mode:** | | | |
| AO | mm | PWd | mm |
| LA | mm | PWs | mm |
| LVIDd | mm | EDV | ml |
| LVIDs | mm | ESV | ml |
| IVSs | mm | LVEF | % |
| IVSd | mm | FS | % |
| **Additional Information**: |  | | |
| No pericardial/Pleural effusion. | | | |
| **Final Diagnosis:** | | | |
| 1. {S, D, D} Levocardia. 2. DORV 3. PFO, L – R Shunt 4. Large Sub aortic VSD, L – R Shunt 5. Mild PS | | | |
| **Remark**: | | | |
| **Recommendation**: | | | |
| SIGNATURE  Done by: Tesfaye T., Pediatrician, Pediatric Cardiologist _______________ 04/11/2014Eth.C | | | |

| Patient Name: **Yabibal Kassaye**. Referring Institute: **FHRH**. SEX/ Age: **M/5years**.  Date of Report: **04/11/14**. Referral Diagnosis: **FTT. AGH8.415** | | | |
| --- | --- | --- | --- |
| **Features** | **Finding** | **Features** | **Finding** |
| **Profile** |  | **Atria** |  |
| Abdominal situs | Solitus | Left atrium | Normal |
| Cardiac position | Levocardia | Right atrium | Normal |
| Systemic venous drainage | Normal. | **Atrioventricular valves** |  |
| Pulmonary venous drainage | Normal | Mitral valve | Annulus = 15mm |
| Atrioventricular connection | Concordant | Tricuspid valve | Annulus = 17mm  TAPSE = mm |
| Ventriculoarterial connection | Concordant | **Ventricles** |  |
| Ventricular loop | d-Loop | Left ventricle | Normal |
|  |  | Right ventricle | Normal |
| **Septae** |  | **Coronary arteries** | ----- |
| Interventricular septum | 6mm PM VSD, L – R Shunt | **Doppler Measurement** |  |
| Interatrial septum | Intact | Mitral | ----- |
| **Semilunar valves** |  | Aortic | ------- |
| Aortic valve | Annulus = 15mm | Tricuspid | ------- |
| Pulmonary valve | Annulus = 17mm | pulmonic | -------- |
| **Great arteries** | NRGA | **Aortic arch** | Left. No CoA. |
| Aorta | ----- | **PDA** | No |
| Pulmonary artery | Normal MPA and Branch PAs. |  |  |
| **M-Mode:**  Normal LV Function on eye balling | | | |
| AO | mm | PWd | mm |
| LA | mm | PWs | mm |
| LVIDd | mm | EDV | ml |
| LVIDs | mm | ESV | ml |
| IVSs | mm | LVEF | % |
| IVSd | mm | FS | % |
| **Additional Information**: |  | | |
| No pericardial/Pleural effusion. | | | |
| **Final Diagnosis:** | | | |
| 1. {S, D, S} Levocardia. 2. Small PM VSD, L – R Shunt 3. Normal LV Systolic Function | | | |
| **Remark**: child was restless during study | | | |
| **Recommendation**: | | | |
| SIGNATURE  Done by: Tesfaye T., Pediatrician, Pediatric Cardiologist _______________ 04/11/2014Eth.C | | | |

| Patient Name: **Helen Melaku**. Referring Institute: **Mekane – Selam H**. SEX/ Age: **F/9months**.  Date of Report: **05/11/14**. Referral Diagnosis: **FTT + Murmur. AGH8.416** | | | |
| --- | --- | --- | --- |
| **Features** | **Finding** | **Features** | **Finding** |
| **Profile** |  | **Atria** |  |
| Abdominal situs | Solitus | Left atrium | Normal |
| Cardiac position | Levocardia | Right atrium | Normal |
| Systemic venous drainage | Normal. | **Atrioventricular valves** |  |
| Pulmonary venous drainage | Normal | Mitral valve | Annulus = 12mm |
| Atrioventricular connection | Concordant | Tricuspid valve | Annulus = 14mm |
| Ventriculoarterial connection | Concordant | **Ventricles** |  |
| Ventricular loop | d-Loop | Left ventricle | Normal |
|  |  | Right ventricle | Normal |
| **Septae** |  | **Coronary arteries** | ----- |
| Interventricular septum | 5mm PM VSD, L – R Shunt | **Doppler Measurement** |  |
| Interatrial septum | Intact | Mitral | ----- |
| **Semilunar valves** |  | Aortic | ------- |
| Aortic valve | Annulus = 11mm | Tricuspid | ------- |
| Pulmonary valve | Annulus = 13mm | pulmonic | -------- |
| **Great arteries** | NRGA | **Aortic arch** | Left. No CoA. |
| Aorta | ----- | **PDA** | No |
| Pulmonary artery | Normal MPA and Branch PAs. |  |  |
| **M-Mode:**  Normal LV Function on eye balling | | | |
| AO | mm | PWd | mm |
| LA | mm | PWs | mm |
| LVIDd | mm | EDV | ml |
| LVIDs | mm | ESV | ml |
| IVSs | mm | LVEF | % |
| IVSd | mm | FS | % |
| **Additional Information**: |  | | |
| No pericardial/Pleural effusion. | | | |
| **Final Diagnosis:** | | | |
| 1. {S, D, S} Levocardia. 2. Moderate PM VSD, L – R Shunt 3. Normal LV Systolic Function | | | |
| **Remark**: | | | |
| **Recommendation**: | | | |
| SIGNATURE  Done by: Tesfaye T., Pediatrician, Pediatric Cardiologist _______________ 05/11/2014Eth.C | | | |

| Patient Name: **Muluken Eyeberu** Referring Institute: **FHRH**. SEX/ Age: **M/4months**.  Date of Report: **06/11/14**. Referral Diagnosis: **RD. AGH8.417** | | | |
| --- | --- | --- | --- |
| **Features** | **Finding** | **Features** | **Finding** |
| **Profile** |  | **Atria** |  |
| Abdominal situs | Solitus | Left atrium | Normal |
| Cardiac position | Levocardia | Right atrium | Normal |
| Systemic venous drainage | Normal. | **Atrioventricular valves** |  |
| Pulmonary venous drainage | Normal | Mitral valve | Annulus = 11mm |
| Atrioventricular connection | Concordant | Tricuspid valve | Annulus = 12mm |
| Ventriculoarterial connection | Concordant | **Ventricles** |  |
| Ventricular loop | d-Loop | Left ventricle | Normal |
|  |  | Right ventricle | Normal |
| **Septae** |  | **Coronary arteries** | ----- |
| Interventricular septum | Intact | **Doppler Measurement** |  |
| Interatrial septum | Intact | Mitral | ----- |
| **Semilunar valves** |  | Aortic | ------- |
| Aortic valve | Annulus = 8mm | Tricuspid | ------- |
| Pulmonary valve | Annulus = 9mm | pulmonic | -------- |
| **Great arteries** | NRGA | **Aortic arch** | Left. No CoA. |
| Aorta | ----- | **PDA** | No |
| Pulmonary artery | Normal MPA and Branch PAs. |  |  |
| **M-Mode:**  Normal LV Function on eye balling | | | |
| AO | mm | PWd | mm |
| LA | mm | PWs | mm |
| LVIDd | mm | EDV | ml |
| LVIDs | mm | ESV | ml |
| IVSs | mm | LVEF | % |
| IVSd | mm | FS | % |
| **Additional Information**: |  | | |
| No pericardial/Pleural effusion. | | | |
| **Final Diagnosis:** | | | |
| 1. Normal Echocardiography Study. | | | |
| **Remark**: | | | |
| **Recommendation**: | | | |
| SIGNATURE  Done by: Tesfaye T., Pediatrician, Pediatric Cardiologist _______________ 06/11/2014Eth.C | | | |

| Patient Name: **Abibalech Habitamu**. Referring Institute: **Addis Alem PH**. SEX/ Age: **F/11years**.  Date of Report: **06/11/14**. Referral Diagnosis: **Chest Pain. AGH8.418** | | | |
| --- | --- | --- | --- |
| **Features** | **Finding** | **Features** | **Finding** |
| **Profile** |  | **Atria** |  |
| Abdominal situs | Solitus | Left atrium | Normal |
| Cardiac position | Levocardia | Right atrium | Normal |
| Systemic venous drainage | Normal. | **Atrioventricular valves** |  |
| Pulmonary venous drainage | Normal | Mitral valve | Annulus = 18mm |
| Atrioventricular connection | Concordant | Tricuspid valve | Annulus = 18mm  TAPSE = 21mm |
| Ventriculoarterial connection | Concordant | **Ventricles** |  |
| Ventricular loop | d-Loop | Left ventricle | Normal |
|  |  | Right ventricle | Normal |
| **Septae** |  | **Coronary arteries** | ----- |
| Interventricular septum | Intact | **Doppler Measurement** |  |
| Interatrial septum | Intact | Mitral | ----- |
| **Semilunar valves** |  | Aortic | ------- |
| Aortic valve | Annulus = 17mm | Tricuspid | ------- |
| Pulmonary valve | Annulus = 18mm | pulmonic | -------- |
| **Great arteries** | NRGA | **Aortic arch** | Left. No CoA. |
| Aorta | ----- | **PDA** | No |
| Pulmonary artery | Normal MPA and Branch PAs. |  |  |
| **M-Mode:** | | | |
| AO | mm | PWd | mm |
| LA | mm | PWs | mm |
| LVIDd | mm | EDV | ml |
| LVIDs | mm | ESV | ml |
| IVSs | mm | LVEF | 61% |
| IVSd | mm | FS | 32% |
| **Additional Information**: |  | | |
| No pericardial/Pleural effusion. | | | |
| **Final Diagnosis:** | | | |
| 1. Normal Echocardiography Study. | | | |
| **Remark**: | | | |
| **Recommendation**: | | | |
| SIGNATURE  Done by: Tesfaye T., Pediatrician, Pediatric Cardiologist _______________ 06/11/2014Eth.C | | | |

| Patient Name: **Wubalem Tarko**. Referring Institute: **TGSH**. SEX/ Age: **F/12years**.  Date of Report: **06/11/14**. Referral Diagnosis: **DOE + easy fatigability. AGH8.419** | | | |
| --- | --- | --- | --- |
| **Features** | **Finding** | **Features** | **Finding** |
| **Profile** |  | **Atria** |  |
| Abdominal situs | Solitus | Left atrium | Normal |
| Cardiac position | Levocardia | Right atrium | Normal |
| Systemic venous drainage | Normal. | **Atrioventricular valves** |  |
| Pulmonary venous drainage | Normal | Mitral valve | Annulus = 20mm |
| Atrioventricular connection | Concordant | Tricuspid valve | Annulus = 21mm  TAPSE = 19mm |
| Ventriculoarterial connection | Concordant | **Ventricles** |  |
| Ventricular loop | d-Loop | Left ventricle | Normal |
|  |  | Right ventricle | Normal |
| **Septae** |  | **Coronary arteries** | ----- |
| Interventricular septum | Intact | **Doppler Measurement** |  |
| Interatrial septum | Intact | Mitral | ----- |
| **Semilunar valves** |  | Aortic | ------- |
| Aortic valve | Annulus = 19mm | Tricuspid | ------- |
| Pulmonary valve | Annulus = 20mm | pulmonic | -------- |
| **Great arteries** | NRGA | **Aortic arch** | Left. No CoA. |
| Aorta | ----- | **PDA** | No |
| Pulmonary artery | Normal MPA and Branch PAs. |  |  |
| **M-Mode:** | | | |
| AO | mm | PWd | mm |
| LA | mm | PWs | mm |
| LVIDd | mm | EDV | ml |
| LVIDs | mm | ESV | ml |
| IVSs | mm | LVEF | 60% |
| IVSd | mm | FS | 32% |
| **Additional Information**: |  | | |
| No pericardial/Pleural effusion. | | | |
| **Final Diagnosis:** | | | |
| 1. Normal Echocardiography Study. | | | |
| **Remark**: | | | |
| **Recommendation**: | | | |
| SIGNATURE  Done by: Tesfaye T., Pediatrician, Pediatric Cardiologist _______________ 06/11/2014Eth.C | | | |

| Patient Name: **Hanna Chalie**. Referring Institute: **Debre – Tabour RH**. SEX/ Age: **F/2years**.  Date of Report: **06/11/14**. Referral Diagnosis: **Incidental Murmur Funding (G-II systolic). AGH8.420** | | | |
| --- | --- | --- | --- |
| **Features** | **Finding** | **Features** | **Finding** |
| **Profile** |  | **Atria** |  |
| Abdominal situs | Solitus | Left atrium | Normal |
| Cardiac position | Levocardia | Right atrium | Normal |
| Systemic venous drainage | Normal. | **Atrioventricular valves** |  |
| Pulmonary venous drainage | Normal | Mitral valve | Annulus = 13mm |
| Atrioventricular connection | Concordant | Tricuspid valve | Annulus = 14mm  TAPSE = 16mm |
| Ventriculoarterial connection | Concordant | **Ventricles** |  |
| Ventricular loop | d-Loop | Left ventricle | Normal |
|  |  | Right ventricle | Normal |
| **Septae** |  | **Coronary arteries** | ----- |
| Interventricular septum | Intact | **Doppler Measurement** |  |
| Interatrial septum | Intact | Mitral | ----- |
| **Semilunar valves** |  | Aortic | ------- |
| Aortic valve | Annulus = 11mm | Tricuspid | ------- |
| Pulmonary valve | Annulus = 11mm | pulmonic | -------- |
| **Great arteries** | NRGA | **Aortic arch** | Left. No CoA. |
| Aorta | ----- | **PDA** | No |
| Pulmonary artery | Normal MPA and Branch PAs. |  |  |
| **M-Mode:**  Normal LV Function on eye balling | | | |
| AO | mm | PWd | mm |
| LA | mm | PWs | mm |
| LVIDd | mm | EDV | ml |
| LVIDs | mm | ESV | ml |
| IVSs | mm | LVEF | % |
| IVSd | mm | FS | % |
| **Additional Information**: |  | | |
| No pericardial/Pleural effusion. | | | |
| **Final Diagnosis:** | | | |
| 1. Normal Echocardiography Study. | | | |
| **Remark**: Grade II Vibrant Systolic Murmur is appreciated. Can consider Innocent Murmur | | | |
| **Recommendation**: | | | |
| SIGNATURE  Done by: Tesfaye T., Pediatrician, Pediatric Cardiologist _______________ 06/11/2014Eth.C | | | |

| Patient Name: **Melkamu Belayneh**. Referring Institute: **FHRH**. SEX/ Age: **M/12years**.  Date of Report: **06/11/14**. Referral Diagnosis: **RD + CHF. AGH8.421** | | | |
| --- | --- | --- | --- |
| **Features** | **Finding** | **Features** | **Finding** |
| **Profile** |  | **Atria** |  |
| Abdominal situs | Solitus | Left atrium | Mildly dilated |
| Cardiac position | Levocardia | Right atrium | Normal |
| Systemic venous drainage | Normal. | **Atrioventricular valves** |  |
| Pulmonary venous drainage | Normal | Mitral valve | Annulus = 21mm |
| Atrioventricular connection | Concordant | Tricuspid valve | Annulus = 18mm |
| Ventriculoarterial connection | Concordant | **Ventricles** |  |
| Ventricular loop | d-Loop | Left ventricle | Mildly dilated |
|  |  | Right ventricle | Normal |
| **Septae** |  | **Coronary arteries** | ----- |
| Interventricular septum | 16mm Inlet VSD, Partially closed by STL, L – R Shunt | **Doppler Measurement** |  |
| Interatrial septum | Intact | Mitral | ----- |
| **Semilunar valves** |  | Aortic | ------- |
| Aortic valve | Annulus = 19mm | Tricuspid | ------- |
| Pulmonary valve | Annulus = 23mm | pulmonic | -------- |
| **Great arteries** | NRGA | **Aortic arch** | Left. No CoA. |
| Aorta | ----- | **PDA** | No |
| Pulmonary artery | Normal MPA and Branch PAs. |  |  |
| **M-Mode:**  Normal LV Function on eye balling | | | |
| AO | mm | PWd | mm |
| LA | mm | PWs | mm |
| LVIDd | mm | EDV | ml |
| LVIDs | mm | ESV | ml |
| IVSs | mm | LVEF | % |
| IVSd | mm | FS | % |
| **Additional Information**: |  | | |
| Circumferential Pericardial effusion with maximum depth of 8mm on RV Side. | | | |
| **Final Diagnosis:** | | | |
| 1. {S, D, S} Levocardia. 2. LA/LV Mildly dilated 3. Large Inlet VSD, Partially closed by STL, L – R Shunt 4. Normal LV Systolic Function | | | |
| **Remark**: Child was restless during study | | | |
| **Recommendation**: | | | |
| SIGNATURE  Done by: Tesfaye T., Pediatrician, Pediatric Cardiologist _______________ 06/11/2014Eth.C | | | |

| Patient Name: **Amen Abat**. Referring Institute: **FHRH**. SEX/ Age: **M/7months**.  Date of Report: **07/11/14**. Referral Diagnosis: **DS. AGH8.422** | | | |
| --- | --- | --- | --- |
| **Features** | **Finding** | **Features** | **Finding** |
| **Profile** |  | **Atria** |  |
| Abdominal situs | Solitus | Left atrium | Normal |
| Cardiac position | Levocardia | Right atrium | Normal |
| Systemic venous drainage | Normal. | **Atrioventricular valves** |  |
| Pulmonary venous drainage | Normal | Mitral valve | Annulus = 14mm |
| Atrioventricular connection | Concordant | Tricuspid valve | Annulus = 14mm |
| Ventriculoarterial connection | Concordant | **Ventricles** |  |
| Ventricular loop | d-Loop | Left ventricle | Normal |
|  |  | Right ventricle | Normal |
| **Septae** |  | **Coronary arteries** | ----- |
| Interventricular septum | Intact | **Doppler Measurement** |  |
| Interatrial septum | Intact | Mitral | ----- |
| **Semilunar valves** |  | Aortic | ------- |
| Aortic valve | Annulus = 11mm | Tricuspid | ------- |
| Pulmonary valve | Annulus = 12mm | pulmonic | -------- |
| **Great arteries** | NRGA | **Aortic arch** | Left. No CoA. |
| Aorta | ----- | **PDA** | No |
| Pulmonary artery | Normal MPA and Branch PAs. |  |  |
| **M-Mode:**  Normal LV Function on eye balling | | | |
| AO | mm | PWd | mm |
| LA | mm | PWs | mm |
| LVIDd | mm | EDV | ml |
| LVIDs | mm | ESV | ml |
| IVSs | mm | LVEF | % |
| IVSd | mm | FS | % |
| **Additional Information**: |  | | |
| Pericardial effusion with maximum depth of 4mm on RV Side. | | | |
| **Final Diagnosis:** | | | |
| 1. {S, D, S} Levocardia. 2. Trace Pericardial effusion 20 to ? | | | |
| **Remark**: | | | |
| **Recommendation**: | | | |
| SIGNATURE  Done by: Tesfaye T., Pediatrician, Pediatric Cardiologist _______________ 07/11/2014Eth.C | | | |

| Patient Name: **Amde-Werk Ghion**. Referring Institute: **TGSH**. SEX/ Age: **M/1 8/12**.  Date of Report: **07/11/14**. Referral Diagnosis: **FTT. AGH8.423** | | | |
| --- | --- | --- | --- |
| **Features** | **Finding** | **Features** | **Finding** |
| **Profile** |  | **Atria** |  |
| Abdominal situs | Solitus | Left atrium | Normal |
| Cardiac position | Levocardia | Right atrium | Dilated |
| Systemic venous drainage | Normal. | **Atrioventricular valves** |  |
| Pulmonary venous drainage | Normal | Mitral valve | Annulus = 11mm |
| Atrioventricular connection | Concordant | Tricuspid valve | Annulus = 16mm |
| Ventriculoarterial connection | Concordant | **Ventricles** |  |
| Ventricular loop | d-Loop | Left ventricle | Normal |
|  |  | Right ventricle | Dilated |
| **Septae** |  | **Coronary arteries** | ----- |
| Interventricular septum | Intact | **Doppler Measurement** |  |
| Interatrial septum | 10 X 12mm OS ASD, L – R Shunt | Mitral | ----- |
| **Semilunar valves** |  | Aortic | ------- |
| Aortic valve | Annulus = 12mm | Tricuspid | ------- |
| Pulmonary valve | Annulus = 13mm | pulmonic | -------- |
| **Great arteries** | NRGA | **Aortic arch** | Left. No CoA. |
| Aorta | ----- | **PDA** | No |
| Pulmonary artery | Normal MPA and Branch PAs. |  |  |
| **M-Mode:**  Normal LV Function on eye balling | | | |
| AO | mm | PWd | mm |
| LA | mm | PWs | mm |
| LVIDd | mm | EDV | ml |
| LVIDs | mm | ESV | ml |
| IVSs | mm | LVEF | % |
| IVSd | mm | FS | % |
| **Additional Information**: |  | | |
| No pericardial/Pleural effusion. | | | |
| **Final Diagnosis:** | | | |
| 1. {S, D, S} Levocardia. 2. RA/RV Dilated 3. Large OS ASD, L – R Shunt 4. Normal LV Systolic Function | | | |
| **Remark**: | | | |
| **Recommendation**: | | | |
| SIGNATURE  Done by: Tesfaye T., Pediatrician, Pediatric Cardiologist _______________ 07/11/2014Eth.C | | | |

| Patient Name: **Eyuel Birhanu**. Referring Institute: **TGSH**. SEX/ Age: **M/8years**.  Date of Report: **07/11/14**. Referral Diagnosis: **ARF. AGH8.424** | | | |
| --- | --- | --- | --- |
| **Features** | **Finding** | **Features** | **Finding** |
| **Profile** |  | **Atria** |  |
| Abdominal situs | Solitus | Left atrium | Normal |
| Cardiac position | Levocardia | Right atrium | Normal |
| Systemic venous drainage | Normal. | **Atrioventricular valves** |  |
| Pulmonary venous drainage | Normal | Mitral valve | Annulus = 15mm. Patulous elongated MVL |
| Atrioventricular connection | Concordant | Tricuspid valve | Annulus = 16mm  TAPSE = 19mm |
| Ventriculoarterial connection | Concordant | **Ventricles** |  |
| Ventricular loop | d-Loop | Left ventricle | Normal |
|  |  | Right ventricle | Normal |
| **Septae** |  | **Coronary arteries** | ----- |
| Interventricular septum | Intact | **Doppler Measurement** |  |
| Interatrial septum | Intact | Mitral | Mild MR, Holosystolic, Jet velocity = 3.5m/sec |
| **Semilunar valves** |  | Aortic | ------- |
| Aortic valve | Annulus = 16mm | Tricuspid | ------- |
| Pulmonary valve | Annulus = 16mm | pulmonic | -------- |
| **Great arteries** | NRGA | **Aortic arch** | Left. No CoA. |
| Aorta | ----- | **PDA** | No |
| Pulmonary artery | Normal MPA and Branch PAs. |  |  |
| **M-Mode:** | | | |
| AO | mm | PWd | mm |
| LA | mm | PWs | mm |
| LVIDd | mm | EDV | ml |
| LVIDs | mm | ESV | ml |
| IVSs | mm | LVEF | % |
| IVSd | mm | FS | % |
| **Additional Information**: |  | | |
| No pericardial/Pleural effusion. | | | |
| **Final Diagnosis:** | | | |
| 1. {S, D, S} Levocardia. 2. Patulous elongated MVL 3. Mild MR 4. Normal Biventricular Function | | | |
| **Remark**: Poor echo window. | | | |
| **Recommendation**: Needs repeat echo. | | | |
| SIGNATURE  Done by: Tesfaye T., Pediatrician, Pediatric Cardiologist _______________ 07/11/2014Eth.C | | | |

| Patient Name: **Eshetu Derib**. Referring Institute: **Adinas GH**. SEX/ Age: **M/2 1/12**.  Date of Report: **12/11/14**. Referral Diagnosis: **Incidental Murmur Finding. AGH8.425** | | | |
| --- | --- | --- | --- |
| **Features** | **Finding** | **Features** | **Finding** |
| **Profile** |  | **Atria** |  |
| Abdominal situs | Solitus | Left atrium | Normal |
| Cardiac position | Levocardia | Right atrium | Normal |
| Systemic venous drainage | Normal. | **Atrioventricular valves** |  |
| Pulmonary venous drainage | Normal | Mitral valve | Annulus = 13mm |
| Atrioventricular connection | Concordant | Tricuspid valve | Annulus = 15mm  TAPSE = 15mm |
| Ventriculoarterial connection | Concordant | **Ventricles** |  |
| Ventricular loop | d-Loop | Left ventricle | Normal |
|  |  | Right ventricle | Normal |
| **Septae** |  | **Coronary arteries** | ----- |
| Interventricular septum | Intact | **Doppler Measurement** |  |
| Interatrial septum | Intact | Mitral | ----- |
| **Semilunar valves** |  | Aortic | ------- |
| Aortic valve | Annulus = 11mm | Tricuspid | ------- |
| Pulmonary valve | Annulus = 12mm | pulmonic | Mild PS, PPG = 35mmHg |
| **Great arteries** | NRGA | **Aortic arch** | Left. No CoA. |
| Aorta | ----- | **PDA** | No |
| Pulmonary artery | Normal MPA and Branch PAs. |  |  |
| **M-Mode:**  Normal LV Function on eye balling | | | |
| AO | mm | PWd | mm |
| LA | mm | PWs | mm |
| LVIDd | mm | EDV | ml |
| LVIDs | mm | ESV | ml |
| IVSs | mm | LVEF | % |
| IVSd | mm | FS | % |
| **Additional Information**: |  | | |
| No pericardial/Pleural effusion. | | | |
| **Final Diagnosis:** | | | |
| 1. {S, D, S} Levocardia. 2. Mild PS | | | |
| **Remark**: yearly echocardiography follow up | | | |
| **Recommendation**: | | | |
| SIGNATURE  Done by: Tesfaye T., Pediatrician, Pediatric Cardiologist _______________ 12/11/2014Eth.C | | | |

| Patient Name: **Baby of Rahel Molla**. Referring Institute: **MSI-Ethiopia, Bahir Dar**. SEX/ Age: **M/5days**.  Date of Report: **12/11/14**. Referral Diagnosis: **RD. AGH8.426** | | | |
| --- | --- | --- | --- |
| **Features** | **Finding** | **Features** | **Finding** |
| **Profile** |  | **Atria** |  |
| Abdominal situs | Solitus | Left atrium | Normal |
| Cardiac position | Levocardia | Right atrium | Normal |
| Systemic venous drainage | Normal. | **Atrioventricular valves** |  |
| Pulmonary venous drainage | Normal | Mitral valve | Annulus = 9mm |
| Atrioventricular connection | Concordant | Tricuspid valve | Annulus = 9mm |
| Ventriculoarterial connection | Concordant | **Ventricles** |  |
| Ventricular loop | d-Loop | Left ventricle | Normal |
|  |  | Right ventricle | Normal |
| **Septae** |  | **Coronary arteries** | ----- |
| Interventricular septum | Intact | **Doppler Measurement** |  |
| Interatrial septum | PFO, L – R Shunt | Mitral | ----- |
| **Semilunar valves** |  | Aortic | ------- |
| Aortic valve | Annulus = 8mm | Tricuspid | ------- |
| Pulmonary valve | Annulus = 8mm | pulmonic | -------- |
| **Great arteries** | NRGA | **Aortic arch** | Left. No CoA. |
| Aorta | ----- | **PDA** | No |
| Pulmonary artery | Normal MPA and Branch PAs. |  |  |
| **M-Mode:**  Normal LV Function on eye balling | | | |
| AO | mm | PWd | mm |
| LA | mm | PWs | mm |
| LVIDd | mm | EDV | ml |
| LVIDs | mm | ESV | ml |
| IVSs | mm | LVEF | % |
| IVSd | mm | FS | % |
| **Additional Information**: |  | | |
| No pericardial/Pleural effusion. | | | |
| **Final Diagnosis:** | | | |
| 1. {S, D, S} Levocardia. 2. PFO, L – R Shunt | | | |
| **Remark**: | | | |
| **Recommendation**: | | | |
| SIGNATURE  Done by: Tesfaye T., Pediatrician, Pediatric Cardiologist _______________ 12/11/2014Eth.C | | | |

| Patient Name: **Teninet Alemu**. Referring Institute: **FHRH**. SEX/ Age: **F/4 1/12**.  Date of Report: **12/11/14**. Referral Diagnosis: **FTT. AGH8.427** | | | |
| --- | --- | --- | --- |
| **Features** | **Finding** | **Features** | **Finding** |
| **Profile** |  | **Atria** |  |
| Abdominal situs | Solitus | Left atrium | Normal |
| Cardiac position | Levocardia | Right atrium | Normal |
| Systemic venous drainage | Normal. | **Atrioventricular valves** |  |
| Pulmonary venous drainage | Normal | Mitral valve | Annulus = 15mm |
| Atrioventricular connection | Concordant | Tricuspid valve | Annulus = 17mm |
| Ventriculoarterial connection | Concordant | **Ventricles** |  |
| Ventricular loop | d-Loop | Left ventricle | Normal |
|  |  | Right ventricle | Normal |
| **Septae** |  | **Coronary arteries** | ----- |
| Interventricular septum | Intact | **Doppler Measurement** |  |
| Interatrial septum | Intact | Mitral | ----- |
| **Semilunar valves** |  | Aortic | ------- |
| Aortic valve | Annulus = 13mm | Tricuspid | ------- |
| Pulmonary valve | Annulus = 13mm | pulmonic | -------- |
| **Great arteries** | NRGA | **Aortic arch** | Left. No CoA. |
| Aorta | ----- | **PDA** | No |
| Pulmonary artery | Normal MPA and Branch PAs. |  |  |
| **M-Mode:**  Normal LV Function on eye balling | | | |
| AO | mm | PWd | mm |
| LA | mm | PWs | mm |
| LVIDd | mm | EDV | ml |
| LVIDs | mm | ESV | ml |
| IVSs | mm | LVEF | % |
| IVSd | mm | FS | % |
| **Additional Information**: |  | | |
| No pericardial/Pleural effusion. | | | |
| **Final Diagnosis:** | | | |
| 1. Normal Echocardiography Study. | | | |
| **Remark**: | | | |
| **Recommendation**: | | | |
| SIGNATURE  Done by: Tesfaye T., Pediatrician, Pediatric Cardiologist _______________ 12/11/2014Eth.C | | | |

| Patient Name: **Birtukan Baye**. Referring Institute: **TGSH**. SEX/ Age: **F/7years**.  Date of Report: **12/11/14**. Referral Diagnosis: **Easy fatigability. AGH8.428** | | | |
| --- | --- | --- | --- |
| **Features** | **Finding** | **Features** | **Finding** |
| **Profile** |  | **Atria** |  |
| Abdominal situs | Solitus | Left atrium | Normal |
| Cardiac position | Levocardia | Right atrium | Normal |
| Systemic venous drainage | Normal. | **Atrioventricular valves** |  |
| Pulmonary venous drainage | Normal | Mitral valve | Annulus = 17mm |
| Atrioventricular connection | Concordant | Tricuspid valve | Annulus = 19mm |
| Ventriculoarterial connection | Concordant | **Ventricles** |  |
| Ventricular loop | d-Loop | Left ventricle | Normal |
|  |  | Right ventricle | Normal |
| **Septae** |  | **Coronary arteries** | ----- |
| Interventricular septum | Intact | **Doppler Measurement** |  |
| Interatrial septum | Intact | Mitral | ----- |
| **Semilunar valves** |  | Aortic | ------- |
| Aortic valve | Annulus = 15mm | Tricuspid | Trivial TR, PPG = 21mmHg |
| Pulmonary valve | Annulus = 16mm | pulmonic | -------- |
| **Great arteries** | NRGA | **Aortic arch** | Left. No CoA. |
| Aorta | ----- | **PDA** | No |
| Pulmonary artery | Normal MPA and Branch PAs. |  |  |
| **M-Mode:**  Normal Echocardiography Study | | | |
| AO | mm | PWd | mm |
| LA | mm | PWs | mm |
| LVIDd | mm | EDV | ml |
| LVIDs | mm | ESV | ml |
| IVSs | mm | LVEF | % |
| IVSd | mm | FS | % |
| **Additional Information**: |  | | |
| No pericardial/Pleural effusion. | | | |
| **Final Diagnosis:** | | | |
| 1. Normal Echocardiography Study. | | | |
| **Remark**: | | | |
| **Recommendation**: | | | |
| SIGNATURE  Done by: Tesfaye T., Pediatrician, Pediatric Cardiologist _______________ 12/11/2014Eth.C | | | |

| Patient Name: **Hilina Wuletaw**. Referring Institute: **Adinas GH**. SEX/ Age: **F/7months**.  Date of Report: **12/11/14**. Referral Diagnosis: **Incidental Murmur Finding. AGH8.429 (TGSH2)** | | | |
| --- | --- | --- | --- |
| **Features** | **Finding** | **Features** | **Finding** |
| **Profile** |  | **Atria** |  |
| Abdominal situs | Solitus | Left atrium | Normal |
| Cardiac position | Levocardia | Right atrium | Normal |
| Systemic venous drainage | Normal. | **Atrioventricular valves** |  |
| Pulmonary venous drainage | Normal | Mitral valve | Annulus = 13mm |
| Atrioventricular connection | Concordant | Tricuspid valve | Annulus = 13mm |
| Ventriculoarterial connection | Concordant | **Ventricles** |  |
| Ventricular loop | d-Loop | Left ventricle | Normal |
|  |  | Right ventricle | Normal |
| **Septae** |  | **Coronary arteries** | ----- |
| Interventricular septum | Intact | **Doppler Measurement** |  |
| Interatrial septum | 7mm X 8mm Fenestrated ASD  Septal Aneurysm protruding 11mm to RA and 8mm to LA | Mitral | ----- |
| **Semilunar valves** |  | Aortic | ------- |
| Aortic valve | Annulus = 10mm | Tricuspid | ------- |
| Pulmonary valve | Annulus = 11mm | pulmonic | -------- |
| **Great arteries** | NRGA | **Aortic arch** | Left. No CoA. |
| Aorta | ----- | **PDA** | 1.5mm PDA, L – R Shunt |
| Pulmonary artery | Normal MPA and Branch PAs. |  |  |
| **M-Mode:**  Normal LV Function on eye balling | | | |
| AO | mm | PWd | mm |
| LA | mm | PWs | mm |
| LVIDd | mm | EDV | ml |
| LVIDs | mm | ESV | ml |
| IVSs | mm | LVEF | % |
| IVSd | mm | FS | % |
| **Additional Information**: |  | | |
| Circumferential Pericardial effusion with maximum depth of 11mm on RA/RV Side. | | | |
| **Final Diagnosis:** | | | |
| 1. {S, D, S} Levocardia. 2. Moderate Fenestrated OS ASD, L – R Shunt 3. Atrial Septal Aneurysm, type I 4. Moderate PDA, L – R Shunt 5. Moderate Circumferential Pericardial effusion 6. Normal Systolic function | | | |
| **Remark**: | | | |
| **Recommendation**: | | | |
| SIGNATURE  Done by: Tesfaye T., Pediatrician, Pediatric Cardiologist _______________ 12/11/2014Eth.C | | | |

| Patient Name: **Eyob Simachew**. Referring Institute: **FHRH**. SEX/ Age: **M/4Months**.  Date of Report: **14/11/14**. Referral Diagnosis: **RD. AGH8.430** | | | |
| --- | --- | --- | --- |
| **Features** | **Finding** | **Features** | **Finding** |
| **Profile** |  | **Atria** |  |
| Abdominal situs | Solitus | Left atrium | Normal |
| Cardiac position | Levocardia | Right atrium | Normal |
| Systemic venous drainage | Normal. | **Atrioventricular valves** |  |
| Pulmonary venous drainage | Normal | Mitral valve | Annulus = 9mm |
| Atrioventricular connection | Concordant | Tricuspid valve | Annulus = 10mm |
| Ventriculoarterial connection | Concordant | **Ventricles** |  |
| Ventricular loop | d-Loop | Left ventricle | Normal |
|  |  | Right ventricle | Normal |
| **Septae** |  | **Coronary arteries** | ----- |
| Interventricular septum | Intact | **Doppler Measurement** |  |
| Interatrial septum | Intact | Mitral | ----- |
| **Semilunar valves** |  | Aortic | ------- |
| Aortic valve | Annulus = 9mm | Tricuspid | ------- |
| Pulmonary valve | Annulus = 9mm | pulmonic | -------- |
| **Great arteries** | NRGA | **Aortic arch** | Left. No CoA. |
| Aorta | ----- | **PDA** | No |
| Pulmonary artery | Normal MPA and Branch PAs. |  |  |
| **M-Mode:** | | | |
| AO | mm | PWd | mm |
| LA | mm | PWs | mm |
| LVIDd | mm | EDV | ml |
| LVIDs | mm | ESV | ml |
| IVSs | mm | LVEF | % |
| IVSd | mm | FS | % |
| **Additional Information**: |  | | |
| No pericardial/Pleural effusion. | | | |
| **Final Diagnosis:** | | | |
| 1. Normal Echocardiography Study. | | | |
| **Remark**: | | | |
| **Recommendation**: | | | |
| SIGNATURE  Done by: Tesfaye T., Pediatrician, Pediatric Cardiologist _______________ 14/11/2014Eth.C | | | |

| Patient Name: **Dagim Yenealem**. Referring Institute: **MSI-Ethiopia, Bahir Dar**. SEX/ Age: **M/6MONTHs**.  Date of Report: **14/11/14**. Referral Diagnosis: **CHF. AGH8.431** | | | |
| --- | --- | --- | --- |
| **Features** | **Finding** | **Features** | **Finding** |
| **Profile** |  | **Atria** |  |
| Abdominal situs | Solitus | Left atrium | Dilated |
| Cardiac position | Levocardia | Right atrium | Dilated |
| Systemic venous drainage | Normal. | **Atrioventricular valves** |  |
| Pulmonary venous drainage | Normal | Mitral valve | Annulus = 13mm |
| Atrioventricular connection | Concordant | Tricuspid valve | Annulus = 15mm |
| Ventriculoarterial connection | Concordant | **Ventricles** |  |
| Ventricular loop | d-Loop | Left ventricle | Dilated |
|  |  | Right ventricle | Dilated |
| **Septae** |  | **Coronary arteries** | ----- |
| Interventricular septum | 7mm Inlet VSD, L – R Shunt | **Doppler Measurement** |  |
| Interatrial septum | 7mm Primum defect, l – r sHUNT | Mitral | Mild MR |
| **Semilunar valves** |  | Aortic | ------- |
| Aortic valve | Annulus = 9mm | Tricuspid | Mild TR |
| Pulmonary valve | Annulus = 11mm | pulmonic | Mild PR, PPG = 50mmHg |
| **Great arteries** | NRGA | **Aortic arch** | Left. No CoA. |
| Aorta | ----- | **PDA** | No |
| Pulmonary artery | Normal MPA and Branch PAs. |  |  |
| **M-Mode:**  Normal LV Function on eye balling | | | |
| AO | mm | PWd | mm |
| LA | mm | PWs | mm |
| LVIDd | mm | EDV | ml |
| LVIDs | mm | ESV | ml |
| IVSs | mm | LVEF | % |
| IVSd | mm | FS | % |
| **Additional Information**: |  | | |
| Circumferential Pericardial effusion with maximum depth of 11mm on LV Side. | | | |
| **Final Diagnosis:** | | | |
| 1. {S, D, S} Levocardia. 2. All chambers dilated 3. Intermediate AVSD, L – R Shunt 4. Mild MR 5. Mild TR 6. Mild PR 7. Moderate Pulmonary Hypertension 8. Moderate Pericardial effusion | | | |
| **Remark**: | | | |
| **Recommendation**: | | | |
| SIGNATURE  Done by: Tesfaye T., Pediatrician, Pediatric Cardiologist _______________ 14/11/2014Eth.C | | | |

| Patient Name: **Baby of Hiwet Geletie**. Referring Institute: **FHRH**. SEX/ Age: **F/2days**.  Date of Report: **14/11/14**. Referral Diagnosis: **Incidental Murmur Finding. AGH8.432** | | | |
| --- | --- | --- | --- |
| **Features** | **Finding** | **Features** | **Finding** |
| **Profile** |  | **Atria** |  |
| Abdominal situs | Solitus | Left atrium | Normal |
| Cardiac position | Levocardia | Right atrium | Normal |
| Systemic venous drainage | Normal. | **Atrioventricular valves** |  |
| Pulmonary venous drainage | Normal | Mitral valve | Annulus = 8mm |
| Atrioventricular connection | Concordant | Tricuspid valve | Annulus = 8mm |
| Ventriculoarterial connection | Concordant | **Ventricles** |  |
| Ventricular loop | d-Loop | Left ventricle | Normal |
|  |  | Right ventricle | Normal |
| **Septae** |  | **Coronary arteries** | ----- |
| Interventricular septum | Intact | **Doppler Measurement** |  |
| Interatrial septum | PFO, L – R Shunt | Mitral | ----- |
| **Semilunar valves** |  | Aortic | ------- |
| Aortic valve | Annulus = 6mm | Tricuspid | ------- |
| Pulmonary valve | Annulus = 7mm | pulmonic | -------- |
| **Great arteries** | NRGA | **Aortic arch** | Left. No CoA. |
| Aorta | ----- | **PDA** | 1mm PDA, L – R Shunt |
| Pulmonary artery | Normal MPA and Branch PAs. |  |  |
| **M-Mode:**  Normal LV Function on eye balling | | | |
| AO | mm | PWd | mm |
| LA | mm | PWs | mm |
| LVIDd | mm | EDV | ml |
| LVIDs | mm | ESV | ml |
| IVSs | mm | LVEF | % |
| IVSd | mm | FS | % |
| **Additional Information**: |  | | |
| No pericardial/Pleural effusion. | | | |
| **Final Diagnosis:** | | | |
| 1. {S, D, S} Levocardia. 2. PFO, L – R Shunt 3. Small PDA, L – R Shunt 4. Normal LV Systolic Function | | | |
| **Remark**: | | | |
| **Recommendation**: | | | |
| SIGNATURE  Done by: Tesfaye T., Pediatrician, Pediatric Cardiologist _______________ 14/11/2014Eth.C | | | |

| Patient Name: **Yared Werkneh**. Referring Institute: **Dr. Addisu PSC**. SEX/ Age: **M/4months**.  Date of Report: **14/11/14**. Referral Diagnosis: **Murmur. AGH8.433** | | | |
| --- | --- | --- | --- |
| **Features** | **Finding** | **Features** | **Finding** |
| **Profile** |  | **Atria** |  |
| Abdominal situs | Solitus | Left atrium | Normal |
| Cardiac position | Levocardia | Right atrium | Normal |
| Systemic venous drainage | Normal. | **Atrioventricular valves** |  |
| Pulmonary venous drainage | Normal | Mitral valve | Annulus = 13mm |
| Atrioventricular connection | Concordant | Tricuspid valve | Annulus = 14mm  TAPSE = 12mm |
| Ventriculoarterial connection | Concordant | **Ventricles** |  |
| Ventricular loop | d-Loop | Left ventricle | Normal |
|  |  | Right ventricle | Normal |
| **Septae** |  | **Coronary arteries** | ----- |
| Interventricular septum | 4mm PM VSD, Partially covered by STL, L – R Shunt | **Doppler Measurement** |  |
| Interatrial septum | PFO, L – R Shunt | Mitral | ----- |
| **Semilunar valves** |  | Aortic | ------- |
| Aortic valve | Annulus = 12mm | Tricuspid | ------- |
| Pulmonary valve | Annulus = 15mm | pulmonic | -------- |
| **Great arteries** | NRGA | **Aortic arch** | Left. No CoA. |
| Aorta | ----- | **PDA** | No |
| Pulmonary artery | MPA =14mm. |  |  |
| **M-Mode:** | | | |
| AO | mm | PWd | mm |
| LA | mm | PWs | mm |
| LVIDd | mm | EDV | ml |
| LVIDs | mm | ESV | ml |
| IVSs | mm | LVEF | 72% |
| IVSd | mm | FS | 39% |
| **Additional Information**: |  | | |
| No pericardial/Pleural effusion. | | | |
| **Final Diagnosis:** | | | |
| 1. {S, D, S} Levocardia. 2. PFO, L – R Shunt 3. Small PM VSD, Partially covered by STL, L – R Shunt 4. Normal LV Systolic Function | | | |
| **Remark**: | | | |
| **Recommendation**: | | | |
| SIGNATURE  Done by: Tesfaye T., Pediatrician, Pediatric Cardiologist _______________ 14/11/2014Eth.C | | | |

| Patient Name: **Lidya Habtamu**. Referring Institute: **Eyasta MS**. SEX/ Age: **F/1year**.  Date of Report: **14/11/14**. Referral Diagnosis: **Incidental Murmur. AGH8.434** | | | |
| --- | --- | --- | --- |
| **Features** | **Finding** | **Features** | **Finding** |
| **Profile** |  | **Atria** |  |
| Abdominal situs | Solitus | Left atrium | Normal |
| Cardiac position | Levocardia | Right atrium | Normal |
| Systemic venous drainage | Normal. | **Atrioventricular valves** |  |
| Pulmonary venous drainage | Normal | Mitral valve | Annulus = 14mm |
| Atrioventricular connection | Concordant | Tricuspid valve | Annulus = 16mm |
| Ventriculoarterial connection | Concordant | **Ventricles** |  |
| Ventricular loop | d-Loop | Left ventricle | Normal |
|  |  | Right ventricle | Normal |
| **Septae** |  | **Coronary arteries** | ----- |
| Interventricular septum | 4mm PM VSD, L – R Shunt with a gradient of 50mmHg | **Doppler Measurement** |  |
| Interatrial septum | Intact | Mitral | ----- |
| **Semilunar valves** |  | Aortic | ------- |
| Aortic valve | Annulus = 14mm | Tricuspid | ------- |
| Pulmonary valve | Annulus = 14mm | pulmonic | -------- |
| **Great arteries** | NRGA | **Aortic arch** | Left. No CoA. |
| Aorta | ----- | **PDA** | No |
| Pulmonary artery | Normal MPA and Branch PAs. |  |  |
| **M-Mode:**  Normal LV Systolic Function on eye balling | | | |
| AO | mm | PWd | mm |
| LA | mm | PWs | mm |
| LVIDd | mm | EDV | ml |
| LVIDs | mm | ESV | ml |
| IVSs | mm | LVEF | % |
| IVSd | mm | FS | % |
| **Additional Information**: |  | | |
| No pericardial/Pleural effusion. | | | |
| **Final Diagnosis:** | | | |
| 1. {S, D, S} Levocardia. 2. Small Restrictive PM VSD, L – R Shunt 3. Normal LV Systolic Function | | | |
| **Remark**: | | | |
| **Recommendation**: | | | |
| SIGNATURE  Done by: Tesfaye T., Pediatrician, Pediatric Cardiologist _______________ 14/11/2014Eth.C | | | |

| Patient Name: **Amaru Gedefaw**. Referring Institute: **FHRH**. SEX/ Age: **F/5years**.  Date of Report: **15/11/14**. Referral Diagnosis: **Recurrent chest infection. AGH8.435** | | | |
| --- | --- | --- | --- |
| **Features** | **Finding** | **Features** | **Finding** |
| **Profile** |  | **Atria** |  |
| Abdominal situs | Solitus | Left atrium | Normal |
| Cardiac position | Levocardia | Right atrium | Normal |
| Systemic venous drainage | Normal. | **Atrioventricular valves** |  |
| Pulmonary venous drainage | Normal | Mitral valve | Annulus = 16mm |
| Atrioventricular connection | Concordant | Tricuspid valve | Annulus = 18mm  TAPSE = 16mm |
| Ventriculoarterial connection | Concordant | **Ventricles** |  |
| Ventricular loop | d-Loop | Left ventricle | Normal |
|  |  | Right ventricle | Normal |
| **Septae** |  | **Coronary arteries** | ----- |
| Interventricular septum | Intact | **Doppler Measurement** |  |
| Interatrial septum | Intact | Mitral | ----- |
| **Semilunar valves** |  | Aortic | ------- |
| Aortic valve | Annulus = 12mm | Tricuspid | ------- |
| Pulmonary valve | Annulus = 13mm | pulmonic | -------- |
| **Great arteries** | NRGA | **Aortic arch** | Left. No CoA. |
| Aorta | ----- | **PDA** | No |
| Pulmonary artery | Normal MPA and Branch PAs. |  |  |
| **M-Mode:**  Normal Echocardiography Study | | | |
| AO | mm | PWd | mm |
| LA | mm | PWs | mm |
| LVIDd | mm | EDV | ml |
| LVIDs | mm | ESV | ml |
| IVSs | mm | LVEF | % |
| IVSd | mm | FS | % |
| **Additional Information**: |  | | |
| No pericardial/Pleural effusion. | | | |
| **Final Diagnosis:** | | | |
| 1. Normal Echocardiography Study. | | | |
| **Remark**: | | | |
| **Recommendation**: | | | |
| SIGNATURE  Done by: Tesfaye T., Pediatrician, Pediatric Cardiologist _______________ 15/11/2014Eth.C | | | |

| Patient Name: **Bezawit Marye**. Referring Institute: **FHRH**. SEX/ Age: **F/6months**.  Date of Report: **16/11/14**. Referral Diagnosis: **CHF + DS. AGH8.436** | | | |
| --- | --- | --- | --- |
| **Features** | **Finding** | **Features** | **Finding** |
| **Profile** |  | **Atria** |  |
| Abdominal situs | Solitus | Left atrium | Normal |
| Cardiac position | Levocardia | Right atrium | Dilated |
| Systemic venous drainage | Normal. | **Atrioventricular valves** |  |
| Pulmonary venous drainage | Normal | Mitral valve | Common Complete AVSD. |
| Atrioventricular connection | Concordant | Tricuspid valve |  |
| Ventriculoarterial connection | Concordant | **Ventricles** |  |
| Ventricular loop | d-Loop | Left ventricle | Normal |
|  |  | Right ventricle | Dilated |
| **Septae** |  | **Coronary arteries** | ----- |
| Interventricular septum | Common Complete AVSD, L – R Shunt.  Additional 5mm OS ASD, L – R Shunt | **Doppler Measurement** |  |
| Interatrial septum |  | Mitral | ----- |
| **Semilunar valves** |  | Aortic | ------- |
| Aortic valve | Annulus = 12mm | Tricuspid | ------- |
| Pulmonary valve | Annulus = 13mm | pulmonic | -------- |
| **Great arteries** | NRGA | **Aortic arch** | Left. No CoA. |
| Aorta | ----- | **PDA** | No |
| Pulmonary artery | Normal MPA and Branch PAs. |  |  |
| **M-Mode:**  Normal LV Function on eye balling | | | |
| AO | mm | PWd | mm |
| LA | mm | PWs | mm |
| LVIDd | mm | EDV | ml |
| LVIDs | mm | ESV | ml |
| IVSs | mm | LVEF | % |
| IVSd | mm | FS | % |
| **Additional Information**: |  | | |
| Pericardial effusion measuring maximum depth of 2mm on RV Side. | | | |
| **Final Diagnosis:** | | | |
| 1. {S, D, S} Levocardia. 2. RA/RV Dilated 3. Common Complete Balanced AVSD, L – R Shunt 4. Additional Small OS ASD, L – R Shunt 5. Normal LV Systolic Function | | | |
| **Remark**: | | | |
| **Recommendation**: | | | |
| SIGNATURE  Done by: Tesfaye T., Pediatrician, Pediatric Cardiologist _______________ 16/11/2014Eth.C | | | |

| Patient Name: **Kindnew Getachew**. Referring Institute: **Addis Alem PH**. SEX/ Age: **M/11days**.  Date of Report: **18/11/14**. Referral Diagnosis: **DS. AGH8.437** | | | |
| --- | --- | --- | --- |
| **Features** | **Finding** | **Features** | **Finding** |
| **Profile** |  | **Atria** |  |
| Abdominal situs | Solitus | Left atrium | Normal |
| Cardiac position | Levocardia | Right atrium | Normal |
| Systemic venous drainage | Normal. | **Atrioventricular valves** |  |
| Pulmonary venous drainage | Normal | Mitral valve | Annulus = 8mm |
| Atrioventricular connection | Concordant | Tricuspid valve | Annulus = 9mm |
| Ventriculoarterial connection | Concordant | **Ventricles** |  |
| Ventricular loop | d-Loop | Left ventricle | Normal |
|  |  | Right ventricle | Normal |
| **Septae** |  | **Coronary arteries** | ----- |
| Interventricular septum | Intact | **Doppler Measurement** |  |
| Interatrial septum | PFO, L – R Shunt | Mitral | ----- |
| **Semilunar valves** |  | Aortic | ------- |
| Aortic valve | Annulus = 8mm | Tricuspid | ------- |
| Pulmonary valve | Annulus = 8mm | pulmonic | -------- |
| **Great arteries** | NRGA | **Aortic arch** | Left. No CoA. |
| Aorta | ----- | **PDA** | No |
| Pulmonary artery | Normal MPA and Branch PAs. |  |  |
| **M-Mode:**  Normal LV Function on eye balling. | | | |
| AO | mm | PWd | mm |
| LA | mm | PWs | mm |
| LVIDd | mm | EDV | ml |
| LVIDs | mm | ESV | ml |
| IVSs | mm | LVEF | % |
| IVSd | mm | FS | % |
| **Additional Information**: |  | | |
| No pericardial/Pleural effusion. | | | |
| **Final Diagnosis:** | | | |
| 1. {S, D, S} Levocardia. 2. PFO, L – R Shunt | | | |
| **Remark**: | | | |
| **Recommendation**: | | | |
| SIGNATURE  Done by: Tesfaye T., Pediatrician, Pediatric Cardiologist _______________ 18/11/2014Eth.C | | | |

| Patient Name: **Baby of Birtukan Engida**. Referring Institute: **FHRH**. SEX/ Age: **F/51days**.  Date of Report: **18/11/14**. Referral Diagnosis: **RD. AGH8.438** | | | |
| --- | --- | --- | --- |
| **Features** | **Finding** | **Features** | **Finding** |
| **Profile** |  | **Atria** |  |
| Abdominal situs | Solitus | Left atrium | Normal |
| Cardiac position | Levocardia | Right atrium | Normal |
| Systemic venous drainage | Normal. | **Atrioventricular valves** |  |
| Pulmonary venous drainage | Normal | Mitral valve | Annulus = 10mm |
| Atrioventricular connection | Concordant | Tricuspid valve | Annulus = 11mm |
| Ventriculoarterial connection | Concordant | **Ventricles** |  |
| Ventricular loop | d-Loop | Left ventricle | Normal |
|  |  | Right ventricle | Normal |
| **Septae** |  | **Coronary arteries** | ----- |
| Interventricular septum | Intact | **Doppler Measurement** |  |
| Interatrial septum | Intact | Mitral | ----- |
| **Semilunar valves** |  | Aortic | ------- |
| Aortic valve | Annulus = 9mm | Tricuspid | ------- |
| Pulmonary valve | Annulus = 9mm | pulmonic | -------- |
| **Great arteries** | NRGA | **Aortic arch** | Left. No CoA. |
| Aorta | ----- | **PDA** | No |
| Pulmonary artery | Normal MPA and Branch PAs. |  |  |
| **M-Mode:**  Normal LV Function on eye balling | | | |
| AO | mm | PWd | mm |
| LA | mm | PWs | mm |
| LVIDd | mm | EDV | ml |
| LVIDs | mm | ESV | ml |
| IVSs | mm | LVEF | % |
| IVSd | mm | FS | % |
| **Additional Information**: |  | | |
| No pericardial/Pleural effusion. | | | |
| **Final Diagnosis:** | | | |
| 1. Normal Echocardiography Study. | | | |
| **Remark**: | | | |
| **Recommendation**: | | | |
| SIGNATURE  Done by: Tesfaye T., Pediatrician, Pediatric Cardiologist _______________ 18/11/2014Eth.C | | | |

| Patient Name: **Robel Getu**. Referring Institute: **FHRH**. SEX/ Age: **M/5years**.  Date of Report: **18/11/14**. Referral Diagnosis: **Easy fatigability. AGH8.439** | | | |
| --- | --- | --- | --- |
| **Features** | **Finding** | **Features** | **Finding** |
| **Profile** |  | **Atria** |  |
| Abdominal situs | Solitus | Left atrium | Normal |
| Cardiac position | Levocardia | Right atrium | Normal |
| Systemic venous drainage | Normal. | **Atrioventricular valves** |  |
| Pulmonary venous drainage | Normal | Mitral valve | Annulus = 14mm |
| Atrioventricular connection | Concordant | Tricuspid valve | Annulus = 16mm  TAPSE = 17mm |
| Ventriculoarterial connection | Concordant | **Ventricles** |  |
| Ventricular loop | d-Loop | Left ventricle | Normal |
|  |  | Right ventricle | Normal |
| **Septae** |  | **Coronary arteries** | ----- |
| Interventricular septum | 3mm PM VSD, Closed by STL. | **Doppler Measurement** |  |
| Interatrial septum | Intact | Mitral | ----- |
| **Semilunar valves** |  | Aortic | ------- |
| Aortic valve | Annulus = 13mm | Tricuspid | ------- |
| Pulmonary valve | Annulus = 14mm | pulmonic | -------- |
| **Great arteries** | NRGA | **Aortic arch** | Left. No CoA. |
| Aorta | ----- | **PDA** | No |
| Pulmonary artery | Normal MPA and Branch PAs. |  |  |
| **M-Mode:** | | | |
| AO | mm | PWd | mm |
| LA | mm | PWs | mm |
| LVIDd | mm | EDV | ml |
| LVIDs | mm | ESV | ml |
| IVSs | mm | LVEF | % |
| IVSd | mm | FS | % |
| **Additional Information**: |  | | |
| No pericardial/Pleural effusion. | | | |
| **Final Diagnosis:** | | | |
| 1. {S, D, S} Levocardia. 2. Small PM VSD, Closed by STL | | | |
| **Remark**: | | | |
| **Recommendation**:   1. No need to start any cardiac medicine 2. No hemodynamic Impact 3. Follow up | | | |
| SIGNATURE  Done by: Tesfaye T., Pediatrician, Pediatric Cardiologist _______________ 18/11/2014Eth.C | | | |

| Patient Name: **Yohannes Ferede**. Referring Institute: **FHRH**. SEX/ Age: **M/1 4/12**.  Date of Report: **18/11/14**. Referral Diagnosis: **Recurrent Chest Infection. AGH8.440** | | | |
| --- | --- | --- | --- |
| **Features** | **Finding** | **Features** | **Finding** |
| **Profile** |  | **Atria** |  |
| Abdominal situs | Solitus | Left atrium | Normal |
| Cardiac position | Levocardia | Right atrium | Normal |
| Systemic venous drainage | Normal. | **Atrioventricular valves** |  |
| Pulmonary venous drainage | Normal | Mitral valve | Annulus = 12mm |
| Atrioventricular connection | Concordant | Tricuspid valve | Annulus = 13mm  TAPSE = 14mm |
| Ventriculoarterial connection | Concordant | **Ventricles** |  |
| Ventricular loop | d-Loop | Left ventricle | Normal |
|  |  | Right ventricle | Normal |
| **Septae** |  | **Coronary arteries** | ----- |
| Interventricular septum | Intact | **Doppler Measurement** |  |
| Interatrial septum | Intact | Mitral | ----- |
| **Semilunar valves** |  | Aortic | ------- |
| Aortic valve | Annulus = 12mm | Tricuspid | ------- |
| Pulmonary valve | Annulus = 12mm | pulmonic | -------- |
| **Great arteries** | NRGA | **Aortic arch** | Left. No CoA. |
| Aorta | ----- | **PDA** | No |
| Pulmonary artery | Normal MPA and Branch PAs. |  |  |
| **M-Mode:**  Normal LV Function on eye balling | | | |
| AO | mm | PWd | mm |
| LA | mm | PWs | mm |
| LVIDd | mm | EDV | ml |
| LVIDs | mm | ESV | ml |
| IVSs | mm | LVEF | % |
| IVSd | mm | FS | % |
| **Additional Information**: |  | | |
| No pericardial/Pleural effusion. | | | |
| **Final Diagnosis:** | | | |
| 1. Normal Echocardiography Study. | | | |
| **Remark**: | | | |
| **Recommendation**: | | | |
| SIGNATURE  Done by: Tesfaye T., Pediatrician, Pediatric Cardiologist _______________ 18/11/2014Eth.C | | | |

| Patient Name: **Baby of Banchayehu Terefe**. Referring Institute: **TGSH**. SEX/ Age: **M/16days**.  Date of Report: **18/11/14**. Referral Diagnosis: **DS. AGH8.441** | | | |
| --- | --- | --- | --- |
| **Features** | **Finding** | **Features** | **Finding** |
| **Profile** |  | **Atria** |  |
| Abdominal situs | Solitus | Left atrium | Normal |
| Cardiac position | Levocardia | Right atrium | Normal |
| Systemic venous drainage | Normal. | **Atrioventricular valves** |  |
| Pulmonary venous drainage | Normal | Mitral valve | Annulus = 9mm |
| Atrioventricular connection | Concordant | Tricuspid valve | Annulus = 10mm |
| Ventriculoarterial connection | Concordant | **Ventricles** |  |
| Ventricular loop | d-Loop | Left ventricle | Normal |
|  |  | Right ventricle | Normal |
| **Septae** |  | **Coronary arteries** | ----- |
| Interventricular septum | Intact | **Doppler Measurement** |  |
| Interatrial septum | PFO, L – R Shunt | Mitral | ----- |
| **Semilunar valves** |  | Aortic | ------- |
| Aortic valve | Annulus = 8mm | Tricuspid | ------- |
| Pulmonary valve | Annulus = 9mm | pulmonic | -------- |
| **Great arteries** | NRGA | **Aortic arch** | Left. No CoA. |
| Aorta | ----- | **PDA** | No |
| Pulmonary artery | Normal MPA and Branch PAs. |  |  |
| **M-Mode:**  Normal LV Function on eye balling | | | |
| AO | mm | PWd | mm |
| LA | mm | PWs | mm |
| LVIDd | mm | EDV | ml |
| LVIDs | mm | ESV | ml |
| IVSs | mm | LVEF | % |
| IVSd | mm | FS | % |
| **Additional Information**: |  | | |
| No pericardial/Pleural effusion. | | | |
| **Final Diagnosis:** | | | |
| 1. {S, D, S} Levocardia. 2. PFO, L – R Shunt | | | |
| **Remark**: | | | |
| **Recommendation**: | | | |
| SIGNATURE  Done by: Tesfaye T., Pediatrician, Pediatric Cardiologist _______________ 18/11/2014Eth.C | | | |

| Patient Name: **Darek Zemenu**. Referring Institute: **Adinas GH**. SEX/ Age: **M/9 4/12**.  Date of Report: **18/11/14**. Referral Diagnosis: **Easy fatigability. AGH8.442** | | | |
| --- | --- | --- | --- |
| **Features** | **Finding** | **Features** | **Finding** |
| **Profile** |  | **Atria** |  |
| Abdominal situs | Solitus | Left atrium | Normal |
| Cardiac position | Levocardia | Right atrium | Normal |
| Systemic venous drainage | Normal. | **Atrioventricular valves** |  |
| Pulmonary venous drainage | Normal | Mitral valve | Annulus = 18mm |
| Atrioventricular connection | Concordant | Tricuspid valve | Annulus = 21mm  TAPSE = 21mm |
| Ventriculoarterial connection | Concordant | **Ventricles** |  |
| Ventricular loop | d-Loop | Left ventricle | Normal |
|  |  | Right ventricle | Normal |
| **Septae** |  | **Coronary arteries** | ----- |
| Interventricular septum | Intact | **Doppler Measurement** |  |
| Interatrial septum | Intact | Mitral | ----- |
| **Semilunar valves** |  | Aortic | ------- |
| Aortic valve | Annulus = 18mm | Tricuspid | ------- |
| Pulmonary valve | Annulus = 19mm | pulmonic | -------- |
| **Great arteries** | NRGA | **Aortic arch** | Left. No CoA. |
| Aorta | ----- | **PDA** | No |
| Pulmonary artery | Normal MPA and Branch PAs. |  |  |
| **M-Mode:** | | | |
| AO | mm | PWd | mm |
| LA | mm | PWs | mm |
| LVIDd | mm | EDV | ml |
| LVIDs | mm | ESV | ml |
| IVSs | mm | LVEF | 67% |
| IVSd | mm | FS | 36% |
| **Additional Information**: |  | | |
| No pericardial/Pleural effusion. | | | |
| **Final Diagnosis:** | | | |
| 1. Normal Echocardiography Study. | | | |
| **Remark**: | | | |
| **Recommendation**: | | | |
| SIGNATURE  Done by: Tesfaye T., Pediatrician, Pediatric Cardiologist _______________ 18/11/2014Eth.C | | | |

| Patient Name: **Bisrat Desalegn**. Referring Institute: **TGSH**. SEX/ Age: **F/3months**.  Date of Report: **19/11/14**. Referral Diagnosis: **DS. AGH8.443** | | | |
| --- | --- | --- | --- |
| **Features** | **Finding** | **Features** | **Finding** |
| **Profile** |  | **Atria** |  |
| Abdominal situs | Solitus | Left atrium | Normal |
| Cardiac position | Levocardia | Right atrium | Normal |
| Systemic venous drainage | Normal. | **Atrioventricular valves** |  |
| Pulmonary venous drainage | Normal | Mitral valve | Common Complete AVSD.  TAPSE = 14mm |
| Atrioventricular connection | Concordant | Tricuspid valve |  |
| Ventriculoarterial connection | Concordant | **Ventricles** |  |
| Ventricular loop | d-Loop | Left ventricle | Normal |
|  |  | Right ventricle | Normal |
| **Septae** |  | **Coronary arteries** | ----- |
| Interventricular septum | Common Complete AVSD, L – R Shunt | **Doppler Measurement** |  |
| Interatrial septum |  | Mitral | ----- |
| **Semilunar valves** |  | Aortic | ------- |
| Aortic valve | Annulus = 9mm | Tricuspid | ------- |
| Pulmonary valve | Annulus = 10mm | pulmonic | -------- |
| **Great arteries** | NRGA | **Aortic arch** | Left. No CoA. |
| Aorta | ----- | **PDA** | No |
| Pulmonary artery | Normal MPA and Branch PAs. |  |  |
| **M-Mode:**  Normal LV Function on eye balling. | | | |
| AO | mm | PWd | mm |
| LA | mm | PWs | mm |
| LVIDd | mm | EDV | ml |
| LVIDs | mm | ESV | ml |
| IVSs | mm | LVEF | % |
| IVSd | mm | FS | % |
| **Additional Information**: |  | | |
| No pericardial/Pleural effusion. | | | |
| **Final Diagnosis:** | | | |
| 1. {S, D, S} Levocardia. 2. Common Complete AVSD, L – R Shunt | | | |
| **Remark**: | | | |
| **Recommendation**: | | | |
| SIGNATURE  Done by: Tesfaye T., Pediatrician, Pediatric Cardiologist _______________ 19/11/2014Eth.C | | | |

| Patient Name: **Tariku Fentie**. Referring Institute: **TGSH**. SEX/ Age: **M/10months**.  Date of Report: **19/11/14**. Referral Diagnosis: **Pre-op screening. AGH8.444** | | | |
| --- | --- | --- | --- |
| **Features** | **Finding** | **Features** | **Finding** |
| **Profile** |  | **Atria** |  |
| Abdominal situs | Solitus | Left atrium | Normal |
| Cardiac position | Levocardia | Right atrium | Normal |
| Systemic venous drainage | Normal. | **Atrioventricular valves** |  |
| Pulmonary venous drainage | Normal | Mitral valve | Annulus = 13mm |
| Atrioventricular connection | Concordant | Tricuspid valve | Annulus = 14mm |
| Ventriculoarterial connection | Concordant | **Ventricles** |  |
| Ventricular loop | d-Loop | Left ventricle | Normal |
|  |  | Right ventricle | Normal |
| **Septae** |  | **Coronary arteries** | ----- |
| Interventricular septum | Intact | **Doppler Measurement** |  |
| Interatrial septum | Intact | Mitral | ----- |
| **Semilunar valves** |  | Aortic | ------- |
| Aortic valve | Annulus = 10mm | Tricuspid | ------- |
| Pulmonary valve | Annulus = 11mm | pulmonic | -------- |
| **Great arteries** | NRGA | **Aortic arch** | Left. No CoA. |
| Aorta | ----- | **PDA** | No |
| Pulmonary artery | Normal MPA and Branch PAs. |  |  |
| **M-Mode:**  Normal LV Function on eye balling | | | |
| AO | mm | PWd | mm |
| LA | mm | PWs | mm |
| LVIDd | mm | EDV | ml |
| LVIDs | mm | ESV | ml |
| IVSs | mm | LVEF | % |
| IVSd | mm | FS | % |
| **Additional Information**: |  | | |
| No pericardial/Pleural effusion. | | | |
| **Final Diagnosis:** | | | |
| 1. Normal Echocardiography Study. | | | |
| **Remark**: | | | |
| **Recommendation**: | | | |
| SIGNATURE  Done by: Tesfaye T., Pediatrician, Pediatric Cardiologist _______________ 19/11/2014Eth.C | | | |

| Patient Name: **Fikirte Dagnachew**. Referring Institute: **FHRH**. SEX/ Age: **F/12 6/12**.  Date of Report: **19/11/14**. Referral Diagnosis: **Palpitation. AGH8.445** | | | |
| --- | --- | --- | --- |
| **Features** | **Finding** | **Features** | **Finding** |
| **Profile** |  | **Atria** |  |
| Abdominal situs | Solitus | Left atrium | Normal |
| Cardiac position | Levocardia | Right atrium | Normal |
| Systemic venous drainage | Normal. | **Atrioventricular valves** |  |
| Pulmonary venous drainage | Normal | Mitral valve | Annulus = 22mm |
| Atrioventricular connection | Concordant | Tricuspid valve | Annulus = 22mm  TAPSE = 18mm |
| Ventriculoarterial connection | Concordant | **Ventricles** |  |
| Ventricular loop | d-Loop | Left ventricle | Normal |
|  |  | Right ventricle | Normal |
| **Septae** |  | **Coronary arteries** | ----- |
| Interventricular septum | Intact | **Doppler Measurement** |  |
| Interatrial septum | Intact | Mitral | ----- |
| **Semilunar valves** |  | Aortic | ------- |
| Aortic valve | Annulus = 16mm | Tricuspid | ------- |
| Pulmonary valve | Annulus = 20mm | pulmonic | Trivial PR, PPG = 8mmHg |
| **Great arteries** | NRGA | **Aortic arch** | Left. No CoA. |
| Aorta | ----- | **PDA** | No |
| Pulmonary artery | Normal MPA and Branch PAs. |  |  |
| **M-Mode:** | | | |
| AO | mm | PWd | mm |
| LA | mm | PWs | mm |
| LVIDd | mm | EDV | ml |
| LVIDs | mm | ESV | ml |
| IVSs | mm | LVEF | 68% |
| IVSd | mm | FS | 37% |
| **Additional Information**: |  | | |
| No pericardial/Pleural effusion. | | | |
| **Final Diagnosis:** | | | |
| 1. Normal Echocardiography Study. | | | |
| **Remark**: | | | |
| **Recommendation**: | | | |
| SIGNATURE  Done by: Tesfaye T., Pediatrician, Pediatric Cardiologist _______________ 19/11/2014Eth.C | | | |

| Patient Name: **Baby of Mebrat Tadesse**. Referring Institute: **TGSH**. SEX/ Age: **F/1 3/12**.  Date of Report: **19/11/14**. Referral Diagnosis: **Cardiomegaly on CXR. AGH8.446** | | | |
| --- | --- | --- | --- |
| **Features** | **Finding** | **Features** | **Finding** |
| **Profile** |  | **Atria** |  |
| Abdominal situs | Solitus | Left atrium | Normal |
| Cardiac position | Levocardia | Right atrium | Normal |
| Systemic venous drainage | Normal. | **Atrioventricular valves** |  |
| Pulmonary venous drainage | Normal | Mitral valve | Annulus = 12mm |
| Atrioventricular connection | Concordant | Tricuspid valve | Annulus = 13mm |
| Ventriculoarterial connection | Concordant | **Ventricles** |  |
| Ventricular loop | d-Loop | Left ventricle | Normal |
|  |  | Right ventricle | Normal |
| **Septae** |  | **Coronary arteries** | ----- |
| Interventricular septum | Intact | **Doppler Measurement** |  |
| Interatrial septum | Intact | Mitral | ----- |
| **Semilunar valves** |  | Aortic | ------- |
| Aortic valve | Annulus = 11mm | Tricuspid | ------- |
| Pulmonary valve | Annulus = 11mm | pulmonic | -------- |
| **Great arteries** | NRGA | **Aortic arch** | Left. No CoA. |
| Aorta | ----- | **PDA** | No |
| Pulmonary artery | Normal MPA and Branch PAs. |  |  |
| **M-Mode:**  Normal LV Function on eye balling | | | |
| AO | mm | PWd | mm |
| LA | mm | PWs | mm |
| LVIDd | mm | EDV | ml |
| LVIDs | mm | ESV | ml |
| IVSs | mm | LVEF | % |
| IVSd | mm | FS | % |
| **Additional Information**: |  | | |
| No pericardial/Pleural effusion. | | | |
| **Final Diagnosis:** | | | |
| 1. Normal Echocardiography Study. | | | |
| **Remark**: | | | |
| **Recommendation**: | | | |
| SIGNATURE  Done by: Tesfaye T., Pediatrician, Pediatric Cardiologist _______________ 19/11/2014Eth.C | | | |

| Patient Name: **Fikrte Dereje**. Referring Institute: **FHRH**. SEX/ Age: **F/3years**. Date of Report: **20/11/14**.  Referral Diagnosis: **Cyanosis. AGH8.447** | | | |
| --- | --- | --- | --- |
| **Features** | **Finding** | **Features** | **Finding** |
| **Profile** |  | **Atria** |  |
| Abdominal situs | Solitus | Left atrium | Dilated |
| Cardiac position | Levocardia | Right atrium | Normal |
| Systemic venous drainage | Normal. | **Atrioventricular valves** |  |
| Pulmonary venous drainage | Normal | Mitral valve | Annulus = 15mm |
| Atrioventricular connection | Concordant | Tricuspid valve | Atretic |
| Ventriculoarterial connection | DORV | **Ventricles** |  |
| Ventricular loop | d-Loop | Left ventricle | Dilated |
|  |  | Right ventricle | Smallish |
| **Septae** |  | **Coronary arteries** | ----- |
| Interventricular septum | 10mm outlet/subpulmonic VSD, BD Shunt | **Doppler Measurement** |  |
| Interatrial septum | 23mm OS ASD, amounting to single atrium, R – L Shunt | Mitral | ----- |
| **Semilunar valves** |  | Aortic | ------- |
| Aortic valve | Annulus = 14mm | Tricuspid | ------- |
| Pulmonary valve | Annulus = 10mm | pulmonic | Infundibular PS, PPG = 26mmHg |
| **Great arteries** | NRGA | **Aortic arch** | Left. No CoA. |
| Aorta | Anterior and to the right. From RV | **PDA** | No |
| Pulmonary artery | Posterior and to the right. From RV |  |  |
| **M-Mode:** | | | |
| AO | mm | PWd | mm |
| LA | mm | PWs | mm |
| LVIDd | mm | EDV | ml |
| LVIDs | mm | ESV | ml |
| IVSs | mm | LVEF | % |
| IVSd | mm | FS | % |
| **Additional Information**: |  | | |
| 3mm Circumferential pericardial effusion. | | | |
| **Final Diagnosis:** | | | |
| 1. {S, D, D} Levocardia. 2. LA/LV Dilated 3. Large OS ASD, amounting to single atrium, R – L Shunt 4. DORV 5. d-TGA 6. Type IIB Tricuspid atresia 7. Large Outlet/subpulmonic VSD, BD Shunt 8. Mild PS 9. Smallish RV 10. Smallish MPA and Branch PAs 11. Trace circumferential Pericardial effusion | | | |
| SIGNATURE  Done by: Tesfaye T., Pediatrician, Pediatric Cardiologist _______________ 20/11/2014Eth.C | | | |

| Patient Name: **Huzeifa Hashim**. Referring Institute: **Mekane-Selam PH**. SEX/ Age: **M/2 5/12**.  Date of Report: **20/11/14**. Referral Diagnosis: **Incidental Murmur Finding. AGH8.448** | | | |
| --- | --- | --- | --- |
| **Features** | **Finding** | **Features** | **Finding** |
| **Profile** |  | **Atria** |  |
| Abdominal situs | Solitus | Left atrium | Normal |
| Cardiac position | Levocardia | Right atrium | Dilated |
| Systemic venous drainage | Normal. | **Atrioventricular valves** |  |
| Pulmonary venous drainage | Normal | Mitral valve | Annulus = 14mm |
| Atrioventricular connection | Concordant | Tricuspid valve | Annulus = 15mm  TAPSE = 15mm |
| Ventriculoarterial connection | Concordant | **Ventricles** |  |
| Ventricular loop | d-Loop | Left ventricle | Normal |
|  |  | Right ventricle | Dilated |
| **Septae** |  | **Coronary arteries** | ----- |
| Interventricular septum | 2mm Supra cristal VSD, L – R Shunt | **Doppler Measurement** |  |
| Interatrial septum | 11mm Fenestrated ASD, L – R Shunt | Mitral | ----- |
| **Semilunar valves** |  | Aortic | Mild AR, PHT = 534ms |
| Aortic valve | Annulus = 13mm. Prolapsing AV cusp to the VSD | Tricuspid | ------- |
| Pulmonary valve | Annulus = 11mm | pulmonic | Severe PS, PPG = 62mmHg |
| **Great arteries** | NRGA | **Aortic arch** | Left. No CoA. |
| Aorta | ----- | **PDA** | No |
| Pulmonary artery | Normal MPA and Branch PAs. |  |  |
| **M-Mode:**  Normal LV Function on eye balling | | | |
| AO | mm | PWd | mm |
| LA | mm | PWs | mm |
| LVIDd | mm | EDV | ml |
| LVIDs | mm | ESV | ml |
| IVSs | mm | LVEF | % |
| IVSd | mm | FS | % |
| **Additional Information**: |  | | |
| No pericardial/Pleural effusion. | | | |
| **Final Diagnosis:** | | | |
| 1. {S, D, S} Levocardia. 2. Large Fenestrated ASD, L – R Shunt 3. Small Supracristal VSD, L – R Shunt with Prolapsing Aortic cusp 4. Mild AR 5. Severe PS 6. Normal Biventricular Systolic Function | | | |
| **Remark**: | | | |
| **Recommendation**: A candidate for surgical intervention | | | |
| SIGNATURE  Done by: Tesfaye T., Pediatrician, Pediatric Cardiologist _______________ 20/11/2014Eth.C | | | |

| Patient Name: **Befekadu Asaye**. Referring Institute: **Adinas GH**. SEX/ Age: **M/4 6/12**. Date of Report: **20/11/14**.  Referral Diagnosis: **CHF. AGH8.449** | | | |
| --- | --- | --- | --- |
| **Features** | **Finding** | **Features** | **Finding** |
| **Profile** |  | **Atria** |  |
| Abdominal situs | Solitus | Left atrium | Dilated |
| Cardiac position | Levocardia | Right atrium | Normal |
| Systemic venous drainage | Normal. | **Atrioventricular valves** |  |
| Pulmonary venous drainage | Normal | Mitral valve | Annulus = 20mm. thickened MVL |
| Atrioventricular connection | Concordant | Tricuspid valve | Annulus = 18mm  TAPSE = 19mm |
| Ventriculoarterial connection | Concordant | **Ventricles** |  |
| Ventricular loop | d-Loop | Left ventricle | Dilated |
|  |  | Right ventricle | Normal |
| **Septae** |  | **Coronary arteries** | ----- |
| Interventricular septum | Intact | **Doppler Measurement** |  |
| Interatrial septum | Intact | Mitral | Severe MR, Holosystolic, posterior projection, seen in two planes with jet velocity = 4m/sec. |
| **Semilunar valves** |  | Aortic | ------- |
| Aortic valve | Annulus = 15mm | Tricuspid | Moderate TR, PPG = 53mmHg |
| Pulmonary valve | Annulus = 15mm | pulmonic | -------- |
| **Great arteries** | NRGA | **Aortic arch** | Left. No CoA. |
| Aorta | ----- | **PDA** | No |
| Pulmonary artery | Normal MPA and Branch PAs. |  |  |
| **M-Mode:** | | | |
| AO | mm | PWd | mm |
| LA | mm | PWs | mm |
| LVIDd | mm | EDV | ml |
| LVIDs | mm | ESV | ml |
| IVSs | mm | LVEF | 71% |
| IVSd | mm | FS | 40% |
| **Additional Information**: |  | | |
| Pericardial effusion on RA/RV Side with a maximum depth of 10mm. | | | |
| **Final Diagnosis:** | | | |
| 1. {S, D, S} Levocardia. 2. LA/LV Dilated 3. Thickened MVL 4. Severe MR 5. Moderate TR 6. Moderate Pulmonary Hypertension 7. Moderate Pericardial effusion 8. Normal Biventricular Systolic Function | | | |
| **Remark**: | | | |
| **Recommendation**: | | | |
| SIGNATURE  Done by: Tesfaye T., Pediatrician, Pediatric Cardiologist _______________ 20/11/2014Eth.C | | | |

| Patient Name: **Habtamu Bihon**. Referring Institute: **TGSH**. SEX/ Age: **M/6years**.  Date of Report: **20/11/14**. Referral Diagnosis: **Preop screening for ATH surgery. AGH8.450** | | | |
| --- | --- | --- | --- |
| **Features** | **Finding** | **Features** | **Finding** |
| **Profile** |  | **Atria** |  |
| Abdominal situs | Solitus | Left atrium | Normal |
| Cardiac position | Levocardia | Right atrium | Normal |
| Systemic venous drainage | Normal. | **Atrioventricular valves** |  |
| Pulmonary venous drainage | Normal | Mitral valve | Annulus = 17mm |
| Atrioventricular connection | Concordant | Tricuspid valve | Annulus = 17mm  TAPSE = 19mm |
| Ventriculoarterial connection | Concordant | **Ventricles** |  |
| Ventricular loop | d-Loop | Left ventricle | Normal |
|  |  | Right ventricle | Normal |
| **Septae** |  | **Coronary arteries** | ----- |
| Interventricular septum | Intact | **Doppler Measurement** |  |
| Interatrial septum | Intact | Mitral | ----- |
| **Semilunar valves** |  | Aortic | ------- |
| Aortic valve | Annulus = 16mm | Tricuspid | ------- |
| Pulmonary valve | Annulus = 17mm | pulmonic | -------- |
| **Great arteries** | NRGA | **Aortic arch** | Left. No CoA. |
| Aorta | ----- | **PDA** | No |
| Pulmonary artery | Normal MPA and Branch PAs. |  |  |
| **M-Mode:**  Normal LV Function on eye balling | | | |
| AO | mm | PWd | mm |
| LA | mm | PWs | mm |
| LVIDd | mm | EDV | ml |
| LVIDs | mm | ESV | ml |
| IVSs | mm | LVEF | % |
| IVSd | mm | FS | % |
| **Additional Information**: |  | | |
| No pericardial/Pleural effusion. | | | |
| **Final Diagnosis:** | | | |
| 1. Normal Echocardiography Study. | | | |
| **Remark**: | | | |
| **Recommendation**: | | | |
| SIGNATURE  Done by: Tesfaye T., Pediatrician, Pediatric Cardiologist _______________ 20/11/2014Eth.C | | | |

| Patient Name: **Haymanot Admasu**. Referring Institute: **Tefera Hailu**. SEX/ Age: **F/10years**.  Date of Report: **21/11/14**. Referral Diagnosis: **ARF.AGH8.451** | | | |
| --- | --- | --- | --- |
| **Features** | **Finding** | **Features** | **Finding** |
| **Profile** |  | **Atria** |  |
| Abdominal situs | Solitus | Left atrium | Normal |
| Cardiac position | Levocardia | Right atrium | Normal |
| Systemic venous drainage | Normal. | **Atrioventricular valves** |  |
| Pulmonary venous drainage | Normal | Mitral valve | Annulus = 21mm. elongated, patulous anterior MVL |
| Atrioventricular connection | Concordant | Tricuspid valve | Annulus = 20mm  TAPSE = 23mm |
| Ventriculoarterial connection | Concordant | **Ventricles** |  |
| Ventricular loop | d-Loop | Left ventricle | Normal |
|  |  | Right ventricle | Normal |
| **Septae** |  | **Coronary arteries** | ----- |
| Interventricular septum | Intact | **Doppler Measurement** |  |
| Interatrial septum | Intact | Mitral | Mild MR, Holosystolic, posterior projection, seen in two planes with jet velocity = 4.2m/sec |
| **Semilunar valves** |  | Aortic | ------- |
| Aortic valve | Annulus = 18mm | Tricuspid | ------- |
| Pulmonary valve | Annulus = 20mm | pulmonic | -------- |
| **Great arteries** | NRGA | **Aortic arch** | Left. No CoA. |
| Aorta | ----- | **PDA** | No |
| Pulmonary artery | Normal MPA and Branch PAs. |  |  |
| **M-Mode:** | | | |
| AO | mm | PWd | mm |
| LA | mm | PWs | mm |
| LVIDd | mm | EDV | ml |
| LVIDs | mm | ESV | ml |
| IVSs | mm | LVEF | 66% |
| IVSd | mm | FS | 36% |
| **Additional Information**: |  | | |
| No pericardial/Pleural effusion. | | | |
| **Final Diagnosis:** | | | |
| 1. {S, D, S} Levocardia. 2. Mild MR 3. Patulous, elongated Anterior MVL 4. Normal Biventricular Systolic Function | | | |
| **Remark**: | | | |
| **Recommendation**: Manage as RHD | | | |
| SIGNATURE  Done by: Tesfaye T., Pediatrician, Pediatric Cardiologist _______________ 21/11/2014Eth.C | | | |

| Patient Name: **Mezid Abdela**. Referring Institute: **Chagni Hospital**. SEX/ Age: **M/1year**.  Date of Report: **21/11/14**. Referral Diagnosis: **FTT. AGH8.452** | | | |
| --- | --- | --- | --- |
| **Features** | **Finding** | **Features** | **Finding** |
| **Profile** |  | **Atria** |  |
| Abdominal situs | Solitus | Left atrium | Normal |
| Cardiac position | Levocardia | Right atrium | Normal |
| Systemic venous drainage | Normal. | **Atrioventricular valves** |  |
| Pulmonary venous drainage | Normal | Mitral valve | Annulus = 12mm |
| Atrioventricular connection | Concordant | Tricuspid valve | Annulus = 14mm  TAPSE = 18mm |
| Ventriculoarterial connection | Concordant | **Ventricles** |  |
| Ventricular loop | d-Loop | Left ventricle | Normal |
|  |  | Right ventricle | Normal |
| **Septae** |  | **Coronary arteries** | ----- |
| Interventricular septum | Intact | **Doppler Measurement** |  |
| Interatrial septum | Intact | Mitral | ----- |
| **Semilunar valves** |  | Aortic | ------- |
| Aortic valve | Annulus = 13mm | Tricuspid | ------- |
| Pulmonary valve | Annulus = 13mm | pulmonic | -------- |
| **Great arteries** | NRGA | **Aortic arch** | Left. No CoA. |
| Aorta | ----- | **PDA** | No |
| Pulmonary artery | Normal MPA and Branch PAs. |  |  |
| **M-Mode:**  Normal LV Function on eye balling | | | |
| AO | mm | PWd | mm |
| LA | mm | PWs | mm |
| LVIDd | mm | EDV | ml |
| LVIDs | mm | ESV | ml |
| IVSs | mm | LVEF | % |
| IVSd | mm | FS | % |
| **Additional Information**: |  | | |
| No pericardial/Pleural effusion. | | | |
| **Final Diagnosis:** | | | |
| 1. Normal Echocardiography Study. | | | |
| **Remark**: | | | |
| **Recommendation**: | | | |
| SIGNATURE  Done by: Tesfaye T., Pediatrician, Pediatric Cardiologist _______________ 21/11/2014Eth.C | | | |

| Patient Name: **Fasikaw Gashaw**. Referring Institute: **TGSH**. SEX/ Age: **M/3 3/12**.  Date of Report: **21/11/14**. Referral Diagnosis: **DS. AGH8.453** | | | |
| --- | --- | --- | --- |
| **Features** | **Finding** | **Features** | **Finding** |
| **Profile** |  | **Atria** |  |
| Abdominal situs | Solitus | Left atrium | Normal |
| Cardiac position | Levocardia | Right atrium | Normal |
| Systemic venous drainage | Normal. | **Atrioventricular valves** |  |
| Pulmonary venous drainage | Normal | Mitral valve | Annulus = 14mm |
| Atrioventricular connection | Concordant | Tricuspid valve | Annulus = 13mm |
| Ventriculoarterial connection | Concordant | **Ventricles** |  |
| Ventricular loop | d-Loop | Left ventricle | Normal |
|  |  | Right ventricle | Normal |
| **Septae** |  | **Coronary arteries** | ----- |
| Interventricular septum | Intact | **Doppler Measurement** |  |
| Interatrial septum | Intact | Mitral | ----- |
| **Semilunar valves** |  | Aortic | ------- |
| Aortic valve | Annulus = 13mm | Tricuspid | ------- |
| Pulmonary valve | Annulus = 14mm | pulmonic | -------- |
| **Great arteries** | NRGA | **Aortic arch** | Left. No CoA. |
| Aorta | ----- | **PDA** | No |
| Pulmonary artery | Normal MPA and Branch PAs. |  |  |
| **M-Mode:**  Normal LV Function on eye balling | | | |
| AO | mm | PWd | mm |
| LA | mm | PWs | mm |
| LVIDd | mm | EDV | ml |
| LVIDs | mm | ESV | ml |
| IVSs | mm | LVEF | % |
| IVSd | mm | FS | % |
| **Additional Information**: |  | | |
| Pericardial effusion with maximum depth of 3mm on RV Side. | | | |
| **Final Diagnosis:** | | | |
| 1. {S, D, S} Levocardia. 2. Trace pericardial effusion 3. Normal LV Systolic Function | | | |
| **Remark**: Child was crying during study and only sub costal window is accessible | | | |
| **Recommendation**: | | | |
| SIGNATURE  Done by: Tesfaye T., Pediatrician, Pediatric Cardiologist _______________ 21/11/2014Eth.C | | | |

| Patient Name: **Ye’ab – nat Habtamu**. Referring Institute: **FHRH**. SEX/ Age: **F/6years**.  Date of Report: **21/11/14**. Referral Diagnosis: **Follow up echo for PDA. Incidental. AGH8.454** | | | |
| --- | --- | --- | --- |
| **Features** | **Finding** | **Features** | **Finding** |
| **Profile** |  | **Atria** |  |
| Abdominal situs | Solitus | Left atrium | Mildly Dilated |
| Cardiac position | Levocardia | Right atrium | Normal |
| Systemic venous drainage | Normal. | **Atrioventricular valves** |  |
| Pulmonary venous drainage | Normal | Mitral valve | Annulus = 23mm |
| Atrioventricular connection | Concordant | Tricuspid valve | Annulus = 18mm  TAPSE = 23mm |
| Ventriculoarterial connection | Concordant | **Ventricles** |  |
| Ventricular loop | d-Loop | Left ventricle | Mildly Dilated |
|  |  | Right ventricle | Normal |
| **Septae** |  | **Coronary arteries** | ----- |
| Interventricular septum | Intact | **Doppler Measurement** |  |
| Interatrial septum | Intact | Mitral | Trivial MR, Incomplete signal, seen in apical view with jet velocity = 3m/sec. |
| **Semilunar valves** |  | Aortic | ------- |
| Aortic valve | Annulus = 16mm | Tricuspid | ------- |
| Pulmonary valve | Annulus = 17mm | pulmonic | -------- |
| **Great arteries** | NRGA | **Aortic arch** | Left. No CoA. |
| Aorta | ----- | **PDA** | 2mm PDA, L – R Shunt |
| Pulmonary artery | Normal MPA and Branch PAs. |  |  |
| **M-Mode:** | | | |
| AO | mm | PWd | mm |
| LA | mm | PWs | mm |
| LVIDd | mm | EDV | ml |
| LVIDs | mm | ESV | ml |
| IVSs | mm | LVEF | 69% |
| IVSd | mm | FS | 39% |
| **Additional Information**: |  | | |
| No pericardial/Pleural effusion. | | | |
| **Final Diagnosis:** | | | |
| 1. {S, D, S} Levocardia. 2. LA/LV Dilated 3. Trivial MR 4. Small to Moderate PDA, L – R Shunt 5. Normal Biventricular Systolic Function | | | |
| **Remark**: Size has apparently decreased from previous report | | | |
| **Recommendation**: | | | |
| SIGNATURE  Done by: Tesfaye T., Pediatrician, Pediatric Cardiologist _______________ 21/11/2014Eth.C | | | |

| Patient Name: **Makbel Tesfa**. Referring Institute: **Adinas GH**. SEX/ Age: **M/5months**.  Date of Report: **21/11/14**. Referral Diagnosis: **Cyanosis. AGH8.455** | | | |
| --- | --- | --- | --- |
| **Features** | **Finding** | **Features** | **Finding** |
| **Profile** |  | **Atria** |  |
| Abdominal situs | Solitus | Left atrium | Mildly Dilated |
| Cardiac position | Levocardia | Right atrium | Normal |
| Systemic venous drainage | Normal. | **Atrioventricular valves** |  |
| Pulmonary venous drainage | Normal | Mitral valve | Annulus = 16mm |
| Atrioventricular connection | Concordant | Tricuspid valve | Annulus = 14mm |
| Ventriculoarterial connection | DORV | **Ventricles** |  |
| Ventricular loop | d-Loop | Left ventricle | Mildly Dilated |
|  |  | Right ventricle | Normal |
| **Septae** |  | **Coronary arteries** | ----- |
| Interventricular septum | 9mm Sub-aortic VSD, L – R Shunt | **Doppler Measurement** |  |
| Interatrial septum | Intact | Mitral | ----- |
| **Semilunar valves** |  | Aortic | ------- |
| Aortic valve | Annulus = 9mm | Tricuspid | ------- |
| Pulmonary valve | Annulus = 12mm | pulmonic | Mild PR, PPG = 41mmHg |
| **Great arteries** | NRGA | **Aortic arch** | Left. No CoA. |
| Aorta | Posterior, to the right and >50% Over-ride to RV | **PDA** | No |
| Pulmonary artery | Normal MPA and Branch PAs. from RV |  |  |
| **M-Mode:**  Normal LV Function on eye balling. | | | |
| AO | mm | PWd | mm |
| LA | mm | PWs | mm |
| LVIDd | mm | EDV | ml |
| LVIDs | mm | ESV | ml |
| IVSs | mm | LVEF | % |
| IVSd | mm | FS | % |
| **Additional Information**: | **Aorto – Mitral fibrous Discontinuity.** | | |
| No pericardial/Pleural effusion. | | | |
| **Final Diagnosis:** | | | |
| 1. {S, D, D} Levocardia. 2. Mildly Dilated LA/LV 3. DORV 4. Large Sub-Aortic VSD, L – R Shunt 5. Mild Pulmonary Hypertension 6. Normal LV Systolic Function | | | |
| **Remark**: | | | |
| **Recommendation**: | | | |
| SIGNATURE  Done by: Tesfaye T., Pediatrician, Pediatric Cardiologist _______________ 21/11/2014Eth.C | | | |

| Patient Name: **Kidus Mezgebu**. Referring Institute: **Amaris PSC**. SEX/ Age: **M/45days**.  Date of Report: **21/11/14**. Referral Diagnosis: **RD. AGH8.456** | | | |
| --- | --- | --- | --- |
| **Features** | **Finding** | **Features** | **Finding** |
| **Profile** |  | **Atria** |  |
| Abdominal situs | Solitus | Left atrium | Normal |
| Cardiac position | Levocardia | Right atrium | Normal |
| Systemic venous drainage | Normal. | **Atrioventricular valves** |  |
| Pulmonary venous drainage | Normal | Mitral valve | Annulus = 9mm |
| Atrioventricular connection | Concordant | Tricuspid valve | Annulus = 9mm |
| Ventriculoarterial connection | Concordant | **Ventricles** |  |
| Ventricular loop | d-Loop | Left ventricle | Normal |
|  |  | Right ventricle | Normal |
| **Septae** |  | **Coronary arteries** | ----- |
| Interventricular septum | Intact | **Doppler Measurement** |  |
| Interatrial septum | PFO, L – R Shunt | Mitral | ----- |
| **Semilunar valves** |  | Aortic | ------- |
| Aortic valve | Annulus = 8mm | Tricuspid | ------- |
| Pulmonary valve | Annulus = 9mm | pulmonic | -------- |
| **Great arteries** | NRGA | **Aortic arch** | Left. No CoA. |
| Aorta | ----- | **PDA** | No |
| Pulmonary artery | Normal MPA and Branch PAs. |  |  |
| **M-Mode:**  Normal LV Function on eye balling | | | |
| AO | mm | PWd | mm |
| LA | mm | PWs | mm |
| LVIDd | mm | EDV | ml |
| LVIDs | mm | ESV | ml |
| IVSs | mm | LVEF | % |
| IVSd | mm | FS | % |
| **Additional Information**: |  | | |
| No pericardial/Pleural effusion. | | | |
| **Final Diagnosis:** | | | |
| 1. {S, D, S} Levocardia. 2. PFO, L – R Shunt | | | |
| **Remark**: | | | |
| **Recommendation**: | | | |
| SIGNATURE  Done by: Tesfaye T., Pediatrician, Pediatric Cardiologist _______________ 21/11/2014Eth.C | | | |

| Patient Name: **Semira Mustofa**. Referring Institute: **Dr. Addisu PSC**. SEX/ Age: **F/13years**.  Date of Report: **22/11/14**. Referral Diagnosis: **Incidental Murmur. AGH8.457** | | | |
| --- | --- | --- | --- |
| **Features** | **Finding** | **Features** | **Finding** |
| **Profile** |  | **Atria** |  |
| Abdominal situs | Solitus | Left atrium | Normal |
| Cardiac position | Levocardia | Right atrium | Normal |
| Systemic venous drainage | Normal. | **Atrioventricular valves** |  |
| Pulmonary venous drainage | Normal | Mitral valve | Annulus = 20mm. anterior MVL thickeness = 2mm. |
| Atrioventricular connection | Concordant | Tricuspid valve | Annulus = 19mm |
| Ventriculoarterial connection | Concordant | **Ventricles** |  |
| Ventricular loop | d-Loop | Left ventricle | Normal |
|  |  | Right ventricle | Normal |
| **Septae** |  | **Coronary arteries** | ----- |
| Interventricular septum | Intact | **Doppler Measurement** |  |
| Interatrial septum | Intact | Mitral | Mild MR, Incomplete Signal, central Projection with jet velocity = 2.3m/sec. |
| **Semilunar valves** |  | Aortic | ------- |
| Aortic valve | Annulus = 19mm | Tricuspid | ------- |
| Pulmonary valve | Annulus = 19mm | pulmonic | -------- |
| **Great arteries** | NRGA | **Aortic arch** | Left. No CoA. |
| Aorta | ----- | **PDA** | No |
| Pulmonary artery | Normal MPA and Branch PAs. |  |  |
| **M-Mode:**  Normal LV Function on eye balling. | | | |
| AO | mm | PWd | mm |
| LA | mm | PWs | mm |
| LVIDd | mm | EDV | ml |
| LVIDs | mm | ESV | ml |
| IVSs | mm | LVEF | % |
| IVSd | mm | FS | % |
| **Additional Information**: |  | | |
| No pericardial/Pleural effusion. | | | |
| **Final Diagnosis:** | | | |
| 1. {S, D, S} Levocardia. 2. Mild MR 3. Normal LV Systolic Function | | | |
| **Remark**: MR is mild with central projection, incomplete signal and jet velocity = 2.3m/sec | | | |
| **Recommendation**: Corelate with the other clinical findings to reach to a highly likely diagnosis. | | | |
| SIGNATURE  Done by: Tesfaye T., Pediatrician, Pediatric Cardiologist _______________ 22/11/2014Eth.C | | | |

| Patient Name: **Bitanya Bazezew**. Referring Institute: **Amaris PSC**. SEX/ Age: **F/2years**.  Date of Report: **24/11/14**. Referral Diagnosis: **IE. AGH8.458** | | | |
| --- | --- | --- | --- |
| **Features** | **Finding** | **Features** | **Finding** |
| **Profile** |  | **Atria** |  |
| Abdominal situs | Solitus | Left atrium | Normal |
| Cardiac position | Levocardia | Right atrium | Normal |
| Systemic venous drainage | Normal. | **Atrioventricular valves** |  |
| Pulmonary venous drainage | Normal | Mitral valve | Annulus = 14mm |
| Atrioventricular connection | Concordant | Tricuspid valve | Annulus = 14mm |
| Ventriculoarterial connection | Concordant | **Ventricles** |  |
| Ventricular loop | d-Loop | Left ventricle | Normal |
|  |  | Right ventricle | Normal |
| **Septae** |  | **Coronary arteries** | ----- |
| Interventricular septum | 2mm PM VSD, L – R Shunt | **Doppler Measurement** |  |
| Interatrial septum | Intact | Mitral | ----- |
| **Semilunar valves** |  | Aortic | ------- |
| Aortic valve | Annulus = 13mm | Tricuspid | ------- |
| Pulmonary valve | Annulus = 14mm | pulmonic | -------- |
| **Great arteries** | NRGA | **Aortic arch** | Left. No CoA. |
| Aorta | ----- | **PDA** | No |
| Pulmonary artery | Normal MPA and Branch PAs. |  |  |
| **M-Mode:**  Normal LV Function on eye balling | | | |
| AO | mm | PWd | mm |
| LA | mm | PWs | mm |
| LVIDd | mm | EDV | ml |
| LVIDs | mm | ESV | ml |
| IVSs | mm | LVEF | % |
| IVSd | mm | FS | % |
| **Additional Information**: |  | | |
| No pericardial/Pleural effusion. | | | |
| **Final Diagnosis:** | | | |
| 1. {S, D, S} Levocardia. 2. Small PM VSD, L – R Shunt 3. Normal LV Systolic Function | | | |
| **Remark**: No gross vegetation seen across the valves, defect, mural area, ……. | | | |
| **Recommendation**: Corelate with the clinical finding | | | |
| SIGNATURE  Done by: Tesfaye T., Pediatrician, Pediatric Cardiologist _______________ 24/11/2014Eth.C | | | |

| Patient Name: **Amanuel Agegnew**. Referring Institute: **TGSH**. SEX/ Age: **M/8years**.  Date of Report: **25/11/14**. Referral Diagnosis: **DOE. AGH8.459** | | | |
| --- | --- | --- | --- |
| **Features** | **Finding** | **Features** | **Finding** |
| **Profile** |  | **Atria** |  |
| Abdominal situs | Solitus | Left atrium | Normal |
| Cardiac position | Levocardia | Right atrium | Normal |
| Systemic venous drainage | Normal. | **Atrioventricular valves** |  |
| Pulmonary venous drainage | Normal | Mitral valve | Annulus = 18mm |
| Atrioventricular connection | Concordant | Tricuspid valve | Annulus = 17mm  TAPSE = 19mm |
| Ventriculoarterial connection | Concordant | **Ventricles** |  |
| Ventricular loop | d-Loop | Left ventricle | Normal |
|  |  | Right ventricle | Normal |
| **Septae** |  | **Coronary arteries** | ----- |
| Interventricular septum | Intact | **Doppler Measurement** |  |
| Interatrial septum | Intact | Mitral | ----- |
| **Semilunar valves** |  | Aortic | ------- |
| Aortic valve | Annulus = 13mm | Tricuspid | ------- |
| Pulmonary valve | Annulus = mm | pulmonic | -------- |
| **Great arteries** | NRGA | **Aortic arch** | Left. No CoA. |
| Aorta | ----- | **PDA** | No |
| Pulmonary artery | Normal MPA and Branch PAs. |  |  |
| **M-Mode:** | | | |
| AO | mm | PWd | mm |
| LA | mm | PWs | mm |
| LVIDd | mm | EDV | ml |
| LVIDs | mm | ESV | ml |
| IVSs | mm | LVEF | 56% |
| IVSd | mm | FS | 28% |
| **Additional Information**: |  | | |
| No pericardial/Pleural effusion. | | | |
| **Final Diagnosis:** | | | |
| 1. Normal Echocardiography Study. | | | |
| **Remark**: | | | |
| **Recommendation**: | | | |
| SIGNATURE  Done by: Tesfaye T., Pediatrician, Pediatric Cardiologist _______________ 25/11/2014Eth.C | | | |

| Patient Name: **Amanuel Abrham**. Referring Institute: **Dangila PH**. SEX/ Age: **M/1year**.  Date of Report: **25/11/14**. Referral Diagnosis: **FTT. AGH8.460** | | | |
| --- | --- | --- | --- |
| **Features** | **Finding** | **Features** | **Finding** |
| **Profile** |  | **Atria** |  |
| Abdominal situs | Solitus | Left atrium | Normal |
| Cardiac position | Levocardia | Right atrium | Normal |
| Systemic venous drainage | Normal. | **Atrioventricular valves** |  |
| Pulmonary venous drainage | Normal | Mitral valve | Annulus = 10mm |
| Atrioventricular connection | Concordant | Tricuspid valve | Annulus = 11mm |
| Ventriculoarterial connection | Concordant | **Ventricles** |  |
| Ventricular loop | d-Loop | Left ventricle | Normal |
|  |  | Right ventricle | Normal |
| **Septae** |  | **Coronary arteries** | ----- |
| Interventricular septum | Intact | **Doppler Measurement** |  |
| Interatrial septum | Intact | Mitral | ----- |
| **Semilunar valves** |  | Aortic | ------- |
| Aortic valve | Annulus = 11mm | Tricuspid | ------- |
| Pulmonary valve | Annulus = 11mm | pulmonic | -------- |
| **Great arteries** | NRGA | **Aortic arch** | Left. No CoA. |
| Aorta | ----- | **PDA** | No |
| Pulmonary artery | Normal MPA and Branch PAs. |  |  |
| **M-Mode:**  Normal LV Function on eye balling | | | |
| AO | mm | PWd | mm |
| LA | mm | PWs | mm |
| LVIDd | mm | EDV | ml |
| LVIDs | mm | ESV | ml |
| IVSs | mm | LVEF | % |
| IVSd | mm | FS | % |
| **Additional Information**: |  | | |
| No pericardial/Pleural effusion. | | | |
| **Final Diagnosis:** | | | |
| 1. Normal LV Function on eye balling. | | | |
| **Remark**: | | | |
| **Recommendation**: | | | |
| SIGNATURE  Done by: Tesfaye T., Pediatrician, Pediatric Cardiologist _______________ 25/11/2014Eth.C | | | |

| Patient Name: **Tsedenya Engidaw**. Referring Institute: **FHRH**. SEX/ Age: **F/11years**.  Date of Report: **25/11/14**. Referral Diagnosis: **ARF. AGH8.461** | | | |
| --- | --- | --- | --- |
| **Features** | **Finding** | **Features** | **Finding** |
| **Profile** |  | **Atria** |  |
| Abdominal situs | Solitus | Left atrium | Normal |
| Cardiac position | Levocardia | Right atrium | Normal |
| Systemic venous drainage | Normal. | **Atrioventricular valves** |  |
| Pulmonary venous drainage | Normal | Mitral valve | Annulus = 17mm |
| Atrioventricular connection | Concordant | Tricuspid valve | Annulus = 19mm  TAPSE = 21mm |
| Ventriculoarterial connection | Concordant | **Ventricles** |  |
| Ventricular loop | d-Loop | Left ventricle | Normal |
|  |  | Right ventricle | Normal |
| **Septae** |  | **Coronary arteries** | ----- |
| Interventricular septum | Intact | **Doppler Measurement** |  |
| Interatrial septum | Intact | Mitral | ----- |
| **Semilunar valves** |  | Aortic | ------- |
| Aortic valve | Annulus = 19mm | Tricuspid | ------- |
| Pulmonary valve | Annulus = 20mm | pulmonic | -------- |
| **Great arteries** | NRGA | **Aortic arch** | Left. No CoA. |
| Aorta | ----- | **PDA** | No |
| Pulmonary artery | Normal MPA and Branch PAs. |  |  |
| **M-Mode:** | | | |
| AO | mm | PWd | mm |
| LA | mm | PWs | mm |
| LVIDd | mm | EDV | ml |
| LVIDs | mm | ESV | ml |
| IVSs | mm | LVEF | 69% |
| IVSd | mm | FS | 38% |
| **Additional Information**: |  | | |
| No pericardial/Pleural effusion. | | | |
| **Final Diagnosis:** | | | |
| 1. {S, D, S} Levocardia. | | | |
| **Remark**: Normal Echocardiography doesn’t rule out Acute Rheumatic Fever. Clinical correlation do have paramount importance for conclusion and decision. | | | |
| **Recommendation**: | | | |
| SIGNATURE  Done by: Tesfaye T., Pediatrician, Pediatric Cardiologist _______________ 25/11/2014Eth.C | | | |

| Patient Name: **Rahel Abrham**. Referring Institute: **Addis Alem PH**. SEX/ Age: **F/3years**.  Date of Report: **25/11/14**. Referral Diagnosis: **DS. AGH8.462** | | | |
| --- | --- | --- | --- |
| **Features** | **Finding** | **Features** | **Finding** |
| **Profile** |  | **Atria** |  |
| Abdominal situs | Solitus | Left atrium | Normal |
| Cardiac position | Levocardia | Right atrium | Normal |
| Systemic venous drainage | Normal. | **Atrioventricular valves** |  |
| Pulmonary venous drainage | Normal | Mitral valve | Annulus = 16mm |
| Atrioventricular connection | Concordant | Tricuspid valve | Annulus = 15mm |
| Ventriculoarterial connection | Concordant | **Ventricles** |  |
| Ventricular loop | d-Loop | Left ventricle | Normal |
|  |  | Right ventricle | Normal |
| **Septae** |  | **Coronary arteries** | ----- |
| Interventricular septum | 9mm Inlet VSD, L – R Shunt | **Doppler Measurement** |  |
| Interatrial septum | 15mm Primum defect, L – R Shunt | Mitral | Mild MR |
| **Semilunar valves** |  | Aortic | ------- |
| Aortic valve | Annulus = 12mm | Tricuspid | ------- |
| Pulmonary valve | Annulus = 14mm | pulmonic | -------- |
| **Great arteries** | NRGA | **Aortic arch** | Left. No CoA. |
| Aorta | ----- | **PDA** | No |
| Pulmonary artery | Normal MPA and Branch PAs. |  |  |
| **M-Mode:**  Normal LV Function on eye balling | | | |
| AO | mm | PWd | mm |
| LA | mm | PWs | mm |
| LVIDd | mm | EDV | ml |
| LVIDs | mm | ESV | ml |
| IVSs | mm | LVEF | % |
| IVSd | mm | FS | % |
| **Additional Information**: |  | | |
| No pericardial/Pleural effusion. | | | |
| **Final Diagnosis:** | | | |
| 1. {S, D, S} Levocardia. 2. Intermediate AVSD, L – R Shunt 3. Normal LV Function on eye balling | | | |
| **Remark**: | | | |
| **Recommendation**: | | | |
| SIGNATURE  Done by: Tesfaye T., Pediatrician, Pediatric Cardiologist _______________ 25/11/2014Eth.C | | | |

| Patient Name: **Maritu Legesse**. Referring Institute: **FHRH**. SEX/ Age: **F/14years**.  Date of Report: **26/11/14**. Referral Diagnosis: **ARF. AGH8.463** | | | |
| --- | --- | --- | --- |
| **Features** | **Finding** | **Features** | **Finding** |
| **Profile** |  | **Atria** |  |
| Abdominal situs | Solitus | Left atrium | Normal |
| Cardiac position | Levocardia | Right atrium | Normal |
| Systemic venous drainage | Normal. | **Atrioventricular valves** |  |
| Pulmonary venous drainage | Normal | Mitral valve | Annulus = 21mm  Patulous MVL |
| Atrioventricular connection | Concordant | Tricuspid valve | Annulus = 21mm  TAPSE = 24mm |
| Ventriculoarterial connection | Concordant | **Ventricles** |  |
| Ventricular loop | d-Loop | Left ventricle | Normal |
|  |  | Right ventricle | Normal |
| **Septae** |  | **Coronary arteries** | ----- |
| Interventricular septum | Intact | **Doppler Measurement** |  |
| Interatrial septum | Intact | Mitral | Trivial MR, Incomplete signal, seen in two planes with Jet velocity = 1.6m/sec. |
| **Semilunar valves** |  | Aortic | ------- |
| Aortic valve | Annulus = 15mm | Tricuspid | ------- |
| Pulmonary valve | Annulus = 18mm | pulmonic | -------- |
| **Great arteries** | NRGA | **Aortic arch** | Left. No CoA. |
| Aorta | ----- | **PDA** | No |
| Pulmonary artery | Normal MPA and Branch PAs. |  |  |
| **M-Mode:** | | | |
| AO | mm | PWd | mm |
| LA | mm | PWs | mm |
| LVIDd | mm | EDV | ml |
| LVIDs | mm | ESV | ml |
| IVSs | mm | LVEF | 71% |
| IVSd | mm | FS | 40% |
| **Additional Information**: |  | | |
| No pericardial/Pleural effusion. | | | |
| **Final Diagnosis:** | | | |
| 1. {S, D, S} Levocardia. 2. Trivial MR 3. Patulous MVL | | | |
| **Remark**: Cerrelate with the clinical and other investigation findings | | | |
| **Recommendation**: Manage as Boarderline RHD Case (to be on patient’s benefit side) | | | |
| SIGNATURE  Done by: Tesfaye T., Pediatrician, Pediatric Cardiologist _______________ 26/11/2014Eth.C | | | |

| Patient Name: **Yohannes Derso**. Referring Institute: **FHRH**. SEX/ Age: **M/5 8/12**.  Date of Report: **26/11/14**. Referral Diagnosis: **Easy Fatigability. AGH8.464** | | | |
| --- | --- | --- | --- |
| **Features** | **Finding** | **Features** | **Finding** |
| **Profile** |  | **Atria** |  |
| Abdominal situs | Solitus | Left atrium | Normal |
| Cardiac position | Levocardia | Right atrium | Normal |
| Systemic venous drainage | Normal. | **Atrioventricular valves** |  |
| Pulmonary venous drainage | Normal | Mitral valve | Annulus = 19mm |
| Atrioventricular connection | Concordant | Tricuspid valve | Annulus = 19mm  TAPSE = 17mm |
| Ventriculoarterial connection | Concordant | **Ventricles** |  |
| Ventricular loop | d-Loop | Left ventricle | Normal |
|  |  | Right ventricle | Normal |
| **Septae** |  | **Coronary arteries** | ----- |
| Interventricular septum | Intact | **Doppler Measurement** |  |
| Interatrial septum | Intact | Mitral | ----- |
| **Semilunar valves** |  | Aortic | ------- |
| Aortic valve | Annulus = 15mm | Tricuspid | ------- |
| Pulmonary valve | Annulus = 15mm | pulmonic | -------- |
| **Great arteries** | NRGA | **Aortic arch** | Left. No CoA. |
| Aorta | ----- | **PDA** | No |
| Pulmonary artery | Normal MPA and Branch PAs. |  |  |
| **M-Mode:** | | | |
| AO | mm | PWd | mm |
| LA | mm | PWs | mm |
| LVIDd | mm | EDV | ml |
| LVIDs | mm | ESV | ml |
| IVSs | mm | LVEF | 72% |
| IVSd | mm | FS | 40% |
| **Additional Information**: |  | | |
| No pericardial/Pleural effusion. | | | |
| **Final Diagnosis:** | | | |
| 1. Normal Echocardiography Study. | | | |
| **Remark**: | | | |
| **Recommendation**: | | | |
| SIGNATURE  Done by: Tesfaye T., Pediatrician, Pediatric Cardiologist _______________ 26/11/2014Eth.C | | | |

| Patient Name: **Sosina Tesfalem**. Referring Institute: **FHRH**. SEX/ Age: **F/6years**.  Date of Report: **26/11/14**. Referral Diagnosis: **Easy fatigability. AGH8.465** | | | |
| --- | --- | --- | --- |
| **Features** | **Finding** | **Features** | **Finding** |
| **Profile** |  | **Atria** |  |
| Abdominal situs | Solitus | Left atrium | Normal |
| Cardiac position | Levocardia | Right atrium | Normal |
| Systemic venous drainage | Normal. | **Atrioventricular valves** |  |
| Pulmonary venous drainage | Normal | Mitral valve | Annulus = 20mm |
| Atrioventricular connection | Concordant | Tricuspid valve | Annulus = 20mm  TAPSE = 23mm |
| Ventriculoarterial connection | Concordant | **Ventricles** |  |
| Ventricular loop | d-Loop | Left ventricle | Normal |
|  |  | Right ventricle | Normal |
| **Septae** |  | **Coronary arteries** | ----- |
| Interventricular septum | Intact | **Doppler Measurement** |  |
| Interatrial septum | Intact | Mitral | ----- |
| **Semilunar valves** |  | Aortic | ------- |
| Aortic valve | Annulus = 16mm | Tricuspid | ------- |
| Pulmonary valve | Annulus = 18mm | pulmonic | -------- |
| **Great arteries** | NRGA | **Aortic arch** | Left. No CoA. |
| Aorta | ----- | **PDA** | No |
| Pulmonary artery | Normal MPA and Branch PAs. |  |  |
| **M-Mode:**  Normal LV Function on eye balling | | | |
| AO | mm | PWd | mm |
| LA | mm | PWs | mm |
| LVIDd | mm | EDV | ml |
| LVIDs | mm | ESV | ml |
| IVSs | mm | LVEF | % |
| IVSd | mm | FS | % |
| **Additional Information**: |  | | |
| No pericardial/Pleural effusion. | | | |
| **Final Diagnosis:** | | | |
| 1. Normal Echocardiography Study. | | | |
| **Remark**: | | | |
| **Recommendation**: | | | |
| SIGNATURE  Done by: Tesfaye T., Pediatrician, Pediatric Cardiologist _______________ 26/11/2014Eth.C | | | |

| Patient Name: **Yohana Yibeltal**. Referring Institute: **FHRH**. SEX/ Age: **F/3 6/12**.  Date of Report: **26/11/14**. Referral Diagnosis: **RD. AGH8.466** | | | |
| --- | --- | --- | --- |
| **Features** | **Finding** | **Features** | **Finding** |
| **Profile** |  | **Atria** |  |
| Abdominal situs | Solitus | Left atrium | Normal |
| Cardiac position | Levocardia | Right atrium | Normal |
| Systemic venous drainage | Normal. | **Atrioventricular valves** |  |
| Pulmonary venous drainage | Normal | Mitral valve | Annulus = 15mm |
| Atrioventricular connection | Concordant | Tricuspid valve | Annulus = 17mm  TAPSE= 16mm |
| Ventriculoarterial connection | Concordant | **Ventricles** |  |
| Ventricular loop | d-Loop | Left ventricle | Normal |
|  |  | Right ventricle | Normal |
| **Septae** |  | **Coronary arteries** | ----- |
| Interventricular septum | Intact | **Doppler Measurement** |  |
| Interatrial septum | Intact | Mitral | ----- |
| **Semilunar valves** |  | Aortic | ------- |
| Aortic valve | Annulus = 15mm | Tricuspid | Trivial TR, PPG = 15mmHg |
| Pulmonary valve | Annulus = 16mm | pulmonic | -------- |
| **Great arteries** | NRGA | **Aortic arch** | Left. No CoA. |
| Aorta | ----- | **PDA** | No |
| Pulmonary artery | Normal MPA and Branch PAs. |  |  |
| **M-Mode:** | | | |
| AO | mm | PWd | mm |
| LA | mm | PWs | mm |
| LVIDd | mm | EDV | ml |
| LVIDs | mm | ESV | ml |
| IVSs | mm | LVEF | 56% |
| IVSd | mm | FS | 28% |
| **Additional Information**: |  | | |
| No pericardial/Pleural effusion. | | | |
| **Final Diagnosis:** | | | |
| 1. Normal Echocardiography Study. | | | |
| **Remark**: | | | |
| **Recommendation**: | | | |
| SIGNATURE  Done by: Tesfaye T., Pediatrician, Pediatric Cardiologist _______________ 26/11/2014Eth.C | | | |

| Patient Name: **Redeat Yiketel**. Referring Institute: **FHRH**. SEX/ Age: **F/55days**.  Date of Report: **26/11/14**. Referral Diagnosis: **RD. AGH8.467** | | | |
| --- | --- | --- | --- |
| **Features** | **Finding** | **Features** | **Finding** |
| **Profile** |  | **Atria** |  |
| Abdominal situs | Solitus | Left atrium | Normal |
| Cardiac position | Levocardia | Right atrium | Normal |
| Systemic venous drainage | Normal. | **Atrioventricular valves** |  |
| Pulmonary venous drainage | Normal | Mitral valve | Annulus = 9mm |
| Atrioventricular connection | Concordant | Tricuspid valve | Annulus = 10mm |
| Ventriculoarterial connection | Concordant | **Ventricles** |  |
| Ventricular loop | d-Loop | Left ventricle | Normal |
|  |  | Right ventricle | Normal |
| **Septae** |  | **Coronary arteries** | ----- |
| Interventricular septum | Intact | **Doppler Measurement** |  |
| Interatrial septum | Intact | Mitral | ----- |
| **Semilunar valves** |  | Aortic | ------- |
| Aortic valve | Annulus = 8mm | Tricuspid | ------- |
| Pulmonary valve | Annulus = 8mm | pulmonic | -------- |
| **Great arteries** | NRGA | **Aortic arch** | Left. No CoA. |
| Aorta | ----- | **PDA** | No |
| Pulmonary artery | Normal MPA and Branch PAs. |  |  |
| **M-Mode:**  Normal LV Function on eye balling | | | |
| AO | mm | PWd | mm |
| LA | mm | PWs | mm |
| LVIDd | mm | EDV | ml |
| LVIDs | mm | ESV | ml |
| IVSs | mm | LVEF | % |
| IVSd | mm | FS | % |
| **Additional Information**: |  | | |
| No pericardial/Pleural effusion. | | | |
| **Final Diagnosis:** | | | |
| 1. Normal Echocardiography study. | | | |
| **Remark**: Poor apical and parasternal window | | | |
| **Recommendation**: Do Chest X - Ray | | | |
| SIGNATURE  Done by: Tesfaye T., Pediatrician, Pediatric Cardiologist _______________ 26/11/2014Eth.C | | | |

| Patient Name: **Almaz Demelash**. Referring Institute: **TGSH**. SEX/ Age: **F/10years**.  Date of Report: **26/11/14**. Referral Diagnosis: **Pre-op screening. AGH8.468** | | | |
| --- | --- | --- | --- |
| **Features** | **Finding** | **Features** | **Finding** |
| **Profile** |  | **Atria** |  |
| Abdominal situs | Solitus | Left atrium | Normal |
| Cardiac position | Levocardia | Right atrium | Normal |
| Systemic venous drainage | Normal. | **Atrioventricular valves** |  |
| Pulmonary venous drainage | Normal | Mitral valve | Annulus = 19mm |
| Atrioventricular connection | Concordant | Tricuspid valve | Annulus = 19mm  TAPSE = 17mm |
| Ventriculoarterial connection | Concordant | **Ventricles** |  |
| Ventricular loop | d-Loop | Left ventricle | Normal |
|  |  | Right ventricle | Normal |
| **Septae** |  | **Coronary arteries** | ----- |
| Interventricular septum | Intact | **Doppler Measurement** |  |
| Interatrial septum | Intact | Mitral | ----- |
| **Semilunar valves** |  | Aortic | ------- |
| Aortic valve | Annulus = 15mm | Tricuspid | ------- |
| Pulmonary valve | Annulus = 17mm | pulmonic | -------- |
| **Great arteries** | NRGA | **Aortic arch** | Left. No CoA. |
| Aorta | ----- | **PDA** | No |
| Pulmonary artery | Normal MPA and Branch PAs. |  |  |
| **M-Mode:** | | | |
| AO | mm | PWd | mm |
| LA | mm | PWs | mm |
| LVIDd | mm | EDV | ml |
| LVIDs | mm | ESV | ml |
| IVSs | mm | LVEF | 66% |
| IVSd | mm | FS | 36% |
| **Additional Information**: |  | | |
| No pericardial/Pleural effusion. | | | |
| **Final Diagnosis:** | | | |
| 1. Normal Echocardiography Study. | | | |
| **Remark**: | | | |
| **Recommendation**: | | | |
| SIGNATURE  Done by: Tesfaye T., Pediatrician, Pediatric Cardiologist _______________ 26/11/2014Eth.C | | | |

| Patient Name: **Hikma Alqadir**. Referring Institute: **FHRH**. SEX/ Age: **F/5 6/12**.  Date of Report: **26/11/14**. Referral Diagnosis: **Easy fatigability. AGH8.469** | | | |
| --- | --- | --- | --- |
| **Features** | **Finding** | **Features** | **Finding** |
| **Profile** |  | **Atria** |  |
| Abdominal situs | Solitus | Left atrium | Normal |
| Cardiac position | Levocardia | Right atrium | Normal |
| Systemic venous drainage | Normal. | **Atrioventricular valves** |  |
| Pulmonary venous drainage | Normal | Mitral valve | Annulus = 16mm |
| Atrioventricular connection | Concordant | Tricuspid valve | Annulus = 16mm  TAPSE = 18mm |
| Ventriculoarterial connection | Concordant | **Ventricles** |  |
| Ventricular loop | d-Loop | Left ventricle | Normal |
|  |  | Right ventricle | Normal |
| **Septae** |  | **Coronary arteries** | ----- |
| Interventricular septum | Intact | **Doppler Measurement** |  |
| Interatrial septum | Intact | Mitral | ----- |
| **Semilunar valves** |  | Aortic | ------- |
| Aortic valve | Annulus = 13mm | Tricuspid | ------- |
| Pulmonary valve | Annulus = 14mm | pulmonic | -------- |
| **Great arteries** | NRGA | **Aortic arch** | Left. No CoA. |
| Aorta | ----- | **PDA** | No |
| Pulmonary artery | Normal MPA and Branch PAs. |  |  |
| **M-Mode:**  Normal LV Function on eye balling | | | |
| AO | mm | PWd | mm |
| LA | mm | PWs | mm |
| LVIDd | mm | EDV | ml |
| LVIDs | mm | ESV | ml |
| IVSs | mm | LVEF | % |
| IVSd | mm | FS | % |
| **Additional Information**: |  | | |
| No pericardial/Pleural effusion. | | | |
| **Final Diagnosis:** | | | |
| 1. Normal Echocardiography Study. | | | |
| **Remark**: | | | |
| **Recommendation**: | | | |
| SIGNATURE  Done by: Tesfaye T., Pediatrician, Pediatric Cardiologist _______________ 26/11/2014Eth.C | | | |

| Patient Name: **Seifu Mulugeta**. Referring Institute: **Mekane – Selam PH**. SEX/ Age: **M/1 8/12**.  Date of Report: **27/11/14**. Referral Diagnosis: **Recurrent Chest Infection. AGH8.470** | | | |
| --- | --- | --- | --- |
| **Features** | **Finding** | **Features** | **Finding** |
| **Profile** |  | **Atria** |  |
| Abdominal situs | Solitus | Left atrium | Normal |
| Cardiac position | Levocardia | Right atrium | Normal |
| Systemic venous drainage | Normal. | **Atrioventricular valves** |  |
| Pulmonary venous drainage | Normal | Mitral valve | Annulus = 15mm |
| Atrioventricular connection | Concordant | Tricuspid valve | Annulus = 15mm  TAPSE = 17mm |
| Ventriculoarterial connection | Concordant | **Ventricles** |  |
| Ventricular loop | d-Loop | Left ventricle | Normal |
|  |  | Right ventricle | Normal |
| **Septae** |  | **Coronary arteries** | ----- |
| Interventricular septum | Intact | **Doppler Measurement** |  |
| Interatrial septum | Intact | Mitral | ----- |
| **Semilunar valves** |  | Aortic | ------- |
| Aortic valve | Annulus = 13mm | Tricuspid | ------- |
| Pulmonary valve | Annulus = 14mm | pulmonic | -------- |
| **Great arteries** | NRGA | **Aortic arch** | Left. No CoA. |
| Aorta | ----- | **PDA** | No |
| Pulmonary artery | Normal MPA and Branch PAs. |  |  |
| **M-Mode:**  Normal LV Function on eye balling | | | |
| AO | mm | PWd | mm |
| LA | mm | PWs | mm |
| LVIDd | mm | EDV | ml |
| LVIDs | mm | ESV | ml |
| IVSs | mm | LVEF | % |
| IVSd | mm | FS | % |
| **Additional Information**: |  | | |
| No pericardial/Pleural effusion. | | | |
| **Final Diagnosis:** | | | |
| 1. Normal Echocardiography Study. | | | |
| **Remark**: | | | |
| **Recommendation**: | | | |
| SIGNATURE  Done by: Tesfaye T., Pediatrician, Pediatric Cardiologist _______________ 27/11/2014Eth.C | | | |

| Patient Name: **Yikeber Endeshaw**. Referring Institute: **FHRH**. SEX/ Age: **M/6 2/12**.  Date of Report: **27/11/14**. Referral Diagnosis: **RD. AGH8.471** | | | |
| --- | --- | --- | --- |
| **Features** | **Finding** | **Features** | **Finding** |
| **Profile** |  | **Atria** |  |
| Abdominal situs | Solitus | Left atrium | Normal |
| Cardiac position | Levocardia | Right atrium | Normal |
| Systemic venous drainage | Normal. | **Atrioventricular valves** |  |
| Pulmonary venous drainage | Normal | Mitral valve | Annulus = 19mm |
| Atrioventricular connection | Concordant | Tricuspid valve | Annulus = 20mm  TAPSE = 20mm |
| Ventriculoarterial connection | Concordant | **Ventricles** |  |
| Ventricular loop | d-Loop | Left ventricle | Normal |
|  |  | Right ventricle | Normal |
| **Septae** |  | **Coronary arteries** | ----- |
| Interventricular septum | Intact | **Doppler Measurement** |  |
| Interatrial septum | Intact | Mitral | ----- |
| **Semilunar valves** |  | Aortic | ------- |
| Aortic valve | Annulus = 15mm | Tricuspid | ------- |
| Pulmonary valve | Annulus = 16mm | pulmonic | -------- |
| **Great arteries** | NRGA | **Aortic arch** | Left. No CoA. |
| Aorta | ----- | **PDA** | No |
| Pulmonary artery | Normal MPA and Branch PAs. |  |  |
| **M-Mode:** | | | |
| AO | mm | PWd | mm |
| LA | mm | PWs | mm |
| LVIDd | mm | EDV | ml |
| LVIDs | mm | ESV | ml |
| IVSs | mm | LVEF | 70% |
| IVSd | mm | FS | 38% |
| **Additional Information**: |  | | |
| No pericardial effusion. Right Pleural effusion with maximum depth of 20mm. | | | |
| **Final Diagnosis:** | | | |
| 1. {S, D, S} Levocardia. 2. Large Right Pleural Effusion 20 to ? | | | |
| **Remark**: | | | |
| **Recommendation**: | | | |
| SIGNATURE  Done by: Tesfaye T., Pediatrician, Pediatric Cardiologist _______________ 27/11/2014Eth.C | | | |

| Patient Name: **Mestayet Marew**. Referring Institute: **Debre – Tabour Hospital**. SEX/ Age: **F/4 1/12**.  Date of Report: **27/11/14**. Referral Diagnosis: **Recurrent Chest Infection. AGH8.472** | | | |
| --- | --- | --- | --- |
| **Features** | **Finding** | **Features** | **Finding** |
| **Profile** |  | **Atria** |  |
| Abdominal situs | Solitus | Left atrium | Normal |
| Cardiac position | Levocardia | Right atrium | Normal |
| Systemic venous drainage | Normal. | **Atrioventricular valves** |  |
| Pulmonary venous drainage | Normal | Mitral valve | Annulus = 18mm |
| Atrioventricular connection | Concordant | Tricuspid valve | Annulus = 19mm  TAPSE = 18mm |
| Ventriculoarterial connection | Concordant | **Ventricles** |  |
| Ventricular loop | d-Loop | Left ventricle | Normal |
|  |  | Right ventricle | Normal |
| **Septae** |  | **Coronary arteries** | ----- |
| Interventricular septum | Intact | **Doppler Measurement** |  |
| Interatrial septum | Intact | Mitral | ----- |
| **Semilunar valves** |  | Aortic | ------- |
| Aortic valve | Annulus = 13mm | Tricuspid | ------- |
| Pulmonary valve | Annulus = 15mm | pulmonic | -------- |
| **Great arteries** | NRGA | **Aortic arch** | Left. No CoA. |
| Aorta | ----- | **PDA** | No |
| Pulmonary artery | Normal MPA and Branch PAs. |  |  |
| **M-Mode:** | | | |
| AO | mm | PWd | mm |
| LA | mm | PWs | mm |
| LVIDd | mm | EDV | ml |
| LVIDs | mm | ESV | ml |
| IVSs | mm | LVEF | 63% |
| IVSd | mm | FS | 32% |
| **Additional Information**: |  | | |
| No pericardial/Pleural effusion. | | | |
| **Final Diagnosis:** | | | |
| 1. Normal Echocardiography Study. | | | |
| **Remark**: | | | |
| **Recommendation**: | | | |
| SIGNATURE  Done by: Tesfaye T., Pediatrician, Pediatric Cardiologist _______________ 27/11/2014Eth.C | | | |

| Patient Name: **Maru Werku**. Referring Institute: **FHRH**. SEX/ Age: **M/12years**.  Date of Report: **27/11/14**. Referral Diagnosis: **Easy fatigability. AGH8.473** | | | |
| --- | --- | --- | --- |
| **Features** | **Finding** | **Features** | **Finding** |
| **Profile** |  | **Atria** |  |
| Abdominal situs | Solitus | Left atrium | Normal |
| Cardiac position | Levocardia | Right atrium | Normal |
| Systemic venous drainage | Normal. | **Atrioventricular valves** |  |
| Pulmonary venous drainage | Normal | Mitral valve | Annulus = 19mm |
| Atrioventricular connection | Concordant | Tricuspid valve | Annulus = 22mm  TAPSE = 21mm |
| Ventriculoarterial connection | Concordant | **Ventricles** |  |
| Ventricular loop | d-Loop | Left ventricle | Normal |
|  |  | Right ventricle | Normal |
| **Septae** |  | **Coronary arteries** | ----- |
| Interventricular septum | Intact | **Doppler Measurement** |  |
| Interatrial septum | Intact | Mitral | ----- |
| **Semilunar valves** |  | Aortic | ------- |
| Aortic valve | Annulus = 18mm. Trileaflet | Tricuspid | ------- |
| Pulmonary valve | Annulus = 22mm | pulmonic | -------- |
| **Great arteries** | NRGA | **Aortic arch** | Left. No CoA. |
| Aorta | ----- | **PDA** | No |
| Pulmonary artery | Normal MPA and Branch PAs. |  |  |
| **M-Mode:** | | | |
| AO | mm | PWd | mm |
| LA | mm | PWs | mm |
| LVIDd | mm | EDV | ml |
| LVIDs | mm | ESV | ml |
| IVSs | mm | LVEF | 64% |
| IVSd | mm | FS | 34% |
| **Additional Information**: |  | | |
| No pericardial/Pleural effusion. | | | |
| **Final Diagnosis:** | | | |
| 1. Normal Echocardiography Study. | | | |
| **Remark**: | | | |
| **Recommendation**: | | | |
| SIGNATURE  Done by: Tesfaye T., Pediatrician, Pediatric Cardiologist _______________ 27/11/2014Eth.C | | | |

| Patient Name: **Baby of yengusie Alamrie**. Referring Institute: **TGSH**. SEX/ Age: **M/20days**.  Date of Report: **27/11/14**. Referral Diagnosis: **DS. AGH8.474** | | | |
| --- | --- | --- | --- |
| **Features** | **Finding** | **Features** | **Finding** |
| **Profile** |  | **Atria** |  |
| Abdominal situs | Solitus | Left atrium | Normal |
| Cardiac position | Levocardia | Right atrium | Normal |
| Systemic venous drainage | Normal. | **Atrioventricular valves** |  |
| Pulmonary venous drainage | Normal | Mitral valve | Annulus = 11mm |
| Atrioventricular connection | Concordant | Tricuspid valve | Annulus = 11mm  TAPSE = 13mm |
| Ventriculoarterial connection | Concordant | **Ventricles** |  |
| Ventricular loop | d-Loop | Left ventricle | Normal |
|  |  | Right ventricle | Normal |
| **Septae** |  | **Coronary arteries** | ----- |
| Interventricular septum | Intact | **Doppler Measurement** |  |
| Interatrial septum | PFO, L – R Shunt | Mitral | ----- |
| **Semilunar valves** |  | Aortic | ------- |
| Aortic valve | Annulus = 9mm | Tricuspid | ------- |
| Pulmonary valve | Annulus = 9mm | pulmonic | -------- |
| **Great arteries** | NRGA | **Aortic arch** | Left. No CoA. |
| Aorta | ----- | **PDA** | No |
| Pulmonary artery | Normal MPA and Branch PAs. |  |  |
| **M-Mode:**  Normal LV Function on eye balling | | | |
| AO | mm | PWd | mm |
| LA | mm | PWs | mm |
| LVIDd | mm | EDV | ml |
| LVIDs | mm | ESV | ml |
| IVSs | mm | LVEF | % |
| IVSd | mm | FS | % |
| **Additional Information**: |  | | |
| No pericardial/Pleural effusion. | | | |
| **Final Diagnosis:** | | | |
| 1. {S, D, S} Levocardia. 2. PFO, L – R Shunt | | | |
| **Remark**: | | | |
| **Recommendation**: | | | |
| SIGNATURE  Done by: Tesfaye T., Pediatrician, Pediatric Cardiologist _______________ 27/11/2014Eth.C | | | |

| Patient Name: **Tsion Gebeyehu**. Referring Institute: **Guzara SC_**. SEX/ Age: **F/5 11/12**.  Date of Report: **28/11/14**. Referral Diagnosis: **Recurrent Chest Infection. AGH8.475** | | | |
| --- | --- | --- | --- |
| **Features** | **Finding** | **Features** | **Finding** |
| **Profile** |  | **Atria** |  |
| Abdominal situs | Solitus | Left atrium | Normal |
| Cardiac position | Levocardia | Right atrium | Normal |
| Systemic venous drainage | Normal. | **Atrioventricular valves** |  |
| Pulmonary venous drainage | Normal | Mitral valve | Annulus = 18mm |
| Atrioventricular connection | Concordant | Tricuspid valve | Annulus = 18mm  TAPSE = 18mm |
| Ventriculoarterial connection | Concordant | **Ventricles** |  |
| Ventricular loop | d-Loop | Left ventricle | Normal |
|  |  | Right ventricle | Normal |
| **Septae** |  | **Coronary arteries** | ----- |
| Interventricular septum | Intact | **Doppler Measurement** |  |
| Interatrial septum | Intact | Mitral | ----- |
| **Semilunar valves** |  | Aortic | ------- |
| Aortic valve | Annulus = 14mm | Tricuspid | ------- |
| Pulmonary valve | Annulus = 16mm | pulmonic | -------- |
| **Great arteries** | NRGA | **Aortic arch** | Left. No CoA. |
| Aorta | ----- | **PDA** | No |
| Pulmonary artery | Normal MPA and Branch PAs. |  |  |
| **M-Mode:** | | | |
| AO | mm | PWd | mm |
| LA | mm | PWs | mm |
| LVIDd | mm | EDV | ml |
| LVIDs | mm | ESV | ml |
| IVSs | mm | LVEF | 64% |
| IVSd | mm | FS | 34% |
| **Additional Information**: |  | | |
| No pericardial/Pleural effusion. | | | |
| **Final Diagnosis:** | | | |
| 1. Normal Echocardiography Study. | | | |
| **Remark**: | | | |
| **Recommendation**: | | | |
| SIGNATURE  Done by: Tesfaye T., Pediatrician, Pediatric Cardiologist _______________ 28/11/2014Eth.C | | | |

| Patient Name: **Baby of Yalem – Tsehay Tewachew**. Referring Institute: **MSI – Ethiopia, Bahir Dar**. SEX/ Age: **M/3/12**. Date of Report: **28/11/14**. Referral Diagnosis: **Incidental. AGH8.476** | | | |
| --- | --- | --- | --- |
| **Features** | **Finding** | **Features** | **Finding** |
| **Profile** |  | **Atria** |  |
| Abdominal situs | Solitus | Left atrium | Normal |
| Cardiac position | Levocardia | Right atrium | Normal |
| Systemic venous drainage | Normal. | **Atrioventricular valves** |  |
| Pulmonary venous drainage | Normal | Mitral valve | Annulus = 11mm |
| Atrioventricular connection | Concordant | Tricuspid valve | Annulus = 12mm |
| Ventriculoarterial connection | Concordant | **Ventricles** |  |
| Ventricular loop | d-Loop | Left ventricle | Normal |
|  |  | Right ventricle | Normal |
| **Septae** |  | **Coronary arteries** | ----- |
| Interventricular septum | Intact | **Doppler Measurement** |  |
| Interatrial septum | PFO, L – R Shunt | Mitral | ----- |
| **Semilunar valves** |  | Aortic | ------- |
| Aortic valve | Annulus = 8mm | Tricuspid | ------- |
| Pulmonary valve | Annulus = 9mm | pulmonic | -------- |
| **Great arteries** | NRGA | **Aortic arch** | Left. No CoA. |
| Aorta | ----- | **PDA** | 1mm PDA, L – R Shunt |
| Pulmonary artery | Normal MPA and Branch PAs. |  |  |
| **M-Mode:**  Normal LV Function on eye balling | | | |
| AO | mm | PWd | mm |
| LA | mm | PWs | mm |
| LVIDd | mm | EDV | ml |
| LVIDs | mm | ESV | ml |
| IVSs | mm | LVEF | % |
| IVSd | mm | FS | % |
| **Additional Information**: |  | | |
| No pericardial/Pleural effusion. | | | |
| **Final Diagnosis:** | | | |
| 1. {S, D, S} Levocardia. 2. PFO, L – R Shunt 3. Small PDA, L – R Shunt (can be considered as Silent PDA, if no murmur) | | | |
| **Remark**: | | | |
| **Recommendation**: | | | |
| SIGNATURE  Done by: Tesfaye T., Pediatrician, Pediatric Cardiologist _______________ 28/11/2014Eth.C | | | |

| Patient Name: **Eyerus Asmamaw**. Referring Institute: **FHRH**. SEX/ Age: **F/6years**.  Date of Report: **28/11/14**. Referral Diagnosis: **ARF + CHF. AGH8.477** | | | |
| --- | --- | --- | --- |
| **Features** | **Finding** | **Features** | **Finding** |
| **Profile** |  | **Atria** |  |
| Abdominal situs | Solitus | Left atrium | Dilated |
| Cardiac position | Levocardia | Right atrium | Normal |
| Systemic venous drainage | Normal. | **Atrioventricular valves** |  |
| Pulmonary venous drainage | Normal | Mitral valve | Annulus = 29mm. Thickened MVL. |
| Atrioventricular connection | Concordant | Tricuspid valve | Annulus = 21mm |
| Ventriculoarterial connection | Concordant | **Ventricles** |  |
| Ventricular loop | d-Loop | Left ventricle | Dilated |
|  |  | Right ventricle | Normal |
| **Septae** |  | **Coronary arteries** | ----- |
| Interventricular septum | Intact | **Doppler Measurement** |  |
| Interatrial septum | Intact | Mitral | Severe MR, Holosystolic, Posterior projection, seen in two planes with jet velocity = 4.2m/sec. |
| **Semilunar valves** |  | Aortic | Moderate AR, PHT = 240ms. |
| Aortic valve | Annulus = 16mm | Tricuspid | Mild TR, PPG = 39mmHg |
| Pulmonary valve | Annulus = 20mm | pulmonic | -------- |
| **Great arteries** | NRGA | **Aortic arch** | Left. No CoA. |
| Aorta | ----- | **PDA** | No |
| Pulmonary artery | Normal MPA and Branch PAs. |  |  |
| **M-Mode:** | | | |
| AO | mm | PWd | mm |
| LA | mm | PWs | mm |
| LVIDd | mm | EDV | ml |
| LVIDs | mm | ESV | ml |
| IVSs | mm | LVEF | 57% |
| IVSd | mm | FS | 30% |
| **Additional Information**: |  | | |
| No pericardial/Pleural effusion. | | | |
| **Final Diagnosis:** | | | |
| 1. {S, D, S} Levocardia. 2. LA/LV Dilated 3. Thickened MVL 4. Severe MR 5. Mild TR 6. Moderate AR 7. Mild Pulmonary Hypertension 8. Normal LV Systolic Function | | | |
| **Remark**: | | | |
| **Recommendation**: | | | |
| SIGNATURE  Done by: Tesfaye T., Pediatrician, Pediatric Cardiologist _______________ 28/11/2014Eth.C | | | |

| Patient Name: **Enaya Muhammed**. Referring Institute: **FHRH**. SEX/ Age: **F/1 3/12**.  Date of Report: **28/11/14**. Referral Diagnosis: **Cardiomegaly on CXR. AGH8.478** | | | |
| --- | --- | --- | --- |
| **Features** | **Finding** | **Features** | **Finding** |
| **Profile** |  | **Atria** |  |
| Abdominal situs | Solitus | Left atrium | Normal |
| Cardiac position | Levocardia | Right atrium | Normal |
| Systemic venous drainage | Normal. | **Atrioventricular valves** |  |
| Pulmonary venous drainage | Normal | Mitral valve | Annulus = 13mm |
| Atrioventricular connection | Concordant | Tricuspid valve | Annulus = 13mm |
| Ventriculoarterial connection | Concordant | **Ventricles** |  |
| Ventricular loop | d-Loop | Left ventricle | Normal |
|  |  | Right ventricle | Normal |
| **Septae** |  | **Coronary arteries** | ----- |
| Interventricular septum | Intact | **Doppler Measurement** |  |
| Interatrial septum | Intact | Mitral | ----- |
| **Semilunar valves** |  | Aortic | ------- |
| Aortic valve | Annulus = 11mm | Tricuspid | ------- |
| Pulmonary valve | Annulus = 13mm | pulmonic | -------- |
| **Great arteries** | NRGA | **Aortic arch** | Left. No CoA. |
| Aorta | ----- | **PDA** | No |
| Pulmonary artery | Normal MPA and Branch PAs. |  |  |
| **M-Mode:**  Normal LV Function on eye balling | | | |
| AO | mm | PWd | mm |
| LA | mm | PWs | mm |
| LVIDd | mm | EDV | ml |
| LVIDs | mm | ESV | ml |
| IVSs | mm | LVEF | % |
| IVSd | mm | FS | % |
| **Additional Information**: |  | | |
| No pericardial/Pleural effusion. | | | |
| **Final Diagnosis:** | | | |
| 1. Normal Echocardiography Study. | | | |
| **Remark**: | | | |
| **Recommendation**: | | | |
| SIGNATURE  Done by: Tesfaye T., Pediatrician, Pediatric Cardiologist _______________ 28/11/2014Eth.C | | | |

| Patient Name: **Messele Dessie**. Referring Institute: **TGSH**. SEX/ Age: **M/14years**.  Date of Report: **29/11/14**. Referral Diagnosis: **Easy fatigability. AGH8.479** | | | |
| --- | --- | --- | --- |
| **Features** | **Finding** | **Features** | **Finding** |
| **Profile** |  | **Atria** |  |
| Abdominal situs | Solitus | Left atrium | Normal |
| Cardiac position | Levocardia | Right atrium | Dilated |
| Systemic venous drainage | Normal. | **Atrioventricular valves** |  |
| Pulmonary venous drainage | Normal | Mitral valve | Annulus = 21mm |
| Atrioventricular connection | Concordant | Tricuspid valve | Annulus = 28mm. 11mm downward displacement of the STL. Both anterior and septal leaflets are freely mobile.  TAPSE = 23mm |
| Ventriculoarterial connection | Concordant | **Ventricles** |  |
| Ventricular loop | d-Loop | Left ventricle | Normal |
|  |  | Right ventricle | Dilated |
| **Septae** |  | **Coronary arteries** | ----- |
| Interventricular septum | Intact | **Doppler Measurement** |  |
| Interatrial septum | Intact | Mitral | ----- |
| **Semilunar valves** |  | Aortic | ------- |
| Aortic valve | Annulus = 17mm | Tricuspid | Moderate TR, PPG = 22mmHg |
| Pulmonary valve | Annulus = 18mm | pulmonic | -------- |
| **Great arteries** | NRGA | **Aortic arch** | Left. No CoA. |
| Aorta | ----- | **PDA** | No |
| Pulmonary artery | Normal MPA and Branch PAs. |  |  |
| **M-Mode:** | | | |
| AO | mm | PWd | mm |
| LA | mm | PWs | mm |
| LVIDd | mm | EDV | ml |
| LVIDs | mm | ESV | ml |
| IVSs | mm | LVEF | 70% |
| IVSd | mm | FS | 39% |
| **Additional Information**: |  | | |
| No pericardial/Pleural effusion. | | | |
| **Final Diagnosis:** | | | |
| 1. {S, D, S} Levocardia. 2. RA/RV Dilated 3. Moderate TR 4. Type A Ebstein Anomaly 5. Normal Biventricular Systolic Function | | | |
| **Remark**: | | | |
| **Recommendation**: | | | |
| SIGNATURE  Done by: Tesfaye T., Pediatrician, Pediatric Cardiologist _______________ 29/11/2014Eth.C | | | |

| Patient Name: **Baby of Kore Asegie**. Referring Institute: **TGSH**. SEX/ Age: **F/3days**.  Date of Report: **29/11/14**. Referral Diagnosis: **DS. AGH8.480** | | | |
| --- | --- | --- | --- |
| **Features** | **Finding** | **Features** | **Finding** |
| **Profile** |  | **Atria** |  |
| Abdominal situs | Solitus | Left atrium | Normal |
| Cardiac position | Levocardia | Right atrium | Normal |
| Systemic venous drainage | Normal. | **Atrioventricular valves** |  |
| Pulmonary venous drainage | Normal | Mitral valve | Annulus = 10mm |
| Atrioventricular connection | Concordant | Tricuspid valve | Annulus = 10mm |
| Ventriculoarterial connection | Concordant | **Ventricles** |  |
| Ventricular loop | d-Loop | Left ventricle | Normal |
|  |  | Right ventricle | Normal |
| **Septae** |  | **Coronary arteries** | ----- |
| Interventricular septum | 3mm PM VSD, L – R Shunt | **Doppler Measurement** |  |
| Interatrial septum | PFO, L – R Shunt | Mitral | ----- |
| **Semilunar valves** |  | Aortic | ------- |
| Aortic valve | Annulus = 9mm | Tricuspid | ------- |
| Pulmonary valve | Annulus = 8mm | pulmonic | -------- |
| **Great arteries** | NRGA | **Aortic arch** | Left. No CoA. |
| Aorta | ----- | **PDA** | 1.5mm PDA, L – R Shunt |
| Pulmonary artery | Normal MPA and Branch PAs. |  |  |
| **M-Mode:**  Normal LV Function on eye balling | | | |
| AO | mm | PWd | mm |
| LA | mm | PWs | mm |
| LVIDd | mm | EDV | ml |
| LVIDs | mm | ESV | ml |
| IVSs | mm | LVEF | % |
| IVSd | mm | FS | % |
| **Additional Information**: |  | | |
| No pericardial/Pleural effusion. | | | |
| **Final Diagnosis:** | | | |
| 1. {S, D, S} Levocardia. 2. PFO, L – R Shunt 3. Small PM VSD, L – R Shunt 4. Small PDA, L – R Shunt 5. Normal LV Systolic Function | | | |
| **Remark**: | | | |
| **Recommendation**: | | | |
| SIGNATURE  Done by: Tesfaye T., Pediatrician, Pediatric Cardiologist _______________ 29/11/2014Eth.C | | | |
[truncated: 487,373 more chars]
