# Supplementary material for: Two–Dimensional and Doppler trans-thoracic echocardiographic patterns of suspected pediatric heart diseases at Tibebe-—Ghion specialized Teaching Hospital and Adinas General Hospital, Bahir Dar, North-west Ethiopia:–An experience from an LMIC
Source: PLoS One. 2024 Mar 11;19(3):e0292694. doi: 10.1371/journal.pone.0292694 (PMC10927071; doi:10.1371/journal.pone.0292694)
Supplement: S2 File — (ZIP) [file pone.0292694.s003.zip › AGH10 Pediatric Transthoracic Echocardiography Report February 2022 SPSS.docx]

| Patient Name: **Zelalem Addisu**. Patient ID: **Alef Clinic**. SEX/ Age: M/8Years. Date of Report: 16/**07/2013**.  BP: ___ Weight: ___ Height: ___ BSA: ___. Referral Diagnosis: **Recurrence. AGH10.734** | | | |
| --- | --- | --- | --- |
| **Features** | **Finding** | **Features** | **Finding** |
| **Profile** |  | **Atria** |  |
| Abdominal situs | Solitus | Left atrium | Mildly Dilated |
| Cardiac position | Levocardia | Right atrium | Normal |
| Systemic venous drainage | Normal. | **Atrioventricular valves** |  |
| Pulmonary venous drainage | Normal | Mitral valve | Annulus = 23mm. Thickened MVL |
| Atrioventricular connection | Concordant | Tricuspid valve | Annulus = 17mm  TAPSE = 21mm |
| Ventriculoarterial connection | Concordant | **Ventricles** |  |
| Ventricular loop | d-Loop | Left ventricle | Mildly Dilated |
|  |  | Right ventricle | Normal |
| **Septae** |  | **Coronary arteries** | ----- |
| Interventricular septum | Intact | **Doppler Measurement** |  |
| Interatrial septum | Intact | Mitral | Moderate MR, Holosystolic, posterior Projection, seen in two planes with jet velocity = 4.5m/sec |
| **Semilunal valves** |  | Aortic | Moderate AR, PHT = 227ms |
| Aortic valve | Annulus = 18mm | Tricuspid | ------- |
| Pulmonary valve | Annulus = 18mm | pulmonic | -------- |
| **Great arteries** | NRGA | **Aortic arch** | Left |
| Aorta | ----- | **PDA** | No |
| Pulmonary artery | Normal MPA and Branch PAs. |  |  |
| **M-Mode:** | | | |
| AO | mm | PWd | mm |
| LA | mm | EDV | ml |
| LVIDd | mm | ESV | ml |
| LVIDs | mm | LVEF | 72% |
| IVSd | mm | FS | 40% |
| **Additional Information**: |  | | |
| No pericardial/Pleural effusion. | | | |
| **Final Diagnosis:** | | | |
| 1. {S, D, S} Levocardia. 2. Mildly dilated LA/LV 3. Mildly thickened MVL 4. Moderate MR 5. Moderate AR 6. Normal Biventricular Function | | | |
| SIGNATURE  Done by: Tesfaye T., Pediatrician, Pediatric Cardiologist _______________ 16/07/2013Eth.C | | | |

| Patient Name: **Henock Million**. Patient ID: **Guzara Clinic**. SEX/ Age: M/11/12. Date of Report: 16/**07/2013**.  BP: ____ Weight: ____ Height: _____ BSA: ___. Referral Diagnosis: **RD. AGH10.735** | | | |
| --- | --- | --- | --- |
| **Features** | **Finding** | **Features** | **Finding** |
| **Profile** |  | **Atria** |  |
| Abdominal situs | Solitus | Left atrium | Normal |
| Cardiac position | Levocardia | Right atrium | Normal |
| Systemic venous drainage | Normal. | **Atrioventricular valves** |  |
| Pulmonary venous drainage | Normal | Mitral valve | Annulus = 13mm |
| Atrioventricular connection | Concordant | Tricuspid valve | Annulus = 13mm |
| Ventriculoarterial connection | Concordant | **Ventricles** |  |
| Ventricular loop | d-Loop | Left ventricle | Normal |
|  |  | Right ventricle | Normal |
| **Septae** |  | **Coronary arteries** | ----- |
| Interventricular septum | Intact | **Doppler Measurement** |  |
| Interatrial septum | Intact | Mitral | ----- |
| **Semilunal valves** |  | Aortic | ------- |
| Aortic valve | Annulus = 11mm | Tricuspid | ------- |
| Pulmonary valve | Annulus = 11mm | pulmonic | -------- |
| **Great arteries** | NRGA | **Aortic arch** | Left |
| Aorta | ----- | **PDA** | No |
| Pulmonary artery | Normal MPA and Branch PAs. |  |  |
| **M-Mode:** Normal LV Function on eye balling. | | | |
| AO | mm | PWd | mm |
| LA | mm | EDV | ml |
| LVIDd | mm | ESV | ml |
| LVIDs | mm | LVEF | % |
| IVSd | mm | FS | % |
| **Additional Information**: |  | | |
| No pericardial/Pleural effusion. | | | |
| **Final Diagnosis:** | | | |
| 1. Normal Echocardiography Study. | | | |
| **Remark**: | | | |
| **Recommendation**: | | | |
| SIGNATURE  Done by: Tesfaye T., Pediatrician, Pediatric Cardiologist _______________ 16/07/2013Eth.C | | | |

| Patient Name: **Yididiya Kerebih**. Patient ID: **SBPSC**. SEX/ Age: F/6/12. Date of Report: 16/**07/2013**.  BP: ____ Weight: ___ Height: ____ BSA: ____. Referral Dx: **CHF + G-III ESM. AGH10.736** | | | |
| --- | --- | --- | --- |
| **Features** | **Finding** | **Features** | **Finding** |
| **Profile** |  | **Atria** |  |
| Abdominal situs | Solitus | Left atrium | Normal |
| Cardiac position | Levocardia | Right atrium | Dilated |
| Systemic venous drainage | Normal. | **Atrioventricular valves** |  |
| Pulmonary venous drainage | Normal | Mitral valve | Annulus = mm |
| Atrioventricular connection | Concordant | Tricuspid valve | Annulus = 15mm  TAPSE = 6mm |
| Ventriculoarterial connection | Concordant | **Ventricles** |  |
| Ventricular loop | d-Loop | Left ventricle | Normal |
|  |  | Right ventricle | Dilated, Hypertrophied, Dysfunctional |
| **Septae** |  | **Coronary arteries** | ----- |
| Interventricular septum | Intact | **Doppler Measurement** |  |
| Interatrial septum | Probe patent PFO | Mitral | ----- |
| **Semilunal valves** |  | Aortic | ------- |
| Aortic valve | Annulus = 11mm | Tricuspid | Mild to moderate TR |
| Pulmonary valve | Annulus = 6mm | pulmonic | Severe PS, PPG = 80mmHg |
| **Great arteries** | NRGA | **Aortic arch** | Left |
| Aorta | ----- | **PDA** | No |
| Pulmonary artery | Normal MPA and Branch PAs. |  |  |
| **M-Mode:** | | | |
| AO | mm | PWd | mm |
| LA | mm | EDV | ml |
| LVIDd | mm | ESV | ml |
| LVIDs | mm | LVEF | % |
| IVSd | mm | FS | % |
| **Additional Information**: |  | | |
| No pericardial/Pleural effusion. | | | |
| **Final Diagnosis:** | | | |
| 1. {S, D, S} Levocardia. 2. Probe Patent PFO 3. Severe PS 4. RV Dilated, Hypertrophied and Dysfunctional | | | |
| **Remark**: | | | |
| **Recommendation**: Needs Urgent Intervention | | | |
| SIGNATURE  Done by: Tesfaye T., Pediatrician, Pediatric Cardiologist _______________ 16/07/2013Eth.C | | | |

| Patient Name: **Yerom Birhanu**. Patient ID: **Enjibara GH**. SEX/ Age: F/32days. Date of Report: 16/**07/2013**.  BP: _______ Weight: ______ Height: ____________ BSA: ________. R.Dx: **DS. AGH10.737** | | | |
| --- | --- | --- | --- |
| **Features** | **Finding** | **Features** | **Finding** |
| **Profile** |  | **Atria** |  |
| Abdominal situs | Solitus | Left atrium | Normal |
| Cardiac position | Levocardia | Right atrium | Normal |
| Systemic venous drainage | Normal. | **Atrioventricular valves** |  |
| Pulmonary venous drainage | Normal | Mitral valve | Annulus = 8mm |
| Atrioventricular connection | Concordant | Tricuspid valve | Annulus = 10mm |
| Ventriculoarterial connection | Concordant | **Ventricles** |  |
| Ventricular loop | d-Loop | Left ventricle | Normal |
|  |  | Right ventricle | Normal |
| **Septae** |  | **Coronary arteries** | ----- |
| Interventricular septum | Intact | **Doppler Measurement** |  |
| Interatrial septum | Intact | Mitral | ----- |
| **Semilunal valves** |  | Aortic | ------- |
| Aortic valve | Annulus = 7mm | Tricuspid | ------- |
| Pulmonary valve | Annulus = 8mm | pulmonic | -------- |
| **Great arteries** | NRGA | **Aortic arch** | Left |
| Aorta | ----- | **PDA** | No |
| Pulmonary artery | Normal MPA and Branch PAs. |  |  |
| **M-Mode:** Normal LV Function on eye balling. | | | |
| AO | mm | PWd | mm |
| LA | mm | EDV | ml |
| LVIDd | mm | ESV | ml |
| LVIDs | mm | LVEF | % |
| IVSd | mm | FS | % |
| **Additional Information**: |  | | |
| No pericardial/Pleural effusion. | | | |
| **Final Diagnosis:** | | | |
| 1. Normal Echocardiography Study. | | | |
| **Remark**: | | | |
| **Recommendation**: | | | |
| SIGNATURE  Done by: Tesfaye T., Pediatrician, Pediatric Cardiologist _______________ 16/07/2013Eth.C | | | |

| Patient Name: **Muluhabit Genie**. Patient ID: **FHRH**. SEX/ Age: F/14Years. Date of Report: 16/**07/2013**.  R.Dx: **Rheumatic Recurrence. AGH10.738** | | | |
| --- | --- | --- | --- |
| **Features** | **Finding** | **Features** | **Finding** |
| **Profile** |  | **Atria** |  |
| Abdominal situs | Solitus | Left atrium | Dilated |
| Cardiac position | Levocardia | Right atrium | Dilated |
| Systemic venous drainage | Normal. | **Atrioventricular valves** |  |
| Pulmonary venous drainage | Normal | Mitral valve | Annulus = 21mm. Thickened MVL |
| Atrioventricular connection | Concordant | Tricuspid valve | Annulus = 23mm. Non coapting TVL  TAPSE = 15mm |
| Ventriculoarterial connection | Concordant | **Ventricles** |  |
| Ventricular loop | d-Loop | Left ventricle | Dilated |
|  |  | Right ventricle | Dilated |
| **Septae** |  | **Coronary arteries** | ----- |
| Interventricular septum | Intact | **Doppler Measurement** |  |
| Interatrial septum | Intact | Mitral | Severe MR, Holosystolic, posterior projection, seen in two planes with jet velocity = 4.5m/sec |
| **Semilunal valves** |  | Aortic | Mild AR, PHT = 510ms |
| Aortic valve | Annulus = 16mm. Thickened AVL | Tricuspid | Severe TR, PPG = 63mmHg |
| Pulmonary valve | Annulus = 22mm | pulmonic | -------- |
| **Great arteries** | NRGA | **Aortic arch** | Left |
| Aorta | ----- | **PDA** | No |
| Pulmonary artery | Normal MPA and Branch PAs. |  |  |
| **M-Mode:** | | | |
| AO | mm | PWd | mm |
| LA | mm | EDV | ml |
| LVIDd | mm | ESV | ml |
| LVIDs | mm | LVEF | 50% |
| IVSd | mm | FS | 25% |
| **Additional Information**: |  | | |
| Trace pericardial effusion. | | | |
| **Final Diagnosis:** | | | |
| 1. {S, D, S} Levocardia. 2. All Chambers Dilated 3. Thickened MVL and AVL 4. Non Coapting TVL 5. Severe MR 6. Severe TR 7. Mild AR 8. Severe Pulmonary Hypertension 9. Mildly Reduced LV Function | | | |
| SIGNATURE  Done by: Tesfaye T., Pediatrician, Pediatric Cardiologist _______________ 16/07/2013Eth.C | | | |

| Patient Name: **Tiruye Shimelash**. Patient ID: **Adinas GH**. SEX/ Age: F/13Years. Date of Report: 17/**07/2013**.  R.Dx: **IE + CHF. AGH10.739** | | | |
| --- | --- | --- | --- |
| **Features** | **Finding** | **Features** | **Finding** |
| **Profile** |  | **Atria** |  |
| Abdominal situs | Solitus | Left atrium | Dilated |
| Cardiac position | Levocardia | Right atrium | Normal |
| Systemic venous drainage | Normal. | **Atrioventricular valves** |  |
| Pulmonary venous drainage | Normal | Mitral valve | Annulus = 24mm. thickened, clubbed MVL. MVA = 0.5cm2. |
| Atrioventricular connection | Concordant | Tricuspid valve | Annulus = 20mm |
| Ventriculoarterial connection | Concordant | **Ventricles** |  |
| Ventricular loop | d-Loop | Left ventricle | Dilated. Echogenic Mass on the LVOT. |
|  |  | Right ventricle | Normal |
| **Septae** |  | **Coronary arteries** | ----- |
| Interventricular septum | Intact | **Doppler Measurement** |  |
| Interatrial septum | Intact | Mitral | Severe MR, Holosystolic, posterior projection, seen in two planes with jet velocity = 5.3m/sec. Severe MS, PPG/MPG = 29/18mmHg |
| **Semilunal valves** |  | Aortic | Moderate AR, PHT = 484ms. |
| Aortic valve | Annulus = 19mm | Tricuspid | ------- |
| Pulmonary valve | Annulus = 22mm | pulmonic | Trivial PR, PPG = 15mmHg |
| **Great arteries** | NRGA | **Aortic arch** | Left |
| Aorta | ----- | **PDA** | No |
| Pulmonary artery | Normal MPA and Branch PAs. |  |  |
| **M-Mode:** | | | |
| AO | mm | PWd | mm |
| LA | mm | EDV | ml |
| LVIDd | mm | ESV | ml |
| LVIDs | mm | LVEF | 54% |
| IVSd | mm | FS | 28% |
| **Additional Information**: |  | | |
| No pericardial/Pleural effusion. | | | |
| **Final Diagnosis:** | | | |
| 1. {S, D, S} Levocardia. 2. LA/LV Dilated 3. Thickened, Clubbed MVL 4. Echogenic mass on the LVOT 5. Severe MR 6. Severe MS 7. Mildly reduced LV Function | | | |
| SIGNATURE  Done by: Tesfaye T., Pediatrician, Pediatric Cardiologist _______________ 17/07/2013Eth.C | | | |

| Patient Name: **Yared Alebachew**. Patient ID: **Addis Alem PH**. SEX/ Age: M/2/12. Date of Report: 18/**07/2013**.  BP: _______ Weight: ______ Height: ____________ BSA: ________. R.Dx: **DS. AGH10.740** | | | |
| --- | --- | --- | --- |
| **Features** | **Finding** | **Features** | **Finding** |
| **Profile** |  | **Atria** |  |
| Abdominal situs | Solitus | Left atrium | Normal |
| Cardiac position | Levocardia | Right atrium | Normal |
| Systemic venous drainage | Normal. | **Atrioventricular valves** |  |
| Pulmonary venous drainage | Normal | Mitral valve | Annulus = 10mm |
| Atrioventricular connection | Concordant | Tricuspid valve | Annulus = 10mm  TAPSE = 13mm |
| Ventriculoarterial connection | Concordant | **Ventricles** |  |
| Ventricular loop | d-Loop | Left ventricle | Normal |
|  |  | Right ventricle | Normal |
| **Septae** |  | **Coronary arteries** | ----- |
| Interventricular septum | Intact | **Doppler Measurement** |  |
| Interatrial septum | Intact | Mitral | ----- |
| **Semilunal valves** |  | Aortic | ------- |
| Aortic valve | Annulus = 8mm | Tricuspid | ------- |
| Pulmonary valve | Annulus = 8mm | pulmonic | -------- |
| **Great arteries** | NRGA | **Aortic arch** | Left |
| Aorta | ----- | **PDA** | No |
| Pulmonary artery | Normal MPA and Branch PAs. |  |  |
| **M-Mode:** Normal LV Function on eye balling. | | | |
| AO | mm | PWd | mm |
| LA | mm | EDV | ml |
| LVIDd | mm | ESV | ml |
| LVIDs | mm | LVEF | % |
| IVSd | mm | FS | % |
| **Additional Information**: |  | | |
| No pericardial/Pleural effusion. | | | |
| **Final Diagnosis:** | | | |
| 1. Normal Echocardiography Study. | | | |
| **Remark**: | | | |
| **Recommendation**: | | | |
| SIGNATURE  Done by: Tesfaye T., Pediatrician, Pediatric Cardiologist _______________ 18/07/2013Eth.C | | | |

| Patient Name: **Arsema Melesse**. Patient ID: **Adinas GH**. SEX/ Age: F/40days. Date of Report: 18/**07/2013**.  BP: _______ Weight: ______ Height: ____________ BSA: ________ Incidental Murmur Finding | | | |
| --- | --- | --- | --- |
| **Features** | **Finding** | **Features** | **Finding** |
| **Profile** |  | **Atria** |  |
| Abdominal situs | Solitus | Left atrium | Normal |
| Cardiac position | Levocardia | Right atrium | Normal |
| Systemic venous drainage | Normal. | **Atrioventricular valves** |  |
| Pulmonary venous drainage | Normal | Mitral valve | Annulus = 10mm |
| Atrioventricular connection | Concordant | Tricuspid valve | Annulus = 11mm  TAPSE = 12mm |
| Ventriculoarterial connection | Concordant | **Ventricles** |  |
| Ventricular loop | d-Loop | Left ventricle | Normal |
|  |  | Right ventricle | Normal |
| **Septae** |  | **Coronary arteries** | ----- |
| Interventricular septum | 3mm PM VSD, L – R Shunt. | **Doppler Measurement** |  |
| Interatrial septum | Intact | Mitral | ----- |
| **Semilunal valves** |  | Aortic | ------- |
| Aortic valve | Annulus = 11mm | Tricuspid | ------- |
| Pulmonary valve | Annulus = 12mm | pulmonic | -------- |
| **Great arteries** | NRGA | **Aortic arch** | Left |
| Aorta | ----- | **PDA** | No |
| Pulmonary artery | Normal MPA and Branch PAs. |  |  |
| **M-Mode:** Normal LV Function on eye balling. | | | |
| AO | mm | PWd | mm |
| LA | mm | EDV | ml |
| LVIDd | mm | ESV | ml |
| LVIDs | mm | LVEF | % |
| IVSd | mm | FS | % |
| **Additional Information**: |  | | |
| No pericardial/Pleural effusion. | | | |
| **Final Diagnosis:** | | | |
| 1. {S, D, S} Levocardia. 2. Small PM VSD, L – R Shunt 3. Normal Biventricular Function | | | |
| **Remark**: | | | |
| **Recommendation**: | | | |
| SIGNATURE  Done by: Tesfaye T., Pediatrician, Pediatric Cardiologist _______________ 18/07/2013Eth.C | | | |

| Patient Name: **Temesgen Tigabu**. Patient ID: **FHRH**. SEX/ Age: M/2/12. Date of Report: 18/**07/2013**.  BP: _______ Weight: ______ Height: ____________ BSA: ________. R.Dx: **Cyanosis + RD. AGH10.741** | | | |
| --- | --- | --- | --- |
| **Features** | **Finding** | **Features** | **Finding** |
| **Profile** |  | **Atria** |  |
| Abdominal situs | Solitus | Left atrium | Normal |
| Cardiac position | Levocardia | Right atrium | Dilated |
| Systemic venous drainage | Normal. | **Atrioventricular valves** |  |
| Pulmonary venous drainage | Normal | Mitral valve | Annulus = 5mm |
| Atrioventricular connection | Concordant | Tricuspid valve | Annulus = 11mm |
| Ventriculoarterial connection | Concordant | **Ventricles** |  |
| Ventricular loop | d-Loop | Left ventricle | Smallish hypertrophied |
|  |  | Right ventricle | Dilated |
| **Septae** |  | **Coronary arteries** | ----- |
| Interventricular septum | Intact | **Doppler Measurement** |  |
| Interatrial septum | 5 X 8mm OS ASD, L – R Shunt | Mitral | ----- |
| **Semilunal valves** |  | Aortic | ------- |
| Aortic valve | ? Atretic | Tricuspid | ------- |
| Pulmonary valve | Annulus = 14mm | pulmonic | Severe PR, PPG = 71mmHg |
| **Great arteries** | NRGA | **Aortic arch** | Not visualized |
| Aorta | Not visualized | **PDA** | 2mm PDA, R – L Shunt |
| Pulmonary artery | Dilated MPA and Branch PAs. |  |  |
| **M-Mode:** | | | |
| AO | mm | PWd | mm |
| LA | mm | EDV | ml |
| LVIDd | mm | ESV | ml |
| LVIDs | mm | LVEF | % |
| IVSd | mm | FS | % |
| **Additional Information**: |  | | |
| No pericardial/Pleural effusion. | | | |
| **Final Diagnosis:** | | | |
| 1. {S, D, S} Levocardia. 2. RA/RV Dilated 3. LV Smallish and Hypertrophied 4. Moderate OS ASD, L – R Shunt 5. ? aortic interruption 6. Severe Pulmonary Hypertension | | | |
| **Remark**: Infant was crying throughout the study | | | |
| **Recommendation**: HLHS | | | |
| SIGNATURE  Done by: Tesfaye T., Pediatrician, Pediatric Cardiologist _______________ 18/07/2013Eth.C | | | |

| Patient Name: **Tena Yibeltal**. Patient ID: **FHRH**. SEX/ Age: F/4Years. Date of Report: 18/**07/2013**.  BP: ____ Weight: ____ Height: ____ BSA: _____. R.Dx: **easyfatigability + FTT + Murmur. AGH10.742** | | | |
| --- | --- | --- | --- |
| **Features** | **Finding** | **Features** | **Finding** |
| **Profile** |  | **Atria** |  |
| Abdominal situs | Solitus | Left atrium | Normal |
| Cardiac position | Levocardia | Right atrium | Dilated |
| Systemic venous drainage | Normal. | **Atrioventricular valves** |  |
| Pulmonary venous drainage | Normal | Mitral valve | Annulus = 14mm |
| Atrioventricular connection | Concordant | Tricuspid valve | Annulus = 16mm  TAPSE = 18mm |
| Ventriculoarterial connection | Concordant | **Ventricles** |  |
| Ventricular loop | d-Loop | Left ventricle | Normal |
|  |  | Right ventricle | Dilated |
| **Septae** |  | **Coronary arteries** | ----- |
| Interventricular septum | Intact | **Doppler Measurement** |  |
| Interatrial septum | 9 X 13mm OS ASD, L – R Shunt | Mitral | ----- |
| **Semilunal valves** |  | Aortic | ------- |
| Aortic valve | Annulus = 13mm | Tricuspid | ------- |
| Pulmonary valve | Annulus = 15mm | pulmonic | -------- |
| **Great arteries** | NRGA | **Aortic arch** | Left |
| Aorta | ----- | **PDA** | No |
| Pulmonary artery | Normal MPA and Branch PAs. |  |  |
| **M-Mode:** | | | |
| AO | mm | PWd | mm |
| LA | mm | EDV | ml |
| LVIDd | mm | ESV | ml |
| LVIDs | mm | LVEF | 57% |
| IVSd | mm | FS | 28% |
| **Additional Information**: |  | | |
| No pericardial/Pleural effusion. | | | |
| **Final Diagnosis:** | | | |
| 1. {S, D, S} Levocardia. 2. RA/RV Dilated 3. Large OS ASD, L – R Shunt 4. Normal Biventricular Function | | | |
| **Remark**: abnormal beat detected during echocardiography study | | | |
| **Recommendation**:   1. do ECG 2. Candidate for closure. | | | |
| SIGNATURE  Done by: Tesfaye T., Pediatrician, Pediatric Cardiologist _______________ 18/07/2013Eth.C | | | |

| Patient Name: **Getachew Simegnew**. Patient ID: **FHRH**. SEX/ Age: M/2Years. Date of Report: 20/**07/2013**.  BP: ____ Weight: ____ Height: _____ BSA: _____. R.DX: **Recurrent Chest Infection. AGH10.743** | | | |
| --- | --- | --- | --- |
| **Features** | **Finding** | **Features** | **Finding** |
| **Profile** |  | **Atria** |  |
| Abdominal situs | Solitus | Left atrium | Normal |
| Cardiac position | Levocardia | Right atrium | Normal |
| Systemic venous drainage | Normal. | **Atrioventricular valves** |  |
| Pulmonary venous drainage | Normal | Mitral valve | Annulus = 13mm |
| Atrioventricular connection | Concordant | Tricuspid valve | Annulus = 15mm |
| Ventriculoarterial connection | Concordant | **Ventricles** |  |
| Ventricular loop | d-Loop | Left ventricle | Normal |
|  |  | Right ventricle | Normal |
| **Septae** |  | **Coronary arteries** | ----- |
| Interventricular septum | Intact | **Doppler Measurement** |  |
| Interatrial septum | Intact | Mitral | ----- |
| **Semilunal valves** |  | Aortic | ------- |
| Aortic valve | Annulus = 12mm | Tricuspid | ------- |
| Pulmonary valve | Annulus = 14mm | pulmonic | -------- |
| **Great arteries** | NRGA | **Aortic arch** | Left |
| Aorta | ----- | **PDA** | No |
| Pulmonary artery | Normal MPA and Branch PAs. |  |  |
| **M-Mode:** | | | |
| AO | mm | PWd | mm |
| LA | mm | EDV | ml |
| LVIDd | mm | ESV | ml |
| LVIDs | mm | LVEF | % |
| IVSd | mm | FS | % |
| **Additional Information**: |  | | |
| No pericardial/Pleural effusion. | | | |
| **Final Diagnosis:** | | | |
| 1. Normal Echocardiography Study. | | | |
| **Remark**: | | | |
| **Recommendation**: | | | |
| SIGNATURE  Done by: Tesfaye T., Pediatrician, Pediatric Cardiologist _______________ 20/07/2013Eth.C | | | |

| Patient Name: **Biruk Alemnew**. Patient ID: **Enjibara GH**. SEX/ Age: M/1 7/12. Date of Report: 20/**07/2013**.  BP: _______ Weight: ______ Height: ____________ BSA: ________. **R.Dx: _____. AGH10.744** | | | |
| --- | --- | --- | --- |
| **Features** | **Finding** | **Features** | **Finding** |
| **Profile** |  | **Atria** |  |
| Abdominal situs | Solitus | Left atrium | Normal |
| Cardiac position | Levocardia | Right atrium | Mildly Dilated |
| Systemic venous drainage | Normal. | **Atrioventricular valves** |  |
| Pulmonary venous drainage | Normal | Mitral valve | Annulus = 13mm |
| Atrioventricular connection | Concordant | Tricuspid valve | Annulus = 14mm |
| Ventriculoarterial connection | Concordant | **Ventricles** |  |
| Ventricular loop | d-Loop | Left ventricle | Normal |
|  |  | Right ventricle | Mildly Dilated |
| **Septae** |  | **Coronary arteries** | ----- |
| Interventricular septum | Intact | **Doppler Measurement** |  |
| Interatrial septum | Intact | Mitral | ----- |
| **Semilunal valves** |  | Aortic | ------- |
| Aortic valve | Annulus = 12mm | Tricuspid | ------- |
| Pulmonary valve | Annulus = 11mm | pulmonic | Valvar PS, PPG = 36mmHg |
| **Great arteries** | NRGA | **Aortic arch** | Left |
| Aorta | ----- | **PDA** | No |
| Pulmonary artery | Normal MPA and Branch PAs. |  |  |
| **M-Mode:** Normal LV Function on eye balling. | | | |
| AO | mm | PWd | mm |
| LA | mm | EDV | ml |
| LVIDd | mm | ESV | ml |
| LVIDs | mm | LVEF | % |
| IVSd | mm | FS | % |
| **Additional Information**: |  | | |
| No pericardial/Pleural effusion. | | | |
| **Final Diagnosis:** | | | |
| 1. {S, D, S} Levocardia. 2. Mild Valvar PS | | | |
| **Remark**: Child was crying through out Study. | | | |
| **Recommendation**: | | | |
| SIGNATURE  Done by: Tesfaye T., Pediatrician, Pediatric Cardiologist _______________ 20/07/2013Eth.C | | | |

| Patient Name: **Birhanu Alehegn**. Patient ID: **FHRH**. SEX/ Age: M/7Years. Date of Report: 21/**07/2013**.  BP: _______ Weight: ______ Height: ____________ BSA: ________. R.Dx: **ARF + Murmur. AGH10.745** | | | |
| --- | --- | --- | --- |
| **Features** | **Finding** | **Features** | **Finding** |
| **Profile** |  | **Atria** |  |
| Abdominal situs | Solitus | Left atrium | Normal |
| Cardiac position | Levocardia | Right atrium | Normal |
| Systemic venous drainage | Normal. | **Atrioventricular valves** |  |
| Pulmonary venous drainage | Normal | Mitral valve | Annulus = 15mm. mildly Thickened MVL. |
| Atrioventricular connection | Concordant | Tricuspid valve | Annulus = 17mm |
| Ventriculoarterial connection | Concordant | **Ventricles** |  |
| Ventricular loop | d-Loop | Left ventricle | Normal |
|  |  | Right ventricle | Normal |
| **Septae** |  | **Coronary arteries** | ----- |
| Interventricular septum | Intact | **Doppler Measurement** |  |
| Interatrial septum | Intact | Mitral | Mild MR, Holosystolic, seen in two planes, posterior projection with jet velocity = 4.1m/sec |
| **Semilunal valves** |  | Aortic | ------- |
| Aortic valve | Annulus = 15mm | Tricuspid | ------- |
| Pulmonary valve | Annulus = 16mm | pulmonic | -------- |
| **Great arteries** | NRGA | **Aortic arch** | Left |
| Aorta | ----- | **PDA** | No |
| Pulmonary artery | Normal MPA and Branch PAs. |  |  |
| **M-Mode:** | | | |
| AO | mm | PWd | mm |
| LA | mm | EDV | ml |
| LVIDd | mm | ESV | ml |
| LVIDs | mm | LVEF | 55% |
| IVSd | mm | FS | 28% |
| **Additional Information**: |  | | |
| No pericardial/Pleural effusion. | | | |
| **Final Diagnosis:** | | | |
| 1. {S, D, S} Levocardia. 2. Mildly Thickened MVL 3. Mild MR 4. Normal Function | | | |
| **Remark**: | | | |
| **Recommendation**: | | | |
| SIGNATURE  Done by: Tesfaye T., Pediatrician, Pediatric Cardiologist _______________ 21/07/2013Eth.C | | | |

| Patient Name: **Zemenay Nigus**. Patient ID: **Adinas GH**. SEX/ Age: F/11Years. Date of Report: 21/**07/2013**.  BP: _______ Weight: ______ Height: ____________ BSA: ________. R.Dx: **Palpitation. AGH10.746** | | | |
| --- | --- | --- | --- |
| **Features** | **Finding** | **Features** | **Finding** |
| **Profile** |  | **Atria** |  |
| Abdominal situs | Solitus | Left atrium | Normal |
| Cardiac position | Levocardia | Right atrium | Normal |
| Systemic venous drainage | Normal. | **Atrioventricular valves** |  |
| Pulmonary venous drainage | Normal | Mitral valve | Annulus = 18mm |
| Atrioventricular connection | Concordant | Tricuspid valve | Annulus = 21mm  TAPSE = 22mm |
| Ventriculoarterial connection | Concordant | **Ventricles** |  |
| Ventricular loop | d-Loop | Left ventricle | Normal |
|  |  | Right ventricle | Normal |
| **Septae** |  | **Coronary arteries** | ----- |
| Interventricular septum | Intact | **Doppler Measurement** |  |
| Interatrial septum | Intact | Mitral | ----- |
| **Semilunal valves** |  | Aortic | ------- |
| Aortic valve | Annulus = 16mm | Tricuspid | ------- |
| Pulmonary valve | Annulus = 17mm | pulmonic | -------- |
| **Great arteries** | NRGA | **Aortic arch** | Left |
| Aorta | ----- | **PDA** | No |
| Pulmonary artery | Normal MPA and Branch PAs. |  |  |
| **M-Mode:** | | | |
| AO | mm | PWd | mm |
| LA | mm | EDV | ml |
| LVIDd | mm | ESV | ml |
| LVIDs | mm | LVEF | 65% |
| IVSd | mm | FS | 35% |
| **Additional Information**: |  | | |
| No pericardial/Pleural effusion. | | | |
| **Final Diagnosis:** | | | |
| 1. Normal Echocardiography Study. | | | |
| **Remark**: | | | |
| **Recommendation**: | | | |
| SIGNATURE  Done by: Tesfaye T., Pediatrician, Pediatric Cardiologist _______________ 21/07/2013Eth.C | | | |

| Patient Name: **Esubalew Yeshiwas**. Patient ID: **TGSH**. SEX/ Age: M/11Years. Date of Report: 22/**07/2013**.  BP: _______ Weight: ______ Height: ____________ BSA: ________. R.Dx: **Pre-Op screening. AGH10.747** | | | |
| --- | --- | --- | --- |
| **Features** | **Finding** | **Features** | **Finding** |
| **Profile** |  | **Atria** |  |
| Abdominal situs | Solitus | Left atrium | Normal |
| Cardiac position | Levocardia | Right atrium | Normal |
| Systemic venous drainage | Normal. | **Atrioventricular valves** |  |
| Pulmonary venous drainage | Normal | Mitral valve | Annulus = 25mm |
| Atrioventricular connection | Concordant | Tricuspid valve | Annulus = 25mm  TAPSE = 23mm |
| Ventriculoarterial connection | Concordant | **Ventricles** |  |
| Ventricular loop | d-Loop | Left ventricle | Normal |
|  |  | Right ventricle | Normal |
| **Septae** |  | **Coronary arteries** | ----- |
| Interventricular septum | Intact | **Doppler Measurement** |  |
| Interatrial septum | Intact | Mitral | ----- |
| **Semilunal valves** |  | Aortic | ------- |
| Aortic valve | Annulus = 18mm | Tricuspid | ------- |
| Pulmonary valve | Annulus = 19mm | pulmonic | -------- |
| **Great arteries** | NRGA | **Aortic arch** | Left |
| Aorta | ----- | **PDA** | No |
| Pulmonary artery | Normal MPA and Branch PAs. |  |  |
| **M-Mode:** | | | |
| AO | mm | PWd | mm |
| LA | mm | EDV | ml |
| LVIDd | mm | ESV | ml |
| LVIDs | mm | LVEF | 59% |
| IVSd | mm | FS | 32% |
| **Additional Information**: |  | | |
| No pericardial/Pleural effusion. | | | |
| **Final Diagnosis:** | | | |
| 1. Normal Echocardiography Study. | | | |
| **Remark**: | | | |
| **Recommendation**: | | | |
| SIGNATURE  Done by: Tesfaye T., Pediatrician, Pediatric Cardiologist _______________ 22/07/2013Eth.C | | | |

| Patient Name: **Tamiru Gizachew**. Patient ID: **Dangila PH**. SEX/ Age: M/1Year. Date of Report: 22/**07/2013**.  BP: _______ Weight: ______ Height: ____________ BSA: ________. R.Dx: **DS. AGH10.748** | | | |
| --- | --- | --- | --- |
| **Features** | **Finding** | **Features** | **Finding** |
| **Profile** |  | **Atria** |  |
| Abdominal situs | Solitus | Left atrium | Normal |
| Cardiac position | Levocardia | Right atrium | Normal |
| Systemic venous drainage | Normal. | **Atrioventricular valves** |  |
| Pulmonary venous drainage | Normal | Mitral valve | Annulus = 11mm |
| Atrioventricular connection | Concordant | Tricuspid valve | Annulus = 12mm |
| Ventriculoarterial connection | Concordant | **Ventricles** |  |
| Ventricular loop | d-Loop | Left ventricle | Normal |
|  |  | Right ventricle | Normal |
| **Septae** |  | **Coronary arteries** | ----- |
| Interventricular septum | Intact | **Doppler Measurement** |  |
| Interatrial septum | PFO, L – R Shunt | Mitral | ----- |
| **Semilunal valves** |  | Aortic | ------- |
| Aortic valve | Annulus = 11mm | Tricuspid | ------- |
| Pulmonary valve | Annulus = 12mm | pulmonic | -------- |
| **Great arteries** | NRGA | **Aortic arch** | Left |
| Aorta | ----- | **PDA** | No |
| Pulmonary artery | Normal MPA and Branch PAs. |  |  |
| **M-Mode:** Normal LV Function on eye balling. | | | |
| AO | mm | PWd | mm |
| LA | mm | EDV | ml |
| LVIDd | mm | ESV | ml |
| LVIDs | mm | LVEF | % |
| IVSd | mm | FS | % |
| **Additional Information**: |  | | |
| No pericardial/Pleural effusion. | | | |
| **Final Diagnosis:** | | | |
| 1. {S, D, S} Levocardia. 2. PFO, L – R Shunt | | | |
| **Remark**: | | | |
| **Recommendation**: | | | |
| SIGNATURE  Done by: Tesfaye T., Pediatrician, Pediatric Cardiologist _______________ 22/07/2013Eth.C | | | |

| Patient Name: **Gedefaw Wetetu**. Patient ID: **Merawi PH**. SEX/ Age: M/7Years. Date of Report: 23/**07/2013**.  BP: ____ Weight: ____ Height: _____ BSA: _____. R.Dx: **Cyanosis + Clubbing + Stroke. AGH10.749** | | | |
| --- | --- | --- | --- |
| **Features** | **Finding** | **Features** | **Finding** |
| **Profile** |  | **Atria** |  |
| Abdominal situs | Solitus | Left atrium | Normal |
| Cardiac position | Levocardia | Right atrium | DILATED |
| Systemic venous drainage | Normal. | **Atrioventricular valves** |  |
| Pulmonary venous drainage | Normal | Mitral valve | Annulus = 14mm |
| Atrioventricular connection | Concordant | Tricuspid valve | Annulus = 18mm  TAPSE = 15mm |
| Ventriculoarterial connection | Concordant | **Ventricles** |  |
| Ventricular loop | d-Loop | Left ventricle | Normal |
|  |  | Right ventricle | DILATED |
| **Septae** |  | **Coronary arteries** | ----- |
| Interventricular septum | Mal Aligned sub Aortic VSD, R – L Shunt | **Doppler Measurement** |  |
| Interatrial septum | Intact | Mitral | ----- |
| **Semilunal valves** |  | Aortic | ------- |
| Aortic valve | Annulus = 16mm | Tricuspid | ------- |
| Pulmonary valve | Annulus = 10mm | pulmonic | Severe PS, PPG = 89mmHg |
| **Great arteries** | NRGA | **Aortic arch** | Left |
| Aorta | ----- | **PDA** | No |
| Pulmonary artery | Normal MPA and Branch PAs. |  |  |
| **M-Mode:** Normal LV Function on eye balling. | | | |
| AO | mm | PWd | mm |
| LA | mm | EDV | ml |
| LVIDd | mm | ESV | ml |
| LVIDs | mm | LVEF | % |
| IVSd | mm | FS | % |
| **Additional Information**: |  | | |
| No pericardial/Pleural effusion. | | | |
| **Final Diagnosis:** | | | |
| 1. {S, D, S} Levocardia. 2. TOF | | | |
| **Remark**: | | | |
| **Recommendation**: | | | |
| SIGNATURE  Done by: Tesfaye T., Pediatrician, Pediatric Cardiologist _______________ 23/07/2013Eth.C | | | |

| Patient Name: **Baby of Birtukan Setegn**. Patient ID: **FHRH**. SEX/ Age: M/25days. Date of Report: 23/**07/2013**.  BP: _______ Weight: ______ Height: ____________ BSA: ________. R.Dx: **RD. AGH10.750** | | | |
| --- | --- | --- | --- |
| **Features** | **Finding** | **Features** | **Finding** |
| **Profile** |  | **Atria** |  |
| Abdominal situs | Solitus | Left atrium | Normal |
| Cardiac position | Levocardia | Right atrium | Normal |
| Systemic venous drainage | Normal. | **Atrioventricular valves** |  |
| Pulmonary venous drainage | Normal | Mitral valve | Annulus = 10mm |
| Atrioventricular connection | Concordant | Tricuspid valve | Annulus = 10mm |
| Ventriculoarterial connection | Concordant | **Ventricles** |  |
| Ventricular loop | d-Loop | Left ventricle | Normal |
|  |  | Right ventricle | Normal |
| **Septae** |  | **Coronary arteries** | ----- |
| Interventricular septum | Intact | **Doppler Measurement** |  |
| Interatrial septum | Intact | Mitral | ----- |
| **Semilunal valves** |  | Aortic | ------- |
| Aortic valve | Annulus = 10mm | Tricuspid | ------- |
| Pulmonary valve | Annulus = 9mm | pulmonic | -------- |
| **Great arteries** | NRGA | **Aortic arch** | Left |
| Aorta | ----- | **PDA** | No |
| Pulmonary artery | Normal MPA and Branch PAs. |  |  |
| **M-Mode:** Normal LV Function on eye balling. | | | |
| AO | mm | PWd | mm |
| LA | mm | EDV | ml |
| LVIDd | mm | ESV | ml |
| LVIDs | mm | LVEF | % |
| IVSd | mm | FS | % |
| **Additional Information**: |  | | |
| No pericardial/Pleural effusion. | | | |
| **Final Diagnosis:** | | | |
| 1. Normal Echocardiography Study. | | | |
| **Remark**: | | | |
| **Recommendation**: | | | |
| SIGNATURE  Done by: Tesfaye T., Pediatrician, Pediatric Cardiologist _______________ 23/07/2013Eth.C | | | |

| Patient Name: **Kidus Grace**. Patient ID: **Afilas GH**. SEX/ Age: M/3 5/12. Date of Report: 23/**07/2013**.  BP: _______ Weight: ______ Height: ____________ BSA: ________. **R.Dx: FTT. AGH10.751** | | | |
| --- | --- | --- | --- |
| **Features** | **Finding** | **Features** | **Finding** |
| **Profile** |  | **Atria** |  |
| Abdominal situs | Solitus | Left atrium | Normal |
| Cardiac position | Levocardia | Right atrium | Normal |
| Systemic venous drainage | Normal. | **Atrioventricular valves** |  |
| Pulmonary venous drainage | Normal | Mitral valve | Annulus = 16mm |
| Atrioventricular connection | Concordant | Tricuspid valve | Annulus = 16mm  TAPSE = 17mm |
| Ventriculoarterial connection | Concordant | **Ventricles** |  |
| Ventricular loop | d-Loop | Left ventricle | Normal |
|  |  | Right ventricle | Normal |
| **Septae** |  | **Coronary arteries** | ----- |
| Interventricular septum | Intact | **Doppler Measurement** |  |
| Interatrial septum | Intact | Mitral | ----- |
| **Semilunal valves** |  | Aortic | ------- |
| Aortic valve | Annulus = 13mm | Tricuspid | ------- |
| Pulmonary valve | Annulus = 16mm | pulmonic | -------- |
| **Great arteries** | NRGA | **Aortic arch** | Left |
| Aorta | ----- | **PDA** | No |
| Pulmonary artery | Normal MPA and Branch PAs. |  |  |
| **M-Mode:** | | | |
| AO | mm | PWd | mm |
| LA | mm | EDV | ml |
| LVIDd | mm | ESV | ml |
| LVIDs | mm | LVEF | 73% |
| IVSd | mm | FS | 40% |
| **Additional Information**: |  | | |
| No pericardial/Pleural effusion. | | | |
| **Final Diagnosis:** | | | |
| 1. Normal Echocardiography Study. | | | |
| **Remark**: | | | |
| **Recommendation**: | | | |
| SIGNATURE  Done by: Tesfaye T., Pediatrician, Pediatric Cardiologist _______________ 23/07/2013Eth.C | | | |

| Patient Name: **Baby of Alemnesh Alehegn**. Patient ID: **FHRH**. SEX/ Age: F/4/12. Date of Report: 23/**07/2013**.  BP: _______ Weight: ______ Height: ____________ BSA: ________. R.Dx: **____. AGH10.752** | | | |
| --- | --- | --- | --- |
| **Follow up Echo** | Small PDA, small ASD (23/03/2013, 8days/F, AGH6) DS | | |
| **Features** | **Finding** | **Features** | **Finding** |
| **Profile** |  | **Atria** |  |
| Abdominal situs | Solitus | Left atrium | Normal |
| Cardiac position | Levocardia | Right atrium | Normal |
| Systemic venous drainage | Normal. | **Atrioventricular valves** |  |
| Pulmonary venous drainage | Normal | Mitral valve | Annulus = 11mm |
| Atrioventricular connection | Concordant | Tricuspid valve | Annulus = 10mm |
| Ventriculoarterial connection | Concordant | **Ventricles** |  |
| Ventricular loop | d-Loop | Left ventricle | Normal |
|  |  | Right ventricle | Normal |
| **Septae** |  | **Coronary arteries** | ----- |
| Interventricular septum | Intact | **Doppler Measurement** |  |
| Interatrial septum | Intact | Mitral | ----- |
| **Semilunal valves** |  | Aortic | ------- |
| Aortic valve | Annulus = 11mm | Tricuspid | ------- |
| Pulmonary valve | Annulus = 11mm | pulmonic | -------- |
| **Great arteries** | NRGA | **Aortic arch** | Left |
| Aorta | ----- | **PDA** | No |
| Pulmonary artery | Normal MPA and Branch PAs. |  |  |
| **M-Mode:** Normal LV Function on eye balling. | | | |
| AO | mm | PWd | mm |
| LA | mm | EDV | ml |
| LVIDd | mm | ESV | ml |
| LVIDs | mm | LVEF | % |
| IVSd | mm | FS | % |
| **Additional Information**: |  | | |
| No pericardial/Pleural effusion. | | | |
| **Final Diagnosis:** | | | |
| 1. Normal Echocardiography Study. | | | |
| **Remark**: | | | |
| **Recommendation**: | | | |
| SIGNATURE  Done by: Tesfaye T., Pediatrician, Pediatric Cardiologist _______________ 23/07/2013Eth.C | | | |

| Patient Name: **Baby of Fire weini Tasew**. Patient ID: **FHRH**. SEX/ Age: M/7days. Date of Report: 23/**07/2013**.  BP: _______ Weight: ______ Height: ____________ BSA: ________. R.Dx: **DS. AGH10.753** | | | |
| --- | --- | --- | --- |
| **Features** | **Finding** | **Features** | **Finding** |
| **Profile** |  | **Atria** |  |
| Abdominal situs | Solitus | Left atrium | Normal |
| Cardiac position | Levocardia | Right atrium | Normal |
| Systemic venous drainage | Normal. | **Atrioventricular valves** |  |
| Pulmonary venous drainage | Normal | Mitral valve | Annulus = 11mm |
| Atrioventricular connection | Concordant | Tricuspid valve | Annulus = 11mm |
| Ventriculoarterial connection | Concordant | **Ventricles** |  |
| Ventricular loop | d-Loop | Left ventricle | Normal |
|  |  | Right ventricle | Normal |
| **Septae** |  | **Coronary arteries** | ----- |
| Interventricular septum | Intact | **Doppler Measurement** |  |
| Interatrial septum | 5mm X 5mm OS ASD, L – R Shunt | Mitral | ----- |
| **Semilunal valves** |  | Aortic | ------- |
| Aortic valve | Annulus = 9mm | Tricuspid | ------- |
| Pulmonary valve | Annulus = 10mm | pulmonic | -------- |
| **Great arteries** | NRGA | **Aortic arch** | Left |
| Aorta | ----- | **PDA** | No |
| Pulmonary artery | Normal MPA and Branch PAs. |  |  |
| **M-Mode:** | | | |
| AO | mm | PWd | mm |
| LA | mm | EDV | ml |
| LVIDd | mm | ESV | ml |
| LVIDs | mm | LVEF | % |
| IVSd | mm | FS | % |
| **Additional Information**: |  | | |
| No pericardial/Pleural effusion. | | | |
| **Final Diagnosis:** | | | |
| 1. {S, D, S} Levocardia. 2. Small OS ASD, L – R Shunt | | | |
| **Remark**: | | | |
| **Recommendation**: | | | |
| SIGNATURE  Done by: Tesfaye T., Pediatrician, Pediatric Cardiologist _______________ 23/07/2013Eth.C | | | |

| Patient Name: **Melkam Nega**. Patient ID: **Finote Selam GH**. SEX/ Age: F/12Years. Date of Report: 24/**07/2013**.  BP: _____ Weight: ____ Height: _____ BSA: ____. R.Dx: **DOE + Easy fatigability + Murmur. AGH10.754** | | | |
| --- | --- | --- | --- |
| **Features** | **Finding** | **Features** | **Finding** |
| **Profile** |  | **Atria** |  |
| Abdominal situs | Solitus | Left atrium | Normal |
| Cardiac position | Levocardia | Right atrium | Dilated |
| Systemic venous drainage | Normal. | **Atrioventricular valves** |  |
| Pulmonary venous drainage | Normal | Mitral valve | Annulus = 17mm |
| Atrioventricular connection | Concordant | Tricuspid valve | Annulus = 19mm  TAPSE = 15mm |
| Ventriculoarterial connection | Concordant | **Ventricles** |  |
| Ventricular loop | d-Loop | Left ventricle | Normal |
|  |  | Right ventricle | Hypertrophied, Dilated |
| **Septae** |  | **Coronary arteries** | ----- |
| Interventricular septum | Intact | **Doppler Measurement** |  |
| Interatrial septum | Intact | Mitral | ----- |
| **Semilunal valves** |  | Aortic | ------- |
| Aortic valve | Annulus = 16mm | Tricuspid | Mild MR |
| Pulmonary valve | Annulus = 14mm | pulmonic | Severe PS, PPG = 128mmHg. |
| **Great arteries** | NRGA | **Aortic arch** | Left |
| Aorta | ----- | **PDA** | No |
| Pulmonary artery | Mildly dilated MPA. |  |  |
| **M-Mode:** | | | |
| AO | mm | PWd | mm |
| LA | mm | EDV | ml |
| LVIDd | mm | ESV | ml |
| LVIDs | mm | LVEF | % |
| IVSd | mm | FS | % |
| **Additional Information**: |  | | |
| No pericardial/Pleural effusion. | | | |
| **Final Diagnosis:** | | | |
| 1. {S, D, S} Levocardia. 2. Severe PS 3. Dilated, Hypertrophied Dysfunctional RV 4. Normal LV Function | | | |
| **Remark**: | | | |
| **Recommendation**: Needs urgent RVOTO Release. | | | |
| SIGNATURE  Done by: Tesfaye T., Pediatrician, Pediatric Cardiologist _______________ 24/07/2013Eth.C | | | |

| Patient Name: **Nardos Ashenafi**. Patient ID: **TGSH**. SEX/ Age: F/1 8/12. Date of Report: 24/**07/2013**.  BP: _______ Weight: ______ Height: _______ BSA: ______. R.Dx: **Recurrent Chest Infectio. AGH10.755** | | | |
| --- | --- | --- | --- |
| **Features** | **Finding** | **Features** | **Finding** |
| **Profile** |  | **Atria** |  |
| Abdominal situs | Solitus | Left atrium | Normal |
| Cardiac position | Levocardia | Right atrium | Normal |
| Systemic venous drainage | Normal. | **Atrioventricular valves** |  |
| Pulmonary venous drainage | Normal | Mitral valve | Annulus = 13mm |
| Atrioventricular connection | Concordant | Tricuspid valve | Annulus = 13mm |
| Ventriculoarterial connection | Concordant | **Ventricles** |  |
| Ventricular loop | d-Loop | Left ventricle | Normal |
|  |  | Right ventricle | Normal |
| **Septae** |  | **Coronary arteries** | ----- |
| Interventricular septum | Intact | **Doppler Measurement** |  |
| Interatrial septum | Intact | Mitral | ----- |
| **Semilunal valves** |  | Aortic | ------- |
| Aortic valve | Annulus = 12mm | Tricuspid | ------- |
| Pulmonary valve | Annulus = 11mm | pulmonic | -------- |
| **Great arteries** | NRGA | **Aortic arch** | Left |
| Aorta | ----- | **PDA** | No |
| Pulmonary artery | Normal MPA and Branch PAs. |  |  |
| **M-Mode:** Normal LV Function on eye balling. | | | |
| AO | mm | PWd | mm |
| LA | mm | EDV | ml |
| LVIDd | mm | ESV | ml |
| LVIDs | mm | LVEF | % |
| IVSd | mm | FS | % |
| **Additional Information**: |  | | |
| No pericardial/Pleural effusion. | | | |
| **Final Diagnosis:** | | | |
| 1. Normal Echocardiography Study. | | | |
| **Remark**: Only subcostal window. | | | |
| **Recommendation**: | | | |
| SIGNATURE  Done by: Tesfaye T., Pediatrician, Pediatric Cardiologist _______________ 24/07/2013Eth.C | | | |

| Patient Name: **Shalom Gizachew**. Patient ID: **FHRH**. SEX/ Age: F/8Years. Date of Report: 25/**07/2013**.  BP: _______ Weight: ______ Height: ____________ BSA:_____. R.Dx: **Murmur + FTT . AGH10.756** | | | |
| --- | --- | --- | --- |
| **Features** | **Finding** | **Features** | **Finding** |
| **Profile** |  | **Atria** |  |
| Abdominal situs | Solitus | Left atrium | Normal |
| Cardiac position | Levocardia | Right atrium | Normal |
| Systemic venous drainage | Normal. | **Atrioventricular valves** |  |
| Pulmonary venous drainage | Normal | Mitral valve | Annulus = 18mm |
| Atrioventricular connection | Concordant | Tricuspid valve | Annulus = 18mm  TAPSE = 16mm |
| Ventriculoarterial connection | Concordant | **Ventricles** |  |
| Ventricular loop | d-Loop | Left ventricle | Normal |
|  |  | Right ventricle | Normal |
| **Septae** |  | **Coronary arteries** | ----- |
| Interventricular septum | Large VSD amounting to single Ventricle (No IVS seen) (Inlet | **Doppler Measurement** |  |
| Interatrial septum | Intact | Mitral | ----- |
| **Semilunal valves** |  | Aortic | ------- |
| Aortic valve | Annulus = 18mm | Tricuspid | ------- |
| Pulmonary valve | Annulus = 13mm | pulmonic | Moderate PS, PPG = 48mmHg |
| **Great arteries** | NRGA | **Aortic arch** | Left |
| Aorta | ----- | **PDA** | No |
| Pulmonary artery | Normal MPA and Branch PAs. |  |  |
| **M-Mode:** | | | |
| AO | mm | PWd | mm |
| LA | mm | EDV | ml |
| LVIDd | mm | ESV | ml |
| LVIDs | mm | LVEF | % |
| IVSd | mm | FS | % |
| **Additional Information**: |  | | |
| No pericardial/Pleural effusion. | | | |
| **Final Diagnosis:** | | | |
| 1. {S, D, S} Levocardia. 2. Large VSD amounting to single ventricle (No IVS) 3. Moderate PS | | | |
| **Remark**: | | | |
| **Recommendation**: | | | |
| SIGNATURE  Done by: Tesfaye T., Pediatrician, Pediatric Cardiologist _______________ 25/07/2013Eth.C | | | |

| Patient Name: **Demelash Tilahun**. Patient ID: **FHRH**. SEX/ Age: M/5Years. Date of Report: 27/**07/2013**.  BP: _______ Weight: ______ Height: ____________ BSA: ________. R.Dx: **CHF + RD. AGH10.757** | | | |
| --- | --- | --- | --- |
| **Features** | **Finding** | **Features** | **Finding** |
| **Profile** |  | **Atria** |  |
| Abdominal situs | Solitus | Left atrium | More dilated |
| Cardiac position | Levocardia | Right atrium | Dilated |
| Systemic venous drainage | Normal. | **Atrioventricular valves** |  |
| Pulmonary venous drainage | Normal | Mitral valve | Annulus = 25mm |
| Atrioventricular connection | Concordant | Tricuspid valve | Annulus = 23mm  TAPSE = 26mm |
| Ventriculoarterial connection | Concordant | **Ventricles** |  |
| Ventricular loop | d-Loop | Left ventricle | More dilated |
|  |  | Right ventricle | Dilated |
| **Septae** |  | **Coronary arteries** | ----- |
| Interventricular septum | 30mm Inlet VSD, L – R Shunt | **Doppler Measurement** |  |
| Interatrial septum | Intact | Mitral | Moderate MR, Holosystolic, posterior projection, seen in two planes with jet velocity = 5.5m/sec |
| **Semilunal valves** |  | Aortic | ------- |
| Aortic valve | Annulus = 12mm | Tricuspid | ------- |
| Pulmonary valve | Annulus = 25mm | pulmonic | Moderate PR, PPG = 70mmHg |
| **Great arteries** | NRGA | **Aortic arch** | Left |
| Aorta | ----- | **PDA** | No |
| Pulmonary artery | MPA =33mm. |  |  |
| **M-Mode:** | | | |
| AO | mm | PWd | mm |
| LA | mm | EDV | ml |
| LVIDd | mm | ESV | ml |
| LVIDs | mm | LVEF | 65% |
| IVSd | mm | FS | 35% |
| **Additional Information**: |  | | |
| 5mmpericardial effusion on RA Side. | | | |
| **Final Diagnosis:** | | | |
| 1. {S, D, S} Levocardia. 2. All chambers dilated 3. Large Inlet VSD, L – R Shunt 4. Moderate MR 5. Moderate PR 6. Severe Pulmonary Hypertension 7. Small Pericardial effusion 8. Normal Biventricular Function | | | |
| SIGNATURE  Done by: Tesfaye T., Pediatrician, Pediatric Cardiologist _______________ 27/07/2013Eth.C | | | |

| Patient Name: **Birhanu Kefale**. Patient ID: **FHRH**. SEX/ Age: M/9/12. Date of Report: 27/**07/2013**.  BP: _______ Weight: ______ Height: ____________ BSA: ________. R.Dx: **DS. AGH10.758** | | | |
| --- | --- | --- | --- |
| **Features** | **Finding** | **Features** | **Finding** |
| **Profile** |  | **Atria** |  |
| Abdominal situs | Solitus | Left atrium | Normal |
| Cardiac position | Levocardia | Right atrium | Dilated |
| Systemic venous drainage | Normal. | **Atrioventricular valves** |  |
| Pulmonary venous drainage | Normal | Mitral valve | Common Complete AVSD, L – R Shunt |
| Atrioventricular connection | Common Complete AVSD | Tricuspid valve |  |
| Ventriculoarterial connection | Concordant | **Ventricles** |  |
| Ventricular loop | d-Loop | Left ventricle | Normal |
|  |  | Right ventricle | Dilated |
| **Septae** |  | **Coronary arteries** | ----- |
| Interventricular septum | Common Complete AVSD, L – R Shunt | **Doppler Measurement** |  |
| Interatrial septum |  | Mitral | ----- |
| **Semilunal valves** |  | Aortic | ------- |
| Aortic valve | Annulus = 11mm | Tricuspid | ------- |
| Pulmonary valve | Annulus = 12mm | pulmonic | -------- |
| **Great arteries** | NRGA | **Aortic arch** | Left |
| Aorta | ----- | **PDA** | No |
| Pulmonary artery | Normal MPA and Branch PAs. |  |  |
| **M-Mode:** Normal LV Function on eye balling. | | | |
| AO | mm | PWd | mm |
| LA | mm | EDV | ml |
| LVIDd | mm | ESV | ml |
| LVIDs | mm | LVEF | % |
| IVSd | mm | FS | % |
| **Additional Information**: |  | | |
| 5mm pericardial effusion on RA/RV Side. | | | |
| **Final Diagnosis:** | | | |
| 1. {S, D, S} Levocardia. 2. Common Complete Balanced AVSD, L – R Shunt 3. Normal Function | | | |
| **Remark**: | | | |
| **Recommendation**: | | | |
| SIGNATURE  Done by: Tesfaye T., Pediatrician, Pediatric Cardiologist _______________ 27/07/2013Eth.C | | | |

| Patient Name: **Tayu Kefale**. Patient ID: **Adinas GH**. SEX/ Age: M/12Years. Date of Report: 28/**07/2013**.  BP: ____ Weight: ___ Height: _____ BSA: _____. R.Dx: **Rheumatic Recurrence + Murmur + CHF. AGH10.759** | | | |
| --- | --- | --- | --- |
| **Features** | **Finding** | **Features** | **Finding** |
| **Profile** |  | **Atria** |  |
| Abdominal situs | Solitus | Left atrium | More dilated |
| Cardiac position | Levocardia | Right atrium | dilated |
| Systemic venous drainage | Normal. | **Atrioventricular valves** |  |
| Pulmonary venous drainage | Normal | Mitral valve | Annulus = 21mm. Thickened MVL |
| Atrioventricular connection | Concordant | Tricuspid valve | Annulus = 20mm  TAPSE = 17mm |
| Ventriculoarterial connection | Concordant | **Ventricles** |  |
| Ventricular loop | d-Loop | Left ventricle | More dilated |
|  |  | Right ventricle | Dilated |
| **Septae** |  | **Coronary arteries** | ----- |
| Interventricular septum | Intact | **Doppler Measurement** |  |
| Interatrial septum | Intact | Mitral | Severe MR, Holosystolic, posterior projection, seen in two planes with jet velocity = 5.2m/sec |
| **Semilunal valves** |  | Aortic | Moderate AR |
| Aortic valve | Annulus = 14mm | Tricuspid | Moderate TR, PPG = 49mmHg |
| Pulmonary valve | Annulus = 20mm | pulmonic | -------- |
| **Great arteries** | NRGA | **Aortic arch** | Left |
| Aorta | ----- | **PDA** | No |
| Pulmonary artery | Normal MPA and Branch PAs. |  |  |
| **M-Mode:** | | | |
| AO | mm | PWd | mm |
| LA | mm | EDV | ml |
| LVIDd | mm | ESV | ml |
| LVIDs | mm | LVEF | % |
| IVSd | mm | FS | % |
| **Additional Information**: |  | | |
| No pericardial/Pleural effusion. | | | |
| **Final Diagnosis:** | | | |
| 1. {S, D, S} Levocardia. 2. All chambers dilated 3. Severe MR 4. Moderate TR 5. Moderate AR 6. Moderate Pulmonary Hypertension 7. Reduced LV Function | | | |
| **Remark**: | | | |
| **Recommendation**: | | | |
| SIGNATURE  Done by: Tesfaye T., Pediatrician, Pediatric Cardiologist _______________ 28/07/2013Eth.C | | | |

| Patient Name: **Dawit Ayenew**. Patient ID: **FHRH**. SEX/ Age: M/7Years. Date of Report: 28/**07/2013**.  BP: _______ Weight: ______ Height: ______ BSA: ____. R.Dx: **Incidental Murmur + Palpitation. AGH10.760** | | | |
| --- | --- | --- | --- |
| **Follow up Echocardiography** | | | |
| **Features** | **Finding** | **Features** | **Finding** |
| **Profile** |  | **Atria** |  |
| Abdominal situs | Solitus | Left atrium | Normal |
| Cardiac position | Levocardia | Right atrium | Normal |
| Systemic venous drainage | Normal. | **Atrioventricular valves** |  |
| Pulmonary venous drainage | Normal | Mitral valve | Annulus = 20mm |
| Atrioventricular connection | Concordant | Tricuspid valve | Annulus = 22mm |
| Ventriculoarterial connection | Concordant | **Ventricles** |  |
| Ventricular loop | d-Loop | Left ventricle | Normal |
|  |  | Right ventricle | Normal |
| **Septae** |  | **Coronary arteries** | ----- |
| Interventricular septum | Intact | **Doppler Measurement** |  |
| Interatrial septum | Intact | Mitral | ----- |
| **Semilunal valves** |  | Aortic | Moderate AR, PHT = 325ms. |
| Aortic valve | Annulus = 18mm | Tricuspid | ------- |
| Pulmonary valve | Annulus = 18mm | pulmonic | -------- |
| **Great arteries** | NRGA | **Aortic arch** | Left |
| Aorta | ----- | **PDA** | No |
| Pulmonary artery | Normal MPA and Branch PAs. |  |  |
| **M-Mode:** Normal LV Function on eye balling. | | | |
| AO | mm | PWd | mm |
| LA | mm | EDV | ml |
| LVIDd | mm | ESV | ml |
| LVIDs | mm | LVEF | % |
| IVSd | mm | FS | % |
| **Additional Information**: |  | | |
| No pericardial/Pleural effusion. | | | |
| **Final Diagnosis:** | | | |
| 1. {S, D, S} Levocardia. 2. Moderate AR 3. Normal LV Function | | | |
| **Remark**: | | | |
| **Recommendation**: | | | |
| SIGNATURE  Done by: Tesfaye T., Pediatrician, Pediatric Cardiologist _______________ 28/07/2013Eth.C | | | |

| Patient Name: **Baby of Genet Mekonen**. Patient ID: **FHRH**. SEX/ Age: M/20days. Date of Report: 28/**07/2013**.  BP: _______ Weight: ______ Height: ____________ BSA: ________. R.Dx: **Cyanosis. AGH10.761** | | | |
| --- | --- | --- | --- |
| **Features** | **Finding** | **Features** | **Finding** |
| **Profile** |  | **Atria** |  |
| Abdominal situs | Solitus | Left atrium | Normal |
| Cardiac position | Levocardia | Right atrium | Normal |
| Systemic venous drainage | Normal. | **Atrioventricular valves** |  |
| Pulmonary venous drainage | Normal | Mitral valve | Annulus = 8mm |
| Atrioventricular connection | Concordant | Tricuspid valve | Annulus = 8mm |
| Ventriculoarterial connection | Concordant | **Ventricles** |  |
| Ventricular loop | d-Loop | Left ventricle | Normal |
|  |  | Right ventricle | Hypertrophied |
| **Septae** |  | **Coronary arteries** | ----- |
| Interventricular septum | Malaligned Subaortic VSD, R – L Shunt | **Doppler Measurement** |  |
| Interatrial septum | Intact | Mitral | ----- |
| **Semilunal valves** |  | Aortic | ------- |
| Aortic valve | Annulus = 7mm | Tricuspid | ------- |
| Pulmonary valve | Annulus = 6mm | pulmonic | Severe PS, PPG = 66mmHg |
| **Great arteries** | NRGA | **Aortic arch** | Left |
| Aorta | Overriding aorta | **PDA** | No |
| Pulmonary artery | Normal MPA and Branch PAs. |  |  |
| **M-Mode:** | | | |
| AO | mm | PWd | mm |
| LA | mm | EDV | ml |
| LVIDd | mm | ESV | ml |
| LVIDs | mm | LVEF | 72% |
| IVSd | mm | FS | 38% |
| **Additional Information**: |  | | |
| No pericardial/Pleural effusion. | | | |
| **Final Diagnosis:** | | | |
| 1. {S, D, S} Levocardia. 2. TOF | | | |
| **Remark**: | | | |
| **Recommendation**: | | | |
| SIGNATURE  Done by: Tesfaye T., Pediatrician, Pediatric Cardiologist _______________ 28/07/2013Eth.C | | | |

| Patient Name: **Silabat Dessie**. Patient ID: **Adiss Alem H**. SEX/ Age: M/9Years. Date of Report: 29/**07/2013**.  BP: _______ Weight: ______ Height: ____________ BSA: ________. R.Dx: **Easy Fatigability. AGH10.762** | | | |
| --- | --- | --- | --- |
| **Features** | **Finding** | **Features** | **Finding** |
| **Profile** |  | **Atria** |  |
| Abdominal situs | Solitus | Left atrium | Normal |
| Cardiac position | Levocardia | Right atrium | Normal |
| Systemic venous drainage | Normal. | **Atrioventricular valves** |  |
| Pulmonary venous drainage | Normal | Mitral valve | Annulus = 17mm |
| Atrioventricular connection | Concordant | Tricuspid valve | Annulus = 17mm  TAPSE = 19mm |
| Ventriculoarterial connection | Concordant | **Ventricles** |  |
| Ventricular loop | d-Loop | Left ventricle | Normal |
|  |  | Right ventricle | Normal |
| **Septae** |  | **Coronary arteries** | ----- |
| Interventricular septum | Intact | **Doppler Measurement** |  |
| Interatrial septum | Intact | Mitral | ----- |
| **Semilunal valves** |  | Aortic | ------- |
| Aortic valve | Annulus = 16mm | Tricuspid | ------- |
| Pulmonary valve | Annulus = 17mm | pulmonic | Trivial PR, PPG = 10mmHg |
| **Great arteries** | NRGA | **Aortic arch** | Left |
| Aorta | ----- | **PDA** | No |
| Pulmonary artery | Normal MPA and Branch PAs. |  |  |
| **M-Mode:** | | | |
| AO | mm | PWd | mm |
| LA | mm | EDV | ml |
| LVIDd | mm | ESV | ml |
| LVIDs | mm | LVEF | 57% |
| IVSd | mm | FS | 29% |
| **Additional Information**: |  | | |
| No pericardial/Pleural effusion. | | | |
| **Final Diagnosis:** | | | |
| 1. {S, D, S} Levocardia. 2. Normal Echocardiography Study | | | |
| **Remark**: | | | |
| **Recommendation**: | | | |
| SIGNATURE  Done by: Tesfaye T., Pediatrician, Pediatric Cardiologist _______________ 29/07/2013Eth.C | | | |

| Patient Name: **Ashebir Endeshaw**. Patient ID: **FHRH**. SEX/ Age: M/8Years. Date of Report: 29/**07/2013**.  BP: _______ Weight: ______ Height: ____________ BSA: ________. R.Dx: **Rheumatic Recurrence. AGH10.763** | | | |
| --- | --- | --- | --- |
| **Features** | **Finding** | **Features** | **Finding** |
| **Profile** |  | **Atria** |  |
| Abdominal situs | Solitus | Left atrium | Dilated |
| Cardiac position | Levocardia | Right atrium | Normal |
| Systemic venous drainage | Normal. | **Atrioventricular valves** |  |
| Pulmonary venous drainage | Normal | Mitral valve | Annulus = 20mm. Thickened MVL |
| Atrioventricular connection | Concordant | Tricuspid valve | Annulus = 20mm |
| Ventriculoarterial connection | Concordant | **Ventricles** |  |
| Ventricular loop | d-Loop | Left ventricle | Dilated |
|  |  | Right ventricle | Normal |
| **Septae** |  | **Coronary arteries** | ----- |
| Interventricular septum | Intact | **Doppler Measurement** |  |
| Interatrial septum | Intact | Mitral | Moderate MR, Holosystolic, posterior projection, seen in two planes with jet velocity = 5.2m/sec. mild MS, PPG/MPG = 8/5mmHg |
| **Semilunal valves** |  | Aortic | Mild AR, PHT = 522ms |
| Aortic valve | Annulus = 16mm | Tricuspid | ------- |
| Pulmonary valve | Annulus = 15mm | pulmonic | -------- |
| **Great arteries** | NRGA | **Aortic arch** | Left |
| Aorta | ----- | **PDA** | No |
| Pulmonary artery | Normal MPA and Branch PAs. |  |  |
| **M-Mode:** | | | |
| AO | mm | PWd | mm |
| LA | mm | EDV | ml |
| LVIDd | mm | ESV | ml |
| LVIDs | mm | LVEF | 66% |
| IVSd | mm | FS | 36% |
| **Additional Information**: |  | | |
| 8mm pericardial effusion on RA Side. | | | |
| **Final Diagnosis:** | | | |
| 1. {S, D, S} Levocardia. 2. LA/LV Dilated 3. Thickened MVL 4. Moderate MR 5. Mild MS (Increased blood flow) 6. Mild AR 7. Normal LV Function | | | |
| SIGNATURE  Done by: Tesfaye T., Pediatrician, Pediatric Cardiologist _______________ 29/07/2013Eth.C | | | |

| Patient Name: **Mohamed amin Yahya**. Patient ID: **Adinas GH**. SEX/ Age: M/12Years. Date of Report: 29/**07/2013**.  BP: _______ Weight: ______ Height: ____________ BSA: ________. R.Dx: **Easy Fatigability. AGH10.764** | | | |
| --- | --- | --- | --- |
| **Features** | **Finding** | **Features** | **Finding** |
| **Profile** |  | **Atria** |  |
| Abdominal situs | Solitus | Left atrium | Normal |
| Cardiac position | Levocardia | Right atrium | Normal |
| Systemic venous drainage | Normal. | **Atrioventricular valves** |  |
| Pulmonary venous drainage | Normal | Mitral valve | Annulus = 18mm |
| Atrioventricular connection | Concordant | Tricuspid valve | Annulus = 20mm  TAPSE = 24mm |
| Ventriculoarterial connection | Concordant | **Ventricles** |  |
| Ventricular loop | d-Loop | Left ventricle | Normal |
|  |  | Right ventricle | Normal |
| **Septae** |  | **Coronary arteries** | ----- |
| Interventricular septum | Intact | **Doppler Measurement** |  |
| Interatrial septum | Intact | Mitral | ----- |
| **Semilunal valves** |  | Aortic | ------- |
| Aortic valve | Annulus = 18mm | Tricuspid | Trivial TR, Incomplete signal, PPG = 21mmHg |
| Pulmonary valve | Annulus = 21mm | pulmonic | -------- |
| **Great arteries** | NRGA | **Aortic arch** | Left |
| Aorta | ----- | **PDA** | No |
| Pulmonary artery | Normal MPA and Branch PAs. |  |  |
| **M-Mode:** | | | |
| AO | mm | PWd | mm |
| LA | mm | EDV | ml |
| LVIDd | mm | ESV | ml |
| LVIDs | mm | LVEF | 67% |
| IVSd | mm | FS | 36% |
| **Additional Information**: |  | | |
| No pericardial/Pleural effusion. | | | |
| **Final Diagnosis:** | | | |
| 1. Normal Echocardiography Study. | | | |
| **Remark**: | | | |
| **Recommendation**: | | | |
| SIGNATURE  Done by: Tesfaye T., Pediatrician, Pediatric Cardiologist _______________ 29/07/2013Eth.C | | | |

| Patient Name: **Elsabet Yihunie**. Patient ID: **Adinas GH**. SEX/ Age: F/11Years. Date of Report: 29/**07/2013**.  BP: _______ Weight: ______ Height: ____________ BSA: ________. R.Dx: **Easy fatigability. AGH10.765** | | | |
| --- | --- | --- | --- |
| **Features** | **Finding** | **Features** | **Finding** |
| **Profile** |  | **Atria** |  |
| Abdominal situs | Solitus | Left atrium | Normal |
| Cardiac position | Levocardia | Right atrium | Normal |
| Systemic venous drainage | Normal. | **Atrioventricular valves** |  |
| Pulmonary venous drainage | Normal | Mitral valve | Annulus = 21mm |
| Atrioventricular connection | Concordant | Tricuspid valve | Annulus = 21mm |
| Ventriculoarterial connection | Concordant | **Ventricles** |  |
| Ventricular loop | d-Loop | Left ventricle | Normal |
|  |  | Right ventricle | Normal |
| **Septae** |  | **Coronary arteries** | ----- |
| Interventricular septum | Intact | **Doppler Measurement** |  |
| Interatrial septum | Intact | Mitral | ----- |
| **Semilunal valves** |  | Aortic | ------- |
| Aortic valve | Annulus = 17mm | Tricuspid | ------- |
| Pulmonary valve | Annulus = 20mm | pulmonic | -------- |
| **Great arteries** | NRGA | **Aortic arch** | Left |
| Aorta | ----- | **PDA** | <1mm PDA, L – R Shunt |
| Pulmonary artery | Normal MPA and Branch PAs. |  |  |
| **M-Mode:** | | | |
| AO | mm | PWd | mm |
| LA | mm | EDV | ml |
| LVIDd | mm | ESV | ml |
| LVIDs | mm | LVEF | 56% |
| IVSd | mm | FS | 29% |
| **Additional Information**: |  | | |
| No pericardial/Pleural effusion. | | | |
| **Final Diagnosis:** | | | |
| 1. {S, D, S} Levocardia. 2. Silent PDA | | | |
| **Remark**: | | | |
| **Recommendation**: No need of any form of intervention | | | |
| SIGNATURE  Done by: Tesfaye T., Pediatrician, Pediatric Cardiologist _______________ 29/07/2013Eth.C | | | |

| Patient Name: **Negasu Aweke**. Patient ID: **Adinas GH**. SEX/ Age: M/8Years. Date of Report: 30/**07/2013**.  BP: _______ Weight: ______ Height: ____________ BSA: ________. R.Dx: **Sydenham’s Chorea. AGH10.766** | | | |
| --- | --- | --- | --- |
| **Features** | **Finding** | **Features** | **Finding** |
| **Profile** |  | **Atria** |  |
| Abdominal situs | Solitus | Left atrium | Normal |
| Cardiac position | Levocardia | Right atrium | Normal |
| Systemic venous drainage | Normal. | **Atrioventricular valves** |  |
| Pulmonary venous drainage | Normal | Mitral valve | Annulus = 20mm |
| Atrioventricular connection | Concordant | Tricuspid valve | Annulus = 21mm |
| Ventriculoarterial connection | Concordant | **Ventricles** |  |
| Ventricular loop | d-Loop | Left ventricle | Normal |
|  |  | Right ventricle | Normal |
| **Septae** |  | **Coronary arteries** | ----- |
| Interventricular septum | Intact | **Doppler Measurement** |  |
| Interatrial septum | Intact | Mitral | ----- |
| **Semilunal valves** |  | Aortic | ------- |
| Aortic valve | Annulus = 17mm | Tricuspid | ------- |
| Pulmonary valve | Annulus = 18mm | pulmonic | -------- |
| **Great arteries** | NRGA | **Aortic arch** | Left |
| Aorta | ----- | **PDA** | No |
| Pulmonary artery | Normal MPA and Branch PAs. |  |  |
| **M-Mode:** | | | |
| AO | mm | PWd | mm |
| LA | mm | EDV | ml |
| LVIDd | mm | ESV | ml |
| LVIDs | mm | LVEF | 59% |
| IVSd | mm | FS | 31% |
| **Additional Information**: |  | | |
| No pericardial/Pleural effusion. | | | |
| **Final Diagnosis:** | | | |
| 1. Normal Echocardiography Study. | | | |
| **Remark**: | | | |
| **Recommendation**: | | | |
| SIGNATURE  Done by: Tesfaye T., Pediatrician, Pediatric Cardiologist _______________ 30/07/2013Eth.C | | | |

| Patient Name: **Melaku Wendie**. Patient ID: **FHRH**. SEX/ Age: M/1Year. Date of Report: 30/**07/2013**.  BP: _______ Weight: ______ Height: ____________ BSA: ________. R.Dx: **CHF. AGH10.767** | | | |
| --- | --- | --- | --- |
| **Features** | **Finding** | **Features** | **Finding** |
| **Profile** |  | **Atria** |  |
| Abdominal situs | Solitus | Left atrium | Dilated |
| Cardiac position | Levocardia | Right atrium | Dilated |
| Systemic venous drainage | Normal. | **Atrioventricular valves** |  |
| Pulmonary venous drainage | Normal | Mitral valve | Annulus = 15mm |
| Atrioventricular connection | Concordant | Tricuspid valve | Annulus = 14mm  TAPSE = 12mm |
| Ventriculoarterial connection | Concordant | **Ventricles** |  |
| Ventricular loop | d-Loop | Left ventricle | Dilated |
|  |  | Right ventricle | Dilated |
| **Septae** |  | **Coronary arteries** | ----- |
| Interventricular septum | Intact | **Doppler Measurement** |  |
| Interatrial septum | Intact | Mitral | ----- |
| **Semilunal valves** |  | Aortic | ------- |
| Aortic valve | Annulus = 12mm | Tricuspid | Moderate TR, PPG = 61mmHg. |
| Pulmonary valve | Annulus = 14mm | pulmonic | -------- |
| **Great arteries** | NRGA | **Aortic arch** | Left |
| Aorta | ----- | **PDA** | 3mm PDA, L – R Shunt |
| Pulmonary artery | Dilated MPA |  |  |
| **M-Mode:** | | | |
| AO | mm | PWd | mm |
| LA | mm | EDV | ml |
| LVIDd | mm | ESV | ml |
| LVIDs | mm | LVEF | 59% |
| IVSd | mm | FS | 30% |
| **Additional Information**: |  | | |
| No pericardial/Pleural effusion. | | | |
| **Final Diagnosis:** | | | |
| 1. {S, D, S} Levocardia. 2. Large PDA, L – R Shunt 3. Severe Pulmonary Hypertension 4. Normal LV Function | | | |
| **Remark**: | | | |
| **Recommendation**: | | | |
| SIGNATURE  Done by: Tesfaye T., Pediatrician, Pediatric Cardiologist _______________ 30/07/2013Eth.C | | | |

| Patient Name: **Baby of Mulu-Gojam Adamtie**. Patient ID: **FHRH**. SEX/ Age: F/8days. Date of Report: 30/**07/2013**.  BP: _______ Weight: ______ Height: ____________ BSA: ________. R.Dx: **RD + Incidental Murmur. AGH10.768** | | | |
| --- | --- | --- | --- |
| **Features** | **Finding** | **Features** | **Finding** |
| **Profile** |  | **Atria** |  |
| Abdominal situs | Solitus | Left atrium | Normal |
| Cardiac position | Levocardia | Right atrium | Normal |
| Systemic venous drainage | Normal. | **Atrioventricular valves** |  |
| Pulmonary venous drainage | Normal | Mitral valve | Annulus = 9mm |
| Atrioventricular connection | Concordant | Tricuspid valve | Annulus = 10mm |
| Ventriculoarterial connection | Concordant | **Ventricles** |  |
| Ventricular loop | d-Loop | Left ventricle | Normal |
|  |  | Right ventricle | Normal |
| **Septae** |  | **Coronary arteries** | ----- |
| Interventricular septum | 8mm Inlet VSD with PM Extension, L – R Shunt | **Doppler Measurement** |  |
| Interatrial septum | 5mm OS ASD, L – R Shunt | Mitral | ----- |
| **Semilunal valves** |  | Aortic | ------- |
| Aortic valve | Annulus = 8mm | Tricuspid | Trivial TR |
| Pulmonary valve | Annulus = 8mm | pulmonic | -------- |
| **Great arteries** | NRGA | **Aortic arch** | Left |
| Aorta | ----- | **PDA** | No |
| Pulmonary artery | Normal MPA and Branch PAs. |  |  |
| **M-Mode:** | | | |
| AO | mm | PWd | mm |
| LA | mm | EDV | ml |
| LVIDd | mm | ESV | ml |
| LVIDs | mm | LVEF | 74% |
| IVSd | mm | FS | 39% |
| **Additional Information**: |  | | |
| No pericardial/Pleural effusion. | | | |
| **Final Diagnosis:** | | | |
| 1. {S, D, S} Levocardia. 2. Small OS ASD, L – R Shunt 3. Large Inlet VSD with PM extension, L – R Shunt 4. Normal LV Function | | | |
| **Remark**: | | | |
| **Recommendation**: | | | |
| SIGNATURE  Done by: Tesfaye T., Pediatrician, Pediatric Cardiologist _______________ 30/07/2013Eth.C | | | |

| Patient Name: **Asmra Antehun**. Patient ID: **FHRH**. SEX/ Age: F/12Years. Date of Report: 30/**07/2013**.  BP: _______ Weight: ______ Height: ____________ BSA: ________. R.Dx: **palpitation. AGH10.769** | | | |
| --- | --- | --- | --- |
| **Features** | **Finding** | **Features** | **Finding** |
| **Profile** |  | **Atria** |  |
| Abdominal situs | Solitus | Left atrium | Normal |
| Cardiac position | Levocardia | Right atrium | Normal |
| Systemic venous drainage | Normal. | **Atrioventricular valves** |  |
| Pulmonary venous drainage | Normal | Mitral valve | Annulus = 17mm |
| Atrioventricular connection | Concordant | Tricuspid valve | Annulus = 17mm |
| Ventriculoarterial connection | Concordant | **Ventricles** |  |
| Ventricular loop | d-Loop | Left ventricle | Normal |
|  |  | Right ventricle | Normal |
| **Septae** |  | **Coronary arteries** | ----- |
| Interventricular septum | Intact | **Doppler Measurement** |  |
| Interatrial septum | Intact | Mitral | ----- |
| **Semilunal valves** |  | Aortic | ------- |
| Aortic valve | Annulus = 16mm | Tricuspid | ------- |
| Pulmonary valve | Annulus = 18mm | pulmonic | -------- |
| **Great arteries** | NRGA | **Aortic arch** | Left |
| Aorta | ----- | **PDA** | No |
| Pulmonary artery | Normal MPA and Branch PAs. |  |  |
| **M-Mode:** | | | |
| AO | mm | PWd | mm |
| LA | mm | EDV | ml |
| LVIDd | mm | ESV | ml |
| LVIDs | mm | LVEF | 59% |
| IVSd | mm | FS | 31% |
| **Additional Information**: |  | | |
| No pericardial/Pleural effusion. | | | |
| **Final Diagnosis:** | | | |
| 1. Normal Echocardiography Study. | | | |
| **Remark**: | | | |
| **Recommendation**: | | | |
| SIGNATURE  Done by: Tesfaye T., Pediatrician, Pediatric Cardiologist _______________ 30/07/2013Eth.C | | | |

| Patient Name: **Robel Tessema.** Patient ID: **Amaris PSC**. SEX/ Age: M/8/12. Date of Report: 01/**08/2013**.  BP: _______ Weight: ______ Height: ____________ BSA: ________. R.Dx: **Recurrent Chest Infection. AGH10.770** | | | |
| --- | --- | --- | --- |
| **Features** | **Finding** | **Features** | **Finding** |
| **Profile** |  | **Atria** |  |
| Abdominal situs | Solitus | Left atrium | Normal |
| Cardiac position | Levocardia | Right atrium | Normal |
| Systemic venous drainage | Normal. | **Atrioventricular valves** |  |
| Pulmonary venous drainage | Normal | Mitral valve | Annulus = 13mm |
| Atrioventricular connection | Concordant | Tricuspid valve | Annulus = 15mm  TAPSE = 15mm |
| Ventriculoarterial connection | Concordant | **Ventricles** |  |
| Ventricular loop | d-Loop | Left ventricle | Normal |
|  |  | Right ventricle | Normal |
| **Septae** |  | **Coronary arteries** | ----- |
| Interventricular septum | Intact | **Doppler Measurement** |  |
| Interatrial septum | Intact | Mitral | ----- |
| **Semilunal valves** |  | Aortic | ------- |
| Aortic valve | Annulus = 10mm | Tricuspid | ------- |
| Pulmonary valve | Annulus = 11mm | pulmonic | -------- |
| **Great arteries** | NRGA | **Aortic arch** | Left |
| Aorta | ----- | **PDA** | No |
| Pulmonary artery | Normal MPA and Branch PAs. |  |  |
| **M-Mode:** Normal LV Function on eye balling. | | | |
| AO | mm | PWd | mm |
| LA | mm | EDV | ml |
| LVIDd | mm | ESV | ml |
| LVIDs | mm | LVEF | % |
| IVSd | mm | FS | % |
| **Additional Information**: |  | | |
| No pericardial/Pleural effusion. | | | |
| **Final Diagnosis:** | | | |
| 1. Normal Echocardiography Study. | | | |
| **Remark**: | | | |
| **Recommendation**: | | | |
| SIGNATURE  Done by: Tesfaye T., Pediatrician, Pediatric Cardiologist _______________ 01/08/2013Eth.C | | | |

| Patient Name: **Serk-addis Amsale**. Patient ID: **FHRH**. SEX/ Age: F/7Years. Date of Report: 01/**08/2013**.  BP: _______ Weight: ______ Height: ________ BSA: ______. R.Dx: **Rheumatic Recurrence + CHF. AGH10.771** | | | |
| --- | --- | --- | --- |
| **Features** | **Finding** | **Features** | **Finding** |
| **Profile** |  | **Atria** |  |
| Abdominal situs | Solitus | Left atrium | Dilated |
| Cardiac position | Levocardia | Right atrium | Normal |
| Systemic venous drainage | Normal. IVC Dilated. | **Atrioventricular valves** |  |
| Pulmonary venous drainage | Normal | Mitral valve | Annulus = 21mm. thickened, non coapting MVL. Shortened PMVL. MVA = 1.33cm2. |
| Atrioventricular connection | Concordant | Tricuspid valve | Annulus = 20mm |
| Ventriculoarterial connection | Concordant | **Ventricles** |  |
| Ventricular loop | d-Loop | Left ventricle | Dilated |
|  |  | Right ventricle | Normal |
| **Septae** |  | **Coronary arteries** | ----- |
| Interventricular septum | Intact | **Doppler Measurement** |  |
| Interatrial septum | Intact | Mitral | Severe MR, Holosystolic, posterior projection, seen in two planes with jet velocity = 3.9m/sec. Moderate MS, PPG/MPG = 11/8mmHg. |
| **Semilunal valves** |  | Aortic | ------- |
| Aortic valve | Annulus = 16mm | Tricuspid | Mild TR, PPG = 30mmHg |
| Pulmonary valve | Annulus = 20mm | pulmonic | Mild PR, PPG = 44mmHg |
| **Great arteries** | NRGA | **Aortic arch** | Left |
| Aorta | ----- | **PDA** | No |
| Pulmonary artery | MPA = 18mm. |  |  |
| **M-Mode:** | | | |
| AO | mm | PWd | mm |
| LA | mm | EDV | ml |
| LVIDd | mm | ESV | ml |
| LVIDs | mm | LVEF | 67% |
| IVSd | mm | FS | 37% |
| **Additional Information**: |  | | |
| pericardial effusion 10mm on RA Side, 6mm on LV Side. | | | |
| **Final Diagnosis:** | | | |
| 1. {S, D, S} Levocardia. 2. LA/LV Dilated 3. Thickened, non coapting MVL. Shortened PMVL 4. Severe MR 5. Moderate MS 6. Small Pericardial Effusion 7. Normal LV Function | | | |
| SIGNATURE  Done by: Tesfaye T., Pediatrician, Pediatric Cardiologist _______________ 01/08/2013Eth.C | | | |

| Patient Name: **Michael Fasil**. Patient ID: **Adinas GH**. SEX/ Age: M/4Years. Date of Report: 01/**08/2013**.  BP: _______ Weight: ______ Height: ____________ BSA: ________. R.Dx: **Pre-op screening. AGH10.772** | | | |
| --- | --- | --- | --- |
| **Features** | **Finding** | **Features** | **Finding** |
| **Profile** |  | **Atria** |  |
| Abdominal situs | Solitus | Left atrium | Normal |
| Cardiac position | Levocardia | Right atrium | Normal |
| Systemic venous drainage | Normal. | **Atrioventricular valves** |  |
| Pulmonary venous drainage | Normal | Mitral valve | Annulus = 19mm |
| Atrioventricular connection | Concordant | Tricuspid valve | Annulus = 19mm  TAPSE = 18mm |
| Ventriculoarterial connection | Concordant | **Ventricles** |  |
| Ventricular loop | d-Loop | Left ventricle | Normal |
|  |  | Right ventricle | Normal |
| **Septae** |  | **Coronary arteries** | ----- |
| Interventricular septum | Intact | **Doppler Measurement** |  |
| Interatrial septum | Intact | Mitral | ----- |
| **Semilunal valves** |  | Aortic | ------- |
| Aortic valve | Annulus = 15mm | Tricuspid | Trivial TR, PPG = 15mmHg |
| Pulmonary valve | Annulus = 17mm | pulmonic | -------- |
| **Great arteries** | NRGA | **Aortic arch** | Left |
| Aorta | ----- | **PDA** | No |
| Pulmonary artery | Normal MPA and Branch PAs. |  |  |
| **M-Mode:** | | | |
| AO | mm | PWd | mm |
| LA | mm | EDV | ml |
| LVIDd | mm | ESV | ml |
| LVIDs | mm | LVEF | 69% |
| IVSd | mm | FS | 38% |
| **Additional Information**: |  | | |
| No pericardial/Pleural effusion. | | | |
| **Final Diagnosis:** | | | |
| 1. Normal Echocardiography Study. | | | |
| **Remark**: | | | |
| **Recommendation**: | | | |
| SIGNATURE  Done by: Tesfaye T., Pediatrician, Pediatric Cardiologist _______________ 01/08/2013Eth.C | | | |

| Patient Name: **Mita Gubilie**. Patient ID: **FHRH**. SEX/ Age: F/9/12. Date of Report: 04/**08/2013**.  BP: _______ Weight: ______ Height: ____________ BSA: ________. R.Dx: **CHF + Murmur. AGH10.773** | | | |
| --- | --- | --- | --- |
| **Features** | **Finding** | **Features** | **Finding** |
| **Profile** |  | **Atria** |  |
| Abdominal situs | Solitus | Left atrium | More Dilated |
| Cardiac position | Levocardia | Right atrium | Dilated |
| Systemic venous drainage | Normal. | **Atrioventricular valves** |  |
| Pulmonary venous drainage | Normal | Mitral valve | Annulus = 16mm |
| Atrioventricular connection | Concordant | Tricuspid valve | Annulus = 15mm |
| Ventriculoarterial connection | Concordant | **Ventricles** |  |
| Ventricular loop | d-Loop | Left ventricle | More Dilated |
|  |  | Right ventricle | Dilated |
| **Septae** |  | **Coronary arteries** | ----- |
| Interventricular septum | Intact | **Doppler Measurement** |  |
| Interatrial septum | Intact | Mitral | Mild MR |
| **Semilunal valves** |  | Aortic | ------- |
| Aortic valve | Annulus = 13mm | Tricuspid | ------- |
| Pulmonary valve | Annulus = 13mm | pulmonic | -------- |
| **Great arteries** | NRGA | **Aortic arch** | Left |
| Aorta | ----- | **PDA** | 3mm PDA, L – R Shunt |
| Pulmonary artery | MPA = 19mm. |  |  |
| **M-Mode:** Normal LV Function on eye balling. | | | |
| AO | mm | PWd | mm |
| LA | mm | EDV | ml |
| LVIDd | mm | ESV | ml |
| LVIDs | mm | LVEF | % |
| IVSd | mm | FS | % |
| **Additional Information**: |  | | |
| No pericardial/Pleural effusion. | | | |
| **Final Diagnosis:** | | | |
| 1. {S, D, S} Levocardia. 2. Large PDA, L – R Shunt 3. Moderate Pulmonary Hypertension 4. Normal LV Function | | | |
| **Remark**: Infant was crying during study. | | | |
| **Recommendation**: | | | |
| SIGNATURE  Done by: Tesfaye T., Pediatrician, Pediatric Cardiologist _______________ 04/08/2013Eth.C | | | |

| Patient Name: **Hiryakos Getachew**. Patient ID: **Adinas GH**. SEX/ Age: M/_6 9/12. Date of Report: 04/**08/2013**.  BP: _______ Weight: ______ Height: ____________ BSA: ________. R.Dx: **ARF. AGH10.774** | | | |
| --- | --- | --- | --- |
| **Features** | **Finding** | **Features** | **Finding** |
| **Profile** |  | **Atria** |  |
| Abdominal situs | Solitus | Left atrium | Mildly dilated |
| Cardiac position | Levocardia | Right atrium | Normal |
| Systemic venous drainage | Normal. | **Atrioventricular valves** |  |
| Pulmonary venous drainage | Normal | Mitral valve | Annulus = 21mm. Mildly thickened MVL. |
| Atrioventricular connection | Concordant | Tricuspid valve | Annulus = 17mm |
| Ventriculoarterial connection | Concordant | **Ventricles** |  |
| Ventricular loop | d-Loop | Left ventricle | Mildly Dilated |
|  |  | Right ventricle | Normal |
| **Septae** |  | **Coronary arteries** | ----- |
| Interventricular septum | Intact | **Doppler Measurement** |  |
| Interatrial septum | Intact | Mitral | Mild MR, Holosystolic, Posterior projection, seen in two planes with jet velocity = 3.6m/sec |
| **Semilunal valves** |  | Aortic | ------- |
| Aortic valve | Annulus = 16mm | Tricuspid | ------- |
| Pulmonary valve | Annulus = 19mm | pulmonic | -------- |
| **Great arteries** | NRGA | **Aortic arch** | Left |
| Aorta | ----- | **PDA** | No |
| Pulmonary artery | Normal MPA and Branch PAs. |  |  |
| **M-Mode:** | | | |
| AO | mm | PWd | mm |
| LA | mm | EDV | ml |
| LVIDd | mm | ESV | ml |
| LVIDs | mm | LVEF | 58% |
| IVSd | mm | FS | 30% |
| **Additional Information**: |  | | |
| No pericardial/Pleural effusion. | | | |
| **Final Diagnosis:** | | | |
| 1. {S, D, S} Levocardia. 2. Mildly dilated LA/LV 3. Mildly thickened MVL 4. Mild MR 5. Normal LV Function | | | |
| **Remark**: Significant improvement from previous Echo | | | |
| **Recommendation**: | | | |
| SIGNATURE  Done by: Tesfaye T., Pediatrician, Pediatric Cardiologist _______________ 04/08/2013Eth.C | | | |

| Patient Name: **Rakeb Wallelign**. Patient ID: **Amaris PSC**. SEX/ Age: F/1 3/12. Date of Report: 04/**08/2013**.  BP: _______ Weight: ______ Height: ____________ BSA: ________. R.Dx: **DS. AGH10.775** | | | |
| --- | --- | --- | --- |
| **Features** | **Finding** | **Features** | **Finding** |
| **Profile** |  | **Atria** |  |
| Abdominal situs | Solitus | Left atrium | Normal |
| Cardiac position | Levocardia | Right atrium | Normal |
| Systemic venous drainage | Normal. | **Atrioventricular valves** |  |
| Pulmonary venous drainage | Normal | Mitral valve | Annulus = 12mm |
| Atrioventricular connection | Concordant | Tricuspid valve | Annulus = 13mm |
| Ventriculoarterial connection | Concordant | **Ventricles** |  |
| Ventricular loop | d-Loop | Left ventricle | Normal |
|  |  | Right ventricle | Normal |
| **Septae** |  | **Coronary arteries** | ----- |
| Interventricular septum | Intact | **Doppler Measurement** |  |
| Interatrial septum | Intact | Mitral | ----- |
| **Semilunal valves** |  | Aortic | ------- |
| Aortic valve | Annulus = 11mm | Tricuspid | ------- |
| Pulmonary valve | Annulus = 11mm | pulmonic | -------- |
| **Great arteries** | NRGA | **Aortic arch** | Left |
| Aorta | ----- | **PDA** | No |
| Pulmonary artery | Normal MPA and Branch PAs. |  |  |
| **M-Mode:** Normal LV Function on eye balling | | | |
| AO | mm | PWd | mm |
| LA | mm | EDV | ml |
| LVIDd | mm | ESV | ml |
| LVIDs | mm | LVEF | % |
| IVSd | mm | FS | % |
| **Additional Information**: |  | | |
| No pericardial/Pleural effusion. | | | |
| **Final Diagnosis:** | | | |
| 1. Normal Echocardiography Study. | | | |
| **Remark**: | | | |
| **Recommendation**: | | | |
| SIGNATURE  Done by: Tesfaye T., Pediatrician, Pediatric Cardiologist _______________ 04/08/2013Eth.C | | | |

| Patient Name: **Gedeon Alemu**. Patient ID: **Adinas GH**. SEX/ Age: M/9/12. Date of Report: 04/**08/2013**.  BP: _______ Weight: ______ Height: ____________ BSA: ________. R.Dx: **RD + CHF + DS. AGH10.776** | | | |
| --- | --- | --- | --- |
| **Features** | **Finding** | **Features** | **Finding** |
| **Profile** |  | **Atria** |  |
| Abdominal situs | Solitus | Left atrium | Normal |
| Cardiac position | Levocardia | Right atrium | Markedly dilated |
| Systemic venous drainage | Normal. | **Atrioventricular valves** |  |
| Pulmonary venous drainage | Normal | Mitral valve | Annulus = 10mm |
| Atrioventricular connection | Concordant | Tricuspid valve | Annulus = 15mm  TAPSE = 8mm |
| Ventriculoarterial connection | Concordant | **Ventricles** |  |
| Ventricular loop | d-Loop | Left ventricle | Normal |
|  |  | Right ventricle | Markedly dilated |
| **Septae** | Abnormal Motion | **Coronary arteries** | ----- |
| Interventricular septum | Intact | **Doppler Measurement** |  |
| Interatrial septum | Intact | Mitral | ----- |
| **Semilunal valves** |  | Aortic | ------- |
| Aortic valve | Annulus = 9mm | Tricuspid | Mild to moderate TR, PPG = 57mmHg |
| Pulmonary valve | Annulus = 13mm | pulmonic | Moderate PR, PPG = 54mmHg |
| **Great arteries** | NRGA | **Aortic arch** | Left |
| Aorta | ----- | **PDA** | No |
| Pulmonary artery | Normal MPA and Branch PAs. |  |  |
| **M-Mode:** | | | |
| AO | mm | PWd | mm |
| LA | mm | EDV | ml |
| LVIDd | mm | ESV | ml |
| LVIDs | mm | LVEF | 58% |
| IVSd | mm | FS | 29% |
| **Additional Information**: |  | | |
| 3mm pericardial effusion on RA Side. | | | |
| **Final Diagnosis:** | | | |
| 1. {S, D, S} Levocardia. 2. RA/RV Dilated 3. Moderate PR 4. Mild to moderate TR 5. Abnormal Septal Motion 6. RV Dysfunctional 7. Severe Pulmonary Hypertension 8. Normal LV Function 9. Trace Pericardial effusion | | | |
| **Remark**: | | | |
| **Recommendation**: | | | |
| SIGNATURE  Done by: Tesfaye T., Pediatrician, Pediatric Cardiologist _______________ 04/08/2013Eth.C | | | |

| Patient Name: **Tigist Muluken**. Patient ID: **Adinas GH**. SEX/ Age: F/7/12. Date of Report: 05/**08/2013**.  BP: _______ Weight: ______ Height: ____________ BSA: ________. R.Dx: **Cyanosis. AGH10.777** | | | |
| --- | --- | --- | --- |
| **Features** | **Finding** | **Features** | **Finding** |
| **Profile** |  | **Atria** |  |
| Abdominal situs | Solitus | Left atrium | Normal |
| Cardiac position | Levocardia | Right atrium | Normal |
| Systemic venous drainage | Normal. | **Atrioventricular valves** |  |
| Pulmonary venous drainage | Normal | Mitral valve | Annulus = 16mm |
| Atrioventricular connection | Concordant | Tricuspid valve | Atretic |
| Ventriculoarterial connection | Concordant | **Ventricles** |  |
| Ventricular loop | d-Loop | Left ventricle | Normal |
|  |  | Right ventricle | Normal |
| **Septae** |  | **Coronary arteries** | ----- |
| Interventricular septum | 5mm Inlet VSD with PM extension, L – R Shunt | **Doppler Measurement** |  |
| Interatrial septum | 7mm OS ASD, R – L Shunt | Mitral | ----- |
| **Semilunal valves** |  | Aortic | ------- |
| Aortic valve | Annulus = 12mm | Tricuspid | ------- |
| Pulmonary valve | Annulus = 6mm | pulmonic | Moderate PS, PPG = 47mmHg |
| **Great arteries** | NRGA | **Aortic arch** | Left |
| Aorta | ----- | **PDA** | No |
| Pulmonary artery | Smallish MPA and Branch PAs. |  |  |
| **M-Mode:** | | | |
| AO | mm | PWd | mm |
| LA | mm | EDV | ml |
| LVIDd | mm | ESV | ml |
| LVIDs | mm | LVEF | % |
| IVSd | mm | FS | % |
| **Additional Information**: |  | | |
| No pericardial/Pleural effusion. | | | |
| **Final Diagnosis:** | | | |
| 1. {S, D, S} Levocardia. 2. Tricuspid Atresia Type IB. | | | |
| **Remark**: | | | |
| **Recommendation**: | | | |
| SIGNATURE  Done by: Tesfaye T., Pediatrician, Pediatric Cardiologist _______________ 05/08/2013Eth.C | | | |

| Patient Name: **Liqinaw Ayele**. Patient ID: **FHRH**. SEX/ Age: M/4 6/12. Date of Report: 05/**08/2013**.  BP: _______ Weight: ______ Height: ____________ BSA: ________. R.Dx: **Chest Pain. AGH10.778** | | | |
| --- | --- | --- | --- |
| **Features** | **Finding** | **Features** | **Finding** |
| **Profile** |  | **Atria** |  |
| Abdominal situs | Solitus | Left atrium | Normal |
| Cardiac position | Levocardia | Right atrium | Normal |
| Systemic venous drainage | Normal. | **Atrioventricular valves** |  |
| Pulmonary venous drainage | Normal | Mitral valve | Annulus = 20mm |
| Atrioventricular connection | Concordant | Tricuspid valve | Annulus = 21mm  TAPSE = 18mm |
| Ventriculoarterial connection | Concordant | **Ventricles** |  |
| Ventricular loop | d-Loop | Left ventricle | Normal |
|  |  | Right ventricle | Normal |
| **Septae** |  | **Coronary arteries** | ----- |
| Interventricular septum | Intact | **Doppler Measurement** |  |
| Interatrial septum | Intact | Mitral | ----- |
| **Semilunal valves** |  | Aortic | ------- |
| Aortic valve | Annulus = 16mm | Tricuspid | Trivial TR, PPG = 17mmHg |
| Pulmonary valve | Annulus = 17mm | pulmonic | -------- |
| **Great arteries** | NRGA | **Aortic arch** | Left |
| Aorta | ----- | **PDA** | No |
| Pulmonary artery | Normal MPA and Branch PAs. |  |  |
| **M-Mode:** | | | |
| AO | mm | PWd | mm |
| LA | mm | EDV | ml |
| LVIDd | mm | ESV | ml |
| LVIDs | mm | LVEF | 59% |
| IVSd | mm | FS | 31% |
| **Additional Information**: |  | | |
| pericardial effusion 8mm on RA Side and 6mm on LV Side. | | | |
| **Final Diagnosis:** | | | |
| 1. {S, D, S} Levocardia. 2. Small Pericardial effusion secondary to ? | | | |
| **Remark**: | | | |
| **Recommendation**: Pericarditis | | | |
| SIGNATURE  Done by: Tesfaye T., Pediatrician, Pediatric Cardiologist _______________ 05/08/2013Eth.C | | | |

| Patient Name: **Sefiw Yohannes.** Patient ID: **FHRH**. SEX/ Age: M/13Years. Date of Report: 07/**08/2013**.  BP: _______ Weight: ______ Height: ____________ BSA: ________. R.Dx: **DOE + Murmur. AGH10.779** | | | |
| --- | --- | --- | --- |
| **Features** | **Finding** | **Features** | **Finding** |
| **Profile** |  | **Atria** |  |
| Abdominal situs | Solitus | Left atrium | Dilated |
| Cardiac position | Levocardia | Right atrium | Normal |
| Systemic venous drainage | Normal. | **Atrioventricular valves** |  |
| Pulmonary venous drainage | Normal | Mitral valve | Annulus = 22mm |
| Atrioventricular connection | Concordant | Tricuspid valve | Annulus = 22mm  TAPSE = 17mm |
| Ventriculoarterial connection | Concordant | **Ventricles** |  |
| Ventricular loop | d-Loop | Left ventricle | Dilated |
|  |  | Right ventricle | Normal |
| **Septae** |  | **Coronary arteries** | ----- |
| Interventricular septum | 10mm Sub aortic VSD, L – R Shunt with Gradient 24mmHg | **Doppler Measurement** |  |
| Interatrial septum | Intact | Mitral | ----- |
| **Semilunal valves** |  | Aortic | ------- |
| Aortic valve | Annulus = 21mm | Tricuspid | ------- |
| Pulmonary valve | Annulus = 19mm | pulmonic | -------- |
| **Great arteries** | NRGA | **Aortic arch** | Left |
| Aorta | ----- | **PDA** | No |
| Pulmonary artery | Normal MPA and Branch PAs. |  |  |
| **M-Mode:** | | | |
| AO | mm | PWd | mm |
| LA | mm | EDV | ml |
| LVIDd | mm | ESV | ml |
| LVIDs | mm | LVEF | 61% |
| IVSd | mm | FS | 33% |
| **Additional Information**: |  | | |
| No pericardial/Pleural effusion. | | | |
| **Final Diagnosis:** | | | |
| 1. {S, D, S} Levocardia. 2. Non-Restrictive Moderate Sub Aortic VSD, L – R Shunt 3. Normal Biventricular Function | | | |
| **Remark**: | | | |
| **Recommendation**: | | | |
| SIGNATURE  Done by: Tesfaye T., Pediatrician, Pediatric Cardiologist _______________ 07/08/2013Eth.C | | | |

| Patient Name: **Hanna Simeneh**. Patient ID: **Injibara GH**. SEX/ Age: F/14Years. Date of Report: 07/**08/2013**.  BP: _______ Weight: ______ Height: ____________ BSA: ________. R.Dx: **easy fatigability. AGH10.780** | | | |
| --- | --- | --- | --- |
| **Features** | **Finding** | **Features** | **Finding** |
| **Profile** |  | **Atria** |  |
| Abdominal situs | Solitus | Left atrium | Normal |
| Cardiac position | Levocardia | Right atrium | Normal |
| Systemic venous drainage | Normal. | **Atrioventricular valves** |  |
| Pulmonary venous drainage | Normal | Mitral valve | Annulus = 20mm |
| Atrioventricular connection | Concordant | Tricuspid valve | Annulus = 20mm  TAPSE = 20mm |
| Ventriculoarterial connection | Concordant | **Ventricles** |  |
| Ventricular loop | d-Loop | Left ventricle | Normal |
|  |  | Right ventricle | Normal |
| **Septae** |  | **Coronary arteries** | ----- |
| Interventricular septum | Intact | **Doppler Measurement** |  |
| Interatrial septum | Intact | Mitral | ----- |
| **Semilunal valves** |  | Aortic | ------- |
| Aortic valve | Annulus = 19mm | Tricuspid | ------- |
| Pulmonary valve | Annulus = 21mm | pulmonic | -------- |
| **Great arteries** | NRGA | **Aortic arch** | Left |
| Aorta | ----- | **PDA** | No |
| Pulmonary artery | Normal MPA and Branch PAs. |  |  |
| **M-Mode:** | | | |
| AO | mm | PWd | mm |
| LA | mm | EDV | ml |
| LVIDd | mm | ESV | ml |
| LVIDs | mm | LVEF | 67% |
| IVSd | mm | FS | 37% |
| **Additional Information**: |  | | |
| No pericardial/Pleural effusion. | | | |
| **Final Diagnosis:** | | | |
| 1. Normal Echocardiography Study. | | | |
| **Remark**: | | | |
| **Recommendation**: | | | |
| SIGNATURE  Done by: Tesfaye T., Pediatrician, Pediatric Cardiologist _______________ 07/08/2013Eth.C | | | |

| Patient Name: **Desta Kassa**. Patient ID: **Adinas GH**. SEX/ Age: M/4Years. Date of Report: 08/**08/2013**.  BP: _______ Weight: ______ Height: ____________ BSA: ________. R.Dx: **Familiy Hx. AGH10.781** | | | |
| --- | --- | --- | --- |
| **Features** | **Finding** | **Features** | **Finding** |
| **Profile** |  | **Atria** |  |
| Abdominal situs | Solitus | Left atrium | Dilated |
| Cardiac position | Levocardia | Right atrium | Normal |
| Systemic venous drainage | Normal. | **Atrioventricular valves** |  |
| Pulmonary venous drainage | Normal | Mitral valve | Annulus = 25mm. 7mm superior displacement of MVL s in to LA |
| Atrioventricular connection | Concordant | Tricuspid valve | Annulus = 20mm |
| Ventriculoarterial connection | Concordant | **Ventricles** |  |
| Ventricular loop | d-Loop | Left ventricle | Dilated |
|  |  | Right ventricle | Normal |
| **Septae** |  | **Coronary arteries** | ----- |
| Interventricular septum | Intact | **Doppler Measurement** |  |
| Interatrial septum | Intact | Mitral | Mild MR, Incomplete Signal, seen in two planes with jet velocity = 4.4m/sec |
| **Semilunal valves** |  | Aortic | ------- |
| Aortic valve | Annulus = 17mm | Tricuspid | ------- |
| Pulmonary valve | Annulus = 20mm | pulmonic | -------- |
| **Great arteries** | NRGA | **Aortic arch** | Left |
| Aorta | SoV = 24mm, ASc.Ao = 15mm | **PDA** | No |
| Pulmonary artery | MPA =21mm. |  |  |
| **M-Mode:** | | | |
| AO | mm | PWd | mm |
| LA | mm | EDV | ml |
| LVIDd | mm | ESV | ml |
| LVIDs | mm | LVEF | 59% |
| IVSd | mm | FS | 31% |
| **Additional Information**: |  | | |
| No pericardial/Pleural effusion. | | | |
| **Final Diagnosis:** | | | |
| 1. {S, D, S} Levocardia. 2. Mild MR 3. MVP 4. Normal LV Function | | | |
| **Remark**: | | | |
| **Recommendation**: Follow up echo yearly | | | |
| SIGNATURE  Done by: Tesfaye T., Pediatrician, Pediatric Cardiologist _______________ 08/08/2013Eth.C | | | |

| Patient Name: **Hirut Atrsaw**. Patient ID: **TGSH**. SEX/ Age: F/28days. Date of Report: 08/**08/2013**.  BP: _______ Weight: ______ Height: ____________ BSA: ________. R.Dx: **DS. AGH10.782** | | | |
| --- | --- | --- | --- |
| **Features** | **Finding** | **Features** | **Finding** |
| **Profile** |  | **Atria** |  |
| Abdominal situs | Solitus | Left atrium | Normal |
| Cardiac position | Levocardia | Right atrium | Normal |
| Systemic venous drainage | Normal. | **Atrioventricular valves** |  |
| Pulmonary venous drainage | Normal | Mitral valve | Annulus = 9mm |
| Atrioventricular connection | Concordant | Tricuspid valve | Annulus = 10mm |
| Ventriculoarterial connection | Concordant | **Ventricles** |  |
| Ventricular loop | d-Loop | Left ventricle | Normal |
|  |  | Right ventricle | Normal |
| **Septae** |  | **Coronary arteries** | ----- |
| Interventricular septum | Intact | **Doppler Measurement** |  |
| Interatrial septum | PFO, L – R Shunt | Mitral | ----- |
| **Semilunal valves** |  | Aortic | ------- |
| Aortic valve | Annulus = 9mm | Tricuspid | ------- |
| Pulmonary valve | Annulus = 9mm | pulmonic | -------- |
| **Great arteries** | NRGA | **Aortic arch** | Left |
| Aorta | ----- | **PDA** | No |
| Pulmonary artery | Normal MPA and Branch PAs. |  |  |
| **M-Mode:** Normal LV Function on eye balling. | | | |
| AO | mm | PWd | mm |
| LA | mm | EDV | ml |
| LVIDd | mm | ESV | ml |
| LVIDs | mm | LVEF | % |
| IVSd | mm | FS | % |
| **Additional Information**: |  | | |
| No pericardial/Pleural effusion. | | | |
| **Final Diagnosis:** | | | |
| 1. {S, D, S} Levocardia. 2. PFO, L – R Shunt | | | |
| **Remark**: | | | |
| **Recommendation**: | | | |
| SIGNATURE  Done by: Tesfaye T., Pediatrician, Pediatric Cardiologist _______________ 08/08/2013Eth.C | | | |

| Patient Name: **Baby of Fasika Jenber**. Patient ID: **Adinas GH**. SEX/ Age: M/6days. Date of Report: 09/**08/2013**.  BP: _______ Weight: ______ Height: ____________ BSA: ________. R.Dx: **PPHTN. AGH10.783** | | | |
| --- | --- | --- | --- |
| **Features** | **Finding** | **Features** | **Finding** |
| **Profile** |  | **Atria** |  |
| Abdominal situs | Solitus | Left atrium | Normal |
| Cardiac position | Levocardia | Right atrium | Normal |
| Systemic venous drainage | Normal. | **Atrioventricular valves** |  |
| Pulmonary venous drainage | Normal | Mitral valve | Annulus = 8mm |
| Atrioventricular connection | Concordant | Tricuspid valve | Annulus = 10mm |
| Ventriculoarterial connection | Concordant | **Ventricles** |  |
| Ventricular loop | d-Loop | Left ventricle | Normal |
|  |  | Right ventricle | Normal |
| **Septae** |  | **Coronary arteries** | ----- |
| Interventricular septum | Intact | **Doppler Measurement** |  |
| Interatrial septum | PFO, L – R Shunt | Mitral | ----- |
| **Semilunal valves** |  | Aortic | ------- |
| Aortic valve | Annulus = 9mm | Tricuspid | ------- |
| Pulmonary valve | Annulus = 8mm | pulmonic | -------- |
| **Great arteries** | NRGA | **Aortic arch** | Left |
| Aorta | ----- | **PDA** | No |
| Pulmonary artery | Normal MPA and Branch PAs. |  |  |
| **M-Mode:** Normal LV Function on eye balling. | | | |
| AO | mm | PWd | mm |
| LA | mm | EDV | ml |
| LVIDd | mm | ESV | ml |
| LVIDs | mm | LVEF | % |
| IVSd | mm | FS | % |
| **Additional Information**: |  | | |
| No pericardial/Pleural effusion. | | | |
| **Final Diagnosis:** | | | |
| 1. {S, D, S} Levocardia. 2. PFO, L – R Shunt | | | |
| **Remark**: | | | |
| **Recommendation**: | | | |
| SIGNATURE  Done by: Tesfaye T., Pediatrician, Pediatric Cardiologist _______________ 09/08/2013Eth.C | | | |

| Patient Name: **Asfaw Wendale**. Patient ID: **Adinas GH**. SEX/ Age: M/15Years. Date of Report: 10/**08/2013**.  BP: _______ Weight: ______ Height: _______ BSA: _____. R.Dx: **DOE + Chest Pain + Palpitation. AGH10.784** | | | |
| --- | --- | --- | --- |
| **Features** | **Finding** | **Features** | **Finding** |
| **Profile** |  | **Atria** |  |
| Abdominal situs | Solitus | Left atrium | Mildly dilated |
| Cardiac position | Levocardia | Right atrium | Normal |
| Systemic venous drainage | Normal. | **Atrioventricular valves** |  |
| Pulmonary venous drainage | Normal | Mitral valve | Annulus = 24mm. Thickened MVL |
| Atrioventricular connection | Concordant | Tricuspid valve | Annulus = 20mm  TAPSE = 24mm |
| Ventriculoarterial connection | Concordant | **Ventricles** |  |
| Ventricular loop | d-Loop | Left ventricle | Mildly dilated |
|  |  | Right ventricle | Normal |
| **Septae** |  | **Coronary arteries** | ----- |
| Interventricular septum | Intact | **Doppler Measurement** |  |
| Interatrial septum | Intact | Mitral | Mild MR, Holosystolic, posterior projection, seen in two planes with jet velocity = 3.7m/sec |
| **Semilunal valves** |  | Aortic | Moderate AR, PHT = 357ms |
| Aortic valve | Annulus = 23mm | Tricuspid | ------- |
| Pulmonary valve | Annulus = 24mm | pulmonic | -------- |
| **Great arteries** | NRGA | **Aortic arch** | Left |
| Aorta | ----- | **PDA** | No |
| Pulmonary artery | Normal MPA and Branch PAs. |  |  |
| **M-Mode:** | | | |
| AO | mm | PWd | mm |
| LA | mm | EDV | ml |
| LVIDd | mm | ESV | ml |
| LVIDs | mm | LVEF | 62% |
| IVSd | mm | FS | 34% |
| **Additional Information**: |  | | |
| No pericardial/Pleural effusion. | | | |
| **Final Diagnosis:** | | | |
| 1. {S, D, S} Levocardia. 2. Thickened MVL 3. Mild MR 4. Moderate AR 5. Normal Biventricular Function | | | |
| **Remark**: | | | |
| **Recommendation**: | | | |
| SIGNATURE  Done by: Tesfaye T., Pediatrician, Pediatric Cardiologist _______________ 10/08/2013Eth.C | | | |

| Patient Name: **Hibist Mandefro**. Patient ID: **FHRH**. SEX/ Age: F/2Years. Date of Report: 11/**08/2013**.  BP: _______ Weight: ______ Height: ____________ BSA: ________. R.Dx: **Incidental Murmur. AGH10.785** | | | |
| --- | --- | --- | --- |
| **Features** | **Finding** | **Features** | **Finding** |
| **Profile** |  | **Atria** |  |
| Abdominal situs | Solitus | Left atrium | Dilated |
| Cardiac position | Levocardia | Right atrium | Normal |
| Systemic venous drainage | Normal. | **Atrioventricular valves** |  |
| Pulmonary venous drainage | Normal | Mitral valve | Annulus = 20mm |
| Atrioventricular connection | Concordant | Tricuspid valve | Annulus = 16mm |
| Ventriculoarterial connection | Concordant | **Ventricles** |  |
| Ventricular loop | d-Loop | Left ventricle | Dilated |
|  |  | Right ventricle | Normal |
| **Septae** |  | **Coronary arteries** | ----- |
| Interventricular septum | Intact | **Doppler Measurement** |  |
| Interatrial septum | Intact | Mitral | Mild MR, Holosystolic with jet velocity= 4.8m/sec |
| **Semilunal valves** |  | Aortic | ------- |
| Aortic valve | Annulus = 15mm | Tricuspid | ------- |
| Pulmonary valve | Annulus = 13mm | pulmonic | -------- |
| **Great arteries** | NRGA | **Aortic arch** | Left |
| Aorta | ----- | **PDA** | 2mm PDA, L – R Shunt |
| Pulmonary artery | Normal MPA and Branch PAs. |  |  |
| **M-Mode:** | | | |
| AO | mm | PWd | mm |
| LA | mm | EDV | ml |
| LVIDd | mm | ESV | ml |
| LVIDs | mm | LVEF | 59% |
| IVSd | mm | FS | 31% |
| **Additional Information**: |  | | |
| No pericardial/Pleural effusion. | | | |
| **Final Diagnosis:** | | | |
| 1. {S, D, S} Levocardia. 2. Moderate PDA, L – R Shunt | | | |
| **Remark**:  Child was crying during study.  Limited Echo window. | | | |
| **Recommendation**: | | | |
| SIGNATURE  Done by: Tesfaye T., Pediatrician, Pediatric Cardiologist _______________ 11/08/2013Eth.C | | | |

| Patient Name: **Birtukan Tsehay**. Patient ID: **FHRH**. SEX/ Age: F/14Years. Date of Report: 11/**08/2013**.  BP: _______ Weight: ______ Height: _______ BSA: _____. R.Dx: **CHF + DOE + Easy fatigability. AGH10.786** | | | |
| --- | --- | --- | --- |
| **Features** | **Finding** | **Features** | **Finding** |
| **Profile** |  | **Atria** |  |
| Abdominal situs | Solitus | Left atrium | Markedly Dilated |
| Cardiac position | Levocardia | Right atrium | Markedly Dilated |
| Systemic venous drainage | Normal. | **Atrioventricular valves** |  |
| Pulmonary venous drainage | Normal | Mitral valve | Annulus = 32mm. MVA = 2.5cm2. MV E/A Ratio = 2.7 |
| Atrioventricular connection | Concordant | Tricuspid valve | Annulus = 30mm  TAPSE = 11mm |
| Ventriculoarterial connection | Concordant | **Ventricles** |  |
| Ventricular loop | d-Loop | Left ventricle | Normal |
|  |  | Right ventricle | Normal |
| **Septae** |  | **Coronary arteries** | ----- |
| Interventricular septum | Intact | **Doppler Measurement** |  |
| Interatrial septum | Intact | Mitral | Moderate MR, Holosystolic, posterior projection, seen in two planes with jet velocity = 4.7m/sec. |
| **Semilunal valves** |  | Aortic | ------- |
| Aortic valve | Annulus = 15mm | Tricuspid | Moderate TR, PPG = 75mmHg |
| Pulmonary valve | Annulus = 20mm | pulmonic | -------- |
| **Great arteries** | NRGA | **Aortic arch** | Left |
| Aorta | ----- | **PDA** | No |
| Pulmonary artery | Normal MPA. |  |  |
| **M-Mode:** | | | |
| AO | mm | PWd | mm |
| LA | mm | EDV | ml |
| LVIDd | mm | ESV | ml |
| LVIDs | mm | LVEF | 48% |
| IVSd | mm | FS | 25% |
| **Additional Information**: | No pericardial/Pleural effusion. | | |
| **Final Diagnosis:** | | | |
| 1. {S, D, S} Levocardia. 2. Markedly Dilated RA/LA 3. Moderate TR 4. Moderate MR 5. Severe Pulmonary Hypertension 6. Moderate LV Systolic Dysfunction 7. Diastolic Dysfunction 8. Dysfunctional RV | | | |
| **Remark**: Restrictive Cardiomyopathy is highly likely (Dwarf sign). DDx: Constrictive. Rhythm abnormality during ech | | | |
| **Recommendation**: Additional Imaging would help for Dx settlement. | | | |
| SIGNATURE  Done by: Tesfaye T., Pediatrician, Pediatric Cardiologist _______________ 11/08/2013Eth.C | | | |

| Patient Name: **Meklit Shimelash**. Patient ID: **FHCSRH**. SEX/ Age: F/42days. Date of Report: 12/**08/2013**.  BP: _______ Weight: ______ Height: ____________ BSA: ________. R.Dx: **Incidental Murmur. AGH10.787** | | | |
| --- | --- | --- | --- |
| **Features** | **Finding** | **Features** | **Finding** |
| **Profile** |  | **Atria** |  |
| Abdominal situs | Solitus | Left atrium | Normal |
| Cardiac position | Levocardia | Right atrium | Normal |
| Systemic venous drainage | Normal. | **Atrioventricular valves** |  |
| Pulmonary venous drainage | Normal | Mitral valve | Annulus = 11mm |
| Atrioventricular connection | Concordant | Tricuspid valve | Annulus = 11mm |
| Ventriculoarterial connection | Concordant | **Ventricles** |  |
| Ventricular loop | d-Loop | Left ventricle | Normal |
|  |  | Right ventricle | Normal |
| **Septae** |  | **Coronary arteries** | ----- |
| Interventricular septum | 3mm Inlet VSD, Partially covered by STL, L – R Shunt | **Doppler Measurement** |  |
| Interatrial septum | Intact | Mitral | ----- |
| **Semilunal valves** |  | Aortic | ------- |
| Aortic valve | Annulus = 10mm | Tricuspid | ------- |
| Pulmonary valve | Annulus = 10mm | pulmonic | -------- |
| **Great arteries** | NRGA | **Aortic arch** | Left |
| Aorta | ----- | **PDA** | No |
| Pulmonary artery | Normal MPA and Branch PAs. |  |  |
| **M-Mode: Normal LV Function on eye balling** | | | |
| AO | mm | PWd | mm |
| LA | mm | EDV | ml |
| LVIDd | mm | ESV | ml |
| LVIDs | mm | LVEF | % |
| IVSd | mm | FS | % |
| **Additional Information**: |  | | |
| No pericardial/Pleural effusion. | | | |
| **Final Diagnosis:** | | | |
| 1. {S, D, S} Levocardia. 2. Small Inlet VSD, L – R Shunt 3. Normal Ventricular Systolic Function | | | |
| **Remark**: | | | |
| **Recommendation**: | | | |
| SIGNATURE  Done by: Tesfaye T., Pediatrician, Pediatric Cardiologist _______________ 12/08/2013Eth.C | | | |

| Patient Name: **Baby of Helen Yitayew**. Patient ID: **FHRH**. SEX/ Age: M/23days. Date of Report: 12/**08/2013**.  BP: _______ Weight: ______ Height: ____________ BSA: ________. R.Dx: **DS. AGH10.788** | | | |
| --- | --- | --- | --- |
| **Features** | **Finding** | **Features** | **Finding** |
| **Profile** |  | **Atria** |  |
| Abdominal situs | Solitus | Left atrium | Normal |
| Cardiac position | Levocardia | Right atrium | Normal |
| Systemic venous drainage | Normal. | **Atrioventricular valves** |  |
| Pulmonary venous drainage | Normal | Mitral valve | Annulus = 8mm |
| Atrioventricular connection | Concordant | Tricuspid valve | Annulus = 11mm  TAPSE = 10mm |
| Ventriculoarterial connection | Concordant | **Ventricles** |  |
| Ventricular loop | d-Loop | Left ventricle | Normal |
|  |  | Right ventricle | Normal |
| **Septae** |  | **Coronary arteries** | ----- |
| Interventricular septum | Intact | **Doppler Measurement** |  |
| Interatrial septum | Intact | Mitral | ----- |
| **Semilunal valves** |  | Aortic | ------- |
| Aortic valve | Annulus = 9mm | Tricuspid | Trivial TR, PPG = 24mmHg |
| Pulmonary valve | Annulus = 8mm | pulmonic | -------- |
| **Great arteries** | NRGA | **Aortic arch** | Left |
| Aorta | ----- | **PDA** | No |
| Pulmonary artery | Normal MPA and Branch PAs. |  |  |
| **M-Mode:** Normal LV Function on eye balling | | | |
| AO | mm | PWd | mm |
| LA | mm | EDV | ml |
| LVIDd | mm | ESV | ml |
| LVIDs | mm | LVEF | % |
| IVSd | mm | FS | % |
| **Additional Information**: |  | | |
| No pericardial/Pleural effusion. | | | |
| **Final Diagnosis:** | | | |
| 1. Normal Echocardiography Study. | | | |
| **Remark**: | | | |
| **Recommendation**: | | | |
| SIGNATURE  Done by: Tesfaye T., Pediatrician, Pediatric Cardiologist _______________ 12/08/2013Eth.C | | | |

| Patient Name: **Ehitnesh Walle**. Patient ID: TGS**H**. SEX/ Age: F/8/12. Date of Report: 12/**08/2013**.  BP: _______ Weight: ______ Height: ____________ BSA: ________. R.Dx: **Incidental Murmur. AGH10.789** | | | |
| --- | --- | --- | --- |
| **Features** | **Finding** | **Features** | **Finding** |
| **Profile** |  | **Atria** |  |
| Abdominal situs | Solitus | Left atrium | Dilated |
| Cardiac position | Levocardia | Right atrium | Normal |
| Systemic venous drainage | Normal. | **Atrioventricular valves** |  |
| Pulmonary venous drainage | Normal | Mitral valve | Annulus = 13mm |
| Atrioventricular connection | Concordant | Tricuspid valve | Annulus = 15mm |
| Ventriculoarterial connection | Concordant | **Ventricles** |  |
| Ventricular loop | d-Loop | Left ventricle | Dilated |
|  |  | Right ventricle | Normal |
| **Septae** |  | **Coronary arteries** | ----- |
| Interventricular septum | 6mm PM VSD, L – R Shunt | **Doppler Measurement** |  |
| Interatrial septum | Intact | Mitral | ----- |
| **Semilunal valves** |  | Aortic | ------- |
| Aortic valve | Annulus = 11mm | Tricuspid | ------- |
| Pulmonary valve | Annulus = 12mm | pulmonic | Mild PR, PPG = 50mmHg |
| **Great arteries** | NRGA | **Aortic arch** | Left |
| Aorta | ----- | **PDA** | No |
| Pulmonary artery | Normal MPA and Branch PAs. |  |  |
| **M-Mode:** Normal LV Function on eye balling. | | | |
| AO | mm | PWd | mm |
| LA | mm | EDV | ml |
| LVIDd | mm | ESV | ml |
| LVIDs | mm | LVEF | % |
| IVSd | mm | FS | % |
| **Additional Information**: |  | | |
| No pericardial/Pleural effusion. | | | |
| **Final Diagnosis:** | | | |
| 1. {S, D, S} Levocardia. 2. Moderate PM VSD, L – R Shunt 3. Moderate Pulmonary Hypertension 4. Normal LV Function | | | |
| **Remark**: | | | |
| **Recommendation**: | | | |
| SIGNATURE  Done by: Tesfaye T., Pediatrician, Pediatric Cardiologist _______________ 12/08/2013Eth.C | | | |

| Patient Name: **Banchayehu Kerie**. Patient ID: **TGSH**. SEX/ Age: F/11Years. Date of Report: 12/**08/2013**.  BP: _______ Weight: ______ Height: _______ BSA: ________. R.Dx: **Cyanosis + clubbing + DOE. AGH10.790** | | | |
| --- | --- | --- | --- |
| **Features** | **Finding** | **Features** | **Finding** |
| **Profile** |  | **Atria** |  |
| Abdominal situs | Solitus | Left atrium | Normal |
| Cardiac position | Levocardia | Right atrium | Dilated |
| Systemic venous drainage | Normal. | **Atrioventricular valves** |  |
| Pulmonary venous drainage | Normal | Mitral valve | Annulus = 15mm |
| Atrioventricular connection | Concordant | Tricuspid valve | Annulus = 22mm  TAPSE = 11mm |
| Ventriculoarterial connection | Concordant | **Ventricles** |  |
| Ventricular loop | d-Loop | Left ventricle | Normal |
|  |  | Right ventricle | Dilated, Hypertrophied and Dysfunctional |
| **Septae** |  | **Coronary arteries** | ----- |
| Interventricular septum | Mal aligned non restrictive Sub Aortic VSD, R – L Shunt. | **Doppler Measurement** |  |
| Interatrial septum | 8mm OS ASD, R – L Shunt | Mitral | ----- |
| **Semilunal valves** |  | Aortic | ------- |
| Aortic valve | Annulus = 17mm | Tricuspid | ------- |
| Pulmonary valve | Annulus = 9mm | pulmonic | Severe PS, PPG = 61mmHg |
| **Great arteries** | NRGA | **Aortic arch** | Left |
| Aorta | Over riding | **PDA** | No |
| Pulmonary artery | Smallish MPA and Branch PAs. |  |  |
| **M-Mode:** Normal LV Function on eye balling. | | | |
| AO | mm | PWd | mm |
| LA | mm | EDV | ml |
| LVIDd | mm | ESV | ml |
| LVIDs | mm | LVEF | % |
| IVSd | mm | FS | % |
| **Additional Information**: |  | | |
| No pericardial/Pleural effusion. | | | |
| **Final Diagnosis:** | | | |
| 1. {S, D, S} Levocardia. 2. Moderate OS ASD, R – L Shunt 3. TOF 4. Smallish MPA and Branch PAs. 5. RV Hypertrophied, Dilated and Dysfunctional | | | |
| **Remark**: | | | |
| **Recommendation**: | | | |
| SIGNATURE  Done by: Tesfaye T., Pediatrician, Pediatric Cardiologist _______________ 12/08/2013Eth.C | | | |

| Patient Name: **Nardos Birhanu**. Patient ID: **FHRH**. SEX/ Age: F/3Years. Date of Report: 12/**08/2013**.  BP: _______ Weight: ______ Height: ____________ BSA: ________. R.Dx: **DS + RD. AGH10.791** | | | |
| --- | --- | --- | --- |
| **Features** | **Finding** | **Features** | **Finding** |
| **Profile** |  | **Atria** |  |
| Abdominal situs | Solitus | Left atrium | Dilated |
| Cardiac position | Levocardia | Right atrium | Dilated |
| Systemic venous drainage | Normal. | **Atrioventricular valves** |  |
| Pulmonary venous drainage | Normal | Mitral valve | Annulus = mm |
| Atrioventricular connection | Concordant | Tricuspid valve | Annulus = 22mm  TAPSE = 19mm |
| Ventriculoarterial connection | Concordant | **Ventricles** |  |
| Ventricular loop | d-Loop | Left ventricle | Dilated |
|  |  | Right ventricle | Dilated |
| **Septae** |  | **Coronary arteries** | ----- |
| Interventricular septum | 3mm Inlet VSD, L – R Shunt | **Doppler Measurement** |  |
| Interatrial septum | Large Primum Defect, L – R Shunt | Mitral | ----- |
| **Semilunal valves** |  | Aortic | ------- |
| Aortic valve | Annulus = 13mm | Tricuspid | Moderate TR |
| Pulmonary valve | Annulus = 16mm | pulmonic | -------- |
| **Great arteries** | NRGA | **Aortic arch** | Left |
| Aorta | ----- | **PDA** | No |
| Pulmonary artery | MPA =22mm. |  |  |
| **M-Mode:** | | | |
| AO | mm | PWd | mm |
| LA | mm | EDV | ml |
| LVIDd | mm | ESV | ml |
| LVIDs | mm | LVEF | 61% |
| IVSd | mm | FS | 32% |
| **Additional Information**: |  | | |
| 10mm pericardial effusion on RA Side. | | | |
| **Final Diagnosis:** | | | |
| 1. {S, D, S} Levocardia. 2. Transitional AVSD, L – R Shunt 3. Severe Pulmonary Hypertension 4. Normal Biventricular Function | | | |
| **Remark**: | | | |
| **Recommendation**: | | | |
| SIGNATURE  Done by: Tesfaye T., Pediatrician, Pediatric Cardiologist _______________ 12/08/2013Eth.C | | | |

| Patient Name: **Selamawit G/Hiwet**. Patient ID: **Amaris PSC**. SEX/ Age: F/12Years. Date of Report: 13/**08/2013**.  BP: _______ Weight: ______ Height: ____________ BSA: ________. R.Dx: **Palpitation. AGH10.792** | | | |
| --- | --- | --- | --- |
| **Features** | **Finding** | **Features** | **Finding** |
| **Profile** |  | **Atria** |  |
| Abdominal situs | Solitus | Left atrium | Normal |
| Cardiac position | Levocardia | Right atrium | Normal |
| Systemic venous drainage | Normal. | **Atrioventricular valves** |  |
| Pulmonary venous drainage | Normal | Mitral valve | Annulus = 18mm |
| Atrioventricular connection | Concordant | Tricuspid valve | Annulus = 19mm  TAPSE = 17mm |
| Ventriculoarterial connection | Concordant | **Ventricles** |  |
| Ventricular loop | d-Loop | Left ventricle | Normal |
|  |  | Right ventricle | Normal |
| **Septae** |  | **Coronary arteries** | ----- |
| Interventricular septum | Intact | **Doppler Measurement** |  |
| Interatrial septum | Intact | Mitral | ----- |
| **Semilunal valves** |  | Aortic | ------- |
| Aortic valve | Annulus = 16mm | Tricuspid | ------- |
| Pulmonary valve | Annulus = 17mm | pulmonic | -------- |
| **Great arteries** | NRGA | **Aortic arch** | Left. No CoA |
| Aorta | ----- | **PDA** | No |
| Pulmonary artery | Normal MPA and Branch PAs. |  |  |
| **M-Mode:** | | | |
| AO | mm | PWd | mm |
| LA | mm | EDV | ml |
| LVIDd | mm | ESV | ml |
| LVIDs | mm | LVEF | 57% |
| IVSd | mm | FS | 29% |
| **Additional Information**: |  | | |
| No pericardial/Pleural effusion. | | | |
| **Final Diagnosis:** | | | |
| 1. Normal Echocardiography Study. | | | |
| **Remark**: | | | |
| **Recommendation**: | | | |
| SIGNATURE  Done by: Tesfaye T., Pediatrician, Pediatric Cardiologist _______________ 13/08/2013Eth.C | | | |

| Patient Name: **Baby of Tiru Alemayehu**. Patient ID: **FHRH**. SEX/ Age: M/19Days. Date of Report: 13/**08/2013**.  BP: _______ Weight: ______ Height: ____________ BSA: ________. R.Dx: **RD. AGH10.793** | | | |
| --- | --- | --- | --- |
| **Features** | **Finding** | **Features** | **Finding** |
| **Profile** |  | **Atria** |  |
| Abdominal situs | Solitus | Left atrium | Normal |
| Cardiac position | Levocardia | Right atrium | Normal |
| Systemic venous drainage | Normal. | **Atrioventricular valves** |  |
| Pulmonary venous drainage | Normal | Mitral valve | Annulus = 8mm |
| Atrioventricular connection | Concordant | Tricuspid valve | Annulus = 10mm  TAPSE = 10mm |
| Ventriculoarterial connection | Concordant | **Ventricles** |  |
| Ventricular loop | d-Loop | Left ventricle | Normal |
|  |  | Right ventricle | Normal |
| **Septae** |  | **Coronary arteries** | ----- |
| Interventricular septum | Intact | **Doppler Measurement** |  |
| Interatrial septum | Intact | Mitral | ----- |
| **Semilunal valves** |  | Aortic | ------- |
| Aortic valve | Annulus = 9mm | Tricuspid | ------- |
| Pulmonary valve | Annulus = 9mm | pulmonic | -------- |
| **Great arteries** | NRGA | **Aortic arch** | Left. No CoA |
| Aorta | ----- | **PDA** | No |
| Pulmonary artery | Normal MPA and Branch PAs. |  |  |
| **M-Mode:** Normal LV Function on eye balling. | | | |
| AO | mm | PWd | mm |
| LA | mm | EDV | ml |
| LVIDd | mm | ESV | ml |
| LVIDs | mm | LVEF | % |
| IVSd | mm | FS | % |
| **Additional Information**: |  | | |
| No pericardial/Pleural effusion. | | | |
| **Final Diagnosis:** | | | |
| 1. Normal Echocardiography Study. | | | |
| **Remark**: | | | |
| **Recommendation**: | | | |
| SIGNATURE  Done by: Tesfaye T., Pediatrician, Pediatric Cardiologist _______________ 13/08/2013Eth.C | | | |

| Patient Name: **Nigusu Mekonen**. Patient ID: **FHCSH**. SEX/ Age: M/6/12. Date of Report: 13/**08/2013**.  BP: _______ Weight: ______ Height: ____________ BSA: ________. R.Dx: **FTT. AGH10.794** | | | |
| --- | --- | --- | --- |
| **Features** | **Finding** | **Features** | **Finding** |
| **Profile** |  | **Atria** |  |
| Abdominal situs | Solitus | Left atrium | Normal |
| Cardiac position | Levocardia | Right atrium | Normal |
| Systemic venous drainage | Normal. | **Atrioventricular valves** |  |
| Pulmonary venous drainage | Normal | Mitral valve | Annulus = 11mm |
| Atrioventricular connection | Concordant | Tricuspid valve | Annulus = 13mm |
| Ventriculoarterial connection | Concordant | **Ventricles** |  |
| Ventricular loop | d-Loop | Left ventricle | Normal |
|  |  | Right ventricle | Normal |
| **Septae** |  | **Coronary arteries** | ----- |
| Interventricular septum | 6mm PM VSD, L – R Shunt. | **Doppler Measurement** |  |
| Interatrial septum | Intact | Mitral | ----- |
| **Semilunal valves** |  | Aortic | ------- |
| Aortic valve | Annulus = 11mm | Tricuspid | ------- |
| Pulmonary valve | Annulus = 10mm | pulmonic | -------- |
| **Great arteries** | NRGA | **Aortic arch** | Left |
| Aorta | ----- | **PDA** | No |
| Pulmonary artery | Normal MPA and Branch PAs. |  |  |
| **M-Mode:** Normal LV Function on eye balling. | | | |
| AO | mm | PWd | mm |
| LA | mm | EDV | ml |
| LVIDd | mm | ESV | ml |
| LVIDs | mm | LVEF | % |
| IVSd | mm | FS | % |
| **Additional Information**: |  | | |
| No pericardial/Pleural effusion. | | | |
| **Final Diagnosis:** | | | |
| 1. {S, D, S} Levocardia. 2. Moderate PM VSD, L – R Shunt 3. Normal Function | | | |
| **Remark**: | | | |
| **Recommendation**: | | | |
| SIGNATURE  Done by: Tesfaye T., Pediatrician, Pediatric Cardiologist _______________ 13/08/2013Eth.C | | | |

| Patient Name: **Getinet Aynewa**. Patient ID: **FHRH**. SEX/ Age: M/13Years. Date of Report: 16/**08/2013**.  BP: _______ Weight: ______ Height: ____________ BSA: ________. R.Dx: **DOE. AGH10.795** | | | |
| --- | --- | --- | --- |
| **Features** | **Finding** | **Features** | **Finding** |
| **Profile** |  | **Atria** |  |
| Abdominal situs | Solitus | Left atrium | Normal |
| Cardiac position | Levocardia | Right atrium | Dilated |
| Systemic venous drainage | Normal. | **Atrioventricular valves** |  |
| Pulmonary venous drainage | Normal | Mitral valve | Annulus = 19mm |
| Atrioventricular connection | Concordant | Tricuspid valve | Annulus = 29mm. Echogenic mass on the RA Side of the anterior TVL, mobile.  TAPSE = 23mm |
| Ventriculoarterial connection | Concordant | **Ventricles** |  |
| Ventricular loop | d-Loop | Left ventricle | Normal |
|  |  | Right ventricle | Dilated |
| **Septae** | Abnormal Septal motion. | **Coronary arteries** | ----- |
| Interventricular septum | Intact | **Doppler Measurement** |  |
| Interatrial septum | Intact | Mitral | ----- |
| **Semilunal valves** |  | Aortic | ------- |
| Aortic valve | Annulus = 17mm | Tricuspid | Moderate TR, PPG = 37mmHg |
| Pulmonary valve | Annulus = 20mm | pulmonic | -------- |
| **Great arteries** | NRGA | **Aortic arch** | Left |
| Aorta | ----- | **PDA** | No |
| Pulmonary artery | Normal MPA and Branch PAs. |  |  |
| **M-Mode:** | | | |
| AO | mm | PWd | mm |
| LA | mm | EDV | ml |
| LVIDd | mm | ESV | ml |
| LVIDs | mm | LVEF | 47% |
| IVSd | mm | FS | 23% |
| **Additional Information**: |  | | |
| pericardial effusion 8mm on RV side and 7mm on LV Side. | | | |
| **Final Diagnosis:** | | | |
| 1. {S, D, S} Levocardia. 2. RA/RV Dilated 3. Moderate TR 4. Echogenic mass on the RA Side of the anterior TVL 5. LV Systolic Dysfunction 6. Mild Pulmonary Hypertension 7. Small Pericardial effusion | | | |
| **Remark**: | | | |
| **Recommendation**: Corelate the finding with the clinical data | | | |
| SIGNATURE  Done by: Tesfaye T., Pediatrician, Pediatric Cardiologist _______________ 16/08/2013Eth.C | | | |

| Patient Name: **Haile Bogale**. Patient ID: **Adinas GH**. SEX/ Age: M/11Years. Date of Report: 16/**08/2013**.  BP: _______ Weight: ______ Height: ____________ BSA: ________. R.Dx: **easy fatigability. AGH10.796** | | | |
| --- | --- | --- | --- |
| **Features** | **Finding** | **Features** | **Finding** |
| **Profile** |  | **Atria** |  |
| Abdominal situs | Solitus | Left atrium | Normal |
| Cardiac position | Levocardia | Right atrium | Normal |
| Systemic venous drainage | Normal. | **Atrioventricular valves** |  |
| Pulmonary venous drainage | Normal | Mitral valve | Annulus = 19mm |
| Atrioventricular connection | Concordant | Tricuspid valve | Annulus = 19mm  TAPSE = 24mm |
| Ventriculoarterial connection | Concordant | **Ventricles** |  |
| Ventricular loop | d-Loop | Left ventricle | Normal |
|  |  | Right ventricle | Normal |
| **Septae** |  | **Coronary arteries** | ----- |
| Interventricular septum | Intact | **Doppler Measurement** |  |
| Interatrial septum | Intact | Mitral | ----- |
| **Semilunal valves** |  | Aortic | ------- |
| Aortic valve | Annulus = 15mm | Tricuspid | ------- |
| Pulmonary valve | Annulus = 20mm | pulmonic | -------- |
| **Great arteries** | NRGA | **Aortic arch** | Left |
| Aorta | ----- | **PDA** | No |
| Pulmonary artery | Normal MPA and Branch PAs. |  |  |
| **M-Mode:** | | | |
| AO | mm | PWd | mm |
| LA | mm | EDV | ml |
| LVIDd | mm | ESV | ml |
| LVIDs | mm | LVEF | 66% |
| IVSd | mm | FS | 36% |
| **Additional Information**: |  | | |
| No pericardial/Pleural effusion. | | | |
| **Final Diagnosis:** | | | |
| 1. Normal Echocardiography Study. | | | |
| **Remark**: | | | |
| **Recommendation**: | | | |
| SIGNATURE  Done by: Tesfaye T., Pediatrician, Pediatric Cardiologist _______________ 16/08/2013Eth.C | | | |

| Patient Name: **Wudsew Getinet**. Patient ID: **FHRH**. SEX/ Age: M/11Years. Date of Report: 18/**08/2013**.  BP: _______ Weight: ______ Height: ____________ BSA: ________. R.Dx: **Incidental Murmur. AGH10.797** | | | |
| --- | --- | --- | --- |
| **Features** | **Finding** | **Features** | **Finding** |
| **Profile** |  | **Atria** |  |
| Abdominal situs | Solitus | Left atrium | Normal |
| Cardiac position | Levocardia | Right atrium | Normal |
| Systemic venous drainage | Normal. | **Atrioventricular valves** |  |
| Pulmonary venous drainage | Normal | Mitral valve | Annulus = 21mm  Patulous MVL |
| Atrioventricular connection | Concordant | Tricuspid valve | Annulus = 21mm  TAPSE = 21mm |
| Ventriculoarterial connection | Concordant | **Ventricles** |  |
| Ventricular loop | d-Loop | Left ventricle | Normal |
|  |  | Right ventricle | Normal |
| **Septae** |  | **Coronary arteries** | ----- |
| Interventricular septum | Intact | **Doppler Measurement** |  |
| Interatrial septum | Intact | Mitral | Trivial MR, Incomplete signal, seen In two planes with jet velocity = 2m/sec |
| **Semilunal valves** |  | Aortic | ------- |
| Aortic valve | Annulus = 20mm | Tricuspid | ------- |
| Pulmonary valve | Annulus = 21mm | pulmonic | -------- |
| **Great arteries** | NRGA | **Aortic arch** | Left |
| Aorta | ----- | **PDA** | No |
| Pulmonary artery | Normal MPA and Branch PAs. |  |  |
| **M-Mode:** | | | |
| AO | mm | PWd | mm |
| LA | mm | EDV | ml |
| LVIDd | mm | ESV | ml |
| LVIDs | mm | LVEF | 64% |
| IVSd | mm | FS | 34% |
| **Additional Information**: |  | | |
| No pericardial/Pleural effusion. | | | |
| **Final Diagnosis:** | | | |
| 1. {S, D, S} Levocardia. 2. Trivial MR 3. Normal Biventricular Function | | | |
| **Remark**: | | | |
| **Recommendation**: | | | |
| SIGNATURE  Done by: Tesfaye T., Pediatrician, Pediatric Cardiologist _______________ 18/08/2013Eth.C | | | |

| Patient Name: **Baby of Emebet Asmamaw**. Patient ID: **FHRH**. SEX/ Age: M/8days. Date of Report: 18/**08/2013**.  BP: _______ Weight: ______ Height: ____________ BSA: ________. R.Dx: **DS. AGH10.798** | | | |
| --- | --- | --- | --- |
| **Features** | **Finding** | **Features** | **Finding** |
| **Profile** |  | **Atria** |  |
| Abdominal situs | Solitus | Left atrium | Normal |
| Cardiac position | Levocardia | Right atrium | Normal |
| Systemic venous drainage | Normal. | **Atrioventricular valves** |  |
| Pulmonary venous drainage | Normal | Mitral valve | Annulus = 11mm |
| Atrioventricular connection | Concordant | Tricuspid valve | Annulus = 10mm |
| Ventriculoarterial connection | Concordant | **Ventricles** |  |
| Ventricular loop | d-Loop | Left ventricle | Normal |
|  |  | Right ventricle | Normal |
| **Septae** |  | **Coronary arteries** | ----- |
| Interventricular septum | Intact | **Doppler Measurement** |  |
| Interatrial septum | Intact | Mitral | ----- |
| **Semilunal valves** |  | Aortic | ------- |
| Aortic valve | Annulus = 8mm | Tricuspid | ------- |
| Pulmonary valve | Annulus = 8mm | pulmonic | -------- |
| **Great arteries** | NRGA | **Aortic arch** | Left |
| Aorta | ----- | **PDA** | No |
| Pulmonary artery | Normal MPA and Branch PAs. |  |  |
| **M-Mode:** Normal LV Function on eye balling. | | | |
| AO | mm | PWd | mm |
| LA | mm | EDV | ml |
| LVIDd | mm | ESV | ml |
| LVIDs | mm | LVEF | % |
| IVSd | mm | FS | % |
| **Additional Information**: |  | | |
| No pericardial/Pleural effusion. | | | |
| **Final Diagnosis:** | | | |
| 1. Normal Echocardiography Study. | | | |
| **Remark**: | | | |
| **Recommendation**: | | | |
| SIGNATURE  Done by: Tesfaye T., Pediatrician, Pediatric Cardiologist _______________ 18/08/2013Eth.C | | | |

| Patient Name: **Chalachew Yeshiwas**. Patient ID: **TGSH**. SEX/ Age: M/10Years. Date of Report: 19/**08/2013**.  BP: _______ Weight: ______ Height: ____________ BSA: ________. R.Dx: **easy fatigability. AGH10.799** | | | |
| --- | --- | --- | --- |
| **Features** | **Finding** | **Features** | **Finding** |
| **Profile** |  | **Atria** |  |
| Abdominal situs | Solitus | Left atrium | Normal |
| Cardiac position | Levocardia | Right atrium | Normal |
| Systemic venous drainage | Normal. | **Atrioventricular valves** |  |
| Pulmonary venous drainage | Normal | Mitral valve | Annulus = 20mm |
| Atrioventricular connection | Concordant | Tricuspid valve | Annulus = 21mm  TAPSE = 25mm |
| Ventriculoarterial connection | Concordant | **Ventricles** |  |
| Ventricular loop | d-Loop | Left ventricle | Normal |
|  |  | Right ventricle | Normal |
| **Septae** |  | **Coronary arteries** | ----- |
| Interventricular septum | Intact | **Doppler Measurement** |  |
| Interatrial septum | Intact | Mitral | ----- |
| **Semilunal valves** |  | Aortic | ------- |
| Aortic valve | Annulus = 17mm | Tricuspid | ------- |
| Pulmonary valve | Annulus = 21mm | pulmonic | -------- |
| **Great arteries** | NRGA | **Aortic arch** | Left |
| Aorta | ----- | **PDA** | No |
| Pulmonary artery | Normal MPA and Branch PAs. |  |  |
| **M-Mode:** | | | |
| AO | mm | PWd | mm |
| LA | mm | EDV | ml |
| LVIDd | mm | ESV | ml |
| LVIDs | mm | LVEF | 69% |
| IVSd | mm | FS | 38% |
| **Additional Information**: |  | | |
| No pericardial/Pleural effusion. | | | |
| **Final Diagnosis:** | | | |
| 1. Normal Echocardiography Study. | | | |
| **Remark**: | | | |
| **Recommendation**: | | | |
| SIGNATURE  Done by: Tesfaye T., Pediatrician, Pediatric Cardiologist _______________ 19/08/2013Eth.C | | | |

| Patient Name: **Sualihat Mohamed**. Patient ID: **FHRH**. SEX/ Age: F/9/12. Date of Report: 19/**08/2013**.  BP: _______ Weight: ______ Height: ____________ BSA: ________. R.Dx: **Incidental Murmur. AGH10.800** | | | |
| --- | --- | --- | --- |
| **Features** | **Finding** | **Features** | **Finding** |
| **Profile** |  | **Atria** |  |
| Abdominal situs | Solitus | Left atrium | Normal |
| Cardiac position | Levocardia | Right atrium | Normal |
| Systemic venous drainage | Normal. | **Atrioventricular valves** |  |
| Pulmonary venous drainage | Normal | Mitral valve | Annulus = 11mm |
| Atrioventricular connection | Concordant | Tricuspid valve | Annulus = 13mm |
| Ventriculoarterial connection | Concordant | **Ventricles** |  |
| Ventricular loop | d-Loop | Left ventricle | Normal |
|  |  | Right ventricle | Normal |
| **Septae** |  | **Coronary arteries** | ----- |
| Interventricular septum | 2mm PM VSD, L – R Shunt | **Doppler Measurement** |  |
| Interatrial septum | Intact | Mitral | ----- |
| **Semilunal valves** |  | Aortic | ------- |
| Aortic valve | Annulus = 12mm | Tricuspid | ------- |
| Pulmonary valve | Annulus = 12mm | pulmonic | -------- |
| **Great arteries** | NRGA | **Aortic arch** | Left |
| Aorta | ----- | **PDA** | No |
| Pulmonary artery | Normal MPA and Branch PAs. |  |  |
| **M-Mode:** | | | |
| AO | mm | PWd | mm |
| LA | mm | EDV | ml |
| LVIDd | mm | ESV | ml |
| LVIDs | mm | LVEF | 65% |
| IVSd | mm | FS | 33% |
| **Additional Information**: |  | | |
| No pericardial/Pleural effusion. | | | |
| **Final Diagnosis:** | | | |
| 1. {S, D, S} Levocardia. 2. Small PM VSD, L – R Shunt 3. Normal LV Function | | | |
| **Remark**: Follow up echo for PFO and small PM VSD | | | |
| **Recommendation**:  No need to put on any form of medication | | | |
| SIGNATURE  Done by: Tesfaye T., Pediatrician, Pediatric Cardiologist _______________ 19/08/2013Eth.C | | | |

| Patient Name: **Bethelihem Melash**. Patient ID: **FHCSH**. SEX/ Age: F/12Years. Date of Report: 19/**08/2013**.  BP: _______ Weight: ______ Height: ____________ BSA: ________. R.Dx: **Arrhythmia10.801** | | | |
| --- | --- | --- | --- |
| **Features** | **Finding** | **Features** | **Finding** |
| **Profile** |  | **Atria** |  |
| Abdominal situs | Solitus | Left atrium | Normal |
| Cardiac position | Levocardia | Right atrium | Normal |
| Systemic venous drainage | Normal. | **Atrioventricular valves** |  |
| Pulmonary venous drainage | Normal | Mitral valve | Annulus = 15mm |
| Atrioventricular connection | Concordant | Tricuspid valve | Annulus = 15mm  TAPSE = 18mm |
| Ventriculoarterial connection | Concordant | **Ventricles** |  |
| Ventricular loop | d-Loop | Left ventricle | Normal |
|  |  | Right ventricle | Normal |
| **Septae** |  | **Coronary arteries** | ----- |
| Interventricular septum | Intact | **Doppler Measurement** |  |
| Interatrial septum | Intact | Mitral | ----- |
| **Semilunal valves** |  | Aortic | ------- |
| Aortic valve | Annulus = 15mm | Tricuspid | ------- |
| Pulmonary valve | Annulus = 16mm | pulmonic | -------- |
| **Great arteries** | NRGA | **Aortic arch** | Left |
| Aorta | ----- | **PDA** | No |
| Pulmonary artery | Normal MPA and Branch PAs. |  |  |
| **M-Mode:** | | | |
| AO | mm | PWd | mm |
| LA | mm | EDV | ml |
| LVIDd | mm | ESV | ml |
| LVIDs | mm | LVEF | 57% |
| IVSd | mm | FS | 29% |
| **Additional Information**: |  | | |
| No pericardial/Pleural effusion. | | | |
| **Final Diagnosis:** | | | |
| 1. Normal Echocardiography Study. | | | |
| **Remark**: Rhythm abnormality during study | | | |
| **Recommendation**: ECG | | | |
| SIGNATURE  Done by: Tesfaye T., Pediatrician, Pediatric Cardiologist _______________ 19/08/2013Eth.C | | | |

| Patient Name: **Seble-werk Agmassie**. Patient ID: **FHCSH**. SEX/ Age: F/1 1/12. Date of Report: 19/**08/2013**.  BP: _______ Weight: ______ Height: ____________ BSA: ______. R.Dx: **Recurrent Chest Infection. AGH10.802** | | | |
| --- | --- | --- | --- |
| **Features** | **Finding** | **Features** | **Finding** |
| **Profile** |  | **Atria** |  |
| Abdominal situs | Solitus | Left atrium | Normal |
| Cardiac position | Levocardia | Right atrium | Normal |
| Systemic venous drainage | Normal. | **Atrioventricular valves** |  |
| Pulmonary venous drainage | Normal | Mitral valve | Annulus = 12mm |
| Atrioventricular connection | Concordant | Tricuspid valve | Annulus = 12mm |
| Ventriculoarterial connection | Concordant | **Ventricles** |  |
| Ventricular loop | d-Loop | Left ventricle | Normal |
|  |  | Right ventricle | Normal |
| **Septae** |  | **Coronary arteries** | ----- |
| Interventricular septum | Intact | **Doppler Measurement** |  |
| Interatrial septum | Intact | Mitral | ----- |
| **Semilunal valves** |  | Aortic | ------- |
| Aortic valve | Annulus = 11mm | Tricuspid | ------- |
| Pulmonary valve | Annulus = 11mm | pulmonic | -------- |
| **Great arteries** | NRGA | **Aortic arch** | Left |
| Aorta | ----- | **PDA** | No |
| Pulmonary artery | Normal MPA and Branch PAs. |  |  |
| **M-Mode:** Normal LV Function on eye balling. | | | |
| AO | mm | PWd | mm |
| LA | mm | EDV | ml |
| LVIDd | mm | ESV | ml |
| LVIDs | mm | LVEF | % |
| IVSd | mm | FS | % |
| **Additional Information**: |  | | |
| No pericardial/Pleural effusion. | | | |
| **Final Diagnosis:** | | | |
| 1. Normal Echocardiography Study. | | | |
| **Remark**: | | | |
| **Recommendation**: | | | |
| SIGNATURE  Done by: Tesfaye T., Pediatrician, Pediatric Cardiologist _______________ 19/08/2013Eth.C | | | |

| Patient Name: **Libona Tewuhbo**. Patient ID: **TGSH**. SEX/ Age: F/9Years. Date of Report: 19/**08/2013**.  BP: _______ Weight: ______ Height: ____________ BSA: ________. R.Dx: **Sydenham’s Chorea. AGH10.803** | | | |
| --- | --- | --- | --- |
| **Features** | **Finding** | **Features** | **Finding** |
| **Profile** |  | **Atria** |  |
| Abdominal situs | Solitus | Left atrium | Mildly Dilated |
| Cardiac position | Levocardia | Right atrium | Normal |
| Systemic venous drainage | Normal. | **Atrioventricular valves** |  |
| Pulmonary venous drainage | Normal | Mitral valve | Annulus = 20mm. Thickened MVL |
| Atrioventricular connection | Concordant | Tricuspid valve | Annulus = 18mm  TAPSE = 22mm |
| Ventriculoarterial connection | Concordant | **Ventricles** |  |
| Ventricular loop | d-Loop | Left ventricle | Mildly Dilated |
|  |  | Right ventricle | Normal |
| **Septae** |  | **Coronary arteries** | ----- |
| Interventricular septum | Intact | **Doppler Measurement** |  |
| Interatrial septum | Intact | Mitral | Mild MR, Holosystolic, posterior projection, seen in two planes with jet velocity = 4.6m/sec |
| **Semilunal valves** |  | Aortic | ------- |
| Aortic valve | Annulus = 18mm | Tricuspid | ------- |
| Pulmonary valve | Annulus = 20mm | pulmonic | -------- |
| **Great arteries** | NRGA | **Aortic arch** | Left |
| Aorta | ----- | **PDA** | No |
| Pulmonary artery | Normal MPA and Branch PAs. |  |  |
| **M-Mode:** | | | |
| AO | mm | PWd | mm |
| LA | mm | EDV | ml |
| LVIDd | mm | ESV | ml |
| LVIDs | mm | LVEF | 65% |
| IVSd | mm | FS | 35% |
| **Additional Information**: |  | | |
| No pericardial/Pleural effusion. | | | |
| **Final Diagnosis:** | | | |
| 1. {S, D, S} Levocardia. 2. Thickened MVL 3. Mild MR 4. Normal Biventricular Function | | | |
| **Remark**: | | | |
| **Recommendation**: | | | |
| SIGNATURE  Done by: Tesfaye T., Pediatrician, Pediatric Cardiologist _______________ 19/08/2013Eth.C | | | |

| Patient Name: **Baby of Tirualem Fenta**. Patient ID: **FHRH**. SEX/ Age: M/23days. Date of Report: 19/**08/2013**.  BP: _______ Weight: ______ Height: ____________ BSA: ________. R.Dx: **DS. AGH10.804** | | | |
| --- | --- | --- | --- |
| **Features** | **Finding** | **Features** | **Finding** |
| **Profile** |  | **Atria** |  |
| Abdominal situs | Solitus | Left atrium | Normal |
| Cardiac position | Levocardia | Right atrium | Normal |
| Systemic venous drainage | Normal. | **Atrioventricular valves** |  |
| Pulmonary venous drainage | Normal | Mitral valve | Annulus = 11mm |
| Atrioventricular connection | Concordant | Tricuspid valve | Annulus = 11mm |
| Ventriculoarterial connection | Concordant | **Ventricles** |  |
| Ventricular loop | d-Loop | Left ventricle | Normal |
|  |  | Right ventricle | Normal |
| **Septae** |  | **Coronary arteries** | ----- |
| Interventricular septum | Intact | **Doppler Measurement** |  |
| Interatrial septum | Intact | Mitral | ----- |
| **Semilunal valves** |  | Aortic | ------- |
| Aortic valve | Annulus = 11mm | Tricuspid | ------- |
| Pulmonary valve | Annulus = 11mm | pulmonic | -------- |
| **Great arteries** | NRGA | **Aortic arch** | Left |
| Aorta | ----- | **PDA** | No |
| Pulmonary artery | Normal MPA and Branch PAs. |  |  |
| **M-Mode:** Normal LV Function on eye balling. | | | |
| AO | mm | PWd | mm |
| LA | mm | EDV | ml |
| LVIDd | mm | ESV | ml |
| LVIDs | mm | LVEF | % |
| IVSd | mm | FS | % |
| **Additional Information**: |  | | |
| No pericardial/Pleural effusion. | | | |
| **Final Diagnosis:** | | | |
| 1. Normal Echocardiography Study. | | | |
| **Remark**: Limited Echo window (Only Subcostal window) | | | |
| **Recommendation**: | | | |
| SIGNATURE  Done by: Tesfaye T., Pediatrician, Pediatric Cardiologist _______________ 19/08/2013Eth.C | | | |

| Patient Name: **Tsion Haregu**. Patient ID: **Adinas GH**. SEX/ Age: F/15Years. Date of Report: 20/**08/2013**.  BP: _______ Weight: ______ Height: ____________ BSA: ________. R.Dx: **easy fatigability. AGH10.805** | | | |
| --- | --- | --- | --- |
| **Features** | **Finding** | **Features** | **Finding** |
| **Profile** |  | **Atria** |  |
| Abdominal situs | Solitus | Left atrium | Normal |
| Cardiac position | Levocardia | Right atrium | Normal |
| Systemic venous drainage | Normal. | **Atrioventricular valves** |  |
| Pulmonary venous drainage | Normal | Mitral valve | Annulus = 21mm |
| Atrioventricular connection | Concordant | Tricuspid valve | Annulus = 21mm |
| Ventriculoarterial connection | Concordant | **Ventricles** |  |
| Ventricular loop | d-Loop | Left ventricle | Normal |
|  |  | Right ventricle | Normal |
| **Septae** |  | **Coronary arteries** | ----- |
| Interventricular septum | Intact | **Doppler Measurement** |  |
| Interatrial septum | Intact | Mitral | ----- |
| **Semilunal valves** |  | Aortic | ------- |
| Aortic valve | Annulus = 19mm | Tricuspid | ------- |
| Pulmonary valve | Annulus = 19mm | pulmonic | -------- |
| **Great arteries** | NRGA | **Aortic arch** | Left |
| Aorta | ----- | **PDA** | No |
| Pulmonary artery | Normal MPA and Branch PAs. |  |  |
| **M-Mode:** Normal LV Function on eye balling. | | | |
| AO | mm | PWd | mm |
| LA | mm | EDV | ml |
| LVIDd | mm | ESV | ml |
| LVIDs | mm | LVEF | % |
| IVSd | mm | FS | % |
| **Additional Information**: |  | | |
| No pericardial/Pleural effusion. | | | |
| **Final Diagnosis:** | | | |
| 1. Normal Echocardiography Study. | | | |
| **Remark**: | | | |
| **Recommendation**: | | | |
| SIGNATURE  Done by: Tesfaye T., Pediatrician, Pediatric Cardiologist _______________ 20/08/2013Eth.C | | | |

| Patient Name: **Baby of Wubalech Melkie**. Patient ID: **FHRH**. SEX/ Age: F/6Days. Date of Report: 20/**08/2013**.  BP: _______ Weight: ______ Height: ________ BSA: ____. R.Dx: **Incidental Murmur. AGH10.806** | | | |
| --- | --- | --- | --- |
| **Features** | **Finding** | **Features** | **Finding** |
| **Profile** |  | **Atria** |  |
| Abdominal situs | Solitus | Left atrium | Normal |
| Cardiac position | Levocardia | Right atrium | Normal |
| Systemic venous drainage | Normal. | **Atrioventricular valves** |  |
| Pulmonary venous drainage | Normal | Mitral valve | Annulus = 9mm |
| Atrioventricular connection | Concordant | Tricuspid valve | Annulus = 9mm  TAPSE = 10mm |
| Ventriculoarterial connection | Concordant | **Ventricles** |  |
| Ventricular loop | d-Loop | Left ventricle | Normal |
|  |  | Right ventricle | Normal |
| **Septae** |  | **Coronary arteries** | ----- |
| Interventricular septum | Intact | **Doppler Measurement** |  |
| Interatrial septum | 5mm OS ASD, L – R Shunt | Mitral | ----- |
| **Semilunal valves** |  | Aortic | ------- |
| Aortic valve | Annulus = 8mm | Tricuspid | ------- |
| Pulmonary valve | Annulus = 9mm | pulmonic | -------- |
| **Great arteries** | NRGA | **Aortic arch** | Left |
| Aorta | ----- | **PDA** | 1mm PDA, L – R Shunt |
| Pulmonary artery | Normal MPA and Branch PAs. |  |  |
| **M-Mode:** Normal LV Function on eye balling. | | | |
| AO | mm | PWd | mm |
| LA | mm | EDV | ml |
| LVIDd | mm | ESV | ml |
| LVIDs | mm | LVEF | % |
| IVSd | mm | FS | % |
| **Additional Information**: |  | | |
| No pericardial/Pleural effusion. | | | |
| **Final Diagnosis:** | | | |
| 1. {S, D, S} Levocardia. 2. Small OS ASD, L – R Shunt 3. Small PDA, L – R Shunt 4. Normal Biventricular Function | | | |
| **Remark**: | | | |
| **Recommendation**: | | | |
| SIGNATURE  Done by: Tesfaye T., Pediatrician, Pediatric Cardiologist _______________ 20/08/2013Eth.C | | | |

| Patient Name: **Atinkut Dagne**. Patient ID: **FHCSH**. SEX/ Age: M/13Years. Date of Report: 21/**08/2013**.  BP: _______ Weight: ______ Height: ____________ BSA: ________. R.Dx: **Sydenham’s Chorea. AGH10.807** | | | |
| --- | --- | --- | --- |
| **Features** | **Finding** | **Features** | **Finding** |
| **Profile** |  | **Atria** |  |
| Abdominal situs | Solitus | Left atrium | Normal |
| Cardiac position | Levocardia | Right atrium | Normal |
| Systemic venous drainage | Normal. | **Atrioventricular valves** |  |
| Pulmonary venous drainage | Normal | Mitral valve | Annulus = 20mm. patulous MVL |
| Atrioventricular connection | Concordant | Tricuspid valve | Annulus = 20mm  TAPSE = 18mm |
| Ventriculoarterial connection | Concordant | **Ventricles** |  |
| Ventricular loop | d-Loop | Left ventricle | Normal |
|  |  | Right ventricle | Normal |
| **Septae** |  | **Coronary arteries** | ----- |
| Interventricular septum | Intact | **Doppler Measurement** |  |
| Interatrial septum | Intact | Mitral | Trivial MR, Incomplete signal, seen in apical view with jet velocity = 4m/sec |
| **Semilunal valves** |  | Aortic | ------- |
| Aortic valve | Annulus = 20mm | Tricuspid | ------- |
| Pulmonary valve | Annulus = 23mm | pulmonic | -------- |
| **Great arteries** | NRGA | **Aortic arch** | Left |
| Aorta | ----- | **PDA** | No |
| Pulmonary artery | Normal MPA and Branch PAs. |  |  |
| **M-Mode:** | | | |
| AO | mm | PWd | mm |
| LA | mm | EDV | ml |
| LVIDd | mm | ESV | ml |
| LVIDs | mm | LVEF | 58% |
| IVSd | mm | FS | 31% |
| **Additional Information**: |  | | |
| No pericardial/Pleural effusion. | | | |
| **Final Diagnosis:** | | | |
| 1. {S, D, S} Levocardia. 2. Trivial MR 3. Normal Biventricular Function | | | |
| **Remark**: Borderline RHD | | | |
| **Recommendation**: | | | |
| SIGNATURE  Done by: Tesfaye T., Pediatrician, Pediatric Cardiologist _______________ 21/08/2013Eth.C | | | |

| Patient Name: **Tigist Adane**. Patient ID: **TGSH**. SEX/ Age: F/12Years. Date of Report: 21/**08/2013**.  BP: _______ Weight: ______ Height: ____________ BSA: ________. R.Dx: **arrhythmia. AGH10.808** | | | |
| --- | --- | --- | --- |
| **Features** | **Finding** | **Features** | **Finding** |
| **Profile** |  | **Atria** |  |
| Abdominal situs | Solitus | Left atrium | Normal |
| Cardiac position | Levocardia | Right atrium | Normal |
| Systemic venous drainage | Normal. | **Atrioventricular valves** |  |
| Pulmonary venous drainage | Normal | Mitral valve | Annulus = 19mm |
| Atrioventricular connection | Concordant | Tricuspid valve | Annulus = 20mm  TAPSE = 21mm |
| Ventriculoarterial connection | Concordant | **Ventricles** |  |
| Ventricular loop | d-Loop | Left ventricle | Normal |
|  |  | Right ventricle | Normal |
| **Septae** |  | **Coronary arteries** | ----- |
| Interventricular septum | Intact | **Doppler Measurement** |  |
| Interatrial septum | Intact | Mitral | ----- |
| **Semilunal valves** |  | Aortic | ------- |
| Aortic valve | Annulus = 17mm | Tricuspid | ------- |
| Pulmonary valve | Annulus = 18mm | pulmonic | -------- |
| **Great arteries** | NRGA | **Aortic arch** | Left. No CoA. |
| Aorta | ----- | **PDA** | No |
| Pulmonary artery | Normal MPA and Branch PAs. |  |  |
| **M-Mode:** | | | |
| AO | mm | PWd | mm |
| LA | mm | EDV | ml |
| LVIDd | mm | ESV | ml |
| LVIDs | mm | LVEF | 65% |
| IVSd | mm | FS | 35% |
| **Additional Information**: |  | | |
| No pericardial/Pleural effusion. | | | |
| **Final Diagnosis:** | | | |
| 1. Normal Echocardiography Study. | | | |
| **Remark**: | | | |
| **Recommendation**: Do ECG | | | |
| SIGNATURE  Done by: Tesfaye T., Pediatrician, Pediatric Cardiologist _______________ 21/08/2013Eth.C | | | |

| Patient Name: **Tseganat Yenie-eshet**. Patient ID: **Adet PH**. SEX/ Age: F/10Years. Date of Report: 21/**08/2013**.  BP: _______ Weight: ______ Height: ____________ BSA: ________. R.Dx: **chest pain. AGH10. 809** | | | |
| --- | --- | --- | --- |
| **Features** | **Finding** | **Features** | **Finding** |
| **Profile** |  | **Atria** |  |
| Abdominal situs | Solitus | Left atrium | Normal |
| Cardiac position | Levocardia | Right atrium | Normal |
| Systemic venous drainage | Normal. | **Atrioventricular valves** |  |
| Pulmonary venous drainage | Normal | Mitral valve | Annulus = 18mm |
| Atrioventricular connection | Concordant | Tricuspid valve | Annulus = 18mm  TAPSE = 25mm |
| Ventriculoarterial connection | Concordant | **Ventricles** |  |
| Ventricular loop | d-Loop | Left ventricle | Normal |
|  |  | Right ventricle | Normal |
| **Septae** |  | **Coronary arteries** | ----- |
| Interventricular septum | Intact | **Doppler Measurement** |  |
| Interatrial septum | Intact | Mitral | ----- |
| **Semilunal valves** |  | Aortic | ------- |
| Aortic valve | Annulus = 14mm | Tricuspid | ------- |
| Pulmonary valve | Annulus = 15mm | pulmonic | -------- |
| **Great arteries** | NRGA | **Aortic arch** | Left |
| Aorta | ----- | **PDA** | No |
| Pulmonary artery | Normal MPA and Branch PAs. |  |  |
| **M-Mode:** | | | |
| AO | mm | PWd | mm |
| LA | mm | EDV | ml |
| LVIDd | mm | ESV | ml |
| LVIDs | mm | LVEF | 65% |
| IVSd | mm | FS | 35% |
| **Additional Information**: |  | | |
| No pericardial/Pleural effusion. | | | |
| **Final Diagnosis:** | | | |
| 1. Normal Echocardiography Study. | | | |
| **Remark**: | | | |
| **Recommendation**: | | | |
| SIGNATURE  Done by: Tesfaye T., Pediatrician, Pediatric Cardiologist _______________ 21/08/2013Eth.C | | | |

| Patient Name: **Zelalem Misgan**. Patient ID: **FHRH**. SEX/ Age: M/14Years. Date of Report: 25/**08/2013**.  BP: _______ Weight: ______ Height: _____ BSA: _____. R.Dx: **Rheumatic Recurrence. AGH10.810** | | | |
| --- | --- | --- | --- |
| **Features** | **Finding** | **Features** | **Finding** |
| **Profile** |  | **Atria** |  |
| Abdominal situs | Solitus | Left atrium | Dilated |
| Cardiac position | Levocardia | Right atrium | Normal |
| Systemic venous drainage | Normal. | **Atrioventricular valves** |  |
| Pulmonary venous drainage | Normal | Mitral valve | Annulus = 30mm. thickened MVL. Shortened PMVL. |
| Atrioventricular connection | Concordant | Tricuspid valve | Annulus = 19mm  TAPSE = 18mm |
| Ventriculoarterial connection | Concordant | **Ventricles** |  |
| Ventricular loop | d-Loop | Left ventricle | Dilated |
|  |  | Right ventricle | Normal |
| **Septae** |  | **Coronary arteries** | ----- |
| Interventricular septum | Intact | **Doppler Measurement** |  |
| Interatrial septum | Intact | Mitral | Moderate MR, Holosystolic, posterior projection, seen in two planes, jet velocity = 4.8m/sec. |
| **Semilunal valves** |  | Aortic | Mild AR, PHT = 508ms. |
| Aortic valve | Annulus = 19mm | Tricuspid | ------- |
| Pulmonary valve | Annulus = 23mm | pulmonic | -------- |
| **Great arteries** | NRGA | **Aortic arch** | Left |
| Aorta | ----- | **PDA** | No |
| Pulmonary artery | Normal MPA and Branch PAs. |  |  |
| **M-Mode:** | | | |
| AO | mm | PWd | mm |
| LA | mm | EDV | ml |
| LVIDd | mm | ESV | ml |
| LVIDs | mm | LVEF | 70% |
| IVSd | mm | FS | 40% |
| **Additional Information**: |  | | |
| No pericardial/Pleural effusion. | | | |
| **Final Diagnosis:** | | | |
| 1. {S, D, S} Levocardia. 2. LA/LV Dilated 3. Thickened, shortened PMVL 4. Moderate MR 5. Mild AR 6. Normal Biventricular Function | | | |
| **Remark**: | | | |
| **Recommendation**: | | | |
| SIGNATURE  Done by: Tesfaye T., Pediatrician, Pediatric Cardiologist _______________ 25/08/2013Eth.C | | | |

| Patient Name: **Baby of Wubalem Endalew**. Patient ID: **FHRH**. SEX/ Age: F/29days. Date of Report: 25/**08/2013**.  BP: _______ Weight: ______ Height: ____________ BSA: ________. R.Dx: **RD. AGH10.811** | | | |
| --- | --- | --- | --- |
| **Features** | **Finding** | **Features** | **Finding** |
| **Profile** |  | **Atria** |  |
| Abdominal situs | Solitus | Left atrium | Normal |
| Cardiac position | Levocardia | Right atrium | Normal |
| Systemic venous drainage | Normal. | **Atrioventricular valves** |  |
| Pulmonary venous drainage | Normal | Mitral valve | Annulus = 9mm |
| Atrioventricular connection | Concordant | Tricuspid valve | Annulus = 8mm |
| Ventriculoarterial connection | Concordant | **Ventricles** |  |
| Ventricular loop | d-Loop | Left ventricle | Normal |
|  |  | Right ventricle | Normal |
| **Septae** |  | **Coronary arteries** | ----- |
| Interventricular septum | Intact | **Doppler Measurement** |  |
| Interatrial septum | Intact | Mitral | ----- |
| **Semilunal valves** |  | Aortic | ------- |
| Aortic valve | Annulus = 8mm | Tricuspid | ------- |
| Pulmonary valve | Annulus = 8mm | pulmonic | -------- |
| **Great arteries** | NRGA | **Aortic arch** | Left |
| Aorta | ----- | **PDA** | No |
| Pulmonary artery | Normal MPA and Branch PAs. |  |  |
| **M-Mode:** Normal LV Function on eye balling. | | | |
| AO | mm | PWd | mm |
| LA | mm | EDV | ml |
| LVIDd | mm | ESV | ml |
| LVIDs | mm | LVEF | % |
| IVSd | mm | FS | % |
| **Additional Information**: |  | | |
| No pericardial/Pleural effusion. | | | |
| **Final Diagnosis:** | | | |
| 1. Normal Echocardiography Study. | | | |
| **Remark**: Limited Echo window | | | |
| **Recommendation**: | | | |
| SIGNATURE  Done by: Tesfaye T., Pediatrician, Pediatric Cardiologist _______________ 25/08/2013Eth.C | | | |

| Patient Name: **Kedir Seid**. Patient ID: **FHCSH**. SEX/ Age: M/14Years. Date of Report: 26/**08/2013**.  BP: _______ Weight: ______ Height: ____________ BSA: ________. R.Dx: **DOE. AGH10.812** | | | |
| --- | --- | --- | --- |
| **Features** | **Finding** | **Features** | **Finding** |
| **Profile** |  | **Atria** |  |
| Abdominal situs | Solitus | Left atrium | Normal |
| Cardiac position | Levocardia | Right atrium | Dilated |
| Systemic venous drainage | Normal. | **Atrioventricular valves** |  |
| Pulmonary venous drainage | Normal | Mitral valve | Annulus = 17mm |
| Atrioventricular connection | Concordant | Tricuspid valve | Annulus = 22mm  TAPSE = 24mm |
| Ventriculoarterial connection | Concordant | **Ventricles** |  |
| Ventricular loop | d-Loop | Left ventricle | Normal |
|  |  | Right ventricle | Dilated |
| **Septae** | Abnormal Septal Motion | **Coronary arteries** | ----- |
| Interventricular septum | Intact | **Doppler Measurement** |  |
| Interatrial septum | 35mm X 39mm OS ASD amounting to single atrium, BD Shunt. | Mitral | ----- |
| **Semilunal valves** |  | Aortic | ------- |
| Aortic valve | Annulus = 15mm | Tricuspid | ------- |
| Pulmonary valve | Annulus = 25mm | pulmonic | -------- |
| **Great arteries** | NRGA | **Aortic arch** | Left |
| Aorta | ----- | **PDA** | No |
| Pulmonary artery | **MPA =36mm.** |  |  |
| **M-Mode:** | | | |
| AO | mm | PWd | mm |
| LA | mm | EDV | ml |
| LVIDd | mm | ESV | ml |
| LVIDs | mm | LVEF | 60% |
| IVSd | mm | FS | 31% |
| **Additional Information**: |  | | |
| 4mm pericardial effusion on RV Side. | | | |
| **Final Diagnosis:** | | | |
| 1. {S, D, S} Levocardia. 2. RA/RV Dilated 3. Large OS ASD, BD Shunt amounting to single atrium, BD Shunt 4. Severe Pulmonary Hypertension | | | |
| **Remark**: | | | |
| **Recommendation**: Candidate for catheterization study and intervention (surgical) after cath. result | | | |
| SIGNATURE  Done by: Tesfaye T., Pediatrician, Pediatric Cardiologist _______________ 26/08/2013Eth.C | | | |

| Patient Name: **Etenat Ambaye**. Patient ID: **FHCSH**. SEX/ Age: __F/12Years. Date of Report: 26/**08/2013**.  BP: _______ Weight: ______ Height: _______ BSA: _____. R.Dx: **CHF + Rheumatic Recurrence. AGH10.813** | | | |
| --- | --- | --- | --- |
| **Features** | **Finding** | **Features** | **Finding** |
| **Profile** |  | **Atria** |  |
| Abdominal situs | Solitus | Left atrium | Dilated |
| Cardiac position | Levocardia | Right atrium | Normal |
| Systemic venous drainage | Normal. | **Atrioventricular valves** |  |
| Pulmonary venous drainage | Normal | Mitral valve | Annulus = 31mm. Thickened MVL. MVA = 1.3cm2. |
| Atrioventricular connection | Concordant | Tricuspid valve | Annulus = 30mm. Thickened TVL.  TAPSE = 21mm |
| Ventriculoarterial connection | Concordant | **Ventricles** |  |
| Ventricular loop | d-Loop | Left ventricle | Dilated |
|  |  | Right ventricle | Normal |
| **Septae** |  | **Coronary arteries** | ----- |
| Interventricular septum | Intact | **Doppler Measurement** |  |
| Interatrial septum | Intact | Mitral | Severe MR, Holosystolic, posterior projection, seen in 2 planes with jet velocity = 3.9m/sec. Moderate MS, PPG/MPG = 12/9mmHg. |
| **Semilunal valves** |  | Aortic | ------- |
| Aortic valve | Annulus = 17mm | Tricuspid | Severe TR, PPG = 54mmHg. Mild TS, PPG = 6/5mmHg. |
| Pulmonary valve | Annulus = 23mm | pulmonic | -------- |
| **Great arteries** | NRGA | **Aortic arch** | Left |
| Aorta | ----- | **PDA** | No |
| Pulmonary artery | Normal MPA and Branch PAs. |  |  |
| **M-Mode:** | | | |
| AO | mm | PWd | mm |
| LA | mm | EDV | ml |
| LVIDd | mm | ESV | ml |
| LVIDs | mm | LVEF | 59% |
| IVSd | mm | FS | 31% |
| **Final Diagnosis:** | | | |
| 1. {S, D, S} Levocardia. 2. LA/LV Dilated 3. Thickened MVL and TVL 4. Severe MR 5. Severe TR 6. Moderate MS 7. Mild TS 8. Moderate Pulmonary Hypertension 9. Normal Function | | | |
| SIGNATURE  Done by: Tesfaye T., Pediatrician, Pediatric Cardiologist _______________ 26/08/2013Eth.C | | | |

| Patient Name: **Hikma Dawd**. Patient ID: **GAMBY GH**. SEX/ Age: F/7/12. Date of Report: 27/**08/2013**.  BP: _______ Weight: ______ Height: ____________ BSA: ________. R.Dx: **_Incidental Murmur. AGH10.814** | | | |
| --- | --- | --- | --- |
| **Features** | **Finding** | **Features** | **Finding** |
| **Profile** |  | **Atria** |  |
| Abdominal situs | Solitus | Left atrium | Normal |
| Cardiac position | Levocardia | Right atrium | Normal |
| Systemic venous drainage | Normal. | **Atrioventricular valves** |  |
| Pulmonary venous drainage | Normal | Mitral valve | Annulus = 11mm |
| Atrioventricular connection | Concordant | Tricuspid valve | Annulus = 11mm  TAPSE = 15mm |
| Ventriculoarterial connection | Concordant | **Ventricles** |  |
| Ventricular loop | d-Loop | Left ventricle | Normal |
|  |  | Right ventricle | Normal |
| **Septae** |  | **Coronary arteries** | ----- |
| Interventricular septum | Intact | **Doppler Measurement** |  |
| Interatrial septum | Intact | Mitral | ----- |
| **Semilunal valves** |  | Aortic | ------- |
| Aortic valve | Annulus = 11mm | Tricuspid | ------- |
| Pulmonary valve | Annulus = 10mm | pulmonic | Mild PS, PPG = 22mmHg. |
| **Great arteries** | NRGA | **Aortic arch** | Left |
| Aorta | ----- | **PDA** | No |
| Pulmonary artery | Normal MPA and Branch PAs. |  |  |
| **M-Mode:** | | | |
| AO | mm | PWd | mm |
| LA | mm | EDV | ml |
| LVIDd | mm | ESV | ml |
| LVIDs | mm | LVEF | 68% |
| IVSd | mm | FS | 35% |
| **Additional Information**: |  | | |
| No pericardial/Pleural effusion. | | | |
| **Final Diagnosis:** | | | |
| 1. {S, D, S} Levocardia. 2. Mild PS 3. Normal Biventricular Function | | | |
| **Remark**: | | | |
| **Recommendation**: No need of intervention, medication…...  Follow up echocardiography every year (Spontanous resolution or progression is expected) | | | |
| SIGNATURE  Done by: Tesfaye T., Pediatrician, Pediatric Cardiologist _______________ 27/08/2013Eth.C | | | |

| Patient Name: **Baby of Mebrate Tadesse**. Patient ID: **TGSH**. SEX/ Age: F/3days. Date of Report: 27/**08/2013**.  BP: _______ Weight: ______ Height: ____________ BSA: ____. R.Dx: **DS + RD. AGH10.815** | | | |
| --- | --- | --- | --- |
| **Features** | **Finding** | **Features** | **Finding** |
| **Profile** |  | **Atria** |  |
| Abdominal situs | Solitus | Left atrium | Normal |
| Cardiac position | Levocardia | Right atrium | Dilated |
| Systemic venous drainage | Normal. | **Atrioventricular valves** |  |
| Pulmonary venous drainage | Normal | Mitral valve | Annulus = 9mm |
| Atrioventricular connection | Concordant | Tricuspid valve | Annulus = 11mm  TAPSE = 10mm |
| Ventriculoarterial connection | Concordant | **Ventricles** |  |
| Ventricular loop | d-Loop | Left ventricle | Normal |
|  |  | Right ventricle | Dilated |
| **Septae** |  | **Coronary arteries** | ----- |
| Interventricular septum | Intact | **Doppler Measurement** |  |
| Interatrial septum | PFO, L – R Shunt | Mitral | ----- |
| **Semilunal valves** |  | Aortic | ------- |
| Aortic valve | Annulus = 7mm | Tricuspid | Moderate TR, PPG = 75mmHg |
| Pulmonary valve | Annulus = 8mm | pulmonic | -------- |
| **Great arteries** | NRGA | **Aortic arch** | Left |
| Aorta | ----- | **PDA** | No |
| Pulmonary artery | Normal MPA and Branch PAs. |  |  |
| **M-Mode:** Normal LV Function on eye balling. | | | |
| AO | mm | PWd | mm |
| LA | mm | EDV | ml |
| LVIDd | mm | ESV | ml |
| LVIDs | mm | LVEF | % |
| IVSd | mm | FS | % |
| **Additional Information**: |  | | |
| 3mm pericardial effusion on RV Side. | | | |
| **Final Diagnosis:** | | | |
| 1. {S, D, S} Levocardia. 2. RA/RV Dilated 3. PFO, L – R Shunt 4. Moderate TR 5. Severe Pulmonary Hypertension secondary to ? 6. Trace pericardial effusion. | | | |
| **Remark**: | | | |
| **Recommendation**: Repeat echo after a month. | | | |
| SIGNATURE  Done by: Tesfaye T., Pediatrician, Pediatric Cardiologist _______________ 27/08/2013Eth.C | | | |

| Patient Name: **Bereket Dubale.** Patient ID: **FHRH**. SEX/ Age: M/4/12. Date of Report: 28/**08/2013**.  BP: _______ Weight: ______ Height: ____________ BSA: ________. R.Dx: **Recurrent Chest Infection. AGH10.816** | | | |
| --- | --- | --- | --- |
| **Features** | **Finding** | **Features** | **Finding** |
| **Profile** |  | **Atria** |  |
| Abdominal situs | Solitus | Left atrium | Normal |
| Cardiac position | Levocardia | Right atrium | Normal |
| Systemic venous drainage | Normal. | **Atrioventricular valves** |  |
| Pulmonary venous drainage | Normal | Mitral valve | Annulus = 11mm |
| Atrioventricular connection | Concordant | Tricuspid valve | Annulus = 12mm |
| Ventriculoarterial connection | Concordant | **Ventricles** |  |
| Ventricular loop | d-Loop | Left ventricle | Normal |
|  |  | Right ventricle | Normal |
| **Septae** |  | **Coronary arteries** | ----- |
| Interventricular septum | Intact | **Doppler Measurement** |  |
| Interatrial septum | Intact | Mitral | ----- |
| **Semilunal valves** |  | Aortic | ------- |
| Aortic valve | Annulus = 9mm | Tricuspid | ------- |
| Pulmonary valve | Annulus = 9mm | pulmonic | -------- |
| **Great arteries** | NRGA | **Aortic arch** | Left |
| Aorta | ----- | **PDA** | No |
| Pulmonary artery | Normal MPA and Branch PAs. |  |  |
| **M-Mode:** Normal LV Function on eye balling. | | | |
| AO | mm | PWd | mm |
| LA | mm | EDV | ml |
| LVIDd | mm | ESV | ml |
| LVIDs | mm | LVEF | % |
| IVSd | mm | FS | % |
| **Additional Information**: |  | | |
| No pericardial/Pleural effusion. | | | |
| **Final Diagnosis:** | | | |
| 1. Normal Echocardiography Study. | | | |
| **Remark**: | | | |
| **Recommendation**: | | | |
| SIGNATURE  Done by: Tesfaye T., Pediatrician, Pediatric Cardiologist _______________ 28/08/2013Eth.C | | | |

| Patient Name: **Yechale Mollalign**. Patient ID: **FHRH**. SEX/ Age: M/2/12. Date of Report: 28/**08/2013**.  BP: _______ Weight: ______ Height: ____________ BSA: ________. R.Dx: **DS + RD. AGH10.817** | | | |
| --- | --- | --- | --- |
| **Features** | **Finding** | **Features** | **Finding** |
| **Profile** |  | **Atria** |  |
| Abdominal situs | Solitus | Left atrium | Normal |
| Cardiac position | Levocardia | Right atrium | Dilated |
| Systemic venous drainage | Normal. | **Atrioventricular valves** |  |
| Pulmonary venous drainage | Normal | Mitral valve | Annulus = 9mm |
| Atrioventricular connection | Concordant | Tricuspid valve | Annulus = 9mm  TAPSE = 10mm |
| Ventriculoarterial connection | Concordant | **Ventricles** |  |
| Ventricular loop | d-Loop | Left ventricle | Normal |
|  |  | Right ventricle | Dilated |
| **Septae** |  | **Coronary arteries** | ----- |
| Interventricular septum | Intact | **Doppler Measurement** |  |
| Interatrial septum | Intact | Mitral | ----- |
| **Semilunal valves** |  | Aortic | ------- |
| Aortic valve | Annulus = 8mm | Tricuspid | Mild TR, PPG = 50mmHg |
| Pulmonary valve | Annulus = 10mm | pulmonic | Moderate PR, PPG = 45mmHg |
| **Great arteries** | NRGA | **Aortic arch** | Left |
| Aorta | ----- | **PDA** | No |
| Pulmonary artery | MPA =11mm. |  |  |
| **M-Mode:** Normal LV Function on eye balling. | | | |
| AO | mm | PWd | mm |
| LA | mm | EDV | ml |
| LVIDd | mm | ESV | ml |
| LVIDs | mm | LVEF | % |
| IVSd | mm | FS | % |
| **Additional Information**: |  | | |
| 3mm pericardial effusion on RV Side. | | | |
| **Final Diagnosis:** | | | |
| 1. {S, D, S} Levocardia. 2. RA/RV Dilated 3. Moderate Pulmonary Hypertension secondary to ? 4. Trace pericardial effusion 5. Normal Biventricular Function | | | |
| **Remark**: Only Subcostal window | | | |
| **Recommendation**: Repeat echo after 3 months | | | |
| SIGNATURE  Done by: Tesfaye T., Pediatrician, Pediatric Cardiologist _______________ 28/08/2013Eth.C | | | |

| Patient Name: **Alemwerk Takele**. Patient ID: **FHRH**. SEX/ Age: F/2Years. Date of Report: 28/**08/2013**.  BP: _______ Weight: ______ Height: ____________ BSA: ________. R.Dx: **CHF + RD. AGH10.818** | | | |
| --- | --- | --- | --- |
| **Features** | **Finding** | **Features** | **Finding** |
| **Profile** |  | **Atria** |  |
| Abdominal situs | Solitus | Left atrium | Dilated |
| Cardiac position | Levocardia | Right atrium | Markedly Dilated |
| Systemic venous drainage | Normal. | **Atrioventricular valves** |  |
| Pulmonary venous drainage | Normal | Mitral valve | Annulus = 11mm |
| Atrioventricular connection | Concordant | Tricuspid valve | Annulus = 22mm  TAPSE = 19mm |
| Ventriculoarterial connection | Concordant | **Ventricles** |  |
| Ventricular loop | d-Loop | Left ventricle | Dilated |
|  |  | Right ventricle | Markedly Dilated |
| **Septae** | Abnormal Septal Motion | **Coronary arteries** | ----- |
| Interventricular septum | 13mm PM VSD, BD Shunt predominantly R – L. | **Doppler Measurement** |  |
| Interatrial septum | Intact | Mitral | Mild MR |
| **Semilunal valves** |  | Aortic | ------- |
| Aortic valve | Annulus = 12mm | Tricuspid | Severe TR.? Fenestrated TVL (anterior) |
| Pulmonary valve | Annulus = 19mm | pulmonic | Severe PR, PPG = 80mmHg. |
| **Great arteries** | NRGA | **Aortic arch** | Left |
| Aorta | ----- | **PDA** | No |
| Pulmonary artery | MPA =21mm. |  |  |
| **M-Mode:** Reduced LV Function (Abnormal Septal Motion). | | | |
| AO | mm | PWd | mm |
| LA | mm | EDV | ml |
| LVIDd | mm | ESV | ml |
| LVIDs | mm | LVEF | % |
| IVSd | mm | FS | % |
| **Additional Information**: |  | | |
| No pericardial/Pleural effusion. | | | |
| **Final Diagnosis:** | | | |
| 1. {S, D, S} Levocardia. 2. All chambers dilated (Marked RA/RV) 3. Large PM VSD, BD Shunt predominantly R – L Shunt 4. Severe TR 5. Severe PR 6. Mild MR 7. Severe Pulmonary Hypertension 8. Reduced LV Function | | | |
| **Remark**: | | | |
| **Recommendation**: | | | |
| SIGNATURE  Done by: Tesfaye T., Pediatrician, Pediatric Cardiologist _______________ 28/08/2013Eth.C | | | |

| Patient Name: **Hawulet Mohamed**. Patient ID: **Efrata PSC**. SEX/ Age: F/6/12. Date of Report: 29/**08/2013**.  BP: _______ Weight: ______ Height: ____________ BSA: ________. R.Dx: **CHF. AGH10.819** | | | |
| --- | --- | --- | --- |
| **Features** | **Finding** | **Features** | **Finding** |
| **Profile** |  | **Atria** |  |
| Abdominal situs | Solitus | Left atrium | Dilated |
| Cardiac position | Levocardia | Right atrium | Dilated |
| Systemic venous drainage | Normal. | **Atrioventricular valves** |  |
| Pulmonary venous drainage | Normal | Mitral valve | Annulus = 17mm |
| Atrioventricular connection | Concordant | Tricuspid valve | Annulus = 13mm  TAPSE = 15mm |
| Ventriculoarterial connection | Concordant | **Ventricles** |  |
| Ventricular loop | d-Loop | Left ventricle | Dilated |
|  |  | Right ventricle | Dilated |
| **Septae** |  | **Coronary arteries** | ----- |
| Interventricular septum | 8mm Inlet VSD with PM extension, L – R Shunt | **Doppler Measurement** |  |
| Interatrial septum | Intact | Mitral | Mild MR |
| **Semilunal valves** |  | Aortic | ------- |
| Aortic valve | Annulus = 10mm | Tricuspid | ------- |
| Pulmonary valve | Annulus = 14mm | pulmonic | -------- |
| **Great arteries** | NRGA | **Aortic arch** | Left |
| Aorta | ----- | **PDA** | No |
| Pulmonary artery | MPA =16mm. |  |  |
| **M-Mode:** | | | |
| AO | mm | PWd | mm |
| LA | mm | EDV | ml |
| LVIDd | mm | ESV | ml |
| LVIDs | mm | LVEF | 68% |
| IVSd | mm | FS | 37% |
| **Additional Information**: |  | | |
| 3mm pericardial effusion on RA/RV Side. | | | |
| **Final Diagnosis:** | | | |
| 1. {S, D, S} Levocardia. 2. Large Inlet VSD with PM extension, L – R Shunt 3. Trace pericardial effusion 4. Severe Pulmonary Hypertension 5. Normal Biventricular Function | | | |
| **Remark**: | | | |
| **Recommendation**: Manage the CHF and is a candidate for surgical closure. | | | |
| SIGNATURE  Done by: Tesfaye T., Pediatrician, Pediatric Cardiologist _______________ 29/08/2013Eth.C | | | |

| Patient Name: **Tsedey Desalegn**. Patient ID: **Adinas GH**. SEX/ Age: F/8Years. Date of Report: 29/**08/2013**.  BP: _______ Weight: ______ Height: ____________ BSA: ________. R.Dx: **Easy fatigability. AGH10.820** | | | |
| --- | --- | --- | --- |
| **Features** | **Finding** | **Features** | **Finding** |
| **Profile** |  | **Atria** |  |
| Abdominal situs | Solitus | Left atrium | Normal |
| Cardiac position | Levocardia | Right atrium | Normal |
| Systemic venous drainage | Normal. | **Atrioventricular valves** |  |
| Pulmonary venous drainage | Normal | Mitral valve | Annulus = 16mm |
| Atrioventricular connection | Concordant | Tricuspid valve | Annulus = 16mm  TAPSE = 18mm |
| Ventriculoarterial connection | Concordant | **Ventricles** |  |
| Ventricular loop | d-Loop | Left ventricle | Normal |
|  |  | Right ventricle | Normal |
| **Septae** |  | **Coronary arteries** | ----- |
| Interventricular septum | Intact | **Doppler Measurement** |  |
| Interatrial septum | Intact | Mitral | ----- |
| **Semilunal valves** |  | Aortic | ------- |
| Aortic valve | Annulus = 14mm | Tricuspid | ------- |
| Pulmonary valve | Annulus = 15mm | pulmonic | -------- |
| **Great arteries** | NRGA | **Aortic arch** | Left |
| Aorta | ----- | **PDA** | No |
| Pulmonary artery | Normal MPA and Branch PAs. |  |  |
| **M-Mode:** | | | |
| AO | mm | PWd | mm |
| LA | mm | EDV | ml |
| LVIDd | mm | ESV | ml |
| LVIDs | mm | LVEF | 64% |
| IVSd | mm | FS | 33% |
| **Additional Information**: |  | | |
| No pericardial/Pleural effusion. | | | |
| **Final Diagnosis:** | | | |
| 1. Normal Echocardiography Study. | | | |
| **Remark**: | | | |
| **Recommendation**: | | | |
| SIGNATURE  Done by: Tesfaye T., Pediatrician, Pediatric Cardiologist _______________ 29/08/2013Eth.C | | | |

| Patient Name: **Mekdes Aysheshim**. Patient ID: **Adinas GH**. SEX/ Age: F/9Years. Date of Report: 30/**08/2013**.  BP: _______ Weight: ______ Height: ____________ BSA: ________. R.Dx: **Rheumatic Recurrence. AGH10.821** | | | |
| --- | --- | --- | --- |
| **Features** | **Finding** | **Features** | **Finding** |
| **Profile** |  | **Atria** |  |
| Abdominal situs | Solitus | Left atrium | Markedly dilated |
| Cardiac position | Levocardia | Right atrium | Dilated |
| Systemic venous drainage | Normal. IVC Dilated | **Atrioventricular valves** |  |
| Pulmonary venous drainage | Normal | Mitral valve | Annulus = 31mm. Thickened MVL. |
| Atrioventricular connection | Concordant | Tricuspid valve | Annulus = 25mm |
| Ventriculoarterial connection | Concordant | **Ventricles** |  |
| Ventricular loop | d-Loop | Left ventricle | Markedly dilated |
|  |  | Right ventricle | Dilated |
| **Septae** |  | **Coronary arteries** | ----- |
| Interventricular septum | Intact | **Doppler Measurement** |  |
| Interatrial septum | Intact | Mitral | Severe MR, Holosystolic, posterior projection, seen in two planes with jet velocity = 5m/sec. |
| **Semilunal valves** |  | Aortic | ------- |
| Aortic valve | Annulus = 13mm | Tricuspid | Severe TR, PPG = 65mmHg. |
| Pulmonary valve | Annulus = 22mm | pulmonic | -------- |
| **Great arteries** | NRGA | **Aortic arch** | Left |
| Aorta | ----- | **PDA** | No |
| Pulmonary artery | Dilated MPA. |  |  |
| **M-Mode:** | | | |
| AO | mm | PWd | mm |
| LA | mm | EDV | ml |
| LVIDd | mm | ESV | ml |
| LVIDs | mm | LVEF | 62% |
| IVSd | mm | FS | 34% |
| **Additional Information**: |  | | |
| 8mm pericardial effusion, Circumferential. | | | |
| **Final Diagnosis:** | | | |
| 1. {S, D, S} Levocardia. 2. All chambers dilated 3. Thickened MVL 4. Severe MR 5. Severe TR 6. Mild Pericardial effusion 7. Severe Pulmonary Hypertension 8. Normal LV Function | | | |
| **Remark**: | | | |
| **Recommendation**: | | | |
| SIGNATURE  Done by: Tesfaye T., Pediatrician, Pediatric Cardiologist _______________ 30/08/2013Eth.C | | | |

| Patient Name: **Yohana Are’aya**. Patient ID: **Adinas GH**. SEX/ Age: F/11Years. Date of Report: 30/**08/2013**.  BP: _______ Weight: ______ Height: ____________ BSA: ________. R.Dx: **Easy fatigability. AGH10.822** | | | |
| --- | --- | --- | --- |
| **Features** | **Finding** | **Features** | **Finding** |
| **Profile** |  | **Atria** |  |
| Abdominal situs | Solitus | Left atrium | Normal |
| Cardiac position | Levocardia | Right atrium | Normal |
| Systemic venous drainage | Normal. | **Atrioventricular valves** |  |
| Pulmonary venous drainage | Normal | Mitral valve | Annulus = 20mm |
| Atrioventricular connection | Concordant | Tricuspid valve | Annulus = 20mm  TAPSE = 21mm |
| Ventriculoarterial connection | Concordant | **Ventricles** |  |
| Ventricular loop | d-Loop | Left ventricle | Normal |
|  |  | Right ventricle | Normal |
| **Septae** |  | **Coronary arteries** | ----- |
| Interventricular septum | Intact | **Doppler Measurement** |  |
| Interatrial septum | Intact | Mitral | ----- |
| **Semilunal valves** |  | Aortic | ------- |
| Aortic valve | Annulus = 18mm | Tricuspid | ------- |
| Pulmonary valve | Annulus = 21mm | pulmonic | -------- |
| **Great arteries** | NRGA | **Aortic arch** | Left, No CoA. |
| Aorta | ----- | **PDA** | No |
| Pulmonary artery | Normal MPA and Branch PAs. |  |  |
| **M-Mode:** | | | |
| AO | mm | PWd | mm |
| LA | mm | EDV | ml |
| LVIDd | mm | ESV | ml |
| LVIDs | mm | LVEF | 72% |
| IVSd | mm | FS | 40% |
| **Additional Information**: |  | | |
| No pericardial/Pleural effusion. | | | |
| **Final Diagnosis:** | | | |
| 1. Normal Echocardiography Study. | | | |
| **Remark**: | | | |
| **Recommendation**: | | | |
| SIGNATURE  Done by: Tesfaye T., Pediatrician, Pediatric Cardiologist _______________ 30/08/2013Eth.C | | | |

| Patient Name: **Amen Fantahun**. Patient ID: **FHRH**. SEX/ Age: F/3/12. Date of Report: 30/**08/2013**.  BP: _______ Weight: ______ Height: ____________ BSA: ________. R.Dx: **Incidental Murmur. AGH10.823** | | | |
| --- | --- | --- | --- |
| **Features** | **Finding** | **Features** | **Finding** |
| **Profile** |  | **Atria** |  |
| Abdominal situs | Solitus | Left atrium | Normal |
| Cardiac position | Levocardia | Right atrium | Normal |
| Systemic venous drainage | Normal. | **Atrioventricular valves** |  |
| Pulmonary venous drainage | Normal | Mitral valve | Annulus = 9mm |
| Atrioventricular connection | Concordant | Tricuspid valve | Annulus = 10mm  TAPSE = 10mm |
| Ventriculoarterial connection | Concordant | **Ventricles** |  |
| Ventricular loop | d-Loop | Left ventricle | Normal |
|  |  | Right ventricle | Normal |
| **Septae** |  | **Coronary arteries** | ----- |
| Interventricular septum | Intact | **Doppler Measurement** |  |
| Interatrial septum | PFO, L – R Shunt | Mitral | ----- |
| **Semilunal valves** |  | Aortic | ------- |
| Aortic valve | Annulus = 8mm | Tricuspid | ------- |
| Pulmonary valve | Annulus = 9mm | pulmonic | Mild PS, PPG = 25mmHg |
| **Great arteries** | NRGA | **Aortic arch** | Left |
| Aorta | ----- | **PDA** | No |
| Pulmonary artery | Normal MPA and Branch PAs. |  |  |
| **M-Mode:** Normal LV Function on eye balling. | | | |
| AO | mm | PWd | mm |
| LA | mm | EDV | ml |
| LVIDd | mm | ESV | ml |
| LVIDs | mm | LVEF | % |
| IVSd | mm | FS | % |
| **Additional Information**: |  | | |
| No pericardial/Pleural effusion. | | | |
| **Final Diagnosis:** | | | |
| 1. {S, D, S} Levocardia. 2. PFO, L – R Shunt 3. Mild PS | | | |
| **Remark**: Only Subcostal and apical view | | | |
| **Recommendation**: Repeat echo after 6months. No need of medication/intervention for the cardiac finding | | | |
| SIGNATURE  Done by: Tesfaye T., Pediatrician, Pediatric Cardiologist _______________ 30/08/2013Eth.C | | | |

| Patient Name: **Chilot Alemnew**. Patient ID: **FHRH**. SEX/ Age: M/1 1/12. Date of Report: 02/**09/2013**.  BP: _______ Weight: ______ Height: ____________ BSA: ________. R.Dx: **DS. AGH10.824** | | | |
| --- | --- | --- | --- |
| **Features** | **Finding** | **Features** | **Finding** |
| **Profile** |  | **Atria** |  |
| Abdominal situs | Solitus | Left atrium | Normal |
| Cardiac position | Levocardia | Right atrium | Normal |
| Systemic venous drainage | Normal. | **Atrioventricular valves** |  |
| Pulmonary venous drainage | Normal | Mitral valve | Annulus = 13mm |
| Atrioventricular connection | Concordant | Tricuspid valve | Annulus = 14mm  TAPSE = 17mm |
| Ventriculoarterial connection | Concordant | **Ventricles** |  |
| Ventricular loop | d-Loop | Left ventricle | Normal |
|  |  | Right ventricle | Normal |
| **Septae** |  | **Coronary arteries** | ----- |
| Interventricular septum | Intact | **Doppler Measurement** |  |
| Interatrial septum | Intact | Mitral | ----- |
| **Semilunal valves** |  | Aortic | ------- |
| Aortic valve | Annulus = 12mm | Tricuspid | ------- |
| Pulmonary valve | Annulus = 13mm | pulmonic | -------- |
| **Great arteries** | NRGA | **Aortic arch** | Left |
| Aorta | ----- | **PDA** | No |
| Pulmonary artery | Normal MPA and Branch PAs. |  |  |
| **M-Mode:** Normal LV Function on eye balling. | | | |
| AO | mm | PWd | mm |
| LA | mm | EDV | ml |
| LVIDd | mm | ESV | ml |
| LVIDs | mm | LVEF | % |
| IVSd | mm | FS | % |
| **Additional Information**: |  | | |
| No pericardial/Pleural effusion. | | | |
| **Final Diagnosis:** | | | |
| 1. Normal Echocardiography Study. | | | |
| **Remark**: Subcostal window only | | | |
| **Recommendation**: | | | |
| SIGNATURE  Done by: Tesfaye T., Pediatrician, Pediatric Cardiologist _______________ 02/09/2013Eth.C | | | |

| Patient Name: **Ketemaw Bogale**. Patient ID: **FHCSRH**. SEX/ Age: M/5/12. Date of Report: 02/**09/2013**.  BP: _______ Weight: ______ Height: ____________ BSA: ________. R.Dx: **DS. AGH10.825** | | | |
| --- | --- | --- | --- |
| **Features** | **Finding** | **Features** | **Finding** |
| **Profile** |  | **Atria** |  |
| Abdominal situs | Solitus | Left atrium | Normal |
| Cardiac position | Levocardia | Right atrium | Normal |
| Systemic venous drainage | Normal. | **Atrioventricular valves** |  |
| Pulmonary venous drainage | Normal | Mitral valve | Common Complete AVSD |
| Atrioventricular connection | Common Complete AVSD | Tricuspid valve |  |
| Ventriculoarterial connection | Concordant | **Ventricles** |  |
| Ventricular loop | d-Loop | Left ventricle | Normal |
|  |  | Right ventricle | Normal |
| **Septae** |  | **Coronary arteries** | ----- |
| Interventricular septum | Common Complete AVSD, L – R Shunt | **Doppler Measurement** |  |
| Interatrial septum |  | Mitral | Mild left AVVR |
| **Semilunal valves** |  | Aortic | ------- |
| Aortic valve | Annulus = 9mm | Tricuspid | ------- |
| Pulmonary valve | Annulus = 13mm | pulmonic | -------- |
| **Great arteries** | NRGA | **Aortic arch** | Left |
| Aorta | ----- | **PDA** | No |
| Pulmonary artery | Normal MPA and Branch PAs. |  |  |
| **M-Mode:** Normal LV Function on eye balling. | | | |
| AO | mm | PWd | mm |
| LA | mm | EDV | ml |
| LVIDd | mm | ESV | ml |
| LVIDs | mm | LVEF | % |
| IVSd | mm | FS | % |
| **Additional Information**: |  | | |
| No pericardial/Pleural effusion. | | | |
| **Final Diagnosis:** | | | |
| 1. {S, D, S} Levocardia. 2. Common Complete Balanced AVSD, L – R Shunt 3. Mild left AVVR 4. Normal LV Function | | | |
| **Remark**: | | | |
| **Recommendation**: | | | |
| SIGNATURE  Done by: Tesfaye T., Pediatrician, Pediatric Cardiologist _______________ 02/09/2013Eth.C | | | |

| Patient Name: **Ab-Lenie** **Abebe**. Patient ID: **Adinas GH**. SEX/ Age: M/8Years. Date of Report: 02/**09/2013**.  BP: _______ Weight: ______ Height: ______ BSA: _____. R.Dx: **Systemic Hypertension. AGH10.826** | | | |
| --- | --- | --- | --- |
| **Features** | **Finding** | **Features** | **Finding** |
| **Profile** |  | **Atria** |  |
| Abdominal situs | Solitus | Left atrium | Normal |
| Cardiac position | Levocardia | Right atrium | Normal |
| Systemic venous drainage | Normal. | **Atrioventricular valves** |  |
| Pulmonary venous drainage | Normal | Mitral valve | Annulus = 17mm |
| Atrioventricular connection | Concordant | Tricuspid valve | Annulus = 20mm  TAPSE = 24mm |
| Ventriculoarterial connection | Concordant | **Ventricles** |  |
| Ventricular loop | d-Loop | Left ventricle | Normal |
|  |  | Right ventricle | Normal |
| **Septae** |  | **Coronary arteries** | ----- |
| Interventricular septum | Intact | **Doppler Measurement** |  |
| Interatrial septum | Intact | Mitral | ----- |
| **Semilunal valves** |  | Aortic | ------- |
| Aortic valve | Annulus = 17mm | Tricuspid | Trivial TR, PPG = 29mmHg. |
| Pulmonary valve | Annulus = 18mm | pulmonic | -------- |
| **Great arteries** | NRGA | **Aortic arch** | Left |
| Aorta | ----- | **PDA** | No |
| Pulmonary artery | Normal MPA and Branch PAs. |  |  |
| **M-Mode:** | | | |
| AO | mm | PWd | 8.5mm |
| LA | mm | EDV | 58ml |
| LVIDd | 37mm | ESV | 22ml |
| LVIDs | 25mm | LVEF | 62% |
| IVSd | 10mm | FS | 33% |
| **Additional Information**: |  | | |
| No pericardial/Pleural effusion. | | | |
| **Final Diagnosis:** | | | |
| 1. {S, D, S} Levocardia. 2. LVH | | | |
| **Remark**: | | | |
| **Recommendation**: | | | |
| SIGNATURE  Done by: Tesfaye T., Pediatrician, Pediatric Cardiologist _______________ 02/09/2013Eth.C | | | |

| Patient Name: **Meron Getinet**. Patient ID: **Adet PH**. SEX/ Age: F/4 5/12. Date of Report: 03/**09/2013**.  BP: _______ Weight: ______ Height: ____________ BSA: ________. R.Dx: **FTT. AGH10.827** | | | |
| --- | --- | --- | --- |
| **Features** | **Finding** | **Features** | **Finding** |
| **Profile** |  | **Atria** |  |
| Abdominal situs | Solitus | Left atrium | Normal |
| Cardiac position | Levocardia | Right atrium | Normal |
| Systemic venous drainage | Normal. | **Atrioventricular valves** |  |
| Pulmonary venous drainage | Normal | Mitral valve | Annulus = 16mm |
| Atrioventricular connection | Concordant | Tricuspid valve | Annulus = 17mm  TAPSE = 19mm |
| Ventriculoarterial connection | Concordant | **Ventricles** |  |
| Ventricular loop | d-Loop | Left ventricle | Normal |
|  |  | Right ventricle | Normal |
| **Septae** |  | **Coronary arteries** | ----- |
| Interventricular septum | Intact | **Doppler Measurement** |  |
| Interatrial septum | Intact | Mitral | ----- |
| **Semilunal valves** |  | Aortic | ------- |
| Aortic valve | Annulus = 13mm | Tricuspid | Trivial TR, PPG = 29mmHg |
| Pulmonary valve | Annulus = 16mm | pulmonic | -------- |
| **Great arteries** | NRGA | **Aortic arch** | Left |
| Aorta | ----- | **PDA** | No |
| Pulmonary artery | Normal MPA and Branch PAs. |  |  |
| **M-Mode:** | | | |
| AO | mm | PWd | mm |
| LA | mm | EDV | ml |
| LVIDd | mm | ESV | ml |
| LVIDs | mm | LVEF | 68% |
| IVSd | mm | FS | 37% |
| **Additional Information**: |  | | |
| No pericardial/Pleural effusion. | | | |
| **Final Diagnosis:** | | | |
| 1. Normal Echocardiography Study. | | | |
| **Remark**: | | | |
| **Recommendation**: | | | |
| SIGNATURE  Done by: Tesfaye T., Pediatrician, Pediatric Cardiologist _______________ 03/09/2013Eth.C | | | |

| Patient Name: **Yabibal Aschalew**. Patient ID: **FHRH**. SEX/ Age: M/10Years. Date of Report: 04/**09/2013**.  BP: ____ Weight: ___ Height: ___ BSA: _____. R.Dx: **Rheumatic Recurrence + RD + DOE + CHF. AGH10.828** | | | |
| --- | --- | --- | --- |
| **Features** | **Finding** | **Features** | **Finding** |
| **Profile** |  | **Atria** |  |
| Abdominal situs | Solitus | Left atrium | Dilated |
| Cardiac position | Levocardia | Right atrium | Dilated |
| Systemic venous drainage | Normal. | **Atrioventricular valves** |  |
| Pulmonary venous drainage | Normal | Mitral valve | Annulus = 25mm. Thickened, shortened PMVL |
| Atrioventricular connection | Concordant | Tricuspid valve | Annulus = 25mm  TAPSE = 16mm |
| Ventriculoarterial connection | Concordant | **Ventricles** |  |
| Ventricular loop | d-Loop | Left ventricle | Dilated |
|  |  | Right ventricle | Dilated |
| **Septae** |  | **Coronary arteries** | ----- |
| Interventricular septum | Intact | **Doppler Measurement** |  |
| Interatrial septum | Intact | Mitral | Severe MR, Holosystolic, posterior projection, seen in two planes with jet velocity = 4.3m/sec |
| **Semilunal valves** |  | Aortic | ------- |
| Aortic valve | Annulus = 14mm | Tricuspid | Severe TR, PPG = 78mmHg |
| Pulmonary valve | Annulus = 24mm | pulmonic | -------- |
| **Great arteries** | NRGA | **Aortic arch** | Left |
| Aorta | ----- | **PDA** | No |
| Pulmonary artery | Normal MPA and Branch PAs. |  |  |
| **M-Mode:** | | | |
| AO | mm | PWd | mm |
| LA | mm | EDV | ml |
| LVIDd | mm | ESV | ml |
| LVIDs | mm | LVEF | 45% |
| IVSd | mm | FS | 23% |
| **Additional Information**: |  | | |
| 6mm pericardial effusion on RV Side. | | | |
| **Final Diagnosis:** | | | |
| 1. {S, D, S} Levocardia. 2. All chambers dilated 3. Thickened shortened PMVL 4. Severe MR 5. Severe TR 6. Severe Pulmonary Hypertension 7. Reduced LV Function | | | |
| SIGNATURE  Done by: Tesfaye T., Pediatrician, Pediatric Cardiologist _______________ 04/09/2013Eth.C | | | |

| Patient Name: **Aschila Yeshambel**. Patient ID: **FHRH**. SEX/ Age: F/2 8/12. Date of Report: 04/**09/2013**.  BP: _______ Weight: ______ Height: ____________ BSA: ________. R.Dx: **CHF + Cyanosis. AGH10.829** | | | |
| --- | --- | --- | --- |
| **Features** | **Finding** | **Features** | **Finding** |
| **Profile** |  | **Atria** |  |
| Abdominal situs | Ambiguous | Left atrium | dilated |
| Cardiac position | Dextrocardia | Right atrium | Dilated |
| Systemic venous drainage | Normal. | **Atrioventricular valves** |  |
| Pulmonary venous drainage | Normal | Mitral valve | Annulus = 14mm |
| Atrioventricular connection | Concordant | Tricuspid valve | Annulus = 12mm |
| Ventriculoarterial connection | DORV | **Ventricles** |  |
| Ventricular loop | l-Loop | Left ventricle | On the right side, dilated |
|  |  | Right ventricle | On the left side. dilated |
| **Septae** |  | **Coronary arteries** | ----- |
| Interventricular septum | 12mm Doubly committed Inlet VSD, from Right side LV to Left side RV | **Doppler Measurement** |  |
| Interatrial septum | 12mm OS ASD, BD Shunt | Mitral | ----- |
| **Semilunal valves** |  | Aortic | ------- |
| Aortic valve | Annulus = 12mm | Tricuspid | ------- |
| Pulmonary valve | Annulus = 7mm | pulmonic | -------- |
| **Great arteries** | l-TGA | **Aortic arch** | Left |
| Aorta | From left side RV | **PDA** | No |
| Pulmonary artery | From Left side RV. MPA dilated | |  |
| **M-Mode: Reduced ventricular function on eye balling** | | | |
| AO | mm | PWd | mm |
| LA | mm | EDV | ml |
| LVIDd | mm | ESV | ml |
| LVIDs | mm | LVEF | % |
| IVSd | mm | FS | % |
| **Additional Information**: |  | | |
| No pericardial/Pleural effusion. | | | |
| **Final Diagnosis:** | | | |
| 1. {I, L, L} Dextrocardia. 2. DORV 3. Large OS ASD, BD Shunt 4. Large inlet VSD, from right side LV to left side RV 5. Reduced Ventricular Function 6. Severe Pulmonary HTN | | | |
| **Remark**: Complex Cyanotic Congenital Heart Disease | | | |
| **Recommendation**: Put on anti-congestive management. | | | |
| SIGNATURE  Done by: Tesfaye T., Pediatrician, Pediatric Cardiologist _______________ 04/09/2013Eth.C | | | |

| Patient Name: **Emebet Abebe**. Patient ID: **Adinas GH**. SEX/ Age: F/2 6/12. Date of Report: 05/**09/2013**.  BP: _______ Weight: ______ Height: ____________ BSA: ________. R.Dx: **Recurrent Chest Infection. AGH10.830** | | | |
| --- | --- | --- | --- |
| **Features** | **Finding** | **Features** | **Finding** |
| **Profile** |  | **Atria** |  |
| Abdominal situs | Solitus | Left atrium | Normal |
| Cardiac position | Levocardia | Right atrium | Normal |
| Systemic venous drainage | Normal. | **Atrioventricular valves** |  |
| Pulmonary venous drainage | Normal | Mitral valve | Annulus = 15mm |
| Atrioventricular connection | Concordant | Tricuspid valve | Annulus = 19mm  TAPSE = 17mm |
| Ventriculoarterial connection | Concordant | **Ventricles** |  |
| Ventricular loop | d-Loop | Left ventricle | Normal |
|  |  | Right ventricle | Normal |
| **Septae** |  | **Coronary arteries** | ----- |
| Interventricular septum | Intact | **Doppler Measurement** |  |
| Interatrial septum | Intact | Mitral | ----- |
| **Semilunal valves** |  | Aortic | ------- |
| Aortic valve | Annulus = 14mm | Tricuspid | ------- |
| Pulmonary valve | Annulus = 16mm | pulmonic | -------- |
| **Great arteries** | NRGA | **Aortic arch** | Left |
| Aorta | ----- | **PDA** | No |
| Pulmonary artery | Normal MPA and Branch PAs. |  |  |
| **M-Mode:** | | | |
| AO | mm | PWd | mm |
| LA | mm | EDV | ml |
| LVIDd | mm | ESV | ml |
| LVIDs | mm | LVEF | 61% |
| IVSd | mm | FS | 32% |
| **Additional Information**: |  | | |
| No pericardial/Pleural effusion. | | | |
| **Final Diagnosis:** | | | |
| 1. Normal Echocardiography Study. | | | |
| **Remark**: Tachycardia | | | |
| **Recommendation**: | | | |
| SIGNATURE  Done by: Tesfaye T., Pediatrician, Pediatric Cardiologist _______________ 05/09/2013Eth.C | | | |

| Patient Name: **Zufan Belete**. Patient ID: **FHRH**. SEX/ Age: F/13Years. Date of Report: 05/**09/2013**.  BP: _______ Weight: ______ Height: ____________ BSA: ________. R.Dx: **DOE. AGH10.831** | | | |
| --- | --- | --- | --- |
| **Features** | **Finding** | **Features** | **Finding** |
| **Profile** |  | **Atria** |  |
| Abdominal situs | Solitus | Left atrium | Normal |
| Cardiac position | Levocardia | Right atrium | Mildly dilated |
| Systemic venous drainage | Normal. | **Atrioventricular valves** |  |
| Pulmonary venous drainage | Normal | Mitral valve | Annulus = 21mm |
| Atrioventricular connection | Concordant | Tricuspid valve | Annulus = 21mm  TAPSE = 20mm |
| Ventriculoarterial connection | Concordant | **Ventricles** |  |
| Ventricular loop | d-Loop | Left ventricle | Normal |
|  |  | Right ventricle | Mildly Dilated |
| **Septae** |  | **Coronary arteries** | ----- |
| Interventricular septum | Intact | **Doppler Measurement** |  |
| Interatrial septum | Intact | Mitral | ----- |
| **Semilunal valves** |  | Aortic | ------- |
| Aortic valve | Annulus = 17mm | Tricuspid | ------- |
| Pulmonary valve | Annulus = 17mm. Doming PV | pulmonic | Severe Valvar PS, PPG = 80mmHg |
| **Great arteries** | NRGA | **Aortic arch** | Left |
| Aorta | ----- | **PDA** | No |
| Pulmonary artery | Normal MPA and Branch PAs. |  |  |
| **M-Mode:** | | | |
| AO | mm | PWd | mm |
| LA | mm | EDV | ml |
| LVIDd | mm | ESV | ml |
| LVIDs | mm | LVEF | 65% |
| IVSd | mm | FS | 35% |
| **Additional Information**: |  | | |
| No pericardial/Pleural effusion. | | | |
| **Final Diagnosis:** | | | |
| 1. {S, D, S} Levocardia. 2. Severe Valvular PS 3. Doming Pulmonary Valve 4. Normal Biventricular Function | | | |
| **Remark**: | | | |
| **Recommendation**: Needs urgent BPV. | | | |
| SIGNATURE  Done by: Tesfaye T., Pediatrician, Pediatric Cardiologist _______________ 05/09/2013Eth.C | | | |

| Patient Name: **Bereket Chalachew**. Patient ID: **FHRH**. SEX/ Age: M/1Year. Date of Report: 06/**09/2013**.  BP: _______ Weight: ______ Height: ____________ BSA: ________. R.Dx: **Diaphoresis. AGH10.832** | | | |
| --- | --- | --- | --- |
| **Features** | **Finding** | **Features** | **Finding** |
| **Profile** |  | **Atria** |  |
| Abdominal situs | Solitus | Left atrium | Normal |
| Cardiac position | Levocardia | Right atrium | Normal |
| Systemic venous drainage | Normal. | **Atrioventricular valves** |  |
| Pulmonary venous drainage | Normal | Mitral valve | Annulus = 13mm |
| Atrioventricular connection | Concordant | Tricuspid valve | Annulus = 12mm |
| Ventriculoarterial connection | Concordant | **Ventricles** |  |
| Ventricular loop | d-Loop | Left ventricle | Normal |
|  |  | Right ventricle | Normal |
| **Septae** |  | **Coronary arteries** | ----- |
| Interventricular septum | Intact | **Doppler Measurement** |  |
| Interatrial septum | Intact | Mitral | ------- |
| **Semilunal valves** |  | Aortic | ------- |
| Aortic valve | Annulus = 12mm | Tricuspid | Trivial TR, PPG = 26mmHg |
| Pulmonary valve | Annulus = 11mm | pulmonic | -------- |
| **Great arteries** | NRGA | **Aortic arch** | Left |
| Aorta | ----- | **PDA** | No |
| Pulmonary artery | Normal MPA and Branch PAs. |  |  |
| **M-Mode:** Normal LV Function on eye balling. | | | |
| AO | mm | PWd | mm |
| LA | mm | EDV | ml |
| LVIDd | mm | ESV | ml |
| LVIDs | mm | LVEF | % |
| IVSd | mm | FS | % |
| **Additional Information**: |  | | |
| No pericardial/Pleural effusion. | | | |
| **Final Diagnosis:** | | | |
| 1. Normal Echocardiography Study. | | | |
| **Remark**: Limited Echo window. | | | |
| **Recommendation**: | | | |
| SIGNATURE  Done by: Tesfaye T., Pediatrician, Pediatric Cardiologist _______________ 06/09/2013Eth.C | | | |

| Patient Name: **Bisrat Debash**. Patient ID: **TGSH**. SEX/ Age: F/1 7/12. Date of Report: 06/**09/2013**.  BP: _____ Weight: ____ Height: ______ BSA: ____. R.Dx: **Recurrent Chest Infection. AGH10.833** | | | |
| --- | --- | --- | --- |
| **Features** | **Finding** | **Features** | **Finding** |
| **Profile** |  | **Atria** |  |
| Abdominal situs | Solitus | Left atrium | Dilated |
| Cardiac position | Levocardia | Right atrium | Normal |
| Systemic venous drainage | Normal. | **Atrioventricular valves** |  |
| Pulmonary venous drainage | Normal | Mitral valve | Annulus = 19mm |
| Atrioventricular connection | Concordant | Tricuspid valve | Annulus = 15mm |
| Ventriculoarterial connection | Concordant | **Ventricles** |  |
| Ventricular loop | d-Loop | Left ventricle | Dilated |
|  |  | Right ventricle | Normal |
| **Septae** |  | **Coronary arteries** | ----- |
| Interventricular septum | 11mm Upper Muscular VSD, L – R Shunt | **Doppler Measurement** |  |
| Interatrial septum | Intact | Mitral | ----- |
| **Semilunal valves** |  | Aortic | ------- |
| Aortic valve | Annulus = 11mm | Tricuspid | ------- |
| Pulmonary valve | Annulus = 15mm | pulmonic | -------- |
| **Great arteries** | NRGA | **Aortic arch** | Left |
| Aorta | ----- | **PDA** | No |
| Pulmonary artery | Normal MPA and Branch PAs. |  |  |
| **M-Mode:** | | | |
| AO | mm | PWd | mm |
| LA | mm | EDV | ml |
| LVIDd | mm | ESV | ml |
| LVIDs | mm | LVEF | 56% |
| IVSd | mm | FS | 29% |
| **Additional Information**: |  | | |
| No pericardial/Pleural effusion. | | | |
| **Final Diagnosis:** | | | |
| 1. {S, D, S} Levocardia. 2. Large Upper Muscular VSD, L – R Shunt 3. Normal LV Function | | | |
| **Remark**: | | | |
| **Recommendation**: | | | |
| SIGNATURE  Done by: Tesfaye T., Pediatrician, Pediatric Cardiologist _______________ 06/09/2013Eth.C | | | |

| Patient Name: **Abrham Temesgen**. Patient ID: **TGSH**. SEX/ Age: M/5/12. Date of Report: 06/**09/2013**.  BP: _______ Weight: ______ Height: ____________ BSA: ________. R.Dx: **DS. AGH10.834** | | | |
| --- | --- | --- | --- |
| **Features** | **Finding** | **Features** | **Finding** |
| **Profile** |  | **Atria** |  |
| Abdominal situs | Solitus | Left atrium | Normal |
| Cardiac position | Levocardia | Right atrium | Normal |
| Systemic venous drainage | Normal. | **Atrioventricular valves** |  |
| Pulmonary venous drainage | Normal | Mitral valve | Annulus = 11mm |
| Atrioventricular connection | Concordant | Tricuspid valve | Annulus = 12mm  TAPSE = 14mm |
| Ventriculoarterial connection | Concordant | **Ventricles** |  |
| Ventricular loop | d-Loop | Left ventricle | Normal |
|  |  | Right ventricle | Normal |
| **Septae** |  | **Coronary arteries** | ----- |
| Interventricular septum | Intact | **Doppler Measurement** |  |
| Interatrial septum | Intact | Mitral | ----- |
| **Semilunal valves** |  | Aortic | ------- |
| Aortic valve | Annulus = 10mm | Tricuspid | ------- |
| Pulmonary valve | Annulus = 11mm | pulmonic | -------- |
| **Great arteries** | NRGA | **Aortic arch** | Left |
| Aorta | ----- | **PDA** | No |
| Pulmonary artery | Normal MPA and Branch PAs. |  |  |
| **M-Mode:** | | | |
| AO | mm | PWd | mm |
| LA | mm | EDV | ml |
| LVIDd | mm | ESV | ml |
| LVIDs | mm | LVEF | 66% |
| IVSd | mm | FS | 34% |
| **Additional Information**: |  | | |
| No pericardial/Pleural effusion. | | | |
| **Final Diagnosis:** | | | |
| 1. Normal Echocardiography Study. | | | |
| **Remark**: | | | |
| **Recommendation**: | | | |
| SIGNATURE  Done by: Tesfaye T., Pediatrician, Pediatric Cardiologist _______________ 06/09/2013Eth.C | | | |

| Patient Name: **Senayit Mogninet**. Patient ID: **FHRH**. SEX/ Age: F/5/12. Date of Report: 06/**09/2013**.  BP: _______ Weight: ______ Height: ____________ BSA: ________. R.Dx: **RD. AGH10.835** | | | |
| --- | --- | --- | --- |
| **Features** | **Finding** | **Features** | **Finding** |
| **Profile** |  | **Atria** |  |
| Abdominal situs | Solitus | Left atrium | Normal |
| Cardiac position | Levocardia | Right atrium | Normal |
| Systemic venous drainage | Normal. | **Atrioventricular valves** |  |
| Pulmonary venous drainage | Normal | Mitral valve | Annulus = 10mm |
| Atrioventricular connection | Concordant | Tricuspid valve | Annulus = 12mm  TAPSE = 15mm |
| Ventriculoarterial connection | Concordant | **Ventricles** |  |
| Ventricular loop | d-Loop | Left ventricle | Normal |
|  |  | Right ventricle | Normal |
| **Septae** |  | **Coronary arteries** | ----- |
| Interventricular septum | Intact | **Doppler Measurement** |  |
| Interatrial septum | PFO, L – R Shunt | Mitral | ----- |
| **Semilunal valves** |  | Aortic | ------- |
| Aortic valve | Annulus = 9mm | Tricuspid | ------- |
| Pulmonary valve | Annulus = 11mm | pulmonic | -------- |
| **Great arteries** | NRGA | **Aortic arch** | Left |
| Aorta | ----- | **PDA** | No |
| Pulmonary artery | Normal MPA and Branch PAs. |  |  |
| **M-Mode:** Normal LV Function on eye balling. | | | |
| AO | mm | PWd | mm |
| LA | mm | EDV | ml |
| LVIDd | mm | ESV | ml |
| LVIDs | mm | LVEF | % |
| IVSd | mm | FS | % |
| **Additional Information**: |  | | |
| No pericardial/Pleural effusion. | | | |
| **Final Diagnosis:** | | | |
| 1. {S, D, S} Levocardia. 2. PFO, L – R Shunt 3. Normal Biventricular Function | | | |
| **Remark**: | | | |
| **Recommendation**: | | | |
| SIGNATURE  Done by: Tesfaye T., Pediatrician, Pediatric Cardiologist _______________ 06/09/2013Eth.C | | | |

| Patient Name: **Amanuel Melesse**. Patient ID: **FHRH**. SEX/ Age: M/1 5/12. Date of Report: 07/**09/2013**.  BP: _______ Weight: ______ Height: ____________ BSA: ________R.Dx: **Incidental Murmur. AGH10.836** | | | |
| --- | --- | --- | --- |
| **Features** | **Finding** | **Features** | **Finding** |
| **Profile** |  | **Atria** |  |
| Abdominal situs | Solitus | Left atrium | Normal |
| Cardiac position | Levocardia | Right atrium | Normal |
| Systemic venous drainage | Normal. | **Atrioventricular valves** |  |
| Pulmonary venous drainage | Normal | Mitral valve | Annulus = 15mm |
| Atrioventricular connection | Concordant | Tricuspid valve | Annulus = 15mm  TAPSE = 14mm |
| Ventriculoarterial connection | Concordant | **Ventricles** |  |
| Ventricular loop | d-Loop | Left ventricle | Normal |
|  |  | Right ventricle | Normal |
| **Septae** |  | **Coronary arteries** | ----- |
| Interventricular septum | Intact | **Doppler Measurement** |  |
| Interatrial septum | PFO, L – R Shunt | Mitral | ----- |
| **Semilunal valves** |  | Aortic | ------- |
| Aortic valve | Annulus = 14mm | Tricuspid | Trivial TR, PPG = 17mmHg |
| Pulmonary valve | Annulus = 15mm | pulmonic | -------- |
| **Great arteries** | NRGA | **Aortic arch** | Left |
| Aorta | ----- | **PDA** | 1.5mm PDA, L – R Shunt |
| Pulmonary artery | Normal MPA and Branch PAs. |  |  |
| **M-Mode:** | | | |
| AO | mm | PWd | mm |
| LA | mm | EDV | ml |
| LVIDd | mm | ESV | ml |
| LVIDs | mm | LVEF | 66% |
| IVSd | mm | FS | 35% |
| **Additional Information**: |  | | |
| 7mm pericardial effusion on RA/RV Side. | | | |
| **Final Diagnosis:** | | | |
| 1. {S, D, S} Levocardia. 2. PFO, L – R Shunt 3. Small PDA, L – R Shunt 4. Trace Pericardial effusion 5. Normal Biventricular Function | | | |
| **Remark**: | | | |
| **Recommendation**: No need to put on any form of medication. | | | |
| SIGNATURE  Done by: Tesfaye T., Pediatrician, Pediatric Cardiologist _______________ 07/09/2013Eth.C | | | |

| Patient Name: **Dawit Baye**. Patient ID: **FHRH**. SEX/ Age: M/1 5/12. Date of Report: 16/**09/2013**.  BP: _______ Weight: ______ Height: ____________ BSA: ________. R.Dx: **Cyanosis. AGH10.837** | | | |
| --- | --- | --- | --- |
| **Features** | **Finding** | **Features** | **Finding** |
| **Profile** |  | **Atria** |  |
| Abdominal situs | Solitus | Left atrium | Normal |
| Cardiac position | Levocardia | Right atrium | Dilated |
| Systemic venous drainage | Normal. | **Atrioventricular valves** |  |
| Pulmonary venous drainage | Normal | Mitral valve | Annulus = 12mm |
| Atrioventricular connection | Concordant | Tricuspid valve | Annulus = 17mm  TAPSE = 15mm |
| Ventriculoarterial connection | DORV | **Ventricles** |  |
| Ventricular loop | d-Loop | Left ventricle | Normal |
|  |  | Right ventricle | Dilated, Hypertrophied |
| **Septae** |  | **Coronary arteries** | ----- |
| Interventricular septum | Subaortic VSD, L – R Shunt. | **Doppler Measurement** |  |
| Interatrial septum | Intact | Mitral | ----- |
| **Semilunal valves** | Aorto mitral discontinuity | Aortic | ------- |
| Aortic valve | Annulus = 20mm | Tricuspid | Trivial TR, PPG = 15mmHg |
| Pulmonary valve | Annulus = 7mm | pulmonic | Severe Valvar and supra valvar PS, PPG = 82mmHg |
| **Great arteries** | NRGA | **Aortic arch** | Left |
| Aorta | To the right and posterior. From RV | **PDA** | No |
| Pulmonary artery | To the left and anterior. From RV. Smallish MPA and Branch PAs |  |  |
| **M-Mode:** | | | |
| AO | mm | PWd | mm |
| LA | mm | EDV | ml |
| LVIDd | mm | ESV | ml |
| LVIDs | mm | LVEF | 71% |
| IVSd | mm | FS | 38% |
| **Additional Information**: |  | | |
| No pericardial/Pleural effusion. | | | |
| **Final Diagnosis:** | | | |
| 1. {S, D, D} Levocardia. 2. DORV (TOF Variant) 3. Malaligned Nonrestrictive VSD, L – R Shunt 4. Severe PS 5. Smallish MPA and Branch PAs | | | |
| SIGNATURE  Done by: Tesfaye T., Pediatrician, Pediatric Cardiologist _______________ 16/09/2013Eth.C | | | |

| Patient Name: **Redeat Abayneh**. Patient ID: **Injibara GH**. SEX/ Age: F/1 2/12. Date of Report: 16/**09/2013**.  BP: _______ Weight: ______ Height: ____________ BSA: ________. R.Dx: **Down Syndrome. AGH10.838** | | | |
| --- | --- | --- | --- |
| **Features** | **Finding** | **Features** | **Finding** |
| **Profile** |  | **Atria** |  |
| Abdominal situs | Solitus | Left atrium | Normal |
| Cardiac position | Levocardia | Right atrium | Normal |
| Systemic venous drainage | Normal. | **Atrioventricular valves** |  |
| Pulmonary venous drainage | Normal | Mitral valve | Annulus = 13mm |
| Atrioventricular connection | Concordant | Tricuspid valve | Annulus = 14mm  TAPSE = 16mm |
| Ventriculoarterial connection | Concordant | **Ventricles** |  |
| Ventricular loop | d-Loop | Left ventricle | Normal |
|  |  | Right ventricle | Normal |
| **Septae** |  | **Coronary arteries** | ----- |
| Interventricular septum | Intact | **Doppler Measurement** |  |
| Interatrial septum | Intact | Mitral | ----- |
| **Semilunal valves** |  | Aortic | ------- |
| Aortic valve | Annulus = 12mm | Tricuspid | ------- |
| Pulmonary valve | Annulus = 14mm | pulmonic | -------- |
| **Great arteries** | NRGA | **Aortic arch** | Left |
| Aorta | ----- | **PDA** | No |
| Pulmonary artery | Normal MPA and Branch PAs. |  |  |
| **M-Mode:** Normal LV Function on eye balling | | | |
| AO | mm | PWd | mm |
| LA | mm | EDV | ml |
| LVIDd | mm | ESV | ml |
| LVIDs | mm | LVEF | % |
| IVSd | mm | FS | % |
| **Additional Information**: |  | | |
| No pericardial/Pleural effusion. | | | |
| **Final Diagnosis:** | | | |
| 1. Normal Echocardiography Study. | | | |
| **Remark**: | | | |
| **Recommendation**: | | | |
| SIGNATURE  Done by: Tesfaye T., Pediatrician, Pediatric Cardiologist _______________ 16/09/2013Eth.C | | | |

| Patient Name: **Baby of Enanye Nigussie**. Patient ID: **FHRH**. SEX/ Age: M/30days. Date of Report: 16/**09/2013**.  BP: _______ Weight: ______ Height: ____________ BSA: ________. R.Dx: **RD. AGH10.839** | | | |
| --- | --- | --- | --- |
| **Features** | **Finding** | **Features** | **Finding** |
| **Profile** |  | **Atria** |  |
| Abdominal situs | Solitus | Left atrium | Normal |
| Cardiac position | Levocardia | Right atrium | Normal |
| Systemic venous drainage | Normal. | **Atrioventricular valves** |  |
| Pulmonary venous drainage | Normal | Mitral valve | Annulus = 8mm |
| Atrioventricular connection | Concordant | Tricuspid valve | Annulus = 9mm |
| Ventriculoarterial connection | Concordant | **Ventricles** |  |
| Ventricular loop | d-Loop | Left ventricle | Normal |
|  |  | Right ventricle | Normal |
| **Septae** |  | **Coronary arteries** | ----- |
| Interventricular septum | Intact | **Doppler Measurement** |  |
| Interatrial septum | Intact | Mitral | ----- |
| **Semilunal valves** |  | Aortic | ------- |
| Aortic valve | Annulus = 6mm | Tricuspid | ------- |
| Pulmonary valve | Annulus = 7mm | pulmonic | -------- |
| **Great arteries** | NRGA | **Aortic arch** | Left |
| Aorta | ----- | **PDA** | No |
| Pulmonary artery | Normal MPA and Branch PAs. |  |  |
| **M-Mode:** Normal LV Function on eye balling | | | |
| AO | mm | PWd | mm |
| LA | mm | EDV | ml |
| LVIDd | mm | ESV | ml |
| LVIDs | mm | LVEF | % |
| IVSd | mm | FS | % |
| **Additional Information**: |  | | |
| No pericardial/Pleural effusion. | | | |
| **Final Diagnosis:** | | | |
| 1. Normal echocardiography Study. | | | |
| **Remark**: only subcostal windo | | | |
| **Recommendation**: | | | |
| SIGNATURE  Done by: Tesfaye T., Pediatrician, Pediatric Cardiologist _______________ 16/09/2013Eth.C | | | |

| Patient Name: **Dawit Haile**. Patient ID: **Amaris PSC.** SEX/ Age: M/4 11/12. Date of Report: 16/**09/2013**.  BP: _______ Weight: ___ Height: ______ BSA: ______. R.Dx: **Incidental Murmur. AGH10.840 (AGH10)** | | | |
| --- | --- | --- | --- |
| **Features** | **Finding** | **Features** | **Finding** |
| **Profile** |  | **Atria** |  |
| Abdominal situs | Solitus | Left atrium | Normal |
| Cardiac position | Levocardia | Right atrium | Normal |
| Systemic venous drainage | Normal. | **Atrioventricular valves** |  |
| Pulmonary venous drainage | Normal | Mitral valve | Annulus = 17mm |
| Atrioventricular connection | Concordant | Tricuspid valve | Annulus = 18mm |
| Ventriculoarterial connection | Concordant | **Ventricles** |  |
| Ventricular loop | d-Loop | Left ventricle | Normal |
|  |  | Right ventricle | Normal |
| **Septae** |  | **Coronary arteries** | ----- |
| Interventricular septum | Intact, SAM+ | **Doppler Measurement** |  |
| Interatrial septum | Intact | Mitral | ----- |
| **Semilunal valves** |  | Aortic | Mild LVOTO, PPG/MPG = 32/20mmHg |
| Aortic valve | Annulus = 14mm | Tricuspid | ------- |
| Pulmonary valve | Annulus = 14mm | pulmonic | -------- |
| **Great arteries** | NRGA | **Aortic arch** | Left |
| Aorta | ----- | **PDA** | No |
| Pulmonary artery | Normal MPA and Branch PAs. |  |  |
| **M-Mode:** Normal LV Function on eye balling. | | | |
| AO | mm | PWd | mm |
| LA | mm | EDV | ml |
| LVIDd | mm | ESV | ml |
| LVIDs | mm | LVEF | % |
| IVSd | mm | FS | % |
| **Additional Information**: |  | | |
| No pericardial/Pleural effusion. | | | |
| **Final Diagnosis:** | | | |
| 1. {S, D, S} Levocardia. 2. SAM+ 3. Mild LVOTO 4. Normal LV Function | | | |
| **Remark**: | | | |
| **Recommendation**: Follow up echo yearly | | | |
| SIGNATURE  Done by: Tesfaye T., Pediatrician, Pediatric Cardiologist _______________ 16/09/2013Eth.C | | | |

| Patient Name: **Kirkos Habtamu**. Patient ID: **FHRH**. SEX/ Age: M/6Years. Date of Report: 16/**09/2013**.  BP: _______ Weight: ______ Height: ____________ BSA: ________. R.Dx: **easy fatigability. AGH10.841** | | | |
| --- | --- | --- | --- |
| **Features** | **Finding** | **Features** | **Finding** |
| **Profile** |  | **Atria** |  |
| Abdominal situs | Solitus | Left atrium | Normal |
| Cardiac position | Levocardia | Right atrium | Normal |
| Systemic venous drainage | Normal. | **Atrioventricular valves** |  |
| Pulmonary venous drainage | Normal | Mitral valve | Annulus = 19mm |
| Atrioventricular connection | Concordant | Tricuspid valve | Annulus = 20mm  TAPSE = 24mm |
| Ventriculoarterial connection | Concordant | **Ventricles** |  |
| Ventricular loop | d-Loop | Left ventricle | Normal |
|  |  | Right ventricle | Normal |
| **Septae** |  | **Coronary arteries** | ----- |
| Interventricular septum | Intact | **Doppler Measurement** |  |
| Interatrial septum | Intact | Mitral | ----- |
| **Semilunal valves** |  | Aortic | ------- |
| Aortic valve | Annulus = 14mm | Tricuspid | Trivial TR, PPG = 18mmHg |
| Pulmonary valve | Annulus = 16mm | pulmonic | -------- |
| **Great arteries** | NRGA | **Aortic arch** | Left |
| Aorta | ----- | **PDA** | No |
| Pulmonary artery | Normal MPA and Branch PAs. |  |  |
| **M-Mode:** | | | |
| AO | mm | PWd | mm |
| LA | mm | EDV | ml |
| LVIDd | mm | ESV | ml |
| LVIDs | mm | LVEF | % |
| IVSd | mm | FS | % |
| **Additional Information**: |  | | |
| No pericardial/Pleural effusion. | | | |
| **Final Diagnosis:** | | | |
| 1. Normal Echocardiography Study. | | | |
| **Remark**: | | | |
| **Recommendation**: | | | |
| SIGNATURE  Done by: Tesfaye T., Pediatrician, Pediatric Cardiologist _______________ 16/09/2013Eth.C | | | |

| Patient Name: **Nuhamin Dereje**. Patient ID: **FHRH**. SEX/ Age: F/7/12. Date of Report: 17/**09/2013**.  BP: _______ Weight: ______ Height: ____________ BSA: ________. R.Dx: **Rhythm abnormality. AGH10.842** | | | |
| --- | --- | --- | --- |
| **Features** | **Finding** | **Features** | **Finding** |
| **Profile** |  | **Atria** |  |
| Abdominal situs | Solitus | Left atrium | Normal |
| Cardiac position | Levocardia | Right atrium | Normal |
| Systemic venous drainage | Normal. | **Atrioventricular valves** |  |
| Pulmonary venous drainage | Normal | Mitral valve | Annulus = 12mm |
| Atrioventricular connection | Concordant | Tricuspid valve | Annulus = 12mm |
| Ventriculoarterial connection | Concordant | **Ventricles** |  |
| Ventricular loop | d-Loop | Left ventricle | Normal |
|  |  | Right ventricle | Normal |
| **Septae** |  | **Coronary arteries** | ----- |
| Interventricular septum | Intact | **Doppler Measurement** |  |
| Interatrial septum | Intact | Mitral | ----- |
| **Semilunal valves** |  | Aortic | ------- |
| Aortic valve | Annulus = 10mm | Tricuspid | ------- |
| Pulmonary valve | Annulus = 10mm | pulmonic | -------- |
| **Great arteries** | NRGA | **Aortic arch** | Left |
| Aorta | ----- | **PDA** | No |
| Pulmonary artery | Normal MPA and Branch PAs. |  |  |
| **M-Mode:** Normal LV Function on eye balling | | | |
| AO | mm | PWd | mm |
| LA | mm | EDV | ml |
| LVIDd | mm | ESV | ml |
| LVIDs | mm | LVEF | % |
| IVSd | mm | FS | % |
| **Additional Information**: |  | | |
| No pericardial/Pleural effusion. | | | |
| **Final Diagnosis:** | | | |
| 1. Normal Echocardiography Study. | | | |
| **Remark**: Tachycardia during Study | | | |
| **Recommendation**: | | | |
| SIGNATURE  Done by: Tesfaye T., Pediatrician, Pediatric Cardiologist _______________ 17/09/2013Eth.C | | | |

| Patient Name: **Betsinat Getie**. Patient ID: **Addis Alem PH**. SEX/ Age: M/1 10/12. Date of Report: 17/**09/2013**.  BP: _______ Weight: ______ Height: ____________ BSA: ________. R.Dx: **FTT. AGH10.843** | | | |
| --- | --- | --- | --- |
| **Features** | **Finding** | **Features** | **Finding** |
| **Profile** |  | **Atria** |  |
| Abdominal situs | Solitus | Left atrium | Normal |
| Cardiac position | Levocardia | Right atrium | Normal |
| Systemic venous drainage | Normal. | **Atrioventricular valves** |  |
| Pulmonary venous drainage | Normal | Mitral valve | Annulus = 13mm |
| Atrioventricular connection | Concordant | Tricuspid valve | Annulus = 15mm |
| Ventriculoarterial connection | Concordant | **Ventricles** |  |
| Ventricular loop | d-Loop | Left ventricle | Normal |
|  |  | Right ventricle | Normal |
| **Septae** |  | **Coronary arteries** | ----- |
| Interventricular septum | Intact | **Doppler Measurement** |  |
| Interatrial septum | Intact | Mitral | ----- |
| **Semilunal valves** |  | Aortic | ------- |
| Aortic valve | Annulus = 13mm | Tricuspid | ------- |
| Pulmonary valve | Annulus = 13mm | pulmonic | -------- |
| **Great arteries** | NRGA | **Aortic arch** | Left |
| Aorta | ----- | **PDA** | No |
| Pulmonary artery | Normal MPA and Branch PAs. |  |  |
| **M-Mode:** Normal LV Function on eye balling. | | | |
| AO | mm | PWd | mm |
| LA | mm | EDV | ml |
| LVIDd | mm | ESV | ml |
| LVIDs | mm | LVEF | % |
| IVSd | mm | FS | % |
| **Additional Information**: |  | | |
| No pericardial/Pleural effusion. | | | |
| **Final Diagnosis:** | | | |
| 1. Normal Echocardiography Study. | | | |
| **Remark**: Child was restless during study | | | |
| **Recommendation**: | | | |
| SIGNATURE  Done by: Tesfaye T., Pediatrician, Pediatric Cardiologist _______________ 17/09/2013Eth.C | | | |

| Patient Name: **Amlak Yibeltal (Baby of Ethiopia Mengist)**. Patient ID: **MSI**. SEX/ Age: M/6/12. Date of Report: 17/**09/2013**. R.Dx: **RD. AGH10.844** | | | |
| --- | --- | --- | --- |
| **Follow up echocardiography for PFO and small PDA (3/12) AGH7 (3days/M)** | | | |
| **Features** | **Finding** | **Features** | **Finding** |
| **Profile** |  | **Atria** |  |
| Abdominal situs | Solitus | Left atrium | Normal |
| Cardiac position | Levocardia | Right atrium | Normal |
| Systemic venous drainage | Normal. | **Atrioventricular valves** |  |
| Pulmonary venous drainage | Normal | Mitral valve | Annulus = 13mm |
| Atrioventricular connection | Concordant | Tricuspid valve | Annulus = 13mm |
| Ventriculoarterial connection | Concordant | **Ventricles** |  |
| Ventricular loop | d-Loop | Left ventricle | Normal |
|  |  | Right ventricle | Normal |
| **Septae** |  | **Coronary arteries** | ----- |
| Interventricular septum | Intact | **Doppler Measurement** |  |
| Interatrial septum | 4mm OS ASD, L – R Shunt | Mitral | ----- |
| **Semilunal valves** |  | Aortic | ------- |
| Aortic valve | Annulus = 12mm | Tricuspid | ------- |
| Pulmonary valve | Annulus = 13mm | pulmonic | -------- |
| **Great arteries** | NRGA | **Aortic arch** | Left |
| Aorta | ----- | **PDA** | No |
| Pulmonary artery | Normal MPA and Branch PAs. |  |  |
| **M-Mode:** Normal LV Function on eye balling. | | | |
| AO | mm | PWd | mm |
| LA | mm | EDV | ml |
| LVIDd | mm | ESV | ml |
| LVIDs | mm | LVEF | % |
| IVSd | mm | FS | % |
| **Additional Information**: |  | | |
| No pericardial/Pleural effusion. | | | |
| **Final Diagnosis:** | | | |
| 1. {S, D, S} Levocardia. 2. Small OS ASD, L – R Shunt 3. Normal LV Function | | | |
| **Remark**: PDA has closed | | | |
| **Recommendation**: Follow up only (Highly likely to close spontaneously) | | | |
| SIGNATURE  Done by: Tesfaye T., Pediatrician, Pediatric Cardiologist _______________ 17/09/2013Eth.C | | | |

| Patient Name: **Mariamawit Fantahun**. Patient ID: **Addis Alem PH**. SEX/ Age: F/11/12. Date of Report: 17/**09/2013**.  BP: _______ Weight: ______ Height: ____________ BSA: ________. R.Dx: **DS. AGH10.845** | | | |
| --- | --- | --- | --- |
| **Features** | **Finding** | **Features** | **Finding** |
| **Profile** |  | **Atria** |  |
| Abdominal situs | Solitus | Left atrium | Normal |
| Cardiac position | Levocardia | Right atrium | Normal |
| Systemic venous drainage | Normal. | **Atrioventricular valves** |  |
| Pulmonary venous drainage | Normal | Mitral valve | Annulus = 11mm |
| Atrioventricular connection | Concordant | Tricuspid valve | Annulus = 12mm |
| Ventriculoarterial connection | Concordant | **Ventricles** |  |
| Ventricular loop | d-Loop | Left ventricle | Normal |
|  |  | Right ventricle | Normal |
| **Septae** |  | **Coronary arteries** | ----- |
| Interventricular septum | Intact | **Doppler Measurement** |  |
| Interatrial septum | Intact | Mitral | ----- |
| **Semilunal valves** |  | Aortic | ------- |
| Aortic valve | Annulus = 11mm | Tricuspid | ------- |
| Pulmonary valve | Annulus = 12mm | pulmonic | -------- |
| **Great arteries** | NRGA | **Aortic arch** | Left |
| Aorta | ----- | **PDA** | <1mm PDA, L – R Shunt |
| Pulmonary artery | Normal MPA and Branch PAs. |  |  |
| **M-Mode:** Normal LV Function on eye balling. | | | |
| AO | mm | PWd | mm |
| LA | mm | EDV | ml |
| LVIDd | mm | ESV | ml |
| LVIDs | mm | LVEF | % |
| IVSd | mm | FS | % |
| **Additional Information**: |  | | |
| 5mm pericardial effusion on RA/RV Side. | | | |
| **Final Diagnosis:** | | | |
| 1. {S, D, S} Levocardia. 2. Silent PDA, L – R Shunt 3. Small Pericardial effusion | | | |
| **Remark**: | | | |
| **Recommendation**: Conservative management (No need of intervention for the silent PDA, ECHO yearly) | | | |
| SIGNATURE  Done by: Tesfaye T., Pediatrician, Pediatric Cardiologist _______________ 17/09/2013Eth.C | | | |

| Patient Name: **Haymanot Mezigebu**. Patient ID: **TGSH**. SEX/ Age: M/11Years. Date of Report: 17/**09/2013**.  BP: _______ Weight: ______ Height: ____________ BSA: ________. R.Dx: **Sydenham’s Chorea. AGH10.846** | | | |
| --- | --- | --- | --- |
| **Features** | **Finding** | **Features** | **Finding** |
| **Profile** |  | **Atria** |  |
| Abdominal situs | Solitus | Left atrium | Normal |
| Cardiac position | Levocardia | Right atrium | Normal |
| Systemic venous drainage | Normal. | **Atrioventricular valves** |  |
| Pulmonary venous drainage | Normal | Mitral valve | Annulus = 18mm. Mildly Thickened MVL |
| Atrioventricular connection | Concordant | Tricuspid valve | Annulus = 18mm  TAPSE = 21mm |
| Ventriculoarterial connection | Concordant | **Ventricles** |  |
| Ventricular loop | d-Loop | Left ventricle | Normal |
|  |  | Right ventricle | Normal |
| **Septae** |  | **Coronary arteries** | ----- |
| Interventricular septum | Intact | **Doppler Measurement** |  |
| Interatrial septum | Intact | Mitral | Mild MR, Holosystolic, posterior projection, seen in two planes with jet velocity = 4.4m/sec |
| **Semilunal valves** |  | Aortic | ------- |
| Aortic valve | Annulus = 15mm | Tricuspid | ------- |
| Pulmonary valve | Annulus = 17mm | pulmonic | -------- |
| **Great arteries** | NRGA | **Aortic arch** | Left |
| Aorta | ----- | **PDA** | No |
| Pulmonary artery | Normal MPA and Branch PAs. |  |  |
| **M-Mode:** | | | |
| AO | mm | PWd | mm |
| LA | mm | EDV | ml |
| LVIDd | mm | ESV | ml |
| LVIDs | mm | LVEF | 61% |
| IVSd | mm | FS | 32% |
| **Additional Information**: |  | | |
| No pericardial/Pleural effusion. | | | |
| **Final Diagnosis:** | | | |
| 1. {S, D, S} Levocardia. 2. Mildly thickened MVL 3. Mild MR 4. Normal Biventricular Function | | | |
| **Remark**: | | | |
| **Recommendation**: Manage in the line of Rheumatic Carditis. | | | |
| SIGNATURE  Done by: Tesfaye T., Pediatrician, Pediatric Cardiologist _______________ 17/09/2013Eth.C | | | |

| Patient Name: **Muhamedamin Mussa**. Patient ID: **Amaris PSC**. SEX/ Age: M/1Year. Date of Report: 18/**09/2013**.  BP: _______ Weight: ______ Height: _______ BSA: ________. R.Dx: **recurrent chest infection. AGH10.847** | | | |
| --- | --- | --- | --- |
| **Features** | **Finding** | **Features** | **Finding** |
| **Profile** |  | **Atria** |  |
| Abdominal situs | Solitus | Left atrium | Normal |
| Cardiac position | Levocardia | Right atrium | Normal |
| Systemic venous drainage | Normal. | **Atrioventricular valves** |  |
| Pulmonary venous drainage | Normal | Mitral valve | Annulus = 16mm |
| Atrioventricular connection | Concordant | Tricuspid valve | Annulus = 16mm  TAPSE = 19mm |
| Ventriculoarterial connection | Concordant | **Ventricles** |  |
| Ventricular loop | d-Loop | Left ventricle | Normal |
|  |  | Right ventricle | Normal |
| **Septae** |  | **Coronary arteries** | ----- |
| Interventricular septum | Intact | **Doppler Measurement** |  |
| Interatrial septum | Intact | Mitral | ----- |
| **Semilunal valves** |  | Aortic | ------- |
| Aortic valve | Annulus = 13mm | Tricuspid | ------- |
| Pulmonary valve | Annulus = 14mm | pulmonic | -------- |
| **Great arteries** | NRGA | **Aortic arch** | Left |
| Aorta | ----- | **PDA** | No |
| Pulmonary artery | Normal MPA and Branch PAs. |  |  |
| **M-Mode:** | | | |
| AO | mm | PWd | mm |
| LA | mm | EDV | ml |
| LVIDd | mm | ESV | ml |
| LVIDs | mm | LVEF | 72% |
| IVSd | mm | FS | 39% |
| **Additional Information**: |  | | |
| No pericardial/Pleural effusion. | | | |
| **Final Diagnosis:** | | | |
| 1. Normal Echocardiography Study. | | | |
| **Remark**: | | | |
| **Recommendation**: | | | |
| SIGNATURE  Done by: Tesfaye T., Pediatrician, Pediatric Cardiologist _______________ 18/09/2013Eth.C | | | |

| Patient Name: **Mirtzer Eshetie**. Patient ID: **TGSH**. SEX/ Age: F/9Years. Date of Report: 18/**09/2013**.  BP: _______ Weight: ______ Height: ____________ BSA: ________. R.Dx: **Easy fatigability. AGH10.848** | | | |
| --- | --- | --- | --- |
| **Features** | **Finding** | **Features** | **Finding** |
| **Profile** |  | **Atria** |  |
| Abdominal situs | Solitus | Left atrium | Normal |
| Cardiac position | Levocardia | Right atrium | Normal |
| Systemic venous drainage | Normal. | **Atrioventricular valves** |  |
| Pulmonary venous drainage | Normal | Mitral valve | Annulus = 19mm |
| Atrioventricular connection | Concordant | Tricuspid valve | Annulus = 20mm  TAPSE = 23mm |
| Ventriculoarterial connection | Concordant | **Ventricles** |  |
| Ventricular loop | d-Loop | Left ventricle | Normal |
|  |  | Right ventricle | Normal |
| **Septae** |  | **Coronary arteries** | ----- |
| Interventricular septum | Intact | **Doppler Measurement** |  |
| Interatrial septum | Intact | Mitral | ----- |
| **Semilunal valves** |  | Aortic | ------- |
| Aortic valve | Annulus = 15mm | Tricuspid | ------- |
| Pulmonary valve | Annulus = 17mm | pulmonic | Trivial PR, Incomplete signal, PPG = 22mmHg |
| **Great arteries** | NRGA | **Aortic arch** | Left |
| Aorta | ----- | **PDA** | No |
| Pulmonary artery | Normal MPA and Branch PAs. |  |  |
| **M-Mode:** | | | |
| AO | mm | PWd | mm |
| LA | mm | EDV | ml |
| LVIDd | mm | ESV | ml |
| LVIDs | mm | LVEF | 64% |
| IVSd | mm | FS | 34% |
| **Additional Information**: |  | | |
| No pericardial/Pleural effusion. | | | |
| **Final Diagnosis:** | | | |
| 1. Normal Echocardiography Study. | | | |
| **Remark**: | | | |
| **Recommendation**: | | | |
| SIGNATURE  Done by: Tesfaye T., Pediatrician, Pediatric Cardiologist _______________ 18/09/2013Eth.C | | | |

| Patient Name: **Amanuel Berihun**. Patient ID: **FHRH**. SEX/ Age: M/2 3/12. Date of Report: 18/**09/2013**.  BP: _______ Weight: ______ Height: ____________ BSA: ________. R.Dx: **Murmur. AGH10.849** | | | |
| --- | --- | --- | --- |
| **Features** | **Finding** | **Features** | **Finding** |
| **Profile** |  | **Atria** |  |
| Abdominal situs | Solitus | Left atrium | Normal |
| Cardiac position | Levocardia | Right atrium | Normal |
| Systemic venous drainage | Normal. | **Atrioventricular valves** |  |
| Pulmonary venous drainage | Normal | Mitral valve | Annulus = 13mm |
| Atrioventricular connection | Concordant | Tricuspid valve | Annulus = 15mm  TAPSE = 14mm |
| Ventriculoarterial connection | Concordant | **Ventricles** |  |
| Ventricular loop | d-Loop | Left ventricle | Normal |
|  |  | Right ventricle | Normal |
| **Septae** |  | **Coronary arteries** | ----- |
| Interventricular septum | Intact | **Doppler Measurement** |  |
| Interatrial septum | 5mm OS ASD, L – R Shunt | Mitral | ----- |
| **Semilunal valves** |  | Aortic | ------- |
| Aortic valve | Annulus = 13mm | Tricuspid | ------- |
| Pulmonary valve | Annulus = 14mm | pulmonic | -------- |
| **Great arteries** | NRGA | **Aortic arch** | Left |
| Aorta | ----- | **PDA** | No |
| Pulmonary artery | Normal MPA and Branch PAs. |  |  |
| **M-Mode:** Normal LV Function on eye balling. | | | |
| AO | mm | PWd | mm |
| LA | mm | EDV | ml |
| LVIDd | mm | ESV | ml |
| LVIDs | mm | LVEF | % |
| IVSd | mm | FS | % |
| **Additional Information**: |  | | |
| No pericardial/Pleural effusion. | | | |
| **Final Diagnosis:** | | | |
| 1. {S, D, S} Levocardia. 2. Small OS ASD, L – R Shunt 3. Normal Function | | | |
| **Remark**: Only subcostal and apical window | | | |
| **Recommendation**: | | | |
| SIGNATURE  Done by: Tesfaye T., Pediatrician, Pediatric Cardiologist _______________ 18/09/2013Eth.C | | | |

| Patient Name: **Addis Adane**. Patient ID: **TGSH**. SEX/ Age: F/14Years. Date of Report: 18/**09/2013**.  R.Dx: **Rheumatic recurrence. AGH10.850** | | | |
| --- | --- | --- | --- |
| **Features** | **Finding** | **Features** | **Finding** |
| **Profile** |  | **Atria** |  |
| Abdominal situs | Solitus | Left atrium | Dilated |
| Cardiac position | Levocardia | Right atrium | Dilated |
| Systemic venous drainage | Normal. | **Atrioventricular valves** |  |
| Pulmonary venous drainage | Normal | Mitral valve | Annulus = 32mm. Thickened MVL. MVA = 2.9cm2. |
| Atrioventricular connection | Concordant | Tricuspid valve | Annulus = 35mm. Non coapting TV  TAPSE = 31mm |
| Ventriculoarterial connection | Concordant | **Ventricles** |  |
| Ventricular loop | d-Loop | Left ventricle | Dilated |
|  |  | Right ventricle | Dilated |
| **Septae** |  | **Coronary arteries** | ----- |
| Interventricular septum | Intact | **Doppler Measurement** |  |
| Interatrial septum | Intact | Mitral | Severe MR, Holosystolic, posterior projection, seen in two planes with jet velocity = 4.7m/sec. Mitral Inflow PPG/MPG = 14/6mmHg |
| **Semilunal valves** |  | Aortic | ------- |
| Aortic valve | Annulus = 15mm | Tricuspid | Severe TR, PPG = 33mmHg |
| Pulmonary valve | Annulus = 20mm | pulmonic | -------- |
| **Great arteries** | NRGA | **Aortic arch** | Left |
| Aorta | ----- | **PDA** | No |
| Pulmonary artery | Normal MPA and Branch PAs. |  |  |
| **M-Mode:** | | | |
| AO | mm | PWd | mm |
| LA | mm | EDV | ml |
| LVIDd | mm | ESV | ml |
| LVIDs | mm | LVEF | 69% |
| IVSd | mm | FS | 38% |
| **Additional Information**: |  | | |
| No pericardial/Pleural effusion. | | | |
| **Final Diagnosis:** | | | |
| 1. {S, D, S} Levocardia. 2. All chambers dilated 3. Thickened MVL 4. Non coapting TVL 5. Severe MR 6. Severe TR 7. Normal Biventricular Function | | | |
| SIGNATURE  Done by: Tesfaye T., Pediatrician, Pediatric Cardiologist _______________ 18/09/2013Eth.C | | | |

| Patient Name: **Arsema Mequanint**. Patient ID: **Adinas GH**. SEX/ Age: F/13days. Date of Report: 18/**09/2013**.  BP: _______ Weight: ______ Height: ____________ BSA: ________. R.Dx: **Incidental Murmur. AGH10.851** | | | |
| --- | --- | --- | --- |
| **Features** | **Finding** | **Features** | **Finding** |
| **Profile** |  | **Atria** |  |
| Abdominal situs | Solitus | Left atrium | Normal |
| Cardiac position | Levocardia | Right atrium | Normal |
| Systemic venous drainage | Normal. | **Atrioventricular valves** |  |
| Pulmonary venous drainage | Normal | Mitral valve | Annulus = 9mm |
| Atrioventricular connection | Concordant | Tricuspid valve | Annulus = 10mm |
| Ventriculoarterial connection | Concordant | **Ventricles** |  |
| Ventricular loop | d-Loop | Left ventricle | Normal |
|  |  | Right ventricle | Normal |
| **Septae** |  | **Coronary arteries** | ----- |
| Interventricular septum | Intact | **Doppler Measurement** |  |
| Interatrial septum | Intact | Mitral | ----- |
| **Semilunal valves** |  | Aortic | ------- |
| Aortic valve | Annulus = 8mm | Tricuspid | Mild TR, PPG = 37mmHg |
| Pulmonary valve | Annulus = 8mm | pulmonic | -------- |
| **Great arteries** | NRGA | **Aortic arch** | Left |
| Aorta | ----- | **PDA** | 1mm PDA, L – R Shunt |
| Pulmonary artery | Normal MPA and Branch PAs. |  |  |
| **M-Mode:** Normal LV Function on eye balling. | | | |
| AO | mm | PWd | mm |
| LA | mm | EDV | ml |
| LVIDd | mm | ESV | ml |
| LVIDs | mm | LVEF | % |
| IVSd | mm | FS | % |
| **Additional Information**: |  | | |
| No pericardial/Pleural effusion. | | | |
| **Final Diagnosis:** | | | |
| 1. {S, D, S} Levocardia. 2. Small PDA, L – R Shunt 3. Mild Pulmonary Hypertension 4. Normal LV Function | | | |
| **Remark**: | | | |
| **Recommendation**: | | | |
| SIGNATURE  Done by: Tesfaye T., Pediatrician, Pediatric Cardiologist _______________ 18/09/2013Eth.C | | | |

| Patient Name: **Tigist Sawkachew**. Patient ID: **FHRH**. SEX/ Age: F/7Years. Date of Report: 19/**09/2013**.  R.Dx: **CHF. AGH10.852** | | | |
| --- | --- | --- | --- |
| **Features** | **Finding** | **Features** | **Finding** |
| **Profile** |  | **Atria** |  |
| Abdominal situs | Solitus | Left atrium | Dilated |
| Cardiac position | Levocardia | Right atrium | Normal |
| Systemic venous drainage | Normal. | **Atrioventricular valves** |  |
| Pulmonary venous drainage | Normal | Mitral valve | Annulus = 22mm. Thickened MVL. MVA = 1.5cm2. |
| Atrioventricular connection | Concordant | Tricuspid valve | Annulus = 16mm  TAPSE = 21mm |
| Ventriculoarterial connection | Concordant | **Ventricles** |  |
| Ventricular loop | d-Loop | Left ventricle | Dilated |
|  |  | Right ventricle | Normal |
| **Septae** |  | **Coronary arteries** | ----- |
| Interventricular septum | Intact | **Doppler Measurement** |  |
| Interatrial septum | Intact | Mitral | Severe MR, Holosystolic, posterior projection seen in two planes with jet velocity = 4.2m/sec. mild MS, PPG/MPG = 10/5mmHg |
| **Semilunal valves** |  | Aortic | Moderate AR, PHT = 225ms. |
| Aortic valve | Annulus = 13mm | Tricuspid | Mild TR, PPG = 38mmHg |
| Pulmonary valve | Annulus = 16mm | pulmonic | -------- |
| **Great arteries** | NRGA | **Aortic arch** | Left |
| Aorta | ----- | **PDA** | No |
| Pulmonary artery | Normal MPA and Branch PAs. |  |  |
| **M-Mode:** | | | |
| AO | mm | PWd | mm |
| LA | mm | EDV | ml |
| LVIDd | mm | ESV | ml |
| LVIDs | mm | LVEF | 65% |
| IVSd | mm | FS | 36% |
| **Additional Information**: |  | | |
| 11mm pericardial effusion on RA/RV Side. | | | |
| **Final Diagnosis:** | | | |
| 1. {S, D, S} Levocardia. 2. LA/LV Dilated 3. Thickened MVL 4. Severe MR 5. Mild MS 6. Moderate AR 7. Mild TR 8. Mild Pulmonary Hypertension 9. Moderate Pericardial effusion 10. Normal Biventricular Function | | | |
| SIGNATURE  Done by: Tesfaye T., Pediatrician, Pediatric Cardiologist _______________ 19/09/2013Eth.C | | | |

| Patient Name: **Baby of Kidist Shibeshi**. Patient ID: **FHRH**. SEX/ Age: M/23days. Date of Report: 19/**09/2013**.  BP: _______ Weight: ______ Height: ____________ BSA: ________. R.Dx: **Cyanosis. AGH10.853** | | | |
| --- | --- | --- | --- |
| **Features** | **Finding** | **Features** | **Finding** |
| **Profile** |  | **Atria** |  |
| Abdominal situs | Solitus | Left atrium | Normal |
| Cardiac position | Levocardia | Right atrium | Normal |
| Systemic venous drainage | Normal. | **Atrioventricular valves** |  |
| Pulmonary venous drainage | Normal | Mitral valve | Annulus = 7mm |
| Atrioventricular connection | Concordant | Tricuspid valve | Annulus = 8mm |
| Ventriculoarterial connection | Discordant | **Ventricles** |  |
| Ventricular loop | d-Loop | Left ventricle | Normal |
|  |  | Right ventricle | Normal |
| **Septae** |  | **Coronary arteries** | ----- |
| Interventricular septum | 5mm sub arterial VSD, BD Shunt | **Doppler Measurement** |  |
| Interatrial septum | PFO, L – R Shunt | Mitral | ----- |
| **Semilunal valves** |  | Aortic | ------- |
| Aortic valve | Annulus = 7mm | Tricuspid | ------- |
| Pulmonary valve | Annulus = 6mm | pulmonic | Mild LVOTO (PS), PPG = 35mmHg |
| **Great arteries** | d-TGA | **Aortic arch** | Left |
| Aorta | From RV, anterior and to the right | **PDA** | No |
| Pulmonary artery | From LV, posterior and to the left |  |  |
| **M-Mode:** Normal Function on eye balling. | | | |
| AO | mm | PWd | mm |
| LA | mm | EDV | ml |
| LVIDd | mm | ESV | ml |
| LVIDs | mm | LVEF | % |
| IVSd | mm | FS | % |
| **Additional Information**: |  | | |
| No pericardial/Pleural effusion. | | | |
| **Final Diagnosis:** | | | |
| 1. {S, D, D} Levocardia. 2. PFO, L- R Shunt 3. d-TGA with Sub arterial VSD, BD Shunt 4. Mild LVOTO (PS) 5. Normal Function | | | |
| **Remark**: | | | |
| **Recommendation**: | | | |
| SIGNATURE  Done by: Tesfaye T., Pediatrician, Pediatric Cardiologist _______________ 19/09/2013Eth.C | | | |

| Patient Name: **Kidus Esuyawkal**. Patient ID: **Adinas GH**. SEX/ Age: M/3/12. Date of Report: 19/**09/2013**.  BP: _______ Weight: ______ Height: ____________ BSA: ________ | | | |
| --- | --- | --- | --- |
| **Features** | **Finding** | **Features** | **Finding** |
| **Profile** |  | **Atria** |  |
| Abdominal situs | Solitus | Left atrium | Normal |
| Cardiac position | Levocardia | Right atrium | Normal |
| Systemic venous drainage | Normal. | **Atrioventricular valves** |  |
| Pulmonary venous drainage | Normal | Mitral valve | Annulus = 12mm |
| Atrioventricular connection | Concordant | Tricuspid valve | Annulus = 13mm |
| Ventriculoarterial connection | Concordant | **Ventricles** |  |
| Ventricular loop | d-Loop | Left ventricle | Normal |
|  |  | Right ventricle | Normal |
| **Septae** |  | **Coronary arteries** | ----- |
| Interventricular septum | Intact | **Doppler Measurement** |  |
| Interatrial septum | Intact | Mitral | ----- |
| **Semilunal valves** |  | Aortic | ------- |
| Aortic valve | Annulus = 10mm | Tricuspid | ------- |
| Pulmonary valve | Annulus = 9mm.  ? Dysplastic | pulmonic | Mild PS, PPG = 21mmHg |
| **Great arteries** | NRGA | **Aortic arch** | Left |
| Aorta | ----- | **PDA** | No |
| Pulmonary artery | Normal MPA and Branch PAs. |  |  |
| **M-Mode:** | | | |
| AO | mm | PWd | mm |
| LA | mm | EDV | ml |
| LVIDd | mm | ESV | ml |
| LVIDs | mm | LVEF | % |
| IVSd | mm | FS | % |
| **Additional Information**: |  | | |
| No pericardial/Pleural effusion. | | | |
| **Final Diagnosis:** | | | |
| 1. {S, D, S} Levocardia. 2. Mild PS 3. Normal Function | | | |
| **Remark**: | | | |
| **Recommendation**: Yearly Echocardiographic Follow up | | | |
| SIGNATURE  Done by: Tesfaye T., Pediatrician, Pediatric Cardiologist _______________ 19/09/2013Eth.C | | | |

| Patient Name: **Baby of Laway Mengistu**. Patient ID: **TGSH**. SEX/ Age: F/18days. Date of Report: 19/**09/2013**.  BP: _______ Weight: ______ Height: ____________ BSA: ________. R.Dx: **Incidental Murmur. AGH10.854** | | | |
| --- | --- | --- | --- |
| **Features** | **Finding** | **Features** | **Finding** |
| **Profile** |  | **Atria** |  |
| Abdominal situs | Solitus | Left atrium | Normal |
| Cardiac position | Levocardia | Right atrium | Normal |
| Systemic venous drainage | Normal. | **Atrioventricular valves** |  |
| Pulmonary venous drainage | Normal | Mitral valve | Annulus = 10mm |
| Atrioventricular connection | Concordant | Tricuspid valve | Annulus = 10mm  TAPSE = 10mm |
| Ventriculoarterial connection | Concordant | **Ventricles** |  |
| Ventricular loop | d-Loop | Left ventricle | Normal |
|  |  | Right ventricle | Normal |
| **Septae** |  | **Coronary arteries** | ----- |
| Interventricular septum | 4mm PM VSD, L – R Shunt | **Doppler Measurement** |  |
| Interatrial septum | Intact | Mitral | ----- |
| **Semilunal valves** |  | Aortic | ------- |
| Aortic valve | Annulus = 7mm | Tricuspid | ------- |
| Pulmonary valve | Annulus = 8mm | pulmonic | Flow acceleration across the PV, PPG = 18mmHg |
| **Great arteries** | NRGA | **Aortic arch** | Left |
| Aorta | ----- | **PDA** | No |
| Pulmonary artery | Normal MPA and Branch PAs. |  |  |
| **M-Mode:** Normal LV Function on eye balling. | | | |
| AO | mm | PWd | mm |
| LA | mm | EDV | ml |
| LVIDd | mm | ESV | ml |
| LVIDs | mm | LVEF | % |
| IVSd | mm | FS | % |
| **Additional Information**: |  | | |
| No pericardial/Pleural effusion. | | | |
| **Final Diagnosis:** | | | |
| 1. {S, D, S} Levocardia. 2. Moderate PM VSD, L – R Shunt 3. Normal Biventricular Function | | | |
| **Remark**: | | | |
| **Recommendation**: | | | |
| SIGNATURE  Done by: Tesfaye T., Pediatrician, Pediatric Cardiologist _______________ 19/09/2013Eth.C | | | |

| Patient Name: Temesgen Agegn. Patient ID: **FHRH**. SEX/ Age: M/6/12. Date of Report: 20/**09/2013**.  BP: _______ Weight: ______ Height: ____________ BSA: ________. R.Dx: **RD + DS. AGH10.855** | | | |
| --- | --- | --- | --- |
| **Features** | **Finding** | **Features** | **Finding** |
| **Profile** |  | **Atria** |  |
| Abdominal situs | Solitus | Left atrium | Normal |
| Cardiac position | Levocardia | Right atrium | Normal |
| Systemic venous drainage | Normal. | **Atrioventricular valves** |  |
| Pulmonary venous drainage | Normal | Mitral valve | Annulus = 13mm |
| Atrioventricular connection | Concordant | Tricuspid valve | Annulus = 13mm |
| Ventriculoarterial connection | Concordant | **Ventricles** |  |
| Ventricular loop | d-Loop | Left ventricle | Normal |
|  |  | Right ventricle | Normal |
| **Septae** |  | **Coronary arteries** | ----- |
| Interventricular septum | 8mm Inlet VSD with PM extension, Predominantly L – R Shunt | **Doppler Measurement** |  |
| Interatrial septum | 4mm OS ASD, L – R Shunt | Mitral | ----- |
| **Semilunal valves** |  | Aortic | ------- |
| Aortic valve | Annulus = 11mm | Tricuspid | ------- |
| Pulmonary valve | Annulus = 13mm | pulmonic | -------- |
| **Great arteries** | NRGA | **Aortic arch** | Left |
| Aorta | ----- | **PDA** | No |
| Pulmonary artery | Normal MPA and Branch PAs. |  |  |
| **M-Mode:** | | | |
| AO | mm | PWd | mm |
| LA | mm | EDV | ml |
| LVIDd | mm | ESV | ml |
| LVIDs | mm | LVEF | 70% |
| IVSd | mm | FS | 38% |
| **Additional Information**: |  | | |
| No pericardial/Pleural effusion. | | | |
| **Final Diagnosis:** | | | |
| 1. {S, D, S} Levocardia. 2. Small OS ASD, L – R Shunt 3. Moderate to large Inlet VSD with PM extension, predominantly L – R Shunt 4. Normal LV Function | | | |
| **Remark**: | | | |
| **Recommendation**: | | | |
| SIGNATURE  Done by: Tesfaye T., Pediatrician, Pediatric Cardiologist _______________ 20/09/2013Eth.C | | | |

| Patient Name: **Medina Ibrahim**. Patient ID: **Adinas GH**. SEX/ Age: F/14Years. Date of Report: 21/**09/2013**.  BP: _______ Weight: ______ Height: ____________ BSA: ________. R.Dx: **?Marfan Syndrome. AGH10.856** | | | |
| --- | --- | --- | --- |
| **Features** | **Finding** | **Features** | **Finding** |
| **Profile** |  | **Atria** |  |
| Abdominal situs | Solitus | Left atrium | Normal |
| Cardiac position | Levocardia | Right atrium | Normal |
| Systemic venous drainage | Normal. | **Atrioventricular valves** |  |
| Pulmonary venous drainage | Normal | Mitral valve | Annulus = 21mm. Bowing of anterior mitral valve to LA with coaptation of valves in the LA. thickened |
| Atrioventricular connection | Concordant | Tricuspid valve | Annulus = 23mm |
| Ventriculoarterial connection | Concordant | **Ventricles** |  |
| Ventricular loop | d-Loop | Left ventricle | Normal |
|  |  | Right ventricle | Normal |
| **Septae** |  | **Coronary arteries** | ----- |
| Interventricular septum | Intact | **Doppler Measurement** |  |
| Interatrial septum | Intact | Mitral | Trivial MR, Incomplete signal, seen in two planes with Jet Velocity = 3.5m/sec |
| **Semilunal valves** |  | Aortic | ------- |
| Aortic valve | Annulus = 18mm | Tricuspid | Trivial TR, PPG = 15mmHg |
| Pulmonary valve | Annulus = 19mm | pulmonic | -------- |
| **Great arteries** | NRGA | **Aortic arch** | Left |
| Aorta | ----- | **PDA** | No |
| Pulmonary artery | Normal MPA and Branch PAs. |  |  |
| **M-Mode:** | | | |
| AO | mm | PWd | mm |
| LA | mm | EDV | ml |
| LVIDd | mm | ESV | ml |
| LVIDs | mm | LVEF | 59% |
| IVSd | mm | FS | 31% |
| **Additional Information**: |  | | |
| No pericardial/Pleural effusion. | | | |
| **Final Diagnosis:** | | | |
| 1. {S, D, S} Levocardia. 2. Mitral Valve Prolapse 3. Trivial MR 4. Normal LV Function | | | |
| **Remark**: | | | |
| **Recommendation**: | | | |
| SIGNATURE  Done by: Tesfaye T., Pediatrician, Pediatric Cardiologist _______________ 21/09/2013Eth.C | | | |

| Patient Name: **Netsanet Muluneh**. Patient ID: **Adinas GH**. SEX/ Age: F/9/12. Date of Report: 21/**09/2013**.  BP: _______ Weight: ______ Height: ____________ BSA: ________. R.Dx: **RD + CHF. AGH10.857** | | | |
| --- | --- | --- | --- |
| **Follow up echo** | | | |
| **Features** | **Finding** | **Features** | **Finding** |
| **Profile** |  | **Atria** |  |
| Abdominal situs | Solitus | Left atrium | Dilated |
| Cardiac position | Levocardia | Right atrium | Normal |
| Systemic venous drainage | Normal. | **Atrioventricular valves** |  |
| Pulmonary venous drainage | Normal | Mitral valve | Annulus = 14mm |
| Atrioventricular connection | Concordant | Tricuspid valve | Annulus = 15mm |
| Ventriculoarterial connection | Concordant | **Ventricles** |  |
| Ventricular loop | d-Loop | Left ventricle | Dilated |
|  |  | Right ventricle | Normal |
| **Septae** |  | **Coronary arteries** | ----- |
| Interventricular septum | Intact | **Doppler Measurement** |  |
| Interatrial septum | Intact | Mitral | ----- |
| **Semilunal valves** |  | Aortic | ------- |
| Aortic valve | Annulus = 12mm | Tricuspid | ------- |
| Pulmonary valve | Annulus = 13mm | pulmonic | -------- |
| **Great arteries** | NRGA | **Aortic arch** | Left |
| Aorta | ----- | **PDA** | 3mm PDA, L – R Shunt |
| Pulmonary artery | Normal MPA and Branch PAs. |  |  |
| **M-Mode:** Normal LV Function on eye balling. | | | |
| AO | mm | PWd | mm |
| LA | mm | EDV | ml |
| LVIDd | mm | ESV | ml |
| LVIDs | mm | LVEF | % |
| IVSd | mm | FS | % |
| **Additional Information**: |  | | |
| No pericardial/Pleural effusion. | | | |
| **Final Diagnosis:** | | | |
| 1. {S, D, S} Levocardia. 2. Large PDA, L – R Shunt 3. Normal LV Function | | | |
| **Remark**: | | | |
| **Recommendation**: Needs definitive management | | | |
| SIGNATURE  Done by: Tesfaye T., Pediatrician, Pediatric Cardiologist _______________ 21/09/2013Eth.C | | | |

| Patient Name: **Addis Alem Getachew**. Patient ID: **FHRH**. SEX/ Age: F/6Years. Date of Report: 23/**09/2013**.  BP: _______ Weight: ______ Height: _______ BSA: ________. R.Dx: **Rheumatic Recurrence. AGH10.858** | | | |
| --- | --- | --- | --- |
| **Features** | **Finding** | **Features** | **Finding** |
| **Profile** |  | **Atria** |  |
| Abdominal situs | Solitus | Left atrium | Normal |
| Cardiac position | Levocardia | Right atrium | Normal |
| Systemic venous drainage | Normal. | **Atrioventricular valves** |  |
| Pulmonary venous drainage | Normal | Mitral valve | Annulus = 21mm. Thickened MVL with shortened PMVL. |
| Atrioventricular connection | Concordant | Tricuspid valve | Annulus = 21mm  TAPSE = 25mm |
| Ventriculoarterial connection | Concordant | **Ventricles** |  |
| Ventricular loop | d-Loop | Left ventricle | Normal |
|  |  | Right ventricle | Normal |
| **Septae** |  | **Coronary arteries** | ----- |
| Interventricular septum | Intact | **Doppler Measurement** |  |
| Interatrial septum | Intact | Mitral | Moderate MR, Holosystolic, posterior projection, seen in two planes with jet velocity = 4.1m/sec. |
| **Semilunal valves** |  | Aortic | ------- |
| Aortic valve | Annulus = 15mm | Tricuspid | ------- |
| Pulmonary valve | Annulus = 19mm | pulmonic | -------- |
| **Great arteries** | NRGA | **Aortic arch** | Left |
| Aorta | ----- | **PDA** | No |
| Pulmonary artery | Normal MPA and Branch PAs. |  |  |
| **M-Mode:** | | | |
| AO | mm | PWd | mm |
| LA | mm | EDV | ml |
| LVIDd | mm | ESV | ml |
| LVIDs | mm | LVEF | 69% |
| IVSd | mm | FS | 38% |
| **Additional Information**: |  | | |
| No pericardial/Pleural effusion. | | | |
| **Final Diagnosis:** | | | |
| 1. {S, D, S} Levocardia. 2. Thickened MVL with shortening of PMVL 3. Moderate MR 4. Normal Biventricular Function | | | |
| **Remark**: | | | |
| **Recommendation**: in line with Rheumatic Carditis | | | |
| SIGNATURE  Done by: Tesfaye T., Pediatrician, Pediatric Cardiologist _______________ 23/09/2013Eth.C | | | |

| Patient Name: **Nathanem Getahun**. Patient ID: **Amaris PSC**. SEX/ Age: M/4 10/12. Date of Report: 23/**09/2013**.  BP: _______ Weight: ______ Height: _______ BSA: ________. R.Dx:**Recurrent chest infection. AGH10.859** | | | |
| --- | --- | --- | --- |
| **Features** | **Finding** | **Features** | **Finding** |
| **Profile** |  | **Atria** |  |
| Abdominal situs | Solitus | Left atrium | Normal |
| Cardiac position | Levocardia | Right atrium | Normal |
| Systemic venous drainage | Normal. | **Atrioventricular valves** |  |
| Pulmonary venous drainage | Normal | Mitral valve | Annulus = 18mm |
| Atrioventricular connection | Concordant | Tricuspid valve | Annulus = 19mm  TAPSE = 19mm |
| Ventriculoarterial connection | Concordant | **Ventricles** |  |
| Ventricular loop | d-Loop | Left ventricle | Normal |
|  |  | Right ventricle | Normal |
| **Septae** |  | **Coronary arteries** | ----- |
| Interventricular septum | Intact | **Doppler Measurement** |  |
| Interatrial septum | Intact | Mitral | ----- |
| **Semilunal valves** |  | Aortic | ------- |
| Aortic valve | Annulus = 16mm | Tricuspid | Trivial TR, PPG = 18mmHg |
| Pulmonary valve | Annulus = 18mm | pulmonic | Trivial PR, PPG = 13mmHg. |
| **Great arteries** | NRGA | **Aortic arch** | Left |
| Aorta | ----- | **PDA** | No |
| Pulmonary artery | Normal MPA and Branch PAs. |  |  |
| **M-Mode:** | | | |
| AO | mm | PWd | mm |
| LA | mm | EDV | ml |
| LVIDd | mm | ESV | ml |
| LVIDs | mm | LVEF | 61% |
| IVSd | mm | FS | 32% |
| **Additional Information**: |  | | |
| No pericardial/Pleural effusion. | | | |
| **Final Diagnosis:** | | | |
| 1. Normal Echocardiography Study. | | | |
| **Remark**: | | | |
| **Recommendation**: | | | |
| SIGNATURE  Done by: Tesfaye T., Pediatrician, Pediatric Cardiologist _______________ 23/09/2013Eth.C | | | |

| Patient Name: **Fasil Ferede**. Patient ID: **TGSH**. SEX/ Age: M/12Years. Date of Report: 24/**09/2013**.  BP: _____ Weight: ____ Height: _______ BSA: _____. R.Dx: **CHF + Rheumatic Recurrence + DOE. AGH10.860** | | | |
| --- | --- | --- | --- |
| **Features** | **Finding** | **Features** | **Finding** |
| **Profile** |  | **Atria** |  |
| Abdominal situs | Solitus | Left atrium | Dilated |
| Cardiac position | Levocardia | Right atrium | Normal |
| Systemic venous drainage | Normal. | **Atrioventricular valves** |  |
| Pulmonary venous drainage | Normal | Mitral valve | Annulus = 23mm. Thickened, clubbed, deformed MV. MVA = 0.7cm2. |
| Atrioventricular connection | Concordant | Tricuspid valve | Annulus = 19mm  TAPSE = 18mm |
| Ventriculoarterial connection | Concordant | **Ventricles** |  |
| Ventricular loop | d-Loop | Left ventricle | Dilated |
|  |  | Right ventricle | Normal |
| **Septae** |  | **Coronary arteries** | ----- |
| Interventricular septum | Intact | **Doppler Measurement** |  |
| Interatrial septum | Intact | Mitral | Severe MS, PPG/MPG = 20/13mmHg. Mild MR, Holosystolic |
| **Semilunal valves** |  | Aortic | ------- |
| Aortic valve | Annulus = 14mm | Tricuspid | Moderate TR, PPG = 47mmHg |
| Pulmonary valve | Annulus = 18mm | pulmonic | -------- |
| **Great arteries** | NRGA | **Aortic arch** | Left |
| Aorta | ----- | **PDA** | No |
| Pulmonary artery | Normal MPA and Branch PAs. |  |  |
| **M-Mode:** | | | |
| AO | mm | PWd | mm |
| LA | mm | EDV | ml |
| LVIDd | mm | ESV | ml |
| LVIDs | mm | LVEF | 58% |
| IVSd | mm | FS | 30% |
| **Additional Information**: |  | | |
| No pericardial/Pleural effusion. | | | |
| **Final Diagnosis:** | | | |
| 1. {S, D, S} Levocardia. 2. LA/LV Dilated 3. Thickened, clubbed, deformed MVL 4. Severe MS 5. Mild MR 6. Moderate TR 7. Mild to moderate Pulmonary Hypertension 8. Normal Biventricular Function | | | |
| SIGNATURE  Done by: Tesfaye T., Pediatrician, Pediatric Cardiologist _______________ 24/09/2013Eth.C | | | |

| Patient Name: **Meriem Nurhusien**. Patient ID: **Adinas GH**. SEX/ Age: F/3/12. Date of Report: 24/**09/2013**.  BP: _______ Weight: ______ Height: ____________ BSA: ________. R.Dx: **Pre-op screening. AGH10.861** | | | |
| --- | --- | --- | --- |
| **Features** | **Finding** | **Features** | **Finding** |
| **Profile** |  | **Atria** |  |
| Abdominal situs | Solitus | Left atrium | Normal |
| Cardiac position | Levocardia | Right atrium | Normal |
| Systemic venous drainage | Normal. | **Atrioventricular valves** |  |
| Pulmonary venous drainage | Normal | Mitral valve | Annulus = 12mm |
| Atrioventricular connection | Concordant | Tricuspid valve | Annulus = 13mm |
| Ventriculoarterial connection | Concordant | **Ventricles** |  |
| Ventricular loop | d-Loop | Left ventricle | Normal |
|  |  | Right ventricle | Normal |
| **Septae** |  | **Coronary arteries** | ----- |
| Interventricular septum | Intact | **Doppler Measurement** |  |
| Interatrial septum | PFO, L – R Shunt | Mitral | ----- |
| **Semilunal valves** |  | Aortic | ------- |
| Aortic valve | Annulus = 11mm | Tricuspid | ------- |
| Pulmonary valve | Annulus = 10mm | pulmonic | -------- |
| **Great arteries** | NRGA | **Aortic arch** | Left |
| Aorta | ----- | **PDA** | No |
| Pulmonary artery | Normal MPA and Branch PAs. |  |  |
| **M-Mode:** Normal LV Function on eye balling. | | | |
| AO | mm | PWd | mm |
| LA | mm | EDV | ml |
| LVIDd | mm | ESV | ml |
| LVIDs | mm | LVEF | % |
| IVSd | mm | FS | % |
| **Additional Information**: |  | | |
| No pericardial/Pleural effusion. | | | |
| **Final Diagnosis:** | | | |
| 1. {S, D, S} Levocardia. 2. PFO, L – R Shunt | | | |
| **Remark**: | | | |
| **Recommendation**: | | | |
| SIGNATURE  Done by: Tesfaye T., Pediatrician, Pediatric Cardiologist _______________ 24/09/2013Eth.C | | | |

| Patient Name: **Baby of Birkie Negese**. Patient ID: **FHRH**. SEX/ Age: M/47days. Date of Report: 24/**09/2013**.  BP: _______ Weight: ______ Height: ____________ BSA: ________. R.Dx: **DS. AGH10.862** | | | |
| --- | --- | --- | --- |
| **Features** | **Finding** | **Features** | **Finding** |
| **Profile** |  | **Atria** |  |
| Abdominal situs | Solitus | Left atrium | Normal |
| Cardiac position | Levocardia | Right atrium | Normal |
| Systemic venous drainage | Normal. | **Atrioventricular valves** |  |
| Pulmonary venous drainage | Normal | Mitral valve | Annulus = 8mm |
| Atrioventricular connection | Concordant | Tricuspid valve | Annulus = 9mm |
| Ventriculoarterial connection | Concordant | **Ventricles** |  |
| Ventricular loop | d-Loop | Left ventricle | Normal |
|  |  | Right ventricle | Normal |
| **Septae** |  | **Coronary arteries** | ----- |
| Interventricular septum | Intact | **Doppler Measurement** |  |
| Interatrial septum | PFO, L – R Shunt | Mitral | ----- |
| **Semilunal valves** |  | Aortic | ------- |
| Aortic valve | Annulus = 6mm | Tricuspid | ------- |
| Pulmonary valve | Annulus = 7mm | pulmonic | -------- |
| **Great arteries** | NRGA | **Aortic arch** | Left |
| Aorta | ----- | **PDA** | No |
| Pulmonary artery | Normal MPA and Branch PAs. |  |  |
| **M-Mode:** Normal LV Function on eye balling | | | |
| AO | mm | PWd | mm |
| LA | mm | EDV | ml |
| LVIDd | mm | ESV | ml |
| LVIDs | mm | LVEF | % |
| IVSd | mm | FS | % |
| **Additional Information**: |  | | |
| No pericardial/Pleural effusion. | | | |
| **Final Diagnosis:** | | | |
| 1. {S, D, S} Levocardia. 2. PFO, L – R Shunt | | | |
| **Remark**: | | | |
| **Recommendation**: | | | |
| SIGNATURE  Done by: Tesfaye T., Pediatrician, Pediatric Cardiologist _______________ 24/09/2013Eth.C | | | |

| Patient Name: **Habte-Mariam Tenesa**. Patient ID: **TGSH**. SEX/ Age: M/3 8/12. Date of Report: 24/**09/2013**.  BP: _______ Weight: ______ Height: ____________ BSA: ________. R.Dx: **RD + DS. AGH10.863** | | | |
| --- | --- | --- | --- |
| **Features** | **Finding** | **Features** | **Finding** |
| **Profile** |  | **Atria** |  |
| Abdominal situs | Solitus | Left atrium | Normal |
| Cardiac position | Levocardia | Right atrium | Dilated |
| Systemic venous drainage | Normal. | **Atrioventricular valves** |  |
| Pulmonary venous drainage | Normal | Mitral valve | Annulus = 14mm |
| Atrioventricular connection | Concordant | Tricuspid valve | Annulus = 18mm  TAPSE = 19mm |
| Ventriculoarterial connection | Concordant | **Ventricles** |  |
| Ventricular loop | d-Loop | Left ventricle | Normal |
|  |  | Right ventricle | Dilated |
| **Septae** |  | **Coronary arteries** | ----- |
| Interventricular septum | Intact | **Doppler Measurement** |  |
| Interatrial septum | Intact | Mitral | ----- |
| **Semilunal valves** |  | Aortic | ------- |
| Aortic valve | Annulus = 15mm | Tricuspid | Mild TR, PPG = 53mmHg |
| Pulmonary valve | Annulus = 16mm | pulmonic | Moderate PR, PPG = 54mmHg |
| **Great arteries** | NRGA | **Aortic arch** | Left |
| Aorta | ----- | **PDA** | No |
| Pulmonary artery | Normal MPA and Branch PAs. |  |  |
| **M-Mode:** Normal LV Function on eye balling. | | | |
| AO | mm | PWd | mm |
| LA | mm | EDV | ml |
| LVIDd | mm | ESV | ml |
| LVIDs | mm | LVEF | % |
| IVSd | mm | FS | % |
| **Additional Information**: |  | | |
| 4mm pericardial effusion on RA/RV Side. | | | |
| **Final Diagnosis:** | | | |
| 1. {S, D, S} Levocardia. 2. RA/RV Dilated 3. Mild TR 4. Moderate PR 5. Moderate Pulmonary Hypertension 6. Normal Biventricular Function | | | |
| **Remark**: | | | |
| **Recommendation**: | | | |
| SIGNATURE  Done by: Tesfaye T., Pediatrician, Pediatric Cardiologist _______________ 24/09/2013Eth.C | | | |

| Patient Name: **Tiringo Hailu**. Patient ID: **Mekane – Selam H**. SEX/ Age: F/14Years. Date of Report: 27/**09/2013**.  BP: _______ Weight: ______ Height: ____________ BSA: ________. R.Dx: **easy fatigability. AGH10.864** | | | |
| --- | --- | --- | --- |
| **Features** | **Finding** | **Features** | **Finding** |
| **Profile** |  | **Atria** |  |
| Abdominal situs | Solitus | Left atrium | Normal |
| Cardiac position | Levocardia | Right atrium | Normal |
| Systemic venous drainage | Normal. | **Atrioventricular valves** |  |
| Pulmonary venous drainage | Normal | Mitral valve | Annulus = 20mm |
| Atrioventricular connection | Concordant | Tricuspid valve | Annulus = 23mm  TAPSE = 20mm |
| Ventriculoarterial connection | Concordant | **Ventricles** |  |
| Ventricular loop | d-Loop | Left ventricle | Normal |
|  |  | Right ventricle | Normal |
| **Septae** |  | **Coronary arteries** | ----- |
| Interventricular septum | Intact | **Doppler Measurement** |  |
| Interatrial septum | Intact | Mitral | ----- |
| **Semilunal valves** |  | Aortic | ------- |
| Aortic valve | Annulus = 15mm | Tricuspid | Trivial TR, PPG = 10mmHg |
| Pulmonary valve | Annulus = 20mm | pulmonic | -------- |
| **Great arteries** | NRGA | **Aortic arch** | Left |
| Aorta | ----- | **PDA** | No |
| Pulmonary artery | Normal MPA and Branch PAs. |  |  |
| **M-Mode:** | | | |
| AO | mm | PWd | mm |
| LA | mm | EDV | ml |
| LVIDd | mm | ESV | ml |
| LVIDs | mm | LVEF | 58% |
| IVSd | mm | FS | 30% |
| **Additional Information**: |  | | |
| No pericardial/Pleural effusion. | | | |
| **Final Diagnosis:** | | | |
| 1. Normal Echocardiography Study. | | | |
| **Remark**: | | | |
| **Recommendation**: | | | |
| SIGNATURE  Done by: Tesfaye T., Pediatrician, Pediatric Cardiologist _______________ 27/09/2013Eth.C | | | |

N.B: She shall continue her prophylaxis at least for 5 complete years before stopping it (Benzathine penicillin) as she was having Mild carditis in the previous echocardiography study.32

| Patient Name: **Robel Tadege**. Patient ID: **FHRH**. SEX/ Age: M/1Year. Date of Report: 28/**09/2013**.  BP: _______ Weight: ______ Height: ____ BSA: ____. R.Dx: **recurrent chest infection. AGH10.865** | | | |
| --- | --- | --- | --- |
| **Features** | **Finding** | **Features** | **Finding** |
| **Profile** |  | **Atria** |  |
| Abdominal situs | Solitus | Left atrium | Normal |
| Cardiac position | Levocardia | Right atrium | Normal |
| Systemic venous drainage | Normal. | **Atrioventricular valves** |  |
| Pulmonary venous drainage | Normal | Mitral valve | Annulus = 14mm |
| Atrioventricular connection | Concordant | Tricuspid valve | Annulus = 14mm  TAPSE = 18mm |
| Ventriculoarterial connection | Concordant | **Ventricles** |  |
| Ventricular loop | d-Loop | Left ventricle | Normal |
|  |  | Right ventricle | Normal |
| **Septae** |  | **Coronary arteries** | ----- |
| Interventricular septum | Intact | **Doppler Measurement** |  |
| Interatrial septum | Intact | Mitral | ----- |
| **Semilunal valves** |  | Aortic | ------- |
| Aortic valve | Annulus = 12mm | Tricuspid | ------- |
| Pulmonary valve | Annulus = 12mm | pulmonic | -------- |
| **Great arteries** | NRGA | **Aortic arch** | Left |
| Aorta | ----- | **PDA** | No |
| Pulmonary artery | Normal MPA and Branch PAs. |  |  |
| **M-Mode:** | | | |
| AO | mm | PWd | mm |
| LA | mm | EDV | ml |
| LVIDd | mm | ESV | ml |
| LVIDs | mm | LVEF | 65% |
| IVSd | mm | FS | 34% |
| **Additional Information**: |  | | |
| No pericardial/Pleural effusion. | | | |
| **Final Diagnosis:** | | | |
| 1. Normal Echocardiography Study. | | | |
| **Remark**: | | | |
| **Recommendation**: | | | |
| SIGNATURE  Done by: Tesfaye T., Pediatrician, Pediatric Cardiologist _______________ 28/09/2013Eth.C | | | |

| Patient Name: **Hannan Mohammed**. Patient ID: **Mekane Selam H**. SEX/ Age: F/8Years. Date of Report: 28/**09/2013**.  BP: _______ Weight: ______ Height: ____________ BSA: ______. R.Dx: **Incidental Murmur. AGH10.866**__ | | | |
| --- | --- | --- | --- |
| **Features** | **Finding** | **Features** | **Finding** |
| **Profile** |  | **Atria** |  |
| Abdominal situs | Solitus | Left atrium | Normal |
| Cardiac position | Levocardia | Right atrium | Normal |
| Systemic venous drainage | Normal. | **Atrioventricular valves** |  |
| Pulmonary venous drainage | Normal | Mitral valve | Annulus = 21mm. Thickened MVL |
| Atrioventricular connection | Concordant | Tricuspid valve | Annulus = 21mm |
| Ventriculoarterial connection | Concordant | **Ventricles** |  |
| Ventricular loop | d-Loop | Left ventricle | Normal |
|  |  | Right ventricle | Normal |
| **Septae** |  | **Coronary arteries** | ----- |
| Interventricular septum | Intact | **Doppler Measurement** |  |
| Interatrial septum | Intact | Mitral | Mild MR, Seen in two planes, posterior projection, jet velocity = 4.7m/sec. Late systolic |
| **Semilunal valves** |  | Aortic | ------- |
| Aortic valve | Annulus = 16mm | Tricuspid | ------- |
| Pulmonary valve | Annulus = 20mm | pulmonic | -------- |
| **Great arteries** | NRGA | **Aortic arch** | Left |
| Aorta | ----- | **PDA** | No |
| Pulmonary artery | Normal MPA and Branch PAs. |  |  |
| **M-Mode:** | | | |
| AO | mm | PWd | mm |
| LA | mm | EDV | ml |
| LVIDd | mm | ESV | ml |
| LVIDs | mm | LVEF | 61% |
| IVSd | mm | FS | 32% |
| **Additional Information**: |  | | |
| No pericardial/Pleural effusion. | | | |
| **Final Diagnosis:** | | | |
| 1. {S, D, S} Levocardia. 2. Thickened MVL 3. Mild MR 4. Normal LV Function | | | |
| **Remark**: the MR is not Holosystolic rather Late systolic | | | |
| **Recommendation**: | | | |
| SIGNATURE  Done by: Tesfaye T., Pediatrician, Pediatric Cardiologist _______________ 28/09/2013Eth.C | | | |

| Patient Name: **Zemichael Mengistu**. Patient ID: **Adinas GH**. SEX/ Age: M/1 3/12. Date of Report: 28/**09/2013**.  BP: _______ Weight: ______ Height: ____________ BSA: ________. R.Dx: **DS. AGH10.867** | | | |
| --- | --- | --- | --- |
| **Features** | **Finding** | **Features** | **Finding** |
| **Profile** |  | **Atria** |  |
| Abdominal situs | Solitus | Left atrium | Normal |
| Cardiac position | Levocardia | Right atrium | Normal |
| Systemic venous drainage | Normal. | **Atrioventricular valves** |  |
| Pulmonary venous drainage | Normal | Mitral valve | Annulus = 13mm |
| Atrioventricular connection | Concordant | Tricuspid valve | Annulus = 14mm |
| Ventriculoarterial connection | Concordant | **Ventricles** |  |
| Ventricular loop | d-Loop | Left ventricle | Normal |
|  |  | Right ventricle | Normal |
| **Septae** |  | **Coronary arteries** | ----- |
| Interventricular septum | Intact | **Doppler Measurement** |  |
| Interatrial septum | Intact | Mitral | ----- |
| **Semilunal valves** |  | Aortic | ------- |
| Aortic valve | Annulus = 10mm | Tricuspid | ------- |
| Pulmonary valve | Annulus = 12mm | pulmonic | -------- |
| **Great arteries** | NRGA | **Aortic arch** | Left |
| Aorta | ----- | **PDA** | No PDA visible |
| Pulmonary artery | Normal MPA and Branch PAs. |  |  |
| **M-Mode:** | | | |
| AO | mm | PWd | mm |
| LA | mm | EDV | ml |
| LVIDd | mm | ESV | ml |
| LVIDs | mm | LVEF | % |
| IVSd | mm | FS | % |
| **Additional Information**: |  | | |
| trace pericardial effusion. | | | |
| **Final Diagnosis:** | | | |
| 1. {S, D, S} Levocardia. 2. Trace Pericardial Effusion | | | |
| **Remark**: toddler is irritable and only subcostal window is used. | | | |
| **Recommendation**: | | | |
| SIGNATURE  Done by: Tesfaye T., Pediatrician, Pediatric Cardiologist _______________ 28/09/2013Eth.C | | | |

| Patient Name: **Fikre-Selam Mersha**. Patient ID: **FHRH**. SEX/ Age: F/2Years. Date of Report: 30/**09/2013**.  BP: _______ Weight: ______ Height: ____________ BSA: ________. R.Dx: **Incidental Murmur. AGH10.868** | | | |
| --- | --- | --- | --- |
| **Features** | **Finding** | **Features** | **Finding** |
| **Profile** |  | **Atria** |  |
| Abdominal situs | Solitus | Left atrium | Normal |
| Cardiac position | Levocardia | Right atrium | Normal |
| Systemic venous drainage | Normal. | **Atrioventricular valves** |  |
| Pulmonary venous drainage | Normal | Mitral valve | Annulus = 15mm |
| Atrioventricular connection | Concordant | Tricuspid valve | Annulus = 16mm  TAPSE = 14mm |
| Ventriculoarterial connection | Concordant | **Ventricles** |  |
| Ventricular loop | d-Loop | Left ventricle | Normal |
|  |  | Right ventricle | Normal |
| **Septae** |  | **Coronary arteries** | ----- |
| Interventricular septum | 3mm PM VSD Partially covered by septal leaflet of the TV, R- L Shunt | **Doppler Measurement** |  |
| Interatrial septum | Intact | Mitral | ----- |
| **Semilunal valves** |  | Aortic | ------- |
| Aortic valve | Annulus = 13mm | Tricuspid | ------- |
| Pulmonary valve | Annulus = 16mm | pulmonic | -------- |
| **Great arteries** | NRGA | **Aortic arch** | Left |
| Aorta | ----- | **PDA** | No |
| Pulmonary artery | Normal MPA and Branch PAs. |  |  |
| **M-Mode:** | | | |
| AO | mm | PWd | mm |
| LA | mm | EDV | ml |
| LVIDd | mm | ESV | ml |
| LVIDs | mm | LVEF | 67% |
| IVSd | mm | FS | 36% |
| **Additional Information**: |  | | |
| No pericardial/Pleural effusion. | | | |
| **Final Diagnosis:** | | | |
| 1. {S, D, S} Levocardia. 2. Small PM VSD Partially covered by septal leaflet of TV 3. Normal Biventricular Function | | | |
| **Remark**: | | | |
| **Recommendation**: | | | |
| SIGNATURE  Done by: Tesfaye T., Pediatrician, Pediatric Cardiologist _______________ 30/09/2013Eth.C | | | |

| Patient Name: **Balewlet Amare**. Patient ID: FHR**H**. SEX/ Age: M/3/12. Date of Report: 30/**09/2013**.  BP: _______ Weight: ______ Height: ____________ BSA: ________. R.Dx: **DS. AGH10.869** | | | |
| --- | --- | --- | --- |
| **Features** | **Finding** | **Features** | **Finding** |
| **Profile** |  | **Atria** |  |
| Abdominal situs | Solitus | Left atrium | Normal |
| Cardiac position | Levocardia | Right atrium | Dilated |
| Systemic venous drainage | Normal. | **Atrioventricular valves** |  |
| Pulmonary venous drainage | Normal | Mitral valve | Annulus = 9mm |
| Atrioventricular connection | Concordant | Tricuspid valve | Annulus = 13mm |
| Ventriculoarterial connection | Concordant | **Ventricles** |  |
| Ventricular loop | d-Loop | Left ventricle | Normal |
|  |  | Right ventricle | Dilated |
| **Septae** |  | **Coronary arteries** | ----- |
| Interventricular septum | Intact | **Doppler Measurement** |  |
| Interatrial septum | 7mm OS ASD, L – R Shunt | Mitral | ----- |
| **Semilunal valves** |  | Aortic | ------- |
| Aortic valve | Annulus = 9mm | Tricuspid | Trivial TR, PPG = 17mmHg |
| Pulmonary valve | Annulus = 12mm | pulmonic | Trivial PR, PPG = 22mmHg |
| **Great arteries** | NRGA | **Aortic arch** | Left |
| Aorta | ----- | **PDA** | No |
| Pulmonary artery | Normal MPA and Branch PAs. |  |  |
| **M-Mode:** | | | |
| AO | mm | PWd | mm |
| LA | mm | EDV | ml |
| LVIDd | mm | ESV | ml |
| LVIDs | mm | LVEF | 73% |
| IVSd | mm | FS | 38% |
| **Additional Information**: |  | | |
| 4mm pericardial effusion on RA/RV Side. | | | |
| **Final Diagnosis:** | | | |
| 1. {S, D, S} Levocardia. 2. Moderate OS ASD, L – R Shunt 3. Trivial Pericardial effusion 4. Normal LV Function | | | |
| **Remark**: | | | |
| **Recommendation**: | | | |
| SIGNATURE  Done by: Tesfaye T., Pediatrician, Pediatric Cardiologist _______________ 30/09/2013Eth.C | | | |

| Patient Name: **Befikir Bayayibgn**. Patient ID: **FHRH**. SEX/ Age: F/8 7/12. Date of Report: 30/**09/2013**.  BP: _______ Weight: ______ Height: ____________ BSA: ________. R.Dx: **easy fatigability. AGH10.870** | | | |
| --- | --- | --- | --- |
| **Features** | **Finding** | **Features** | **Finding** |
| **Profile** |  | **Atria** |  |
| Abdominal situs | Solitus | Left atrium | Normal |
| Cardiac position | Levocardia | Right atrium | Normal |
| Systemic venous drainage | Normal. | **Atrioventricular valves** |  |
| Pulmonary venous drainage | Normal | Mitral valve | Annulus = 15mm |
| Atrioventricular connection | Concordant | Tricuspid valve | Annulus = 16mm  TAPSE = 19mm |
| Ventriculoarterial connection | Concordant | **Ventricles** |  |
| Ventricular loop | d-Loop | Left ventricle | Normal |
|  |  | Right ventricle | Normal |
| **Septae** |  | **Coronary arteries** | ----- |
| Interventricular septum | Intact | **Doppler Measurement** |  |
| Interatrial septum | Intact | Mitral | ----- |
| **Semilunal valves** |  | Aortic | ------- |
| Aortic valve | Annulus = 14mm | Tricuspid | ------- |
| Pulmonary valve | Annulus = 15mm | pulmonic | -------- |
| **Great arteries** | NRGA | **Aortic arch** | Left |
| Aorta | ----- | **PDA** | No |
| Pulmonary artery | Normal MPA and Branch PAs. |  |  |
| **M-Mode:** Normal LV Function on eye balling. | | | |
| AO | mm | PWd | mm |
| LA | mm | EDV | ml |
| LVIDd | mm | ESV | ml |
| LVIDs | mm | LVEF | % |
| IVSd | mm | FS | % |
| **Additional Information**: |  | | |
| No pericardial/Pleural effusion. | | | |
| **Final Diagnosis:** | | | |
| 1. Normal Echocardiography Study. | | | |
| **Remark**: | | | |
| **Recommendation**: | | | |
| SIGNATURE  Done by: Tesfaye T., Pediatrician, Pediatric Cardiologist _______________ 30/09/2013Eth.C | | | |

| Patient Name: **Solomie Asmamaw**. Patient ID: **Adinas GH**. SEX/ Age: F/1 11/12. Date of Report: 30/**09/2013**.  BP: _______ Weight: ______ Height: ____________ BSA: ________. R.Dx: **_____. AGH10.871 (AGH3, 1YEAR)** | | | |
| --- | --- | --- | --- |
| **Follow up Echo:** Dysfunctional, dilated RV, Severe Pulmonary HTN (on Rx: Lasix, Spironolactone, Sildenafil) | | | |
| **Features** | **Finding** | **Features** | **Finding** |
| **Profile** |  | **Atria** |  |
| Abdominal situs | Solitus | Left atrium | Normal |
| Cardiac position | Levocardia | Right atrium | Normal |
| Systemic venous drainage | Normal. | **Atrioventricular valves** |  |
| Pulmonary venous drainage | Normal | Mitral valve | Annulus = 14mm |
| Atrioventricular connection | Concordant | Tricuspid valve | Annulus = 18mm  TAPSE = 13mm |
| Ventriculoarterial connection | Concordant | **Ventricles** |  |
| Ventricular loop | d-Loop | Left ventricle | Normal |
|  |  | Right ventricle | Normal |
| **Septae** |  | **Coronary arteries** | ----- |
| Interventricular septum | Intact | **Doppler Measurement** |  |
| Interatrial septum | Intact | Mitral | ----- |
| **Semilunal valves** |  | Aortic | ------- |
| Aortic valve | Annulus = 12mm | Tricuspid | Trivial TR, PPG = 24mmHg |
| Pulmonary valve | Annulus = 13mm | pulmonic | -------- |
| **Great arteries** | NRGA | **Aortic arch** | Left |
| Aorta | ----- | **PDA** | No |
| Pulmonary artery | Normal MPA and Branch PAs. |  |  |
| **M-Mode:** | | | |
| AO | mm | PWd | mm |
| LA | mm | EDV | ml |
| LVIDd | mm | ESV | ml |
| LVIDs | mm | LVEF | 73% |
| IVSd | mm | FS | 40% |
| **Additional Information**: |  | | |
| 4mm pericardial effusion on RV Side. | | | |
| **Final Diagnosis:** | | | |
| 1. {S, D, S} Levocardia. 2. RA/RV Dilated 3. Dysfunctional RV 4. Normal LV Function | | | |
| **Remark**: has significant improvement | | | |
| **Recommendation**: Continue same management | | | |
| SIGNATURE  Done by: Tesfaye T., Pediatrician, Pediatric Cardiologist _______________ 30/09/2013Eth.C | | | |

| Patient Name: **Yilkal Kassahun**. Patient ID: **FHRH**. SEX/ Age: M/10Years. Date of Report: 30/**09/2013**.  BP: _______ Weight: ______ Height: ____________ BSA:_____. R.Dx: **Rheumatic Fever . AGH10.872** | | | |
| --- | --- | --- | --- |
| **Features** | **Finding** | **Features** | **Finding** |
| **Profile** |  | **Atria** |  |
| Abdominal situs | Solitus | Left atrium | Normal |
| Cardiac position | Levocardia | Right atrium | Normal |
| Systemic venous drainage | Normal. | **Atrioventricular valves** |  |
| Pulmonary venous drainage | Normal | Mitral valve | Annulus = 20mm. Mildly thickened MVL |
| Atrioventricular connection | Concordant | Tricuspid valve | Annulus = 21mm  TAPSE = 17mm |
| Ventriculoarterial connection | Concordant | **Ventricles** |  |
| Ventricular loop | d-Loop | Left ventricle | Normal |
|  |  | Right ventricle | Normal |
| **Septae** |  | **Coronary arteries** | ----- |
| Interventricular septum | Intact | **Doppler Measurement** |  |
| Interatrial septum | Intact | Mitral | Mild MR, Holosystolic, posterior projection, seen in two planes with jet velocity = 3.6m/sec |
| **Semilunal valves** |  | Aortic | ------- |
| Aortic valve | Annulus = 17mm | Tricuspid | ------- |
| Pulmonary valve | Annulus = 20mm | pulmonic | -------- |
| **Great arteries** | NRGA | **Aortic arch** | Left |
| Aorta | ----- | **PDA** | No |
| Pulmonary artery | Normal MPA and Branch PAs. |  |  |
| **M-Mode:** | | | |
| AO | mm | PWd | mm |
| LA | mm | EDV | ml |
| LVIDd | mm | ESV | ml |
| LVIDs | mm | LVEF | 63% |
| IVSd | mm | FS | 33% |
| **Additional Information**: |  | | |
| No pericardial/Pleural effusion. | | | |
| **Final Diagnosis:** | | | |
| 1. {S, D, S} Levocardia. 2. Mildly thickened MVL 3. Mild MR 4. Normal Biventricular Function | | | |
| **Remark**: | | | |
| **Recommendation**: | | | |
| SIGNATURE  Done by: Tesfaye T., Pediatrician, Pediatric Cardiologist _______________ 30/09/2013Eth.C | | | |

| Patient Name: **Mebe’a Dagne**. Patient ID: **FHRH**. SEX/ Age: M/2/12. Date of Report: 30/**09/2013**.  BP: _______ Weight: ______ Height: ____________ BSA: ________. R.Dx: **Incidental Murmur. AGH10.873** | | | |
| --- | --- | --- | --- |
| **Features** | **Finding** | **Features** | **Finding** |
| **Profile** |  | **Atria** |  |
| Abdominal situs | Solitus | Left atrium | Normal |
| Cardiac position | Levocardia | Right atrium | Normal |
| Systemic venous drainage | Normal. | **Atrioventricular valves** |  |
| Pulmonary venous drainage | Normal | Mitral valve | Annulus = 10mm |
| Atrioventricular connection | Concordant | Tricuspid valve | Annulus = 12mm |
| Ventriculoarterial connection | Concordant | **Ventricles** |  |
| Ventricular loop | d-Loop | Left ventricle | Normal |
|  |  | Right ventricle | Normal |
| **Septae** |  | **Coronary arteries** | ----- |
| Interventricular septum | 3mm PM VSD, L – R Shunt | **Doppler Measurement** |  |
| Interatrial septum | PFO, L – R Shunt | Mitral | ----- |
| **Semilunal valves** |  | Aortic | ------- |
| Aortic valve | Annulus = 9mm | Tricuspid | Trivial TR |
| Pulmonary valve | Annulus = 10mm | pulmonic | -------- |
| **Great arteries** | NRGA | **Aortic arch** | Left |
| Aorta | ----- | **PDA** | 1mm PDA, L – R Shunt |
| Pulmonary artery | Normal MPA and Branch PAs. |  |  |
| **M-Mode:** | | | |
| AO | mm | PWd | mm |
| LA | mm | EDV | ml |
| LVIDd | mm | ESV | ml |
| LVIDs | mm | LVEF | 70% |
| IVSd | mm | FS | 38% |
| **Additional Information**: |  | | |
| No pericardial/Pleural effusion. | | | |
| **Final Diagnosis:** | | | |
| 1. {S, D, S} Levocardia. 2. PFO, L – R Shunt 3. Small PM VSD, L – R Shunt 4. Small PDA, L – R Shunt 5. Normal LV Function | | | |
| **Remark**: | | | |
| **Recommendation**: | | | |
| SIGNATURE  Done by: Tesfaye T., Pediatrician, Pediatric Cardiologist _______________ 30/09/2013Eth.C | | | |

| Patient Name: **Sofonias Desalegn**. Patient ID: **Adinas GH**. SEX/ Age: M/8/12. Date of Report: 30/**09/2013**.  BP: _______ Weight: ______ Height: ____________ BSA: ________. R.Dx: **_____. Filled @ AGH2.070** | | | |
| --- | --- | --- | --- |
| **Follow Up Echo:** S/P ASO for d-TGA, PM VSD Closure and PDA Ligation. | | | |
| **Features** | **Finding** | **Features** | **Finding** |
| **Profile** |  | **Atria** |  |
| Abdominal situs | Solitus | Left atrium | Normal |
| Cardiac position | Levocardia | Right atrium | Normal |
| Systemic venous drainage | Normal. | **Atrioventricular valves** |  |
| Pulmonary venous drainage | Normal | Mitral valve | Annulus = 11mm |
| Atrioventricular connection | Concordant | Tricuspid valve | Annulus = 13mm |
| Ventriculoarterial connection | Concordant | **Ventricles** |  |
| Ventricular loop | d-Loop | Left ventricle | Normal |
|  |  | Right ventricle | Normal |
| **Septae** |  | **Coronary arteries** | ----- |
| Interventricular septum | 2.5mm Residual VSD, L – R Shunt | **Doppler Measurement** |  |
| Interatrial septum | Intact | Mitral | ----- |
| **Semilunal valves** |  | Aortic | Mild Neo AR |
| Aortic valve | Annulus = 11mm | Tricuspid | ------- |
| Pulmonary valve | Annulus = 11mm | pulmonic | Flow acceleration across the PV with PPG = 16mmHg |
| **Great arteries** | NRGA | **Aortic arch** | Left |
| Aorta | ----- | **PDA** | No |
| Pulmonary artery | Normal MPA and Branch PAs. |  |  |
| **M-Mode:** Normal LV Function on eye balling | | | |
| AO | mm | PWd | mm |
| LA | mm | EDV | ml |
| LVIDd | mm | ESV | ml |
| LVIDs | mm | LVEF | % |
| IVSd | mm | FS | % |
| **Additional Information**: |  | | |
| No pericardial/Pleural effusion. | | | |
| **Final Diagnosis:** | | | |
| 1. S/P ASO for d-TGA, PM VSD Closure and PDA Ligation 2. {S, D, S} Levocardia. 3. Mild Neo AR 4. Small Residual PM VSD, L – R Shunt 5. Flow acceleration across the PV (No obstruction) 6. Normal LV Function | | | |
| **Remark**: | | | |
| **Recommendation**: | | | |
| SIGNATURE  Done by: Tesfaye T., Pediatrician, Pediatric Cardiologist _______________ 30/09/2013Eth.C | | | |

| Patient Name: **Nebyu Lakachew**. Patient ID: **Adinas GH**. SEX/ Age: M/5Years. Date of Report: 30/**09/2013**.  BP: _______ Weight: ______ Height: ____________ BSA: ________. R.Dx: **Recurrent Chest Infection. AGH10.874** | | | |
| --- | --- | --- | --- |
| **Features** | **Finding** | **Features** | **Finding** |
| **Profile** |  | **Atria** |  |
| Abdominal situs | Solitus | Left atrium | Normal |
| Cardiac position | Levocardia | Right atrium | Normal |
| Systemic venous drainage | Normal. | **Atrioventricular valves** |  |
| Pulmonary venous drainage | Normal | Mitral valve | Annulus = 16mm |
| Atrioventricular connection | Concordant | Tricuspid valve | Annulus = 19mm  TAPSE = 18mm |
| Ventriculoarterial connection | Concordant | **Ventricles** |  |
| Ventricular loop | d-Loop | Left ventricle | Normal |
|  |  | Right ventricle | Normal |
| **Septae** |  | **Coronary arteries** | ----- |
| Interventricular septum | Intact | **Doppler Measurement** |  |
| Interatrial septum | Intact | Mitral | ----- |
| **Semilunal valves** |  | Aortic | ------- |
| Aortic valve | Annulus = 13mm | Tricuspid | ------- |
| Pulmonary valve | Annulus = 14mm | pulmonic | -------- |
| **Great arteries** | NRGA | **Aortic arch** | Left |
| Aorta | ----- | **PDA** | No |
| Pulmonary artery | Normal MPA and Branch PAs. |  |  |
| **M-Mode:** | | | |
| AO | mm | PWd | mm |
| LA | mm | EDV | ml |
| LVIDd | mm | ESV | ml |
| LVIDs | mm | LVEF | 60% |
| IVSd | mm | FS | 31% |
| **Additional Information**: |  | | |
| No pericardial/Pleural effusion. | | | |
| **Final Diagnosis:** | | | |
| 1. Normal Echocardiography Study. | | | |
| **Remark**: | | | |
| **Recommendation**: | | | |
| SIGNATURE  Done by: Tesfaye T., Pediatrician, Pediatric Cardiologist _______________ 30/09/2013Eth.C | | | |

| Patient Name: **Medhanit Abrham**. Patient ID: **TGSH**. SEX/ Age: F/3 6/12. Date of Report: 01/1**0/2013**.  BP: _______ Weight: ______ Height: ____________ BSA: ________. R.Dx: **recurrent chest infection. AGH10.875** | | | |
| --- | --- | --- | --- |
| **Features** | **Finding** | **Features** | **Finding** |
| **Profile** |  | **Atria** |  |
| Abdominal situs | Solitus | Left atrium | Normal |
| Cardiac position | Levocardia | Right atrium | Normal |
| Systemic venous drainage | Normal. | **Atrioventricular valves** |  |
| Pulmonary venous drainage | Normal | Mitral valve | Annulus = 15mm |
| Atrioventricular connection | Concordant | Tricuspid valve | Annulus = 17mm  TAPSE = 17mm |
| Ventriculoarterial connection | Concordant | **Ventricles** |  |
| Ventricular loop | d-Loop | Left ventricle | Normal |
|  |  | Right ventricle | Normal |
| **Septae** |  | **Coronary arteries** | ----- |
| Interventricular septum | Intact | **Doppler Measurement** |  |
| Interatrial septum | Intact | Mitral | ----- |
| **Semilunal valves** |  | Aortic | ------- |
| Aortic valve | Annulus = 14mm | Tricuspid | ------- |
| Pulmonary valve | Annulus = 16mm | pulmonic | -------- |
| **Great arteries** | NRGA | **Aortic arch** | Left |
| Aorta | ----- | **PDA** | No |
| Pulmonary artery | Normal MPA and Branch PAs. |  |  |
| **M-Mode:** | | | |
| AO | mm | PWd | mm |
| LA | mm | EDV | ml |
| LVIDd | mm | ESV | ml |
| LVIDs | mm | LVEF | 68% |
| IVSd | mm | FS | 36% |
| **Additional Information**: |  | | |
| No pericardial/Pleural effusion. | | | |
| **Final Diagnosis:** | | | |
| 1. Normal Echocardiography Study. | | | |
| **Remark**: | | | |
| **Recommendation**: | | | |
| SIGNATURE  Done by: Tesfaye T., Pediatrician, Pediatric Cardiologist _______________ 01/10/2013Eth.C | | | |

| Patient Name: **Sale-Amlak Degsira**. Patient ID: **TGSH**. SEX/ Age: M/1 3/12. Date of Report: 01/1**0/2013**.  BP: _______ Weight: ______ Height: ____________ BSA: ________. R.Dx: **DS. AGH10.876** | | | |
| --- | --- | --- | --- |
| **Features** | **Finding** | **Features** | **Finding** |
| **Profile** |  | **Atria** |  |
| Abdominal situs | Solitus | Left atrium | Normal |
| Cardiac position | Levocardia | Right atrium | Dilated |
| Systemic venous drainage | Normal. | **Atrioventricular valves** |  |
| Pulmonary venous drainage | Normal | Mitral valve | Annulus = 12mm |
| Atrioventricular connection | Concordant | Tricuspid valve | Annulus = 17mm  TAPSE = 13mm |
| Ventriculoarterial connection | Concordant | **Ventricles** |  |
| Ventricular loop | d-Loop | Left ventricle | Normal |
|  |  | Right ventricle | Dilated |
| **Septae** |  | **Coronary arteries** | ----- |
| Interventricular septum | Intact | **Doppler Measurement** |  |
| Interatrial septum | 13mm Primum defect, L – R Shunt.  Additional 4mm OS ASD, L – R Shunt | Mitral | ----- |
| **Semilunal valves** |  | Aortic | ------- |
| Aortic valve | Annulus = 13mm | Tricuspid | Mild TR |
| Pulmonary valve | Annulus = 14mm | pulmonic | -------- |
| **Great arteries** | NRGA | **Aortic arch** | Left |
| Aorta | ----- | **PDA** | No |
| Pulmonary artery | Normal MPA and Branch PAs. |  |  |
| **M-Mode:** Normal LV Function on eye balling. | | | |
| AO | mm | PWd | mm |
| LA | mm | EDV | ml |
| LVIDd | mm | ESV | ml |
| LVIDs | mm | LVEF | % |
| IVSd | mm | FS | % |
| **Additional Information**: |  | | |
| No pericardial/Pleural effusion. | | | |
| **Final Diagnosis:** | | | |
| 1. {S, D, S} Levocardia. 2. Partial AVSD, L – R Shunt 3. Additional Small OS ASD, L – R Shunt 4. Normal LV Function | | | |
| **Remark**: | | | |
| **Recommendation**: | | | |
| SIGNATURE  Done by: Tesfaye T., Pediatrician, Pediatric Cardiologist _______________ 01/10/2013Eth.C | | | |

| Patient Name: **Sofia Seidu**. Patient ID: **FHRH**. SEX/ Age: **M/4Years**. Date of Report: **01/10/2013**.  BP: _______ Weight: ______ Height: ____________ BSA: ________. R.Dx: **recurrent chest infection. AGH10.877** | | | |
| --- | --- | --- | --- |
| **Features** | **Finding** | **Features** | **Finding** |
| **Profile** |  | **Atria** |  |
| Abdominal situs | Solitus | Left atrium | Normal |
| Cardiac position | Levocardia | Right atrium | Normal |
| Systemic venous drainage | Normal. | **Atrioventricular valves** |  |
| Pulmonary venous drainage | Normal | Mitral valve | Annulus = 13mm |
| Atrioventricular connection | Concordant | Tricuspid valve | Annulus = 16mm  TAPSE = 16mm |
| Ventriculoarterial connection | Concordant | **Ventricles** |  |
| Ventricular loop | d-Loop | Left ventricle | Normal |
|  |  | Right ventricle | Normal |
| **Septae** |  | **Coronary arteries** | ----- |
| Interventricular septum | Intact | **Doppler Measurement** |  |
| Interatrial septum | Intact | Mitral | ----- |
| **Semilunal valves** |  | Aortic | ------- |
| Aortic valve | Annulus = 12mm | Tricuspid | ------- |
| Pulmonary valve | Annulus = 13mm | pulmonic | -------- |
| **Great arteries** | NRGA | **Aortic arch** | Left |
| Aorta | ----- | **PDA** | No |
| Pulmonary artery | Normal MPA and Branch PAs. |  |  |
| **M-Mode:** | | | |
| AO | mm | PWd | mm |
| LA | mm | EDV | ml |
| LVIDd | mm | ESV | ml |
| LVIDs | mm | LVEF | 59% |
| IVSd | mm | FS | 30% |
| **Additional Information**: |  | | |
| No pericardial/Pleural effusion. | | | |
| **Final Diagnosis:** | | | |
| 1. Normal Echocardiography Study. | | | |
| **Remark**: | | | |
| **Recommendation**: | | | |
| SIGNATURE  Done by: Tesfaye T., Pediatrician, Pediatric Cardiologist _______________ 01/10/2013Eth.C | | | |

| Patient Name: **Amanuel Tesfahun**. Patient ID: **TGSH**. SEX/ Age: M**/5/12**. Date of Report: **02/10/2013**.  BP: _______ Weight: ______ Height: ____________ BSA: ________. R.Dx: **DS. AGH10.878** | | | |
| --- | --- | --- | --- |
| **Features** | **Finding** | **Features** | **Finding** |
| **Profile** |  | **Atria** |  |
| Abdominal situs | Solitus | Left atrium | Normal |
| Cardiac position | Levocardia | Right atrium | Normal |
| Systemic venous drainage | Normal. | **Atrioventricular valves** |  |
| Pulmonary venous drainage | Normal | Mitral valve | Annulus = 9mm |
| Atrioventricular connection | Concordant | Tricuspid valve | Annulus = 11mm |
| Ventriculoarterial connection | Concordant | **Ventricles** |  |
| Ventricular loop | d-Loop | Left ventricle | Normal |
|  |  | Right ventricle | Normal |
| **Septae** |  | **Coronary arteries** | ----- |
| Interventricular septum | Intact | **Doppler Measurement** |  |
| Interatrial septum | Intact | Mitral | ----- |
| **Semilunal valves** |  | Aortic | ------- |
| Aortic valve | Annulus = 10mm | Tricuspid | ------- |
| Pulmonary valve | Annulus = 12mm | pulmonic | -------- |
| **Great arteries** | NRGA | **Aortic arch** | Left |
| Aorta | ----- | **PDA** | No |
| Pulmonary artery | Normal MPA and Branch PAs. |  |  |
| **M-Mode:** Normal LV Function on eye balling. | | | |
| AO | mm | PWd | mm |
| LA | mm | EDV | ml |
| LVIDd | mm | ESV | ml |
| LVIDs | mm | LVEF | % |
| IVSd | mm | FS | % |
| **Additional Information**: |  | | |
| No pericardial/Pleural effusion. | | | |
| **Final Diagnosis:** | | | |
| 1. Normal Echocardiography Study. | | | |
| **Remark**: Only Subcostal window (Chest is deformed) | | | |
| **Recommendation**: | | | |
| SIGNATURE  Done by: Tesfaye T., Pediatrician, Pediatric Cardiologist _______________ 02/10/2013Eth.C | | | |

| Patient Name: **Redeat Tizazu**. Referring Institute: **Kokeb Medium Clinic**. SEX/ Age: F**/15Years**.  Date of Report: **03/10/2013**. Referral Diagnosis: **R/O CRVHD. AGH10.879** | | | |
| --- | --- | --- | --- |
| **Features** | **Finding** | **Features** | **Finding** |
| **Profile** |  | **Atria** |  |
| Abdominal situs | Solitus | Left atrium | Normal |
| Cardiac position | Levocardia | Right atrium | Normal |
| Systemic venous drainage | Normal. | **Atrioventricular valves** |  |
| Pulmonary venous drainage | Normal | Mitral valve | Annulus = 24mm |
| Atrioventricular connection | Concordant | Tricuspid valve | Annulus = 24mm  TAPSE = 19mm |
| Ventriculoarterial connection | Concordant | **Ventricles** |  |
| Ventricular loop | d-Loop | Left ventricle | Normal |
|  |  | Right ventricle | Normal |
| **Septae** |  | **Coronary arteries** | ----- |
| Interventricular septum | Intact | **Doppler Measurement** |  |
| Interatrial septum | Intact | Mitral | ----- |
| **Semilunal valves** |  | Aortic | ------- |
| Aortic valve | Annulus = 19mm | Tricuspid | Trivial TR, PPG = 15mmHg |
| Pulmonary valve | Annulus = 24mm | pulmonic | Trivial PR, PPG = 8mmHg |
| **Great arteries** | NRGA | **Aortic arch** | Left |
| Aorta | ----- | **PDA** | No |
| Pulmonary artery | Normal MPA and Branch PAs. |  |  |
| **M-Mode:** | | | |
| AO | mm | PWd | mm |
| LA | mm | EDV | ml |
| LVIDd | mm | ESV | ml |
| LVIDs | mm | LVEF | 59% |
| IVSd | mm | FS | 31% |
| **Additional Information**: |  | | |
| 7mm pericardial effusion on RA/RV Side. | | | |
| **Final Diagnosis:** | | | |
| 1. {S, D, S} Levocardia. 2. Small Pericardial effusion 3. Normal Biventricular Function | | | |
| **Remark**: No evidences of CRVHD | | | |
| **Recommendation**: | | | |
| SIGNATURE  Done by: Tesfaye T., Pediatrician, Pediatric Cardiologist _______________ 03/10/2013Eth.C | | | |

| Patient Name: **Bitanya Addis**. Referring Institute: **Adinas GH**. SEX/ Age: F**/2 10/12**.  Date of Report: **04/10/2013**. Referral Diagnosis: **TOF(Cyanosis). AGH10.880** | | | |
| --- | --- | --- | --- |
| **Features** | **Finding** | **Features** | **Finding** |
| **Profile** |  | **Atria** |  |
| Abdominal situs | Solitus | Left atrium | Normal |
| Cardiac position | Levocardia | Right atrium | Dilated |
| Systemic venous drainage | Normal. | **Atrioventricular valves** |  |
| Pulmonary venous drainage | Normal | Mitral valve | Annulus = 13mm |
| Atrioventricular connection | Concordant | Tricuspid valve | Annulus = 29mm  TAPSE = 7mm |
| Ventriculoarterial connection | Concordant | **Ventricles** |  |
| Ventricular loop | d-Loop | Left ventricle | Normal |
|  |  | Right ventricle | Dilated & Hypertrophied |
| **Septae** |  | **Coronary arteries** | ----- |
| Interventricular septum | Intact | **Doppler Measurement** |  |
| Interatrial septum | PFO, R – L Shunt | Mitral | ----- |
| **Semilunal valves** |  | Aortic | ------- |
| Aortic valve | Annulus = 12mm | Tricuspid | Severe TR |
| Pulmonary valve | Annulus = 5mm | pulmonic | Severe PS, PPG = 105mmHg |
| **Great arteries** | NRGA | **Aortic arch** | Left |
| Aorta | ----- | **PDA** | No |
| Pulmonary artery | Smallish MPA and Branch PAs. |  |  |
| **M-Mode:** Normal LV Function on eye balling | | | |
| AO | mm | PWd | mm |
| LA | mm | EDV | ml |
| LVIDd | mm | ESV | ml |
| LVIDs | mm | LVEF | % |
| IVSd | mm | FS | % |
| **Additional Information**: |  | | |
| 11mm pericardial effusion on RV Side. | | | |
| **Final Diagnosis:** | | | |
| 1. {S, D, S} Levocardia. 2. RA/RV Dilated 3. PFO, R – L Shunt 4. Severe TR 5. Severe PS 6. Smallish MPA and Branch PAs. 7. RV Dilated, Hypertrophied and Dysfunctional | | | |
| **Remark**: | | | |
| **Recommendation**: Needs Urgent Intervention | | | |
| SIGNATURE  Done by: Tesfaye T., Pediatrician, Pediatric Cardiologist _______________ 04/10/2013Eth.C | | | |

| Patient Name: **Abi Abebaw**. Referring Institute: **FHRH**. SEX/ Age: M**/1 3/12**.  Date of Report: **04/10/2013**. Referral Diagnosis: **CHD(RD+CHF). AGH10.881** | | | |
| --- | --- | --- | --- |
| **Features** | **Finding** | **Features** | **Finding** |
| **Profile** |  | **Atria** |  |
| Abdominal situs | Solitus | Left atrium | Mildly Dilated |
| Cardiac position | Levocardia | Right atrium | Mildly Dilated |
| Systemic venous drainage | Normal. | **Atrioventricular valves** |  |
| Pulmonary venous drainage | Normal | Mitral valve | Annulus = 15mm |
| Atrioventricular connection | Concordant | Tricuspid valve | Annulus = 17mm  TAPSE = 17mm |
| Ventriculoarterial connection | Concordant | **Ventricles** |  |
| Ventricular loop | d-Loop | Left ventricle | Mildly Dilated |
|  |  | Right ventricle | Mildly Dilated |
| **Septae** |  | **Coronary arteries** | ----- |
| Interventricular septum | Intact | **Doppler Measurement** |  |
| Interatrial septum | Intact | Mitral | Trivial MR |
| **Semilunal valves** |  | Aortic | ------- |
| Aortic valve | Annulus = 13mm | Tricuspid | Mild TR, PPG = 31mmHg |
| Pulmonary valve | Annulus = 14mm | pulmonic | -------- |
| **Great arteries** | NRGA | **Aortic arch** | Left |
| Aorta | ----- | **PDA** | No |
| Pulmonary artery | Normal MPA and Branch PAs. |  |  |
| **M-Mode:** | | | |
| AO | mm | PWd | mm |
| LA | mm | EDV | ml |
| LVIDd | mm | ESV | ml |
| LVIDs | mm | LVEF | 52% |
| IVSd | mm | FS | 26% |
| **Additional Information**: |  | | |
| 3mm pericardial effusion on RA/RV Side. | | | |
| **Final Diagnosis:** | | | |
| 1. {S, D, S} Levocardia. 2. Trivial MR 3. Trivial TR 4. Trace Pericardial Effusion 5. Mildly Reduced LV Function | | | |
| **Remark**: Pancarditis | | | |
| **Recommendation**:  DDx:   1. Myocarditis 2. Association with micro/macro nutrient deficiency | | | |
| SIGNATURE  Done by: Tesfaye T., Pediatrician, Pediatric Cardiologist _______________ 04/10/2013Eth.C | | | |

| Patient Name: **Mariamawit Eyayu**. Referring Institute: **Adinas GH**. SEX/ Age: F**/1Day**.  Date of Report: **04/10/2013**. Referral Diagnosis: **Pericardial effusion(Cardiomegaly on CXR + DS). AGH10.882** | | | |
| --- | --- | --- | --- |
| **Features** | **Finding** | **Features** | **Finding** |
| **Profile** |  | **Atria** |  |
| Abdominal situs | Solitus | Left atrium | Normal |
| Cardiac position | Levocardia | Right atrium | Normal |
| Systemic venous drainage | Normal. | **Atrioventricular valves** |  |
| Pulmonary venous drainage | Normal | Mitral valve | Annulus = 9mm |
| Atrioventricular connection | Concordant | Tricuspid valve | Annulus = 10mm |
| Ventriculoarterial connection | Concordant | **Ventricles** |  |
| Ventricular loop | d-Loop | Left ventricle | Normal |
|  |  | Right ventricle | Normal |
| **Septae** |  | **Coronary arteries** | ----- |
| Interventricular septum | Intact | **Doppler Measurement** |  |
| Interatrial septum | PFO, L – R Shunt | Mitral | ----- |
| **Semilunal valves** |  | Aortic | ------- |
| Aortic valve | Annulus = 7mm | Tricuspid | ------- |
| Pulmonary valve | Annulus = 8mm | pulmonic | -------- |
| **Great arteries** | NRGA | **Aortic arch** | Left |
| Aorta | ----- | **PDA** | <1mm PDA, L – R Shunt |
| Pulmonary artery | Normal MPA and Branch PAs. |  |  |
| **M-Mode:** | | | |
| AO | mm | PWd | mm |
| LA | mm | EDV | ml |
| LVIDd | mm | ESV | ml |
| LVIDs | mm | LVEF | 65% |
| IVSd | mm | FS | 33% |
| **Additional Information**: |  | | |
| No pericardial/Pleural effusion. | | | |
| **Final Diagnosis:** | | | |
| 1. {S, D, S} Levocardia. 2. PFO, L – R Shunt 3. Silent PDA, L – R Shunt (No murmur on auscultation) 4. Normal LV Function | | | |
| **Remark**: No pericardial effusion | | | |
| **Recommendation**: | | | |
| SIGNATURE  Done by: Tesfaye T., Pediatrician, Pediatric Cardiologist _______________ 04/10/2013Eth.C | | | |

| Patient Name: **Baby of Ethiopia Abeje**. Referring Institute: **FHRH**. SEX/ Age: M**/4days**.  Date of Report: **06/10/2013**. Referral Diagnosis: **CHD ?VSD (RD). AGH10.883** | | | |
| --- | --- | --- | --- |
| **Features** | **Finding** | **Features** | **Finding** |
| **Profile** |  | **Atria** |  |
| Abdominal situs | Solitus | Left atrium | Normal |
| Cardiac position | Levocardia | Right atrium | Normal |
| Systemic venous drainage | Normal. | **Atrioventricular valves** |  |
| Pulmonary venous drainage | Normal | Mitral valve | Annulus = 8mm |
| Atrioventricular connection | Concordant | Tricuspid valve | Annulus = 9mm  TAPSE = 10mm |
| Ventriculoarterial connection | Concordant | **Ventricles** |  |
| Ventricular loop | d-Loop | Left ventricle | Normal |
|  |  | Right ventricle | Normal |
| **Septae** |  | **Coronary arteries** | ----- |
| Interventricular septum | Intact | **Doppler Measurement** |  |
| Interatrial septum | Intact | Mitral | ----- |
| **Semilunal valves** |  | Aortic | ------- |
| Aortic valve | Annulus = 8mm | Tricuspid | ------- |
| Pulmonary valve | Annulus = 8mm | pulmonic | -------- |
| **Great arteries** | NRGA | **Aortic arch** | Left |
| Aorta | ----- | **PDA** | No |
| Pulmonary artery | Normal MPA and Branch PAs. |  |  |
| **M-Mode:** | | | |
| AO | mm | PWd | mm |
| LA | mm | EDV | ml |
| LVIDd | mm | ESV | ml |
| LVIDs | mm | LVEF | 69% |
| IVSd | mm | FS | 36% |
| **Additional Information**: |  | | |
| No pericardial/Pleural effusion. | | | |
| **Final Diagnosis:** | | | |
| 1. Normal Echocardiography Study. | | | |
| **Remark**: | | | |
| **Recommendation**: | | | |
| SIGNATURE  Done by: Tesfaye T., Pediatrician, Pediatric Cardiologist _______________ 06/10/2013Eth.C | | | |

| Patient Name: **Zelalem Temesgen**. Referring Institute: **Chageni H**. SEX/ Age: M**/6days**.  Date of Report: **07/10/2013**. Referral Diagnosis: **Down Syndrome. AGH10.884** | | | |
| --- | --- | --- | --- |
| **Features** | **Finding** | **Features** | **Finding** |
| **Profile** |  | **Atria** |  |
| Abdominal situs | Solitus | Left atrium | Normal |
| Cardiac position | Levocardia | Right atrium | Normal |
| Systemic venous drainage | Normal. | **Atrioventricular valves** |  |
| Pulmonary venous drainage | Normal | Mitral valve | Annulus = 11mm |
| Atrioventricular connection | Concordant | Tricuspid valve | Annulus = 10mm |
| Ventriculoarterial connection | Concordant | **Ventricles** |  |
| Ventricular loop | d-Loop | Left ventricle | Normal |
|  |  | Right ventricle | Normal |
| **Septae** |  | **Coronary arteries** | ----- |
| Interventricular septum | Intact | **Doppler Measurement** |  |
| Interatrial septum | Intact | Mitral | ----- |
| **Semilunal valves** |  | Aortic | ------- |
| Aortic valve | Annulus = 9mm | Tricuspid | ------- |
| Pulmonary valve | Annulus = 9mm | pulmonic | -------- |
| **Great arteries** | NRGA | **Aortic arch** | Left |
| Aorta | ----- | **PDA** | No |
| Pulmonary artery | Normal MPA and Branch PAs. |  |  |
| **M-Mode:** Normal LV Function on eye balling. | | | |
| AO | mm | PWd | mm |
| LA | mm | EDV | ml |
| LVIDd | mm | ESV | ml |
| LVIDs | mm | LVEF | % |
| IVSd | mm | FS | % |
| **Additional Information**: |  | | |
| No pericardial/Pleural effusion. | | | |
| **Final Diagnosis:** | | | |
| 1. Normal Echocardiography Study. | | | |
| **Remark**: | | | |
| **Recommendation**: | | | |
| SIGNATURE  Done by: Tesfaye T., Pediatrician, Pediatric Cardiologist _______________ 07/10/2013Eth.C | | | |

| Patient Name: **Muluken Terefe**. Referring Institute: **Injibara GH**. SEX/ Age: M**/5Months**.  Date of Report: **07/10/2013**. Referral Diagnosis: **Down Syndrome. AGH10.885** | | | |
| --- | --- | --- | --- |
| **Features** | **Finding** | **Features** | **Finding** |
| **Profile** |  | **Atria** |  |
| Abdominal situs | Solitus | Left atrium | Normal |
| Cardiac position | Levocardia | Right atrium | Normal |
| Systemic venous drainage | Normal. | **Atrioventricular valves** |  |
| Pulmonary venous drainage | Normal | Mitral valve | Annulus = 11mm |
| Atrioventricular connection | Concordant | Tricuspid valve | Annulus = 12mm  TAPSE = 14mm |
| Ventriculoarterial connection | Concordant | **Ventricles** |  |
| Ventricular loop | d-Loop | Left ventricle | Normal |
|  |  | Right ventricle | Normal |
| **Septae** |  | **Coronary arteries** | ----- |
| Interventricular septum | Intact | **Doppler Measurement** |  |
| Interatrial septum | PFO, L – R Shunt | Mitral | ----- |
| **Semilunal valves** |  | Aortic | ------- |
| Aortic valve | Annulus = 11mm | Tricuspid | ------- |
| Pulmonary valve | Annulus = 11mm | pulmonic | -------- |
| **Great arteries** | NRGA | **Aortic arch** | Left |
| Aorta | ----- | **PDA** | No |
| Pulmonary artery | Normal MPA and Branch PAs. |  |  |
| **M-Mode:** Normal LV Function on eye balling | | | |
| AO | mm | PWd | mm |
| LA | mm | EDV | ml |
| LVIDd | mm | ESV | ml |
| LVIDs | mm | LVEF | % |
| IVSd | mm | FS | % |
| **Additional Information**: |  | | |
| No pericardial/Pleural effusion. | | | |
| **Final Diagnosis:** | | | |
| 1. {S, D, S} Levocardia. 2. PFO, L – R Shunt 3. Normal Biventricular Function | | | |
| **Remark**: | | | |
| **Recommendation**: | | | |
| SIGNATURE  Done by: Tesfaye T., Pediatrician, Pediatric Cardiologist _______________ 07/10/2013Eth.C | | | |

| Patient Name: **Baby of Beteha Abate**. Referring Institute: **TGSH**. SEX/ Age: F**/4Months**.  Date of Report: **07/10/2013**.  Referral Diagnosis: **Moderate OS ASD, L – R Shunt (Follow up Echo). Previous echo @ TGSH** | | | |
| --- | --- | --- | --- |
| **Features** | **Finding** | **Features** | **Finding** |
| **Profile** |  | **Atria** |  |
| Abdominal situs | Solitus | Left atrium | Normal |
| Cardiac position | Levocardia | Right atrium | Normal |
| Systemic venous drainage | Normal. | **Atrioventricular valves** |  |
| Pulmonary venous drainage | Normal | Mitral valve | Annulus = 12mm |
| Atrioventricular connection | Concordant | Tricuspid valve | Annulus = 13mm |
| Ventriculoarterial connection | Concordant | **Ventricles** |  |
| Ventricular loop | d-Loop | Left ventricle | Normal |
|  |  | Right ventricle | Normal |
| **Septae** |  | **Coronary arteries** | ----- |
| Interventricular septum | Intact | **Doppler Measurement** |  |
| Interatrial septum | Intact | Mitral | ----- |
| **Semilunal valves** |  | Aortic | ------- |
| Aortic valve | Annulus = 10mm | Tricuspid | ------- |
| Pulmonary valve | Annulus = 12mm | pulmonic | -------- |
| **Great arteries** | NRGA | **Aortic arch** | Left |
| Aorta | ----- | **PDA** | No |
| Pulmonary artery | Normal MPA and Branch PAs. |  |  |
| **M-Mode:** Normal LV Function on eye balling. | | | |
| AO | mm | PWd | mm |
| LA | mm | EDV | ml |
| LVIDd | mm | ESV | ml |
| LVIDs | mm | LVEF | % |
| IVSd | mm | FS | % |
| **Additional Information**: |  | | |
| No pericardial/Pleural effusion. | | | |
| **Final Diagnosis:** | | | |
| 1. Normal Echocardiography Study. | | | |
| **Remark**: | | | |
| **Recommendation**: | | | |
| SIGNATURE  Done by: Tesfaye T., Pediatrician, Pediatric Cardiologist _______________ 07/10/2013Eth.C | | | |

| Patient Name: **Tsehaynesh Abebe**. Referring Institute: **TGSH**. SEX/ Age: F**/4Years**.  Date of Report: **07/10/2013**. Referral Diagnosis: **Down Syndrome. AGH10.886** | | | |
| --- | --- | --- | --- |
| **Features** | **Finding** | **Features** | **Finding** |
| **Profile** |  | **Atria** |  |
| Abdominal situs | Solitus | Left atrium | Normal |
| Cardiac position | Levocardia | Right atrium | Normal |
| Systemic venous drainage | Normal. | **Atrioventricular valves** |  |
| Pulmonary venous drainage | Normal | Mitral valve | Annulus = 13mm |
| Atrioventricular connection | Concordant | Tricuspid valve | Annulus = 14mm |
| Ventriculoarterial connection | Concordant | **Ventricles** |  |
| Ventricular loop | d-Loop | Left ventricle | Normal |
|  |  | Right ventricle | Normal |
| **Septae** |  | **Coronary arteries** | ----- |
| Interventricular septum | Intact | **Doppler Measurement** |  |
| Interatrial septum | Intact | Mitral | ----- |
| **Semilunal valves** |  | Aortic | ------- |
| Aortic valve | Annulus = 12mm | Tricuspid | ------- |
| Pulmonary valve | Annulus = 13mm | pulmonic | -------- |
| **Great arteries** | NRGA | **Aortic arch** | Left |
| Aorta | ----- | **PDA** | No |
| Pulmonary artery | Normal MPA and Branch PAs. |  |  |
| **M-Mode:** Normal LV Function on eye balling. | | | |
| AO | mm | PWd | mm |
| LA | mm | EDV | ml |
| LVIDd | mm | ESV | ml |
| LVIDs | mm | LVEF | % |
| IVSd | mm | FS | % |
| **Additional Information**: |  | | |
| No pericardial/Pleural effusion. | | | |
| **Final Diagnosis:** | | | |
| 1. Normal Echocardiography Study. | | | |
| **Remark**: | | | |
| **Recommendation**: | | | |
| SIGNATURE  Done by: Tesfaye T., Pediatrician, Pediatric Cardiologist _______________ 07/10/2013Eth.C | | | |

| Patient Name: **Emran Kedir**. Referring Institute: **Adinas GH**. SEX/ Age: M**/4Months**.  Date of Report: **08/10/2013**. Referral Diagnosis: **Down Syndrome. AGH10.887** | | | |
| --- | --- | --- | --- |
| **Features** | **Finding** | **Features** | **Finding** |
| **Profile** |  | **Atria** |  |
| Abdominal situs | Solitus | Left atrium | Normal |
| Cardiac position | Levocardia | Right atrium | Normal |
| Systemic venous drainage | Normal. | **Atrioventricular valves** |  |
| Pulmonary venous drainage | Normal | Mitral valve | Annulus = 9mm |
| Atrioventricular connection | Concordant | Tricuspid valve | Annulus = 11mm  TAPSE = 14mm |
| Ventriculoarterial connection | Concordant | **Ventricles** |  |
| Ventricular loop | d-Loop | Left ventricle | Normal |
|  |  | Right ventricle | Normal |
| **Septae** |  | **Coronary arteries** | ----- |
| Interventricular septum | Intact | **Doppler Measurement** |  |
| Interatrial septum | PFO, L – R Shunt | Mitral | ----- |
| **Semilunal valves** |  | Aortic | ------- |
| Aortic valve | Annulus = 10mm | Tricuspid | ------- |
| Pulmonary valve | Annulus = 11mm | pulmonic | -------- |
| **Great arteries** | NRGA | **Aortic arch** | Left |
| Aorta | ----- | **PDA** | No |
| Pulmonary artery | Normal MPA and Branch PAs. |  |  |
| **M-Mode:** | | | |
| AO | mm | PWd | mm |
| LA | mm | EDV | ml |
| LVIDd | mm | ESV | ml |
| LVIDs | mm | LVEF | 68% |
| IVSd | mm | FS | 35% |
| **Additional Information**: |  | | |
| No pericardial/Pleural effusion. | | | |
| **Final Diagnosis:** | | | |
| 1. {S, D, S} Levocardia. 2. PFO, L – R Shunt 3. Normal Biventricular Function | | | |
| **Remark**: | | | |
| **Recommendation**: | | | |
| SIGNATURE  Done by: Tesfaye T., Pediatrician, Pediatric Cardiologist _______________ 08/10/2013Eth.C | | | |

| Patient Name: **Elarya Ahmed**. Referring Institute: **Adinas GH**. SEX/ Age: F**/1 6/12**.  Date of Report: **08/10/2013**. Referral Diagnosis: **Silent PDA (Before 1 year) + PDA. AGH10.888** | | | |
| --- | --- | --- | --- |
| **Follow up echocardiography for silent PDA** | | | |
| **Features** | **Finding** | **Features** | **Finding** |
| **Profile** |  | **Atria** |  |
| Abdominal situs | Solitus | Left atrium | Normal |
| Cardiac position | Levocardia | Right atrium | Normal |
| Systemic venous drainage | Normal. | **Atrioventricular valves** |  |
| Pulmonary venous drainage | Normal | Mitral valve | Annulus = 12mm |
| Atrioventricular connection | Concordant | Tricuspid valve | Annulus = 14mm  TAPSE = 16mm |
| Ventriculoarterial connection | Concordant | **Ventricles** |  |
| Ventricular loop | d-Loop | Left ventricle | Normal |
|  |  | Right ventricle | Normal |
| **Septae** |  | **Coronary arteries** | ----- |
| Interventricular septum | Intact | **Doppler Measurement** |  |
| Interatrial septum | Intact | Mitral | ----- |
| **Semilunal valves** |  | Aortic | ------- |
| Aortic valve | Annulus = 11mm | Tricuspid | ------- |
| Pulmonary valve | Annulus = 11mm | pulmonic | -------- |
| **Great arteries** | NRGA | **Aortic arch** | Left |
| Aorta | ----- | **PDA** | <1mm PDA, L – R Shunt |
| Pulmonary artery | Normal MPA and Branch PAs. |  |  |
| **M-Mode:** Normal LV Function on eye balling. | | | |
| AO | mm | PWd | mm |
| LA | mm | EDV | ml |
| LVIDd | mm | ESV | ml |
| LVIDs | mm | LVEF | % |
| IVSd | mm | FS | % |
| **Additional Information**: |  | | |
| No pericardial/Pleural effusion. | | | |
| **Final Diagnosis:** | | | |
| 1. {S, D, S} Levocardia. 2. Silent PDA, L – R Shunt (No Murmur detected on auscultation) 3. Normal Biventricular Function | | | |
| **Remark**: No physical Examination finding. | | | |
| **Recommendation**: | | | |
| SIGNATURE  Done by: Tesfaye T., Pediatrician, Pediatric Cardiologist _______________ 08/10/2013Eth.C | | | |

| Patient Name: **Redeat Getinet**. Referring Institute: **Injibara GH**. SEX/ Age: M**/2Months**.  Date of Report: **09/10/2013**. Referral Diagnosis: **CHD(DS). AGH10.889** | | | |
| --- | --- | --- | --- |
| **Features** | **Finding** | **Features** | **Finding** |
| **Profile** |  | **Atria** |  |
| Abdominal situs | Solitus | Left atrium | Normal |
| Cardiac position | Levocardia | Right atrium | Normal |
| Systemic venous drainage | Normal. | **Atrioventricular valves** |  |
| Pulmonary venous drainage | Normal | Mitral valve | Annulus = 11mm |
| Atrioventricular connection | Concordant | Tricuspid valve | Annulus = 11mm |
| Ventriculoarterial connection | Concordant | **Ventricles** |  |
| Ventricular loop | d-Loop | Left ventricle | Normal |
|  |  | Right ventricle | Normal |
| **Septae** |  | **Coronary arteries** | ----- |
| Interventricular septum | Intact | **Doppler Measurement** |  |
| Interatrial septum | 5mm OS ASD, L – R Shunt | Mitral | ----- |
| **Semilunal valves** |  | Aortic | ------- |
| Aortic valve | Annulus = 8mm | Tricuspid | ------- |
| Pulmonary valve | Annulus = 8mm | pulmonic | -------- |
| **Great arteries** | NRGA | **Aortic arch** | Left |
| Aorta | ----- | **PDA** | No |
| Pulmonary artery | Normal MPA and Branch PAs. |  |  |
| **M-Mode:** Normal LV Function on eye balling. | | | |
| AO | mm | PWd | mm |
| LA | mm | EDV | ml |
| LVIDd | mm | ESV | ml |
| LVIDs | mm | LVEF | % |
| IVSd | mm | FS | % |
| **Additional Information**: |  | | |
| No pericardial/Pleural effusion. | | | |
| **Final Diagnosis:** | | | |
| 1. {S, D, S} Levocardia. 2. Small OS ASD, L – R Shunt 3. Normal LV Function | | | |
| **Remark**: | | | |
| **Recommendation**: | | | |
| SIGNATURE  Done by: Tesfaye T., Pediatrician, Pediatric Cardiologist _______________ 09/10/2013Eth.C | | | |

| Patient Name: **Agegnehush Jenberu**. Referring Institute: **Injibara GH**. SEX/ Age: F**/1 4/12**.  Date of Report: **09/10/2013**. Referral Diagnosis: **CHD 20 ?VSD(Murmur + DS). AGH10.890** | | | |
| --- | --- | --- | --- |
| **Features** | **Finding** | **Features** | **Finding** |
| **Profile** |  | **Atria** |  |
| Abdominal situs | Solitus | Left atrium | Normal |
| Cardiac position | Levocardia | Right atrium | Dilated |
| Systemic venous drainage | Normal. | **Atrioventricular valves** |  |
| Pulmonary venous drainage | Normal | Mitral valve | Common Complete AVSD |
| Atrioventricular connection | Common Complete AVSD | Tricuspid valve | Common Complete AVSD  TAPSE = 14mm |
| Ventriculoarterial connection | Concordant | **Ventricles** |  |
| Ventricular loop | d-Loop | Left ventricle | Normal |
|  |  | Right ventricle | Dilated |
| **Septae** |  | **Coronary arteries** | ----- |
| Interventricular septum | Common Complete AVSD, L – R Shunt | **Doppler Measurement** |  |
| Interatrial septum |  | Mitral | Mild Left AVVR |
| **Semilunal valves** |  | Aortic | ------- |
| Aortic valve | Annulus = 12mm | Tricuspid | Severe Right AVVR |
| Pulmonary valve | Annulus = 12mm | pulmonic | -------- |
| **Great arteries** | NRGA | **Aortic arch** | Left |
| Aorta | ----- | **PDA** | No |
| Pulmonary artery | MPA = 14mm. |  |  |
| **M-Mode: Normal LV Function** | | | |
| AO | mm | PWd | mm |
| LA | mm | EDV | ml |
| LVIDd | mm | ESV | ml |
| LVIDs | mm | LVEF | % |
| IVSd | mm | FS | % |
| **Additional Information**: |  | | |
| No pericardial/Pleural effusion. | | | |
| **Final Diagnosis:** | | | |
| 1. {S, D, S} Levocardia. 2. Common Complete Balanced AVSD, L – R Shunt 3. Pulmonary Hypertension 4. Normal Biventricular Function | | | |
| **Remark**: | | | |
| **Recommendation**: | | | |
| SIGNATURE  Done by: Tesfaye T., Pediatrician, Pediatric Cardiologist _______________ 09/10/2013Eth.C | | | |

| Patient Name: **Asegedech Alebachew**. Referring Institute: **GAMBY GH**. SEX/ Age: F**/3/12**.  Date of Report: **09/10/2013**. Referral Diagnosis: **Cyanosis. AGH10.891** | | | |
| --- | --- | --- | --- |
| **Features** | **Finding** | **Features** | **Finding** |
| **Profile** |  | **Atria** |  |
| Abdominal situs | Solitus | Left atrium | Normal |
| Cardiac position | Levocardia | Right atrium | Normal |
| Systemic venous drainage | Normal. | **Atrioventricular valves** |  |
| Pulmonary venous drainage | Normal | Mitral valve | Annulus = 9mm |
| Atrioventricular connection | Concordant | Tricuspid valve | Annulus = 10mm  TAPSE = 12mm |
| Ventriculoarterial connection | Concordant | **Ventricles** |  |
| Ventricular loop | d-Loop | Left ventricle | Normal |
|  |  | Right ventricle | Normal |
| **Septae** |  | **Coronary arteries** | ----- |
| Interventricular septum | 7mm Subaortic VSD, R – L Shunt | **Doppler Measurement** |  |
| Interatrial septum | PFO, BD Shunt | Mitral | ----- |
| **Semilunal valves** |  | Aortic | ------- |
| Aortic valve | Annulus = 9mm | Tricuspid | ------- |
| Pulmonary valve | Atretic | pulmonic | Atretic |
| **Great arteries** | NRGA | **Aortic arch** | Left |
| Aorta | ----- | **PDA** | 1mm PDA, L – R Shunt |
| Pulmonary artery | MPA and Branch PAs are visible. |  |  |
| **M-Mode: Normal LV Function** | | | |
| AO | mm | PWd | mm |
| LA | mm | EDV | ml |
| LVIDd | mm | ESV | ml |
| LVIDs | mm | LVEF | % |
| IVSd | mm | FS | % |
| **Additional Information**: |  | | |
| No pericardial/Pleural effusion. | | | |
| **Final Diagnosis:** | | | |
| 1. {S, D, S} Levocardia. 2. PFO, BD Shunt 3. Large Subaortic VSD, R – L Shunt 4. Pulmonary Atresia (Valvular) (Extreme variant of TOF) | | | |
| **Remark**: | | | |
| **Recommendation**: | | | |
| SIGNATURE  Done by: Tesfaye T., Pediatrician, Pediatric Cardiologist _______________ 09/10/2013Eth.C | | | |

| Patient Name: **Bethelihem Esubalew**. Referring Institute: **Amaris PSC**. SEX/ Age: F**/4 9/12**.  Date of Report: **09/10/2013**. Referral Diagnosis: **RAAD R/O CHD. AGH10.892** | | | |
| --- | --- | --- | --- |
| **Features** | **Finding** | **Features** | **Finding** |
| **Profile** |  | **Atria** |  |
| Abdominal situs | Solitus | Left atrium | Normal |
| Cardiac position | Levocardia | Right atrium | Normal |
| Systemic venous drainage | Normal. | **Atrioventricular valves** |  |
| Pulmonary venous drainage | Normal | Mitral valve | Annulus = 19mm |
| Atrioventricular connection | Concordant | Tricuspid valve | Annulus = 18mm  TAPSE = 18mm |
| Ventriculoarterial connection | Concordant | **Ventricles** |  |
| Ventricular loop | d-Loop | Left ventricle | Normal |
|  |  | Right ventricle | Normal |
| **Septae** |  | **Coronary arteries** | ----- |
| Interventricular septum | Intact | **Doppler Measurement** |  |
| Interatrial septum | Intact | Mitral | ----- |
| **Semilunal valves** |  | Aortic | ------- |
| Aortic valve | Annulus = 16mm | Tricuspid | ------- |
| Pulmonary valve | Annulus = 18mm | pulmonic | -------- |
| **Great arteries** | NRGA | **Aortic arch** | Left |
| Aorta | ----- | **PDA** | No |
| Pulmonary artery | Normal MPA and Branch PAs. |  |  |
| **M-Mode:** | | | |
| AO | mm | PWd | mm |
| LA | mm | EDV | ml |
| LVIDd | mm | ESV | ml |
| LVIDs | mm | LVEF | 60% |
| IVSd | mm | FS | 31% |
| **Additional Information**: |  | | |
| No pericardial/Pleural effusion. | | | |
| **Final Diagnosis:** | | | |
| 1. Normal Echocardiography Study. | | | |
| **Remark**: | | | |
| **Recommendation**: | | | |
| SIGNATURE  Done by: Tesfaye T., Pediatrician, Pediatric Cardiologist _______________ 09/10/2013Eth.C | | | |

| Patient Name: **Mesfin Eyasu**. Referring Institute: **FHRH**. SEX/ Age: M**/2Years**.  Date of Report: **09/10/2013**. Referral Diagnosis: **CHD 20 ?VSD (Cyanosis). AGH10.893** | | | |
| --- | --- | --- | --- |
| **Features** | **Finding** | **Features** | **Finding** |
| **Profile** |  | **Atria** |  |
| Abdominal situs | Solitus | Left atrium | Normal |
| Cardiac position | Levocardia | Right atrium | Normal |
| Systemic venous drainage | Normal. | **Atrioventricular valves** |  |
| Pulmonary venous drainage | Normal | Mitral valve | Annulus = 12mm |
| Atrioventricular connection | Double Inlet Univentricular | Tricuspid valve | Annulus = 12mm |
| Ventriculoarterial connection | d-TGA | **Ventricles** |  |
| Ventricular loop | X-Loop | Left ventricle | Indeterminate Double Inlet Single ventricle |
|  |  | Right ventricle |
| **Septae** |  | **Coronary arteries** | ----- |
| Interventricular septum | Indeterminate Double Inlet Single Ventricle | **Doppler Measurement** |  |
| Interatrial septum | Intact | Mitral | ----- |
| **Semilunal valves** |  | Aortic | ------- |
| Aortic valve | Annulus = 13mm | Tricuspid | ------- |
| Pulmonary valve | Annulus = 17mm | pulmonic | Severe PR, PPG = 85mmHg |
| **Great arteries** | d-TGA | **Aortic arch** | Left |
| Aorta | Anterior & to right | **PDA** | No |
| Pulmonary artery | Posterior & to left |  |  |
| **M-Mode:** | | | |
| AO | mm | PWd | mm |
| LA | mm | EDV | ml |
| LVIDd | mm | ESV | ml |
| LVIDs | mm | LVEF | % |
| IVSd | mm | FS | % |
| **Additional Information**: |  | | |
| No pericardial/Pleural effusion. | | | |
| **Final Diagnosis:** | | | |
| 1. {S, x, D} Levocardia. 2. d-TGA 3. Indeterminate Double Inlet Univentricle Physiology 4. Severe PR 5. Severe Pulmonary Hypertension | | | |
| **Remark**: | | | |
| **Recommendation**: | | | |
| SIGNATURE  Done by: Tesfaye T., Pediatrician, Pediatric Cardiologist _______________ 09/10/2013Eth.C | | | |

| Patient Name: **Birhane – Meskel Emwedew**. Referring Institute: **Adinas GH**. SEX/ Age: M**/3 2/12**.  Date of Report: **09/10/2013**. Referral Diagnosis: **DS. AGH10.894** | | | |
| --- | --- | --- | --- |
| **Features** | **Finding** | **Features** | **Finding** |
| **Profile** |  | **Atria** |  |
| Abdominal situs | Solitus | Left atrium | Normal |
| Cardiac position | Levocardia | Right atrium | Normal |
| Systemic venous drainage | Normal. | **Atrioventricular valves** |  |
| Pulmonary venous drainage | Normal | Mitral valve | Annulus = 15mm |
| Atrioventricular connection | Concordant | Tricuspid valve | Annulus = 16mm  TAPSE = 18mm |
| Ventriculoarterial connection | Concordant | **Ventricles** |  |
| Ventricular loop | d-Loop | Left ventricle | Normal |
|  |  | Right ventricle | Normal |
| **Septae** |  | **Coronary arteries** | ----- |
| Interventricular septum | Intact | **Doppler Measurement** |  |
| Interatrial septum | Intact | Mitral | ----- |
| **Semilunal valves** |  | Aortic | ------- |
| Aortic valve | Annulus = 12mm | Tricuspid | ------- |
| Pulmonary valve | Annulus = 13mm | pulmonic | -------- |
| **Great arteries** | NRGA | **Aortic arch** | Left |
| Aorta | ----- | **PDA** | No |
| Pulmonary artery | Normal MPA and Branch PAs. |  |  |
| **M-Mode:** Normal LV Function | | | |
| AO | mm | PWd | mm |
| LA | mm | EDV | ml |
| LVIDd | mm | ESV | ml |
| LVIDs | mm | LVEF | % |
| IVSd | mm | FS | % |
| **Additional Information**: |  | | |
| No pericardial/Pleural effusion. | | | |
| **Final Diagnosis:** | | | |
| 1. Normal Echocardiography Study. | | | |
| **Remark**: | | | |
| **Recommendation**: | | | |
| SIGNATURE  Done by: Tesfaye T., Pediatrician, Pediatric Cardiologist _______________ 09/10/2013Eth.C | | | |

| Patient Name: **Anteneh Ewunetu**. Referring Institute: **FHRH**. SEX/ Age: M**/1 7/12**.  Date of Report: 1**0/10/2013**. Referral Diagnosis: **R/O CHD(RD + Recurrent Chest Infection). AGH10.895** | | | |
| --- | --- | --- | --- |
| **Features** | **Finding** | **Features** | **Finding** |
| **Profile** |  | **Atria** |  |
| Abdominal situs | Solitus | Left atrium | Normal |
| Cardiac position | Levocardia | Right atrium | Normal |
| Systemic venous drainage | Normal. | **Atrioventricular valves** |  |
| Pulmonary venous drainage | Normal | Mitral valve | Annulus = 13mm |
| Atrioventricular connection | Concordant | Tricuspid valve | Annulus = 13mm |
| Ventriculoarterial connection | Concordant | **Ventricles** |  |
| Ventricular loop | d-Loop | Left ventricle | Normal |
|  |  | Right ventricle | Normal |
| **Septae** |  | **Coronary arteries** | ----- |
| Interventricular septum | Intact | **Doppler Measurement** |  |
| Interatrial septum | Intact | Mitral | ----- |
| **Semilunal valves** |  | Aortic | ------- |
| Aortic valve | Annulus = 11mm | Tricuspid | ------- |
| Pulmonary valve | Annulus = 12mm | pulmonic | -------- |
| **Great arteries** | NRGA | **Aortic arch** | Left |
| Aorta | ----- | **PDA** | No |
| Pulmonary artery | Normal MPA and Branch PAs. |  |  |
| **M-Mode:** Normal LV Function | | | |
| AO | mm | PWd | mm |
| LA | mm | EDV | ml |
| LVIDd | mm | ESV | ml |
| LVIDs | mm | LVEF | % |
| IVSd | mm | FS | % |
| **Additional Information**: |  | | |
| No pericardial/Pleural effusion. | | | |
| **Final Diagnosis:** | | | |
| 1. Normal Echocardiography Study. | | | |
| **Remark**: | | | |
| **Recommendation**: | | | |
| SIGNATURE  Done by: Tesfaye T., Pediatrician, Pediatric Cardiologist _______________ 10/10/2013Eth.C | | | |

| Patient Name: **Sajin Lulie**. Referring Institute: **TGSH**. SEX/ Age: M**/6Years**.  Date of Report: 1**0/10/2013**. Referral Diagnosis: **Acute Rheumatic Fever + RD + CHF. AGH10.896** | | | |
| --- | --- | --- | --- |
| **Features** | **Finding** | **Features** | **Finding** |
| **Profile** |  | **Atria** |  |
| Abdominal situs | Solitus | Left atrium | Dilated |
| Cardiac position | Levocardia | Right atrium | Normal |
| Systemic venous drainage | Normal. | **Atrioventricular valves** |  |
| Pulmonary venous drainage | Normal | Mitral valve | Annulus = 25mm. Mildly thickened MVL |
| Atrioventricular connection | Concordant | Tricuspid valve | Annulus = 21mm  TAPSE = 20mm |
| Ventriculoarterial connection | Concordant | **Ventricles** |  |
| Ventricular loop | d-Loop | Left ventricle | Dilated |
|  |  | Right ventricle | Normal |
| **Septae** |  | **Coronary arteries** | ----- |
| Interventricular septum | Intact | **Doppler Measurement** |  |
| Interatrial septum | Intact | Mitral | Moderate MR, Holosystolic, posterior projection, seen in two planes with jet velocity = 4.5m/sec |
| **Semilunal valves** |  | Aortic | ------- |
| Aortic valve | Annulus = 16mm | Tricuspid | ------- |
| Pulmonary valve | Annulus = 20mm | pulmonic | -------- |
| **Great arteries** | NRGA | **Aortic arch** | Left |
| Aorta | ----- | **PDA** | No |
| Pulmonary artery | Normal MPA and Branch PAs. |  |  |
| **M-Mode:** | | | |
| AO | mm | PWd | mm |
| LA | mm | EDV | ml |
| LVIDd | mm | ESV | ml |
| LVIDs | mm | LVEF | 56% |
| IVSd | mm | FS | 29% |
| **Additional Information**: |  | | |
| 16mm Right Pleural Effusion. 5mm pericardial effusion on RA/RV Side. | | | |
| **Final Diagnosis:** | | | |
| 1. {S, D, S} Levocardia. 2. LA/LV Dilated 3. Mildly thickened MVL 4. Moderate MR 5. Mild Pericardial effusion 6. Moderate Right Pleural effusion 7. Normal Biventricular Function | | | |
| SIGNATURE  Done by: Tesfaye T., Pediatrician, Pediatric Cardiologist _______________ 10/10/2013Eth.C | | | |

| Patient Name: **Tiruaynet Mulat**. Referring Institute: **Addis Alem PH**. SEX/ Age: F**/2Years**.  Date of Report: 1**0/10/2013**. Referral Diagnosis: **R/O CHD (G-II Systolic Murmur). AGH10.897** | | | |
| --- | --- | --- | --- |
| **Features** | **Finding** | **Features** | **Finding** |
| **Profile** |  | **Atria** |  |
| Abdominal situs | Solitus | Left atrium | Normal |
| Cardiac position | Levocardia | Right atrium | Normal |
| Systemic venous drainage | Normal. | **Atrioventricular valves** |  |
| Pulmonary venous drainage | Normal | Mitral valve | Annulus = 10mm |
| Atrioventricular connection | Concordant | Tricuspid valve | Annulus = 11mm |
| Ventriculoarterial connection | Concordant | **Ventricles** |  |
| Ventricular loop | d-Loop | Left ventricle | Normal |
|  |  | Right ventricle | Normal |
| **Septae** |  | **Coronary arteries** | ----- |
| Interventricular septum | Intact | **Doppler Measurement** |  |
| Interatrial septum | Intact | Mitral | ----- |
| **Semilunal valves** |  | Aortic | ------- |
| Aortic valve | Annulus = 10mm | Tricuspid | ------- |
| Pulmonary valve | Annulus = 11mm | pulmonic | -------- |
| **Great arteries** | NRGA | **Aortic arch** | Left |
| Aorta | ----- | **PDA** | No |
| Pulmonary artery | Normal MPA and Branch PAs. |  |  |
| **M-Mode:** Normal LV Function | | | |
| AO | mm | PWd | mm |
| LA | mm | EDV | ml |
| LVIDd | mm | ESV | ml |
| LVIDs | mm | LVEF | % |
| IVSd | mm | FS | % |
| **Additional Information**: |  | | |
| No pericardial/Pleural effusion. | | | |
| **Final Diagnosis:** | | | |
| 1. Normal Echocardiography Study. | | | |
| **Remark**: Child was irritable during study | | | |
| **Recommendation**: | | | |
| SIGNATURE  Done by: Tesfaye T., Pediatrician, Pediatric Cardiologist _______________ 10/10/2013Eth.C | | | |

| Patient Name: **Arsema Desalegn**. Referring Institute: **FHRH**. SEX/ Age: F**/1 3/12**. (11/12 / F): DS + CHF  Date of Report: 11**/10/2013**. Referral Diagnosis: **AVSD (Follow up Echocardiography). AGH10.898 (AGH6)** | | | |
| --- | --- | --- | --- |
| **Features** | **Finding** | **Features** | **Finding** |
| **Profile** |  | **Atria** |  |
| Abdominal situs | Solitus | Left atrium | Normal |
| Cardiac position | Levocardia | Right atrium | Normal |
| Systemic venous drainage | Normal. | **Atrioventricular valves** |  |
| Pulmonary venous drainage | Normal | Mitral valve | Common Complete AVSD |
| Atrioventricular connection | Concordant | Tricuspid valve |  |
| Ventriculoarterial connection | Concordant | **Ventricles** |  |
| Ventricular loop | d-Loop | Left ventricle | Normal |
|  |  | Right ventricle | Normal |
| **Septae** |  | **Coronary arteries** | ----- |
| Interventricular septum | Common Complete AVSD, L – R Shunt | **Doppler Measurement** |  |
| Interatrial septum |  | Mitral | Mild Left AVVR |
| **Semilunal valves** |  | Aortic | ------- |
| Aortic valve | Annulus = 14mm | Tricuspid | Moderate Right AVVR |
| Pulmonary valve | Annulus = 15mm | pulmonic | -------- |
| **Great arteries** | NRGA | **Aortic arch** | Left |
| Aorta | ----- | **PDA** | 1.5mm PDA, L – R Shunt |
| Pulmonary artery | MPA =17mm. |  |  |
| **M-Mode:** | | | |
| AO | mm | PWd | mm |
| LA | mm | EDV | ml |
| LVIDd | mm | ESV | ml |
| LVIDs | mm | LVEF | 64% |
| IVSd | mm | FS | 34% |
| **Additional Information**: |  | | |
| 9mm circumferential pericardial effusion. | | | |
| **Final Diagnosis:** | | | |
| 1. {S, D, S} Levocardia. 2. Common Complete Balanced AVSD, L – R Shunt 3. Mild Left AVVR 4. Moderate Right AVVR 5. PDA, L – R Shunt 6. Mild circumferential Pericardial effusion 7. Normal LV Function | | | |
| **Remark**: | | | |
| **Recommendation**: Needs surgical Intervention | | | |
| SIGNATURE  Done by: Tesfaye T., Pediatrician, Pediatric Cardiologist _______________ 11/10/2013Eth.C | | | |

| Patient Name: **Hemen Gebrie**. Referring Institute: **Adinas GH**. SEX/ Age: F**/1 4/12**.  Date of Report: **12/10/2013**. Referral Diagnosis: **Small PM VSD(Follow up echo done on 10/10/12Eth.C). AGH10.899 (AGH3, 4/12/F)** | | | |
| --- | --- | --- | --- |
| **Features** | **Finding** | **Features** | **Finding** |
| **Profile** |  | **Atria** |  |
| Abdominal situs | Solitus | Left atrium | Normal |
| Cardiac position | Levocardia | Right atrium | Normal |
| Systemic venous drainage | Normal. | **Atrioventricular valves** |  |
| Pulmonary venous drainage | Normal | Mitral valve | Annulus = 11mm |
| Atrioventricular connection | Concordant | Tricuspid valve | Annulus = 13mm  TAPSE = 16mm |
| Ventriculoarterial connection | Concordant | **Ventricles** |  |
| Ventricular loop | d-Loop | Left ventricle | Normal |
|  |  | Right ventricle | Normal |
| **Septae** |  | **Coronary arteries** | ----- |
| Interventricular septum | 2mm Restrictive PM VSD, L – R Shunt with a Gradient of 70mmHg | **Doppler Measurement** |  |
| Interatrial septum | Intact | Mitral | ----- |
| **Semilunal valves** |  | Aortic | ------- |
| Aortic valve | Annulus = 13mm | Tricuspid | ------- |
| Pulmonary valve | Annulus = 13mm | pulmonic | -------- |
| **Great arteries** | NRGA | **Aortic arch** | Left |
| Aorta | ----- | **PDA** | No |
| Pulmonary artery | Normal MPA and Branch PAs. |  |  |
| **M-Mode:** Normal LV Function (eye balling). | | | |
| AO | mm | PWd | mm |
| LA | mm | EDV | ml |
| LVIDd | mm | ESV | ml |
| LVIDs | mm | LVEF | % |
| IVSd | mm | FS | % |
| **Additional Information**: |  | | |
| No pericardial/Pleural effusion. | | | |
| **Final Diagnosis:** | | | |
| 1. {S, D, S} Levocardia. 2. Small Restrictive PM VSD, L – R Shunt 3. Normal Biventricular Function | | | |
| **Remark**: Size is decreasing | | | |
| **Recommendation**: | | | |
| SIGNATURE  Done by: Tesfaye T., Pediatrician, Pediatric Cardiologist _______________ 12/10/2013Eth.C | | | |

| Patient Name: **Yeabsira Tadesse**. Referring Institute: **FHRH**. SEX/ Age: F**/4/12**.  Date of Report: 15**/10/2013**. Referral Diagnosis: **Preterm, VLBW, PDA (immediately after birth). AGH10.900** | | | |
| --- | --- | --- | --- |
| **Features** | **Finding** | **Features** | **Finding** |
| **Profile** |  | **Atria** |  |
| Abdominal situs | Solitus | Left atrium | Normal |
| Cardiac position | Levocardia | Right atrium | Normal |
| Systemic venous drainage | Normal. | **Atrioventricular valves** |  |
| Pulmonary venous drainage | Normal | Mitral valve | Annulus = 11mm |
| Atrioventricular connection | Concordant | Tricuspid valve | Annulus = 11mm  TAPSE = 16mm |
| Ventriculoarterial connection | Concordant | **Ventricles** |  |
| Ventricular loop | d-Loop | Left ventricle | Normal |
|  |  | Right ventricle | Normal |
| **Septae** |  | **Coronary arteries** | ----- |
| Interventricular septum | Intact | **Doppler Measurement** |  |
| Interatrial septum | Intact | Mitral | ----- |
| **Semilunal valves** |  | Aortic | ------- |
| Aortic valve | Annulus = 9mm | Tricuspid | ------- |
| Pulmonary valve | Annulus = 9mm | pulmonic | -------- |
| **Great arteries** | NRGA | **Aortic arch** | Left |
| Aorta | ----- | **PDA** | No |
| Pulmonary artery | Normal MPA and Branch PAs. |  |  |
| **M-Mode:** Normal LV Function on eye balling | | | |
| AO | mm | PWd | mm |
| LA | mm | EDV | ml |
| LVIDd | mm | ESV | ml |
| LVIDs | mm | LVEF | % |
| IVSd | mm | FS | % |
| **Additional Information**: |  | | |
| No pericardial/Pleural effusion. | | | |
| **Final Diagnosis:** | | | |
| 1. Normal Echocardiography study. | | | |
| **Remark**: the PDA has closed | | | |
| **Recommendation**: | | | |
| SIGNATURE  Done by: Tesfaye T., Pediatrician, Pediatric Cardiologist _______________ 15/10/2013Eth.C | | | |

| Patient Name: **Tinsae Getachew**. Referring Institute: **FHRH**. SEX/ Age: M**/8Years**.  Date of Report: 15**/10/2013**. Referral Diagnosis: **Easy fatigability. AGH10.901** | | | |
| --- | --- | --- | --- |
| **Features** | **Finding** | **Features** | **Finding** |
| **Profile** |  | **Atria** |  |
| Abdominal situs | Solitus | Left atrium | Normal |
| Cardiac position | Levocardia | Right atrium | Normal |
| Systemic venous drainage | Normal. | **Atrioventricular valves** |  |
| Pulmonary venous drainage | Normal | Mitral valve | Annulus = 18mm |
| Atrioventricular connection | Concordant | Tricuspid valve | Annulus = 20mm  TAPSE = 20mm |
| Ventriculoarterial connection | Concordant | **Ventricles** |  |
| Ventricular loop | d-Loop | Left ventricle | Normal |
|  |  | Right ventricle | Normal |
| **Septae** |  | **Coronary arteries** | ----- |
| Interventricular septum | Intact | **Doppler Measurement** |  |
| Interatrial septum | Intact | Mitral | ----- |
| **Semilunal valves** |  | Aortic | ------- |
| Aortic valve | Annulus = 17mm | Tricuspid | Trivial TR, PPG = 20mmHg |
| Pulmonary valve | Annulus = 18mm | pulmonic | -------- |
| **Great arteries** | NRGA | **Aortic arch** | Left |
| Aorta | ----- | **PDA** | No |
| Pulmonary artery | Normal MPA and Branch PAs. |  |  |
| **M-Mode:** | | | |
| AO | mm | PWd | mm |
| LA | mm | EDV | ml |
| LVIDd | mm | ESV | ml |
| LVIDs | mm | LVEF | 71% |
| IVSd | mm | FS | 39% |
| **Additional Information**: |  | | |
| No pericardial/Pleural effusion. | | | |
| **Final Diagnosis:** | | | |
| 1. Normal Echocardiography Study. | | | |
| **Remark**: | | | |
| **Recommendation**: | | | |
| SIGNATURE  Done by: Tesfaye T., Pediatrician, Pediatric Cardiologist _______________ 15/10/2013Eth.C | | | |

| Patient Name: **Abdulkerim Seid**. Referring Institute: **FHRH**. SEX/ Age: M**/7Years**.  Date of Report: 16**/10/2013**. Referral Diagnosis: **Incidental Murmur. AGH10.902** | | | |
| --- | --- | --- | --- |
| **Features** | **Finding** | **Features** | **Finding** |
| **Profile** |  | **Atria** |  |
| Abdominal situs | Solitus | Left atrium | Normal |
| Cardiac position | Levocardia | Right atrium | Normal |
| Systemic venous drainage | Normal. | **Atrioventricular valves** |  |
| Pulmonary venous drainage | Normal | Mitral valve | Annulus = 18mm |
| Atrioventricular connection | Concordant | Tricuspid valve | Annulus = 21mm  TAPSE = 20mm |
| Ventriculoarterial connection | Concordant | **Ventricles** |  |
| Ventricular loop | d-Loop | Left ventricle | Normal |
|  |  | Right ventricle | Normal |
| **Septae** |  | **Coronary arteries** | ----- |
| Interventricular septum | 5mm Sub arterial VSD, L – R Shunt | **Doppler Measurement** |  |
| Interatrial septum | Intact | Mitral | ----- |
| **Semilunal valves** |  | Aortic | ------- |
| Aortic valve | Annulus = 16mm | Tricuspid | ------- |
| Pulmonary valve | Annulus = 19mm | pulmonic | -------- |
| **Great arteries** | NRGA | **Aortic arch** | Left |
| Aorta | ----- | **PDA** | No |
| Pulmonary artery | Normal MPA and Branch PAs. |  |  |
| **M-Mode:** | | | |
| AO | mm | PWd | mm |
| LA | mm | EDV | ml |
| LVIDd | mm | ESV | ml |
| LVIDs | mm | LVEF | 67% |
| IVSd | mm | FS | 37% |
| **Additional Information**: |  | | |
| No pericardial/Pleural effusion. | | | |
| **Final Diagnosis:** | | | |
| 1. {S, D, S} Levocardia. 2. Small Sub arterial VSD, L – R Shunt 3. Normal Biventricular Function | | | |
| **Remark**: | | | |
| **Recommendation**:   1. Yearly echocardiographic evaluation 2. No need to start Medication currently | | | |
| SIGNATURE  Done by: Tesfaye T., Pediatrician, Pediatric Cardiologist _______________ 16/10/2013Eth.C | | | |

| Patient Name: **Fitsum Yegnaneh**. Referring Institute: **FHRH**. SEX/ Age: M**/2Years**.  Date of Report: 17**/10/2013**. Referral Diagnosis: **Diaphoresis + recurrent chest infection. AGH10.903** | | | |
| --- | --- | --- | --- |
| **Features** | **Finding** | **Features** | **Finding** |
| **Profile** |  | **Atria** |  |
| Abdominal situs | Solitus | Left atrium | Normal |
| Cardiac position | Levocardia | Right atrium | Normal |
| Systemic venous drainage | Normal. | **Atrioventricular valves** |  |
| Pulmonary venous drainage | Normal | Mitral valve | Annulus = 12mm |
| Atrioventricular connection | Concordant | Tricuspid valve | Annulus = 14mm |
| Ventriculoarterial connection | Concordant | **Ventricles** |  |
| Ventricular loop | d-Loop | Left ventricle | Normal |
|  |  | Right ventricle | Normal |
| **Septae** |  | **Coronary arteries** | ----- |
| Interventricular septum | 6mm PM VSD, L – R Shunt partially covered by septal leaflet of TV | **Doppler Measurement** |  |
| Interatrial septum | Intact | Mitral | ----- |
| **Semilunal valves** |  | Aortic | ------- |
| Aortic valve | Annulus = 10mm | Tricuspid | ------- |
| Pulmonary valve | Annulus = 10mm | pulmonic | -------- |
| **Great arteries** | NRGA | **Aortic arch** | Left |
| Aorta | ----- | **PDA** | No |
| Pulmonary artery | Normal MPA and Branch PAs. |  |  |
| **M-Mode:** | | | |
| AO | mm | PWd | mm |
| LA | mm | EDV | ml |
| LVIDd | mm | ESV | ml |
| LVIDs | mm | LVEF | 70% |
| IVSd | mm | FS | 38% |
| **Additional Information**: |  | | |
| No pericardial/Pleural effusion. | | | |
| **Final Diagnosis:** | | | |
| 1. {S, D, S} Levocardia. 2. Moderate PM VSD, L – R Shunt Partially covered by septal leaflet of the TV 3. Normal LV Function | | | |
| **Remark**: | | | |
| **Recommendation**: | | | |
| SIGNATURE  Done by: Tesfaye T., Pediatrician, Pediatric Cardiologist _______________ 17/10/2013Eth.C | | | |

| Patient Name: **Baby of Emebet Mitiku**. Referring Institute: **FHRH**. SEX/ Age: M**/11days**.  Date of Report: 17**/10/2013**. Referral Diagnosis: **DS. AGH10.904** | | | |
| --- | --- | --- | --- |
| **Features** | **Finding** | **Features** | **Finding** |
| **Profile** |  | **Atria** |  |
| Abdominal situs | Solitus | Left atrium | Normal |
| Cardiac position | Levocardia | Right atrium | Normal |
| Systemic venous drainage | Normal. | **Atrioventricular valves** |  |
| Pulmonary venous drainage | Normal | Mitral valve | Annulus = 9mm |
| Atrioventricular connection | Concordant | Tricuspid valve | Annulus = 9mm  TAPSE = 10mm |
| Ventriculoarterial connection | Concordant | **Ventricles** |  |
| Ventricular loop | d-Loop | Left ventricle | Normal |
|  |  | Right ventricle | Normal |
| **Septae** |  | **Coronary arteries** | ----- |
| Interventricular septum | Intact | **Doppler Measurement** |  |
| Interatrial septum | Intact | Mitral | ----- |
| **Semilunal valves** |  | Aortic | ------- |
| Aortic valve | Annulus = 8mm | Tricuspid | ------- |
| Pulmonary valve | Annulus = 8mm | pulmonic | -------- |
| **Great arteries** | NRGA | **Aortic arch** | Left |
| Aorta | ----- | **PDA** | No |
| Pulmonary artery | Normal MPA and Branch PAs. |  |  |
| **M-Mode:** Normal LV Function on eye balling. | | | |
| AO | mm | PWd | mm |
| LA | mm | EDV | ml |
| LVIDd | mm | ESV | ml |
| LVIDs | mm | LVEF | % |
| IVSd | mm | FS | % |
| **Additional Information**: |  | | |
| No pericardial/Pleural effusion. | | | |
| **Final Diagnosis:** | | | |
| 1. Normal Echocardiography Study. | | | |
| **Remark**: | | | |
| **Recommendation**: | | | |
| SIGNATURE  Done by: Tesfaye T., Pediatrician, Pediatric Cardiologist _______________ 17/10/2013Eth.C | | | |

| Patient Name: **Sisay Shumet**. Referring Institute: **TGSH**. SEX/ Age: M**/2Years**.  Date of Report: 17**/10/2013**. Referral Diagnosis: **Cardiomegaly on CXR. AGH10.905** | | | |
| --- | --- | --- | --- |
| **Features** | **Finding** | **Features** | **Finding** |
| **Profile** |  | **Atria** |  |
| Abdominal situs | Solitus | Left atrium | Normal |
| Cardiac position | Levocardia | Right atrium | Normal |
| Systemic venous drainage | Normal. | **Atrioventricular valves** |  |
| Pulmonary venous drainage | Normal | Mitral valve | Annulus = 13mm |
| Atrioventricular connection | Concordant | Tricuspid valve | Annulus = 13mm  TAPSE = 15mm |
| Ventriculoarterial connection | Concordant | **Ventricles** |  |
| Ventricular loop | d-Loop | Left ventricle | Normal |
|  |  | Right ventricle | Normal |
| **Septae** |  | **Coronary arteries** | ----- |
| Interventricular septum | Intact | **Doppler Measurement** |  |
| Interatrial septum | Intact | Mitral | ----- |
| **Semilunal valves** |  | Aortic | ------- |
| Aortic valve | Annulus = 11mm | Tricuspid | ------- |
| Pulmonary valve | Annulus = 12mm | pulmonic | -------- |
| **Great arteries** | NRGA | **Aortic arch** | Left |
| Aorta | ----- | **PDA** | No |
| Pulmonary artery | Normal MPA and Branch PAs. |  |  |
| **M-Mode:** Normal LV Function on eye balling. | | | |
| AO | mm | PWd | mm |
| LA | mm | EDV | ml |
| LVIDd | mm | ESV | ml |
| LVIDs | mm | LVEF | % |
| IVSd | mm | FS | % |
| **Additional Information**: |  | | |
| No pericardial/Pleural effusion. | | | |
| **Final Diagnosis:** | | | |
| 1. Normal Echocardiography Study. | | | |
| **Remark**: | | | |
| **Recommendation**: | | | |
| SIGNATURE  Done by: Tesfaye T., Pediatrician, Pediatric Cardiologist _______________ 17/10/2013Eth.C | | | |

| Patient Name: **Leyued Nebyu**. Referring Institute: **Adinas GH**. SEX/ Age: M**/1 8/12**.  Date of Report: 17**/10/2013**. Referral Diagnosis: **Recurrent Chest Infection. AGH10.906** | | | |
| --- | --- | --- | --- |
| **Features** | **Finding** | **Features** | **Finding** |
| **Profile** |  | **Atria** |  |
| Abdominal situs | Solitus | Left atrium | Normal |
| Cardiac position | Levocardia | Right atrium | Normal |
| Systemic venous drainage | Normal. | **Atrioventricular valves** |  |
| Pulmonary venous drainage | Normal | Mitral valve | Annulus = 15mm |
| Atrioventricular connection | Concordant | Tricuspid valve | Annulus = 16mm  TAPSE = 18mm |
| Ventriculoarterial connection | Concordant | **Ventricles** |  |
| Ventricular loop | d-Loop | Left ventricle | Normal |
|  |  | Right ventricle | Normal |
| **Septae** |  | **Coronary arteries** | ----- |
| Interventricular septum | Intact | **Doppler Measurement** |  |
| Interatrial septum | Intact | Mitral | ----- |
| **Semilunal valves** |  | Aortic | ------- |
| Aortic valve | Annulus = 13mm | Tricuspid | Trivial TR, PPG = 15mmHg |
| Pulmonary valve | Annulus = 14mm | pulmonic | -------- |
| **Great arteries** | NRGA | **Aortic arch** | Left |
| Aorta | ----- | **PDA** | No |
| Pulmonary artery | Normal MPA and Branch PAs. |  |  |
| **M-Mode:** | | | |
| AO | mm | PWd | mm |
| LA | mm | EDV | ml |
| LVIDd | mm | ESV | ml |
| LVIDs | mm | LVEF | 56% |
| IVSd | mm | FS | 28% |
| **Additional Information**: |  | | |
| No pericardial/Pleural effusion. | | | |
| **Final Diagnosis:** | | | |
| 1. Normal Echocardiography Study. | | | |
| **Remark**: | | | |
| **Recommendation**: | | | |
| SIGNATURE  Done by: Tesfaye T., Pediatrician, Pediatric Cardiologist _______________ 17/10/2013Eth.C | | | |

| Patient Name: **Belay Tsegaye**. Referring Institute: **FHRH**. SEX/ Age: M**/7Years**.  Date of Report: 18**/10/2013**. Referral Diagnosis: **Incidental Murmur. AGH10.907** | | | |
| --- | --- | --- | --- |
| **Features** | **Finding** | **Features** | **Finding** |
| **Profile** |  | **Atria** |  |
| Abdominal situs | Solitus | Left atrium | Normal |
| Cardiac position | Levocardia | Right atrium | Normal |
| Systemic venous drainage | Normal. | **Atrioventricular valves** |  |
| Pulmonary venous drainage | Normal | Mitral valve | Annulus = 23mm. Mildly thickened MVL |
| Atrioventricular connection | Concordant | Tricuspid valve | Annulus = 21mm  TAPSE = 24mm |
| Ventriculoarterial connection | Concordant | **Ventricles** |  |
| Ventricular loop | d-Loop | Left ventricle | Normal |
|  |  | Right ventricle | Normal |
| **Septae** |  | **Coronary arteries** | ----- |
| Interventricular septum | 6mm PM VSD, L – R Shunt with A gradient of 76mmHg. | **Doppler Measurement** |  |
| Interatrial septum | Intact | Mitral | ----- |
| **Semilunal valves** |  | Aortic | ------- |
| Aortic valve | Annulus = 18mm | Tricuspid | Trivial TR, PPG = 12mmHg |
| Pulmonary valve | Annulus = 19mm | pulmonic | Trivial PR, PPG = 10mmHg |
| **Great arteries** | NRGA | **Aortic arch** | Left |
| Aorta | ----- | **PDA** | No |
| Pulmonary artery | Flow acceleration across the MPA, PPG = 15mmHg |  |  |
| **M-Mode:** | | | |
| AO | mm | PWd | mm |
| LA | mm | EDV | ml |
| LVIDd | mm | ESV | ml |
| LVIDs | mm | LVEF | 56% |
| IVSd | mm | FS | 29% |
| **Additional Information**: |  | | |
| No pericardial/Pleural effusion. | | | |
| **Final Diagnosis:** | | | |
| 1. {S, D, S} Levocardia. 2. Mildly thickened MVL 3. Mild MR 4. Small PM VSD, L – R Shunt 5. Normal Biventricular Function | | | |
| SIGNATURE  Done by: Tesfaye T., Pediatrician, Pediatric Cardiologist _______________ 18/10/2013Eth.C | | | |

| Patient Name: **Aregash Endeshaw**. Referring Institute: **FHRH**. SEX/ Age: F**/3 6/12**.  Date of Report: 18**/10/2013**. Referral Diagnosis: **CHF. AGH10.908** | | | |
| --- | --- | --- | --- |
| **Features** | **Finding** | **Features** | **Finding** |
| **Profile** |  | **Atria** |  |
| Abdominal situs | Solitus | Left atrium | Normal |
| Cardiac position | Levocardia | Right atrium | Markedly Dilated |
| Systemic venous drainage | Normal. | **Atrioventricular valves** |  |
| Pulmonary venous drainage | Normal | Mitral valve | Annulus = 14mm |
| Atrioventricular connection | Concordant | Tricuspid valve | Annulus = 18mm  TAPSE = 5mm |
| Ventriculoarterial connection | Concordant | **Ventricles** |  |
| Ventricular loop | d-Loop | Left ventricle | Banana Shaped |
|  |  | Right ventricle | Markedly dilated, Dysfunctional |
| **Septae** |  | **Coronary arteries** | ----- |
| Interventricular septum | Intact | **Doppler Measurement** |  |
| Interatrial septum | Intact | Mitral | ----- |
| **Semilunal valves** |  | Aortic | ------- |
| Aortic valve | Annulus = 12mm | Tricuspid | Mild TR, PPG = 38mmHg (?Under estimated) |
| Pulmonary valve | Annulus = 20mm | pulmonic | Moderate to severe PR, PPG = 46mmHg (Under estimated) |
| **Great arteries** | NRGA | **Aortic arch** | Left |
| Aorta | ----- | **PDA** | No |
| Pulmonary artery | Normal MPA and Branch PAs. |  |  |
| **M-Mode:** | | | |
| AO | mm | PWd | mm |
| LA | mm | EDV | ml |
| LVIDd | mm | ESV | ml |
| LVIDs | mm | LVEF | 71% |
| IVSd | mm | FS | 39% |
| **Additional Information**: |  | | |
| 10mm Circumferential pericardial effusion. 22mm Right Pleural effusion. | | | |
| **Final Diagnosis:** | | | |
| 1. {S, D, S} Levocardia. 2. Markedly dilated RA/RV 3. Mild TR 4. Moderate to severe PR 5. RV DILATED and Dysfunctional 6. Normal LV Function | | | |
| **Recommendation**: Work in the line of Primary pulmonary Hypertension | | | |
| SIGNATURE  Done by: Tesfaye T., Pediatrician, Pediatric Cardiologist _______________ 18/10/2013Eth.C | | | |

| Patient Name: **Abrham Bahiru**. Referring Institute: **TGSH**. SEX/ Age: M**/46days**.  Date of Report: 18**/10/2013**. Referral Diagnosis: **DS. AGH10.909** | | | |
| --- | --- | --- | --- |
| **Features** | **Finding** | **Features** | **Finding** |
| **Profile** |  | **Atria** |  |
| Abdominal situs | Solitus | Left atrium | Normal |
| Cardiac position | Levocardia | Right atrium | Normal |
| Systemic venous drainage | Normal. | **Atrioventricular valves** |  |
| Pulmonary venous drainage | Normal | Mitral valve | Annulus = 9mm |
| Atrioventricular connection | Concordant | Tricuspid valve | Annulus = 10mm |
| Ventriculoarterial connection | Concordant | **Ventricles** |  |
| Ventricular loop | d-Loop | Left ventricle | Normal |
|  |  | Right ventricle | Normal |
| **Septae** |  | **Coronary arteries** | ----- |
| Interventricular septum | Intact | **Doppler Measurement** |  |
| Interatrial septum | Intact | Mitral | ----- |
| **Semilunal valves** |  | Aortic | ------- |
| Aortic valve | Annulus = 9mm | Tricuspid | ------- |
| Pulmonary valve | Annulus = 9mm | pulmonic | -------- |
| **Great arteries** | NRGA | **Aortic arch** | Left |
| Aorta | ----- | **PDA** | No |
| Pulmonary artery | Normal MPA and Branch PAs. |  |  |
| **M-Mode:** Normal LV Function on eye balling. | | | |
| AO | mm | PWd | mm |
| LA | mm | EDV | ml |
| LVIDd | mm | ESV | ml |
| LVIDs | mm | LVEF | % |
| IVSd | mm | FS | % |
| **Additional Information**: |  | | |
| No pericardial/Pleural effusion. | | | |
| **Final Diagnosis:** | | | |
| 1. Normal Echocardiography Study. | | | |
| **Remark**: | | | |
| **Recommendation**: | | | |
| SIGNATURE  Done by: Tesfaye T., Pediatrician, Pediatric Cardiologist _______________ 18/10/2013Eth.C | | | |

| Patient Name: **Desalegn Kindu**. Referring Institute: **Debre Tabour GH**. SEX/ Age: M**/2 5/12**.  Date of Report: 18**/10/2013**. Referral Diagnosis: **FTT + Recurrent chest infection. AGH10.910** | | | |
| --- | --- | --- | --- |
| **Features** | **Finding** | **Features** | **Finding** |
| **Profile** |  | **Atria** |  |
| Abdominal situs | Solitus | Left atrium | Normal |
| Cardiac position | Levocardia | Right atrium | Mildly dilated |
| Systemic venous drainage | Normal. | **Atrioventricular valves** |  |
| Pulmonary venous drainage | Normal | Mitral valve | Annulus = 16mm |
| Atrioventricular connection | Concordant | Tricuspid valve | Annulus = 17mm  TAPSE = 21mm |
| Ventriculoarterial connection | Concordant | **Ventricles** |  |
| Ventricular loop | d-Loop | Left ventricle | Normal |
|  |  | Right ventricle | Mildly dilated |
| **Septae** |  | **Coronary arteries** | ----- |
| Interventricular septum | 8mm PM VSD, L – R Shunt Partly covered by septal leaflet of TV. | **Doppler Measurement** |  |
| Interatrial septum | Intact | Mitral | ----- |
| **Semilunal valves** |  | Aortic | ------- |
| Aortic valve | Annulus = 12mm | Tricuspid | Trivial TR |
| Pulmonary valve | Annulus = 13mm | pulmonic | Moderate PR, PPG = 50mmHg. Mild Valvular PS, PPG = 27mmHg |
| **Great arteries** | NRGA | **Aortic arch** | Left |
| Aorta | ----- | **PDA** | No |
| Pulmonary artery | Normal MPA and Branch PAs. |  |  |
| **M-Mode:** | | | |
| AO | mm | PWd | mm |
| LA | mm | EDV | ml |
| LVIDd | mm | ESV | ml |
| LVIDs | mm | LVEF | 64% |
| IVSd | mm | FS | 33% |
| **Additional Information**: |  | | |
| No pericardial/Pleural effusion. | | | |
| **Final Diagnosis:** | | | |
| 1. {S, D, S} Levocardia. 2. Moderate PM VSD, L – R Shunt 3. Moderate PR 4. Mild Valvular PS 5. Moderate Pulmonary Hypertension 6. Normal Biventricular Function | | | |
| SIGNATURE  Done by: Tesfaye T., Pediatrician, Pediatric Cardiologist _______________ 18/10/2013Eth.C | | | |

| Patient Name: **Aman Getinet**. Referring Institute: **Adinas GH**. SEX/ Age: M**/3 3/12**.  Date of Report: 19**/10/2013**. Referral Diagnosis: **Small PM VSD (Follow up echo). AGH10.911 (AGH3, 2 2/12/M)** | | | |
| --- | --- | --- | --- |
| **Features** | **Finding** | **Features** | **Finding** |
| **Profile** |  | **Atria** |  |
| Abdominal situs | Solitus | Left atrium | Normal |
| Cardiac position | Levocardia | Right atrium | Normal |
| Systemic venous drainage | Normal. | **Atrioventricular valves** |  |
| Pulmonary venous drainage | Normal | Mitral valve | Annulus = 14mm |
| Atrioventricular connection | Concordant | Tricuspid valve | Annulus = 16mm |
| Ventriculoarterial connection | Concordant | **Ventricles** |  |
| Ventricular loop | d-Loop | Left ventricle | Normal |
|  |  | Right ventricle | Normal |
| **Septae** |  | **Coronary arteries** | ----- |
| Interventricular septum | Intact | **Doppler Measurement** |  |
| Interatrial septum | Intact | Mitral | ----- |
| **Semilunal valves** |  | Aortic | ------- |
| Aortic valve | Annulus = 13mm | Tricuspid | ------- |
| Pulmonary valve | Annulus = 13mm | pulmonic | -------- |
| **Great arteries** | NRGA | **Aortic arch** | Left |
| Aorta | ----- | **PDA** | No |
| Pulmonary artery | Normal MPA and Branch PAs. |  |  |
| **M-Mode:** Normal LV Function (eye balling) | | | |
| AO | mm | PWd | mm |
| LA | mm | EDV | ml |
| LVIDd | mm | ESV | ml |
| LVIDs | mm | LVEF | % |
| IVSd | mm | FS | % |
| **Additional Information**: |  | | |
| No pericardial/Pleural effusion. | | | |
| **Final Diagnosis:** | | | |
| 1. Normal Echocardiography Study. | | | |
| **Remark**: the VSD has closed spontaneously. | | | |
| **Recommendation**: | | | |
| SIGNATURE  Done by: Tesfaye T., Pediatrician, Pediatric Cardiologist _______________ 19/10/2013Eth.C | | | |

| Patient Name: **Bitanya Getinet**. Referring Institute: **Afilas GH**. SEX/ Age: F**/10Years**.  Date of Report: 18**/10/2013**. Referral Diagnosis: **VHD(Easy fatigability + palpitation). AGH10.912** | | | |
| --- | --- | --- | --- |
| **Features** | **Finding** | **Features** | **Finding** |
| **Profile** |  | **Atria** |  |
| Abdominal situs | Solitus | Left atrium | Normal |
| Cardiac position | Levocardia | Right atrium | Normal |
| Systemic venous drainage | Normal. | **Atrioventricular valves** |  |
| Pulmonary venous drainage | Normal | Mitral valve | Annulus = 18mm |
| Atrioventricular connection | Concordant | Tricuspid valve | Annulus = 18mm  TAPSE = 20mm |
| Ventriculoarterial connection | Concordant | **Ventricles** |  |
| Ventricular loop | d-Loop | Left ventricle | Normal |
|  |  | Right ventricle | Normal |
| **Septae** |  | **Coronary arteries** | ----- |
| Interventricular septum | Intact | **Doppler Measurement** |  |
| Interatrial septum | Intact | Mitral | ----- |
| **Semilunal valves** |  | Aortic | ------- |
| Aortic valve | Annulus = 16mm | Tricuspid | ------- |
| Pulmonary valve | Annulus = 18mm | pulmonic | Trivial PR, PPG = 9mmHg |
| **Great arteries** | NRGA | **Aortic arch** | Left |
| Aorta | ----- | **PDA** | No |
| Pulmonary artery | Normal MPA and Branch PAs. |  |  |
| **M-Mode:** | | | |
| AO | mm | PWd | mm |
| LA | mm | EDV | ml |
| LVIDd | mm | ESV | ml |
| LVIDs | mm | LVEF | 67% |
| IVSd | mm | FS | 36% |
| **Additional Information**: |  | | |
| No pericardial/Pleural effusion. | | | |
| **Final Diagnosis:** | | | |
| 1. Normal Echocardiography Study. | | | |
| **Remark**: | | | |
| **Recommendation**: | | | |
| SIGNATURE  Done by: Tesfaye T., Pediatrician, Pediatric Cardiologist _______________ 18/10/2013Eth.C | | | |

| Patient Name: **Kerebat mekie**. Referring Institute: **Adinas GH**. SEX/ Age: F**/5 6/12**.  Date of Report: 20**/10/2013**. R.Dx: **CHF. AGH10.913** | | | |
| --- | --- | --- | --- |
| **Features** | **Finding** | **Features** | **Finding** |
| **Profile** |  | **Atria** |  |
| Abdominal situs | Solitus | Left atrium | Moderately dilated |
| Cardiac position | Levocardia | Right atrium | Normal |
| Systemic venous drainage | Normal. | **Atrioventricular valves** |  |
| Pulmonary venous drainage | Normal | Mitral valve | Annulus = 18mm. Mildly thickened MVL |
| Atrioventricular connection | Concordant | Tricuspid valve | Annulus = 15mm  TAPSE = 20mm |
| Ventriculoarterial connection | Concordant | **Ventricles** |  |
| Ventricular loop | d-Loop | Left ventricle | Moderately dilated |
|  |  | Right ventricle | Normal |
| **Septae** |  | **Coronary arteries** | ----- |
| Interventricular septum | Intact | **Doppler Measurement** |  |
| Interatrial septum | Intact | Mitral | Moderate MR, Holosystolic, posterior projection, seen in two planes with jet velocity = 4.4m/sec |
| **Semilunal valves** |  | Aortic | ------- |
| Aortic valve | Annulus = 15mm | Tricuspid | ------- |
| Pulmonary valve | Annulus = 17mm | pulmonic | -------- |
| **Great arteries** | NRGA | **Aortic arch** | Left |
| Aorta | ----- | **PDA** | No |
| Pulmonary artery | Normal MPA and Branch PAs. |  |  |
| **M-Mode:** | | | |
| AO | mm | PWd | mm |
| LA | mm | EDV | ml |
| LVIDd | mm | ESV | ml |
| LVIDs | mm | LVEF | 65% |
| IVSd | mm | FS | 35% |
| **Additional Information**: |  | | |
| No pericardial/Pleural effusion. | | | |
| **Final Diagnosis:** | | | |
| 1. {S, D, S} Levocardia. 2. LA/LV Dilated 3. Mildly thickened MVL 4. Moderate MR 5. Normal Biventricular Function | | | |
| **Remark**: | | | |
| **Recommendation**: | | | |
| SIGNATURE  Done by: Tesfaye T., Pediatrician, Pediatric Cardiologist _______________ 20/10/2013Eth.C | | | |

| Patient Name: **Dawit Habtam**. Referring Institute: **Adet PH**. SEX/ Age: M**/9Years**.  Date of Report: **21/10/2013**. Referral Diagnosis: **RHD. AGH10.914** | | | |
| --- | --- | --- | --- |
| **Features** | **Finding** | **Features** | **Finding** |
| **Profile** |  | **Atria** |  |
| Abdominal situs | Solitus | Left atrium | Normal |
| Cardiac position | Levocardia | Right atrium | Normal |
| Systemic venous drainage | Normal. | **Atrioventricular valves** |  |
| Pulmonary venous drainage | Normal | Mitral valve | Annulus = 18mm |
| Atrioventricular connection | Concordant | Tricuspid valve | Annulus = 21mm  TAPSE = 22mm |
| Ventriculoarterial connection | Concordant | **Ventricles** |  |
| Ventricular loop | d-Loop | Left ventricle | Normal |
|  |  | Right ventricle | Normal |
| **Septae** |  | **Coronary arteries** | ----- |
| Interventricular septum | Intact | **Doppler Measurement** |  |
| Interatrial septum | Intact | Mitral | ----- |
| **Semilunal valves** |  | Aortic | ------- |
| Aortic valve | Annulus = 14mm | Tricuspid | ------- |
| Pulmonary valve | Annulus = 15mm | pulmonic | -------- |
| **Great arteries** | NRGA | **Aortic arch** | Left |
| Aorta | ----- | **PDA** | No |
| Pulmonary artery | Normal MPA and Branch PAs. |  |  |
| **M-Mode:** | | | |
| AO | mm | PWd | mm |
| LA | mm | EDV | ml |
| LVIDd | mm | ESV | ml |
| LVIDs | mm | LVEF | 65% |
| IVSd | mm | FS | 35% |
| **Additional Information**: |  | | |
| No pericardial/Pleural effusion. | | | |
| **Final Diagnosis:** | | | |
| 1. Normal Echocardiography Study. | | | |
| **Remark**: tachycardia during study | | | |
| **Recommendation**: | | | |
| SIGNATURE  Done by: Tesfaye T., Pediatrician, Pediatric Cardiologist _______________ 21/10/2013Eth.C | | | |

| Patient Name: **Yamrot Muluken**. Referring Institute: **TGSH**. SEX/ Age: F**/11/12**.  Date of Report: 21**/10/2013**. Referral Diagnosis: **Diaphoresis + recurrent chest infection. AGH10.915** | | | |
| --- | --- | --- | --- |
| **Features** | **Finding** | **Features** | **Finding** |
| **Profile** |  | **Atria** |  |
| Abdominal situs | Solitus | Left atrium | Dilated |
| Cardiac position | Levocardia | Right atrium | Normal |
| Systemic venous drainage | Normal. | **Atrioventricular valves** |  |
| Pulmonary venous drainage | Normal | Mitral valve | Annulus = 10mm |
| Atrioventricular connection | Concordant | Tricuspid valve | Annulus = 14mm  TAPSE = 13mm |
| Ventriculoarterial connection | Concordant | **Ventricles** |  |
| Ventricular loop | d-Loop | Left ventricle | Dilated |
|  |  | Right ventricle | Normal |
| **Septae** |  | **Coronary arteries** | ----- |
| Interventricular septum | 7mm PM VSD, L – R Shunt partially covered by septal leaflet of TV | **Doppler Measurement** |  |
| Interatrial septum | Intact | Mitral | ----- |
| **Semilunal valves** |  | Aortic | ------- |
| Aortic valve | Annulus = 10mm | Tricuspid | Trivial TR |
| Pulmonary valve | Annulus = 13mm | pulmonic | -------- |
| **Great arteries** | NRGA | **Aortic arch** | Left |
| Aorta | ----- | **PDA** | No |
| Pulmonary artery | MPA =12mm. |  |  |
| **M-Mode:** | | | |
| AO | mm | PWd | mm |
| LA | mm | EDV | ml |
| LVIDd | mm | ESV | ml |
| LVIDs | mm | LVEF | % |
| IVSd | mm | FS | % |
| **Additional Information**: |  | | |
| 6mm pericardial effusion on RA/RV Side. | | | |
| **Final Diagnosis:** | | | |
| 1. {S, D, S} Levocardia. 2. RA/RV Dilated 3. Moderate PM VSD, L – R Shunt, partially covered by septal leaflet of the TV 4. Small Pericardial effusion 5. Normal Biventricular Function | | | |
| **Remark**: | | | |
| **Recommendation**: | | | |
| SIGNATURE  Done by: Tesfaye T., Pediatrician, Pediatric Cardiologist _______________ 21/10/2013Eth.C | | | |

| Patient Name: **Baby of Etenesh Haile-Mariam**. Referring Institute: **TGSH**. SEX/ Age: F**/24days**.  Date of Report: 21**/10/2013**. Referral Diagnosis: **?CHD(cardiomegaly on CXR). AGH10.916** | | | |
| --- | --- | --- | --- |
| **Features** | **Finding** | **Features** | **Finding** |
| **Profile** |  | **Atria** |  |
| Abdominal situs | Solitus | Left atrium | Normal |
| Cardiac position | Levocardia | Right atrium | Normal |
| Systemic venous drainage | Normal. | **Atrioventricular valves** |  |
| Pulmonary venous drainage | Normal | Mitral valve | Annulus = 10mm |
| Atrioventricular connection | Concordant | Tricuspid valve | Annulus = 12mm  TAPSE = 11mm |
| Ventriculoarterial connection | Concordant | **Ventricles** |  |
| Ventricular loop | d-Loop | Left ventricle | Normal |
|  |  | Right ventricle | Normal |
| **Septae** |  | **Coronary arteries** | ----- |
| Interventricular septum | Intact | **Doppler Measurement** |  |
| Interatrial septum | Intact | Mitral | ----- |
| **Semilunal valves** |  | Aortic | ------- |
| Aortic valve | Annulus = 9mm | Tricuspid | ------- |
| Pulmonary valve | Annulus = 9mm | pulmonic | -------- |
| **Great arteries** | NRGA | **Aortic arch** | Left |
| Aorta | ----- | **PDA** | No |
| Pulmonary artery | Normal MPA and Branch PAs. |  |  |
| **M-Mode:** | | | |
| AO | mm | PWd | mm |
| LA | mm | EDV | ml |
| LVIDd | mm | ESV | ml |
| LVIDs | mm | LVEF | 66% |
| IVSd | mm | FS | 34% |
| **Additional Information**: |  | | |
| No pericardial/Pleural effusion. | | | |
| **Final Diagnosis:** | | | |
| 1. Normal Echocardiography Study. | | | |
| **Remark**: | | | |
| **Recommendation**: | | | |
| SIGNATURE  Done by: Tesfaye T., Pediatrician, Pediatric Cardiologist _______________ 21/10/2013Eth.C | | | |

| Patient Name: **Haile-Mariam Lakachew**. Referring Institute: **FHRH**. SEX/ Age: M**/12Years**.  Date of Report: 22**/10/2013**. Referral Diagnosis: **Incidental Murmur. AGH10.917** | | | |
| --- | --- | --- | --- |
| **Features** | **Finding** | **Features** | **Finding** |
| **Profile** |  | **Atria** |  |
| Abdominal situs | Solitus | Left atrium | Normal |
| Cardiac position | Levocardia | Right atrium | Normal |
| Systemic venous drainage | Normal. | **Atrioventricular valves** |  |
| Pulmonary venous drainage | Normal | Mitral valve | Annulus = 22mm |
| Atrioventricular connection | Concordant | Tricuspid valve | Annulus = 19mm  TAPSE = 18mm |
| Ventriculoarterial connection | Concordant | **Ventricles** |  |
| Ventricular loop | d-Loop | Left ventricle | Normal |
|  |  | Right ventricle | Normal |
| **Septae** |  | **Coronary arteries** | ----- |
| Interventricular septum | 4mm PM VSD, L – R Shunt partially covered by septal leaflet of TV | **Doppler Measurement** |  |
| Interatrial septum | Intact | Mitral | ----- |
| **Semilunal valves** |  | Aortic | ------- |
| Aortic valve | Annulus = 16mm | Tricuspid | ------- |
| Pulmonary valve | Annulus = 21mm | pulmonic | -------- |
| **Great arteries** | NRGA | **Aortic arch** | Left |
| Aorta | ----- | **PDA** | No |
| Pulmonary artery | Normal MPA and Branch PAs. |  |  |
| **M-Mode:** | | | |
| AO | mm | PWd | mm |
| LA | mm | EDV | ml |
| LVIDd | mm | ESV | ml |
| LVIDs | mm | LVEF | 67% |
| IVSd | mm | FS | 37% |
| **Additional Information**: |  | | |
| No pericardial/Pleural effusion. | | | |
| **Final Diagnosis:** | | | |
| 1. {S, D, S} Levocardia. 2. Small PM VSD, L – R Shunt partially covered by septal leaflet of TV 3. Normal Biventricular Function | | | |
| **Remark**: | | | |
| **Recommendation**: | | | |
| SIGNATURE  Done by: Tesfaye T., Pediatrician, Pediatric Cardiologist _______________ 22/10/2013Eth.C | | | |

| Patient Name: **Kale-Hiwet Akilew**. Referring Institute: **Adinas GH**. SEX/ Age: M**/1 1/12**.  Date of Report: 22**/10/2013**. Referral Diagnosis: **Diaphoresis + FTT. AGH10.918** | | | |
| --- | --- | --- | --- |
| **Features** | **Finding** | **Features** | **Finding** |
| **Profile** |  | **Atria** |  |
| Abdominal situs | Solitus | Left atrium | Normal |
| Cardiac position | Levocardia | Right atrium | Normal |
| Systemic venous drainage | Normal. | **Atrioventricular valves** |  |
| Pulmonary venous drainage | Normal | Mitral valve | Annulus = 12mm |
| Atrioventricular connection | Concordant | Tricuspid valve | Annulus = 13mm  TAPSE = 15mm |
| Ventriculoarterial connection | Concordant | **Ventricles** |  |
| Ventricular loop | d-Loop | Left ventricle | Normal |
|  |  | Right ventricle | Normal |
| **Septae** |  | **Coronary arteries** | ----- |
| Interventricular septum | Intact | **Doppler Measurement** |  |
| Interatrial septum | Intact | Mitral | ----- |
| **Semilunal valves** |  | Aortic | ------- |
| Aortic valve | Annulus = 13mm | Tricuspid | ------- |
| Pulmonary valve | Annulus = 13mm | pulmonic | -------- |
| **Great arteries** | NRGA | **Aortic arch** | Left |
| Aorta | ----- | **PDA** | No |
| Pulmonary artery | Normal MPA and Branch PAs. |  |  |
| **M-Mode:** Normal LV Function on eye balling. | | | |
| AO | mm | PWd | mm |
| LA | mm | EDV | ml |
| LVIDd | mm | ESV | ml |
| LVIDs | mm | LVEF | % |
| IVSd | mm | FS | % |
| **Additional Information**: |  | | |
| No pericardial/Pleural effusion. | | | |
| **Final Diagnosis:** | | | |
| 1. Normal Echocardiography Study. | | | |
| **Remark**: | | | |
| **Recommendation**: | | | |
| SIGNATURE  Done by: Tesfaye T., Pediatrician, Pediatric Cardiologist _______________ 22/10/2013Eth.C | | | |

| Patient Name: **Besufekad Minwagaw**. Referring Institute: **TGSH**. SEX/ Age: M**/2Years**.  Date of Report: 23**/10/2013**. Referral Diagnosis: **DS. AGH10.919** | | | |
| --- | --- | --- | --- |
| **Features** | **Finding** | **Features** | **Finding** |
| **Profile** |  | **Atria** |  |
| Abdominal situs | Solitus | Left atrium | Normal |
| Cardiac position | Levocardia | Right atrium | Normal |
| Systemic venous drainage | Normal. | **Atrioventricular valves** |  |
| Pulmonary venous drainage | Normal | Mitral valve | Annulus = 14mm |
| Atrioventricular connection | Concordant | Tricuspid valve | Annulus = 16mm  TAPSE = 17mm |
| Ventriculoarterial connection | Concordant | **Ventricles** |  |
| Ventricular loop | d-Loop | Left ventricle | Normal |
|  |  | Right ventricle | Normal |
| **Septae** |  | **Coronary arteries** | ----- |
| Interventricular septum | Intact | **Doppler Measurement** |  |
| Interatrial septum | Intact | Mitral | ----- |
| **Semilunal valves** |  | Aortic | ------- |
| Aortic valve | Annulus = 13mm | Tricuspid | ------- |
| Pulmonary valve | Annulus = 13mm | pulmonic | -------- |
| **Great arteries** | NRGA | **Aortic arch** | Left |
| Aorta | ----- | **PDA** | No |
| Pulmonary artery | Normal MPA and Branch PAs. |  |  |
| **M-Mode:** | | | |
| AO | mm | PWd | mm |
| LA | mm | EDV | ml |
| LVIDd | mm | ESV | ml |
| LVIDs | mm | LVEF | 59% |
| IVSd | mm | FS | 30% |
| **Additional Information**: |  | | |
| 4mm pericardial effusion on RA/RV Junction. | | | |
| **Final Diagnosis:** | | | |
| 1. {S, D, S} Levocardia. 2. Trace Pericardial effusion | | | |
| **Remark**: | | | |
| **Recommendation**: | | | |
| SIGNATURE  Done by: Tesfaye T., Pediatrician, Pediatric Cardiologist _______________ 23/10/2013Eth.C | | | |

| Patient Name: **Baby of Birtukan Amare**. Referring Institute: **FHRH**. SEX/ Age: F**/18Hours**.  Date of Report: 23**/10/2013**. AGH8.667  Referral Diagnosis: **DS.** | | | |
| --- | --- | --- | --- |
| **Features** | **Finding** | **Features** | **Finding** |
| **Profile** |  | **Atria** |  |
| Abdominal situs | Solitus | Left atrium | Normal |
| Cardiac position | Levocardia | Right atrium | Normal |
| Systemic venous drainage | Normal. | **Atrioventricular valves** |  |
| Pulmonary venous drainage | Normal | Mitral valve | Annulus = 8mm |
| Atrioventricular connection | Concordant | Tricuspid valve | Annulus = 8mm |
| Ventriculoarterial connection | Concordant | **Ventricles** |  |
| Ventricular loop | d-Loop | Left ventricle | Normal |
|  |  | Right ventricle | Normal |
| **Septae** |  | **Coronary arteries** | ----- |
| Interventricular septum | Intact | **Doppler Measurement** |  |
| Interatrial septum | 4mm OS ASD, L – R Shunt | Mitral | ----- |
| **Semilunal valves** |  | Aortic | ------- |
| Aortic valve | Annulus = 8mm | Tricuspid | ------- |
| Pulmonary valve | Annulus = 9mm | pulmonic | -------- |
| **Great arteries** | NRGA | **Aortic arch** | Left |
| Aorta | ----- | **PDA** | <1mm PDA, L – R Shunt |
| Pulmonary artery | Normal MPA and Branch PAs. |  |  |
| **M-Mode:** Normal LV Function on eye balling. | | | |
| AO | mm | PWd | mm |
| LA | mm | EDV | ml |
| LVIDd | mm | ESV | ml |
| LVIDs | mm | LVEF | % |
| IVSd | mm | FS | % |
| **Additional Information**: |  | | |
| No pericardial/Pleural effusion. | | | |
| **Final Diagnosis:** | | | |
| 1. {S, D, S} Levocardia. 2. Small OS ASD, L – R Shunt 3. Silent PDA, L – R Shunt 4. Normal LV Function | | | |
| **Remark**: | | | |
| **Recommendation**: | | | |
| SIGNATURE  Done by: Tesfaye T., Pediatrician, Pediatric Cardiologist _______________ 23/10/2013Eth.C | | | |

| Patient Name: **Gebre-Hiwet Alebel**. Referring Institute: **TGSH**. SEX/ Age: M**/4Years**.  Date of Report: 24**/10/2013**. Referral Diagnosis: **Arrhthmia. AGH10.920** | | | |
| --- | --- | --- | --- |
| **Features** | **Finding** | **Features** | **Finding** |
| **Profile** |  | **Atria** |  |
| Abdominal situs | Solitus | Left atrium | Normal |
| Cardiac position | Levocardia | Right atrium | Normal |
| Systemic venous drainage | Normal. | **Atrioventricular valves** |  |
| Pulmonary venous drainage | Normal | Mitral valve | Annulus = 15mm |
| Atrioventricular connection | Concordant | Tricuspid valve | Annulus = 15mm  TAPSE = 15mm |
| Ventriculoarterial connection | Concordant | **Ventricles** |  |
| Ventricular loop | d-Loop | Left ventricle | Normal |
|  |  | Right ventricle | Normal |
| **Septae** |  | **Coronary arteries** | ----- |
| Interventricular septum | Intact | **Doppler Measurement** |  |
| Interatrial septum | Intact | Mitral | ----- |
| **Semilunal valves** |  | Aortic | ------- |
| Aortic valve | Annulus = 13mm | Tricuspid | ------- |
| Pulmonary valve | Annulus = 14mm | pulmonic | -------- |
| **Great arteries** | NRGA | **Aortic arch** | Left |
| Aorta | ----- | **PDA** | No |
| Pulmonary artery | Normal MPA and Branch PAs. |  |  |
| **M-Mode:** | | | |
| AO | mm | PWd | mm |
| LA | mm | EDV | ml |
| LVIDd | mm | ESV | ml |
| LVIDs | mm | LVEF | 59% |
| IVSd | mm | FS | 30% |
| **Additional Information**: |  | | |
| No pericardial/Pleural effusion. | | | |
| **Final Diagnosis:** | | | |
| 1. Normal Echocardiography Study. | | | |
| **Remark**: | | | |
| **Recommendation**: | | | |
| SIGNATURE  Done by: Tesfaye T., Pediatrician, Pediatric Cardiologist _______________ 24/10/2013Eth.C | | | |

| Patient Name: **Asiya Werku**. Referring Institute: **TGSH**. SEX/ Age: F**/11Years**.  Date of Report: 24**/10/2013**. Referral Diagnosis: **Easy fatigability. AGH10.921** | | | |
| --- | --- | --- | --- |
| **Features** | **Finding** | **Features** | **Finding** |
| **Profile** |  | **Atria** |  |
| Abdominal situs | Solitus | Left atrium | Normal |
| Cardiac position | Levocardia | Right atrium | Normal |
| Systemic venous drainage | Normal. | **Atrioventricular valves** |  |
| Pulmonary venous drainage | Normal | Mitral valve | Annulus = 18mm |
| Atrioventricular connection | Concordant | Tricuspid valve | Annulus = 18mm  TAPSE = 22mm |
| Ventriculoarterial connection | Concordant | **Ventricles** |  |
| Ventricular loop | d-Loop | Left ventricle | Normal |
|  |  | Right ventricle | Normal |
| **Septae** |  | **Coronary arteries** | ----- |
| Interventricular septum | Intact | **Doppler Measurement** |  |
| Interatrial septum | Intact | Mitral | ----- |
| **Semilunal valves** |  | Aortic | ------- |
| Aortic valve | Annulus = 16mm | Tricuspid | ------- |
| Pulmonary valve | Annulus = 18mm | pulmonic | -------- |
| **Great arteries** | NRGA | **Aortic arch** | Left |
| Aorta | ----- | **PDA** | No |
| Pulmonary artery | Normal MPA and Branch PAs. |  |  |
| **M-Mode:** | | | |
| AO | mm | PWd | mm |
| LA | mm | EDV | ml |
| LVIDd | mm | ESV | ml |
| LVIDs | mm | LVEF | 58% |
| IVSd | mm | FS | 30% |
| **Additional Information**: |  | | |
| No pericardial/Pleural effusion. | | | |
| **Final Diagnosis:** | | | |
| 1. Normal Echocardiography Study. | | | |
| **Remark**: | | | |
| **Recommendation**: | | | |
| SIGNATURE  Done by: Tesfaye T., Pediatrician, Pediatric Cardiologist _______________ 24/10/2013Eth.C | | | |

| Patient Name: **Bisrat Wassie**. Referring Institute: **Addis Alem PH**. SEX/ Age: M**/3 7/12**.  Date of Report: 25**/10/2013**. Referral Diagnosis: **Down Syndrome. AGH10.922** | | | |
| --- | --- | --- | --- |
| **Features** | **Finding** | **Features** | **Finding** |
| **Profile** |  | **Atria** |  |
| Abdominal situs | Solitus | Left atrium | Normal |
| Cardiac position | Levocardia | Right atrium | Normal |
| Systemic venous drainage | Normal. | **Atrioventricular valves** |  |
| Pulmonary venous drainage | Normal | Mitral valve | Annulus = 15mm |
| Atrioventricular connection | Concordant | Tricuspid valve | Annulus = 17mm  TAPSE = 21mm |
| Ventriculoarterial connection | Concordant | **Ventricles** |  |
| Ventricular loop | d-Loop | Left ventricle | Normal |
|  |  | Right ventricle | Normal |
| **Septae** |  | **Coronary arteries** | ----- |
| Interventricular septum | Intact | **Doppler Measurement** |  |
| Interatrial septum | Intact | Mitral | ----- |
| **Semilunal valves** |  | Aortic | ------- |
| Aortic valve | Annulus = 14mm | Tricuspid | ------- |
| Pulmonary valve | Annulus = 14mm | pulmonic | -------- |
| **Great arteries** | NRGA | **Aortic arch** | Left |
| Aorta | ----- | **PDA** | No |
| Pulmonary artery | Normal MPA and Branch PAs. |  |  |
| **M-Mode:** | | | |
| AO | mm | PWd | mm |
| LA | mm | EDV | ml |
| LVIDd | mm | ESV | ml |
| LVIDs | mm | LVEF | 63% |
| IVSd | mm | FS | 33% |
| **Additional Information**: |  | | |
| 8mm pericardial effusion on RA/RV Junction. | | | |
| **Final Diagnosis:** | | | |
| 1. {S, D, S} Levocardia. 2. Small Pericardial Effusion 3. Normal Biventricular Function | | | |
| **Remark**: | | | |
| **Recommendation**: | | | |
| SIGNATURE  Done by: Tesfaye T., Pediatrician, Pediatric Cardiologist _______________ 25/10/2013Eth.C | | | |

| Patient Name: **Mekdes Haile**. Referring Institute: **Injibara GH**. SEX/ Age: F**/7/12**.  Date of Report: **29/10/2013**. Referral Diagnosis: **RD + DS. AGH10.923** | | | |
| --- | --- | --- | --- |
| **Features** | **Finding** | **Features** | **Finding** |
| **Profile** |  | **Atria** |  |
| Abdominal situs | Solitus | Left atrium | Normal |
| Cardiac position | Levocardia | Right atrium | Dilated |
| Systemic venous drainage | Normal. | **Atrioventricular valves** |  |
| Pulmonary venous drainage | Normal | Mitral valve | Annulus = 9mm |
| Atrioventricular connection | Concordant | Tricuspid valve | Annulus = 14mm  TAPSE = 14mm |
| Ventriculoarterial connection | Concordant | **Ventricles** |  |
| Ventricular loop | d-Loop | Left ventricle | Normal |
|  |  | Right ventricle | Dilated |
| **Septae** |  | **Coronary arteries** | ----- |
| Interventricular septum | Intact | **Doppler Measurement** |  |
| Interatrial septum | 8mm Primum defect, L – R Shunt. Additional 8mm Fenestrated OS ASD, L – R Shunt | Mitral | Mild MR |
| **Semilunal valves** |  | Aortic | ------- |
| Aortic valve | Annulus = 11mm | Tricuspid | Moderate TR |
| Pulmonary valve | Annulus = 11mm | pulmonic | -------- |
| **Great arteries** | NRGA | **Aortic arch** | Left |
| Aorta | ----- | **PDA** | <1mm PDA, L – R Shunt |
| Pulmonary artery | Normal MPA and Branch PAs. |  |  |
| **M-Mode:** Normal LV Function on eye balling | | | |
| AO | mm | PWd | mm |
| LA | mm | EDV | ml |
| LVIDd | mm | ESV | ml |
| LVIDs | mm | LVEF | % |
| IVSd | mm | FS | % |
| **Additional Information**: |  | | |
| No pericardial/Pleural effusion. | | | |
| **Final Diagnosis:** | | | |
| 1. {S, D, S} Levocardia. 2. RA/RV Dilated 3. Partial AVSD, L – R Shunt 4. Additional Moderate OS ASD, L – R Shunt 5. Moderate TR 6. Mild MT 7. Small PDA, L – R Shunt (?Silent) 8. Normal Biventricular Function | | | |
| SIGNATURE  Done by: Tesfaye T., Pediatrician, Pediatric Cardiologist _______________ 29/10/2013Eth.C | | | |

| Patient Name: **Baby of Habtam Desalegn**. Referring Institute: **FHRH**. SEX/ Age: F**/14days**.  Date of Report: **29/10/2013**. Referral Diagnosis: **RD. AGH10.924** | | | |
| --- | --- | --- | --- |
| **Features** | **Finding** | **Features** | **Finding** |
| **Profile** |  | **Atria** |  |
| Abdominal situs | Solitus | Left atrium | Normal |
| Cardiac position | Levocardia | Right atrium | Normal |
| Systemic venous drainage | Normal. | **Atrioventricular valves** |  |
| Pulmonary venous drainage | Normal | Mitral valve | Annulus = 9mm |
| Atrioventricular connection | Concordant | Tricuspid valve | Annulus = 9mm |
| Ventriculoarterial connection | Concordant | **Ventricles** |  |
| Ventricular loop | d-Loop | Left ventricle | Normal |
|  |  | Right ventricle | Normal |
| **Septae** |  | **Coronary arteries** | ----- |
| Interventricular septum | Intact | **Doppler Measurement** |  |
| Interatrial septum | Intact | Mitral | ----- |
| **Semilunal valves** |  | Aortic | ------- |
| Aortic valve | Annulus = 8mm | Tricuspid | ------- |
| Pulmonary valve | Annulus = 8mm | pulmonic | -------- |
| **Great arteries** | NRGA | **Aortic arch** | Left |
| Aorta | ----- | **PDA** | No |
| Pulmonary artery | Normal MPA and Branch PAs. |  |  |
| **M-Mode:** Normal LV Function on eye balling. | | | |
| AO | mm | PWd | mm |
| LA | mm | EDV | ml |
| LVIDd | mm | ESV | ml |
| LVIDs | mm | LVEF | % |
| IVSd | mm | FS | % |
| **Additional Information**: |  | | |
| No pericardial/Pleural effusion. | | | |
| **Final Diagnosis:** | | | |
| 1. Normal Echocardiography Study. | | | |
| **Remark**: | | | |
| **Recommendation**: | | | |
| SIGNATURE  Done by: Tesfaye T., Pediatrician, Pediatric Cardiologist _______________ 29/10/2013Eth.C | | | |

| Patient Name: **Desta Asmamaw**. Referring Institute: **Adinas GH**. SEX/ Age: F**/7Years**.  Date of Report: **29/10/2013**. Referral Diagnosis: **ARF. AGH10.925** | | | |
| --- | --- | --- | --- |
| **Features** | **Finding** | **Features** | **Finding** |
| **Profile** |  | **Atria** |  |
| Abdominal situs | Solitus | Left atrium | Normal |
| Cardiac position | Levocardia | Right atrium | Normal |
| Systemic venous drainage | Normal. | **Atrioventricular valves** |  |
| Pulmonary venous drainage | Normal | Mitral valve | Annulus = 14mm |
| Atrioventricular connection | Concordant | Tricuspid valve | Annulus = 16mm  TAPSE = 18mm |
| Ventriculoarterial connection | Concordant | **Ventricles** |  |
| Ventricular loop | d-Loop | Left ventricle | Normal |
|  |  | Right ventricle | Normal |
| **Septae** |  | **Coronary arteries** | ----- |
| Interventricular septum | Intact | **Doppler Measurement** |  |
| Interatrial septum | Intact | Mitral | ----- |
| **Semilunal valves** |  | Aortic | ------- |
| Aortic valve | Annulus = 15mm | Tricuspid | ------- |
| Pulmonary valve | Annulus = 15mm | pulmonic | -------- |
| **Great arteries** | NRGA | **Aortic arch** | Left |
| Aorta | ----- | **PDA** | No |
| Pulmonary artery | Normal MPA and Branch PAs. |  |  |
| **M-Mode:** | | | |
| AO | mm | PWd | mm |
| LA | mm | EDV | ml |
| LVIDd | mm | ESV | ml |
| LVIDs | mm | LVEF | 60% |
| IVSd | mm | FS | 31% |
| **Additional Information**: |  | | |
| No pericardial/Pleural effusion. | | | |
| **Final Diagnosis:** | | | |
| 1. Normal Echocardiography Study. | | | |
| **Remark**: | | | |
| **Recommendation**: | | | |
| SIGNATURE  Done by: Tesfaye T., Pediatrician, Pediatric Cardiologist _______________ 29/10/2013Eth.C | | | |

| Patient Name: **Mebe’a Dagne**. Referring Institute: **FHRH**. SEX/ Age: M**/2/12**.  Date of Report: **29/10/2013**.  Referral Diagnosis: **___. AGH10.873** | | | |
| --- | --- | --- | --- |
| **Follow up echo (PFO, Small VSD, PDA (L – R Shunt)** | | | |
| **Features** | **Finding** | **Features** | **Finding** |
| **Profile** |  | **Atria** |  |
| Abdominal situs | Solitus | Left atrium | Normal |
| Cardiac position | Levocardia | Right atrium | Dilated |
| Systemic venous drainage | Normal. | **Atrioventricular valves** |  |
| Pulmonary venous drainage | Normal | Mitral valve | Annulus = 9mm |
| Atrioventricular connection | Concordant | Tricuspid valve | Annulus = 15mm  TAPSE = 12mm |
| Ventriculoarterial connection | Concordant | **Ventricles** |  |
| Ventricular loop | d-Loop | Left ventricle | Normal |
|  |  | Right ventricle | Dilated |
| **Septae** |  | **Coronary arteries** | ----- |
| Interventricular septum | 4mm PM VSD, BD Shunt | **Doppler Measurement** |  |
| Interatrial septum | Intact | Mitral | ----- |
| **Semilunal valves** |  | Aortic | ------- |
| Aortic valve | Annulus = 9mm | Tricuspid | ------- |
| Pulmonary valve | Annulus = 12mm | pulmonic | -------- |
| **Great arteries** | NRGA | **Aortic arch** | Left |
| Aorta | ----- | **PDA** | 1mm PDA, R – L Shunt |
| Pulmonary artery | MPA =14mm. |  |  |
| **M-Mode:** Normal LV Function on eye balling. | | | |
| AO | mm | PWd | mm |
| LA | mm | EDV | ml |
| LVIDd | mm | ESV | ml |
| LVIDs | mm | LVEF | % |
| IVSd | mm | FS | % |
| **Additional Information**: |  | | |
| No pericardial/Pleural effusion. | | | |
| **Final Diagnosis:** | | | |
| 1. {S, D, S} Levocardia. 2. RA/RV Dilated 3. Small PM VSD, BD Shunt 4. Small PDA, R – L Shunt 5. Severe Pulmonary Hypertension 6. Normal Function | | | |
| **Remark**: the pulmonary Hypertension is Less likely to be caused by the underlying cardiac problem. | | | |
| **Recommendation**: Investigate in the line of Pulmonary Hypertension caused by conditions other than underlying Cardiac Problem (10, PPHN,…….. | | | |
| SIGNATURE  Done by: Tesfaye T., Pediatrician, Pediatric Cardiologist _______________ 29/10/2013Eth.C | | | |

| Patient Name: **Mekdes Abebe**. Referring Institute: **FHRH**. SEX/ Age: F**/1/12**.  Date of Report: **29/10/2013**. Referral Diagnosis: **Cyanosis. AGH10.926** | | | |
| --- | --- | --- | --- |
| **Features** | **Finding** | **Features** | **Finding** |
| **Profile** |  | **Atria** |  |
| Abdominal situs | Solitus | Left atrium | Normal |
| Cardiac position | Levocardia | Right atrium | Normal |
| Systemic venous drainage | Normal. | **Atrioventricular valves** |  |
| Pulmonary venous drainage | Normal | Mitral valve | Annulus = 12mm |
| Atrioventricular connection | Concordant | Tricuspid valve | Annulus = 17mm |
| Ventriculoarterial connection | DORV | **Ventricles** |  |
| Ventricular loop | d-Loop | Left ventricle | Normal |
|  |  | Right ventricle | Normal |
| **Septae** |  | **Coronary arteries** | ----- |
| Interventricular septum | Non restrictive Sub arterial (Pulmonic) VSD, L – R Shunt | **Doppler Measurement** |  |
| Interatrial septum | PFO, L – R Shunt | Mitral | ----- |
| **Semilunal valves** |  | Aortic | ------- |
| Aortic valve | Annulus = 9mm | Tricuspid | ------- |
| Pulmonary valve | Annulus = 12mm | pulmonic | -------- |
| **Great arteries** | d-TGA | **Aortic arch** | Left |
| Aorta | Anterior and to the right. From RV | **PDA** | No |
| Pulmonary artery | Posterior and to the left. From RV |  |  |
| **M-Mode:** | | | |
| AO | mm | PWd | mm |
| LA | mm | EDV | ml |
| LVIDd | mm | ESV | ml |
| LVIDs | mm | LVEF | % |
| IVSd | mm | FS | % |
| **Additional Information**: |  | | |
| No pericardial/Pleural effusion. | | | |
| **Final Diagnosis:** | | | |
| 1. {S, D, D} Levocardia. 2. PFO, L – R Shunt 3. DORV with Sub Pulmonic VSD 4. d-TGA | | | |
| **Remark**: Taussig Bing Anomaly | | | |
| **Recommendation**: | | | |
| SIGNATURE  Done by: Tesfaye T., Pediatrician, Pediatric Cardiologist _______________ 29/10/2013Eth.C | | | |

| Patient Name: **Yosef Meseret**. Referring Institute: **Adinas GH**. SEX/ Age: M**/5Years**.  Date of Report: **29/10/2013**. Referral Diagnosis: **Incidental Murmur. AGH10.927** | | | |
| --- | --- | --- | --- |
| **Features** | **Finding** | **Features** | **Finding** |
| **Profile** |  | **Atria** |  |
| Abdominal situs | Solitus | Left atrium | Mildly dilated |
| Cardiac position | Levocardia | Right atrium | Normal |
| Systemic venous drainage | Normal. | **Atrioventricular valves** |  |
| Pulmonary venous drainage | Normal | Mitral valve | Annulus = 19mm |
| Atrioventricular connection | Concordant | Tricuspid valve | Annulus = 17mm  TAPSE = mm |
| Ventriculoarterial connection | Concordant | **Ventricles** |  |
| Ventricular loop | d-Loop | Left ventricle | Mildly Dilated |
|  |  | Right ventricle | Normal |
| **Septae** |  | **Coronary arteries** | ----- |
| Interventricular septum | 5mm Sub-Aortic VSD, L – R Shunt | **Doppler Measurement** |  |
| Interatrial septum | Intact | Mitral | ----- |
| **Semilunal valves** |  | Aortic | Mild AR |
| Aortic valve | Annulus = 17mm | Tricuspid | ------- |
| Pulmonary valve | Annulus = 18mm | pulmonic | -------- |
| **Great arteries** | NRGA | **Aortic arch** | Left |
| Aorta | ----- | **PDA** | No |
| Pulmonary artery | Normal MPA and Branch PAs. |  |  |
| **M-Mode:** | | | |
| AO | mm | PWd | mm |
| LA | mm | EDV | ml |
| LVIDd | mm | ESV | ml |
| LVIDs | mm | LVEF | 64% |
| IVSd | mm | FS | 35% |
| **Additional Information**: |  | | |
| No pericardial/Pleural effusion. | | | |
| **Final Diagnosis:** | | | |
| 1. {S, D, S} Levocardia. 2. Small Subaortic VSD, L – R Shunt 3. Mild MR | | | |
| **Remark**: | | | |
| **Recommendation**: | | | |
| SIGNATURE  Done by: Tesfaye T., Pediatrician, Pediatric Cardiologist _______________ 29/10/2013Eth.C | | | |

| Patient Name: **Weineshet Bukayaw**. Referring Institute: **FHRH**. SEX/ Age: F**/1 2/12.**  Date of Report: **30/10/2013**. R.Dx: **RD + Cyanosis. AGH10.928** | | | |
| --- | --- | --- | --- |
| **Features** | **Finding** | **Features** | **Finding** |
| **Profile** |  | **Atria** |  |
| Abdominal situs | Solitus | Left atrium | Smallish |
| Cardiac position | Levocardia | Right atrium | Dilated |
| Systemic venous drainage | Normal. | **Atrioventricular valves** | |
| Pulmonary venous drainage | Create venous confluent posterior to the LA, drain to Innominate to SVC to RA | Mitral valve | Annulus = 12mm. Dysplastic |
| Atrioventricular connection | DIRV | Tricuspid valve | Annulus = 18mm  TAPSE = 11mm |
| Ventriculoarterial connection | DORV | **Ventricles** |  |
| Ventricular loop | d-Loop | Left ventricle | Smallish |
|  |  | Right ventricle | Dilated |
| **Septae** |  | **Coronary arteries** | ----- |
| Interventricular septum | 27mm Inlet VSD, L – R Shunt, amounting to single ventricle | **Doppler Measurement** |  |
| Interatrial septum | 9mm OS ASD, BD Shunt predominantly R – L. | Mitral | Mild MR |
| **Semilunal valves** |  | Aortic | ------- |
| Aortic valve | Annulus = 9mm | Tricuspid | Trivial TR |
| Pulmonary valve | Annulus = 11mm. Doming PV | pulmonic | Severe PS, PPG = 63mmHg |
| **Great arteries** | d-TGA | **Aortic arch** | Left |
| Aorta | Anterior to PA and to the right. From RV | **PDA** | No |
| Pulmonary artery | Posterior to the aorta and to the left. From RV |  |  |
| **M-Mode:** | | | |
| AO | mm | PWd | mm |
| LA | mm | EDV | ml |
| LVIDd | mm | ESV | ml |
| LVIDs | mm | LVEF | % |
| IVSd | mm | FS | % |
| **Additional Information**: |  | | |
| No pericardial/Pleural effusion. | | | |
| **Final Diagnosis:** | | | |
| 1. {S, D, D} Levocardia. 2. ?TAPVC 3. Moderate OS ASD, BD Shunt Predominantly R – L 4. DIRV 5. DORV 6. d-TGA 7. Large Inlet VSD, L – R Shunt amounting to single ventricle 8. Smallish LV 9. Dysplastic MV 10. Severe PS 11. Doming PV | | | |
| **Remark**: | | | |
| **Recommendation**: | | | |
| SIGNATURE  Done by: Tesfaye T., Pediatrician, Pediatric Cardiologist _______________ 30/10/2013Eth.C | | | |

| Patient Name: **Kale-wengel Desalegn**. Referring Institute: **TGSH**. SEX/ Age: M**/3 4/12**.  Date of Report: **01/11/2013**. Referral Diagnosis: **Pre-op screening. AGH10.929** | | | |
| --- | --- | --- | --- |
| **Features** | **Finding** | **Features** | **Finding** |
| **Profile** |  | **Atria** |  |
| Abdominal situs | Solitus | Left atrium | Normal |
| Cardiac position | Levocardia | Right atrium | Normal |
| Systemic venous drainage | Normal. | **Atrioventricular valves** |  |
| Pulmonary venous drainage | Normal | Mitral valve | Annulus = 16mm |
| Atrioventricular connection | Concordant | Tricuspid valve | Annulus = 17mm  TAPSE = 16mm |
| Ventriculoarterial connection | Concordant | **Ventricles** |  |
| Ventricular loop | d-Loop | Left ventricle | Normal |
|  |  | Right ventricle | Normal |
| **Septae** |  | **Coronary arteries** | ----- |
| Interventricular septum | Intact | **Doppler Measurement** |  |
| Interatrial septum | Intact | Mitral | ----- |
| **Semilunal valves** |  | Aortic | ------- |
| Aortic valve | Annulus = 14mm | Tricuspid | ------- |
| Pulmonary valve | Annulus = 16mm | pulmonic | -------- |
| **Great arteries** | NRGA | **Aortic arch** | Left |
| Aorta | ----- | **PDA** | No |
| Pulmonary artery | Normal MPA and Branch PAs. |  |  |
| **M-Mode:** | | | |
| AO | mm | PWd | 5mm |
| LA | mm | EDV | 46ml |
| LVIDd | 34mm | ESV | 14ml |
| LVIDs | 20mm | LVEF | 70% |
| IVSd | 4mm | FS | 38% |
| IVSs | 9mm | PWs | 8mm |
| **Additional Information**: |  | | |
| No pericardial/Pleural effusion. | | | |
| **Final Diagnosis:** | | | |
| 1. Normal Echocardiography Study. | | | |
| **Remark**: | | | |
| **Recommendation**: | | | |
| SIGNATURE  Done by: Tesfaye T., Pediatrician, Pediatric Cardiologist _______________ 01/11/2013Eth.C | | | |

| Patient Name: **Werknesh Demeke**. Referring Institute: **Addis Zemen PH**. SEX/ Age: F**/8Years**.  Date of Report: **06/11/2013**. Referral Diagnosis: **R/O CRVHD. AGH10.930** | | | |
| --- | --- | --- | --- |
| **Features** | **Finding** | **Features** | **Finding** |
| **Profile** |  | **Atria** |  |
| Abdominal situs | Solitus | Left atrium | Normal |
| Cardiac position | Levocardia | Right atrium | Normal |
| Systemic venous drainage | Normal. | **Atrioventricular valves** |  |
| Pulmonary venous drainage | Normal | Mitral valve | Annulus = 17mm |
| Atrioventricular connection | Concordant | Tricuspid valve | Annulus = 18mm  TAPSE = 17mm |
| Ventriculoarterial connection | Concordant | **Ventricles** |  |
| Ventricular loop | d-Loop | Left ventricle | Normal |
|  |  | Right ventricle | Normal |
| **Septae** |  | **Coronary arteries** | ----- |
| Interventricular septum | Intact | **Doppler Measurement** |  |
| Interatrial septum | Intact | Mitral | ----- |
| **Semilunal valves** |  | Aortic | ------- |
| Aortic valve | Annulus = 14mm | Tricuspid | ------- |
| Pulmonary valve | Annulus = 18mm | pulmonic | -------- |
| **Great arteries** | NRGA | **Aortic arch** | Left |
| Aorta | ----- | **PDA** | No |
| Pulmonary artery | Normal MPA and Branch PAs. |  |  |
| **M-Mode:** | | | |
| AO | mm | PWd | mm |
| LA | mm | EDV | ml |
| LVIDd | mm | ESV | ml |
| LVIDs | mm | LVEF | 69% |
| IVSd | mm | FS | 38% |
| **Additional Information**: |  | | |
| No pericardial/Pleural effusion. | | | |
| **Final Diagnosis:** | | | |
| 1. Normal Echocardiography Study. | | | |
| **Remark**: | | | |
| **Recommendation**: | | | |
| SIGNATURE  Done by: Tesfaye T., Pediatrician, Pediatric Cardiologist _______________ 06/11/2013Eth.C | | | |

| Patient Name: **Baby of Banchalem Adella**. Referring Institute: **FHRH**. SEX/ Age: M**/39days**.  Date of Report: **06/11/2013**. Referral Diagnosis: **R/O CHD (? VSD) + Incidental Murmur. AGH10.931** | | | |
| --- | --- | --- | --- |
| **Features** | **Finding** | **Features** | **Finding** |
| **Profile** |  | **Atria** |  |
| Abdominal situs | Solitus | Left atrium | Normal |
| Cardiac position | Levocardia | Right atrium | Normal |
| Systemic venous drainage | Normal. | **Atrioventricular valves** |  |
| Pulmonary venous drainage | Normal | Mitral valve | Annulus = 11mm |
| Atrioventricular connection | Concordant | Tricuspid valve | Annulus = 12mm |
| Ventriculoarterial connection | Concordant | **Ventricles** |  |
| Ventricular loop | d-Loop | Left ventricle | Normal |
|  |  | Right ventricle | Normal |
| **Septae** |  | **Coronary arteries** | ----- |
| Interventricular septum | 4mm PM VSD, L – R Shunt, partially covered by STL | **Doppler Measurement** |  |
| Interatrial septum | Intact | Mitral | ----- |
| **Semilunal valves** |  | Aortic | ------- |
| Aortic valve | Annulus = 10mm | Tricuspid | ------- |
| Pulmonary valve | Annulus = 11mm | pulmonic | Mild PS, PPG = 21mmHg |
| **Great arteries** | NRGA | **Aortic arch** | Left |
| Aorta | ----- | **PDA** | No |
| Pulmonary artery | Normal MPA and Branch PAs. |  |  |
| **M-Mode:** Normal LV Function on eye balling. | | | |
| AO | mm | PWd | mm |
| LA | mm | EDV | ml |
| LVIDd | mm | ESV | ml |
| LVIDs | mm | LVEF | % |
| IVSd | mm | FS | % |
| **Additional Information**: |  | | |
| No pericardial/Pleural effusion. | | | |
| **Final Diagnosis:** | | | |
| 1. {S, D, S} Levocardia. 2. Small PM VSD, L – R Shunt 3. Mild PS 4. Normal LV Function | | | |
| **Remark**: | | | |
| **Recommendation**: | | | |
| SIGNATURE  Done by: Tesfaye T., Pediatrician, Pediatric Cardiologist _______________ 06/11/2013Eth.C | | | |

| Patient Name: **Betsebay Asnake**. Referring Institute: **Adinas GH**. SEX/ Age: F**/8/12**.  Date of Report: **06/11/2013**.  Referral Diagnosis: **CHD (Moderate PM VSD, Doming PV, Mild Valvular PS).** | | | |
| --- | --- | --- | --- |
| **Features** | **Finding** | **Features** | **Finding** |
| **Profile** |  | **Atria** |  |
| Abdominal situs | Solitus | Left atrium | Normal |
| Cardiac position | Levocardia | Right atrium | Normal |
| Systemic venous drainage | Normal. | **Atrioventricular valves** |  |
| Pulmonary venous drainage | Normal | Mitral valve | Annulus = 13mm |
| Atrioventricular connection | Concordant | Tricuspid valve | Annulus = 14mm  TAPSE = 15mm |
| Ventriculoarterial connection | Concordant | **Ventricles** |  |
| Ventricular loop | d-Loop | Left ventricle | Normal |
|  |  | Right ventricle | Normal |
| **Septae** |  | **Coronary arteries** | ----- |
| Interventricular septum | 4mm PM VSD, L – R Shunt. Partially covered by STL | **Doppler Measurement** |  |
| Interatrial septum | Intact | Mitral | ----- |
| **Semilunal valves** |  | Aortic | ------- |
| Aortic valve | Annulus = 12mm | Tricuspid | ------- |
| Pulmonary valve | Annulus = 13mm. Doming. | pulmonic | Mild PS, PPG = 24mmHg |
| **Great arteries** | NRGA | **Aortic arch** | Left |
| Aorta | ----- | **PDA** | No |
| Pulmonary artery | Normal MPA and Branch PAs. |  |  |
| **M-Mode:** | | | |
| AO | mm | PWd | mm |
| LA | mm | EDV | ml |
| LVIDd | mm | ESV | ml |
| LVIDs | mm | LVEF | 74% |
| IVSd | mm | FS | 41% |
| **Additional Information**: |  | | |
| No pericardial/Pleural effusion. | | | |
| **Final Diagnosis:** | | | |
| 1. {S, D, S} Levocardia. 2. Small PM VSD, L – R Shunt 3. Doming Pulmonary Valve 4. Mild Valvular PS 5. Normal Biventricular Function | | | |
| **Remark**: | | | |
| **Recommendation**: | | | |
| SIGNATURE  Done by: Tesfaye T., Pediatrician, Pediatric Cardiologist _______________ 06/11/2013Eth.C | | | |

| Patient Name: **Ikram Abebe**. Referring Institute: **Adinas GH**. SEX/ Age: F**/2 6/12**.  Date of Report: **06/11/2013**. Referral Diagnosis: **CHD (? VSD) + CHF. AGH10.932** | | | |
| --- | --- | --- | --- |
| **Features** | **Finding** | **Features** | **Finding** |
| **Profile** |  | **Atria** |  |
| Abdominal situs | Solitus | Left atrium | Normal |
| Cardiac position | Levocardia | Right atrium | Normal |
| Systemic venous drainage | Normal. | **Atrioventricular valves** |  |
| Pulmonary venous drainage | Normal | Mitral valve | Annulus = 19mm |
| Atrioventricular connection | Concordant | Tricuspid valve | Annulus = 19mm  TAPSE = 24mm |
| Ventriculoarterial connection | Truncus | **Ventricles** |  |
| Ventricular loop | d-Loop | Left ventricle | Normal |
|  |  | Right ventricle | Normal |
| **Septae** |  | **Coronary arteries** | ----- |
| Interventricular septum | Non-Restrictive Malaligned sub arterial VSD, BD Shunt | **Doppler Measurement** |  |
| Interatrial septum | Intact | Mitral | ----- |
| **Semilunal valves** |  | Aortic | ------- |
| Aortic valve | Truncus over-riding VSD. Annulus = 17mm | Tricuspid | Mild TR |
| Pulmonary valve | Arising from a truncus as a trunck | pulmonic | -------- |
| **Great arteries** | -------- | **Aortic arch** | Left |
| Aorta | ----- | **PDA** | No |
| Pulmonary artery | Not visualized |  |  |
| **M-Mode:** | | | |
| AO | mm | PWd | mm |
| LA | mm | EDV | ml |
| LVIDd | mm | ESV | ml |
| LVIDs | mm | LVEF | % |
| IVSd | mm | FS | % |
| **Additional Information**: |  | | |
| No pericardial/Pleural effusion. | | | |
| **Final Diagnosis:** | | | |
| 1. {S, D, S} Levocardia. 2. Non-Restrictive Malaligned Sub arterial VSD, BD Shunt 3. Truncus arteriosus | | | |
| **Remark**: Child was restless and crying during study. Deformed chest | | | |
| **Recommendation**: Needs additional Imaging | | | |
| SIGNATURE  Done by: Tesfaye T., Pediatrician, Pediatric Cardiologist _______________ 06/11/2013Eth.C | | | |

| Patient Name: **Halid Tesfaw**. Referring Institute: **Mekane-Selam PH**. SEX/ Age: M**/60days**.  Date of Report: **07/11/2013**. Referral Diagnosis: **DS. AGH10.933** | | | |
| --- | --- | --- | --- |
| **Features** | **Finding** | **Features** | **Finding** |
| **Profile** |  | **Atria** |  |
| Abdominal situs | Solitus | Left atrium | Normal |
| Cardiac position | Levocardia | Right atrium | Normal |
| Systemic venous drainage | Normal. | **Atrioventricular valves** |  |
| Pulmonary venous drainage | Normal | Mitral valve | Annulus = 10mm |
| Atrioventricular connection | Concordant | Tricuspid valve | Annulus = 10mm  TAPSE = 11mm |
| Ventriculoarterial connection | Concordant | **Ventricles** |  |
| Ventricular loop | d-Loop | Left ventricle | Normal |
|  |  | Right ventricle | Normal |
| **Septae** |  | **Coronary arteries** | ----- |
| Interventricular septum | Intact | **Doppler Measurement** |  |
| Interatrial septum | PFO, L – R Shunt | Mitral | ----- |
| **Semilunal valves** |  | Aortic | ------- |
| Aortic valve | Annulus = 9mm | Tricuspid | ------- |
| Pulmonary valve | Annulus = 10mm | pulmonic | -------- |
| **Great arteries** | NRGA | **Aortic arch** | Left |
| Aorta | ----- | **PDA** | No |
| Pulmonary artery | Normal MPA and Branch PAs. |  |  |
| **M-Mode:** | | | |
| AO | mm | PWd | mm |
| LA | mm | EDV | ml |
| LVIDd | mm | ESV | ml |
| LVIDs | mm | LVEF | 69% |
| IVSd | mm | FS | 36% |
| **Additional Information**: |  | | |
| No pericardial/Pleural effusion. | | | |
| **Final Diagnosis:** | | | |
| 1. {S, D, S} Levocardia. 2. PFO, L – R Shunt 3. Normal Biventricular Function | | | |
| **Remark**: | | | |
| **Recommendation**: | | | |
| SIGNATURE  Done by: Tesfaye T., Pediatrician, Pediatric Cardiologist _______________ 07/11/2013Eth.C | | | |

| Patient Name: **Lidya Melkamu**. Referring Institute: **Finote-Selam GH**. SEX/ Age: F**/3/12**.  Date of Report: **07/11/2013**. Referral Diagnosis: **RD + CHF. AGH10.934** | | | |
| --- | --- | --- | --- |
| **Features** | **Finding** | **Features** | **Finding** |
| **Profile** |  | **Atria** |  |
| Abdominal situs | Solitus | Left atrium | Normal |
| Cardiac position | Levocardia | Right atrium | Normal |
| Systemic venous drainage | Normal. | **Atrioventricular valves** |  |
| Pulmonary venous drainage | Normal | Mitral valve | Annulus = 11mm |
| Atrioventricular connection | Concordant | Tricuspid valve | Annulus = 11mm  TAPSE = 13mm |
| Ventriculoarterial connection | Concordant | **Ventricles** |  |
| Ventricular loop | d-Loop | Left ventricle | Normal |
|  |  | Right ventricle | Normal |
| **Septae** |  | **Coronary arteries** | ----- |
| Interventricular septum | 8mm PM VSD, L – R Shunt, partially covered by STL. | **Doppler Measurement** |  |
| Interatrial septum | Intact | Mitral | ----- |
| **Semilunal valves** |  | Aortic | ------- |
| Aortic valve | Annulus = 11mm | Tricuspid | Trivial TR |
| Pulmonary valve | Annulus = 11mm | pulmonic | Mild PR, PPG = 43mmHg |
| **Great arteries** | NRGA | **Aortic arch** | Left |
| Aorta | ----- | **PDA** | <1mm PDA, L – R Shunt |
| Pulmonary artery | Normal MPA and Branch PAs. |  |  |
| **M-Mode:** | | | |
| AO | mm | PWd | mm |
| LA | mm | EDV | ml |
| LVIDd | mm | ESV | ml |
| LVIDs | mm | LVEF | 75% |
| IVSd | mm | FS | 41% |
| **Additional Information**: |  | | |
| No pericardial/Pleural effusion. | | | |
| **Final Diagnosis:** | | | |
| 1. {S, D, S} Levocardia. 2. Moderate to Large PM VSD, L – R Shunt 3. Small (? Silent) PDA, L – R Shunt 4. Mild Pulmonary Hypertension 5. Normal Biventricular Function | | | |
| **Remark**: | | | |
| **Recommendation**: | | | |
| SIGNATURE  Done by: Tesfaye T., Pediatrician, Pediatric Cardiologist _______________ 07/11/2013Eth.C | | | |

| Patient Name: **Meklit Shimelash**. Referring Institute: **FHRH**. SEX/ Age: F**/4months**.  Date of Report: **07/11/2013**. Referral Diagnosis: **RD. AGH10.935** | | | |
| --- | --- | --- | --- |
| **Features** | **Finding** | **Features** | **Finding** |
| **Profile** |  | **Atria** |  |
| Abdominal situs | Solitus | Left atrium | Dilated |
| Cardiac position | Levocardia | Right atrium | Normal |
| Systemic venous drainage | Normal. | **Atrioventricular valves** |  |
| Pulmonary venous drainage | Normal | Mitral valve | Annulus = 14mm |
| Atrioventricular connection | Concordant | Tricuspid valve | Annulus = 13mm |
| Ventriculoarterial connection | Concordant | **Ventricles** |  |
| Ventricular loop | d-Loop | Left ventricle | Dilated |
|  |  | Right ventricle | Normal |
| **Septae** |  | **Coronary arteries** | ----- |
| Interventricular septum | 4mm Inlet VSD with PM extension, L – R Shunt with a gradient of 60mmHg | **Doppler Measurement** |  |
| Interatrial septum | Intact | Mitral | ----- |
| **Semilunal valves** |  | Aortic | ------- |
| Aortic valve | Annulus = 8mm | Tricuspid | ------- |
| Pulmonary valve | Annulus = 12mm | pulmonic | -------- |
| **Great arteries** | NRGA | **Aortic arch** | Left |
| Aorta | ----- | **PDA** | No |
| Pulmonary artery | Normal MPA and Branch PAs. |  |  |
| **M-Mode:** | | | |
| AO | mm | PWd | mm |
| LA | mm | EDV | ml |
| LVIDd | mm | ESV | ml |
| LVIDs | mm | LVEF | 67% |
| IVSd | mm | FS | 36% |
| **Additional Information**: |  | | |
| No pericardial/Pleural effusion. | | | |
| **Final Diagnosis:** | | | |
| 1. {S, D, S} Levocardia. 2. LA/LV Dilated 3. Moderate Inlet VSD with PM extension, L – R Shunt 4. Normal LV Function | | | |
| **Remark**: | | | |
| **Recommendation**: | | | |
| SIGNATURE  Done by: Tesfaye T., Pediatrician, Pediatric Cardiologist _______________ 07/11/2013Eth.C | | | |

| Patient Name: **Baby of Addisie Mersha**. Referring Institute: **FHRH**. SEX/ Age: M**/13days**.  Date of Report: **07/11/2013**. Referral Diagnosis: **DS. AGH10.936** | | | |
| --- | --- | --- | --- |
| **Features** | **Finding** | **Features** | **Finding** |
| **Profile** |  | **Atria** |  |
| Abdominal situs | Solitus | Left atrium | Normal |
| Cardiac position | Levocardia | Right atrium | Normal |
| Systemic venous drainage | Normal. | **Atrioventricular valves** |  |
| Pulmonary venous drainage | Normal | Mitral valve | Annulus = 10mm |
| Atrioventricular connection | Concordant | Tricuspid valve | Annulus = 11mm |
| Ventriculoarterial connection | Concordant | **Ventricles** |  |
| Ventricular loop | d-Loop | Left ventricle | Normal |
|  |  | Right ventricle | Normal |
| **Septae** |  | **Coronary arteries** | ----- |
| Interventricular septum | Intact | **Doppler Measurement** |  |
| Interatrial septum | 4mm OS ASD, L – R Shunt | Mitral | ----- |
| **Semilunal valves** |  | Aortic | ------- |
| Aortic valve | Annulus = 8mm | Tricuspid | ------- |
| Pulmonary valve | Annulus = 9mm | pulmonic | -------- |
| **Great arteries** | NRGA | **Aortic arch** | Left |
| Aorta | ----- | **PDA** | No |
| Pulmonary artery | Normal MPA and Branch PAs. |  |  |
| **M-Mode:** Normal LV Function on eye balling. | | | |
| AO | mm | PWd | mm |
| LA | mm | EDV | ml |
| LVIDd | mm | ESV | ml |
| LVIDs | mm | LVEF | % |
| IVSd | mm | FS | % |
| **Additional Information**: |  | | |
| No pericardial/Pleural effusion. | | | |
| **Final Diagnosis:** | | | |
| 1. {S, D, S} Levocardia. 2. Small OS ASD, L – R Shunt | | | |
| **Remark**: | | | |
| **Recommendation**: | | | |
| SIGNATURE  Done by: Tesfaye T., Pediatrician, Pediatric Cardiologist _______________ 07/11/2013Eth.C | | | |

| Patient Name: **Marelign Desalew**. Referring Institute: **FHRH**. SEX/ Age: M**/3Years**.  Date of Report: **07/11/2013**. Referral Diagnosis: **Incidental Murmur. AGH10.937** | | | |
| --- | --- | --- | --- |
| **Features** | **Finding** | **Features** | **Finding** |
| **Profile** |  | **Atria** |  |
| Abdominal situs | Solitus | Left atrium | Normal |
| Cardiac position | Levocardia | Right atrium | Normal |
| Systemic venous drainage | Normal. | **Atrioventricular valves** |  |
| Pulmonary venous drainage | Normal | Mitral valve | Annulus = 18mm |
| Atrioventricular connection | Concordant | Tricuspid valve | Annulus = 18mm  TAPSE = 19mm |
| Ventriculoarterial connection | Concordant | **Ventricles** |  |
| Ventricular loop | d-Loop | Left ventricle | Normal |
|  |  | Right ventricle | Normal |
| **Septae** |  | **Coronary arteries** | ----- |
| Interventricular septum | 3mm Mid Muscular VSD, L – R Shunt, PPG = 60mmHg | **Doppler Measurement** |  |
| Interatrial septum | Intact | Mitral | ----- |
| **Semilunal valves** |  | Aortic | ------- |
| Aortic valve | Annulus = 15mm | Tricuspid | ------- |
| Pulmonary valve | Annulus = 18mm | pulmonic | -------- |
| **Great arteries** | NRGA | **Aortic arch** | Left |
| Aorta | ----- | **PDA** | No |
| Pulmonary artery | Normal MPA and Branch PAs. |  |  |
| **M-Mode:** Normal LV Function on eye balling | | | |
| AO | mm | PWd | mm |
| LA | mm | EDV | ml |
| LVIDd | mm | ESV | ml |
| LVIDs | mm | LVEF | % |
| IVSd | mm | FS | % |
| **Additional Information**: |  | | |
| No pericardial/Pleural effusion. | | | |
| **Final Diagnosis:** | | | |
| 1. {S, D, S} Levocardia. 2. Small Restrictive Mid Muscular VSD, L – R Shunt 3. Normal Biventricular Function | | | |
| **Remark**: | | | |
| **Recommendation**: | | | |
| SIGNATURE  Done by: Tesfaye T., Pediatrician, Pediatric Cardiologist _______________ 07/11/2013Eth.C | | | |

| Patient Name: **Fikir Temesgen**. Referring Institute: **Adinas GH**. SEX/ Age: M**/1 1/12**.  Date of Report: **07/11/2013**.  Referral Diagnosis: **Incidental.** | | | |
| --- | --- | --- | --- |
| **Features** | **Finding** | **Features** | **Finding** |
| **Profile** |  | **Atria** |  |
| Abdominal situs | Solitus | Left atrium | Normal |
| Cardiac position | Levocardia | Right atrium | Normal |
| Systemic venous drainage | Normal. | **Atrioventricular valves** |  |
| Pulmonary venous drainage | Normal | Mitral valve | Annulus = 14mm |
| Atrioventricular connection | Concordant | Tricuspid valve | Annulus = 16mm |
| Ventriculoarterial connection | Concordant | **Ventricles** |  |
| Ventricular loop | d-Loop | Left ventricle | Normal |
|  |  | Right ventricle | Normal |
| **Septae** |  | **Coronary arteries** | ----- |
| Interventricular septum | 3mm PM VSD, L – R Shunt with gradient of 60mmHg | **Doppler Measurement** |  |
| Interatrial septum | Intact | Mitral | ----- |
| **Semilunal valves** |  | Aortic | ------- |
| Aortic valve | Annulus = 12mm | Tricuspid | ------- |
| Pulmonary valve | Annulus = 13mm | pulmonic | -------- |
| **Great arteries** | NRGA | **Aortic arch** | Left |
| Aorta | ----- | **PDA** | No |
| Pulmonary artery | Normal MPA and Branch PAs. |  |  |
| **M-Mode:** Normal LV Function on eye balling | | | |
| AO | mm | PWd | mm |
| LA | mm | EDV | ml |
| LVIDd | mm | ESV | ml |
| LVIDs | mm | LVEF | % |
| IVSd | mm | FS | % |
| **Additional Information**: |  | | |
| No pericardial/Pleural effusion. | | | |
| **Final Diagnosis:** | | | |
| 1. {S, D, S} Levocardia. 2. Small Restrictive PM VSD, L – R Shunt 3. Normal LV Function | | | |
[truncated: 1,091,933 more chars]
