# Supplementary material for: Two–Dimensional and Doppler trans-thoracic echocardiographic patterns of suspected pediatric heart diseases at Tibebe-—Ghion specialized Teaching Hospital and Adinas General Hospital, Bahir Dar, North-west Ethiopia:–An experience from an LMIC
Source: PLoS One. 2024 Mar 11;19(3):e0292694. doi: 10.1371/journal.pone.0292694 (PMC10927071; doi:10.1371/journal.pone.0292694)
Supplement: S2 File — (ZIP) [file pone.0292694.s003.zip › AGH12 Paediatric Echocardiography Report March 2023 - June 2023.docx]

| Patient Name: **Abebech Azanaw**. Referring Institute: **Debre – Tabour GH**. SEX/ Age: **F/9years**. Date of Report: **30/06/15**.  Referral Diagnosis: **NYHA Class IV CHF + Rheumatic Recurrence. AGH12.3344** | | | |
| --- | --- | --- | --- |
| **Features** | **Finding** | **Features** | **Finding** |
| **Profile** |  | **Atria** |  |
| Abdominal situs | Solitus | Left atrium | Dilated |
| Cardiac position | Levocardia | Right atrium | Normal |
| Systemic venous drainage | Normal. | **Atrioventricular valves** |  |
| Pulmonary venous drainage | Normal | Mitral valve | Annulus = 26mm. Patulous MVL. |
| Atrioventricular connection | Concordant | Tricuspid valve | Annulus = 22mm TAPSE = 20mm |
| Ventriculoarterial connection | Concordant | **Ventricles** |  |
| Ventricular loop | d-Loop | Left ventricle | Dilated |
|  |  | Right ventricle | Normal |
| **Septae** |  | **Coronary arteries** | ----- |
| Interventricular septum | Intact | **Doppler Measurement** |  |
| Interatrial septum | Intact | Mitral | Severe MR, Holosystolic, posterior projection, seen in two planes with jet velocity = 4m/sec. |
| **Semilunar valves** |  | Aortic | Moderate AR, PHT = 215ms. |
| Aortic valve | Annulus = 16mm | Tricuspid | Moderate TR, PPG = 38mmHg. |
| Pulmonary valve | Annulus = 18mm | pulmonic | -------- |
| **Great arteries** | NRGA | **Aortic arch** | Left. No CoA. |
| Aorta | ----- | **PDA** | No |
| Pulmonary artery | Normal MPA and Branch PAs. |  |  |
| **M-Mode:** | | | |
| AO | mm | PWd | mm |
| LA | mm | PWs | mm |
| LVIDd | mm | EDV | ml |
| LVIDs | mm | ESV | ml |
| IVSs | mm | LVEF | 65% |
| IVSd | mm | FS | 35% |
| **Additional Information**: |  | | |
| No pericardial/Pleural effusion. | | | |
| **Final Diagnosis:** | | | |
| 1. {S, D, S} Levocardia. 2. LA/LV Dilated 3. Patulous MVL 4. Severe MR 5. Moderate AR 6. Moderate TR 7. Mild Pulmonary Hypertension 8. Normal Biventricular Systolic Function | | | |
| **Remark**: | | | |
| **Recommendation**: | | | |
| SIGNATURE  Done by: Tesfaye T., Pediatrician, Pediatric Cardiologist _______________ 30/06/2015Eth.C | | | |

| Patient Name: **Meklit Yilkal**. Referring Institute: **Addis –Alem PH**. SEX/ Age: **F/6years**. Date of Report: **30/06/15**.  Referral Diagnosis: **Easy Fatigability. AGH12.3345** | | | |
| --- | --- | --- | --- |
| **Features** | **Finding** | **Features** | **Finding** |
| **Profile** |  | **Atria** |  |
| Abdominal situs | Solitus | Left atrium | Normal |
| Cardiac position | Levocardia | Right atrium | Normal |
| Systemic venous drainage | Normal. | **Atrioventricular valves** |  |
| Pulmonary venous drainage | Normal | Mitral valve | Annulus = 19mm |
| Atrioventricular connection | Concordant | Tricuspid valve | Annulus = 20mm  TAPSE = 17mm |
| Ventriculoarterial connection | Concordant | **Ventricles** |  |
| Ventricular loop | d-Loop | Left ventricle | Normal |
|  |  | Right ventricle | Normal |
| **Septae** |  | **Coronary arteries** | ----- |
| Interventricular septum | Intact | **Doppler Measurement** |  |
| Interatrial septum | Intact | Mitral | ----- |
| **Semilunar valves** |  | Aortic | ------- |
| Aortic valve | Annulus = 15mm | Tricuspid | ------- |
| Pulmonary valve | Annulus = 18mm | pulmonic | -------- |
| **Great arteries** | NRGA | **Aortic arch** | Left. No CoA. |
| Aorta | ----- | **PDA** | No |
| Pulmonary artery | Normal MPA and Branch PAs. |  |  |
| **M-Mode:** | | | |
| AO | mm | PWd | mm |
| LA | mm | PWs | mm |
| LVIDd | mm | EDV | ml |
| LVIDs | mm | ESV | ml |
| IVSs | mm | LVEF | 63% |
| IVSd | mm | FS | 33% |
| **Additional Information**: |  | | |
| No pericardial/Pleural effusion. | | | |
| **Final Diagnosis:** | | | |
| 1. Normal Echocardiography Study. | | | |
| **Remark**: | | | |
| **Recommendation**: | | | |
| SIGNATURE  Done by: Tesfaye T., Pediatrician, Pediatric Cardiologist _______________ 30/06/2015Eth.C | | | |

| Patient Name: **Baby of Bethelihem Adam**. Referring Institute: **TGSH**. SEX/ Age: **M/41days**. Date of Report: **30/06/15**.  Referral Diagnosis: **RD. AGH12.3346** | | | |
| --- | --- | --- | --- |
| **Features** | **Finding** | **Features** | **Finding** |
| **Profile** |  | **Atria** |  |
| Abdominal situs | Solitus | Left atrium | Normal |
| Cardiac position | Levocardia | Right atrium | Normal |
| Systemic venous drainage | Normal. | **Atrioventricular valves** |  |
| Pulmonary venous drainage | Normal | Mitral valve | Annulus = 11mm |
| Atrioventricular connection | Concordant | Tricuspid valve | Annulus = 11mm |
| Ventriculoarterial connection | Concordant | **Ventricles** |  |
| Ventricular loop | d-Loop | Left ventricle | Normal |
|  |  | Right ventricle | Normal |
| **Septae** |  | **Coronary arteries** | ----- |
| Interventricular septum | Intact | **Doppler Measurement** |  |
| Interatrial septum | Intact | Mitral | ----- |
| **Semilunar valves** |  | Aortic | ------- |
| Aortic valve | Annulus = 10mm | Tricuspid | ------- |
| Pulmonary valve | Annulus = 10mm | pulmonic | -------- |
| **Great arteries** | NRGA | **Aortic arch** | Left. No CoA. |
| Aorta | ----- | **PDA** | No |
| Pulmonary artery | Normal MPA and Branch PAs. |  |  |
| **M-Mode:**  Normal LV Function on eye balling. | | | |
| AO | mm | PWd | mm |
| LA | mm | PWs | mm |
| LVIDd | mm | EDV | ml |
| LVIDs | mm | ESV | ml |
| IVSs | mm | LVEF | % |
| IVSd | mm | FS | % |
| **Additional Information**: |  | | |
| No pericardial/Pleural effusion. | | | |
| **Final Diagnosis:** | | | |
| 1. Normal Echocardiography Study. | | | |
| **Remark**: | | | |
| **Recommendation**: | | | |
| SIGNATURE  Done by: Tesfaye T., Pediatrician, Pediatric Cardiologist _______________ 30/06/2015Eth.C | | | |

| Patient Name: **Ayinaddis Ademe**. Referring Institute: **TGSH**. SEX/ Age: **F/7months**. Date of Report: **30/06/15**.  Referral Diagnosis: **Down Syndrome + G-III HSM @ LLSB. AGH12.3347** | | | |
| --- | --- | --- | --- |
| **Features** | **Finding** | **Features** | **Finding** |
| **Profile** |  | **Atria** |  |
| Abdominal situs | Solitus | Left atrium | Normal |
| Cardiac position | Levocardia | Right atrium | Dilated |
| Systemic venous drainage | Normal. | **Atrioventricular valves** |  |
| Pulmonary venous drainage | Normal | Mitral valve | Common Complete AVSD.  TAPSE = 19mm |
| Atrioventricular connection | Concordant | Tricuspid valve |  |
| Ventriculoarterial connection | Concordant | **Ventricles** |  |
| Ventricular loop | d-Loop | Left ventricle | Normal |
|  |  | Right ventricle | Dilated |
| **Septae** |  | **Coronary arteries** | ----- |
| Interventricular septum | Common Complete AVSD, L – R Shunt | **Doppler Measurement** |  |
| Interatrial septum |  | Mitral | ----- |
| **Semilunar valves** |  | Aortic | ------- |
| Aortic valve | Annulus = 11mm | Tricuspid | Moderate Right AVVR |
| Pulmonary valve | Annulus = 15mm. Doming PV. | pulmonic | Mild PS, PPG = 23mmHg |
| **Great arteries** | NRGA | **Aortic arch** | Left. No CoA. |
| Aorta | ----- | **PDA** | No |
| Pulmonary artery | MPA = 15mm. |  |  |
| **M-Mode:**  Normal LV Function on eye balling | | | |
| AO | mm | PWd | mm |
| LA | mm | PWs | mm |
| LVIDd | mm | EDV | ml |
| LVIDs | mm | ESV | ml |
| IVSs | mm | LVEF | % |
| IVSd | mm | FS | % |
| **Additional Information**: |  | | |
| Pericardial effusion with maximum depth of 7mm on RA Side. | | | |
| **Final Diagnosis:** | | | |
| 1. {S, D, S} Levocardia. 2. RA/RV Dilated 3. Common Complete Balanced AVSD, L – R Shunt 4. Doming PV 5. Mild Valvular PS 6. Normal Biventricular Systolic Function 7. Small Pericardial effusion | | | |
| **Remark**: | | | |
| **Recommendation**: | | | |
| SIGNATURE  Done by: Tesfaye T., Pediatrician, Pediatric Cardiologist _______________ 30/06/2015Eth.C | | | |

| Patient Name: **Yonas Asresie**. Referring Institute: **TGSH**. SEX/ Age: **M/9years**. Date of Report: **30/06/15**.  Referral Diagnosis: **DOE + Murmur.** | | | |
| --- | --- | --- | --- |
| **Features** | **Finding** | **Features** | **Finding** |
| **Profile** |  | **Atria** |  |
| Abdominal situs | Solitus | Left atrium | Normal |
| Cardiac position | Levocardia | Right atrium | Dilated |
| Systemic venous drainage | Normal. | **Atrioventricular valves** |  |
| Pulmonary venous drainage | Normal | Mitral valve | Annulus = 17mm |
| Atrioventricular connection | Concordant | Tricuspid valve | Annulus = 25mm  TAPSE = 19mm |
| Ventriculoarterial connection | Concordant | **Ventricles** |  |
| Ventricular loop | d-Loop | Left ventricle | Normal |
|  |  | Right ventricle | Dilated |
| **Septae** |  | **Coronary arteries** | ----- |
| Interventricular septum | Intact | **Doppler Measurement** |  |
| Interatrial septum | 20mm OS ASD, L – R Shunt | Mitral | ----- |
| **Semilunar valves** |  | Aortic | ------- |
| Aortic valve | Annulus = 19mm | Tricuspid | Trivial TR, PPG = 29mmHg |
| Pulmonary valve | Annulus = 20mm | pulmonic | -------- |
| **Great arteries** | NRGA | **Aortic arch** | Left. No CoA. |
| Aorta | ----- | **PDA** | 1mm PDA, L – R Shunt |
| Pulmonary artery | Normal MPA and Branch PAs. |  |  |
| **M-Mode:** | | | |
| AO | mm | PWd | mm |
| LA | mm | PWs | mm |
| LVIDd | mm | EDV | ml |
| LVIDs | mm | ESV | ml |
| IVSs | mm | LVEF | 66% |
| IVSd | mm | FS | 35% |
| **Additional Information**: |  | | |
| No pericardial/Pleural effusion. | | | |
| **Final Diagnosis:** | | | |
| 1. {S, D, S} Levocardia. 2. RA/RV Dilated 3. Large OS ASD, L – R Shunt 4. Small PDA, L – R Shunt 5. Norma Biventricular Systolic Function | | | |
| **Remark**: | | | |
| **Recommendation**: | | | |
| SIGNATURE  Done by: Tesfaye T., Pediatrician, Pediatric Cardiologist _______________ 30/06/2015Eth.C | | | |

| Patient Name: **Sifelig Gebrie**. Referring Institute: **Addis Alem PH**. SEX/ Age: **F/7months**. Date of Report: **01/07/15**.  Referral Diagnosis: **Down Syndrome (Screening). AGH12.3349** | | | |
| --- | --- | --- | --- |
| **Features** | **Finding** | **Features** | **Finding** |
| **Profile** |  | **Atria** |  |
| Abdominal situs | Solitus | Left atrium | Normal |
| Cardiac position | Levocardia | Right atrium | Normal |
| Systemic venous drainage | Normal. | **Atrioventricular valves** |  |
| Pulmonary venous drainage | Normal | Mitral valve | Annulus = 12mm |
| Atrioventricular connection | Concordant | Tricuspid valve | Annulus = 12mm |
| Ventriculoarterial connection | Concordant | **Ventricles** |  |
| Ventricular loop | d-Loop | Left ventricle | Normal |
|  |  | Right ventricle | Normal |
| **Septae** | Tongue of tissue in b/n the defects | **Coronary arteries** | ----- |
| Interventricular septum | 6mm Inlet VSD, L – R Shunt | **Doppler Measurement** |  |
| Interatrial septum | 12mm Primum defect, L – R Shunt | Mitral | ----- |
| **Semilunar valves** |  | Aortic | ------- |
| Aortic valve | Annulus = 10mm | Tricuspid | Mild TR |
| Pulmonary valve | Annulus = 12mm | pulmonic | -------- |
| **Great arteries** | NRGA | **Aortic arch** | Left. No CoA. |
| Aorta | ----- | **PDA** | No |
| Pulmonary artery | Normal MPA and Branch PAs. |  |  |
| **M-Mode:**  Normal LV Function on eye balling | | | |
| AO | mm | PWd | mm |
| LA | mm | PWs | mm |
| LVIDd | mm | EDV | ml |
| LVIDs | mm | ESV | ml |
| IVSs | mm | LVEF | % |
| IVSd | mm | FS | % |
| **Additional Information**: |  | | |
| No pericardial/Pleural effusion. | | | |
| **Final Diagnosis:** | | | |
| 1. {S, D, S} Levocardia. 2. Intermediate AVSD, L – R Shunt 3. Mild TR 4. Normal LV Systolic Function | | | |
| **Remark**: | | | |
| **Recommendation**: | | | |
| SIGNATURE  Done by: Tesfaye T., Pediatrician, Pediatric Cardiologist _______________ 01/07/2015Eth.C | | | |

| Patient Name: **Baby of Gebeyanesh Muche**. Referring Institute: **FHRH**. SEX/ Age: **M/15days**. Date of Report: **01/07/15**.  Referral Diagnosis: **Cyanosis + Murmur. AGH12.3350** | | | |
| --- | --- | --- | --- |
| **Features** | **Finding** | **Features** | **Finding** |
| **Profile** |  | **Atria** |  |
| Abdominal situs | Solitus | Left atrium | Normal |
| Cardiac position | Levocardia | Right atrium | Normal |
| Systemic venous drainage | Normal. | **Atrioventricular valves** |  |
| Pulmonary venous drainage | Normal | Mitral valve | Annulus = 11mm |
| Atrioventricular connection | Concordant | Tricuspid valve | Annulus = 12mm |
| Ventriculoarterial connection | DORV | **Ventricles** |  |
| Ventricular loop | d-Loop | Left ventricle | Hypoplastic LV |
|  |  | Right ventricle | Normal |
| **Septae** |  | **Coronary arteries** | ----- |
| Interventricular septum | 10mm Inlet VSD, L – R Shunt. | **Doppler Measurement** |  |
| Interatrial septum | PFO, L – R Shunt | Mitral | ----- |
| **Semilunar valves** |  | Aortic | ------- |
| Aortic valve | Annulus = 11mm | Tricuspid | ------- |
| Pulmonary valve | Annulus = 15mm | pulmonic | -------- |
| **Great arteries** | d-TGA | **Aortic arch** | Left. No CoA. |
| Aorta | Anterior and to the right. From RV | **PDA** | No |
| Pulmonary artery | Posterior & to the left. From RV |  |  |
| **M-Mode:** | | | |
| AO | mm | PWd | mm |
| LA | mm | PWs | mm |
| LVIDd | mm | EDV | ml |
| LVIDs | mm | ESV | ml |
| IVSs | mm | LVEF | % |
| IVSd | mm | FS | % |
| **Additional Information**: |  | | |
| No pericardial/Pleural effusion. | | | |
| **Final Diagnosis:** | | | |
| 1. {S, D, D} Levocardia. 2. DORV 3. d-TGA 4. PFO, L – R Shunt 5. Large Inlet VSD, L – R Shunt 6. Hypoplastic LV | | | |
| **Remark**: | | | |
| **Recommendation**: | | | |
| SIGNATURE  Done by: Tesfaye T., Pediatrician, Pediatric Cardiologist _______________ 01/07/2015Eth.C | | | |

| Patient Name: **Elarya Ahmed**. Referring Institute: **Adinas GH**. SEX/ Age: **F/3 2/12**. Date of Report: **01/07/15**.  Referral Diagnosis: **Follow up echocardiography for Silent PDA. REGISTERED IN THE SPSS** | | | |
| --- | --- | --- | --- |
| **Features** | **Finding** | **Features** | **Finding** |
| **Profile** |  | **Atria** |  |
| Abdominal situs | Solitus | Left atrium | Normal |
| Cardiac position | Levocardia | Right atrium | Normal |
| Systemic venous drainage | Normal. | **Atrioventricular valves** |  |
| Pulmonary venous drainage | Normal | Mitral valve | Annulus = 15mm |
| Atrioventricular connection | Concordant | Tricuspid valve | Annulus = 16mm |
| Ventriculoarterial connection | Concordant | **Ventricles** |  |
| Ventricular loop | d-Loop | Left ventricle | Normal |
|  |  | Right ventricle | Normal |
| **Septae** |  | **Coronary arteries** | ----- |
| Interventricular septum | Intact | **Doppler Measurement** |  |
| Interatrial septum | Intact | Mitral | ----- |
| **Semilunar valves** |  | Aortic | ------- |
| Aortic valve | Annulus = 12mm | Tricuspid | ------- |
| Pulmonary valve | Annulus = 13mm | pulmonic | -------- |
| **Great arteries** | NRGA | **Aortic arch** | Left. No CoA. |
| Aorta | ----- | **PDA** | 1mm PDA, L – R Shunt |
| Pulmonary artery | Normal MPA and Branch PAs. |  |  |
| **M-Mode:**  Normal LV Function on eye balling | | | |
| AO | mm | PWd | mm |
| LA | mm | PWs | mm |
| LVIDd | mm | EDV | ml |
| LVIDs | mm | ESV | ml |
| IVSs | mm | LVEF | % |
| IVSd | mm | FS | % |
| **Additional Information**: |  | | |
| No pericardial/Pleural effusion. | | | |
| **Final Diagnosis:** | | | |
| 1. {S, D, S} Levocardia. 2. Small PDA, L – R Shunt | | | |
| **Remark**: Can be considered as Silent, as there is no Murmur clinically appreciated | | | |
| **Recommendation**: | | | |
| SIGNATURE  Done by: Tesfaye T., Pediatrician, Pediatric Cardiologist _______________ 01/07/2015Eth.C | | | |

| Patient Name: **Mekdes Molla**. Referring Institute: **FHRH**. SEX/ Age: **F/13years**. Date of Report: **01/07/15**.  Referral Diagnosis: **Palpitation and easy fatigability. AGH12.3351.** | | | |
| --- | --- | --- | --- |
| **Features** | **Finding** | **Features** | **Finding** |
| **Profile** |  | **Atria** |  |
| Abdominal situs | Solitus | Left atrium | Dilated |
| Cardiac position | Levocardia | Right atrium | Dilated |
| Systemic venous drainage | Normal. | **Atrioventricular valves** |  |
| Pulmonary venous drainage | Normal | Mitral valve | Annulus = 26mm |
| Atrioventricular connection | Concordant | Tricuspid valve | Annulus = 26mm  TAPSE = 23mm |
| Ventriculoarterial connection | Concordant | **Ventricles** |  |
| Ventricular loop | d-Loop | Left ventricle | Dilated |
|  |  | Right ventricle | Dilated |
| **Septae** |  | **Coronary arteries** | ----- |
| Interventricular septum | 13mm PM VSD, Partially closed by STL, L – R Shunt. | **Doppler Measurement** |  |
| Interatrial septum | Intact | Mitral | ----- |
| **Semilunar valves** |  | Aortic | ------- |
| Aortic valve | Annulus = 18mm | Tricuspid | Severe TR |
| Pulmonary valve | Annulus = 26mm | pulmonic | -------- |
| **Great arteries** | NRGA | **Aortic arch** | Left. No CoA. |
| Aorta | ----- | **PDA** | 4mm PDA, L – R Shunt |
| Pulmonary artery | Normal MPA and Branch PAs. |  |  |
| **M-Mode:** | | | |
| AO | mm | PWd | mm |
| LA | mm | PWs | mm |
| LVIDd | mm | EDV | ml |
| LVIDs | mm | ESV | ml |
| IVSs | mm | LVEF | 61% |
| IVSd | mm | FS | 33% |
| **Additional Information**: |  | | |
| No pericardial/Pleural effusion. | | | |
| **Final Diagnosis:** | | | |
| 1. {S, D, S} Levocardia. 2. All chambers dilated 3. Large PM VSD, Partially closed by STL, L – R Shunt 4. Large PDA, L – R Shunt 5. Severe TR 6. Pulmonary Hypertension 7. Normal Biventricular Systolic Function | | | |
| **Remark**: | | | |
| **Recommendation**: | | | |
| SIGNATURE  Done by: Tesfaye T., Pediatrician, Pediatric Cardiologist _______________ 01/07/2015Eth.C | | | |

| Patient Name: **Baby of Meseret Limenih**. Referring Institute: **FHRH**. SEX/ Age: **M/3days**. Date of Report: **02/07/15**.  Referral Diagnosis: **Incidental Murmur. AGH12.3352.** | | | |
| --- | --- | --- | --- |
| **Features** | **Finding** | **Features** | **Finding** |
| **Profile** |  | **Atria** |  |
| Abdominal situs | Solitus | Left atrium | Normal |
| Cardiac position | Levocardia | Right atrium | Normal |
| Systemic venous drainage | Normal. | **Atrioventricular valves** |  |
| Pulmonary venous drainage | Normal | Mitral valve | Annulus = 11mm |
| Atrioventricular connection | Concordant | Tricuspid valve | Annulus = 12mm  TAPSE = 13mm |
| Ventriculoarterial connection | Concordant | **Ventricles** |  |
| Ventricular loop | d-Loop | Left ventricle | Normal |
|  |  | Right ventricle | Normal |
| **Septae** |  | **Coronary arteries** | ----- |
| Interventricular septum | Intact | **Doppler Measurement** |  |
| Interatrial septum | 6 X 7mm OS ASD, L – R Shunt. | Mitral | ----- |
| **Semilunar valves** |  | Aortic | ------- |
| Aortic valve | Annulus = 9mm | Tricuspid | ------- |
| Pulmonary valve | Annulus = 10mm | pulmonic | -------- |
| **Great arteries** | NRGA | **Aortic arch** | Left. No CoA. |
| Aorta | ----- | **PDA** | 1.5mm PDA, L – R Shunt |
| Pulmonary artery | Normal MPA and Branch PAs. |  |  |
| **M-Mode:**  Normal LV Function on eye balling | | | |
| AO | mm | PWd | mm |
| LA | mm | PWs | mm |
| LVIDd | mm | EDV | ml |
| LVIDs | mm | ESV | ml |
| IVSs | mm | LVEF | % |
| IVSd | mm | FS | % |
| **Additional Information**: |  | | |
| No pericardial/Pleural effusion. | | | |
| **Final Diagnosis:** | | | |
| 1. {S, D, S} Levocardia. 2. Small OS ASD, L – R Shunt 3. Small PDA, L – R Shunt 4. Normal Biventricular Systolic Function | | | |
| **Remark**: | | | |
| **Recommendation**: | | | |
| SIGNATURE  Done by: Tesfaye T., Pediatrician, Pediatric Cardiologist _______________ 02/07/2015Eth.C | | | |

| Patient Name: **Mebe’a-Tsion Abebaw**. Referring Institute: **Adinas GH**. SEX/ Age: **F/4 5/12**. Date of Report: **02/07/15**.  Referral Diagnosis: **Follow up echocardiography for trivial TR.** | | | |
| --- | --- | --- | --- |
| **Features** | **Finding** | **Features** | **Finding** |
| **Profile** |  | **Atria** |  |
| Abdominal situs | Solitus | Left atrium | Normal |
| Cardiac position | Levocardia | Right atrium | Normal |
| Systemic venous drainage | Normal. | **Atrioventricular valves** |  |
| Pulmonary venous drainage | Normal | Mitral valve | Annulus = 20mm |
| Atrioventricular connection | Concordant | Tricuspid valve | Annulus = 19mm |
| Ventriculoarterial connection | Concordant | **Ventricles** |  |
| Ventricular loop | d-Loop | Left ventricle | Normal |
|  |  | Right ventricle | Normal |
| **Septae** |  | **Coronary arteries** | ----- |
| Interventricular septum | Intact | **Doppler Measurement** |  |
| Interatrial septum | Intact | Mitral | ----- |
| **Semilunar valves** |  | Aortic | ------- |
| Aortic valve | Annulus = 15mm | Tricuspid | Trivial TR, PPG = 14mmHg |
| Pulmonary valve | Annulus = 17mm | pulmonic | -------- |
| **Great arteries** | NRGA | **Aortic arch** | Left. No CoA. |
| Aorta | ----- | **PDA** | No |
| Pulmonary artery | Normal MPA and Branch PAs. |  |  |
| **M-Mode:** | | | |
| AO | mm | PWd | mm |
| LA | mm | PWs | mm |
| LVIDd | mm | EDV | ml |
| LVIDs | mm | ESV | ml |
| IVSs | mm | LVEF | 58% |
| IVSd | mm | FS | 30% |
| **Additional Information**: |  | | |
| No pericardial/Pleural effusion. | | | |
| **Final Diagnosis:** | | | |
| 1. Normal Echocardiography Study. | | | |
| **Remark**: | | | |
| **Recommendation**: | | | |
| SIGNATURE  Done by: Tesfaye T., Pediatrician, Pediatric Cardiologist _______________ 02/07/2015Eth.C | | | |

| Patient Name: **Fikir Yibeltal**. Referring Institute: **Adinas GH**. SEX/ Age: **F/8 6/12**. Date of Report: **02/07/15**.  Referral Diagnosis: **excessive sweating + recurrent cough. AGH12.3353.** | | | |
| --- | --- | --- | --- |
| **Features** | **Finding** | **Features** | **Finding** |
| **Profile** |  | **Atria** |  |
| Abdominal situs | Solitus | Left atrium | Normal |
| Cardiac position | Levocardia | Right atrium | Normal |
| Systemic venous drainage | Normal. | **Atrioventricular valves** |  |
| Pulmonary venous drainage | Normal | Mitral valve | Annulus = 20mm |
| Atrioventricular connection | Concordant | Tricuspid valve | Annulus = 22mm  TAPSE = 19mm |
| Ventriculoarterial connection | Concordant | **Ventricles** |  |
| Ventricular loop | d-Loop | Left ventricle | Normal |
|  |  | Right ventricle | Normal |
| **Septae** |  | **Coronary arteries** | ----- |
| Interventricular septum | Intact | **Doppler Measurement** |  |
| Interatrial septum | Intact | Mitral | ----- |
| **Semilunar valves** |  | Aortic | ------- |
| Aortic valve | Annulus = 15mm | Tricuspid | ------- |
| Pulmonary valve | Annulus = 17mm | pulmonic | -------- |
| **Great arteries** | NRGA | **Aortic arch** | Left. No CoA. |
| Aorta | ----- | **PDA** | No |
| Pulmonary artery | Normal MPA and Branch PAs. |  |  |
| **M-Mode:** | | | |
| AO | mm | PWd | mm |
| LA | mm | PWs | mm |
| LVIDd | mm | EDV | ml |
| LVIDs | mm | ESV | ml |
| IVSs | mm | LVEF | 75% |
| IVSd | mm | FS | 40% |
| **Additional Information**: |  | | |
| No pericardial/Pleural effusion. | | | |
| **Final Diagnosis:** | | | |
| 1. Normal Echocardiography Study. | | | |
| **Remark**: | | | |
| **Recommendation**: | | | |
| SIGNATURE  Done by: Tesfaye T., Pediatrician, Pediatric Cardiologist _______________ 02/07/2015Eth.C | | | |

| Patient Name: **Bisrat Nega**. Referring Institute: **Adinas GH**. SEX/ Age: **M/5 3/12**. Date of Report: **02/07/15**.  Referral Diagnosis: **Easy fatiguability. AGH12.3354.** | | | |
| --- | --- | --- | --- |
| **Features** | **Finding** | **Features** | **Finding** |
| **Profile** |  | **Atria** |  |
| Abdominal situs | Solitus | Left atrium | Normal |
| Cardiac position | Levocardia | Right atrium | Normal |
| Systemic venous drainage | Normal. | **Atrioventricular valves** |  |
| Pulmonary venous drainage | Normal | Mitral valve | Annulus = 19mm |
| Atrioventricular connection | Concordant | Tricuspid valve | Annulus = 18mm |
| Ventriculoarterial connection | Concordant | **Ventricles** |  |
| Ventricular loop | d-Loop | Left ventricle | Normal |
|  |  | Right ventricle | Normal |
| **Septae** |  | **Coronary arteries** | ----- |
| Interventricular septum | Intact | **Doppler Measurement** |  |
| Interatrial septum | Intact | Mitral | ----- |
| **Semilunar valves** |  | Aortic | ------- |
| Aortic valve | Annulus = 15mm | Tricuspid | ------- |
| Pulmonary valve | Annulus = 17mm | pulmonic | -------- |
| **Great arteries** | NRGA | **Aortic arch** | Left. No CoA. |
| Aorta | ----- | **PDA** | No |
| Pulmonary artery | Normal MPA and Branch PAs. |  |  |
| **M-Mode:**  Normal LV Function on eye balling | | | |
| AO | mm | PWd | mm |
| LA | mm | PWs | mm |
| LVIDd | mm | EDV | ml |
| LVIDs | mm | ESV | ml |
| IVSs | mm | LVEF | % |
| IVSd | mm | FS | % |
| **Additional Information**: |  | | |
| No pericardial/Pleural effusion. | | | |
| **Final Diagnosis:** | | | |
| 1. Normal Echocardiography Study. | | | |
| **Remark**: | | | |
| **Recommendation**: | | | |
| SIGNATURE  Done by: Tesfaye T., Pediatrician, Pediatric Cardiologist _______________ 02/07/2015Eth.C | | | |

| Patient Name: **Simegnew Kassahun**. Referring Institute: **FHRH**. SEX/ Age: **M/8years**. Date of Report: **02/07/15**.  Referral Diagnosis: **?Acute Rheumatic Fever. AGH12.3355.** | | | |
| --- | --- | --- | --- |
| **Features** | **Finding** | **Features** | **Finding** |
| **Profile** |  | **Atria** |  |
| Abdominal situs | Solitus | Left atrium | Normal |
| Cardiac position | Levocardia | Right atrium | Normal |
| Systemic venous drainage | Normal. | **Atrioventricular valves** |  |
| Pulmonary venous drainage | Normal | Mitral valve | Annulus = 18mm. Patulous MVL. |
| Atrioventricular connection | Concordant | Tricuspid valve | Annulus = 19mm  TAPSE = 15mm |
| Ventriculoarterial connection | Concordant | **Ventricles** |  |
| Ventricular loop | d-Loop | Left ventricle | Normal |
|  |  | Right ventricle | Normal |
| **Septae** |  | **Coronary arteries** | ----- |
| Interventricular septum | Intact | **Doppler Measurement** |  |
| Interatrial septum | Intact | Mitral | Mild MR, Holosystolic, posterior projection, seen in two planes with jet velocity = 3.3m/sec. |
| **Semilunar valves** |  | Aortic | ------- |
| Aortic valve | Annulus = 15mm | Tricuspid | ------- |
| Pulmonary valve | Annulus = 16mm | pulmonic | -------- |
| **Great arteries** | NRGA | **Aortic arch** | Left. No CoA. |
| Aorta | ----- | **PDA** | No |
| Pulmonary artery | Normal MPA and Branch PAs. |  |  |
| **M-Mode:** | | | |
| AO | mm | PWd | mm |
| LA | mm | PWs | mm |
| LVIDd | mm | EDV | ml |
| LVIDs | mm | ESV | ml |
| IVSs | mm | LVEF | 54% |
| IVSd | mm | FS | 37% |
| **Additional Information**: |  | | |
| Circumferential pericardial effusion with maximum depth of 12mm on LV Side. | | | |
| **Final Diagnosis:** | | | |
| 1. {S, D, S} Levocardia. 2. Patulous MVL 3. Mild MR 4. Moderate Circumferential Pericardial effusion 5. Reduced Biventricular Systolic Function | | | |
| **Remark**: Pancarditis (can be considered as hallmark of Rheumatic Carditis) | | | |
| **Recommendation**: | | | |
| SIGNATURE  Done by: Tesfaye T., Pediatrician, Pediatric Cardiologist _______________ 02/07/2015Eth.C | | | |

| Patient Name: **Mita Terefe**. Referring Institute: **FHRH**. SEX/ Age: **F/1 3/12**. Date of Report: **04/07/15**.  Referral Diagnosis: **Down Syndrome. AGH12.3356.** | | | |
| --- | --- | --- | --- |
| **Features** | **Finding** | **Features** | **Finding** |
| **Profile** |  | **Atria** |  |
| Abdominal situs | Solitus | Left atrium | Normal |
| Cardiac position | Levocardia | Right atrium | Dilated |
| Systemic venous drainage | Normal. | **Atrioventricular valves** |  |
| Pulmonary venous drainage | Normal | Mitral valve | Annulus = 14mm |
| Atrioventricular connection | Concordant | Tricuspid valve | Annulus = 15mm |
| Ventriculoarterial connection | Concordant | **Ventricles** |  |
| Ventricular loop | d-Loop | Left ventricle | Normal |
|  |  | Right ventricle | Dilated |
| **Septae** |  | **Coronary arteries** | ----- |
| Interventricular septum | Intact | **Doppler Measurement** |  |
| Interatrial septum | 9 X 11mm OS ASD, L – R Shunt | Mitral | ----- |
| **Semilunar valves** |  | Aortic | ------- |
| Aortic valve | Annulus = 13mm | Tricuspid | ------- |
| Pulmonary valve | Annulus = 15mm | pulmonic | -------- |
| **Great arteries** | NRGA | **Aortic arch** | Left. No CoA. |
| Aorta | ----- | **PDA** | No |
| Pulmonary artery | Normal MPA and Branch PAs. |  |  |
| **M-Mode:**  Normal LV Function on eye balling | | | |
| AO | mm | PWd | mm |
| LA | mm | PWs | mm |
| LVIDd | mm | EDV | ml |
| LVIDs | mm | ESV | ml |
| IVSs | mm | LVEF | % |
| IVSd | mm | FS | % |
| **Additional Information**: |  | | |
| Circumferential pericardial effusion with maximum depth of 10mm on RV Side. | | | |
| **Final Diagnosis:** | | | |
| 1. {S, D, S} Levocardia. 2. RA/RV Dilated 3. Moderate to Large OS ASD, L – R Shunt 4. Normal LV Function | | | |
| **Remark**: | | | |
| **Recommendation**: | | | |
| SIGNATURE  Done by: Tesfaye T., Pediatrician, Pediatric Cardiologist _______________ 04/07/2015Eth.C | | | |

| Patient Name: **Memar Lake**. Referring Institute: **Amaris PSC**. SEX/ Age: **M/2 6/12**. Date of Report: **04/07/15**.  Referral Diagnosis: **Down Syndrome. AGH12.3357.** | | | |
| --- | --- | --- | --- |
| **Features** | **Finding** | **Features** | **Finding** |
| **Profile** |  | **Atria** |  |
| Abdominal situs | Solitus | Left atrium | Dilated |
| Cardiac position | Levocardia | Right atrium | Normal |
| Systemic venous drainage | Normal. | **Atrioventricular valves** |  |
| Pulmonary venous drainage | Normal | Mitral valve | Annulus = 15mm |
| Atrioventricular connection | Concordant | Tricuspid valve | Annulus = 16mm |
| Ventriculoarterial connection | Concordant | **Ventricles** |  |
| Ventricular loop | d-Loop | Left ventricle | Dilated |
|  |  | Right ventricle | Normal |
| **Septae** |  | **Coronary arteries** | ----- |
| Interventricular septum | 12mm PM VSD, Partially closed by STL, L – R Shunt. | **Doppler Measurement** |  |
| Interatrial septum | Intact | Mitral | ----- |
| **Semilunar valves** |  | Aortic | ------- |
| Aortic valve | Annulus = 14mm | Tricuspid | ------- |
| Pulmonary valve | Annulus = 16mm | pulmonic | -------- |
| **Great arteries** | NRGA | **Aortic arch** | Left. No CoA. |
| Aorta | ----- | **PDA** | No |
| Pulmonary artery | Normal MPA and Branch PAs. |  |  |
| **M-Mode:**  Normal LV Function on eye balling | | | |
| AO | mm | PWd | mm |
| LA | mm | PWs | mm |
| LVIDd | mm | EDV | ml |
| LVIDs | mm | ESV | ml |
| IVSs | mm | LVEF | % |
| IVSd | mm | FS | % |
| **Additional Information**: |  | | |
| Pericardial effusion with maximum depth of 4mm on RV Side. | | | |
| **Final Diagnosis:** | | | |
| 1. {S, D, S} Levocardia. 2. LA/LV Dilated 3. Large PM VSD, Partially closed by STL, L – R Shunt 4. Normal LV Function 5. Trace pericardial effusion | | | |
| **Remark**: | | | |
| **Recommendation**: | | | |
| SIGNATURE  Done by: Tesfaye T., Pediatrician, Pediatric Cardiologist _______________ 04/07/2015Eth.C | | | |

| Patient Name: **Fasil Agegnehu**. Referring Institute: **Amaris PSC**. SEX/ Age: **M/10years**. Date of Report: **04/07/15**.  Referral Diagnosis: **CVHD (?MS) (Incidental Murmur). AGH12.3358.** | | | |
| --- | --- | --- | --- |
| **Features** | **Finding** | **Features** | **Finding** |
| **Profile** |  | **Atria** |  |
| Abdominal situs | Solitus | Left atrium | Normal |
| Cardiac position | Levocardia | Right atrium | Normal |
| Systemic venous drainage | Normal. | **Atrioventricular valves** |  |
| Pulmonary venous drainage | Normal | Mitral valve | Annulus = 21mm |
| Atrioventricular connection | Concordant | Tricuspid valve | Annulus = 21mm  TAPSE = 21mm |
| Ventriculoarterial connection | Concordant | **Ventricles** |  |
| Ventricular loop | d-Loop | Left ventricle | Normal |
|  |  | Right ventricle | Normal |
| **Septae** |  | **Coronary arteries** | ----- |
| Interventricular septum | Intact | **Doppler Measurement** |  |
| Interatrial septum | Intact | Mitral | ----- |
| **Semilunar valves** |  | Aortic | ------- |
| Aortic valve | Annulus = 16mm | Tricuspid | ------- |
| Pulmonary valve | Annulus = 18mm | pulmonic | -------- |
| **Great arteries** | NRGA | **Aortic arch** | Left. No CoA. |
| Aorta | ----- | **PDA** | No |
| Pulmonary artery | Normal MPA and Branch PAs. |  |  |
| **M-Mode:** | | | |
| AO | mm | PWd | mm |
| LA | mm | PWs | mm |
| LVIDd | mm | EDV | ml |
| LVIDs | mm | ESV | ml |
| IVSs | mm | LVEF | 69% |
| IVSd | mm | FS | 38% |
| **Additional Information**: |  | | |
| No pericardial/Pleural effusion. | | | |
| **Final Diagnosis:** | | | |
| 1. Normal Echocardiography Study. | | | |
| **Remark**: | | | |
| **Recommendation**: | | | |
| SIGNATURE  Done by: Tesfaye T., Pediatrician, Pediatric Cardiologist _______________ 04/07/2015Eth.C | | | |

| Patient Name: **Mahider Kifle-Mariam**. Referring Institute: **Amaris PSC**. SEX/ Age: **F/7months**. Date of Report: **04/07/15**.  Referral Diagnosis: **Down Syndrome. AGH12.3359.** | | | |
| --- | --- | --- | --- |
| **Features** | **Finding** | **Features** | **Finding** |
| **Profile** |  | **Atria** |  |
| Abdominal situs | Solitus | Left atrium | Normal |
| Cardiac position | Levocardia | Right atrium | Normal |
| Systemic venous drainage | Normal. | **Atrioventricular valves** |  |
| Pulmonary venous drainage | Normal | Mitral valve | Annulus = 11mm |
| Atrioventricular connection | Concordant | Tricuspid valve | Annulus = 12mm |
| Ventriculoarterial connection | Concordant | **Ventricles** |  |
| Ventricular loop | d-Loop | Left ventricle | Normal |
|  |  | Right ventricle | Normal |
| **Septae** |  | **Coronary arteries** | ----- |
| Interventricular septum | Intact | **Doppler Measurement** |  |
| Interatrial septum | PFO, L – R Shunt | Mitral | ----- |
| **Semilunar valves** |  | Aortic | ------- |
| Aortic valve | Annulus = 10mm | Tricuspid | ------- |
| Pulmonary valve | Annulus = 12mm | pulmonic | -------- |
| **Great arteries** | NRGA | **Aortic arch** | Left. No CoA. |
| Aorta | ----- | **PDA** | No |
| Pulmonary artery | Normal MPA and Branch PAs. |  |  |
| **M-Mode:**  Normal LV Function on eye balling | | | |
| AO | mm | PWd | mm |
| LA | mm | PWs | mm |
| LVIDd | mm | EDV | ml |
| LVIDs | mm | ESV | ml |
| IVSs | mm | LVEF | % |
| IVSd | mm | FS | % |
| **Additional Information**: |  | | |
| No pericardial/Pleural effusion. | | | |
| **Final Diagnosis:** | | | |
| 1. {S, D, S} Levocardia. 2. PFO, L – R Shunt | | | |
| **Remark**: | | | |
| **Recommendation**: | | | |
| SIGNATURE  Done by: Tesfaye T., Pediatrician, Pediatric Cardiologist _______________ 04/07/2015Eth.C | | | |

| Patient Name: **Aynalem Mihret**. Referring Institute: **FHRH**. SEX/ Age: **F/10years**. Date of Report: **04/07/15**.  Referral Diagnosis: **Palpitation + easy fatigability. AGH12.3360.** | | | |
| --- | --- | --- | --- |
| **Features** | **Finding** | **Features** | **Finding** |
| **Profile** |  | **Atria** |  |
| Abdominal situs | Solitus | Left atrium | Dilated |
| Cardiac position | Levocardia | Right atrium | Dilated |
| Systemic venous drainage | Normal. | **Atrioventricular valves** |  |
| Pulmonary venous drainage | Normal | Mitral valve | Annulus = 26mm |
| Atrioventricular connection | Concordant | Tricuspid valve | Annulus = 26mm  TAPSE = 22mm |
| Ventriculoarterial connection | Truncus Arteriosus | **Ventricles** |  |
| Ventricular loop | d-Loop | Left ventricle | Dilated |
|  |  | Right ventricle | Dilated |
| **Septae** |  | **Coronary arteries** | ----- |
| Interventricular septum | Non Restrictive Sub-Arterial VSD, L – R Shunt | **Doppler Measurement** |  |
| Interatrial septum | Intact | Mitral | ----- |
| **Semilunar valves** |  | Aortic | Moderate Truncal Regurgitation |
| Aortic valve | Truncal Annulus = 29mm | Tricuspid | ------- |
| Pulmonary valve | Pulmonary Branch opening Annulus = 16mm | pulmonic | -------- |
| **Great arteries** | Truncus | **Aortic arch** | Left. No CoA. |
| Aorta | ----- | **PDA** | No |
| Pulmonary artery | PA arising from the Left side of the Truncus. |  |  |
| **M-Mode:** | | | |
| AO | mm | PWd | mm |
| LA | mm | PWs | mm |
| LVIDd | mm | EDV | ml |
| LVIDs | mm | ESV | ml |
| IVSs | mm | LVEF | 63% |
| IVSd | mm | FS | 34% |
| **Additional Information**: |  | | |
| No pericardial/Pleural effusion. | | | |
| **Final Diagnosis:** | | | |
| 1. {S, D, S} Levocardia. 2. All chambers dilated 3. Truncus Arteriosus 4. Non – Restrictive Sub-Arterial VSD, L – R Shunt 5. Moderate Truncal Regurgitation 6. Normal Biventricular Systolic Function | | | |
| **Remark**: | | | |
| **Recommendation**: | | | |
| SIGNATURE  Done by: Tesfaye T., Pediatrician, Pediatric Cardiologist _______________ 04/07/2015Eth.C | | | |

| Patient Name: **Baby of Zewuditu Ayana**. Referring Institute: **TGSH**. SEX/ Age: **M/11days**. Date of Report: **04/07/15**.  Referral Diagnosis: **Pre-operative evaluation. AGH12.3361.** | | | |
| --- | --- | --- | --- |
| **Features** | **Finding** | **Features** | **Finding** |
| **Profile** |  | **Atria** |  |
| Abdominal situs | Solitus | Left atrium | Normal |
| Cardiac position | Levocardia | Right atrium | Normal |
| Systemic venous drainage | Normal. | **Atrioventricular valves** |  |
| Pulmonary venous drainage | Normal | Mitral valve | Annulus = 9mm |
| Atrioventricular connection | Concordant | Tricuspid valve | Annulus = 10mm |
| Ventriculoarterial connection | Concordant | **Ventricles** |  |
| Ventricular loop | d-Loop | Left ventricle | Normal |
|  |  | Right ventricle | Normal |
| **Septae** |  | **Coronary arteries** | ----- |
| Interventricular septum | Intact | **Doppler Measurement** |  |
| Interatrial septum | Intact | Mitral | ----- |
| **Semilunar valves** |  | Aortic | ------- |
| Aortic valve | Annulus = 9mm | Tricuspid | ------- |
| Pulmonary valve | Annulus = 9mm | pulmonic | -------- |
| **Great arteries** | NRGA | **Aortic arch** | Left. No CoA. |
| Aorta | ----- | **PDA** | <1mm PDA, L – R Shunt |
| Pulmonary artery | Normal MPA and Branch PAs. |  |  |
| **M-Mode:**  Normal LV Function on eye balling | | | |
| AO | mm | PWd | mm |
| LA | mm | PWs | mm |
| LVIDd | mm | EDV | ml |
| LVIDs | mm | ESV | ml |
| IVSs | mm | LVEF | % |
| IVSd | mm | FS | % |
| **Additional Information**: |  | | |
| No pericardial/Pleural effusion. | | | |
| **Final Diagnosis:** | | | |
| 1. {S, D, S} Levocardia. 2. Small PDA, L – R Shunt | | | |
| **Remark**: Can be considered as silent if no Murmur appreciated. | | | |
| **Recommendation**: Can proceed with the planned surgery. | | | |
| SIGNATURE  Done by: Tesfaye T., Pediatrician, Pediatric Cardiologist _______________ 04/07/2015Eth.C | | | |

| Patient Name: **Bereket Mersha**. Referring Institute: **Adinas GH**. SEX/ Age: **M/9years**. Date of Report: **04/07/15**.  Referral Diagnosis: **Chest Pain. AGH12.3362.** | | | |
| --- | --- | --- | --- |
| **Features** | **Finding** | **Features** | **Finding** |
| **Profile** |  | **Atria** |  |
| Abdominal situs | Solitus | Left atrium | Normal |
| Cardiac position | Levocardia | Right atrium | Normal |
| Systemic venous drainage | Normal. | **Atrioventricular valves** |  |
| Pulmonary venous drainage | Normal | Mitral valve | Annulus = 20mm |
| Atrioventricular connection | Concordant | Tricuspid valve | Annulus = 21mm  TAPSE = 20mm |
| Ventriculoarterial connection | Concordant | **Ventricles** |  |
| Ventricular loop | d-Loop | Left ventricle | Normal |
|  |  | Right ventricle | Normal |
| **Septae** |  | **Coronary arteries** | ----- |
| Interventricular septum | Intact | **Doppler Measurement** |  |
| Interatrial septum | Intact | Mitral | ----- |
| **Semilunar valves** |  | Aortic | ------- |
| Aortic valve | Annulus = 16mm | Tricuspid | ------- |
| Pulmonary valve | Annulus = 20mm | pulmonic | -------- |
| **Great arteries** | NRGA | **Aortic arch** | Left. No CoA. |
| Aorta | ----- | **PDA** | No |
| Pulmonary artery | Normal MPA and Branch PAs. |  |  |
| **M-Mode:** | | | |
| AO | mm | PWd | mm |
| LA | mm | PWs | mm |
| LVIDd | mm | EDV | ml |
| LVIDs | mm | ESV | ml |
| IVSs | mm | LVEF | 59% |
| IVSd | mm | FS | 31% |
| **Additional Information**: |  | | |
| No pericardial/Pleural effusion. | | | |
| **Final Diagnosis:** | | | |
| 1. Normal Echocardiography Study. | | | |
| **Remark**: | | | |
| **Recommendation**: | | | |
| SIGNATURE  Done by: Tesfaye T., Pediatrician, Pediatric Cardiologist _______________ 04/07/2015Eth.C | | | |

| Patient Name: **Addisu Derebe**. Referring Institute: **Dr. Addisu PSC**. SEX/ Age: **M/4 4/12**. Date of Report: **05/07/15**.  Referral Diagnosis: **Down Syndrome. AGH12.3363.** | | | |
| --- | --- | --- | --- |
| **Features** | **Finding** | **Features** | **Finding** |
| **Profile** |  | **Atria** |  |
| Abdominal situs | Solitus | Left atrium | Normal |
| Cardiac position | Levocardia | Right atrium | Normal |
| Systemic venous drainage | Normal. | **Atrioventricular valves** |  |
| Pulmonary venous drainage | Normal | Mitral valve | Annulus = 14mm |
| Atrioventricular connection | Concordant | Tricuspid valve | Annulus = 15mm |
| Ventriculoarterial connection | Concordant | **Ventricles** |  |
| Ventricular loop | d-Loop | Left ventricle | Normal |
|  |  | Right ventricle | Normal |
| **Septae** |  | **Coronary arteries** | ----- |
| Interventricular septum | Intact | **Doppler Measurement** |  |
| Interatrial septum | Intact | Mitral | ----- |
| **Semilunar valves** |  | Aortic | ------- |
| Aortic valve | Annulus = 12mm | Tricuspid | ------- |
| Pulmonary valve | Annulus = 15mm | pulmonic | -------- |
| **Great arteries** | NRGA | **Aortic arch** | Left. No CoA. |
| Aorta | ----- | **PDA** | No |
| Pulmonary artery | Normal MPA and Branch PAs. |  |  |
| **M-Mode:**  Normal LV Function on eye balling. | | | |
| AO | mm | PWd | mm |
| LA | mm | PWs | mm |
| LVIDd | mm | EDV | ml |
| LVIDs | mm | ESV | ml |
| IVSs | mm | LVEF | % |
| IVSd | mm | FS | % |
| **Additional Information**: |  | | |
| No pericardial/Pleural effusion. | | | |
| **Final Diagnosis:** | | | |
| 1. Normal Echocardiography Study. | | | |
| **Remark**: | | | |
| **Recommendation**: | | | |
| SIGNATURE  Done by: Tesfaye T., Pediatrician, Pediatric Cardiologist _______________ 05/07/2015Eth.C | | | |

| Patient Name: **Baby of Abeba Wendimu**. Referring Institute: **TGSH**. SEX/ Age: **M/4months**. Date of Report: **05/07/15**.  Referral Diagnosis: **Down Syndrome + ARM. AGH12.3364.** | | | |
| --- | --- | --- | --- |
| **Features** | **Finding** | **Features** | **Finding** |
| **Profile** |  | **Atria** |  |
| Abdominal situs | Solitus | Left atrium | Normal |
| Cardiac position | Levocardia | Right atrium | Normal |
| Systemic venous drainage | Normal. | **Atrioventricular valves** |  |
| Pulmonary venous drainage | Normal | Mitral valve | Annulus = 10mm |
| Atrioventricular connection | Concordant | Tricuspid valve | Annulus = 12mm |
| Ventriculoarterial connection | Concordant | **Ventricles** |  |
| Ventricular loop | d-Loop | Left ventricle | Normal |
|  |  | Right ventricle | Normal |
| **Septae** |  | **Coronary arteries** | ----- |
| Interventricular septum | Intact | **Doppler Measurement** |  |
| Interatrial septum | PFO, L – R Shunt.  5mm Ostium Primum Defect, L – R Shunt | Mitral | ----- |
| **Semilunar valves** |  | Aortic | ------- |
| Aortic valve | Annulus = 12mm | Tricuspid | ------- |
| Pulmonary valve | Annulus = 12mm | pulmonic | -------- |
| **Great arteries** | NRGA | **Aortic arch** | Left. No CoA. |
| Aorta | ----- | **PDA** | No |
| Pulmonary artery | Normal MPA and Branch PAs. |  |  |
| **M-Mode:**  Normal LV Function on eye balling. | | | |
| AO | mm | PWd | mm |
| LA | mm | PWs | mm |
| LVIDd | mm | EDV | ml |
| LVIDs | mm | ESV | ml |
| IVSs | mm | LVEF | % |
| IVSd | mm | FS | % |
| **Additional Information**: |  | | |
| No pericardial/Pleural effusion. | | | |
| **Final Diagnosis:** | | | |
| 1. {S, D, S} Levocardia. 2. PFO, L – R Shunt 3. Small Ostium Primum Defect, L – R Shunt | | | |
| **Remark**: | | | |
| **Recommendation**: | | | |
| SIGNATURE  Done by: Tesfaye T., Pediatrician, Pediatric Cardiologist _______________ 05/07/2015Eth.C | | | |

| Patient Name: **Baby of Werknesh Tsega**. Referring Institute: **TGSH**. SEX/ Age: **M/12days**. Date of Report: **05/07/15**.  Referral Diagnosis: **Fast breathing. AGH12.3365.** | | | |
| --- | --- | --- | --- |
| **Features** | **Finding** | **Features** | **Finding** |
| **Profile** |  | **Atria** |  |
| Abdominal situs | Solitus | Left atrium | Normal |
| Cardiac position | Levocardia | Right atrium | Normal |
| Systemic venous drainage | Normal. | **Atrioventricular valves** |  |
| Pulmonary venous drainage | Normal | Mitral valve | Annulus = 12mm |
| Atrioventricular connection | Concordant | Tricuspid valve | Annulus = 11mm |
| Ventriculoarterial connection | Concordant | **Ventricles** |  |
| Ventricular loop | d-Loop | Left ventricle | Normal |
|  |  | Right ventricle | Normal |
| **Septae** |  | **Coronary arteries** | ----- |
| Interventricular septum | Intact | **Doppler Measurement** |  |
| Interatrial septum | Intact | Mitral | ----- |
| **Semilunar valves** |  | Aortic | ------- |
| Aortic valve | Annulus = 8mm | Tricuspid | ------- |
| Pulmonary valve | Annulus = 11mm | pulmonic | -------- |
| **Great arteries** | NRGA | **Aortic arch** | Left. No CoA. |
| Aorta | ----- | **PDA** | No |
| Pulmonary artery | Normal MPA and Branch PAs. |  |  |
| **M-Mode:**  Normal LV Function on eye balling. | | | |
| AO | mm | PWd | mm |
| LA | mm | PWs | mm |
| LVIDd | mm | EDV | ml |
| LVIDs | mm | ESV | ml |
| IVSs | mm | LVEF | % |
| IVSd | mm | FS | % |
| **Additional Information**: |  | | |
| No pericardial/Pleural effusion. | | | |
| **Final Diagnosis:** | | | |
| 1. Normal Echocardiography Study. | | | |
| **Remark**: | | | |
| **Recommendation**: | | | |
| SIGNATURE  Done by: Tesfaye T., Pediatrician, Pediatric Cardiologist _______________ 05/07/2015Eth.C | | | |

| Patient Name: **Baby of Birhan Admasu**. Referring Institute: **TGSH**. SEX/ Age: **F/5days**. Date of Report: **05/07/15**.  Referral Diagnosis: **?Pat au Syndrome. AGH12.3366.** | | | |
| --- | --- | --- | --- |
| **Features** | **Finding** | **Features** | **Finding** |
| **Profile** |  | **Atria** |  |
| Abdominal situs | Solitus | Left atrium | Normal |
| Cardiac position | Levocardia | Right atrium | Dilated |
| Systemic venous drainage | Normal. | **Atrioventricular valves** |  |
| Pulmonary venous drainage | Normal | Mitral valve | Annulus = 8mm |
| Atrioventricular connection | Concordant | Tricuspid valve | Annulus = 11mm  TAPSE = 12mm |
| Ventriculoarterial connection | DORV | **Ventricles** |  |
| Ventricular loop | d-Loop | Left ventricle | Normal |
|  |  | Right ventricle | Dilated |
| **Septae** |  | **Coronary arteries** | ----- |
| Interventricular septum | Non Restrictive Sub-aortic VSD, L – R Shunt. | **Doppler Measurement** |  |
| Interatrial septum | 9mm OS ASD, L – R Shunt | Mitral | ----- |
| **Semilunar valves** |  | Aortic | ------- |
| Aortic valve | Annulus = 8mm | Tricuspid | ------- |
| Pulmonary valve | Annulus = 6mm | pulmonic | Severe Valvular and supra valvular PS, PPG = 61mmHg. |
| **Great arteries** | NRGA | **Aortic arch** | Left. No CoA. |
| Aorta | Posterior, to the right and from RV | **PDA** | No |
| Pulmonary artery | Smallish MPA and Branch PAs. Anterior to the left and from RV. |  |  |
| **M-Mode:** | | | |
| AO | mm | PWd | mm |
| LA | mm | PWs | mm |
| LVIDd | mm | EDV | ml |
| LVIDs | mm | ESV | ml |
| IVSs | mm | LVEF | % |
| IVSd | mm | FS | % |
| **Additional Information**: |  | | |
| No pericardial/Pleural effusion. | | | |
| **Final Diagnosis:** | | | |
| 1. {S, D, D} Levocardia. 2. DORV 3. Moderate OS ASD, L – R Shunt 4. Non – Restrictive Sub-aortic VSD, L – R Shunt 5. Severe Valvular and supra valvular PS | | | |
| **Recommendation**: | | | |
| SIGNATURE  Done by: Tesfaye T., Pediatrician, Pediatric Cardiologist _______________ 05/07/2015Eth.C | | | |

| Patient Name: **Baby of Birtukan Yihun**. Referring Institute: **TGSH**. SEX/ Age: **M/1 6/12**. Date of Report: **06/07/15**.  Referral Diagnosis: **Follow up echo for Severe PHTN (PPHTN, age = 8days), Moderate PHTN (age = 27days) & Mild PHTN (age = 4months).** | | | |
| --- | --- | --- | --- |
| **Features** | **Finding** | **Features** | **Finding** |
| **Profile** |  | **Atria** |  |
| Abdominal situs | Solitus | Left atrium | Normal |
| Cardiac position | Levocardia | Right atrium | Normal |
| Systemic venous drainage | Normal. | **Atrioventricular valves** |  |
| Pulmonary venous drainage | Normal | Mitral valve | Annulus = 17mm |
| Atrioventricular connection | Concordant | Tricuspid valve | Annulus = 18mm  TAPSE = 18mm |
| Ventriculoarterial connection | Concordant | **Ventricles** |  |
| Ventricular loop | d-Loop | Left ventricle | Normal |
|  |  | Right ventricle | Normal |
| **Septae** |  | **Coronary arteries** | ----- |
| Interventricular septum | Intact | **Doppler Measurement** |  |
| Interatrial septum | Intact | Mitral | ----- |
| **Semilunar valves** |  | Aortic | ------- |
| Aortic valve | Annulus = 13mm | Tricuspid | Trivial TR, PPG = 25mmHg. |
| Pulmonary valve | Annulus = 15mm | pulmonic | -------- |
| **Great arteries** | NRGA | **Aortic arch** | Left. No CoA. |
| Aorta | ----- | **PDA** | No |
| Pulmonary artery | Normal MPA and Branch PAs. |  |  |
| **M-Mode:** | | | |
| AO | mm | PWd | mm |
| LA | mm | PWs | mm |
| LVIDd | mm | EDV | ml |
| LVIDs | mm | ESV | ml |
| IVSs | mm | LVEF | 70% |
| IVSd | mm | FS | 38% |
| **Additional Information**: |  | | |
| No pericardial/Pleural effusion. | | | |
| **Final Diagnosis:** | | | |
| 1. Normal Echocardiography Study. | | | |
| **Remark**: The PPHTN has resolved. | | | |
| **Recommendation**:   1. No need to have cardiac follow up and treatment for Pulmonary Hypertension. 2. Discharge from follow up | | | |
| SIGNATURE  Done by: Tesfaye T., Pediatrician, Pediatric Cardiologist _______________ 06/07/2015Eth.C | | | |

| Patient Name: **Baby of** **Wubie Kassie**. Referring Institute: **FHRH**. SEX/ Age: **F/20days**. Date of Report: **07/07/15**.  Referral Diagnosis: **?CHD (RD). AGH12.3367.** | | | |
| --- | --- | --- | --- |
| **Features** | **Finding** | **Features** | **Finding** |
| **Profile** |  | **Atria** |  |
| Abdominal situs | Solitus | Left atrium | Normal |
| Cardiac position | Levocardia | Right atrium | Normal |
| Systemic venous drainage | Normal. | **Atrioventricular valves** |  |
| Pulmonary venous drainage | Normal | Mitral valve | Annulus = 8mm |
| Atrioventricular connection | Concordant | Tricuspid valve | Annulus = 8mm  TAPSE = mm |
| Ventriculoarterial connection | Concordant | **Ventricles** |  |
| Ventricular loop | d-Loop | Left ventricle | Normal |
|  |  | Right ventricle | Normal |
| **Septae** |  | **Coronary arteries** | ----- |
| Interventricular septum | Intact | **Doppler Measurement** |  |
| Interatrial septum | Intact | Mitral | ----- |
| **Semilunar valves** |  | Aortic | ------- |
| Aortic valve | Annulus = 9mm | Tricuspid | ------- |
| Pulmonary valve | Annulus = 8mm | pulmonic | -------- |
| **Great arteries** | NRGA | **Aortic arch** | Left. No CoA. |
| Aorta | ----- | **PDA** | No |
| Pulmonary artery | Normal MPA and Branch PAs. |  |  |
| **M-Mode:**  Normal LV Function on eye balling. | | | |
| AO | mm | PWd | mm |
| LA | mm | PWs | mm |
| LVIDd | mm | EDV | ml |
| LVIDs | mm | ESV | ml |
| IVSs | mm | LVEF | % |
| IVSd | mm | FS | % |
| **Additional Information**: |  | | |
| No pericardial/Pleural effusion. | | | |
| **Final Diagnosis:** | | | |
| 1. Normal Echocardiography Study. | | | |
| **Remark**: | | | |
| **Recommendation**: | | | |
| SIGNATURE  Done by: Tesfaye T., Pediatrician, Pediatric Cardiologist _______________ 07/07/2015Eth.C | | | |

| Patient Name: **Tsion Muluken**. Referring Institute: **Addis Alem PH**. SEX/ Age: **F/7years**. Date of Report: **08/07/15**.  Referral Diagnosis: **Easy Fatigability. AGH12.3368.** | | | |
| --- | --- | --- | --- |
| **Features** | **Finding** | **Features** | **Finding** |
| **Profile** |  | **Atria** |  |
| Abdominal situs | Solitus | Left atrium | Normal |
| Cardiac position | Levocardia | Right atrium | Normal |
| Systemic venous drainage | Normal. | **Atrioventricular valves** |  |
| Pulmonary venous drainage | Normal | Mitral valve | Annulus = 21mm |
| Atrioventricular connection | Concordant | Tricuspid valve | Annulus = 20mm  TAPSE = 18mm |
| Ventriculoarterial connection | Concordant | **Ventricles** |  |
| Ventricular loop | d-Loop | Left ventricle | Normal |
|  |  | Right ventricle | Normal |
| **Septae** |  | **Coronary arteries** | ----- |
| Interventricular septum | Intact | **Doppler Measurement** |  |
| Interatrial septum | Intact | Mitral | ----- |
| **Semilunar valves** |  | Aortic | ------- |
| Aortic valve | Annulus = 15mm | Tricuspid | ------- |
| Pulmonary valve | Annulus = 19mm | pulmonic | -------- |
| **Great arteries** | NRGA | **Aortic arch** | Left. No CoA. |
| Aorta | ----- | **PDA** | No |
| Pulmonary artery | Normal MPA and Branch PAs. |  |  |
| **M-Mode:** | | | |
| AO | mm | PWd | mm |
| LA | mm | PWs | mm |
| LVIDd | mm | EDV | ml |
| LVIDs | mm | ESV | ml |
| IVSs | mm | LVEF | 65% |
| IVSd | mm | FS | 35% |
| **Additional Information**: |  | | |
| No pericardial/Pleural effusion. | | | |
| **Final Diagnosis:** | | | |
| 1. Normal Echocardiography Study. | | | |
| **Remark**: | | | |
| **Recommendation**: | | | |
| SIGNATURE  Done by: Tesfaye T., Pediatrician, Pediatric Cardiologist _______________ 08/07/2015Eth.C | | | |

| Patient Name: **Wuyzir Adane**. Referring Institute: **FHRH**. SEX/ Age: **F/11years**. Date of Report: **08/07/15**.  Referral Diagnosis: **R/O CRVHD + Chest Pain.** | | | |
| --- | --- | --- | --- |
| **Features** | **Finding** | **Features** | **Finding** |
| **Profile** |  | **Atria** |  |
| Abdominal situs | Solitus | Left atrium | Normal |
| Cardiac position | Levocardia | Right atrium | Normal |
| Systemic venous drainage | Normal. | **Atrioventricular valves** |  |
| Pulmonary venous drainage | Normal | Mitral valve | Annulus = 18mm |
| Atrioventricular connection | Concordant | Tricuspid valve | Annulus = 17mm |
| Ventriculoarterial connection | Concordant | **Ventricles** |  |
| Ventricular loop | d-Loop | Left ventricle | Normal |
|  |  | Right ventricle | Normal |
| **Septae** |  | **Coronary arteries** | ----- |
| Interventricular septum | Intact | **Doppler Measurement** |  |
| Interatrial septum | Intact | Mitral | ----- |
| **Semilunar valves** |  | Aortic | ------- |
| Aortic valve | Annulus = 17mm | Tricuspid | ------- |
| Pulmonary valve | Annulus = 19mm | pulmonic | -------- |
| **Great arteries** | NRGA | **Aortic arch** | Left. No CoA. |
| Aorta | ----- | **PDA** | No |
| Pulmonary artery | Normal MPA and Branch PAs. |  |  |
| **M-Mode:** | | | |
| AO | mm | PWd | mm |
| LA | mm | PWs | mm |
| LVIDd | mm | EDV | ml |
| LVIDs | mm | ESV | ml |
| IVSs | mm | LVEF | % |
| IVSd | mm | FS | % |
| **Additional Information**: |  | | |
| Pericardial effusion with maximum depth of 29mm on LV Side and 4mm on RV Side. Echodebris seen. | | | |
| **Final Diagnosis:** | | | |
| 1. {S, D, S} Levocardia. 2. Large Pericardial effusion. ? Purulent | | | |
| **Remark**: | | | |
| **Recommendation**: | | | |
| SIGNATURE  Done by: Tesfaye T., Pediatrician, Pediatric Cardiologist _______________ 08/07/2015Eth.C | | | |

| Patient Name: **Belay Mihretie**. Referring Institute: **FHRH**. SEX/ Age: **M/10years**. Date of Report: **08/07/15**.  Referral Diagnosis: **ARF + Murmur.** | | | |
| --- | --- | --- | --- |
| **Features** | **Finding** | **Features** | **Finding** |
| **Profile** |  | **Atria** |  |
| Abdominal situs | Solitus | Left atrium | Normal |
| Cardiac position | Levocardia | Right atrium | Normal |
| Systemic venous drainage | Normal. | **Atrioventricular valves** |  |
| Pulmonary venous drainage | Normal | Mitral valve | Annulus = 22mm. Thickened MVL |
| Atrioventricular connection | Concordant | Tricuspid valve | Annulus = 20mm |
| Ventriculoarterial connection | Concordant | **Ventricles** |  |
| Ventricular loop | d-Loop | Left ventricle | Normal |
|  |  | Right ventricle | Normal |
| **Septae** |  | **Coronary arteries** | ----- |
| Interventricular septum | Intact | **Doppler Measurement** |  |
| Interatrial septum | Intact | Mitral | Moderate MR, Holosystolic, seen in two planes with jet velocity = 4m/sec. |
| **Semilunar valves** |  | Aortic | Mild AR |
| Aortic valve | Annulus = 15mm | Tricuspid | ------- |
| Pulmonary valve | Annulus = 16mm | pulmonic | -------- |
| **Great arteries** | NRGA | **Aortic arch** | Left. No CoA. |
| Aorta | ----- | **PDA** | No |
| Pulmonary artery | Normal MPA and Branch PAs. |  |  |
| **M-Mode:** | | | |
| AO | mm | PWd | mm |
| LA | mm | PWs | mm |
| LVIDd | mm | EDV | ml |
| LVIDs | mm | ESV | ml |
| IVSs | mm | LVEF | 70% |
| IVSd | mm | FS | 39% |
| **Additional Information**: |  | | |
| Pericardial effusion with maximum depth of 4mm. | | | |
| **Final Diagnosis:** | | | |
| 1. {S, D, S} Levocardia. 2. Thickened MVL 3. Moderate MR 4. Mild AR 5. Normal LV Systolic Function | | | |
| **Remark**: | | | |
| **Recommendation**: | | | |
| SIGNATURE  Done by: Tesfaye T., Pediatrician, Pediatric Cardiologist _______________ 08/07/2015Eth.C | | | |

| Patient Name: **Eleni Begosew**. Referring Institute: **TGSH**. SEX/ Age: **F/4years**. Date of Report: **08/07/15**.  Referral Diagnosis: **Acute Rheumatic Fever with Carditis. AGH12.3371.** | | | |
| --- | --- | --- | --- |
| **Features** | **Finding** | **Features** | **Finding** |
| **Profile** |  | **Atria** |  |
| Abdominal situs | Solitus | Left atrium | Mildly Dilated |
| Cardiac position | Levocardia | Right atrium | Normal |
| Systemic venous drainage | **IVC Dilated** | **Atrioventricular valves** |  |
| Pulmonary venous drainage | Normal | Mitral valve | Annulus = 21mm. Thickened MVL. |
| Atrioventricular connection | Concordant | Tricuspid valve | Annulus = 22mm  TAPSE = 18mm |
| Ventriculoarterial connection | Concordant | **Ventricles** |  |
| Ventricular loop | d-Loop | Left ventricle | Mildly Dilated |
|  |  | Right ventricle | Normal |
| **Septae** |  | **Coronary arteries** | ----- |
| Interventricular septum | Intact | **Doppler Measurement** |  |
| Interatrial septum | Intact | Mitral | Moderate MR, Holosystolic, Posterior projection, seen in two planes with jet velocity = 3.7m/sec. |
| **Semilunar valves** |  | Aortic | ------- |
| Aortic valve | Annulus = 16mm | Tricuspid | Moderate TR, PPG = 41mmHg |
| Pulmonary valve | Annulus = 20mm | pulmonic | -------- |
| **Great arteries** | NRGA | **Aortic arch** | Left. No CoA. |
| Aorta | ----- | **PDA** | No |
| Pulmonary artery | Normal |  |  |
| **M-Mode:** | | | |
| AO | mm | PWd | mm |
| LA | mm | PWs | mm |
| LVIDd | mm | EDV | ml |
| LVIDs | mm | ESV | ml |
| IVSs | mm | LVEF | 58% |
| IVSd | mm | FS | 29% |
| **Additional Information**: |  | | |
| No pericardial/Pleural effusion. | | | |
| **Final Diagnosis:** | | | |
| 1. {S, D, S} Levocardia. 2. LA/LV Mildly Dilated 3. Thickened MVL 4. Moderate MR 5. Moderate TR 6. Mild Pulmonary Hypertension 7. Normal Biventricular Systolic Function | | | |
| **Remark**: In Congestive Heart Failure | | | |
| **Recommendation**: Admit her | | | |
| SIGNATURE  Done by: Tesfaye T., Pediatrician, Pediatric Cardiologist _______________ 08/07/2015Eth.C | | | |

| Patient Name: **Atitegeb Tazeb**. Referring Institute: **TGSH**. SEX/ Age: **F/12years**. Date of Report: **08/07/15**.  Referral Diagnosis: **?CRVHD. AGH12.3372.** | | | |
| --- | --- | --- | --- |
| **Features** | **Finding** | **Features** | **Finding** |
| **Profile** |  | **Atria** |  |
| Abdominal situs | Solitus | Left atrium | Markedly Dilated |
| Cardiac position | Levocardia | Right atrium | Normal |
| Systemic venous drainage | Normal. | **Atrioventricular valves** |  |
| Pulmonary venous drainage | Normal | Mitral valve | Annulus = 26mm. Thickened MVL. |
| Atrioventricular connection | Concordant | Tricuspid valve | Annulus = 23mm  TAPSE = 19mm |
| Ventriculoarterial connection | Concordant | **Ventricles** |  |
| Ventricular loop | d-Loop | Left ventricle | Markedly Dilated & Dysfunctional |
|  |  | Right ventricle | Normal |
| **Septae** |  | **Coronary arteries** | ----- |
| Interventricular septum | Intact | **Doppler Measurement** |  |
| Interatrial septum | Intact | Mitral | Moderate MR, Holosystolic, posterior projection, seen in two planes with jet velocity = 3.2m/sec. |
| **Semilunar valves** |  | Aortic | Severe AR |
| Aortic valve | Annulus = 20mm | Tricuspid | ------- |
| Pulmonary valve | Annulus = 22mm | pulmonic | -------- |
| **Great arteries** | NRGA | **Aortic arch** | Left. No CoA. |
| Aorta | ----- | **PDA** | No |
| Pulmonary artery | Normal MPA and Branch PAs. |  |  |
| **M-Mode:** | | | |
| AO | mm | PWd | mm |
| LA | mm | PWs | mm |
| LVIDd | mm | EDV | ml |
| LVIDs | mm | ESV | ml |
| IVSs | mm | LVEF | 36% |
| IVSd | mm | FS | 18% |
| **Additional Information**: |  | | |
| No pericardial/Pleural effusion. | | | |
| **Final Diagnosis:** | | | |
| 1. {S, D, S} Levocardia. 2. LA/LV Markedly Dilated 3. Thickened MVL 4. Moderate MR 5. Severe AR 6. Reduced LV Systolic Function | | | |
| SIGNATURE  Done by: Tesfaye T., Pediatrician, Pediatric Cardiologist _______________ 08/07/2015Eth.C | | | |

| Patient Name: **Endesew Fekad**. Referring Institute: **FHRH**. SEX/ Age: **M/2years**. Date of Report: **08/07/15**.  Referral Diagnosis: **CHF + FTT + RD + Murmur. AGH12.3373.** | | | |
| --- | --- | --- | --- |
| **Features** | **Finding** | **Features** | **Finding** |
| **Profile** |  | **Atria** |  |
| Abdominal situs | Solitus | Left atrium | Dilated |
| Cardiac position | Levocardia | Right atrium | Dilated |
| Systemic venous drainage | Normal. | **Atrioventricular valves** |  |
| Pulmonary venous drainage | Normal | Mitral valve | Annulus = 21mm |
| Atrioventricular connection | Concordant | Tricuspid valve | Annulus = 19mm  TAPSE = 19mm |
| Ventriculoarterial connection | Truncus Arteriosus | **Ventricles** |  |
| Ventricular loop | d-Loop | Left ventricle | Dilated |
|  |  | Right ventricle | Dilated |
| **Septae** |  | **Coronary arteries** | ----- |
| Interventricular septum | Non-Restrictive Sub Truncal VSD, R – L Shunt | **Doppler Measurement** |  |
| Interatrial septum | Intact | Mitral | ----- |
| **Semilunar valves** |  | Aortic | ------- |
| Aortic valve | Truncal Annulus = 20mm | Tricuspid | ------- |
| Pulmonary valve |  | pulmonic | -------- |
| **Great arteries** | Truncus | **Aortic arch** | Left. No CoA. |
| Aorta | ----- | **PDA** | No |
| Pulmonary artery | The right and left Branch PAs arise directly from Truncus at the same plane.  Right annulus = 8mm  Left annulus = 8mm. |  |  |
| **M-Mode:** | | | |
| AO | mm | PWd | mm |
| LA | mm | PWs | mm |
| LVIDd | mm | EDV | ml |
| LVIDs | mm | ESV | ml |
| IVSs | mm | LVEF | 71% |
| IVSd | mm | FS | 38% |
| **Additional Information**: |  | | |
| No pericardial/Pleural effusion. | | | |
| **Final Diagnosis:** | | | |
| 1. {S, D, S} Levocardia. 2. All chambers dilated 3. Truncus Arteriosus type II 4. Normal LV Systolic Function | | | |
| SIGNATURE  Done by: Tesfaye T., Pediatrician, Pediatric Cardiologist _______________ 08/07/2015Eth.C | | | |

| Patient Name: **Amanuel Ambachew**. Referring Institute: **FHRH**. SEX/ Age: **M/1 9/12**. Date of Report: **11/07/15**.  Referral Diagnosis: **Follow up echo for Small OS ASD.** | | | |
| --- | --- | --- | --- |
| **Features** | **Finding** | **Features** | **Finding** |
| **Profile** |  | **Atria** |  |
| Abdominal situs | Solitus | Left atrium | Normal |
| Cardiac position | Levocardia | Right atrium | Normal |
| Systemic venous drainage | Normal. | **Atrioventricular valves** |  |
| Pulmonary venous drainage | Normal | Mitral valve | Annulus = 13mm |
| Atrioventricular connection | Concordant | Tricuspid valve | Annulus = 14mm |
| Ventriculoarterial connection | Concordant | **Ventricles** |  |
| Ventricular loop | d-Loop | Left ventricle | Normal |
|  |  | Right ventricle | Normal |
| **Septae** |  | **Coronary arteries** | ----- |
| Interventricular septum | intact | **Doppler Measurement** |  |
| Interatrial septum | Intact | Mitral | ----- |
| **Semilunar valves** |  | Aortic | ------- |
| Aortic valve | Annulus = 11mm | Tricuspid | ------- |
| Pulmonary valve | Annulus = 12mm | pulmonic | -------- |
| **Great arteries** | NRGA | **Aortic arch** | Left. No CoA. |
| Aorta | ----- | **PDA** | No |
| Pulmonary artery | Normal MPA and Branch PAs. |  |  |
| **M-Mode:**  Normal LV Function on eye balling | | | |
| AO | mm | PWd | mm |
| LA | mm | PWs | mm |
| LVIDd | mm | EDV | ml |
| LVIDs | mm | ESV | ml |
| IVSs | mm | LVEF | % |
| IVSd | mm | FS | % |
| **Additional Information**: |  | | |
| No pericardial/Pleural effusion. | | | |
| **Final Diagnosis:** | | | |
| 1. Normal Echocardiography Study. | | | |
| **Remark**: | | | |
| **Recommendation**: | | | |
| SIGNATURE  Done by: Tesfaye T., Pediatrician, Pediatric Cardiologist _______________ 11/07/2015Eth.C | | | |

| Patient Name: **Hayat Abdela**. Referring Institute: **APSC**. SEX/ Age: **F/3years**. Date of Report: **11/07/15**.  Referral Diagnosis: **Incidental Finding ?PDA. AGH12.3374.** | | | |
| --- | --- | --- | --- |
| **Features** | **Finding** | **Features** | **Finding** |
| **Profile** |  | **Atria** |  |
| Abdominal situs | Solitus | Left atrium | Mildly Dilated |
| Cardiac position | Levocardia | Right atrium | Normal |
| Systemic venous drainage | Normal. | **Atrioventricular valves** |  |
| Pulmonary venous drainage | Normal | Mitral valve | Annulus = 17mm |
| Atrioventricular connection | Concordant | Tricuspid valve | Annulus = 16mm |
| Ventriculoarterial connection | Concordant | **Ventricles** |  |
| Ventricular loop | d-Loop | Left ventricle | Mildly Dilated |
|  |  | Right ventricle | Normal |
| **Septae** |  | **Coronary arteries** | ----- |
| Interventricular septum | Intact | **Doppler Measurement** |  |
| Interatrial septum | Intact | Mitral | ----- |
| **Semilunar valves** |  | Aortic | ------- |
| Aortic valve | Annulus = 14mm | Tricuspid | ------- |
| Pulmonary valve | Annulus = 16mm | pulmonic | -------- |
| **Great arteries** | NRGA | **Aortic arch** | Left. No CoA. |
| Aorta | ----- | **PDA** | 1.5mm PDA, L – R Shunt. Funnel Shaped |
| Pulmonary artery | Normal MPA and Branch PAs. |  |  |
| **M-Mode:**  Normal LV Function on eye balling | | | |
| AO | mm | PWd | mm |
| LA | mm | PWs | mm |
| LVIDd | mm | EDV | ml |
| LVIDs | mm | ESV | ml |
| IVSs | mm | LVEF | % |
| IVSd | mm | FS | % |
| **Additional Information**: |  | | |
| No pericardial/Pleural effusion. | | | |
| **Final Diagnosis:** | | | |
| 1. {S, D, S} Levocardia. 2. Small PDA, L – R Shunt | | | |
| **Remark**: | | | |
| **Recommendation**: | | | |
| SIGNATURE  Done by: Tesfaye T., Pediatrician, Pediatric Cardiologist _______________ 11/07/2015Eth.C | | | |

| Patient Name: **Fikir Zelalem**. Referring Institute: **Adinas GH**. SEX/ Age: **M/2 5/12**. Date of Report: **11/07/15**.  Referral Diagnosis: **Follow up echo for Large PDA(4mm), L – R Shunt.** | | | |
| --- | --- | --- | --- |
| **Features** | **Finding** | **Features** | **Finding** |
| **Profile** |  | **Atria** |  |
| Abdominal situs | Solitus | Left atrium | Mildly Dilated |
| Cardiac position | Levocardia | Right atrium | Normal |
| Systemic venous drainage | Normal. | **Atrioventricular valves** |  |
| Pulmonary venous drainage | Normal | Mitral valve | Annulus = 19mm |
| Atrioventricular connection | Concordant | Tricuspid valve | Annulus = 18mm |
| Ventriculoarterial connection | Concordant | **Ventricles** |  |
| Ventricular loop | d-Loop | Left ventricle | Mildly Dilated |
|  |  | Right ventricle | Normal |
| **Septae** |  | **Coronary arteries** | ----- |
| Interventricular septum | Intact | **Doppler Measurement** |  |
| Interatrial septum | Intact | Mitral | ----- |
| **Semilunar valves** |  | Aortic | ------- |
| Aortic valve | Annulus = 15mm | Tricuspid | ------- |
| Pulmonary valve | Annulus = 19mm | pulmonic | -------- |
| **Great arteries** | NRGA | **Aortic arch** | Left. No CoA. |
| Aorta | ----- | **PDA** | 4mm PDA, L – R Shunt |
| Pulmonary artery | Normal MPA and Branch PAs. |  |  |
| **M-Mode:** | | | |
| AO | mm | PWd | mm |
| LA | mm | PWs | mm |
| LVIDd | mm | EDV | ml |
| LVIDs | mm | ESV | ml |
| IVSs | mm | LVEF | 74% |
| IVSd | mm | FS | 40% |
| **Additional Information**: |  | | |
| No pericardial/Pleural effusion. | | | |
| **Final Diagnosis:** | | | |
| 1. {S, D, S} Levocardia. 2. LA/LV Mildly Dilated 3. Large PDA, L – R Shunt | | | |
| **Remark**: | | | |
| **Recommendation**: | | | |
| SIGNATURE  Done by: Tesfaye T., Pediatrician, Pediatric Cardiologist _______________ 11/07/2015Eth.C | | | |

| Patient Name: **Rifan Muhammed**. Referring Institute: **Adinas GH**. SEX/ Age: **F/4 2/12**. Date of Report: **11/07/15**.  Referral Diagnosis: **Incidental Finding. AGH12.3375.** | | | |
| --- | --- | --- | --- |
| **Features** | **Finding** | **Features** | **Finding** |
| **Profile** |  | **Atria** |  |
| Abdominal situs | Solitus | Left atrium | Normal |
| Cardiac position | Levocardia | Right atrium | Dilated |
| Systemic venous drainage | Normal. | **Atrioventricular valves** |  |
| Pulmonary venous drainage | Normal | Mitral valve | Annulus = 16mm |
| Atrioventricular connection | Concordant | Tricuspid valve | Annulus = 17mm  TAPSE = 18mm |
| Ventriculoarterial connection | Concordant | **Ventricles** |  |
| Ventricular loop | d-Loop | Left ventricle | Normal |
|  |  | Right ventricle | Dilated |
| **Septae** |  | **Coronary arteries** | ----- |
| Interventricular septum | Intact | **Doppler Measurement** |  |
| Interatrial septum | 13mm Fenestrated OS ASD, L – R Shunt | Mitral | ----- |
| **Semilunar valves** |  | Aortic | ------- |
| Aortic valve | Annulus = mm | Tricuspid | ------- |
| Pulmonary valve | Annulus = 17mm. Doming PV. | pulmonic | Moderate Valvular PS, PPG = 53mmHg. |
| **Great arteries** | NRGA | **Aortic arch** | Left. No CoA. |
| Aorta | ----- | **PDA** | No |
| Pulmonary artery | Normal MPA and Branch PAs. |  |  |
| **M-Mode:** | | | |
| AO | mm | PWd | mm |
| LA | mm | PWs | mm |
| LVIDd | mm | EDV | ml |
| LVIDs | mm | ESV | ml |
| IVSs | mm | LVEF | 67% |
| IVSd | mm | FS | 35% |
| **Additional Information**: |  | | |
| No pericardial/Pleural effusion. | | | |
| **Final Diagnosis:** | | | |
| 1. {S, D, S} Levocardia. 2. RA/RV Dilated 3. Large Fenestrated OS ASD, L – R Shunt 4. Doming Pulmonary Valve 5. Moderate Valvular PS 6. Normal Biventricular Systolic Function | | | |
| **Recommendation**: | | | |
| SIGNATURE  Done by: Tesfaye T., Pediatrician, Pediatric Cardiologist _______________ 11/07/2015Eth.C | | | |

| Patient Name: **Filmon Pawulos**. Referring Institute: **Adinas GH**. SEX/ Age: **M/5 4/12**. Date of Report: **11/07/15**.  Referral Diagnosis: **ARF. AGH12.3376.** | | | |
| --- | --- | --- | --- |
| **Features** | **Finding** | **Features** | **Finding** |
| **Profile** |  | **Atria** |  |
| Abdominal situs | Solitus | Left atrium | Normal |
| Cardiac position | Levocardia | Right atrium | Normal |
| Systemic venous drainage | Normal. | **Atrioventricular valves** |  |
| Pulmonary venous drainage | Normal | Mitral valve | Annulus = 20mm |
| Atrioventricular connection | Concordant | Tricuspid valve | Annulus = 21mm  TAPSE = 19mm |
| Ventriculoarterial connection | Concordant | **Ventricles** |  |
| Ventricular loop | d-Loop | Left ventricle | Normal |
|  |  | Right ventricle | Normal |
| **Septae** |  | **Coronary arteries** | ----- |
| Interventricular septum | Intact | **Doppler Measurement** |  |
| Interatrial septum | Intact | Mitral | ----- |
| **Semilunar valves** |  | Aortic | ------- |
| Aortic valve | Annulus = 17mm | Tricuspid | ------- |
| Pulmonary valve | Annulus = 17mm | pulmonic | -------- |
| **Great arteries** | NRGA | **Aortic arch** | Left. No CoA. |
| Aorta | ----- | **PDA** | No |
| Pulmonary artery | Normal MPA and Branch PAs. |  |  |
| **M-Mode:** | | | |
| AO | mm | PWd | mm |
| LA | mm | PWs | mm |
| LVIDd | mm | EDV | ml |
| LVIDs | mm | ESV | ml |
| IVSs | mm | LVEF | 69% |
| IVSd | mm | FS | 38% |
| **Additional Information**: |  | | |
| No pericardial/Pleural effusion. | | | |
| **Final Diagnosis:** | | | |
| 1. Normal Echocardiography Study. | | | |
| **Remark**: No features of carditis | | | |
| **Recommendation**: | | | |
| SIGNATURE  Done by: Tesfaye T., Pediatrician, Pediatric Cardiologist _______________ 11/07/2015Eth.C | | | |

| Patient Name: **Wubnesh Fentie**. Referring Institute: **FHRH**. SEX/ Age: **F/9 9/12**. Date of Report: **12/07/15**.  Referral Diagnosis: **Sydenham’s Chorea. AGH12.3377.** | | | |
| --- | --- | --- | --- |
| **Features** | **Finding** | **Features** | **Finding** |
| **Profile** |  | **Atria** |  |
| Abdominal situs | Solitus | Left atrium | Normal |
| Cardiac position | Levocardia | Right atrium | Normal |
| Systemic venous drainage | Normal. | **Atrioventricular valves** |  |
| Pulmonary venous drainage | Normal | Mitral valve | Annulus = 18mm |
| Atrioventricular connection | Concordant | Tricuspid valve | Annulus = 18mm  TAPSE = 18mm |
| Ventriculoarterial connection | Concordant | **Ventricles** |  |
| Ventricular loop | d-Loop | Left ventricle | Normal |
|  |  | Right ventricle | Normal |
| **Septae** |  | **Coronary arteries** | ----- |
| Interventricular septum | Intact | **Doppler Measurement** |  |
| Interatrial septum | Intact | Mitral | ----- |
| **Semilunar valves** |  | Aortic | ------- |
| Aortic valve | Annulus = 16mm | Tricuspid | ------- |
| Pulmonary valve | Annulus = 16mm | pulmonic | -------- |
| **Great arteries** | NRGA | **Aortic arch** | Left. No CoA. |
| Aorta | ----- | **PDA** | No |
| Pulmonary artery | Normal MPA and Branch PAs. |  |  |
| **M-Mode:** | | | |
| AO | mm | PWd | mm |
| LA | mm | PWs | mm |
| LVIDd | mm | EDV | ml |
| LVIDs | mm | ESV | ml |
| IVSs | mm | LVEF | 67% |
| IVSd | mm | FS | 35% |
| **Additional Information**: |  | | |
| No pericardial/Pleural effusion. | | | |
| **Final Diagnosis:** | | | |
| 1. Normal Echocardiography Study. | | | |
| **Remark**: Sydenham’s chorea can present in the absence of Carditis | | | |
| **Recommendation**: | | | |
| SIGNATURE  Done by: Tesfaye T., Pediatrician, Pediatric Cardiologist _______________ 12/07/2015Eth.C | | | |

| Patient Name: **Baby of Bete-khinet Lake**. Referring Institute: **TGSH**. SEX/ Age: **F/12days**. Date of Report: **12/07/15**.  Referral Diagnosis: **RD. AGH12.3378.** | | | |
| --- | --- | --- | --- |
| **Features** | **Finding** | **Features** | **Finding** |
| **Profile** |  | **Atria** |  |
| Abdominal situs | Solitus | Left atrium | Normal |
| Cardiac position | Levocardia | Right atrium | Normal |
| Systemic venous drainage | Normal. | **Atrioventricular valves** |  |
| Pulmonary venous drainage | Normal | Mitral valve | Annulus = 11mm |
| Atrioventricular connection | Concordant | Tricuspid valve | Annulus = 11mm |
| Ventriculoarterial connection | Concordant | **Ventricles** |  |
| Ventricular loop | d-Loop | Left ventricle | Normal |
|  |  | Right ventricle | Normal |
| **Septae** |  | **Coronary arteries** | ----- |
| Interventricular septum | Intact | **Doppler Measurement** |  |
| Interatrial septum | PFO, L – R Shunt | Mitral | ----- |
| **Semilunar valves** |  | Aortic | ------- |
| Aortic valve | Annulus = 10mm | Tricuspid | ------- |
| Pulmonary valve | Annulus = 10mm | pulmonic | -------- |
| **Great arteries** | NRGA | **Aortic arch** | Left. No CoA. |
| Aorta | ----- | **PDA** | No |
| Pulmonary artery | Normal MPA and Branch PAs. |  |  |
| **M-Mode:**  Normal LV Function on eye balling | | | |
| AO | mm | PWd | mm |
| LA | mm | PWs | mm |
| LVIDd | mm | EDV | ml |
| LVIDs | mm | ESV | ml |
| IVSs | mm | LVEF | % |
| IVSd | mm | FS | % |
| **Additional Information**: |  | | |
| No pericardial/Pleural effusion. | | | |
| **Final Diagnosis:** | | | |
| 1. {S, D, S} Levocardia. 2. PFO, L – R Shunt | | | |
| **Remark**: | | | |
| **Recommendation**: | | | |
| SIGNATURE  Done by: Tesfaye T., Pediatrician, Pediatric Cardiologist _______________ 12/07/2015Eth.C | | | |

| Patient Name: **Yetsedaw Arega**. Referring Institute: **FHRH**. SEX/ Age: **M/6months**. Date of Report: **12/07/15**.  Referral Diagnosis: **Recurrent chest infection. AGH12.3379.** | | | |
| --- | --- | --- | --- |
| **Features** | **Finding** | **Features** | **Finding** |
| **Profile** |  | **Atria** |  |
| Abdominal situs | Solitus | Left atrium | Normal |
| Cardiac position | Levocardia | Right atrium | Normal |
| Systemic venous drainage | Normal. | **Atrioventricular valves** |  |
| Pulmonary venous drainage | Normal | Mitral valve | Annulus = 14mm |
| Atrioventricular connection | Concordant | Tricuspid valve | Annulus = 15mm |
| Ventriculoarterial connection | Concordant | **Ventricles** |  |
| Ventricular loop | d-Loop | Left ventricle | Normal |
|  |  | Right ventricle | Hypertrophied |
| **Septae** |  | **Coronary arteries** | ----- |
| Interventricular septum | Non-Restrictive Sub-aortic SVD, R – L Shunt | **Doppler Measurement** |  |
| Interatrial septum | Intact | Mitral | ----- |
| **Semilunar valves** |  | Aortic | ------- |
| Aortic valve | Annulus = 13mm | Tricuspid | ------- |
| Pulmonary valve | Annulus = mm | pulmonic | Moderate Valvular, Supra Valvular and Supra-valvular PS, PPG = 50mmHg |
| **Great arteries** | NRGA | **Aortic arch** | Left. No CoA. |
| Aorta | Over-riding aorta | **PDA** | No |
| Pulmonary artery | Smallish MPA and Branch PAs. |  |  |
| **M-Mode:** | | | |
| AO | mm | PWd | mm |
| LA | mm | PWs | mm |
| LVIDd | mm | EDV | ml |
| LVIDs | mm | ESV | ml |
| IVSs | mm | LVEF | % |
| IVSd | mm | FS | % |
| **Additional Information**: |  | | |
| No pericardial/Pleural effusion. | | | |
| **Final Diagnosis:** | | | |
| 1. {S, D, S} Levocardia. 2. TOF | | | |
| **Remark**: | | | |
| **Recommendation**: | | | |
| SIGNATURE  Done by: Tesfaye T., Pediatrician, Pediatric Cardiologist _______________ 12/07/2015Eth.C | | | |

| Patient Name: **Ayalnesh Tarekegn**. Referring Institute: **FHRH**. SEX/ Age: **F/1 1/12**. Date of Report: **12/07/15**.  Referral Diagnosis: **SOB + Unable to crawl. AGH12.3380.** | | | |
| --- | --- | --- | --- |
| **Features** | **Finding** | **Features** | **Finding** |
| **Profile** |  | **Atria** |  |
| Abdominal situs | Solitus | Left atrium | Normal |
| Cardiac position | Levocardia | Right atrium | Normal |
| Systemic venous drainage | Normal. | **Atrioventricular valves** |  |
| Pulmonary venous drainage | Normal | Mitral valve | Annulus = 12mm |
| Atrioventricular connection | Concordant | Tricuspid valve | Annulus = 12mm |
| Ventriculoarterial connection | Concordant | **Ventricles** |  |
| Ventricular loop | d-Loop | Left ventricle | Normal |
|  |  | Right ventricle | Normal |
| **Septae** |  | **Coronary arteries** | ----- |
| Interventricular septum | Intact | **Doppler Measurement** |  |
| Interatrial septum | Intact | Mitral | ----- |
| **Semilunar valves** |  | Aortic | ------- |
| Aortic valve | Annulus = 12mm | Tricuspid | ------- |
| Pulmonary valve | Annulus = 11mm | pulmonic | -------- |
| **Great arteries** | NRGA | **Aortic arch** | Left. No CoA. |
| Aorta | ----- | **PDA** | No |
| Pulmonary artery | Normal MPA and Branch PAs. |  |  |
| **M-Mode:** | | | |
| AO | mm | PWd | mm |
| LA | mm | PWs | mm |
| LVIDd | mm | EDV | ml |
| LVIDs | mm | ESV | ml |
| IVSs | mm | LVEF | % |
| IVSd | mm | FS | % |
| **Additional Information**: |  | | |
| No pericardial/Pleural effusion. | | | |
| **Final Diagnosis:** | | | |
| 1. Normal Echocardiography Study. | | | |
| **Remark**: | | | |
| **Recommendation**: | | | |
| SIGNATURE  Done by: Tesfaye T., Pediatrician, Pediatric Cardiologist _______________ 12/07/2015Eth.C | | | |

| Patient Name: **Sefiw Delie**. Referring Institute: **FHRH**. SEX/ Age: **M/8years**. Date of Report: **12/07/15**.  Referral Diagnosis: **SOB (DOE). AGH12.3381.** | | | |
| --- | --- | --- | --- |
| **Features** | **Finding** | **Features** | **Finding** |
| **Profile** |  | **Atria** |  |
| Abdominal situs | Solitus | Left atrium | Normal |
| Cardiac position | Levocardia | Right atrium | Normal |
| Systemic venous drainage | Normal. | **Atrioventricular valves** |  |
| Pulmonary venous drainage | Normal | Mitral valve | Annulus = 19mm |
| Atrioventricular connection | Concordant | Tricuspid valve | Annulus = 21mm  TAPSE = 19mm |
| Ventriculoarterial connection | Concordant | **Ventricles** |  |
| Ventricular loop | d-Loop | Left ventricle | Normal |
|  |  | Right ventricle | Normal |
| **Septae** |  | **Coronary arteries** | ----- |
| Interventricular septum | Intact | **Doppler Measurement** |  |
| Interatrial septum | Intact | Mitral | ----- |
| **Semilunar valves** |  | Aortic | ------- |
| Aortic valve | Annulus = 17mm | Tricuspid | Trivial TR, PPG = 31mmHg |
| Pulmonary valve | Annulus = 15mm | pulmonic | Trivial PR, PPG = 23mmHg |
| **Great arteries** | NRGA | **Aortic arch** | Left. No CoA. |
| Aorta | ----- | **PDA** | No |
| Pulmonary artery | Normal MPA and Branch PAs. |  |  |
| **M-Mode:**  Normal LV Function on eye balling | | | |
| AO | mm | PWd | mm |
| LA | mm | PWs | mm |
| LVIDd | mm | EDV | ml |
| LVIDs | mm | ESV | ml |
| IVSs | mm | LVEF | % |
| IVSd | mm | FS | % |
| **Additional Information**: |  | | |
| No pericardial/Pleural effusion. | | | |
| **Final Diagnosis:** | | | |
| 1. Normal Echocardiography Study. | | | |
| **Remark**: | | | |
| **Recommendation**: | | | |
| SIGNATURE  Done by: Tesfaye T., Pediatrician, Pediatric Cardiologist _______________ 12/07/2015Eth.C | | | |

| Patient Name: **Michael Tesfaye**. Referring Institute: **Adinas GH**. SEX/ Age: **M/28days**. Date of Report: **12/07/15**.  Referral Diagnosis: **Incidental Murmur. AGH12.3382.** | | | |
| --- | --- | --- | --- |
| **Features** | **Finding** | **Features** | **Finding** |
| **Profile** |  | **Atria** |  |
| Abdominal situs | Solitus | Left atrium | Normal |
| Cardiac position | Levocardia | Right atrium | Normal |
| Systemic venous drainage | Normal. | **Atrioventricular valves** |  |
| Pulmonary venous drainage | Normal | Mitral valve | Annulus = 12mm |
| Atrioventricular connection | Concordant | Tricuspid valve | Annulus = 12mm  TAPSE = 10mm |
| Ventriculoarterial connection | Concordant | **Ventricles** |  |
| Ventricular loop | d-Loop | Left ventricle | Normal |
|  |  | Right ventricle | Normal |
| **Septae** |  | **Coronary arteries** | ----- |
| Interventricular septum | Intact | **Doppler Measurement** |  |
| Interatrial septum | Intact | Mitral | ----- |
| **Semilunar valves** |  | Aortic | ------- |
| Aortic valve | Annulus = 8mm | Tricuspid | ------- |
| Pulmonary valve | Annulus = 8mm | pulmonic | -------- |
| **Great arteries** | NRGA | **Aortic arch** | Left. No CoA. |
| Aorta | ----- | **PDA** | 1.5mm PDA, L – R Shunt |
| Pulmonary artery | Normal MPA and Branch PAs. |  |  |
| **M-Mode:**  Normal LV Function on eye balling | | | |
| AO | mm | PWd | mm |
| LA | mm | PWs | mm |
| LVIDd | mm | EDV | ml |
| LVIDs | mm | ESV | ml |
| IVSs | mm | LVEF | % |
| IVSd | mm | FS | % |
| **Additional Information**: |  | | |
| No pericardial/Pleural effusion. | | | |
| **Final Diagnosis:** | | | |
| 1. {S, D, S} Levocardia. 2. Small PDA, L – R Shunt 3. Normal Biventricular Systolic Function | | | |
| **Remark**: | | | |
| **Recommendation**: | | | |
| SIGNATURE  Done by: Tesfaye T., Pediatrician, Pediatric Cardiologist _______________ 12/07/2015Eth.C | | | |

| Patient Name: **Kalkidan Dagninet**. Referring Institute: **TGSH**. SEX/ Age: **F/10months**. Date of Report: **13/07/15**.  Referral Diagnosis: **_Syndromic. AGH12.3383.** | | | |
| --- | --- | --- | --- |
| **Features** | **Finding** | **Features** | **Finding** |
| **Profile** |  | **Atria** |  |
| Abdominal situs | Solitus | Left atrium | Normal |
| Cardiac position | Levocardia | Right atrium | Normal |
| Systemic venous drainage | Normal. | **Atrioventricular valves** |  |
| Pulmonary venous drainage | Normal | Mitral valve | Annulus = 10mm |
| Atrioventricular connection | Concordant | Tricuspid valve | Annulus = 10mm |
| Ventriculoarterial connection | Concordant | **Ventricles** |  |
| Ventricular loop | d-Loop | Left ventricle | Normal |
|  |  | Right ventricle | Normal |
| **Septae** |  | **Coronary arteries** | ----- |
| Interventricular septum | Intact | **Doppler Measurement** |  |
| Interatrial septum | Intact | Mitral | ----- |
| **Semilunar valves** |  | Aortic | ------- |
| Aortic valve | Annulus = 10mm | Tricuspid | ------- |
| Pulmonary valve | Annulus = 9mm | pulmonic | -------- |
| **Great arteries** | NRGA | **Aortic arch** | Left. No CoA. |
| Aorta | ----- | **PDA** | No |
| Pulmonary artery | Normal MPA and Branch PAs. |  |  |
| **M-Mode:**  Normal LV Function on eye balling | | | |
| AO | mm | PWd | mm |
| LA | mm | PWs | mm |
| LVIDd | mm | EDV | ml |
| LVIDs | mm | ESV | ml |
| IVSs | mm | LVEF | % |
| IVSd | mm | FS | % |
| **Additional Information**: |  | | |
| No pericardial/Pleural effusion. | | | |
| **Final Diagnosis:** | | | |
| 1. Normal Echocardiography Study. | | | |
| **Remark**: | | | |
| **Recommendation**: | | | |
| SIGNATURE  Done by: Tesfaye T., Pediatrician, Pediatric Cardiologist _______________ 13/07/2015Eth.C | | | |

| Patient Name: **Yohannes Alebel**. Referring Institute: **Adinas GH**. SEX/ Age: **M/5years**. Date of Report: **13/07/15**.  Referral Diagnosis: **DS. AGH12.3384.** | | | |
| --- | --- | --- | --- |
| **Features** | **Finding** | **Features** | **Finding** |
| **Profile** |  | **Atria** |  |
| Abdominal situs | Solitus | Left atrium | Normal |
| Cardiac position | Levocardia | Right atrium | Normal |
| Systemic venous drainage | Normal. | **Atrioventricular valves** |  |
| Pulmonary venous drainage | Normal | Mitral valve | Annulus = 13mm |
| Atrioventricular connection | Concordant | Tricuspid valve | Annulus = 14mm |
| Ventriculoarterial connection | Concordant | **Ventricles** |  |
| Ventricular loop | d-Loop | Left ventricle | Normal |
|  |  | Right ventricle | Normal |
| **Septae** |  | **Coronary arteries** | ----- |
| Interventricular septum | Intact | **Doppler Measurement** |  |
| Interatrial septum | Intact | Mitral | ----- |
| **Semilunar valves** |  | Aortic | ------- |
| Aortic valve | Annulus = 14mm | Tricuspid | ------- |
| Pulmonary valve | Annulus = 13mm | pulmonic | -------- |
| **Great arteries** | NRGA | **Aortic arch** | Left. No CoA. |
| Aorta | ----- | **PDA** | No |
| Pulmonary artery | Normal MPA and Branch PAs. |  |  |
| **M-Mode:**  Normal LV Function on eye balling | | | |
| AO | mm | PWd | mm |
| LA | mm | PWs | mm |
| LVIDd | mm | EDV | ml |
| LVIDs | mm | ESV | ml |
| IVSs | mm | LVEF | % |
| IVSd | mm | FS | % |
| **Additional Information**: |  | | |
| No pericardial/Pleural effusion. | | | |
| **Final Diagnosis:** | | | |
| 1. Normal Echocardiography Study | | | |
| **Remark**: | | | |
| **Recommendation**: | | | |
| SIGNATURE  Done by: Tesfaye T., Pediatrician, Pediatric Cardiologist _______________ 13/07/2015Eth.C | | | |

| Patient Name: **Sewhareg Belete**. Referring Institute: **Pawe Hospital**. SEX/ Age: **F/10years**. Date of Report: **13/07/15**.  Referral Diagnosis: **CRVHD. AGH12.3385.** | | | |
| --- | --- | --- | --- |
| **Features** | **Finding** | **Features** | **Finding** |
| **Profile** |  | **Atria** |  |
| Abdominal situs | Solitus | Left atrium | Markedly Dilated |
| Cardiac position | Levocardia | Right atrium | Dilated |
| Systemic venous drainage | Normal. | **Atrioventricular valves** |  |
| Pulmonary venous drainage | Normal | Mitral valve | Annulus = 33mm. Thickened MVL. |
| Atrioventricular connection | Concordant | Tricuspid valve | Annulus = 26mm  TAPSE = 25mm |
| Ventriculoarterial connection | Concordant | **Ventricles** |  |
| Ventricular loop | d-Loop | Left ventricle | Markedly Dilated |
|  |  | Right ventricle | Dilated |
| **Septae** |  | **Coronary arteries** | ----- |
| Interventricular septum | Intact | **Doppler Measurement** |  |
| Interatrial septum | Intact | Mitral | Severe MR, Holosystolic, posterior projection, seen in two planes with jet velocity = 4.4m/sec. |
| **Semilunar valves** |  | Aortic | Mild AR |
| Aortic valve | Annulus = 19mm | Tricuspid | Moderate TR, PPG = 53mmHg. |
| Pulmonary valve | Annulus = 22mm | pulmonic | -------- |
| **Great arteries** | NRGA | **Aortic arch** | Left. No CoA. |
| Aorta | ----- | **PDA** | No |
| Pulmonary artery | Normal MPA. |  |  |
| **M-Mode:** | | | |
| AO | mm | PWd | mm |
| LA | mm | PWs | mm |
| LVIDd | mm | EDV | ml |
| LVIDs | mm | ESV | ml |
| IVSs | mm | LVEF | 55% |
| IVSd | mm | FS | 29% |
| **Additional Information**: |  | | |
| Circumferential pericardial effusion with maximum depth of 3mm. | | | |
| **Final Diagnosis:** | | | |
| 1. {S, D, S} Levocardia. 2. All chambers are dilated 3. Thickened MVL 4. Severe MR 5. Mild AR 6. Moderate TR 7. Moderate Pulmonary Hypertension 8. Normal Biventricular Systolic Function (LV Function is borderline) 9. Trace Circumferential Pericardial effusion | | | |
| SIGNATURE  Done by: Tesfaye T., Pediatrician, Pediatric Cardiologist _______________ 13/07/2015Eth.C | | | |

| Patient Name: **Mahider Alehegn**. Referring Institute: **Dr. Addisu PSC**. SEX/ Age: **F/4years**. Date of Report: **13/07/15**.  Referral Diagnosis: **?Pulmonary Hypertension R/O CHD. AGH12.3386.** | | | |
| --- | --- | --- | --- |
| **Features** | **Finding** | **Features** | **Finding** |
| **Profile** |  | **Atria** |  |
| Abdominal situs | Solitus | Left atrium | Normal |
| Cardiac position | Levocardia | Right atrium | Normal |
| Systemic venous drainage | Normal. | **Atrioventricular valves** |  |
| Pulmonary venous drainage | Normal | Mitral valve | Annulus = 13mm |
| Atrioventricular connection | Concordant | Tricuspid valve | Annulus = 15mm  TAPSE = 16mm |
| Ventriculoarterial connection | Concordant | **Ventricles** |  |
| Ventricular loop | d-Loop | Left ventricle | Normal |
|  |  | Right ventricle | Normal |
| **Septae** |  | **Coronary arteries** | ----- |
| Interventricular septum | Intact | **Doppler Measurement** |  |
| Interatrial septum | Intact | Mitral | ----- |
| **Semilunar valves** |  | Aortic | ------- |
| Aortic valve | Annulus = 12mm | Tricuspid | ------- |
| Pulmonary valve | Annulus = 13mm | pulmonic | -------- |
| **Great arteries** | NRGA | **Aortic arch** | Left. No CoA. |
| Aorta | ----- | **PDA** | No |
| Pulmonary artery | Normal MPA and Branch PAs. |  |  |
| **M-Mode:**  Normal LV Function on eye balling | | | |
| AO | mm | PWd | mm |
| LA | mm | PWs | mm |
| LVIDd | mm | EDV | ml |
| LVIDs | mm | ESV | ml |
| IVSs | mm | LVEF | % |
| IVSd | mm | FS | % |
| **Additional Information**: |  | | |
| No pericardial/Pleural effusion. | | | |
| **Final Diagnosis:** | | | |
| 1. Normal Echocardiography Study. | | | |
| **Remark**: | | | |
| **Recommendation**: | | | |
| SIGNATURE  Done by: Tesfaye T., Pediatrician, Pediatric Cardiologist _______________ 13/07/2015Eth.C | | | |

| Patient Name: **Haymanot Belete**. Referring Institute: **Addis Alem PH**. SEX/ Age: **F/2months**. Date of Report: **13/07/15**.  Referral Diagnosis: **Incidental Murmur finding. AGH12.3387.** | | | |
| --- | --- | --- | --- |
| **Features** | **Finding** | **Features** | **Finding** |
| **Profile** |  | **Atria** |  |
| Abdominal situs | Solitus | Left atrium | Normal |
| Cardiac position | Levocardia | Right atrium | Normal |
| Systemic venous drainage | Normal. | **Atrioventricular valves** |  |
| Pulmonary venous drainage | Normal | Mitral valve | Annulus = 12mm |
| Atrioventricular connection | Concordant | Tricuspid valve | Annulus = 11mm |
| Ventriculoarterial connection | Concordant | **Ventricles** |  |
| Ventricular loop | d-Loop | Left ventricle | Normal |
|  |  | Right ventricle | Normal |
| **Septae** |  | **Coronary arteries** | ----- |
| Interventricular septum | Intact | **Doppler Measurement** |  |
| Interatrial septum | Intact | Mitral | ----- |
| **Semilunar valves** |  | Aortic | ------- |
| Aortic valve | Annulus = 9mm | Tricuspid | ------- |
| Pulmonary valve | Annulus = 10mm | pulmonic | -------- |
| **Great arteries** | NRGA | **Aortic arch** | Left. No CoA. |
| Aorta | ----- | **PDA** | 1mm PDA, L – R Shunt |
| Pulmonary artery | Normal MPA and Branch PAs. |  |  |
| **M-Mode:**  Normal LV Function on eye balling | | | |
| AO | mm | PWd | mm |
| LA | mm | PWs | mm |
| LVIDd | mm | EDV | ml |
| LVIDs | mm | ESV | ml |
| IVSs | mm | LVEF | % |
| IVSd | mm | FS | % |
| **Additional Information**: |  | | |
| No pericardial/Pleural effusion. | | | |
| **Final Diagnosis:** | | | |
| 1. {S, D, S} Levocardia. 2. Small PDA, L – R Shunt 3. Normal LV Systolic Function | | | |
| **Remark**: | | | |
| **Recommendation**: | | | |
| SIGNATURE  Done by: Tesfaye T., Pediatrician, Pediatric Cardiologist _______________ 13/07/2015Eth.C | | | |

| Patient Name: **Bezawit Sew-tenaw**. Referring Institute: **Adinas GH**. SEX/ Age: **F/8years**. Date of Report: **13/07/15**.  Referral Diagnosis: **Left side recurrent anterior chest pain. AGH12.3388.** | | | |
| --- | --- | --- | --- |
| **Features** | **Finding** | **Features** | **Finding** |
| **Profile** |  | **Atria** |  |
| Abdominal situs | Solitus | Left atrium | Normal |
| Cardiac position | Levocardia | Right atrium | Normal |
| Systemic venous drainage | Normal. | **Atrioventricular valves** |  |
| Pulmonary venous drainage | Normal | Mitral valve | Annulus = 18mm |
| Atrioventricular connection | Concordant | Tricuspid valve | Annulus = 17mm  TAPSE = 20mm |
| Ventriculoarterial connection | Concordant | **Ventricles** |  |
| Ventricular loop | d-Loop | Left ventricle | Normal |
|  |  | Right ventricle | Normal |
| **Septae** |  | **Coronary arteries** | ----- |
| Interventricular septum | Intact | **Doppler Measurement** |  |
| Interatrial septum | Intact | Mitral | ----- |
| **Semilunar valves** |  | Aortic | ------- |
| Aortic valve | Annulus = 15mm | Tricuspid | ------- |
| Pulmonary valve | Annulus = 15mm | pulmonic | -------- |
| **Great arteries** | NRGA | **Aortic arch** | Left. No CoA. |
| Aorta | ----- | **PDA** | No |
| Pulmonary artery | Normal MPA and Branch PAs. |  |  |
| **M-Mode:** | | | |
| AO | mm | PWd | mm |
| LA | mm | PWs | mm |
| LVIDd | mm | EDV | ml |
| LVIDs | mm | ESV | ml |
| IVSs | mm | LVEF | 72% |
| IVSd | mm | FS | 40% |
| **Additional Information**: |  | | |
| No pericardial/Pleural effusion. | | | |
| **Final Diagnosis:** | | | |
| 1. Normal Echocardiography Study. | | | |
| **Remark**: | | | |
| **Recommendation**: | | | |
| SIGNATURE  Done by: Tesfaye T., Pediatrician, Pediatric Cardiologist _______________ 13/07/2015Eth.C | | | |

| Patient Name: **Yohannes Simegnew**. Referring Institute: **TGSH**. SEX/ Age: **M/8months**. Date of Report: **13/07/15**.  Referral Diagnosis: **Down Syndrome. AGH12.3389.** | | | |
| --- | --- | --- | --- |
| **Features** | **Finding** | **Features** | **Finding** |
| **Profile** |  | **Atria** |  |
| Abdominal situs | Solitus | Left atrium | Normal |
| Cardiac position | Levocardia | Right atrium | Dilated |
| Systemic venous drainage | Normal. | **Atrioventricular valves** |  |
| Pulmonary venous drainage | Normal | Mitral valve | Annulus = 13mm |
| Atrioventricular connection | Concordant | Tricuspid valve | Annulus = 19mm  TAPSE = 15mm |
| Ventriculoarterial connection | Concordant | **Ventricles** |  |
| Ventricular loop | d-Loop | Left ventricle | Normal |
|  |  | Right ventricle | Dilated |
| **Septae** |  | **Coronary arteries** | ----- |
| Interventricular septum | Intact | **Doppler Measurement** |  |
| Interatrial septum | 8mm OS ASD, L – R Shunt | Mitral | ----- |
| **Semilunar valves** |  | Aortic | ------- |
| Aortic valve | Annulus = 12mm | Tricuspid | Trivial TR, PPG = 30mmHg |
| Pulmonary valve | Annulus = 13mm | pulmonic | -------- |
| **Great arteries** | NRGA | **Aortic arch** | Left. No CoA. |
| Aorta | ----- | **PDA** | 1.5mm PDA, L – R Shunt |
| Pulmonary artery | Normal MPA and Branch PAs. |  |  |
| **M-Mode:**  Normal LV Function on eye balling | | | |
| AO | mm | PWd | mm |
| LA | mm | PWs | mm |
| LVIDd | mm | EDV | ml |
| LVIDs | mm | ESV | ml |
| IVSs | mm | LVEF | % |
| IVSd | mm | FS | % |
| **Additional Information**: |  | | |
| No pericardial/Pleural effusion. | | | |
| **Final Diagnosis:** | | | |
| 1. {S, D, S} Levocardia. 2. RA/RV Dilated 3. Moderate OS ASD, L – R Shunt 4. Small PDA, L – R Shunt 5. Normal Biventricular Systolic Function | | | |
| **Remark**: | | | |
| **Recommendation**: | | | |
| SIGNATURE  Done by: Tesfaye T., Pediatrician, Pediatric Cardiologist _______________ 13/07/2015Eth.C | | | |

| Patient Name: **Meseret Amare**. Referring Institute: **FHRH**. SEX/ Age: **F/12years**. Date of Report: **13/07/15**.  Referral Diagnosis: **ARF + Bradycardia. AGH12.3390.** | | | |
| --- | --- | --- | --- |
| **Features** | **Finding** | **Features** | **Finding** |
| **Profile** |  | **Atria** |  |
| Abdominal situs | Solitus | Left atrium | Normal |
| Cardiac position | Levocardia | Right atrium | Normal |
| Systemic venous drainage | Normal. | **Atrioventricular valves** |  |
| Pulmonary venous drainage | Normal | Mitral valve | Annulus = 21mm |
| Atrioventricular connection | Concordant | Tricuspid valve | Annulus = 20mm  TAPSE = 20mm |
| Ventriculoarterial connection | Concordant | **Ventricles** |  |
| Ventricular loop | d-Loop | Left ventricle | Normal |
|  |  | Right ventricle | Normal |
| **Septae** |  | **Coronary arteries** | ----- |
| Interventricular septum | Intact | **Doppler Measurement** |  |
| Interatrial septum | Intact | Mitral | ----- |
| **Semilunar valves** |  | Aortic | ------- |
| Aortic valve | Annulus = 15mm | Tricuspid | ------- |
| Pulmonary valve | Annulus = 19mm | pulmonic | -------- |
| **Great arteries** | NRGA | **Aortic arch** | Left. No CoA. |
| Aorta | ----- | **PDA** | No |
| Pulmonary artery | Normal MPA and Branch PAs. |  |  |
| **M-Mode:** | | | |
| AO | mm | PWd | mm |
| LA | mm | PWs | mm |
| LVIDd | mm | EDV | ml |
| LVIDs | mm | ESV | 34ml |
| IVSs | mm | LVEF | 65% |
| IVSd | mm | FS | % |
| **Additional Information**: |  | | |
| No pericardial/Pleural effusion. | | | |
| **Final Diagnosis:** | | | |
| 1. Normal Echocardiography Study. | | | |
| **Remark**: | | | |
| **Recommendation**: | | | |
| SIGNATURE  Done by: Tesfaye T., Pediatrician, Pediatric Cardiologist _______________ 13/07/2015Eth.C | | | |

| Patient Name: **Betselot Mikir**. Referring Institute: **TGSH**. SEX/ Age: **F/1year**. Date of Report: **14/07/15**.  Referral Diagnosis: **Down Syndrome, screening. AGH12.3391.** | | | |
| --- | --- | --- | --- |
| **Features** | **Finding** | **Features** | **Finding** |
| **Profile** |  | **Atria** |  |
| Abdominal situs | Solitus | Left atrium | Normal |
| Cardiac position | Levocardia | Right atrium | Normal |
| Systemic venous drainage | Normal. | **Atrioventricular valves** |  |
| Pulmonary venous drainage | Normal | Mitral valve | Annulus = 12mm |
| Atrioventricular connection | Concordant | Tricuspid valve | Annulus = 12mm |
| Ventriculoarterial connection | Concordant | **Ventricles** |  |
| Ventricular loop | d-Loop | Left ventricle | Normal |
|  |  | Right ventricle | Normal |
| **Septae** |  | **Coronary arteries** | ----- |
| Interventricular septum | Intact | **Doppler Measurement** |  |
| Interatrial septum | Intact | Mitral | ----- |
| **Semilunar valves** |  | Aortic | ------- |
| Aortic valve | Annulus = 11mm | Tricuspid | ------- |
| Pulmonary valve | Annulus = 11mm | pulmonic | -------- |
| **Great arteries** | NRGA | **Aortic arch** | Left. No CoA. |
| Aorta | ----- | **PDA** | No |
| Pulmonary artery | Normal MPA and Branch PAs. |  |  |
| **M-Mode:** | | | |
| AO | mm | PWd | mm |
| LA | mm | PWs | mm |
| LVIDd | mm | EDV | ml |
| LVIDs | mm | ESV | ml |
| IVSs | mm | LVEF | % |
| IVSd | mm | FS | % |
| **Additional Information**: |  | | |
| No pericardial/Pleural effusion. | | | |
| **Final Diagnosis:** | | | |
| 1. Normal Echocardiography Study. | | | |
| **Remark**: | | | |
| **Recommendation**: | | | |
| SIGNATURE  Done by: Tesfaye T., Pediatrician, Pediatric Cardiologist _______________ 14/07/2015Eth.C | | | |

| Patient Name: **Tsion Mesfin**. Referring Institute: **TGSH**. SEX/ Age: **F/12years**. Date of Report: **14/07/15**.  Referral Diagnosis: **Syncope + easy fatigability + palpitation. AGH12.3392.** | | | |
| --- | --- | --- | --- |
| **Features** | **Finding** | **Features** | **Finding** |
| **Profile** |  | **Atria** |  |
| Abdominal situs | Solitus | Left atrium | Normal |
| Cardiac position | Levocardia | Right atrium | Normal |
| Systemic venous drainage | Normal. | **Atrioventricular valves** |  |
| Pulmonary venous drainage | Normal | Mitral valve | Annulus = 25mm. Patulous MVL |
| Atrioventricular connection | Concordant | Tricuspid valve | Annulus = 25mm  TAPSE = 19mm |
| Ventriculoarterial connection | Concordant | **Ventricles** |  |
| Ventricular loop | d-Loop | Left ventricle | Normal |
|  |  | Right ventricle | Normal |
| **Septae** |  | **Coronary arteries** | ----- |
| Interventricular septum | Intact | **Doppler Measurement** |  |
| Interatrial septum | Intact | Mitral | Trivial MR, Holosystolic, seen in two planes with jet velocity = 2m/sec. |
| **Semilunar valves** |  | Aortic | ------- |
| Aortic valve | Annulus = 16mm | Tricuspid | ------- |
| Pulmonary valve | Annulus = 22mm | pulmonic | -------- |
| **Great arteries** | NRGA | **Aortic arch** | Left. No CoA. |
| Aorta | ----- | **PDA** | No |
| Pulmonary artery | Normal |  |  |
| **M-Mode:** | | | |
| AO | mm | PWd | mm |
| LA | mm | PWs | mm |
| LVIDd | mm | EDV | ml |
| LVIDs | mm | ESV | ml |
| IVSs | mm | LVEF | 56% |
| IVSd | mm | FS | 29% |
| **Additional Information**: |  | | |
| No pericardial/Pleural effusion. | | | |
| **Final Diagnosis:** | | | |
| 1. {S, D, S} Levocardia. 2. Patulous MVL 3. Trivial MR 4. Normal Biventricular Systolic Function | | | |
| **Remark**: Consider Borderline RHD | | | |
| **Recommendation**:   1. Secondary Prophylaxis for two years. 2. Repeat echocardiography after a year | | | |
| SIGNATURE  Done by: Tesfaye T., Pediatrician, Pediatric Cardiologist _______________ 14/07/2015Eth.C | | | |

| Patient Name: **Bamlak Birhanu**. Referring Institute: **TGSH**. SEX/ Age: **M/49days**. Date of Report: **14/07/15**.  Referral Diagnosis: **FB and Grunting. AGH12.3393.** | | | |
| --- | --- | --- | --- |
| **Features** | **Finding** | **Features** | **Finding** |
| **Profile** |  | **Atria** |  |
| Abdominal situs | Solitus | Left atrium | Dilated |
| Cardiac position | Levocardia | Right atrium | Mildly Dilated |
| Systemic venous drainage | Normal. | **Atrioventricular valves** |  |
| Pulmonary venous drainage | Normal | Mitral valve | Annulus = 13mm |
| Atrioventricular connection | Concordant | Tricuspid valve | Annulus = 13mm  TAPSE = 8mm |
| Ventriculoarterial connection | Concordant | **Ventricles** |  |
| Ventricular loop | d-Loop | Left ventricle | Mildly Dilated |
|  |  | Right ventricle | Dilated |
| **Septae** |  | **Coronary arteries** | ----- |
| Interventricular septum | Intact | **Doppler Measurement** |  |
| Interatrial septum | Intact | Mitral | ----- |
| **Semilunar valves** |  | Aortic | ------- |
| Aortic valve | Annulus = 11mm | Tricuspid | ------- |
| Pulmonary valve | Annulus = 12mm | pulmonic | -------- |
| **Great arteries** | NRGA | **Aortic arch** | Left. No CoA. |
| Aorta | ----- | **PDA** | No |
| Pulmonary artery | Normal MPA and Branch PAs. |  |  |
| **M-Mode:** | | | |
| AO | mm | PWd | mm |
| LA | mm | PWs | mm |
| LVIDd | mm | EDV | ml |
| LVIDs | mm | ESV | ml |
| IVSs | mm | LVEF | 49% |
| IVSd | mm | FS | 24% |
| **Additional Information**: |  | | |
| No pericardial/Pleural effusion. | | | |
| **Final Diagnosis:** | | | |
| 1. {S, D, S} Levocardia. 2. Mildly Dilated LV/RV 3. Reduced Biventricular Systolic Function secondary to ? | | | |
| **Remark**: | | | |
| **Recommendation**: | | | |
| SIGNATURE  Done by: Tesfaye T., Pediatrician, Pediatric Cardiologist _______________ 14/07/2015Eth.C | | | |

| Patient Name: **Yemikir Endawek**. Referring Institute: **FHRH**. SEX/ Age: **F/14years**. Date of Report: **14/07/15**.  Referral Diagnosis: **Palpitation + easy fatigability. AGH12.3394.** | | | |
| --- | --- | --- | --- |
| **Features** | **Finding** | **Features** | **Finding** |
| **Profile** |  | **Atria** |  |
| Abdominal situs | Solitus | Left atrium | Normal |
| Cardiac position | Levocardia | Right atrium | Normal. Late diastolic collapse of RA. |
| Systemic venous drainage | Normal. | **Atrioventricular valves** |  |
| Pulmonary venous drainage | Normal | Mitral valve | Annulus = 28mm |
| Atrioventricular connection | Concordant | Tricuspid valve | Annulus = 28mm  TAPSE = mm |
| Ventriculoarterial connection | Concordant | **Ventricles** |  |
| Ventricular loop | d-Loop | Left ventricle | Normal |
|  |  | Right ventricle | Normal. Early diastolic collapse of the RV. |
| **Septae** | Abnormal Ventricular septal motion. | **Coronary arteries** | ----- |
| Interventricular septum | Intact | **Doppler Measurement** |  |
| Interatrial septum | Intact | Mitral | Respiratory variability in Mitral inflow velocity = 30% |
| **Semilunar valves** |  | Aortic | Pulsus paradoxus. |
| Aortic valve | Annulus = 20mm | Tricuspid | ------- |
| Pulmonary valve | Annulus = 21mm | pulmonic | -------- |
| **Great arteries** | NRGA | **Aortic arch** | Left. No CoA. |
| Aorta | ----- | **PDA** | No |
| Pulmonary artery | Normal MPA and Branch PAs. |  |  |
| **M-Mode:** | | | |
| AO | mm | PWd | mm |
| LA | mm | PWs | mm |
| LVIDd | mm | EDV | ml |
| LVIDs | mm | ESV | ml |
| IVSs | mm | LVEF | 55% |
| IVSd | mm | FS | 28% |
| **Additional Information**: |  | | |
| 1. Circumferential pericardial effusion with maximum depth of 21mm. septated. 2. Swinging heart | | | |
| **Final Diagnosis:** | | | |
| 1. {S, D, S} Levocardia. 2. Large septated Pericardial effusion with features of cardiac tamponade 3. Normal LV Systolic Function | | | |
| **Remark**: | | | |
| **Recommendation**: | | | |
| SIGNATURE  Done by: Tesfaye T., Pediatrician, Pediatric Cardiologist _______________ 14/07/2015Eth.C | | | |

| Patient Name: **Desalegn Shegaw**. Referring Institute: **FHRH**. SEX/ Age: **M/3months**. Date of Report: **14/07/15**.  Referral Diagnosis: **FB and grunting + Stridor. AGH12.3395.** | | | |
| --- | --- | --- | --- |
| **Features** | **Finding** | **Features** | **Finding** |
| **Profile** |  | **Atria** |  |
| Abdominal situs | Solitus | Left atrium | Normal |
| Cardiac position | Levocardia | Right atrium | Normal |
| Systemic venous drainage | Normal. | **Atrioventricular valves** |  |
| Pulmonary venous drainage | Normal | Mitral valve | Annulus = 13mm |
| Atrioventricular connection | Concordant | Tricuspid valve | Annulus = 13mm |
| Ventriculoarterial connection | Concordant | **Ventricles** |  |
| Ventricular loop | d-Loop | Left ventricle | Normal |
|  |  | Right ventricle | Normal |
| **Septae** |  | **Coronary arteries** | ----- |
| Interventricular septum | Intact | **Doppler Measurement** |  |
| Interatrial septum | Intact | Mitral | ----- |
| **Semilunar valves** |  | Aortic | ------- |
| Aortic valve | Annulus = 10mm | Tricuspid | ------- |
| Pulmonary valve | Annulus = 11mm | pulmonic | -------- |
| **Great arteries** | NRGA | **Aortic arch** | Left. No CoA. |
| Aorta | ----- | **PDA** | 1mm PDA, L – R Shunt |
| Pulmonary artery | Normal MPA and Branch PAs. |  |  |
| **M-Mode:**  Normal LV Function on eye balling | | | |
| AO | mm | PWd | mm |
| LA | mm | PWs | mm |
| LVIDd | mm | EDV | ml |
| LVIDs | mm | ESV | ml |
| IVSs | mm | LVEF | % |
| IVSd | mm | FS | % |
| **Additional Information**: |  | | |
| No pericardial/Pleural effusion. | | | |
| **Final Diagnosis:** | | | |
| 1. {S, D, S} Levocardia. 2. Small PDA, L – R Shunt 3. Normal LV Systolic Function | | | |
| **Remark**: | | | |
| **Recommendation**: | | | |
| SIGNATURE  Done by: Tesfaye T., Pediatrician, Pediatric Cardiologist _______________ 14/07/2015Eth.C | | | |

| Patient Name: **Tiruayehu Simegn**. Referring Institute: **TGSH**. SEX/ Age: **F/14years**. Date of Report: **14/07/15**.  Referral Diagnosis: **CRVHD. AGH12.3396.** | | | |
| --- | --- | --- | --- |
| **Features** | **Finding** | **Features** | **Finding** |
| **Profile** |  | **Atria** |  |
| Abdominal situs | Solitus | Left atrium | Markedly dilated |
| Cardiac position | Levocardia | Right atrium | Dilated |
| Systemic venous drainage | Normal. | **Atrioventricular valves** |  |
| Pulmonary venous drainage | Normal | Mitral valve | Annulus = 27mm. thickened, clubbed MVL. MVA = 1.1cm**2**. |
| Atrioventricular connection | Concordant | Tricuspid valve | Annulus = 27mm  TAPSE = 26mm |
| Ventriculoarterial connection | Concordant | **Ventricles** |  |
| Ventricular loop | d-Loop | Left ventricle | Markedly Dilated |
|  |  | Right ventricle | Dilated |
| **Septae** |  | **Coronary arteries** | ----- |
| Interventricular septum | Intact | **Doppler Measurement** |  |
| Interatrial septum | Intact | Mitral | Severe MR, Holosystolic, posterior projection, seen in two planes with jet velocity = 3.8m/sec. Moderate MS, PPG/MPG = 25/9mmHg. |
| **Semilunar valves** |  | Aortic | Severe AR |
| Aortic valve | Annulus = 16mm | Tricuspid | Severe TR, PPG = 60mmHg |
| Pulmonary valve | Annulus = 21mm | pulmonic | -------- |
| **Great arteries** | NRGA | **Aortic arch** | Left. No CoA. |
| Aorta | ----- | **PDA** | No |
| Pulmonary artery | Normal |  |  |
| **M-Mode:** | | | |
| AO | mm | PWd | mm |
| LA | mm | PWs | mm |
| LVIDd | mm | EDV | ml |
| LVIDs | mm | ESV | ml |
| IVSs | mm | LVEF | 63% |
| IVSd | mm | FS | 34% |
| **Additional Information**: |  | | |
| Pericardial effusion with maximum depth of 3mm on RA Side. | | | |
| **Final Diagnosis:** | | | |
| 1. {S, D, S} Levocardia. 2. All chambers dilated 3. Thickened, clubbed MVL 4. Severe MR 5. Moderate MS 6. Severe AR 7. Severe TR 8. Severe Pulmonary Hypertension 9. Normal Biventricular Systolic Function 10. Trace Pericardial effusion | | | |
| SIGNATURE  Done by: Tesfaye T., Pediatrician, Pediatric Cardiologist _______________ 14/07/2015Eth.C | | | |

| Patient Name: **Agernesh Misganaw**. Referring Institute: **TGSH**. SEX/ Age: **F/1year**. Date of Report: **14/07/15**.  Referral Diagnosis: **FTT + Syndromic (?Noonan). AGH12.3397.** | | | |
| --- | --- | --- | --- |
| **Features** | **Finding** | **Features** | **Finding** |
| **Profile** |  | **Atria** |  |
| Abdominal situs | Solitus | Left atrium | Normal |
| Cardiac position | Levocardia | Right atrium | Normal |
| Systemic venous drainage | Normal. | **Atrioventricular valves** |  |
| Pulmonary venous drainage | Normal | Mitral valve | Annulus = 12mm |
| Atrioventricular connection | Concordant | Tricuspid valve | Annulus = 11mm |
| Ventriculoarterial connection | Concordant | **Ventricles** |  |
| Ventricular loop | d-Loop | Left ventricle | Normal |
|  |  | Right ventricle | Normal |
| **Septae** |  | **Coronary arteries** | ----- |
| Interventricular septum | Intact | **Doppler Measurement** |  |
| Interatrial septum | Intact | Mitral | ----- |
| **Semilunar valves** |  | Aortic | ------- |
| Aortic valve | Annulus = 9mm | Tricuspid | ------- |
| Pulmonary valve | Annulus = 9mm | pulmonic | Valvular PS, PPG = 24mmHg |
| **Great arteries** | NRGA | **Aortic arch** | Left. No CoA. |
| Aorta | ----- | **PDA** | No |
| Pulmonary artery | Normal MPA and Branch PAs. |  |  |
| **M-Mode:**  Normal LV Function on eye balling | | | |
| AO | mm | PWd | mm |
| LA | mm | PWs | mm |
| LVIDd | mm | EDV | ml |
| LVIDs | mm | ESV | ml |
| IVSs | mm | LVEF | % |
| IVSd | mm | FS | % |
| **Additional Information**: |  | | |
| No pericardial/Pleural effusion. | | | |
| **Final Diagnosis:** | | | |
| 1. {S, D, S} Levocardia. 2. Mild Valvular PS | | | |
| **Remark**: | | | |
| **Recommendation**: | | | |
| SIGNATURE  Done by: Tesfaye T., Pediatrician, Pediatric Cardiologist _______________ 14/07/2015Eth.C | | | |

| Patient Name: **Abrham Gulma**. Referring Institute: **Addis Alem PH**. SEX/ Age: **M/8years**. Date of Report: **15/07/15**.  Referral Diagnosis: **Easy fatigability + RHD. AGH12.3398.** | | | |
| --- | --- | --- | --- |
| **Features** | **Finding** | **Features** | **Finding** |
| **Profile** |  | **Atria** |  |
| Abdominal situs | Solitus | Left atrium | Normal |
| Cardiac position | Levocardia | Right atrium | Normal |
| Systemic venous drainage | Normal. | **Atrioventricular valves** |  |
| Pulmonary venous drainage | Normal | Mitral valve | Annulus = 18mm |
| Atrioventricular connection | Concordant | Tricuspid valve | Annulus = 21mm  TAPSE = 24mm |
| Ventriculoarterial connection | Concordant | **Ventricles** |  |
| Ventricular loop | d-Loop | Left ventricle | Normal |
|  |  | Right ventricle | Normal |
| **Septae** |  | **Coronary arteries** | ----- |
| Interventricular septum | Intact | **Doppler Measurement** |  |
| Interatrial septum | Intact | Mitral | ----- |
| **Semilunar valves** |  | Aortic | ------- |
| Aortic valve | Annulus = 18mm | Tricuspid | ------- |
| Pulmonary valve | Annulus = 21mm | pulmonic | -------- |
| **Great arteries** | NRGA | **Aortic arch** | Left. No CoA. |
| Aorta | ----- | **PDA** | No |
| Pulmonary artery | Normal MPA and Branch PAs. |  |  |
| **M-Mode:** | | | |
| AO | mm | PWd | mm |
| LA | mm | PWs | mm |
| LVIDd | mm | EDV | ml |
| LVIDs | mm | ESV | ml |
| IVSs | mm | LVEF | 60% |
| IVSd | mm | FS | 31% |
| **Additional Information**: |  | | |
| No pericardial/Pleural effusion. | | | |
| **Final Diagnosis:** | | | |
| 1. Normal Echocardiography Study. | | | |
| **Remark**: | | | |
| **Recommendation**: | | | |
| SIGNATURE  Done by: Tesfaye T., Pediatrician, Pediatric Cardiologist _______________ 15/07/2015Eth.C | | | |

| Patient Name: **Adel Melkamu**. Referring Institute: **Adinas GH**. SEX/ Age: **F/8years**. Date of Report: **15/07/15**.  Referral Diagnosis: **Follow up echo for Mild Ebstein anomaly (Incidental finding).** | | | |
| --- | --- | --- | --- |
| **Features** | **Finding** | **Features** | **Finding** |
| **Profile** |  | **Atria** |  |
| Abdominal situs | Solitus | Left atrium | Normal |
| Cardiac position | Levocardia | Right atrium | Normal |
| Systemic venous drainage | Normal. | **Atrioventricular valves** |  |
| Pulmonary venous drainage | Normal | Mitral valve | Annulus = 19mm |
| Atrioventricular connection | Concordant | Tricuspid valve | Annulus = 23mm. 11mm apical displacement of STL from mitral insertion point. |
| Ventriculoarterial connection | Concordant | **Ventricles** |  |
| Ventricular loop | d-Loop | Left ventricle | Normal |
|  |  | Right ventricle | Normal |
| **Septae** |  | **Coronary arteries** | ----- |
| Interventricular septum | Intact | **Doppler Measurement** |  |
| Interatrial septum | Intact | Mitral | ----- |
| **Semilunar valves** |  | Aortic | ------- |
| Aortic valve | Annulus = 15mm | Tricuspid | Mild TR, PPG = 25mmHg |
| Pulmonary valve | Annulus = 18mm | pulmonic | -------- |
| **Great arteries** | NRGA | **Aortic arch** | Left. No CoA. |
| Aorta | ----- | **PDA** | No |
| Pulmonary artery | Normal MPA and Branch PAs. |  |  |
| **M-Mode:** | | | |
| AO | mm | PWd | mm |
| LA | mm | PWs | mm |
| LVIDd | mm | EDV | ml |
| LVIDs | mm | ESV | ml |
| IVSs | mm | LVEF | 68% |
| IVSd | mm | FS | 37% |
| **Additional Information**: |  | | |
| No pericardial/Pleural effusion. | | | |
| **Final Diagnosis:** | | | |
| 1. {S, D, S} Levocardia. 2. Mild TR 3. Mild Ebstein anomaly | | | |
| **Remark**: | | | |
| **Recommendation**: | | | |
| SIGNATURE  Done by: Tesfaye T., Pediatrician, Pediatric Cardiologist _______________ 15/07/2015Eth.C | | | |

| Patient Name: **Bethel Tibebu**. Referring Institute: **Adinas GH**. SEX/ Age: **F/6months**. Date of Report: **16/07/15**.  Referral Diagnosis: **Incidental Murmur. AGH12.3399.** | | | |
| --- | --- | --- | --- |
| **Features** | **Finding** | **Features** | **Finding** |
| **Profile** |  | **Atria** |  |
| Abdominal situs | Solitus | Left atrium | Normal |
| Cardiac position | Levocardia | Right atrium | Normal |
| Systemic venous drainage | Normal. | **Atrioventricular valves** |  |
| Pulmonary venous drainage | Normal | Mitral valve | Annulus = 13mm |
| Atrioventricular connection | Concordant | Tricuspid valve | Annulus = 14mm  TAPSE = 17mm |
| Ventriculoarterial connection | Concordant | **Ventricles** |  |
| Ventricular loop | d-Loop | Left ventricle | Normal |
|  |  | Right ventricle | Normal |
| **Septae** |  | **Coronary arteries** | ----- |
| Interventricular septum | 1mm Mid –Muscular VSD, L – R Shunt | **Doppler Measurement** |  |
| Interatrial septum | Intact | Mitral | ----- |
| **Semilunar valves** |  | Aortic | ------- |
| Aortic valve | Annulus = 11mm | Tricuspid | ------- |
| Pulmonary valve | Annulus = 12mm | pulmonic | -------- |
| **Great arteries** | NRGA | **Aortic arch** | Left. No CoA. |
| Aorta | ----- | **PDA** | No |
| Pulmonary artery | Normal MPA and Branch PAs. |  |  |
| **M-Mode:**  Normal LV Function on eye balling | | | |
| AO | mm | PWd | mm |
| LA | mm | PWs | mm |
| LVIDd | mm | EDV | ml |
| LVIDs | mm | ESV | ml |
| IVSs | mm | LVEF | % |
| IVSd | mm | FS | % |
| **Additional Information**: |  | | |
| No pericardial/Pleural effusion. | | | |
| **Final Diagnosis:** | | | |
| 1. {S, D, S} Levocardia. 2. Tiny Mid – Muscular VSD, L – R Shunt 3. Normal Biventricular Systolic Function | | | |
| **Remark**: | | | |
| **Recommendation**: | | | |
| SIGNATURE  Done by: Tesfaye T., Pediatrician, Pediatric Cardiologist _______________ 16/07/2015Eth.C | | | |

| Patient Name: **Yeshambel Lakachew**. Referring Institute: **FHRH**. SEX/ Age: **M/14years**. Date of Report: **18/07/15**.  Referral Diagnosis: Follow up for **CRVHD ( MR + AR + Thickened MVL + LA/LV Dilated + Reduced LV Function).** | | | |
| --- | --- | --- | --- |
| **Features** | **Finding** | **Features** | **Finding** |
| **Profile** |  | **Atria** |  |
| Abdominal situs | Solitus | Left atrium | Dilated |
| Cardiac position | Levocardia | Right atrium | Normal |
| Systemic venous drainage | Normal. | **Atrioventricular valves** |  |
| Pulmonary venous drainage | Normal | Mitral valve | Annulus = 29mm. Thickened MVL |
| Atrioventricular connection | Concordant | Tricuspid valve | Annulus = 25mm |
| Ventriculoarterial connection | Concordant | **Ventricles** |  |
| Ventricular loop | d-Loop | Left ventricle | Globularly dilated |
|  |  | Right ventricle | Normal |
| **Septae** |  | **Coronary arteries** | ----- |
| Interventricular septum | Intact | **Doppler Measurement** |  |
| Interatrial septum | Intact | Mitral | Moderate MR, Holosystolic, posterior projection, seen in two planes with jet velocity = 4.8m/sec |
| **Semilunar valves** |  | Aortic | Severe AR, PHT = 164ms |
| Aortic valve | Annulus = 24mm | Tricuspid | ------- |
| Pulmonary valve | Annulus = 24mm | pulmonic | -------- |
| **Great arteries** | NRGA | **Aortic arch** | Left. No CoA. |
| Aorta | ----- | **PDA** | No |
| Pulmonary artery | Normal MPA and Branch PAs. |  |  |
| **M-Mode:** | | | |
| AO | mm | PWd | mm |
| LA | mm | PWs | mm |
| LVIDd | mm | EDV | ml |
| LVIDs | mm | ESV | ml |
| IVSs | mm | LVEF | 42% |
| IVSd | mm | FS | 21% |
| **Additional Information**: |  | | |
| No pericardial/Pleural effusion. | | | |
| **Final Diagnosis:** | | | |
| 1. {S, D, S} Levocardia. 2. LA/LV Dilated 3. Thickened MVL 4. Mild MR 5. Severe AR 6. Reduced LV Systolic Function | | | |
| **Remark**: | | | |
| **Recommendation**: | | | |
| SIGNATURE  Done by: Tesfaye T., Pediatrician, Pediatric Cardiologist _______________ 18/07/2015Eth.C | | | |

| Patient Name: **Wellela Alebachew**. Referring Institute: **FHRH**. SEX/ Age: **F/11years**. Date of Report: **18/07/15**.  Referral Diagnosis: **CHF. AGH12.3400.** | | | |
| --- | --- | --- | --- |
| **Features** | **Finding** | **Features** | **Finding** |
| **Profile** |  | **Atria** |  |
| Abdominal situs | Solitus | Left atrium | Normal |
| Cardiac position | Levocardia | Right atrium | Normal |
| Systemic venous drainage | Normal. | **Atrioventricular valves** |  |
| Pulmonary venous drainage | Normal | Mitral valve | Annulus = 20mm |
| Atrioventricular connection | Concordant | Tricuspid valve | Annulus = 19mm  TAPSE = 19mm |
| Ventriculoarterial connection | Concordant | **Ventricles** |  |
| Ventricular loop | d-Loop | Left ventricle | Normal |
|  |  | Right ventricle | Normal |
| **Septae** |  | **Coronary arteries** | ----- |
| Interventricular septum | Intact | **Doppler Measurement** |  |
| Interatrial septum | 9mm OS ASD, L – R Shunt | Mitral | ----- |
| **Semilunar valves** |  | Aortic | ------- |
| Aortic valve | Annulus = 15mm | Tricuspid | Trivial TR, PPG = 14mmHg |
| Pulmonary valve | Annulus = 17mm | pulmonic | -------- |
| **Great arteries** | NRGA | **Aortic arch** | Left. No CoA. |
| Aorta | ----- | **PDA** | No |
| Pulmonary artery | Normal MPA and Branch PAs. |  |  |
| **M-Mode:** | | | |
| AO | mm | PWd | **8.5mm** |
| LA | mm | PWs | **10mm** |
| LVIDd | **31mm** | EDV | 38ml |
| LVIDs | **20mm** | ESV | 13ml |
| IVSs | **13mm** | LVEF | 66% |
| IVSd | **8.5mm** | FS | 35% |
| **Additional Information**: |  | | |
| Circumferential Pericardial effusion with maximum depth of 4mm. | | | |
| **Final Diagnosis:** | | | |
| 1. {S, D, S} Levocardia. 2. Moderate OS ASD, L – R Shunt 3. Trace Circumferential pericardial effusion 4. Normal LV Systolic Function | | | |
| **Remark**: The M-Mode finding needs standardization based on weight and Height. | | | |
| **Recommendation**: | | | |
| SIGNATURE  Done by: Tesfaye T., Pediatrician, Pediatric Cardiologist _______________ 18/07/2015Eth.C | | | |

| Patient Name: **Yohannes Tesfu**. Referring Institute: **Finote-Selam GH**. SEX/ Age: **M/6months**. Date of Report: **18/07/15**.  Referral Diagnosis: **RD + CHF. AGH12.3401.** | | | |
| --- | --- | --- | --- |
| **Features** | **Finding** | **Features** | **Finding** |
| **Profile** |  | **Atria** |  |
| Abdominal situs | Solitus | Left atrium | Dilated |
| Cardiac position | Levocardia | Right atrium | Normal |
| Systemic venous drainage | Normal. | **Atrioventricular valves** |  |
| Pulmonary venous drainage | Normal | Mitral valve | Annulus = 17mm |
| Atrioventricular connection | Concordant | Tricuspid valve | Annulus = 16mm |
| Ventriculoarterial connection | Concordant | **Ventricles** |  |
| Ventricular loop | d-Loop | Left ventricle | Globularly Dilated & Dysfunctional |
|  |  | Right ventricle | Normal |
| **Septae** |  | **Coronary arteries** | ----- |
| Interventricular septum | Intact | **Doppler Measurement** |  |
| Interatrial septum | Intact | Mitral | Moderate MR, Holosystolic, posterior projection, seen in two planes with jet velocity = 4.2m/sec |
| **Semilunar valves** |  | Aortic | ------- |
| Aortic valve | Annulus = 13mm | Tricuspid | Mild TR, PPG = 22mmHg |
| Pulmonary valve | Annulus = 15mm | pulmonic | -------- |
| **Great arteries** | NRGA | **Aortic arch** | Left. No CoA. |
| Aorta | ----- | **PDA** | No |
| Pulmonary artery | Normal MPA and Branch PAs. | **Coronaries** | No ALCAPA |
| **M-Mode:** | | | |
| AO | mm | PWd | mm |
| LA | mm | PWs | mm |
| LVIDd | mm | EDV | ml |
| LVIDs | mm | ESV | ml |
| IVSs | mm | LVEF | 30% |
| IVSd | mm | FS | 14% |
| **Additional Information**: |  | | |
| No pericardial/Pleural effusion. | | | |
| **Final Diagnosis:** | | | |
| 1. {S, D, S} Levocardia. 2. LA/LV Dilated 3. Moderate MR 4. Mild TR 5. Severe LV Systolic Dysfunction | | | |
| **Remark**: | | | |
| **Recommendation**: | | | |
| SIGNATURE  Done by: Tesfaye T., Pediatrician, Pediatric Cardiologist _______________ 18/07/2015Eth.C | | | |

| Patient Name: **Baby of Meseret Melak**. Referring Institute: **FHRH**. SEX/ Age: **M/7months**. Date of Report: **18/07/15**.  Referral Diagnosis: **Incidental Murmur finding (G-II). AGH12.3402.** | | | |
| --- | --- | --- | --- |
| **Features** | **Finding** | **Features** | **Finding** |
| **Profile** |  | **Atria** |  |
| Abdominal situs | Solitus | Left atrium | Normal |
| Cardiac position | Levocardia | Right atrium | Normal |
| Systemic venous drainage | Normal. | **Atrioventricular valves** |  |
| Pulmonary venous drainage | Normal | Mitral valve | Annulus = 13mm |
| Atrioventricular connection | Concordant | Tricuspid valve | Annulus = 14mm |
| Ventriculoarterial connection | Concordant | **Ventricles** |  |
| Ventricular loop | d-Loop | Left ventricle | Normal |
|  |  | Right ventricle | Normal |
| **Septae** |  | **Coronary arteries** | ----- |
| Interventricular septum | Intact | **Doppler Measurement** |  |
| Interatrial septum | Intact | Mitral | ----- |
| **Semilunar valves** |  | Aortic | ------- |
| Aortic valve | Annulus = 10mm | Tricuspid | ------- |
| Pulmonary valve | Annulus = 11mm | pulmonic | -------- |
| **Great arteries** | NRGA | **Aortic arch** | Left. No CoA. |
| Aorta | ----- | **PDA** | No |
| Pulmonary artery | Normal MPA and Branch PAs. |  |  |
| **M-Mode:** | | | |
| AO | mm | PWd | mm |
| LA | mm | PWs | mm |
| LVIDd | mm | EDV | ml |
| LVIDs | mm | ESV | ml |
| IVSs | mm | LVEF | % |
| IVSd | mm | FS | % |
| **Additional Information**: |  | | |
| No pericardial/Pleural effusion. | | | |
| **Final Diagnosis:** | | | |
| 1. Normal Echocardiography Study. | | | |
| **Remark**: | | | |
| **Recommendation**: | | | |
| SIGNATURE  Done by: Tesfaye T., Pediatrician, Pediatric Cardiologist _______________ 18/07/2015Eth.C | | | |

| Patient Name: **Ersu-yawukal Derso**. Referring Institute: **FHRH**. SEX/ Age: **M/1 6/12**. Date of Report: **18/07/15**.  Referral Diagnosis: **FTT + Cough and fast breathing. AGH12.3403.** | | | |
| --- | --- | --- | --- |
| **Features** | **Finding** | **Features** | **Finding** |
| **Profile** |  | **Atria** |  |
| Abdominal situs | Solitus | Left atrium | Normal |
| Cardiac position | Levocardia | Right atrium | Dilated |
| Systemic venous drainage | Normal. | **Atrioventricular valves** |  |
| Pulmonary venous drainage | Normal | Mitral valve | Annulus = 12mm |
| Atrioventricular connection | Concordant | Tricuspid valve | Annulus = 17mm  TAPSE = 13mm |
| Ventriculoarterial connection | Concordant | **Ventricles** |  |
| Ventricular loop | d-Loop | Left ventricle | Normal |
|  |  | Right ventricle | Dilated and Hypertrophied |
| **Septae** |  | **Coronary arteries** | ----- |
| Interventricular septum | Mal-aligned Sub-aortic Non-Restrictive VSD, R - L Shunt | **Doppler Measurement** |  |
| Interatrial septum | Intact | Mitral | ----- |
| **Semilunar valves** |  | Aortic | ------- |
| Aortic valve | Annulus = 14mm | Tricuspid | ------- |
| Pulmonary valve | Annulus = 11mm | pulmonic | Moderate PS, PPG = 56mmHg |
| **Great arteries** | NRGA | **Aortic arch** | Left. No CoA. |
| Aorta | ----- | **PDA** | 1mm PDA, L – R Shunt |
| Pulmonary artery | Normal MPA and Branch PAs. |  |  |
| **M-Mode:**  Normal LV Function on eye balling | | | |
| AO | mm | PWd | mm |
| LA | mm | PWs | mm |
| LVIDd | mm | EDV | ml |
| LVIDs | mm | ESV | ml |
| IVSs | mm | LVEF | % |
| IVSd | mm | FS | % |
| **Additional Information**: |  | | |
| No pericardial/Pleural effusion. | | | |
| **Final Diagnosis:** | | | |
| 1. {S, D, S} Levocardia. 2. RA/RV Dilated 3. TOF 4. Small PDA, L – R Shunt | | | |
| **Remark**: | | | |
| **Recommendation**: | | | |
| SIGNATURE  Done by: Tesfaye T., Pediatrician, Pediatric Cardiologist _______________ 18/07/2015Eth.C | | | |

| Patient Name: **Biruk Estifanos**. Institute: **Adinas GH**. SEX/ Age: **M/12years**. Date of Report: **18/07/15**.  Referral Diagnosis: **ARF. AGH12.3404.** | | | |
| --- | --- | --- | --- |
| **Features** | **Finding** | **Features** | **Finding** |
| **Profile** |  | **Atria** |  |
| Abdominal situs | Solitus | Left atrium | Mildly Dilated |
| Cardiac position | Levocardia | Right atrium | Normal |
| Systemic venous drainage | Normal. | **Atrioventricular valves** |  |
| Pulmonary venous drainage | Normal | Mitral valve | Annulus = 22mm. Thickened AMVL. |
| Atrioventricular connection | Concordant | Tricuspid valve | Annulus = 19mm  TAPSE = 20mm |
| Ventriculoarterial connection | Concordant | **Ventricles** |  |
| Ventricular loop | d-Loop | Left ventricle | Mildly Dilated |
|  |  | Right ventricle | Normal |
| **Septae** |  | **Coronary arteries** | ----- |
| Interventricular septum | Intact | **Doppler Measurement** |  |
| Interatrial septum | Intact | Mitral | Moderate MR, Holosystolic, posterior projection, seen In two planes with jet velocity = 4.5m/sec |
| **Semilunar valves** |  | Aortic | Mild AR, PHT = 555ms |
| Aortic valve | Annulus = 16mm | Tricuspid | Trivial TR, PPG = 12mmHg |
| Pulmonary valve | Annulus = 19mm | pulmonic | -------- |
| **Great arteries** | NRGA | **Aortic arch** | Left. No CoA. |
| Aorta | ----- | **PDA** | No |
| Pulmonary artery | Normal MPA and Branch PAs. |  |  |
| **M-Mode:** | | | |
| AO | mm | PWd | mm |
| LA | mm | PWs | mm |
| LVIDd | mm | EDV | ml |
| LVIDs | mm | ESV | ml |
| IVSs | mm | LVEF | 59% |
| IVSd | mm | FS | 31% |
| **Additional Information**: |  | | |
| No pericardial/Pleural effusion. | | | |
| **Final Diagnosis:** | | | |
| 1. {S, D, S} Levocardia. 2. LA/LV Mildly Dilated 3. Thickened AMVL 4. Moderate MR 5. Mild AR 6. Normal Biventricular Systolic Function | | | |
| **Recommendation**: | | | |
| SIGNATURE  Done by: Tesfaye T., Pediatrician, Pediatric Cardiologist _______________ 18/07/2015Eth.C | | | |

| Patient Name: **Yihun Wereda**. Referring Institute: **Addis Alem PH**. SEX/ Age: **M/5 7/12**. Date of Report: **19/07/15**.  Referral Diagnosis: **Cough and easy fatigability. AGH12.3405.** | | | |
| --- | --- | --- | --- |
| **Features** | **Finding** | **Features** | **Finding** |
| **Profile** |  | **Atria** |  |
| Abdominal situs | Solitus | Left atrium | Dilated |
| Atrial Situs | Solitus | Right atrium | Dilated |
| Cardiac position | Levocardia | **Atrioventricular valves** |  |
| Systemic venous drainage | Normal. | Mitral valve | Annulus = 22mm |
| Pulmonary venous drainage | Normal | Tricuspid valve | Annulus = 22mm |
| Atrioventricular connection | Concordant |  | TAPSE = 20mm |
| Ventriculoarterial connection | Concordant | **Ventricles** |  |
| Ventricular loop | d-Loop | Left ventricle | Dilated |
|  |  | Right ventricle | Dilated |
| **Septae** |  | **Coronary arteries** | ----- |
| Interventricular septum | Intact | **Doppler Measurement** |  |
| Interatrial septum | Intact | Mitral | ----- |
| **Semilunar valves** |  | Aortic | ------- |
| Aortic valve | Annulus = 17mm | Tricuspid | ------- |
| Pulmonary valve | Annulus = 20mm | pulmonic | -------- |
| **Great arteries** | NRGA | **Aortic arch** | Left. No CoA. |
| Aorta | ----- | **PDA** | 3mm PDA, L – R Shunt |
| Pulmonary artery | Normal MPA and Branch PAs. |  |  |
| **M-Mode:** | | | |
| AO | mm | PWd | mm |
| LA | mm | PWs | mm |
| LVIDd | mm | EDV | ml |
| LVIDs | mm | ESV | ml |
| IVSs | mm | LVEF | 60% |
| IVSd | mm | FS | 32% |
| **Additional Information**: |  | | |
| No pericardial/Pleural effusion. | | | |
| **Final Diagnosis:** | | | |
| 1. {S, D, S} Levocardia. 2. All chambers dilated 3. Large PDA, L – R Shunt 4. Normal Biventricular Systolic Function | | | |
| **Remark**: | | | |
| **Recommendation**: | | | |
| SIGNATURE  Done by: Tesfaye T., Pediatrician, Pediatric Cardiologist _______________ 19/07/2015Eth.C | | | |

| Patient Name: **Alebachew Gebrie**. Referring Institute: **FHRH**. SEX/ Age: **M/1year**. Date of Report: **19/07/15**.  Referral Diagnosis: **DS. AGH12.3406.** | | | |
| --- | --- | --- | --- |
| **Features** | **Finding** | **Features** | **Finding** |
| **Profile** |  | **Atria** |  |
| Abdominal situs | Solitus | Left atrium | Normal |
| Atrial Situs | Solitus | Right atrium | Normal |
| Cardiac position | Levocardia | **Atrioventricular valves** |  |
| Systemic venous drainage | Normal. | Mitral valve | Annulus = 14mm |
| Pulmonary venous drainage | Normal | Tricuspid valve | Annulus = 15mm |
| Atrioventricular connection | Concordant |  |  |
| Ventriculoarterial connection | Concordant | **Ventricles** |  |
| Ventricular loop | d-Loop | Left ventricle | Normal |
|  |  | Right ventricle | Normal |
| **Septae** |  | **Coronary arteries** | ----- |
| Interventricular septum | Intact | **Doppler Measurement** |  |
| Interatrial septum | Intact | Mitral | ----- |
| **Semilunar valves** |  | Aortic | ------- |
| Aortic valve | Annulus = 11mm | Tricuspid | ------- |
| Pulmonary valve | Annulus = 11mm | pulmonic | -------- |
| **Great arteries** | NRGA | **Aortic arch** | Left. No CoA. |
| Aorta | ----- | **PDA** | No |
| Pulmonary artery | Normal MPA and Branch PAs. |  |  |
| **M-Mode:**  Normal LV Function on eye balling | | | |
| AO | mm | PWd | mm |
| LA | mm | PWs | mm |
| LVIDd | mm | EDV | ml |
| LVIDs | mm | ESV | ml |
| IVSs | mm | LVEF | % |
| IVSd | mm | FS | % |
| **Additional Information**: |  | | |
| No pericardial/Pleural effusion. | | | |
| **Final Diagnosis:** | | | |
| 1. Normal Echocardiography Study. | | | |
| **Remark**: | | | |
| **Recommendation**: | | | |
| SIGNATURE  Done by: Tesfaye T., Pediatrician, Pediatric Cardiologist _______________ 19/07/2015Eth.C | | | |

| Patient Name: **Nibretu Tewachew**. Referring Institute: **TGSH**. SEX/ Age: **M/1 8/12**. Date of Report: **19/07/15**.  Referral Diagnosis: **Down Syndrome. AGH12.3407.** | | | |
| --- | --- | --- | --- |
| **Features** | **Finding** | **Features** | **Finding** |
| **Profile** |  | **Atria** |  |
| Abdominal situs | Solitus | Left atrium | Normal |
| Atrial Situs | Solitus | Right atrium | Normal |
| Cardiac position | Levocardia | **Atrioventricular valves** |  |
| Systemic venous drainage | Normal. | Mitral valve | Annulus = 11mm |
| Pulmonary venous drainage | Normal | Tricuspid valve | Annulus = 13mm |
| Atrioventricular connection | Concordant |  | TAPSE = 15mm |
| Ventriculoarterial connection | Concordant | **Ventricles** |  |
| Ventricular loop | d-Loop | Left ventricle | Normal |
|  |  | Right ventricle | Normal |
| **Septae** |  | **Coronary arteries** | ----- |
| Interventricular septum | Intact | **Doppler Measurement** |  |
| Interatrial septum | Intact | Mitral | ----- |
| **Semilunar valves** |  | Aortic | ------- |
| Aortic valve | Annulus = 12mm | Tricuspid | ------- |
| Pulmonary valve | Annulus = 14mm | pulmonic | -------- |
| **Great arteries** | NRGA | **Aortic arch** | Left. No CoA. |
| Aorta | ----- | **PDA** | No |
| Pulmonary artery | Normal MPA and Branch PAs. |  |  |
| **M-Mode:**  Normal LV Function on eye balling | | | |
| AO | mm | PWd | mm |
| LA | mm | PWs | mm |
| LVIDd | mm | EDV | ml |
| LVIDs | mm | ESV | ml |
| IVSs | mm | LVEF | % |
| IVSd | mm | FS | % |
| **Additional Information**: |  | | |
| No pericardial/Pleural effusion. | | | |
| **Final Diagnosis:** | | | |
| 1. Normal Echocardiography Study. | | | |
| **Remark**: | | | |
| **Recommendation**: | | | |
| SIGNATURE  Done by: Tesfaye T., Pediatrician, Pediatric Cardiologist _______________ 19/07/2015Eth.C | | | |

| Patient Name: **Hiwet Enideg**. Referring Institute: **TGSH**. SEX/ Age: **F/5months**. Date of Report: **20/07/15**.  Referral Diagnosis: **Diaphoresis and feeding interruption. AGH12.3408.** | | | |
| --- | --- | --- | --- |
| **Features** | **Finding** | **Features** | **Finding** |
| **Profile** |  | **Atria** |  |
| Abdominal situs | Solitus | Left atrium | Normal |
| Atrial Situs | Solitus | Right atrium | Normal |
| Cardiac position | Levocardia | **Atrioventricular valves** |  |
| Systemic venous drainage | Normal. | Mitral valve | Annulus = 14mm |
| Pulmonary venous drainage | Normal | Tricuspid valve | Annulus = 15mm |
| Atrioventricular connection | Concordant |  |  |
| Ventriculoarterial connection | Concordant | **Ventricles** |  |
| Ventricular loop | d-Loop | Left ventricle | Normal |
|  |  | Right ventricle | Normal |
| **Septae** |  | **Coronary arteries** | ----- |
| Interventricular septum | 5mm Sub aortic VSD, Non-Restrictive, L – R Shunt. | **Doppler Measurement** |  |
| Interatrial septum | 1mm Gerbode defect, LV - RA | Mitral | ----- |
| **Semilunar valves** |  | Aortic | ------- |
| Aortic valve | Annulus = 11mm | Tricuspid | ------- |
| Pulmonary valve | Annulus = 12mm | pulmonic | -------- |
| **Great arteries** | NRGA | **Aortic arch** | Left. No CoA. |
| Aorta | ----- | **PDA** | No |
| Pulmonary artery | Normal MPA and Branch PAs. |  |  |
| **M-Mode:**  Normal LV Function on eye balling | | | |
| AO | mm | PWd | mm |
| LA | mm | PWs | mm |
| LVIDd | mm | EDV | ml |
| LVIDs | mm | ESV | ml |
| IVSs | mm | LVEF | % |
| IVSd | mm | FS | % |
| **Additional Information**: |  | | |
| No pericardial/Pleural effusion. | | | |
| **Final Diagnosis:** | | | |
| 1. {S, D, S} Levocardia. 2. Congenital Gerbode Defect, LV – RA 3. Small TO Moderate Sub- Aortic VSD, L – R Shunt 4. Normal LV Systolic Function | | | |
| **Remark**: | | | |
| **Recommendation**: | | | |
| SIGNATURE  Done by: Tesfaye T., Pediatrician, Pediatric Cardiologist _______________ 20/07/2015Eth.C | | | |

| Patient Name: **Hana – werk Fekad**. Referring Institute: **TGSH**. SEX/ Age: **F/9months**. Date of Report: **20/07/15**.  Referral Diagnosis: **FTT + DS. AGH12.3409.** | | | |
| --- | --- | --- | --- |
| **Features** | **Finding** | **Features** | **Finding** |
| **Profile** |  | **Atria** |  |
| Abdominal situs | Solitus | Left atrium | Normal |
| Atrial Situs | Solitus | Right atrium | Dilated |
| Cardiac position | Levocardia | **Atrioventricular valves** |  |
| Systemic venous drainage | Normal. | Mitral valve | Annulus = 10mm |
| Pulmonary venous drainage | Normal | Tricuspid valve | Annulus = 18mm |
| Atrioventricular connection | Concordant |  | TAPSE = 13mm |
| Ventriculoarterial connection | Concordant | **Ventricles** |  |
| Ventricular loop | d-Loop | Left ventricle | Normal |
|  |  | Right ventricle | Dilated |
| **Septae** |  | **Coronary arteries** | ----- |
| Interventricular septum | Intact | **Doppler Measurement** |  |
| Interatrial septum | 8mm OS ASD, L – R Shunt. The septum is bowing to LA and RA 7.5mm each making total of 15mm. | Mitral | ----- |
| **Semilunar valves** |  | Aortic | ------- |
| Aortic valve | Annulus = 12mm | Tricuspid | ------- |
| Pulmonary valve | Annulus = 15mm | pulmonic | Mild PR, PPG = 36mmHg |
| **Great arteries** | NRGA | **Aortic arch** | Left. No CoA. |
| Aorta | ----- | **PDA** | No |
| Pulmonary artery | Normal MPA and Branch PAs. |  |  |
| **M-Mode:** | | | |
| AO | mm | PWd | mm |
| LA | mm | PWs | mm |
| LVIDd | mm | EDV | ml |
| LVIDs | mm | ESV | ml |
| IVSs | mm | LVEF | 71% |
| IVSd | mm | FS | 38% |
| **Additional Information**: |  | | |
| No pericardial/Pleural effusion. | | | |
| **Final Diagnosis:** | | | |
| 1. {S, D, S} Levocardia. 2. RA/RV Dilated 3. Moderate OS ASD, L – R Shunt 4. Atrial Septal Aneurysm 5. Normal Biventricular Systolic Function | | | |
| **Remark**: | | | |
| **Recommendation**: | | | |
| SIGNATURE  Done by: Tesfaye T., Pediatrician, Pediatric Cardiologist _______________ 20/07/2015Eth.C | | | |

| Patient Name: **Akiya Gizaw**. Referring Institute: **FHRH**. SEX/ Age: **M/6months**. Date of Report: **20/07/15**.  Referral Diagnosis: **Diaphoresis during breast feeding + G-III HSM @ LLSB. AGH12.3410.** | | | |
| --- | --- | --- | --- |
| **Features** | **Finding** | **Features** | **Finding** |
| **Profile** |  | **Atria** |  |
| Abdominal situs | Solitus | Left atrium | Normal |
| Atrial Situs | Solitus | Right atrium | Normal |
| Cardiac position | Levocardia | **Atrioventricular valves** |  |
| Systemic venous drainage | Normal. | Mitral valve | Annulus = 13mm |
| Pulmonary venous drainage | Normal | Tricuspid valve | Annulus = 13mm |
| Atrioventricular connection | Concordant |  | TAPSE = 14mm |
| Ventriculoarterial connection | Concordant | **Ventricles** |  |
| Ventricular loop | d-Loop | Left ventricle | Normal |
|  |  | Right ventricle | Normal |
| **Septae** |  | **Coronary arteries** | ----- |
| Interventricular septum | Intact | **Doppler Measurement** |  |
| Interatrial septum | Intact | Mitral | ----- |
| **Semilunar valves** |  | Aortic | ------- |
| Aortic valve | Annulus = 11mm | Tricuspid | ------- |
| Pulmonary valve | Annulus = 11mm | pulmonic | -------- |
| **Great arteries** | NRGA | **Aortic arch** | Left. No CoA. |
| Aorta | ----- | **PDA** | No |
| Pulmonary artery | Normal MPA and Branch PAs. |  |  |
| **M-Mode:**  Normal LV Function on eye balling | | | |
| AO | mm | PWd | mm |
| LA | mm | PWs | mm |
| LVIDd | mm | EDV | ml |
| LVIDs | mm | ESV | ml |
| IVSs | mm | LVEF | % |
| IVSd | mm | FS | % |
| **Additional Information**: |  | | |
| No pericardial/Pleural effusion. | | | |
| **Final Diagnosis:** | | | |
| 1. Normal Echocardiography Study. | | | |
| **Remark**: | | | |
| **Recommendation**: | | | |
| SIGNATURE  Done by: Tesfaye T., Pediatrician, Pediatric Cardiologist _______________ 20/07/2015Eth.C | | | |

| Patient Name: **Tamagnu Min-Lehon**. Referring Institute: **Guzara SC**. SEX/ Age: **M/7months**. Date of Report: **20/07/15**.  Referral Diagnosis: **CHD/Incidental. AGH12.3411.** | | | |
| --- | --- | --- | --- |
| **Features** | **Finding** | **Features** | **Finding** |
| **Profile** |  | **Atria** |  |
| Abdominal situs | Solitus | Left atrium | Normal |
| Atrial Situs | Solitus | Right atrium | Normal |
| Cardiac position | Levocardia | **Atrioventricular valves** |  |
| Systemic venous drainage | Normal. | Mitral valve | Annulus = 13mm |
| Pulmonary venous drainage | Normal | Tricuspid valve | Annulus = 14mm |
| Atrioventricular connection | Concordant |  |  |
| Ventriculoarterial connection | Concordant | **Ventricles** |  |
| Ventricular loop | d-Loop | Left ventricle | Normal |
|  |  | Right ventricle | Normal |
| **Septae** |  | **Coronary arteries** | ----- |
| Interventricular septum | 4mm PM VSD, L – R Shunt | **Doppler Measurement** |  |
| Interatrial septum | Intact | Mitral | ----- |
| **Semilunar valves** |  | Aortic | ------- |
| Aortic valve | Annulus = 12mm | Tricuspid | ------- |
| Pulmonary valve | Annulus = 14mm | pulmonic | -------- |
| **Great arteries** | NRGA | **Aortic arch** | Left. No CoA. |
| Aorta | ----- | **PDA** | No |
| Pulmonary artery | Normal MPA and Branch PAs. |  |  |
| **M-Mode:**  Normal LV Function on eye balling | | | |
| AO | mm | PWd | mm |
| LA | mm | PWs | mm |
| LVIDd | mm | EDV | ml |
| LVIDs | mm | ESV | ml |
| IVSs | mm | LVEF | % |
| IVSd | mm | FS | % |
| **Additional Information**: |  | | |
| No pericardial/Pleural effusion. | | | |
| **Final Diagnosis:** | | | |
| 1. {S, D, S} Levocardia. 2. Small PM VSD, L – R Shunt 3. Normal LV Systolic Function | | | |
| **Remark**: | | | |
| **Recommendation**: | | | |
| SIGNATURE  Done by: Tesfaye T., Pediatrician, Pediatric Cardiologist _______________ 20/07/2015Eth.C | | | |

| Patient Name: **Bereket Wubet**. Referring Institute: **FHRH**. SEX/ Age: **M/1 6/12**. Date of Report: **20/07/15**.  Referral Diagnosis: **FTT + Recurrent Pneumonia. AGH12.3412.** | | | |
| --- | --- | --- | --- |
| **Features** | **Finding** | **Features** | **Finding** |
| **Profile** |  | **Atria** |  |
| Abdominal situs | Solitus | Left atrium | Normal |
| Atrial Situs | Solitus | Right atrium | Normal |
| Cardiac position | Levocardia | **Atrioventricular valves** |  |
| Systemic venous drainage | Normal. | Mitral valve | Annulus = 13mm |
| Pulmonary venous drainage | Normal | Tricuspid valve | Annulus = 15mm |
| Atrioventricular connection | Concordant |  | TAPSE = 15mm |
| Ventriculoarterial connection | Concordant | **Ventricles** |  |
| Ventricular loop | d-Loop | Left ventricle | Normal |
|  |  | Right ventricle | Normal |
| **Septae** |  | **Coronary arteries** | ----- |
| Interventricular septum | Intact | **Doppler Measurement** |  |
| Interatrial septum | Intact | Mitral | ----- |
| **Semilunar valves** |  | Aortic | ------- |
| Aortic valve | Annulus = 11 | Tricuspid | ------- |
| Pulmonary valve | Annulus = 12mm | pulmonic | -------- |
| **Great arteries** | NRGA | **Aortic arch** | Left. No CoA. |
| Aorta | ----- | **PDA** | No |
| Pulmonary artery | Normal MPA and Branch PAs. |  |  |
| **M-Mode:** | | | |
| AO | mm | PWd | mm |
| LA | mm | PWs | mm |
| LVIDd | mm | EDV | ml |
| LVIDs | mm | ESV | ml |
| IVSs | mm | LVEF | 71% |
| IVSd | mm | FS | 38% |
| **Additional Information**: |  | | |
| No pericardial/Pleural effusion. | | | |
| **Final Diagnosis:** | | | |
| 1. Normal Echocardiography Study. | | | |
| **Remark**: | | | |
| **Recommendation**: | | | |
| SIGNATURE  Done by: Tesfaye T., Pediatrician, Pediatric Cardiologist _______________ 20/07/2015Eth.C | | | |

| Patient Name: **Yibeltal Mulat**. Referring Institute: **Eyasta Medical Services**. SEX/ Age: **M/5years**. Date of Report: **20/07/15**. Referral Diagnosis: **TGSH10.2802.** | | | |
| --- | --- | --- | --- |
| **Features** | **Finding** | **Features** | **Finding** |
| **Profile** |  | **Atria** |  |
| Abdominal situs | Solitus | Left atrium | Compressed Pericardial fluid |
| Atrial Situs | Solitus | Right atrium | Normal |
| Cardiac position | Levocardia | **Atrioventricular valves** |  |
| Systemic venous drainage | Normal. | Mitral valve | Annulus = 16mm |
| Pulmonary venous drainage | Normal | Tricuspid valve | Annulus = 17mm |
| Atrioventricular connection | Concordant |  |  |
| Ventriculoarterial connection | Concordant | **Ventricles** |  |
| Ventricular loop | d-Loop | Left ventricle | Normal |
|  |  | Right ventricle | Normal |
| **Septae** |  | **Coronary arteries** | ----- |
| Interventricular septum | Intact | **Doppler Measurement** |  |
| Interatrial septum | Intact | Mitral | No Mitral inflow gradient |
| **Semilunar valves** |  | Aortic | ------- |
| Aortic valve | Annulus = 13mm | Tricuspid | ------- |
| Pulmonary valve | Annulus = 17mm | pulmonic | -------- |
| **Great arteries** | NRGA | **Aortic arch** | Left. No CoA. |
| Aorta | ----- | **PDA** | No |
| Pulmonary artery | Normal MPA and Branch PAs. |  |  |
| **M-Mode:** | | | |
| AO | mm | PWd | mm |
| LA | mm | PWs | mm |
| LVIDd | mm | EDV | ml |
| LVIDs | mm | ESV | ml |
| IVSs | mm | LVEF | % |
| IVSd | mm | FS | % |
| **Additional Information**: |  | | |
| Pericardial Fluid measuring maximum depth of 38mm on LV Side communication with LV with a defect of 6mm. debris visualized. | | | |
| **Final Diagnosis:** | | | |
| 1. {S, D, S} Levocardia. 2. LA Compressed with No Mitral inflow obliteration 3. Large Posterior Pericardial collection communication with LV posteriorly with a defect of 6mm, Bidirectional flow across the defect. | | | |
| **Remark**: LV Pseudoaneurysm | | | |
| **Recommendation**: Needs Surgical Intervention | | | |
| SIGNATURE  Done by: Tesfaye T., Pediatrician, Pediatric Cardiologist _______________ 20/07/2015Eth.C | | | |

| Patient Name: **Abel Gebre-Mariam**. Referring Institute: **TGSH**. SEX/ Age: **M/5 4/12**. Date of Report: **20/07/15**.  Referral Diagnosis: **ARF. AGH12.3413.** | | | |
| --- | --- | --- | --- |
| **Features** | **Finding** | **Features** | **Finding** |
| **Profile** |  | **Atria** |  |
| Abdominal situs | Solitus | Left atrium | Normal |
| Atrial Situs | Solitus | Right atrium | Normal |
| Cardiac position | Levocardia | **Atrioventricular valves** |  |
| Systemic venous drainage | Normal. | Mitral valve | Annulus = 17mm |
| Pulmonary venous drainage | Normal | Tricuspid valve | Annulus = 18mm |
| Atrioventricular connection | Concordant |  | TAPSE = 18mm |
| Ventriculoarterial connection | Concordant | **Ventricles** |  |
| Ventricular loop | d-Loop | Left ventricle | Normal |
|  |  | Right ventricle | Normal |
| **Septae** |  | **Coronary arteries** | ----- |
| Interventricular septum | Intact | **Doppler Measurement** |  |
| Interatrial septum | Intact | Mitral | ----- |
| **Semilunar valves** |  | Aortic | ------- |
| Aortic valve | Annulus = 13mm | Tricuspid | ------- |
| Pulmonary valve | Annulus = 14mm | pulmonic | -------- |
| **Great arteries** | NRGA | **Aortic arch** | Left. No CoA. |
| Aorta | ----- | **PDA** | No |
| Pulmonary artery | Normal MPA and Branch PAs. |  |  |
| **M-Mode:** | | | |
| AO | mm | PWd | mm |
| LA | mm | PWs | mm |
| LVIDd | mm | EDV | ml |
| LVIDs | mm | ESV | ml |
| IVSs | mm | LVEF | 65% |
| IVSd | mm | FS | 34% |
| **Additional Information**: |  | | |
| No pericardial/Pleural effusion. | | | |
| **Final Diagnosis:** | | | |
| 1. Normal Echocardiography Study. | | | |
| **Remark**: Normal Echocardiography doesn’t rule our Acute Rheumatic Fever | | | |
| **Recommendation**: | | | |
| SIGNATURE  Done by: Tesfaye T., Pediatrician, Pediatric Cardiologist _______________ 20/07/2015Eth.C | | | |

| Patient Name: **Zeleke Alemu**. Referring Institute: **Adinas GH**. SEX/ Age: **M/11years**. Date of Report: **21/07/15**.  Referral Diagnosis: **CRHD. AGH12.3414.** | | | |
| --- | --- | --- | --- |
| **Features** | **Finding** | **Features** | **Finding** |
| **Profile** |  | **Atria** |  |
| Abdominal situs | Solitus | Left atrium | Normal |
| Atrial Situs | Solitus | Right atrium | Normal |
| Cardiac position | Levocardia | **Atrioventricular valves** |  |
| Systemic venous drainage | Normal. | Mitral valve | Annulus = 18mm |
| Pulmonary venous drainage | Normal | Tricuspid valve | Annulus = 18mm |
| Atrioventricular connection | Concordant |  | TAPSE = 17mm |
| Ventriculoarterial connection | Concordant | **Ventricles** |  |
| Ventricular loop | d-Loop | Left ventricle | Normal |
|  |  | Right ventricle | Normal |
| **Septae** |  | **Coronary arteries** | ----- |
| Interventricular septum | Intact | **Doppler Measurement** |  |
| Interatrial septum | Intact | Mitral | ----- |
| **Semilunar valves** |  | Aortic | ------- |
| Aortic valve | Annulus = 17mm | Tricuspid | ------- |
| Pulmonary valve | Annulus = 18mm | pulmonic | -------- |
| **Great arteries** | NRGA | **Aortic arch** | Left. No CoA. |
| Aorta | ----- | **PDA** | No |
| Pulmonary artery | Normal MPA and Branch PAs. |  |  |
| **M-Mode:**  Normal LV Function on eye balling | | | |
| AO | mm | PWd | mm |
| LA | mm | PWs | mm |
| LVIDd | mm | EDV | ml |
| LVIDs | mm | ESV | ml |
| IVSs | mm | LVEF | % |
| IVSd | mm | FS | % |
| **Additional Information**: |  | | |
| No pericardial/Pleural effusion. | | | |
| **Final Diagnosis:** | | | |
| 1. Normal Echocardiography Study. | | | |
| **Remark**: | | | |
| **Recommendation**: | | | |
| SIGNATURE  Done by: Tesfaye T., Pediatrician, Pediatric Cardiologist _______________ 21/07/2015Eth.C | | | |

| Patient Name: **Werkneh Wendie**. Referring Institute: **FHRH**. SEX/ Age: **M/3months**. Date of Report: **21/07/15**.  Referral Diagnosis: **Cough + Fast Breathing + G-III HSM @ LLSB + DS. AGH12.3415.** | | | |
| --- | --- | --- | --- |
| **Features** | **Finding** | **Features** | **Finding** |
| **Profile** |  | **Atria** |  |
| Abdominal situs | Solitus | Left atrium | Dilated |
| Atrial Situs | Solitus | Right atrium | Dilated |
| Cardiac position | Levocardia | **Atrioventricular valves** |  |
| Systemic venous drainage | Normal. | Mitral valve | Annulus = 12mm |
| Pulmonary venous drainage | Normal | Tricuspid valve | Annulus = 13mm |
| Atrioventricular connection | Concordant |  |  |
| Ventriculoarterial connection | Concordant | **Ventricles** |  |
| Ventricular loop | d-Loop | Left ventricle | Dilated |
|  |  | Right ventricle | Dilated |
| **Septae** | Tongue of tissue in b/n the defects | **Coronary arteries** | ----- |
| Interventricular septum | 8mm Inlet VSD, L – R Shunt | **Doppler Measurement** |  |
| Interatrial septum | 9mm Primum defect, L – R Shunt | Mitral | ----- |
| **Semilunar valves** |  | Aortic | ------- |
| Aortic valve | Annulus = 10mm | Tricuspid | Mild TR |
| Pulmonary valve | Annulus = 14mm | pulmonic | Mild PS, PPG = 20mmHg |
| **Great arteries** | NRGA | **Aortic arch** | Left. No CoA. |
| Aorta | ----- | **PDA** | No |
| Pulmonary artery | Normal MPA & Branch PAs. |  |  |
| **M-Mode:**  Normal LV Function (on eye balling) | | | |
| AO | mm | PWd | mm |
| LA | mm | PWs | mm |
| LVIDd | mm | EDV | ml |
| LVIDs | mm | ESV | ml |
| IVSs | mm | LVEF | % |
| IVSd | mm | FS | % |
| **Additional Information**: |  | | |
| No pericardial/Pleural effusion. | | | |
| **Final Diagnosis:** | | | |
| 1. {S, D, S} Levocardia. 2. All Chambers dilated 3. Intermediate AVSD, L – R Shunt 4. Pulmonary Hypertension 5. Normal LV Function | | | |
| **Remark**: | | | |
| **Recommendation**: | | | |
| SIGNATURE  Done by: Tesfaye T., Pediatrician, Pediatric Cardiologist _______________ 21/07/2015Eth.C | | | |

| Patient Name: **Desalegn Mezgebu**. Referring Institute: **FHRH**. SEX/ Age: **M/12years**. Date of Report: **22/07/15**.  Referral Diagnosis: **Duchenne Muscular Dystrophy. AGH12.3416.** | | | |
| --- | --- | --- | --- |
| **Features** | **Finding** | **Features** | **Finding** |
| **Profile** |  | **Atria** |  |
| Abdominal situs | Solitus | Left atrium | Normal |
| Atrial Situs | Solitus | Right atrium | Normal |
| Cardiac position | Levocardia | **Atrioventricular valves** |  |
| Systemic venous drainage | Normal. | Mitral valve | Annulus = 21mm |
| Pulmonary venous drainage | Normal | Tricuspid valve | Annulus = 23mm |
| Atrioventricular connection | Concordant |  | TAPSE = 18mm |
| Ventriculoarterial connection | Concordant | **Ventricles** |  |
| Ventricular loop | d-Loop | Left ventricle | Normal |
|  |  | Right ventricle | Normal |
| **Septae** |  | **Coronary arteries** | ----- |
| Interventricular septum | Intact | **Doppler Measurement** |  |
| Interatrial septum | Intact | Mitral | ----- |
| **Semilunar valves** |  | Aortic | ------- |
| Aortic valve | Annulus = 17mm | Tricuspid | ------- |
| Pulmonary valve | Annulus = 19mm | pulmonic | -------- |
| **Great arteries** | NRGA | **Aortic arch** | Left. No CoA. |
| Aorta | ----- | **PDA** | No |
| Pulmonary artery | Normal MPA and Branch PAs. |  |  |
| **M-Mode:** | | | |
| AO | mm | PWd | mm |
| LA | mm | PWs | mm |
| LVIDd | mm | EDV | ml |
| LVIDs | mm | ESV | ml |
| IVSs | mm | LVEF | 63% |
| IVSd | mm | FS | 34% |
| **Additional Information**: |  | | |
| No pericardial/Pleural effusion. | | | |
| **Final Diagnosis:** | | | |
| 1. Normal Echocardiography Study. | | | |
| **Remark**: | | | |
| **Recommendation**: Needs serial clinical, ECG and Echocardiography assessment; at least annually for early detection and treatment of cardiac involvement. | | | |
| SIGNATURE  Done by: Tesfaye T., Pediatrician, Pediatric Cardiologist _______________ 22/07/2015Eth.C | | | |

| Patient Name: **Baby of Degie Haile**. Referring Institute: **Pawe GH**. SEX/ Age: **F/27days**. Date of Report: **22/07/15**.  Referral Diagnosis: **FB + HSM @ Apex. AGH12.3417.** | | | |
| --- | --- | --- | --- |
| **Features** | **Finding** | **Features** | **Finding** |
| **Profile** |  | **Atria** |  |
| Abdominal situs | Solitus | Left atrium | Normal |
| Atrial Situs | Solitus | Right atrium | Dilated |
| Cardiac position | Levocardia | **Atrioventricular valves** |  |
| Systemic venous drainage | Normal. | Mitral valve | Annulus = 12mm |
| Pulmonary venous drainage | Normal | Tricuspid valve | Annulus = 16mm |
| Atrioventricular connection | Concordant |  | TAPSE = 8mm |
| Ventriculoarterial connection | Discordant | **Ventricles** |  |
| Ventricular loop | d-Loop | Left ventricle | Regressed |
|  |  | Right ventricle | Dilated & Hypertrophied |
| **Septae** |  | **Coronary arteries** | ----- |
| Interventricular septum | Intact | **Doppler Measurement** |  |
| Interatrial septum | Restrictive PFO | Mitral | Mild MR |
| **Semilunar valves** |  | Aortic | Mild AR |
| Aortic valve | Annulus = 8mm | Tricuspid | Severe TR |
| Pulmonary valve | Annulus = 6mm | pulmonic | Severe PS (LVOTO), PPG = 67mmHg |
| **Great arteries** | d-TGA | **Aortic arch** | Left. No CoA. |
| Aorta | Anterior & to the right. From RV | **PDA** | No |
| Pulmonary artery | Posterior & to the left. From LV |  |  |
| **M-Mode:** | | | |
| AO | mm | PWd | mm |
| LA | mm | PWs | mm |
| LVIDd | mm | EDV | ml |
| LVIDs | mm | ESV | ml |
| IVSs | mm | LVEF | % |
| IVSd | mm | FS | % |
| **Additional Information**: |  | | |
| Pericardial effusion with maximum depth of 5mm on RA Side. | | | |
| **Final Diagnosis:** | | | |
| 1. {S, D, D} Levocardia. 2. RA/RV Dilated, RV Hypertrophied 3. d-TGA with Intact IVS 4. Regressed LV 5. Small Pericardial effusion | | | |
| **Remark**: | | | |
| **Recommendation**: | | | |
| SIGNATURE  Done by: Tesfaye T., Pediatrician, Pediatric Cardiologist _______________ 22/07/2015Eth.C | | | |

| Patient Name: **Baby of Birtukan Mareshet**. Referring Institute: **MSI- Ethiopia**. SEX/ Age: **M/24days**. Date of Report: **22/07/15**. Referral Diagnosis: **FB + G-II HSM @LLSB. AGH12.3418.** | | | |
| --- | --- | --- | --- |
| **Features** | **Finding** | **Features** | **Finding** |
| **Profile** |  | **Atria** |  |
| Abdominal situs | Solitus | Left atrium | Normal |
| Atrial Situs | Solitus | Right atrium | Normal |
| Cardiac position | Levocardia | **Atrioventricular valves** |  |
| Systemic venous drainage | Normal. | Mitral valve | Annulus = 11mm |
| Pulmonary venous drainage | Normal | Tricuspid valve | Annulus = 13mm |
| Atrioventricular connection | Concordant |  | TAPSE = 13mm |
| Ventriculoarterial connection | Concordant | **Ventricles** |  |
| Ventricular loop | d-Loop | Left ventricle | Normal |
|  |  | Right ventricle | Normal |
| **Septae** |  | **Coronary arteries** | ----- |
| Interventricular septum | 7mm Inlet VSD, L – R Shunt | **Doppler Measurement** |  |
| Interatrial septum | Intact | Mitral | ----- |
| **Semilunar valves** |  | Aortic | ------- |
| Aortic valve | Annulus = 8mm | Tricuspid | ------- |
| Pulmonary valve | Annulus = 9mm | pulmonic | -------- |
| **Great arteries** | NRGA | **Aortic arch** | Left. No CoA. |
| Aorta | ----- | **PDA** | No |
| Pulmonary artery | Normal MPA and Branch PAs. |  |  |
| **M-Mode:** | | | |
| AO | mm | PWd | mm |
| LA | mm | PWs | mm |
| LVIDd | mm | EDV | ml |
| LVIDs | mm | ESV | ml |
| IVSs | mm | LVEF | % |
| IVSd | mm | FS | % |
| **Additional Information**: |  | | |
| No pericardial/Pleural effusion. | | | |
| **Final Diagnosis:** | | | |
| 1. {S, D, S} Levocardia. 2. Large Inlet VSD, L – R Shunt | | | |
| **Remark**: | | | |
| **Recommendation**: | | | |
| SIGNATURE  Done by: Tesfaye T., Pediatrician, Pediatric Cardiologist _______________ 22/07/2015Eth.C | | | |

| Patient Name: **Teju Abate**. Referring Institute: **TGSH**. SEX/ Age: **M/4years**. Date of Report: **22/07/15**.  Referral Diagnosis: **ARF. AGH12.3419.** | | | |
| --- | --- | --- | --- |
| **Features** | **Finding** | **Features** | **Finding** |
| **Profile** |  | **Atria** |  |
| Abdominal situs | Solitus | Left atrium | Normal |
| Atrial Situs | Solitus | Right atrium | Normal |
| Cardiac position | Levocardia | **Atrioventricular valves** |  |
| Systemic venous drainage | Normal. | Mitral valve | Annulus = 14mm |
| Pulmonary venous drainage | Normal | Tricuspid valve | Annulus = 14mm |
| Atrioventricular connection | Concordant |  | TAPSE = 17mm |
| Ventriculoarterial connection | Concordant | **Ventricles** |  |
| Ventricular loop | d-Loop | Left ventricle | Normal |
|  |  | Right ventricle | Normal |
| **Septae** |  | **Coronary arteries** | ----- |
| Interventricular septum | Intact | **Doppler Measurement** |  |
| Interatrial septum | Intact | Mitral | ----- |
| **Semilunar valves** |  | Aortic | ------- |
| Aortic valve | Annulus = 13mm | Tricuspid | ------- |
| Pulmonary valve | Annulus = 15mm | pulmonic | -------- |
| **Great arteries** | NRGA | **Aortic arch** | Left. No CoA. |
| Aorta | ----- | **PDA** | No |
| Pulmonary artery | Normal MPA and Branch PAs. |  |  |
| **M-Mode:**  Normal LV Function on eye balling | | | |
| AO | mm | PWd | mm |
| LA | mm | PWs | mm |
| LVIDd | mm | EDV | ml |
| LVIDs | mm | ESV | ml |
| IVSs | mm | LVEF | % |
| IVSd | mm | FS | % |
| **Additional Information**: |  | | |
| No pericardial/Pleural effusion. | | | |
| **Final Diagnosis:** | | | |
| 1. Normal Echocardiography Study. | | | |
| **Remark**: | | | |
| **Recommendation**: | | | |
| SIGNATURE  Done by: Tesfaye T., Pediatrician, Pediatric Cardiologist _______________ 22/07/2015Eth.C | | | |

| Patient Name: **Nahom Addis**. Referring Institute: **FHRH**. SEX/ Age: **M/3 10/12**. Date of Report: **23/07/15**.  Referral Diagnosis: **DS. AGH12.3420.** | | | |
| --- | --- | --- | --- |
| **Features** | **Finding** | **Features** | **Finding** |
| **Profile** |  | **Atria** |  |
| Abdominal situs | Solitus | Left atrium | Normal |
| Atrial Situs | Solitus | Right atrium | Normal |
| Cardiac position | Levocardia | **Atrioventricular valves** |  |
| Systemic venous drainage | Normal. | Mitral valve | Annulus = 15mm |
| Pulmonary venous drainage | Normal | Tricuspid valve | Annulus = 17mm |
| Atrioventricular connection | Concordant |  | TAPSE = 18mm |
| Ventriculoarterial connection | Concordant | **Ventricles** |  |
| Ventricular loop | d-Loop | Left ventricle | Normal |
|  |  | Right ventricle | Normal |
| **Septae** |  | **Coronary arteries** | ----- |
| Interventricular septum | Intact | **Doppler Measurement** |  |
| Interatrial septum | Intact | Mitral | ----- |
| **Semilunar valves** |  | Aortic | ------- |
| Aortic valve | Annulus = 13mm | Tricuspid | ------- |
| Pulmonary valve | Annulus = 15mm | pulmonic | -------- |
| **Great arteries** | NRGA | **Aortic arch** | Left. No CoA. |
| Aorta | ----- | **PDA** | No |
| Pulmonary artery | Normal MPA and Branch PAs. |  |  |
| **M-Mode:**  Normal LV Function on eye balling | | | |
| AO | mm | PWd | mm |
| LA | mm | PWs | mm |
| LVIDd | mm | EDV | ml |
| LVIDs | mm | ESV | ml |
| IVSs | mm | LVEF | % |
| IVSd | mm | FS | % |
| **Additional Information**: |  | | |
| No pericardial/Pleural effusion. | | | |
| **Final Diagnosis:** | | | |
| 1. Normal Echocardiography Study. | | | |
| **Remark**: | | | |
| **Recommendation**: | | | |
| SIGNATURE  Done by: Tesfaye T., Pediatrician, Pediatric Cardiologist _______________ 23/07/2015Eth.C | | | |

| Patient Name: **Tsion Fekadu**. Referring Institute: **FHRH**. SEX/ Age: **F/1year**. Date of Report: **23/07/15**.  Referral Diagnosis: **FB and Grunting. AGH12.3421.** | | | |
| --- | --- | --- | --- |
| **Features** | **Finding** | **Features** | **Finding** |
| **Profile** |  | **Atria** |  |
| Abdominal situs | Solitus | Left atrium | Normal |
| Atrial Situs | Solitus | Right atrium | Normal |
| Cardiac position | Levocardia | **Atrioventricular valves** |  |
| Systemic venous drainage | Normal. | Mitral valve | Annulus = 13mm |
| Pulmonary venous drainage | Normal | Tricuspid valve | Annulus = 15mm |
| Atrioventricular connection | Concordant |  | TAPSE = mm |
| Ventriculoarterial connection | Concordant | **Ventricles** |  |
| Ventricular loop | d-Loop | Left ventricle | Normal |
|  |  | Right ventricle | Normal |
| **Septae** |  | **Coronary arteries** | ----- |
| Interventricular septum | Intact | **Doppler Measurement** |  |
| Interatrial septum | Intact | Mitral | ----- |
| **Semilunar valves** |  | Aortic | ------- |
| Aortic valve | Annulus = 13mm | Tricuspid | ------- |
| Pulmonary valve | Annulus = 13mm | pulmonic | -------- |
| **Great arteries** | NRGA | **Aortic arch** | Left. No CoA. |
| Aorta | ----- | **PDA** | No |
| Pulmonary artery | Normal MPA and Branch PAs. |  |  |
| **M-Mode:**  Normal LV Function on eye balling | | | |
| AO | mm | PWd | mm |
| LA | mm | PWs | mm |
| LVIDd | mm | EDV | ml |
| LVIDs | mm | ESV | ml |
| IVSs | mm | LVEF | % |
| IVSd | mm | FS | % |
| **Additional Information**: |  | | |
| No pericardial/Pleural effusion. | | | |
| **Final Diagnosis:** | | | |
| 1. Normal Echocardiography Study. | | | |
| **Remark**: | | | |
| **Recommendation**: | | | |
| SIGNATURE  Done by: Tesfaye T., Pediatrician, Pediatric Cardiologist _______________ 23/07/2015Eth.C | | | |

| Patient Name: **Mahlet Temesgen**. Referring Institute: **Addis Alem PH**. SEX/ Age: **F/2 1/12**. Date of Report: **25/07/15**.  Referral Diagnosis: **Down Syndrome. AGH12.3422.** | | | |
| --- | --- | --- | --- |
| **Features** | **Finding** | **Features** | **Finding** |
| **Profile** |  | **Atria** |  |
| Abdominal situs | Solitus | Left atrium | Normal |
| Atrial Situs | Solitus | Right atrium | Normal |
| Cardiac position | Levocardia | **Atrioventricular valves** |  |
| Systemic venous drainage | Normal. | Mitral valve | Annulus = 12mm |
| Pulmonary venous drainage | Normal | Tricuspid valve | Annulus = 13mm |
| Atrioventricular connection | Concordant |  | TAPSE = 15mm |
| Ventriculoarterial connection | Concordant | **Ventricles** |  |
| Ventricular loop | d-Loop | Left ventricle | Normal |
|  |  | Right ventricle | Normal |
| **Septae** |  | **Coronary arteries** | ----- |
| Interventricular septum | Intact | **Doppler Measurement** |  |
| Interatrial septum | Intact | Mitral | ----- |
| **Semilunar valves** |  | Aortic | ------- |
| Aortic valve | Annulus = 12mm | Tricuspid | ------- |
| Pulmonary valve | Annulus = 12mm | pulmonic | -------- |
| **Great arteries** | NRGA | **Aortic arch** | Left. No CoA. |
| Aorta | ----- | **PDA** | No |
| Pulmonary artery | Normal MPA and Branch PAs. |  |  |
| **M-Mode:**  Normal LV Function on eye balling | | | |
| AO | mm | PWd | mm |
| LA | mm | PWs | mm |
| LVIDd | mm | EDV | ml |
| LVIDs | mm | ESV | ml |
| IVSs | mm | LVEF | % |
| IVSd | mm | FS | % |
| **Additional Information**: |  | | |
| No pericardial/Pleural effusion. | | | |
| **Final Diagnosis:** | | | |
| 1. Normal Echocardiography Study. | | | |
| **Remark**: | | | |
| **Recommendation**: | | | |
| SIGNATURE  Done by: Tesfaye T., Pediatrician, Pediatric Cardiologist _______________ 25/07/2015Eth.C | | | |

| Patient Name: **Birhanu Alie**. Referring Institute: **FHRH**. SEX/ Age: **M/10years**. Date of Report: **25/07/15**.  Referral Diagnosis: **Palpitation, easy fatigability + Cough. AGH12.3423.** | | | |
| --- | --- | --- | --- |
| **Features** | **Finding** | **Features** | **Finding** |
| **Profile** |  | **Atria** |  |
| Abdominal situs | Solitus | Left atrium | Normal |
| Atrial Situs | Solitus | Right atrium | Normal |
| Cardiac position | Levocardia | **Atrioventricular valves** |  |
| Systemic venous drainage | Normal. | Mitral valve | Annulus = 19mm |
| Pulmonary venous drainage | Normal | Tricuspid valve | Annulus = 20mm |
| Atrioventricular connection | Concordant |  | TAPSE = 16mm |
| Ventriculoarterial connection | Concordant | **Ventricles** |  |
| Ventricular loop | d-Loop | Left ventricle | Normal |
|  |  | Right ventricle | Normal |
| **Septae** |  | **Coronary arteries** | ----- |
| Interventricular septum | Intact | **Doppler Measurement** |  |
| Interatrial septum | Intact | Mitral | ----- |
| **Semilunar valves** |  | Aortic | ------- |
| Aortic valve | Annulus = 15mm | Tricuspid | ------- |
| Pulmonary valve | Annulus = 19mm | pulmonic | -------- |
| **Great arteries** | NRGA | **Aortic arch** | Left. No CoA. |
| Aorta | ----- | **PDA** | No |
| Pulmonary artery | Normal MPA and Branch PAs. |  |  |
| **M-Mode:** | | | |
| AO | mm | PWd | mm |
| LA | mm | PWs | mm |
| LVIDd | mm | EDV | ml |
| LVIDs | mm | ESV | ml |
| IVSs | mm | LVEF | 65% |
| IVSd | mm | FS | 34% |
| **Additional Information**: |  | | |
| No pericardial/Pleural effusion. | | | |
| **Final Diagnosis:** | | | |
| 1. Normal Echocardiography Study. | | | |
| **Remark**: | | | |
| **Recommendation**: | | | |
| SIGNATURE  Done by: Tesfaye T., Pediatrician, Pediatric Cardiologist _______________ 25/07/2015Eth.C | | | |

| Patient Name: **Ahlam Adem**. Referring Institute: **Amaris PSC**. SEX/ Age: **F/8years**. Date of Report: **25/07/15**.  Referral Diagnosis: **Palpitation + easy fatigability. AGH12.3424.** | | | |
| --- | --- | --- | --- |
| **Features** | **Finding** | **Features** | **Finding** |
| **Profile** |  | **Atria** |  |
| Abdominal situs | Solitus | Left atrium | Normal |
| Atrial Situs | Solitus | Right atrium | Normal |
| Cardiac position | Levocardia | **Atrioventricular valves** |  |
| Systemic venous drainage | Normal. | Mitral valve | Annulus = 18mm |
| Pulmonary venous drainage | Normal | Tricuspid valve | Annulus = 18mm |
| Atrioventricular connection | Concordant |  | TAPSE = 20mm |
| Ventriculoarterial connection | Concordant | **Ventricles** |  |
| Ventricular loop | d-Loop | Left ventricle | Normal |
|  |  | Right ventricle | Normal |
| **Septae** |  | **Coronary arteries** | ----- |
| Interventricular septum | Intact | **Doppler Measurement** |  |
| Interatrial septum | Intact | Mitral | ----- |
| **Semilunar valves** |  | Aortic | ------- |
| Aortic valve | Annulus = 14mm | Tricuspid | ------- |
| Pulmonary valve | Annulus = 16mm | pulmonic | -------- |
| **Great arteries** | NRGA | **Aortic arch** | Left. No CoA. |
| Aorta | ----- | **PDA** | No |
| Pulmonary artery | Normal MPA and Branch PAs. |  |  |
| **M-Mode:** | | | |
| AO | mm | PWd | mm |
| LA | mm | PWs | mm |
| LVIDd | mm | EDV | ml |
| LVIDs | mm | ESV | ml |
| IVSs | mm | LVEF | 69% |
| IVSd | mm | FS | 38% |
| **Additional Information**: |  | | |
| No pericardial/Pleural effusion. | | | |
| **Final Diagnosis:** | | | |
| 1. Normal Echocardiography Study. | | | |
| **Remark**: | | | |
| **Recommendation**: | | | |
| SIGNATURE  Done by: Tesfaye T., Pediatrician, Pediatric Cardiologist _______________ 25/07/2015Eth.C | | | |

| Patient Name: **Baby of Merkeb Birara**. Referring Institute: **FHRH**. SEX/ Age: **M/7days**. Date of Report: **25/07/15**.  Referral Diagnosis: **FB + Cardiomegaly on CXR. AGH12.3425.** | | | |
| --- | --- | --- | --- |
| **Features** | **Finding** | **Features** | **Finding** |
| **Profile** |  | **Atria** |  |
| Abdominal situs | Solitus | Left atrium | Normal |
| Atrial Situs | Solitus | Right atrium | Normal |
| Cardiac position | Levocardia | **Atrioventricular valves** |  |
| Systemic venous drainage | Normal. | Mitral valve | Annulus = 10mm |
| Pulmonary venous drainage | Normal | Tricuspid valve | Annulus = 12mm |
| Atrioventricular connection | Concordant |  | TAPSE = 12mm |
| Ventriculoarterial connection | Concordant | **Ventricles** |  |
| Ventricular loop | d-Loop | Left ventricle | Normal |
|  |  | Right ventricle | Normal |
| **Septae** |  | **Coronary arteries** | ----- |
| Interventricular septum | Intact | **Doppler Measurement** |  |
| Interatrial septum | Intact | Mitral | ----- |
| **Semilunar valves** |  | Aortic | ------- |
| Aortic valve | Annulus = 9mm | Tricuspid | Trivial TR, PPG = 7mmHg |
| Pulmonary valve | Annulus = 9mm | pulmonic | -------- |
| **Great arteries** | NRGA | **Aortic arch** | Left. No CoA. |
| Aorta | ----- | **PDA** | No |
| Pulmonary artery | Normal MPA and Branch PAs. |  |  |
| **M-Mode:**  Normal LV Function on eye balling | | | |
| AO | mm | PWd | mm |
| LA | mm | PWs | mm |
| LVIDd | mm | EDV | ml |
| LVIDs | mm | ESV | ml |
| IVSs | mm | LVEF | % |
| IVSd | mm | FS | % |
| **Additional Information**: |  | | |
| No pericardial/Pleural effusion. | | | |
| **Final Diagnosis:** | | | |
| 1. Normal Echocardiography Study. | | | |
| **Remark**: | | | |
| **Recommendation**: | | | |
| SIGNATURE  Done by: Tesfaye T., Pediatrician, Pediatric Cardiologist _______________ 25/07/2015Eth.C | | | |

| Patient Name: **Arsema Nigusu**. Referring Institute: **FHRH**. SEX/ Age: **F/11months**. Date of Report: **26/07/15**.  Referral Diagnosis: **RD + Cough. AGH12.3426.** | | | |
| --- | --- | --- | --- |
| **Features** | **Finding** | **Features** | **Finding** |
| **Profile** |  | **Atria** |  |
| Abdominal situs | Solitus | Left atrium | Dilated |
| Atrial Situs | Solitus | Right atrium | Dilated |
| Cardiac position | Levocardia | **Atrioventricular valves** |  |
| Systemic venous drainage | Normal. | Mitral valve | Annulus = 18mm |
| Pulmonary venous drainage | Normal | Tricuspid valve | Annulus = 19mm |
| Atrioventricular connection | Concordant |  | TAPSE = 17mm |
| Ventriculoarterial connection | Concordant | **Ventricles** |  |
| Ventricular loop | d-Loop | Left ventricle | Dilated |
|  |  | Right ventricle | Dilated |
| **Septae** |  | **Coronary arteries** | ----- |
| Interventricular septum | 10mm Inlet VSD, L – R Shunt | **Doppler Measurement** |  |
| Interatrial septum | 6mm OS ASD, L – R Shunt | Mitral | Trivial MR |
| **Semilunar valves** |  | Aortic | ------- |
| Aortic valve | Annulus = 13mm | Tricuspid | Trivial TR |
| Pulmonary valve | Annulus = 19mm | pulmonic | -------- |
| **Great arteries** | NRGA | **Aortic arch** | Left. No CoA. |
| Aorta | ----- | **PDA** | 1mm PDA, L – R Shunt |
| Pulmonary artery | Normal MPA and Branch PAs. |  |  |
| **M-Mode:** | | | |
| AO | mm | PWd | mm |
| LA | mm | PWs | mm |
| LVIDd | mm | EDV | ml |
| LVIDs | mm | ESV | ml |
| IVSs | mm | LVEF | 72% |
| IVSd | mm | FS | 40% |
| **Additional Information**: |  | | |
| No pericardial/Pleural effusion. | | | |
| **Final Diagnosis:** | | | |
| 1. {S, D, S} Levocardia. 2. All chambers Dilated 3. Small OS ASD, L – R Shunt 4. Large Inlet VSD, L – R Shunt 5. Small PDA, L – R Shunt 6. Normal Biventricular Systolic Function | | | |
| SIGNATURE  Done by: Tesfaye T., Pediatrician, Pediatric Cardiologist _______________ 26/07/2015Eth.C | | | |

| Patient Name: **Asmira Tamyalew**. Referring Institute: **FHRH**. SEX/ Age: **F/11years**. Date of Report: **26/07/15**.  Referral Diagnosis: **Dyspnea, Orthopnea, PND (CHF). AGH12.3427.** | | | |
| --- | --- | --- | --- |
| **Features** | **Finding** | **Features** | **Finding** |
| **Profile** |  | **Atria** |  |
| Abdominal situs | Solitus | Left atrium | Dilated |
| Atrial Situs | Solitus | Right atrium | Dilated |
| Cardiac position | Levocardia | **Atrioventricular valves** |  |
| Systemic venous drainage | Normal. | Mitral valve | Annulus = 26mm. Thickened, Clubbed MVL. MVA = 0.87cm**2**. |
| Pulmonary venous drainage | Normal | Tricuspid valve | Annulus = 22mm |
| Atrioventricular connection | Concordant |  | TAPSE = 18mm |
| Ventriculoarterial connection | Concordant | **Ventricles** |  |
| Ventricular loop | d-Loop | Left ventricle | Dilated |
|  |  | Right ventricle | Dilated |
| **Septae** |  | **Coronary arteries** | ----- |
| Interventricular septum | Intact | **Doppler Measurement** |  |
| Interatrial septum | Intact | Mitral | Severe MS, PPG/MPG = 23/13mmHg |
| **Semilunar valves** |  | Aortic | Mild AR, PHT = 504ms |
| Aortic valve | Annulus = 14mm | Tricuspid | Moderate TR, PPG = 57mmHg |
| Pulmonary valve | Annulus = 19mm | pulmonic | -------- |
| **Great arteries** | NRGA | **Aortic arch** | Left. No CoA. |
| Aorta | ----- | **PDA** | No |
| Pulmonary artery | Normal MPA |  |  |
| **M-Mode:** | | | |
| AO | mm | PWd | mm |
| LA | mm | PWs | mm |
| LVIDd | mm | EDV | ml |
| LVIDs | mm | ESV | ml |
| IVSs | mm | LVEF | 51% |
| IVSd | mm | FS | 25% |
| **Additional Information**: |  | | |
| No pericardial/Pleural effusion. | | | |
| **Final Diagnosis:** | | | |
| 1. {S, D, S} Levocardia. 2. All chambers dilated 3. Thickened, clubbed MVL 4. Severe MS 5. Mild AR 6. Moderate TR 7. Moderate Pulmonary Hypertension 8. Mildly Reduced LV Systolic Function | | | |
| SIGNATURE  Done by: Tesfaye T., Pediatrician, Pediatric Cardiologist _______________ 26/07/2015Eth.C | | | |

| Patient Name: **Mebe’a Birhanu_**. Referring Institute: **TGSH**. SEX/ Age: **F/8months**. Date of Report: **26/07/15**.  Referral Diagnosis: **OSA 20 to ATH. AGH12.3428.** | | | |
| --- | --- | --- | --- |
| **Features** | **Finding** | **Features** | **Finding** |
| **Profile** |  | **Atria** |  |
| Abdominal situs | Solitus | Left atrium | Normal |
| Atrial Situs | Solitus | Right atrium | Normal |
| Cardiac position | Levocardia | **Atrioventricular valves** |  |
| Systemic venous drainage | Normal. | Mitral valve | Annulus = 12mm |
| Pulmonary venous drainage | Normal | Tricuspid valve | Annulus = 14mm |
| Atrioventricular connection | Concordant |  |  |
| Ventriculoarterial connection | Concordant | **Ventricles** |  |
| Ventricular loop | d-Loop | Left ventricle | Normal |
|  |  | Right ventricle | Normal |
| **Septae** |  | **Coronary arteries** | ----- |
| Interventricular septum | Intact | **Doppler Measurement** |  |
| Interatrial septum | Intact | Mitral | ----- |
| **Semilunar valves** |  | Aortic | ------- |
| Aortic valve | Annulus = 12mm | Tricuspid | ------- |
| Pulmonary valve | Annulus = 14mm | pulmonic | -------- |
| **Great arteries** | NRGA | **Aortic arch** | Left. No CoA. |
| Aorta | ----- | **PDA** | No |
| Pulmonary artery | Normal MPA and Branch PAs. |  |  |
| **M-Mode:**  Normal LV Function on eye balling | | | |
| AO | mm | PWd | mm |
| LA | mm | PWs | mm |
| LVIDd | mm | EDV | ml |
| LVIDs | mm | ESV | ml |
| IVSs | mm | LVEF | % |
| IVSd | mm | FS | % |
| **Additional Information**: |  | | |
| No pericardial/Pleural effusion. | | | |
| **Final Diagnosis:** | | | |
| 1. Normal Echocardiography Study. | | | |
| **Remark**: | | | |
| **Recommendation**: | | | |
| SIGNATURE  Done by: Tesfaye T., Pediatrician, Pediatric Cardiologist _______________ 26/07/2015Eth.C | | | |

| Patient Name: **Fire-Hiwet Belsti**. Referring Institute: **FHRH**. SEX/ Age: **F/1 9/12**. Date of Report: **26/07/15**.  Referral Diagnosis: **Recurrent Chest Infection. AGH12.3429.** | | | |
| --- | --- | --- | --- |
| **Features** | **Finding** | **Features** | **Finding** |
| **Profile** |  | **Atria** |  |
| Abdominal situs | Solitus | Left atrium | Normal |
| Atrial Situs | Solitus | Right atrium | Normal |
| Cardiac position | Levocardia | **Atrioventricular valves** |  |
| Systemic venous drainage | Normal. | Mitral valve | Annulus = 15mm |
| Pulmonary venous drainage | Normal | Tricuspid valve | Annulus = 16mm |
| Atrioventricular connection | Concordant |  | TAPSE = 17mm |
| Ventriculoarterial connection | Concordant | **Ventricles** |  |
| Ventricular loop | d-Loop | Left ventricle | Normal |
|  |  | Right ventricle | Normal |
| **Septae** |  | **Coronary arteries** | ----- |
| Interventricular septum | Intact | **Doppler Measurement** |  |
| Interatrial septum | Intact | Mitral | ----- |
| **Semilunar valves** |  | Aortic | ------- |
| Aortic valve | Annulus = 12mm | Tricuspid | ------- |
| Pulmonary valve | Annulus = 15mm | pulmonic | -------- |
| **Great arteries** | NRGA | **Aortic arch** | Left. No CoA. |
| Aorta | ----- | **PDA** | No |
| Pulmonary artery | Normal MPA and Branch PAs. |  |  |
| **M-Mode:**  Normal LV Function on eye balling | | | |
| AO | mm | PWd | mm |
| LA | mm | PWs | mm |
| LVIDd | mm | EDV | ml |
| LVIDs | mm | ESV | ml |
| IVSs | mm | LVEF | % |
| IVSd | mm | FS | % |
| **Additional Information**: |  | | |
| No pericardial/Pleural effusion. | | | |
| **Final Diagnosis:** | | | |
| 1. Normal Echocardiography Study. | | | |
| **Remark**: | | | |
| **Recommendation**: | | | |
| SIGNATURE  Done by: Tesfaye T., Pediatrician, Pediatric Cardiologist _______________ 26/07/2015Eth.C | | | |

| Patient Name: **Baby of Zinash Tessera**. Referring Institute: **TGSH**. SEX/ Age: **M/26days**. Date of Report: **26/07/15**.  Referral Diagnosis: **Down Syndrome. AGH12.3430.** | | | |
| --- | --- | --- | --- |
| **Features** | **Finding** | **Features** | **Finding** |
| **Profile** |  | **Atria** |  |
| Abdominal situs | Solitus | Left atrium | Normal |
| Atrial Situs | Solitus | Right atrium | Normal |
| Cardiac position | Levocardia | **Atrioventricular valves** |  |
| Systemic venous drainage | Normal. | Mitral valve | Annulus = 10mm |
| Pulmonary venous drainage | Normal | Tricuspid valve | Annulus = 11mm |
| Atrioventricular connection | Concordant |  |  |
| Ventriculoarterial connection | Concordant | **Ventricles** |  |
| Ventricular loop | d-Loop | Left ventricle | Normal |
|  |  | Right ventricle | Normal |
| **Septae** |  | **Coronary arteries** | ----- |
| Interventricular septum | Intact | **Doppler Measurement** |  |
| Interatrial septum | 4mm OS ASD, L – R Shunt | Mitral | ----- |
| **Semilunar valves** |  | Aortic | ------- |
| Aortic valve | Annulus = 9mm | Tricuspid | ------- |
| Pulmonary valve | Annulus = 12mm | pulmonic | -------- |
| **Great arteries** | NRGA | **Aortic arch** | Left. No CoA. |
| Aorta | ----- | **PDA** | No |
| Pulmonary artery | Normal MPA and Branch PAs. |  |  |
| **M-Mode:**  Normal LV Function | | | |
| AO | mm | PWd | mm |
| LA | mm | PWs | mm |
| LVIDd | mm | EDV | ml |
| LVIDs | mm | ESV | ml |
| IVSs | mm | LVEF | % |
| IVSd | mm | FS | % |
| **Additional Information**: |  | | |
| No pericardial/Pleural effusion. | | | |
| **Final Diagnosis:** | | | |
| 1. {S, D, S} Levocardia. 2. Small OS ASD, L – R Shunt | | | |
| **Remark**: | | | |
| **Recommendation**: | | | |
| SIGNATURE  Done by: Tesfaye T., Pediatrician, Pediatric Cardiologist _______________ 26/07/2015Eth.C | | | |

| Patient Name: **Abel Dagnachew**. Referring Institute: **FHRH**. SEX/ Age: **M/2months**. Date of Report: **26/07/15**.  Referral Diagnosis: **Fast Breathing + Cardiomegaly on CXR. AGH12.3431.** | | | |
| --- | --- | --- | --- |
| **Features** | **Finding** | **Features** | **Finding** |
| **Profile** |  | **Atria** |  |
| Abdominal situs | Solitus | Left atrium | Normal |
| Atrial Situs | Solitus | Right atrium | Normal |
| Cardiac position | Levocardia | **Atrioventricular valves** |  |
| Systemic venous drainage | Normal. | Mitral valve | Annulus = 12mm |
| Pulmonary venous drainage | Normal | Tricuspid valve | Annulus = 11mm |
| Atrioventricular connection | Concordant |  | TAPSE = 13mm |
| Ventriculoarterial connection | Concordant | **Ventricles** |  |
| Ventricular loop | d-Loop | Left ventricle | Normal |
|  |  | Right ventricle | Normal |
| **Septae** |  | **Coronary arteries** | ----- |
| Interventricular septum | Intact | **Doppler Measurement** |  |
| Interatrial septum | PFO, L - R Shunt | Mitral | ----- |
| **Semilunar valves** |  | Aortic | ------- |
| Aortic valve | Annulus = 10mm | Tricuspid | ------- |
| Pulmonary valve | Annulus = 11mm | pulmonic | -------- |
| **Great arteries** | NRGA | **Aortic arch** | Left. No CoA. |
| Aorta | ----- | **PDA** | No |
| Pulmonary artery | Normal MPA and Branch PAs. |  |  |
| **M-Mode:** | | | |
| AO | mm | PWd | mm |
| LA | mm | PWs | mm |
| LVIDd | mm | EDV | ml |
| LVIDs | mm | ESV | ml |
| IVSs | mm | LVEF | % |
| IVSd | mm | FS | % |
| **Additional Information**: |  | | |
| No pericardial/Pleural effusion. | | | |
| **Final Diagnosis:** | | | |
| 1. {S, D, S} Levocardia. 2. PFO, L – R Shunt | | | |
| **Remark**: | | | |
| **Recommendation**: | | | |
| SIGNATURE  Done by: Tesfaye T., Pediatrician, Pediatric Cardiologist _______________ 26/07/2015Eth.C | | | |

| Patient Name: **Solomie Techalew**. Referring Institute: **Amaris PSC**. SEX/ Age: **F/4 8/12**. Date of Report: **26/07/15**.  Referral Diagnosis: **Palpitation. AGH12.3432.** | | | |
| --- | --- | --- | --- |
| **Features** | **Finding** | **Features** | **Finding** |
| **Profile** |  | **Atria** |  |
| Abdominal situs | Solitus | Left atrium | Normal |
| Atrial Situs | Solitus | Right atrium | Normal |
| Cardiac position | Levocardia | **Atrioventricular valves** |  |
| Systemic venous drainage | Normal. | Mitral valve | Annulus = 17mm |
| Pulmonary venous drainage | Normal | Tricuspid valve | Annulus = 17mm |
| Atrioventricular connection | Concordant |  | TAPSE = 18mm |
| Ventriculoarterial connection | Concordant | **Ventricles** |  |
| Ventricular loop | d-Loop | Left ventricle | Normal |
|  |  | Right ventricle | Normal |
| **Septae** |  | **Coronary arteries** | ----- |
| Interventricular septum | Intact | **Doppler Measurement** |  |
| Interatrial septum | Intact | Mitral | ----- |
| **Semilunar valves** |  | Aortic | ------- |
| Aortic valve | Annulus = 15mm | Tricuspid | ------- |
| Pulmonary valve | Annulus = 16mm | pulmonic | -------- |
| **Great arteries** | NRGA | **Aortic arch** | Left. No CoA. |
| Aorta | ----- | **PDA** | No |
| Pulmonary artery | Normal MPA and Branch PAs. |  |  |
| **M-Mode:**  Normal LV Function on eye balling | | | |
| AO | mm | PWd | mm |
| LA | mm | PWs | mm |
| LVIDd | mm | EDV | ml |
| LVIDs | mm | ESV | ml |
| IVSs | mm | LVEF | % |
| IVSd | mm | FS | % |
| **Additional Information**: |  | | |
| No pericardial/Pleural effusion. | | | |
| **Final Diagnosis:** | | | |
| 1. Normal Echocardiography. | | | |
| **Remark**: | | | |
| **Recommendation**: | | | |
| SIGNATURE  Done by: Tesfaye T., Pediatrician, Pediatric Cardiologist _______________ 26/07/2015Eth.C | | | |

| Patient Name: **Hiwet Ayenew**. Referring Institute: **Adinas GH**. SEX/ Age: **F/12years**. Date of Report: **26/07/15**.  Referral Diagnosis: **CHF + CRHD + IE. AGH12.3433.** | | | |
| --- | --- | --- | --- |
| **Features** | **Finding** | **Features** | **Finding** |
| **Profile** |  | **Atria** |  |
| Abdominal situs | Solitus | Left atrium | Dilated |
| Atrial Situs | Solitus | Right atrium | Dilated |
| Cardiac position | Levocardia | **Atrioventricular valves** |  |
| Systemic venous drainage | Normal. | Mitral valve | Annulus = 25mm. Thickened MVL |
| Pulmonary venous drainage | Normal | Tricuspid valve | Annulus = 23mm |
| Atrioventricular connection | Concordant |  | TAPSE = 23mm |
| Ventriculoarterial connection | Concordant | **Ventricles** |  |
| Ventricular loop | d-Loop | Left ventricle | Dilated |
|  |  | Right ventricle | Dilated |
| **Septae** |  | **Coronary arteries** | ----- |
| Interventricular septum | Intact | **Doppler Measurement** |  |
| Interatrial septum | Intact | Mitral | Severe MR, Holosystolic, posterior projection, seen in two planes with jet velocity= 3.8m/sec |
| **Semilunar valves** |  | Aortic | Mild AR |
| Aortic valve | Annulus = 16mm | Tricuspid | Moderate TR, PPG = 23mmHg |
| Pulmonary valve | Annulus = 21mm | pulmonic | -------- |
| **Great arteries** | NRGA | **Aortic arch** | Left. No CoA. |
| Aorta | ----- | **PDA** | No |
| Pulmonary artery | Normal |  |  |
| **M-Mode:** | | | |
| AO | mm | PWd | mm |
| LA | mm | PWs | mm |
| LVIDd | mm | EDV | ml |
| LVIDs | mm | ESV | ml |
| IVSs | mm | LVEF | 51% |
| IVSd | mm | FS | 26% |
| **Additional Information**: |  | | |
| Circumferential pericardial effusion with maximum depth of 6mm. | | | |
| **Final Diagnosis:** | | | |
| 1. {S, D, S} Levocardia. 2. All chambers dilated 3. Thickened MVL 4. Severe MR 5. Moderate TR 6. Mild AR 7. Mildly Reduced LV Systolic Function 8. Small Circumferential Pericardial effusion | | | |
| SIGNATURE  Done by: Tesfaye T., Pediatrician, Pediatric Cardiologist _______________ 26/07/2015Eth.C | | | |

| Patient Name: **Alebachew Werku**. Referring Institute: **FHRH**. SEX/ Age: **M/13years**. Date of Report: **26/07/15**.  Referral Diagnosis: **Rheumatic recurrence. AGH12.3434.** | | | |
| --- | --- | --- | --- |
| **Features** | **Finding** | **Features** | **Finding** |
| **Profile** |  | **Atria** |  |
| Abdominal situs | Solitus | Left atrium | Dilated |
| Atrial Situs | Solitus | Right atrium | Normal |
| Cardiac position | Levocardia | **Atrioventricular valves** |  |
| Systemic venous drainage | Normal. | Mitral valve | Annulus = 21mm. Thickened, Clubbed MVL. MVA = 1.9cm**2**. |
| Pulmonary venous drainage | Normal | Tricuspid valve | Annulus = 22mm |
| Atrioventricular connection | Concordant |  | TAPSE = 23mm |
| Ventriculoarterial connection | Concordant | **Ventricles** |  |
| Ventricular loop | d-Loop | Left ventricle | Normal |
|  |  | Right ventricle | Normal |
| **Septae** |  | **Coronary arteries** | ----- |
| Interventricular septum | Intact | **Doppler Measurement** |  |
| Interatrial septum | Intact | Mitral | Mild MR, Holosystolic, posterior projection, seen in two planes with jet velocity = 4.4m/sec. Mild MS, PPG/MPG = 13/6mmHg |
| **Semilunar valves** |  | Aortic | Moderate AR, PHT = 357ms |
| Aortic valve | Annulus = 20mm | Tricuspid | ------- |
| Pulmonary valve | Annulus = 22mm | pulmonic | -------- |
| **Great arteries** | NRGA | **Aortic arch** | Left. No CoA. |
| Aorta | ----- | **PDA** | No |
| Pulmonary artery | Normal MPA and Branch PAs. |  |  |
| **M-Mode:** | | | |
| AO | mm | PWd | mm |
| LA | mm | PWs | mm |
| LVIDd | mm | EDV | ml |
| LVIDs | mm | ESV | ml |
| IVSs | mm | LVEF | 65% |
| IVSd | mm | FS | 35% |
| **Final Diagnosis:** | | | |
| 1. {S, D, S} Levocardia. 2. LA/LV Dilated 3. Thickened, Clubbed MVL 4. Mild MR 5. Mild MS 6. Moderate AR 7. Normal Biventricular Systolic Function | | | |
| SIGNATURE  Done by: Tesfaye T., Pediatrician, Pediatric Cardiologist _______________ 26/07/2015Eth.C | | | |

| Patient Name: **Nahom Abiyot**. Referring Institute: **Adinas GH**. SEX/ Age: **M/8years**. Date of Report: **26/07/15**.  Referral Diagnosis: ?**ARF + ?JIA. AGH12.3435.** | | | |
| --- | --- | --- | --- |
| **Features** | **Finding** | **Features** | **Finding** |
| **Profile** |  | **Atria** |  |
| Abdominal situs | Solitus | Left atrium | Normal |
| Atrial Situs | Solitus | Right atrium | Normal |
| Cardiac position | Levocardia | **Atrioventricular valves** |  |
| Systemic venous drainage | Normal. | Mitral valve | Annulus = 20mm |
| Pulmonary venous drainage | Normal | Tricuspid valve | Annulus = 22mm |
| Atrioventricular connection | Concordant |  | TAPSE = 22mm |
| Ventriculoarterial connection | Concordant | **Ventricles** |  |
| Ventricular loop | d-Loop | Left ventricle | Normal |
|  |  | Right ventricle | Normal |
| **Septae** |  | **Coronary arteries** | ----- |
| Interventricular septum | Intact | **Doppler Measurement** |  |
| Interatrial septum | Intact | Mitral | ----- |
| **Semilunar valves** |  | Aortic | ------- |
| Aortic valve | Annulus = 17mm | Tricuspid | ------- |
| Pulmonary valve | Annulus = 20mm | pulmonic | Trivial PR, PPG = 8mmHg |
| **Great arteries** | NRGA | **Aortic arch** | Left. No CoA. |
| Aorta | ----- | **PDA** | No |
| Pulmonary artery | Normal MPA and Branch PAs. |  |  |
| **M-Mode:** | | | |
| AO | mm | PWd | mm |
| LA | mm | PWs | mm |
| LVIDd | mm | EDV | ml |
| LVIDs | mm | ESV | ml |
| IVSs | mm | LVEF | 62% |
| IVSd | mm | FS | 33% |
| **Additional Information**: |  | | |
| No pericardial/Pleural effusion. | | | |
| **Final Diagnosis:** | | | |
| 1. Normal Echocardiography Study. | | | |
| **Remark**: | | | |
| **Recommendation**: | | | |
| SIGNATURE  Done by: Tesfaye T., Pediatrician, Pediatric Cardiologist _______________ 26/07/2015Eth.C | | | |

| Patient Name: **Yihun Zewdu**. Referring Institute: **FHRH**. SEX/ Age: **M/45days**. Date of Report: **27/07/15**.  Referral Diagnosis: **RD. AGH12.3436.** | | | |
| --- | --- | --- | --- |
| **Features** | **Finding** | **Features** | **Finding** |
| **Profile** |  | **Atria** |  |
| Abdominal situs | Solitus | Left atrium | Normal |
| Atrial Situs | Solitus | Right atrium | Normal |
| Cardiac position | Levocardia | **Atrioventricular valves** |  |
| Systemic venous drainage | Normal. | Mitral valve | Annulus = 11mm |
| Pulmonary venous drainage | Normal | Tricuspid valve | Annulus = 11mm |
| Atrioventricular connection | Concordant |  |  |
| Ventriculoarterial connection | Concordant | **Ventricles** |  |
| Ventricular loop | d-Loop | Left ventricle | Normal |
|  |  | Right ventricle | Normal |
| **Septae** |  | **Coronary arteries** | ----- |
| Interventricular septum | Intact | **Doppler Measurement** |  |
| Interatrial septum | PFO, L – R Shunt | Mitral | ----- |
| **Semilunar valves** |  | Aortic | ------- |
| Aortic valve | Annulus = 10mm | Tricuspid | ------- |
| Pulmonary valve | Annulus = 9mm | pulmonic | -------- |
| **Great arteries** | NRGA | **Aortic arch** | Left. No CoA. |
| Aorta | ----- | **PDA** | No |
| Pulmonary artery | Normal MPA and Branch PAs. |  |  |
| **M-Mode:**  Normal LV Function | | | |
| AO | mm | PWd | mm |
| LA | mm | PWs | mm |
| LVIDd | mm | EDV | ml |
| LVIDs | mm | ESV | ml |
| IVSs | mm | LVEF | % |
| IVSd | mm | FS | % |
| **Additional Information**: |  | | |
| No pericardial/Pleural effusion. | | | |
| **Final Diagnosis:** | | | |
| 1. {S, D, S} Levocardia. 2. PFO, L – R Shunt | | | |
| **Remark**: | | | |
| **Recommendation**: | | | |
| SIGNATURE  Done by: Tesfaye T., Pediatrician, Pediatric Cardiologist _______________ 27/07/2015Eth.C | | | |

| Patient Name: **Enyew Lakew**. Referring Institute: **FHRH**. SEX/ Age: **M/13years**. Date of Report: **27/07/15**.  Referral Diagnosis: **RHD. AGH12.3437.** | | | |
| --- | --- | --- | --- |
| **Features** | **Finding** | **Features** | **Finding** |
| **Profile** |  | **Atria** |  |
| Abdominal situs | Solitus | Left atrium | Normal |
| Atrial Situs | Solitus | Right atrium | Normal |
| Cardiac position | Levocardia | **Atrioventricular valves** |  |
| Systemic venous drainage | Normal. | Mitral valve | Annulus = 21mm. Thickened MVL. |
| Pulmonary venous drainage | Normal | Tricuspid valve | Annulus = 20mm |
| Atrioventricular connection | Concordant |  | TAPSE = 20mm |
| Ventriculoarterial connection | Concordant | **Ventricles** |  |
| Ventricular loop | d-Loop | Left ventricle | Normal |
|  |  | Right ventricle | Normal |
| **Septae** |  | **Coronary arteries** | ----- |
| Interventricular septum | Intact | **Doppler Measurement** |  |
| Interatrial septum | PFO, L – R Shunt | Mitral | Mild MR, Holosystolic, posterior projection, seen in two planes with jet velocity = 3.6m/sec. |
| **Semilunar valves** |  | Aortic | ------- |
| Aortic valve | Annulus = 17mm | Tricuspid | ------- |
| Pulmonary valve | Annulus = 17mm | pulmonic | -------- |
| **Great arteries** | NRGA | **Aortic arch** | Left. No CoA. |
| Aorta | ----- | **PDA** | No |
| Pulmonary artery | Normal |  |  |
| **M-Mode:** | | | |
| AO | mm | PWd | mm |
| LA | mm | PWs | mm |
| LVIDd | mm | EDV | ml |
| LVIDs | mm | ESV | ml |
| IVSs | mm | LVEF | 59% |
| IVSd | mm | FS | 31% |
| **Additional Information**: |  | | |
| No pericardial/Pleural effusion. | | | |
| **Final Diagnosis:** | | | |
| 1. {S, D, S} Levocardia. 2. PFO, L – R Shunt 3. Thickened MVL 4. Mild MR 5. Normal Biventricular Systolic Function | | | |
| **Remark**: | | | |
| **Recommendation**: | | | |
| SIGNATURE  Done by: Tesfaye T., Pediatrician, Pediatric Cardiologist _______________ 27/07/2015Eth.C | | | |

| Patient Name: **Getanew Tilaye**. Referring Institute: **FHRH**. SEX/ Age: **M/6years**. Date of Report: **27/07/15**.  Referral Diagnosis: **G-II Clubbing. AGH12.3438.** | | | |
| --- | --- | --- | --- |
| **Features** | **Finding** | **Features** | **Finding** |
| **Profile** |  | **Atria** |  |
| Abdominal situs | Solitus | Left atrium | Dilated |
| Atrial Situs | Solitus | Right atrium | Dilated |
| Cardiac position | Levocardia | **Atrioventricular valves** |  |
| Systemic venous drainage | Normal. | Mitral valve | Annulus = 22mm |
| Pulmonary venous drainage | Normal | Tricuspid valve | Annulus = 21mm. Redundant TVL |
| Atrioventricular connection | Concordant |  | TAPSE = mm |
| Ventriculoarterial connection | Discordant | **Ventricles** |  |
| Ventricular loop | d-Loop | Left ventricle | Dilated |
|  |  | Right ventricle | Dilated & Hypertrophied |
| **Septae** |  | **Coronary arteries** | ----- |
| Interventricular septum | 18mm Upper Muscular VSD, BD Shunt | **Doppler Measurement** |  |
| Interatrial septum | Intact | Mitral | ----- |
| **Semilunar valves** |  | Aortic | ------- |
| Aortic valve | Annulus = 20mm | Tricuspid | Trivial TR |
| Pulmonary valve | Annulus = 8mm | pulmonic | Severe PS, PPG = 68mmHg. |
| **Great arteries** | d-TGA | **Aortic arch** | Left. No CoA. |
| Aorta | Anterior & to the right. From RV | **PDA** | No |
| Pulmonary artery | Posterior & to the left. From LV. MPA = 8mm, RPA = 4mm & LPA = 6mm. |  |  |
| **M-Mode:** | | | |
| AO | mm | PWd | mm |
| LA | mm | PWs | mm |
| LVIDd | mm | EDV | ml |
| LVIDs | mm | ESV | ml |
| IVSs | mm | LVEF | % |
| IVSd | mm | FS | % |
| **Additional Information**: |  | | |
| No pericardial/Pleural effusion. | | | |
| **Final Diagnosis:** | | | |
| 1. {S, D, D} Levocardia. 2. All chambers Dilated 3. RV Hypertrophied 4. d-TGA 5. Large Upper Muscular VSD, BD Shunt 6. Severe PS 7. Smallish MPA and Branch PAs. | | | |
| **Recommendation**: No need to put him on diuretics and after load reducers | | | |
| SIGNATURE  Done by: Tesfaye T., Pediatrician, Pediatric Cardiologist _______________ 27/07/2015Eth.C | | | |

| Patient Name: **Yosef Meseret**. Referring Institute: **Adinas GH**. SEX/ Age: **M/6 9/12**. Date of Report: **27/07/15**.  Referral Diagnosis: **S/P Surgical VSD Closure + AV Prolapse + AR.** | | | |
| --- | --- | --- | --- |
| **Features** | **Finding** | **Features** | **Finding** |
| **Profile** |  | **Atria** |  |
| Abdominal situs | Solitus | Left atrium | Normal |
| Atrial Situs | Solitus | Right atrium | Normal |
| Cardiac position | Levocardia | **Atrioventricular valves** |  |
| Systemic venous drainage | Normal. | Mitral valve | Annulus = 20mm |
| Pulmonary venous drainage | Normal | Tricuspid valve | Annulus = 21mm |
| Atrioventricular connection | Concordant |  | TAPSE = 17mm |
| Ventriculoarterial connection | Concordant | **Ventricles** |  |
| Ventricular loop | d-Loop | Left ventricle | Normal |
|  |  | Right ventricle | Normal |
| **Septae** |  | **Coronary arteries** | ----- |
| Interventricular septum | VSD Patch Intact, No Residual VSD | **Doppler Measurement** |  |
| Interatrial septum | Intact | Mitral | ----- |
| **Semilunar valves** |  | Aortic | Trivial AR |
| Aortic valve | Annulus = 19mm | Tricuspid | ------- |
| Pulmonary valve | Annulus = 19mm | pulmonic | -------- |
| **Great arteries** | NRGA | **Aortic arch** | Left. No CoA. |
| Aorta | ----- | **PDA** | No |
| Pulmonary artery | Normal MPA and Branch PAs. |  |  |
| **M-Mode:** | | | |
| AO | mm | PWd | mm |
| LA | mm | PWs | mm |
| LVIDd | mm | EDV | ml |
| LVIDs | mm | ESV | ml |
| IVSs | mm | LVEF | 62% |
| IVSd | mm | FS | 33% |
| **Additional Information**: |  | | |
| No pericardial/Pleural effusion. | | | |
| **Final Diagnosis:** | | | |
| 1. {S, D, S} Levocardia. 2. S/P VSD Surgical Closure 3. VSD Patch Intact 4. No Residual VSD 5. Normal Biventricular Systolic Function | | | |
| **Remark**: | | | |
| **Recommendation**: | | | |
| SIGNATURE  Done by: Tesfaye T., Pediatrician, Pediatric Cardiologist _______________ 27/07/2015Eth.C | | | |

| Patient Name: **Tesfa-Mariam Sefinew**. Referring Institute: **FHRH**. SEX/ Age: **M/7years**. Date of Report: **28/07/15**.  Referral Diagnosis: **Palpitation + Cough + Dyspnea. AGH12.3439.** | | | |
| --- | --- | --- | --- |
| **Features** | **Finding** | **Features** | **Finding** |
| **Profile** |  | **Atria** |  |
| Abdominal situs | Solitus | Left atrium | Normal |
| Atrial Situs | Solitus | Right atrium | Normal |
| Cardiac position | Levocardia | **Atrioventricular valves** |  |
| Systemic venous drainage | Normal. | Mitral valve | Annulus = 18mm |
| Pulmonary venous drainage | Normal | Tricuspid valve | Annulus = 19mm |
| Atrioventricular connection | Concordant |  | TAPSE = 20mm |
| Ventriculoarterial connection | Concordant | **Ventricles** |  |
| Ventricular loop | d-Loop | Left ventricle | Normal |
|  |  | Right ventricle | Normal |
| **Septae** |  | **Coronary arteries** | ----- |
| Interventricular septum | Intact | **Doppler Measurement** |  |
| Interatrial septum | Intact | Mitral | ----- |
| **Semilunar valves** |  | Aortic | ------- |
| Aortic valve | Annulus = 15mm | Tricuspid | Trivial TR, PPG = 16mmHg |
| Pulmonary valve | Annulus = 17mm | pulmonic | Trivial PR, PPG = 10mmHg |
| **Great arteries** | NRGA | **Aortic arch** | Left. No CoA. |
| Aorta | ----- | **PDA** | No |
| Pulmonary artery | Normal MPA and Branch PAs. |  |  |
| **M-Mode:** | | | |
| AO | mm | PWd | mm |
| LA | mm | PWs | mm |
| LVIDd | mm | EDV | ml |
| LVIDs | mm | ESV | ml |
| IVSs | mm | LVEF | 65% |
| IVSd | mm | FS | 35% |
| **Additional Information**: |  | | |
| No pericardial/Pleural effusion. | | | |
| **Final Diagnosis:** | | | |
| 1. Normal Echocardiography Study. | | | |
| **Remark**: | | | |
| **Recommendation**: | | | |
| SIGNATURE  Done by: Tesfaye T., Pediatrician, Pediatric Cardiologist _______________ 28/07/2015Eth.C | | | |

| Patient Name: **Esubalew Gashaw**. Referring Institute: **Addis Alem PH**. SEX/ Age: **M/9months**. Date of Report: **28/07/15**.  Referral Diagnosis: **G – II Systolic Murmur @ LMSB. AGH12.3440.** | | | |
| --- | --- | --- | --- |
| **Features** | **Finding** | **Features** | **Finding** |
| **Profile** |  | **Atria** |  |
| Abdominal situs | Solitus | Left atrium | Normal |
| Atrial Situs | Solitus | Right atrium | Normal |
| Cardiac position | Levocardia | **Atrioventricular valves** |  |
| Systemic venous drainage | Normal. | Mitral valve | Annulus = 12mm |
| Pulmonary venous drainage | Normal | Tricuspid valve | Annulus = 12mm |
| Atrioventricular connection | Concordant |  | TAPSE = 14mm |
| Ventriculoarterial connection | Concordant | **Ventricles** |  |
| Ventricular loop | d-Loop | Left ventricle | Normal |
|  |  | Right ventricle | Normal |
| **Septae** |  | **Coronary arteries** | ----- |
| Interventricular septum | Intact | **Doppler Measurement** |  |
| Interatrial septum | Intact | Mitral | ----- |
| **Semilunar valves** |  | Aortic | ------- |
| Aortic valve | Annulus = 11mm | Tricuspid | ------- |
| Pulmonary valve | Annulus = 12mm | pulmonic | -------- |
| **Great arteries** | NRGA | **Aortic arch** | Left. No CoA. |
| Aorta | ----- | **PDA** | No |
| Pulmonary artery | Normal MPA and Branch PAs. |  |  |
| **M-Mode:**  Normal LV Function on eye balling | | | |
| AO | mm | PWd | mm |
| LA | mm | PWs | mm |
| LVIDd | mm | EDV | ml |
| LVIDs | mm | ESV | ml |
| IVSs | mm | LVEF | % |
| IVSd | mm | FS | % |
| **Additional Information**: |  | | |
| No pericardial/Pleural effusion. | | | |
| **Final Diagnosis:** | | | |
| 1. Normal Echocardiography Study. | | | |
| **Remark**: | | | |
| **Recommendation**: | | | |
| SIGNATURE  Done by: Tesfaye T., Pediatrician, Pediatric Cardiologist _______________ 28/07/2015Eth.C | | | |

| Patient Name: **Jiregna Terefe**. Referring Institute: **TGSH**. SEX/ Age: **M/4years**. Date of Report: **30/07/15**.  Referral Diagnosis: **Cardiomegaly on CXR + G-II HSM + Easy fatigability + palpitation. AGH12.3441.** | | | |
| --- | --- | --- | --- |
| **Features** | **Finding** | **Features** | **Finding** |
| **Profile** |  | **Atria** |  |
| Abdominal situs | Solitus | Left atrium | Normal |
| Atrial Situs | Solitus | Right atrium | Normal |
| Cardiac position | Levocardia | **Atrioventricular valves** |  |
| Systemic venous drainage | Normal. | Mitral valve | Annulus = 15mm |
| Pulmonary venous drainage | Normal | Tricuspid valve | Annulus = 16mm |
| Atrioventricular connection | Concordant |  | TAPSE = 18mm |
| Ventriculoarterial connection | Concordant | **Ventricles** |  |
| Ventricular loop | d-Loop | Left ventricle | Normal |
|  |  | Right ventricle | Normal |
| **Septae** |  | **Coronary arteries** | ----- |
| Interventricular septum | Intact | **Doppler Measurement** |  |
| Interatrial septum | Intact | Mitral | ----- |
| **Semilunar valves** |  | Aortic | ------- |
| Aortic valve | Annulus = 13mm | Tricuspid | ------- |
| Pulmonary valve | Annulus = 16mm | pulmonic | -------- |
| **Great arteries** | NRGA | **Aortic arch** | Left. No CoA. |
| Aorta | ----- | **PDA** | No |
| Pulmonary artery | Normal MPA and Branch PAs. |  |  |
| **M-Mode:**  Normal LV Function on eye balling | | | |
| AO | mm | PWd | mm |
| LA | mm | PWs | mm |
| LVIDd | mm | EDV | ml |
| LVIDs | mm | ESV | ml |
| IVSs | mm | LVEF | % |
| IVSd | mm | FS | % |
| **Additional Information**: |  | | |
| No pericardial/Pleural effusion. | | | |
| **Final Diagnosis:** | | | |
| 1. Normal Echocardiography Study. | | | |
| **Remark**: | | | |
| **Recommendation**: | | | |
| SIGNATURE  Done by: Tesfaye T., Pediatrician, Pediatric Cardiologist _______________ 30/07/2015Eth.C | | | |

| Patient Name: **Haile-Mariam Gizaw**. Referring Institute: **Adinas GH**. SEX/ Age: **M/7 10/12**. Date of Report: **30/07/15**.  Referral Diagnosis: **Follow up echo for Persistent LSVC + DOE. (08/2014)** | | | |
| --- | --- | --- | --- |
| **Features** | **Finding** | **Features** | **Finding** |
| **Profile** |  | **Atria** |  |
| Abdominal situs | Solitus | Left atrium | Normal |
| Atrial Situs | Solitus | Right atrium | Dilated |
| Cardiac position | Levocardia | **Atrioventricular valves** |  |
| Systemic venous drainage | IVC to RA. SVC to RA. LSVC to CS to RA | Mitral valve | Annulus = 18mm |
| Pulmonary venous drainage | Normal | Tricuspid valve | Annulus = 24mm |
| Atrioventricular connection | Concordant |  | TAPSE = 19mm |
| Ventriculoarterial connection | Concordant | **Ventricles** |  |
| Ventricular loop | d-Loop | Left ventricle | Normal |
|  |  | Right ventricle | Dilated |
| **Septae** |  | **Coronary arteries** | ----- |
| Interventricular septum | Intact | **Doppler Measurement** |  |
| Interatrial septum | Intact | Mitral | ----- |
| **Semilunar valves** |  | Aortic | ------- |
| Aortic valve | Annulus = 14mm | Tricuspid | ------- |
| Pulmonary valve | Annulus = 19mm | pulmonic | -------- |
| **Great arteries** | NRGA | **Aortic arch** | Left. No CoA. |
| Aorta | ----- | **PDA** | No |
| Pulmonary artery | Normal MPA and Branch PAs. |  |  |
| **M-Mode:** | | | |
| AO | mm | PWd | mm |
| LA | mm | PWs | mm |
| LVIDd | mm | EDV | ml |
| LVIDs | mm | ESV | ml |
| IVSs | mm | LVEF | 73% |
| IVSd | mm | FS | 41% |
| **Additional Information**: |  | | |
| No pericardial/Pleural effusion. | | | |
| **Final Diagnosis:** | | | |
| 1. {S, D, S} Levocardia. 2. RA/RV Dilated 3. Persistent LSVC 4. Normal Biventricular Systolic Function | | | |
| **Remark**: | | | |
| **Recommendation**: | | | |
| SIGNATURE  Done by: Tesfaye T., Pediatrician, Pediatric Cardiologist _______________ 30/07/2015Eth.C | | | |

| Patient Name: **Derbew Derso**. Referring Institute: **FHRH**. SEX/ Age: **M/7 11/12**. Date of Report: **30/07/15**.  Referral Diagnosis: **Acute Rheumatic Fever. AGH12.3442.** | | | |
| --- | --- | --- | --- |
| **Features** | **Finding** | **Features** | **Finding** |
| **Profile** |  | **Atria** |  |
| Abdominal situs | Solitus | Left atrium | Normal |
| Atrial Situs | Solitus | Right atrium | Normal |
| Cardiac position | Levocardia | **Atrioventricular valves** |  |
| Systemic venous drainage | Normal. | Mitral valve | Annulus = 18mm |
| Pulmonary venous drainage | Normal | Tricuspid valve | Annulus = 18mm |
| Atrioventricular connection | Concordant |  | TAPSE = 20mm |
| Ventriculoarterial connection | Concordant | **Ventricles** |  |
| Ventricular loop | d-Loop | Left ventricle | Normal |
|  |  | Right ventricle | Normal |
| **Septae** |  | **Coronary arteries** | ----- |
| Interventricular septum | Intact | **Doppler Measurement** |  |
| Interatrial septum | Intact | Mitral | ----- |
| **Semilunar valves** |  | Aortic | ------- |
| Aortic valve | Annulus = 16mm | Tricuspid | ------- |
| Pulmonary valve | Annulus = 18mm | pulmonic | -------- |
| **Great arteries** | NRGA | **Aortic arch** | Left. No CoA. |
| Aorta | ----- | **PDA** | No |
| Pulmonary artery | Normal MPA and Branch PAs. |  |  |
| **M-Mode:** | | | |
| AO | mm | PWd | mm |
| LA | mm | PWs | mm |
| LVIDd | mm | EDV | ml |
| LVIDs | mm | ESV | ml |
| IVSs | mm | LVEF | 66% |
| IVSd | mm | FS | 36% |
| **Additional Information**: |  | | |
| No pericardial/Pleural effusion. | | | |
| **Final Diagnosis:** | | | |
| 1. Normal Echocardiography Study. | | | |
| **Remark**: Normal Echocardiography Study doesn’t Rule out Acute Rheumatic Feverui | | | |
| **Recommendation**: | | | |
| SIGNATURE  Done by: Tesfaye T., Pediatrician, Pediatric Cardiologist _______________ 30/07/2015Eth.C | | | |

| Patient Name: **Ruth Basazinew**. Referring Institute: **Adinas GH.**  SEX/ Age: **F/12years**. Date of Report: **30/07/15**.  Referral Diagnosis: **Easy fatigability. AGH12.3443.** | | | |
| --- | --- | --- | --- |
| **Features** | **Finding** | **Features** | **Finding** |
| **Profile** |  | **Atria** |  |
| Abdominal situs | Solitus | Left atrium | Normal |
| Atrial Situs | Solitus | Right atrium | Normal |
| Cardiac position | Levocardia | **Atrioventricular valves** |  |
| Systemic venous drainage | Normal. | Mitral valve | Annulus = 21mm |
| Pulmonary venous drainage | Normal | Tricuspid valve | Annulus = 22mm |
| Atrioventricular connection | Concordant |  | TAPSE = 22mm |
| Ventriculoarterial connection | Concordant | **Ventricles** |  |
| Ventricular loop | d-Loop | Left ventricle | Normal |
|  |  | Right ventricle | Normal |
| **Septae** |  | **Coronary arteries** | ----- |
| Interventricular septum | Intact | **Doppler Measurement** |  |
| Interatrial septum | Intact | Mitral | ----- |
| **Semilunar valves** |  | Aortic | ------- |
| Aortic valve | Annulus = 18mm | Tricuspid | ------- |
| Pulmonary valve | Annulus = 18mm | pulmonic | -------- |
| **Great arteries** | NRGA | **Aortic arch** | Left. No CoA. |
| Aorta | ----- | **PDA** | No |
| Pulmonary artery | Normal MPA and Branch PAs. |  |  |
| **M-Mode:** | | | |
| AO | mm | PWd | mm |
| LA | mm | PWs | mm |
| LVIDd | mm | EDV | ml |
| LVIDs | mm | ESV | ml |
| IVSs | mm | LVEF | 67% |
| IVSd | mm | FS | 37% |
| **Additional Information**: |  | | |
| No pericardial/Pleural effusion. | | | |
| **Final Diagnosis:** | | | |
| 1. Normal Echocardiography Study. | | | |
| **Remark**: | | | |
| **Recommendation**: | | | |
| SIGNATURE  Done by: Tesfaye T., Pediatrician, Pediatric Cardiologist _______________ 30/07/2015Eth.C | | | |

| Patient Name: **Baby of Sintayehu Agumas**. Referring Institute: **FHRH**. SEX/ Age: **F/21days**. Date of Report: **02/08/15**.  Referral Diagnosis: **CHD (Murmur). AGH12.3444.** | | | |
| --- | --- | --- | --- |
| **Features** | **Finding** | **Features** | **Finding** |
| **Profile** |  | **Atria** |  |
| Abdominal situs | Solitus | Left atrium | Normal |
| Atrial Situs | Solitus | Right atrium | Normal |
| Cardiac position | Levocardia | **Atrioventricular valves** |  |
| Systemic venous drainage | Normal. | Mitral valve | Annulus = 8mm |
| Pulmonary venous drainage | Normal | Tricuspid valve | Annulus = 9mm |
| Atrioventricular connection | Concordant |  |  |
| Ventriculoarterial connection | Concordant | **Ventricles** |  |
| Ventricular loop | d-Loop | Left ventricle | Normal |
|  |  | Right ventricle | Normal |
| **Septae** |  | **Coronary arteries** | ----- |
| Interventricular septum | Intact | **Doppler Measurement** |  |
| Interatrial septum | 5mm OS ASD, L – R Shunt | Mitral | ----- |
| **Semilunar valves** |  | Aortic | ------- |
| Aortic valve | Annulus = 7mm | Tricuspid | ------- |
| Pulmonary valve | Annulus = 8mm | pulmonic | -------- |
| **Great arteries** | NRGA | **Aortic arch** | Left. No CoA. |
| Aorta | ----- | **PDA** | No |
| Pulmonary artery | Normal MPA and Branch PAs. |  |  |
| **M-Mode:** | | | |
| AO | mm | PWd | mm |
| LA | mm | PWs | mm |
| LVIDd | mm | EDV | ml |
| LVIDs | mm | ESV | ml |
| IVSs | mm | LVEF | % |
| IVSd | mm | FS | % |
| **Additional Information**: |  | | |
| No pericardial/Pleural effusion. | | | |
| **Final Diagnosis:** | | | |
| 1. {S, D, S} Levocardia. 2. Small OS ASD, L – R Shunt | | | |
| **Remark**: | | | |
| **Recommendation**: | | | |
| SIGNATURE  Done by: Tesfaye T., Pediatrician, Pediatric Cardiologist _______________ 02/08/2015Eth.C | | | |

| Patient Name: **Baby of Birkie Dires_**. Referring Institute: **TGSH**. SEX/ Age: **M/14days**. Date of Report: **05/08/15**.  Referral Diagnosis: **RD + EONS + TEF (Screening). AGH12.3445.** | | | |
| --- | --- | --- | --- |
| **Features** | **Finding** | **Features** | **Finding** |
| **Profile** |  | **Atria** |  |
| Abdominal situs | Solitus | Left atrium | Normal |
| Atrial Situs | Solitus | Right atrium | Normal |
| Cardiac position | Levocardia | **Atrioventricular valves** |  |
| Systemic venous drainage | Normal. | Mitral valve | Annulus = 11mm |
| Pulmonary venous drainage | Normal | Tricuspid valve | Annulus = 10mm |
| Atrioventricular connection | Concordant |  | TAPSE = mm |
| Ventriculoarterial connection | Concordant | **Ventricles** |  |
| Ventricular loop | d-Loop | Left ventricle | Normal |
|  |  | Right ventricle | Normal |
| **Septae** |  | **Coronary arteries** | ----- |
| Interventricular septum | Intact | **Doppler Measurement** |  |
| Interatrial septum | Intact | Mitral | ----- |
| **Semilunar valves** |  | Aortic | ------- |
| Aortic valve | Annulus = 9mm | Tricuspid | ------- |
| Pulmonary valve | Annulus = 10mm | pulmonic | -------- |
| **Great arteries** | NRGA | **Aortic arch** | Left. No CoA. |
| Aorta | ----- | **PDA** | No |
| Pulmonary artery | Normal MPA and Branch PAs. |  |  |
| **M-Mode:** | | | |
| AO | mm | PWd | mm |
| LA | mm | PWs | mm |
| LVIDd | mm | EDV | ml |
| LVIDs | mm | ESV | ml |
| IVSs | mm | LVEF | % |
| IVSd | mm | FS | % |
| **Additional Information**: |  | | |
| No pericardial/Pleural effusion. | | | |
| **Final Diagnosis:** | | | |
| 1. Normal Echocardiography Study. | | | |
| **Remark**: | | | |
| **Recommendation**: | | | |
| SIGNATURE  Done by: Tesfaye T., Pediatrician, Pediatric Cardiologist _______________ 05/08/2015Eth.C | | | |

| Patient Name: **Bisrat Amare**. Referring Institute: **Adinas GH**. SEX/ Age: **M/6 5/12**. Date of Report: **05/08/15**.  Referral Diagnosis: **Follow up echo for Mild AR (incidental finding, 04/05/2014Eth.C).** | | | |
| --- | --- | --- | --- |
| **Features** | **Finding** | **Features** | **Finding** |
| **Profile** |  | **Atria** |  |
| Abdominal situs | Solitus | Left atrium | Normal |
| Atrial Situs | Solitus | Right atrium | Normal |
| Cardiac position | Levocardia | **Atrioventricular valves** |  |
| Systemic venous drainage | Normal. | Mitral valve | Annulus = 17mm |
| Pulmonary venous drainage | Normal | Tricuspid valve | Annulus = 17mm |
| Atrioventricular connection | Concordant |  | TAPSE = 20mm |
| Ventriculoarterial connection | Concordant | **Ventricles** |  |
| Ventricular loop | d-Loop | Left ventricle | Normal |
|  |  | Right ventricle | Normal |
| **Septae** |  | **Coronary arteries** | ----- |
| Interventricular septum | Intact | **Doppler Measurement** |  |
| Interatrial septum | Intact | Mitral | ----- |
| **Semilunar valves** |  | Aortic | Mild AR, PHT = 521ms |
| Aortic valve | Annulus = 17mm. Trileaflet, Thickened | Tricuspid | ------- |
| Pulmonary valve | Annulus = 18mm | pulmonic | -------- |
| **Great arteries** | NRGA | **Aortic arch** | Left. No CoA. |
| Aorta | ----- | **PDA** | No |
| Pulmonary artery | Normal MPA and Branch PAs. |  |  |
| **M-Mode:** | | | |
| AO | mm | PWd | mm |
| LA | mm | PWs | mm |
| LVIDd | mm | EDV | ml |
| LVIDs | mm | ESV | ml |
| IVSs | mm | LVEF | 68% |
| IVSd | mm | FS | 37% |
| **Additional Information**: |  | | |
| No pericardial/Pleural effusion. | | | |
| **Final Diagnosis:** | | | |
| 1. {S, D, S} Levocardia. 2. Trileaflet, Thickened AVL 3. Mild AR 4. Normal Biventricular Systolic Function | | | |
| **Remark**: | | | |
| **Recommendation**: | | | |
| SIGNATURE  Done by: Tesfaye T., Pediatrician, Pediatric Cardiologist _______________ 05/08/2015Eth.C | | | |

| Patient Name: **Yohana Netsanet**. Referring Institute: **Dr. Addisu PSC**. SEX/ Age: **F/10months**. Date of Report: **05/08/15**.  Referral Diagnosis: **Myocarditis + RD + Cardiogenic Shock. AGH12.3446.** | | | |
| --- | --- | --- | --- |
| **Features** | **Finding** | **Features** | **Finding** |
| **Profile** |  | **Atria** |  |
| Abdominal situs | Solitus | Left atrium | Dilated |
| Atrial Situs | Solitus | Right atrium | Dilated |
| Cardiac position | Levocardia | **Atrioventricular valves** |  |
| Systemic venous drainage | Normal. | Mitral valve | Annulus = 15mm |
| Pulmonary venous drainage | Normal | Tricuspid valve | Annulus = 15mm |
| Atrioventricular connection | Concordant |  | TAPSE = 9mm |
| Ventriculoarterial connection | Concordant | **Ventricles** |  |
| Ventricular loop | d-Loop | Left ventricle | Dilated |
|  |  | Right ventricle | Dilated |
| **Septae** |  | **Coronary arteries** | ----- |
| Interventricular septum | Intact | **Doppler Measurement** |  |
| Interatrial septum | Intact | Mitral | Moderate MR |
| **Semilunar valves** |  | Aortic | ------- |
| Aortic valve | Annulus = 12mm | Tricuspid | ------- |
| Pulmonary valve | Annulus = 12mm | pulmonic | -------- |
| **Great arteries** | NRGA | **Aortic arch** | Left. No CoA. |
| Aorta | ----- | **PDA** | No |
| Pulmonary artery | Normal MPA and Branch PAs. |  |  |
| **M-Mode:** | | | |
| AO | mm | PWd | mm |
| LA | mm | PWs | mm |
| LVIDd | mm | EDV | ml |
| LVIDs | mm | ESV | ml |
| IVSs | mm | LVEF | 30% |
| IVSd | mm | FS | 14% |
| **Additional Information**: |  | | |
| No pericardial/Pleural effusion. | | | |
| **Final Diagnosis:** | | | |
| 1. {S, D, S} Levocardia. 2. All chambers dilated 3. Moderate MR 4. Severe Biventricular Dysfunction | | | |
| **Remark**: Fulminant Myocarditis | | | |
| **Recommendation**: | | | |
| SIGNATURE  Done by: Tesfaye T., Pediatrician, Pediatric Cardiologist _______________ 05/08/2015Eth.C | | | |

| Patient Name: **Mihretu Wubet**. Referring Institute: **FHRH**. SEX/ Age: **M/11years**. Date of Report: **07/08/15**.  Referral Diagnosis: **Palpitation. AGH12.3447. (AGH12)** | | | |
| --- | --- | --- | --- |
| **Features** | **Finding** | **Features** | **Finding** |
| **Profile** |  | **Atria** |  |
| Abdominal situs | Solitus | Left atrium | Normal |
| Atrial Situs | Solitus | Right atrium | Dilated |
| Cardiac position | Levocardia | **Atrioventricular valves** |  |
| Systemic venous drainage | Normal. IVC Dilated | Mitral valve | Annulus = 19mm. Patulous MVL |
| Pulmonary venous drainage | Normal | Tricuspid valve | Annulus = 24mm |
| Atrioventricular connection | Concordant |  | TAPSE = 25mm |
| Ventriculoarterial connection | Concordant | **Ventricles** |  |
| Ventricular loop | d-Loop | Left ventricle | Normal |
|  |  | Right ventricle | Dilated |
| **Septae** |  | **Coronary arteries** | ----- |
| Interventricular septum | Intact | **Doppler Measurement** |  |
| Interatrial septum | 22mm X 24mm OS ASD, L – R Shunt | Mitral | Moderate MR, Holosystolic, posterior projection, seen in two planes with jet velocity = 3.7m/sec. |
| **Semilunar valves** |  | Aortic | Mild AR, PHT = 510ms |
| Aortic valve | Annulus = 18mm | Tricuspid | Trivial TR, PPG = 40mmHg |
| Pulmonary valve | Annulus = 29mm | pulmonic | -------- |
| **Great arteries** | NRGA | **Aortic arch** | Left. No CoA. |
| Aorta | ----- | **PDA** | No |
| Pulmonary artery | MPA = 32mm. |  |  |
| **M-Mode:** | | | |
| AO | mm | PWd | mm |
| LA | mm | PWs | mm |
| LVIDd | mm | EDV | ml |
| LVIDs | mm | ESV | ml |
| IVSs | mm | LVEF | 62% |
| IVSd | mm | FS | 33% |
| **Additional Information**: |  | | |
| Pericardial effusion with maximum depth of 7mm on RA Side. 13mm Right Pleural effusion. | | | |
| **Final Diagnosis:** | | | |
| 1. {S, D, S} Levocardia. 2. RA/RV Dilated 3. Large OS ASD, L – R Shunt 4. Patulous MVL 5. Moderate MR 6. Mild AR 7. Trivial TR 8. Mild Pulmonary Hypertension 9. Small Pericardial effusion 10. Moderate Right Pleural Effusion 11. Normal Biventricular Systolic Function | | | |
| SIGNATURE  Done by: Tesfaye T., Pediatrician, Pediatric Cardiologist _______________ 07/08/2015Eth.C | | | |

| Patient Name: **Ozian Yalfal**. Referring Institute: **Amaris PSC**. SEX/ Age: **F/5months**. Date of Report: **07/08/15**.  Referral Diagnosis: **R/O CHD(Diaphoresis during breast feeding). AGH12.3448.** | | | |
| --- | --- | --- | --- |
| **Features** | **Finding** | **Features** | **Finding** |
| **Profile** |  | **Atria** |  |
| Abdominal situs | Solitus | Left atrium | Normal |
| Atrial Situs | Solitus | Right atrium | Normal |
| Cardiac position | Levocardia | **Atrioventricular valves** |  |
| Systemic venous drainage | Normal. | Mitral valve | Annulus = 12mm |
| Pulmonary venous drainage | Normal | Tricuspid valve | Annulus = 13mm |
| Atrioventricular connection | Concordant |  | TAPSE = mm |
| Ventriculoarterial connection | Concordant | **Ventricles** |  |
| Ventricular loop | d-Loop | Left ventricle | Normal |
|  |  | Right ventricle | Normal |
| **Septae** |  | **Coronary arteries** | ----- |
| Interventricular septum | Intact | **Doppler Measurement** |  |
| Interatrial septum | Intact | Mitral | ----- |
| **Semilunar valves** |  | Aortic | ------- |
| Aortic valve | Annulus = 12mm | Tricuspid | ------- |
| Pulmonary valve | Annulus = 13mm | pulmonic | -------- |
| **Great arteries** | NRGA | **Aortic arch** | Left. No CoA. |
| Aorta | ----- | **PDA** | No |
| Pulmonary artery | Normal MPA and Branch PAs. |  |  |
| **M-Mode:**  Normal LV Function on eye balling | | | |
| AO | mm | PWd | mm |
| LA | mm | PWs | mm |
| LVIDd | mm | EDV | ml |
| LVIDs | mm | ESV | ml |
| IVSs | mm | LVEF | % |
| IVSd | mm | FS | % |
| **Additional Information**: |  | | |
| No pericardial/Pleural effusion. | | | |
| **Final Diagnosis:** | | | |
| 1. Normal Echocardiography Study. | | | |
| **Remark**: | | | |
| **Recommendation**: | | | |
| SIGNATURE  Done by: Tesfaye T., Pediatrician, Pediatric Cardiologist _______________ 07/08/2015Eth.C | | | |

| Patient Name: **Kalkidan Desta**. Referring Institute: **FHRH**. SEX/ Age: **F/2 3/12**. Date of Report: **07/08/15**.  Referral Diagnosis: **RD. AGH12.3449.** | | | |
| --- | --- | --- | --- |
| **Features** | **Finding** | **Features** | **Finding** |
| **Profile** |  | **Atria** |  |
| Abdominal situs | Solitus | Left atrium | Normal |
| Atrial Situs | Solitus | Right atrium | Normal |
| Cardiac position | Levocardia | **Atrioventricular valves** |  |
| Systemic venous drainage | Normal. | Mitral valve | Annulus = 13mm |
| Pulmonary venous drainage | Normal | Tricuspid valve | Annulus = 16mm |
| Atrioventricular connection | Concordant |  | TAPSE = 17mm |
| Ventriculoarterial connection | Concordant | **Ventricles** |  |
| Ventricular loop | d-Loop | Left ventricle | Normal |
|  |  | Right ventricle | Normal |
| **Septae** |  | **Coronary arteries** | ----- |
| Interventricular septum | Intact | **Doppler Measurement** |  |
| Interatrial septum | Intact | Mitral | ----- |
| **Semilunar valves** |  | Aortic | ------- |
| Aortic valve | Annulus = 13mm | Tricuspid | ------- |
| Pulmonary valve | Annulus = 14mm | pulmonic | -------- |
| **Great arteries** | NRGA | **Aortic arch** | Left. No CoA. |
| Aorta | ----- | **PDA** | No |
| Pulmonary artery | Normal MPA and Branch PAs. |  |  |
| **M-Mode:** | | | |
| AO | mm | PWd | mm |
| LA | mm | PWs | mm |
| LVIDd | mm | EDV | ml |
| LVIDs | mm | ESV | ml |
| IVSs | mm | LVEF | 69% |
| IVSd | mm | FS | 37% |
| **Additional Information**: |  | | |
| No pericardial/Pleural effusion. | | | |
| **Final Diagnosis:** | | | |
| 1. Normal Echocardiography Study. | | | |
| **Remark**: | | | |
| **Recommendation**: | | | |
| SIGNATURE  Done by: Tesfaye T., Pediatrician, Pediatric Cardiologist _______________ 07/08/2015Eth.C | | | |

| Patient Name: **Birhanu Nega**. Referring Institute: **TGSH**. SEX/ Age: **M/14years**. Date of Report: **07/08/15**.  Referral Diagnosis: **Sydenham’s Chorea. AGH12.3450.** | | | |
| --- | --- | --- | --- |
| **Features** | **Finding** | **Features** | **Finding** |
| **Profile** |  | **Atria** |  |
| Abdominal situs | Solitus | Left atrium | Normal |
| Atrial Situs | Solitus | Right atrium | Normal |
| Cardiac position | Levocardia | **Atrioventricular valves** |  |
| Systemic venous drainage | Normal. | Mitral valve | Annulus = 22mm. Patulous MVL |
| Pulmonary venous drainage | Normal | Tricuspid valve | Annulus = 23mm |
| Atrioventricular connection | Concordant |  | TAPSE = 21mm |
| Ventriculoarterial connection | Concordant | **Ventricles** |  |
| Ventricular loop | d-Loop | Left ventricle | Normal |
|  |  | Right ventricle | Normal |
| **Septae** |  | **Coronary arteries** | ----- |
| Interventricular septum | Intact | **Doppler Measurement** |  |
| Interatrial septum | Intact | Mitral | Moderate MR, Holosystolic, Posterior projection, seen in two planes with jet velocity = 3.7m/sec. |
| **Semilunar valves** |  | Aortic | Moderate AR, PHT = 436ms |
| Aortic valve | Annulus = 18mm | Tricuspid | Trivial TR, PPG = 15mmHg |
| Pulmonary valve | Annulus = 19mm | pulmonic | -------- |
| **Great arteries** | NRGA | **Aortic arch** | Left. No CoA. |
| Aorta | ----- | **PDA** | No |
| Pulmonary artery | Normal MPA and Branch PAs. |  |  |
| **M-Mode:** | | | |
| AO | mm | PWd | mm |
| LA | mm | PWs | mm |
| LVIDd | mm | EDV | ml |
| LVIDs | mm | ESV | ml |
| IVSs | mm | LVEF | 59% |
| IVSd | mm | FS | 32% |
| **Additional Information**: |  | | |
| No pericardial/Pleural effusion. | | | |
| **Final Diagnosis:** | | | |
| 1. {S, D, S} Levocardia. 2. Patulous MVL 3. Moderate MR 4. Moderate AR 5. Normal Biventricular Systolic Function | | | |
| SIGNATURE  Done by: Tesfaye T., Pediatrician, Pediatric Cardiologist _______________ 07/08/2015Eth.C | | | |

| Patient Name: **Solomie Abiyu**. Referring Institute: **Adinas GH**. SEX/ Age: **F/9 10/12**. Date of Report: **10/08/15**.  Referral Diagnosis: **Follow up echo for Mild MR + Thickened MVL.** | | | |
| --- | --- | --- | --- |
| **Features** | **Finding** | **Features** | **Finding** |
| **Profile** |  | **Atria** |  |
| Abdominal situs | Solitus | Left atrium | Mildly Dilated |
| Atrial Situs | Solitus | Right atrium | Normal |
| Cardiac position | Levocardia | **Atrioventricular valves** |  |
| Systemic venous drainage | Normal. | Mitral valve | Annulus = 25mm. Thickened MVL. |
| Pulmonary venous drainage | Normal | Tricuspid valve | Annulus = 21mm |
| Atrioventricular connection | Concordant |  | TAPSE = 19mm |
| Ventriculoarterial connection | Concordant | **Ventricles** |  |
| Ventricular loop | d-Loop | Left ventricle | Mildly Dilated |
|  |  | Right ventricle | Normal |
| **Septae** |  | **Coronary arteries** | ----- |
| Interventricular septum | Intact | **Doppler Measurement** |  |
| Interatrial septum | Intact | Mitral | Mild MR, Holosystolic, posterior Projection, seen in two planes with jet velocity = 4.8m/sec. |
| **Semilunar valves** |  | Aortic | ------- |
| Aortic valve | Annulus = 16mm | Tricuspid | ------- |
| Pulmonary valve | Annulus = 20mm | pulmonic | -------- |
| **Great arteries** | NRGA | **Aortic arch** | Left. No CoA. |
| Aorta | ----- | **PDA** | No |
| Pulmonary artery | Normal. |  |  |
| **M-Mode:** | | | |
| AO | mm | PWd | mm |
| LA | mm | PWs | mm |
| LVIDd | mm | EDV | ml |
| LVIDs | mm | ESV | ml |
| IVSs | mm | LVEF | 68% |
| IVSd | mm | FS | 37% |
| **Additional Information**: |  | | |
| No pericardial/Pleural effusion. | | | |
| **Final Diagnosis:** | | | |
| 1. {S, D, S} Levocardia. 2. Mildly Dilated LA/LV 3. Thickened MVL 4. Mild MR 5. Normal Biventricular Systolic Function | | | |
| **Recommendation**: | | | |
| SIGNATURE  Done by: Tesfaye T., Pediatrician, Pediatric Cardiologist _______________ 10/08/2015Eth.C | | | |

| Patient Name: **Etaferaw Yalew**. Referring Institute: **FHRH**. SEX/ Age: **F/8years**. Date of Report: **11/08/15**.  Referral Diagnosis: **Sydenham’s Chorea. AGH12.3451.** | | | |
| --- | --- | --- | --- |
| **Features** | **Finding** | **Features** | **Finding** |
| **Profile** |  | **Atria** |  |
| Abdominal situs | Solitus | Left atrium | Normal |
| Atrial Situs | Solitus | Right atrium | Normal |
| Cardiac position | Levocardia | **Atrioventricular valves** |  |
| Systemic venous drainage | Normal. | Mitral valve | Annulus = 19mm. Thickened MVL |
| Pulmonary venous drainage | Normal | Tricuspid valve | Annulus = 19mm |
| Atrioventricular connection | Concordant |  | TAPSE = 17mm |
| Ventriculoarterial connection | Concordant | **Ventricles** |  |
| Ventricular loop | d-Loop | Left ventricle | Normal |
|  |  | Right ventricle | Normal |
| **Septae** |  | **Coronary arteries** | ----- |
| Interventricular septum | Intact | **Doppler Measurement** |  |
| Interatrial septum | Intact | Mitral | Mild MR, Holosystolic, Posterior projection, seen in two planes with jet velocity = 4.5m/sec |
| **Semilunar valves** |  | Aortic | ------- |
| Aortic valve | Annulus = 16mm | Tricuspid | ------- |
| Pulmonary valve | Annulus = 18mm | pulmonic | -------- |
| **Great arteries** | NRGA | **Aortic arch** | Left. No CoA. |
| Aorta | ----- | **PDA** | No |
| Pulmonary artery | Normal MPA and Branch PAs. |  |  |
| **M-Mode:** | | | |
| AO | mm | PWd | mm |
| LA | mm | PWs | mm |
| LVIDd | mm | EDV | ml |
| LVIDs | mm | ESV | ml |
| IVSs | mm | LVEF | 62% |
| IVSd | mm | FS | 32% |
| **Additional Information**: |  | | |
| No pericardial/Pleural effusion. | | | |
| **Final Diagnosis:** | | | |
| 1. {S, D, S} Levocardia. 2. Thickened MVL 3. Mild MR 4. Normal Biventricular Systolic Function | | | |
| **Remark**: | | | |
| **Recommendation**: | | | |
| SIGNATURE  Done by: Tesfaye T., Pediatrician, Pediatric Cardiologist _______________ 11/08/2015Eth.C | | | |

| Patient Name: **Estifanos Tewodros**. Referring Institute: **TGSH**. SEX/ Age: **M/4years**. Date of Report: **11/08/15**.  Referral Diagnosis: **Pulmonary HTN 20 ATH + Cardiomegaly on CXR. AGH12.3452.** | | | |
| --- | --- | --- | --- |
| **Features** | **Finding** | **Features** | **Finding** |
| **Profile** |  | **Atria** |  |
| Abdominal situs | Solitus | Left atrium | Normal |
| Atrial Situs | Solitus | Right atrium | Dilated |
| Cardiac position | Levocardia | **Atrioventricular valves** |  |
| Systemic venous drainage | Normal. | Mitral valve | Annulus = 15mm |
| Pulmonary venous drainage | Normal | Tricuspid valve | Annulus = 23mm |
| Atrioventricular connection | Concordant |  | TAPSE = 17mm |
| Ventriculoarterial connection | Concordant | **Ventricles** |  |
| Ventricular loop | d-Loop | Left ventricle | Normal |
|  |  | Right ventricle | Dilated & Hypertrophied |
| **Septae** |  | **Coronary arteries** | ----- |
| Interventricular septum | Intact | **Doppler Measurement** |  |
| Interatrial septum | Intact | Mitral | ----- |
| **Semilunar valves** |  | Aortic | ------- |
| Aortic valve | Annulus = 13mm | Tricuspid | Mild TR, PPG = 65mmHg |
| Pulmonary valve | Annulus = 19mm | pulmonic | -------- |
| **Great arteries** | NRGA | **Aortic arch** | Left. No CoA. |
| Aorta | ----- | **PDA** | No |
| Pulmonary artery | Normal MPA and Branch PAs. |  |  |
| **M-Mode:** | | | |
| AO | mm | PWd | mm |
| LA | mm | PWs | mm |
| LVIDd | mm | EDV | ml |
| LVIDs | mm | ESV | ml |
| IVSs | mm | LVEF | 40% |
| IVSd | mm | FS | 72% |
| **Additional Information**: |  | | |
| No pericardial/Pleural effusion. | | | |
| **Final Diagnosis:** | | | |
| 1. {S, D, S} Levocardia. 2. RA/RV Dilated & RV Hypertrophied 3. Mild TR 4. Severe Pulmonary Hypertension 5. Normal Biventricular Systolic Function | | | |
| **Remark**: secondary causes shall be ruled out and managed | | | |
| **Recommendation**: | | | |
| SIGNATURE  Done by: Tesfaye T., Pediatrician, Pediatric Cardiologist _______________ 11/08/2015Eth.C | | | |

| Patient Name: **Baby of Rahel Belete**. Referring Institute: **Adinas GH**. SEX/ Age: **M/1Hour**. Date of Report: **11/08/15**.  Referral Diagnosis: **Screening echo (?Dandy walker). AGH12.3453.** | | | |
| --- | --- | --- | --- |
| **Features** | **Finding** | **Features** | **Finding** |
| **Profile** |  | **Atria** |  |
| Abdominal situs | Solitus | Left atrium | Normal |
| Atrial Situs | Solitus | Right atrium | Normal |
| Cardiac position | Levocardia | **Atrioventricular valves** |  |
| Systemic venous drainage | Normal. | Mitral valve | Annulus = 10mm |
| Pulmonary venous drainage | Normal | Tricuspid valve | Annulus = 11mm |
| Atrioventricular connection | Concordant |  | TAPSE = mm |
| Ventriculoarterial connection | Concordant | **Ventricles** |  |
| Ventricular loop | d-Loop | Left ventricle | Normal |
|  |  | Right ventricle | Normal |
| **Septae** |  | **Coronary arteries** | ----- |
| Interventricular septum | Intact | **Doppler Measurement** |  |
| Interatrial septum | 4mm OS ASD, L – R Shunt | Mitral | ----- |
| **Semilunar valves** |  | Aortic | ------- |
| Aortic valve | Annulus = 10mm | Tricuspid | ------- |
| Pulmonary valve | Annulus = 11mm | pulmonic | -------- |
| **Great arteries** | NRGA | **Aortic arch** | Left. No CoA. |
| Aorta | ----- | **PDA** | <1mm PDA, L – R Shunt |
| Pulmonary artery | Normal MPA and Branch PAs. |  |  |
| **M-Mode:**  Normal LV Function on eye balling | | | |
| AO | mm | PWd | mm |
| LA | mm | PWs | mm |
| LVIDd | mm | EDV | ml |
| LVIDs | mm | ESV | ml |
| IVSs | mm | LVEF | % |
| IVSd | mm | FS | % |
| **Additional Information**: |  | | |
| No pericardial/Pleural effusion. | | | |
| **Final Diagnosis:** | | | |
| 1. {S, D, S} Levocardia. 2. Small OS ASD, L – R Shunt 3. Silent PDA, L – R Shunt | | | |
| **Remark**: | | | |
| **Recommendation**: | | | |
| SIGNATURE  Done by: Tesfaye T., Pediatrician, Pediatric Cardiologist _______________ 11/08/2015Eth.C | | | |

| Patient Name: **Bereket Tayachew**. Referring Institute: **Adinas GH**. SEX/ Age: **M/8years**. Date of Report: **11/08/15**.  Referral Diagnosis: **ARF R/O Carditis. AGH12.3454.** | | | |
| --- | --- | --- | --- |
| **Features** | **Finding** | **Features** | **Finding** |
| **Profile** |  | **Atria** |  |
| Abdominal situs | Solitus | Left atrium | Normal |
| Atrial Situs | Solitus | Right atrium | Normal |
| Cardiac position | Levocardia | **Atrioventricular valves** |  |
| Systemic venous drainage | Normal. | Mitral valve | Annulus = 19mm |
| Pulmonary venous drainage | Normal | Tricuspid valve | Annulus = 18mm |
| Atrioventricular connection | Concordant |  | TAPSE = 18mm |
| Ventriculoarterial connection | Concordant | **Ventricles** |  |
| Ventricular loop | d-Loop | Left ventricle | Normal |
|  |  | Right ventricle | Normal |
| **Septae** |  | **Coronary arteries** | ----- |
| Interventricular septum | Intact | **Doppler Measurement** |  |
| Interatrial septum | Intact | Mitral | ----- |
| **Semilunar valves** |  | Aortic | ------- |
| Aortic valve | Annulus = 15mm | Tricuspid | ------- |
| Pulmonary valve | Annulus = 18mm | pulmonic | -------- |
| **Great arteries** | NRGA | **Aortic arch** | Left. No CoA. |
| Aorta | ----- | **PDA** | No |
| Pulmonary artery | Normal MPA and Branch PAs. |  |  |
| **M-Mode:**  Normal LV Function on eye balling | | | |
| AO | mm | PWd | mm |
| LA | mm | PWs | mm |
| LVIDd | mm | EDV | ml |
| LVIDs | mm | ESV | ml |
| IVSs | mm | LVEF | % |
| IVSd | mm | FS | % |
| **Additional Information**: |  | | |
| No pericardial/Pleural effusion. | | | |
| **Final Diagnosis:** | | | |
| 1. Normal Echocardiography Study. | | | |
| **Remark**: | | | |
| **Recommendation**: | | | |
| SIGNATURE  Done by: Tesfaye T., Pediatrician, Pediatric Cardiologist _______________ 11/08/2015Eth.C | | | |

| Patient Name: **Baby of Hamelmal Wubie**. Referring Institute: **FHRH**. SEX/ Age: **F/7days**. Date of Report: **11/08/15**.  Referral Diagnosis: **Persistent tachypnea + G- III HSM. AGH12.3455.** | | | |
| --- | --- | --- | --- |
| **Features** | **Finding** | **Features** | **Finding** |
| **Profile** |  | **Atria** |  |
| Abdominal situs | Solitus | Left atrium | Normal |
| Atrial Situs | Solitus | Right atrium | Normal |
| Cardiac position | Levocardia | **Atrioventricular valves** |  |
| Systemic venous drainage | Normal. | Mitral valve | Annulus = 8mm |
| Pulmonary venous drainage | Normal | Tricuspid valve | Annulus = 9mm |
| Atrioventricular connection | Concordant |  | TAPSE = mm |
| Ventriculoarterial connection | Concordant | **Ventricles** |  |
| Ventricular loop | d-Loop | Left ventricle | Normal |
|  |  | Right ventricle | Normal |
| **Septae** |  | **Coronary arteries** | ----- |
| Interventricular septum | Intact | **Doppler Measurement** |  |
| Interatrial septum | 4mm OS ASD, L – R Shunt | Mitral | ----- |
| **Semilunar valves** |  | Aortic | ------- |
| Aortic valve | Annulus = 9mm | Tricuspid | ------- |
| Pulmonary valve | Annulus = 8mm | pulmonic | -------- |
| **Great arteries** | NRGA | **Aortic arch** | Left. No CoA. |
| Aorta | ----- | **PDA** | 1mm PDA, L – R Shunt |
| Pulmonary artery | Normal MPA and Branch PAs. |  |  |
| **M-Mode:**  Normal LV Function on eye balling | | | |
| AO | mm | PWd | mm |
| LA | mm | PWs | mm |
| LVIDd | mm | EDV | ml |
| LVIDs | mm | ESV | ml |
| IVSs | mm | LVEF | % |
| IVSd | mm | FS | % |
| **Additional Information**: |  | | |
| No pericardial/Pleural effusion. | | | |
| **Final Diagnosis:** | | | |
| 1. {S, D, S} Levocardia. 2. Small OS ASD, L – R Shunt 3. Small PDA, L – R Shunt | | | |
| **Remark**: | | | |
| **Recommendation**: | | | |
| SIGNATURE  Done by: Tesfaye T., Pediatrician, Pediatric Cardiologist _______________ 11/08/2015Eth.C | | | |

| Patient Name: **Baby of Banchayehu Tiruneh**. Referring Institute: **FHRH**. SEX/ Age: **F/14days**. Date of Report: **11/08/15**.  Referral Diagnosis: **FB Since birth + Cardiomegaly on CXR. AGH12.3456.** | | | |
| --- | --- | --- | --- |
| **Features** | **Finding** | **Features** | **Finding** |
| **Profile** |  | **Atria** |  |
| Abdominal situs | Solitus | Left atrium | Normal |
| Atrial Situs | Solitus | Right atrium | Normal |
| Cardiac position | Levocardia | **Atrioventricular valves** |  |
| Systemic venous drainage | Normal. | Mitral valve | Annulus = 10mm |
| Pulmonary venous drainage | Normal | Tricuspid valve | Annulus = 11mm |
| Atrioventricular connection | Concordant |  | TAPSE = mm |
| Ventriculoarterial connection | Concordant | **Ventricles** |  |
| Ventricular loop | d-Loop | Left ventricle | Normal |
|  |  | Right ventricle | Normal |
| **Septae** |  | **Coronary arteries** | ----- |
| Interventricular septum | Intact | **Doppler Measurement** |  |
| Interatrial septum | Intact | Mitral | ----- |
| **Semilunar valves** |  | Aortic | ------- |
| Aortic valve | Annulus = 10mm | Tricuspid | ------- |
| Pulmonary valve | Annulus = 8mm | pulmonic | -------- |
| **Great arteries** | NRGA | **Aortic arch** | Left. No CoA. |
| Aorta | ----- | **PDA** | No |
| Pulmonary artery | Normal MPA and Branch PAs. |  |  |
| **M-Mode:**  Normal LV Function on eye balling | | | |
| AO | mm | PWd | mm |
| LA | mm | PWs | mm |
| LVIDd | mm | EDV | ml |
| LVIDs | mm | ESV | ml |
| IVSs | mm | LVEF | % |
| IVSd | mm | FS | % |
| **Additional Information**: |  | | |
| No pericardial/Pleural effusion. | | | |
| **Final Diagnosis:** | | | |
| 1. Normal Echocardiography Study. | | | |
| **Remark**: | | | |
| **Recommendation**: | | | |
| SIGNATURE  Done by: Tesfaye T., Pediatrician, Pediatric Cardiologist _______________ 11/08/2015Eth.C | | | |

| Patient Name: **Baby of Alemitu Tesfahun**. Referring Institute: **FHRH**. SEX/ Age: **F/14days**. Date of Report: **11/08/15**.  Referral Diagnosis: **G-II DM. AGH12.3457.** | | | |
| --- | --- | --- | --- |
| **Features** | **Finding** | **Features** | **Finding** |
| **Profile** |  | **Atria** |  |
| Abdominal situs | Solitus | Left atrium | Normal |
| Atrial Situs | Solitus | Right atrium | Normal |
| Cardiac position | Levocardia | **Atrioventricular valves** |  |
| Systemic venous drainage | Normal. | Mitral valve | Annulus = 11mm |
| Pulmonary venous drainage | Normal | Tricuspid valve | Annulus = 10mm |
| Atrioventricular connection | Concordant |  | TAPSE = mm |
| Ventriculoarterial connection | Concordant | **Ventricles** |  |
| Ventricular loop | d-Loop | Left ventricle | Normal |
|  |  | Right ventricle | Normal |
| **Septae** |  | **Coronary arteries** | ----- |
| Interventricular septum | Intact | **Doppler Measurement** |  |
| Interatrial septum | Intact | Mitral | ----- |
| **Semilunar valves** |  | Aortic | ------- |
| Aortic valve | Annulus = 9mm | Tricuspid | ------- |
| Pulmonary valve | Annulus = 9mm | pulmonic | Mild PS, PPG = 21mmHg |
| **Great arteries** | NRGA | **Aortic arch** | Left. No CoA. |
| Aorta | ----- | **PDA** | No |
| Pulmonary artery | Normal MPA and Branch PAs. |  |  |
| **M-Mode:**  Normal LV Function on eye balling | | | |
| AO | mm | PWd | mm |
| LA | mm | PWs | mm |
| LVIDd | mm | EDV | ml |
| LVIDs | mm | ESV | ml |
| IVSs | mm | LVEF | % |
| IVSd | mm | FS | % |
| **Additional Information**: |  | | |
| No pericardial/Pleural effusion. | | | |
| **Final Diagnosis:** | | | |
| 1. {S, D, S} Levocardia. 2. Mild Valvular PS | | | |
| **Remark**: | | | |
| **Recommendation**: | | | |
| SIGNATURE  Done by: Tesfaye T., Pediatrician, Pediatric Cardiologist _______________ 11/08/2015Eth.C | | | |

| Patient Name: **Dimetros Dawit**. Referring Institute: **Adinas GH**. SEX/ Age: **M/3years**. Date of Report: **11/08/15**.  Referral Diagnosis: **Incidental Murmur. AGH12.3458.** | | | |
| --- | --- | --- | --- |
| **Features** | **Finding** | **Features** | **Finding** |
| **Profile** |  | **Atria** |  |
| Abdominal situs | Solitus | Left atrium | Normal |
| Atrial Situs | Solitus | Right atrium | Normal |
| Cardiac position | Levocardia | **Atrioventricular valves** |  |
| Systemic venous drainage | Normal. | Mitral valve | Annulus = 15mm |
| Pulmonary venous drainage | Normal | Tricuspid valve | Annulus = 16mm |
| Atrioventricular connection | Concordant |  | TAPSE = 16mm |
| Ventriculoarterial connection | Concordant | **Ventricles** |  |
| Ventricular loop | d-Loop | Left ventricle | Normal |
|  |  | Right ventricle | Normal |
| **Septae** |  | **Coronary arteries** | ----- |
| Interventricular septum | Intact | **Doppler Measurement** |  |
| Interatrial septum | PFO, L – R Shunt | Mitral | ----- |
| **Semilunar valves** |  | Aortic | ------- |
| Aortic valve | Annulus = 12mm | Tricuspid | Trivial TR, PPG = 15mmHg |
| Pulmonary valve | Annulus = 16mm | pulmonic | -------- |
| **Great arteries** | NRGA | **Aortic arch** | Left. No CoA. |
| Aorta | ----- | **PDA** | No |
| Pulmonary artery | Normal MPA and Branch PAs. |  |  |
| **M-Mode:**  Normal LV Function on eye balling | | | |
| AO | mm | PWd | mm |
| LA | mm | PWs | mm |
| LVIDd | mm | EDV | ml |
| LVIDs | mm | ESV | ml |
| IVSs | mm | LVEF | % |
| IVSd | mm | FS | % |
| **Additional Information**: |  | | |
| No pericardial/Pleural effusion. | | | |
| **Final Diagnosis:** | | | |
| 1. {S, D, S} Levocardia. 2. PFO, L – R Shunt 3. Trivial TR | | | |
| **Remark**: | | | |
| **Recommendation**: | | | |
| SIGNATURE  Done by: Tesfaye T., Pediatrician, Pediatric Cardiologist _______________ 11/08/2015Eth.C | | | |

| Patient Name: **Kirubel Eskemech**. Referring Institute: **FHRH**. SEX/ Age: **M/1 11/12**. Date of Report: **12/08/15**.  Referral Diagnosis: **Breast feeding interruption. AGH12.3459.** | | | |
| --- | --- | --- | --- |
| **Features** | **Finding** | **Features** | **Finding** |
| **Profile** |  | **Atria** |  |
| Abdominal situs | Solitus | Left atrium | Normal |
| Atrial Situs | Solitus | Right atrium | Normal |
| Cardiac position | Levocardia | **Atrioventricular valves** |  |
| Systemic venous drainage | Normal. | Mitral valve | Annulus = 15mm |
| Pulmonary venous drainage | Normal | Tricuspid valve | Annulus = 15mm |
| Atrioventricular connection | Concordant |  | TAPSE = 17mm |
| Ventriculoarterial connection | Concordant | **Ventricles** |  |
| Ventricular loop | d-Loop | Left ventricle | Normal |
|  |  | Right ventricle | Normal |
| **Septae** |  | **Coronary arteries** | ----- |
| Interventricular septum | Intact | **Doppler Measurement** |  |
| Interatrial septum | Intact | Mitral | ----- |
| **Semilunar valves** |  | Aortic | ------- |
| Aortic valve | Annulus = 12mm | Tricuspid | Trivial TR, Incomplete signal, PPG = 12mmHg |
| Pulmonary valve | Annulus = 14mm | pulmonic | -------- |
| **Great arteries** | NRGA | **Aortic arch** | Left. No CoA. |
| Aorta | ----- | **PDA** | No |
| Pulmonary artery | Normal MPA and Branch PAs. |  |  |
| **M-Mode:**  Normal LV Function on eye balling | | | |
| AO | mm | PWd | mm |
| LA | mm | PWs | mm |
| LVIDd | mm | EDV | ml |
| LVIDs | mm | ESV | ml |
| IVSs | mm | LVEF | % |
| IVSd | mm | FS | % |
| **Additional Information**: |  | | |
| No pericardial/Pleural effusion. | | | |
| **Final Diagnosis:** | | | |
| 1. Normal Echocardiography Study. | | | |
| **Remark**: | | | |
| **Recommendation**: | | | |
| SIGNATURE  Done by: Tesfaye T., Pediatrician, Pediatric Cardiologist _______________ 12/08/2015Eth.C | | | |

| Patient Name: **Baby of Birtukan Azene**. Referring Institute: **TGSH**. SEX/ Age: **M/14days**. Date of Report: **12/08/15**.  Referral Diagnosis: **Cleft lip and palate. AGH12.3460.** | | | |
| --- | --- | --- | --- |
| **Features** | **Finding** | **Features** | **Finding** |
| **Profile** |  | **Atria** |  |
| Abdominal situs | Solitus | Left atrium | Normal |
| Atrial Situs | Solitus | Right atrium | Normal |
| Cardiac position | Levocardia | **Atrioventricular valves** |  |
| Systemic venous drainage | Normal. | Mitral valve | Annulus = 11mm |
| Pulmonary venous drainage | Normal | Tricuspid valve | Annulus = 10mm |
| Atrioventricular connection | Concordant |  | TAPSE = mm |
| Ventriculoarterial connection | Concordant | **Ventricles** |  |
| Ventricular loop | d-Loop | Left ventricle | Normal |
|  |  | Right ventricle | Normal |
| **Septae** |  | **Coronary arteries** | ----- |
| Interventricular septum | Intact | **Doppler Measurement** |  |
| Interatrial septum | Intact | Mitral | ----- |
| **Semilunar valves** |  | Aortic | ------- |
| Aortic valve | Annulus = 9mm | Tricuspid | ------- |
| Pulmonary valve | Annulus = 8mm | pulmonic | -------- |
| **Great arteries** | NRGA | **Aortic arch** | Left. No CoA. |
| Aorta | ----- | **PDA** | No |
| Pulmonary artery | Normal MPA and Branch PAs. |  |  |
| **M-Mode:** | | | |
| AO | mm | PWd | mm |
| LA | mm | PWs | mm |
| LVIDd | mm | EDV | ml |
| LVIDs | mm | ESV | ml |
| IVSs | mm | LVEF | % |
| IVSd | mm | FS | % |
| **Additional Information**: |  | | |
| No pericardial/Pleural effusion. | | | |
| **Final Diagnosis:** | | | |
| 1. Normal Echocardiography Study. | | | |
| **Remark**: | | | |
| **Recommendation**: | | | |
| SIGNATURE  Done by: Tesfaye T., Pediatrician, Pediatric Cardiologist _______________ 12/08/2015Eth.C | | | |

| Patient Name: **Makbel Tesfa**. Referring Institute: **Adinas GH**. SEX/ Age: **M/1 1/12**. Date of Report: **12/08/15**.  Referral Diagnosis: **Increased Perspiration + BF Interruption. AGH12.3461.** | | | |
| --- | --- | --- | --- |
| **Features** | **Finding** | **Features** | **Finding** |
| **Profile** |  | **Atria** |  |
| Abdominal situs | Solitus | Left atrium | Mildly Dilated |
| Atrial Situs | Solitus | Right atrium | Normal |
| Cardiac position | Levocardia | **Atrioventricular valves** |  |
| Systemic venous drainage | Normal. | Mitral valve | Annulus = 17mm. Aorto-mitral discontinuity. |
| Pulmonary venous drainage | Normal | Tricuspid valve | Annulus = 16mm |
| Atrioventricular connection | Concordant |  | TAPSE = mm |
| Ventriculoarterial connection | DORV | **Ventricles** |  |
| Ventricular loop | d-Loop | Left ventricle | Mildly Dilated |
|  |  | Right ventricle | Normal |
| **Septae** |  | **Coronary arteries** | ----- |
| Interventricular septum | Sub-aortic VSD, L – R Shunt | **Doppler Measurement** |  |
| Interatrial septum | Intact | Mitral | ----- |
| **Semilunar valves** |  | Aortic | ------- |
| Aortic valve | Annulus = 13mm | Tricuspid | ------- |
| Pulmonary valve | Annulus = 15mm | pulmonic | -------- |
| **Great arteries** | NRGA | **Aortic arch** | Left. No CoA. |
| Aorta | Aortic over-riding greater than 50% | **PDA** | No |
| Pulmonary artery | Normal MPA and Branch PAs. |  |  |
| **M-Mode:**  Normal LV Function | | | |
| AO | mm | PWd | mm |
| LA | mm | PWs | mm |
| LVIDd | mm | EDV | ml |
| LVIDs | mm | ESV | ml |
| IVSs | mm | LVEF | % |
| IVSd | mm | FS | % |
| **Additional Information**: |  | | |
| No pericardial/Pleural effusion. | | | |
| **Final Diagnosis:** | | | |
| 1. {S, D, D} Levocardia. 2. Mildly Dilated LA/LV 3. DORV 4. Non-Restrictive Sub-aortic VSD, L – R Shunt 5. Normal LV Systolic Function | | | |
| **Remark**: | | | |
| **Recommendation**: | | | |
| SIGNATURE  Done by: Tesfaye T., Pediatrician, Pediatric Cardiologist _______________ 12/08/2015Eth.C | | | |

| Patient Name: **Getahun Asmare**. Referring Institute: **FHRH_**. SEX/ Age: **M/11years**. Date of Report: **13/08/15**.  Referral Diagnosis: **Easy Fatigability + FB and grunting. AGH12.3462.** | | | |
| --- | --- | --- | --- |
| **Features** | **Finding** | **Features** | **Finding** |
| **Profile** |  | **Atria** |  |
| Abdominal situs | Solitus | Left atrium | Normal |
| Atrial Situs | Solitus | Right atrium | Normal |
| Cardiac position | Levocardia | **Atrioventricular valves** |  |
| Systemic venous drainage | Normal. | Mitral valve | Annulus = 20mm. Patulous MVL |
| Pulmonary venous drainage | Normal | Tricuspid valve | Annulus = mm |
| Atrioventricular connection | Concordant |  | TAPSE = 21mm |
| Ventriculoarterial connection | Concordant | **Ventricles** |  |
| Ventricular loop | d-Loop | Left ventricle | Normal |
|  |  | Right ventricle | Normal |
| **Septae** |  | **Coronary arteries** | ----- |
| Interventricular septum | Intact | **Doppler Measurement** |  |
| Interatrial septum | Intact | Mitral | Trivial MR, Holosystolic, posterior projection, seen in two planes with jet velocity = 3.5m/sec |
| **Semilunar valves** |  | Aortic | ------- |
| Aortic valve | Annulus = 14mm | Tricuspid | Mild TR. PPG = 26mmHg |
| Pulmonary valve | Annulus = 18mm | pulmonic | -------- |
| **Great arteries** | NRGA | **Aortic arch** | Left. No CoA. |
| Aorta | ----- | **PDA** | No |
| Pulmonary artery | Normal |  |  |
| **M-Mode:** | | | |
| AO | mm | PWd | mm |
| LA | mm | PWs | mm |
| LVIDd | mm | EDV | ml |
| LVIDs | mm | ESV | ml |
| IVSs | mm | LVEF | 68% |
| IVSd | mm | FS | 37% |
| **Additional Information**: |  | | |
| Pericardial effusion with maximum depth of 6mm on RA.RV Side. 32mm right Pleural effusion. | | | |
| **Final Diagnosis:** | | | |
| 1. {S, D, S} Levocardia. 2. Patulous MVL 3. Trivial MR 4. Mild TR 5. Small Pericardial effusion 6. Large Right Pleural effusion 7. Normal Biventricular Systolic Function | | | |
| **Remark**: Can be considered as Borderline RHD | | | |
| **Recommendation**: Secondary prophylaxis recommended | | | |
| SIGNATURE  Done by: Tesfaye T., Pediatrician, Pediatric Cardiologist _______________ 13/08/2015Eth.C | | | |

| Patient Name: **Meseret Liyew**. Referring Institute: **Finote-Selam GH**. SEX/ Age: **F/5years**. Date of Report: **13/08/15**.  Referral Diagnosis: **Diaphoresis. AGH12.3463.** | | | |
| --- | --- | --- | --- |
| **Features** | **Finding** | **Features** | **Finding** |
| **Profile** |  | **Atria** |  |
| Abdominal situs | Solitus | Left atrium | Normal |
| Atrial Situs | Solitus | Right atrium | Dilated |
| Cardiac position | Levocardia | **Atrioventricular valves** |  |
| Systemic venous drainage | Normal. | Mitral valve | Annulus = 13mm |
| Pulmonary venous drainage | Normal | Tricuspid valve | Annulus = 19mm |
| Atrioventricular connection | Concordant |  | TAPSE = 18mm |
| Ventriculoarterial connection | Concordant | **Ventricles** |  |
| Ventricular loop | d-Loop | Left ventricle | Normal |
|  |  | Right ventricle | Dilated & Hypertrophied |
| **Septae** |  | **Coronary arteries** | ----- |
| Interventricular septum | Restrictive Mal-aligned Sub-aortic VSD, R – L Shunt | **Doppler Measurement** |  |
| Interatrial septum | Intact | Mitral | ----- |
| **Semilunar valves** |  | Aortic | ------- |
| Aortic valve | Annulus = 16mm | Tricuspid | ------- |
| Pulmonary valve | Annulus = 8mm | pulmonic | Severe Valvular & Supra-Valvular PS, PPG = 138mmHg |
| **Great arteries** | NRGA | **Aortic arch** | No CoA. |
| Aorta | Over-riding aorta | **PDA** | No |
| Pulmonary artery | Smallish MPA and Branch PAs. |  |  |
| **M-Mode:**  Normal LV Function on eye balling | | | |
| AO | mm | PWd | mm |
| LA | mm | PWs | mm |
| LVIDd | mm | EDV | ml |
| LVIDs | mm | ESV | ml |
| IVSs | mm | LVEF | % |
| IVSd | mm | FS | % |
| **Additional Information**: |  | | |
| No pericardial/Pleural effusion. | | | |
| **Final Diagnosis:** | | | |
| 1. {S, D, S} Levocardia. 2. TOF 3. Smallish MPA and Branch PAs. | | | |
| **Remark**: | | | |
| **Recommendation**: | | | |
| SIGNATURE  Done by: Tesfaye T., Pediatrician, Pediatric Cardiologist _______________ 13/08/2015Eth.C | | | |

| Patient Name: **Baby of Tigist Alene**. Referring Institute: **FHRH**. SEX/ Age: **M/21days**. Date of Report: **13/08/15**.  Referral Diagnosis: **FB since birth. AGH12.3464.** | | | |
| --- | --- | --- | --- |
| **Features** | **Finding** | **Features** | **Finding** |
| **Profile** |  | **Atria** |  |
| Abdominal situs | Solitus | Left atrium | Normal |
| Atrial Situs | Solitus | Right atrium | Normal |
| Cardiac position | Levocardia | **Atrioventricular valves** |  |
| Systemic venous drainage | Normal. | Mitral valve | Annulus = 9mm |
| Pulmonary venous drainage | Normal | Tricuspid valve | Annulus = 9mm |
| Atrioventricular connection | Concordant |  | TAPSE = mm |
| Ventriculoarterial connection | Concordant | **Ventricles** |  |
| Ventricular loop | d-Loop | Left ventricle | Normal |
|  |  | Right ventricle | Normal |
| **Septae** |  | **Coronary arteries** | ----- |
| Interventricular septum | Intact | **Doppler Measurement** |  |
| Interatrial septum | Intact | Mitral | ----- |
| **Semilunar valves** |  | Aortic | ------- |
| Aortic valve | Annulus = 7mm | Tricuspid | ------- |
| Pulmonary valve | Annulus = 7mm | pulmonic | -------- |
| **Great arteries** | NRGA | **Aortic arch** | Left. No CoA. |
| Aorta | ----- | **PDA** | No |
| Pulmonary artery | Normal MPA and Branch PAs. |  |  |
| **M-Mode:** | | | |
| AO | mm | PWd | mm |
| LA | mm | PWs | mm |
| LVIDd | mm | EDV | ml |
| LVIDs | mm | ESV | ml |
| IVSs | mm | LVEF | % |
| IVSd | mm | FS | % |
| **Additional Information**: |  | | |
| No pericardial/Pleural effusion. | | | |
| **Final Diagnosis:** | | | |
| 1. Normal Echocardiography Study. | | | |
| **Remark**: | | | |
| **Recommendation**: | | | |
| SIGNATURE  Done by: Tesfaye T., Pediatrician, Pediatric Cardiologist _______________ 13/08/2015Eth.C | | | |

| Patient Name: **Hanna Aderaw**. Referring Institute: **Durbete PH**. SEX/ Age: **F/11years**. Date of Report: **13/08/15**.  Referral Diagnosis: **Migratory joint pain, SOB, Cough. AGH12.3465.** | | | | | | |
| --- | --- | --- | --- | --- | --- | --- |
| **Features** | **Finding** | | **Features** | | **Finding** | |
| **Profile** |  | | **Atria** | |  | |
| Abdominal situs | Solitus | | Left atrium | | Markedly Dilated | |
| Atrial Situs | Solitus | | Right atrium | | Dilated | |
| Cardiac position | Levocardia | | **Atrioventricular valves** | |  | |
| Systemic venous drainage | Normal. | | Mitral valve | | Annulus = 24mm. Thickened MVL | |
| Pulmonary venous drainage | Normal | | Tricuspid valve | | Annulus = 21mm | |
| Atrioventricular connection | Concordant | |  | | TAPSE = 20mm | |
| Ventriculoarterial connection | Concordant | | **Ventricles** | |  | |
| Ventricular loop | d-Loop | | Left ventricle | | Markedly Dilated | |
|  |  | | Right ventricle | | Dilated | |
| **Septae** |  | | **Coronary arteries** | | ----- | |
| Interventricular septum | Intact | | **Doppler Measurement** | |  | |
| Interatrial septum | Intact | | Mitral | | Severe MR, Holosystolic, Posterior projection, seen in two planes with jet velocity = 4.3mmHg. | |
| **Semilunar valves** |  | | Aortic | | ------- | |
| Aortic valve | Annulus = 16mm | | Tricuspid | | Moderate TR, PPG = 39mmHg. | |
| Pulmonary valve | Annulus = 18mm | | pulmonic | | Mild PR, PPG = 35mmHg | |
| **Great arteries** | NRGA | | **Aortic arch** | | Left. No CoA. | |
| Aorta | ----- | | **PDA** | | No | |
| Pulmonary artery | Normal | |  | |  | |
| **M-Mode:** | | | | | | |
| AO | | mm | | PWd | | mm |
| LA | | mm | | PWs | | mm |
| LVIDd | | mm | | EDV | | ml |
| LVIDs | | mm | | ESV | | ml |
| IVSs | | mm | | LVEF | | 60% |
| IVSd | | mm | | FS | | 32% |
| **Additional Information**: | |  | | | | |
| pericardial effusion with maximum depth of 4mm. | | | | | | |
| **Final Diagnosis:** | | | | | | |
| 1. {S, D, S} Levocardia. 2. All chambers Dilated 3. Thickened MVL 4. Severe MR 5. Moderate TR 6. Mild PR 7. Normal Biventricular Systolic Function | | | | | | |
| **Remark**: | | | | | | |
| SIGNATURE  Done by: Tesfaye T., Pediatrician, Pediatric Cardiologist _______________ 13/08/2015Eth.C | | | | | | |

| Patient Name: **Alene Delie**. Referring Institute: **FHRH**. SEX/ Age: **M/1year**. Date of Report: **13/08/15**.  Referral Diagnosis: **FB and Grunting + ? DS. AGH12.3466.** | | | |
| --- | --- | --- | --- |
| **Features** | **Finding** | **Features** | **Finding** |
| **Profile** |  | **Atria** |  |
| Abdominal situs | Solitus | Left atrium | Dilated |
| Atrial Situs | Solitus | Right atrium | More dilated |
| Cardiac position | Levocardia | **Atrioventricular valves** |  |
| Systemic venous drainage | Normal. | Mitral valve | Annulus = 14mm |
| Pulmonary venous drainage | Normal | Tricuspid valve | Annulus = 14mm |
| Atrioventricular connection | Concordant |  | TAPSE = 13mm |
| Ventriculoarterial connection | Concordant | **Ventricles** |  |
| Ventricular loop | d-Loop | Left ventricle | Dilated |
|  |  | Right ventricle | More dilated |
| **Septae** | Tongue of tissue in b/n the defects | **Coronary arteries** | ----- |
| Interventricular septum | 8mm Inlet VSD, L – R Shunt | **Doppler Measurement** |  |
| Interatrial septum | 13mm primum defect, L – R Shunt | Mitral | ----- |
| **Semilunar valves** |  | Aortic | ------- |
| Aortic valve | Annulus = 11mm | Tricuspid | Mild TR |
| Pulmonary valve | Annulus = 16mm | pulmonic | -------- |
| **Great arteries** | NRGA | **Aortic arch** | Left. No CoA. |
| Aorta | ----- | **PDA** | No |
| Pulmonary artery | Normal MPA and Branch PAs. |  |  |
| **M-Mode:**  Normal LV Function on eye balling | | | |
| AO | mm | PWd | mm |
| LA | mm | PWs | mm |
| LVIDd | mm | EDV | ml |
| LVIDs | mm | ESV | ml |
| IVSs | mm | LVEF | % |
| IVSd | mm | FS | % |
| **Additional Information**: |  | | |
| Pericardial effusion with maximum depth of 3mm. | | | |
| **Final Diagnosis:** | | | |
| 1. {S, D, S} Levocardia. 2. All chambers dilated 3. Intermediate AVSD, L – R Shunt 4. Mild TR 5. Pulmonary Hypertension 6. Normal Biventricular Systolic Function | | | |
| **Remark**: | | | |
| **Recommendation**: | | | |
| SIGNATURE  Done by: Tesfaye T., Pediatrician, Pediatric Cardiologist _______________ 13/08/2015Eth.C | | | |

| Patient Name: **Tadesse Gebrie**. Referring Institute: **Adinas GH**. SEX/ Age: **M/1year**. Date of Report: **13/08/15**.  Referral Diagnosis: **?Myocarditis (RD). AGH12.3467.** | | | |
| --- | --- | --- | --- |
| **Features** | **Finding** | **Features** | **Finding** |
| **Profile** |  | **Atria** |  |
| Abdominal situs | Solitus | Left atrium | Normal |
| Atrial Situs | Solitus | Right atrium | Normal |
| Cardiac position | Levocardia | **Atrioventricular valves** |  |
| Systemic venous drainage | Normal. | Mitral valve | Annulus = 12mm |
| Pulmonary venous drainage | Normal | Tricuspid valve | Annulus = 15mm |
| Atrioventricular connection | Concordant |  | TAPSE = mm |
| Ventriculoarterial connection | Concordant | **Ventricles** |  |
| Ventricular loop | d-Loop | Left ventricle | Normal |
|  |  | Right ventricle | Normal |
| **Septae** |  | **Coronary arteries** | ----- |
| Interventricular septum | Intact | **Doppler Measurement** |  |
| Interatrial septum | Intact | Mitral | ----- |
| **Semilunar valves** |  | Aortic | ------- |
| Aortic valve | Annulus = 12mm | Tricuspid | ------- |
| Pulmonary valve | Annulus = 13mm | pulmonic | -------- |
| **Great arteries** | NRGA | **Aortic arch** | Left. No CoA. |
| Aorta | ----- | **PDA** | No |
| Pulmonary artery | Normal MPA and Branch PAs. |  |  |
| **M-Mode:** | | | |
| AO | mm | PWd | mm |
| LA | mm | PWs | mm |
| LVIDd | mm | EDV | ml |
| LVIDs | mm | ESV | ml |
| IVSs | mm | LVEF | 73% |
| IVSd | mm | FS | 39% |
| **Additional Information**: |  | | |
| No pericardial/Pleural effusion. | | | |
| **Final Diagnosis:** | | | |
| 1. Normal Echocardiography Study. | | | |
| **Remark**: | | | |
| **Recommendation**: | | | |
| SIGNATURE  Done by: Tesfaye T., Pediatrician, Pediatric Cardiologist _______________ 13/08/2015Eth.C | | | |

| Patient Name: **Cherinet Bihon**. Referring Institute: **FHRH**. SEX/ Age: **M/13years**. Date of Report: **14/08/15**.  Referral Diagnosis: **IE Treatment Completion.** | | | |
| --- | --- | --- | --- |
| **Features** | **Finding** | **Features** | **Finding** |
| **Profile** |  | **Atria** |  |
| Abdominal situs | Solitus | Left atrium | Dilated |
| Atrial Situs | Solitus | Right atrium | Dilated |
| Cardiac position | Levocardia | **Atrioventricular valves** |  |
| Systemic venous drainage | Normal. | Mitral valve | Annulus = 24mm. Thickened MVL |
| Pulmonary venous drainage | Normal | Tricuspid valve | Annulus = 27mm |
| Atrioventricular connection | Concordant |  | TAPSE = 19mm |
| Ventriculoarterial connection | Concordant | **Ventricles** |  |
| Ventricular loop | d-Loop | Left ventricle | Dilated |
|  |  | Right ventricle | Dilated |
| **Septae** |  | **Coronary arteries** | ----- |
| Interventricular septum | Intact | **Doppler Measurement** |  |
| Interatrial septum | Intact | Mitral | Mild MR, Holosystolic, Posterior projection, seen in two planes with jet velocity = 4.2m/sec. |
| **Semilunar valves** |  | Aortic | Moderate AR, PHT = 312ms. |
| Aortic valve | Annulus = 21mm | Tricuspid | Mild TR, PPG= 25mmHg. |
| Pulmonary valve | Annulus = 23mm | pulmonic | -------- |
| **Great arteries** | NRGA | **Aortic arch** | Left. No CoA. |
| Aorta | ----- | **PDA** | No |
| Pulmonary artery | Normal. |  |  |
| **M-Mode:** | | | |
| AO | mm | PWd | mm |
| LA | mm | PWs | mm |
| LVIDd | mm | EDV | ml |
| LVIDs | mm | ESV | ml |
| IVSs | mm | LVEF | 53% |
| IVSd | mm | FS | 28% |
| **Additional Information**: |  | | |
| Pericardial effusion with maximum depth of 4mm on RV Side. | | | |
| **Final Diagnosis:** | | | |
| 1. {S, D, S} Levocardia. 2. All chambers dilated 3. Thickened MVL 4. Mild MR 5. Moderate AR 6. Mild TR 7. Mildly Reduced LV Systolic Function 8. Trace Pericardial effusion | | | |
| **Recommendation**: | | | |
| SIGNATURE  Done by: Tesfaye T., Pediatrician, Pediatric Cardiologist _______________ 14/08/2015Eth.C | | | |

| Patient Name: **Bereket Nibretu**. Referring Institute: **FHRH**. SEX/ Age: **M/4years**. Date of Report: **14/08/15**.  Referral Diagnosis: **ARF with Carditis. AGH12.3468.** | | | |
| --- | --- | --- | --- |
| **Features** | **Finding** | **Features** | **Finding** |
| **Profile** |  | **Atria** |  |
| Abdominal situs | Solitus | Left atrium | Normal |
| Atrial Situs | Solitus | Right atrium | Normal |
| Cardiac position | Levocardia | **Atrioventricular valves** |  |
| Systemic venous drainage | Normal. | Mitral valve | Annulus = 19mm |
| Pulmonary venous drainage | Normal | Tricuspid valve | Annulus = 20mm |
| Atrioventricular connection | Concordant |  | TAPSE = 18mm |
| Ventriculoarterial connection | Concordant | **Ventricles** |  |
| Ventricular loop | d-Loop | Left ventricle | Normal |
|  |  | Right ventricle | Normal |
| **Septae** |  | **Coronary arteries** | ----- |
| Interventricular septum | Intact | **Doppler Measurement** |  |
| Interatrial septum | Intact | Mitral | ----- |
| **Semilunar valves** |  | Aortic | ------- |
| Aortic valve | Annulus = 14mm | Tricuspid | ------- |
| Pulmonary valve | Annulus = 18mm | pulmonic | -------- |
| **Great arteries** | NRGA | **Aortic arch** | Left. No CoA. |
| Aorta | ----- | **PDA** | No |
| Pulmonary artery | Normal MPA and Branch PAs. |  |  |
| **M-Mode:** | | | |
| AO | mm | PWd | mm |
| LA | mm | PWs | mm |
| LVIDd | mm | EDV | ml |
| LVIDs | mm | ESV | ml |
| IVSs | mm | LVEF | 72% |
| IVSd | mm | FS | 40% |
| **Additional Information**: |  | | |
| No pericardial/Pleural effusion. | | | |
| **Final Diagnosis:** | | | |
| 1. Normal Echocardiography Study. | | | |
| **Remark**: Normal Echocardiography Study doesn’t rule out Acute Rheumatic Fever | | | |
| **Recommendation**: | | | |
| SIGNATURE  Done by: Tesfaye T., Pediatrician, Pediatric Cardiologist _______________ 14/08/2015Eth.C | | | |

| Patient Name: **Doctor Getachew**. Referring Institute: **FHRH**. SEX/ Age: **M/6 6/12**. Date of Report: **14/08/15**.  Referral Diagnosis: **Cough + Pyogenic Meningitis. AGH12.3469.** | | | |
| --- | --- | --- | --- |
| **Features** | **Finding** | **Features** | **Finding** |
| **Profile** |  | **Atria** |  |
| Abdominal situs | Solitus | Left atrium | Normal |
| Atrial Situs | Solitus | Right atrium | Normal |
| Cardiac position | Levocardia | **Atrioventricular valves** |  |
| Systemic venous drainage | Normal. | Mitral valve | Annulus = 20mm |
| Pulmonary venous drainage | Normal | Tricuspid valve | Annulus = 20mm |
| Atrioventricular connection | Concordant |  | TAPSE = 17mm |
| Ventriculoarterial connection | Concordant | **Ventricles** |  |
| Ventricular loop | d-Loop | Left ventricle | Normal |
|  |  | Right ventricle | Normal |
| **Septae** |  | **Coronary arteries** | ----- |
| Interventricular septum | Intact | **Doppler Measurement** |  |
| Interatrial septum | Intact | Mitral | ----- |
| **Semilunar valves** |  | Aortic | ------- |
| Aortic valve | Annulus = 15mm | Tricuspid | Trivial TR, PPG = 17mmHg |
| Pulmonary valve | Annulus = 17mm | pulmonic | -------- |
| **Great arteries** | NRGA | **Aortic arch** | Left. No CoA. |
| Aorta | ----- | **PDA** | No |
| Pulmonary artery | Normal MPA and Branch PAs. |  |  |
| **M-Mode:** | | | |
| AO | mm | PWd | mm |
| LA | mm | PWs | mm |
| LVIDd | mm | EDV | ml |
| LVIDs | mm | ESV | ml |
| IVSs | mm | LVEF | 67% |
| IVSd | mm | FS | 36% |
| **Additional Information**: |  | | |
| No pericardial/Pleural effusion. | | | |
| **Final Diagnosis:** | | | |
| 1. Normal Echocardiography Study. | | | |
| **Remark**: | | | |
| **Recommendation**: | | | |
| SIGNATURE  Done by: Tesfaye T., Pediatrician, Pediatric Cardiologist _______________ 14/08/2015Eth.C | | | |

| Patient Name: **Shashitu Kibret**. Referring Institute: **FHRH**. SEX/ Age: **F/9months**. Date of Report: **20/08/15**.  Referral Diagnosis: **RD + CHF.** | | | |
| --- | --- | --- | --- |
| **Features** | **Finding** | **Features** | **Finding** |
| **Profile** |  | **Atria** |  |
| Abdominal situs | Solitus | Left atrium | Dilated |
| Atrial Situs | Solitus | Right atrium | Normal |
| Cardiac position | Levocardia | **Atrioventricular valves** |  |
| Systemic venous drainage | Normal. | Mitral valve | Annulus = 18mm |
| Pulmonary venous drainage | Normal | Tricuspid valve | Annulus = 17mm |
| Atrioventricular connection | Concordant |  | TAPSE = 15mm |
| Ventriculoarterial connection | Concordant | **Ventricles** |  |
| Ventricular loop | d-Loop | Left ventricle | Dilated & Dysfunctional |
|  |  | Right ventricle | Normal |
| **Septae** |  | **Coronary arteries** | ----- |
| Interventricular septum | Intact | **Doppler Measurement** |  |
| Interatrial septum | Intact | Mitral | Moderate MR, Holosystolic, Posterior projection, seen in two planes with jet velocity = 3.8m/sec. |
| **Semilunar valves** |  | Aortic | ------- |
| Aortic valve | Annulus = 11mm | Tricuspid | ------- |
| Pulmonary valve | Annulus = 13mm | pulmonic | -------- |
| **Great arteries** | NRGA | **Aortic arch** | Left. No CoA. |
| Aorta | ----- | **PDA** | No |
| Pulmonary artery | Normal MPA and Branch PAs. | **Coronaries** | Proximal coronaries well visualized |
| **M-Mode:** | | | |
| AO | mm | PWd | mm |
| LA | mm | PWs | mm |
| LVIDd | mm | EDV | ml |
| LVIDs | mm | ESV | ml |
| IVSs | mm | LVEF | 37% |
| IVSd | mm | FS | 18% |
| **Additional Information**: |  | | |
| No pericardial/Pleural effusion. | | | |
| **Final Diagnosis:** | | | |
| 1. {S, D, S} Levocardia. 2. LA/LV Dilated 3. Moderate MR 4. Dysfunctional LV | | | |
| **Remark**: DCM is highly likely | | | |
| **Recommendation**: | | | |
| SIGNATURE  Done by: Tesfaye T., Pediatrician, Pediatric Cardiologist _______________ 20/08/2015Eth.C | | | |

| Patient Name: **Leul Melesse**. Referring Institute: **Warka PH**. SEX/ Age: **M/1 4/12**. Date of Report: **20/08/15**.  Referral Diagnosis: **Diaphoresis. AGH12.3471.** | | | |
| --- | --- | --- | --- |
| **Features** | **Finding** | **Features** | **Finding** |
| **Profile** |  | **Atria** |  |
| Abdominal situs | Solitus | Left atrium | Normal |
| Atrial Situs | Solitus | Right atrium | Normal |
| Cardiac position | Levocardia | **Atrioventricular valves** |  |
| Systemic venous drainage | Normal. | Mitral valve | Annulus = 16mm |
| Pulmonary venous drainage | Normal | Tricuspid valve | Annulus = 18mm |
| Atrioventricular connection | Concordant |  | TAPSE = 21mm |
| Ventriculoarterial connection | Concordant | **Ventricles** |  |
| Ventricular loop | d-Loop | Left ventricle | Normal |
|  |  | Right ventricle | Normal |
| **Septae** |  | **Coronary arteries** | ----- |
| Interventricular septum | 3mm PM VSD, Partially closed by STL, L – R Shunt | **Doppler Measurement** |  |
| Interatrial septum | PFO, L – R Shunt | Mitral | ----- |
| **Semilunar valves** |  | Aortic | ------- |
| Aortic valve | Annulus = 11mm | Tricuspid | Mild TR, STL Prolapsing through the VSD |
| Pulmonary valve | Annulus = 14mm | pulmonic | -------- |
| **Great arteries** | NRGA | **Aortic arch** | Left. No CoA. |
| Aorta | ----- | **PDA** | No |
| Pulmonary artery | Normal MPA and Branch PAs. |  |  |
| **M-Mode:** | | | |
| AO | mm | PWd | mm |
| LA | mm | PWs | mm |
| LVIDd | mm | EDV | ml |
| LVIDs | mm | ESV | ml |
| IVSs | mm | LVEF | 74% |
| IVSd | mm | FS | 41% |
| **Additional Information**: |  | | |
| No pericardial/Pleural effusion. | | | |
| **Final Diagnosis:** | | | |
| 1. {S, D, S} Levocardia. 2. PFO, L – R Shunt 3. Small PM VSD, Partially closed by STL, L – R Shunt 4. Normal Biventricular Systolic Function | | | |
| **Remark**: | | | |
| **Recommendation**: | | | |
| SIGNATURE  Done by: Tesfaye T., Pediatrician, Pediatric Cardiologist _______________ 20/08/2015Eth.C | | | |

| Patient Name: **Baby of Se’ada Issa**. Referring Institute: **Adinas GH**. SEX/ Age: **F/18hours**. Date of Report: **20/08/15**.  Referral Diagnosis: **G-IV LLSB MURMUR. AGH12.3472.** | | | |
| --- | --- | --- | --- |
| **Features** | **Finding** | **Features** | **Finding** |
| **Profile** |  | **Atria** |  |
| Abdominal situs | Solitus | Left atrium | Normal |
| Atrial Situs | Solitus | Right atrium | Normal |
| Cardiac position | Levocardia | **Atrioventricular valves** |  |
| Systemic venous drainage | Normal. | Mitral valve | Annulus = 13mm |
| Pulmonary venous drainage | Normal | Tricuspid valve | Annulus = 13mm |
| Atrioventricular connection | Concordant |  | TAPSE = mm |
| Ventriculoarterial connection | Concordant | **Ventricles** |  |
| Ventricular loop | d-Loop | Left ventricle | Normal |
|  |  | Right ventricle | Normal |
| **Septae** |  | **Coronary arteries** | ----- |
| Interventricular septum | Non-Restrictive Sub Truncal VSD, L – R Shunt | **Doppler Measurement** |  |
| Interatrial septum | 4mm OS ASD, L – R Shunt | Mitral | ----- |
| **Semilunar valves** |  | Aortic | ------- |
| Aortic valve | Truncus Arteriosus. MPA arises from the Truncus from left side. Shortly biforcates | Tricuspid | ------- |
| Pulmonary valve |  | pulmonic | -------- |
| **Great arteries** | NRGA | **Aortic arch** | Left. No CoA. |
| Aorta | ----- | **PDA** | No |
| Pulmonary artery | Normal MPA and Branch PAs. |  |  |
| **M-Mode:** | | | |
| AO | mm | PWd | mm |
| LA | mm | PWs | mm |
| LVIDd | mm | EDV | ml |
| LVIDs | mm | ESV | ml |
| IVSs | mm | LVEF | % |
| IVSd | mm | FS | % |
| **Additional Information**: |  | | |
| No pericardial/Pleural effusion. | | | |
| **Final Diagnosis:** | | | |
| 1. {S, D, S} Levocardia. 2. Truncus Arteriosus Type I | | | |
| **Remark**: | | | |
| **Recommendation**: | | | |
| SIGNATURE  Done by: Tesfaye T., Pediatrician, Pediatric Cardiologist _______________ 20/08/2015Eth.C | | | |

| Patient Name: **Debas Bimr**. Referring Institute: **FHRH**. SEX/ Age: **M/12years**. Date of Report: **20/08/15**.  Referral Diagnosis: **Palpitation. AGH12.3473.** | | | |
| --- | --- | --- | --- |
| **Features** | **Finding** | **Features** | **Finding** |
| **Profile** |  | **Atria** |  |
| Abdominal situs | Solitus | Left atrium | Dilated |
| Atrial Situs | Solitus | Right atrium | Normal |
| Cardiac position | Levocardia | **Atrioventricular valves** |  |
| Systemic venous drainage | Normal. | Mitral valve | Annulus = 22mm. Thickened MVL |
| Pulmonary venous drainage | Normal | Tricuspid valve | Annulus = 18mm |
| Atrioventricular connection | Concordant |  | TAPSE = 16mm |
| Ventriculoarterial connection | Concordant | **Ventricles** |  |
| Ventricular loop | d-Loop | Left ventricle | Dilated |
|  |  | Right ventricle | Normal |
| **Septae** |  | **Coronary arteries** | ----- |
| Interventricular septum | Intact | **Doppler Measurement** |  |
| Interatrial septum | Intact | Mitral | Moderate MR, Holosystolic, posterior projection, seen in two planes with jet velocity = 4.5m/sec. |
| **Semilunar valves** |  | Aortic | Mild AR, PHT = 527ms. |
| Aortic valve | Annulus = 17mm | Tricuspid | ------- |
| Pulmonary valve | Annulus = 21mm | pulmonic | -------- |
| **Great arteries** | NRGA | **Aortic arch** | Left. No CoA. |
| Aorta | ----- | **PDA** | No |
| Pulmonary artery | Normal |  |  |
| **M-Mode:** | | | |
| AO | mm | PWd | mm |
| LA | mm | PWs | mm |
| LVIDd | mm | EDV | ml |
| LVIDs | mm | ESV | ml |
| IVSs | mm | LVEF | 61% |
| IVSd | mm | FS | 32% |
| **Additional Information**: |  | | |
| No pericardial/Pleural effusion. | | | |
| **Final Diagnosis:** | | | |
| 1. {S, D, S} Levocardia. 2. LA/LV Dilated + Thickened MVL 3. Moderate MR 4. Mild AR 5. Normal Biventricular Systolic Function | | | |
| **Remark**: | | | |
| **Recommendation**: | | | |
| SIGNATURE  Done by: Tesfaye T., Pediatrician, Pediatric Cardiologist _______________ 20/08/2015Eth.C | | | |

| Patient Name: **Mekides Mihret**. Referring Institute: **TGSH**. SEX/ Age: **F/64days**. Date of Report: **20/08/15**.  Referral Diagnosis: **DS. AGH12.3474.** | | | |
| --- | --- | --- | --- |
| **Features** | **Finding** | **Features** | **Finding** |
| **Profile** |  | **Atria** |  |
| Abdominal situs | Solitus | Left atrium | Normal |
| Atrial Situs | Solitus | Right atrium | Normal |
| Cardiac position | Levocardia | **Atrioventricular valves** |  |
| Systemic venous drainage | Normal. | Mitral valve | Annulus = 13mm |
| Pulmonary venous drainage | Normal | Tricuspid valve | Annulus = 14mm |
| Atrioventricular connection | Concordant |  | TAPSE = mm |
| Ventriculoarterial connection | Concordant | **Ventricles** |  |
| Ventricular loop | d-Loop | Left ventricle | Normal |
|  |  | Right ventricle | Normal |
| **Septae** | Tongue of tissue in b/n the AV Valves | **Coronary arteries** | ----- |
| Interventricular septum | 8mm Inlet VSD, L – R Shunt | **Doppler Measurement** |  |
| Interatrial septum | 6mm OS ASD, L – R Shunt. 7mm primum defect, L – R Shunt | Mitral | Mild MR |
| **Semilunar valves** |  | Aortic | ------- |
| Aortic valve | Annulus = 7mm | Tricuspid | Mild TR |
| Pulmonary valve | Annulus = 12mm | pulmonic | -------- |
| **Great arteries** | NRGA | **Aortic arch** | Left. No CoA. |
| Aorta | ----- | **PDA** | No |
| Pulmonary artery | Normal MPA and Branch PAs. |  |  |
| **M-Mode:** | | | |
| AO | mm | PWd | mm |
| LA | mm | PWs | mm |
| LVIDd | mm | EDV | ml |
| LVIDs | mm | ESV | ml |
| IVSs | mm | LVEF | % |
| IVSd | mm | FS | % |
| **Additional Information**: |  | | |
| No pericardial/Pleural effusion. | | | |
| **Final Diagnosis:** | | | |
| 1. {S, D, S} Levocardia. 2. Intermediate AVSD, L – R Shunt 3. Additional Small OS ASD, L – R Shunt | | | |
| **Remark**: | | | |
| **Recommendation**: | | | |
| SIGNATURE  Done by: Tesfaye T., Pediatrician, Pediatric Cardiologist _______________ 20/08/2015Eth.C | | | |

| Patient Name: **Gedam Amare**. Referring Institute: **Injibara GH**. SEX/ Age: **F/6years**. Date of Report: **20/08/15**.  Referral Diagnosis: **Palpitation. AGH12.3475.** | | | |
| --- | --- | --- | --- |
| **Features** | **Finding** | **Features** | **Finding** |
| **Profile** |  | **Atria** |  |
| Abdominal situs | Solitus | Left atrium | Dilated |
| Atrial Situs | Solitus | Right atrium | Normal |
| Cardiac position | Levocardia | **Atrioventricular valves** |  |
| Systemic venous drainage | Normal. | Mitral valve | Annulus = 25mm |
| Pulmonary venous drainage | Normal | Tricuspid valve | Annulus = 21mm |
| Atrioventricular connection | Concordant |  | TAPSE = 21mm |
| Ventriculoarterial connection | Concordant | **Ventricles** |  |
| Ventricular loop | d-Loop | Left ventricle | Dilated |
|  |  | Right ventricle | Normal |
| **Septae** |  | **Coronary arteries** | ----- |
| Interventricular septum | Intact | **Doppler Measurement** |  |
| Interatrial septum | Intact | Mitral | ----- |
| **Semilunar valves** |  | Aortic | ------- |
| Aortic valve | Annulus = 17mm | Tricuspid | ------- |
| Pulmonary valve | Annulus = 22mm | pulmonic | -------- |
| **Great arteries** | NRGA | **Aortic arch** | Left. No CoA. |
| Aorta | ----- | **PDA** | 4.5mm PDA, L – R Shunt |
| Pulmonary artery | MPA = 23mm. |  |  |
| **M-Mode:** | | | |
| AO | mm | PWd | mm |
| LA | mm | PWs | mm |
| LVIDd | mm | EDV | ml |
| LVIDs | mm | ESV | ml |
| IVSs | mm | LVEF | 67% |
| IVSd | mm | FS | 37% |
| **Additional Information**: |  | | |
| No pericardial/Pleural effusion. | | | |
| **Final Diagnosis:** | | | |
| 1. {S, D, S} Levocardia. 2. LA/LV Dilated 3. Large PDA, L – R Shunt 4. Normal Biventricular Systolic Function | | | |
| **Remark**: | | | |
| **Recommendation**: | | | |
| SIGNATURE  Done by: Tesfaye T., Pediatrician, Pediatric Cardiologist _______________ 20/08/2015Eth.C | | | |

| Patient Name: **Yohannes Minilik**. Referring Institute: **Pawe Hospital**. SEX/ Age: **M/4 6/12**. Date of Report: **20/08/15**.  Referral Diagnosis: **Palpitation. AGH12.3476.** | | | |
| --- | --- | --- | --- |
| **Features** | **Finding** | **Features** | **Finding** |
| **Profile** |  | **Atria** |  |
| Abdominal situs | Solitus | Left atrium | Normal |
| Atrial Situs | Solitus | Right atrium | Dilated |
| Cardiac position | Levocardia | **Atrioventricular valves** |  |
| Systemic venous drainage | Normal. | Mitral valve | Annulus = mm |
| Pulmonary venous drainage | Normal | Tricuspid valve | Annulus = mm |
| Atrioventricular connection | Concordant |  | TAPSE = 25mm |
| Ventriculoarterial connection | Concordant | **Ventricles** |  |
| Ventricular loop | d-Loop | Left ventricle | Normal |
|  |  | Right ventricle | Dilated |
| **Septae** |  | **Coronary arteries** | ----- |
| Interventricular septum | 2.5mm Inlet VSD, L – R Shunt | **Doppler Measurement** |  |
| Interatrial septum | 31mm Primum defect, L – R Shunt | Mitral | Moderate MR |
| **Semilunar valves** |  | Aortic | ------- |
| Aortic valve | Annulus = 15mm | Tricuspid | Moderate TR |
| Pulmonary valve | Annulus = 20mm. Doming PV | pulmonic | Mild PS, PPG = 26mmHg |
| **Great arteries** | NRGA | **Aortic arch** | Left. No CoA. |
| Aorta | ----- | **PDA** | No |
| Pulmonary artery | Normal. |  |  |
| **M-Mode:** | | | |
| AO | mm | PWd | mm |
| LA | mm | PWs | mm |
| LVIDd | mm | EDV | ml |
| LVIDs | mm | ESV | ml |
| IVSs | mm | LVEF | % |
| IVSd | mm | FS | % |
| **Additional Information**: |  | | |
| No pericardial/Pleural effusion. | | | |
| **Final Diagnosis:** | | | |
| 1. {S, D, S} Levocardia. 2. RA/RV Dilated 3. Transitional AVSD, L – R Shunt 4. Doming PV 5. Mild Valvular PS 6. Normal Biventricular Systolic Function | | | |
| SIGNATURE  Done by: Tesfaye T., Pediatrician, Pediatric Cardiologist _______________ 20/08/2015Eth.C | | | |

| Patient Name: **Baby of Serkalem Setie**. Referring Institute: **The Cure Hospital**. SEX/ Age: **M/1 3/12**. Date of Report: **20/08/15**. Referral Diagnosis: **Syndromic (Bilateral radial Deficiency)_. AGH12.3477.** | | | |
| --- | --- | --- | --- |
| **Features** | **Finding** | **Features** | **Finding** |
| **Profile** |  | **Atria** |  |
| Abdominal situs | Solitus | Left atrium | Normal |
| Atrial Situs | Solitus | Right atrium | Normal |
| Cardiac position | Levocardia | **Atrioventricular valves** |  |
| Systemic venous drainage | Normal. | Mitral valve | Annulus = 16mm |
| Pulmonary venous drainage | Normal | Tricuspid valve | Annulus = 16mm |
| Atrioventricular connection | Concordant |  | TAPSE = mm |
| Ventriculoarterial connection | Concordant | **Ventricles** |  |
| Ventricular loop | d-Loop | Left ventricle | Normal |
|  |  | Right ventricle | Normal |
| **Septae** |  | **Coronary arteries** | ----- |
| Interventricular septum | Intact | **Doppler Measurement** |  |
| Interatrial septum | Intact | Mitral | ----- |
| **Semilunar valves** |  | Aortic | ------- |
| Aortic valve | Annulus = 14mm | Tricuspid | ------- |
| Pulmonary valve | Annulus = 16mm | pulmonic | -------- |
| **Great arteries** | NRGA | **Aortic arch** | Left. No CoA. |
| Aorta | ----- | **PDA** | 1.5mm PDA, L – R Shunt |
| Pulmonary artery | Normal MPA and Branch PAs. |  |  |
| **M-Mode:**  Normal LV Function on eye balling | | | |
| AO | mm | PWd | mm |
| LA | mm | PWs | mm |
| LVIDd | mm | EDV | ml |
| LVIDs | mm | ESV | ml |
| IVSs | mm | LVEF | % |
| IVSd | mm | FS | % |
| **Additional Information**: |  | | |
| No pericardial/Pleural effusion. | | | |
| **Final Diagnosis:** | | | |
| 1. {S, D, S} Levocardia. 2. Small PDA, L – R Shunt | | | |
| **Remark**: | | | |
| **Recommendation**: | | | |
| SIGNATURE  Done by: Tesfaye T., Pediatrician, Pediatric Cardiologist _______________ 20/08/2015Eth.C | | | |

| Patient Name: **Dagim Nigussie**. Referring Institute: **FHRH**. SEX/ Age: **M/5months**. Date of Report: **20/08/15**.  Referral Diagnosis: **Fast Breathing and Grunting. AGH12.3478.** | | | |
| --- | --- | --- | --- |
| **Features** | **Finding** | **Features** | **Finding** |
| **Profile** |  | **Atria** |  |
| Abdominal situs | Solitus | Left atrium | Normal |
| Atrial Situs | Solitus | Right atrium | Normal |
| Cardiac position | Levocardia | **Atrioventricular valves** |  |
| Systemic venous drainage | Normal. | Mitral valve | Annulus = 11mm |
| Pulmonary venous drainage | Normal | Tricuspid valve | Annulus = 11mm |
| Atrioventricular connection | Concordant |  | TAPSE = 15mm |
| Ventriculoarterial connection | Concordant | **Ventricles** |  |
| Ventricular loop | d-Loop | Left ventricle | Normal |
|  |  | Right ventricle | Normal |
| **Septae** |  | **Coronary arteries** | ----- |
| Interventricular septum | Intact | **Doppler Measurement** |  |
| Interatrial septum | Intact | Mitral | ----- |
| **Semilunar valves** |  | Aortic | ------- |
| Aortic valve | Annulus = 10mm | Tricuspid | ------- |
| Pulmonary valve | Annulus = 11mm | pulmonic | -------- |
| **Great arteries** | NRGA | **Aortic arch** | Left. No CoA. |
| Aorta | ----- | **PDA** | No |
| Pulmonary artery | Normal MPA and Branch PAs. |  |  |
| **M-Mode:**  Normal LV Function on eye balling | | | |
| AO | mm | PWd | mm |
| LA | mm | PWs | mm |
| LVIDd | mm | EDV | ml |
| LVIDs | mm | ESV | ml |
| IVSs | mm | LVEF | % |
| IVSd | mm | FS | % |
| **Additional Information**: |  | | |
| No pericardial/Pleural effusion. | | | |
| **Final Diagnosis:** | | | |
| 1. Normal Echocardiography Study. | | | |
| **Remark**: | | | |
| **Recommendation**: | | | |
| SIGNATURE  Done by: Tesfaye T., Pediatrician, Pediatric Cardiologist _______________ 20/08/2015Eth.C | | | |

| Patient Name: **Gebre-Kidan Adane**. Referring Institute: **FHRH**. SEX/ Age: **M/4years**. Date of Report: **20/08/15**.  Referral Diagnosis: **FB + GRUNTING + SOB. AGH12.3479.** | | | |
| --- | --- | --- | --- |
| **Features** | **Finding** | **Features** | **Finding** |
| **Profile** |  | **Atria** |  |
| Abdominal situs | Solitus | Left atrium | Dilated |
| Atrial Situs | Solitus | Right atrium | Normal |
| Cardiac position | Levocardia | **Atrioventricular valves** |  |
| Systemic venous drainage | Normal. | Mitral valve | Annulus = 25mm. Patulous MVL. |
| Pulmonary venous drainage | Normal | Tricuspid valve | Annulus = 20mm |
| Atrioventricular connection | Concordant |  | TAPSE = mm |
| Ventriculoarterial connection | Concordant | **Ventricles** |  |
| Ventricular loop | d-Loop | Left ventricle | Dilated |
|  |  | Right ventricle | Normal |
| **Septae** |  | **Coronary arteries** | ----- |
| Interventricular septum | Intact | **Doppler Measurement** |  |
| Interatrial septum | Intact | Mitral | Severe MR, Holosystolic, posterior projection, seen in two planes with jet velocity = 4.3m/sec |
| **Semilunar valves** |  | Aortic | ------- |
| Aortic valve | Annulus = 13mm | Tricuspid | ------- |
| Pulmonary valve | Annulus = 16mm | pulmonic | -------- |
| **Great arteries** | NRGA | **Aortic arch** | Left. No CoA. |
| Aorta | ----- | **PDA** | No |
| Pulmonary artery | Normal MPA and Branch PAs. |  |  |
| **M-Mode:** | | | |
| AO | mm | PWd | mm |
| LA | mm | PWs | mm |
| LVIDd | mm | EDV | ml |
| LVIDs | mm | ESV | ml |
| IVSs | mm | LVEF | 67% |
| IVSd | mm | FS | 37% |
| **Additional Information**: |  | | |
| No pericardial/Pleural effusion. | | | |
| **Final Diagnosis:** | | | |
| 1. {S, D, S} Levocardia. 2. LA/LV Dilated 3. Patulous MVL 4. Severe MR | | | |
| **Remark**: Rheumatic Carditis is top DDx. | | | |
| **Recommendation**: | | | |
| SIGNATURE  Done by: Tesfaye T., Pediatrician, Pediatric Cardiologist _______________ 20/08/2015Eth.C | | | |

| Patient Name: **Bethelihem Agmas**. Referring Institute: **FHRH**. SEX/ Age: **F/10 8/12**. Date of Report: **20/08/15**.  Referral Diagnosis: **Palpitation + Easy Fatigability. AGH12.3480.** | | | |
| --- | --- | --- | --- |
| **Features** | **Finding** | **Features** | **Finding** |
| **Profile** |  | **Atria** |  |
| Abdominal situs | Solitus | Left atrium | Normal |
| Atrial Situs | Solitus | Right atrium | Normal |
| Cardiac position | Levocardia | **Atrioventricular valves** |  |
| Systemic venous drainage | Normal. | Mitral valve | Annulus = 23mm |
| Pulmonary venous drainage | Normal | Tricuspid valve | Annulus = 23mm |
| Atrioventricular connection | Concordant |  | TAPSE = 21mm |
| Ventriculoarterial connection | Concordant | **Ventricles** |  |
| Ventricular loop | d-Loop | Left ventricle | Normal |
|  |  | Right ventricle | Normal |
| **Septae** |  | **Coronary arteries** | ----- |
| Interventricular septum | Intact | **Doppler Measurement** |  |
| Interatrial septum | Intact | Mitral | ----- |
| **Semilunar valves** |  | Aortic | ------- |
| Aortic valve | Annulus = 18mm | Tricuspid | ------- |
| Pulmonary valve | Annulus = 22mm | pulmonic | -------- |
| **Great arteries** | NRGA | **Aortic arch** | Left. No CoA. |
| Aorta | ----- | **PDA** | No |
| Pulmonary artery | Normal MPA and Branch PAs. |  |  |
| **M-Mode:**  Normal LV Function on eye balling | | | |
| AO | mm | PWd | mm |
| LA | mm | PWs | mm |
| LVIDd | mm | EDV | ml |
| LVIDs | mm | ESV | ml |
| IVSs | mm | LVEF | % |
| IVSd | mm | FS | % |
| **Additional Information**: |  | | |
| No pericardial/Pleural effusion. | | | |
| **Final Diagnosis:** | | | |
| 1. Normal Echocardiography Study. | | | |
| **Remark**: | | | |
| **Recommendation**: | | | |
| SIGNATURE  Done by: Tesfaye T., Pediatrician, Pediatric Cardiologist _______________ 20/08/2015Eth.C | | | |

| Patient Name: **Edilawit Mulugeta**. Referring Institute: **Adinas GH**. SEX/ Age: **F/11 4/12**. Date of Report: **20/08/15**.  Referral Diagnosis: **Follow up echo for Sydenham’s Chorea recurrence.** | | | |
| --- | --- | --- | --- |
| **Features** | **Finding** | **Features** | **Finding** |
| **Profile** |  | **Atria** |  |
| Abdominal situs | Solitus | Left atrium | Normal |
| Atrial Situs | Solitus | Right atrium | Normal |
| Cardiac position | Levocardia | **Atrioventricular valves** |  |
| Systemic venous drainage | Normal. | Mitral valve | Annulus = 25mm. Patulous MVL |
| Pulmonary venous drainage | Normal | Tricuspid valve | Annulus = 23mm |
| Atrioventricular connection | Concordant |  | TAPSE = 21mm |
| Ventriculoarterial connection | Concordant | **Ventricles** |  |
| Ventricular loop | d-Loop | Left ventricle | Normal |
|  |  | Right ventricle | Normal |
| **Septae** |  | **Coronary arteries** | ----- |
| Interventricular septum | Intact | **Doppler Measurement** |  |
| Interatrial septum | Intact | Mitral | Mild MR, Holosystolic, Posterior projection, seen in two planes with jet velocity = 3m/sec. |
| **Semilunar valves** |  | Aortic | ------- |
| Aortic valve | Annulus = 18mm | Tricuspid | ------- |
| Pulmonary valve | Annulus = 20mm | pulmonic | -------- |
| **Great arteries** | NRGA | **Aortic arch** | Left. No CoA. |
| Aorta | ----- | **PDA** | No |
| Pulmonary artery | Normal |  |  |
| **M-Mode:**  Normal LV Function on eye balling | | | |
| AO | mm | PWd | mm |
| LA | mm | PWs | mm |
| LVIDd | mm | EDV | ml |
| LVIDs | mm | ESV | ml |
| IVSs | mm | LVEF | % |
| IVSd | mm | FS | % |
| **Additional Information**: |  | | |
| No pericardial/Pleural effusion. | | | |
| **Final Diagnosis:** | | | |
| 1. {S, D, S} Levocardia. 2. Patulous MVL 3. Mild MR | | | |
| **Remark**: | | | |
| **Recommendation**: | | | |
| SIGNATURE  Done by: Tesfaye T., Pediatrician, Pediatric Cardiologist _______________ 20/08/2015Eth.C | | | |

| Patient Name: **Abayneh Hone**. Referring Institute: **TGSH**. SEX/ Age: **M/10years**. Date of Report: **26/08/15**.  Referral Diagnosis: **Incidental Murmur. AGH12.3481.** | | | |
| --- | --- | --- | --- |
| **Features** | **Finding** | **Features** | **Finding** |
| **Profile** |  | **Atria** |  |
| Abdominal situs | Solitus | Left atrium | Normal |
| Atrial Situs | Solitus | Right atrium | Normal |
| Cardiac position | Levocardia | **Atrioventricular valves** |  |
| Systemic venous drainage | Normal. | Mitral valve | Annulus = 20mm. Patulous MVL |
| Pulmonary venous drainage | Normal | Tricuspid valve | Annulus = 21mm |
| Atrioventricular connection | Concordant |  | TAPSE = 22mm |
| Ventriculoarterial connection | Concordant | **Ventricles** |  |
| Ventricular loop | d-Loop | Left ventricle | Normal |
|  |  | Right ventricle | Normal |
| **Septae** |  | **Coronary arteries** | ----- |
| Interventricular septum | Intact | **Doppler Measurement** |  |
| Interatrial septum | Intact | Mitral | Trivial MR, Incomplete Signal, seen in two planes with jet velocity = 2.6m/sec. |
| **Semilunar valves** |  | Aortic | ------- |
| Aortic valve | Annulus = 17mm | Tricuspid | ------- |
| Pulmonary valve | Annulus = 19mm | pulmonic | -------- |
| **Great arteries** | NRGA | **Aortic arch** | Left. No CoA. |
| Aorta | ----- | **PDA** | No |
| Pulmonary artery | Normal MPA and Branch PAs. |  |  |
| **M-Mode:**  Normal LV Function on eye balling | | | |
| AO | mm | PWd | mm |
| LA | mm | PWs | mm |
| LVIDd | mm | EDV | ml |
| LVIDs | mm | ESV | ml |
| IVSs | mm | LVEF | % |
| IVSd | mm | FS | % |
| **Additional Information**: |  | | |
| No pericardial/Pleural effusion. | | | |
| **Final Diagnosis:** | | | |
| 1. {S, D, S} Levocardia. 2. Patulous MVL 3. Trivial MR | | | |
| **Remark**: Consider as Borderline RHD | | | |
| **Recommendation**: Start secondary Prophylaxis and see after a year to decide | | | |
| SIGNATURE  Done by: Tesfaye T., Pediatrician, Pediatric Cardiologist _______________ 26/08/2015Eth.C | | | |

| Patient Name: **Tsion Abebe**. Referring Institute: **Amaris PSC**. SEX/ Age: **F/8 10/12years**. Date of Report: **26/08/15**.  Referral Diagnosis: **Follow up for RHD.** | | | |
| --- | --- | --- | --- |
| **Features** | **Finding** | **Features** | **Finding** |
| **Profile** |  | **Atria** |  |
| Abdominal situs | Solitus | Left atrium | Normal |
| Atrial Situs | Solitus | Right atrium | Normal |
| Cardiac position | Levocardia | **Atrioventricular valves** |  |
| Systemic venous drainage | Normal. | Mitral valve | Annulus = 24mm. Patulous, Thickened MVL. 8mm displacement to LA. |
| Pulmonary venous drainage | Normal | Tricuspid valve | Annulus = 20mm |
| Atrioventricular connection | Concordant |  | TAPSE = 20mm |
| Ventriculoarterial connection | Concordant | **Ventricles** |  |
| Ventricular loop | d-Loop | Left ventricle | Normal |
|  |  | Right ventricle | Normal |
| **Septae** |  | **Coronary arteries** | ----- |
| Interventricular septum | Intact | **Doppler Measurement** |  |
| Interatrial septum | Intact | Mitral | Mild MR, Incomplete signal, seen in two planes with jet velocity = 3.9m/sec. |
| **Semilunar valves** |  | Aortic | ------- |
| Aortic valve | Annulus = 17mm | Tricuspid | ------- |
| Pulmonary valve | Annulus = 18mm | pulmonic | -------- |
| **Great arteries** | NRGA | **Aortic arch** | Left. No CoA. |
| Aorta | ----- | **PDA** | No |
| Pulmonary artery | Normal |  |  |
| **M-Mode:** | | | |
| AO | mm | PWd | mm |
| LA | mm | PWs | mm |
| LVIDd | mm | EDV | ml |
| LVIDs | mm | ESV | ml |
| IVSs | mm | LVEF | % |
| IVSd | mm | FS | % |
| **Additional Information**: |  | | |
| No pericardial/Pleural effusion. | | | |
| **Final Diagnosis:** | | | |
| 1. {S, D, S} Levocardia. 2. Patulous, Thickened MVL 3. Mild MR 4. Normal Biventricular Systolic Function | | | |
| **Remark**: MVP is a highly likely DDx | | | |
| **Recommendation**: Continue Secondary Prophylaxis | | | |
| SIGNATURE  Done by: Tesfaye T., Pediatrician, Pediatric Cardiologist _______________ 26/08/2015Eth.C | | | |

| Patient Name: **Misganaw Agegnew**. Referring Institute: **TGSH**. SEX/ Age: **M/1 10/12**. Date of Report: **26/08/15**.  Referral Diagnosis: **DS + FTT + Diaphoresis + murmur. AGH12.3482.** | | | |
| --- | --- | --- | --- |
| **Features** | **Finding** | **Features** | **Finding** |
| **Profile** |  | **Atria** |  |
| Abdominal situs | Solitus | Left atrium | Normal |
| Atrial Situs | Solitus | Right atrium | Dilated |
| Cardiac position | Levocardia | **Atrioventricular valves** |  |
| Systemic venous drainage | Normal. | Mitral valve | Annulus = 14mm |
| Pulmonary venous drainage | Normal | Tricuspid valve | Annulus = 17mm |
| Atrioventricular connection | Concordant |  | TAPSE = mm |
| Ventriculoarterial connection | Concordant | **Ventricles** |  |
| Ventricular loop | d-Loop | Left ventricle | Normal |
|  |  | Right ventricle | Dilated |
| **Septae** | Tongue of tissue in b/n the defects. | **Coronary arteries** | ----- |
| Interventricular septum | 10mm Inlet VSD, L – R Shunt | **Doppler Measurement** |  |
| Interatrial septum | 9mm Inlet VSD, L – R Shunt | Mitral | ----- |
| **Semilunar valves** |  | Aortic | ------- |
| Aortic valve | Annulus = 13mm | Tricuspid | ------- |
| Pulmonary valve | Annulus = 16mm | pulmonic | -------- |
| **Great arteries** | NRGA | **Aortic arch** | Left. No CoA. |
| Aorta | ----- | **PDA** | No |
| Pulmonary artery | Normal |  |  |
| **M-Mode:**  Normal LV Function on eye balling | | | |
| AO | mm | PWd | mm |
| LA | mm | PWs | mm |
| LVIDd | mm | EDV | ml |
| LVIDs | mm | ESV | ml |
| IVSs | mm | LVEF | % |
| IVSd | mm | FS | % |
| **Additional Information**: |  | | |
| Pericardial effusion on RV Side with maximum depth of 10mm. | | | |
| **Final Diagnosis:** | | | |
| 1. {S, D, S} Levocardia. 2. RA/RV Dilated 3. Intermediate AVSD, L – R Shunt 4. Normal LV Systolic LV Function | | | |
| **Remark**: | | | |
| **Recommendation**: | | | |
| SIGNATURE  Done by: Tesfaye T., Pediatrician, Pediatric Cardiologist _______________ 26/08/2015Eth.C | | | |

| Patient Name: **Bezawit Biadgelign**. Referring Institute: **APSC**. SEX/ Age: **F/1 11/12**. Date of Report: **26/08/15**.  Referral Diagnosis: **CHF. AGH12.3483.** | | | |
| --- | --- | --- | --- |
| **Features** | **Finding** | **Features** | **Finding** |
| **Profile** |  | **Atria** |  |
| Abdominal situs | Solitus | Left atrium | Normal |
| Atrial Situs | Solitus | Right atrium | Normal |
| Cardiac position | Levocardia | **Atrioventricular valves** |  |
| Systemic venous drainage | Normal. | Mitral valve | Annulus = 17mm |
| Pulmonary venous drainage | Normal | Tricuspid valve | Annulus = 16mm |
| Atrioventricular connection | Concordant |  | TAPSE = mm |
| Ventriculoarterial connection | Concordant | **Ventricles** |  |
| Ventricular loop | d-Loop | Left ventricle | Normal |
|  |  | Right ventricle | Normal |
| **Septae** |  | **Coronary arteries** | ----- |
| Interventricular septum | Intact | **Doppler Measurement** |  |
| Interatrial septum | 5mm OS ASD, L – R Shunt | Mitral | ----- |
| **Semilunar valves** |  | Aortic | ------- |
| Aortic valve | Annulus = 14mm | Tricuspid | ------- |
| Pulmonary valve | Annulus = 15mm | pulmonic | -------- |
| **Great arteries** | NRGA | **Aortic arch** | Left. No CoA. |
| Aorta | ----- | **PDA** | No |
| Pulmonary artery | Normal MPA and Branch PAs. |  |  |
| **M-Mode:** | | | |
| AO | mm | PWd | mm |
| LA | mm | PWs | mm |
| LVIDd | mm | EDV | ml |
| LVIDs | mm | ESV | ml |
| IVSs | mm | LVEF | 65% |
| IVSd | mm | FS | 34% |
| **Additional Information**: |  | | |
| No pericardial/Pleural effusion. | | | |
| **Final Diagnosis:** | | | |
| 1. {S, D, S} Levocardia. 2. Small OS ASD, L – R Shunt | | | |
| **Remark**: There seems intra-abdominal mass (Right Side, ? Hepatic ? supra renal) | | | |
| **Recommendation**: Have abdominal imaging | | | |
| SIGNATURE  Done by: Tesfaye T., Pediatrician, Pediatric Cardiologist _______________ 26/08/2015Eth.C | | | |

| Patient Name: **Bitanya Abrham**. Referring Institute: **FHRH**. SEX/ Age: **F/12years**. Date of Report: **26/08/15**.  Referral Diagnosis: **Palpitation + ?Syncope. AGH12.3484.** | | | |
| --- | --- | --- | --- |
| **Features** | **Finding** | **Features** | **Finding** |
| **Profile** |  | **Atria** |  |
| Abdominal situs | Solitus | Left atrium | Normal |
| Atrial Situs | Solitus | Right atrium | Normal |
| Cardiac position | Levocardia | **Atrioventricular valves** |  |
| Systemic venous drainage | Normal. | Mitral valve | Annulus = 22mm |
| Pulmonary venous drainage | Normal | Tricuspid valve | Annulus = 23mm |
| Atrioventricular connection | Concordant |  | TAPSE = 20mm |
| Ventriculoarterial connection | Concordant | **Ventricles** |  |
| Ventricular loop | d-Loop | Left ventricle | Normal |
|  |  | Right ventricle | Normal |
| **Septae** |  | **Coronary arteries** | ----- |
| Interventricular septum | Intact | **Doppler Measurement** |  |
| Interatrial septum | Intact | Mitral | ----- |
| **Semilunar valves** |  | Aortic | ------- |
| Aortic valve | Annulus = 18mm | Tricuspid | ------- |
| Pulmonary valve | Annulus = 21mm | pulmonic | -------- |
| **Great arteries** | NRGA | **Aortic arch** | Left. No CoA. |
| Aorta | ----- | **PDA** | No |
| Pulmonary artery | Normal MPA and Branch PAs. |  |  |
| **M-Mode:**  Normal LV Function on eye balling | | | |
| AO | mm | PWd | mm |
| LA | mm | PWs | mm |
| LVIDd | mm | EDV | ml |
| LVIDs | mm | ESV | ml |
| IVSs | mm | LVEF | % |
| IVSd | mm | FS | % |
| **Additional Information**: |  | | |
| No pericardial/Pleural effusion. | | | |
| **Final Diagnosis:** | | | |
| 1. Normal Echocardiography Study. | | | |
| **Remark**: | | | |
| **Recommendation**: | | | |
| SIGNATURE  Done by: Tesfaye T., Pediatrician, Pediatric Cardiologist _______________ 26/08/2015Eth.C | | | |

| Patient Name: **Amen Alula**. Referring Institute: **FHRH**. SEX/ Age: **M/4months**. Date of Report: **26/08/15**.  Referral Diagnosis: **Down Syndrome. AGH12.3485.** | | | |
| --- | --- | --- | --- |
| **Features** | **Finding** | **Features** | **Finding** |
| **Profile** |  | **Atria** |  |
| Abdominal situs | Solitus | Left atrium | Normal |
| Atrial Situs | Solitus | Right atrium | Normal |
| Cardiac position | Levocardia | **Atrioventricular valves** |  |
| Systemic venous drainage | Normal. | Mitral valve | Annulus = 14mm |
| Pulmonary venous drainage | Normal | Tricuspid valve | Annulus = 15mm |
| Atrioventricular connection | Concordant |  | TAPSE = mm |
| Ventriculoarterial connection | Concordant | **Ventricles** |  |
| Ventricular loop | d-Loop | Left ventricle | Normal |
|  |  | Right ventricle | Normal |
| **Septae** |  | **Coronary arteries** | ----- |
| Interventricular septum | 5mm Sub-aortic VSD, L – R Shunt | **Doppler Measurement** |  |
| Interatrial septum | Intact | Mitral | ----- |
| **Semilunar valves** |  | Aortic | ------- |
| Aortic valve | Annulus = 12mm | Tricuspid | ------- |
| Pulmonary valve | Annulus = 11mm | pulmonic | -------- |
| **Great arteries** | NRGA | **Aortic arch** | Left. No CoA. |
| Aorta | ----- | **PDA** | 1mm PDA, L – R Shunt |
| Pulmonary artery | Normal MPA and Branch PAs. |  |  |
| **M-Mode:** | | | |
| AO | mm | PWd | mm |
| LA | mm | PWs | mm |
| LVIDd | mm | EDV | ml |
| LVIDs | mm | ESV | ml |
| IVSs | mm | LVEF | % |
| IVSd | mm | FS | % |
| **Additional Information**: |  | | |
| No pericardial/Pleural effusion. | | | |
| **Final Diagnosis:** | | | |
| 1. {S, D, S} Levocardia. 2. Small Sub-aortic VSD, L – R Shunt 3. Small PDA, L – R Shunt | | | |
| **Remark**: | | | |
| **Recommendation**: | | | |
| SIGNATURE  Done by: Tesfaye T., Pediatrician, Pediatric Cardiologist _______________ 26/08/2015Eth.C | | | |

| Patient Name: **Dagim Getinet**. Referring Institute: **Amaris PSC**. SEX/ Age: **M/10months**. Date of Report: **26/08/15**.  Referral Diagnosis: **DS. AGH12.3486.** | | | |
| --- | --- | --- | --- |
| **Features** | **Finding** | **Features** | **Finding** |
| **Profile** |  | **Atria** |  |
| Abdominal situs | Solitus | Left atrium | Normal |
| Atrial Situs | Solitus | Right atrium | Normal |
| Cardiac position | Levocardia | **Atrioventricular valves** |  |
| Systemic venous drainage | Normal. | Mitral valve | Annulus = 12mm |
| Pulmonary venous drainage | Normal | Tricuspid valve | Annulus = 12mm |
| Atrioventricular connection | Concordant |  | TAPSE = mm |
| Ventriculoarterial connection | Concordant | **Ventricles** |  |
| Ventricular loop | d-Loop | Left ventricle | Normal |
|  |  | Right ventricle | Normal |
| **Septae** |  | **Coronary arteries** | ----- |
| Interventricular septum | Intact | **Doppler Measurement** |  |
| Interatrial septum | Intact | Mitral | ----- |
| **Semilunar valves** |  | Aortic | ------- |
| Aortic valve | Annulus = 12mm | Tricuspid | ------- |
| Pulmonary valve | Annulus = 14mm | pulmonic | -------- |
| **Great arteries** | NRGA | **Aortic arch** | Left. No CoA. |
| Aorta | ----- | **PDA** | No |
| Pulmonary artery | Normal MPA and Branch PAs. |  |  |
| **M-Mode:** | | | |
| AO | mm | PWd | mm |
| LA | mm | PWs | mm |
| LVIDd | mm | EDV | ml |
| LVIDs | mm | ESV | ml |
| IVSs | mm | LVEF | % |
| IVSd | mm | FS | % |
| **Additional Information**: |  | | |
| No pericardial/Pleural effusion. | | | |
| **Final Diagnosis:** | | | |
| 1. Normal Echocardiography Study. | | | |
| **Remark**: | | | |
| **Recommendation**: | | | |
| SIGNATURE  Done by: Tesfaye T., Pediatrician, Pediatric Cardiologist _______________ 26/08/2015Eth.C | | | |

| Patient Name: **Estibel Dires**. Referring Institute: **FHRH**. SEX/ Age: **M/4years**. Date of Report: **26/08/15**.  Referral Diagnosis: **Bilateral Leg swelling (CHF). AGH12.3487.** | | | |
| --- | --- | --- | --- |
| **Features** | **Finding** | **Features** | **Finding** |
| **Profile** |  | **Atria** |  |
| Abdominal situs | Solitus | Left atrium | Normal |
| Atrial Situs | Solitus | Right atrium | Normal |
| Cardiac position | Levocardia | **Atrioventricular valves** |  |
| Systemic venous drainage | Normal. | Mitral valve | Annulus = 20mm |
| Pulmonary venous drainage | Normal | Tricuspid valve | Annulus = 19mm |
| Atrioventricular connection | Concordant |  | TAPSE = 18mm |
| Ventriculoarterial connection | Concordant | **Ventricles** |  |
| Ventricular loop | d-Loop | Left ventricle | Dilated Globularly & Dysfunctional |
|  |  | Right ventricle | Normal |
| **Septae** |  | **Coronary arteries** | ----- |
| Interventricular septum | Intact | **Doppler Measurement** |  |
| Interatrial septum | Intact | Mitral | Trivial MR, Seen in two planes with jet velocity = 3m/sec. |
| **Semilunar valves** |  | Aortic | ------- |
| Aortic valve | Annulus = 16mm | Tricuspid | ------- |
| Pulmonary valve | Annulus = 18mm | pulmonic | -------- |
| **Great arteries** | NRGA | **Aortic arch** | Left. No CoA. |
| Aorta | ----- | **PDA** | No |
| Pulmonary artery | Normal MPA and Branch PAs. | **Coronaries** | Proximal coronaries are visible |
| **M-Mode:** | | | |
| AO | mm | PWd | mm |
| LA | mm | PWs | mm |
| LVIDd | mm | EDV | ml |
| LVIDs | mm | ESV | ml |
| IVSs | mm | LVEF | 50% |
| IVSd | mm | FS | 25% |
| **Additional Information**: |  | | |
| No pericardial effusion. 13mm Right Pleural effusion. | | | |
| **Final Diagnosis:** | | | |
| 1. {S, D, S} Levocardia. 2. Trivial MR 3. Globularly Dilated and Dysfunctional LV 4. Moderate Right Pleural effusion | | | |
| **Remark**: | | | |
| **Recommendation**: | | | |
| SIGNATURE  Done by: Tesfaye T., Pediatrician, Pediatric Cardiologist _______________ 26/08/2015Eth.C | | | |

| Patient Name: **Metages Adugna**. Referring Institute: **FHRH**. SEX/ Age: **M/3years**. Date of Report: **27/08/15**.  Referral Diagnosis: **Down Syndrome. AGH12.3488.** | | | |
| --- | --- | --- | --- |
| **Features** | **Finding** | **Features** | **Finding** |
| **Profile** |  | **Atria** |  |
| Abdominal situs | Solitus | Left atrium | Normal |
| Atrial Situs | Solitus | Right atrium | Normal |
| Cardiac position | Levocardia | **Atrioventricular valves** |  |
| Systemic venous drainage | Normal. | Mitral valve | Annulus = 17mm |
| Pulmonary venous drainage | Normal | Tricuspid valve | Annulus = 19mm |
| Atrioventricular connection | Concordant |  | TAPSE = 19mm |
| Ventriculoarterial connection | Concordant | **Ventricles** |  |
| Ventricular loop | d-Loop | Left ventricle | Normal |
|  |  | Right ventricle | Normal |
| **Septae** |  | **Coronary arteries** | ----- |
| Interventricular septum | Intact | **Doppler Measurement** |  |
| Interatrial septum | PFO, L – R Shunt | Mitral | Trivial MR, Seen in 2 planes with jet velocity = 3.7m/sec. Incomplete signal |
| **Semilunar valves** |  | Aortic | ------- |
| Aortic valve | Annulus = 13mm | Tricuspid | ------- |
| Pulmonary valve | Annulus = 14mm | pulmonic | -------- |
| **Great arteries** | NRGA | **Aortic arch** | Left. No CoA. |
| Aorta | ----- | **PDA** | No |
| Pulmonary artery | Normal |  |  |
| **M-Mode:** | | | |
| AO | mm | PWd | mm |
| LA | mm | PWs | mm |
| LVIDd | mm | EDV | ml |
| LVIDs | mm | ESV | ml |
| IVSs | mm | LVEF | 62% |
| IVSd | mm | FS | 33% |
| **Additional Information**: |  | | |
| No pericardial effusion. 11mm Right Pleural effusion | | | |
| **Final Diagnosis:** | | | |
| 1. {S, D, S} Levocardia. 2. PFO, L – R Shunt 3. Trivial MR 4. Moderate Right Pleural effusion secondary to ? | | | |
| **Remark**: | | | |
| **Recommendation**: | | | |
| SIGNATURE  Done by: Tesfaye T., Pediatrician, Pediatric Cardiologist _______________ 27/08/2015Eth.C | | | |

| Patient Name: **Hiwet Asmare**. Referring Institute: **FHRH**. SEX/ Age: **F/8years**. Date of Report: **27/08/15**.  Referral Diagnosis: ? **Acute Rheumatic Fever. AGH12.3489.** | | | |
| --- | --- | --- | --- |
| **Features** | **Finding** | **Features** | **Finding** |
| **Profile** |  | **Atria** |  |
| Abdominal situs | Solitus | Left atrium | Normal |
| Atrial Situs | Solitus | Right atrium | Normal |
| Cardiac position | Levocardia | **Atrioventricular valves** |  |
| Systemic venous drainage | Normal. | Mitral valve | Annulus = 20mm |
| Pulmonary venous drainage | Normal | Tricuspid valve | Annulus = 20mm |
| Atrioventricular connection | Concordant |  | TAPSE = 19mm |
| Ventriculoarterial connection | Concordant | **Ventricles** |  |
| Ventricular loop | d-Loop | Left ventricle | Normal |
|  |  | Right ventricle | Normal |
| **Septae** |  | **Coronary arteries** | ----- |
| Interventricular septum | Intact | **Doppler Measurement** |  |
| Interatrial septum | Intact | Mitral | ----- |
| **Semilunar valves** |  | Aortic | ------- |
| Aortic valve | Annulus = 16mm | Tricuspid | ------- |
| Pulmonary valve | Annulus = 18mm | pulmonic | -------- |
| **Great arteries** | NRGA | **Aortic arch** | Left. No CoA. |
| Aorta | ----- | **PDA** | No |
| Pulmonary artery | Normal MPA and Branch PAs. |  |  |
| **M-Mode:** | | | |
| AO | mm | PWd | mm |
| LA | mm | PWs | mm |
| LVIDd | mm | EDV | ml |
| LVIDs | mm | ESV | ml |
| IVSs | mm | LVEF | 62% |
| IVSd | mm | FS | 32% |
| **Additional Information**: |  | | |
| No pericardial/Pleural effusion. | | | |
| **Final Diagnosis:** | | | |
| 1. Normal Echocardiography Study. | | | |
| **Remark**: | | | |
| **Recommendation**: | | | |
| SIGNATURE  Done by: Tesfaye T., Pediatrician, Pediatric Cardiologist _______________ 27/08/2015Eth.C | | | |

| Patient Name: **Kalkidan Ashenafi**. Referring Institute: **Adinas GH**. SEX/ Age: **F/9 8/12**. Date of Report: **27/08/15**.  Referral Diagnosis: **Syncope. AGH12.3490.** | | | |
| --- | --- | --- | --- |
| **Features** | **Finding** | **Features** | **Finding** |
| **Profile** |  | **Atria** |  |
| Abdominal situs | Solitus | Left atrium | Normal |
| Atrial Situs | Solitus | Right atrium | Normal |
| Cardiac position | Levocardia | **Atrioventricular valves** |  |
| Systemic venous drainage | Normal. | Mitral valve | Annulus = 20mm |
| Pulmonary venous drainage | Normal | Tricuspid valve | Annulus = 20mm |
| Atrioventricular connection | Concordant |  | TAPSE = 21mm |
| Ventriculoarterial connection | Concordant | **Ventricles** |  |
| Ventricular loop | d-Loop | Left ventricle | Normal |
|  |  | Right ventricle | Normal |
| **Septae** |  | **Coronary arteries** | ----- |
| Interventricular septum | Intact | **Doppler Measurement** |  |
| Interatrial septum | Intact | Mitral | ----- |
| **Semilunar valves** |  | Aortic | ------- |
| Aortic valve | Annulus = 17mm | Tricuspid | ------- |
| Pulmonary valve | Annulus = 18mm | pulmonic | -------- |
| **Great arteries** | NRGA | **Aortic arch** | Left. No CoA. |
| Aorta | ----- | **PDA** | No |
| Pulmonary artery | Normal MPA and Branch PAs. |  |  |
| **M-Mode:** | | | |
| AO | mm | PWd | mm |
| LA | mm | PWs | mm |
| LVIDd | mm | EDV | ml |
| LVIDs | mm | ESV | ml |
| IVSs | mm | LVEF | 72% |
| IVSd | mm | FS | 40% |
| **Additional Information**: |  | | |
| No pericardial/Pleural effusion. | | | |
| **Final Diagnosis:** | | | |
| 1. Normal Echocardiography Study. | | | |
| **Remark**: | | | |
| **Recommendation**: | | | |
| SIGNATURE  Done by: Tesfaye T., Pediatrician, Pediatric Cardiologist _______________ 27/08/2015Eth.C | | | |

| Patient Name: **Be’emnet Firew**. Referring Institute: **Adinas GH**. SEX/ Age: **F/9 1/12**. Date of Report: **27/08/15**.  Referral Diagnosis: **S/P PDA Ligation.** | | | |
| --- | --- | --- | --- |
| **Features** | **Finding** | **Features** | **Finding** |
| **Profile** |  | **Atria** |  |
| Abdominal situs | Solitus | Left atrium | Normal |
| Atrial Situs | Solitus | Right atrium | Normal |
| Cardiac position | Levocardia | **Atrioventricular valves** |  |
| Systemic venous drainage | Normal. | Mitral valve | Annulus = 20mm |
| Pulmonary venous drainage | Normal | Tricuspid valve | Annulus = 20mm |
| Atrioventricular connection | Concordant |  | TAPSE = 19mm |
| Ventriculoarterial connection | Concordant | **Ventricles** |  |
| Ventricular loop | d-Loop | Left ventricle | Normal |
|  |  | Right ventricle | Normal |
| **Septae** |  | **Coronary arteries** | ----- |
| Interventricular septum | Intact | **Doppler Measurement** |  |
| Interatrial septum | Intact | Mitral | Moderate MR, Holosystolic, Posterior projection, seen in two planes with jet velocity = 4m/sec. |
| **Semilunar valves** |  | Aortic | ------- |
| Aortic valve | Annulus = 18mm | Tricuspid | ------- |
| Pulmonary valve | Annulus = 18mm | pulmonic | -------- |
| **Great arteries** | NRGA | **Aortic arch** | Left. No CoA. |
| Aorta | ----- | **PDA** | No Residual PDA |
| Pulmonary artery | Unobstructed flow across MPA & Branch PAs. |  |  |
| **M-Mode:** | | | |
| AO | mm | PWd | mm |
| LA | mm | PWs | mm |
| LVIDd | mm | EDV | ml |
| LVIDs | mm | ESV | ml |
| IVSs | mm | LVEF | % |
| IVSd | mm | FS | % |
| **Additional Information**: |  | | |
| No pericardial/Pleural effusion. | | | |
| **Final Diagnosis:** | | | |
| 1. S/P PDA Ligation 2. {S, D, S} Levocardia. 3. No Residual PDA 4. Unobstructed flow across the MPA and Branch PAs. | | | |
| **Remark**: | | | |
| **Recommendation**: | | | |
| SIGNATURE  Done by: Tesfaye T., Pediatrician, Pediatric Cardiologist _______________ 27/08/2015Eth.C | | | |

| Patient Name: **Be’emnet Aragaw**. Referring Institute: **Adinas GH**. SEX/ Age: **F/5 6/12**. Date of Report: **27/08/15**.  Referral Diagnosis: **Incidental Murmur. AGH12.3492.** | | | |
| --- | --- | --- | --- |
| **Features** | **Finding** | **Features** | **Finding** |
| **Profile** |  | **Atria** |  |
| Abdominal situs | Solitus | Left atrium | Dilated |
| Atrial Situs | Solitus | Right atrium | More Dilated |
| Cardiac position | Levocardia | **Atrioventricular valves** |  |
| Systemic venous drainage | Normal. | Mitral valve | Annulus = 19mm |
| Pulmonary venous drainage | Normal | Tricuspid valve | Annulus = 22mm |
| Atrioventricular connection | Concordant |  | TAPSE = 17mm |
| Ventriculoarterial connection | Concordant | **Ventricles** |  |
| Ventricular loop | d-Loop | Left ventricle | Dilated |
|  |  | Right ventricle | More Dilated |
| **Septae** |  | **Coronary arteries** | ----- |
| Interventricular septum | Intact | **Doppler Measurement** |  |
| Interatrial septum | Intact | Mitral | ----- |
| **Semilunar valves** |  | Aortic | ------- |
| Aortic valve | Annulus = 17mm | Tricuspid | Mild TR, PPG = 63mmHg. |
| Pulmonary valve | Annulus = 20mm | pulmonic | -------- |
| **Great arteries** | NRGA | **Aortic arch** | Left. No CoA. |
| Aorta | ----- | **PDA** | 3mm PDA, L – R Shunt |
| Pulmonary artery | MPA = 27mm. |  |  |
| **M-Mode:**  Normal LV Function on eye balling | | | |
| AO | mm | PWd | mm |
| LA | mm | PWs | mm |
| LVIDd | mm | EDV | ml |
| LVIDs | mm | ESV | ml |
| IVSs | mm | LVEF | % |
| IVSd | mm | FS | % |
| **Additional Information**: |  | | |
| No pericardial/Pleural effusion. | | | |
| **Final Diagnosis:** | | | |
| 1. {S, D, S} Levocardia. 2. All chambers are dilated 3. Mild TR 4. Large PDA, L – R Shunt 5. Severe Pulmonary Hypertension 6. Normal Biventricular Systolic Function | | | |
| **Remark**: | | | |
| **Recommendation**: | | | |
| SIGNATURE  Done by: Tesfaye T., Pediatrician, Pediatric Cardiologist _______________ 27/08/2015Eth.C | | | |

| Patient Name: **Mekides Endalamaw**. Referring Institute: **FHRH**. SEX/ Age: **F/11years**. Date of Report: **28/08/15**.  Referral Diagnosis: ? **Sydenham’s Chorea. AGH12.3493.** | | | |
| --- | --- | --- | --- |
| **Features** | **Finding** | **Features** | **Finding** |
| **Profile** |  | **Atria** |  |
| Abdominal situs | Solitus | Left atrium | Normal |
| Atrial Situs | Solitus | Right atrium | Normal |
| Cardiac position | Levocardia | **Atrioventricular valves** |  |
| Systemic venous drainage | Normal. | Mitral valve | Annulus = 21mm. Patulous MVL |
| Pulmonary venous drainage | Normal | Tricuspid valve | Annulus = 20mm |
| Atrioventricular connection | Concordant |  | TAPSE = mm |
| Ventriculoarterial connection | Concordant | **Ventricles** |  |
| Ventricular loop | d-Loop | Left ventricle | Normal |
|  |  | Right ventricle | Normal |
| **Septae** |  | **Coronary arteries** | ----- |
| Interventricular septum | Intact | **Doppler Measurement** |  |
| Interatrial septum | Intact | Mitral | Mild MR |
| **Semilunar valves** |  | Aortic | ------- |
| Aortic valve | Annulus = 18mm | Tricuspid | ------- |
| Pulmonary valve | Annulus = 20mm | pulmonic | -------- |
| **Great arteries** | NRGA | **Aortic arch** | Left. No CoA. |
| Aorta | ----- | **PDA** | No |
| Pulmonary artery | Normal MPA and Branch PAs. |  |  |
| **M-Mode:** | | | |
| AO | mm | PWd | mm |
| LA | mm | PWs | mm |
| LVIDd | mm | EDV | ml |
| LVIDs | mm | ESV | ml |
| IVSs | mm | LVEF | % |
| IVSd | mm | FS | % |
| **Additional Information**: |  | | |
| No pericardial/Pleural effusion. | | | |
| **Final Diagnosis:** | | | |
| 1. {S, D, S} Levocardia. 2. Patulous MVL 3. Mild MR | | | |
| **Remark**: Child is restless. Not able to measure velocity, Function | | | |
| **Recommendation**: Strong sedation and steroid. | | | |
| SIGNATURE  Done by: Tesfaye T., Pediatrician, Pediatric Cardiologist _______________ 28/08/2015Eth.C | | | |

| Patient Name: **Amnen Haile-Mariam**. Referring Institute: **Adinas GH**. SEX/ Age: **F/2 7/12**. Date of Report: **28/08/15**.  Referral Diagnosis: **Follow up Echo (CHB + PDA).** | | | |
| --- | --- | --- | --- |
| **Features** | **Finding** | **Features** | **Finding** |
| **Profile** |  | **Atria** |  |
| Abdominal situs | Solitus | Left atrium | Normal |
| Atrial Situs | Solitus | Right atrium | Normal |
| Cardiac position | Levocardia | **Atrioventricular valves** |  |
| Systemic venous drainage | Normal. | Mitral valve | Annulus = 18mm |
| Pulmonary venous drainage | Normal | Tricuspid valve | Annulus = 18mm |
| Atrioventricular connection | Concordant |  | TAPSE = mm |
| Ventriculoarterial connection | Concordant | **Ventricles** |  |
| Ventricular loop | d-Loop | Left ventricle | Normal |
|  |  | Right ventricle | Normal |
| **Septae** |  | **Coronary arteries** | ----- |
| Interventricular septum | Intact | **Doppler Measurement** |  |
| Interatrial septum | Intact | Mitral | ----- |
| **Semilunar valves** |  | Aortic | ------- |
| Aortic valve | Annulus = 14mm | Tricuspid | ------- |
| Pulmonary valve | Annulus = 16mm | pulmonic | -------- |
| **Great arteries** | NRGA | **Aortic arch** | Left. No CoA. |
| Aorta | ----- | **PDA** | No |
| Pulmonary artery | Normal MPA and Branch PAs. |  |  |
| **M-Mode:** | | | |
| AO | mm | PWd | mm |
| LA | mm | PWs | mm |
| LVIDd | mm | EDV | ml |
| LVIDs | mm | ESV | ml |
| IVSs | mm | LVEF | 68% |
| IVSd | mm | FS | 37% |
| **Additional Information**: |  | | |
| No pericardial/Pleural effusion. | | | |
| **Final Diagnosis:** | | | |
| 1. Normal Echocardiography Study. | | | |
| **Remark**: PDA Closed | | | |
| **Recommendation**: Follow up for the CHB | | | |
| SIGNATURE  Done by: Tesfaye T., Pediatrician, Pediatric Cardiologist _______________ 28/08/2015Eth.C | | | |

| Patient Name: **Yonas Tilahun**. Referring Institute: **Debre-Tabour GH**. SEX/ Age: **M/11months**. Date of Report: **30/08/15**.  Referral Diagnosis: **Diaphoresis and Breast feeding Interruption. AGH12.3494.** | | | |
| --- | --- | --- | --- |
| **Features** | **Finding** | **Features** | **Finding** |
| **Profile** |  | **Atria** |  |
| Abdominal situs | Solitus | Left atrium | Normal |
| Atrial Situs | Solitus | Right atrium | Mildly Dilated |
| Cardiac position | Levocardia | **Atrioventricular valves** |  |
| Systemic venous drainage | Normal. | Mitral valve | Annulus = 13mm |
| Pulmonary venous drainage | Normal | Tricuspid valve | Annulus = 13mm |
| Atrioventricular connection | Concordant |  | TAPSE = mm |
| Ventriculoarterial connection | Concordant | **Ventricles** |  |
| Ventricular loop | d-Loop | Left ventricle | Normal |
|  |  | Right ventricle | Mildly Dilated |
| **Septae** |  | **Coronary arteries** | ----- |
| Interventricular septum | Intact | **Doppler Measurement** |  |
| Interatrial septum | 8 X 6mm Fenestrated OS ASD, L – R Shunt | Mitral | ----- |
| **Semilunar valves** |  | Aortic | ------- |
| Aortic valve | Annulus = 12mm | Tricuspid | ------- |
| Pulmonary valve | Annulus = 14mm | pulmonic | -------- |
| **Great arteries** | NRGA | **Aortic arch** | Left. No CoA. |
| Aorta | ----- | **PDA** | No |
| Pulmonary artery | Normal MPA and Branch PAs. |  |  |
| **M-Mode:** | | | |
| AO | mm | PWd | mm |
| LA | mm | PWs | mm |
| LVIDd | mm | EDV | ml |
| LVIDs | mm | ESV | ml |
| IVSs | mm | LVEF | 71% |
| IVSd | mm | FS | 38% |
| **Additional Information**: |  | | |
| No pericardial/Pleural effusion. | | | |
| **Final Diagnosis:** | | | |
| 1. {S, D, S} Levocardia. 2. RA/RV Mildly Dilated 3. Moderate Fenestrated OS ASD, L – R Shunt 4. Normal LV Systolic Function | | | |
| **Remark**: | | | |
| **Recommendation**: | | | |
| SIGNATURE  Done by: Tesfaye T., Pediatrician, Pediatric Cardiologist _______________ 30/08/2015Eth.C | | | |

| Patient Name: **Baby of Shega Ahmed**. Referring Institute: **FHRH**. SEX/ Age: **F/59hours**. Date of Report: **30/08/15**.  Referral Diagnosis: **Persistent desaturation. AGH12.3495.** | | | |
| --- | --- | --- | --- |
| **Features** | **Finding** | **Features** | **Finding** |
| **Profile** |  | **Atria** |  |
| Abdominal situs | Solitus | Left atrium | Normal |
| Atrial Situs | Solitus | Right atrium | Normal |
| Cardiac position | Levocardia | **Atrioventricular valves** |  |
| Systemic venous drainage | Normal. | Mitral valve | Annulus = 9mm |
| Pulmonary venous drainage | Normal | Tricuspid valve | Annulus = 9mm |
| Atrioventricular connection | Concordant |  | TAPSE = mm |
| Ventriculoarterial connection | Concordant | **Ventricles** |  |
| Ventricular loop | d-Loop | Left ventricle | Normal |
|  |  | Right ventricle | Normal |
| **Septae** |  | **Coronary arteries** | ----- |
| Interventricular septum | 9mm Inlet VSD, L – R Shunt | **Doppler Measurement** |  |
| Interatrial septum | PFO, L – R Shunt | Mitral | ----- |
| **Semilunar valves** |  | Aortic | ------- |
| Aortic valve | Annulus = 9mm | Tricuspid | ------- |
| Pulmonary valve | Annulus = 11mm | pulmonic | -------- |
| **Great arteries** | NRGA | **Aortic arch** | Left. No CoA. |
| Aorta | ----- | **PDA** | <1mm PDA, L – R Shunt |
| Pulmonary artery | Normal MPA and Branch PAs. |  |  |
| **M-Mode:**  Normal LV Function on eye balling | | | |
| AO | mm | PWd | mm |
| LA | mm | PWs | mm |
| LVIDd | mm | EDV | ml |
| LVIDs | mm | ESV | ml |
| IVSs | mm | LVEF | % |
| IVSd | mm | FS | % |
| **Additional Information**: |  | | |
| No pericardial/Pleural effusion. | | | |
| **Final Diagnosis:** | | | |
| 1. {S, D, S} Levocardia. 2. PFO, L – R Shunt 3. Large Inlet VSD, L – R Shunt 4. Small/?Silent PDA, L – R Shunt 5. Normal LV Systolic Function | | | |
| **Remark**: | | | |
| **Recommendation**: | | | |
| SIGNATURE  Done by: Tesfaye T., Pediatrician, Pediatric Cardiologist _______________ 30/08/2015Eth.C | | | |

| Patient Name: **Baby of Fentanesh Telake**. Referring Institute: **FHRH**. SEX/ Age: **F/15days**. Date of Report: **30/08/15**.  Referral Diagnosis: **Fast breathing (RD). AGH12.3496.** | | | |
| --- | --- | --- | --- |
| **Features** | **Finding** | **Features** | **Finding** |
| **Profile** |  | **Atria** |  |
| Abdominal situs | Solitus | Left atrium | Normal |
| Atrial Situs | Solitus | Right atrium | Normal |
| Cardiac position | Levocardia | **Atrioventricular valves** |  |
| Systemic venous drainage | Normal. | Mitral valve | Annulus = 7mm |
| Pulmonary venous drainage | Normal | Tricuspid valve | Annulus = 9mm |
| Atrioventricular connection | Concordant |  | TAPSE = mm |
| Ventriculoarterial connection | Concordant | **Ventricles** |  |
| Ventricular loop | d-Loop | Left ventricle | Normal |
|  |  | Right ventricle | Normal |
| **Septae** |  | **Coronary arteries** | ----- |
| Interventricular septum | Intact | **Doppler Measurement** |  |
| Interatrial septum | Intact | Mitral | ----- |
| **Semilunar valves** |  | Aortic | ------- |
| Aortic valve | Annulus = 7mm | Tricuspid | ------- |
| Pulmonary valve | Annulus = 7mm | pulmonic | -------- |
| **Great arteries** | NRGA | **Aortic arch** | Left. No CoA. |
| Aorta | ----- | **PDA** | No |
| Pulmonary artery | Normal MPA and Branch PAs. |  |  |
| **M-Mode:**  Normal LV Function on eye balling | | | |
| AO | mm | PWd | mm |
| LA | mm | PWs | mm |
| LVIDd | mm | EDV | ml |
| LVIDs | mm | ESV | ml |
| IVSs | mm | LVEF | % |
| IVSd | mm | FS | % |
| **Additional Information**: |  | | |
| No pericardial/Pleural effusion. | | | |
| **Final Diagnosis:** | | | |
| 1. Normal Echocardiography Study. | | | |
| **Remark**: | | | |
| **Recommendation**: | | | |
| SIGNATURE  Done by: Tesfaye T., Pediatrician, Pediatric Cardiologist _______________ 30/08/2015Eth.C | | | |

| Patient Name: **Baby of Mintamir Desta**. Referring Institute: **TGSH**. SEX/ Age: **M/6dayss**. Date of Report: **30/08/15**.  Referral Diagnosis: **DS. AGH12.3497.** | | | |
| --- | --- | --- | --- |
| **Features** | **Finding** | **Features** | **Finding** |
| **Profile** |  | **Atria** |  |
| Abdominal situs | Solitus | Left atrium | Normal |
| Atrial Situs | Solitus | Right atrium | Normal |
| Cardiac position | Levocardia | **Atrioventricular valves** |  |
| Systemic venous drainage | Normal. | Mitral valve | Annulus = 9mm |
| Pulmonary venous drainage | Normal | Tricuspid valve | Annulus = 10mm |
| Atrioventricular connection | Concordant |  | TAPSE = mm |
| Ventriculoarterial connection | Concordant | **Ventricles** |  |
| Ventricular loop | d-Loop | Left ventricle | Normal |
|  |  | Right ventricle | Normal |
| **Septae** |  | **Coronary arteries** | ----- |
| Interventricular septum | Intact | **Doppler Measurement** |  |
| Interatrial septum | Intact | Mitral | ----- |
| **Semilunar valves** |  | Aortic | ------- |
| Aortic valve | Annulus = 10mm | Tricuspid | Trivial TR, PPG = 13mmHg |
| Pulmonary valve | Annulus = 11mm | pulmonic | -------- |
| **Great arteries** | NRGA | **Aortic arch** | Left. No CoA. |
| Aorta | ----- | **PDA** | No |
| Pulmonary artery | Normal MPA and Branch PAs. |  |  |
| **M-Mode:** | | | |
| AO | mm | PWd | mm |
| LA | mm | PWs | mm |
| LVIDd | mm | EDV | ml |
| LVIDs | mm | ESV | ml |
| IVSs | mm | LVEF | % |
| IVSd | mm | FS | % |
| **Additional Information**: |  | | |
| No pericardial/Pleural effusion. | | | |
| **Final Diagnosis:** | | | |
| 1. Normal Echocardiography Study. | | | |
| **Remark**: | | | |
| **Recommendation**: | | | |
| SIGNATURE  Done by: Tesfaye T., Pediatrician, Pediatric Cardiologist _______________ 30/08/2015Eth.C | | | |

| Patient Name: **Baby of Fikir-Addis Mihret**. Referring Institute: **TGSH**. SEX/ Age: **F/35days**. Date of Report: **30/08/15**.  Referral Diagnosis: **Incidental Murmur (?PDA). AGH12.3498.** | | | |
| --- | --- | --- | --- |
| **Features** | **Finding** | **Features** | **Finding** |
| **Profile** |  | **Atria** |  |
| Abdominal situs | Solitus | Left atrium | Normal |
| Atrial Situs | Solitus | Right atrium | Normal |
| Cardiac position | Levocardia | **Atrioventricular valves** |  |
| Systemic venous drainage | Normal. | Mitral valve | Annulus = 10mm |
| Pulmonary venous drainage | Normal | Tricuspid valve | Annulus = 10mm |
| Atrioventricular connection | Concordant |  | TAPSE = mm |
| Ventriculoarterial connection | Concordant | **Ventricles** |  |
| Ventricular loop | d-Loop | Left ventricle | Normal |
|  |  | Right ventricle | Normal |
| **Septae** |  | **Coronary arteries** | ----- |
| Interventricular septum | Intact | **Doppler Measurement** |  |
| Interatrial septum | 5mm OS ASD, L – R Shunt | Mitral | ----- |
| **Semilunar valves** |  | Aortic | ------- |
| Aortic valve | Annulus = 8mm | Tricuspid | ------- |
| Pulmonary valve | Annulus = 9mm | pulmonic | -------- |
| **Great arteries** | NRGA | **Aortic arch** | Left. No CoA. |
| Aorta | ----- | **PDA** | 1mm PDA, L – R Shunt |
| Pulmonary artery | Normal MPA and Branch PAs. |  |  |
| **M-Mode:**  Normal LV Function on eye balling | | | |
| AO | mm | PWd | mm |
| LA | mm | PWs | mm |
| LVIDd | mm | EDV | ml |
| LVIDs | mm | ESV | ml |
| IVSs | mm | LVEF | % |
| IVSd | mm | FS | % |
| **Additional Information**: |  | | |
| No pericardial/Pleural effusion. | | | |
| **Final Diagnosis:** | | | |
| 1. {S, D, S} Levocardia. 2. Small OS ASD, L – R Shunt 3. Small PDA, L – R Shunt 4. Normal LV Systolic Function | | | |
| **Remark**: | | | |
| **Recommendation**: | | | |
| SIGNATURE  Done by: Tesfaye T., Pediatrician, Pediatric Cardiologist _______________ 30/08/2015Eth.C | | | |

| Patient Name: **Eldana Muluken**. Referring Institute: **FHRH**. SEX/ Age: **F/11years**. Date of Report: **30/08/15**.  Referral Diagnosis: **?ARF. AGH12.3499.** | | | |
| --- | --- | --- | --- |
| **Features** | **Finding** | **Features** | **Finding** |
| **Profile** |  | **Atria** |  |
| Abdominal situs | Solitus | Left atrium | Normal |
| Atrial Situs | Solitus | Right atrium | Normal |
| Cardiac position | Levocardia | **Atrioventricular valves** |  |
| Systemic venous drainage | Normal. | Mitral valve | Annulus = 21mm |
| Pulmonary venous drainage | Normal | Tricuspid valve | Annulus = 21mm |
| Atrioventricular connection | Concordant |  | TAPSE = 21mm |
| Ventriculoarterial connection | Concordant | **Ventricles** |  |
| Ventricular loop | d-Loop | Left ventricle | Normal |
|  |  | Right ventricle | Normal |
| **Septae** |  | **Coronary arteries** | ----- |
| Interventricular septum | Intact | **Doppler Measurement** |  |
| Interatrial septum | Intact | Mitral | ----- |
| **Semilunar valves** |  | Aortic | ------- |
| Aortic valve | Annulus = 17mm | Tricuspid | ------- |
| Pulmonary valve | Annulus = 19mm | pulmonic | -------- |
| **Great arteries** | NRGA | **Aortic arch** | Left. No CoA. |
| Aorta | ----- | **PDA** | No |
| Pulmonary artery | Normal MPA and Branch PAs. |  |  |
| **M-Mode:** | | | |
| AO | mm | PWd | mm |
| LA | mm | PWs | mm |
| LVIDd | mm | EDV | ml |
| LVIDs | mm | ESV | ml |
| IVSs | mm | LVEF | 66% |
| IVSd | mm | FS | 35% |
| **Additional Information**: |  | | |
| No pericardial/Pleural effusion. | | | |
| **Final Diagnosis:** | | | |
| 1. Normal Echocardiography Study. | | | |
| **Remark**: Normal Echocardiography study doesn’t rule out Acute Rheumatic Fever | | | |
| **Recommendation**: | | | |
| SIGNATURE  Done by: Tesfaye T., Pediatrician, Pediatric Cardiologist _______________ 30/08/2015Eth.C | | | |

| Patient Name: **Bahiru Yayeh**. Referring Institute: **TGSH**. SEX/ Age: **M/7month**. Date of Report: **02/09/15**.  Referral Diagnosis: **Recurrent Chest Infection. AGH12.3500.** | | | |
| --- | --- | --- | --- |
| **Features** | **Finding** | **Features** | **Finding** |
| **Profile** |  | **Atria** |  |
| Abdominal situs | Solitus | Left atrium | Normal |
| Atrial Situs | Solitus | Right atrium | Normal |
| Cardiac position | Levocardia | **Atrioventricular valves** |  |
| Systemic venous drainage | Normal. | Mitral valve | Annulus = 13mm |
| Pulmonary venous drainage | Normal | Tricuspid valve | Annulus = 14mm |
| Atrioventricular connection | Concordant |  | TAPSE = mm |
| Ventriculoarterial connection | Concordant | **Ventricles** |  |
| Ventricular loop | d-Loop | Left ventricle | Normal |
|  |  | Right ventricle | Normal |
| **Septae** |  | **Coronary arteries** | ----- |
| Interventricular septum | Intact | **Doppler Measurement** |  |
| Interatrial septum | PFO, L – R Shunt | Mitral | ----- |
| **Semilunar valves** |  | Aortic | ------- |
| Aortic valve | Annulus = 11mm | Tricuspid | ------- |
| Pulmonary valve | Annulus = 12mm | pulmonic | -------- |
| **Great arteries** | NRGA | **Aortic arch** | Left. No CoA. |
| Aorta | ----- | **PDA** | No |
| Pulmonary artery | Normal MPA and Branch PAs. |  |  |
| **M-Mode:**  Normal LV Function on eye balling | | | |
| AO | mm | PWd | mm |
| LA | mm | PWs | mm |
| LVIDd | mm | EDV | ml |
| LVIDs | mm | ESV | ml |
| IVSs | mm | LVEF | % |
| IVSd | mm | FS | % |
| **Additional Information**: |  | | |
| No pericardial/Pleural effusion. | | | |
| **Final Diagnosis:** | | | |
| 1. {S, D, S} Levocardia. 2. PFO, L – R Shunt 3. Normal LV Systolic Function | | | |
| **Remark**: | | | |
| **Recommendation**: | | | |
| SIGNATURE  Done by: Tesfaye T., Pediatrician, Pediatric Cardiologist _______________ 02/09/2015Eth.C | | | |

| Patient Name: **Sobrina Sualih**. Referring Institute: **TGSH**. SEX/ Age: **F/2months**. Date of Report: **02/09/15**.  Referral Diagnosis: **Down Syndrome. AGH12.3501.** | | | |
| --- | --- | --- | --- |
| **Features** | **Finding** | **Features** | **Finding** |
| **Profile** |  | **Atria** |  |
| Abdominal situs | Solitus | Left atrium | Normal |
| Atrial Situs | Solitus | Right atrium | Normal |
| Cardiac position | Levocardia | **Atrioventricular valves** |  |
| Systemic venous drainage | Normal. | Mitral valve | Annulus = 10mm |
| Pulmonary venous drainage | Normal | Tricuspid valve | Annulus = 11mm |
| Atrioventricular connection | Concordant |  | TAPSE = 14mm |
| Ventriculoarterial connection | Concordant | **Ventricles** |  |
| Ventricular loop | d-Loop | Left ventricle | Normal |
|  |  | Right ventricle | Normal |
| **Septae** |  | **Coronary arteries** | ----- |
| Interventricular septum | Intact | **Doppler Measurement** |  |
| Interatrial septum | Intact | Mitral | ----- |
| **Semilunar valves** |  | Aortic | ------- |
| Aortic valve | Annulus = 10mm | Tricuspid | ------- |
| Pulmonary valve | Annulus = 10mm | pulmonic | -------- |
| **Great arteries** | NRGA | **Aortic arch** | Left. No CoA. |
| Aorta | ----- | **PDA** | No |
| Pulmonary artery | Normal MPA and Branch PAs. |  |  |
| **M-Mode:** | | | |
| AO | mm | PWd | mm |
| LA | mm | PWs | mm |
| LVIDd | mm | EDV | ml |
| LVIDs | mm | ESV | ml |
| IVSs | mm | LVEF | 62% |
| IVSd | mm | FS | 31% |
| **Additional Information**: |  | | |
| Pericardial effusion with maximum depth of 3mm on RA/RV Side. | | | |
| **Final Diagnosis:** | | | |
| 1. {S, D, S} Levocardia. 2. Trace Pericardial effusion 3. Normal Biventricular Systolic Function | | | |
| **Remark**: | | | |
| **Recommendation**: | | | |
| SIGNATURE  Done by: Tesfaye T., Pediatrician, Pediatric Cardiologist _______________ 02/09/2015Eth.C | | | |

| Patient Name: **Muluye Aweke**. Referring Institute: **Debre-Tabour GH**. SEX/ Age: **M/14years**. Date of Report: **02/09/15**.  Referral Diagnosis: **Easy Fatigability + Orthopnea + PND + Palpitation. AGH12.3502.** | | | |
| --- | --- | --- | --- |
| **Features** | **Finding** | **Features** | **Finding** |
| **Profile** |  | **Atria** |  |
| Abdominal situs | Solitus | Left atrium | Dilated |
| Atrial Situs | Solitus | Right atrium | Normal |
| Cardiac position | Levocardia | **Atrioventricular valves** |  |
| Systemic venous drainage | Normal. | Mitral valve | Annulus = 27mm. Thickened MVL |
| Pulmonary venous drainage | Normal | Tricuspid valve | Annulus = 25mm |
| Atrioventricular connection | Concordant |  | TAPSE = 18mm |
| Ventriculoarterial connection | Concordant | **Ventricles** |  |
| Ventricular loop | d-Loop | Left ventricle | Dilated |
|  |  | Right ventricle | Normal |
| **Septae** |  | **Coronary arteries** | ----- |
| Interventricular septum | Intact | **Doppler Measurement** |  |
| Interatrial septum | Intact | Mitral | Severe MR, Holosystolic, posterior projection, seen in two planes with jet velocity= 3.9m/sec. |
| **Semilunar valves** |  | Aortic | Mild AR, PHT = 504ms |
| Aortic valve | Annulus = 20mm | Tricuspid | Mild TR, PPG = 31mmHg |
| Pulmonary valve | Annulus = 23mm | pulmonic | -------- |
| **Great arteries** | NRGA | **Aortic arch** | Left. No CoA. |
| Aorta | ----- | **PDA** | No |
| Pulmonary artery | Normal MPA and Branch PAs. |  |  |
| **M-Mode:** | | | |
| AO | mm | PWd | mm |
| LA | mm | PWs | mm |
| LVIDd | mm | EDV | ml |
| LVIDs | mm | ESV | ml |
| IVSs | mm | LVEF | 59% |
| IVSd | mm | FS | 31% |
| **Additional Information**: |  | | |
| No pericardial/Pleural effusion. | | | |
| **Final Diagnosis:** | | | |
| 1. {S, D, S} Levocardia. 2. LA/LV Dilated 3. Thickened MVL 4. Severe MR 5. Mild AR 6. Mild TR 7. Normal Biventricular Systolic Function | | | |
| SIGNATURE  Done by: Tesfaye T., Pediatrician, Pediatric Cardiologist _______________ 02/09/2015Eth.C | | | |

| Patient Name: **Yimenu Fentie**. Referring Institute: **FHRH**. SEX/ Age: **M/5years**. Date of Report: **02/09/15**.  Referral Diagnosis: **Joint pain + easy fatigability. AGH12.3503.** | | | |
| --- | --- | --- | --- |
| **Features** | **Finding** | **Features** | **Finding** |
| **Profile** |  | **Atria** |  |
| Abdominal situs | Solitus | Left atrium | Normal |
| Atrial Situs | Solitus | Right atrium | Normal |
| Cardiac position | Levocardia | **Atrioventricular valves** |  |
| Systemic venous drainage | Normal. | Mitral valve | Annulus = 17mm |
| Pulmonary venous drainage | Normal | Tricuspid valve | Annulus = 16mm |
| Atrioventricular connection | Concordant |  | TAPSE = 15mm |
| Ventriculoarterial connection | Concordant | **Ventricles** |  |
| Ventricular loop | d-Loop | Left ventricle | Normal |
|  |  | Right ventricle | Normal |
| **Septae** |  | **Coronary arteries** | ----- |
| Interventricular septum | Intact | **Doppler Measurement** |  |
| Interatrial septum | Intact | Mitral | ----- |
| **Semilunar valves** |  | Aortic | ------- |
| Aortic valve | Annulus = 14mm | Tricuspid | ------- |
| Pulmonary valve | Annulus = 16mm | pulmonic | -------- |
| **Great arteries** | NRGA | **Aortic arch** | Left. No CoA. |
| Aorta | ----- | **PDA** | No |
| Pulmonary artery | Normal MPA and Branch PAs. |  |  |
| **M-Mode:** | | | |
| AO | mm | PWd | mm |
| LA | mm | PWs | mm |
| LVIDd | mm | EDV | ml |
| LVIDs | mm | ESV | ml |
| IVSs | mm | LVEF | 69% |
| IVSd | mm | FS | 38% |
| **Additional Information**: |  | | |
| No pericardial/Pleural effusion. | | | |
| **Final Diagnosis:** | | | |
| 1. Normal Echocardiography Study. | | | |
| **Remark**: | | | |
| **Recommendation**: | | | |
| SIGNATURE  Done by: Tesfaye T., Pediatrician, Pediatric Cardiologist _______________ 02/09/2015Eth.C | | | |

| Patient Name: **Meron Amare**. Referring Institute: **FHRH**. SEX/ Age: **F/8years**. Date of Report: **02/09/15**.  Referral Diagnosis: **HGF + Joint pain + Palpitation + sore throat. AGH12.3504.** | | | |
| --- | --- | --- | --- |
| **Features** | **Finding** | **Features** | **Finding** |
| **Profile** |  | **Atria** |  |
| Abdominal situs | Solitus | Left atrium | Normal |
| Atrial Situs | Solitus | Right atrium | Normal |
| Cardiac position | Levocardia | **Atrioventricular valves** |  |
| Systemic venous drainage | Normal. | Mitral valve | Annulus = 19mm |
| Pulmonary venous drainage | Normal | Tricuspid valve | Annulus = 18mm |
| Atrioventricular connection | Concordant |  | TAPSE = 20mm |
| Ventriculoarterial connection | Concordant | **Ventricles** |  |
| Ventricular loop | d-Loop | Left ventricle | Normal |
|  |  | Right ventricle | Normal |
| **Septae** |  | **Coronary arteries** | ----- |
| Interventricular septum | Intact | **Doppler Measurement** |  |
| Interatrial septum | Intact | Mitral | ----- |
| **Semilunar valves** |  | Aortic | ------- |
| Aortic valve | Annulus = 16mm | Tricuspid | ------- |
| Pulmonary valve | Annulus = 20mm | pulmonic | -------- |
| **Great arteries** | NRGA | **Aortic arch** | Left. No CoA. |
| Aorta | ----- | **PDA** | No |
| Pulmonary artery | Normal MPA and Branch PAs. |  |  |
| **M-Mode:** | | | |
| AO | mm | PWd | mm |
| LA | mm | PWs | mm |
| LVIDd | mm | EDV | ml |
| LVIDs | mm | ESV | ml |
| IVSs | mm | LVEF | 58% |
| IVSd | mm | FS | 30% |
| **Additional Information**: |  | | |
| No pericardial/Pleural effusion. | | | |
| **Final Diagnosis:** | | | |
| 1. Normal Echocardiography Study. | | | |
| **Remark**: | | | |
| **Recommendation**: | | | |
| SIGNATURE  Done by: Tesfaye T., Pediatrician, Pediatric Cardiologist _______________ 02/09/2015Eth.C | | | |

| Patient Name: **Amlakie Aderajew**. Referring Institute: **FHRH**. SEX/ Age: **M/1 6/12**. Date of Report: **02/09/15**.  Referral Diagnosis: **Fast Breathing and Grunting/RD. AGH12.3505.** | | | |
| --- | --- | --- | --- |
| **Features** | **Finding** | **Features** | **Finding** |
| **Profile** |  | **Atria** |  |
| Abdominal situs | Solitus | Left atrium | Normal |
| Atrial Situs | Solitus | Right atrium | Normal |
| Cardiac position | Levocardia | **Atrioventricular valves** |  |
| Systemic venous drainage | Normal. | Mitral valve | Annulus = 16mm |
| Pulmonary venous drainage | Normal | Tricuspid valve | Annulus = 17mm |
| Atrioventricular connection | Concordant |  | TAPSE = 19mm |
| Ventriculoarterial connection | Concordant | **Ventricles** |  |
| Ventricular loop | d-Loop | Left ventricle | Normal |
|  |  | Right ventricle | Normal |
| **Septae** |  | **Coronary arteries** | ----- |
| Interventricular septum | Intact | **Doppler Measurement** |  |
| Interatrial septum | PFO, L – R Shunt | Mitral | ----- |
| **Semilunar valves** |  | Aortic | ------- |
| Aortic valve | Annulus = 13mm | Tricuspid | ------- |
| Pulmonary valve | Annulus = 15mm | pulmonic | -------- |
| **Great arteries** | NRGA | **Aortic arch** | Left. No CoA. |
| Aorta | ----- | **PDA** | No |
| Pulmonary artery | Normal MPA and Branch PAs. |  |  |
| **M-Mode:** | | | |
| AO | mm | PWd | mm |
| LA | mm | PWs | mm |
| LVIDd | mm | EDV | ml |
| LVIDs | mm | ESV | ml |
| IVSs | mm | LVEF | 61% |
| IVSd | mm | FS | 31% |
| **Additional Information**: |  | | |
| No pericardial/Pleural effusion. | | | |
| **Final Diagnosis:** | | | |
| 1. {S, D, S} Levocardia. 2. PFO, L – R Shunt | | | |
| **Remark**: | | | |
| **Recommendation**: | | | |
| SIGNATURE  Done by: Tesfaye T., Pediatrician, Pediatric Cardiologist _______________ 02/09/2015Eth.C | | | |

| Patient Name: **Danawit Lingerew**. Referring Institute: **Adinas GH**. SEX/ Age: **F/5 10/12**. Date of Report: **02/09/15**.  Referral Diagnosis: **Palpitation + SOB + FTT. AGH12.3506.** | | | |
| --- | --- | --- | --- |
| **Features** | **Finding** | **Features** | **Finding** |
| **Profile** |  | **Atria** |  |
| Abdominal situs | Solitus | Left atrium | Dilated |
| Atrial Situs | Solitus | Right atrium | Normal |
| Cardiac position | Levocardia | **Atrioventricular valves** |  |
| Systemic venous drainage | Normal. | Mitral valve | Annulus = 24mm |
| Pulmonary venous drainage | Normal | Tricuspid valve | Annulus = 25mm |
| Atrioventricular connection | Concordant |  | TAPSE = 22mm |
| Ventriculoarterial connection | Concordant | **Ventricles** |  |
| Ventricular loop | d-Loop | Left ventricle | Dilated |
|  |  | Right ventricle | Normal |
| **Septae** |  | **Coronary arteries** | ----- |
| Interventricular septum | 10mm Inlet VSD with PM extension, L – R Shunt | **Doppler Measurement** |  |
| Interatrial septum | Intact | Mitral | ----- |
| **Semilunar valves** |  | Aortic | ------- |
| Aortic valve | Annulus = 17mm | Tricuspid | ------- |
| Pulmonary valve | Annulus = 19mm | pulmonic | -------- |
| **Great arteries** | NRGA | **Aortic arch** | Left. No CoA. |
| Aorta | ----- | **PDA** | No |
| Pulmonary artery | MPA =18mm. |  |  |
| **M-Mode:** | | | |
| AO | mm | PWd | mm |
| LA | mm | PWs | mm |
| LVIDd | mm | EDV | ml |
| LVIDs | mm | ESV | ml |
| IVSs | mm | LVEF | 61% |
| IVSd | mm | FS | 32% |
| **Additional Information**: |  | | |
| No pericardial/Pleural effusion. | | | |
| **Final Diagnosis:** | | | |
| 1. {S, D, S} Levocardia. 2. LA/LV Dilated 3. Moderate to Large Inlet VSD with PM extension 4. Normal Biventricular Systolic Function | | | |
| **Remark**: | | | |
| **Recommendation**: | | | |
| SIGNATURE  Done by: Tesfaye T., Pediatrician, Pediatric Cardiologist _______________ 02/09/2015Eth.C | | | |

| Patient Name: **Samrawit Mulusew**. Referring Institute: **TGSH**. SEX/ Age: **F/2 4/12**. Date of Report: **03/09/15**.  Referral Diagnosis: **DS. AGH12.3507.** | | | |
| --- | --- | --- | --- |
| **Features** | **Finding** | **Features** | **Finding** |
| **Profile** |  | **Atria** |  |
| Abdominal situs | Solitus | Left atrium | Normal |
| Atrial Situs | Solitus | Right atrium | Normal |
| Cardiac position | Levocardia | **Atrioventricular valves** |  |
| Systemic venous drainage | Normal. | Mitral valve | Annulus = 15mm |
| Pulmonary venous drainage | Normal | Tricuspid valve | Annulus = 16mm |
| Atrioventricular connection | Concordant |  | TAPSE = mm |
| Ventriculoarterial connection | Concordant | **Ventricles** |  |
| Ventricular loop | d-Loop | Left ventricle | Normal |
|  |  | Right ventricle | Normal |
| **Septae** |  | **Coronary arteries** | ----- |
| Interventricular septum | Intact | **Doppler Measurement** |  |
| Interatrial septum | Intact | Mitral | ----- |
| **Semilunar valves** |  | Aortic | ------- |
| Aortic valve | Annulus = 13mm | Tricuspid | ------- |
| Pulmonary valve | Annulus = 13mm | pulmonic | -------- |
| **Great arteries** | NRGA | **Aortic arch** | Left. No CoA. |
| Aorta | ----- | **PDA** | No |
| Pulmonary artery | Normal MPA and Branch PAs. |  |  |
| **M-Mode:**  Normal LV Function on eye balling | | | |
| AO | mm | PWd | mm |
| LA | mm | PWs | mm |
| LVIDd | mm | EDV | ml |
| LVIDs | mm | ESV | ml |
| IVSs | mm | LVEF | % |
| IVSd | mm | FS | % |
| **Additional Information**: |  | | |
| No pericardial/Pleural effusion. | | | |
| **Final Diagnosis:** | | | |
| 1. Normal Echocardiography Study   . | | | |
| **Remark**: | | | |
| **Recommendation**: | | | |
| SIGNATURE  Done by: Tesfaye T., Pediatrician, Pediatric Cardiologist _______________ 03/09/2015Eth.C | | | |

| Patient Name: **Fasikaw Fikre-Mariam**. Referring Institute: **FHRH**. SEX/ Age: **M/6years**. Date of Report: **03/09/15**.  Referral Diagnosis: **Easy Fatigability. AGH12.3508.** | | | |
| --- | --- | --- | --- |
| **Features** | **Finding** | **Features** | **Finding** |
| **Profile** |  | **Atria** |  |
| Abdominal situs | Solitus | Left atrium | Normal |
| Atrial Situs | Solitus | Right atrium | Normal |
| Cardiac position | Levocardia | **Atrioventricular valves** |  |
| Systemic venous drainage | Normal. | Mitral valve | Annulus = 20mm |
| Pulmonary venous drainage | Normal | Tricuspid valve | Annulus = 21mm |
| Atrioventricular connection | Concordant |  | TAPSE = 19mm |
| Ventriculoarterial connection | Concordant | **Ventricles** |  |
| Ventricular loop | d-Loop | Left ventricle | Normal |
|  |  | Right ventricle | Normal |
| **Septae** |  | **Coronary arteries** | ----- |
| Interventricular septum | Intact | **Doppler Measurement** |  |
| Interatrial septum | Intact | Mitral | ----- |
| **Semilunar valves** |  | Aortic | ------- |
| Aortic valve | Annulus = 16mm | Tricuspid | ------- |
| Pulmonary valve | Annulus = 18mm | pulmonic | -------- |
| **Great arteries** | NRGA | **Aortic arch** | Left. No CoA. |
| Aorta | ----- | **PDA** | No |
| Pulmonary artery | Normal MPA and Branch PAs. |  |  |
| **M-Mode:**  Normal LV Function on eye balling | | | |
| AO | mm | PWd | mm |
| LA | mm | PWs | mm |
| LVIDd | mm | EDV | ml |
| LVIDs | mm | ESV | ml |
| IVSs | mm | LVEF | % |
| IVSd | mm | FS | % |
| **Additional Information**: |  | | |
| No pericardial/Pleural effusion. | | | |
| **Final Diagnosis:** | | | |
| 1. Normal Echocardiography Study. | | | |
| **Remark**: | | | |
| **Recommendation**: | | | |
| SIGNATURE  Done by: Tesfaye T., Pediatrician, Pediatric Cardiologist _______________ 03/09/2015Eth.C | | | |

| Patient Name: **Arefa Yesuf**. Referring Institute: **Pawe Hospital**. SEX/ Age: **F/8months**. Date of Report: **03/09/15**.  Referral Diagnosis: **Down Syndrome + FTT. AGH12.3509.** | | | |
| --- | --- | --- | --- |
| **Features** | **Finding** | **Features** | **Finding** |
| **Profile** |  | **Atria** |  |
| Abdominal situs | Solitus | Left atrium | Mildly Dilated |
| Atrial Situs | Solitus | Right atrium | Normal |
| Cardiac position | Levocardia | **Atrioventricular valves** |  |
| Systemic venous drainage | Normal. | Mitral valve | Annulus = 15mm |
| Pulmonary venous drainage | Normal | Tricuspid valve | Annulus = 15mm |
| Atrioventricular connection | Concordant |  | TAPSE = 18mm |
| Ventriculoarterial connection | Concordant | **Ventricles** |  |
| Ventricular loop | d-Loop | Left ventricle | Mildly Dilated |
|  |  | Right ventricle | Normal |
| **Septae** |  | **Coronary arteries** | ----- |
| Interventricular septum | 7mm PM VSD, Partially closed by STL, L – R Shunt | **Doppler Measurement** |  |
| Interatrial septum | Intact | Mitral | ----- |
| **Semilunar valves** |  | Aortic | ------- |
| Aortic valve | Annulus = 11mm | Tricuspid | ------- |
| Pulmonary valve | Annulus = 13mm | pulmonic | -------- |
| **Great arteries** | NRGA | **Aortic arch** | Left. No CoA. |
| Aorta | ----- | **PDA** | No |
| Pulmonary artery | Normal MPA and Branch PAs. |  |  |
| **M-Mode:**  Normal LV Function on eye balling | | | |
| AO | mm | PWd | mm |
| LA | mm | PWs | mm |
| LVIDd | mm | EDV | ml |
| LVIDs | mm | ESV | ml |
| IVSs | mm | LVEF | % |
| IVSd | mm | FS | % |
| **Additional Information**: |  | | |
| No pericardial/Pleural effusion. | | | |
| **Final Diagnosis:** | | | |
| 1. {S, D, S} Levocardia. 2. LA/LV Mildly Dilated 3. Moderate PM VSD, L – R Shunt 4. Normal Biventricular Systolic Function | | | |
| **Remark**: | | | |
| **Recommendation**: | | | |
| SIGNATURE  Done by: Tesfaye T., Pediatrician, Pediatric Cardiologist _______________ 03/09/2015Eth.C | | | |

| Patient Name: **Kidus Fitsum**. Referring Institute: **FHRH**. SEX/ Age: **M/1 2/12**. Date of Report: **03/09/15**.  Referral Diagnosis: **Diaphoresis during BF + Fast Breathing. AGH12.3510.** | | | |
| --- | --- | --- | --- |
| **Features** | **Finding** | **Features** | **Finding** |
| **Profile** |  | **Atria** |  |
| Abdominal situs | Solitus | Left atrium | Normal |
| Atrial Situs | Solitus | Right atrium | Normal |
| Cardiac position | Levocardia | **Atrioventricular valves** |  |
| Systemic venous drainage | Normal. | Mitral valve | Annulus = 13mm |
| Pulmonary venous drainage | Normal | Tricuspid valve | Annulus = 12mm |
| Atrioventricular connection | Concordant |  | TAPSE = mm |
| Ventriculoarterial connection | Concordant | **Ventricles** |  |
| Ventricular loop | d-Loop | Left ventricle | Normal |
|  |  | Right ventricle | Normal |
| **Septae** |  | **Coronary arteries** | ----- |
| Interventricular septum | Intact | **Doppler Measurement** |  |
| Interatrial septum | Intact | Mitral | ----- |
| **Semilunar valves** |  | Aortic | ------- |
| Aortic valve | Annulus = 11mm | Tricuspid | ------- |
| Pulmonary valve | Annulus = 12mm | pulmonic | -------- |
| **Great arteries** | NRGA | **Aortic arch** | Left. No CoA. |
| Aorta | ----- | **PDA** | No |
| Pulmonary artery | Normal MPA and Branch PAs. |  |  |
| **M-Mode:**  Normal LV Function on eye balling | | | |
| AO | mm | PWd | mm |
| LA | mm | PWs | mm |
| LVIDd | mm | EDV | ml |
| LVIDs | mm | ESV | ml |
| IVSs | mm | LVEF | % |
| IVSd | mm | FS | % |
| **Additional Information**: |  | | |
| No pericardial/Pleural effusion. | | | |
| **Final Diagnosis:** | | | |
| 1. Normal Echocardiography Study. | | | |
| **Remark**: | | | |
| **Recommendation**: | | | |
| SIGNATURE  Done by: Tesfaye T., Pediatrician, Pediatric Cardiologist _______________ 03/09/2015Eth.C | | | |

| Patient Name: **Degisew Eshetu**. Referring Institute: **FHRH**. SEX/ Age: **M/13years**. Date of Report: **04/09/15**.  Referral Diagnosis: **Easy Fatigability + Palpitation. AGH12.3511.** | | | |
| --- | --- | --- | --- |
| **Features** | **Finding** | **Features** | **Finding** |
| **Profile** |  | **Atria** |  |
| Abdominal situs | Solitus | Left atrium | Markedly Dilated |
| Atrial Situs | Solitus | Right atrium | Dilated |
| Cardiac position | Levocardia | **Atrioventricular valves** |  |
| Systemic venous drainage | Normal. | Mitral valve | Annulus = 33mm. Thickened, Clubbed MVL. MVA= 0.7cm2. |
| Pulmonary venous drainage | Normal | Tricuspid valve | Annulus = 24mm |
| Atrioventricular connection | Concordant |  | TAPSE = 25mm |
| Ventriculoarterial connection | Concordant | **Ventricles** |  |
| Ventricular loop | d-Loop | Left ventricle | Markedly Dilated |
|  |  | Right ventricle | Dilated |
| **Septae** |  | **Coronary arteries** | ----- |
| Interventricular septum | Intact | **Doppler Measurement** |  |
| Interatrial septum | Intact | Mitral | Mild MR, Holosystolic, posterior projection, seen in two planes with jet velocity = 3.8m/sec. Severe MS, PPG/MPG = 25/15mmHG. |
| **Semilunar valves** |  | Aortic | Moderate AR, PHT = 300ms |
| Aortic valve | Annulus = 18mm | Tricuspid | Mild TR, PPG = 50mmHg |
| Pulmonary valve | Annulus = 19mm | pulmonic | -------- |
| **Great arteries** | NRGA | **Aortic arch** | Left. No CoA. |
| Aorta | ----- | **PDA** | No |
| Pulmonary artery | Normal |  |  |
| **M-Mode:** | | | |
| AO | mm | PWd | mm |
| LA | mm | PWs | mm |
| LVIDd | mm | EDV | ml |
| LVIDs | mm | ESV | ml |
| IVSs | mm | LVEF | 58% |
| IVSd | mm | FS | 30% |
| **Additional Information**: |  | | |
| No pericardial/Pleural effusion. | | | |
| **Final Diagnosis:** | | | |
| 1. {S, D, S} Levocardia. 2. All chambers dilated 3. Thickened, clubbed MVL 4. Mild MR 5. Severe MS 6. Moderate AR 7. Mild TR 8. Moderate Pulmonary Hypertension 9. Normal Biventricular Systolic Function | | | |
| SIGNATURE  Done by: Tesfaye T., Pediatrician, Pediatric Cardiologist _______________ 04/09/2015Eth.C | | | |

| Patient Name: **Baby of Ageritu Ayalew**. Referring Institute: **TGSH**. SEX/ Age: **F/30days**. Date of Report: **04/09/15**.  Referral Diagnosis: **Syndromic. AGH12.3512.** | | | |
| --- | --- | --- | --- |
| **Features** | **Finding** | **Features** | **Finding** |
| **Profile** |  | **Atria** |  |
| Abdominal situs | Solitus | Left atrium | Normal |
| Atrial Situs | Solitus | Right atrium | Normal |
| Cardiac position | Levocardia | **Atrioventricular valves** |  |
| Systemic venous drainage | Normal. | Mitral valve | Annulus = 12mm |
| Pulmonary venous drainage | Normal | Tricuspid valve | Annulus = 12mm |
| Atrioventricular connection | Concordant |  | TAPSE = mm |
| Ventriculoarterial connection | Concordant | **Ventricles** |  |
| Ventricular loop | d-Loop | Left ventricle | Normal |
|  |  | Right ventricle | Normal |
| **Septae** |  | **Coronary arteries** | ----- |
| Interventricular septum | Intact | **Doppler Measurement** |  |
| Interatrial septum | PFO, L – R Shunt | Mitral | ----- |
| **Semilunar valves** |  | Aortic | ------- |
| Aortic valve | Annulus = 10mm | Tricuspid | ------- |
| Pulmonary valve | Annulus = 9mm | pulmonic | -------- |
| **Great arteries** | NRGA | **Aortic arch** | Left. No CoA. |
| Aorta | ----- | **PDA** | No |
| Pulmonary artery | Normal MPA and Branch PAs. |  |  |
| **M-Mode:**  Normal LV Function on eye balling | | | |
| AO | mm | PWd | mm |
| LA | mm | PWs | mm |
| LVIDd | mm | EDV | ml |
| LVIDs | mm | ESV | ml |
| IVSs | mm | LVEF | % |
| IVSd | mm | FS | % |
| **Additional Information**: |  | | |
| No pericardial/Pleural effusion. | | | |
| **Final Diagnosis:** | | | |
| 1. {S, D, S} Levocardia. 2. PFO, L – R Shunt | | | |
| **Remark**: | | | |
| **Recommendation**: | | | |
| SIGNATURE  Done by: Tesfaye T., Pediatrician, Pediatric Cardiologist _______________ 04/09/2015Eth.C | | | |

| Patient Name: **Natnael Esubalew**. Referring Institute: **Shegaw Motta GH**. SEX/ Age: **M/6 5/12**. Date of Report: **04/09/15**.  Referral Diagnosis: **ARF. AGH12.3513.** | | | |
| --- | --- | --- | --- |
| **Features** | **Finding** | **Features** | **Finding** |
| **Profile** |  | **Atria** |  |
| Abdominal situs | Solitus | Left atrium | Normal |
| Atrial Situs | Solitus | Right atrium | Normal |
| Cardiac position | Levocardia | **Atrioventricular valves** |  |
| Systemic venous drainage | Normal. | Mitral valve | Annulus = 20mm |
| Pulmonary venous drainage | Normal | Tricuspid valve | Annulus = 20mm |
| Atrioventricular connection | Concordant |  | TAPSE = 20mm |
| Ventriculoarterial connection | Concordant | **Ventricles** |  |
| Ventricular loop | d-Loop | Left ventricle | Normal |
|  |  | Right ventricle | Normal |
| **Septae** |  | **Coronary arteries** | ----- |
| Interventricular septum | Intact | **Doppler Measurement** |  |
| Interatrial septum | Intact | Mitral | ----- |
| **Semilunar valves** |  | Aortic | ------- |
| Aortic valve | Annulus = 15mm | Tricuspid | ------- |
| Pulmonary valve | Annulus = 19mm | pulmonic | -------- |
| **Great arteries** | NRGA | **Aortic arch** | Left. No CoA. |
| Aorta | ----- | **PDA** | No |
| Pulmonary artery | Normal MPA and Branch PAs. |  |  |
| **M-Mode:** | | | |
| AO | mm | PWd | mm |
| LA | mm | PWs | mm |
| LVIDd | mm | EDV | ml |
| LVIDs | mm | ESV | ml |
| IVSs | mm | LVEF | 70% |
| IVSd | mm | FS | 38% |
| **Additional Information**: |  | | |
| No pericardial/Pleural effusion. | | | |
| **Final Diagnosis:** | | | |
| 1. Normal Echocardiography Study. | | | |
| **Remark**: Normal Echocardiography Study doesn’t rule out Acute Rheumatic Fever | | | |
| **Recommendation**: | | | |
| SIGNATURE  Done by: Tesfaye T., Pediatrician, Pediatric Cardiologist _______________ 04/09/2015Eth.C | | | |

| Patient Name: **Haset Yawukal**. Referring Institute: **Adinas GH**. SEX/ Age: **F/7months**. Date of Report: **04/09/15**.  Referral Diagnosis: **Recurrent Chest Infection + FTT. AGH12.3514.** | | | |
| --- | --- | --- | --- |
| **Features** | **Finding** | **Features** | **Finding** |
| **Profile** |  | **Atria** |  |
| Abdominal situs | Solitus | Left atrium | Dilated |
| Atrial Situs | Solitus | Right atrium | Normal |
| Cardiac position | Levocardia | **Atrioventricular valves** |  |
| Systemic venous drainage | Normal. | Mitral valve | Annulus = 15mm |
| Pulmonary venous drainage | Normal | Tricuspid valve | Annulus = 13mm |
| Atrioventricular connection | Concordant |  | TAPSE = mm |
| Ventriculoarterial connection | Concordant | **Ventricles** |  |
| Ventricular loop | d-Loop | Left ventricle | Dilated |
|  |  | Right ventricle | Normal |
| **Septae** |  | **Coronary arteries** | ----- |
| Interventricular septum | 9mm PM VSD, Partially covered by STL, L – R Shunt | **Doppler Measurement** |  |
| Interatrial septum | PFO, L – R Shunt | Mitral | ----- |
| **Semilunar valves** |  | Aortic | ------- |
| Aortic valve | Annulus = 11mm | Tricuspid | ------- |
| Pulmonary valve | Annulus = 14mm. Doming PV | pulmonic | Mild Valvular PS, PPG = 21mmHg |
| **Great arteries** | NRGA | **Aortic arch** | Left. No CoA. |
| Aorta | ----- | **PDA** | No |
| Pulmonary artery | Normal |  |  |
| **M-Mode:**  Normal LV Function on eye balling | | | |
| AO | mm | PWd | mm |
| LA | mm | PWs | mm |
| LVIDd | mm | EDV | ml |
| LVIDs | mm | ESV | ml |
| IVSs | mm | LVEF | % |
| IVSd | mm | FS | % |
| **Additional Information**: |  | | |
| No pericardial/Pleural effusion. | | | |
| **Final Diagnosis:** | | | |
| 1. {S, D, S} Levocardia. 2. LA/LV Dilated 3. Large PM VSD, Partially covered by STL, L – R Shunt 4. Doming PV 5. Mild Valvular PS 6. Normal LV Systolic Function | | | |
| **Remark**: | | | |
| **Recommendation**: | | | |
| SIGNATURE  Done by: Tesfaye T., Pediatrician, Pediatric Cardiologist _______________ 04/09/2015Eth.C | | | |

| Patient Name: **Zebu Berihun**. Referring Institute: **Dur-Bete PH**. SEX/ Age: **F/11years**. Date of Report: **05/09/15**.  Referral Diagnosis: **NYHA – IV CHF + Palpitation + Easy Fatigability. AGH12.3515.** | | | |
| --- | --- | --- | --- |
| **Features** | **Finding** | **Features** | **Finding** |
| **Profile** |  | **Atria** |  |
| Abdominal situs | Solitus | Left atrium | Normal |
| Atrial Situs | Solitus | Right atrium | Normal |
| Cardiac position | Levocardia | **Atrioventricular valves** |  |
| Systemic venous drainage | Normal. | Mitral valve | Annulus = 17mm |
| Pulmonary venous drainage | Normal | Tricuspid valve | Annulus = 17mm |
| Atrioventricular connection | Concordant |  | TAPSE = 19mm |
| Ventriculoarterial connection | Concordant | **Ventricles** |  |
| Ventricular loop | d-Loop | Left ventricle | Normal |
|  |  | Right ventricle | Normal |
| **Septae** |  | **Coronary arteries** | ----- |
| Interventricular septum | Intact | **Doppler Measurement** |  |
| Interatrial septum | Intact | Mitral | ----- |
| **Semilunar valves** |  | Aortic | ------- |
| Aortic valve | Annulus = 16mm | Tricuspid | ------- |
| Pulmonary valve | Annulus = 18mm | pulmonic | -------- |
| **Great arteries** | NRGA | **Aortic arch** | Left. No CoA. |
| Aorta | ----- | **PDA** | No |
| Pulmonary artery | Normal MPA and Branch PAs. |  |  |
| **M-Mode:** | | | |
| AO | mm | PWd | mm |
| LA | mm | PWs | mm |
| LVIDd | mm | EDV | ml |
| LVIDs | mm | ESV | ml |
| IVSs | mm | LVEF | 70% |
| IVSd | mm | FS | 38% |
| **Additional Information**: |  | | |
| No pericardial/Pleural effusion. | | | |
| **Final Diagnosis:** | | | |
| 1. Normal Echocardiography Study. | | | |
| **Remark**: Rhythm abnormality was detected during echocardiography Study | | | |
| **Recommendation**: | | | |
| SIGNATURE  Done by: Tesfaye T., Pediatrician, Pediatric Cardiologist _______________ 05/09/2015Eth.C | | | |

| Patient Name: **Addisu Melesse**. Referring Institute: **Eyasta MS.** SEX/ Age: **M/2 7/12**. Date of Report: **05/09/15**.  Referral Diagnosis: **Incidental. AGH12.3516.** | | | |
| --- | --- | --- | --- |
| **Features** | **Finding** | **Features** | **Finding** |
| **Profile** |  | **Atria** |  |
| Abdominal situs | Solitus | Left atrium | Normal |
| Atrial Situs | Solitus | Right atrium | Dilated |
| Cardiac position | Levocardia | **Atrioventricular valves** |  |
| Systemic venous drainage | Normal. | Mitral valve | Annulus = 15mm |
| Pulmonary venous drainage | Normal | Tricuspid valve | Annulus = 20mm |
| Atrioventricular connection | Concordant |  | TAPSE = 20mm |
| Ventriculoarterial connection | Concordant | **Ventricles** |  |
| Ventricular loop | d-Loop | Left ventricle | Normal |
|  |  | Right ventricle | Dilated & Hypertrophied |
| **Septae** |  | **Coronary arteries** | ----- |
| Interventricular septum | Non-Restrictive Mal-aligned Sub-aortic VSD, R – L Shunt | **Doppler Measurement** |  |
| Interatrial septum | Intact | Mitral | ----- |
| **Semilunar valves** |  | Aortic | ------- |
| Aortic valve | Annulus = 16mm | Tricuspid | ------- |
| Pulmonary valve | Annulus = 11mm. Doming PV | pulmonic | Severe Valvular PS, PPG = 76mmHg |
| **Great arteries** | NRGA | **Aortic arch** | Left. No CoA. |
| Aorta | ----- | **PDA** | No |
| Pulmonary artery | Smallish MPA and Branch PAs. |  |  |
| **M-Mode:** | | | |
| AO | mm | PWd | mm |
| LA | mm | PWs | mm |
| LVIDd | mm | EDV | ml |
| LVIDs | mm | ESV | ml |
| IVSs | mm | LVEF | 62% |
| IVSd | mm | FS | 31% |
| **Additional Information**: |  | | |
| No pericardial/Pleural effusion. | | | |
| **Final Diagnosis:** | | | |
| 1. {S, D, S} Levocardia. 2. RA/RV Dilated, RV Hypertrophied 3. TOF | | | |
| SIGNATURE  Done by: Tesfaye T., Pediatrician, Pediatric Cardiologist _______________ 05/09/2015Eth.C | | | |

| Patient Name: **Maritu Abere**. Referring Institute: **Adinas GH**. SEX/ Age: **F/13 5/12**. Date of Report: **05/09/15**.  Referral Diagnosis: **SOB + ?ILD. AGH12.3517.** | | | |
| --- | --- | --- | --- |
| **Features** | **Finding** | **Features** | **Finding** |
| **Profile** |  | **Atria** |  |
| Abdominal situs | Solitus | Left atrium | Normal |
| Atrial Situs | Solitus | Right atrium | Normal |
| Cardiac position | Levocardia | **Atrioventricular valves** |  |
| Systemic venous drainage | Normal. | Mitral valve | Annulus = 20mm |
| Pulmonary venous drainage | Normal | Tricuspid valve | Annulus = 18mm |
| Atrioventricular connection | Concordant |  | TAPSE = 19mm |
| Ventriculoarterial connection | Concordant | **Ventricles** |  |
| Ventricular loop | d-Loop | Left ventricle | Normal |
|  |  | Right ventricle | Normal |
| **Septae** |  | **Coronary arteries** | ----- |
| Interventricular septum | Intact | **Doppler Measurement** |  |
| Interatrial septum | Intact | Mitral | Trivial MR, JET VELOCITY = 3.5m/sec. |
| **Semilunar valves** |  | Aortic | ------- |
| Aortic valve | Annulus = 15mm | Tricuspid | ------- |
| Pulmonary valve | Annulus = 21mm | pulmonic | -------- |
| **Great arteries** | NRGA | **Aortic arch** | Left. No CoA. |
| Aorta | ----- | **PDA** | No |
| Pulmonary artery | Normal MPA and Branch PAs. |  |  |
| **M-Mode:** | | | |
| AO | mm | PWd | mm |
| LA | mm | PWs | mm |
| LVIDd | mm | EDV | ml |
| LVIDs | mm | ESV | ml |
| IVSs | mm | LVEF | 65% |
| IVSd | mm | FS | 35% |
| **Additional Information**: |  | | |
| No pericardial/Pleural effusion. | | | |
| **Final Diagnosis:** | | | |
| 1. Normal Echocardiography Study | | | |
| **Remark**: | | | |
| **Recommendation**: | | | |
| SIGNATURE  Done by: Tesfaye T., Pediatrician, Pediatric Cardiologist _______________ 05/09/2015Eth.C | | | |

| Patient Name: **Tiru-abay Azimeraw**. Referring Institute: **FHRH**. SEX/ Age: **M/6years**. Date of Report: **05/09/15**.  Referral Diagnosis: **HSM @ apex. AGH12.3518.** | | | |
| --- | --- | --- | --- |
| **Features** | **Finding** | **Features** | **Finding** |
| **Profile** |  | **Atria** |  |
| Abdominal situs | Solitus | Left atrium | Normal |
| Atrial Situs | Solitus | Right atrium | Normal |
| Cardiac position | Levocardia | **Atrioventricular valves** |  |
| Systemic venous drainage | Normal. | Mitral valve | Annulus = 17mm |
| Pulmonary venous drainage | Normal | Tricuspid valve | Annulus = 18mm |
| Atrioventricular connection | Concordant |  | TAPSE = 20mm |
| Ventriculoarterial connection | Concordant | **Ventricles** |  |
| Ventricular loop | d-Loop | Left ventricle | Normal |
|  |  | Right ventricle | Normal |
| **Septae** |  | **Coronary arteries** | ----- |
| Interventricular septum | Intact | **Doppler Measurement** |  |
| Interatrial septum | Intact | Mitral | ----- |
| **Semilunar valves** |  | Aortic | ------- |
| Aortic valve | Annulus = 16mm | Tricuspid | ------- |
| Pulmonary valve | Annulus = mm | pulmonic | -------- |
| **Great arteries** | NRGA | **Aortic arch** | Left. No CoA. |
| Aorta | ----- | **PDA** | No |
| Pulmonary artery | Normal MPA and Branch PAs. |  |  |
| **M-Mode:** | | | |
| AO | mm | PWd | mm |
| LA | mm | PWs | mm |
| LVIDd | mm | EDV | ml |
| LVIDs | mm | ESV | ml |
| IVSs | mm | LVEF | 67% |
| IVSd | mm | FS | 36% |
| **Additional Information**: |  | | |
| No pericardial/Pleural effusion. | | | |
| **Final Diagnosis:** | | | |
| 1. Normal Echocardiography Study. | | | |
| **Remark**: | | | |
| **Recommendation**: | | | |
| SIGNATURE  Done by: Tesfaye T., Pediatrician, Pediatric Cardiologist _______________ 05/09/2015Eth.C | | | |

| Patient Name: **Misanesh Tadesse**. Referring Institute: **FHRH**. SEX/ Age: **F/9years**. Date of Report: **05/09/15**.  Referral Diagnosis: **HSM. AGH12.3519.** | | | |
| --- | --- | --- | --- |
| **Features** | **Finding** | **Features** | **Finding** |
| **Profile** |  | **Atria** |  |
| Abdominal situs | Solitus | Left atrium | Normal |
| Atrial Situs | Solitus | Right atrium | Normal |
| Cardiac position | Levocardia | **Atrioventricular valves** |  |
| Systemic venous drainage | Normal. | Mitral valve | Annulus = 21mm |
| Pulmonary venous drainage | Normal | Tricuspid valve | Annulus = 20mm |
| Atrioventricular connection | Concordant |  | TAPSE = 23mm |
| Ventriculoarterial connection | Concordant | **Ventricles** |  |
| Ventricular loop | d-Loop | Left ventricle | Normal |
|  |  | Right ventricle | Normal |
| **Septae** |  | **Coronary arteries** | ----- |
| Interventricular septum | Intact | **Doppler Measurement** |  |
| Interatrial septum | Intact | Mitral | ----- |
| **Semilunar valves** |  | Aortic | ------- |
| Aortic valve | Annulus = 17mm | Tricuspid | ------- |
| Pulmonary valve | Annulus = 17mm | pulmonic | -------- |
| **Great arteries** | NRGA | **Aortic arch** | Left. No CoA. |
| Aorta | ----- | **PDA** | No |
| Pulmonary artery | Normal MPA and Branch PAs. |  |  |
| **M-Mode:** | | | |
| AO | mm | PWd | mm |
| LA | mm | PWs | mm |
| LVIDd | mm | EDV | ml |
| LVIDs | mm | ESV | ml |
| IVSs | mm | LVEF | 61% |
| IVSd | mm | FS | 32% |
| **Additional Information**: |  | | |
| No pericardial/Pleural effusion. | | | |
| **Final Diagnosis:** | | | |
| 1. {S, D, S} Levocardia. | | | |
| **Remark**: Extreme tachycardia during echocardiography Study | | | |
| **Recommendation**: Do ECG | | | |
| SIGNATURE  Done by: Tesfaye T., Pediatrician, Pediatric Cardiologist _______________ 05/09/2015Eth.C | | | |

| Patient Name: **Ketema Atalay**. Referring Institute: **Motta GH**. SEX/ Age: **F/6months**. Date of Report: **05/09/15**.  Referral Diagnosis: **Diaphoresis during BF and BF Interruption. AGH12.3520.** | | | |
| --- | --- | --- | --- |
| **Features** | **Finding** | **Features** | **Finding** |
| **Profile** |  | **Atria** |  |
| Abdominal situs | Solitus | Left atrium | Normal |
| Atrial Situs | Solitus | Right atrium | Normal |
| Cardiac position | Levocardia | **Atrioventricular valves** |  |
| Systemic venous drainage | Normal. | Mitral valve | Annulus = 12mm |
| Pulmonary venous drainage | Normal | Tricuspid valve | Annulus = 13mm |
| Atrioventricular connection | Concordant |  | TAPSE = mm |
| Ventriculoarterial connection | Concordant | **Ventricles** |  |
| Ventricular loop | d-Loop | Left ventricle | Normal |
|  |  | Right ventricle | Normal |
| **Septae** |  | **Coronary arteries** | ----- |
| Interventricular septum | Intact | **Doppler Measurement** |  |
| Interatrial septum | Intact | Mitral | ----- |
| **Semilunar valves** |  | Aortic | ------- |
| Aortic valve | Annulus = 11mm | Tricuspid | ------- |
| Pulmonary valve | Annulus = 11mm | pulmonic | -------- |
| **Great arteries** | NRGA | **Aortic arch** | Left. No CoA. |
| Aorta | ----- | **PDA** | No |
| Pulmonary artery | Normal MPA and Branch PAs. |  |  |
| **M-Mode:**  Normal LV Function on eye balling | | | |
| AO | mm | PWd | mm |
| LA | mm | PWs | mm |
| LVIDd | mm | EDV | ml |
| LVIDs | mm | ESV | ml |
| IVSs | mm | LVEF | % |
| IVSd | mm | FS | % |
| **Additional Information**: |  | | |
| No pericardial/Pleural effusion. | | | |
| **Final Diagnosis:** | | | |
| 1. Normal Echocardiography Study. | | | |
| **Remark**: | | | |
| **Recommendation**: | | | |
| SIGNATURE  Done by: Tesfaye T., Pediatrician, Pediatric Cardiologist _______________ 05/09/2015Eth.C | | | |

| Patient Name: **Bethelihem Amlaku**. Referring Institute: **Amaris PSC**. SEX/ Age: **F/9months**. Date of Report: **05/09/15**.  Referral Diagnosis: **R/O IE. AGH12.3521.** | | | |
| --- | --- | --- | --- |
| **Features** | **Finding** | **Features** | **Finding** |
| **Profile** |  | **Atria** |  |
| Abdominal situs | Solitus | Left atrium | Normal |
| Atrial Situs | Solitus | Right atrium | Normal |
| Cardiac position | Levocardia | **Atrioventricular valves** |  |
| Systemic venous drainage | Normal. | Mitral valve | Annulus = 12mm |
| Pulmonary venous drainage | Normal | Tricuspid valve | Annulus = 14mm |
| Atrioventricular connection | Concordant |  | TAPSE = mm |
| Ventriculoarterial connection | Concordant | **Ventricles** |  |
| Ventricular loop | d-Loop | Left ventricle | Normal |
|  |  | Right ventricle | Normal |
| **Septae** |  | **Coronary arteries** | ----- |
| Interventricular septum | 3mm PM VSD, L – R Shunt with a gradient of 67mmHg | **Doppler Measurement** |  |
| Interatrial septum | Intact | Mitral | ----- |
| **Semilunar valves** |  | Aortic | ------- |
| Aortic valve | Annulus = 11mm | Tricuspid | ------- |
| Pulmonary valve | Annulus = 12mm | pulmonic | -------- |
| **Great arteries** | NRGA | **Aortic arch** | Left. No CoA. |
| Aorta | ----- | **PDA** | No |
| Pulmonary artery | Normal MPA and Branch PAs. |  |  |
| **M-Mode:**  Normal LV Function on eye balling | | | |
| AO | mm | PWd | mm |
| LA | mm | PWs | mm |
| LVIDd | mm | EDV | ml |
| LVIDs | mm | ESV | ml |
| IVSs | mm | LVEF | % |
| IVSd | mm | FS | % |
| **Additional Information**: |  | | |
| No pericardial/Pleural effusion. | | | |
| **Final Diagnosis:** | | | |
| 1. {S, D, S} Levocardia. 2. Restrictive PM VSD, L – R Shunt | | | |
| **Remark**: Echocardiography Study doesn’t rule out IE | | | |
| **Recommendation**: | | | |
| SIGNATURE  Done by: Tesfaye T., Pediatrician, Pediatric Cardiologist _______________ 05/09/2015Eth.C | | | |

| Patient Name: **Abel Getinet**. Referring Institute: **FHRH**. SEX/ Age: **M/13years**. Date of Report: **07/09/15**.  Referral Diagnosis: **Easy Fatigability + Palpitation. AGH12.3522.** | | | |
| --- | --- | --- | --- |
| **Features** | **Finding** | **Features** | **Finding** |
| **Profile** |  | **Atria** |  |
| Abdominal situs | Solitus | Left atrium | Normal |
| Atrial Situs | Solitus | Right atrium | Normal |
| Cardiac position | Levocardia | **Atrioventricular valves** |  |
| Systemic venous drainage | Normal. | Mitral valve | Annulus = 22mm |
| Pulmonary venous drainage | Normal | Tricuspid valve | Annulus = 22mm |
| Atrioventricular connection | Concordant |  | TAPSE = 24mm |
| Ventriculoarterial connection | Concordant | **Ventricles** |  |
| Ventricular loop | d-Loop | Left ventricle | Normal |
|  |  | Right ventricle | Normal |
| **Septae** |  | **Coronary arteries** | ----- |
| Interventricular septum | Intact | **Doppler Measurement** |  |
| Interatrial septum | Intact | Mitral | ----- |
| **Semilunar valves** |  | Aortic | ------- |
| Aortic valve | Annulus = 18mm | Tricuspid | ------- |
| Pulmonary valve | Annulus = 19mm | pulmonic | -------- |
| **Great arteries** | NRGA | **Aortic arch** | Left. No CoA. |
| Aorta | ----- | **PDA** | No |
| Pulmonary artery | Normal MPA and Branch PAs. |  |  |
| **M-Mode:** | | | |
| AO | mm | PWd | mm |
| LA | mm | PWs | mm |
| LVIDd | mm | EDV | ml |
| LVIDs | mm | ESV | ml |
| IVSs | mm | LVEF | 69% |
| IVSd | mm | FS | 39% |
| **Additional Information**: |  | | |
| No pericardial/Pleural effusion. | | | |
| **Final Diagnosis:** | | | |
| 1. Normal Echocardiography Study. | | | |
| **Remark**: Acute Rheumatic Fever cannot be ruled out with Normal Echocardiography Study. | | | |
| **Recommendation**: | | | |
| SIGNATURE  Done by: Tesfaye T., Pediatrician, Pediatric Cardiologist _______________ 07/09/2015Eth.C | | | |

| Patient Name: **Selamawit Shumet**. Referring Institute: **FHRH**. SEX/ Age: **F/10years**. Date of Report: **07/09/15**.  Referral Diagnosis: **Chest Pain + Palpitation + Easy Fatigability. AGH12.3523.** | | | |
| --- | --- | --- | --- |
| **Features** | **Finding** | **Features** | **Finding** |
| **Profile** |  | **Atria** |  |
| Abdominal situs | Solitus | Left atrium | Normal |
| Atrial Situs | Solitus | Right atrium | Normal |
| Cardiac position | Levocardia | **Atrioventricular valves** |  |
| Systemic venous drainage | Normal. | Mitral valve | Annulus = 25mm |
| Pulmonary venous drainage | Normal | Tricuspid valve | Annulus = 25mm |
| Atrioventricular connection | Concordant |  | TAPSE = 23mm |
| Ventriculoarterial connection | Concordant | **Ventricles** |  |
| Ventricular loop | d-Loop | Left ventricle | Normal |
|  |  | Right ventricle | Normal |
| **Septae** |  | **Coronary arteries** | ----- |
| Interventricular septum | Intact | **Doppler Measurement** |  |
| Interatrial septum | 9mm OS ASD, L – R Shunt | Mitral | ----- |
| **Semilunar valves** |  | Aortic | ------- |
| Aortic valve | Annulus = 18mm | Tricuspid | ------- |
| Pulmonary valve | Annulus = 19mm | pulmonic | -------- |
| **Great arteries** | NRGA | **Aortic arch** | Left. No CoA. |
| Aorta | ----- | **PDA** | No |
| Pulmonary artery | Normal MPA and Branch PAs. |  |  |
| **M-Mode:** | | | |
| AO | mm | PWd | mm |
| LA | mm | PWs | mm |
| LVIDd | mm | EDV | ml |
| LVIDs | mm | ESV | ml |
| IVSs | mm | LVEF | 66% |
| IVSd | mm | FS | 36% |
| **Additional Information**: |  | | |
| No pericardial/Pleural effusion. | | | |
| **Final Diagnosis:** | | | |
| 1. {S, D, S} Levocardia. 2. Moderate OS ASD, L – R Shunt | | | |
| **Remark**: | | | |
| **Recommendation**: | | | |
| SIGNATURE  Done by: Tesfaye T., Pediatrician, Pediatric Cardiologist _______________ 07/09/2015Eth.C | | | |

| Patient Name: **Netsanet Mengistu**. Referring Institute: **FHRH**. SEX/ Age: **F/4 6/12**. Date of Report: **08/09/15**.  Referral Diagnosis: **RD. AGH12.3524.** | | | |
| --- | --- | --- | --- |
| **Features** | **Finding** | **Features** | **Finding** |
| **Profile** |  | **Atria** |  |
| Abdominal situs | Solitus | Left atrium | Normal |
| Atrial Situs | Solitus | Right atrium | Normal |
| Cardiac position | Levocardia | **Atrioventricular valves** |  |
| Systemic venous drainage | Normal. | Mitral valve | Annulus = 17mm. Patulous MVL |
| Pulmonary venous drainage | Normal | Tricuspid valve | Annulus = 17mm |
| Atrioventricular connection | Concordant |  | TAPSE = mm |
| Ventriculoarterial connection | Concordant | **Ventricles** |  |
| Ventricular loop | d-Loop | Left ventricle | Normal |
|  |  | Right ventricle | Normal |
| **Septae** |  | **Coronary arteries** | ----- |
| Interventricular septum | Intact | **Doppler Measurement** |  |
| Interatrial septum | Intact | Mitral | Trivial MR, Incomplete signal, seen in two planes with jet velocity = 3m/sec. |
| **Semilunar valves** |  | Aortic | ------- |
| Aortic valve | Annulus = 14mm | Tricuspid | ------- |
| Pulmonary valve | Annulus = 16mm | pulmonic | -------- |
| **Great arteries** | NRGA | **Aortic arch** | Left. No CoA. |
| Aorta | ----- | **PDA** | No |
| Pulmonary artery | Normal MPA and Branch PAs. |  |  |
| **M-Mode:** | | | |
| AO | mm | PWd | mm |
| LA | mm | PWs | mm |
| LVIDd | mm | EDV | ml |
| LVIDs | mm | ESV | ml |
| IVSs | mm | LVEF | 63% |
| IVSd | mm | FS | 34% |
| **Additional Information**: |  | | |
| No pericardial/Pleural effusion. | | | |
| **Final Diagnosis:** | | | |
| 1. {S, D, S} Levocardia. 2. Patulous MVL 3. Trivial MR | | | |
| **Remark**: Borderline RHD Shall be considered | | | |
| **Recommendation**: Secondary prophylaxis for at least a year. Do echo after a year | | | |
| SIGNATURE  Done by: Tesfaye T., Pediatrician, Pediatric Cardiologist _______________ 08/09/2015Eth.C | | | |

| Patient Name: **Atitegeb Simeneh**. Referring Institute: **FHRH**. SEX/ Age: **F/3months**. Date of Report: **08/09/15**.  Referral Diagnosis: **RD. AGH12.3525.** | | | |
| --- | --- | --- | --- |
| **Features** | **Finding** | **Features** | **Finding** |
| **Profile** |  | **Atria** |  |
| Abdominal situs | Solitus | Left atrium | Normal |
| Atrial Situs | Solitus | Right atrium | Normal |
| Cardiac position | Levocardia | **Atrioventricular valves** |  |
| Systemic venous drainage | Normal. | Mitral valve | Annulus = 11mm |
| Pulmonary venous drainage | Normal | Tricuspid valve | Annulus = 11mm |
| Atrioventricular connection | Concordant |  | TAPSE = mm |
| Ventriculoarterial connection | Concordant | **Ventricles** |  |
| Ventricular loop | d-Loop | Left ventricle | Normal |
|  |  | Right ventricle | Normal |
| **Septae** |  | **Coronary arteries** | ----- |
| Interventricular septum | Intact | **Doppler Measurement** |  |
| Interatrial septum | 4mm OS ASD, L – R Shunt | Mitral | ----- |
| **Semilunar valves** |  | Aortic | ------- |
| Aortic valve | Annulus = 9mm | Tricuspid | ------- |
| Pulmonary valve | Annulus = 11mm | pulmonic | -------- |
| **Great arteries** | NRGA | **Aortic arch** | Left. No CoA. |
| Aorta | ----- | **PDA** | No |
| Pulmonary artery | Normal MPA and Branch PAs. |  |  |
| **M-Mode:**  Normal LV Function on eye balling | | | |
| AO | mm | PWd | mm |
| LA | mm | PWs | mm |
| LVIDd | mm | EDV | ml |
| LVIDs | mm | ESV | ml |
| IVSs | mm | LVEF | % |
| IVSd | mm | FS | % |
| **Additional Information**: |  | | |
| No pericardial/Pleural effusion. | | | |
| **Final Diagnosis:** | | | |
| 1. {S, D, S} Levocardia. 2. Small OS ASD, L – R Shunt | | | |
| **Remark**: | | | |
| **Recommendation**: | | | |
| SIGNATURE  Done by: Tesfaye T., Pediatrician, Pediatric Cardiologist _______________ 08/09/2015Eth.C | | | |

| Patient Name: **Abebech Lealem**. Referring Institute: **Dangila PH**. SEX/ Age: **F/1 2/12**. Date of Report: **08/09/15**.  Referral Diagnosis: **FTT + ?CHD. AGH12.3526.** | | | |
| --- | --- | --- | --- |
| **Features** | **Finding** | **Features** | **Finding** |
| **Profile** |  | **Atria** |  |
| Abdominal situs | Solitus | Left atrium | Normal |
| Atrial Situs | Solitus | Right atrium | Normal |
| Cardiac position | Levocardia | **Atrioventricular valves** |  |
| Systemic venous drainage | Normal. | Mitral valve | Annulus = 14mm |
| Pulmonary venous drainage | Normal | Tricuspid valve | Annulus = 14mm |
| Atrioventricular connection | Concordant |  | TAPSE = mm |
| Ventriculoarterial connection | Concordant | **Ventricles** |  |
| Ventricular loop | d-Loop | Left ventricle | Normal |
|  |  | Right ventricle | Normal |
| **Septae** |  | **Coronary arteries** | ----- |
| Interventricular septum | Intact | **Doppler Measurement** |  |
| Interatrial septum | Intact | Mitral | ----- |
| **Semilunar valves** |  | Aortic | ------- |
| Aortic valve | Annulus = 11mm | Tricuspid | ------- |
| Pulmonary valve | Annulus = 12mm | pulmonic | -------- |
| **Great arteries** | NRGA | **Aortic arch** | Left. No CoA. |
| Aorta | ----- | **PDA** | No |
| Pulmonary artery | Normal MPA and Branch PAs. |  |  |
| **M-Mode:** | | | |
| AO | mm | PWd | mm |
| LA | mm | PWs | mm |
| LVIDd | mm | EDV | ml |
| LVIDs | mm | ESV | ml |
| IVSs | mm | LVEF | 61% |
| IVSd | mm | FS | 31% |
| **Additional Information**: |  | | |
| No pericardial/Pleural effusion. | | | |
| **Final Diagnosis:** | | | |
| 1. Normal Echocardiography Study. | | | |
| **Remark**: | | | |
| **Recommendation**: | | | |
| SIGNATURE  Done by: Tesfaye T., Pediatrician, Pediatric Cardiologist _______________ 08/09/2015Eth.C | | | |

| Patient Name: **Meklit Shimelash**. Referring Institute: **FHRH**. SEX/ Age: **F/2years**. Date of Report: **08/09/15**.  Referral Diagnosis: **Follow up echo for VSD.** | | | |
| --- | --- | --- | --- |
| **Features** | **Finding** | **Features** | **Finding** |
| **Profile** |  | **Atria** |  |
| Abdominal situs | Solitus | Left atrium | Mildly Dilated |
| Atrial Situs | Solitus | Right atrium | Normal |
| Cardiac position | Levocardia | **Atrioventricular valves** |  |
| Systemic venous drainage | Normal. | Mitral valve | Annulus = 17mm |
| Pulmonary venous drainage | Normal | Tricuspid valve | Annulus = 16mm |
| Atrioventricular connection | Concordant |  | TAPSE = 14mm |
| Ventriculoarterial connection | Concordant | **Ventricles** |  |
| Ventricular loop | d-Loop | Left ventricle | Mildly Dilated |
|  |  | Right ventricle | Normal |
| **Septae** |  | **Coronary arteries** | ----- |
| Interventricular septum | 6mm PM VSD, L – R Shunt | **Doppler Measurement** |  |
| Interatrial septum | Intact | Mitral | ----- |
| **Semilunar valves** |  | Aortic | ------- |
| Aortic valve | Annulus = 12mm | Tricuspid | ------- |
| Pulmonary valve | Annulus = 14mm | pulmonic | -------- |
| **Great arteries** | NRGA | **Aortic arch** | Left. No CoA. |
| Aorta | ----- | **PDA** | No |
| Pulmonary artery | Normal MPA and Branch PAs. |  |  |
| **M-Mode:** | | | |
| AO | mm | PWd | mm |
| LA | mm | PWs | mm |
| LVIDd | mm | EDV | ml |
| LVIDs | mm | ESV | ml |
| IVSs | mm | LVEF | 63% |
| IVSd | mm | FS | 33% |
| **Additional Information**: |  | | |
| No pericardial/Pleural effusion. | | | |
| **Final Diagnosis:** | | | |
| 1. {S, D, S} Levocardia. 2. Moderate PM VSD, L – R Shunt 3. Normal Biventricular Systolic Function | | | |
| **Remark**: | | | |
| **Recommendation**: | | | |
| SIGNATURE  Done by: Tesfaye T., Pediatrician, Pediatric Cardiologist _______________ 08/09/2015Eth.C | | | |

| Patient Name: **Mizanu Alemu**. Referring Institute: **FHRH**. SEX/ Age: **M/6years**. Date of Report: **08/09/15**.  Referral Diagnosis: **Sydenham’s Chorea. AGH12.3527.** | | | |
| --- | --- | --- | --- |
| **Features** | **Finding** | **Features** | **Finding** |
| **Profile** |  | **Atria** |  |
| Abdominal situs | Solitus | Left atrium | Normal |
| Atrial Situs | Solitus | Right atrium | Normal |
| Cardiac position | Levocardia | **Atrioventricular valves** |  |
| Systemic venous drainage | Normal. | Mitral valve | Annulus = 17mm. Patulous MVL |
| Pulmonary venous drainage | Normal | Tricuspid valve | Annulus = 19mm |
| Atrioventricular connection | Concordant |  | TAPSE = mm |
| Ventriculoarterial connection | Concordant | **Ventricles** |  |
| Ventricular loop | d-Loop | Left ventricle | Normal |
|  |  | Right ventricle | Normal |
| **Septae** |  | **Coronary arteries** | ----- |
| Interventricular septum | Intact | **Doppler Measurement** |  |
| Interatrial septum | Intact | Mitral | Mild MR, Incomplete Signal, seen in two planes with jet velocity = 2.8m/sec. |
| **Semilunar valves** |  | Aortic | Moderate AR, PHT = 317ms. |
| Aortic valve | Annulus = 17mm | Tricuspid | ------- |
| Pulmonary valve | Annulus = 18mm | pulmonic | -------- |
| **Great arteries** | NRGA | **Aortic arch** | Left. No CoA. |
| Aorta | ----- | **PDA** | No |
| Pulmonary artery | Normal MPA and Branch PAs. |  |  |
| **M-Mode:** | | | |
| AO | mm | PWd | mm |
| LA | mm | PWs | mm |
| LVIDd | mm | EDV | ml |
| LVIDs | mm | ESV | ml |
| IVSs | mm | LVEF | 61% |
| IVSd | mm | FS | 32% |
| **Additional Information**: |  | | |
| No pericardial/Pleural effusion. | | | |
| **Final Diagnosis:** | | | |
| 1. {S, D, S} Levocardia. 2. Patulous MVL 3. Mild MR 4. Moderate AR 5. Normal LV Systolic Function | | | |
| **Remark**: | | | |
| **Recommendation**: | | | |
| SIGNATURE  Done by: Tesfaye T., Pediatrician, Pediatric Cardiologist _______________ 08/09/2015Eth.C | | | |

| Patient Name: **Mekdes Zemen**. Referring Institute: **TGSH**. SEX/ Age: **F/1 9/12**. Date of Report: **08/09/15**.  Referral Diagnosis: **DS. AGH12.3528.** | | | |
| --- | --- | --- | --- |
| **Features** | **Finding** | **Features** | **Finding** |
| **Profile** |  | **Atria** |  |
| Abdominal situs | Solitus | Left atrium | Normal |
| Atrial Situs | Solitus | Right atrium | Normal |
| Cardiac position | Levocardia | **Atrioventricular valves** |  |
| Systemic venous drainage | Normal. | Mitral valve | Annulus = 13mm |
| Pulmonary venous drainage | Normal | Tricuspid valve | Annulus = 15mm |
| Atrioventricular connection | Concordant |  | TAPSE = mm |
| Ventriculoarterial connection | Concordant | **Ventricles** |  |
| Ventricular loop | d-Loop | Left ventricle | Normal |
|  |  | Right ventricle | Normal |
| **Septae** |  | **Coronary arteries** | ----- |
| Interventricular septum | Intact | **Doppler Measurement** |  |
| Interatrial septum | Intact | Mitral | ----- |
| **Semilunar valves** |  | Aortic | ------- |
| Aortic valve | Annulus = 13mm | Tricuspid | ------- |
| Pulmonary valve | Annulus = 15mm | pulmonic | -------- |
| **Great arteries** | NRGA | **Aortic arch** | Left. No CoA. |
| Aorta | ----- | **PDA** | No |
| Pulmonary artery | Normal MPA and Branch PAs. |  |  |
| **M-Mode:**  Normal LV Function on eye balling | | | |
| AO | mm | PWd | mm |
| LA | mm | PWs | mm |
| LVIDd | mm | EDV | ml |
| LVIDs | mm | ESV | ml |
| IVSs | mm | LVEF | % |
| IVSd | mm | FS | % |
| **Additional Information**: |  | | |
| No pericardial/Pleural effusion. | | | |
| **Final Diagnosis:** | | | |
| 1. Normal Echocardiography Study. | | | |
| **Remark**: | | | |
| **Recommendation**: | | | |
| SIGNATURE  Done by: Tesfaye T., Pediatrician, Pediatric Cardiologist _______________ 08/09/2015Eth.C | | | |

| Patient Name: **Kale-Ab Abebaw**. Referring Institute: **FHRH**. SEX/ Age: **M/1 6/12**. Date of Report: **09/09/15**.  Referral Diagnosis: **DS + FTT. AGH12.3529.** | | | |
| --- | --- | --- | --- |
| **Features** | **Finding** | **Features** | **Finding** |
| **Profile** |  | **Atria** |  |
| Abdominal situs | Solitus | Left atrium | Normal |
| Atrial Situs | Solitus | Right atrium | Normal |
| Cardiac position | Levocardia | **Atrioventricular valves** |  |
| Systemic venous drainage | Normal. | Mitral valve | Annulus = 12mm |
| Pulmonary venous drainage | Normal | Tricuspid valve | Annulus = 13mm |
| Atrioventricular connection | Concordant |  | TAPSE = mm |
| Ventriculoarterial connection | Concordant | **Ventricles** |  |
| Ventricular loop | d-Loop | Left ventricle | Normal |
|  |  | Right ventricle | Normal |
| **Septae** |  | **Coronary arteries** | ----- |
| Interventricular septum | Intact | **Doppler Measurement** |  |
| Interatrial septum | PFO, L – R Shunt | Mitral | ----- |
| **Semilunar valves** |  | Aortic | ------- |
| Aortic valve | Annulus = 12mm | Tricuspid | ------- |
| Pulmonary valve | Annulus = 14mm | pulmonic | -------- |
| **Great arteries** | NRGA | **Aortic arch** | Left. No CoA. |
| Aorta | ----- | **PDA** | <1mm PDA, L – R Shunt |
| Pulmonary artery | Normal MPA and Branch PAs. |  |  |
| **M-Mode:**  Normal LV Function on eye balling | | | |
| AO | mm | PWd | mm |
| LA | mm | PWs | mm |
| LVIDd | mm | EDV | ml |
| LVIDs | mm | ESV | ml |
| IVSs | mm | LVEF | % |
| IVSd | mm | FS | % |
| **Additional Information**: |  | | |
| No pericardial/Pleural effusion. | | | |
| **Final Diagnosis:** | | | |
| 1. {S, D, S} Levocardia. 2. PFO, L – R Shunt 3. Silent PDA, L – R Shunt 4. Normal LV Systolic Function | | | |
| **Remark**: | | | |
| **Recommendation**: | | | |
| SIGNATURE  Done by: Tesfaye T., Pediatrician, Pediatric Cardiologist _______________ 09/09/2015Eth.C | | | |

| Patient Name: **Eldana Sisay**. Referring Institute: **FHRH**. SEX/ Age: **F/12years**. Date of Report: **09/09/15**.  Referral Diagnosis: **FTT. AGH12.3530.** | | | |
| --- | --- | --- | --- |
| **Features** | **Finding** | **Features** | **Finding** |
| **Profile** |  | **Atria** |  |
| Abdominal situs | Solitus | Left atrium | Normal |
| Atrial Situs | Solitus | Right atrium | Chiari Network |
| Cardiac position | Levocardia | **Atrioventricular valves** |  |
| Systemic venous drainage | Normal. | Mitral valve | Annulus = 16mm |
| Pulmonary venous drainage | Normal | Tricuspid valve | Annulus = 16mm |
| Atrioventricular connection | Concordant |  | TAPSE = mm |
| Ventriculoarterial connection | Concordant | **Ventricles** |  |
| Ventricular loop | d-Loop | Left ventricle | Normal |
|  |  | Right ventricle | Normal |
| **Septae** |  | **Coronary arteries** | ----- |
| Interventricular septum | Intact | **Doppler Measurement** |  |
| Interatrial septum | Intact | Mitral | ----- |
| **Semilunar valves** |  | Aortic | ------- |
| Aortic valve | Annulus = 15mm | Tricuspid | ------- |
| Pulmonary valve | Annulus = 16mm | pulmonic | -------- |
| **Great arteries** | NRGA | **Aortic arch** | Left. No CoA. |
| Aorta | ----- | **PDA** | No |
| Pulmonary artery | Normal MPA and Branch PAs. |  |  |
| **M-Mode:** | | | |
| AO | mm | PWd | mm |
| LA | mm | PWs | mm |
| LVIDd | mm | EDV | ml |
| LVIDs | mm | ESV | ml |
| IVSs | mm | LVEF | 68% |
| IVSd | mm | FS | 36% |
| **Additional Information**: |  | | |
| No pericardial/Pleural effusion. | | | |
| **Final Diagnosis:** | | | |
| 1. {S, D, S} Levocardia. 2. Chiari network | | | |
| **Remark**: Incidental finding. No clinical significance (Risk for thromboembolic events) | | | |
| **Recommendation**: | | | |
| SIGNATURE  Done by: Tesfaye T., Pediatrician, Pediatric Cardiologist _______________ 09/09/2015Eth.C | | | |

| Patient Name: **Yalem-Sira Tessema**. Referring Institute: **FHRH**. SEX/ Age: **F/9months**. Date of Report: **09/09/15**.  Referral Diagnosis: **Fast Breathing + Systolic Murmur. AGH12.3531.** | | | |
| --- | --- | --- | --- |
| **Features** | **Finding** | **Features** | **Finding** |
| **Profile** |  | **Atria** |  |
| Abdominal situs | Solitus | Left atrium | Normal |
| Atrial Situs | Solitus | Right atrium | Normal |
| Cardiac position | Levocardia | **Atrioventricular valves** |  |
| Systemic venous drainage | Normal. | Mitral valve | Annulus = 12mm |
| Pulmonary venous drainage | Normal | Tricuspid valve | Annulus = 12mm |
| Atrioventricular connection | Concordant |  | TAPSE = mm |
| Ventriculoarterial connection | Concordant | **Ventricles** |  |
| Ventricular loop | d-Loop | Left ventricle | Normal |
|  |  | Right ventricle | Normal |
| **Septae** |  | **Coronary arteries** | ----- |
| Interventricular septum | Intact | **Doppler Measurement** |  |
| Interatrial septum | Intact | Mitral | ----- |
| **Semilunar valves** |  | Aortic | ------- |
| Aortic valve | Annulus = 11mm | Tricuspid | ------- |
| Pulmonary valve | Annulus = 12mm | pulmonic | -------- |
| **Great arteries** | NRGA | **Aortic arch** | Left. No CoA. |
| Aorta | ----- | **PDA** | No |
| Pulmonary artery | Normal MPA and Branch PAs. |  |  |
| **M-Mode:** | | | |
| AO | mm | PWd | mm |
| LA | mm | PWs | mm |
| LVIDd | mm | EDV | ml |
| LVIDs | mm | ESV | ml |
| IVSs | mm | LVEF | % |
| IVSd | mm | FS | % |
| **Additional Information**: |  | | |
| No pericardial/Pleural effusion. | | | |
| **Final Diagnosis:** | | | |
| 1. Normal Echocardiography Study. | | | |
| **Remark**: | | | |
| **Recommendation**: | | | |
| SIGNATURE  Done by: Tesfaye T., Pediatrician, Pediatric Cardiologist _______________ 09/09/2015Eth.C | | | |

| Patient Name: **Liya Yibeltal**. Referring Institute: **FHRH**. SEX/ Age: **F/3 4/12**. Date of Report: **09/09/15**.  Referral Diagnosis: **DS. AGH12.3532.** | | | |
| --- | --- | --- | --- |
| **Features** | **Finding** | **Features** | **Finding** |
| **Profile** |  | **Atria** |  |
| Abdominal situs | Solitus | Left atrium | Normal |
| Atrial Situs | Solitus | Right atrium | Normal |
| Cardiac position | Levocardia | **Atrioventricular valves** |  |
| Systemic venous drainage | Normal. | Mitral valve | Annulus = 15mm |
| Pulmonary venous drainage | Normal | Tricuspid valve | Annulus = 16mm |
| Atrioventricular connection | Concordant |  | TAPSE = 14mm |
| Ventriculoarterial connection | Concordant | **Ventricles** |  |
| Ventricular loop | d-Loop | Left ventricle | Normal |
|  |  | Right ventricle | Normal |
| **Septae** |  | **Coronary arteries** | ----- |
| Interventricular septum | Intact | **Doppler Measurement** |  |
| Interatrial septum | Intact | Mitral | ----- |
| **Semilunar valves** |  | Aortic | ------- |
| Aortic valve | Annulus = 11mm | Tricuspid | Trivial TR, PPG = 10mmHg |
| Pulmonary valve | Annulus = 13mm | pulmonic | -------- |
| **Great arteries** | NRGA | **Aortic arch** | Left. No CoA. |
| Aorta | ----- | **PDA** | No |
| Pulmonary artery | Normal MPA and Branch PAs. |  |  |
| **M-Mode:** | | | |
| AO | mm | PWd | mm |
| LA | mm | PWs | mm |
| LVIDd | mm | EDV | ml |
| LVIDs | mm | ESV | ml |
| IVSs | mm | LVEF | 60% |
| IVSd | mm | FS | 31% |
| **Additional Information**: |  | | |
| 4mm circumferential pericardial effusion. | | | |
| **Final Diagnosis:** | | | |
| 1. {S, D, S} Levocardia. 2. Trace Circumferential Pericardial effusion | | | |
| **Remark**: | | | |
| **Recommendation**: | | | |
| SIGNATURE  Done by: Tesfaye T., Pediatrician, Pediatric Cardiologist _______________ 09/09/2015Eth.C | | | |

| Patient Name: **Baby of Tiruye Alemu**. Referring Institute: **FHRH**. SEX/ Age: **M/20days**. Date of Report: **09/09/15**.  Referral Diagnosis: **R/O CHD(RD). AGH12.3533.** | | | |
| --- | --- | --- | --- |
| **Features** | **Finding** | **Features** | **Finding** |
| **Profile** |  | **Atria** |  |
| Abdominal situs | Solitus | Left atrium | Normal |
| Atrial Situs | Solitus | Right atrium | Normal |
| Cardiac position | Levocardia | **Atrioventricular valves** |  |
| Systemic venous drainage | Normal. | Mitral valve | Annulus = 10mm |
| Pulmonary venous drainage | Normal | Tricuspid valve | Annulus = 10mm |
| Atrioventricular connection | Concordant |  | TAPSE = mm |
| Ventriculoarterial connection | Concordant | **Ventricles** |  |
| Ventricular loop | d-Loop | Left ventricle | Normal |
|  |  | Right ventricle | Normal |
| **Septae** |  | **Coronary arteries** | ----- |
| Interventricular septum | Intact | **Doppler Measurement** |  |
| Interatrial septum | 5 X 9mm OS ASD, L – R Shunt | Mitral | ----- |
| **Semilunar valves** |  | Aortic | ------- |
| Aortic valve | Annulus = 8mm | Tricuspid | ------- |
| Pulmonary valve | Annulus = 9mm | pulmonic | -------- |
| **Great arteries** | NRGA | **Aortic arch** | Left. No CoA. |
| Aorta | ----- | **PDA** | 1mm PDA, L – R Shunt |
| Pulmonary artery | Normal MPA and Branch PAs. |  |  |
| **M-Mode:**  Normal LV Function on eye balling | | | |
| AO | mm | PWd | mm |
| LA | mm | PWs | mm |
| LVIDd | mm | EDV | ml |
| LVIDs | mm | ESV | ml |
| IVSs | mm | LVEF | % |
| IVSd | mm | FS | % |
| **Additional Information**: |  | | |
| No pericardial/Pleural effusion. | | | |
| **Final Diagnosis:** | | | |
| 1. {S, D, S} Levocardia. 2. Moderate OS ASD, L – R Shunt 3. Small PDA, L – R Shunt 4. Normal LV Systolic Function | | | |
| **Remark**: | | | |
| **Recommendation**: | | | |
| SIGNATURE  Done by: Tesfaye T., Pediatrician, Pediatric Cardiologist _______________ 09/09/2015Eth.C | | | |

| Patient Name: **Baby of Netsanet Asires**. Referring Institute: **FHRH**. SEX/ Age: **M/20days**. Date of Report: **09/09/15**.  Referral Diagnosis: **R/O CHD (RD). AGH12.3534.** | | | |
| --- | --- | --- | --- |
| **Features** | **Finding** | **Features** | **Finding** |
| **Profile** |  | **Atria** |  |
| Abdominal situs | Solitus | Left atrium | Normal |
| Atrial Situs | Solitus | Right atrium | Normal |
| Cardiac position | Levocardia | **Atrioventricular valves** |  |
| Systemic venous drainage | Normal. | Mitral valve | Annulus = 10mm |
| Pulmonary venous drainage | Normal | Tricuspid valve | Annulus = 11mm |
| Atrioventricular connection | Concordant |  | TAPSE = mm |
| Ventriculoarterial connection | Concordant | **Ventricles** |  |
| Ventricular loop | d-Loop | Left ventricle | Normal |
|  |  | Right ventricle | Normal |
| **Septae** |  | **Coronary arteries** | ----- |
| Interventricular septum | Intact | **Doppler Measurement** |  |
| Interatrial septum | PFO, L – R Shunt | Mitral | ----- |
| **Semilunar valves** |  | Aortic | ------- |
| Aortic valve | Annulus = 8mm | Tricuspid | ------- |
| Pulmonary valve | Annulus = 10mm | pulmonic | -------- |
| **Great arteries** | NRGA | **Aortic arch** | Left. No CoA. |
| Aorta | ----- | **PDA** | No |
| Pulmonary artery | Normal MPA and Branch PAs. |  |  |
| **M-Mode:**  Normal LV Function on eye balling | | | |
| AO | mm | PWd | mm |
| LA | mm | PWs | mm |
| LVIDd | mm | EDV | ml |
| LVIDs | mm | ESV | ml |
| IVSs | mm | LVEF | % |
| IVSd | mm | FS | % |
| **Additional Information**: |  | | |
| No pericardial effusion. | | | |
| **Final Diagnosis:** | | | |
| 1. {S, D, S} Levocardia. 2. PFO, L – R Shunt | | | |
| **Remark**: | | | |
| **Recommendation**: | | | |
| SIGNATURE  Done by: Tesfaye T., Pediatrician, Pediatric Cardiologist _______________ 09/09/2015Eth.C | | | |

| Patient Name: **Zemenay Marew**. Referring Institute: **FHRH**. SEX/ Age: **F/1 6/12**. Date of Report: **09/09/15**.  Referral Diagnosis: **DS. AGH12.3535.** | | | |
| --- | --- | --- | --- |
| **Features** | **Finding** | **Features** | **Finding** |
| **Profile** |  | **Atria** |  |
| Abdominal situs | Solitus | Left atrium | Normal |
| Atrial Situs | Solitus | Right atrium | Normal |
| Cardiac position | Levocardia | **Atrioventricular valves** |  |
| Systemic venous drainage | Normal. | Mitral valve | Annulus = 14mm |
| Pulmonary venous drainage | Normal | Tricuspid valve | Annulus = 15mm |
| Atrioventricular connection | Concordant |  | TAPSE = 16mm |
| Ventriculoarterial connection | Concordant | **Ventricles** |  |
| Ventricular loop | d-Loop | Left ventricle | Normal |
|  |  | Right ventricle | Normal |
| **Septae** |  | **Coronary arteries** | ----- |
[truncated: 232,708 more chars]
