# Supplementary material for: Two–Dimensional and Doppler trans-thoracic echocardiographic patterns of suspected pediatric heart diseases at Tibebe-—Ghion specialized Teaching Hospital and Adinas General Hospital, Bahir Dar, North-west Ethiopia:–An experience from an LMIC
Source: PLoS One. 2024 Mar 11;19(3):e0292694. doi: 10.1371/journal.pone.0292694 (PMC10927071; doi:10.1371/journal.pone.0292694)
Supplement: Attachment — Submitted filename: Response to reviewers.docx [file pone.0292694.s004.docx]

**Response to academic editor’s and reviewers’ comments**

1. Responses to academic editor’s comment/Journal requirements:
   1.1. *Please ensure that your manuscript meets PLOS ONE's style requirements, including those for file naming.*

Thanks for the recommendations provided to abide with the PLOS ONE’s style requirements. The manuscript is revised and edited in such a way that suits with the requirements.

1.2. *We noticed you have some minor occurrence of overlapping text with the following previous publication(s).*

Yes it overlaps with the publications mentioned (probably, it might be because of repetitive referencing of the studies in our day to day clinical practice). Thanks for the in depth evaluation of our manuscript. We have re-iterated both of them in our own ways.

1.3. *We note that you have stated that you will provide repository information for your data at acceptance.*

Our statements regarding data availability are “Yes - all data are fully available without restriction” and “All relevant data are within the manuscript and its Supporting Information files.” We didn’t say “ I will provide repository information at acceptance”. Hence, with due respect, I ask you to see and amend it ASAP.

1.4. *We are unable to open your Supporting Information file [Echo Submitted to PLOS ONE.rar].*

I will Re-upload the supporting Information file. It contains all the back ground data.

Remark: Diagnosis and classification, and operational definition part are revised. They were in table format and changed to plain write up.

2. Response to reviewers comments:

Dear reviewers,

Thanks for the bold comments, complements and feedbacks you provided us. Here below are the responses for each points raised.

2.1. Review Comments to the Author and the responses
     2.1.1. Reviewer 1:
            2.1.1.1. *Aim of the study*: comments well accepted and edited. “Our aim is to describe the trans-thoracic echocardiography pattern of paediatric heart diseases and reasons of referral for echocardiography in children referred to Bahir Dar University Tibebe – Ghion Hospital and Adinas General Hospital.”
            2.1.1.2. *Title length*: comment well accepted. The aim is well explained. “pattern”. The data were collected from two different hospitals with different levels and merging them might assume the two hospitals with same status.
            2.1.1.3. *Punctuation and grammar errors*: Well accepted and edited ASAP.
            2.1.1.4. *Abstract section*: comments well accepted and edited. Both the result and conclusion parts are re-iterated. In addition, key words are included. (see track changes and final manuscript version)
            2.1.1.5. *Introduction section:* I agree with removal of paragraph – 4 on page three. It has nothing to do here at this part. ( references will nourish the methods part). Regarding paragraph – 2 on page – 4, in addition to describing the patterns, we aimed to describe the reasons that led children visit echocardiography evaluation. Hence, we would be very happy if it stays there as it is with minor rearrangement and modification. (see the revised manuscript)
            2.1.1.6. *End of introduction*: we revised the objective statement and is now perfectly matching with your recommendation.
            2.1.1.7. *Methods:* data collection methods and sources are included in the manuscript. In addition, who collected data and the decision on doubtful findings is also elaborated ASAP. Echo was done by pediatric cardiologist and in doubtful conditions, the adult cardiologists and radiologists will have a look at and suggest their recommendation. Any archived data with incomplete documentation was not included in the analysis. (see figure 1) Post intervention clients are included in the study as we are evaluating the echocardiography pattern of children with suspected heart disease. It reflects the magnitude of the intervention gap.
            2.1.1.8. *Results*: comment accepted.
- For clarity, 3672 are all clients who underwent echocardiography evaluation at the two hospitals echocardiography laboratory over the four years period. The hospitals are located in an area where only 2 pediatric cardiologists are providing service. One in Gondar, North west Ethiopia and the other in Bahir Dar, where most of the Pediatric clients with suspected heart diseases are referred to for echocardiography evaluation.
- Regarding the number of tables: Yes I agree with the reduction of number of tables. Table 2 and 3 are removed. It is revised in both the Manuscript and revised manuscript with track change.
- Table 1 Heading is revised in a way it was intended to …….. and converted to fig as it is larger in size to accommodate and insert it as table. The Table is intended to show the proportion of reasons of referral for echocardiography evaluation that end up with cardiac disorders/normal findings.
  2.1.1.9. *Discussion*: comments accepted and revised considering the inputs, comments and complements of the other reviewer too.
  2.1.1.10. Thanks for all the valuable comments and complements.
  2.1.2. Reviewer 2:
         2.1.2.1. Abstract:
                  2.1.2.1.1. The “indications” is replaced by “ reasons of referral for echocardiography”. The numbers might add up to more than the total number of clients. This is because, children might be referred for echocardiography with more than one reason. We put the clarification at the bottom of the table as remark.
                  2.1.2.1.2. Regarding “detection rate”, comments are well accepted. It was intended to show the proportion of reasons of referrals with positive cardiac findings. It is amended and revised that way.
                  2.1.2.1.3. Regarding “Univariate analysis”, once again comments are well taken. It is a descriptive analysis and amended ASAP.
         2.1.2.2. Introduction:
                  2.1.2.2.1. Too long introduction: comment is well accepted and shortened as recommended.
         2.1.2.3. Methods:
                  2.1.2.3.1. Settings: comment accepted. The echocardiography models used for diagnostic assessment are described and mentioned. The capacity of the hospitals is elaborated with the extent of service and where the clients are getting services.
                  2.1.2.3.2. Analysis: comment accepted and it is descriptive analysis. Univariate analysis is used synonymously with descriptive analysis in some references and we found it that it is confusing. For clarity purpose and comforting the readers, we preferred to use the phrase “ descriptive analysis”.
                  2.1.2.3.3. “Ethical approval”: it is mentioned in the study design sub section. and the minute is already uploaded as a separate file during submission. In addition, as per your recommendation, I have added “Institutional Ethics Committee issued approval protocol number: 795/2023. This hospital-based retrospective observational study was approved by the Institutional Review Board of Bahir Dar University College of Medicine and Health sciences and was approved without comments.” In the disclosure part.
                  2.1.2.3.4. Regarding “ WHF 2012 Borderline RHD Criteria Vs. Stage A RHD”: thanks for the update. I included it in the operational definition and discussed it in the discussion part. Tried to show the difference and similarity.
         2.1.2.4. Results:
                  2.1.2.4.1. Figure 1: comment accepted and edited. it is edited and brought with a better one. In some of our tables and figures, the percent and numbers add up more than hundred percent and the total number of cases because a single client might have more than one cardiac lesion. For example Congenital Heart disease and acquired heart disease. So the readers are not expected to add up the numbers.
                  2.1.2.4.2. “Indications” is revised as “reasons of referral for echocardiography”. They can be single or multiple for a single client which in turn will increase both the precent and number more than 100% and total number of cases. Hence, we recommend the readers not to add up them rather to see the proportion of reasons for referral as it appears. Remarks are there at the bottom of the tables and figures.
                  2.1.2.4.3. Regarding “positive predictive value” : Our intention is not to show the “positive predictive value” rather the proportion of reasons of referral for echocardiography and the cardiac findings and to show which reasons are more common and linked with more cardiac abnormalities. Re-iterated.
                  2.1.2.4.4. Table 3 – 1 and Table 3 – 2 are revised and you are right they were Tables 4 – 1 and 4 – 2.
                  2.1.2.4.5. Regarding the recommendations for use of univariate analysis and reporting associations: it is not our intention and descriptive analysis will not show association rather it is just a description.
                  2.1.2.4.6. Discussion, conclusion and recommendations: they all are re-visited and revised as per all the comments and complements given.
                  2.1.2.4.7. once again, thanks for the comments and complements.
