# Supplementary material for: Two–Dimensional and Doppler trans-thoracic echocardiographic patterns of suspected pediatric heart diseases at Tibebe-—Ghion specialized Teaching Hospital and Adinas General Hospital, Bahir Dar, North-west Ethiopia:–An experience from an LMIC
Source: PLoS One. 2024 Mar 11;19(3):e0292694. doi: 10.1371/journal.pone.0292694 (PMC10927071; doi:10.1371/journal.pone.0292694)
Supplement: Attachment — Submitted filename: Response to reviewers 2.docx [file pone.0292694.s005.docx]

# 1. Response to Academic Editor: Thank you for the critical and valuable feedbacks and comments that nourish our work significantly.

## 1.1. Please add “cross-sectional study” in your study design. The terms mentioned represent the type of study (descriptive) and timeline of data collection (retrospective), not necessarily the study design.

### 1.1.1. Response: comment well accepted and added to the methods part.

## 1.2. The data was collected retrospectively, understandably, from the medical records. Why was verbal consent taken from the patients and how did the authors manage this? I would have expected them to mention they got waiver of consent from IRB.

### 1.2.1. Response: comment well accepted and revised (see previous review comment)

## 1.3. The operational definition section, I know this was motivated by the reviewers’ comments on diagnosis of myocarditis. This diagnosis can be defined in the introduction or methods or discussion sections. Otherwise, it’s needless to give operational definition for all congenital or acquired diseases. It’s sufficient if they are contextually discussed where needed.

### 1.3.1. Response: Comment accepted and removed and inserted ASAP.

## 1.4. The statistical section is vague. It needs clarification on what analysis was used for what (e.g., numerical variables were presented as mean±SD or median (interquartile range) based on …, categorical variables were presented …, etc.)

### 1.4.1. Response: comment well accepted and re-visited.

## 1.5. Page 7, last paragraph, “Out of the 3, 647 pediatric clients with complete documentation and first-time visits; 2,273 children had one or more cardiac diseases on echocardiography”. Is it to mean one or more cardiac lesions? Cardiac disease is not the same as cardiac lesion.

### 1.5.1. Response: It is to mean cardiac lesion. Revised as per the recommendation.

## 1.6. Traditionally, table 1 is meant to describe baseline characteristics of study participants, not results. In addition, the classification as neonate, infant, etc. is not appropriate as the newborn is also included in the infant category. For clarity, it’s better to mention the age classification that the authors used (e.g., 0-28 days, 1-12 months, etc). in addition, this table contains a lot of cells with zero values which made the table so redundant. Some small numbers can be lumped together as “others”. This table needs to be contracted to no more than half a page; otherwise, it will be difficult for the readership to capture the message or even to read.

### 1.6.1. Response 1: the baseline characteristics of study participants in our study are age and gender of children assessed. The reason we omit this one is that they are reflected in the narration, table and figures parts in one or another way.

### 1.6.2. Response 2: the comment on the classification of age of children assessed is well accepted and corrected as per the recommendation provided.

### 1.6.3. Response 3: comment regarding the size of the table is well accepted and contracted as much as possible.

## 1.7. Figure 4 is redundant. The information has already been included in table 1.

### 1.7.1. Response: comment accepted and the figure is removed.

## 1.8. Figure 5. The figure legends are given twice, once on the left side of the figure then at the bottom. In my opinion, this figure also doesn’t add much to the message. This figure also indicates that 15 neonates (0.004*3647) were diagnosed for acquired heart diseases. My question would be what were these acquired lesions in the newborns? What was the degree of certainty?

### 1.8.1. Response 1: The figure legends are duplicated. We removed the legend at the bottom.

### 1.8.2. Response 2: The figure’s added value in nourishing our work: It’s added value is not significant as the content in it is already there in table 1. We agree to remove it from the manuscript.

### 1.8.3. Response 3: The number of neonates with acquired heart disease diagnosis: They are only 2 (0.004 *501 = 2). It is from total neonates screened not from total children screened. The acquired heart lesions were Cardiomyopathy associated with perinatal asphyxia and myocarditis. We used operational definition for diagnosis of Cardiomyopathy and myocarditis.

## 1.9. Discussion: I disagree with the last statement of the first paragraph. The authors need to emphasize their results in the context of the objective of the study. Judging the ability and skill of the referring physician was not the objective of the study. There are multitudes of reasons that physicians may refer patients, especially in setting where there are no guidelines. As a matter of fact, the authors mention that the most common reason for referral was detection of murmur, but we don’t in how many of these patients the murmur was substantiated or refuted by the cardiologists.

### 1.9.1. Response 1: comment well accepted and we believe that it has extended out of the objective and it cannot provide a clue for such a judgment to be concluded. It is revised.

### 1.9.2. Response 2: the most common reason of referral: it only provides the documented reason of referral for echocardiography given by any health care provider. As it is a retrospective analysis of documentation at echo lab., it was not possible to get a documentation that substantiated or refuted the finding based on further clinical evaluation by cardiologists and our objective was not to substantiate or refute it. We revised this part of the discussion in such a way to be clear for readers.

## 1.10. Page 12, last paragraph, statement about a US study doesn’t fit the context of your study and I recommend that you remove the statement and the reference.

### 1.10.1. Response: comment well accepted and it is removed.

## 1.11. Page 13, 1st paragraph, mortality should be included as one of the reasons. Newborns with critical CHD may be misdiagnosed or may not even make it to the hospital which complicates your comparison. However, there is also an issue comparing it with a Cameroonian study and cite these reasons as explanations because the facility and infrastructure in Cameroon is not expected to be much different than that of yours. I recommend that you cite literature in a context that supports your arguments.

### 1.11.1. Response: comment well accepted. Reasons for higher proportion of children in the age group from one month to twelve months are updated as recommended. Evidences that doesn’t support our argument are removed.

## 1.12. Page 13, last but not one paragraph, again the cited literatures are from low-income similar settings but when giving explanations, the authors say the difference is because of the setting, facilities, or maternal infections. This doesn’t make sense as compared settings are not that different.

### 1.12.1. Response: evidences that are not substantiating our discussion are removed.

## 1.13. The discussion section needs overhaul. My recommendation and personal preference:

### 1.13.1. Response: comment well accepted and revised as much as possible to suit the recommendations provided.

## 1.14. Journals need to be written using their standard abbreviations and this needs to be applied throughout the reference list.

### 1.14.1. Response: comment accepted and done ASAP.

## 1.15. PLOS ONE does not copy edit manuscripts. Therefore, the authors need to attend to grammatical, syntax and punctuation errors: preferably seek help from native English speakers if possible.

### 1.15.1. Response : comment accepted.

# 2. Response to reviewer 2: thank you very much for tirelessly evaluating our work tirelessly and commenting constructively.

## 2.1. Please stress that your paper is only descriptive and mention that no further analytic analysis was made. This will make it clear for the end users as to the questions they might have while reading your paper. Please include this as the limitation of your study and if possible quote also the reasons for that like data availability issue etc.

### 2.1.1. Response: comment accepted well and revised as per the recommendation.

## 2.2. Please include the interquartile range for the median age of study participants on page 7.

### 2.2.1. Response: comment accepted and IQR is included.

## 2.3. Please discuss the issue of making a diagnosis of myocarditis or cardiomyopathy in the face of an underlying heart disease or mention it as a limitation as you have a group patients who ended up with it.

### 2.3.1. Response: comment accepted and revised.

## 2.4. You still have many figures, can it be reduced? Like what is the main difference between Stable 1 and Figure 5. They have related information. Please drop one of them.

### 2.4.1. Response: comment accepted. Stable 1, figure 4 and figure 5 are removed as they can be reflected in the other tables and figures.

## 2.5. Figure 3 is challenging for end users i.e. proportions vs clinical symptoms. However, as the authors opted out for only descriptive report, then it can be kept as it is. Please move it to supplemental section.

### 2.5.1. Response: the intention of presenting figure three here is to show the proportion of children identified with heart disease against the presenting complaints/reasons of referral for echocardiography. It is only for descriptive report. We pushed it to supplemental section.

## 2.6. Please make the recommendation short and precise and based on your study findings. It is long as it is now.

### 2.6.1. Response: comment well accepted and revised.

## 2.7. Please address the missing capitalization and syntax issues in the paper one more time.

### 2.7.1. Response: comment accepted and revised.
